# Multiple reader comparison of 2D TOF, 3D TOF, and CEMRA in screening of the carotid bifurcations: Time to reconsider routine contrast use?

**DOI:** 10.1371/journal.pone.0237856

**Published:** 2020-09-02

**Authors:** Jeffrey S. Ross, Skye A. Buckner Petty, Waleed Brinjikji, Joseph M. Hoxworth, Vance T. Lehman, Erik H. Middlebrooks, Ameet C. Patel, Christopher P. Wood

**Affiliations:** 1 Department of Radiology, Mayo Clinic Arizona, Phoenix, Arizona, United States of America; 2 Department of Research Biostatistics, Mayo Clinic Arizona, Phoenix, Arizona, United States of America; 3 Department of Radiology, Mayo Clinic, Rochester, Minnesota, United States of America; 4 Department of Radiology, Mayo Clinic Jacksonville, Jacksonville, Florida, United States of America; Brigham and Women's Faulkner Hospital, UNITED STATES

## Abstract

**Background and purpose:**

MR contrast-enhanced techniques are undergoing increased scrutiny since the FDA applied a warning for gadolinium-based MR contrast agents due to gadolinium deposition within multiple organ systems. While CE-MRA provides excellent image quality, is it required in a screening carotid study? This study compares 2D TOF and 3D TOF MRA vs. CE-MRA in defining carotid stenosis in a large clinical patient population, and with multiple readers with varying experience.

**Materials and methods:**

200 consecutive patients had their carotid bifurcations evaluated with 2D TOF, 3D TOF and CE-MRA sequences by 6 board-certified neuroradiologists. Stenosis and quality of examinations were defined for each study. Inter-rater reliability was assessed using two-way random effects intraclass correlation coefficients. Intra-reader reliability was computed via weighted Cohen’s κ. Weighted Cohen’s κ were also computed to assess agreement in stenosis ratings between enhanced images and unenhanced images.

**Results:**

Agreement between unenhanced and enhanced ratings was substantial with a pooled weighted κ of 0.733 (0.628–0.811). For 5 of the 6 readers, the combination of unenhanced 2D TOF and 3D TOF showed better agreement with contrast-enhanced than either 2D TOF or 3D TOF alone. Intra-reader reliability was substantial.

**Conclusions:**

The combination of 2D TOF and 3D TOF MRA showed substantial agreement with CE-MRA regarding degree of carotid stenosis in this large outpatient population across multiple readers of varying experience. Given the scrutiny that GBCA are undergoing due to concerns regarding CNS and soft tissue deposition, it seems prudent to reserve CE-MRA for cases which are not satisfactorily answered by the nonenhanced study or other noninvasive examinations.

## Introduction

2D TOF MRA, 3D TOF MRA and dynamic contrast-enhanced MRA (CE-MRA) have all been used to evaluate the carotid bifurcations for the degree of stenosis to assist with medical and surgical management decisions. Of the many MR based techniques available, CE-MRA is generally considered the gold standard [1–7]. Multiple studies have demonstrated that both 2D TOF and 3D TOF unenhanced techniques are close to CE-MRA and DSA in sensitivity and specificity for carotid stenosis [5,8–10].

All MR contrast-enhanced techniques are now undergoing increased scrutiny since the FDA applied a class warning for gadolinium-based MR contrast agents (GBCA) with the discovery of gadolinium deposition within multiple organ systems in patients’ bodies after receiving these agents, even in patients without severe renal dysfunction [11–15]. This information has caused changes in type of GBCA administered and a reevaluation of the widespread use of GBCA [16–19].

Evaluation of MRA techniques, both enhanced and unenhanced, tend to be limited by small patient numbers, use within a research setting, or limited reader comparisons. This study adds to the voluminous literature of carotid bifurcation MR imaging by comparing 2D TOF and 3D TOF MRA (alone and in combination) vs. CE-MRA in defining carotid stenosis in a large clinical patient population, and with multiple readers with varying experience.

The hypothesis of this study is that the combination of 2D and 3D TOF MRA has equal agreement to CE-MRA for determining carotid stenosis (based on NASCET criteria) [20]. Given the added expense and potential risks of GBCA, the agreement of the unenhanced to the enhanced technique being corroborated in large outpatient population with a variety of stenoses could improve long term patient safety and decrease cost of imaging.

## Materials and methods

This retrospective study was approved by the Mayo Clinic Institutional Review Board, and written consent waived for chart review. Mayo Clinic Arizona radiology database was searched for contrast-enhanced neck MRA, which also includes both 2D TOF and 3D nonenhanced TOF sequences.

200 consecutive patients who had evaluation of their carotid bifurcations with 2D TOF, 3D TOF and CE sequences as part of routine imaging protocols were included in this study. Patients ranged in age from 29–101 years, with an average of 66.2 years. There were 97 males, and 103 females evaluated.

Indications for the studies varied widely and included stroke (n = 63, 31.5%), headache (n = 36, 18%), follow-up of stenosis or occlusion (n = 27, 13.5%), vertigo or dizziness (n = 16, 8%), syncope (n = 12, 6%), transient ischemic attack (n = 11, 5.5%), vascular disease NOS (n = 6, 3%), vision disturbance (n = 5, 2.5%), family history of cerebrovascular disease (n = 5, 2.5%), aneurysm or AVM follow-up (N = 5, 2.5%), weakness (n = 3, 1.5%), numbness or paresthesia (n = 3, 1.5%), aphasia (n = 3, 1.5%) and cognitive change (n = 2), and one each for seizure, head trauma and preoperative screening.

### MR angiography

All patients were examined on one of two 1.5T MR scanners (GE Signa HD, GE Medical Systems, Milwaukee, Wisconsin) between September 2017 and August 2018. All patients were examined with 2D TOF, 3D TOF and CE sequences, with the TOF sequences performed first with an Head-Neck-Spine coil. 2D TOF and 3D TOF MRA studies were acquired in the transverse orientation to cover the carotid bifurcations with axial coverage of 15cm and 6cm respectively. 2D TOF studies were performed with TR 26, TE 5.1–6.3, 1 excitation, 50-degree flip angle, matrix of 128x256, 1.5mm slice thickness, 16cm FOV, 5.5-minute acquisition time, bandwidth 125 Hz/pixel, voxel size 0.625 x 1.25 x 1.5mm. 3D studies were performed with 26/6.3–6.9 (TR/TE), 2 excitations, 20-degree FA, 128x256 matrix, 1.2mm slice thickness, 20cm FOV, 5-minute acquisition time, 5 overlapping volumes, bandwidth 122 Hz/pixel, voxel size 0.78 x 1.5 x 1.2 mm.

CE studies were performed in the coronal plane with 6.6–7.4ms/2.0–2.2ms (TR/TE), 1 excitation, 45-degree FA, 300-320x256 matrix, 0.7mm slice thickness, 30cm FOV, 1.0 minute acquisition time, voxel size 1 x 1.17 x 0.7 mm. Mask subtraction was utilized. The CE study was acquired with bolus tracking after the administration of 0.1mmol/kg bodyweight of gadolinium-based contrast agent (Gadovist), with an injection rate of 2mL/s, which was followed by a 20mL saline flush.

### Image evaluation

Six board-certified neuroradiologists with varying years of practice since fellowship (2, 4, 7, 12, 19, 27 years) evaluated the image sets. 200 patients were evaluated, with a total of 400 carotid bifurcations, and a total of 1200 sequences (2D, 3D, CE).

Each of the 1200 MRA sequences were curated into 2–4 representative views of the carotid bifurcations by the senior author (JR -32 years of experience) who did not participate in the stenosis grading. All MRA images from all sequences were first evaluated by the senior author, and then similar oblique sagittal MIP projections were chosen across the 2D TOF, 3D TOF and CE-MRA acquisitions. Sagittal oblique MIP projections were picked that showed the greatest narrowing of the proximal internal carotid. When flow voids were present due to stenosis/occlusion, then representative axial images were included at the skull base to help differentiate near occlusion from occlusion.

**Fig 1:**
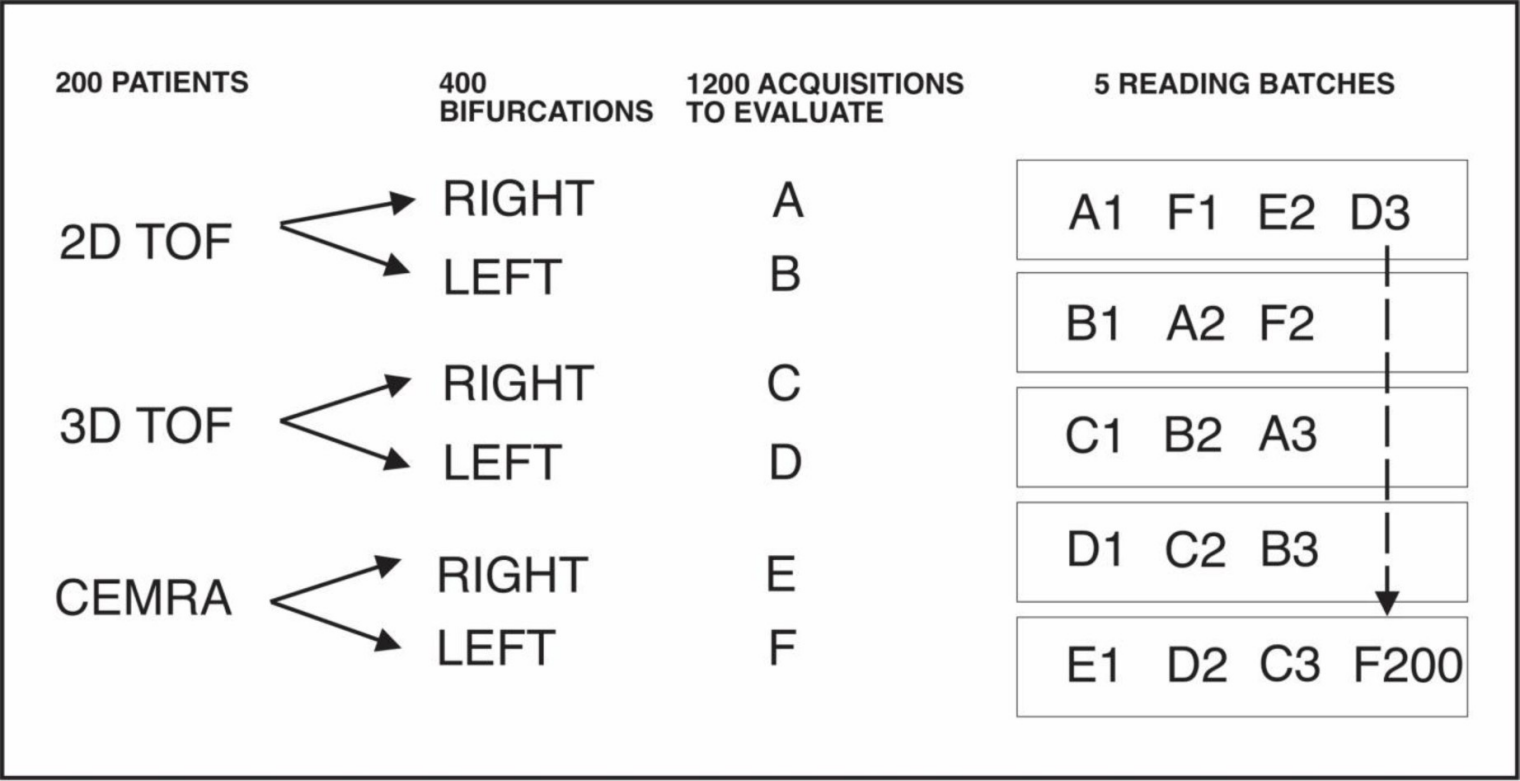
Schematic of image distribution and labeling into the 5 reading batches. Six different MR sequences for each patient were moved into 5 separate reading batches. In any one batch, the same carotid birfurcation was not seen twice.

The sequences were placed into one of five reading batches, with the 5 batches graded sequentially over time by the six readers, with a delay of 1–2 weeks between batch reading to minimize learning. The patient sequences were labeled consecutively as 2D right bifurcation (A), 2D left bifurcation (B), 3D right (C), 3D left (D), CE right (E), CE left (F) (i.e., 1A, 1B, 1C….200E, 200F). Placement of the 6 MRA sequences (A-F) into 5 batches allowed for an offset where each batch had only one MRA sequence of each bifurcation per patient to minimize intra-batch correlations (**Fig 1**). Each reading batch (1–5) contained 240 sequences which were viewed in Adobe Acrobat pdf (Adobe Inc, San Jose, CA) which allowed for direct stenosis measurement. Readers were blinded to other imaging or clinical data. There was no attempt at blinding of the specific sequence types, since they were obvious to these experienced readers.

Stenosis was graded on a six-point scale using NASCET criteria:

Grade 1: 0–30%; grade 2: 31–50%; grade 3: 51–70%; grade 4: >70%; grade 5: Carotid near-occlusion; grade 6: occluded. Near-occlusion was defined by long segment origin signal loss with diminished caliber of the distal cervical internal carotid.

Quality was measured qualitatively on a 5-point scale, 1 being the best and 5 the worst (uninterpretable). Quality was judged based on ideal appearance for the specific sequence being evaluated and not related to quality of 2D TOF vs 3D TOF or CE. The target of the quality interpretation was primarily the carotid system, and not the posterior circulation.

1 = Excellent

2 = Good

3 = Fair

4 = Poor

5 = Uninterpretable

Intra-observer variability was assessed by having a second interpretation of batch 3 (240 sequences) by all six readers greater than 1 week after completing the last batch 5.

### Statistics

To assess inter-rater reliability among the 6 raters, two-way random effects intraclass correlation coefficients (ICC) were computed for ratings of stenosis and quality for each image type. 2D and 3D TOF measurements of each reader were compared against their own measurement on the corresponding CE images for each subject. Thus the ICC is a summary statistic of variation between readers on each carotid, in that the greater variability between readers leads to lower ICC. Based on Cicchetti [21], when the coefficient is below 0.40 the level of significance is poor; when between 0.40 and 0.59 it is fair; when between 0.60 and 0.74 is good; and between 0.75 and 1.00 is excellent.

To assess intra-rater reliability, each rater was randomly assigned 80 of each of the three types of images to provide a second set of ratings. Agreement between first and second ratings for each rater was computed via weighted (quadratic) Cohen’s Kappa (κ) coefficients. Weighted Cohen’s Kappa coefficients were also computed to assess agreement in stenosis ratings between enhanced images and unenhanced images (2D TOF, 3D TOF, and average of 2D TOF and 3D) among the 6 raters. Pooled estimates of the kappa coefficients were computed based on the method suggested by De Vries et al [22]. For each image we computed the mean of the 2D TOF and 3D TOF ordinal ratings and rounded to the nearest whole number. Enhanced images with image quality of 4.5 (uninterpretable) or greater were excluded from this analysis since the CE-MRA was used as the gold standard. Non-parametric bootstrapping was used to compute 95% confidence intervals around all kappa values. We computed sensitivity, specificity, and positive predictive values with 95% confidence intervals for the unenhanced ratings of >70% stenosis using the enhanced ratings as the gold-standard. All analyses were conducted using R version 3.6 (https://www.r-project.org/).

## Results

**Table 1** shows inter-rater reliability (agreement between raters). The 2D TOF stenosis measurements showed an intraclass correlation (ICC) of 0.810 (0.783–0.835 95% CI), 3D TOF stenosis 0.808 (0.783–0.833) and CE-MRA 0.728 (0.693–0.760). Agreement was strong for stenosis ratings despite weaker agreement for quality ratings.

**Table 1:** Inter-rater reliability.

	Reader 1 (N = 400)	2 (N = 400)	3 (N = 400)	4 (N = 400)	5 (N = 400)	6 (N = 400)	Total (N = 2400)	ICC (95% CI)
**STENOSIS 2D TOF**								0.810 (0.783, 0.835)
1 (0–30%)	345 (86.2%)	344 (86.0%)	332 (83.0%)	355 (88.8%)	325 (81.2%)	277 (69.2%)	1978 (82.4%)	
2 (31–50%)	23 (5.8%)	24 (6.0%)	30 (7.5%)	14 (3.5%)	27 (6.8%)	66 (16.5%)	184 (7.7%)	
3 (51–70%)	10 (2.5%)	14 (3.5%)	24 (6.0%)	9 (2.2%)	23 (5.8%)	36 (9.0%)	116 (4.8%)	
4 (>70%)	14 (3.5%)	8 (2.0%)	4 (1.0%)	3 (0.8%)	12 (3.0%)	10 (2.5%)	51 (2.1%)	
5 (near occlusion)	1 (0.2%)	5 (1.2%)	2 (0.5%)	8 (2.0%)	7 (1.8%)	4 (1.0%)	27 (1.1%)	
6 (occluded)	7 (1.8%)	5 (1.2%)	8 (2.0%)	11 (2.8%)	6 (1.5%)	7 (1.8%)	44 (1.8%)	
**QUALITY 2D**								0.481 (0.346, 0.591)
1	142 (35.5%)	34 (8.5%)	125 (31.2%)	114 (28.5%)	153 (38.2%)	14 (3.5%)	582 (24.2%)	
2	161 (40.2%)	198 (49.5%)	192 (48.0%)	145 (36.2%)	175 (43.8%)	107 (26.8%)	978 (40.8%)	
3	57 (14.2%)	130 (32.5%)	52 (13.0%)	99 (24.8%)	55 (13.8%)	146 (36.5%)	539 (22.5%)	
4	31 (7.8%)	35 (8.8%)	27 (6.8%)	33 (8.2%)	15 (3.8%)	91 (22.8%)	232 (9.7%)	
5	9 (2.2%)	3 (0.8%)	4 (1.0%)	9 (2.2%)	2 (0.5%)	42 (10.5%)	69 (2.9%)	
**STENOSIS 3D TOF**								0.808 (0.783, 0.833)
1 (0–30%)	350 (87.5%)	366 (91.5%)	358 (89.5%)	357 (89.2%)	347 (86.8%)	297 (74.2%)	2075 (86.5%)	
2 (31–50%)	17 (4.2%)	13 (3.2%)	16 (4.0%)	14 (3.5%)	20 (5.0%)	62 (15.5%)	142 (5.9%)	
3 (51–70%)	10 (2.5%)	7 (1.8%)	7 (1.8%)	7 (1.8%)	12 (3.0%)	24 (6.0%)	67 (2.8%)	
4 (>70%)	10 (2.5%)	4 (1.0%)	3 (0.8%)	4 (1.0%)	9 (2.2%)	7 (1.8%)	37 (1.5%)	
5 (near occlusion)	2 (0.5%)	5 (1.2%)	5 (1.2%)	6 (1.5%)	5 (1.2%)	4 (1.0%)	27 (1.1%)	
6 (occluded)	11 (2.8%)	5 (1.2%)	11 (2.8%)	12 (3.0%)	7 (1.8%)	6 (1.5%)	52 (2.2%)	
**QUALITY 3D**								0.426 (0.280, 0.548)
1	191 (47.8%)	146 (36.5%)	100 (25.0%)	189 (47.2%)	177 (44.4%)	15 (3.8%)	818 (34.1%)	
2	140 (35.0%)	174 (43.5%)	242 (60.5%)	135 (33.8%)	157 (39.3%)	123 (30.8%)	971 (40.5%)	
3	34 (8.5%)	69 (17.2%)	52 (13.0%)	59 (14.8%)	58 (14.5%)	147 (36.8%)	419 (17.5%)	
4	32 (8.0%)	9 (2.2%)	6 (1.5%)	15 (3.8%)	7 (1.8%)	83 (20.8%)	152 (6.3%)	
5	3 (0.8%)	2 (0.5%)	0 (0.0%)	2 (0.5%)	0 (0.0%)	32 (8.0%)	39 (1.6%)	
**STENOSIS grade CE**								0.728 (0.693, 0.760)
1 (0–30%)	348 (87.0%)	342 (85.5%)	332 (83.0%)	341 (85.2%)	315 (78.8%)	266 (66.5%)	1944 (81.0%)	
2 (31–50%)	21 (5.2%)	34 (8.5%)	30 (7.5%)	26 (6.5%)	27 (6.8%)	70 (17.5%)	208 (8.7%)	
3 (51–70%)	10 (2.5%)	5 (1.2%)	12 (3.0%)	8 (2.0%)	22 (5.5%)	43 (10.8%)	100 (4.2%)	
4 (>70%)	11 (2.8%)	2 (0.5%)	6 (1.5%)	2 (0.5%)	9 (2.2%)	7 (1.8%)	37 (1.5%)	
5 (near occlusion)	3 (0.8%)	9 (2.2%)	5 (1.2%)	5 (1.2%)	13 (3.2%)	4 (1.0%)	39 (1.6%)	
6 (occluded)	7 (1.8%)	8 (2.0%)	15 (3.8%)	18 (4.5%)	14 (3.5%)	10 (2.5%)	72 (3.0%)	
**QUALITY CE**								0.631 (0.582, 0.676)
1	152 (38.0%)	185 (46.2%)	105 (26.2%)	219 (54.8%)	179 (44.8%)	121 (30.2%)	961 (40.0%)	
2	139 (34.8%)	138 (34.5%)	237 (59.2%)	104 (26.0%)	151 (37.8%)	157 (39.2%)	926 (38.6%)	
3	54 (13.5%)	55 (13.8%)	35 (8.8%)	49 (12.2%)	53 (13.2%)	55 (13.8%)	301 (12.5%)	
4	40 (10.0%)	14 (3.5%)	16 (4.0%)	10 (2.5%)	16 (4.0%)	39 (9.8%)	135 (5.6%)	
5	15 (3.8%)	8 (2.0%)	7 (1.8%)	18 (4.5%)	1 (0.2%)	28 (7.0%)	77 (3.2%)	

**Table 2** shows intra-rater reliability (agreement within raters) for each of the 6 readers. While some readers showed better consistency in their ratings than others, agreement was generally strong for most stenosis ratings. Taking all MRA techniques, the intra-rater reliability for stenosis was substantial, with kappa ranging from a low of 0.765 (reader 1, 3D) to a high of 0.986 (reader 4, 3D). Five of 6 readers (except for #3) showed higher intra-rater reliability for unenhanced 2D TOF or 3D TOF relative to the CE-MRA study.

**Table 2:** Intra-rater reliability.

Rater	STENOSIS 2D	QUALITY 2D	STENOSIS 3D	QUALITY 3D	STENOSIS CE	QUALITY CE
**Reader 1** n =	80	80	80	80	80	80
Weighted Cohen’s Kappa	0.955	0.328	0.765	0.521	0.813	0.717
95% CI	(0.835, 0.985)	(0.061, 0.576)	(0.408, 0.968)	(0.334, 0.653)	(0.542, 0.947)	(0.481, 0.852)
**Reader 2** n =	80	80	80	80	80	80
Weighted Cohen’s Kappa	0.946	0.882	0.983	0.943	0.968	0.975
95% CI	(0.772, 1.000)	(0.797, 0.943)	(0.931, 1.000)	(0.889, 0.981)	(0.851, 1.000)	(0.945, 0.994)
**Reader 3** n =	80	80	80	80	80	80
Weighted Cohen’s Kappa	0.886	0.644	0.851	0.710	0.928	0.682
95% CI	(0.709, 0.971)	(0.458, 0.772)	(0.477, 0.989)	(0.538, 0.831)	(0.806, 0.977)	(0.438, 0.806)
**Reader 4** n =	80	80	80	80	80	80
Weighted Cohen’s Kappa	0.918	0.629	0.986	0.787	0.920	0.859
95% CI	(0.710, 0.982)	(0.481, 0.747)	(0.940, 1.000)	(0.681, 0.860)	(0.743, 0.983)	(0.753, 0.925)
**Reader 5** n =	80	80	80	79	80	80
Weighted Cohen’s Kappa	0.923	0.764	0.948	0.670	0.778	0.740
95% CI	(0.797, 0.969)	(0.635, 0.859)	(0.815, 0.992)	(0.530, 0.783)	(0.561, 0.954)	(0.610, 0.832)
**Reader 6** n =	80	80	80	80	80	80
Weighted Cohen’s Kappa	0.850	0.728	0.816	0.752	0.790	0.847
95% CI	(0.639, 0.939)	(0.612, 0.813)	(0.473, 0.919)	(0.623, 0.847)	(0.628, 0.916)	(0.747, 0.910)

**Table 3** shows agreement between unenhanced and enhanced ratings (for each rater and overall). The 4th column compares the average of 2D TOF and 3D TOF against CE-MRA. Agreement was substantial with a pooled weighted κ of 0.733 (0.628–0.811). For 5 of the 6 readers, the combination of unenhanced 2D TOF and 3D TOF showed slightly better agreement with enhanced than either 2D TOF or 3D TOF alone. Reader #1 showed the highest agreement for unenhanced 2D TOF vs CE (0.841), with the combined 2D TOF/3D TOF a close second (0.832).

**Table 3:** Unenhanced vs enhanced agreement.

Rater	STENOSIS 2D vs CE	STENOSIS 3D vs CE	STENOSIS 2D/3D (avg) vs CE
**Overall**			
Pooled Weighted Cohen’s Kappa^1^	0.716	0.693	**0.733**
95% CI	(0.605, 0.8)	(0.566, 0.782)	(0.628, 0.811)
Reader 1			
Weighted Cohen’s Kappa	0.841	0.781	0.832
95% CI	(0.727, 0.908)	(0.625, 0.878)	(0.707, 0.907)
Reader 2			
Weighted Cohen’s Kappa	0.688	0.698	0.709
95% CI	(0.567, 0.787)	(0.564, 0.799)	(0.594, 0.799)
Reader 3			
Weighted Cohen’s Kappa	0.742	0.713	0.774
95% CI	(0.587, 0.846)	(0.549, 0.827)	(0.635, 0.867)
Reader 4			
Weighted Cohen’s Kappa	0.684	0.691	0.711
95% CI	(0.519, 0.808)	(0.509, 0.822)	(0.552, 0.825)
Reader 5			
Weighted Cohen’s Kappa	0.699	0.657	0.711
95% CI	(0.567, 0.801)	(0.509, 0.773)	(0.579, 0.81)
Reader 6			
Weighted Cohen’s Kappa	0.668	0.643	0.679
95% CI	(0.494, 0.786)	(0.447, 0.788)	(0.517, 0.796)

Analysis was also dichotomized into two grades to evaluate the differences in techniques for clinically significant disease: grades 1,2 (stenosis of 0–50%) were evaluated against grades 3–6 (>50% stenosis, occlusion, near occlusion) **(Table 4)**. Agreement for the combined 2D TOF/3D TOF MRA vs CE-MRA continued to be good at 93.4%. Agreement was substantial (κ = 0.607), but decreased from the non-dichotomous evaluation.

**Table 4:** Unenhanced vs enhanced agreement (Dichotomous stenosis rating, grades 1,2 vs 3–6).

Rater	Stenosis 2D TOF vs. CE	Stenosis 3D TOF vs. CE	Stenosis 2D TOF /3D TOF average vs CE
Overall			
% Agreement	92.57	93.47	93.38
Pooled Cohen’s Kappa	0.568	0.577	0.607
95% CI	(0.472, 0.658)	(0.469, 0.669)	(0.516, 0.693)
Reader 1			
% Agreement	94.85	96.39	96.39
Cohen’s Kappa	0.589	0.648	0.677
95% CI	(0.406, 0.741)	(0.443, 0.801)	(0.479, 0.824)
Reader 2			
% Agreement	89.43	90.98	92.01
Cohen’s Kappa	0.575	0.589	0.664
95% CI	(0.452, 0.686)	(0.466, 0.704)	(0.554, 0.763)
Reader 3			
% Agreement	93.04	94.85	94.59
Cohen’s Kappa	0.571	0.628	0.647
95% CI	(0.404, 0.706)	(0.456, 0.768)	(0.495, 0.776)
Reader 4			
% Agreement	94.33	94.59	93.81
Cohen’s Kappa	0.577	0.589	0.553
95% CI	(0.399, 0.721)	(0.413, 0.746)	(0.374, 0.706)
Reader 5			
% Agreement	90.98	90.98	91.75
Cohen’s Kappa	0.580	0.509	0.605
95% CI	(0.444, 0.695)	(0.356, 0.638)	(0.466, 0.718)
Reader 6			
% Agreement	92.78	93.04	91.75
Cohen’s Kappa	0.509	0.534	0.485
95% CI	(0.334, 0.658)	(0.356, 0.685)	(0.315, 0.633)

Regarding quality of the studies, the 2D TOF studies were categorized as good/excellent in 65%, with 2.9% uninterpretable. The 3D TOF studies were categorized as good/excellent in 74.6%, with 1.6% uninterpretable. The CE studies were categorized as good/excellent in 78.6%, with 3.2% uninterpretable.

**Table 5** shows sensitivity, specificity and PPV for each of the readers for stenosis greater or equal to 70%. Sensitivities, specificities and PPV values varied from highs of 0.778, 0.989, 0.750 to lows of 0.5, 0.970, 0.560 respectively.

**Table 5:** Sensitivity/specificity analysis.

READER	2D TOF vs CE-MRA (>70%)	3D TOF vs CE-MRA (>70%)	2D/3D TOF Average vs CE-MRA (>70%)
Reader 1			
Sensitivity (95% CI)	0.812 (0.621, 1.000)	0.688 (0.460, 0.915)	0.750 (0.538, 0.962)
Specificity (95% CI)	0.989 (0.979, 1.000)	0.992 (0.983, 1.000)	0.989 (0.979, 1.000)
PPV (95% CI)	0.765 (0.563, 0.966)	0.786 (0.571, 1.000)	0.750 (0.538, 0.962)
Reader 2			
Sensitivity (95% CI)	0.722 (0.515, 0.929)	0.667 (0.449, 0.884)	0.722 (0.515, 0.929)
Specificity (95% CI)	0.981 (0.967, 0.995)	0.986 (0.975, 0.998)	0.984 (0.971, 0.997)
PPV (95% CI)	0.650 (0.441, 0.859)	0.706 (0.489, 0.922)	0.684 (0.475, 0.893)
Reader 3			
Sensitivity (95% CI)	0.571 (0.360, 0.783)	0.619 (0.411, 0.827)	0.619 (0.411, 0.827)
Specificity (95% CI)	0.997 (0.992, 1.000)	0.984 (0.971, 0.997)	0.984 (0.971, 0.997)
PPV (95% CI)	0.923 (0.778, 1.000)	0.684 (0.475, 0.893)	0.684 (0.475, 0.893)
Reader 4			
Sensitivity (95% CI)	0.667 (0.449, 0.884)	0.778 (0.586, 0.970)	0.778 (0.586, 0.970)
Specificity (95% CI)	0.976 (0.960, 0.991)	0.978 (0.964, 0.993)	0.970 (0.953, 0.988)
PPV (95% CI)	0.571 (0.360, 0.783)	0.636 (0.435, 0.837)	0.560 (0.365, 0.755)
Reader 5			
Sensitivity (95% CI)	0.577 (0.387, 0.767)	0.500 (0.308, 0.692)	0.500 (0.308, 0.692)
Specificity (95% CI)	0.972 (0.955, 0.989)	0.978 (0.963, 0.993)	0.978 (0.963, 0.993)
PPV (95% CI)	0.600 (0.408, 0.792)	0.619 (0.411, 0.827)	0.619 (0.411, 0.827)
Reader 6			
Sensitivity (95% CI)	0.667 (0.465, 0.868)	0.667 (0.465, 0.868)	0.667 (0.465, 0.868)
Specificity (95% CI)	0.978 (0.963, 0.993)	0.975 (0.960, 0.991)	0.975 (0.960, 0.991)
PPV (95% CI)	0.636 (0.435, 0.837)	0.609 (0.409, 0.808)	0.609 (0.409, 0.808)

Contingency tables for CE-MRA ratings vs unenhanced 2D TOF and 3D TOF ratings for overall results and individual readers are present in **S1 Material**. Also included in the Supplement are bubble plots with cell counts to visually show the agreement between readers for the 2D TOF, 3D TOF and CE-MRA studies.

**Fig 2:**
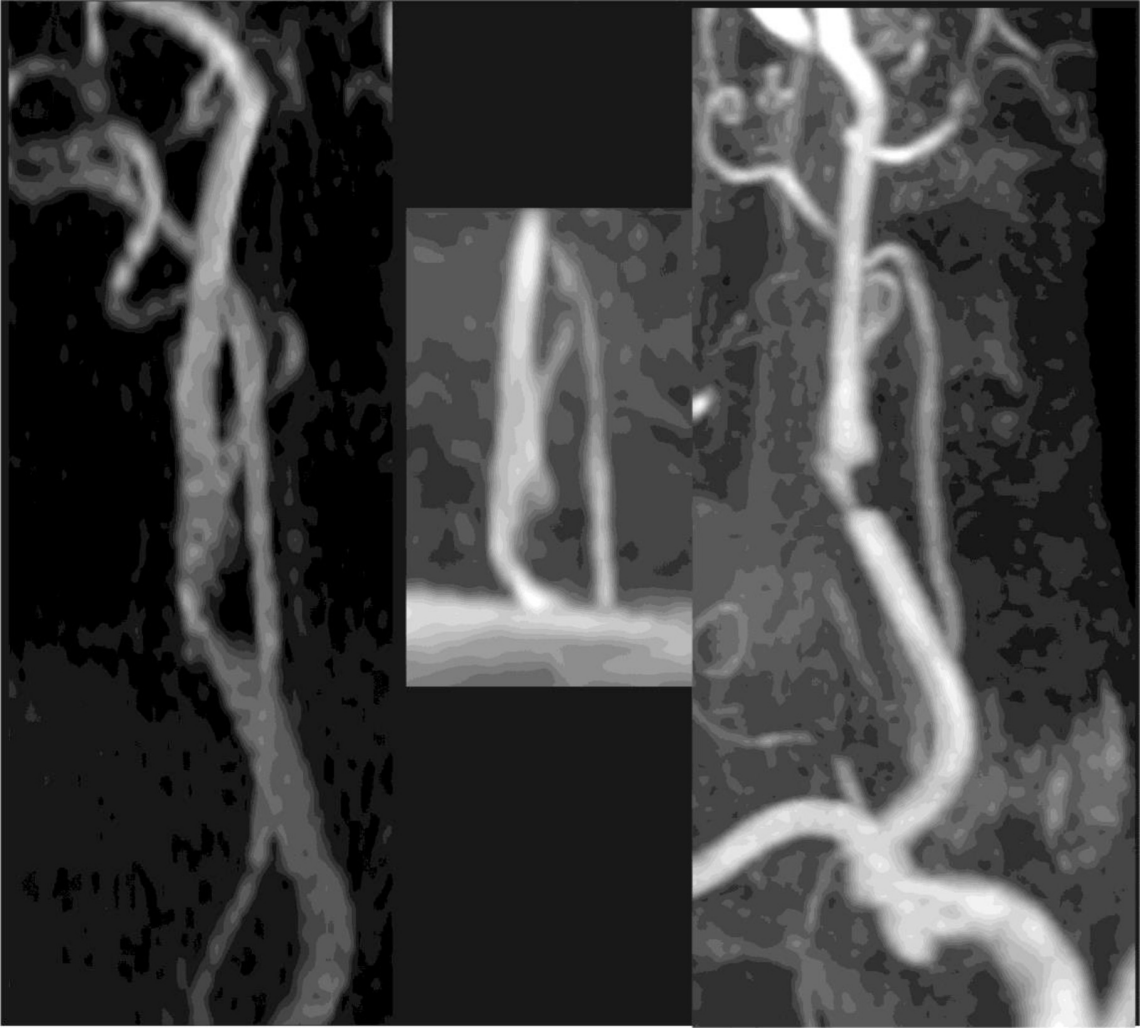
MRA images chosen to show variability of 3D TOF undercalling stenosis relative to CE-MRA and 2D TOF MRA. Variability of interpretation of 2D (left), 3D (middle) and CE-MRA (right) for patient 9. Scores for the 6 readers were: 2D- 3,3,1,1,4,3 (average 2.5); 3D-1,1,1,1,2,2 (1.3); CE- 2,3,2,1,5,3 (2.7) where 1 = 0–30% stenosis, 2 = 31–50%, 3 = 51–70, 4 = >70%, 5 = near occlusion, 6 = occluded. Average quality ratings were 2DTOF = 2.6, 3DTOF = 3.8, CEMRA = 1.8.

**Fig 3:**
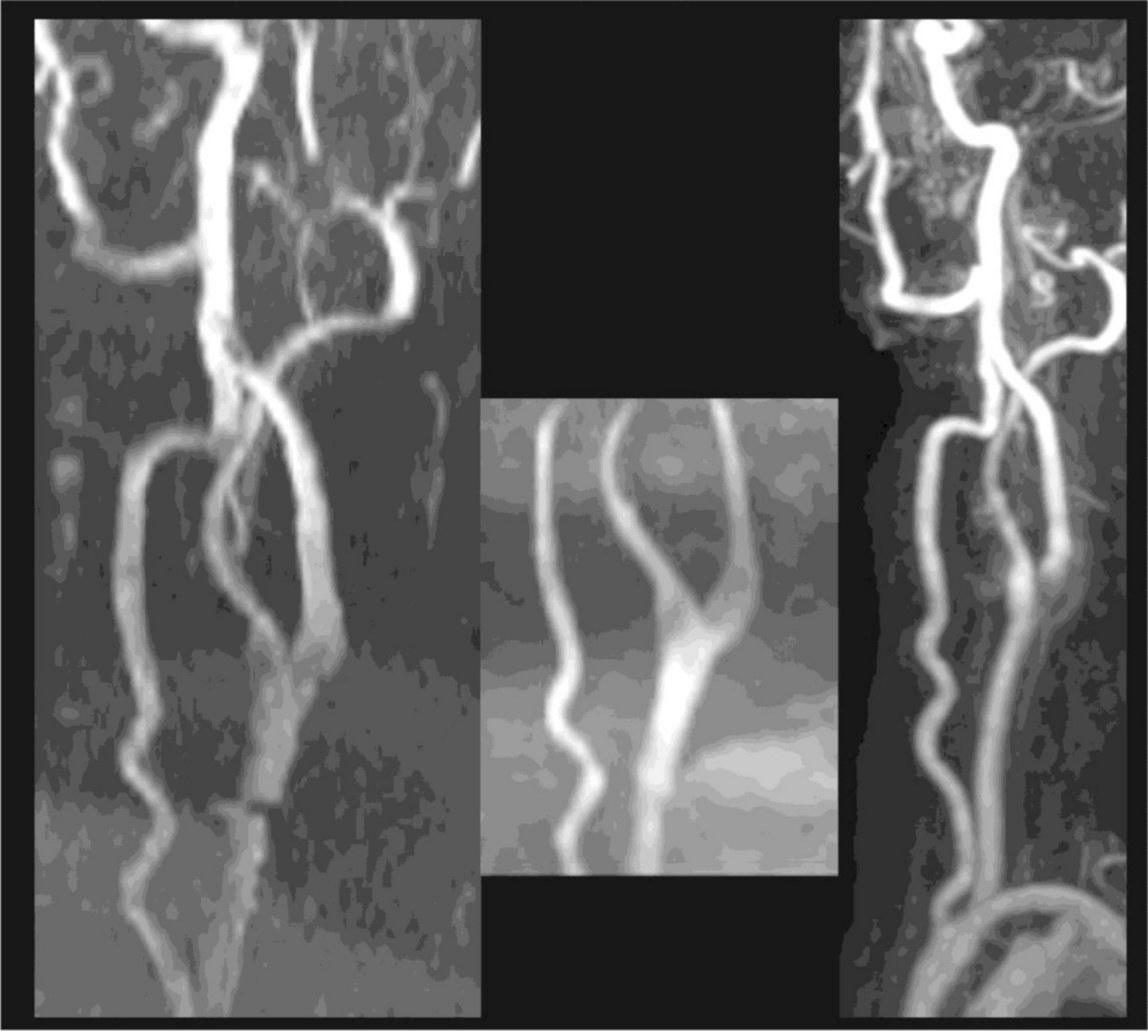
MRA images chosen to show variability of CE-MRA interpretations overcalling stenosis relative to TOF studies. Variability of interpretation of 2D (left), 3D (middle) and CE-MRA (right) for patient 113. Scores for the 6 readers were: 2D- 1,2,1,1,1,1 (average 1.2); 3D-1,1,1,1,1,1 (1.0); CE- 1,3,1,5,5,4 (3.2) where 1 = 0–30% stenosis, 2 = 31–50%, 3 = 51–70, 4 = >70%, 5 = near occlusion, 6 = occluded. Average quality rating were 2DTOF = 2.5, 3DTOF = 1.5, CEMRA = 3.1.

Representative image sets demonstrating the variability of reader interpretations are shown in **Figs 2 and 3.**

## Discussion

This study has shown that the combination of 2D TOF and 3D TOF MRA showed substantial agreement with CE-MRA regarding degree of carotid stenosis in this large outpatient population across multiple readers of varying experience. We observed good agreement between readers despite having varied experience levels. Given the increased scrutiny that GBCA are undergoing due to concerns regarding CNS, soft tissue and bone marrow deposition, it seems prudent to reserve CE-MRA for those cases with are not satisfactorily answered by the nonenhanced study or other noninvasive examination [15,23,24]. That is, recalling the patient for a contrast study in the more limited situations where more information is needed, for vertebrobasilar symptoms or specific problem solving. Patients who are being evaluated preoperatively for symptomatic anterior circulation disease and have additional noninvasive imaging of the carotids (US, CTA) might also benefit from use of a CE-MRA evaluation to better define the vessel origins and proximal common carotid arteries. The European Society for Vascular Surgery guidelines give a class I recommendation that when carotid endarterectomy is being considered, that Duplex ultrasound stenosis estimation be corroborated by computed tomographic angiography or magnetic resonance angiography, or by a repeat Duplex ultrasound performed by a second operator [25]. The “14 Society” guidelines state that for evaluation of symptomatic disease: In patients with acute, focal ischemic neurological symptoms corresponding to the territory supplied by the left or right internal carotid artery, magnetic resonance angiography (MRA) or computed tomography angiography (CTA) is indicated to detect carotid stenosis when sonography either cannot be obtained or yields equivocal or otherwise nondiagnostic results [26].

This study demonstrates that while unenhanced MRA methods may not quite achieve the level of accuracy of CE-MRA, the unenhanced methods are likely satisfactory, particularly if being used as a screening examination in a high-volume outpatient setting with heterogeneous population with variable presenting symptoms. Our study is novel and adds to the extensive body of literature by having a large number of patients (200), multiple MRA sequences (2D TOF, 3D TOF, CE) and multiple readers [6] with varied experience where both intra- and inter-reader variabilities were defined.

High sensitivities ranging from 86% to 90% for detection of severe stenosis are generally reported for unenhanced techniques [27]. The sensitivity and specificity of 3D TOF MRA relative to rotational catheter angiography has been reported as 95.5% and 87.2% [7]. Babiarz et al showed sensitivities and specificities of CE-MRA (relative to CTA) at 84% and 96% respectively and for 2D TOF MRA 80% and 95% and they considered unenhanced 2D TOF MRA is sufficiently accurate to identify patients with >70% ICA stenosis compared with CE-MRA [28]. Wardlaw et al performed a systematic review showing that doppler ultrasound, MRA, CTA, and CEMRA all have high sensitivities and specificities for diagnosing 70–99% carotid stenosis in symptomatic patients [5]. CE-MRA was seen as marginally more accurate, but they cautioned on drawing conclusions from early research and not routine clinical practice. Westwood et al evaluated 26 articles in a systematic review primarily of unenhanced MRA and concluded that MRA was accurate for selecting patients for carotid endarterectomy at the surgical decision thresholds established in the major endarterectomy trials, but the evidence is not robust because of study heterogeneity [29]. Debrey et al performed a meta-analysis involving 37 TOF papers and 21 CE-MRA papers comparing TOF MR to digital subtraction angiography and concluded that TOF MRA and CE-MRA showed high accuracy for the detection of high-grade ICA stenoses and occlusions [30]. In that analysis, CE-MRA had a slight edge over TOF MRA with the sensitivity/specificity of TOF MRA for the detection of >70% to 99% ICA stenoses being 91.2/88.3%, and for CE-MRA 94.6/91.9%.

Several limitations are present in this study. First, the number of clinically significant stenosis was small relative to the normal or mild stenosis categories (0–30% = 1910 vs >50% = 214). These numbers of higher grade stenosis are comparable to prior studies evaluating these techniques [4,7].

Second, CE-MRA is an imperfect gold standard with defined limitations. CE-MRA techniques tended to overestimate the stenoses compared with catheter angiography [31–33]. This occurs both with high spatial resolution CE technique as well as for time-resolved techniques with reduced spatial resolution [1,34,35]. In a study of 177 patients, Babiarz et al found that while CE-MRA better defined the neurovascular anatomy, it did not offer a significant advantage in distinguishing surgically treatable ICA stenosis over 2D TOF MRA [28]. Serfaty et al found that 3D gadolinium-enhanced MR angiography was not accurate enough to replace conventional angiography in the evaluation of extracranial carotid arteries, but requires additional information such as Doppler sonography correlation [34].

A third limitation is that our TOF techniques do not routinely encompass the vertebral origins. However, screening for posterior circulation disease is not typically recommended. The risk of stroke in patients with asymptomatic vertebral artery stenosis is much lower than for symptomatic vertebral artery stenosis. In a study of 3717 patients with atherosclerotic disease, 7.6% had asymptomatic vertebral stenoses by US, with an annual stroke risk of 0.2% [25,36]. The European Society of Vascular Surgery practice guidelines state that population screening for asymptomatic vertebral artery stenosis is not recommended, and asymptomatic vertebral stenoses should not be treated by open or endovascular interventions [25]. A completely different strategy is present if the patient has had a posterior circulation stroke. In that population, the 90-day risk of recurrent stroke is 16% in those with extracranial vertebral artery stenosis and intervention should be undertaken early after symptom onset [25,37]. With posterior fossa symptomatology, imaging solely with unenhanced MRA techniques would not be appropriate.

Fourth, this study does not cover all field strengths nor all potential unenhanced sequences for evaluation of the carotid bifurcations. Newer techniques, such as ungated radial quiescent interval slice-selective imaging (QISS) have shown promise for unenhanced imaging which is comparible to CEMRA, but have not found wide use and are currently vendor specific [37]. While extensive data is available in the literature on the utility and accuracy of both 1.5T and 3.0T MRA, we cannot define the generalizability of this specific data to 3T. Finally, for expediency sake, the data sets were curated so that the blinded readers in different geographic regions could easily avail themselves of the datasets, and the probability of completing the study markedly increased with the more limited imaging evaluation. All images available in the MRA data sets were not evaluated by the readers. The effect of curated views is unknown relative to the evaluation of all images associated with the three different MRA sequences. The presence of a limited data set could potentially improve inter-reader variability, or decrease accuracy for defining stenosis since not all projections of the MIP images are evaluated.

Fifthly, flow dephasing with signal loss was expected to occur with higher grades of stenosis and included in the estimate of stenosis for these experienced readers. General degrees of signal loss were considered to occur at >70% for the 2D TOF, and for >90% for the 3D TOF studies, but were assumed and could not be explicitly measured.

## Conclusion

These results should reassure providers and radiologists that in an outpatient setting, initial evaluation of the carotid bifurcations can be adequately performed with unenhanced 2D TOF and 3D TOF MRA. Contrast enhanced MRA should be considered reserved for those cases not successfully answered by the unenhanced study, for preoperative evaluation, patients with evidence of clinically significant stenosis by other noninvasive modalities or vertebrobasilar symptoms. This has clinical relevance given the shift in clinical practice that is occurring regarding the benefit and risk of GBCA due to systemic gadolinium deposition following GBC administration.

## Supporting information

S1 Material (DOCX)

S1 Data (XLSX)

S1 File (PDF)

S2 File (PDF)

S3 File (PDF)

S4 File (PDF)

S5 File (PDF)
