# Supplementary material for: Multiple reader comparison of 2D TOF, 3D TOF, and CEMRA in screening of the carotid bifurcations: Time to reconsider routine contrast use?
Source: PLoS One. 2020 Sep 2;15(9):e0237856. doi: 10.1371/journal.pone.0237856 (PMC7467222; doi:10.1371/journal.pone.0237856)

**Supplemental material**

**Contingency Tables**

| Crosstab of CE-MRA Ratings vs Unenhanced 2D TOF and 3D TOF Ratings OVERALL | | | | | | |
| --- | --- | --- | --- | --- | --- | --- |
| **CE-MRA Ratings** | **0-30% (N=1910)** | **31-50% (N=204)** | **51-70% (N=94)** | **>70% (N=34)** | **Near occlusion (N=38)** | **Occluded (N=48)** |
| **STENOSIS 2D TOF** |  |  |  |  |  |  |
| 0-30% | 1772 (92.8%) | 102 (50.0%) | 21 (22.3%) | 5 (14.7%) | 7 (18.4%) | 7 (14.6%) |
| 31-50% | 87 (4.6%) | 57 (27.9%) | 31 (33.0%) | 5 (14.7%) | 0 (0.0%) | 1 (2.1%) |
| 51-70% | 29 (1.5%) | 33 (16.2%) | 37 (39.4%) | 10 (29.4%) | 4 (10.5%) | 2 (4.2%) |
| >70% | 13 (0.7%) | 8 (3.9%) | 5 (5.3%) | 11 (32.4%) | 9 (23.7%) | 2 (4.2%) |
| Near occlusion | 3 (0.2%) | 3 (1.5%) | 0 (0.0%) | 0 (0.0%) | 14 (36.8%) | 6 (12.5%) |
| Occluded | 6 (0.3%) | 1 (0.5%) | 0 (0.0%) | 3 (8.8%) | 4 (10.5%) | 30 (62.5%) |
| **STENOSIS 3D TOF** |  |  |  |  |  |  |
| 0-30% | 1825 (95.5%) | 131 (64.2%) | 32 (34.0%) | 7 (20.6%) | 6 (15.8%) | 9 (18.8%) |
| 31-50% | 57 (3.0%) | 42 (20.6%) | 31 (33.0%) | 4 (11.8%) | 4 (10.5%) | 0 (0.0%) |
| 51-70% | 3 (0.2%) | 23 (11.3%) | 25 (26.6%) | 8 (23.5%) | 3 (7.9%) | 2 (4.2%) |
| >70% | 8 (0.4%) | 5 (2.5%) | 5 (5.3%) | 8 (23.5%) | 9 (23.7%) | 2 (4.2%) |
| Near occlusion | 7 (0.4%) | 1 (0.5%) | 1 (1.1%) | 5 (14.7%) | 10 (26.3%) | 3 (6.2%) |
| Occluded | 10 (0.5%) | 2 (1.0%) | 0 (0.0%) | 2 (5.9%) | 6 (15.8%) | 32 (66.7%) |

| **Crosstab of CE-MRA Ratings vs Unenhanced 2D TOF and 3D TOF Ratings by READER** | | | | | | | |
| --- | --- | --- | --- | --- | --- | --- | --- |
| **READER** | **CE-MRA Ratings** | **0-30% (N=1910)** | **31-50% (N=204)** | **51-70% (N=94)** | **>70% (N=34)** | **Near occlusion (N=38)** | **Occluded (N=48)** |
| **READER 1** | **STENOSIS 2D TOF** |  |  |  |  |  |  |
|  | 0-30% | 318 (94.6%) | 7 (33.3%) | 4 (40.0%) | 3 (27.3%) | 1 (33.3%) | 1 (14.3%) |
|  | 31-50% | 7 (2.1%) | 11 (52.4%) | 5 (50.0%) | 0 (0.0%) | 0 (0.0%) | 0 (0.0%) |
|  | 51-70% | 4 (1.2%) | 2 (9.5%) | 1 (10.0%) | 1 (9.1%) | 0 (0.0%) | 1 (14.3%) |
|  | >70% | 6 (1.8%) | 1 (4.8%) | 0 (0.0%) | 5 (45.5%) | 1 (33.3%) | 1 (14.3%) |
|  | Near occlusion | 0 (0.0%) | 0 (0.0%) | 0 (0.0%) | 0 (0.0%) | 1 (33.3%) | 0 (0.0%) |
|  | Occluded | 1 (0.3%) | 0 (0.0%) | 0 (0.0%) | 2 (18.2%) | 0 (0.0%) | 4 (57.1%) |
|  | **STENOSIS 3D TOF** |  |  |  |  |  |  |
|  | 0-30% | 320 (95.2%) | 13 (61.9%) | 2 (20.0%) | 1 (9.1%) | 1 (33.3%) | 2 (28.6%) |
|  | 31-50% | 8 (2.4%) | 2 (9.5%) | 6 (60.0%) | 1 (9.1%) | 0 (0.0%) | 0 (0.0%) |
|  | 51-70% | 1 (0.3%) | 4 (19.0%) | 2 (20.0%) | 2 (18.2%) | 0 (0.0%) | 0 (0.0%) |
|  | >70% | 3 (0.9%) | 2 (9.5%) | 0 (0.0%) | 4 (36.4%) | 1 (33.3%) | 0 (0.0%) |
|  | Near occlusion | 0 (0.0%) | 0 (0.0%) | 0 (0.0%) | 1 (9.1%) | 1 (33.3%) | 0 (0.0%) |
|  | Occluded | 4 (1.2%) | 0 (0.0%) | 0 (0.0%) | 2 (18.2%) | 0 (0.0%) | 5 (71.4%) |
| **READER 2** | **STENOSIS 2D TOF** |  |  |  |  |  |  |
|  | 0-30% | 319 (95.8%) | 12 (35.3%) | 1 (20.0%) | 0 (0.0%) | 1 (11.1%) | 0 (0.0%) |
|  | 31-50% | 8 (2.4%) | 13 (38.2%) | 2 (40.0%) | 1 (50.0%) | 0 (0.0%) | 0 (0.0%) |
|  | 51-70% | 5 (1.5%) | 6 (17.6%) | 2 (40.0%) | 0 (0.0%) | 1 (11.1%) | 0 (0.0%) |
|  | >70% | 1 (0.3%) | 3 (8.8%) | 0 (0.0%) | 1 (50.0%) | 2 (22.2%) | 0 (0.0%) |
|  | Near occlusion | 0 (0.0%) | 0 (0.0%) | 0 (0.0%) | 0 (0.0%) | 5 (55.6%) | 0 (0.0%) |
|  | Occluded | 0 (0.0%) | 0 (0.0%) | 0 (0.0%) | 0 (0.0%) | 0 (0.0%) | 5 (100.0%) |
|  | **STENOSIS 3D TOF** |  |  |  |  |  |  |
|  | 0-30% | 328 (98.5%) | 22 (64.7%) | 3 (60.0%) | 0 (0.0%) | 2 (22.2%) | 0 (0.0%) |
|  | 31-50% | 2 (0.6%) | 8 (23.5%) | 1 (20.0%) | 0 (0.0%) | 1 (11.1%) | 0 (0.0%) |
|  | 51-70% | 0 (0.0%) | 4 (11.8%) | 1 (20.0%) | 1 (50.0%) | 1 (11.1%) | 0 (0.0%) |
|  | >70% | 2 (0.6%) | 0 (0.0%) | 0 (0.0%) | 0 (0.0%) | 2 (22.2%) | 0 (0.0%) |
|  | Near occlusion | 1 (0.3%) | 0 (0.0%) | 0 (0.0%) | 1 (50.0%) | 2 (22.2%) | 1 (20.0%) |
|  | Occluded | 0 (0.0%) | 0 (0.0%) | 0 (0.0%) | 0 (0.0%) | 1 (11.1%) | 4 (80.0%) |
| **READER 3** | **STENOSIS 2D TOF** |  |  |  |  |  |  |
|  | 0-30% | 303 (92.7%) | 12 (41.4%) | 2 (18.2%) | 1 (20.0%) | 1 (20.0%) | 2 (18.2%) |
|  | 31-50% | 16 (4.9%) | 9 (31.0%) | 5 (45.5%) | 0 (0.0%) | 0 (0.0%) | 0 (0.0%) |
|  | 51-70% | 7 (2.1%) | 8 (27.6%) | 4 (36.4%) | 3 (60.0%) | 1 (20.0%) | 1 (9.1%) |
|  | >70% | 0 (0.0%) | 0 (0.0%) | 0 (0.0%) | 1 (20.0%) | 2 (40.0%) | 0 (0.0%) |
|  | Near occlusion | 0 (0.0%) | 0 (0.0%) | 0 (0.0%) | 0 (0.0%) | 1 (20.0%) | 1 (9.1%) |
|  | Occluded | 1 (0.3%) | 0 (0.0%) | 0 (0.0%) | 0 (0.0%) | 0 (0.0%) | 7 (63.6%) |
|  | **STENOSIS 3D TOF** |  |  |  |  |  |  |
|  | 0-30% | 317 (96.9%) | 22 (75.9%) | 4 (36.4%) | 2 (40.0%) | 0 (0.0%) | 2 (18.2%) |
|  | 31-50% | 5 (1.5%) | 5 (17.2%) | 4 (36.4%) | 1 (20.0%) | 0 (0.0%) | 0 (0.0%) |
|  | 51-70% | 1 (0.3%) | 1 (3.4%) | 2 (18.2%) | 0 (0.0%) | 2 (40.0%) | 1 (9.1%) |
|  | >70% | 0 (0.0%) | 0 (0.0%) | 1 (9.1%) | 1 (20.0%) | 1 (20.0%) | 0 (0.0%) |
|  | Near occlusion | 3 (0.9%) | 0 (0.0%) | 0 (0.0%) | 1 (20.0%) | 1 (20.0%) | 0 (0.0%) |
|  | Occluded | 1 (0.3%) | 1 (3.4%) | 0 (0.0%) | 0 (0.0%) | 1 (20.0%) | 8 (72.7%) |
| **READER 4** | **STENOSIS 2D TOF** |  |  |  |  |  |  |
|  | 0-30% | 322 (95.5%) | 16 (64.0%) | 3 (37.5%) | 0 (0.0%) | 2 (40.0%) | 1 (9.1%) |
|  | 31-50% | 8 (2.4%) | 3 (12.0%) | 2 (25.0%) | 1 (50.0%) | 0 (0.0%) | 0 (0.0%) |
|  | 51-70% | 2 (0.6%) | 2 (8.0%) | 3 (37.5%) | 1 (50.0%) | 1 (20.0%) | 0 (0.0%) |
|  | >70% | 1 (0.3%) | 1 (4.0%) | 0 (0.0%) | 0 (0.0%) | 0 (0.0%) | 1 (9.1%) |
|  | Near occlusion | 1 (0.3%) | 2 (8.0%) | 0 (0.0%) | 0 (0.0%) | 2 (40.0%) | 2 (18.2%) |
|  | Occluded | 3 (0.9%) | 1 (4.0%) | 0 (0.0%) | 0 (0.0%) | 0 (0.0%) | 7 (63.6%) |
|  | **STENOSIS 3D TOF** |  |  |  |  |  |  |
|  | 0-30% | 324 (96.1%) | 13 (52.0%) | 7 (87.5%) | 0 (0.0%) | 1 (20.0%) | 1 (9.1%) |
|  | 31-50% | 7 (2.1%) | 6 (24.0%) | 0 (0.0%) | 0 (0.0%) | 0 (0.0%) | 0 (0.0%) |
|  | 51-70% | 0 (0.0%) | 4 (16.0%) | 1 (12.5%) | 1 (50.0%) | 0 (0.0%) | 1 (9.1%) |
|  | >70% | 1 (0.3%) | 1 (4.0%) | 0 (0.0%) | 0 (0.0%) | 1 (20.0%) | 1 (9.1%) |
|  | Near occlusion | 1 (0.3%) | 0 (0.0%) | 0 (0.0%) | 1 (50.0%) | 3 (60.0%) | 1 (9.1%) |
|  | Occluded | 4 (1.2%) | 1 (4.0%) | 0 (0.0%) | 0 (0.0%) | 0 (0.0%) | 7 (63.6%) |
| **READER 5** | **STENOSIS 2D TOF** |  |  |  |  |  |  |
|  | 0-30% | 290 (92.4%) | 14 (51.9%) | 5 (23.8%) | 1 (14.3%) | 2 (16.7%) | 2 (28.6%) |
|  | 31-50% | 16 (5.1%) | 3 (11.1%) | 4 (19.0%) | 3 (42.9%) | 0 (0.0%) | 0 (0.0%) |
|  | 51-70% | 4 (1.3%) | 6 (22.2%) | 10 (47.6%) | 2 (28.6%) | 1 (8.3%) | 0 (0.0%) |
|  | >70% | 2 (0.6%) | 3 (11.1%) | 2 (9.5%) | 1 (14.3%) | 4 (33.3%) | 0 (0.0%) |
|  | Near occlusion | 2 (0.6%) | 1 (3.7%) | 0 (0.0%) | 0 (0.0%) | 3 (25.0%) | 1 (14.3%) |
|  | Occluded | 0 (0.0%) | 0 (0.0%) | 0 (0.0%) | 0 (0.0%) | 2 (16.7%) | 4 (57.1%) |
|  | **STENOSIS 3D TOF** |  |  |  |  |  |  |
|  | 0-30% | 306 (97.5%) | 16 (59.3%) | 7 (33.3%) | 3 (42.9%) | 2 (16.7%) | 2 (28.6%) |
|  | 31-50% | 5 (1.6%) | 4 (14.8%) | 6 (28.6%) | 2 (28.6%) | 3 (25.0%) | 0 (0.0%) |
|  | 51-70% | 0 (0.0%) | 4 (14.8%) | 6 (28.6%) | 1 (14.3%) | 0 (0.0%) | 0 (0.0%) |
|  | >70% | 1 (0.3%) | 2 (7.4%) | 1 (4.8%) | 1 (14.3%) | 3 (25.0%) | 1 (14.3%) |
|  | Near occlusion | 1 (0.3%) | 1 (3.7%) | 1 (4.8%) | 0 (0.0%) | 2 (16.7%) | 0 (0.0%) |
|  | Occluded | 1 (0.3%) | 0 (0.0%) | 0 (0.0%) | 0 (0.0%) | 2 (16.7%) | 4 (57.1%) |
| **READER 6** | **STENOSIS 2D TOF** |  |  |  |  |  |  |
|  | 0-30% | 220 (83.7%) | 41 (60.3%) | 6 (15.4%) | 0 (0.0%) | 0 (0.0%) | 1 (14.3%) |
|  | 31-50% | 32 (12.2%) | 18 (26.5%) | 13 (33.3%) | 0 (0.0%) | 0 (0.0%) | 1 (14.3%) |
|  | 51-70% | 7 (2.7%) | 9 (13.2%) | 17 (43.6%) | 3 (42.9%) | 0 (0.0%) | 0 (0.0%) |
|  | >70% | 3 (1.1%) | 0 (0.0%) | 3 (7.7%) | 3 (42.9%) | 0 (0.0%) | 0 (0.0%) |
|  | Near occlusion | 0 (0.0%) | 0 (0.0%) | 0 (0.0%) | 0 (0.0%) | 2 (50.0%) | 2 (28.6%) |
|  | Occluded | 1 (0.4%) | 0 (0.0%) | 0 (0.0%) | 1 (14.3%) | 2 (50.0%) | 3 (42.9%) |
|  | **STENOSIS 3D TOF** |  |  |  |  |  |  |
|  | 0-30% | 230 (87.5%) | 45 (66.2%) | 9 (23.1%) | 1 (14.3%) | 0 (0.0%) | 2 (28.6%) |
|  | 31-50% | 30 (11.4%) | 17 (25.0%) | 14 (35.9%) | 0 (0.0%) | 0 (0.0%) | 0 (0.0%) |
|  | 51-70% | 1 (0.4%) | 6 (8.8%) | 13 (33.3%) | 3 (42.9%) | 0 (0.0%) | 0 (0.0%) |
|  | >70% | 1 (0.4%) | 0 (0.0%) | 3 (7.7%) | 2 (28.6%) | 1 (25.0%) | 0 (0.0%) |
|  | Near occlusion | 1 (0.4%) | 0 (0.0%) | 0 (0.0%) | 1 (14.3%) | 1 (25.0%) | 1 (14.3%) |
|  | Occluded | 0 (0.0%) | 0 (0.0%) | 0 (0.0%) | 0 (0.0%) | 2 (50.0%) | 4 (57.1%) |

**Supplement**: Bubble plots with cell counts to show agreement between readers

**Stenosis measurement 2D TOF**


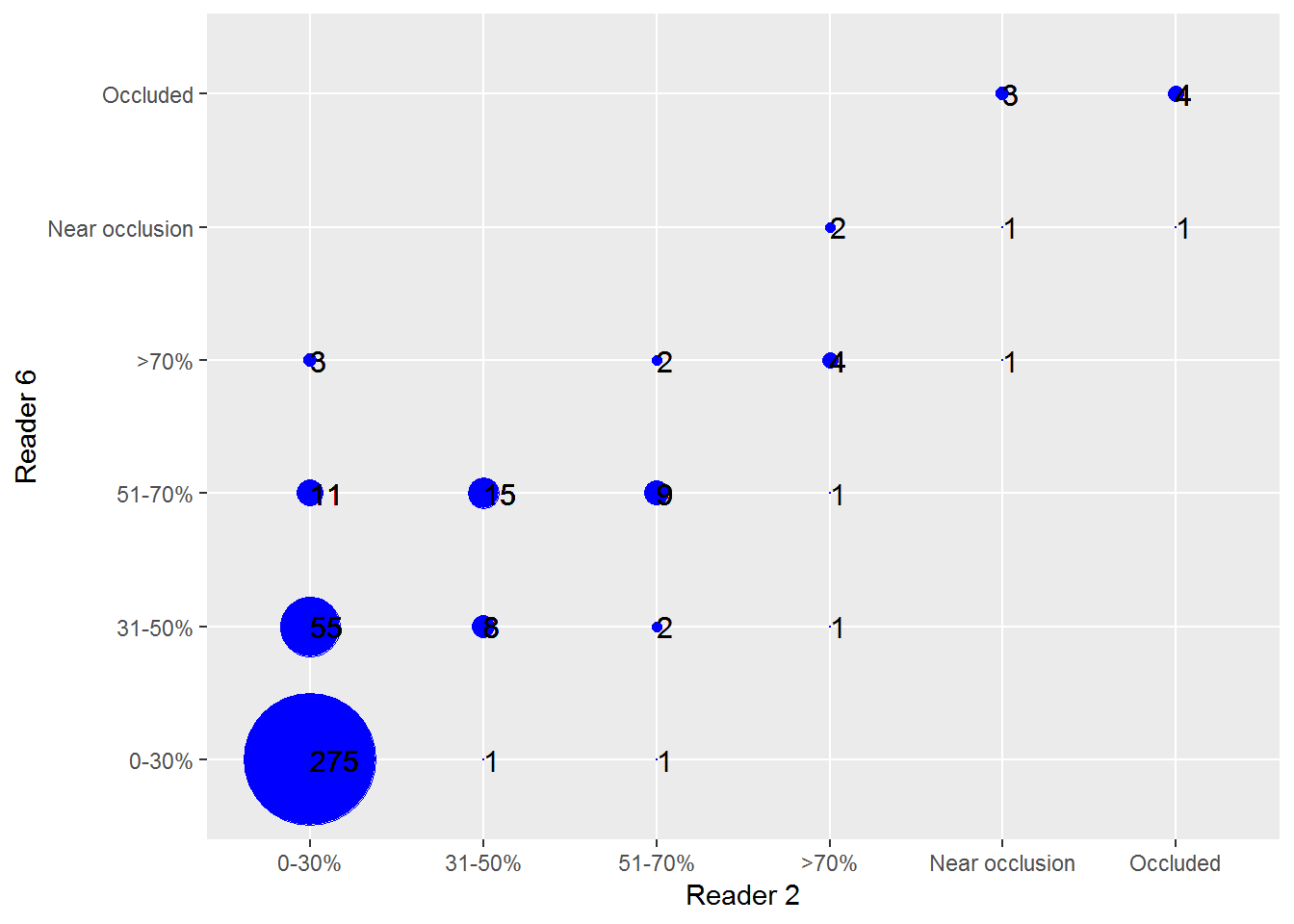


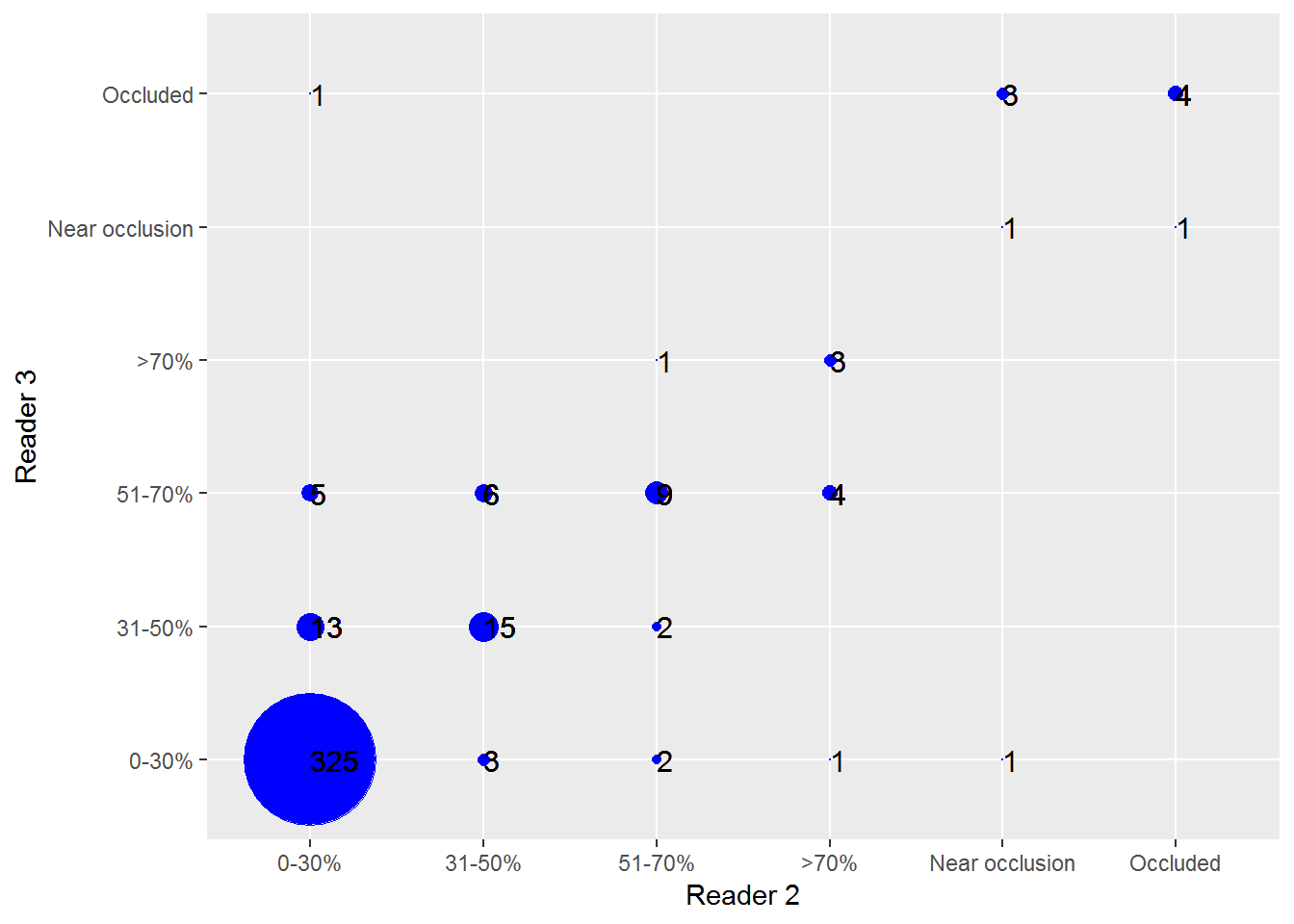


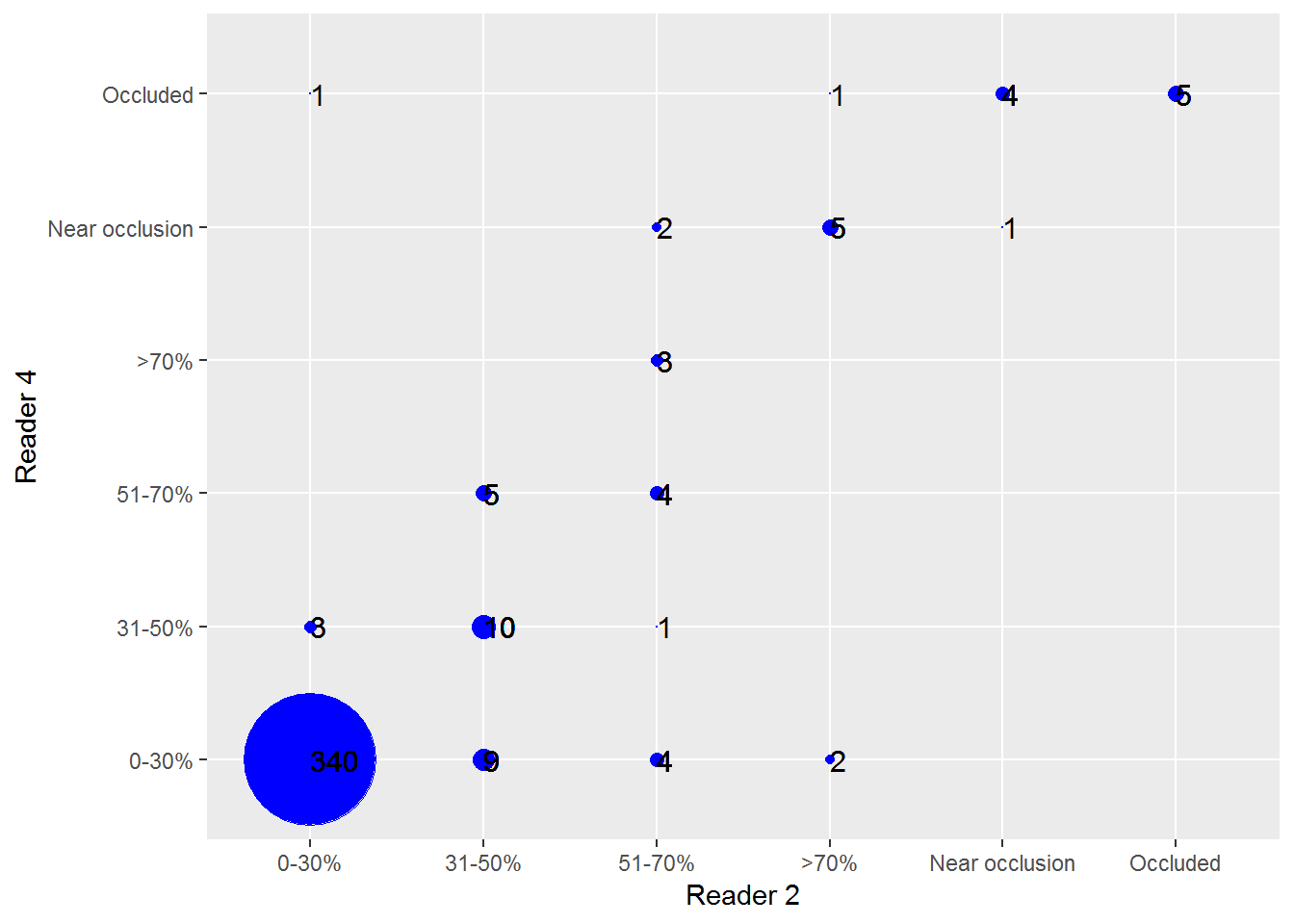


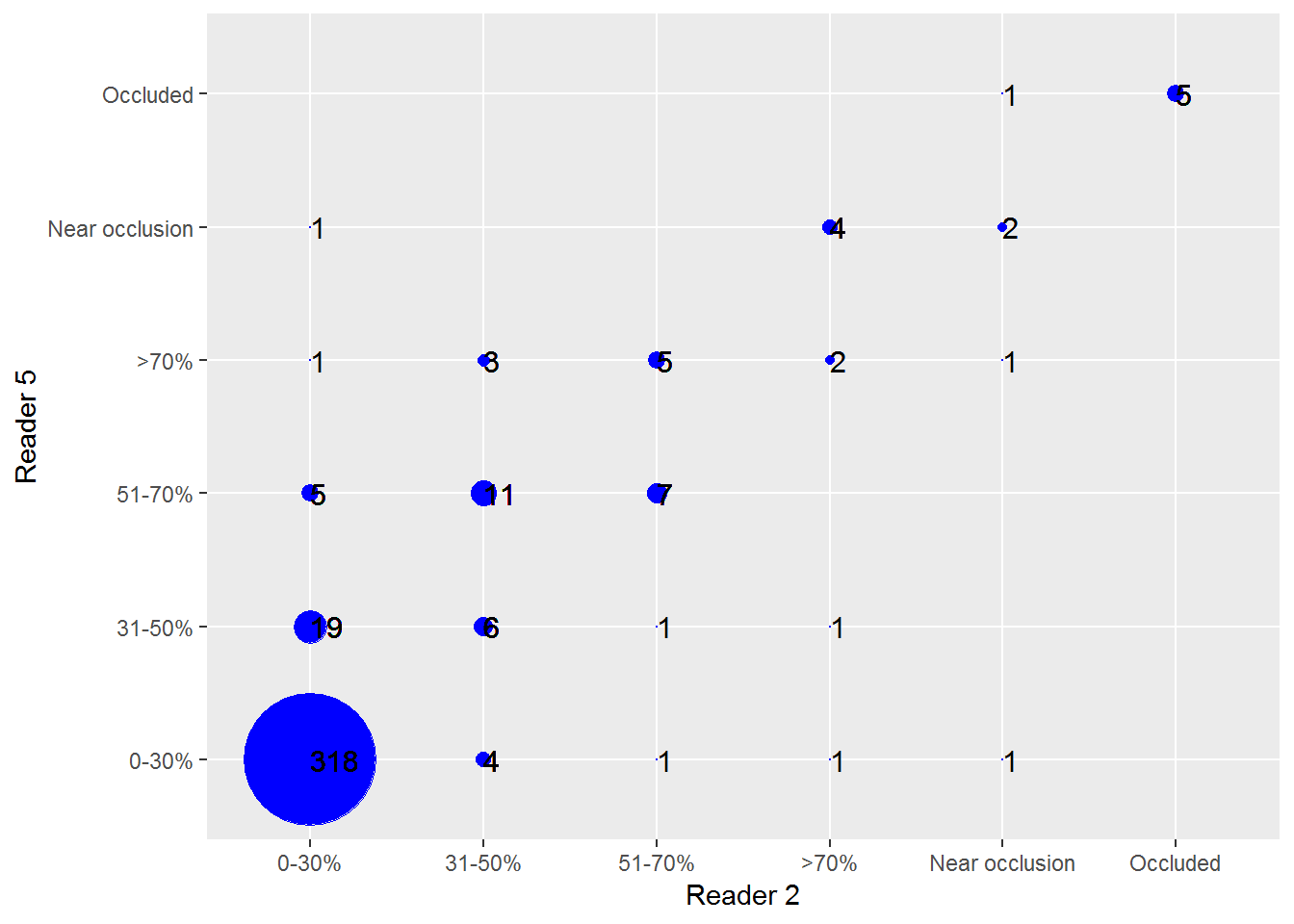


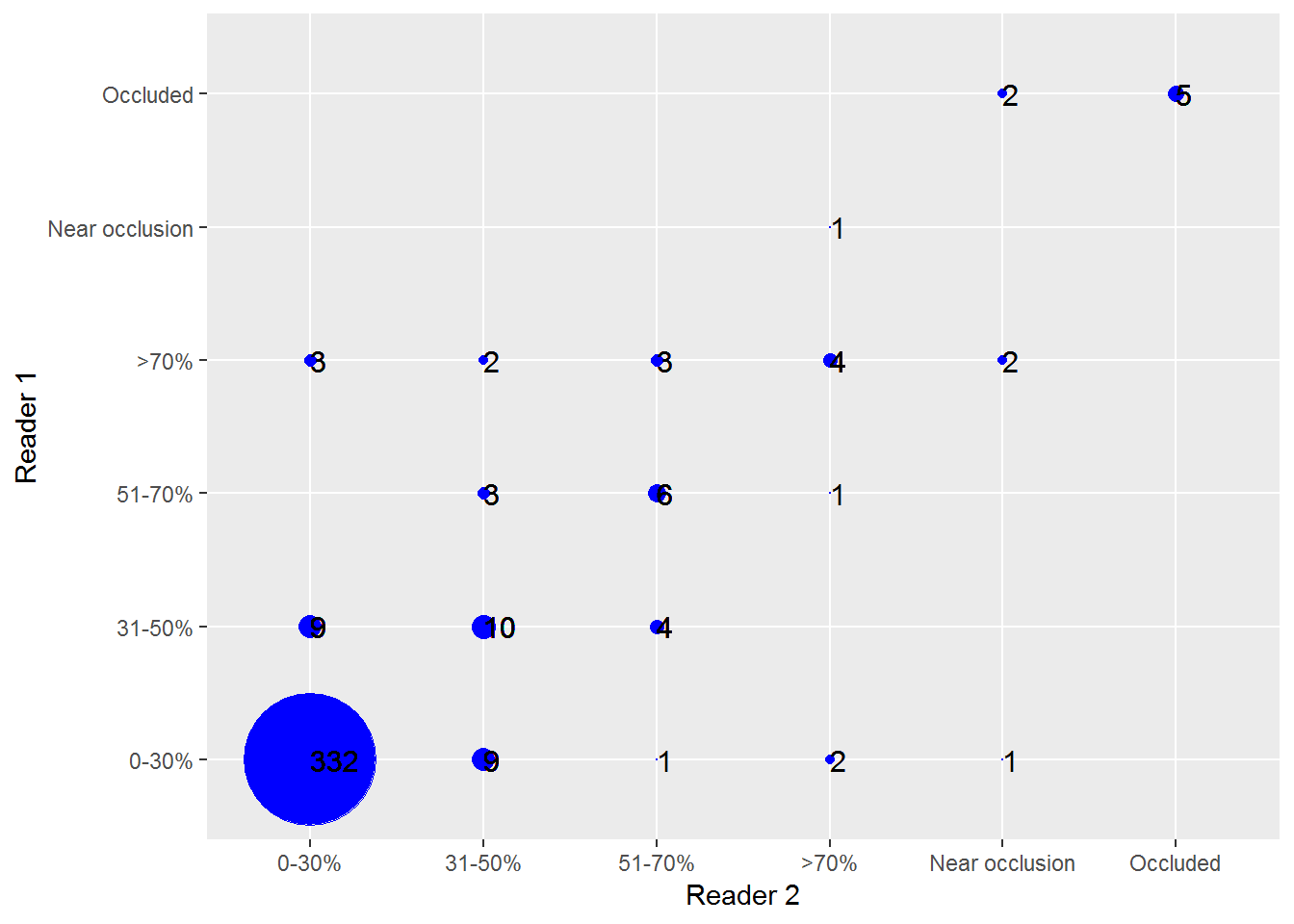


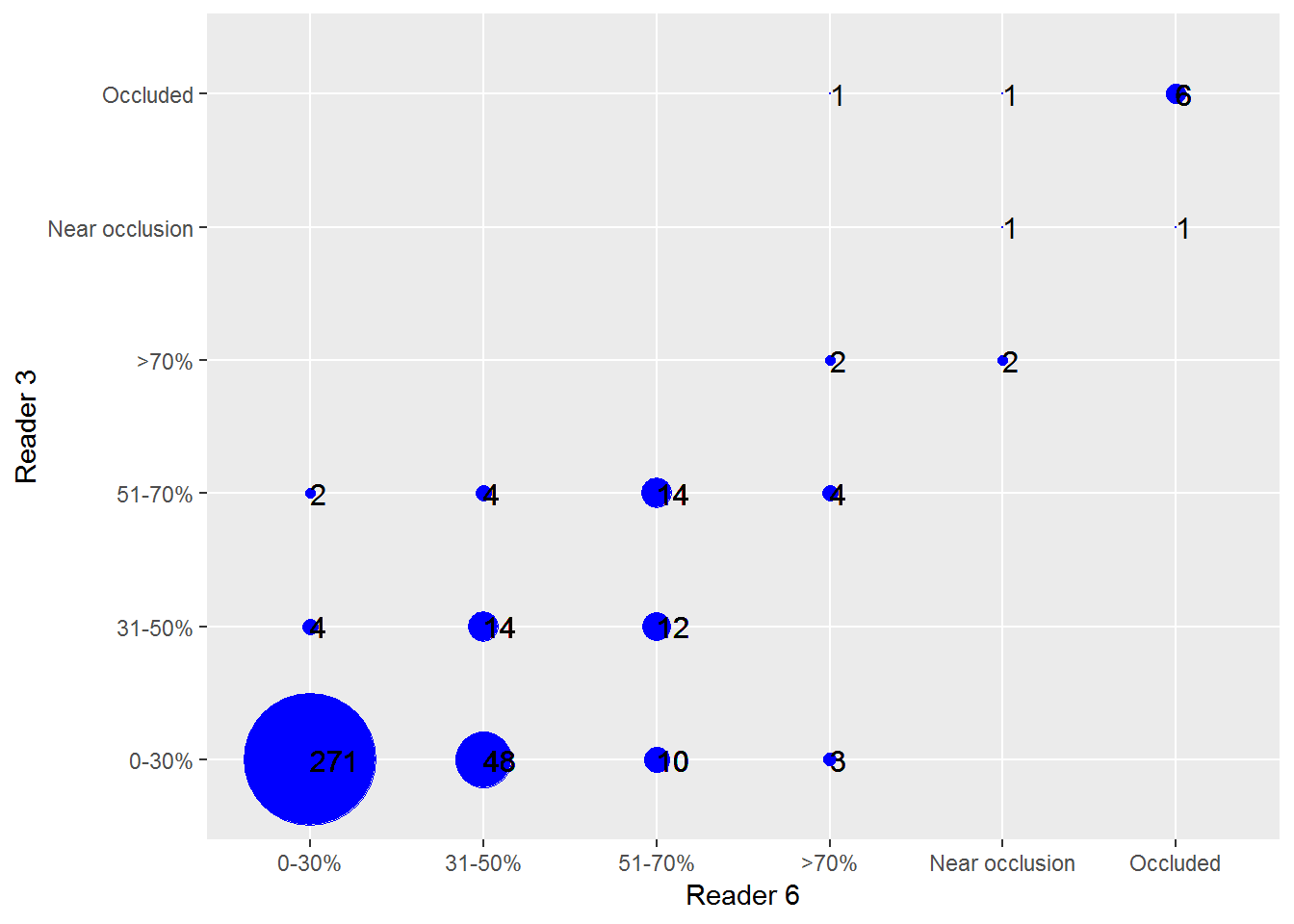


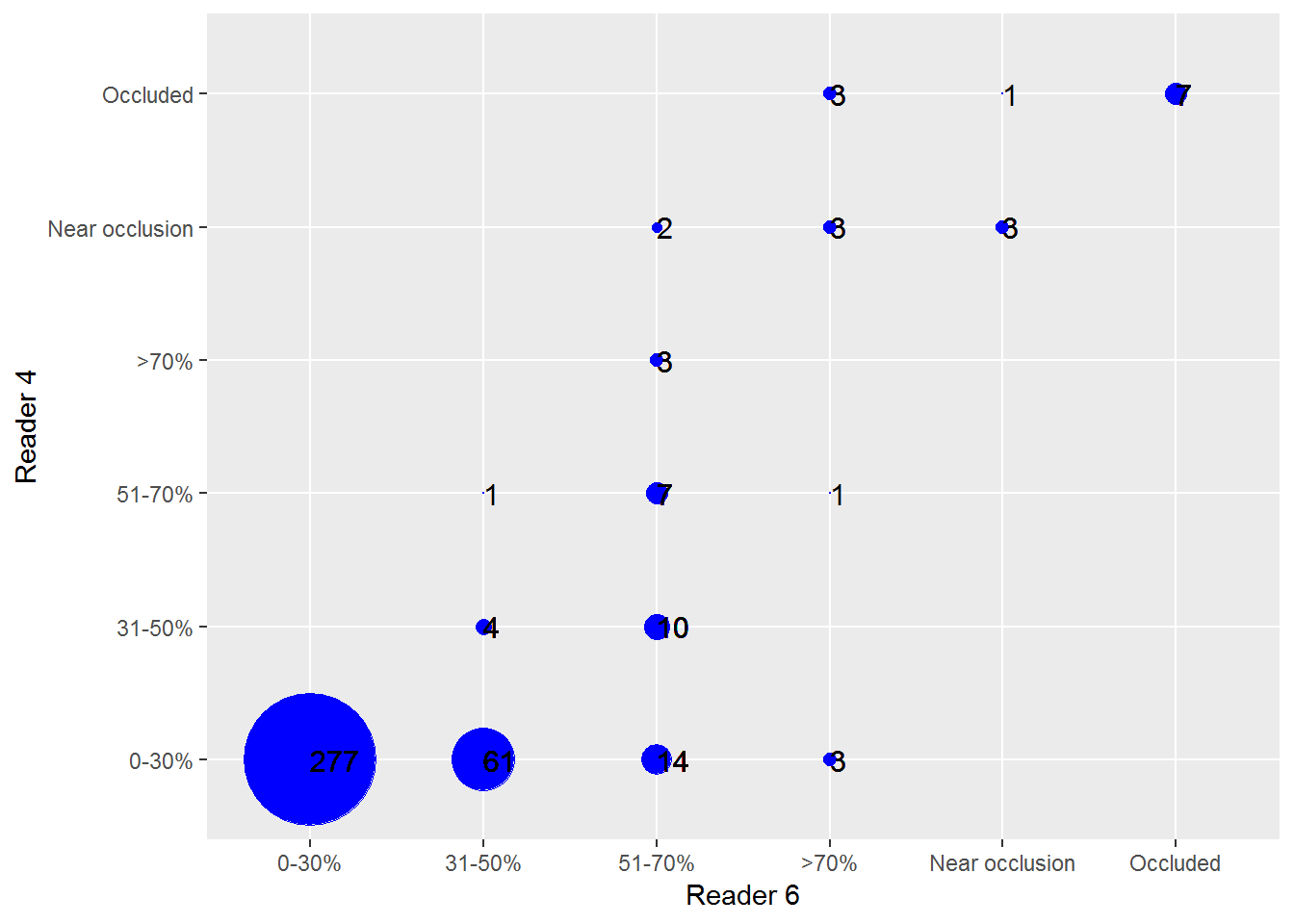


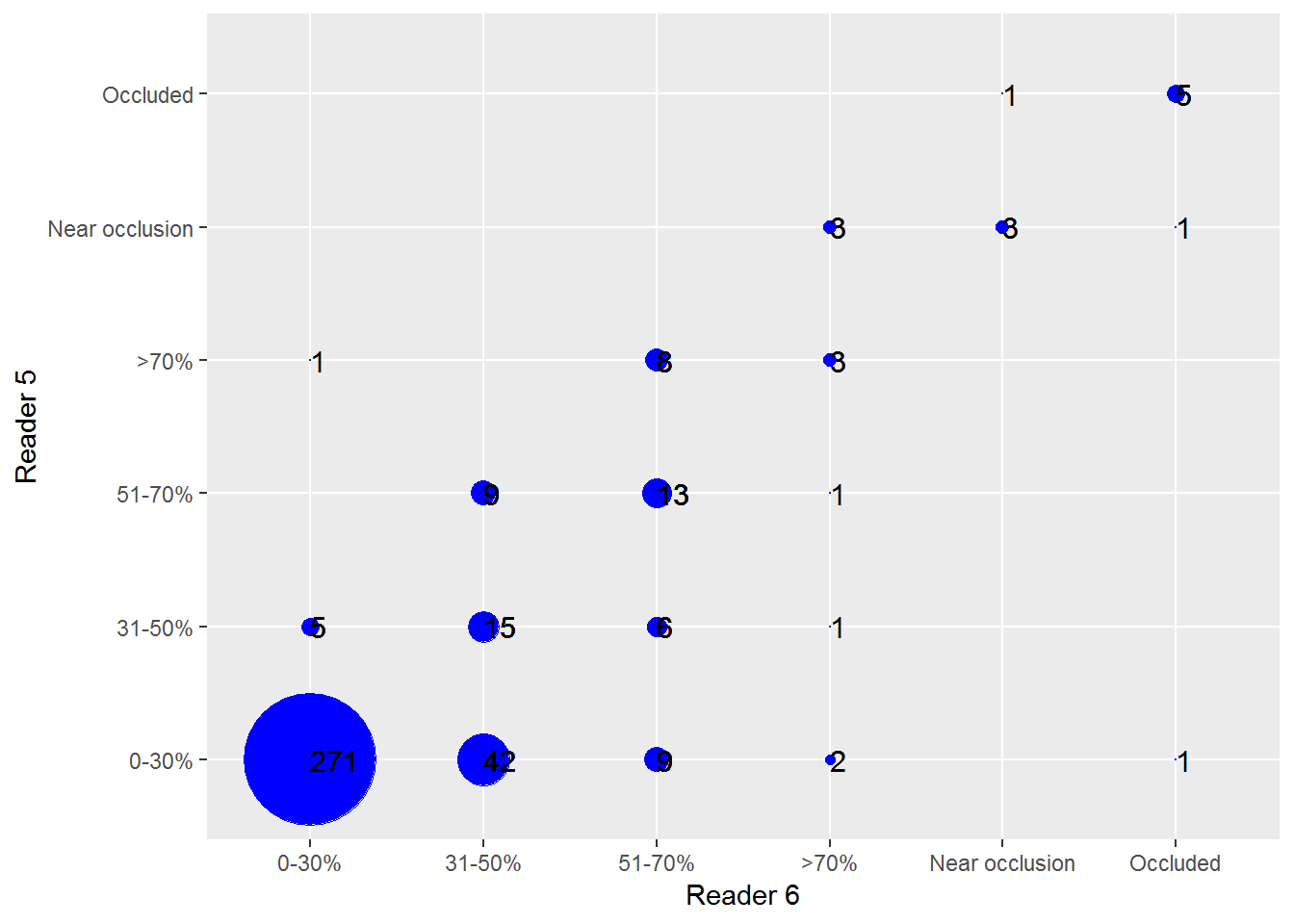


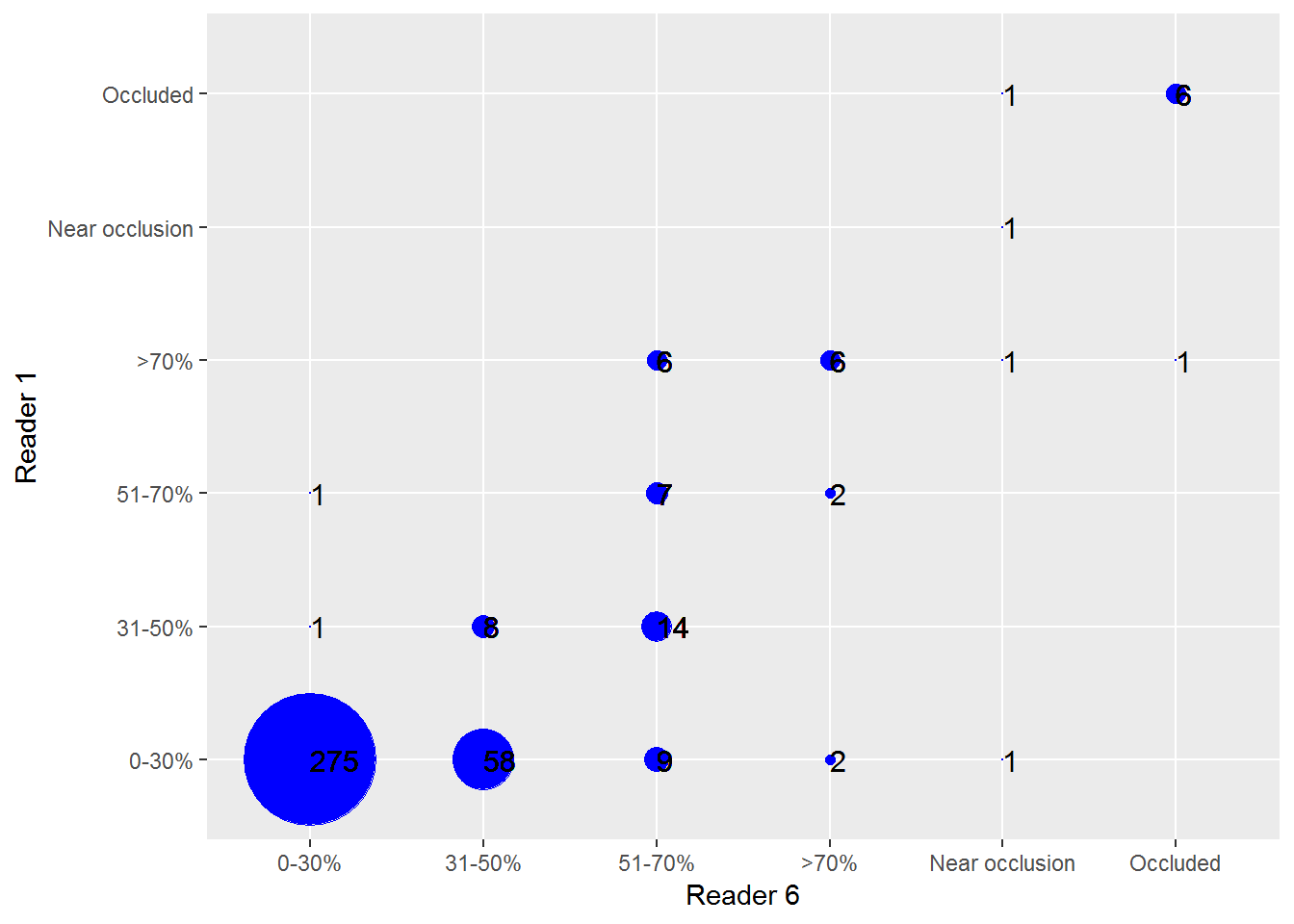


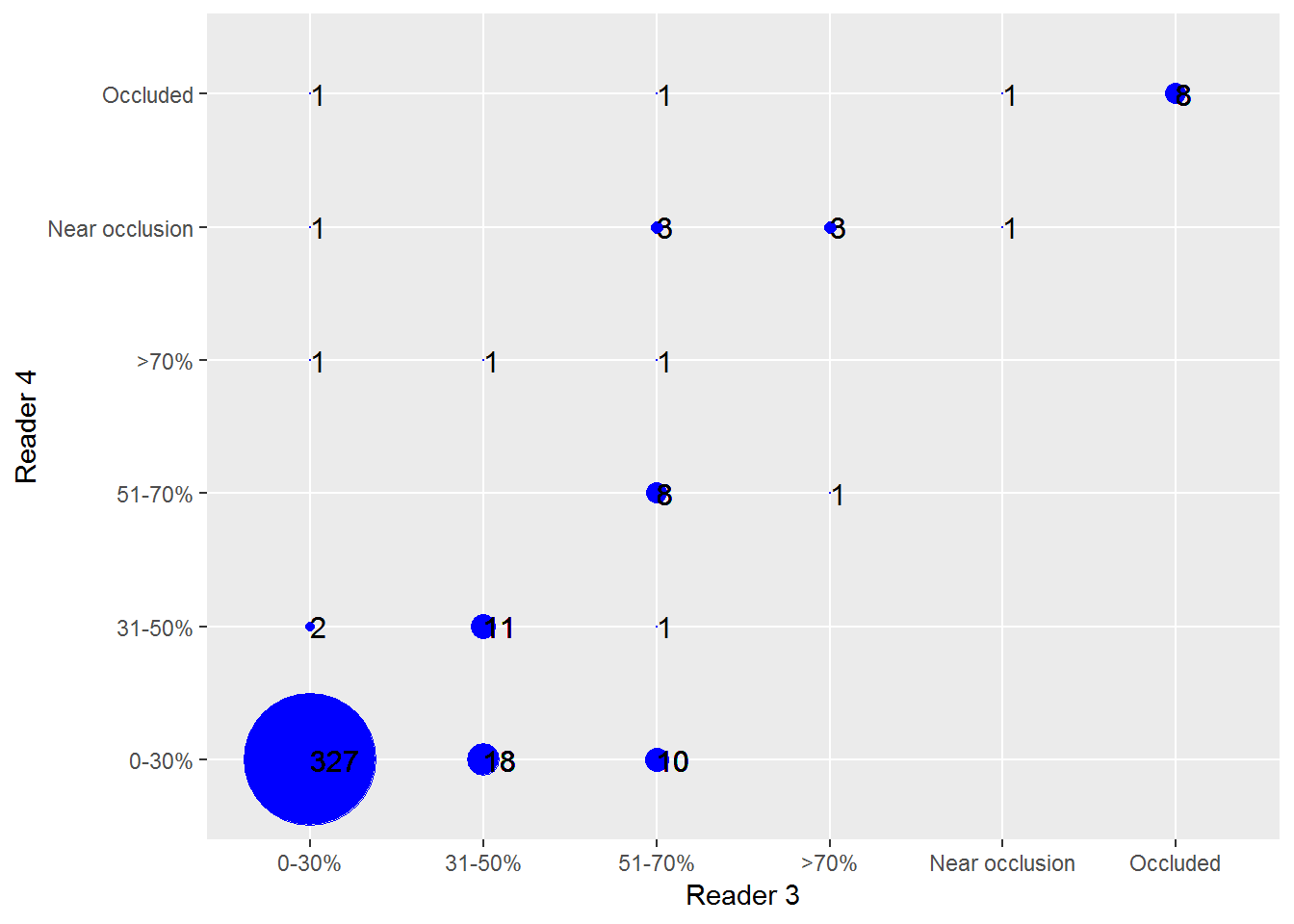


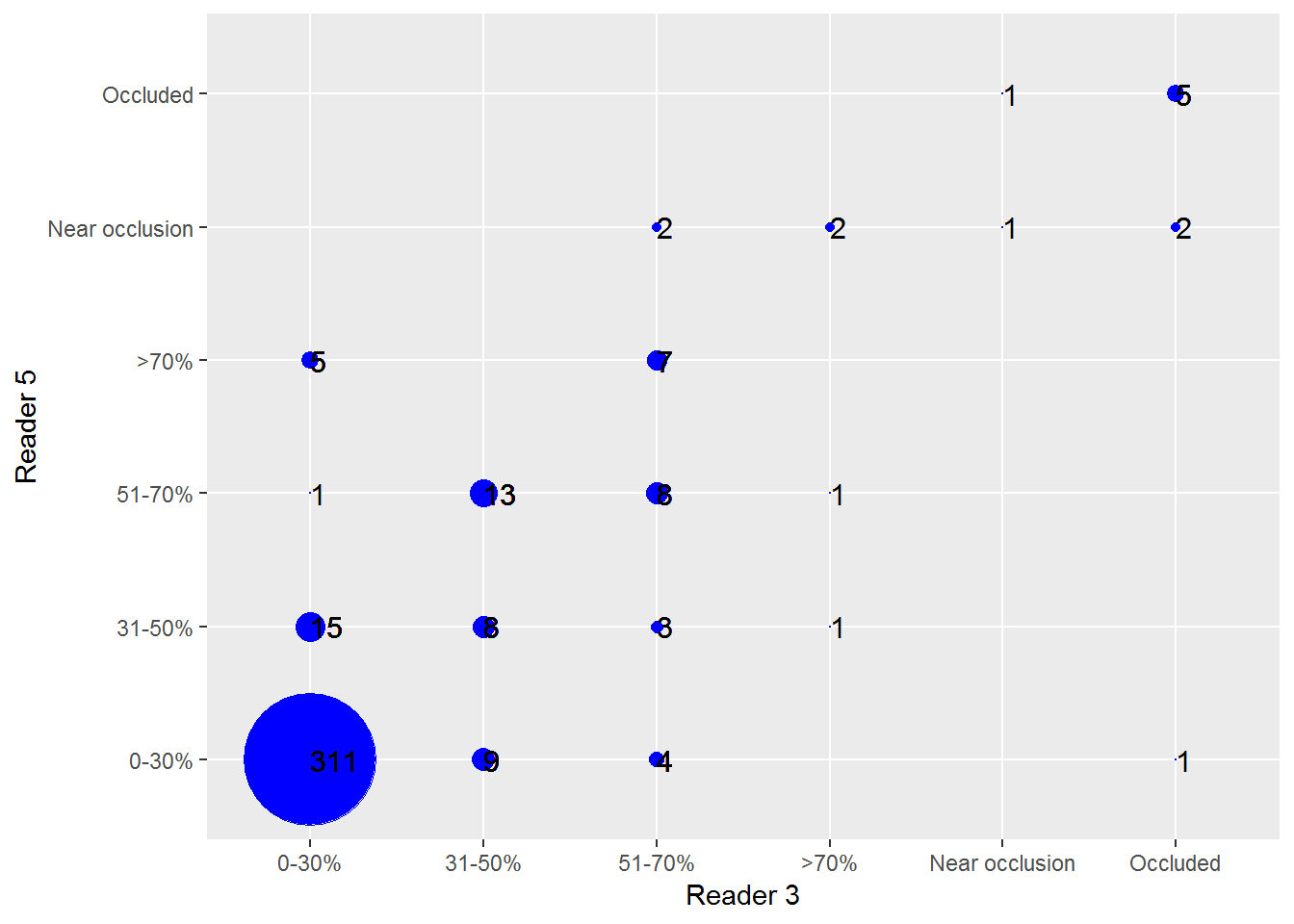


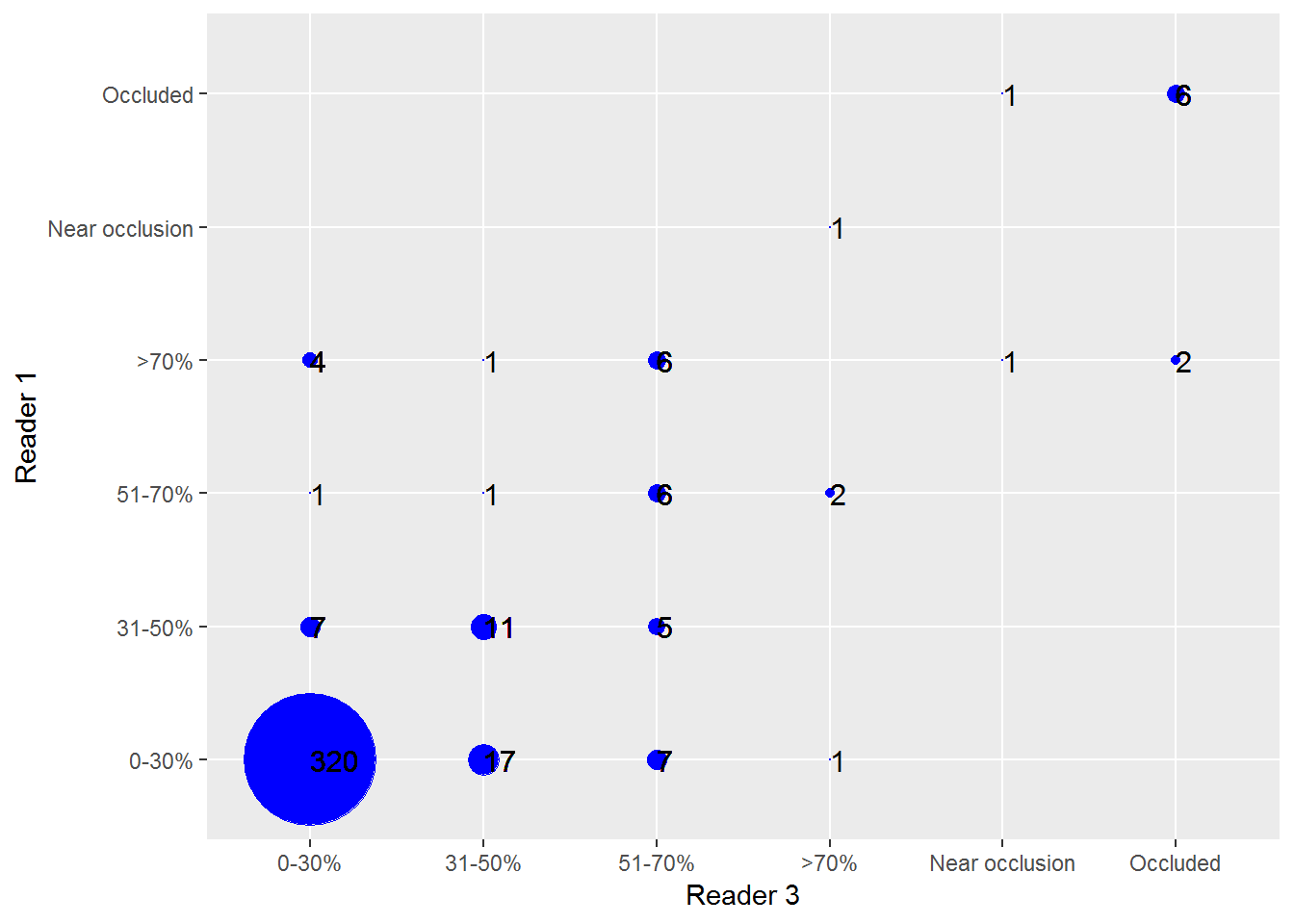


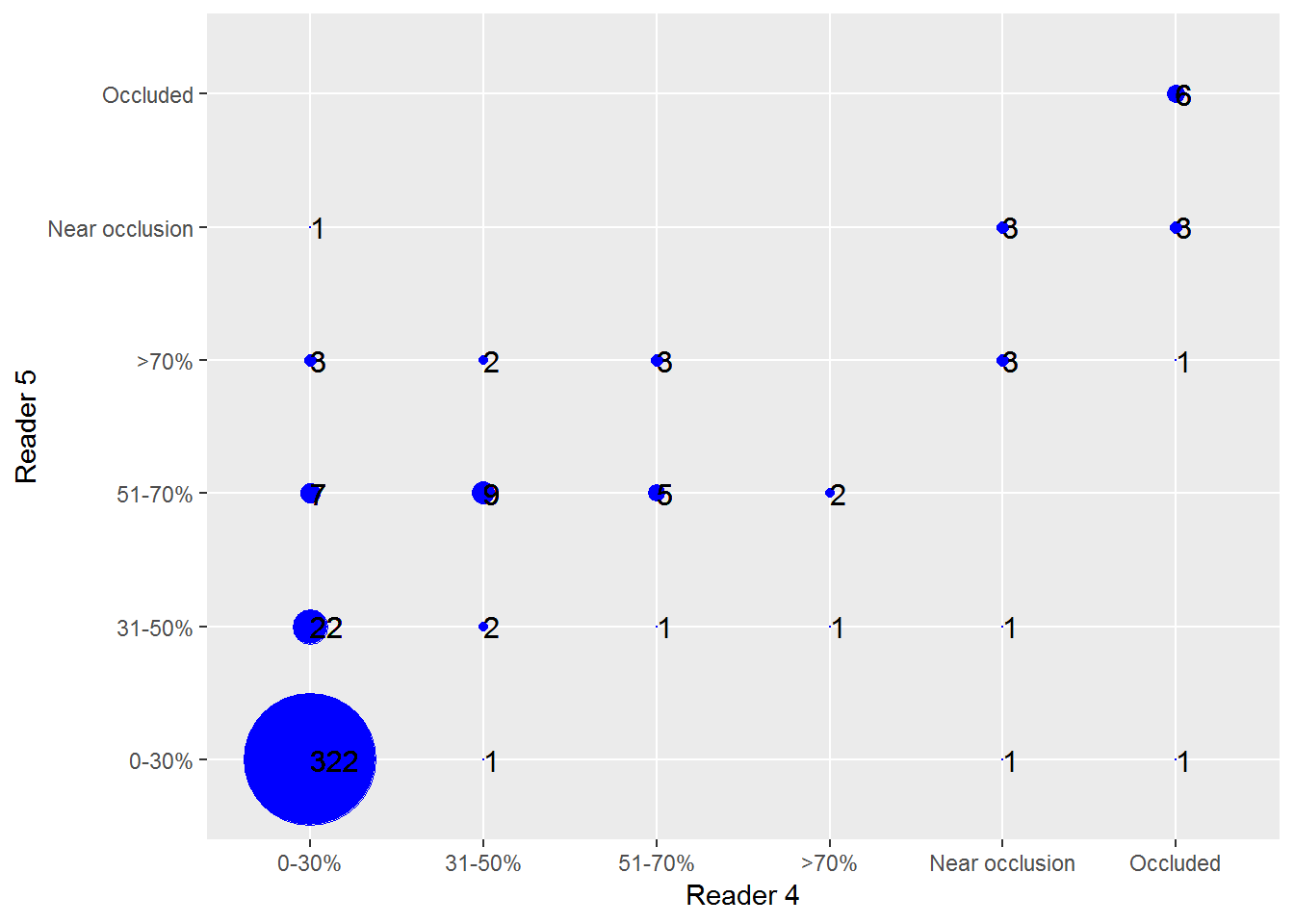


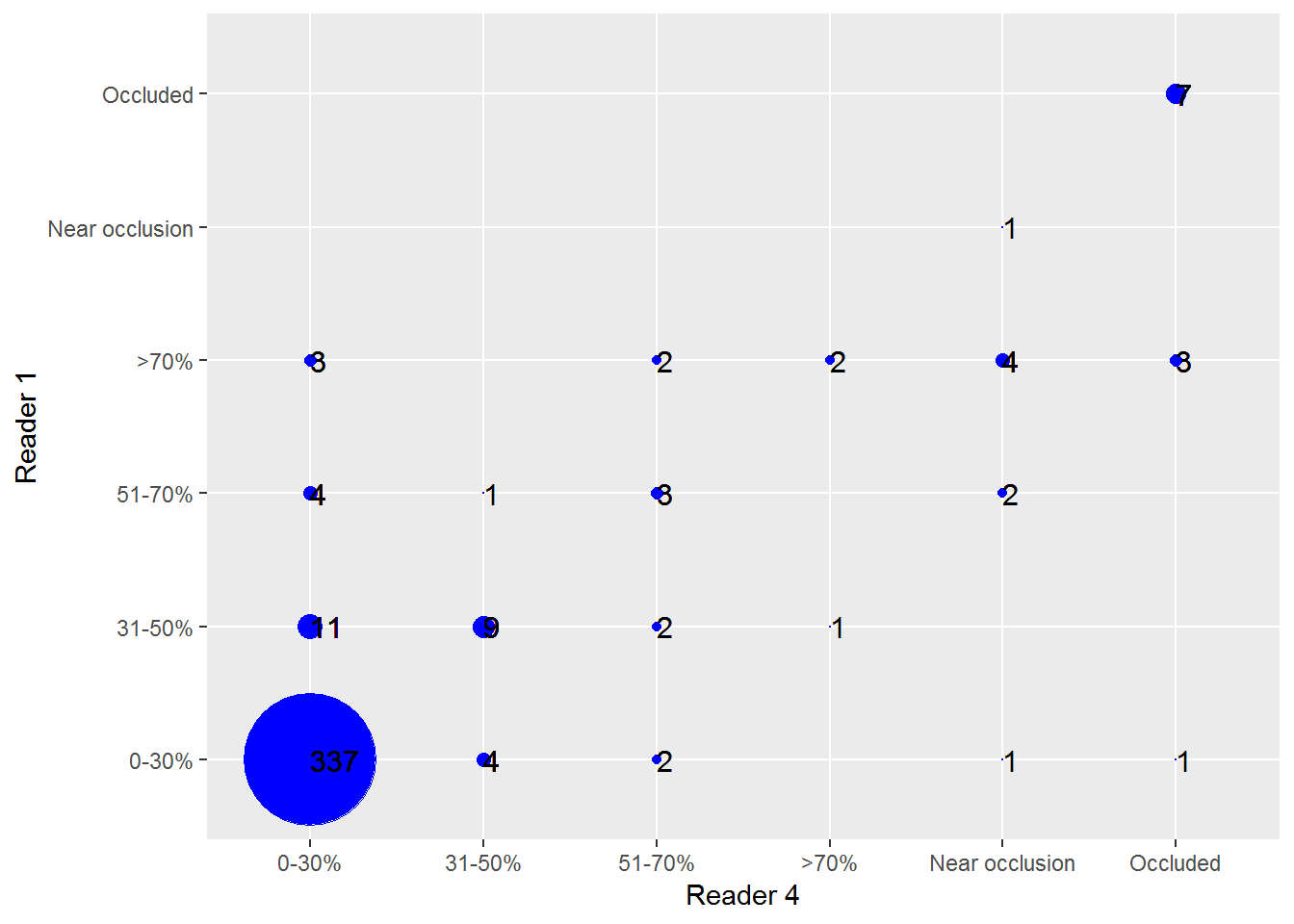


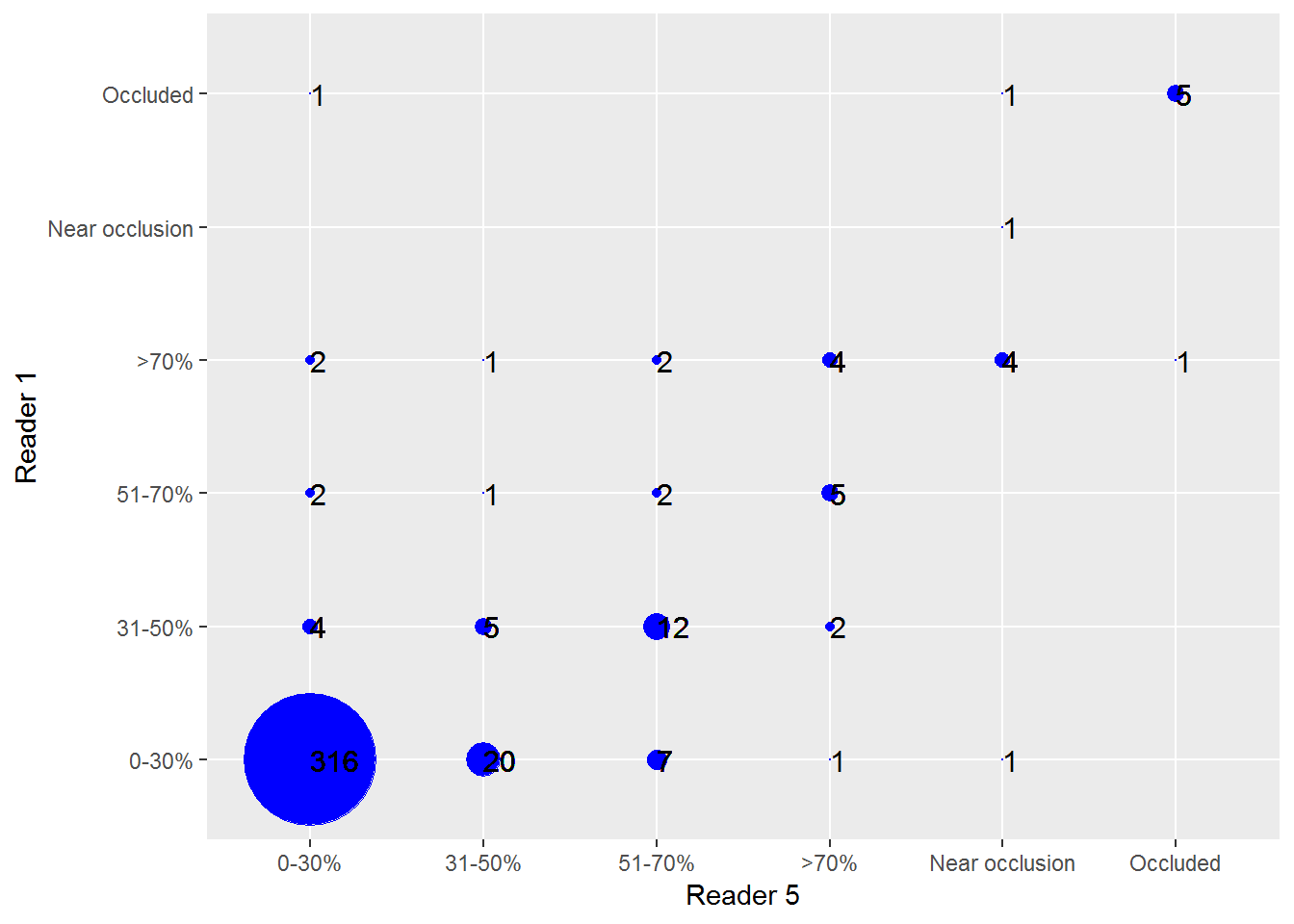


**Stenosis measurement 3D TOF**


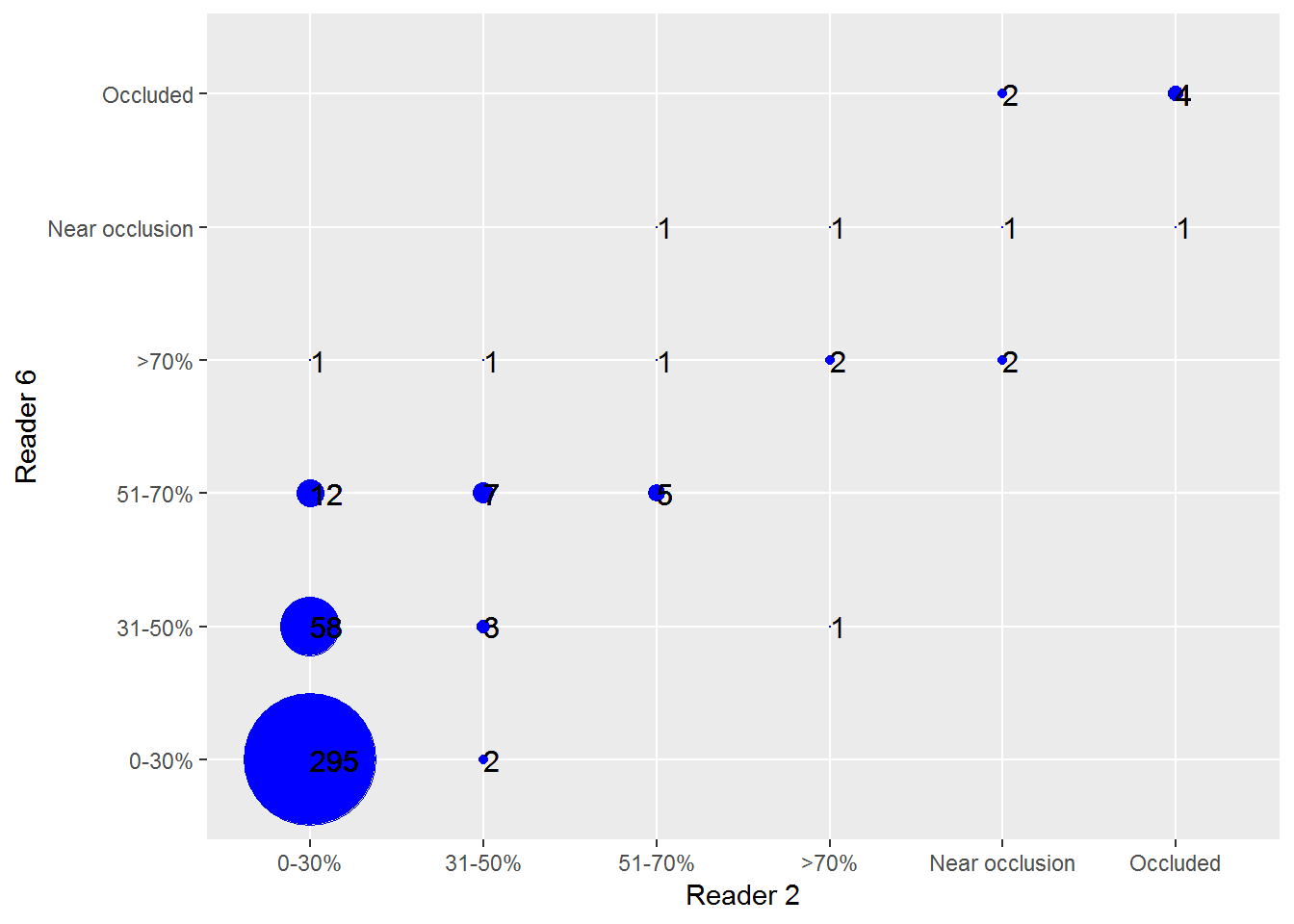


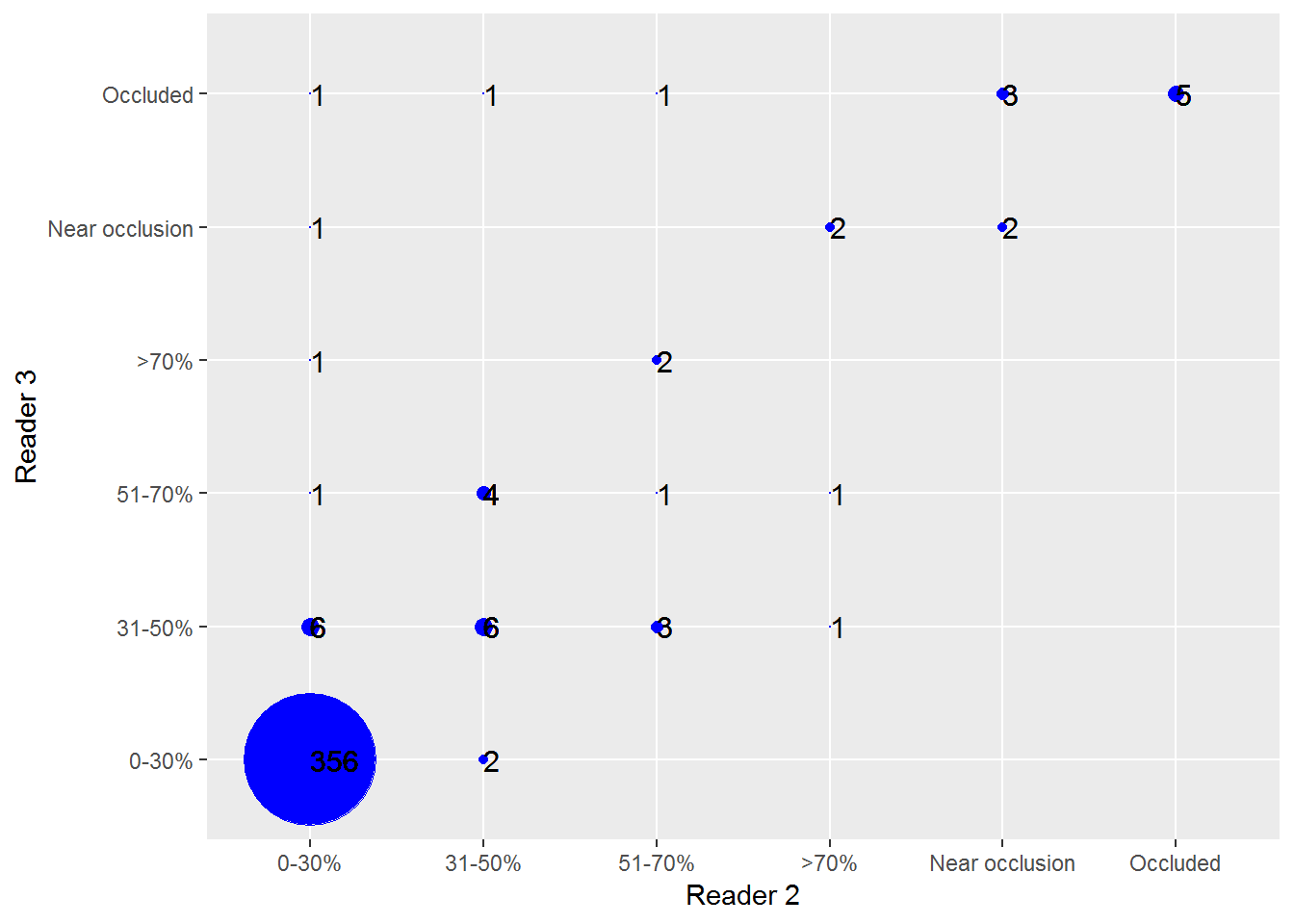


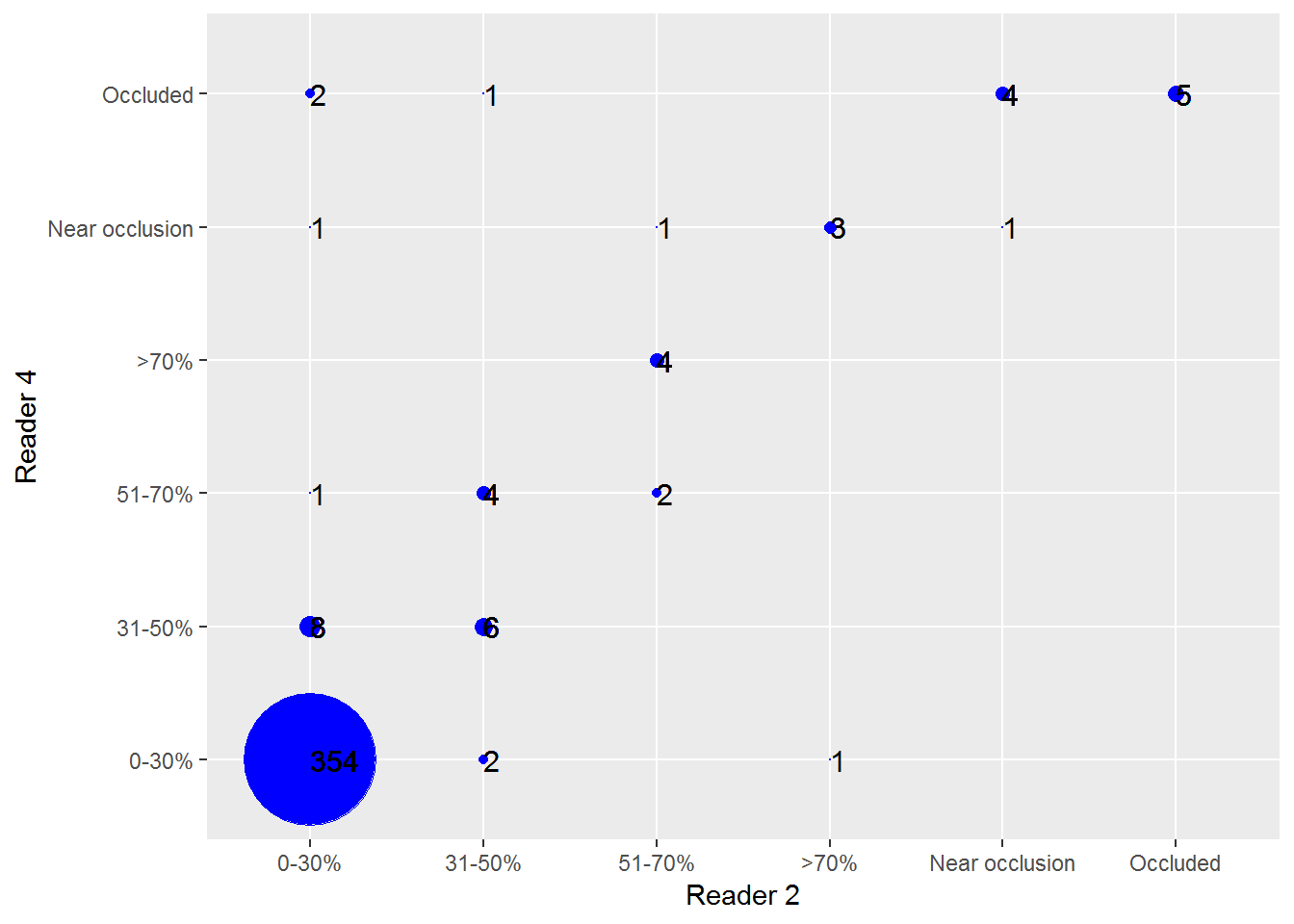


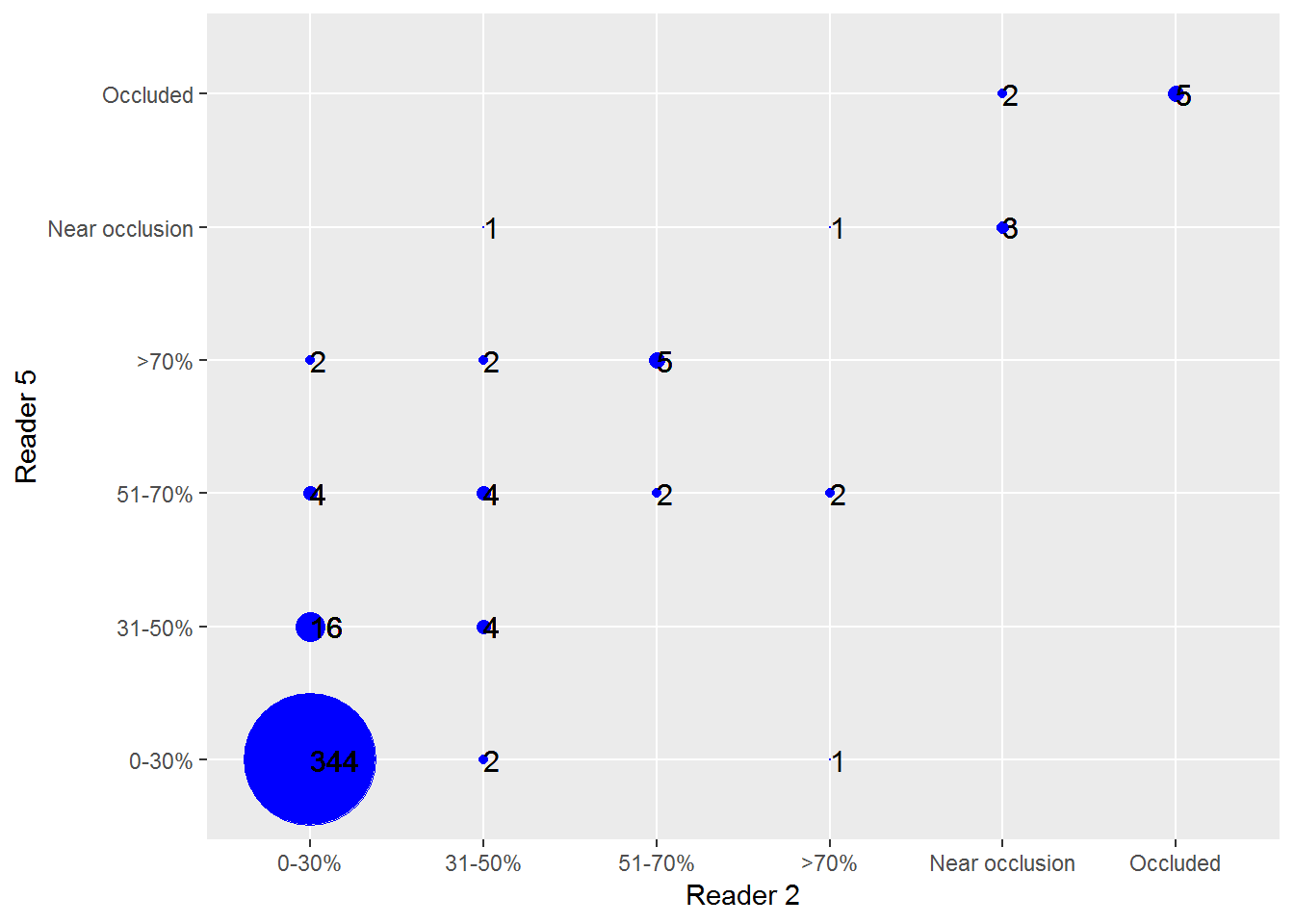


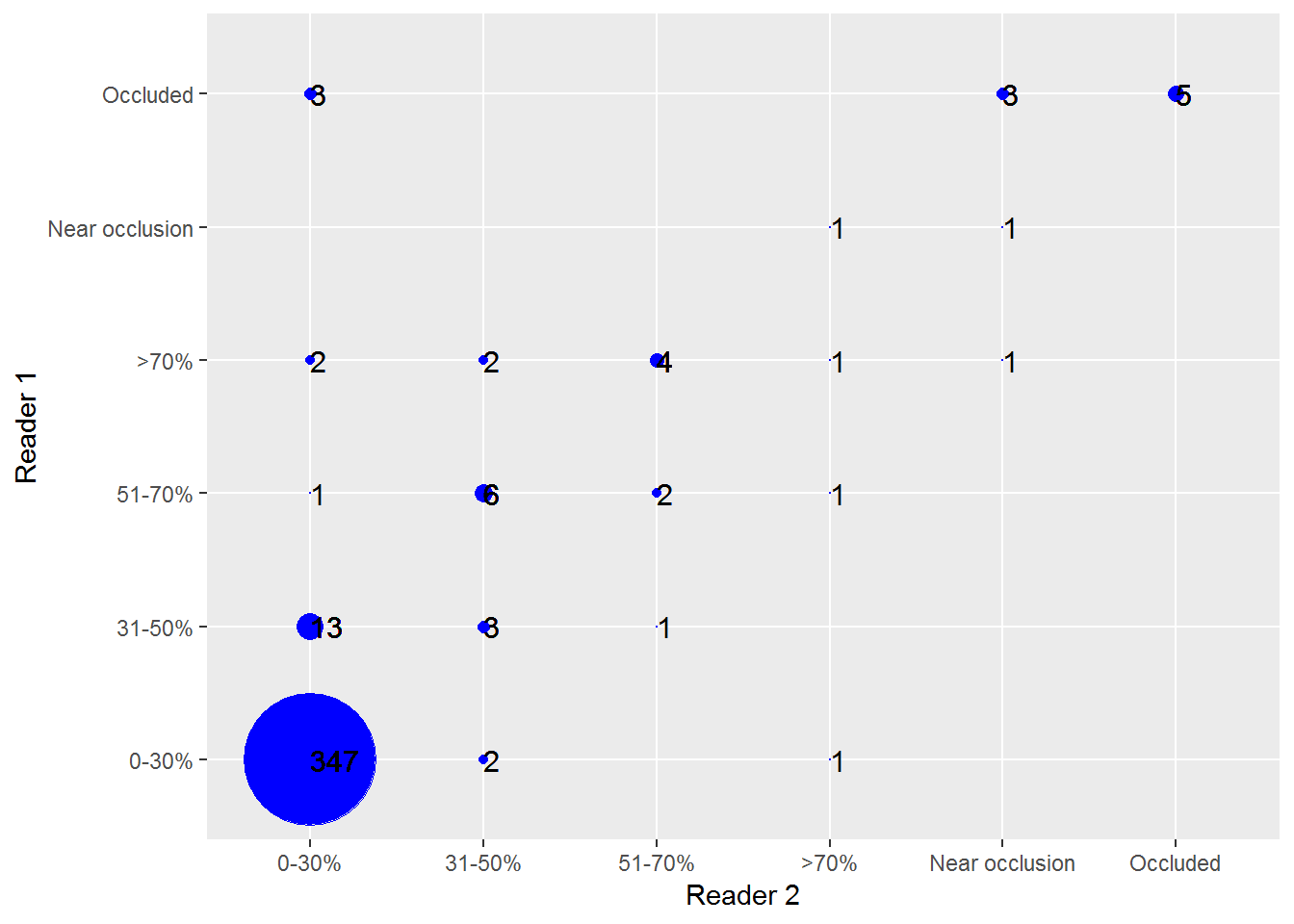


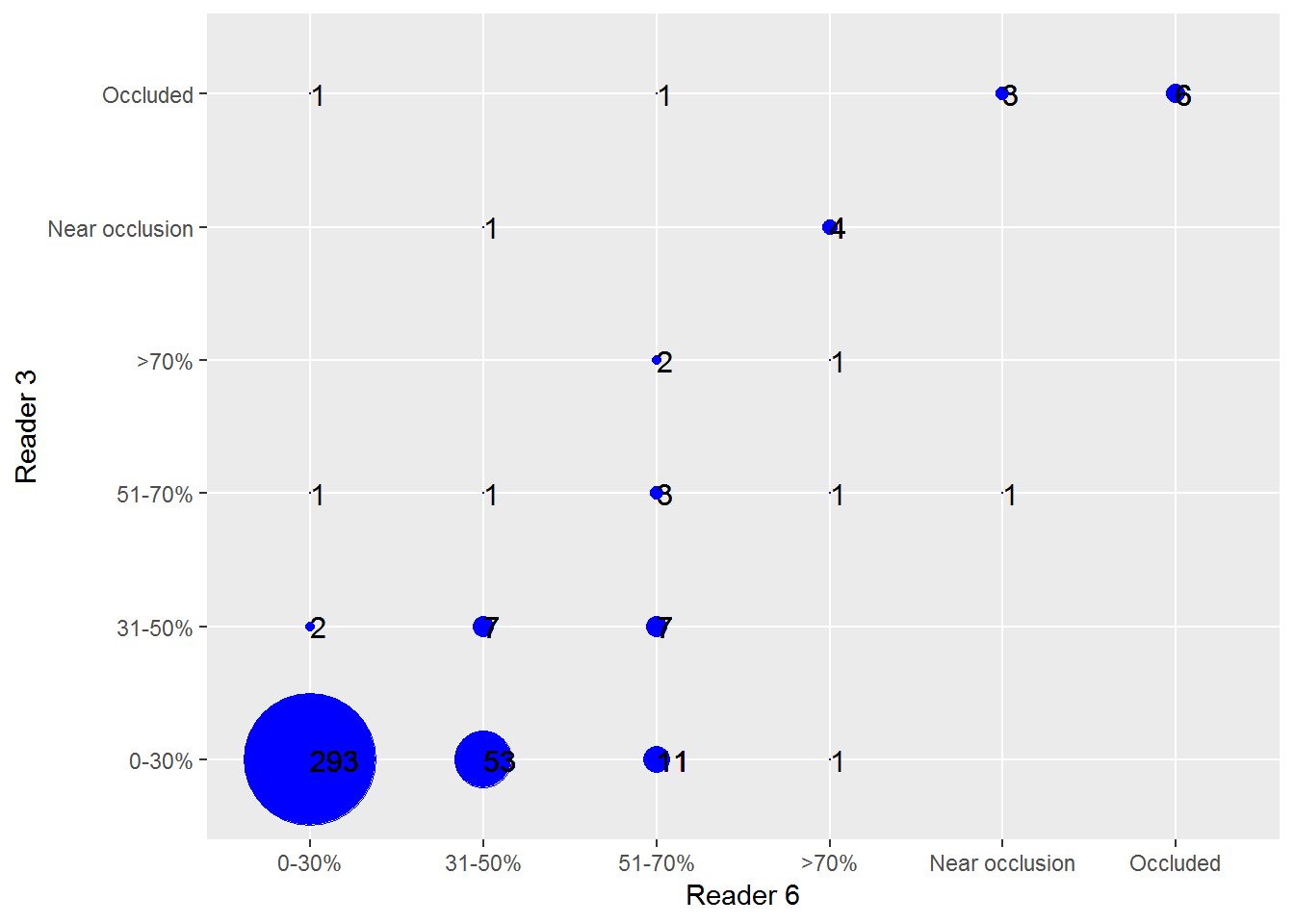


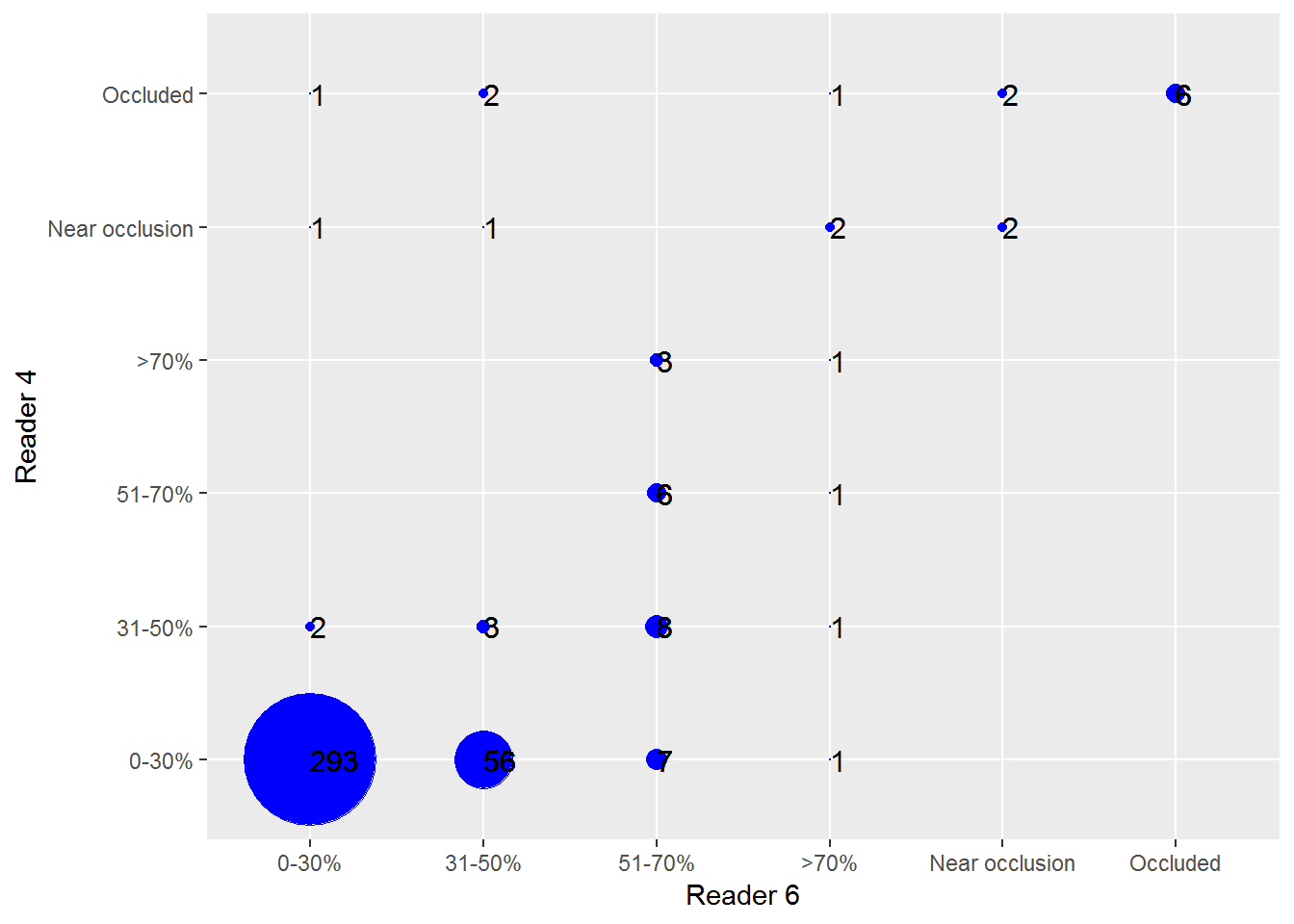


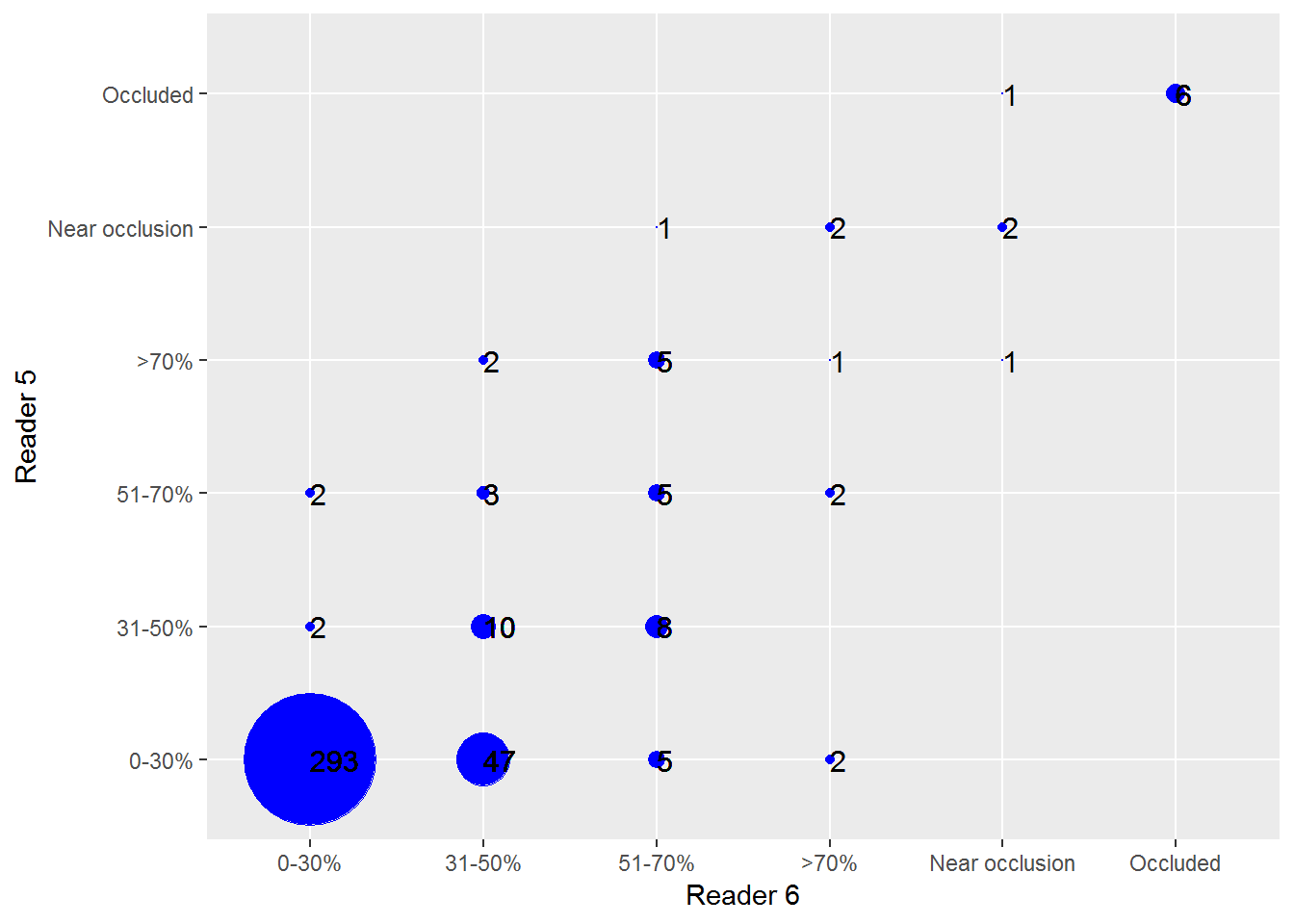


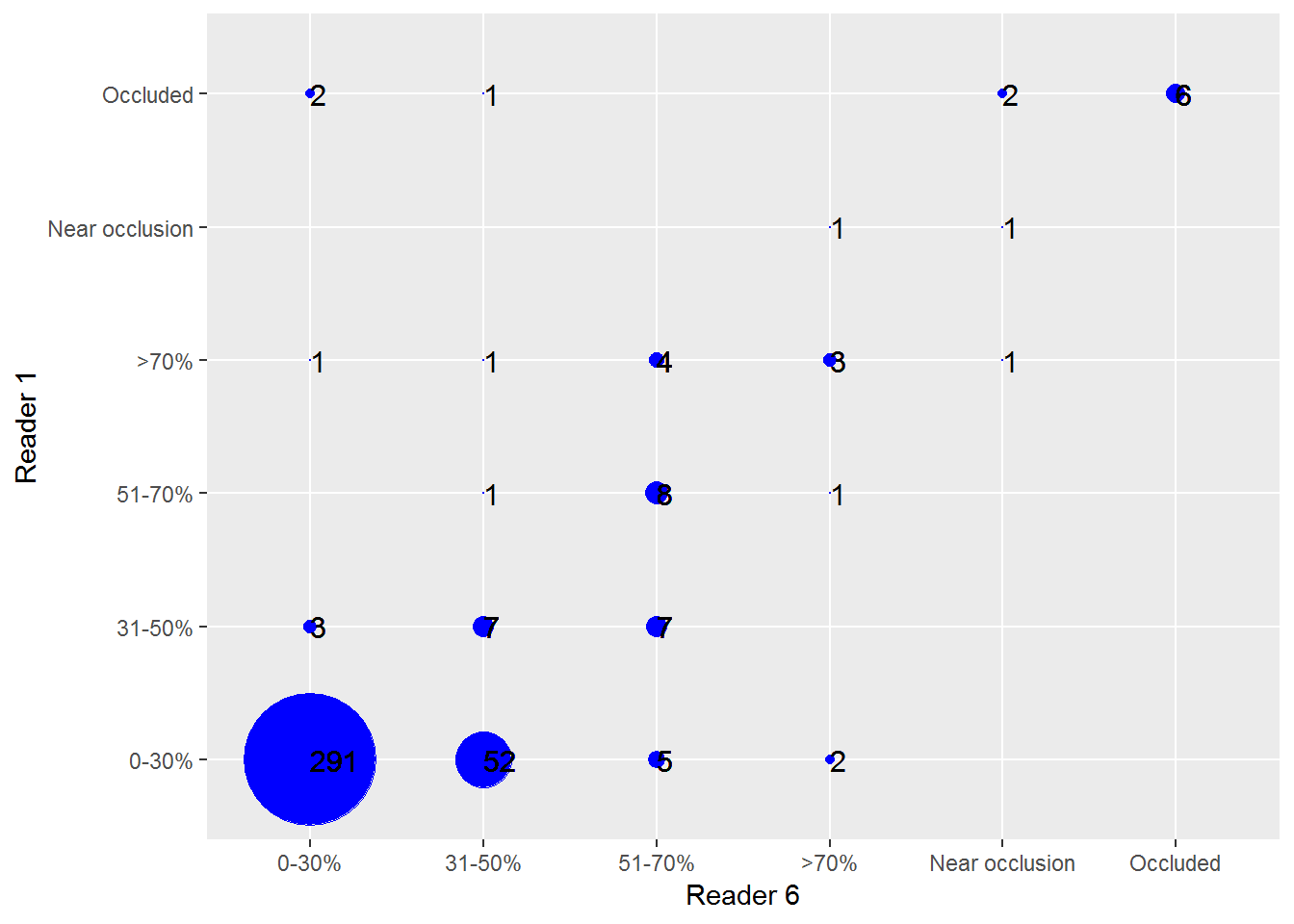


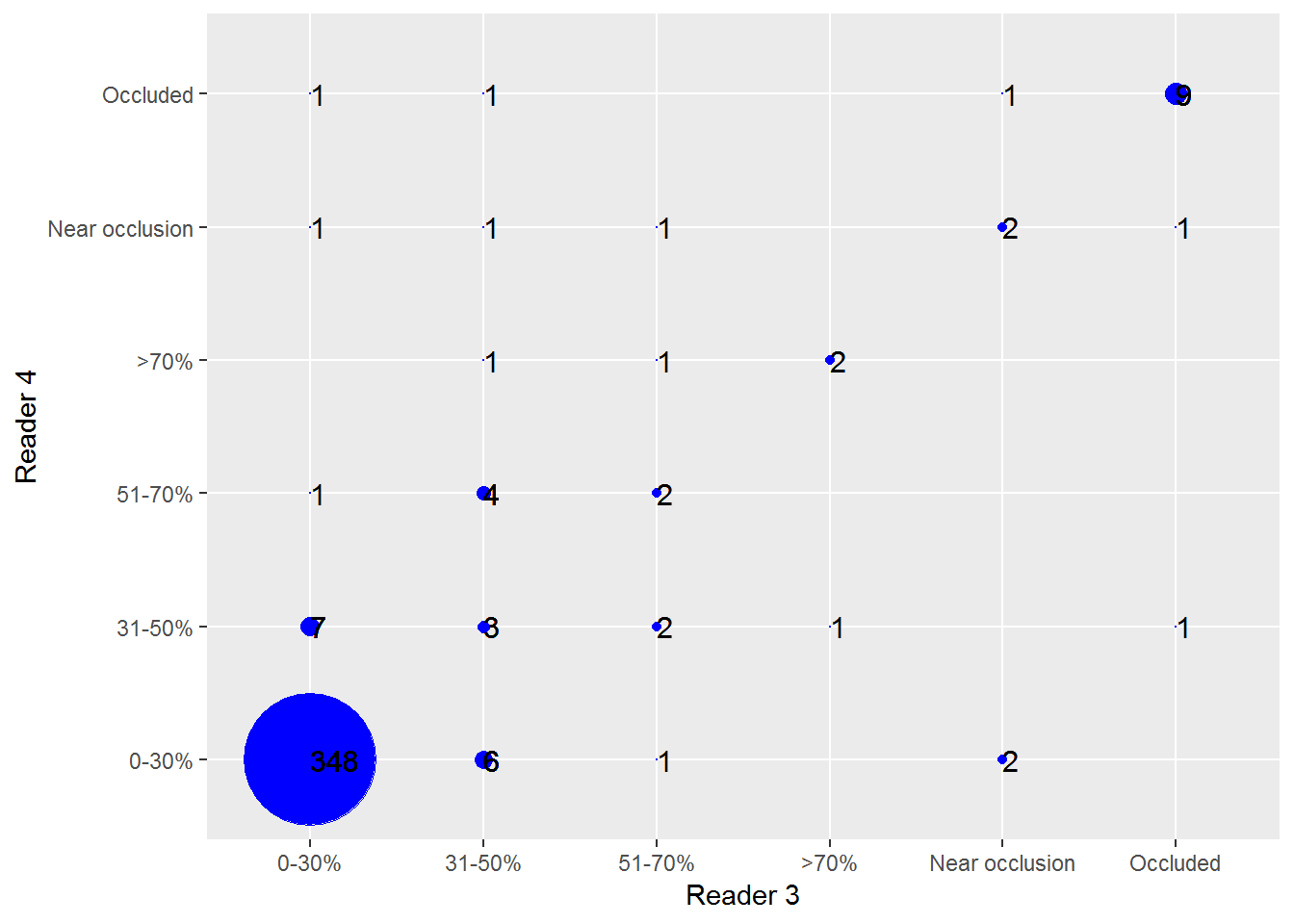


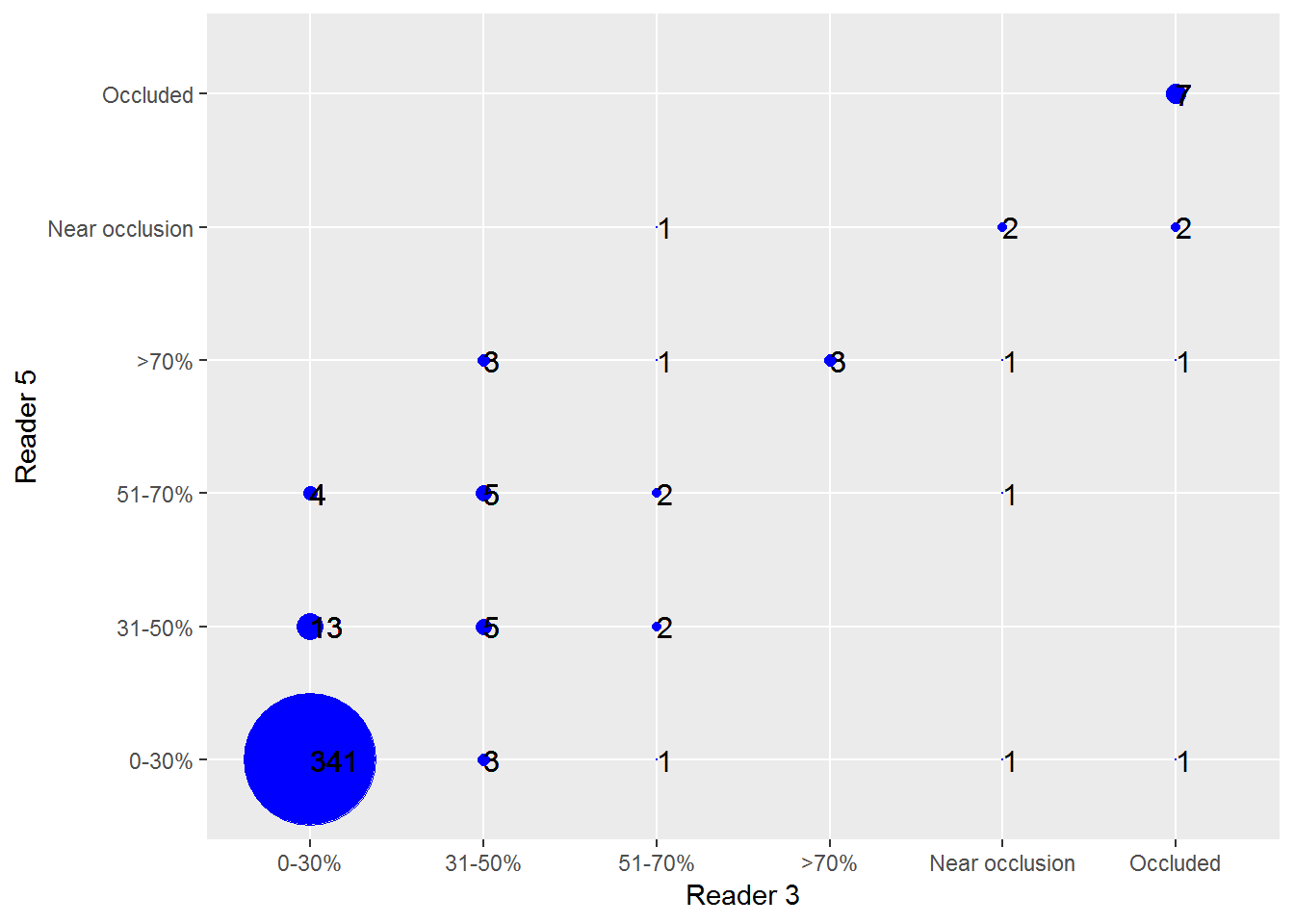


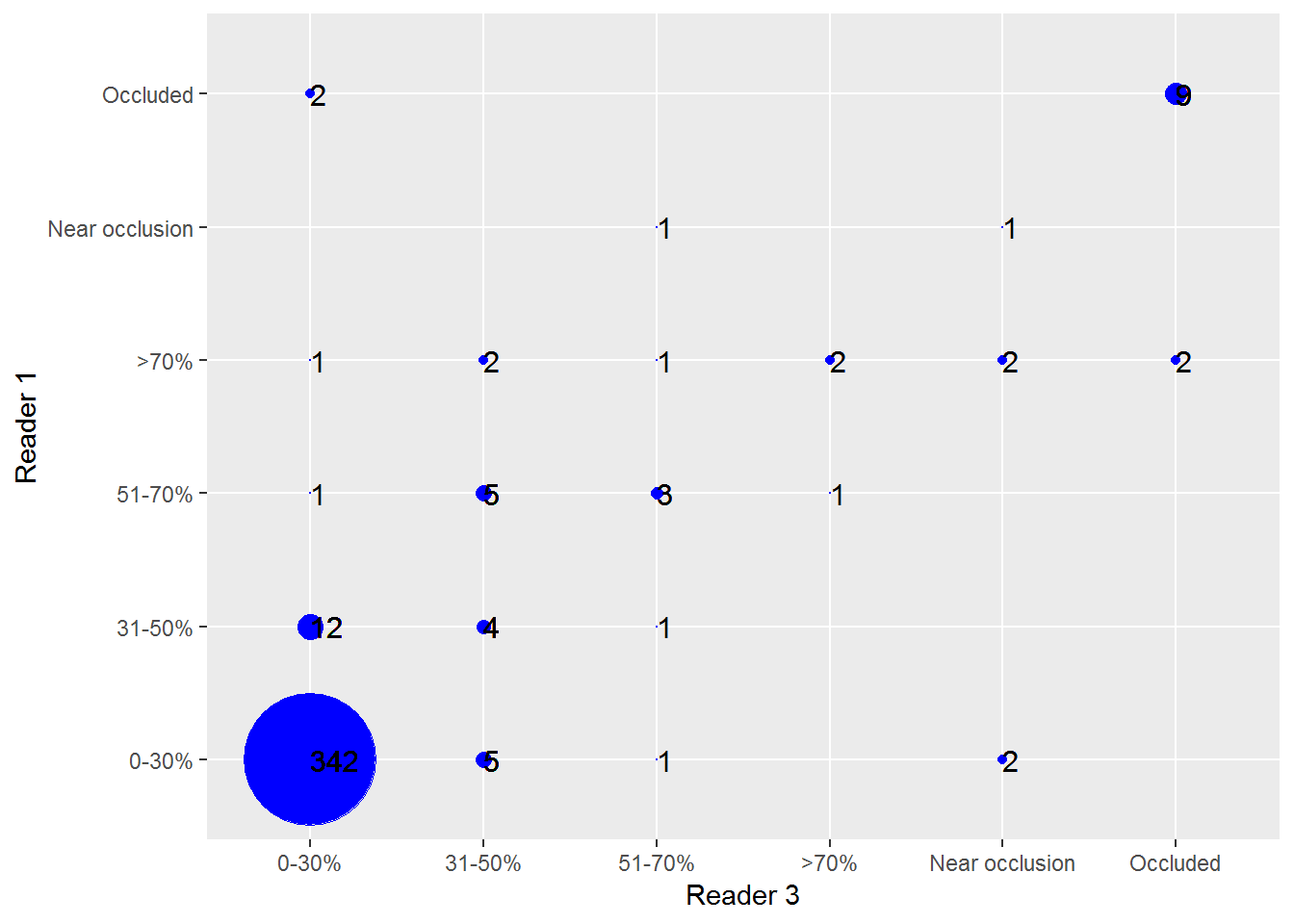


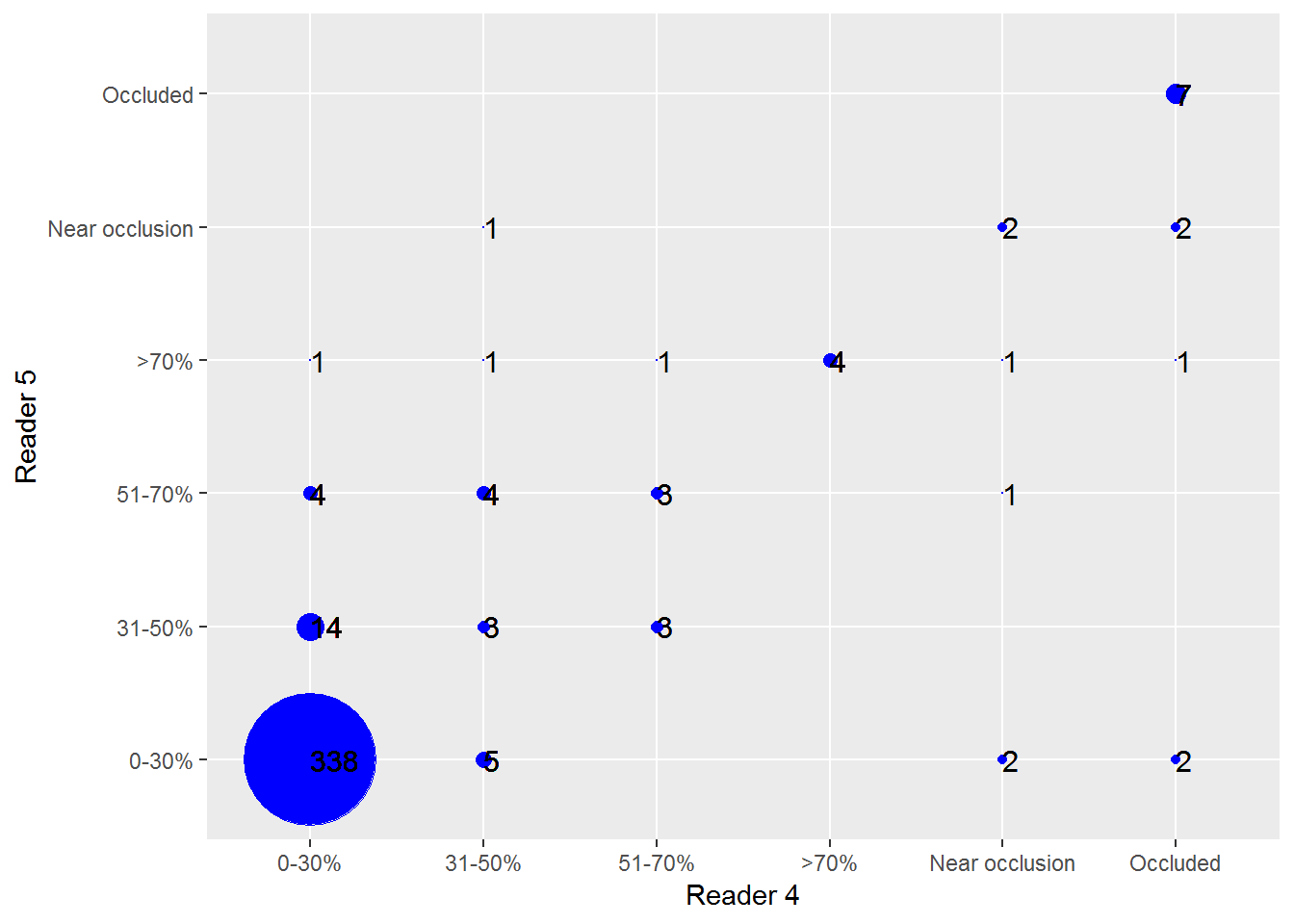


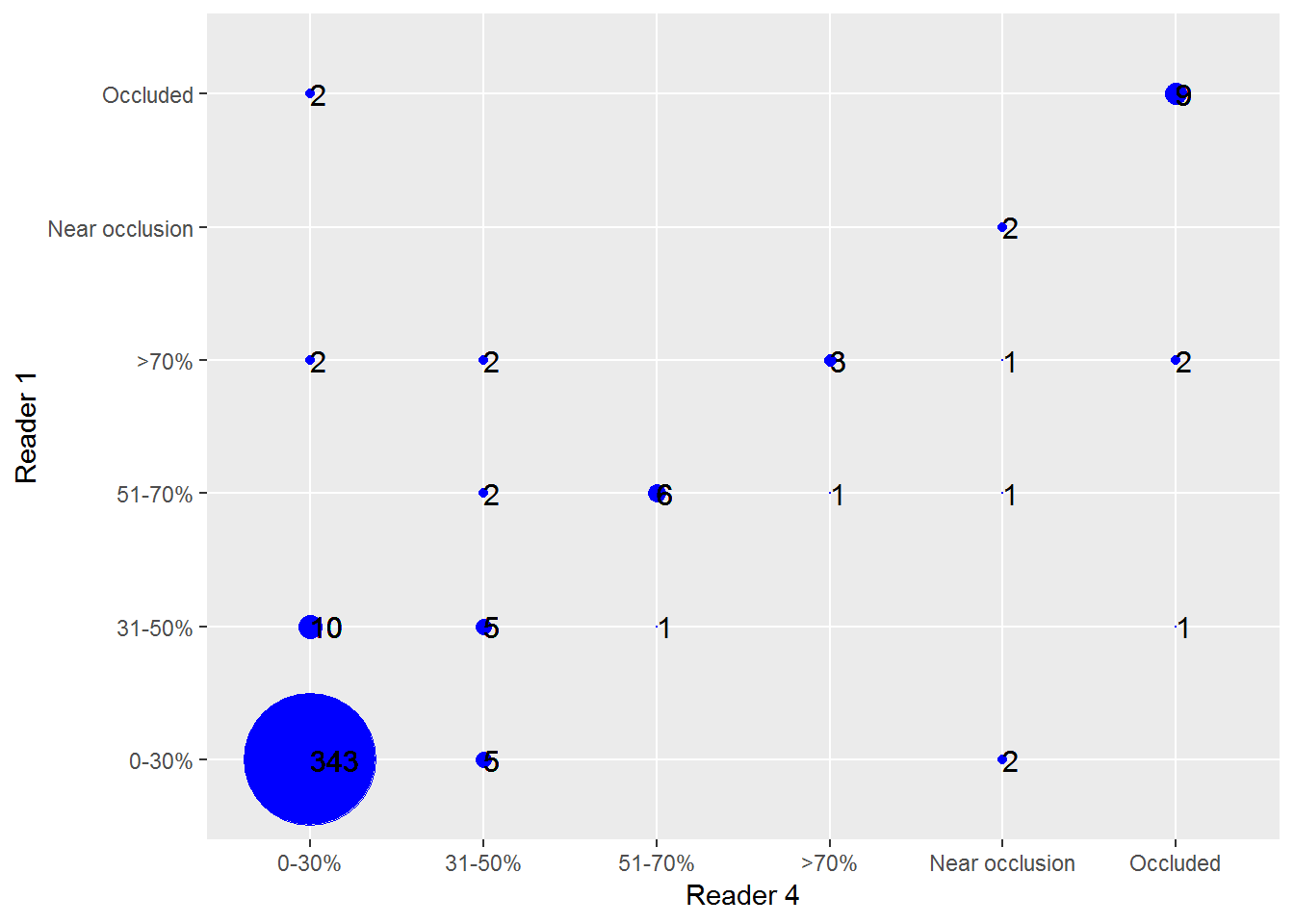


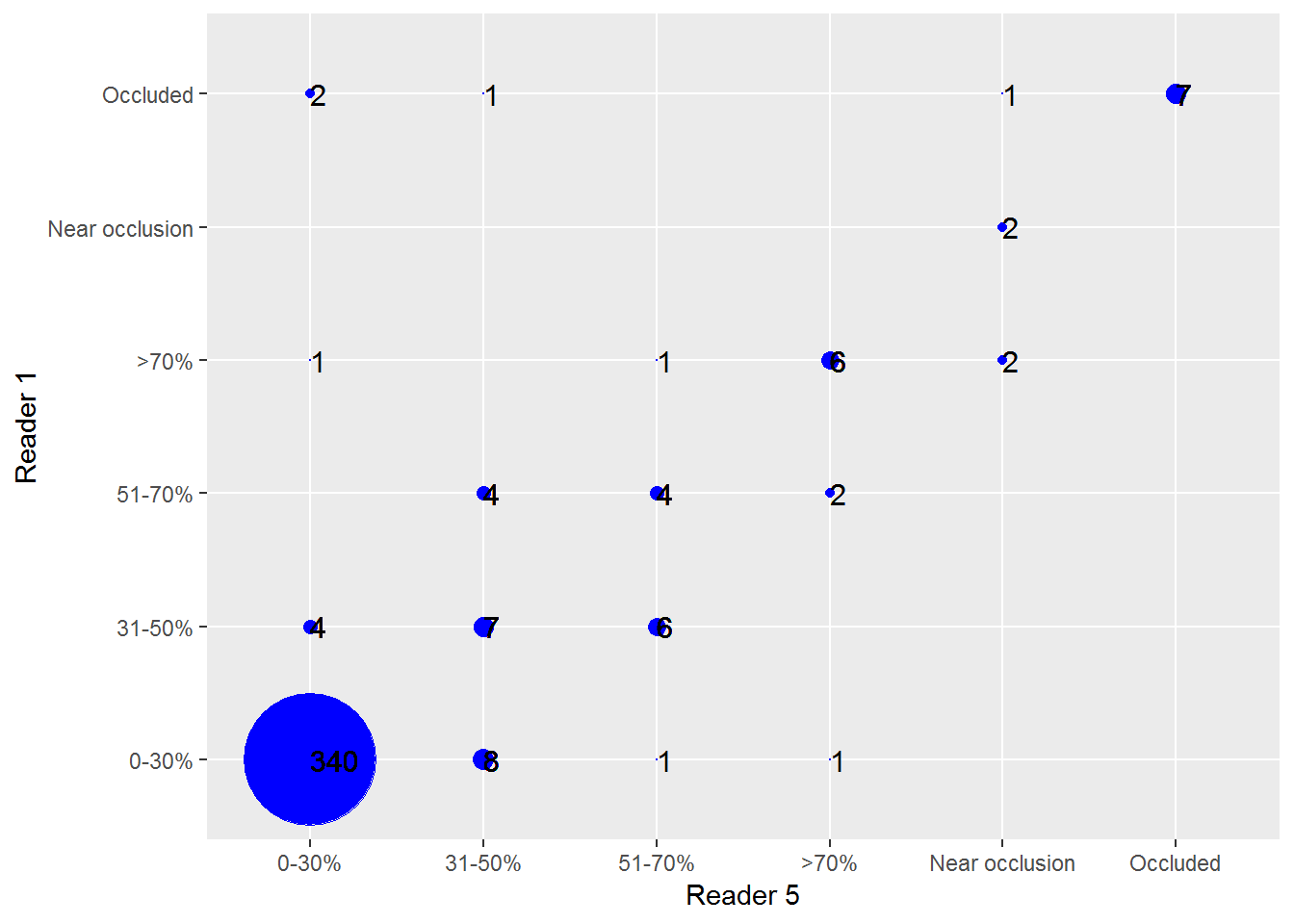


**Stenosis measurement CE-MRA**


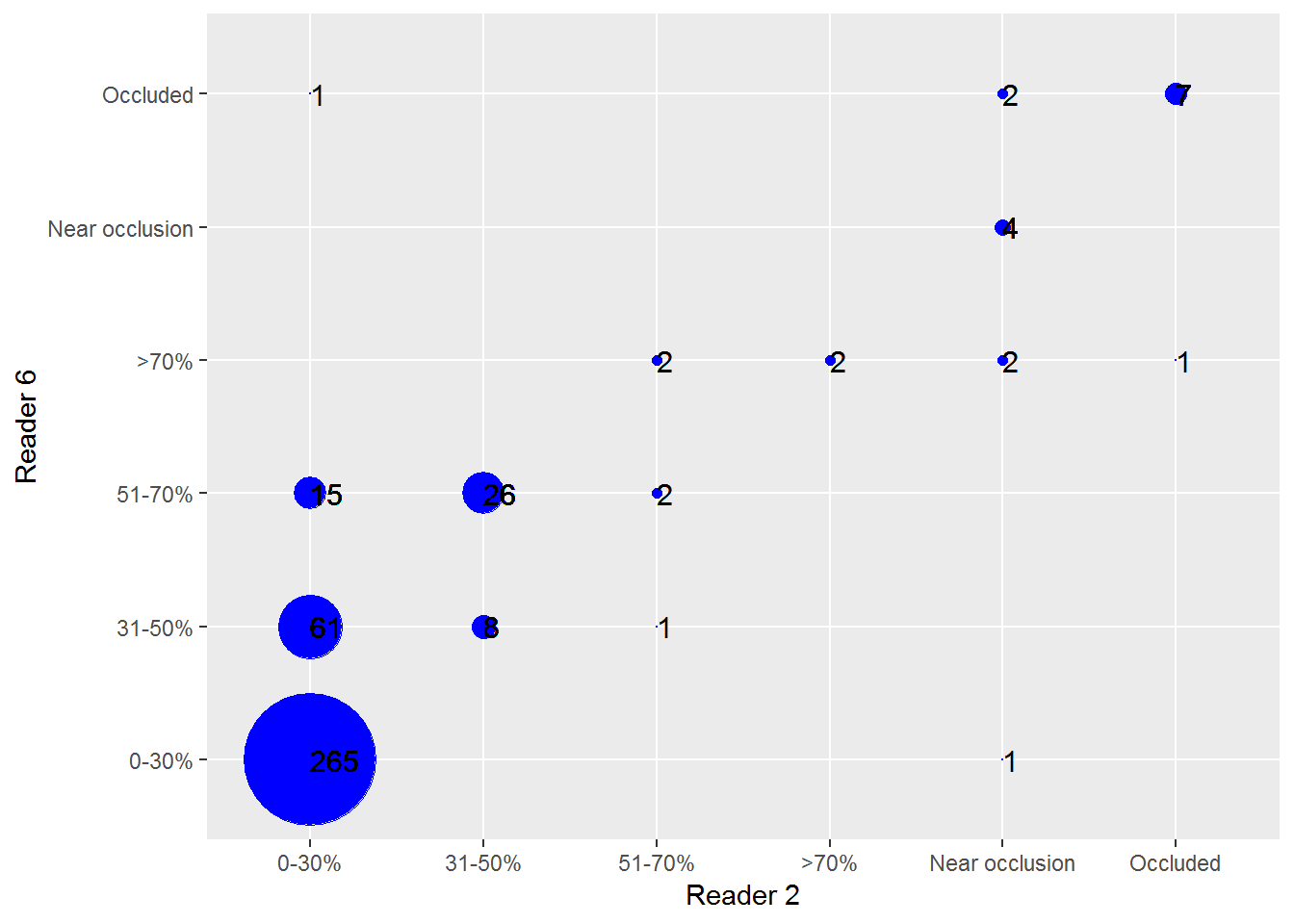


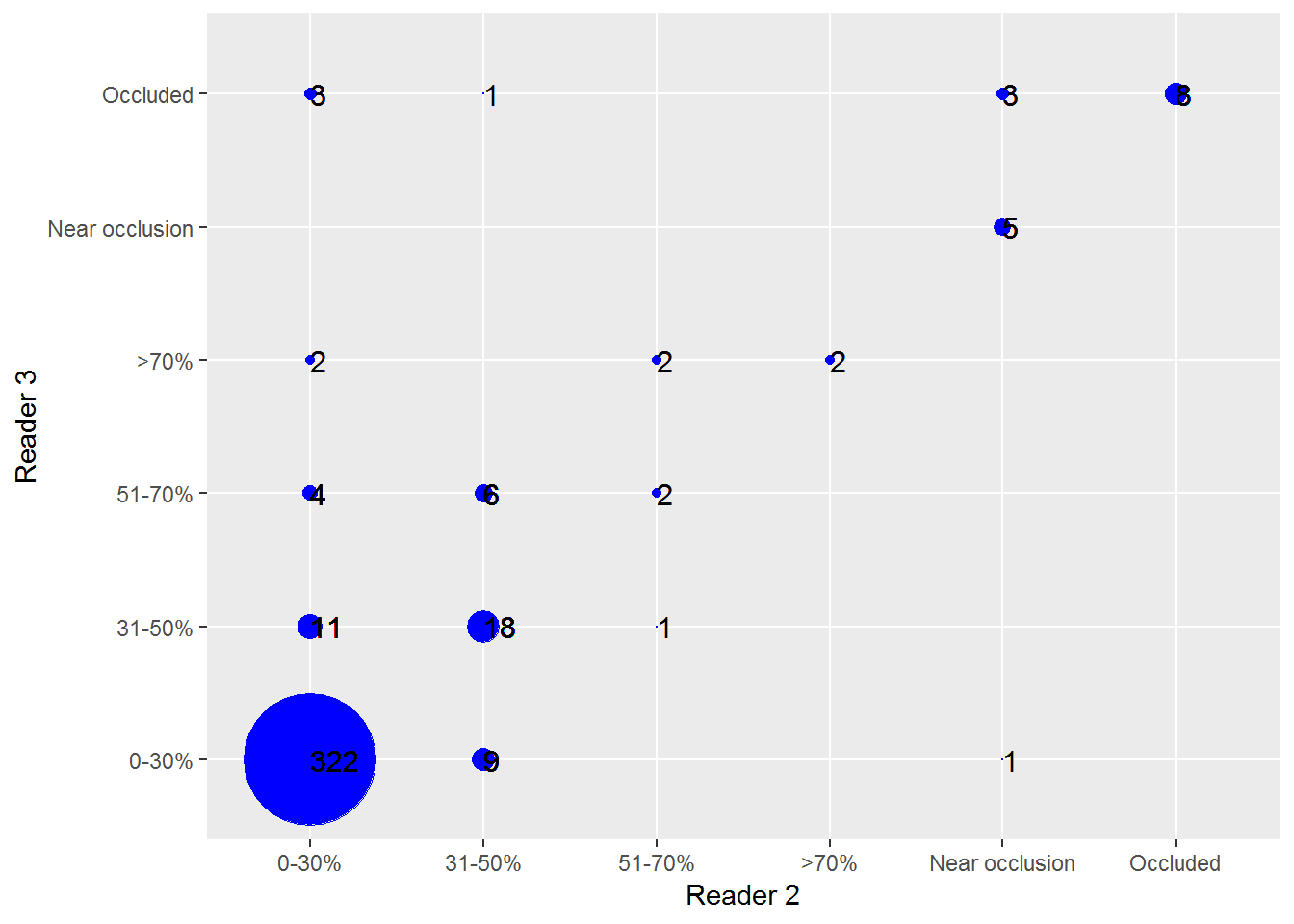


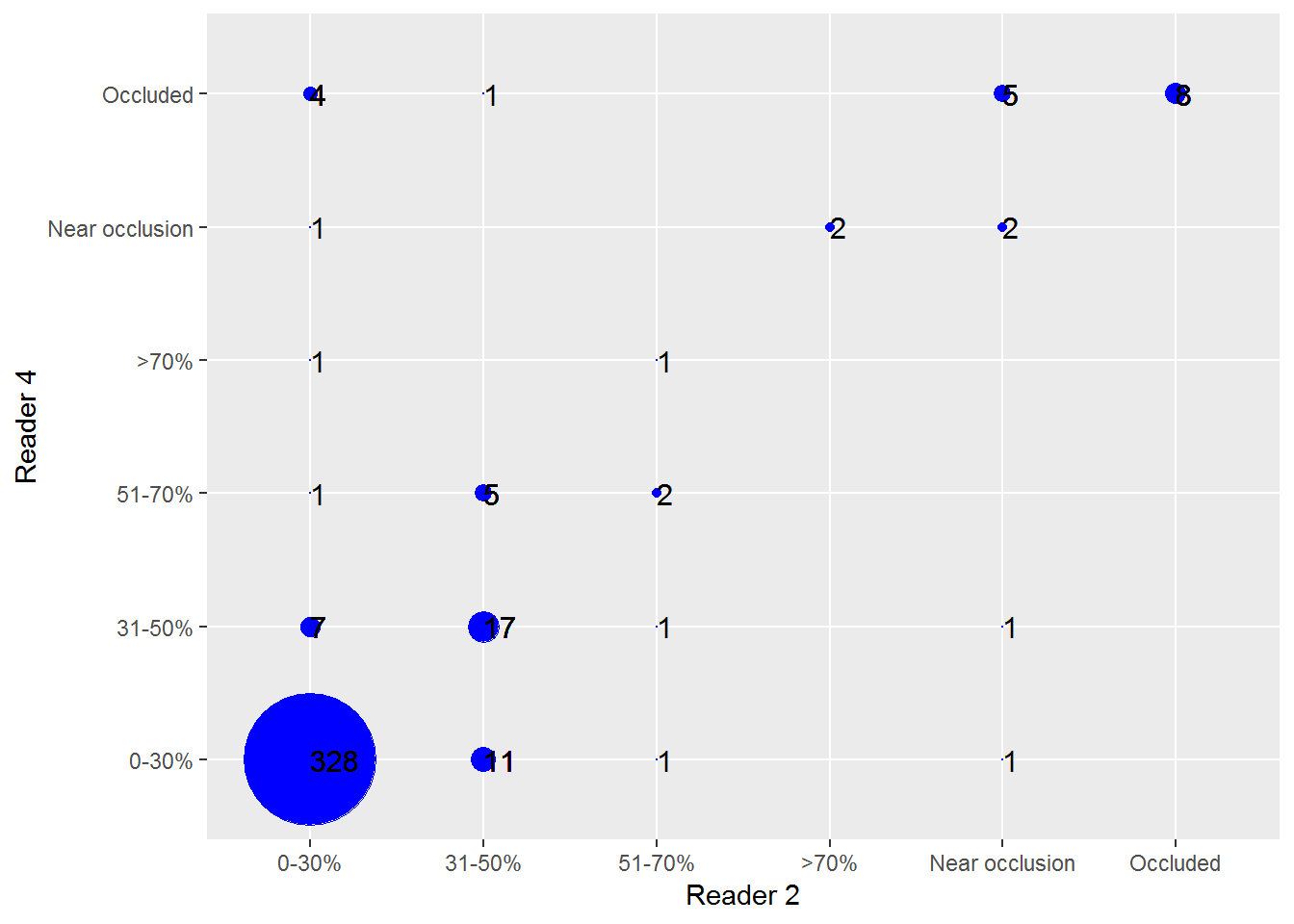


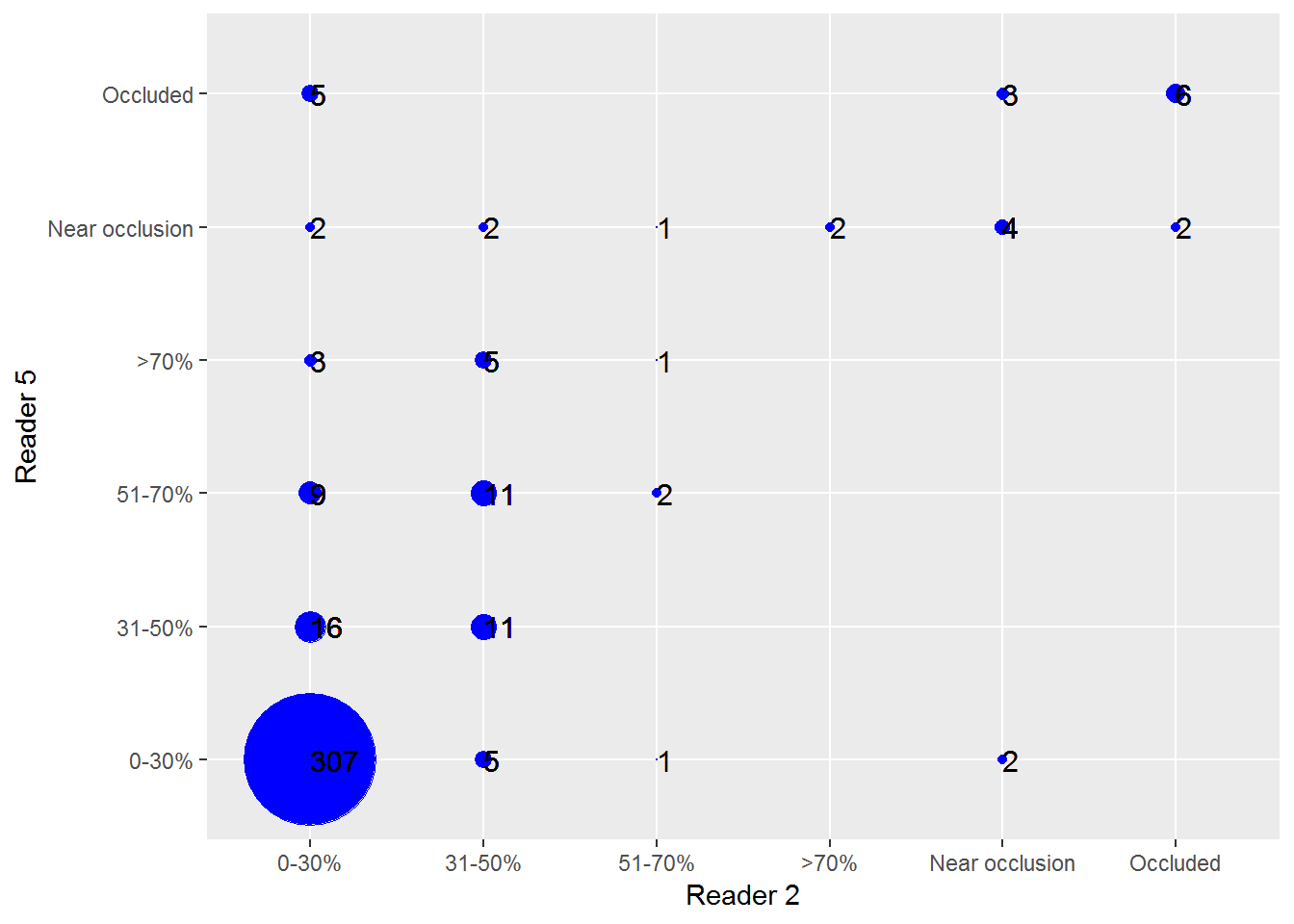


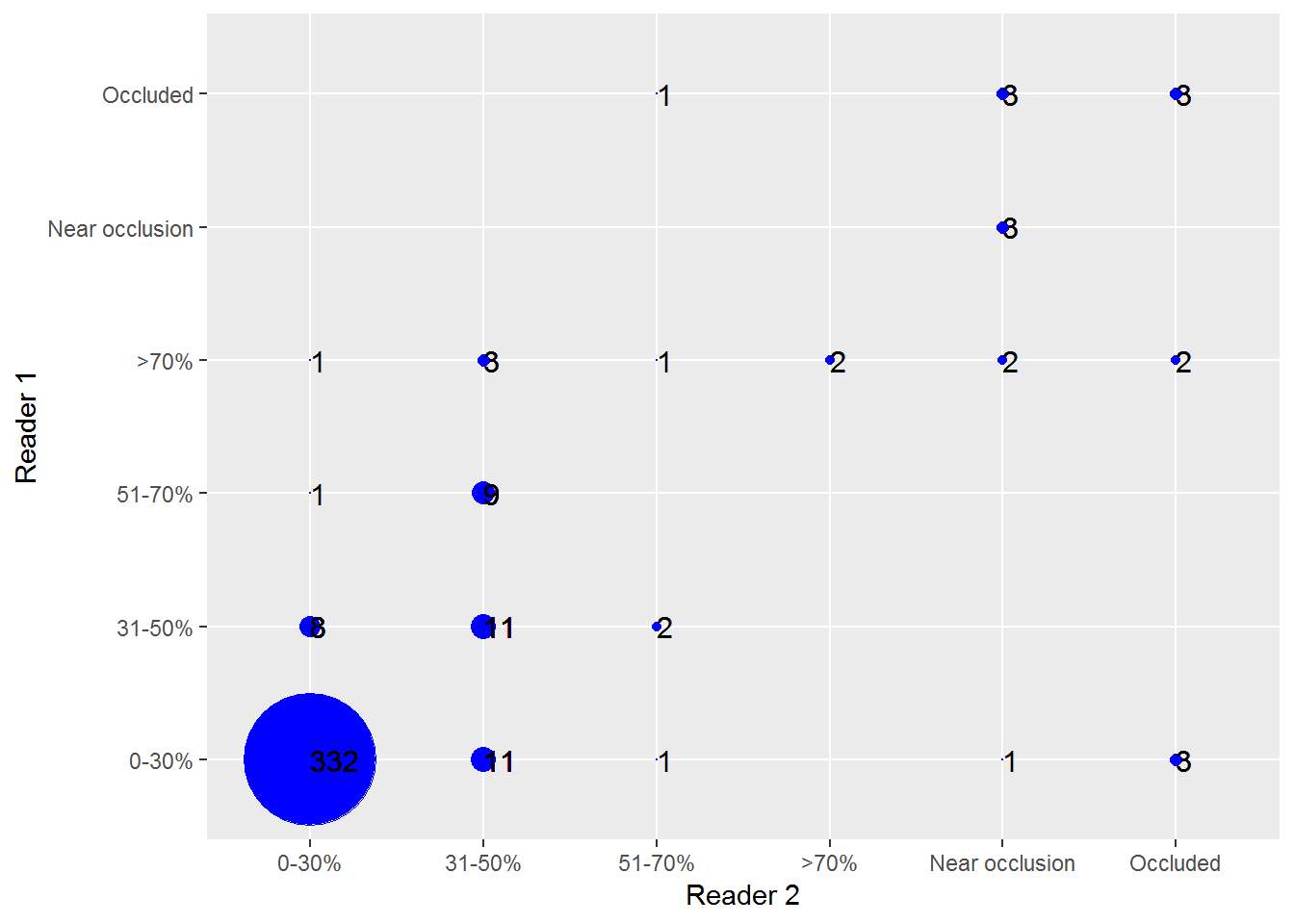


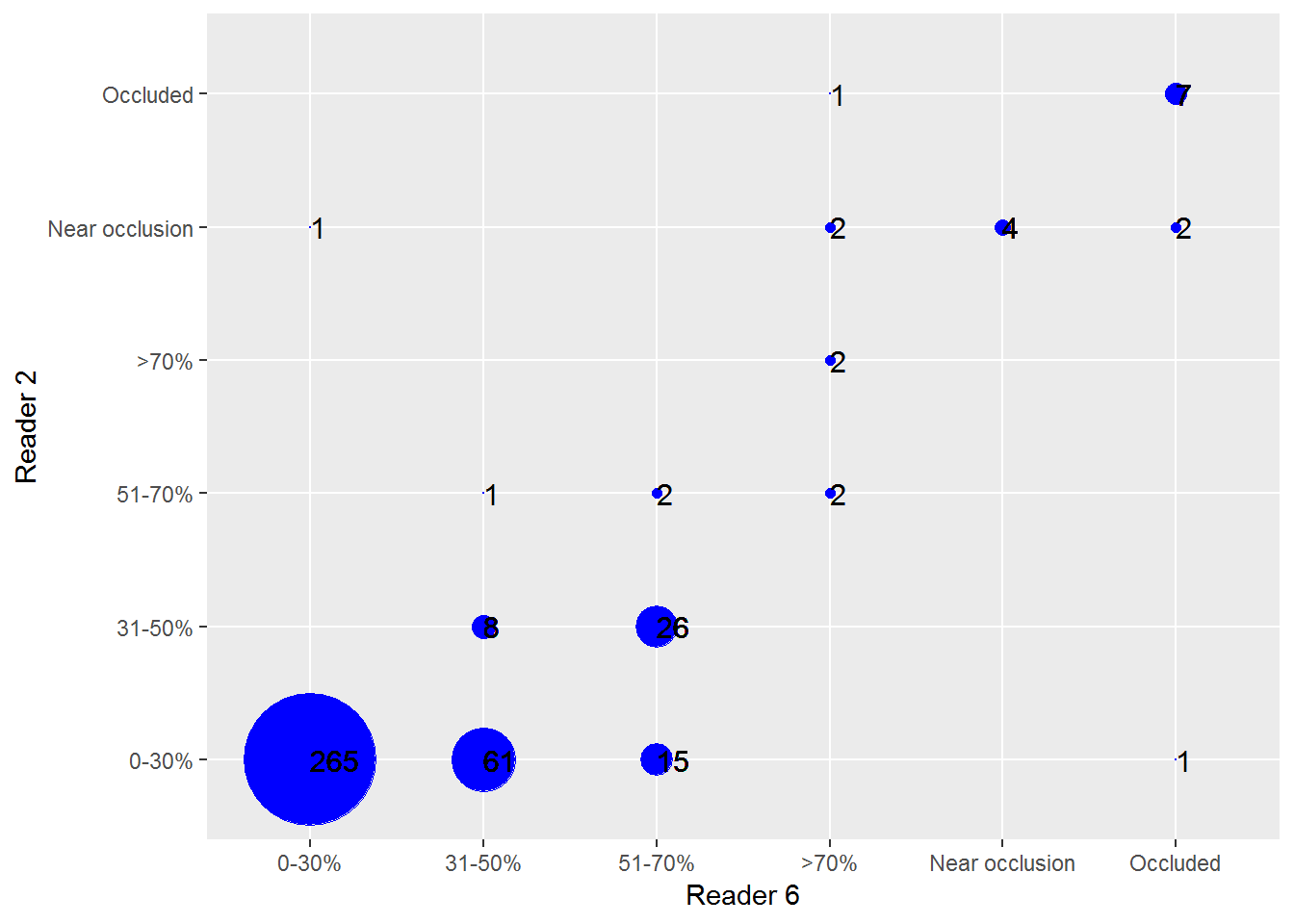


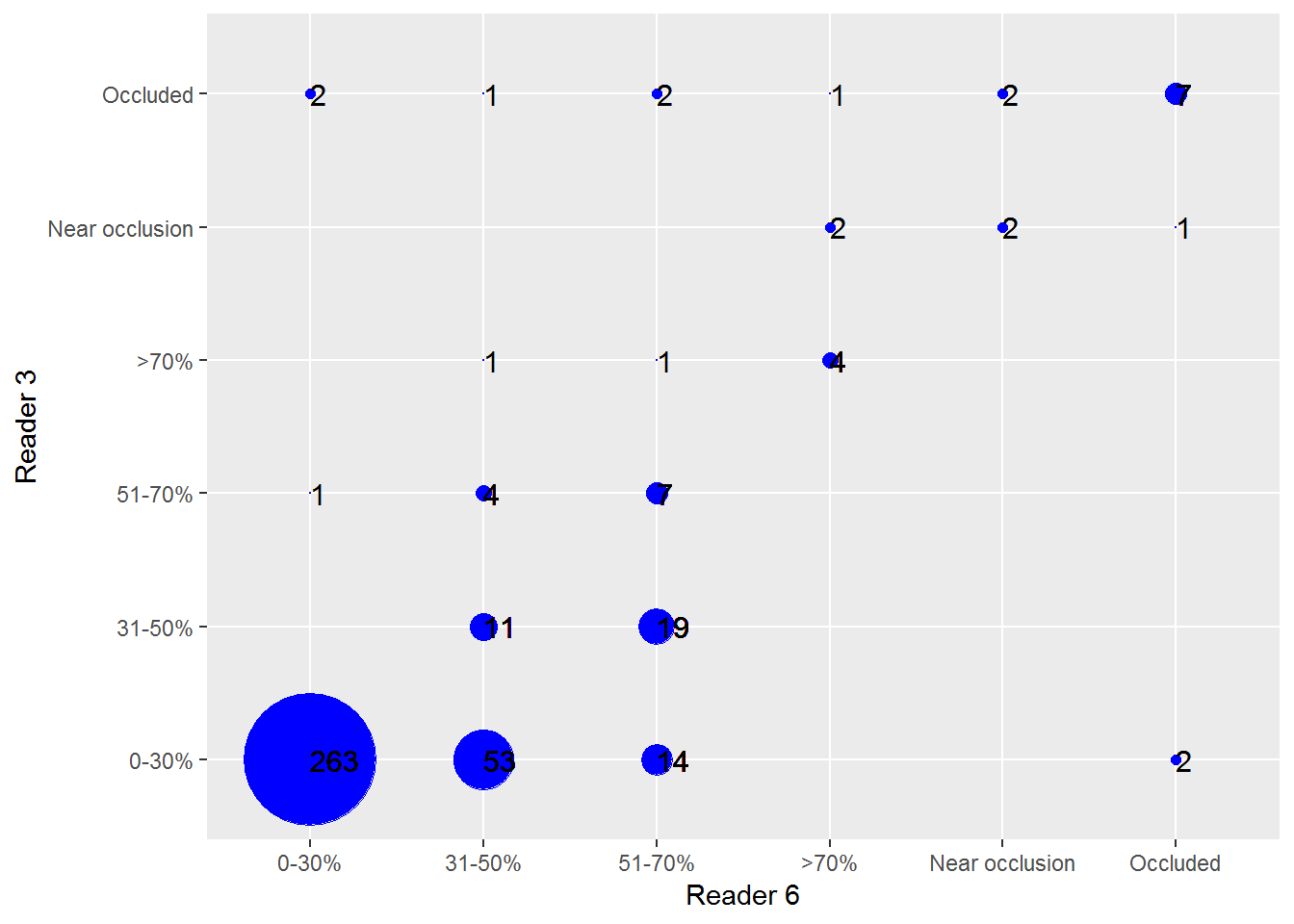


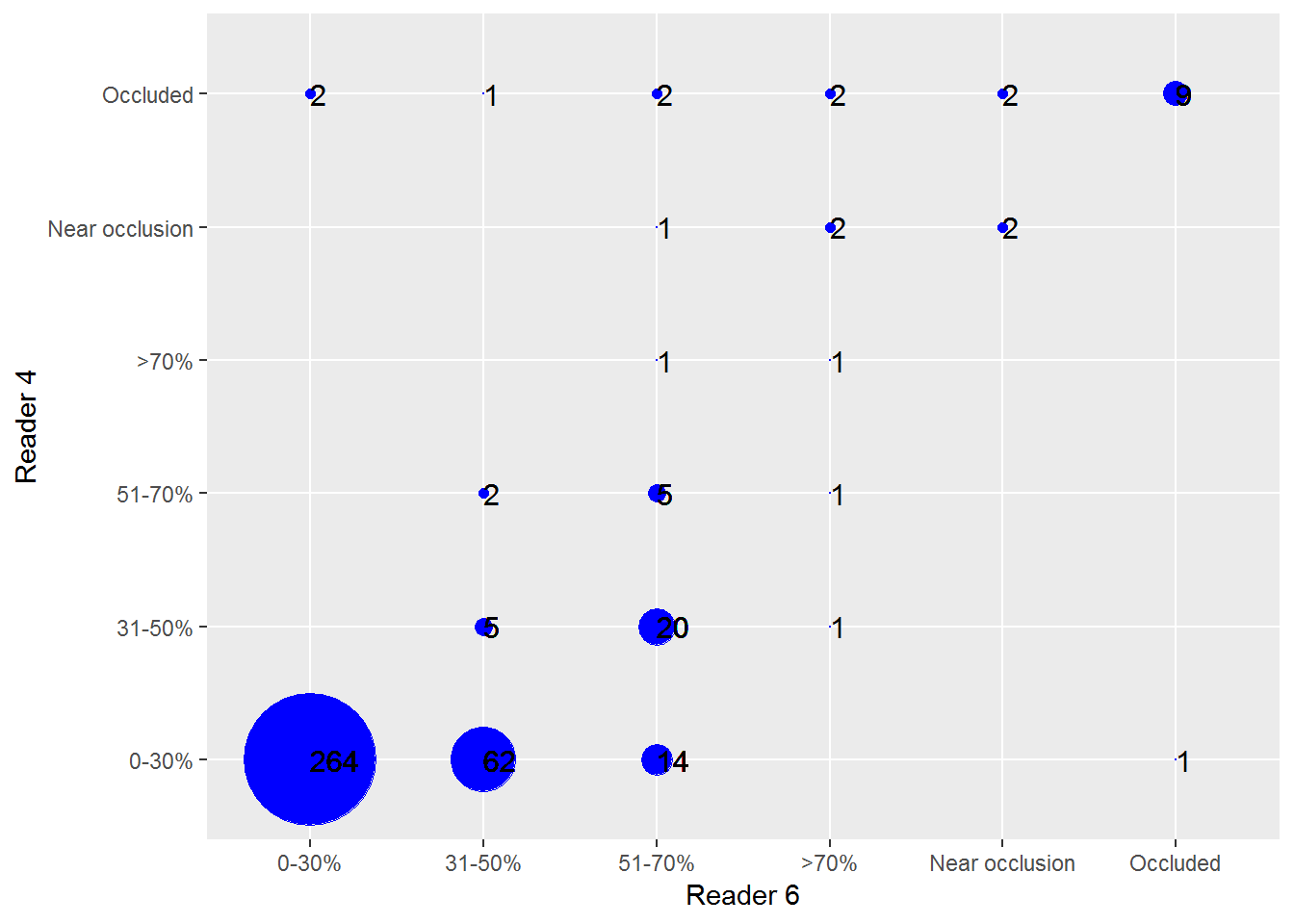


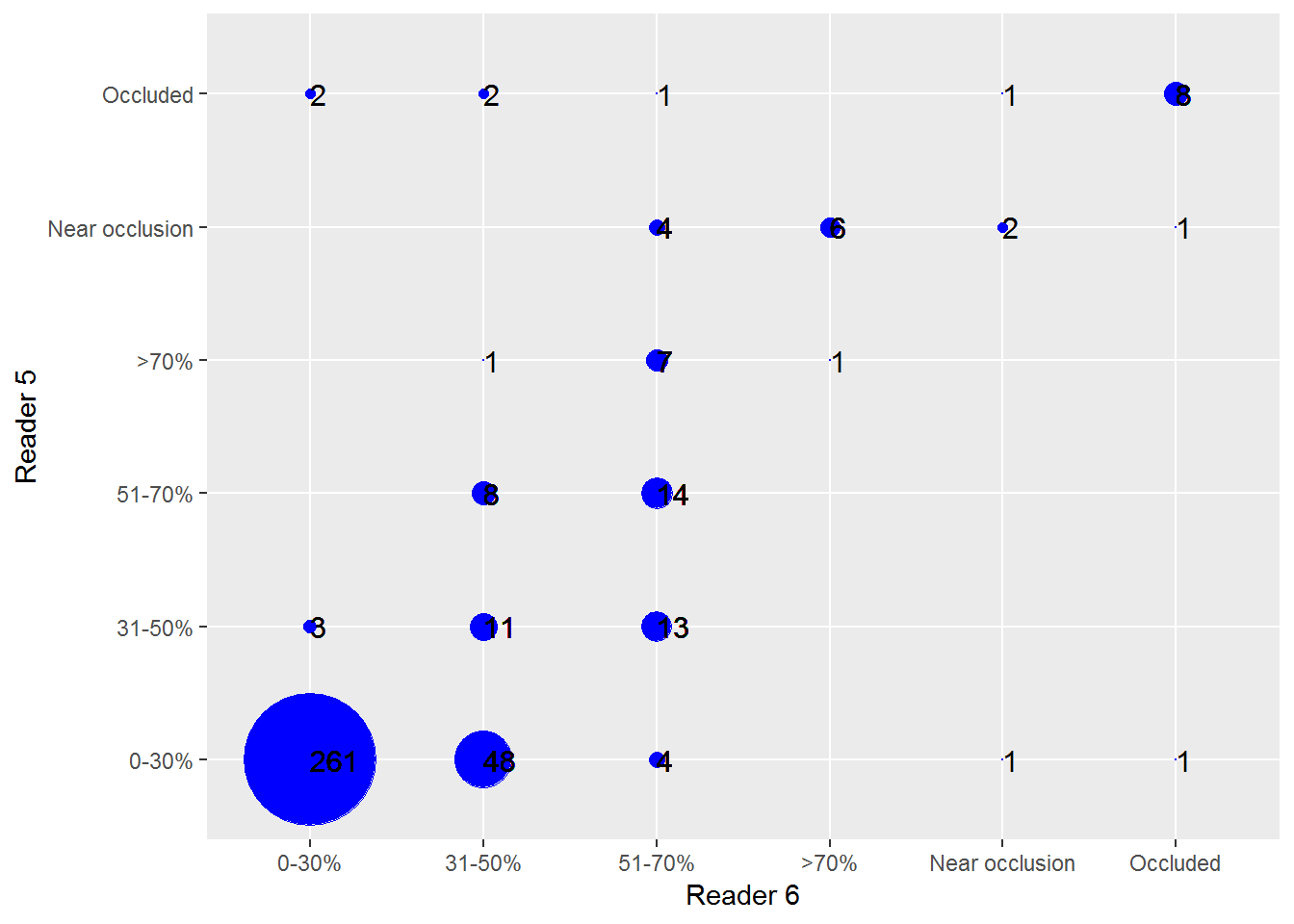


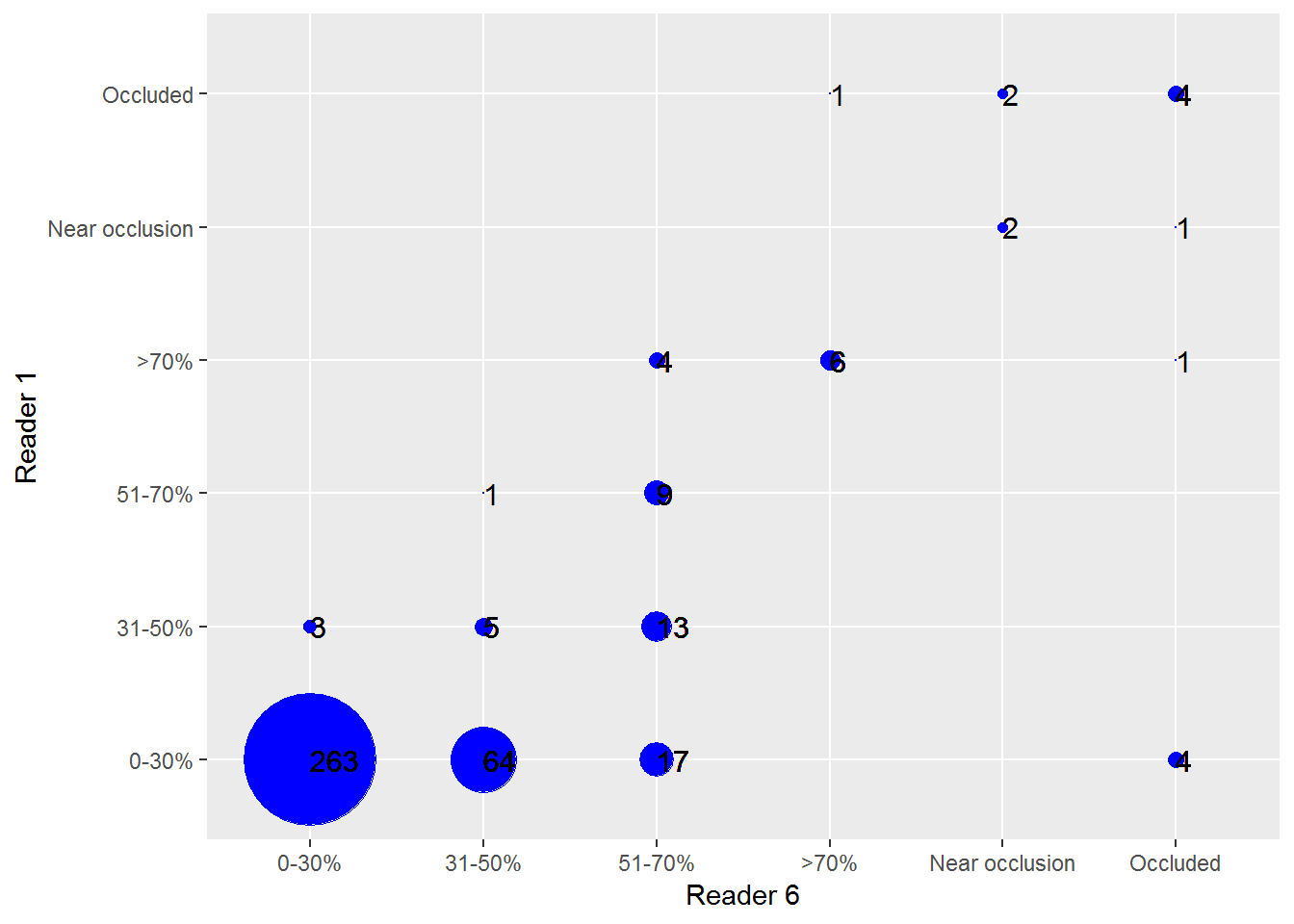


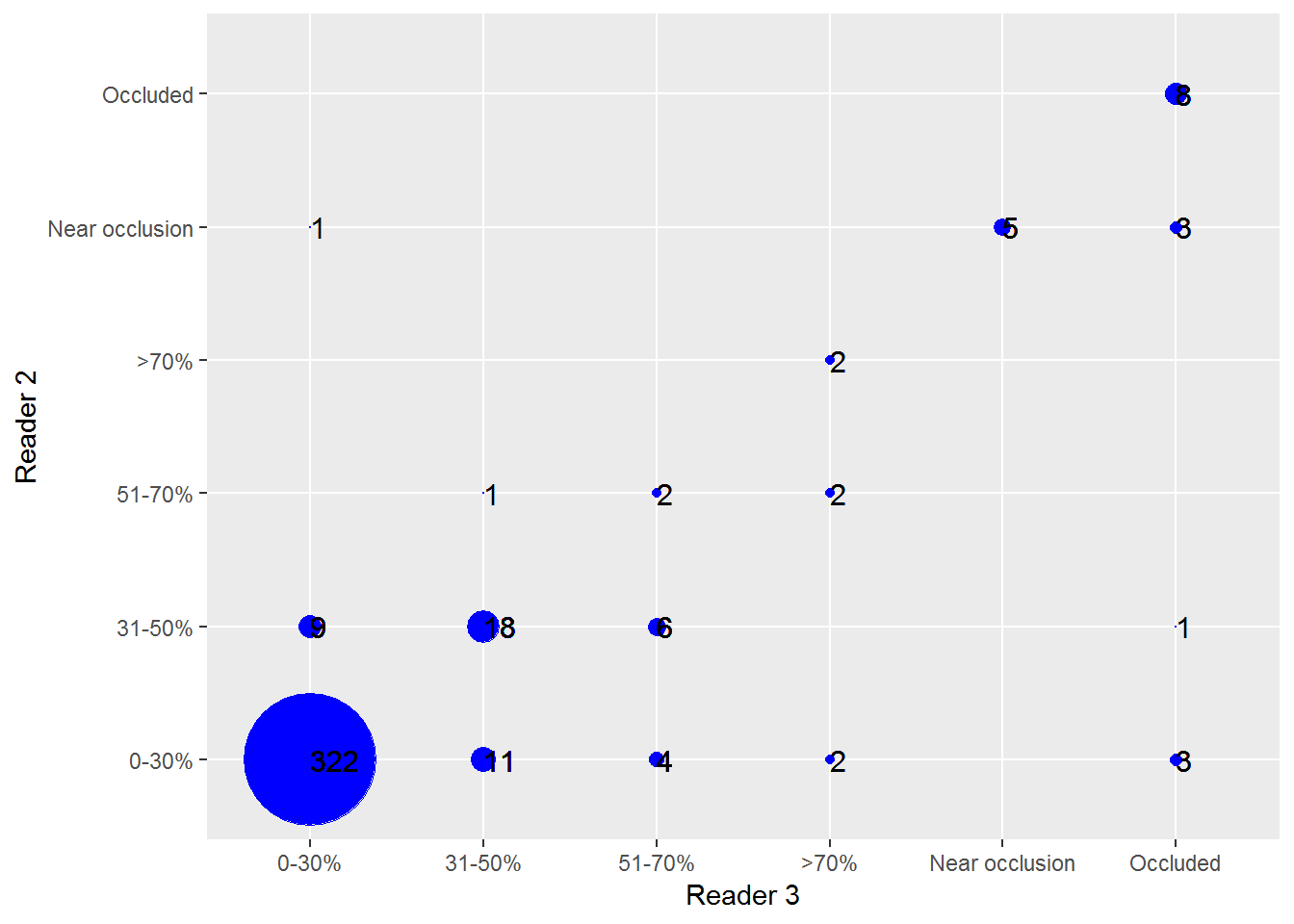


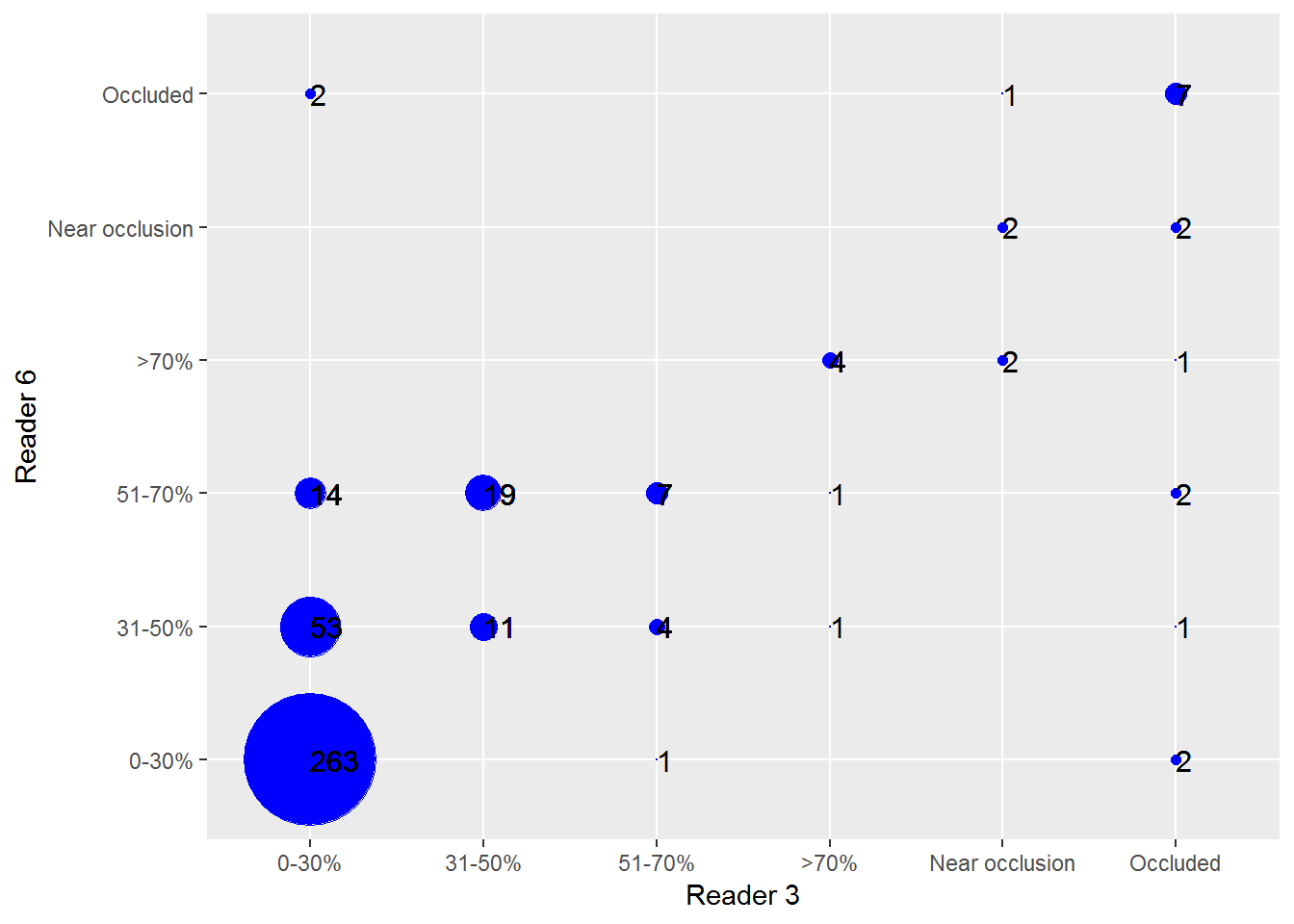


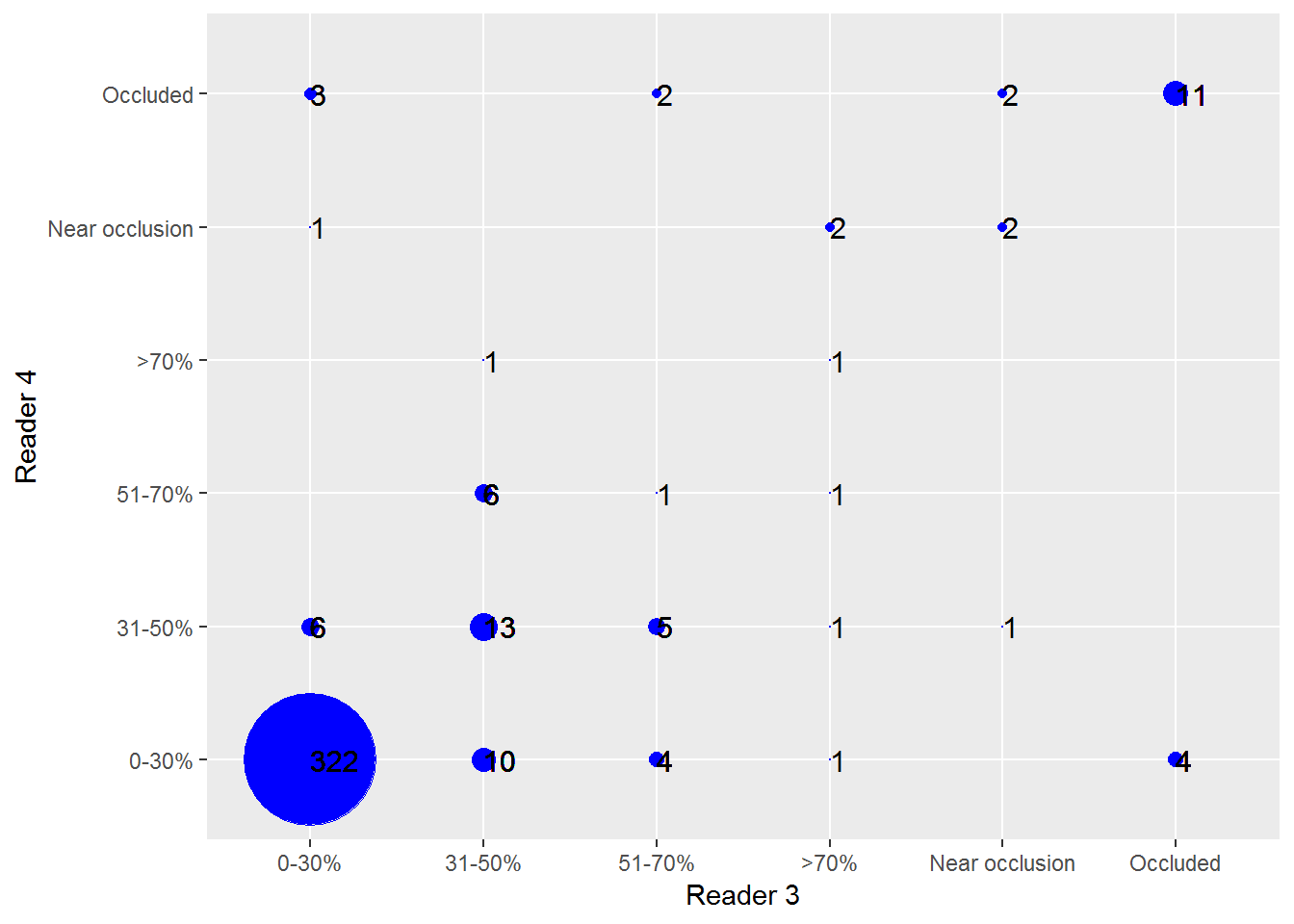


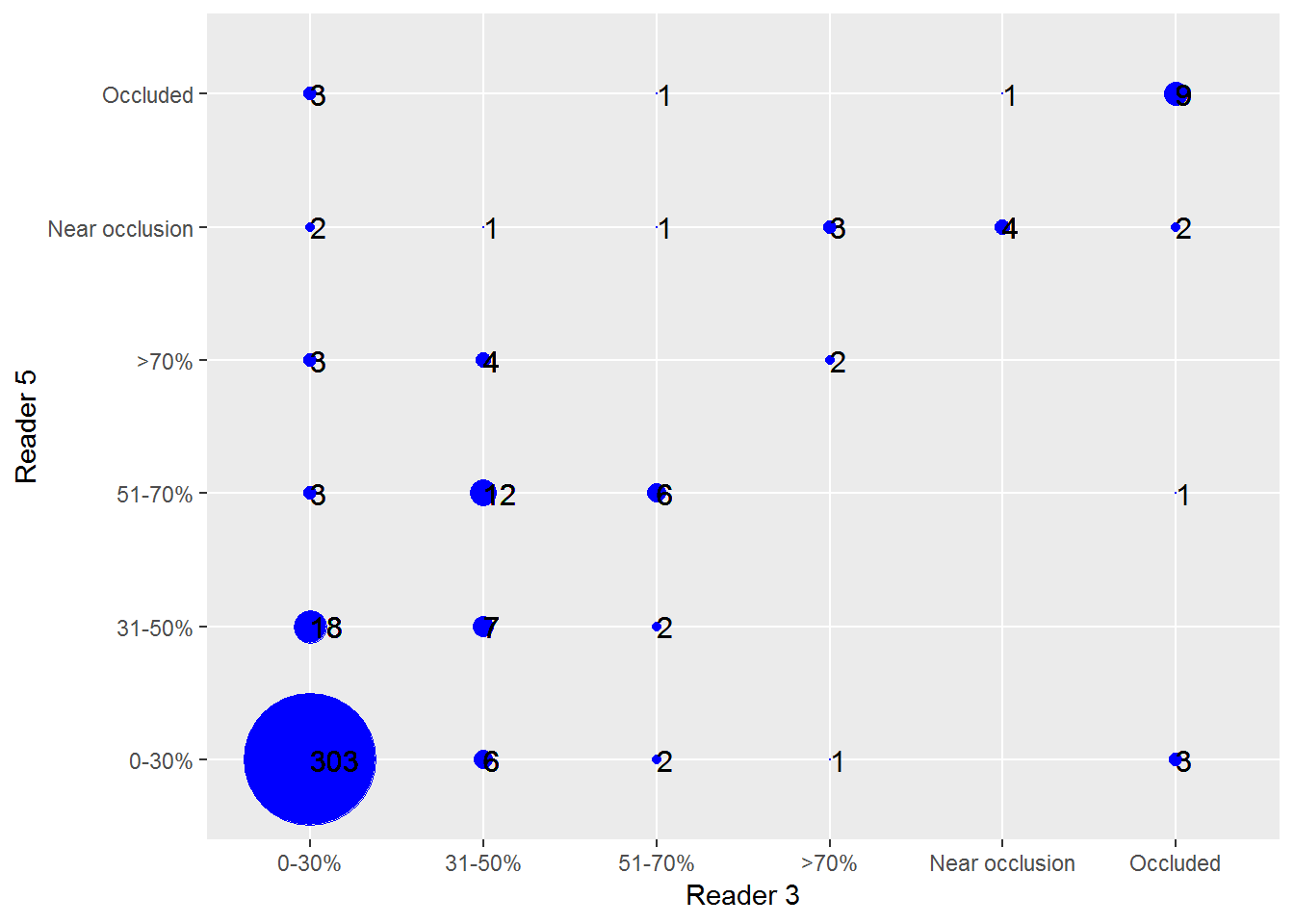


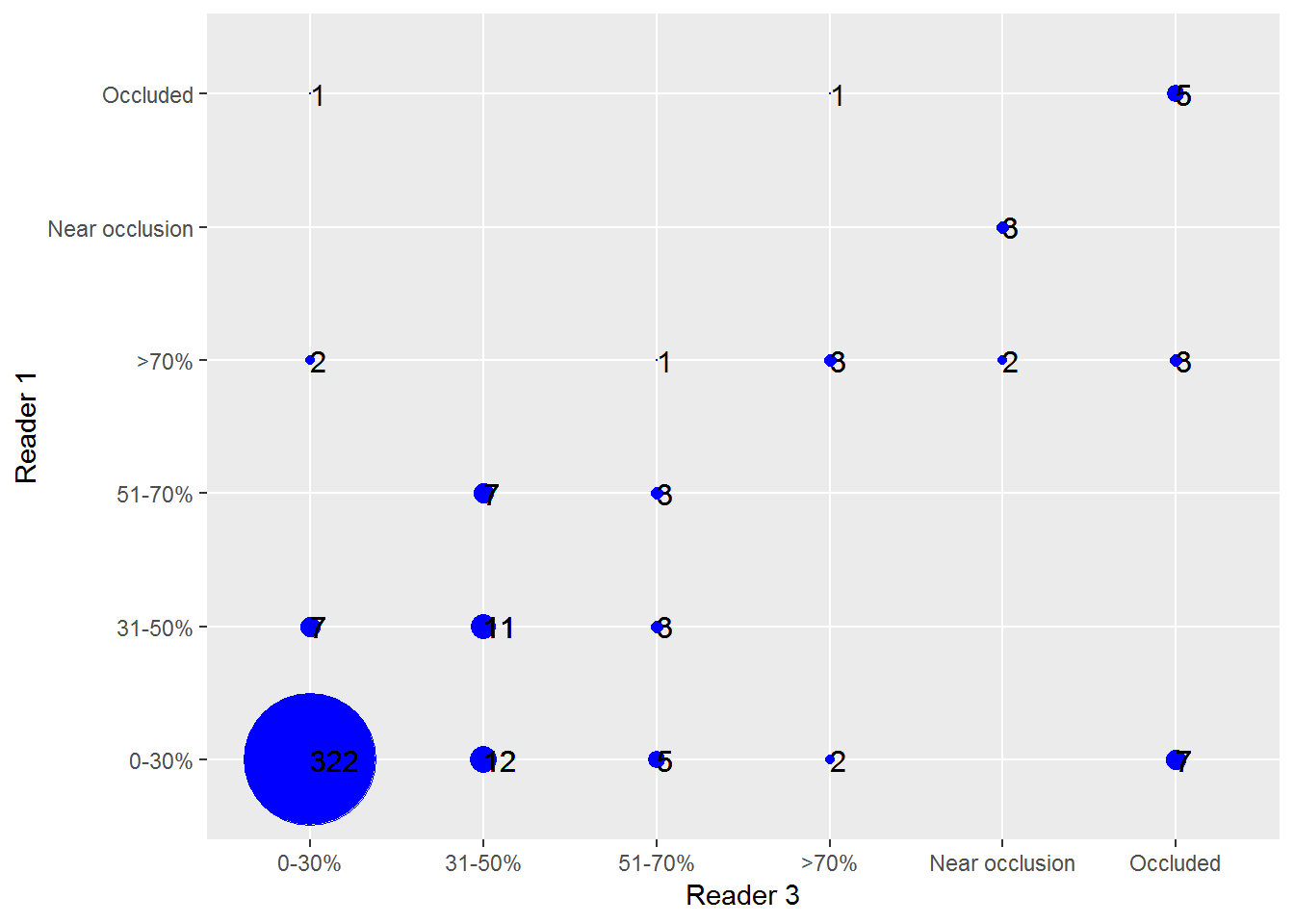

Supplement: S1 Material — (DOCX) [file pone.0237856.s001.docx]
