# Supplementary material for: Multiple reader comparison of 2D TOF, 3D TOF, and CEMRA in screening of the carotid bifurcations: Time to reconsider routine contrast use?
Source: PLoS One. 2020 Sep 2;15(9):e0237856. doi: 10.1371/journal.pone.0237856 (PMC7467222; doi:10.1371/journal.pone.0237856)

1a Score

0-30

31-50

51-70

>70

Near occlusion

Occluded

Quality

1

2

3

4

5

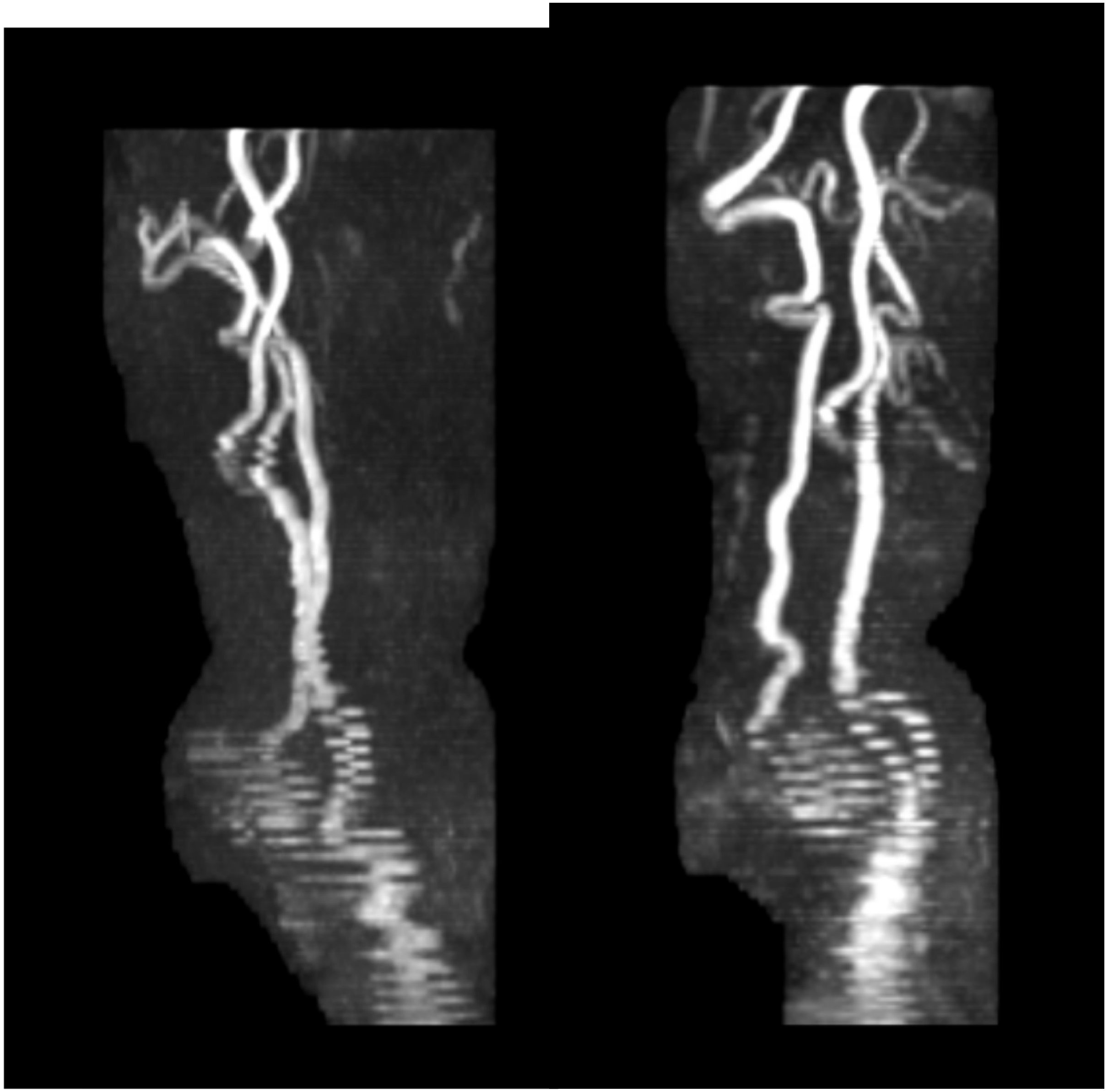

1f Score

0-30

31-50

51-70

>70

Near occlusion

Occluded

Quality

1

2

3

4

5

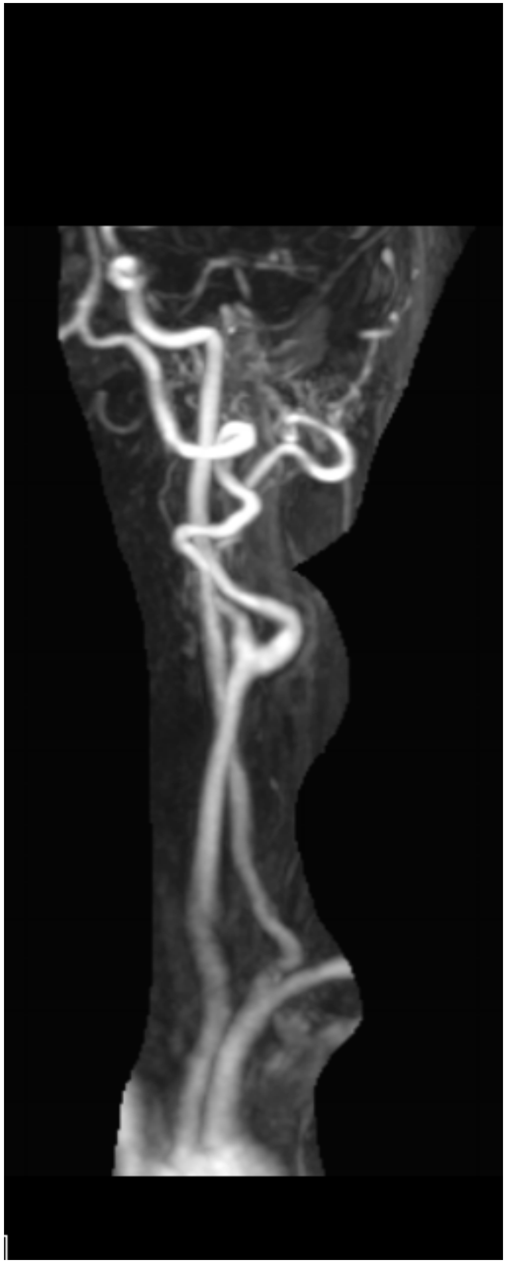

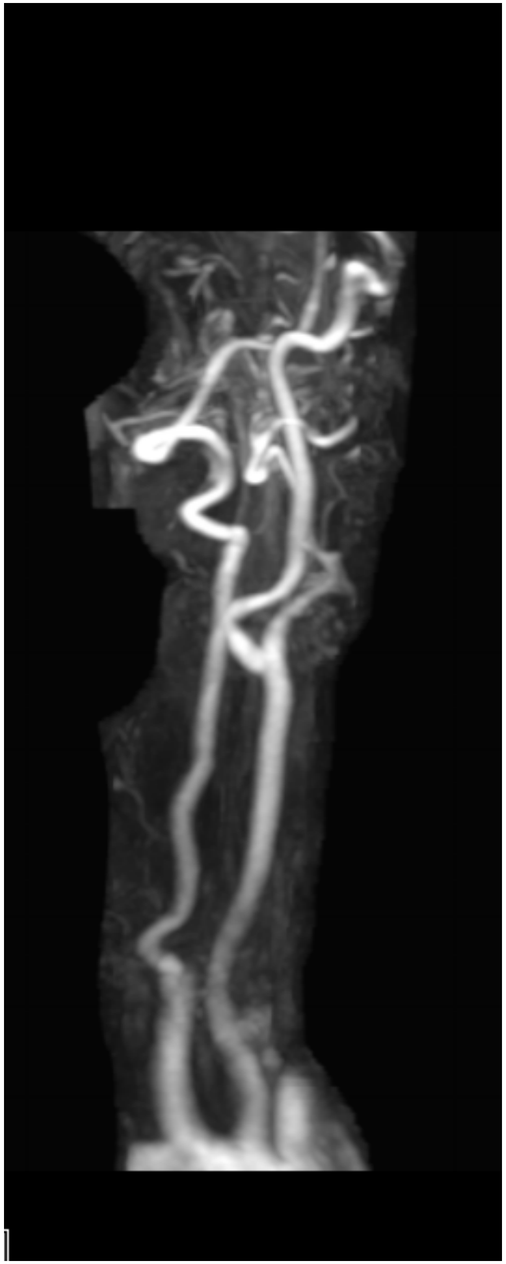

2e Score

0-30

31-50

51-70

>70

Near occlusion

Occluded

Quality

1

2

3

4

5

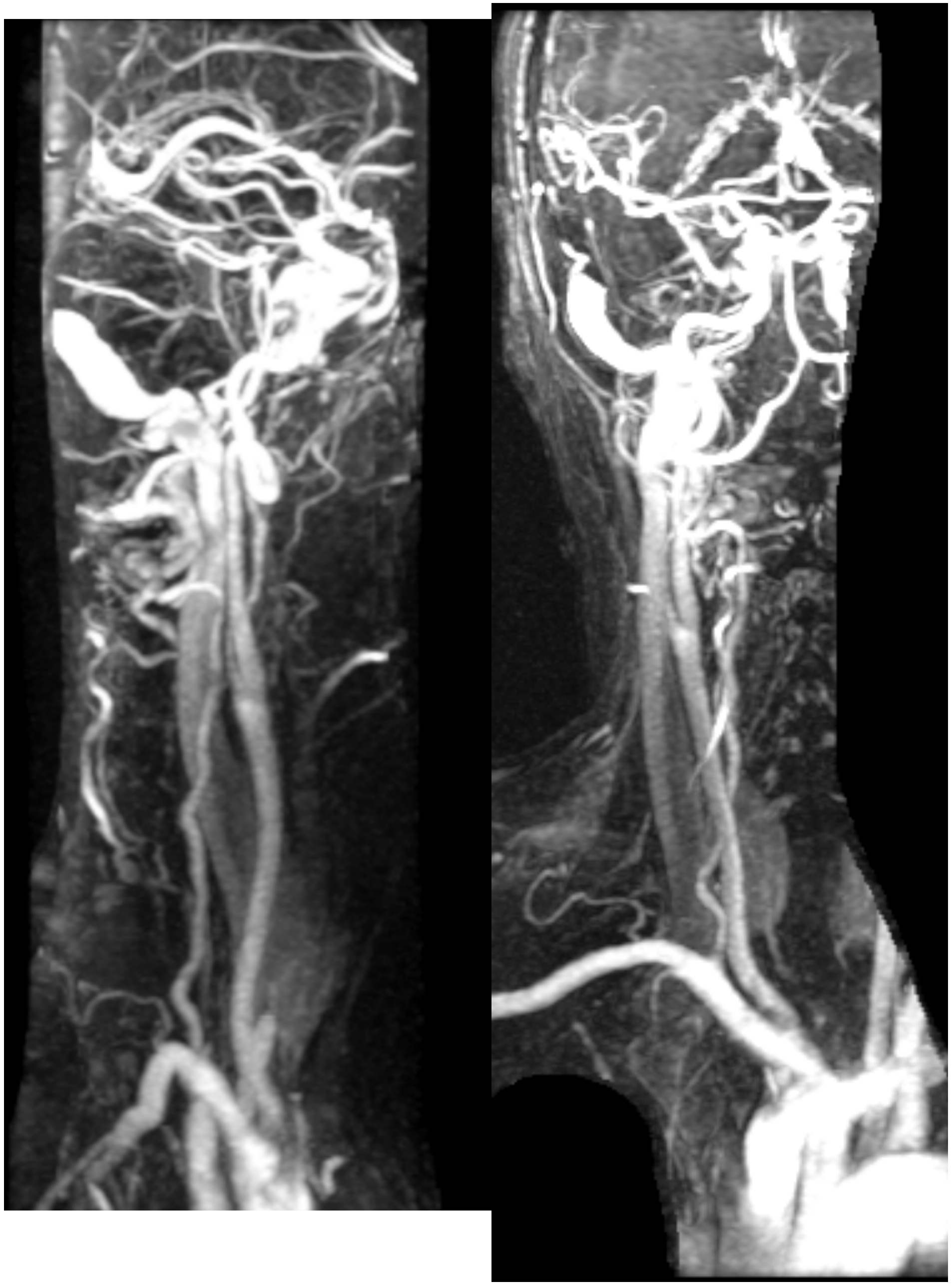

3d Score

0-30

31-50

51-70

>70

Near occlusion

Occluded

Quality

1

2

3

4

5

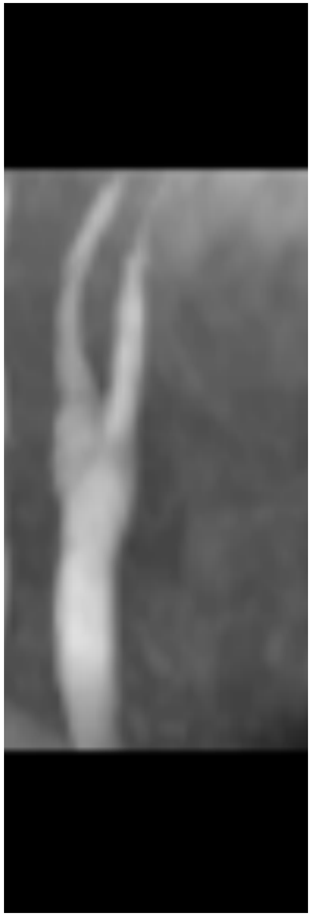

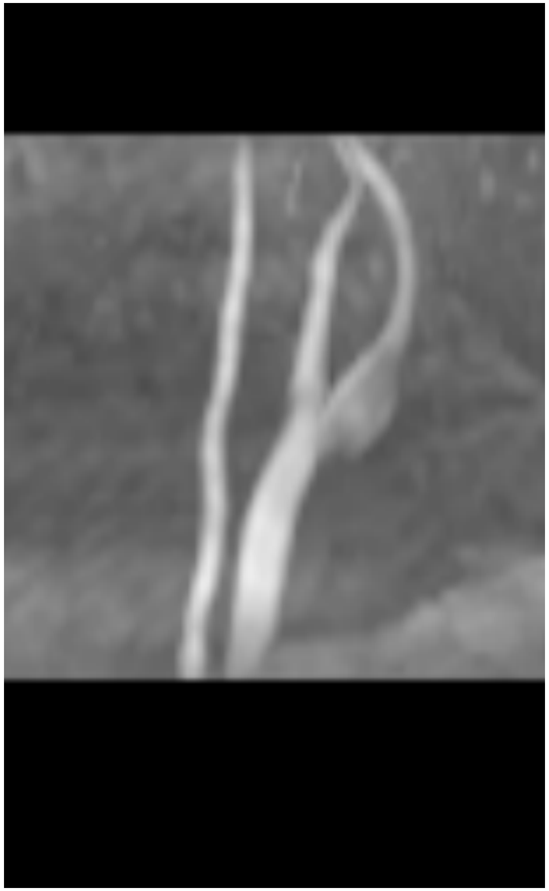

4c Score

0-30

31-50

51-70

>70

Near occlusion

Occluded

Quality

1

2

3

4

5

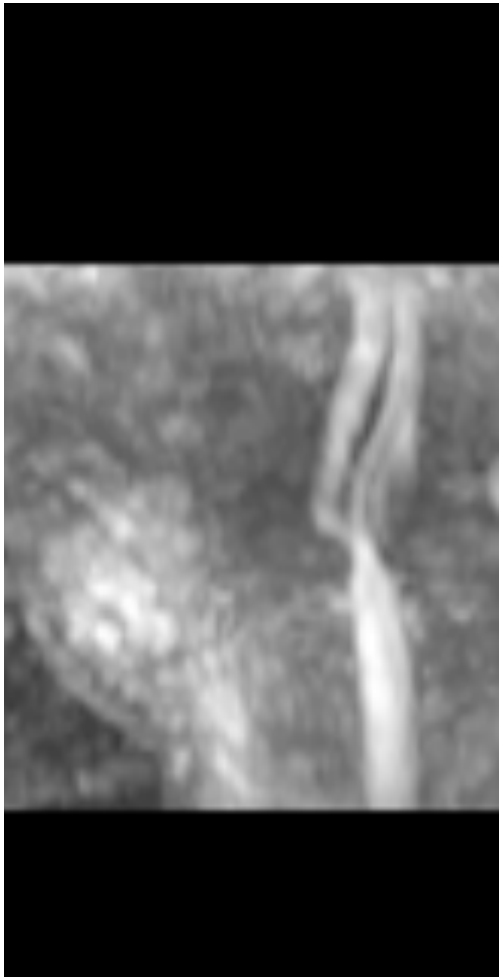

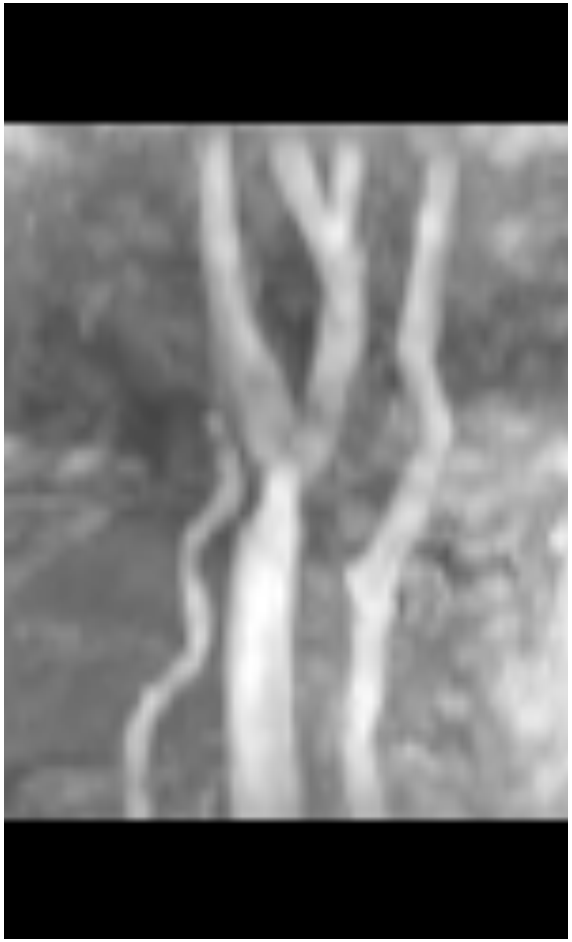

5b Score

0-30

31-50

51-70

>70

Near occlusion

Occluded

Quality

1

2

3

4

5

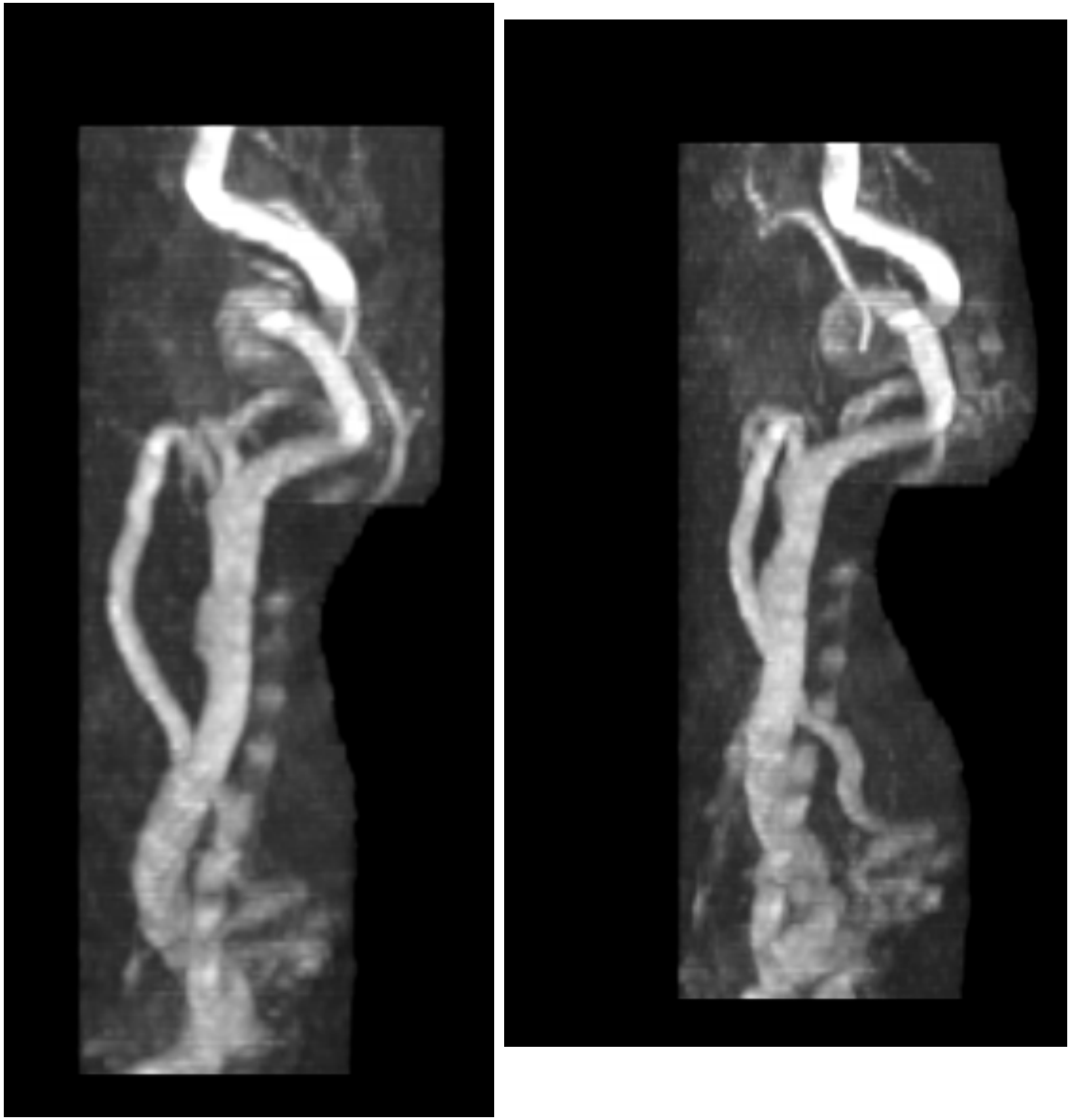

6a Score

0-30

31-50

51-70

>70

Near occlusion

Occluded

Quality

1

2

3

4

5

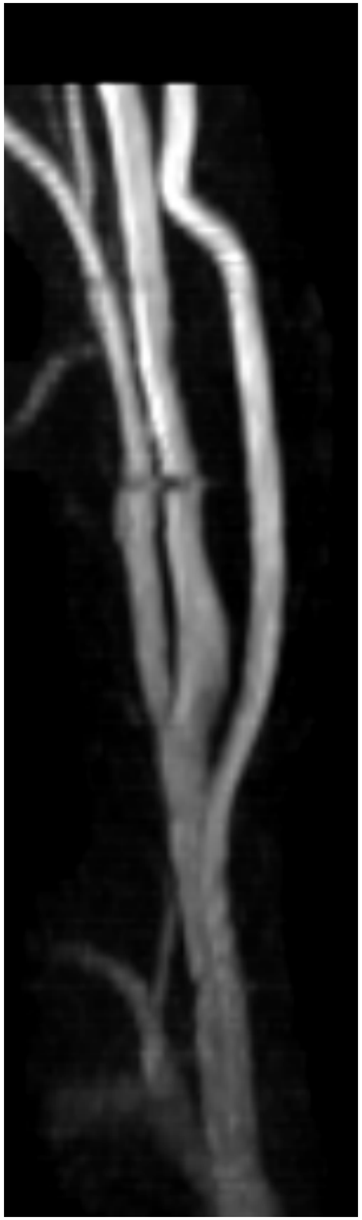

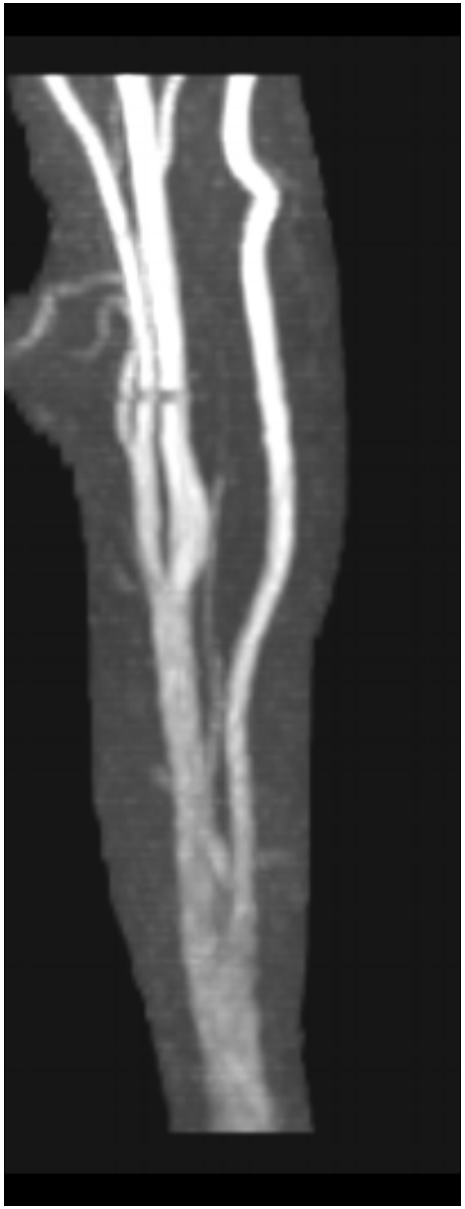

6f Score

0-30

31-50

51-70

>70

Near occlusion

Occluded

Quality

1

2

3

4

5

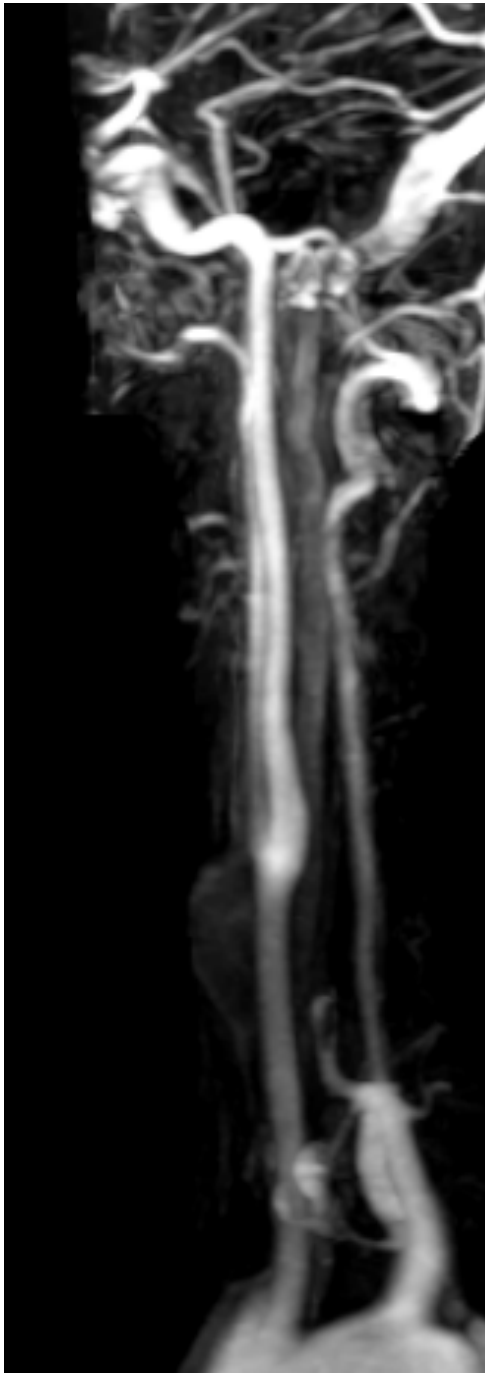

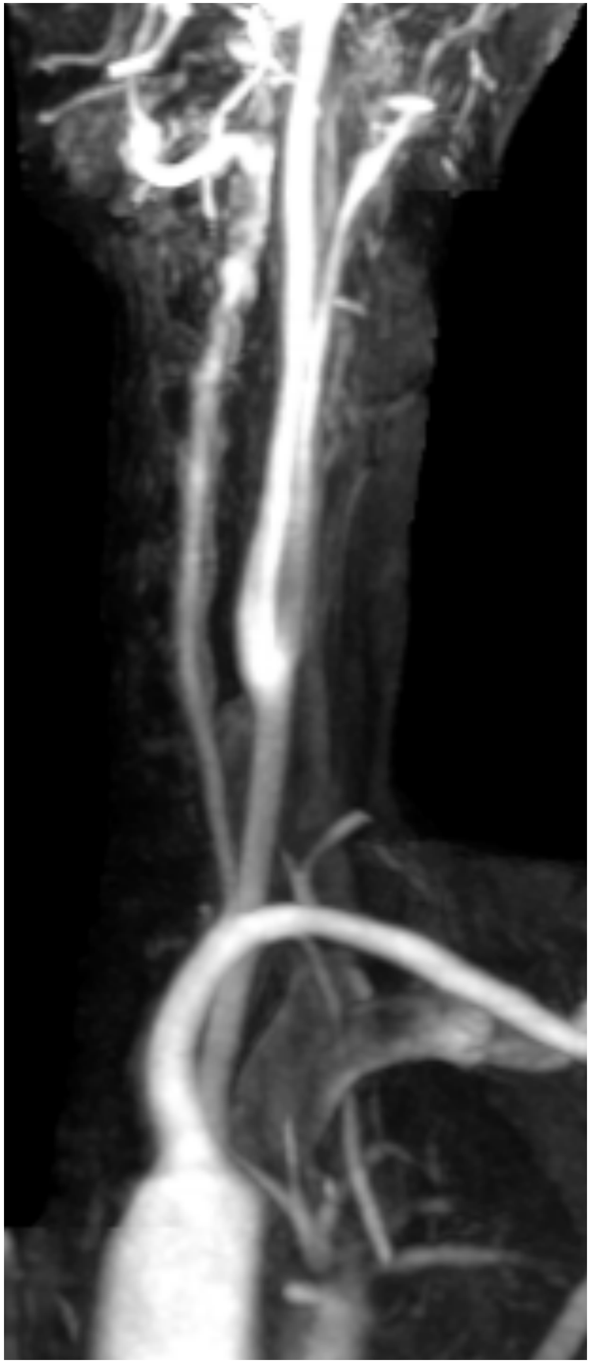

7e Score

0-30

31-50

51-70

>70

Near occlusion

Occluded

Quality

1

2

3

4

5

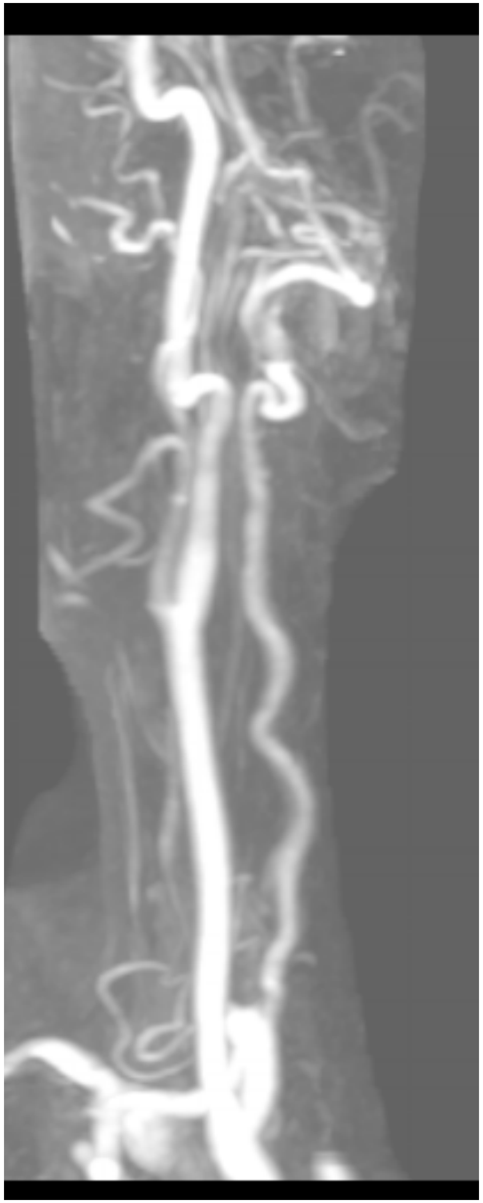

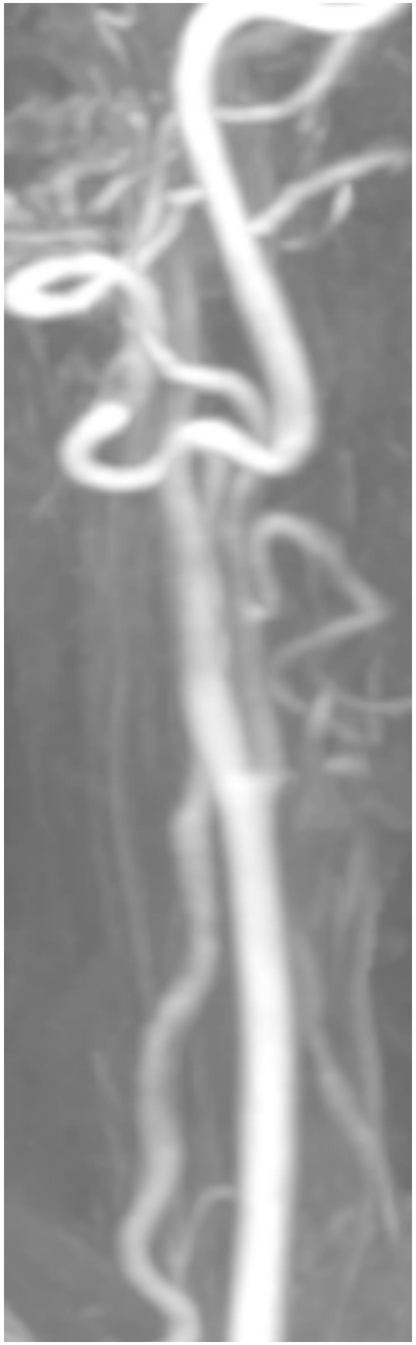

8d Score

0-30

31-50

51-70

>70

Near occlusion

Occluded

Quality

1

2

3

4

5

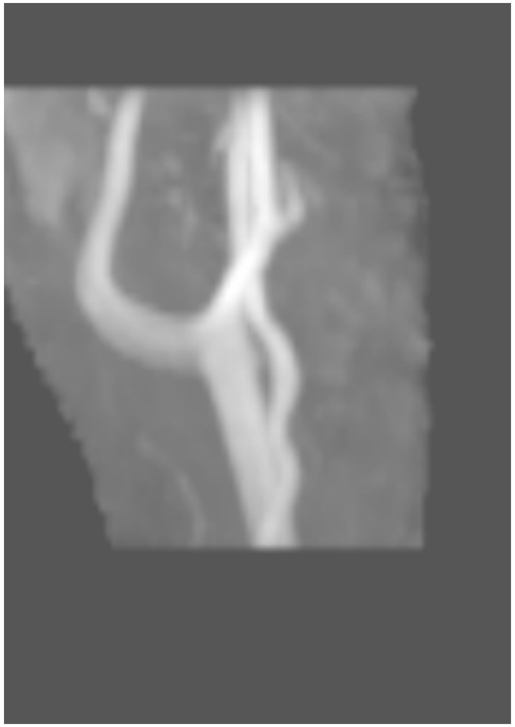

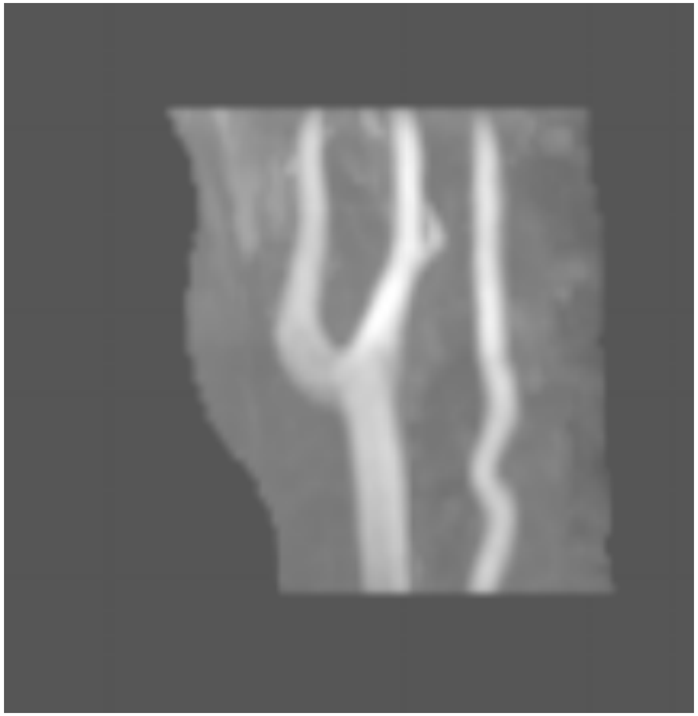

9c Score

0-30

31-50

51-70

>70

Near occlusion

Occluded

Quality

1

2

3

4

5

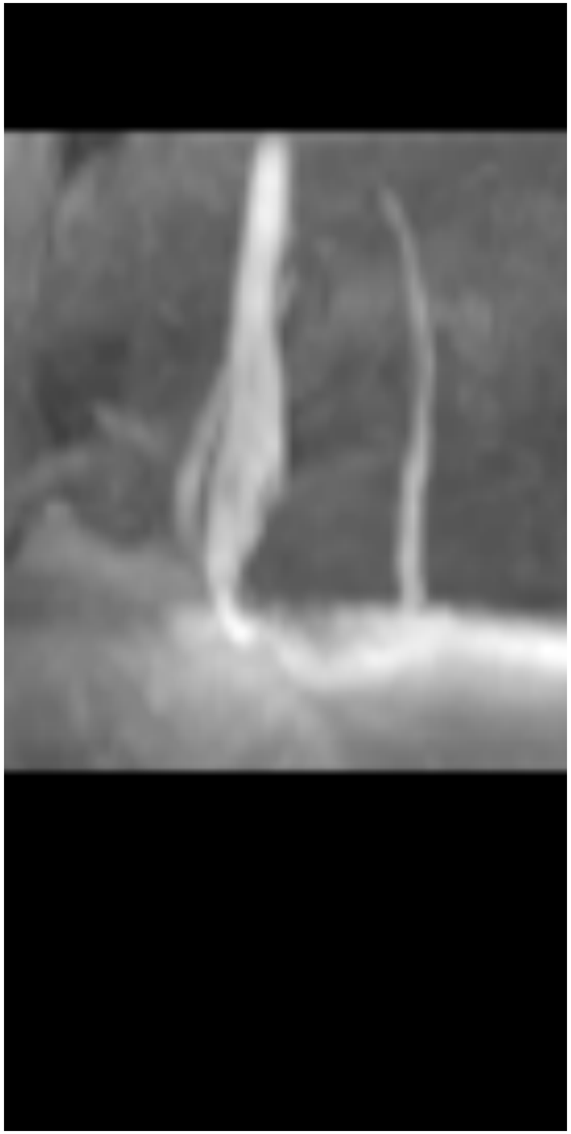

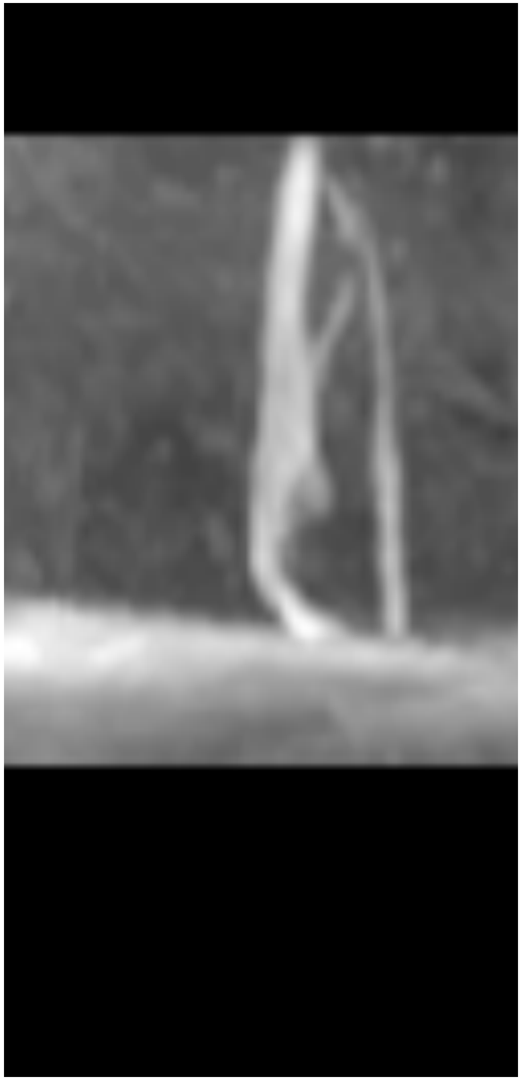

10b Score

0-30

31-50

51-70

>70

Near occlusion

Occluded

Quality

1

2

3

4

5

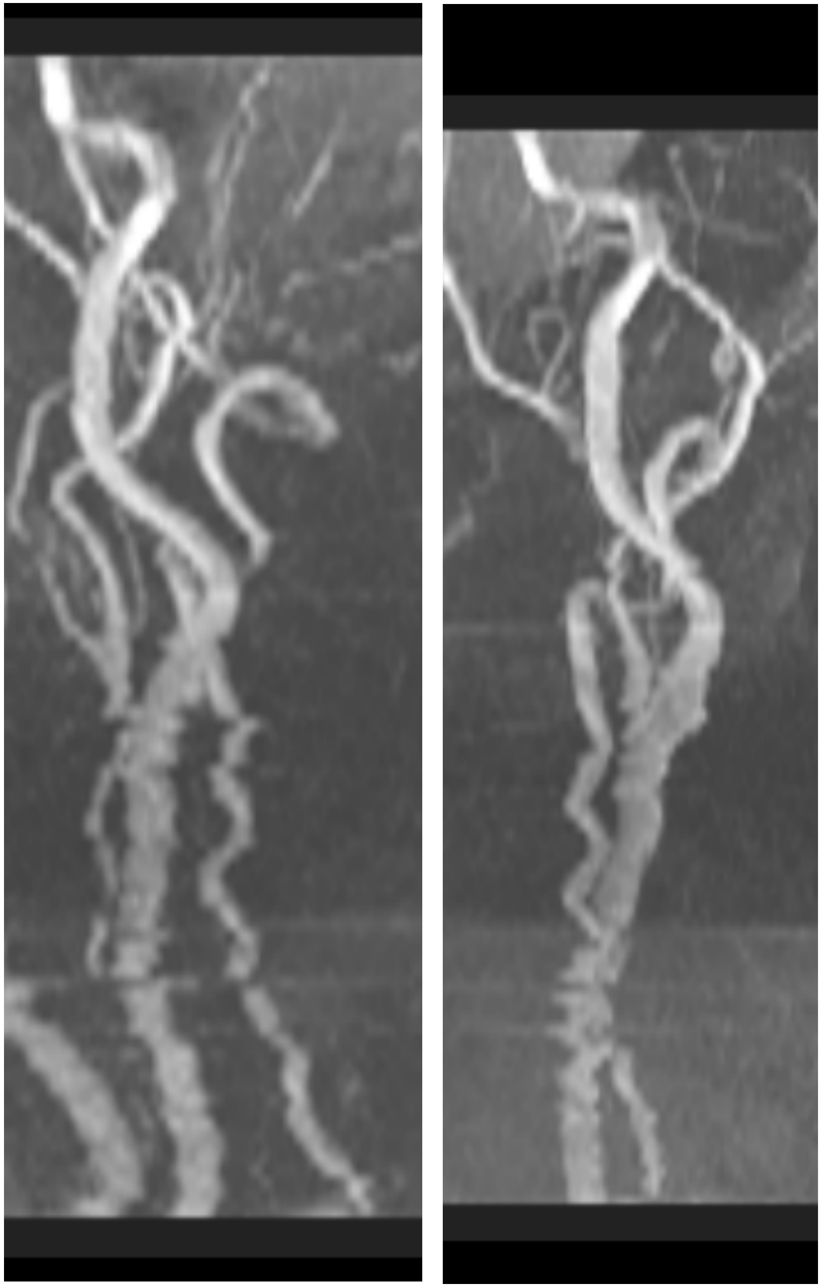

11a Score

0-30

31-50

51-70

>70

Near occlusion

Occluded

Quality

1

2

3

4

5

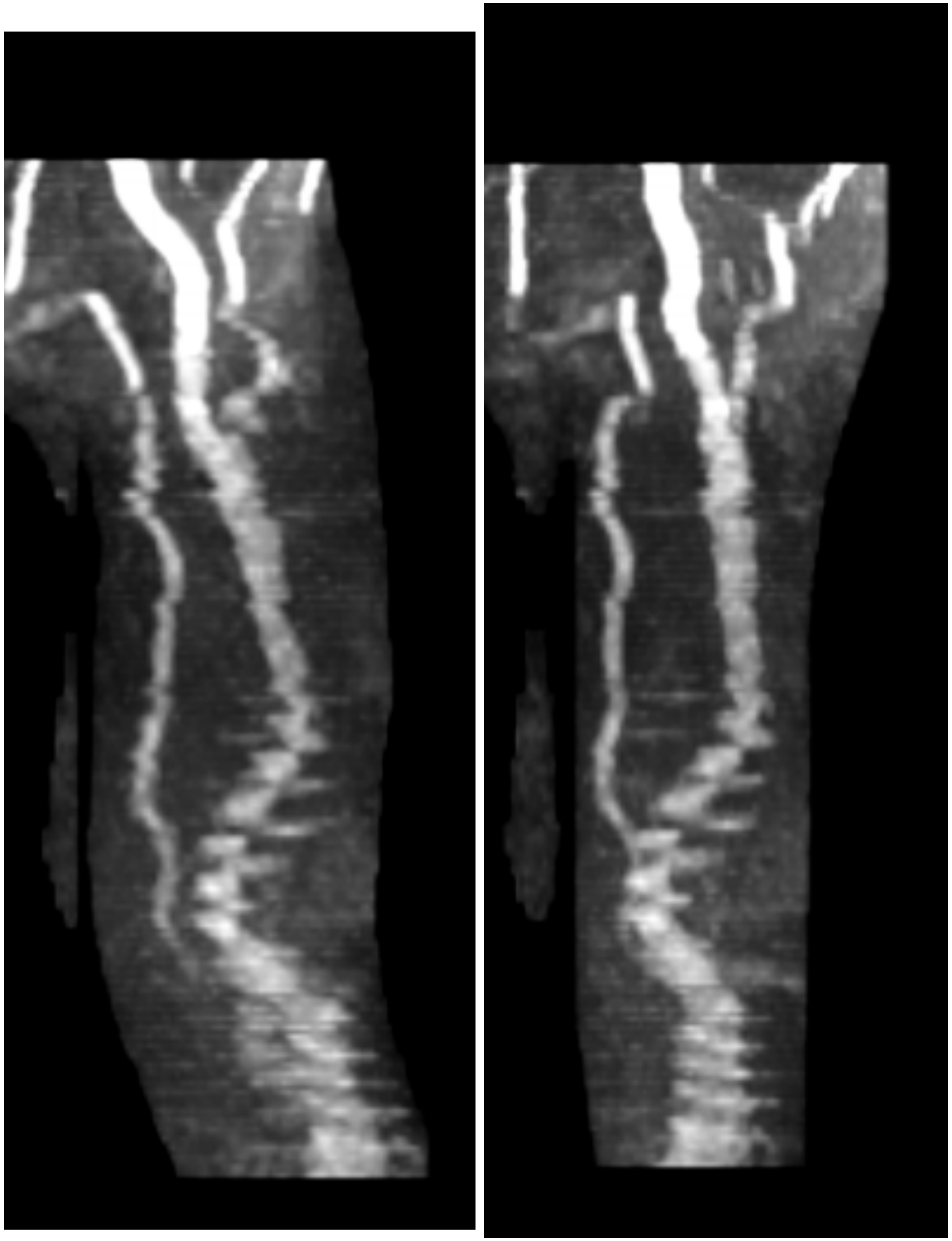

11f Score

0-30

31-50

51-70

>70

Near occlusion

Occluded

Quality

1

2

3

4

5

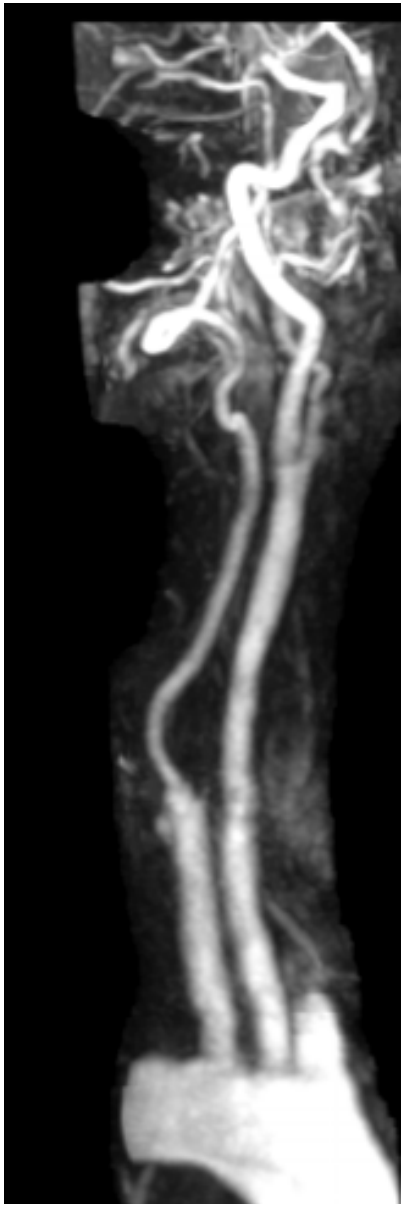

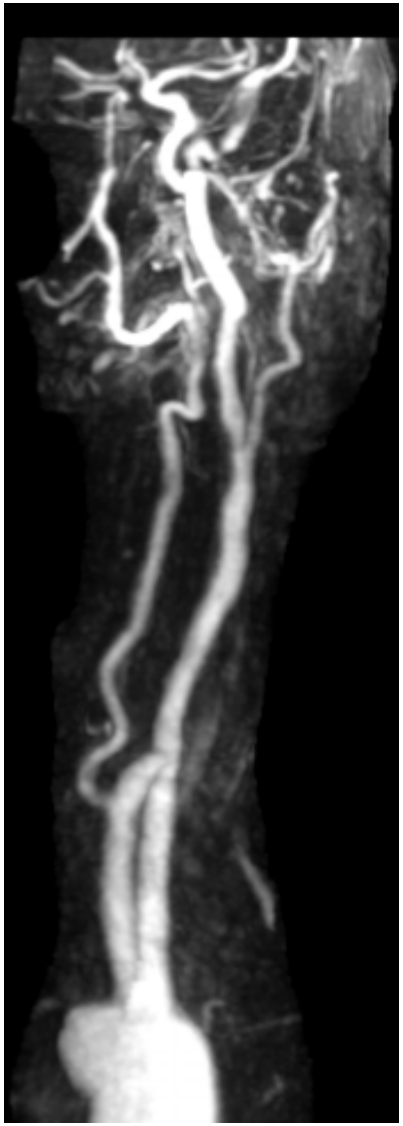

12e Score

0-30

31-50

51-70

>70

Near occlusion

Occluded

Quality

1

2

3

4

5

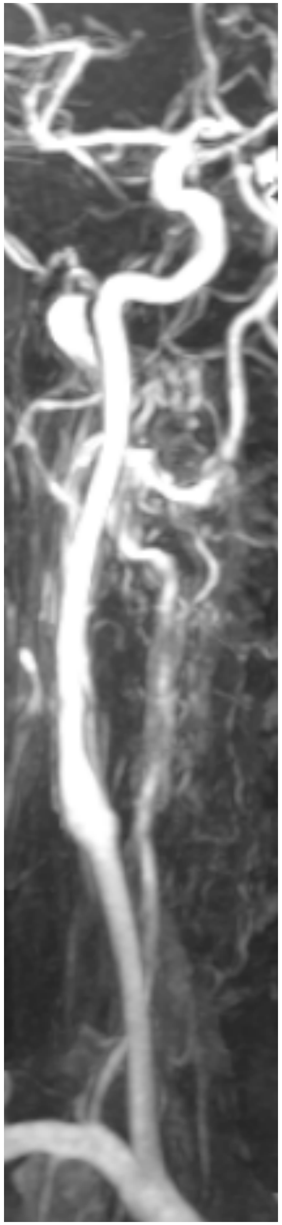

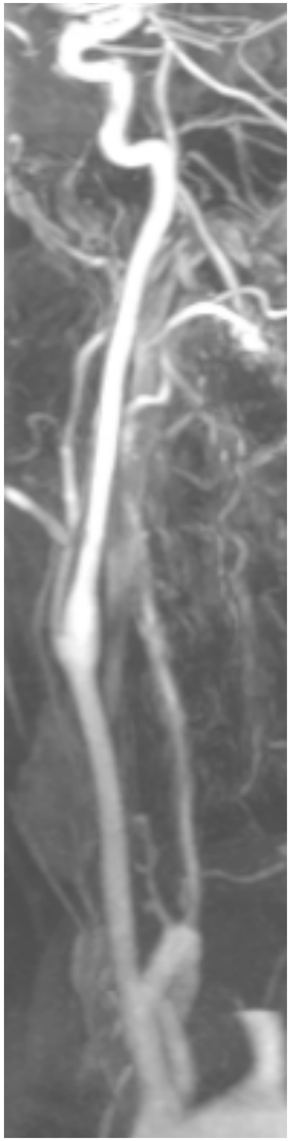

13d Score

0-30

31-50

51-70

>70

Near occlusion

Occluded

Quality

1

2

3

4

5

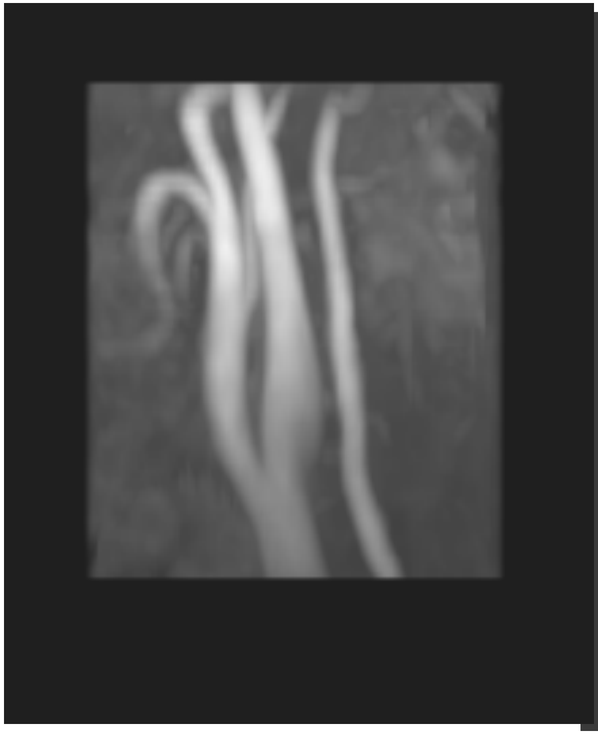

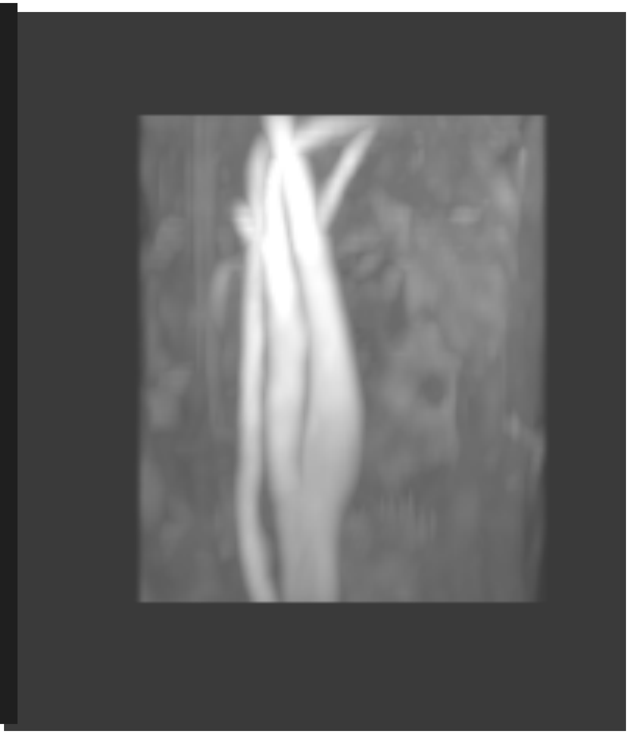

14c Score

0-30

31-50

51-70

>70

Near occlusion

Occluded

Quality

1

2

3

4

5

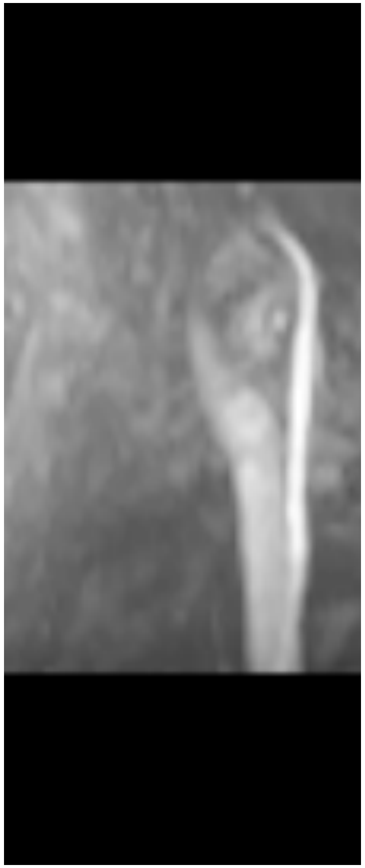

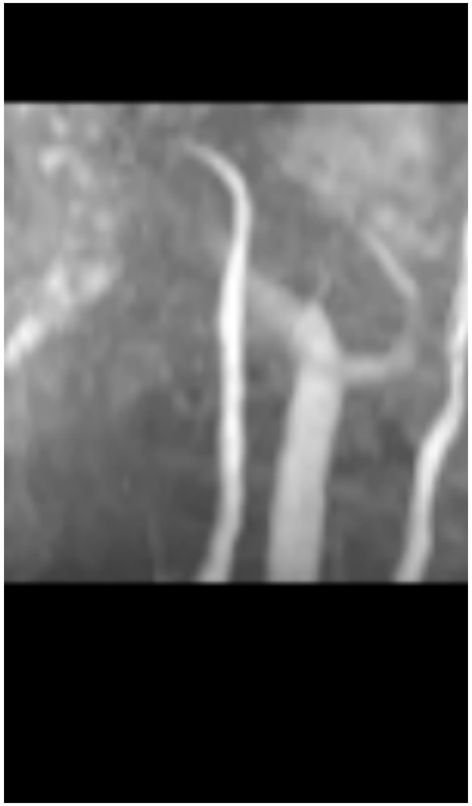

15b Score

0-30

31-50

51-70

>70

Near occlusion

Occluded

Quality

1

2

3

4

5

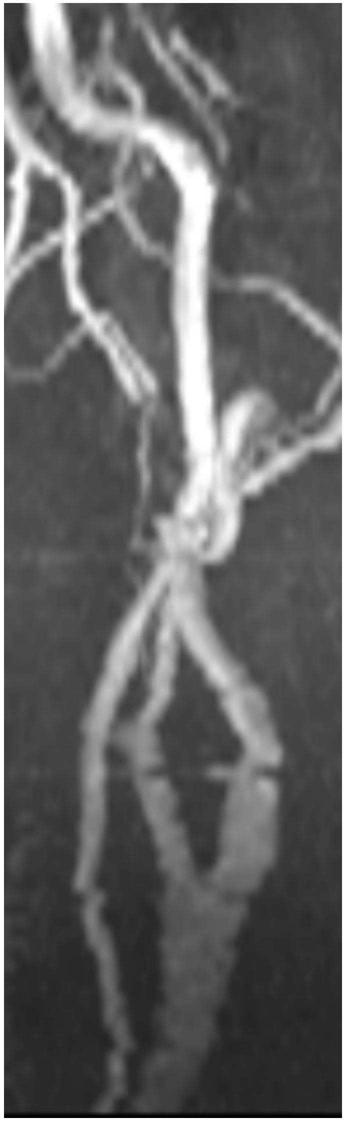

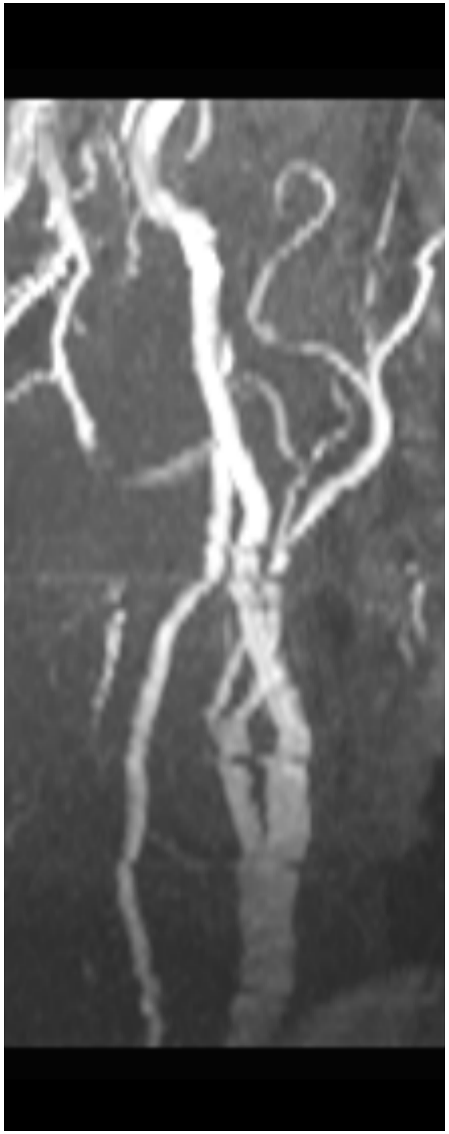

16a Score

0-30

31-50

51-70

>70

Near occlusion

Occluded

Quality

1

2

3

4

5

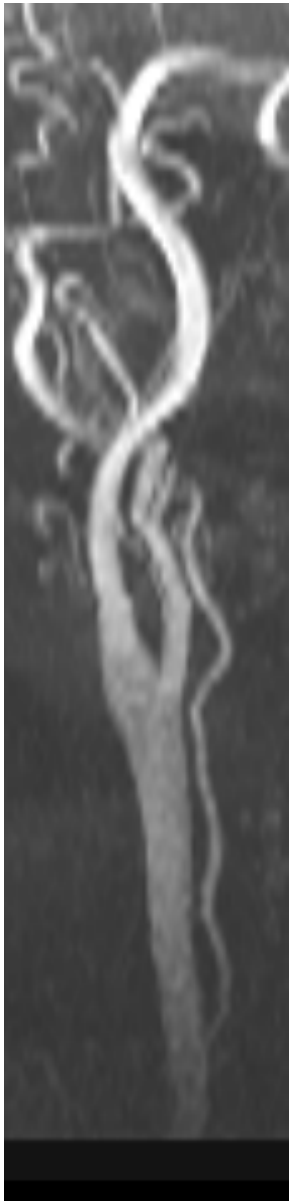

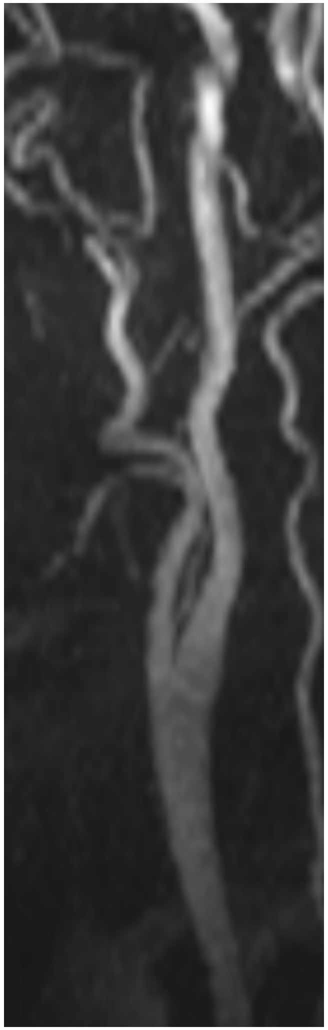

16f Score

0-30

31-50

51-70

>70

Near occlusion

Occluded

Quality

1

2

3

4

5

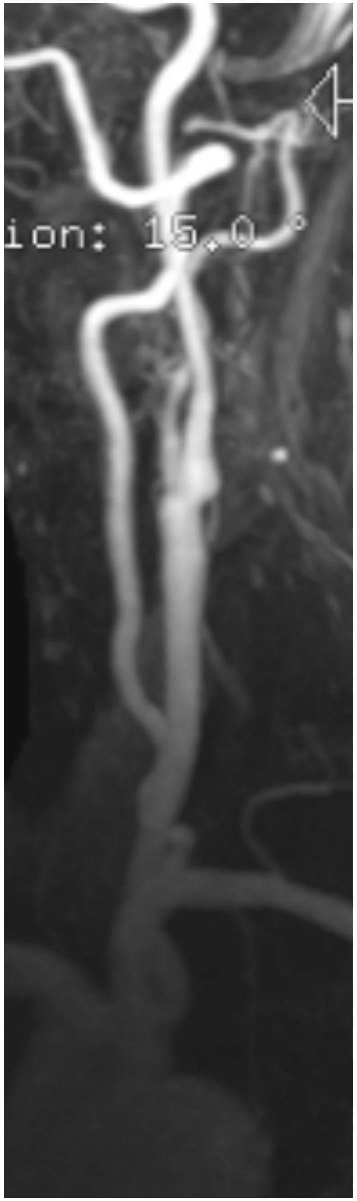

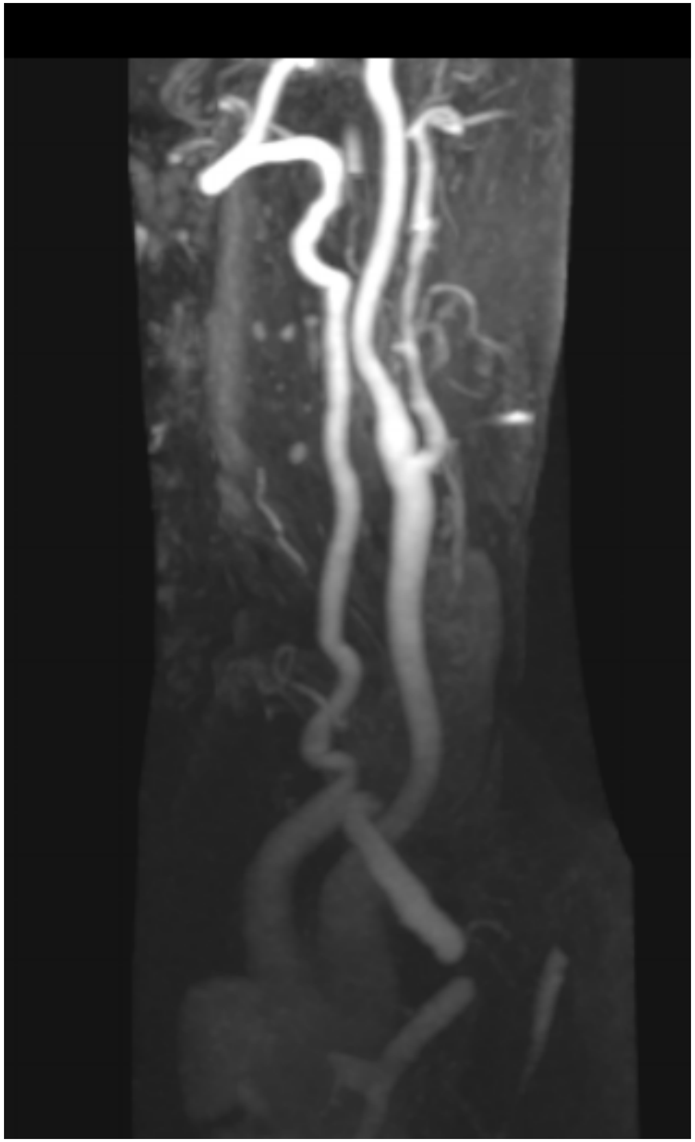

17e Score

0-30

31-50

51-70

>70

Near occlusion

Occluded

Quality

1

2

3

4

5

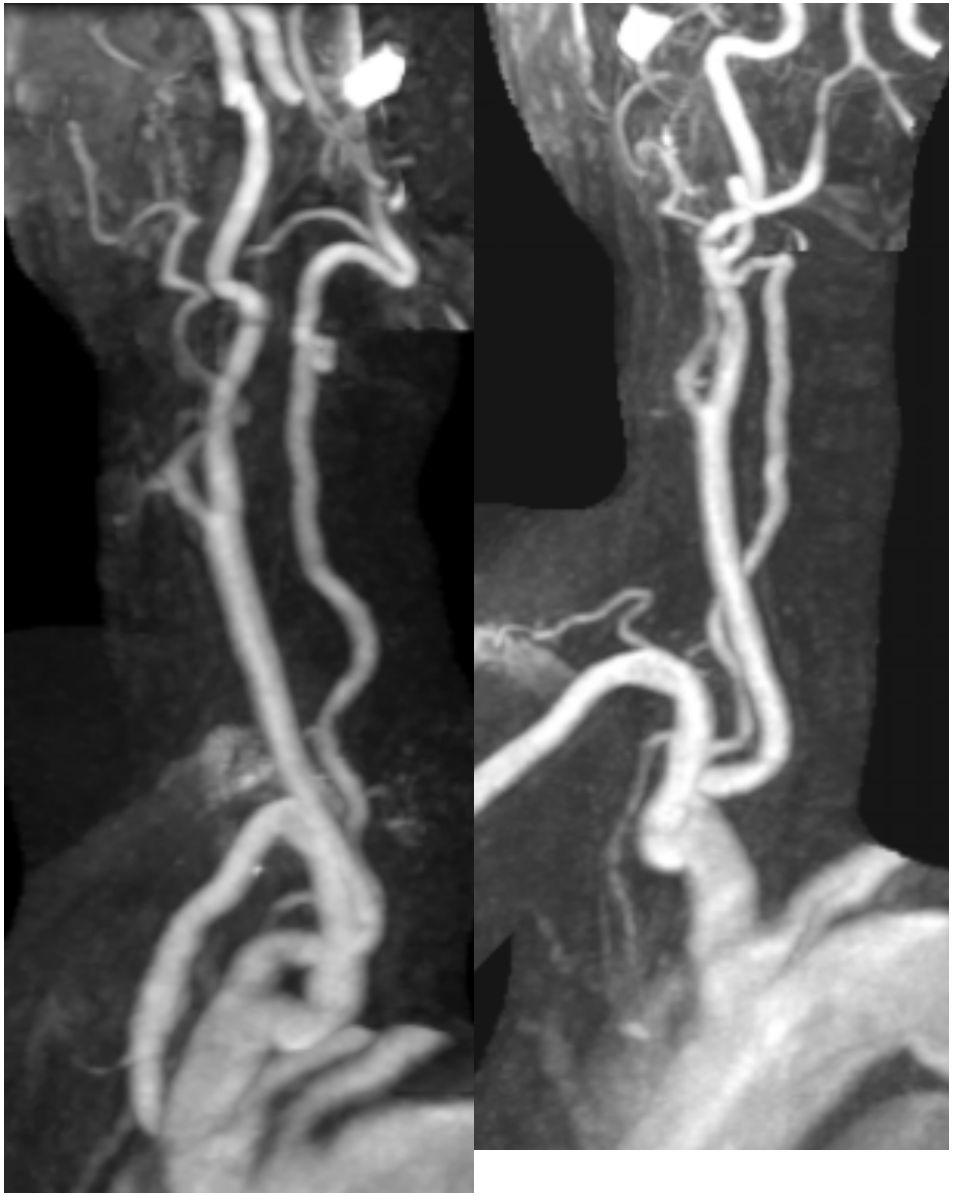

18d Score

0-30

31-50

51-70

>70

Near occlusion

Occluded

Quality

1

2

3

4

5

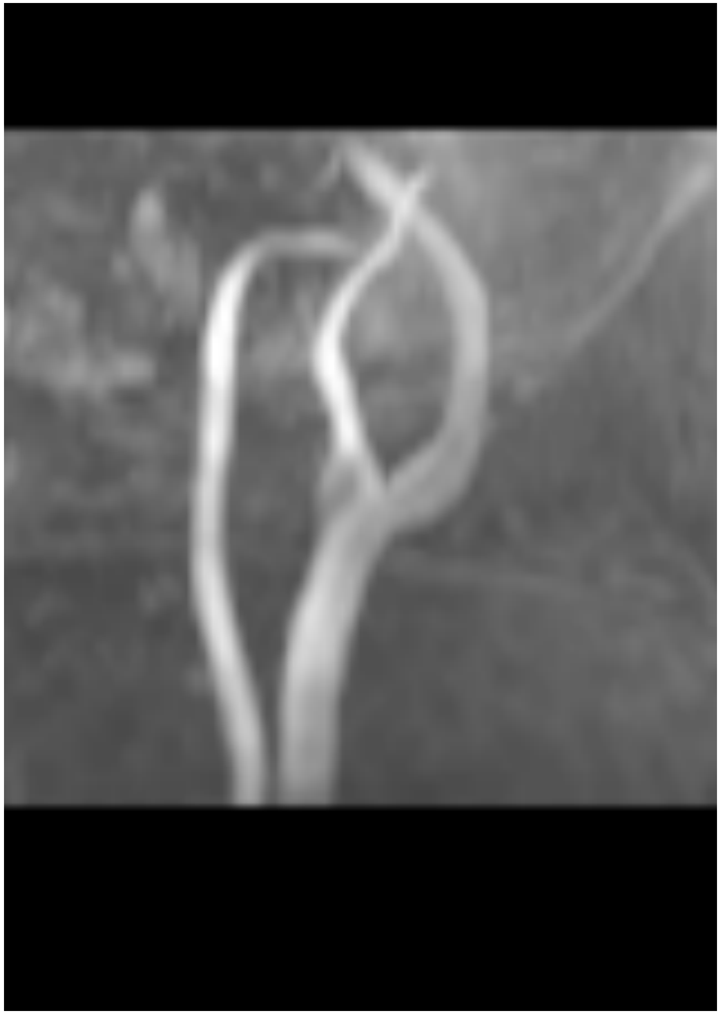

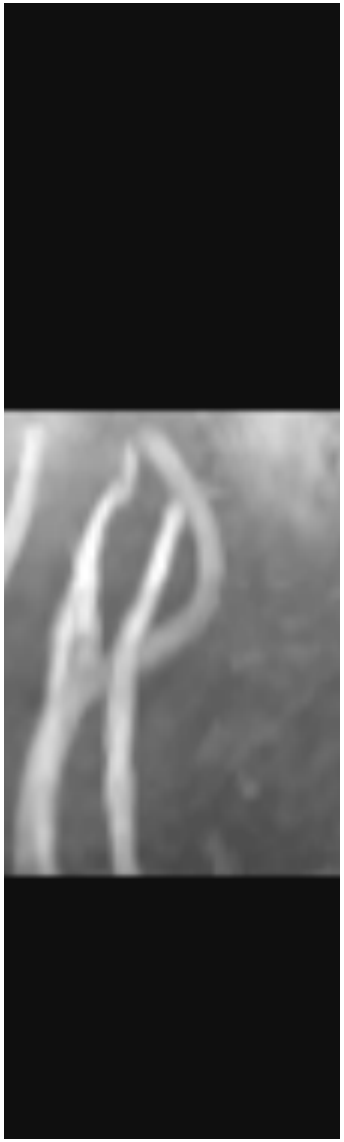

19c Score

0-30

31-50

51-70

>70

Near occlusion

Occluded

Quality

1

2

3

4

5

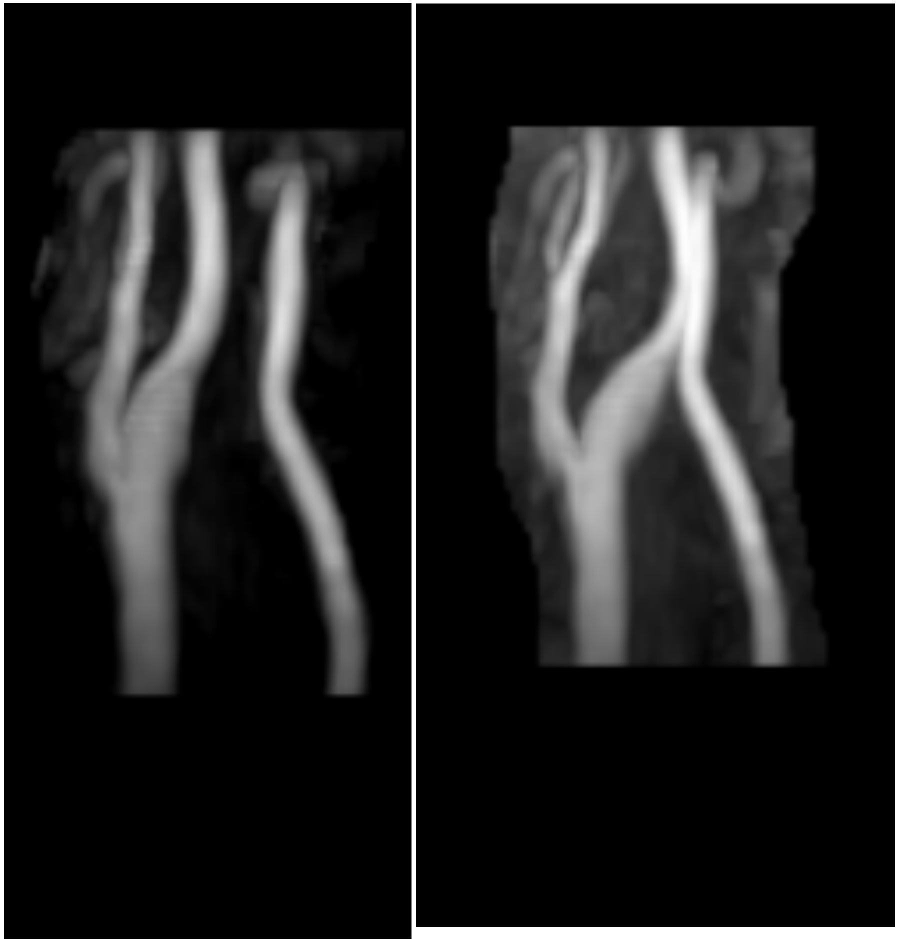

20b Score

0-30

31-50

51-70

>70

Near occlusion

Occluded

Quality

1

2

3

4

5

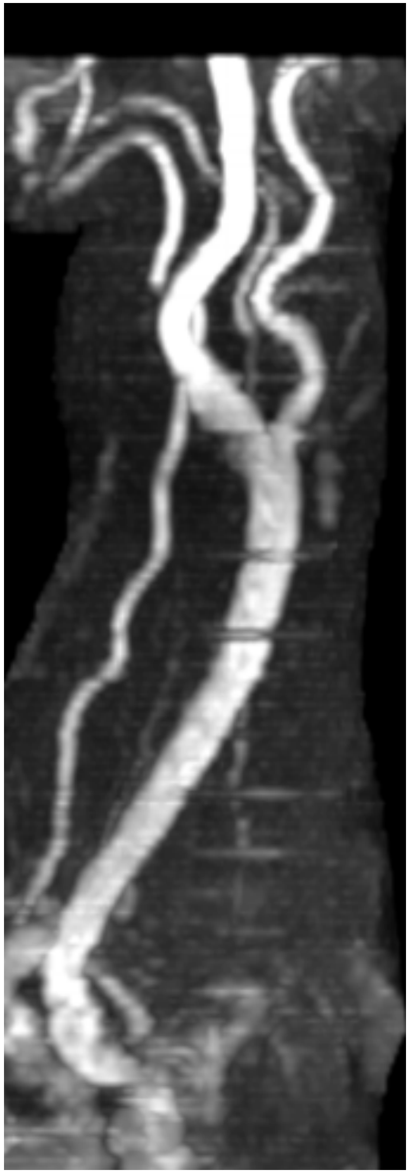

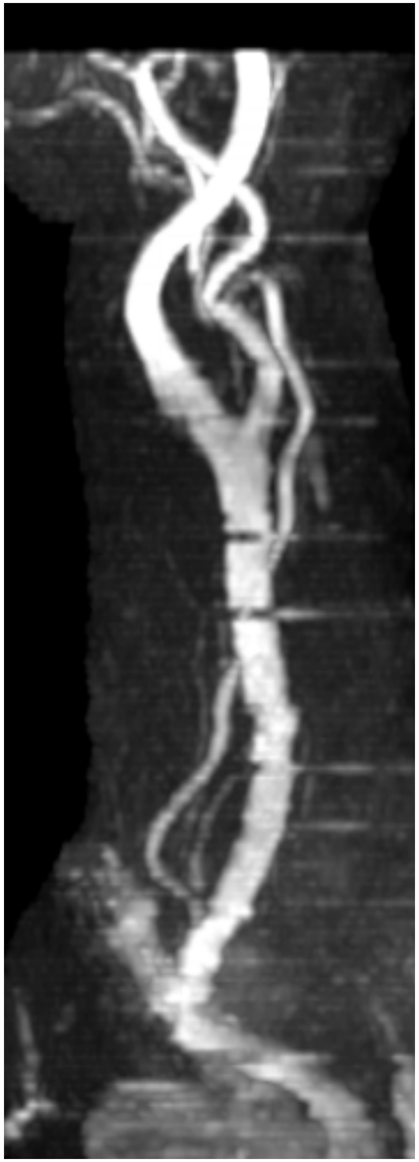

21a Score

0-30

31-50

51-70

>70

Near occlusion

Occluded

Quality

1

2

3

4

5

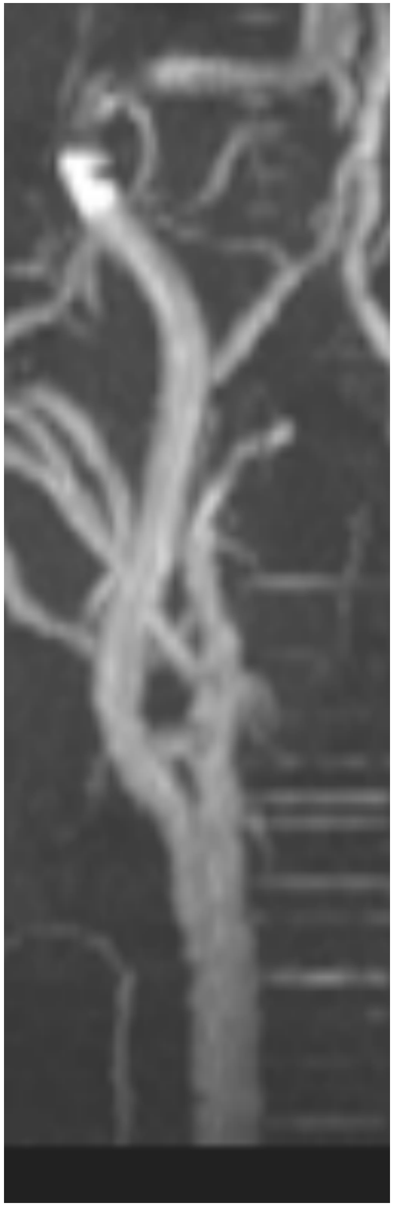

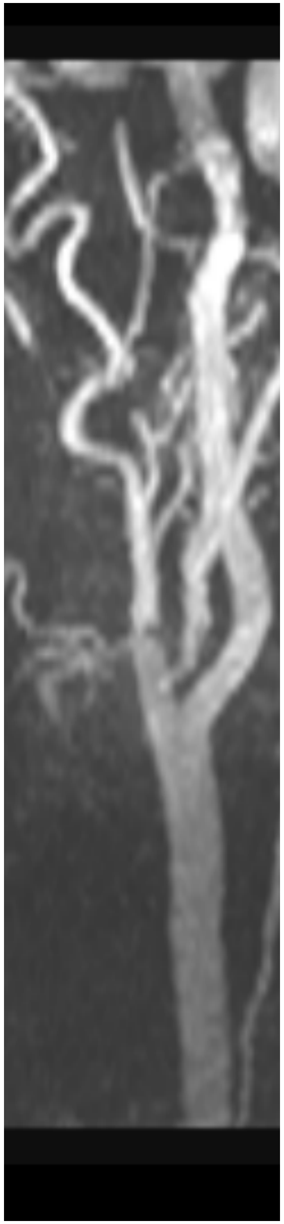

21f Score

0-30

31-50

51-70

>70

Near occlusion

Occluded

Quality

1

2

3

4

5

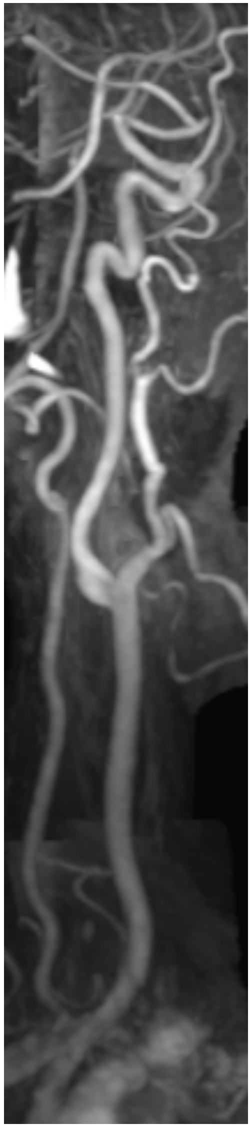

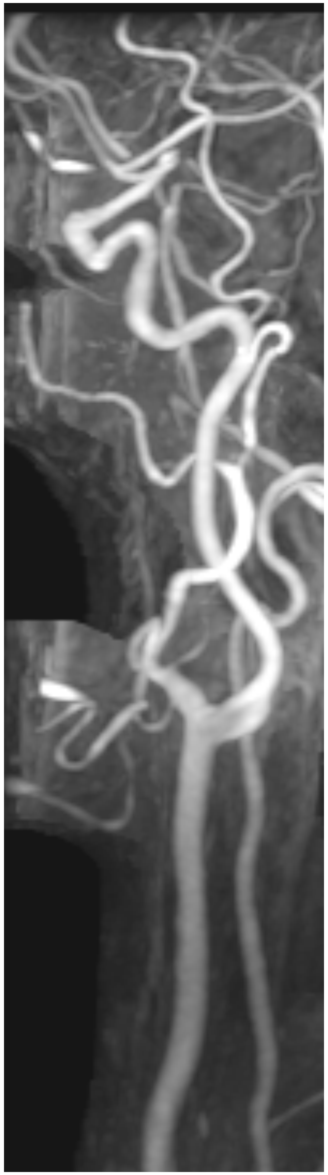

22e Score

0-30

31-50

51-70

>70

Near occlusion

Occluded

Quality

1

2

3

4

5

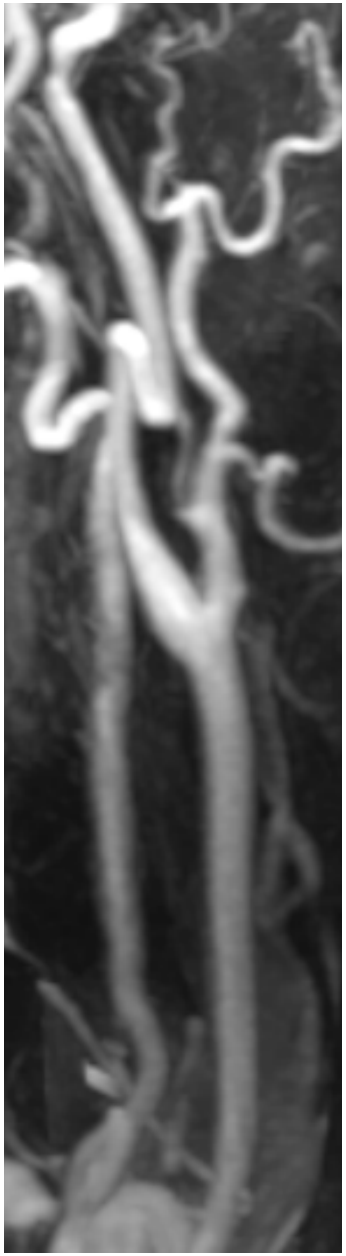

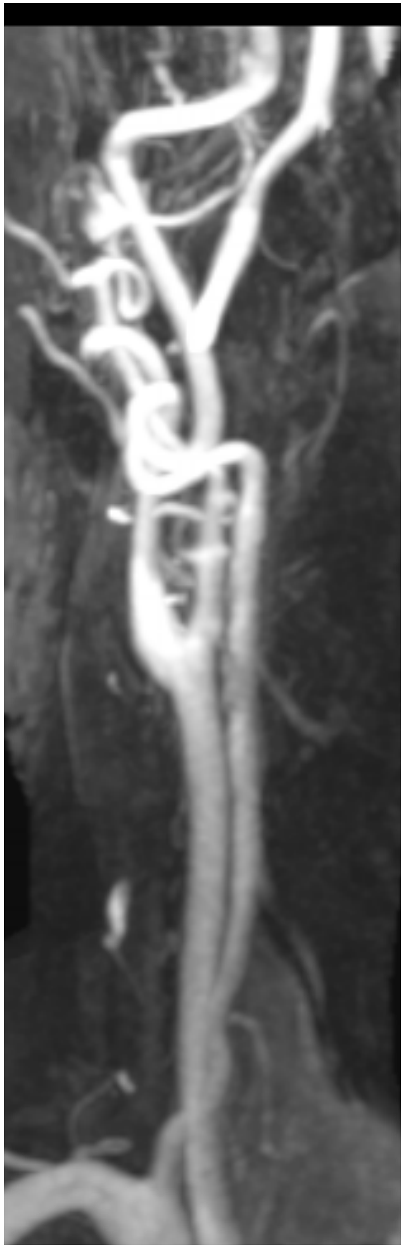

23d Score

0-30

31-50

51-70

>70

Near occlusion

Occluded

Quality

1

2

3

4

5

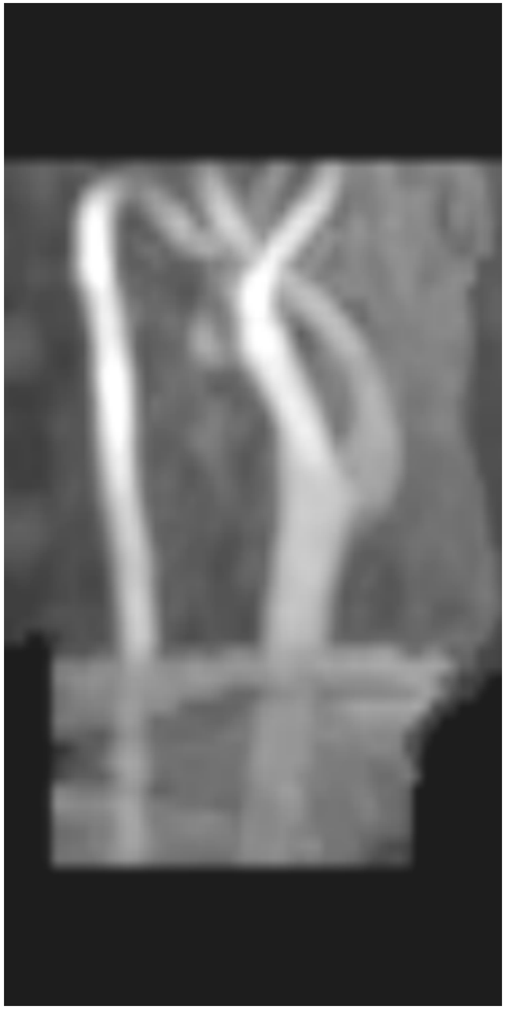

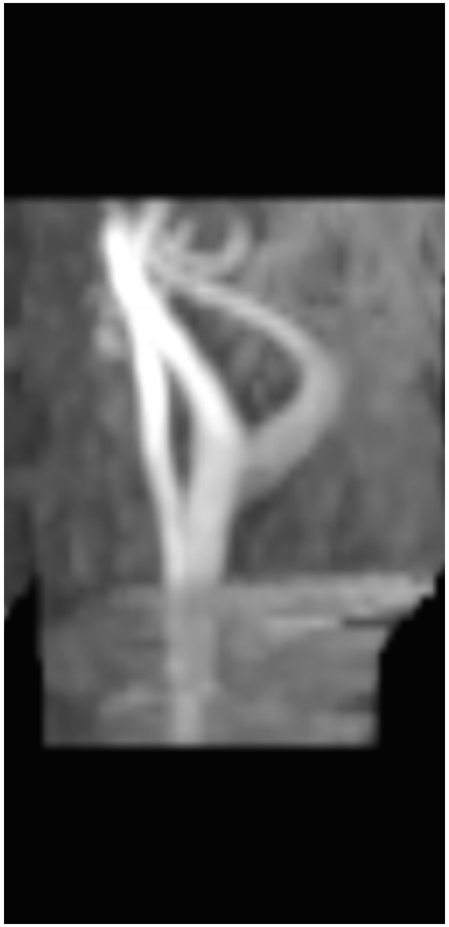

24c Score

0-30

31-50

51-70

>70

Near occlusion

Occluded

Quality

1

2

3

4

5

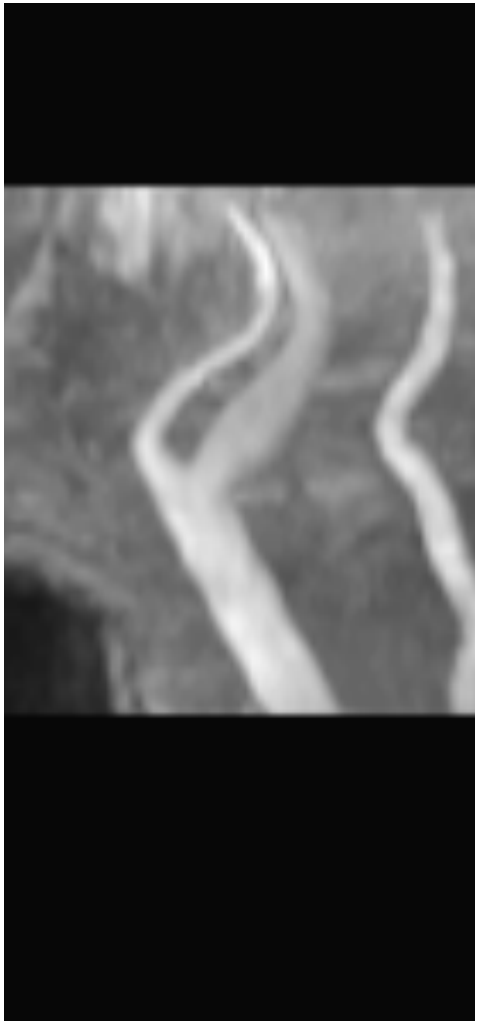

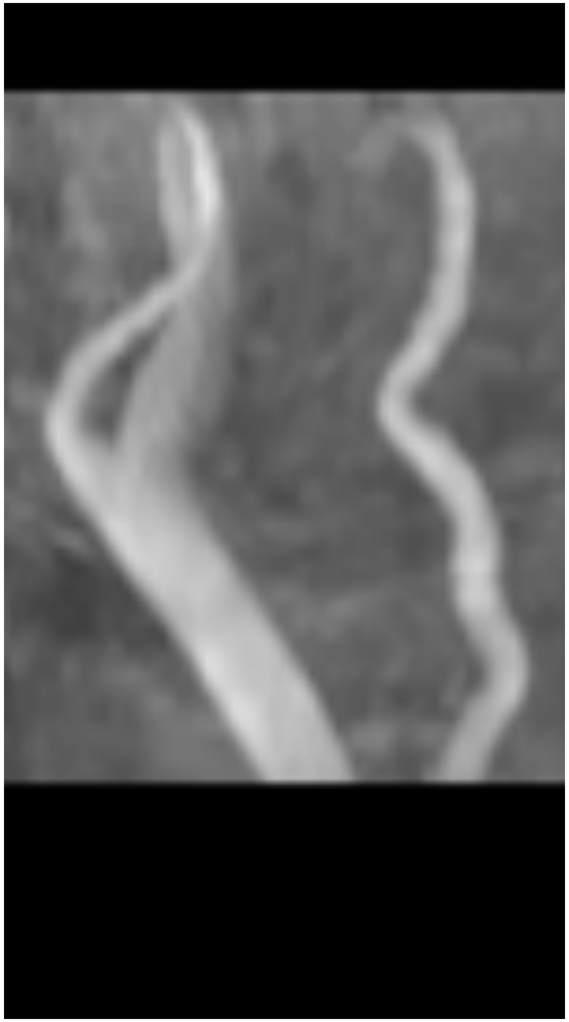

25b Score

0-30

31-50

51-70

>70

Near occlusion

Occluded

Quality

1

2

3

4

5

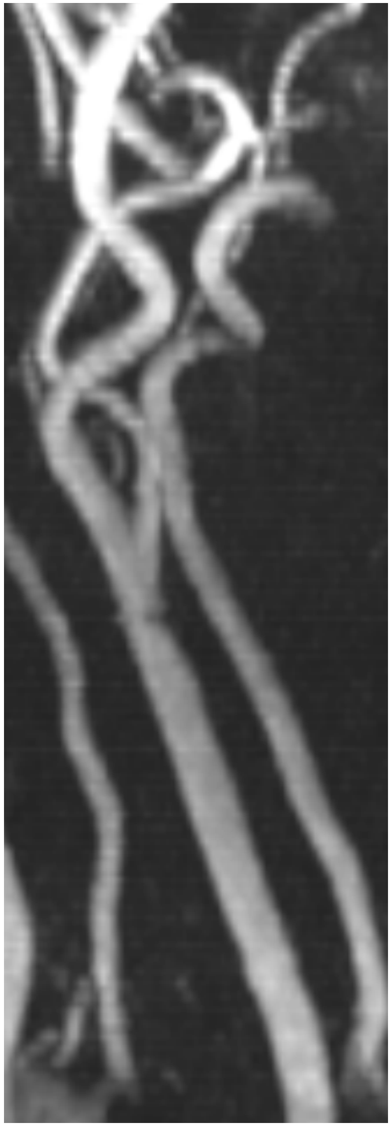

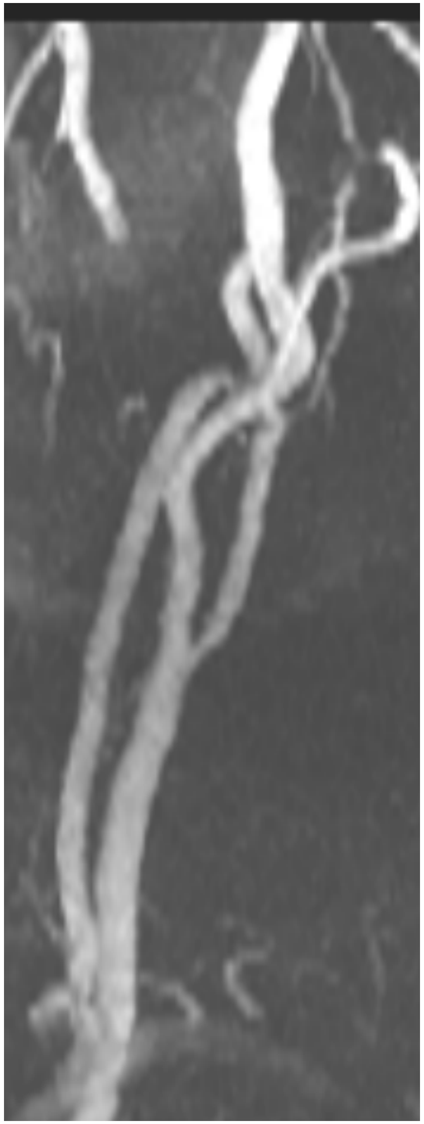

26a Score

0-30

31-50

51-70

>70

Near occlusion

Occluded

Quality

1

2

3

4

5

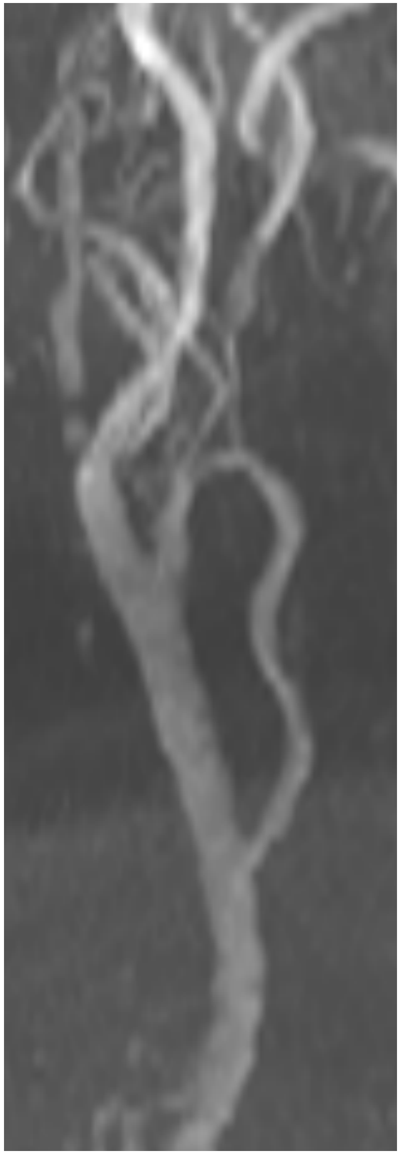

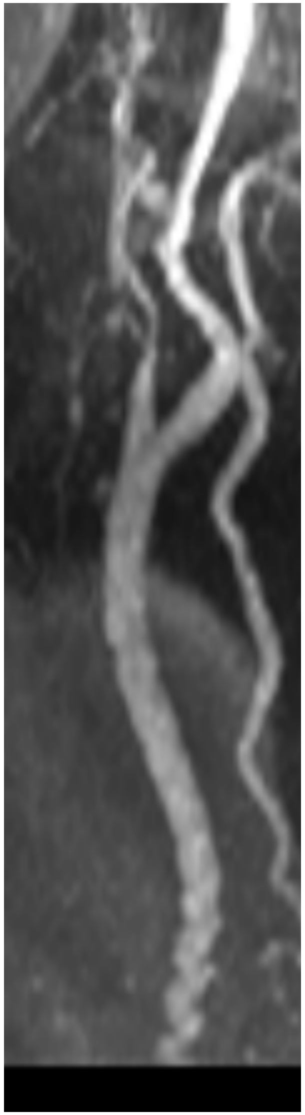

26f Score

0-30

31-50

51-70

>70

Near occlusion

Occluded

Quality

1

2

3

4

5

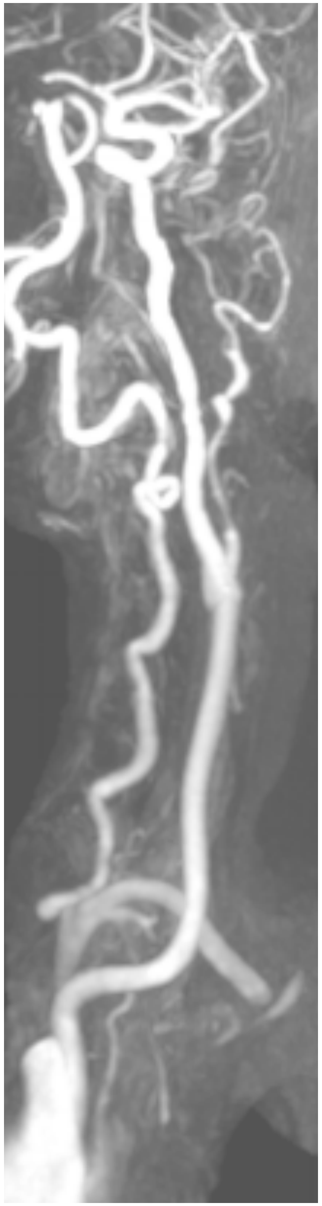

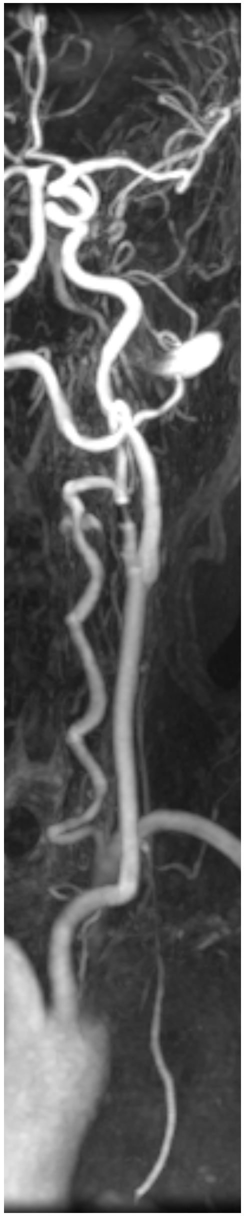

27e Score

0-30

31-50

51-70

>70

Near occlusion

Occluded

Quality

1

2

3

4

5

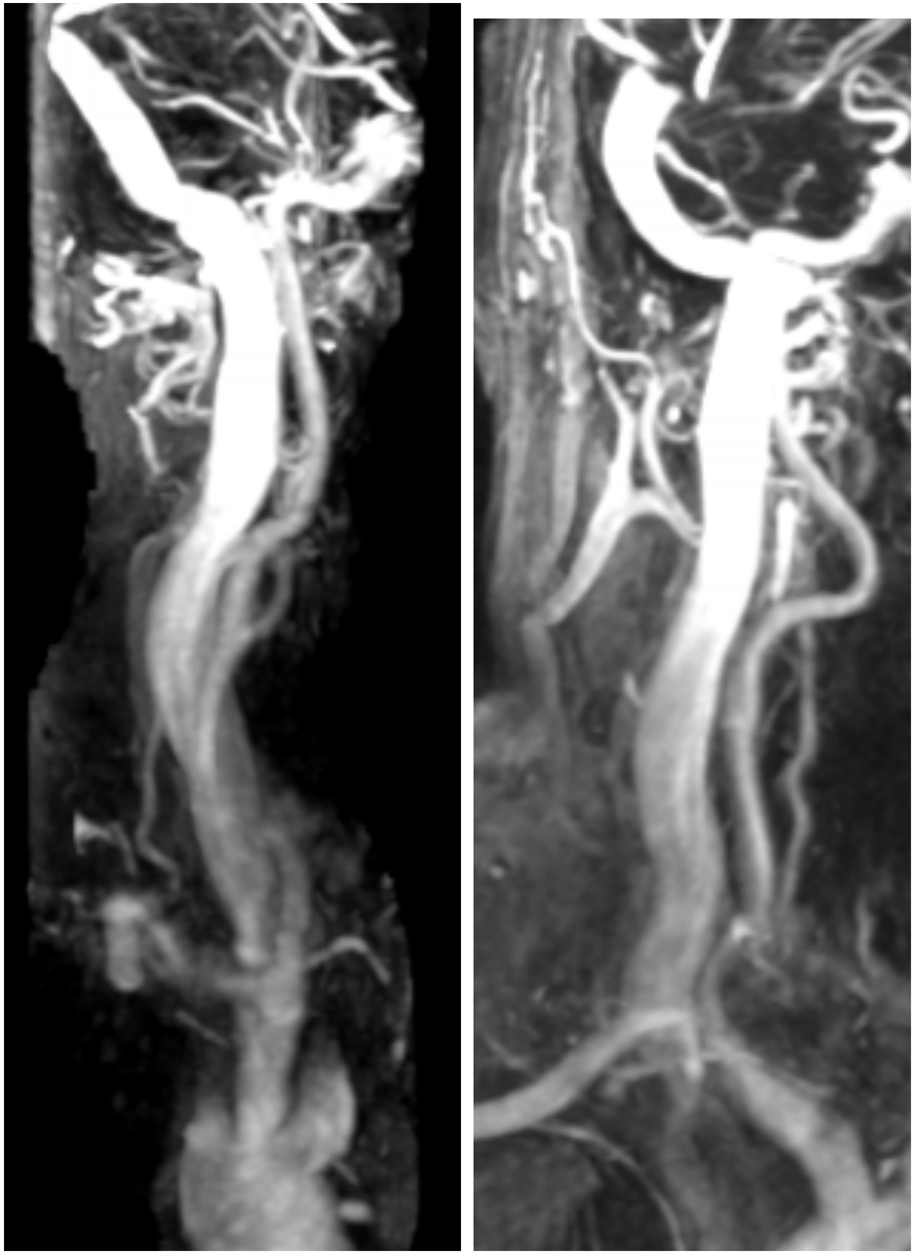

28d Score

0-30

31-50

51-70

>70

Near occlusion

Occluded

Quality

1

2

3

4

5

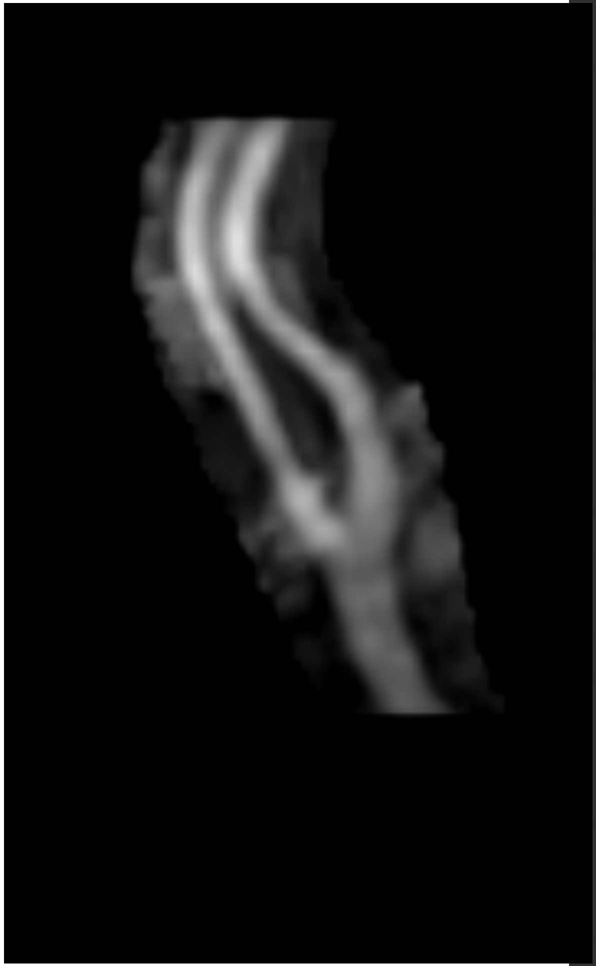

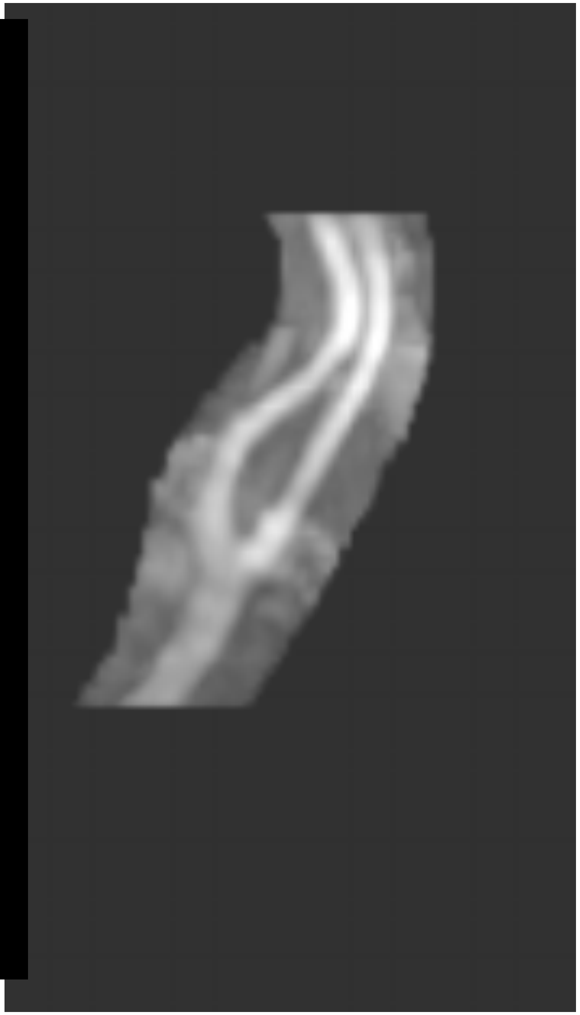

29c Score

0-30

31-50

51-70

>70

Near occlusion

Occluded

Quality

1

2

3

4

5

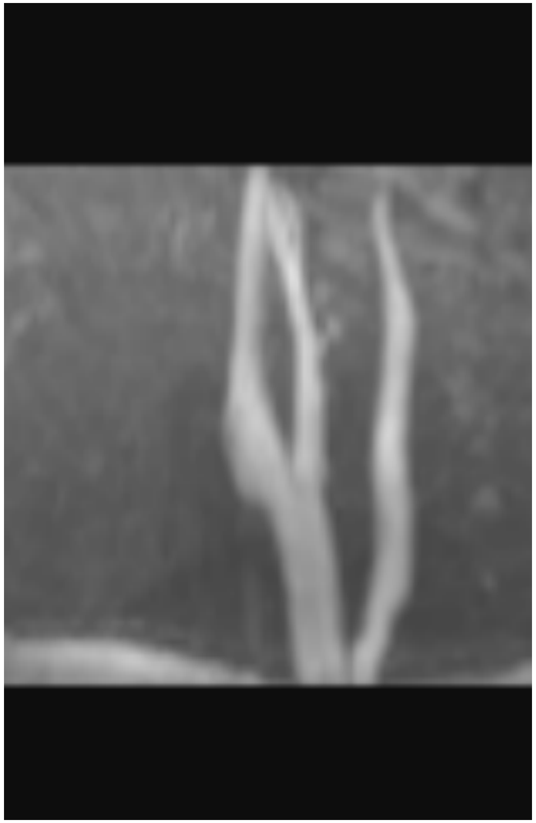

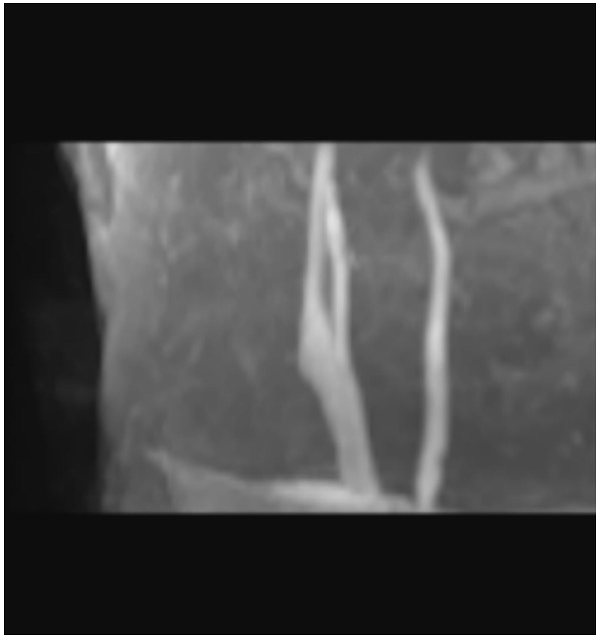

30b Score

0-30

31-50

51-70

>70

Near occlusion

Occluded

Quality

1

2

3

4

5

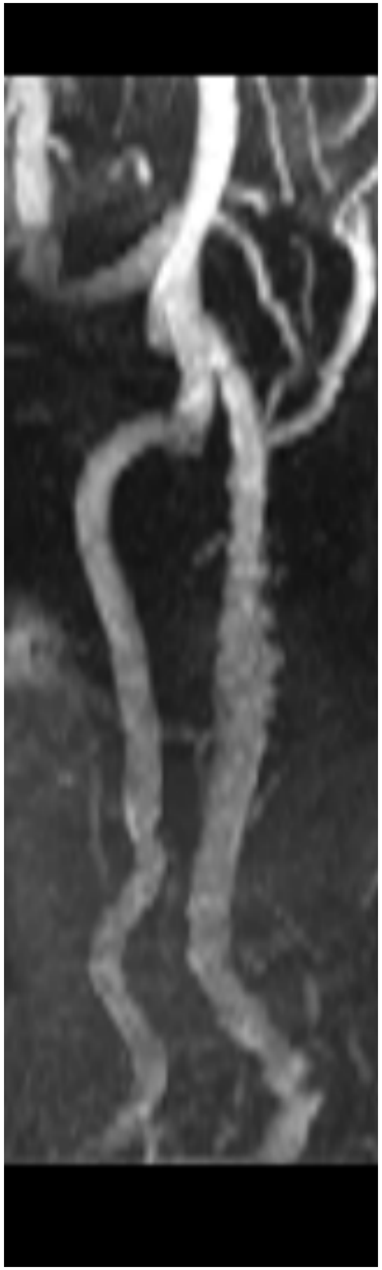

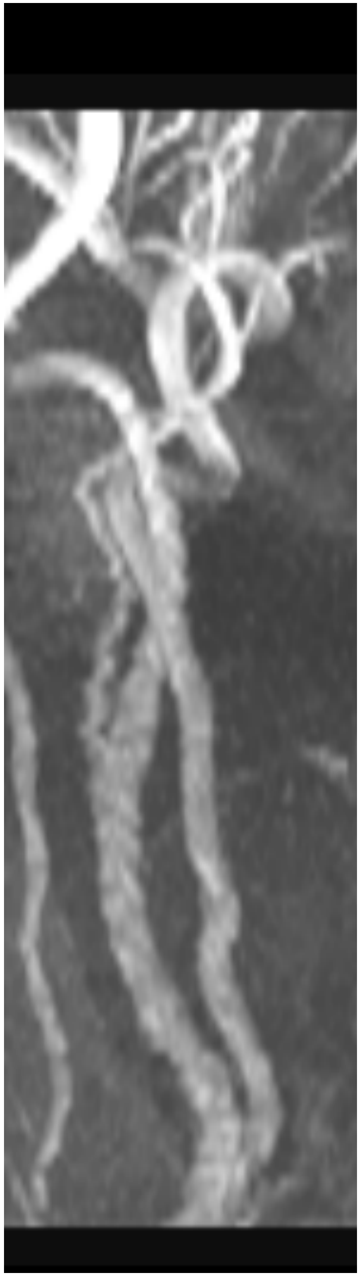

31a Score

0-30

31-50

51-70

>70

Near occlusion

Occluded

Quality

1

2

3

4

5

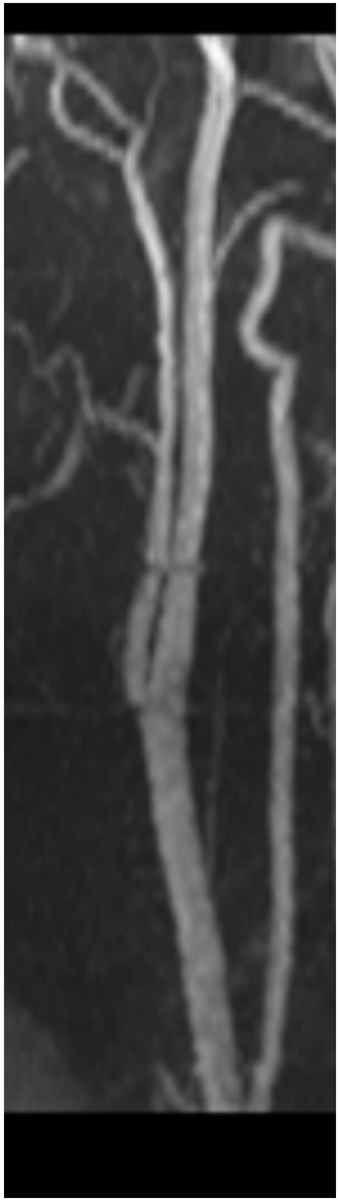

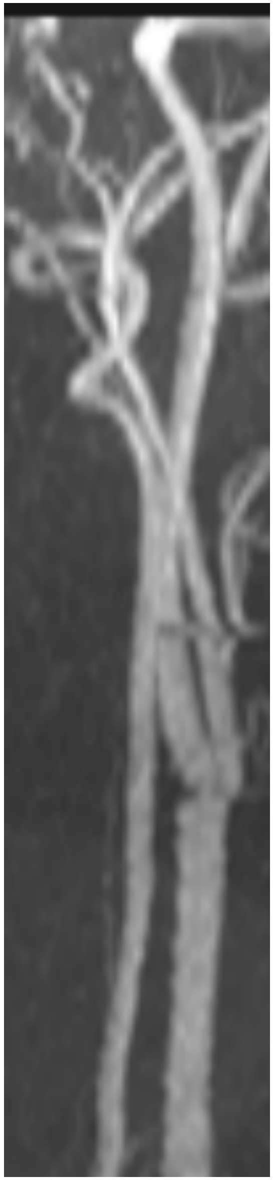

31f Score

0-30

31-50

51-70

>70

Near occlusion

Occluded

Quality

1

2

3

4

5

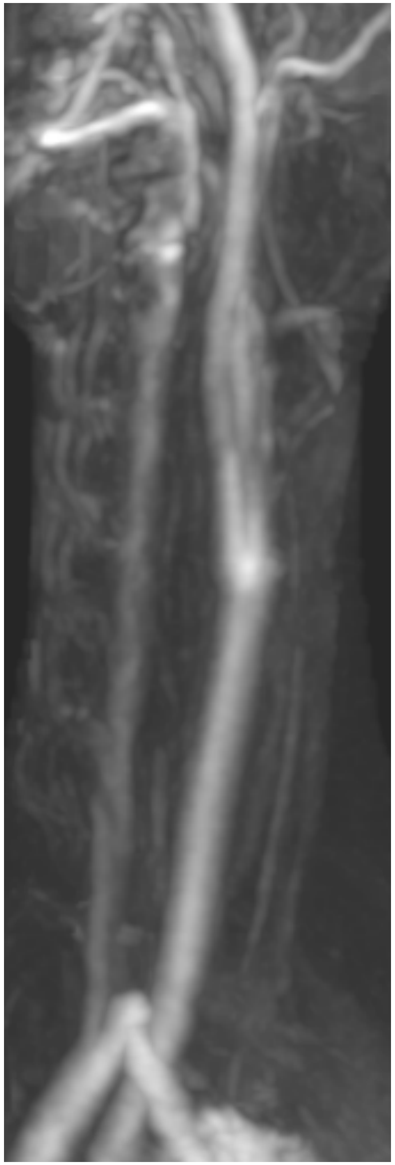

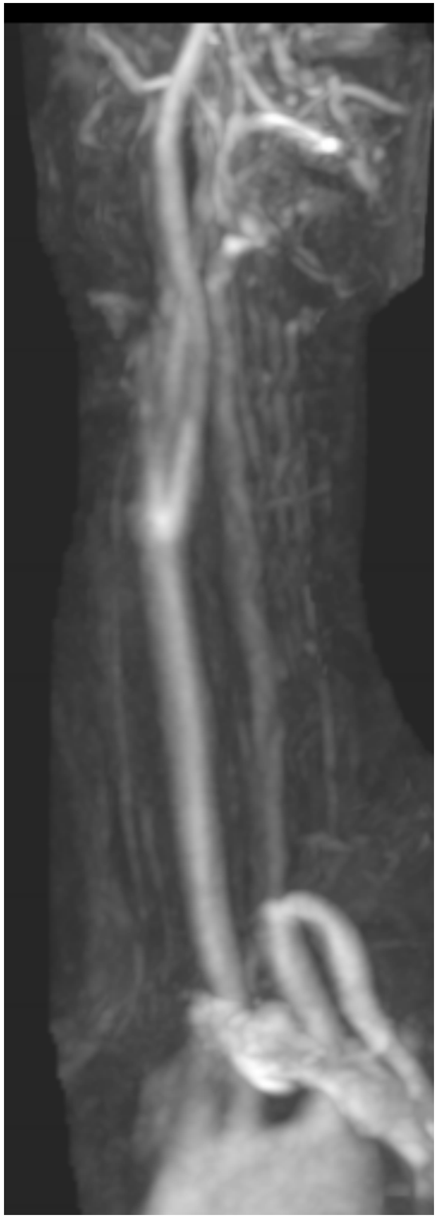

32e Score

0-30

31-50

51-70

>70

Near occlusion

Occluded

Quality

1

2

3

4

5

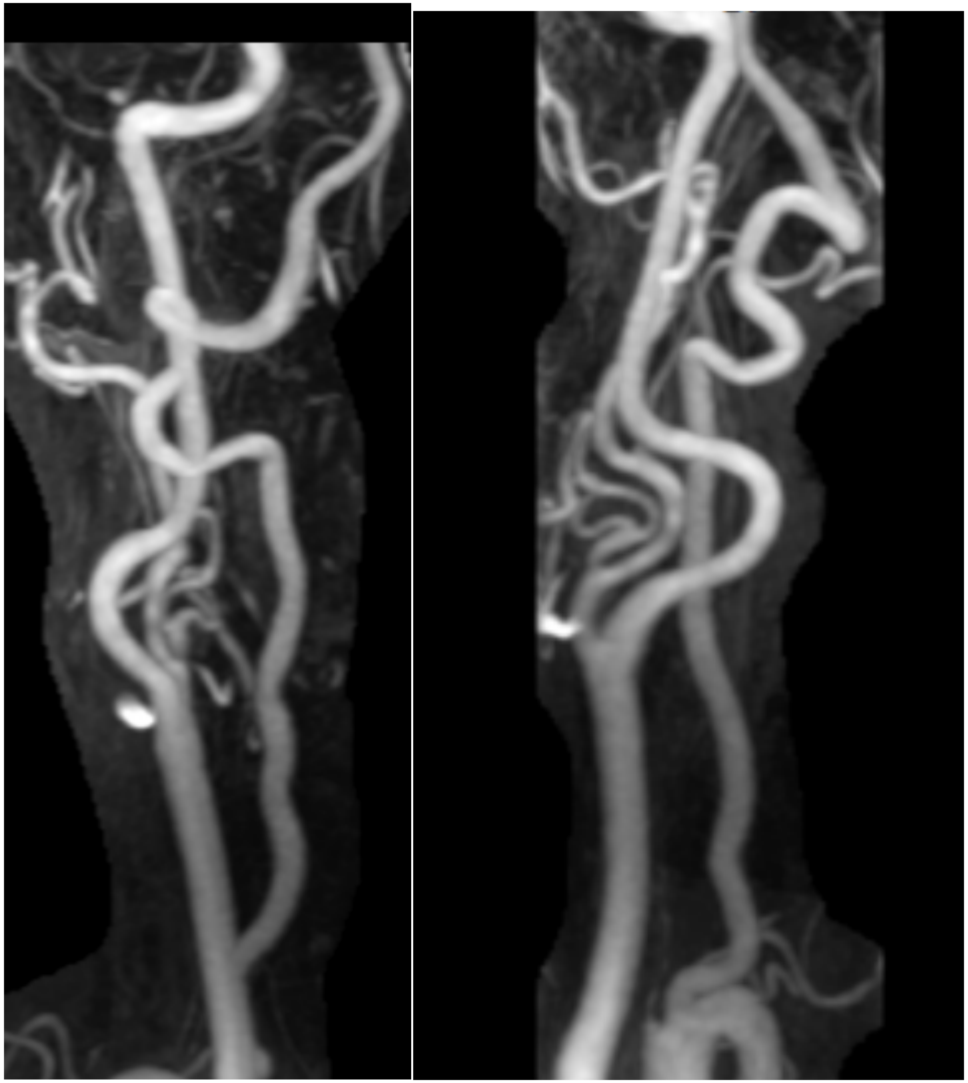

33d Score

0-30

31-50

51-70

>70

Near occlusion

Occluded

Quality

1

2

3

4

5

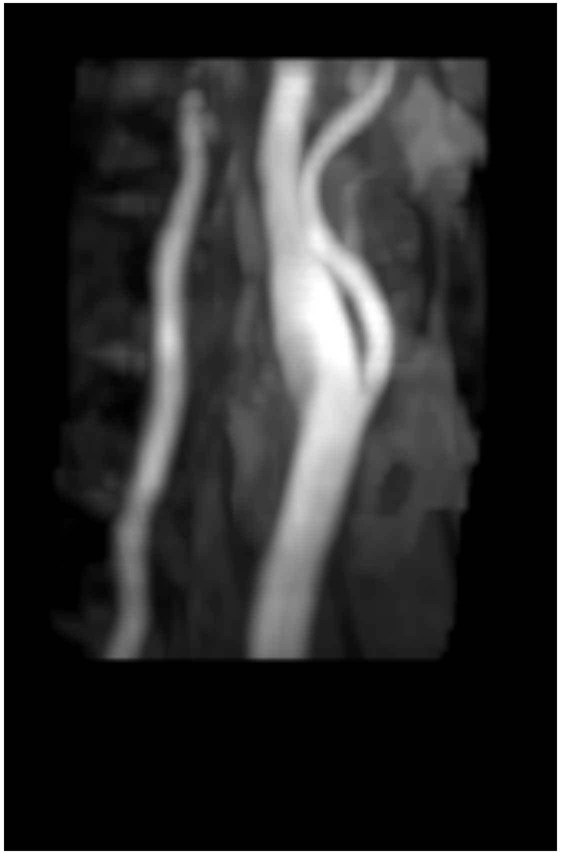

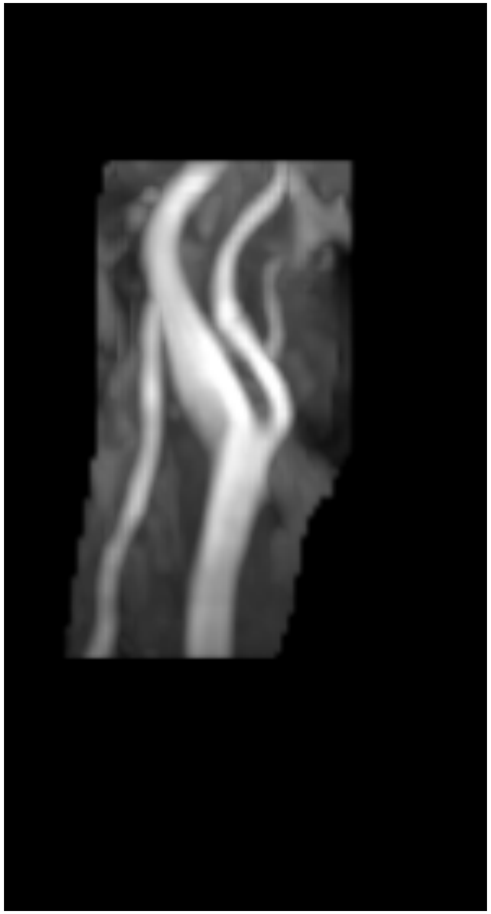

34c Score

0-30

31-50

51-70

>70

Near occlusion

Occluded

Quality

1

2

3

4

5

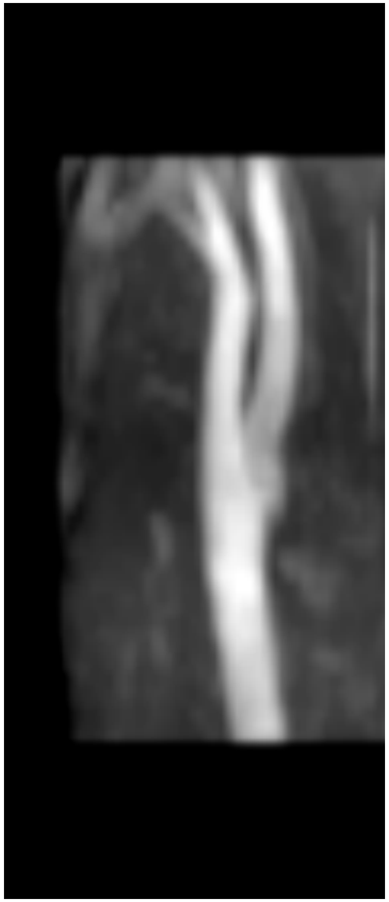

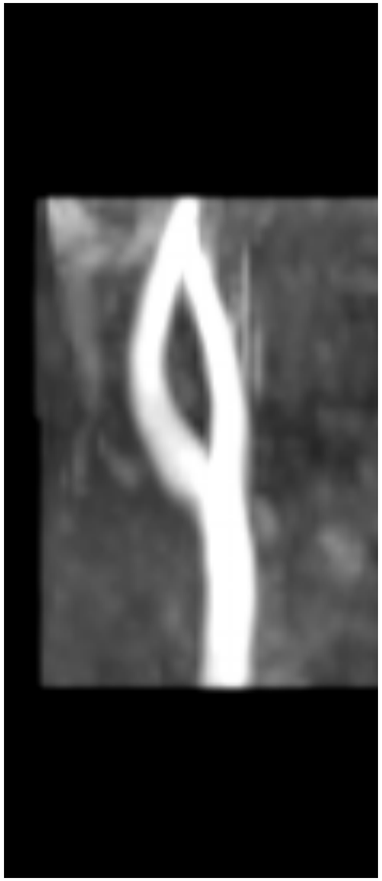

35b Score

0-30

31-50

51-70

>70

Near occlusion

Occluded

Quality

1

2

3

4

5

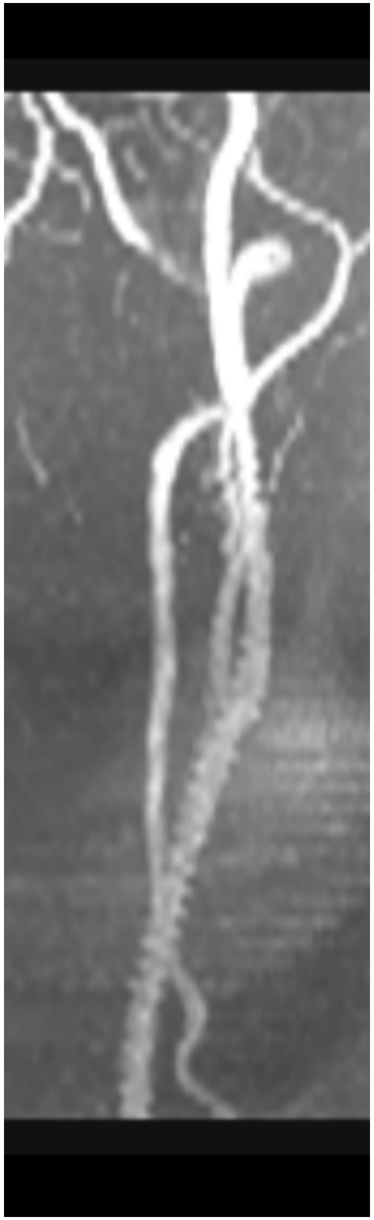

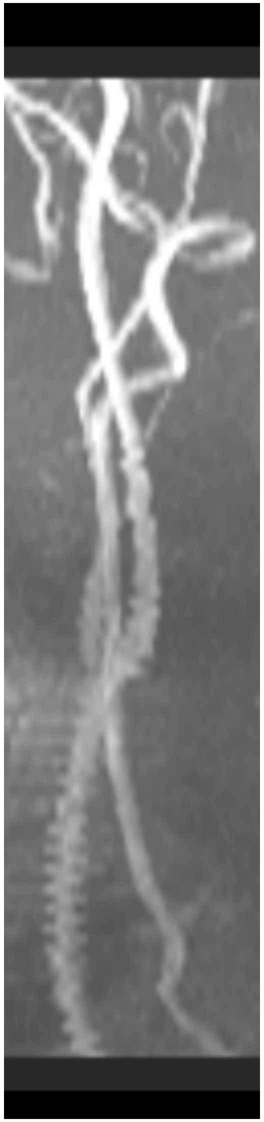

36a Score

0-30

31-50

51-70

>70

Near occlusion

Occluded

Quality

1

2

3

4

5

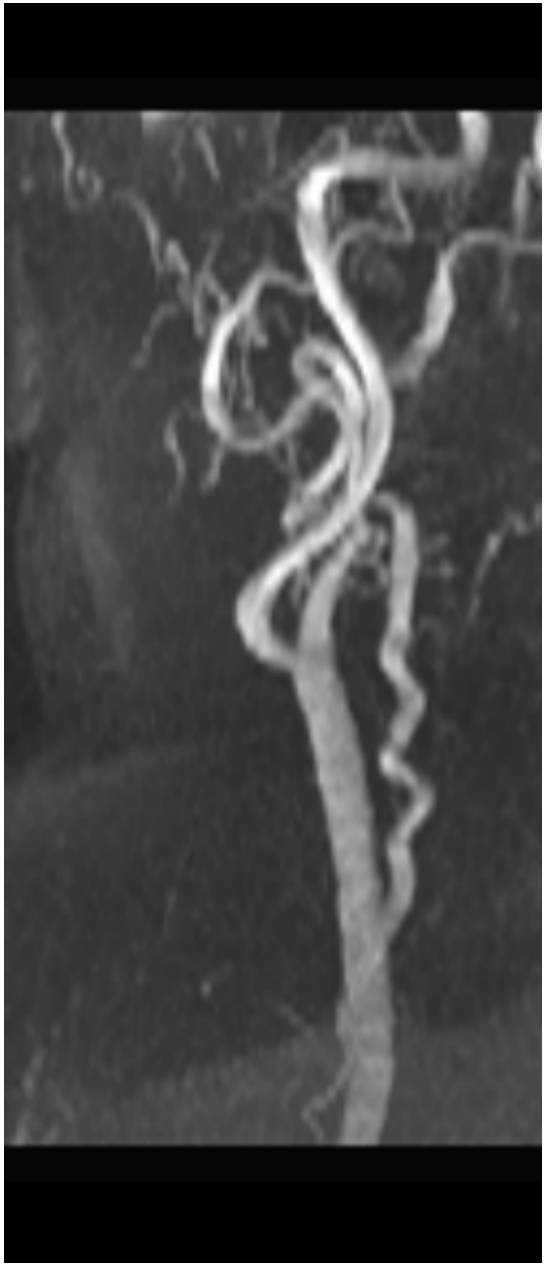

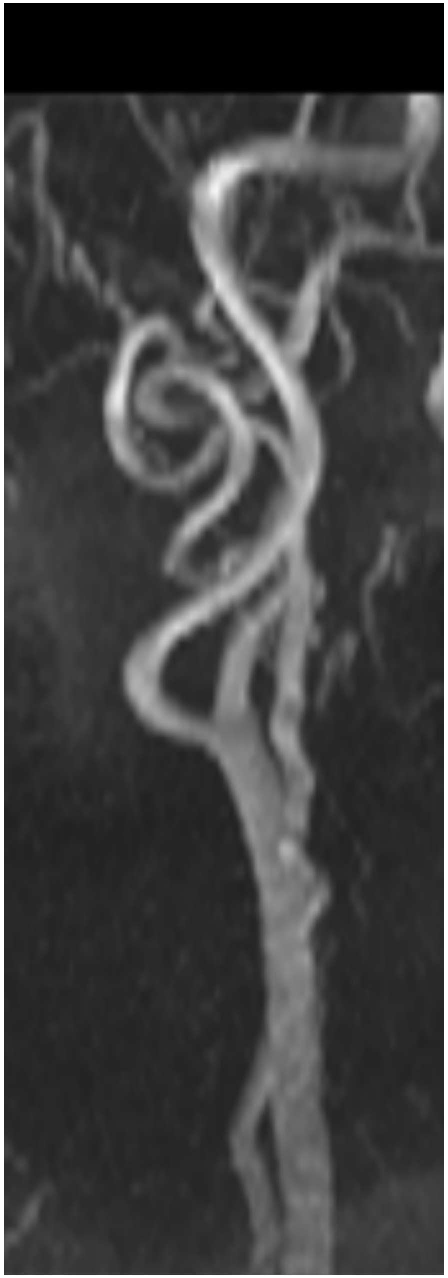

36f Score

0-30

31-50

51-70

>70

Near occlusion

Occluded

Quality

1

2

3

4

5

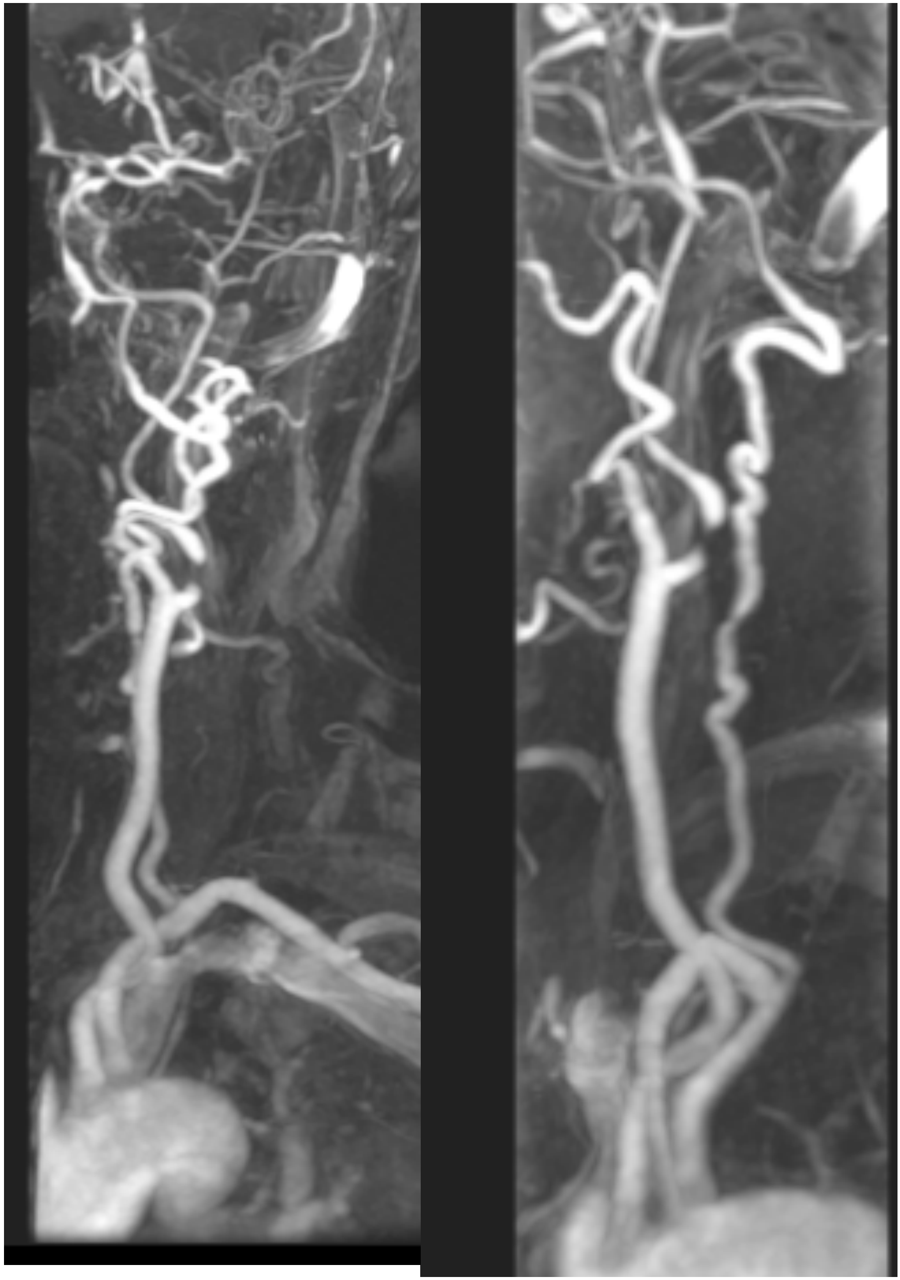

37e Score

0-30

31-50

51-70

>70

Near occlusion

Occluded

Quality

1

2

3

4

5

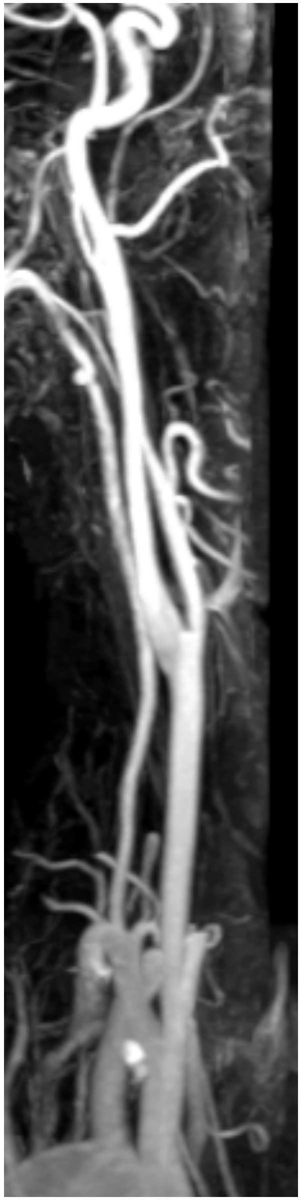

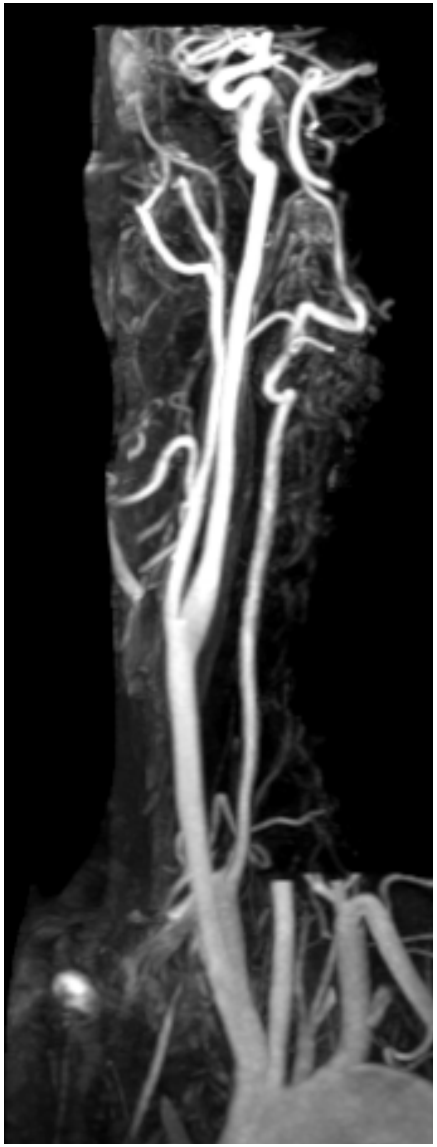

38d Score

0-30

31-50

51-70

>70

Near occlusion

Occluded

Quality

1

2

3

4

5

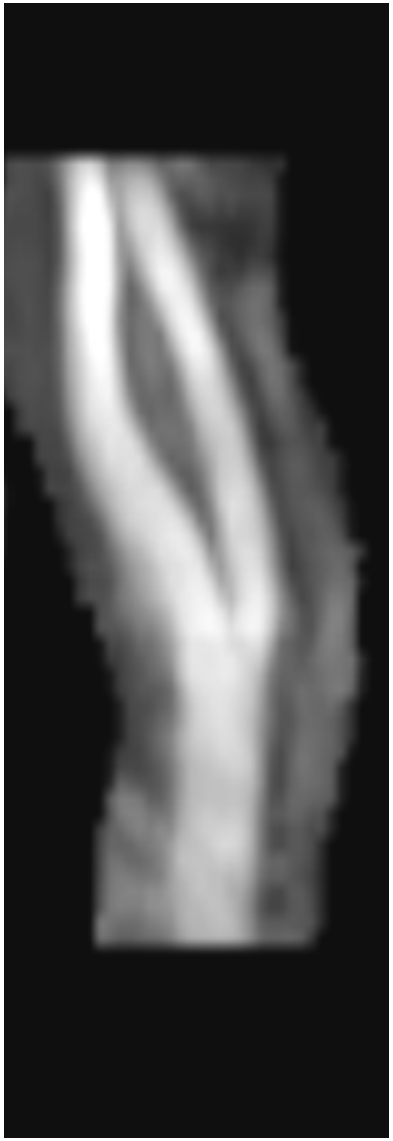

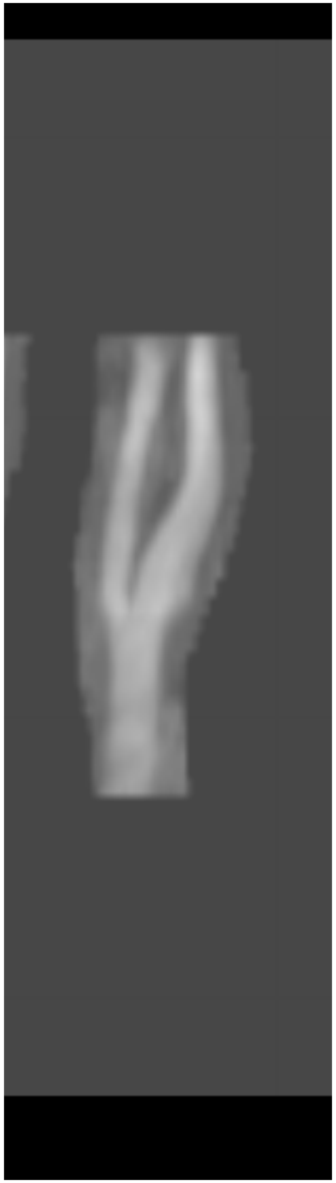

39c Score

0-30

31-50

51-70

>70

Near occlusion

Occluded

Quality

1

2

3

4

5

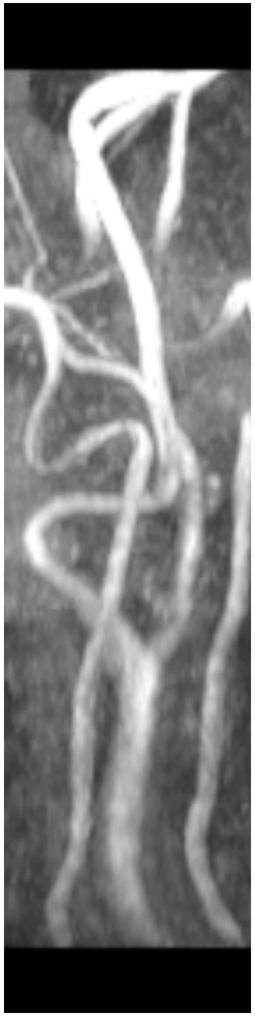

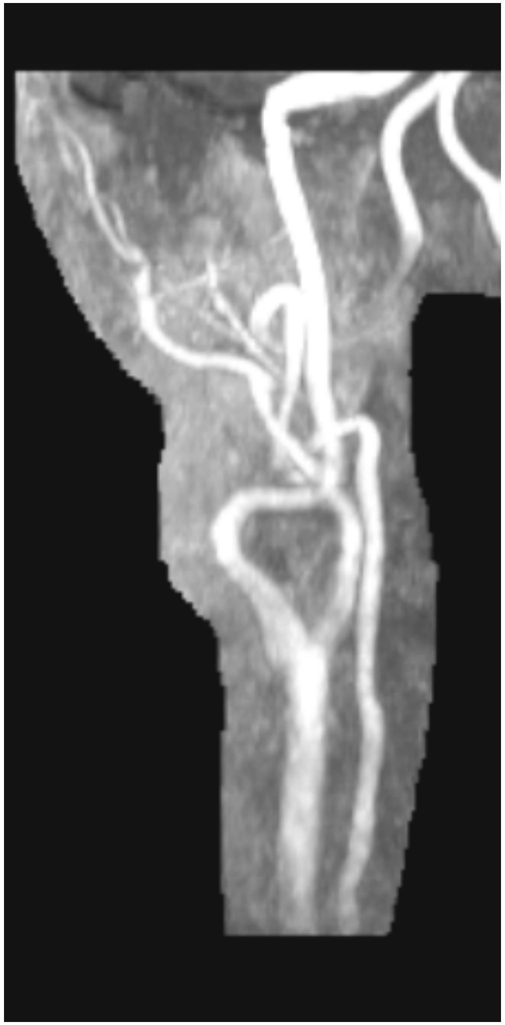

40b Score

0-30

31-50

51-70

>70

Near occlusion

Occluded

Quality

1

2

3

4

5

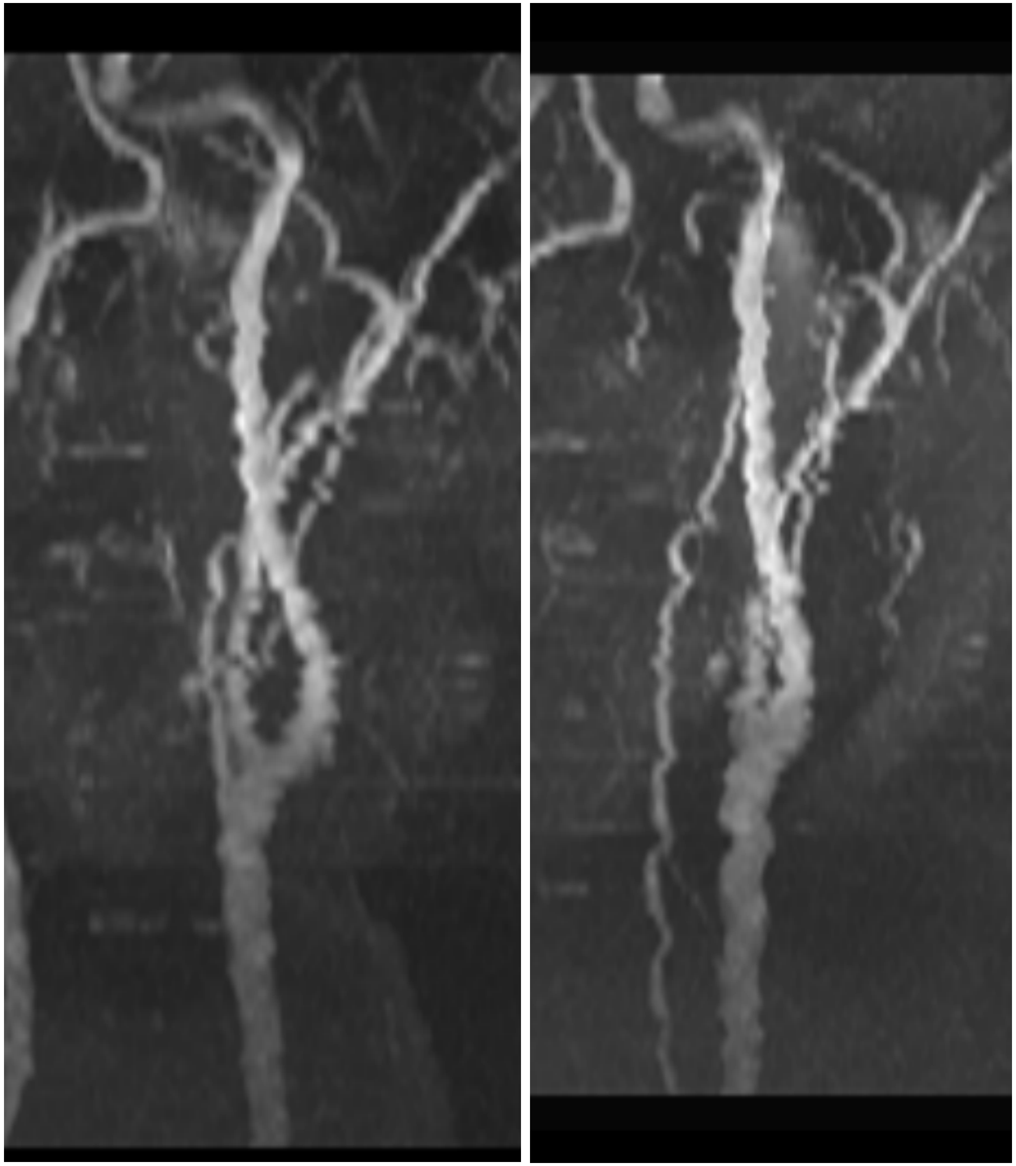

41a Score

0-30

31-50

51-70

>70

Near occlusion

Occluded

Quality

1

2

3

4

5

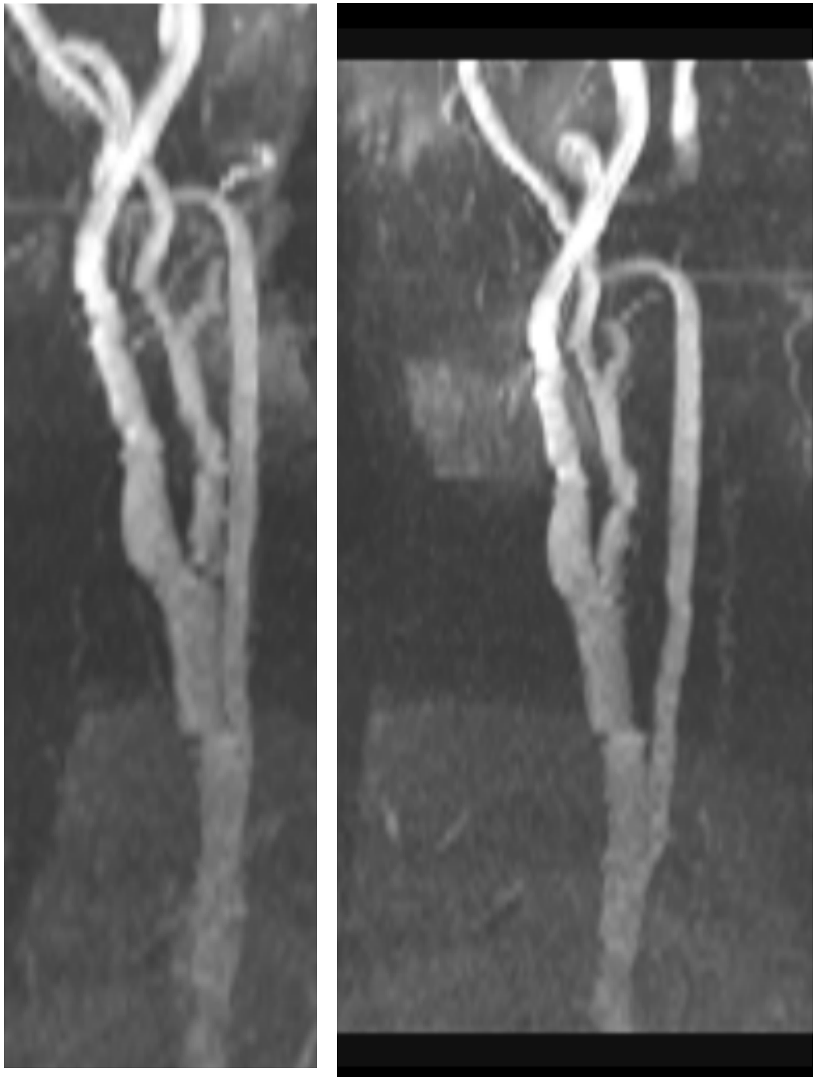

41f Score

0-30

31-50

51-70

>70

Near occlusion

Occluded

Quality

1

2

3

4

5

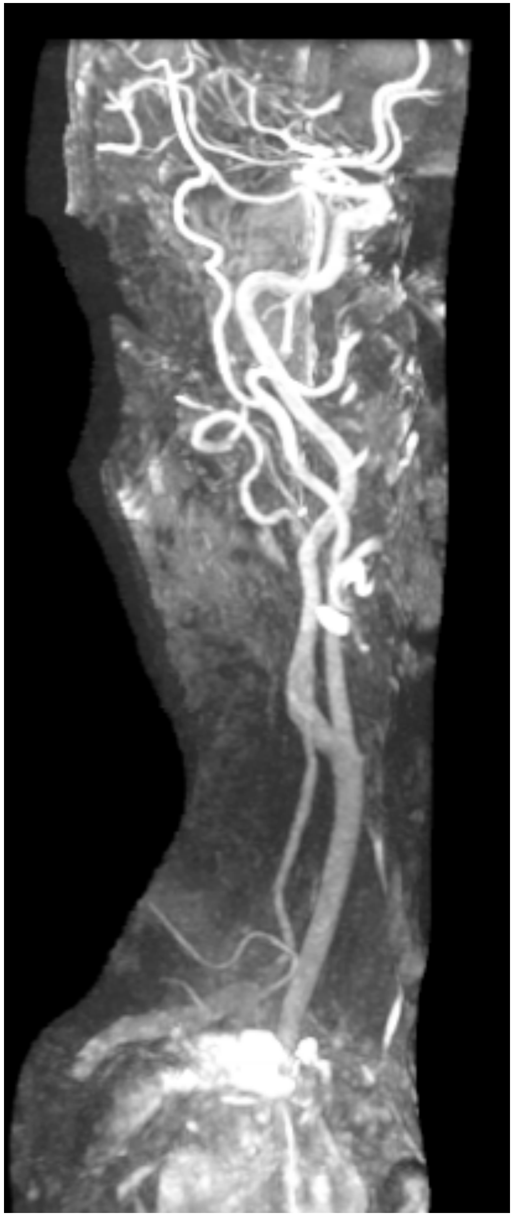

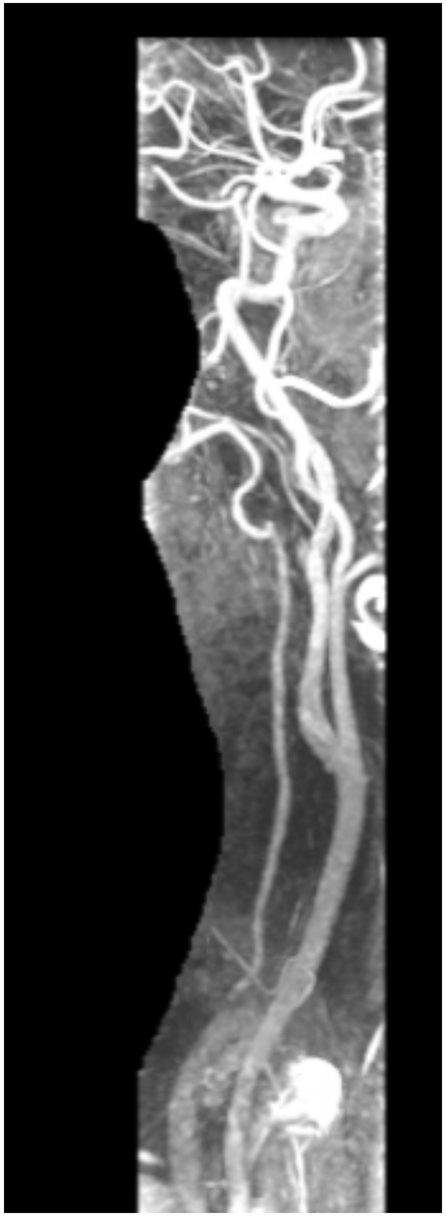

42e Score

0-30

31-50

51-70

>70

Near occlusion

Occluded

Quality

1

2

3

4

5

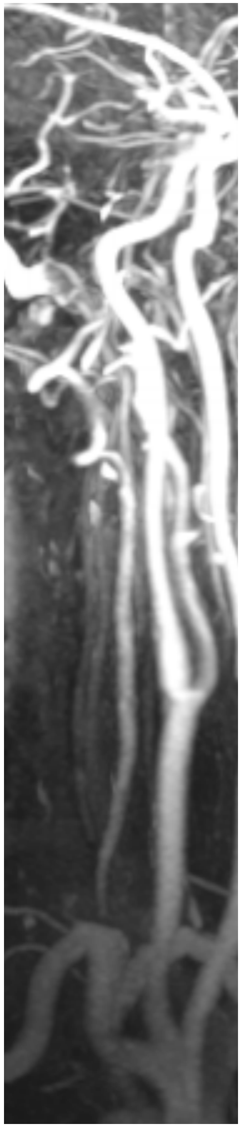

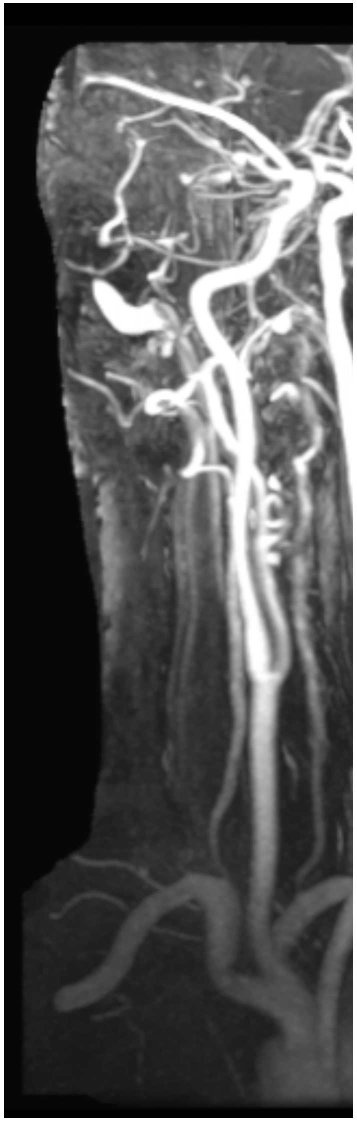

43d Score

0-30

31-50

51-70

>70

Near occlusion

Occluded

Quality

1

2

3

4

5

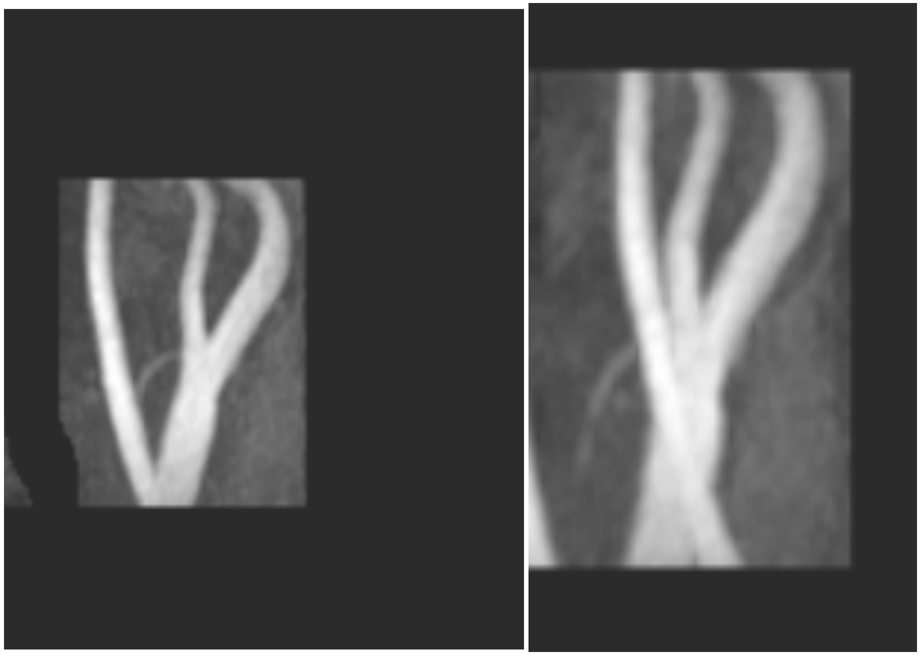

44c Score

0-30

31-50

51-70

>70

Near occlusion

Occluded

Quality

1

2

3

4

5

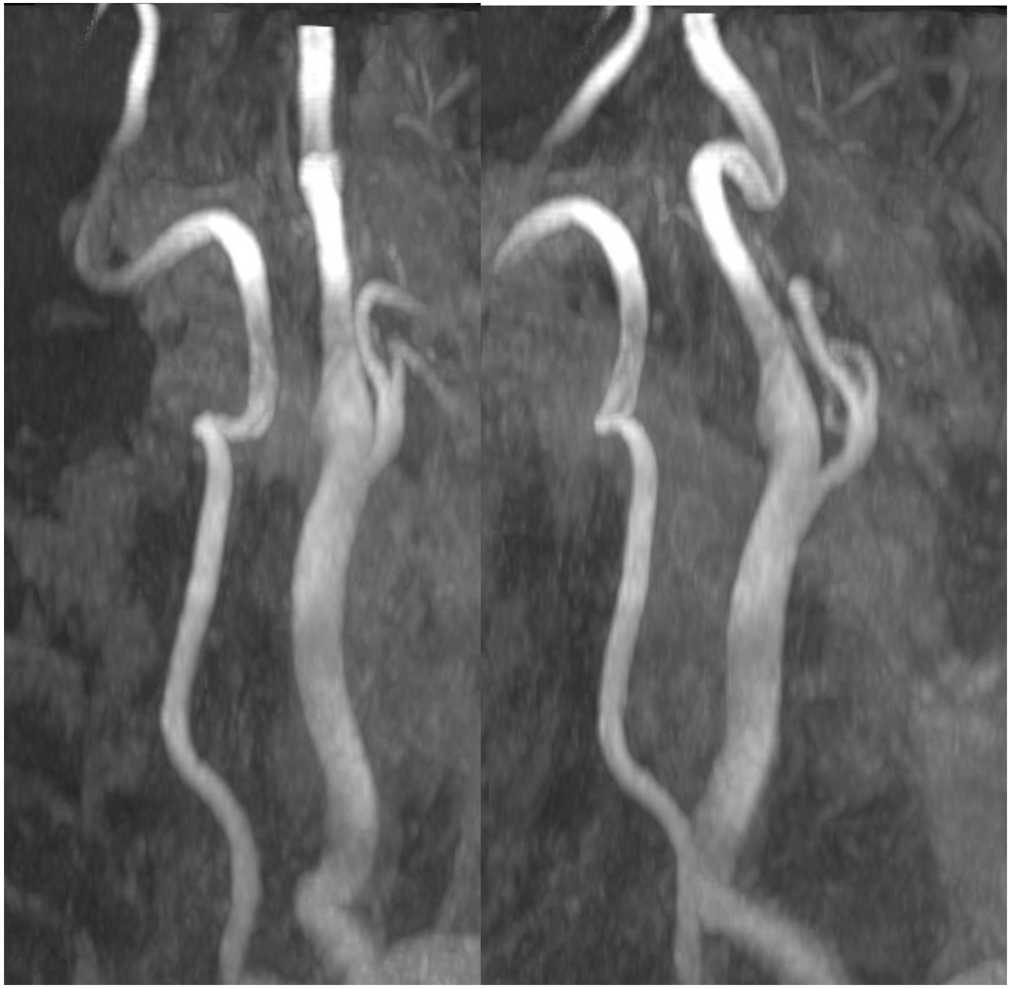

45b Score

0-30

31-50

51-70

>70

Near occlusion

Occluded

Quality

1

2

3

4

5

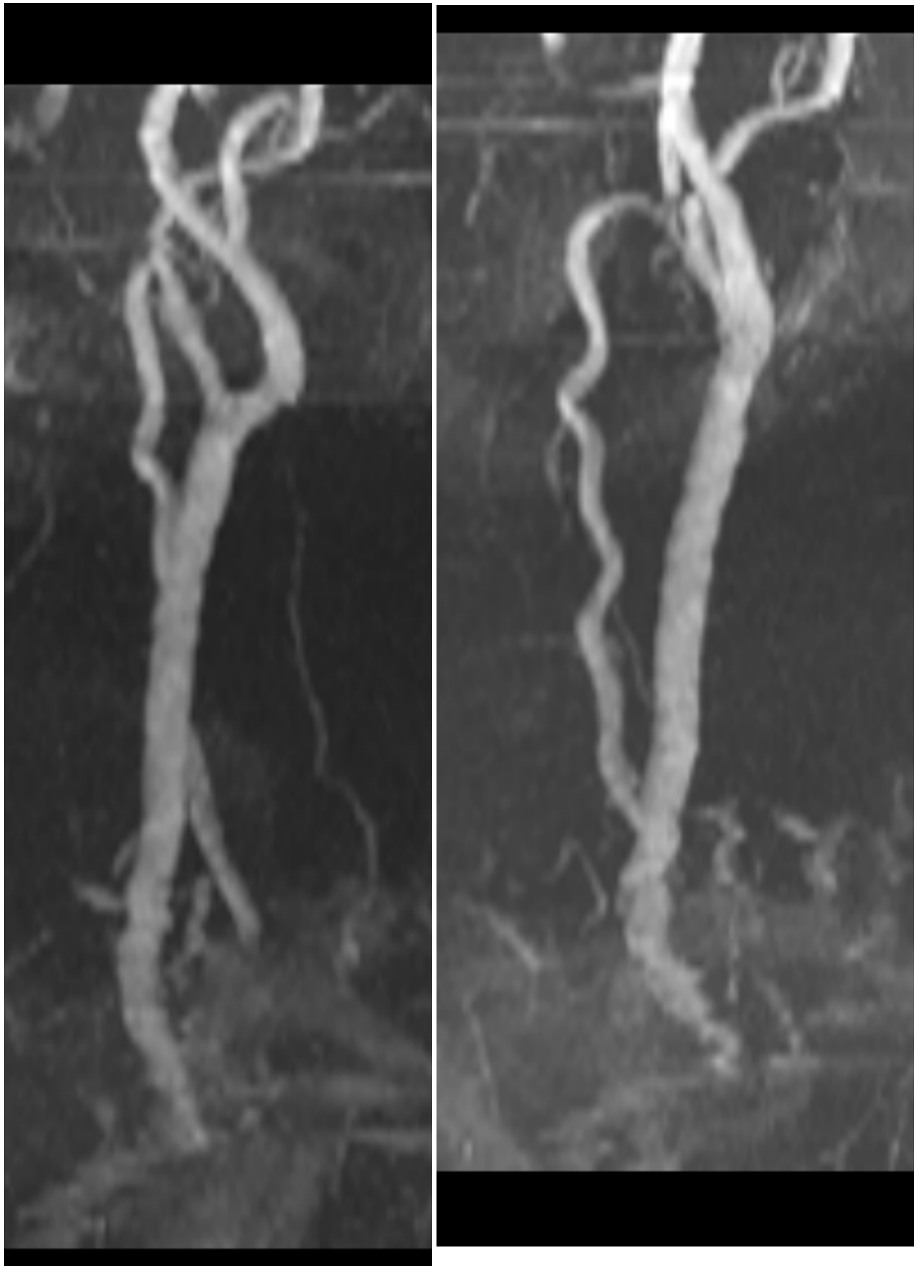

46a Score

0-30

31-50

51-70

>70

Near occlusion

Occluded

Quality

1

2

3

4

5

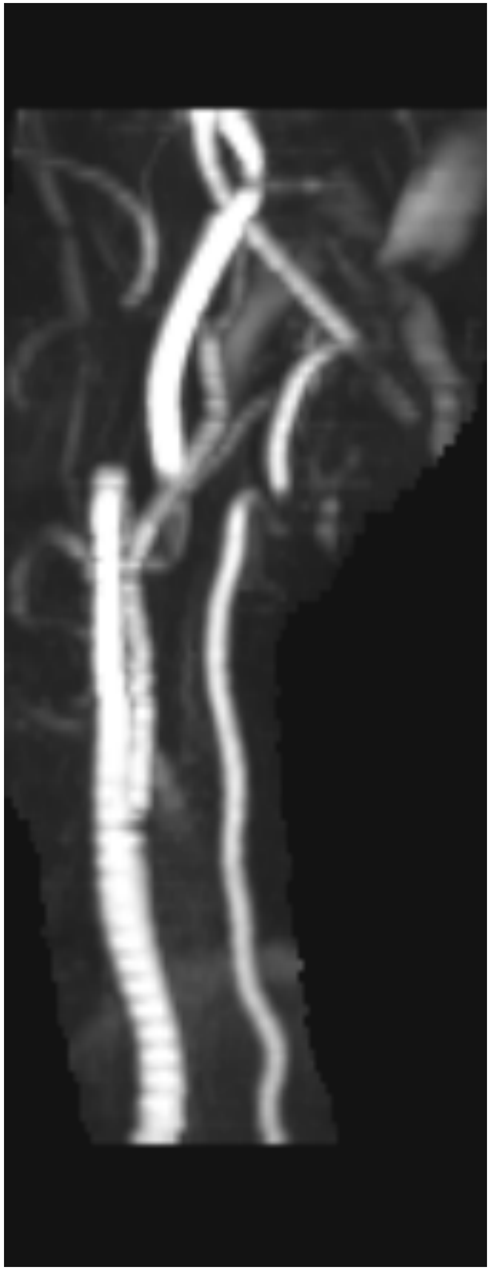

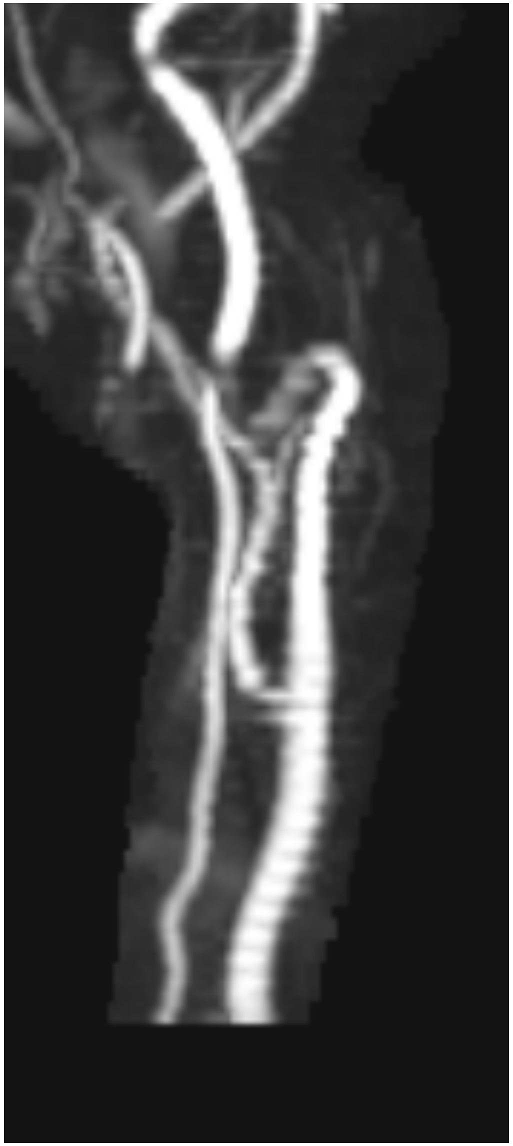

46f Score

0-30

31-50

51-70

>70

Near occlusion

Occluded

Quality

1

2

3

4

5

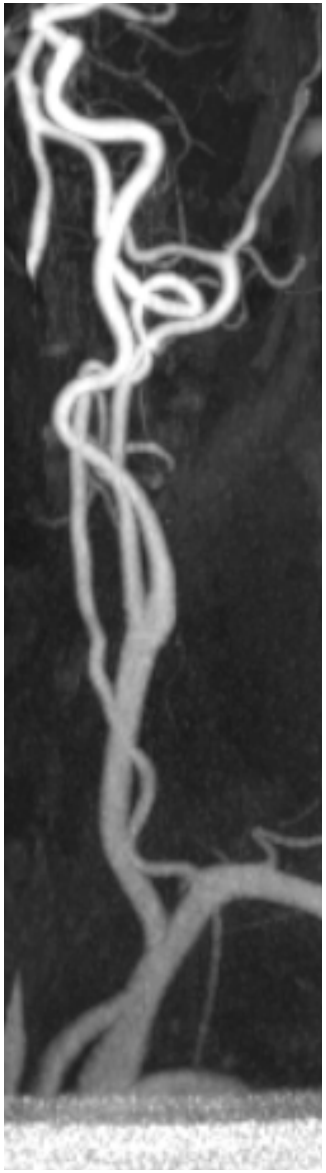

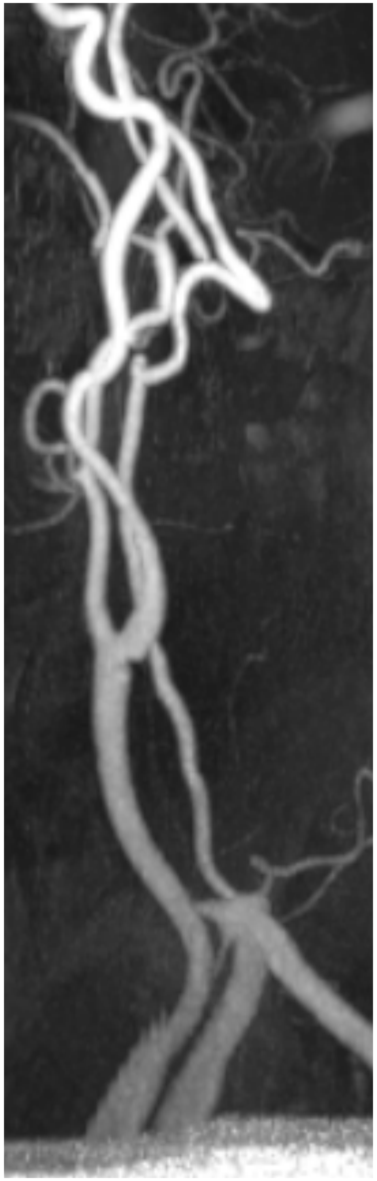

47e Score

0-30

31-50

51-70

>70

Near occlusion

Occluded

Quality

1

2

3

4

5

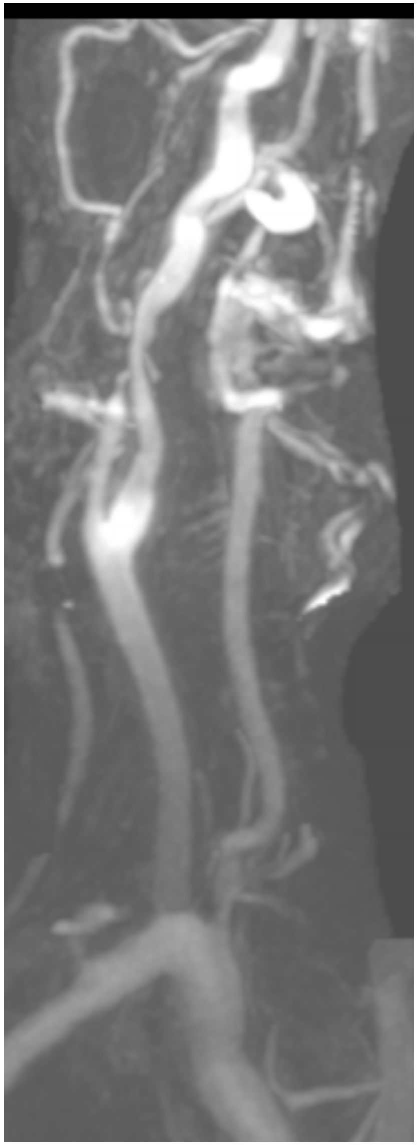

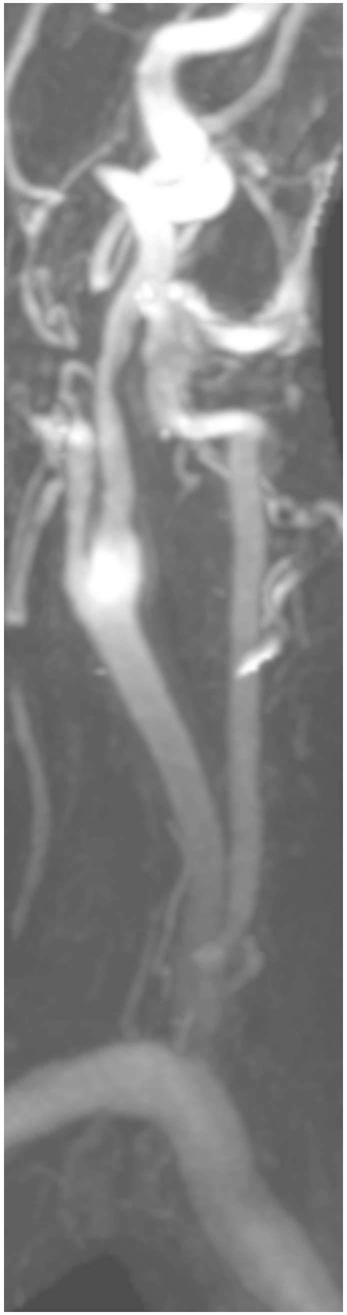

48d Score

0-30

31-50

51-70

>70

Near occlusion

Occluded

Quality

1

2

3

4

5

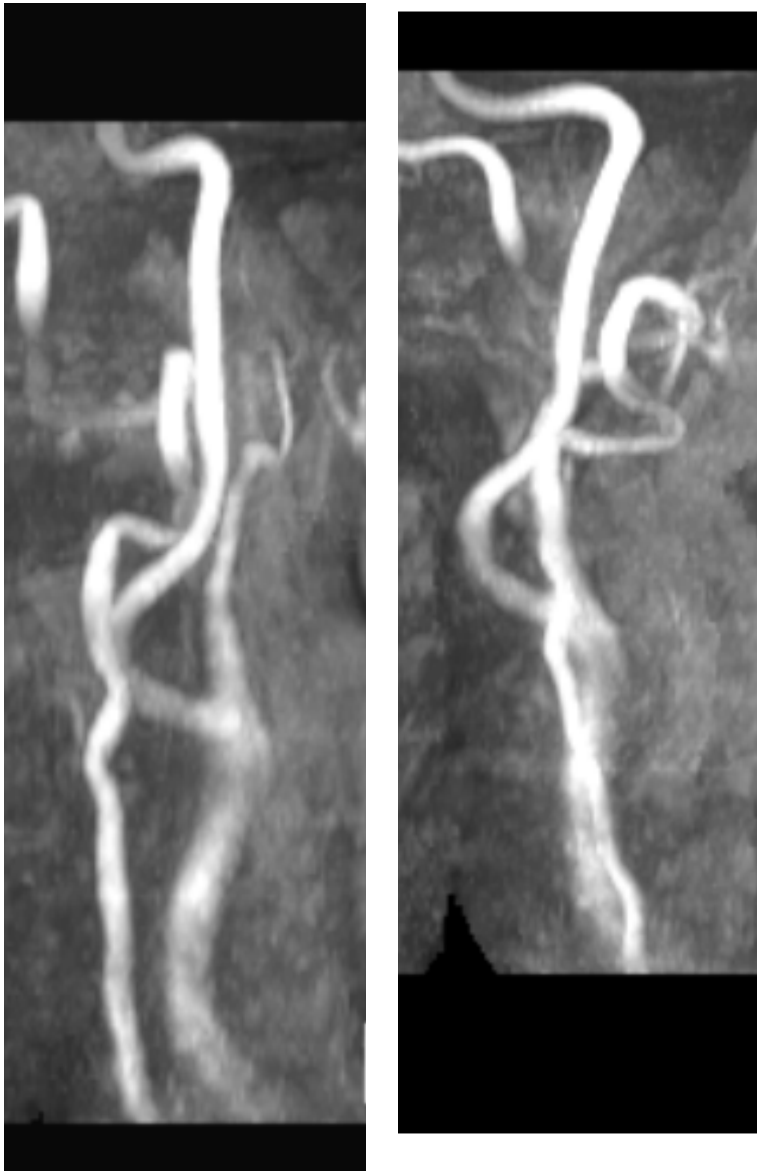

49c Score

0-30

31-50

51-70

>70

Near occlusion

Occluded

Quality

1

2

3

4

5

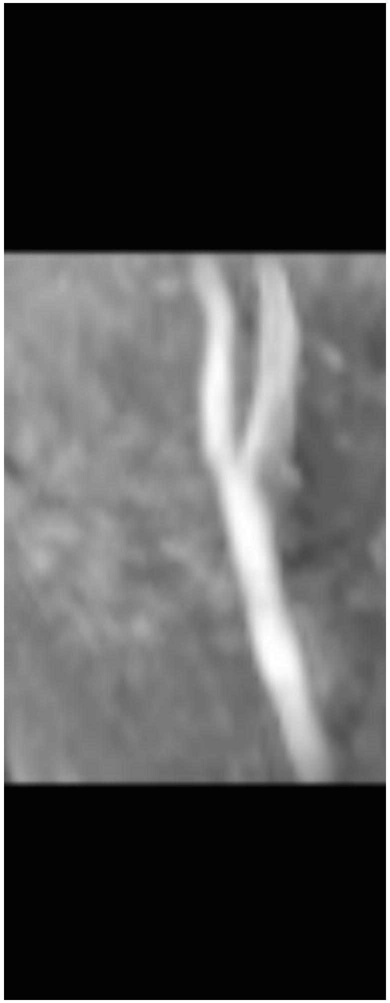

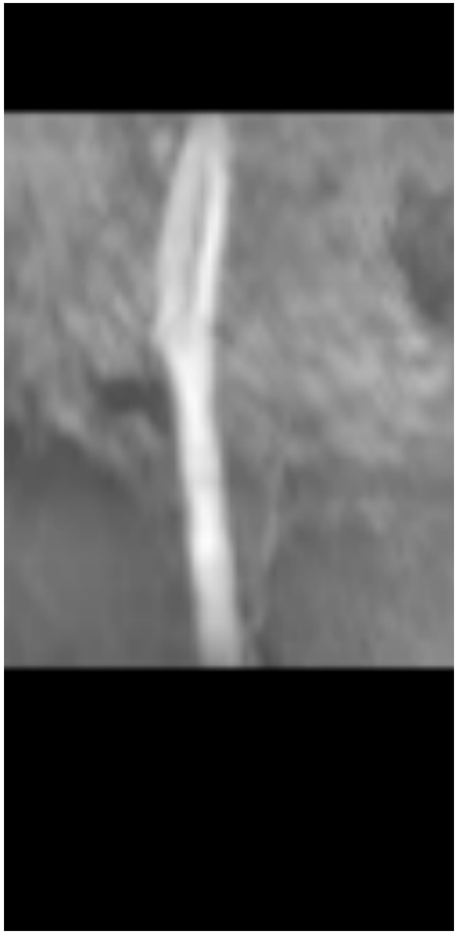

50b Score

0-30

31-50

51-70

>70

Near occlusion

Occluded

Quality

1

2

3

4

5

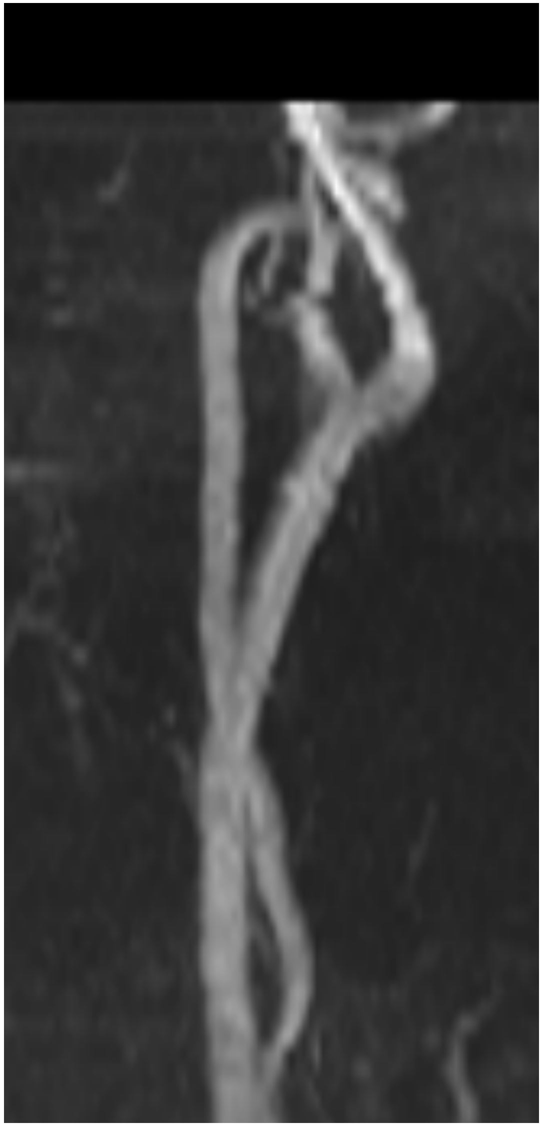

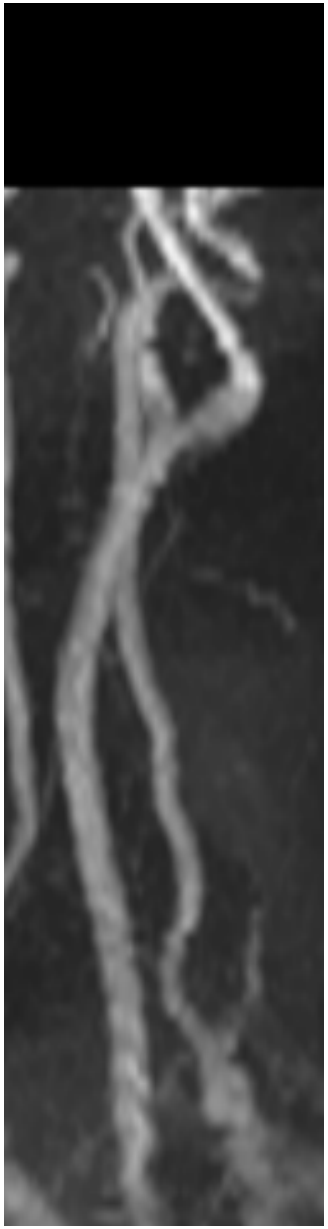

51a Score

0-30

31-50

51-70

>70

Near occlusion

Occluded

Quality

1

2

3

4

5

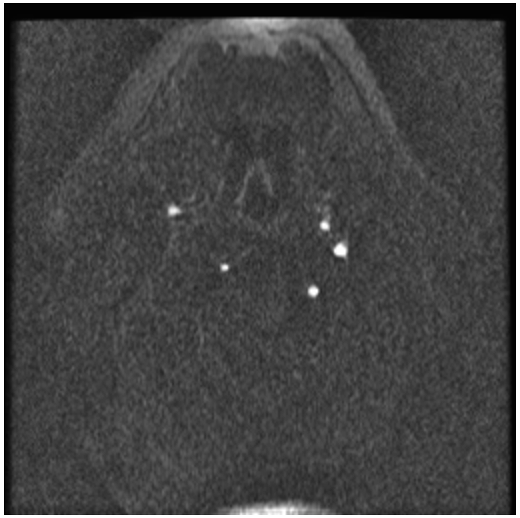

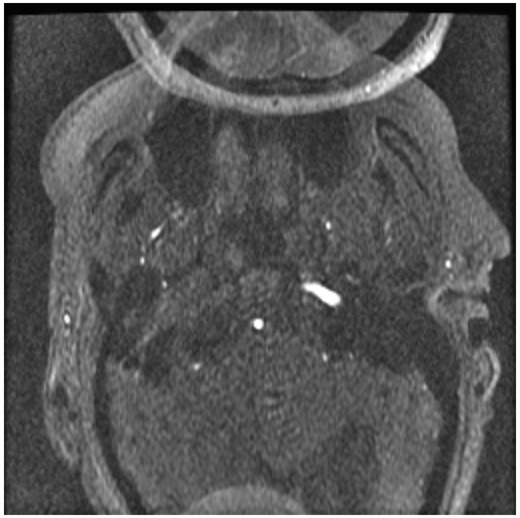

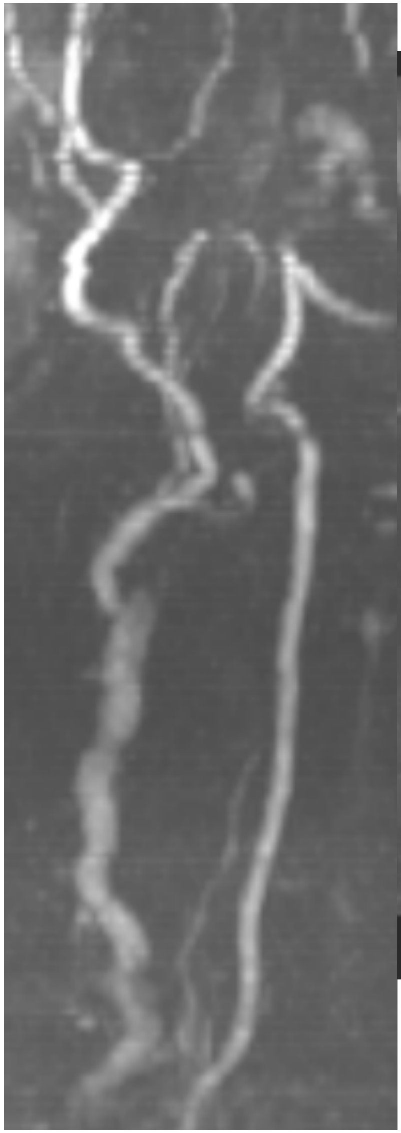

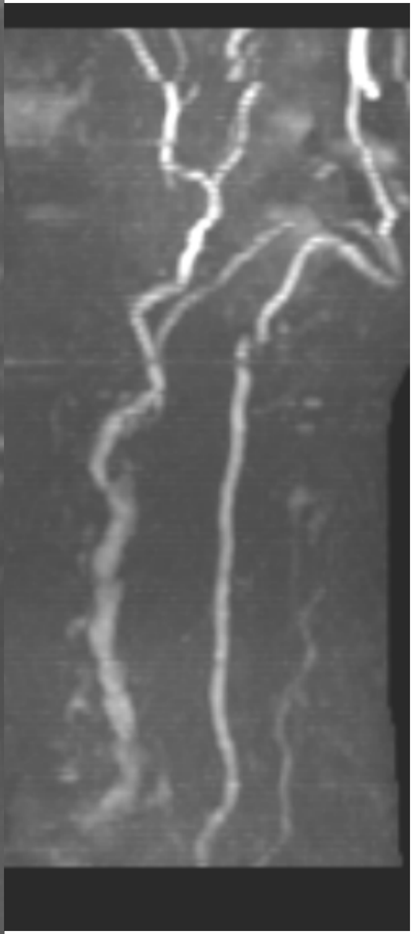

51f Score

0-30

31-50

51-70

>70

Near occlusion

Occluded

Quality

1

2

3

4

5

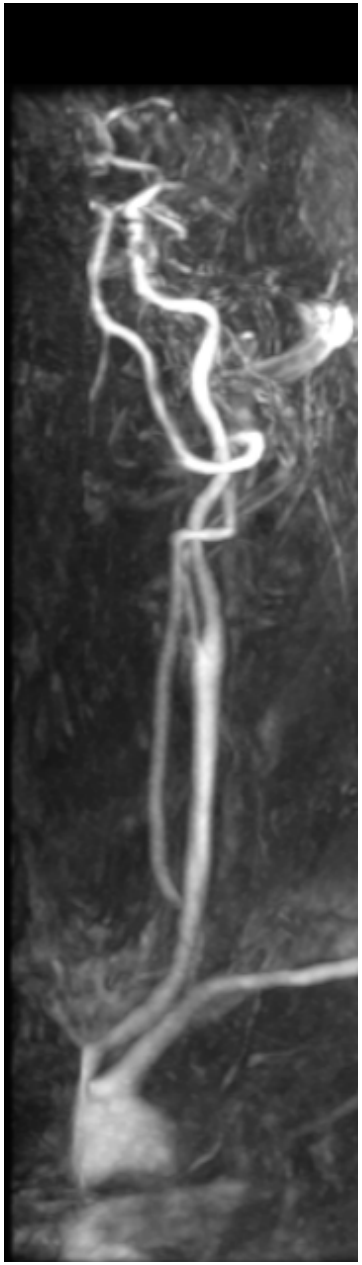

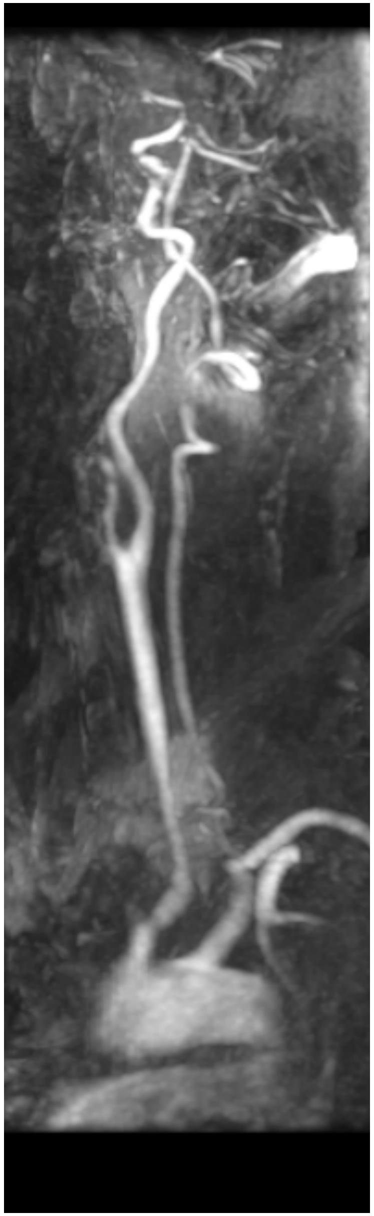

52e Score

0-30

31-50

51-70

>70

Near occlusion

Occluded

Quality

1

2

3

4

5

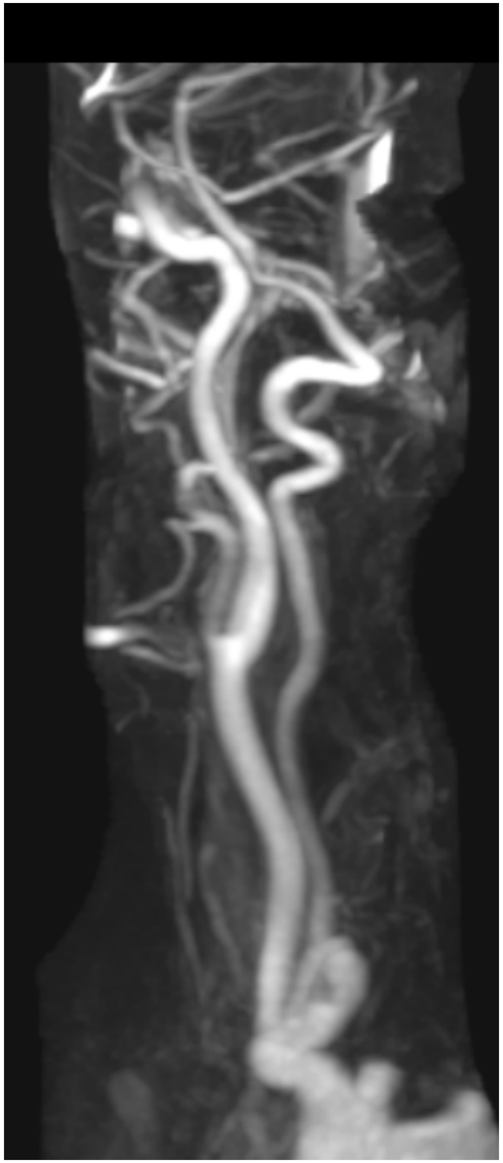

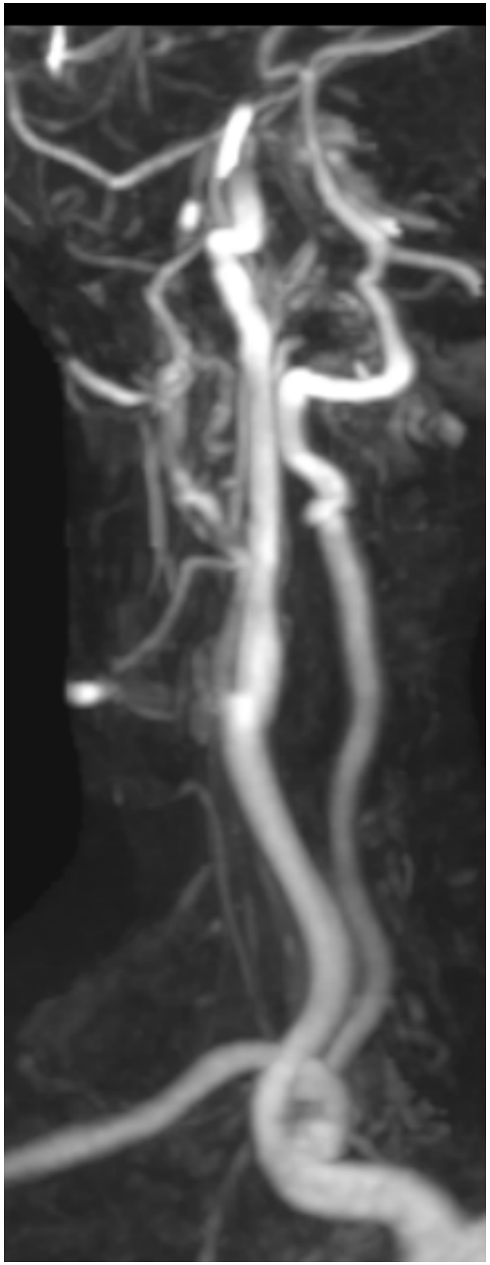

53d Score

0-30

31-50

51-70

>70

Near occlusion

Occluded

Quality

1

2

3

4

5

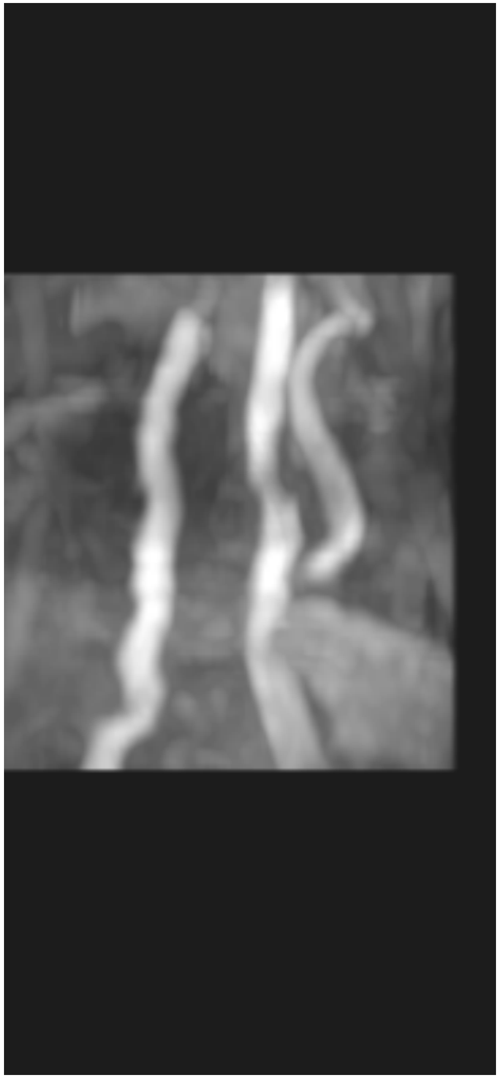

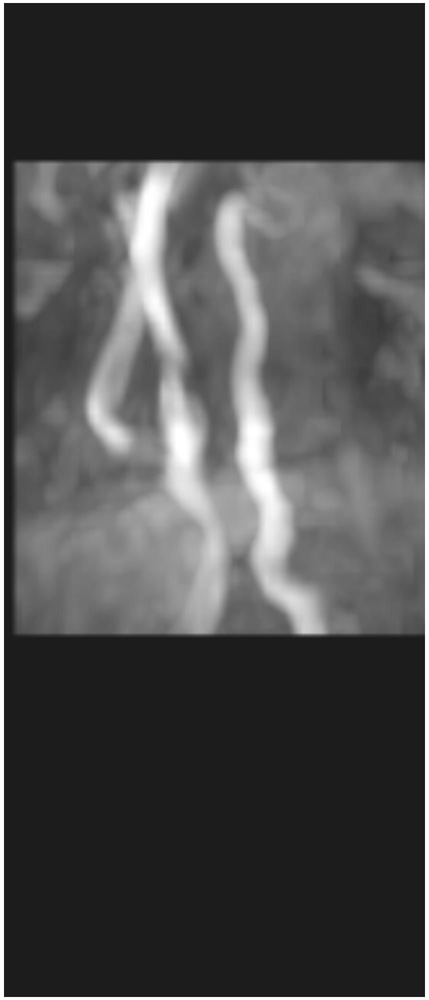

54c Score

0-30

31-50

51-70

>70

Near occlusion

Occluded

Quality

1

2

3

4

5

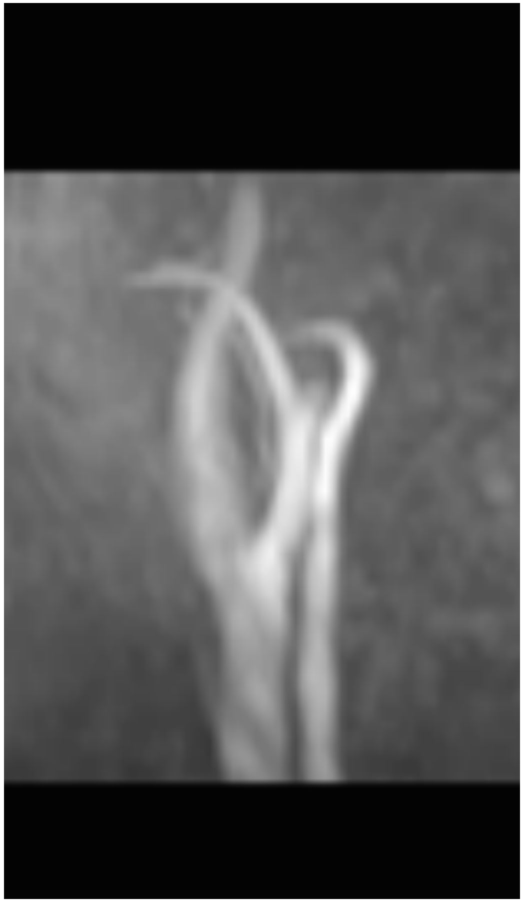

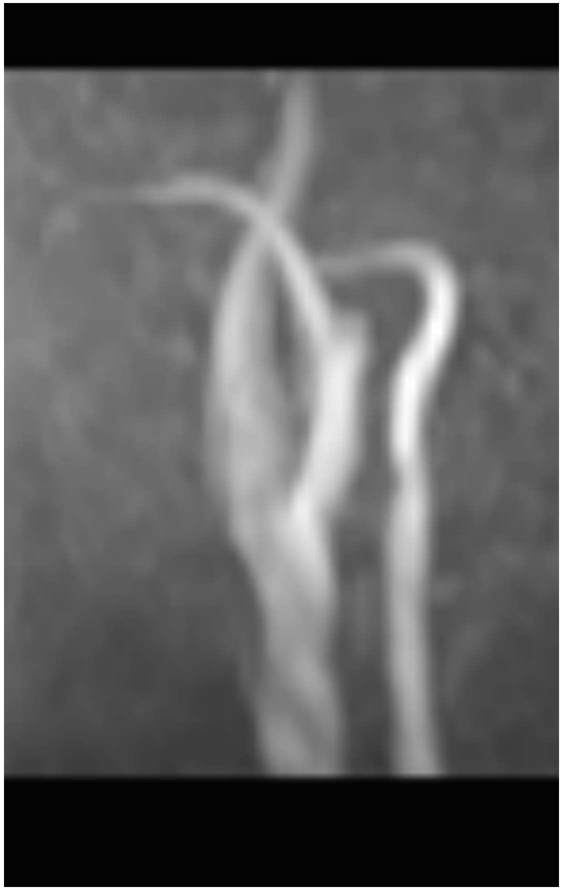

55b Score

0-30

31-50

51-70

>70

Near occlusion

Occluded

Quality

1

2

3

4

5

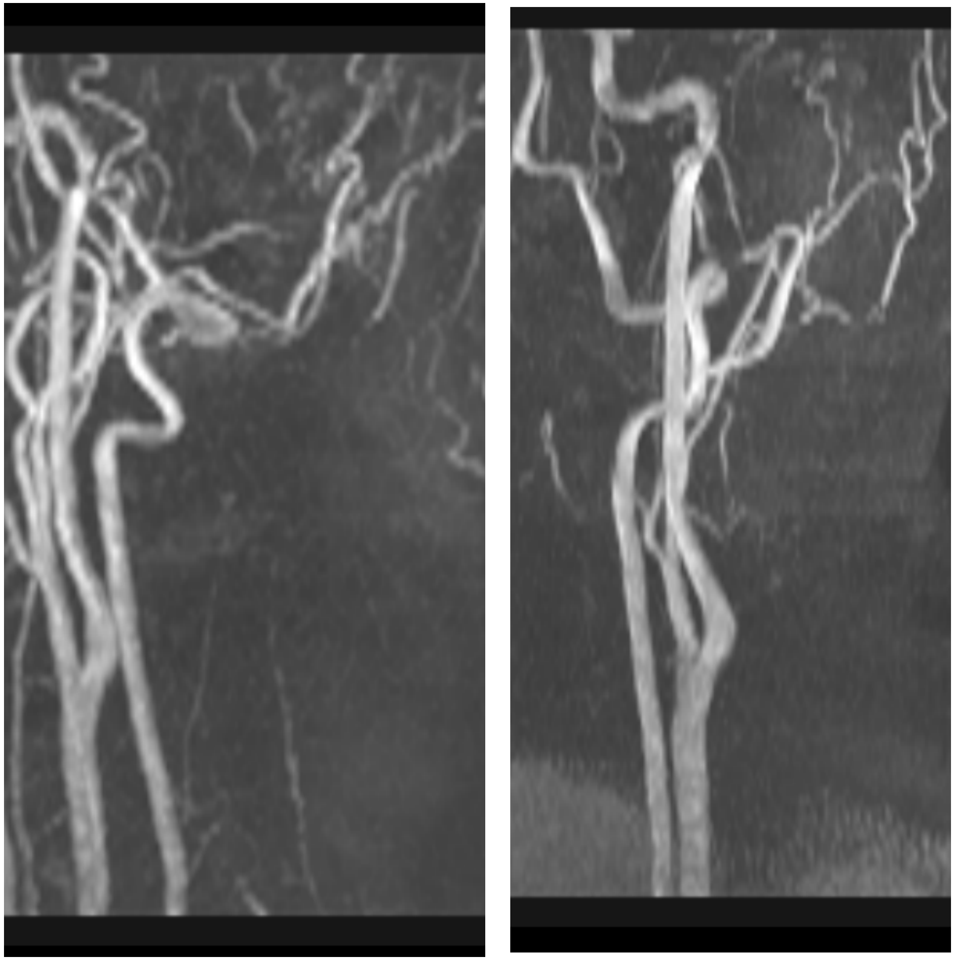

56a Score

0-30

31-50

51-70

>70

Near occlusion

Occluded

Quality

1

2

3

4

5

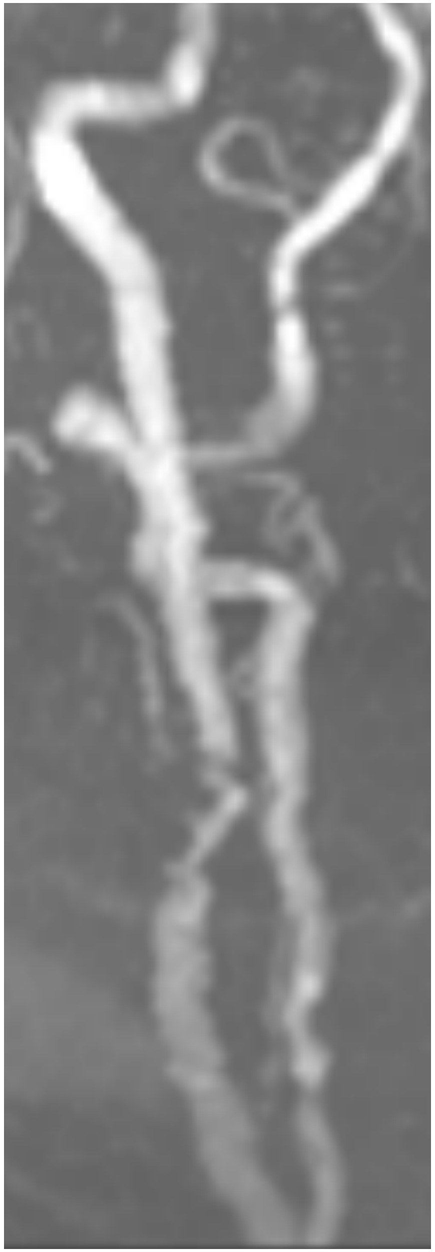

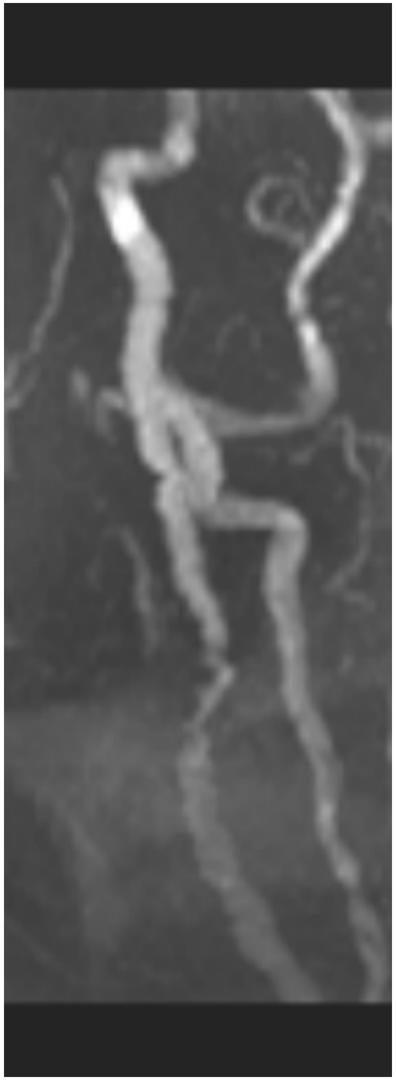

56f Score

0-30

31-50

51-70

>70

Near occlusion

Occluded

Quality

1

2

3

4

5

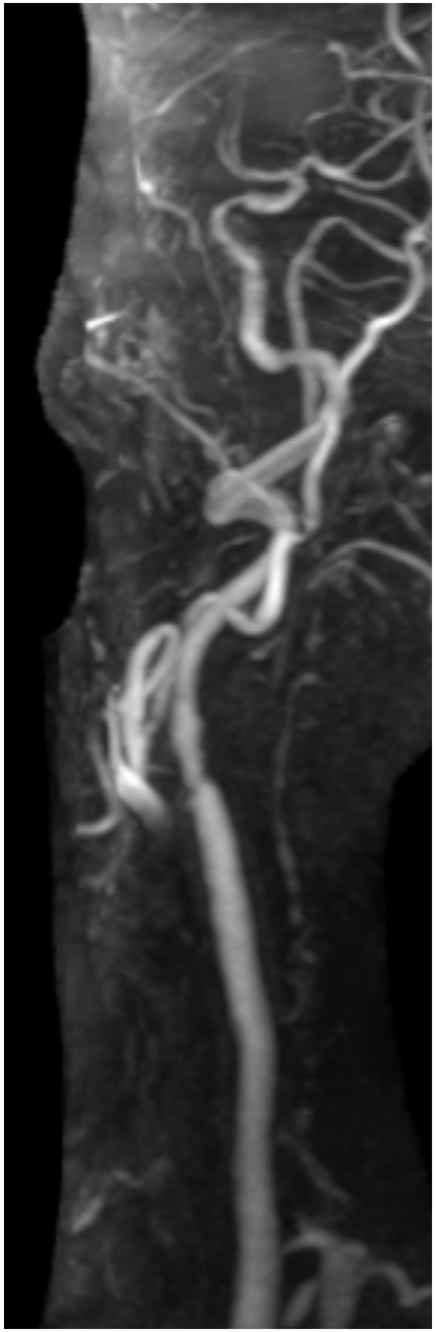

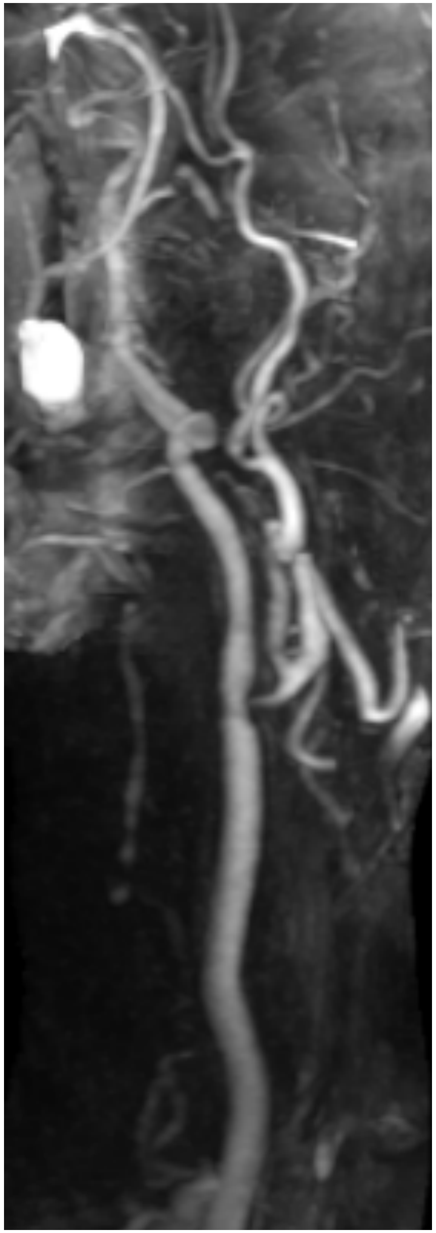

57e Score

0-30

31-50

51-70

>70

Near occlusion

Occluded

Quality

1

2

3

4

5

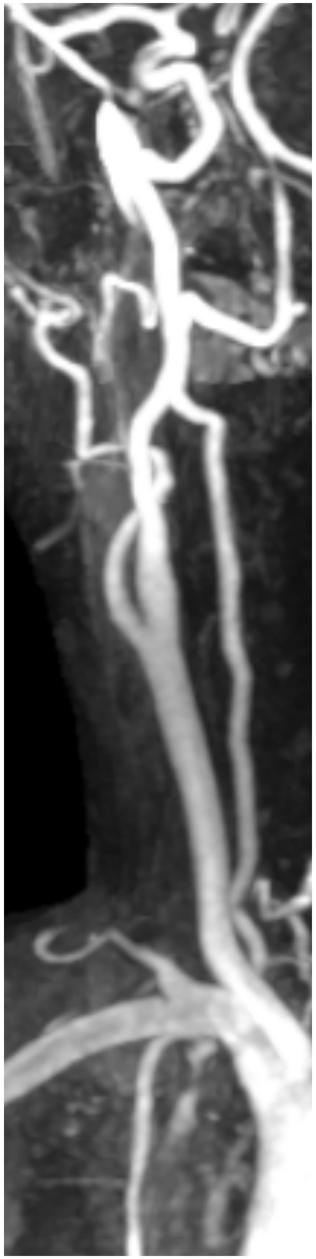

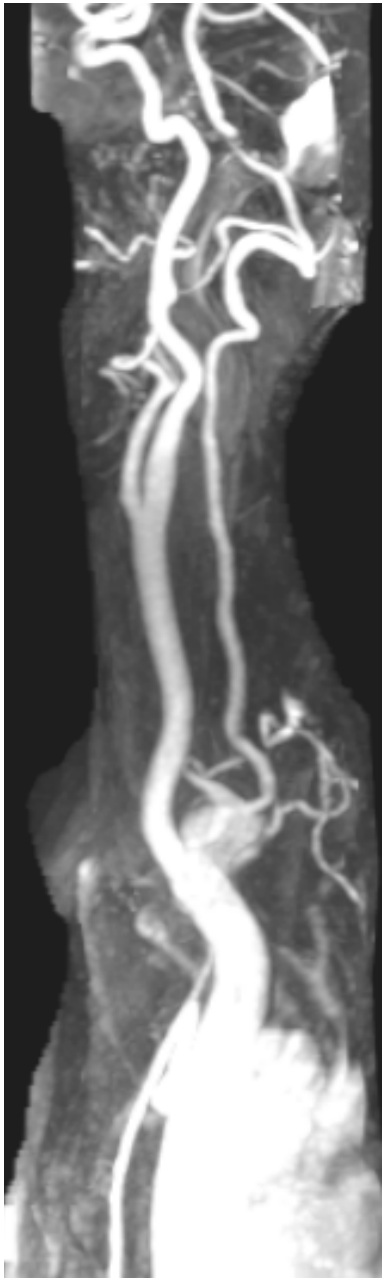

58d Score

0-30

31-50

51-70

>70

Near occlusion

Occluded

Quality

1

2

3

4

5

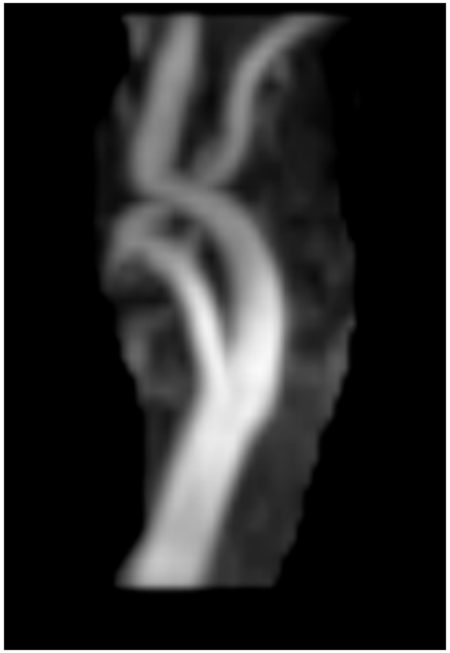

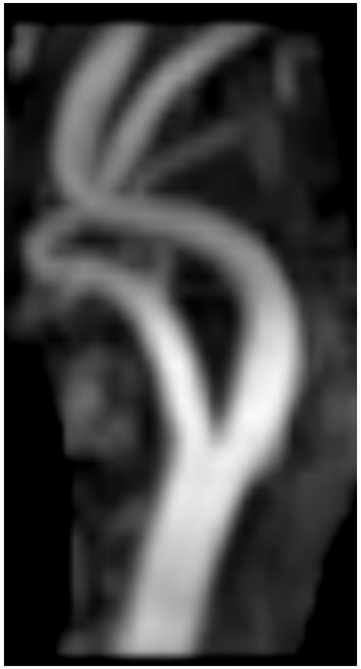

59c Score

0-30

31-50

51-70

>70

Near occlusion

Occluded

Quality

1

2

3

4

5

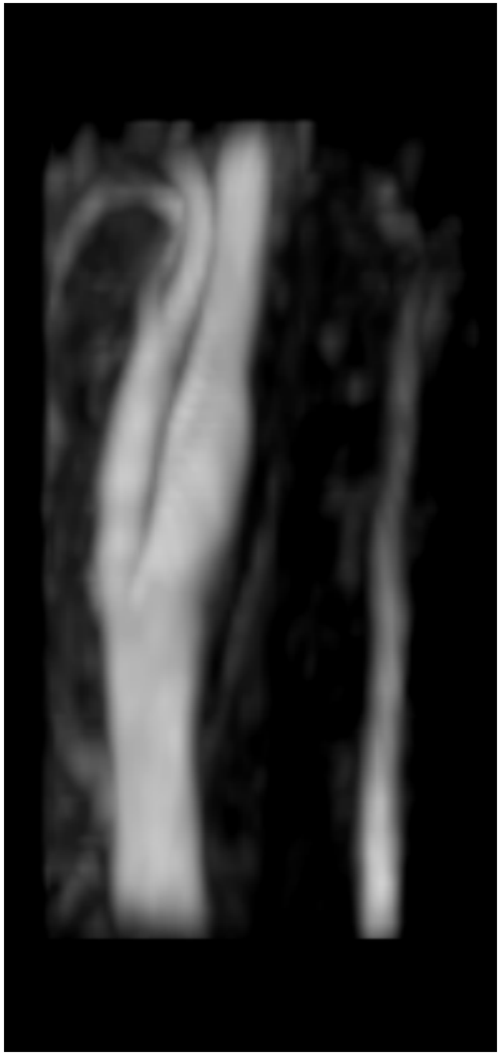

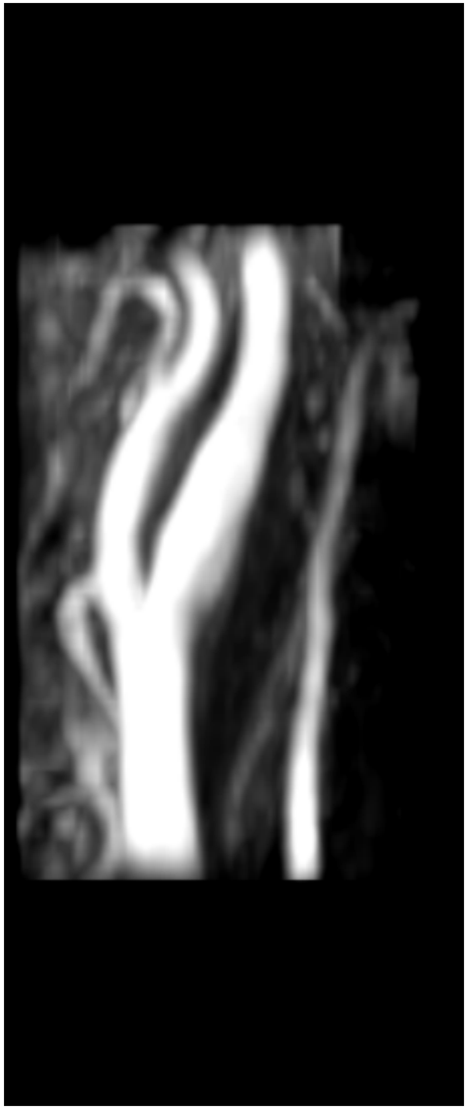

60b Score

0-30

31-50

51-70

>70

Near occlusion

Occluded

Quality

1

2

3

4

5

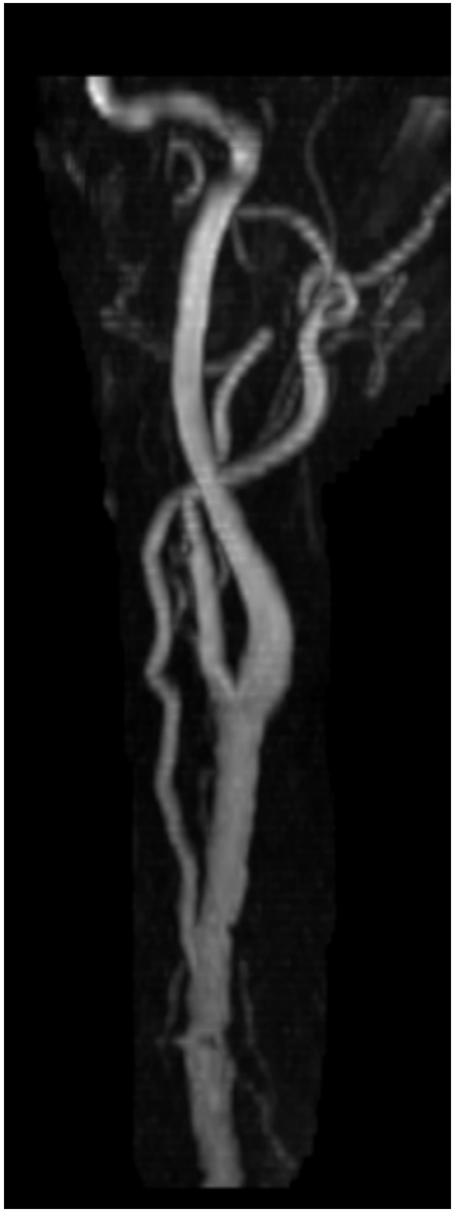

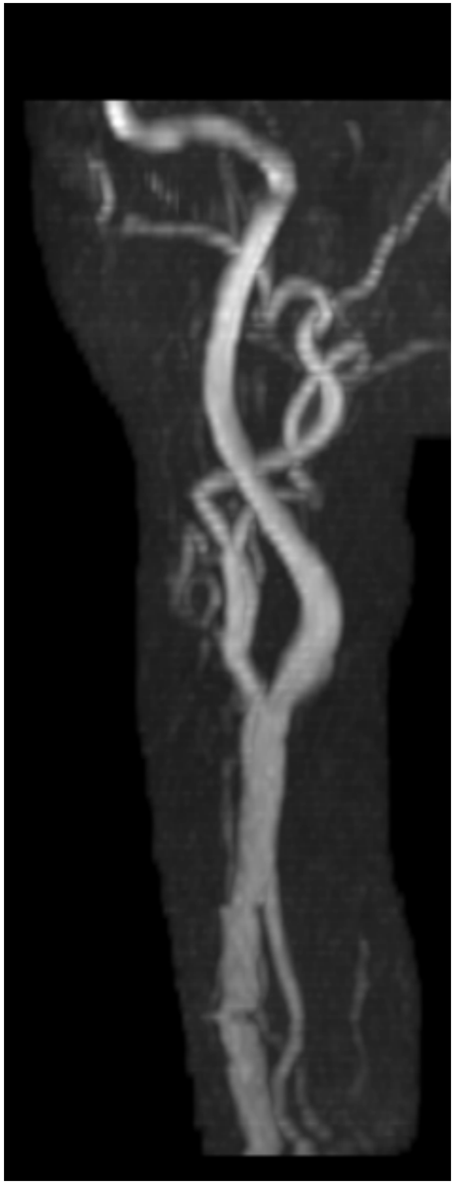

61a Score

0-30

31-50

51-70

>70

Near occlusion

Occluded

Quality

1

2

3

4

5

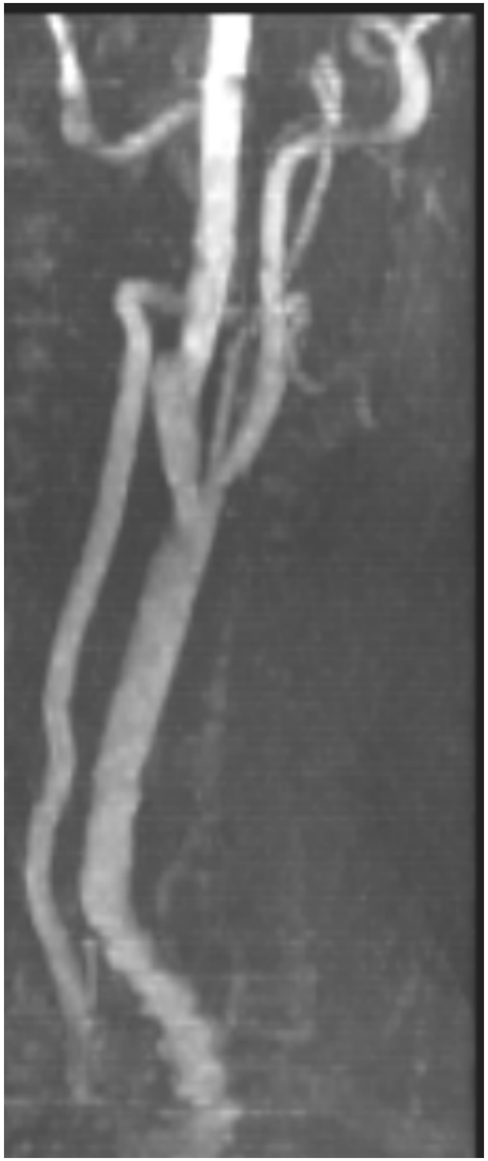

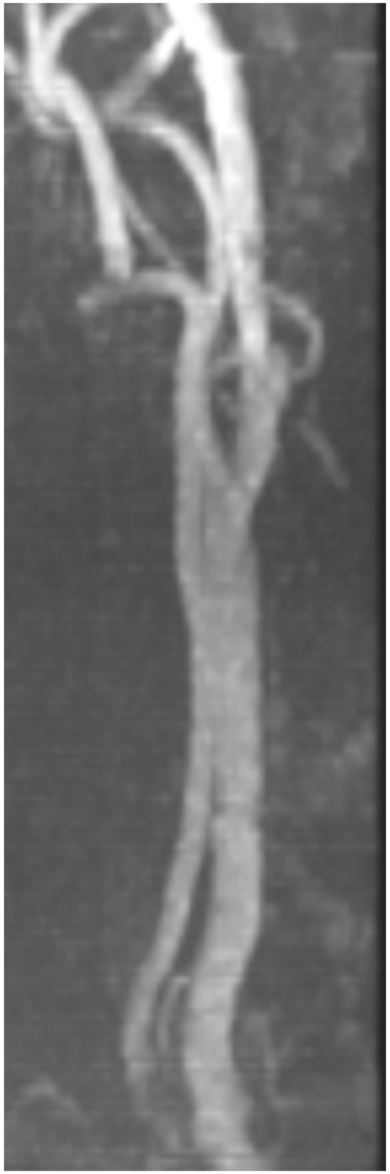

61f Score

0-30

31-50

51-70

>70

Near occlusion

Occluded

Quality

1

2

3

4

5

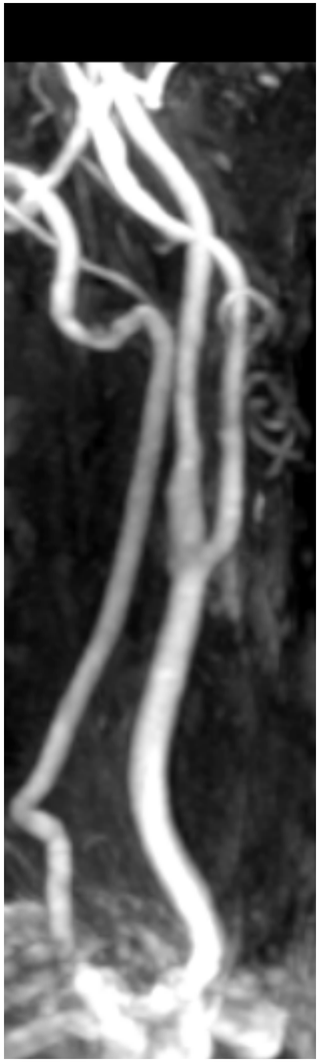

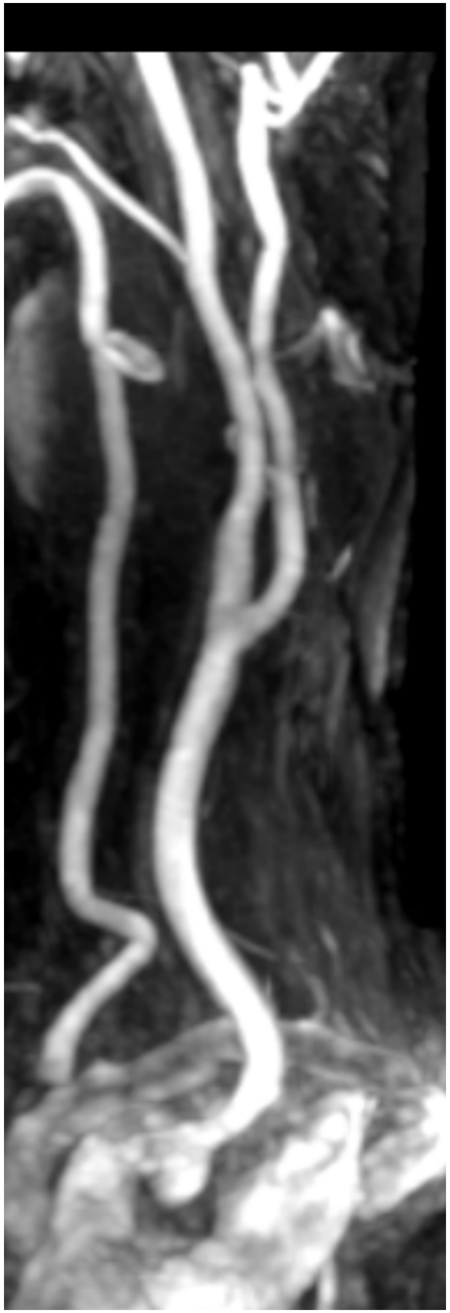

62e Score

0-30

31-50

51-70

>70

Near occlusion

Occluded

Quality

1

2

3

4

5

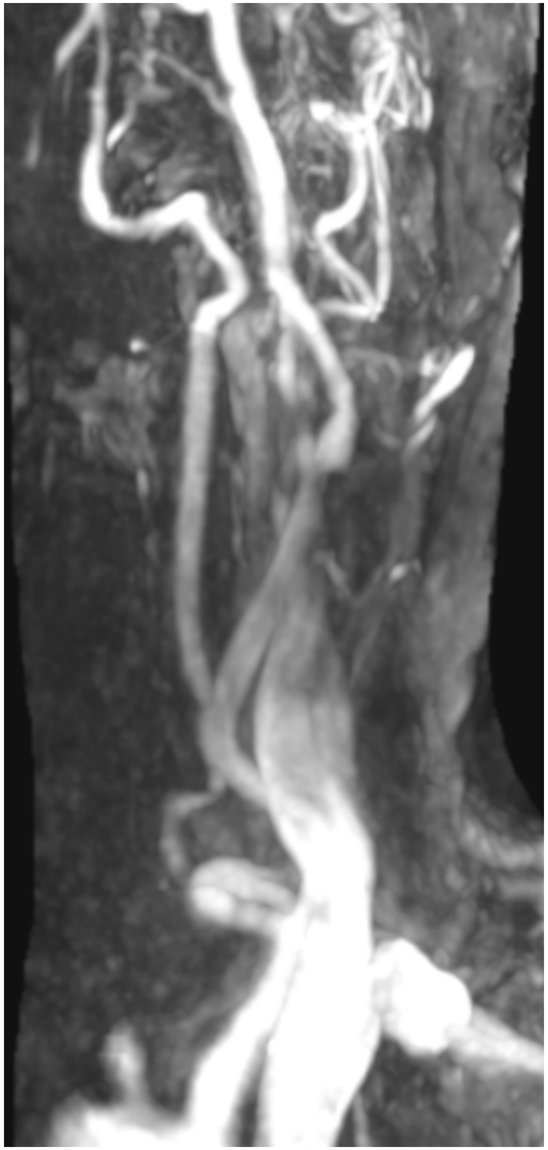

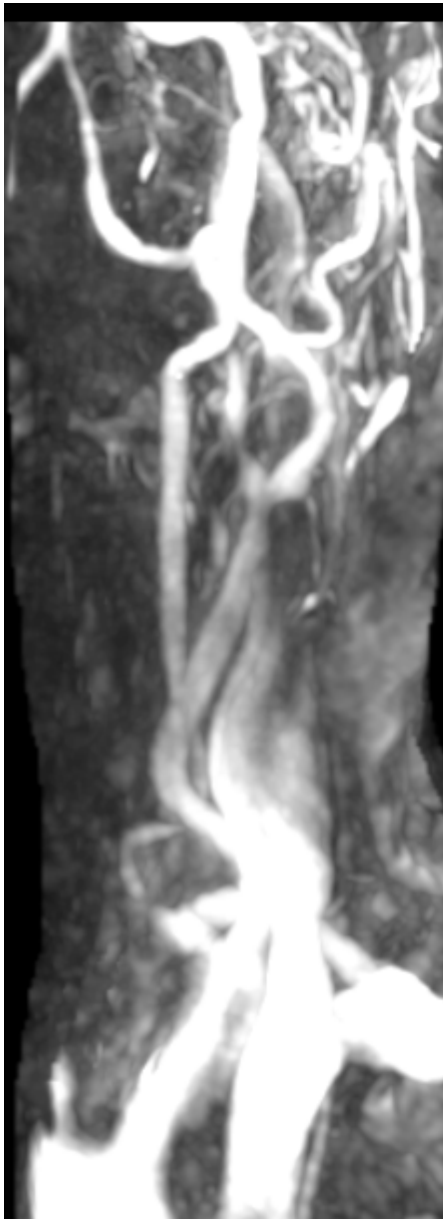

63d Score

0-30

31-50

51-70

>70

Near occlusion

Occluded

Quality

1

2

3

4

5

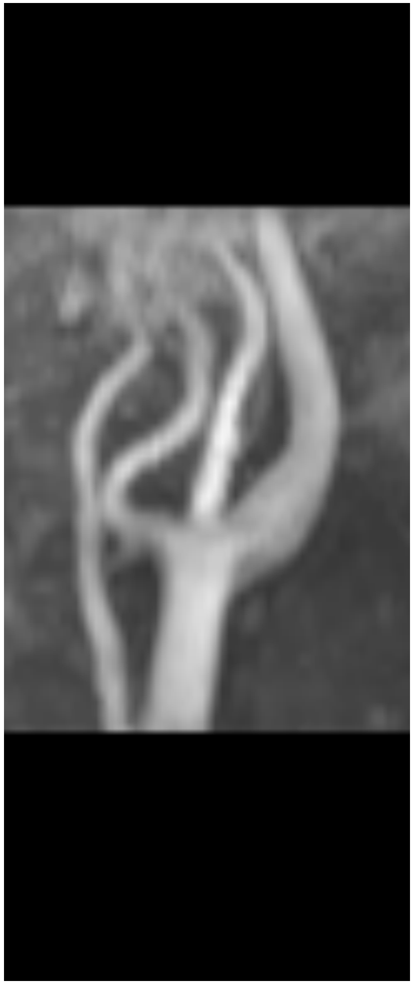

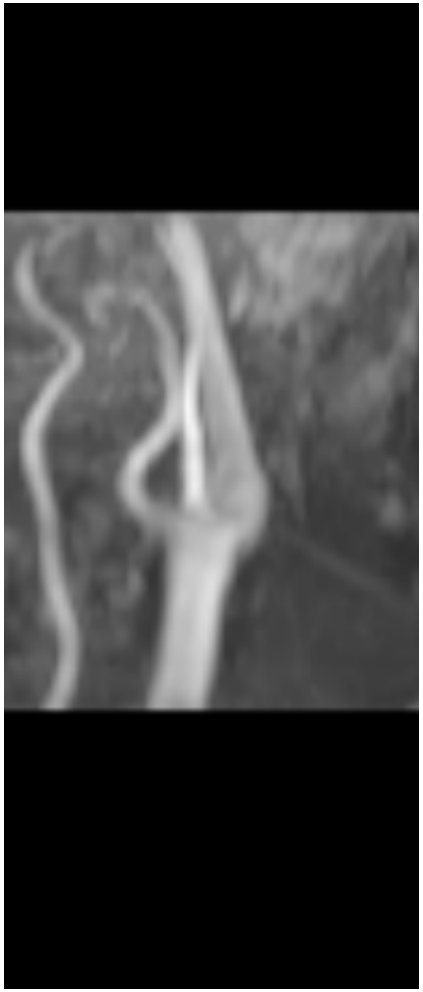

64c Score

0-30

31-50

51-70

>70

Near occlusion

Occluded

Quality

1

2

3

4

5

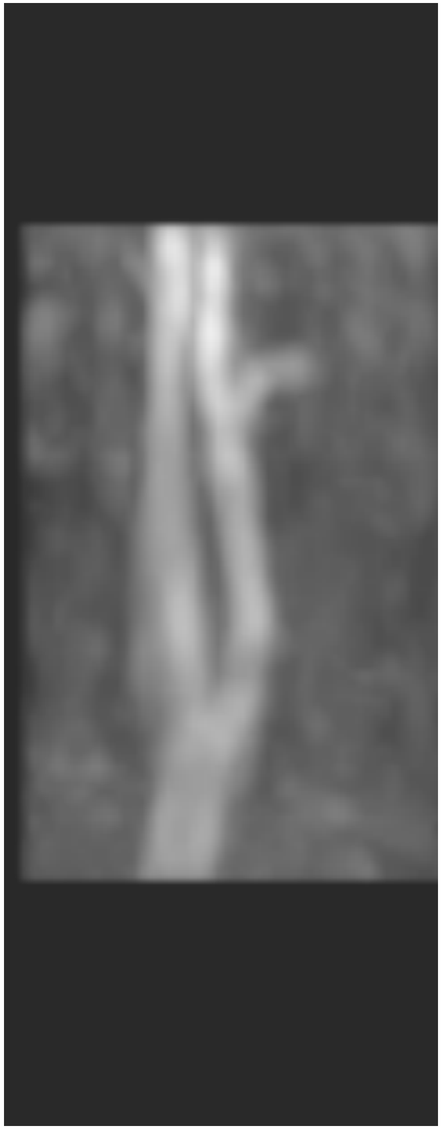

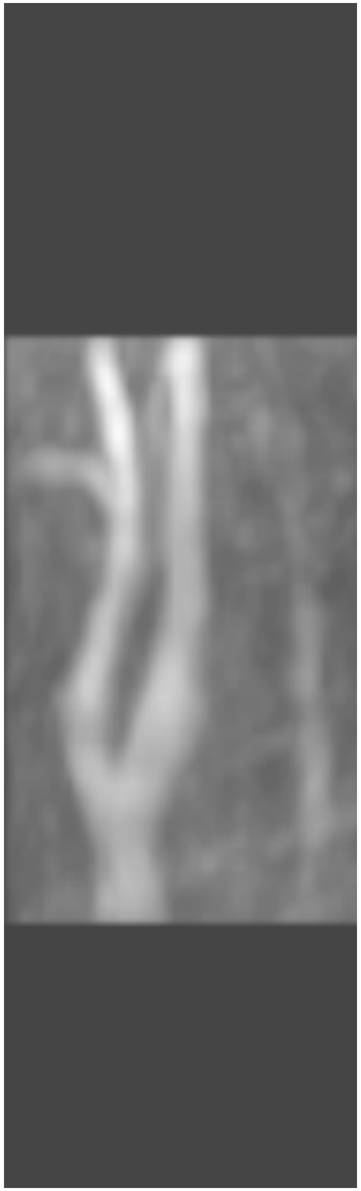

65b Score

0-30

31-50

51-70

>70

Near occlusion

Occluded

Quality

1

2

3

4

5

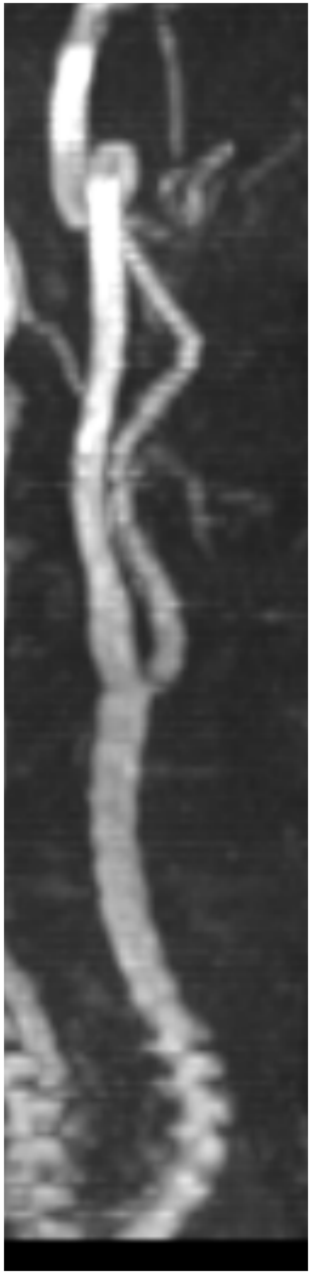

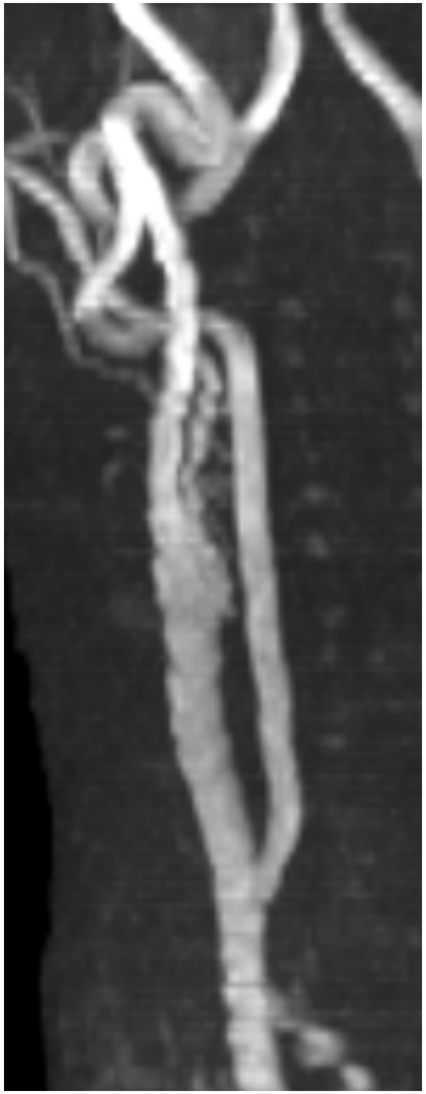

66a Score

0-30

31-50

51-70

>70

Near occlusion

Occluded

Quality

1

2

3

4

5

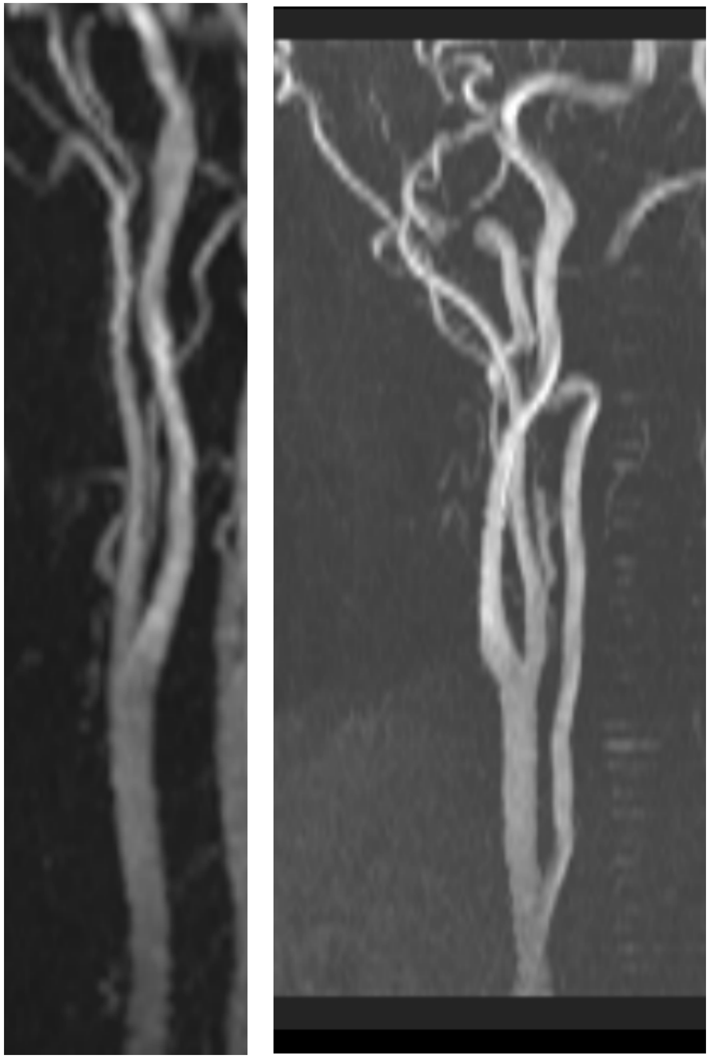

66f Score

0-30

31-50

51-70

>70

Near occlusion

Occluded

Quality

1

2

3

4

5

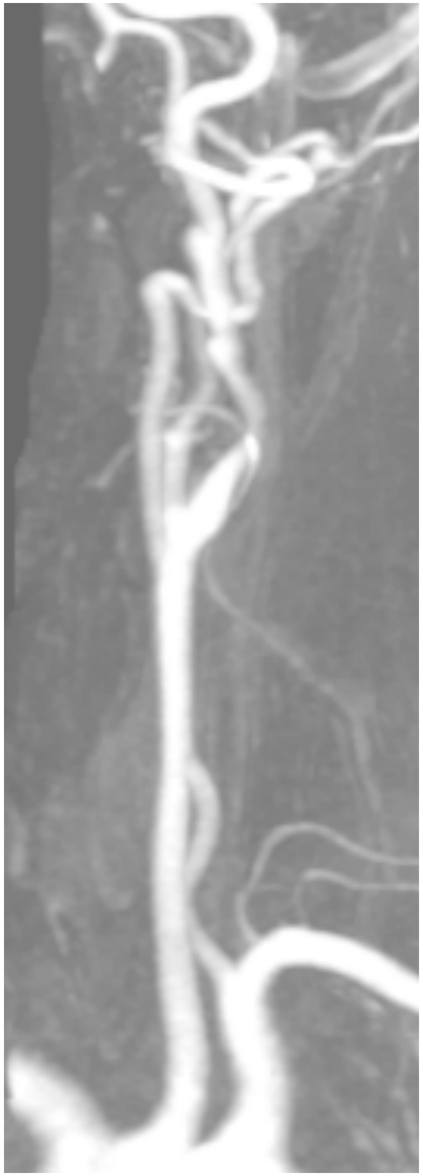

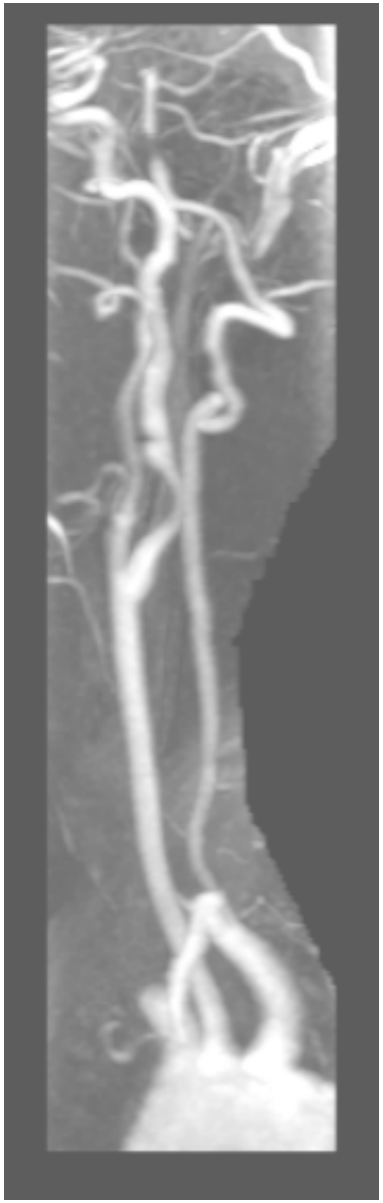

67e Score

0-30

31-50

51-70

>70

Near occlusion

Occluded

Quality

1

2

3

4

5

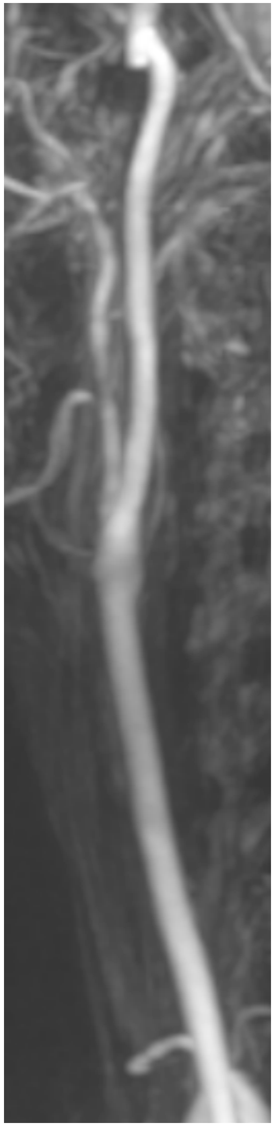

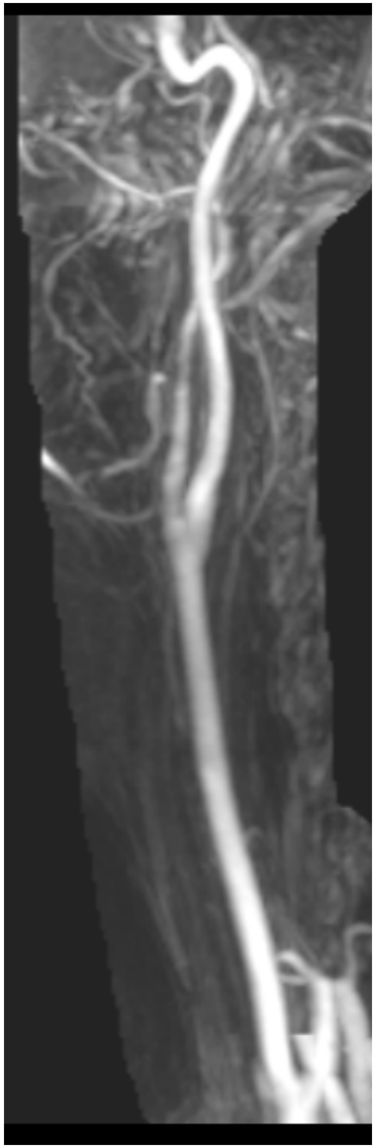

68d Score

0-30

31-50

51-70

>70

Near occlusion

Occluded

Quality

1

2

3

4

5

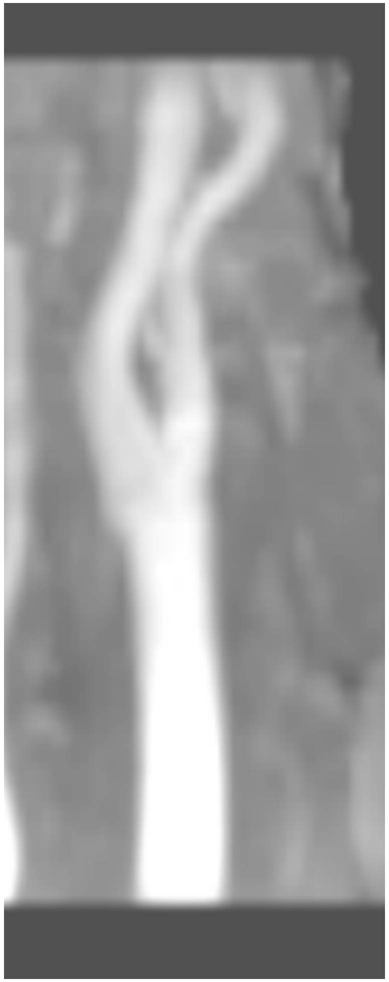

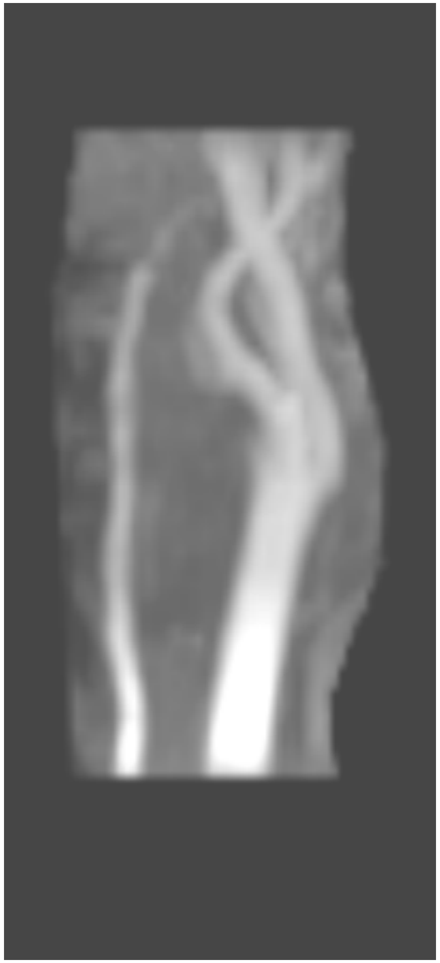

69c Score

0-30

31-50

51-70

>70

Near occlusion

Occluded

Quality

1

2

3

4

5

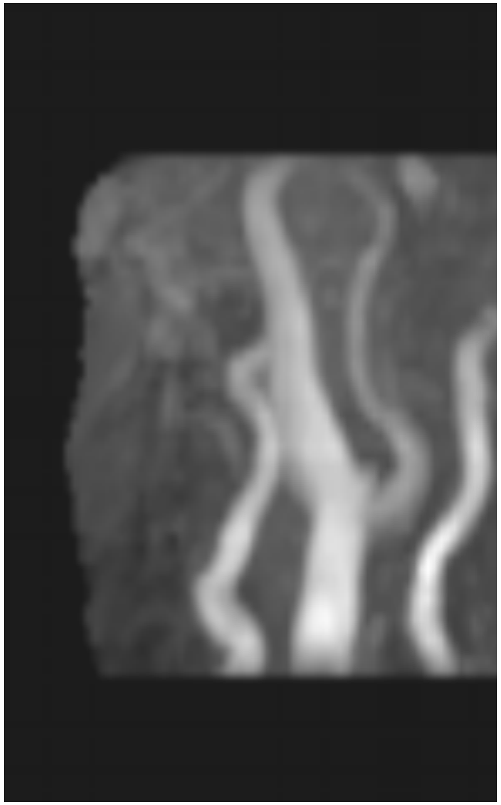

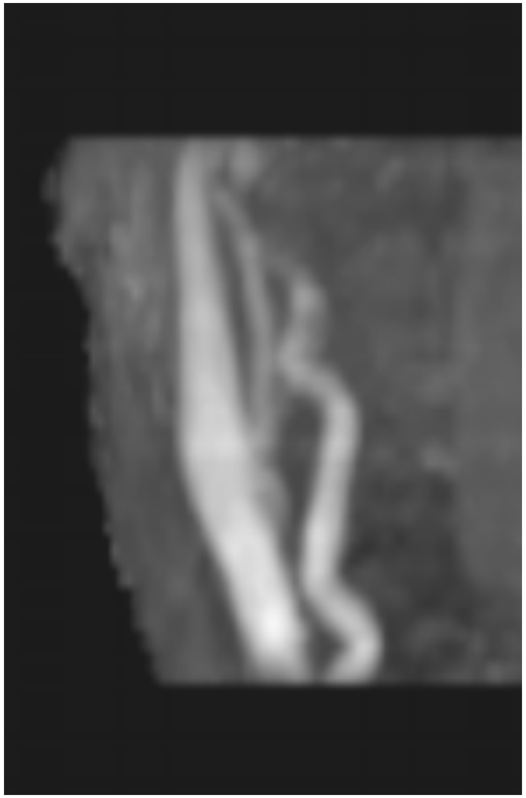

70b Score

0-30

31-50

51-70

>70

Near occlusion

Occluded

Quality

1

2

3

4

5

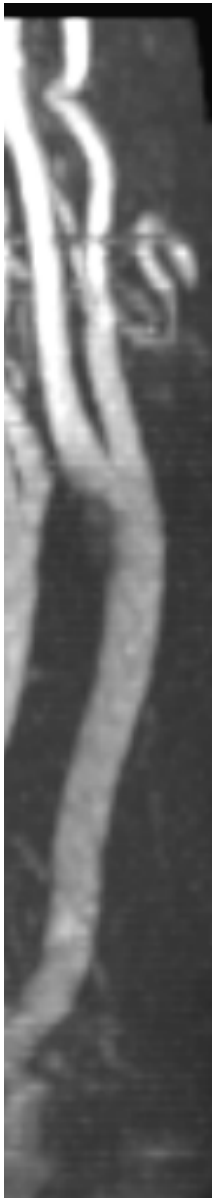

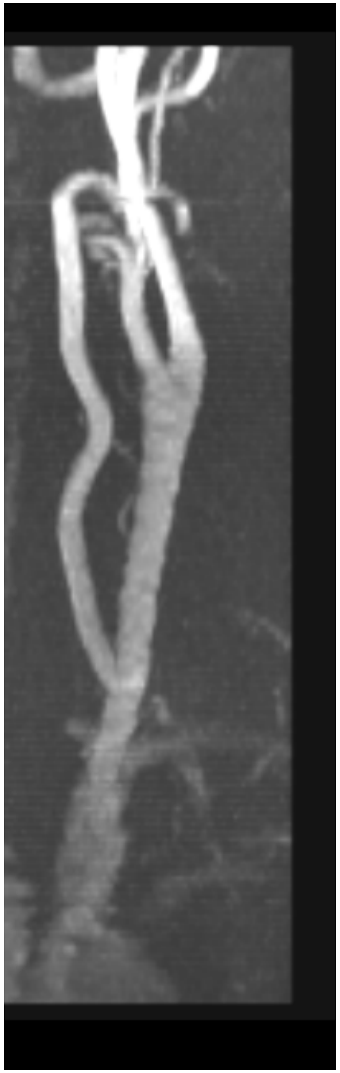

71a Score

0-30

31-50

51-70

>70

Near occlusion

Occluded

Quality

1

2

3

4

5

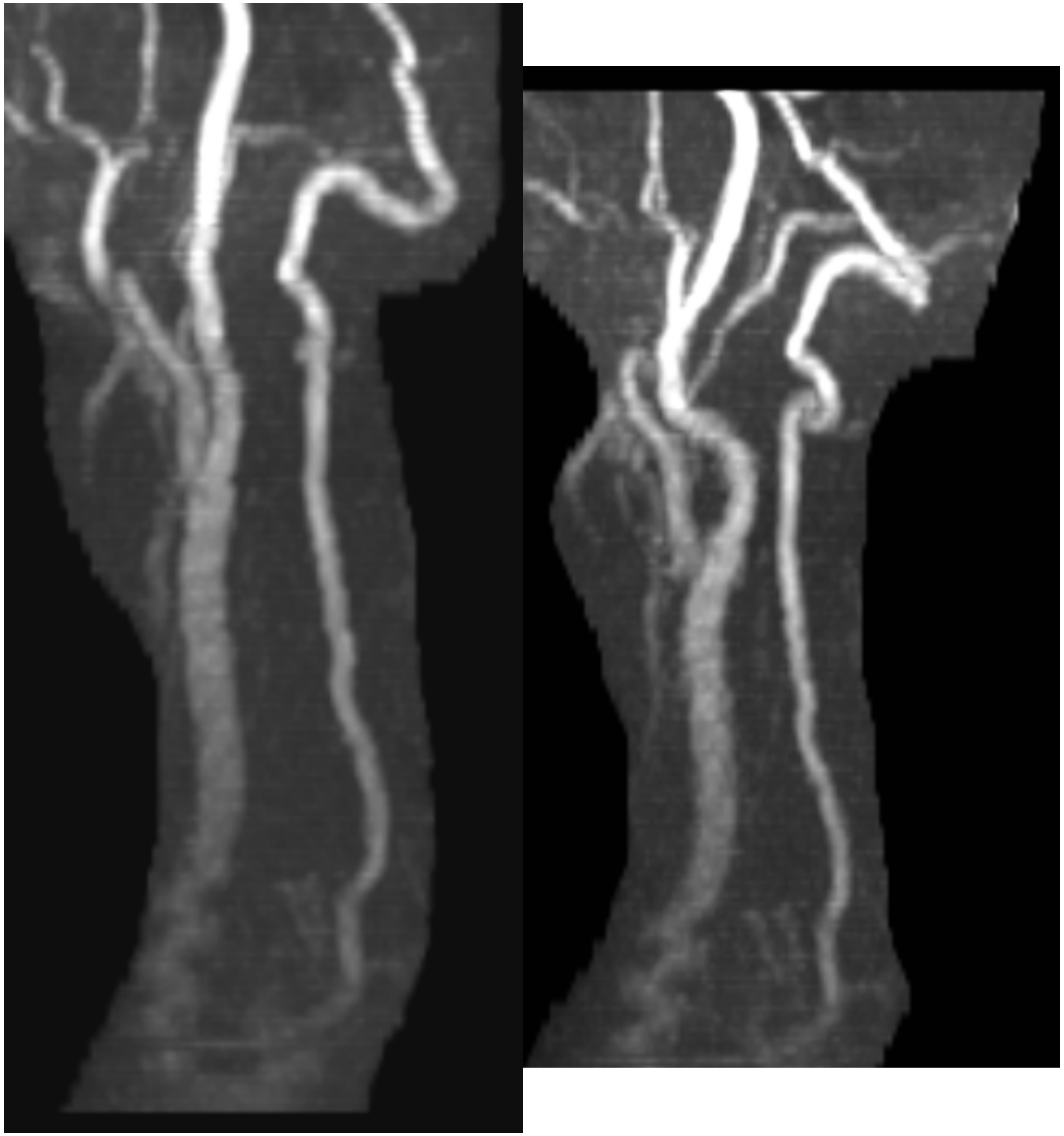

71f Score

0-30

31-50

51-70

>70

Near occlusion

Occluded

Quality

1

2

3

4

5

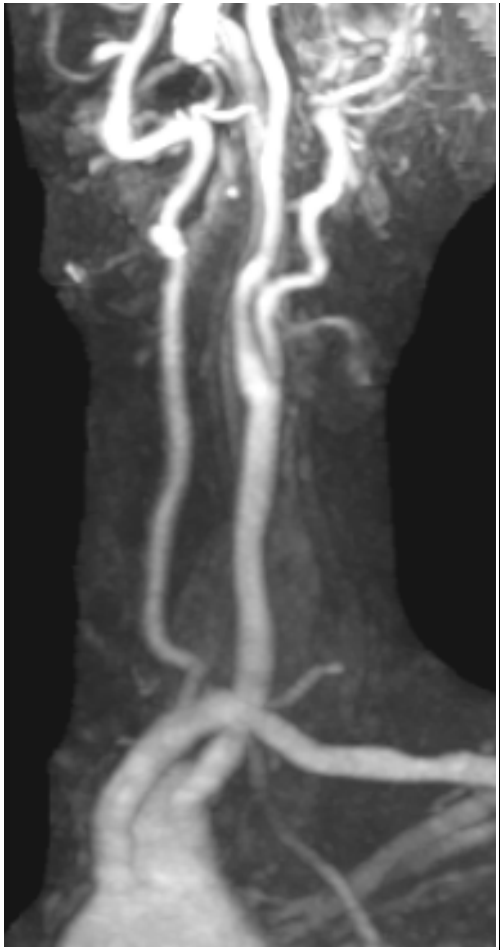

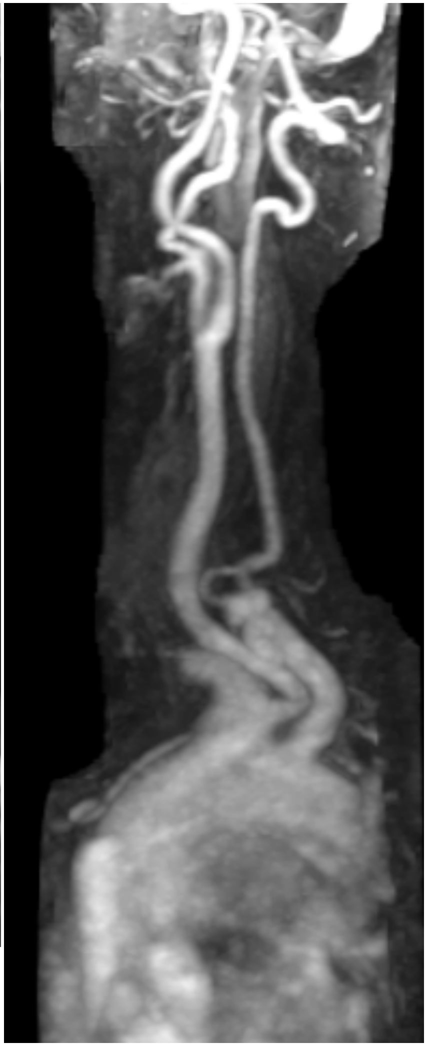

72e Score

0-30

31-50

51-70

>70

Near occlusion

Occluded

Quality

1

2

3

4

5

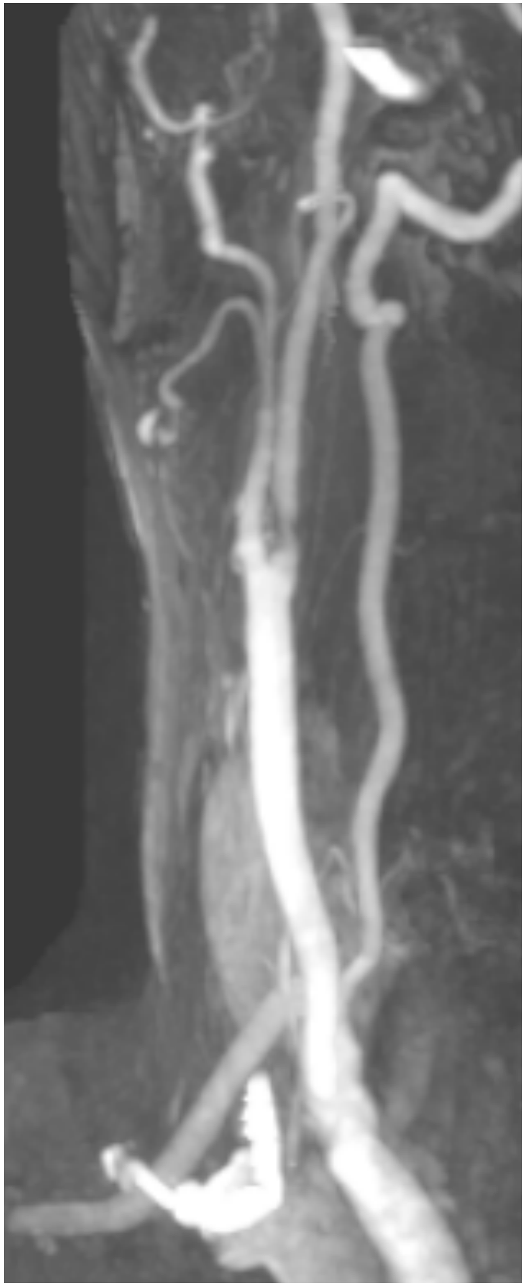

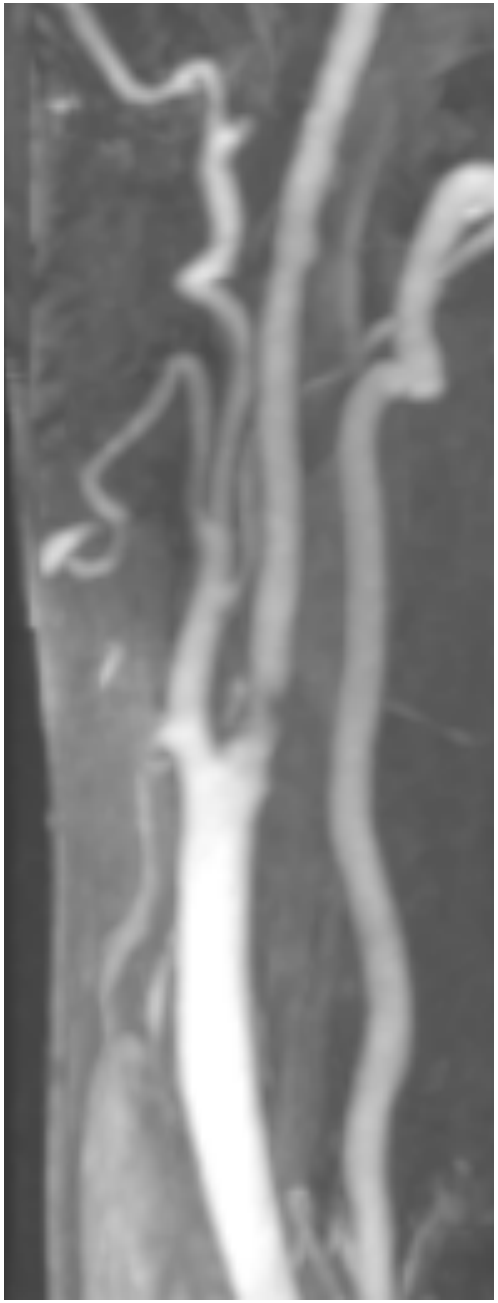

73d Score

0-30

31-50

51-70

>70

Near occlusion

Occluded

Quality

1

2

3

4

5

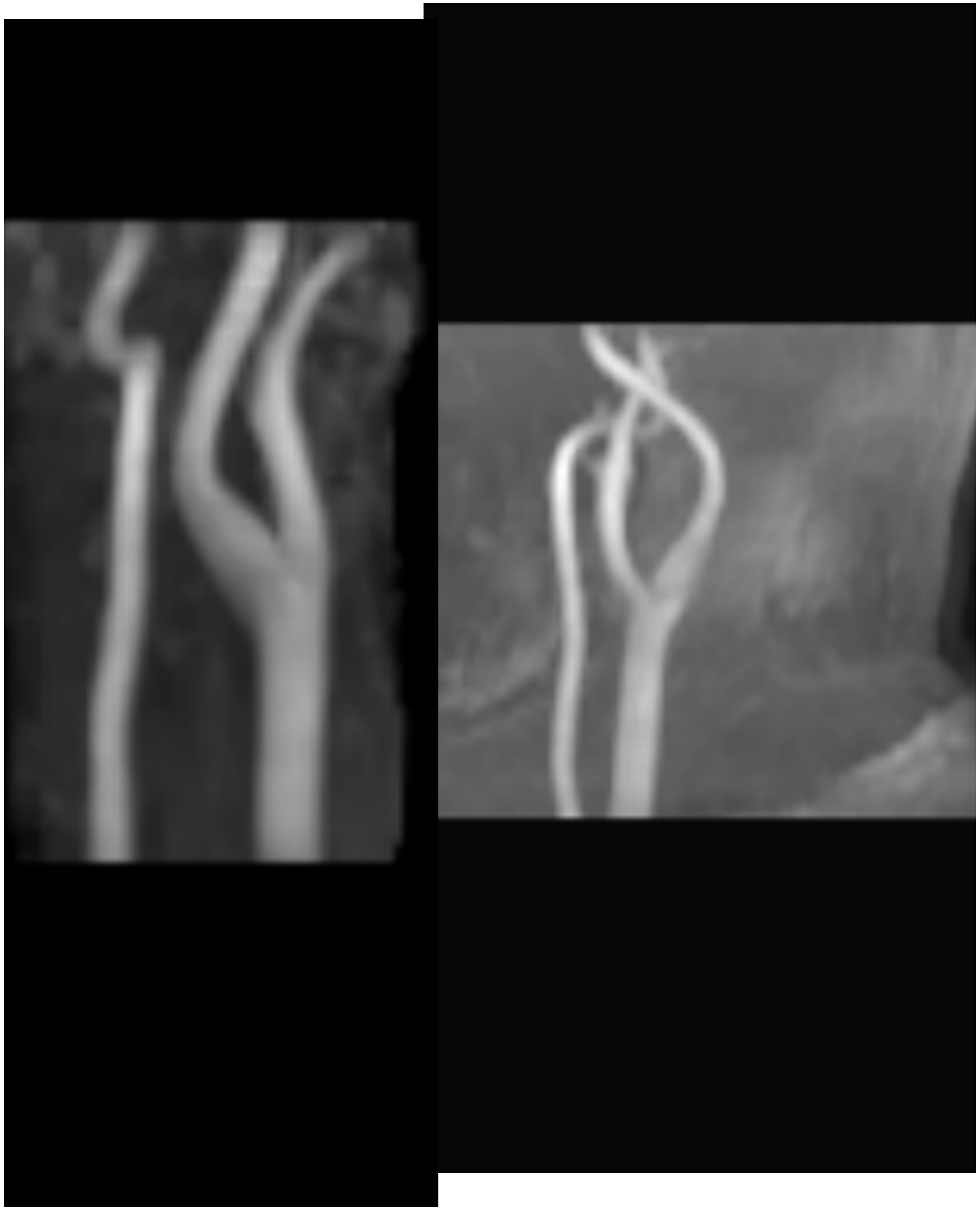

74c Score

0-30

31-50

51-70

>70

Near occlusion

Occluded

Quality

1

2

3

4

5

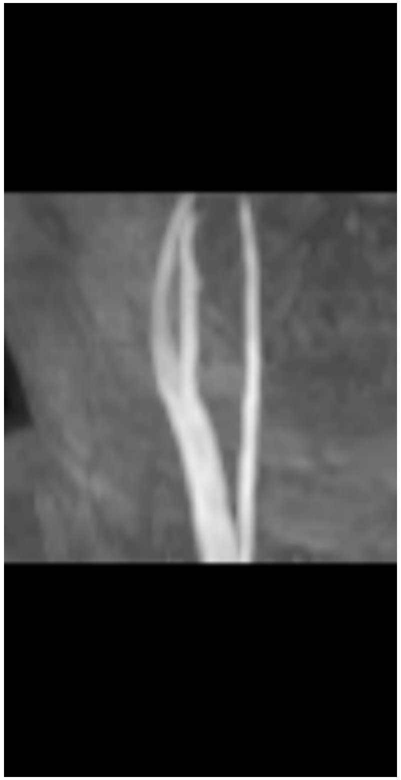

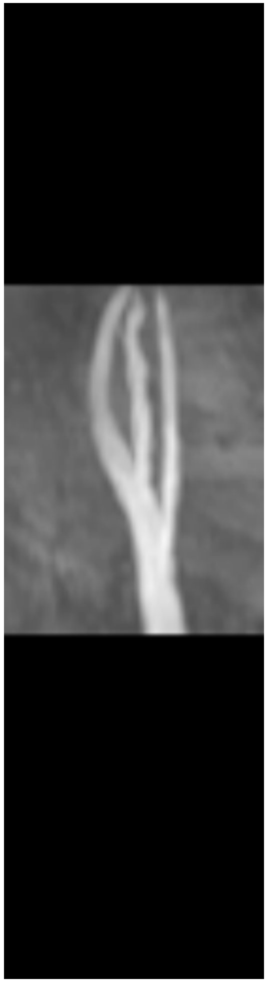

75b Score

0-30

31-50

51-70

>70

Near occlusion

Occluded

Quality

1

2

3

4

5

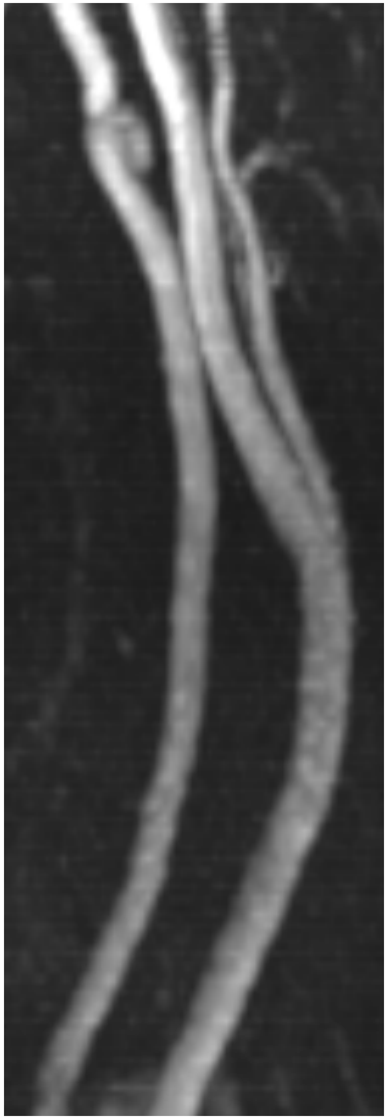

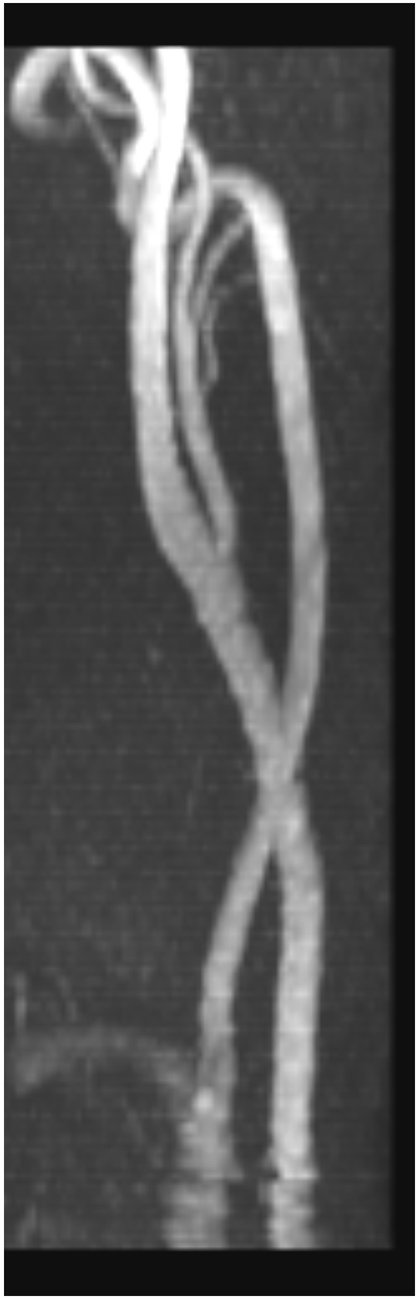

76a Score

0-30

31-50

51-70

>70

Near occlusion

Occluded

Quality

1

2

3

4

5

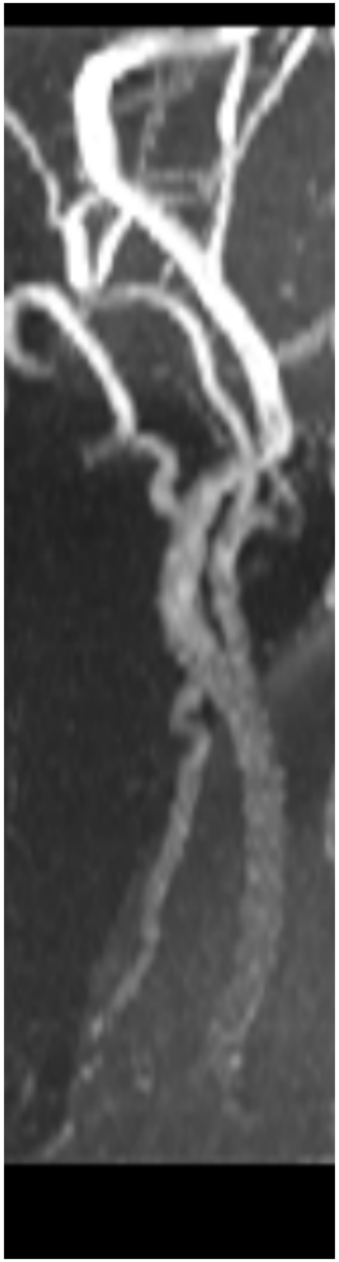

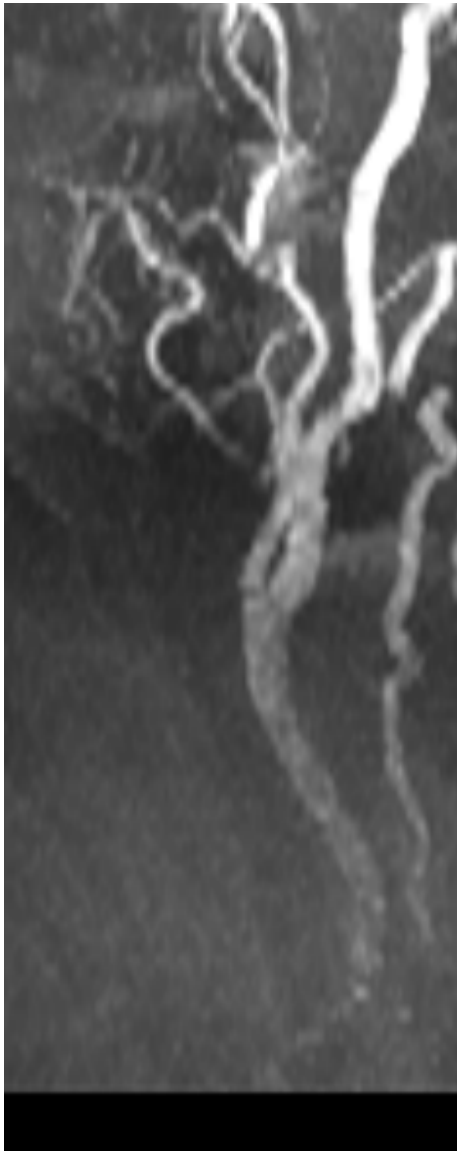

76f Score

0-30

31-50

51-70

>70

Near occlusion

Occluded

Quality

1

2

3

4

5

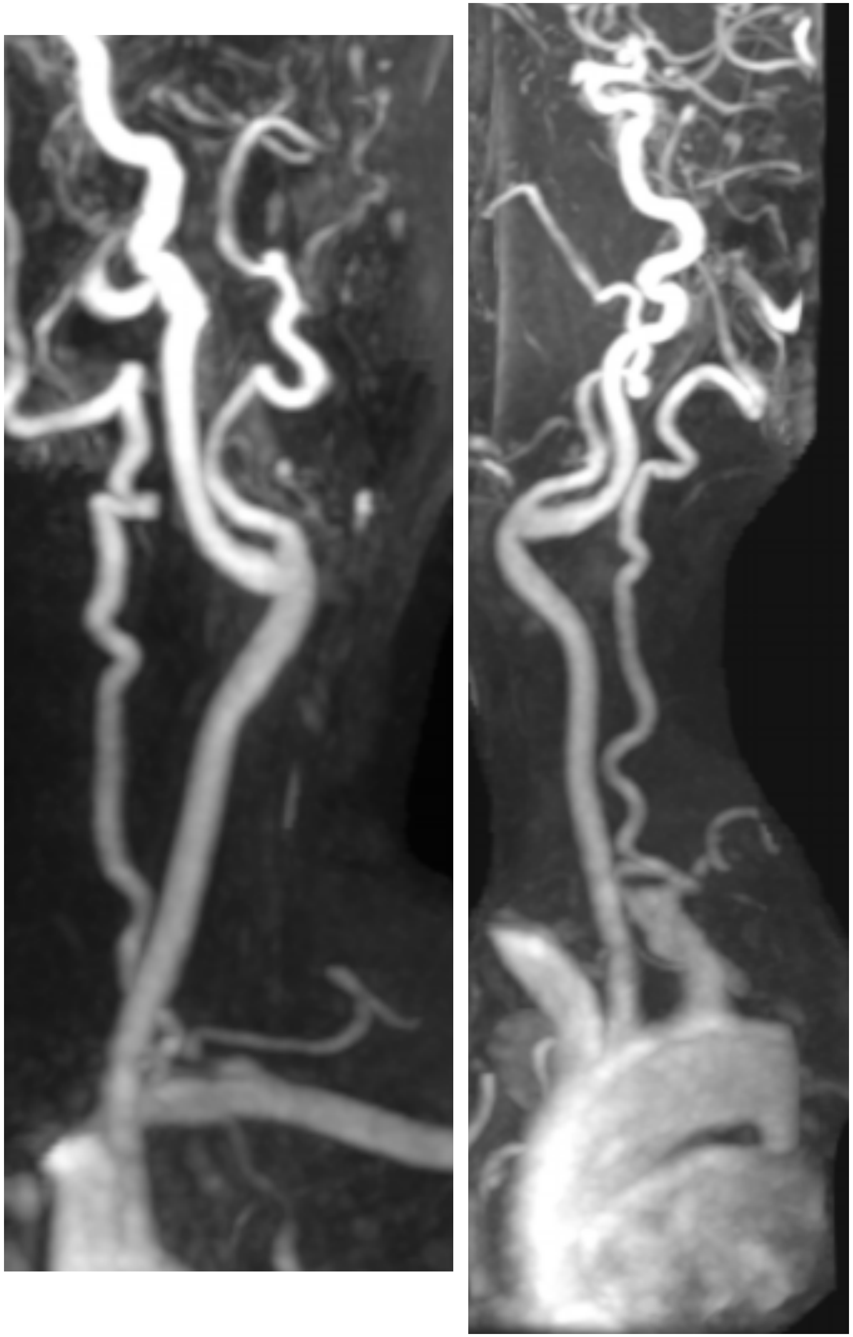

77e Score

0-30

31-50

51-70

>70

Near occlusion

Occluded

Quality

1

2

3

4

5

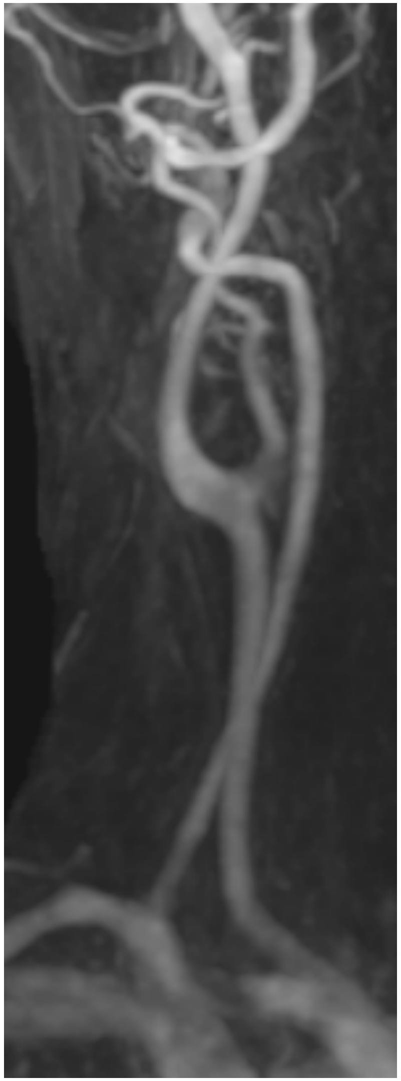

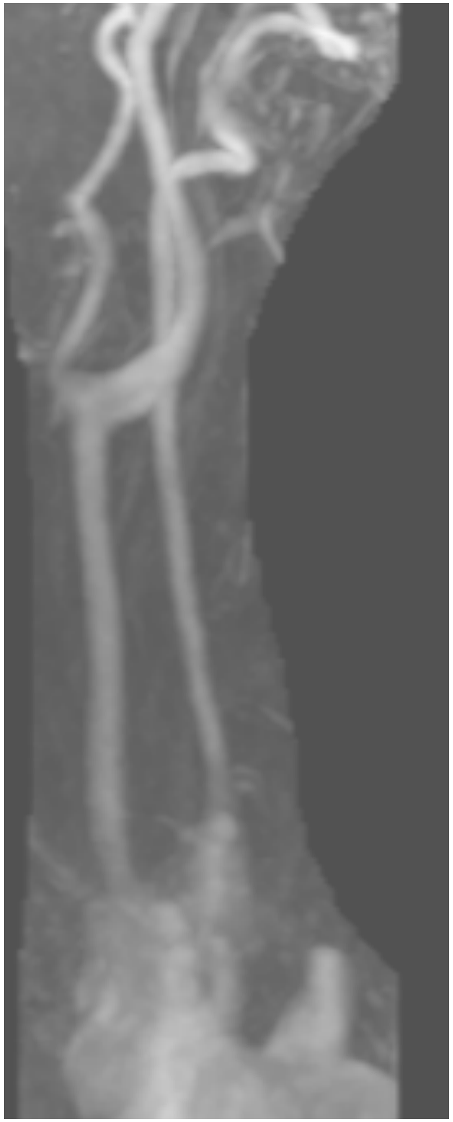

78d Score

0-30

31-50

51-70

>70

Near occlusion

Occluded

Quality

1

2

3

4

5

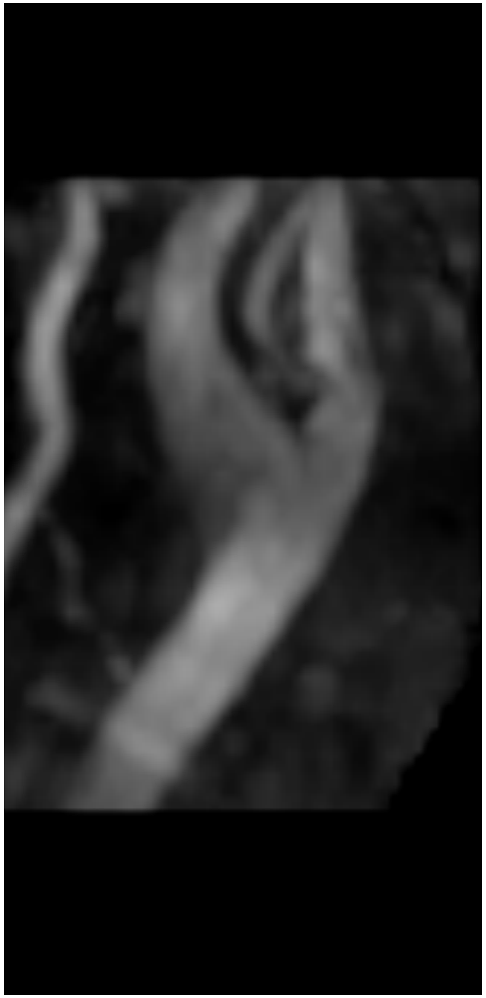

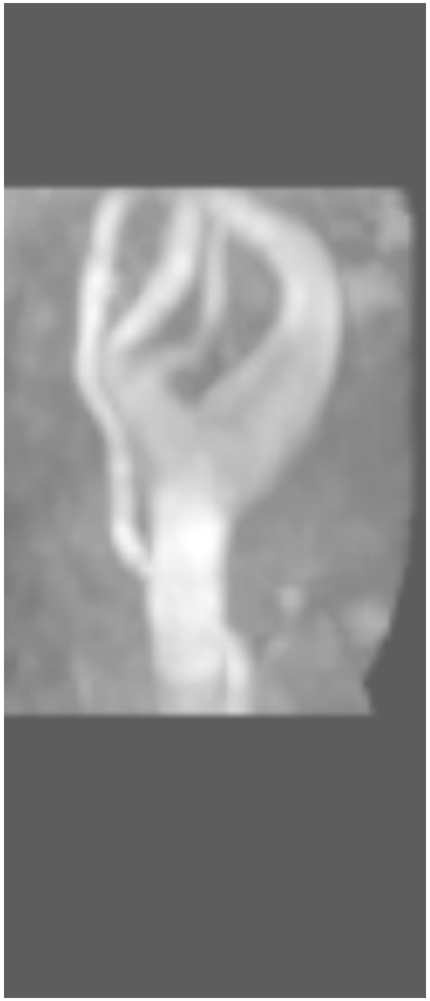

79c Score

0-30

31-50

51-70

>70

Near occlusion

Occluded

Quality

1

2

3

4

5

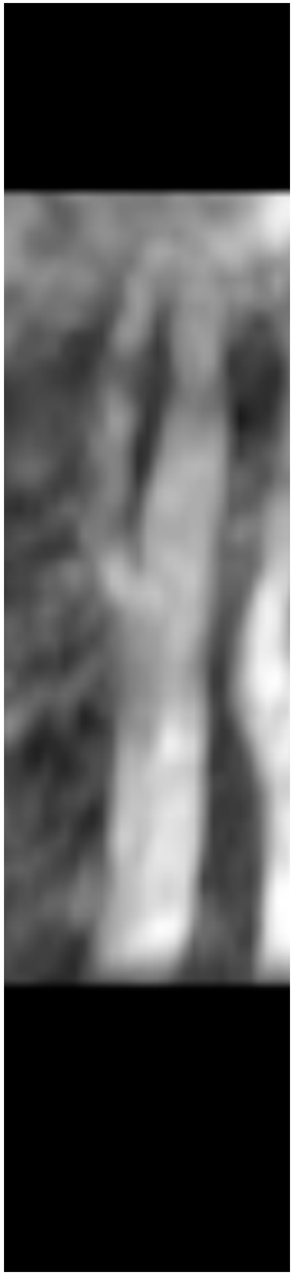

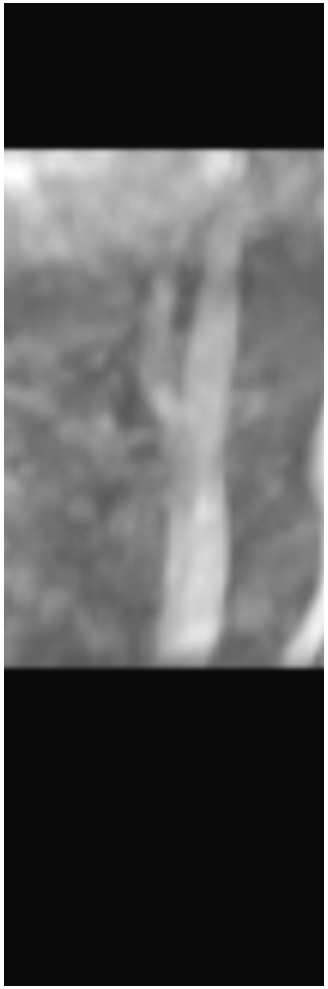

80b Score

0-30

31-50

51-70

>70

Near occlusion

Occluded

Quality

1

2

3

4

5

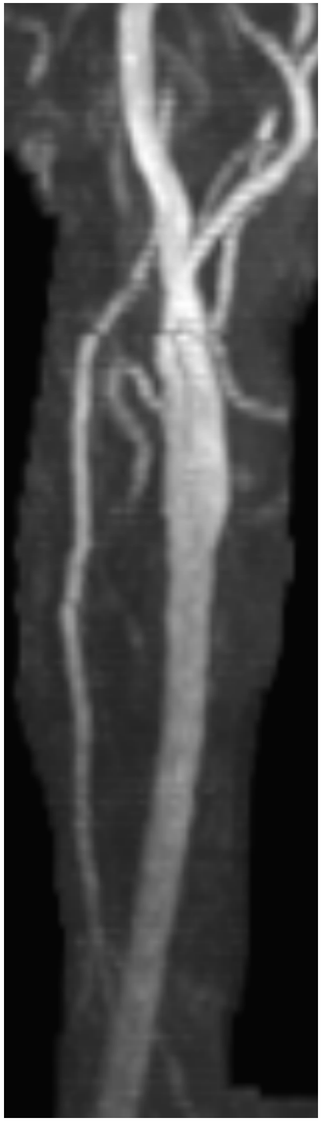

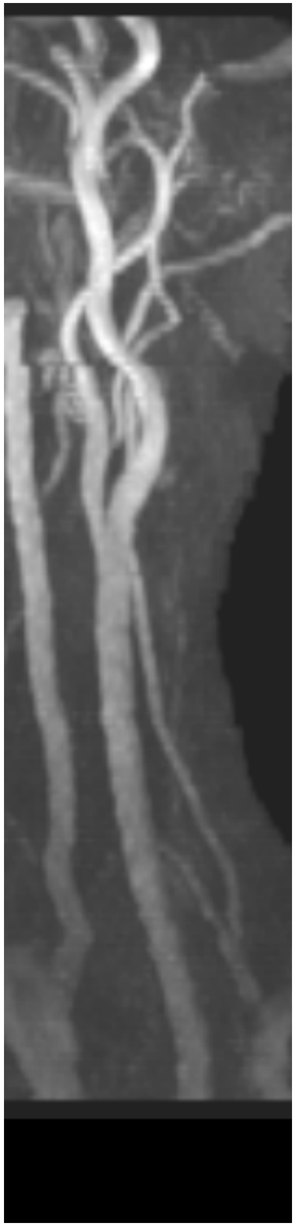

81a Score

0-30

31-50

51-70

>70

Near occlusion

Occluded

Quality

1

2

3

4

5

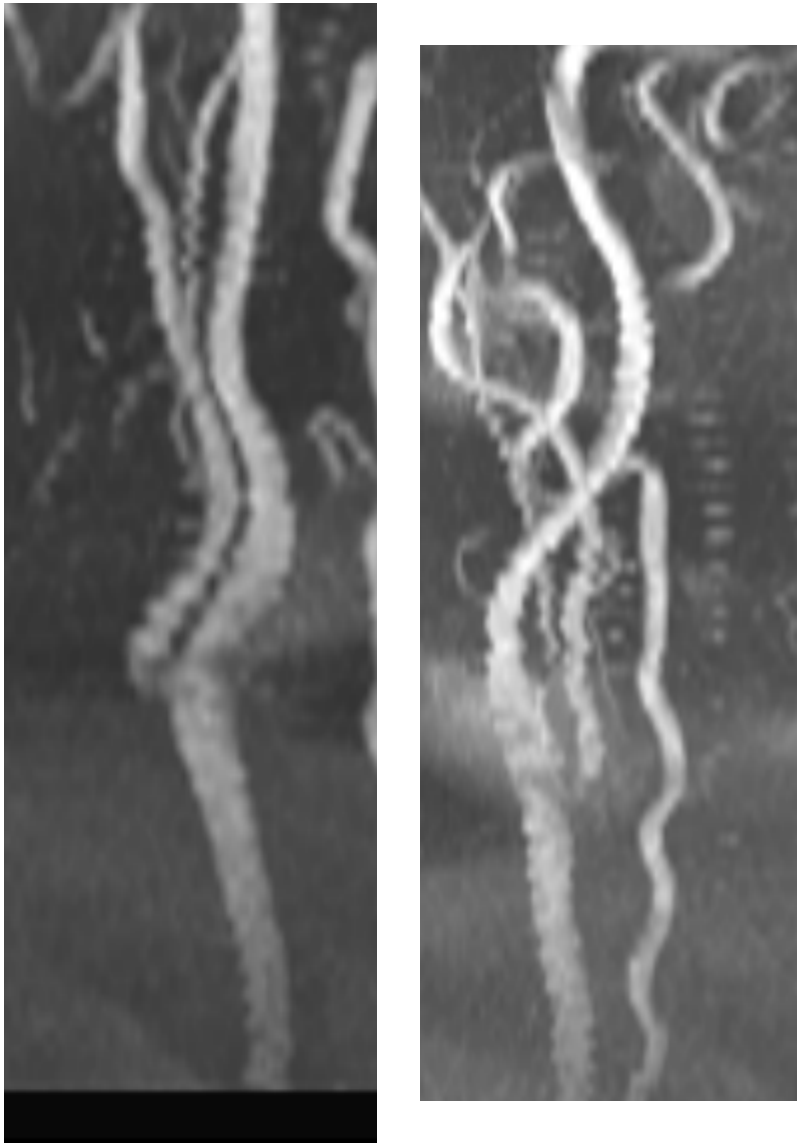

81f Score

0-30

31-50

51-70

>70

Near occlusion

Occluded

Quality

1

2

3

4

5

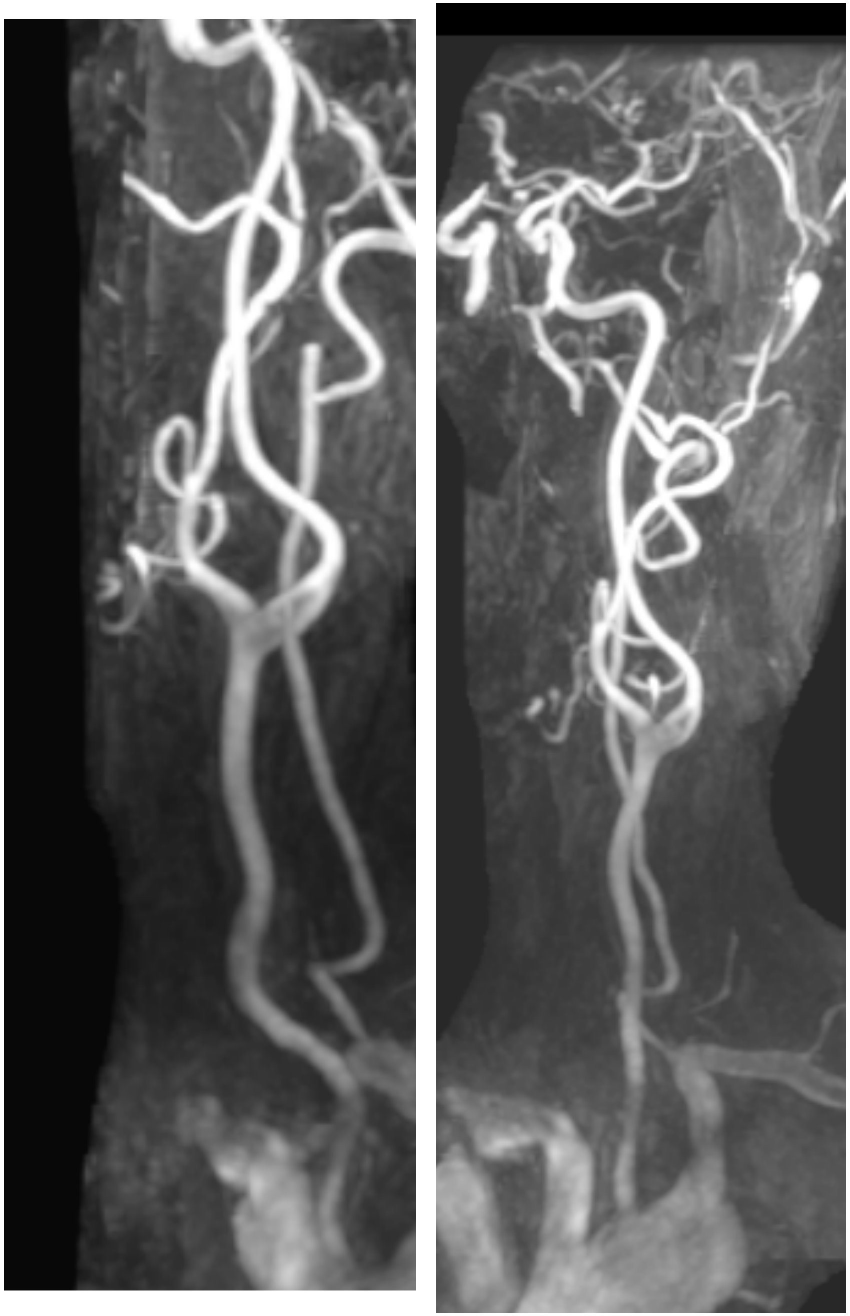

82e Score

0-30

31-50

51-70

>70

Near occlusion

Occluded

Quality

1

2

3

4

5

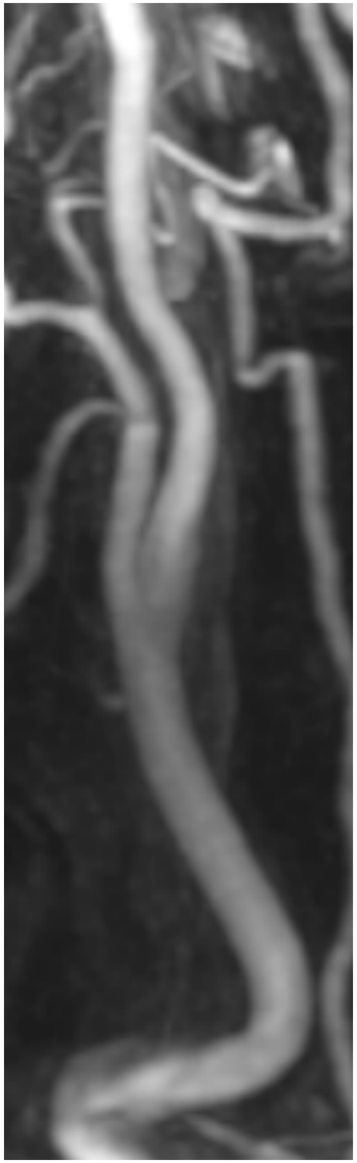

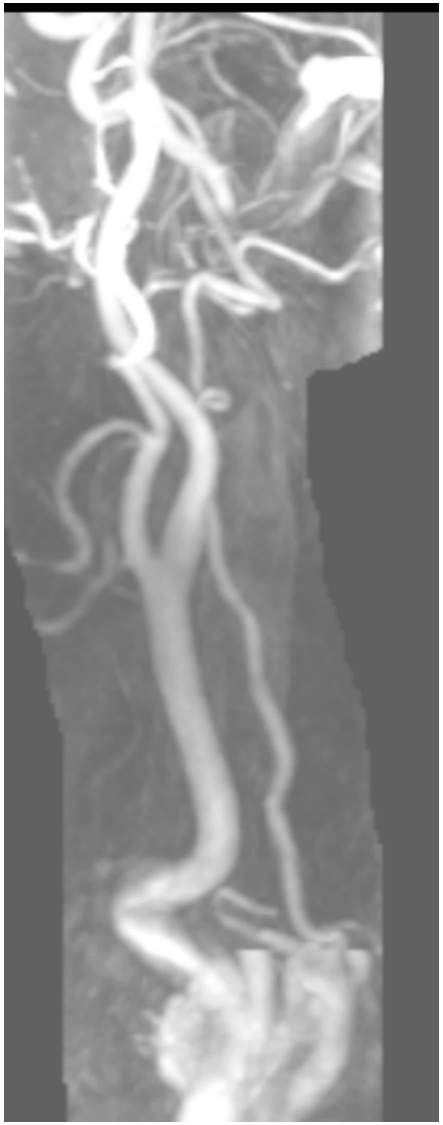

83d Score

0-30

31-50

51-70

>70

Near occlusion

Occluded

Quality

1

2

3

4

5

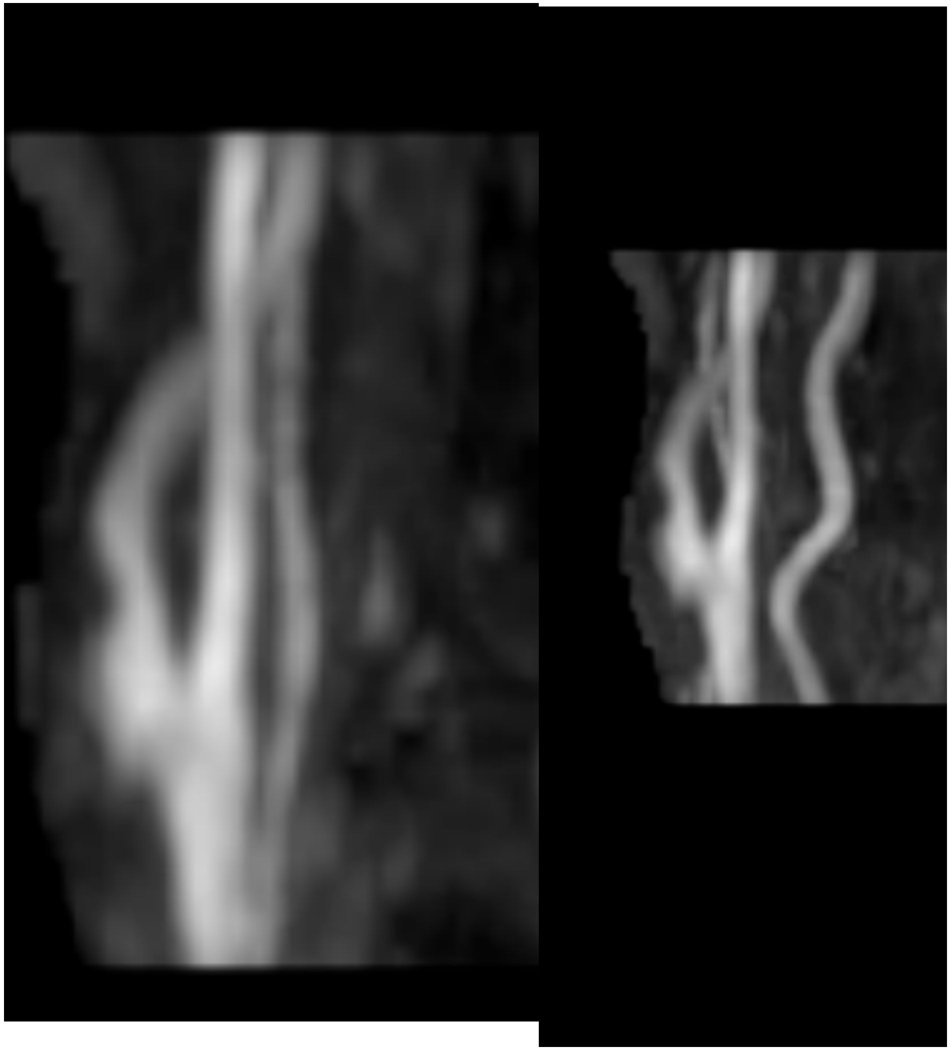

84c Score

0-30

31-50

51-70

>70

Near occlusion

Occluded

Quality

1

2

3

4

5

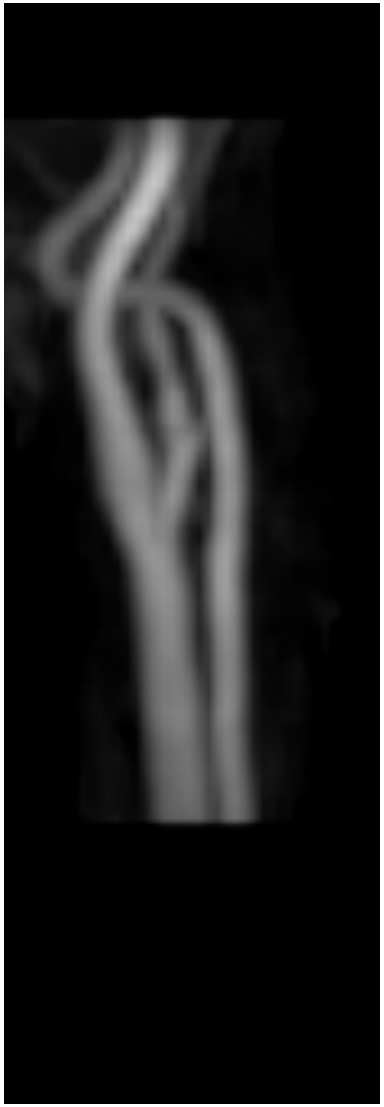

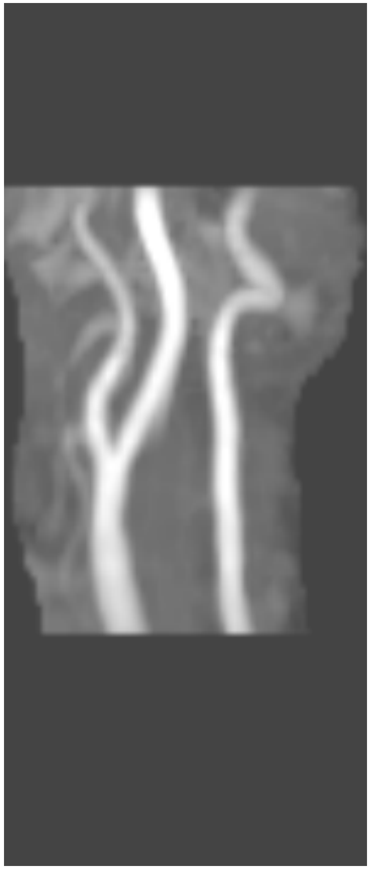

85b Score

0-30

31-50

51-70

>70

Near occlusion

Occluded

Quality

1

2

3

4

5

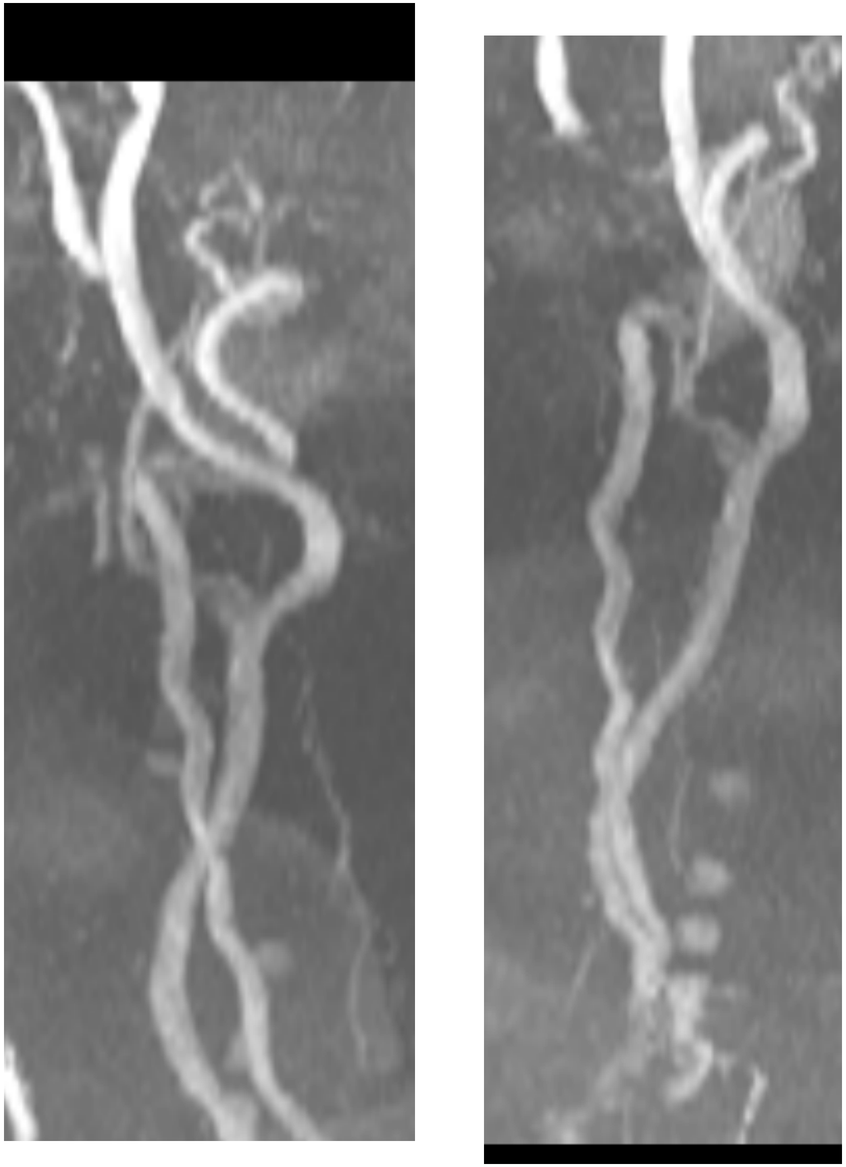

86a Score

0-30

31-50

51-70

>70

Near occlusion

Occluded

Quality

1

2

3

4

5

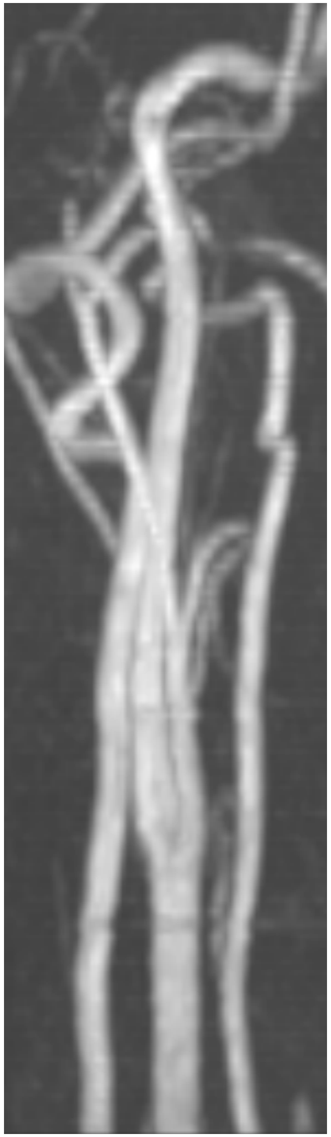

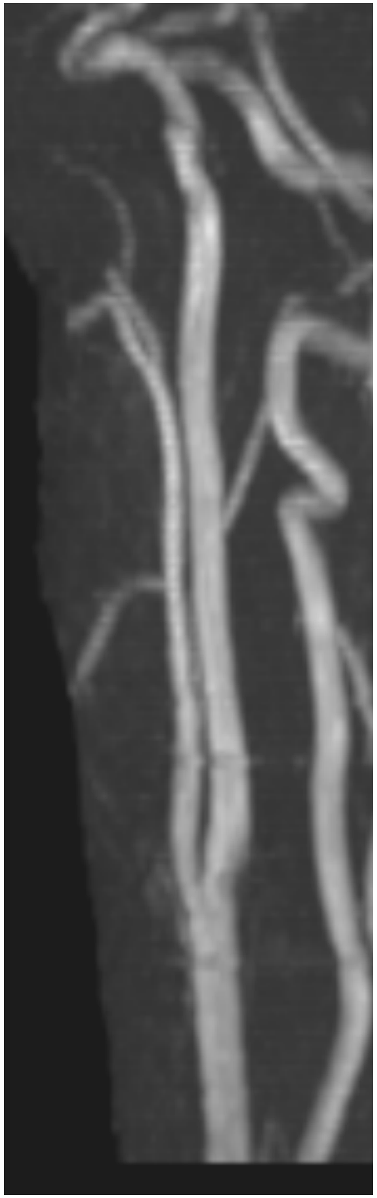

86f Score

0-30

31-50

51-70

>70

Near occlusion

Occluded

Quality

1

2

3

4

5

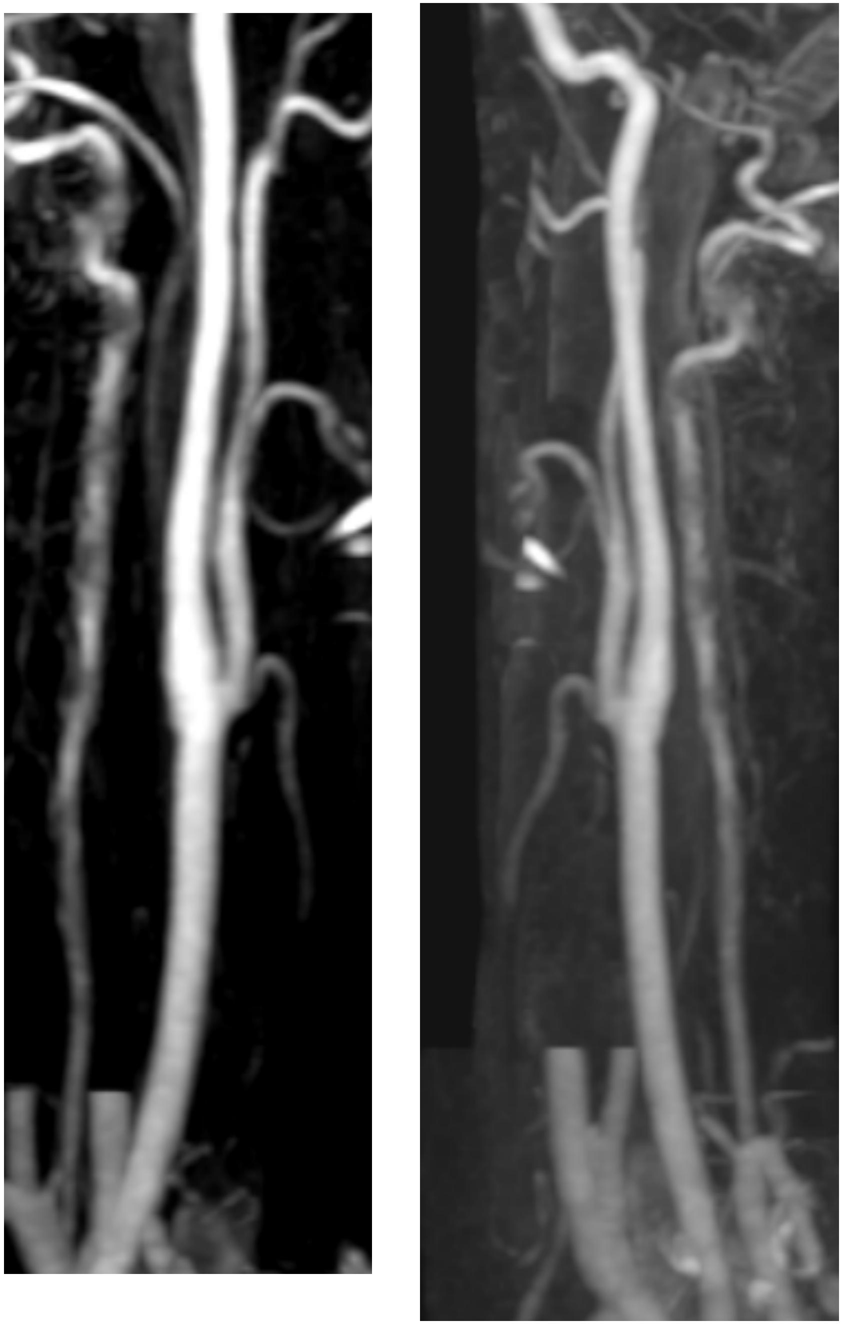

87e Score

0-30

31-50

51-70

>70

Near occlusion

Occluded

Quality

1

2

3

4

5

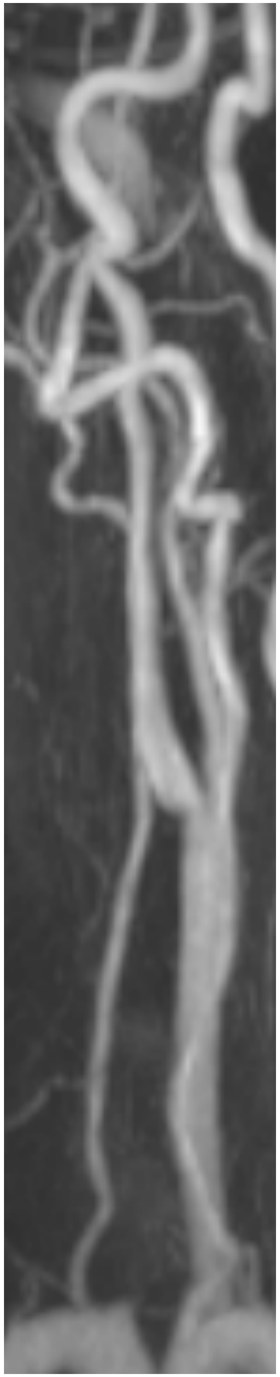

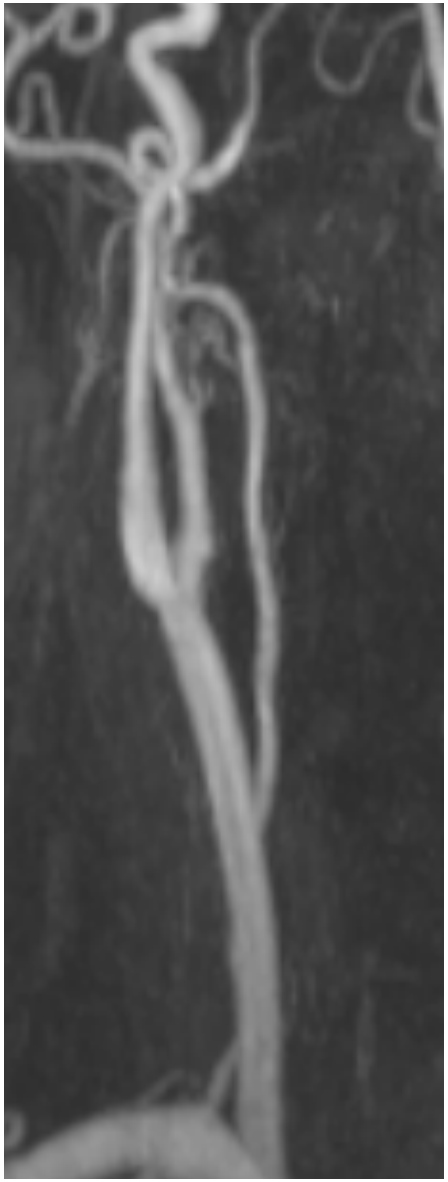

88d Score

0-30

31-50

51-70

>70

Near occlusion

Occluded

Quality

1

2

3

4

5

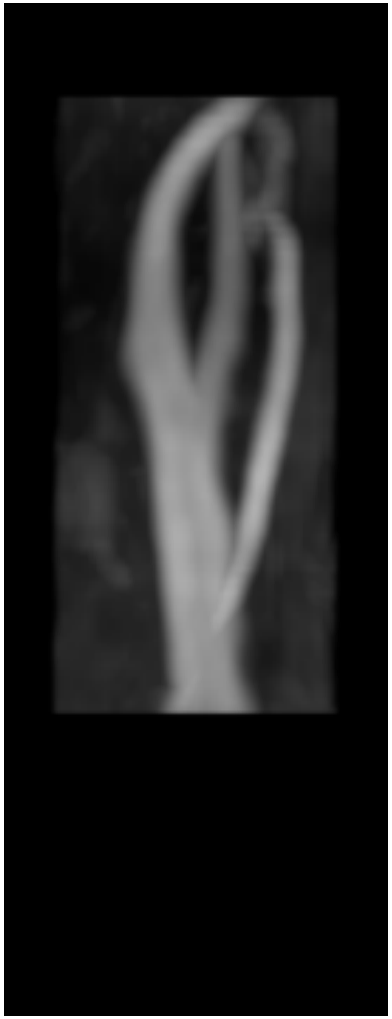

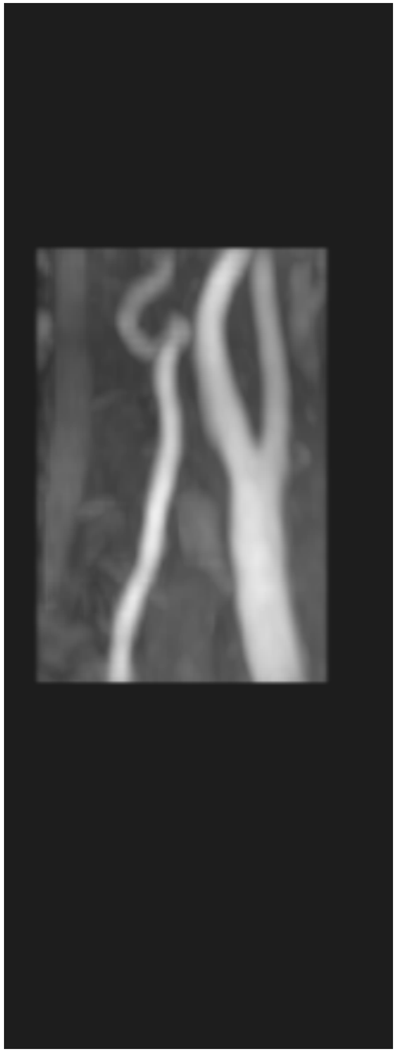

89c Score

0-30

31-50

51-70

>70

Near occlusion

Occluded

Quality

1

2

3

4

5

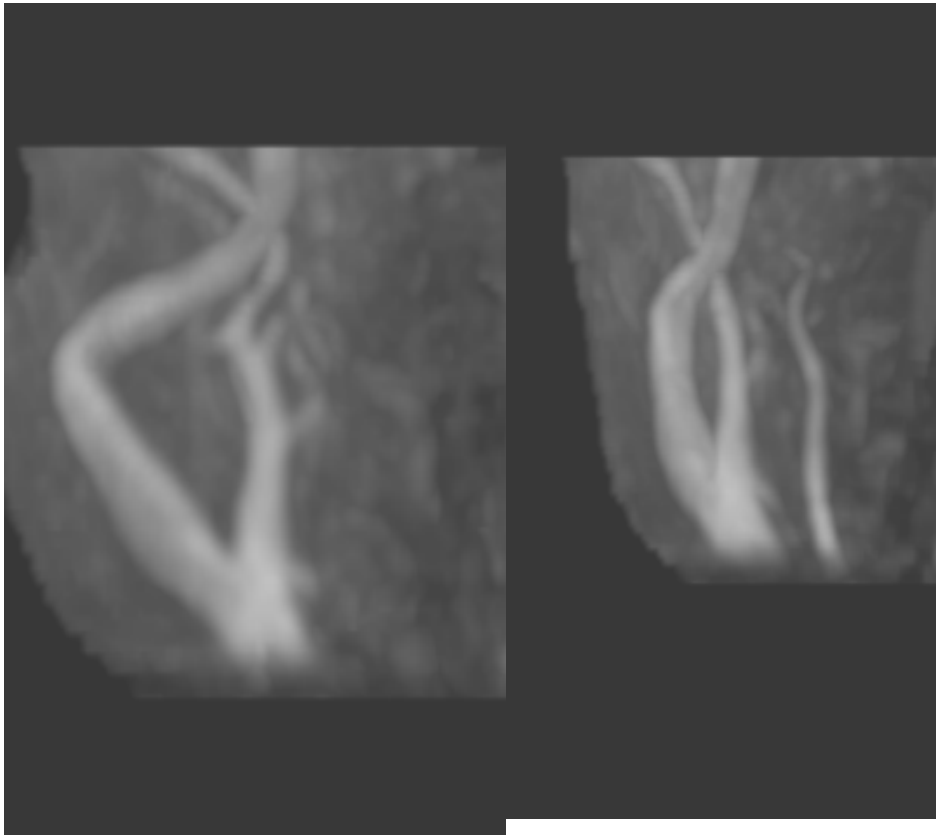

90b Score

0-30

31-50

51-70

>70

Near occlusion

Occluded

Quality

1

2

3

4

5

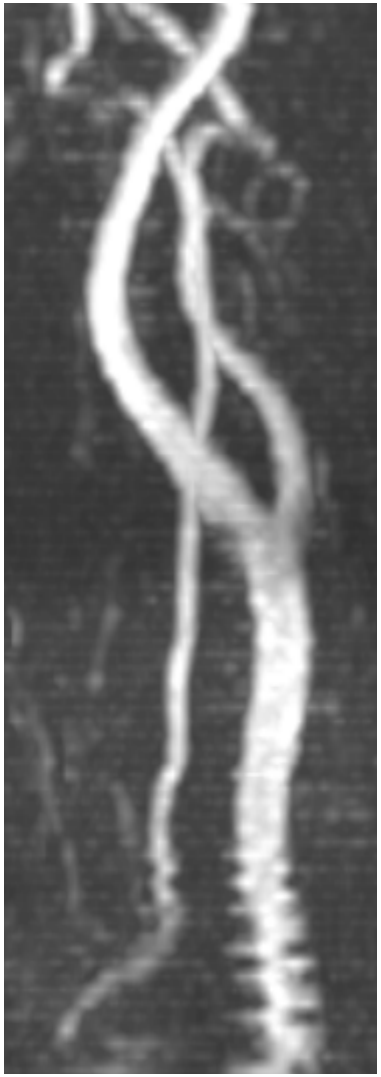

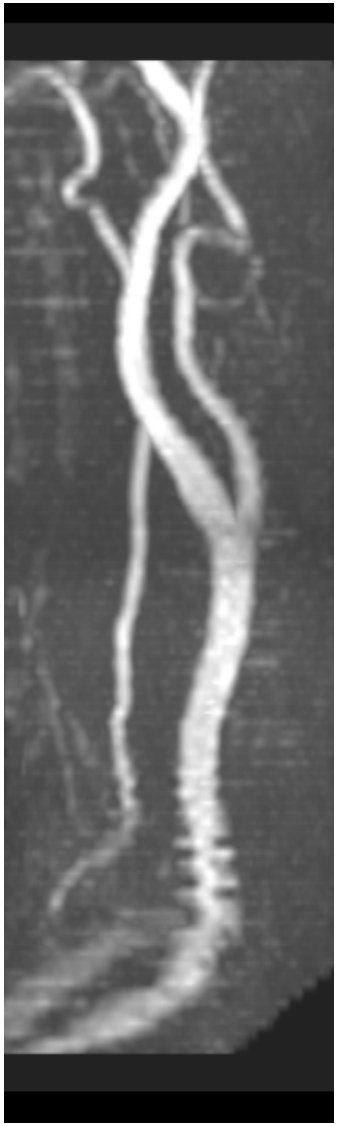

91a Score

0-30

31-50

51-70

>70

Near occlusion

Occluded

Quality

1

2

3

4

5

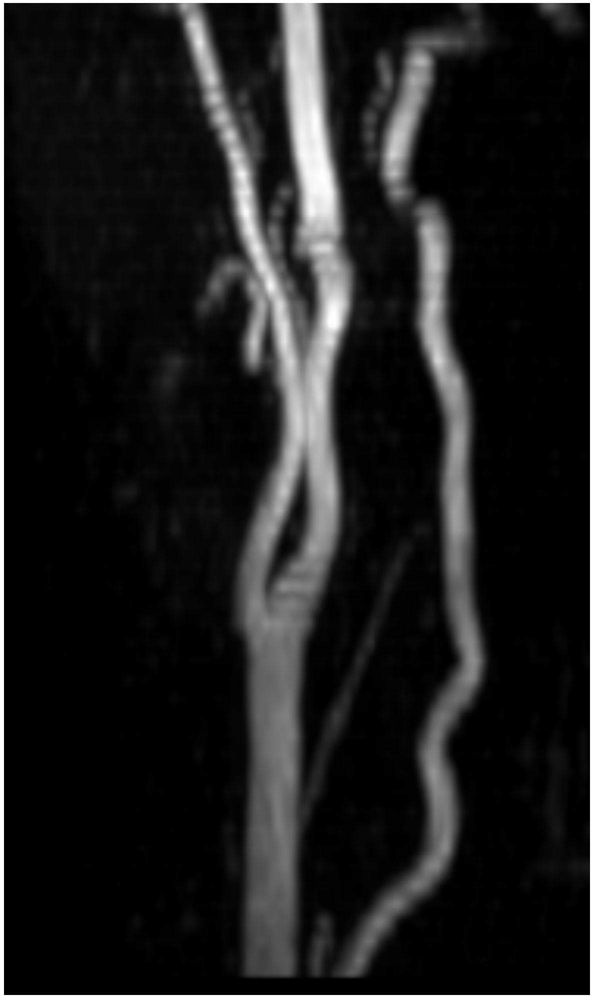

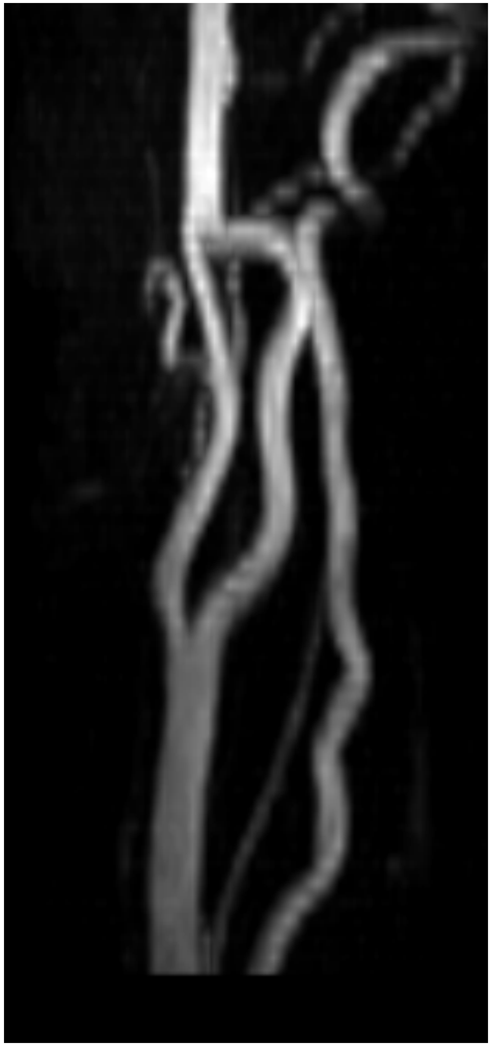

91f Score

0-30

31-50

51-70

>70

Near occlusion

Occluded

Quality

1

2

3

4

5

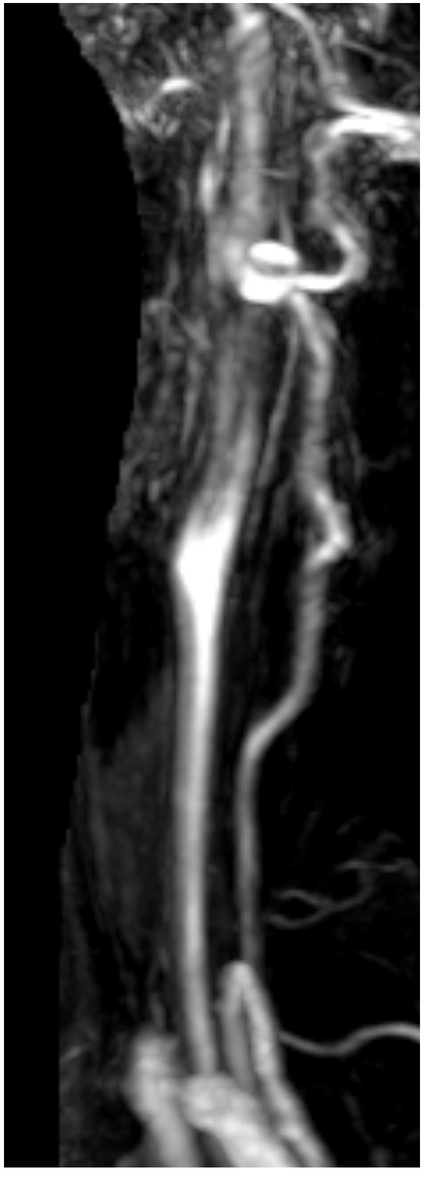

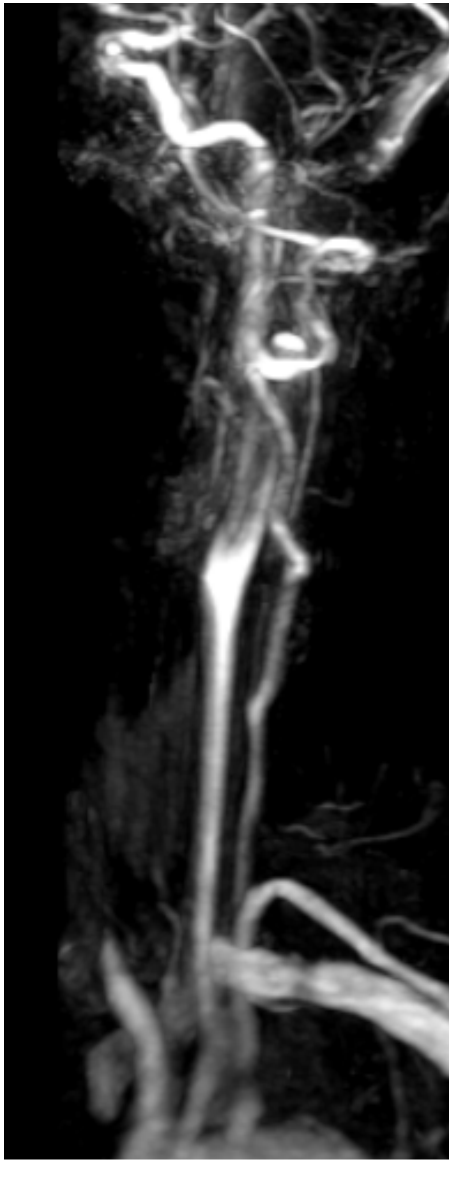

92e Score

0-30

31-50

51-70

>70

Near occlusion

Occluded

Quality

1

2

3

4

5

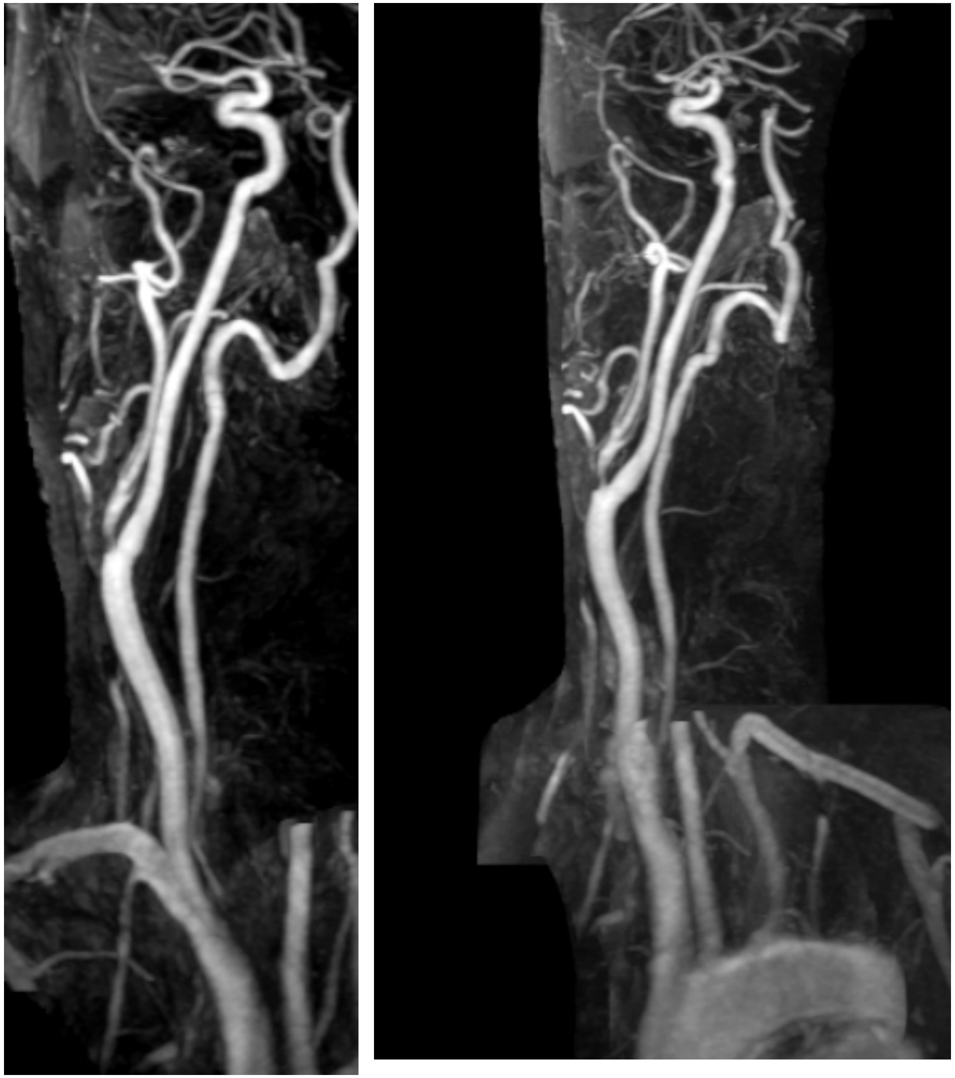

93d Score

0-30

31-50

51-70

>70

Near occlusion

Occluded

Quality

1

2

3

4

5

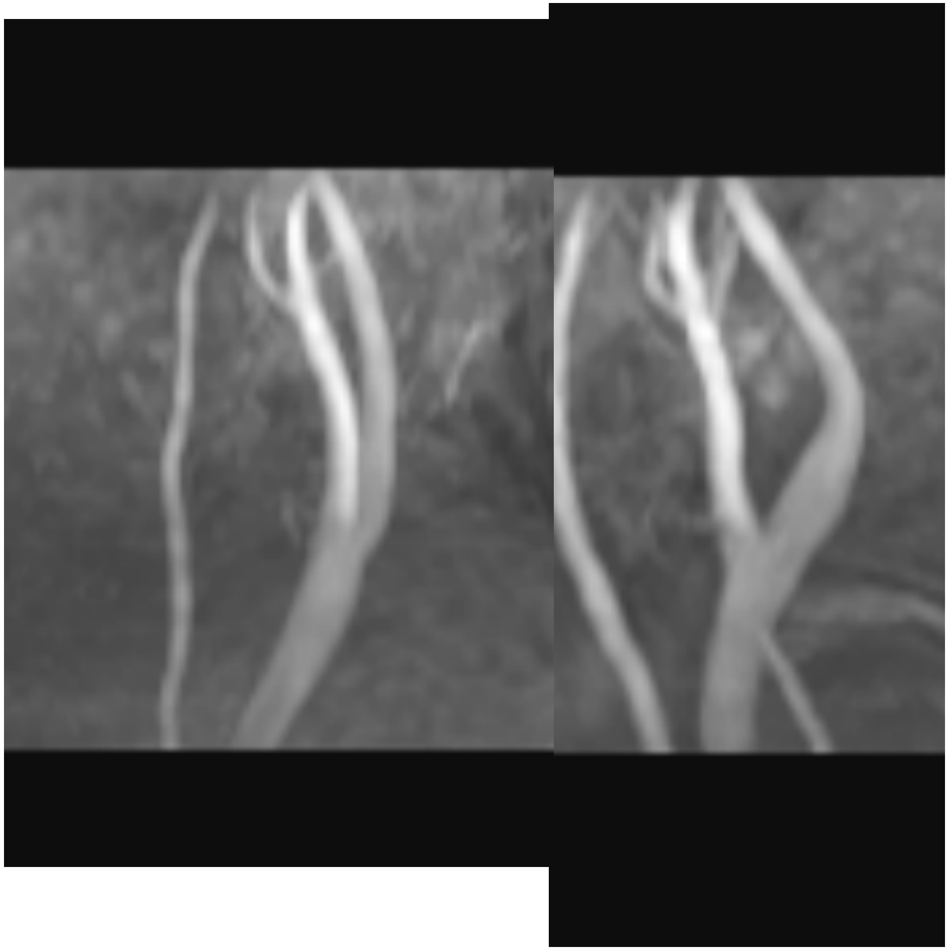

94c Score

0-30

31-50

51-70

>70

Near occlusion

Occluded

Quality

1

2

3

4

5

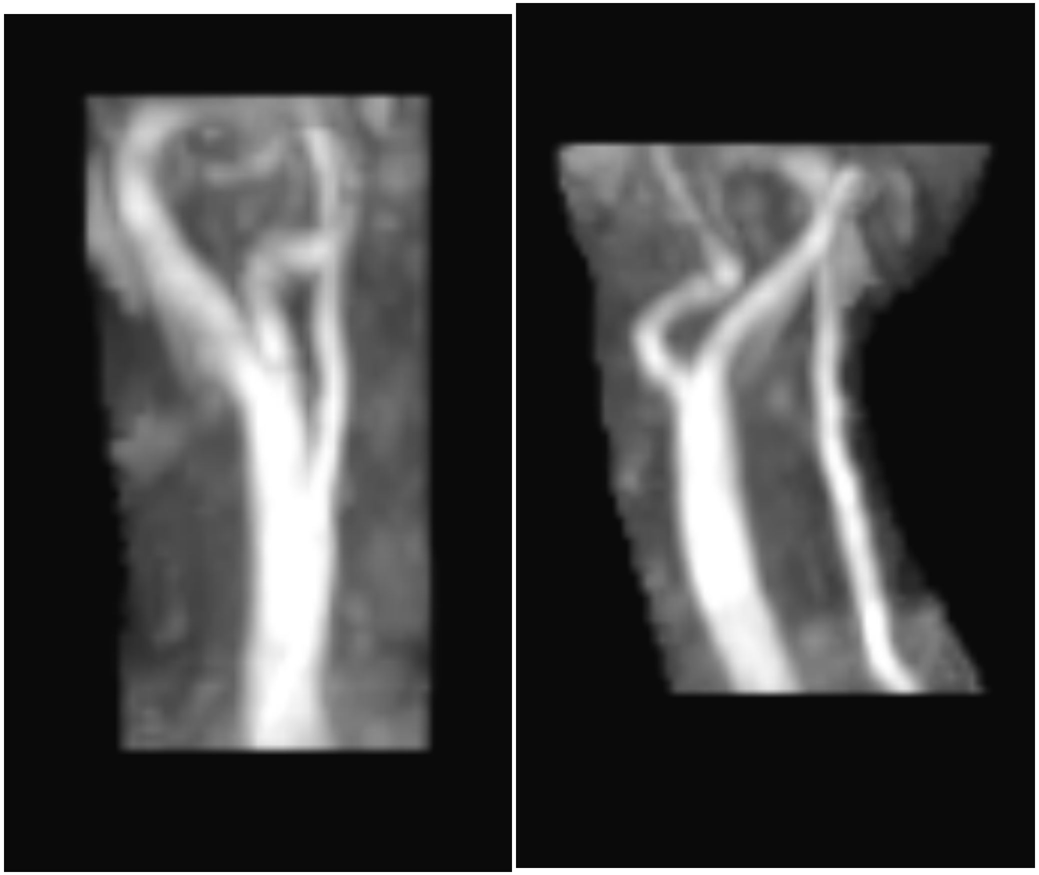

95b Score

0-30

31-50

51-70

>70

Near occlusion

Occluded

Quality

1

2

3

4

5

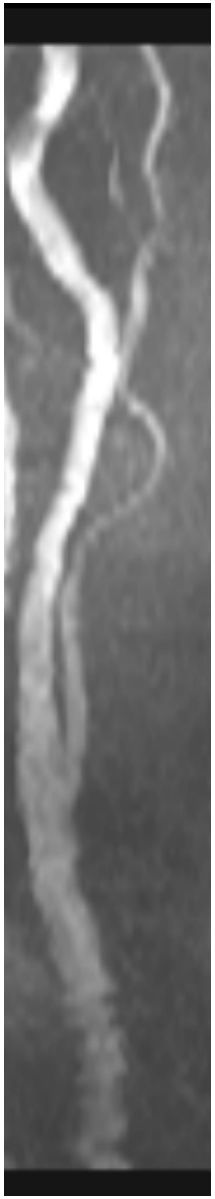

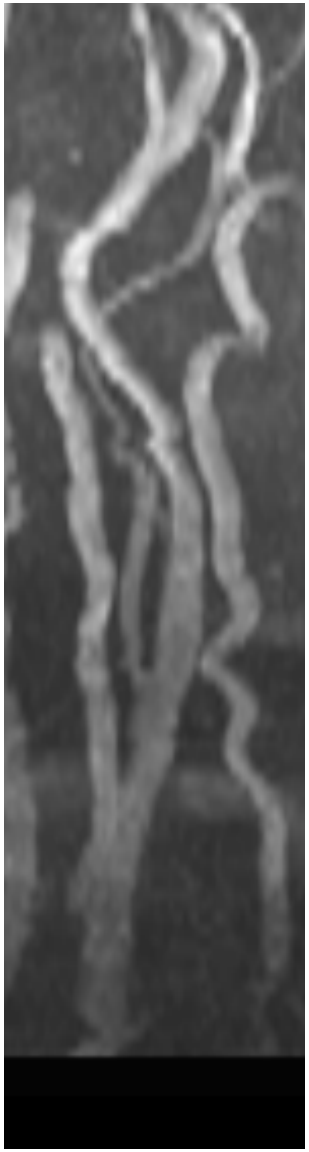

96a Score

0-30

31-50

51-70

>70

Near occlusion

Occluded

Quality

1

2

3

4

5

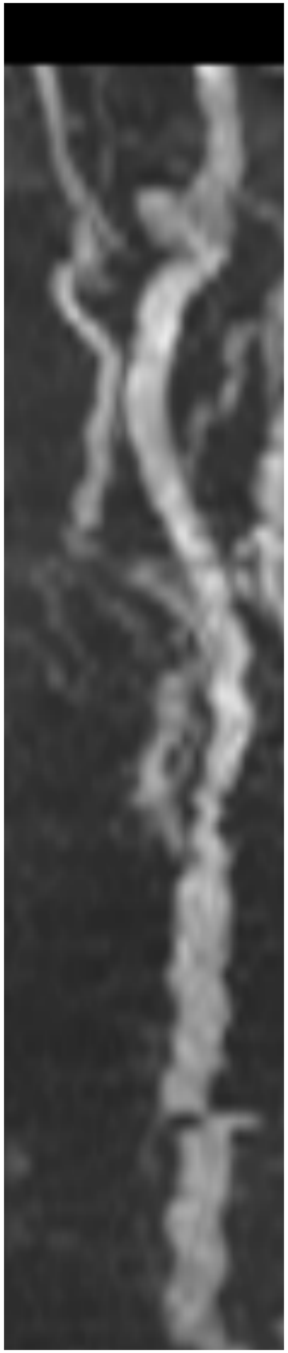

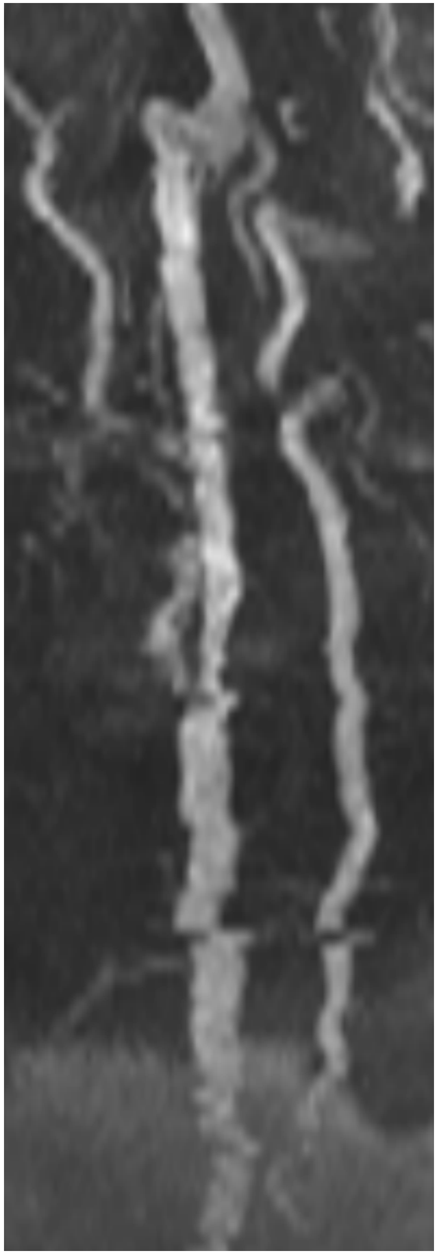

96f Score

0-30

31-50

51-70

>70

Near occlusion

Occluded

Quality

1

2

3

4

5

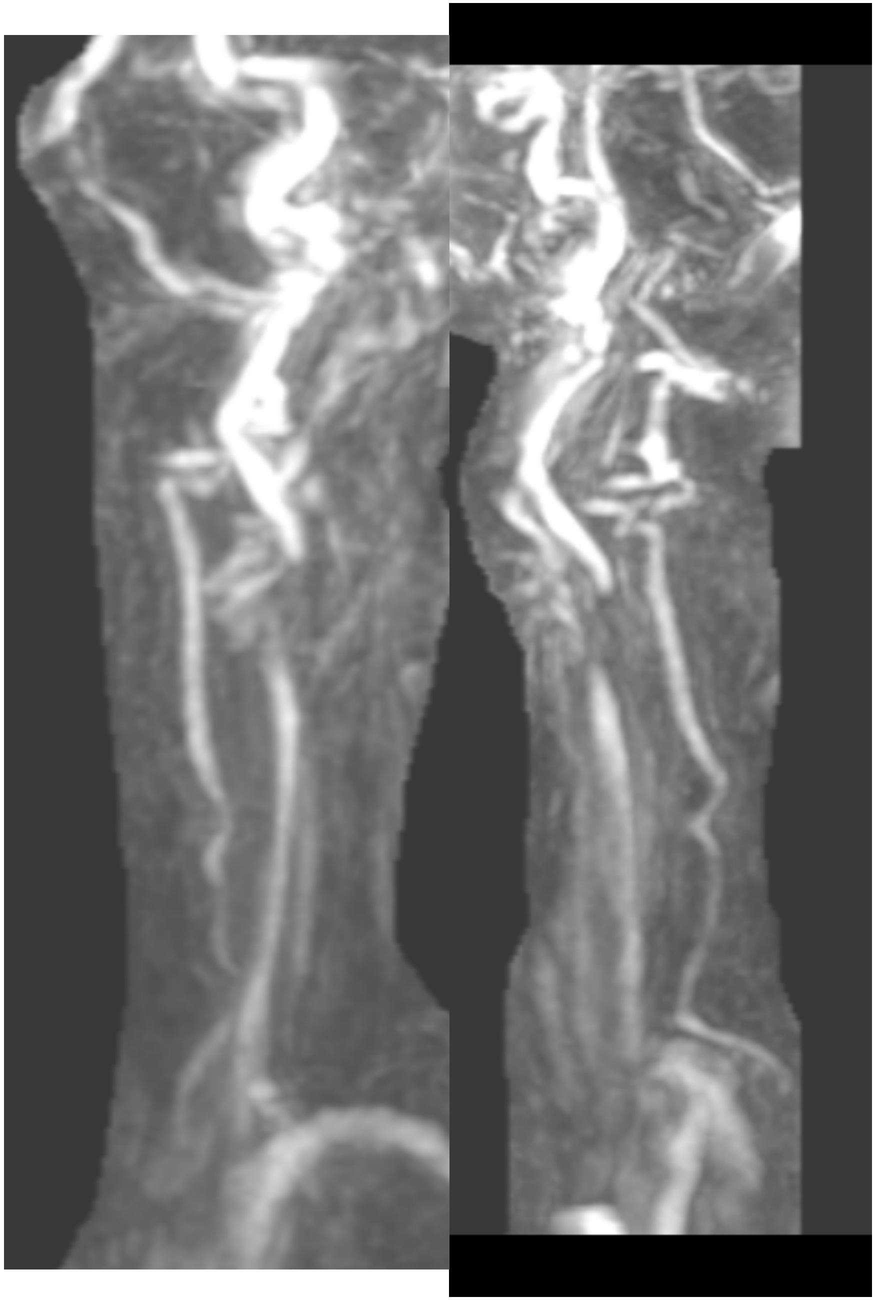

97e Score

0-30

31-50

51-70

>70

Near occlusion

Occluded

Quality

1

2

3

4

5

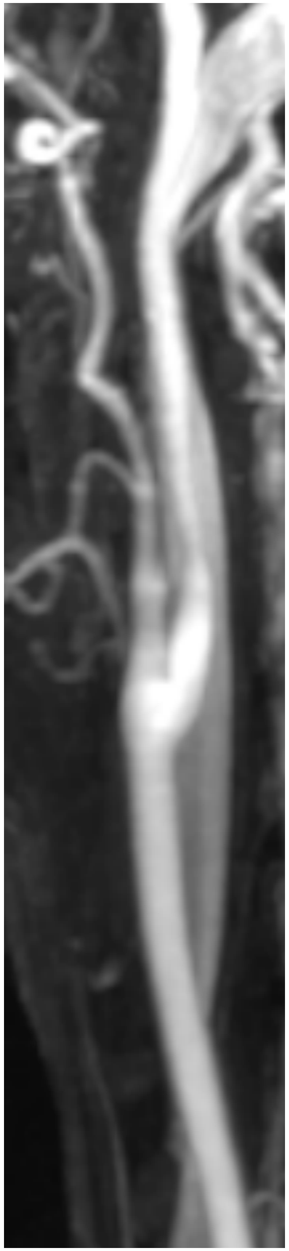

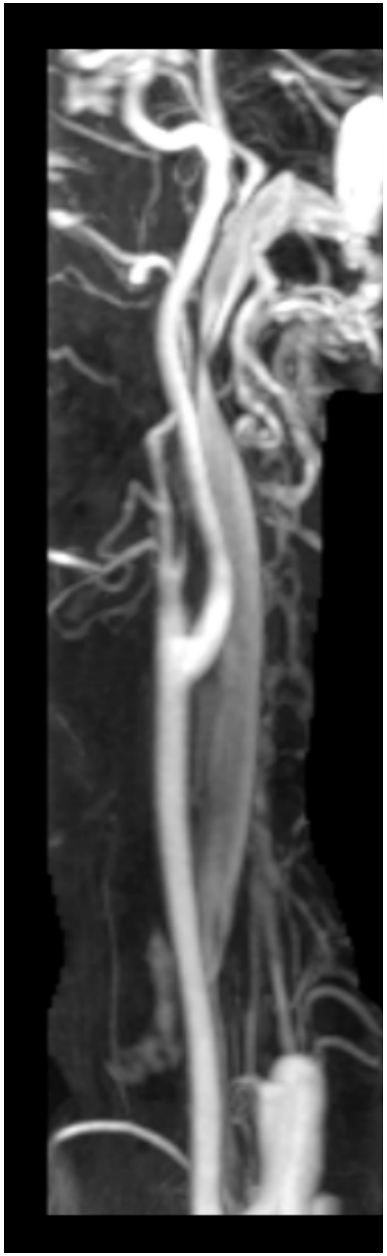

98d Score

0-30

31-50

51-70

>70

Near occlusion

Occluded

Quality

1

2

3

4

5

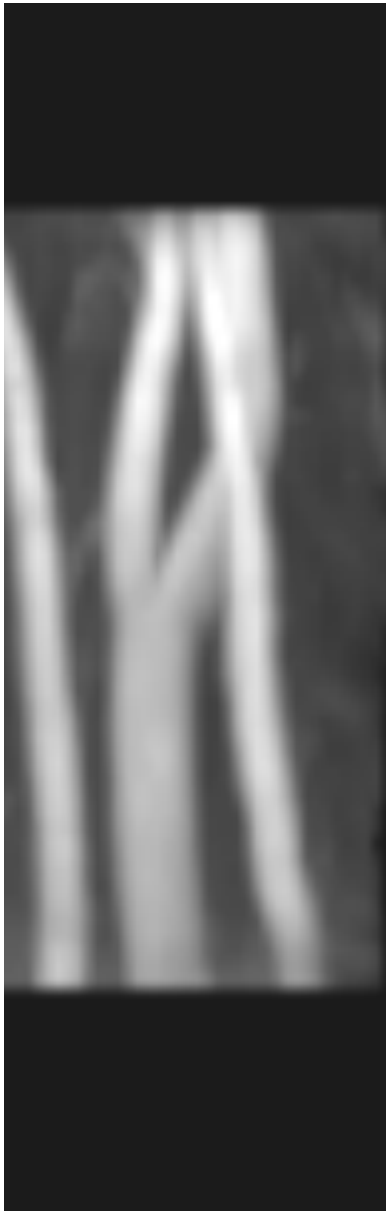

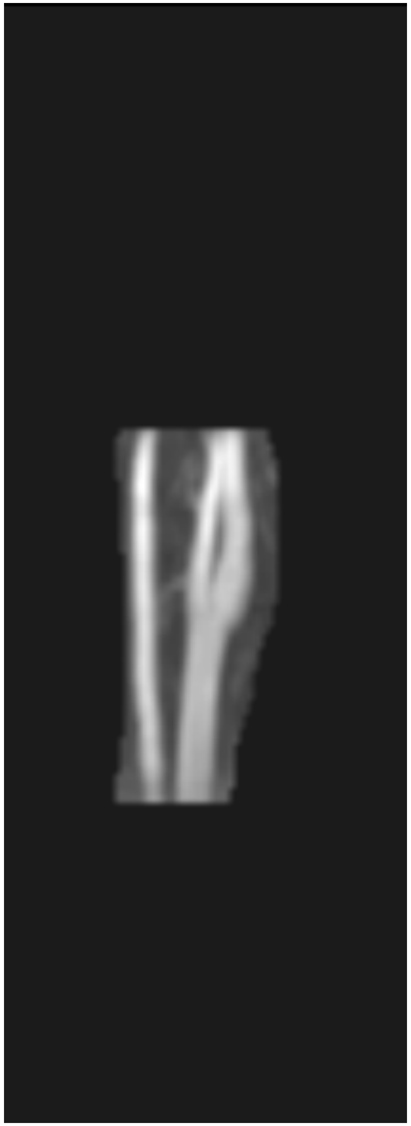

99c Score

0-30

31-50

51-70

>70

Near occlusion

Occluded

Quality

1

2

3

4

5

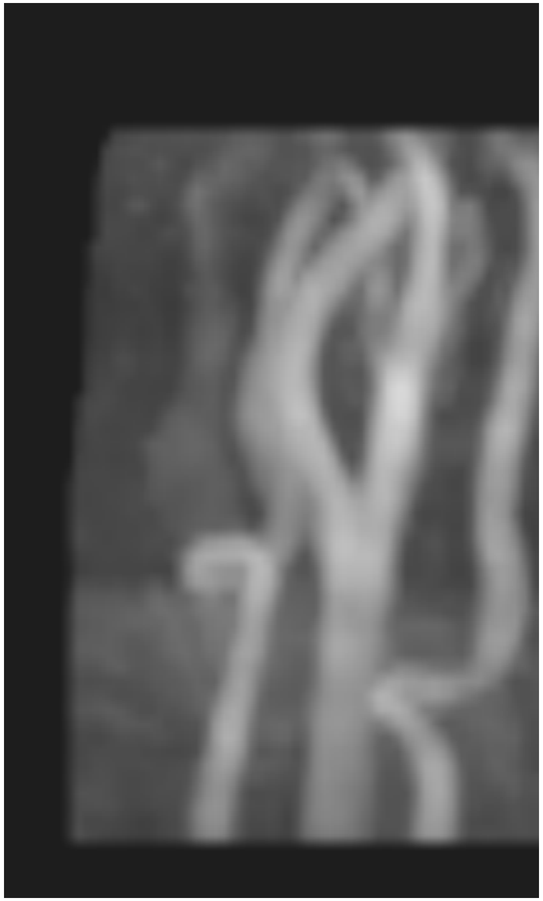

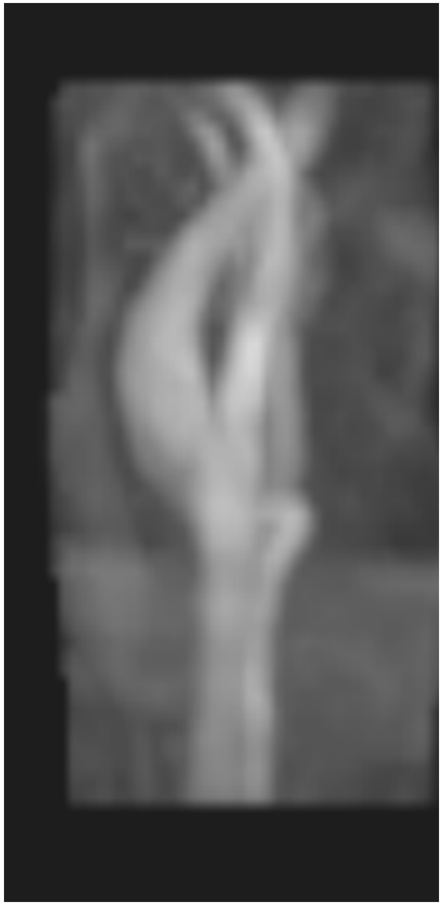

100b Score

0-30

31-50

51-70

>70

Near occlusion

Occluded

Quality

1

2

3

4

5

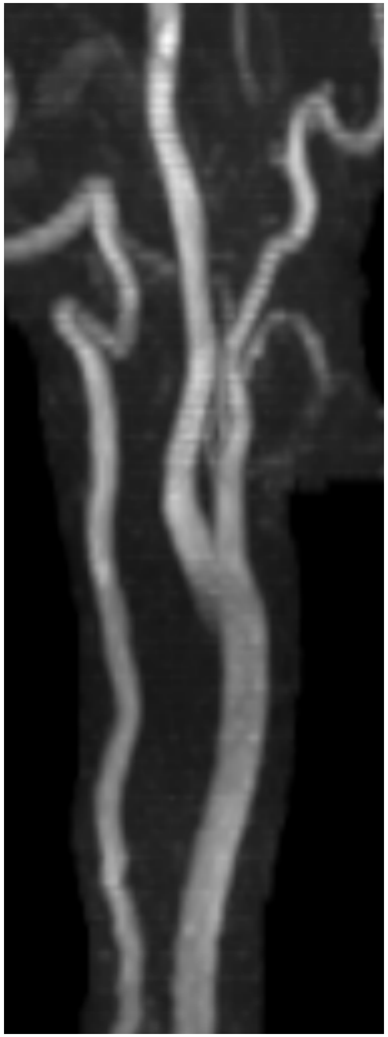

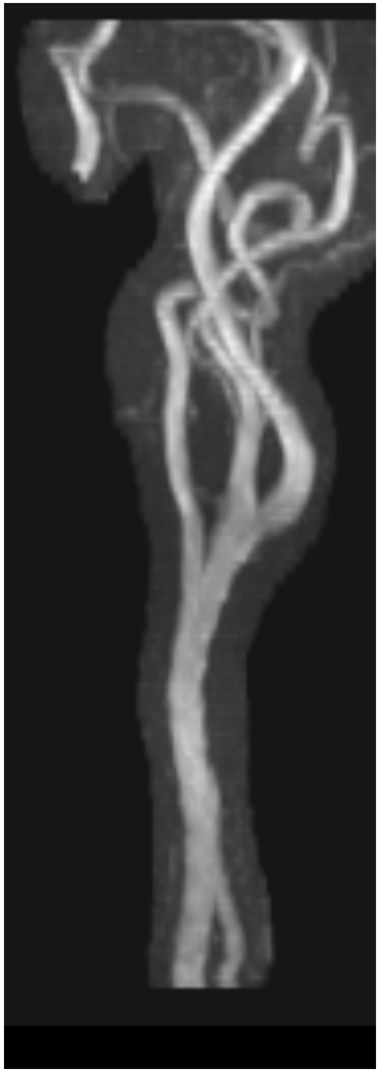

101a Score

0-30

31-50

51-70

>70

Near occlusion

Occluded

Quality

1

2

3

4

5

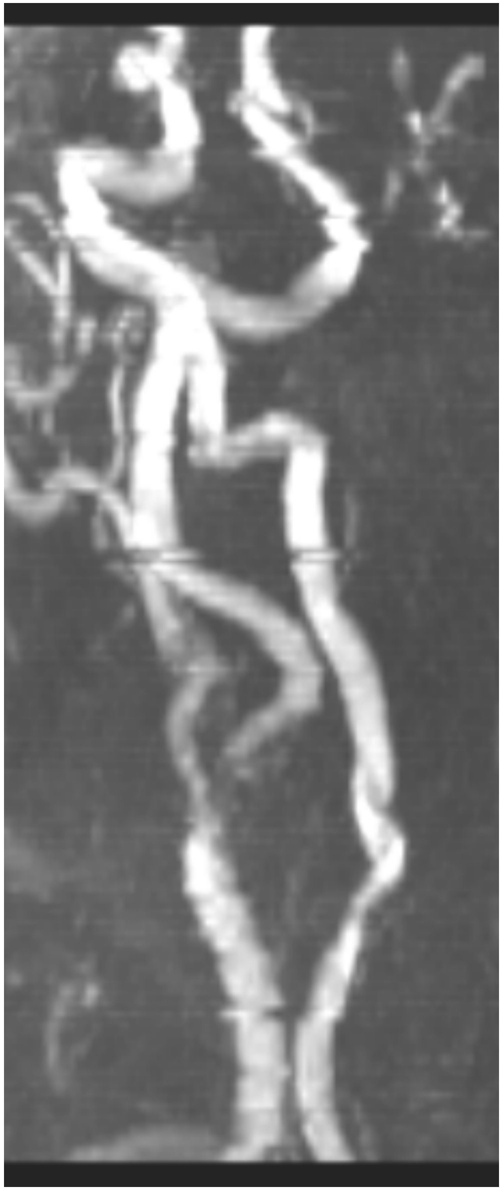

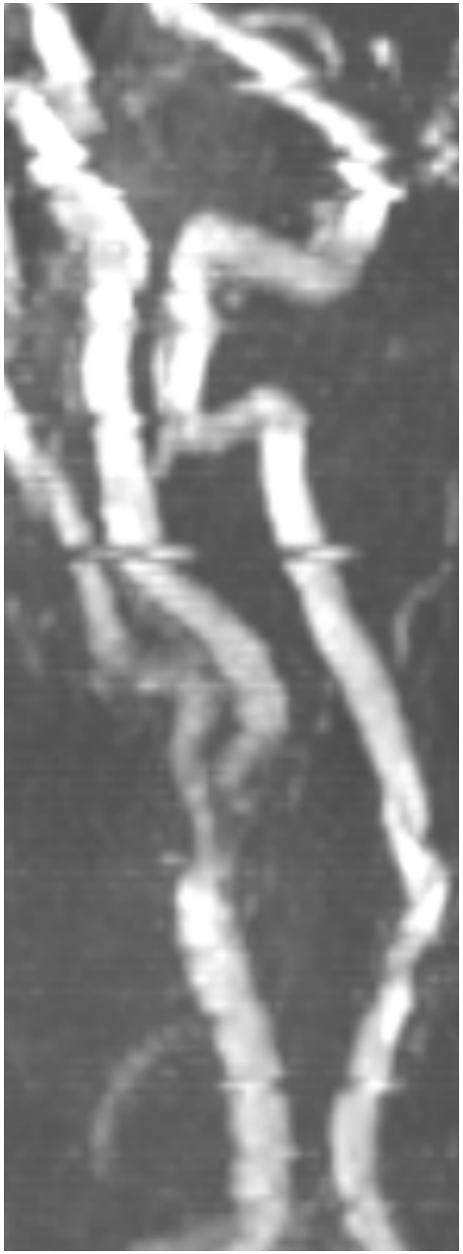

101f Score

0-30

31-50

51-70

>70

Near occlusion

Occluded

Quality

1

2

3

4

5

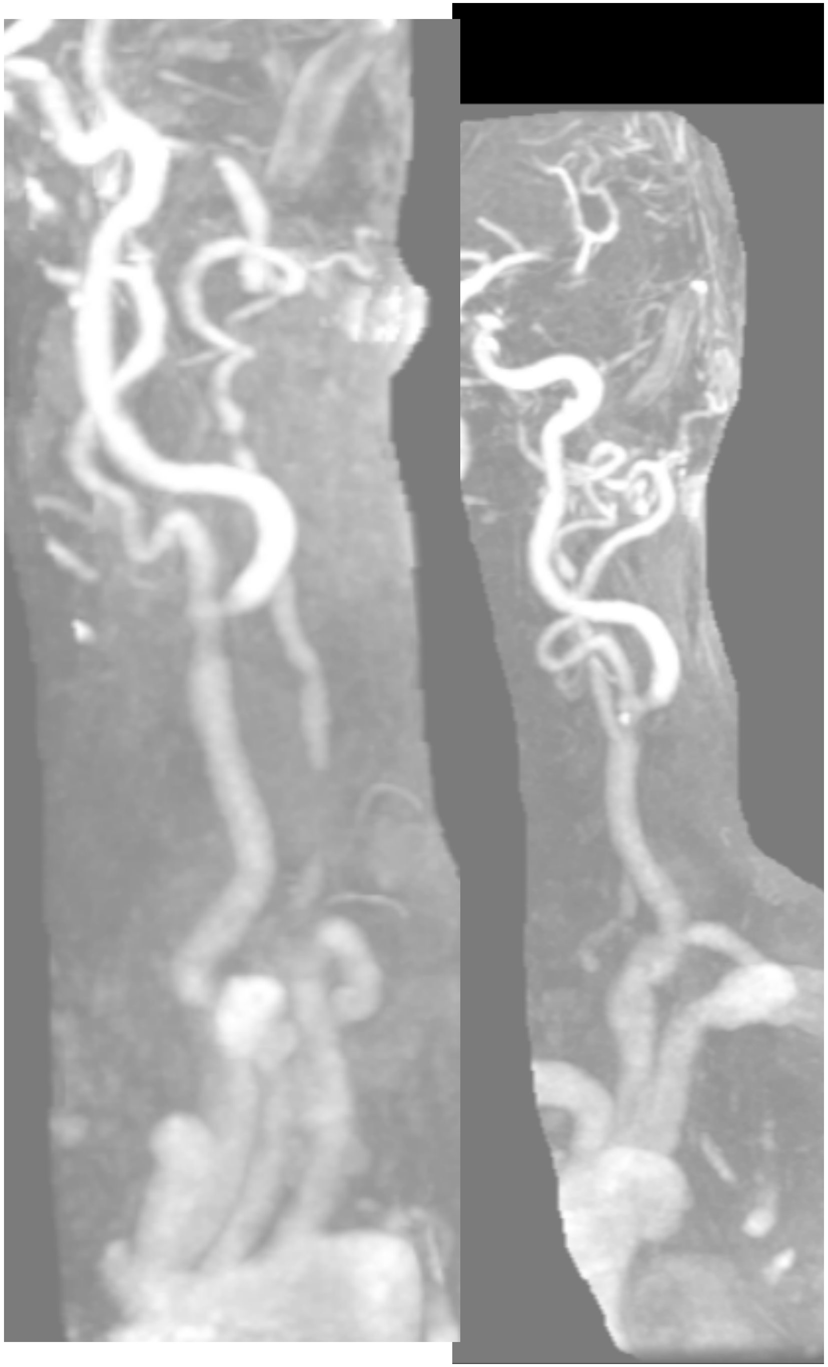

102e Score

0-30

31-50

51-70

>70

Near occlusion

Occluded

Quality

1

2

3

4

5

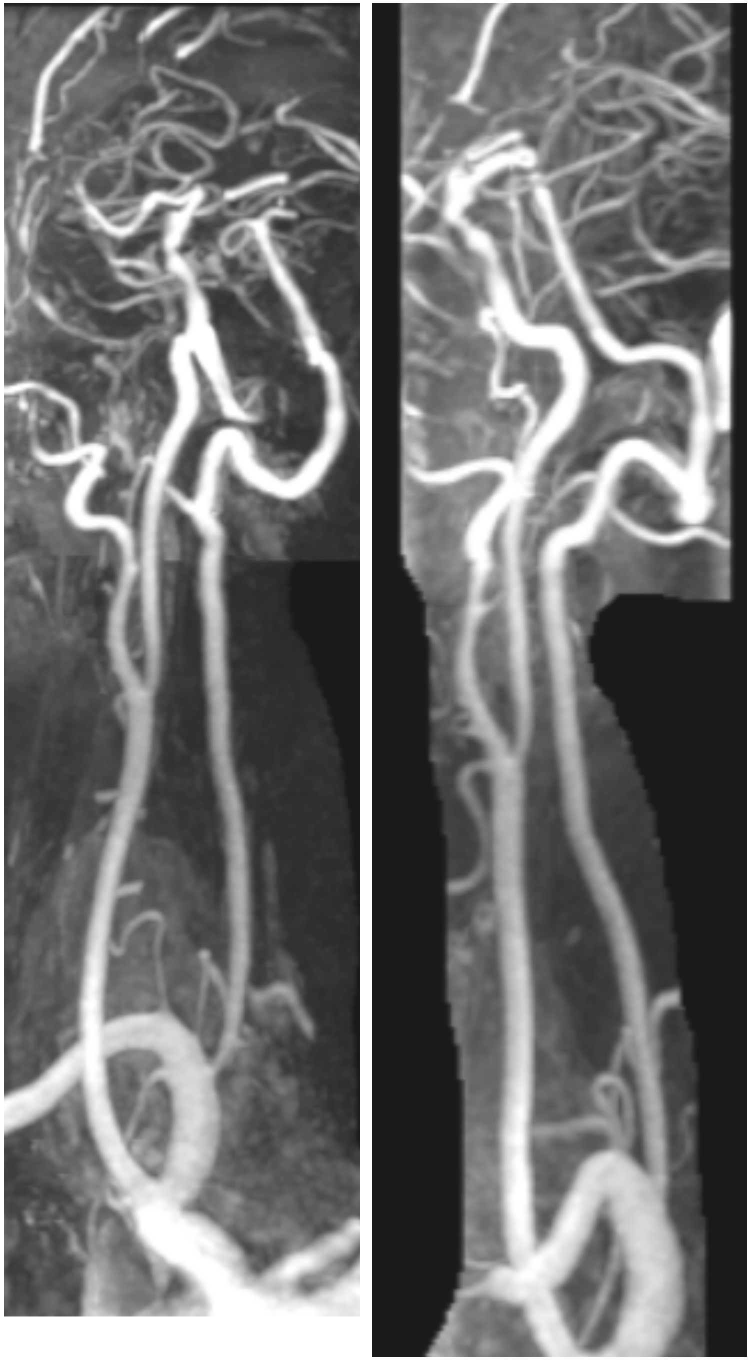

103d Score

0-30

31-50

51-70

>70

Near occlusion

Occluded

Quality

1

2

3

4

5

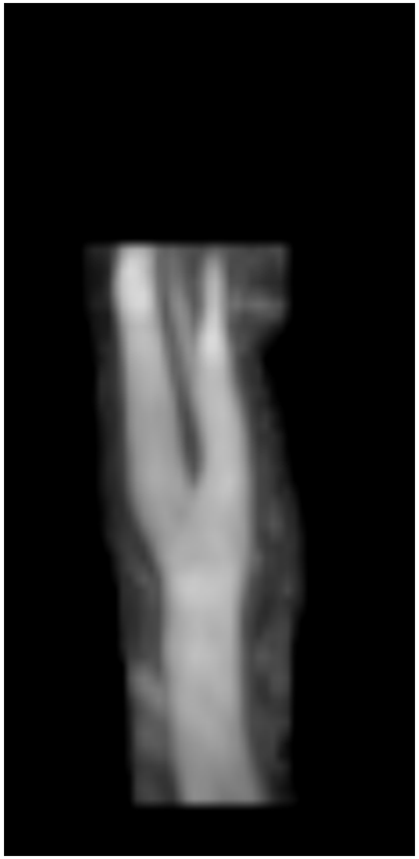

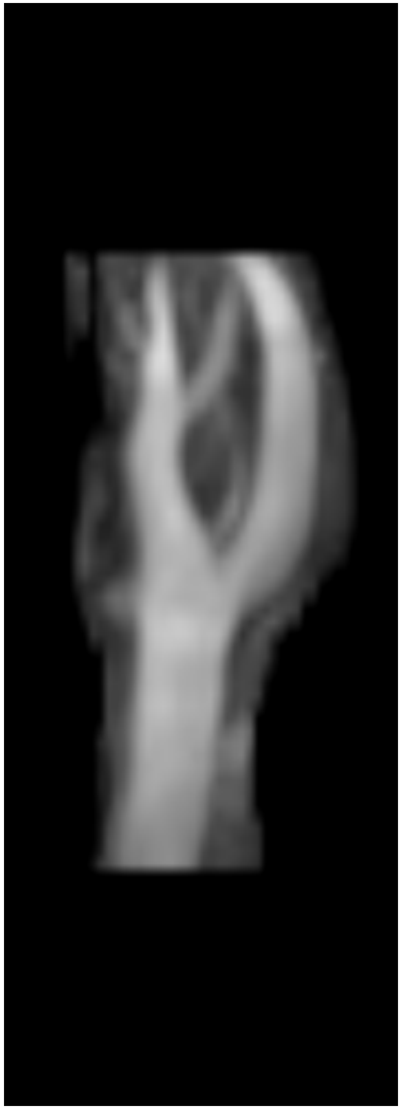

104c Score

0-30

31-50

51-70

>70

Near occlusion

Occluded

Quality

1

2

3

4

5

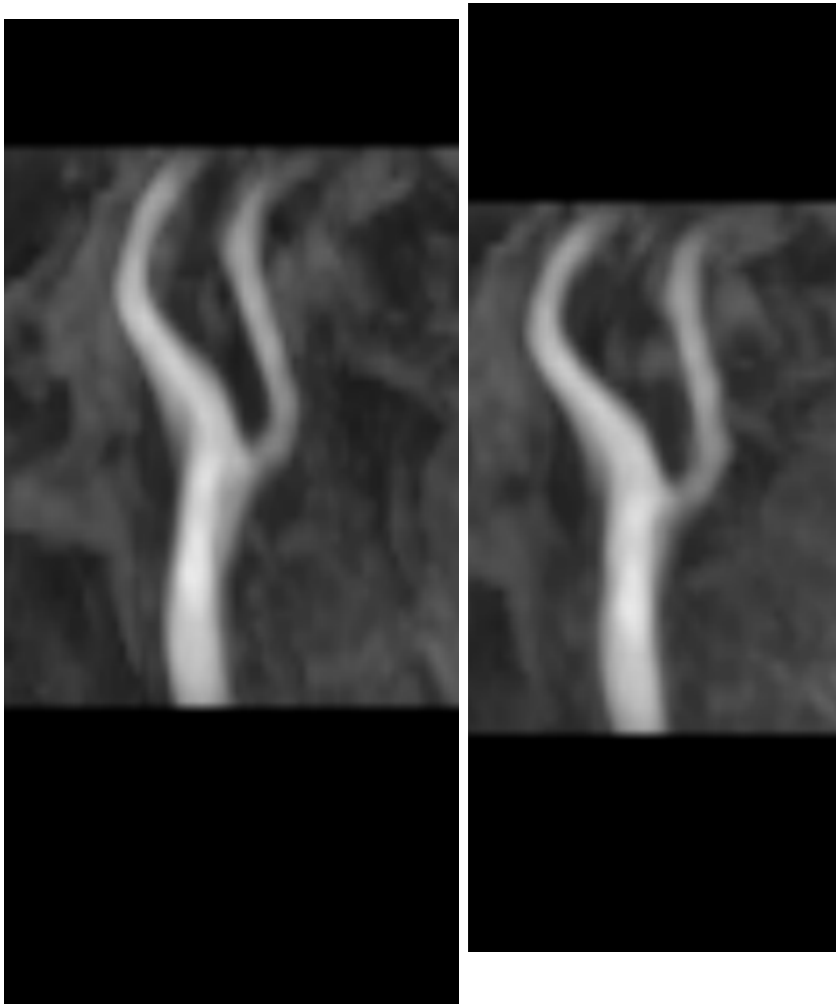

105b Score

0-30

31-50

51-70

>70

Near occlusion

Occluded

Quality

1

2

3

4

5

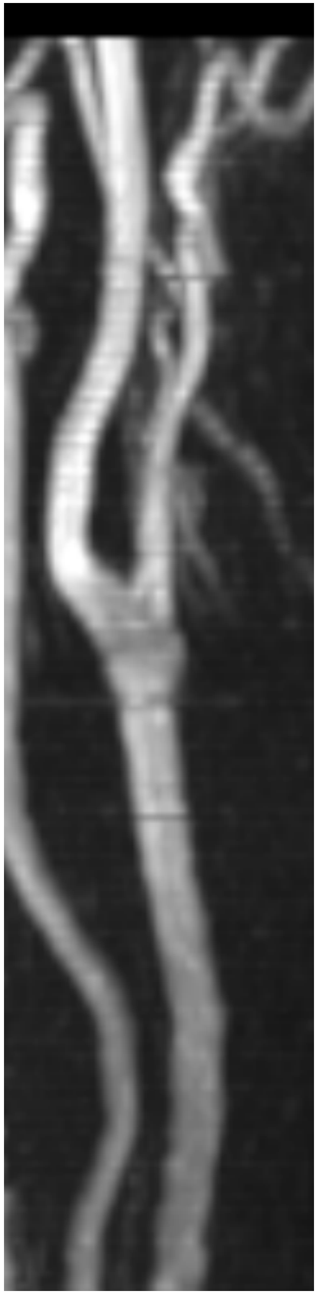

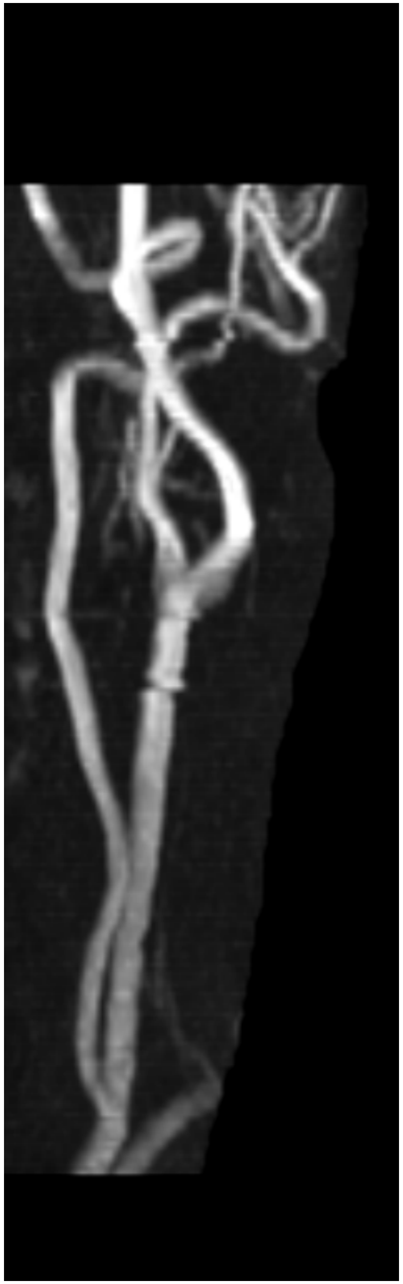

106a Score

0-30

31-50

51-70

>70

Near occlusion

Occluded

Quality

1

2

3

4

5

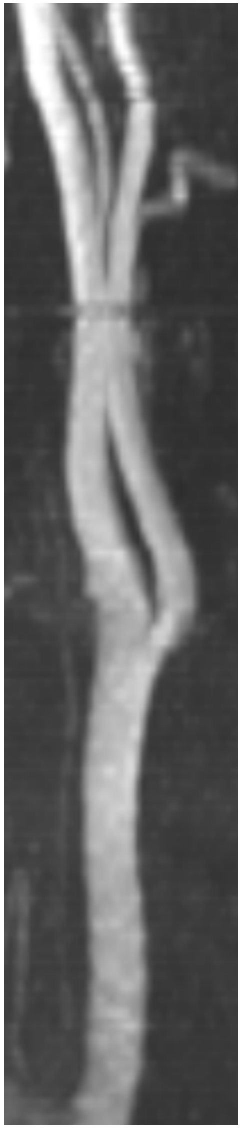

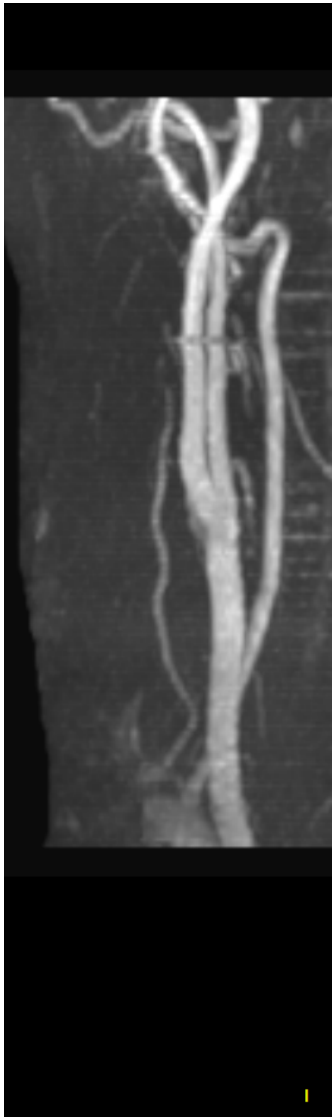

106f Score

0-30

31-50

51-70

>70

Near occlusion

Occluded

Quality

1

2

3

4

5

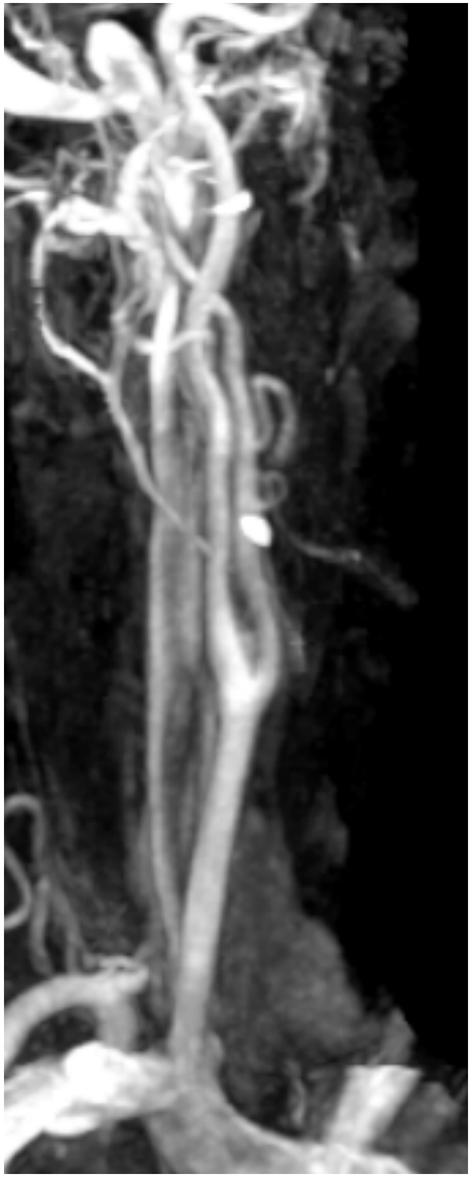

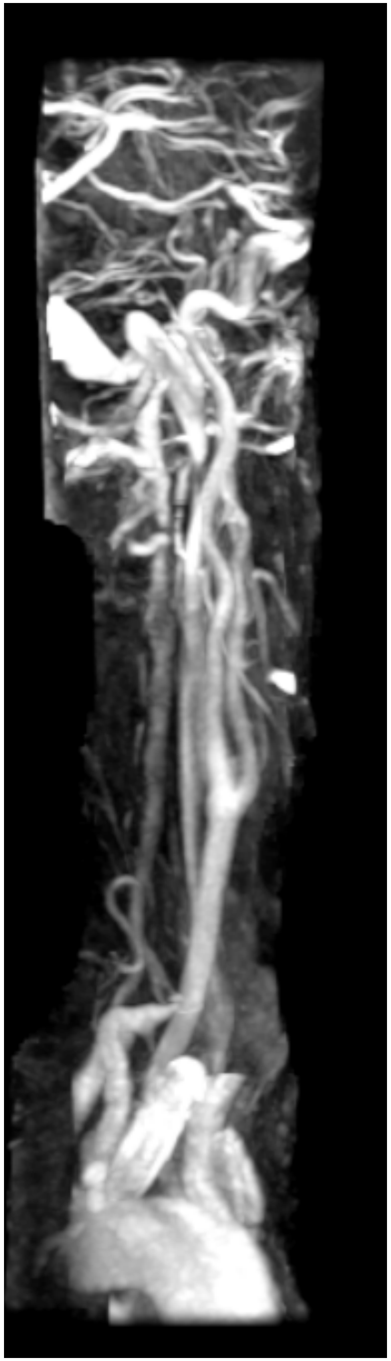

107e Score

0-30

31-50

51-70

>70

Near occlusion

Occluded

Quality

1

2

3

4

5

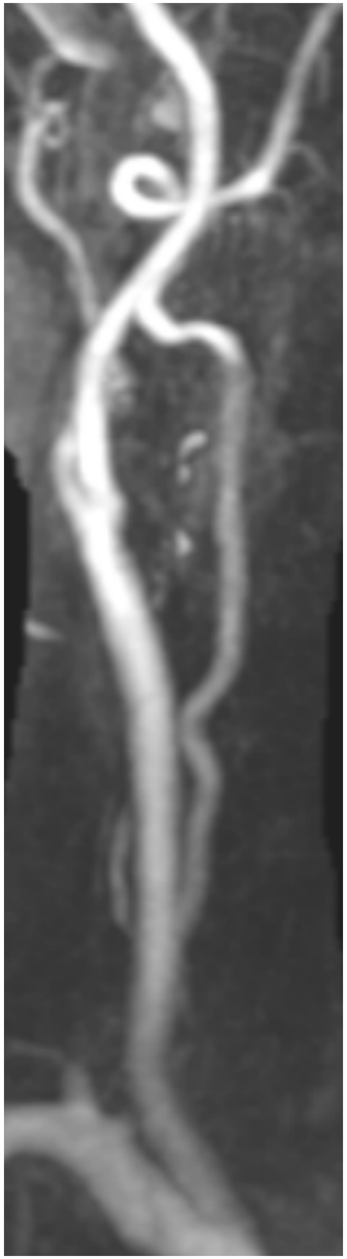

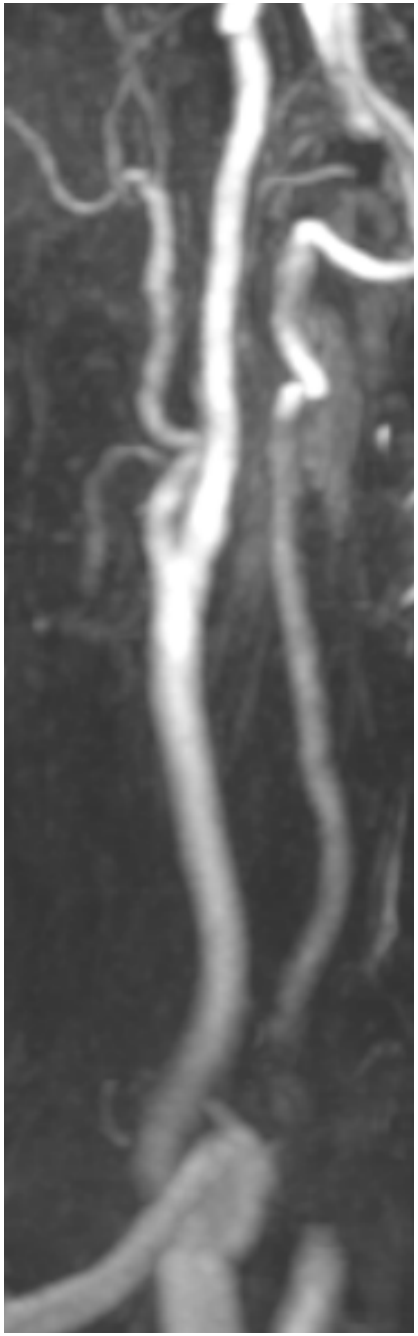

108d Score

0-30

31-50

51-70

>70

Near occlusion

Occluded

Quality

1

2

3

4

5

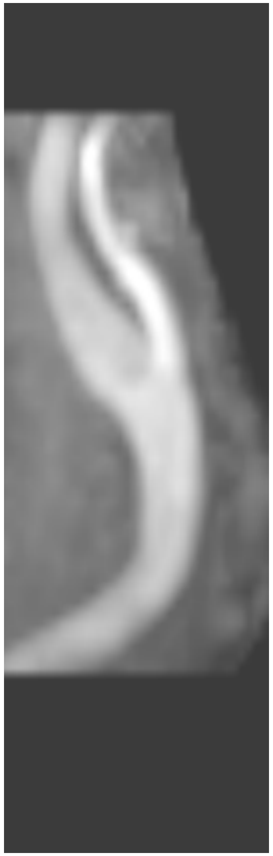

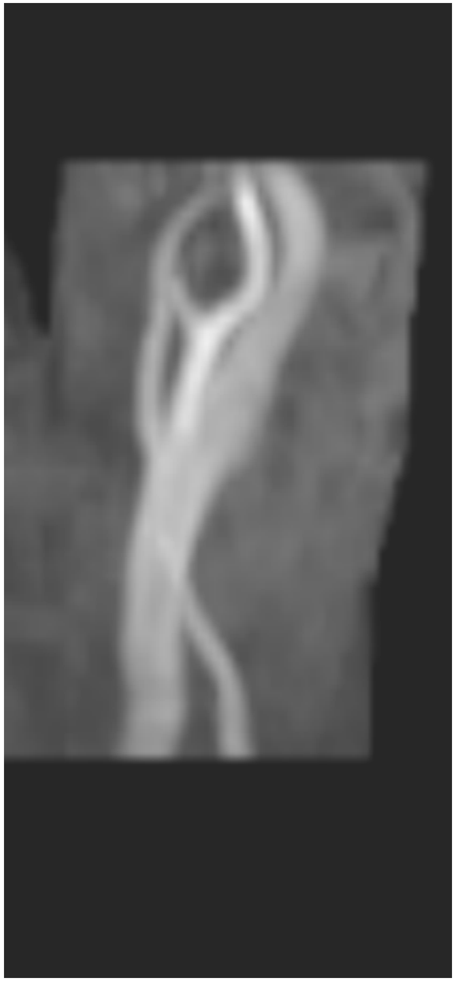

109c Score

0-30

31-50

51-70

>70

Near occlusion

Occluded

Quality

1

2

3

4

5

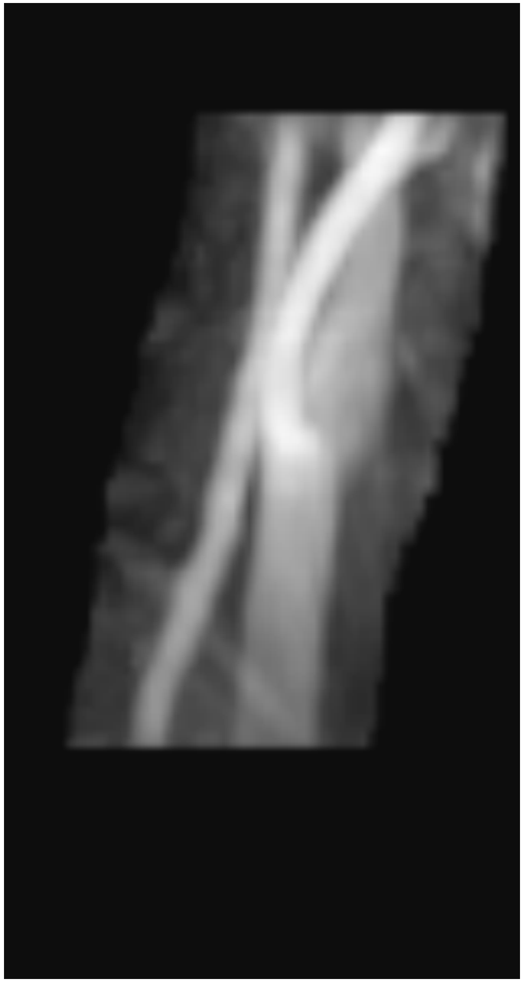

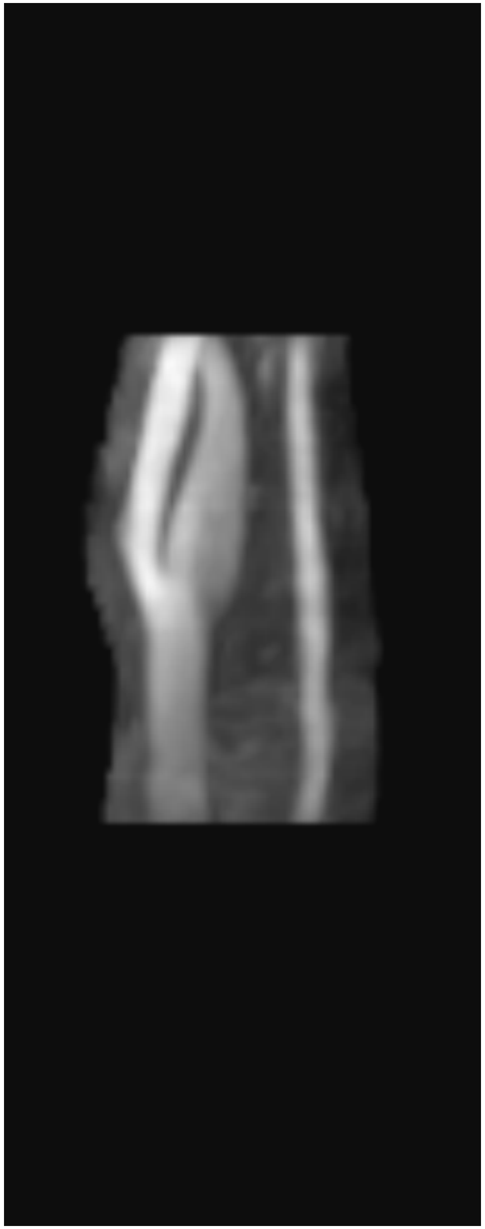

110b Score

0-30

31-50

51-70

>70

Near occlusion

Occluded

Quality

1

2

3

4

5

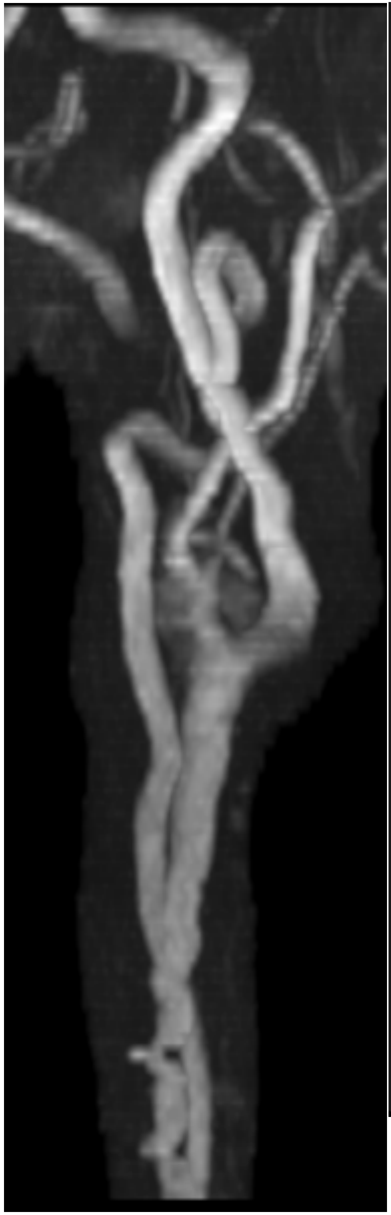

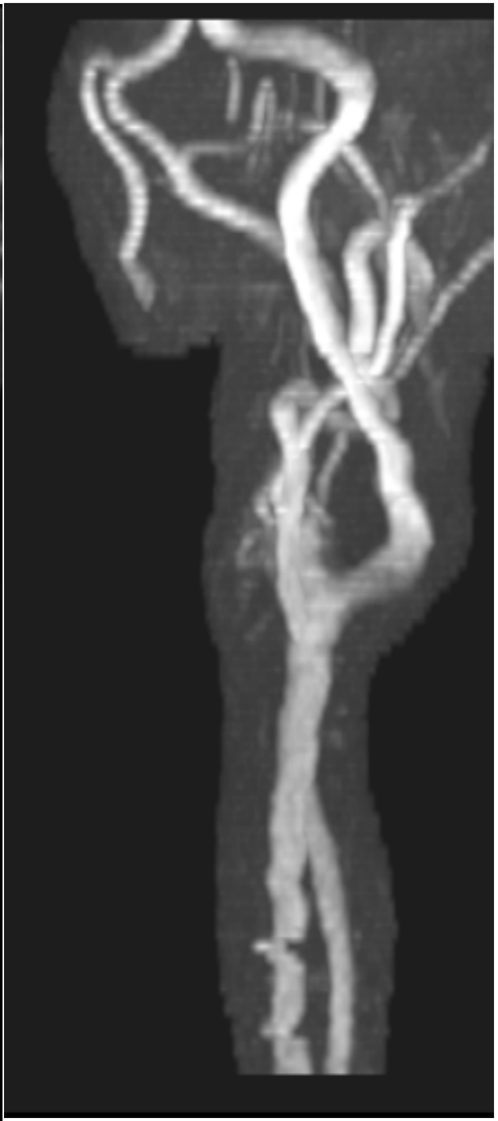

111a Score

0-30

31-50

51-70

>70

Near occlusion

Occluded

Quality

1

2

3

4

5

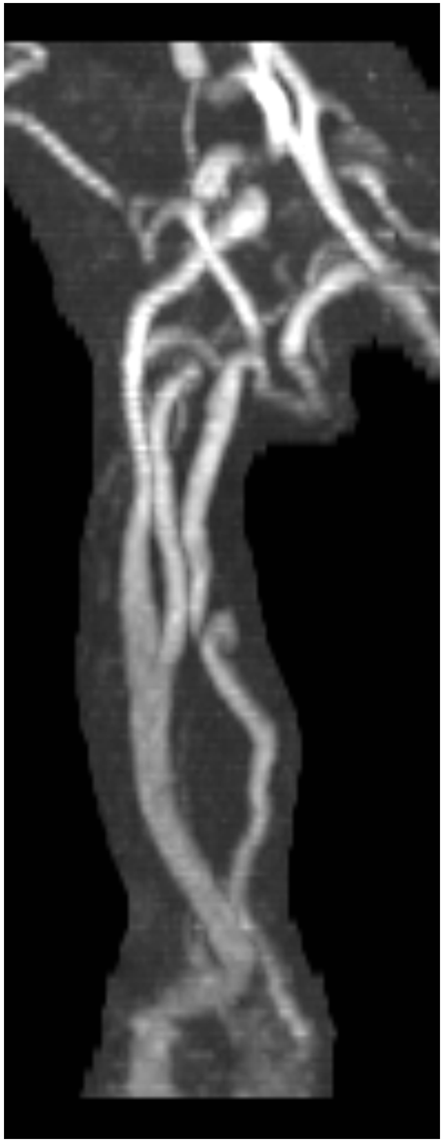

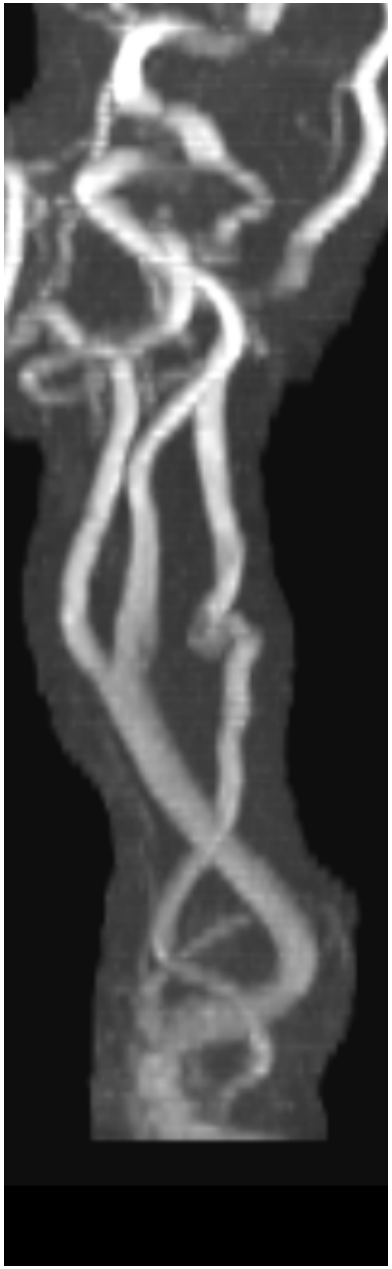

111f Score

0-30

31-50

51-70

>70

Near occlusion

Occluded

Quality

1

2

3

4

5

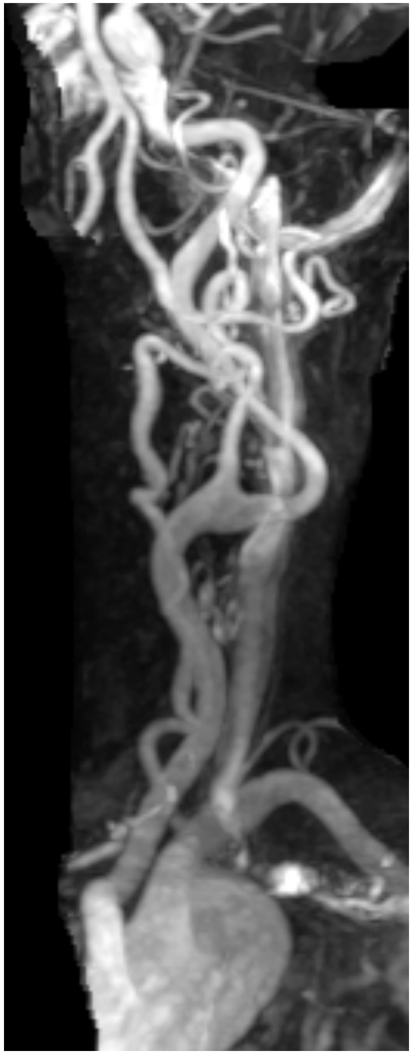

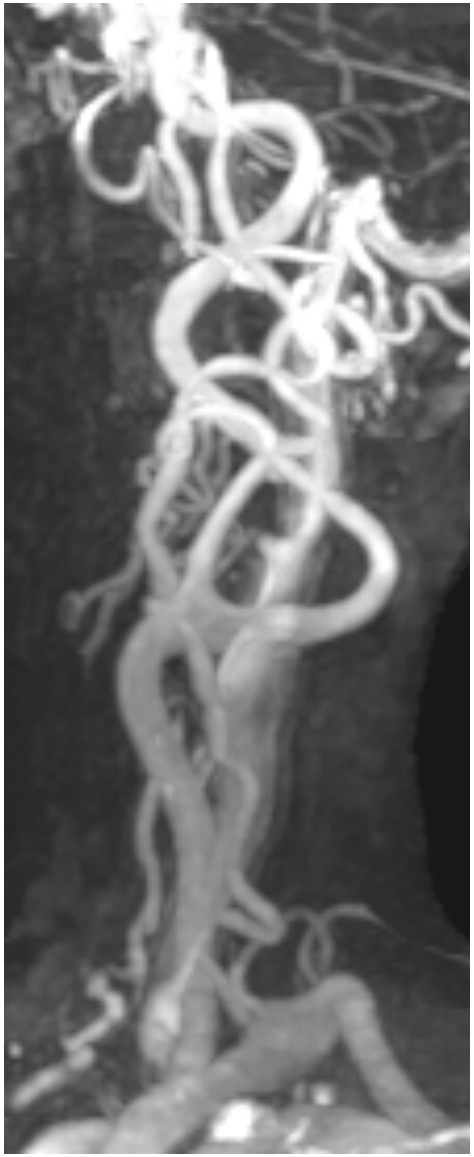

112e Score

0-30

31-50

51-70

>70

Near occlusion

Occluded

Quality

1

2

3

4

5

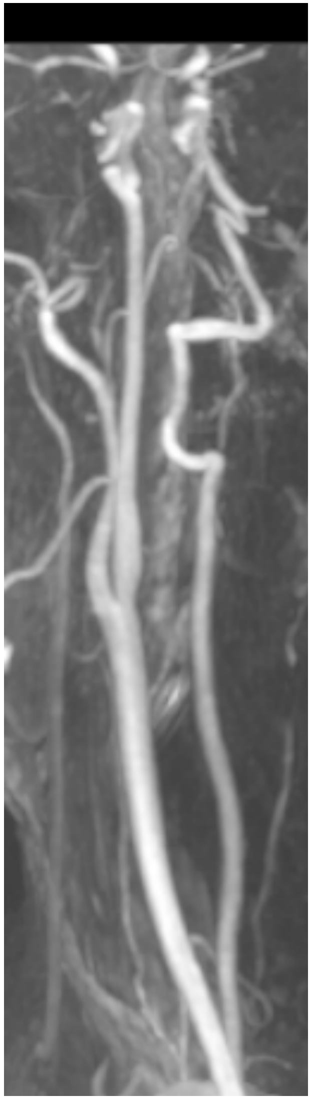

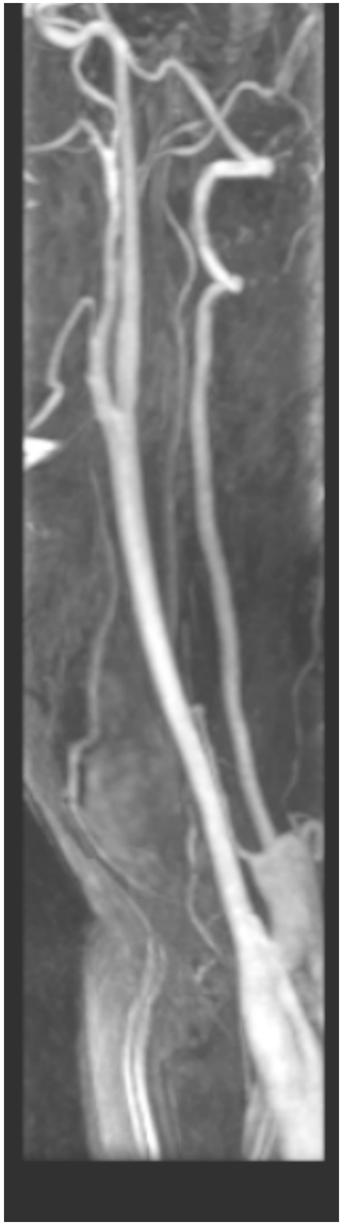

113d Score

0-30

31-50

51-70

>70

Near occlusion

Occluded

Quality

1

2

3

4

5

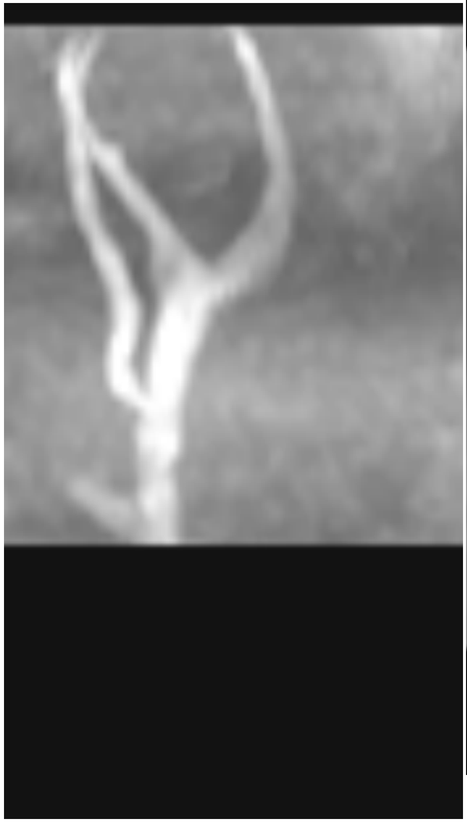

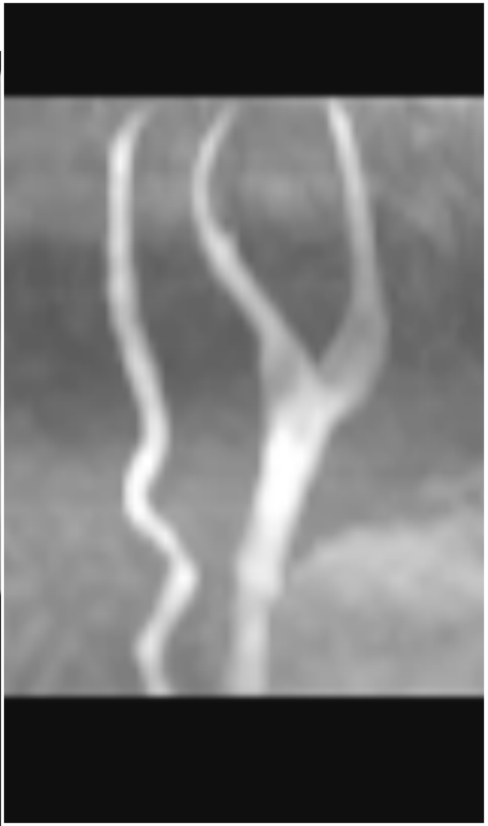

114c Score

0-30

31-50

51-70

>70

Near occlusion

Occluded

Quality

1

2

3

4

5

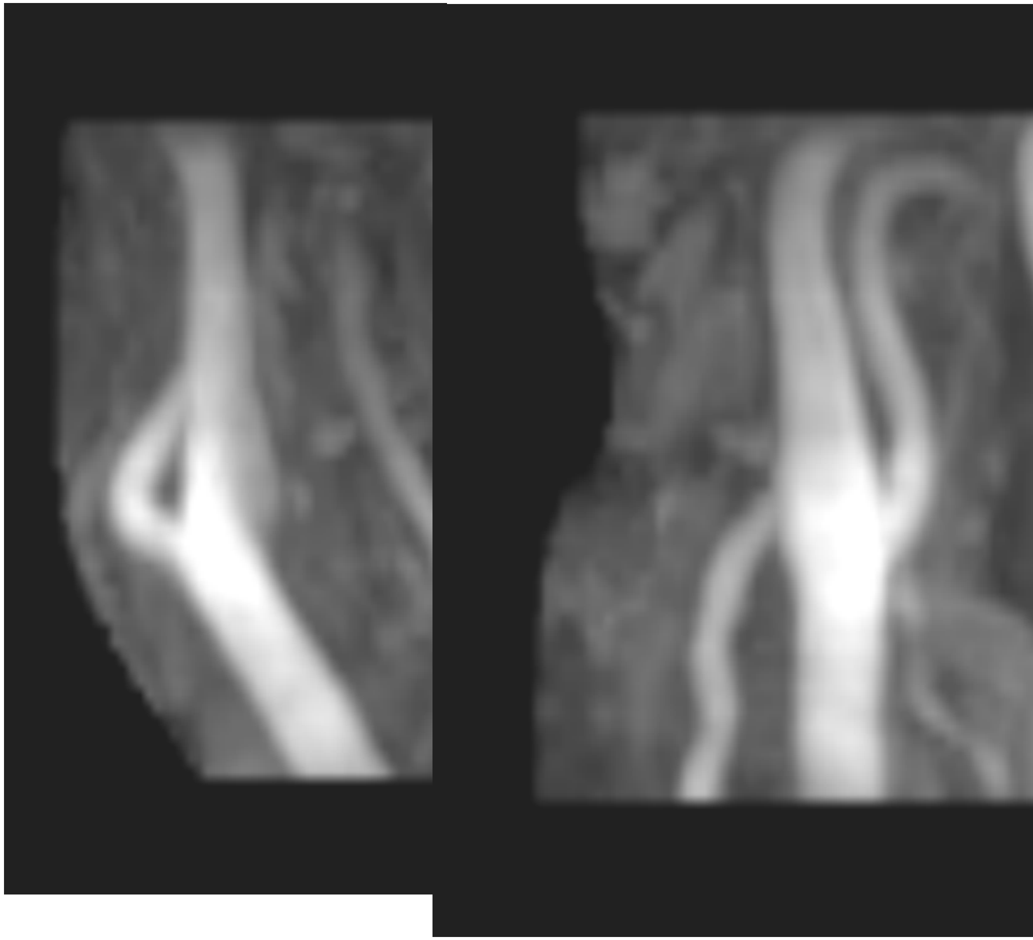

115b Score

0-30

31-50

51-70

>70

Near occlusion

Occluded

Quality

1

2

3

4

5

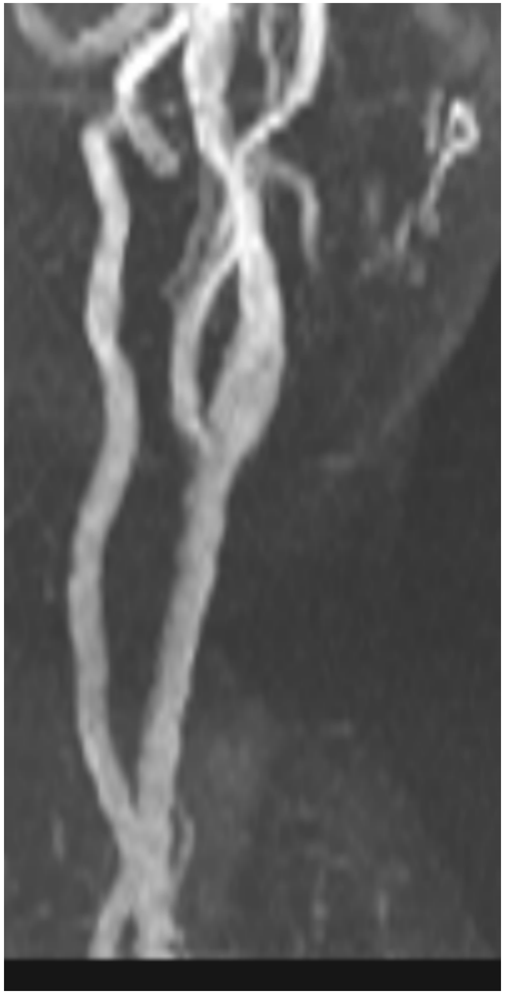

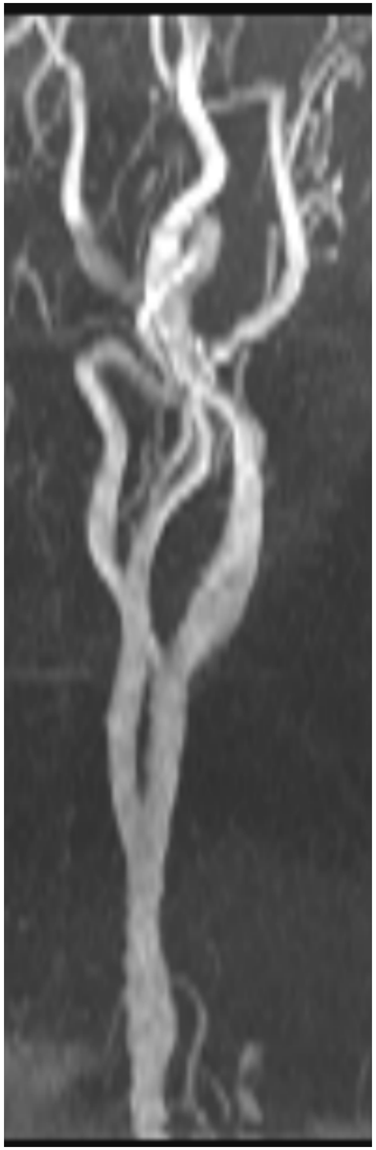

116a Score

0-30

31-50

51-70

>70

Near occlusion

Occluded

Quality

1

2

3

4

5

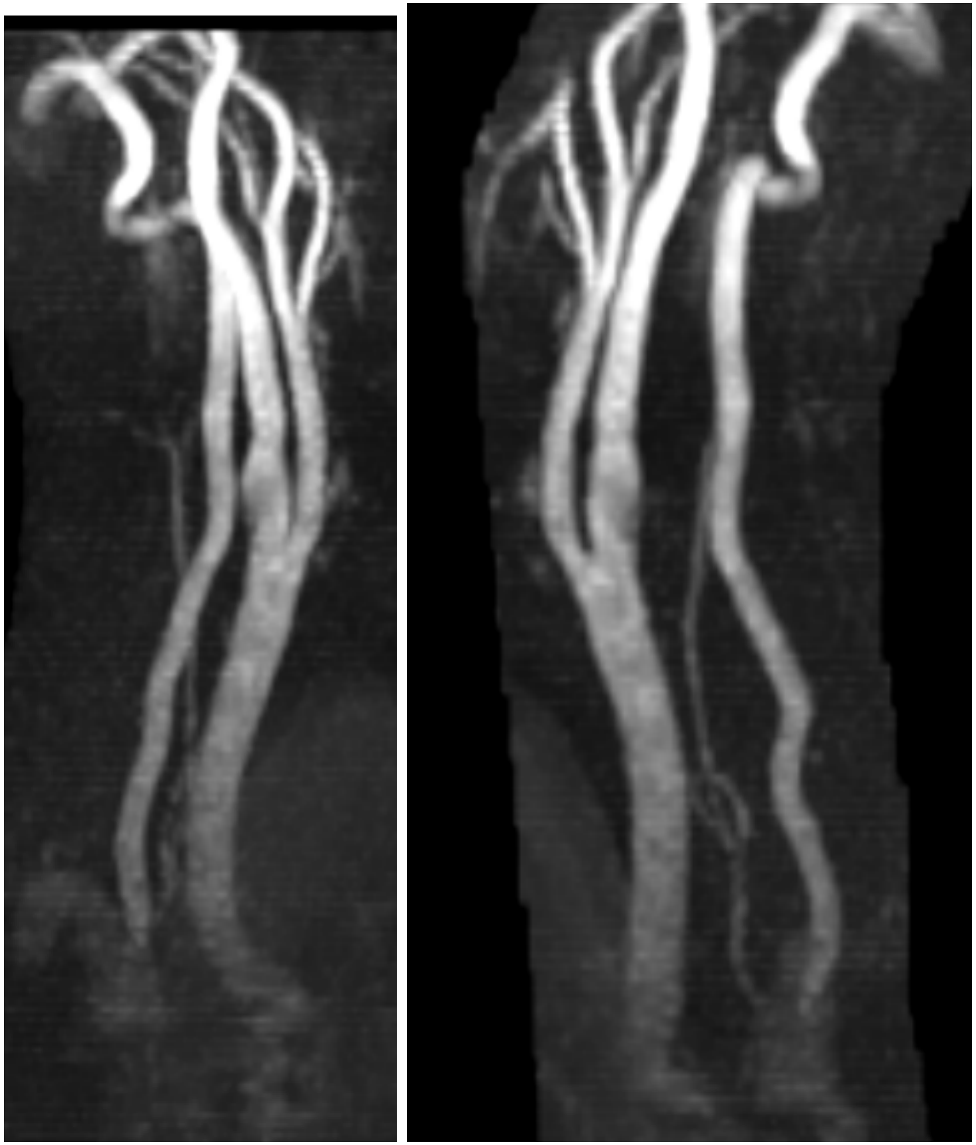

116f Score

0-30

31-50

51-70

>70

Near occlusion

Occluded

Quality

1

2

3

4

5

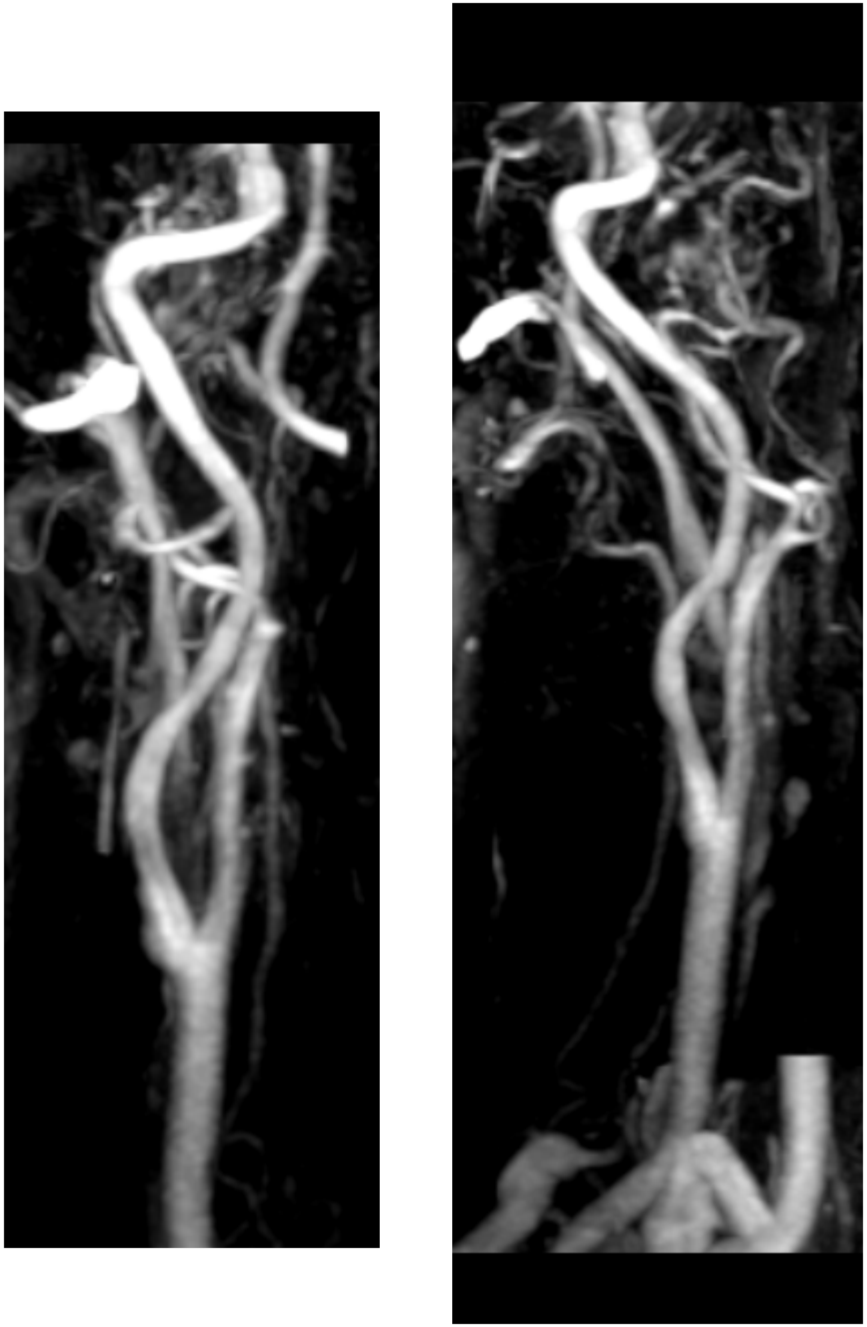

117e Score

0-30

31-50

51-70

>70

Near occlusion

Occluded

Quality

1

2

3

4

5

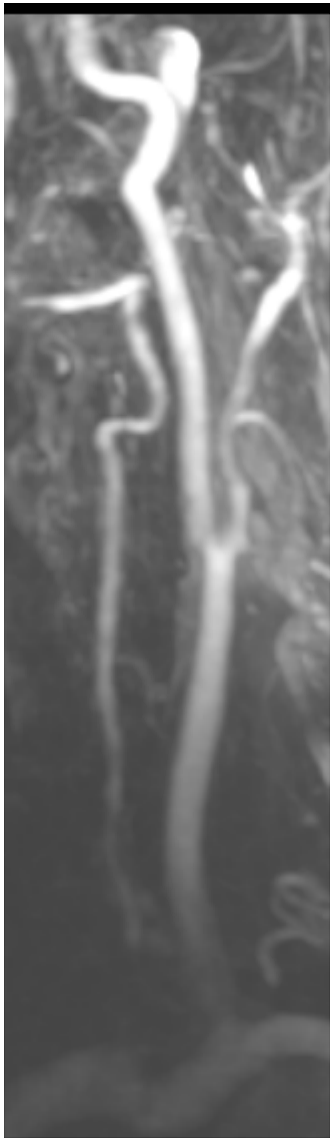

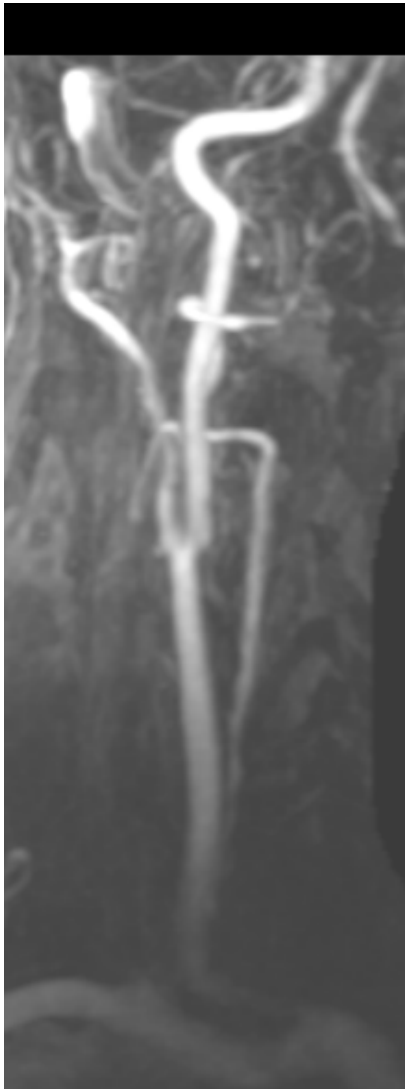

118d Score

0-30

31-50

51-70

>70

Near occlusion

Occluded

Quality

1

2

3

4

5

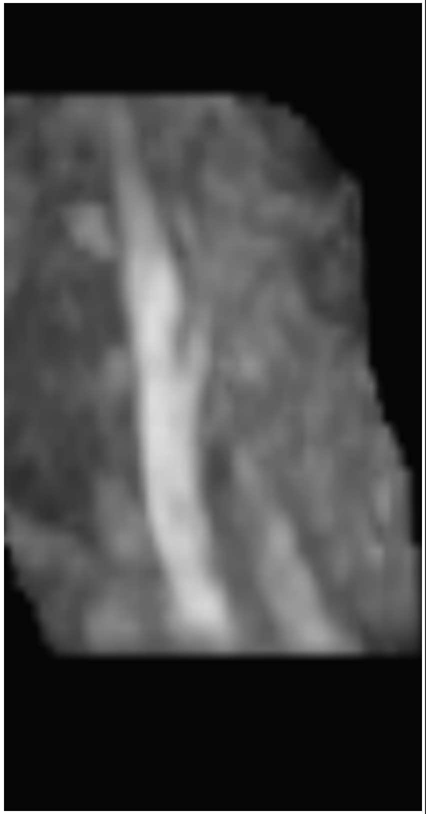

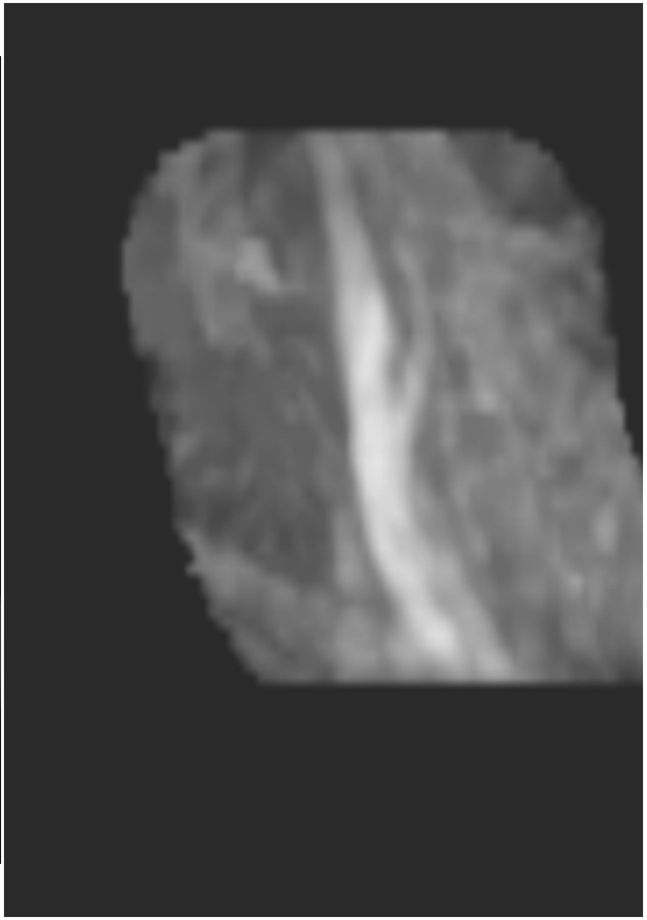

119c Score

0-30

31-50

51-70

>70

Near occlusion

Occluded

Quality

1

2

3

4

5

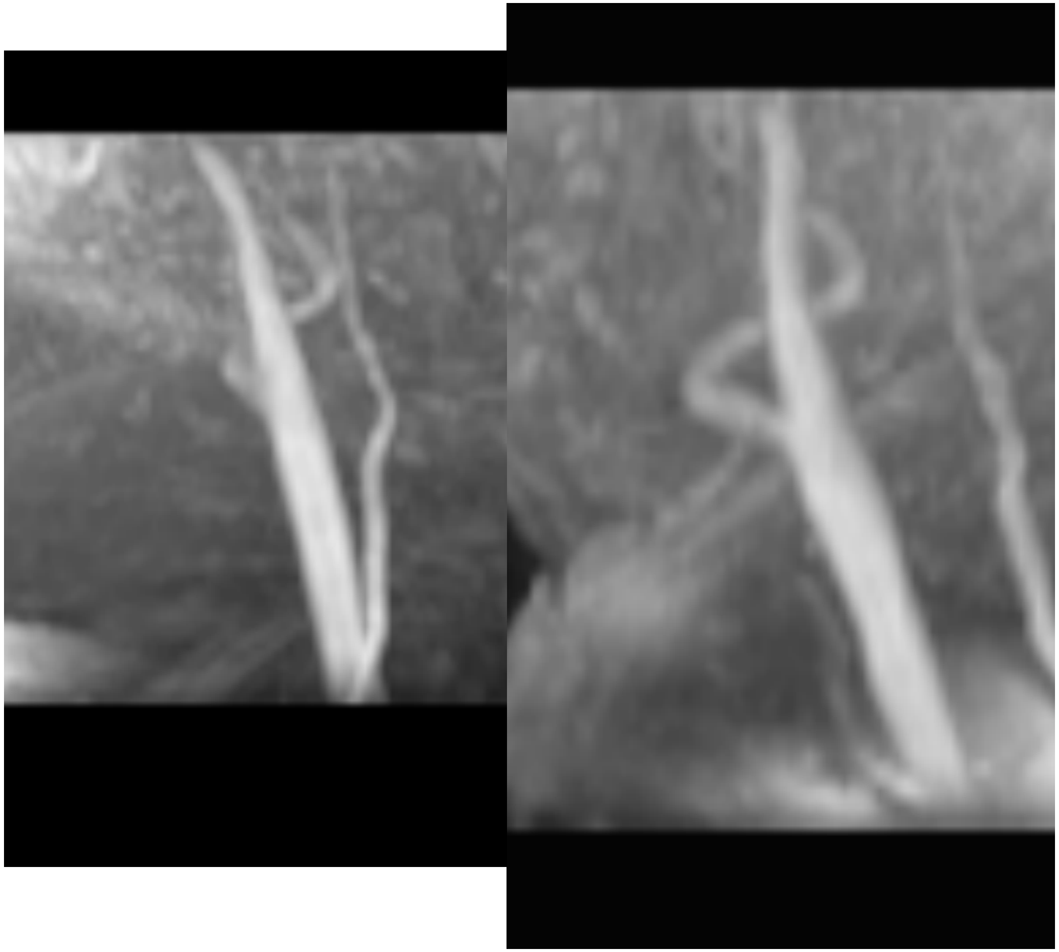

120b Score

0-30

31-50

51-70

>70

Near occlusion

Occluded

Quality

1

2

3

4

5

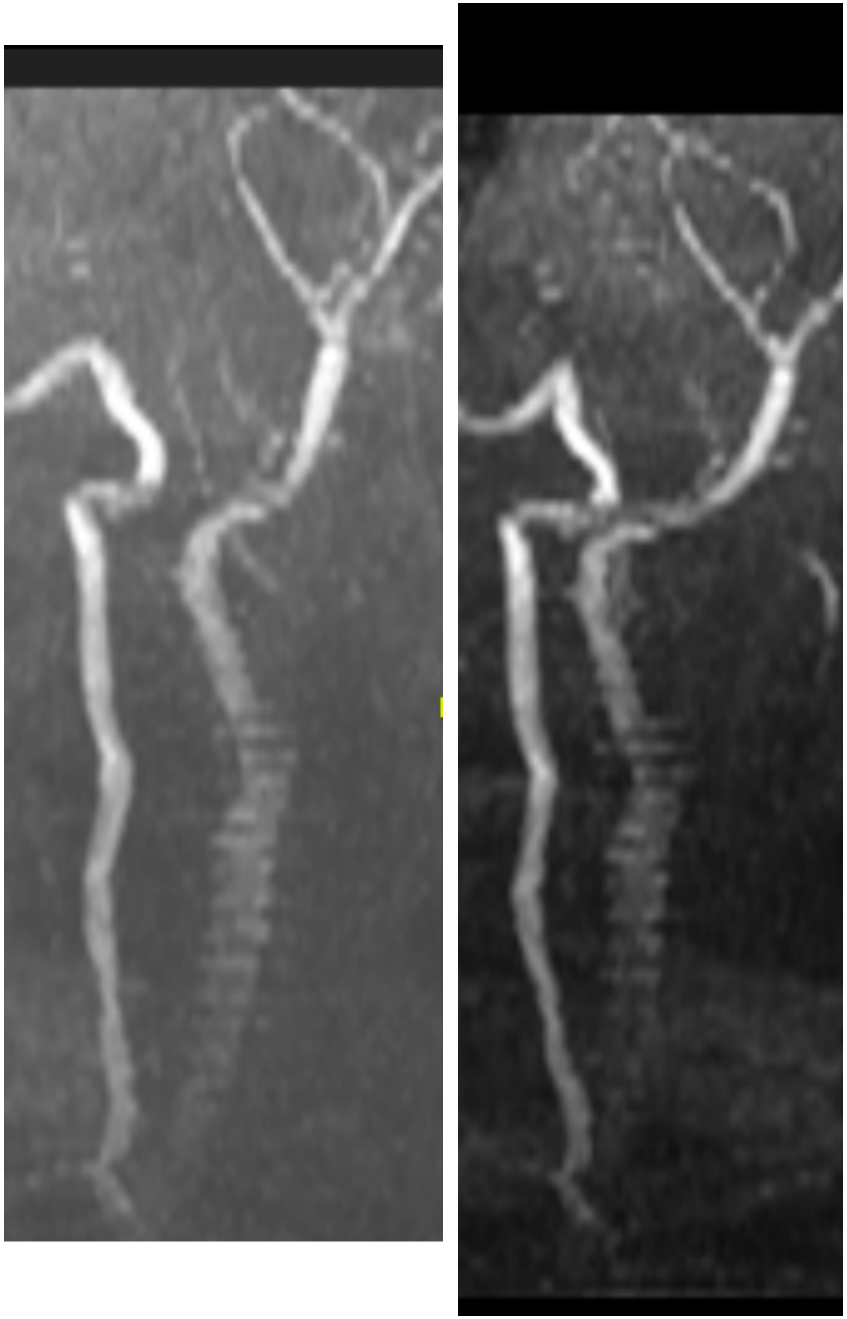

121a Score

0-30

31-50

51-70

>70

Near occlusion

Occluded

Quality

1

2

3

4

5

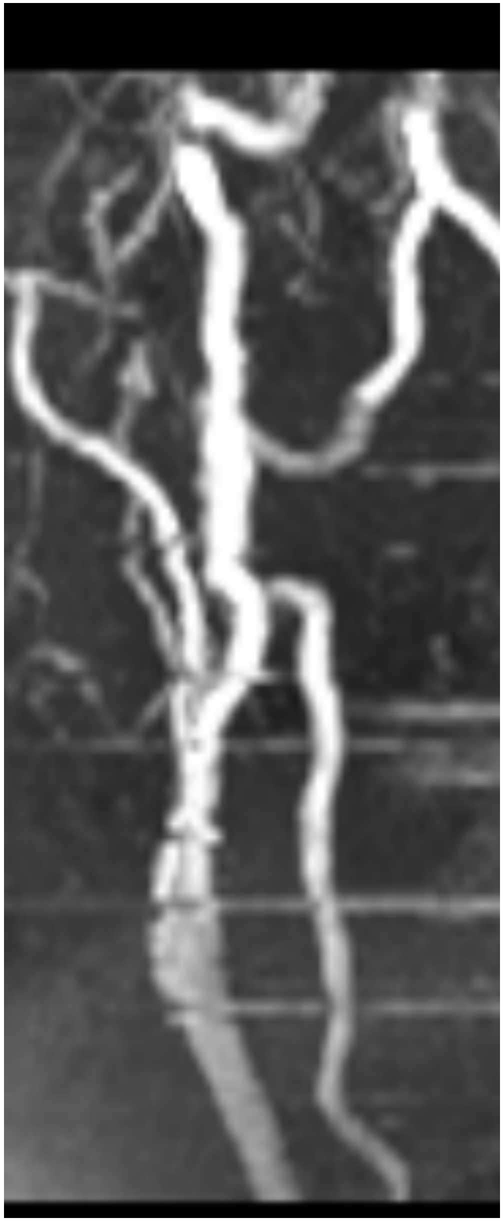

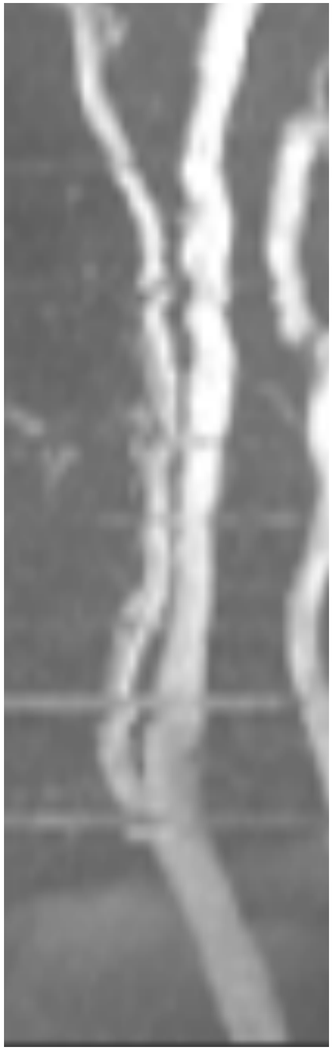

121f Score

0-30

31-50

51-70

>70

Near occlusion

Occluded

Quality

1

2

3

4

5

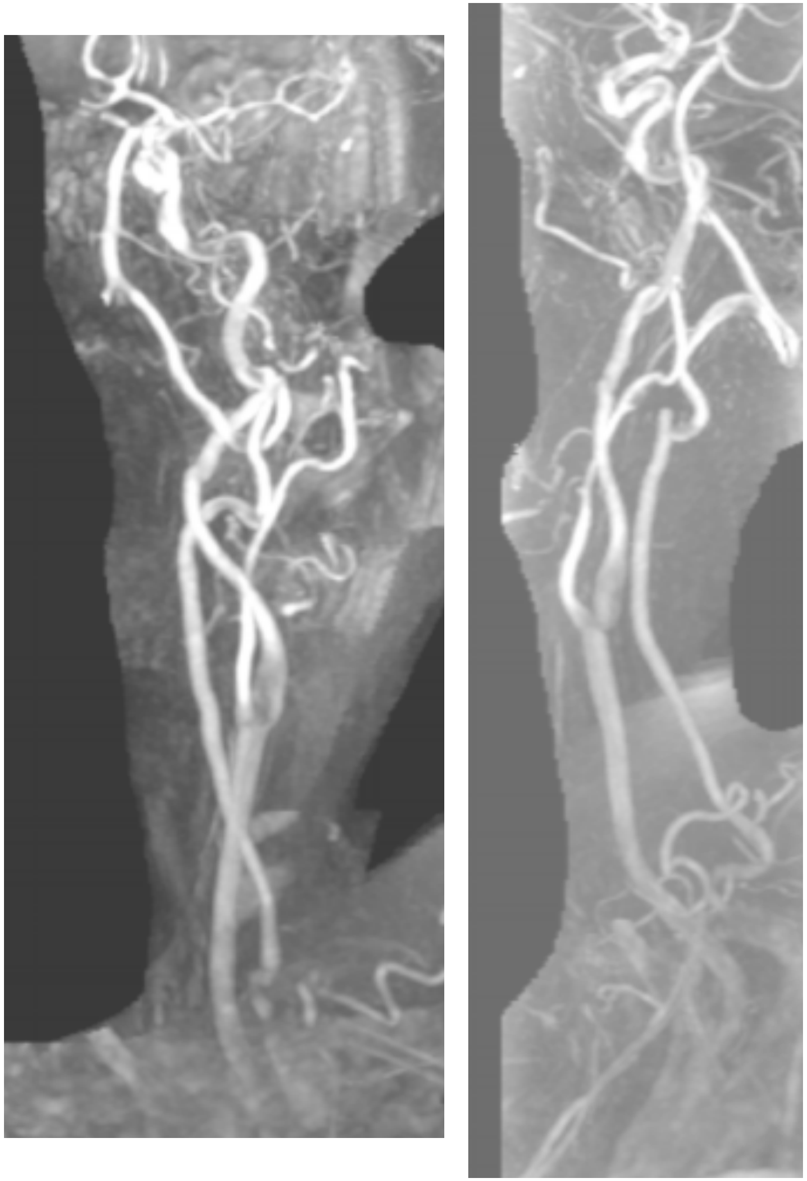

122e Score

0-30

31-50

51-70

>70

Near occlusion

Occluded

Quality

1

2

3

4

5

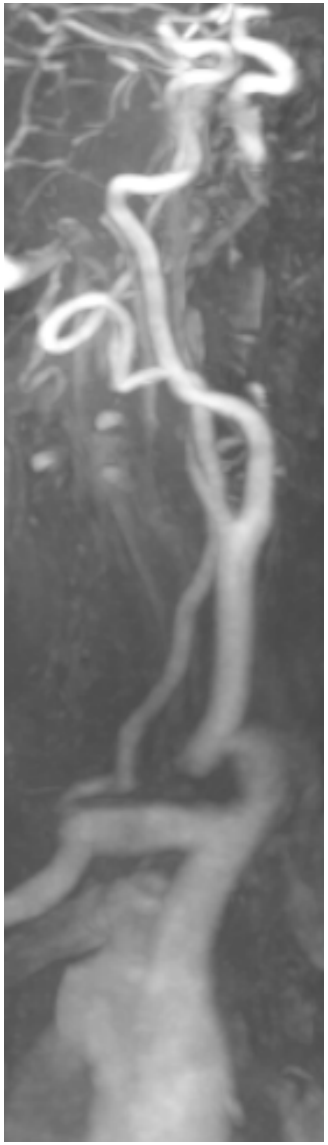

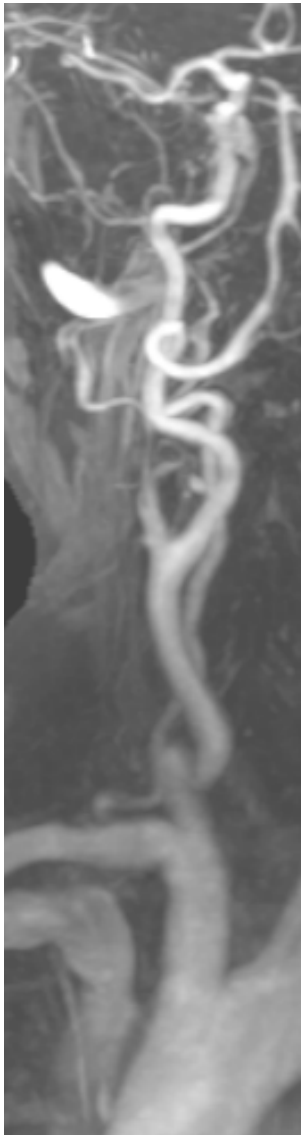

123d Score

0-30

31-50

51-70

>70

Near occlusion

Occluded

Quality

1

2

3

4

5

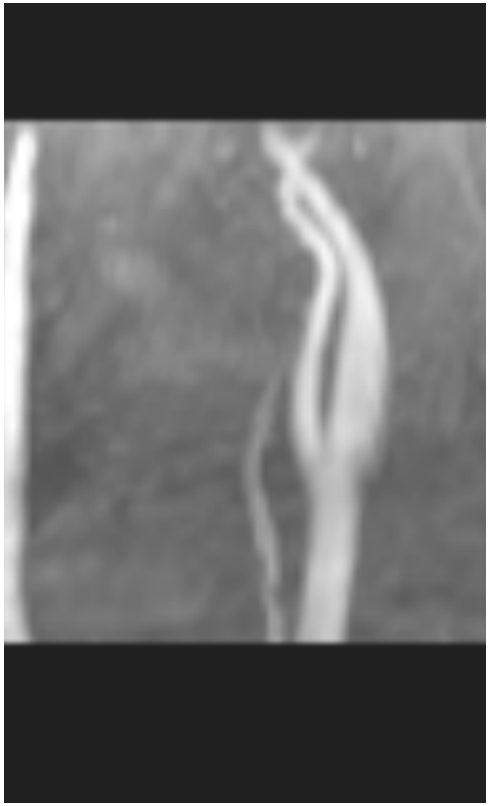

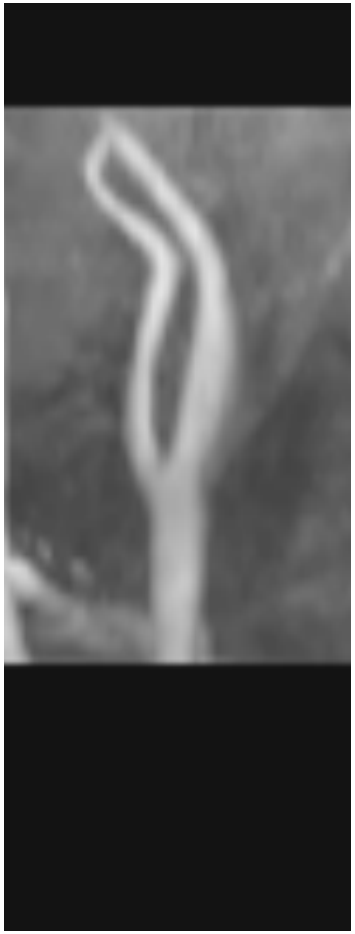

124c Score

0-30

31-50

51-70

>70

Near occlusion

Occluded

Quality

1

2

3

4

5

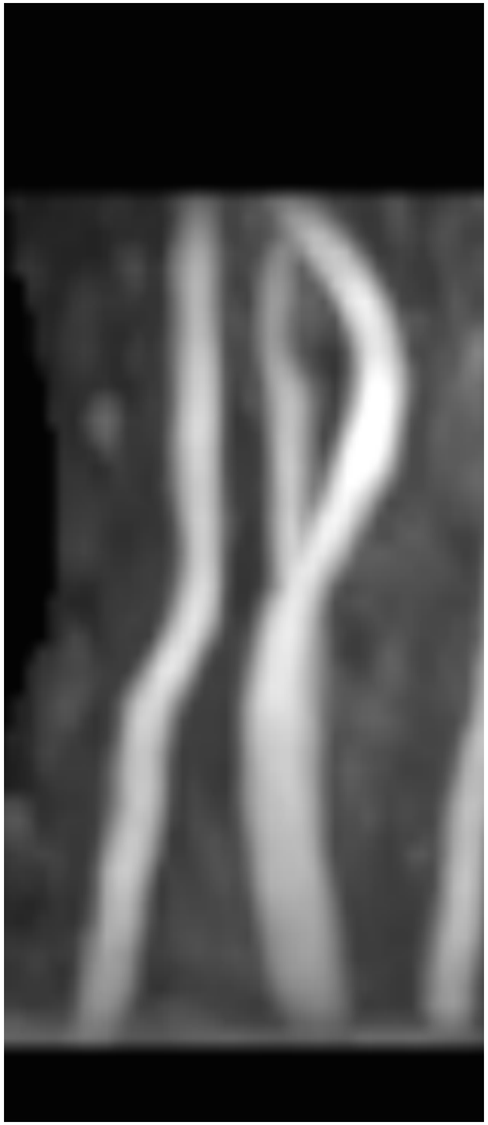

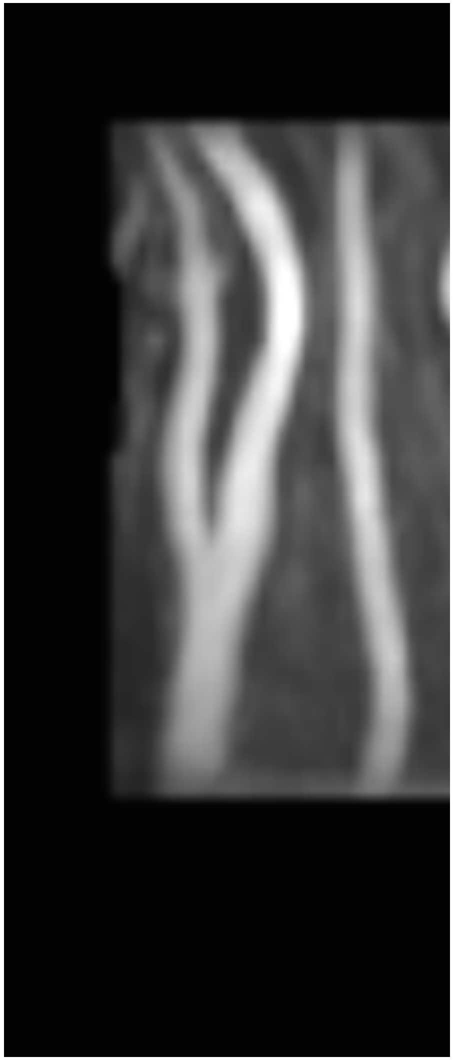

125b Score

0-30

31-50

51-70

>70

Near occlusion

Occluded

Quality

1

2

3

4

5

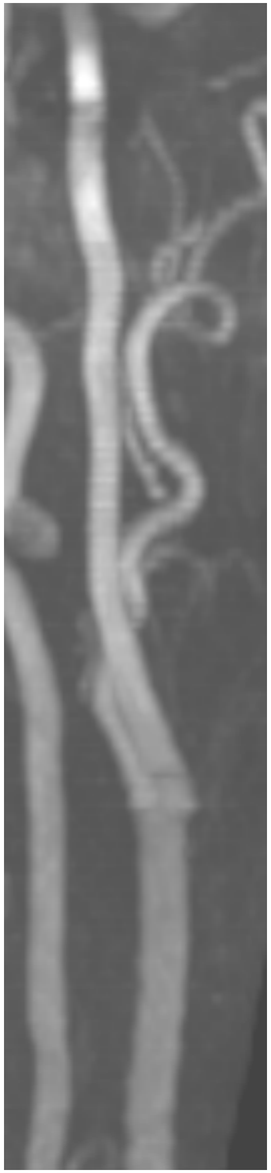

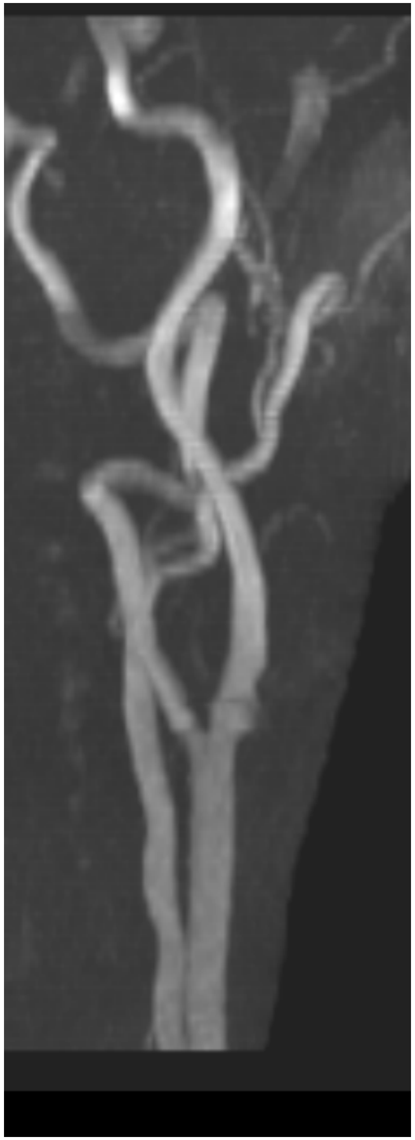

126a Score

0-30

31-50

51-70

>70

Near occlusion

Occluded

Quality

1

2

3

4

5

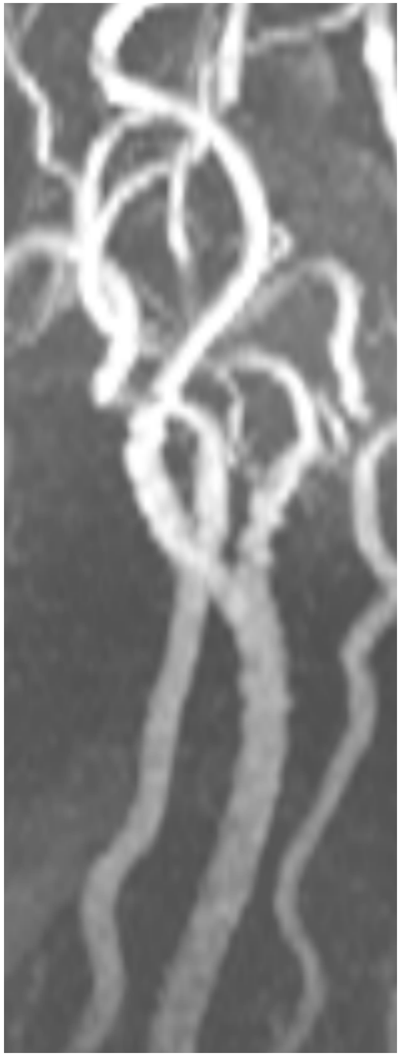

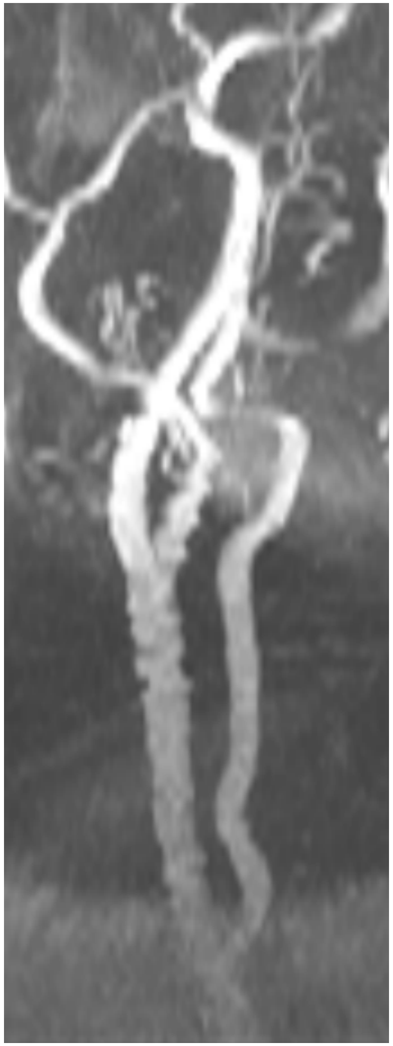

126f Score

0-30

31-50

51-70

>70

Near occlusion

Occluded

Quality

1

2

3

4

5

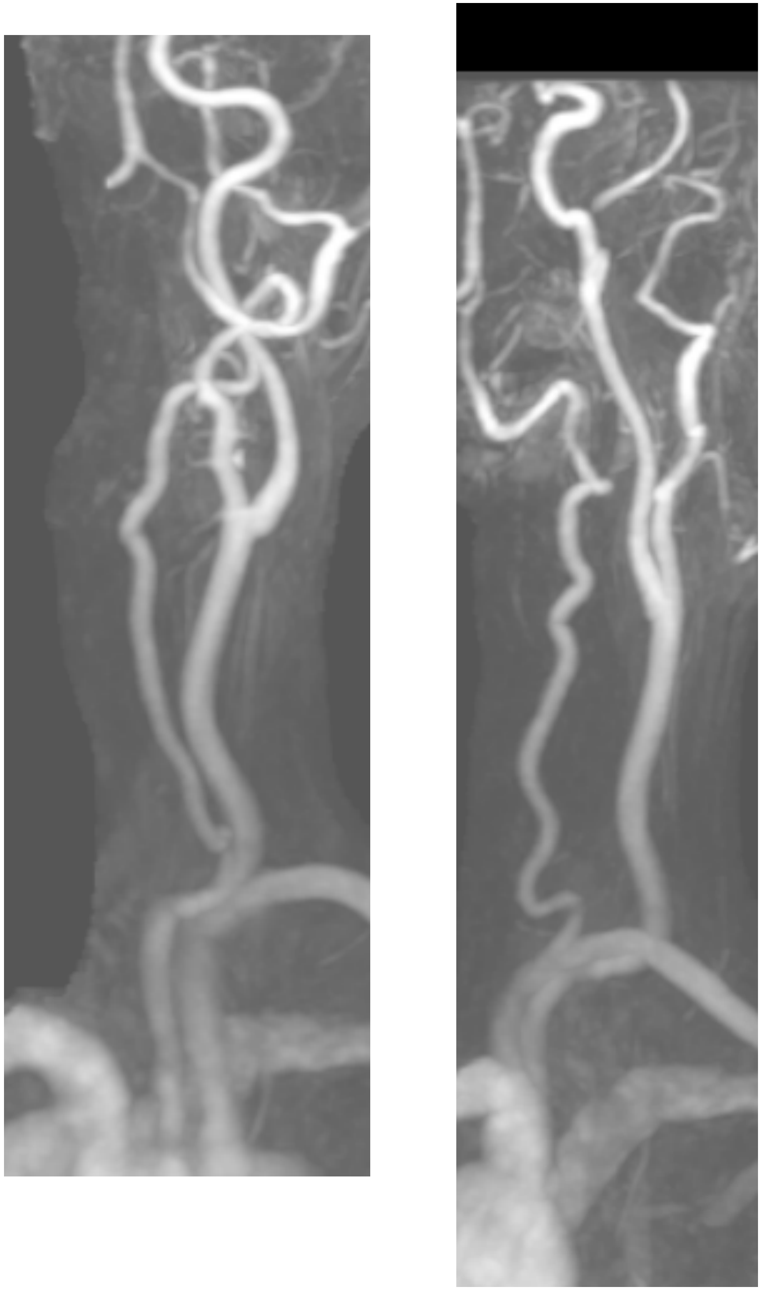

127e Score

0-30

31-50

51-70

>70

Near occlusion

Occluded

Quality

1

2

3

4

5

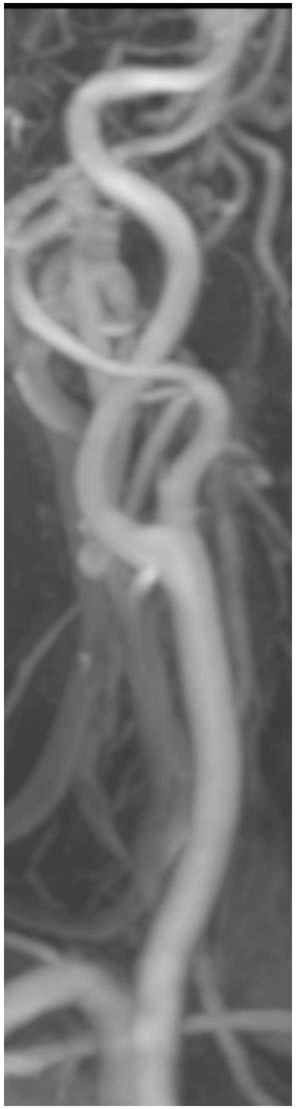

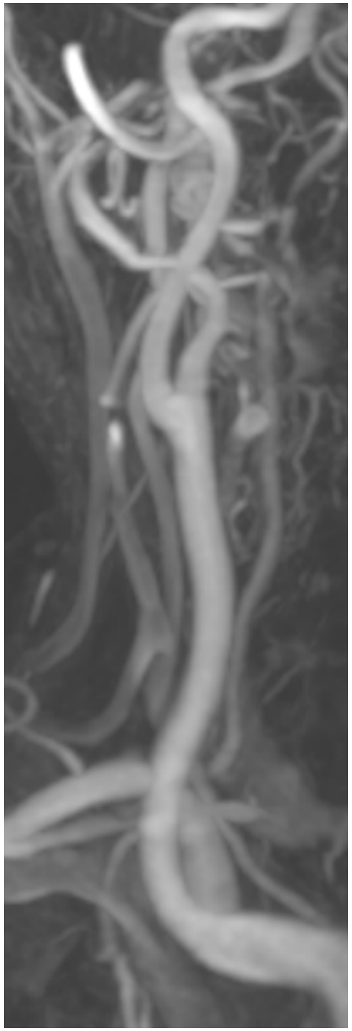

128d Score

0-30

31-50

51-70

>70

Near occlusion

Occluded

Quality

1

2

3

4

5

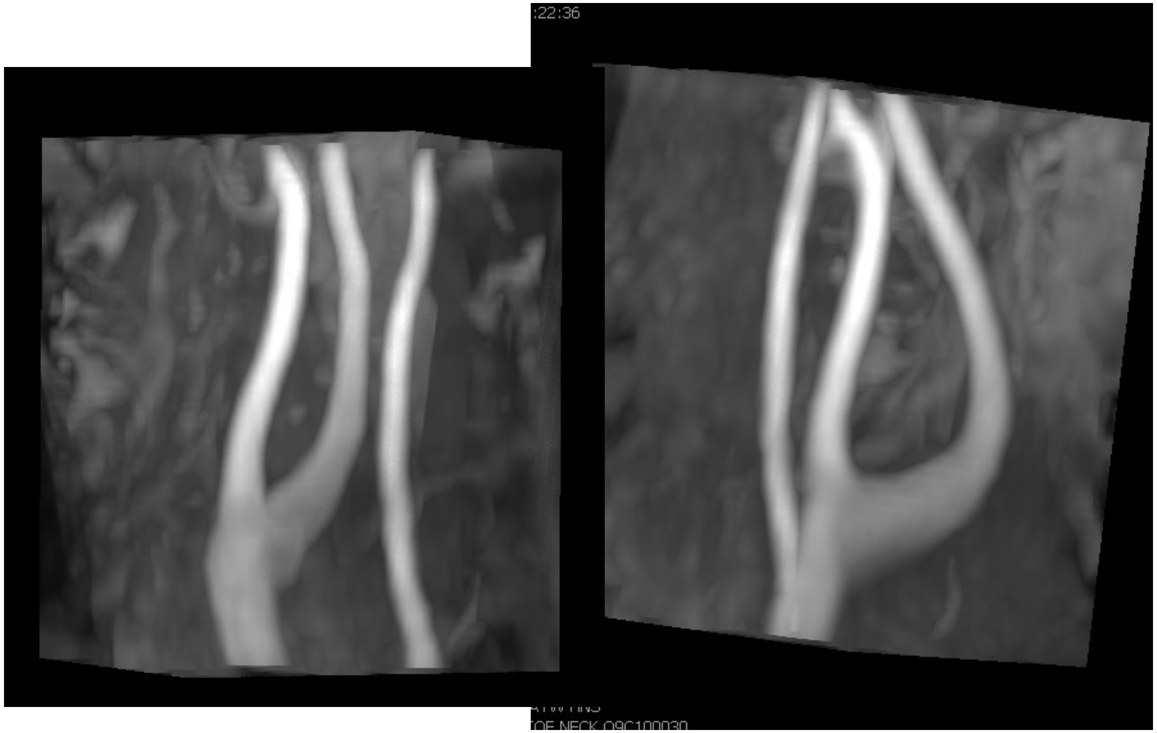

129c Score

0-30

31-50

51-70

>70

Near occlusion

Occluded

Quality

1

2

3

4

5

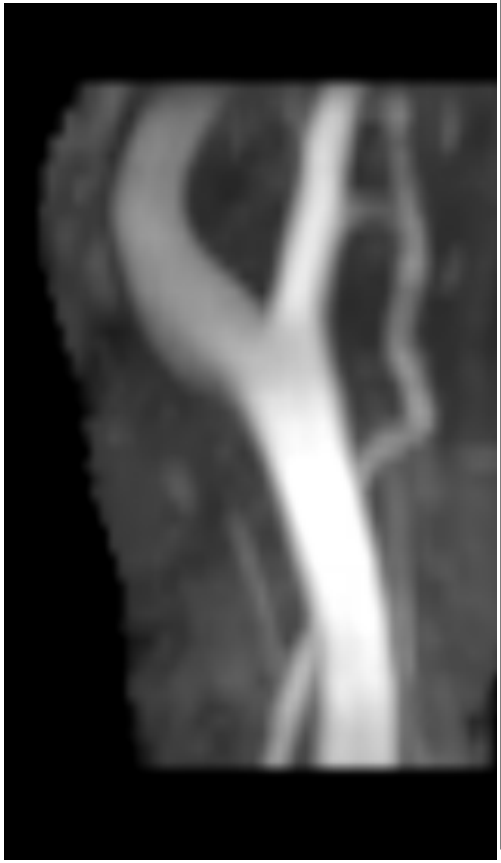

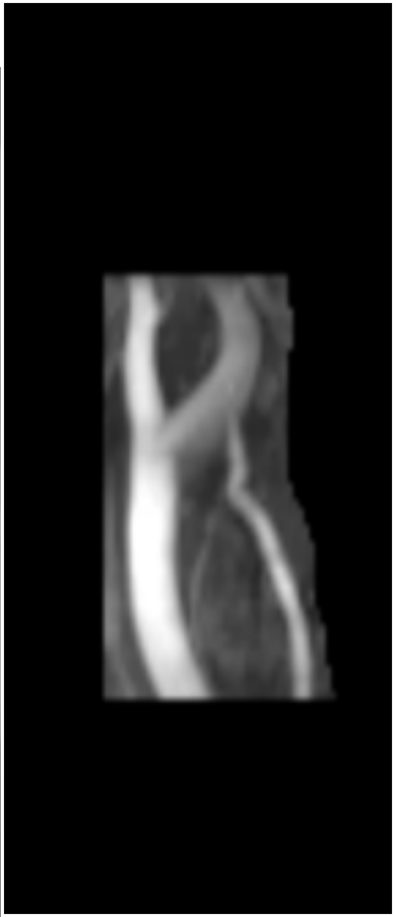

130b Score

0-30

31-50

51-70

>70

Near occlusion

Occluded

Quality

1

2

3

4

5

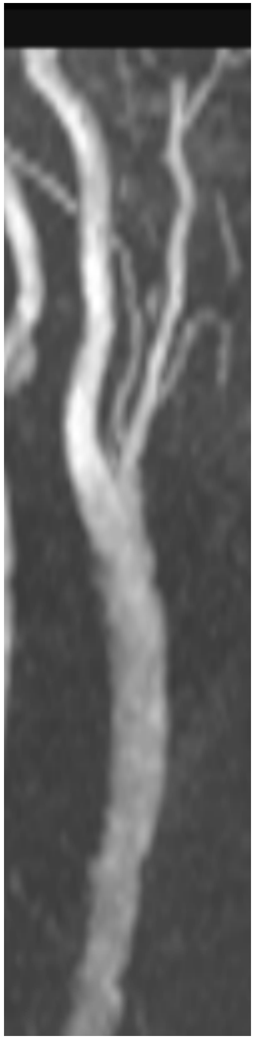

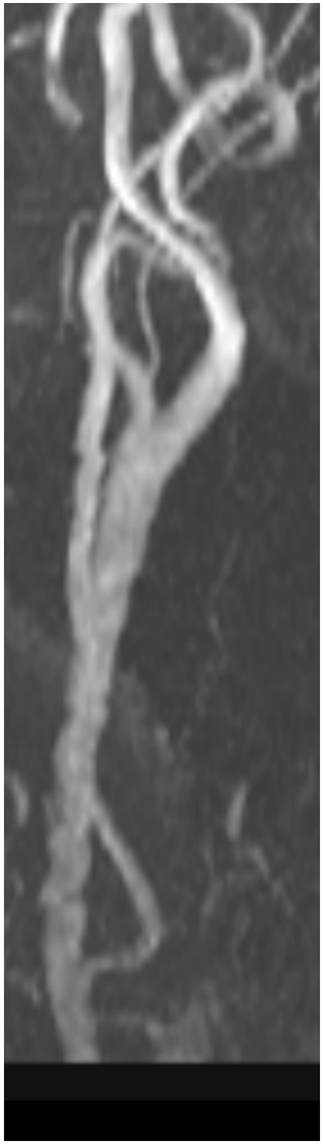

131a Score

0-30

31-50

51-70

>70

Near occlusion

Occluded

Quality

1

2

3

4

5

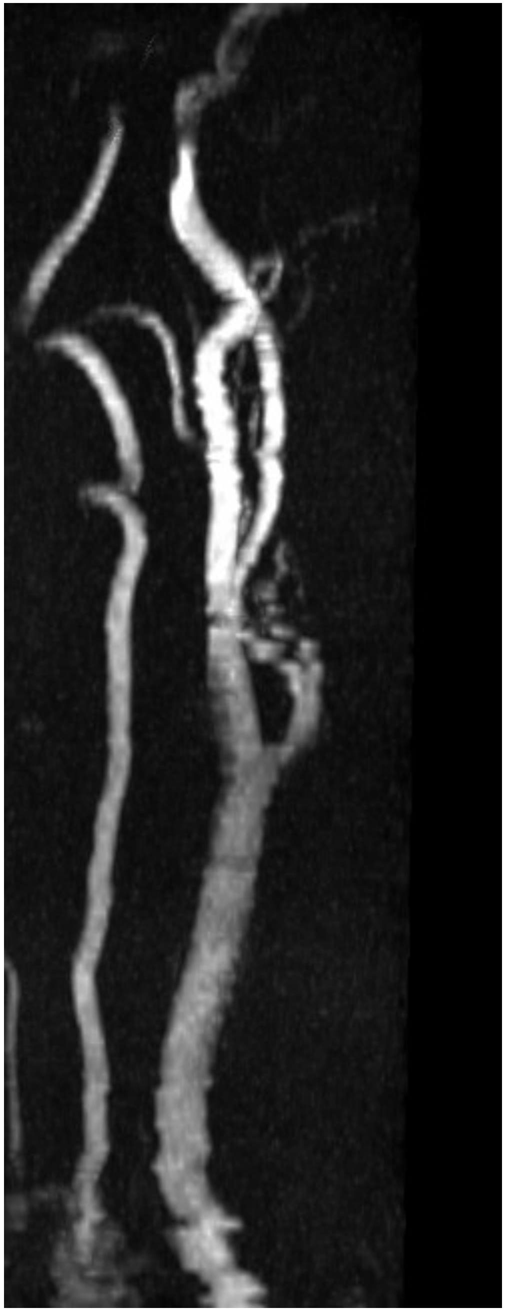

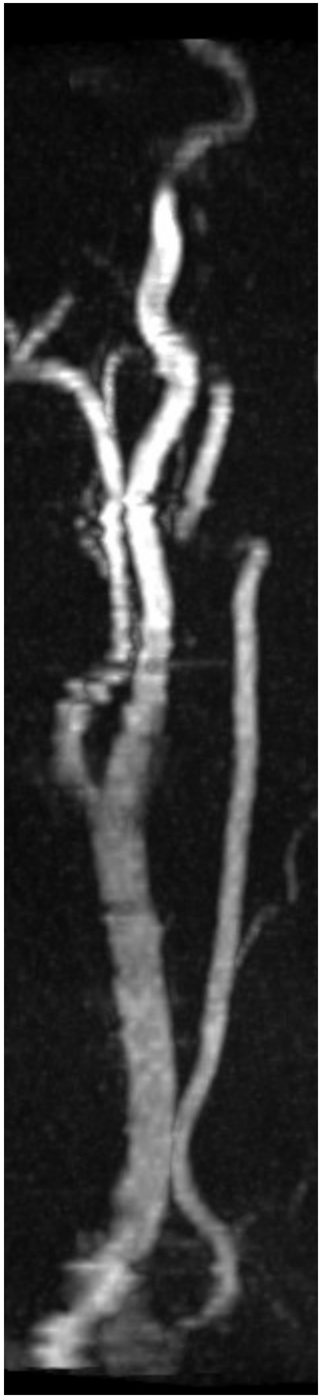

131f Score

0-30

31-50

51-70

>70

Near occlusion

Occluded

Quality

1

2

3

4

5

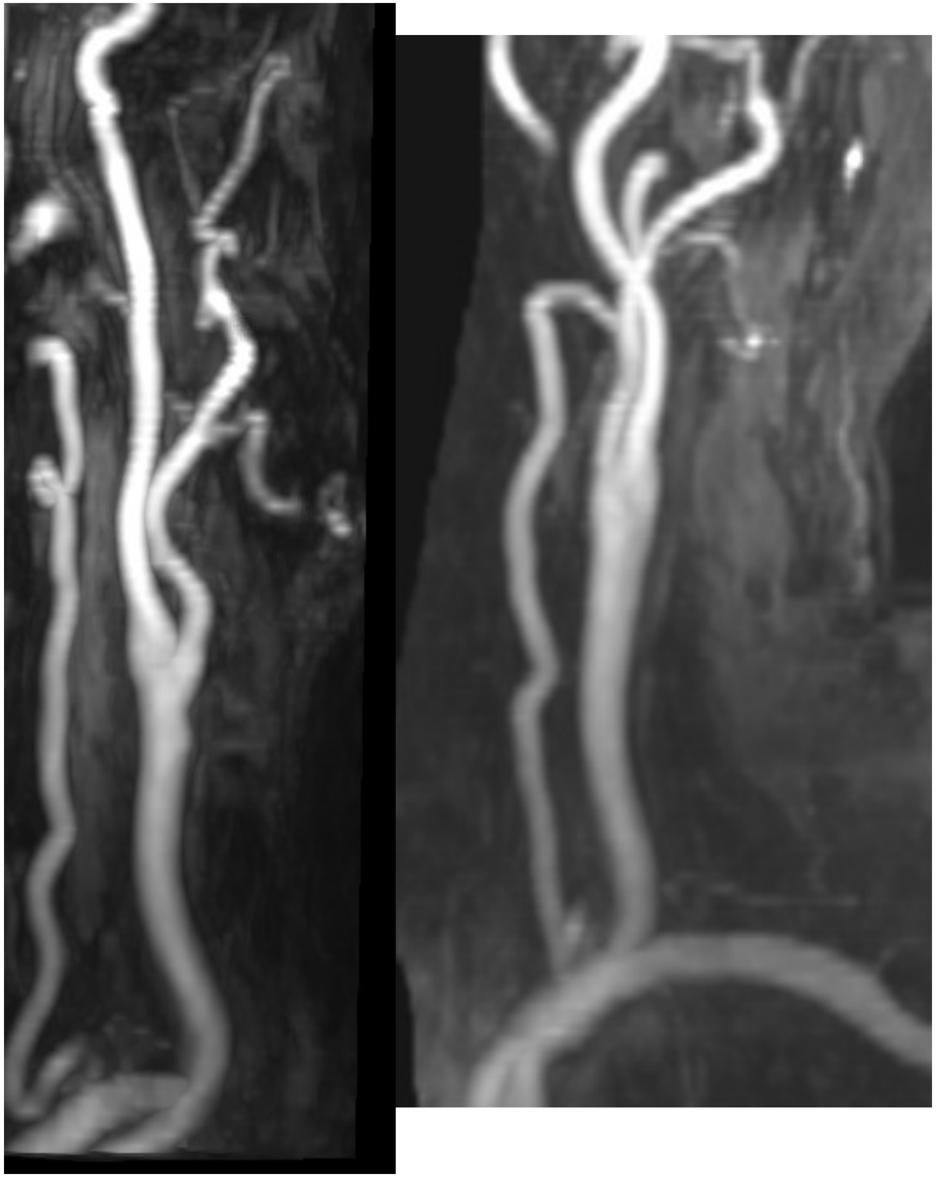

132e Score

0-30

31-50

51-70

>70

Near occlusion

Occluded

Quality

1

2

3

4

5

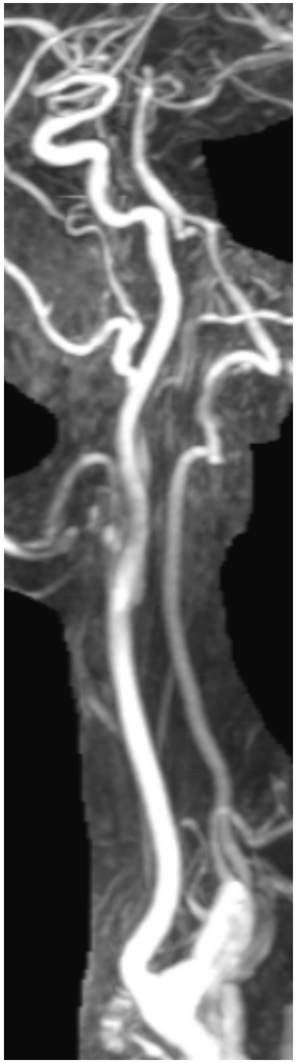

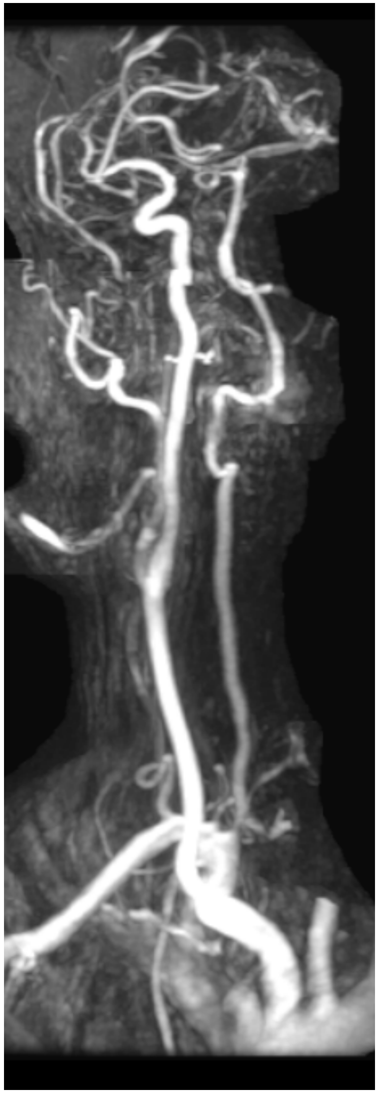

133d Score

0-30

31-50

51-70

>70

Near occlusion

Occluded

Quality

1

2

3

4

5

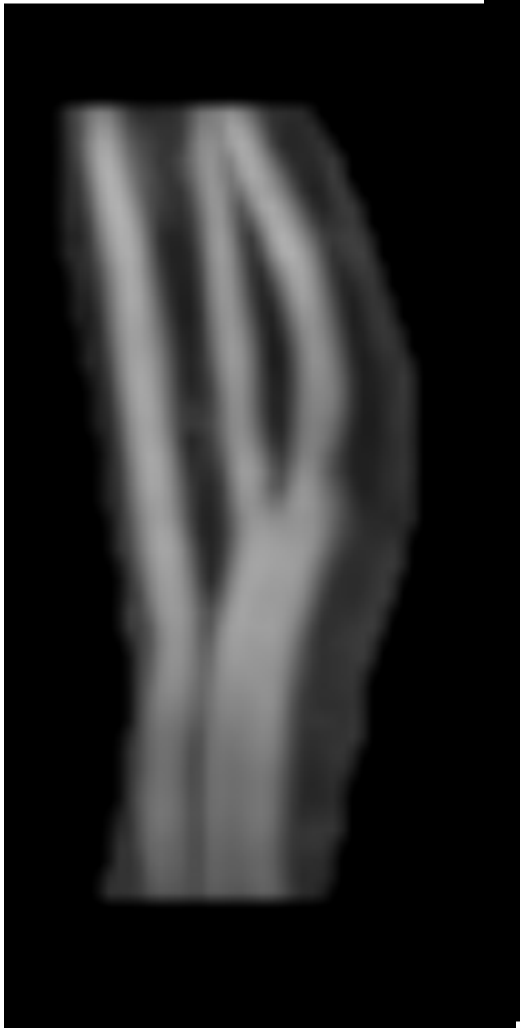

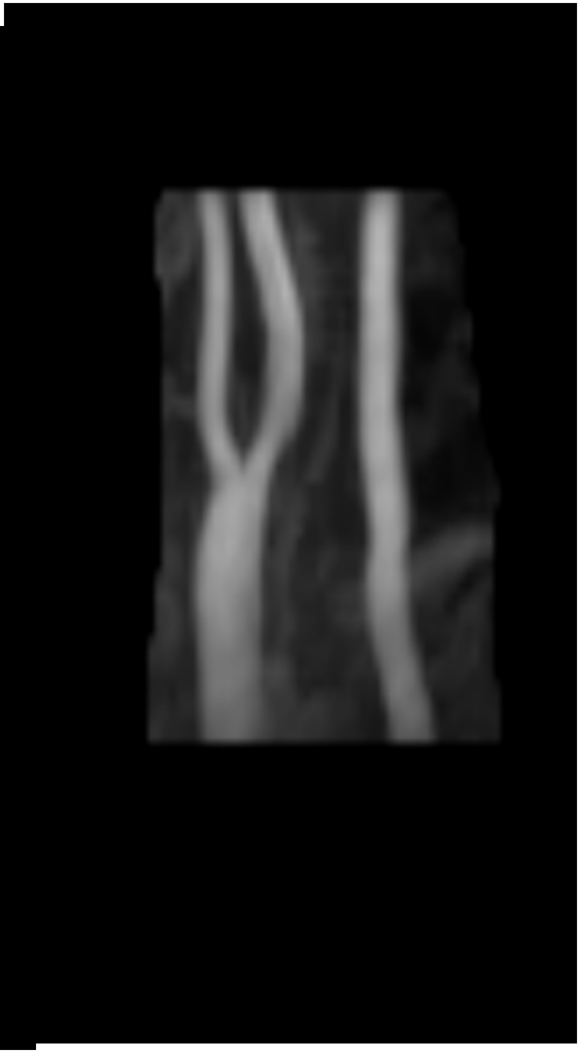

134c Score

0-30

31-50

51-70

>70

Near occlusion

Occluded

Quality

1

2

3

4

5

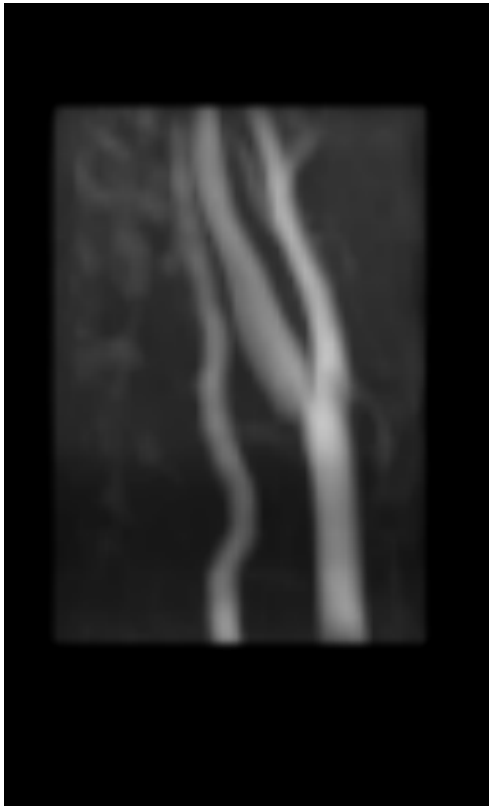

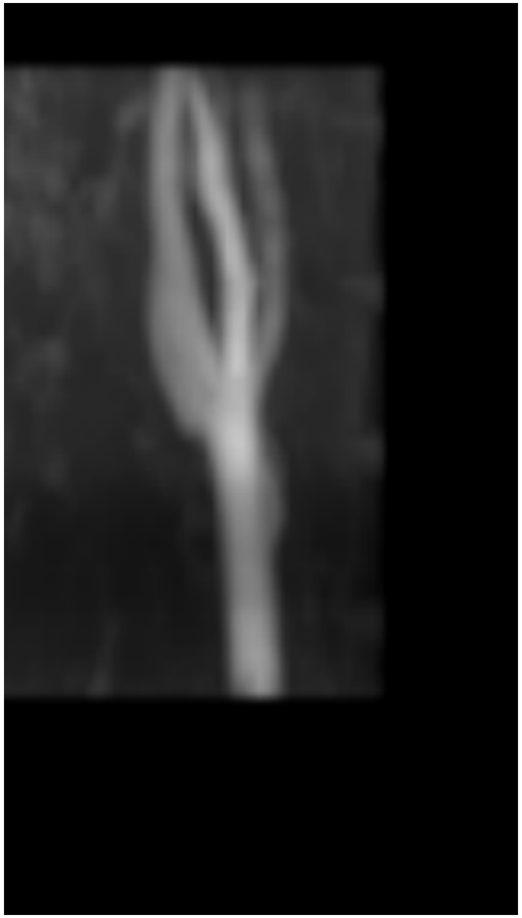

135b Score

0-30

31-50

51-70

>70

Near occlusion

Occluded

Quality

1

2

3

4

5

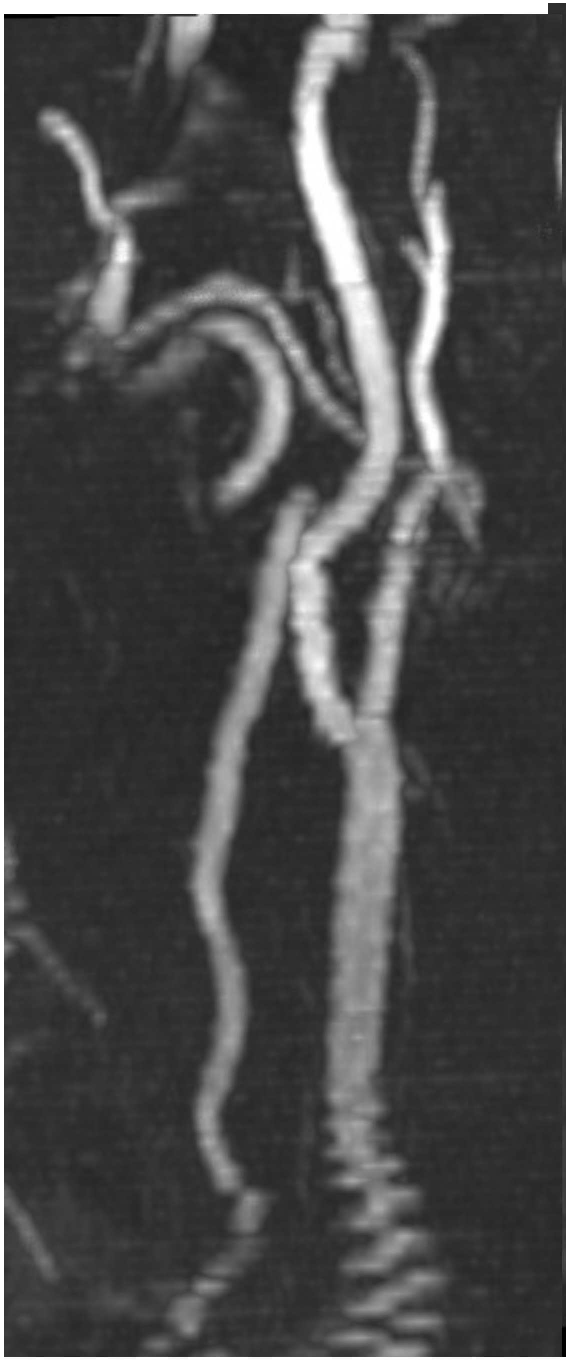

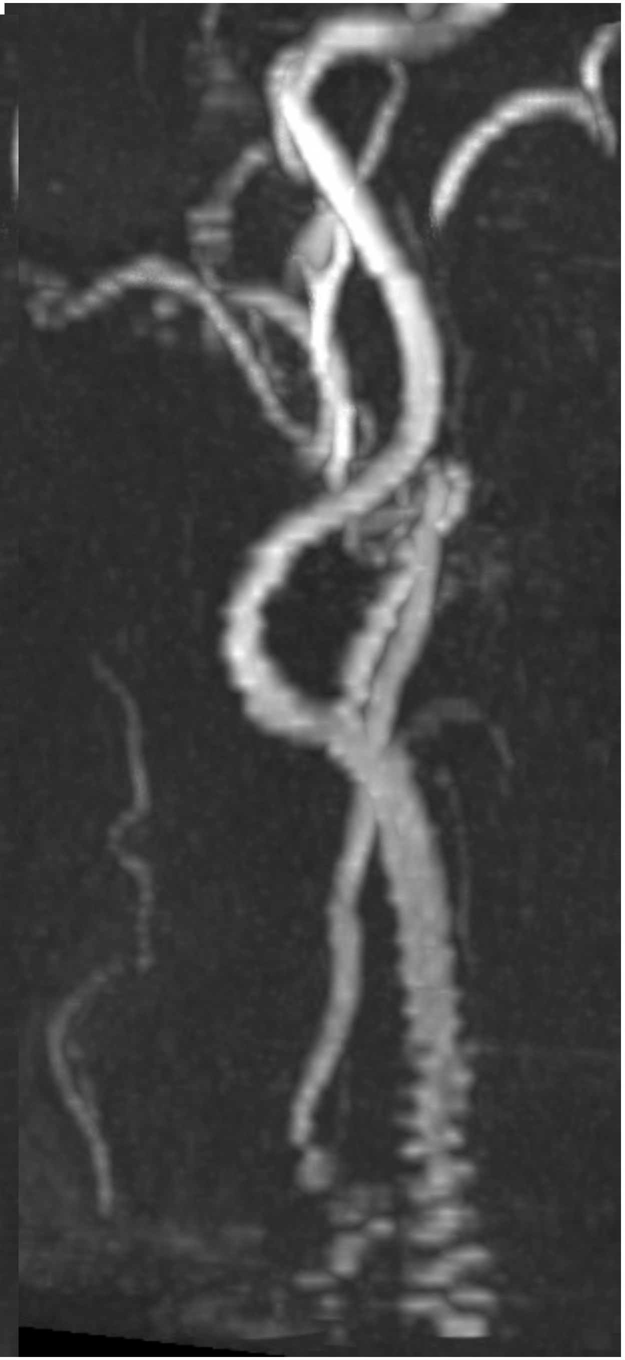

136a Score

0-30

31-50

51-70

>70

Near occlusion

Occluded

Quality

1

2

3

4

5

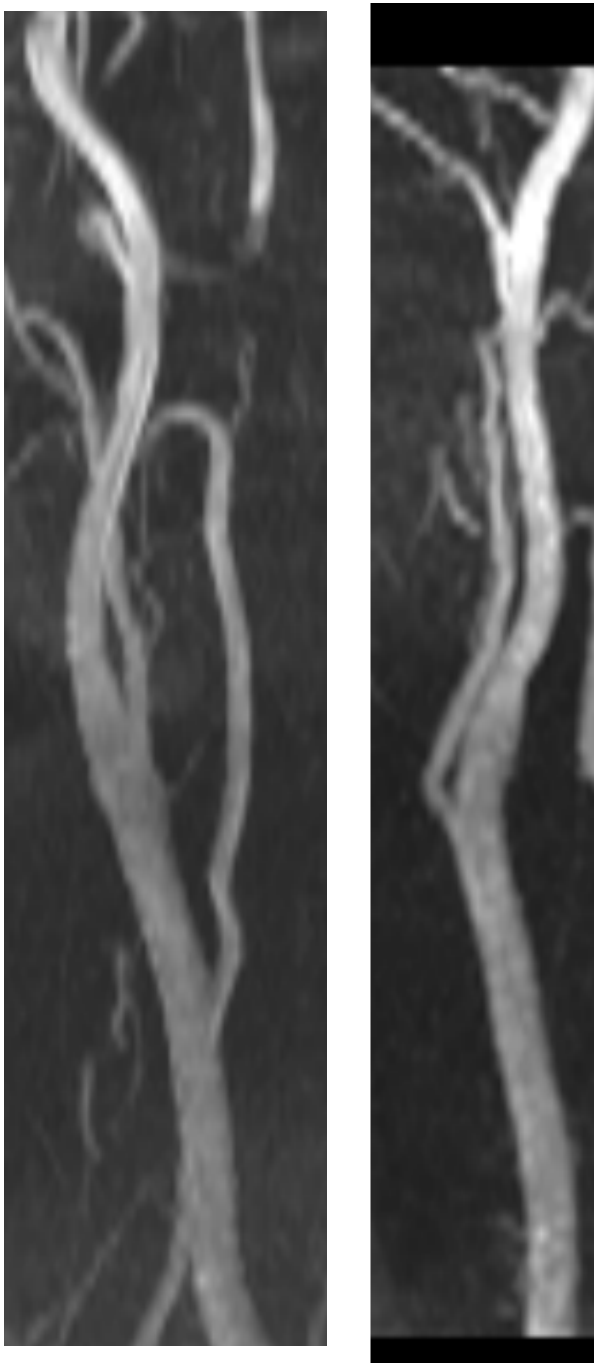

136f Score

0-30

31-50

51-70

>70

Near occlusion

Occluded

Quality

1

2

3

4

5

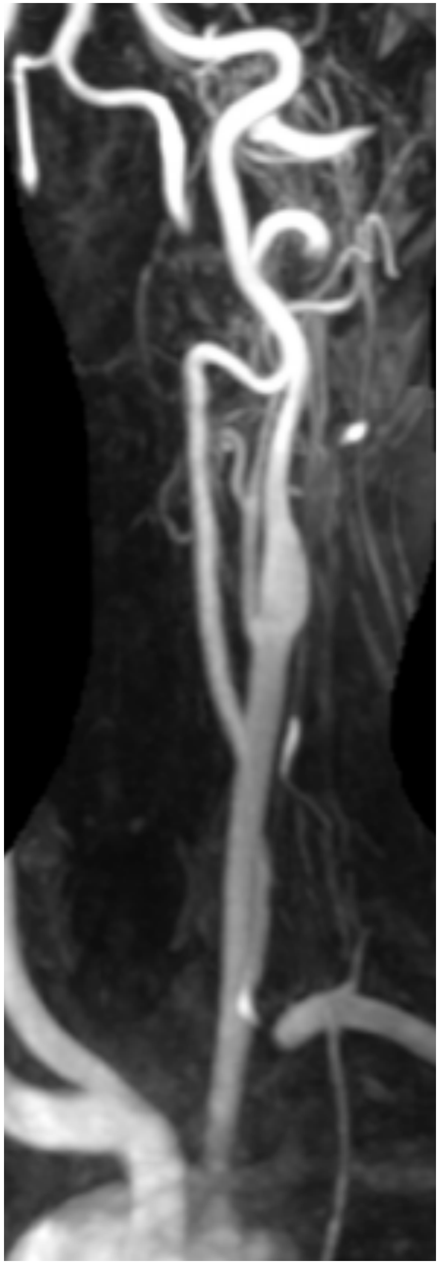

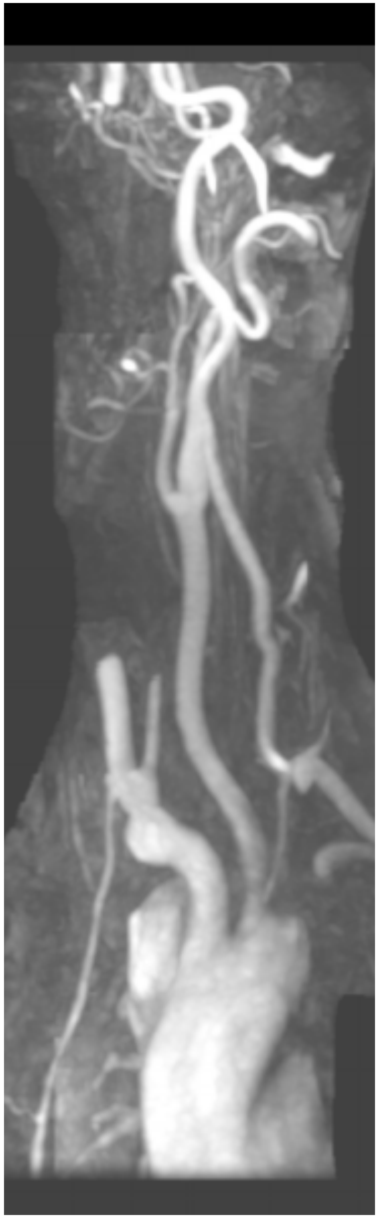

137e Score

0-30

31-50

51-70

>70

Near occlusion

Occluded

Quality

1

2

3

4

5

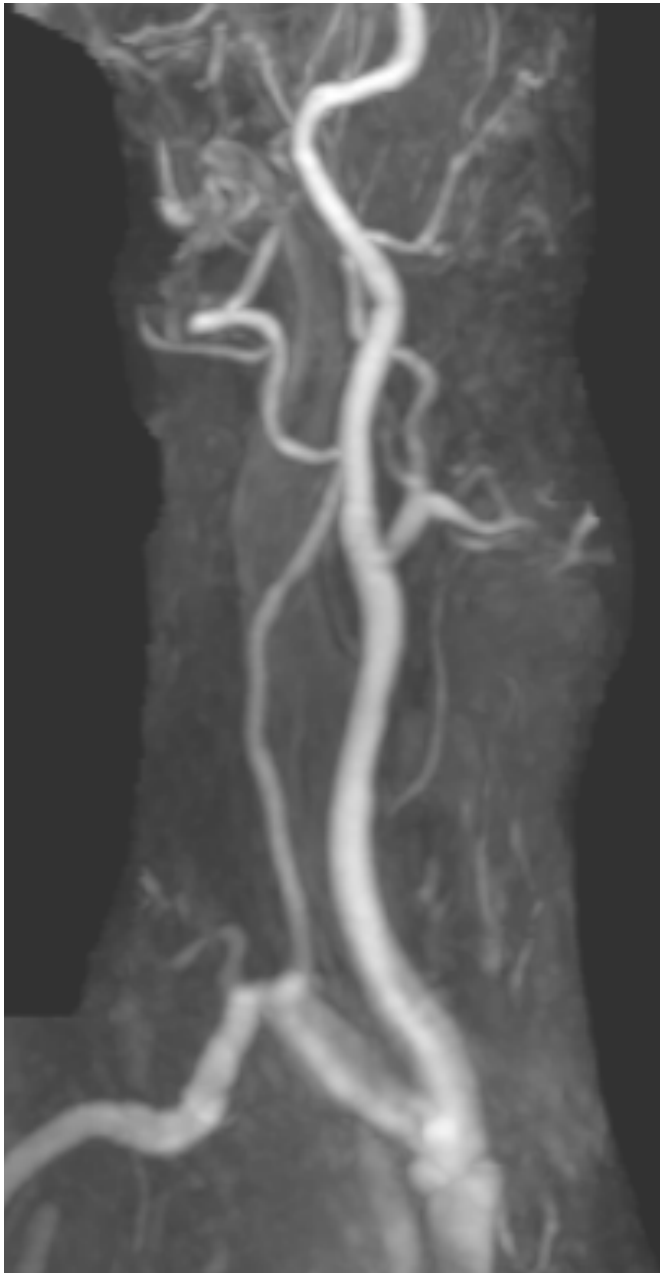

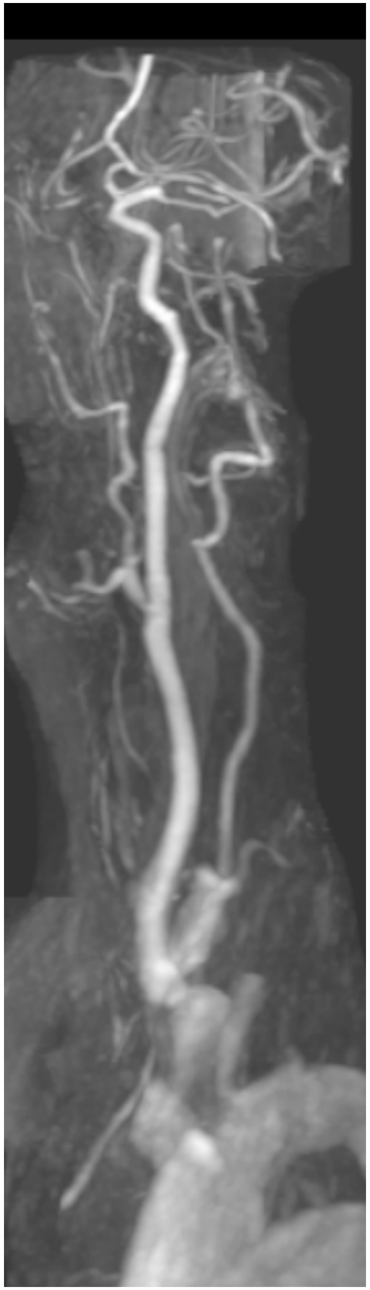

138d Score

0-30

31-50

51-70

>70

Near occlusion

Occluded

Quality

1

2

3

4

5

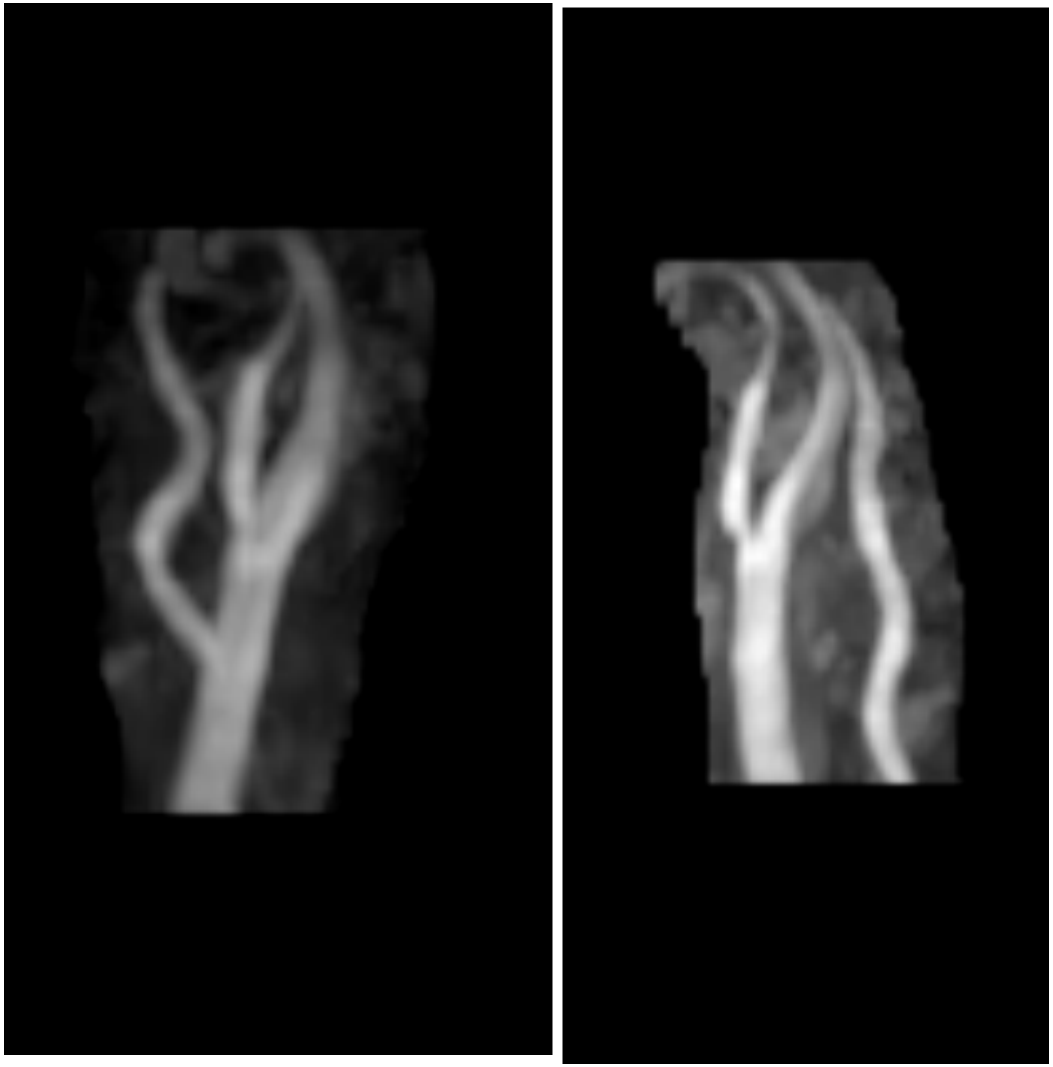

139c Score

0-30

31-50

51-70

>70

Near occlusion

Occluded

Quality

1

2

3

4

5

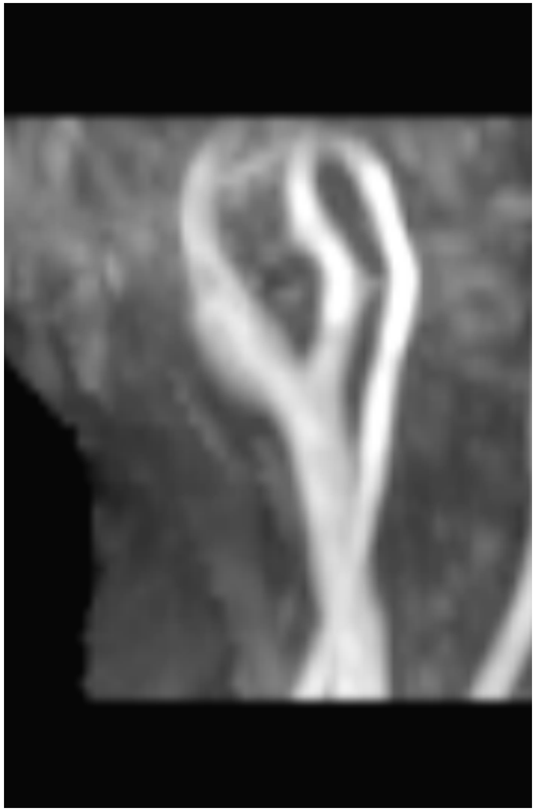

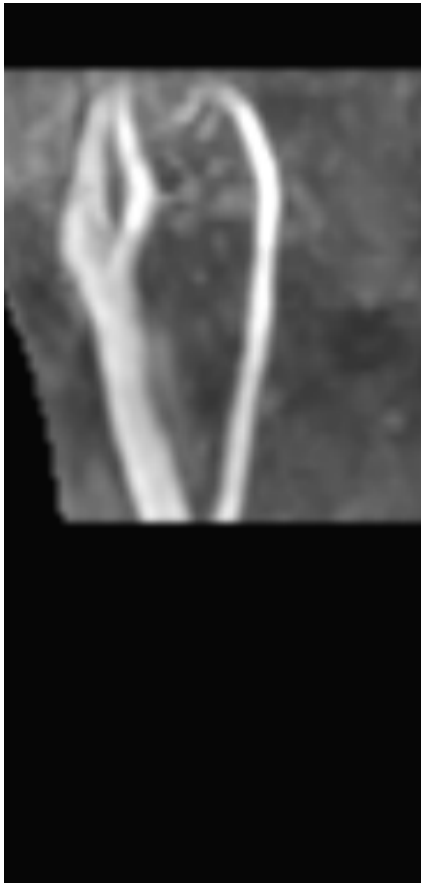

140b Score

0-30

31-50

51-70

>70

Near occlusion

Occluded

Quality

1

2

3

4

5

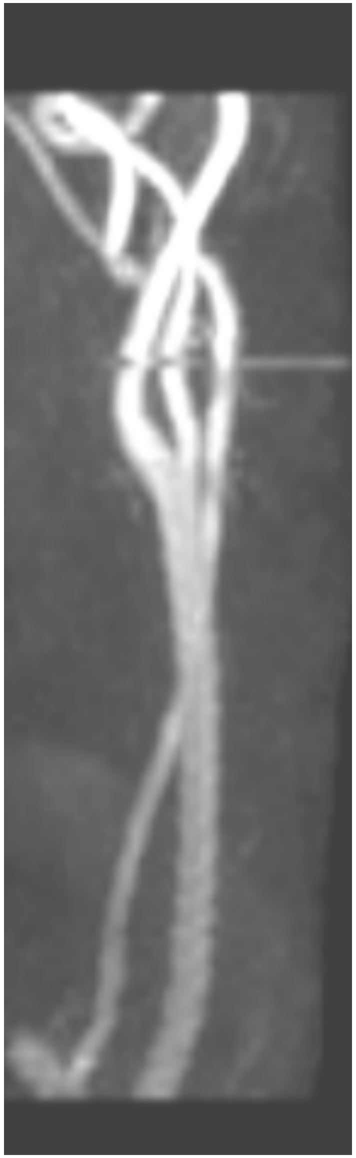

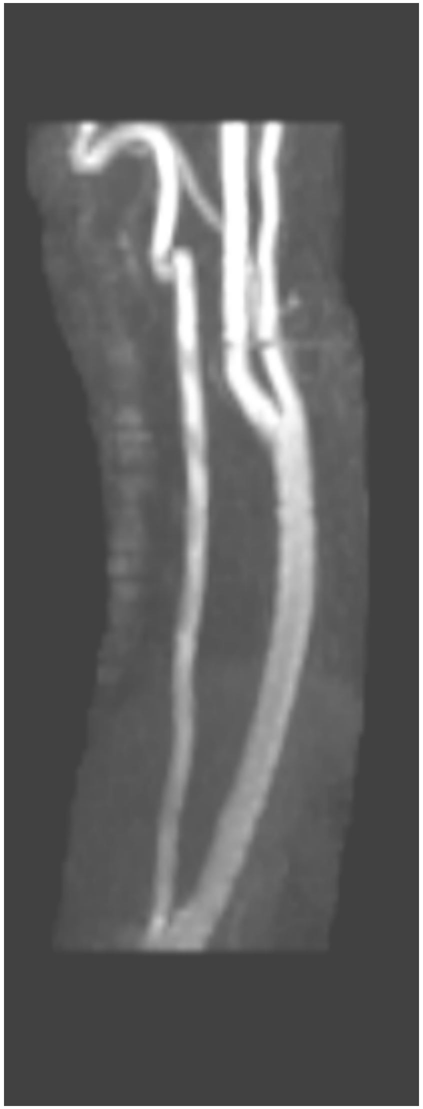

141a Score

0-30

31-50

51-70

>70

Near occlusion

Occluded

Quality

1

2

3

4

5

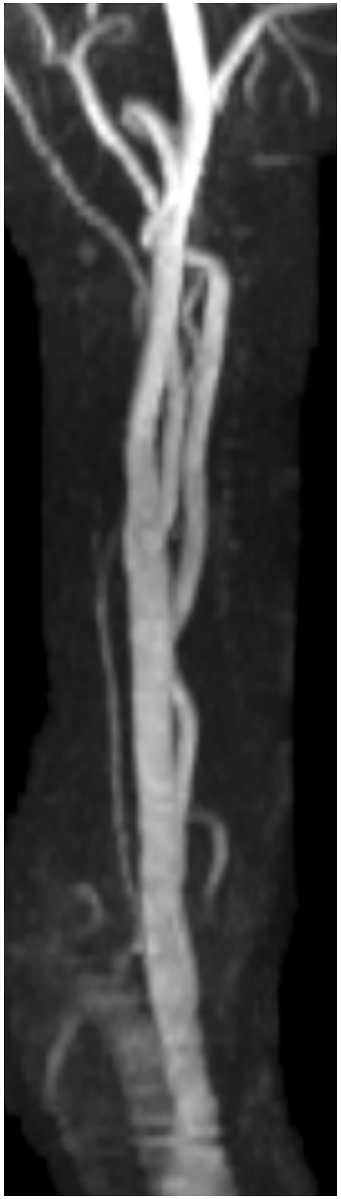

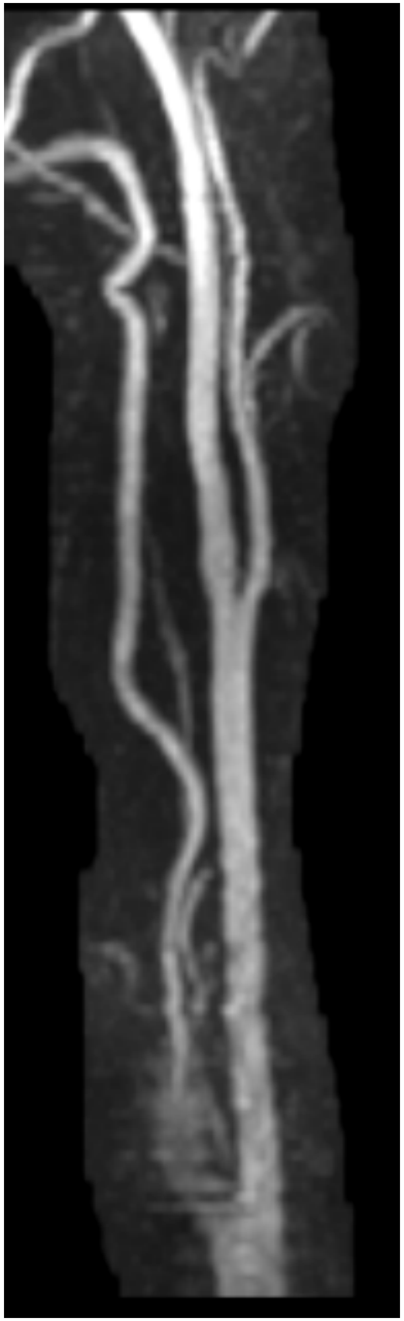

141f Score

0-30

31-50

51-70

>70

Near occlusion

Occluded

Quality

1

2

3

4

5

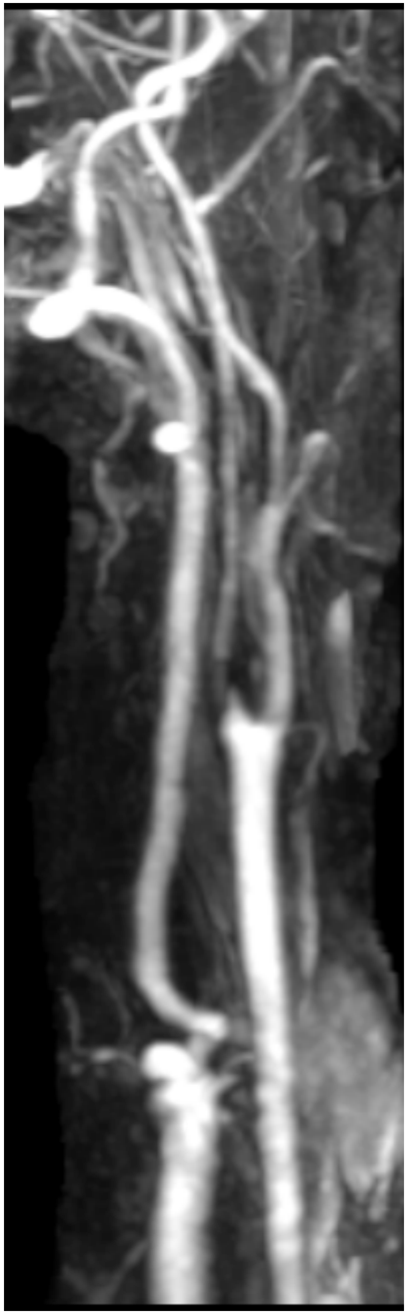

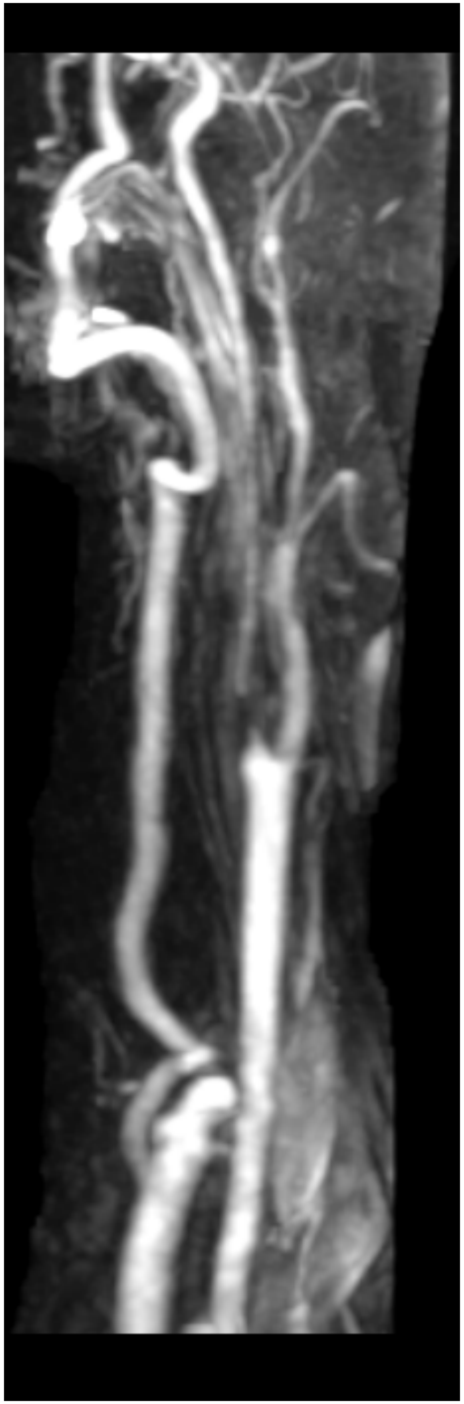

142e Score

0-30

31-50

51-70

>70

Near occlusion

Occluded

Quality

1

2

3

4

5

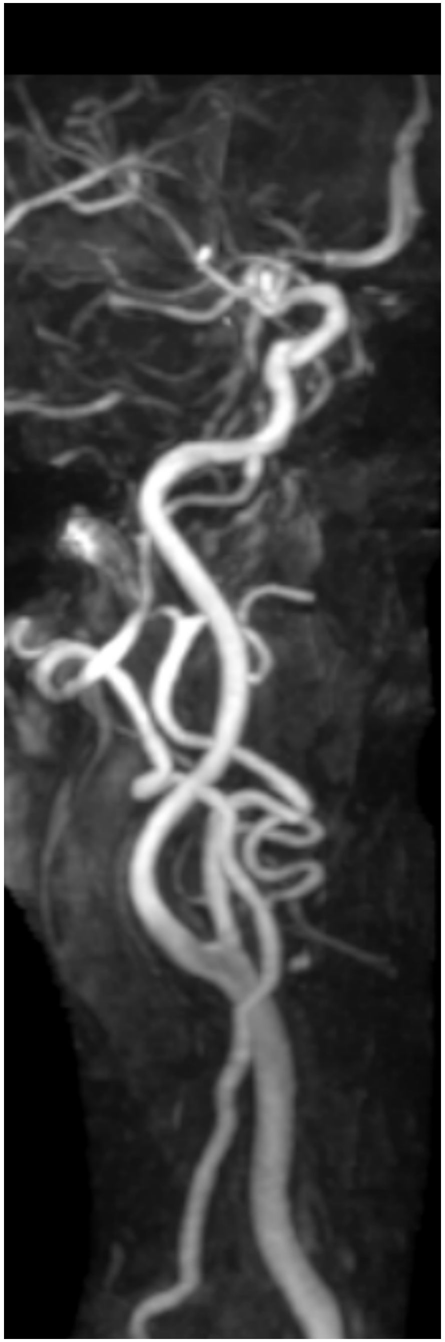

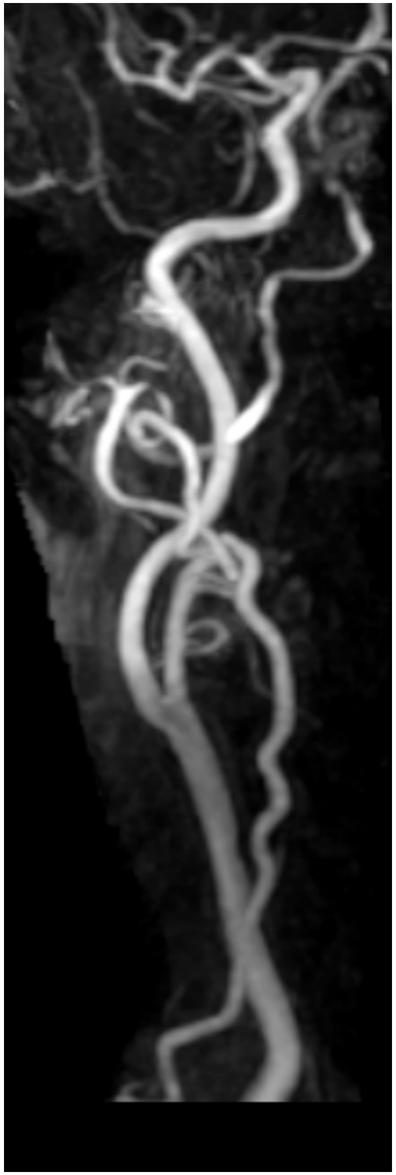

143d Score

0-30

31-50

51-70

>70

Near occlusion

Occluded

Quality

1

2

3

4

5

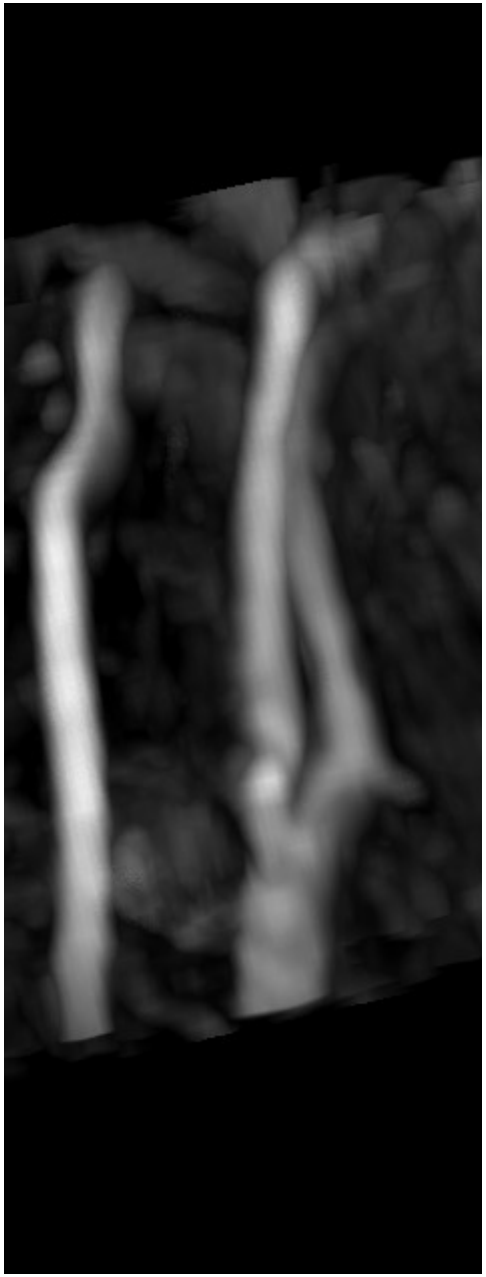

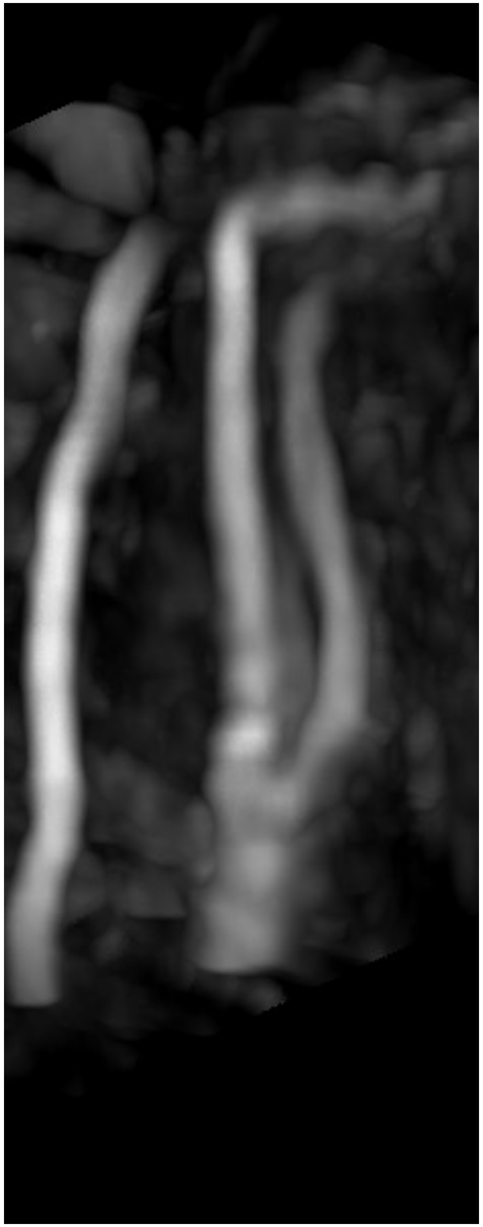

144c Score

0-30

31-50

51-70

>70

Near occlusion

Occluded

Quality

1

2

3

4

5

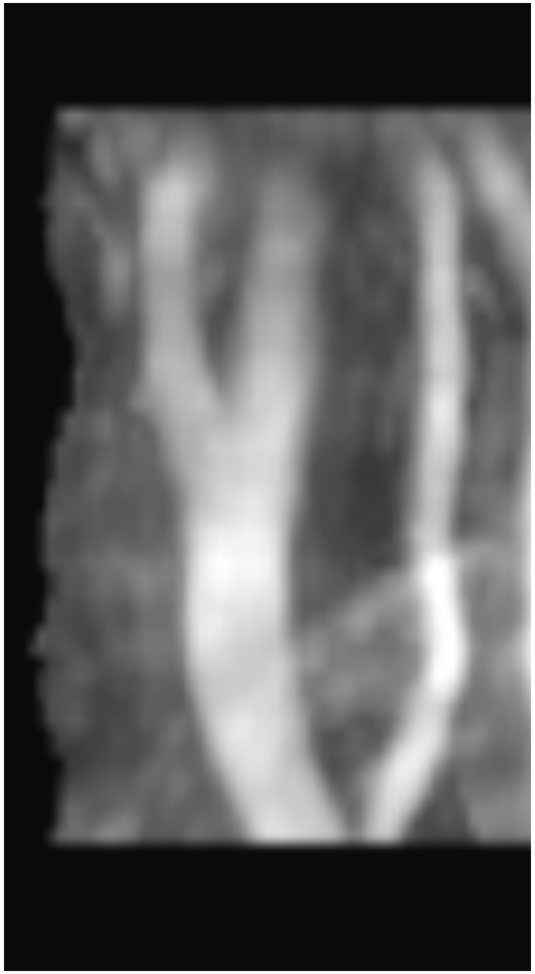

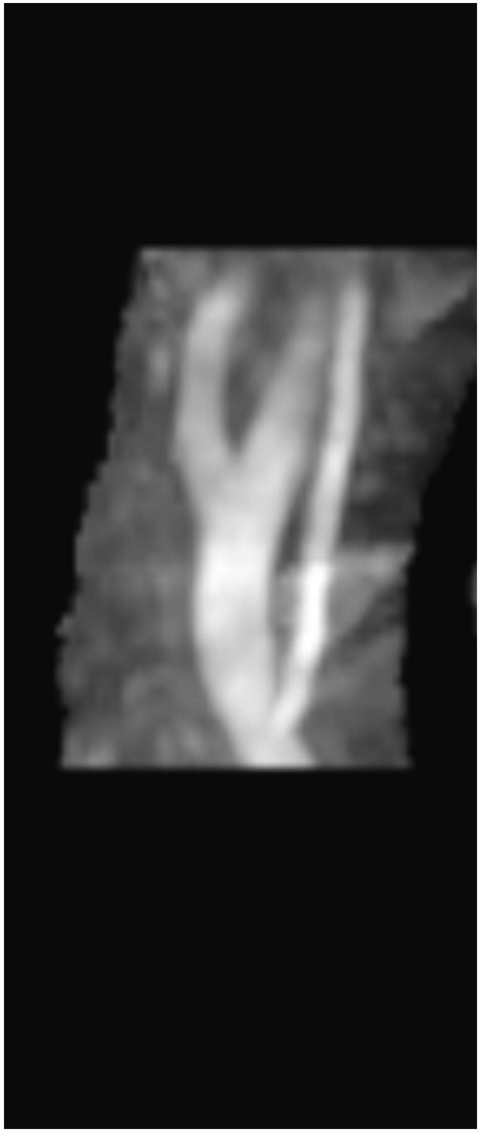

145b Score

0-30

31-50

51-70

>70

Near occlusion

Occluded

Quality

1

2

3

4

5

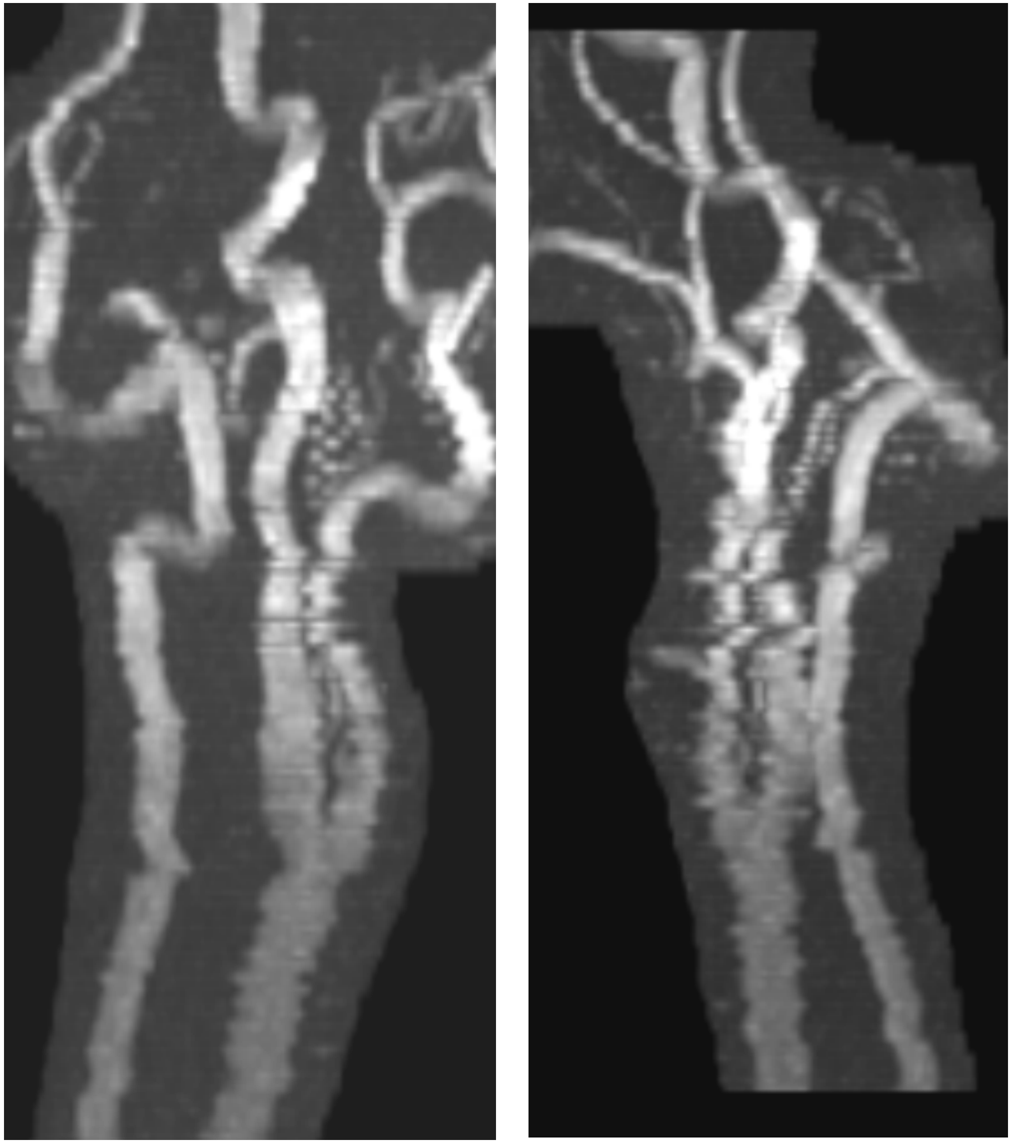

146a Score

0-30

31-50

51-70

>70

Near occlusion

Occluded

Quality

1

2

3

4

5

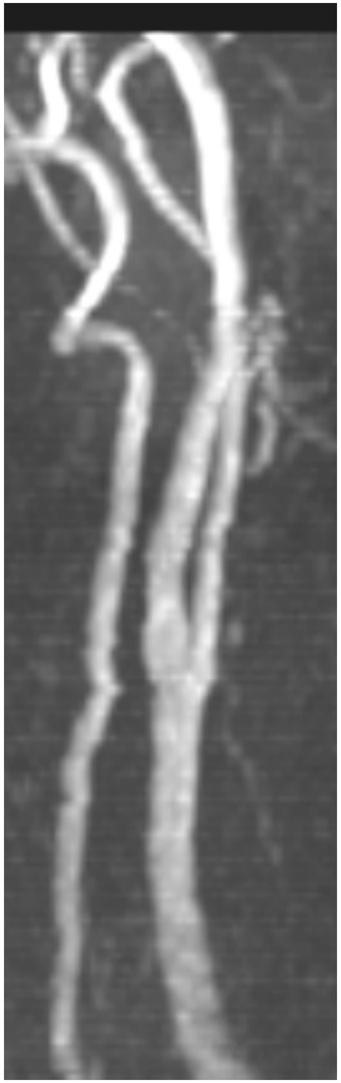

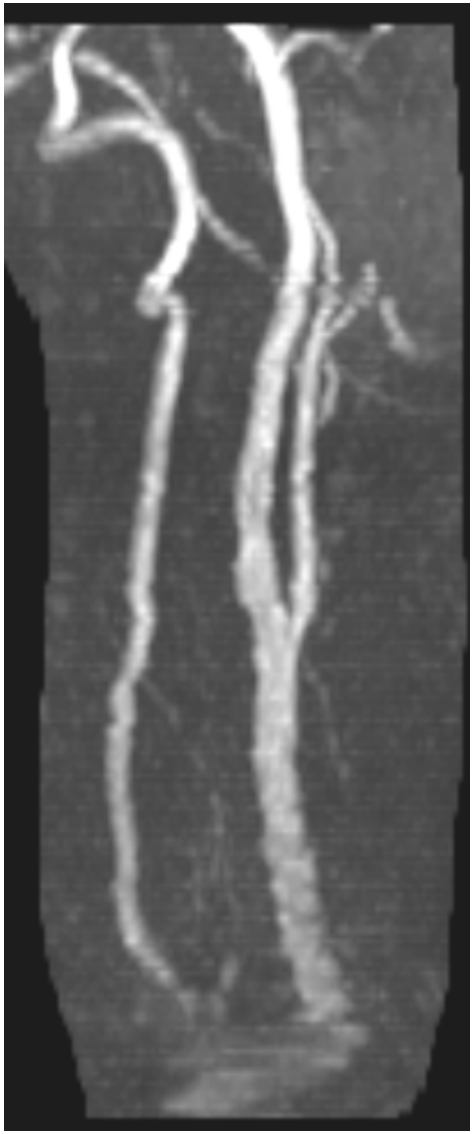

146f Score

0-30

31-50

51-70

>70

Near occlusion

Occluded

Quality

1

2

3

4

5

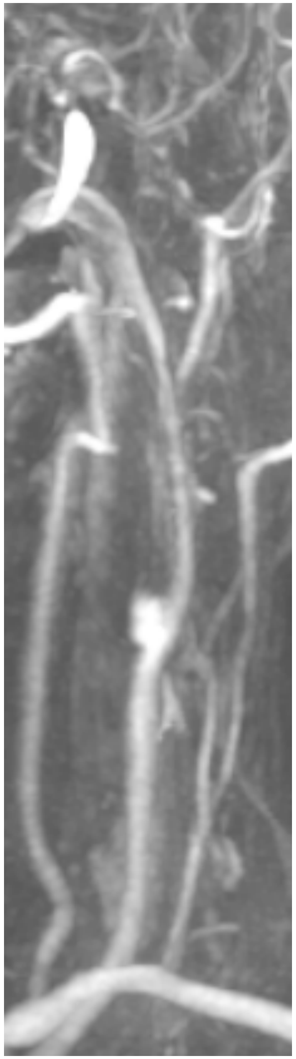

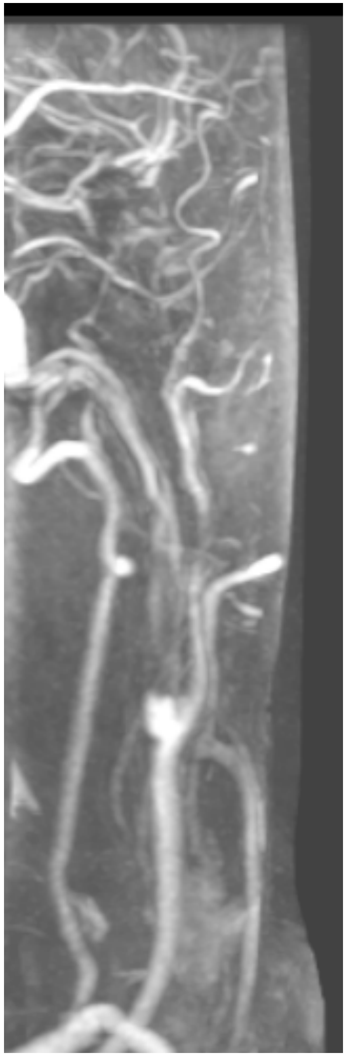

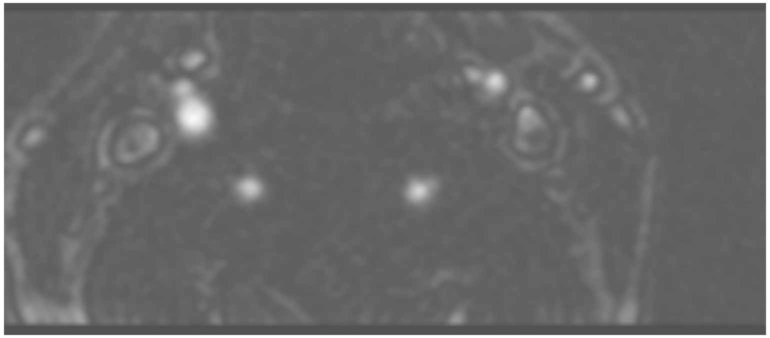

147e Score

0-30

31-50

51-70

>70

Near occlusion

Occluded

Quality

1

2

3

4

5

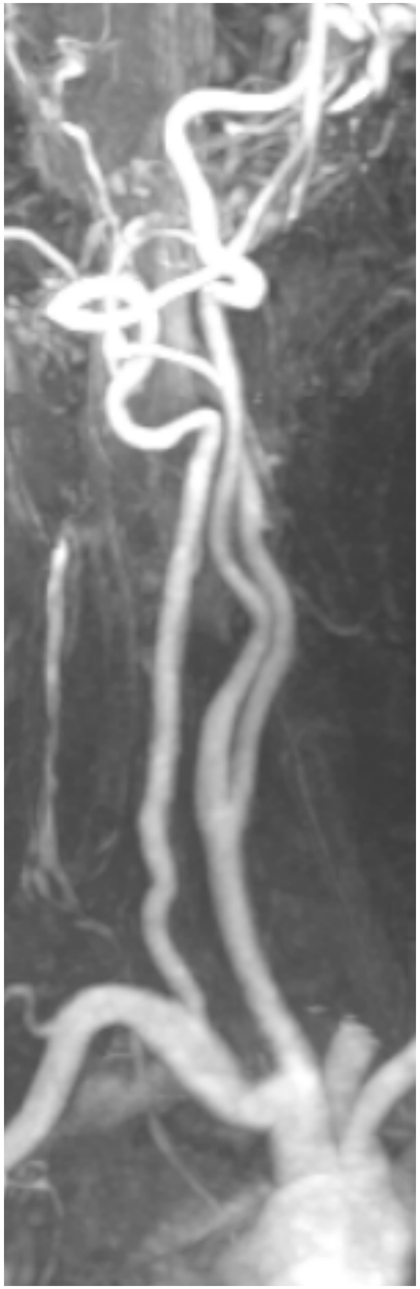

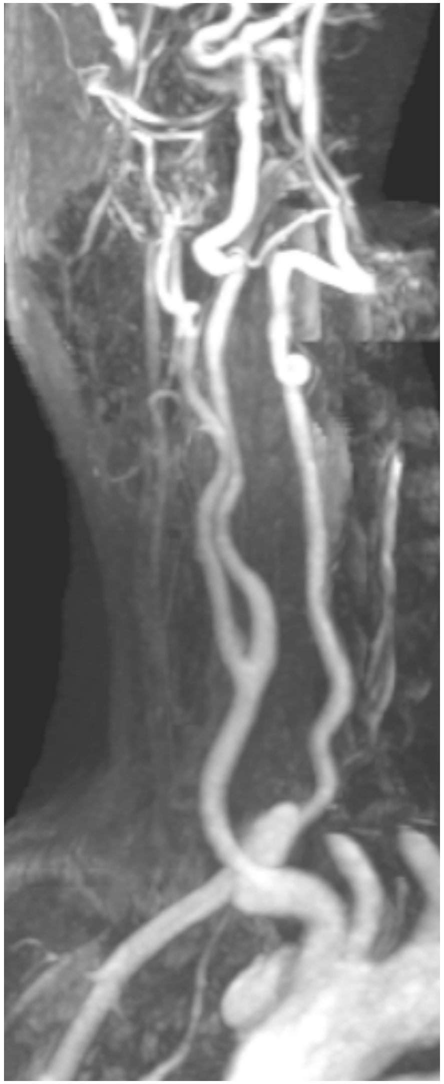

148d Score

0-30

31-50

51-70

>70

Near occlusion

Occluded

Quality

1

2

3

4

5

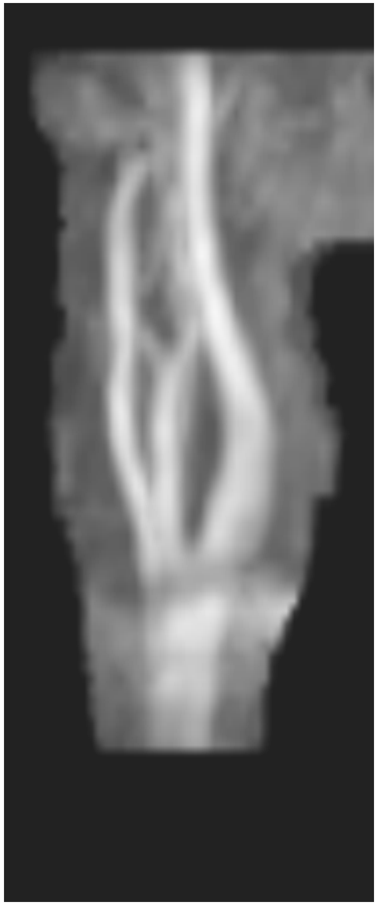

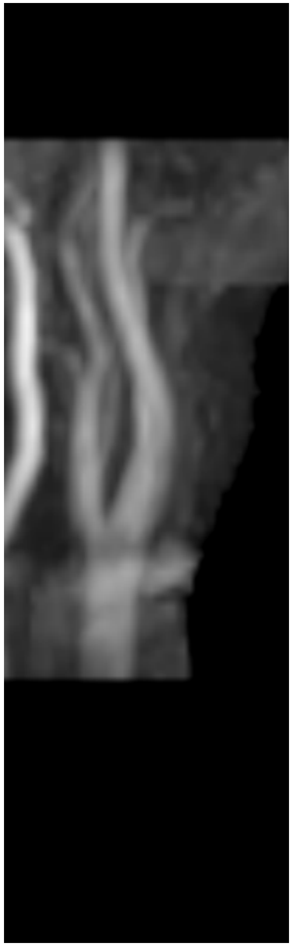

149c Score

0-30

31-50

51-70

>70

Near occlusion

Occluded

Quality

1

2

3

4

5

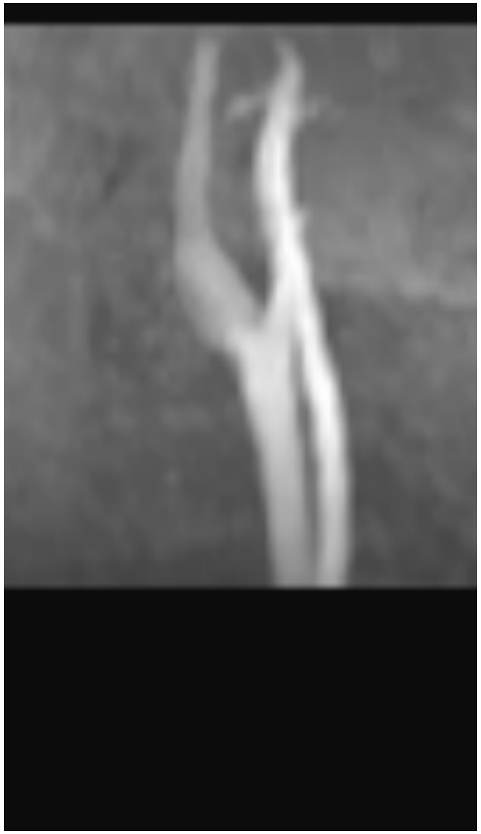

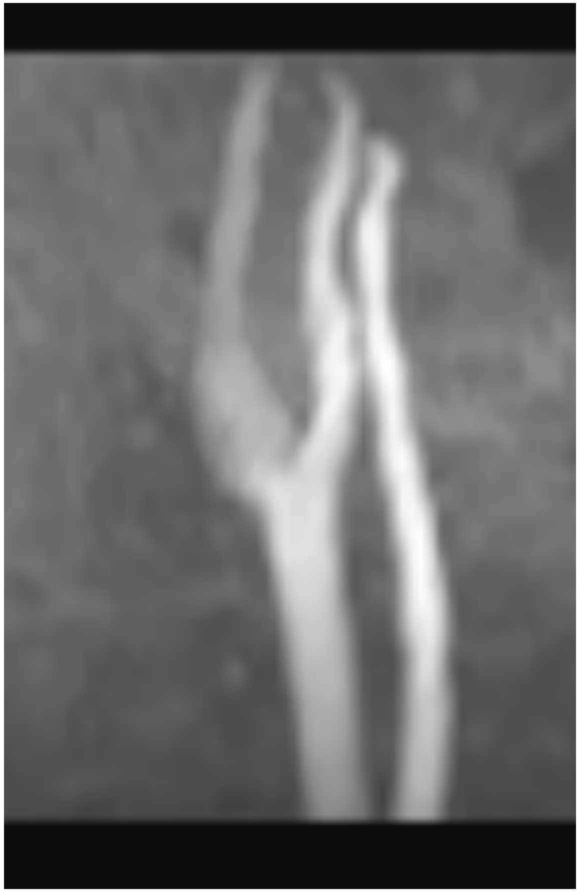

150b Score

0-30

31-50

51-70

>70

Near occlusion

Occluded

Quality

1

2

3

4

5

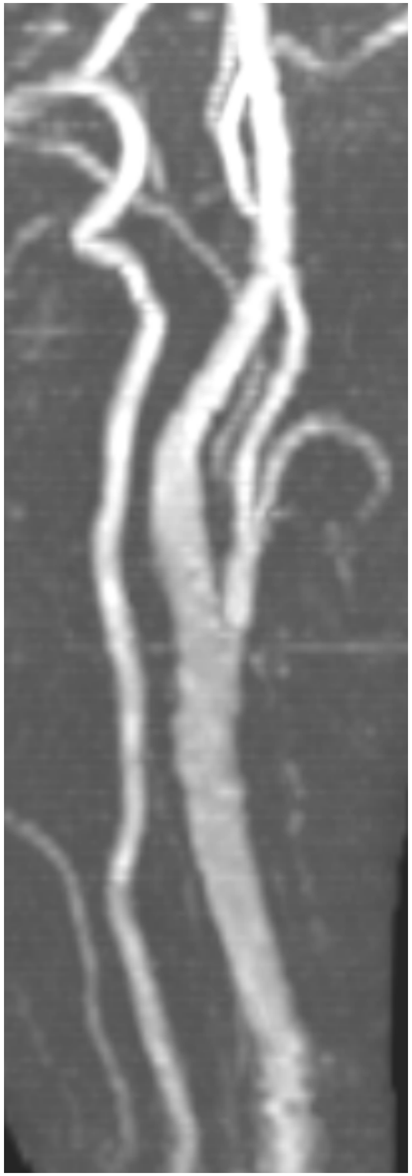

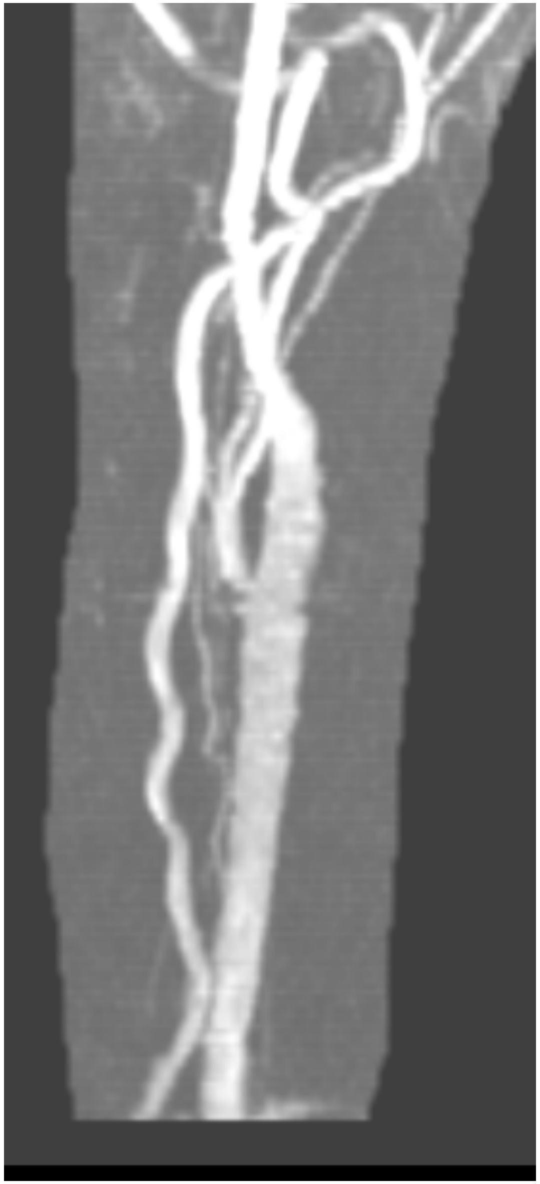

151a Score

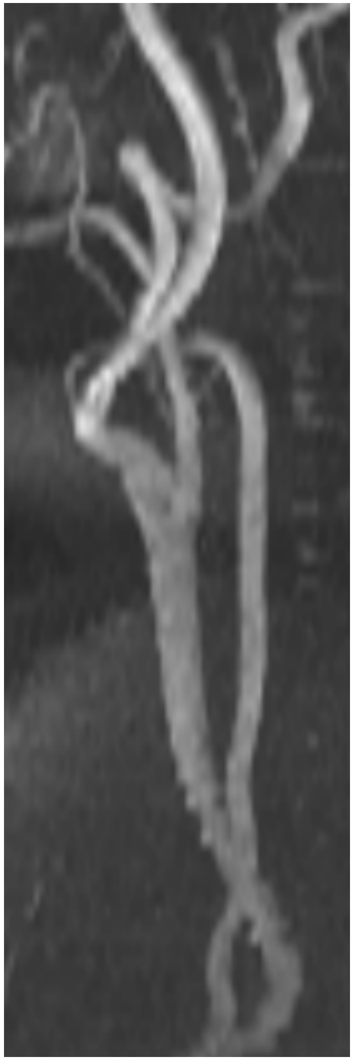

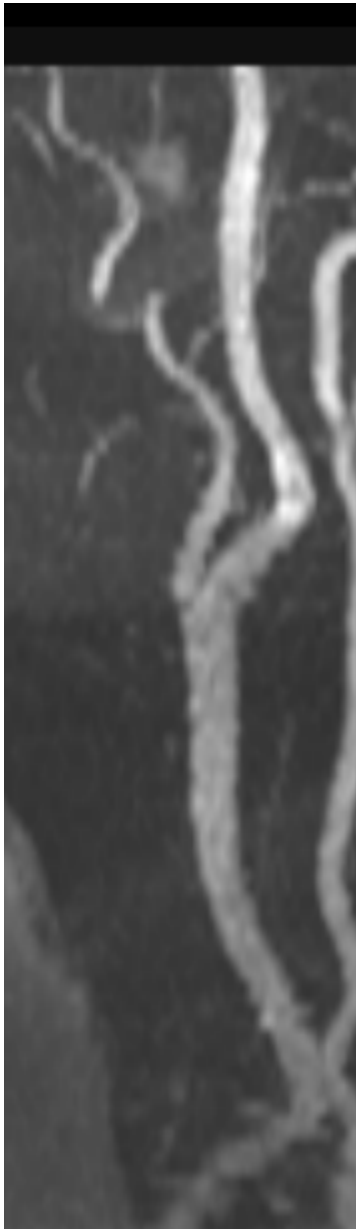

0-30

31-50

51-70

>70

Near occlusion

Occluded

Quality

1

2

3

4

5

151f Score

0-30

31-50

51-70

>70

Near occlusion

Occluded

Quality

1

2

3

4

5

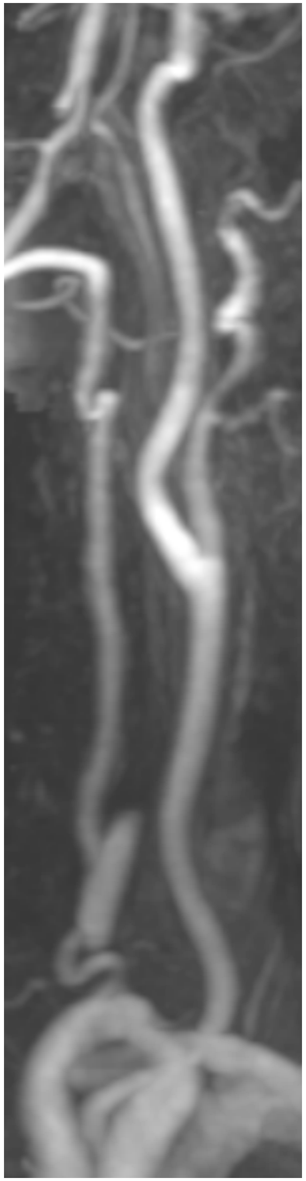

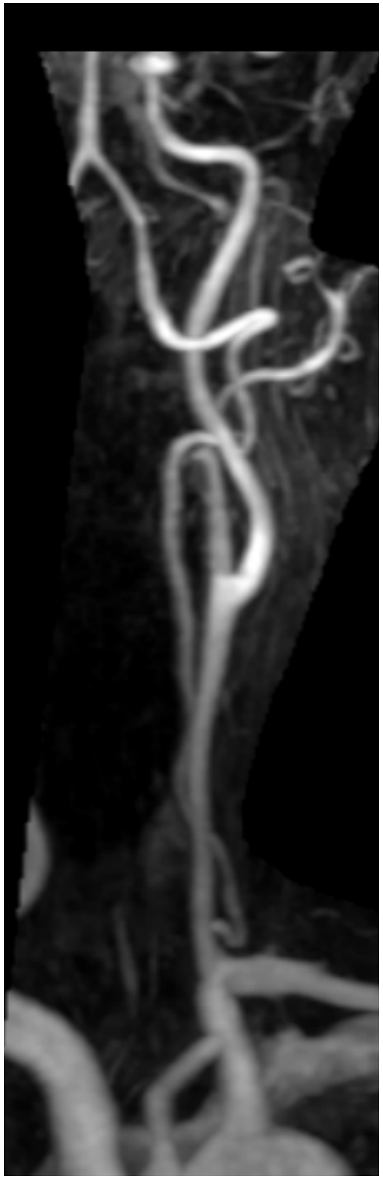

152e Score

0-30

31-50

51-70

>70

Near occlusion

Occluded

Quality

1

2

3

4

5

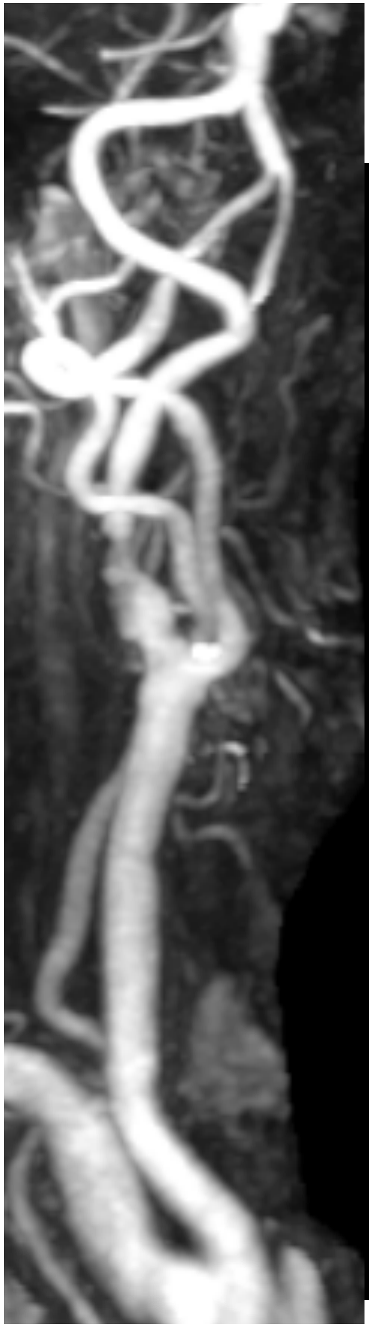

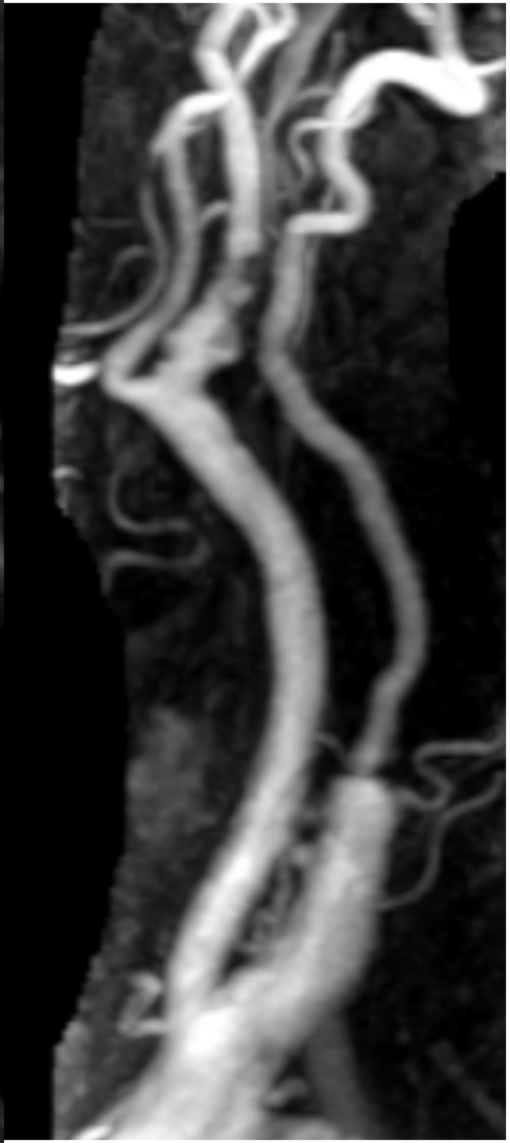

153d Score

0-30

31-50

51-70

>70

Near occlusion

Occluded

Quality

1

2

3

4

5

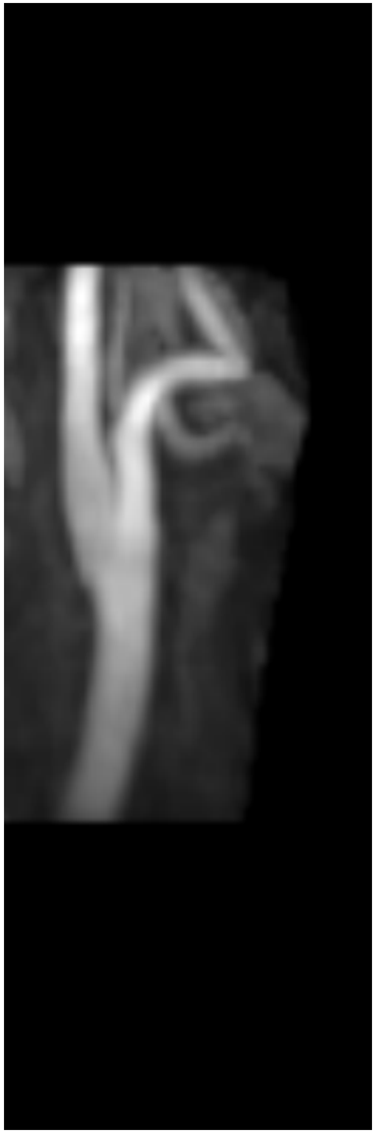

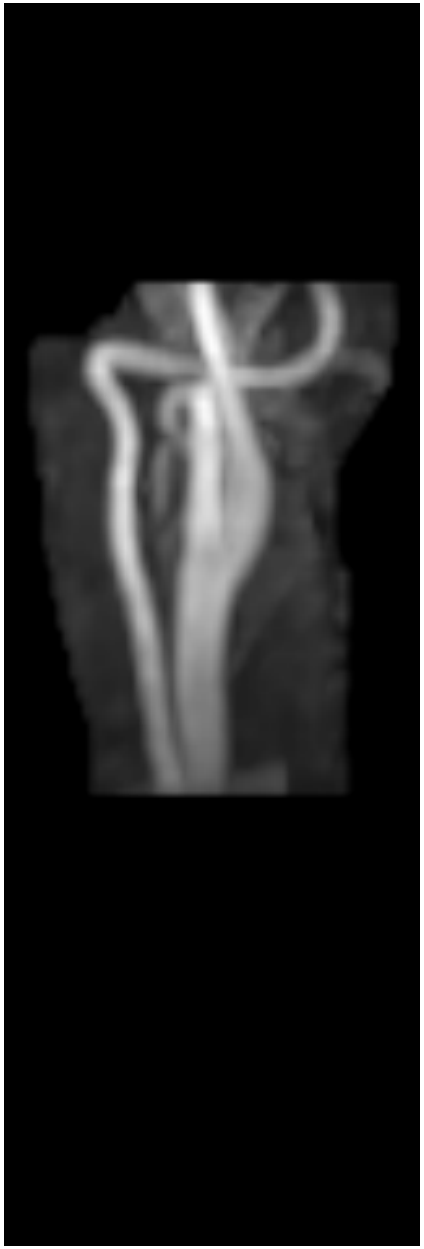

154c Score

0-30

31-50

51-70

>70

Near occlusion

Occluded

Quality

1

2

3

4

5

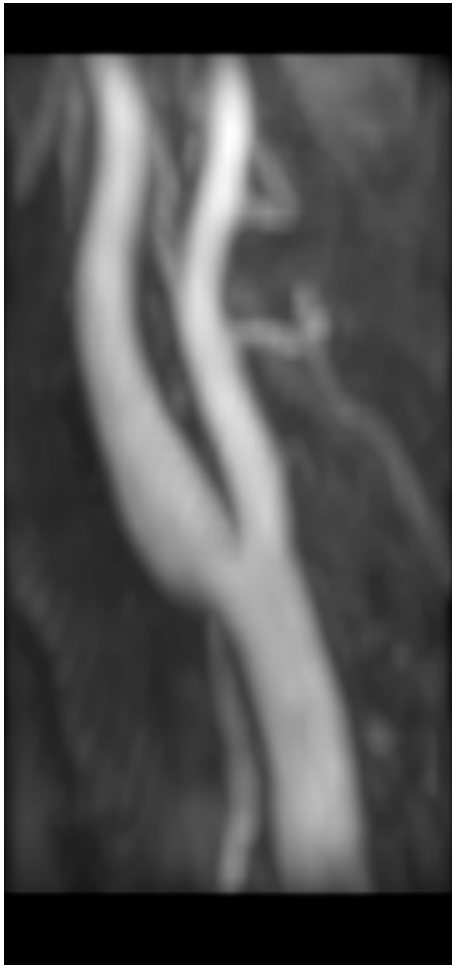

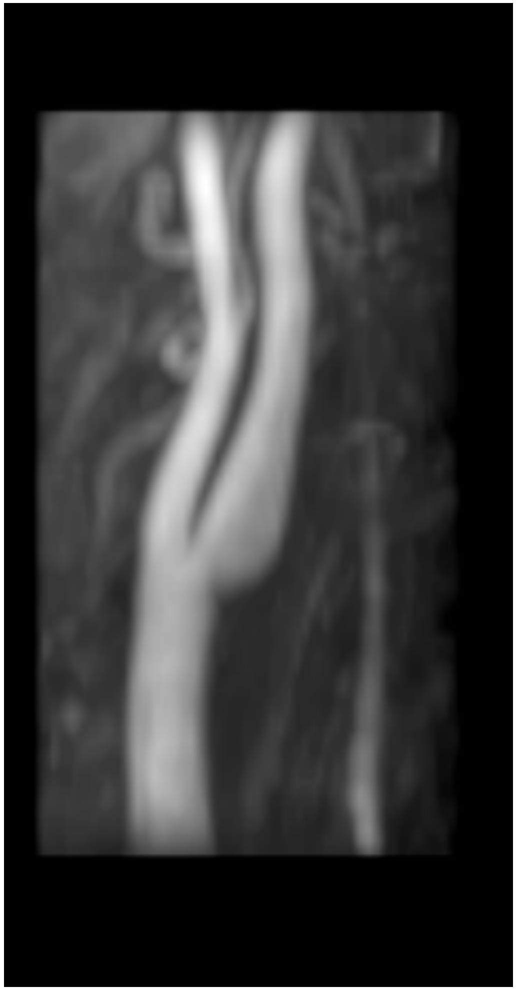

155b Score

0-30

31-50

51-70

>70

Near occlusion

Occluded

Quality

1

2

3

4

5

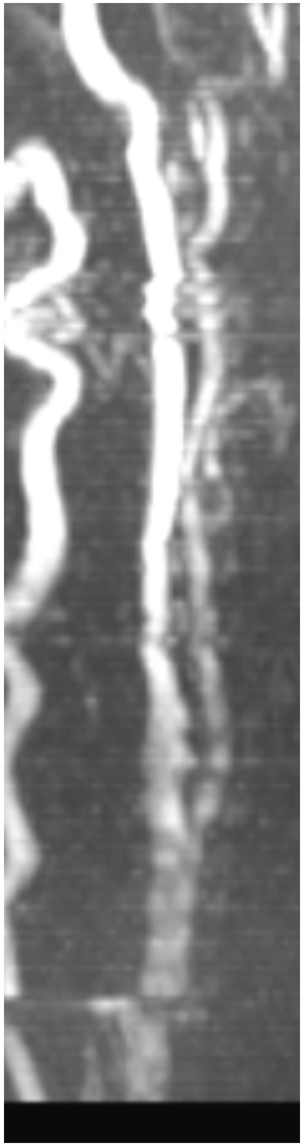

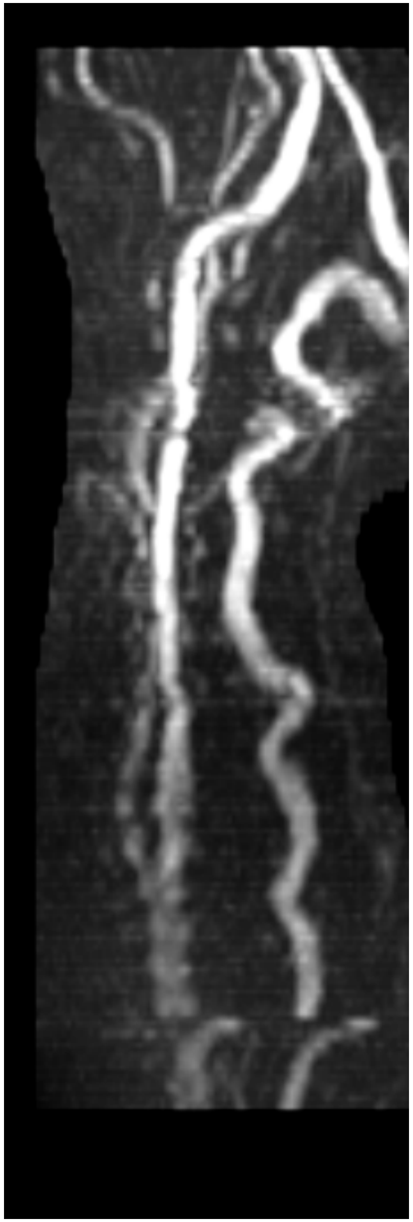

156a Score

0-30

31-50

51-70

>70

Near occlusion

Occluded

Quality

1

2

3

4

5

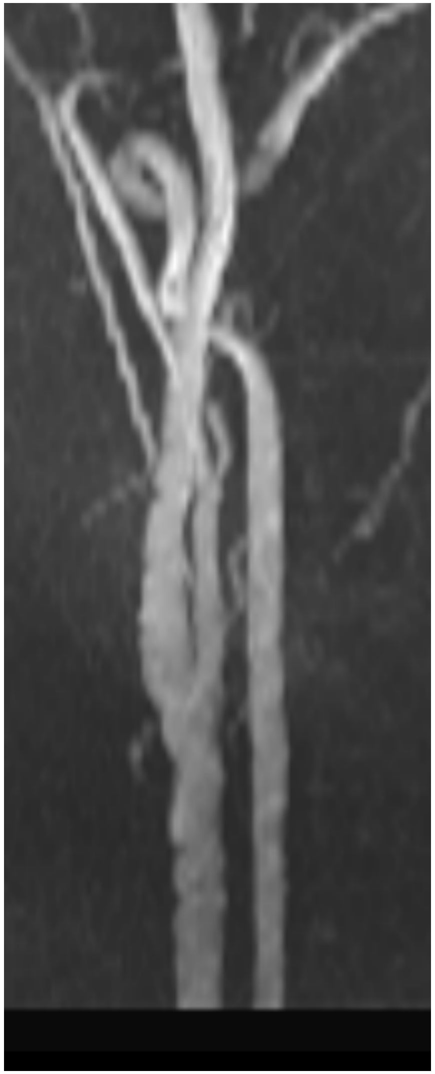

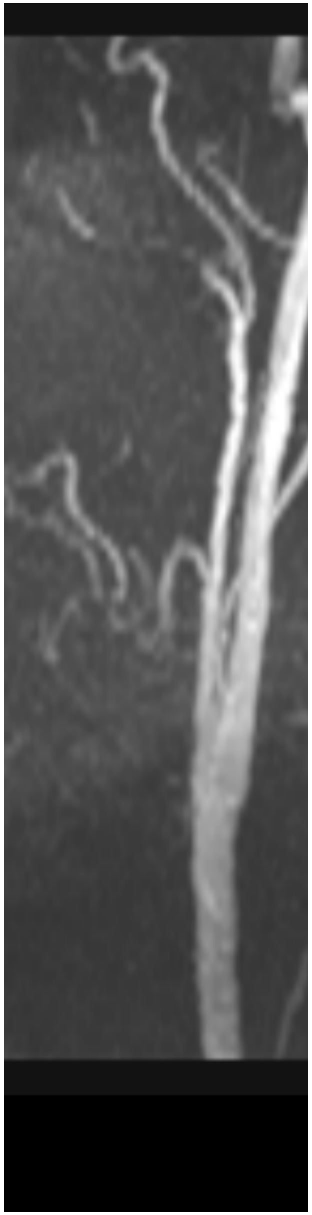

156f Score

0-30

31-50

51-70

>70

Near occlusion

Occluded

Quality

1

2

3

4

5

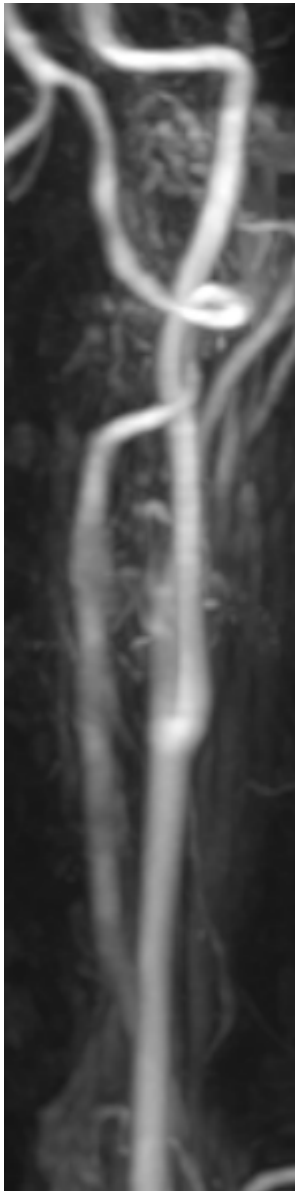

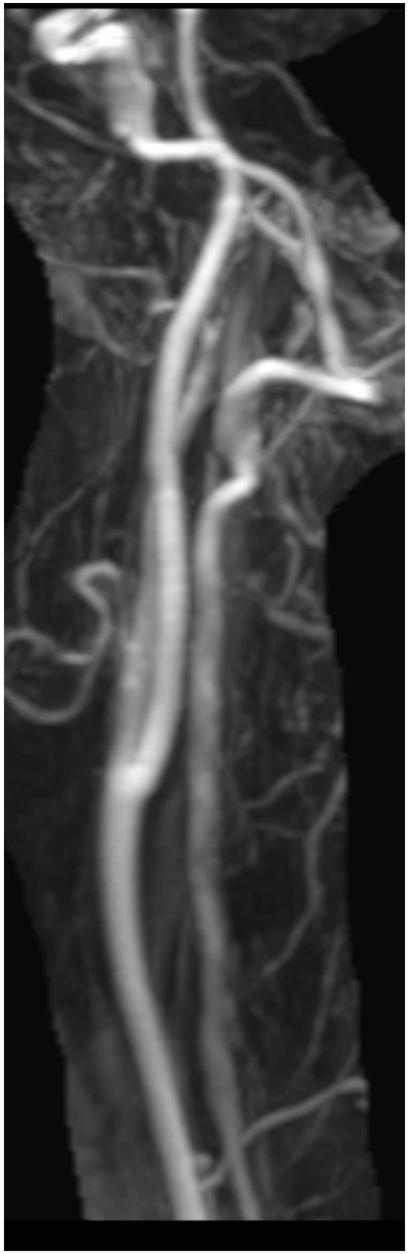

157e Score

0-30

31-50

51-70

>70

Near occlusion

Occluded

Quality

1

2

3

4

5

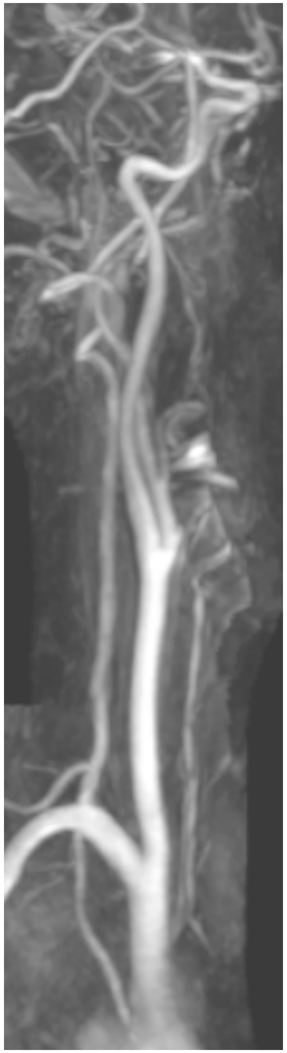

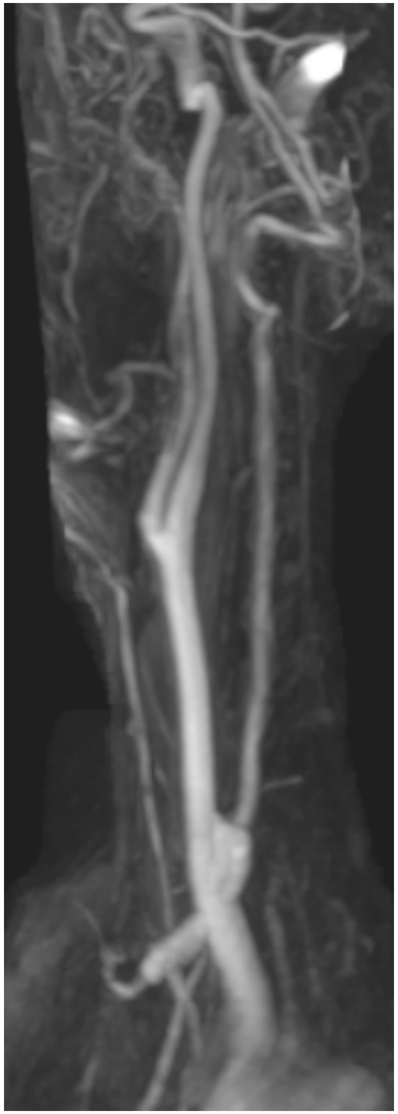

158d Score

0-30

31-50

51-70

>70

Near occlusion

Occluded

Quality

1

2

3

4

5

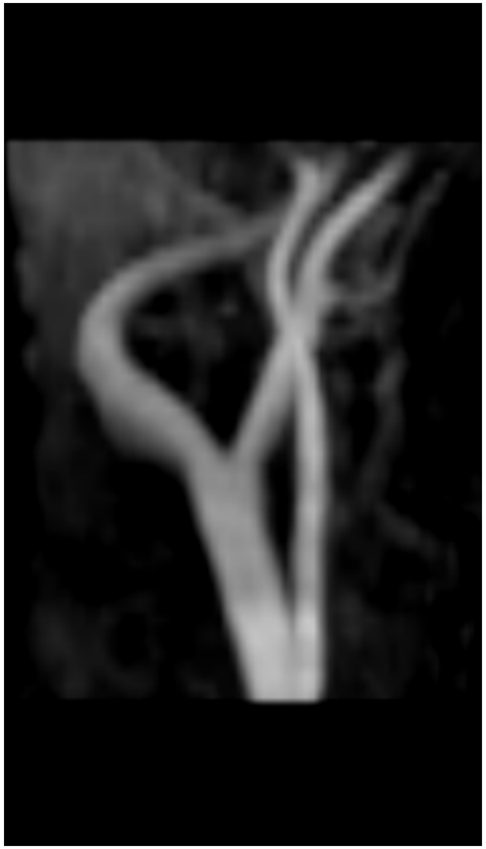

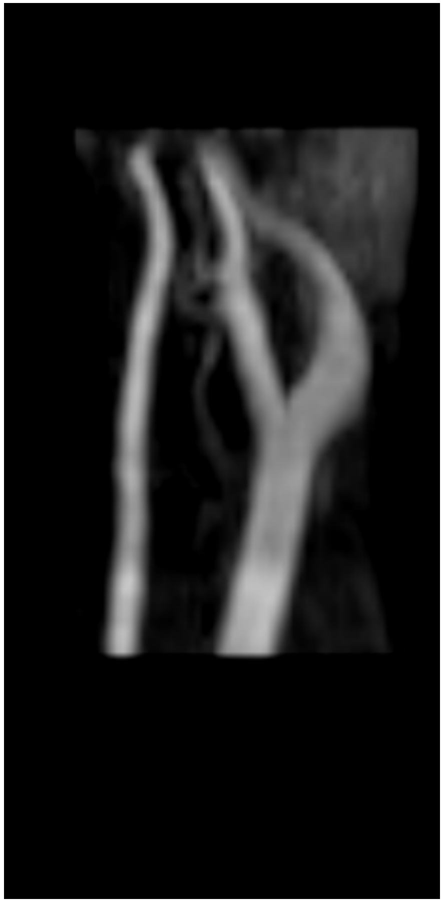

159c Score

0-30

31-50

51-70

>70

Near occlusion

Occluded

Quality

1

2

3

4

5

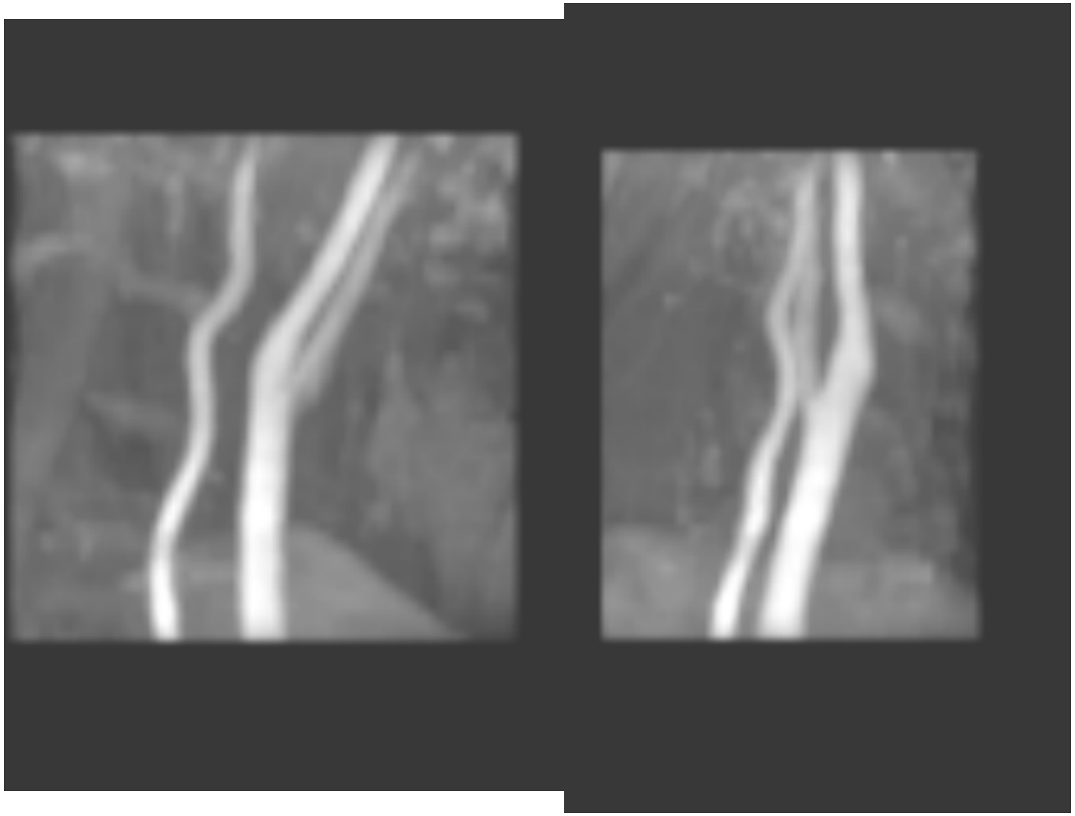

160b Score

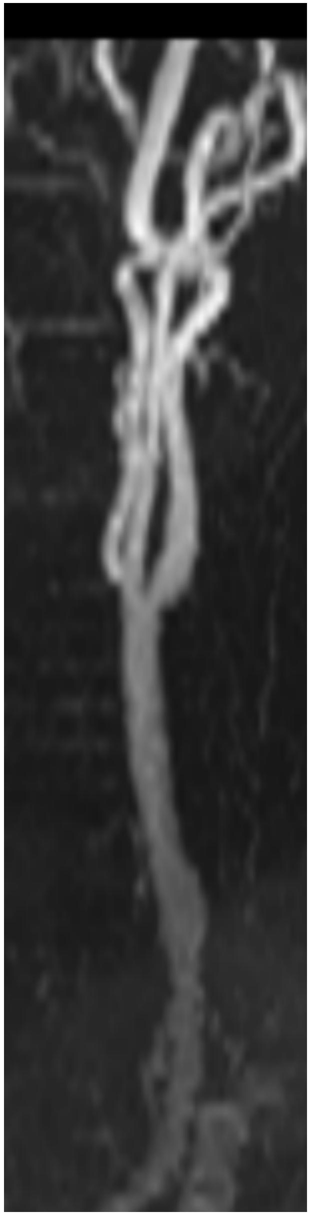

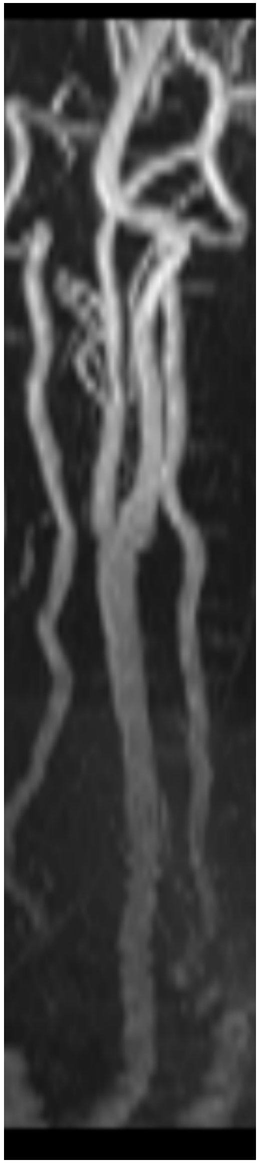

0-30

31-50

51-70

>70

Near occlusion

Occluded

Quality

1

2

3

4

5

161a Score

0-30

31-50

51-70

>70

Near occlusion

Occluded

Quality

1

2

3

4

5

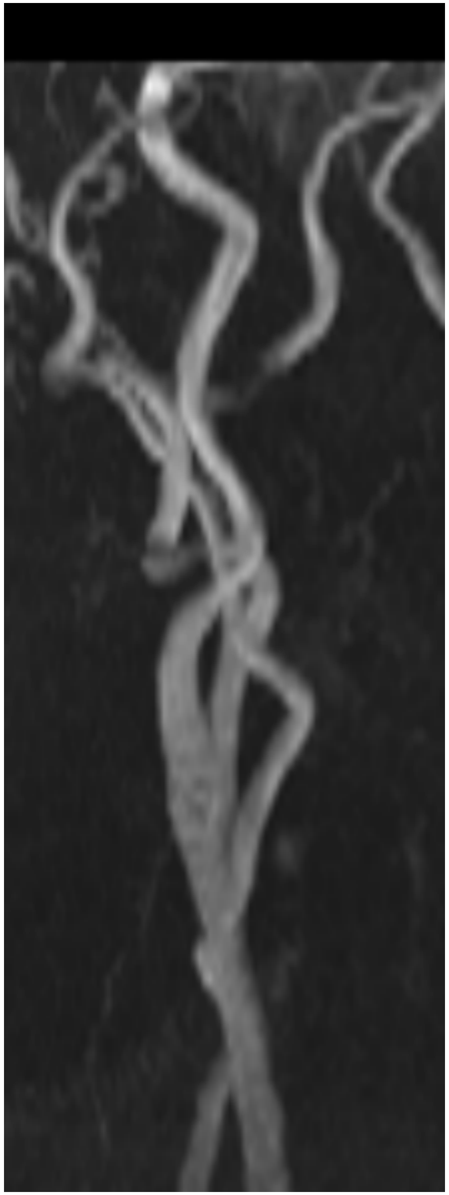

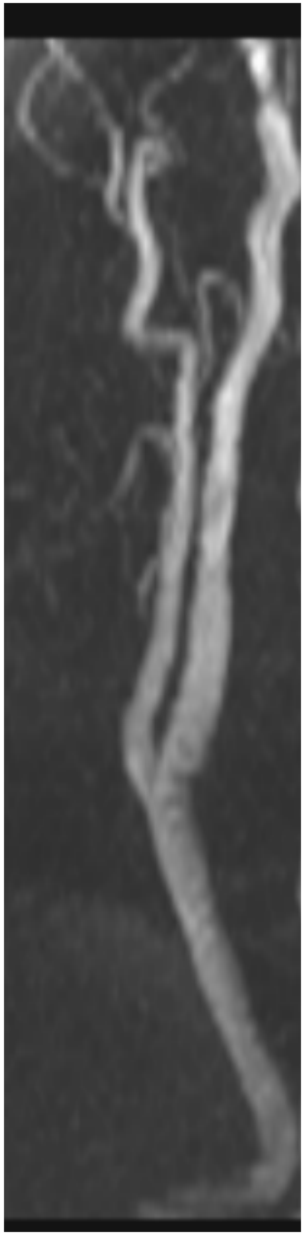

161f Score

0-30

31-50

51-70

>70

Near occlusion

Occluded

Quality

1

2

3

4

5

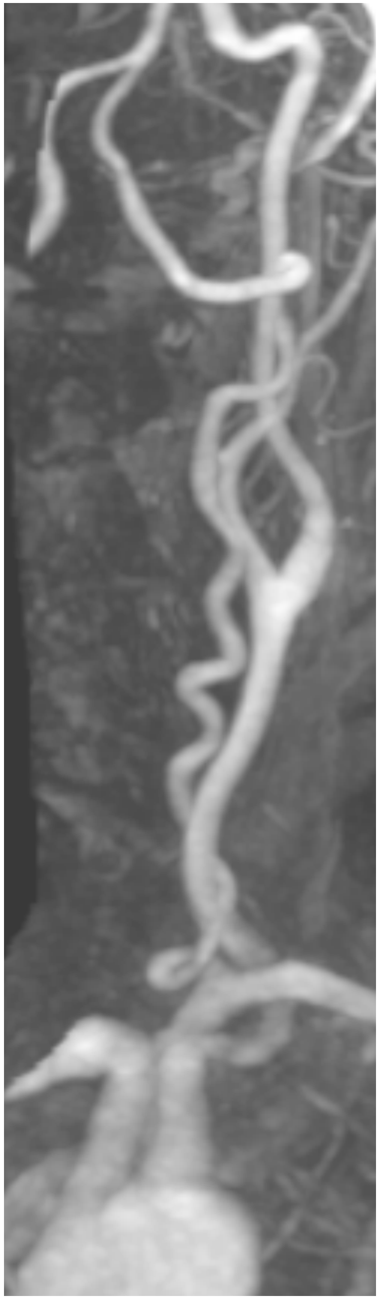

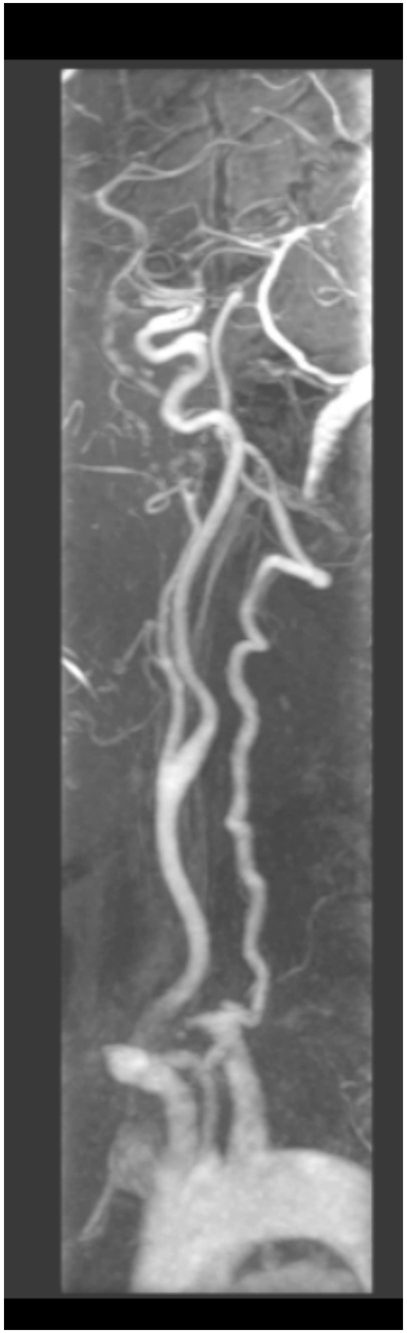

162e Score

0-30

31-50

51-70

>70

Near occlusion

Occluded

Quality

1

2

3

4

5

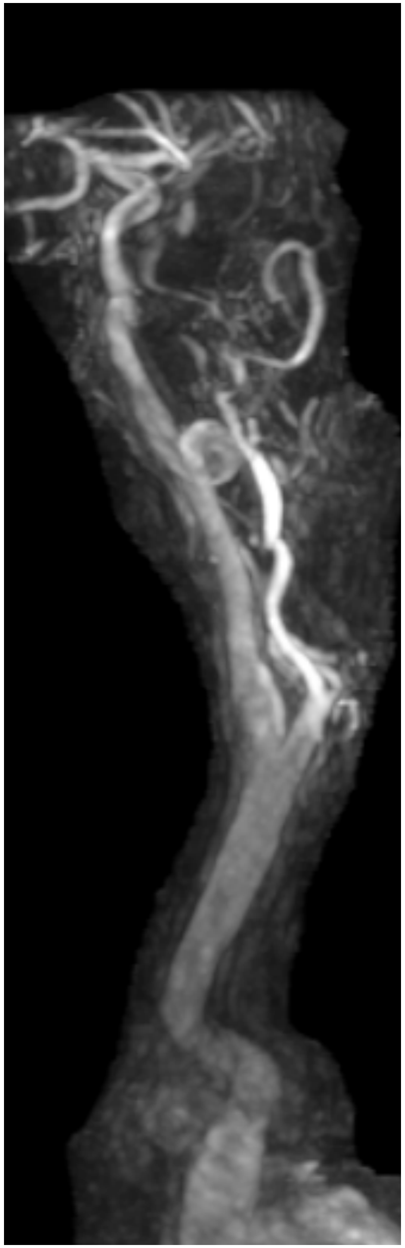

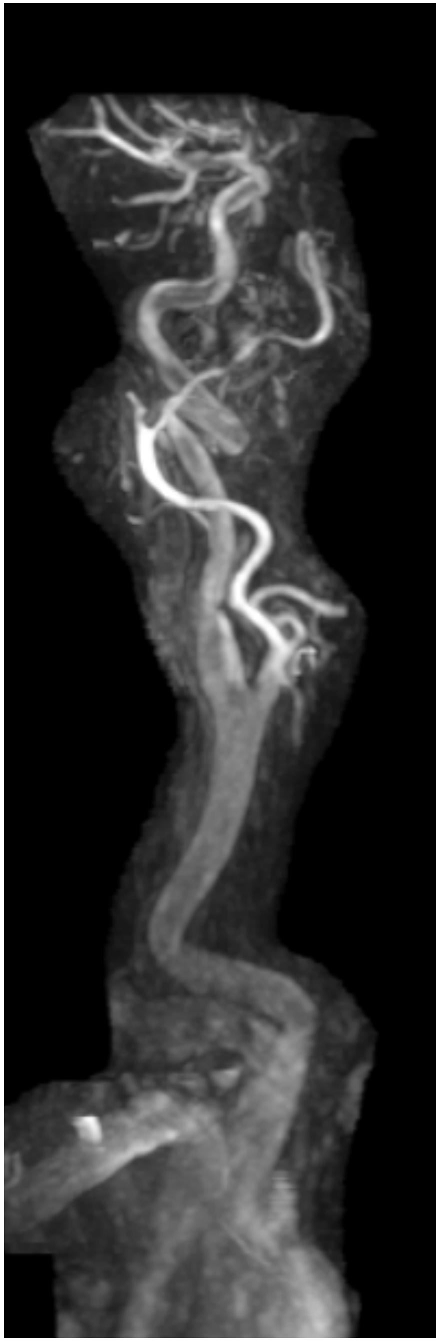

163d Score

0-30

31-50

51-70

>70

Near occlusion

Occluded

Quality

1

2

3

4

5

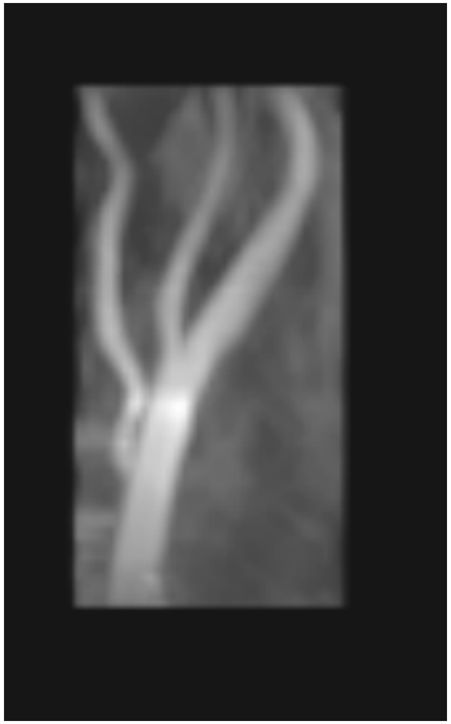

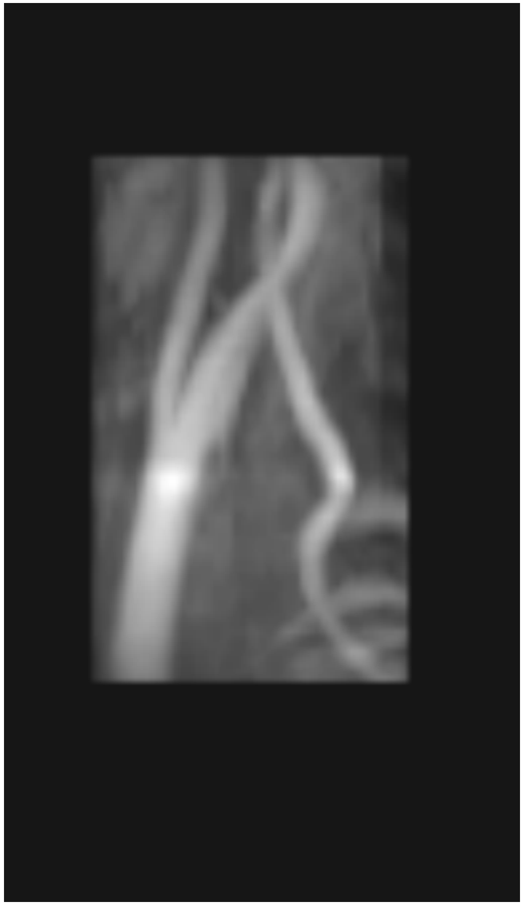

164c Score

0-30

31-50

51-70

>70

Near occlusion

Occluded

Quality

1

2

3

4

5

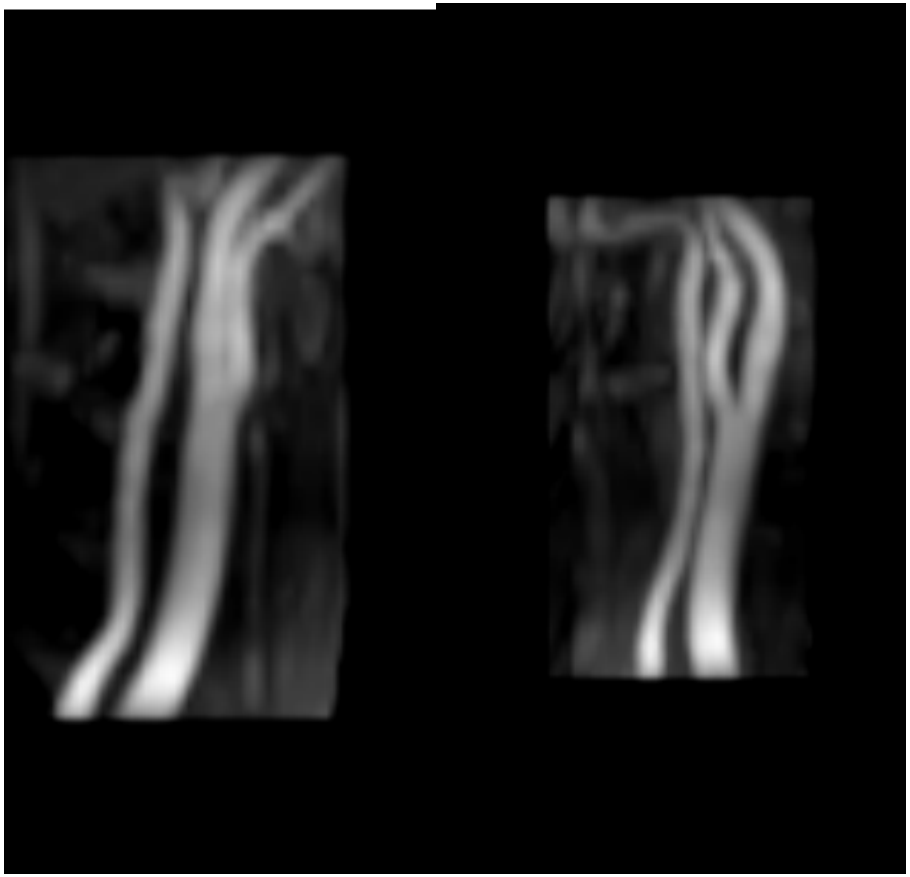

165b Score

0-30

31-50

51-70

>70

Near occlusion

Occluded

Quality

1

2

3

4

5

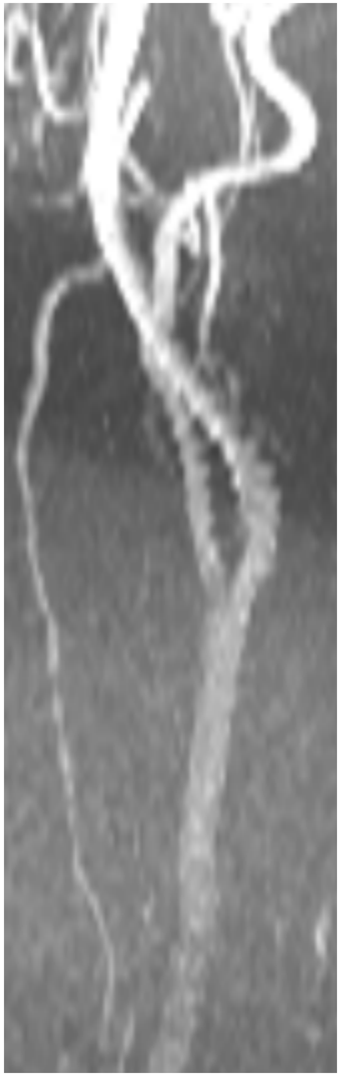

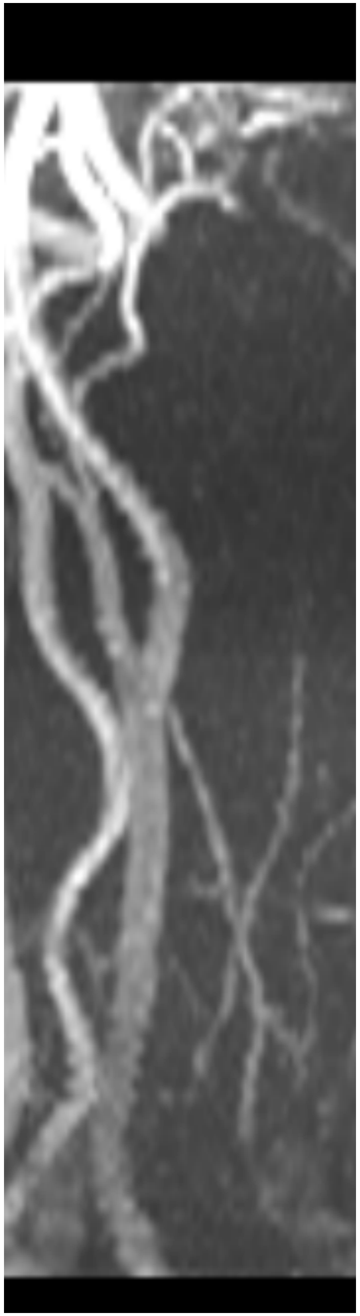

166a Score

0-30

31-50

51-70

>70

Near occlusion

Occluded

Quality

1

2

3

4

5

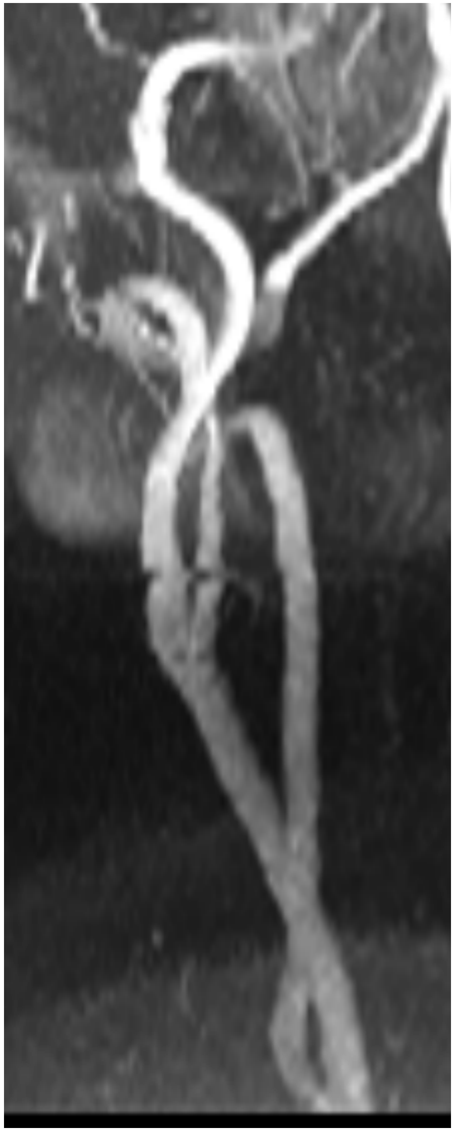

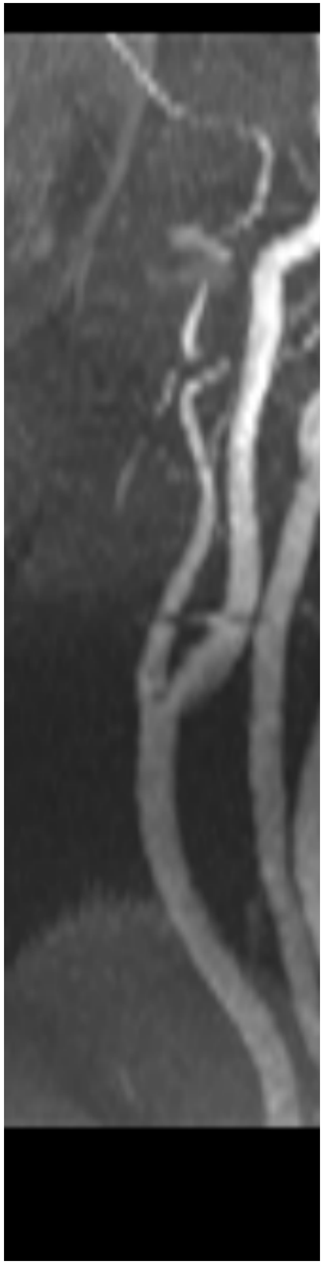

166f Score

0-30

31-50

51-70

>70

Near occlusion

Occluded

Quality

1

2

3

4

5

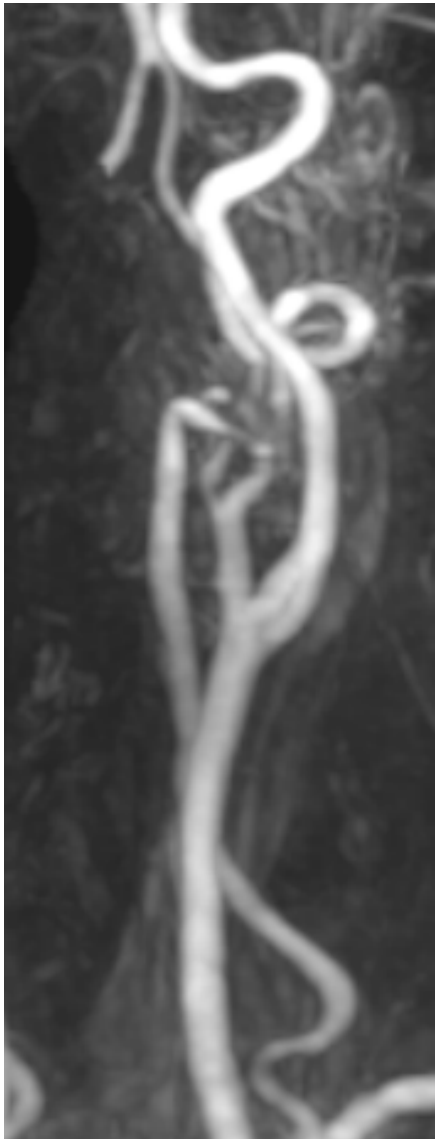

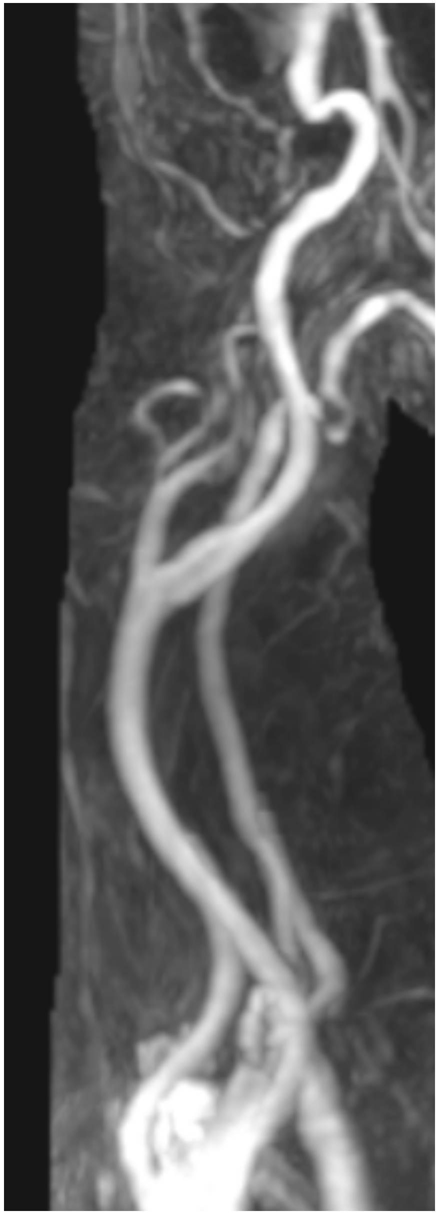

167e Score

0-30

31-50

51-70

>70

Near occlusion

Occluded

Quality

1

2

3

4

5

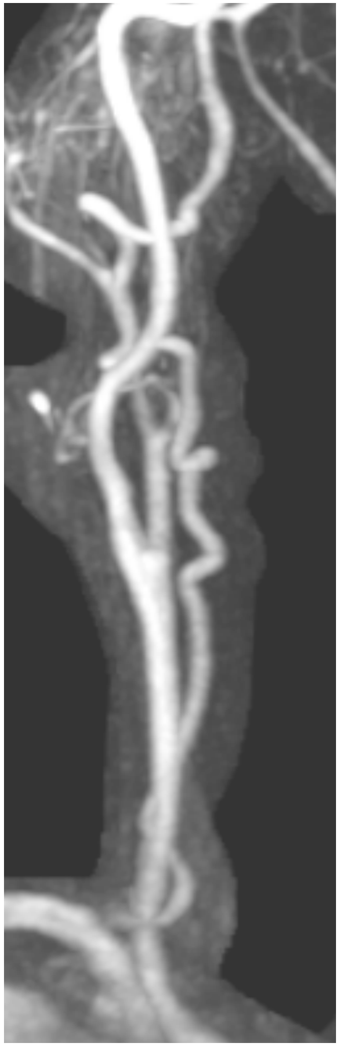

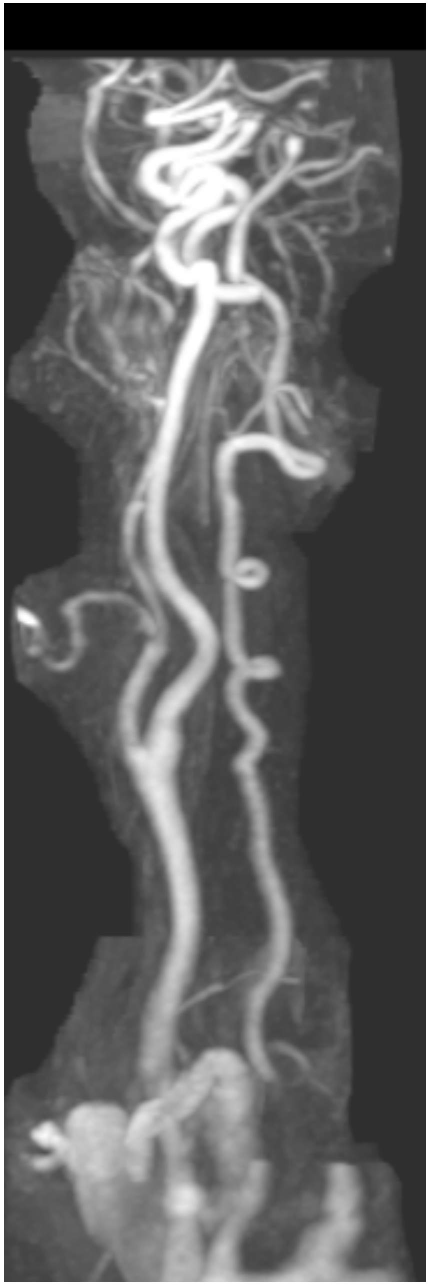

168d Score

0-30

31-50

51-70

>70

Near occlusion

Occluded

Quality

1

2

3

4

5

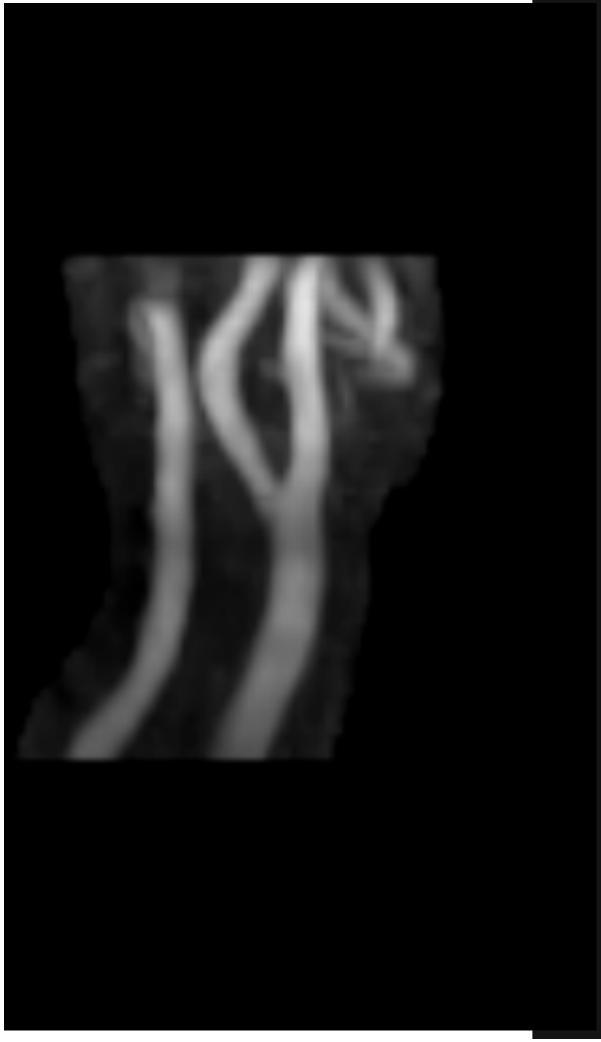

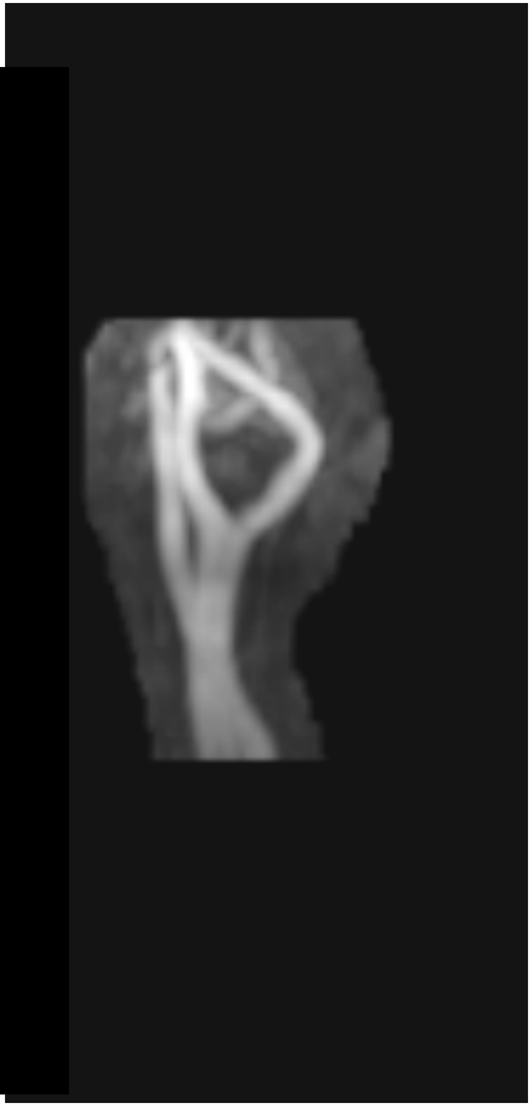

169c Score

0-30

31-50

51-70

>70

Near occlusion

Occluded

Quality

1

2

3

4

5

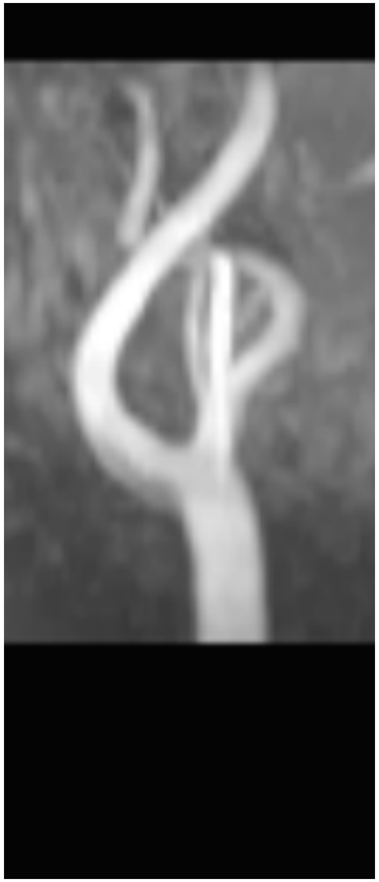

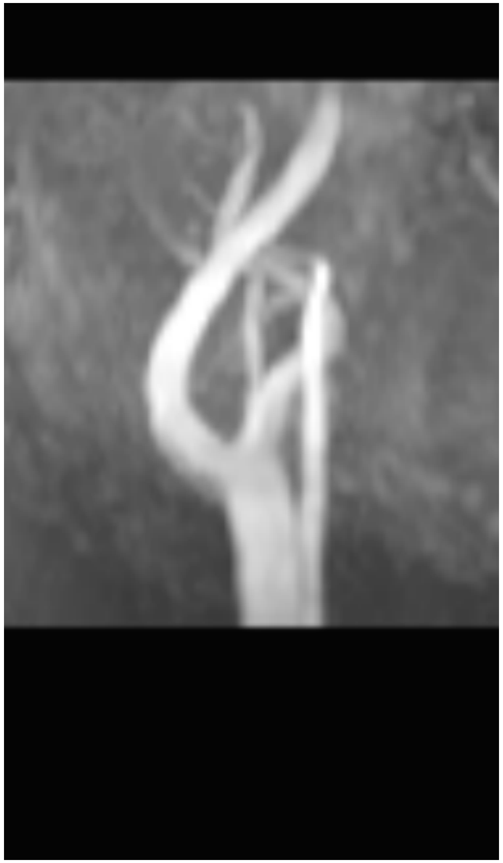

170b Score

0-30

31-50

51-70

>70

Near occlusion

Occluded

Quality

1

2

3

4

5

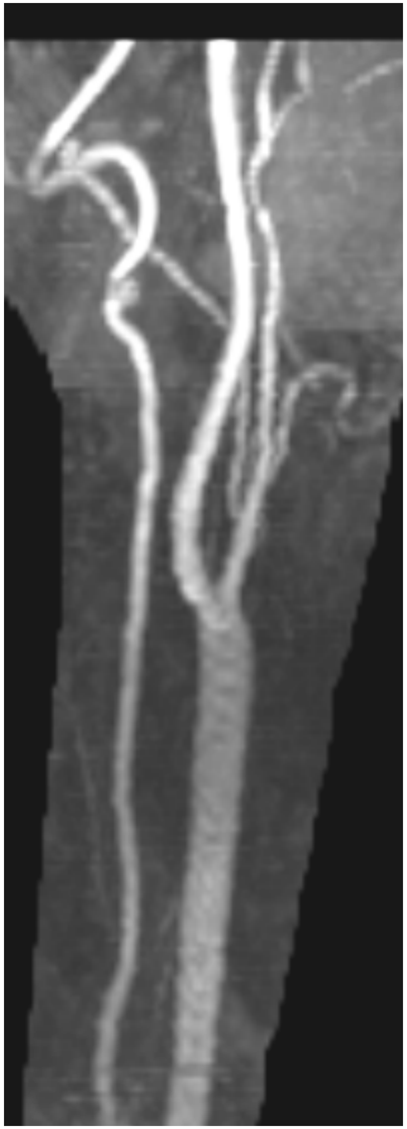

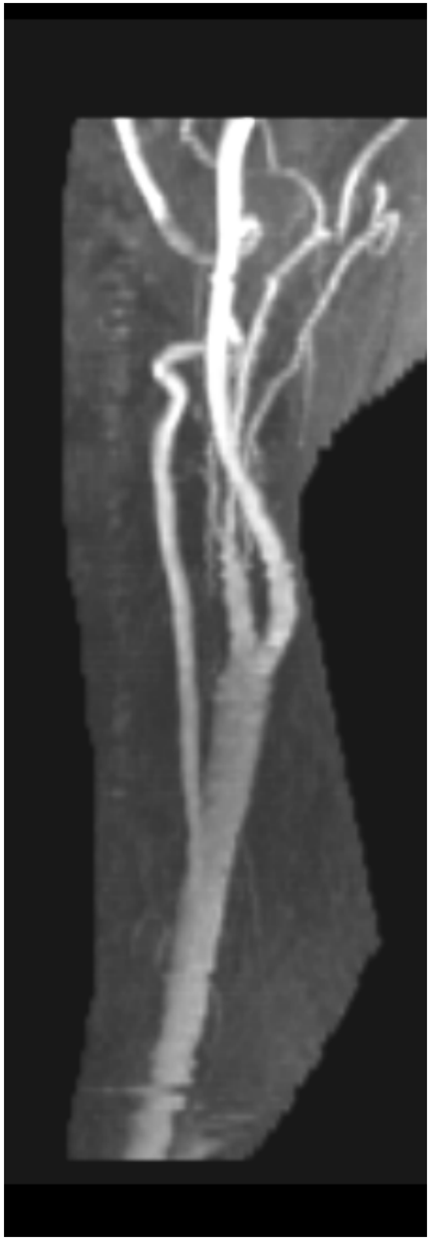

171a Score

0-30

31-50

51-70

>70

Near occlusion

Occluded

Quality

1

2

3

4

5

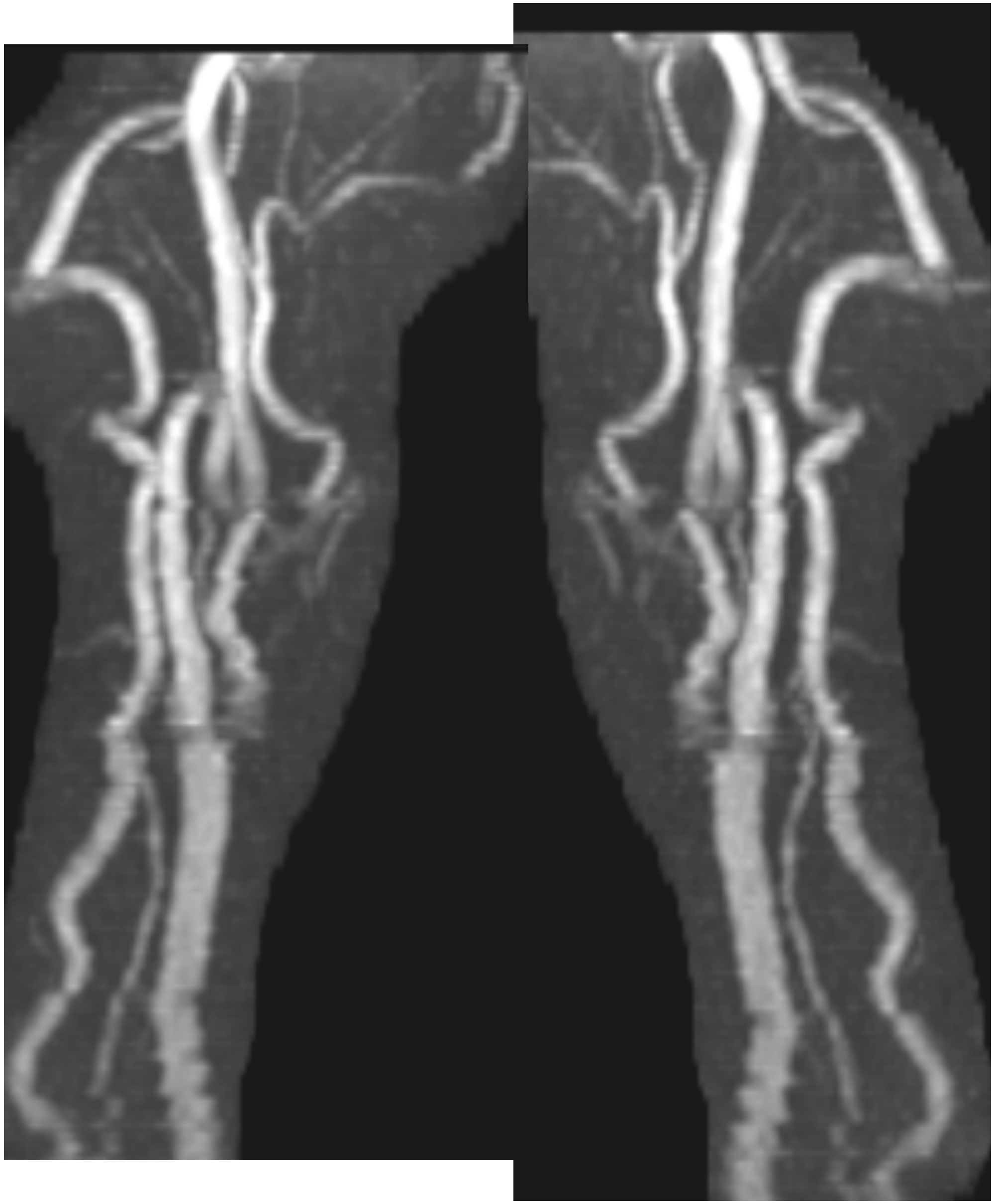

171f Score

0-30

31-50

51-70

>70

Near occlusion

Occluded

Quality

1

2

3

4

5

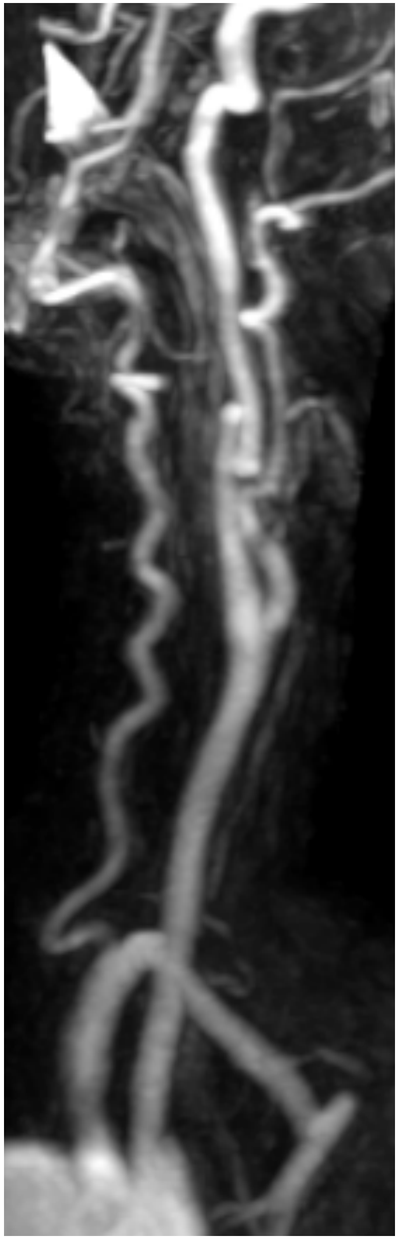

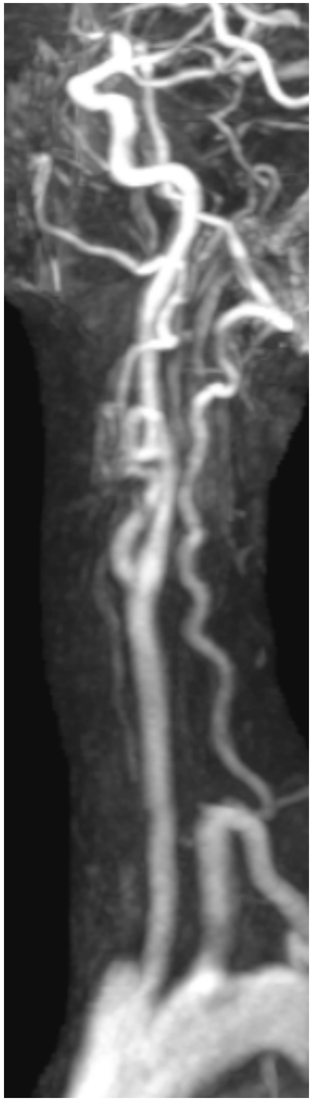

172e Score

0-30

31-50

51-70

>70

Near occlusion

Occluded

Quality

1

2

3

4

5

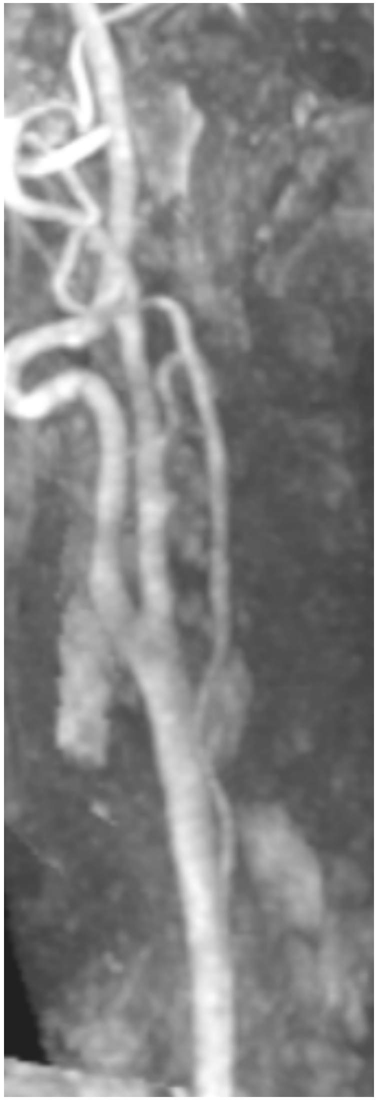

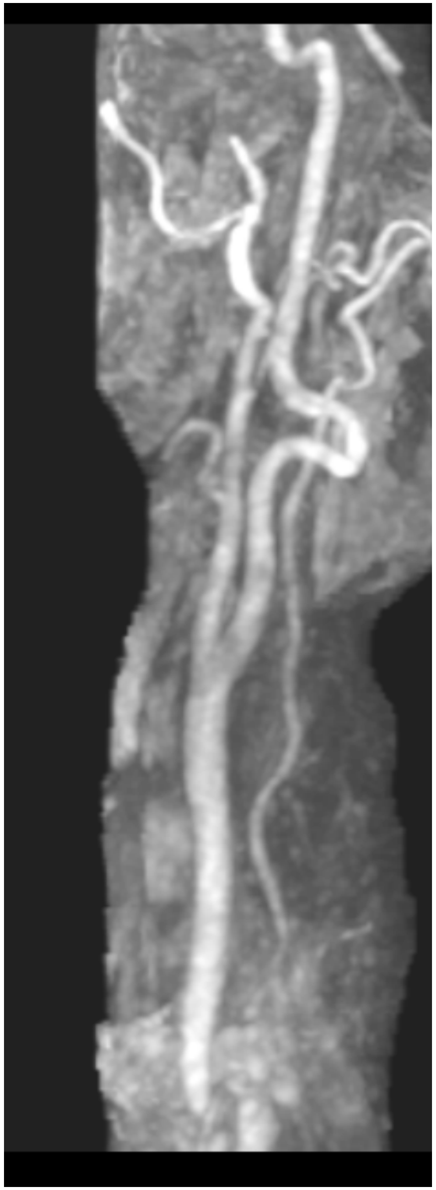

173d Score

0-30

31-50

51-70

>70

Near occlusion

Occluded

Quality

1

2

3

4

5

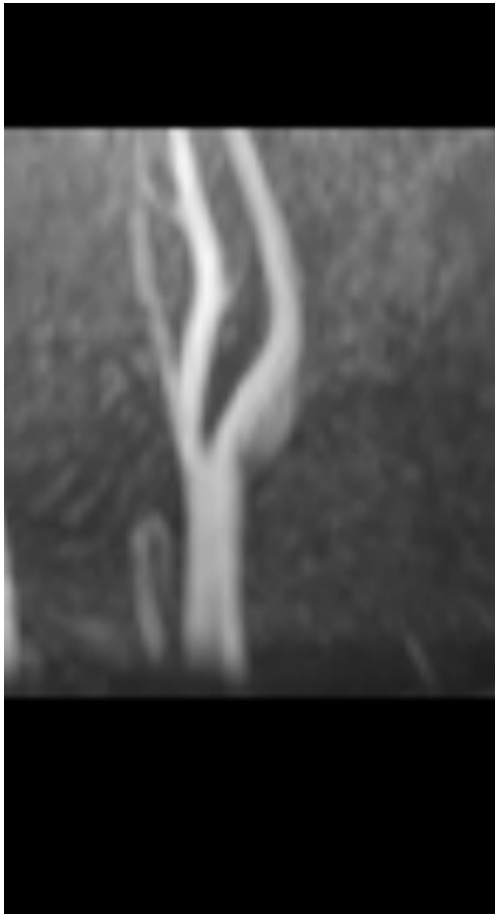

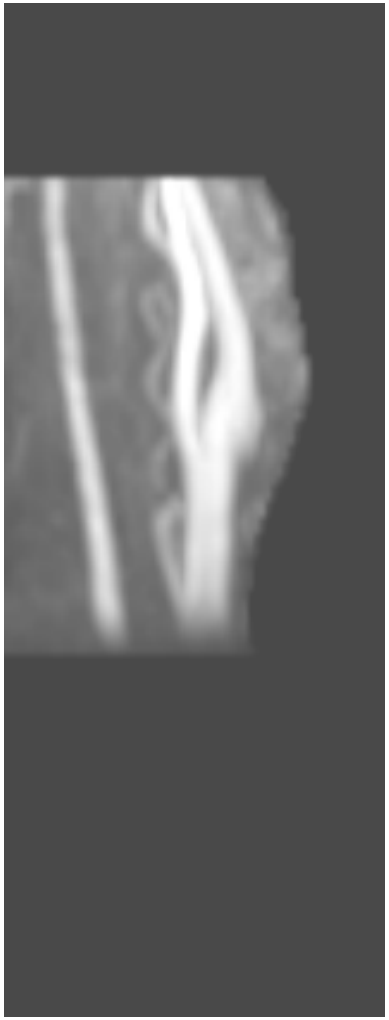

174c Score

0-30

31-50

51-70

>70

Near occlusion

Occluded

Quality

1

2

3

4

5

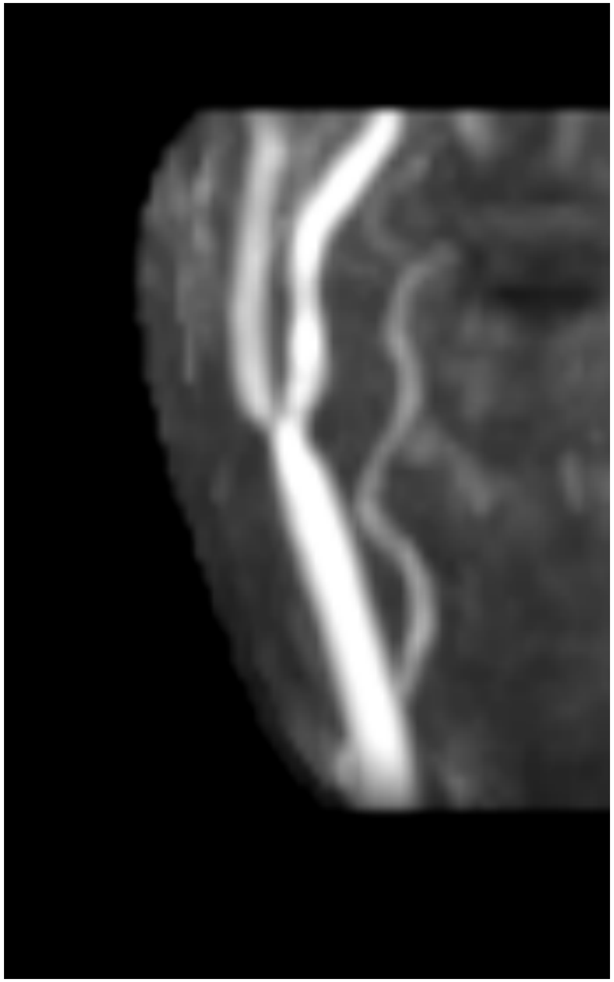

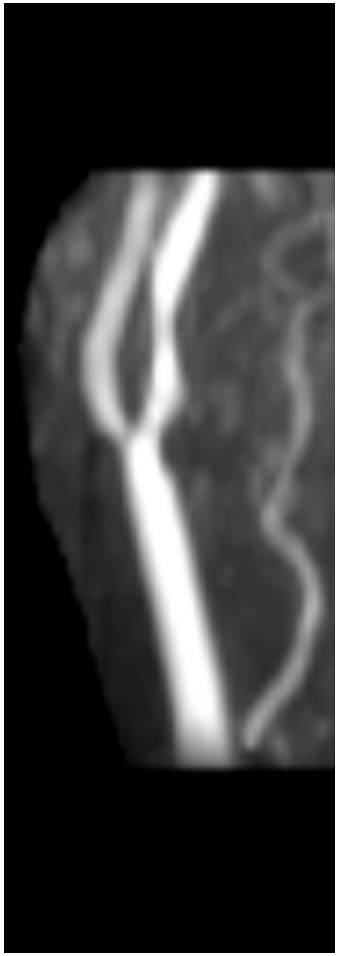

175b Score

0-30

31-50

51-70

>70

Near occlusion

Occluded

Quality

1

2

3

4

5

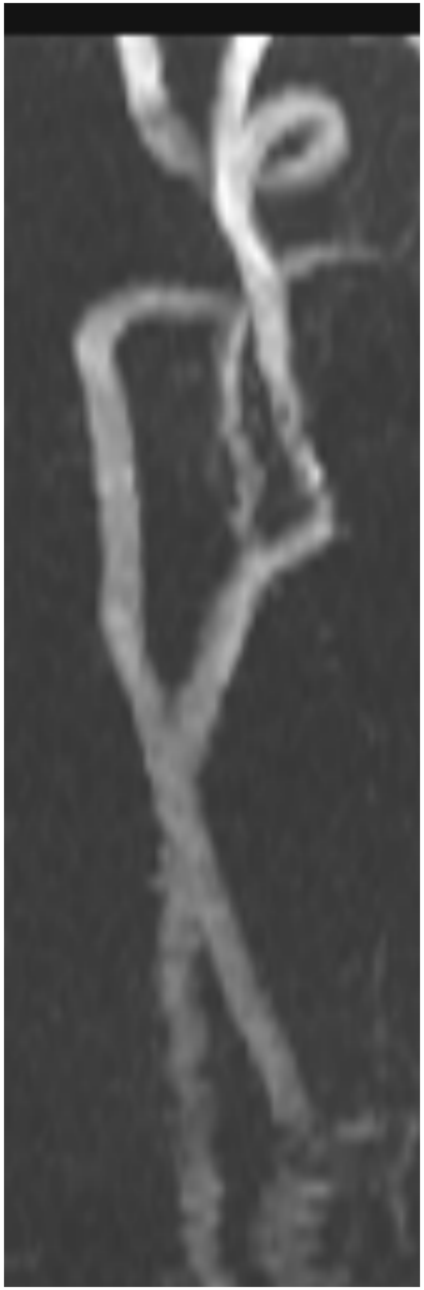

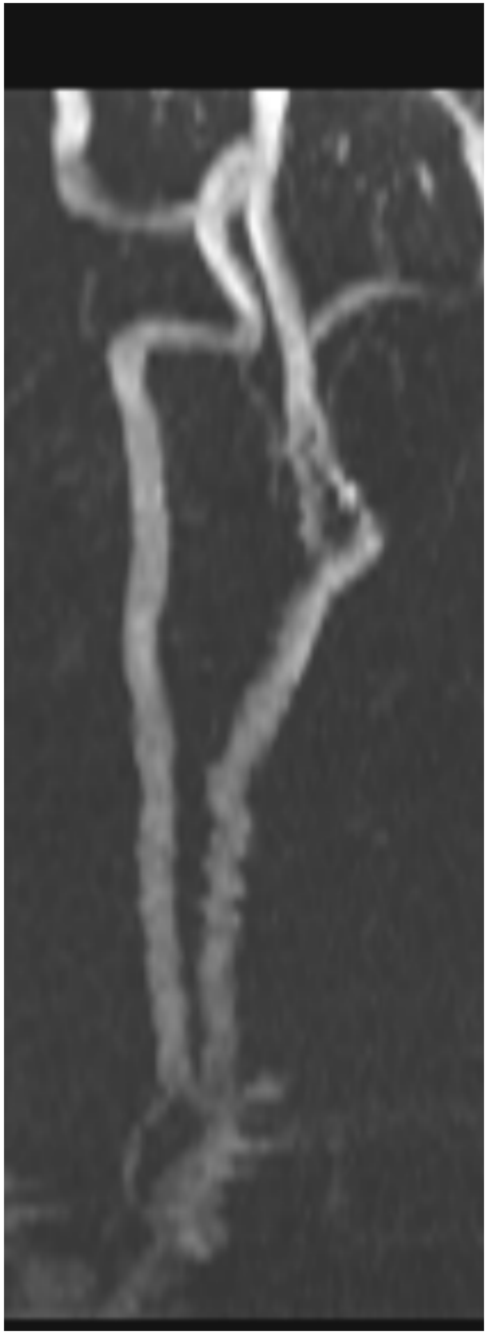

176a Score

0-30

31-50

51-70

>70

Near occlusion

Occluded

Quality

1

2

3

4

5

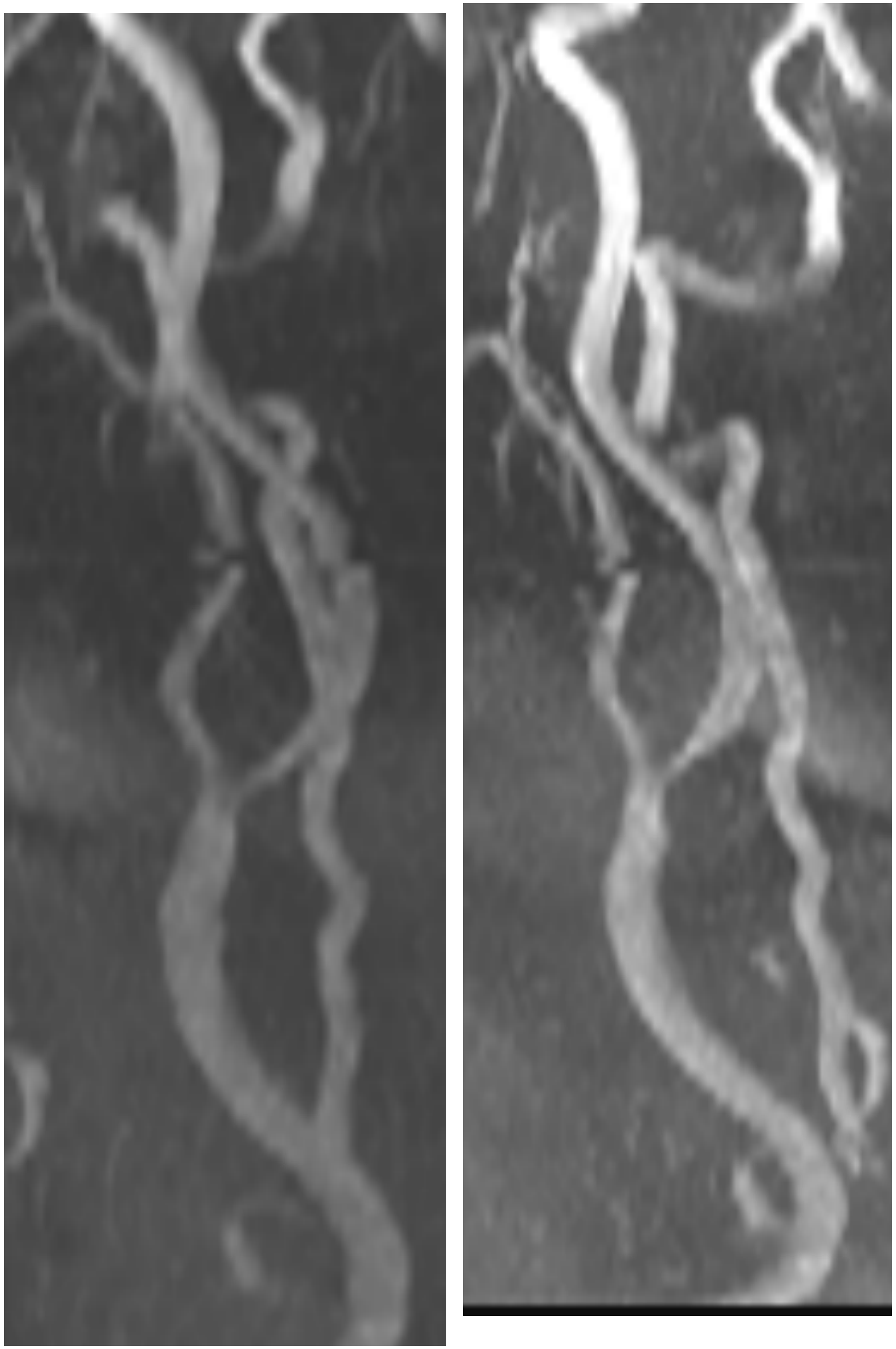

176f Score

0-30

31-50

51-70

>70

Near occlusion

Occluded

Quality

1

2

3

4

5

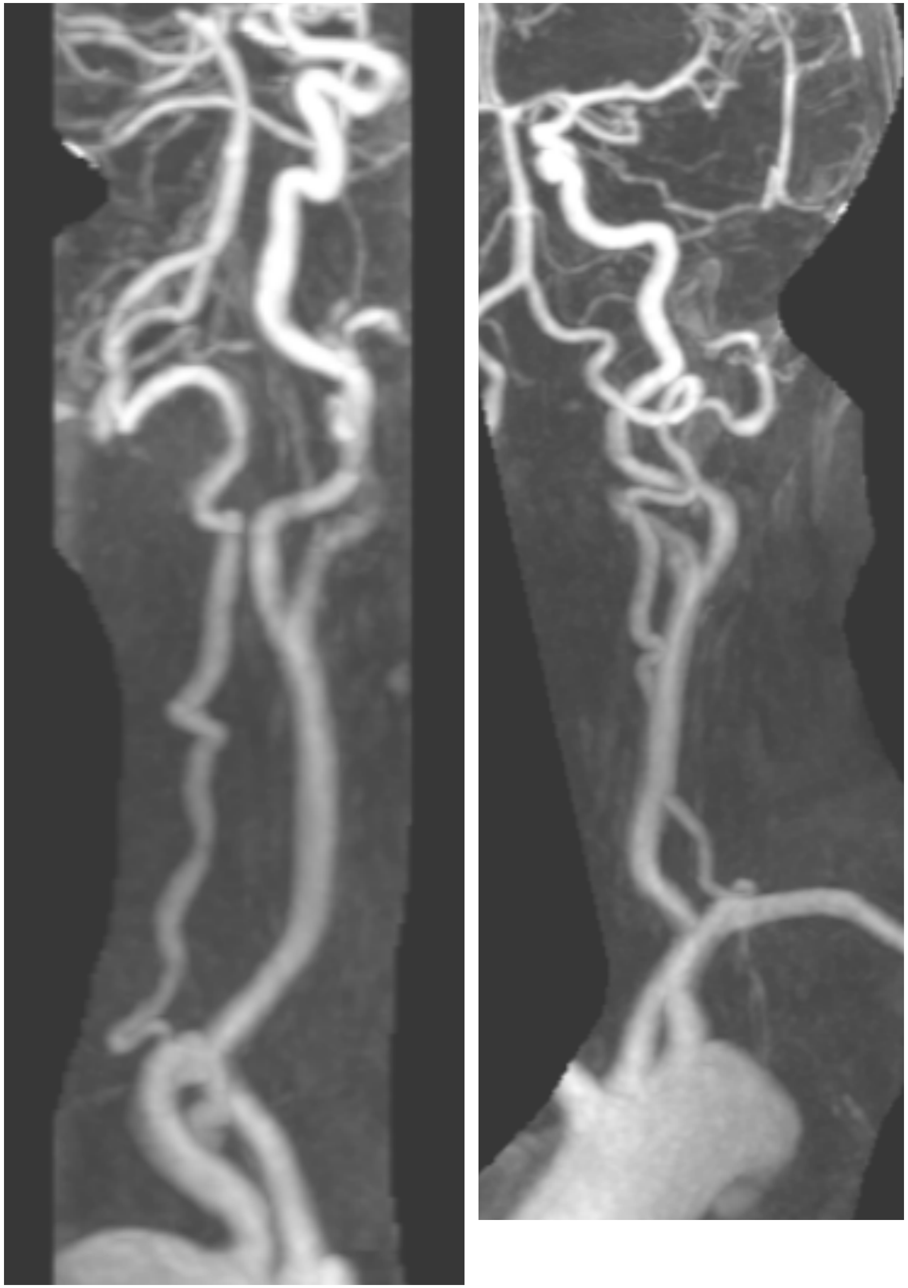

177e Score

0-30

31-50

51-70

>70

Near occlusion

Occluded

Quality

1

2

3

4

5

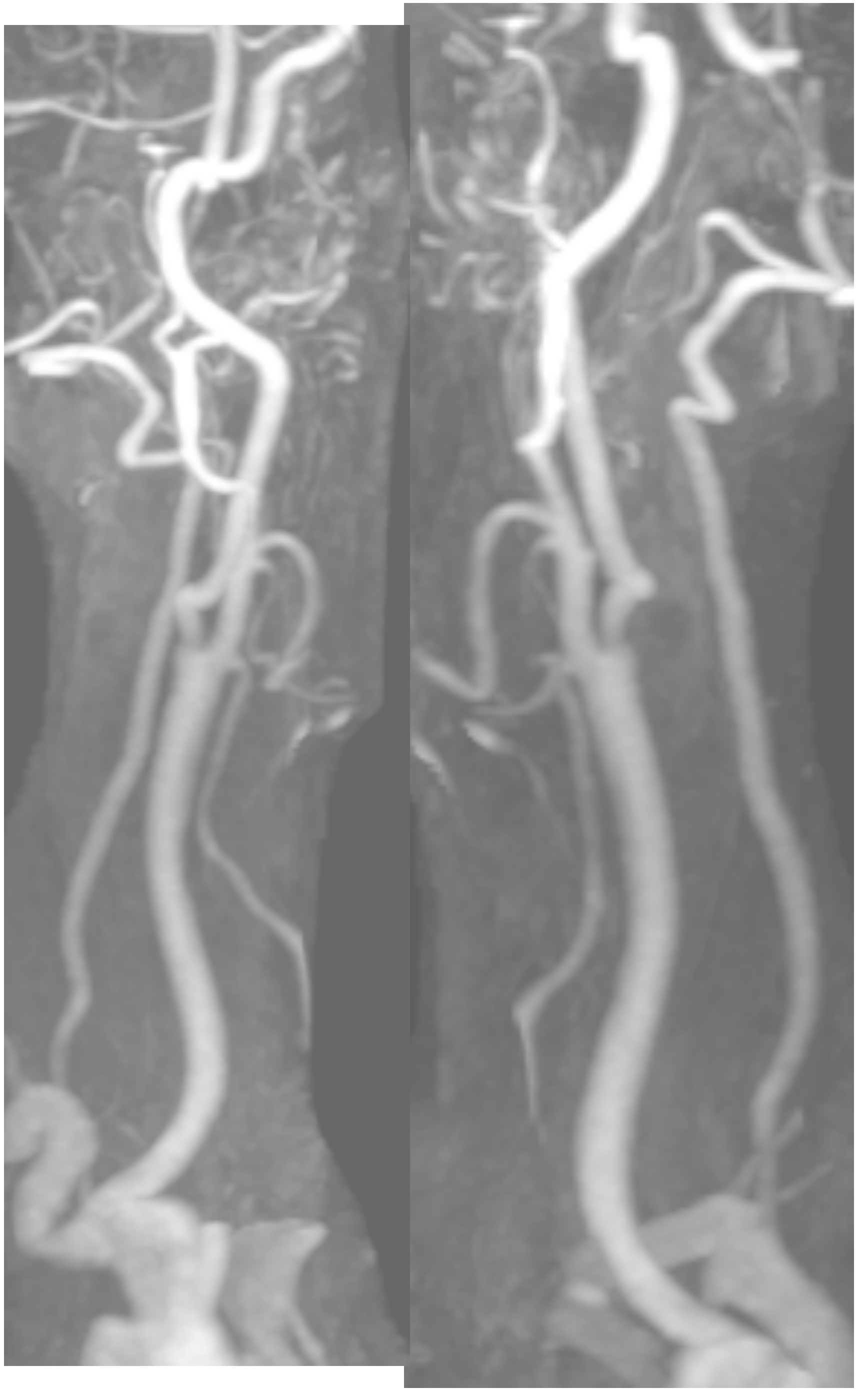

178d Score

0-30

31-50

51-70

>70

Near occlusion

Occluded

Quality

1

2

3

4

5

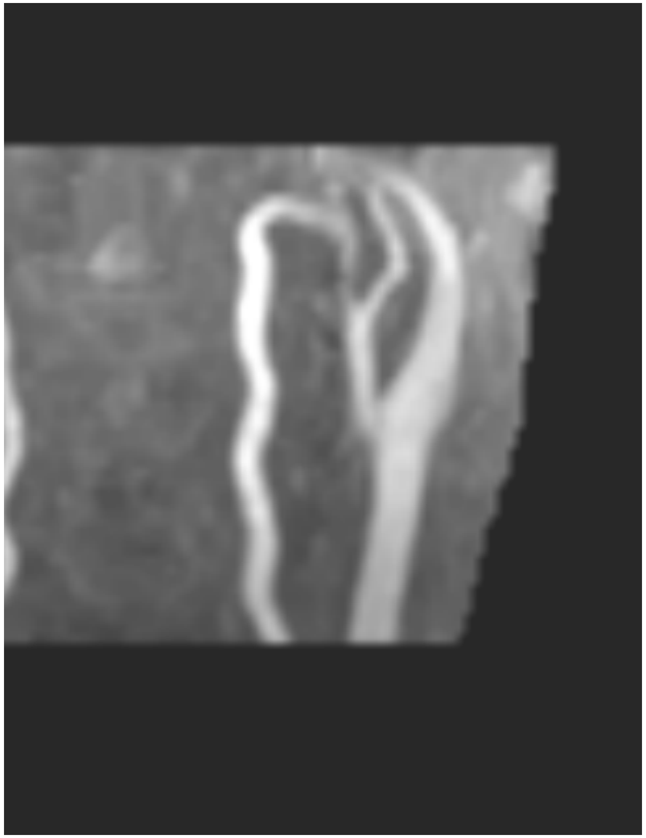

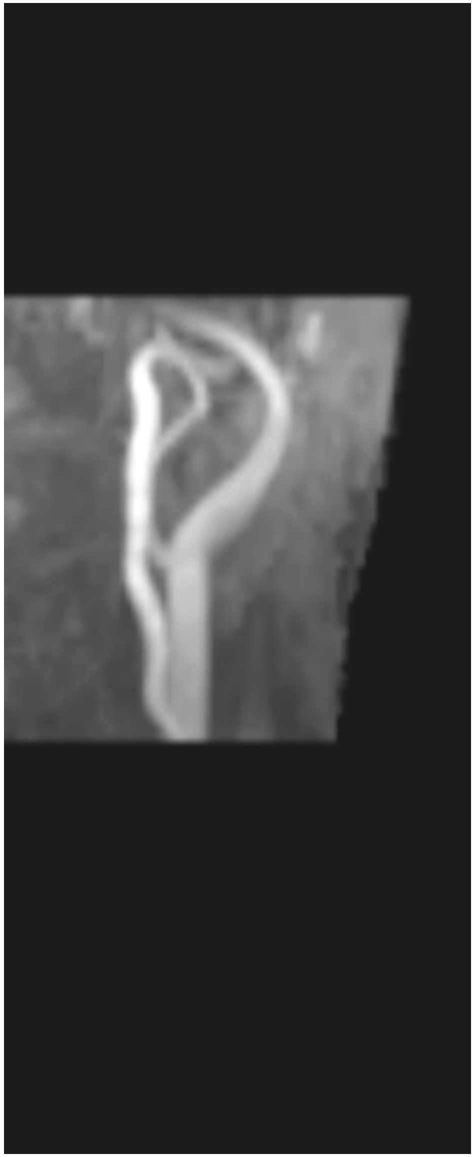

179c Score

0-30

31-50

51-70

>70

Near occlusion

Occluded

Quality

1

2

3

4

5

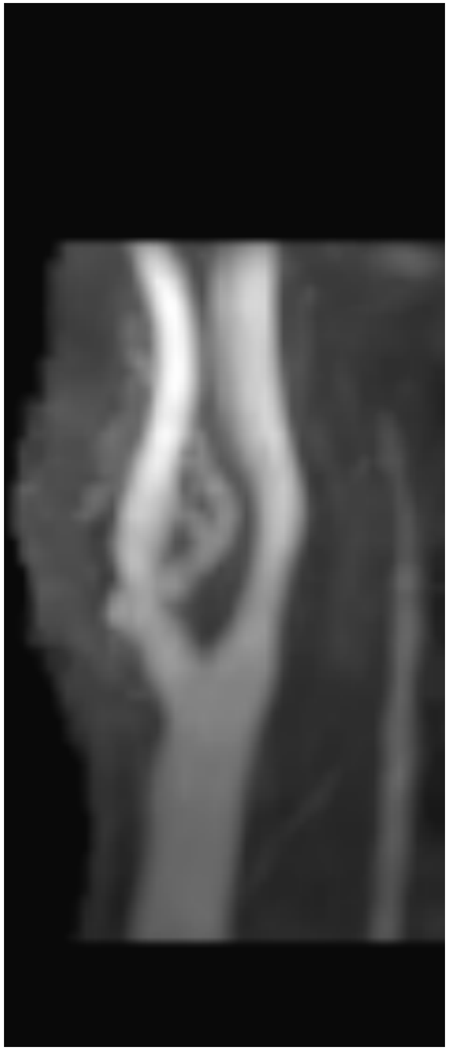

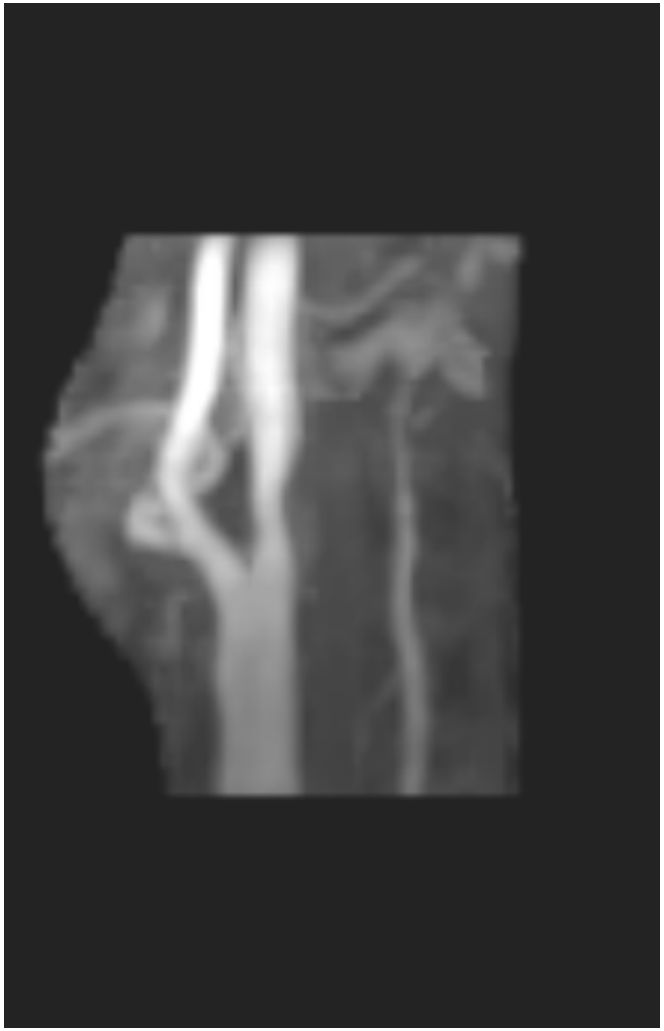

180b Score

0-30

31-50

51-70

>70

Near occlusion

Occluded

Quality

1

2

3

4

5

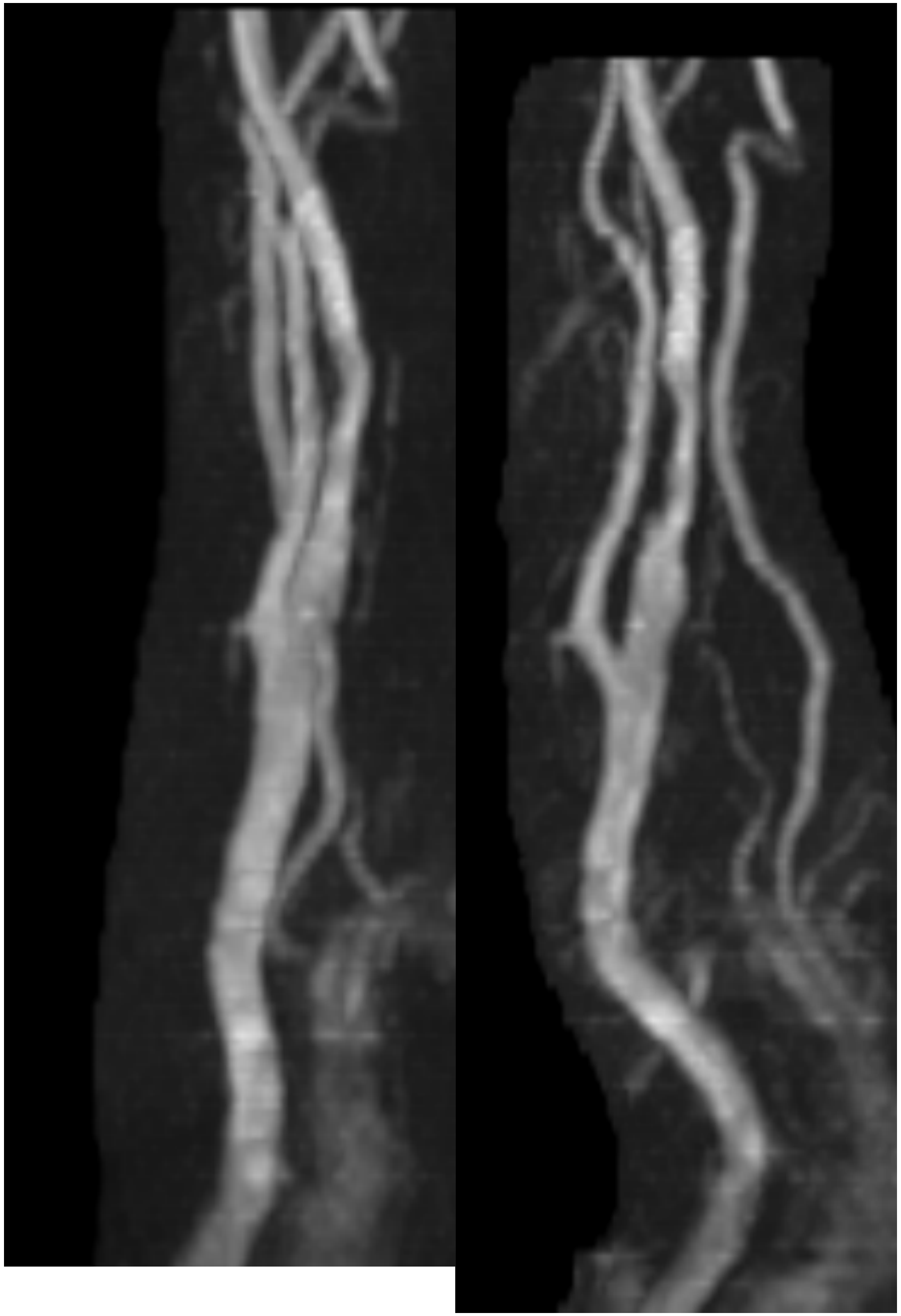

181a Score

0-30

31-50

51-70

>70

Near occlusion

Occluded

Quality

1

2

3

4

5

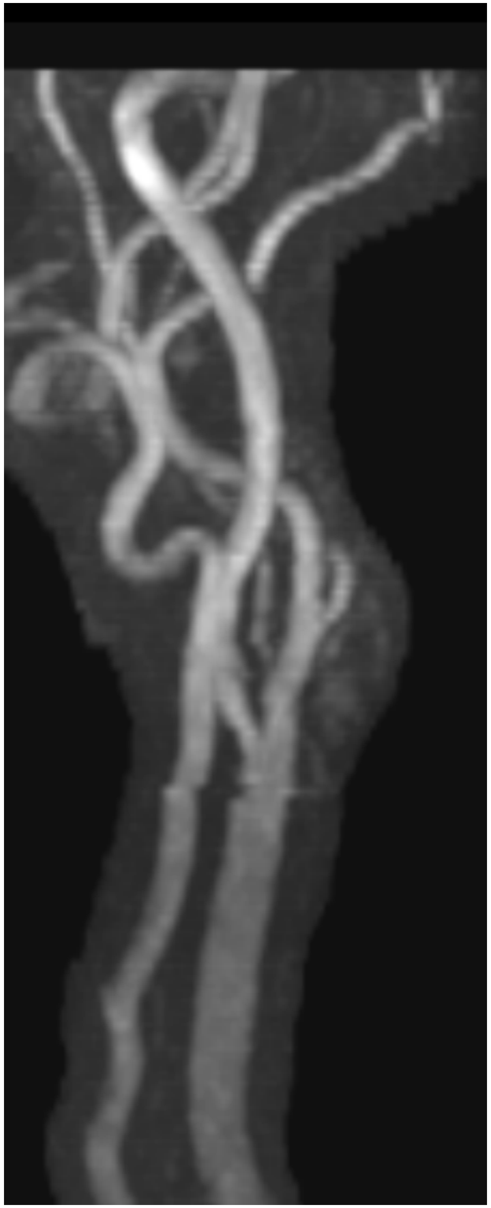

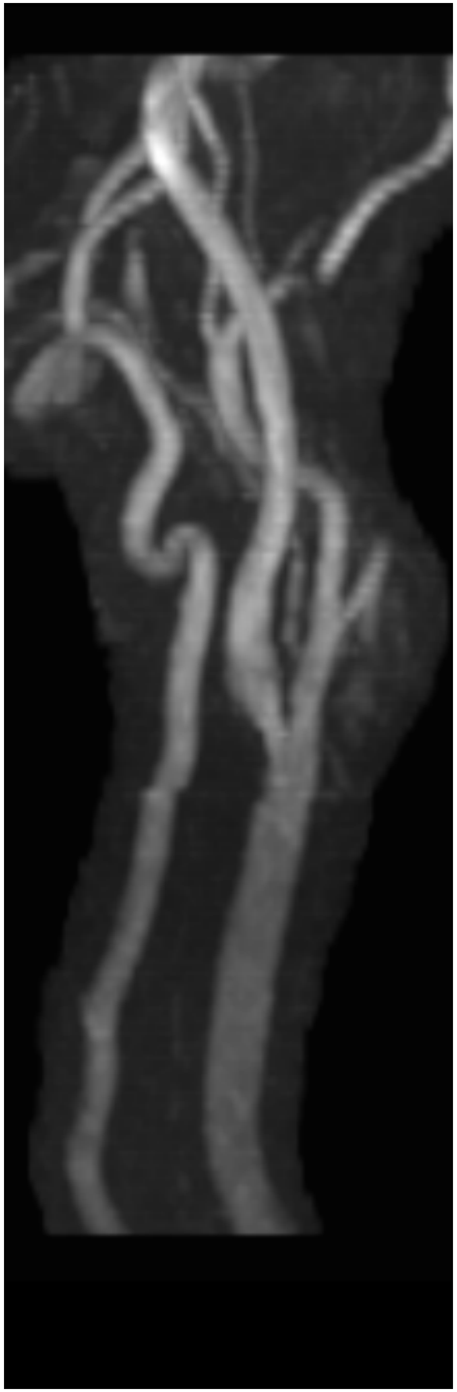

181f Score

0-30

31-50

51-70

>70

Near occlusion

Occluded

Quality

1

2

3

4

5

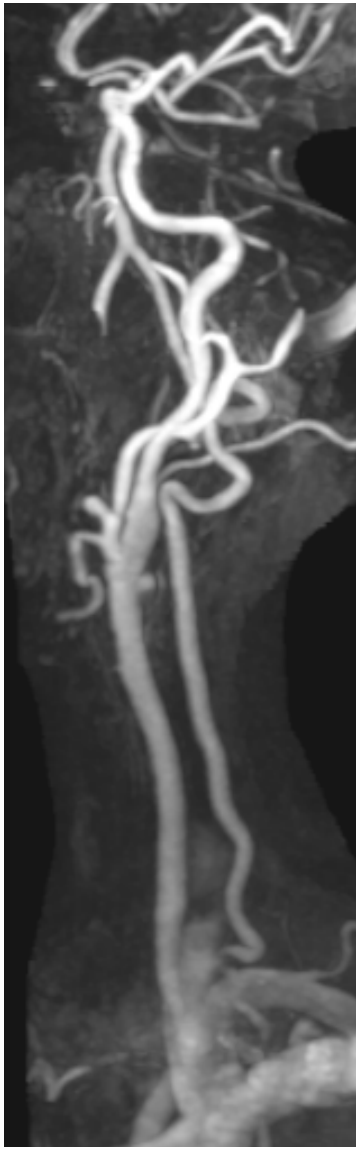

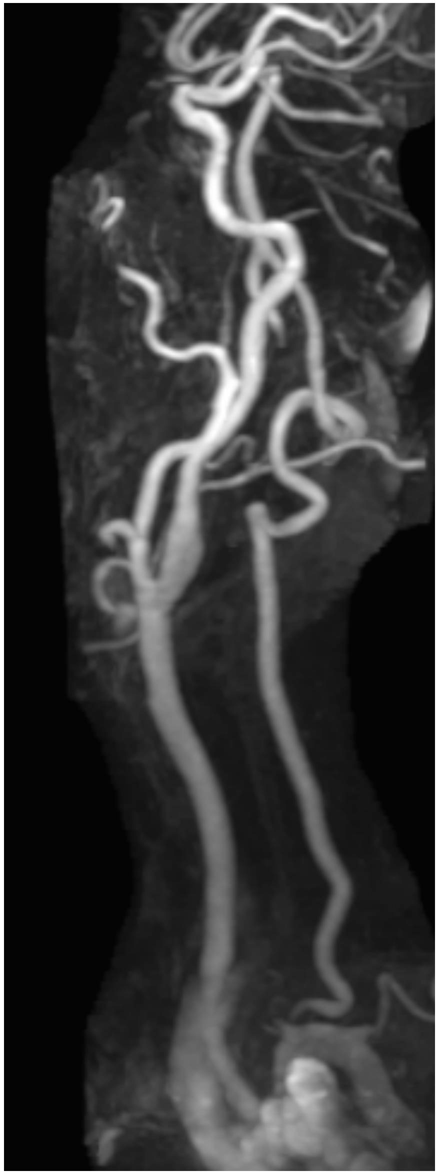

182e Score

0-30

31-50

51-70

>70

Near occlusion

Occluded

Quality

1

2

3

4

5

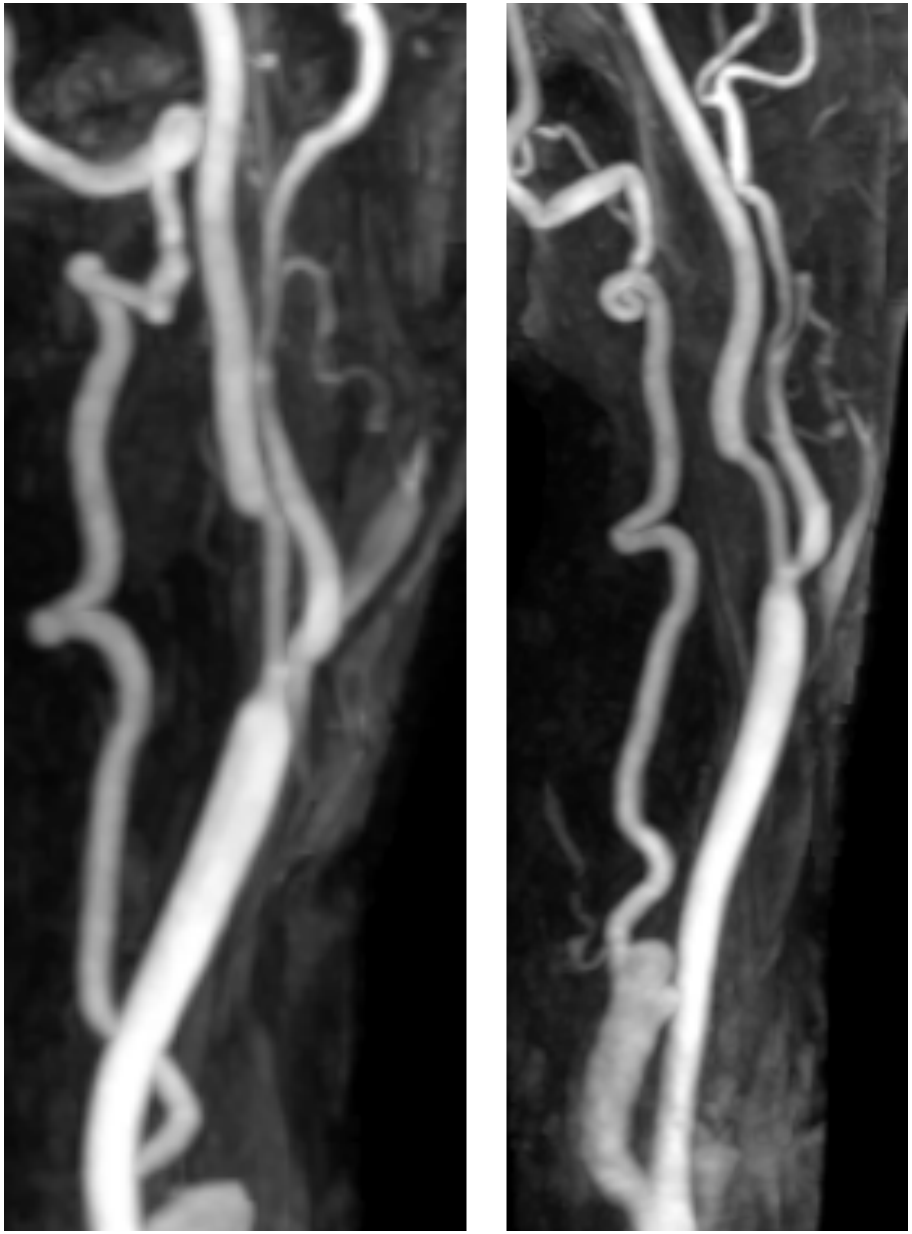

183d Score

0-30

31-50

51-70

>70

Near occlusion

Occluded

Quality

1

2

3

4

5

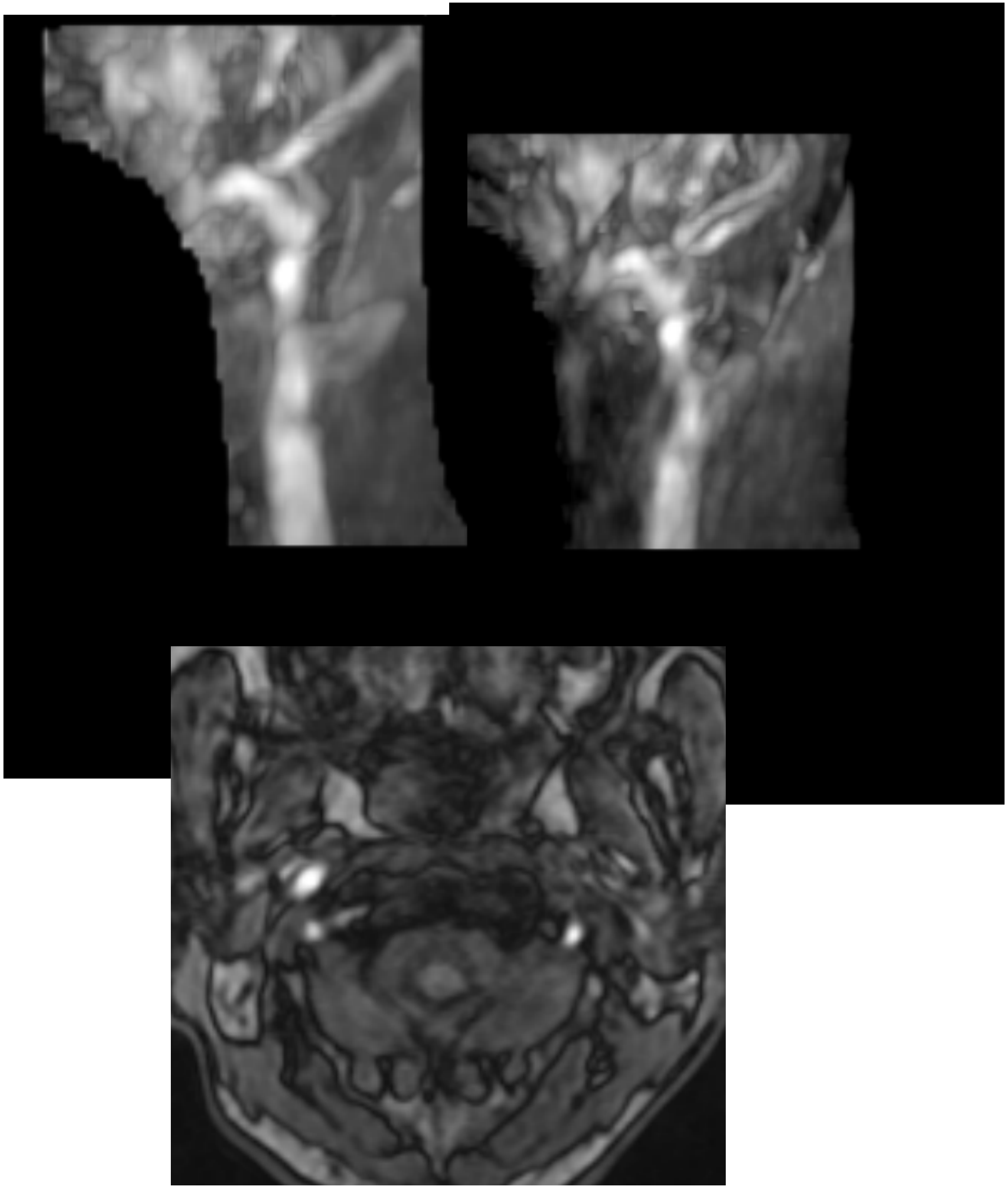

184c Score

0-30

31-50

51-70

>70

Near occlusion

Occluded

Quality

1

2

3

4

5

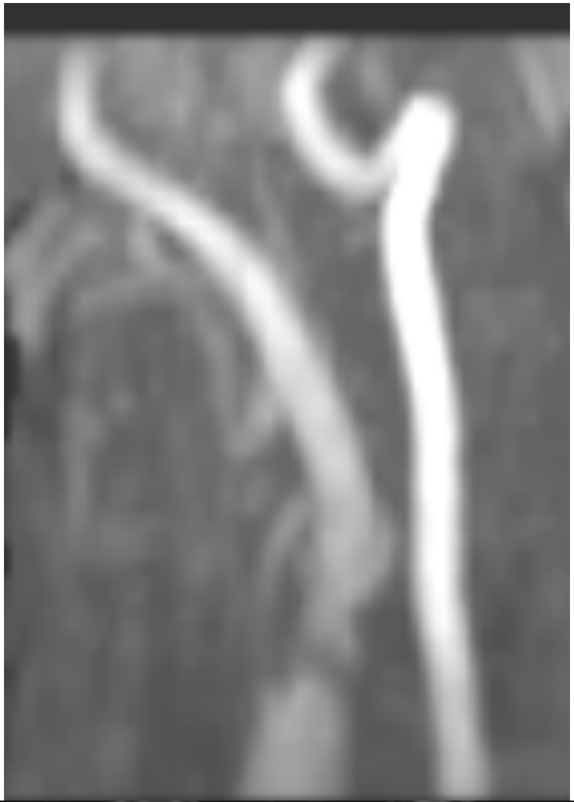

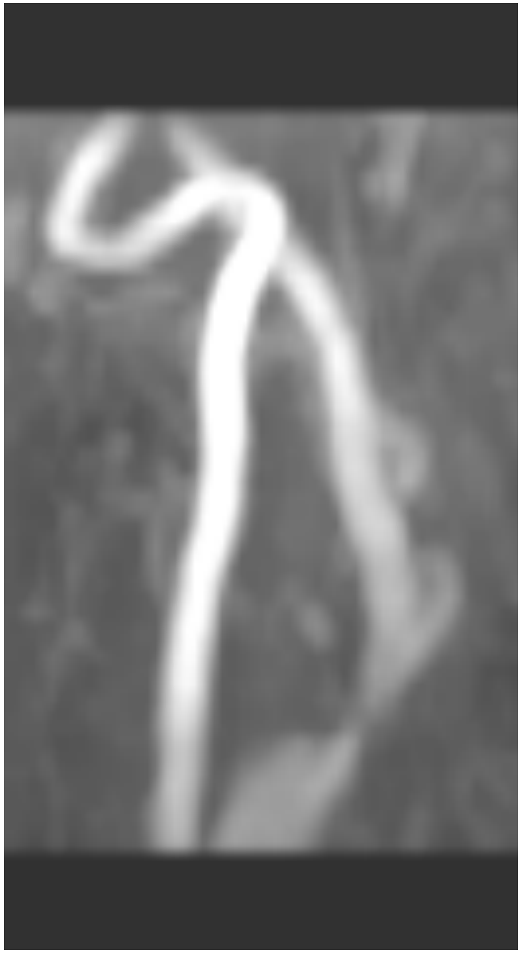

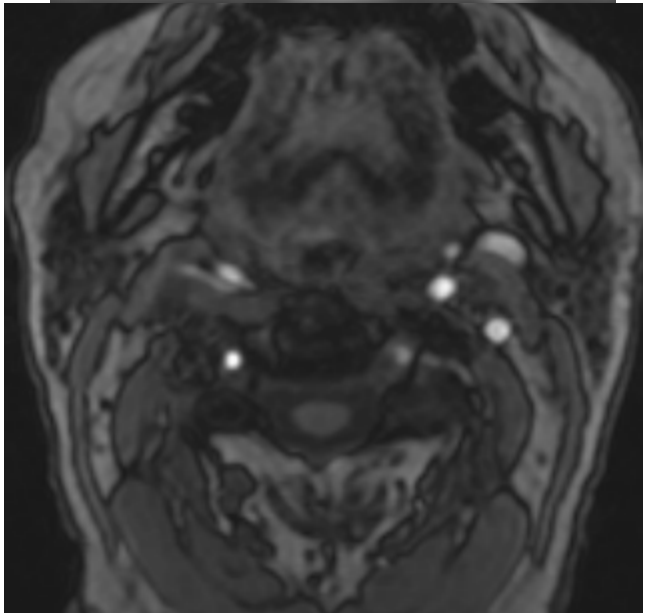

185b Score

0-30

31-50

51-70

>70

Near occlusion

Occluded

Quality

1

2

3

4

5

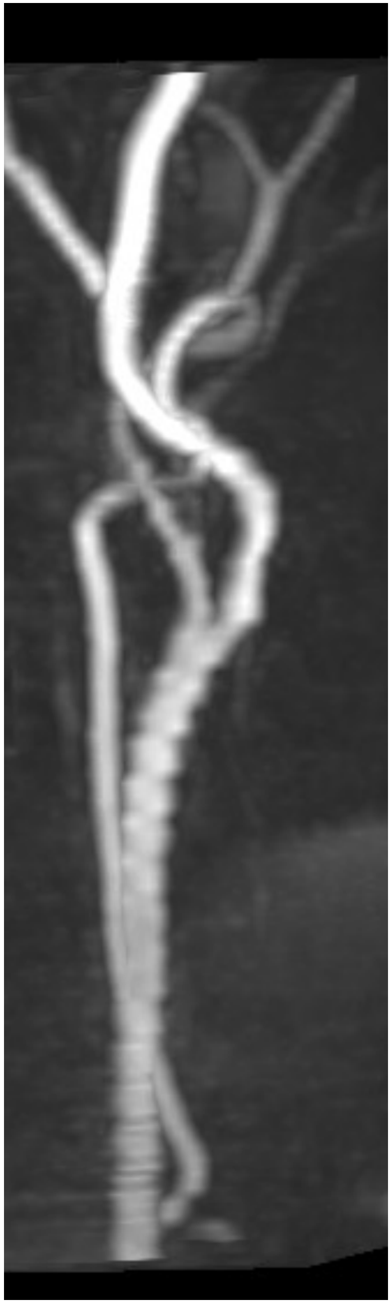

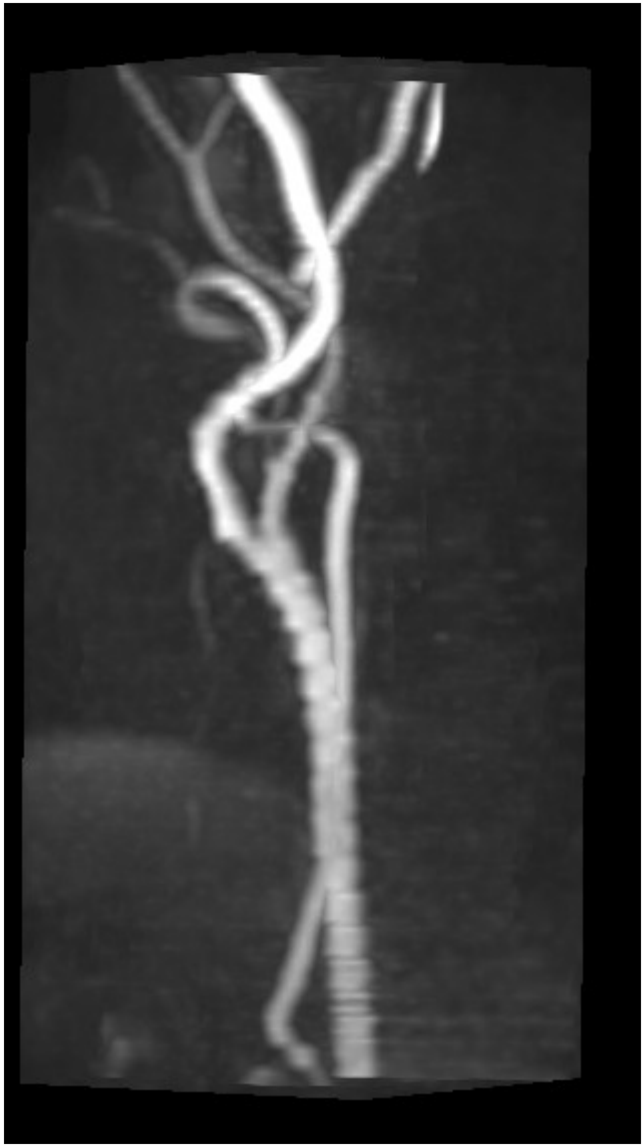

186a Score

0-30

31-50

51-70

>70

Near occlusion

Occluded

Quality

1

2

3

4

5

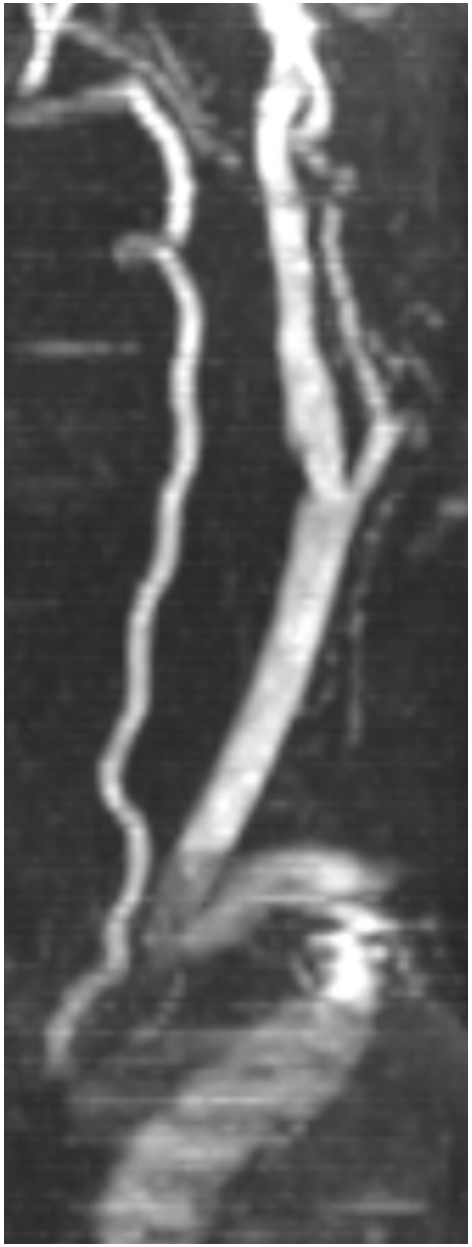

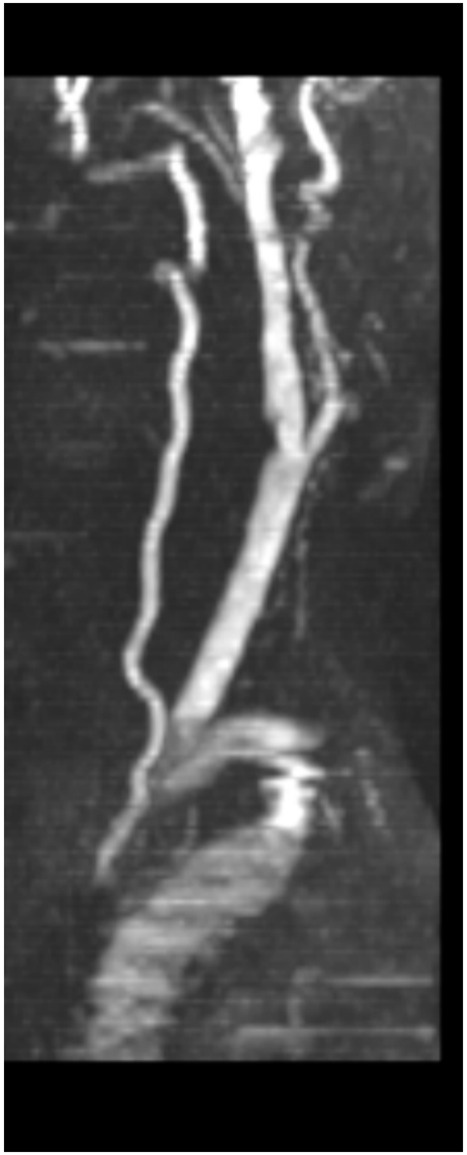

186f Score

0-30

31-50

51-70

>70

Near occlusion

Occluded

Quality

1

2

3

4

5

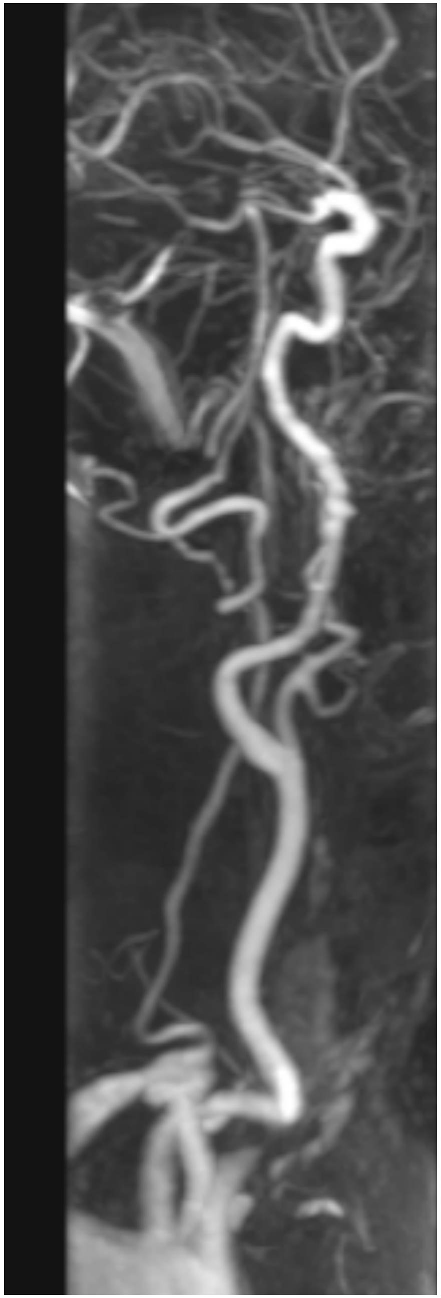

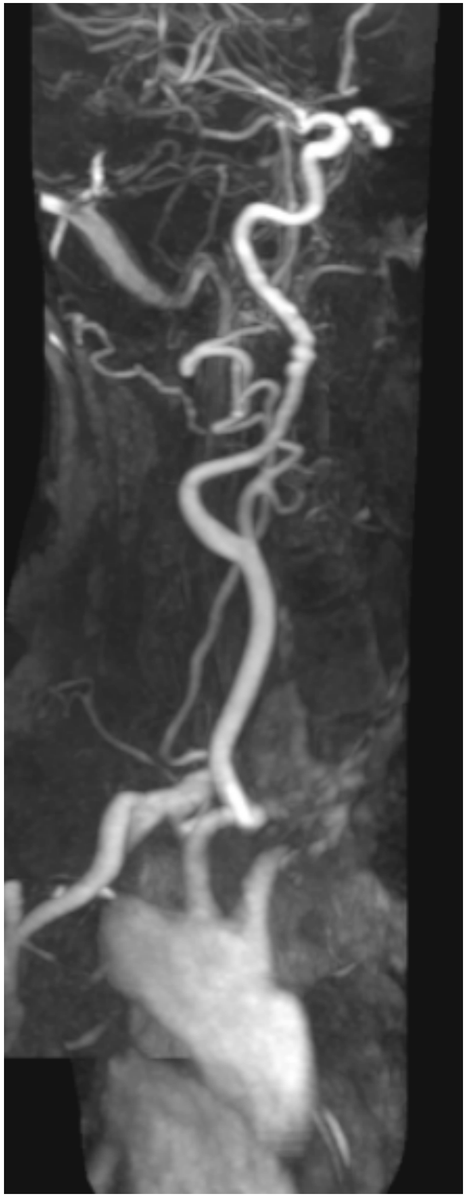

187e Score

0-30

31-50

51-70

>70

Near occlusion

Occluded

Quality

1

2

3

4

5

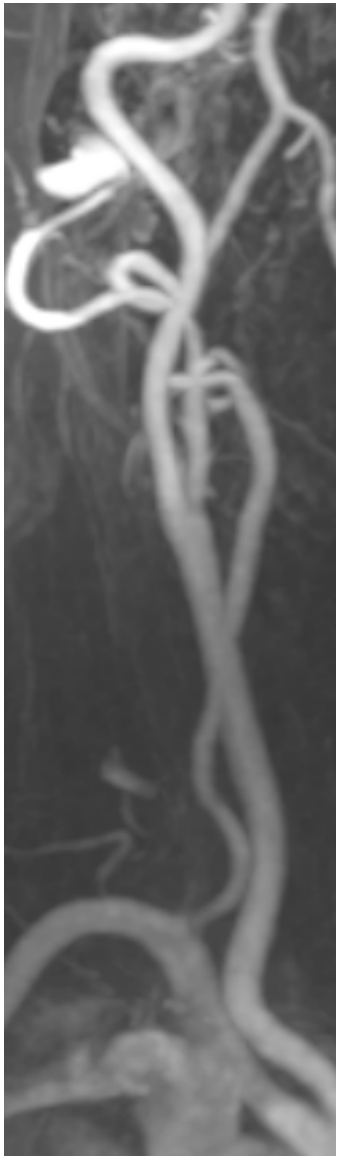

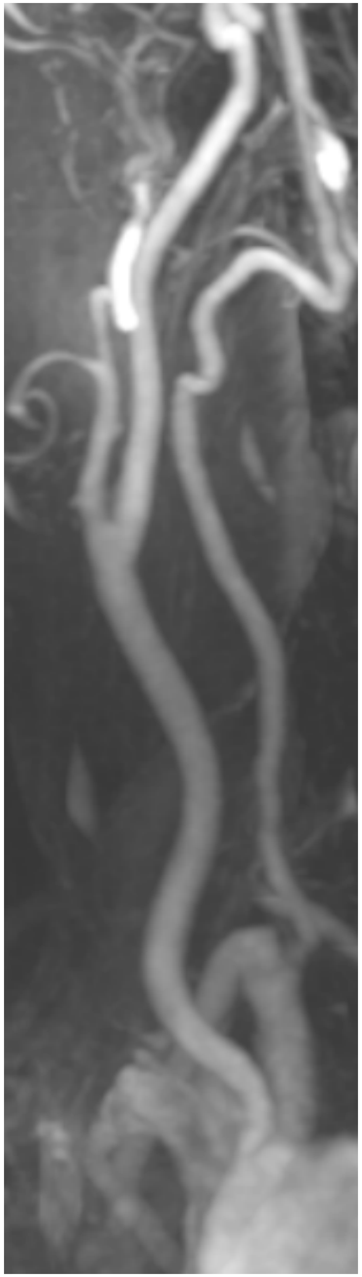

188d Score

0-30

31-50

51-70

>70

Near occlusion

Occluded

Quality

1

2

3

4

5

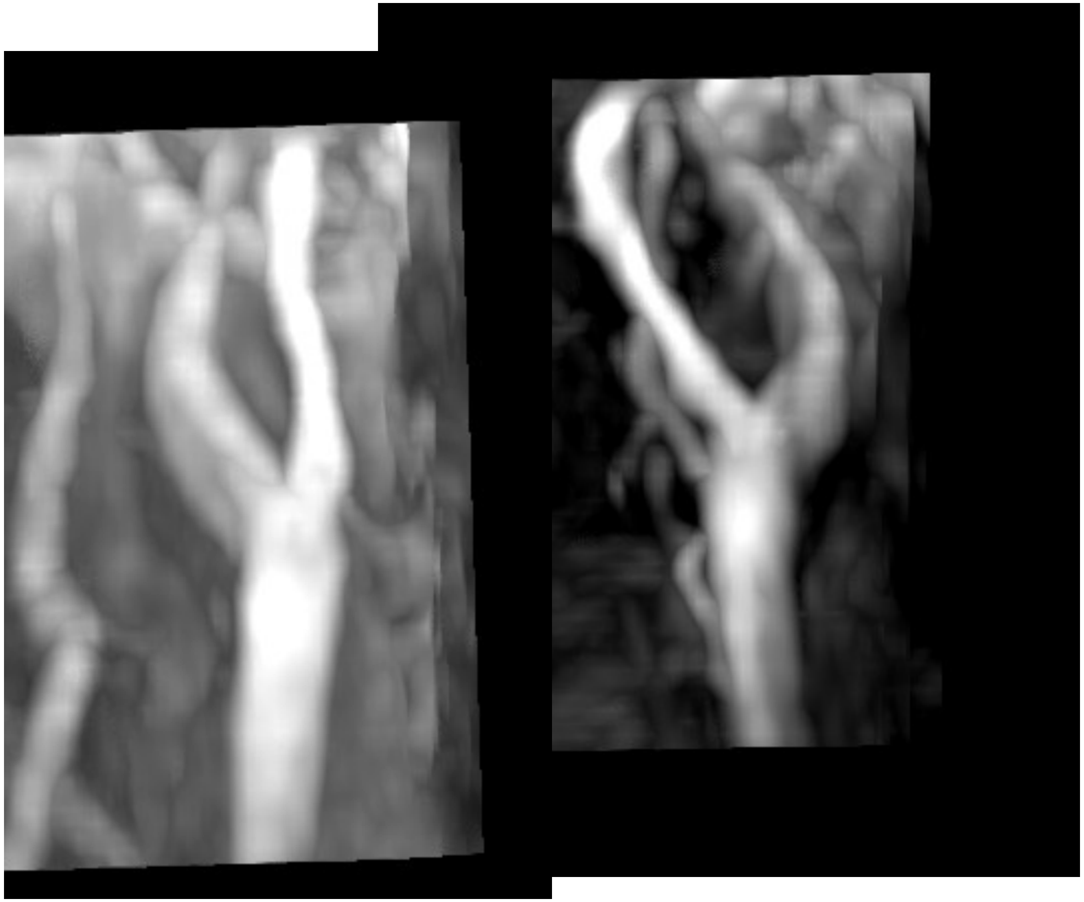

189c Score

0-30

31-50

51-70

>70

Near occlusion

Occluded

Quality

1

2

3

4

5

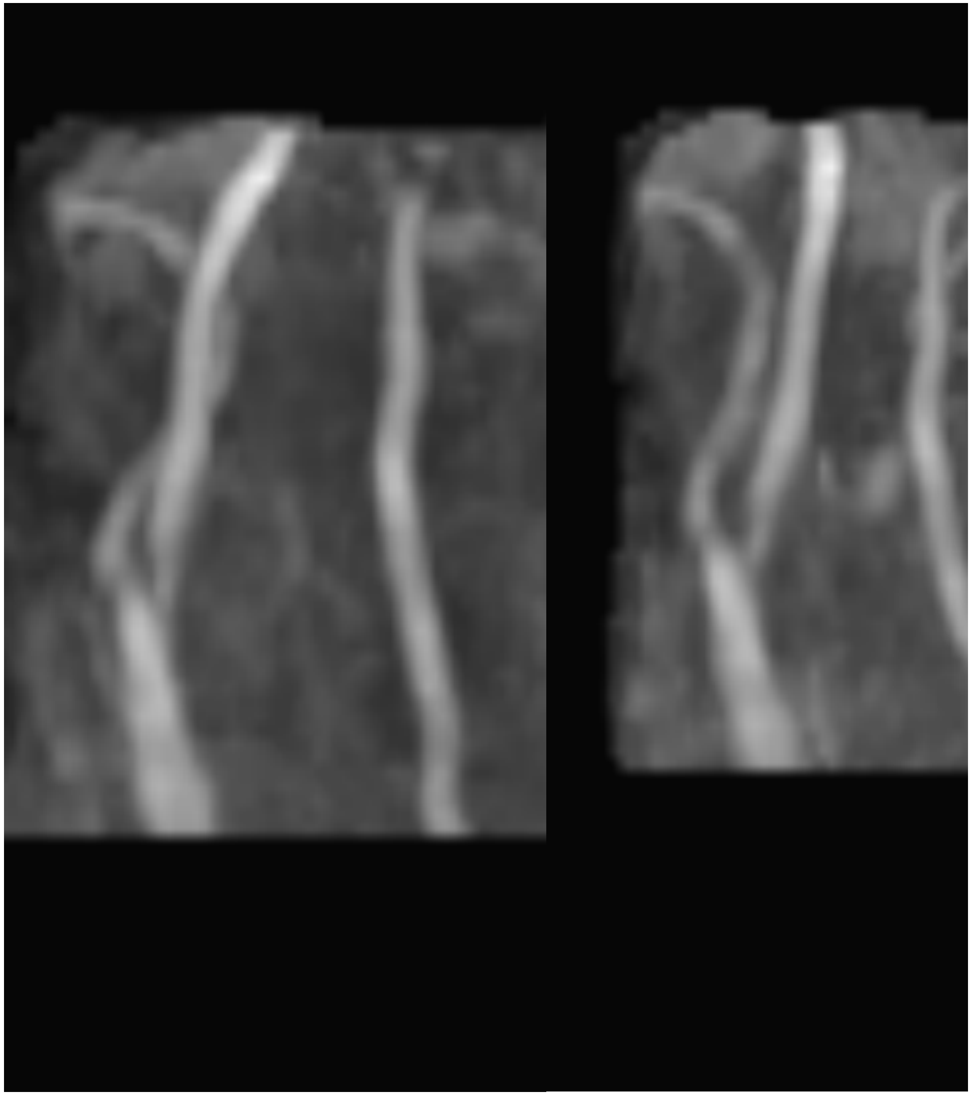

190b Score

0-30

31-50

51-70

>70

Near occlusion

Occluded

Quality

1

2

3

4

5

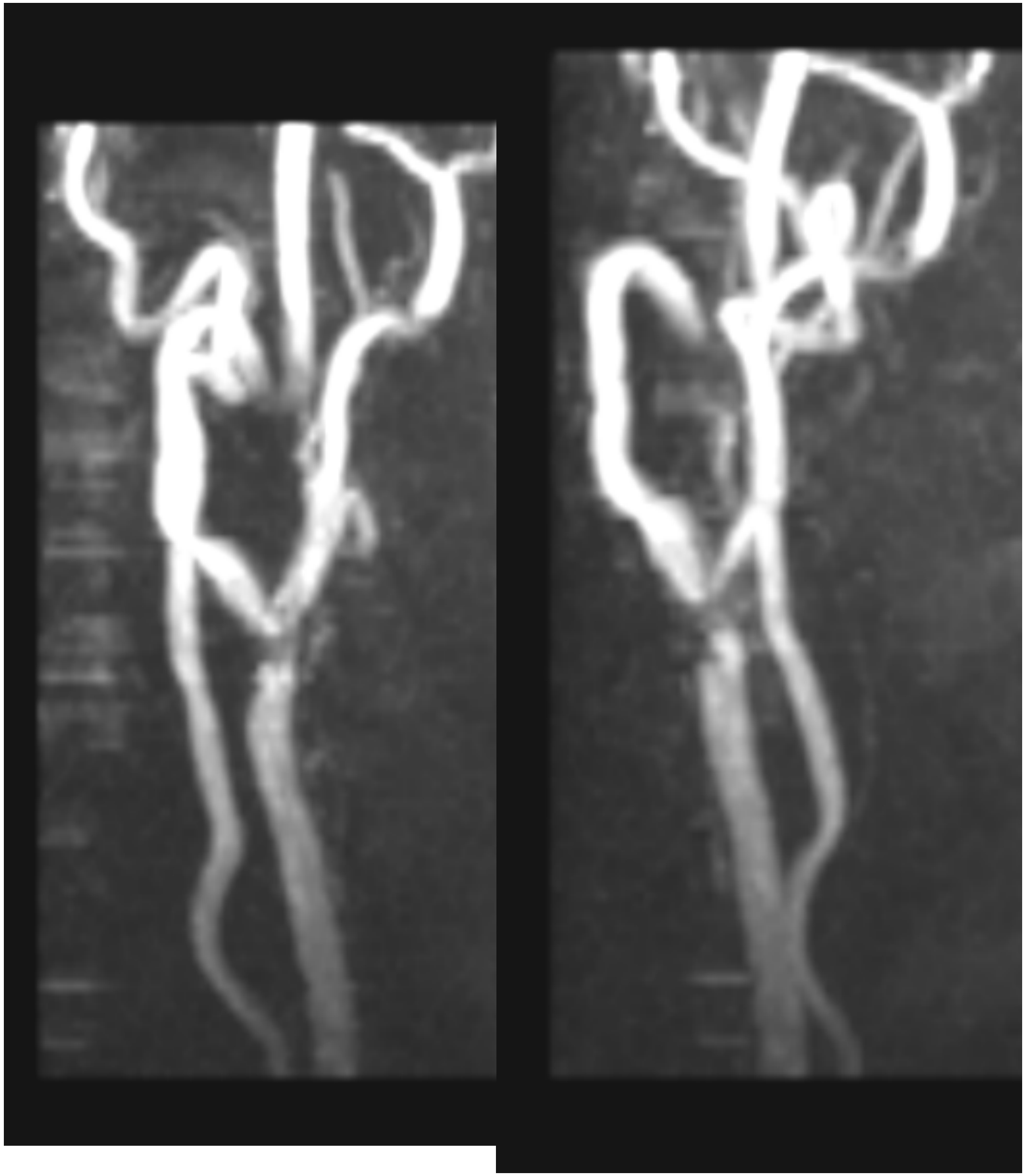

191a Score

0-30

31-50

51-70

>70

Near occlusion

Occluded

Quality

1

2

3

4

5

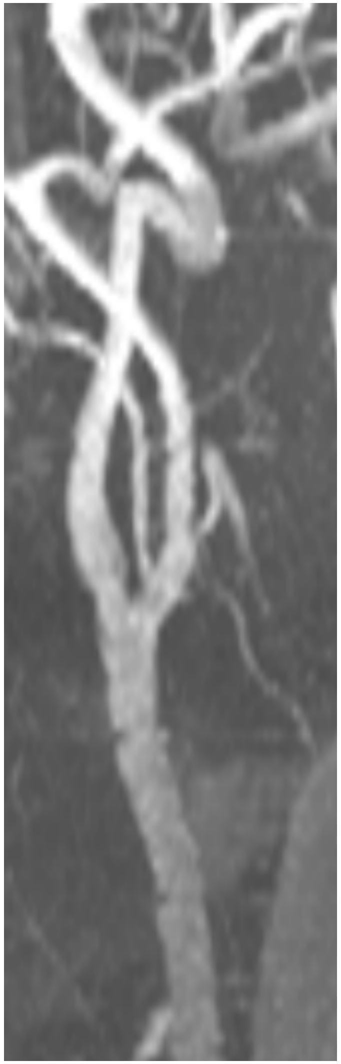

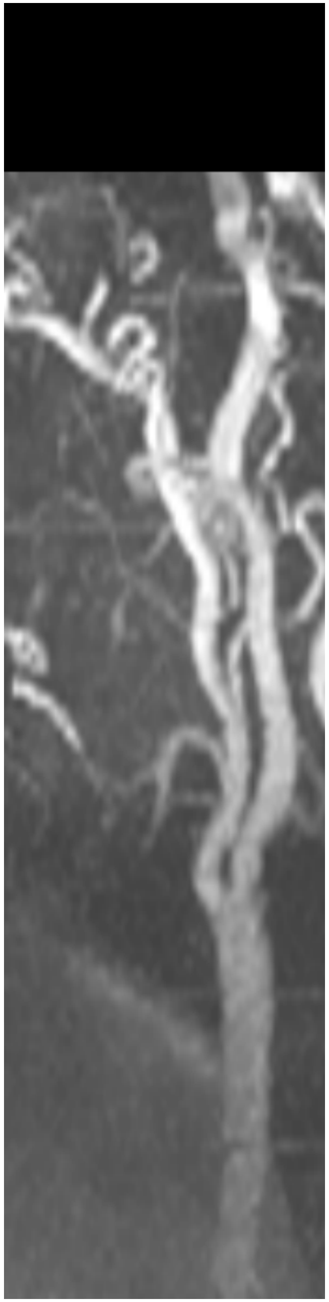

191f Score

0-30

31-50

51-70

>70

Near occlusion

Occluded

Quality

1

2

3

4

5

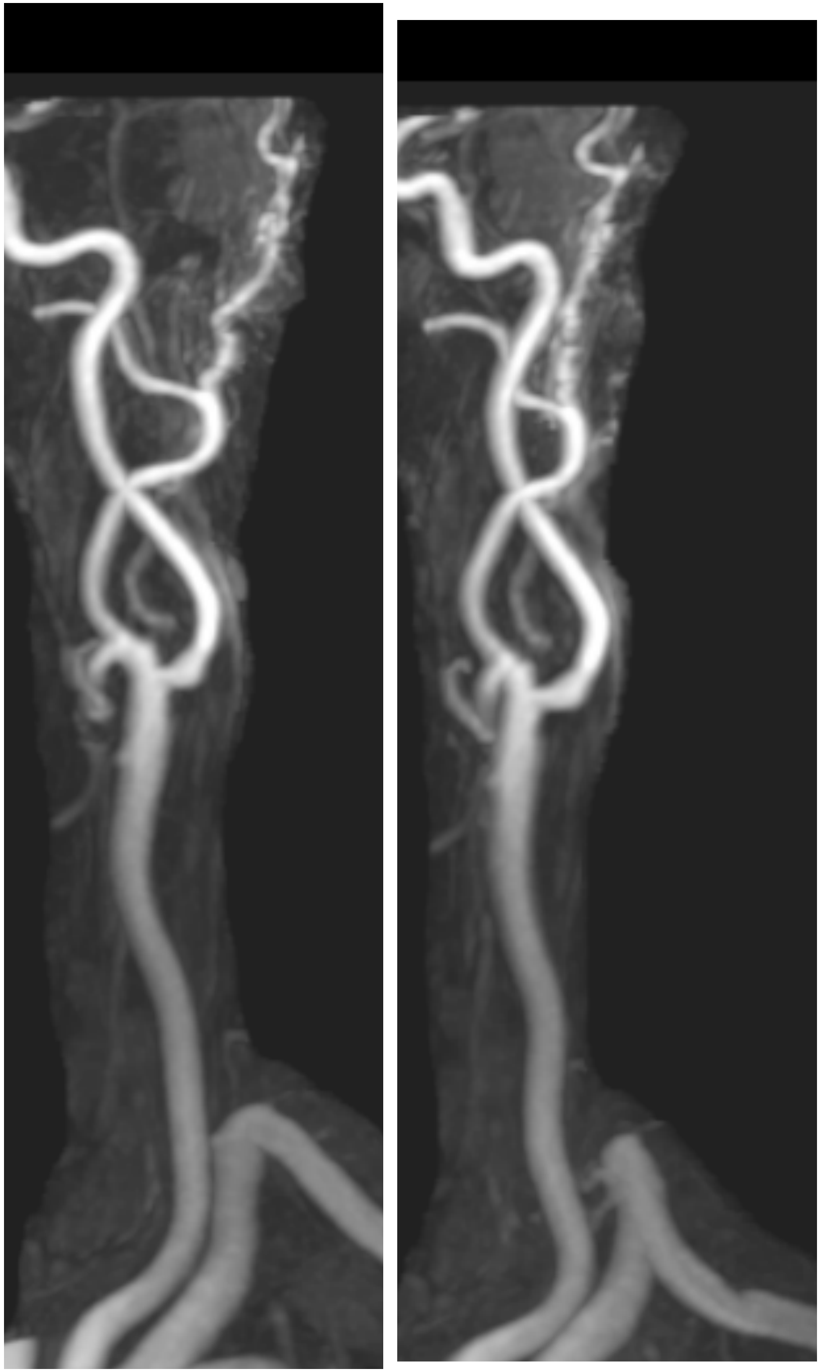

192e Score

0-30

31-50

51-70

>70

Near occlusion

Occluded

Quality

1

2

3

4

5

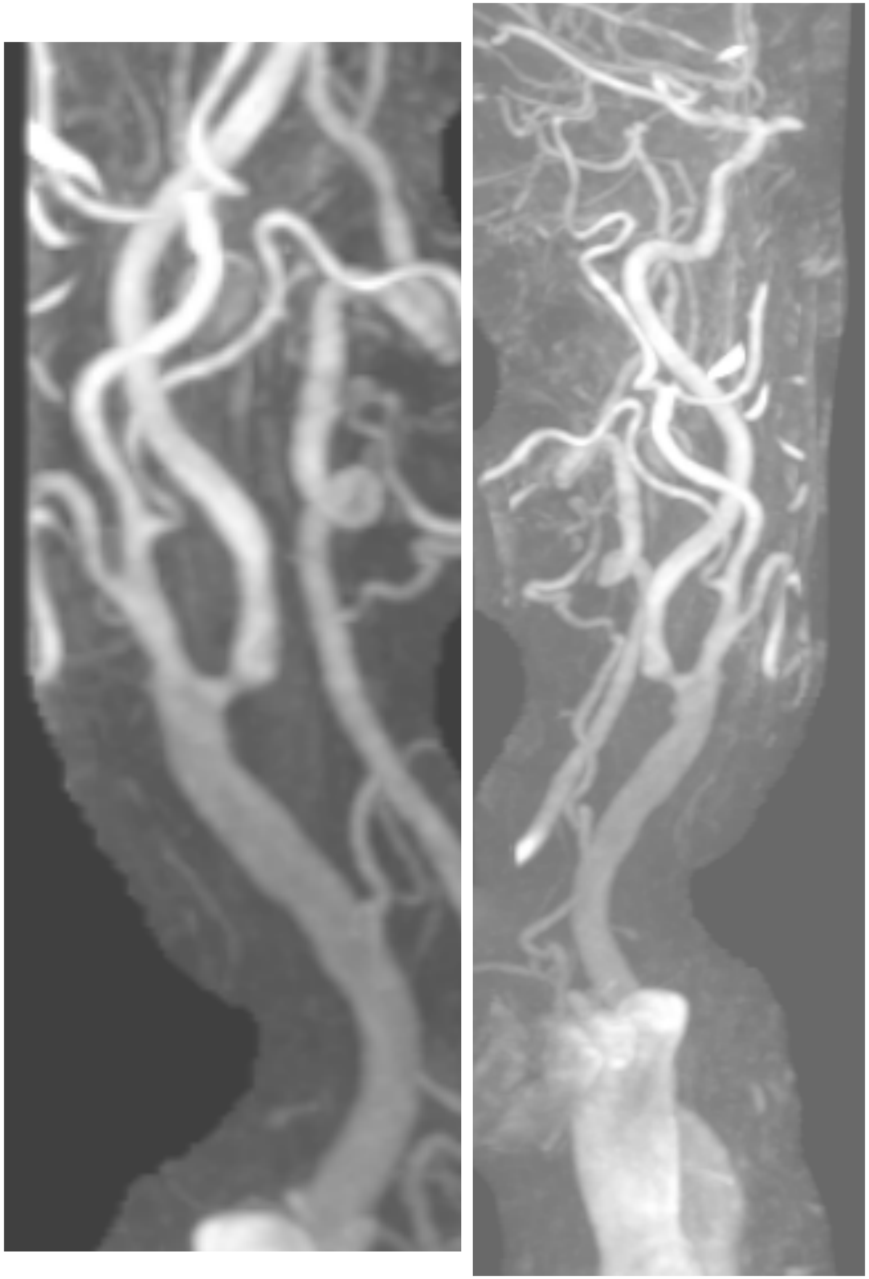

193d Score

0-30

31-50

51-70

>70

Near occlusion

Occluded

Quality

1

2

3

4

5

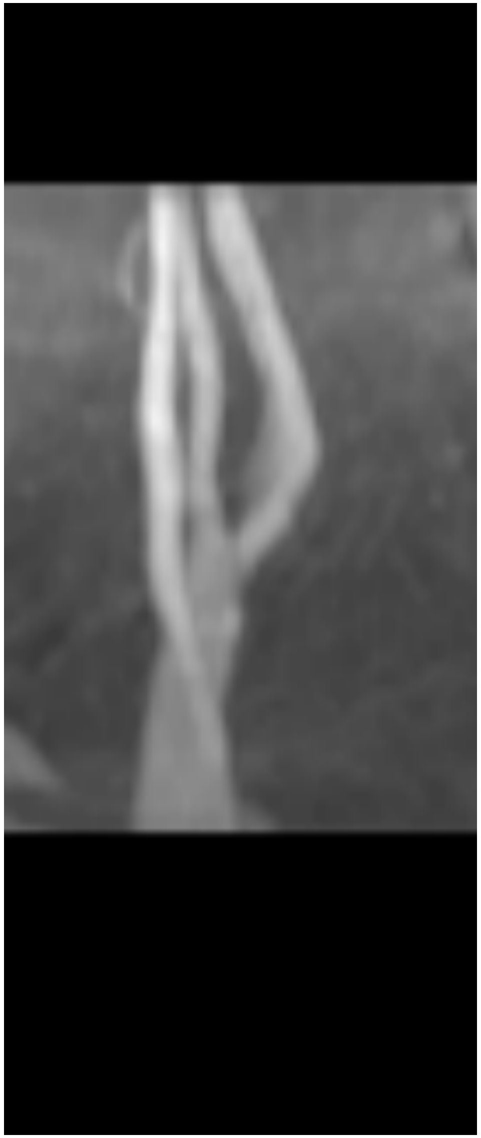

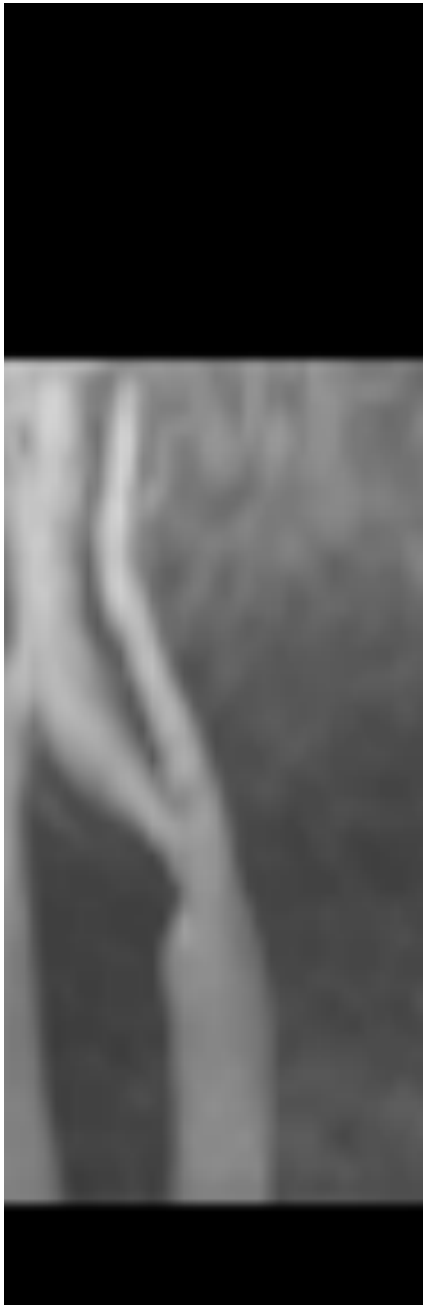

194c Score

0-30

31-50

51-70

>70

Near occlusion

Occluded

Quality

1

2

3

4

5

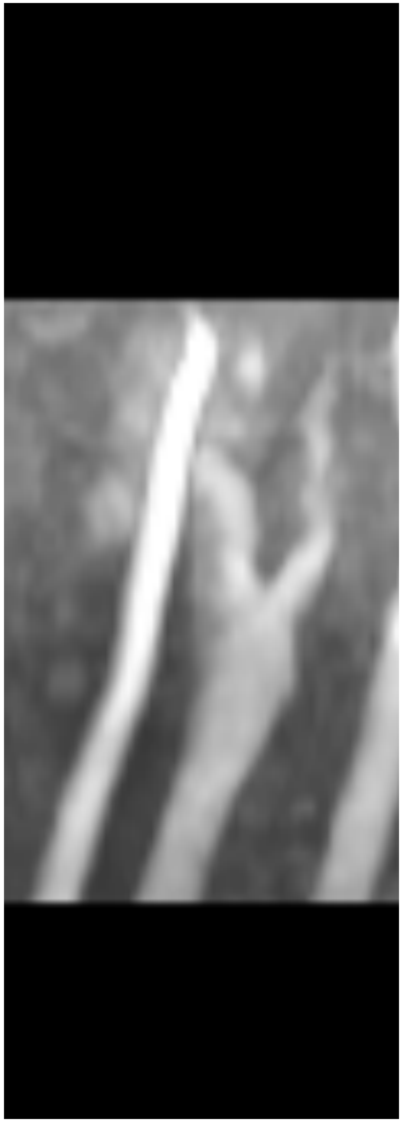

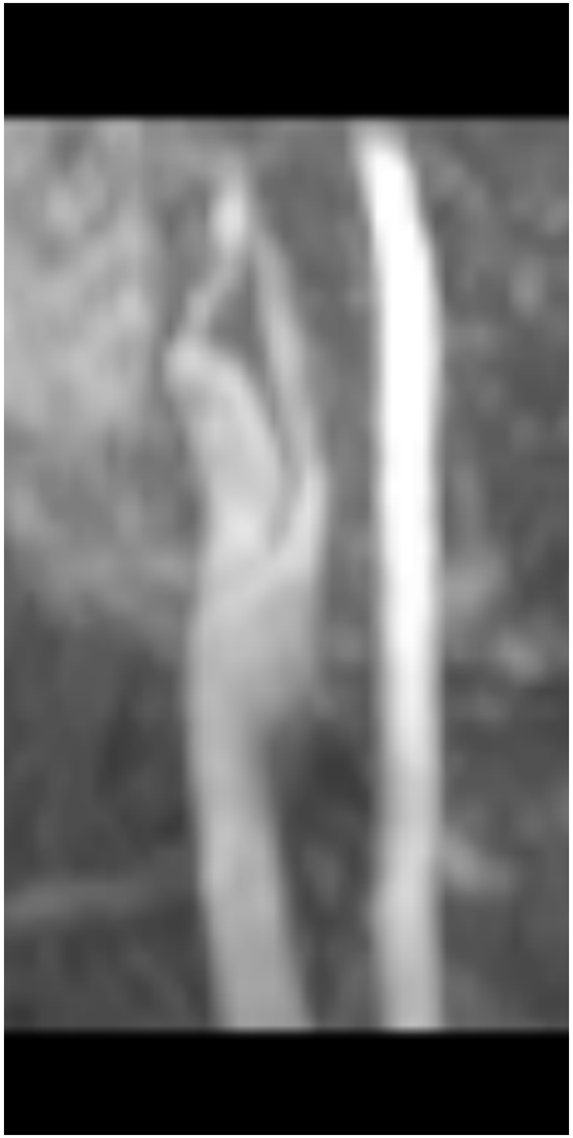

195b Score

0-30

31-50

51-70

>70

Near occlusion

Occluded

Quality

1

2

3

4

5

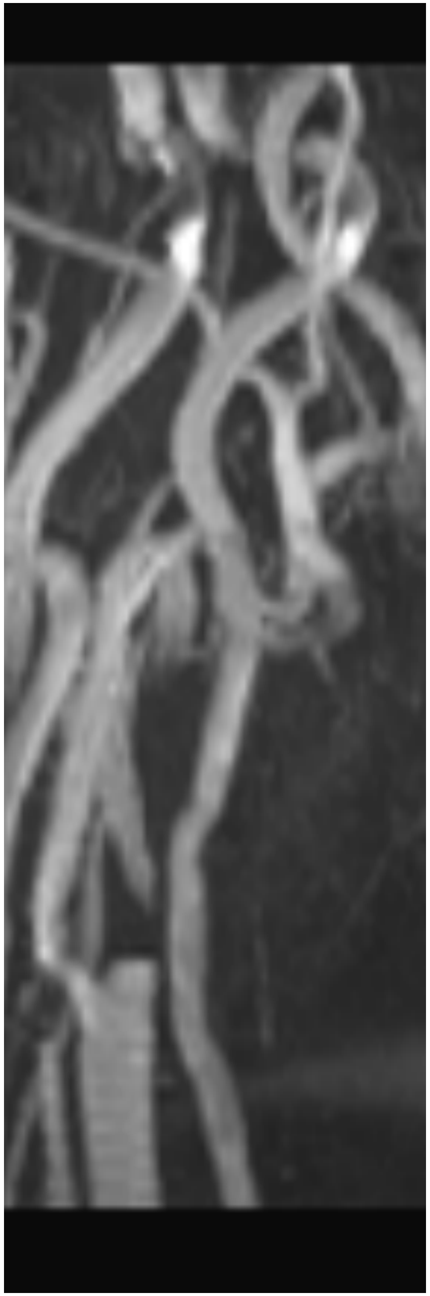

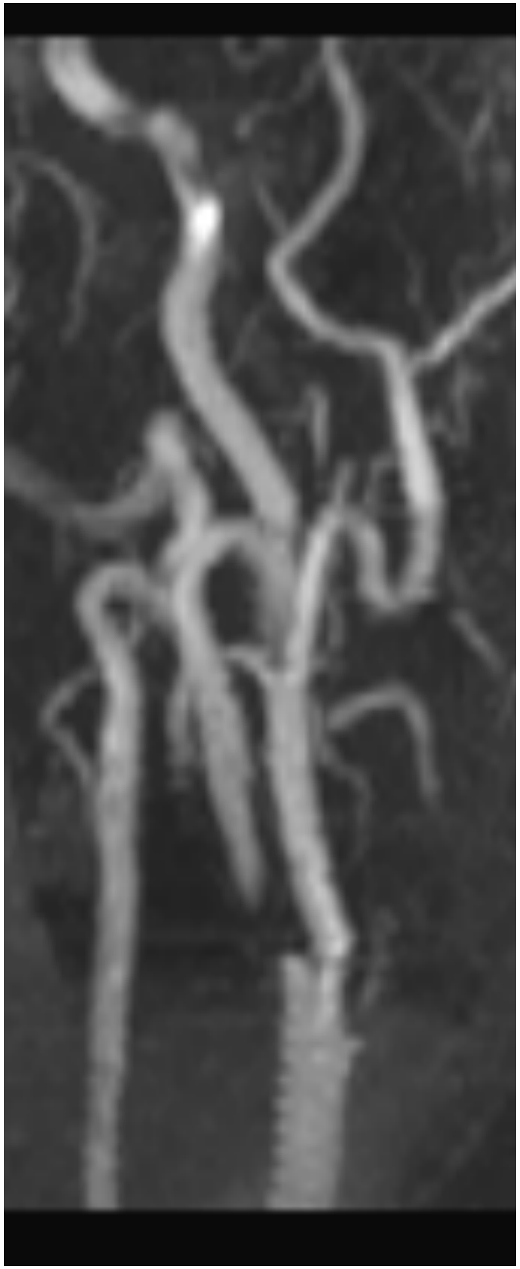

196a Score

0-30

31-50

51-70

>70

Near occlusion

Occluded

Quality

1

2

3

4

5

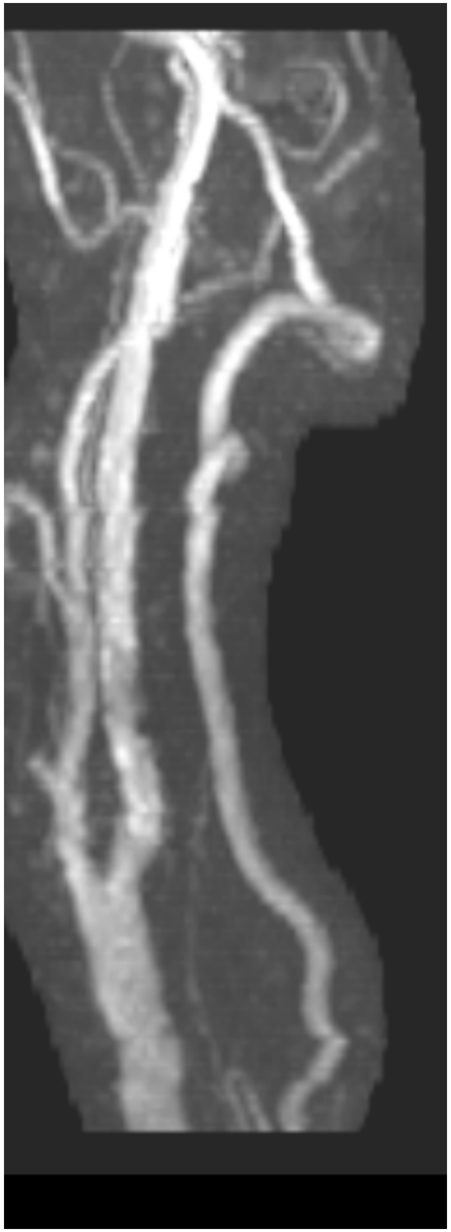

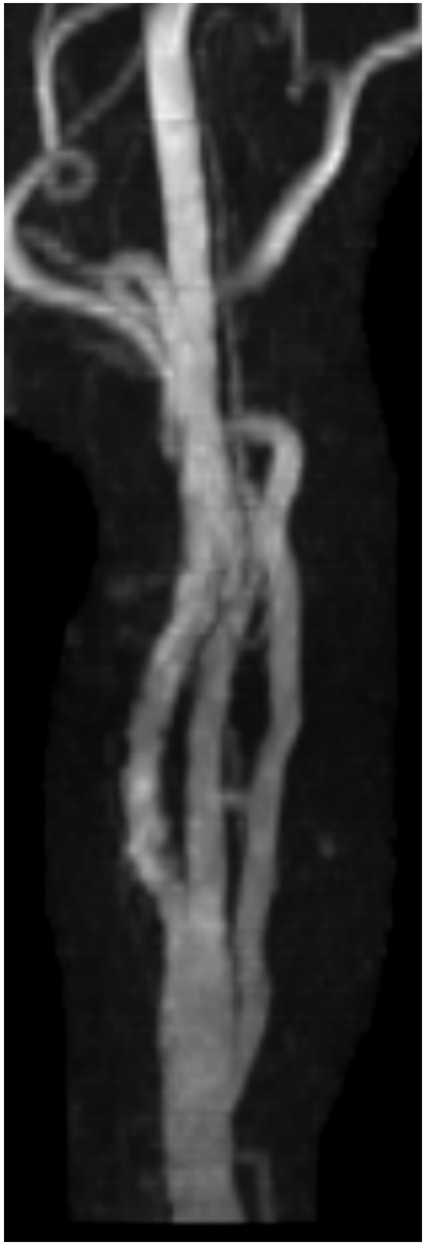

196f Score

0-30

31-50

51-70

>70

Near occlusion

Occluded

Quality

1

2

3

4

5

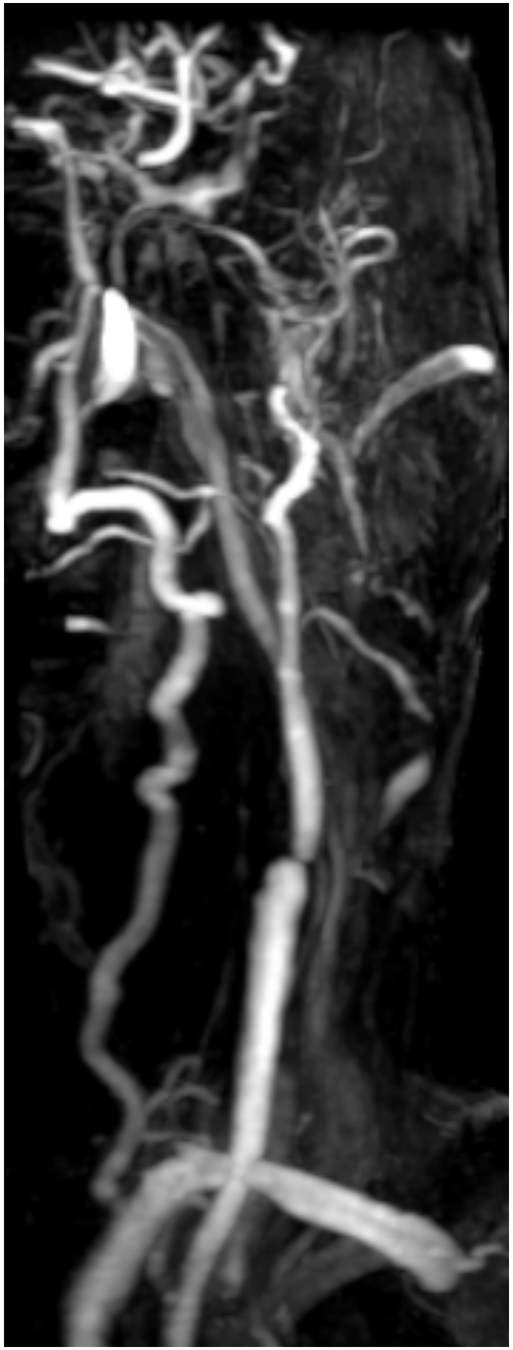

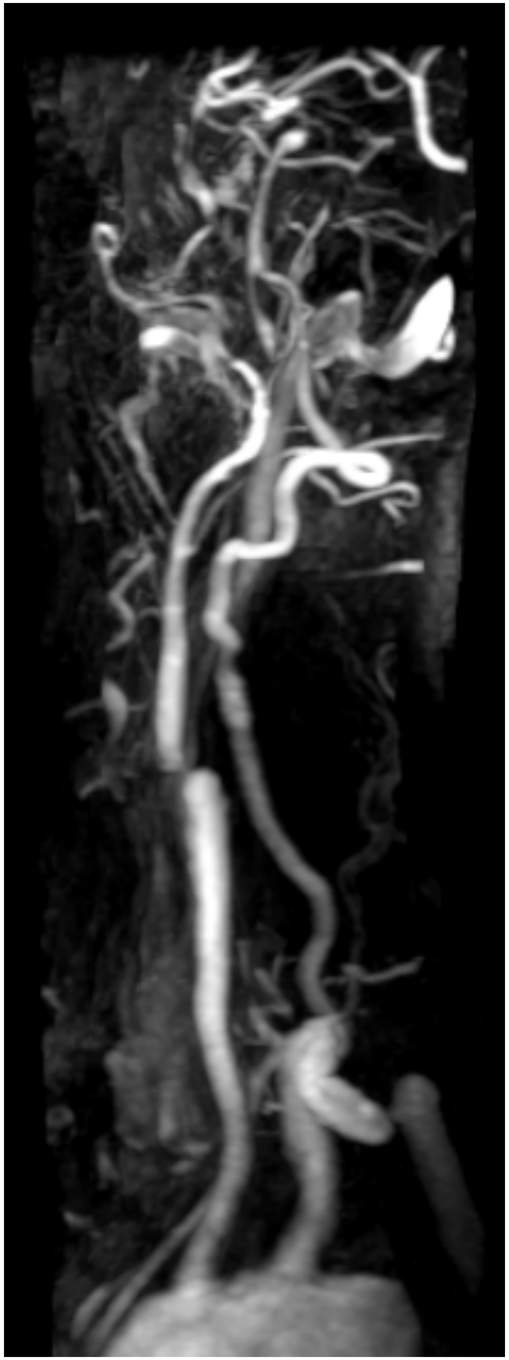

197e Score

0-30

31-50

51-70

>70

Near occlusion

Occluded

Quality

1

2

3

4

5

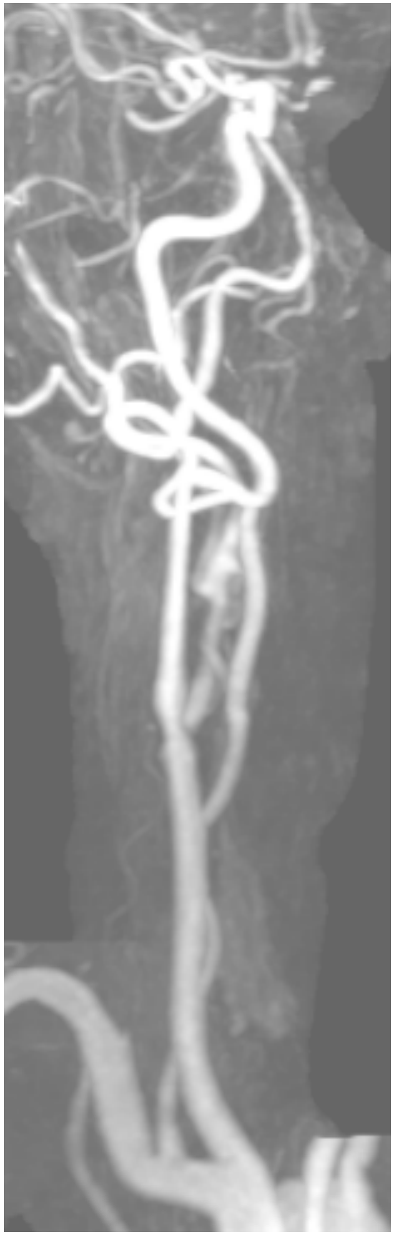

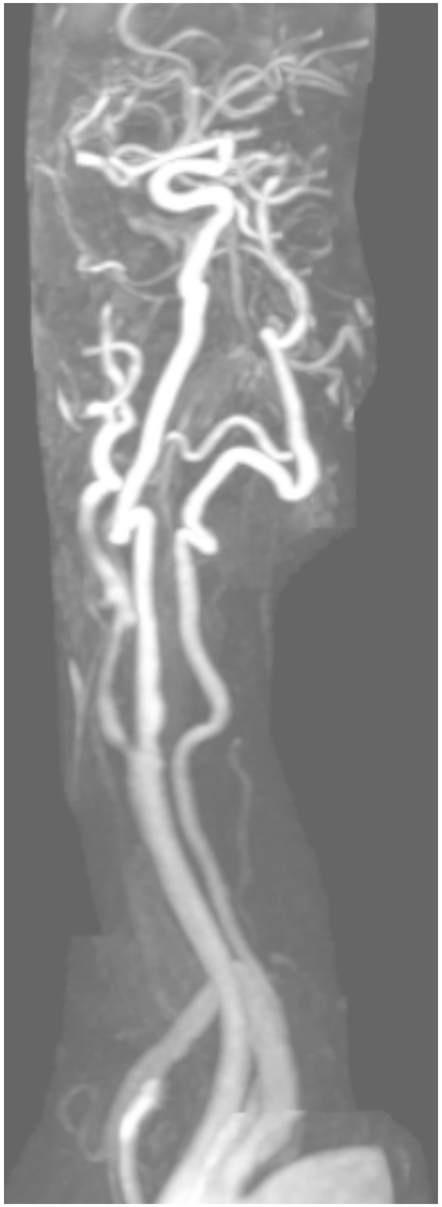

198d Score

0-30

31-50

51-70

>70

Near occlusion

Occluded

Quality

1

2

3

4

5

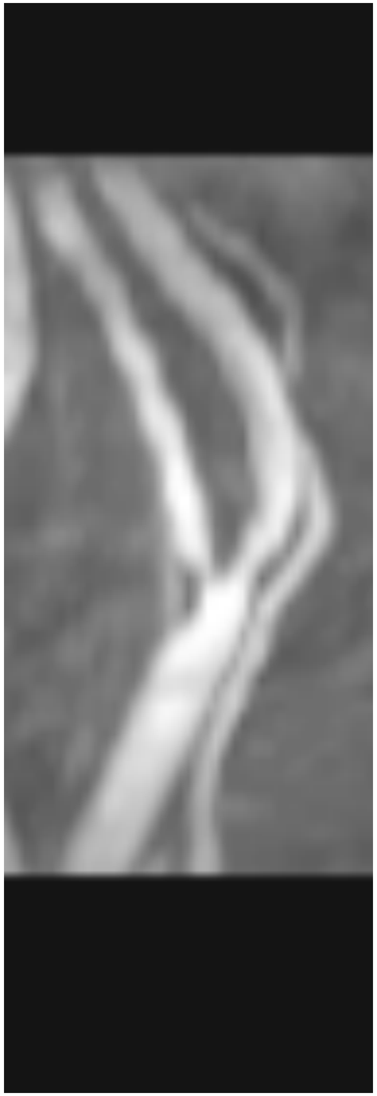

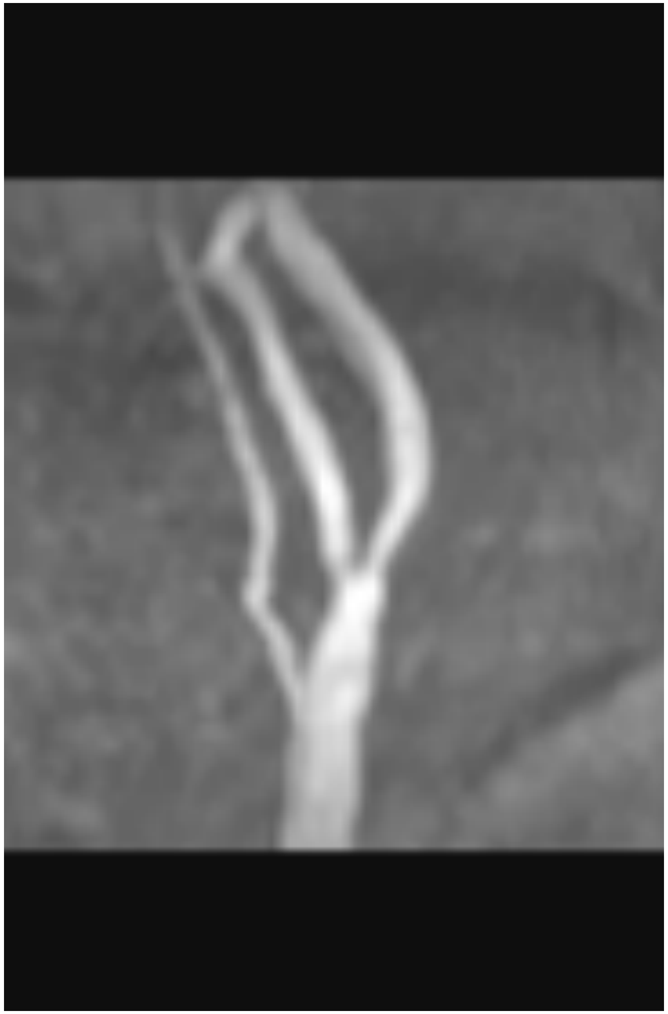

199c Score

0-30

31-50

51-70

>70

Near occlusion

Occluded

Quality

1

2

3

4

5

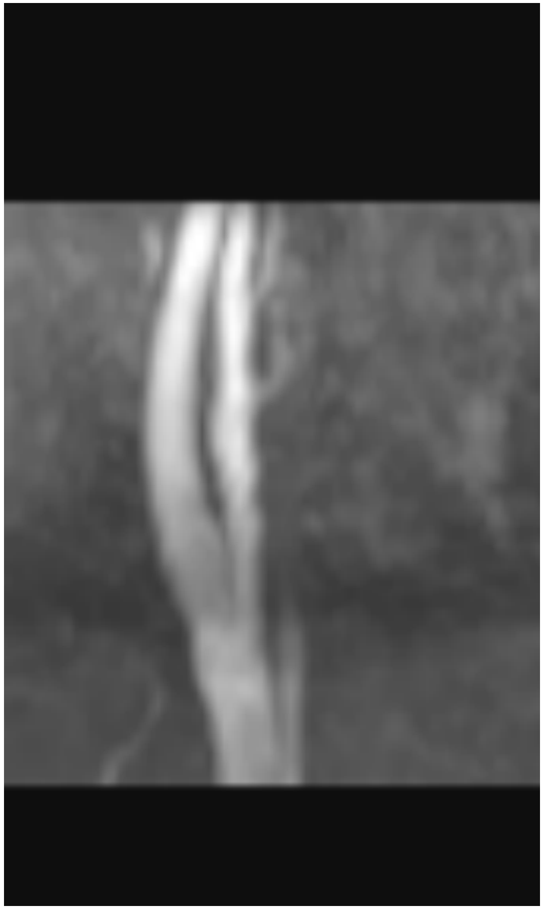

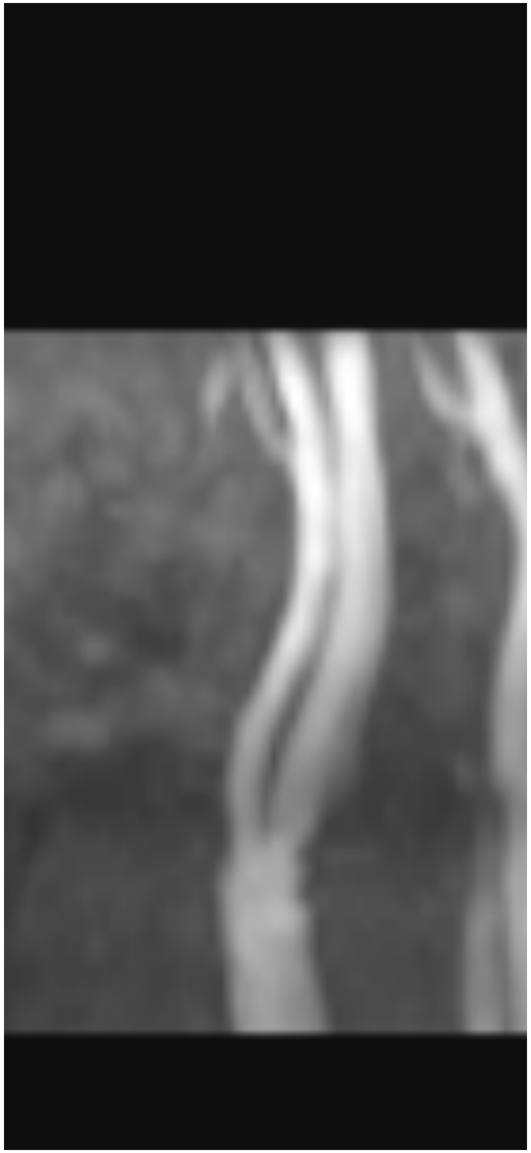

200b Score

0-30

31-50

51-70

>70

Near occlusion

Occluded

Quality

1

2

3

4

5

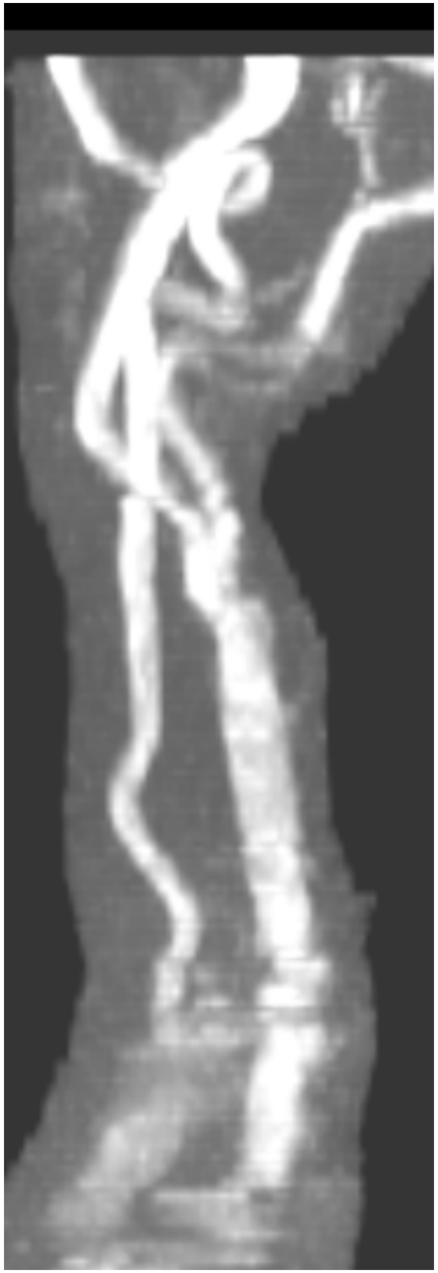

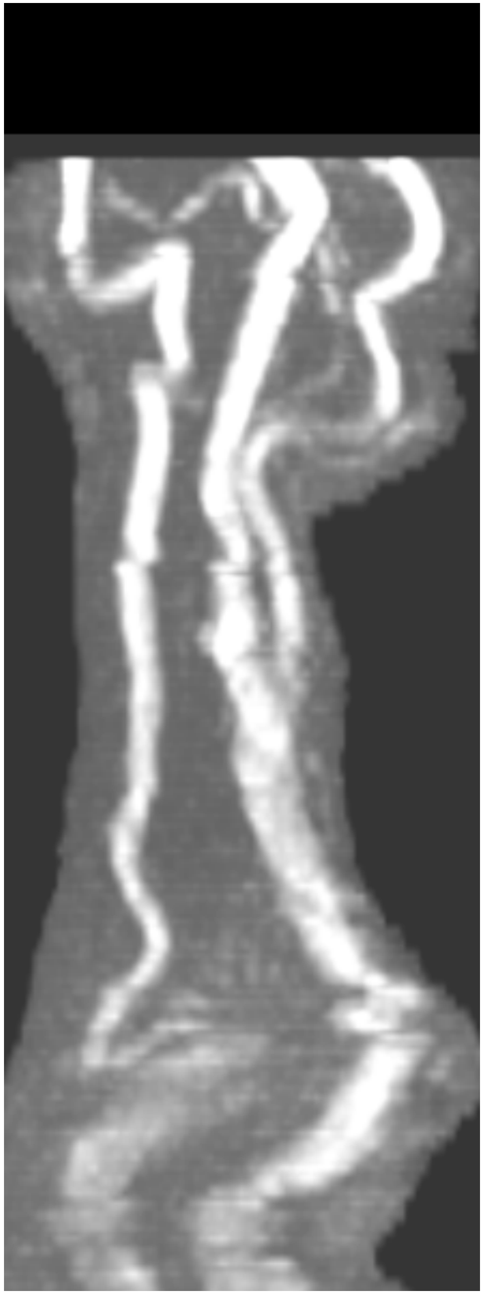

Supplement: S1 File — (PDF) [file pone.0237856.s003.pdf]
