# Supplementary material for: Multiple reader comparison of 2D TOF, 3D TOF, and CEMRA in screening of the carotid bifurcations: Time to reconsider routine contrast use?
Source: PLoS One. 2020 Sep 2;15(9):e0237856. doi: 10.1371/journal.pone.0237856 (PMC7467222; doi:10.1371/journal.pone.0237856)

1b Score

0-30

31-50

51-70

>70

Near occlusion

Occluded

Quality

1

2

3

4

5

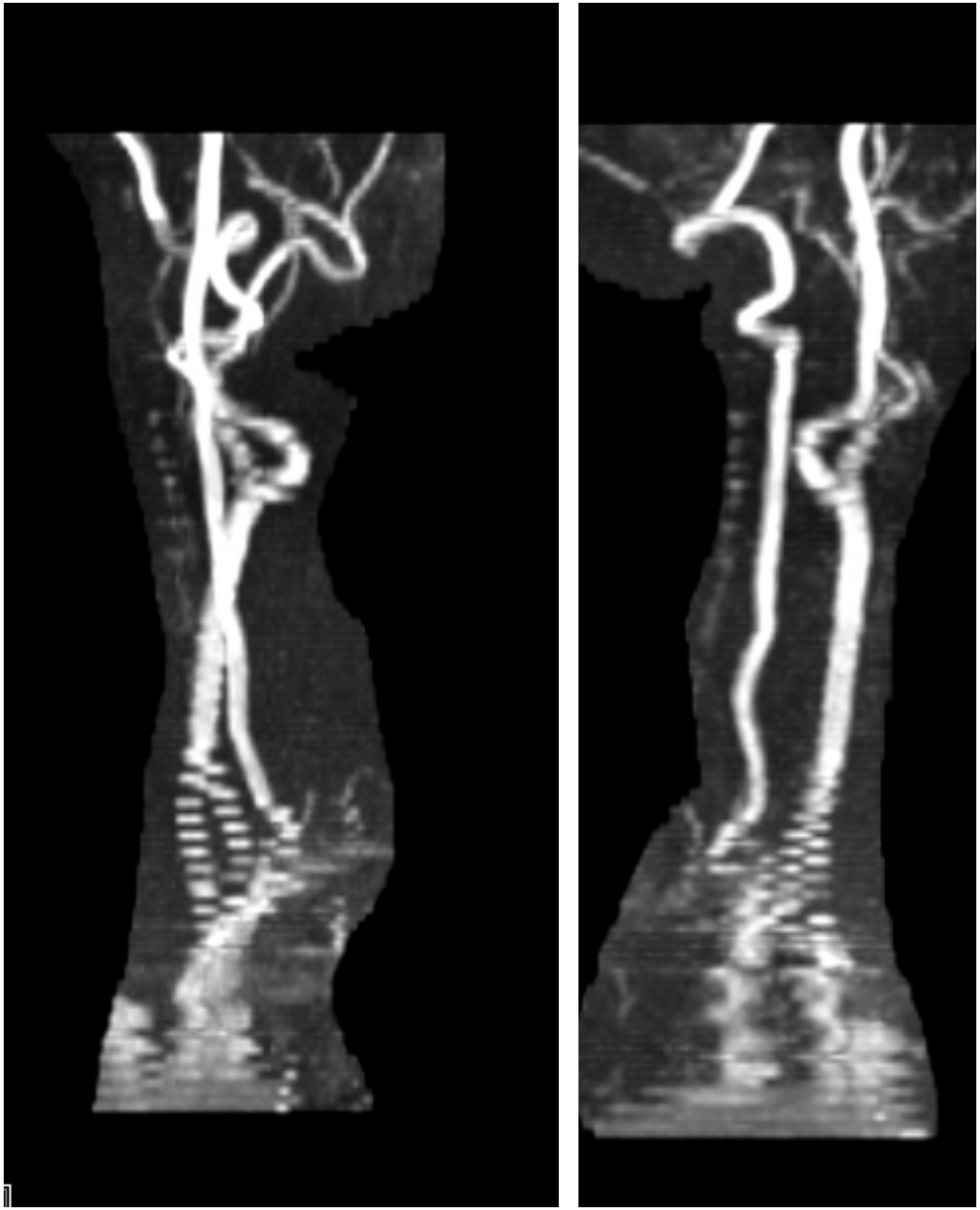

2 a Score

0-30

31-50

51-70

>70

Near occlusion

Occluded

Quality

1

2

3

4

5

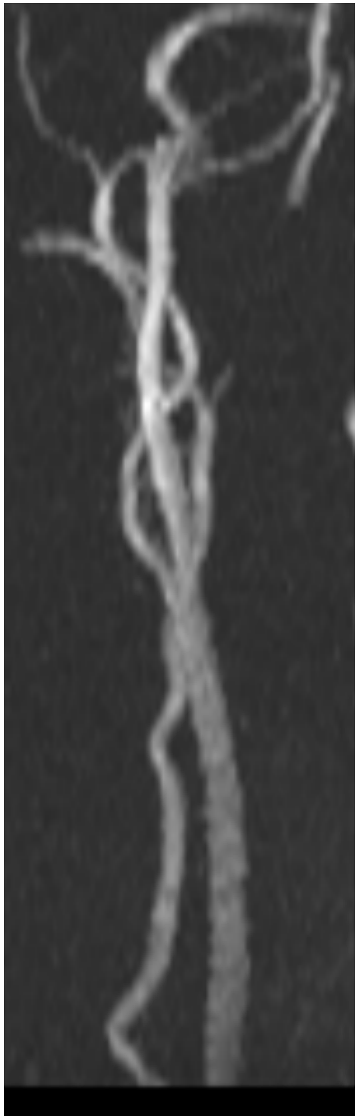

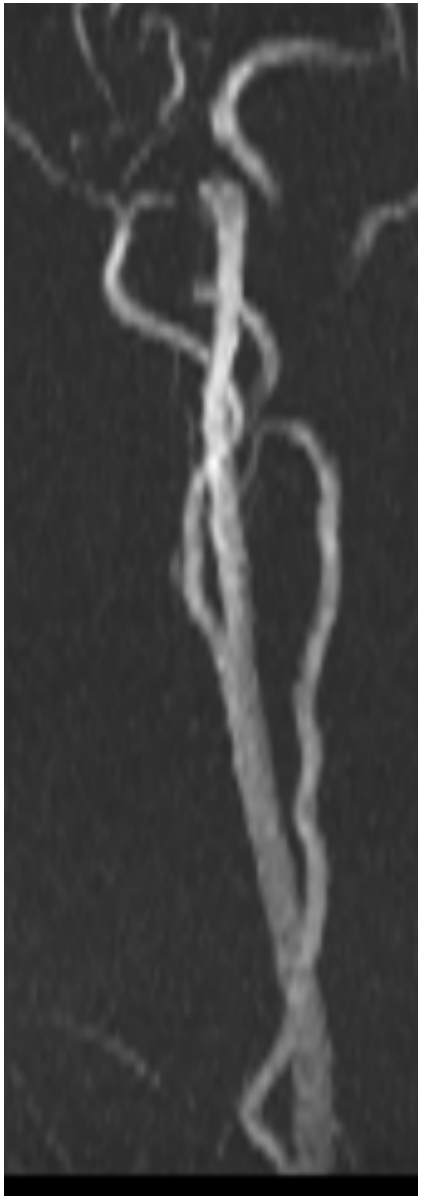

2 f Score

0-30

31-50

51-70

>70

Near occlusion

Occluded

Quality

1

2

3

4

5

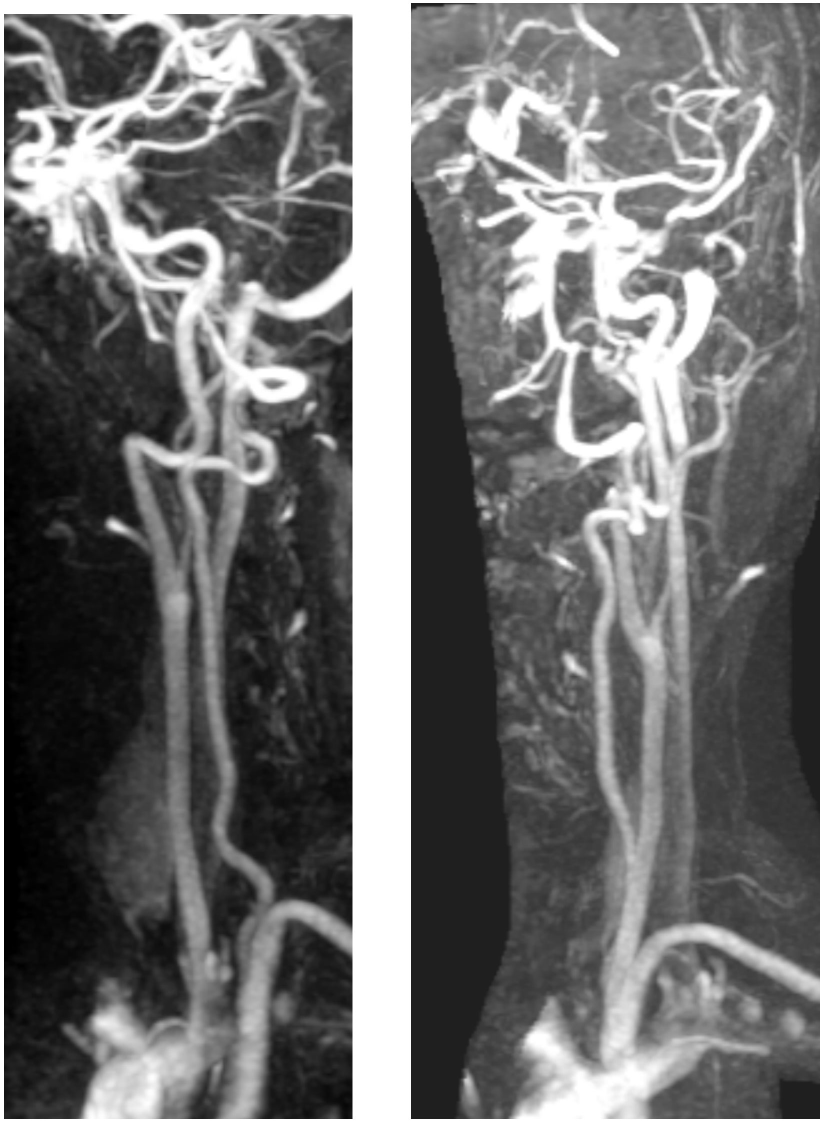

3e Score

0-30

31-50

51-70

>70

Near occlusion

Occluded

Quality

1

2

3

4

5

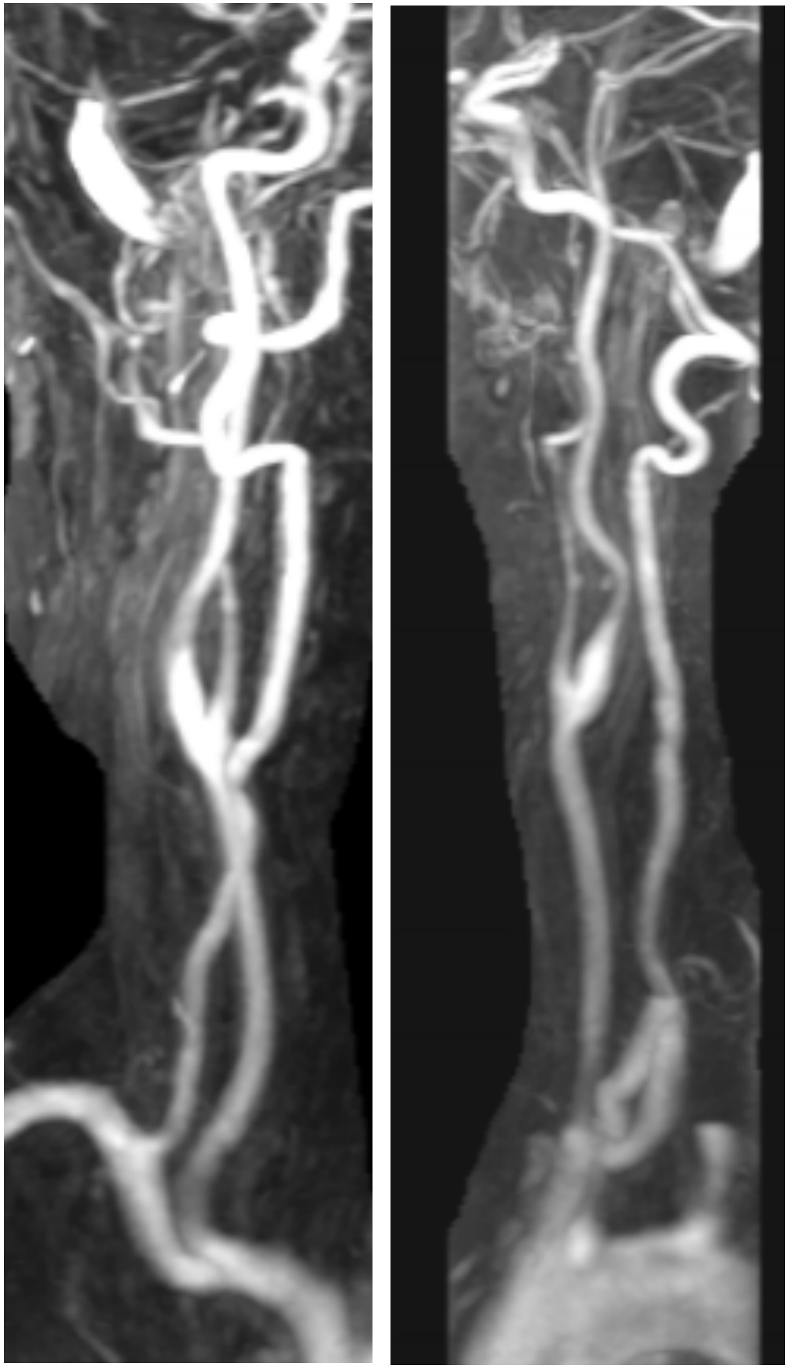

4d Score

0-30

31-50

51-70

>70

Near occlusion

Occluded

Quality

1

2

3

4

5

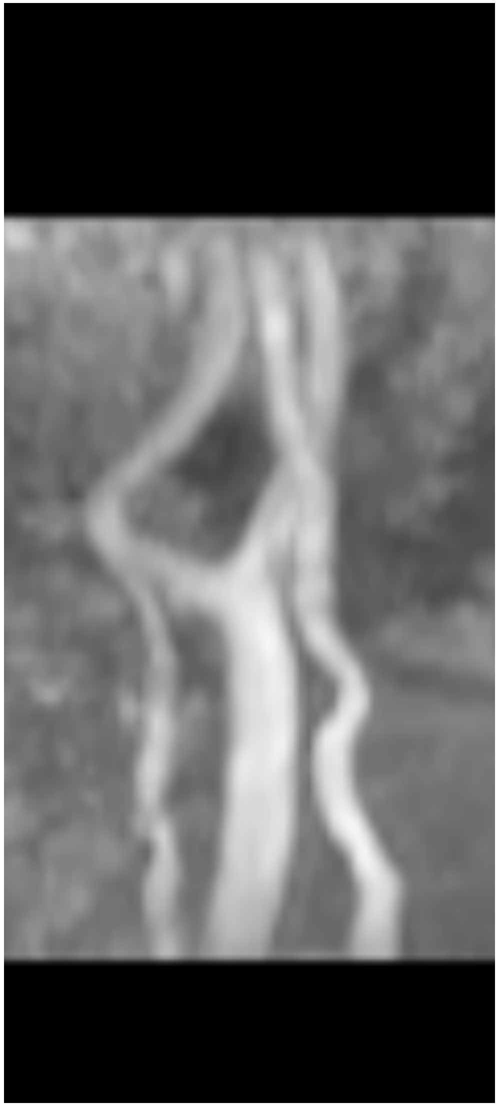

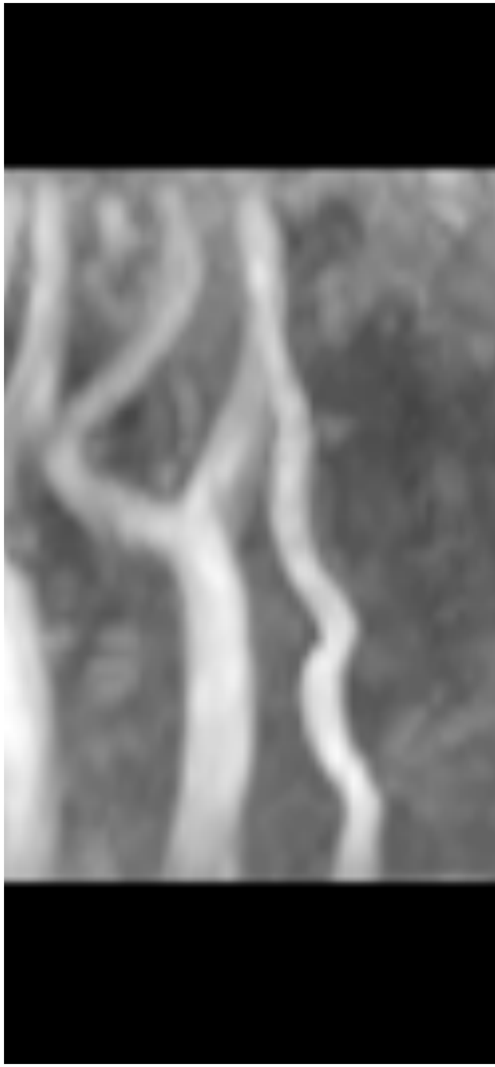

5c Score

0-30

31-50

51-70

>70

Near occlusion

Occluded

Quality

1

2

3

4

5

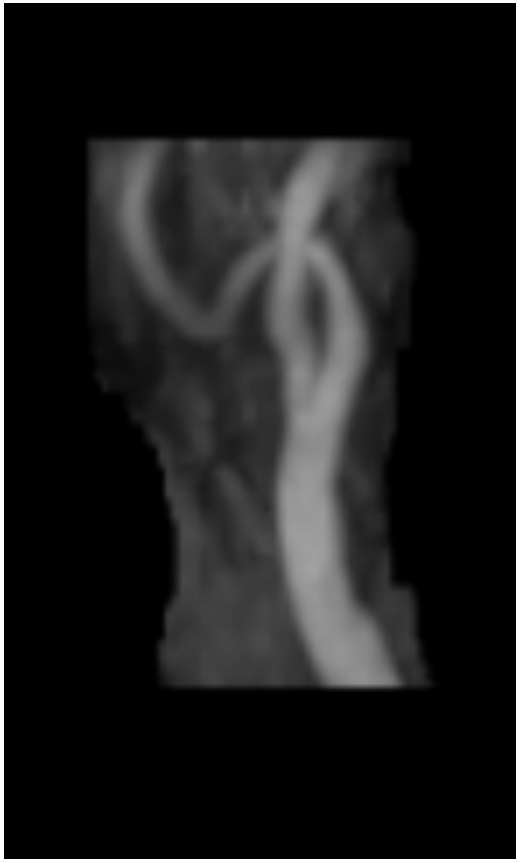

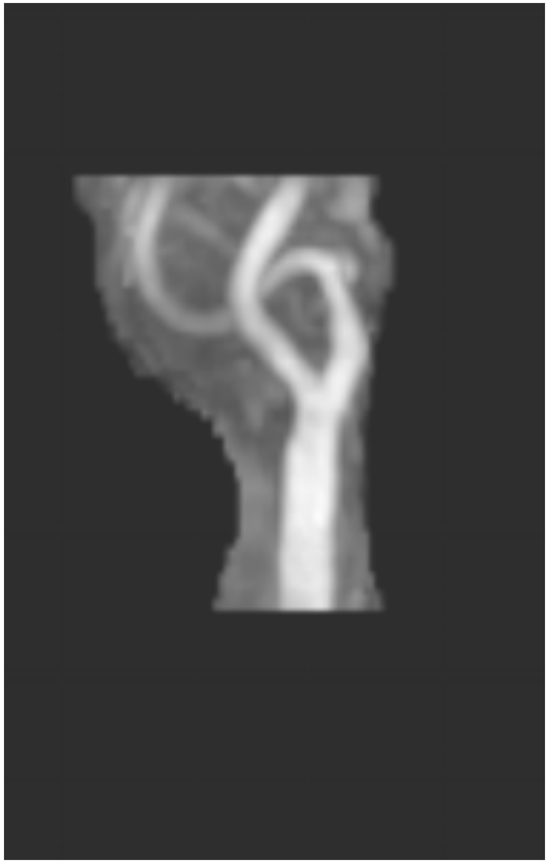

6b Score

0-30

31-50

51-70

>70

Near occlusion

Occluded

Quality

1

2

3

4

5

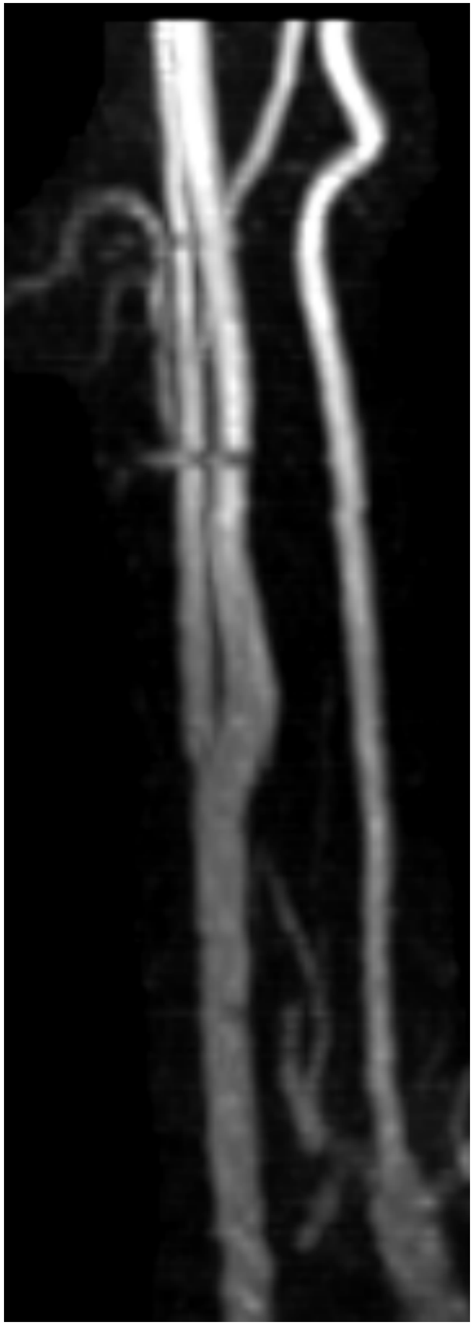

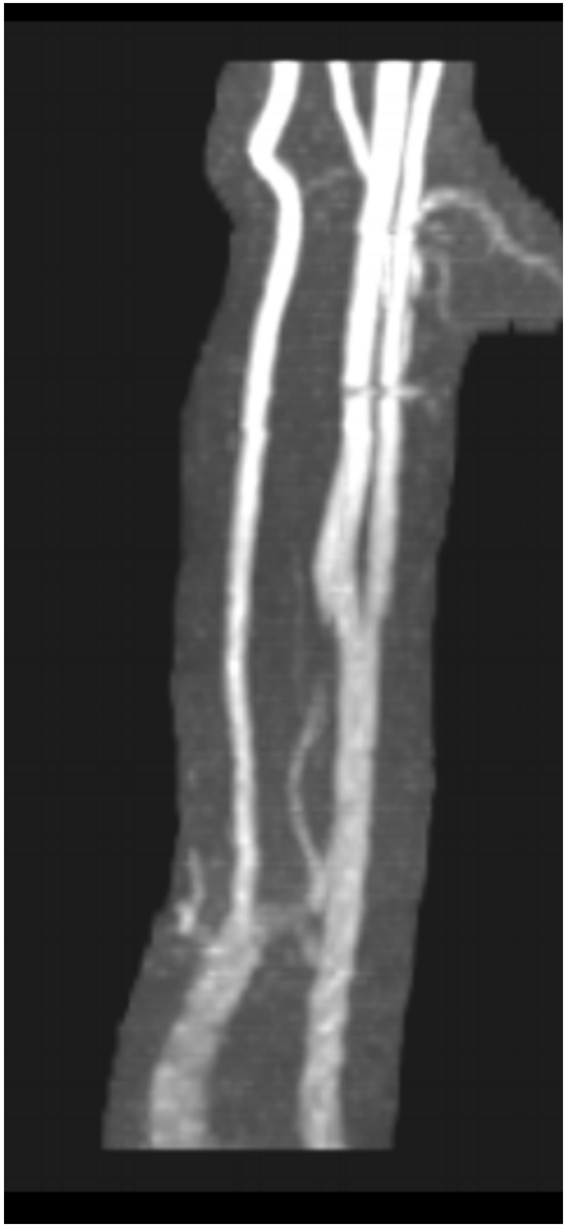

7a Score

0-30

31-50

51-70

>70

Near occlusion

Occluded

Quality

1

2

3

4

5

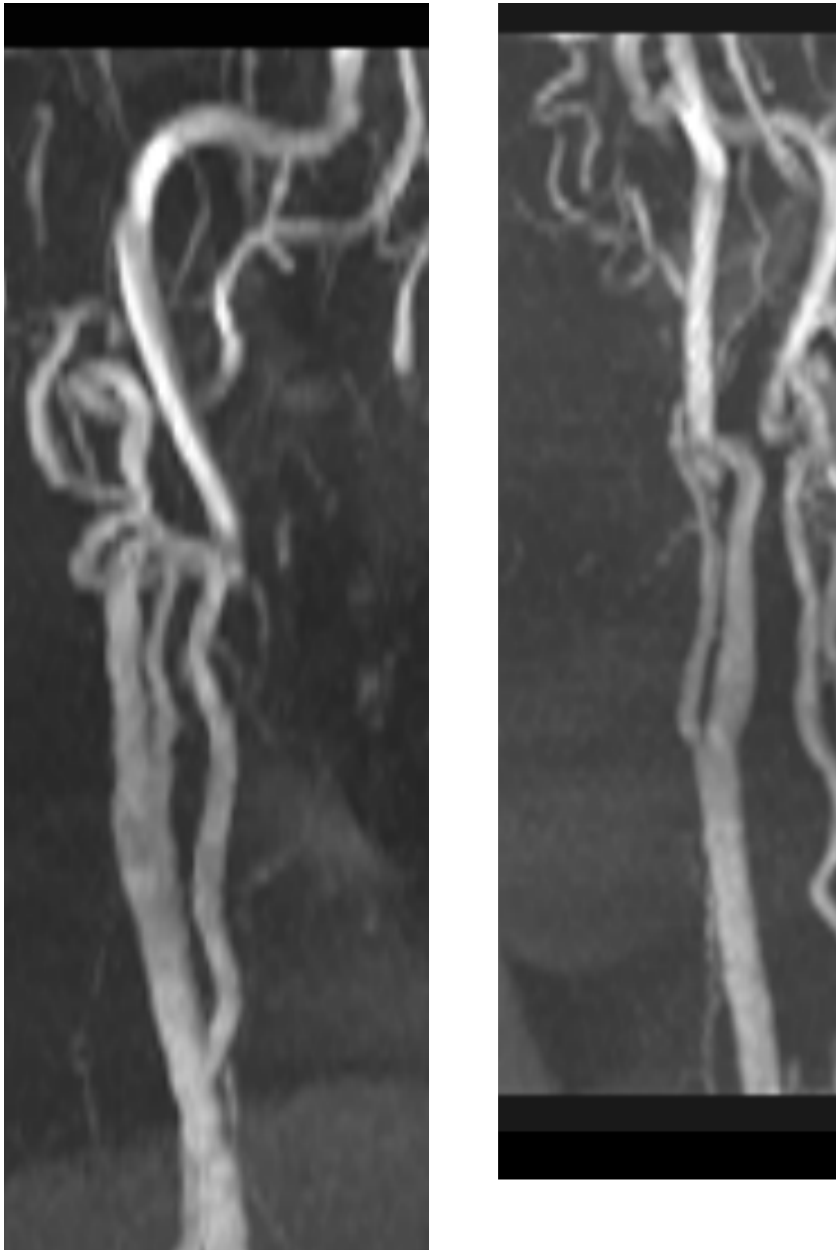

7f Score

0-30

31-50

51-70

>70

Near occlusion

Occluded

Quality

1

2

3

4

5

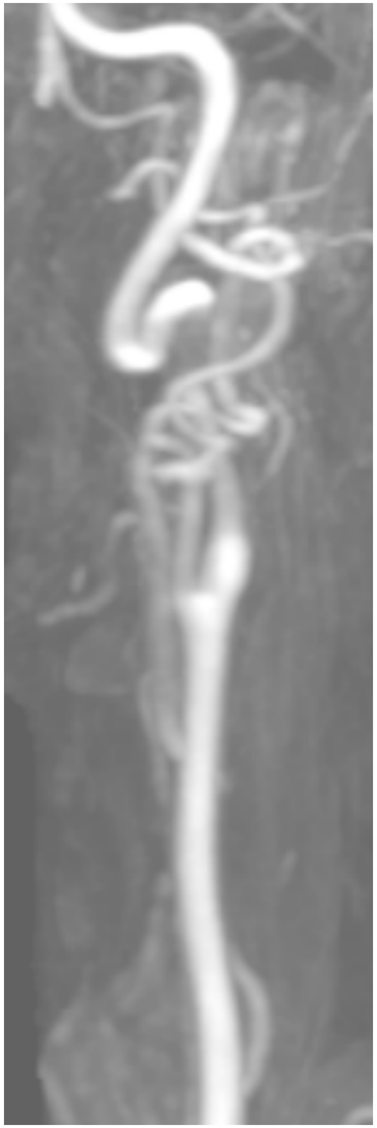

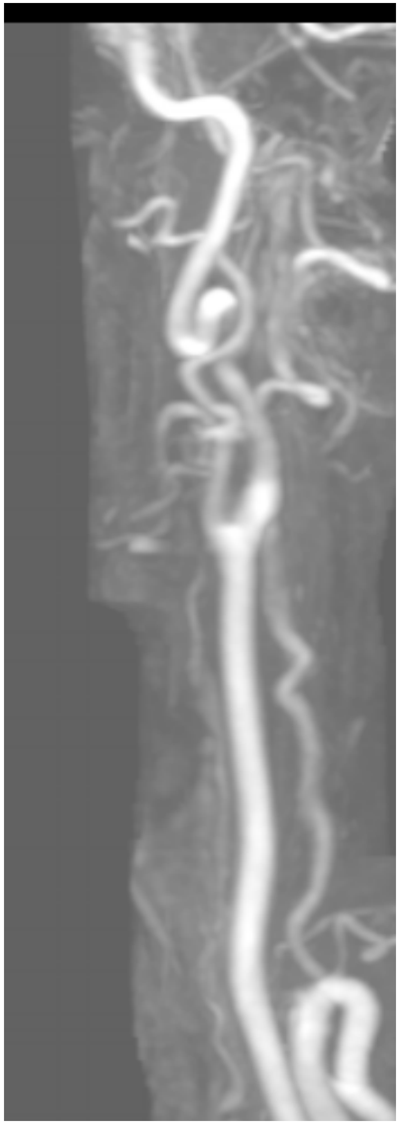

8e Score

0-30

31-50

51-70

>70

Near occlusion

Occluded

Quality

1

2

3

4

5

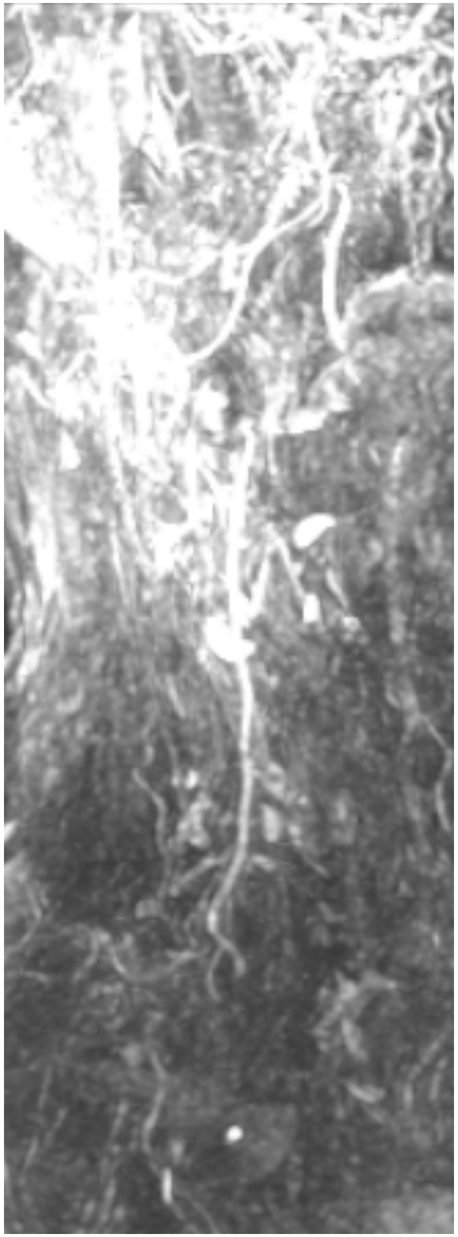

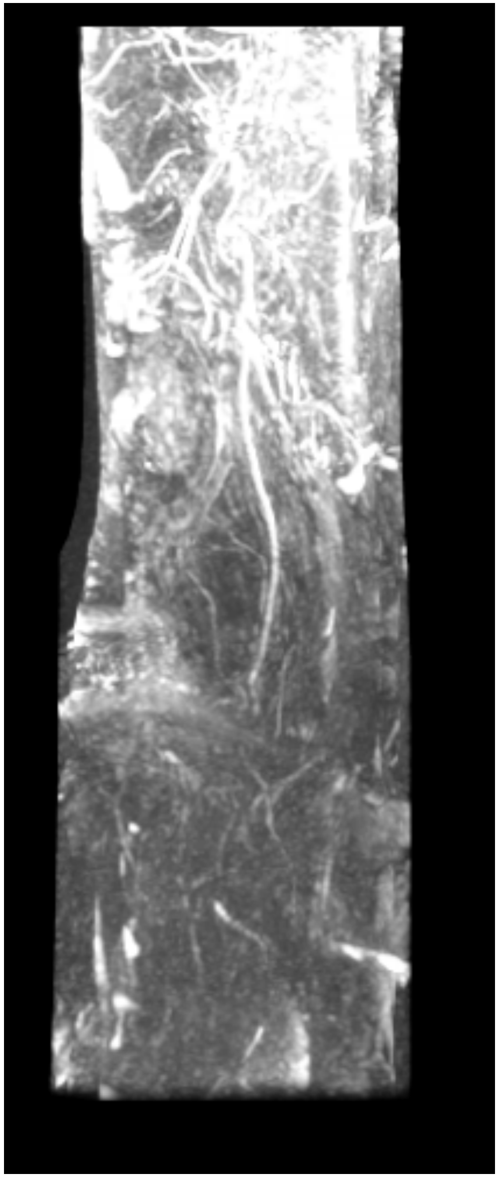

9d Score

0-30

31-50

51-70

>70

Near occlusion

Occluded

Quality

1

2

3

4

5

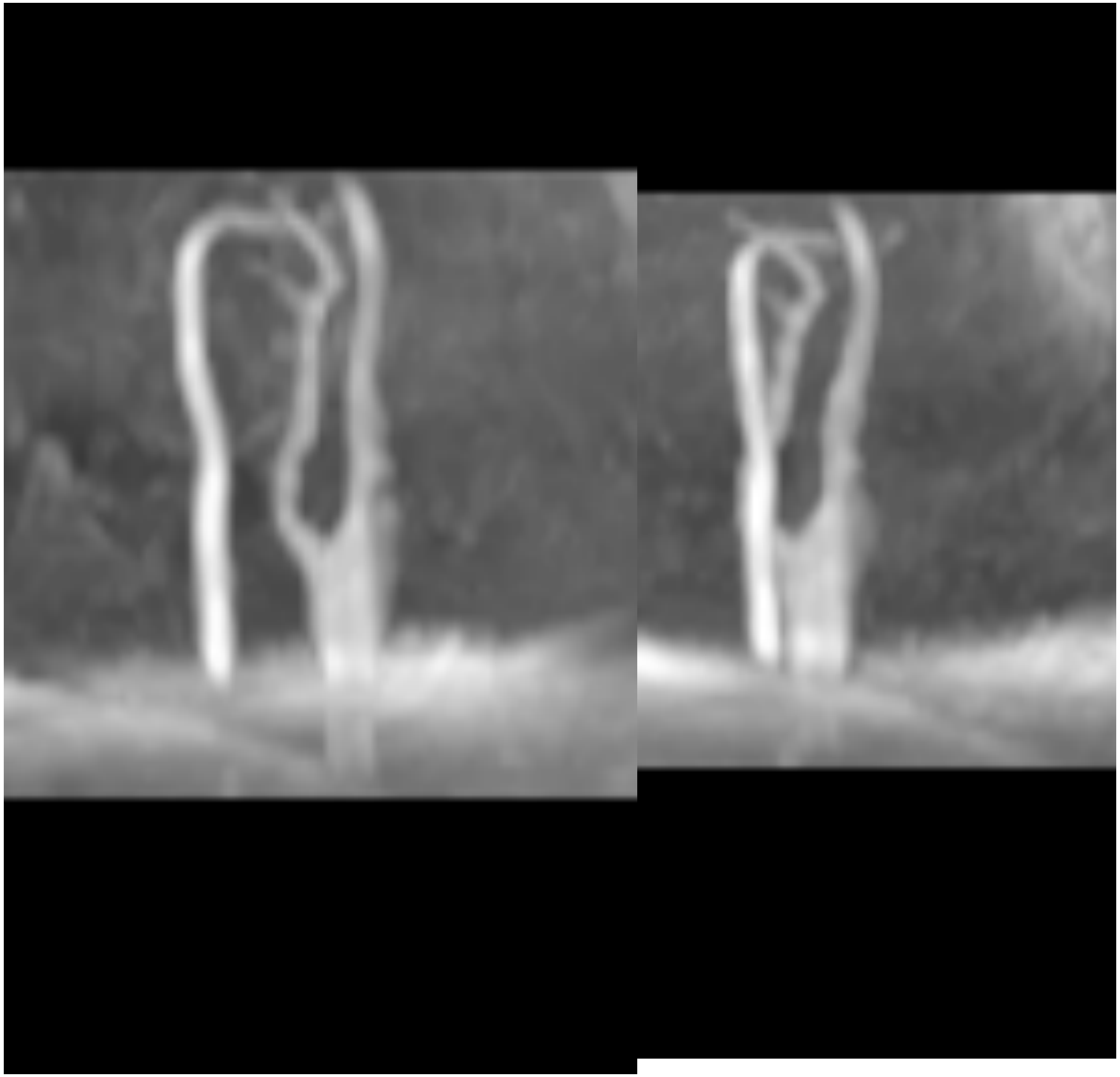

10c Score

0-30

31-50

51-70

>70

Near occlusion

Occluded

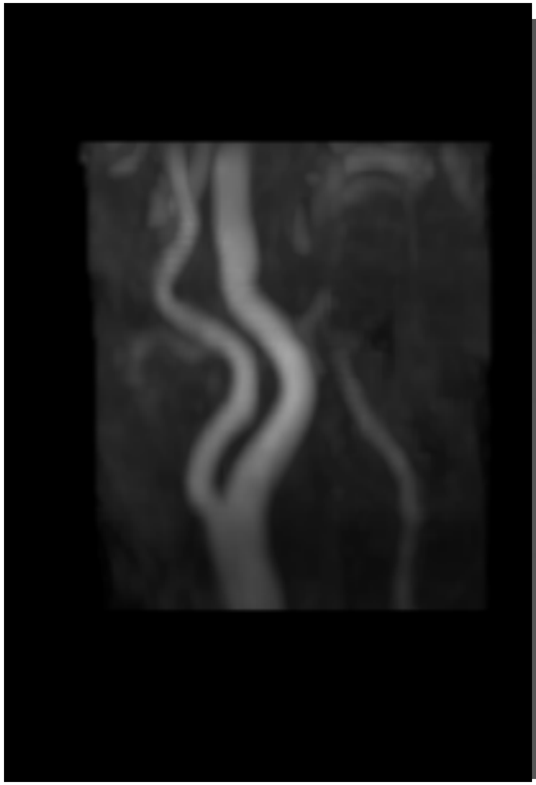

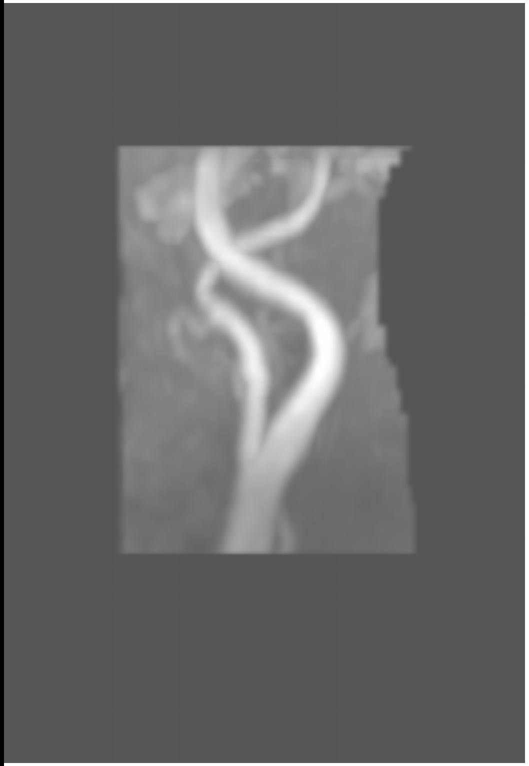

Quality

1

2

3

4

5

11b Score

0-30

31-50

51-70

>70

Near occlusion

Occluded

Quality

1

2

3

4

5

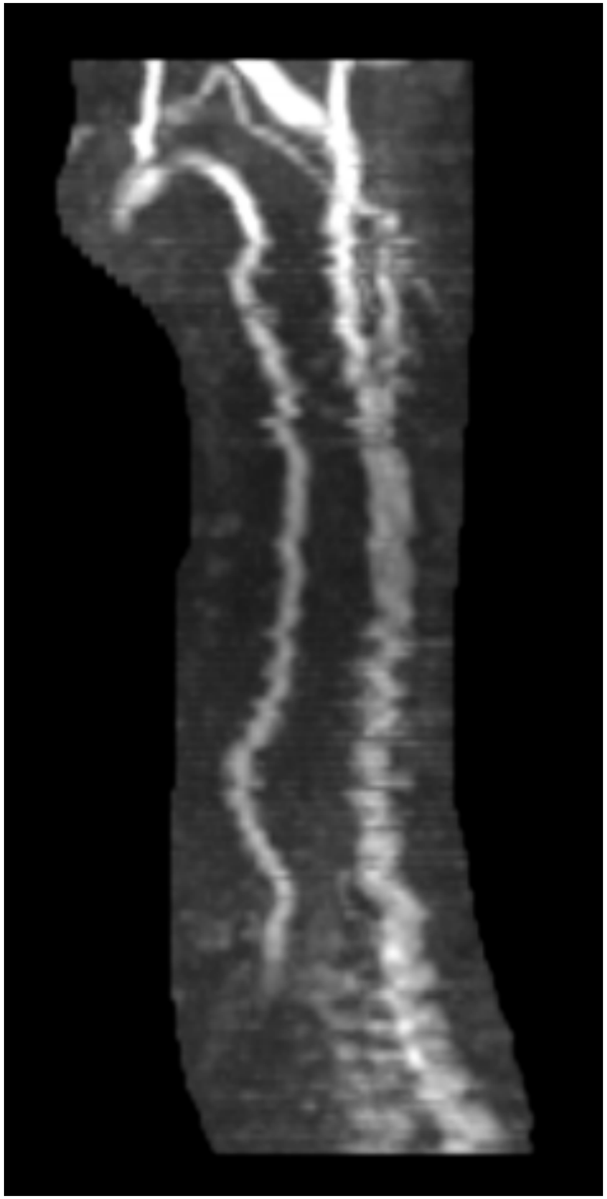

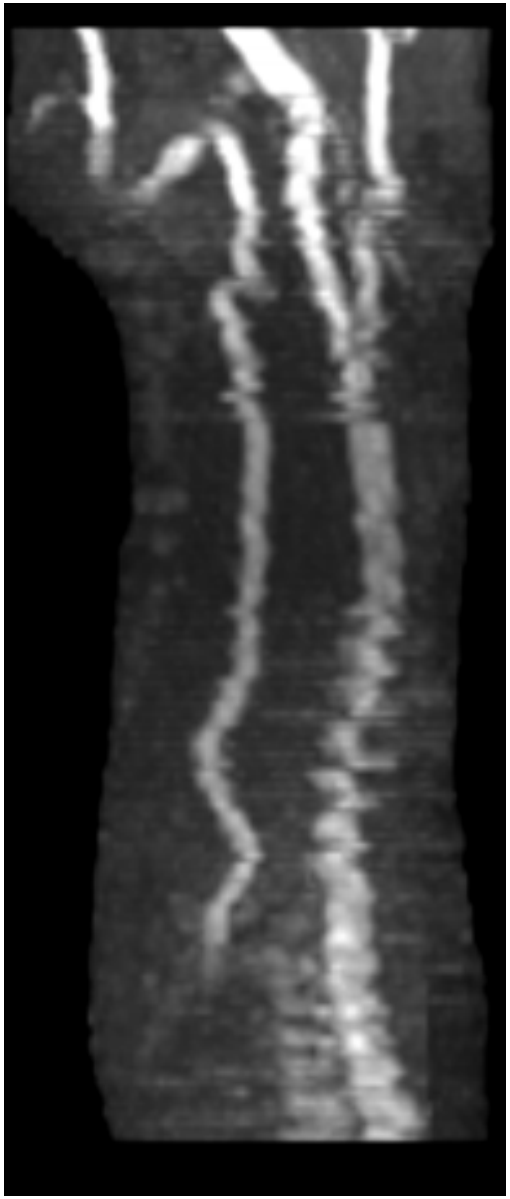

12a Score

0-30

31-50

51-70

>70

Near occlusion

Occluded

Quality

1

2

3

4

5

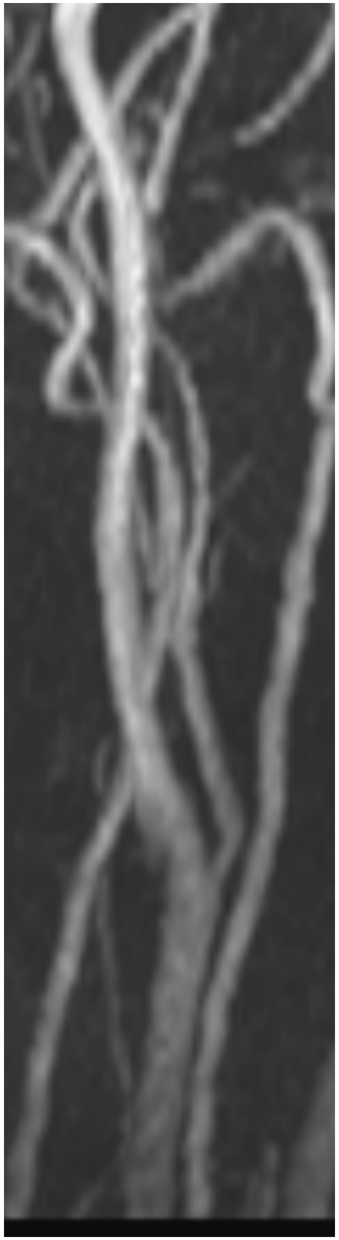

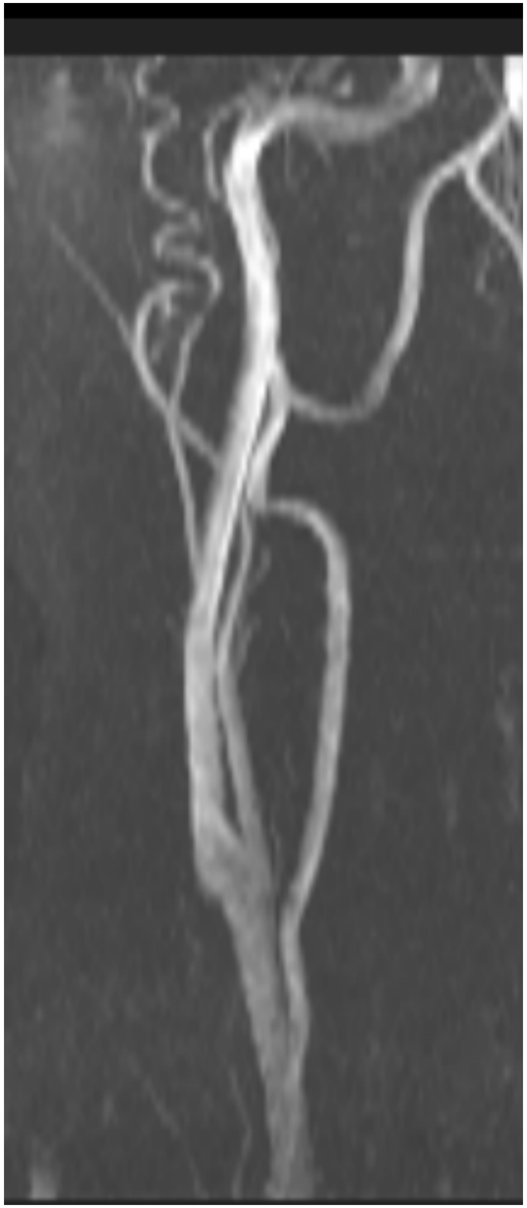

12f Score

0-30

31-50

51-70

>70

Near occlusion

Occluded

Quality

1

2

3

4

5

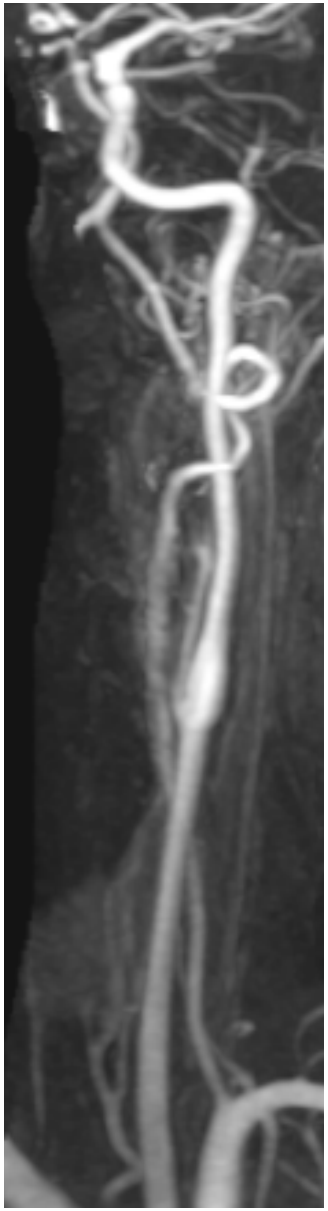

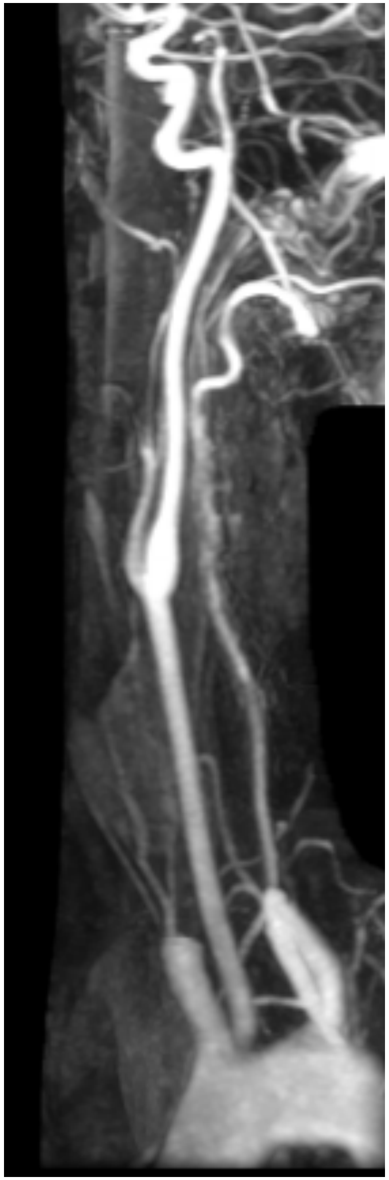

13e Score

0-30

31-50

51-70

>70

Near occlusion

Occluded

Quality

1

2

3

4

5

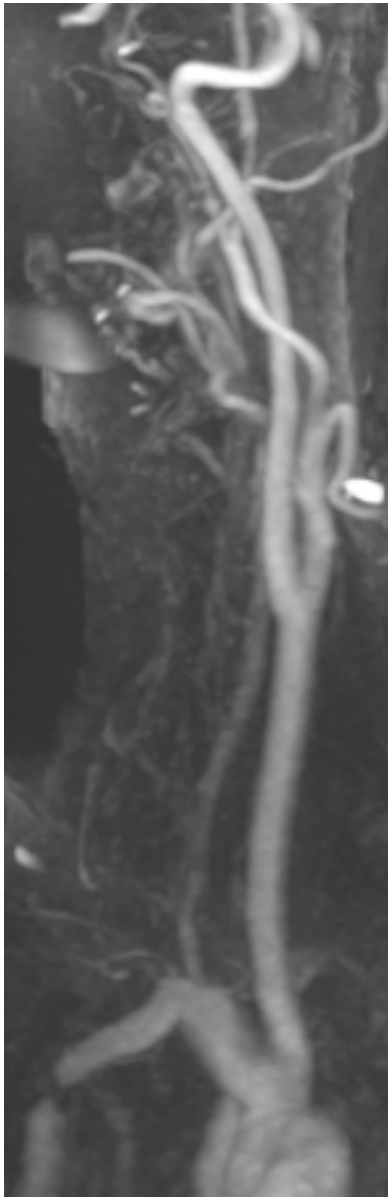

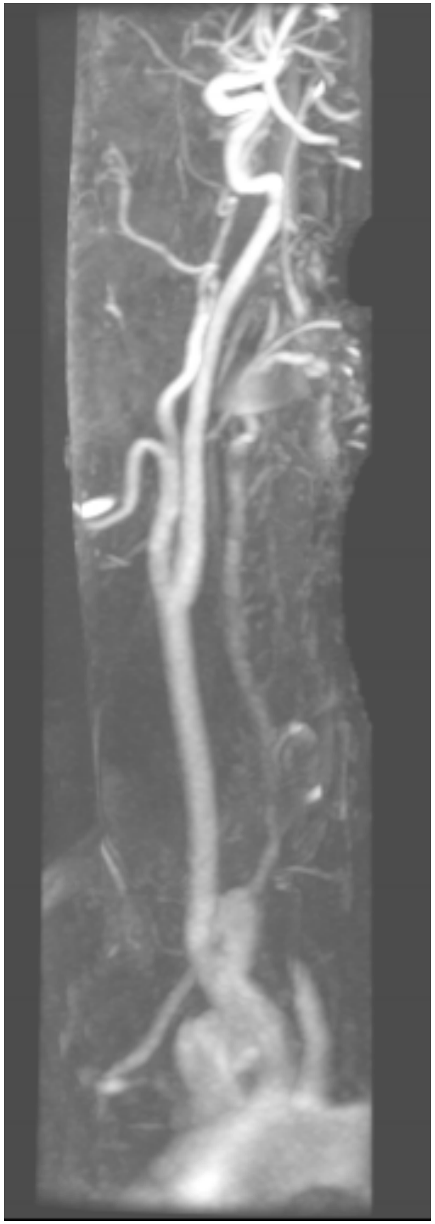

14d Score

0-30

31-50

51-70

>70

Near occlusion

Occluded

Quality

1

2

3

4

5

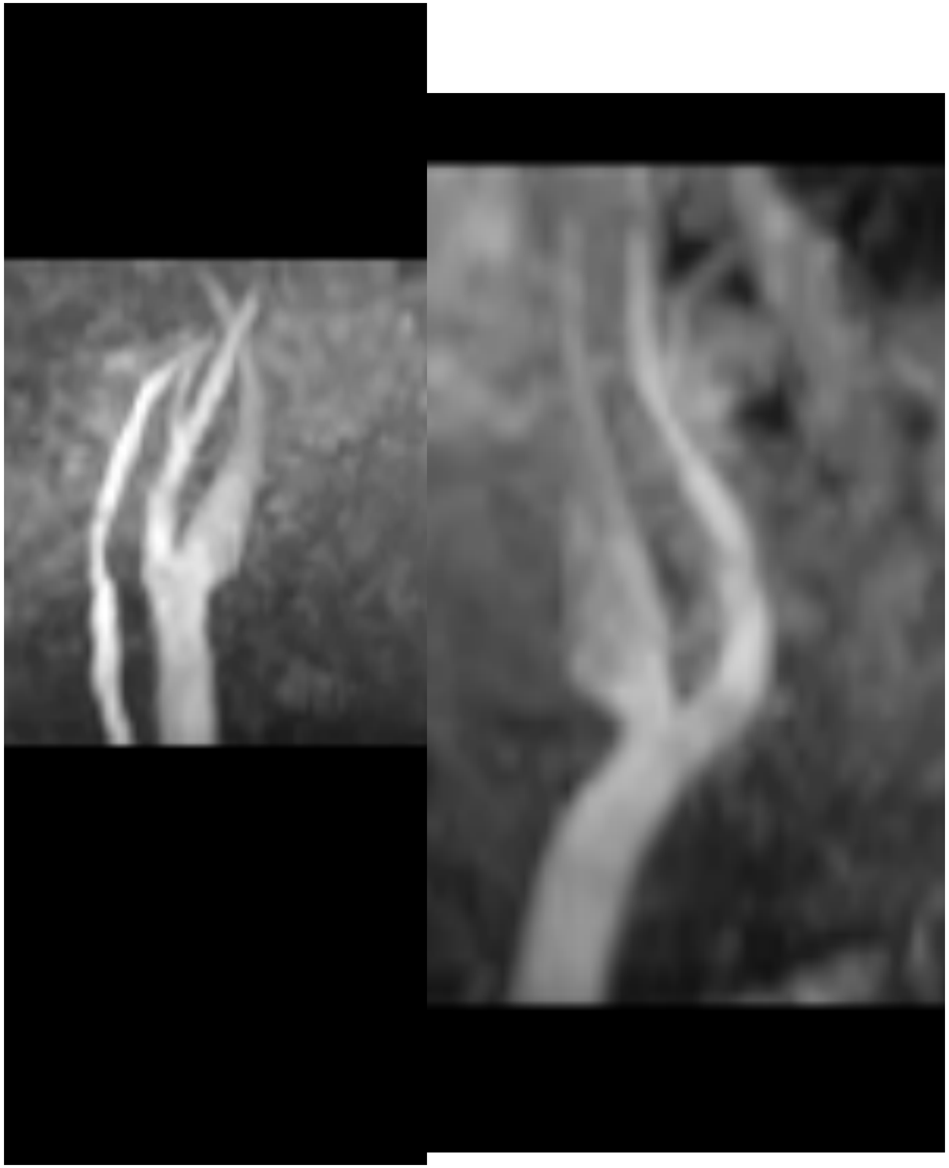

15c Score

0-30

31-50

51-70

>70

Near occlusion

Occluded

Quality

1

2

3

4

5

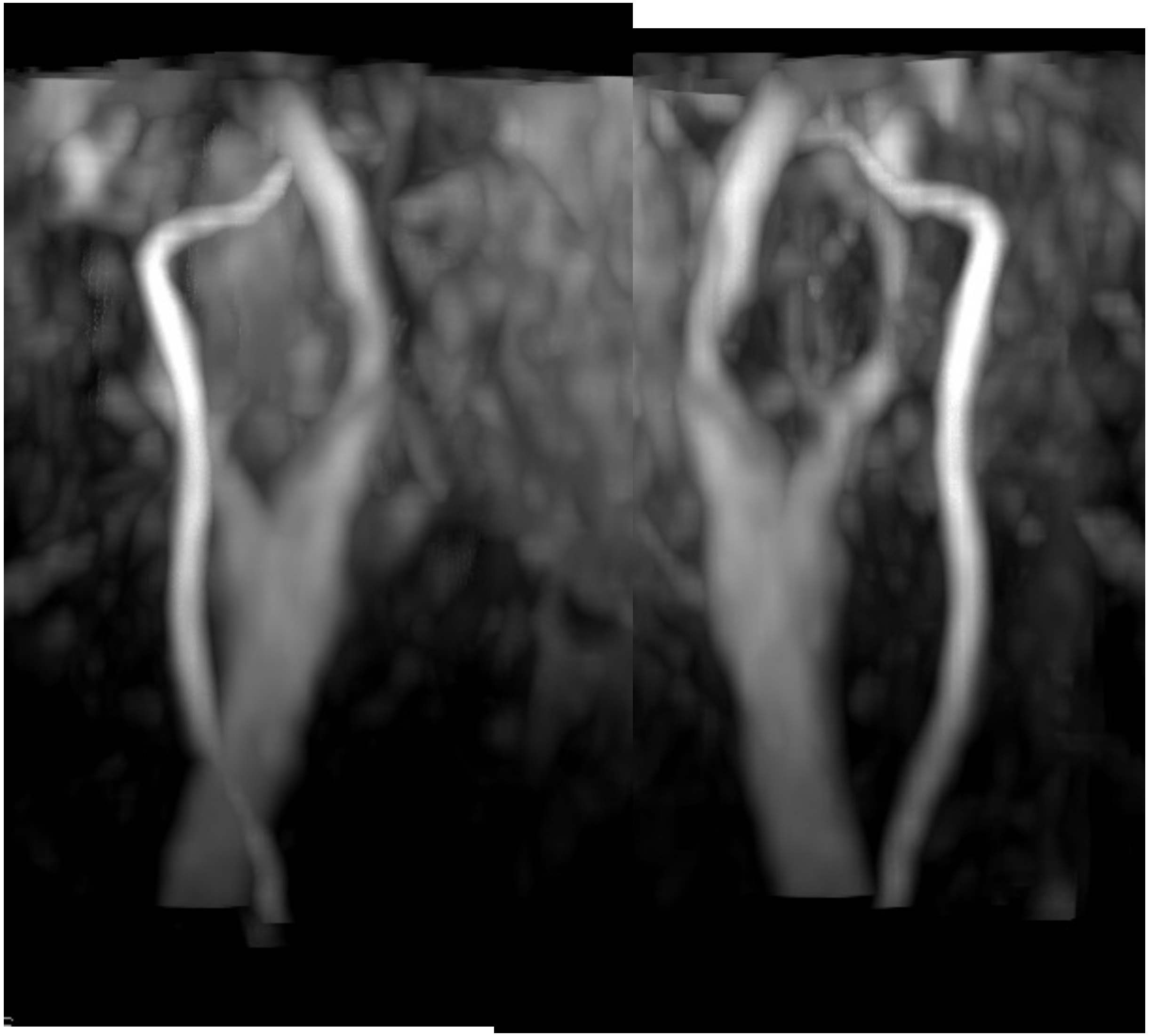

16b Score

0-30

31-50

51-70

>70

Near occlusion

Occluded

Quality

1

2

3

4

5

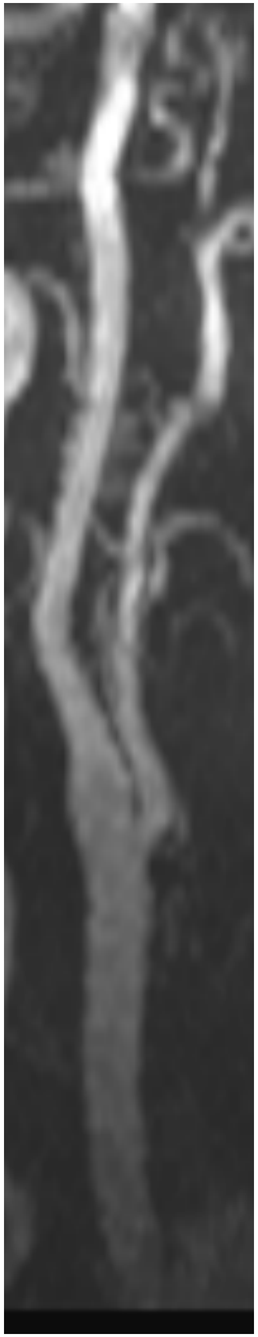

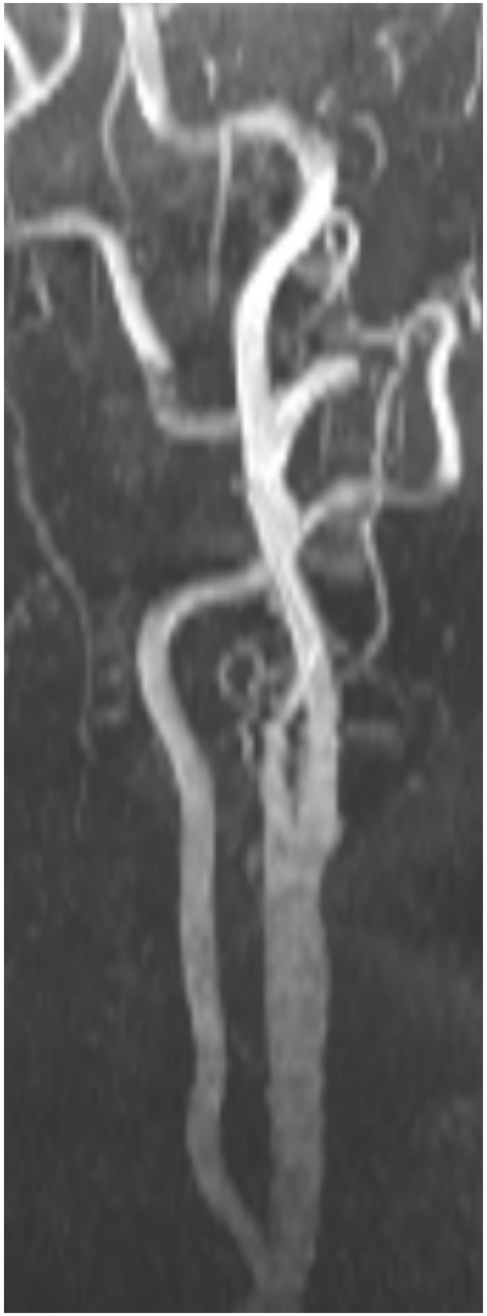

17a Score

0-30

31-50

51-70

>70

Near occlusion

Occluded

Quality

1

2

3

4

5

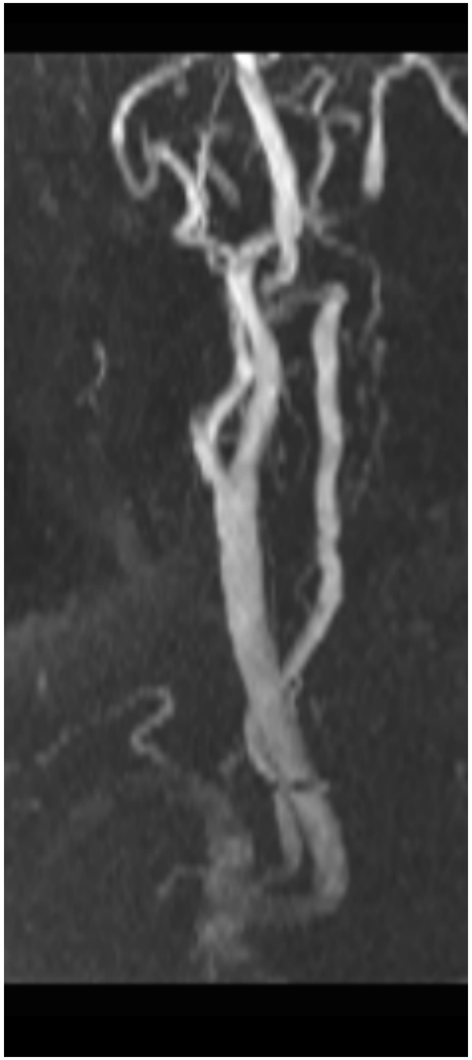

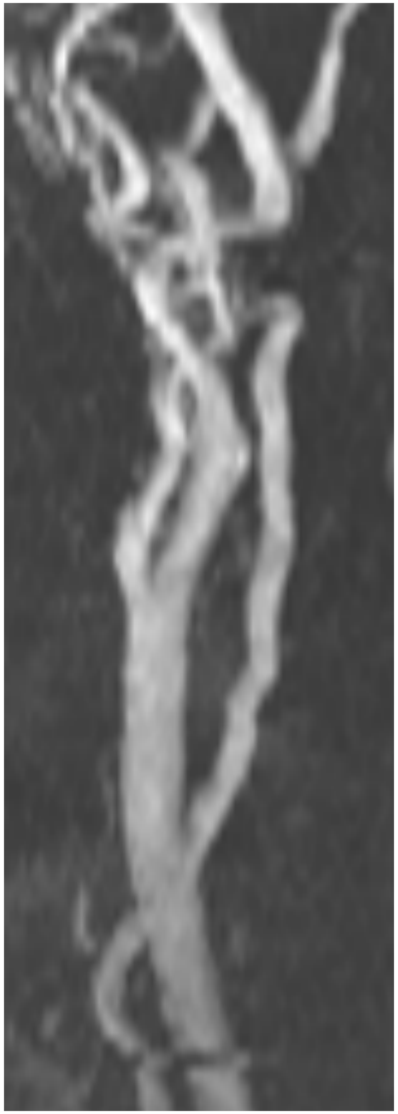

17f Score

0-30

31-50

51-70

>70

Near occlusion

Occluded

Quality

1

2

3

4

5

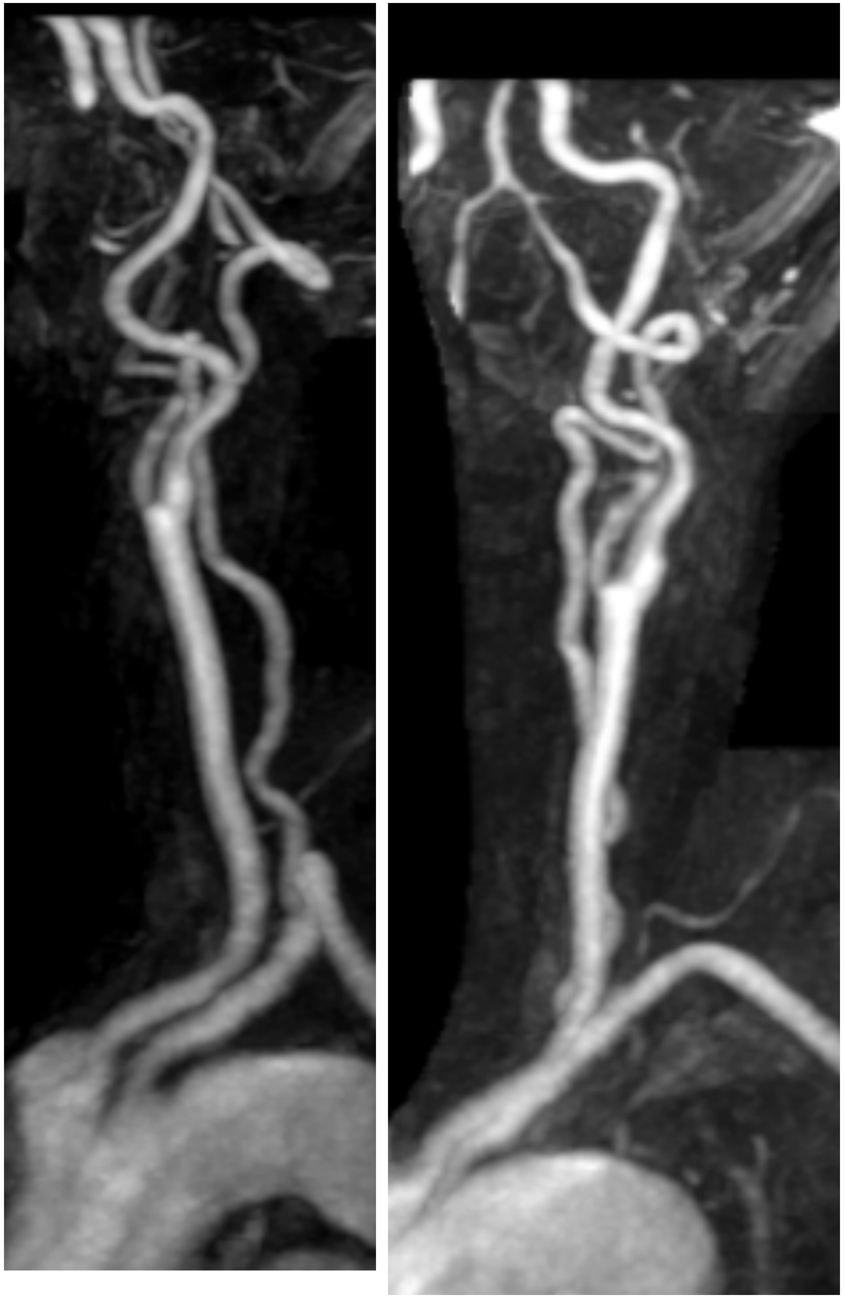

18e Score

0-30

31-50

51-70

>70

Near occlusion

Occluded

Quality

1

2

3

4

5

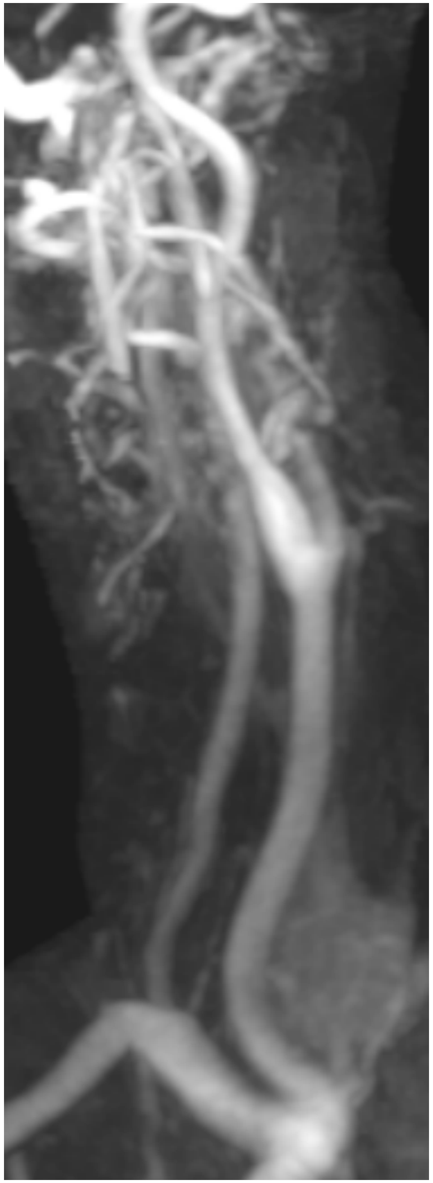

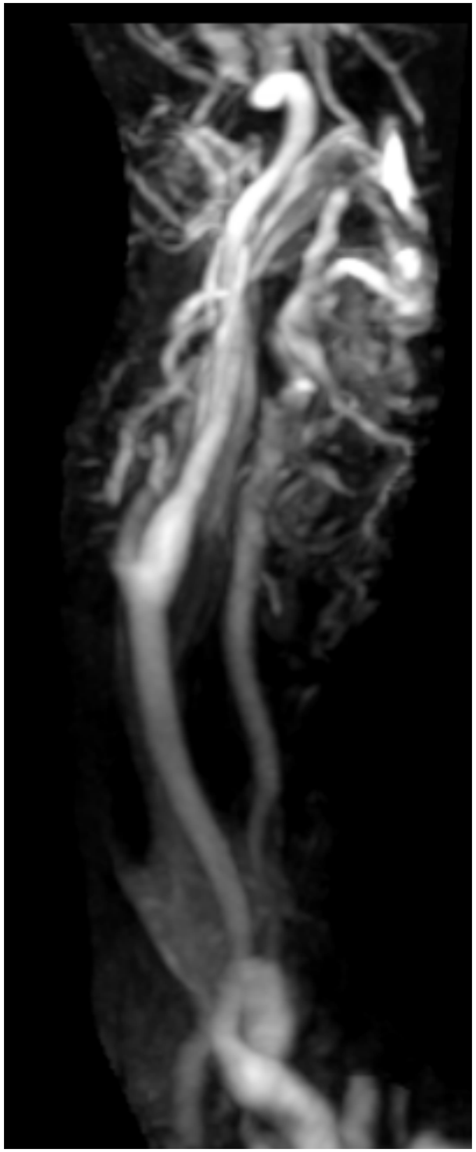

19d Score

0-30

31-50

51-70

>70

Near occlusion

Occluded

Quality

1

2

3

4

5

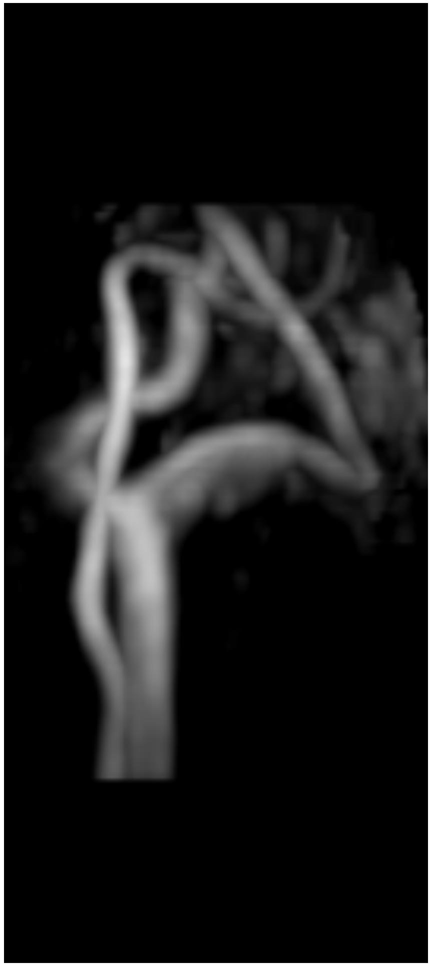

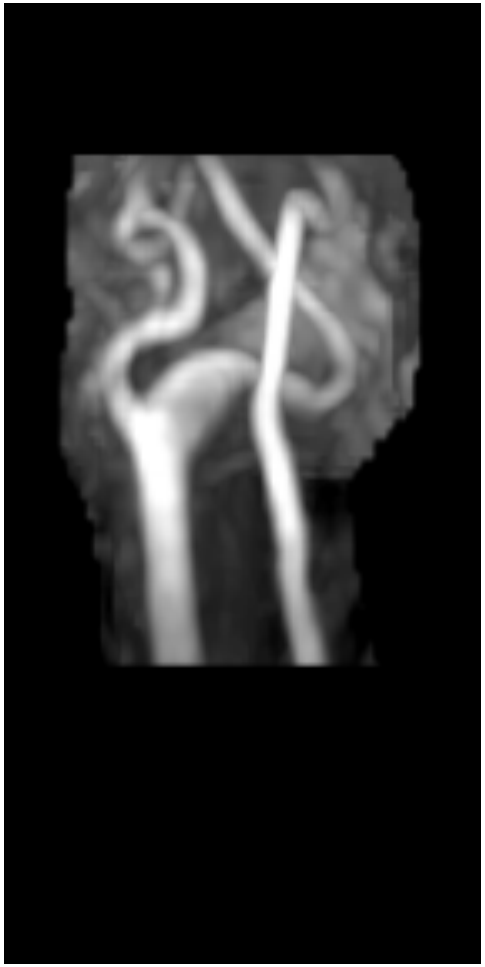

20c Score

0-30

31-50

51-70

>70

Near occlusion

Occluded

Quality

1

2

3

4

5

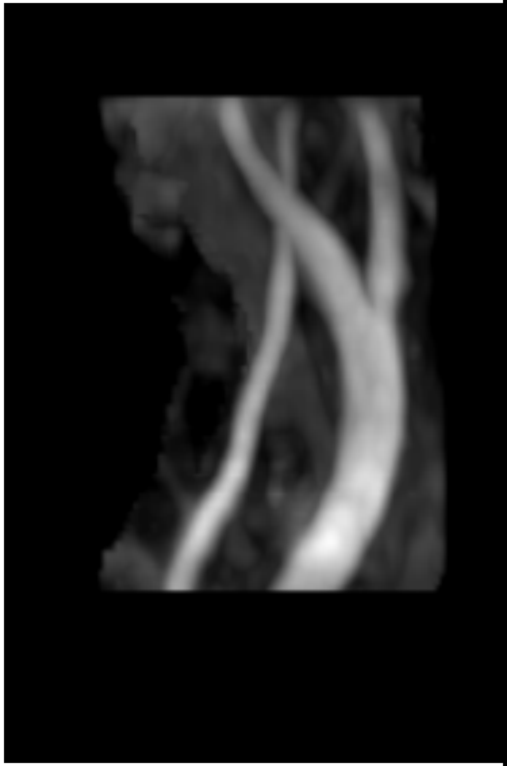

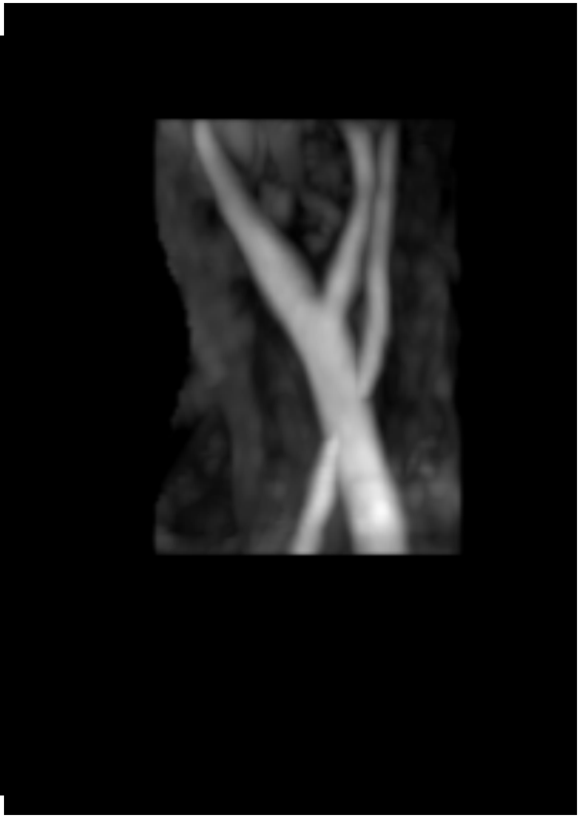

21b Score

0-30

31-50

51-70

>70

Near occlusion

Occluded

Quality

1

2

3

4

5

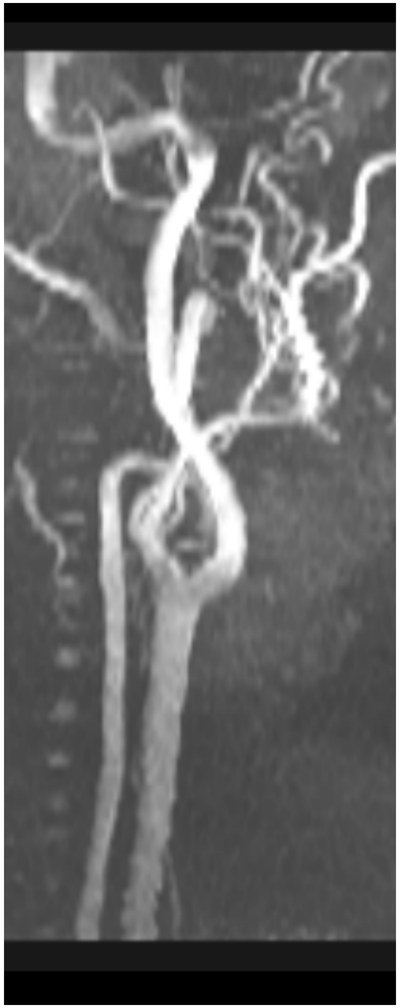

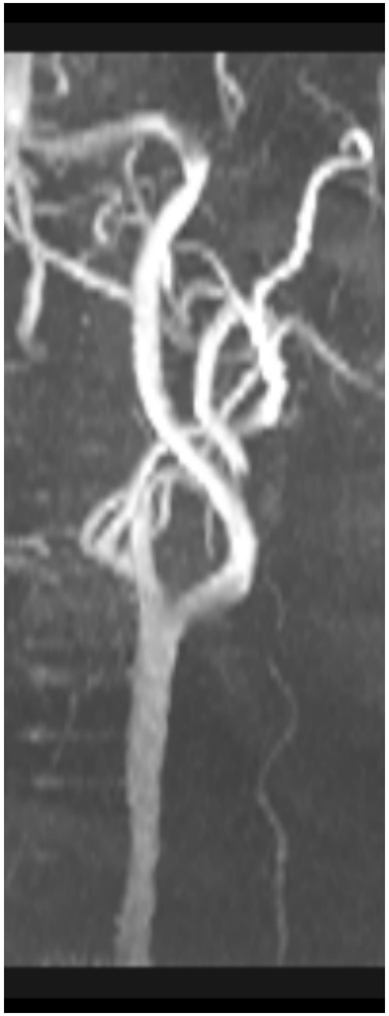

22a Score

0-30

31-50

51-70

>70

Near occlusion

Occluded

Quality

1

2

3

4

5

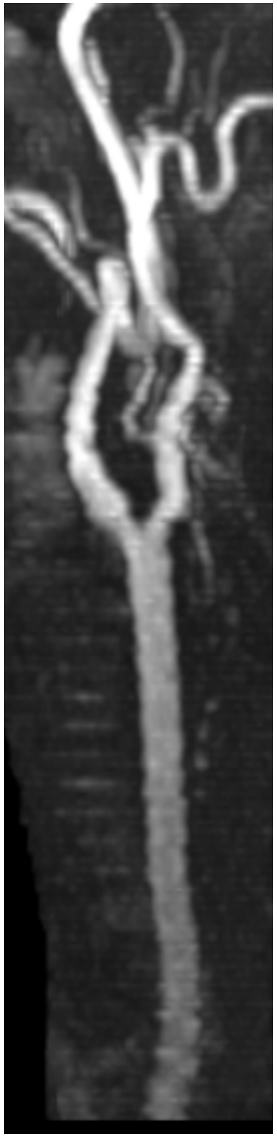

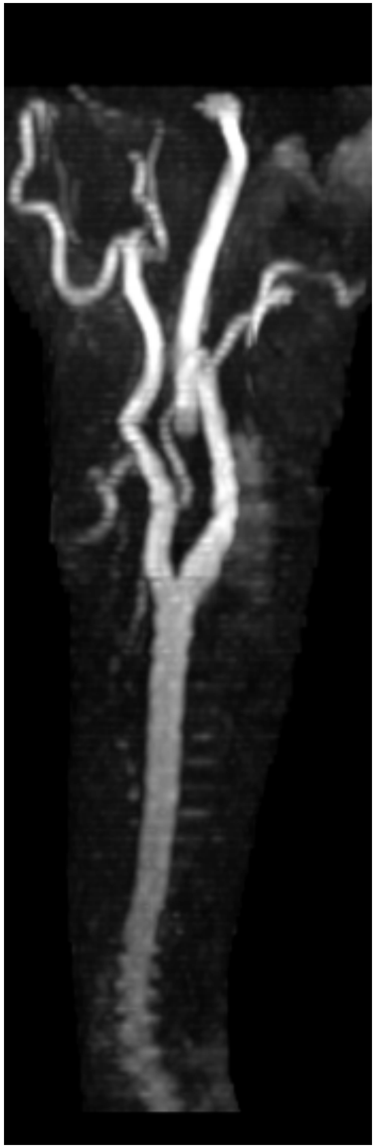

22f Score

0-30

31-50

51-70

>70

Near occlusion

Occluded

Quality

1

2

3

4

5

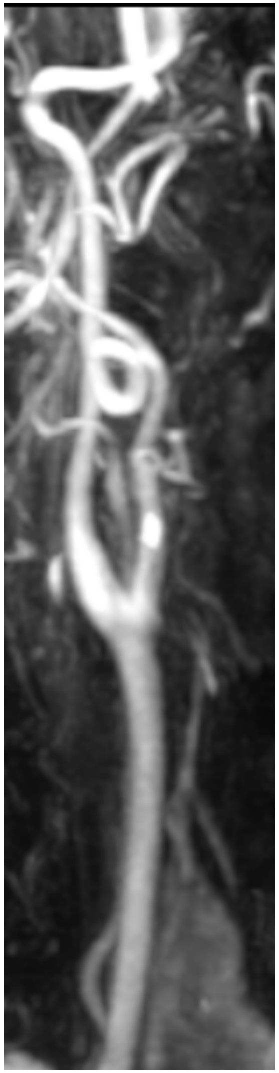

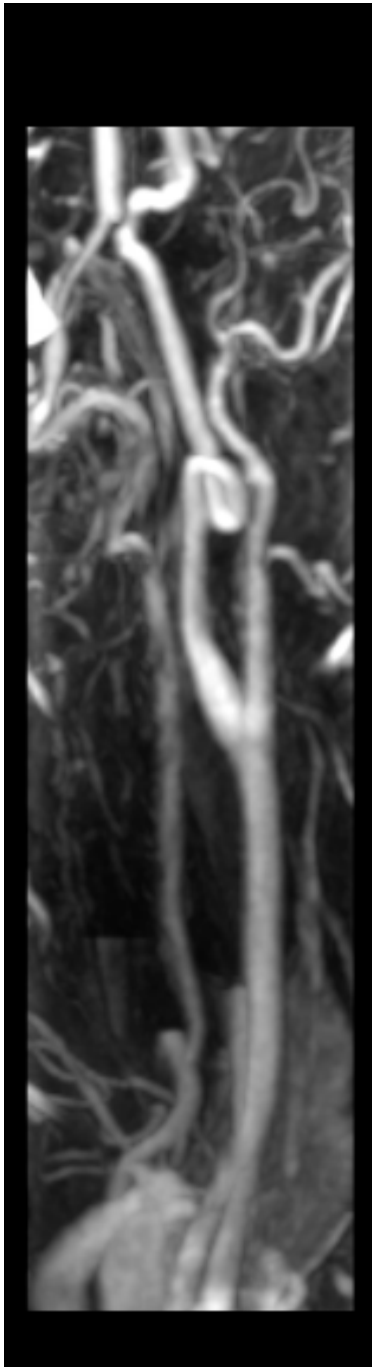

23e Score

0-30

31-50

51-70

>70

Near occlusion

Occluded

Quality

1

2

3

4

5

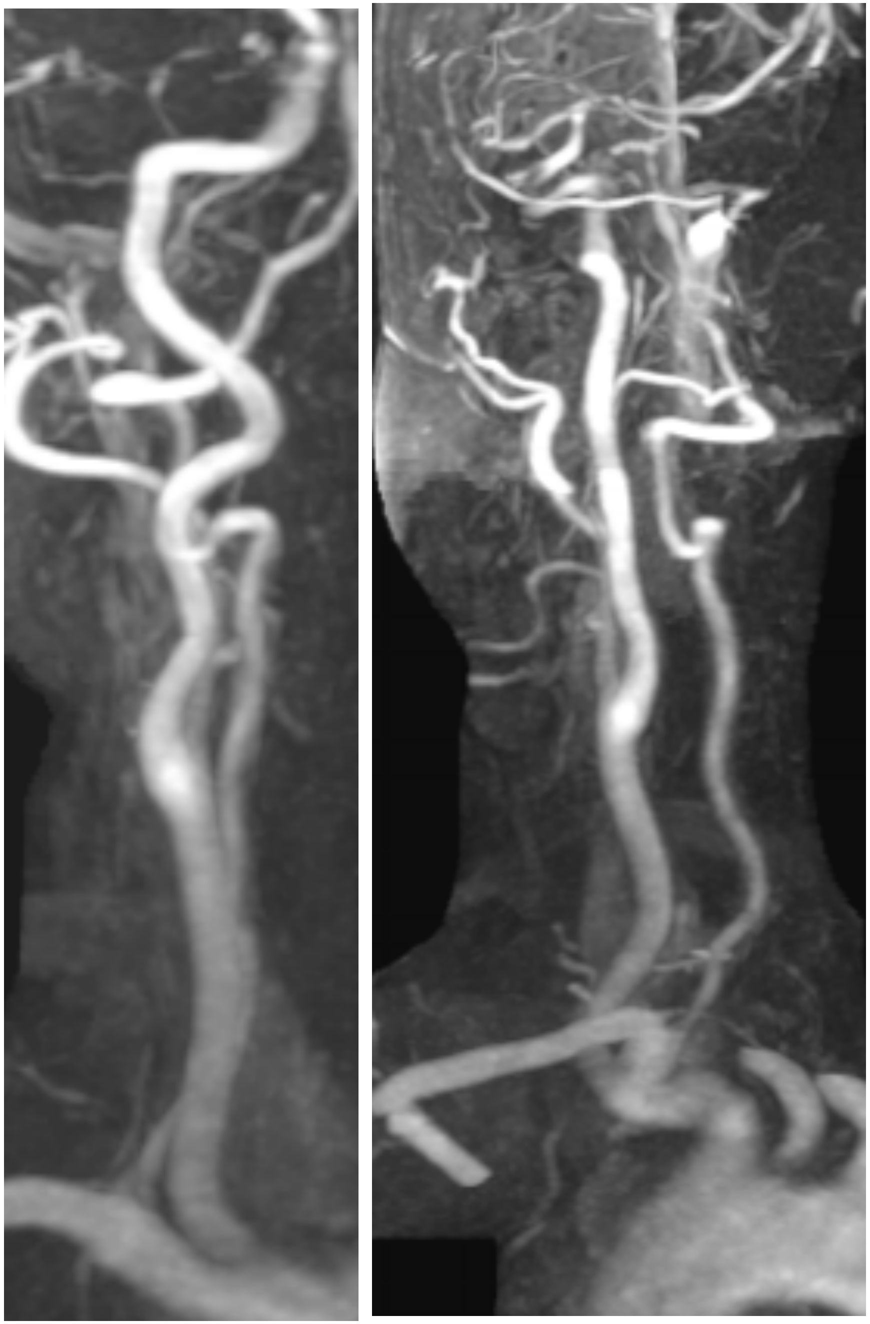

24d Score

0-30

31-50

51-70

>70

Near occlusion

Occluded

Quality

1

2

3

4

5

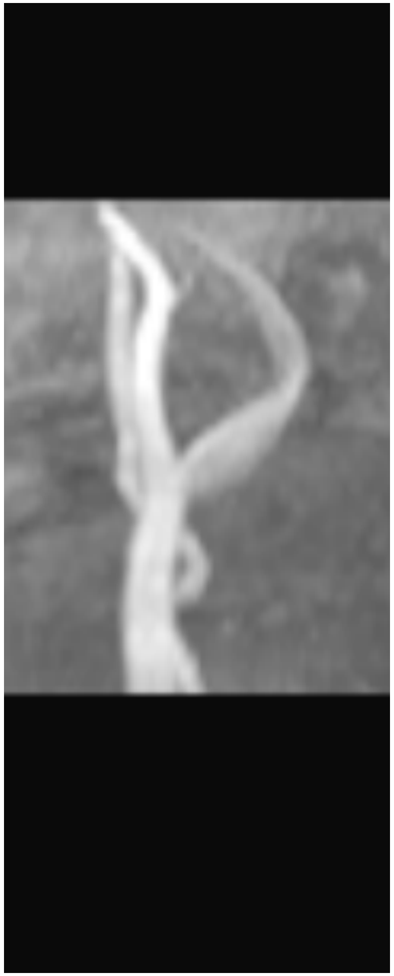

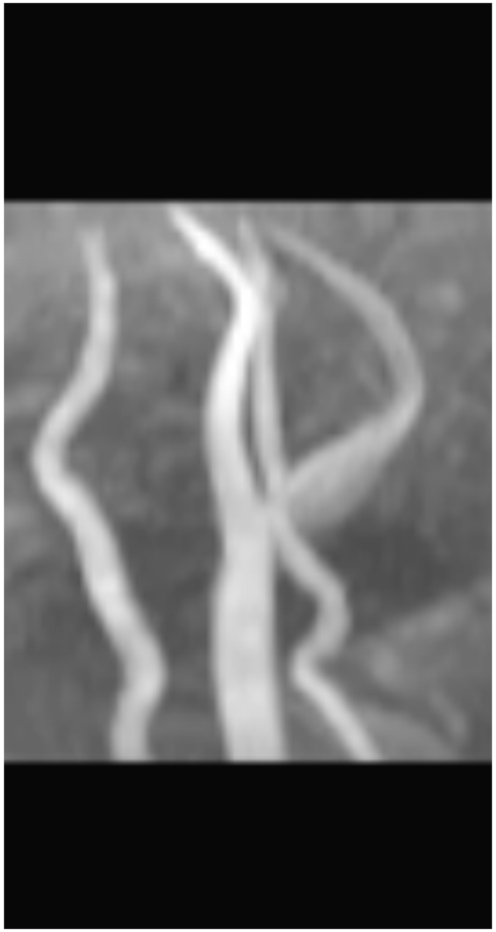

25c Score

0-30

31-50

51-70

>70

Near occlusion

Occluded

Quality

1

2

3

4

5

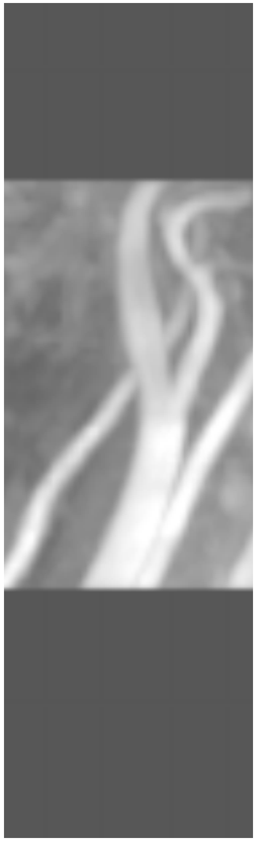

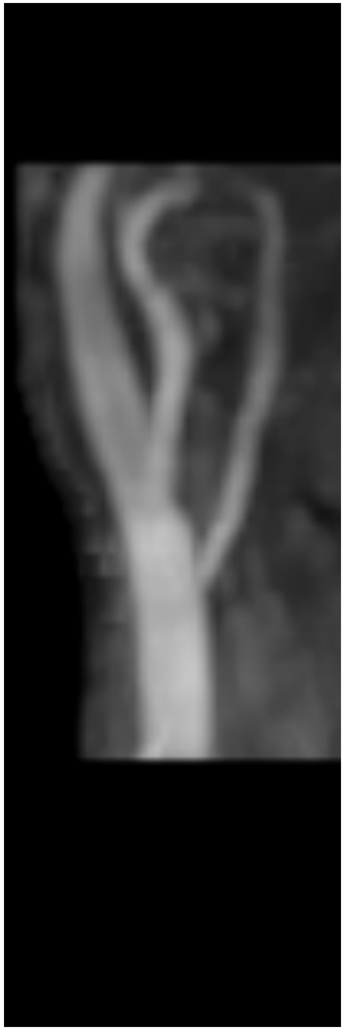

26b Score

0-30

31-50

51-70

>70

Near occlusion

Occluded

Quality

1

2

3

4

5

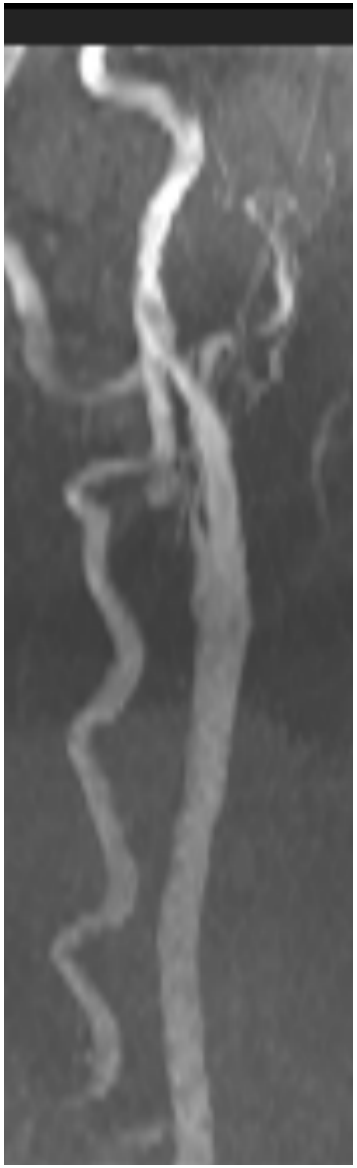

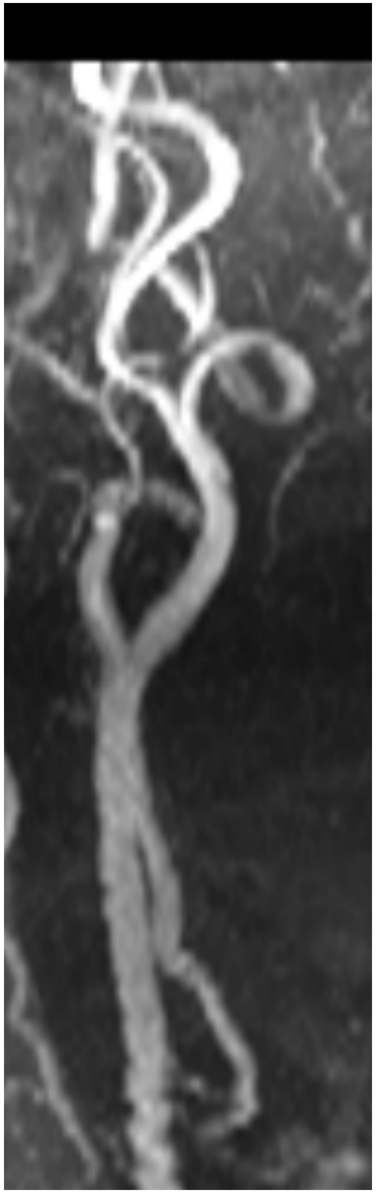

27a Score

0-30

31-50

51-70

>70

Near occlusion

Occluded

Quality

1

2

3

4

5

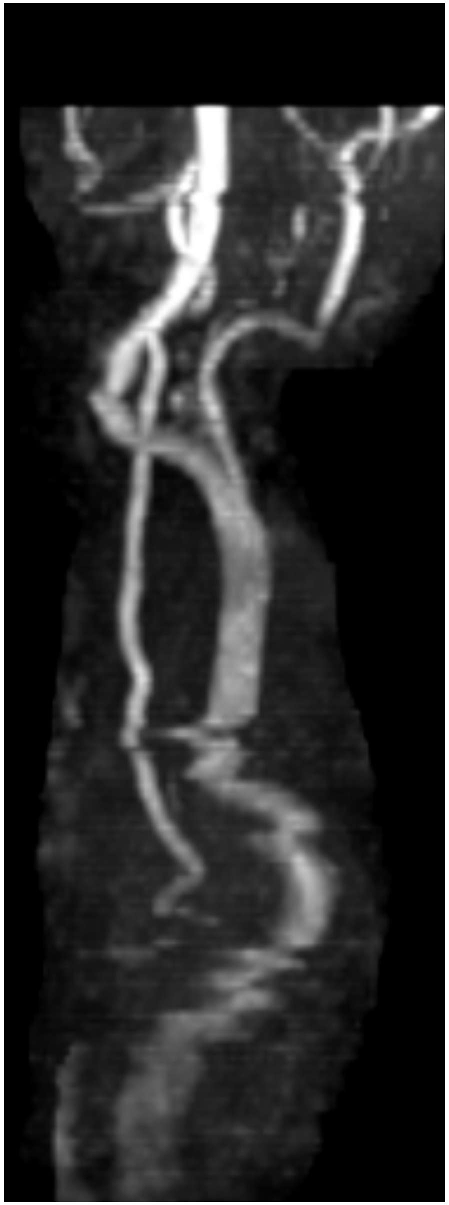

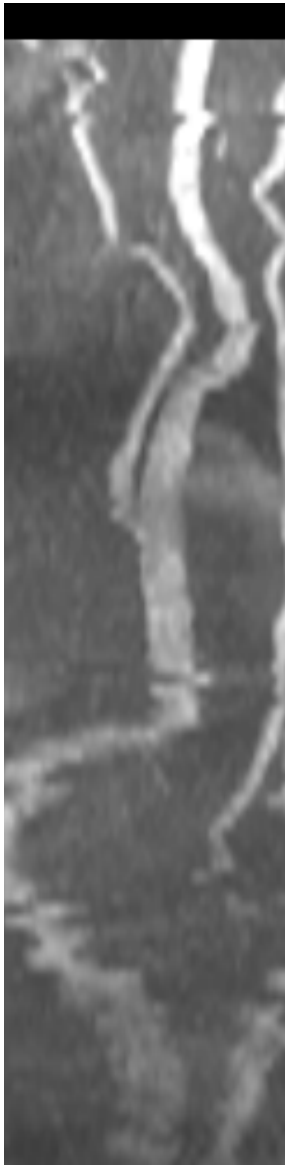

27f Score

0-30

31-50

51-70

>70

Near occlusion

Occluded

Quality

1

2

3

4

5

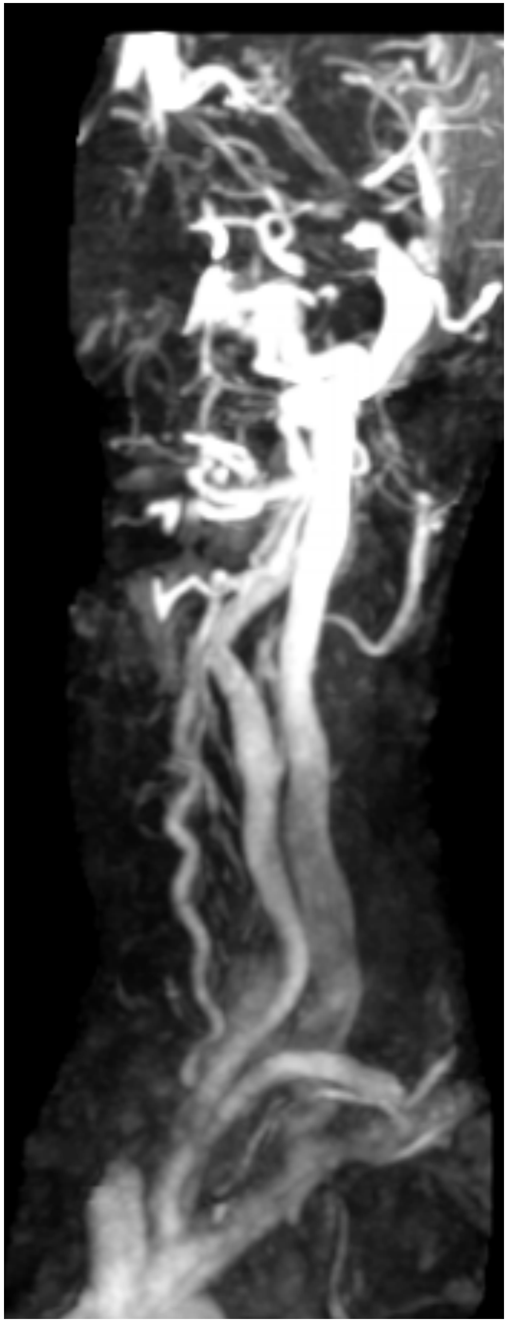

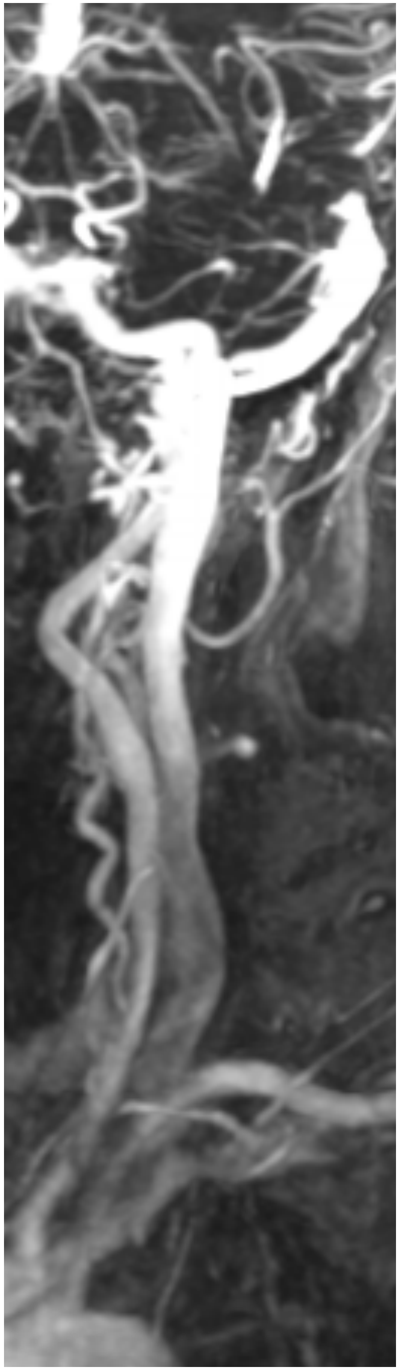

28e Score

0-30

31-50

51-70

>70

Near occlusion

Occluded

Quality

1

2

3

4

5

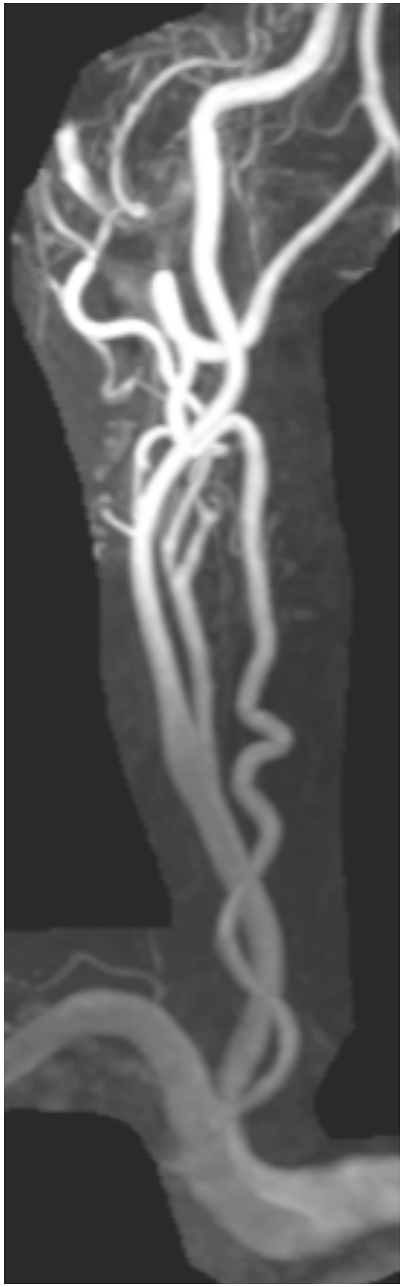

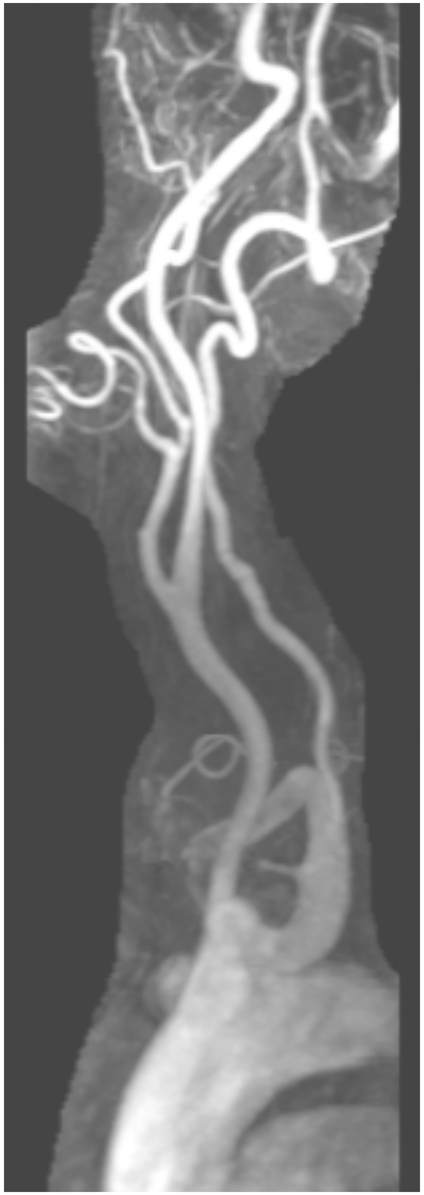

29d Score

0-30

31-50

51-70

>70

Near occlusion

Occluded

Quality

1

2

3

4

5

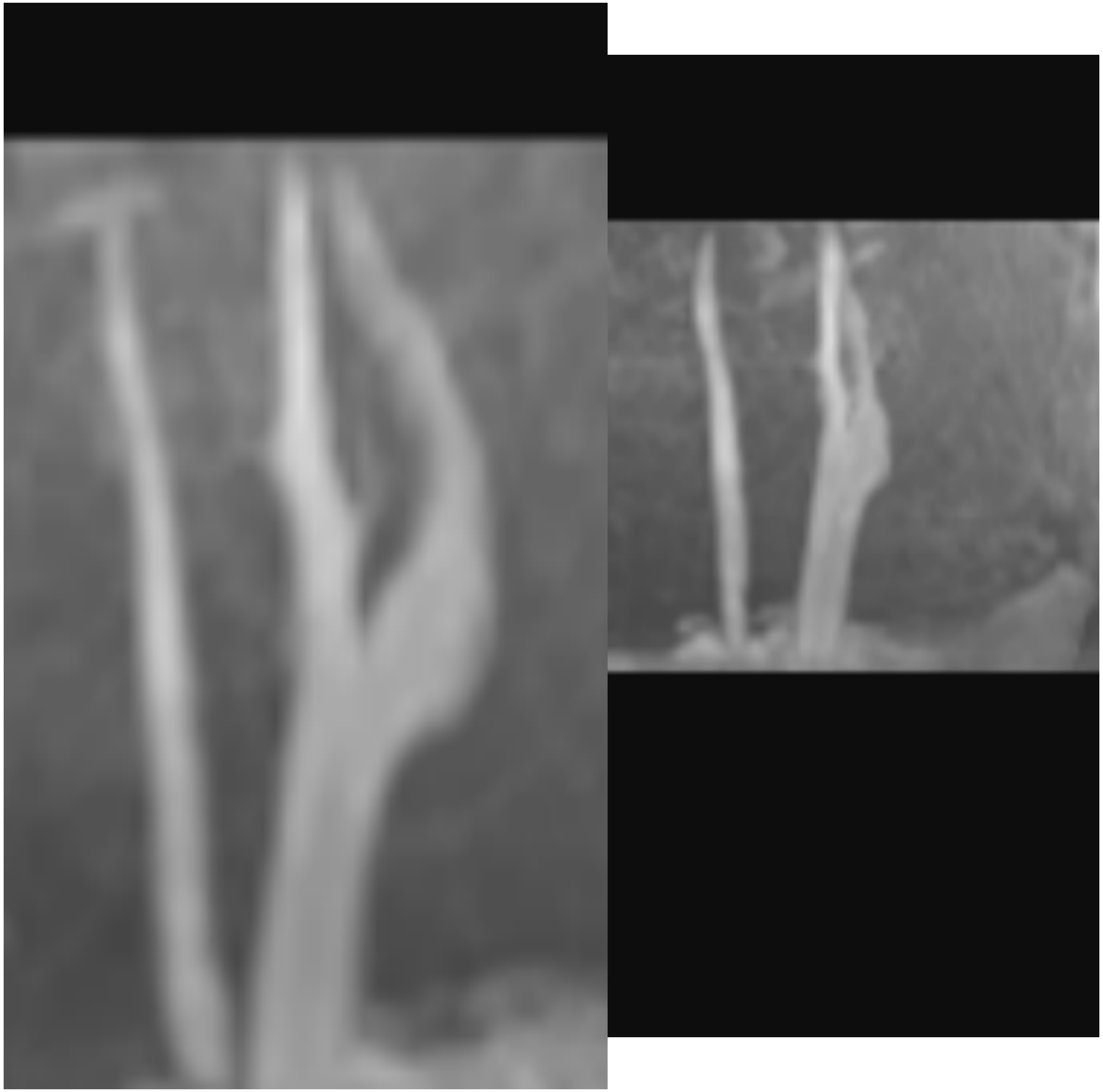

30c Score

0-30

31-50

51-70

>70

Near occlusion

Occluded

Quality

1

2

3

4

5

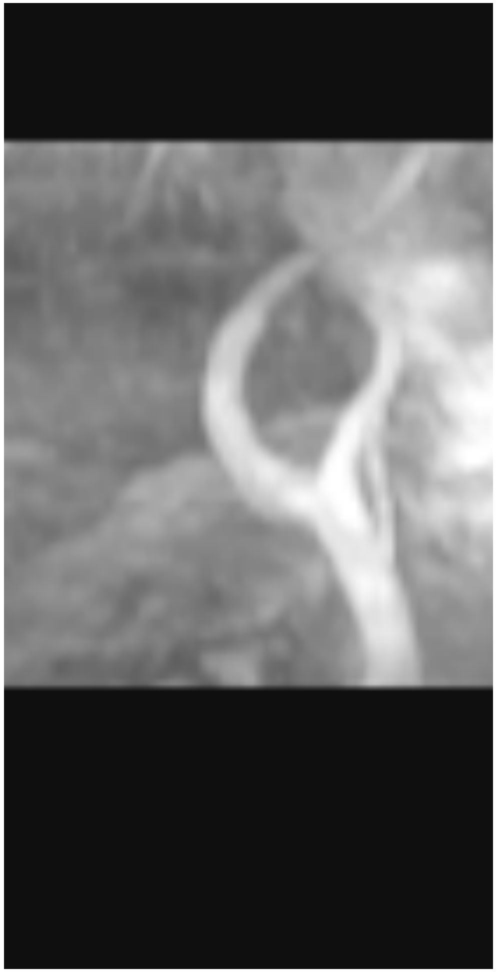

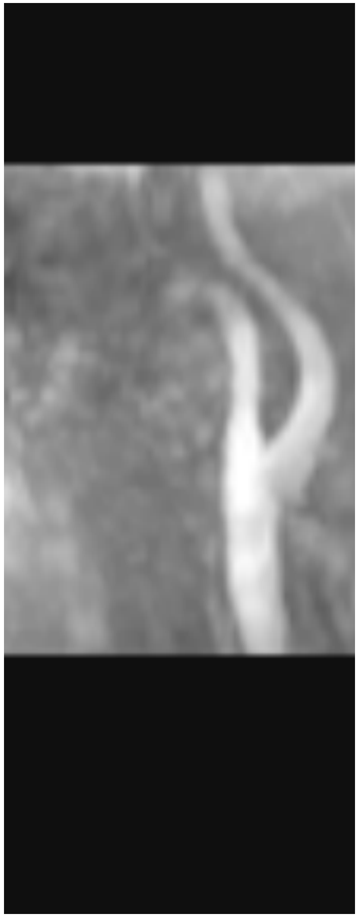

31b Score

0-30

31-50

51-70

>70

Near occlusion

Occluded

Quality

1

2

3

4

5

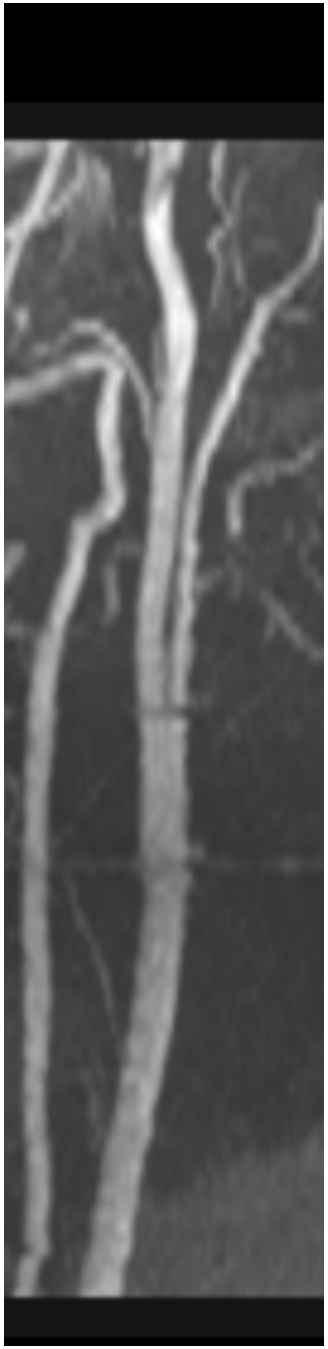

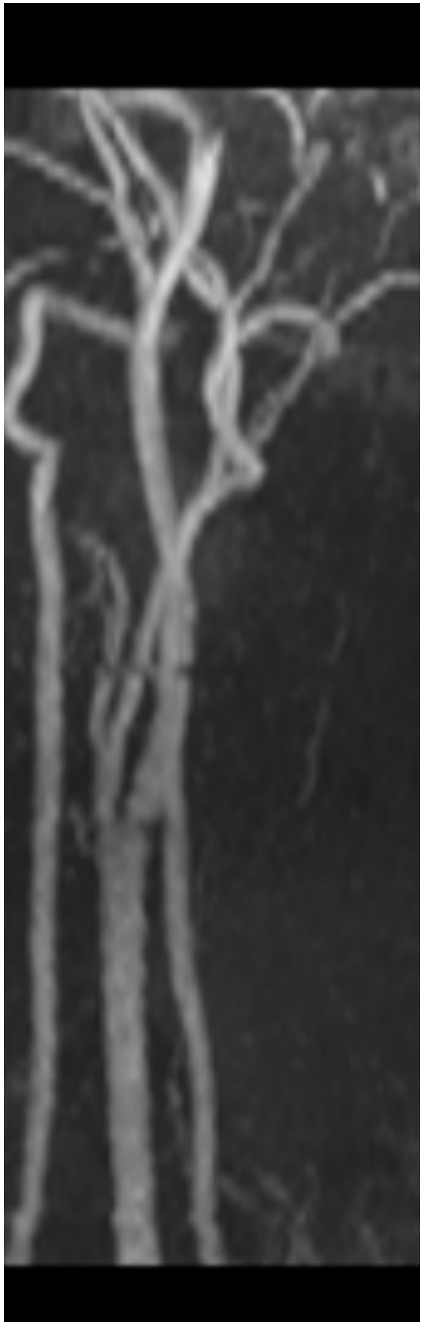

32a Score

0-30

31-50

51-70

>70

Near occlusion

Occluded

Quality

1

2

3

4

5

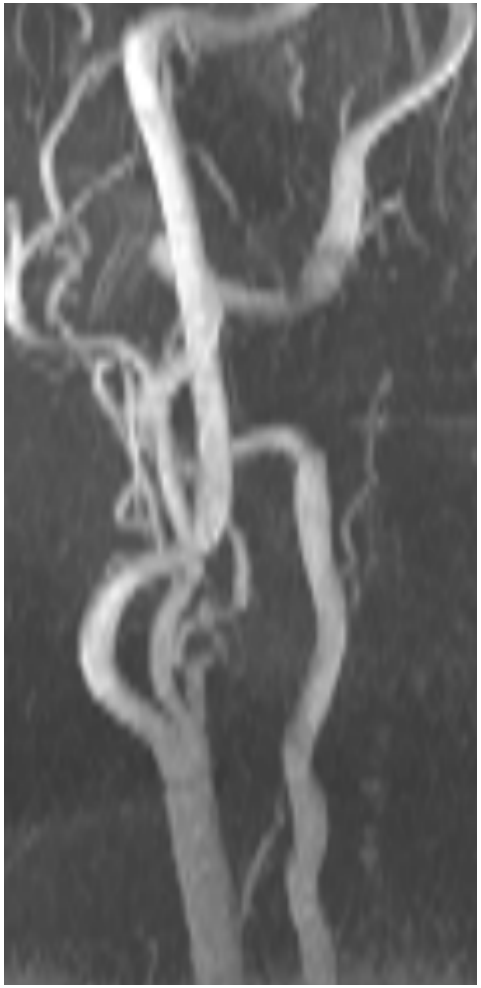

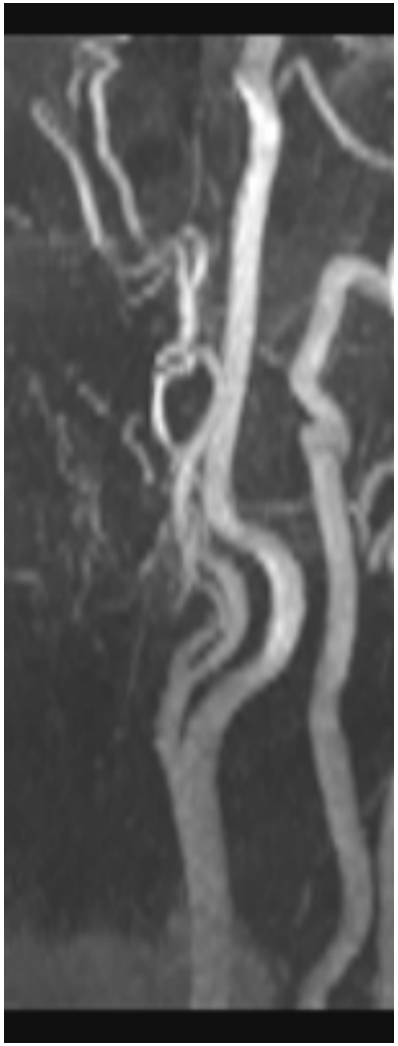

32f Score

0-30

31-50

51-70

>70

Near occlusion

Occluded

Quality

1

2

3

4

5

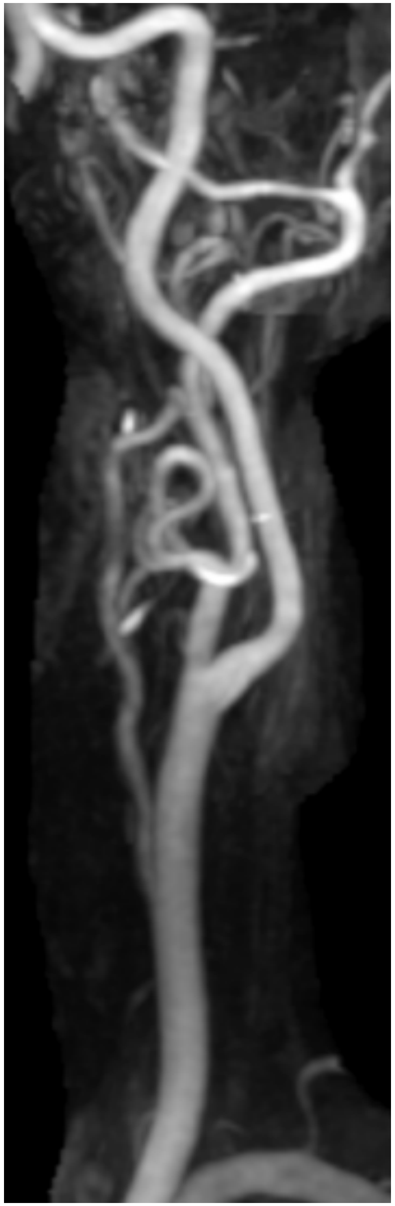

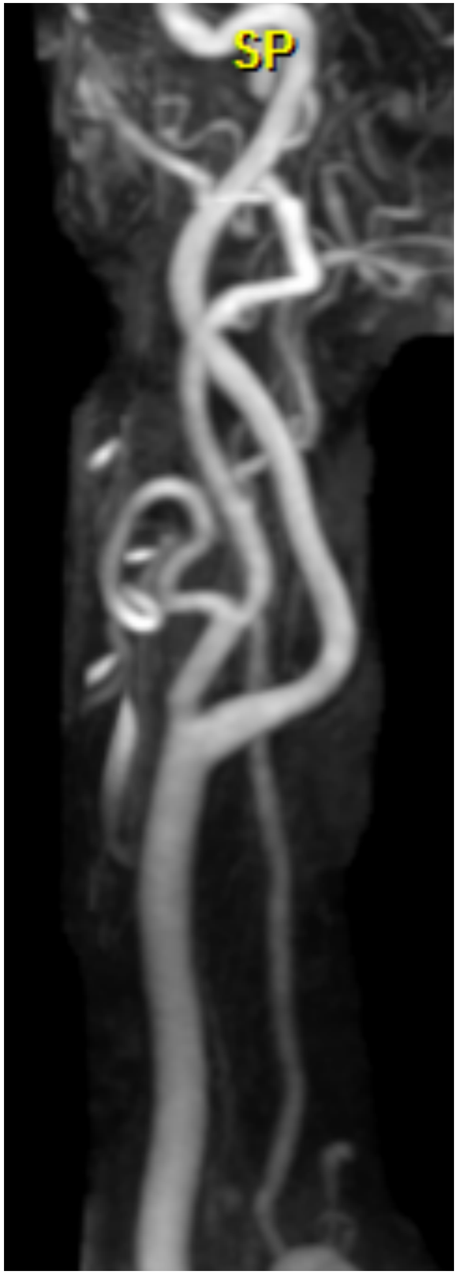

33e Score

0-30

31-50

51-70

>70

Near occlusion

Occluded

Quality

1

2

3

4

5

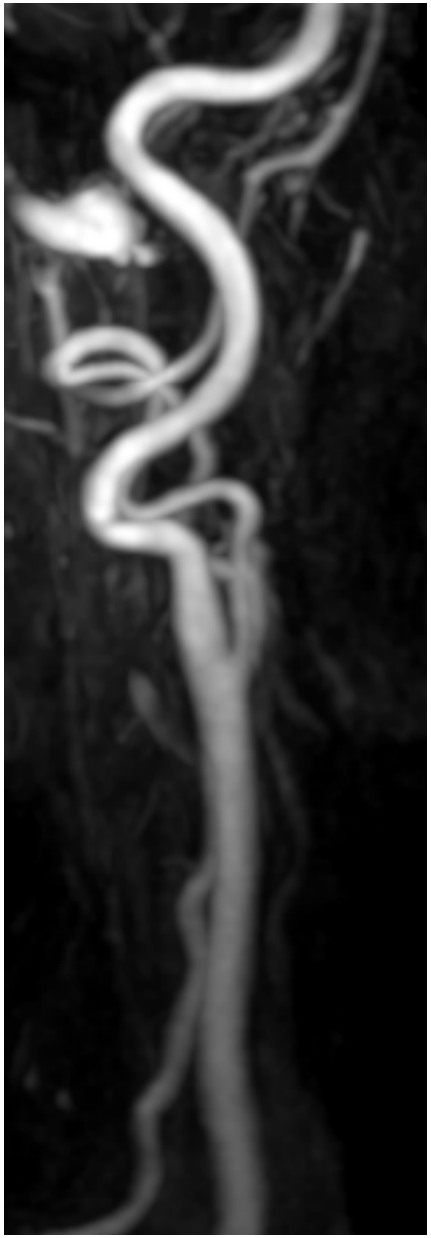

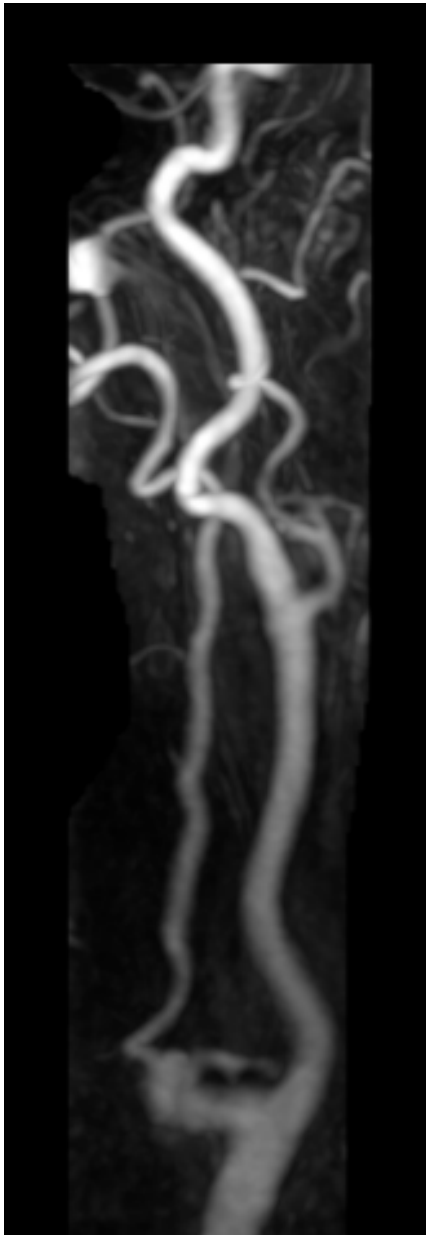

34d Score

0-30

31-50

51-70

>70

Near occlusion

Occluded

Quality

1

2

3

4

5

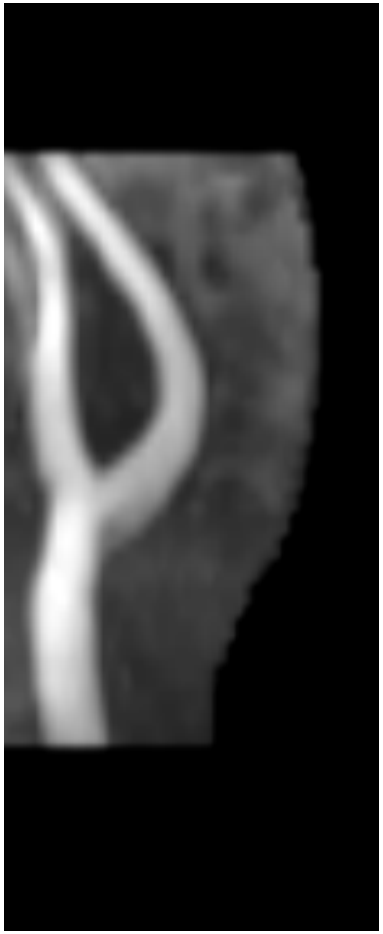

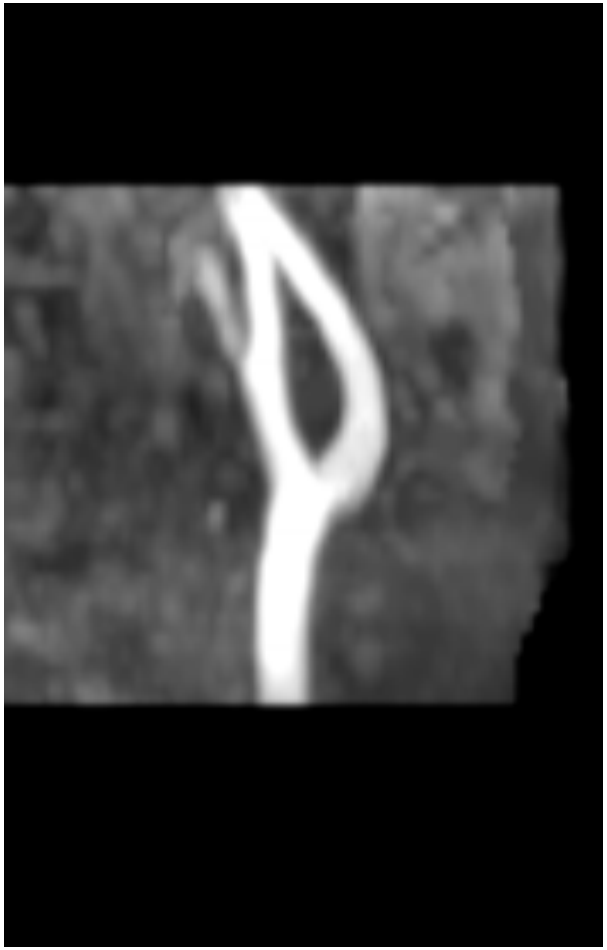

35c Score

0-30

31-50

51-70

>70

Near occlusion

Occluded

Quality

1

2

3

4

5

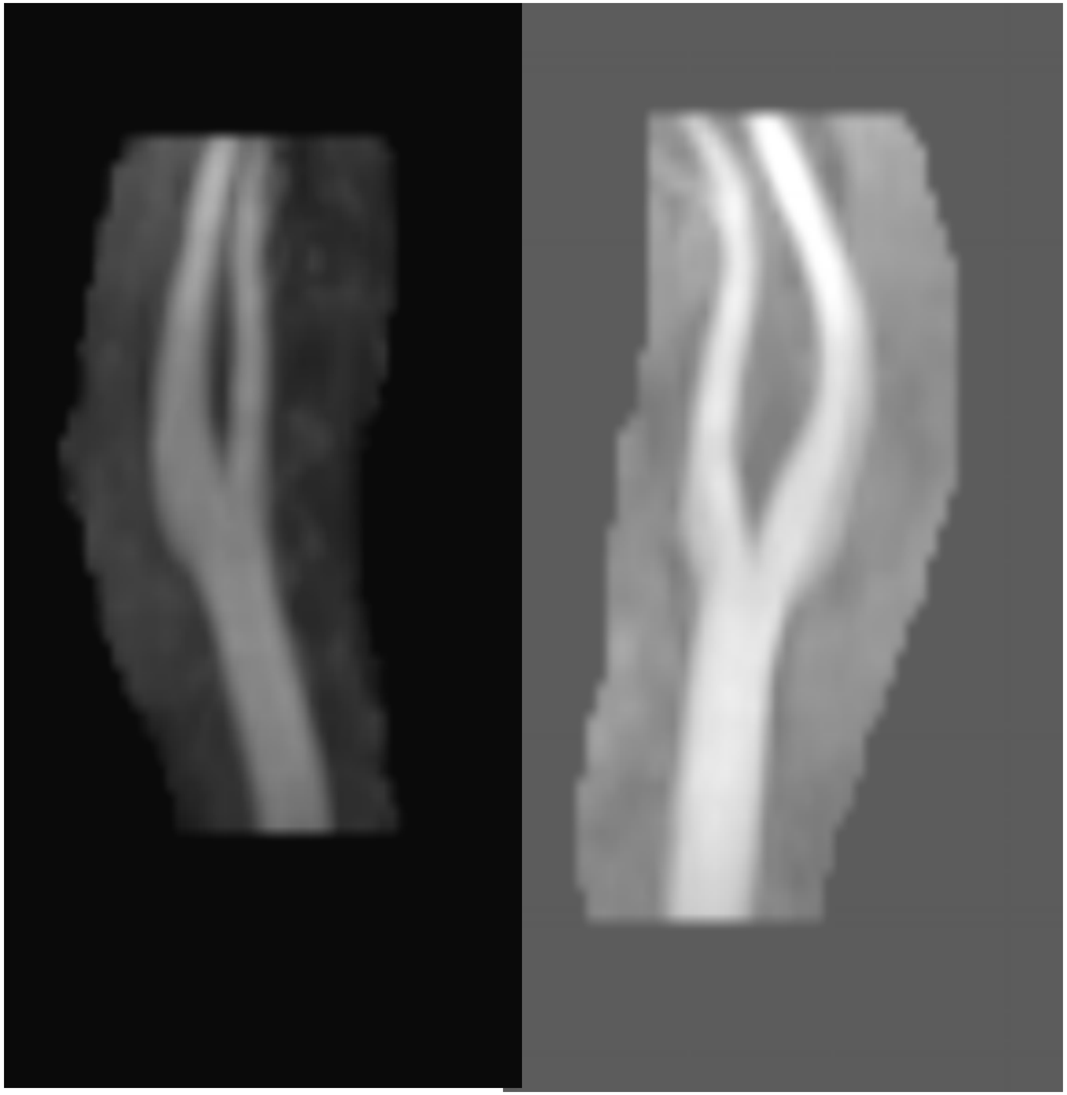

36b Score (LEFT)

0-30

31-50

51-70

>70

Near occlusion

Occluded

Quality

1

2

3

4

5

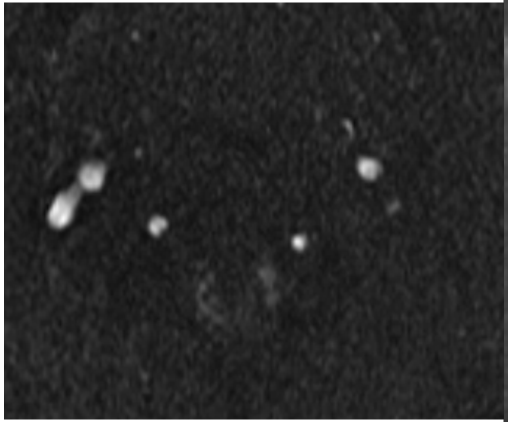

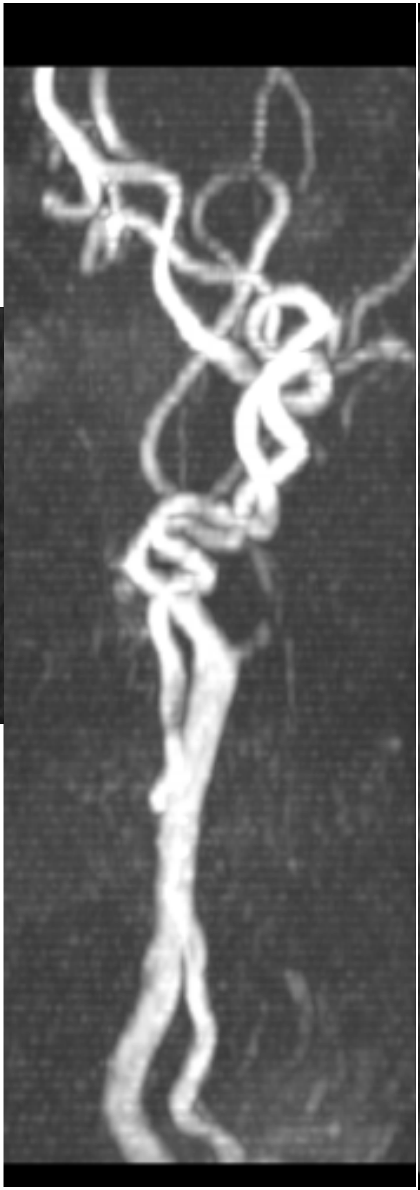

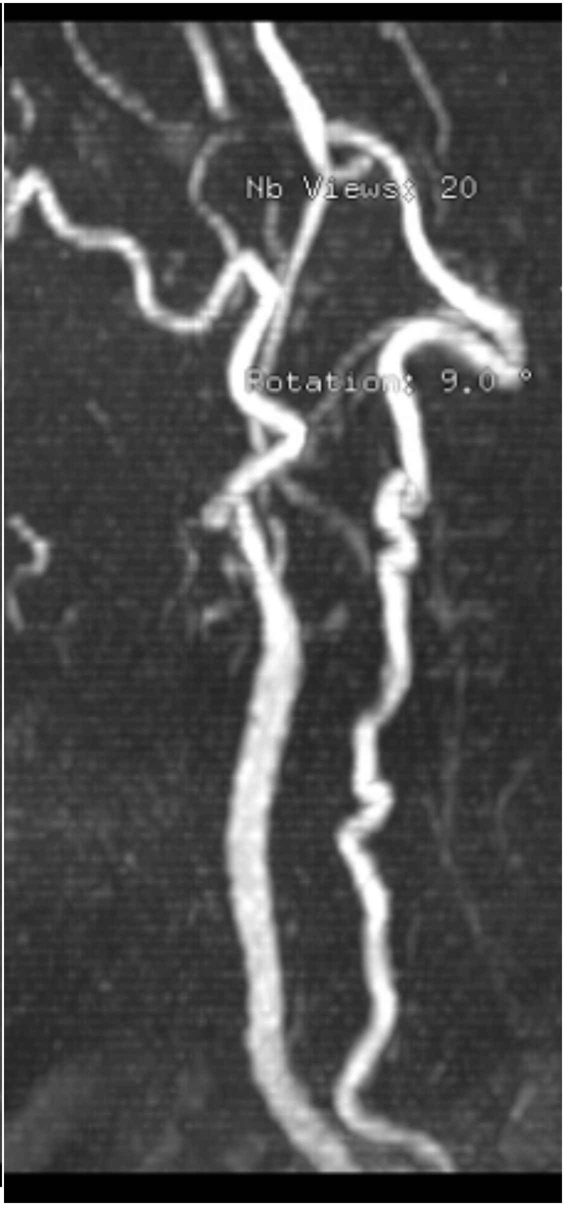

37a Score

0-30

31-50

51-70

>70

Near occlusion

Occluded

Quality

1

2

3

4

5

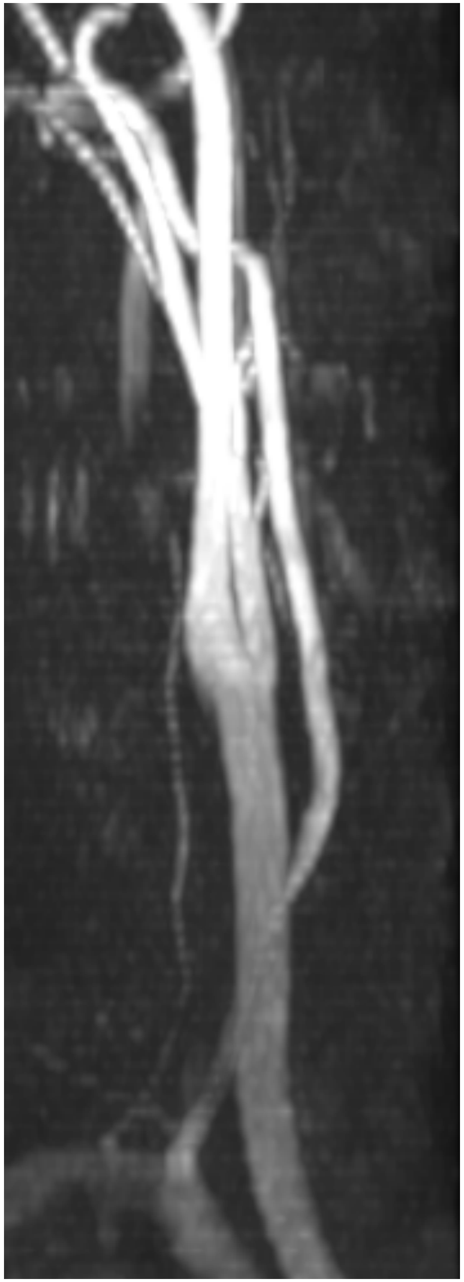

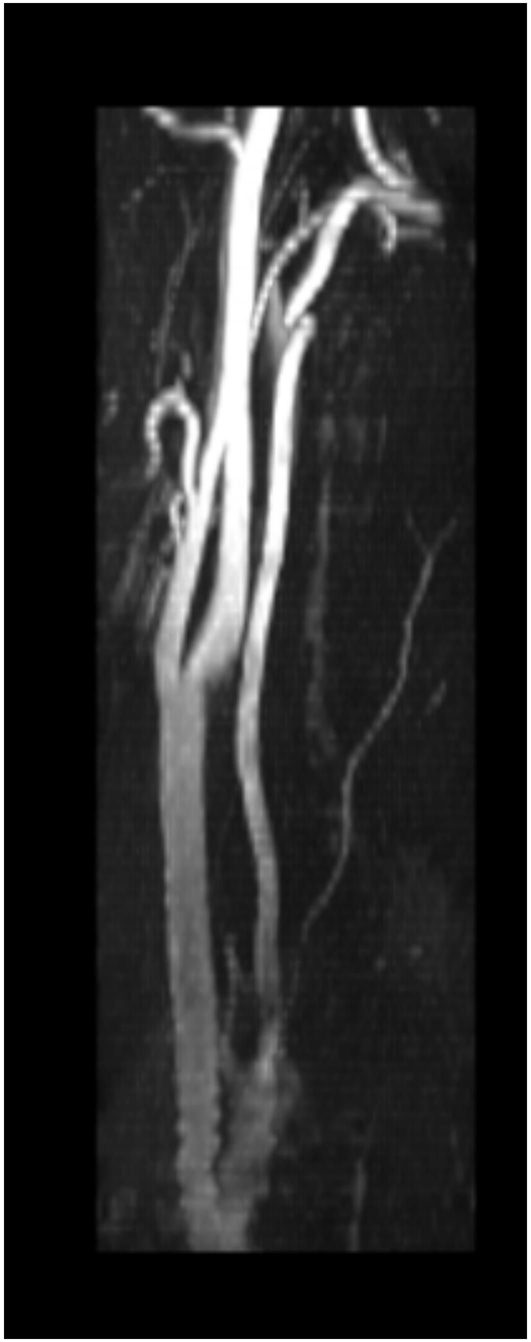

37f Score

0-30

31-50

51-70

>70

Near occlusion

Occluded

Quality

1

2

3

4

5

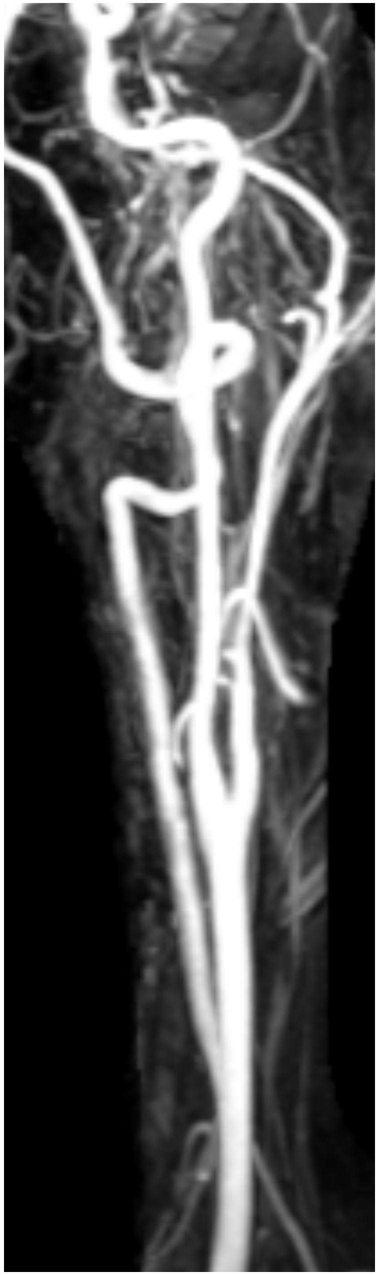

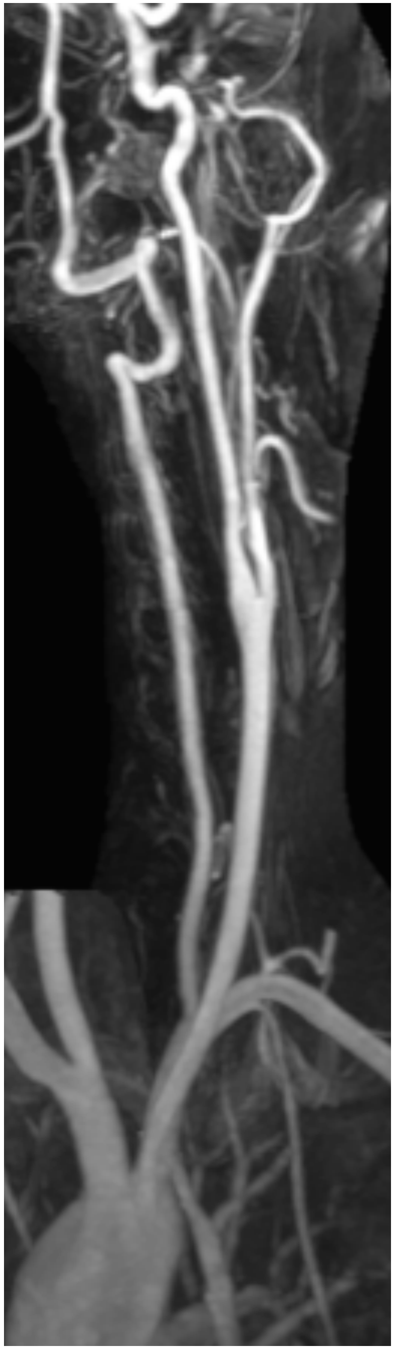

38e Score

0-30

31-50

51-70

>70

Near occlusion

Occluded

Quality

1

2

3

4

5

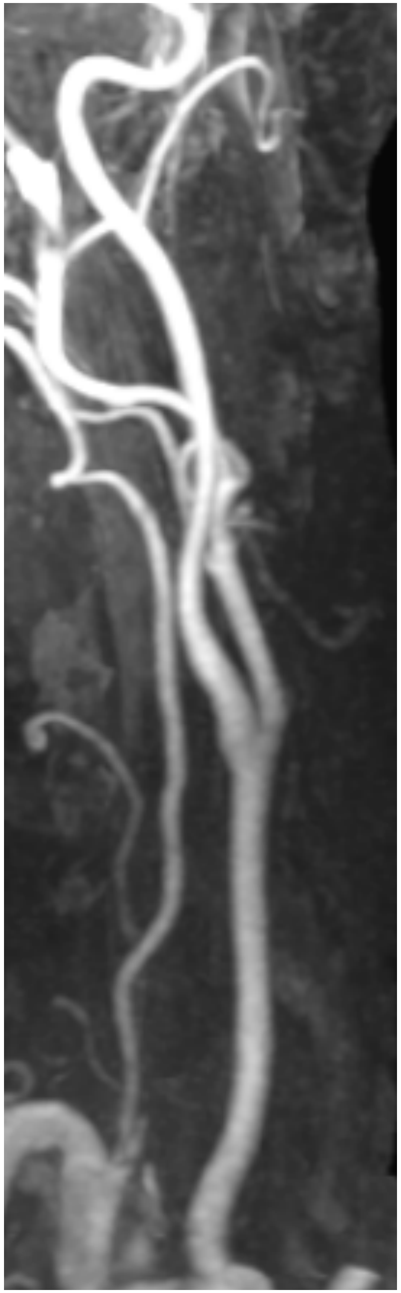

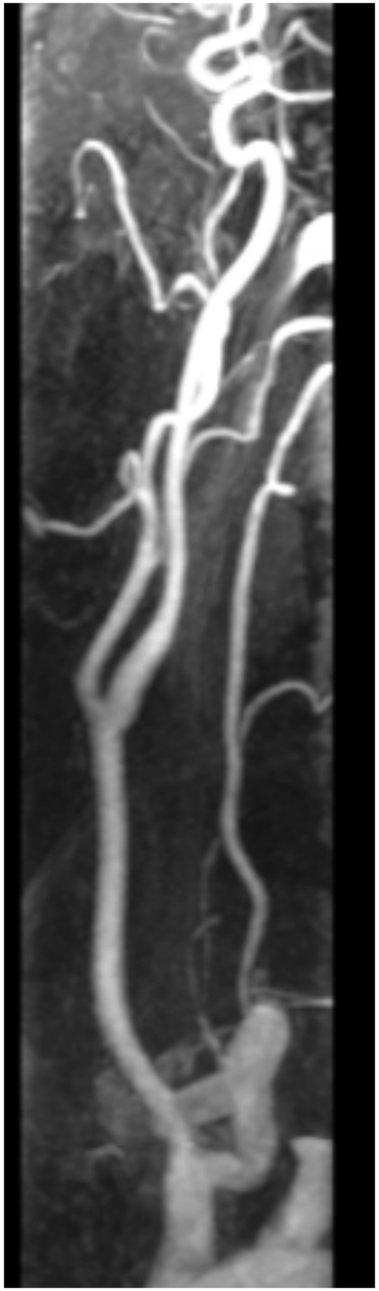

39d Score

0-30

31-50

51-70

>70

Near occlusion

Occluded

Quality

1

2

3

4

5

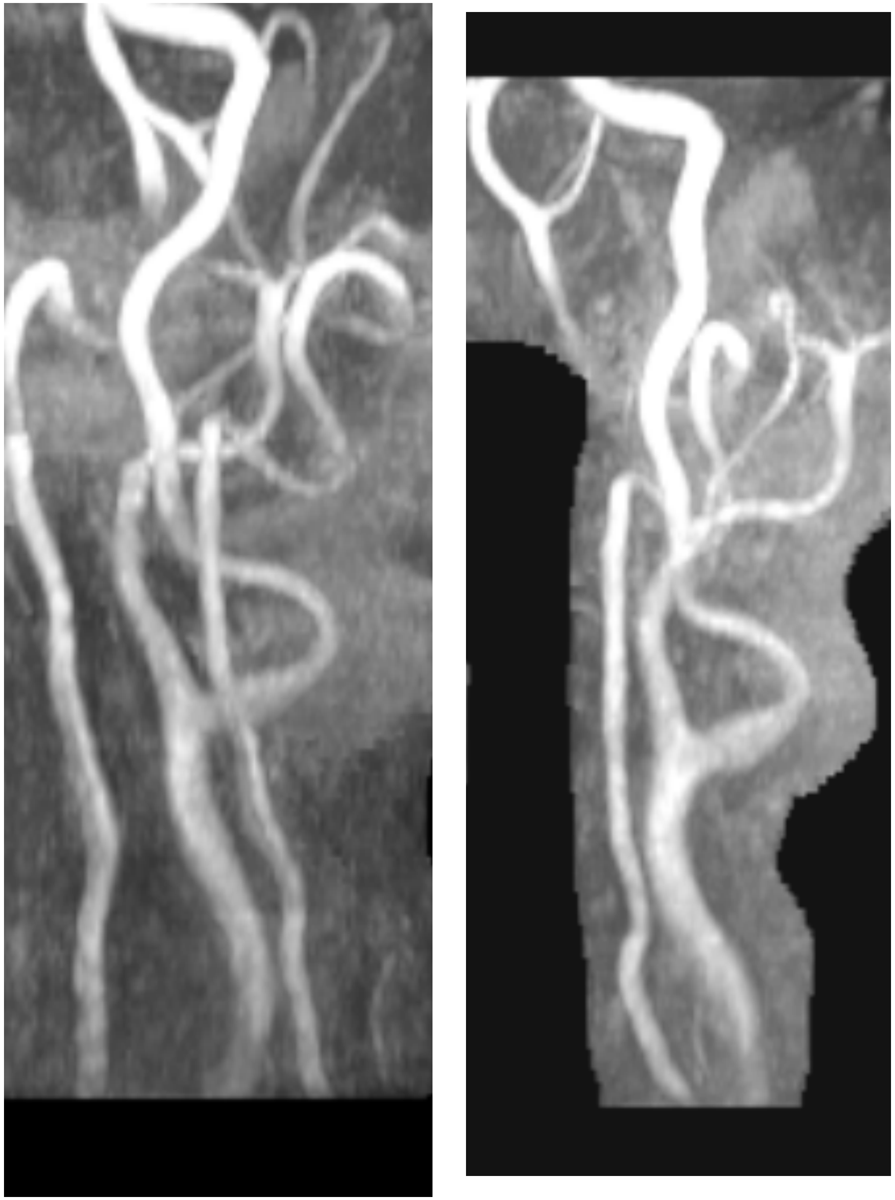

40c Score

0-30

31-50

51-70

>70

Near occlusion

Occluded

Quality

1

2

3

4

5

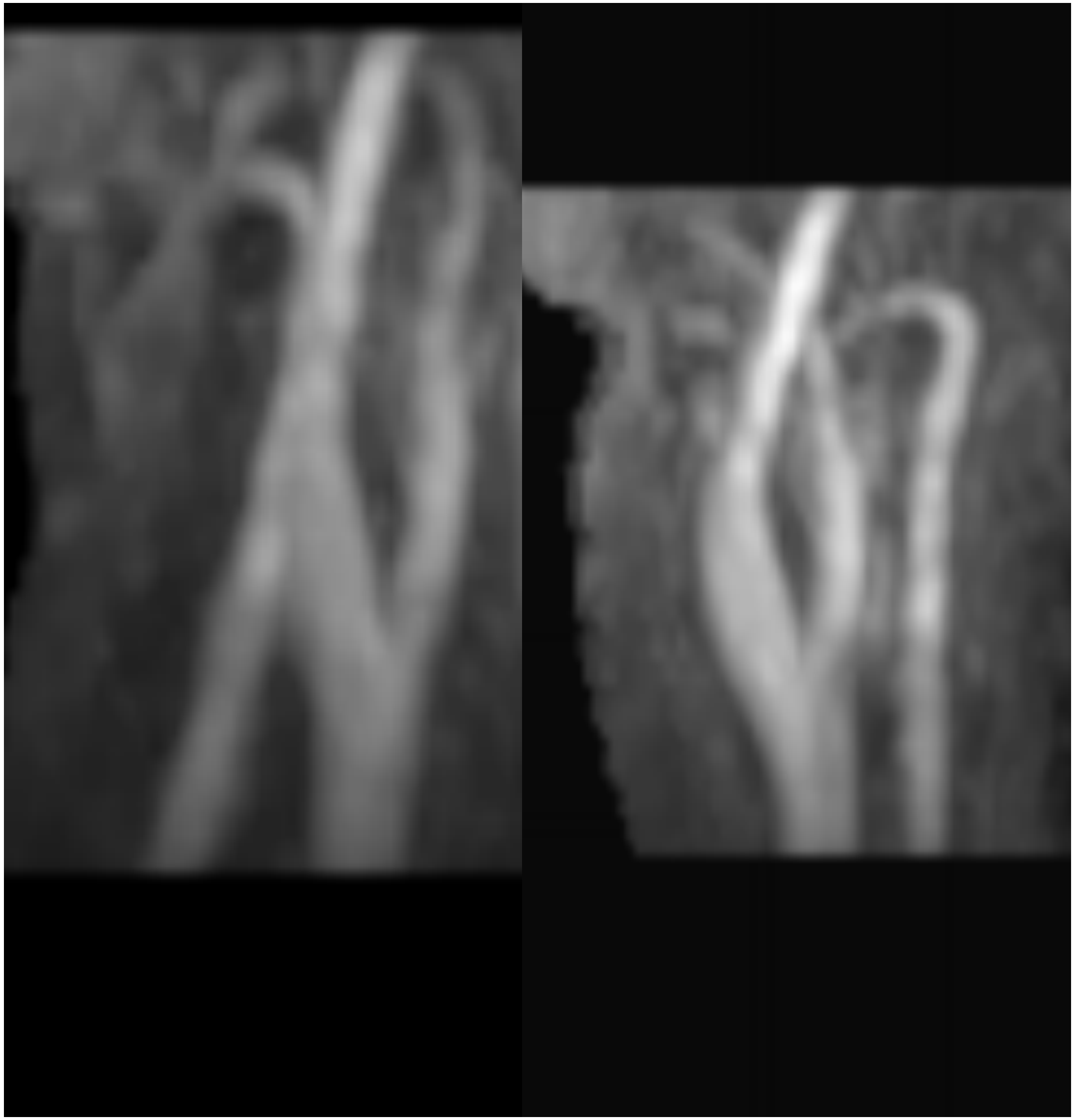

41b Score

0-30

31-50

51-70

>70

Near occlusion

Occluded

Quality

1

2

3

4

5

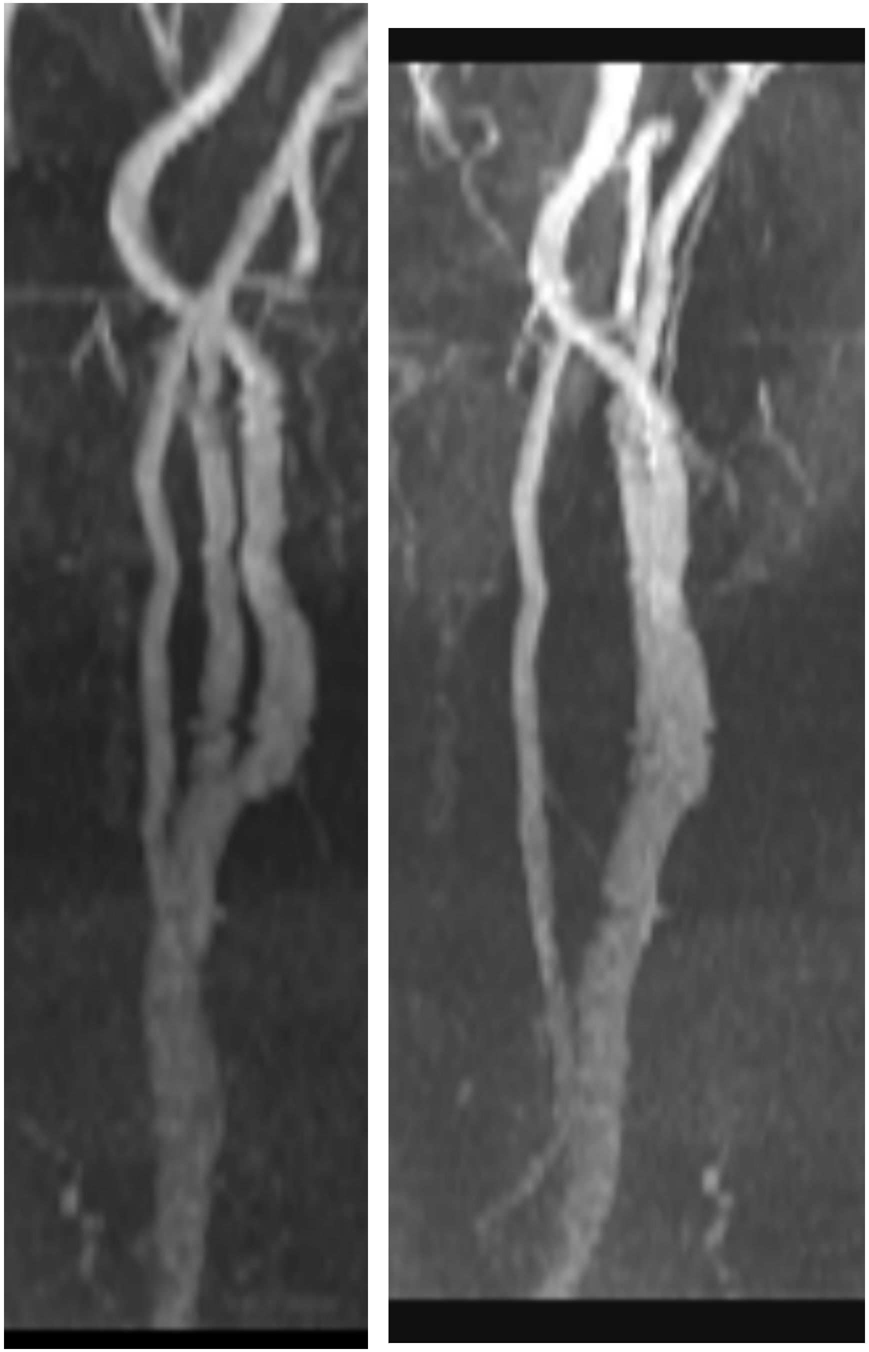

42a Score

0-30

31-50

51-70

>70

Near occlusion

Occluded

Quality

1

2

3

4

5

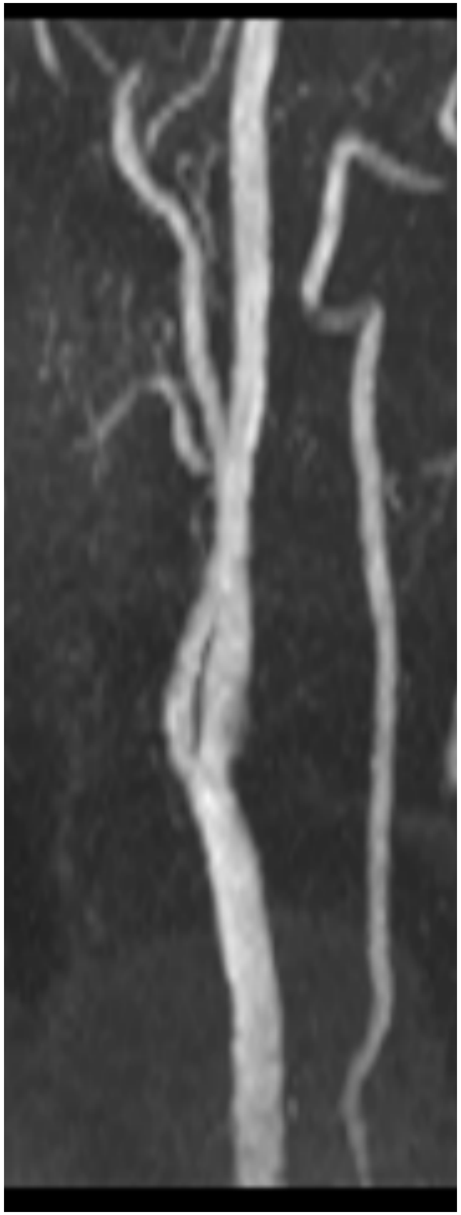

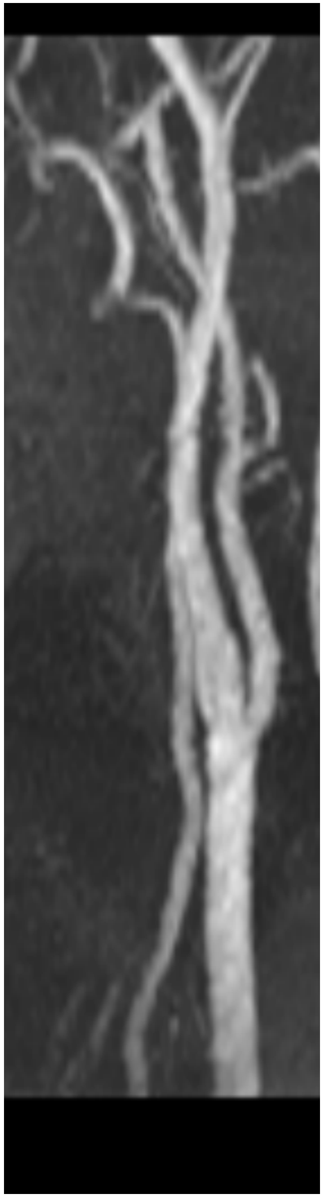

42f Score

0-30

31-50

51-70

>70

Near occlusion

Occluded

Quality

1

2

3

4

5

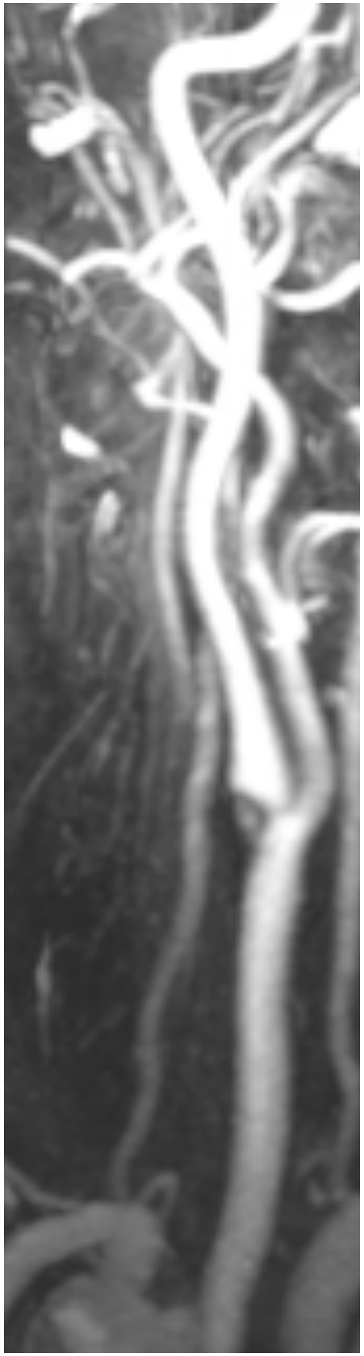

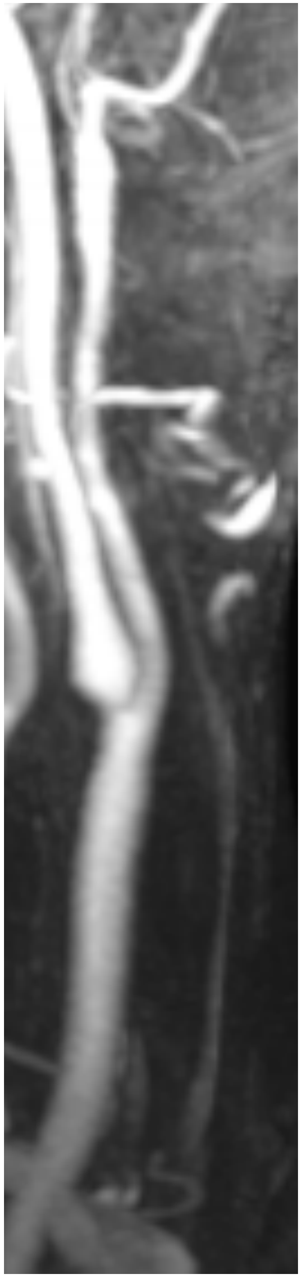

43d Score

0-30

31-50

51-70

>70

Near occlusion

Occluded

Quality

1

2

3

4

5

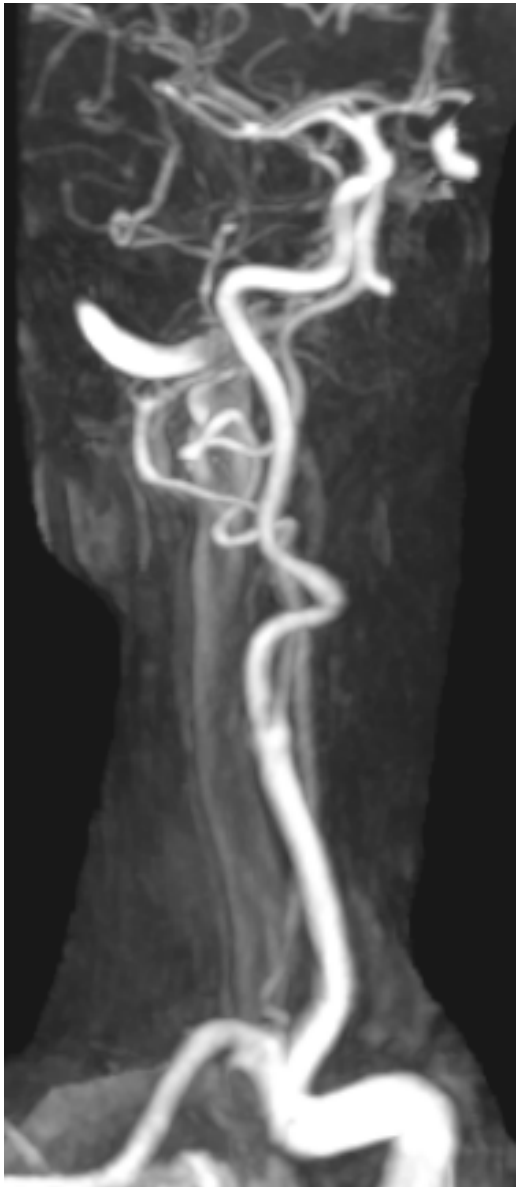

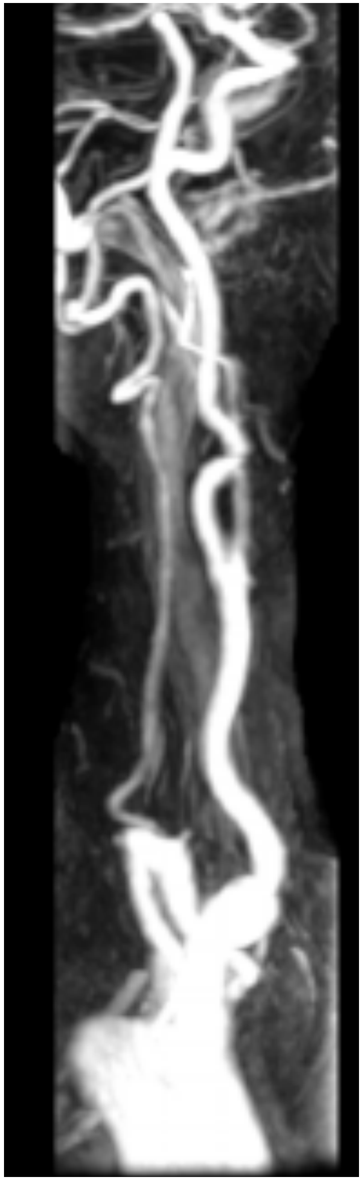

44d Score

0-30

31-50

51-70

>70

Near occlusion

Occluded

Quality

1

2

3

4

5

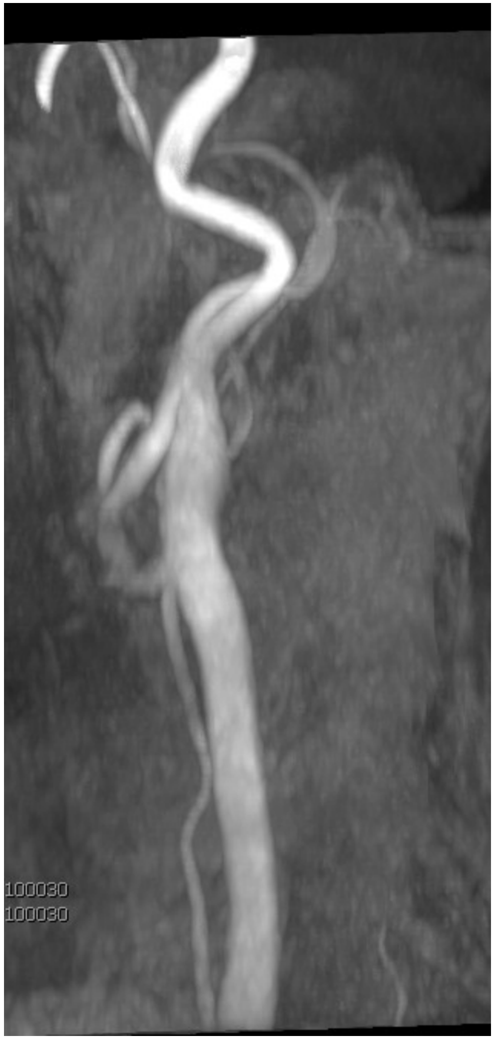

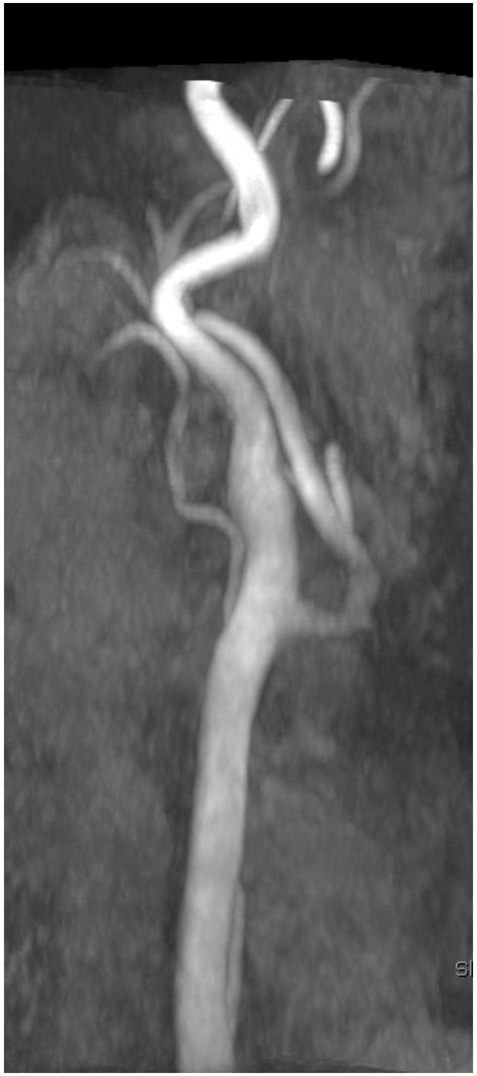

45c Score

0-30

31-50

51-70

>70

Near occlusion

Occluded

Quality

1

2

3

4

5

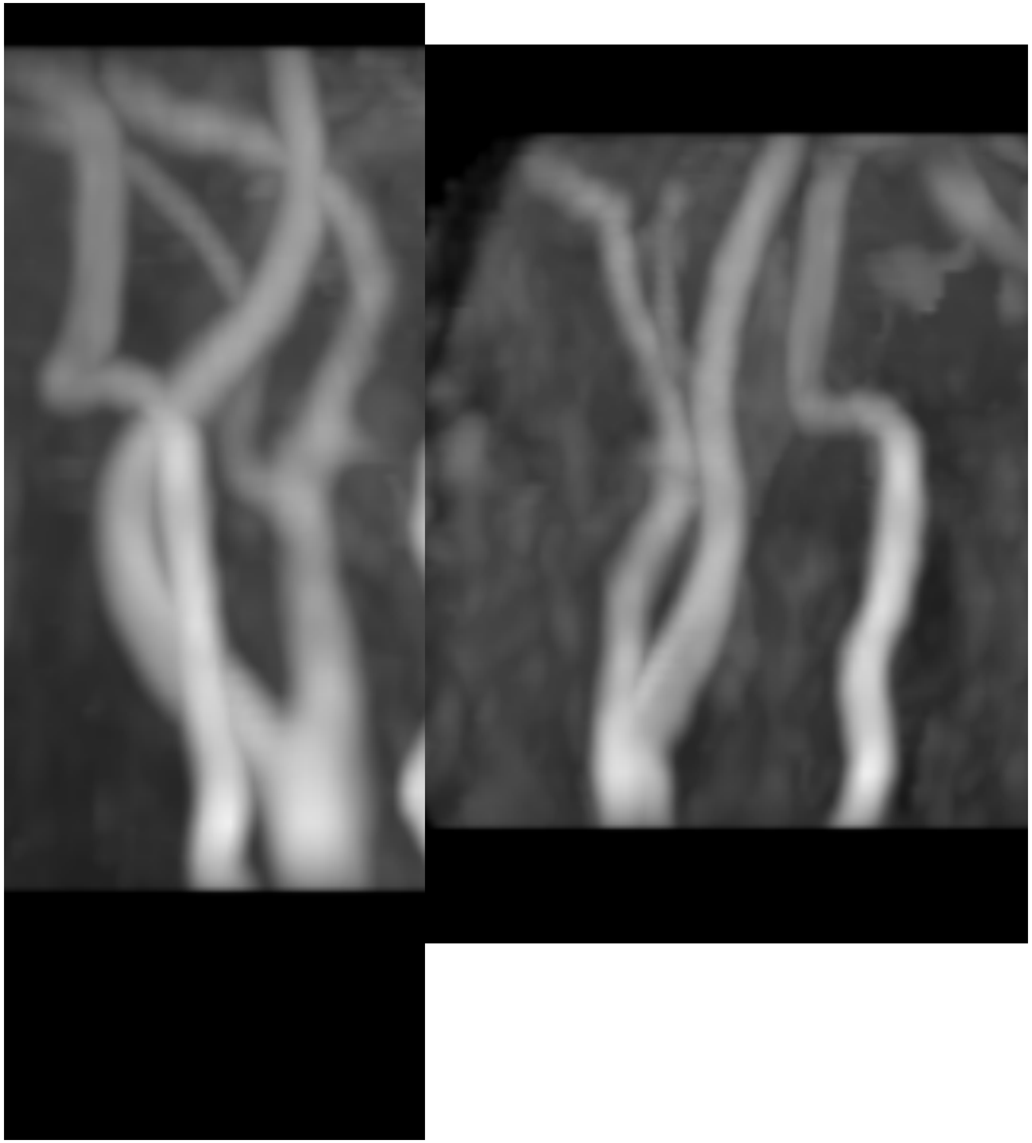

46b Score

0-30

31-50

51-70

>70

Near occlusion

Occluded

Quality

1

2

3

4

5

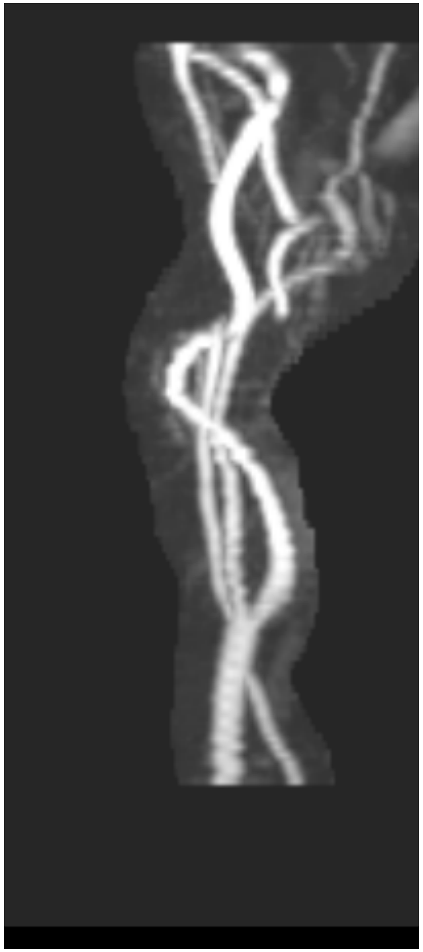

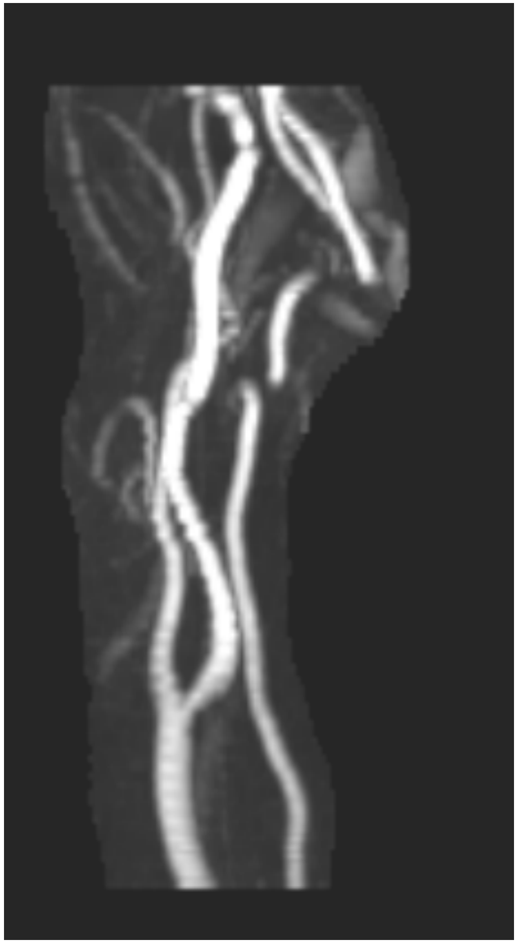

47a Score

0-30

31-50

51-70

>70

Near occlusion

Occluded

Quality

1

2

3

4

5

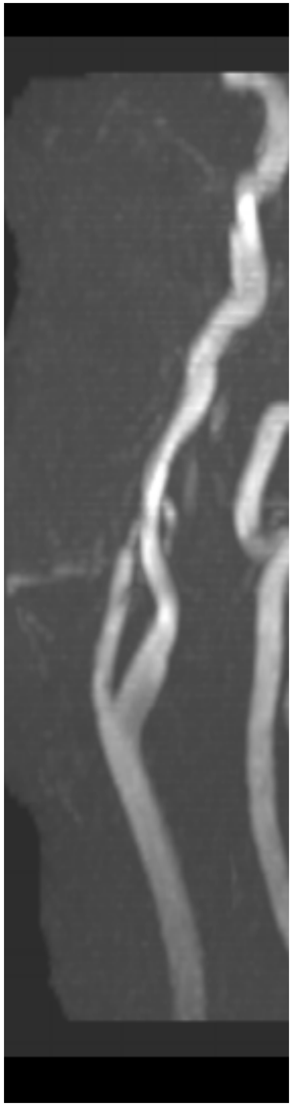

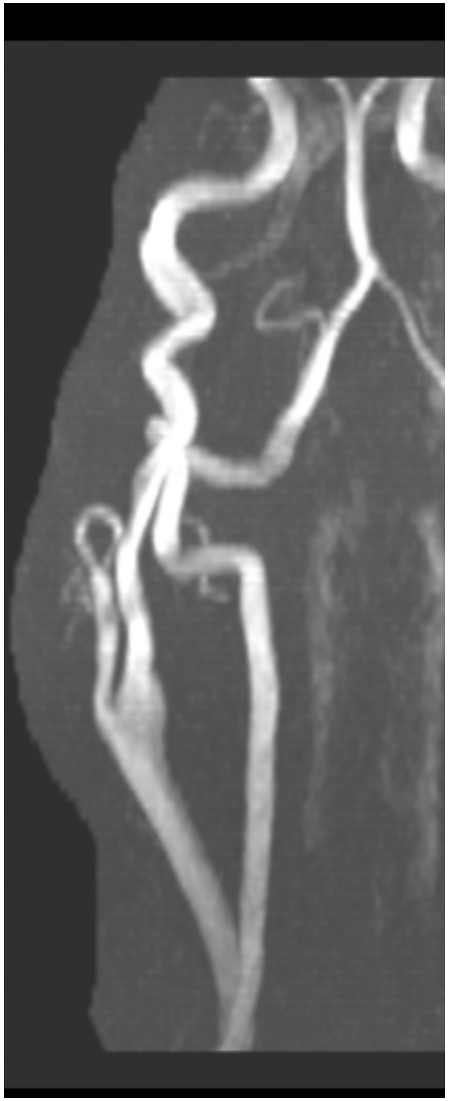

47f Score

0-30

31-50

51-70

>70

Near occlusion

Occluded

Quality

1

2

3

4

5

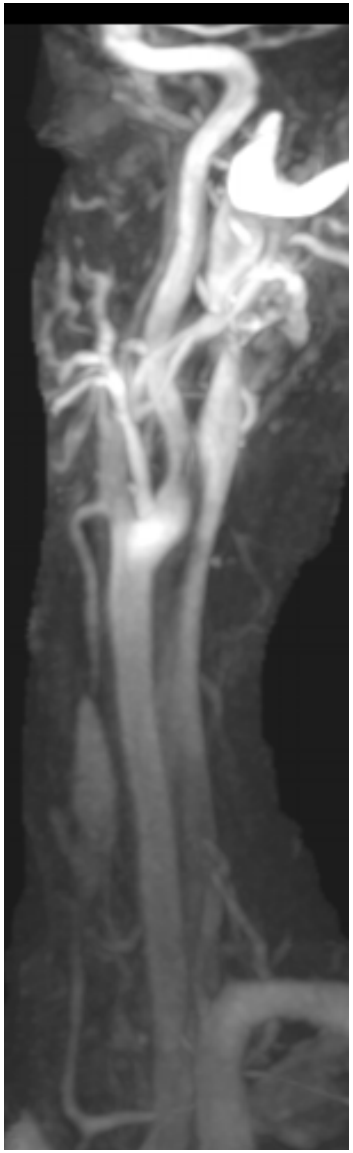

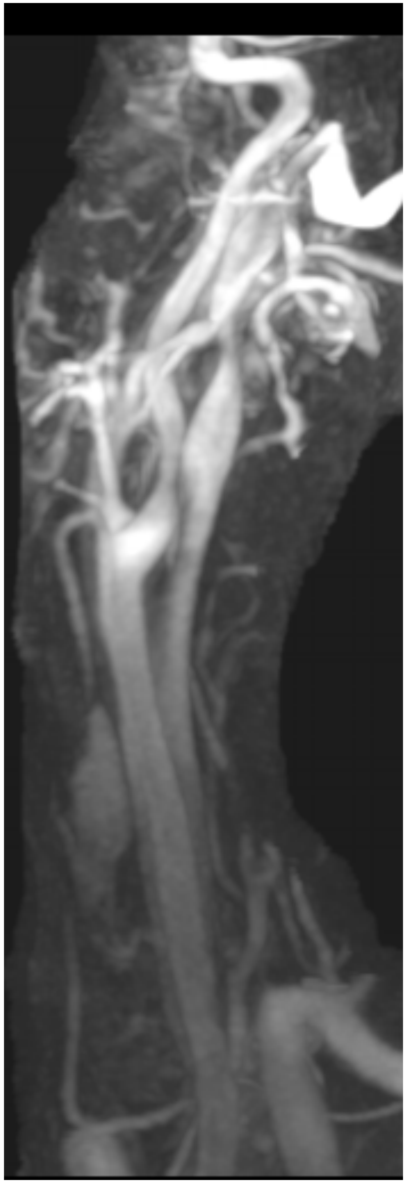

48e Score

0-30

31-50

51-70

>70

Near occlusion

Occluded

Quality

1

2

3

4

5

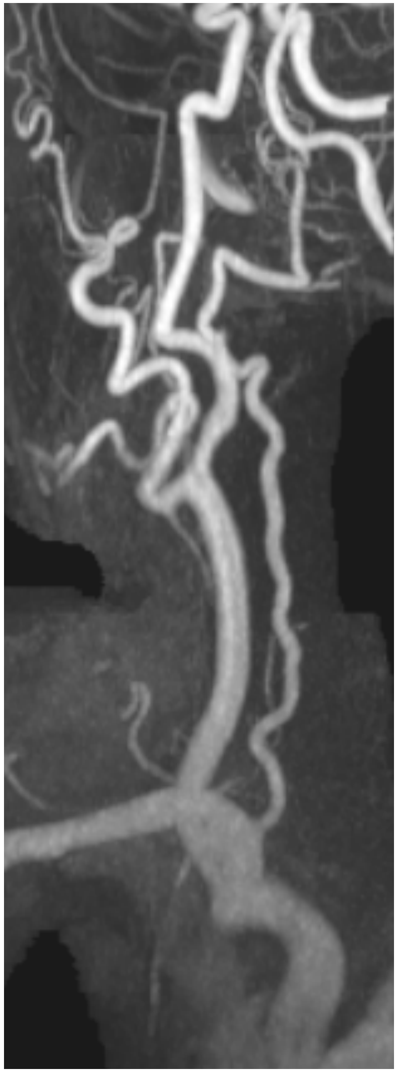

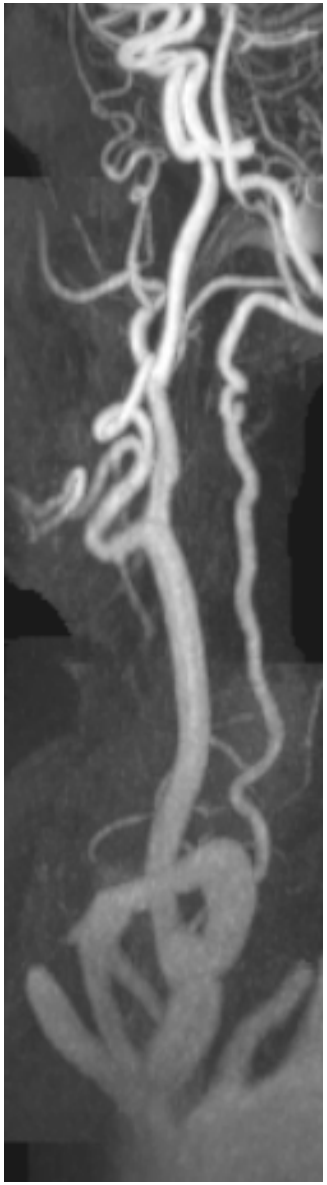

49d Score

0-30

31-50

51-70

>70

Near occlusion

Occluded

Quality

1

2

3

4

5

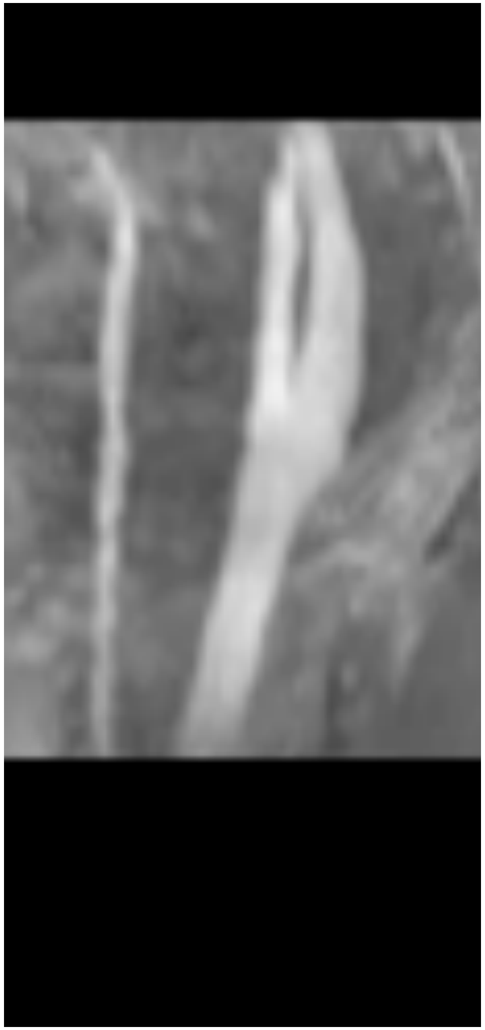

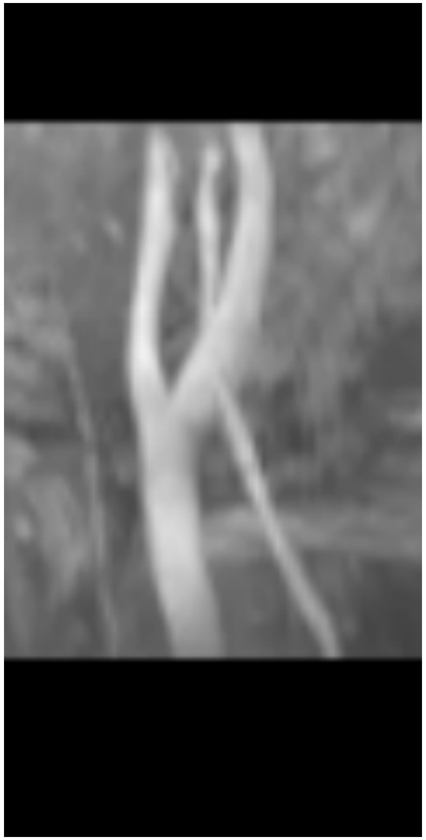

50c Score

0-30

31-50

51-70

>70

Near occlusion

Occluded

Quality

1

2

3

4

5

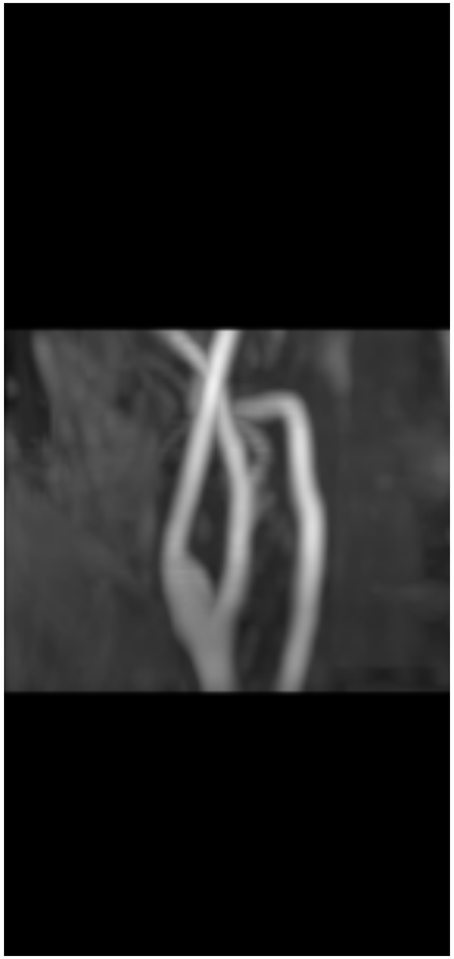

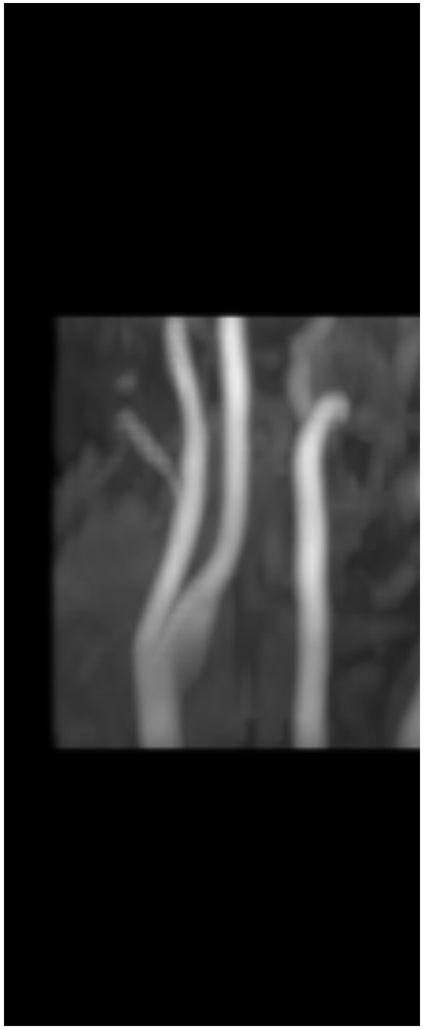

51b Score

0-30

31-50

51-70

>70

Near occlusion

Occluded

Quality

1

2

3

4

5

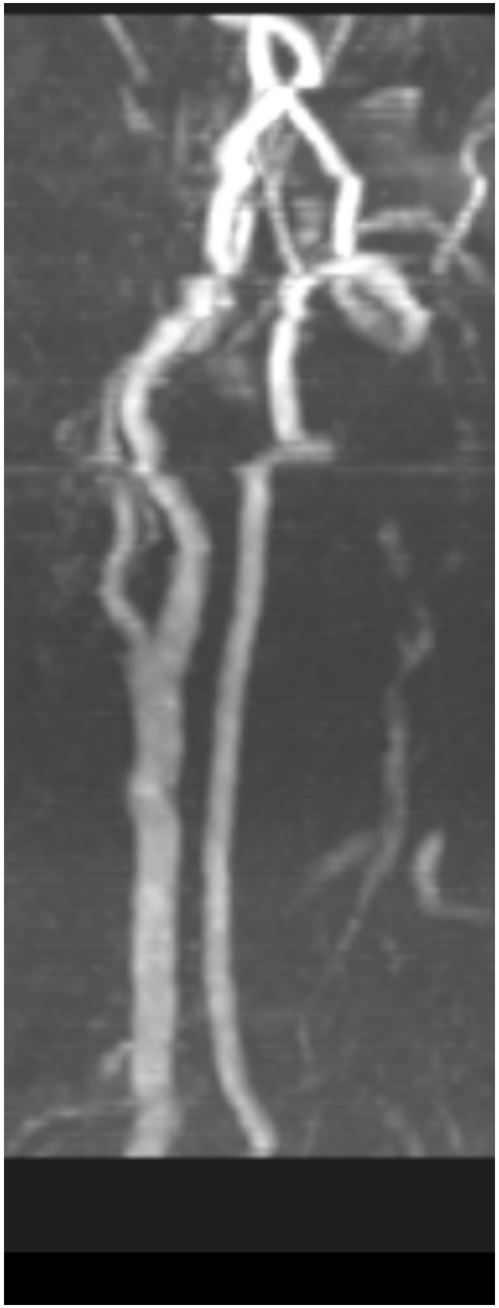

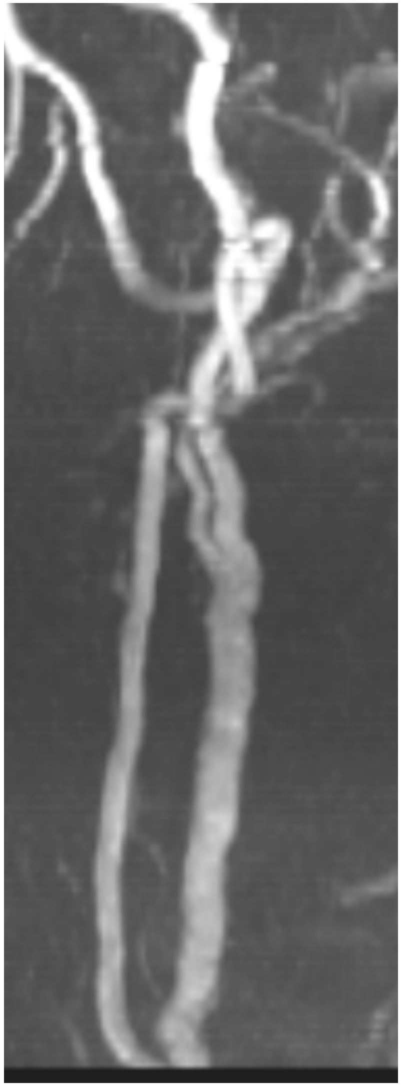

52a Score

0-30

31-50

51-70

>70

Near occlusion

Occluded

Quality

1

2

3

4

5

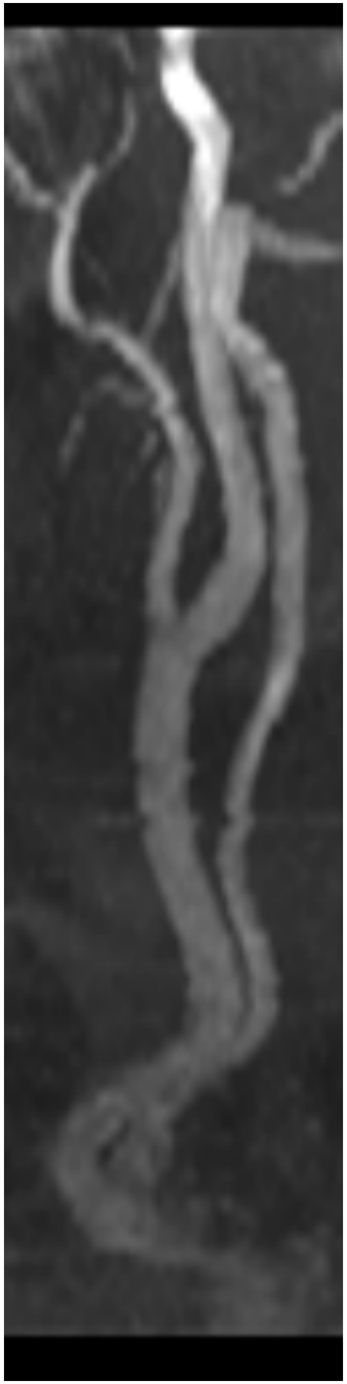

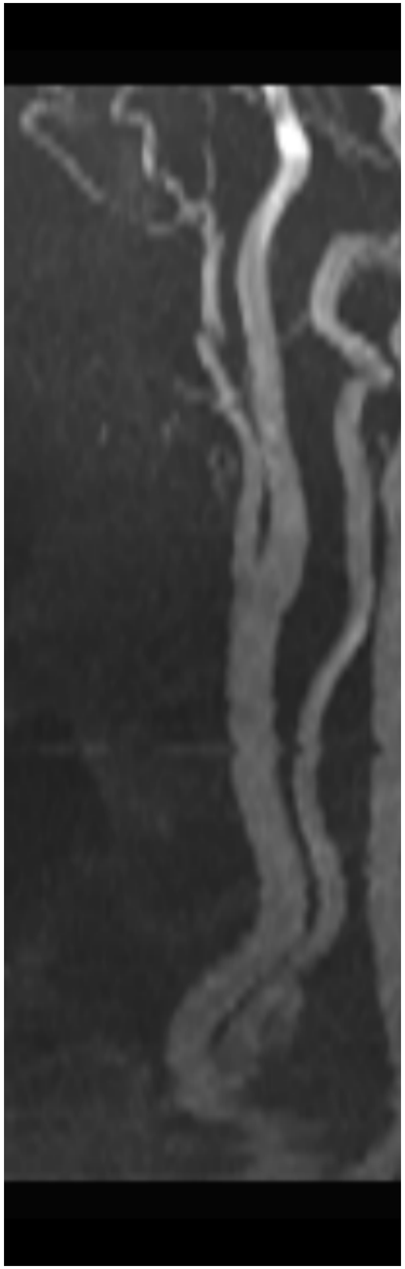

52f Score

0-30

31-50

51-70

>70

Near occlusion

Occluded

Quality

1

2

3

4

5

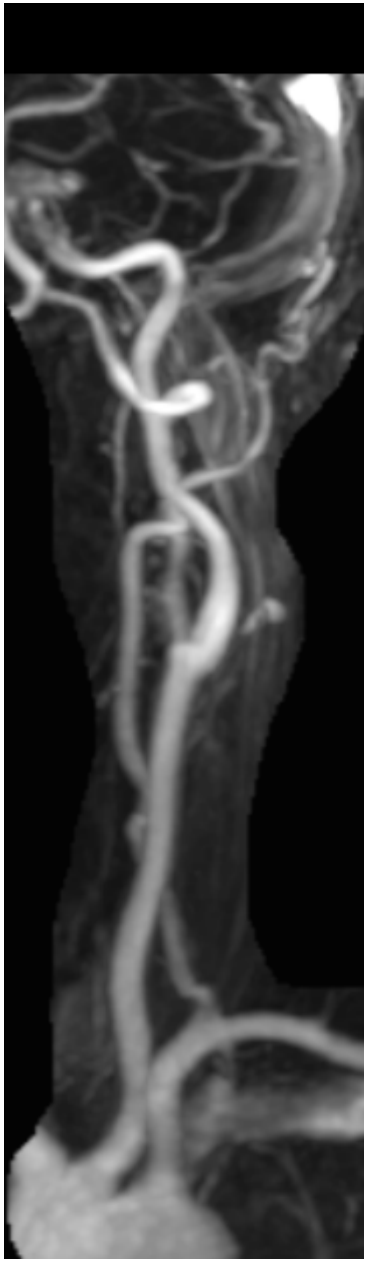

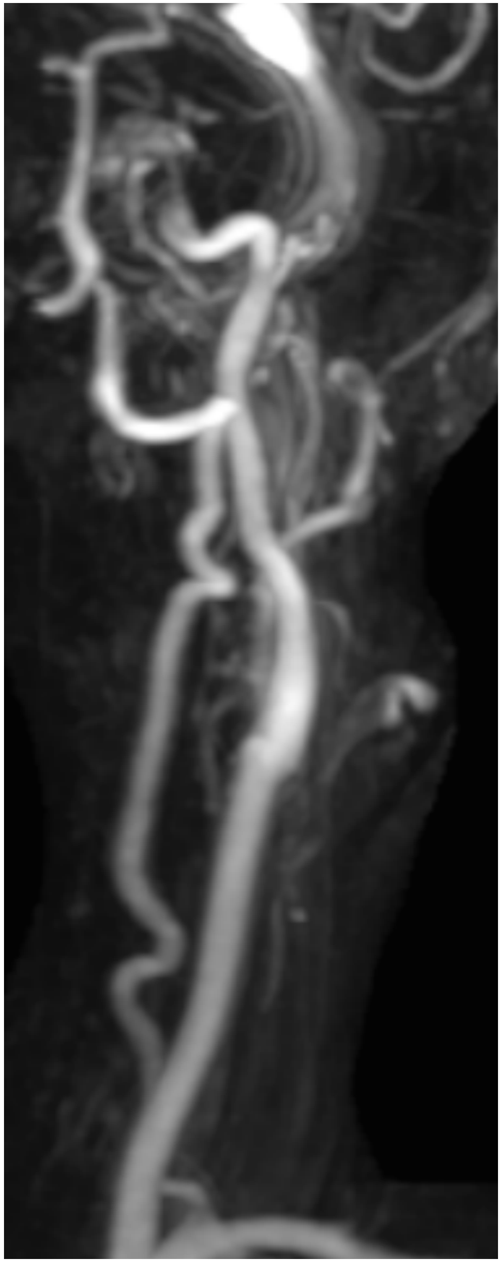

53e Score

0-30

31-50

51-70

>70

Near occlusion

Occluded

Quality

1

2

3

4

5

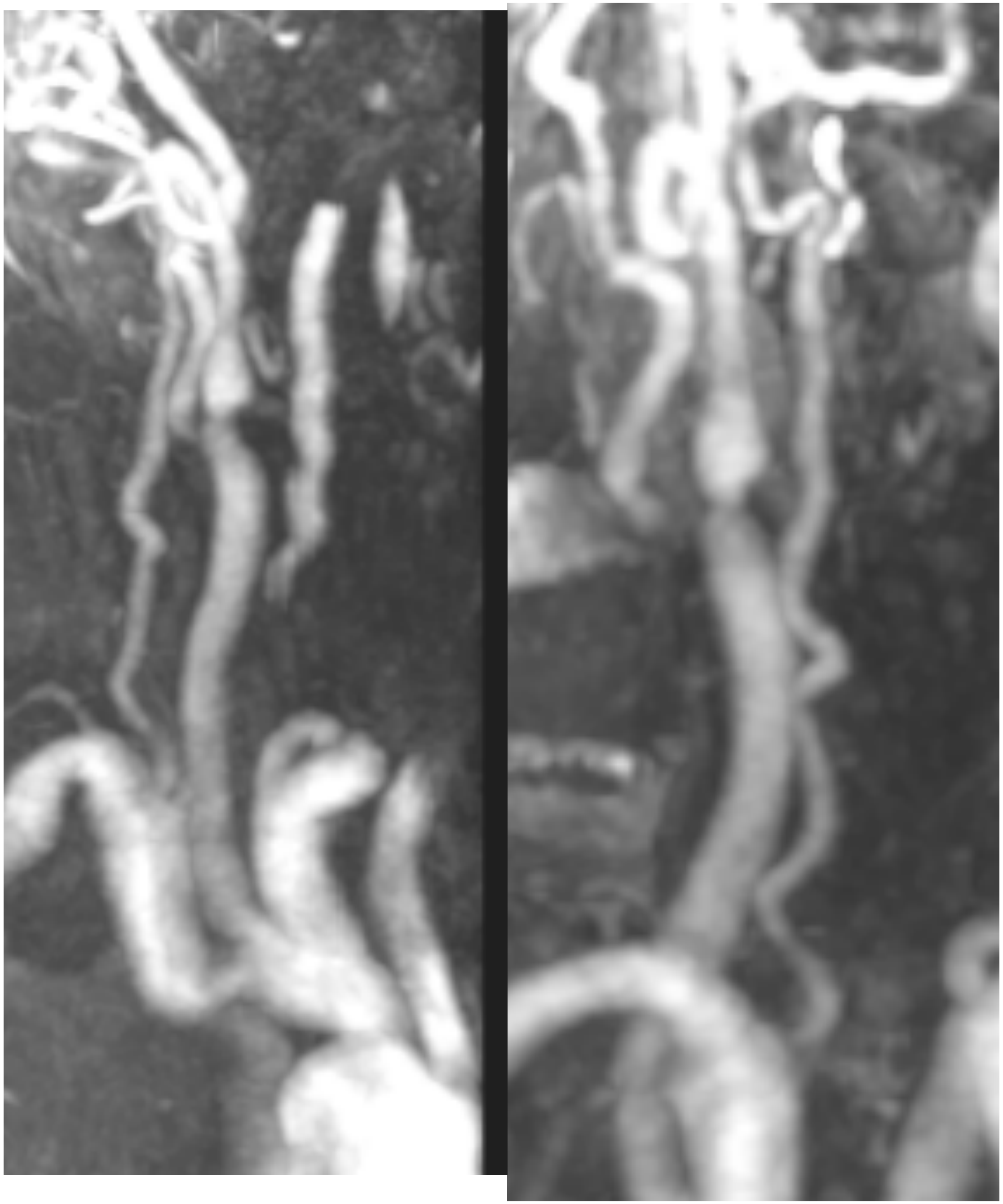

54d Score

0-30

31-50

51-70

>70

Near occlusion

Occluded

Quality

1

2

3

4

5

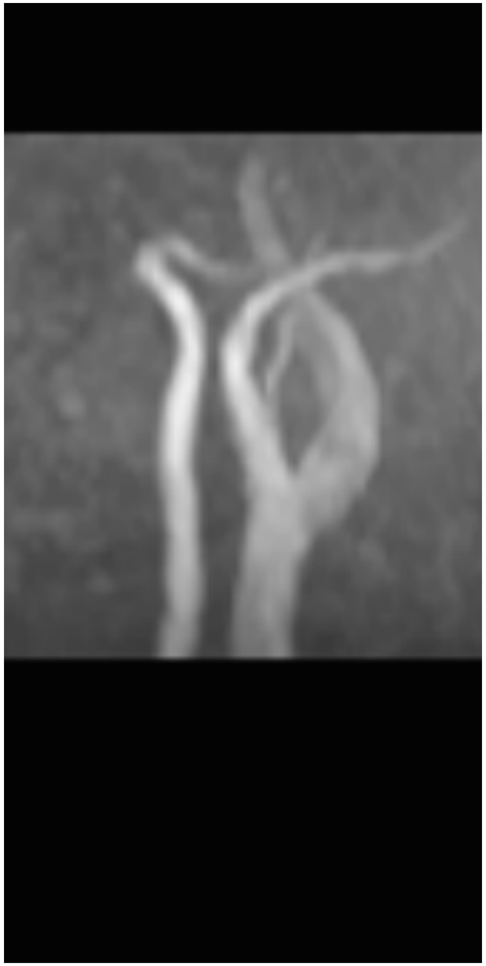

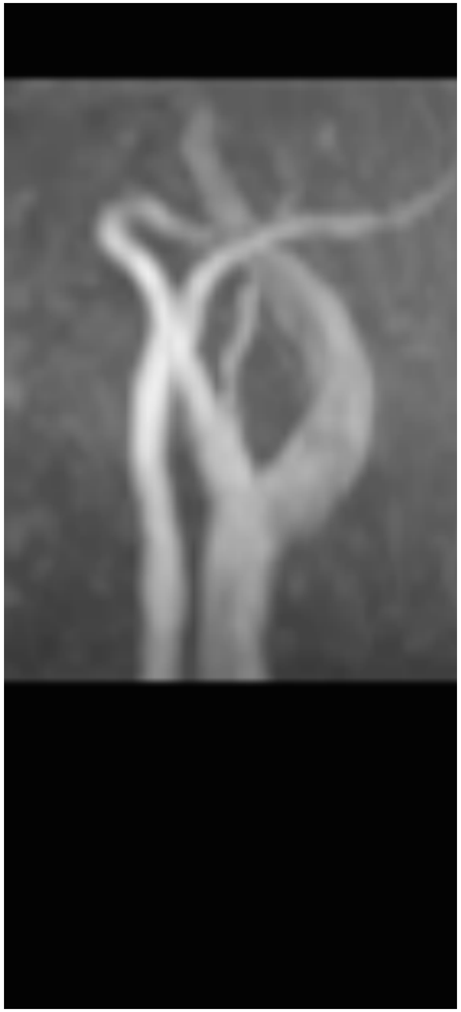

55c Score

0-30

31-50

51-70

>70

Near occlusion

Occluded

Quality

1

2

3

4

5

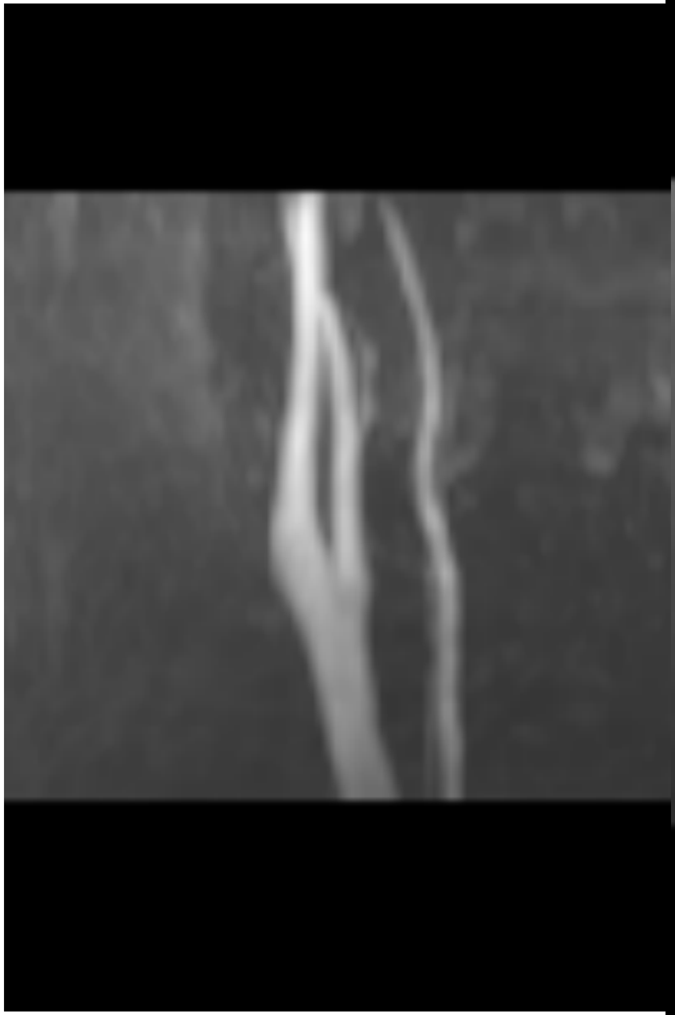

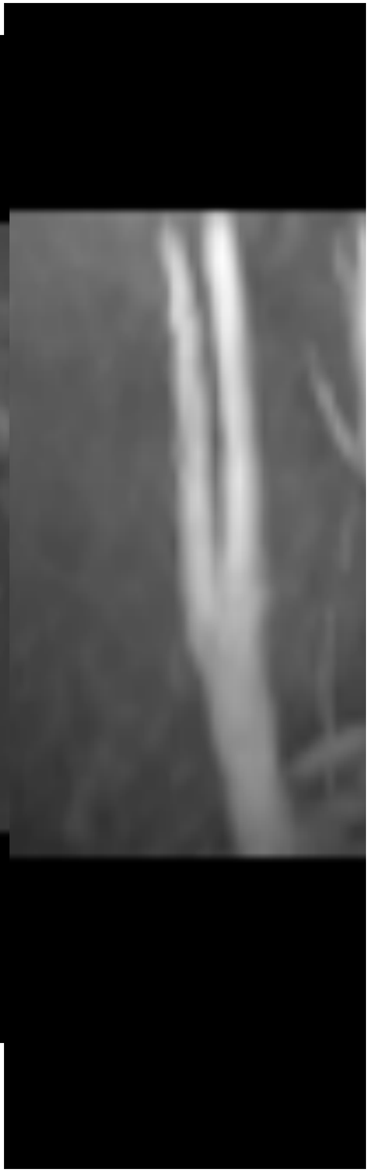

56b Score

0-30

31-50

51-70

>70

Near occlusion

Occluded

Quality

1

2

3

4

5

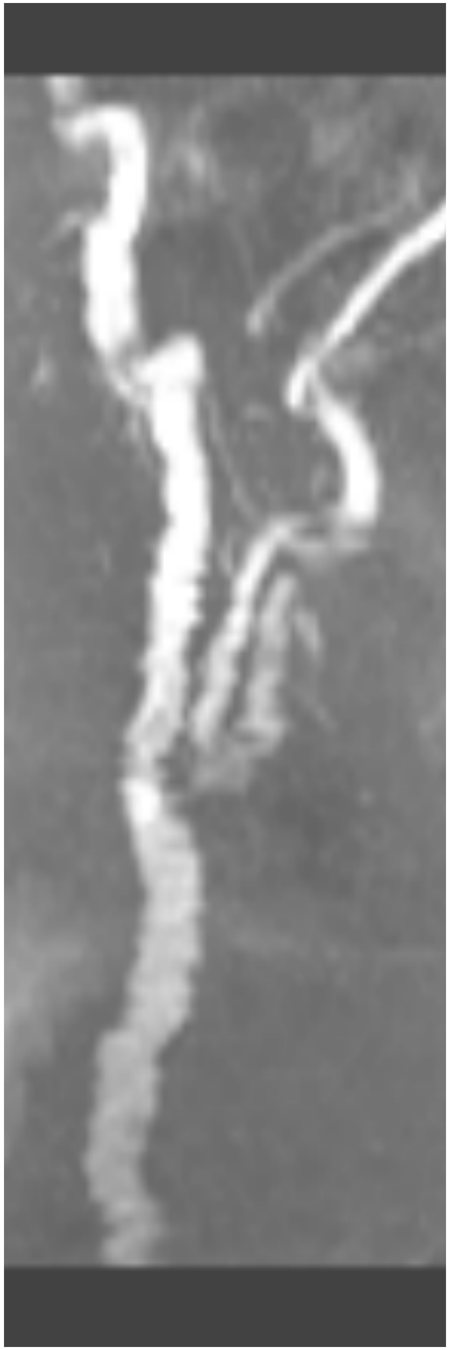

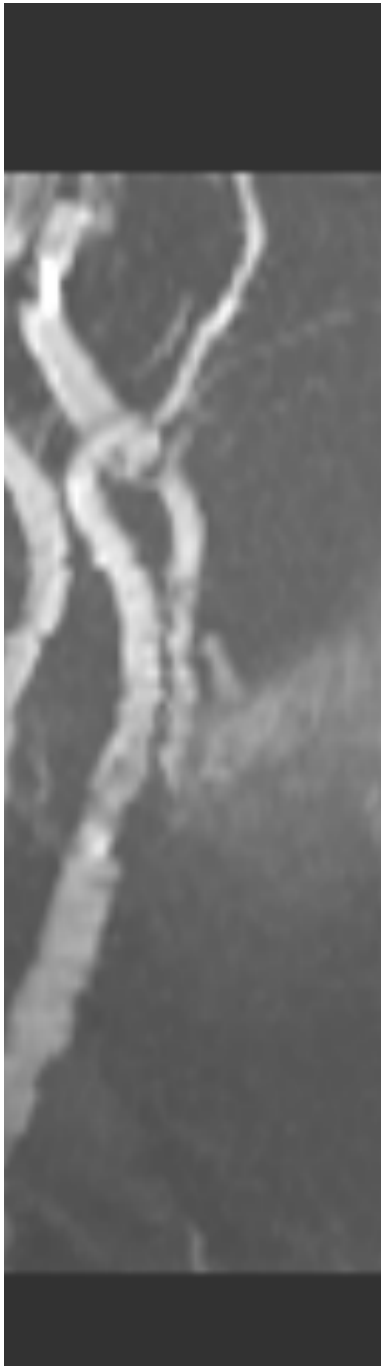

57a Score

0-30

31-50

51-70

>70

Near occlusion

Occluded

Quality

1

2

3

4

5

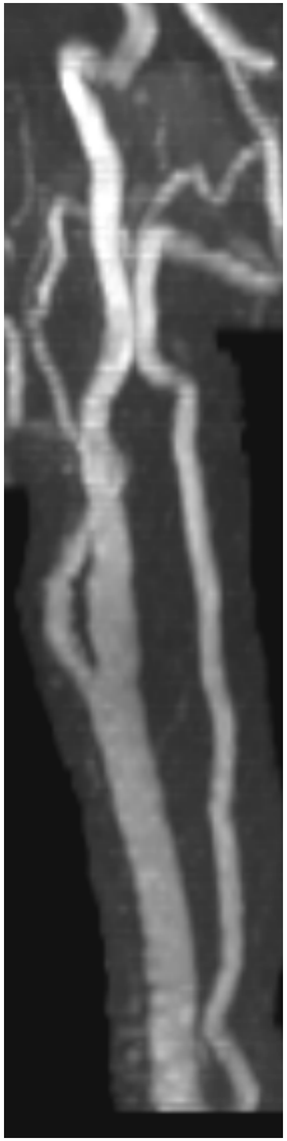

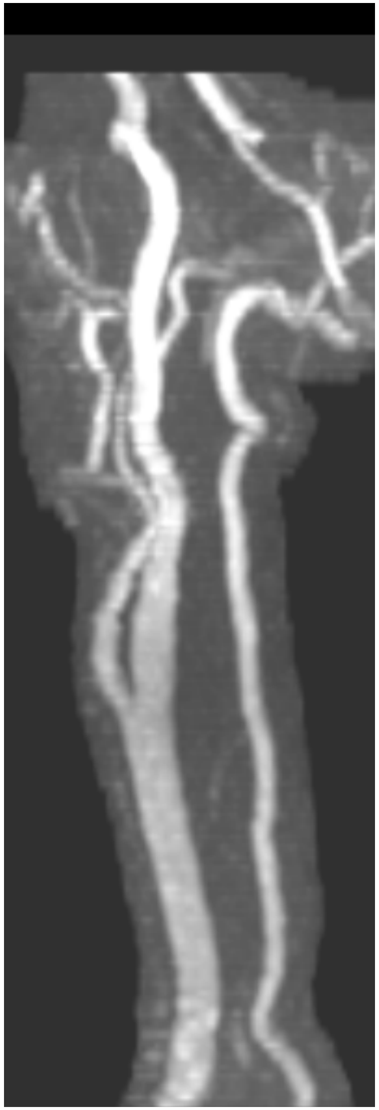

57f Score

0-30

31-50

51-70

>70

Near occlusion

Occluded

Quality

1

2

3

4

5

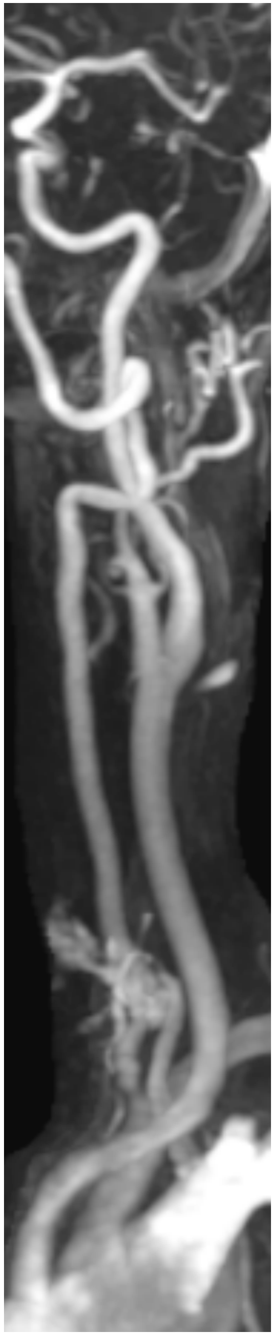

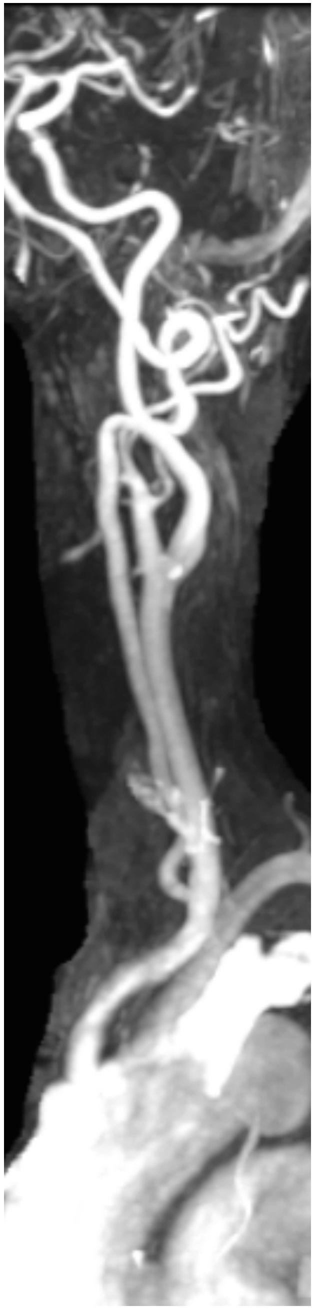

58e Score

0-30

31-50

51-70

>70

Near occlusion

Occluded

Quality

1

2

3

4

5

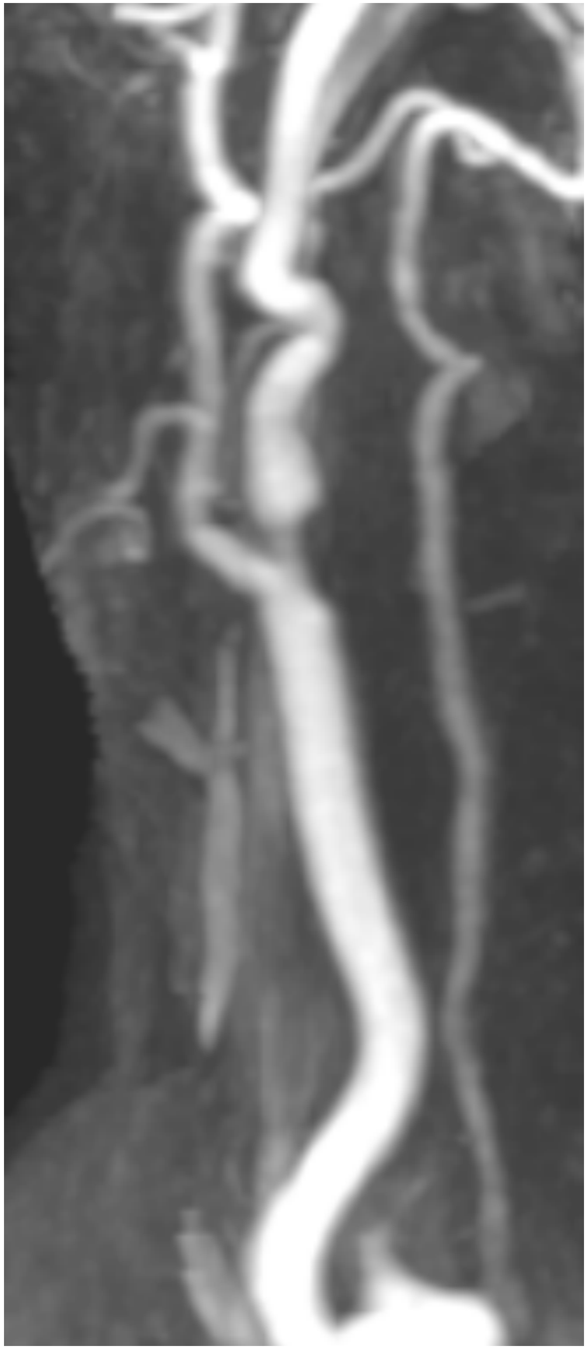

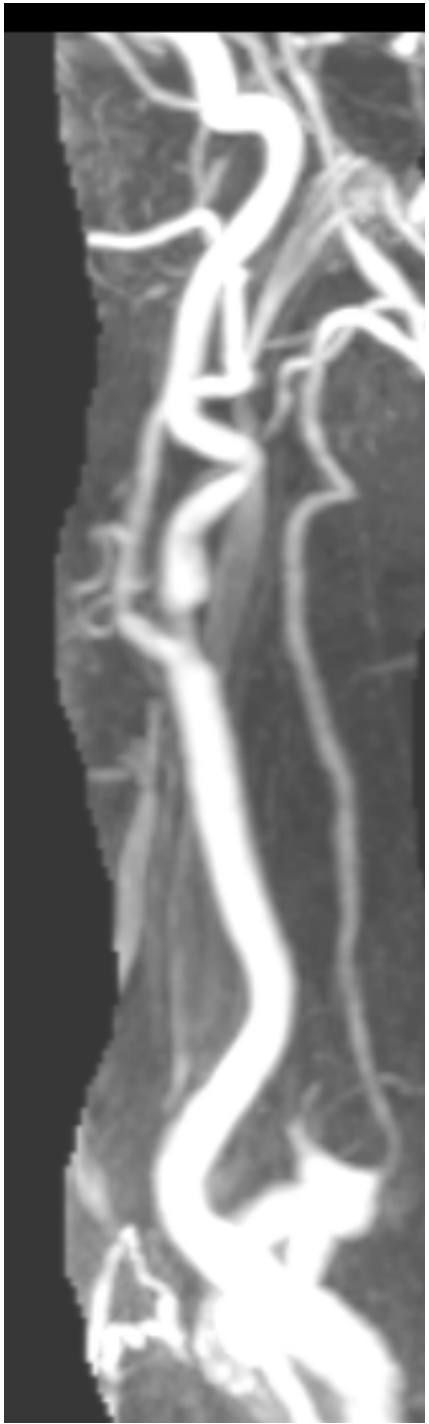

59d Score

0-30

31-50

51-70

>70

Near occlusion

Occluded

Quality

1

2

3

4

5

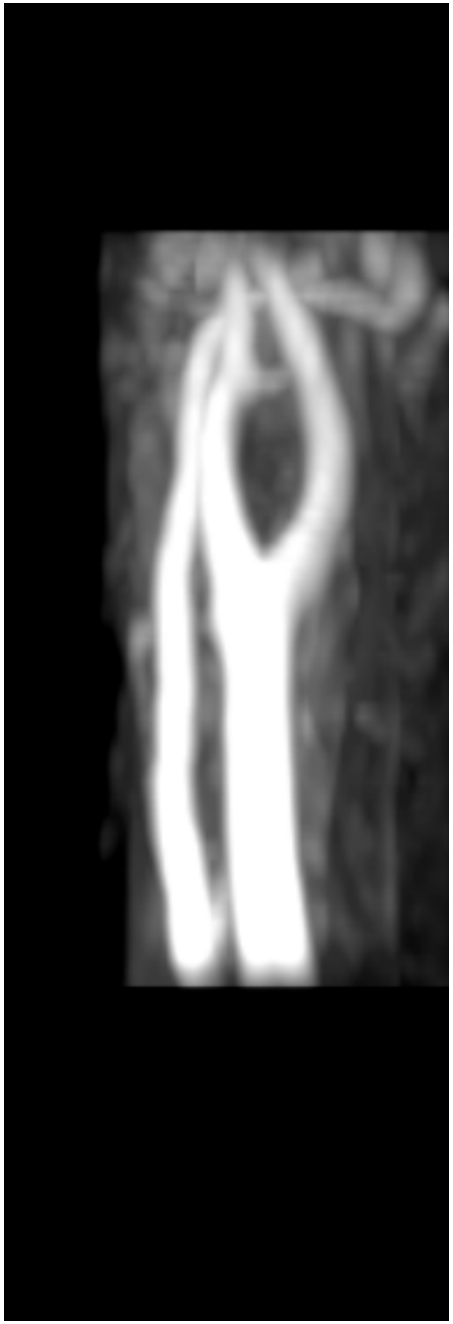

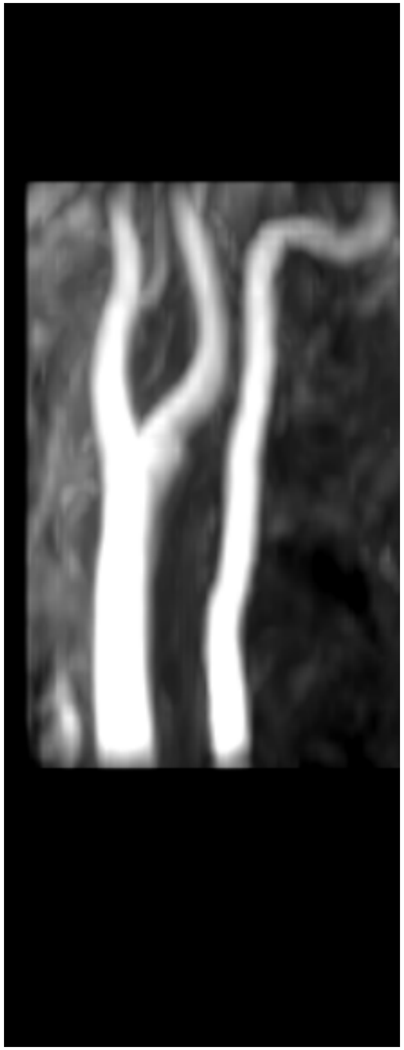

60c Score

0-30

31-50

51-70

>70

Near occlusion

Occluded

Quality

1

2

3

4

5

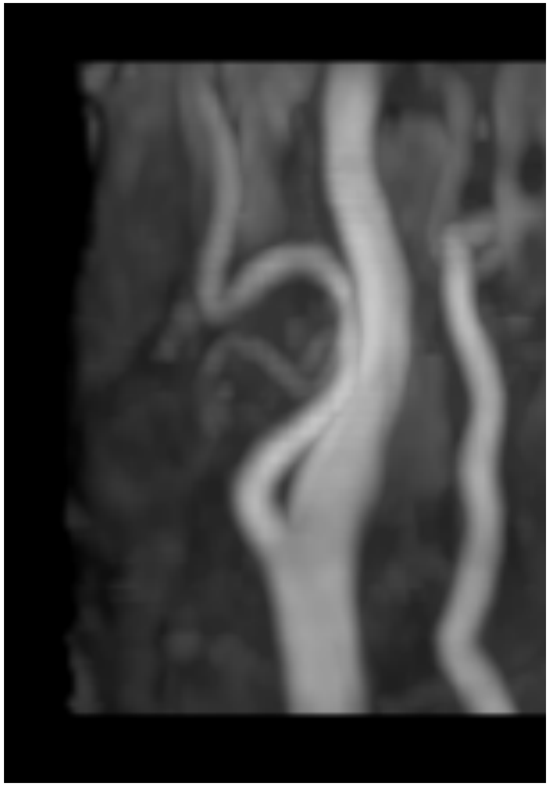

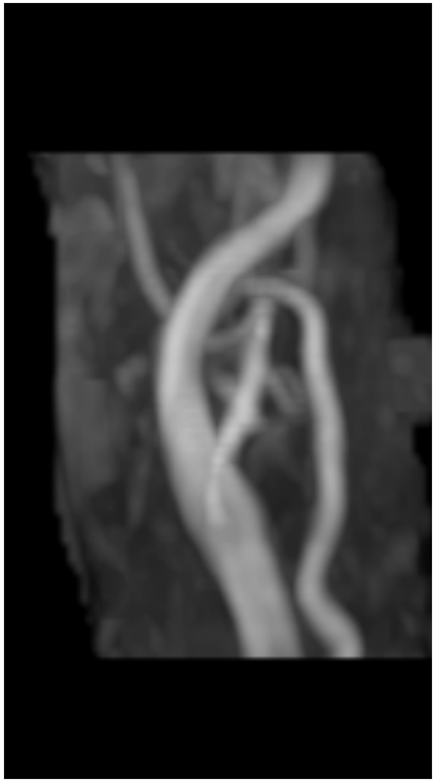

61b Score

0-30

31-50

51-70

>70

Near occlusion

Occluded

Quality

1

2

3

4

5

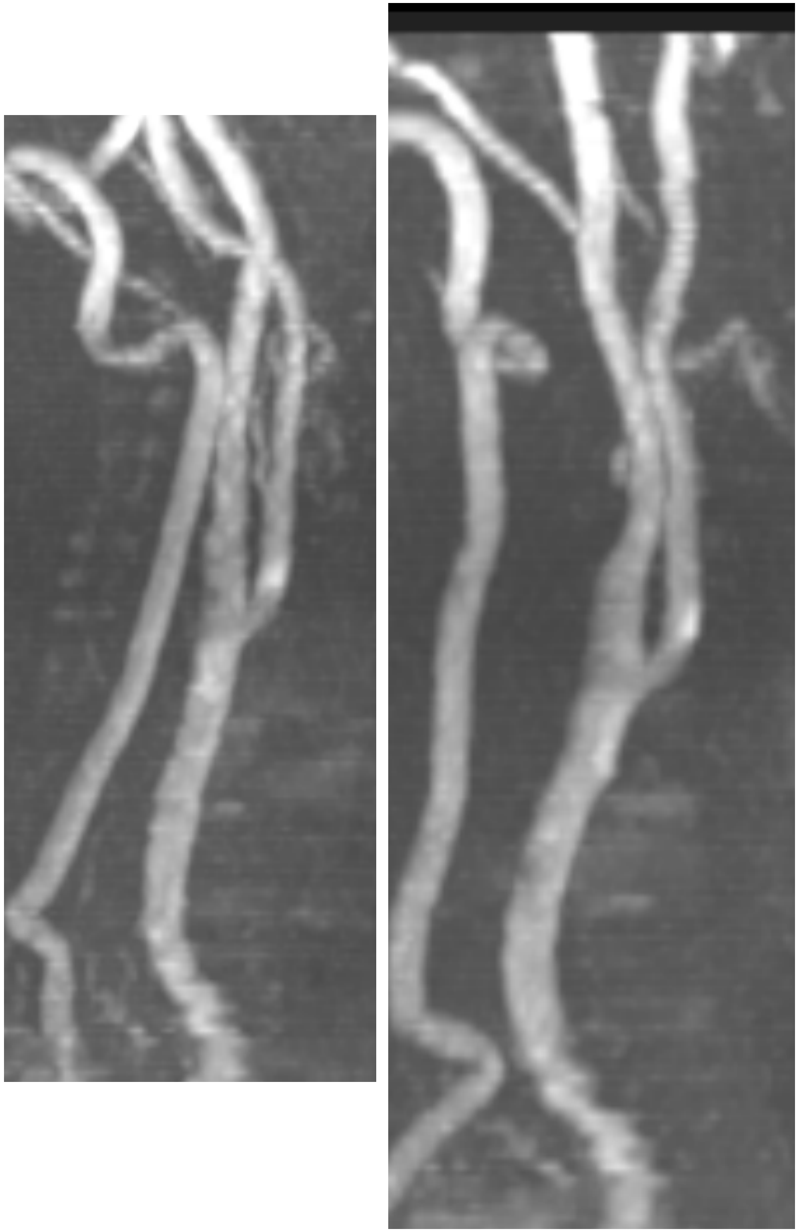

62a Score

0-30

31-50

51-70

>70

Near occlusion

Occluded

Quality

1

2

3

4

5

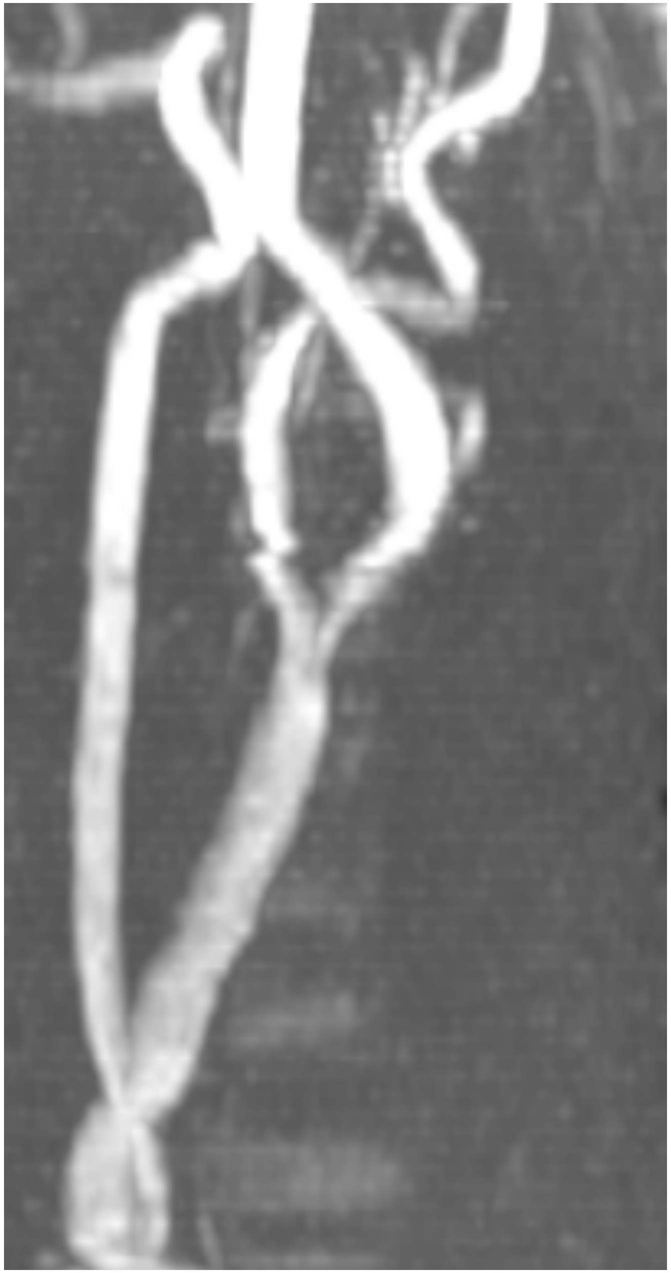

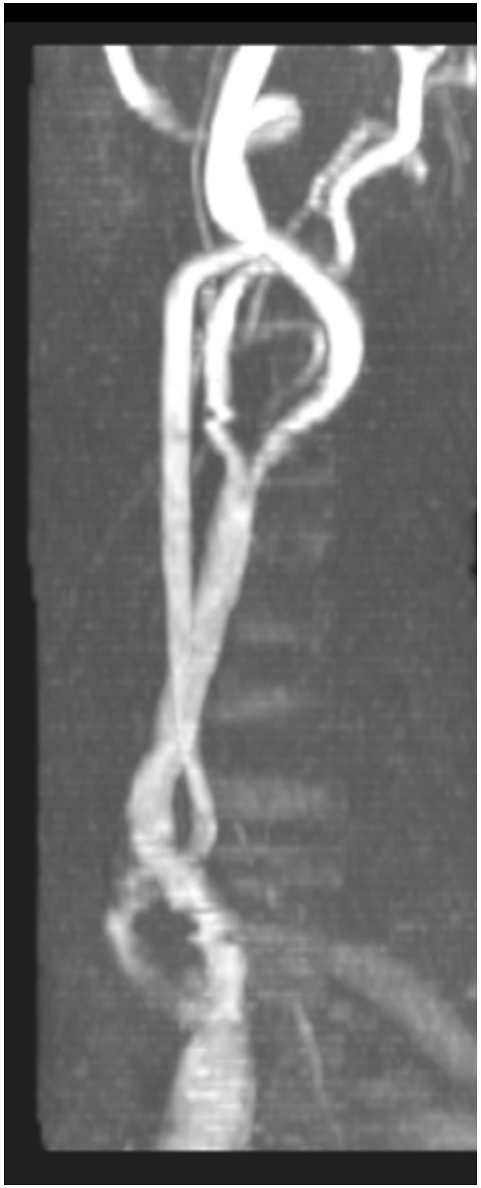

62f Score

0-30

31-50

51-70

>70

Near occlusion

Occluded

Quality

1

2

3

4

5

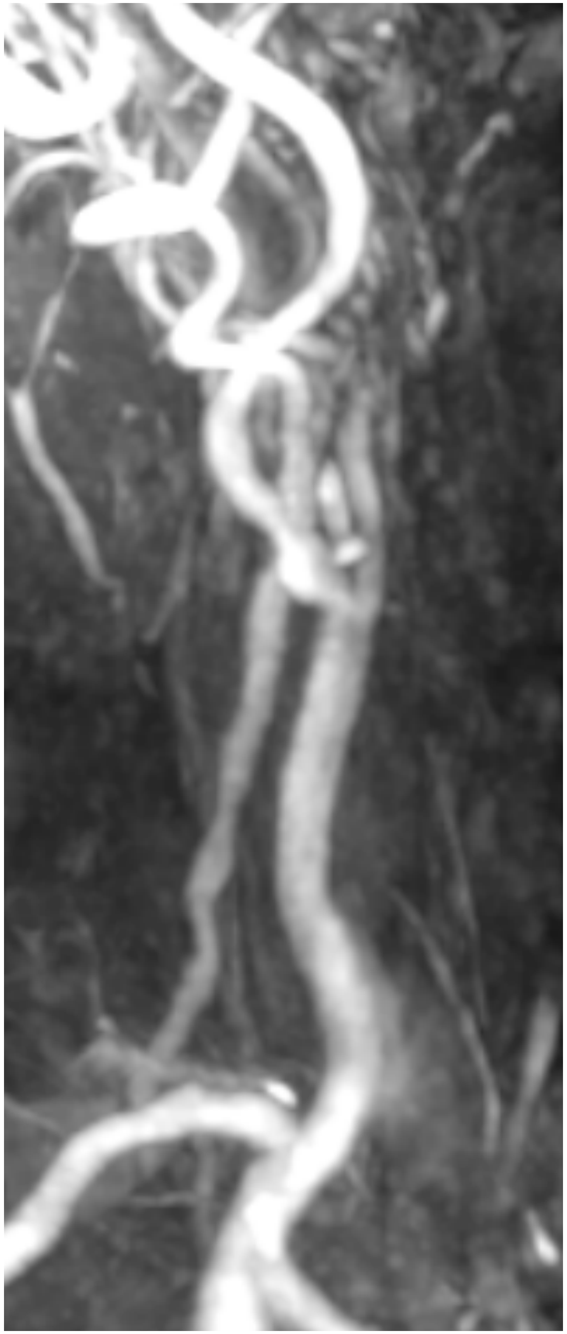

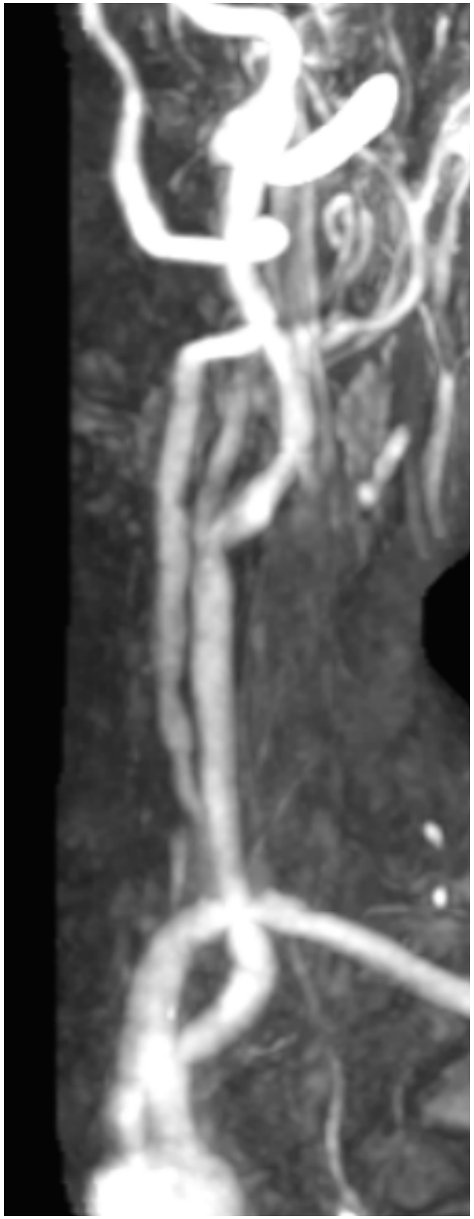

63e Score

0-30

31-50

51-70

>70

Near occlusion

Occluded

Quality

1

2

3

4

5

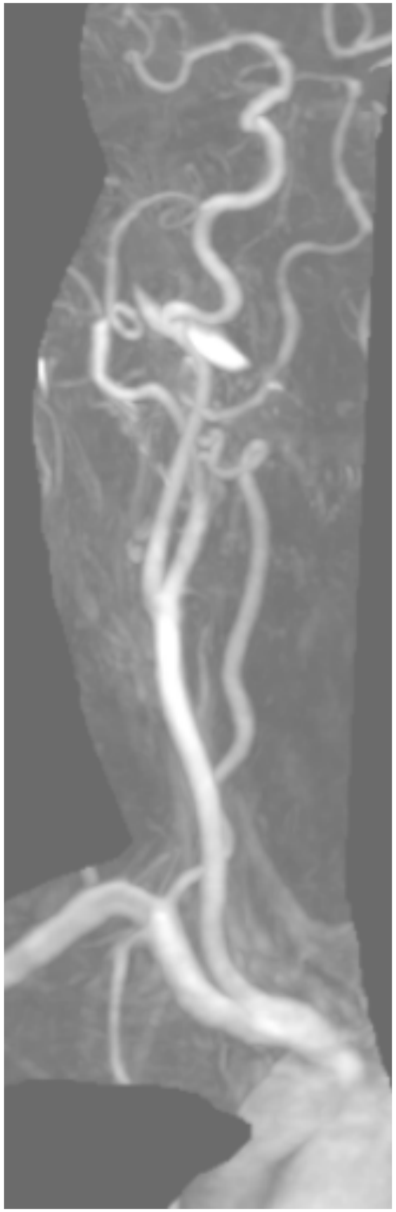

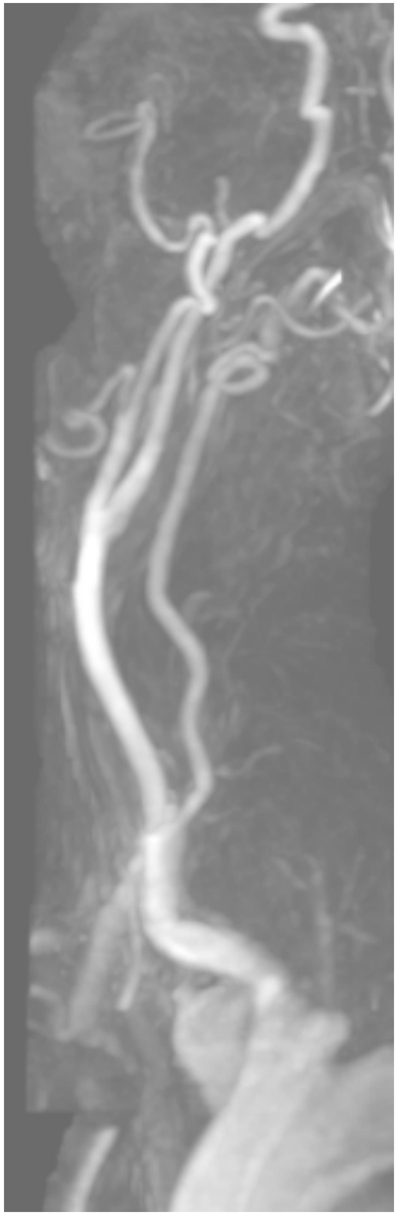

64d Score

0-30

31-50

51-70

>70

Near occlusion

Occluded

Quality

1

2

3

4

5

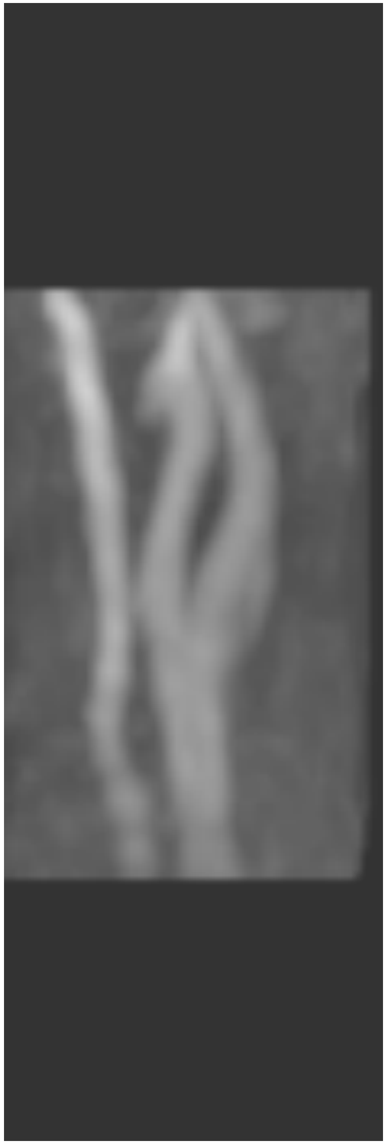

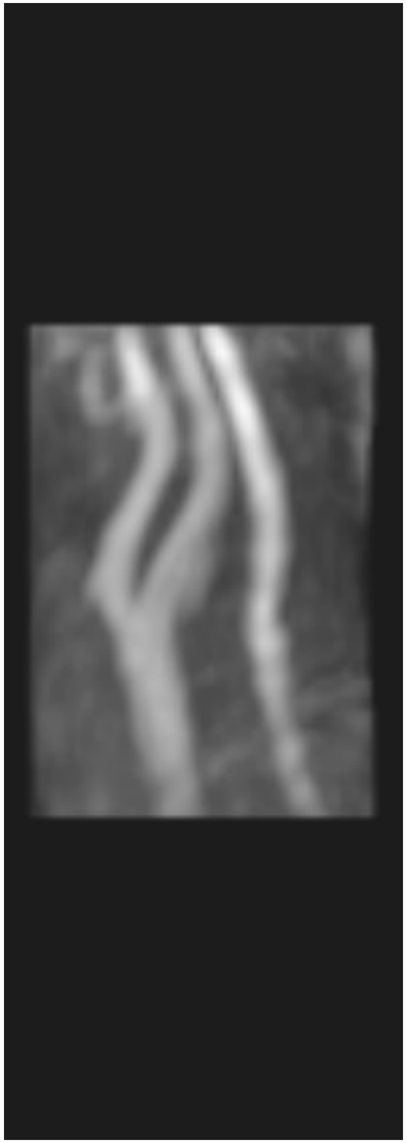

65c Score

0-30

31-50

51-70

>70

Near occlusion

Occluded

Quality

1

2

3

4

5

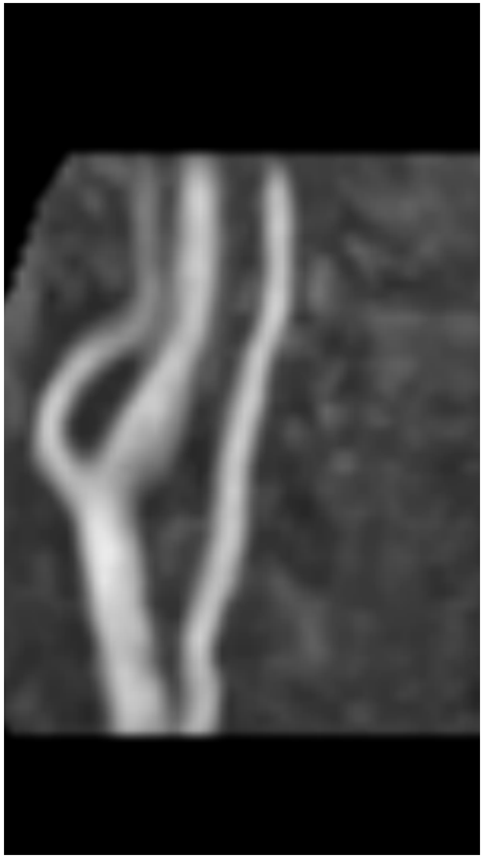

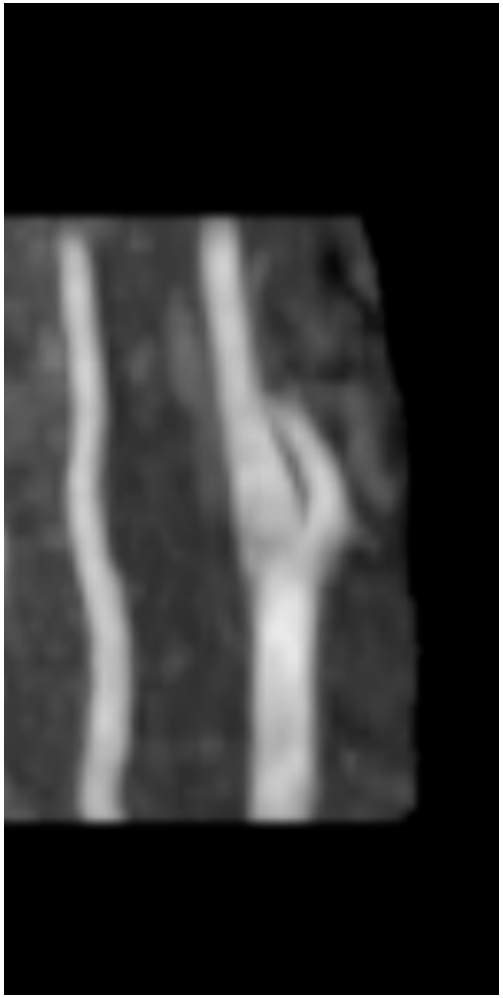

66b Score

0-30

31-50

51-70

>70

Near occlusion

Occluded

Quality

1

2

3

4

5

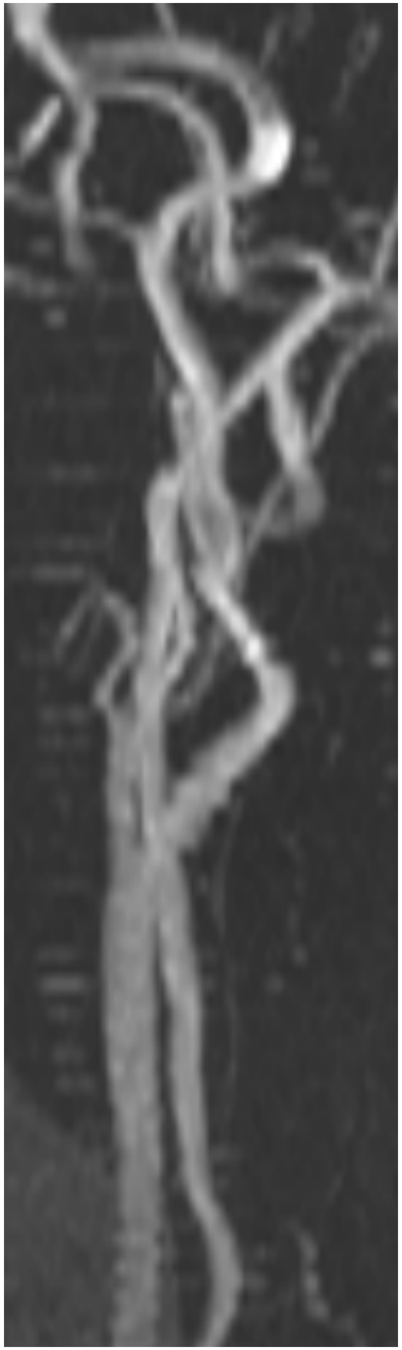

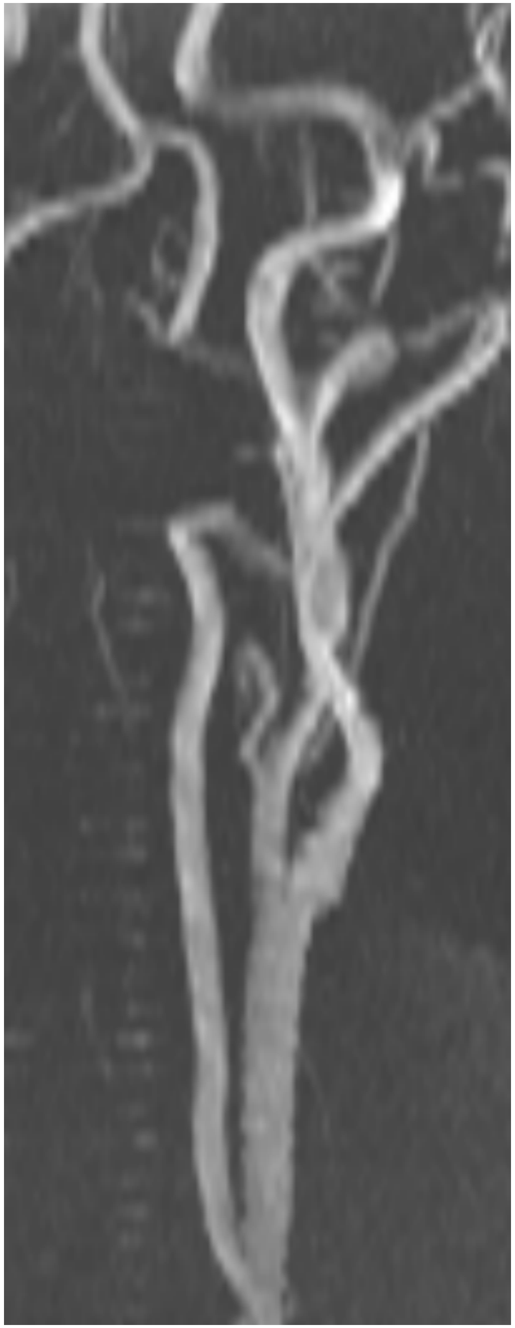

67a Score

0-30

31-50

51-70

>70

Near occlusion

Occluded

Quality

1

2

3

4

5

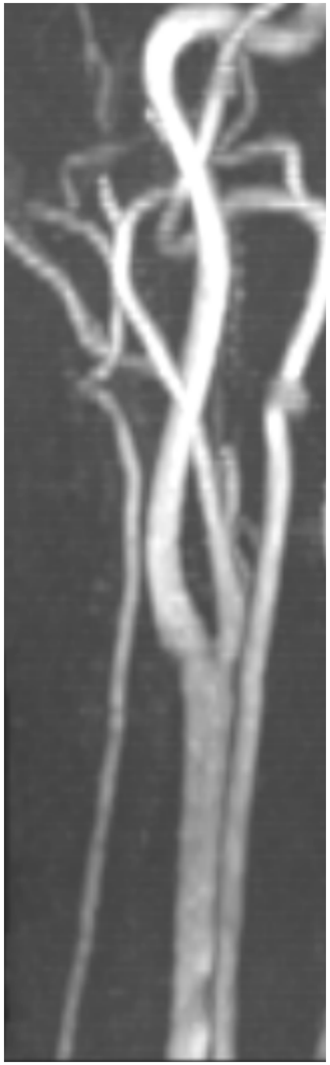

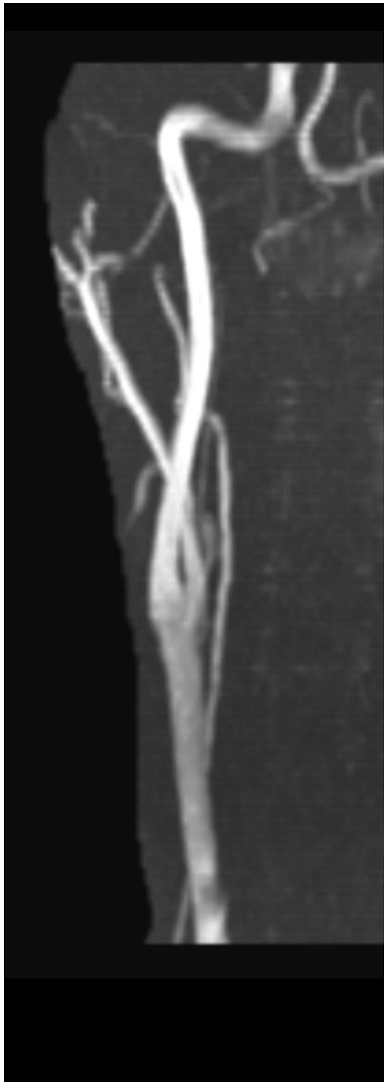

67f Score

0-30

31-50

51-70

>70

Near occlusion

Occluded

Quality

1

2

3

4

5

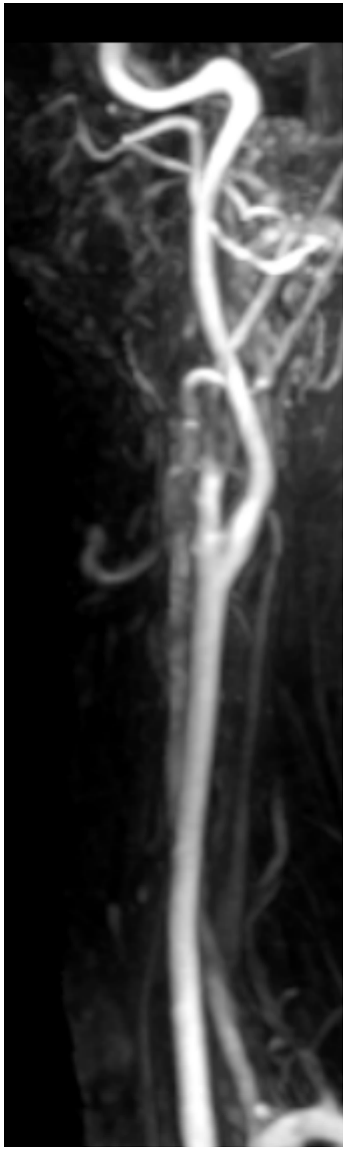

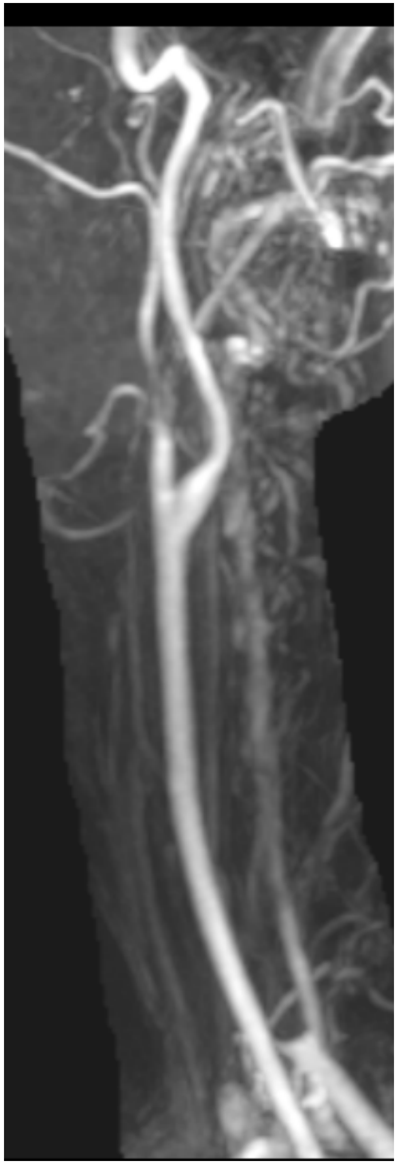

68e Score

0-30

31-50

51-70

>70

Near occlusion

Occluded

Quality

1

2

3

4

5

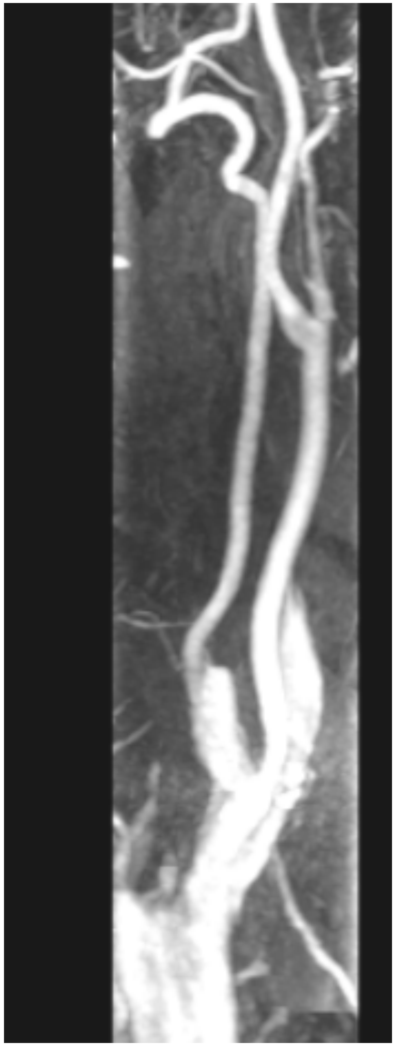

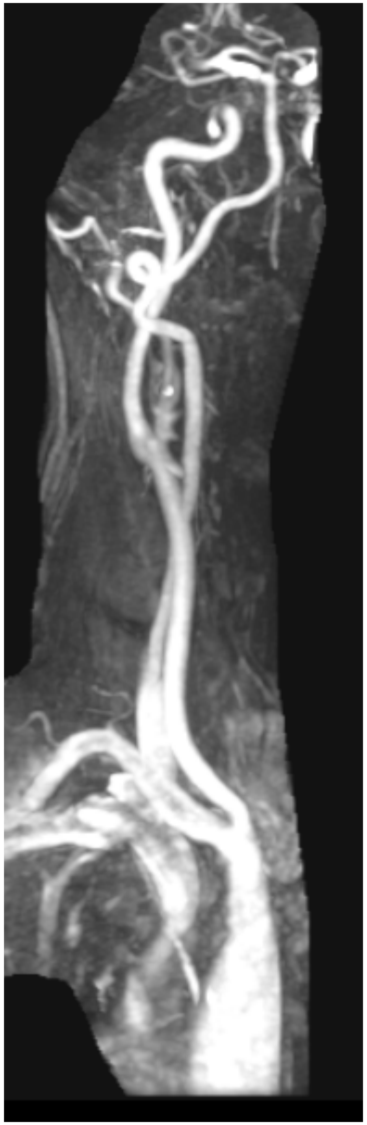

69d Score

0-30

31-50

51-70

>70

Near occlusion

Occluded

Quality

1

2

3

4

5

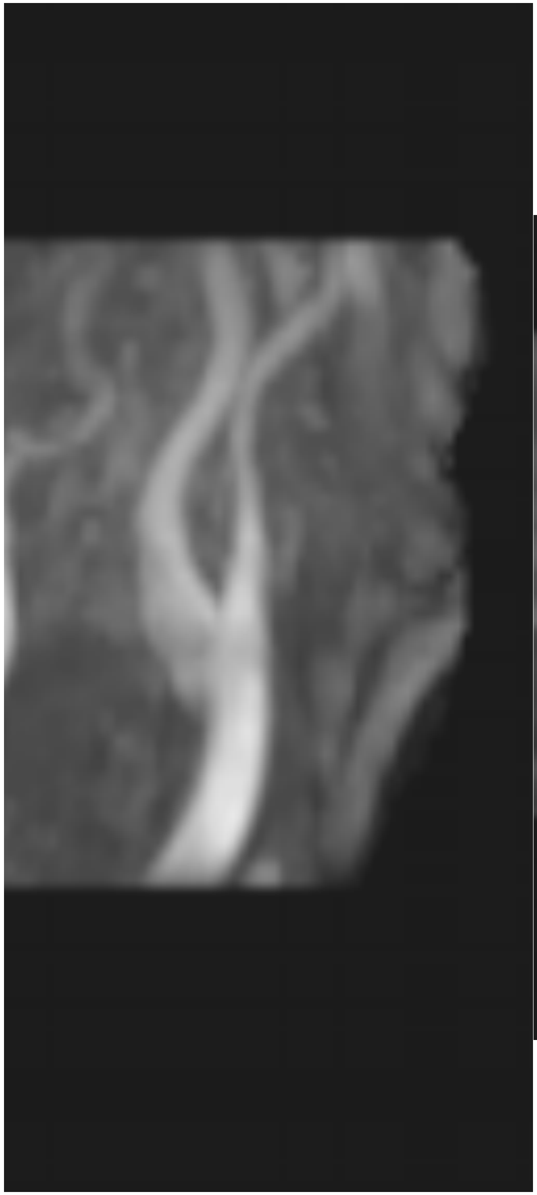

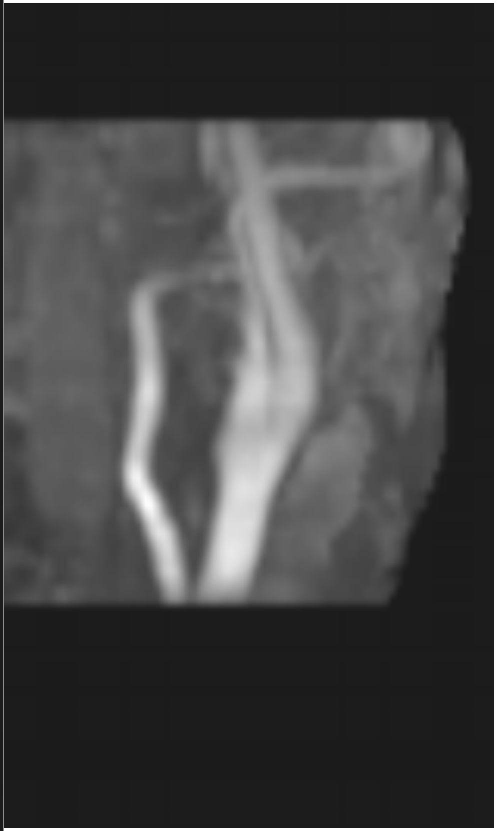

70c Score

0-30

31-50

51-70

>70

Near occlusion

Occluded

Quality

1

2

3

4

5

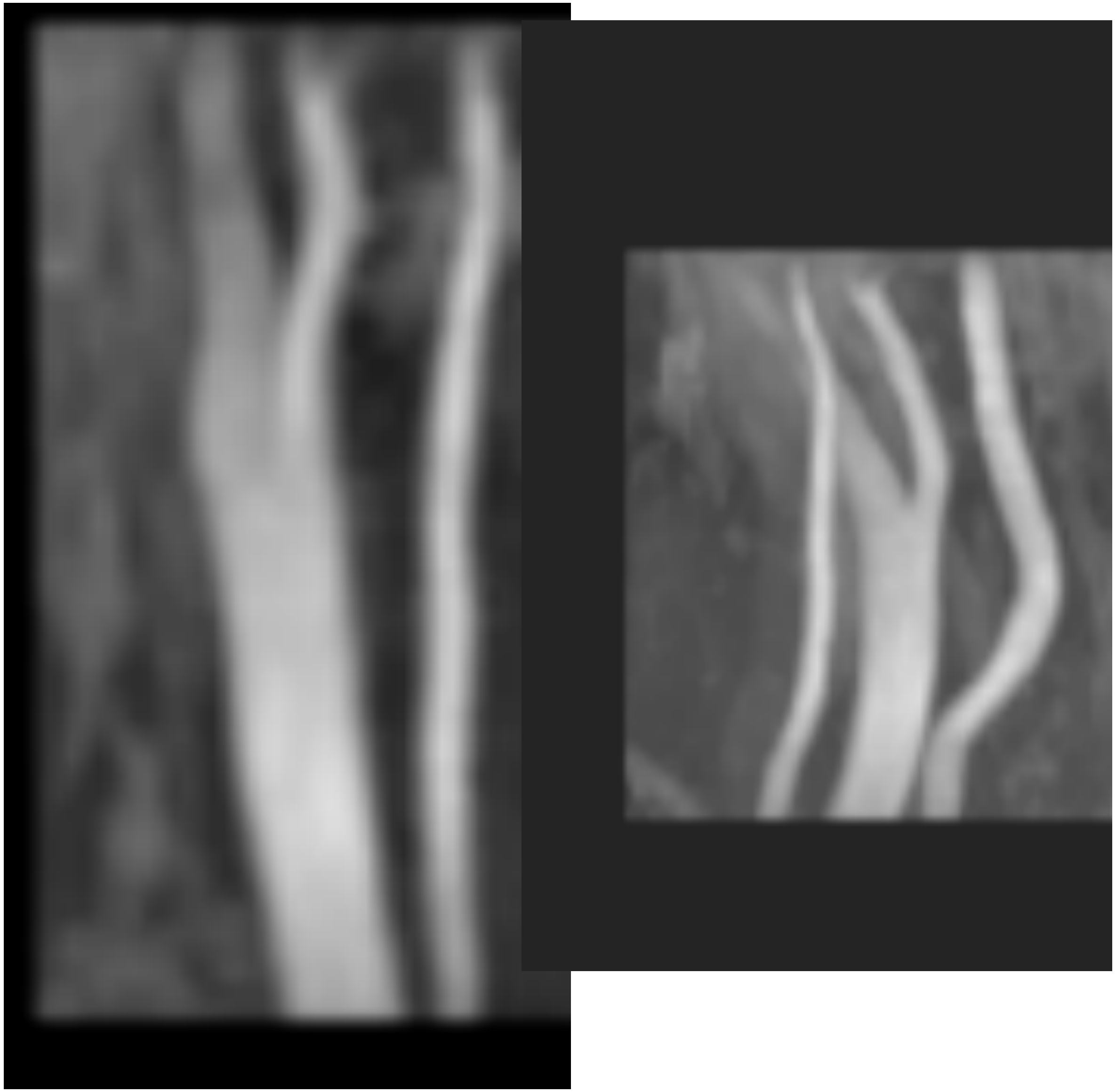

71b Score

0-30

31-50

51-70

>70

Near occlusion

Occluded

Quality

1

2

3

4

5

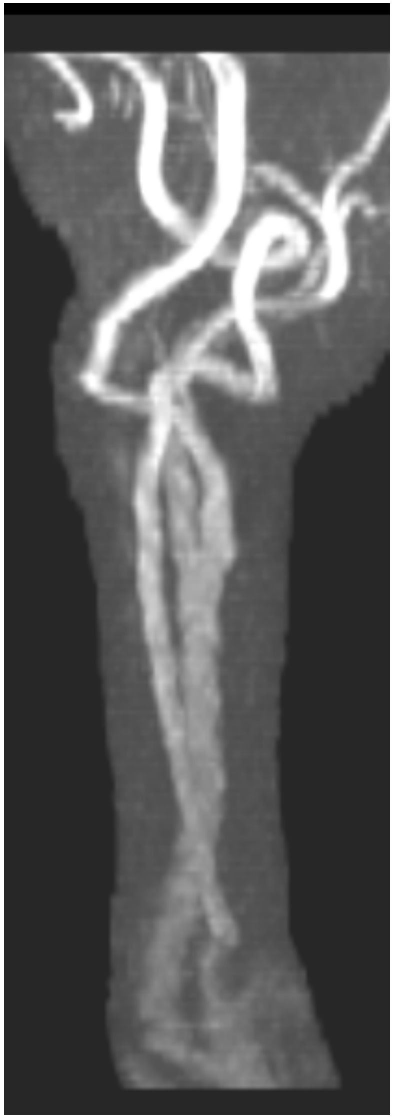

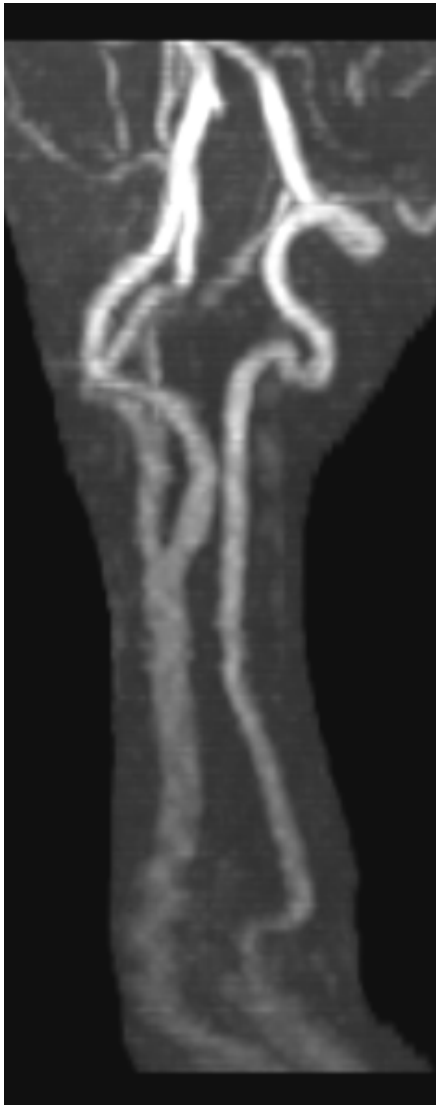

72a Score (right)

0-30

31-50

51-70

>70

Near occlusion

Occluded

Quality

1

2

3

4

5

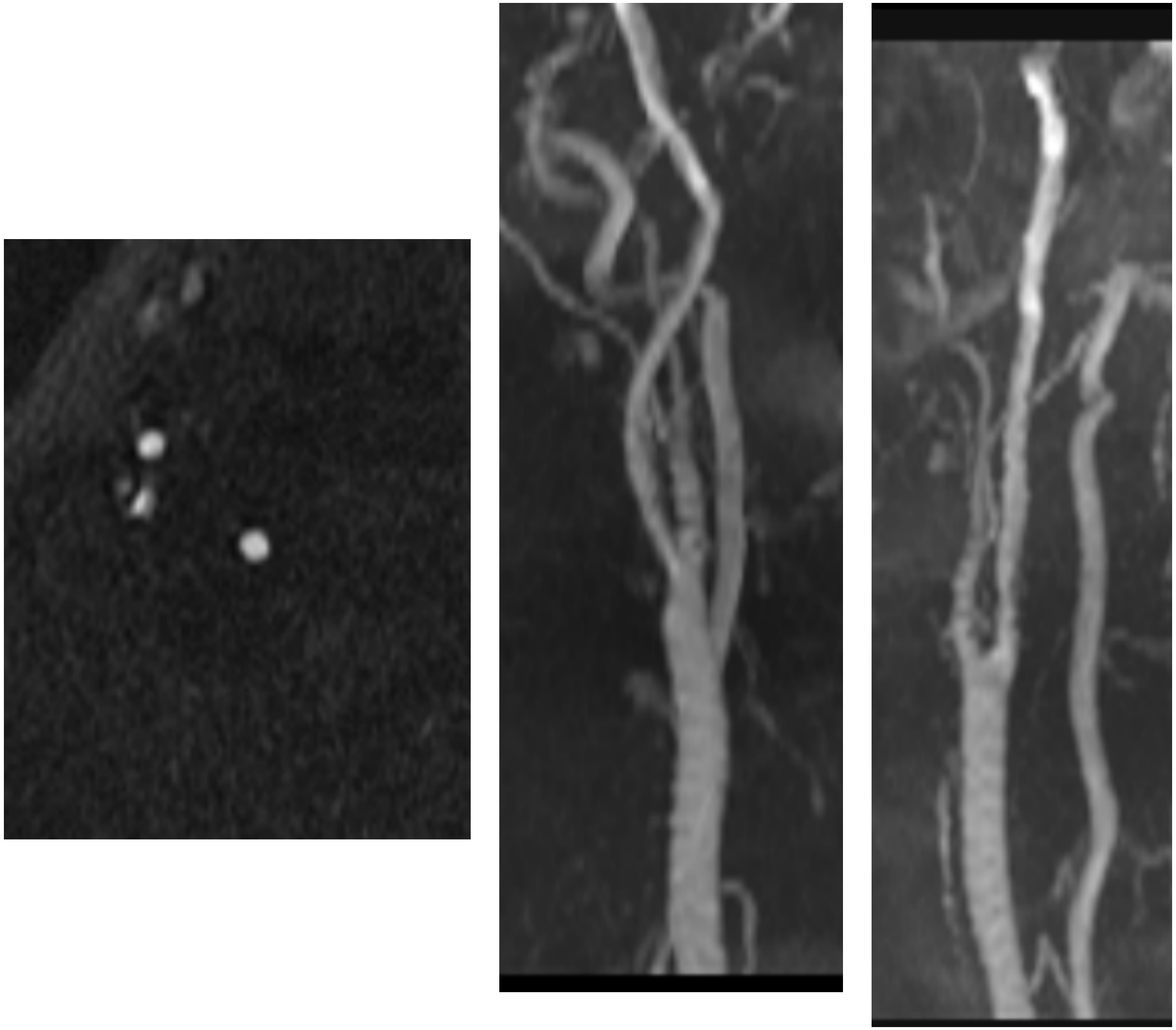

72f Score

0-30

31-50

51-70

>70

Near occlusion

Occluded

Quality

1

2

3

4

5

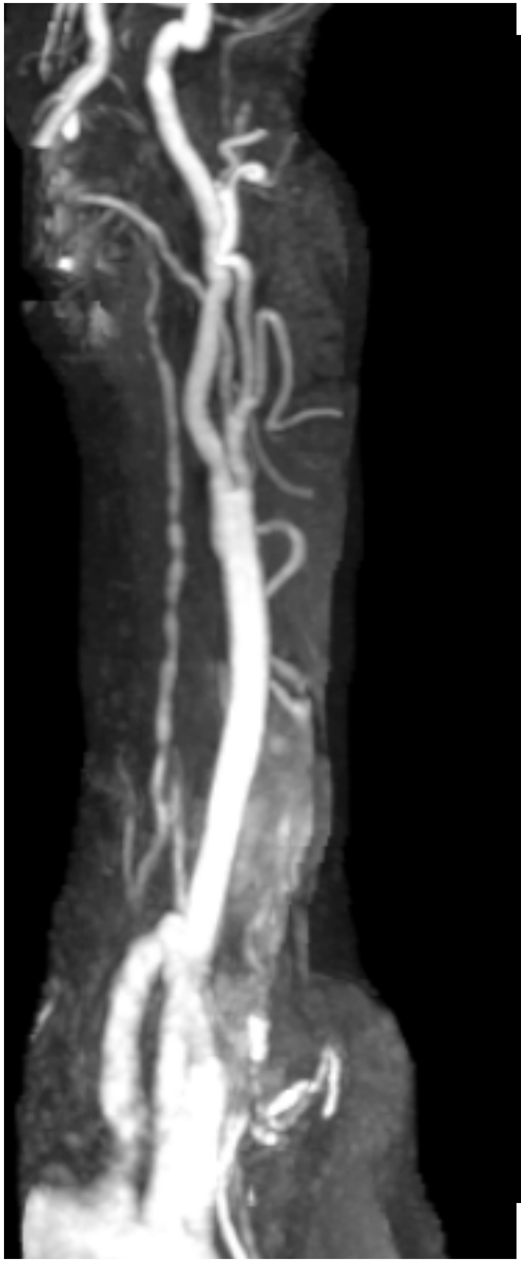

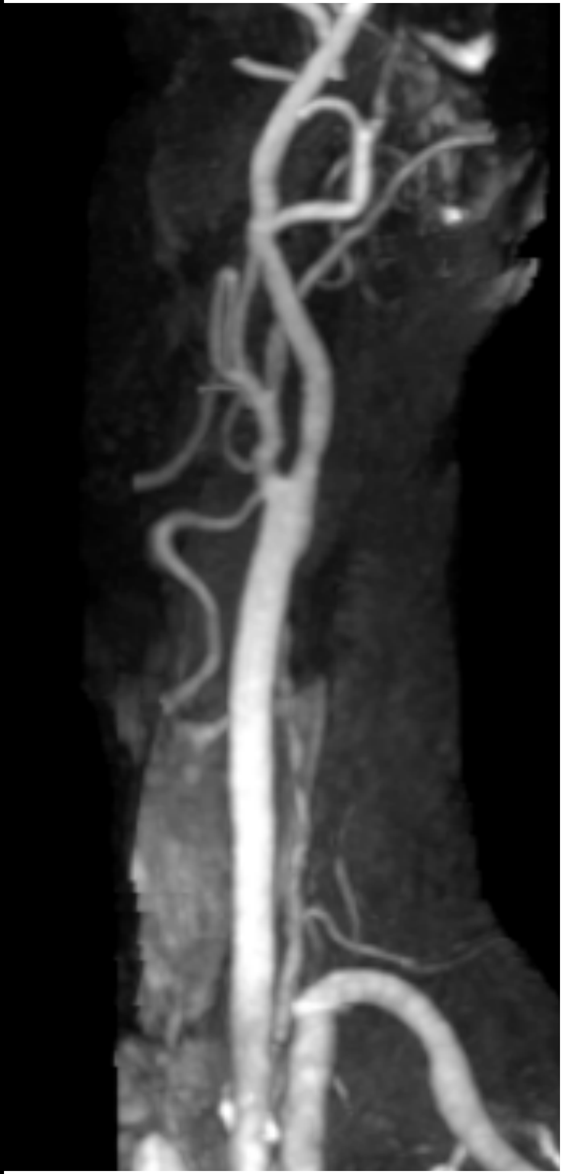

73e Score

0-30

31-50

51-70

>70

Near occlusion

Occluded

Quality

1

2

3

4

5

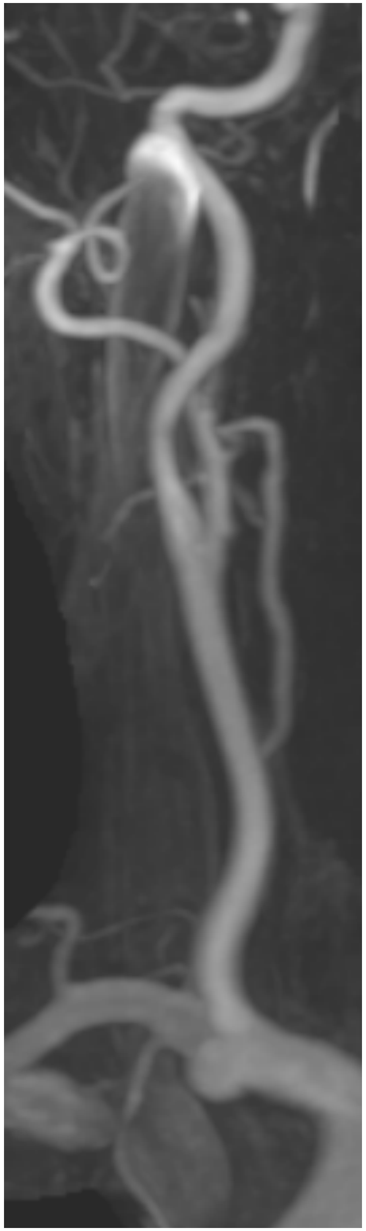

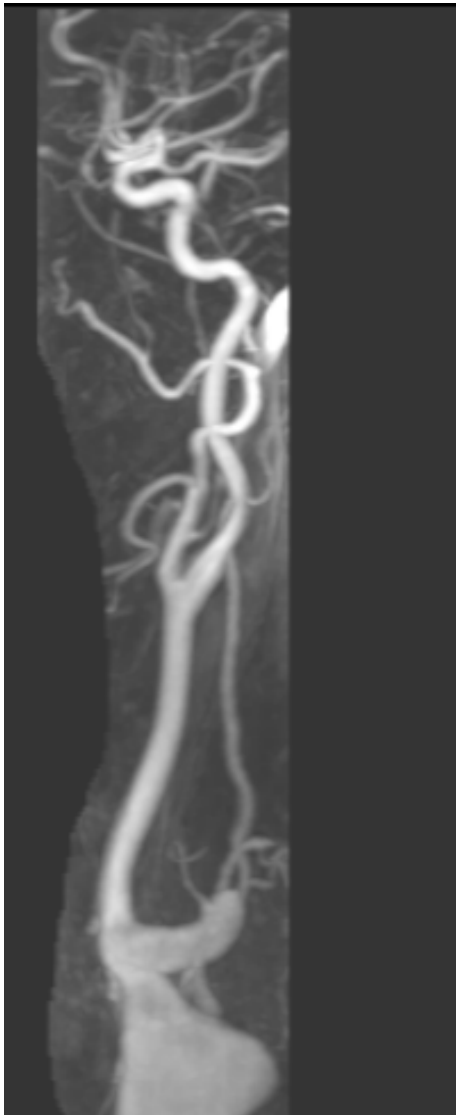

74d Score

0-30

31-50

51-70

>70

Near occlusion

Occluded

Quality

1

2

3

4

5

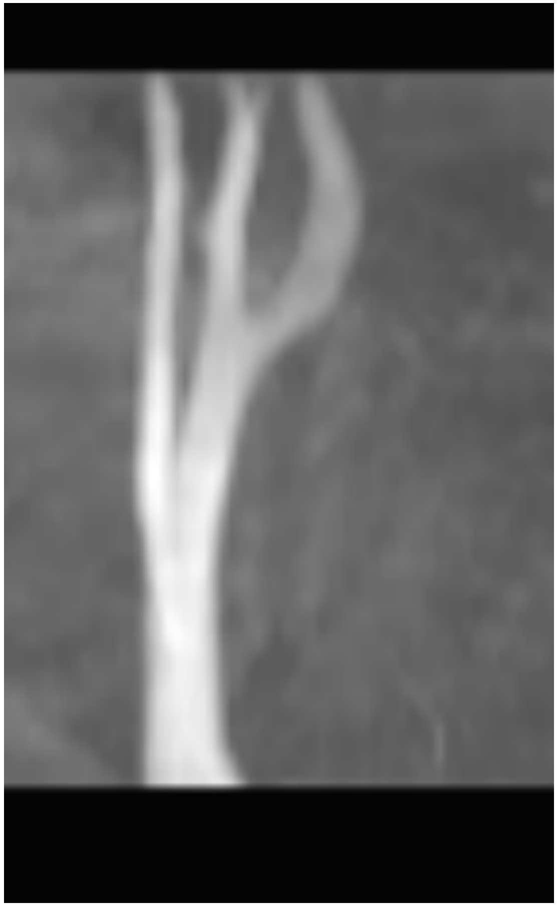

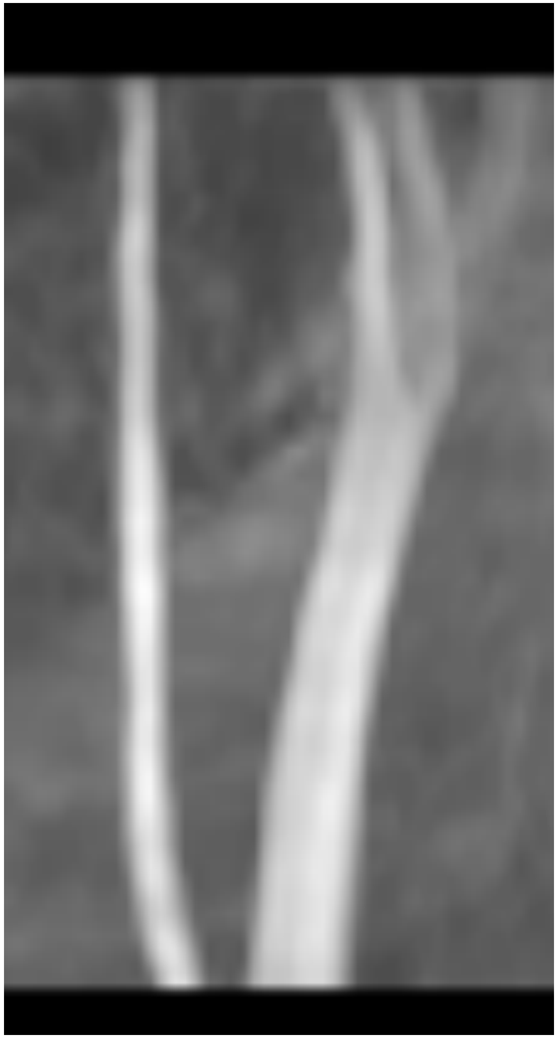

75c Score

0-30

31-50

51-70

>70

Near occlusion

Occluded

Quality

1

2

3

4

5

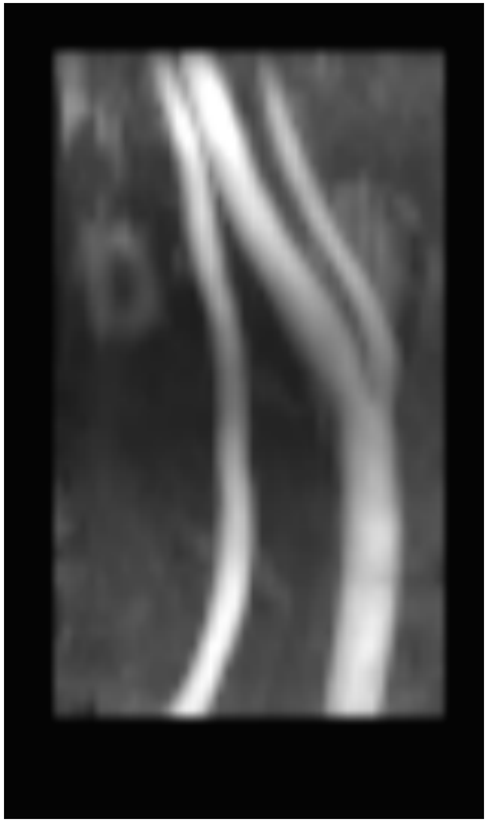

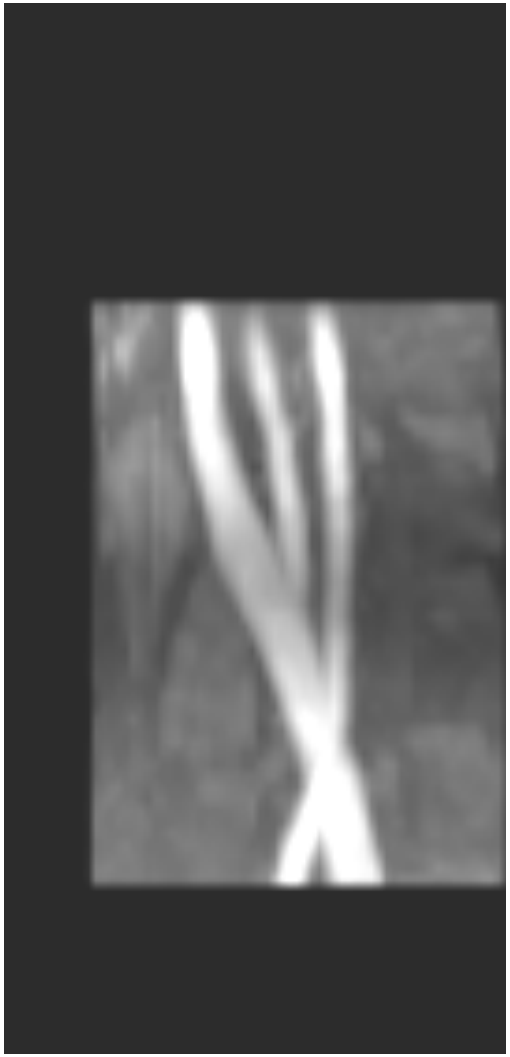

76b Score

0-30

31-50

51-70

>70

Near occlusion

Occluded

Quality

1

2

3

4

5

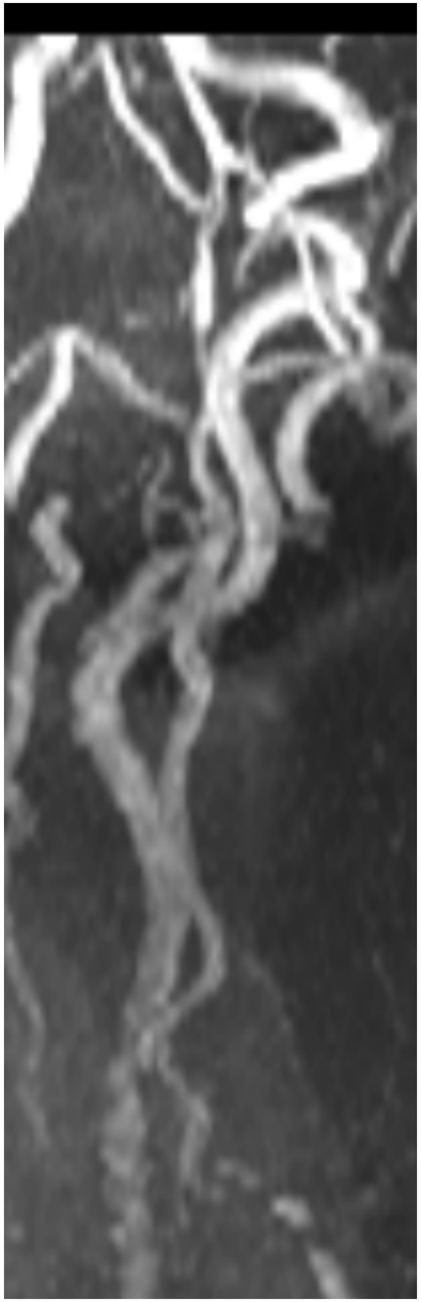

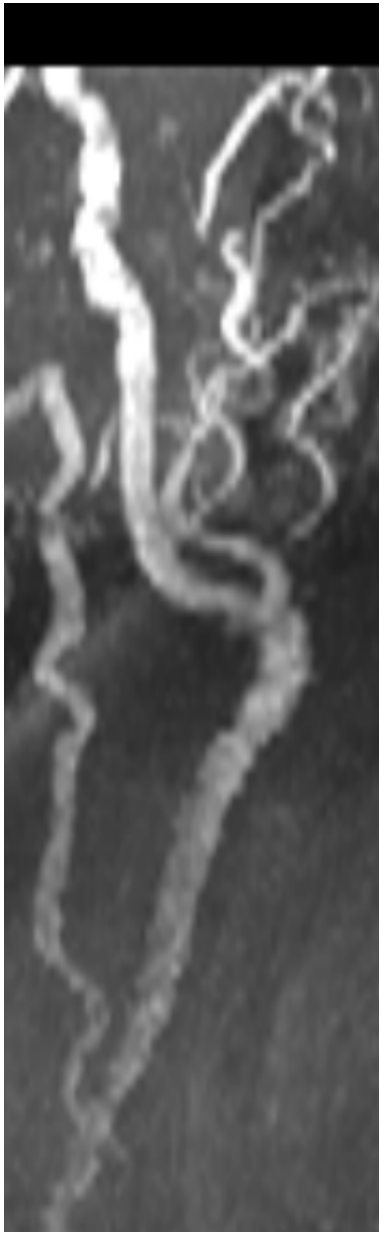

77a Score

0-30

31-50

51-70

>70

Near occlusion

Occluded

Quality

1

2

3

4

5

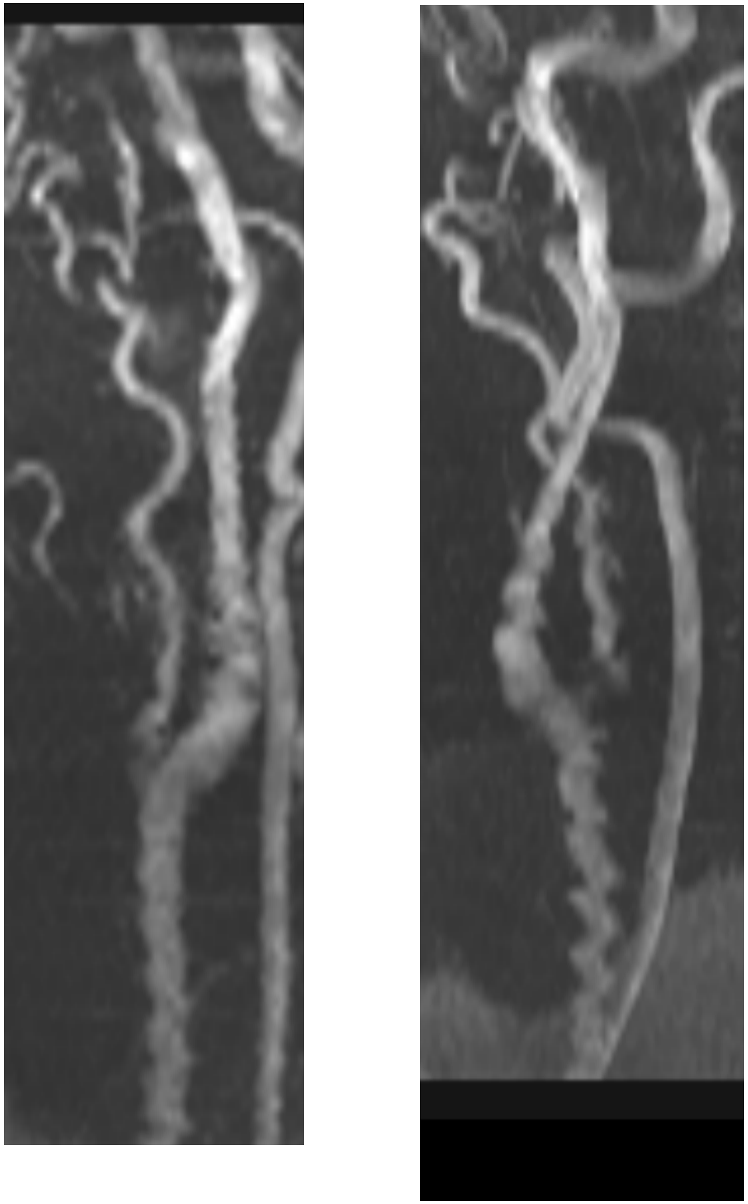

77f Score

0-30

31-50

51-70

>70

Near occlusion

Occluded

Quality

1

2

3

4

5

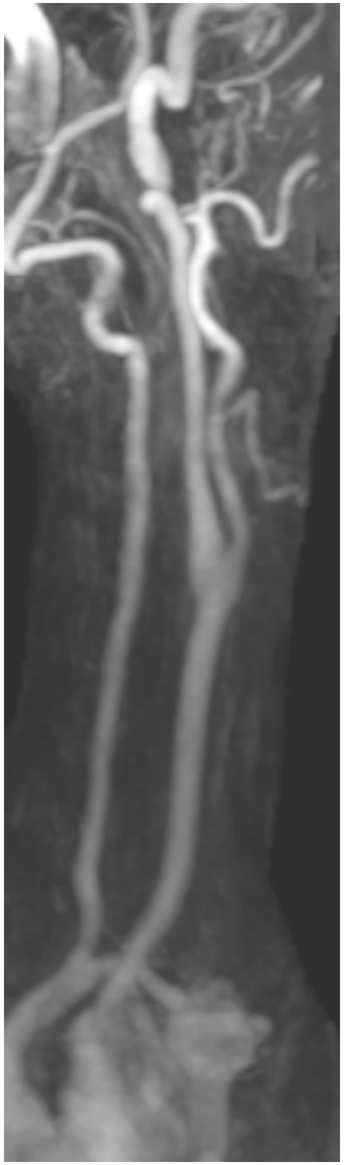

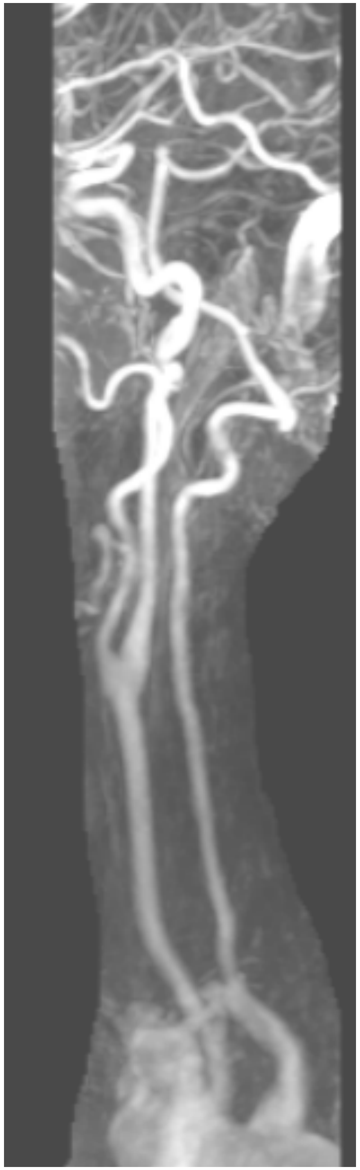

78e Score

0-30

31-50

51-70

>70

Near occlusion

Occluded

Quality

1

2

3

4

5

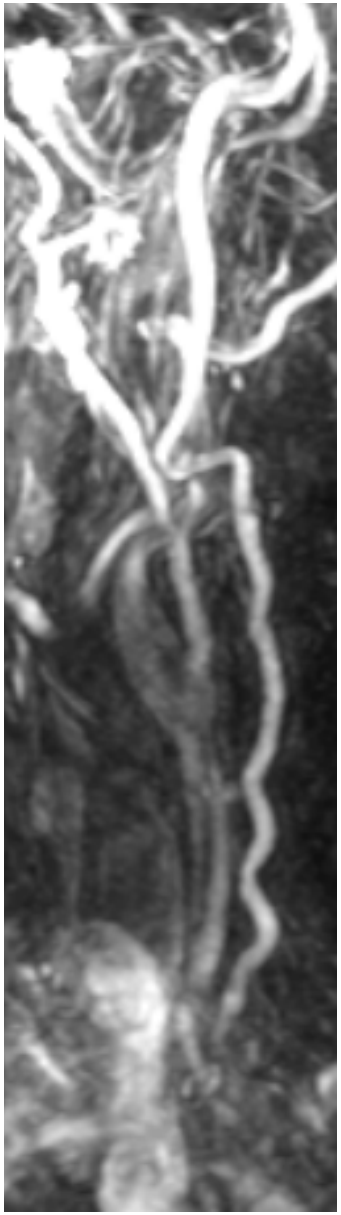

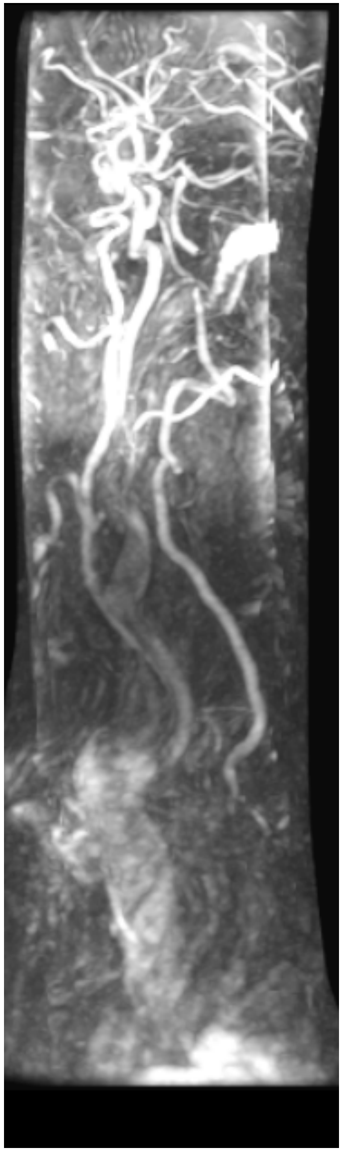

79d Score

0-30

31-50

51-70

>70

Near occlusion

Occluded

Quality

1

2

3

4

5

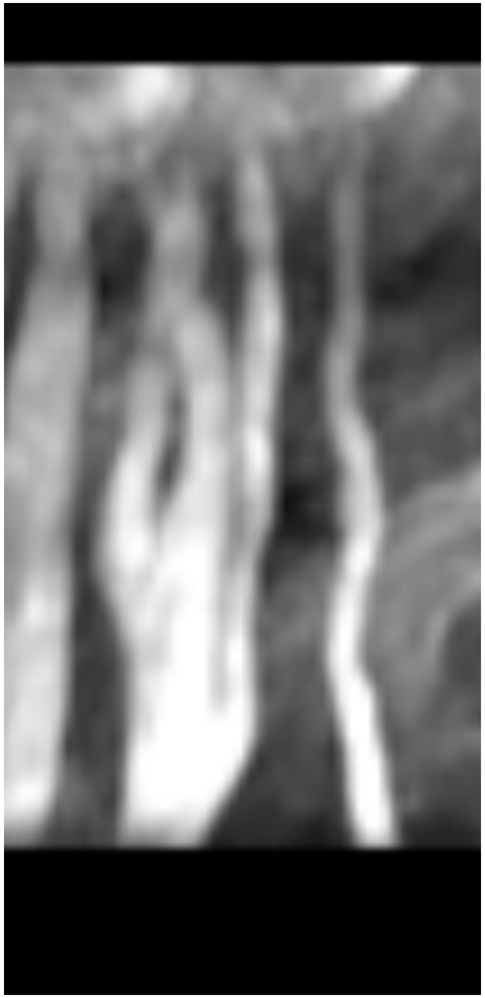

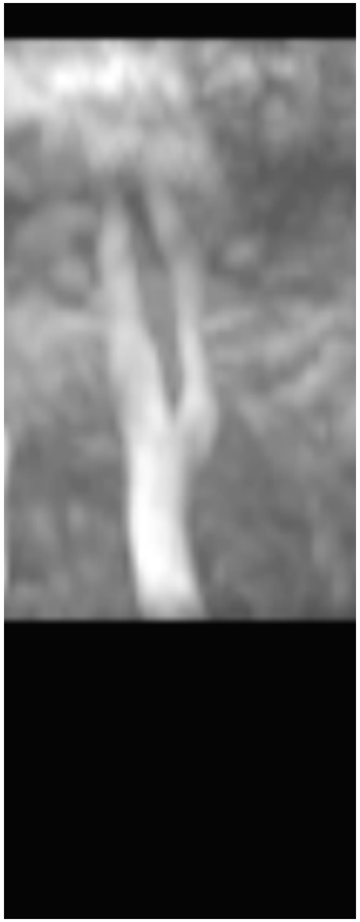

80c Score

0-30

31-50

51-70

>70

Near occlusion

Occluded

Quality

1

2

3

4

5

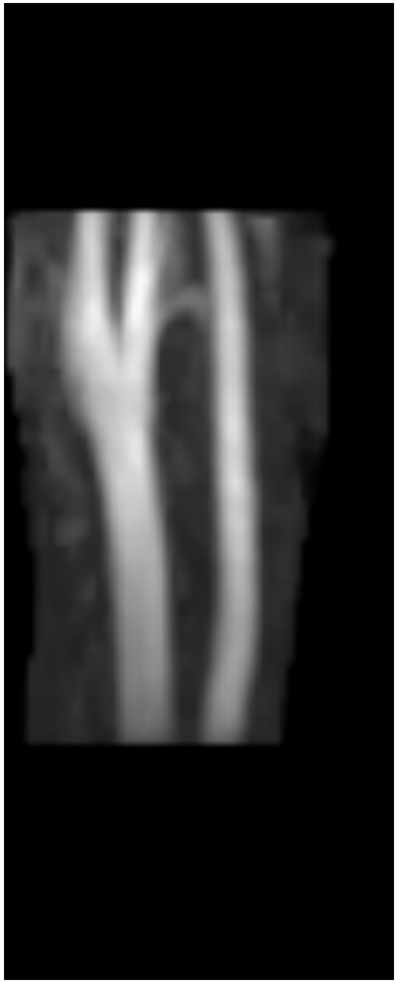

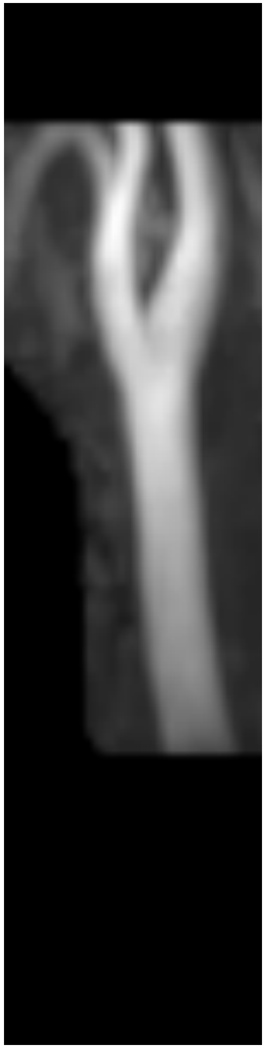

81b Score

0-30

31-50

51-70

>70

Near occlusion

Occluded

Quality

1

2

3

4

5

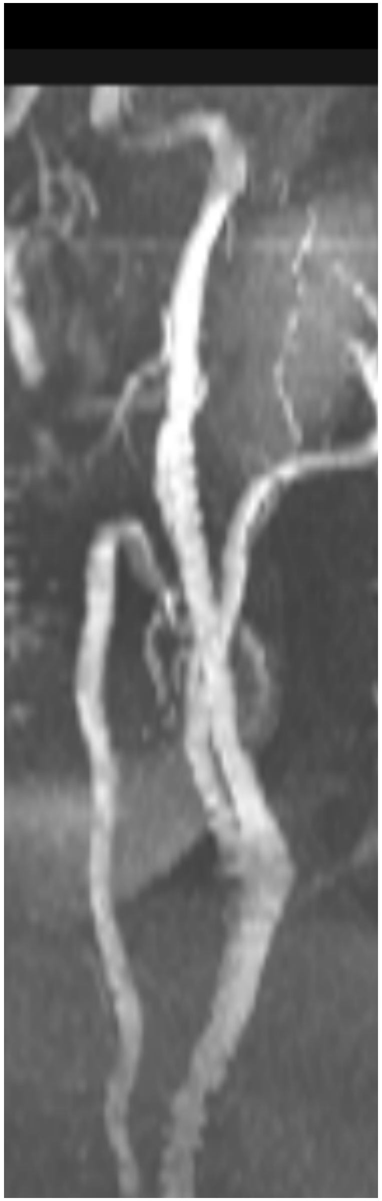

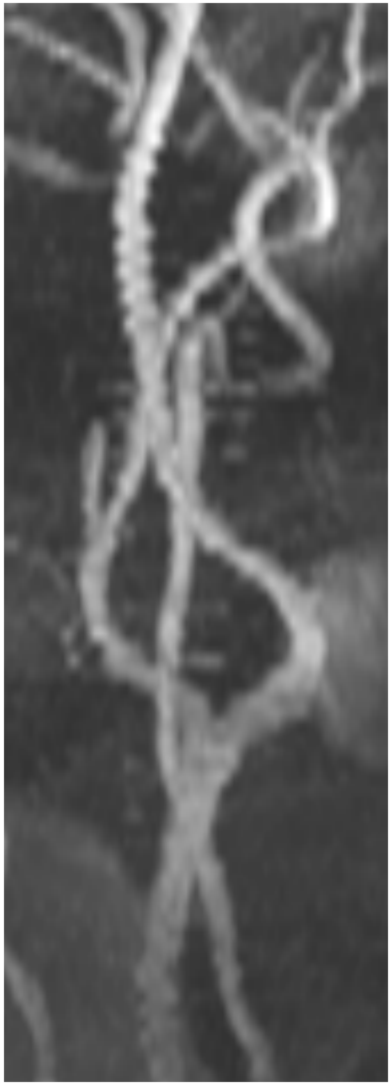

82a Score

0-30

31-50

51-70

>70

Near occlusion

Occluded

Quality

1

2

3

4

5

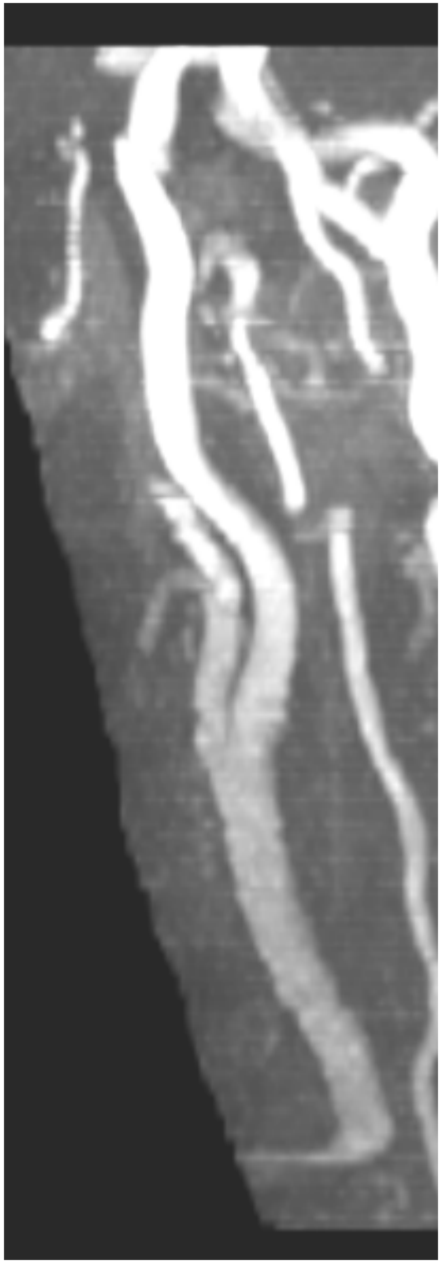

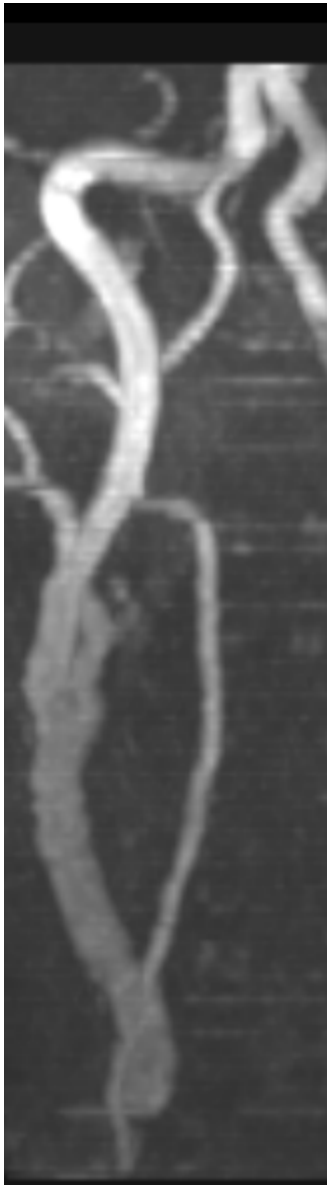

82f Score

0-30

31-50

51-70

>70

Near occlusion

Occluded

Quality

1

2

3

4

5

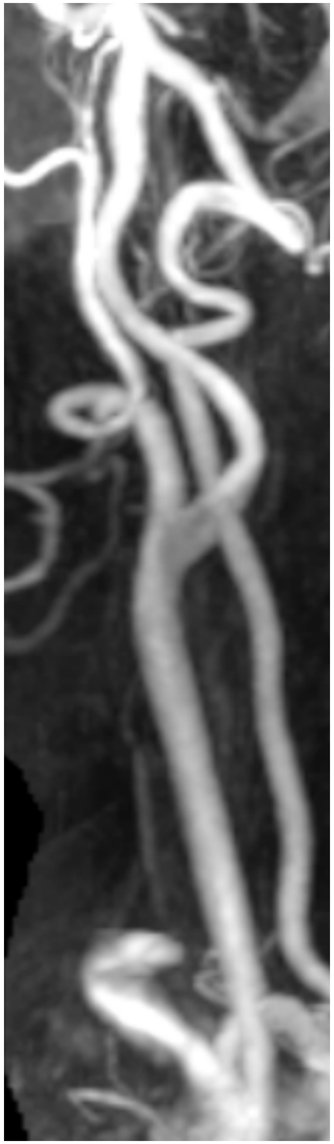

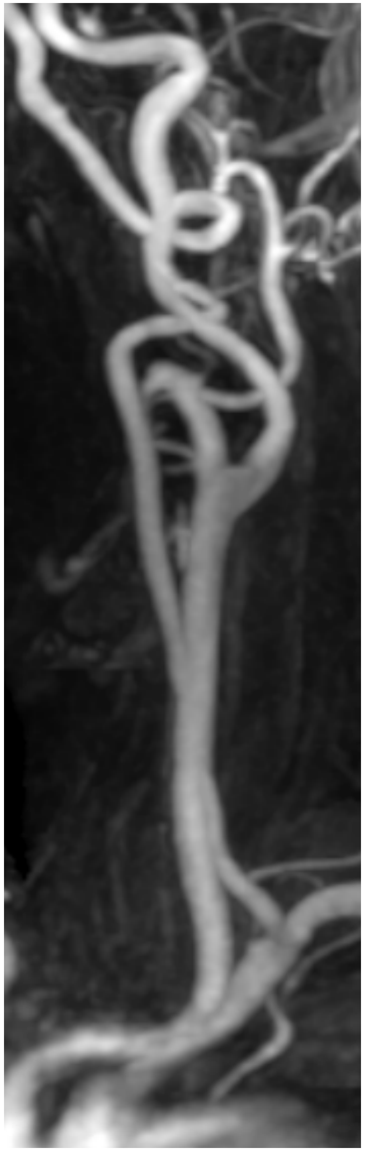

83e Score

0-30

31-50

51-70

>70

Near occlusion

Occluded

Quality

1

2

3

4

5

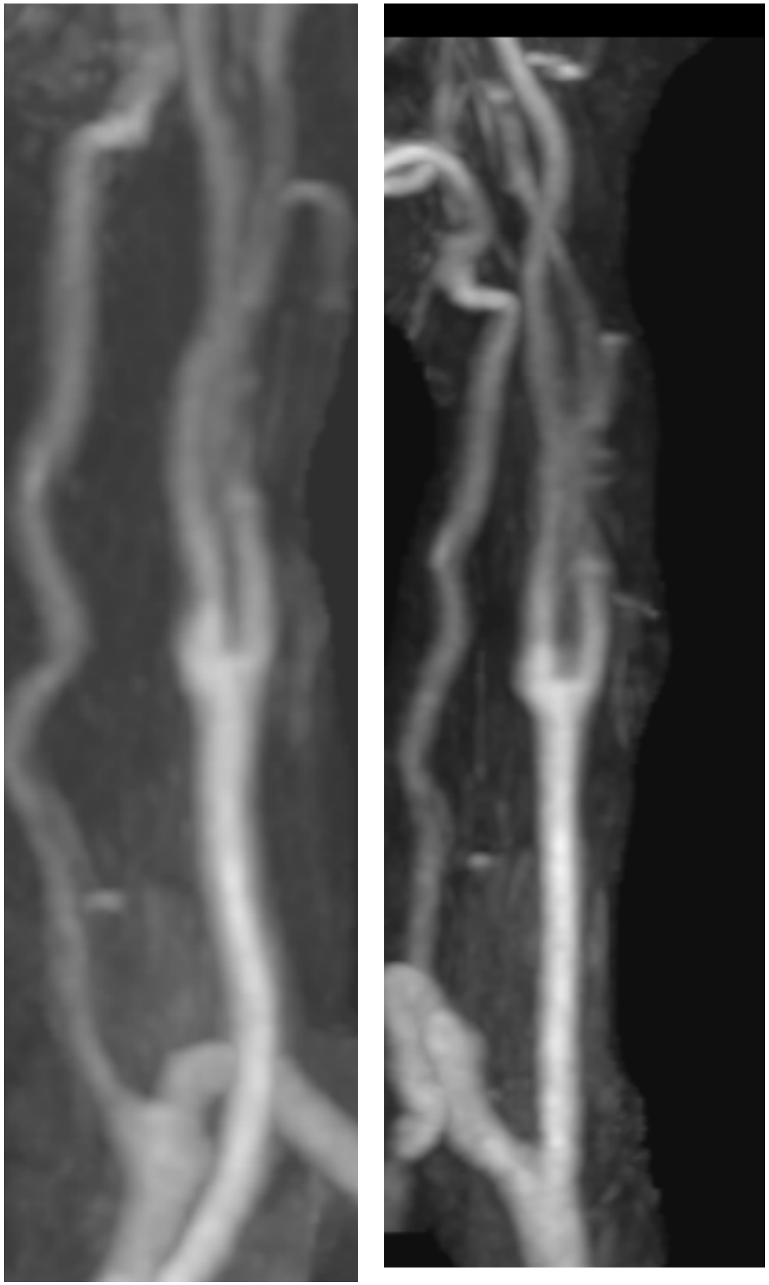

84d Score

0-30

31-50

51-70

>70

Near occlusion

Occluded

Quality

1

2

3

4

5

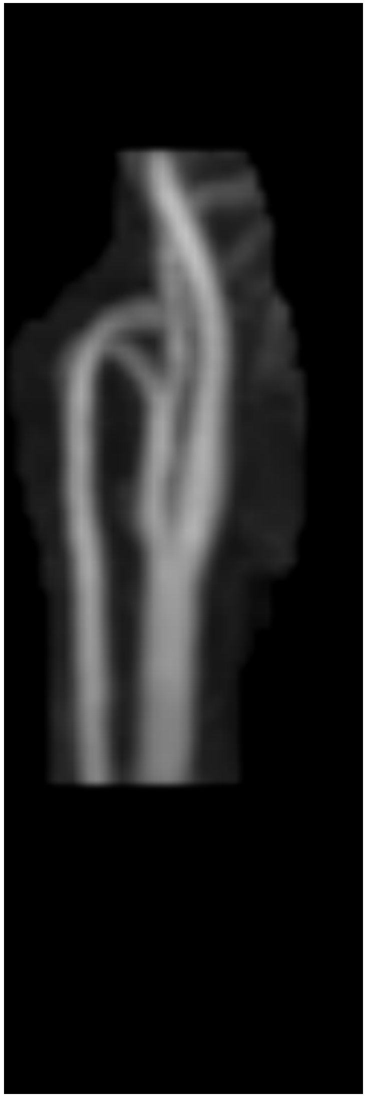

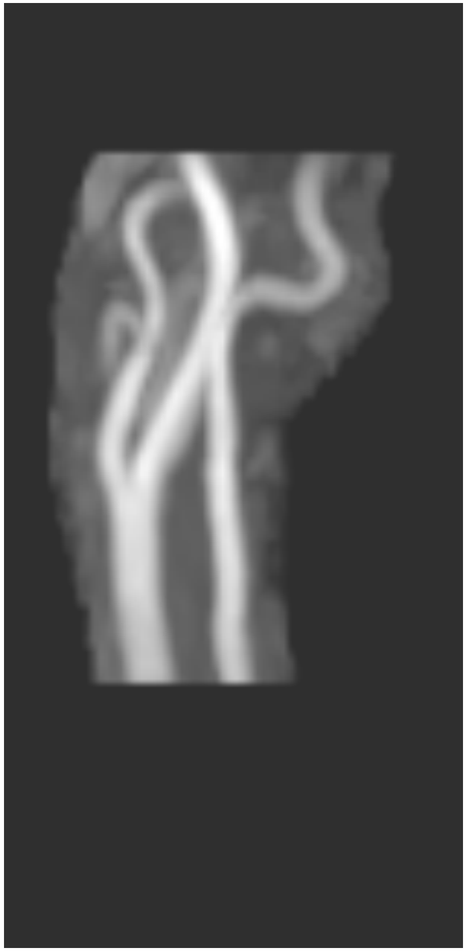

85c Score

0-30

31-50

51-70

>70

Near occlusion

Occluded

Quality

1

2

3

4

5

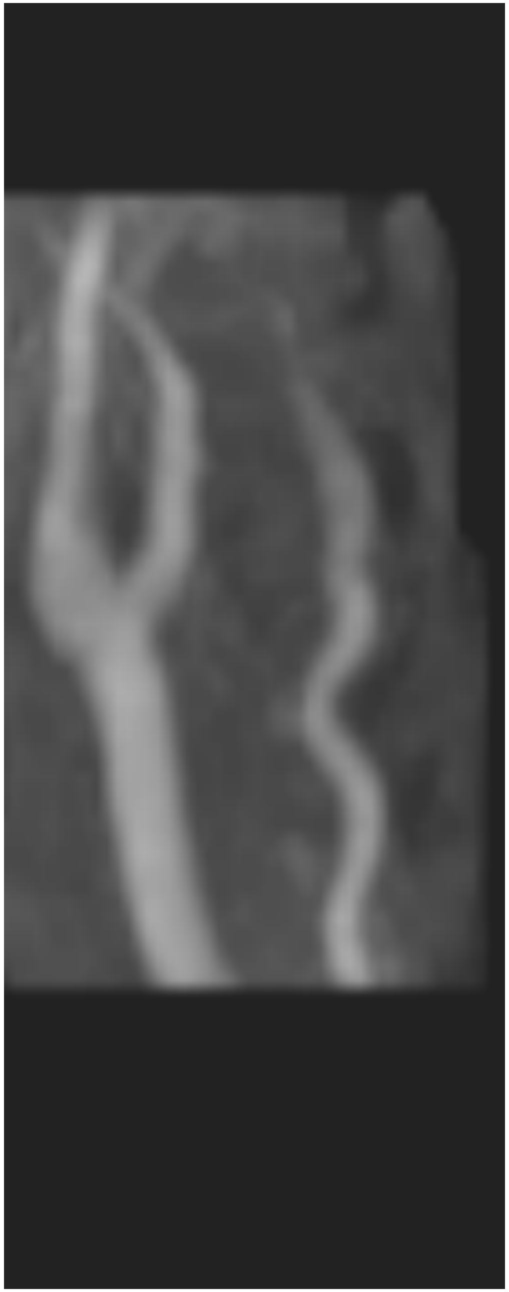

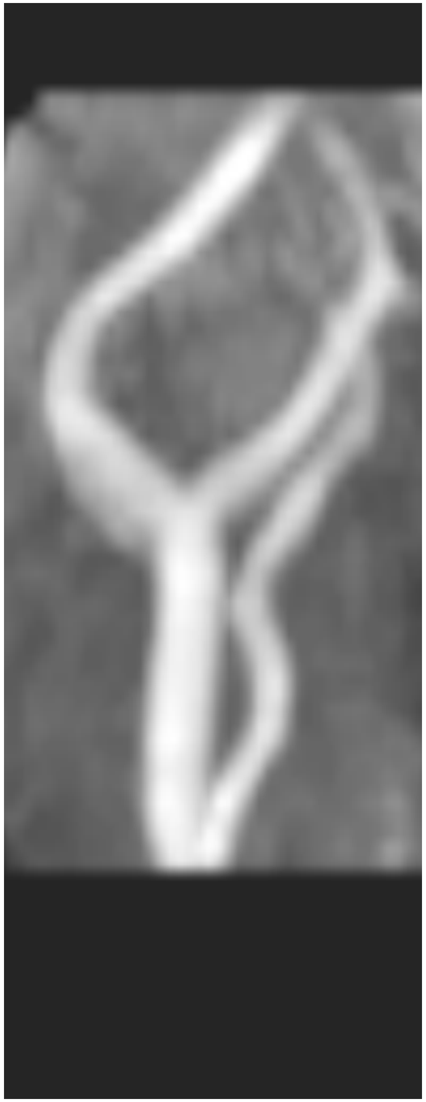

86b Score

0-30

31-50

51-70

>70

Near occlusion

Occluded

Quality

1

2

3

4

5

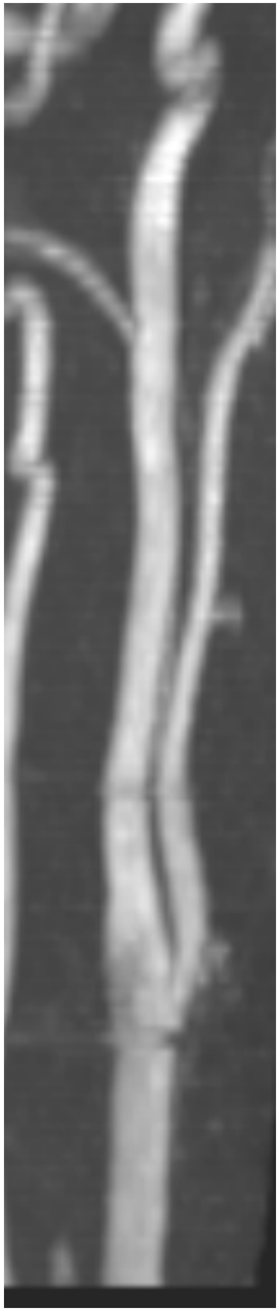

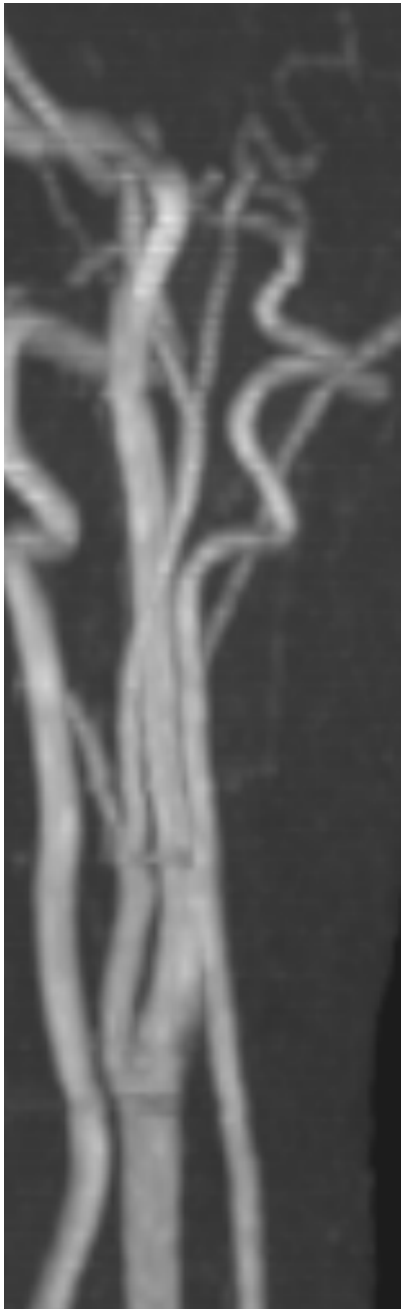

87a Score

0-30

31-50

51-70

>70

Near occlusion

Occluded

Quality

1

2

3

4

5

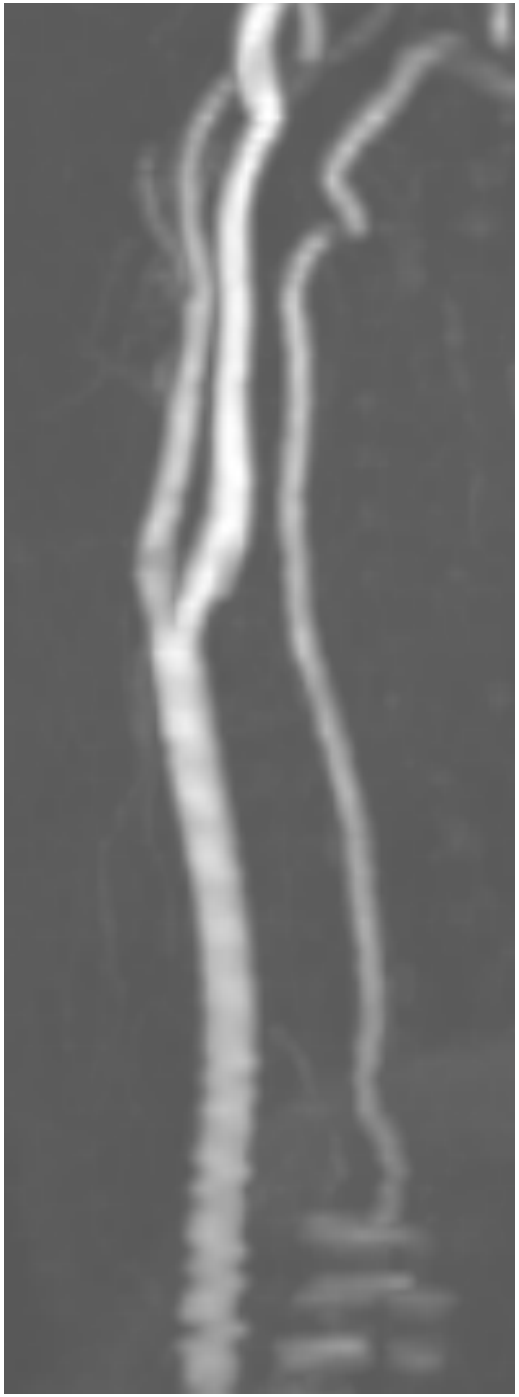

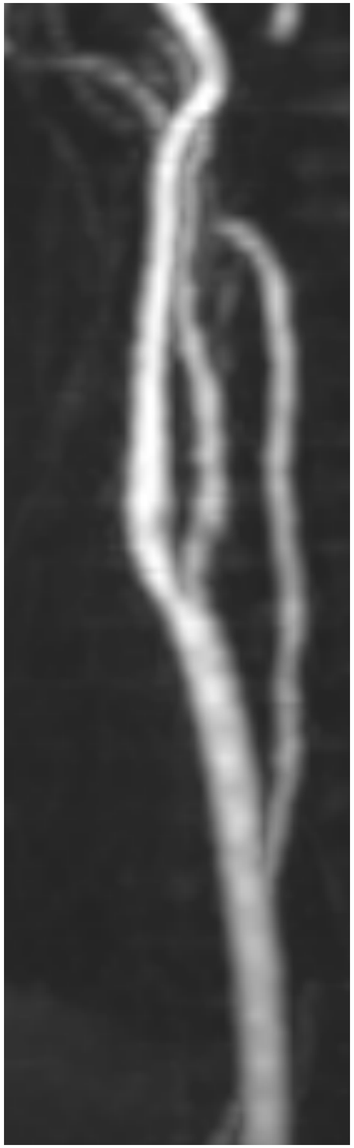

87f Score

0-30

31-50

51-70

>70

Near occlusion

Occluded

Quality

1

2

3

4

5

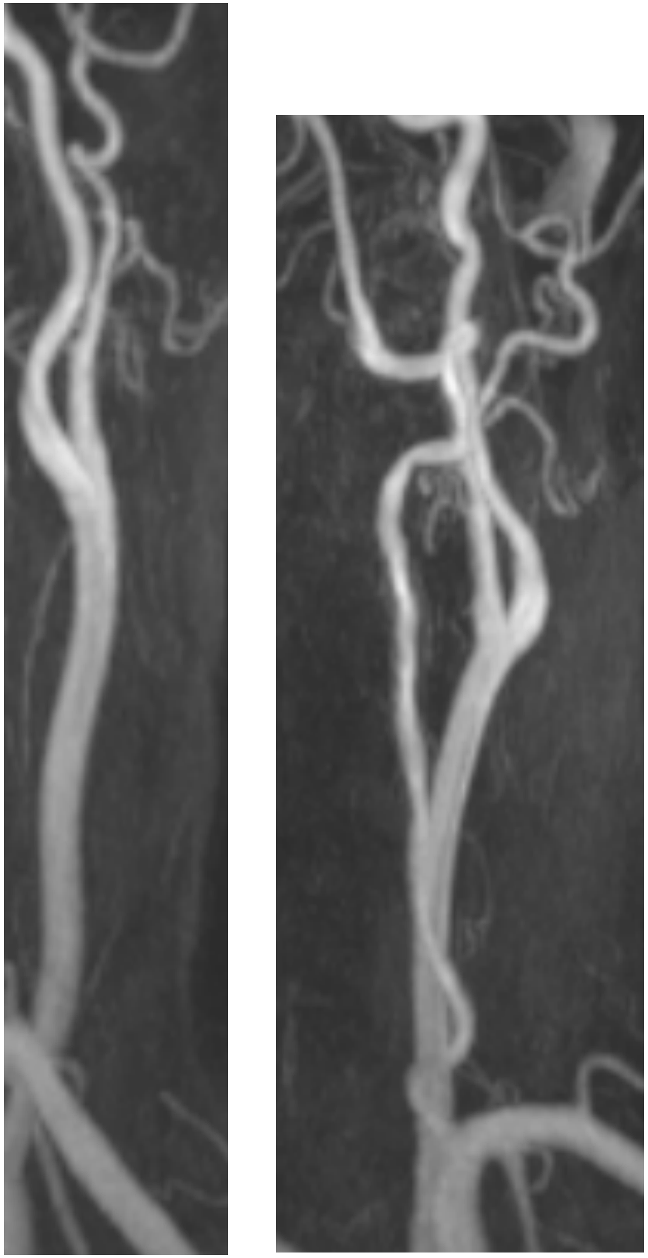

88e Score

0-30

31-50

51-70

>70

Near occlusion

Occluded

Quality

1

2

3

4

5

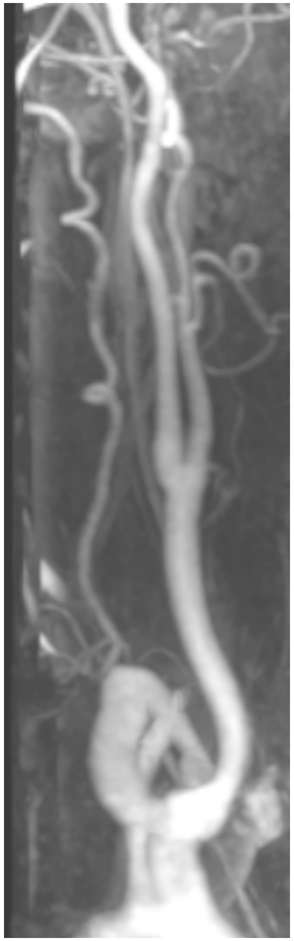

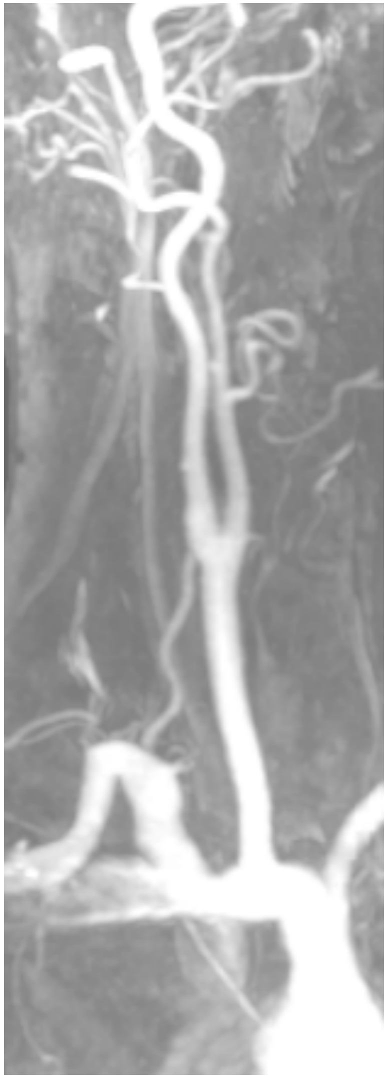

89d Score

0-30

31-50

51-70

>70

Near occlusion

Occluded

Quality

1

2

3

4

5

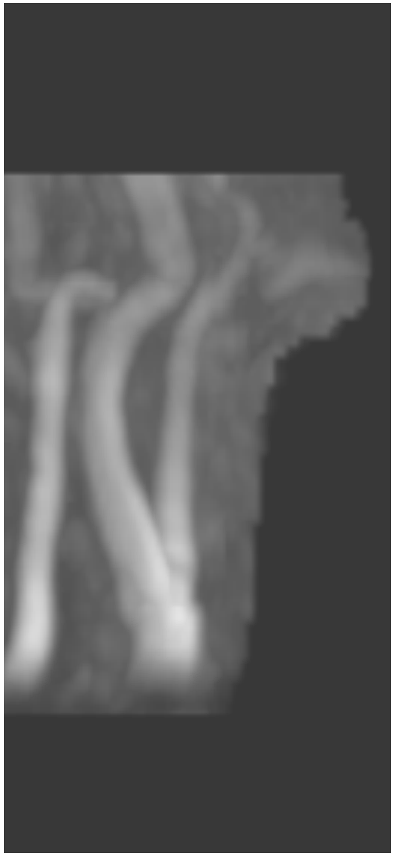

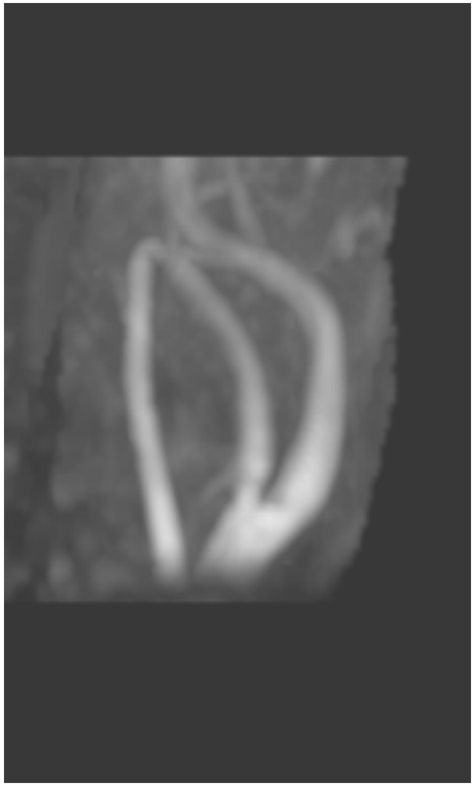

90c Score

0-30

31-50

51-70

>70

Near occlusion

Occluded

Quality

1

2

3

4

5

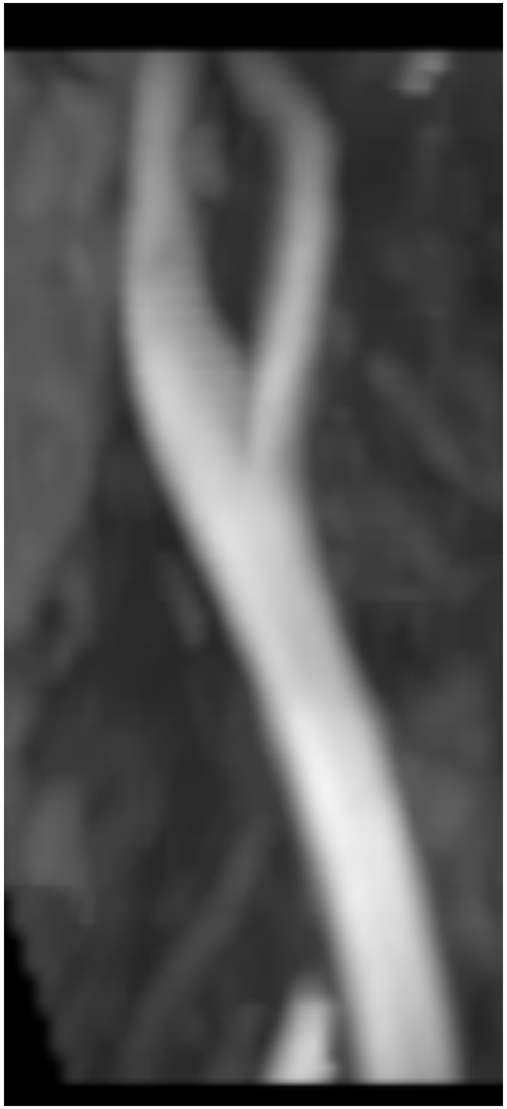

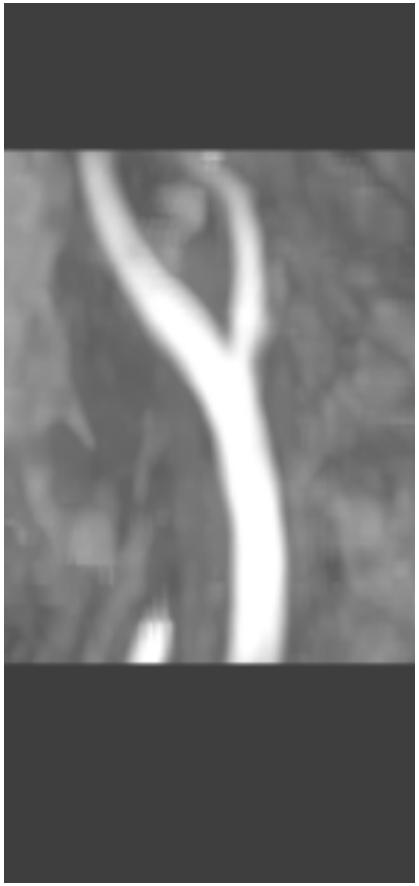

91b Score

0-30

31-50

51-70

>70

Near occlusion

Occluded

Quality

1

2

3

4

5

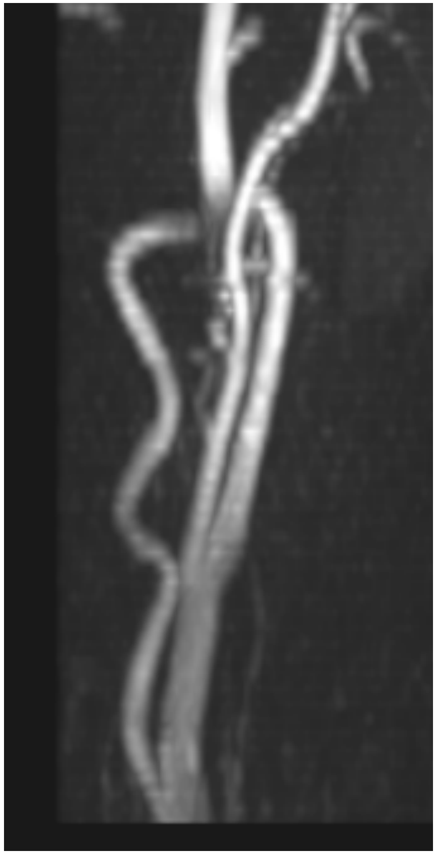

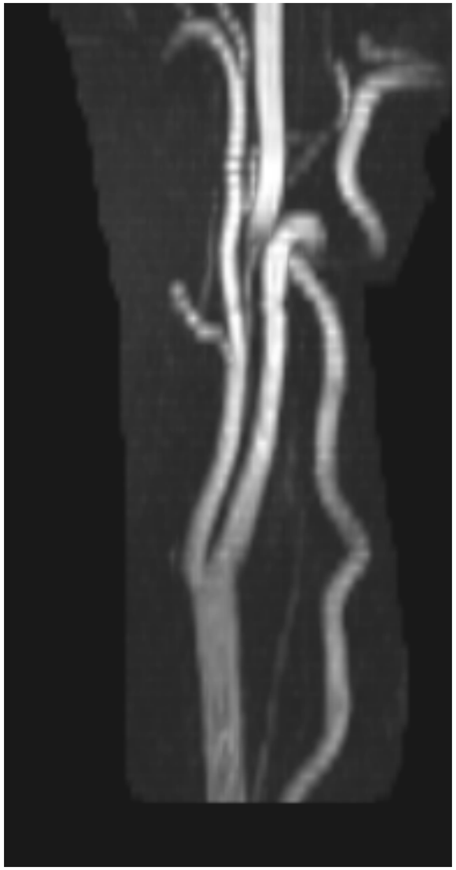

92a Score

0-30

31-50

51-70

>70

Near occlusion

Occluded

Quality

1

2

3

4

5

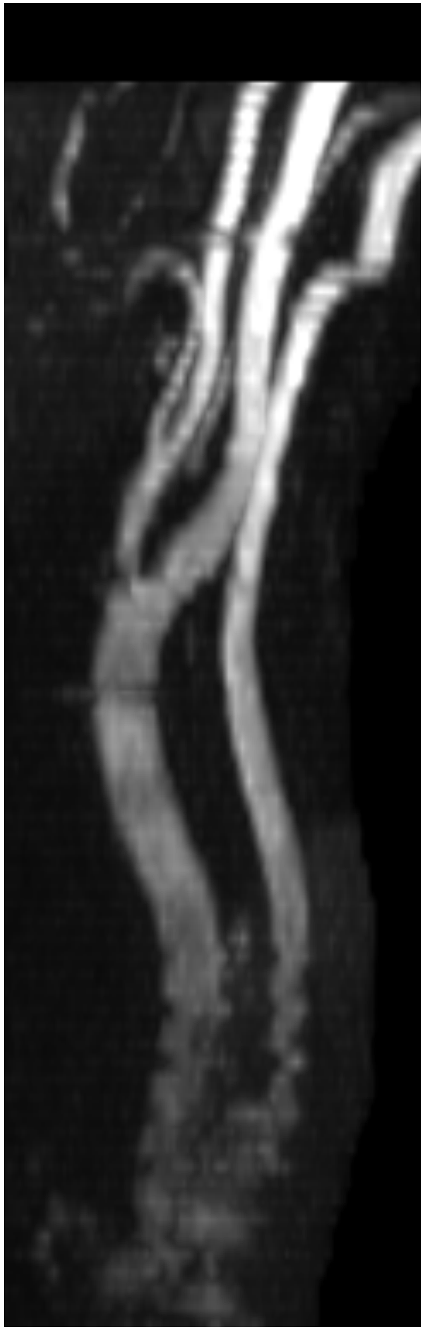

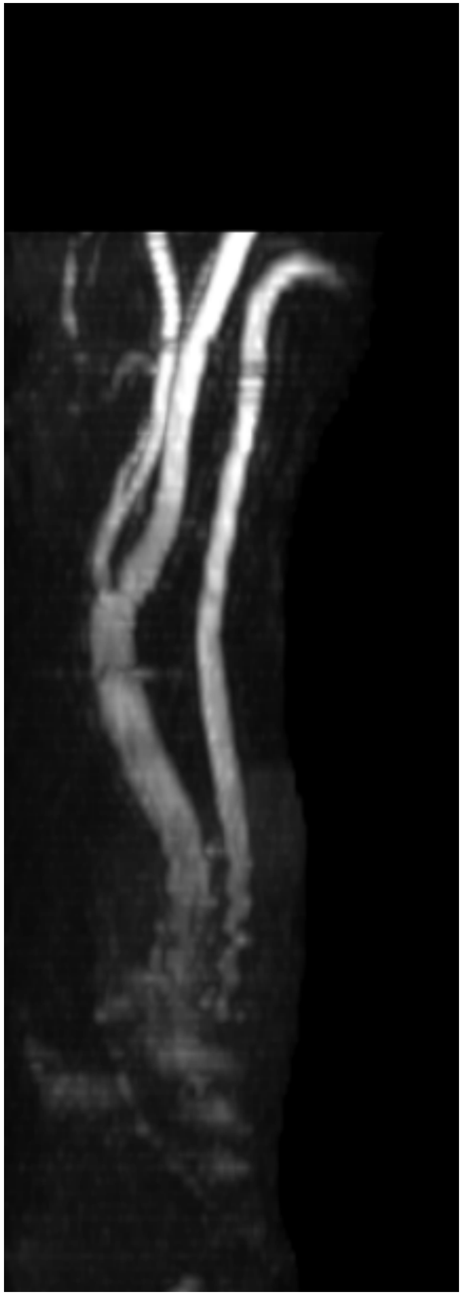

92f Score

0-30

31-50

51-70

>70

Near occlusion

Occluded

Quality

1

2

3

4

5

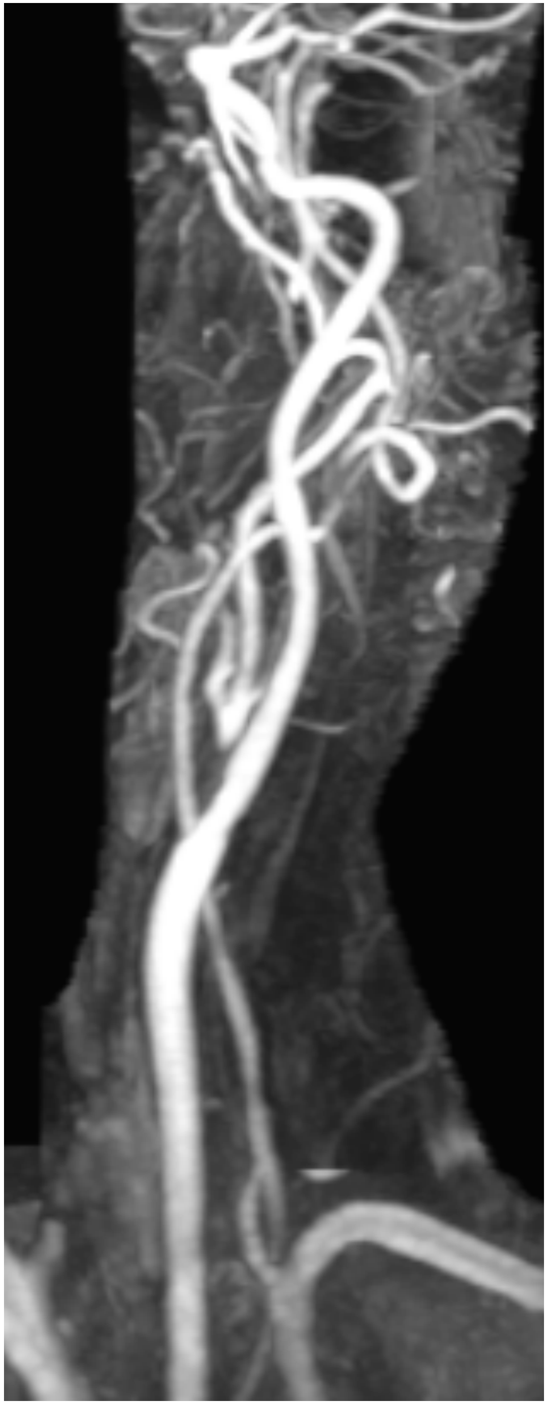

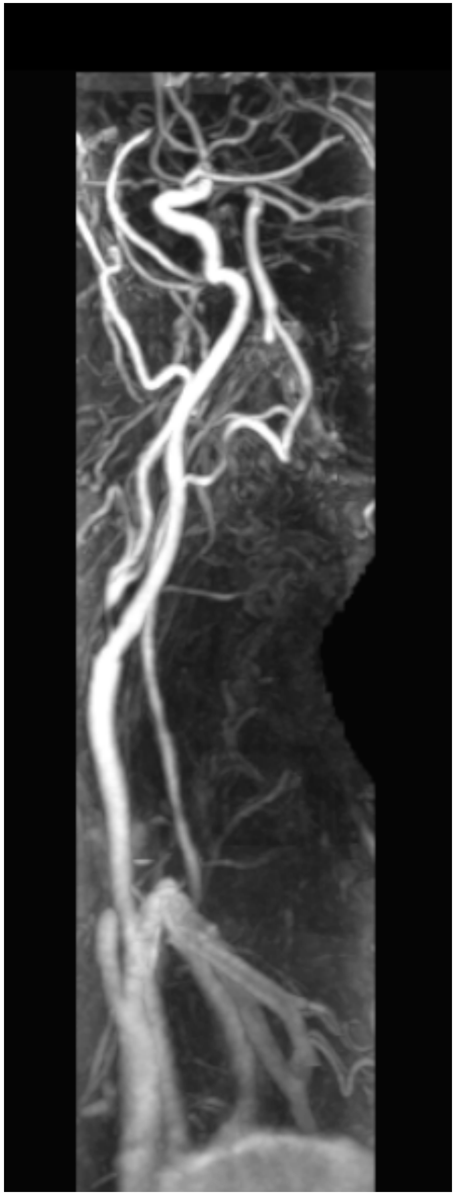

93e Score

0-30

31-50

51-70

>70

Near occlusion

Occluded

Quality

1

2

3

4

5

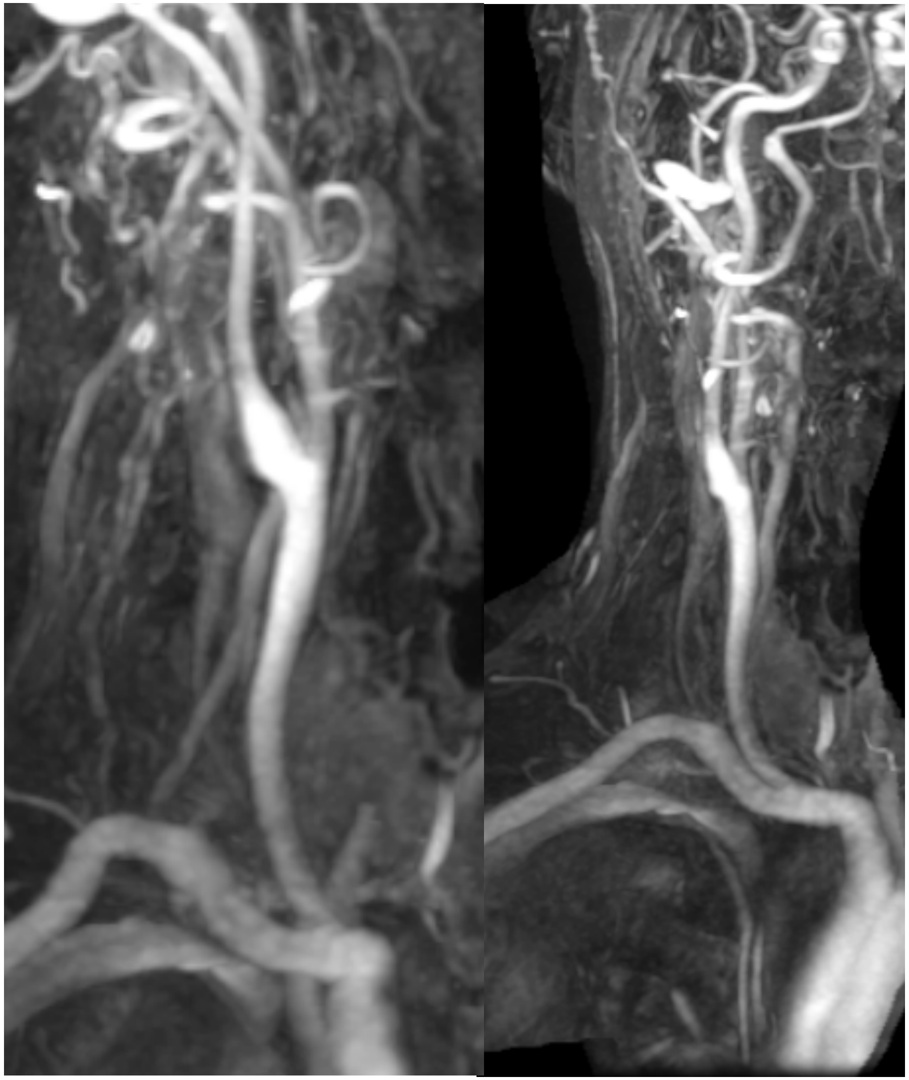

94d Score

0-30

31-50

51-70

>70

Near occlusion

Occluded

Quality

1

2

3

4

5

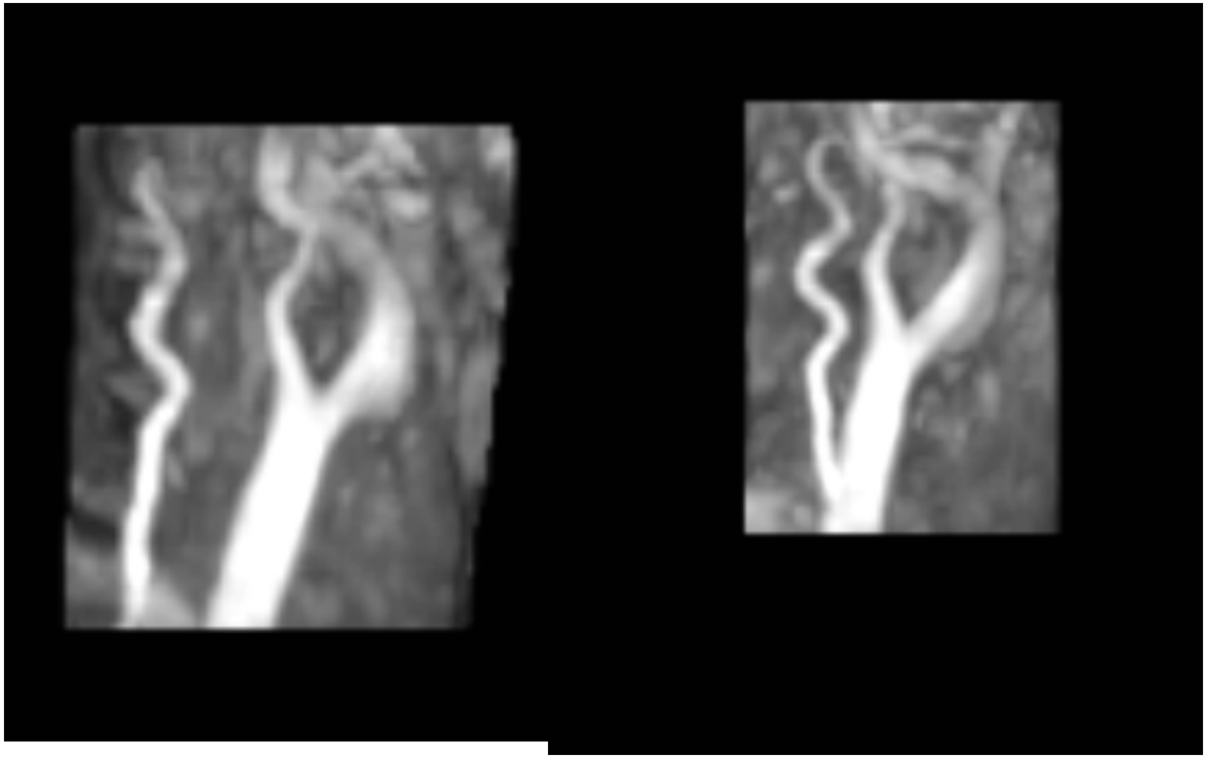

95c Score

0-30

31-50

51-70

>70

Near occlusion

Occluded

Quality

1

2

3

4

5

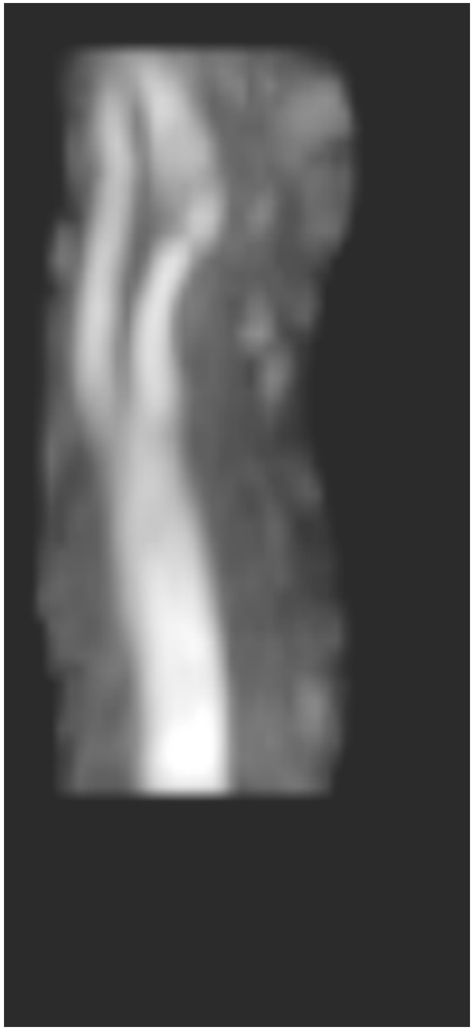

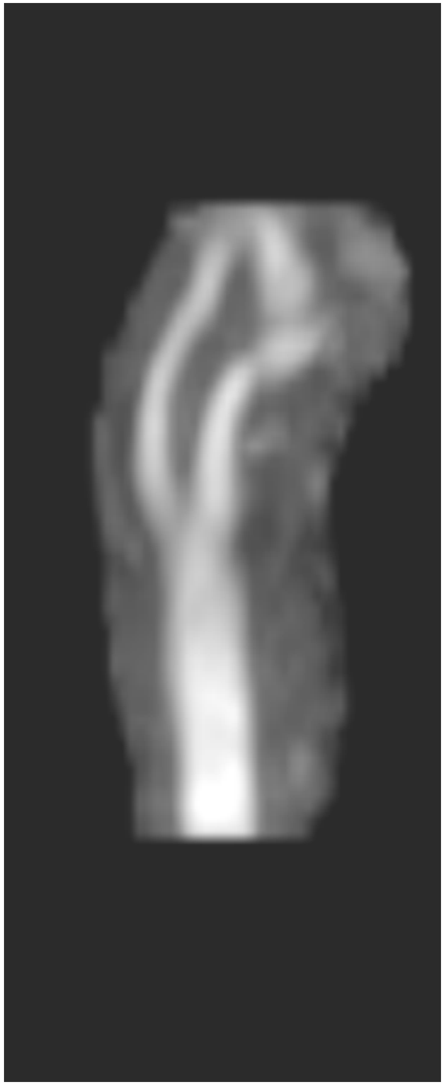

96b Score

0-30

31-50

51-70

>70

Near occlusion

Occluded

Quality

1

2

3

4

5

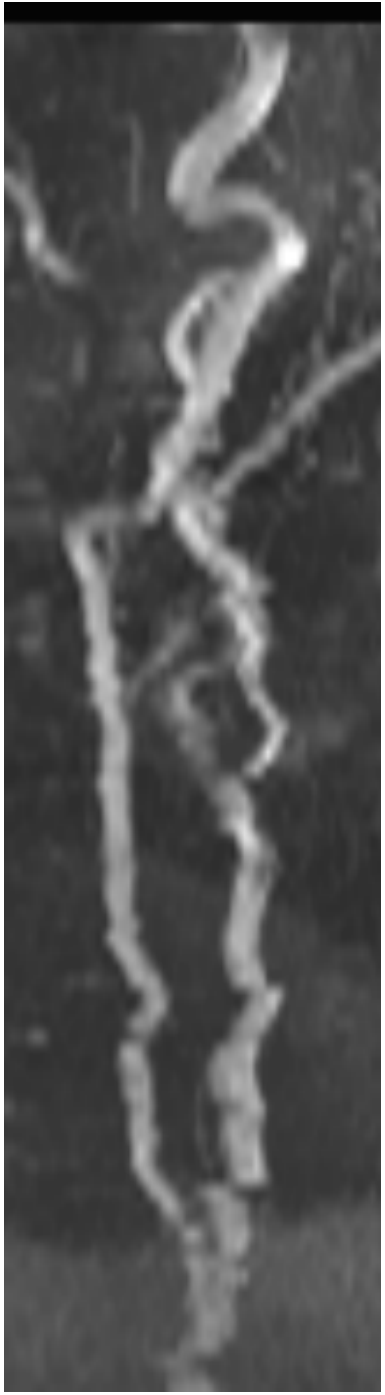

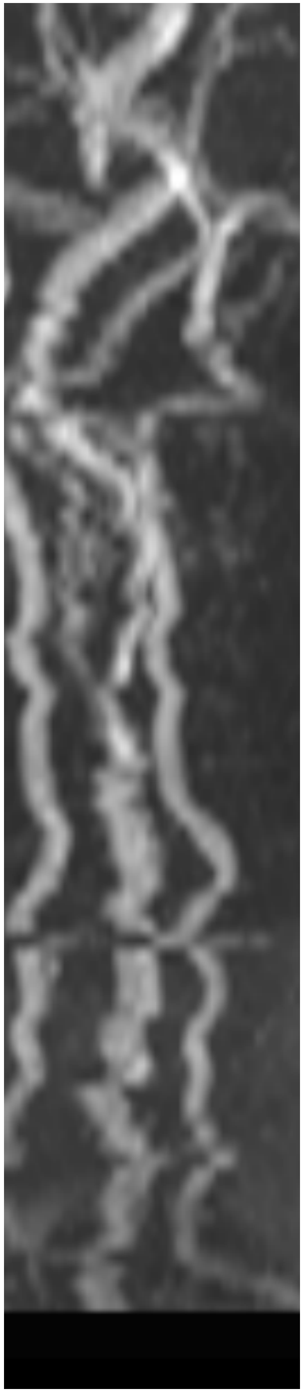

97a Score

0-30

31-50

51-70

>70

Near occlusion

Occluded

Quality

1

2

3

4

5

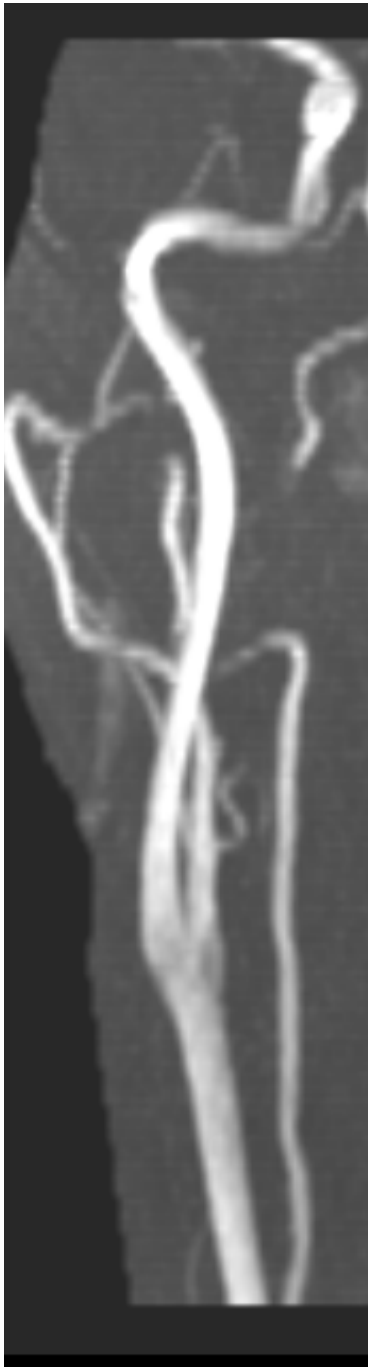

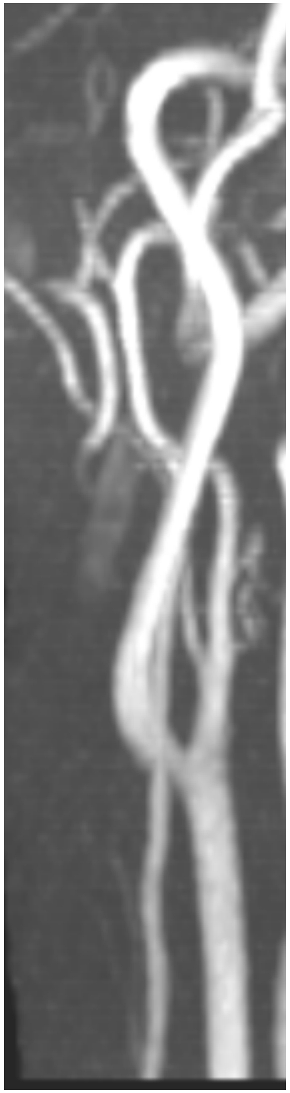

97f Score

0-30

31-50

51-70

>70

Near occlusion

Occluded

Quality

1

2

3

4

5

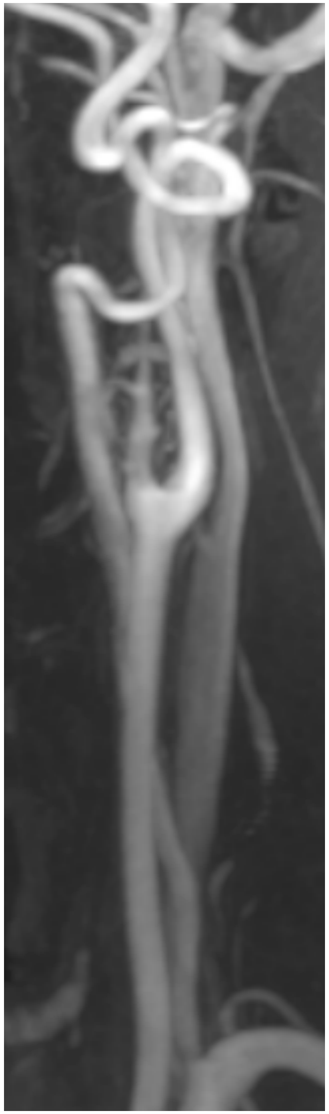

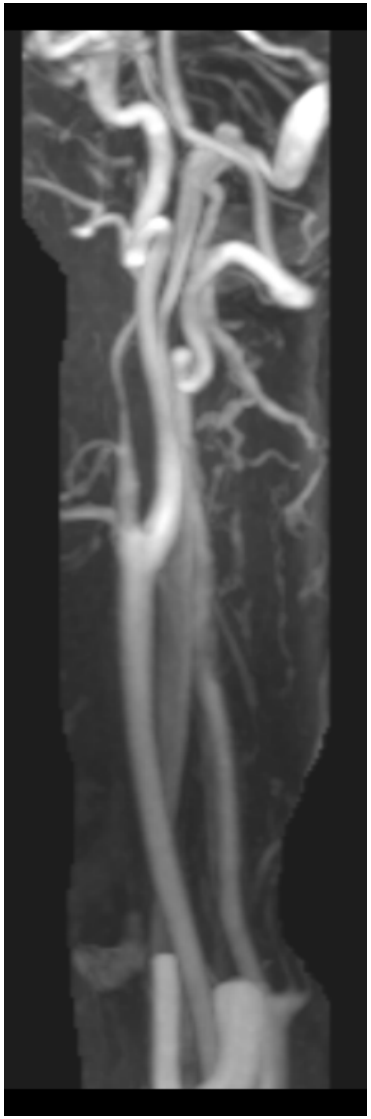

98e Score

0-30

31-50

51-70

>70

Near occlusion

Occluded

Quality

1

2

3

4

5

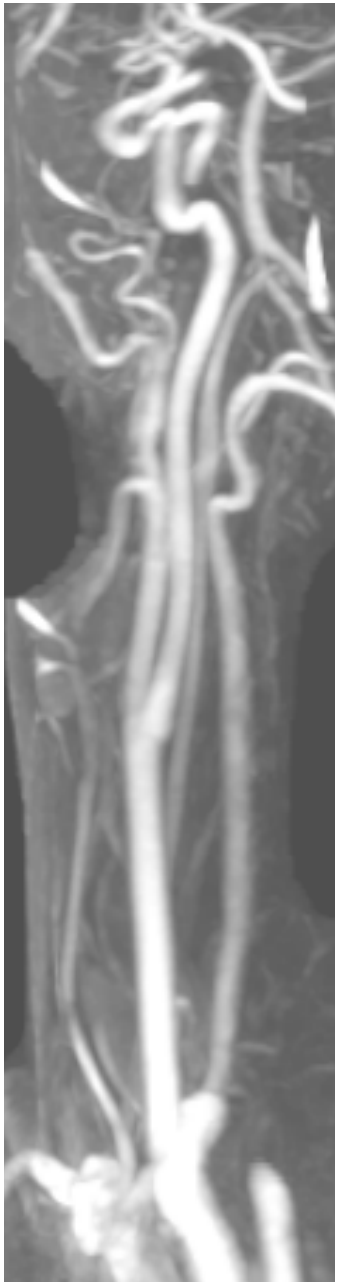

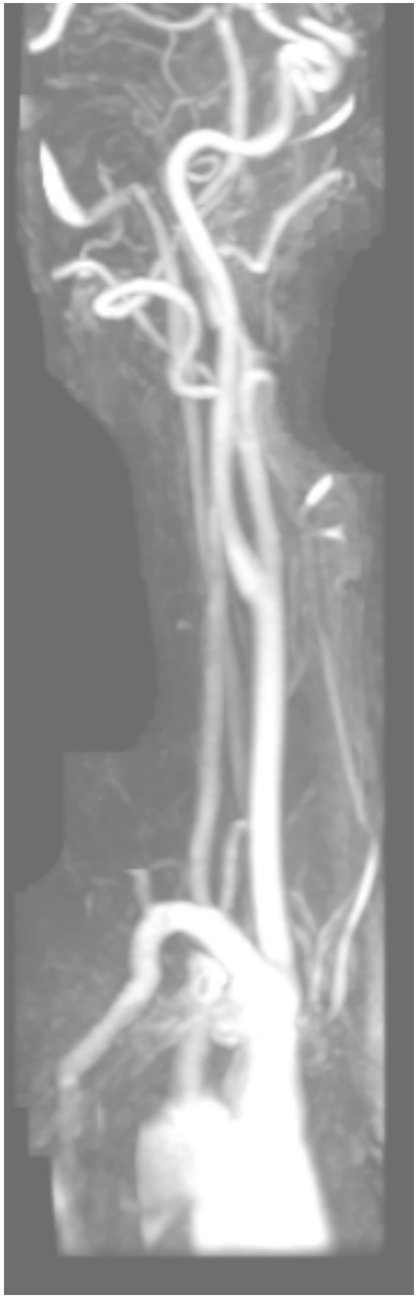

99d Score

0-30

31-50

51-70

>70

Near occlusion

Occluded

Quality

1

2

3

4

5

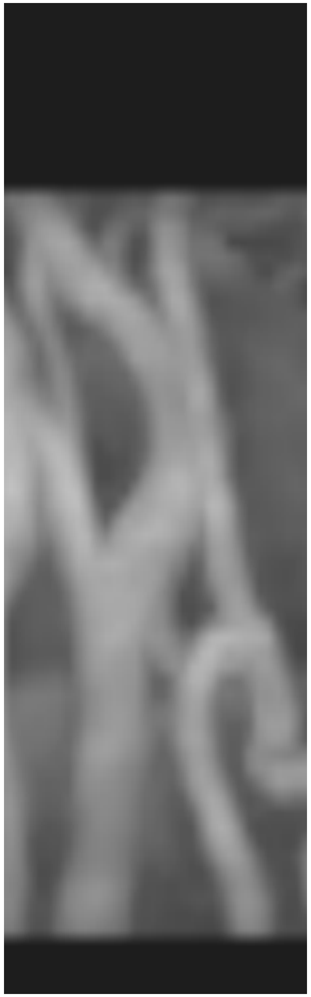

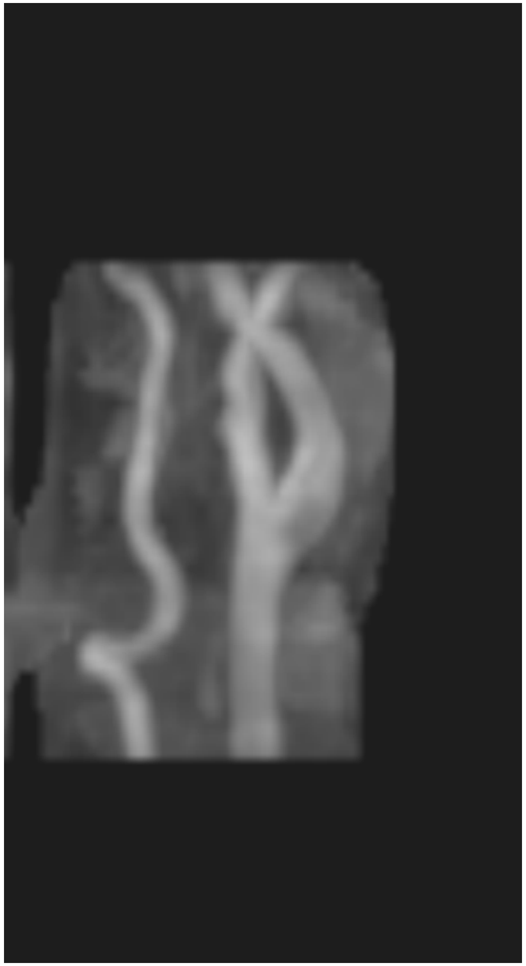

100c Score

0-30

31-50

51-70

>70

Near occlusion

Occluded

Quality

1

2

3

4

5

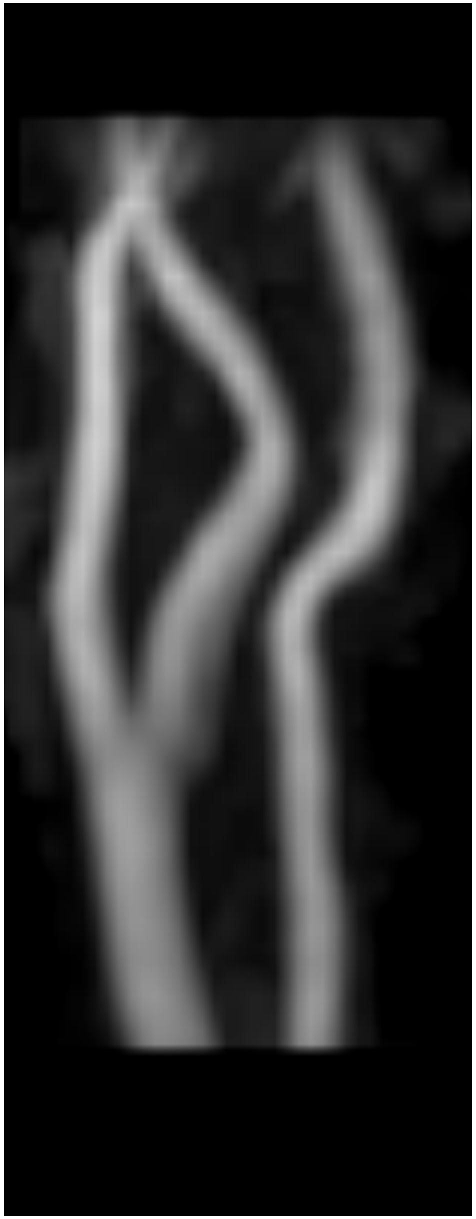

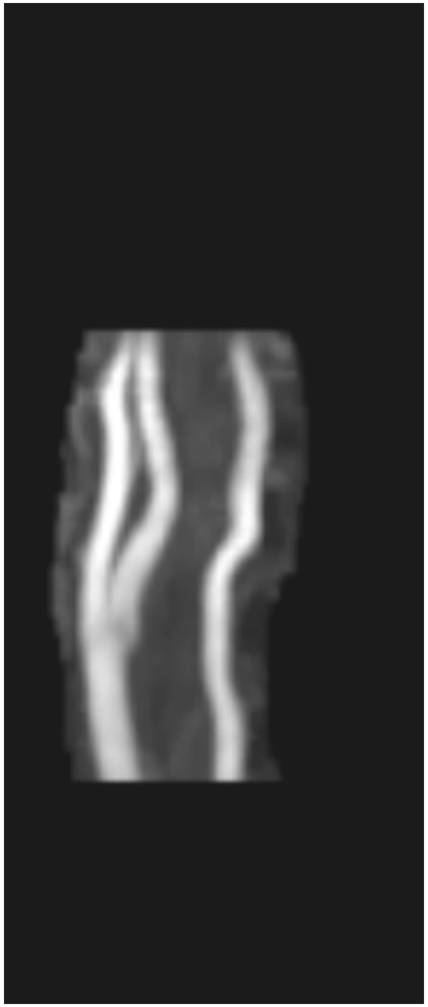

101b Score

0-30

31-50

51-70

>70

Near occlusion

Occluded

Quality

1

2

3

4

5

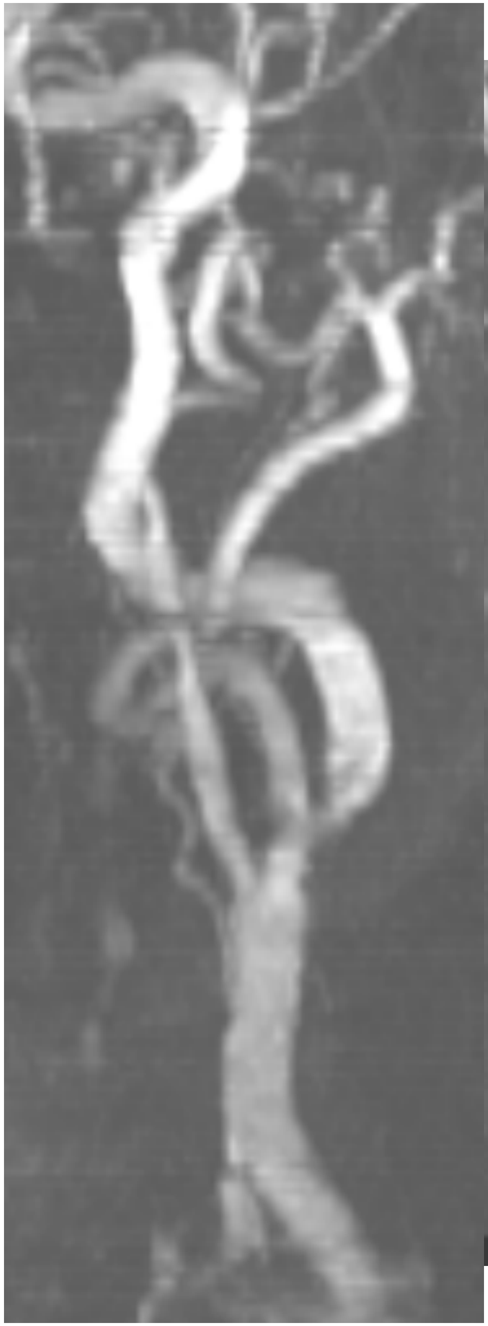

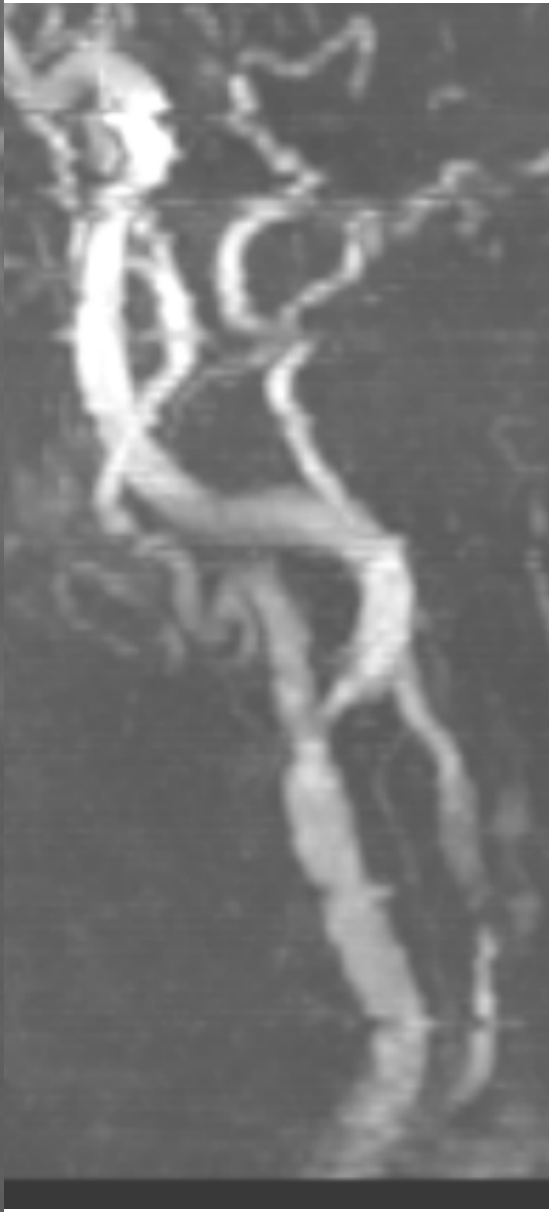

102a Score

0-30

31-50

51-70

>70

Near occlusion

Occluded

Quality

1

2

3

4

5

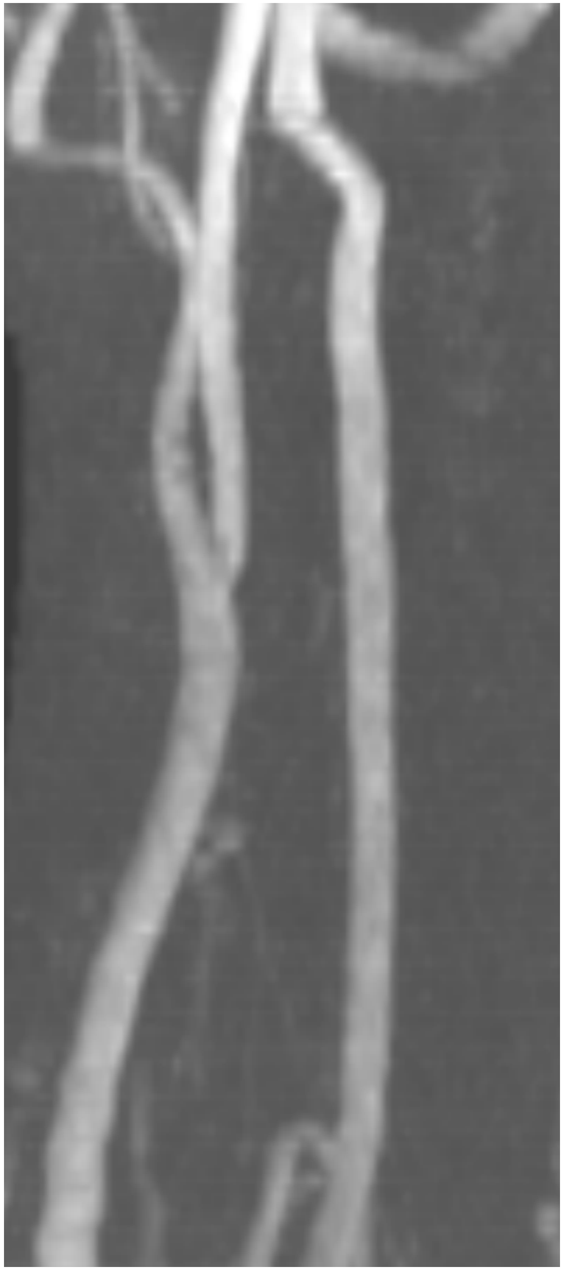

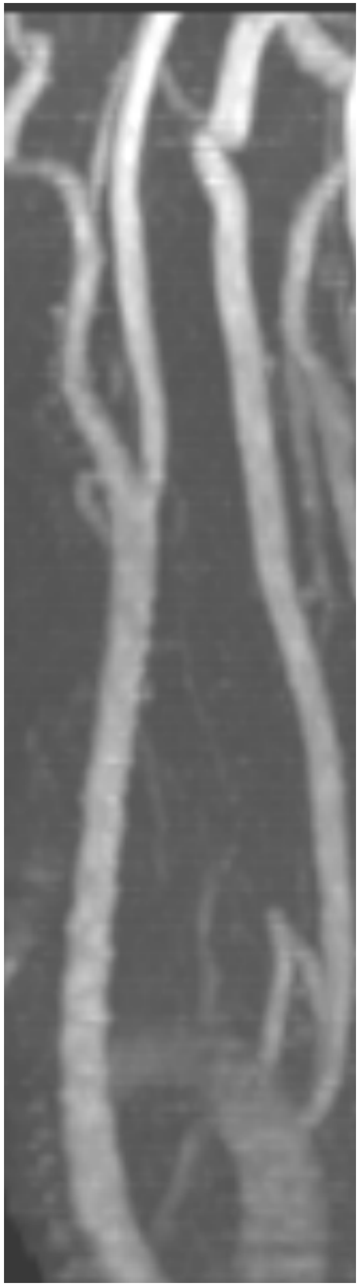

102f Score

0-30

31-50

51-70

>70

Near occlusion

Occluded

Quality

1

2

3

4

5

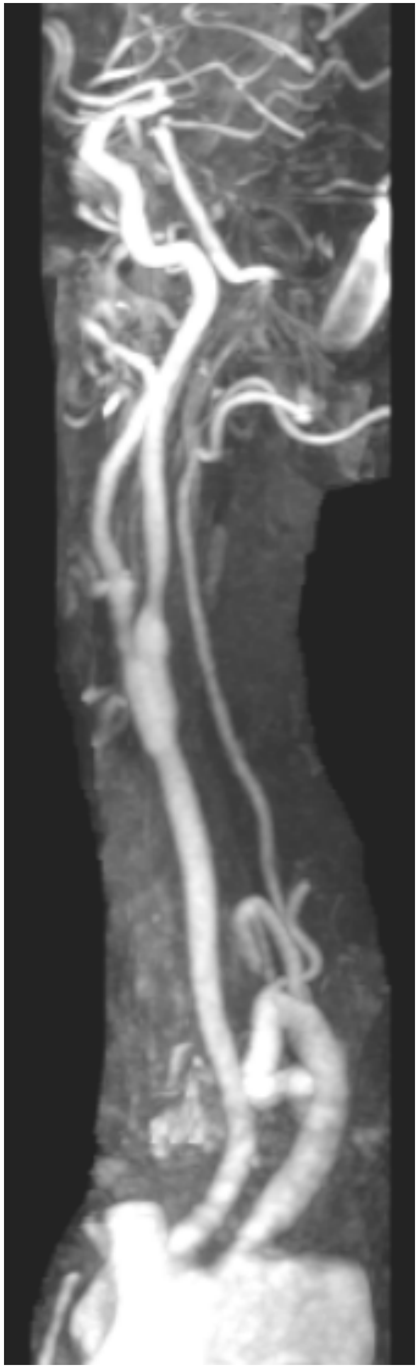

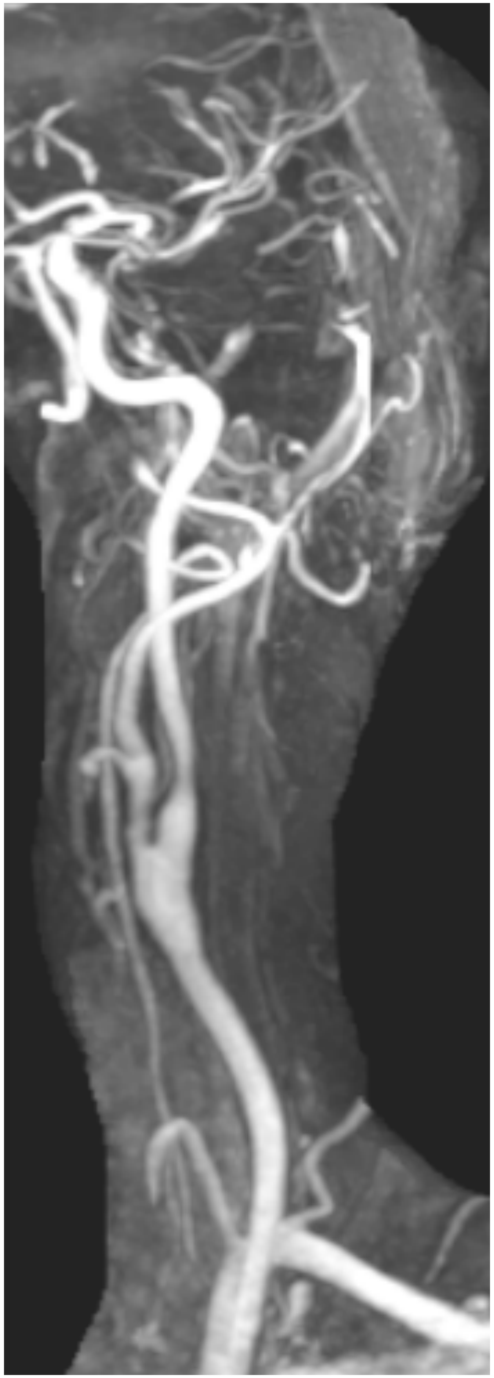

103e Score

0-30

31-50

51-70

>70

Near occlusion

Occluded

Quality

1

2

3

4

5

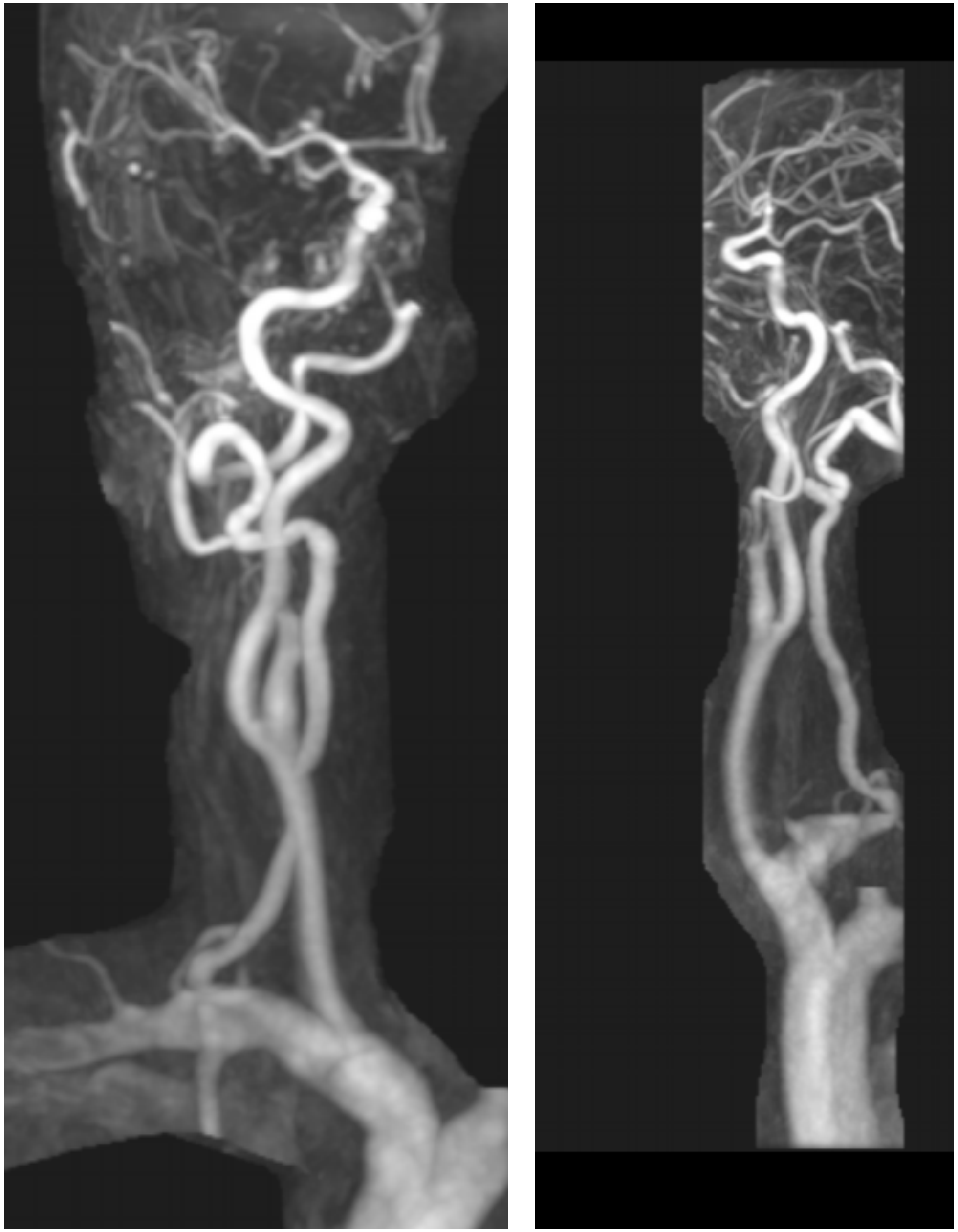

104d Score

0-30

31-50

51-70

>70

Near occlusion

Occluded

Quality

1

2

3

4

5

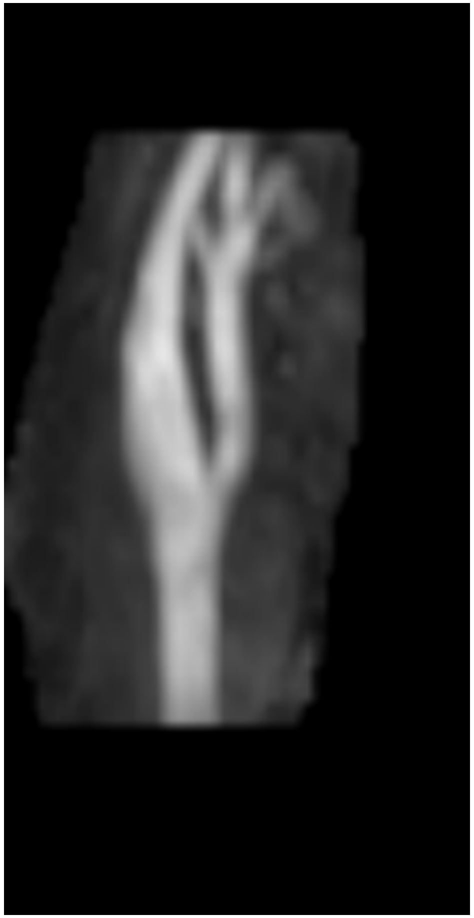

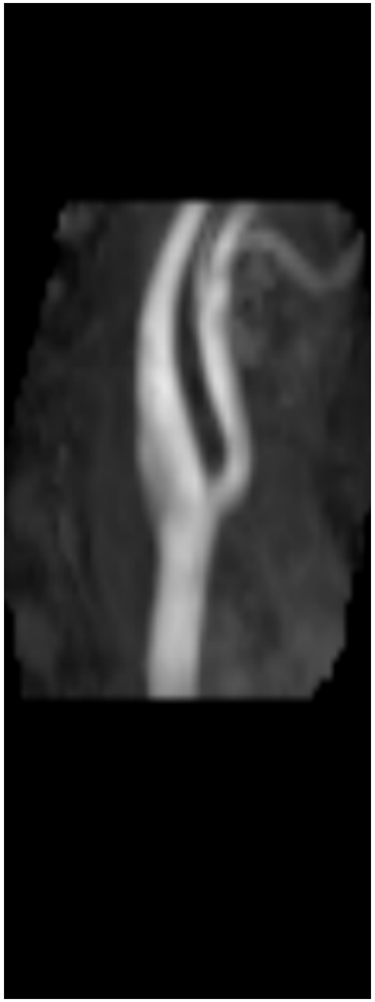

105c Score

0-30

31-50

51-70

>70

Near occlusion

Occluded

Quality

1

2

3

4

5

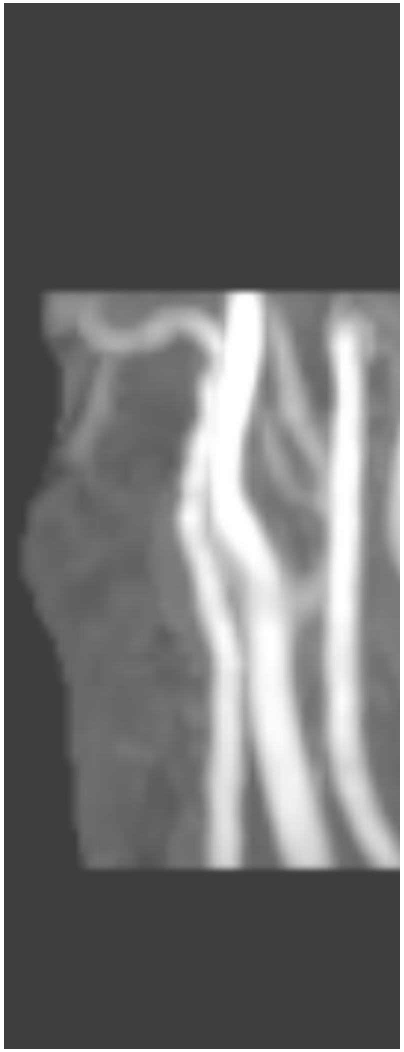

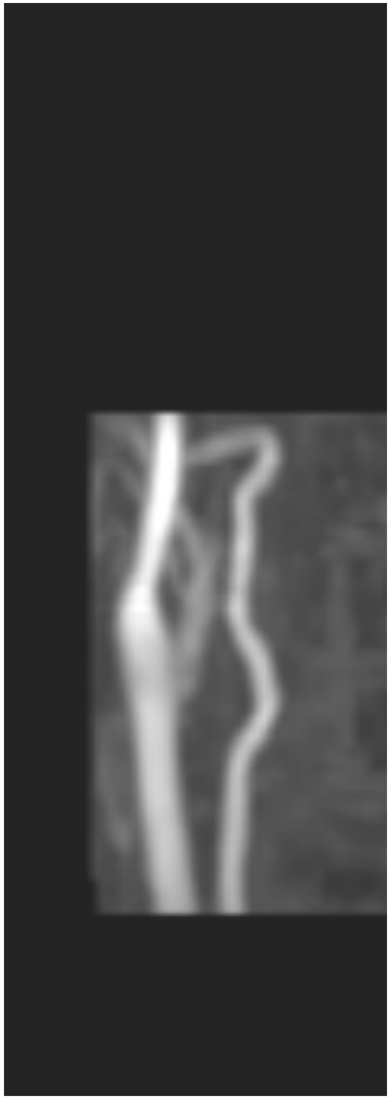

106b Score

0-30

31-50

51-70

>70

Near occlusion

Occluded

Quality

1

2

3

4

5

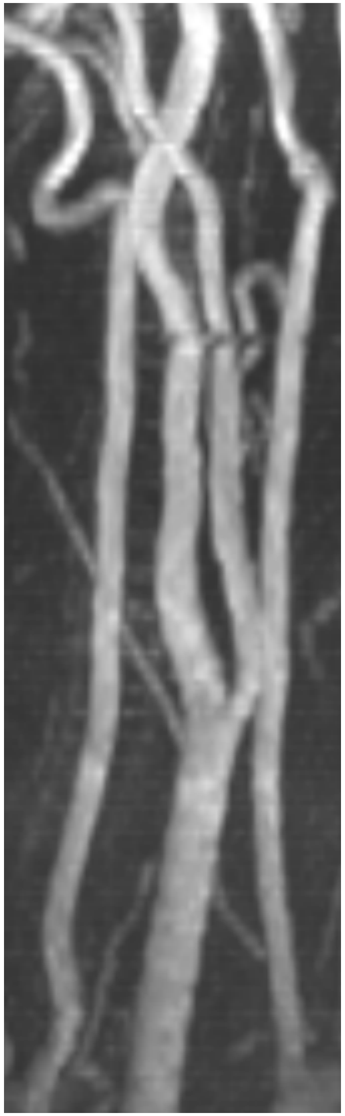

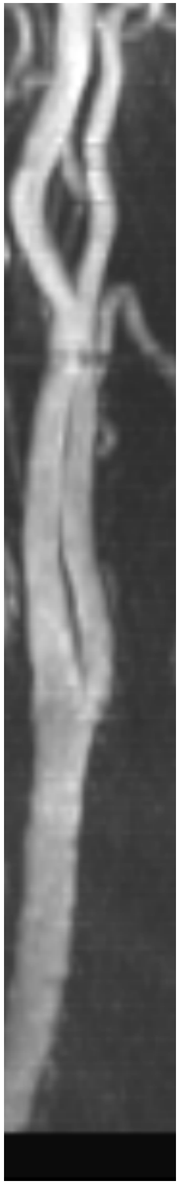

107a Score

0-30

31-50

51-70

>70

Near occlusion

Occluded

Quality

1

2

3

4

5

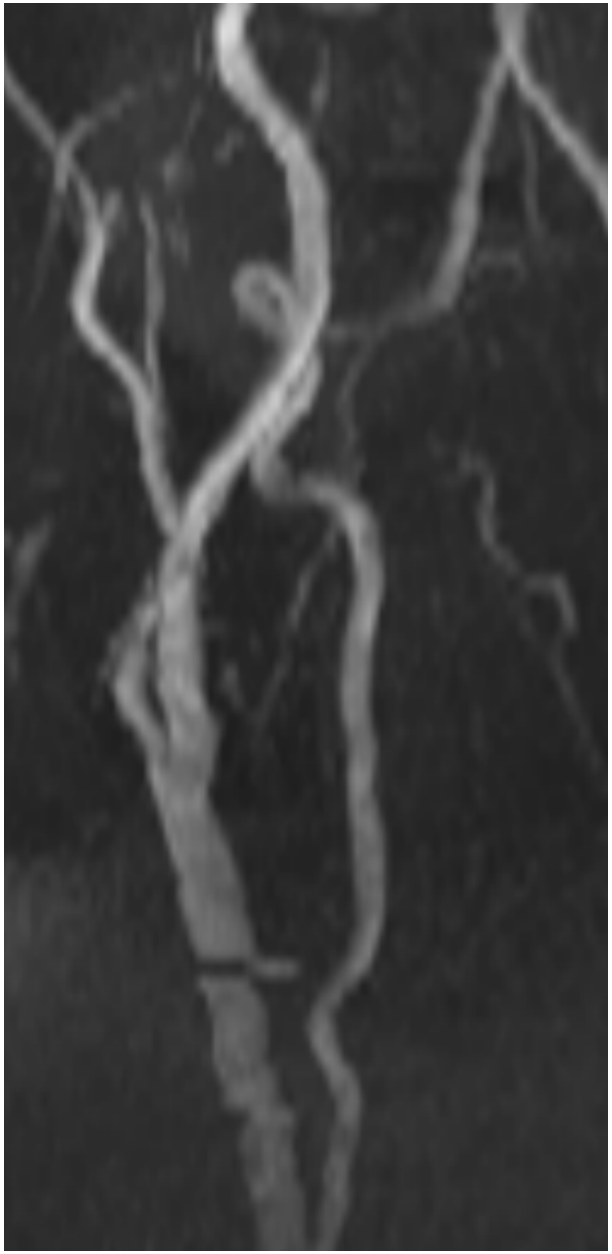

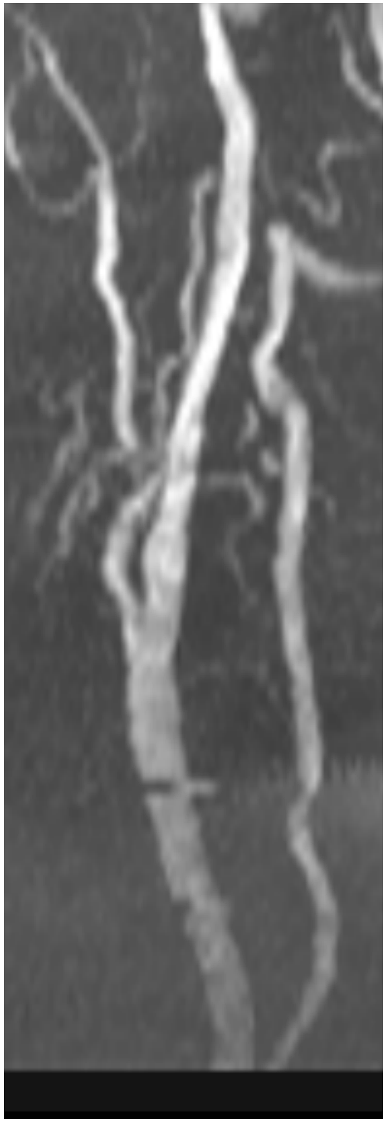

107f Score

0-30

31-50

51-70

>70

Near occlusion

Occluded

Quality

1

2

3

4

5

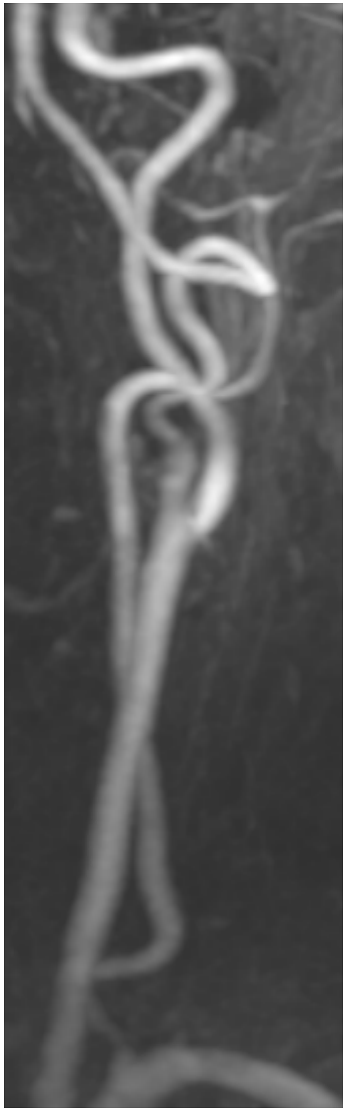

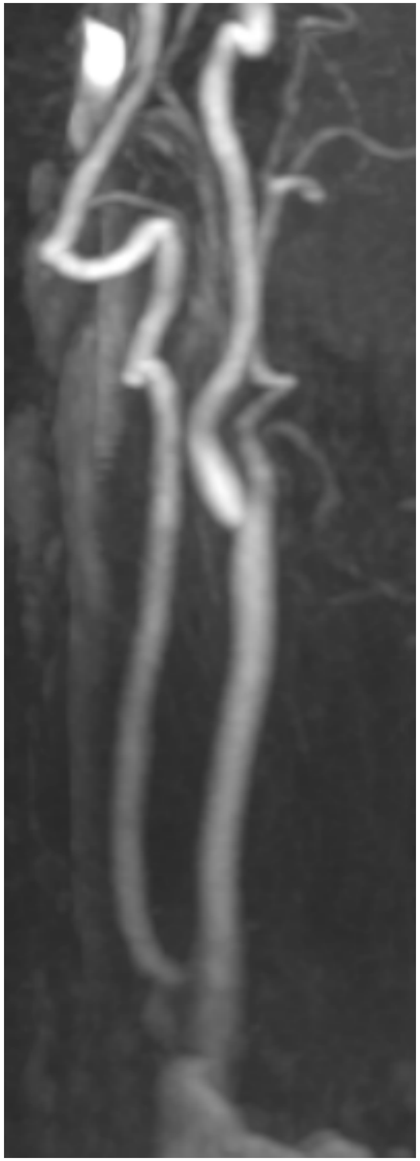

108e Score

0-30

31-50

51-70

>70

Near occlusion

Occluded

Quality

1

2

3

4

5

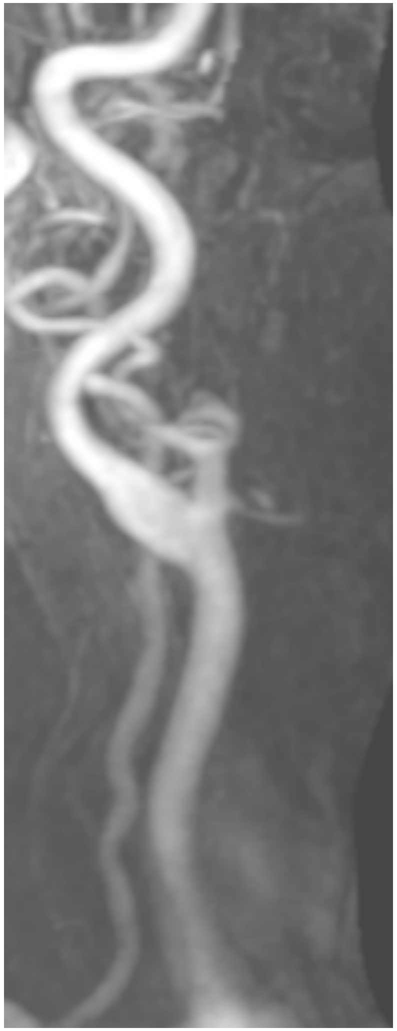

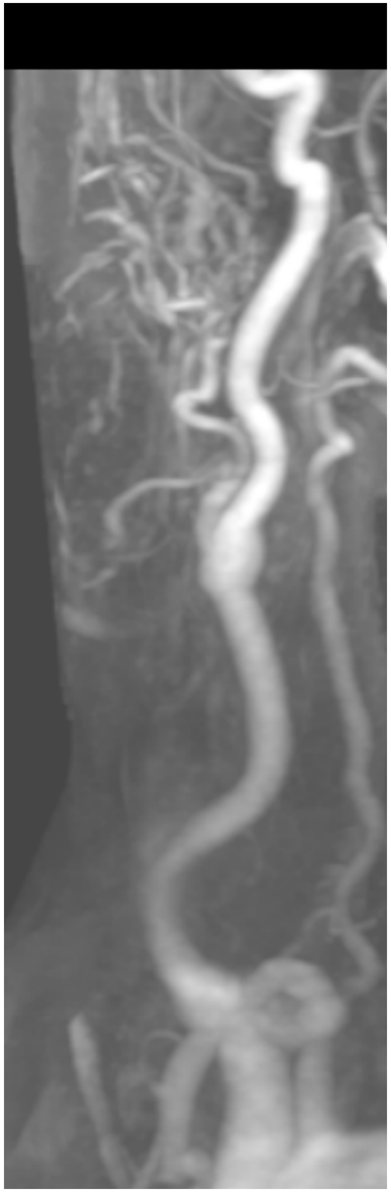

109d Score

0-30

31-50

51-70

>70

Near occlusion

Occluded

Quality

1

2

3

4

5

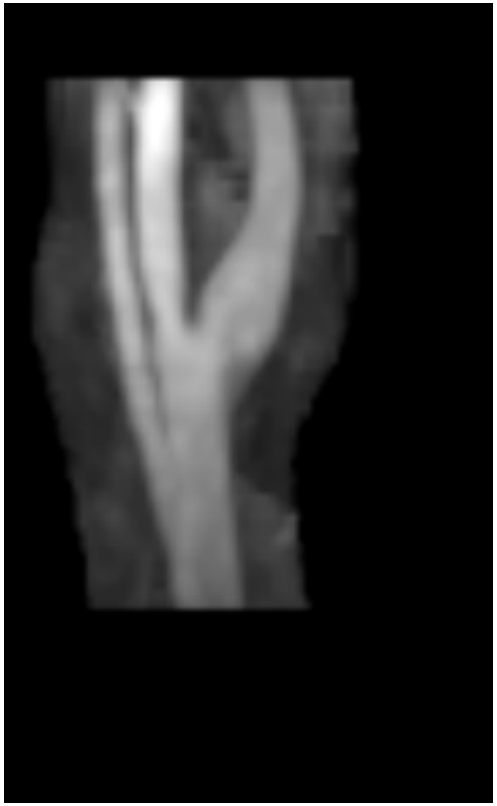

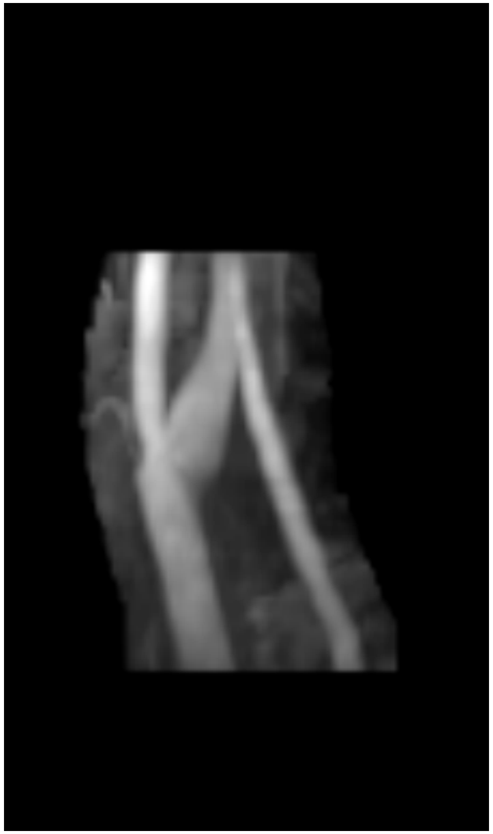

110c Score

0-30

31-50

51-70

>70

Near occlusion

Occluded

Quality

1

2

3

4

5

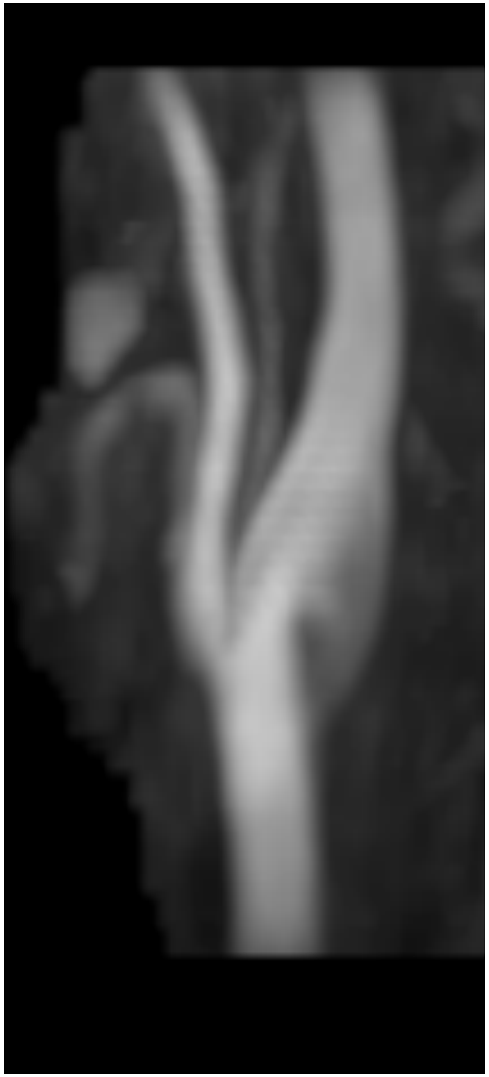

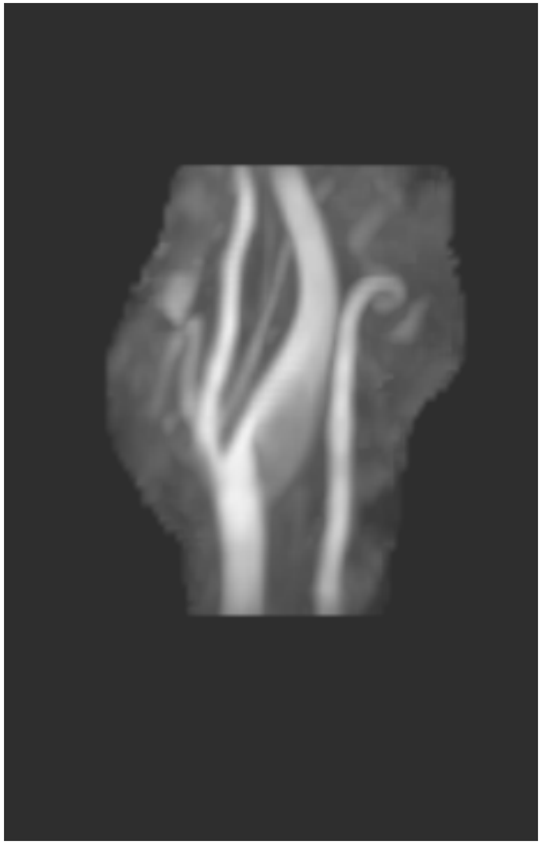

111b Score

0-30

31-50

51-70

>70

Near occlusion

Occluded

Quality

1

2

3

4

5

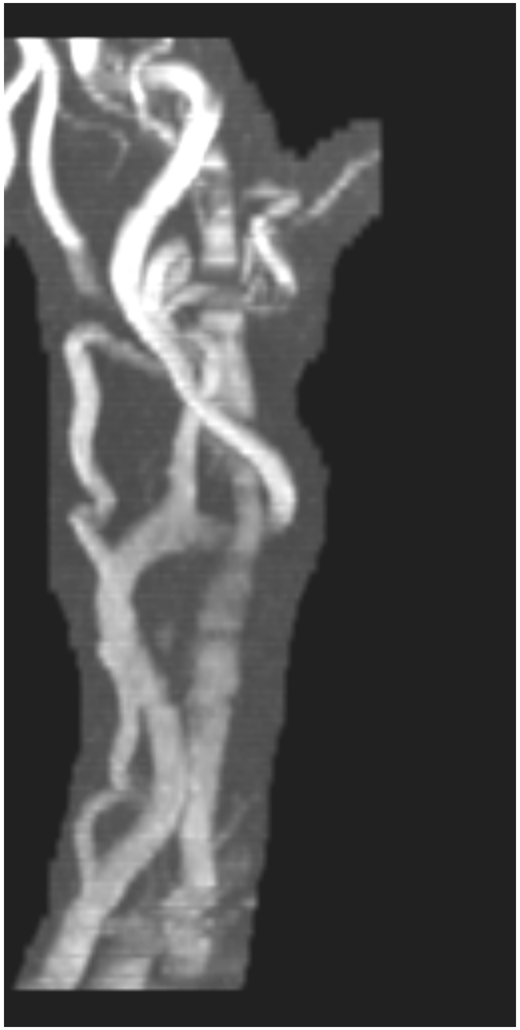

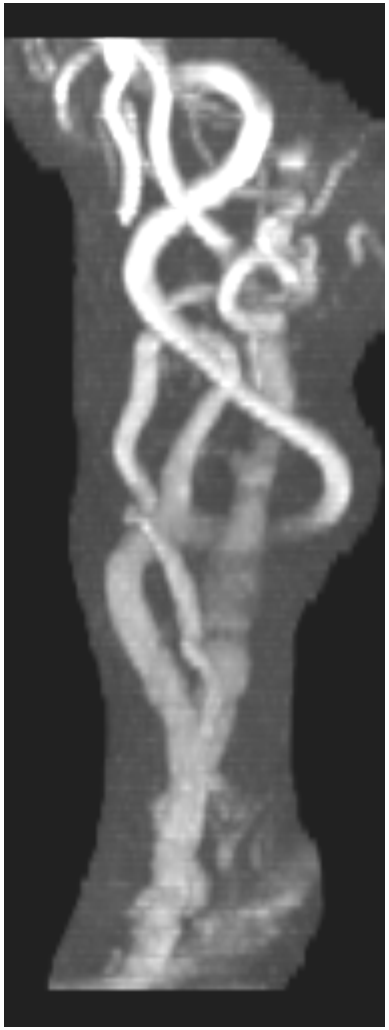

112a Score

0-30

31-50

51-70

>70

Near occlusion

Occluded

Quality

1

2

3

4

5

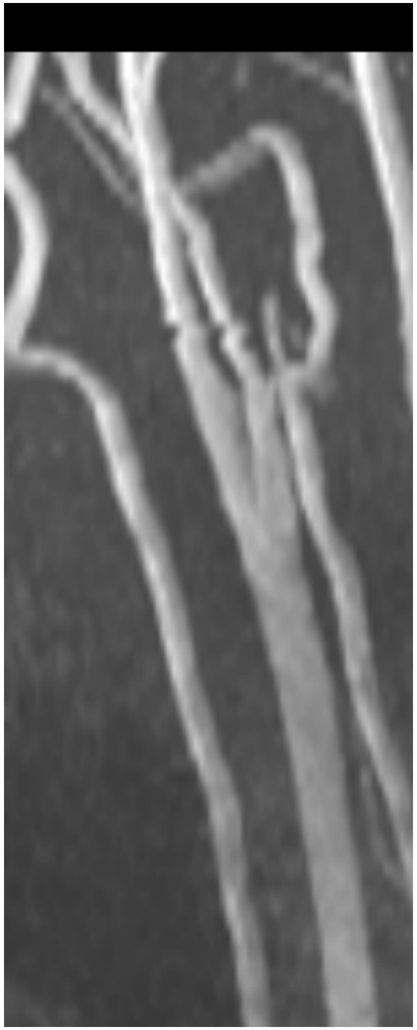

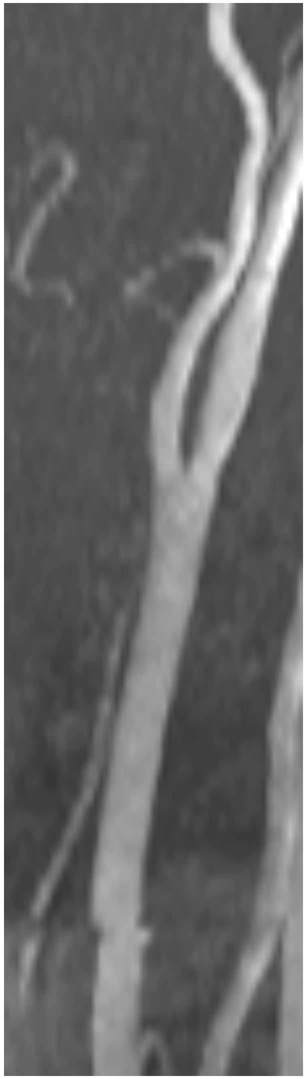

112f Score

0-30

31-50

51-70

>70

Near occlusion

Occluded

Quality

1

2

3

4

5

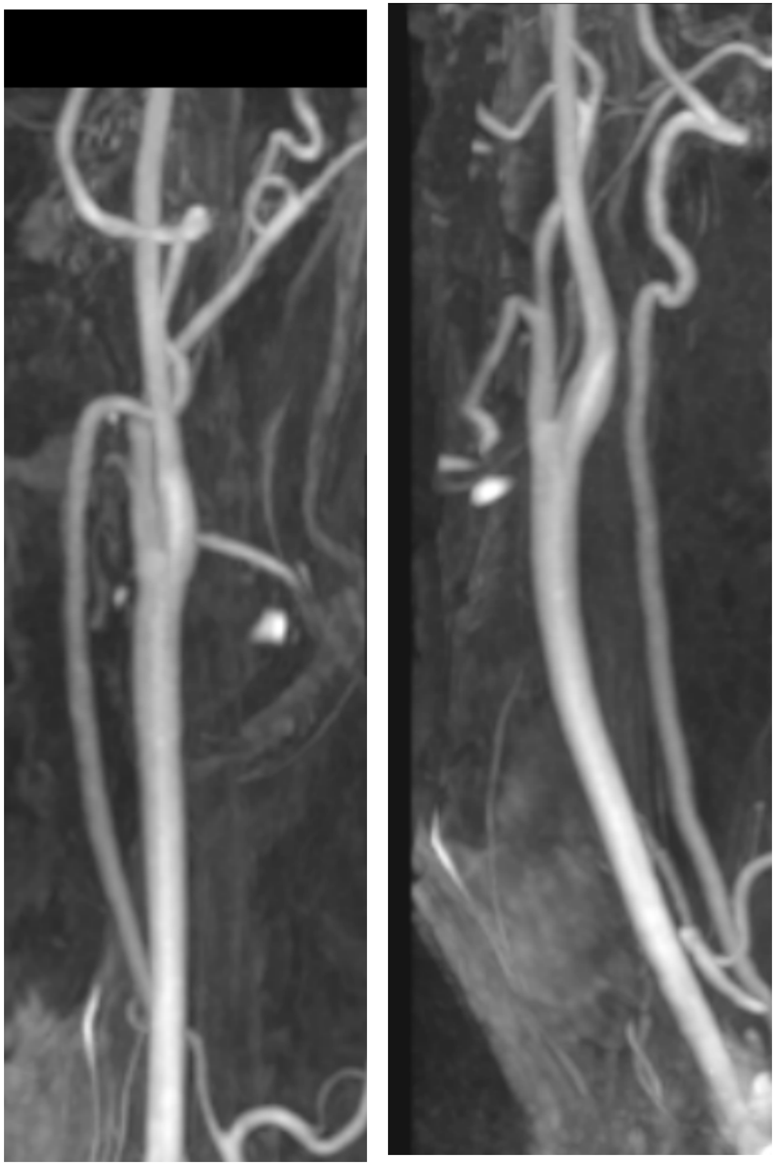

113e Score

0-30

31-50

51-70

>70

Near occlusion

Occluded

Quality

1

2

3

4

5

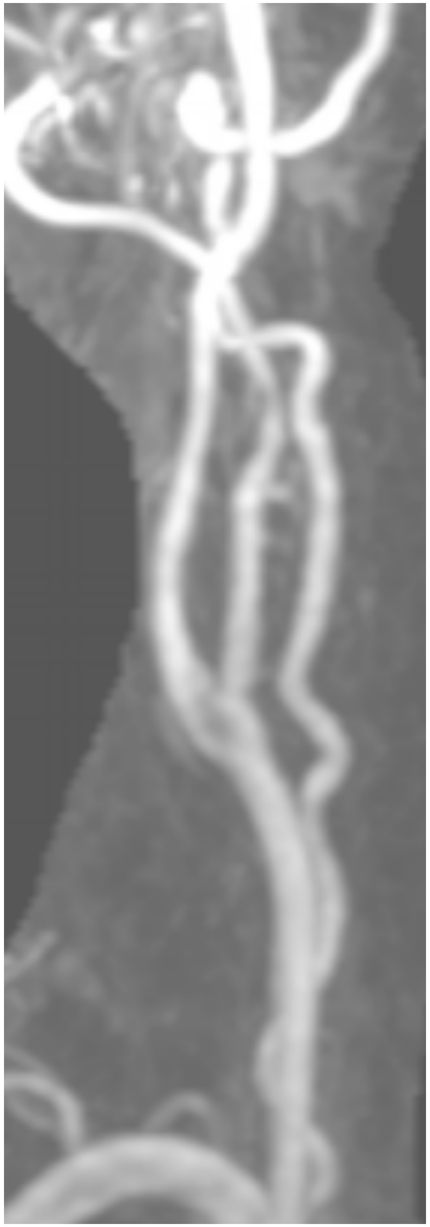

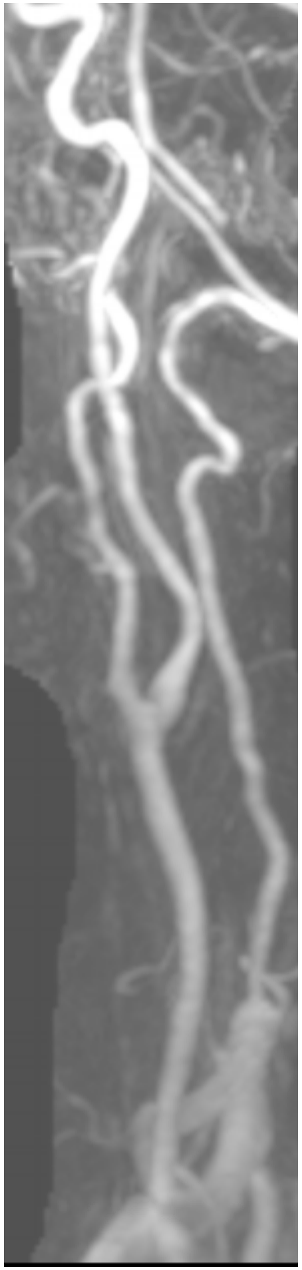

114d Score

0-30

31-50

51-70

>70

Near occlusion

Occluded

Quality

1

2

3

4

5

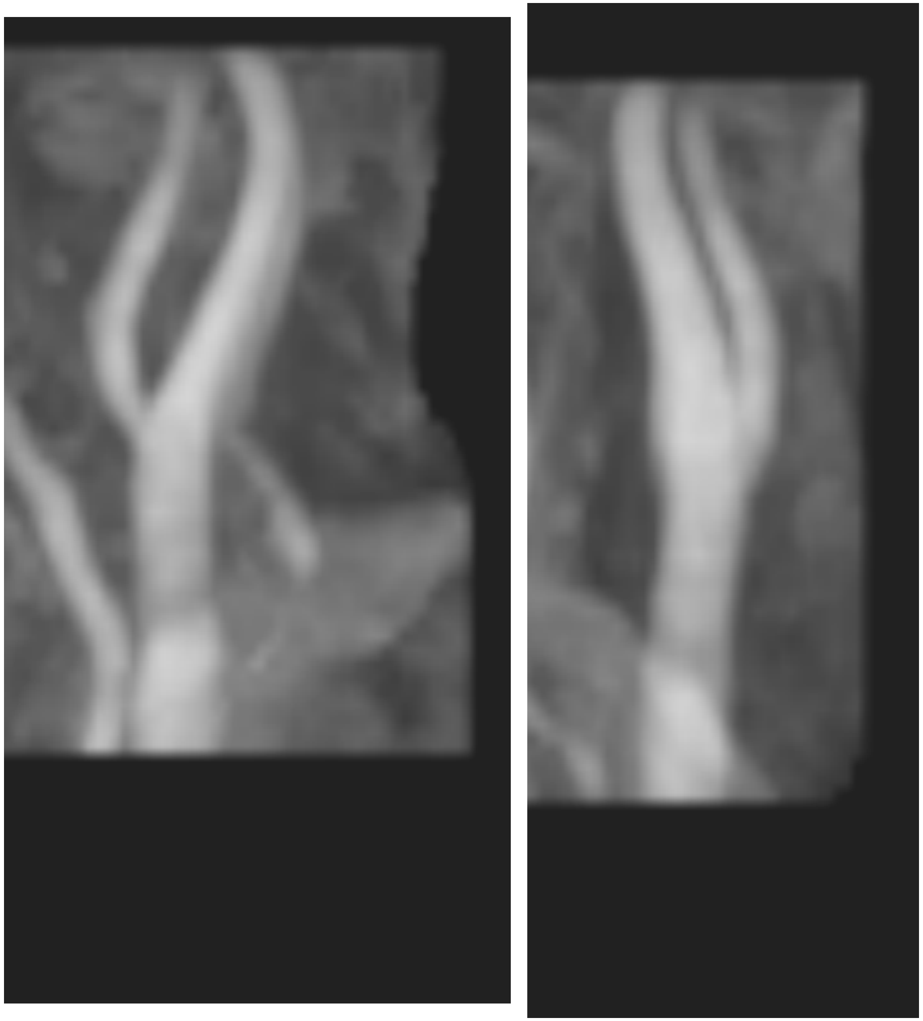

115c Score

0-30

31-50

51-70

>70

Near occlusion

Occluded

Quality

1

2

3

4

5

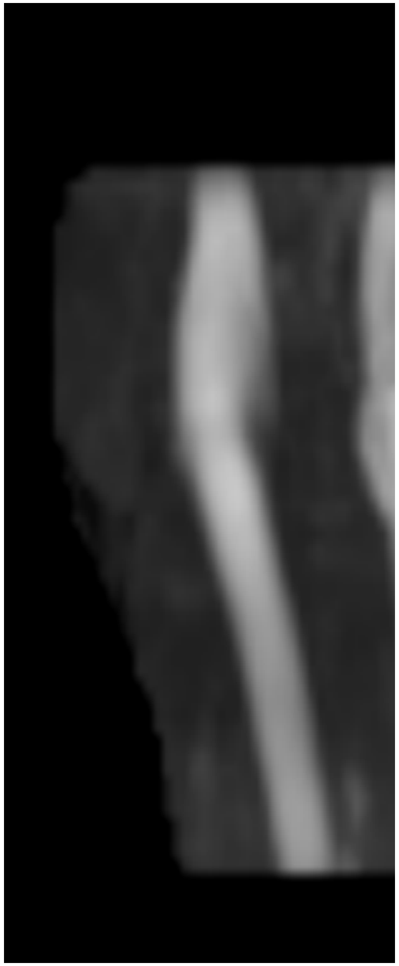

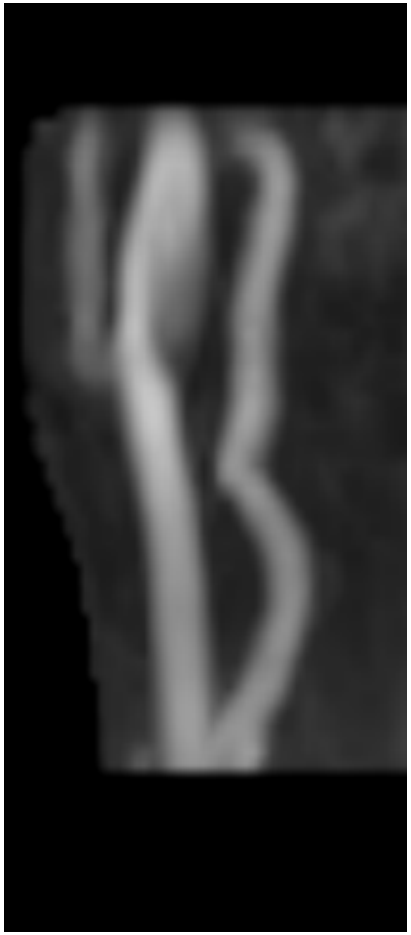

116b Score

0-30

31-50

51-70

>70

Near occlusion

Occluded

Quality

1

2

3

4

5

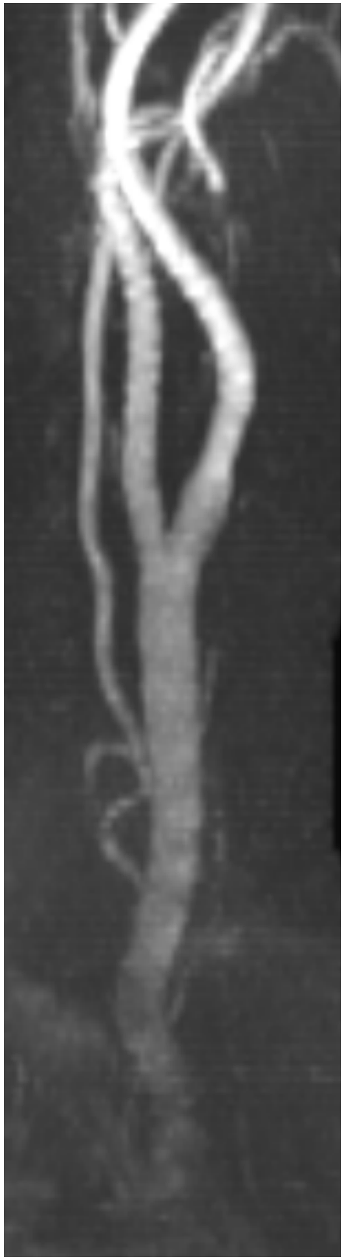

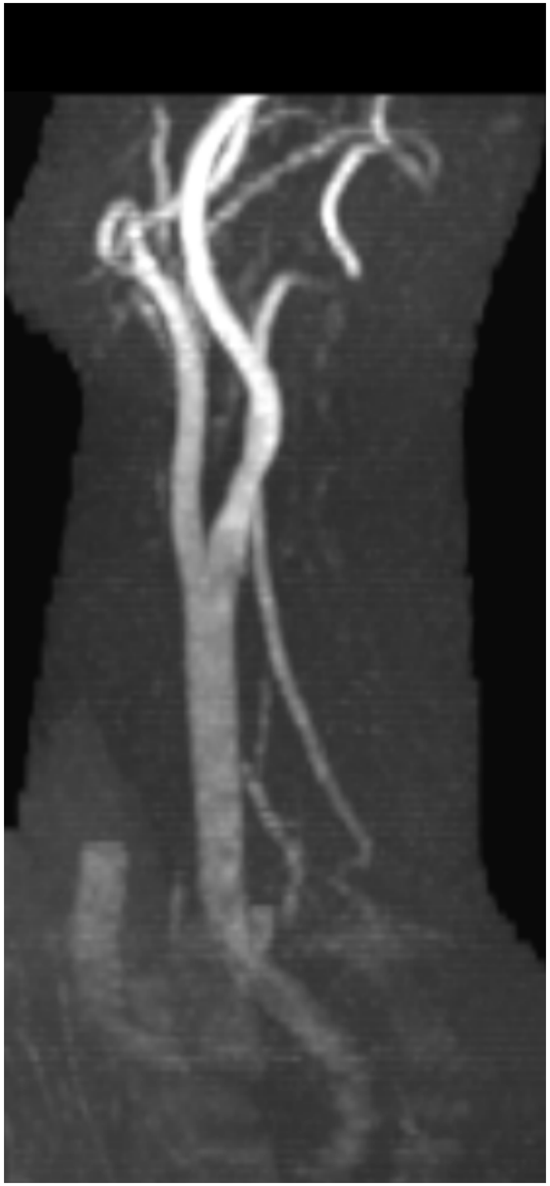

117a Score

0-30

31-50

51-70

>70

Near occlusion

Occluded

Quality

1

2

3

4

5

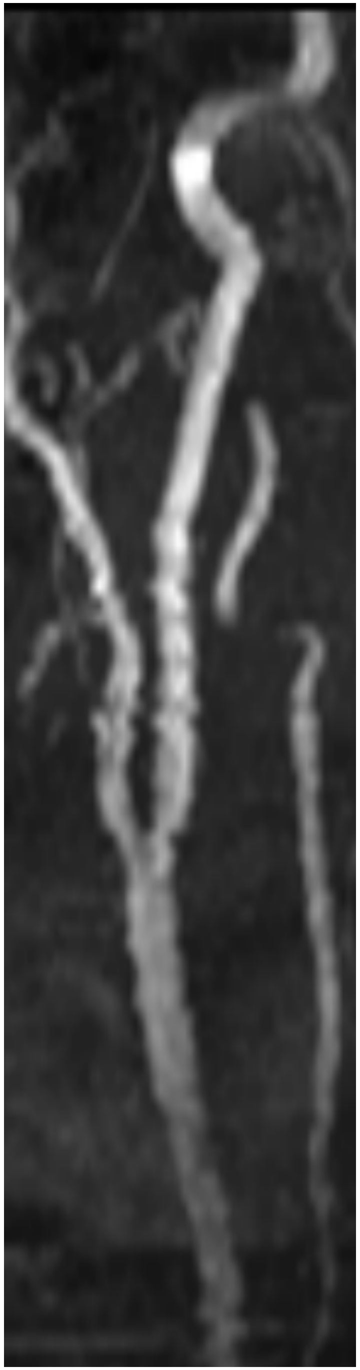

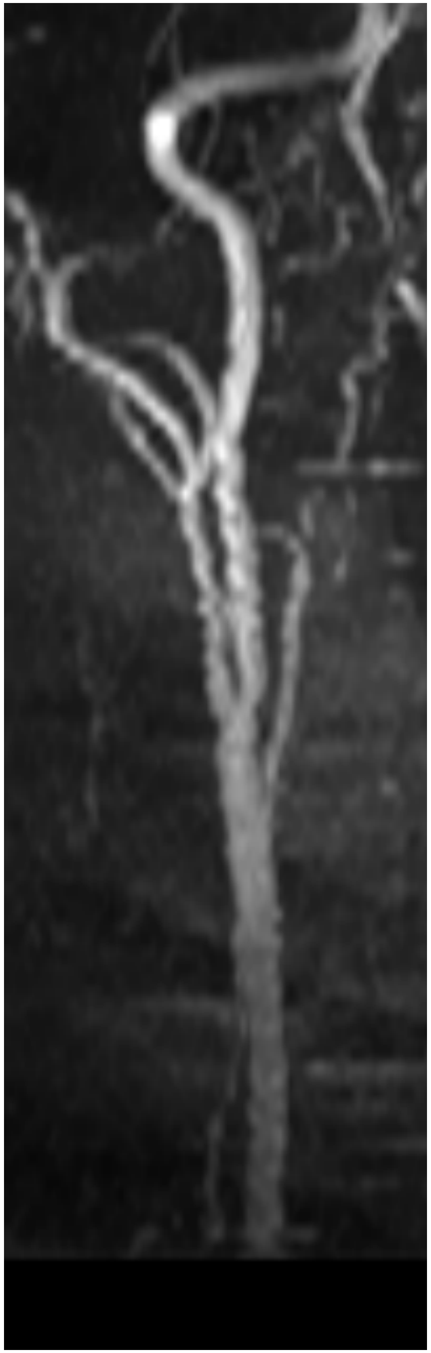

117f Score

0-30

31-50

51-70

>70

Near occlusion

Occluded

Quality

1

2

3

4

5

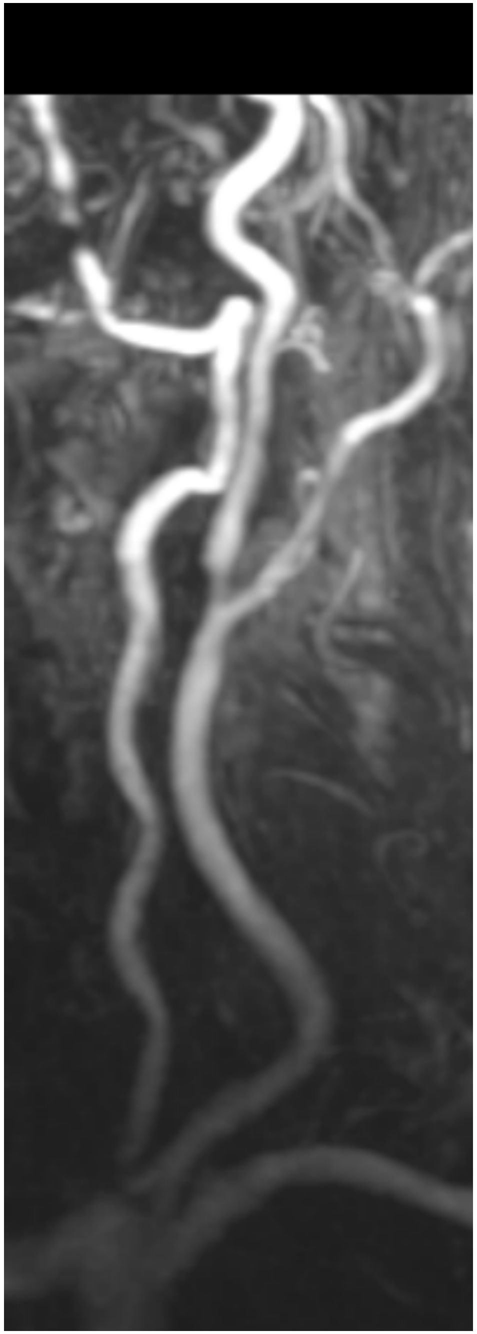

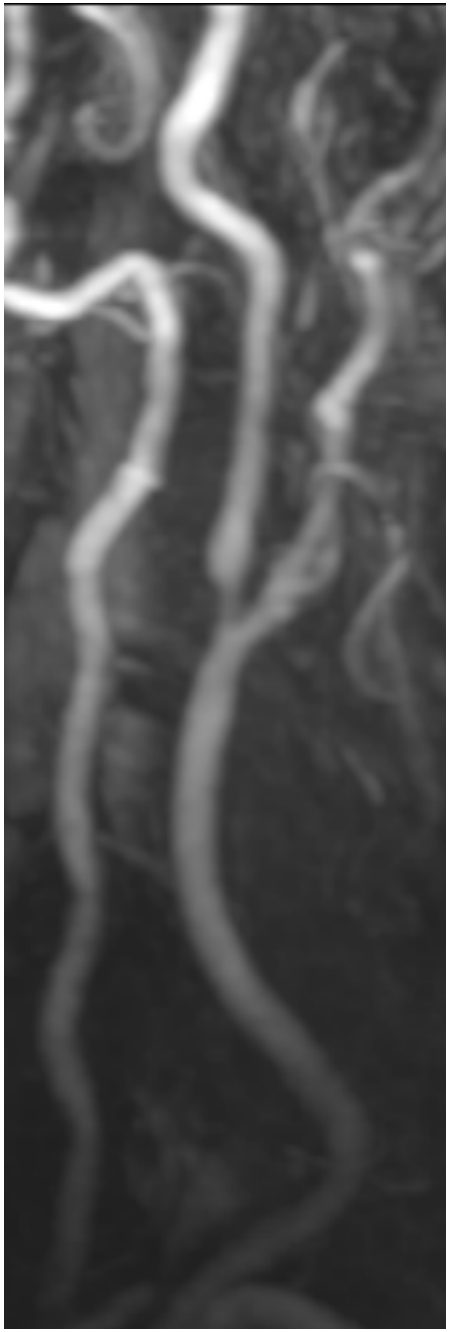

118e Score

0-30

31-50

51-70

>70

Near occlusion

Occluded

Quality

1

2

3

4

5

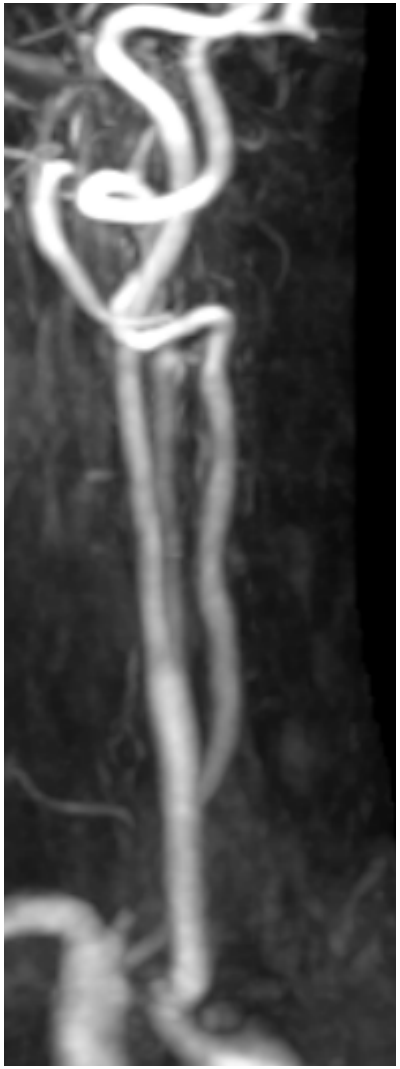

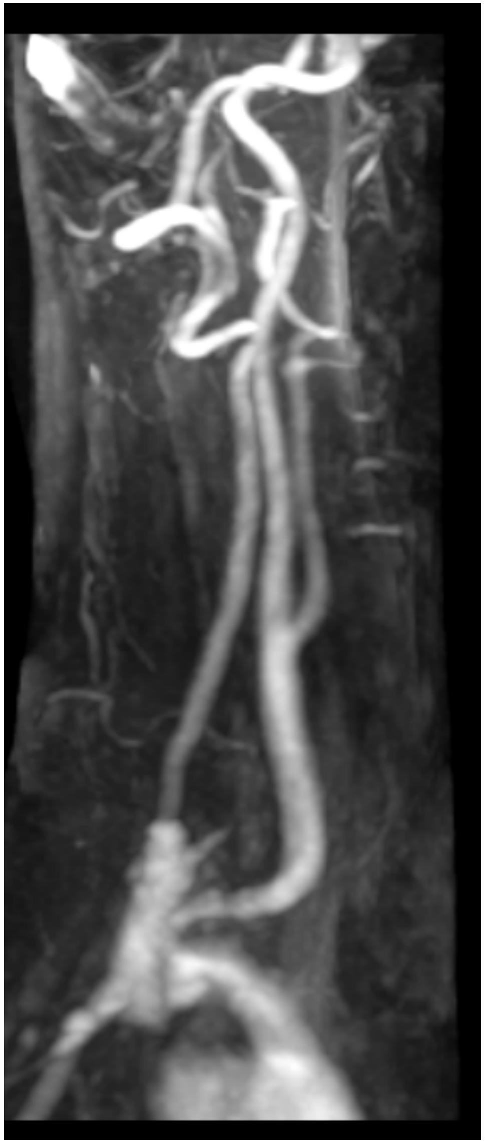

119d Score

0-30

31-50

51-70

>70

Near occlusion

Occluded

Quality

1

2

3

4

5

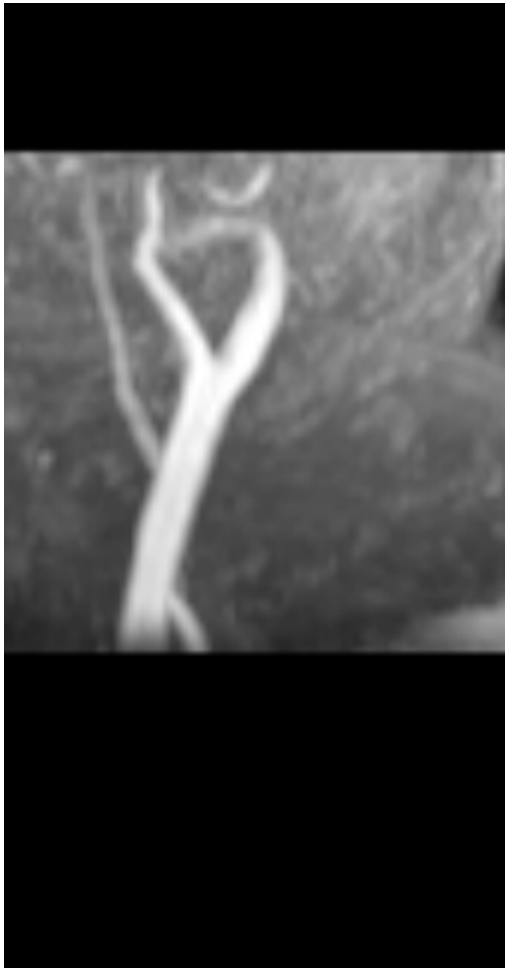

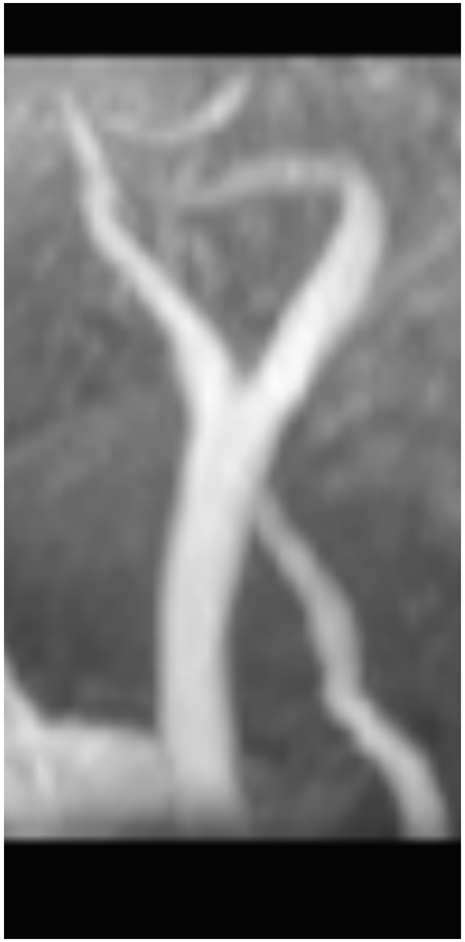

120c Score

0-30

31-50

51-70

>70

Near occlusion

Occluded

Quality

1

2

3

4

5

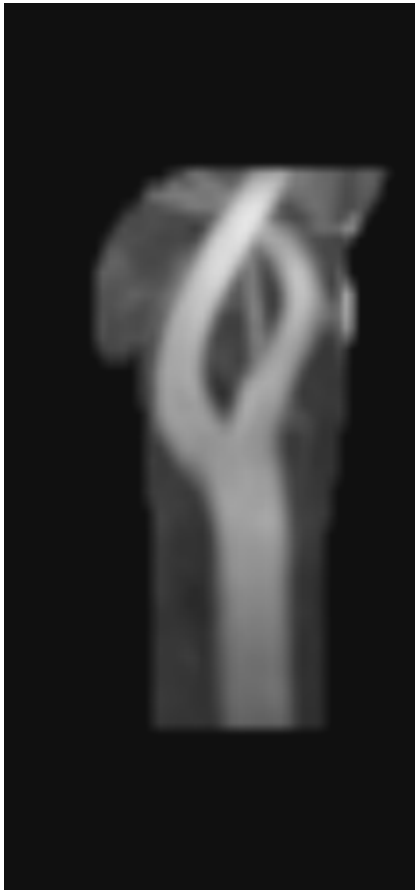

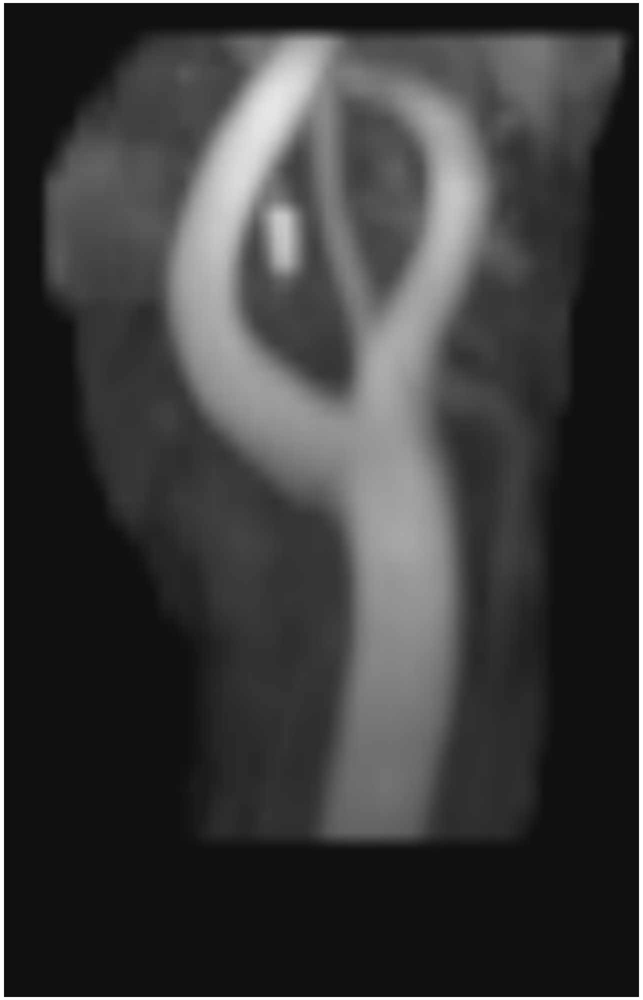

121b Score

0-30

31-50

51-70

>70

Near occlusion

Occluded

Quality

1

2

3

4

5

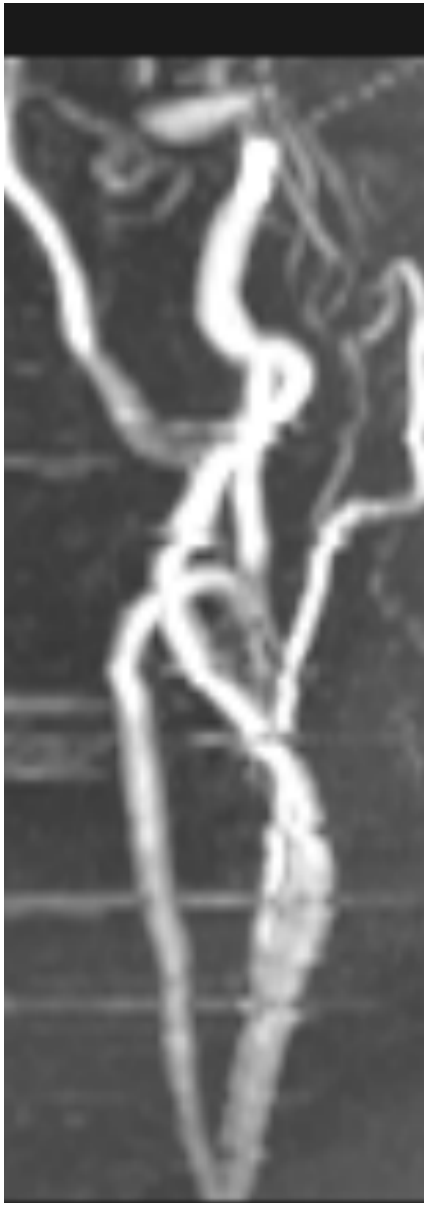

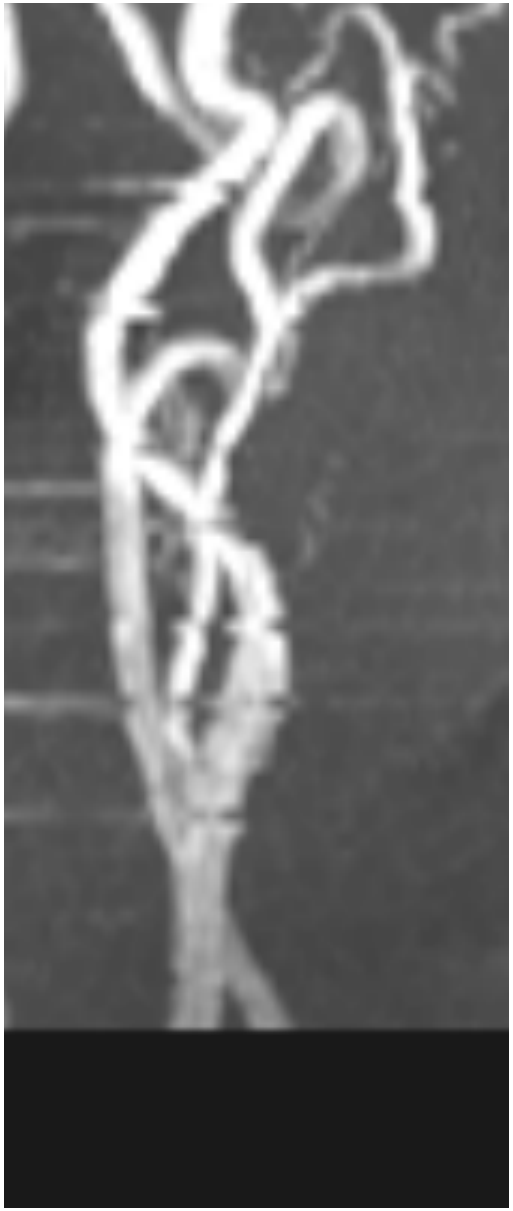

122a Score

0-30

31-50

51-70

>70

Near occlusion

Occluded

Quality

1

2

3

4

5

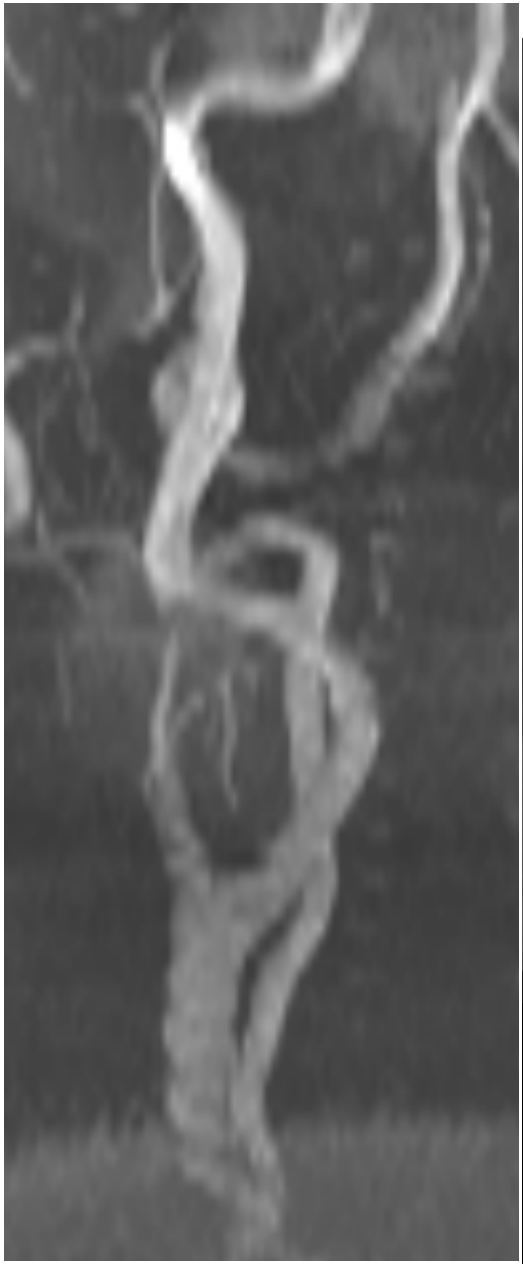

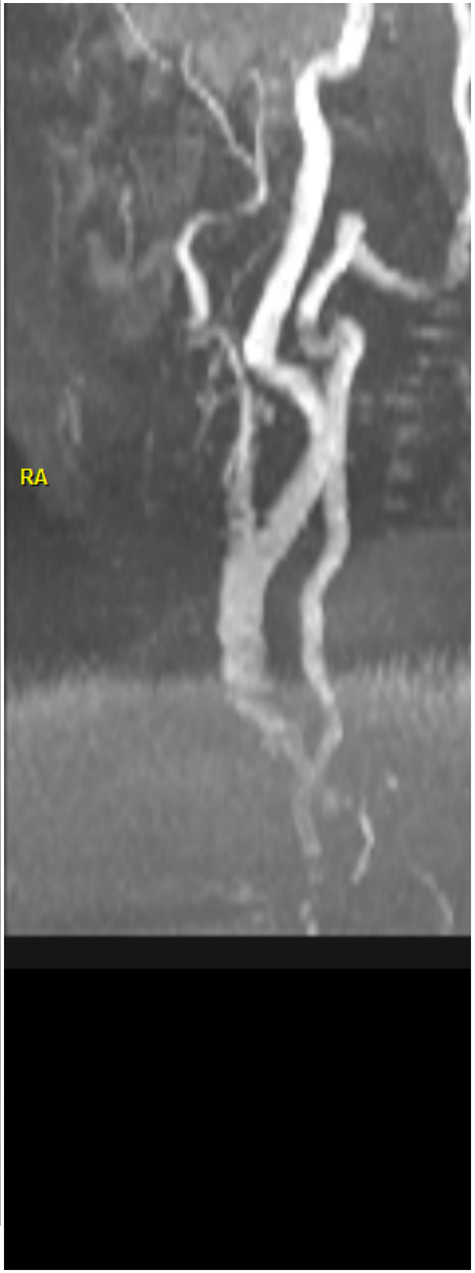

122f Score

0-30

31-50

51-70

>70

Near occlusion

Occluded

Quality

1

2

3

4

5

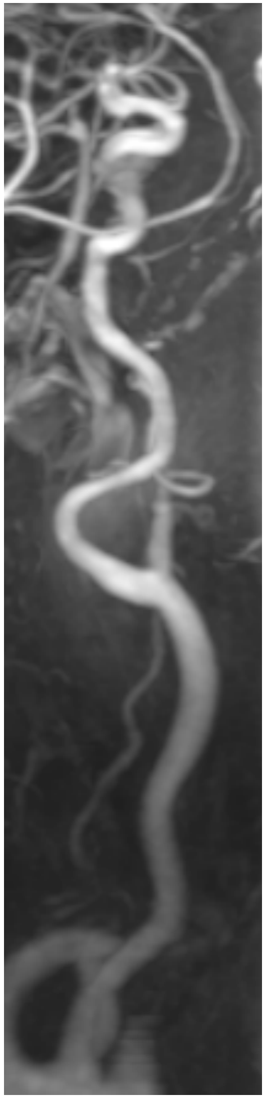

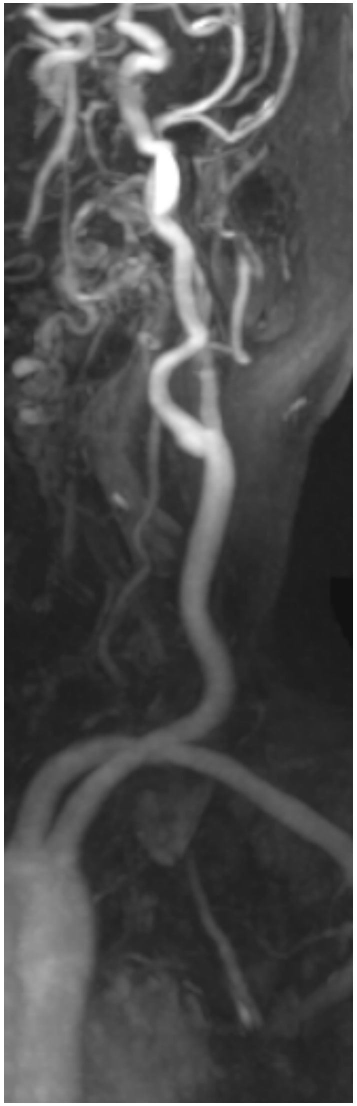

123e Score

0-30

31-50

51-70

>70

Near occlusion

Occluded

Quality

1

2

3

4

5

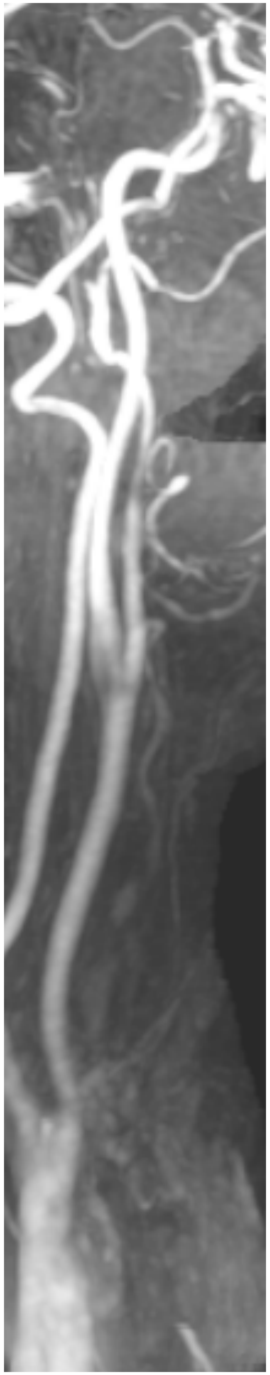

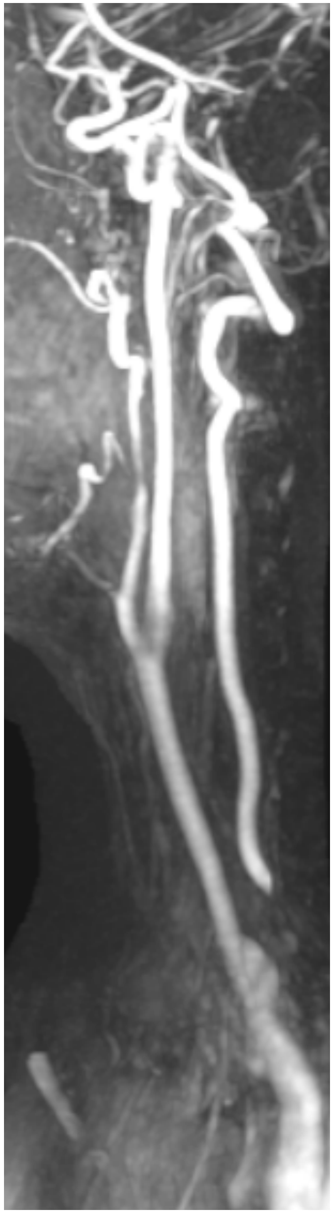

124d Score

0-30

31-50

51-70

>70

Near occlusion

Occluded

Quality

1

2

3

4

5

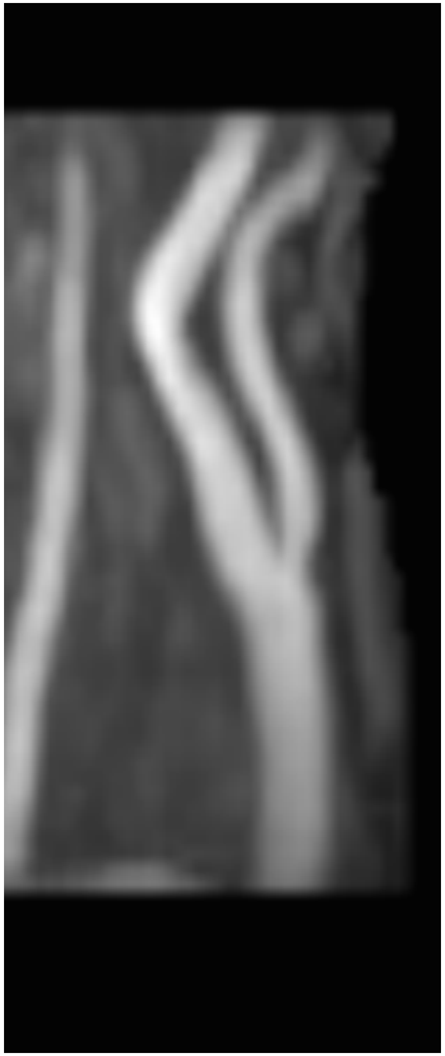

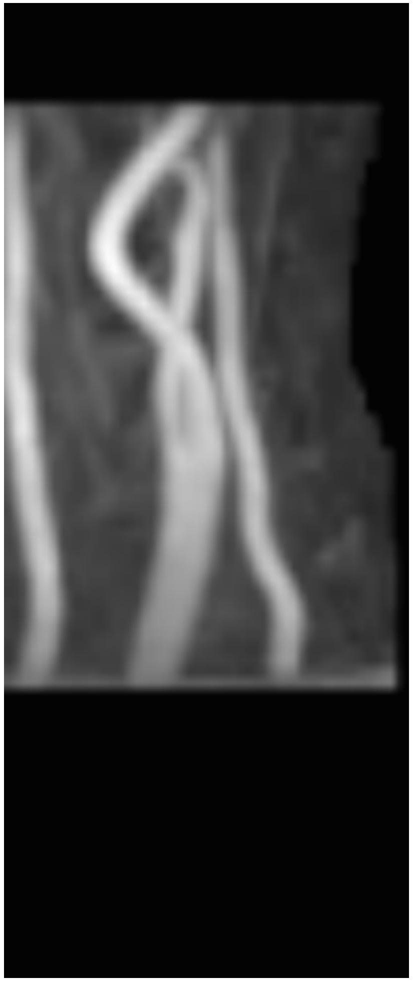

125c Score

0-30

31-50

51-70

>70

Near occlusion

Occluded

Quality

1

2

3

4

5

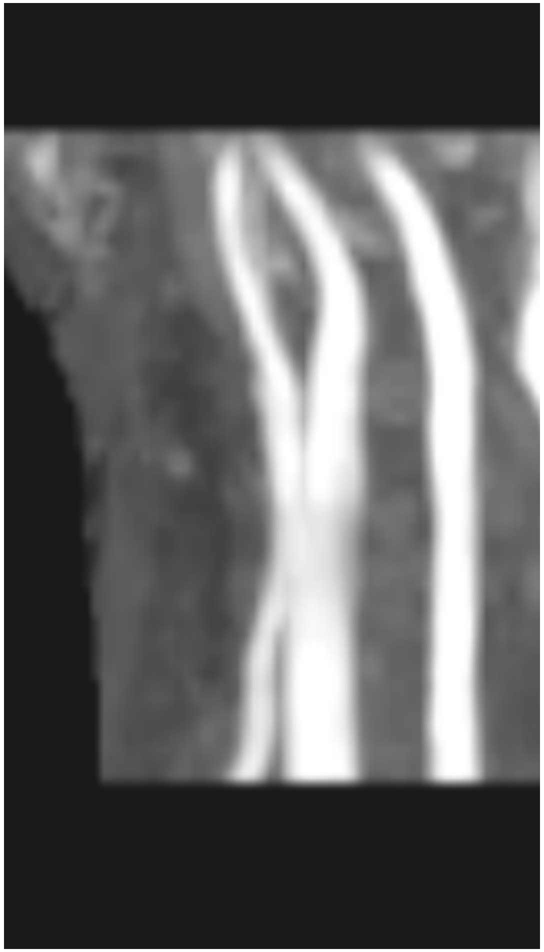

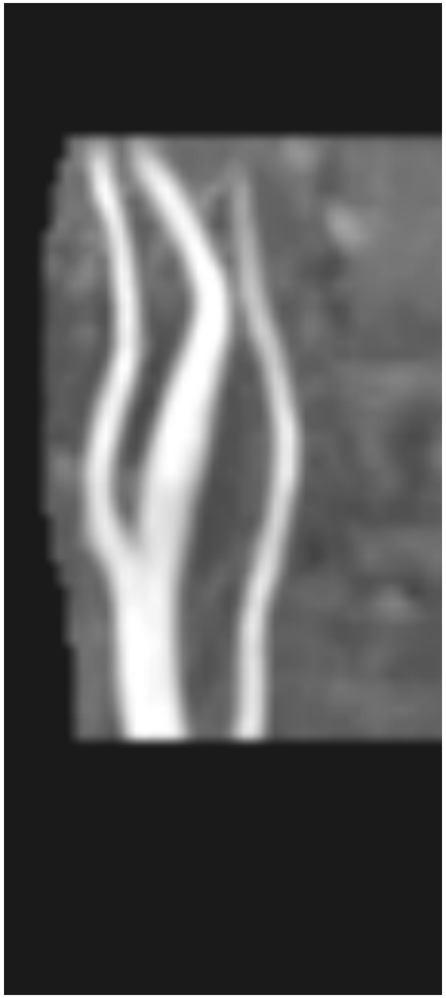

126b Score

0-30

31-50

51-70

>70

Near occlusion

Occluded

Quality

1

2

3

4

5

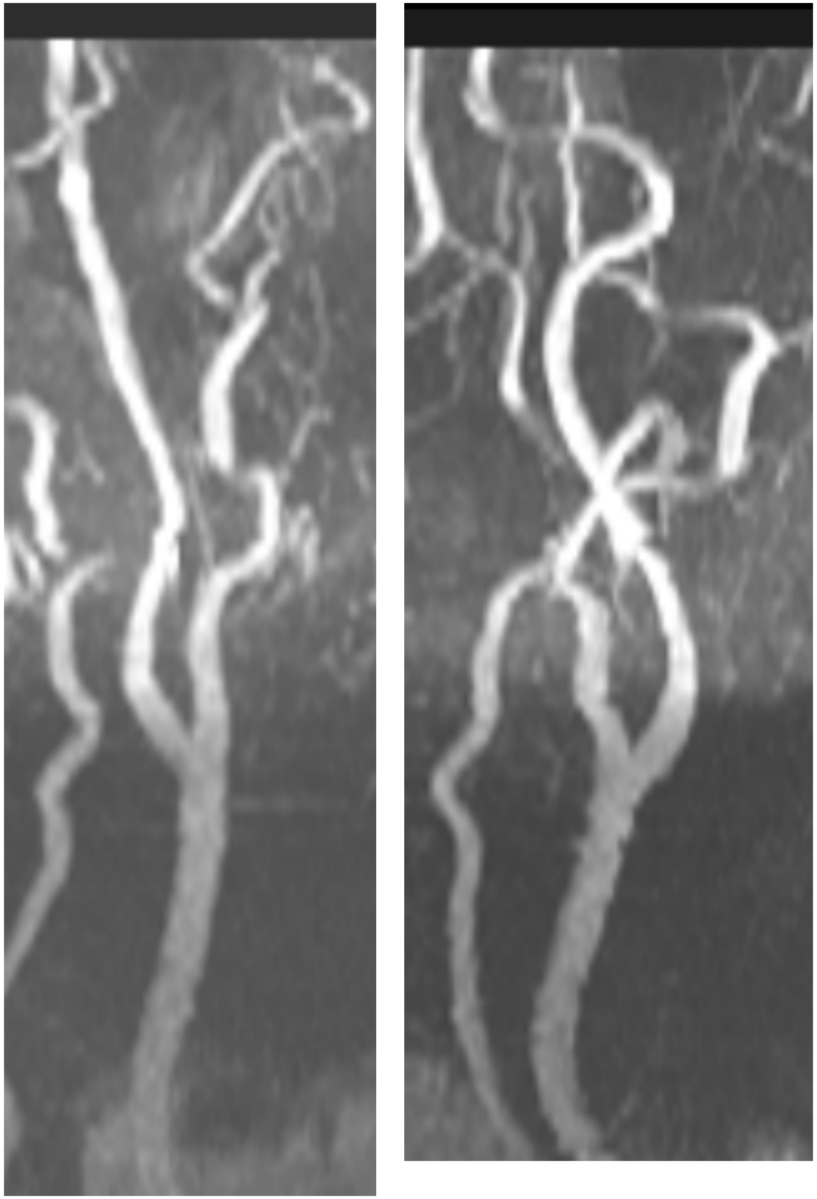

127a Score

0-30

31-50

51-70

>70

Near occlusion

Occluded

Quality

1

2

3

4

5

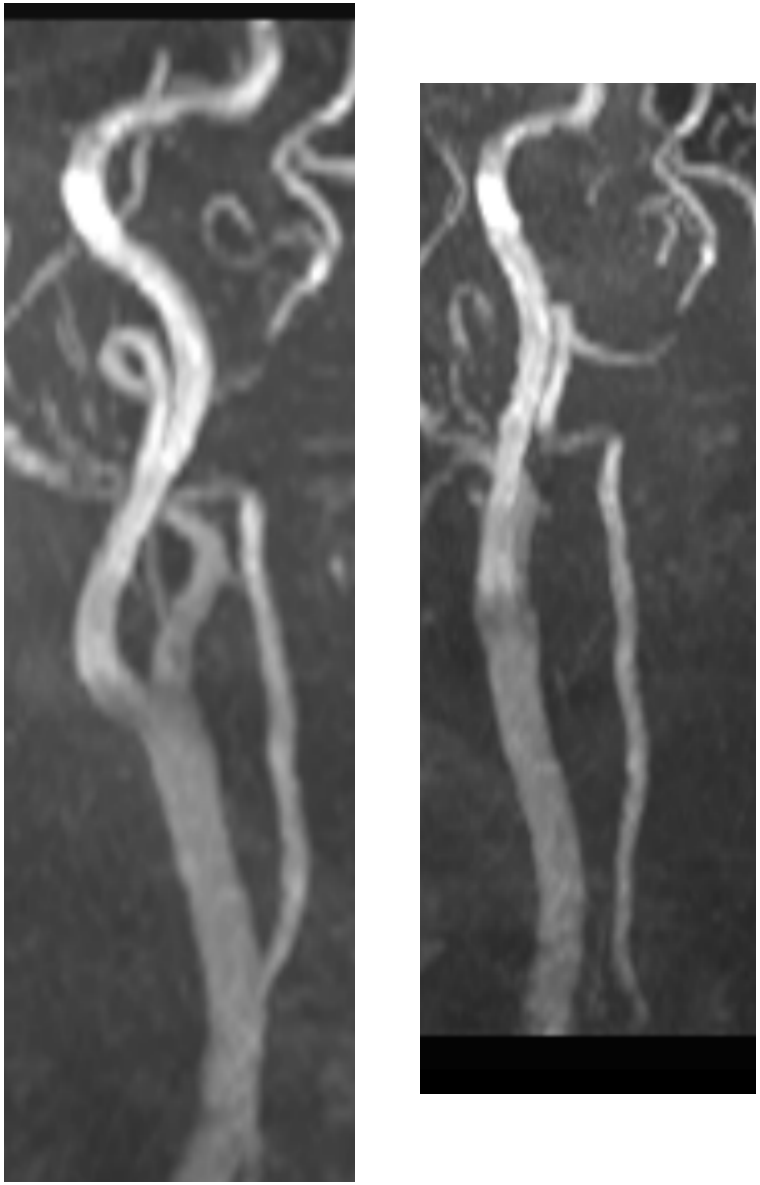

127f Score

0-30

31-50

51-70

>70

Near occlusion

Occluded

Quality

1

2

3

4

5

Axial cav carotid level

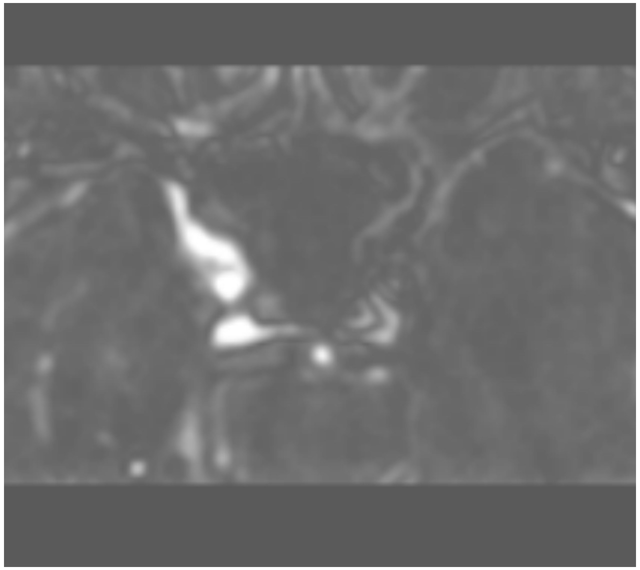

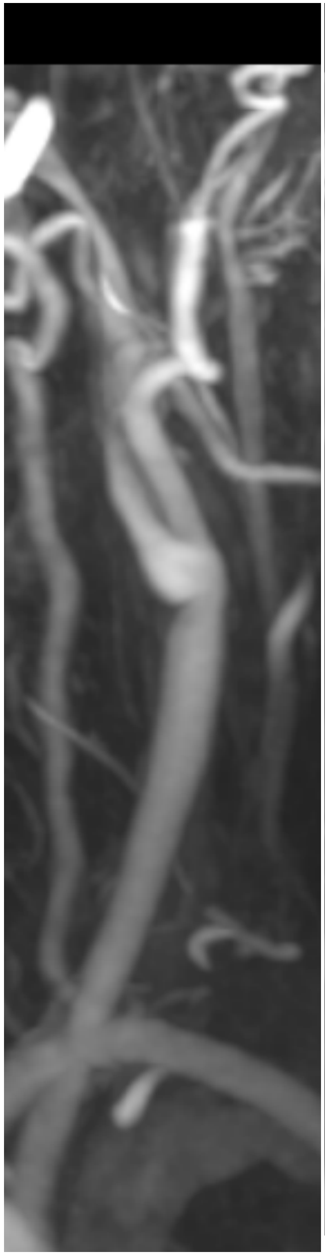

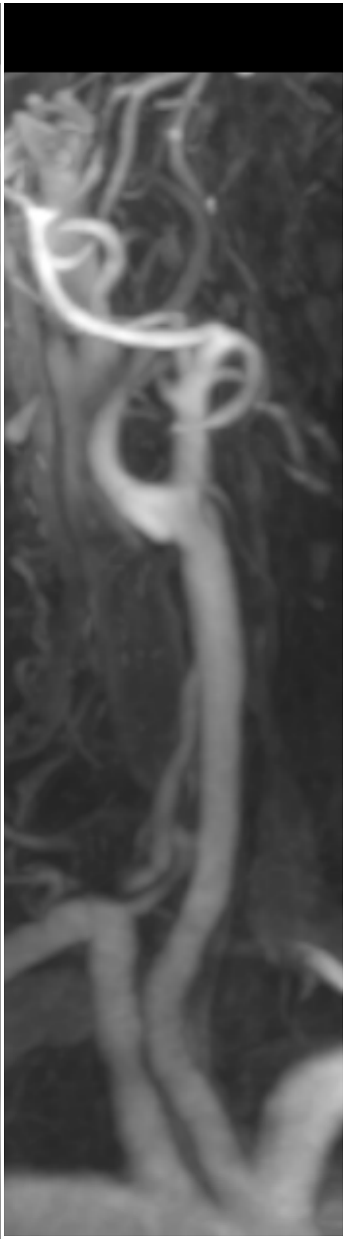

128e Score

0-30

31-50

51-70

>70

Near occlusion

Occluded

Quality

1

2

3

4

5

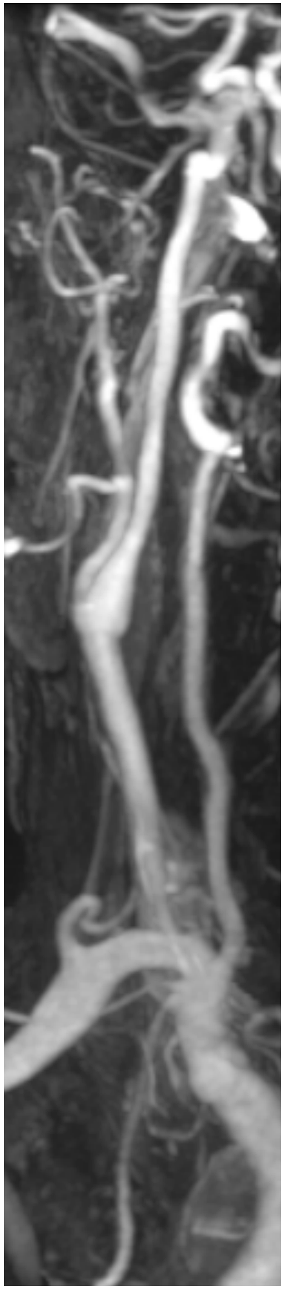

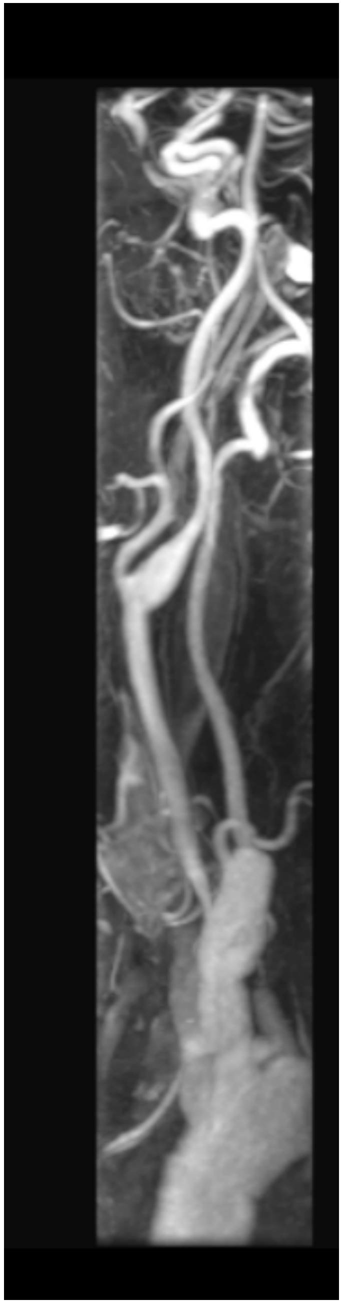

129d Score

0-30

31-50

51-70

>70

Near occlusion

Occluded

Quality

1

2

3

4

5

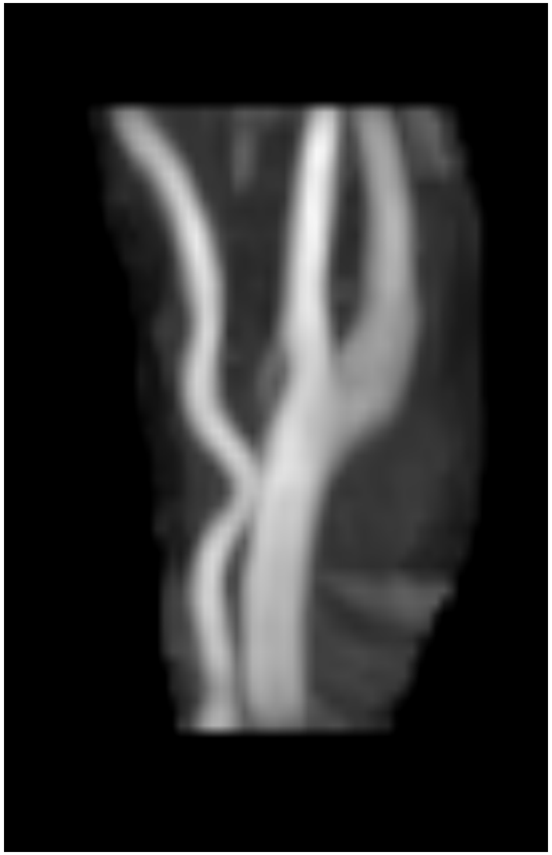

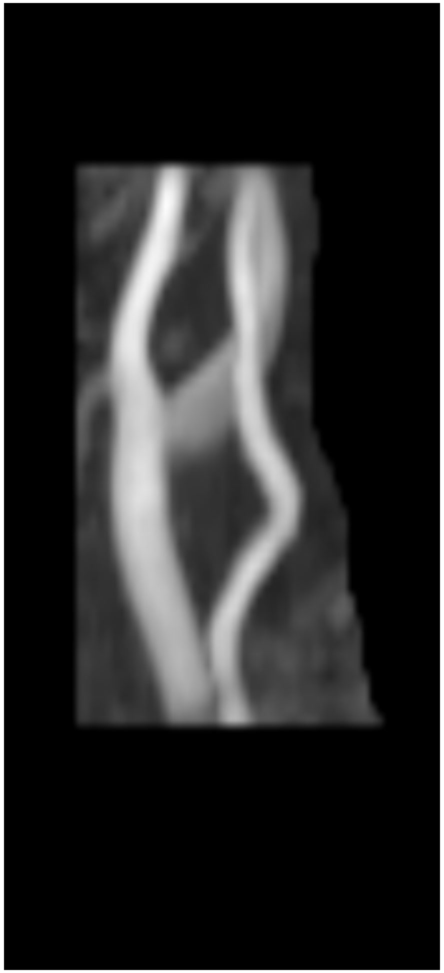

130c Score

0-30

31-50

51-70

>70

Near occlusion

Occluded

Quality

1

2

3

4

5

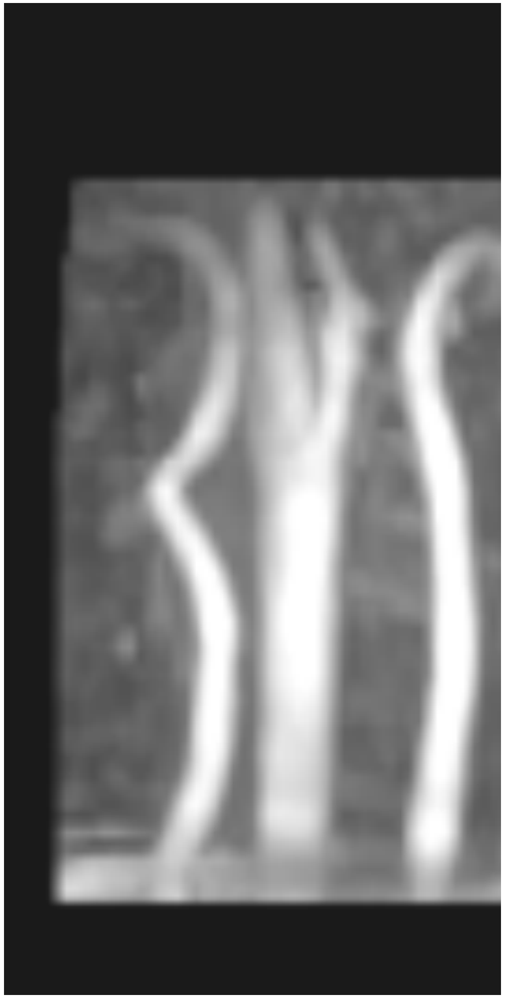

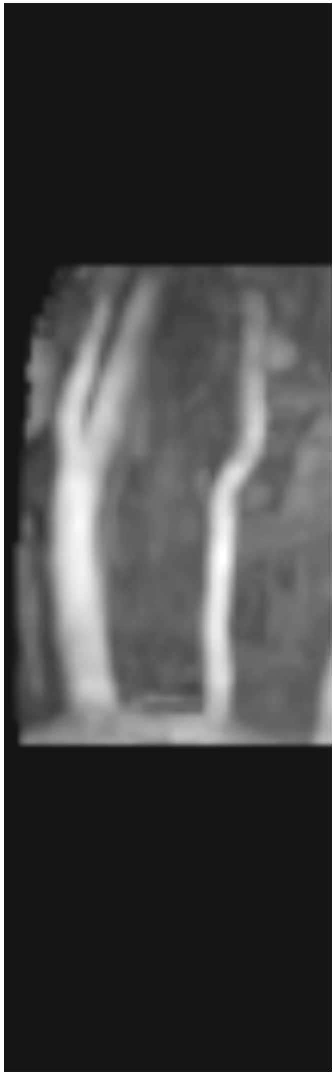

131b Score

0-30

31-50

51-70

>70

Near occlusion

Occluded

Quality

1

2

3

4

5

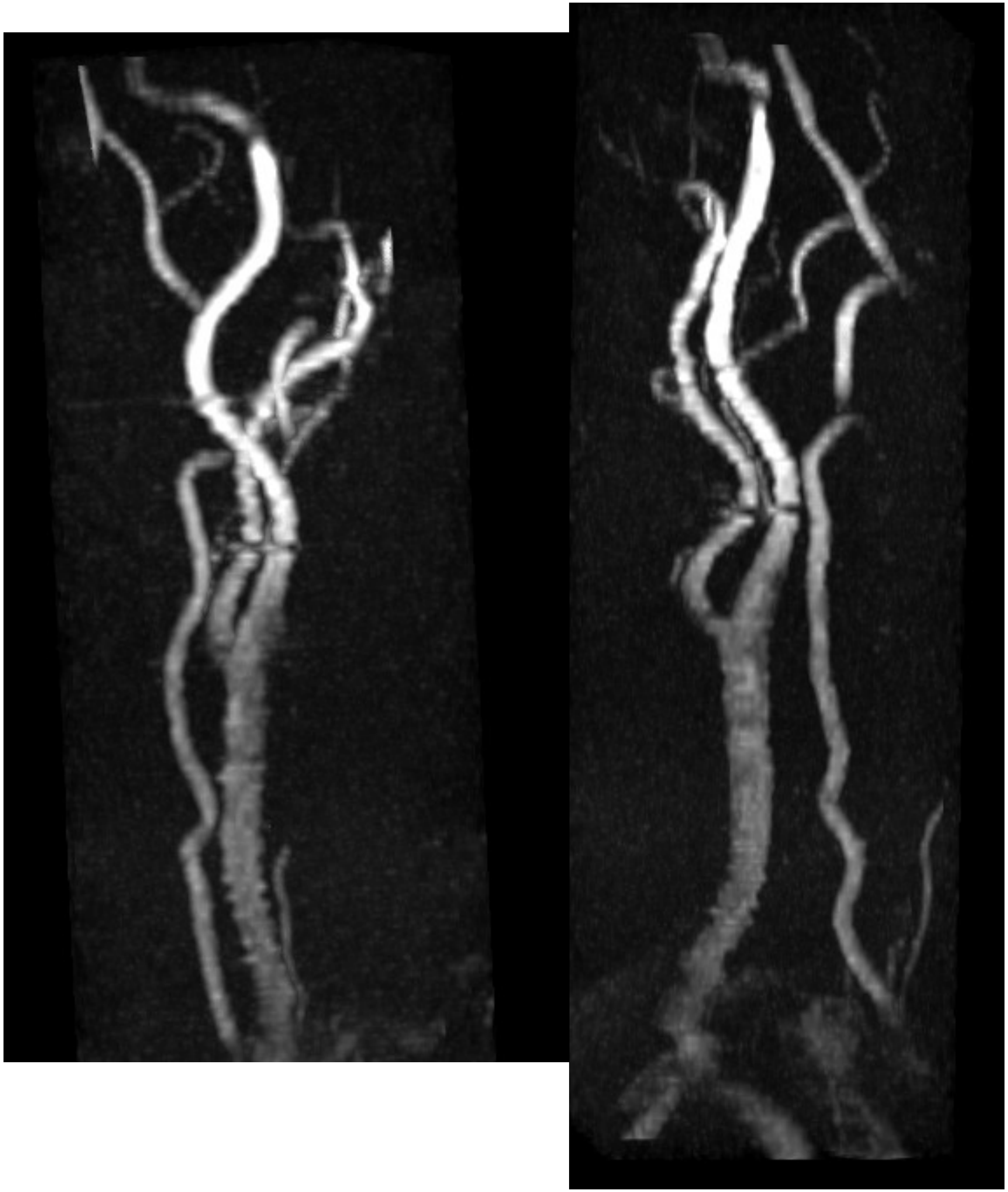

132a Score

0-30

31-50

51-70

>70

Near occlusion

Occluded

Quality

1

2

3

4

5

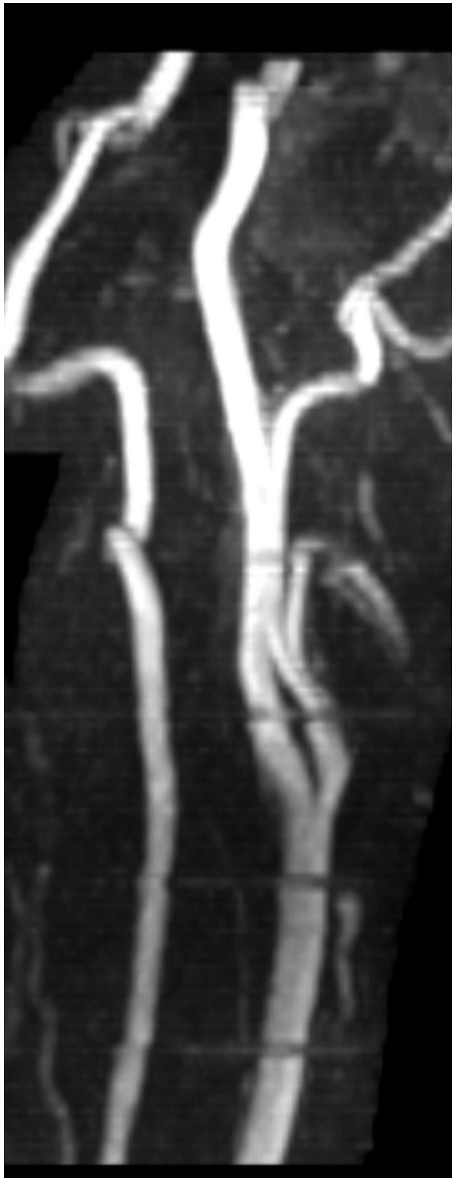

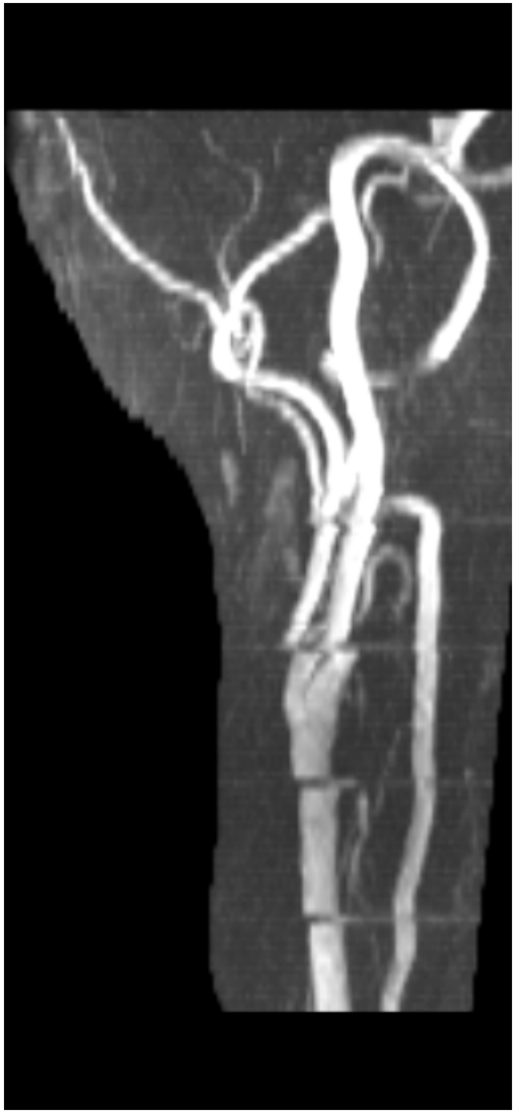

132f Score

0-30

31-50

51-70

>70

Near occlusion

Occluded

Quality

1

2

3

4

5

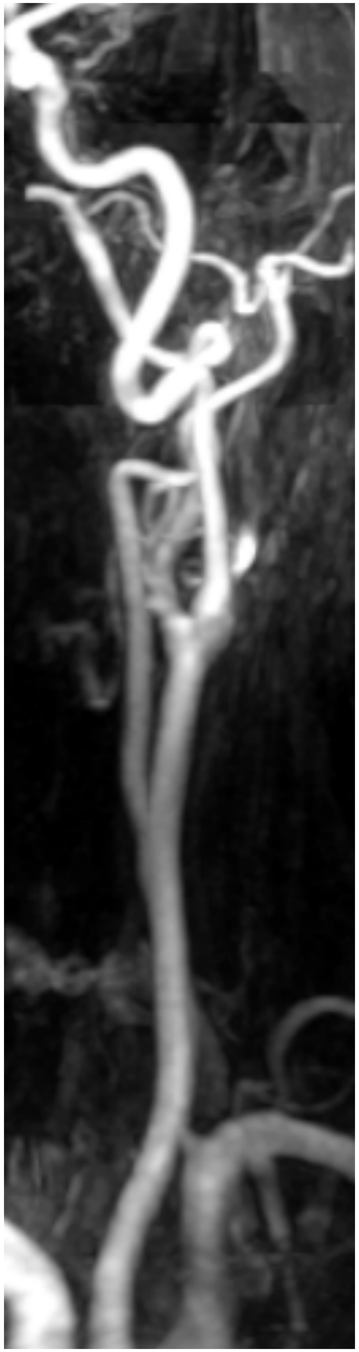

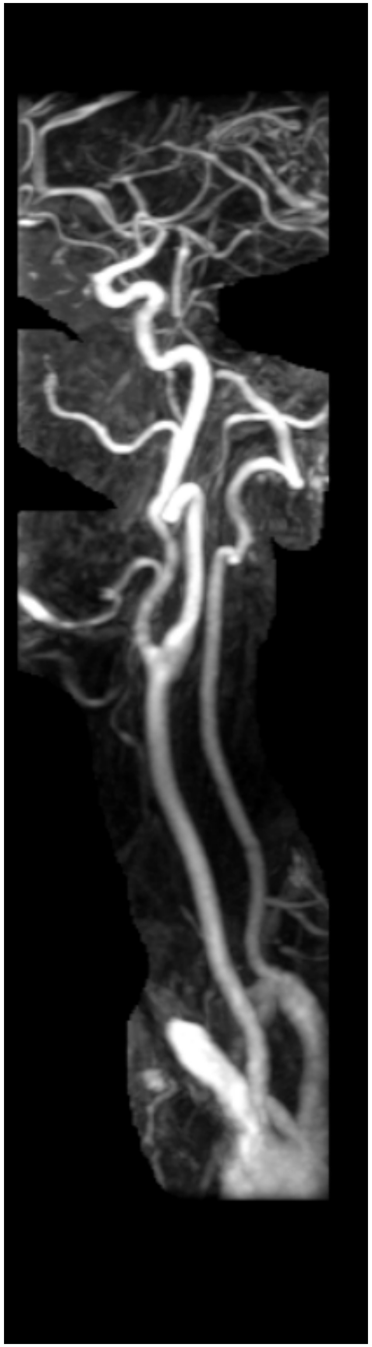

133e Score

0-30

31-50

51-70

>70

Near occlusion

Occluded

Quality

1

2

3

4

5

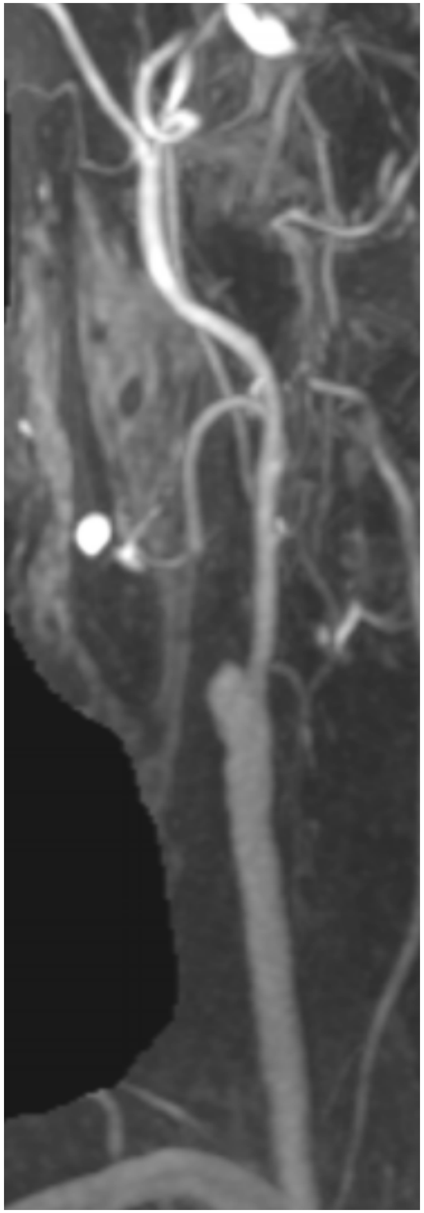

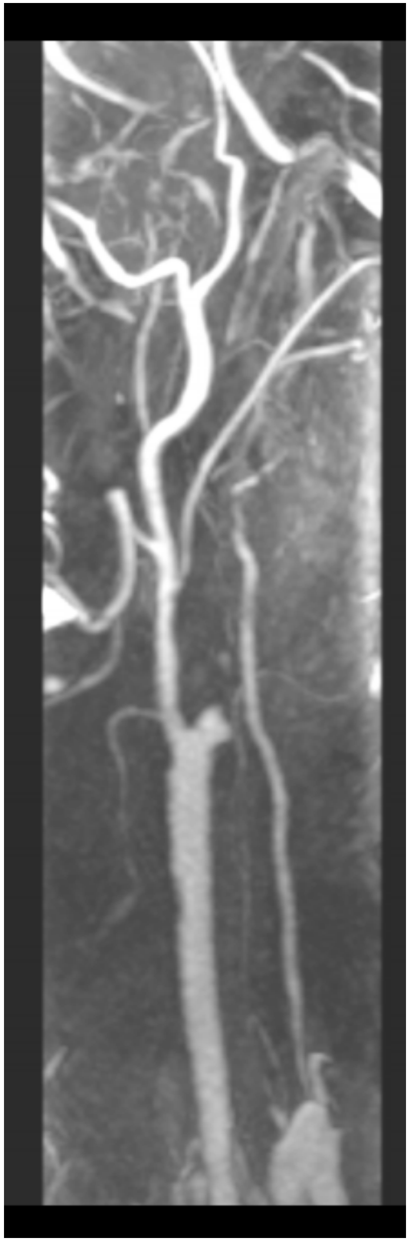

134d Score

0-30

31-50

51-70

>70

Near occlusion

Occluded

Quality

1

2

3

4

5

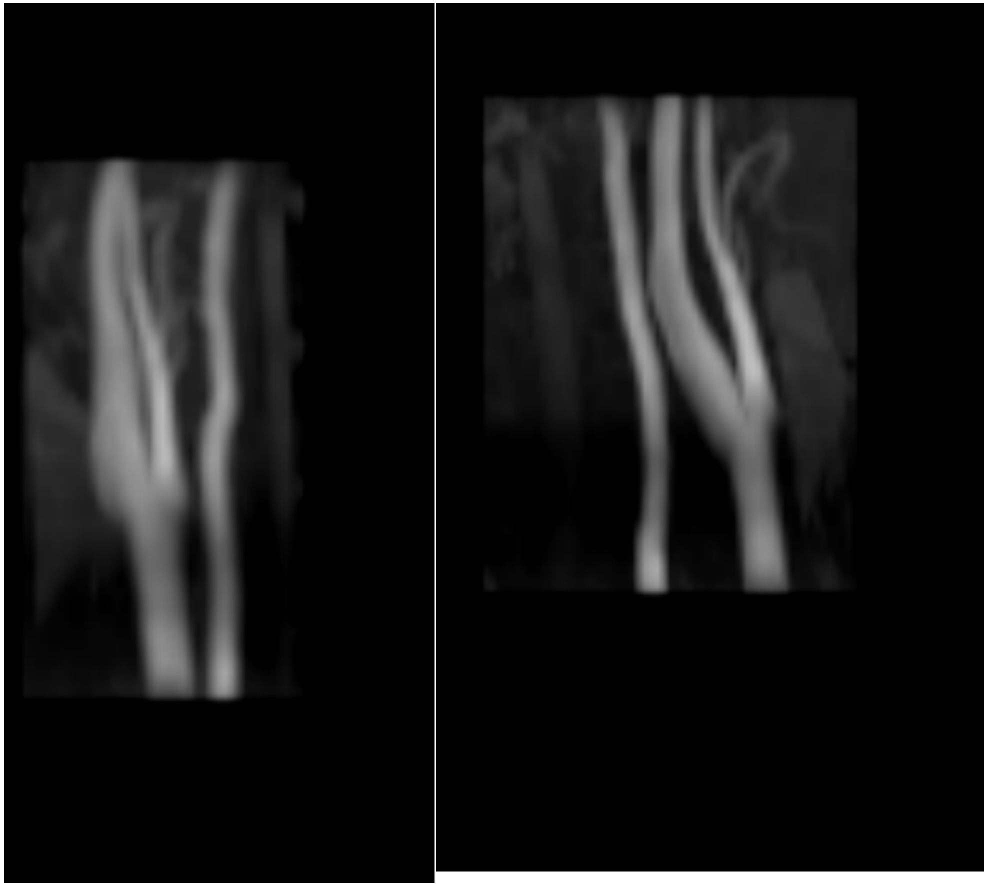

135c Score

0-30

31-50

51-70

>70

Near occlusion

Occluded

Quality

1

2

3

4

5

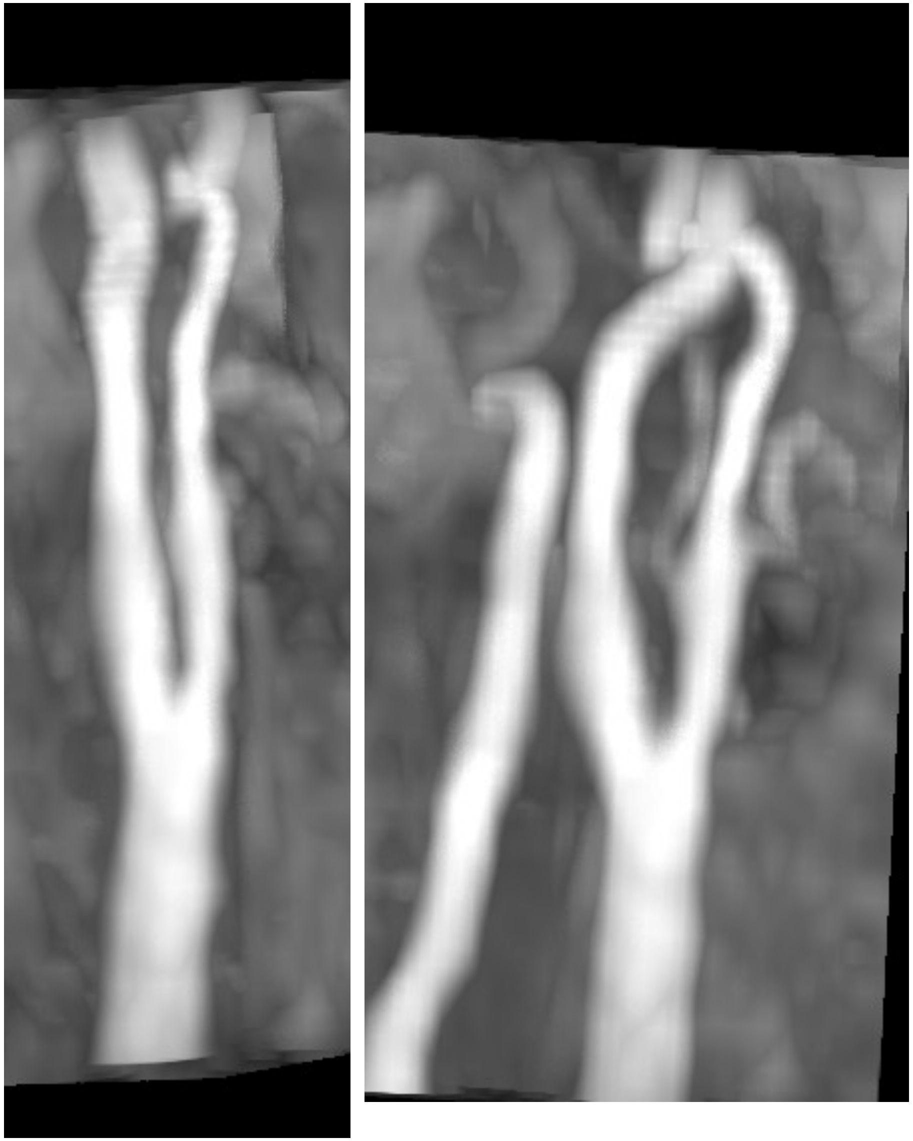

136b Score

0-30

31-50

51-70

>70

Near occlusion

Occluded

Quality

1

2

3

4

5

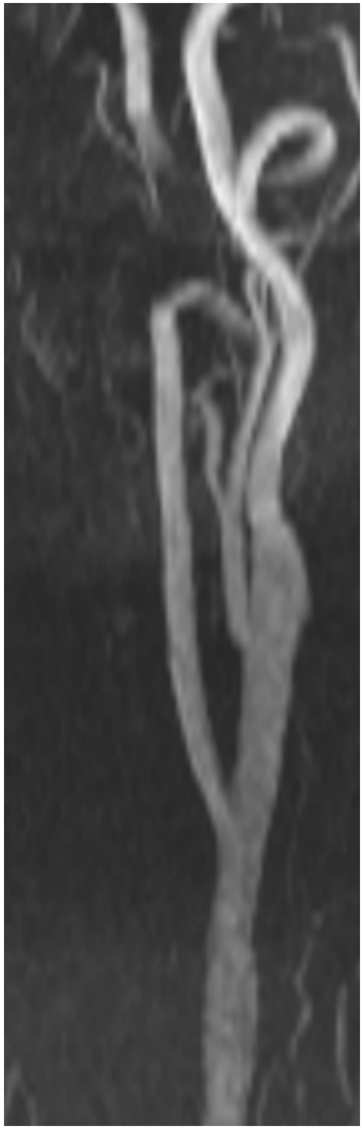

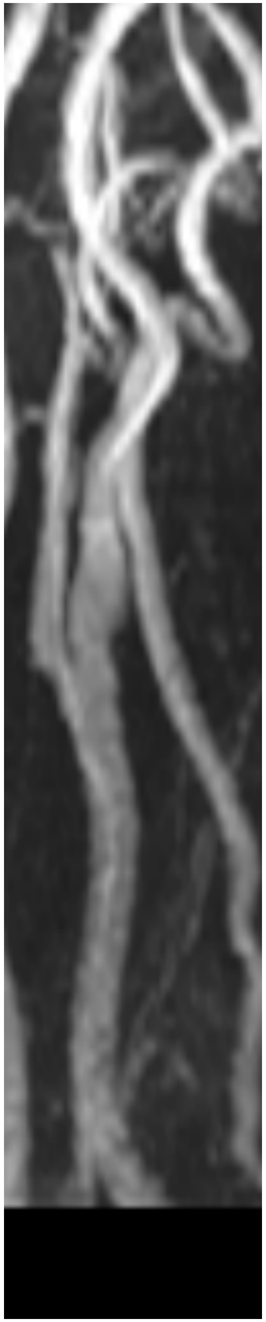

137a Score

0-30

31-50

51-70

>70

Near occlusion

Occluded

Quality

1

2

3

4

5

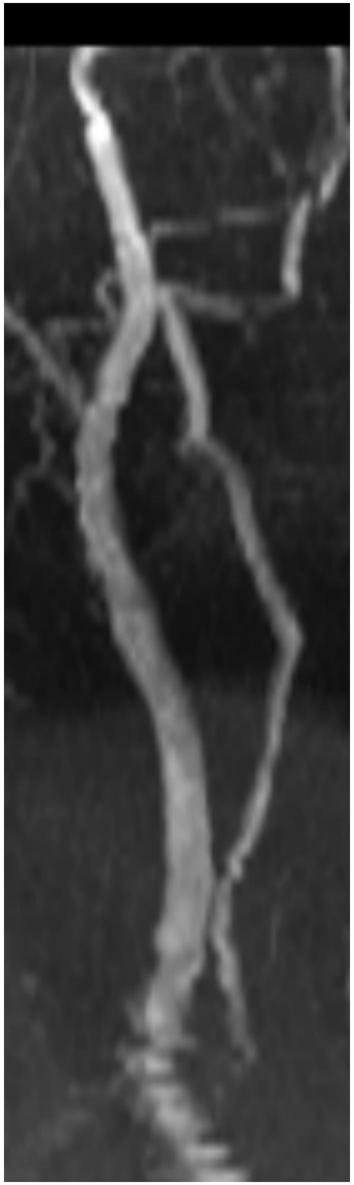

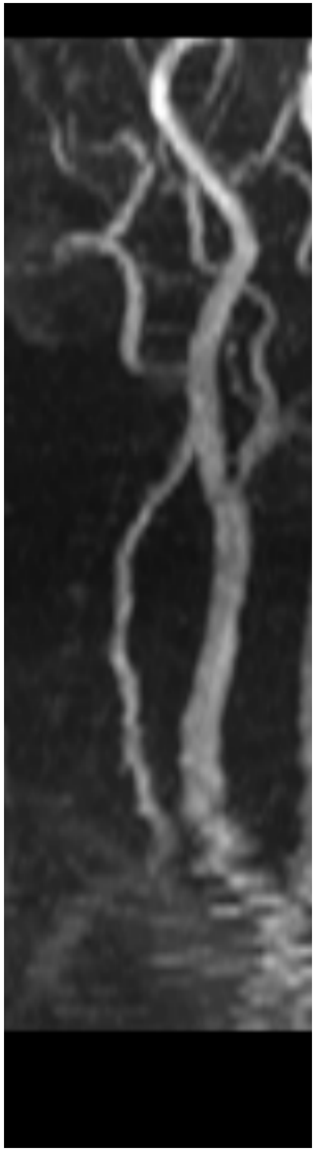

137f Score

0-30

31-50

51-70

>70

Near occlusion

Occluded

Quality

1

2

3

4

5

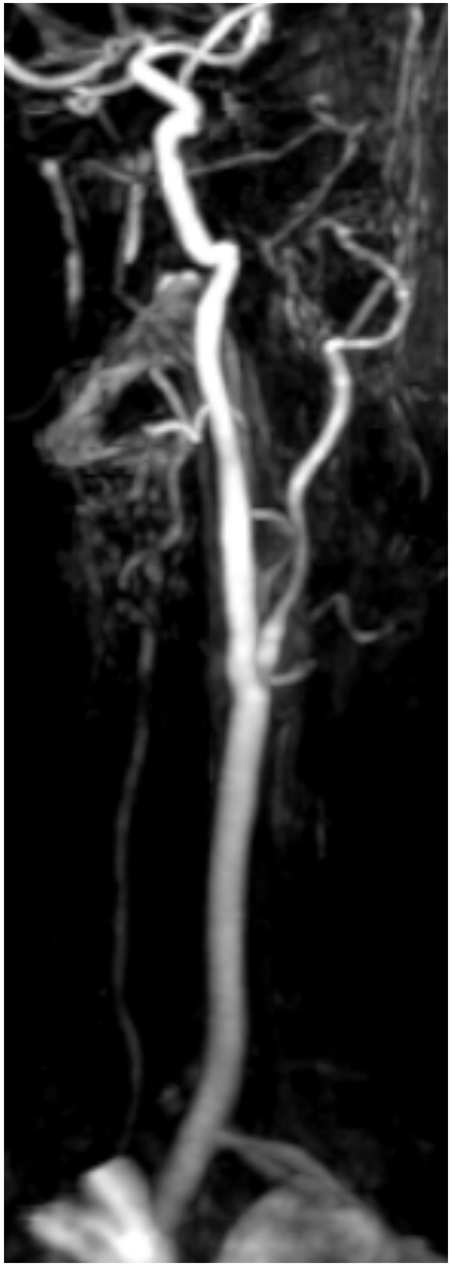

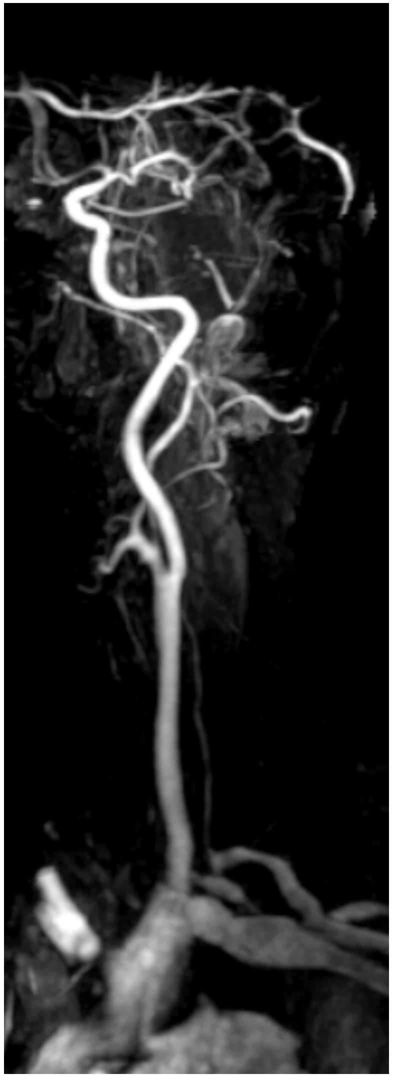

138e Score

0-30

31-50

51-70

>70

Near occlusion

Occluded

Quality

1

2

3

4

5

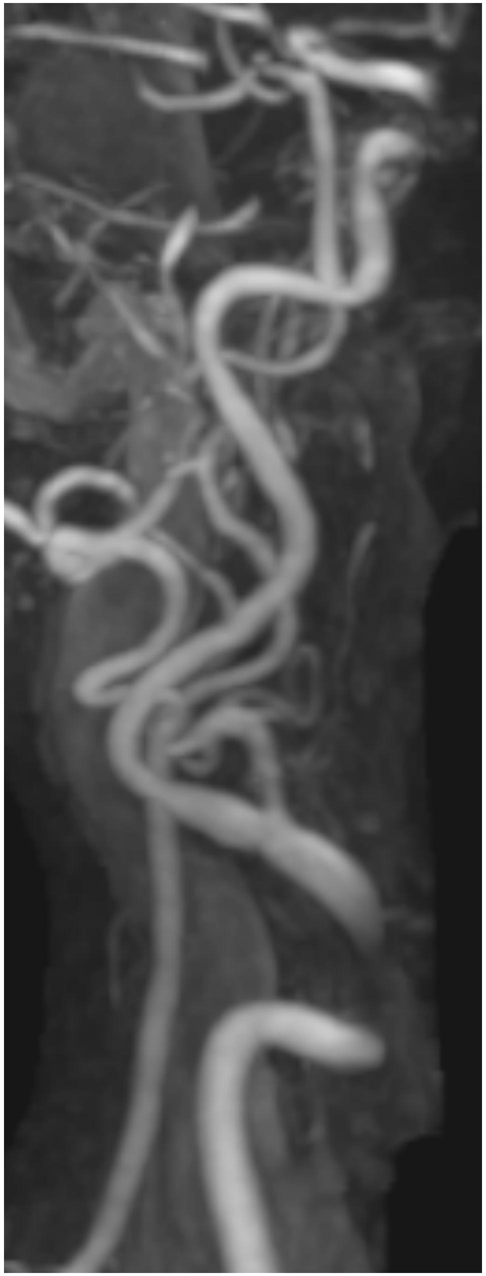

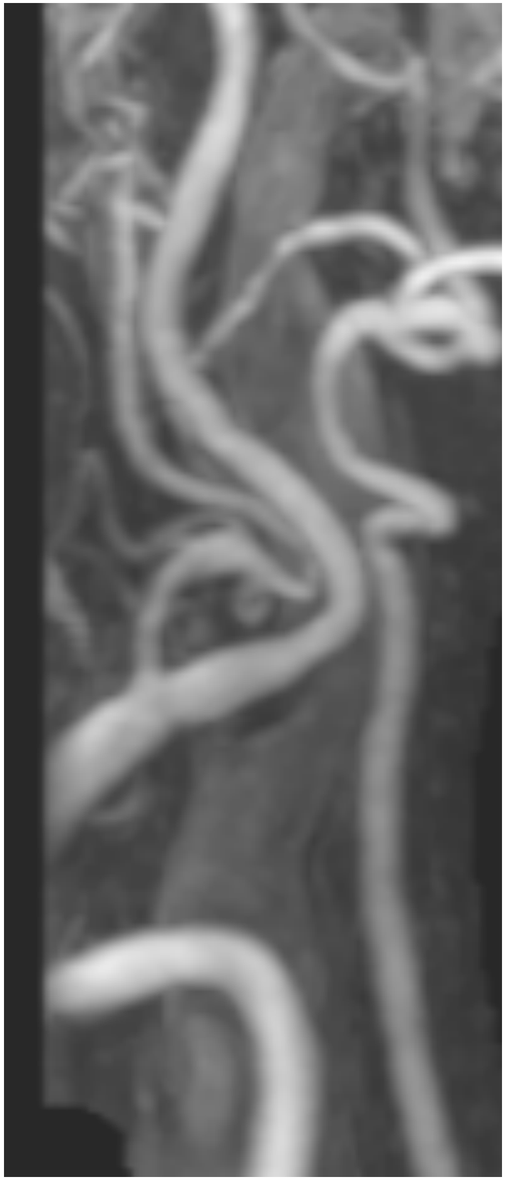

139d Score

0-30

31-50

51-70

>70

Near occlusion

Occluded

Quality

1

2

3

4

5

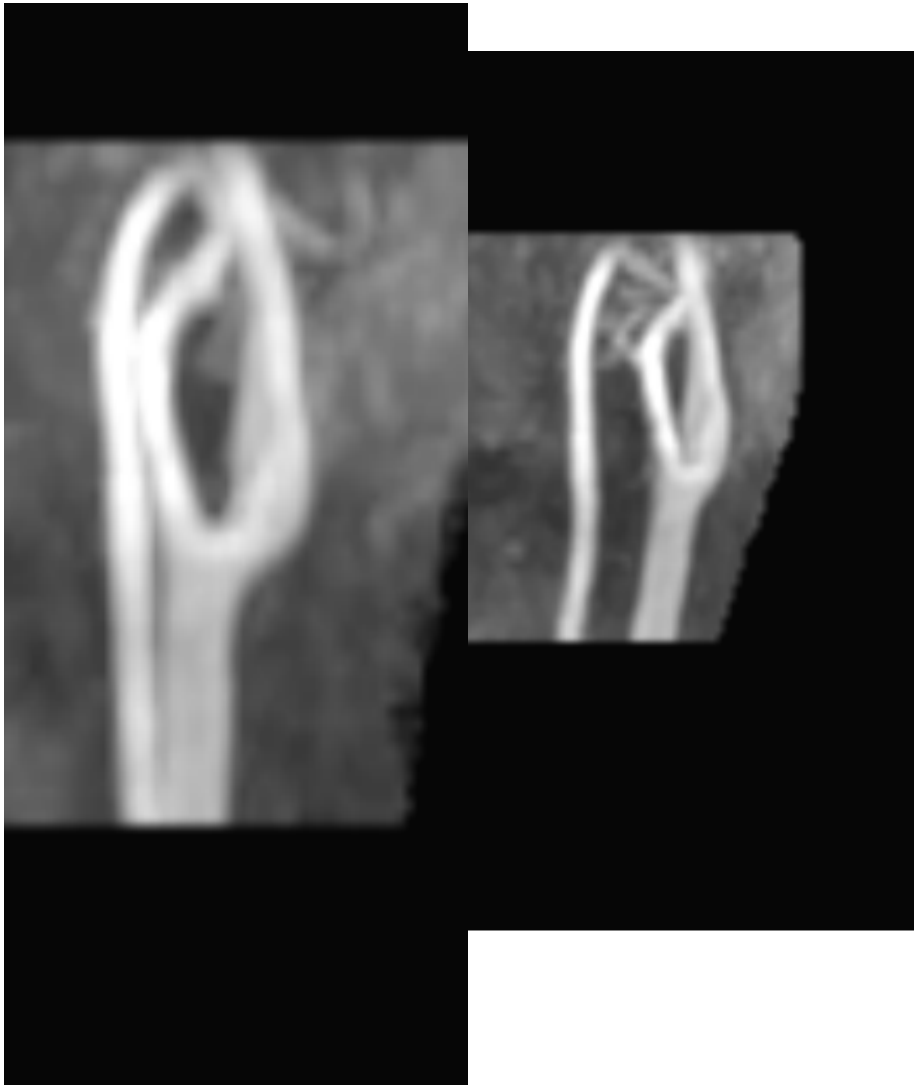

140c Score

0-30

31-50

51-70

>70

Near occlusion

Occluded

Quality

1

2

3

4

5

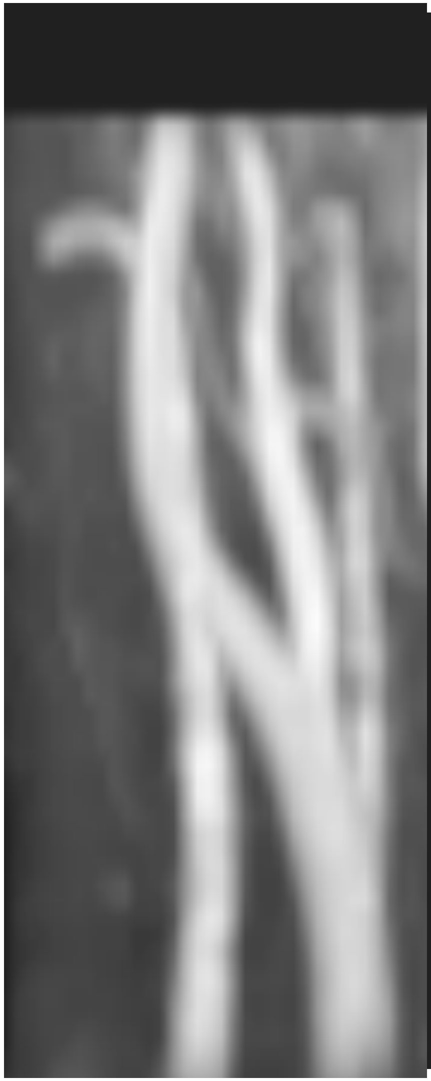

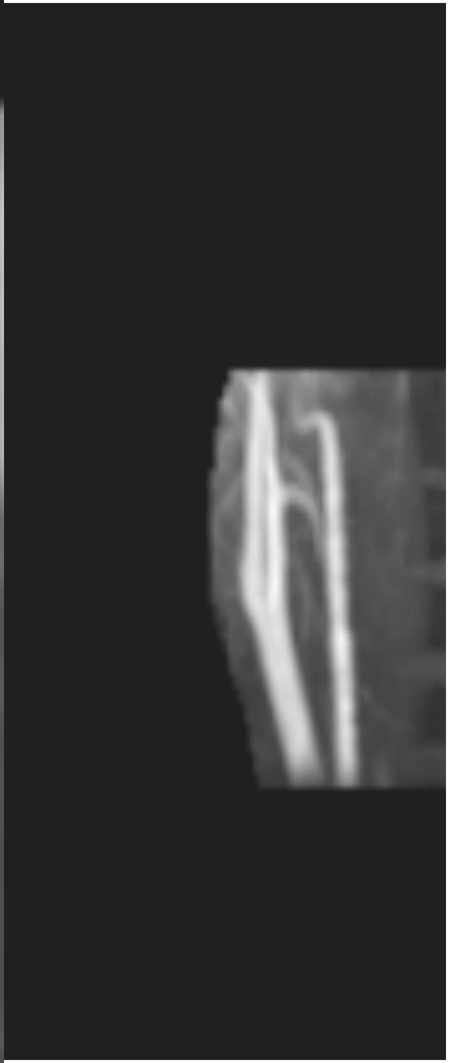

141b Score

0-30

31-50

51-70

>70

Near occlusion

Occluded

Quality

1

2

3

4

5

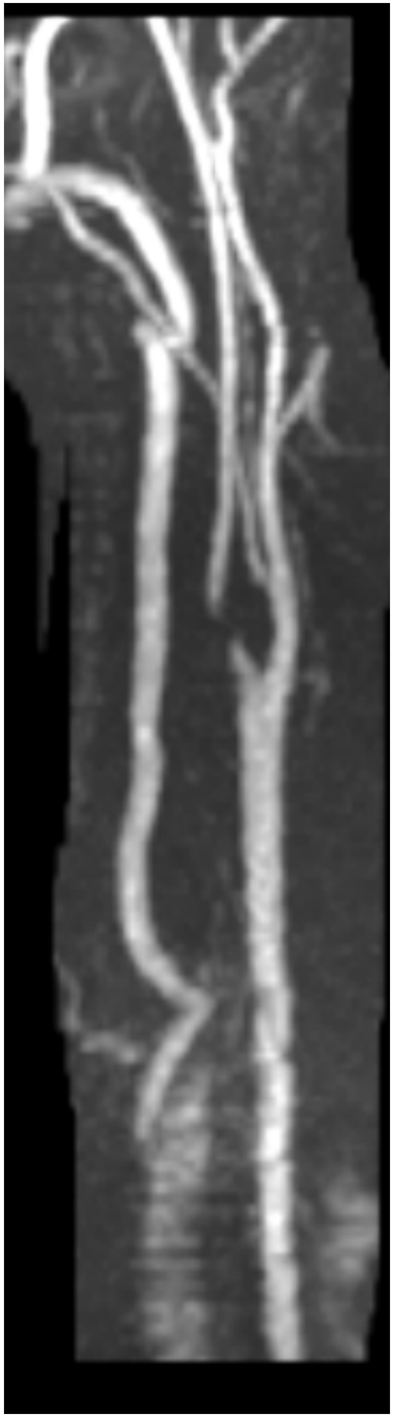

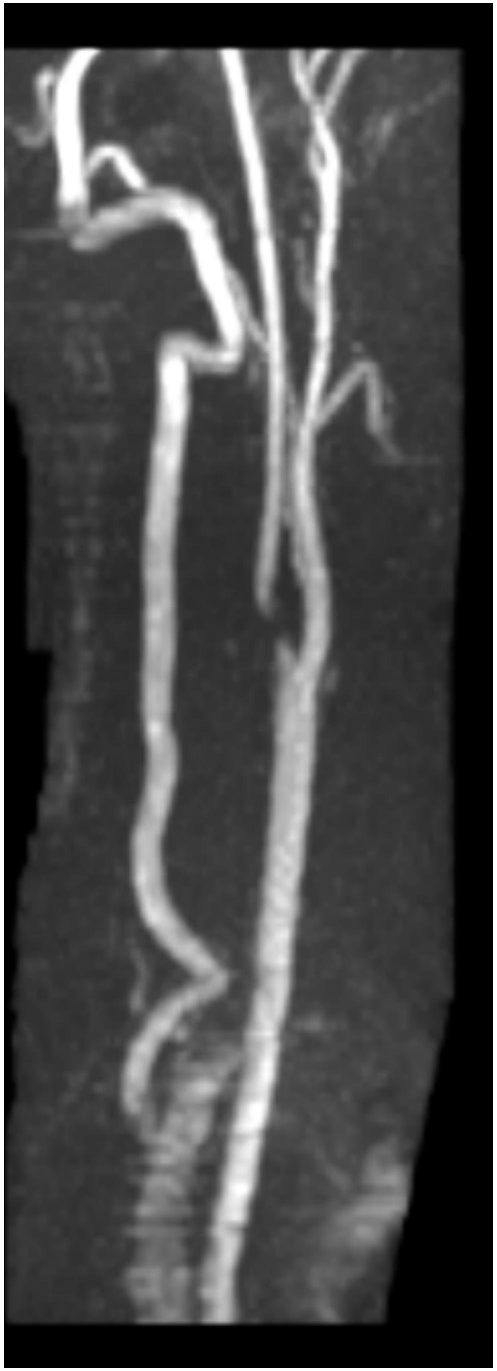

142a Score

0-30

31-50

51-70

>70

Near occlusion

Occluded

Quality

1

2

3

4

5

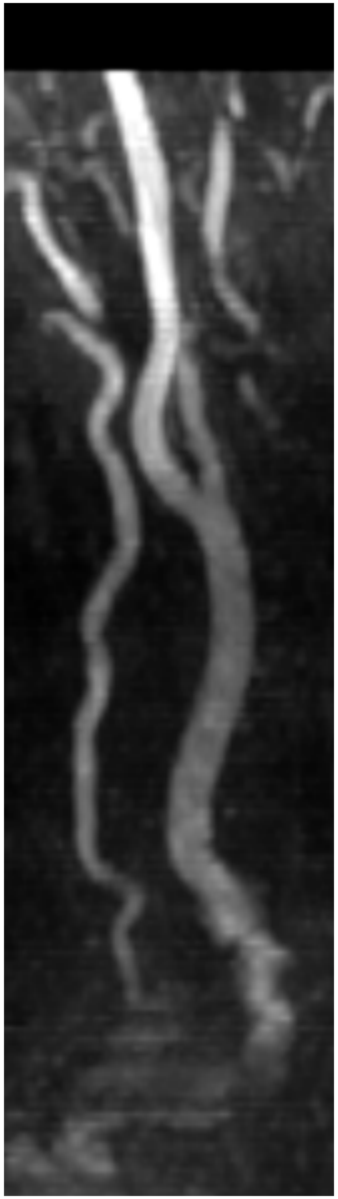

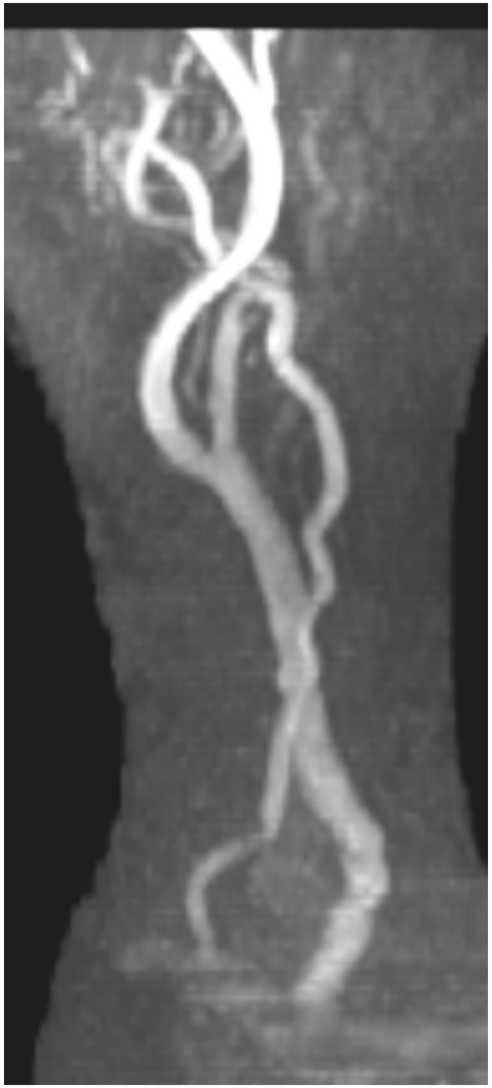

142f Score

0-30

31-50

51-70

>70

Near occlusion

Occluded

Quality

1

2

3

4

5

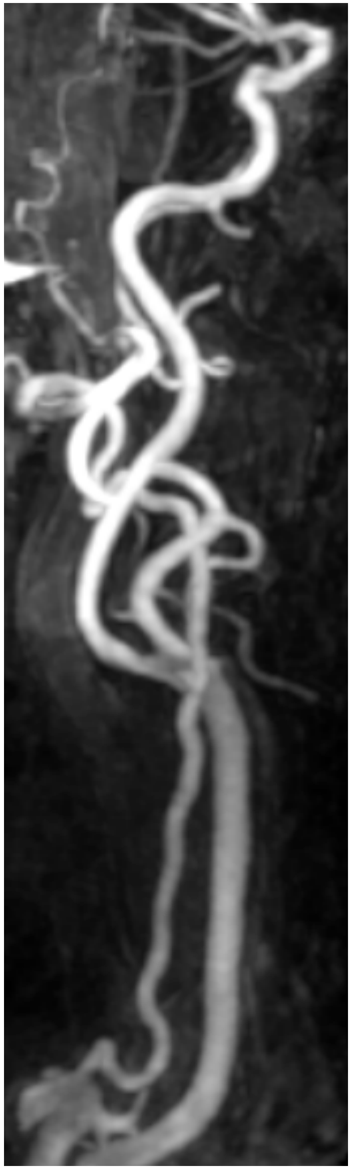

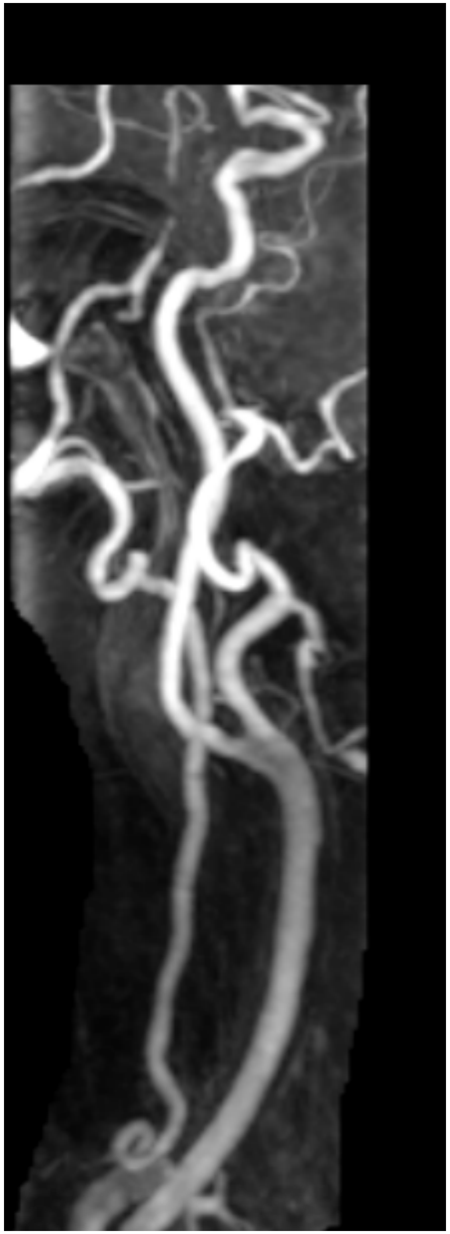

143e Score

0-30

31-50

51-70

>70

Near occlusion

Occluded

Quality

1

2

3

4

5

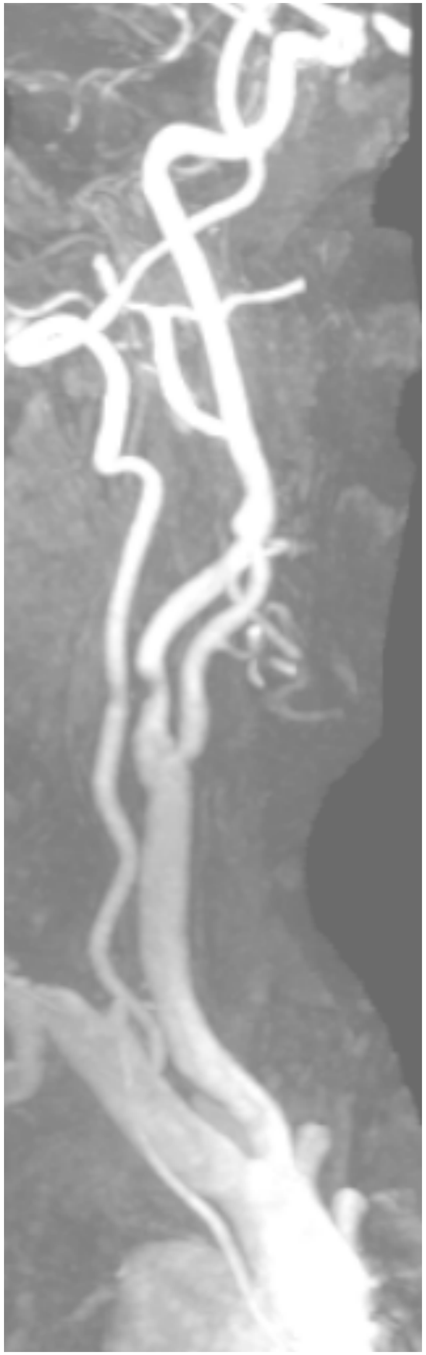

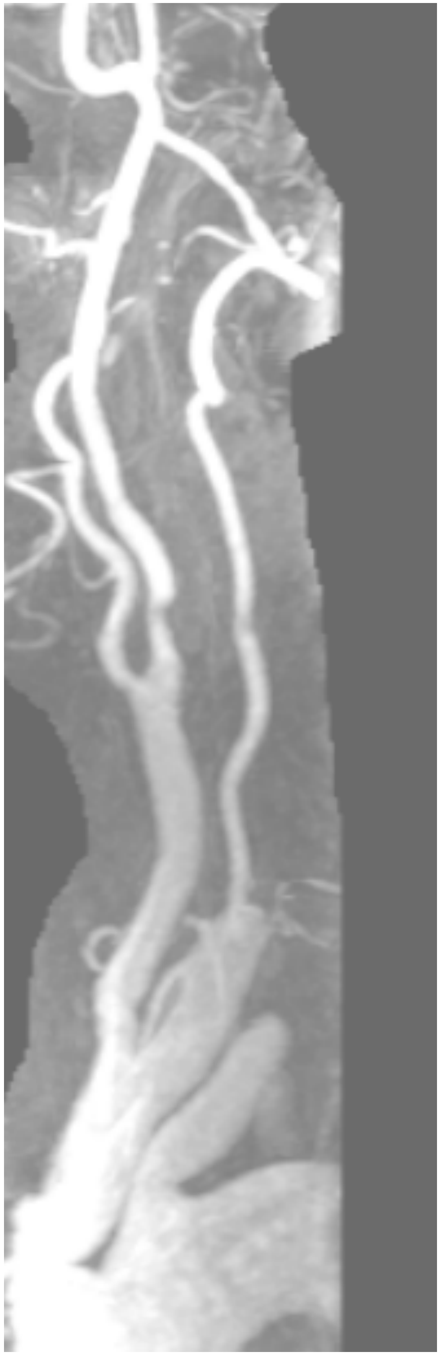

144d Score

0-30

31-50

51-70

>70

Near occlusion

Occluded

Quality

1

2

3

4

5

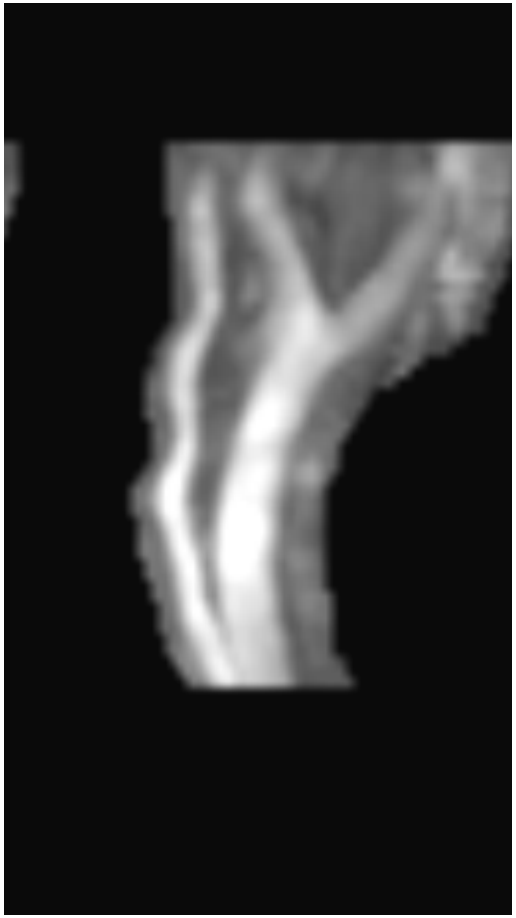

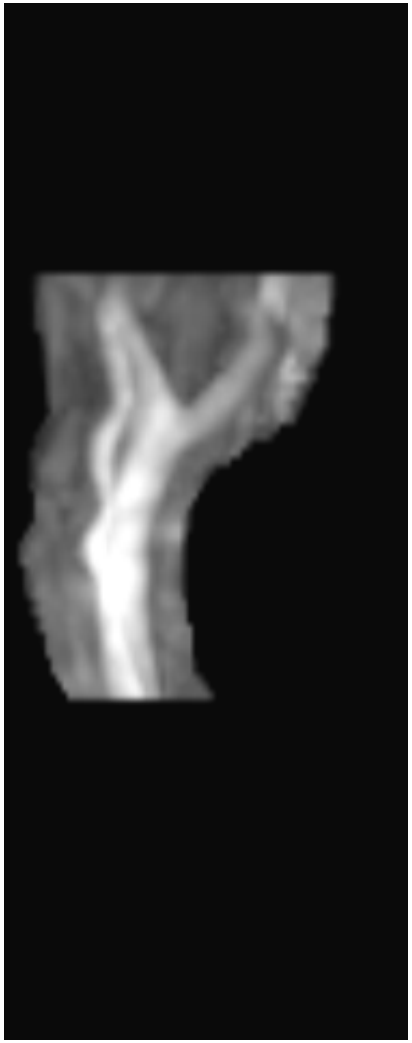

145c Score

0-30

31-50

51-70

>70

Near occlusion

Occluded

Quality

1

2

3

4

5

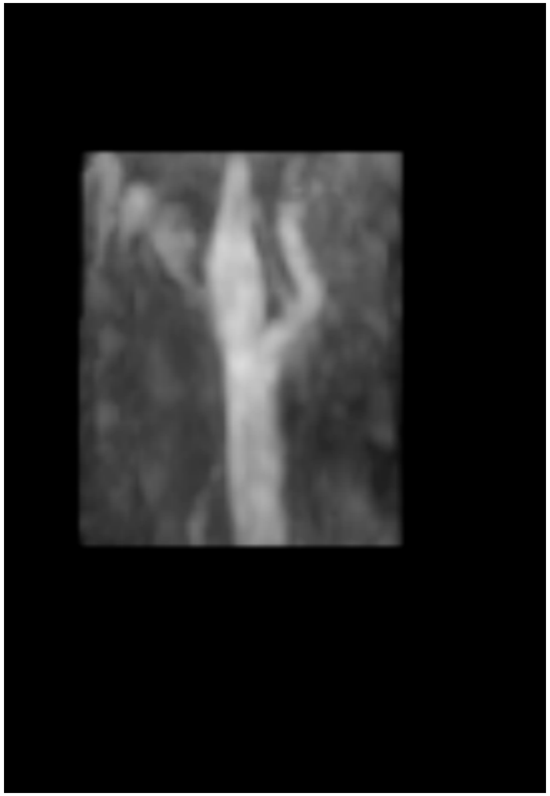

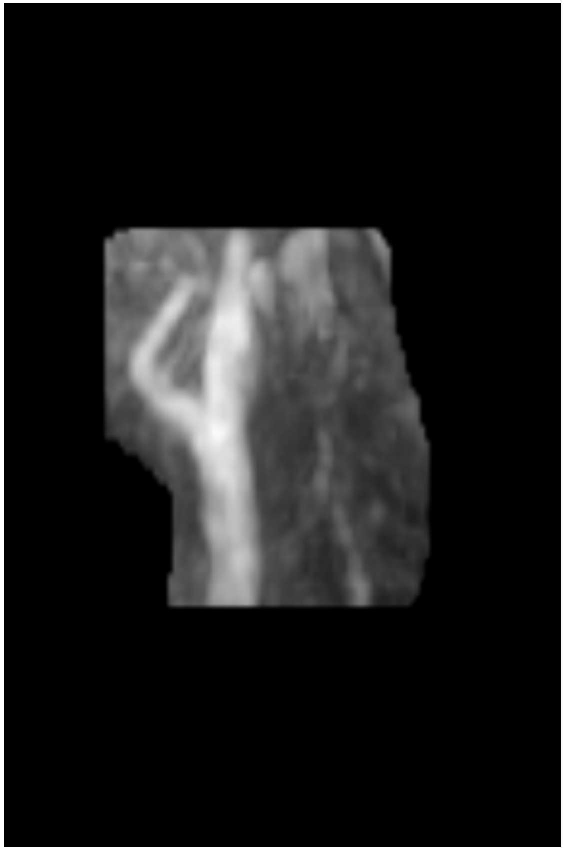

146b Score (left)

0-30

31-50

51-70

>70

Near occlusion

Occluded

Quality

1

2

3

4

5

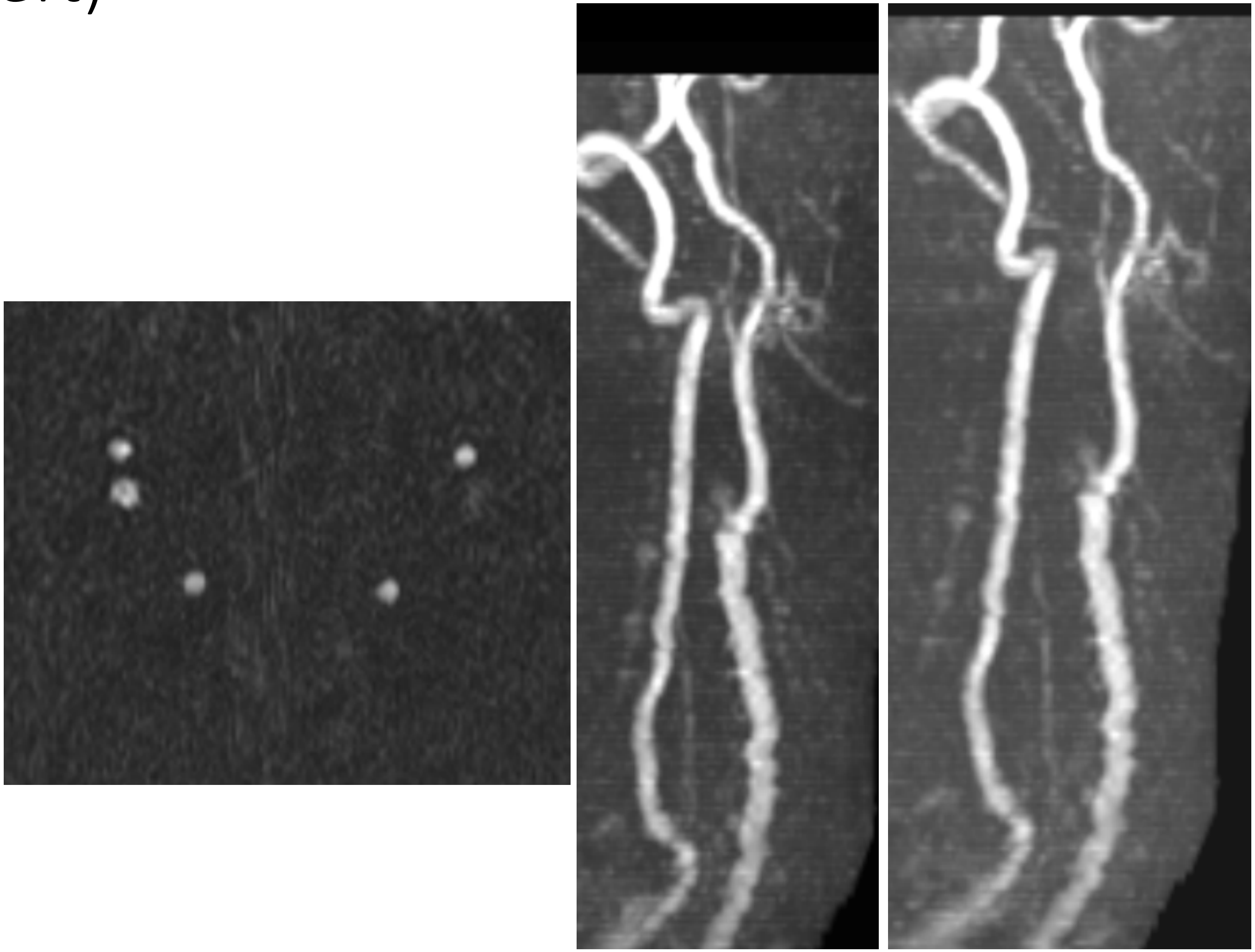

147a Score

0-30

31-50

51-70

>70

Near occlusion

Occluded

Quality

1

2

3

4

5

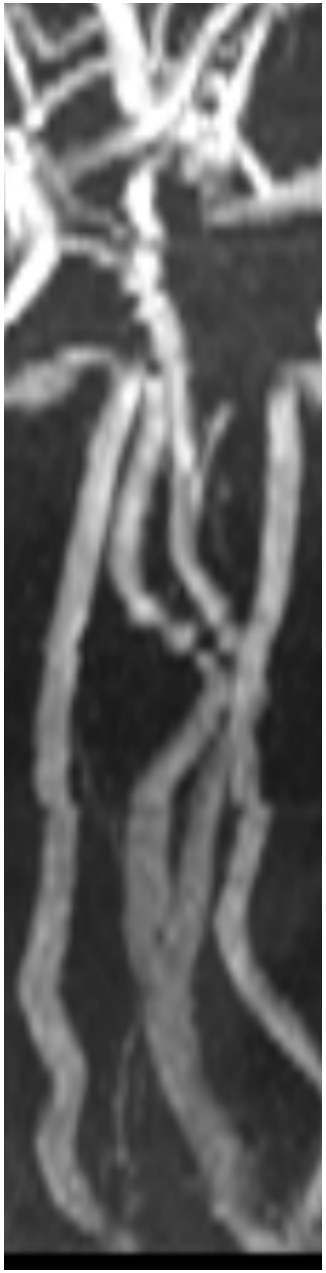

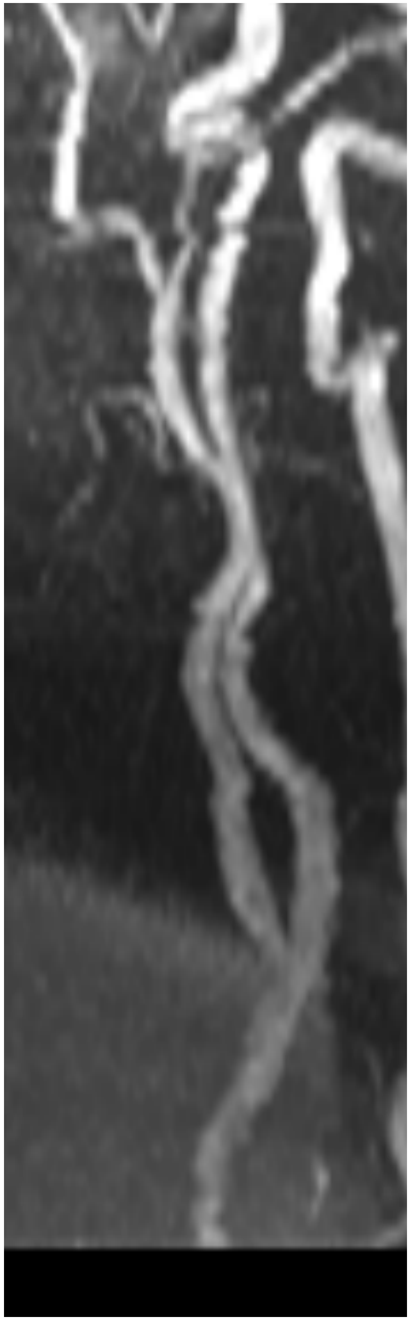

147f Score

0-30

31-50

51-70

>70

Near occlusion

Occluded

Quality

1

2

3

4

5

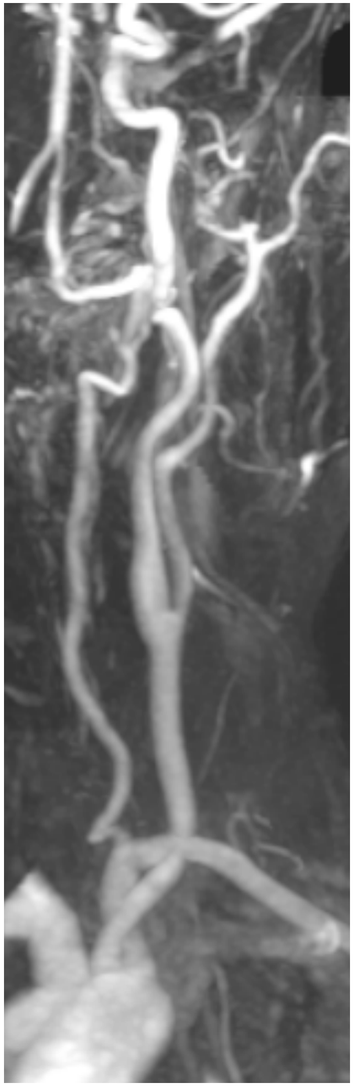

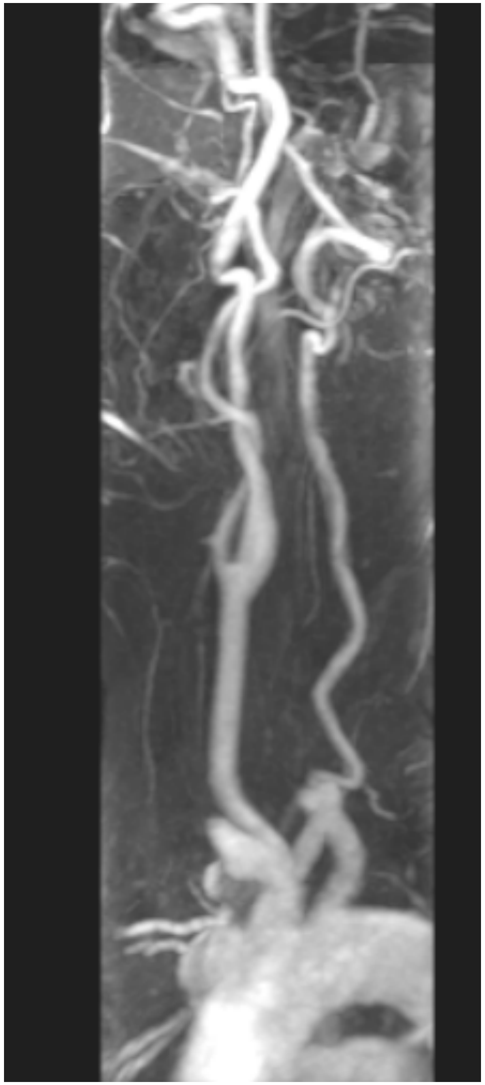

148e Score

0-30

31-50

51-70

>70

Near occlusion

Occluded

Quality

1

2

3

4

5

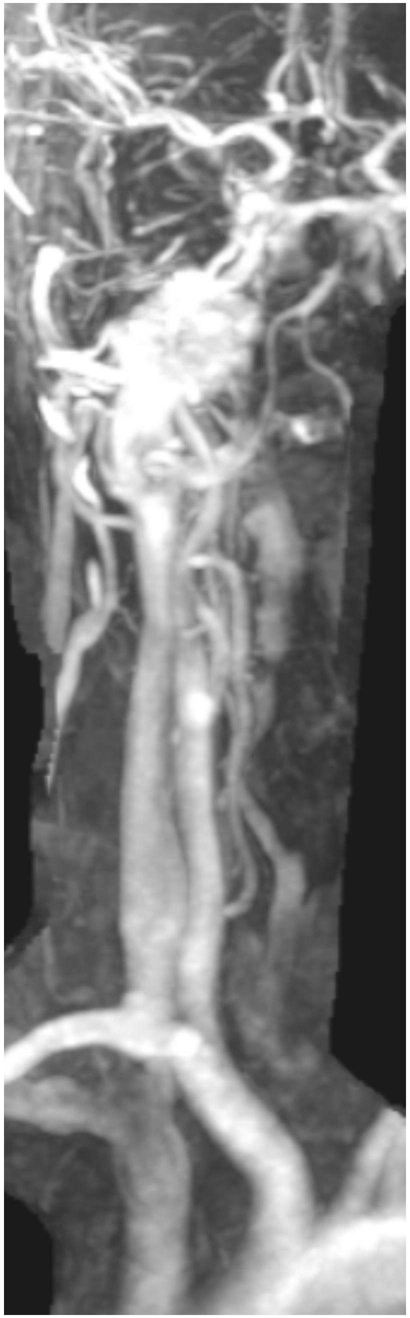

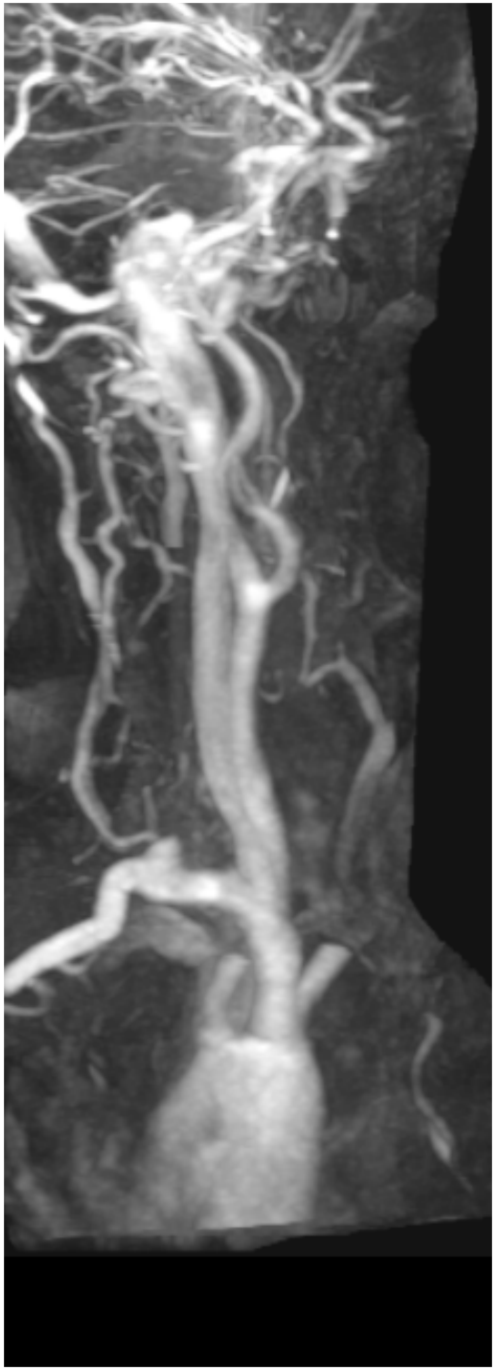

149d Score

0-30

31-50

51-70

>70

Near occlusion

Occluded

Quality

1

2

3

4

5

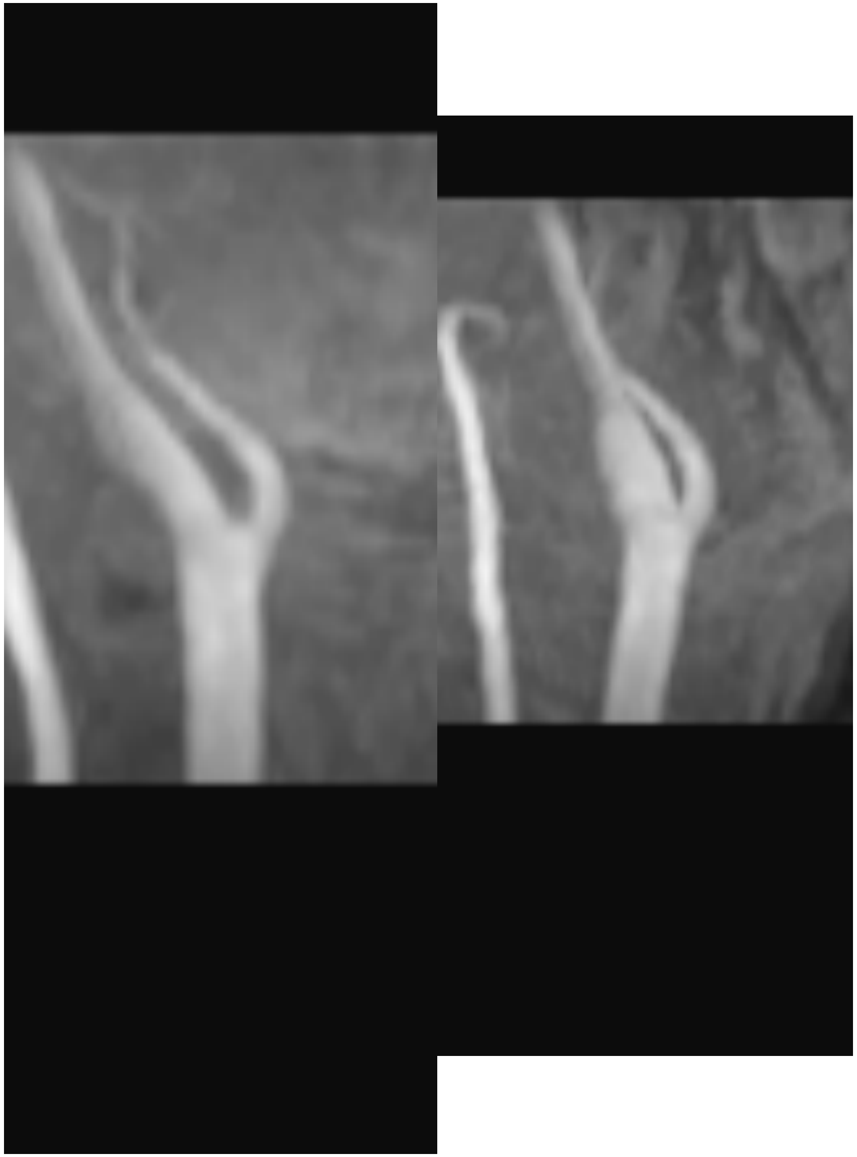

150c Score

0-30

31-50

51-70

>70

Near occlusion

Occluded

Quality

1

2

3

4

5

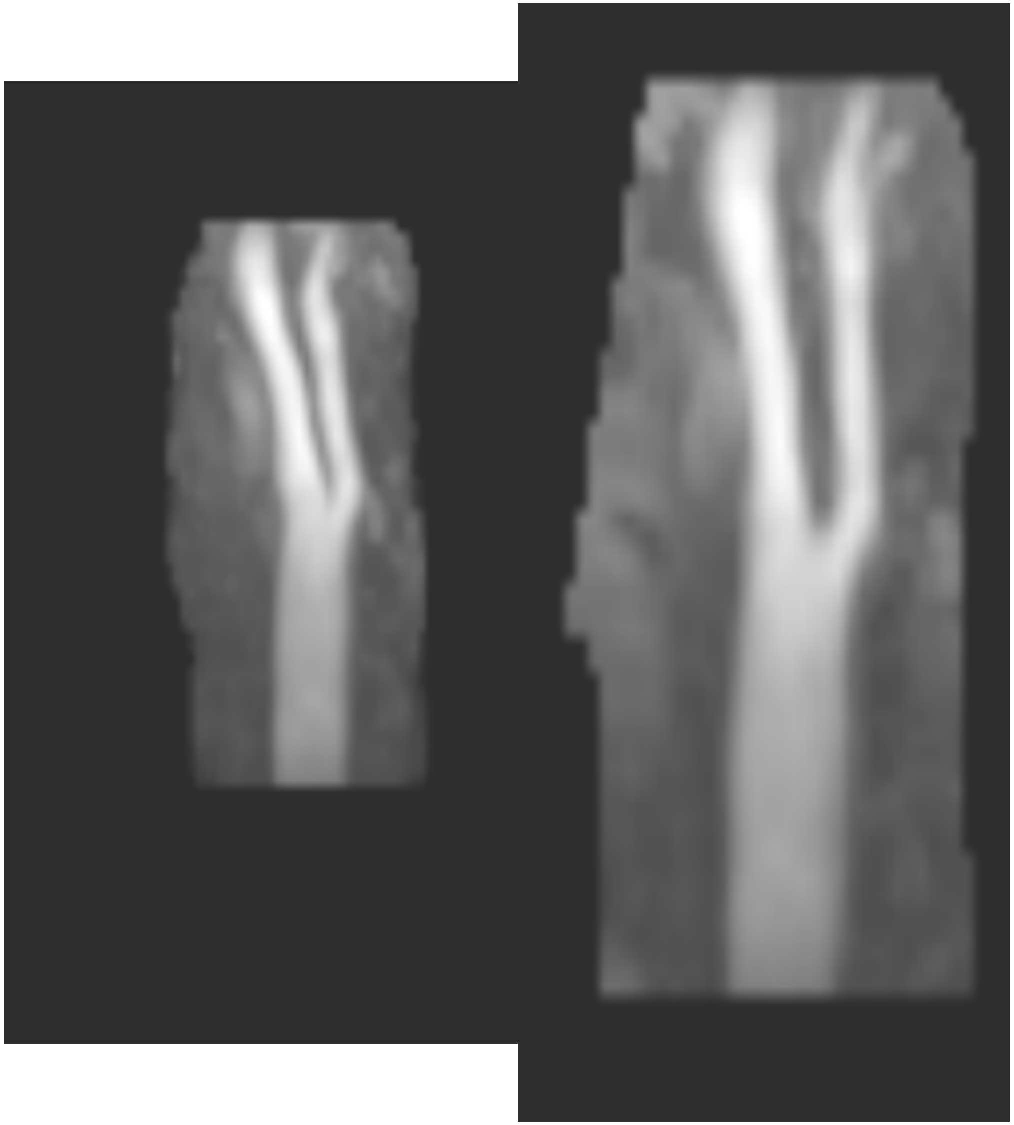

151b Score

0-30

31-50

51-70

>70

Near occlusion

Occluded

Quality

1

2

3

4

5

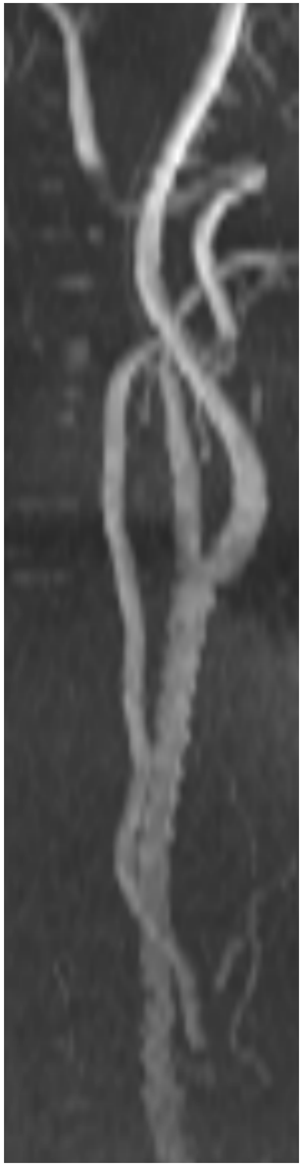

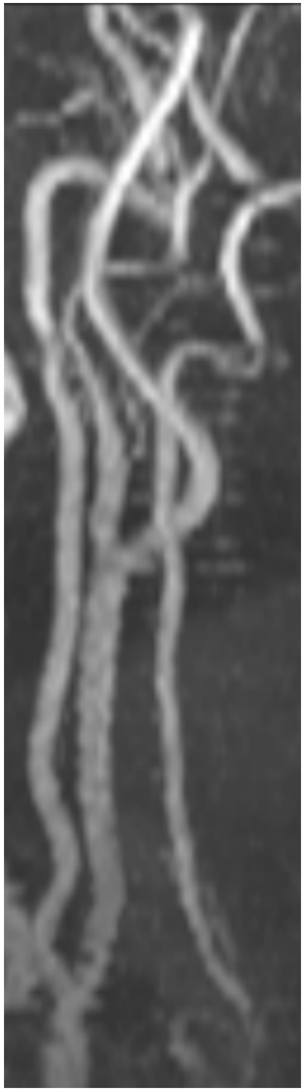

152a Score

0-30

31-50

51-70

>70

Near occlusion

Occluded

Quality

1

2

3

4

5

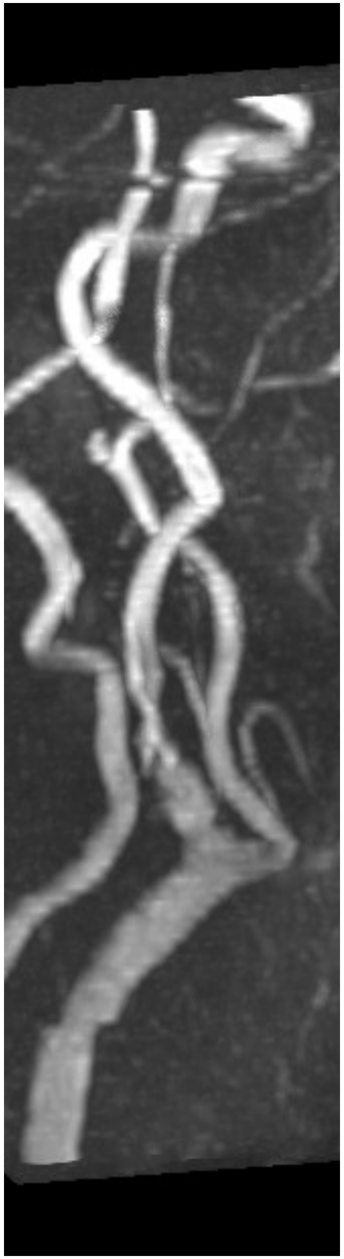

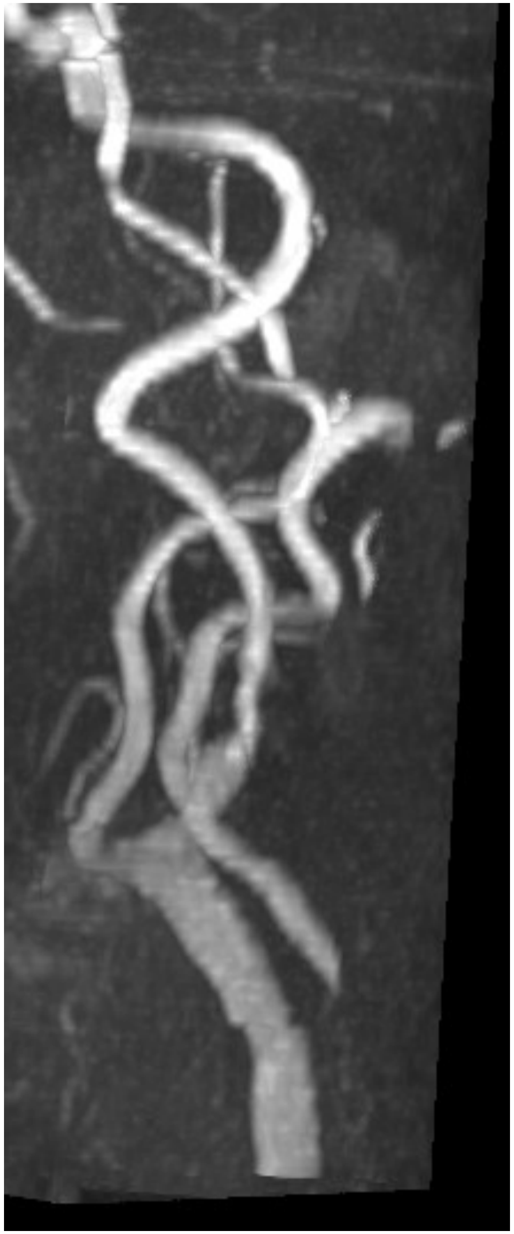

152f Score

0-30

31-50

51-70

>70

Near occlusion

Occluded

Quality

1

2

3

4

5

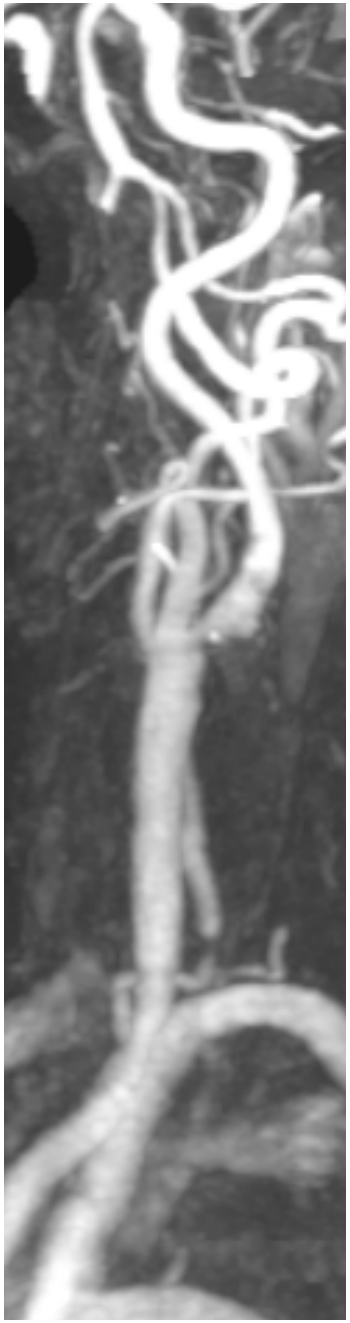

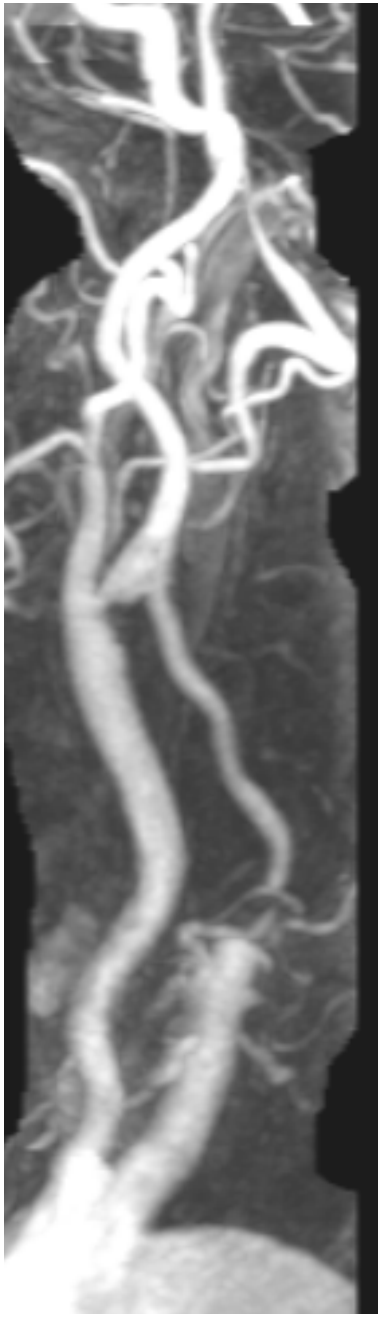

153e Score

0-30

31-50

51-70

>70

Near occlusion

Occluded

Quality

1

2

3

4

5

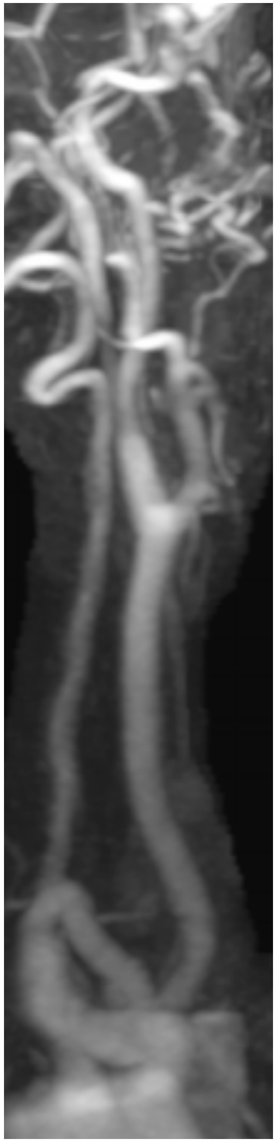

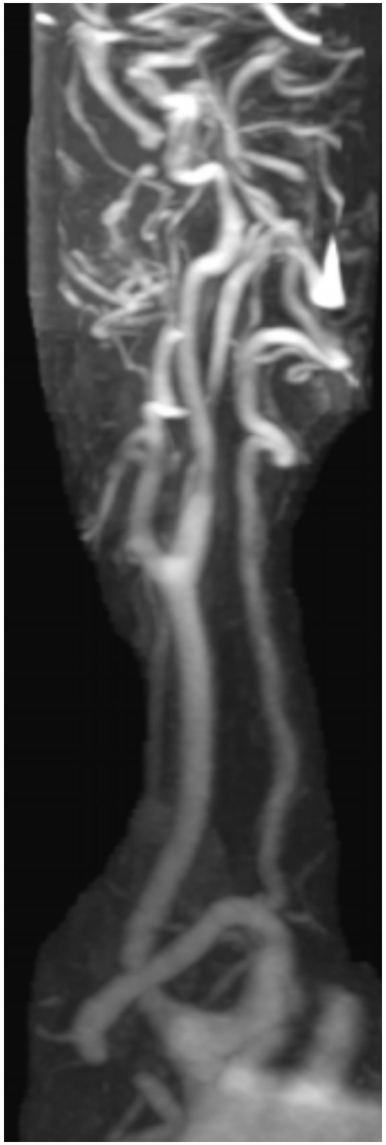

154d Score

0-30

31-50

51-70

>70

Near occlusion

Occluded

Quality

1

2

3

4

5

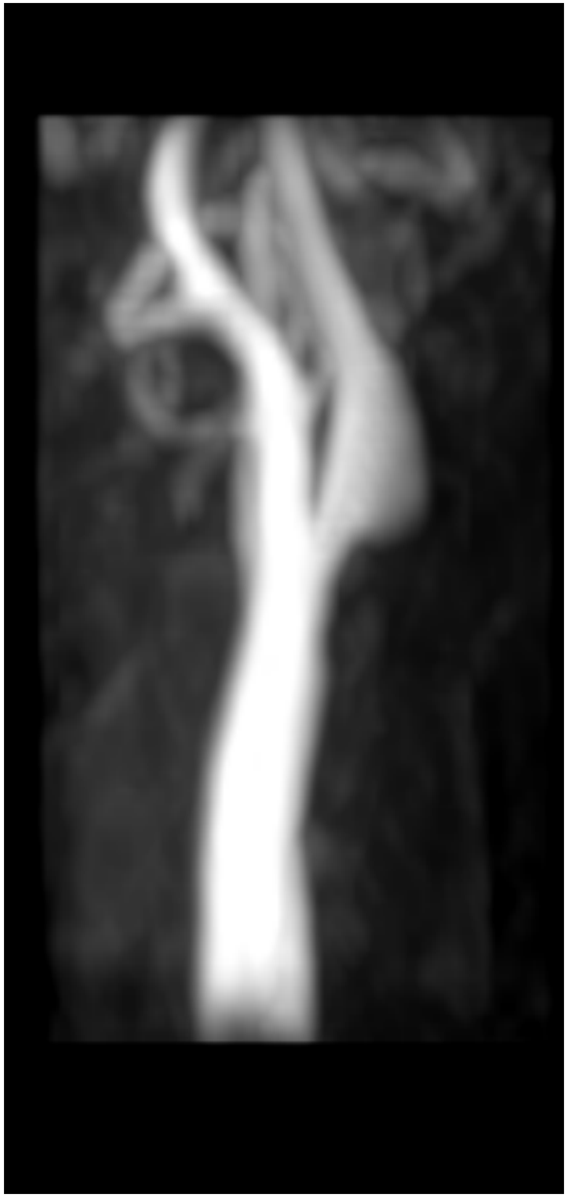

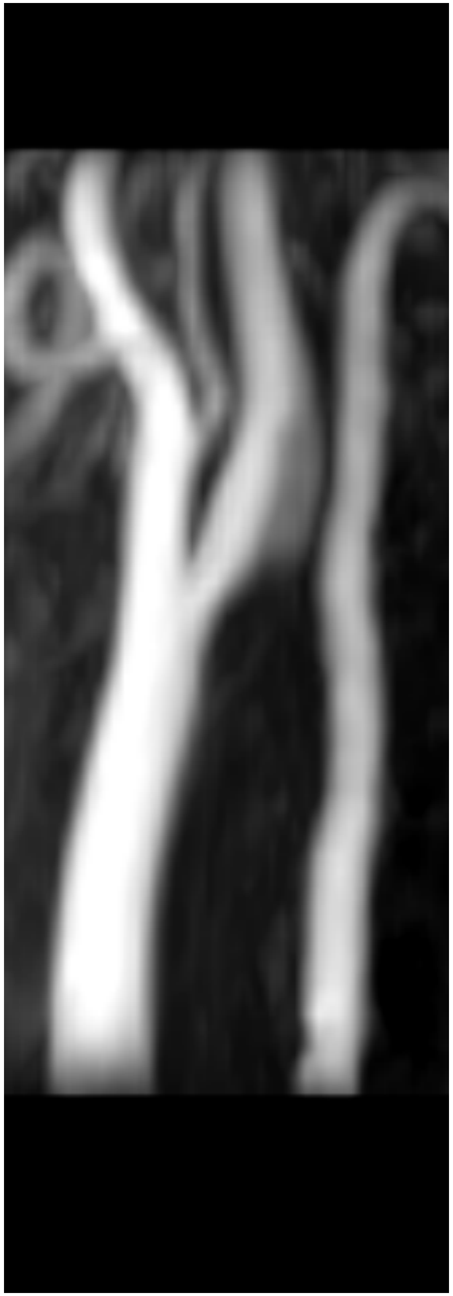

155c Score

0-30

31-50

51-70

>70

Near occlusion

Occluded

Quality

1

2

3

4

5

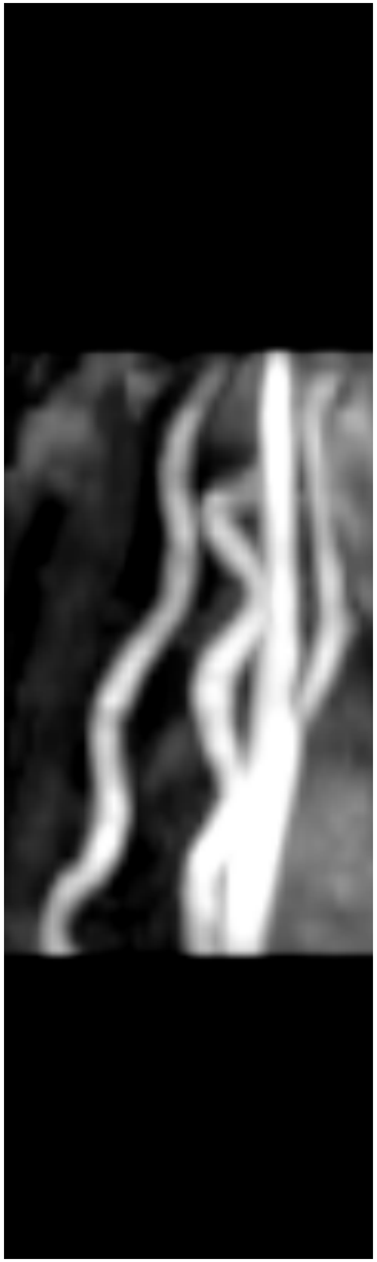

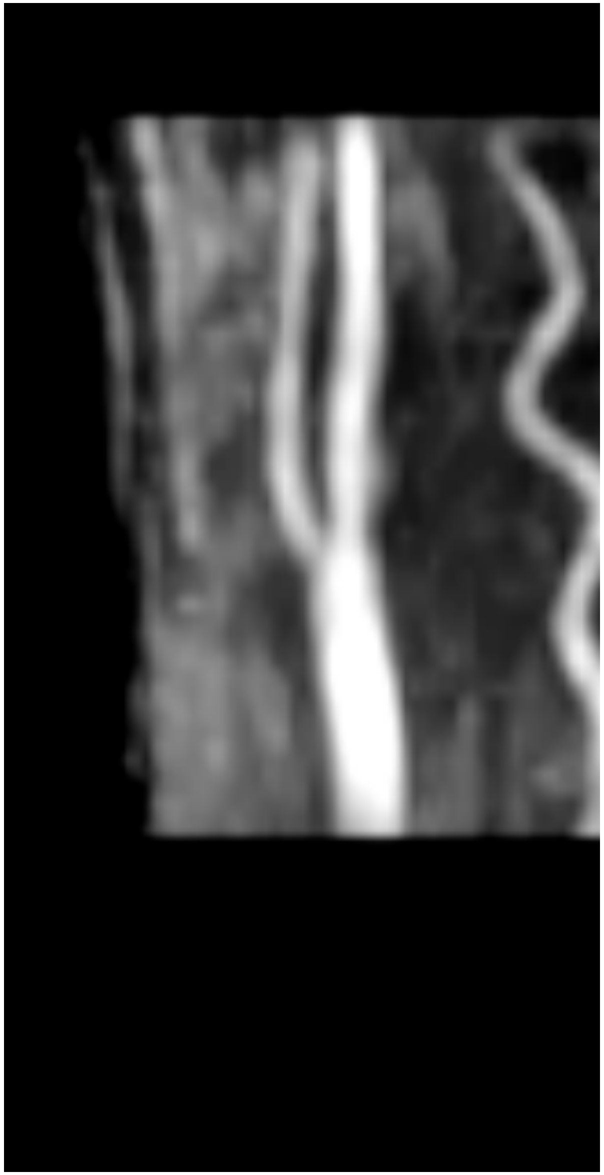

156b Score

0-30

31-50

51-70

>70

Near occlusion

Occluded

Quality

1

2

3

4

5

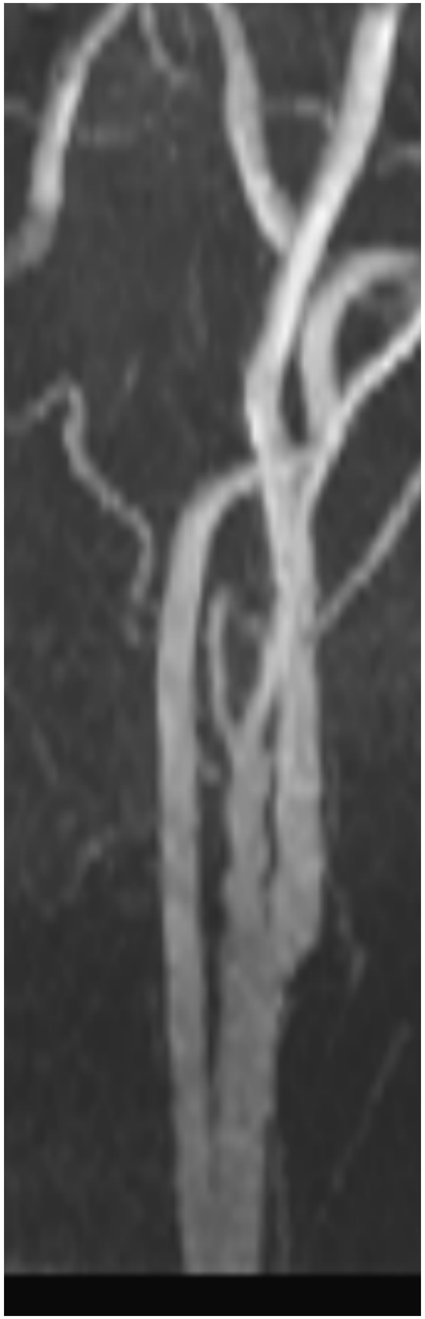

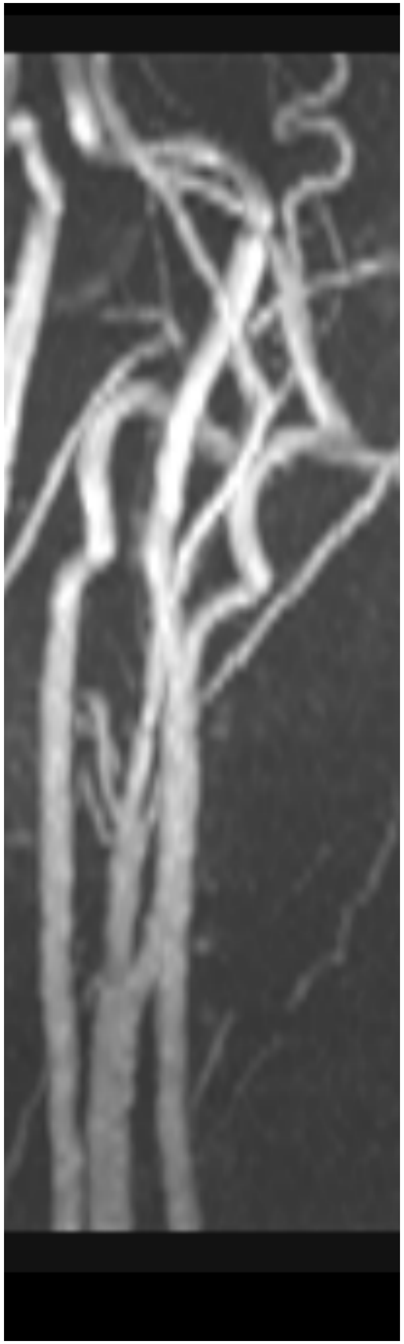

157a Score

0-30

31-50

51-70

>70

Near occlusion

Occluded

Quality

1

2

3

4

5

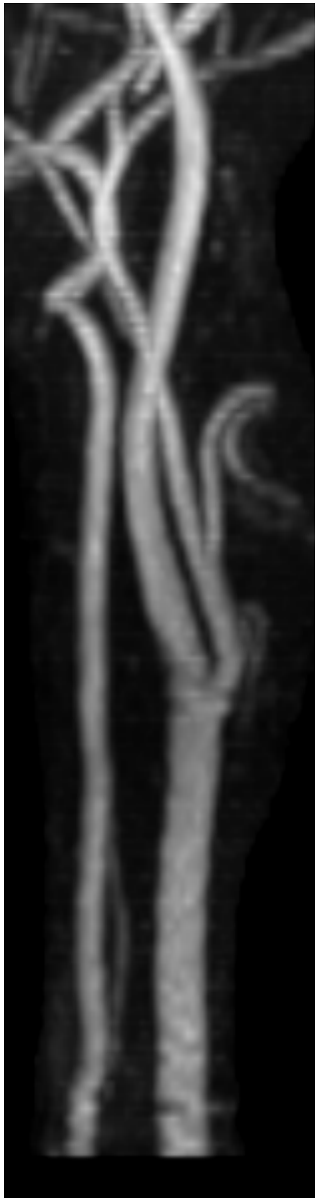

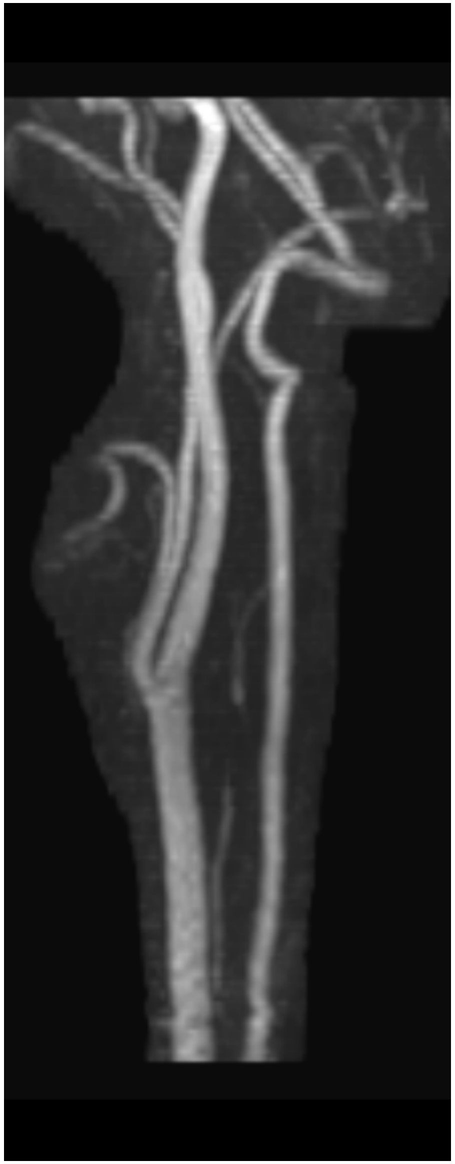

157f Score

0-30

31-50

51-70

>70

Near occlusion

Occluded

Quality

1

2

3

4

5

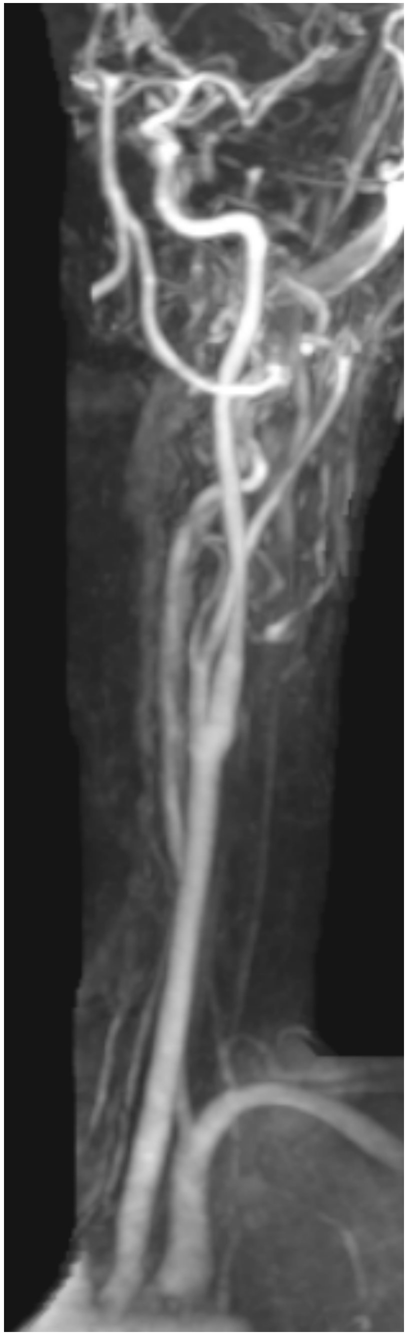

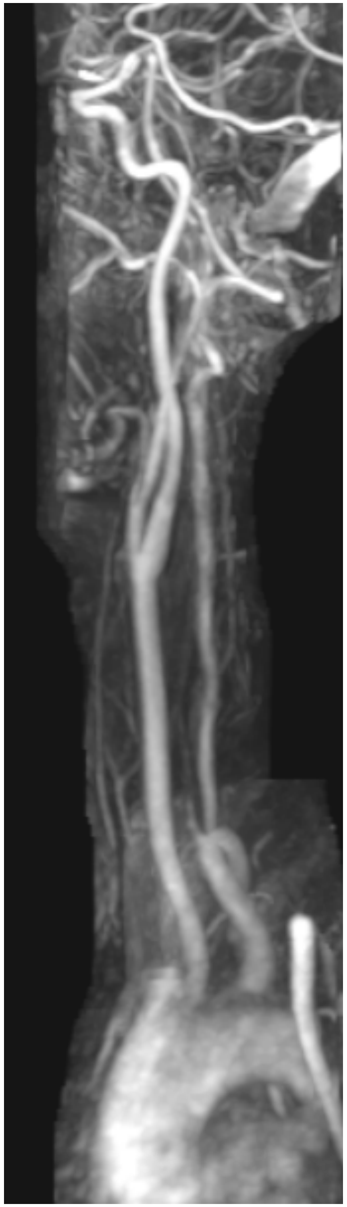

158e Score

0-30

31-50

51-70

>70

Near occlusion

Occluded

Quality

1

2

3

4

5

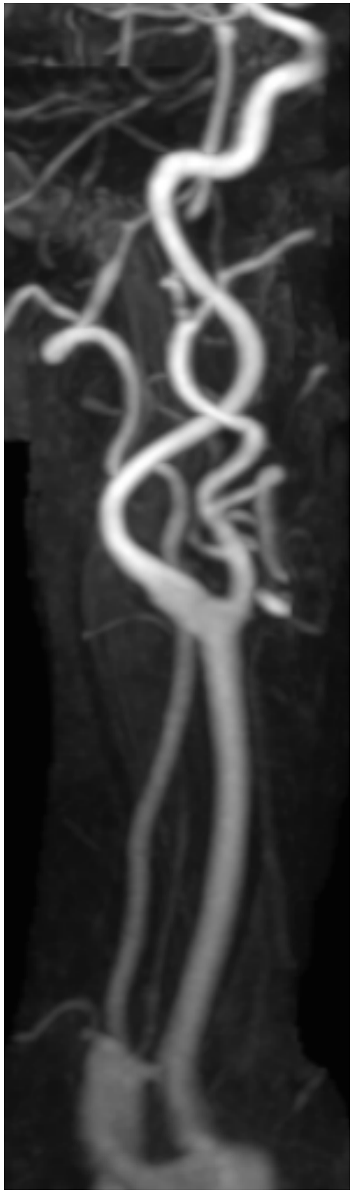

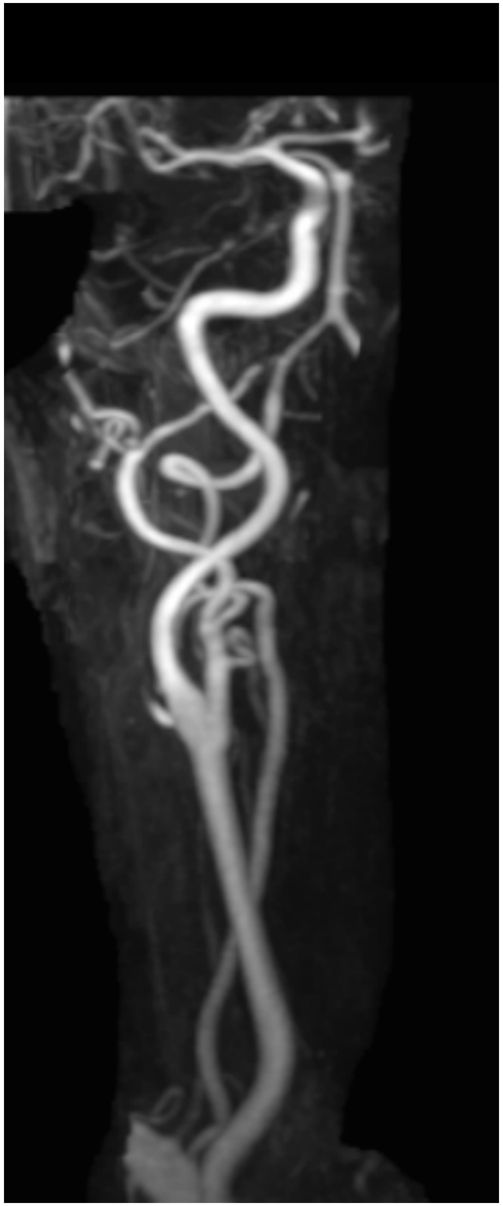

159d Score

0-30

31-50

51-70

>70

Near occlusion

Occluded

Quality

1

2

3

4

5

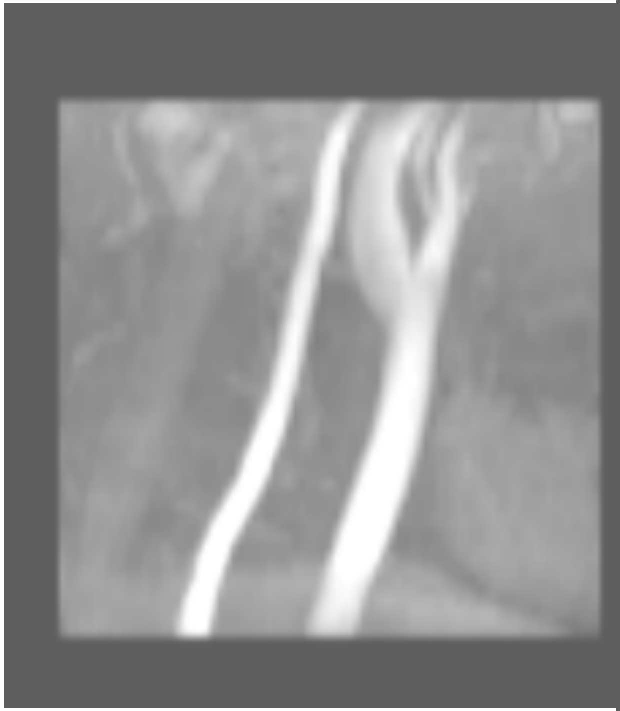

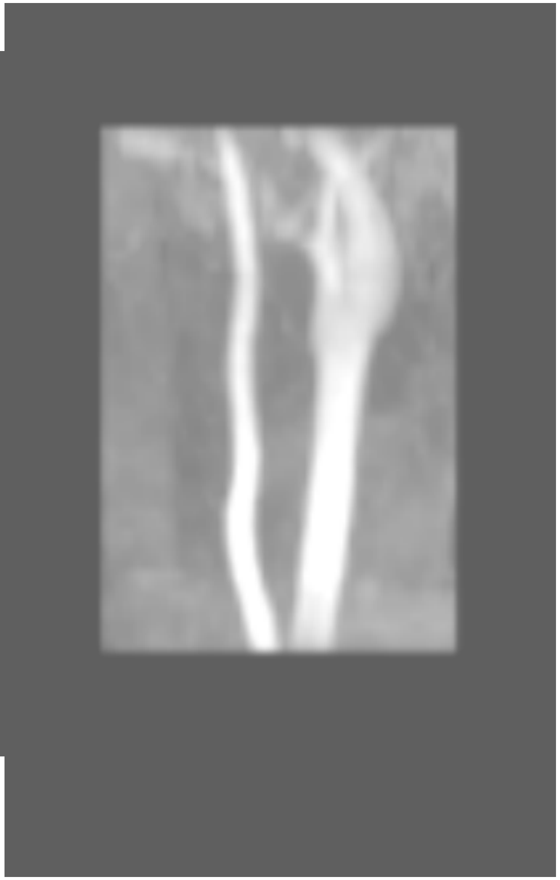

160c Score

0-30

31-50

51-70

>70

Near occlusion

Occluded

Quality

1

2

3

4

5

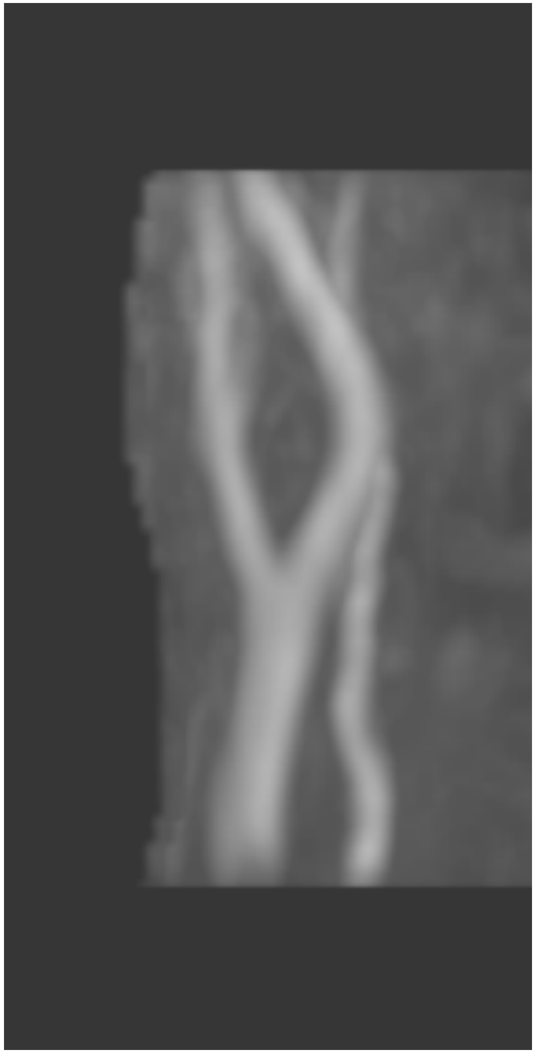

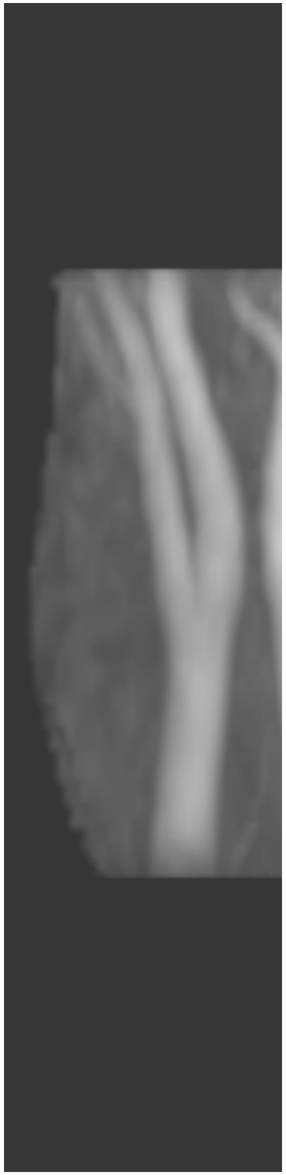

161b Score

0-30

31-50

51-70

>70

Near occlusion

Occluded

Quality

1

2

3

4

5

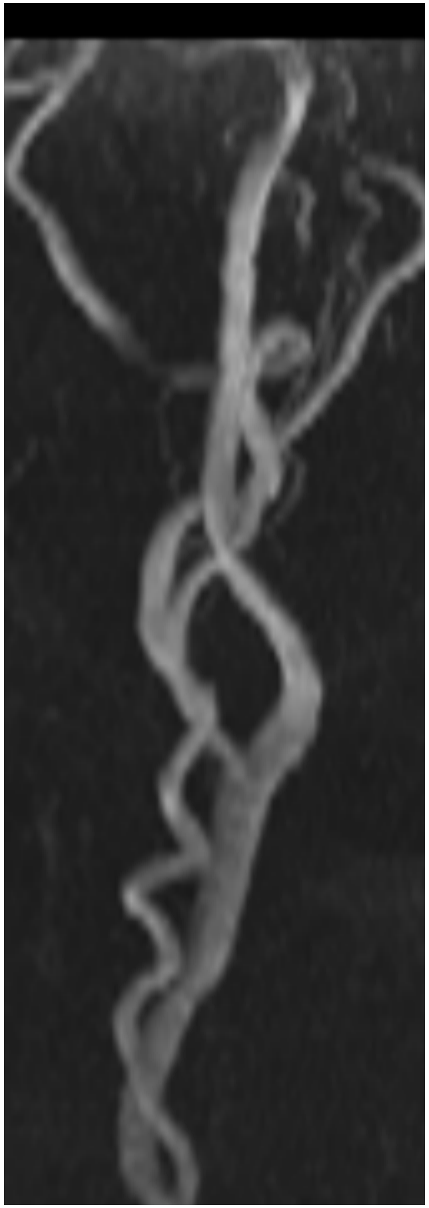

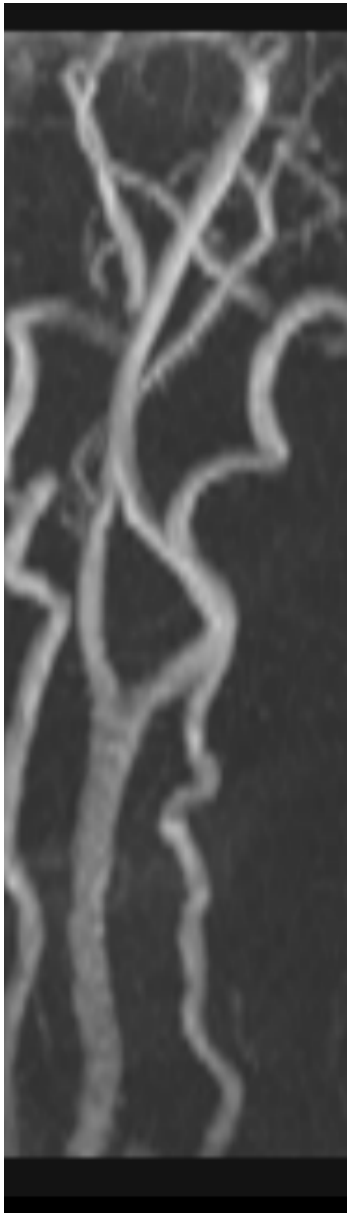

162a Score

0-30

31-50

51-70

>70

Near occlusion

Occluded

Quality

1

2

3

4

5

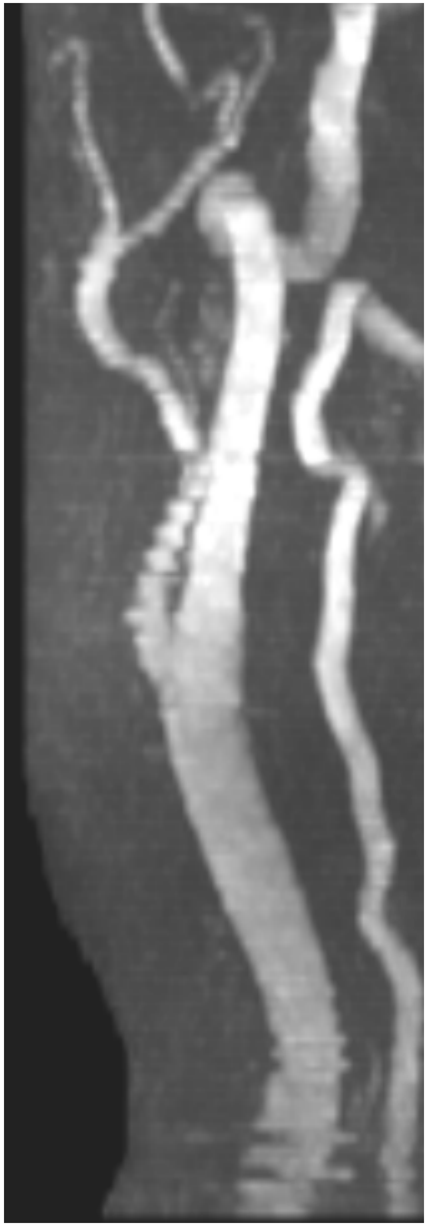

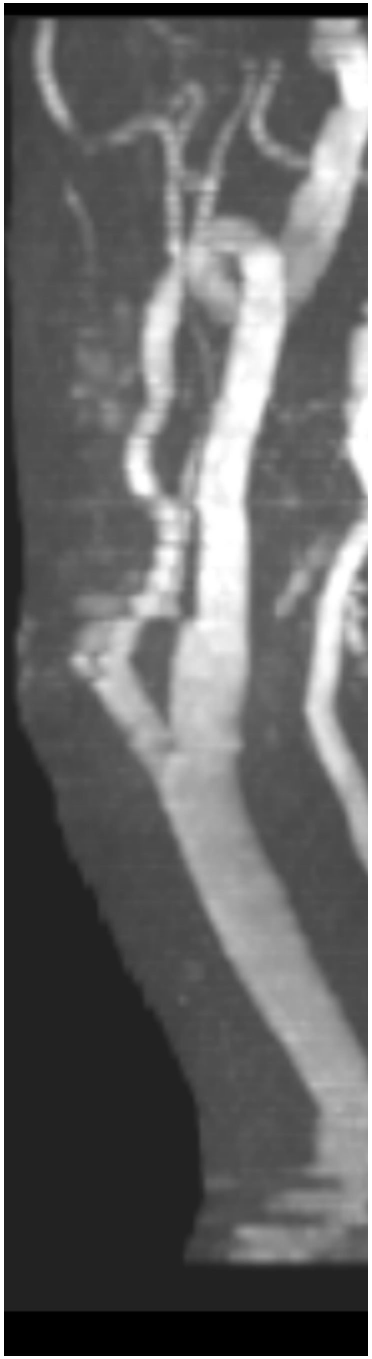

162f Score

0-30

31-50

51-70

>70

Near occlusion

Occluded

Quality

1

2

3

4

5

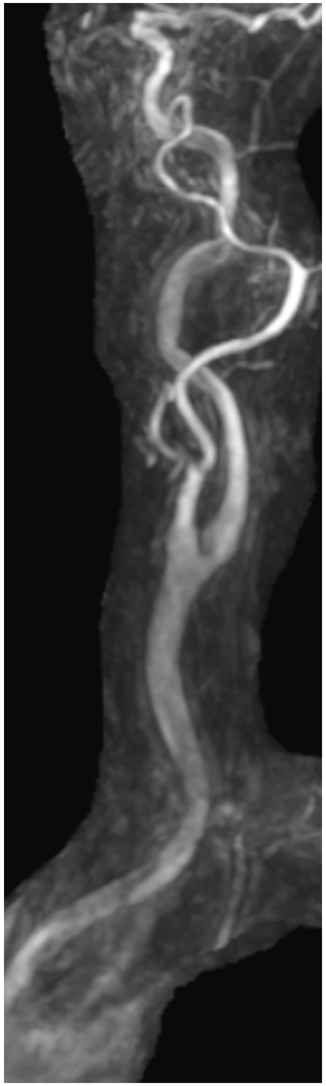

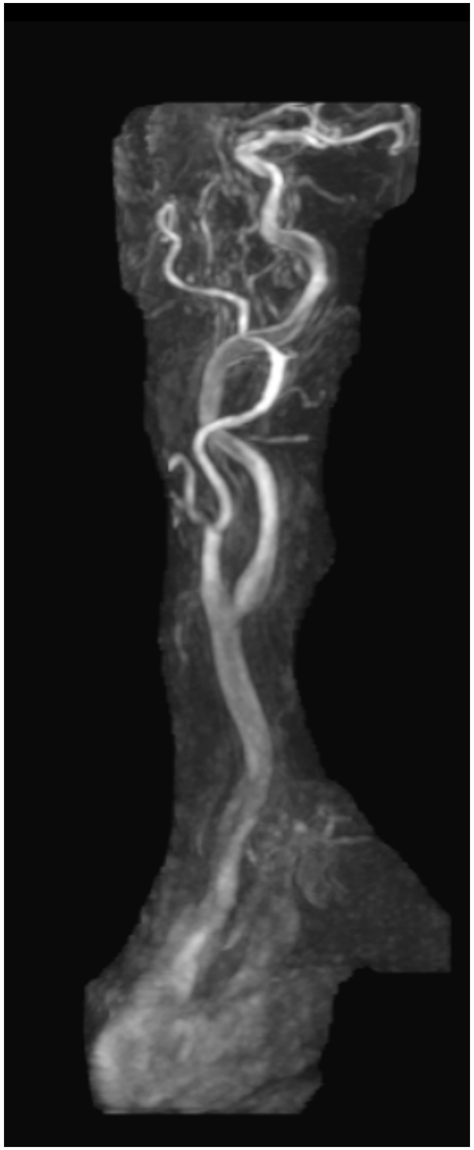

163e Score

0-30

31-50

51-70

>70

Near occlusion

Occluded

Quality

1

2

3

4

5

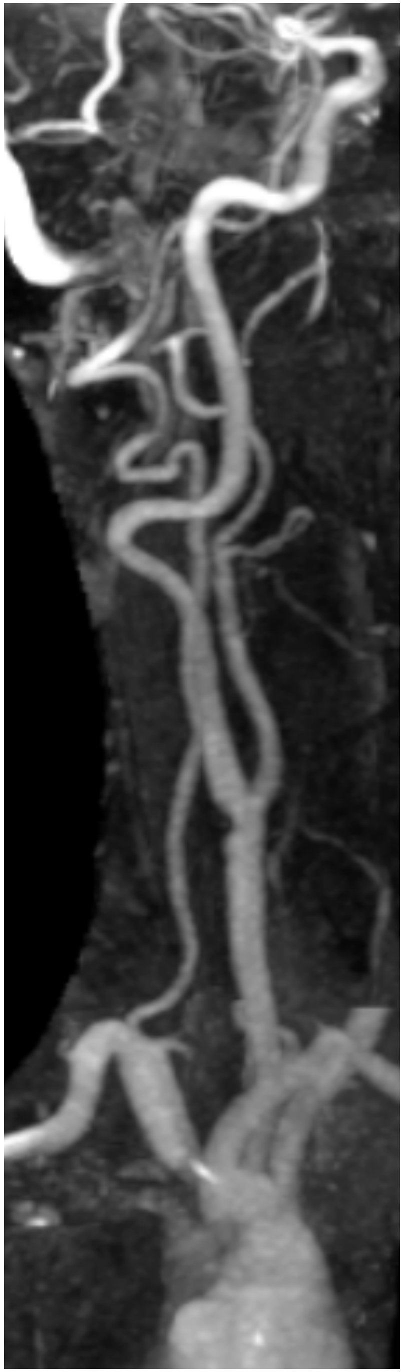

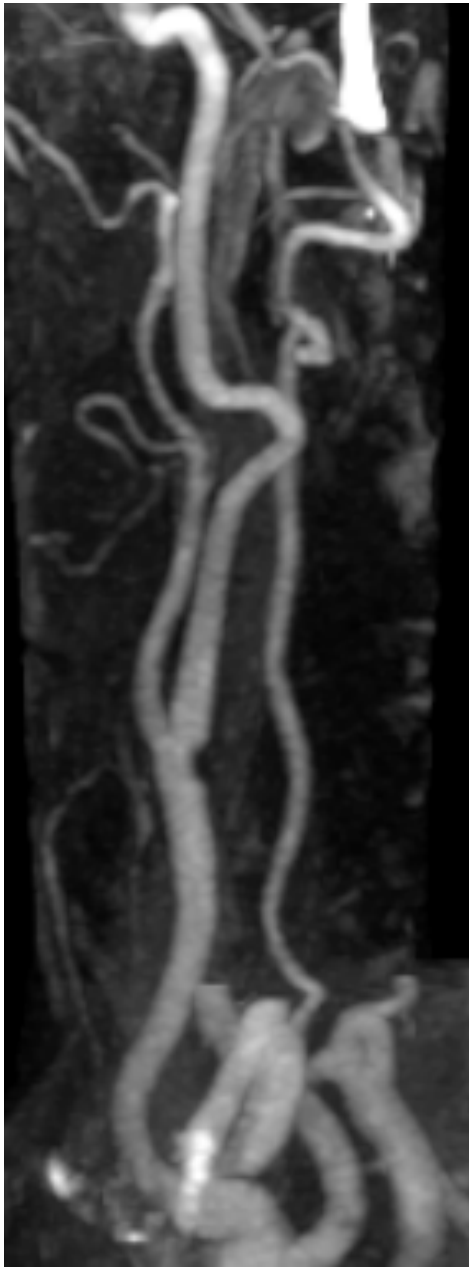

164d Score

0-30

31-50

51-70

>70

Near occlusion

Occluded

Quality

1

2

3

4

5

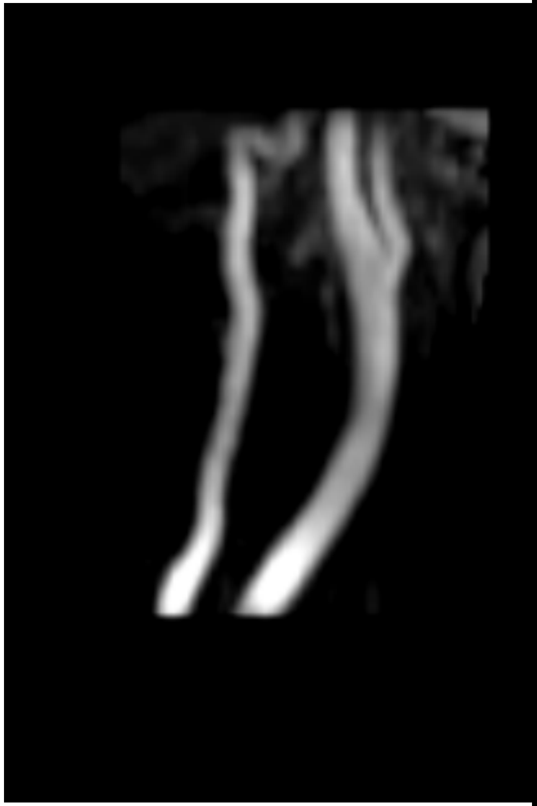

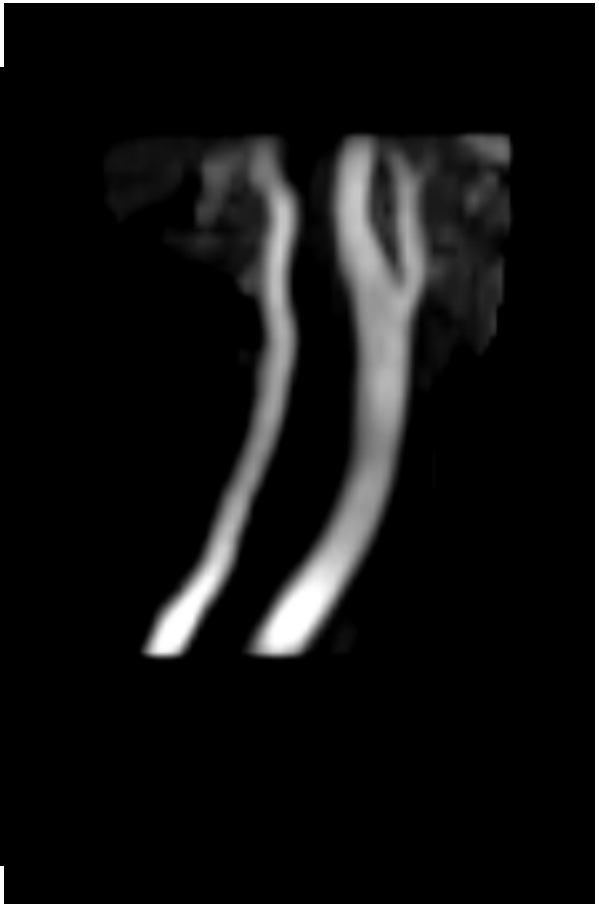

165c Score

0-30

31-50

51-70

>70

Near occlusion

Occluded

Quality

1

2

3

4

5

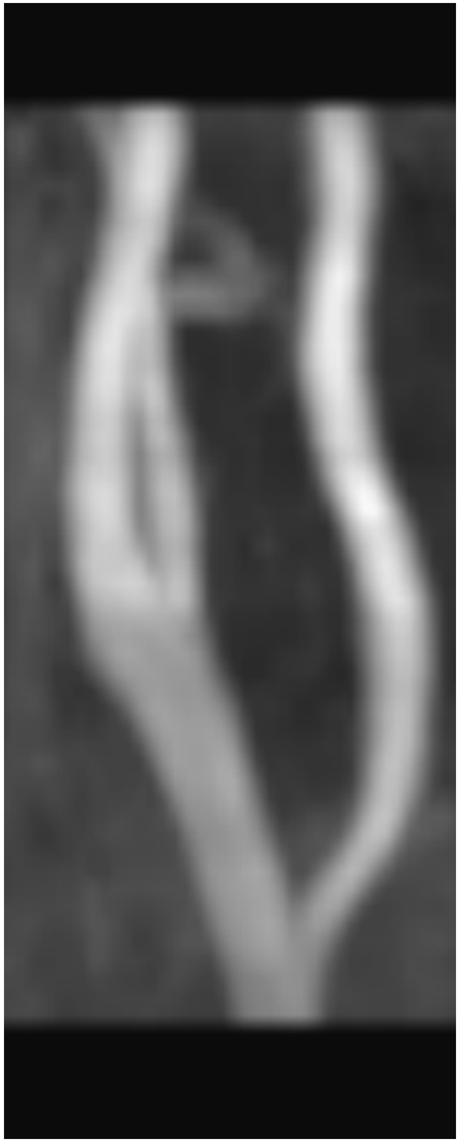

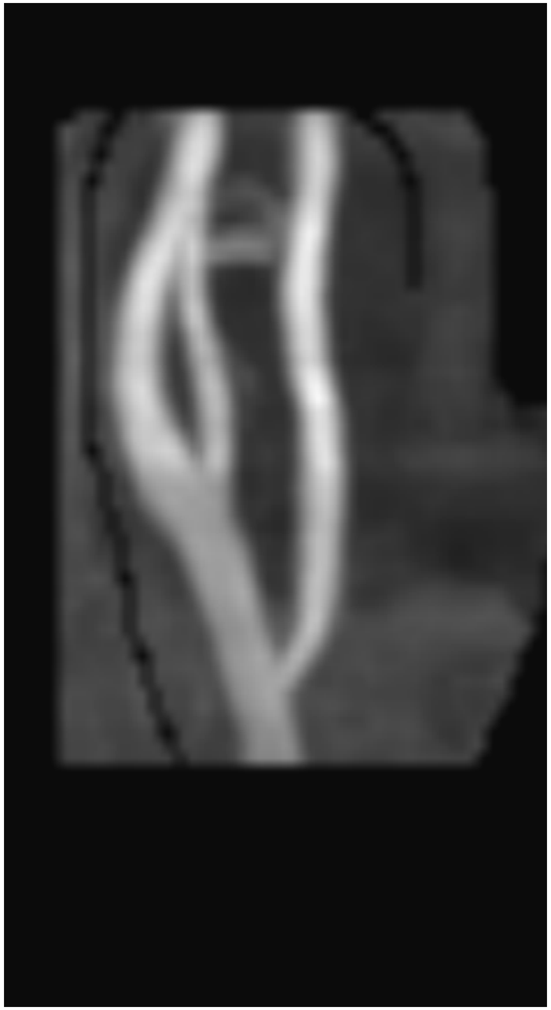

166b Score

0-30

31-50

51-70

>70

Near occlusion

Occluded

Quality

1

2

3

4

5

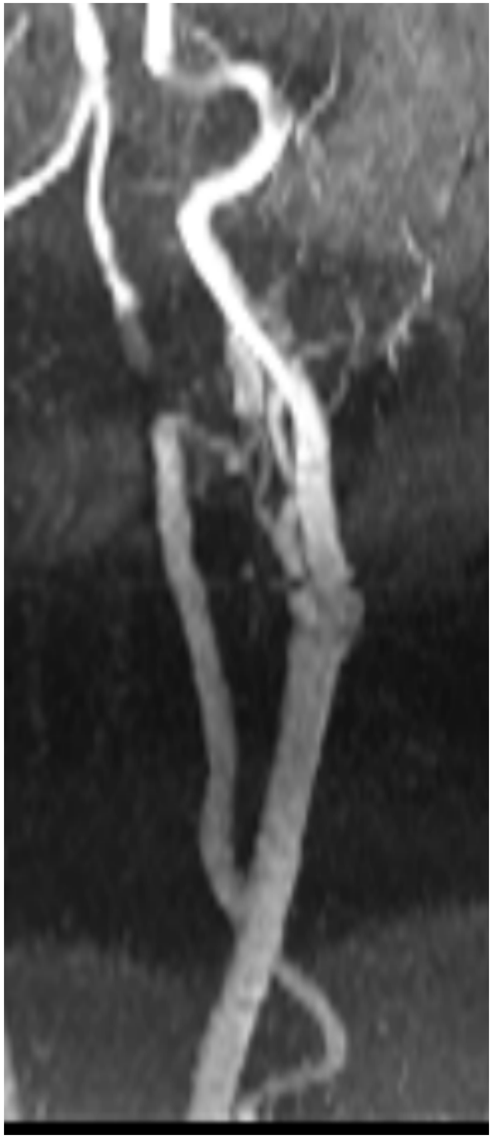

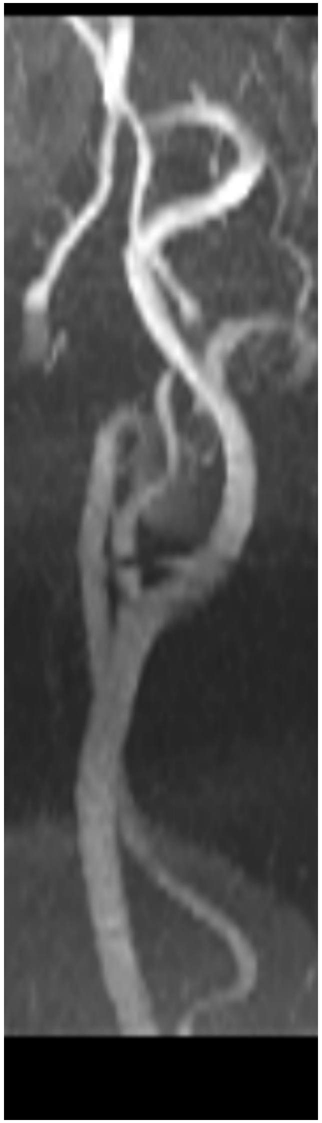

167a Score

0-30

31-50

51-70

>70

Near occlusion

Occluded

Quality

1

2

3

4

5

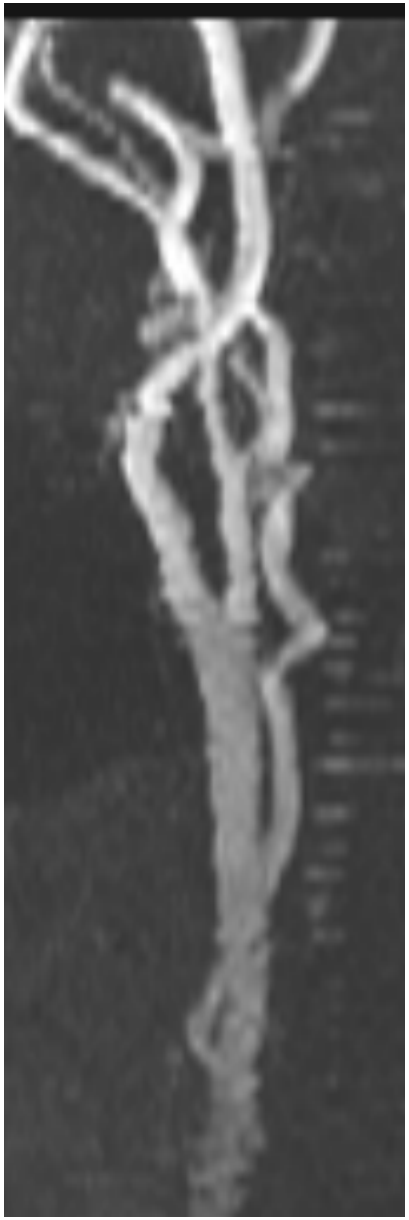

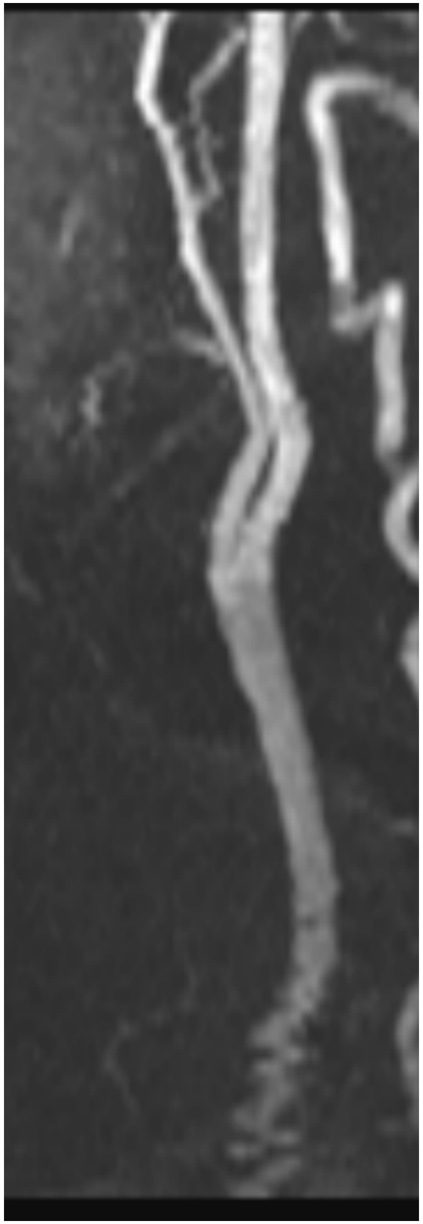

167f Score

0-30

31-50

51-70

>70

Near occlusion

Occluded

Quality

1

2

3

4

5

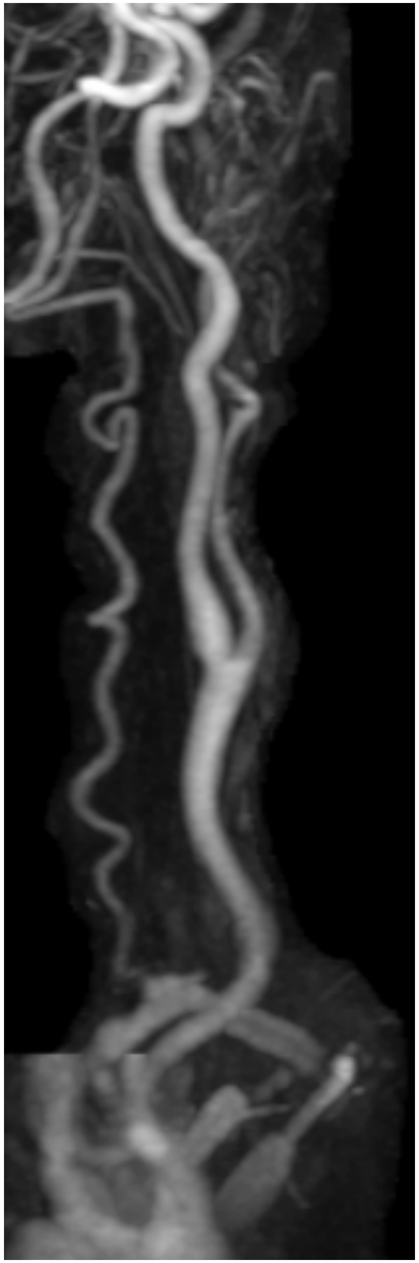

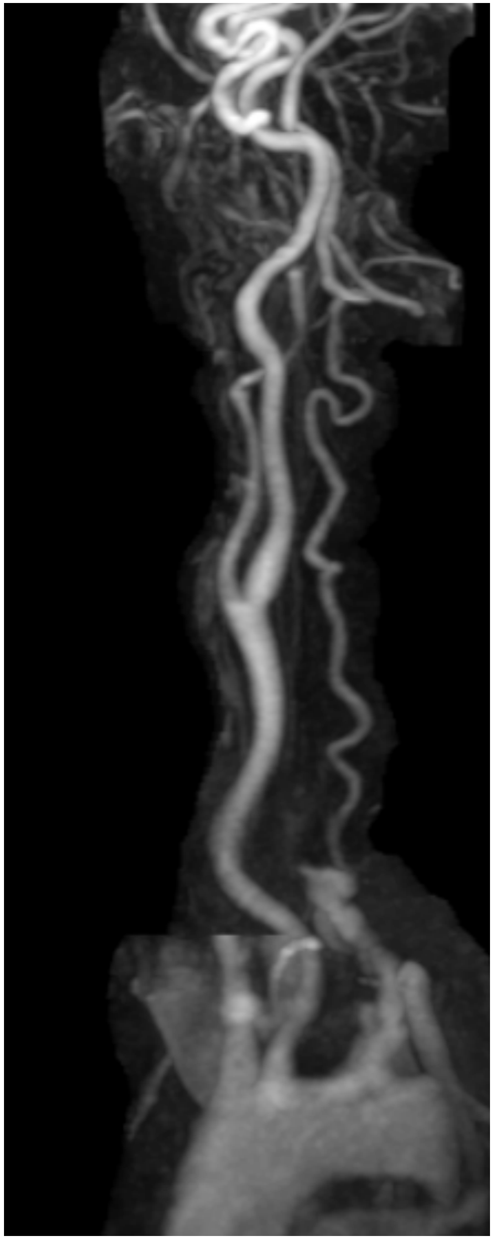

168e Score

0-30

31-50

51-70

>70

Near occlusion

Occluded

Quality

1

2

3

4

5

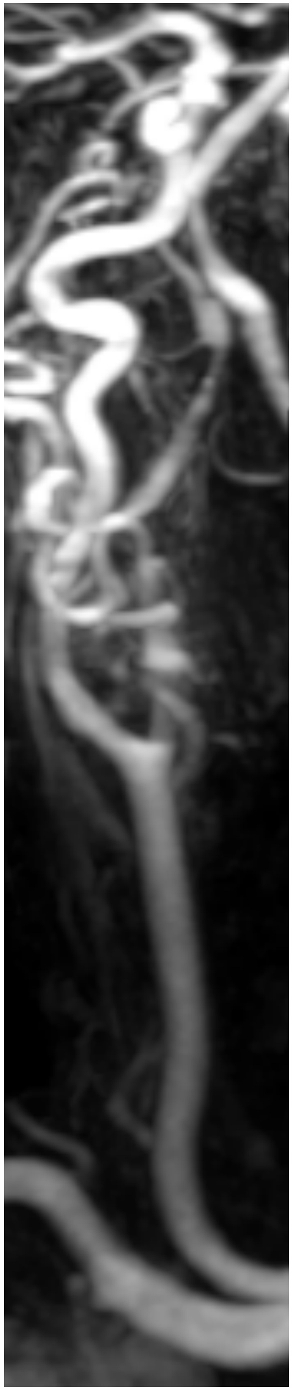

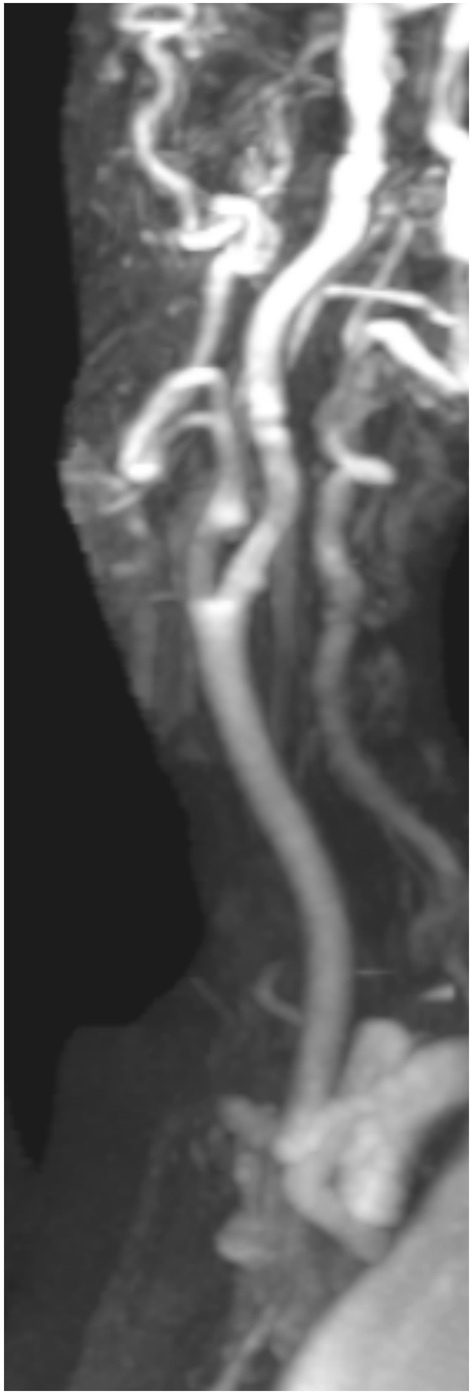

169d Score (left)

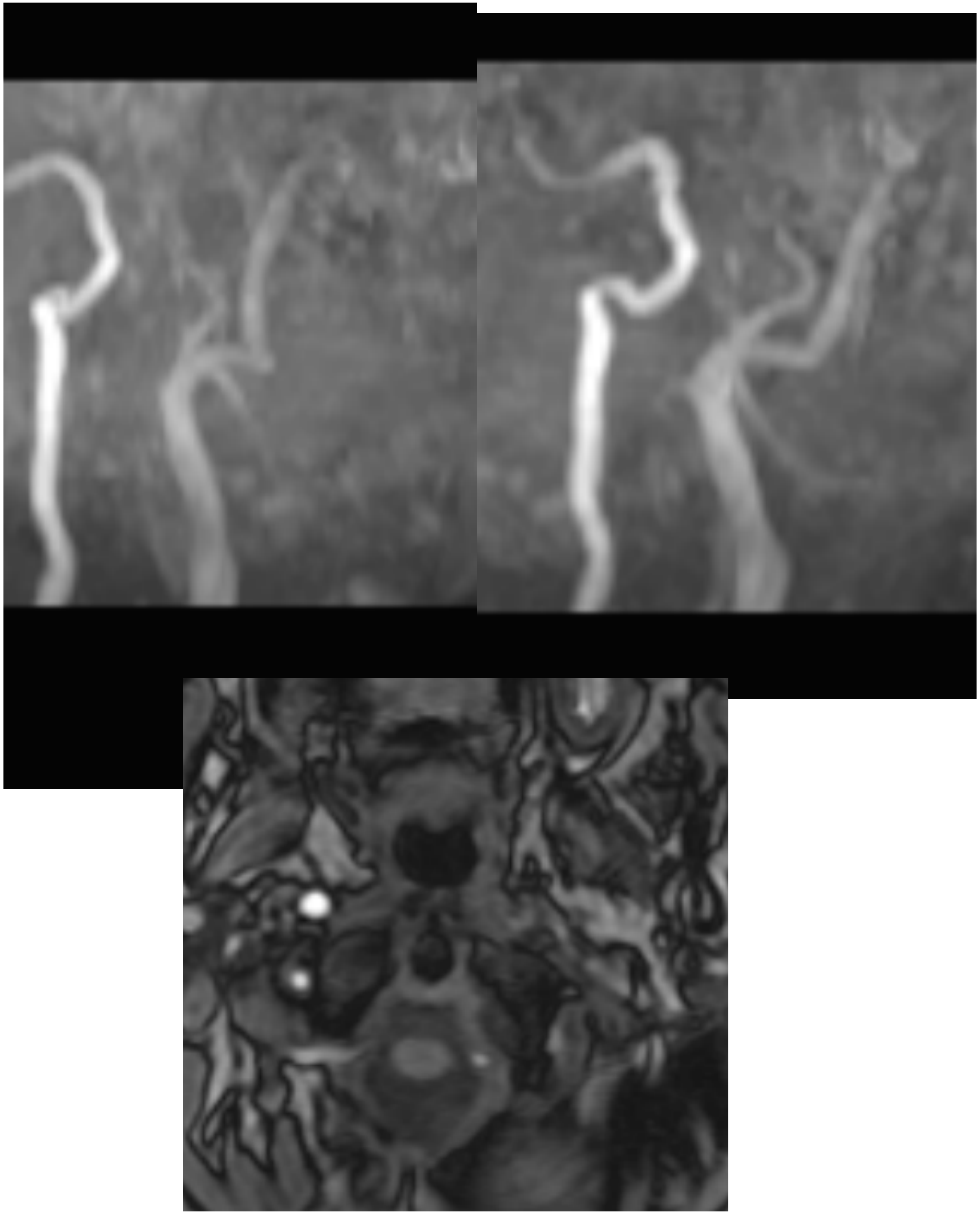

0-30

31-50

51-70

>70

Near occlusion

Occluded

Quality

1

2

3

4

5

170c Score

0-30

31-50

51-70

>70

Near occlusion

Occluded

Quality

1

2

3

4

5

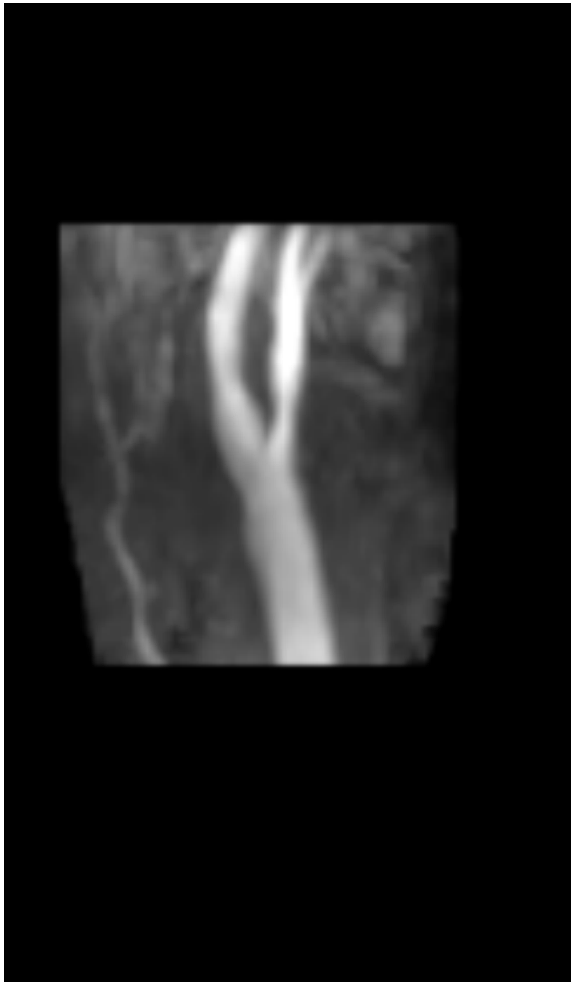

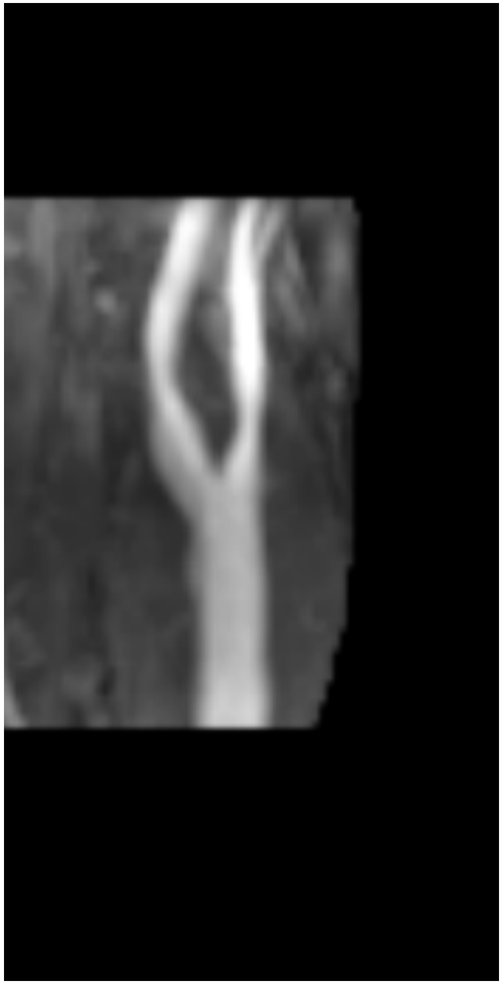

171b Score

0-30

31-50

51-70

>70

Near occlusion

Occluded

Quality

1

2

3

4

5

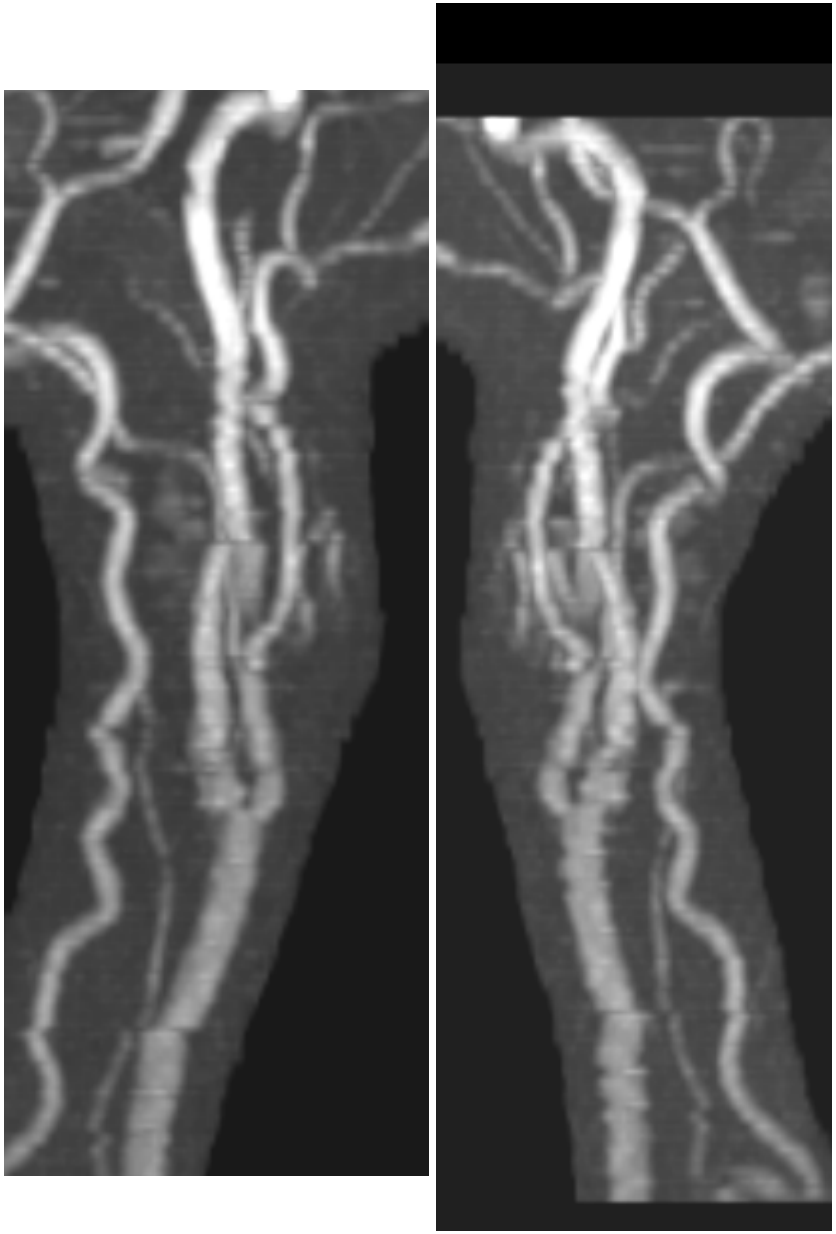

172a Score

0-30

31-50

51-70

>70

Near occlusion

Occluded

Quality

1

2

3

4

5

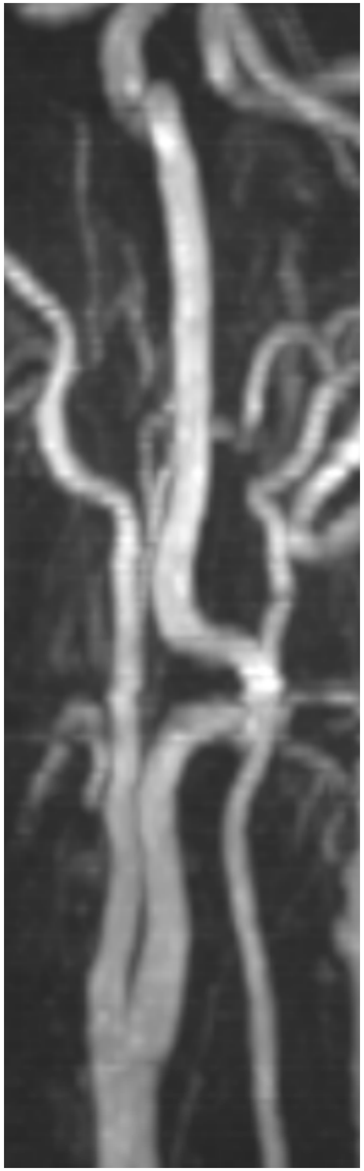

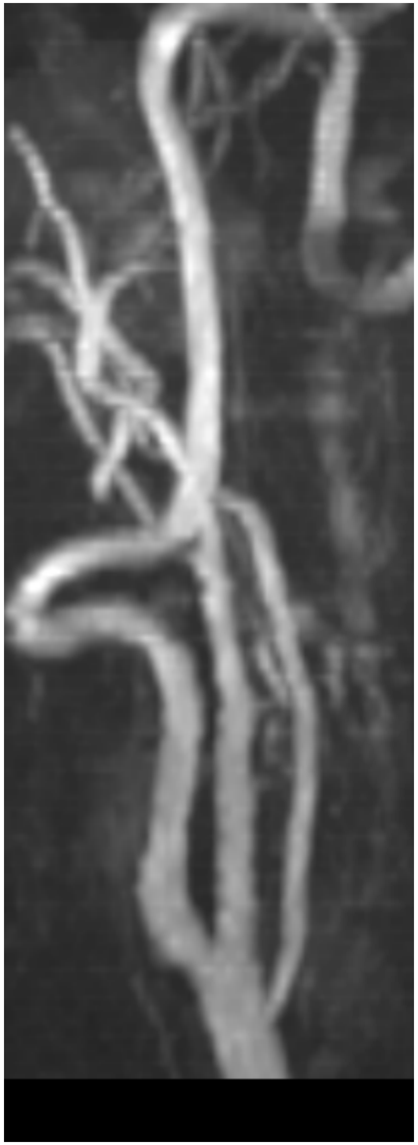

172f Score

0-30

31-50

51-70

>70

Near occlusion

Occluded

Quality

1

2

3

4

5

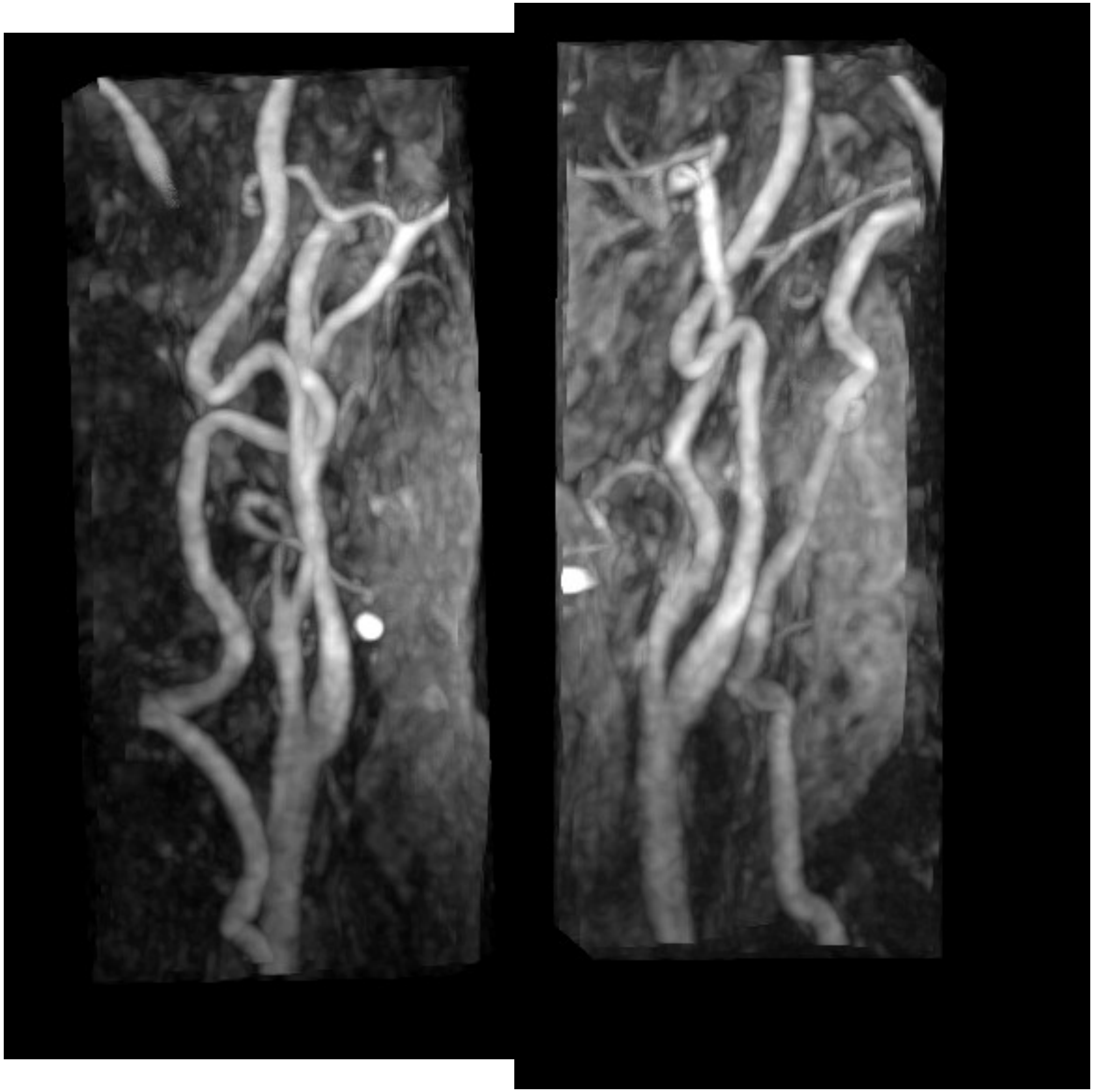

173e Score

0-30

31-50

51-70

>70

Near occlusion

Occluded

Quality

1

2

3

4

5

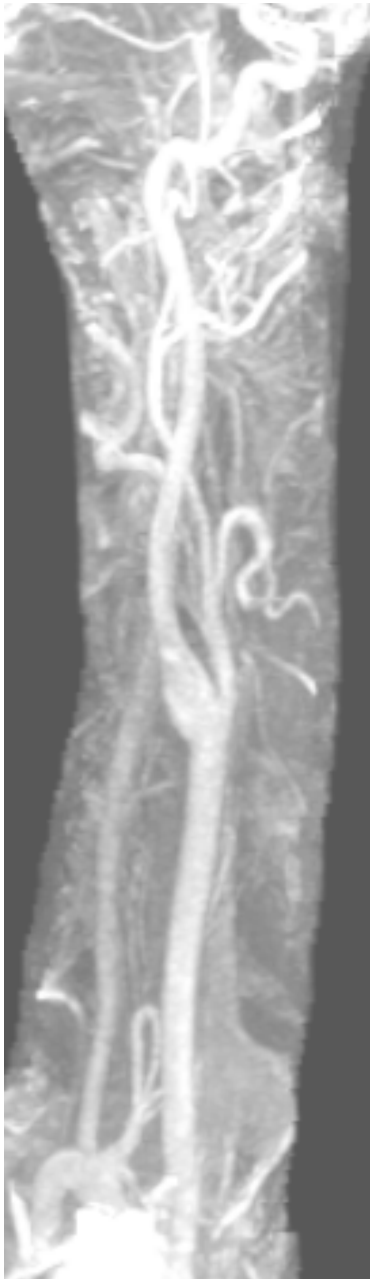

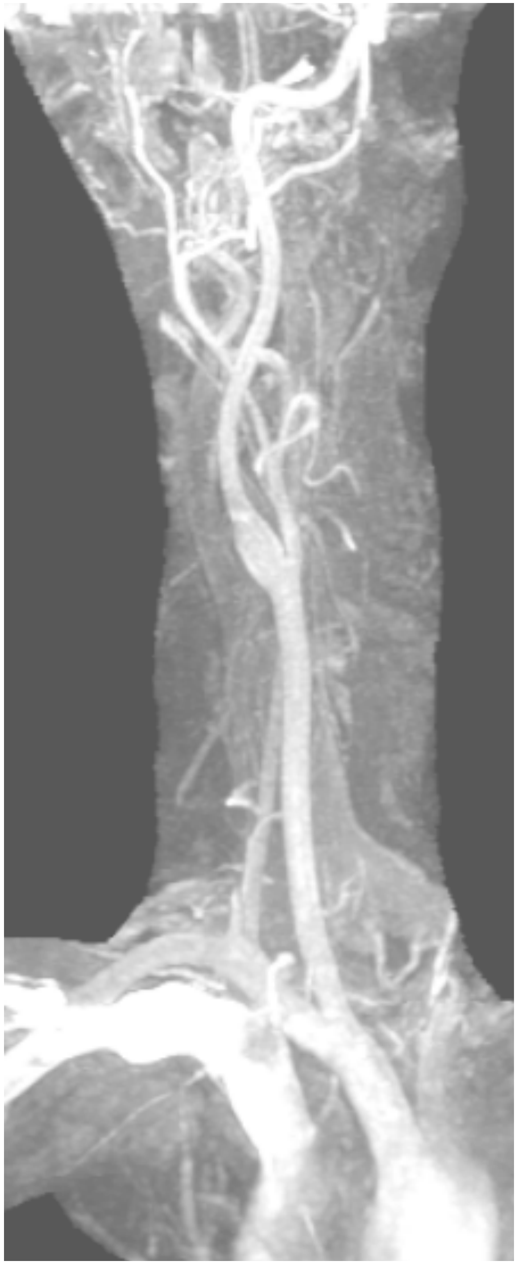

174d Score

0-30

31-50

51-70

>70

Near occlusion

Occluded

Quality

1

2

3

4

5

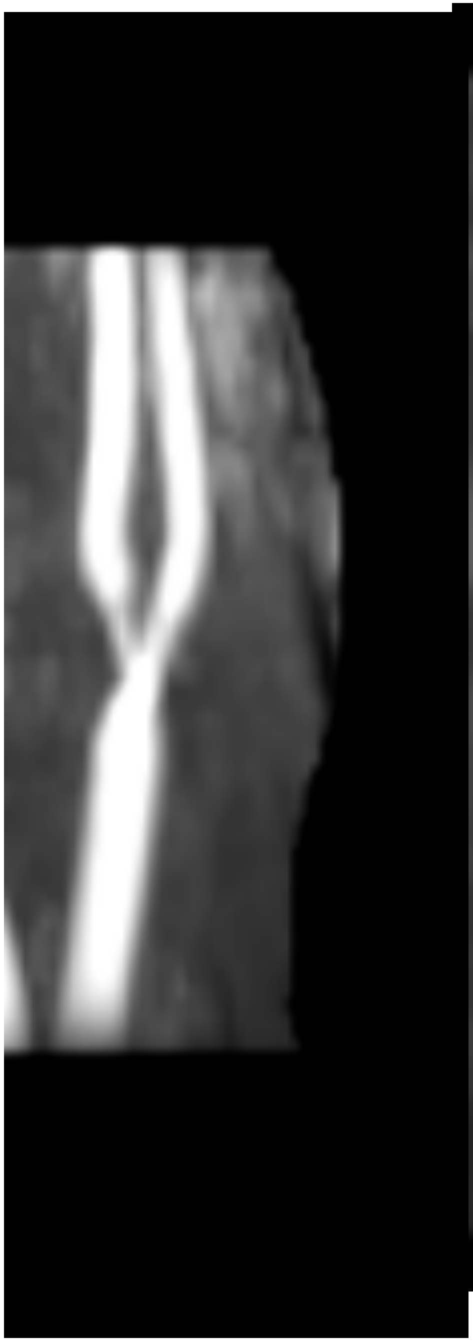

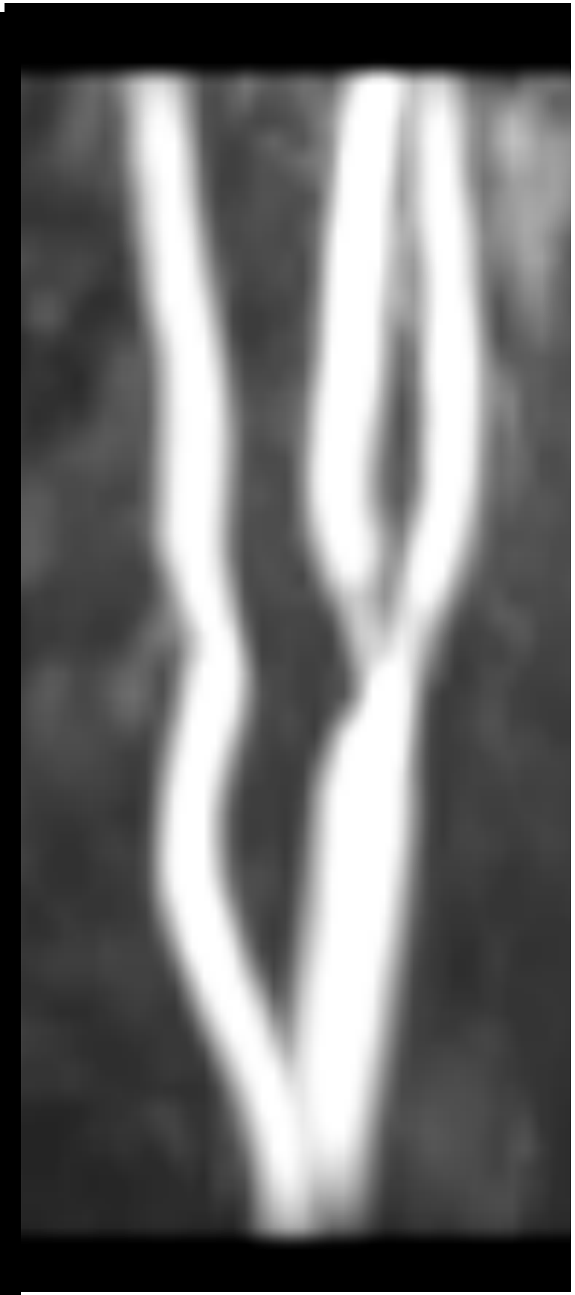

175c Score

0-30

31-50

51-70

>70

Near occlusion

Occluded

Quality

1

2

3

4

5

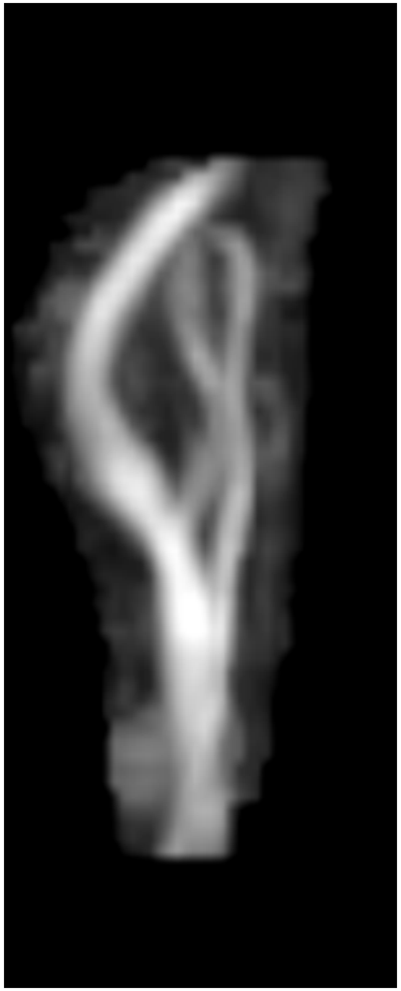

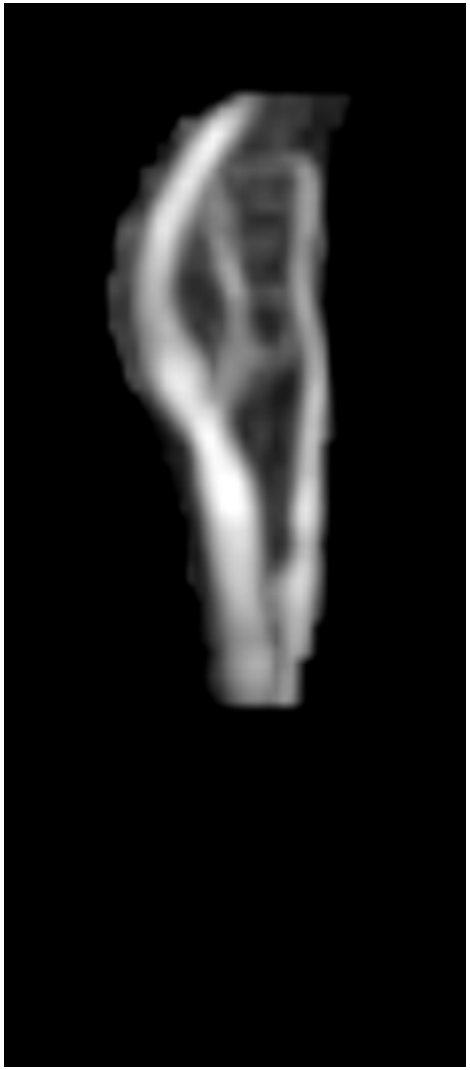

176b Score

0-30

31-50

51-70

>70

Near occlusion

Occluded

Quality

1

2

3

4

5

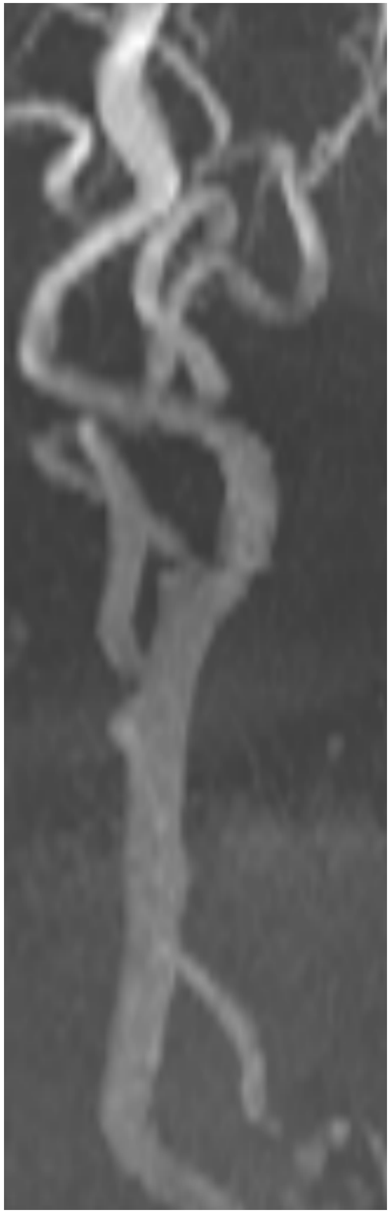

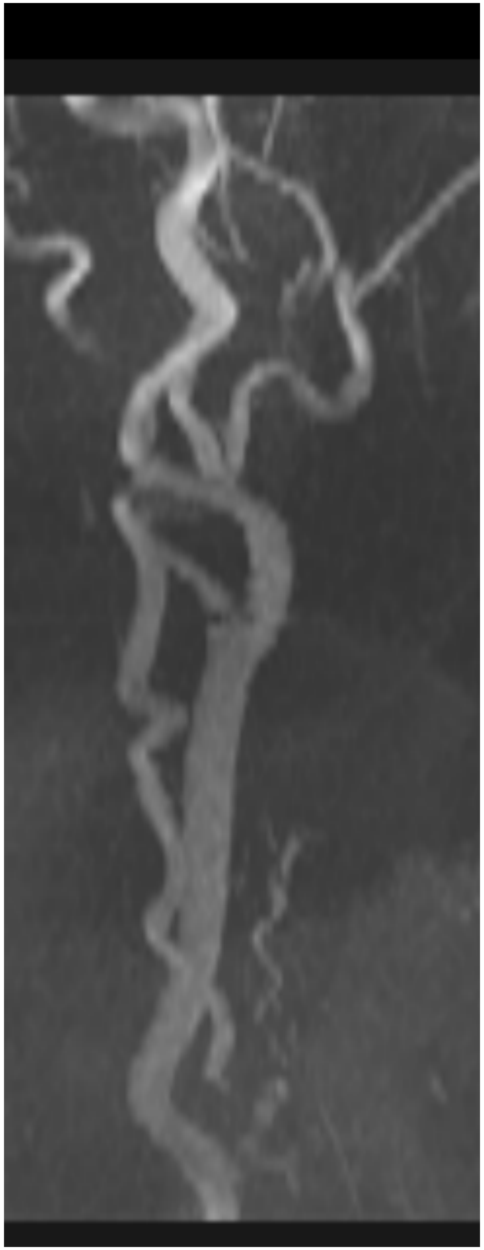

177a Score

0-30

31-50

51-70

>70

Near occlusion

Occluded

Quality

1

2

3

4

5

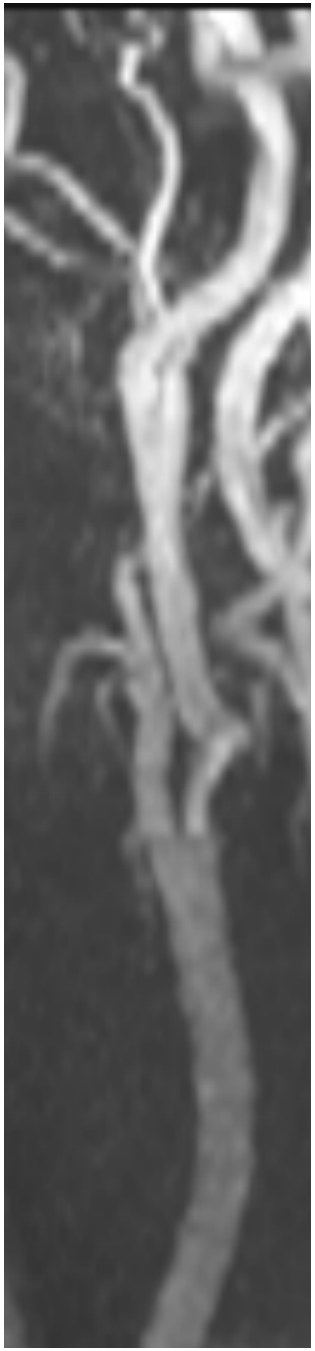

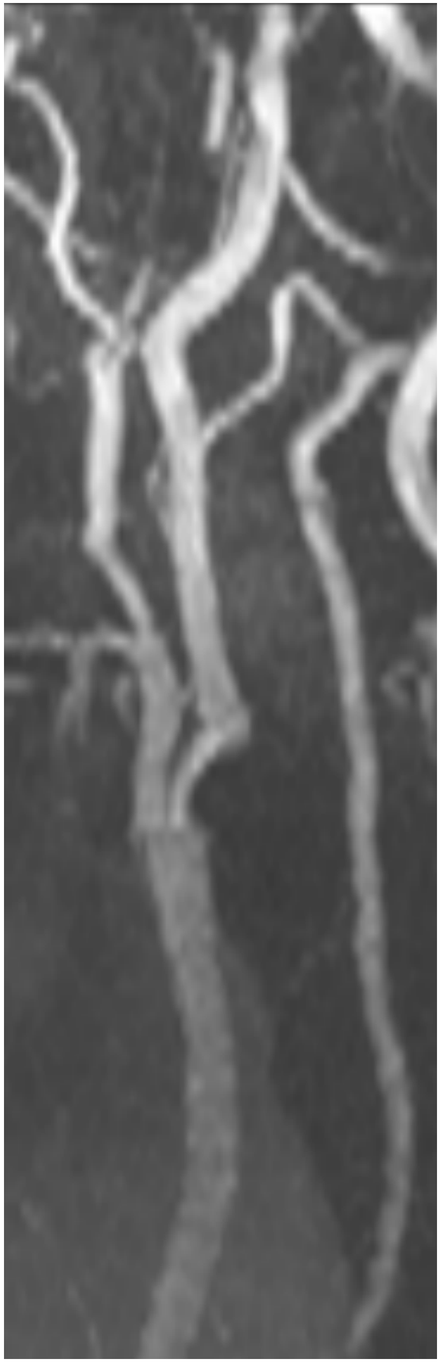

177f Score

0-30

31-50

51-70

>70

Near occlusion

Occluded

Quality

1

2

3

4

5

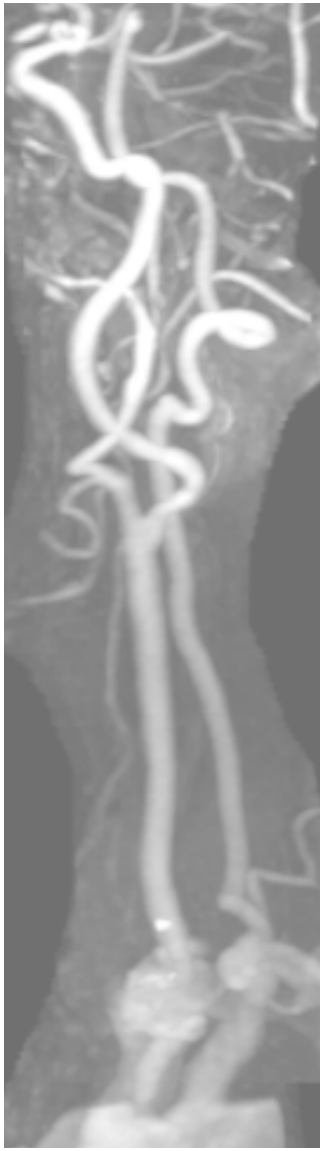

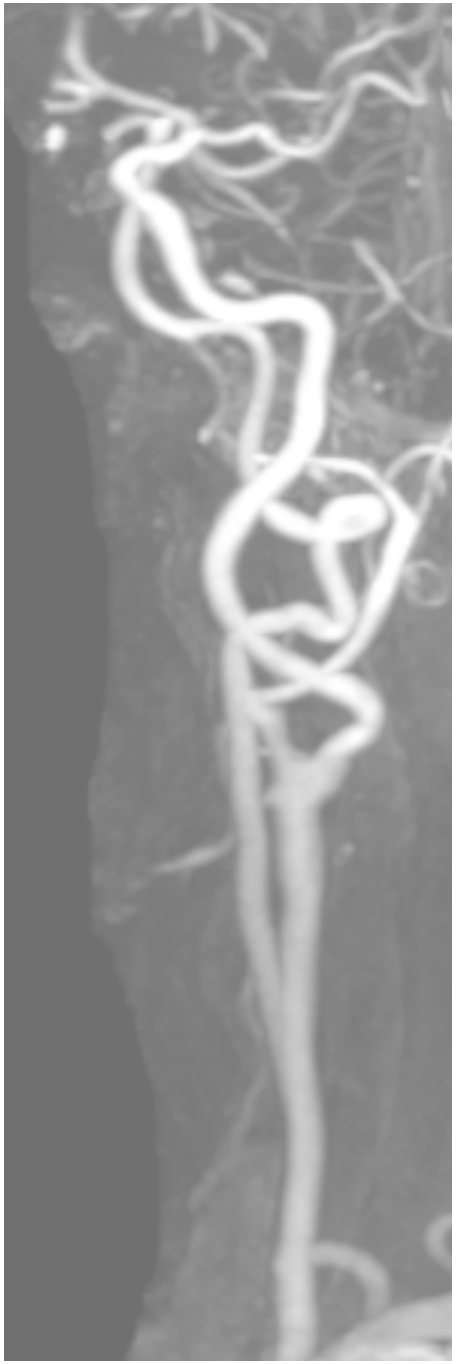

178e Score

0-30

31-50

51-70

>70

Near occlusion

Occluded

Quality

1

2

3

4

5

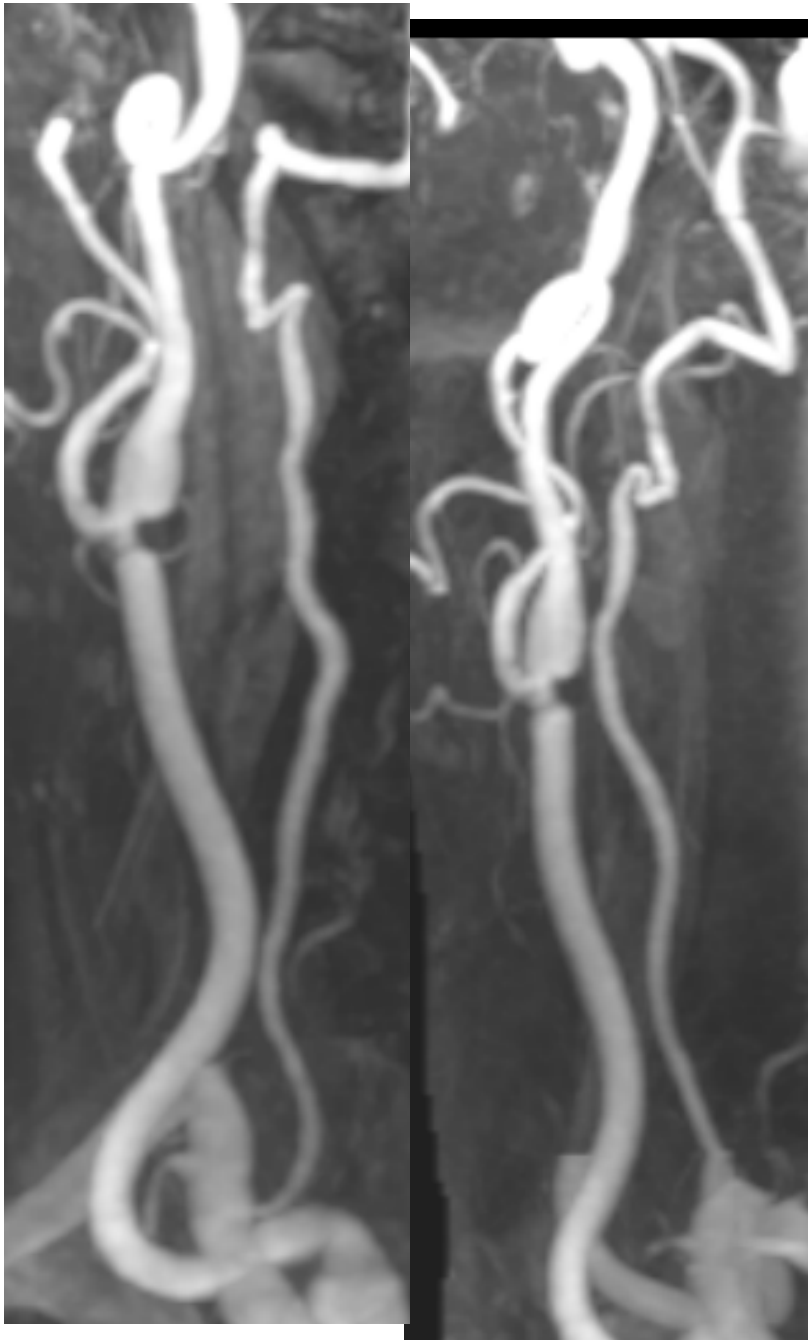

179d Score

0-30

31-50

51-70

>70

Near occlusion

Occluded

Quality

1

2

3

4

5

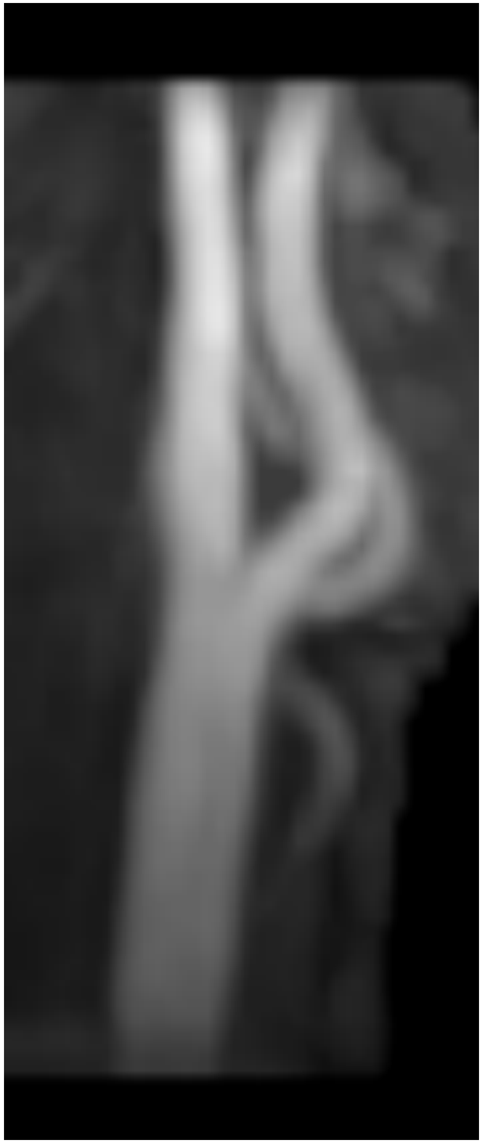

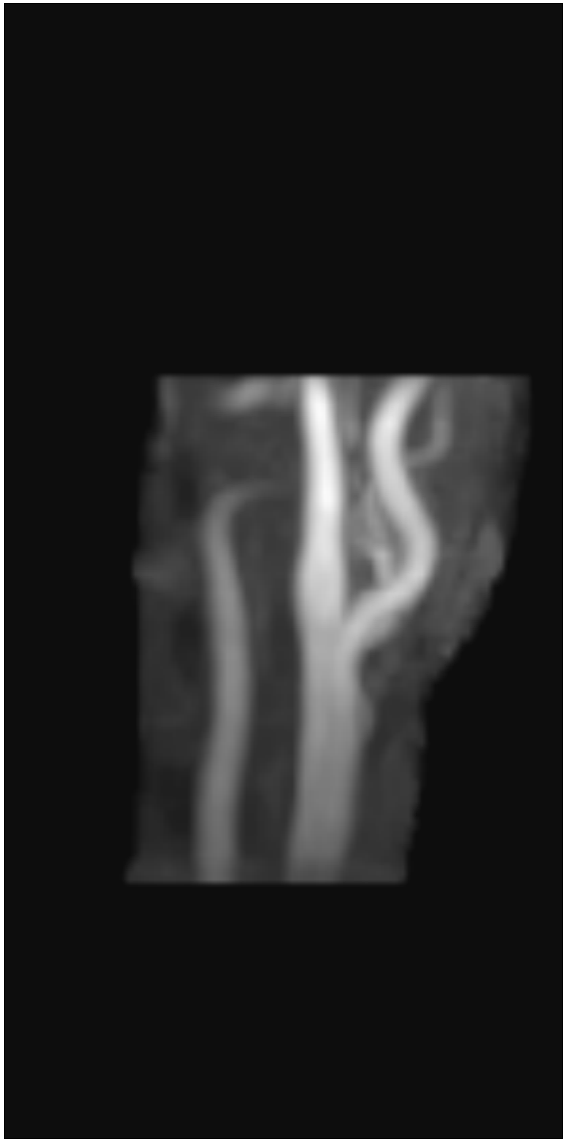

180c Score

0-30

31-50

51-70

>70

Near occlusion

Occluded

Quality

1

2

3

4

5

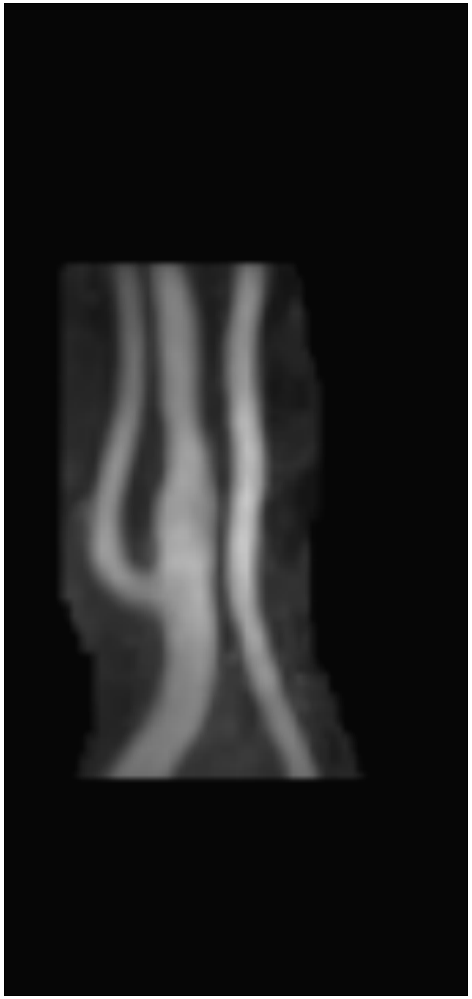

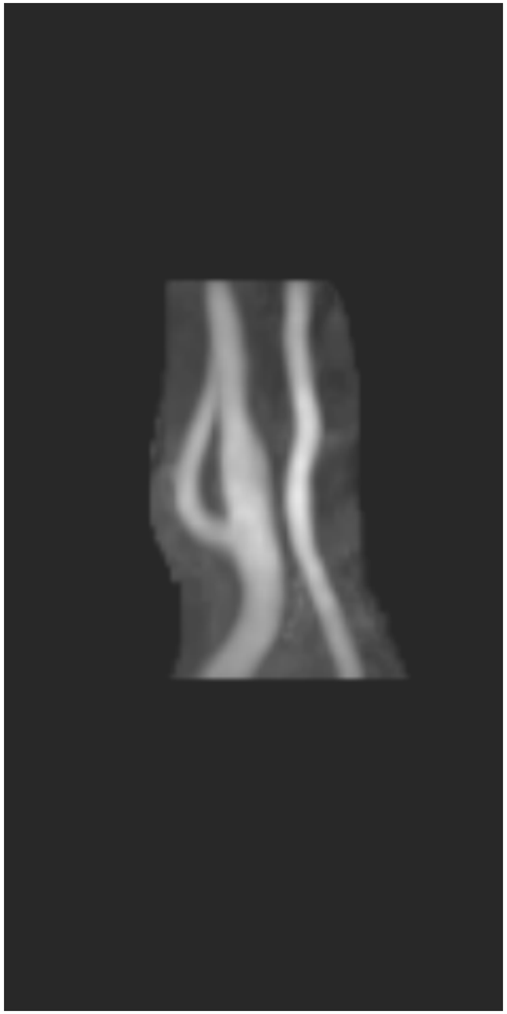

181b Score

0-30

31-50

51-70

>70

Near occlusion

Occluded

Quality

1

2

3

4

5

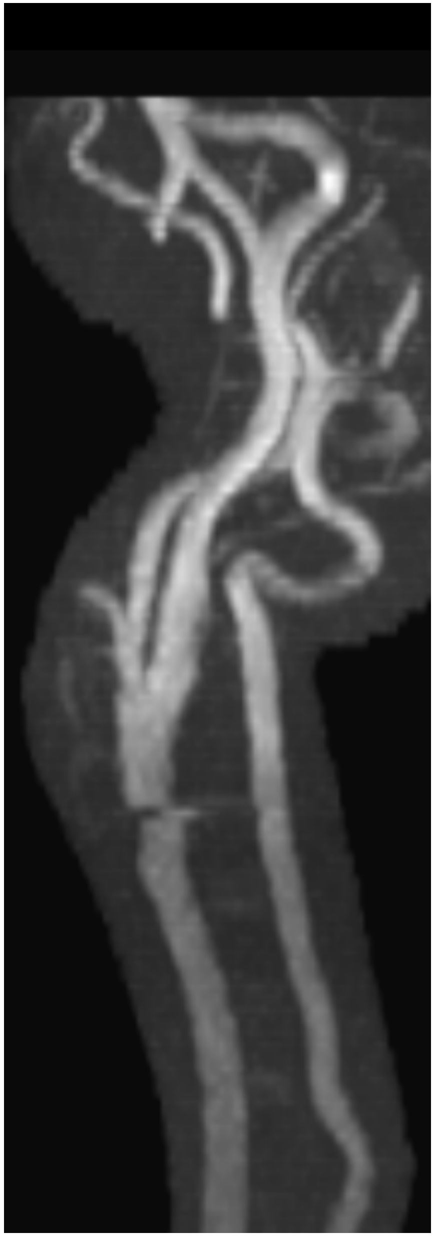

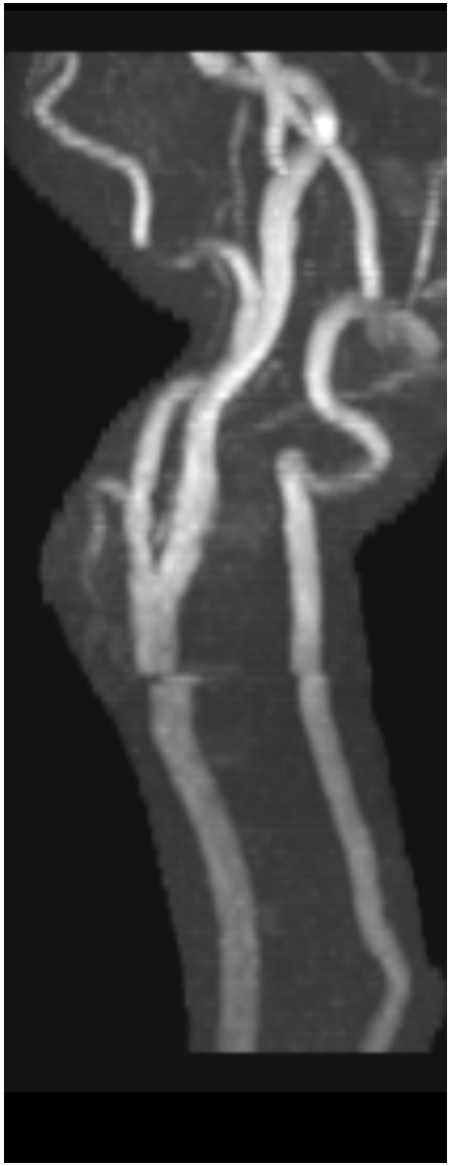

182a Score

0-30

31-50

51-70

>70

Near occlusion

Occluded

Quality

1

2

3

4

5

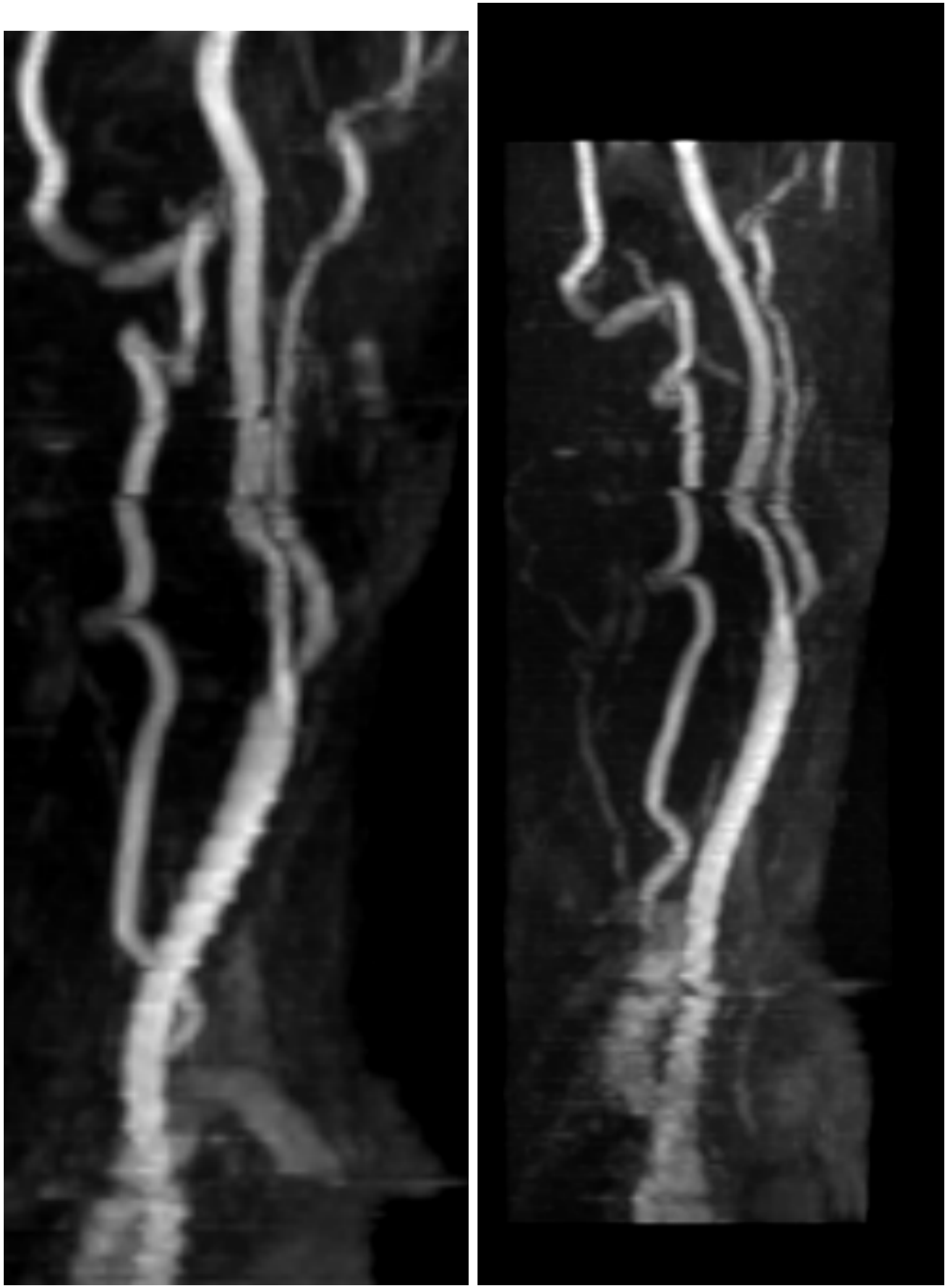

182f Score

0-30

31-50

51-70

>70

Near occlusion

Occluded

Quality

1

2

3

4

5

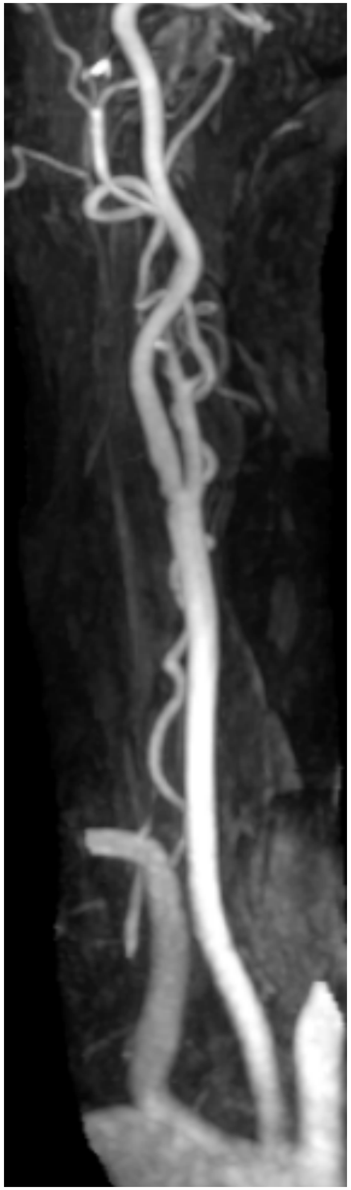

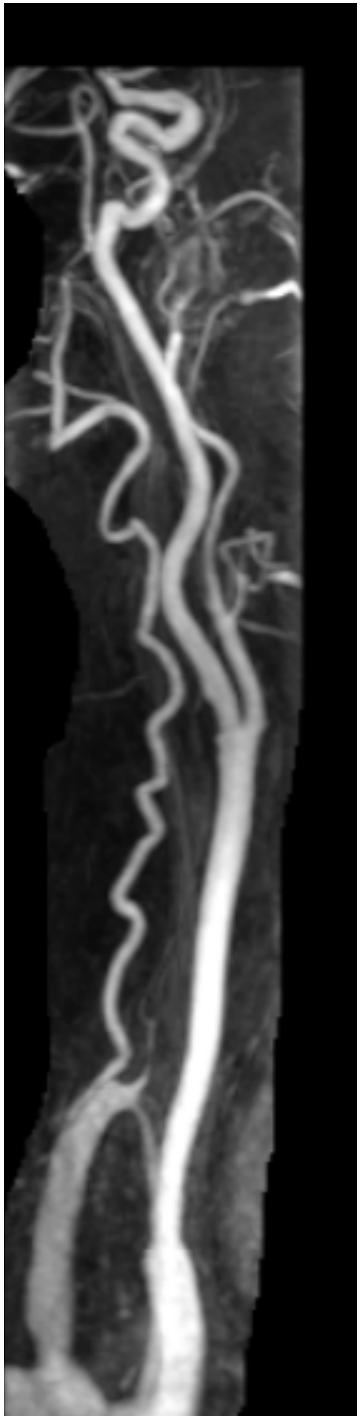

183e Score

0-30

31-50

51-70

>70

Near occlusion

Occluded

Quality

1

2

3

4

5

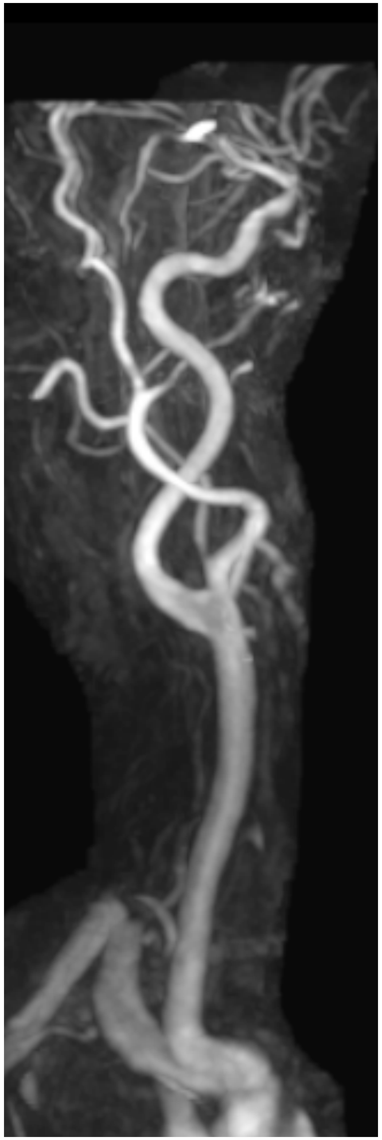

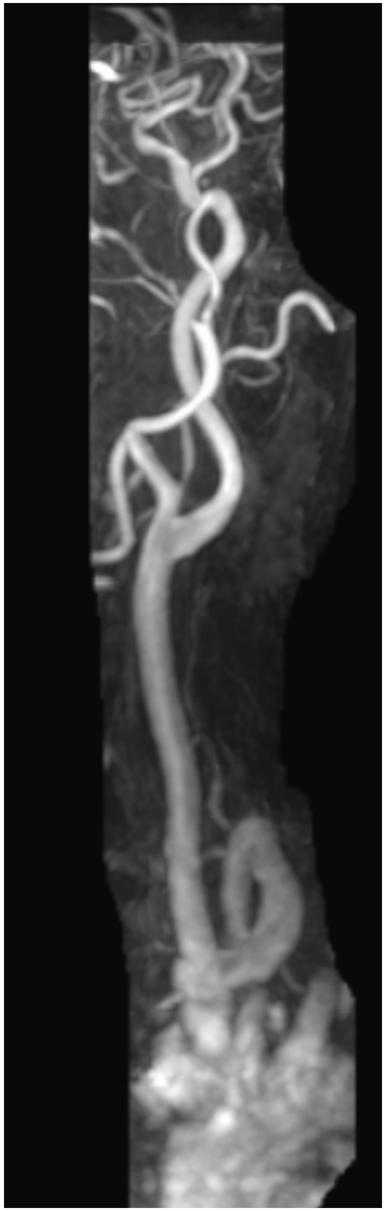

184d Score

0-30

31-50

51-70

>70

Near occlusion

Occluded

Quality

1

2

3

4

5

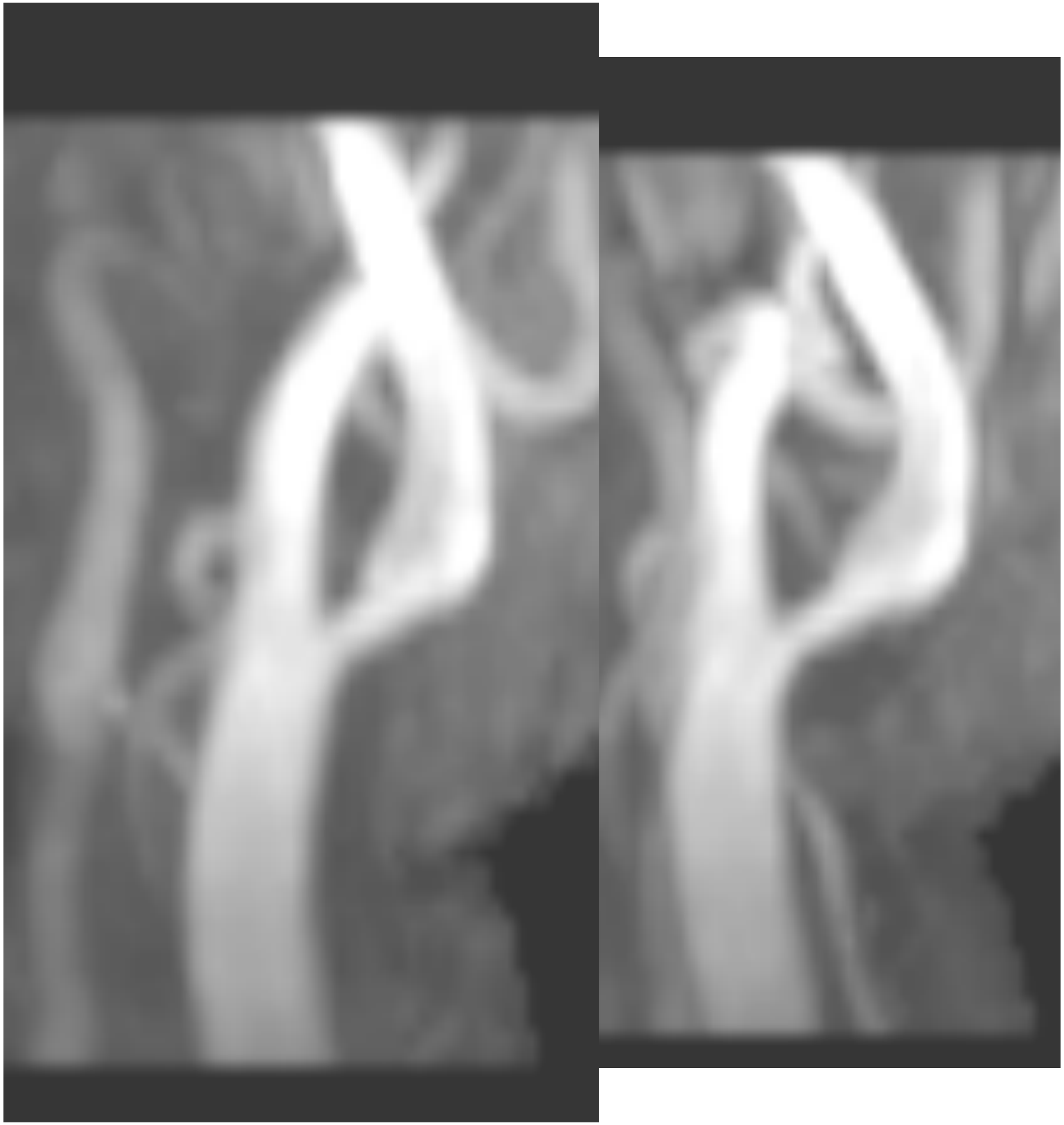

185c Score

0-30

31-50

51-70

>70

Near occlusion

Occluded

Quality

1

2

3

4

5

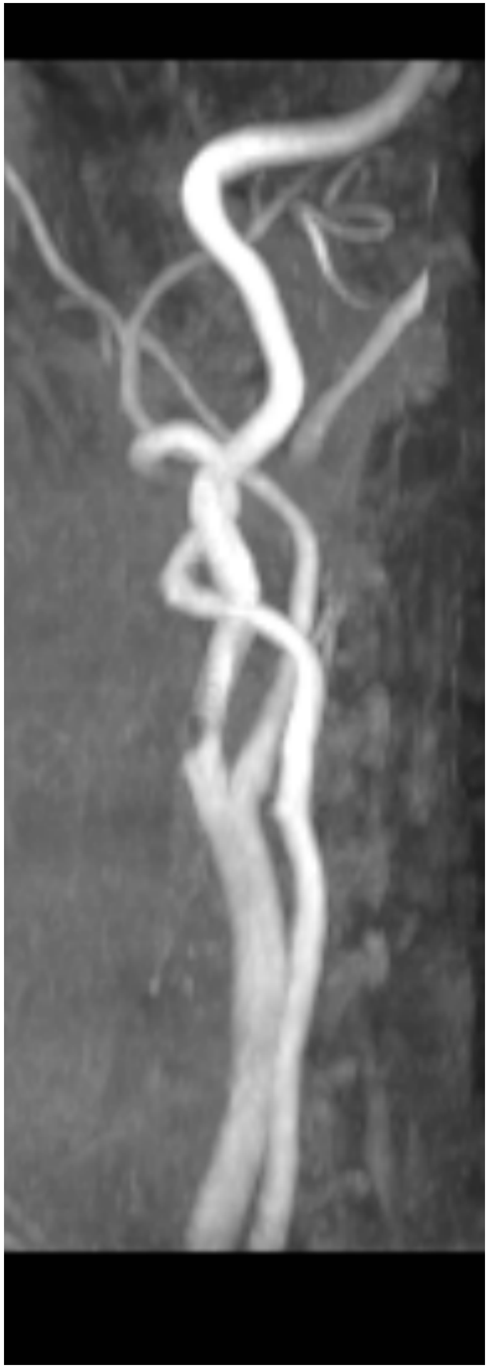

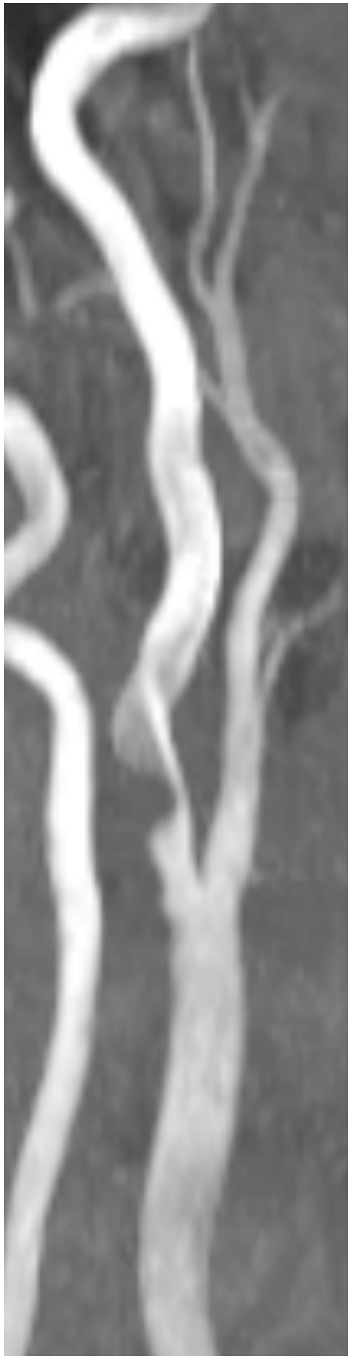

186b Score

0-30

31-50

51-70

>70

Near occlusion

Occluded

Quality

1

2

3

4

5

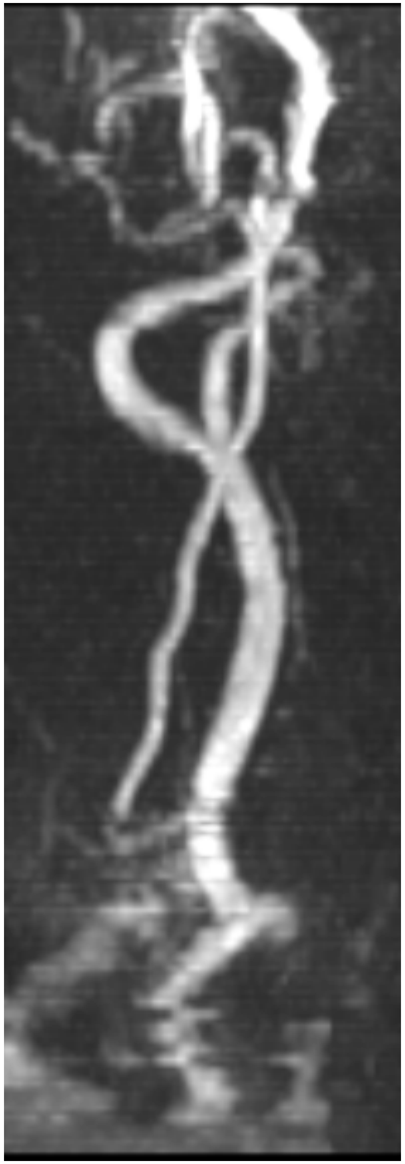

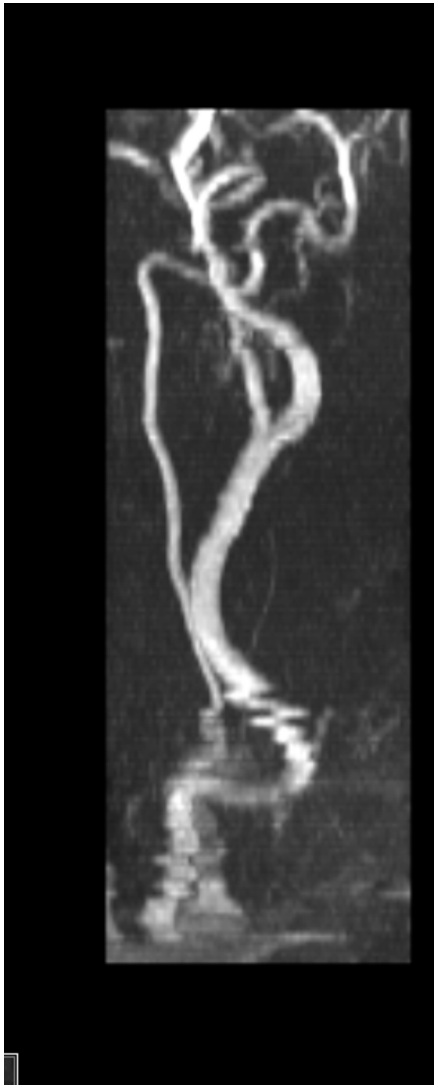

187a Score

0-30

31-50

51-70

>70

Near occlusion

Occluded

Quality

1

2

3

4

5

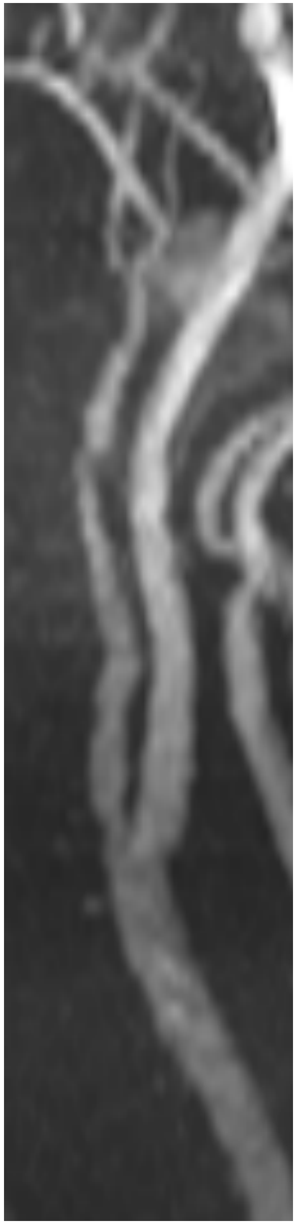

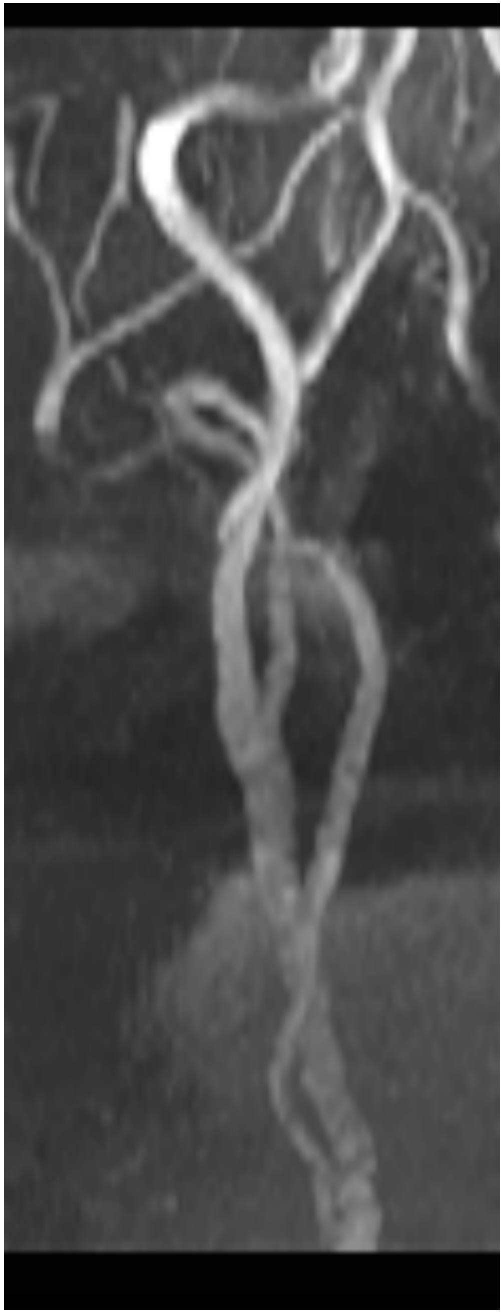

187f Score

0-30

31-50

51-70

>70

Near occlusion

Occluded

Quality

1

2

3

4

5

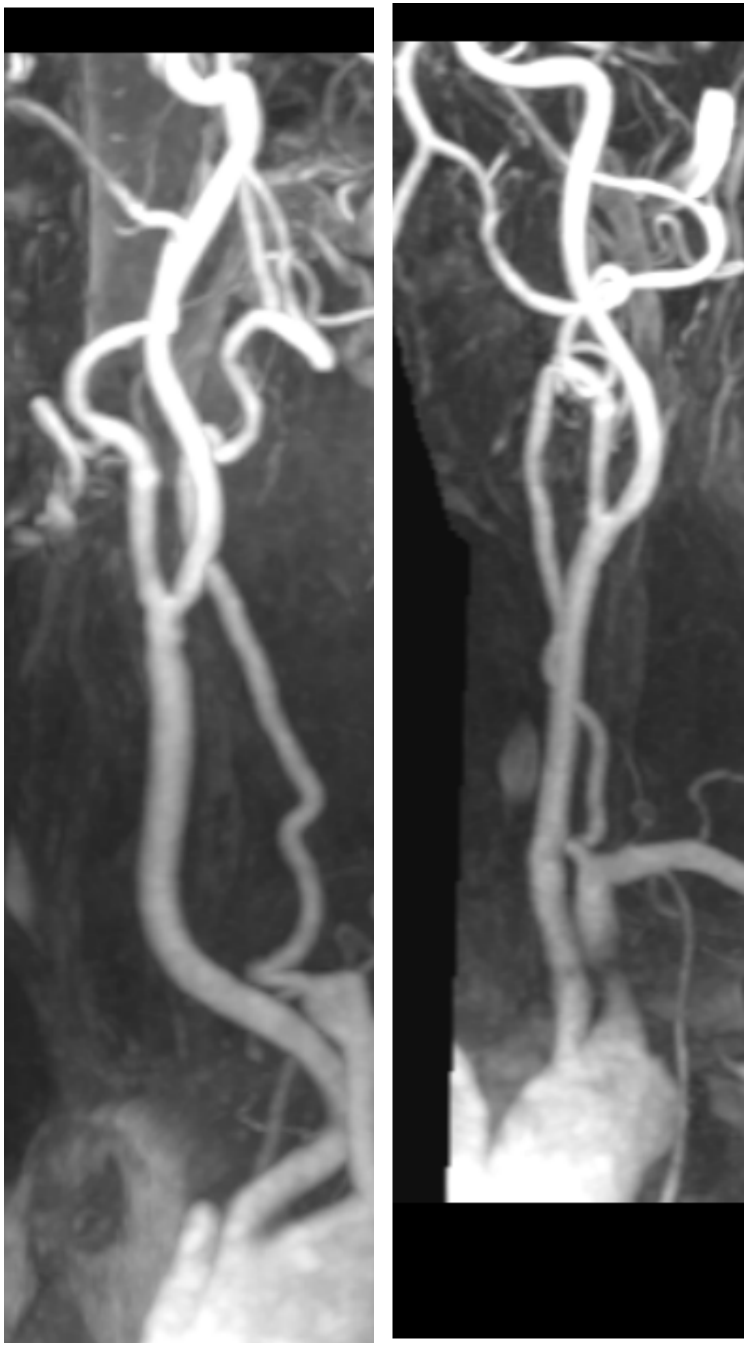

188e Score

0-30

31-50

51-70

>70

Near occlusion

Occluded

Quality

1

2

3

4

5

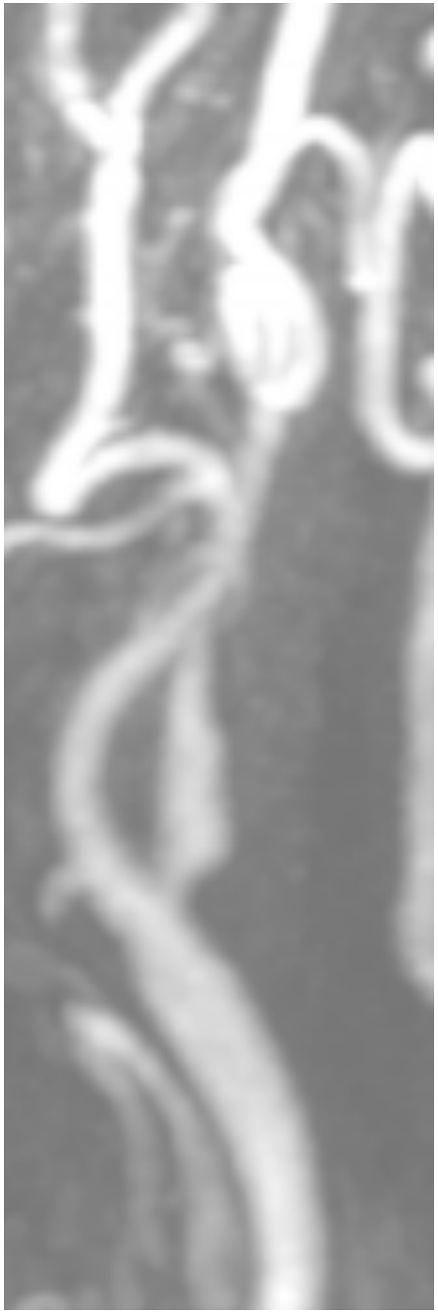

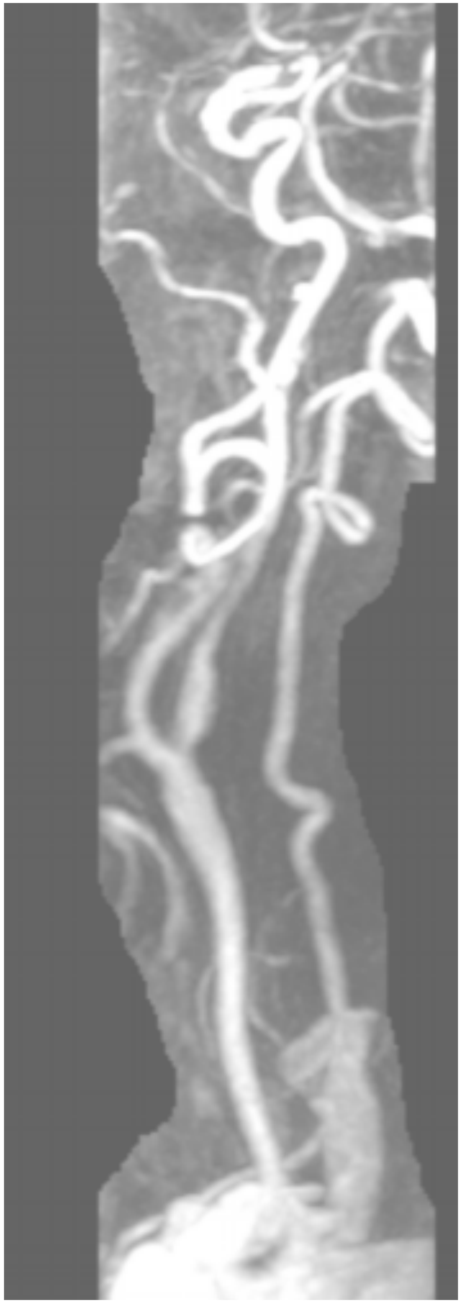

189d Score

0-30

31-50

51-70

>70

Near occlusion

Occluded

Quality

1

2

3

4

5

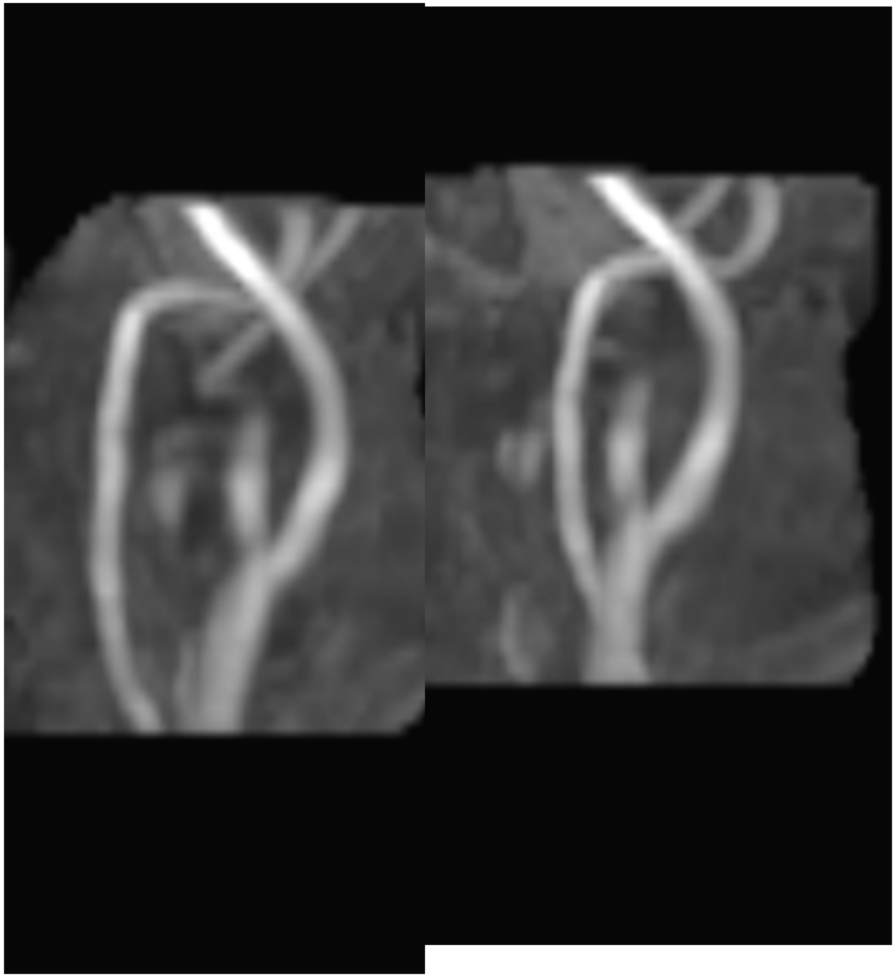

190c Score

0-30

31-50

51-70

>70

Near occlusion

Occluded

Quality

1

2

3

4

5

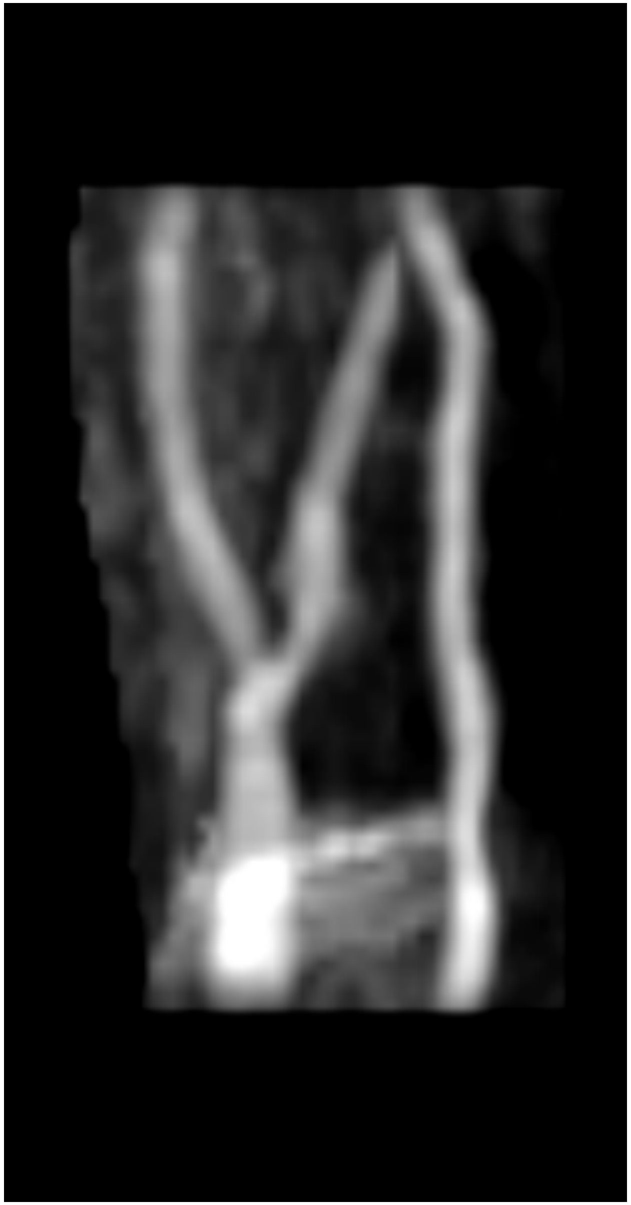

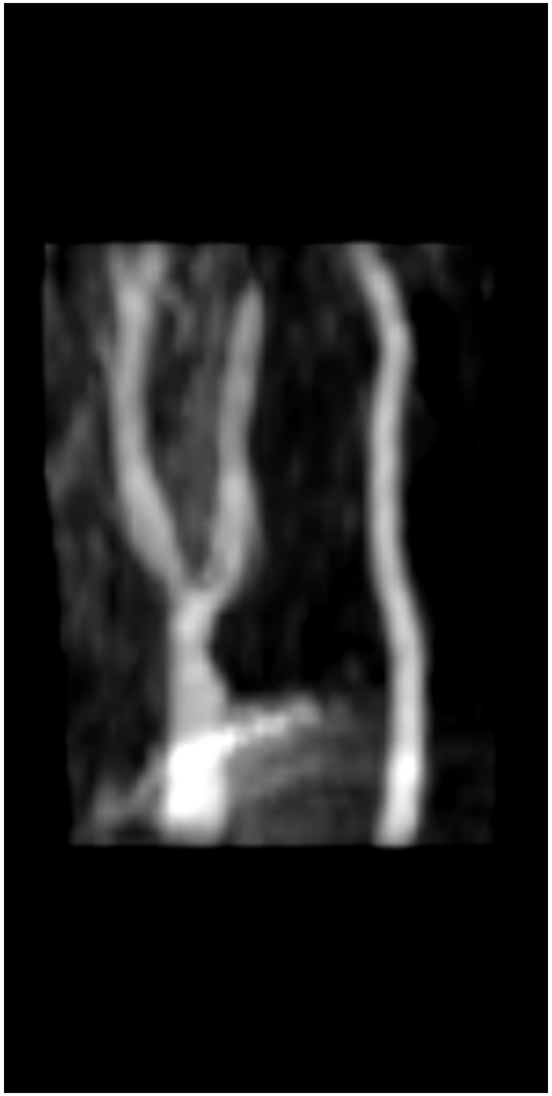

191b Score

0-30

31-50

51-70

>70

Near occlusion

Occluded

Quality

1

2

3

4

5

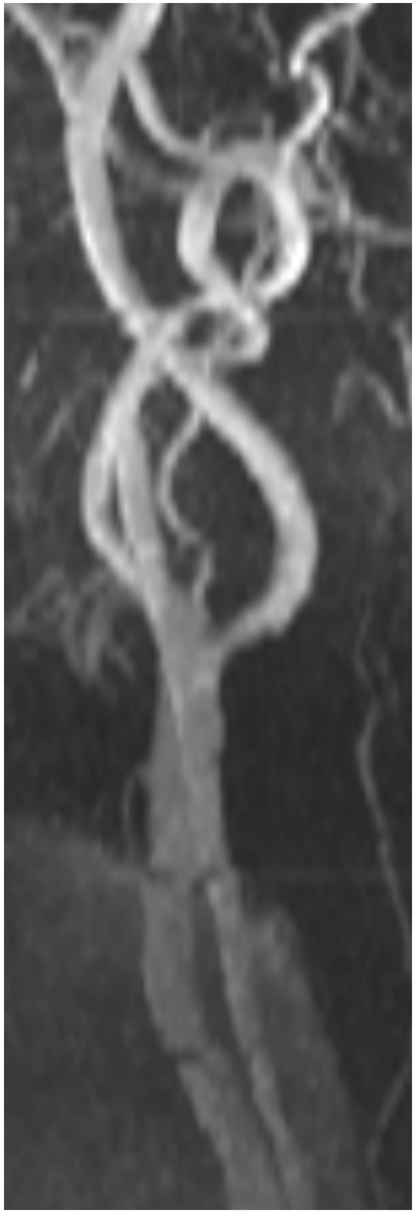

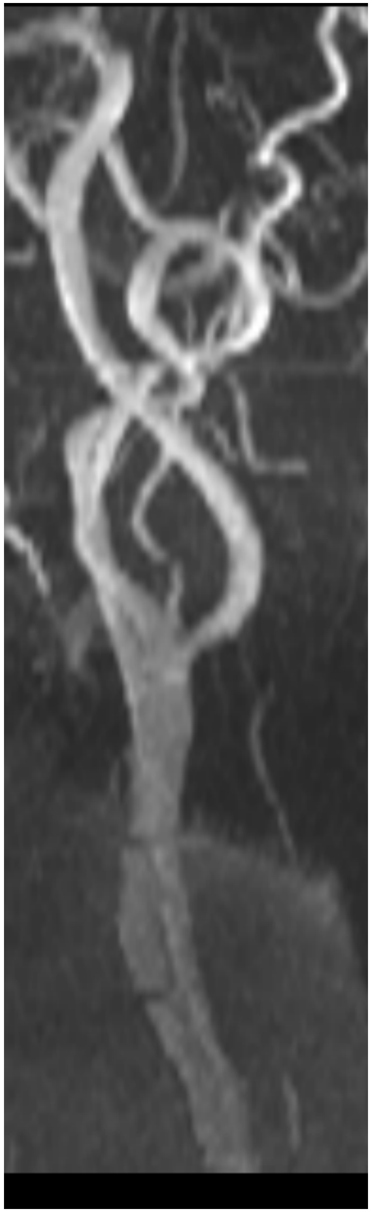

192a Score

0-30

31-50

51-70

>70

Near occlusion

Occluded

Quality

1

2

3

4

5

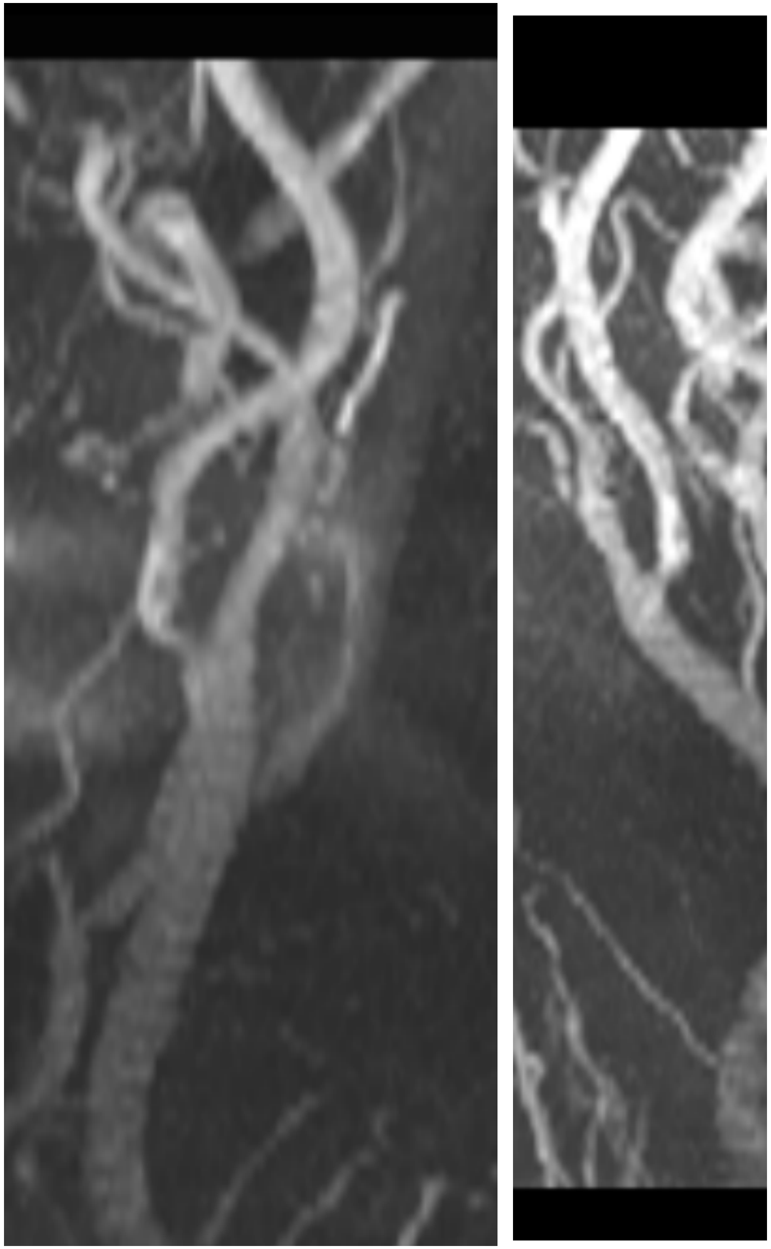

192f Score

0-30

31-50

51-70

>70

Near occlusion

Occluded

Quality

1

2

3

4

5

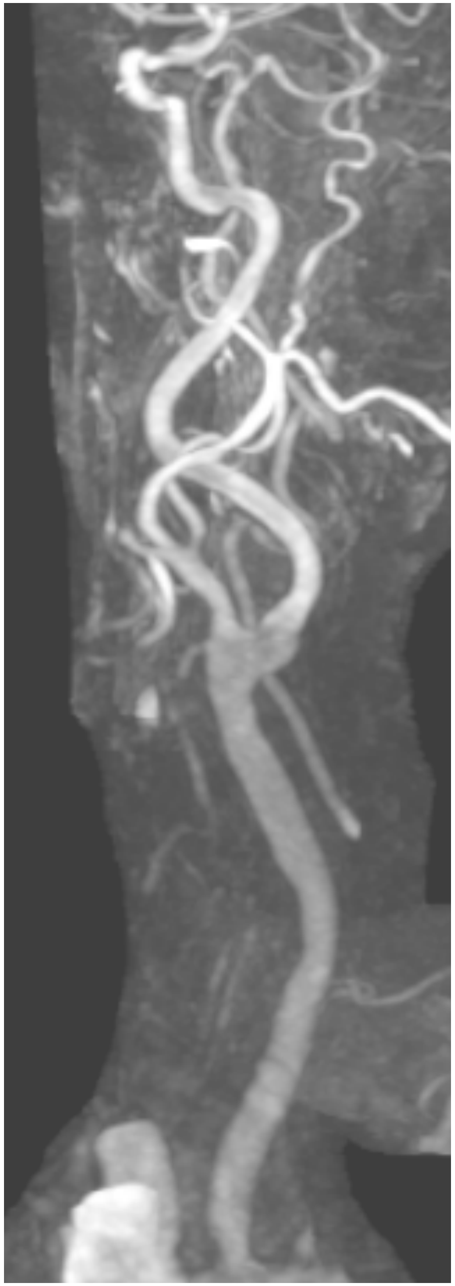

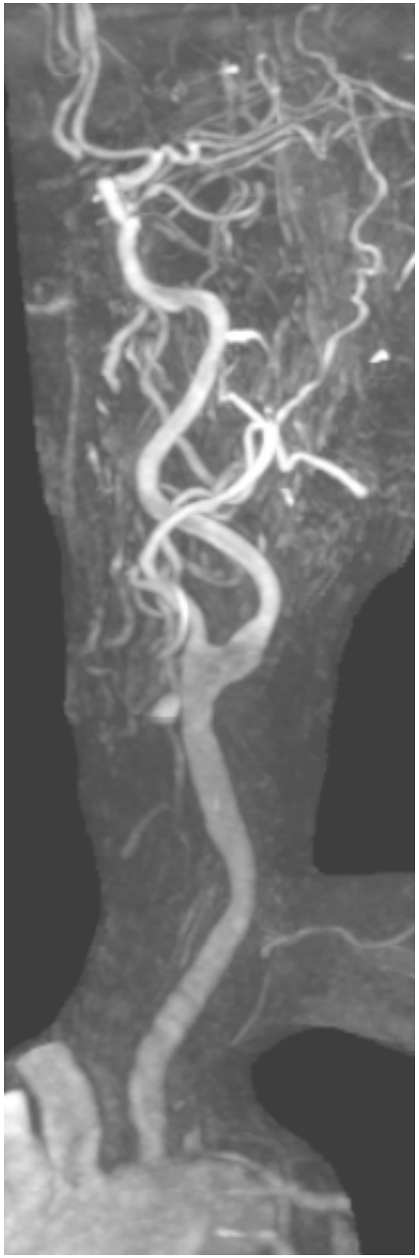

193e Score

0-30

31-50

51-70

>70

Near occlusion

Occluded

Quality

1

2

3

4

5

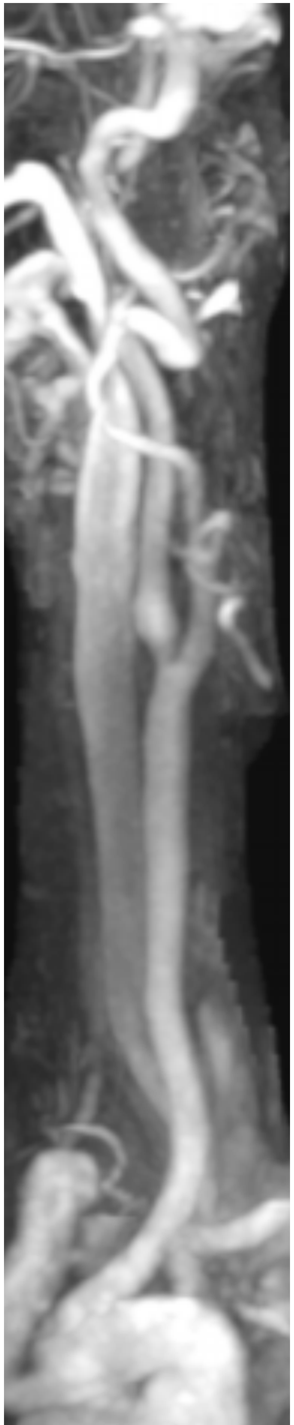

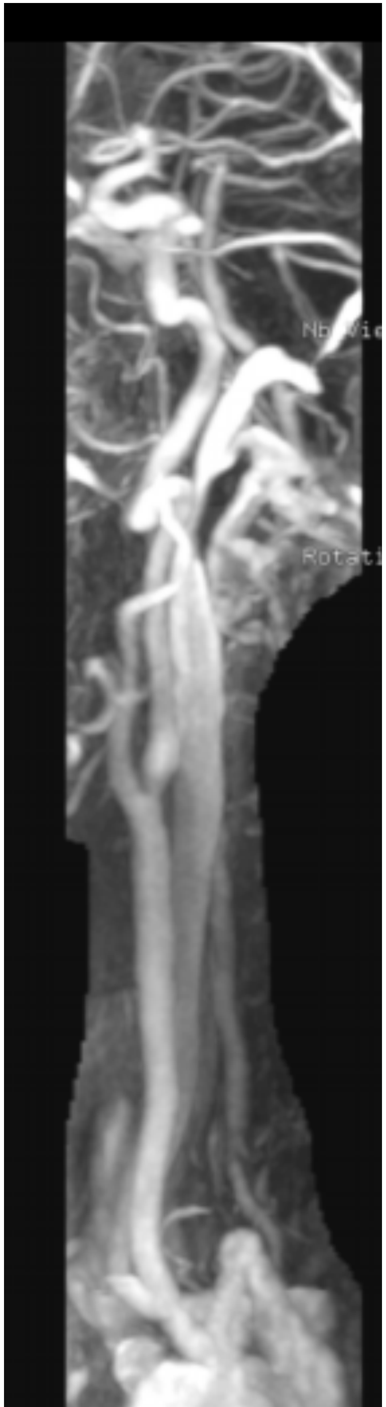

194d Score

0-30

31-50

51-70

>70

Near occlusion

Occluded

Quality

1

2

3

4

5

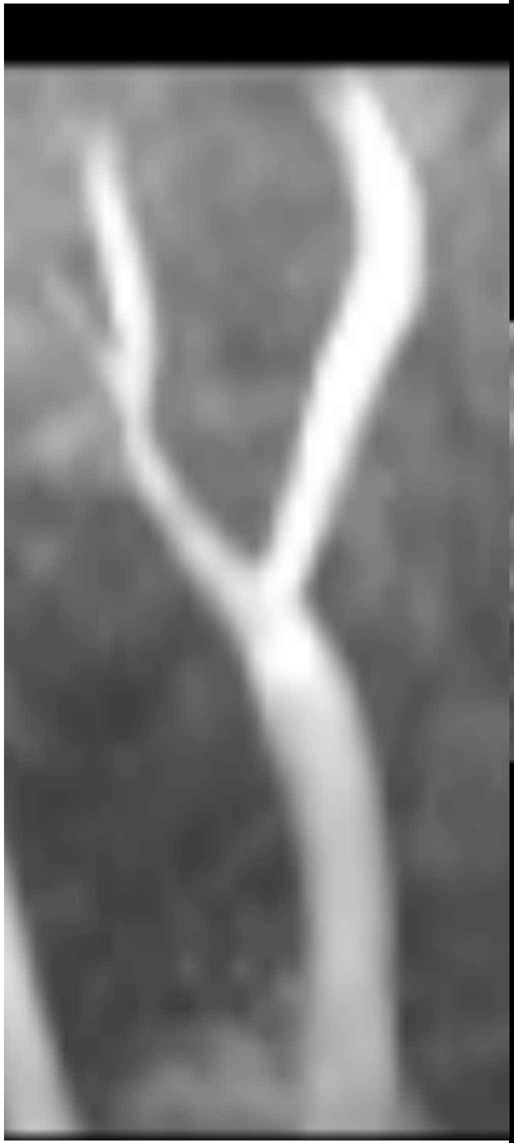

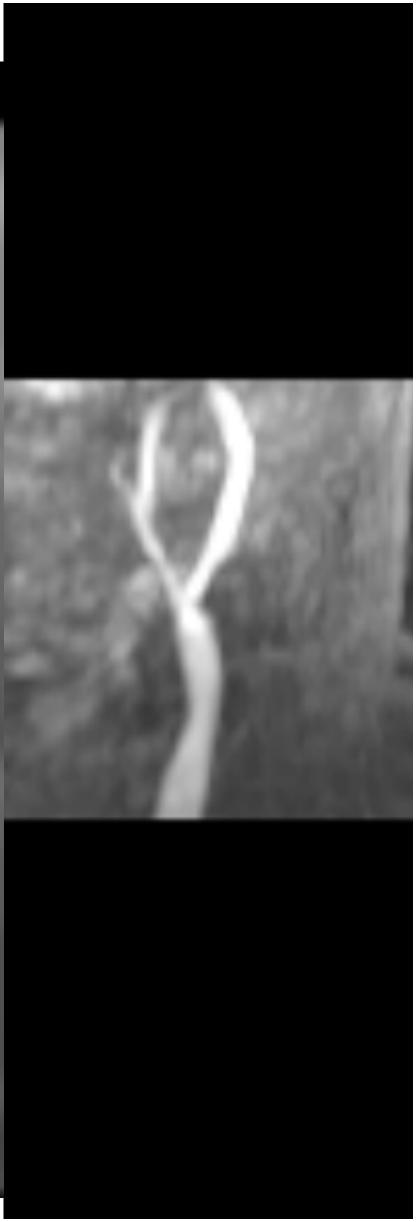

195c Score

0-30

31-50

51-70

>70

Near occlusion

Occluded

Quality

1

2

3

4

5

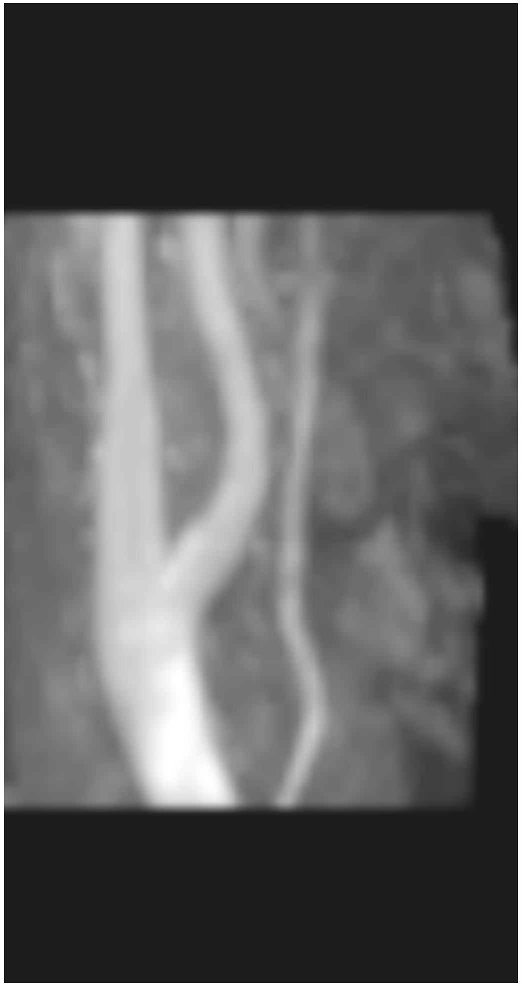

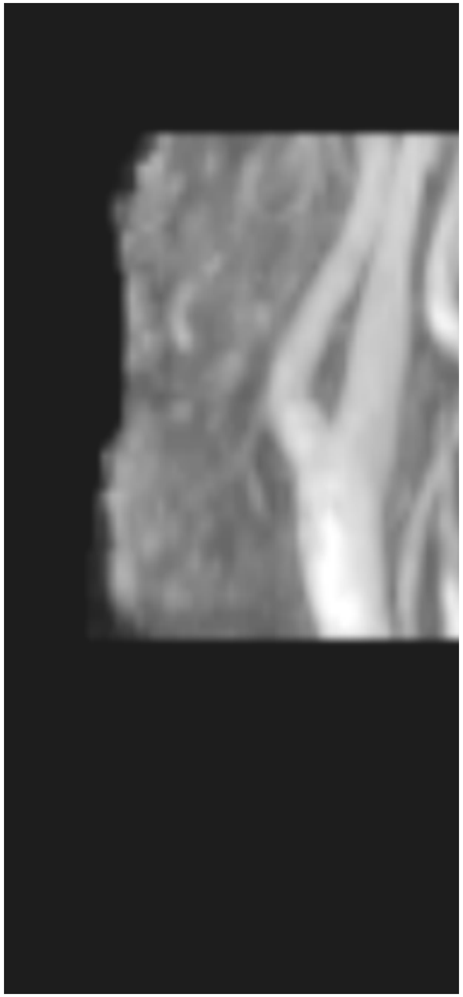

196b Score (left)

0-30

31-50

51-70

>70

Near occlusion

Occluded

Quality

1

2

3

4

5

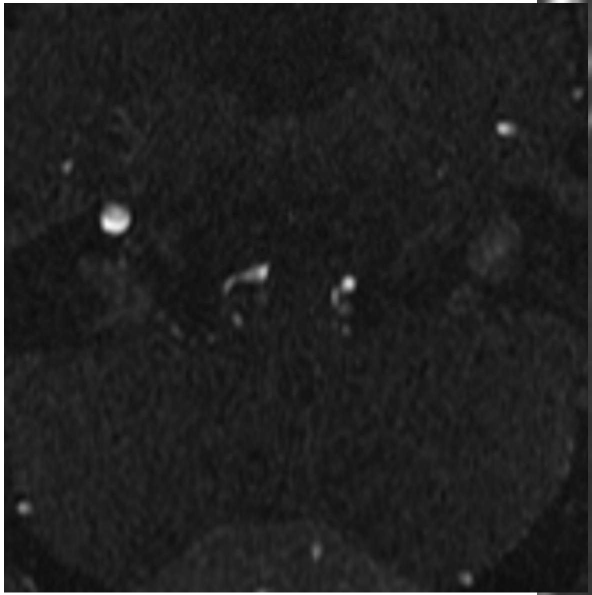

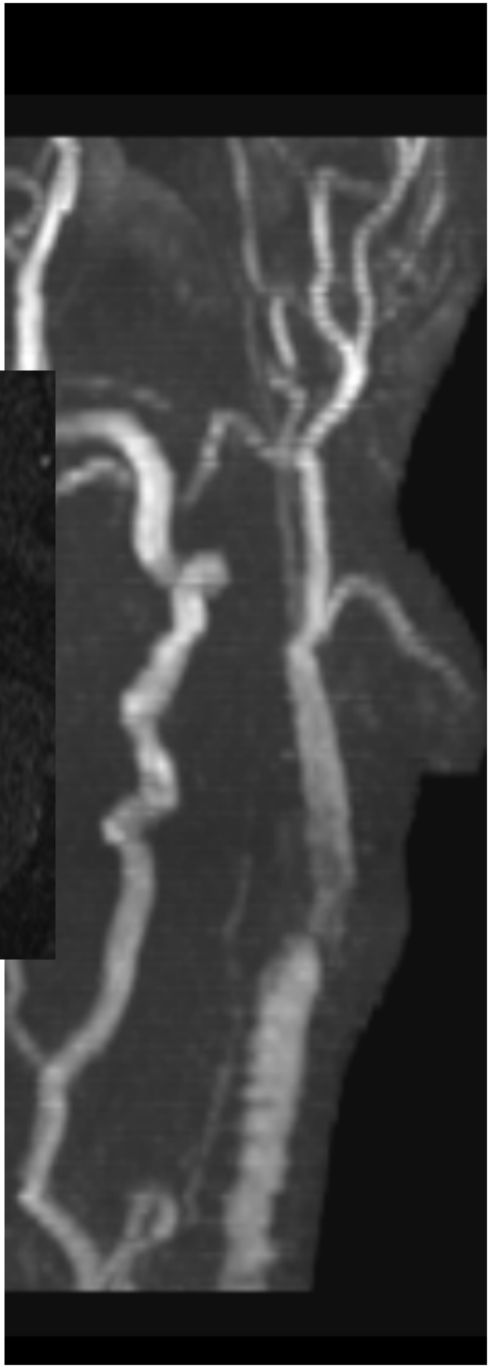

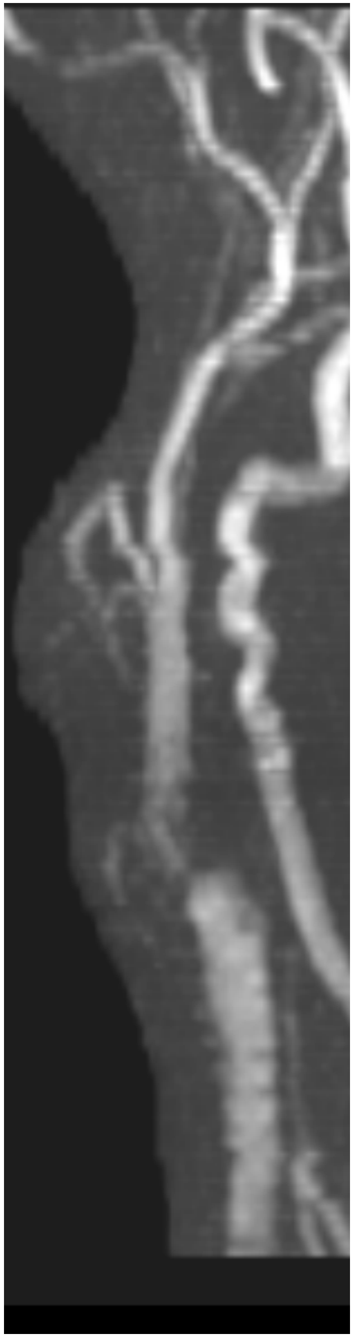

197a Score

0-30

31-50

51-70

>70

Near occlusion

Occluded

Quality

1

2

3

4

5

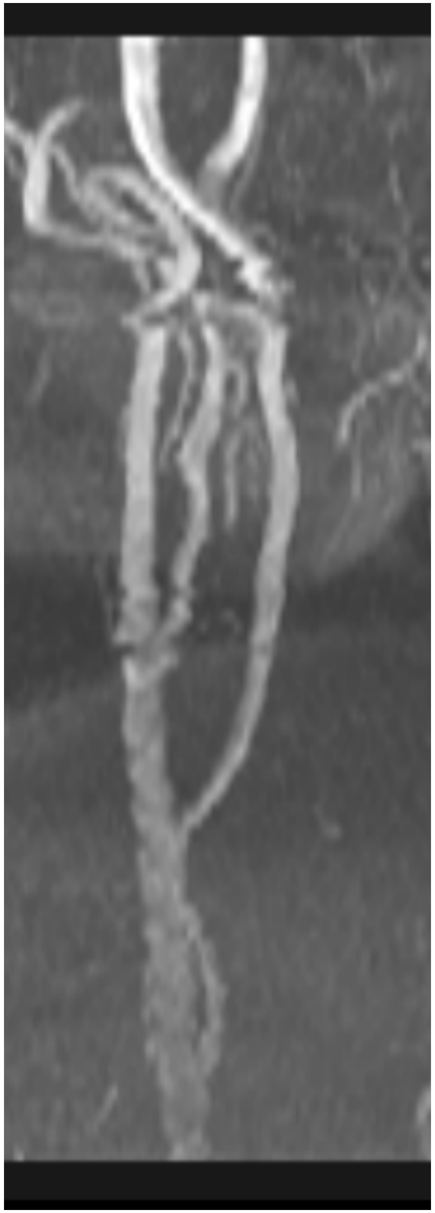

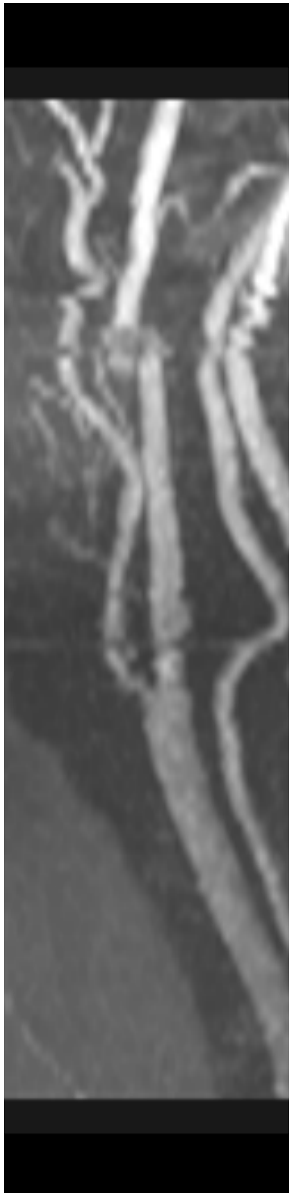

197f Score

0-30

31-50

51-70

>70

Near occlusion

Occluded

Quality

1

2

3

4

5

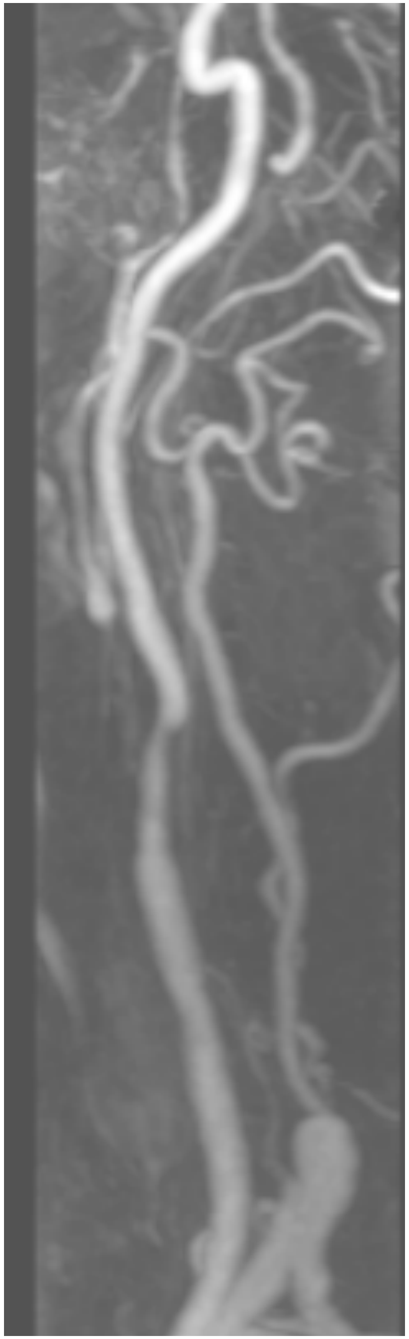

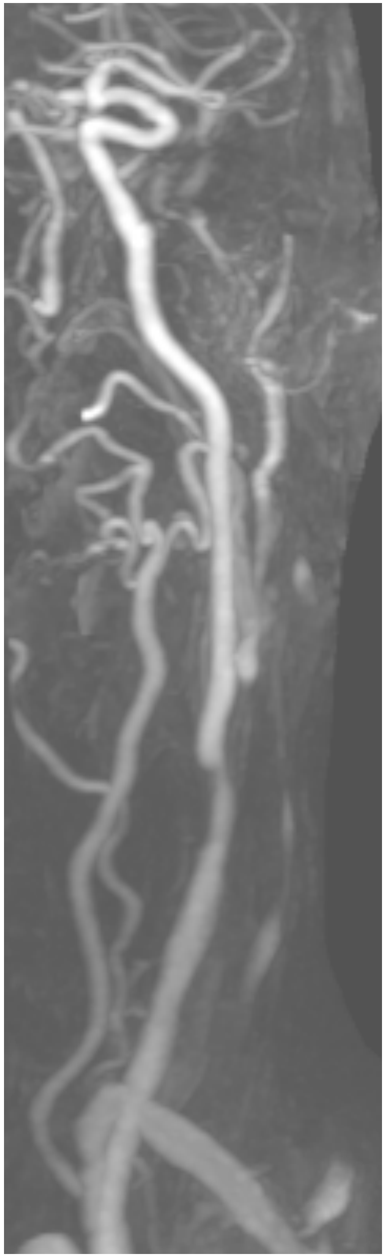

198e Score

0-30

31-50

51-70

>70

Near occlusion

Occluded

Quality

1

2

3

4

5

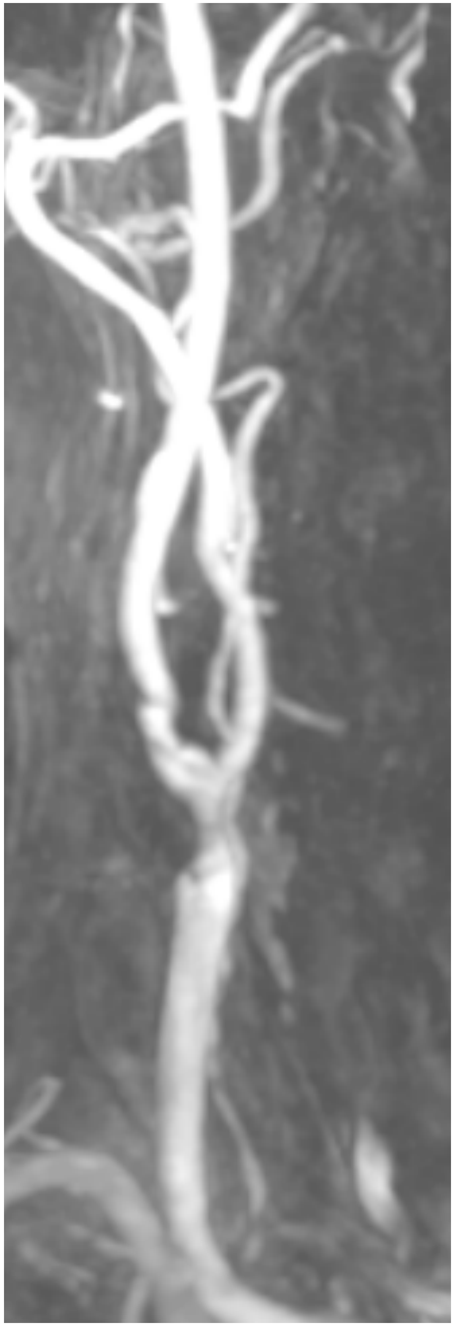

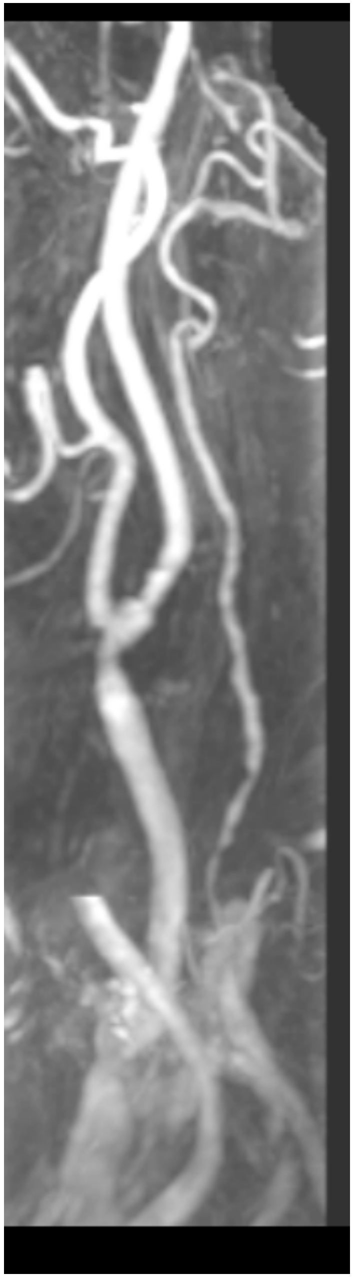

199d Score

0-30

31-50

51-70

>70

Near occlusion

Occluded

Quality

1

2

3

4

5

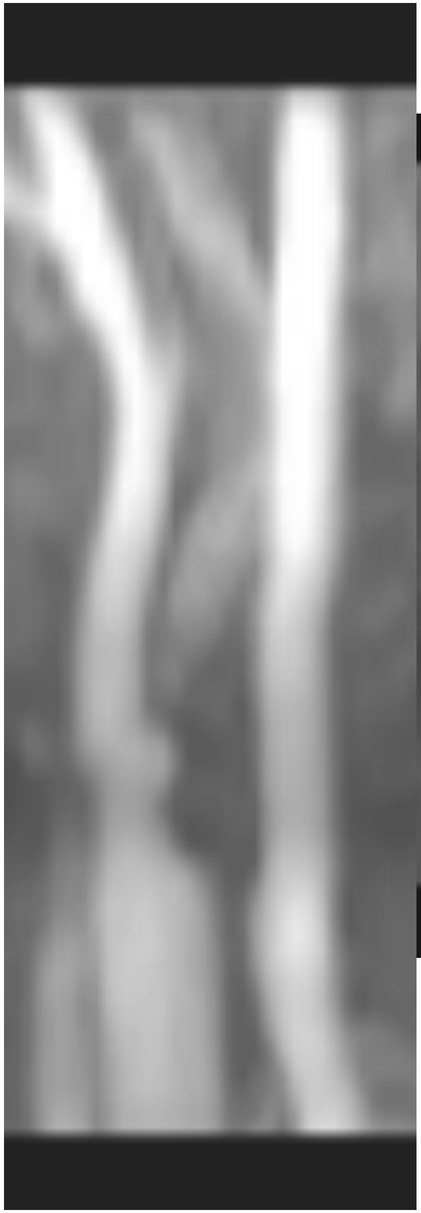

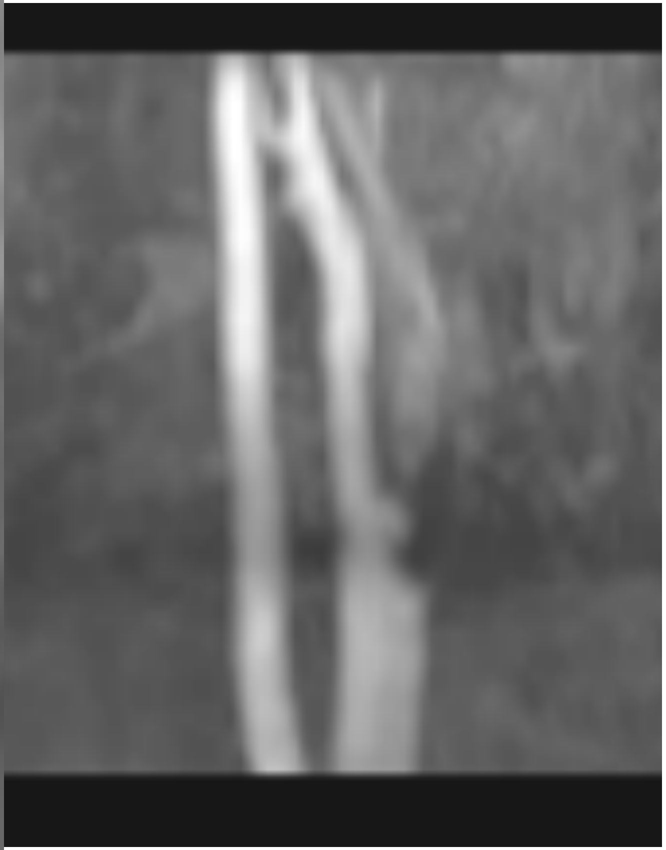

200c Score

0-30

31-50

51-70

>70

Near occlusion

Occluded

Quality

1

2

3

4

5

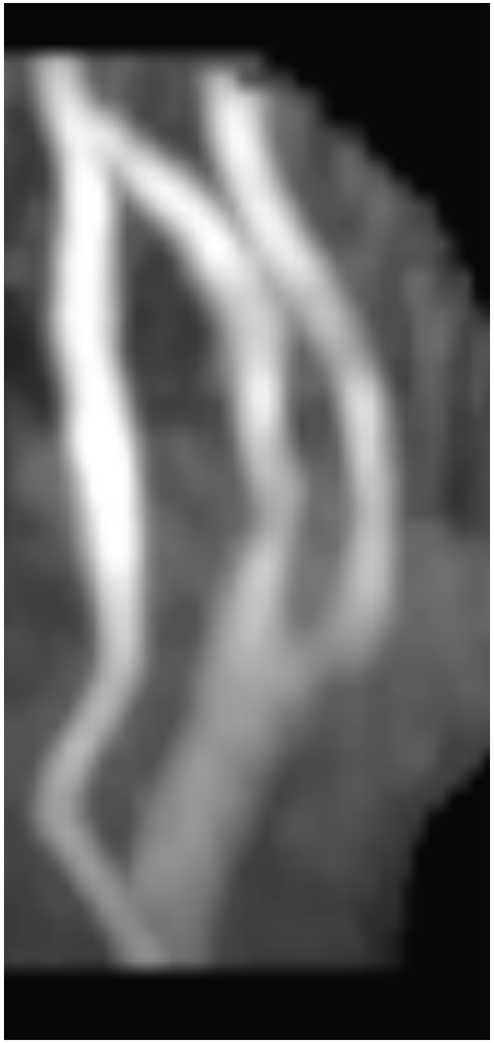

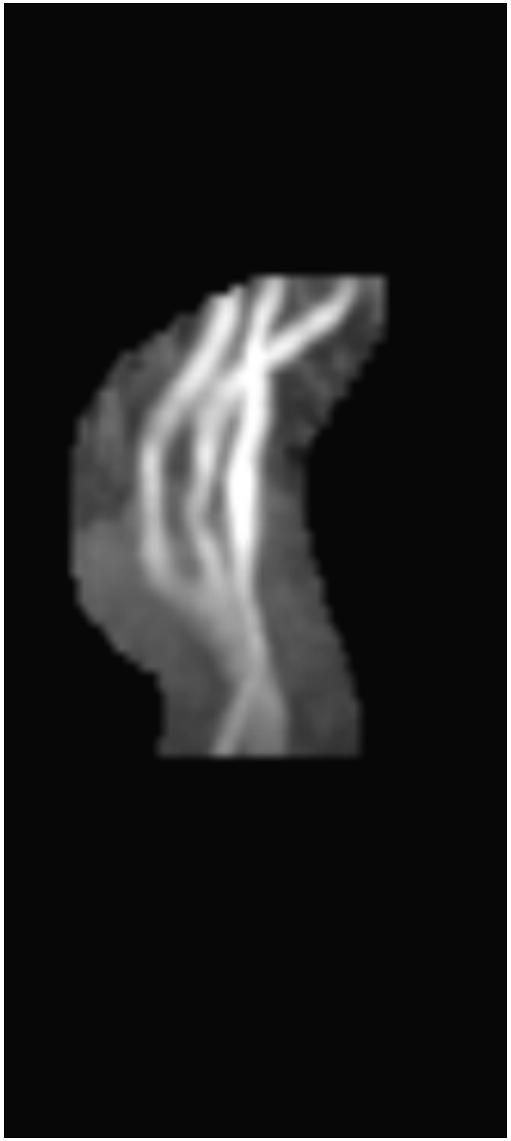

Supplement: S2 File — (PDF) [file pone.0237856.s004.pdf]
