# Supplementary material for: Multiple reader comparison of 2D TOF, 3D TOF, and CEMRA in screening of the carotid bifurcations: Time to reconsider routine contrast use?
Source: PLoS One. 2020 Sep 2;15(9):e0237856. doi: 10.1371/journal.pone.0237856 (PMC7467222; doi:10.1371/journal.pone.0237856)

1c Score

0-30

31-50

51-70

>70

Near occlusion

Occluded

Quality

1

2

3

4

5

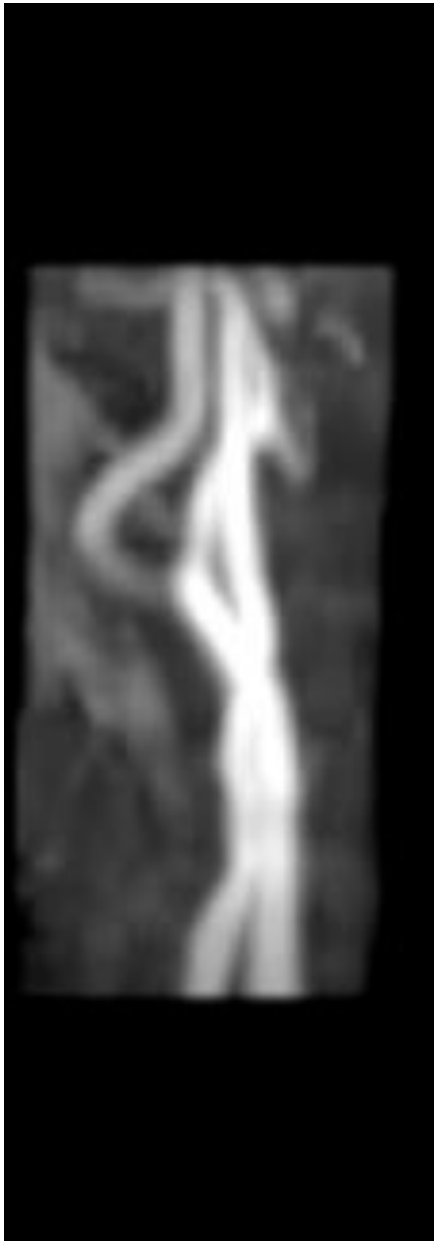

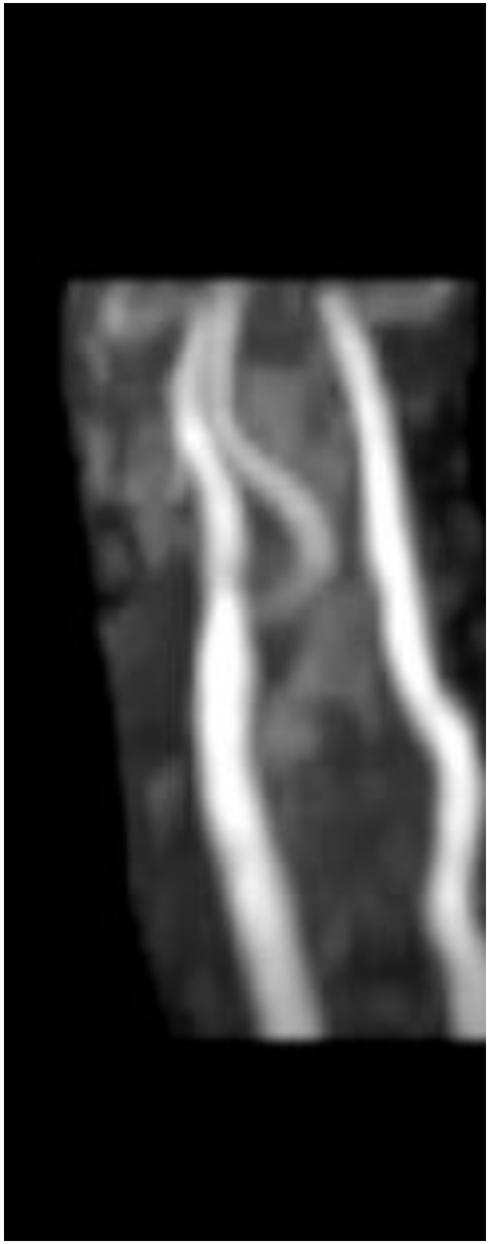

2b Score

0-30

31-50

51-70

>70

Near occlusion

Occluded

Quality

1

2

3

4

5

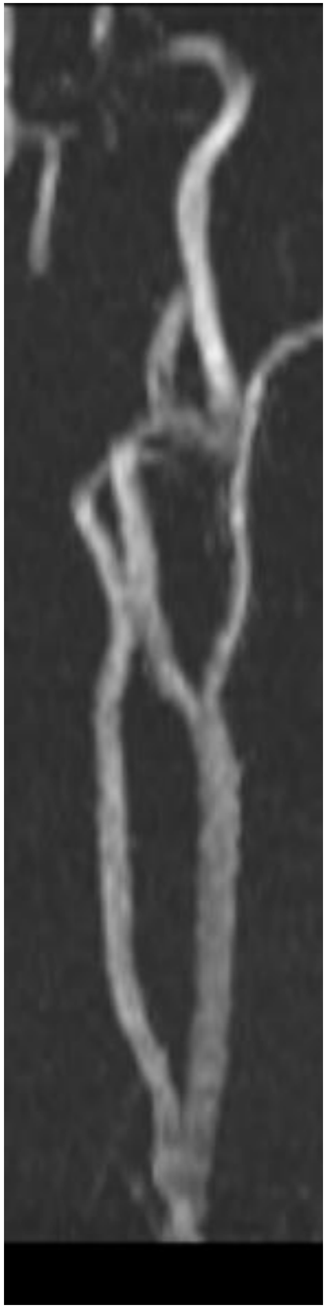

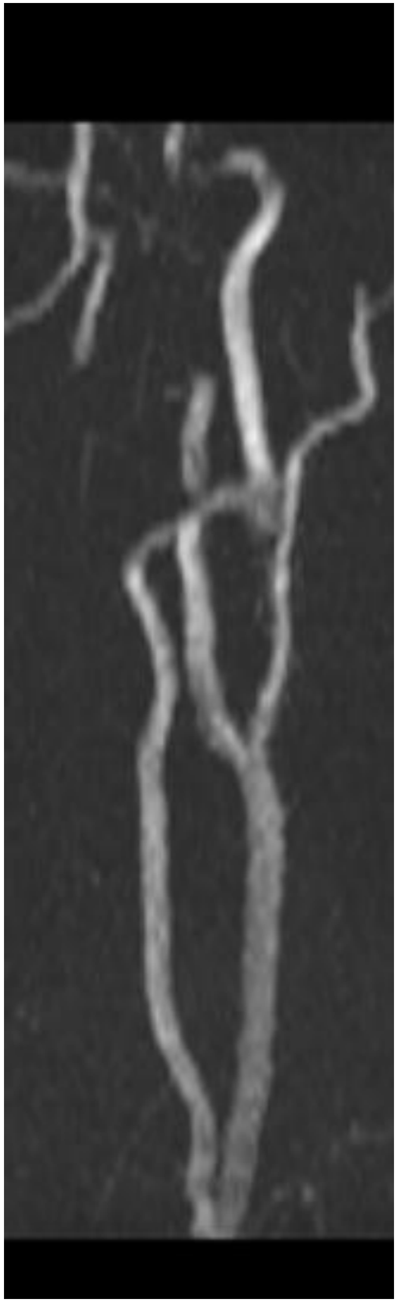

3a Score

0-30

31-50

51-70

>70

Near occlusion

Occluded

Quality

1

2

3

4

5

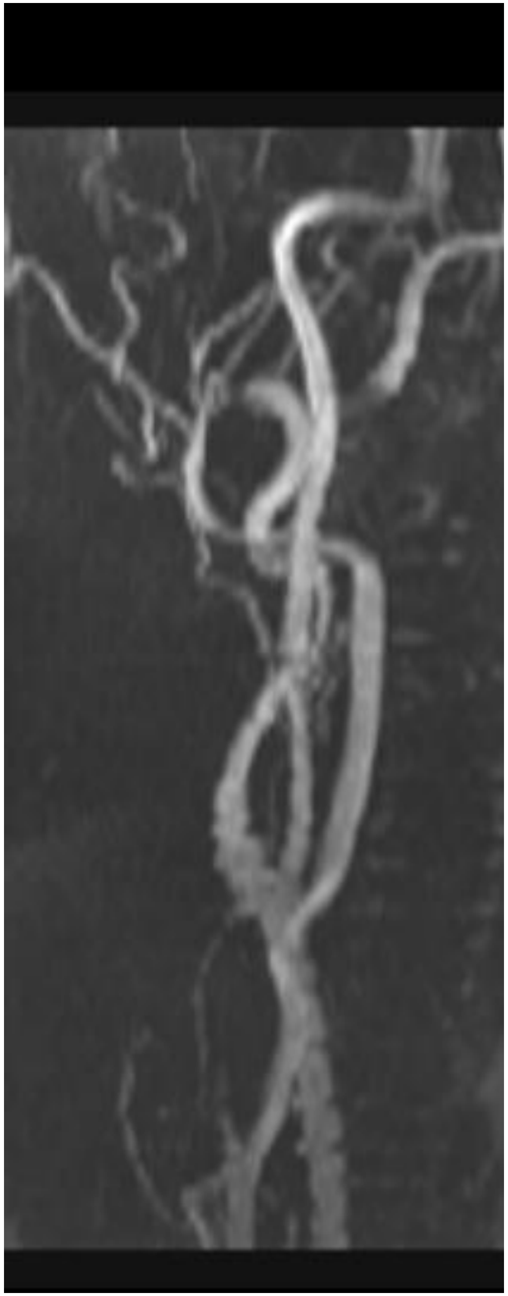

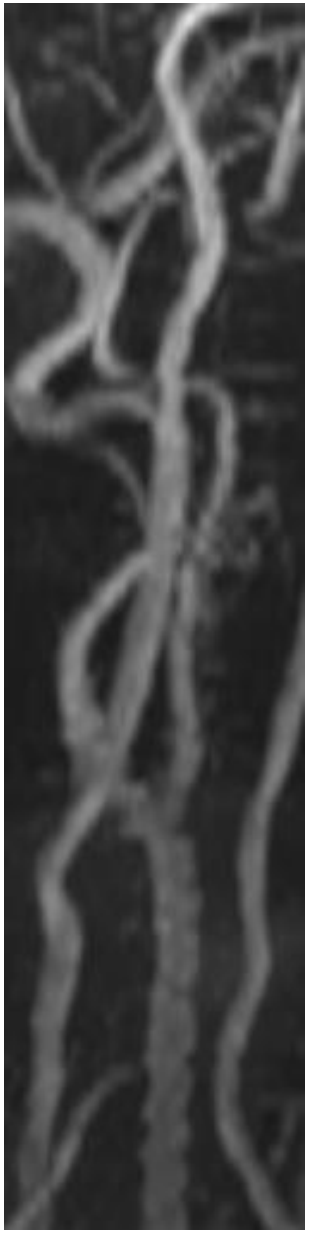

3f Score

0-30

31-50

51-70

>70

Near occlusion

Occluded

Quality

1

2

3

4

5

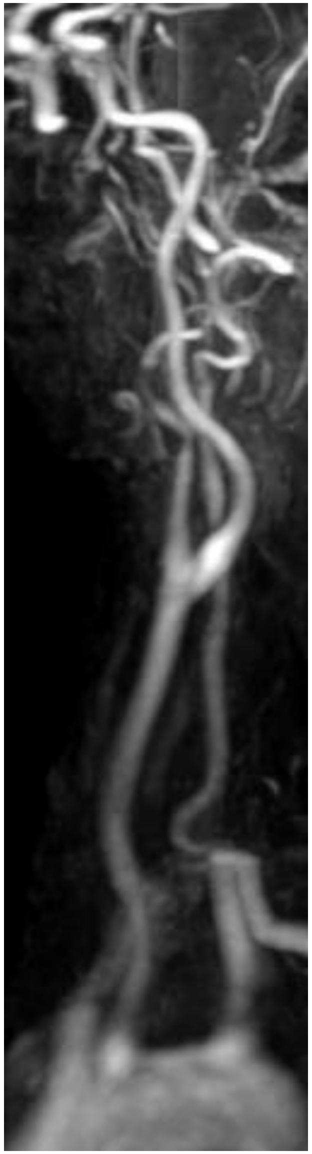

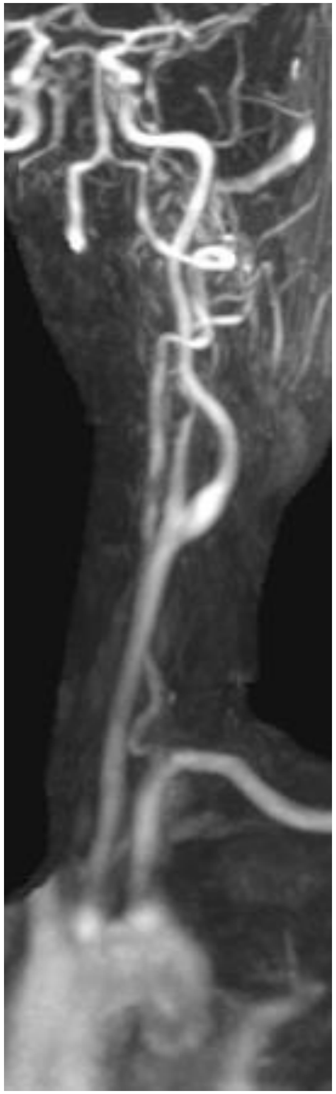

4e Score

0-30

31-50

51-70

>70

Near occlusion

Occluded

Quality

1

2

3

4

5

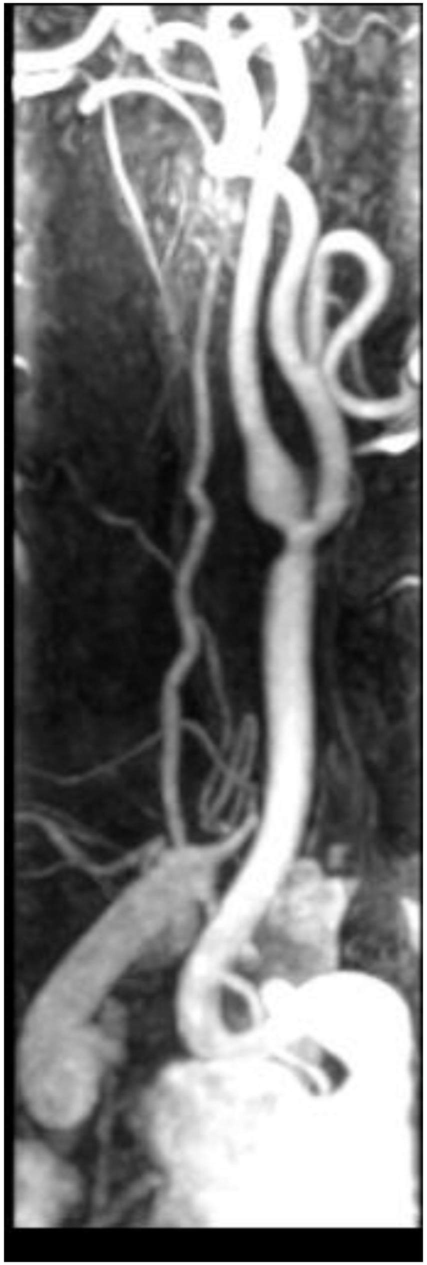

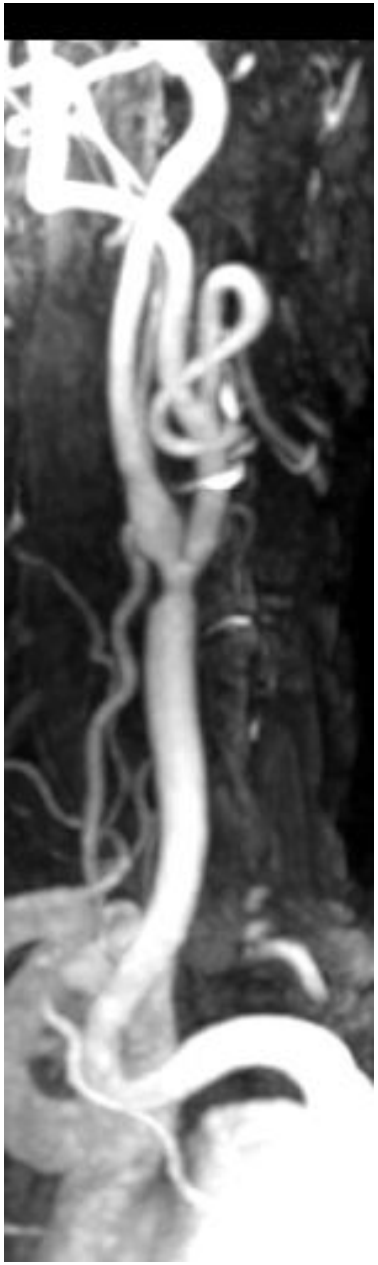

5d Score

0-30

31-50

51-70

>70

Near occlusion

Occluded

Quality

1

2

3

4

5

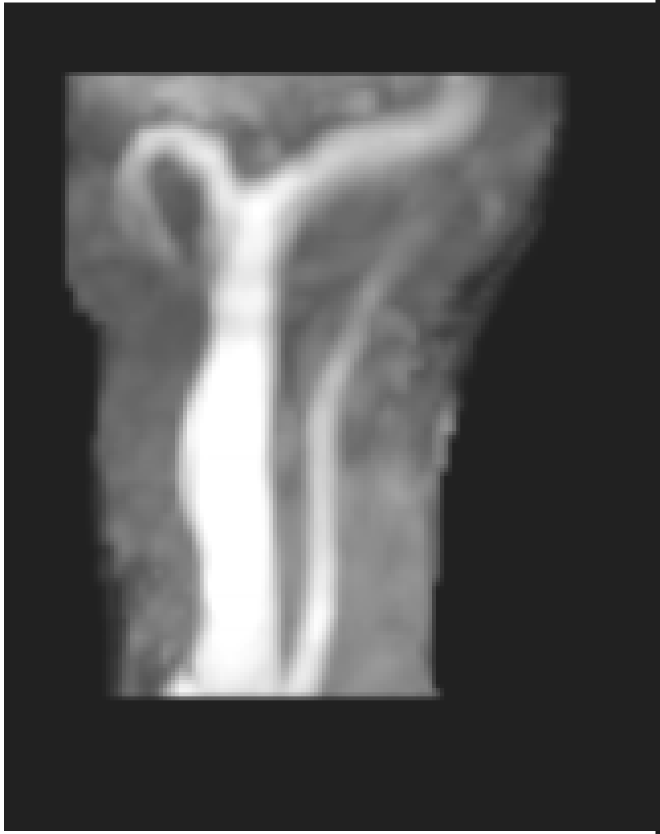

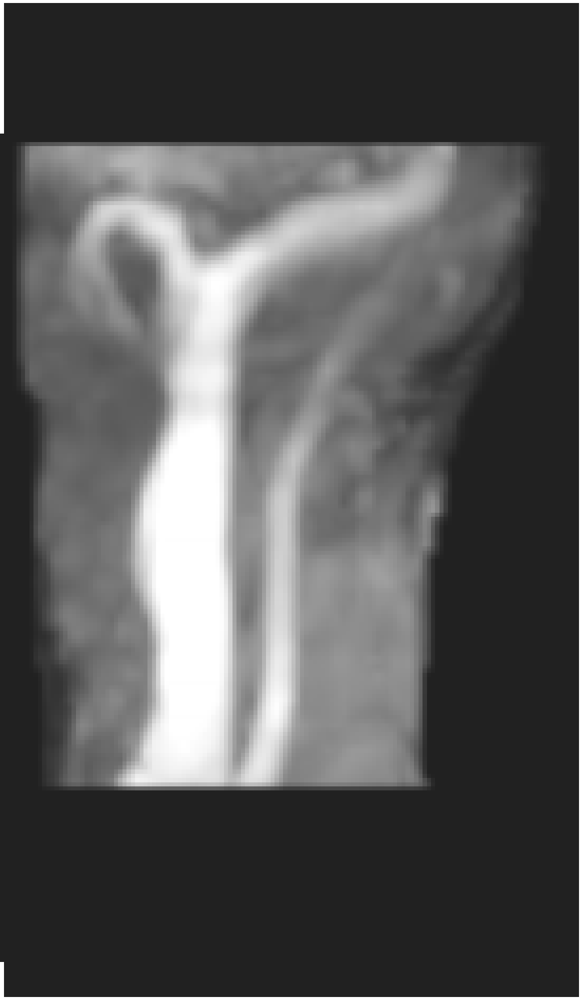

6c Score

0-30

31-50

51-70

>70

Near occlusion

Occluded

Quality

1

2

3

4

5

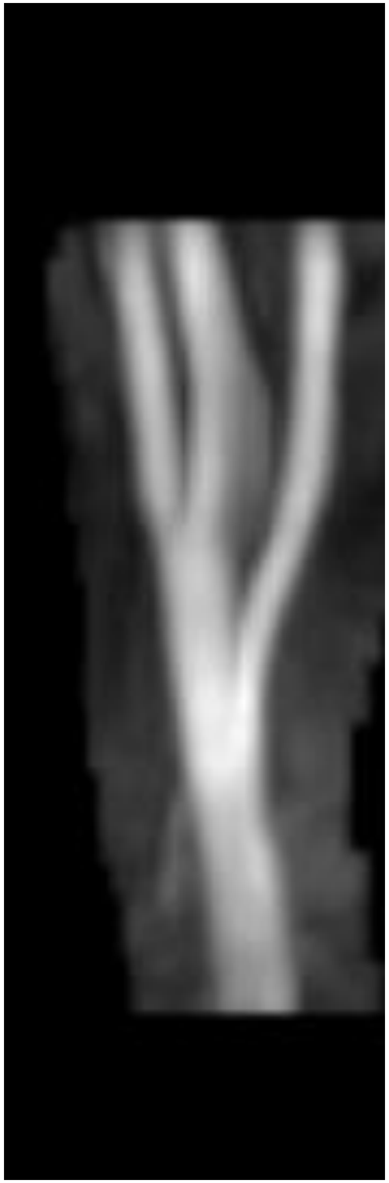

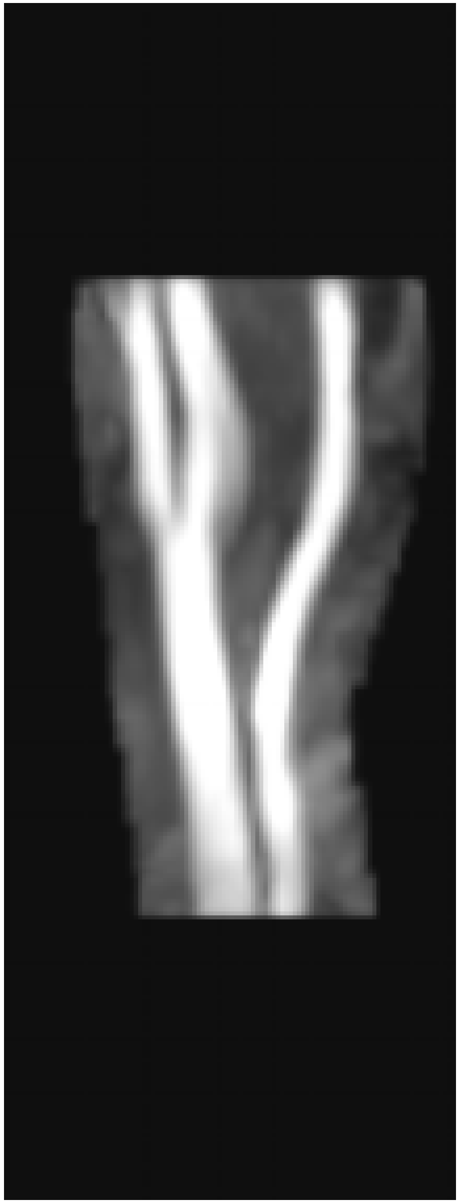

7b Score

0-30

31-50

51-70

>70

Near occlusion

Occluded

Quality

1

2

3

4

5

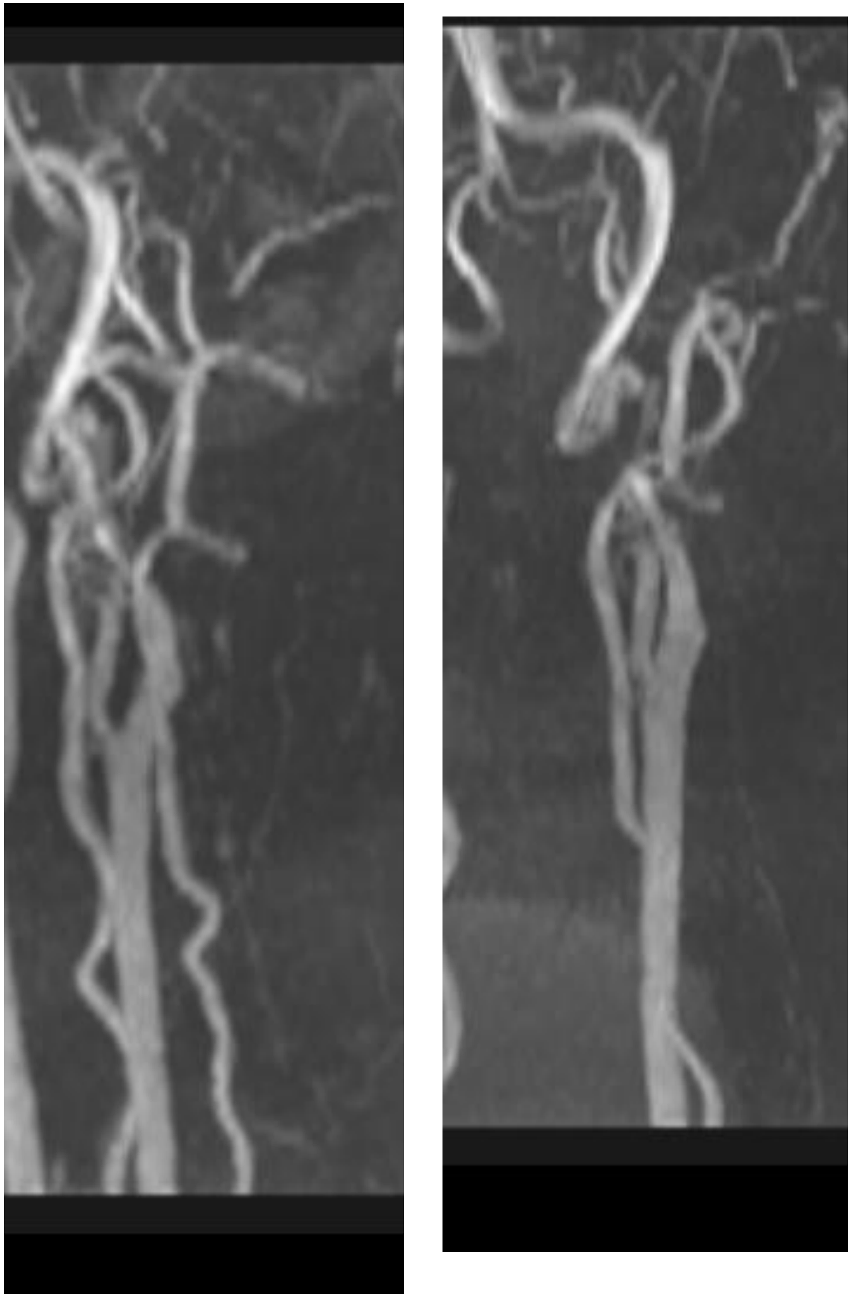

8a Score

0-30

31-50

51-70

>70

Near occlusion

Occluded

Quality

1

2

3

4

5

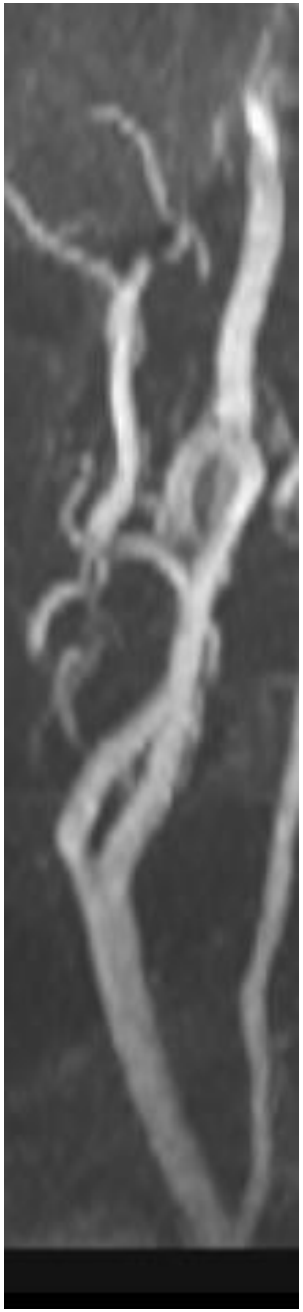

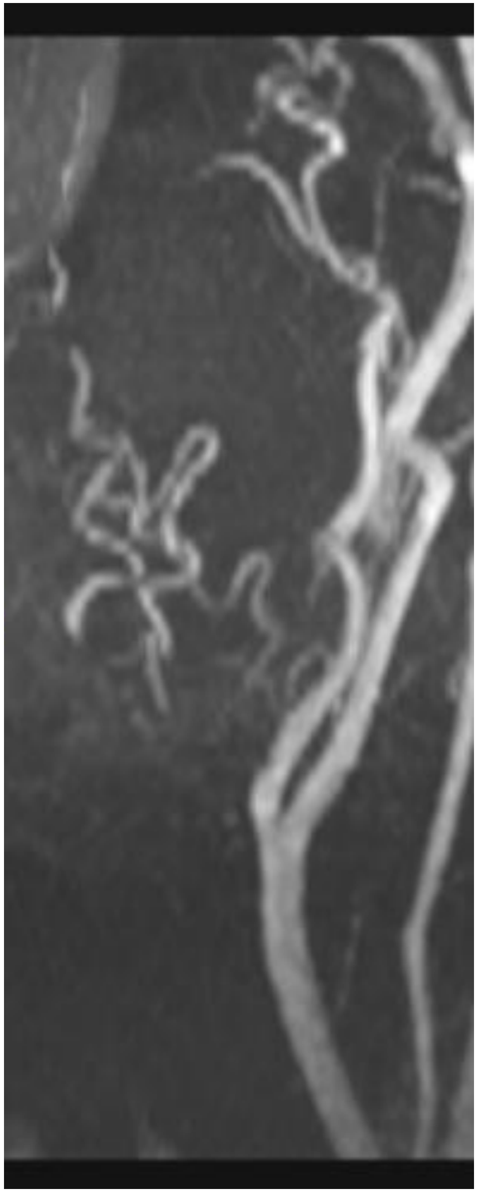

8f Score

0-30

31-50

51-70

>70

Near occlusion

Occluded

Quality

1

2

3

4

5

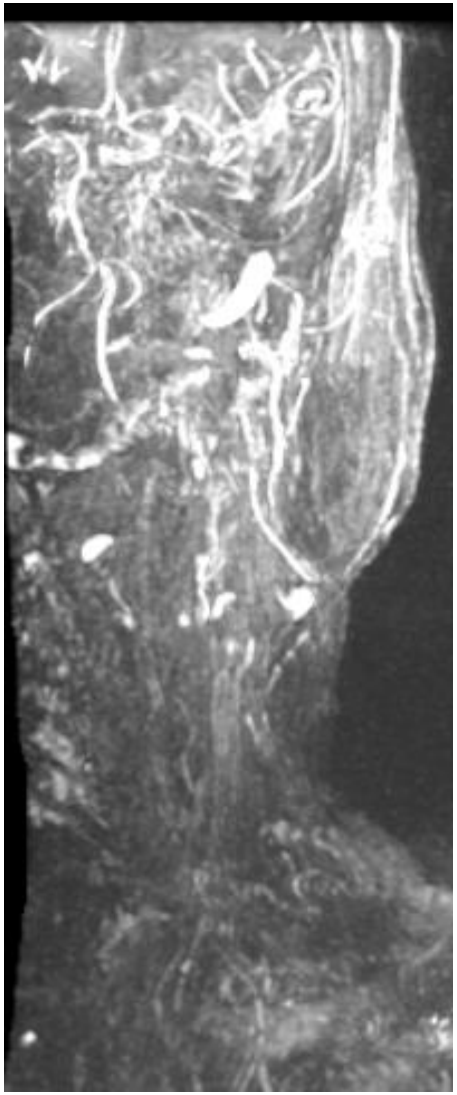

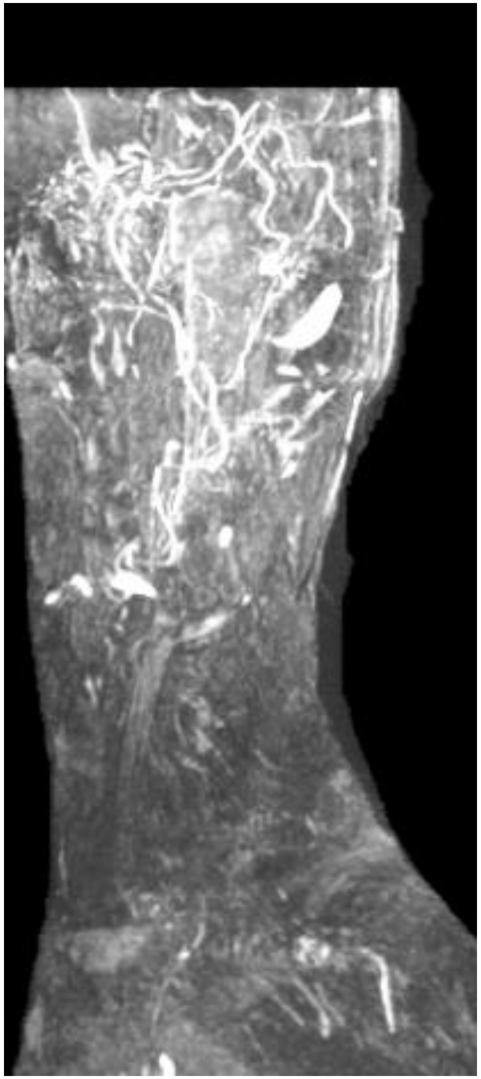

9e Score

0-30

31-50

51-70

>70

Near occlusion

Occluded

Quality

1

2

3

4

5

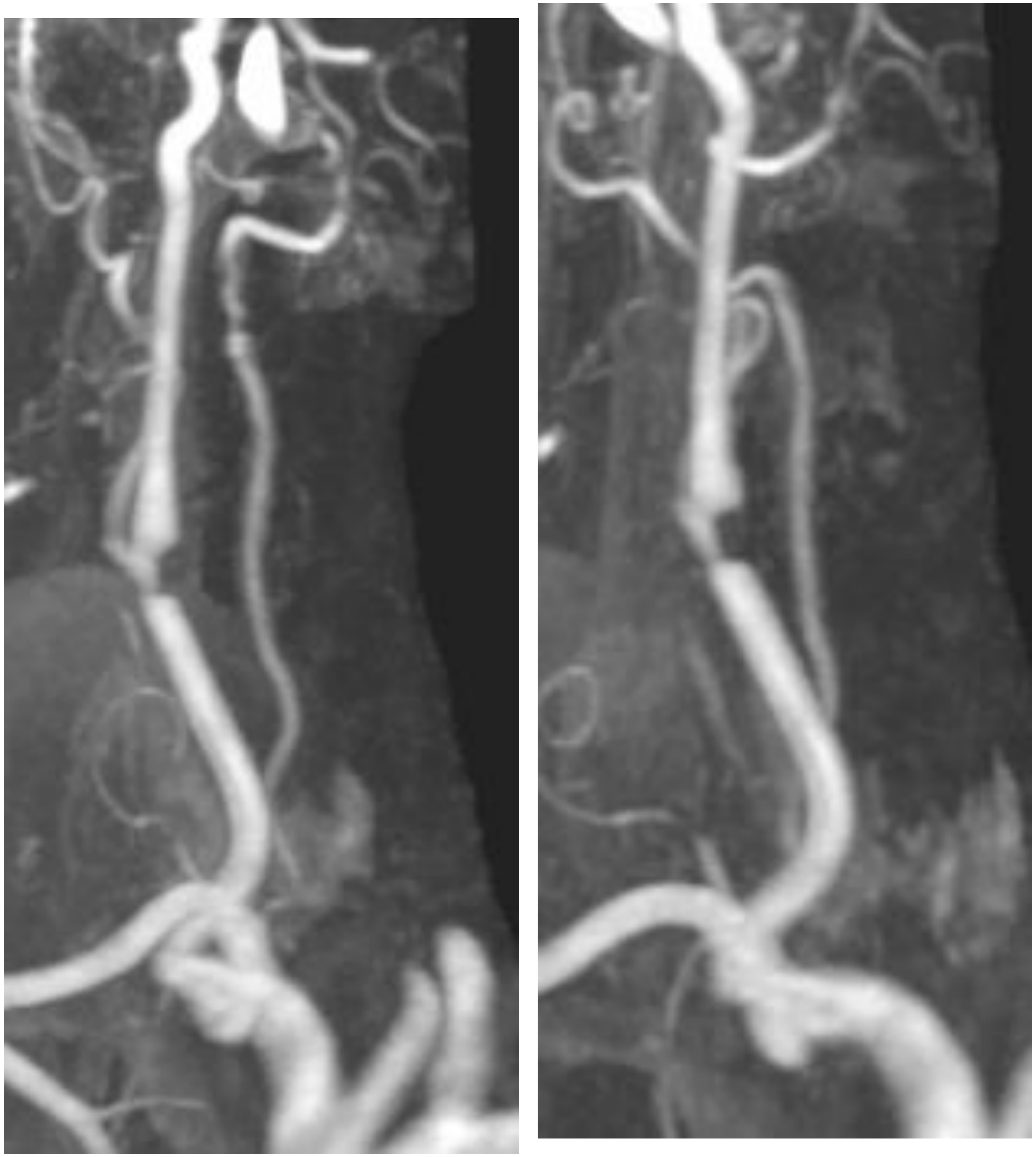

10d Score

0-30

31-50

51-70

>70

Near occlusion

Occluded

Quality

1

2

3

4

5

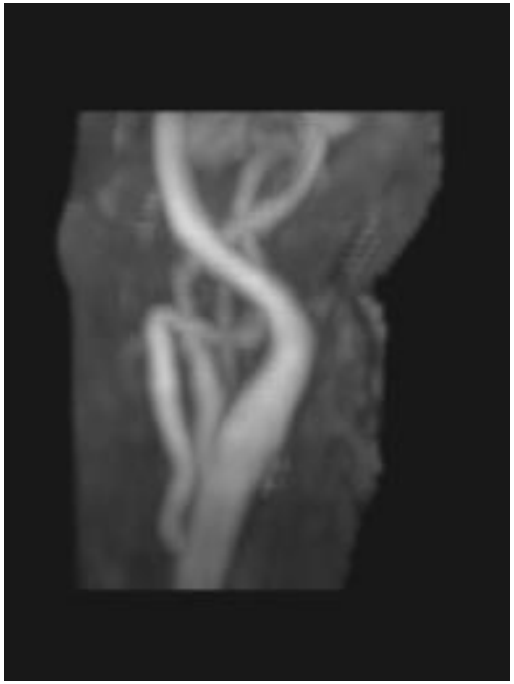

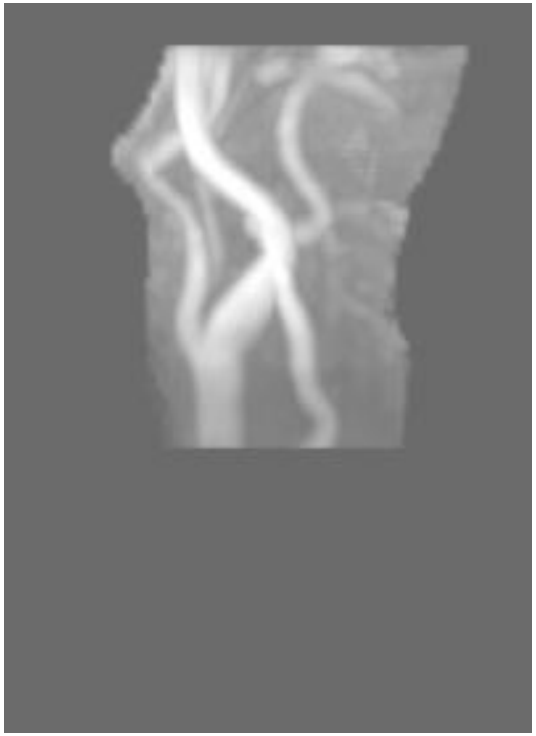

11c Score

0-30

31-50

51-70

>70

Near occlusion

Occluded

Quality

1

2

3

4

5

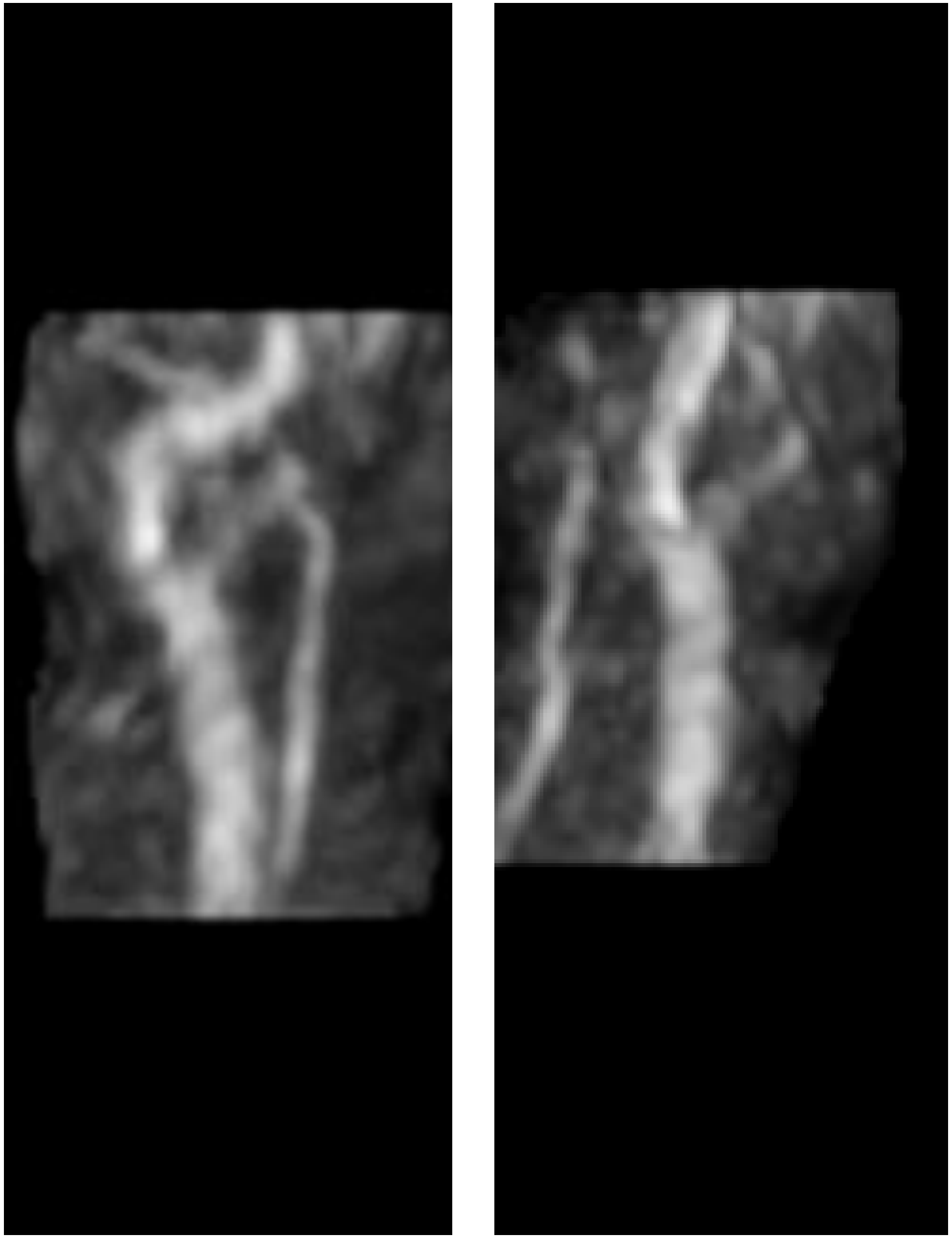

12b Score

0-30

31-50

51-70

>70

Near occlusion

Occluded

Quality

1

2

3

4

5

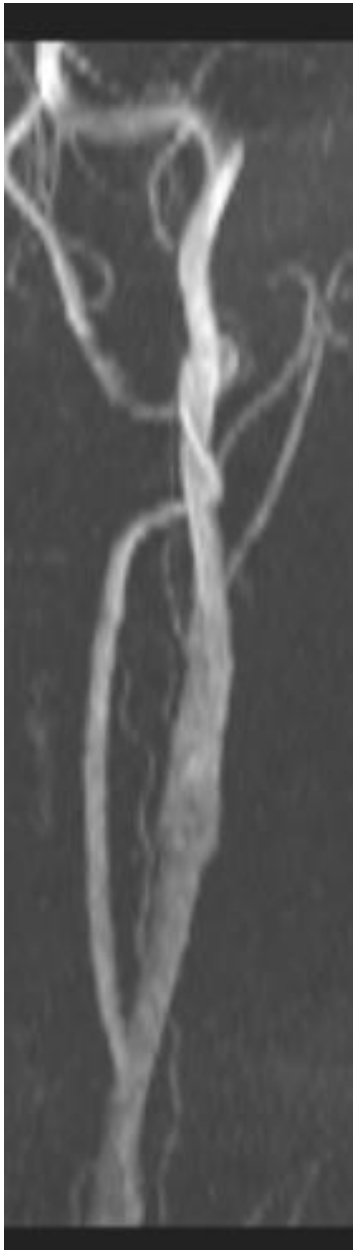

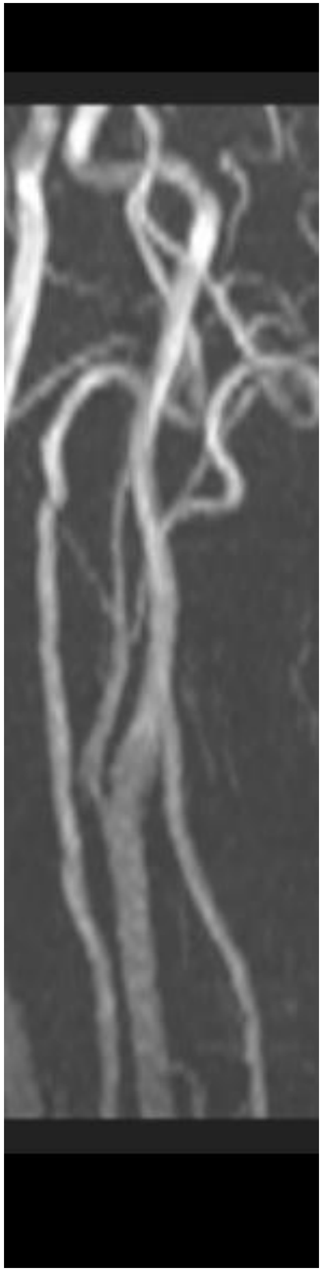

13a Score

0-30

31-50

51-70

>70

Near occlusion

Occluded

Quality

1

2

3

4

5

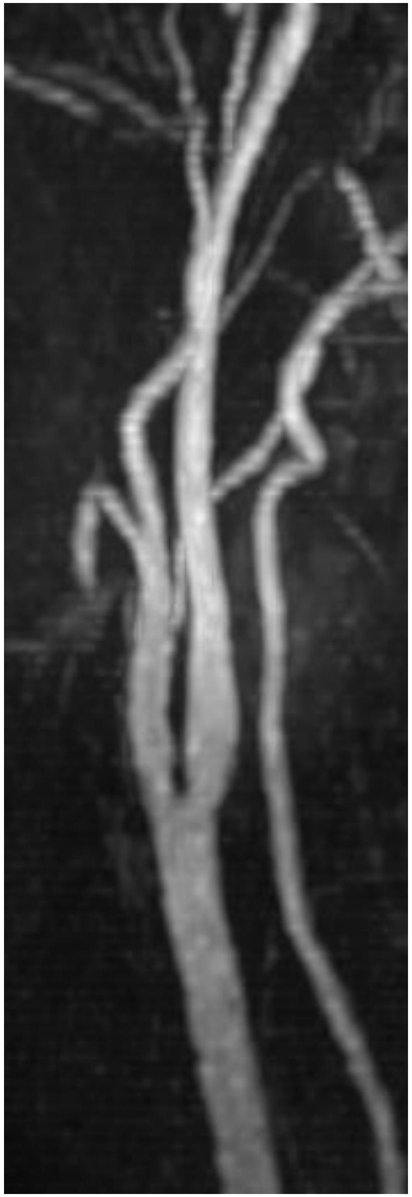

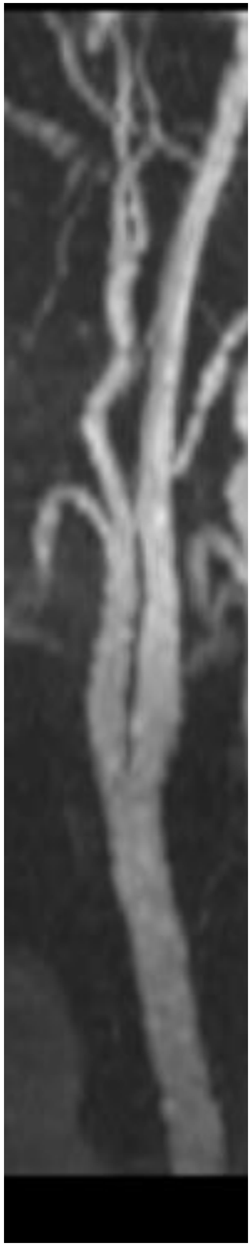

13f Score

0-30

31-50

51-70

>70

Near occlusion

Occluded

Quality

1

2

3

4

5

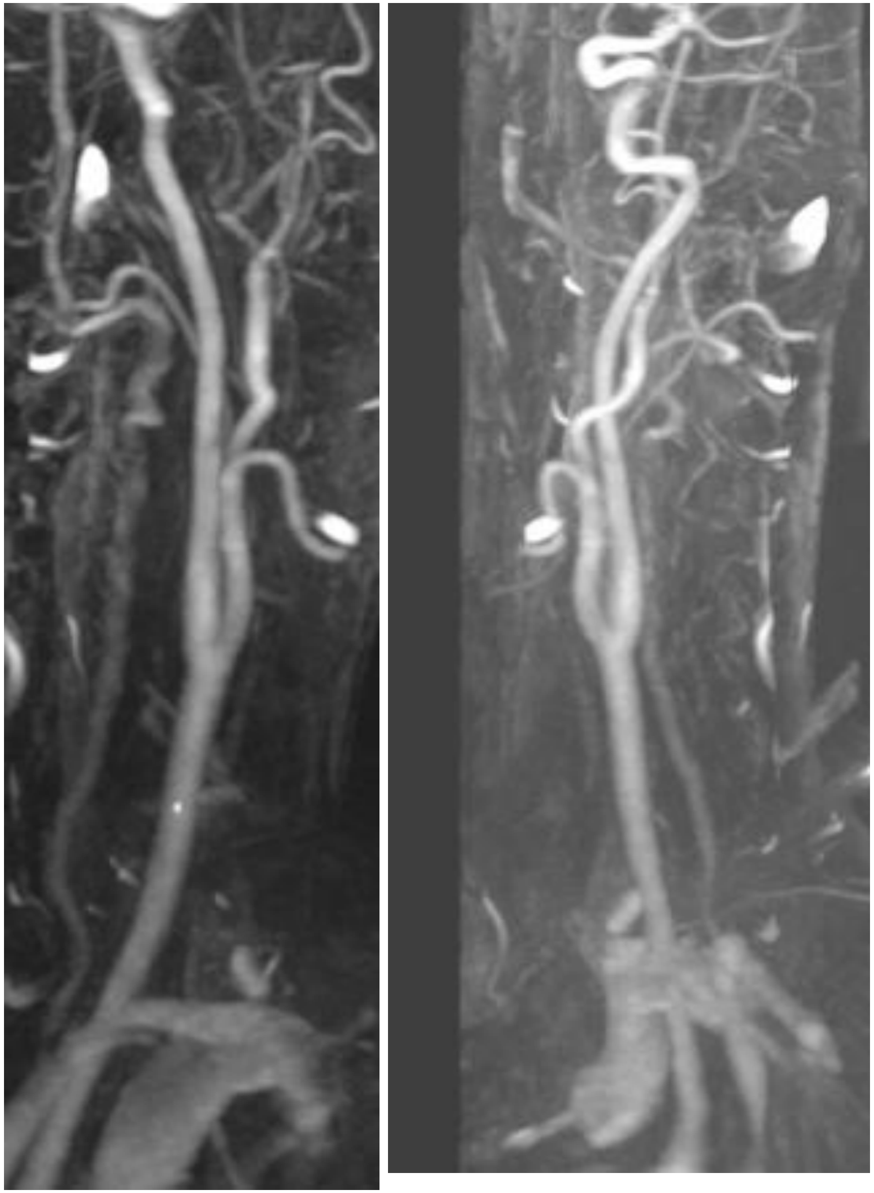

14e Score

0-30

31-50

51-70

>70

Near occlusion

Occluded

Quality

1

2

3

4

5

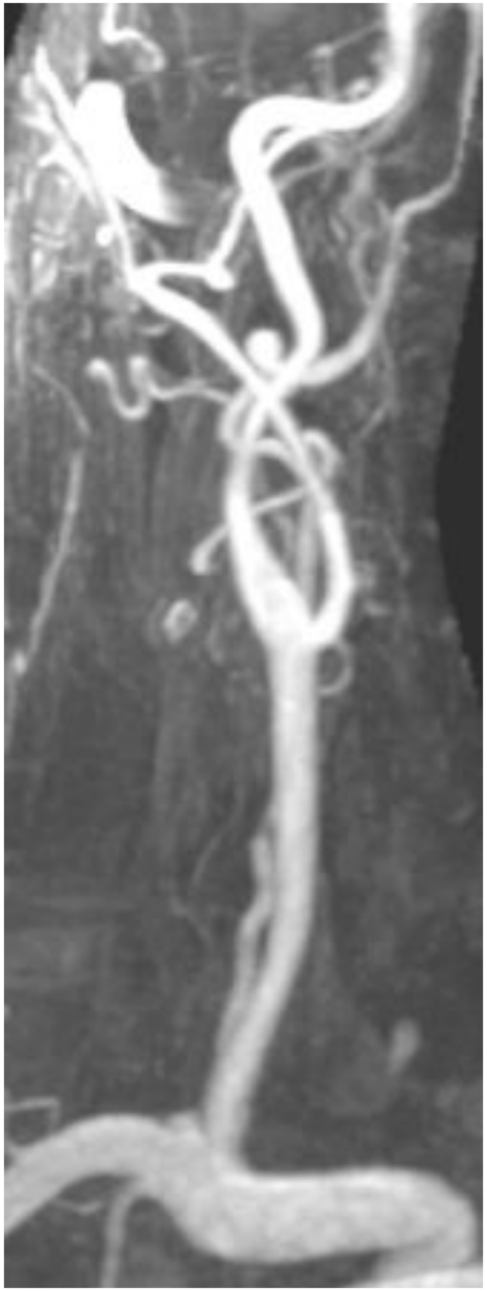

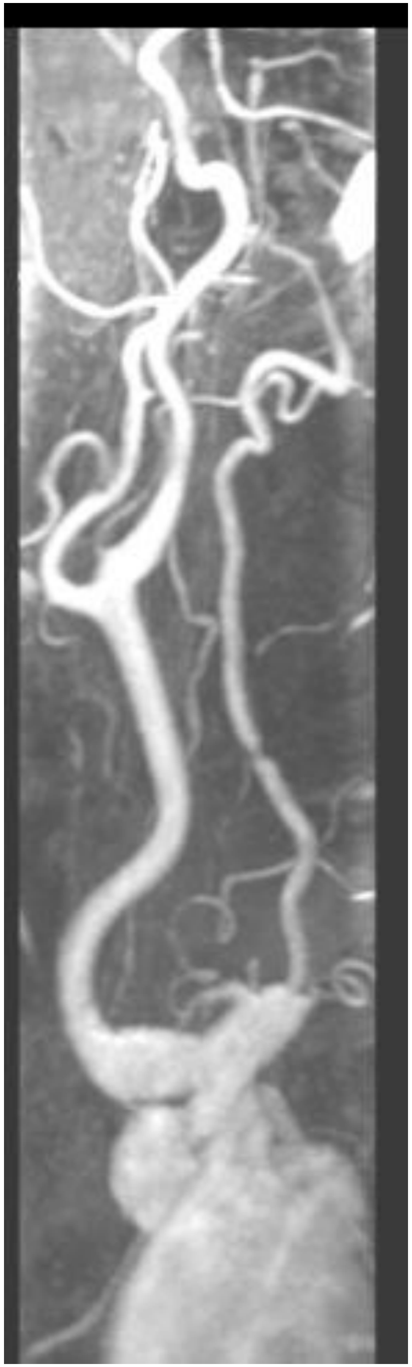

15d Score

0-30

31-50

51-70

>70

Near occlusion

Occluded

Quality

1

2

3

4

5

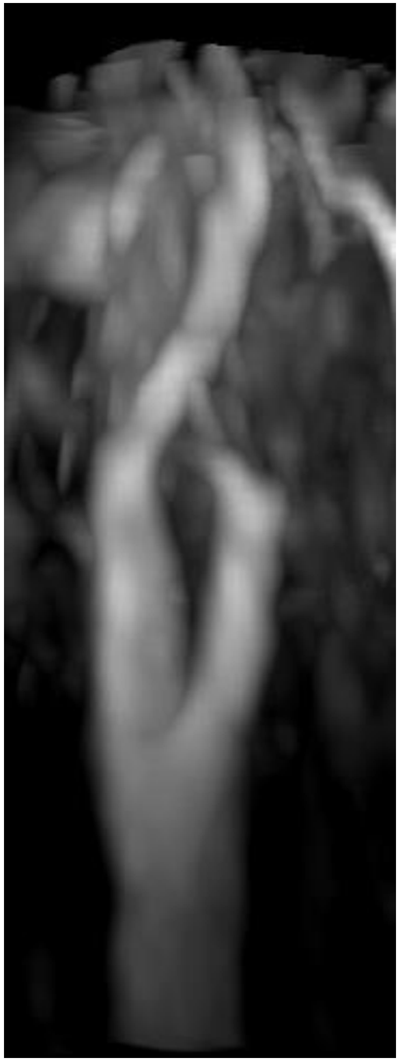

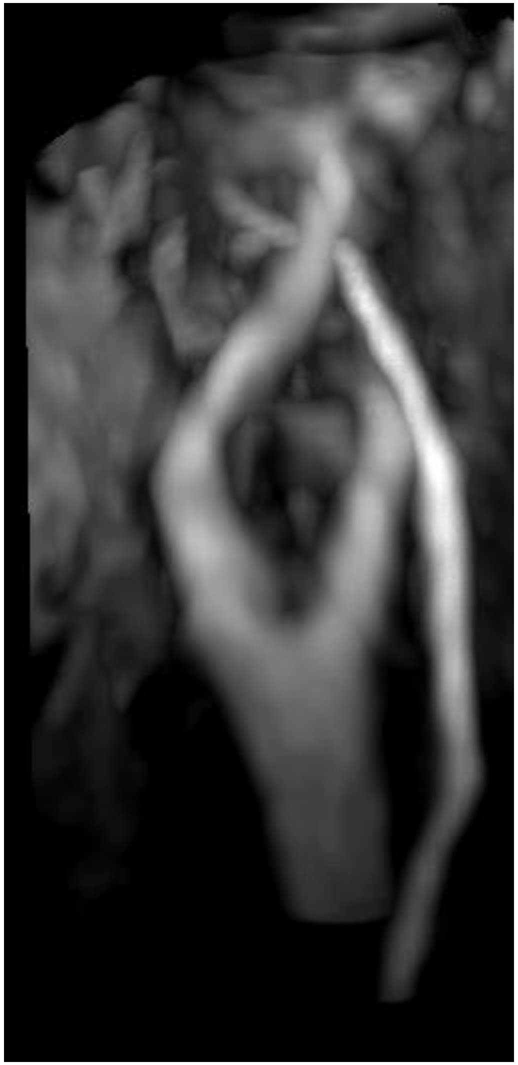

16c Score

0-30

31-50

51-70

>70

Near occlusion

Occluded

Quality

1

2

3

4

5

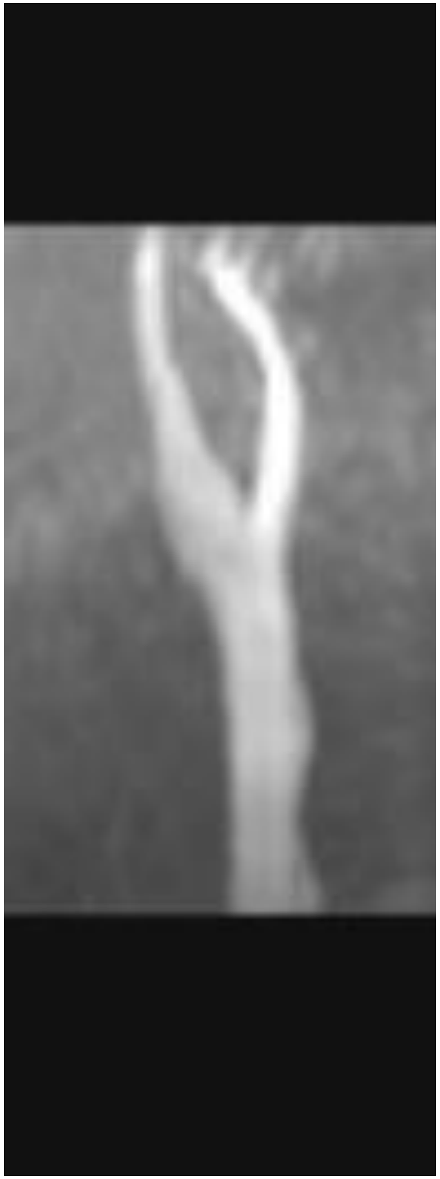

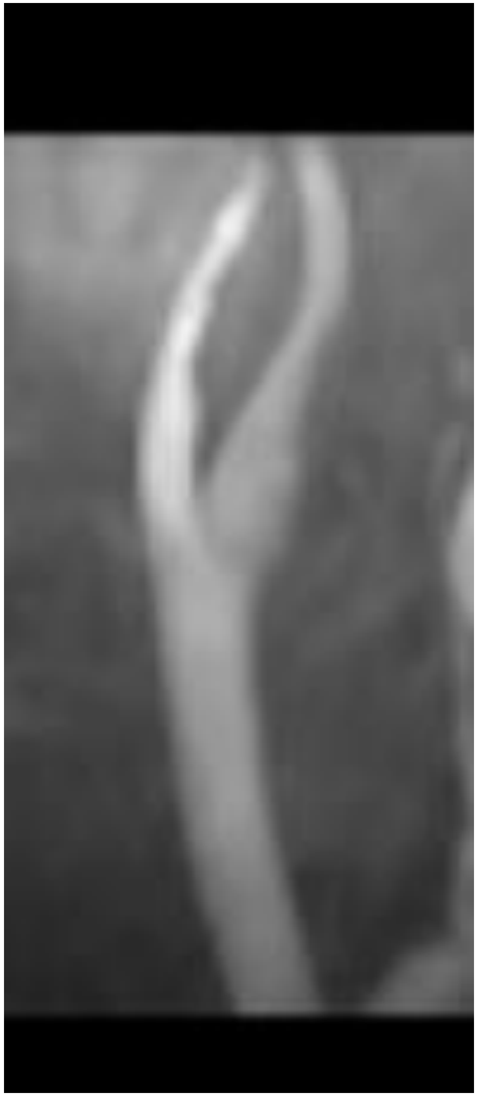

17b Score

0-30

31-50

51-70

>70

Near occlusion

Occluded

Quality

1

2

3

4

5

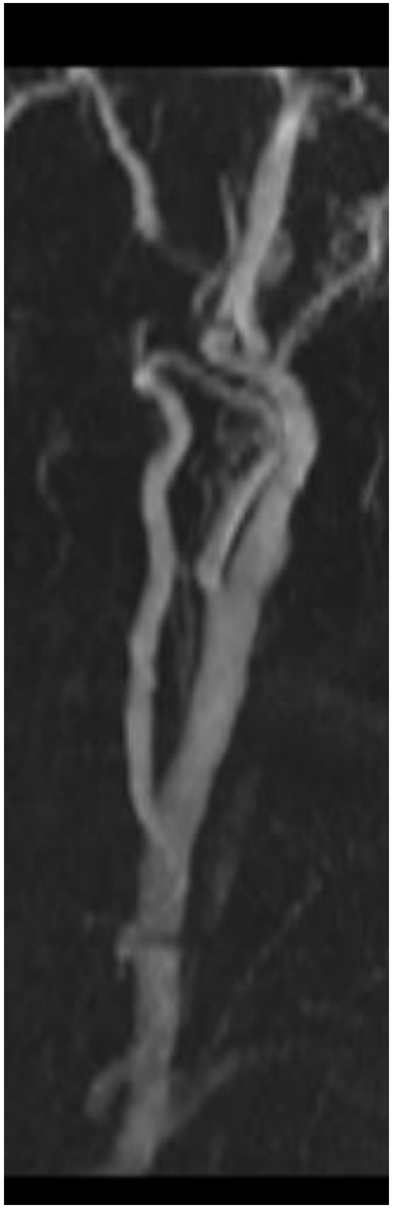

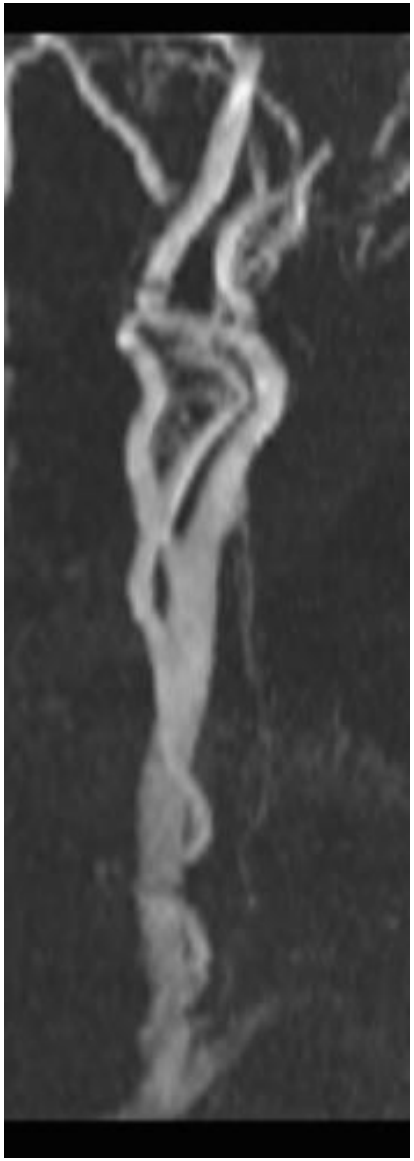

18a Score

0-30

31-50

51-70

>70

Near occlusion

Occluded

Quality

1

2

3

4

5

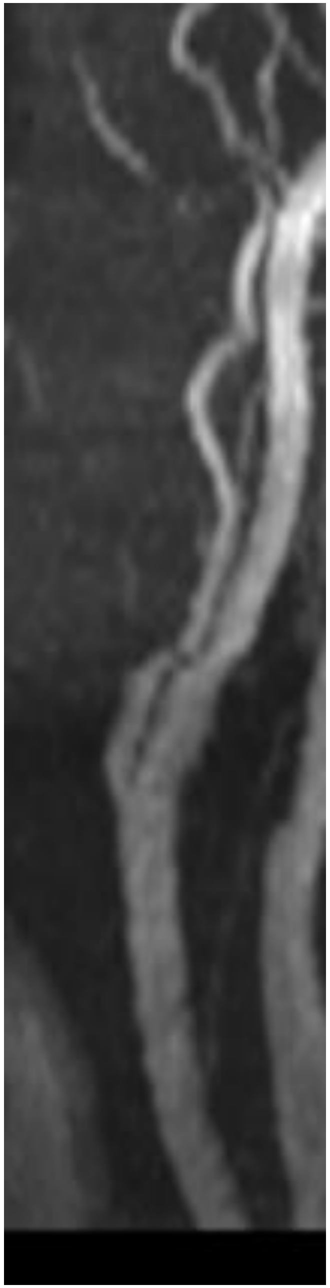

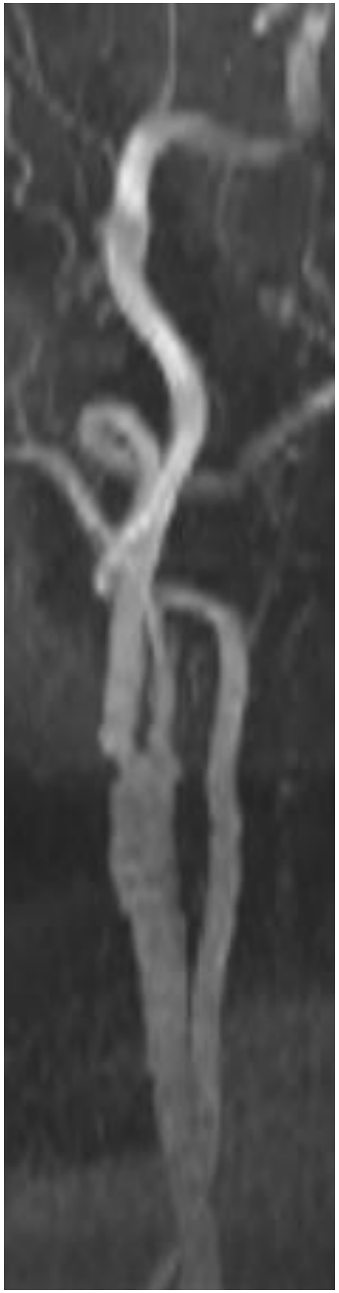

18f Score

0-30

31-50

51-70

>70

Near occlusion

Occluded

Quality

1

2

3

4

5

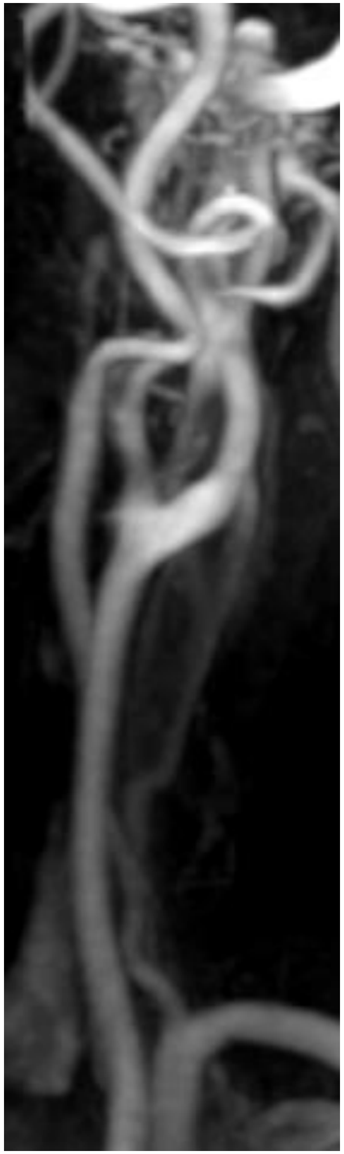

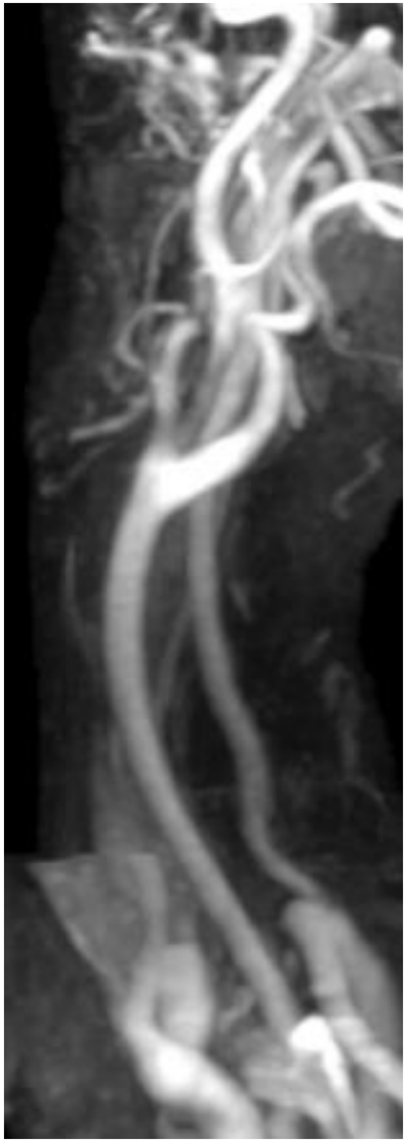

19e Score

0-30

31-50

51-70

>70

Near occlusion

Occluded

Quality

1

2

3

4

5

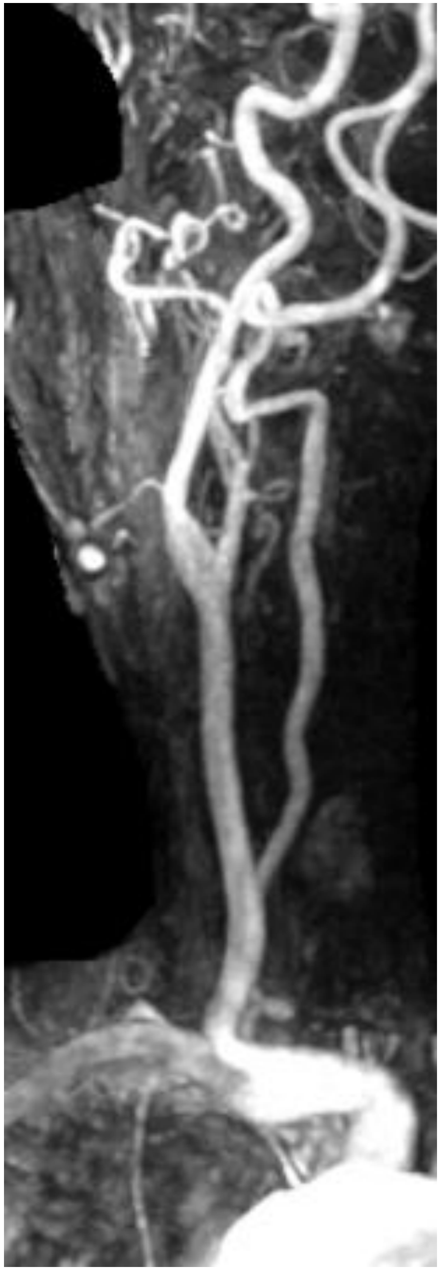

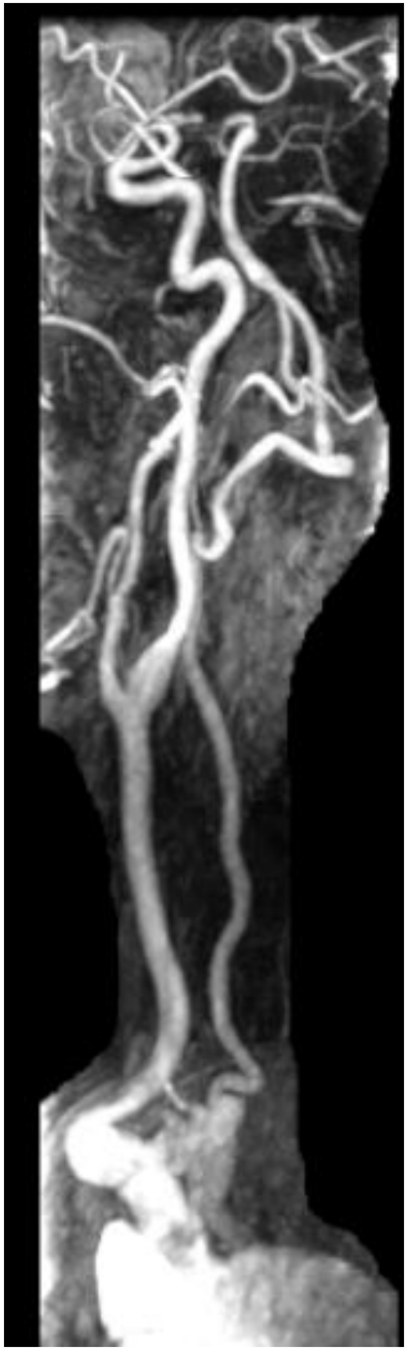

20d Score

0-30

31-50

51-70

>70

Near occlusion

Occluded

Quality

1

2

3

4

5

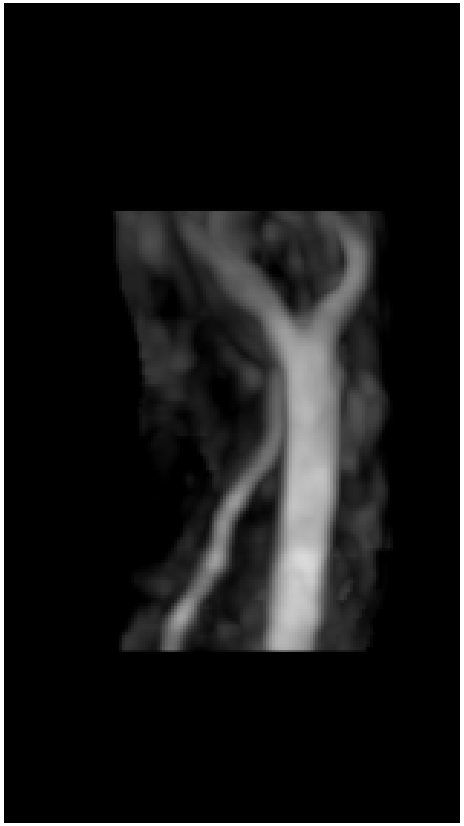

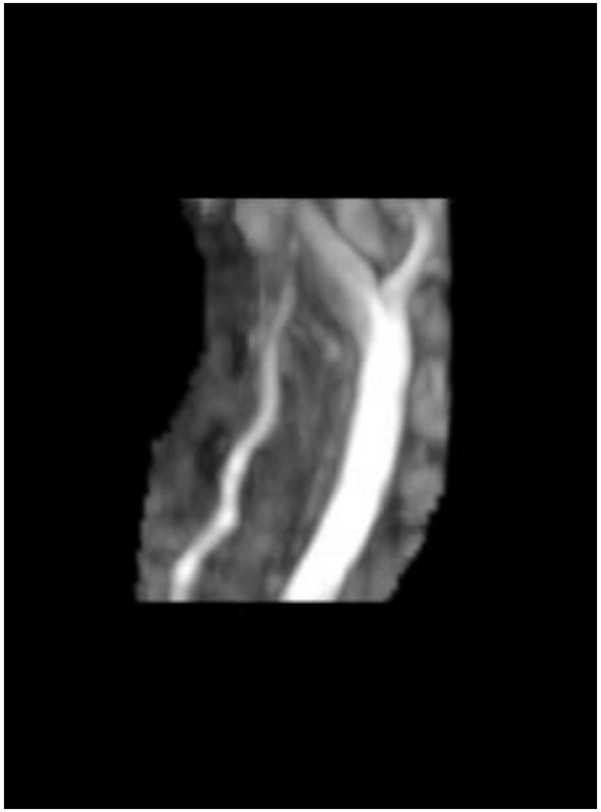

21c Score

0-30

31-50

51-70

>70

Near occlusion

Occluded

Quality

1

2

3

4

5

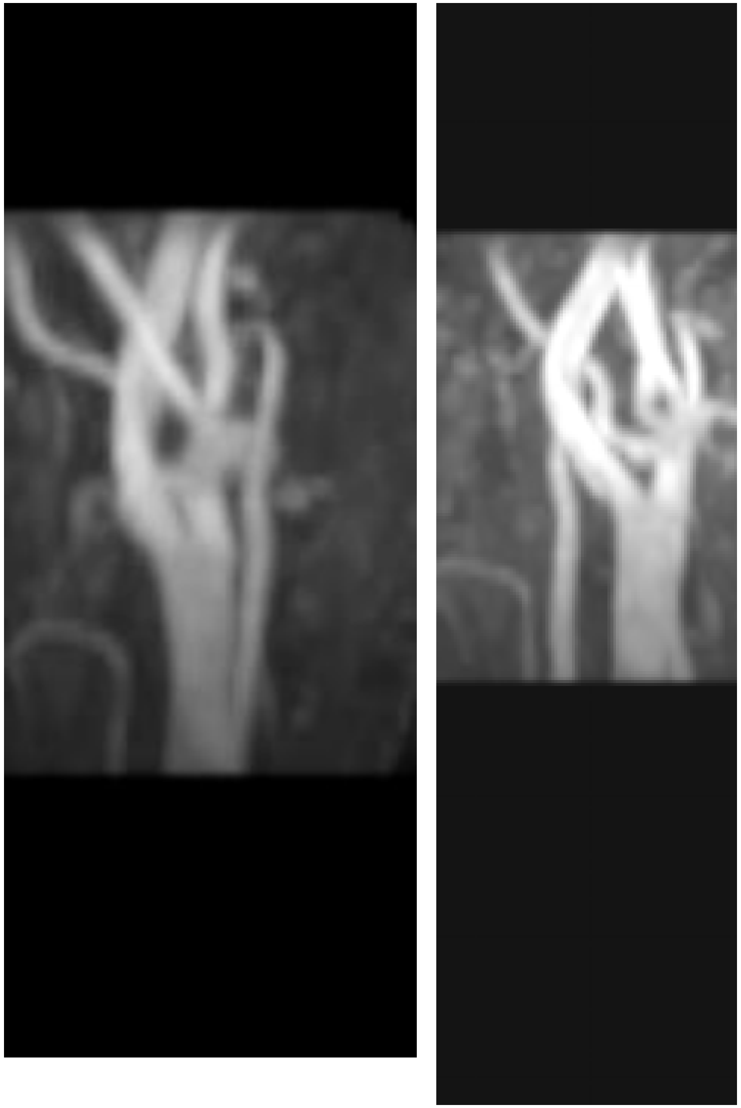

22b Score

0-30

31-50

51-70

>70

Near occlusion

Occluded

Quality

1

2

3

4

5

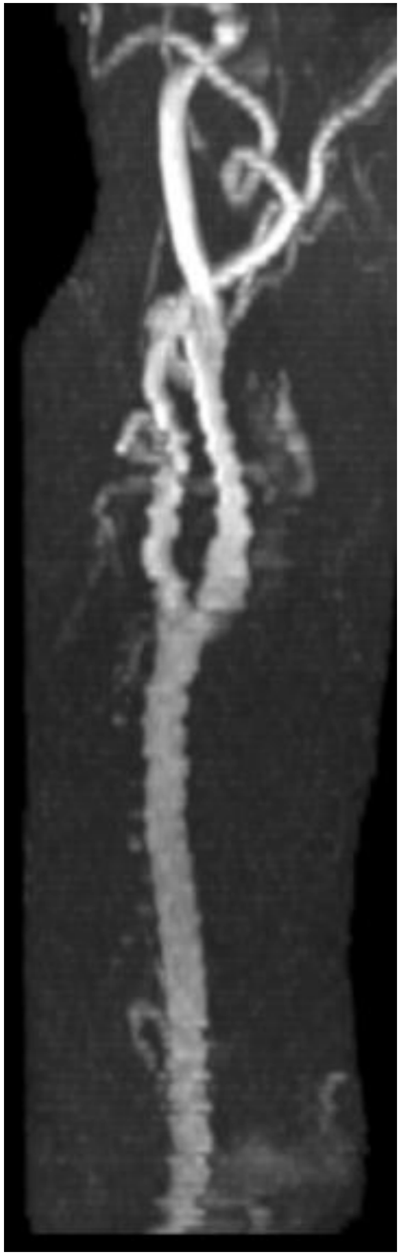

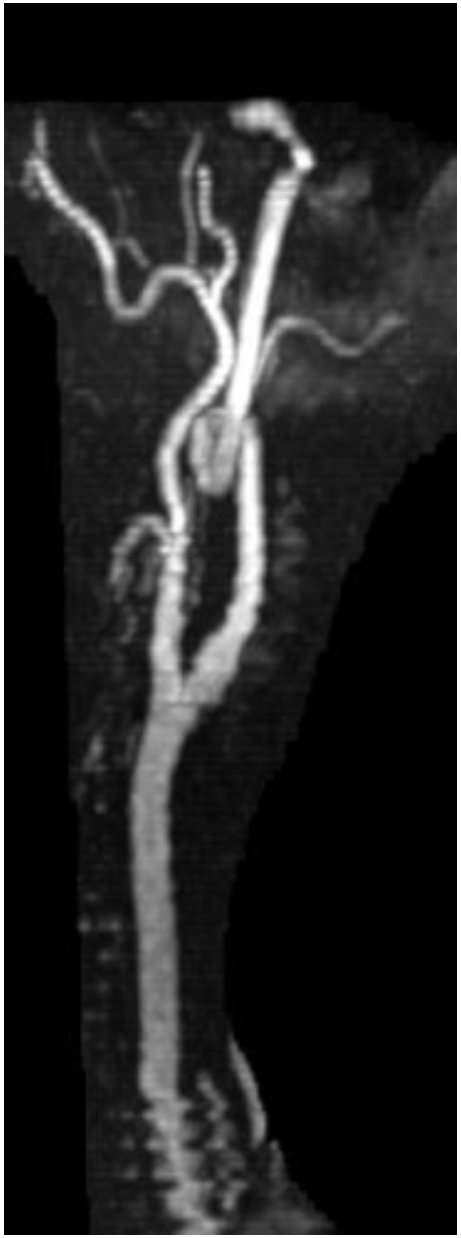

23a Score

0-30

31-50

51-70

>70

Near occlusion

Occluded

Quality

1

2

3

4

5

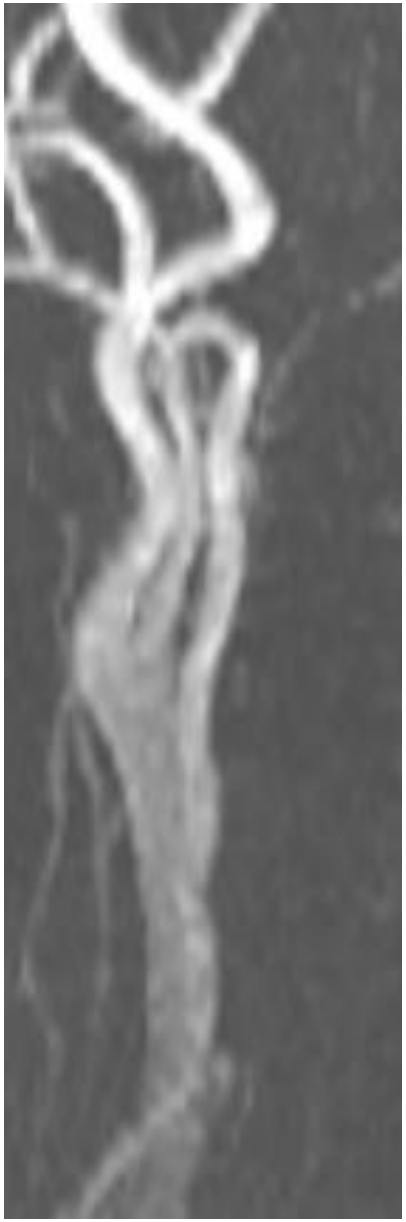

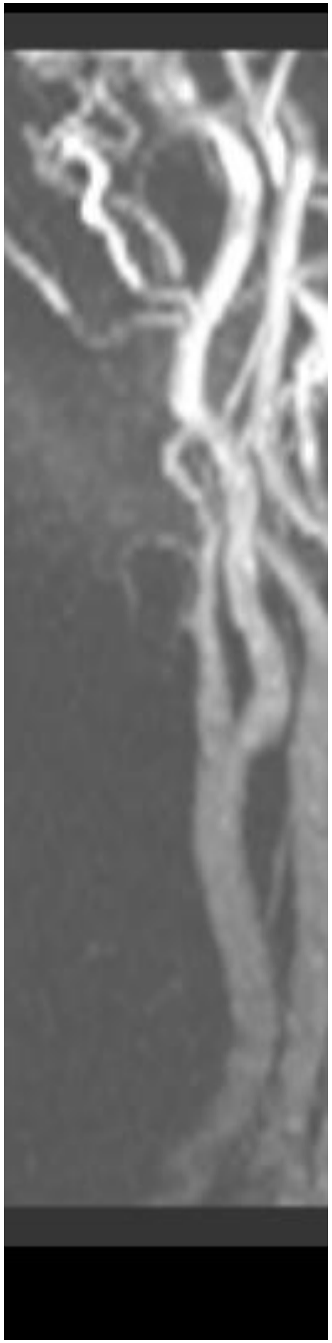

23f Score

0-30

31-50

51-70

>70

Near occlusion

Occluded

Quality

1

2

3

4

5

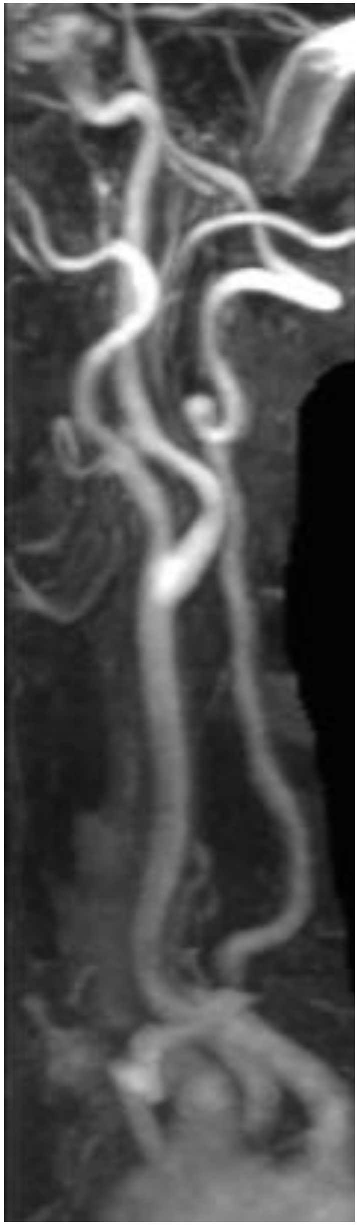

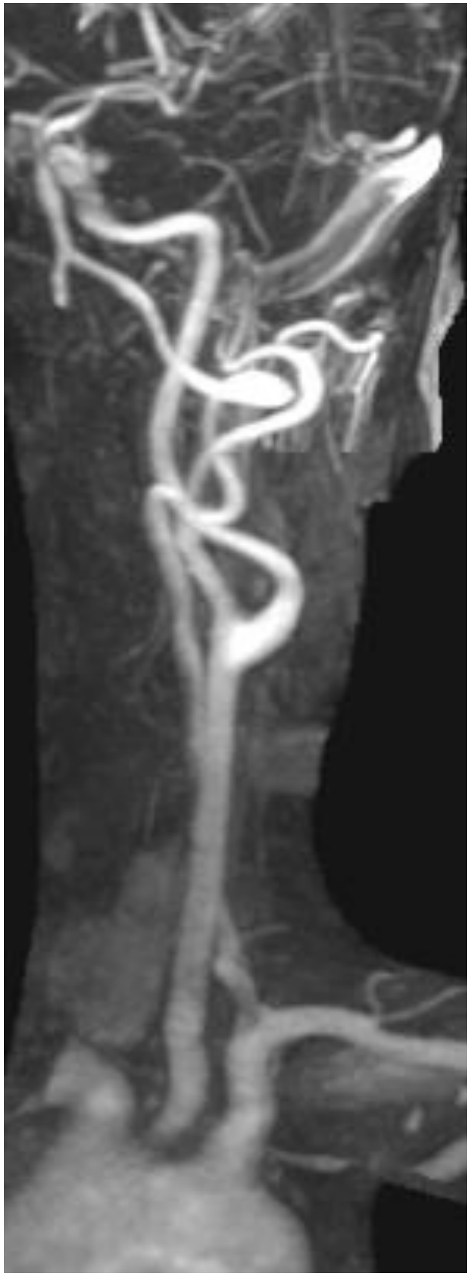

24e Score

0-30

31-50

51-70

>70

Near occlusion

Occluded

Quality

1

2

3

4

5

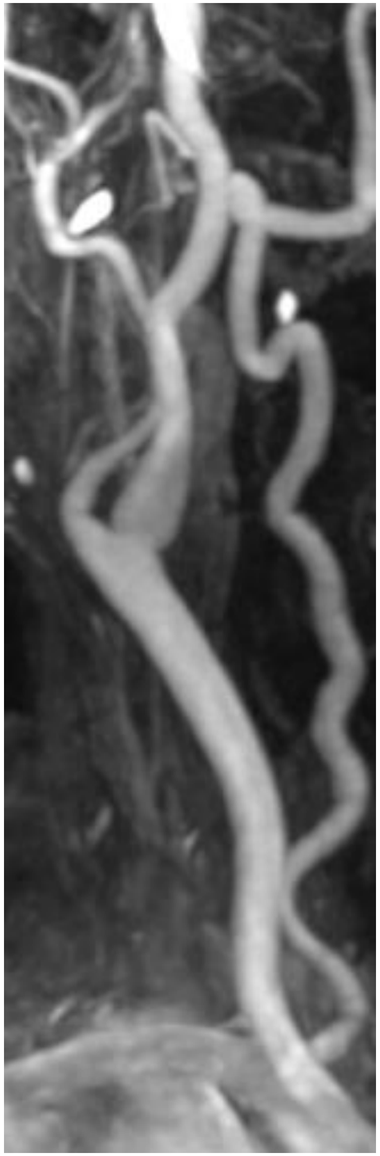

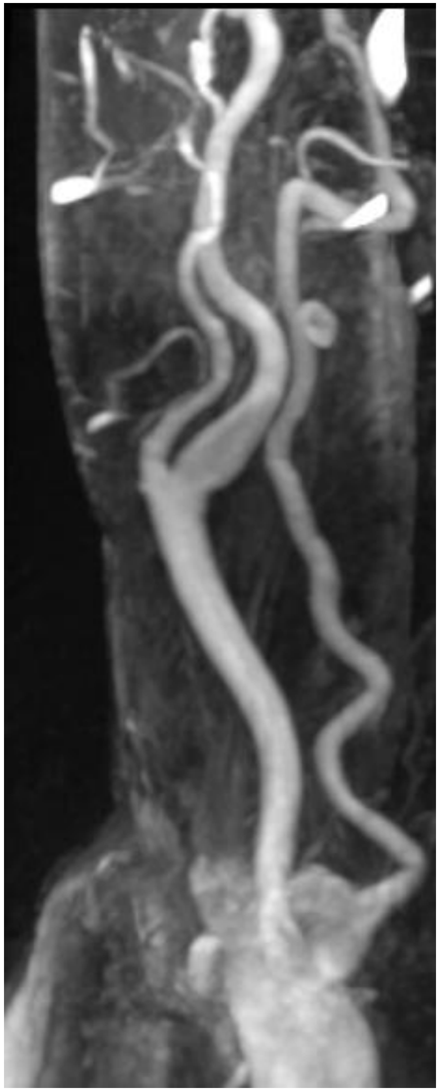

25d Score

0-30

31-50

51-70

>70

Near occlusion

Occluded

Quality

1

2

3

4

5

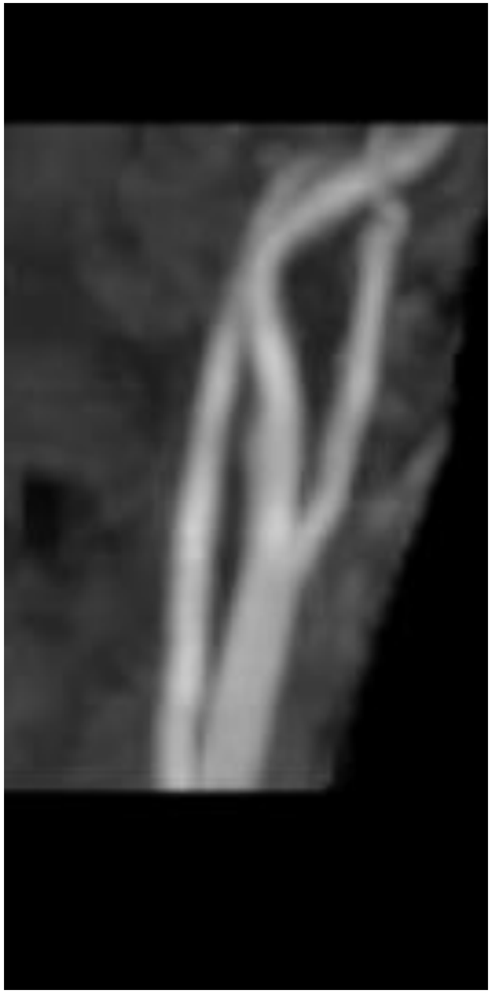

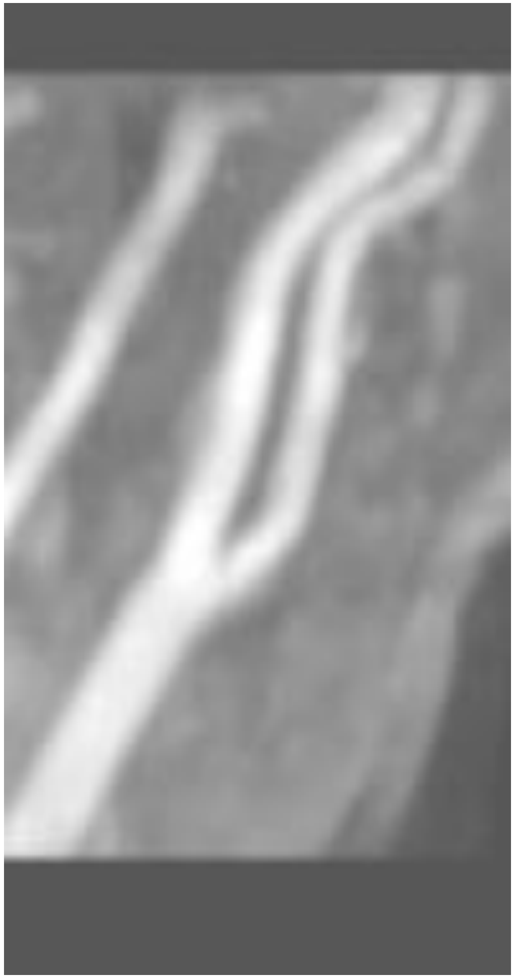

26c Score

0-30

31-50

51-70

>70

Near occlusion

Occluded

Quality

1

2

3

4

5

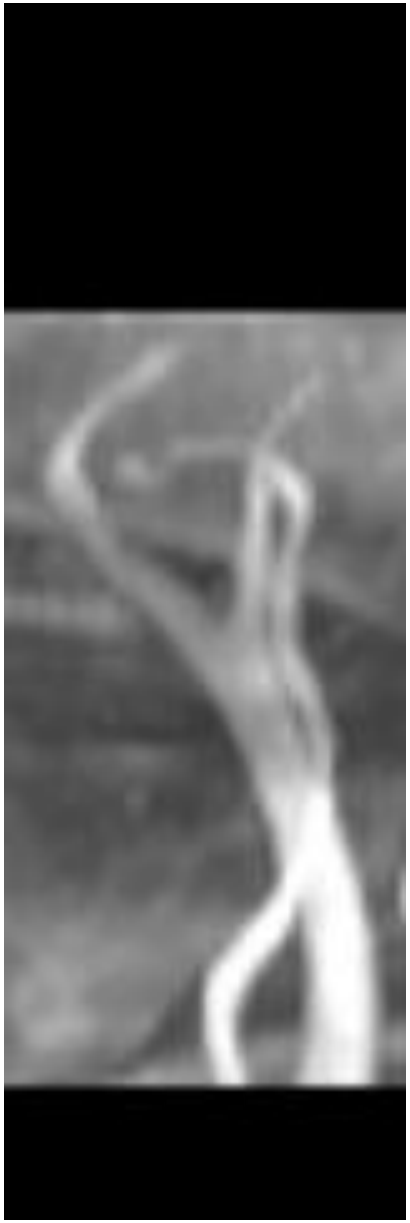

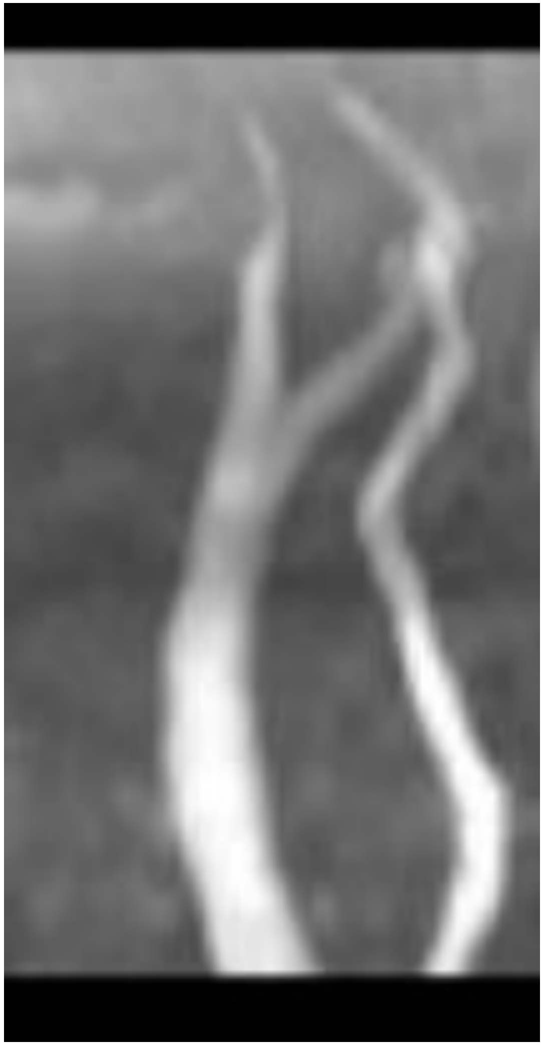

27b Score

0-30

31-50

51-70

>70

Near occlusion

Occluded

Quality

1

2

3

4

5

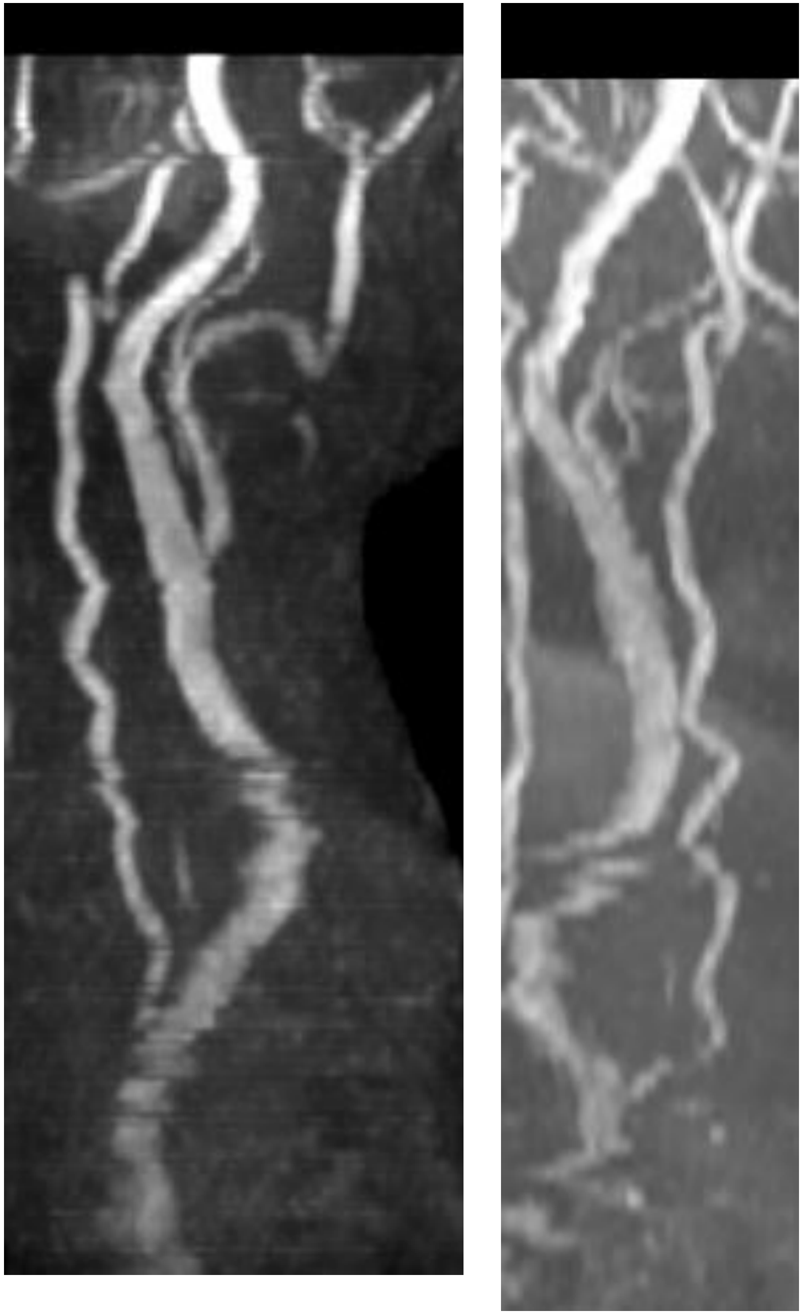

28a Score

0-30

31-50

51-70

>70

Near occlusion

Occluded

Quality

1

2

3

4

5

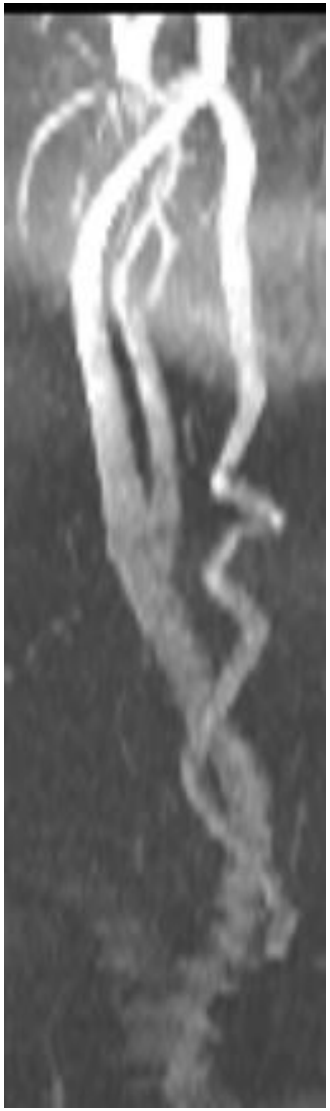

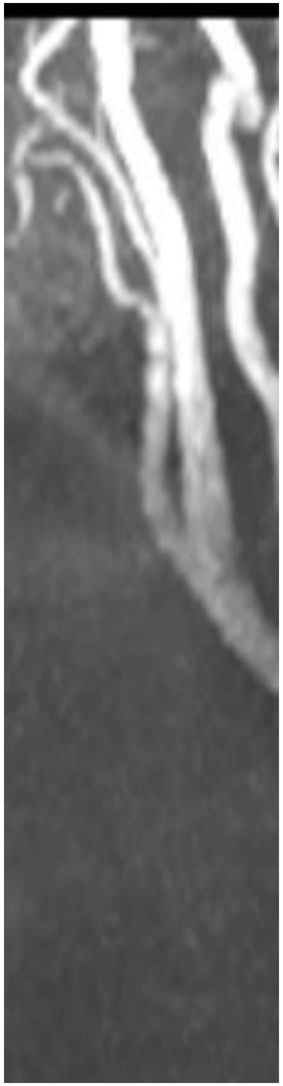

28f Score

0-30

31-50

51-70

>70

Near occlusion

Occluded

Quality

1

2

3

4

5

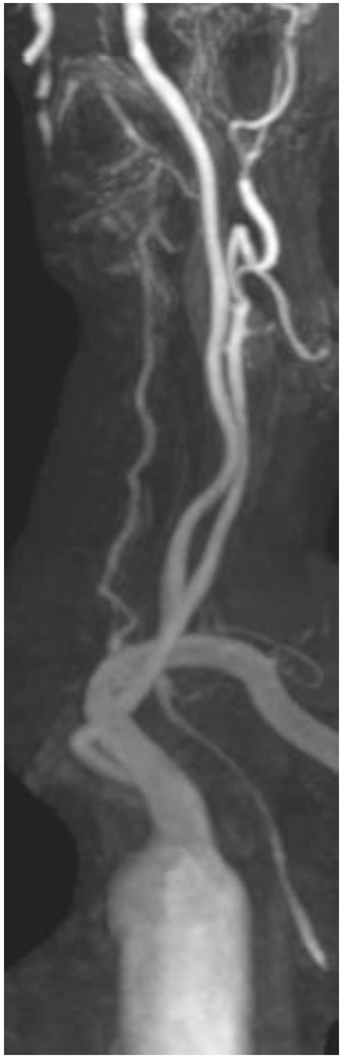

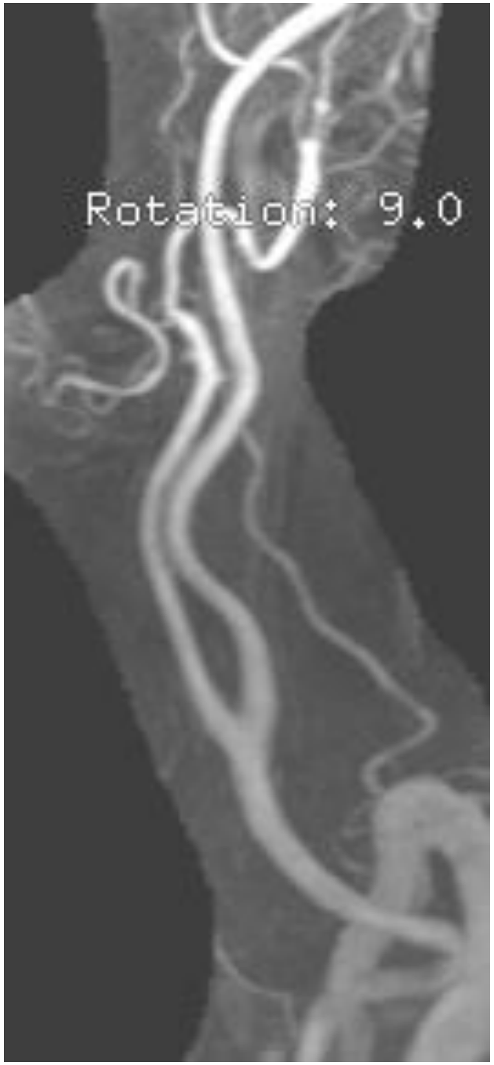

29e Score

0-30

31-50

51-70

>70

Near occlusion

Occluded

Quality

1

2

3

4

5

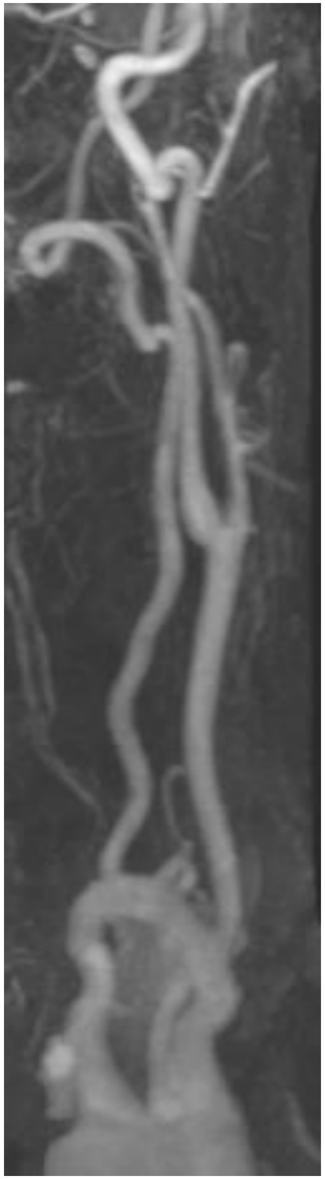

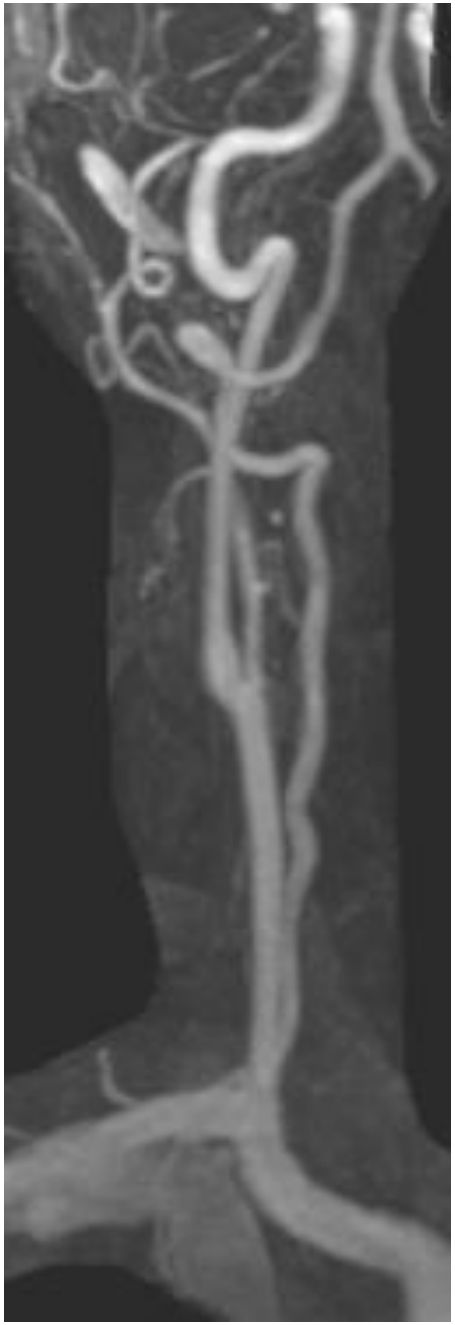

30d Score

0-30

31-50

51-70

>70

Near occlusion

Occluded

Quality

1

2

3

4

5

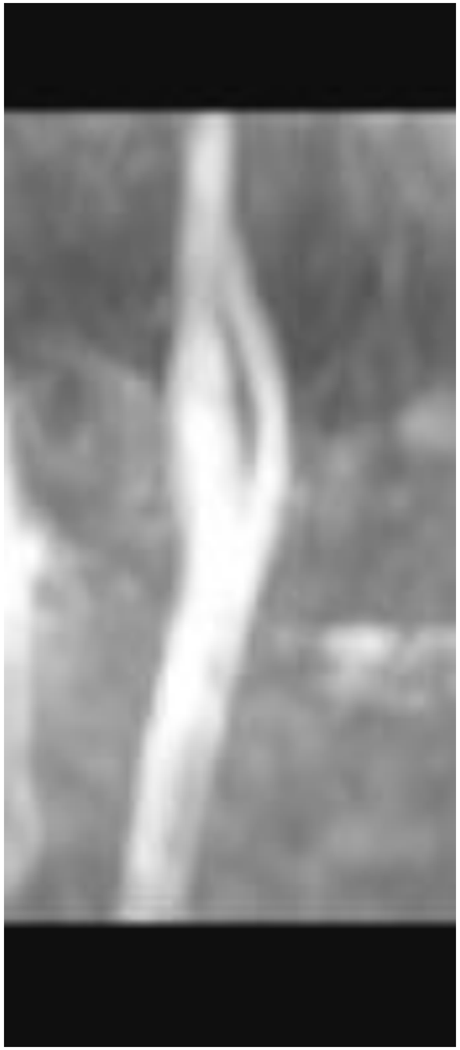

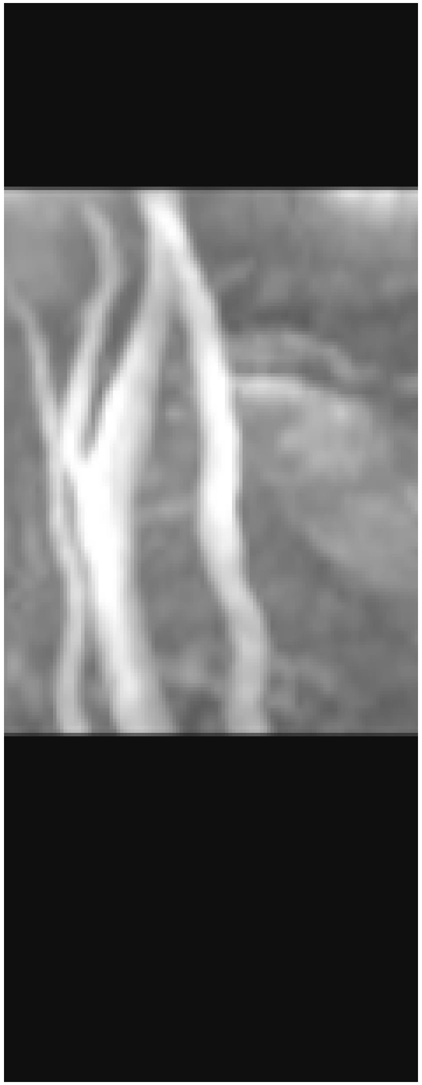

31c Score

0-30

31-50

51-70

>70

Near occlusion

Occluded

Quality

1

2

3

4

5

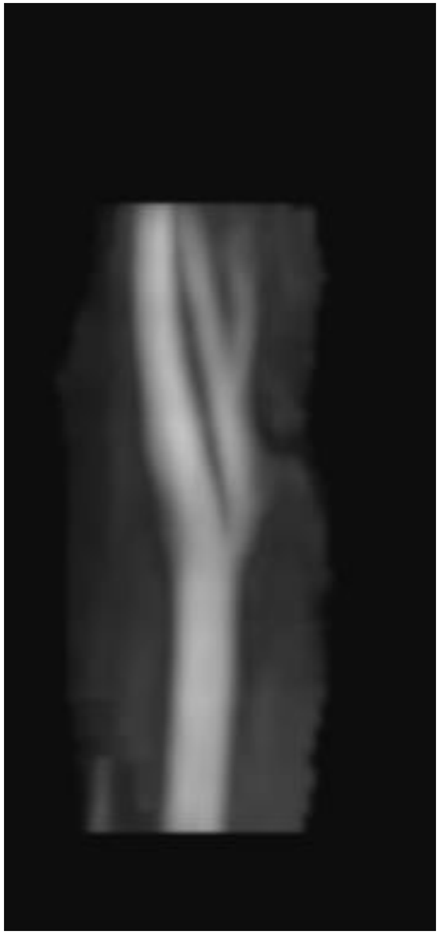

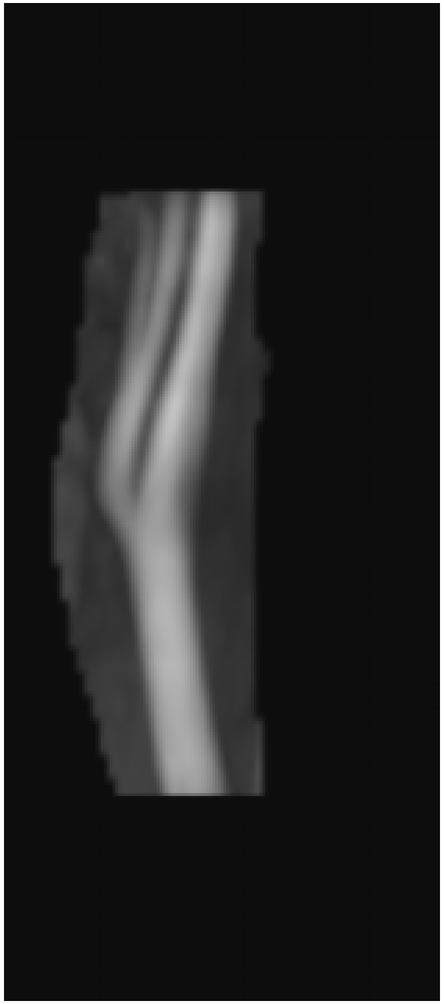

32b Score

0-30

31-50

51-70

>70

Near occlusion

Occluded

Quality

1

2

3

4

5

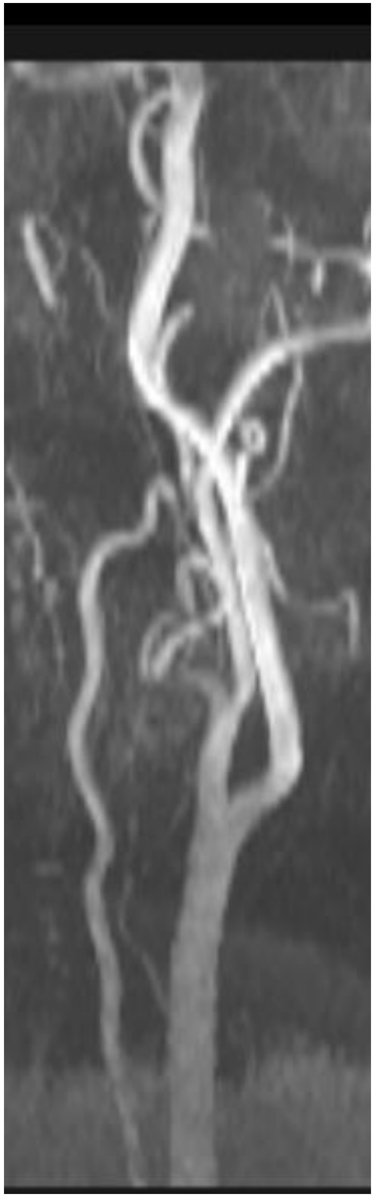

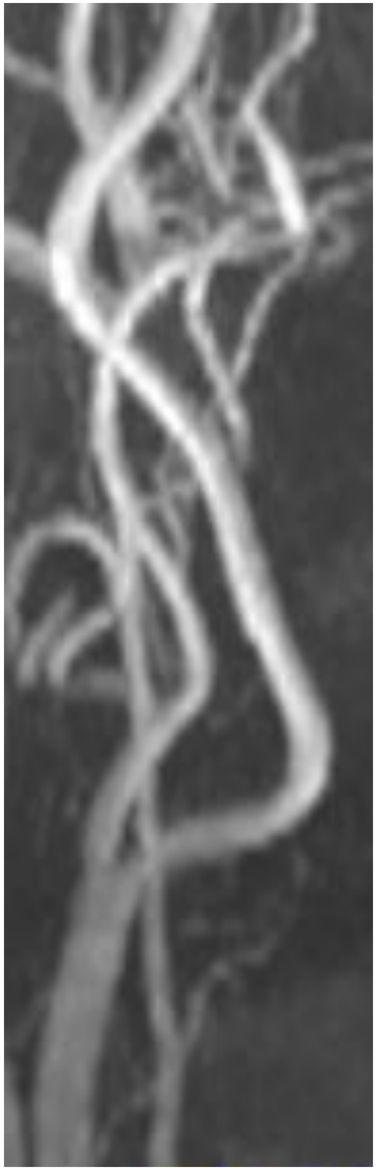

33a Score

0-30

31-50

51-70

>70

Near occlusion

Occluded

Quality

1

2

3

4

5

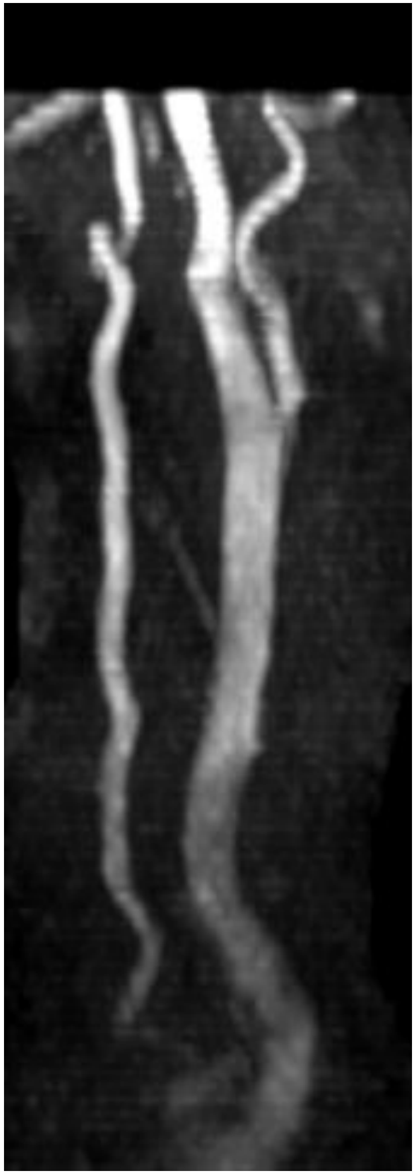

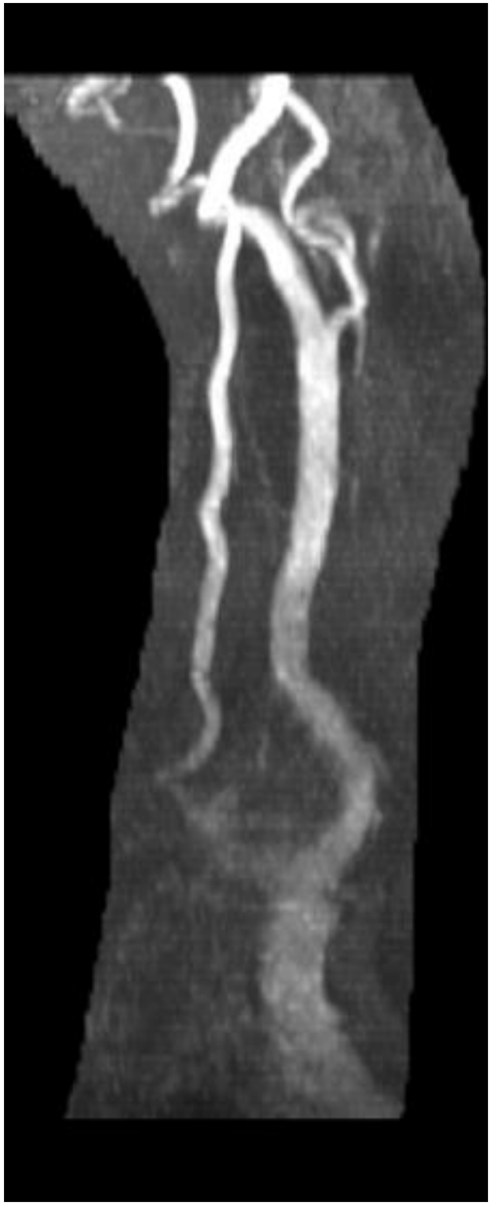

33f Score

0-30

31-50

51-70

>70

Near occlusion

Occluded

Quality

1

2

3

4

5

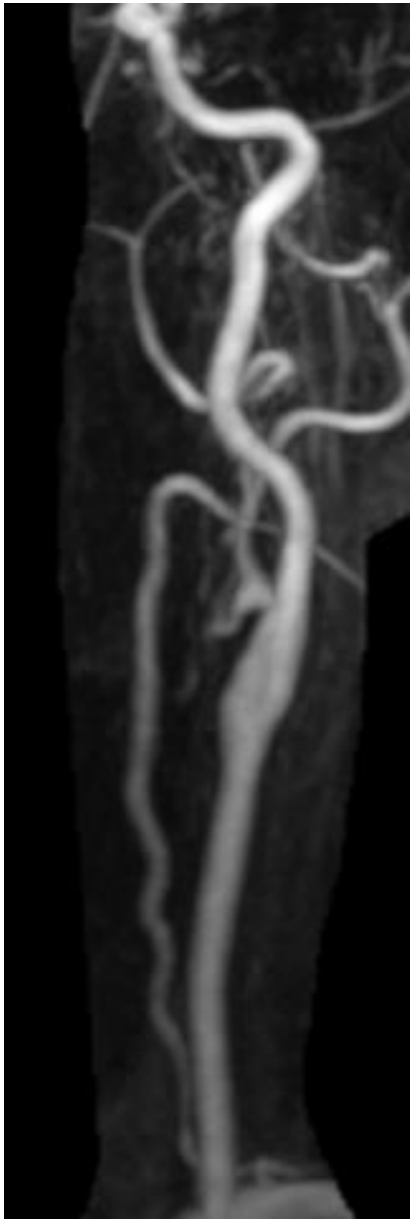

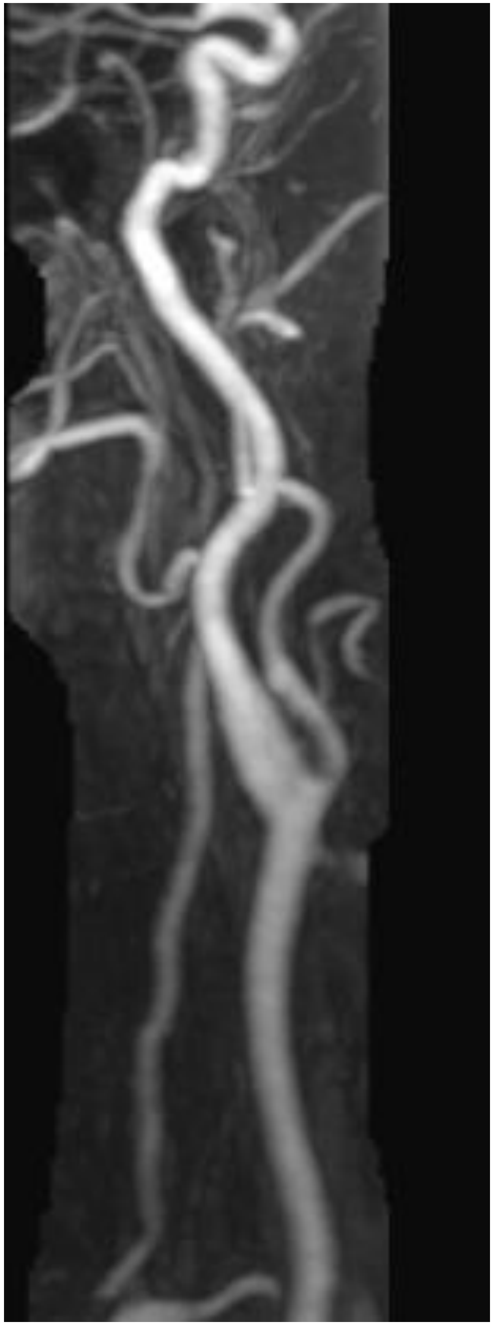

34e Score

0-30

31-50

51-70

>70

Near occlusion

Occluded

Quality

1

2

3

4

5

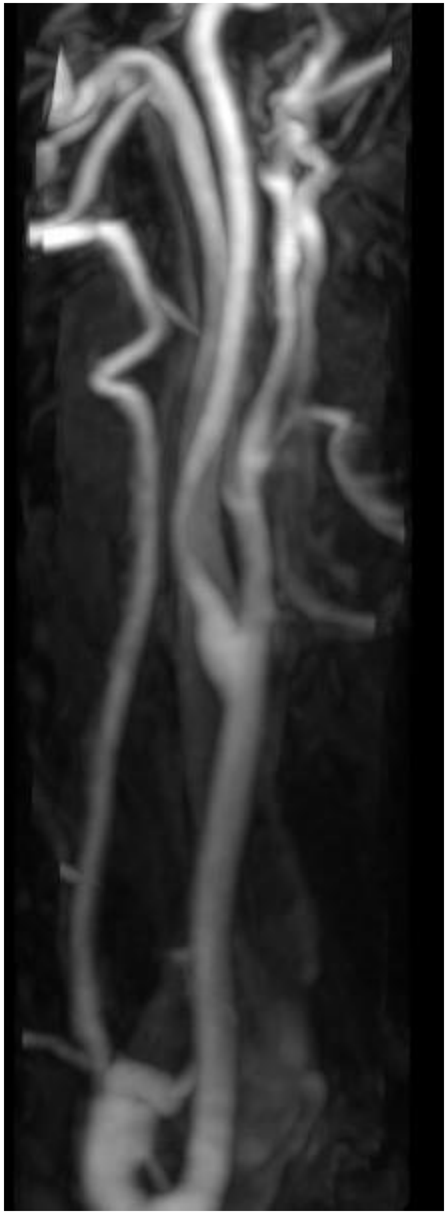

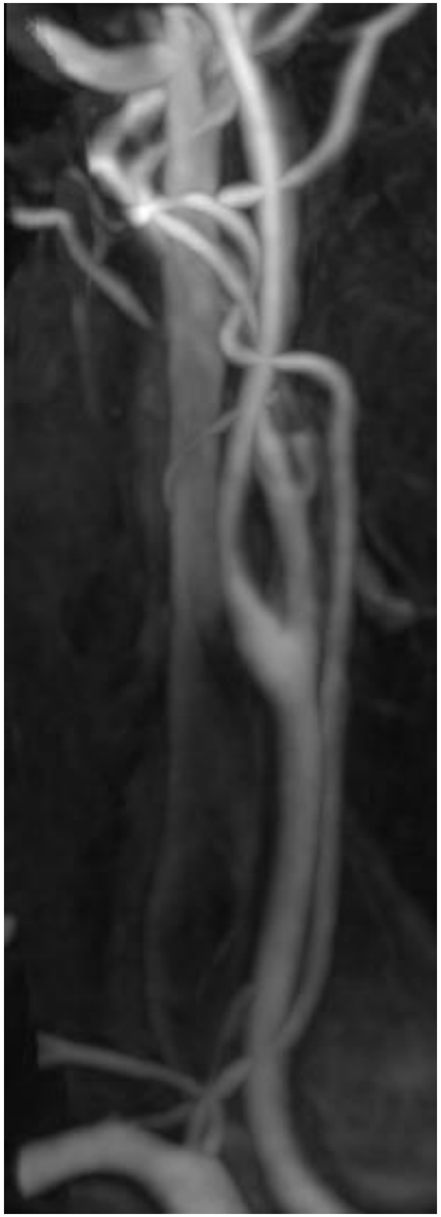

35d Score

0-30

31-50

51-70

>70

Near occlusion

Occluded

Quality

1

2

3

4

5

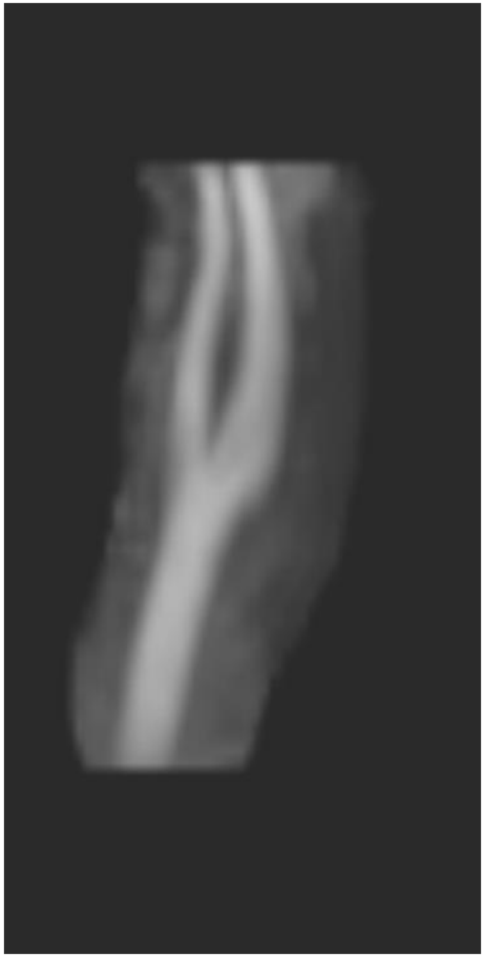

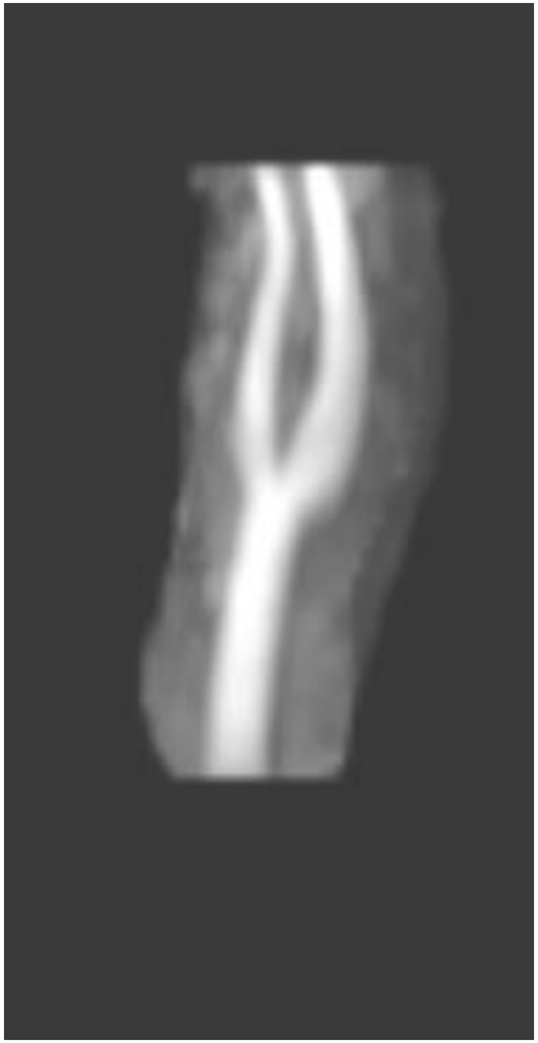

36c Score

0-30

31-50

51-70

>70

Near occlusion

Occluded

Quality

1

2

3

4

5

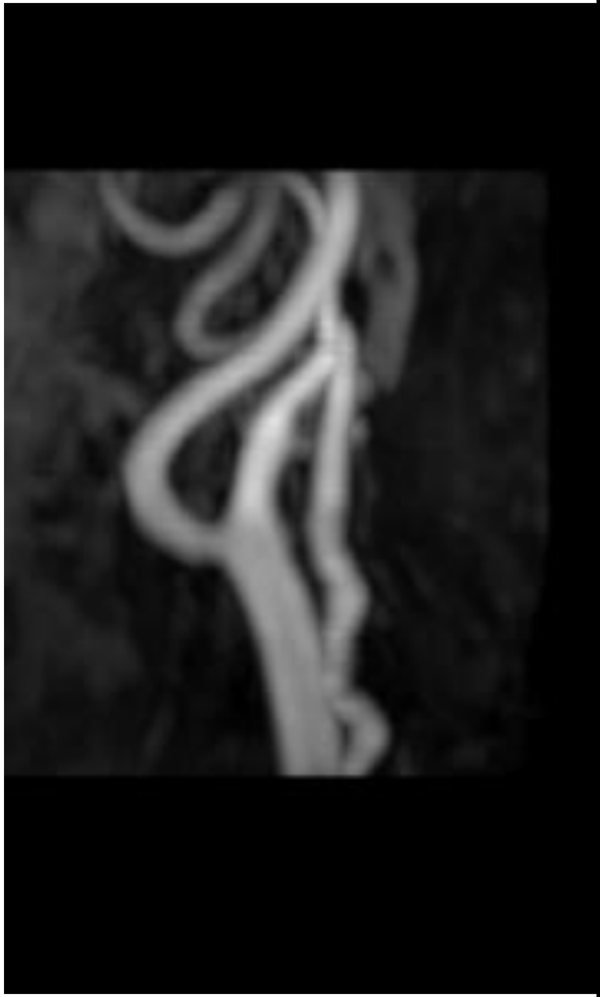

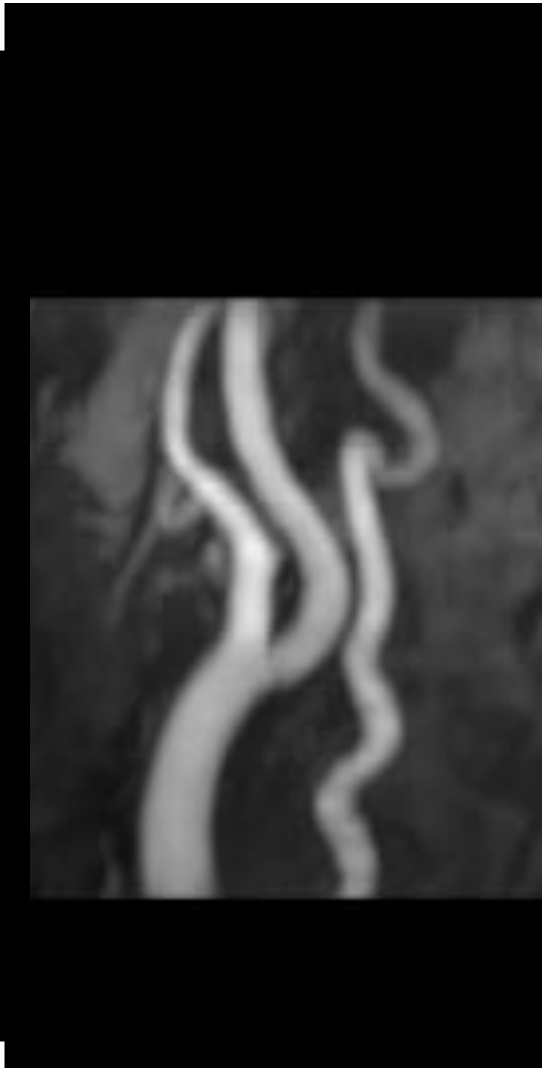

37b Score

0-30

31-50

51-70

>70

Near occlusion

Occluded

Quality

1

2

3

4

5

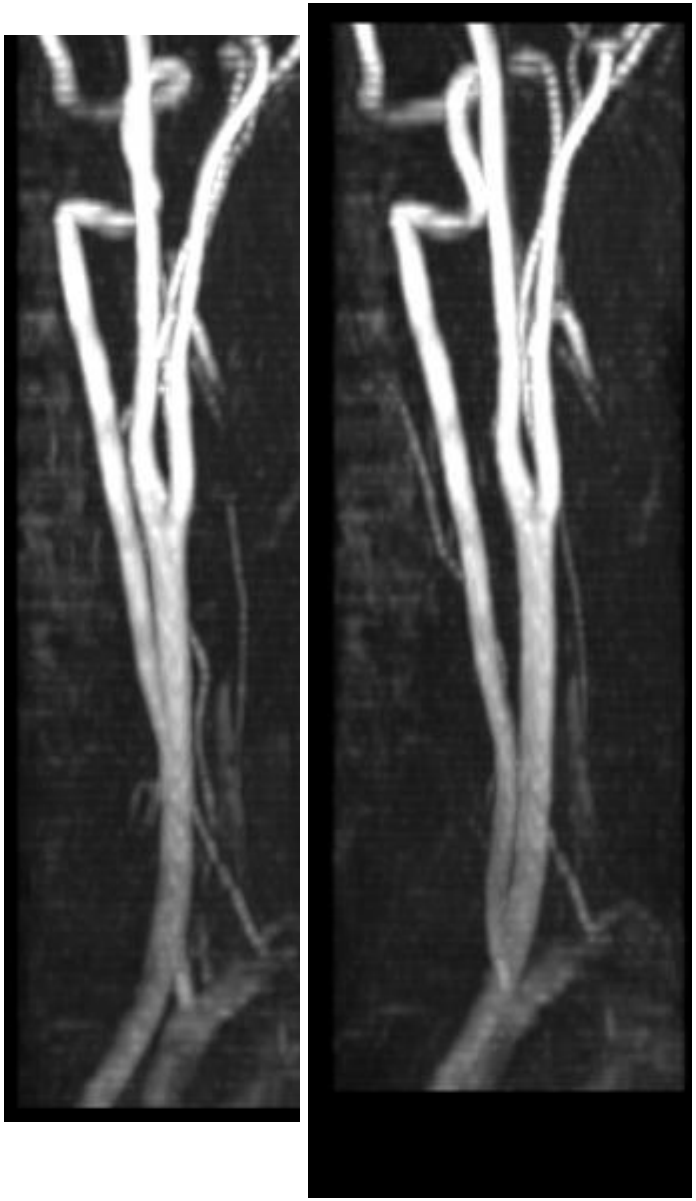

38a Score

0-30

31-50

51-70

>70

Near occlusion

Occluded

Quality

1

2

3

4

5

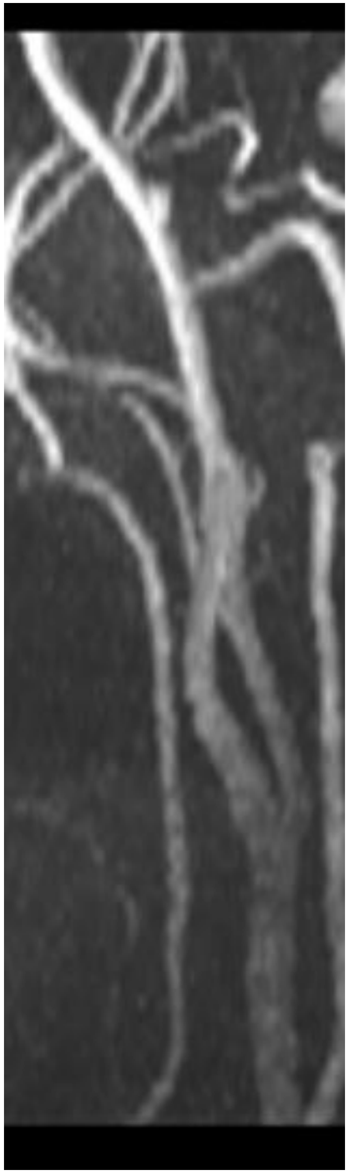

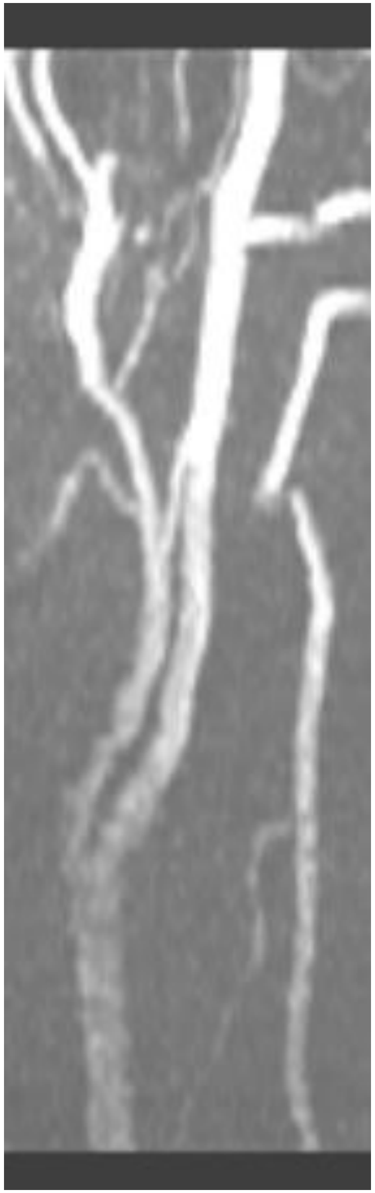

38f Score

0-30

31-50

51-70

>70

Near occlusion

Occluded

Quality

1

2

3

4

5

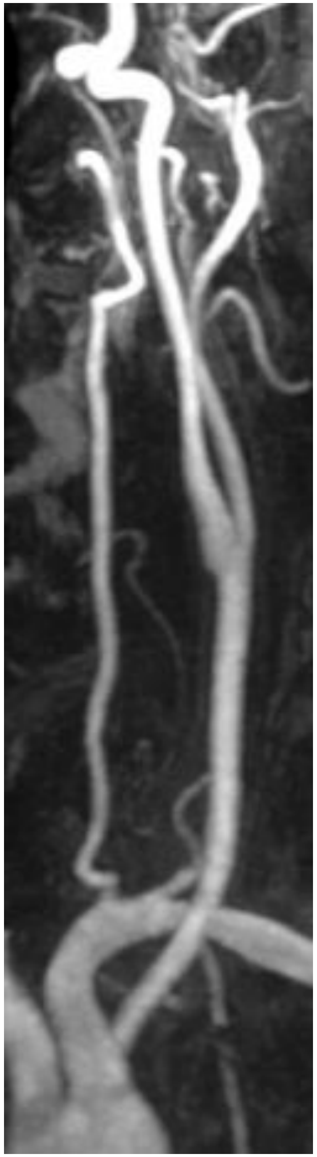

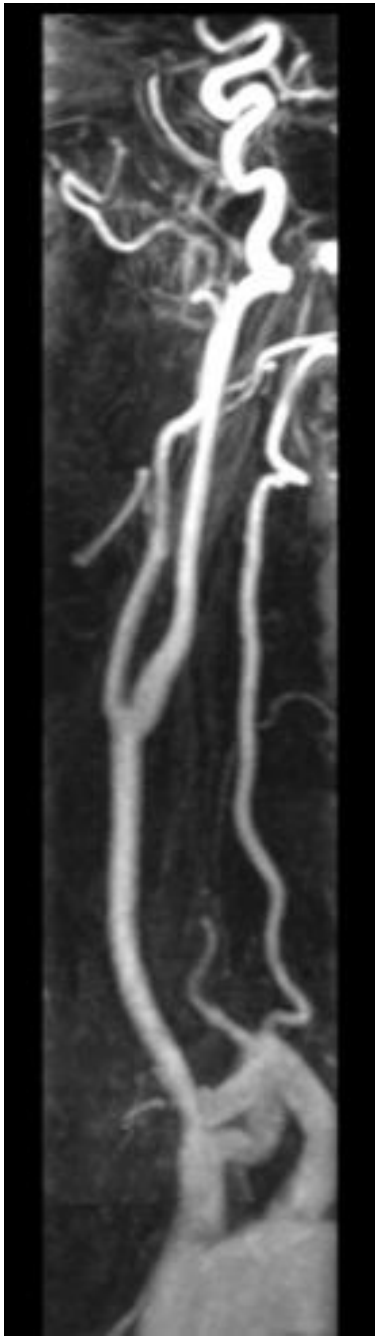

39e Score

0-30

31-50

51-70

>70

Near occlusion

Occluded

Quality

1

2

3

4

5

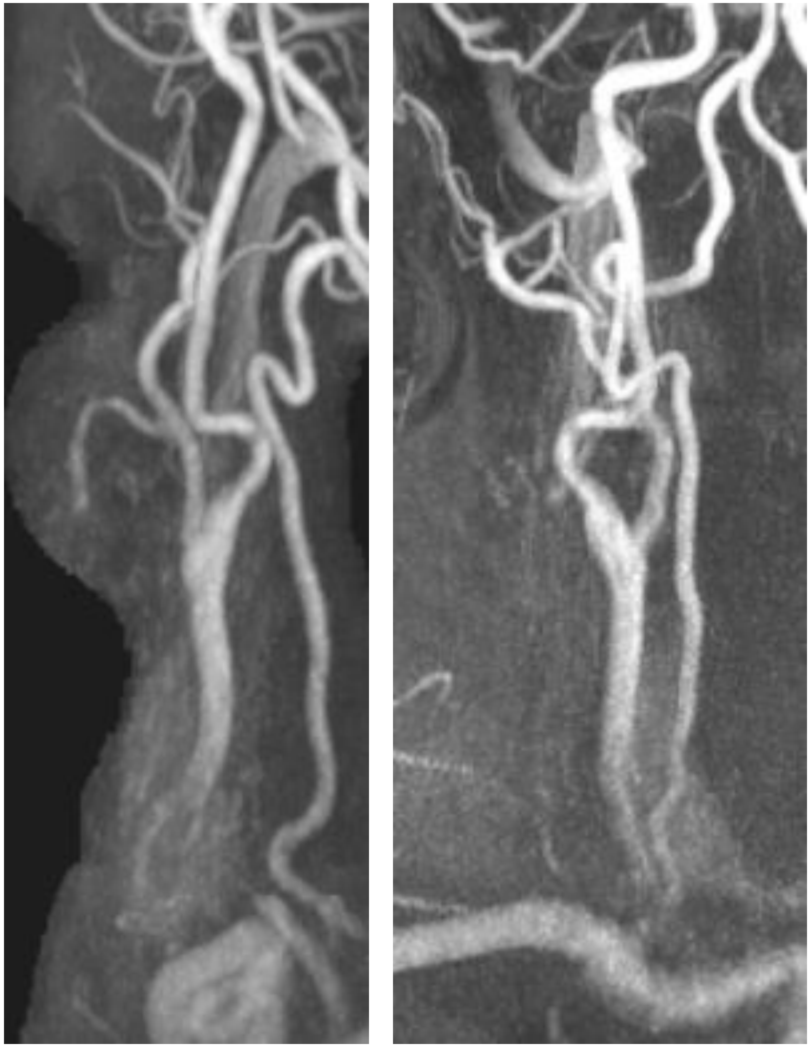

40d Score

0-30

31-50

51-70

>70

Near occlusion

Occluded

Quality

1

2

3

4

5

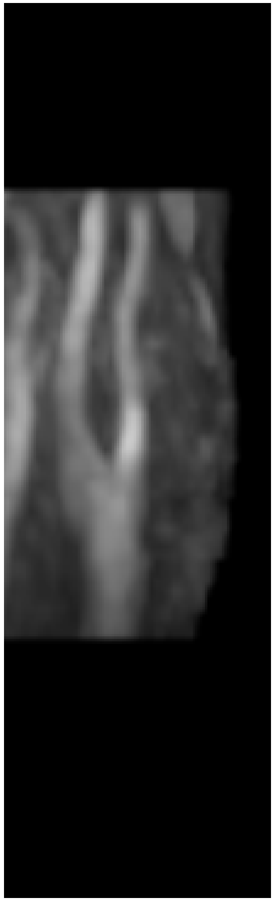

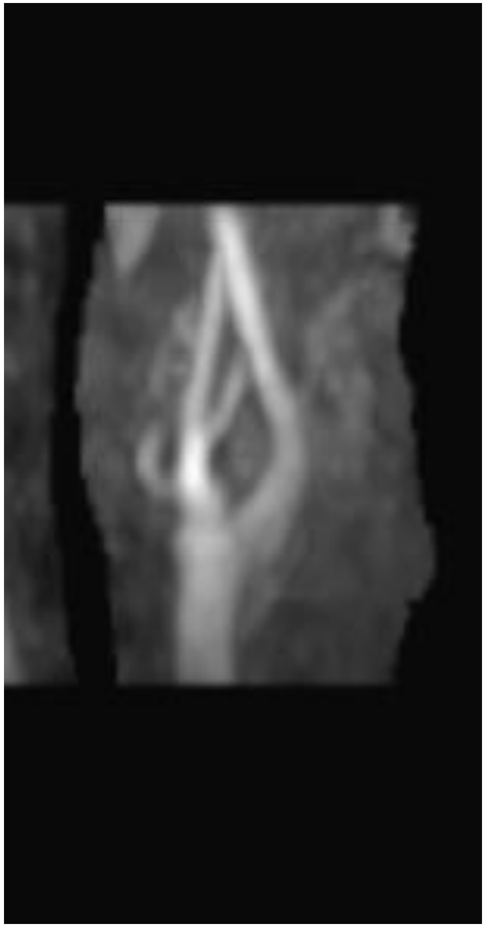

41c Score

0-30

31-50

51-70

>70

Near occlusion

Occluded

Quality

1

2

3

4

5

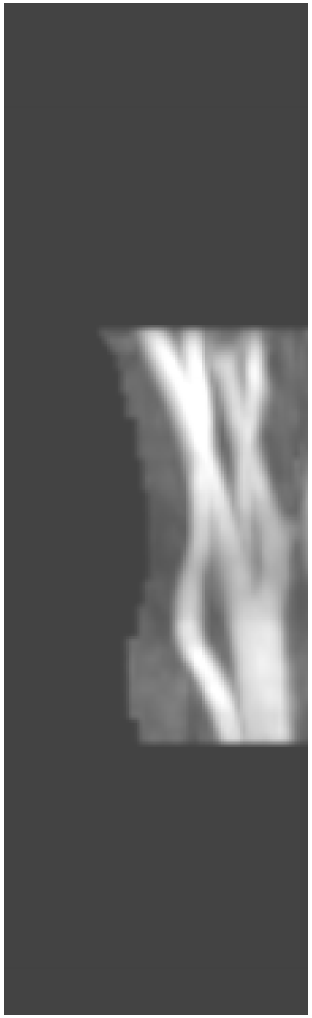

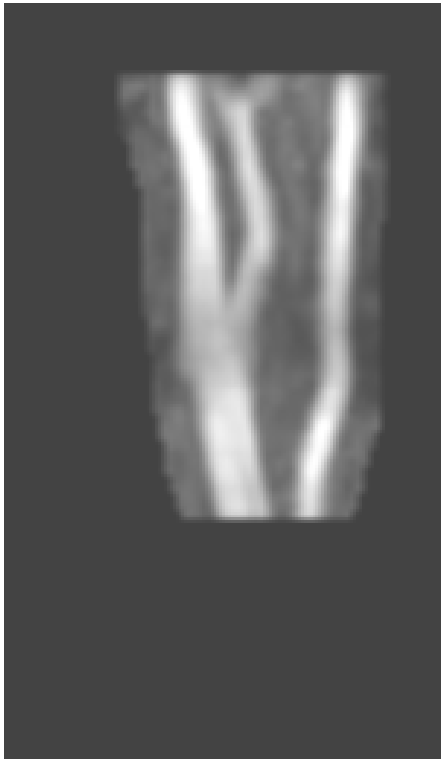

42b Score

0-30

31-50

51-70

>70

Near occlusion

Occluded

Quality

1

2

3

4

5

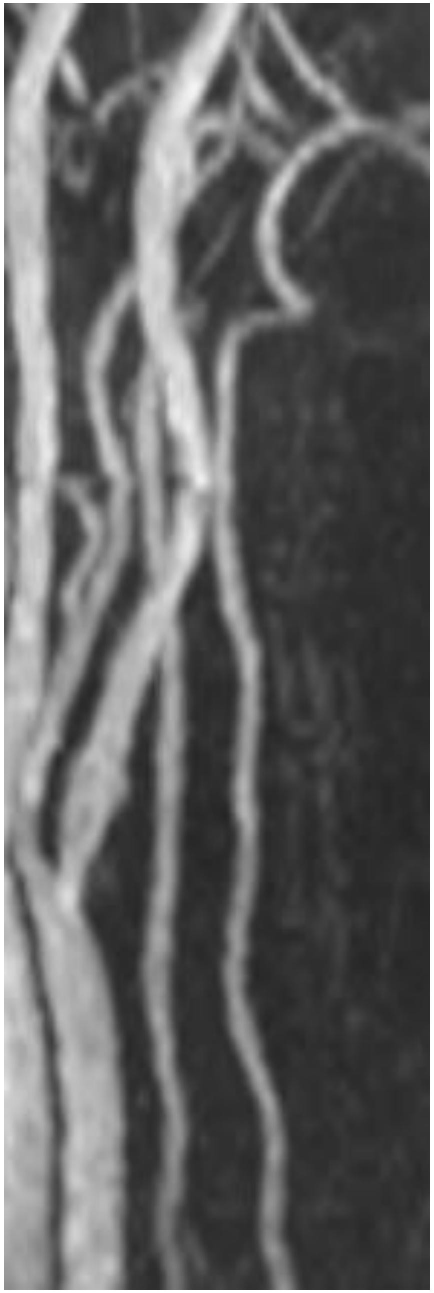

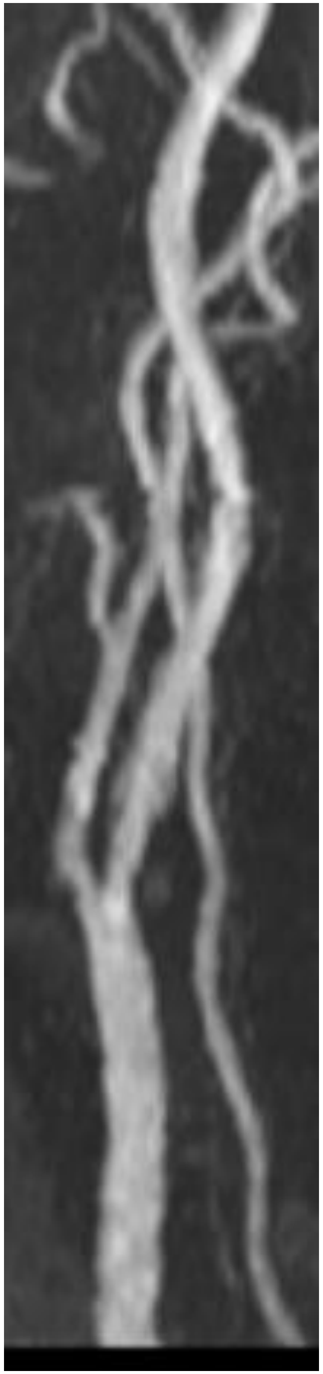

43a Score

0-30

31-50

51-70

>70

Near occlusion

Occluded

Quality

1

2

3

4

5

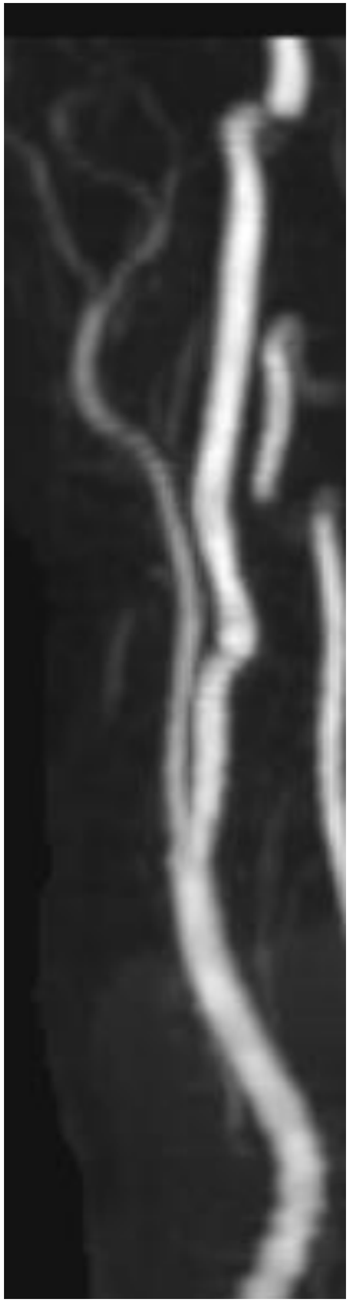

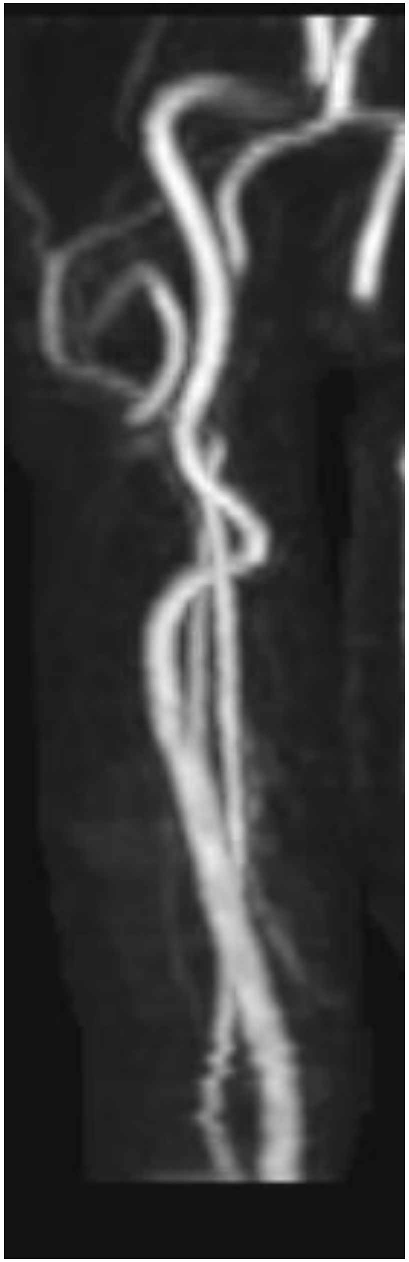

43f Score

0-30

31-50

51-70

>70

Near occlusion

Occluded

Quality

1

2

3

4

5

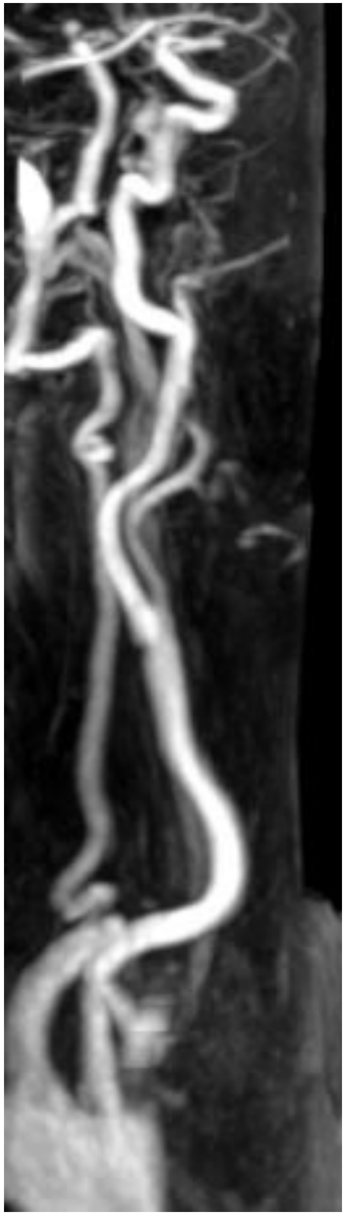

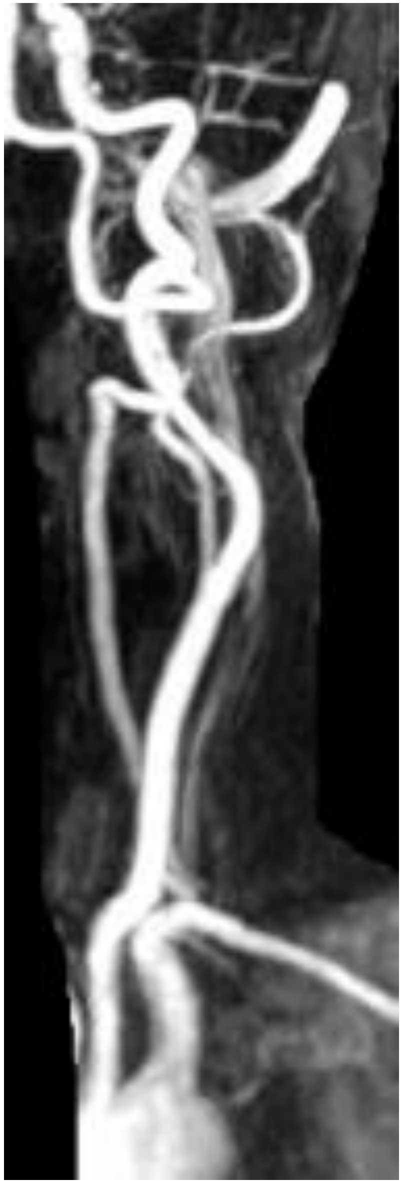

44e Score

0-30

31-50

51-70

>70

Near occlusion

Occluded

Quality

1

2

3

4

5

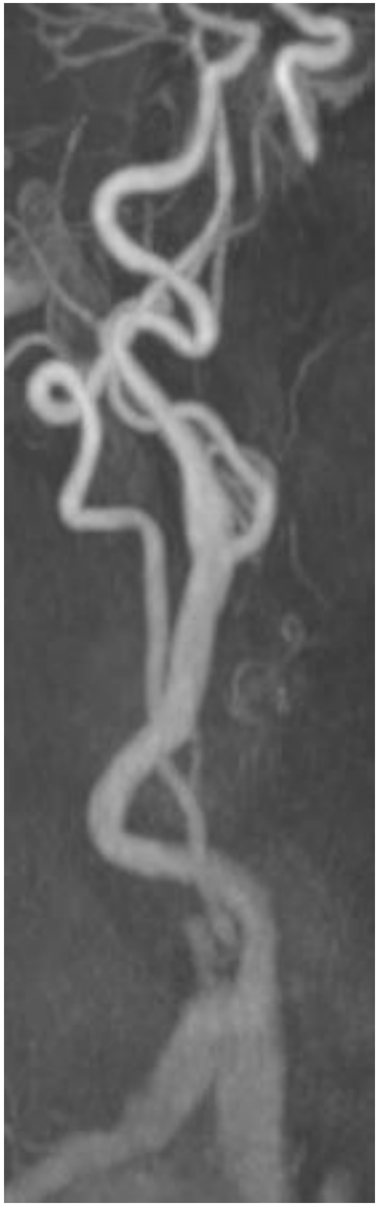

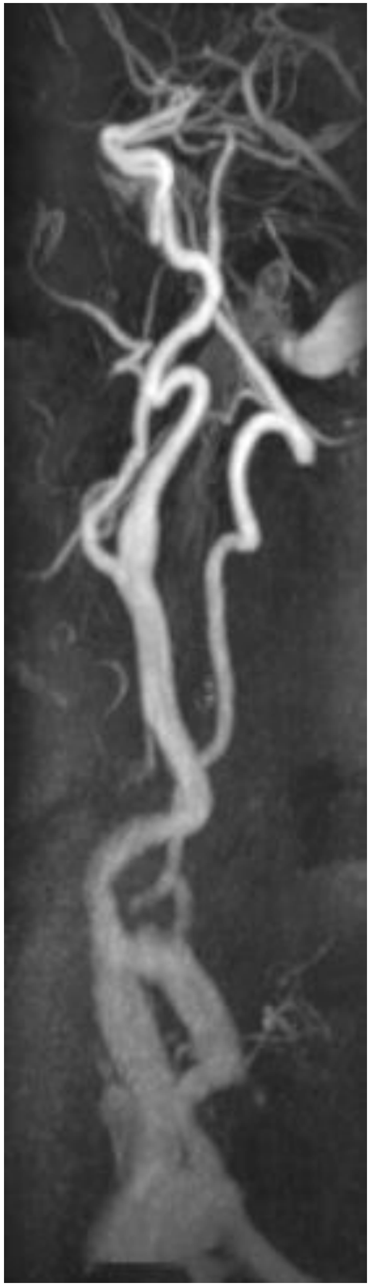

45d Score

0-30

31-50

51-70

>70

Near occlusion

Occluded

Quality

1

2

3

4

5

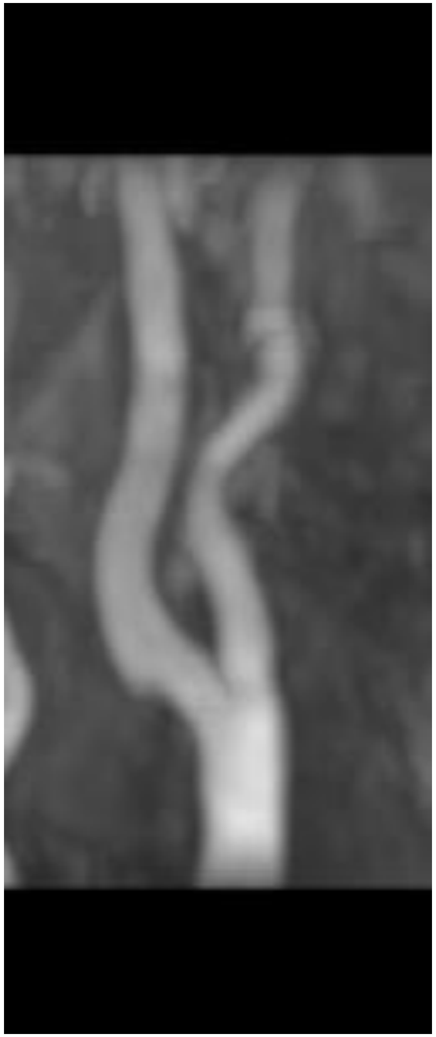

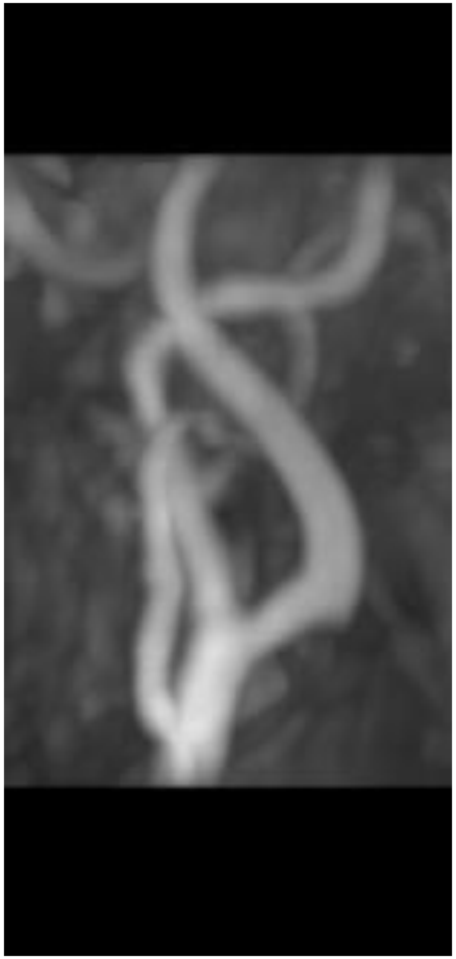

46c Score

0-30

31-50

51-70

>70

Near occlusion

Occluded

Quality

1

2

3

4

5

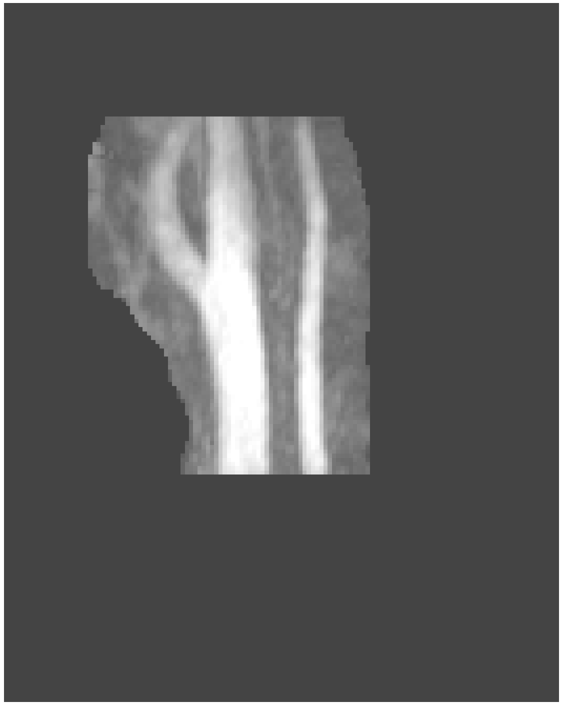

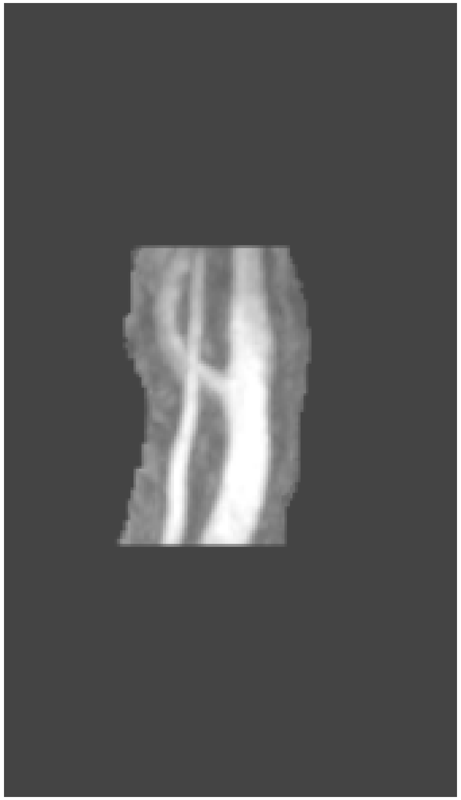

47b Score

0-30

31-50

51-70

>70

Near occlusion

Occluded

Quality

1

2

3

4

5

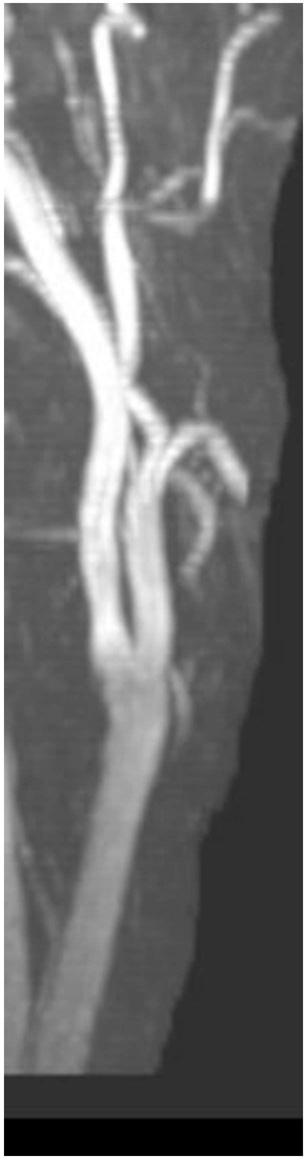

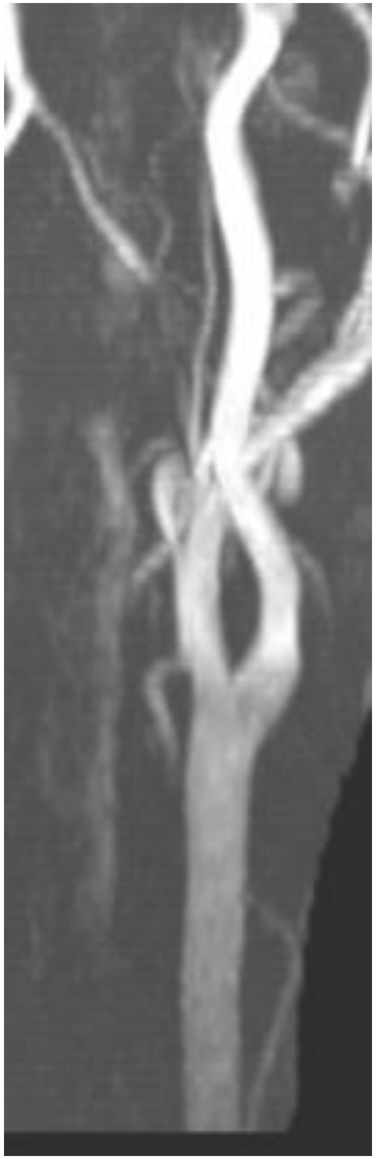

48a Score

0-30

31-50

51-70

>70

Near occlusion

Occluded

Quality

1

2

3

4

5

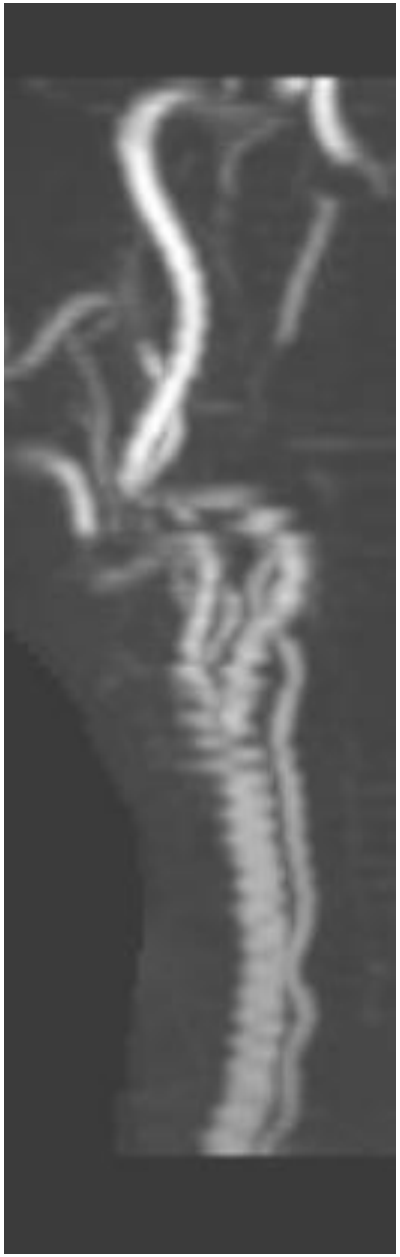

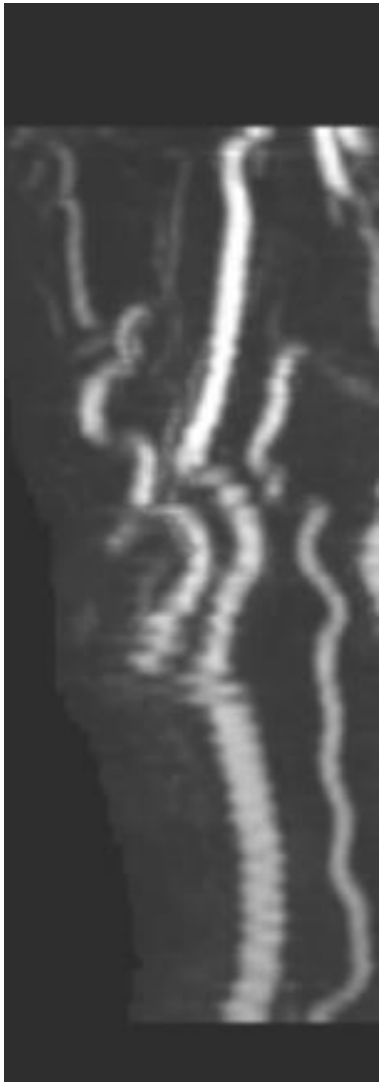

48f Score

0-30

31-50

51-70

>70

Near occlusion

Occluded

Quality

1

2

3

4

5

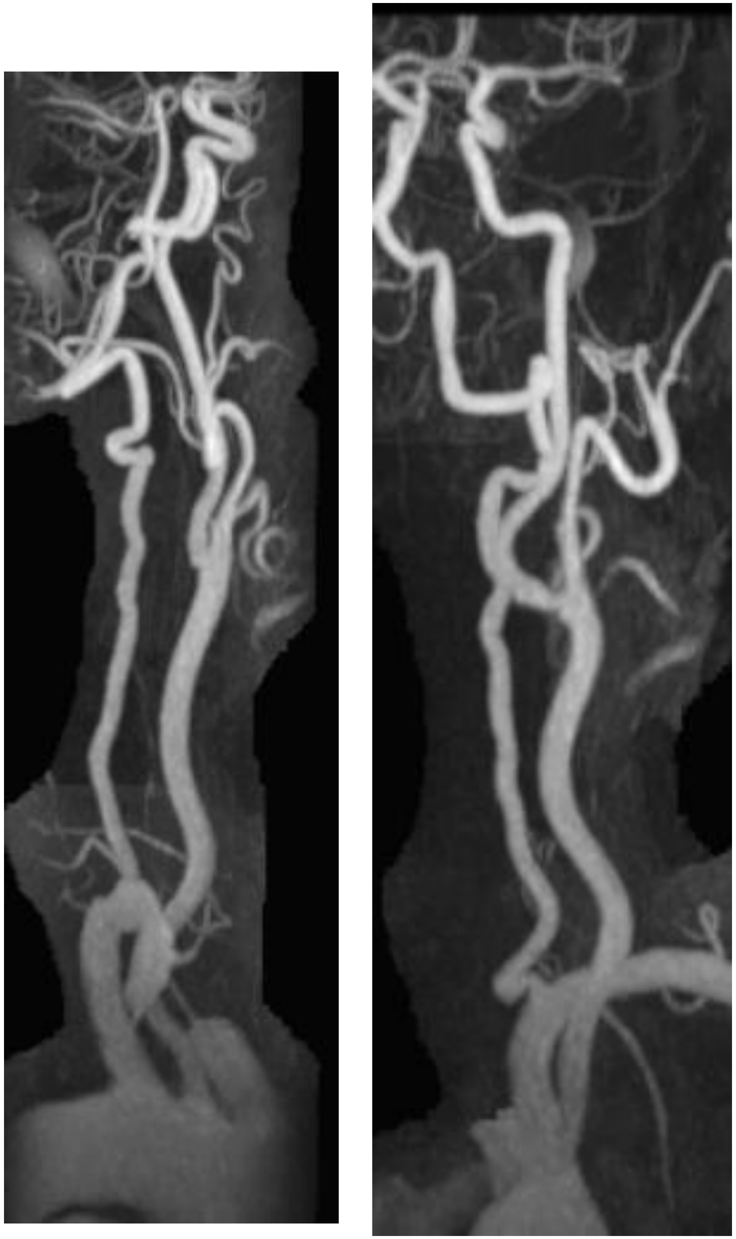

49e Score

0-30

31-50

51-70

>70

Near occlusion

Occluded

Quality

1

2

3

4

5

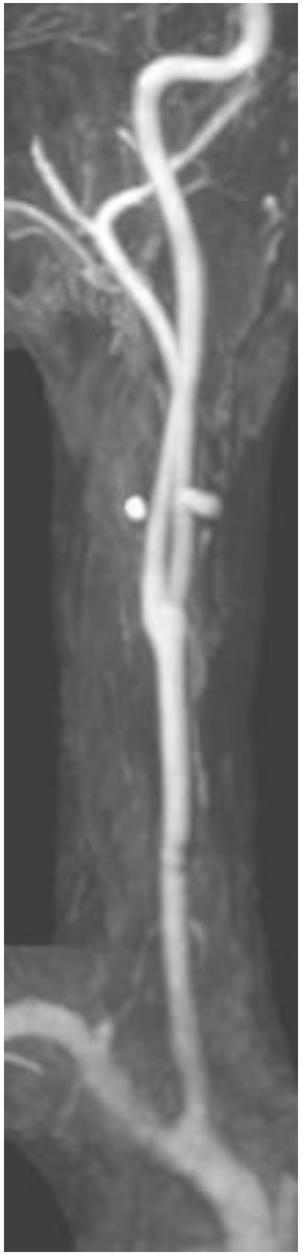

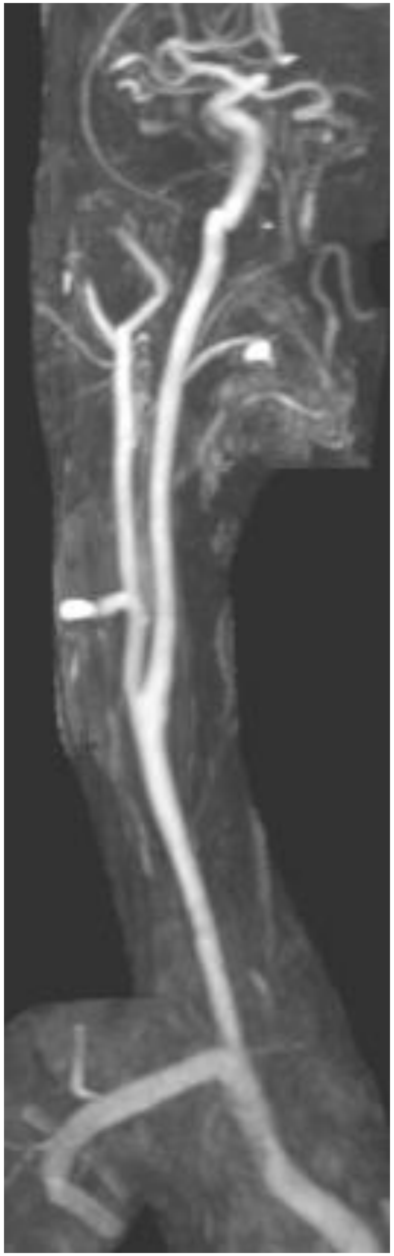

50d Score

0-30

31-50

51-70

>70

Near occlusion

Occluded

Quality

1

2

3

4

5

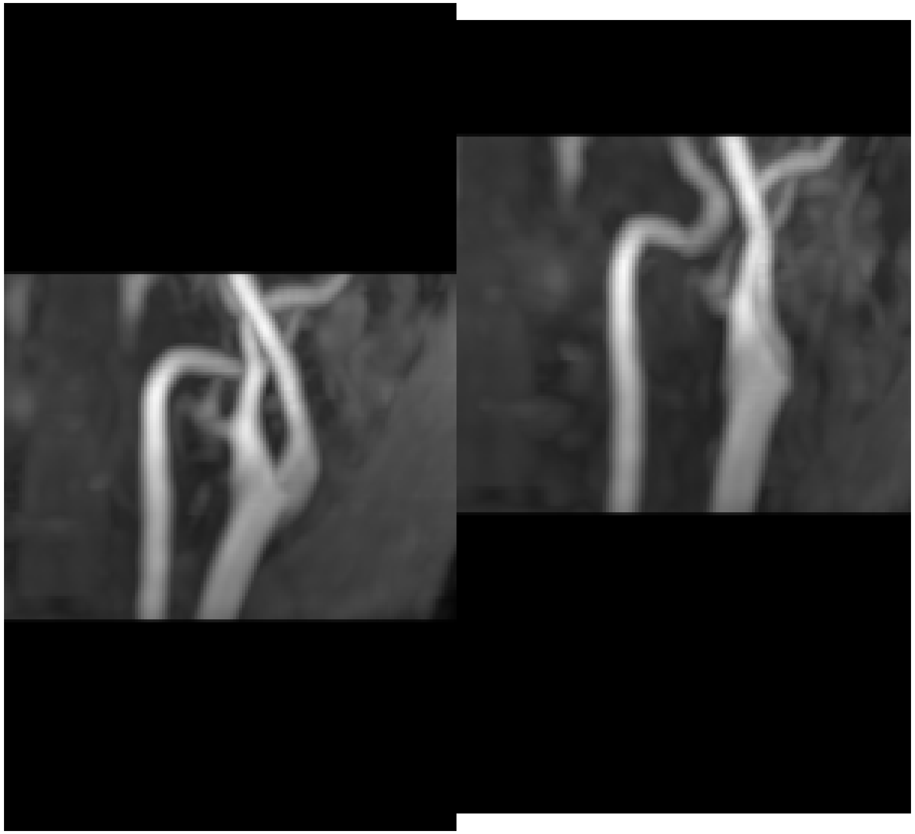

51c Score

0-30

31-50

51-70

>70

Near occlusion

Occluded

Quality

1

2

3

4

5

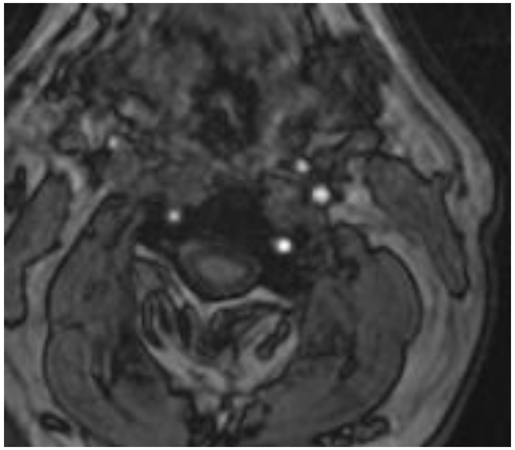

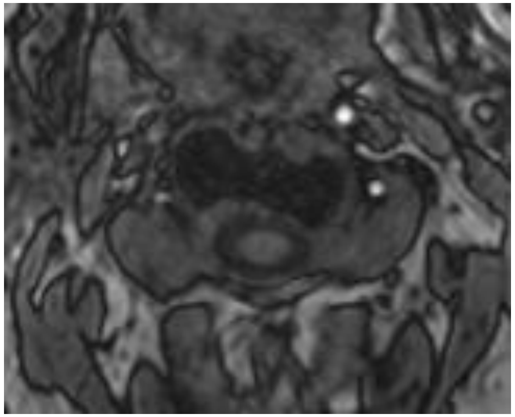

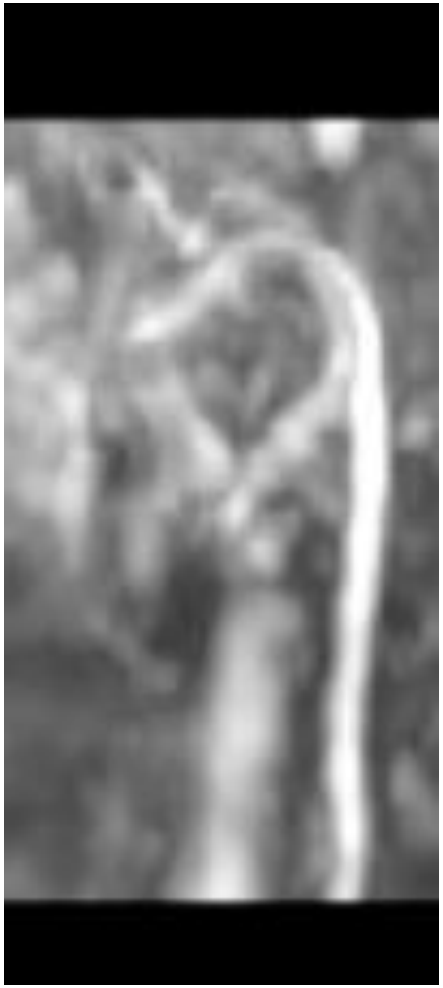

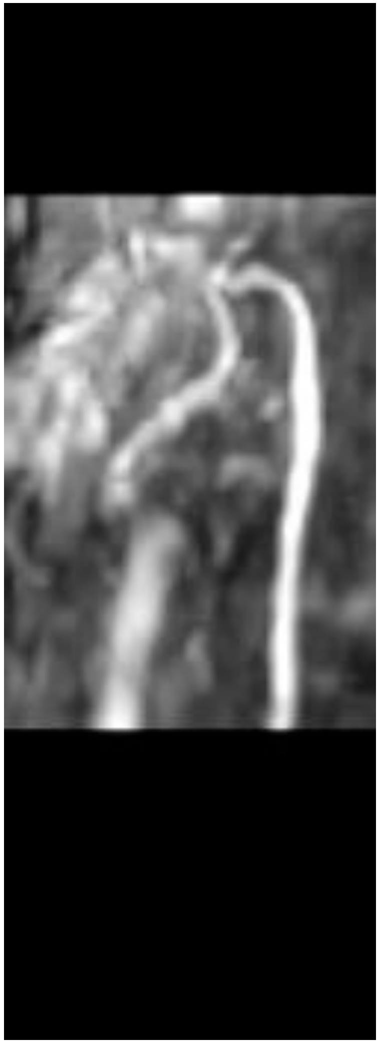

52b Score

0-30

31-50

51-70

>70

Near occlusion

Occluded

Quality

1

2

3

4

5

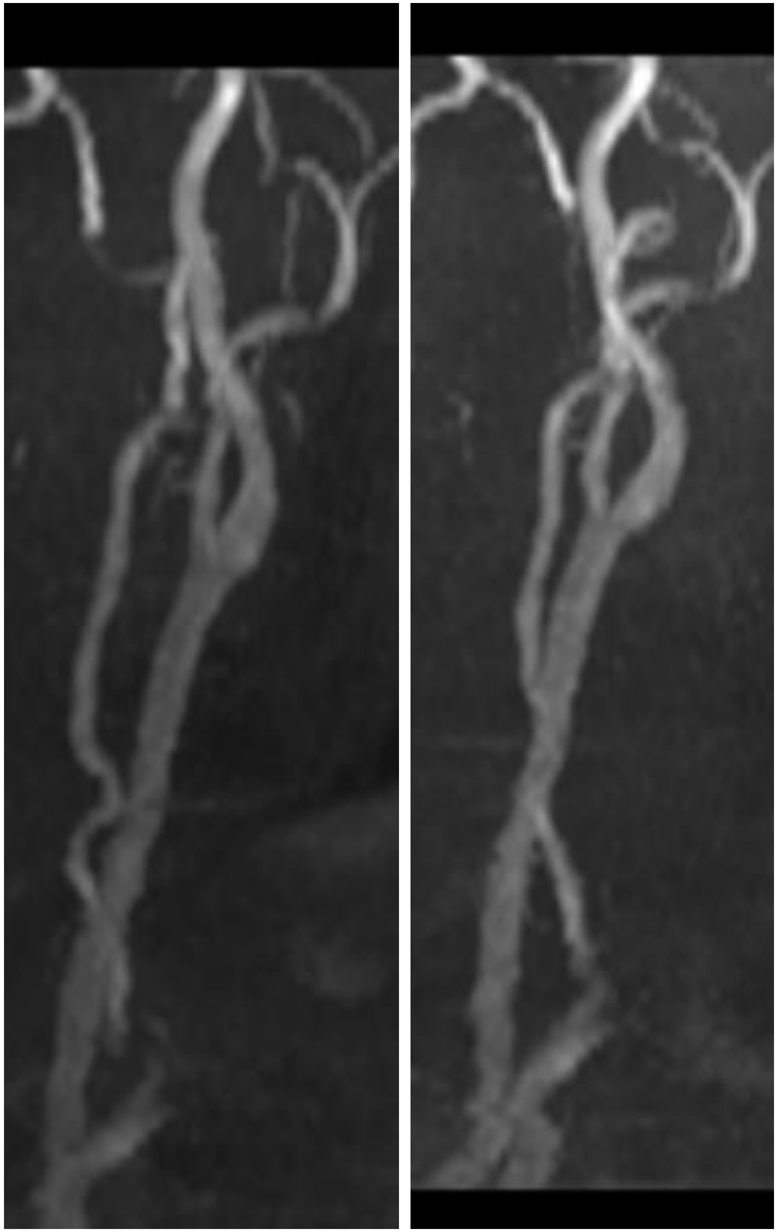

53a Score

0-30

31-50

51-70

>70

Near occlusion

Occluded

Quality

1

2

3

4

5

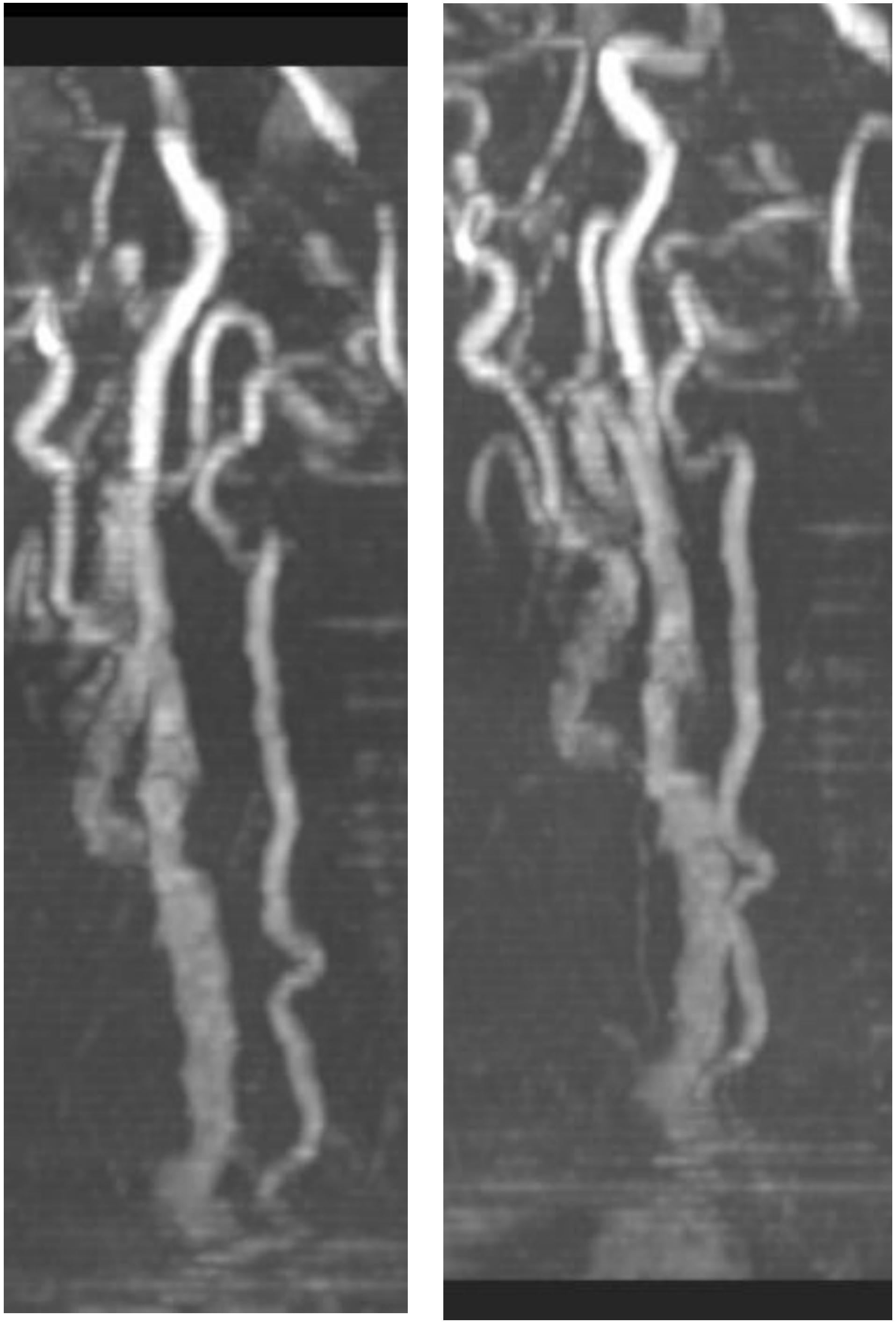

53f Score

0-30

31-50

51-70

>70

Near occlusion

Occluded

Quality

1

2

3

4

5

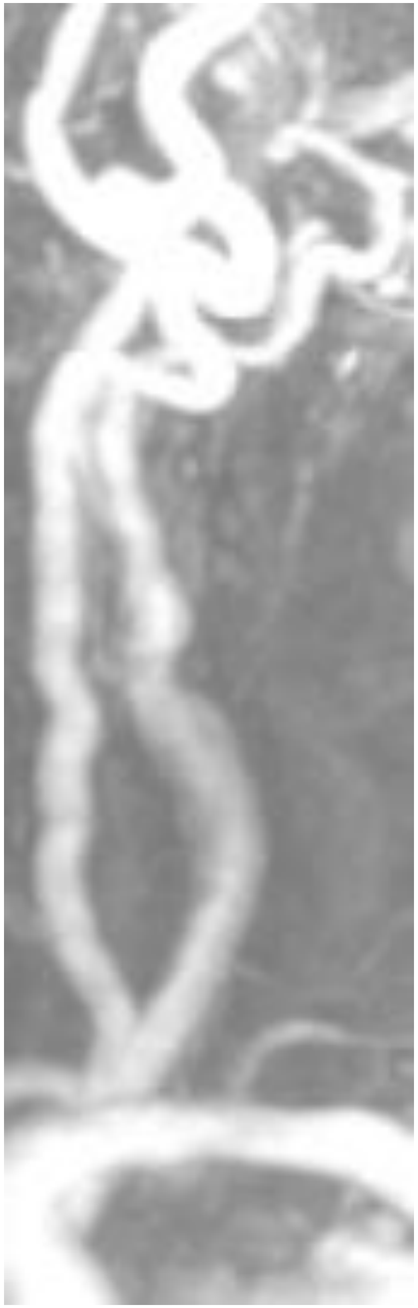

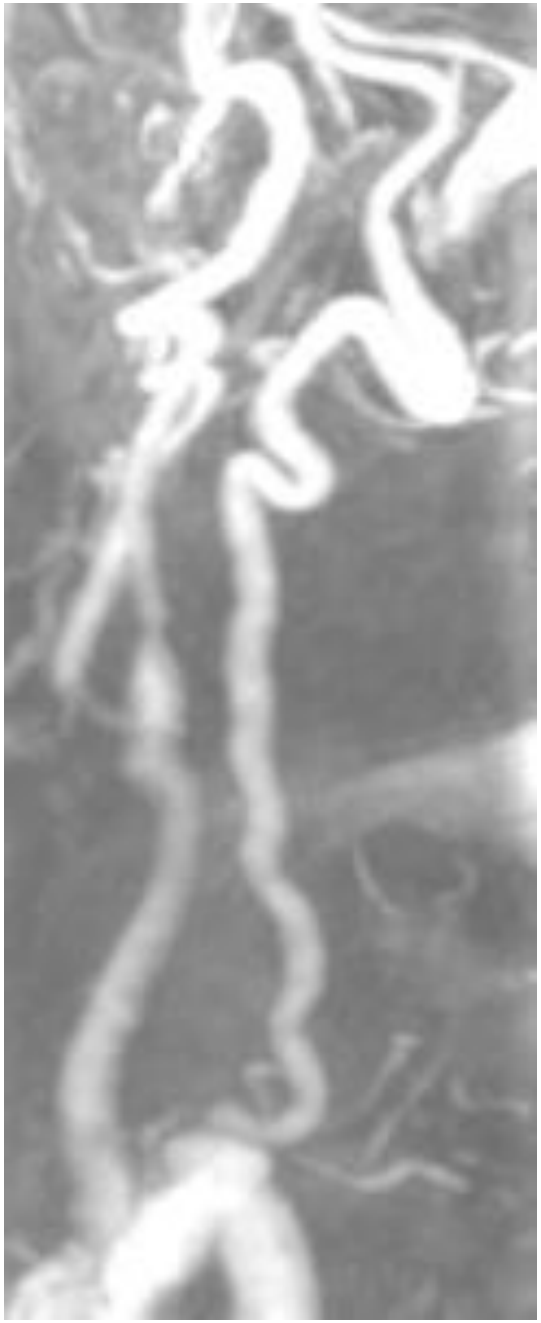

54e Score

0-30

31-50

51-70

>70

Near occlusion

Occluded

Quality

1

2

3

4

5

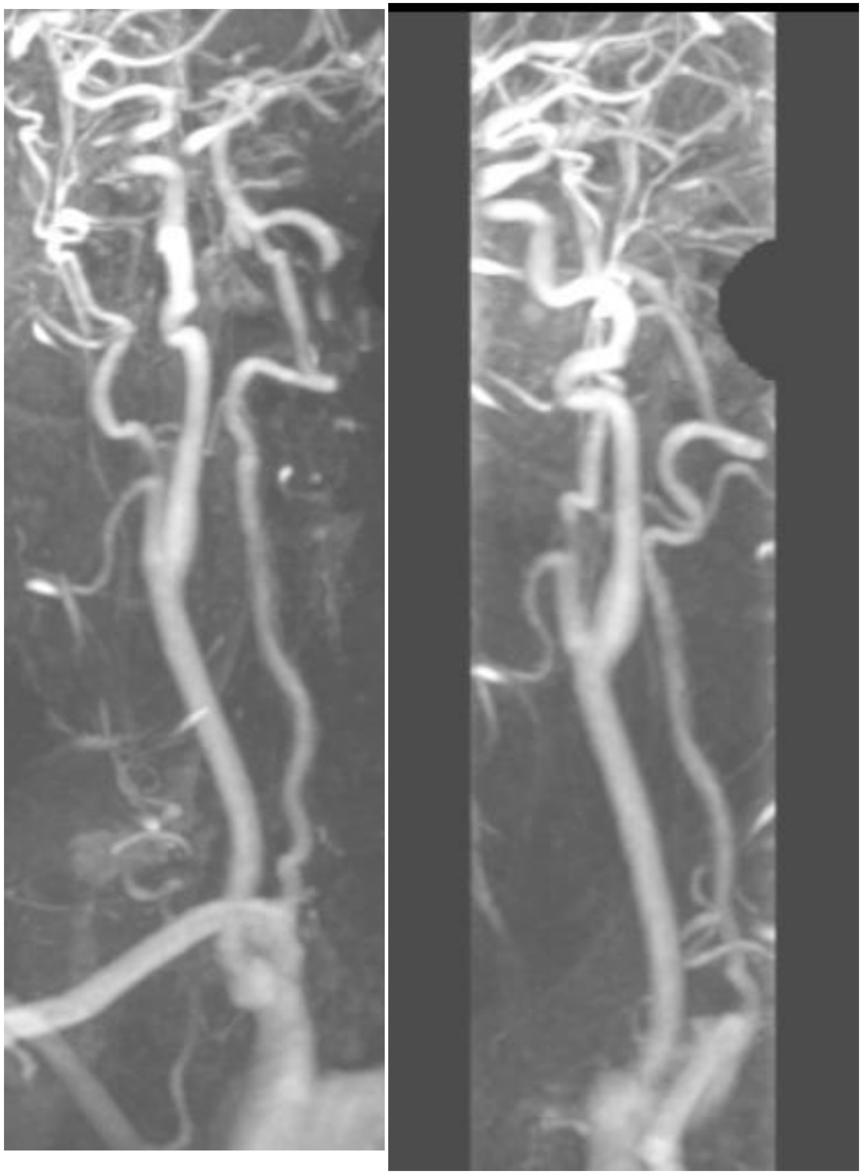

55d Score

0-30

31-50

51-70

>70

Near occlusion

Occluded

Quality

1

2

3

4

5

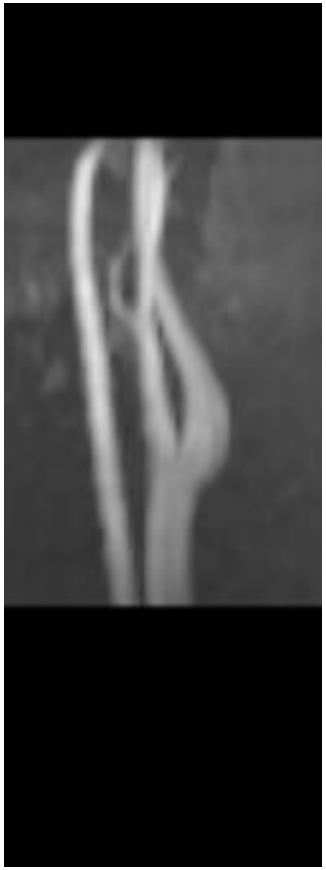

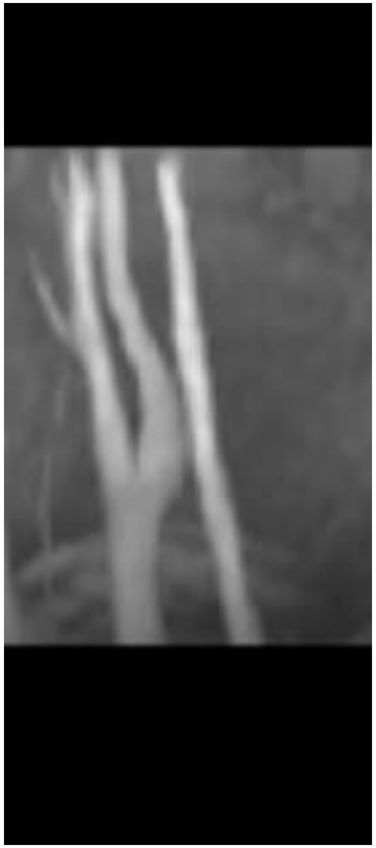

56c Score

0-30

31-50

51-70

>70

Near occlusion

Occluded

Quality

1

2

3

4

5

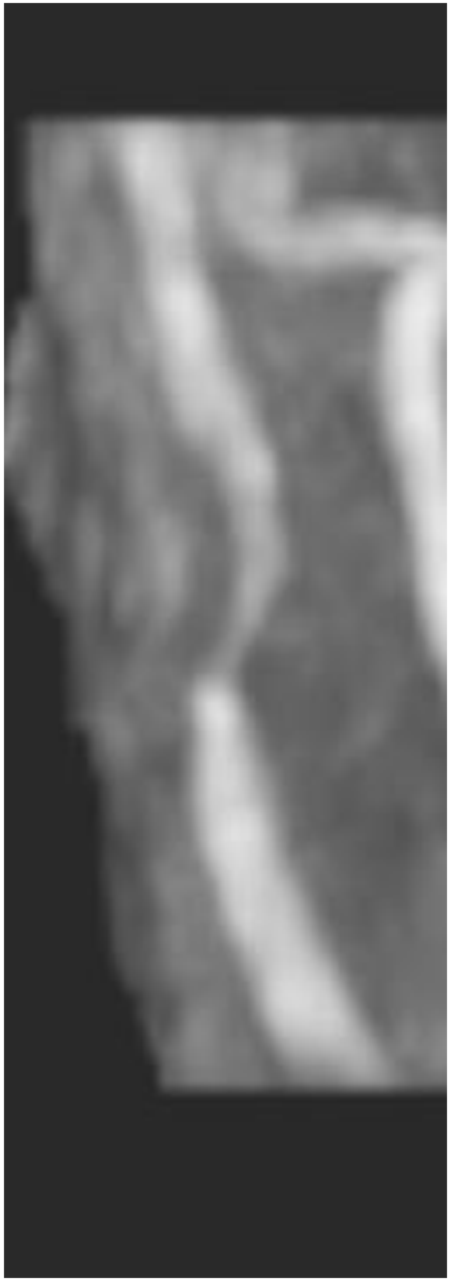

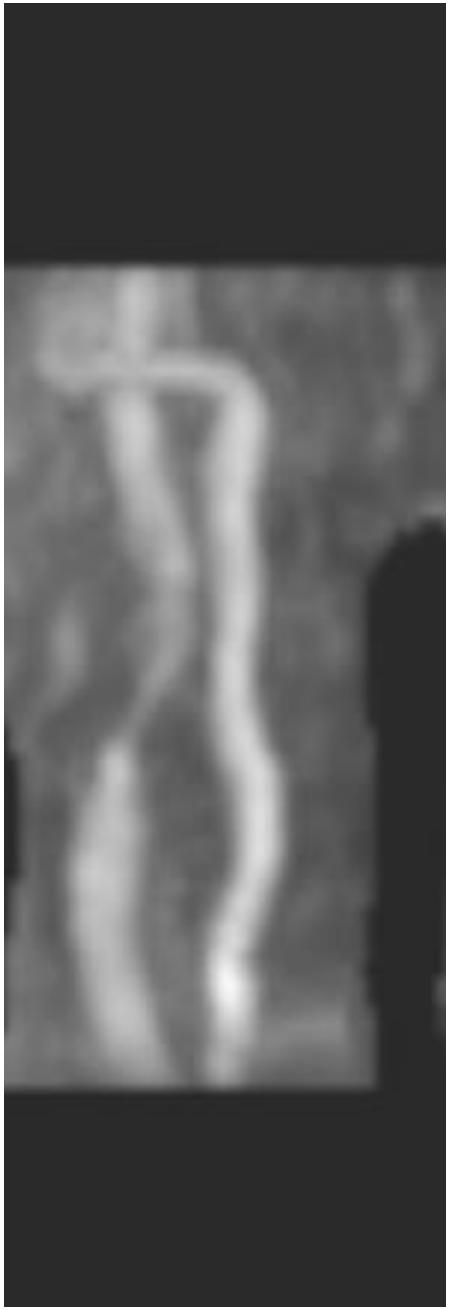

57b Score

0-30

31-50

51-70

>70

Near occlusion

Occluded

Quality

1

2

3

4

5

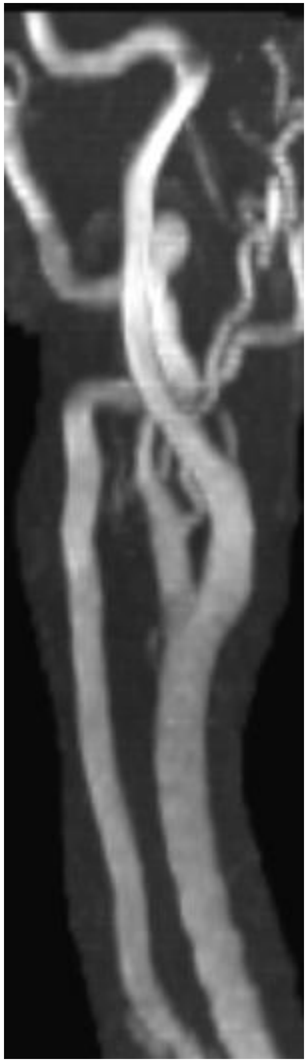

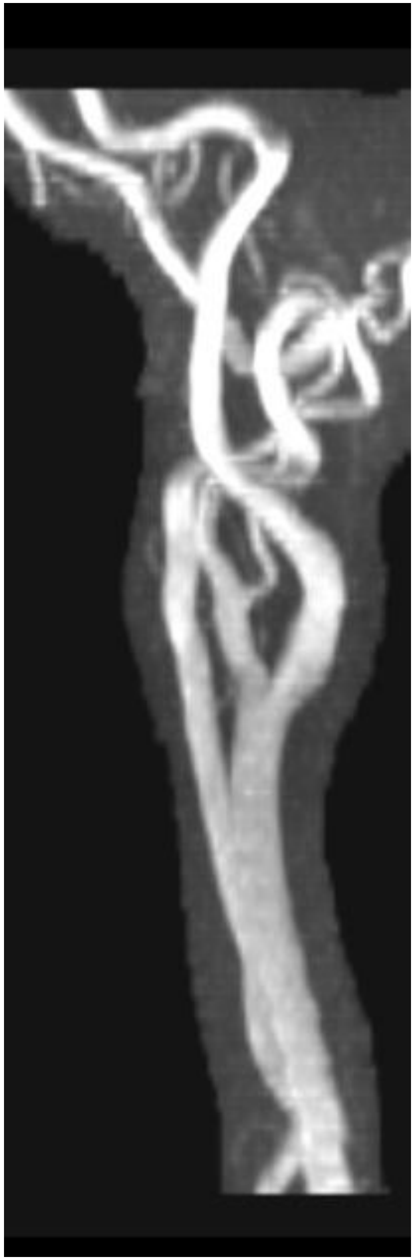

58a Score

0-30

31-50

51-70

>70

Near occlusion

Occluded

Quality

1

2

3

4

5

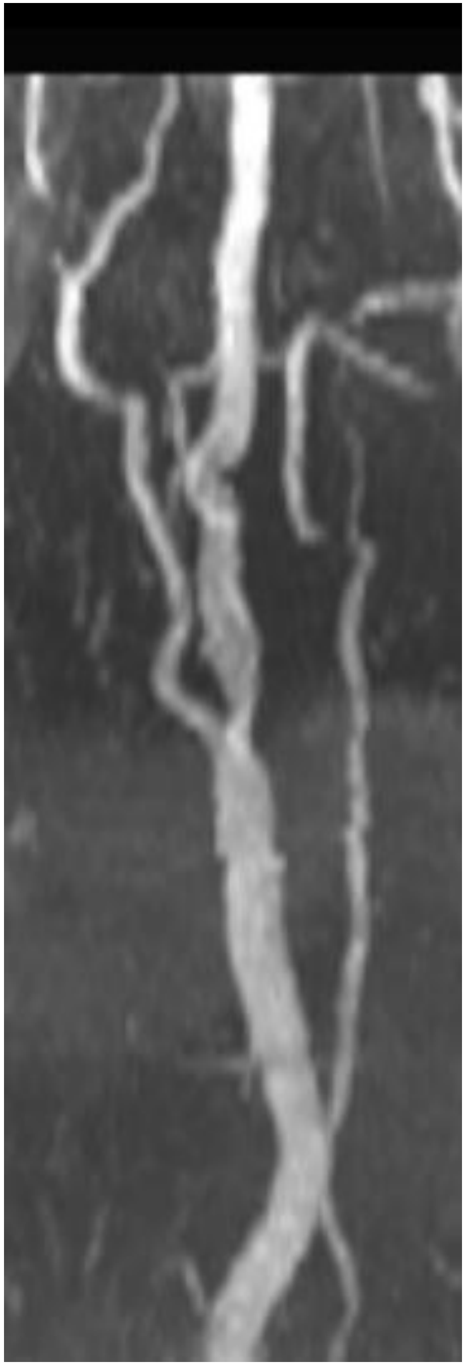

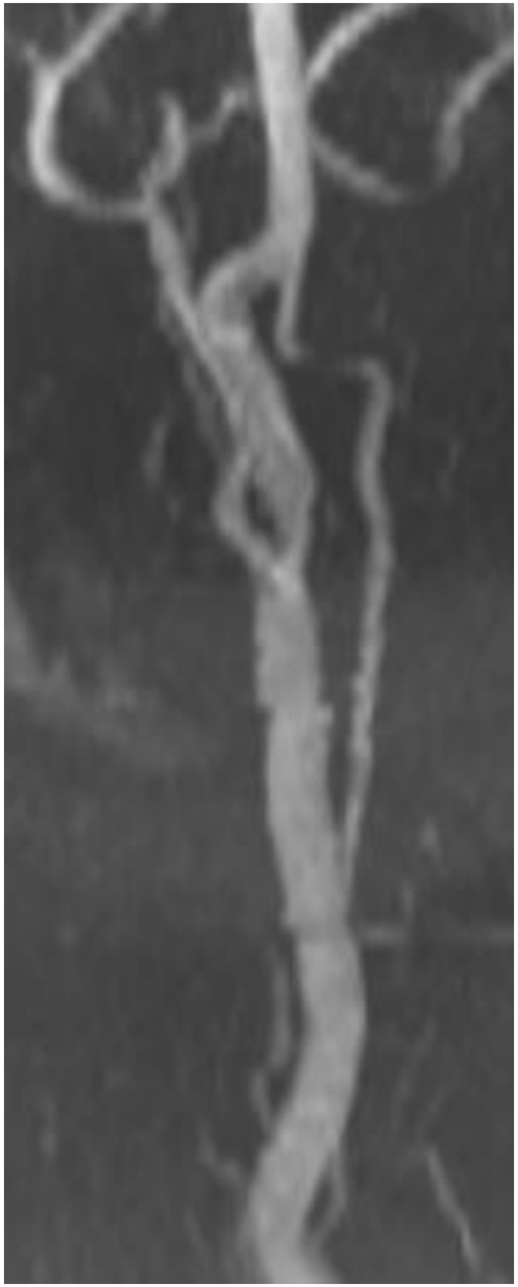

58f Score

0-30

31-50

51-70

>70

Near occlusion

Occluded

Quality

1

2

3

4

5

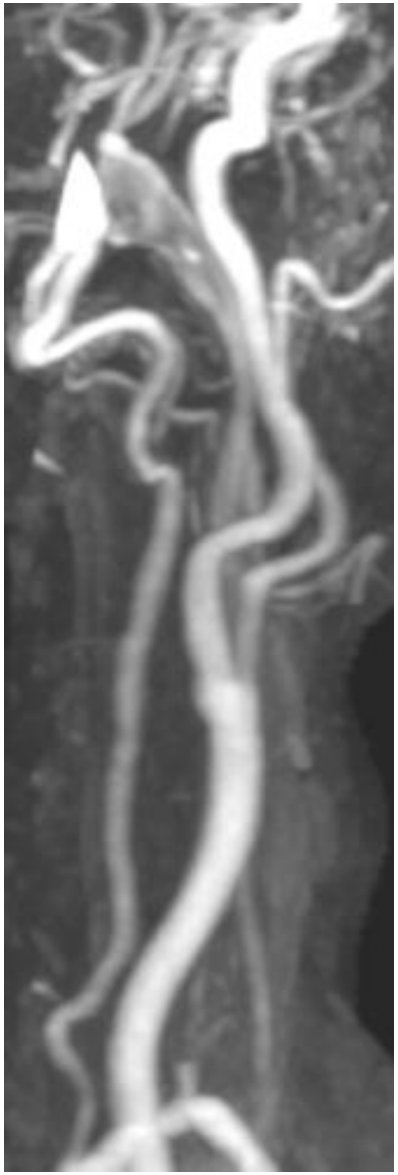

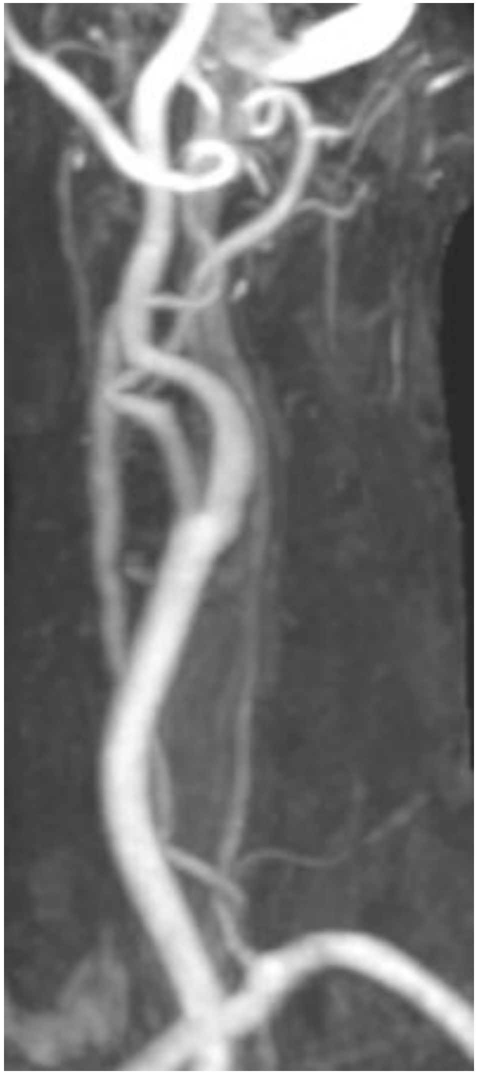

59e Score

0-30

31-50

51-70

>70

Near occlusion

Occluded

Quality

1

2

3

4

5

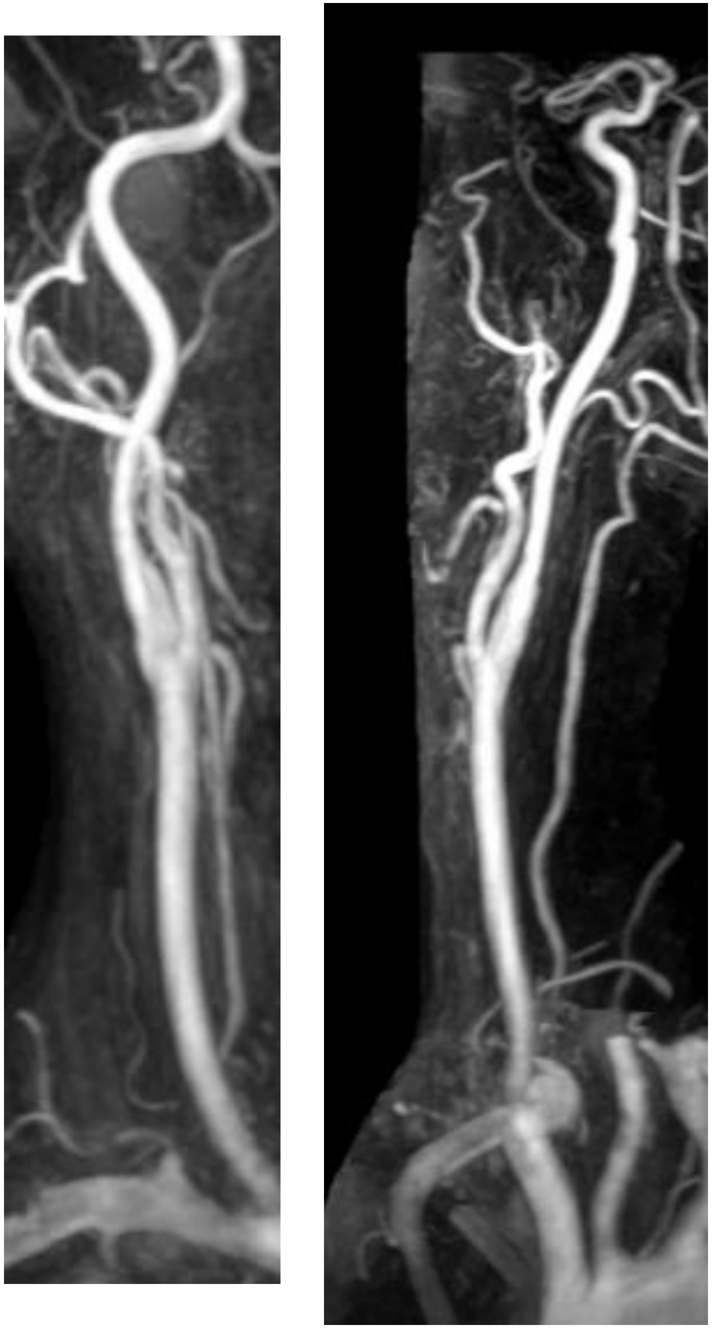

60d Score

0-30

31-50

51-70

>70

Near occlusion

Occluded

Quality

1

2

3

4

5

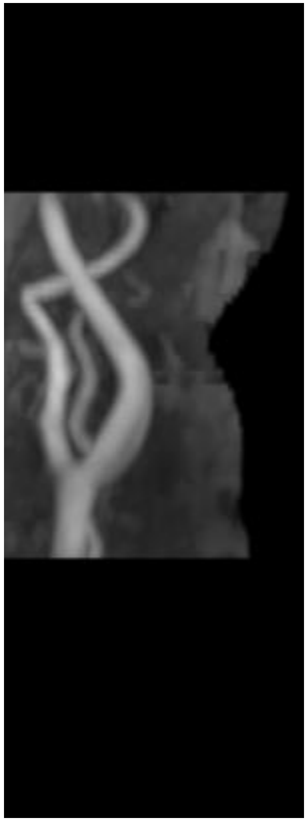

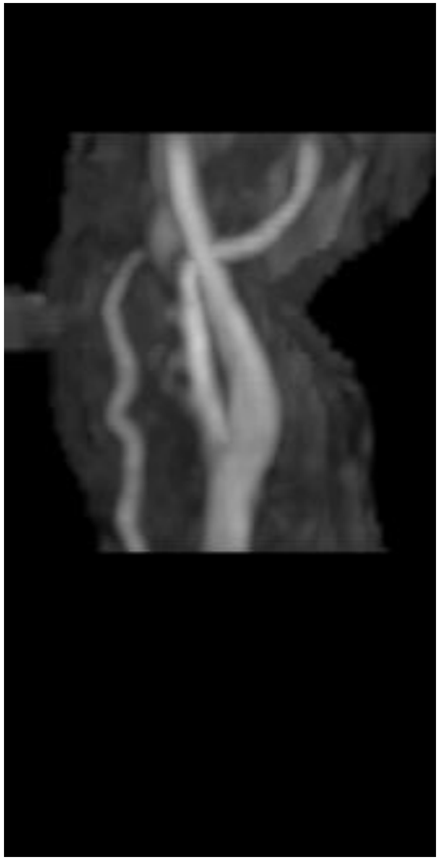

61c Score

0-30

31-50

51-70

>70

Near occlusion

Occluded

Quality

1

2

3

4

5

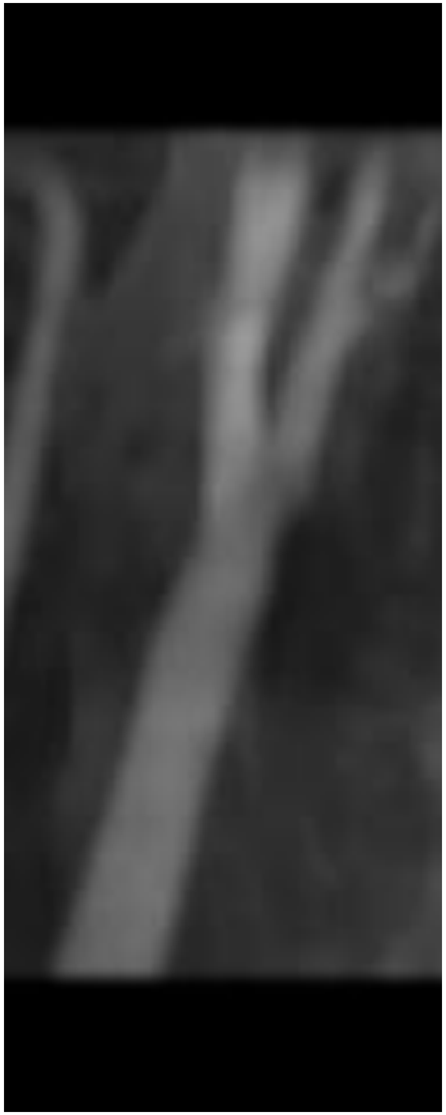

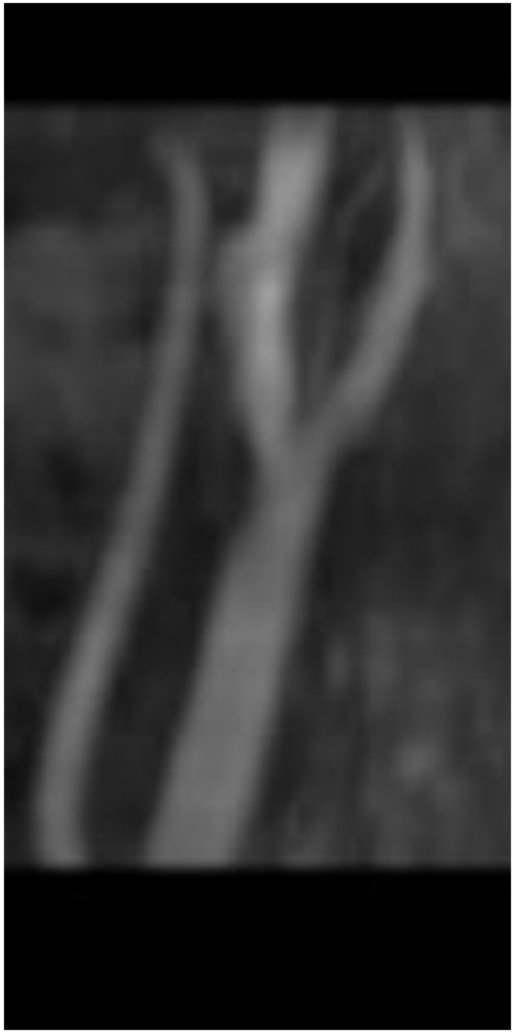

62b Score

0-30

31-50

51-70

>70

Near occlusion

Occluded

Quality

1

2

3

4

5

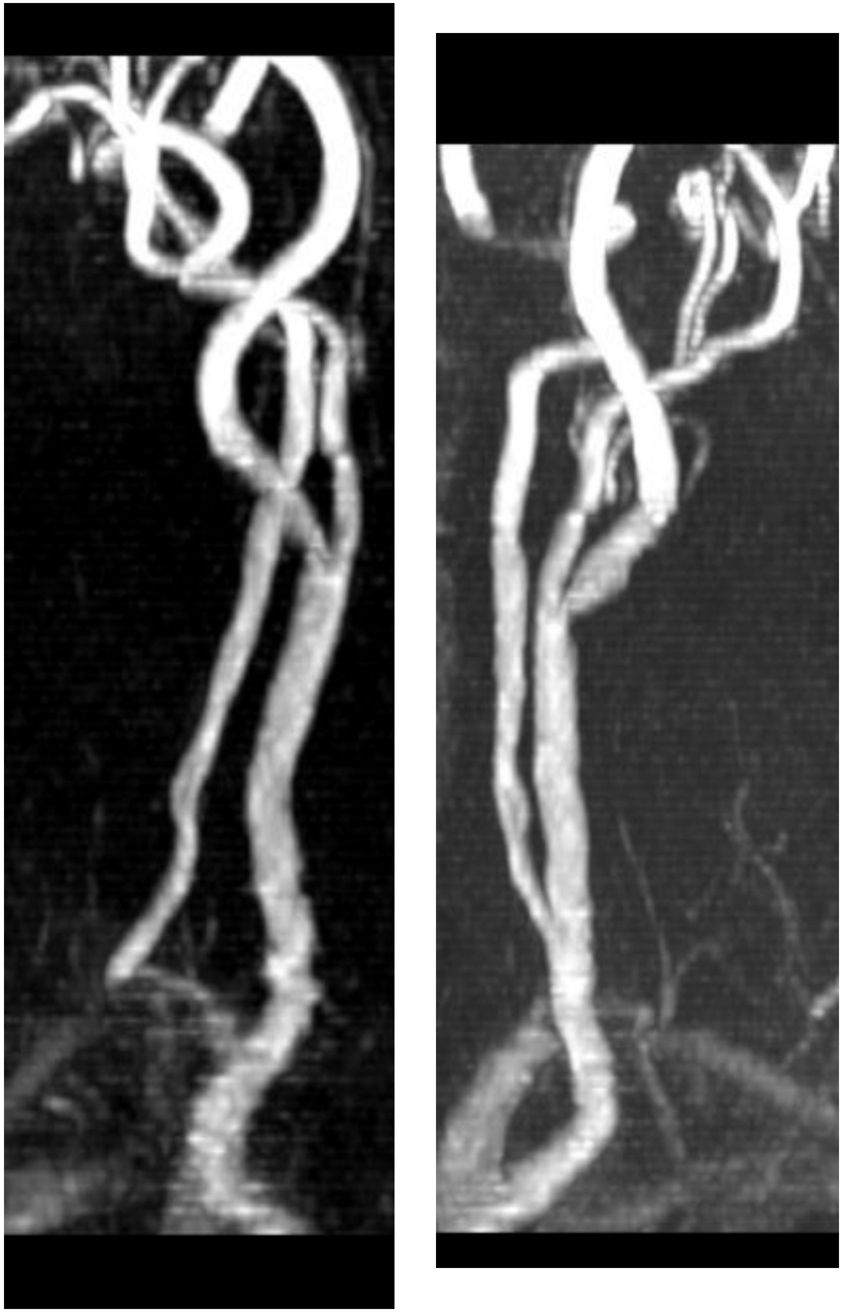

63a Score

0-30

31-50

51-70

>70

Near occlusion

Occluded

Quality

1

2

3

4

5

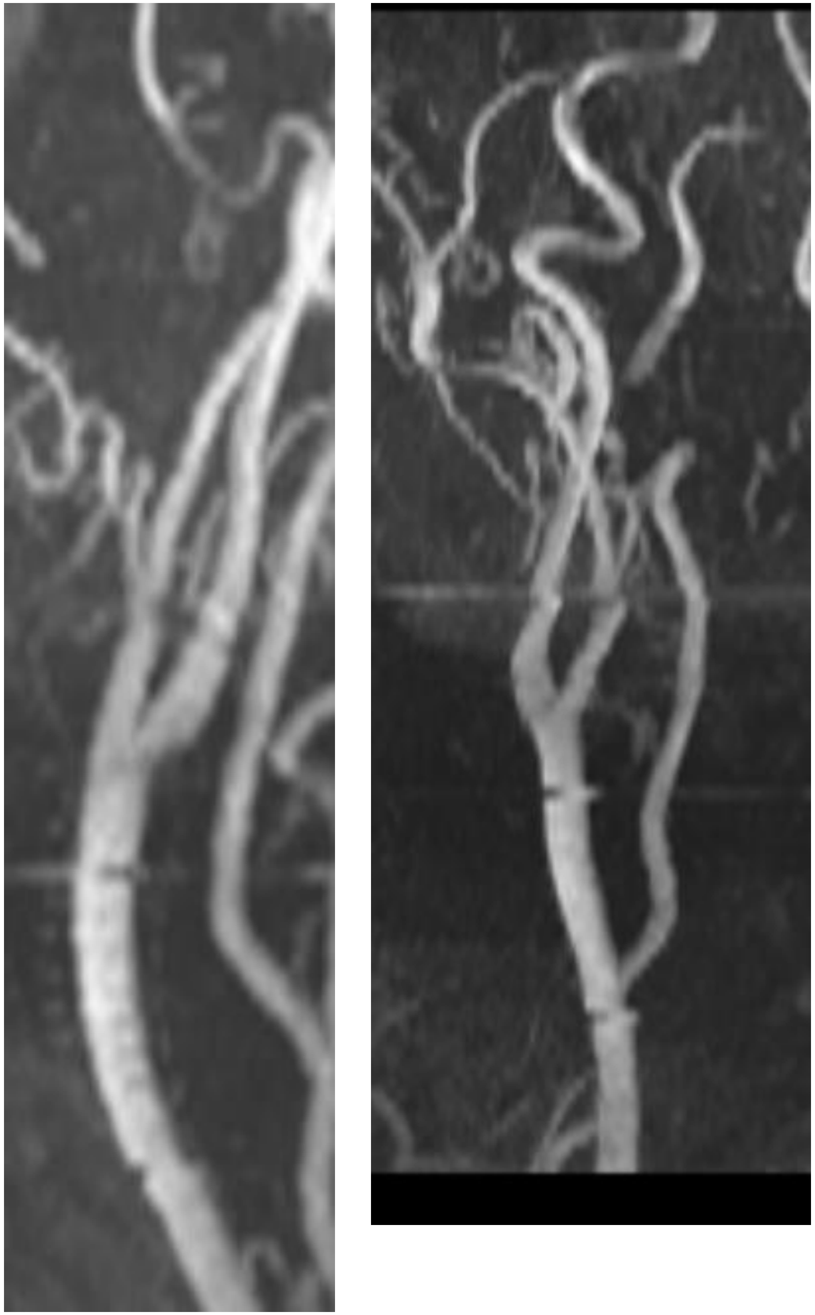

63f Score

0-30

31-50

51-70

>70

Near occlusion

Occluded

Quality

1

2

3

4

5

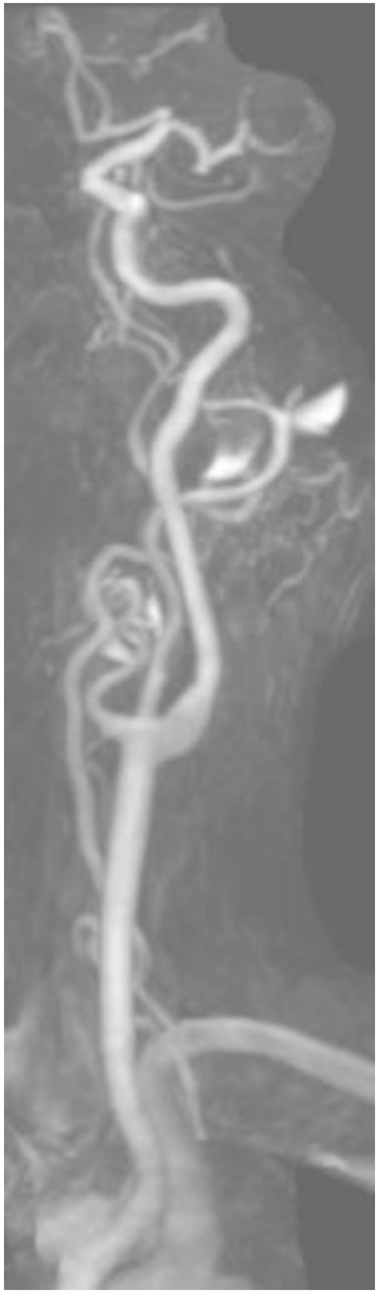

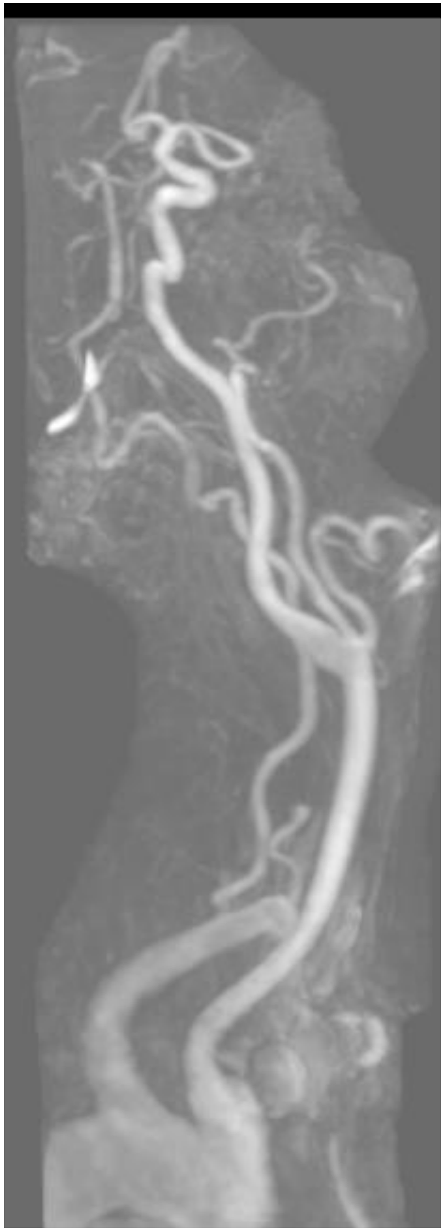

64e Score

0-30

31-50

51-70

>70

Near occlusion

Occluded

Quality

1

2

3

4

5

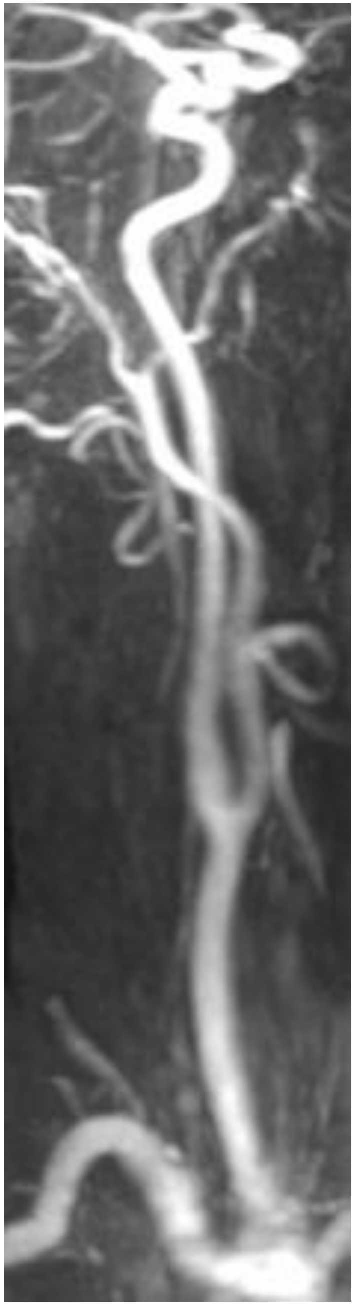

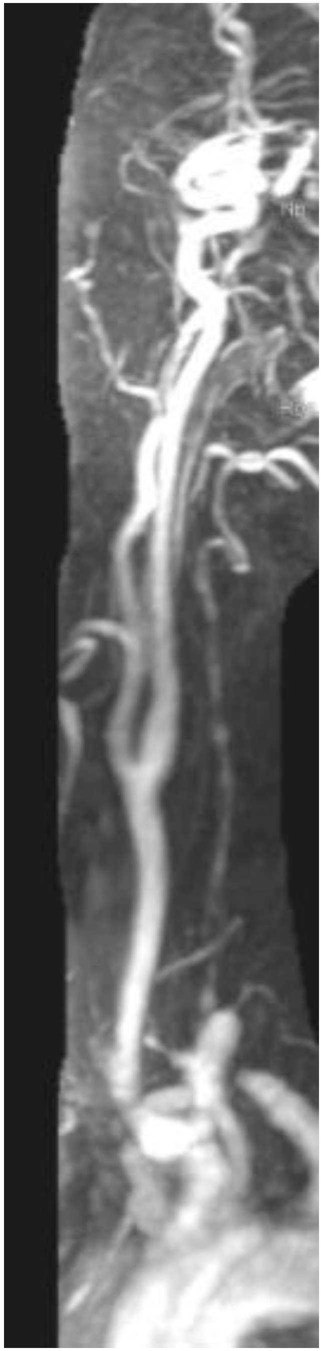

65d Score

0-30

31-50

51-70

>70

Near occlusion

Occluded

Quality

1

2

3

4

5

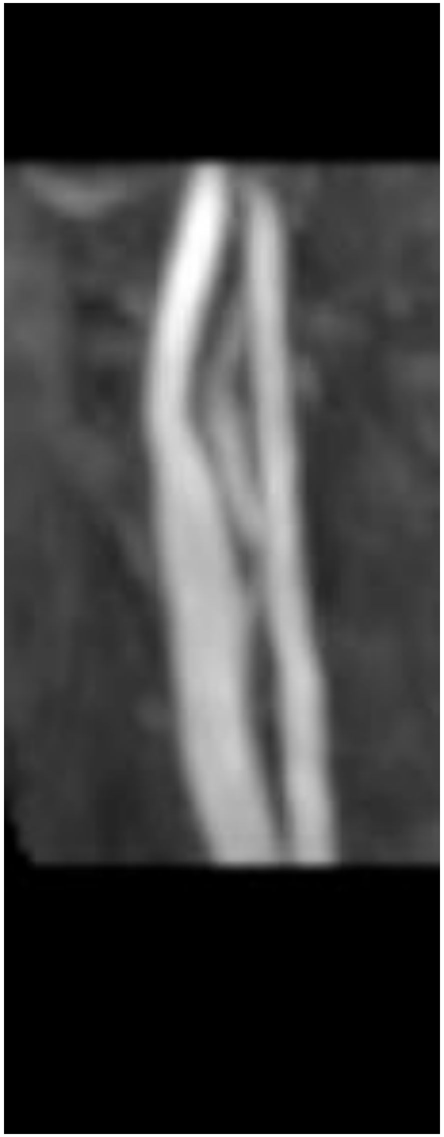

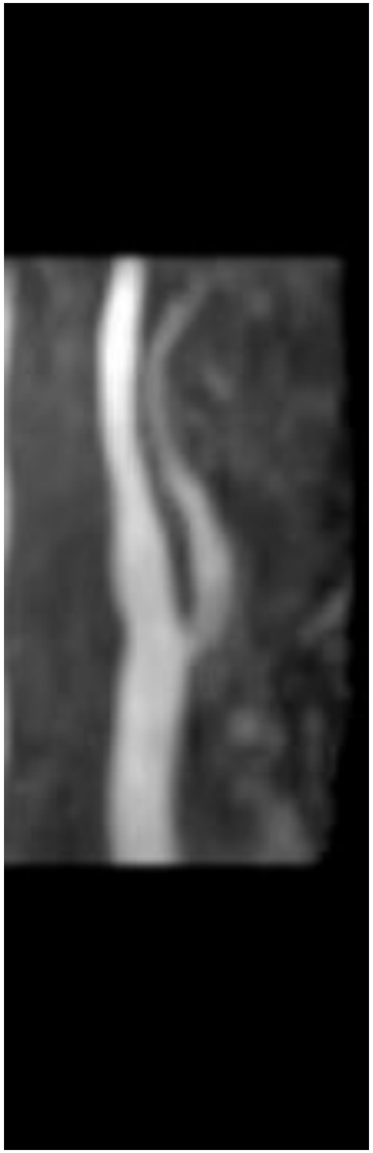

66c Score

0-30

31-50

51-70

>70

Near occlusion

Occluded

Quality

1

2

3

4

5

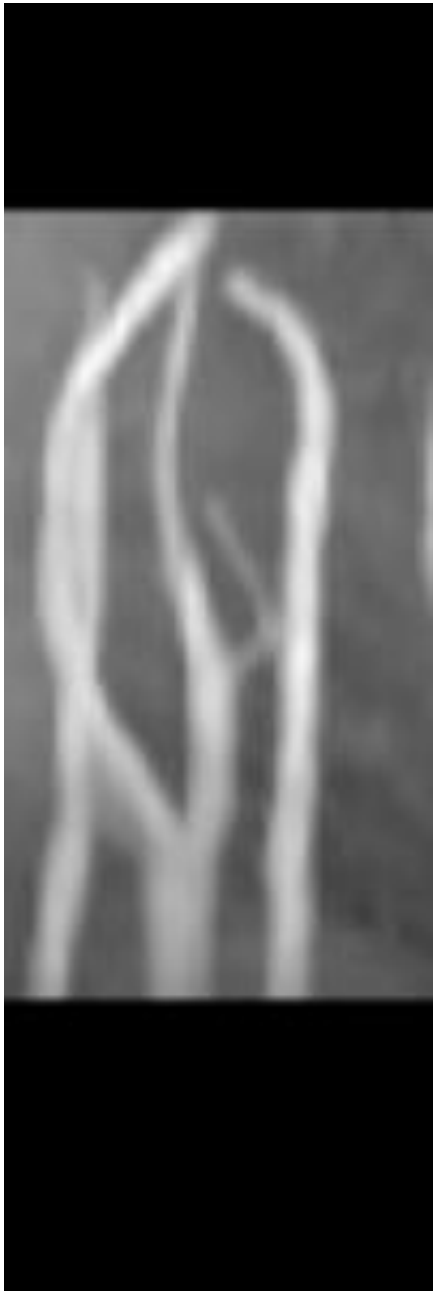

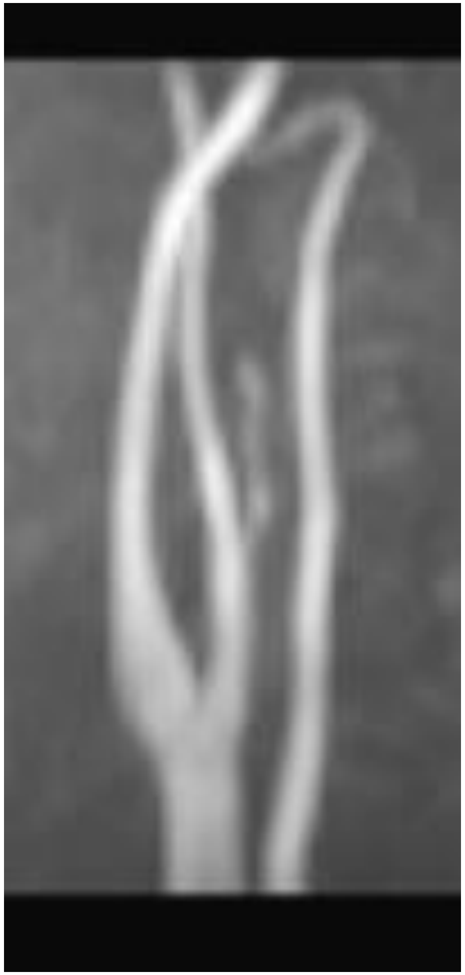

67b Score

0-30

31-50

51-70

>70

Near occlusion

Occluded

Quality

1

2

3

4

5

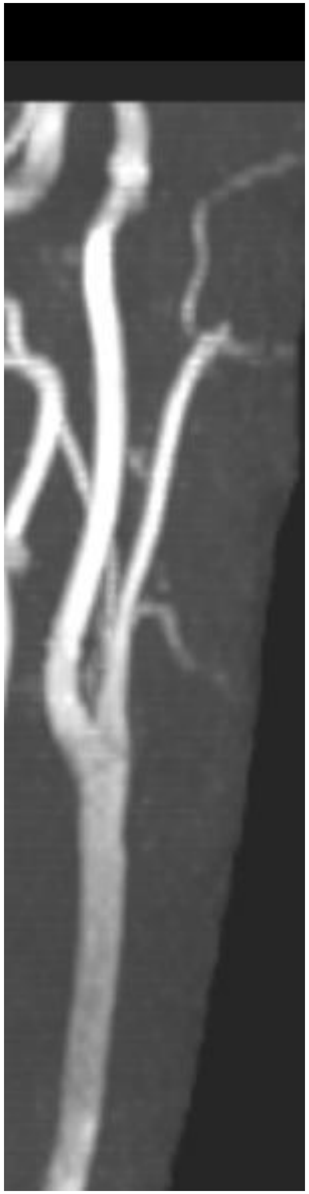

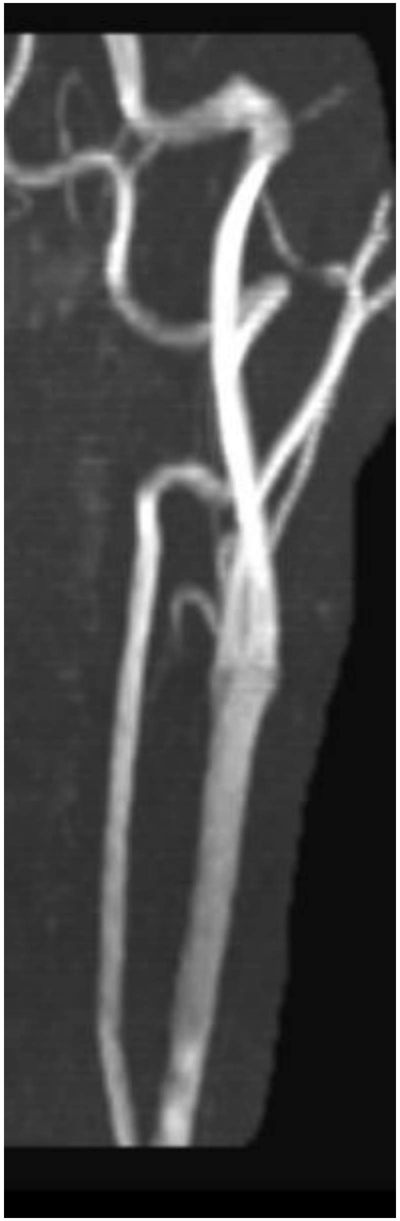

68a Score

0-30

31-50

51-70

>70

Near occlusion

Occluded

Quality

1

2

3

4

5

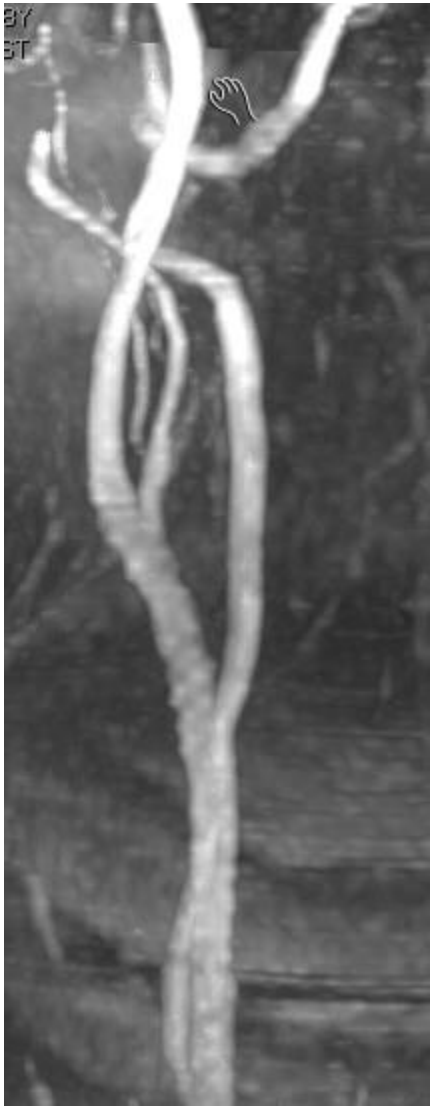

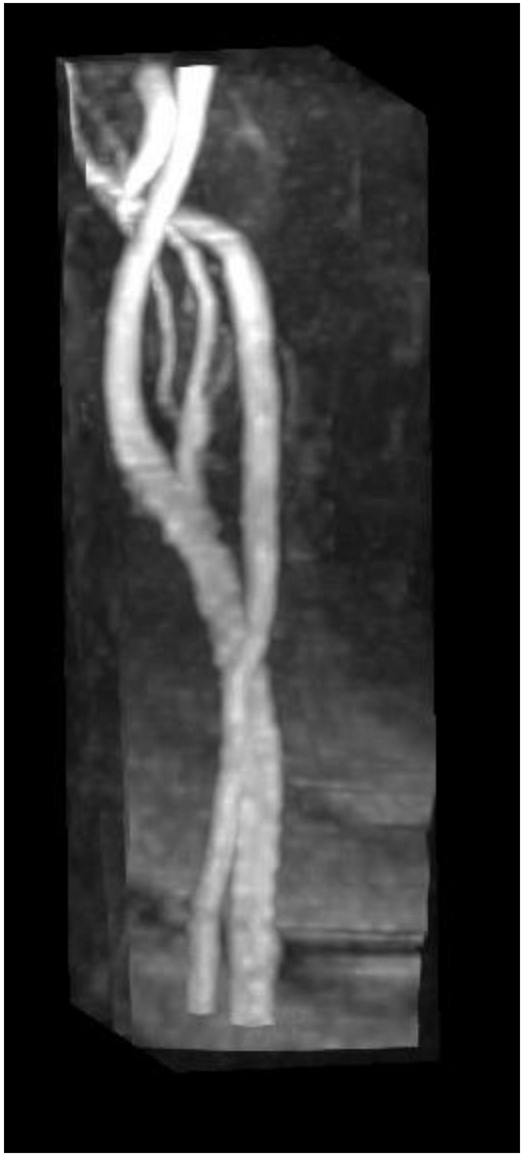

68f Score

0-30

31-50

51-70

>70

Near occlusion

Occluded

Quality

1

2

3

4

5

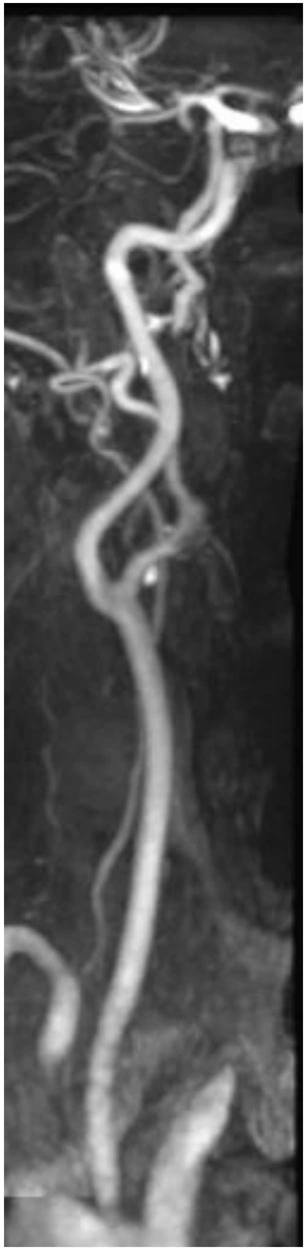

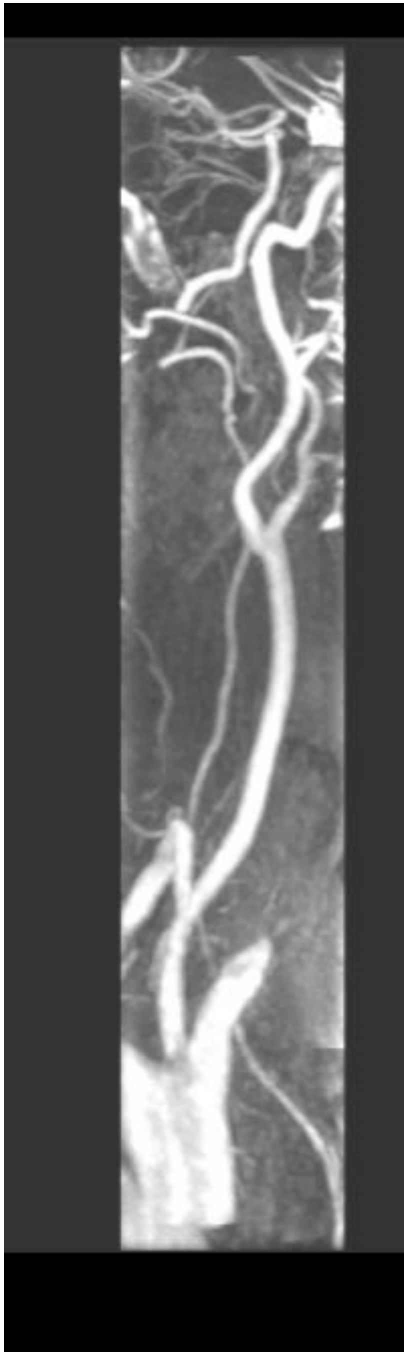

69e Score

0-30

31-50

51-70

>70

Near occlusion

Occluded

Quality

1

2

3

4

5

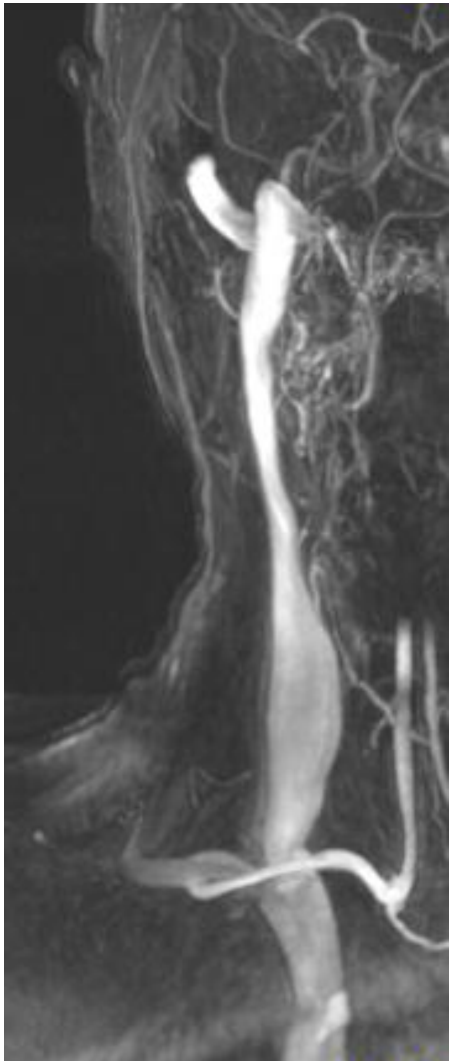

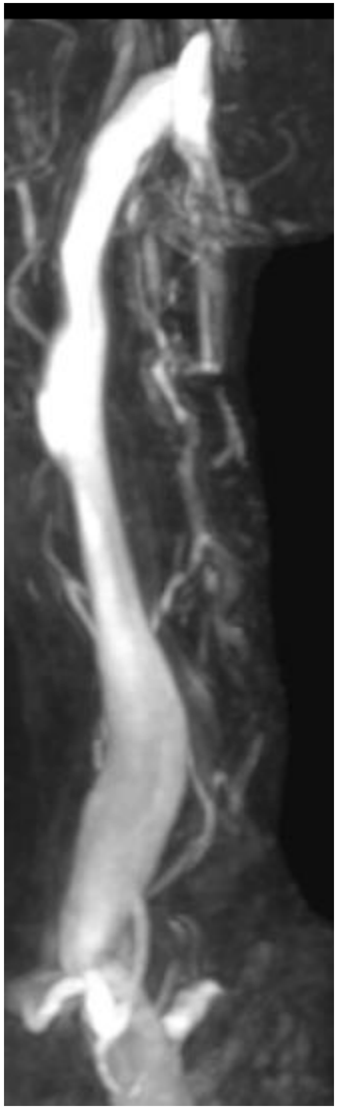

70d Score

0-30

31-50

51-70

>70

Near occlusion

Occluded

Quality

1

2

3

4

5

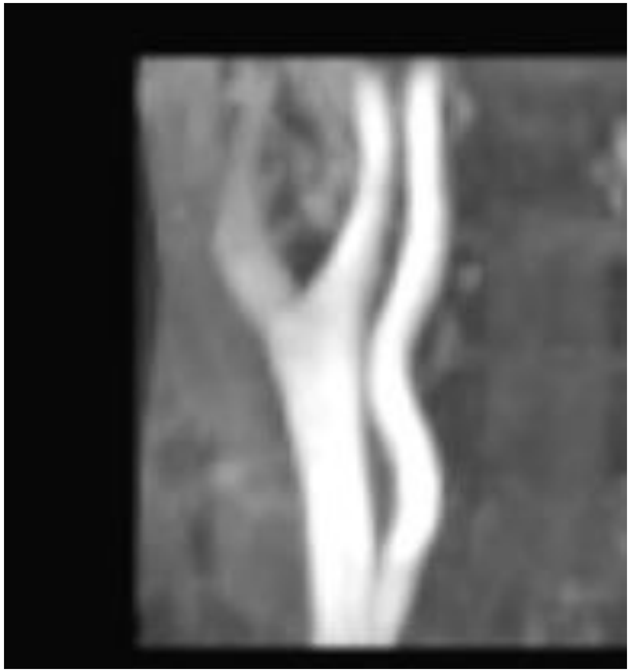

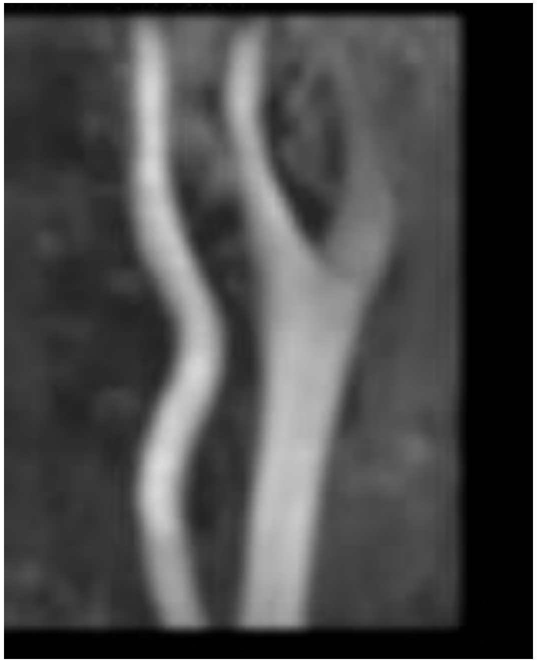

71c Score

0-30

31-50

51-70

>70

Near occlusion

Occluded

Quality

1

2

3

4

5

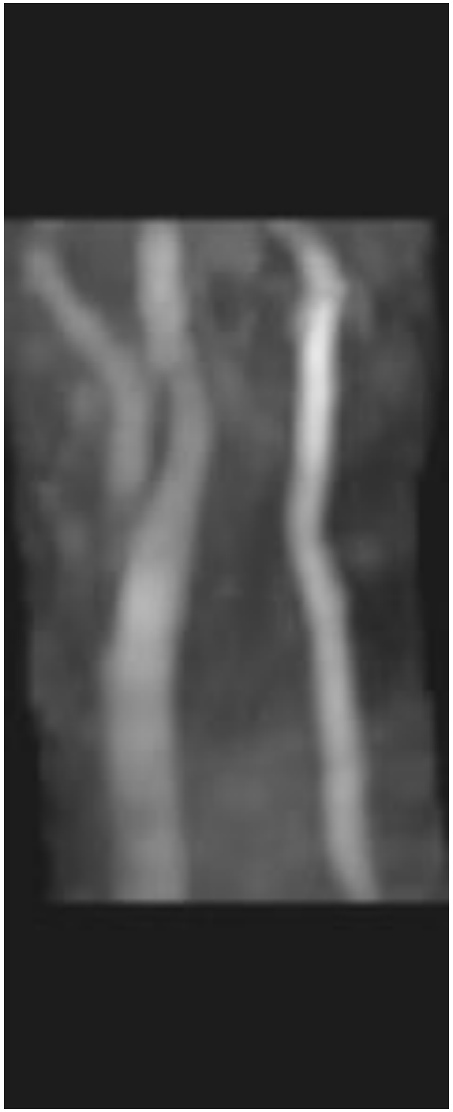

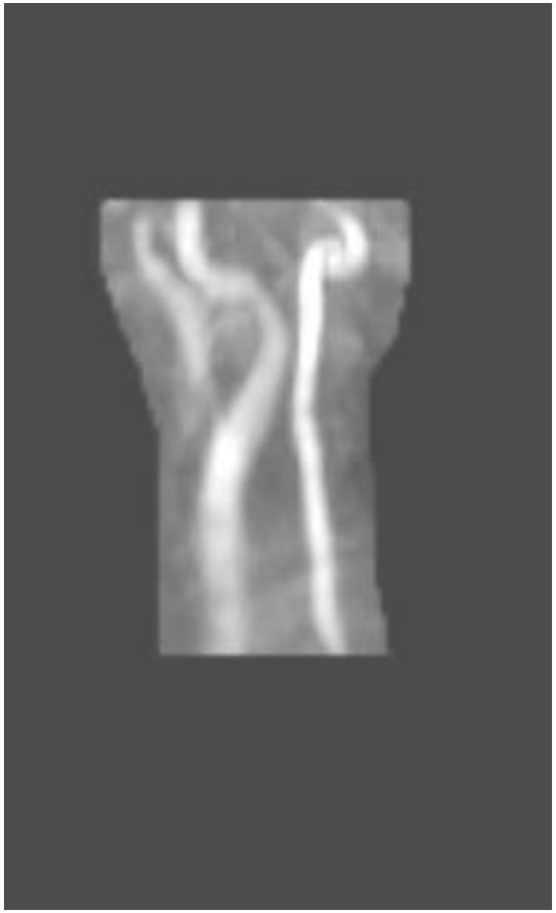

72b Score

0-30

31-50

51-70

>70

Near occlusion

Occluded

Quality

1

2

3

4

5

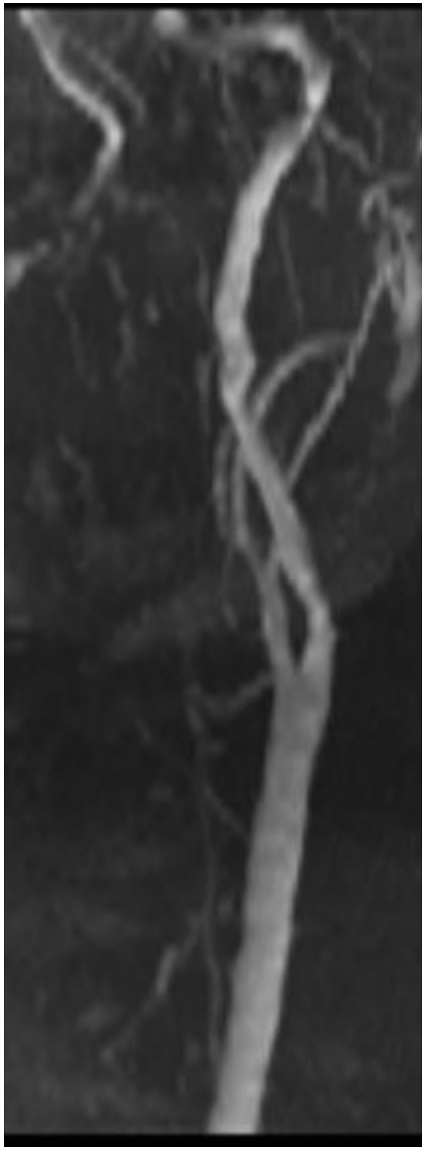

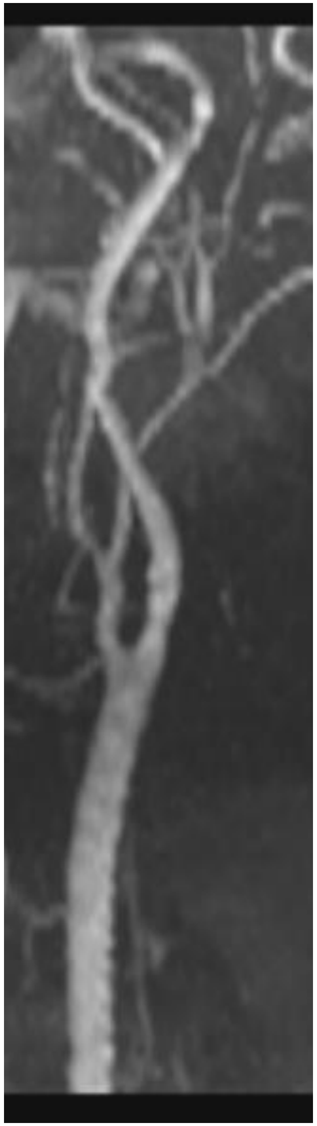

73a Score

0-30

31-50

51-70

>70

Near occlusion

Occluded

Quality

1

2

3

4

5

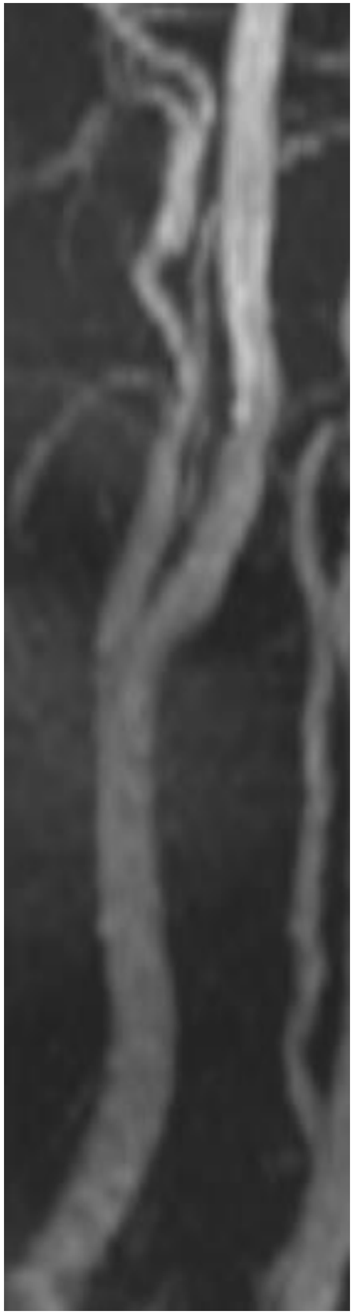

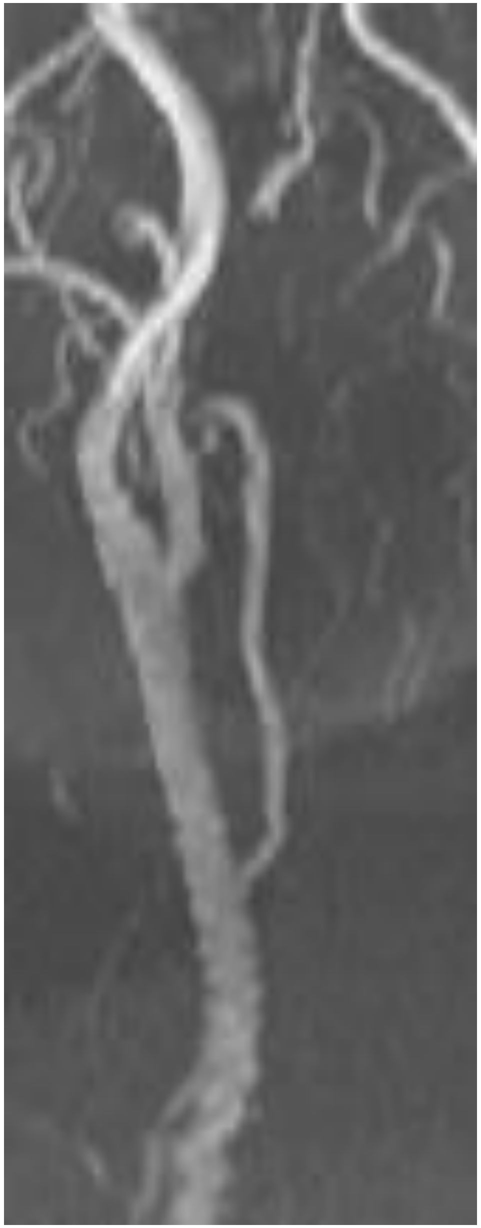

73f Score

0-30

31-50

51-70

>70

Near occlusion

Occluded

Quality

1

2

3

4

5

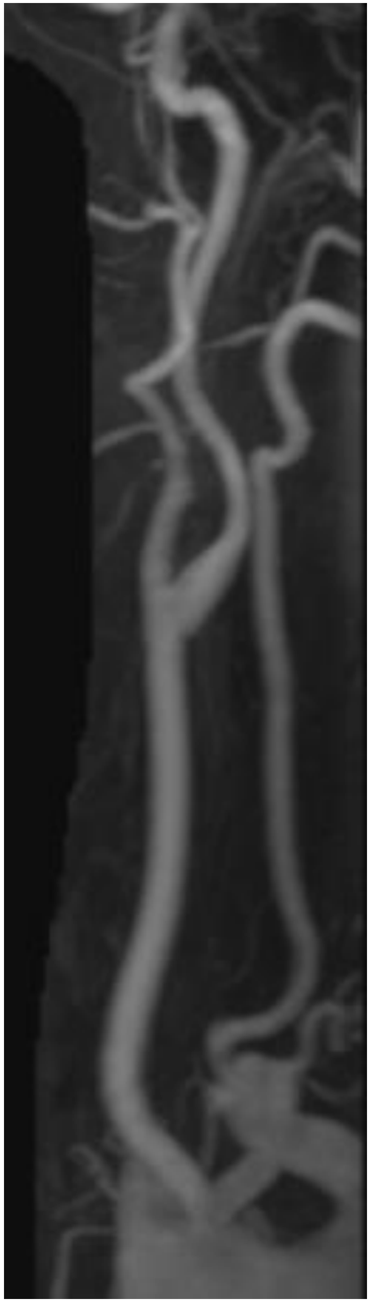

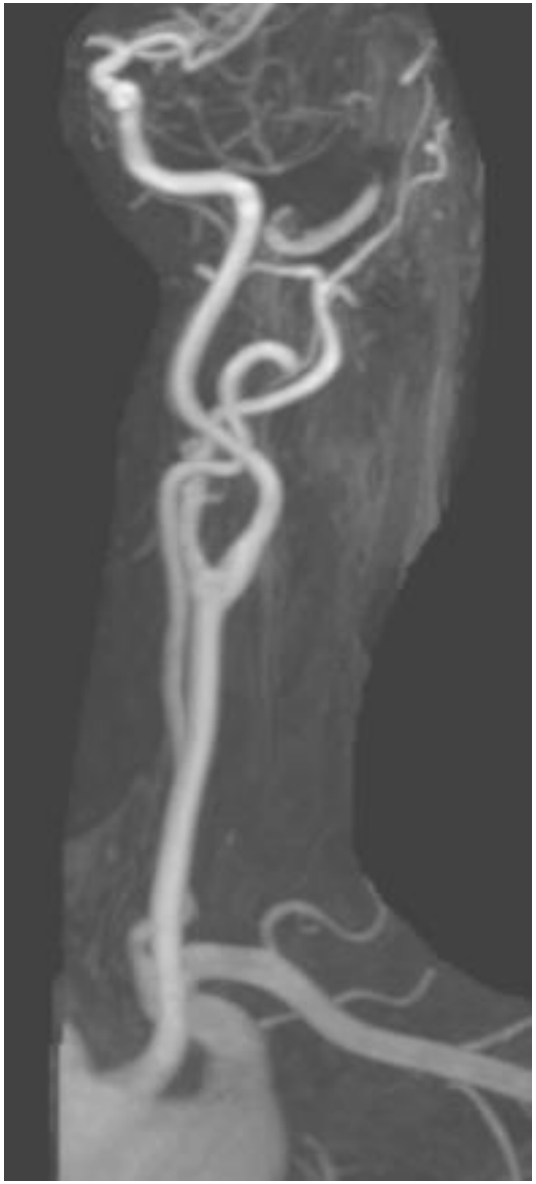

74e Score

0-30

31-50

51-70

>70

Near occlusion

Occluded

Quality

1

2

3

4

5

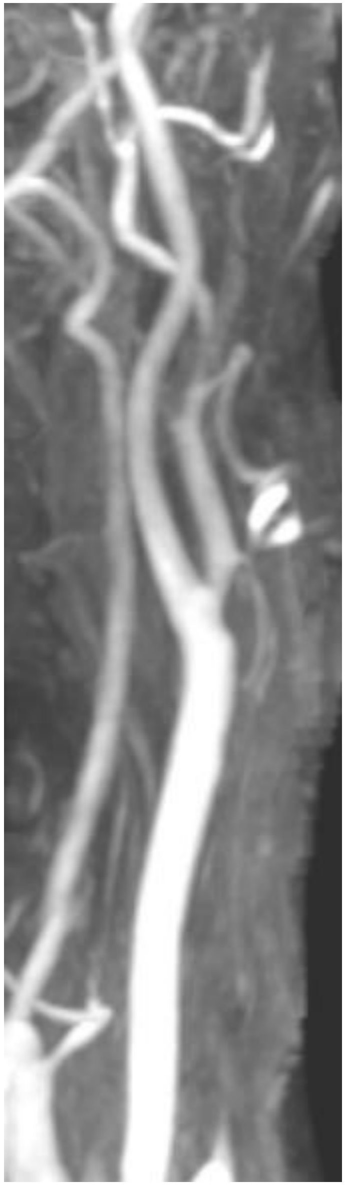

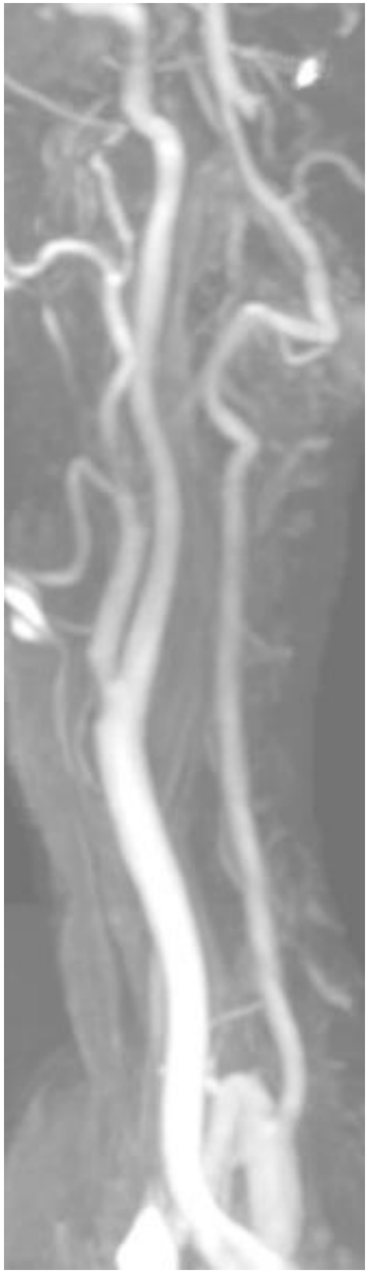

75d Score

0-30

31-50

51-70

>70

Near occlusion

Occluded

Quality

1

2

3

4

5

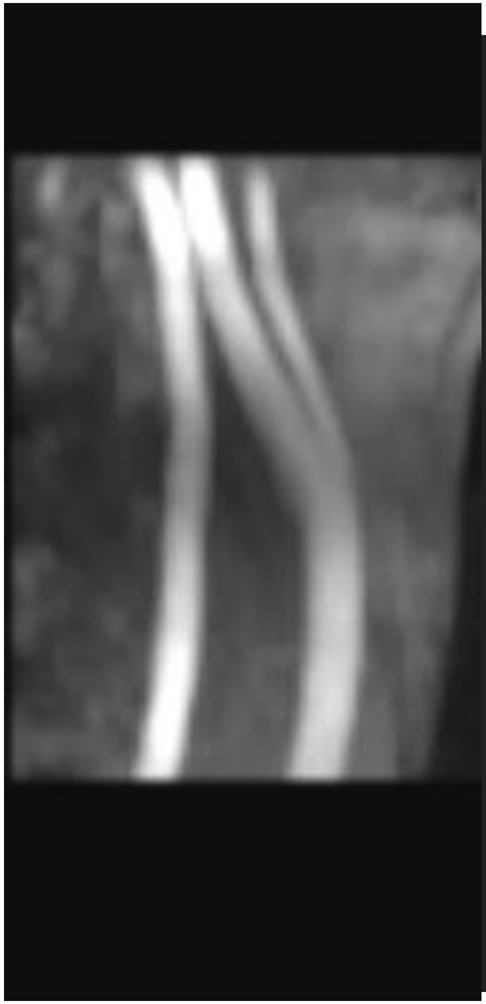

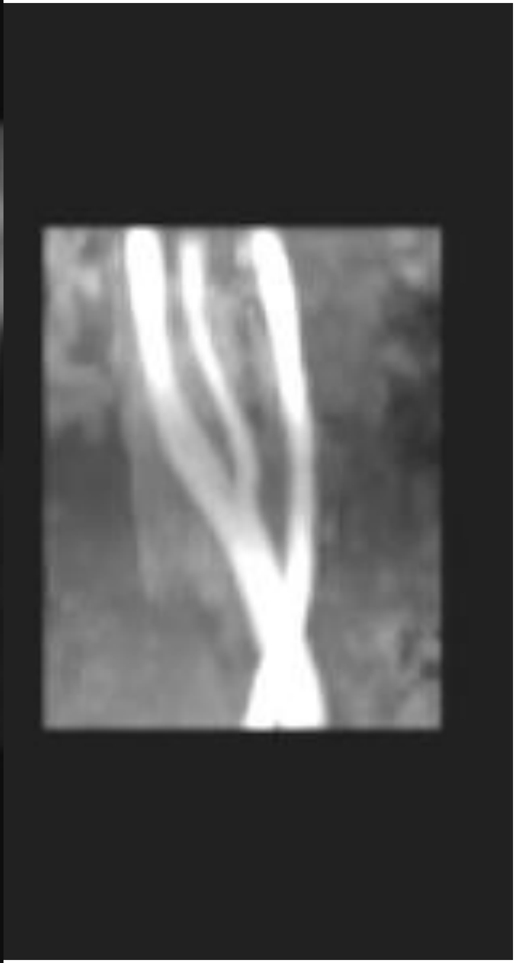

76c Score

0-30

31-50

51-70

>70

Near occlusion

Occluded

Quality

1

2

3

4

5

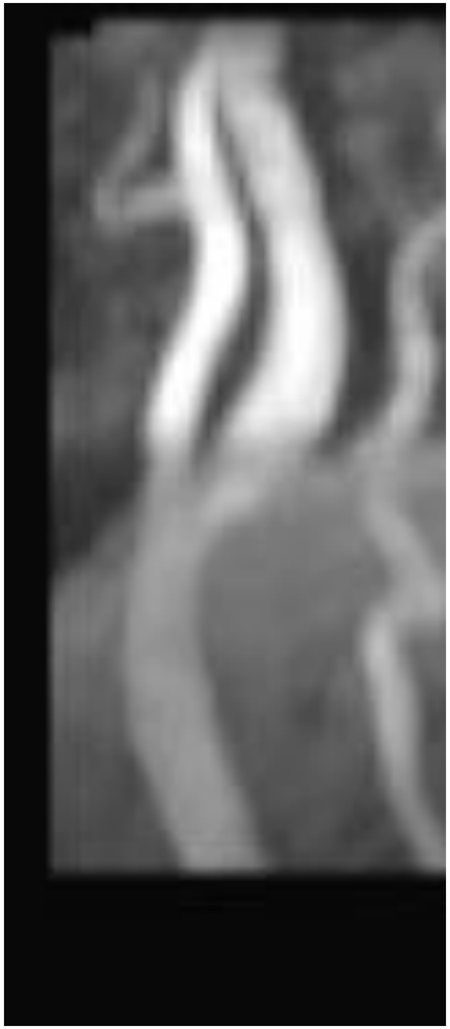

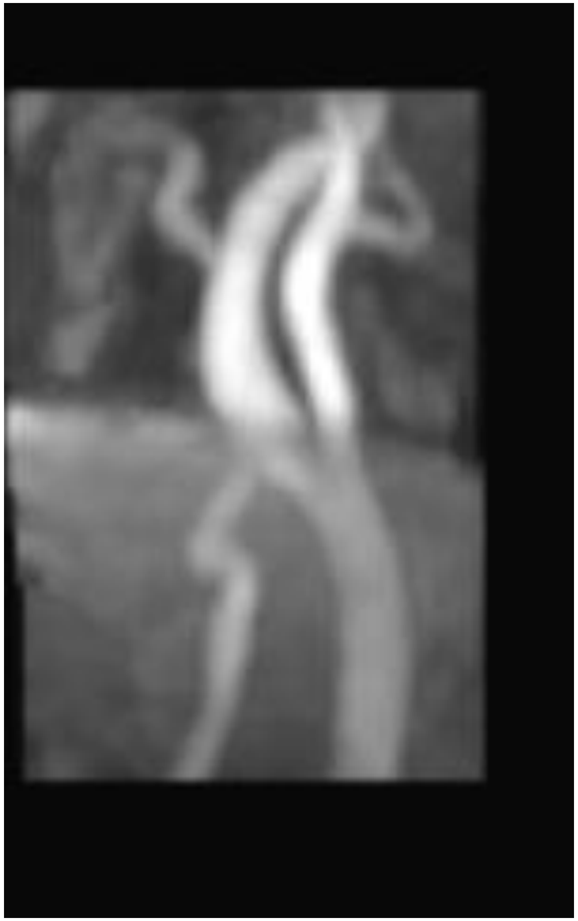

77b Score

0-30

31-50

51-70

>70

Near occlusion

Occluded

Quality

1

2

3

4

5

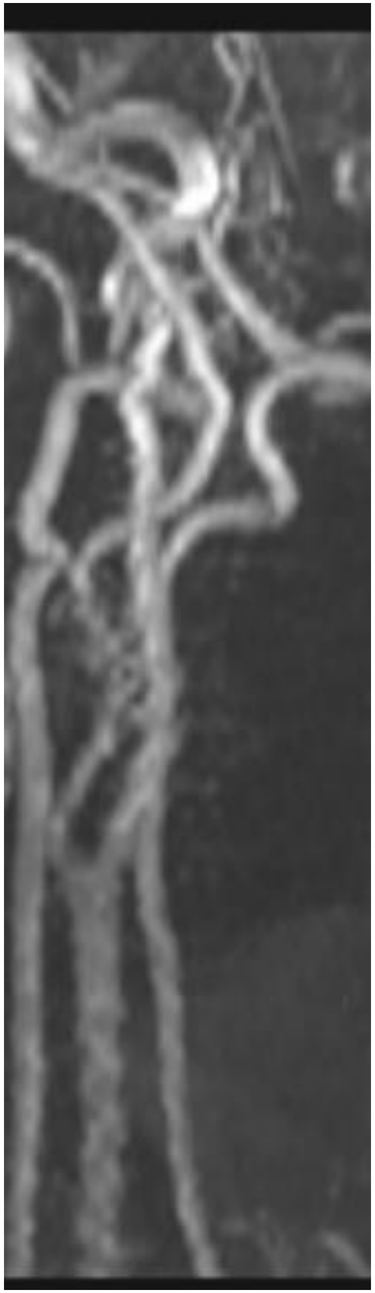

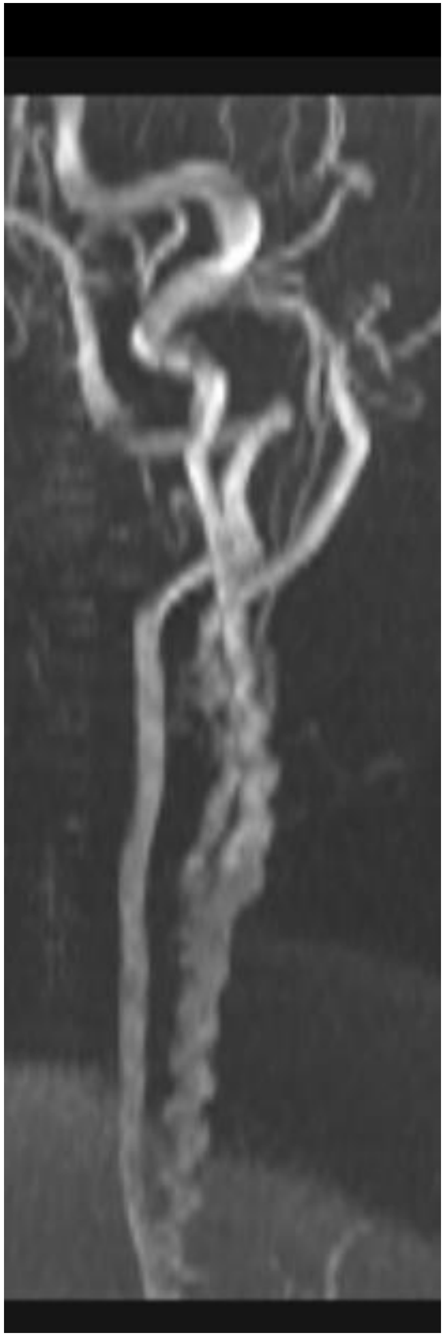

78a Score

0-30

31-50

51-70

>70

Near occlusion

Occluded

Quality

1

2

3

4

5

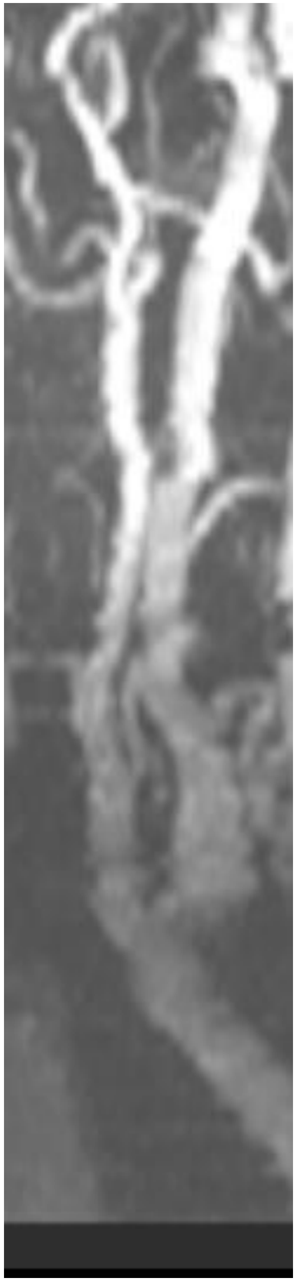

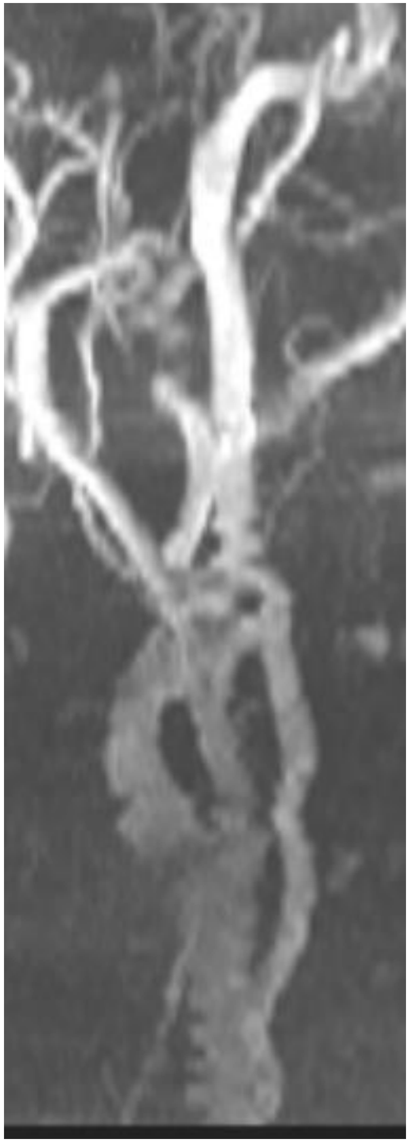

78f Score

0-30

31-50

51-70

>70

Near occlusion

Occluded

Quality

1

2

3

4

5

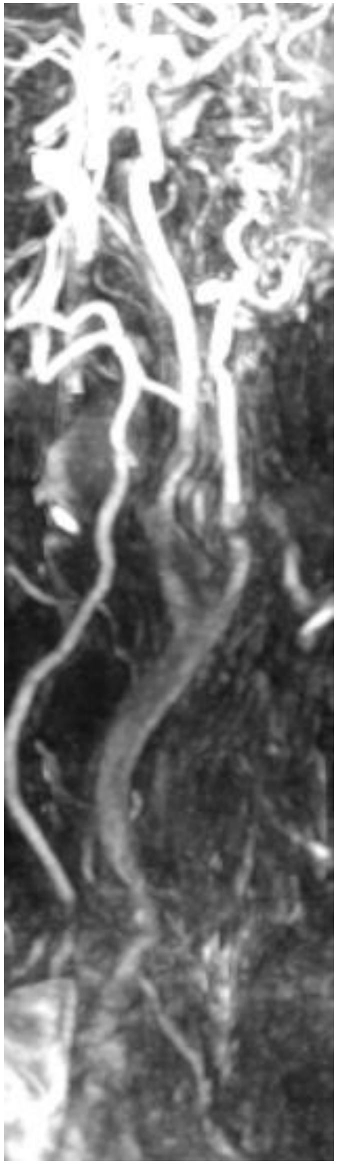

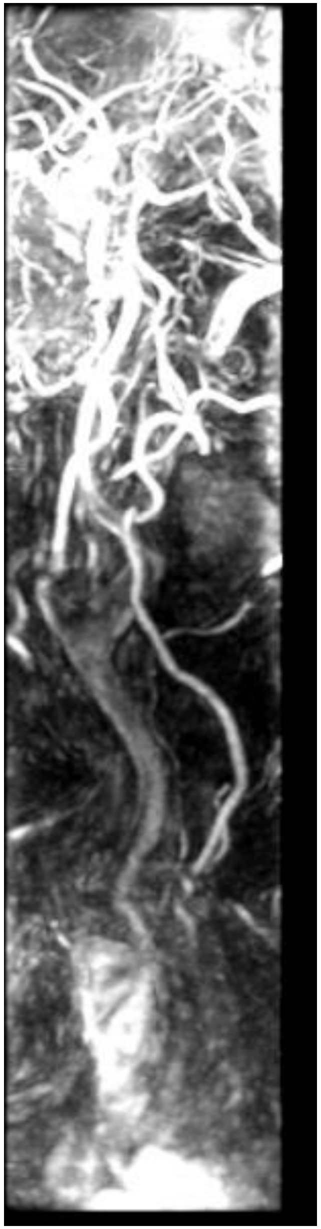

79e Score

0-30

31-50

51-70

>70

Near occlusion

Occluded

Quality

1

2

3

4

5

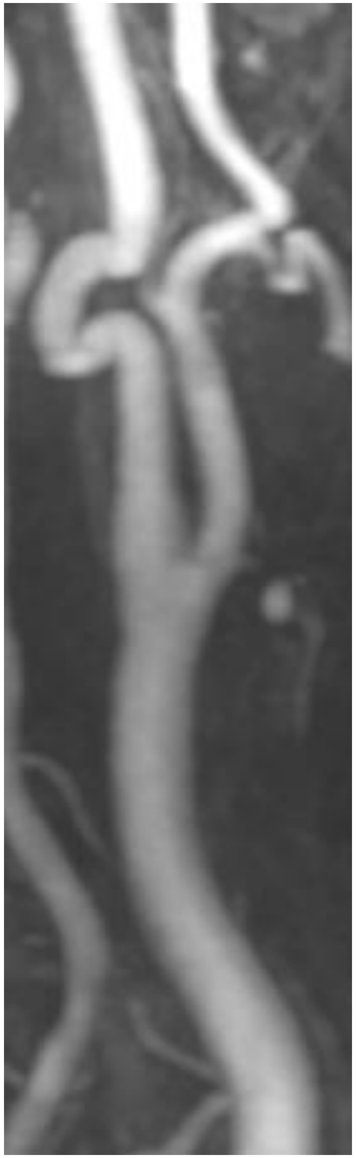

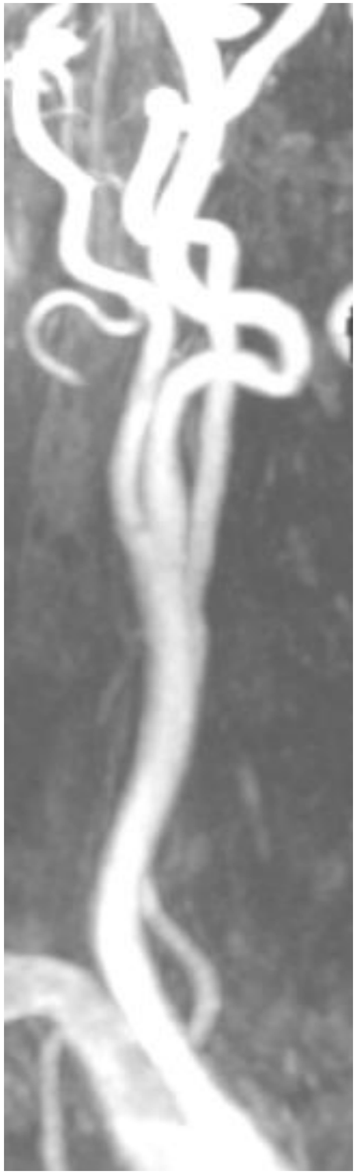

80d Score

0-30

31-50

51-70

>70

Near occlusion

Occluded

Quality

1

2

3

4

5

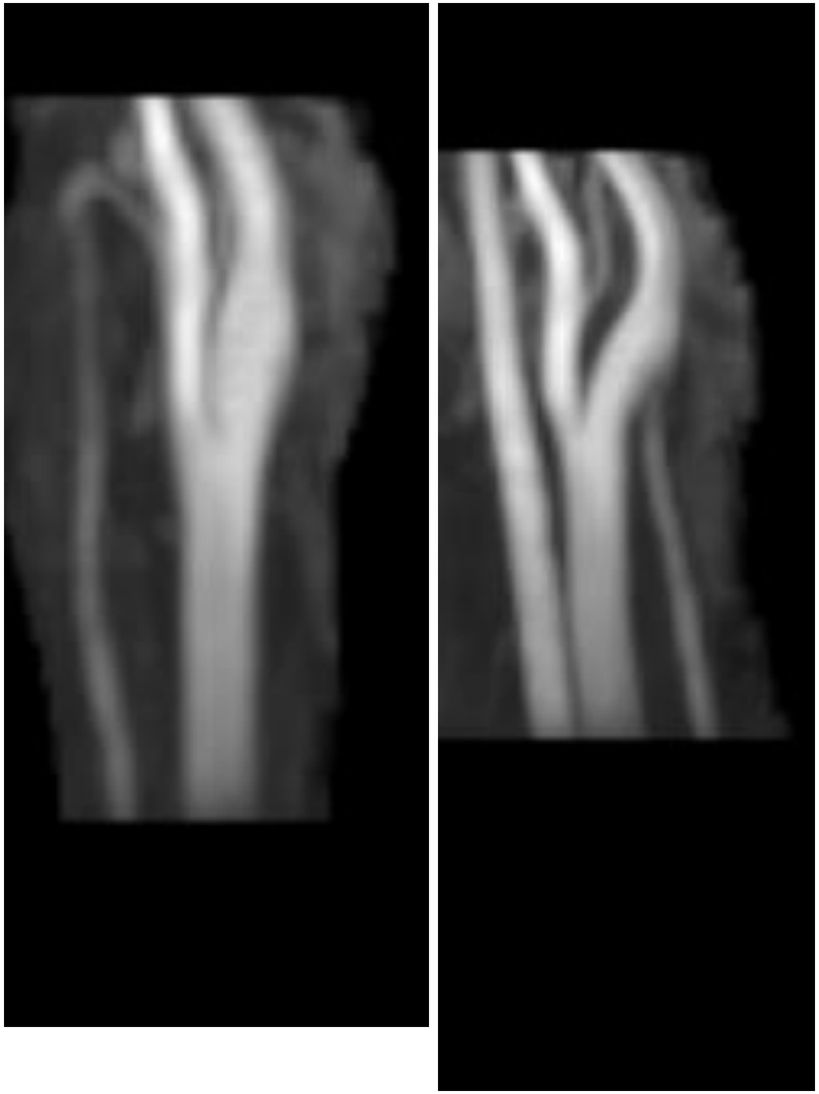

81c Score

0-30

31-50

51-70

>70

Near occlusion

Occluded

Quality

1

2

3

4

5

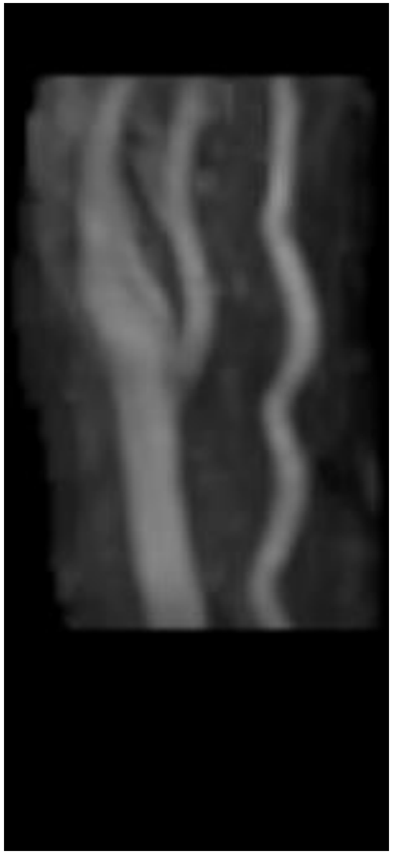

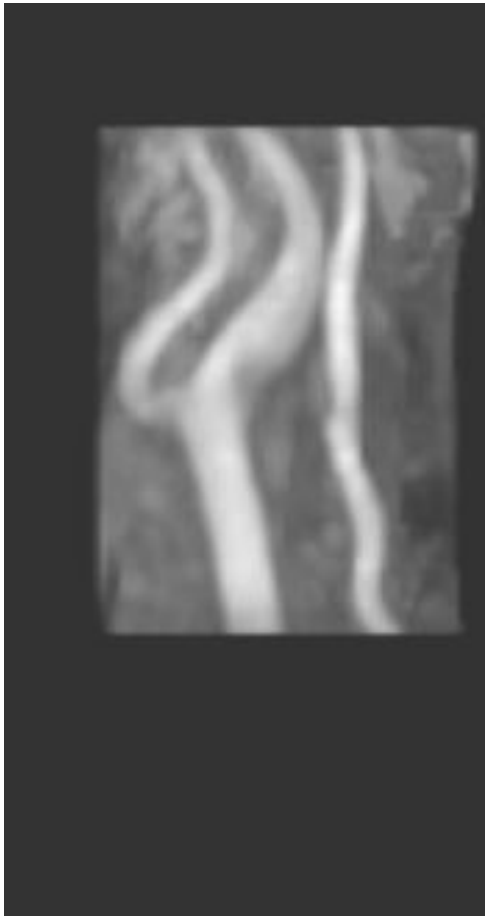

82b Score

0-30

31-50

51-70

>70

Near occlusion

Occluded

Quality

1

2

3

4

5

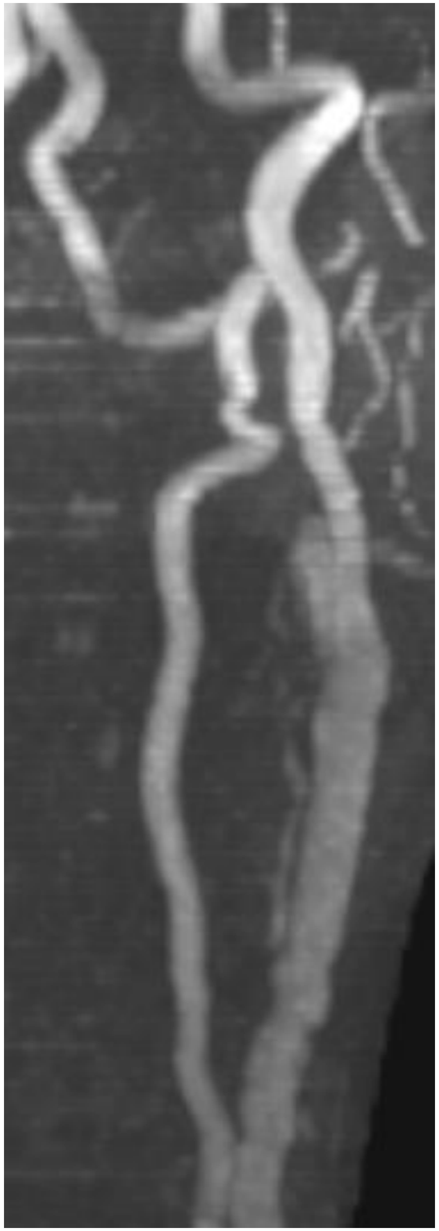

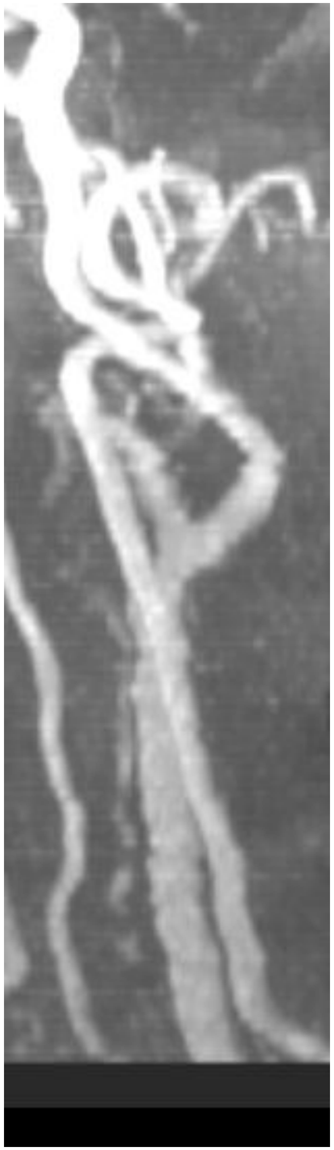

83a Score

0-30

31-50

51-70

>70

Near occlusion

Occluded

Quality

1

2

3

4

5

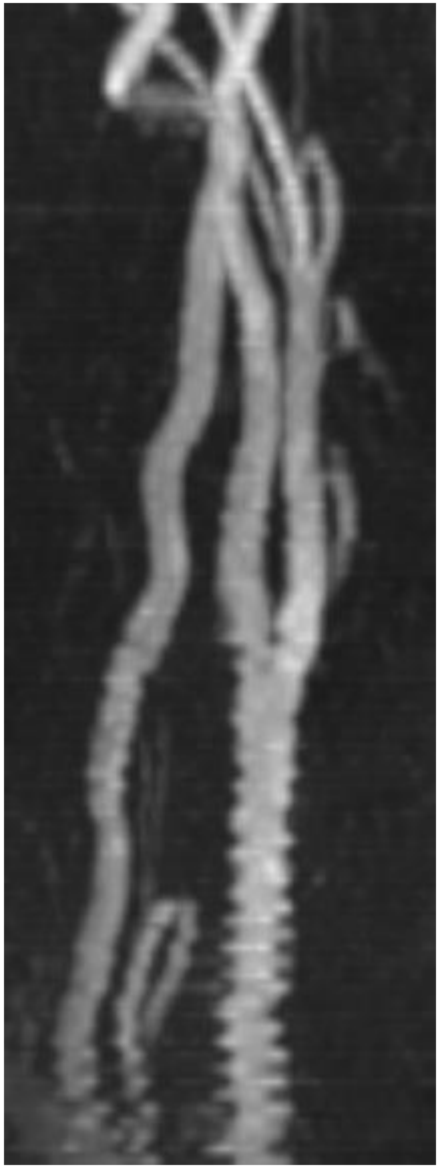

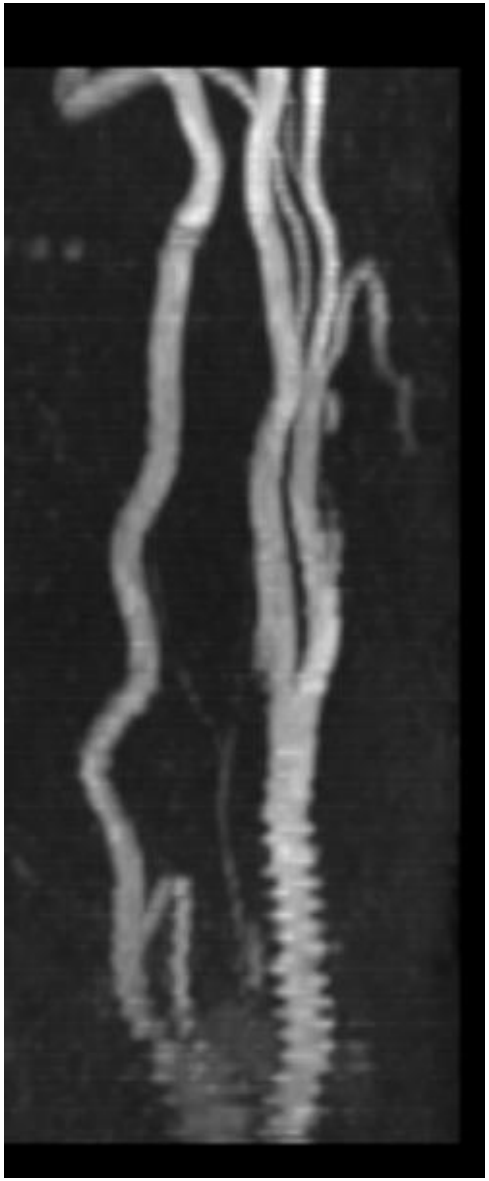

83f Score

0-30

31-50

51-70

>70

Near occlusion

Occluded

Quality

1

2

3

4

5

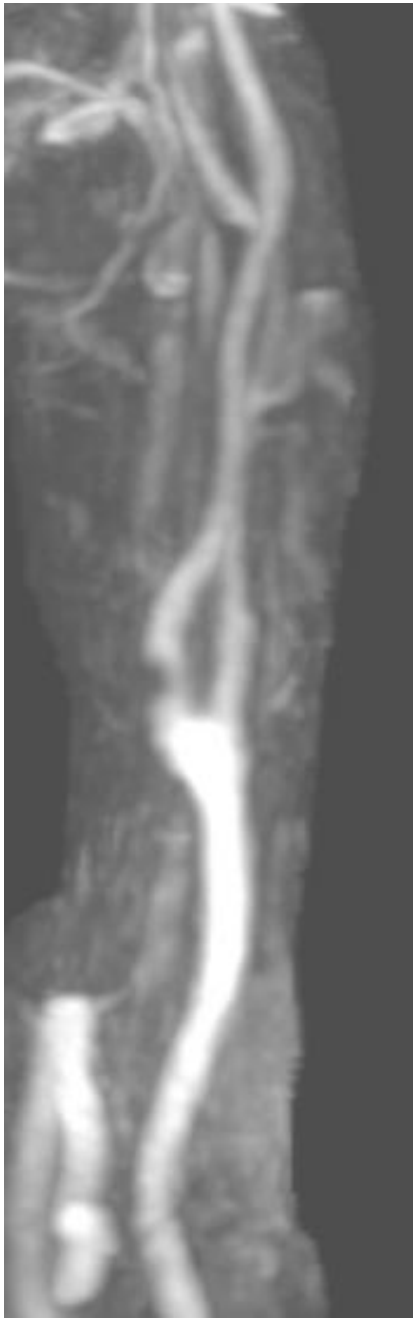

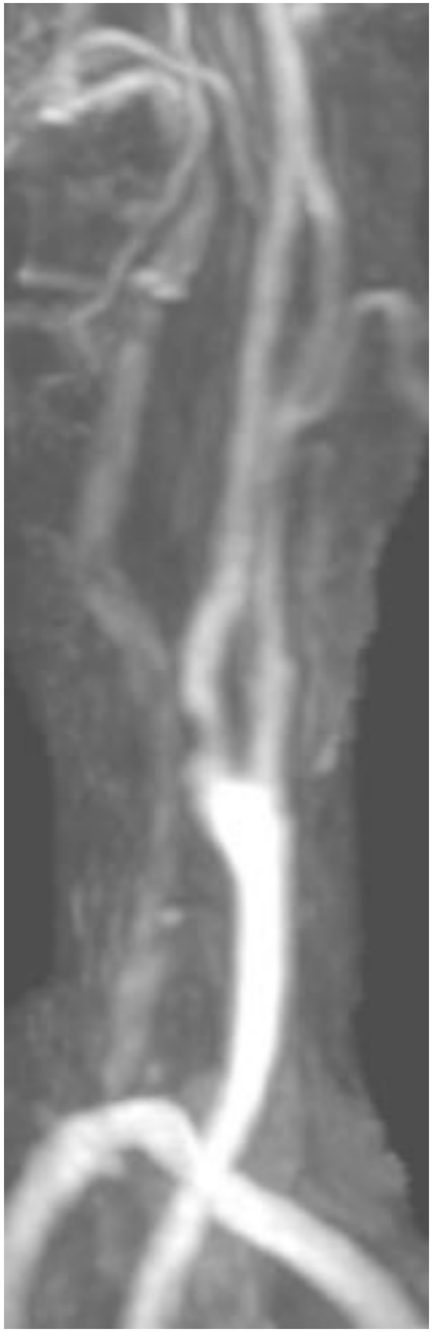

84e Score

0-30

31-50

51-70

>70

Near occlusion

Occluded

Quality

1

2

3

4

5

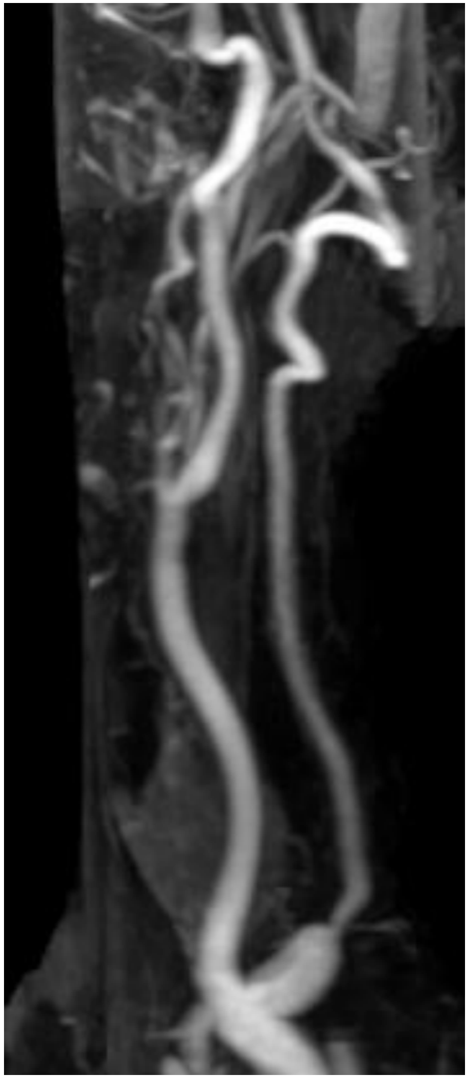

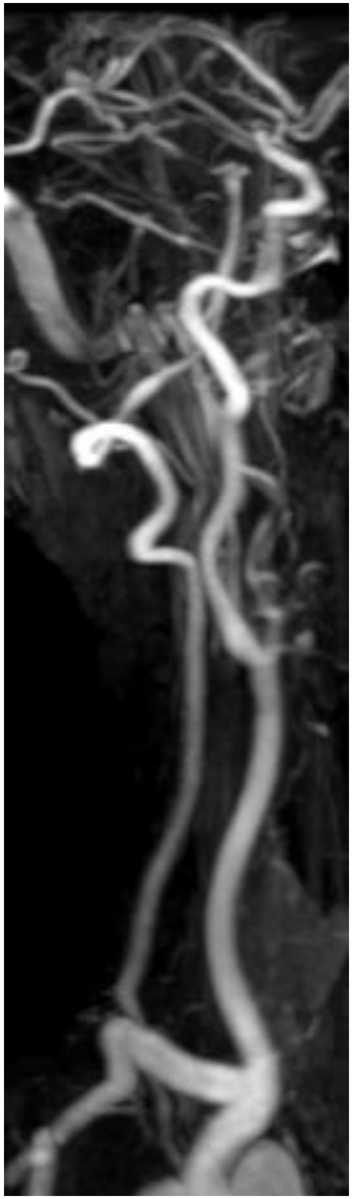

85d Score

0-30

31-50

51-70

>70

Near occlusion

Occluded

Quality

1

2

3

4

5

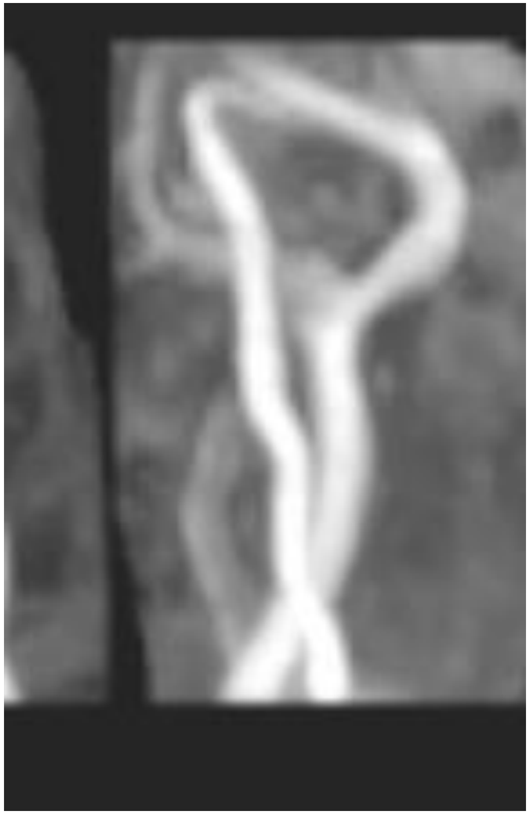

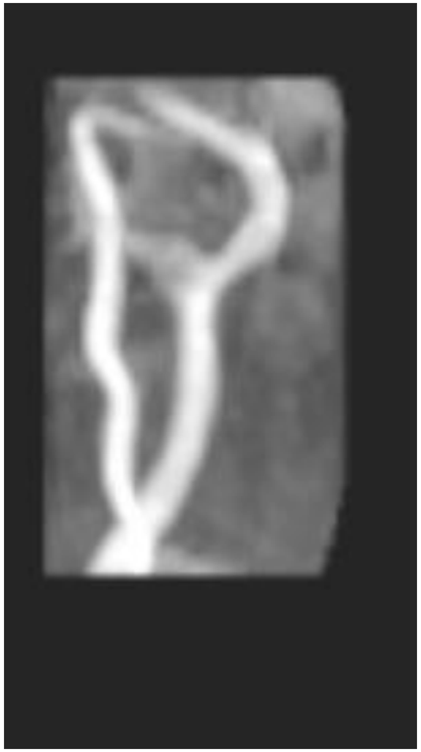

86c Score

0-30

31-50

51-70

>70

Near occlusion

Occluded

Quality

1

2

3

4

5

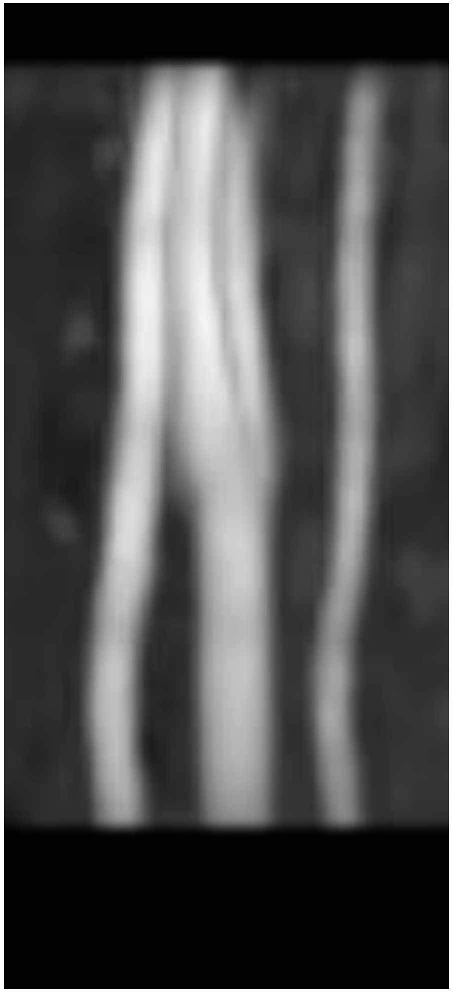

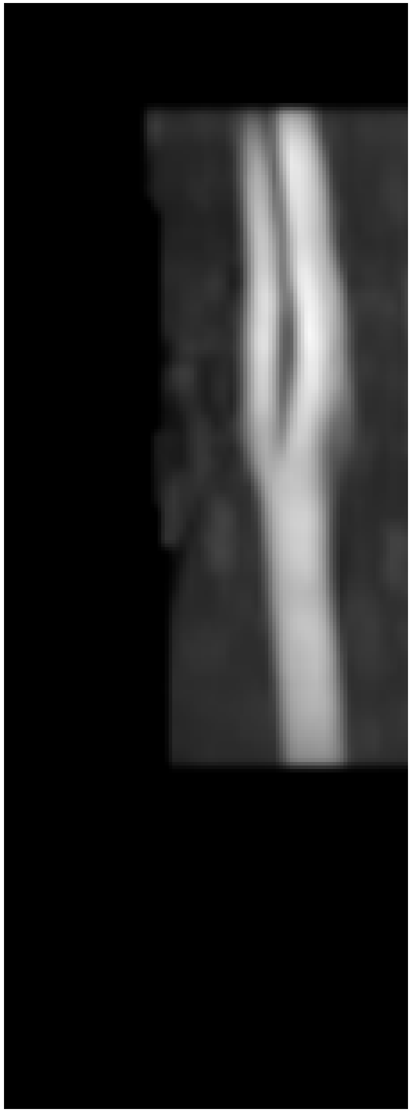

87b Score

0-30

31-50

51-70

>70

Near occlusion

Occluded

Quality

1

2

3

4

5

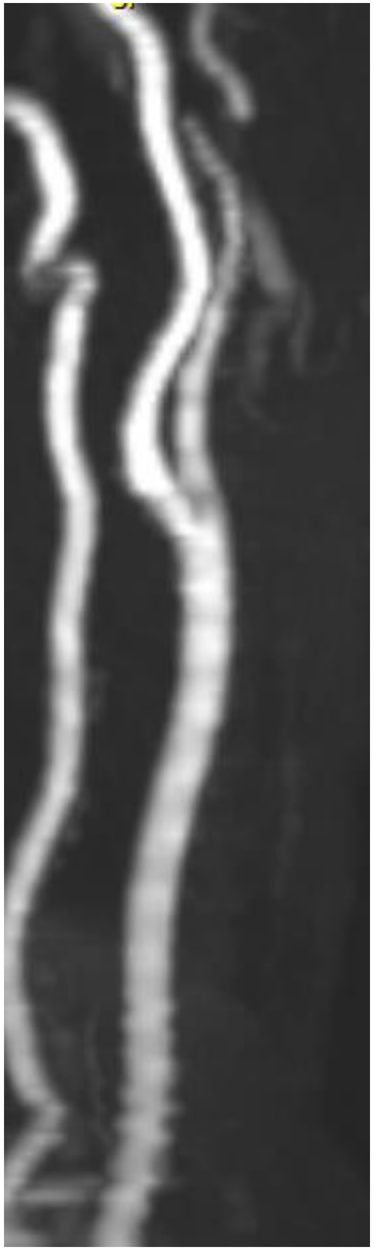

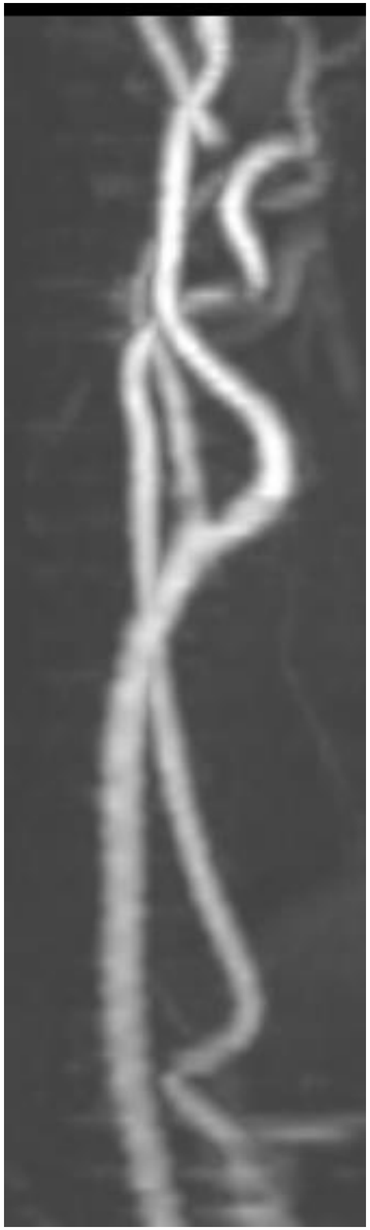

88a Score

0-30

31-50

51-70

>70

Near occlusion

Occluded

Quality

1

2

3

4

5

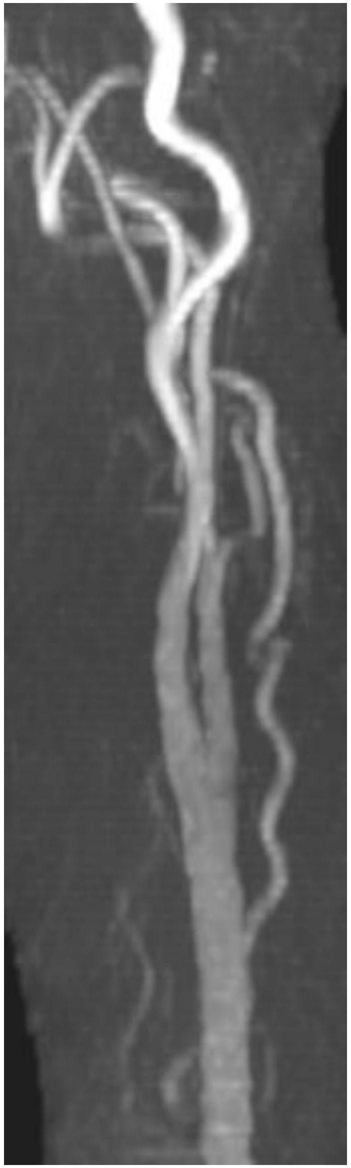

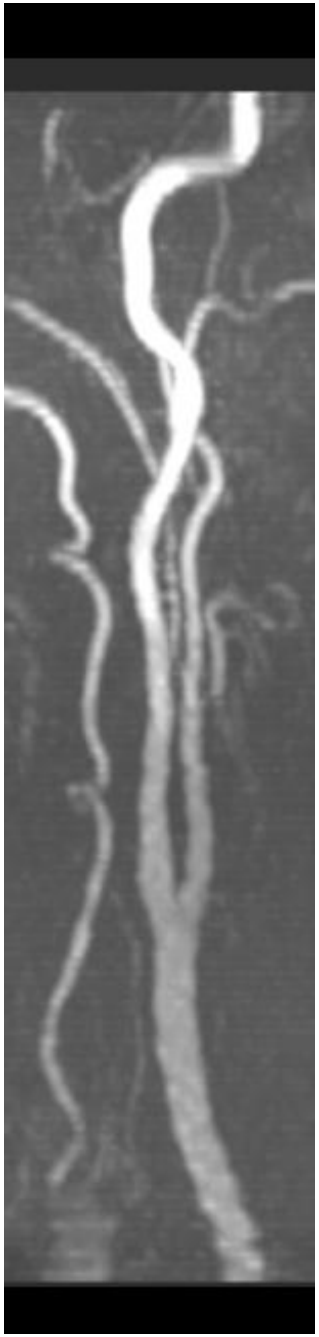

88f Score

0-30

31-50

51-70

>70

Near occlusion

Occluded

Quality

1

2

3

4

5

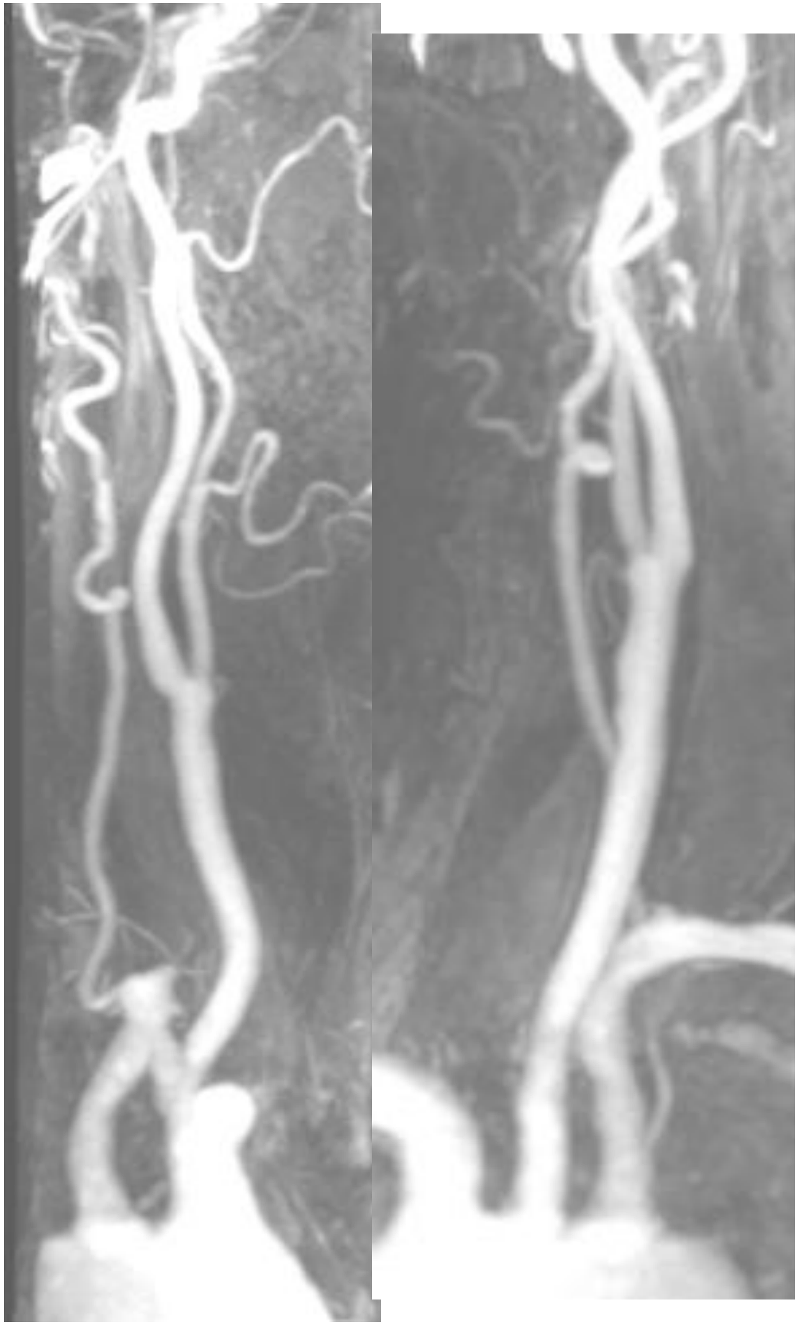

89e Score

0-30

31-50

51-70

>70

Near occlusion

Occluded

Quality

1

2

3

4

5

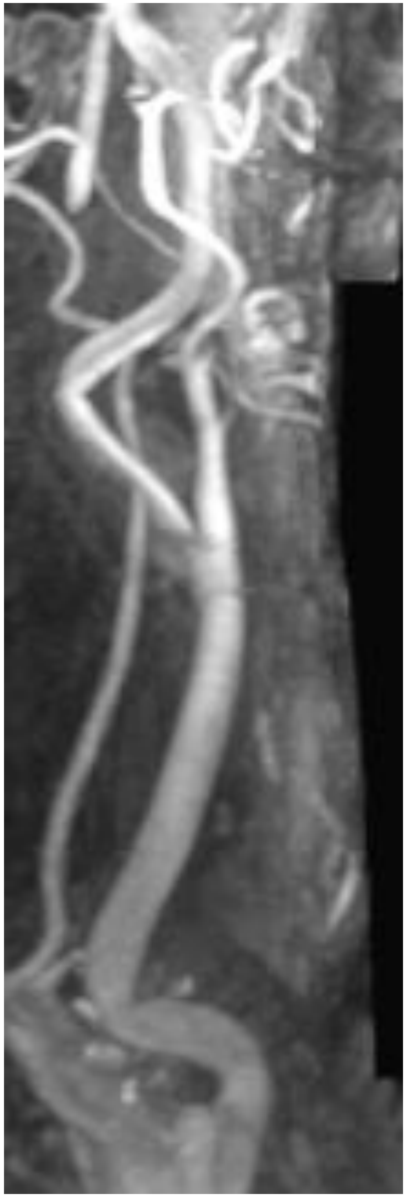

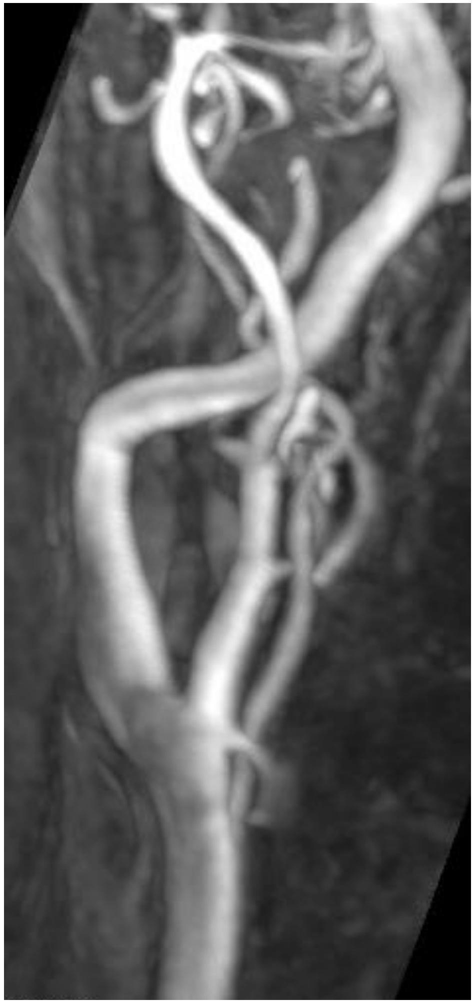

90d Score

0-30

31-50

51-70

>70

Near occlusion

Occluded

Quality

1

2

3

4

5

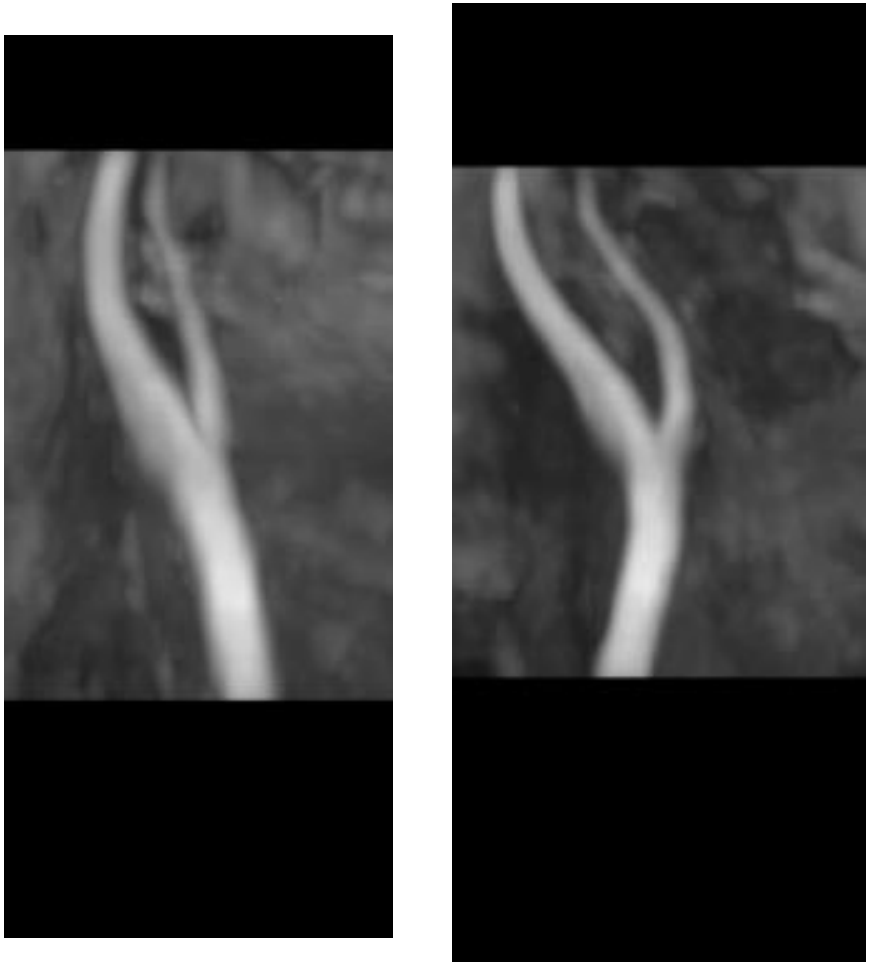

91c Score

0-30

31-50

51-70

>70

Near occlusion

Occluded

Quality

1

2

3

4

5

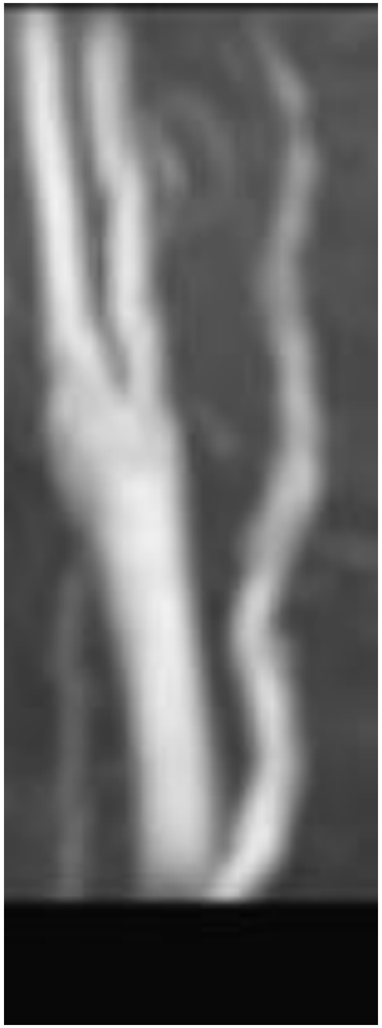

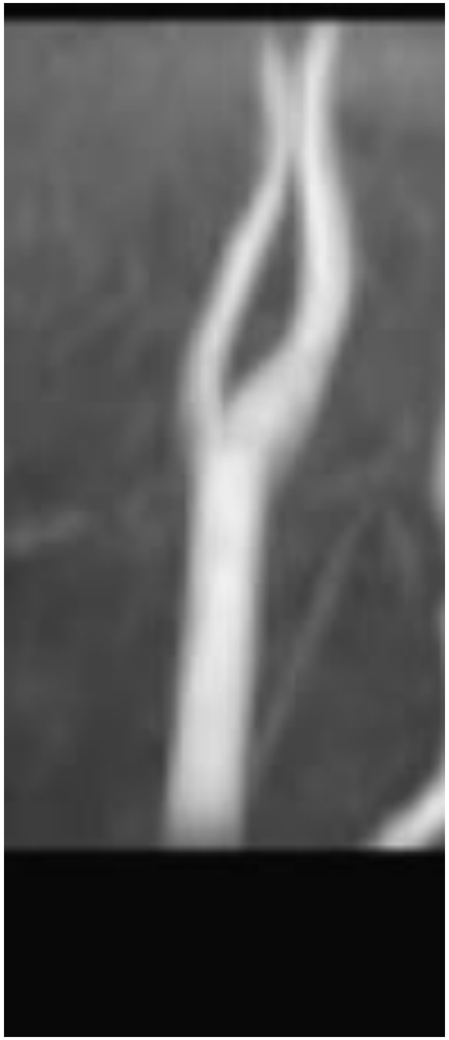

92b Score

0-30

31-50

51-70

>70

Near occlusion

Occluded

Quality

1

2

3

4

5

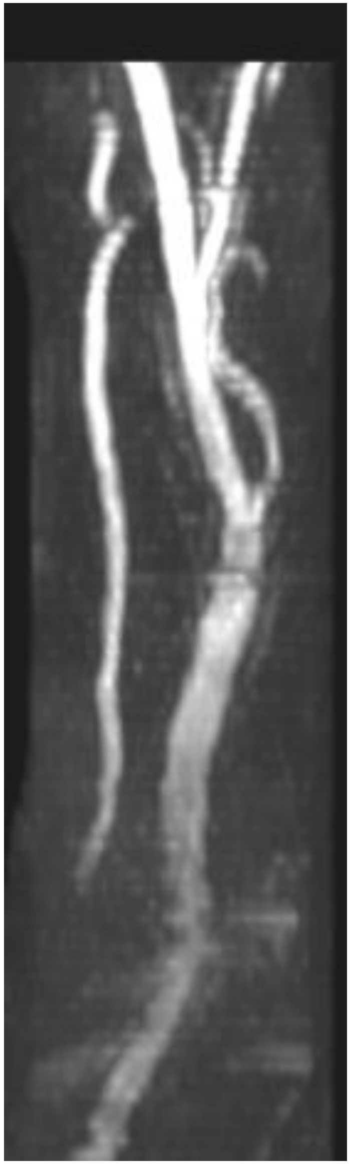

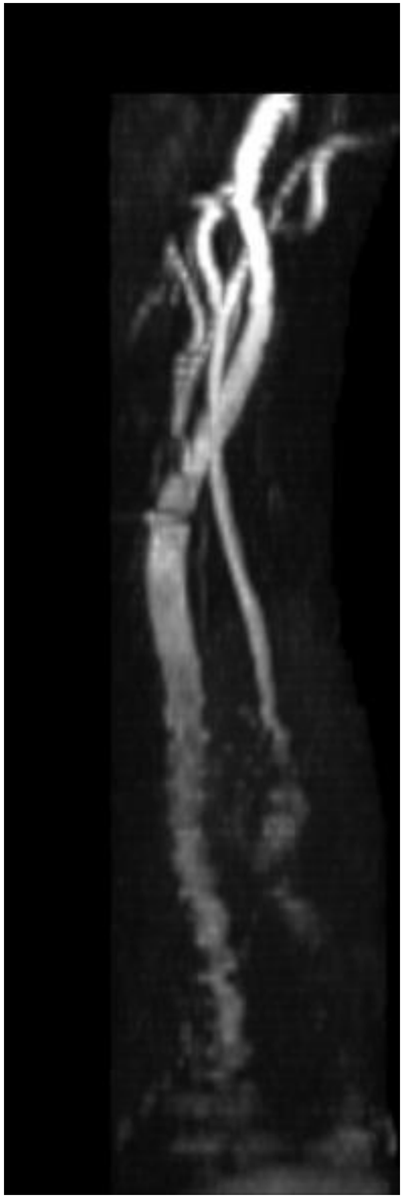

93a Score

0-30

31-50

51-70

>70

Near occlusion

Occluded

Quality

1

2

3

4

5

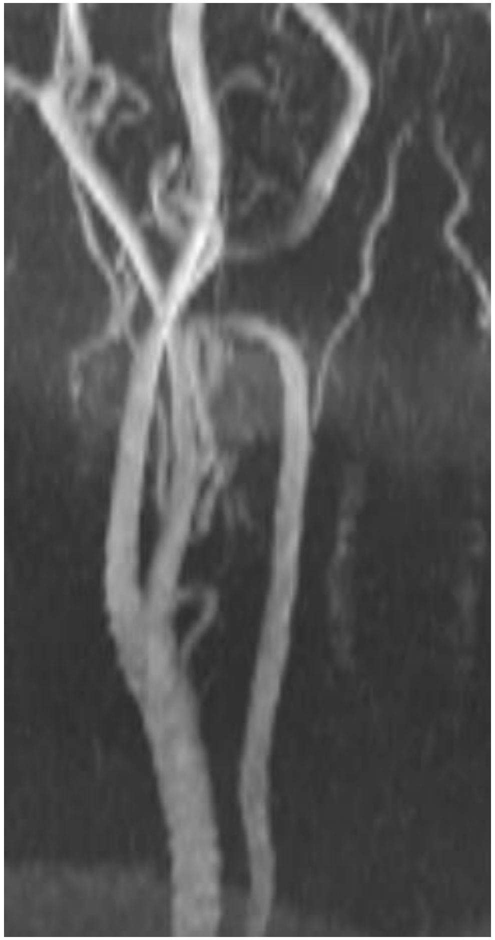

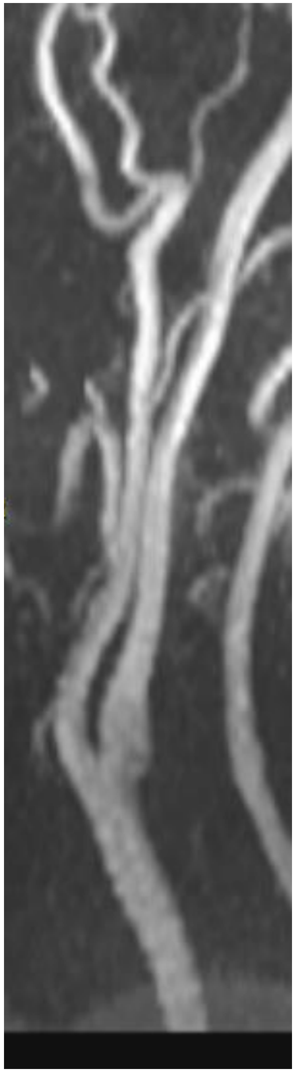

93f Score

0-30

31-50

51-70

>70

Near occlusion

Occluded

Quality

1

2

3

4

5

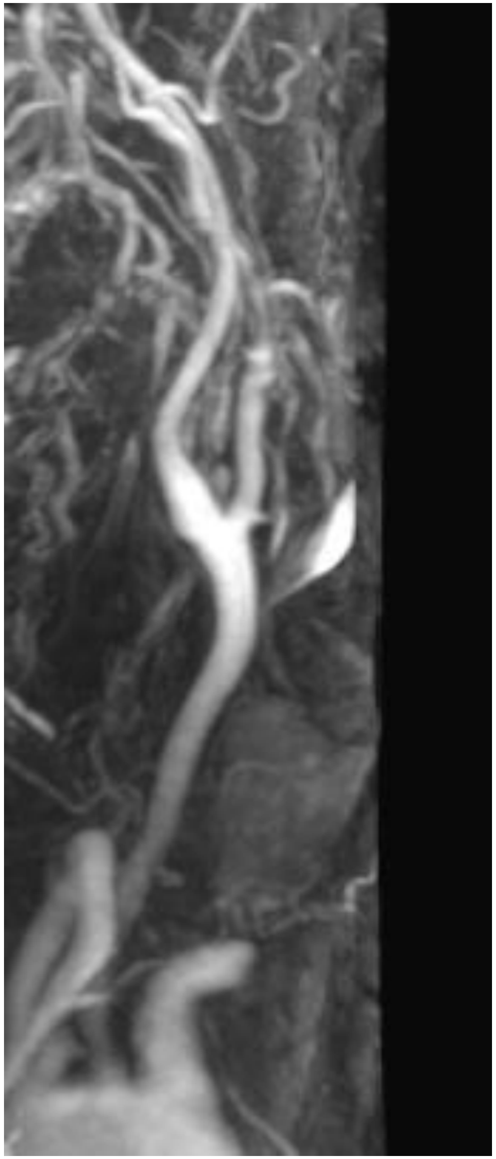

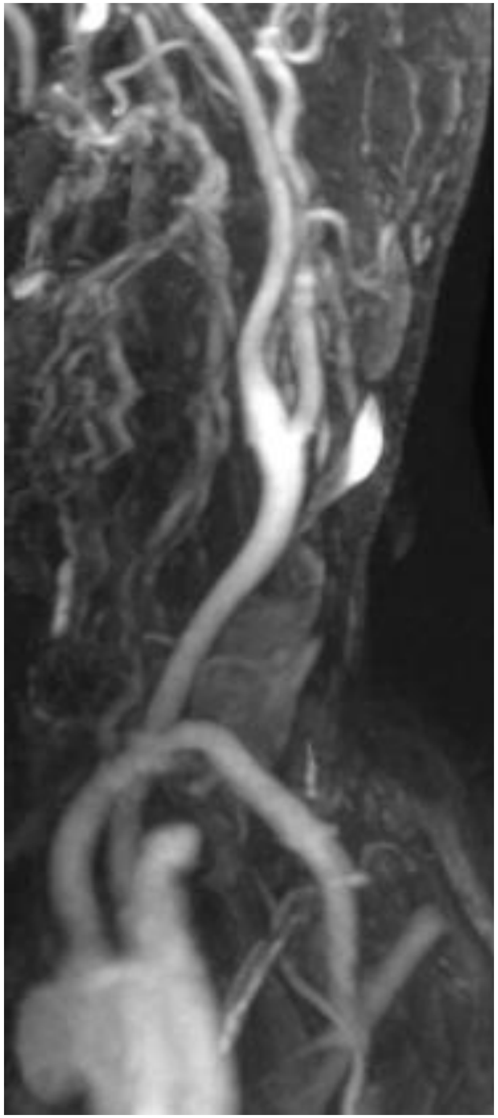

94e Score

0-30

31-50

51-70

>70

Near occlusion

Occluded

Quality

1

2

3

4

5

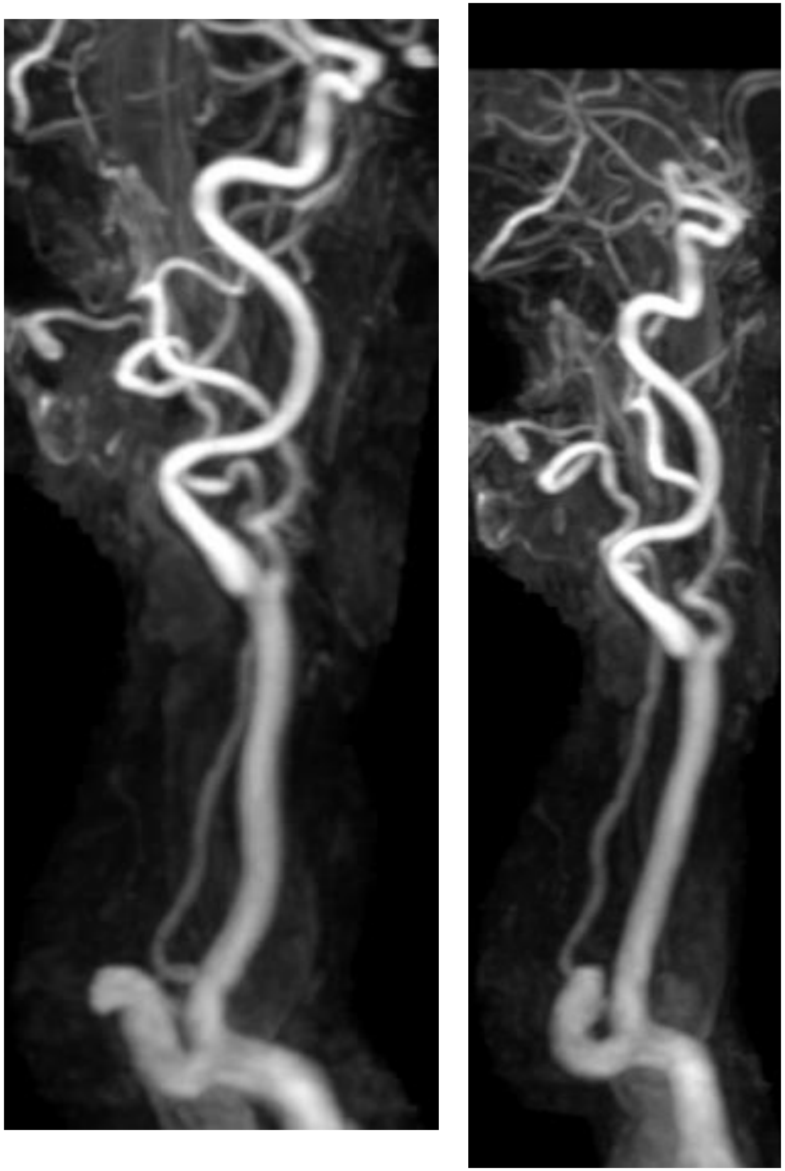

95d Score

0-30

31-50

51-70

>70

Near occlusion

Occluded

Quality

1

2

3

4

5

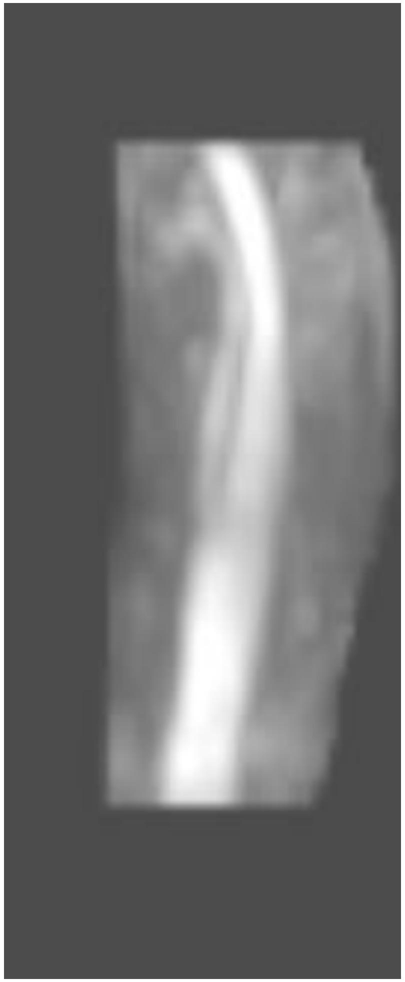

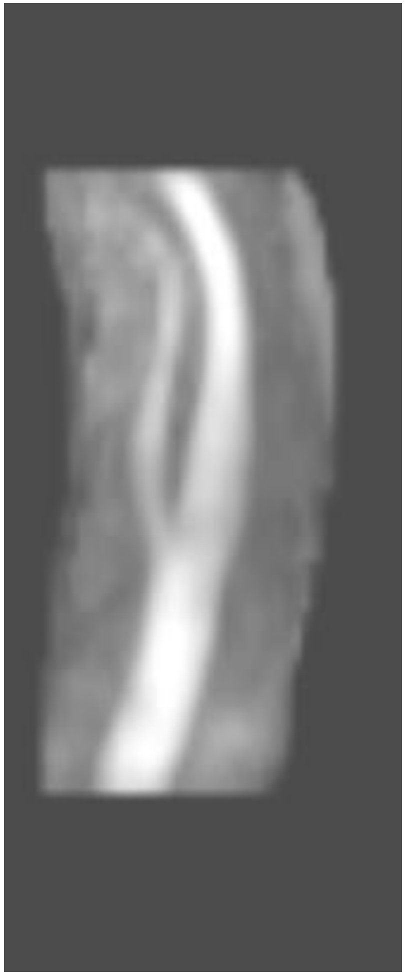

96c Score

0-30

31-50

51-70

>70

Near occlusion

Occluded

Quality

1

2

3

4

5

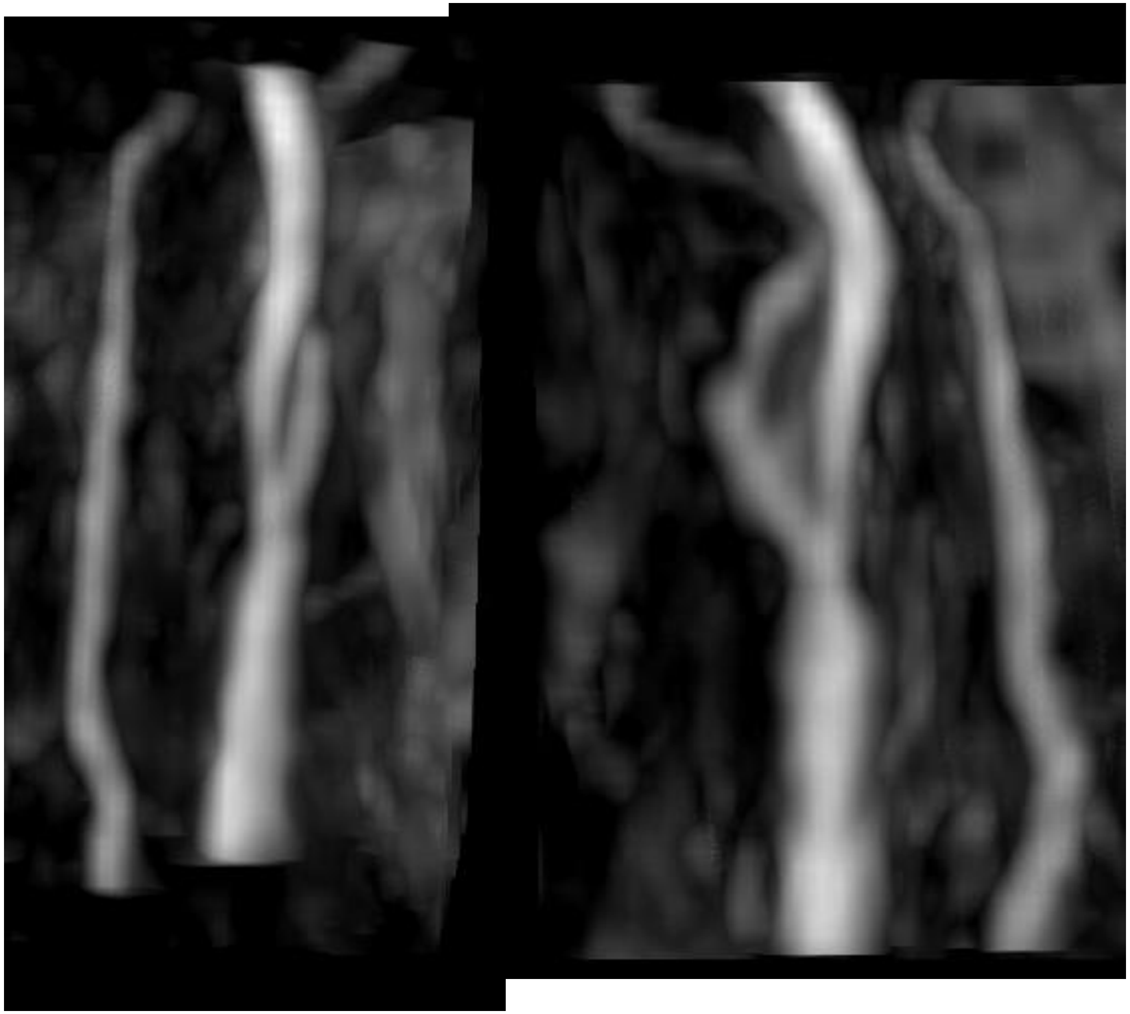

97b Score

0-30

31-50

51-70

>70

Near occlusion

Occluded

Quality

1

2

3

4

5

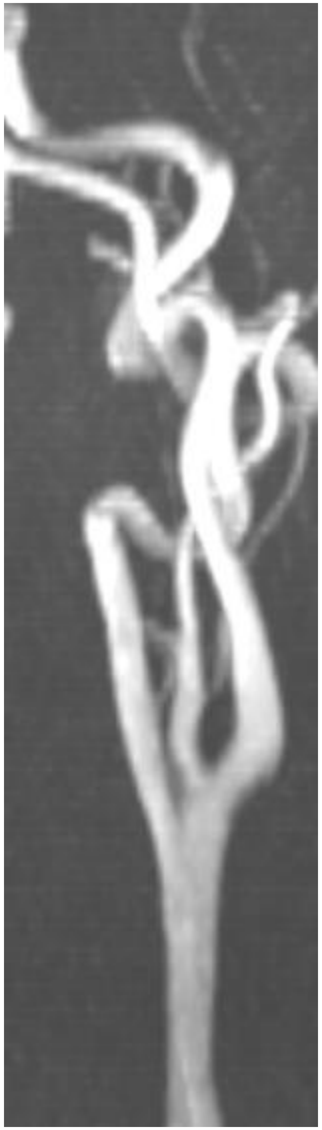

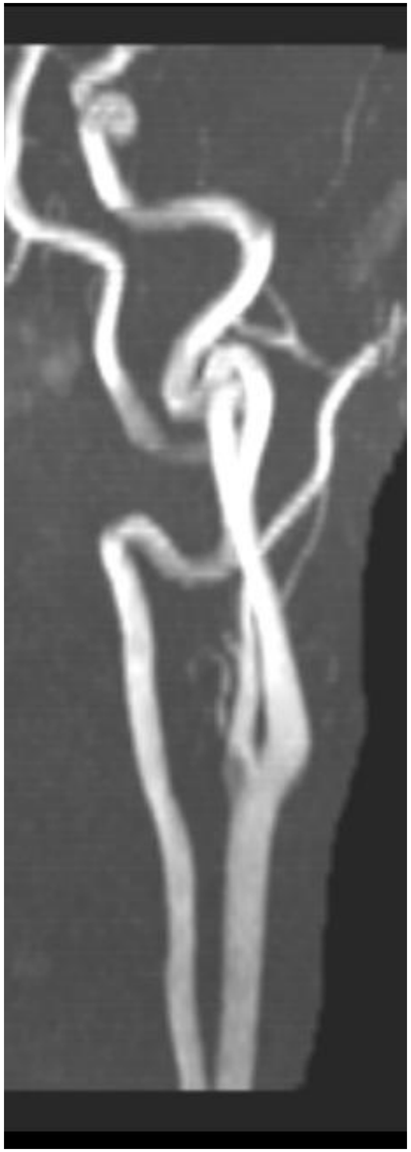

98a Score

0-30

31-50

51-70

>70

Near occlusion

Occluded

Quality

1

2

3

4

5

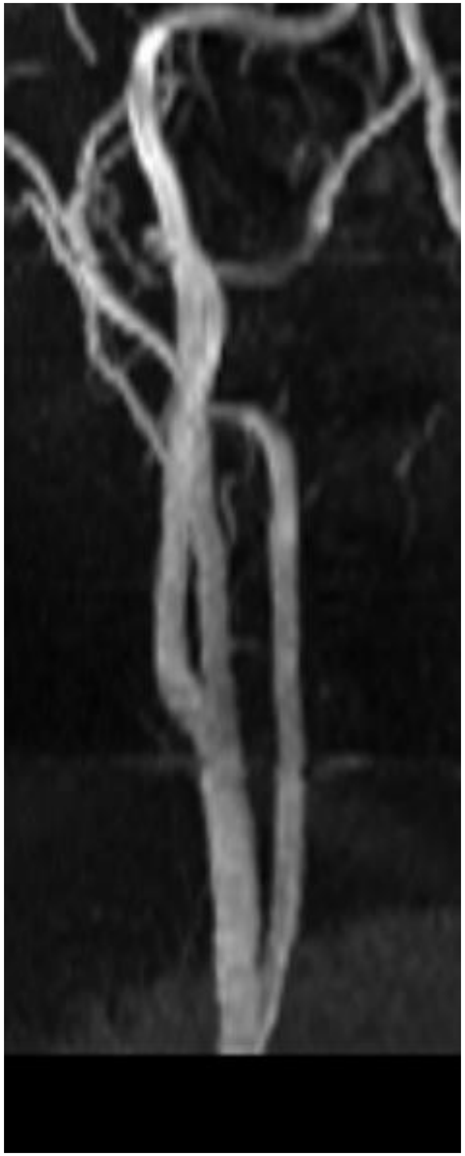

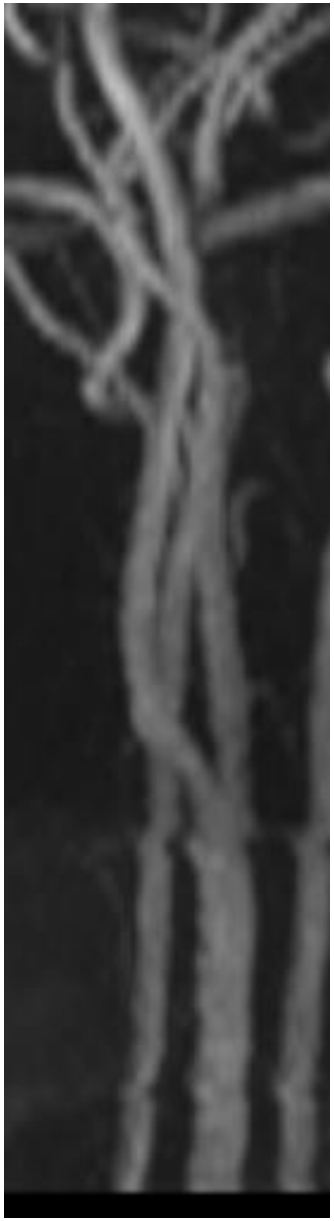

98f Score

0-30

31-50

51-70

>70

Near occlusion

Occluded

Quality

1

2

3

4

5

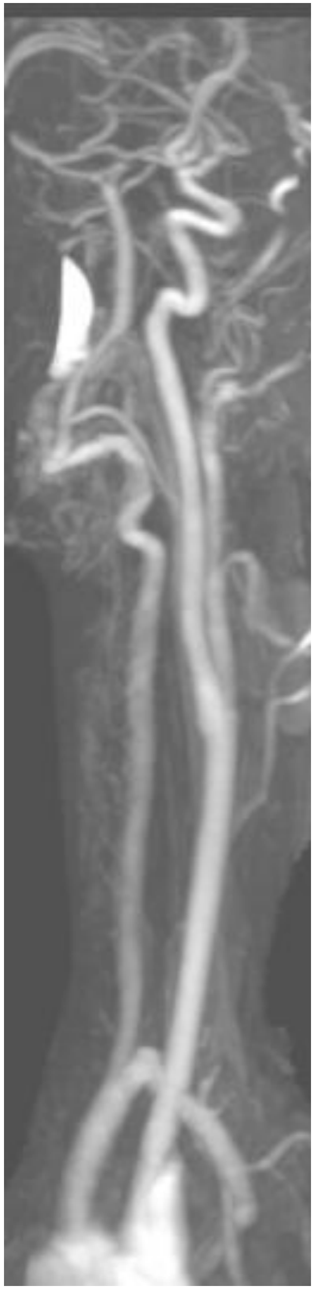

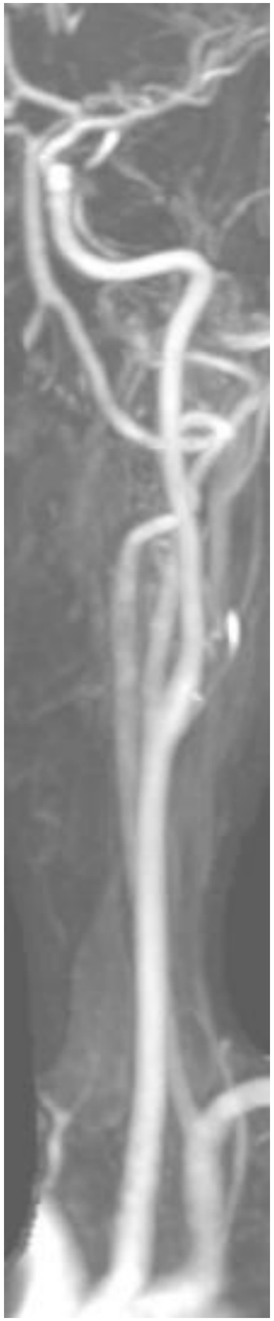

99e Score

0-30

31-50

51-70

>70

Near occlusion

Occluded

Quality

1

2

3

4

5

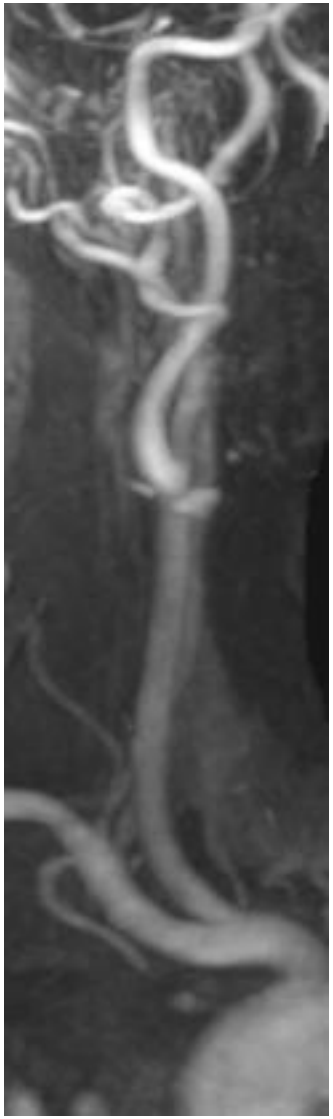

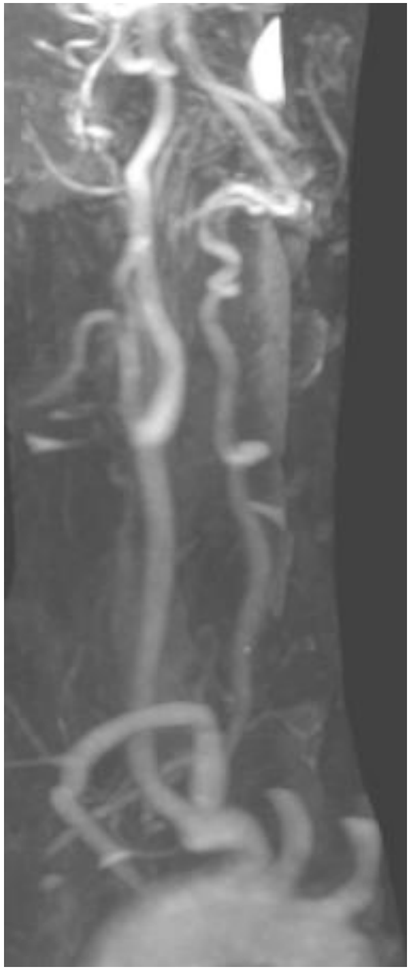

100d Score

0-30

31-50

51-70

>70

Near occlusion

Occluded

Quality

1

2

3

4

5

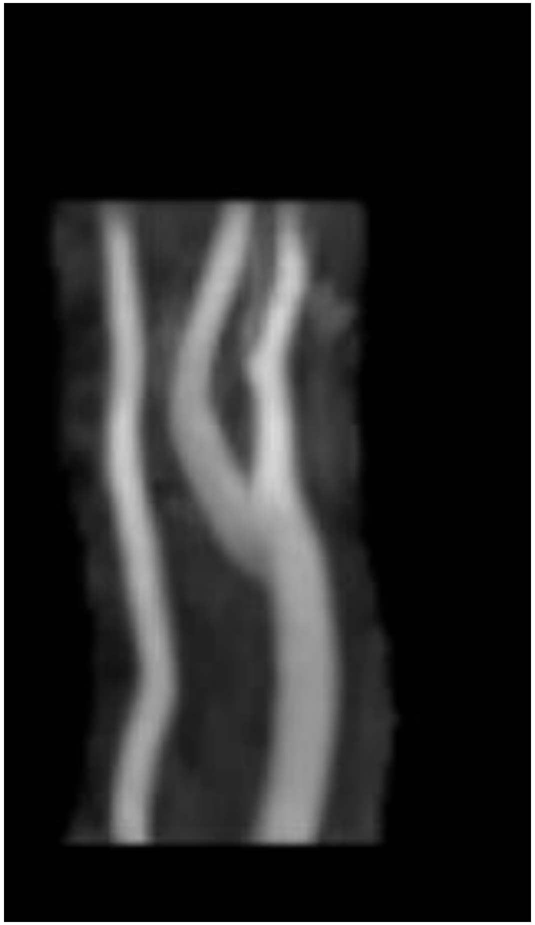

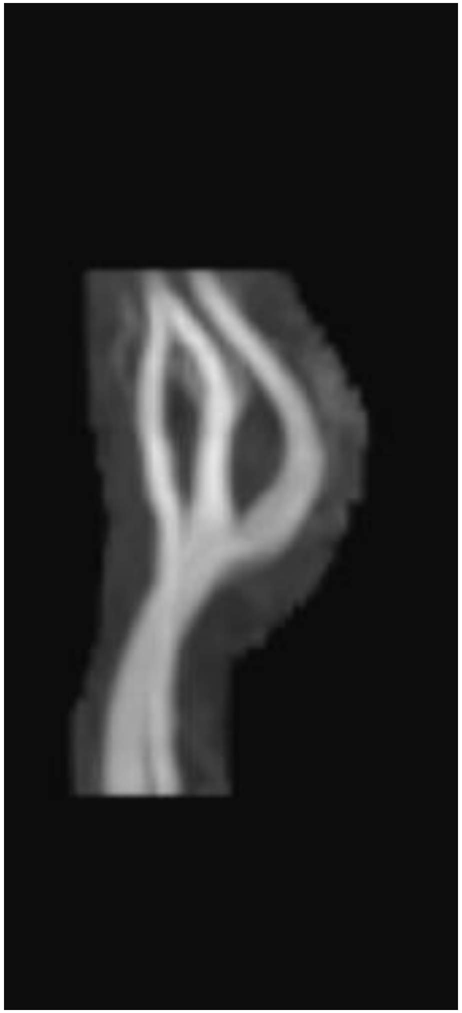

101c Score

0-30

31-50

51-70

>70

Near occlusion

Occluded

Quality

1

2

3

4

5

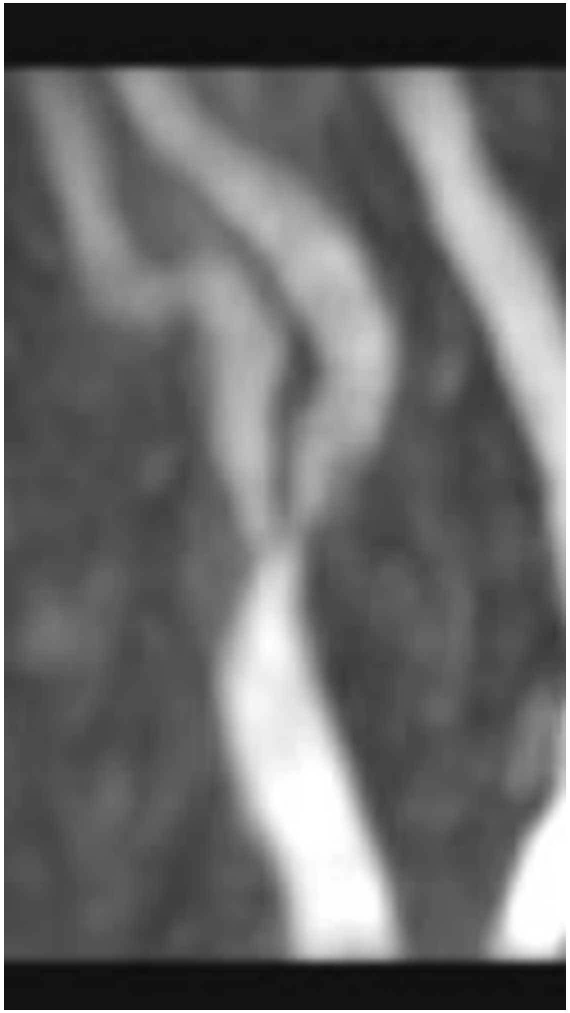

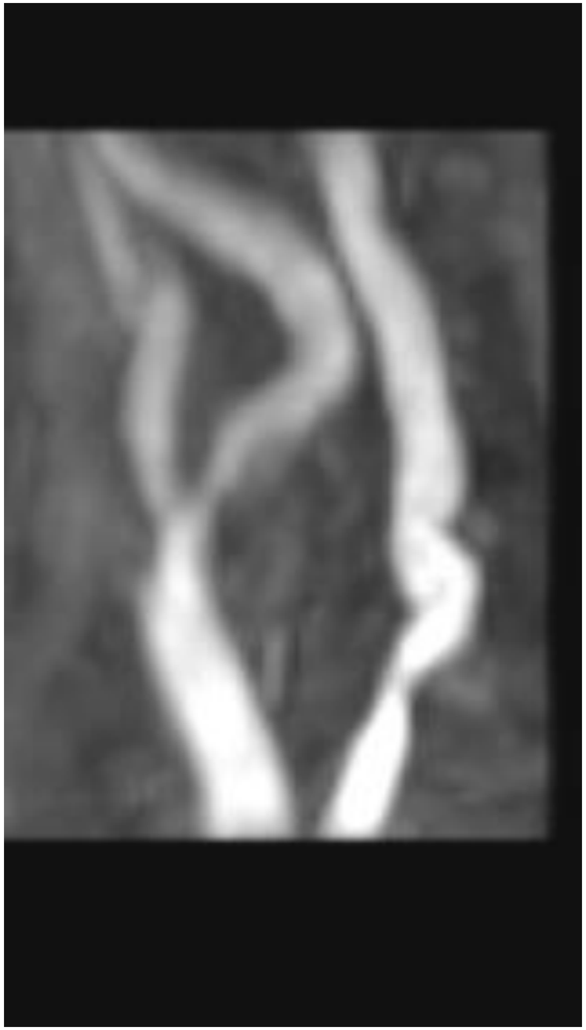

102b Score

0-30

31-50

51-70

>70

Near occlusion

Occluded

Quality

1

2

3

4

5

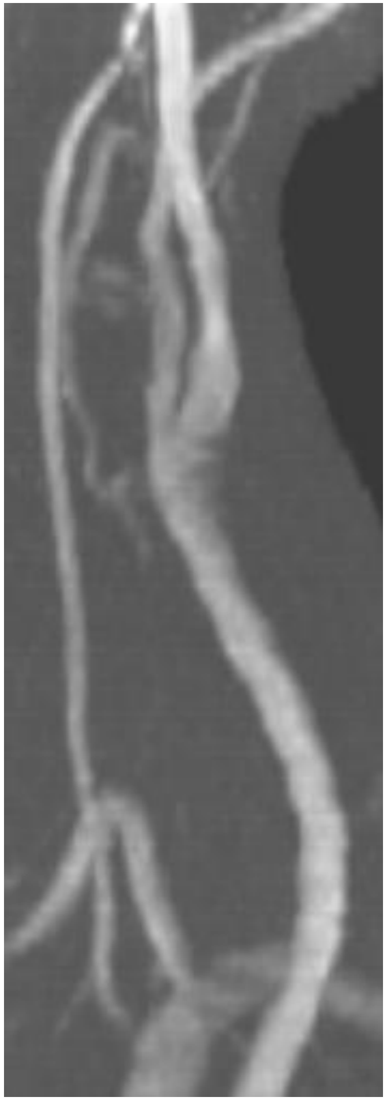

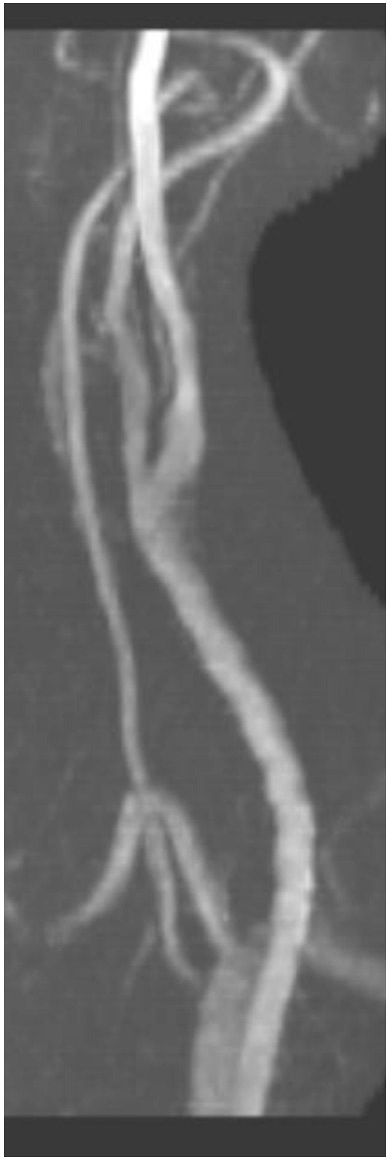

103a Score

0-30

31-50

51-70

>70

Near occlusion

Occluded

Quality

1

2

3

4

5

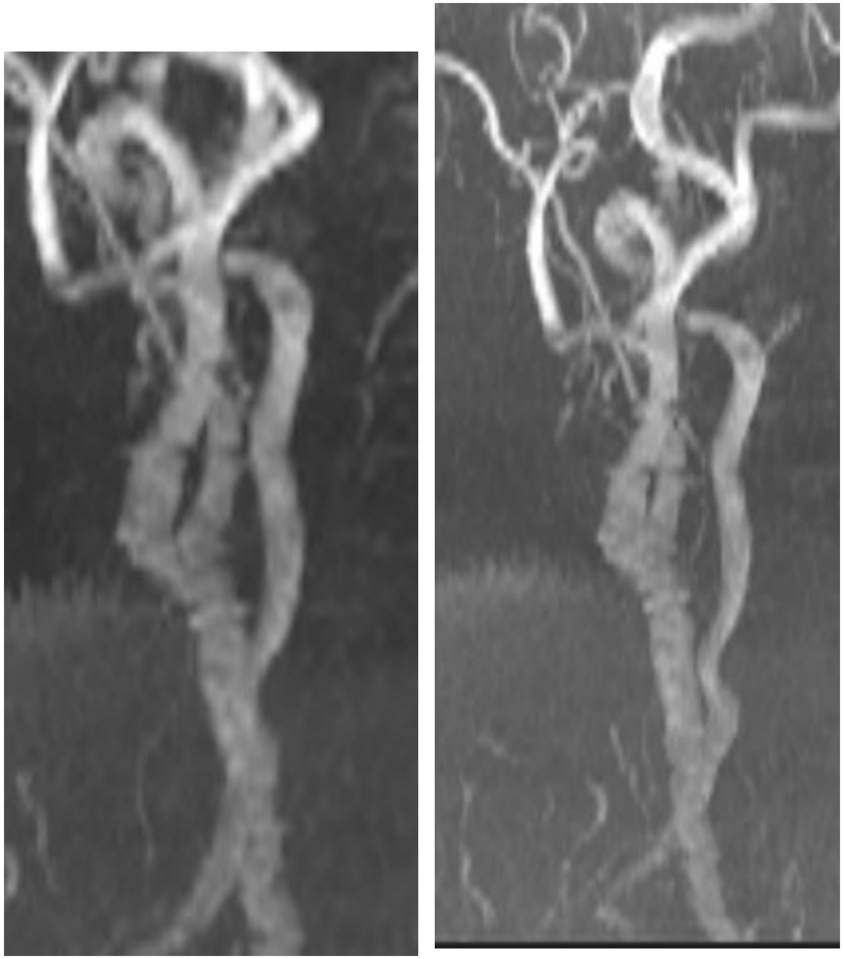

103f Score

0-30

31-50

51-70

>70

Near occlusion

Occluded

Quality

1

2

3

4

5

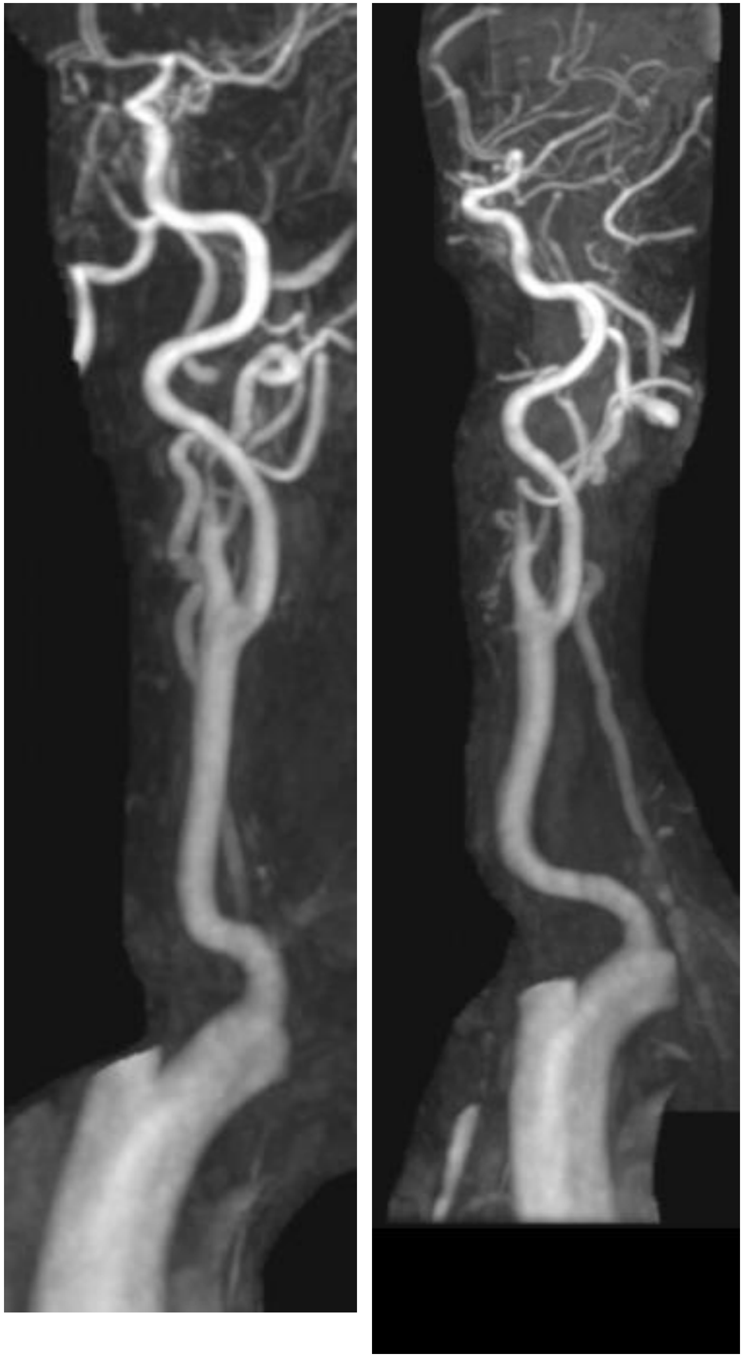

104e Score

0-30

31-50

51-70

>70

Near occlusion

Occluded

Quality

1

2

3

4

5

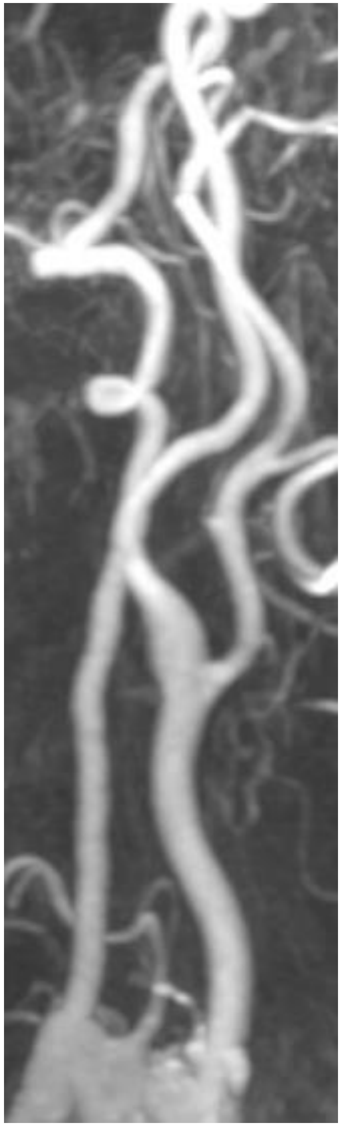

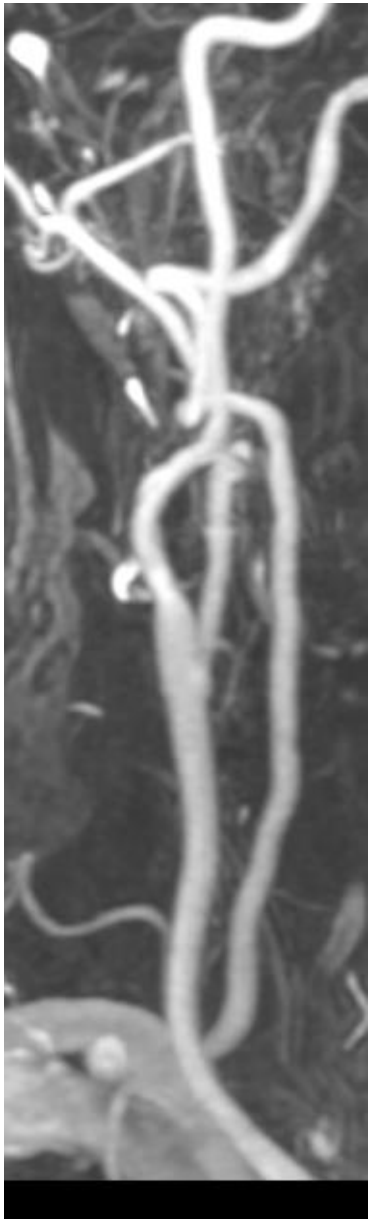

105d Score

0-30

31-50

51-70

>70

Near occlusion

Occluded

Quality

1

2

3

4

5

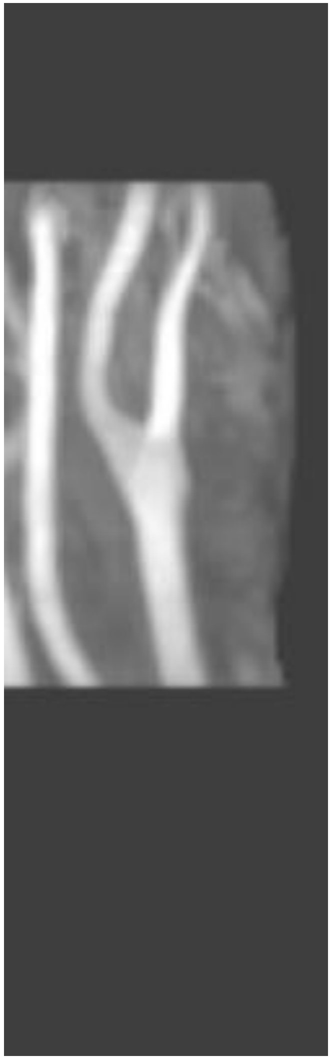

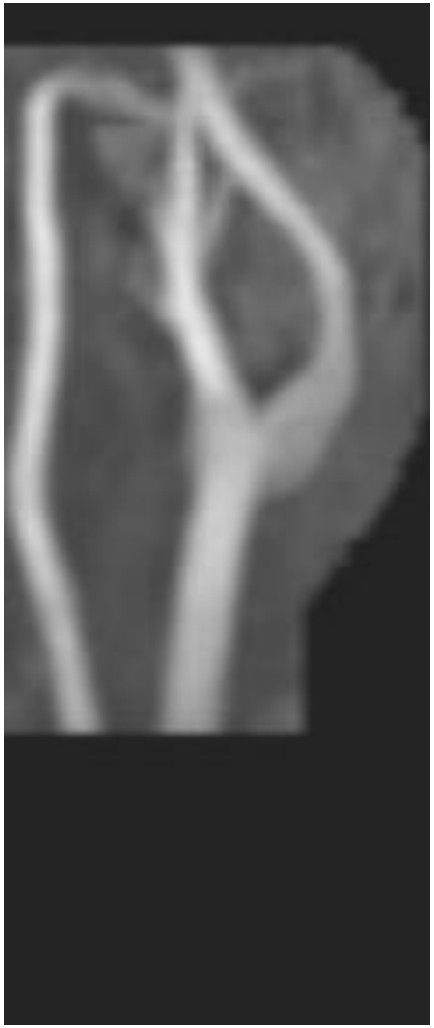

106c Score

0-30

31-50

51-70

>70

Near occlusion

Occluded

Quality

1

2

3

4

5

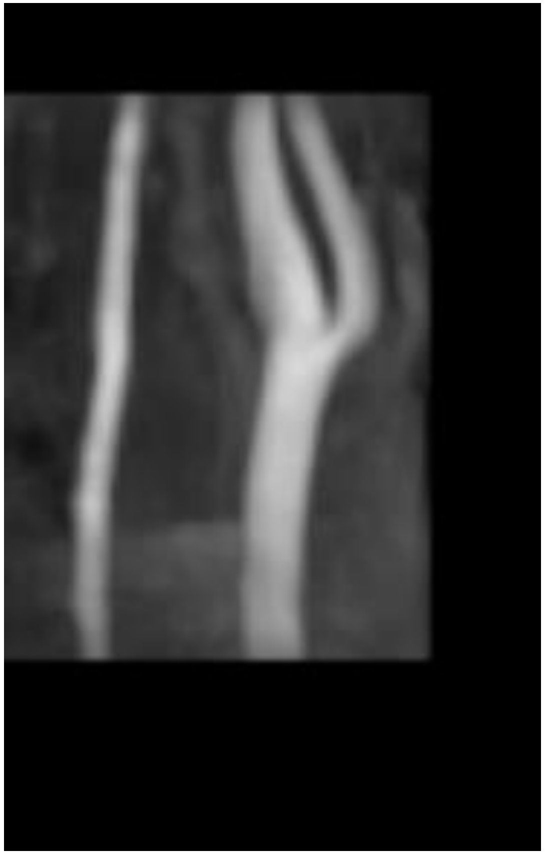

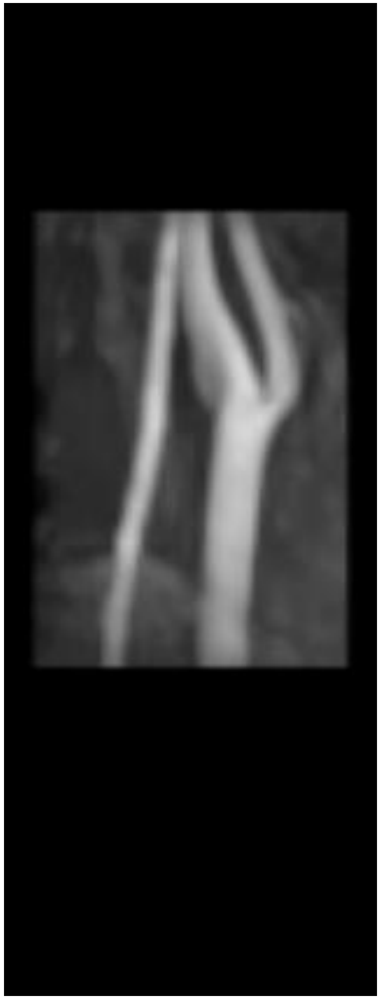

107b Score

0-30

31-50

51-70

>70

Near occlusion

Occluded

Quality

1

2

3

4

5

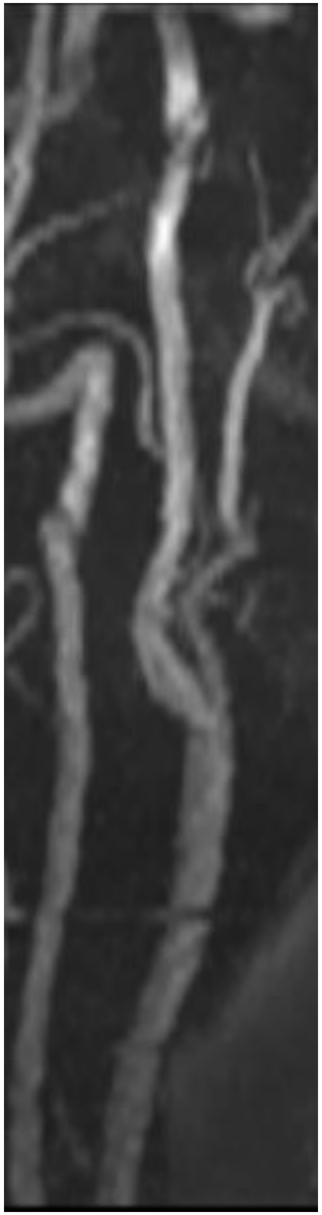

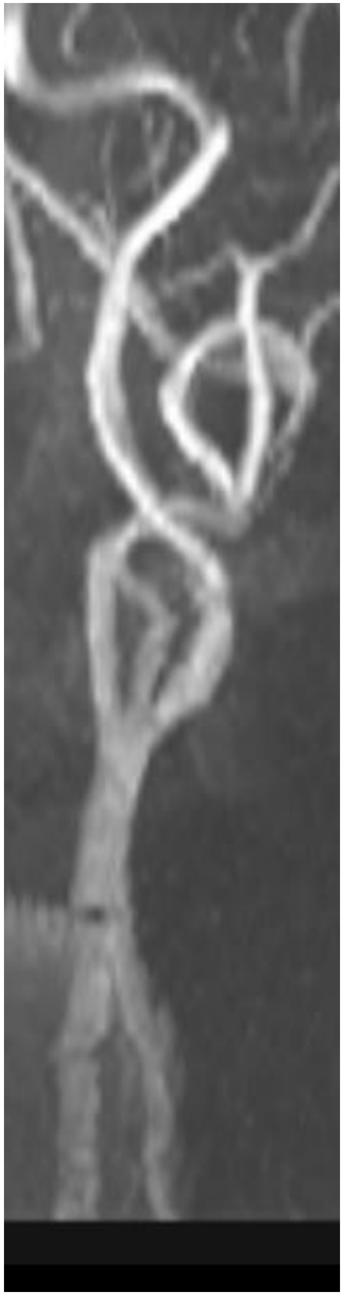

108a Score

0-30

31-50

51-70

>70

Near occlusion

Occluded

Quality

1

2

3

4

5

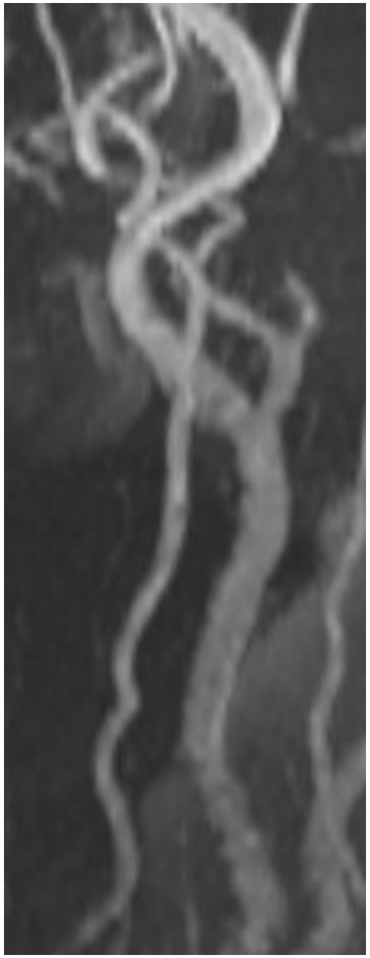

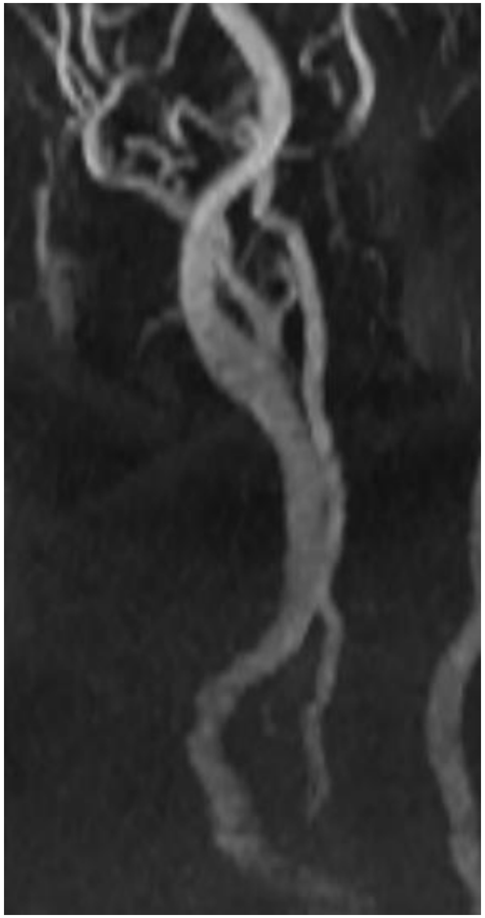

108f Score

0-30

31-50

51-70

>70

Near occlusion

Occluded

Quality

1

2

3

4

5

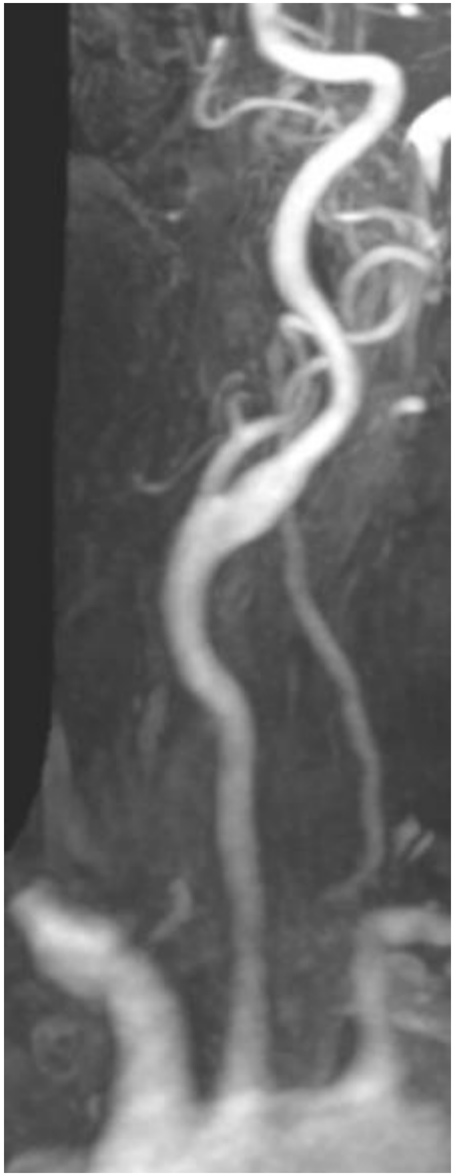

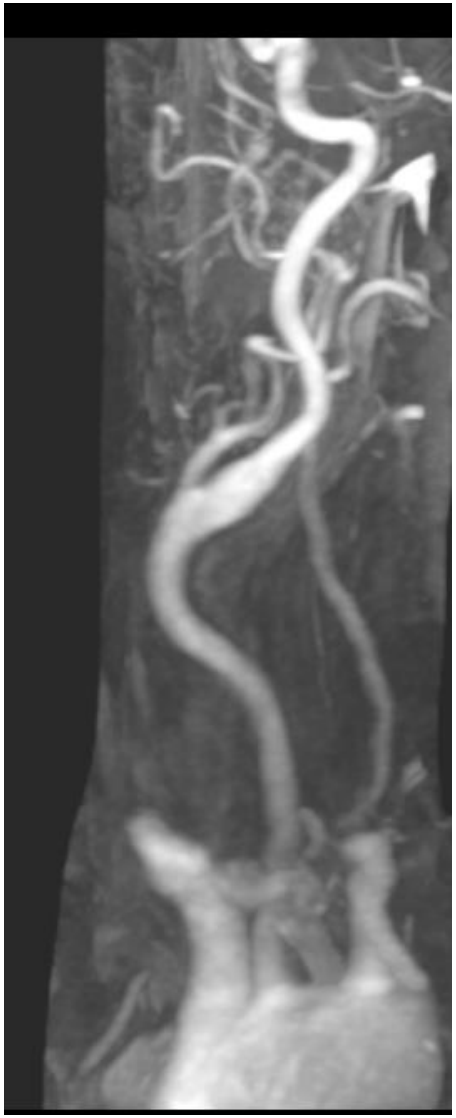

109e Score

0-30

31-50

51-70

>70

Near occlusion

Occluded

Quality

1

2

3

4

5

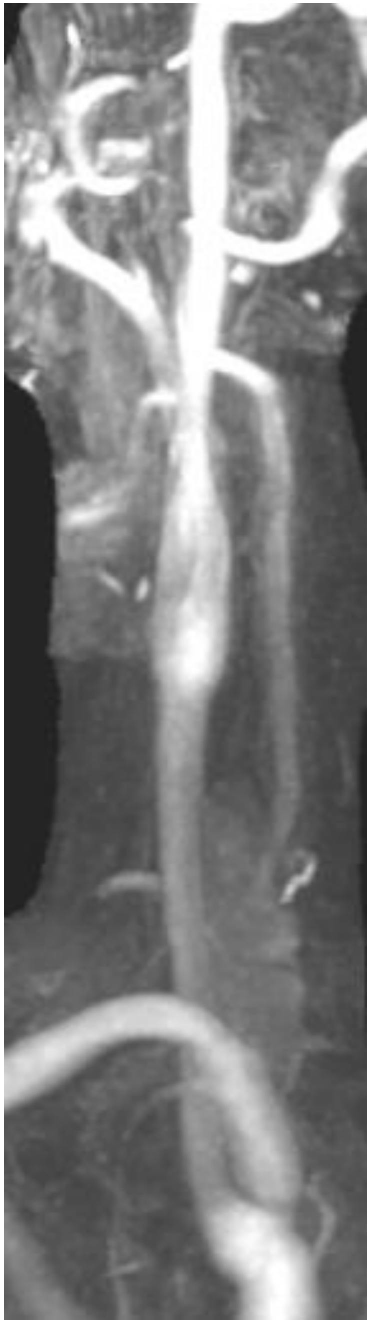

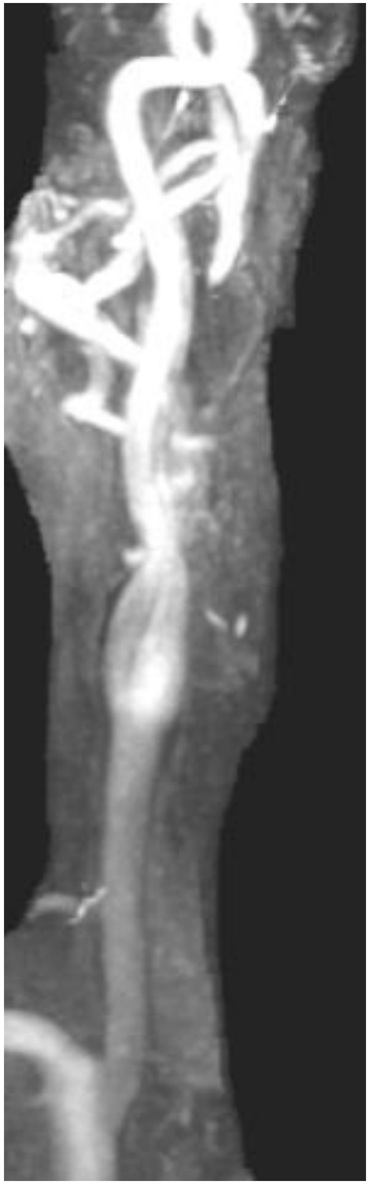

110d Score

0-30

31-50

51-70

>70

Near occlusion

Occluded

Quality

1

2

3

4

5

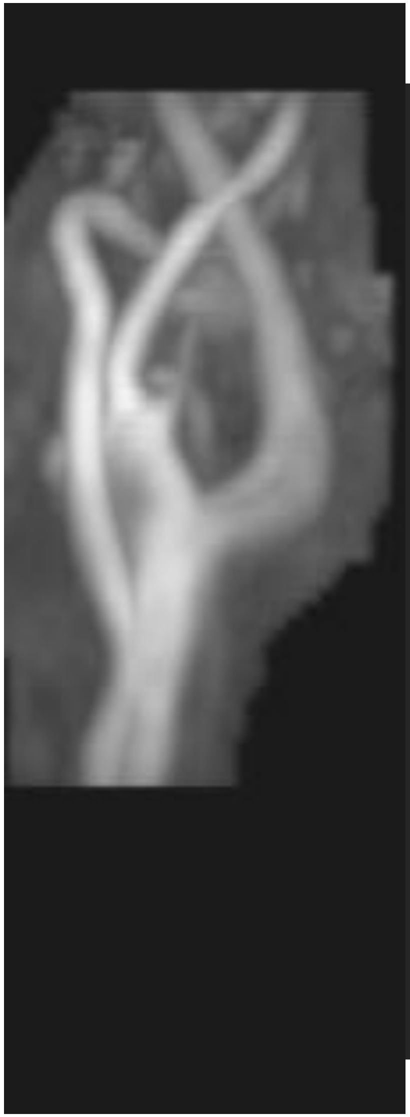

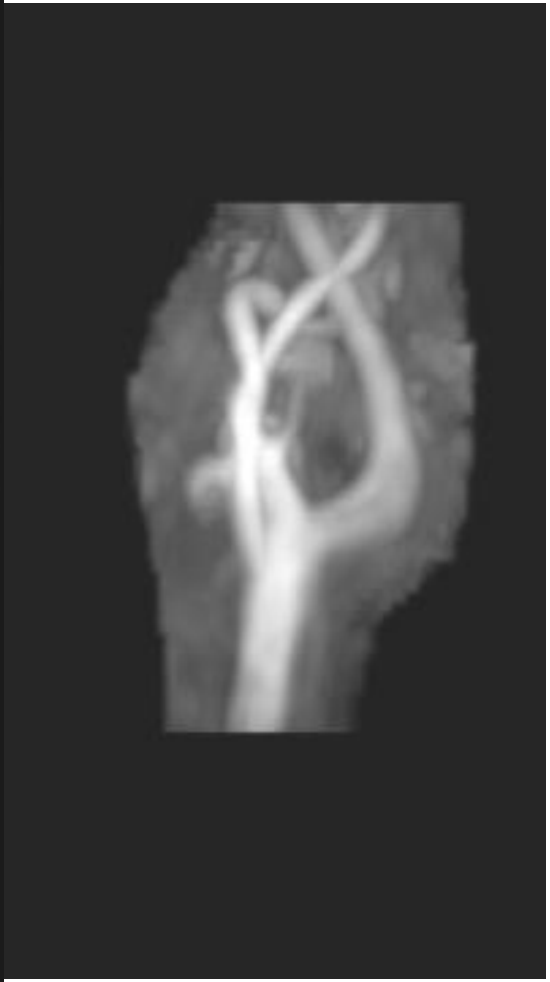

111c Score

0-30

31-50

51-70

>70

Near occlusion

Occluded

Quality

1

2

3

4

5

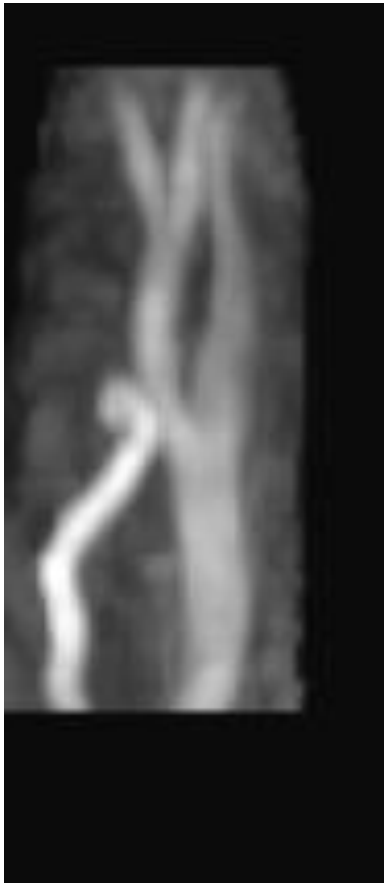

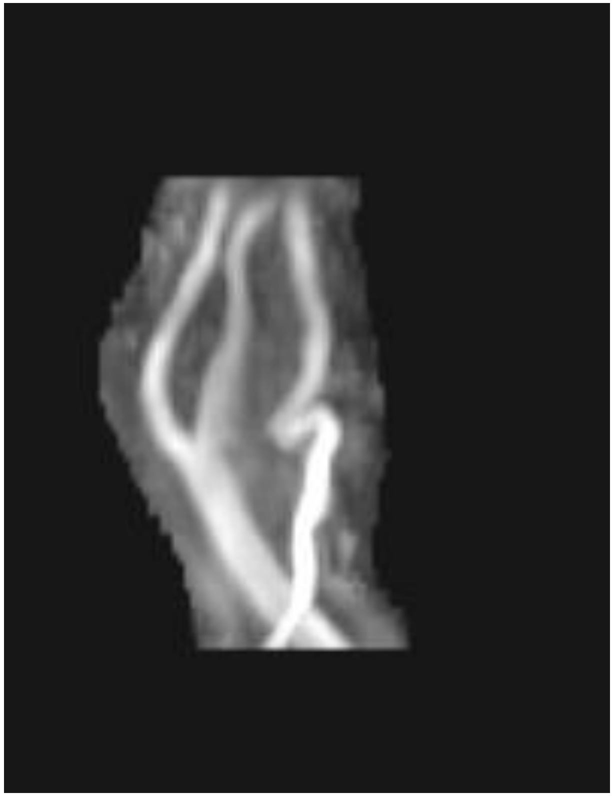

112b Score

0-30

31-50

51-70

>70

Near occlusion

Occluded

Quality

1

2

3

4

5

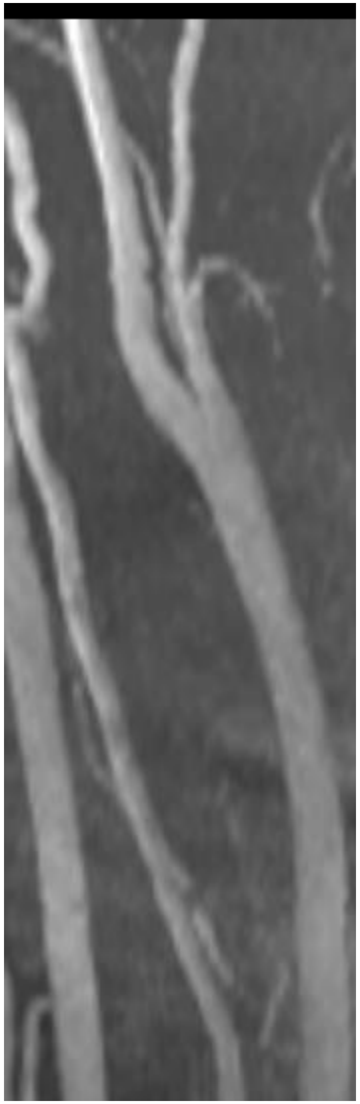

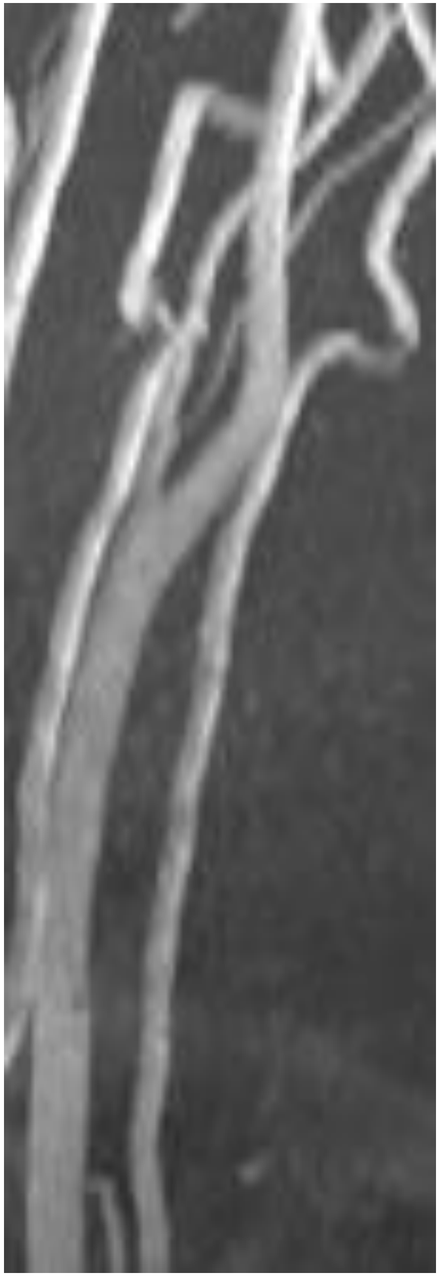

113a Score

0-30

31-50

51-70

>70

Near occlusion

Occluded

Quality

1

2

3

4

5

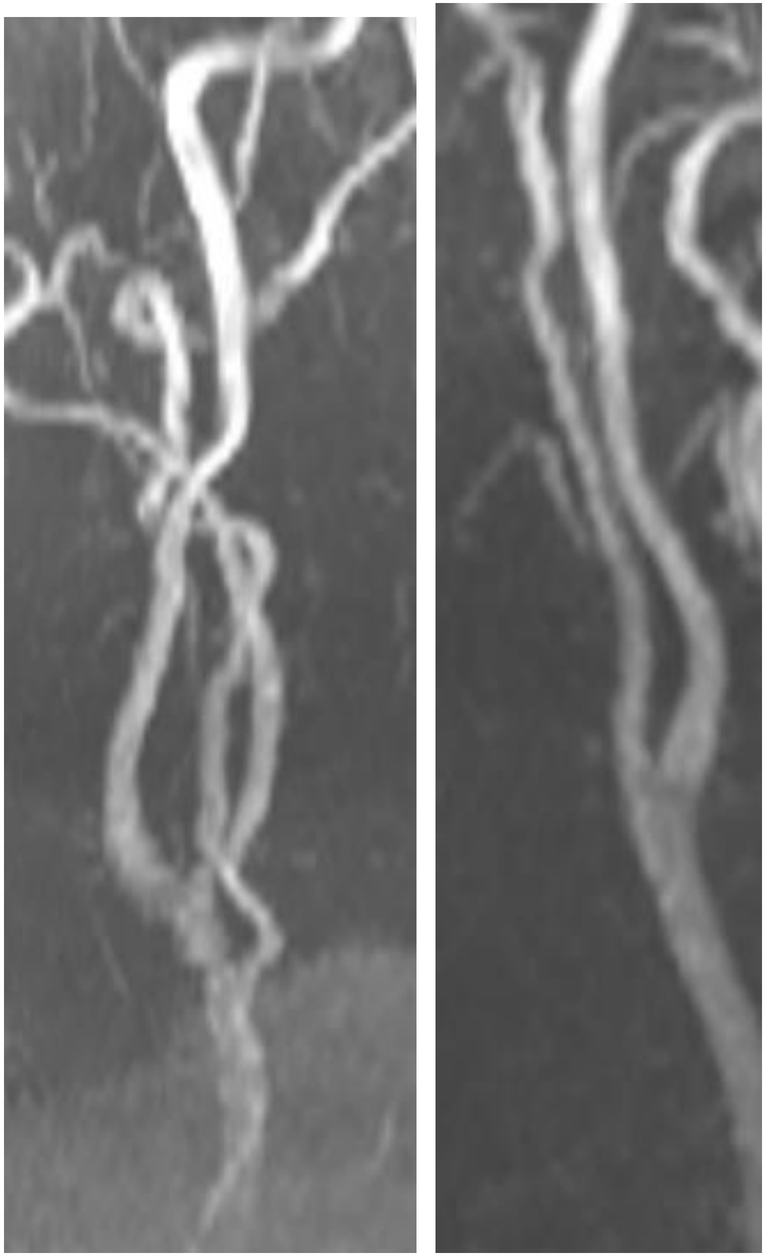

113f Score

0-30

31-50

51-70

>70

Near occlusion

Occluded

Quality

1

2

3

4

5

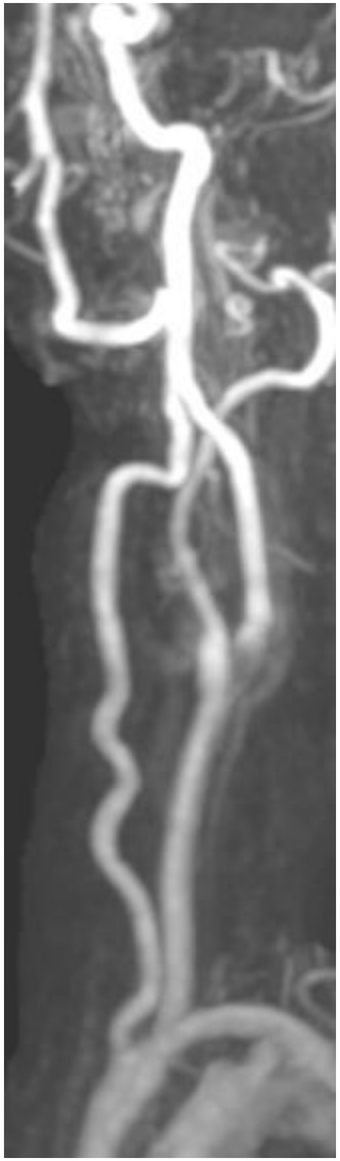

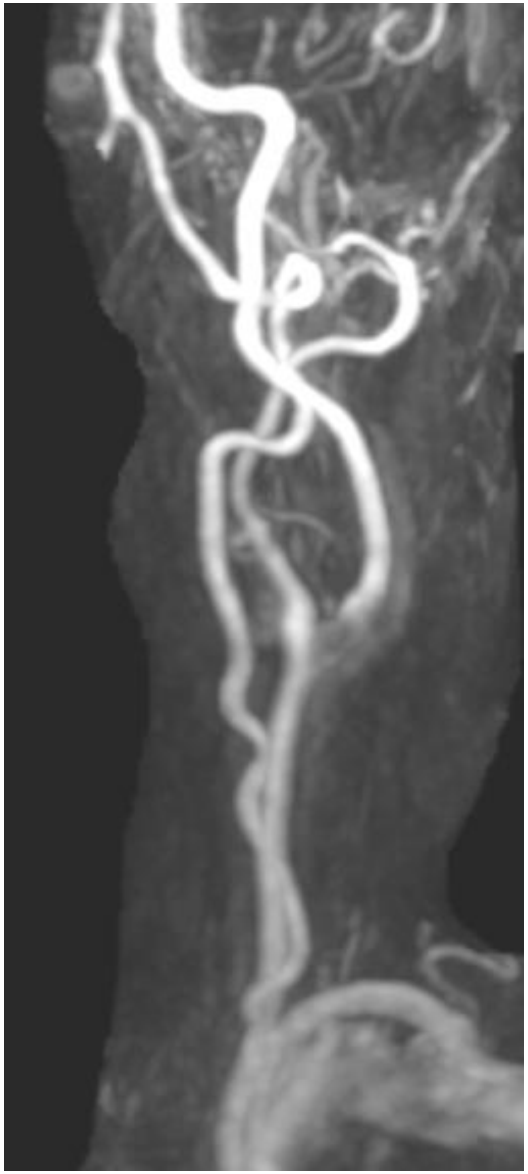

114e Score

0-30

31-50

51-70

>70

Near occlusion

Occluded

Quality

1

2

3

4

5

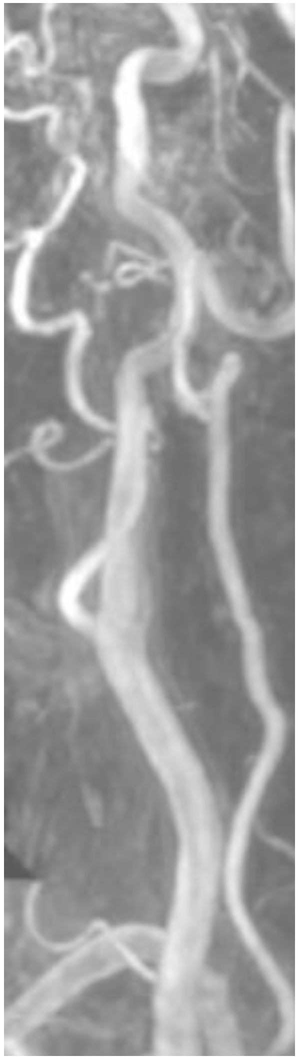

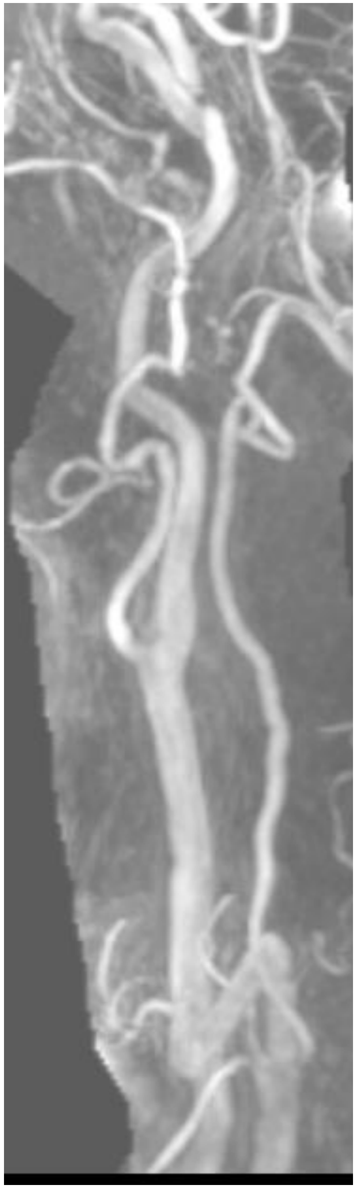

115d Score

0-30

31-50

51-70

>70

Near occlusion

Occluded

Quality

1

2

3

4

5

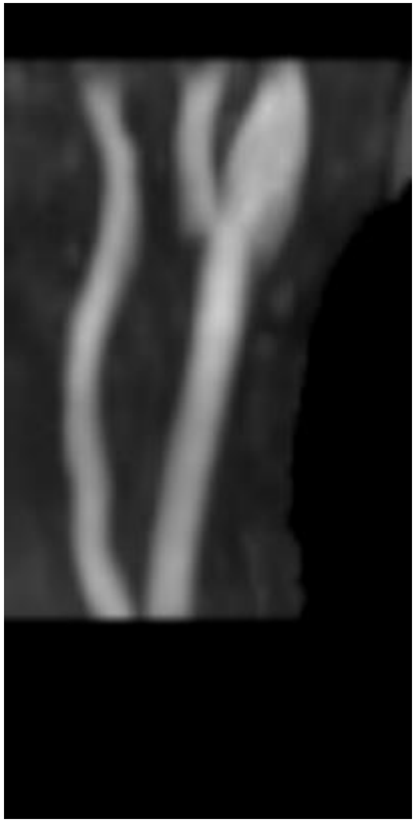

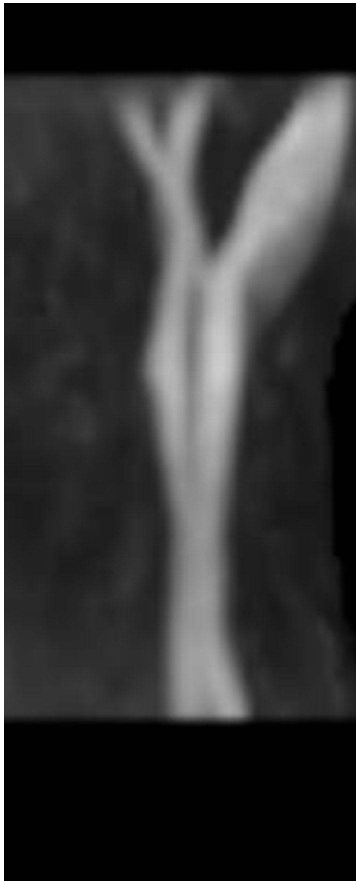

116c Score

0-30

31-50

51-70

>70

Near occlusion

Occluded

Quality

1

2

3

4

5

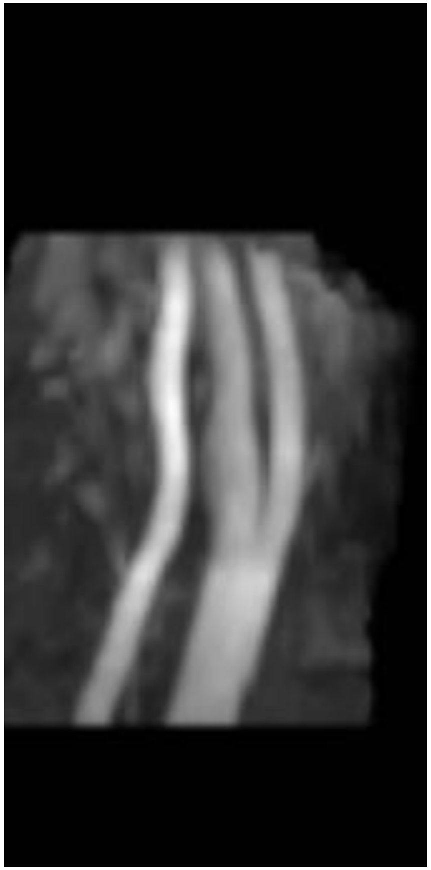

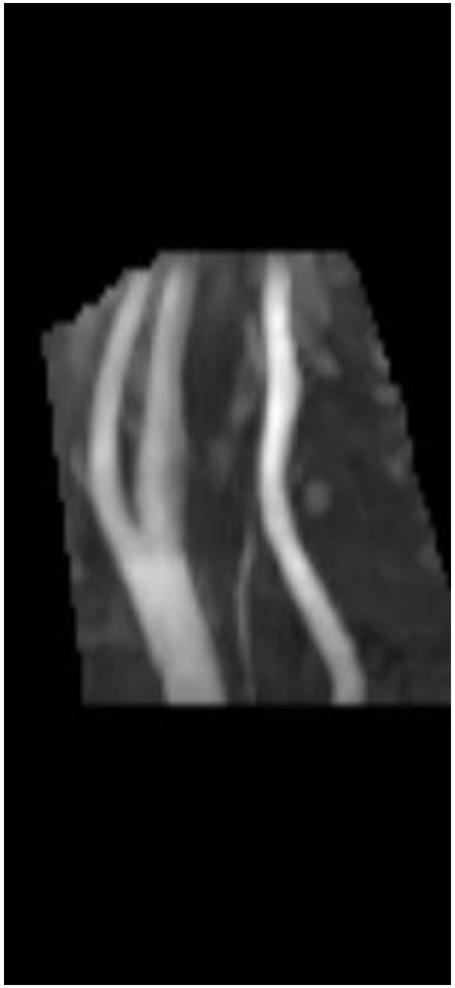

117b Score

0-30

31-50

51-70

>70

Near occlusion

Occluded

Quality

1

2

3

4

5

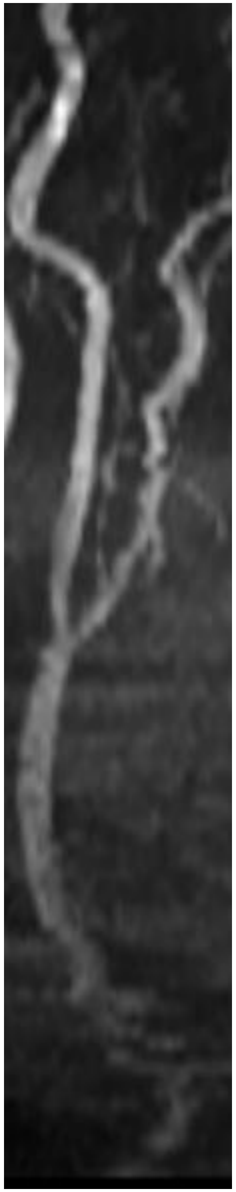

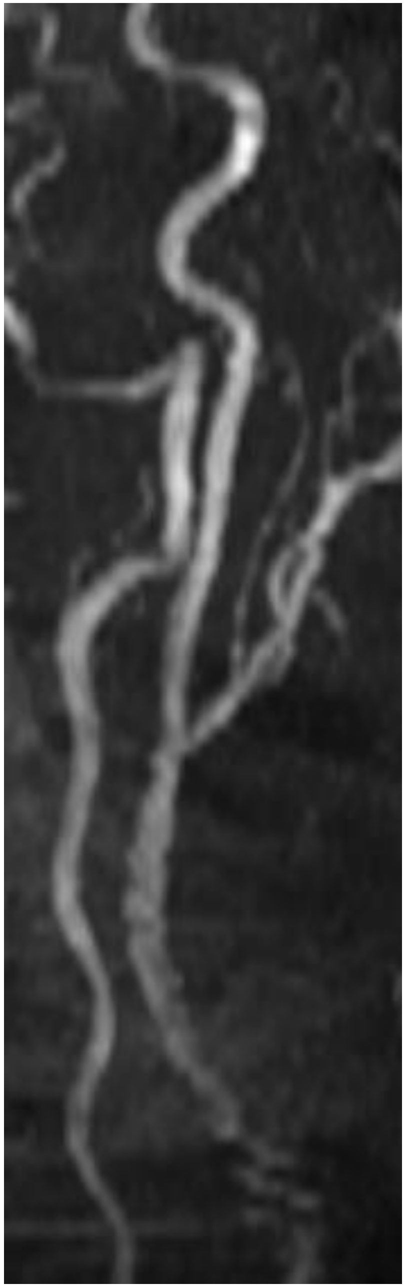

118a Score

0-30

31-50

51-70

>70

Near occlusion

Occluded

Quality

1

2

3

4

5

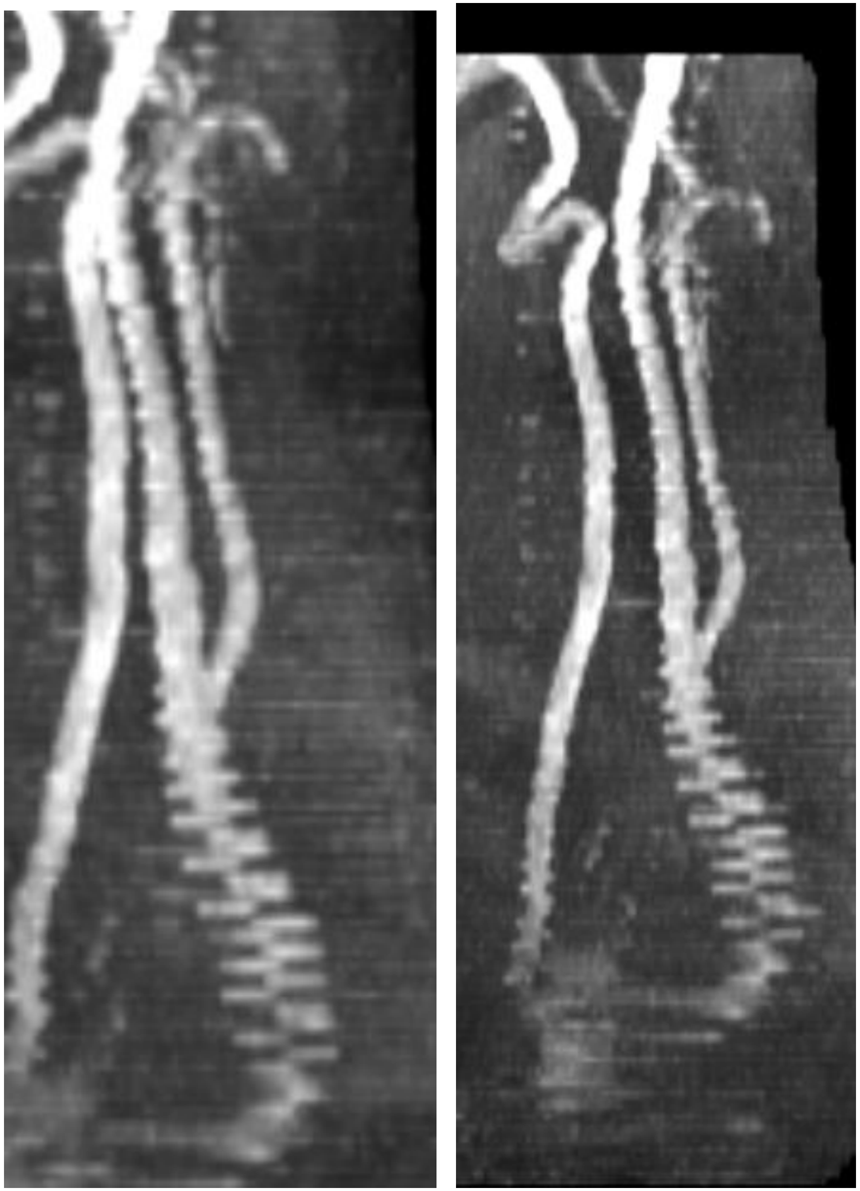

118f Score

0-30

31-50

51-70

>70

Near occlusion

Occluded

Quality

1

2

3

4

5

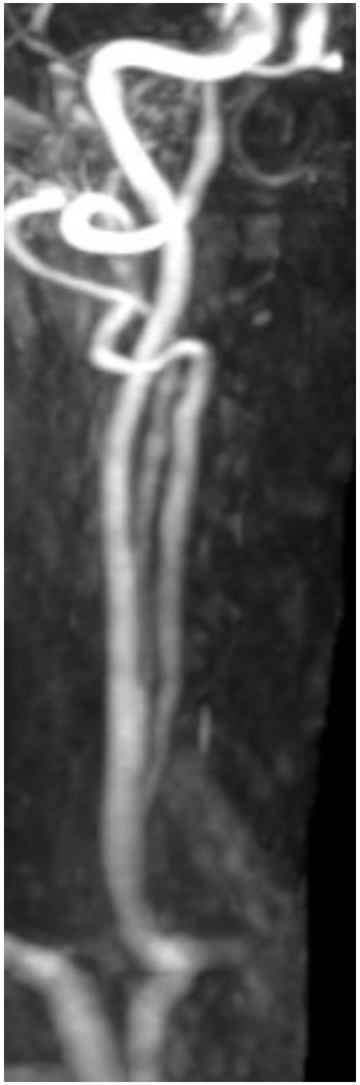

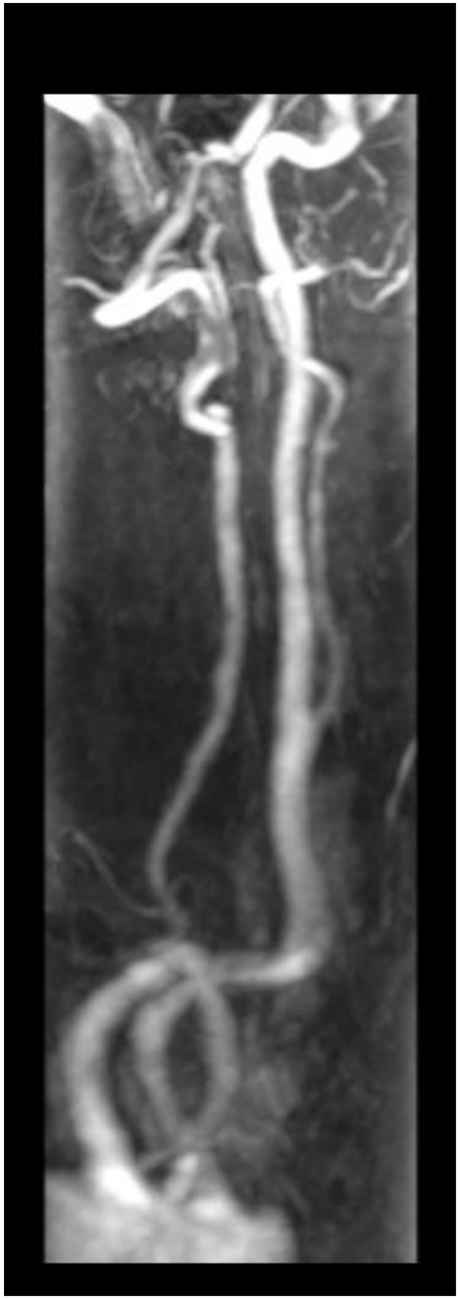

119e Score

0-30

31-50

51-70

>70

Near occlusion

Occluded

Quality

1

2

3

4

5

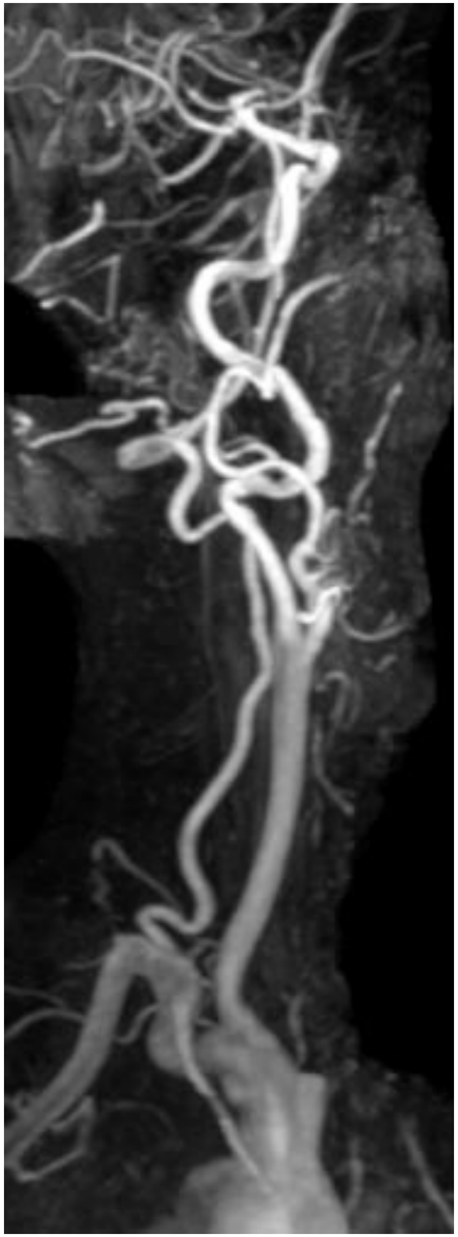

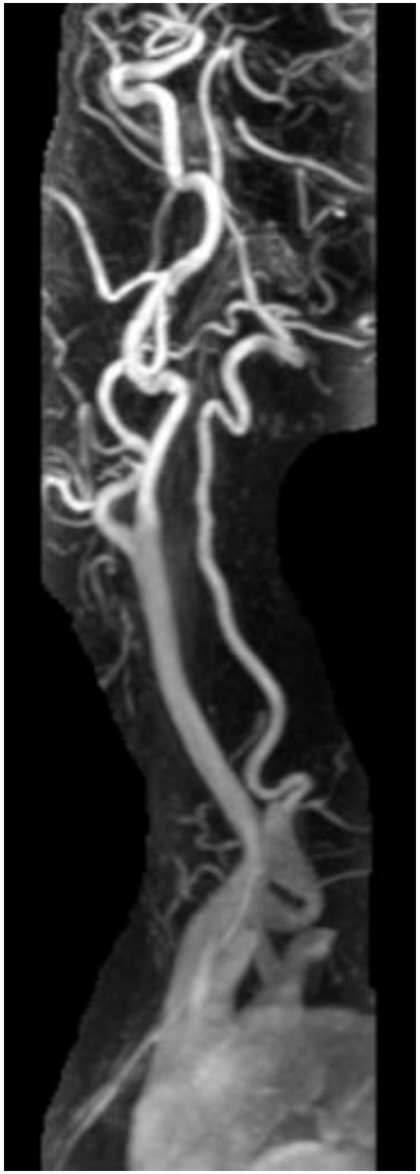

120d Score

0-30

31-50

51-70

>70

Near occlusion

Occluded

Quality

1

2

3

4

5

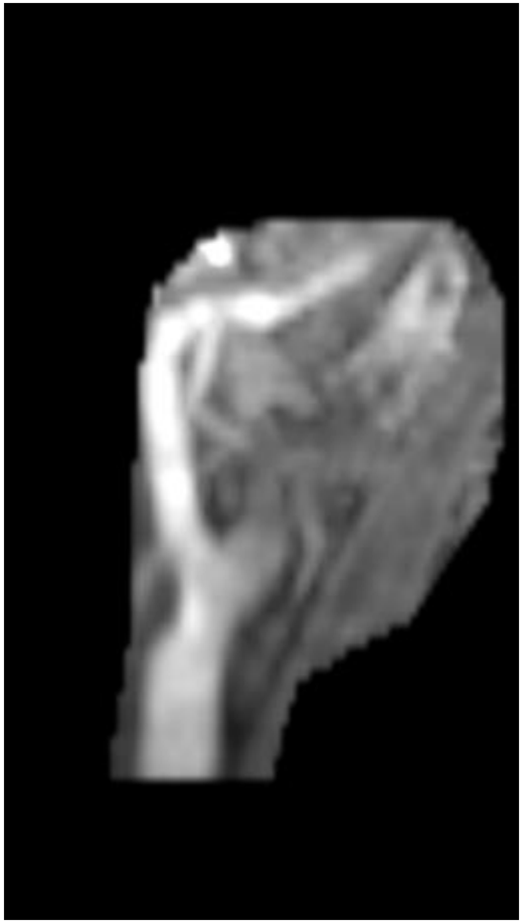

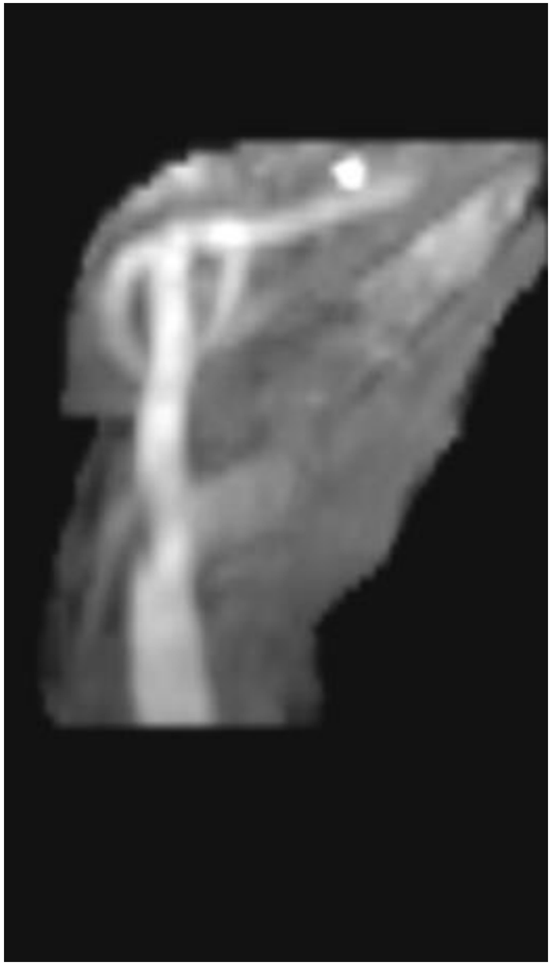

121c Score

0-30

31-50

51-70

>70

Near occlusion

Occluded

Quality

1

2

3

4

5

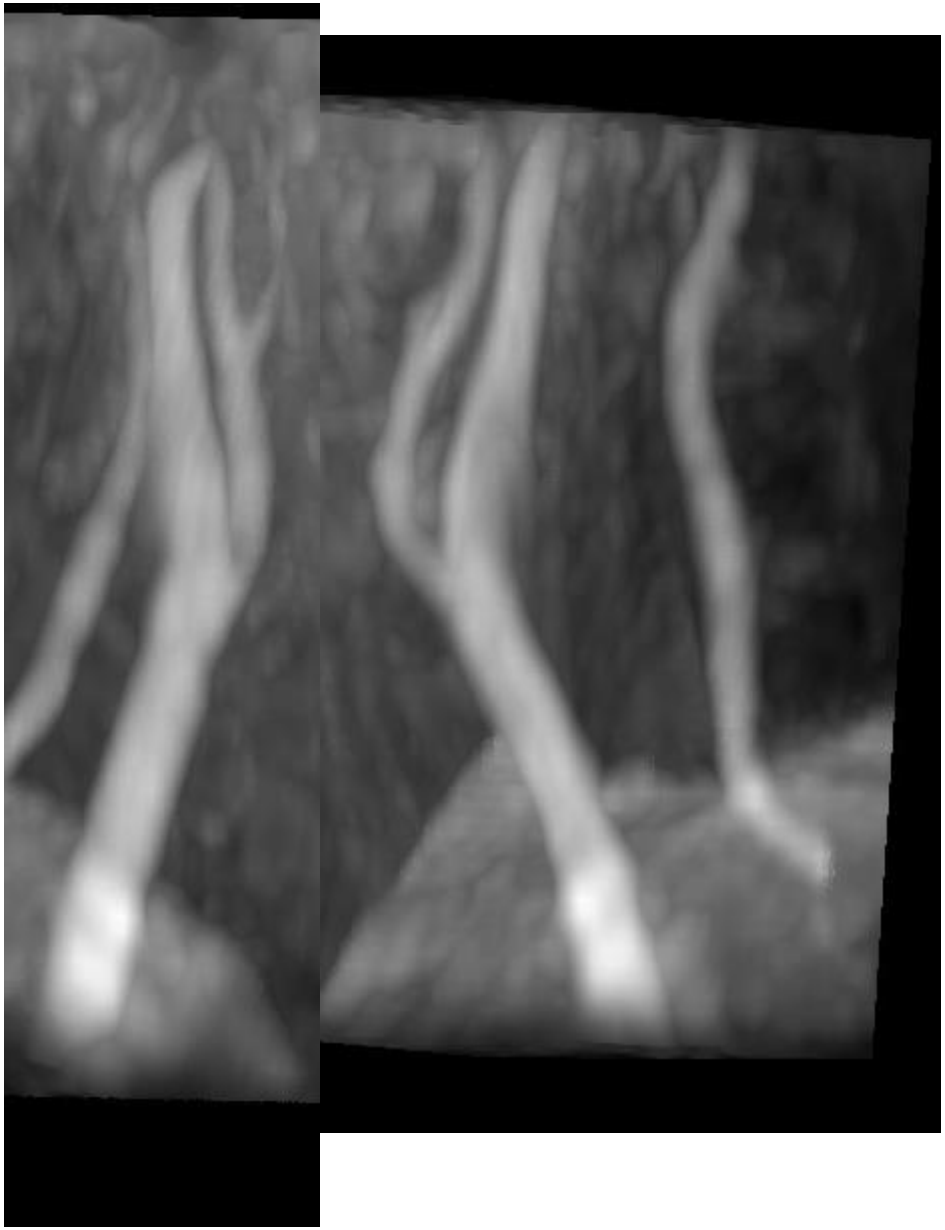

122b Score

0-30

31-50

51-70

>70

Near occlusion

Occluded

Quality

1

2

3

4

5

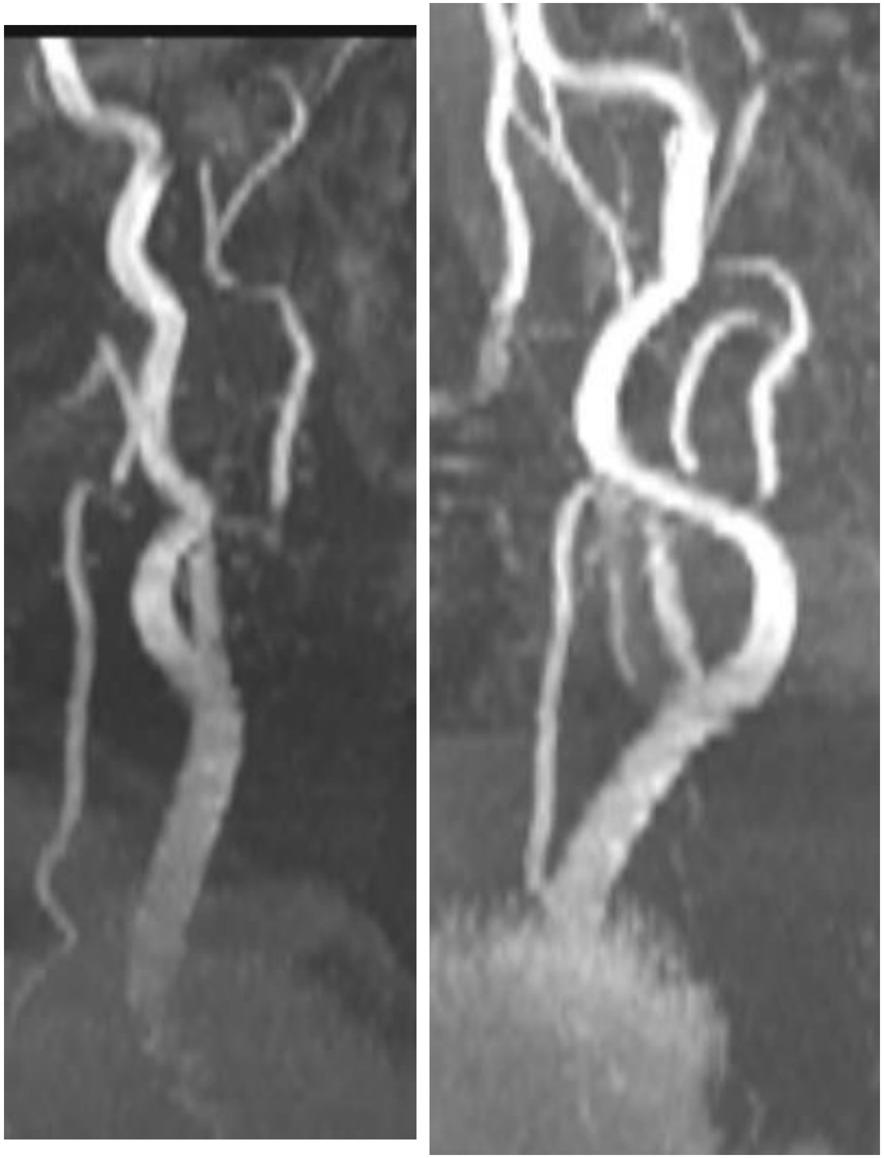

123a Score

0-30

31-50

51-70

>70

Near occlusion

Occluded

Quality

1

2

3

4

5

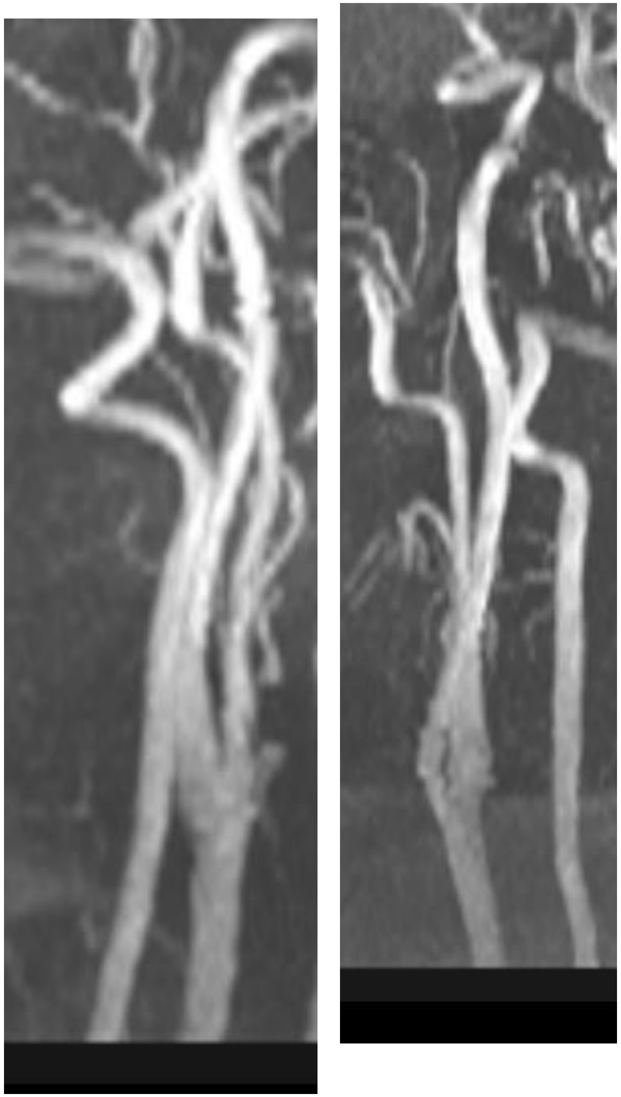

123f Score

0-30

31-50

51-70

>70

Near occlusion

Occluded

Quality

1

2

3

4

5

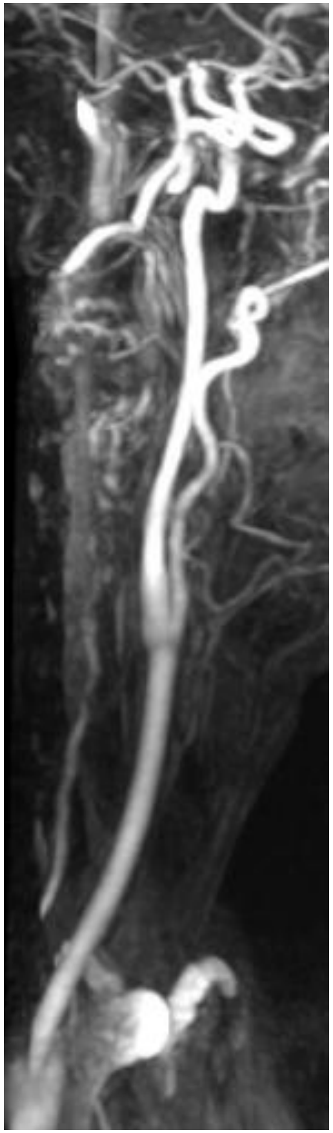

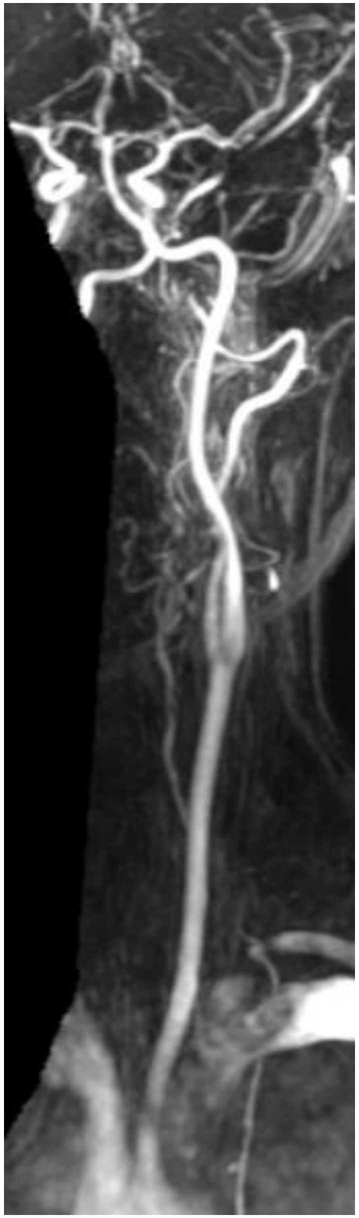

124e Score

0-30

31-50

51-70

>70

Near occlusion

Occluded

Quality

1

2

3

4

5

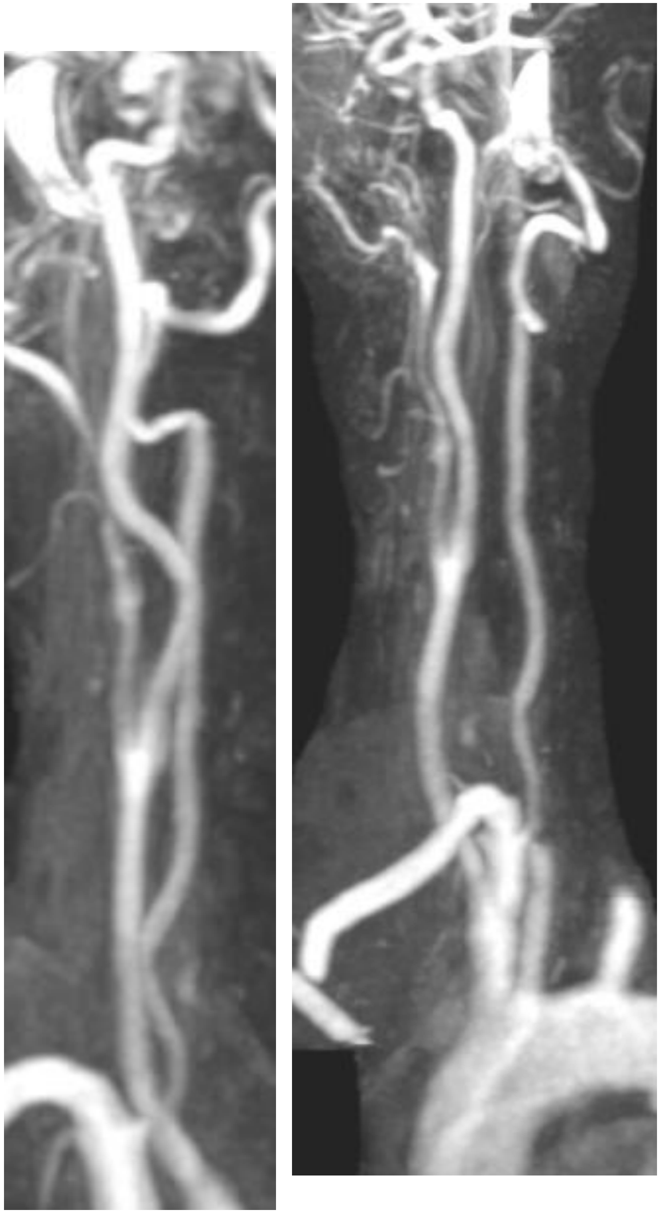

125d Score

0-30

31-50

51-70

>70

Near occlusion

Occluded

Quality

1

2

3

4

5

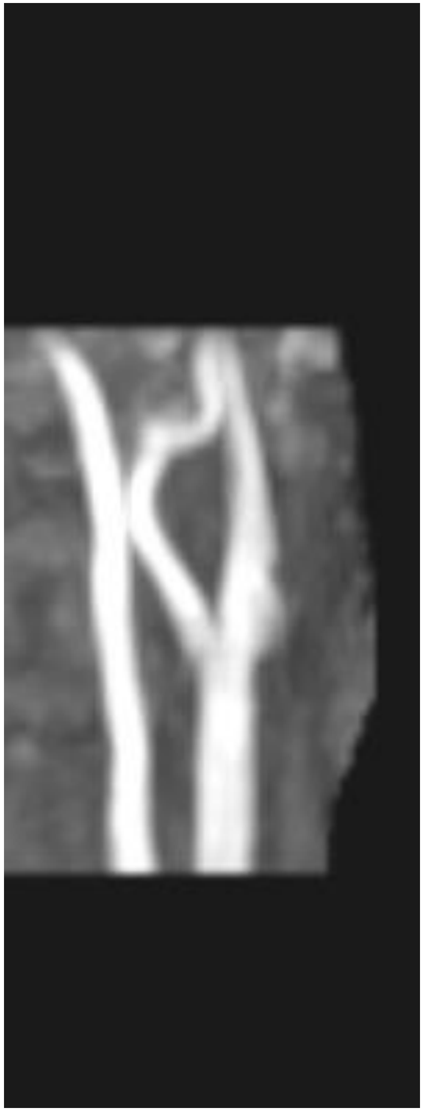

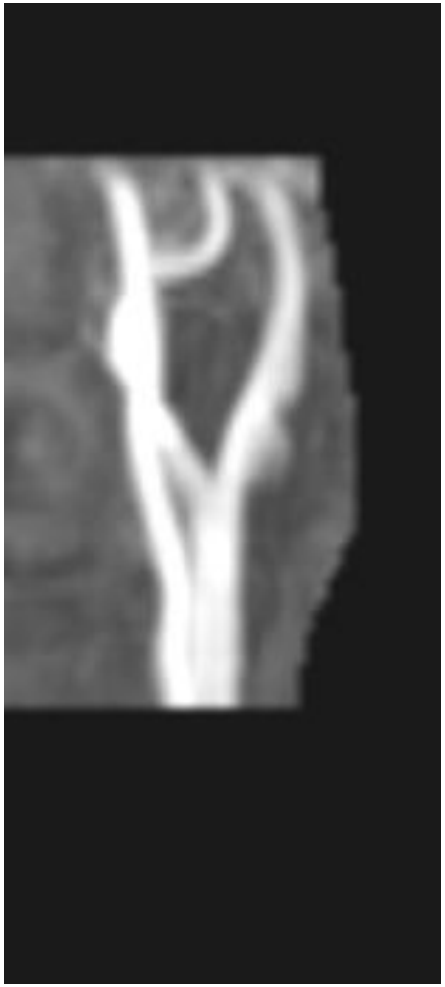

126c Score

0-30

31-50

51-70

>70

Near occlusion

Occluded

Quality

1

2

3

4

5

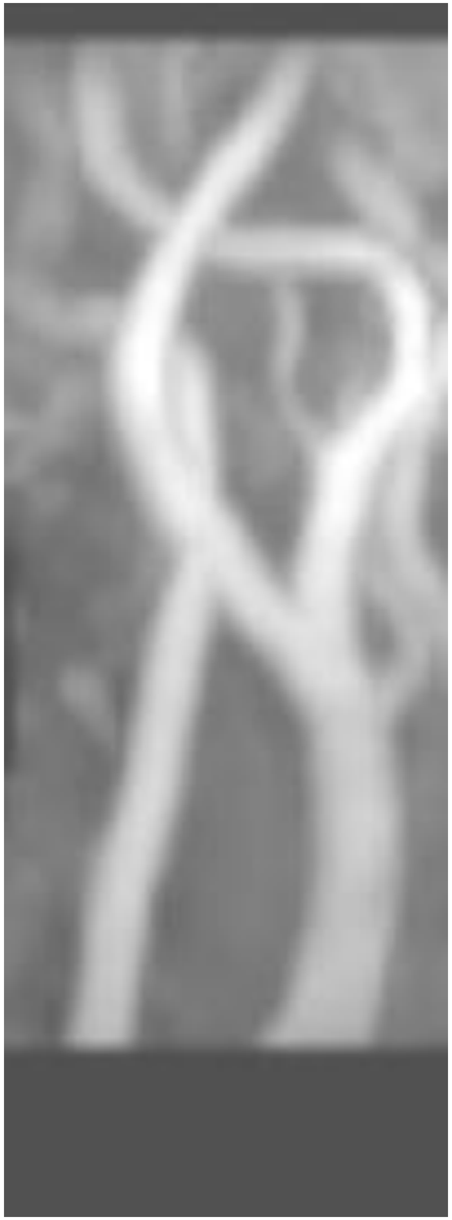

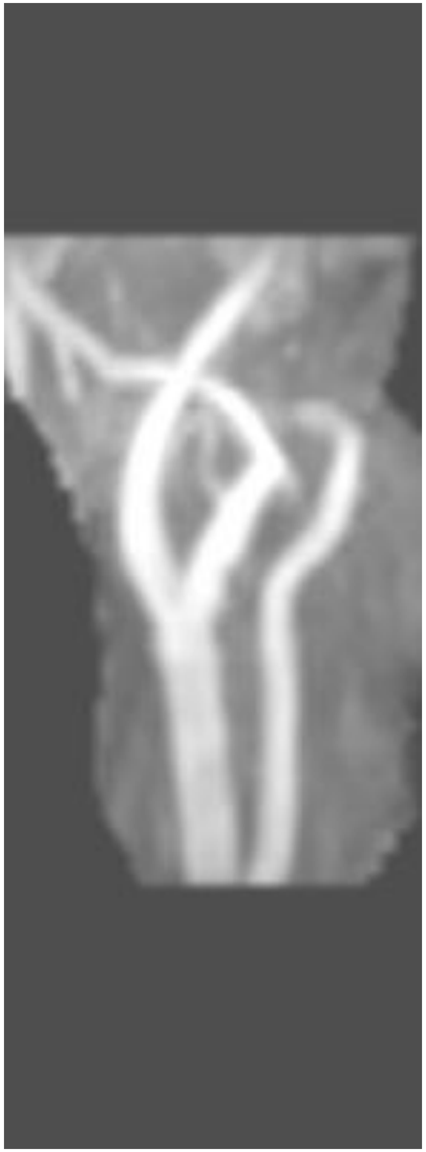

127b Score

0-30

31-50

51-70

>70

Near occlusion

Occluded

Quality

1

2

3

4

5

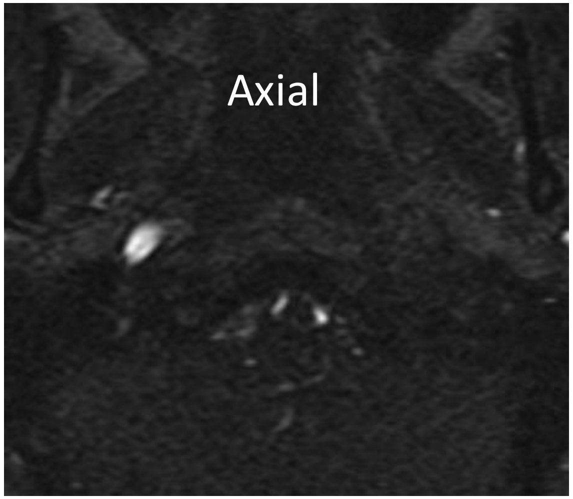

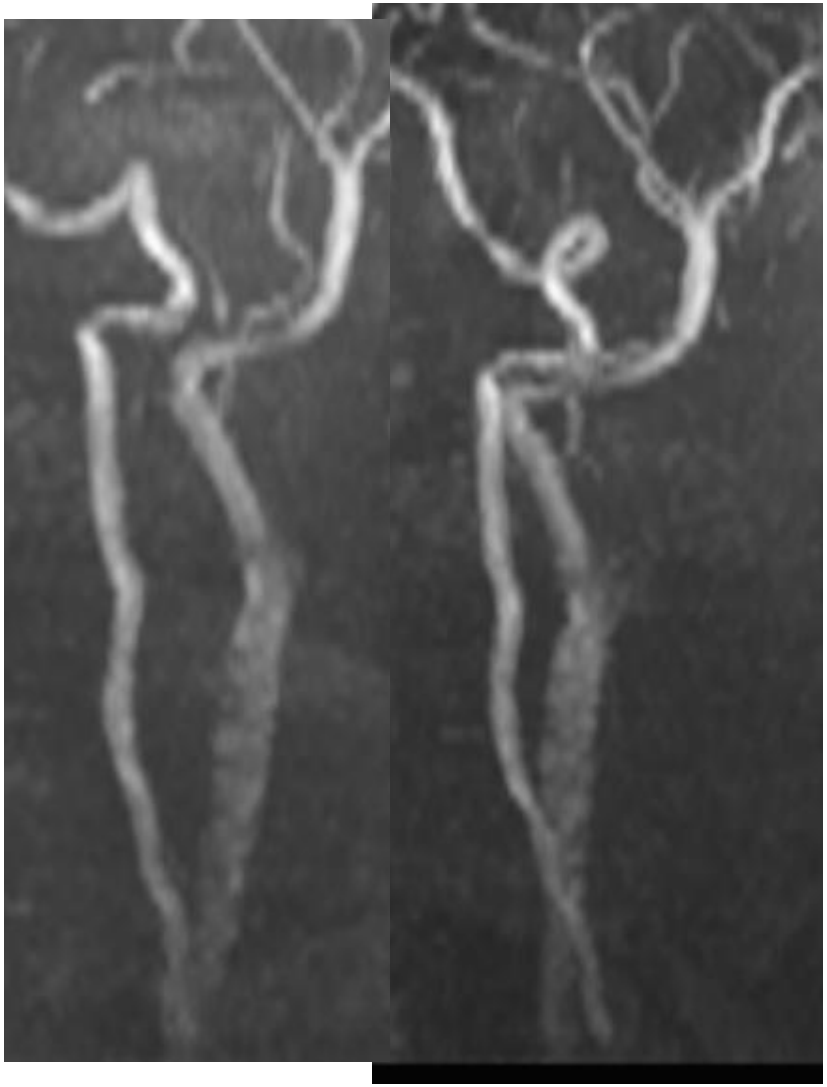

128a Score

0-30

31-50

51-70

>70

Near occlusion

Occluded

Quality

1

2

3

4

5

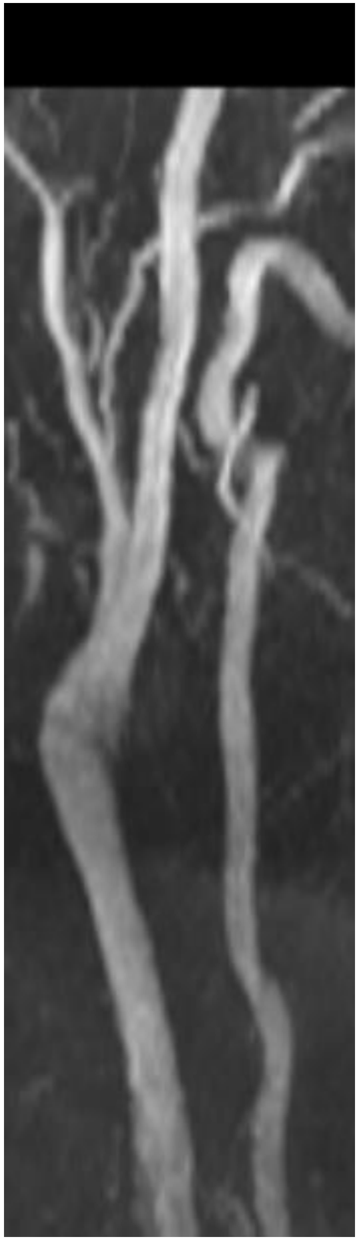

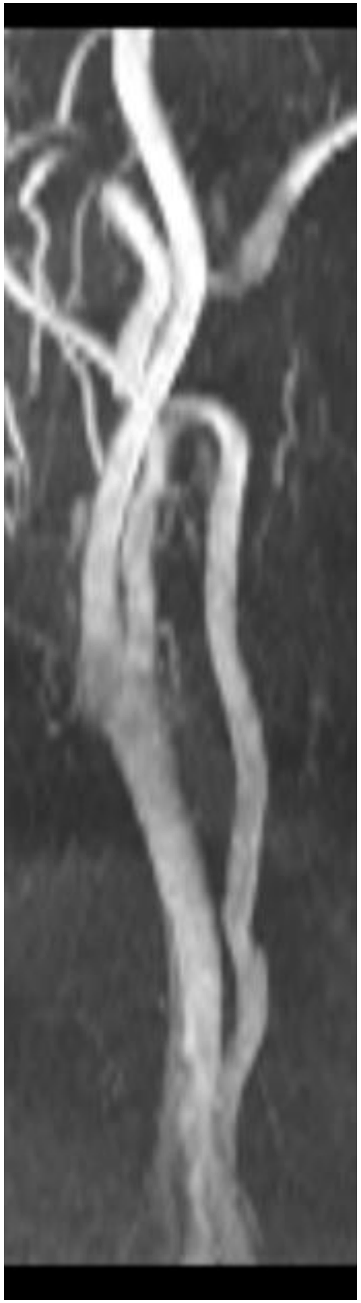

128f Score

0-30

31-50

51-70

>70

Near occlusion

Occluded

Quality

1

2

3

4

5

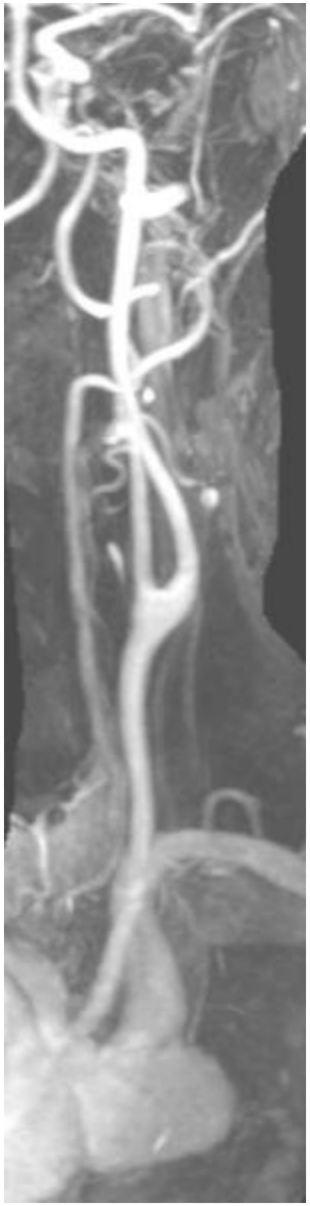

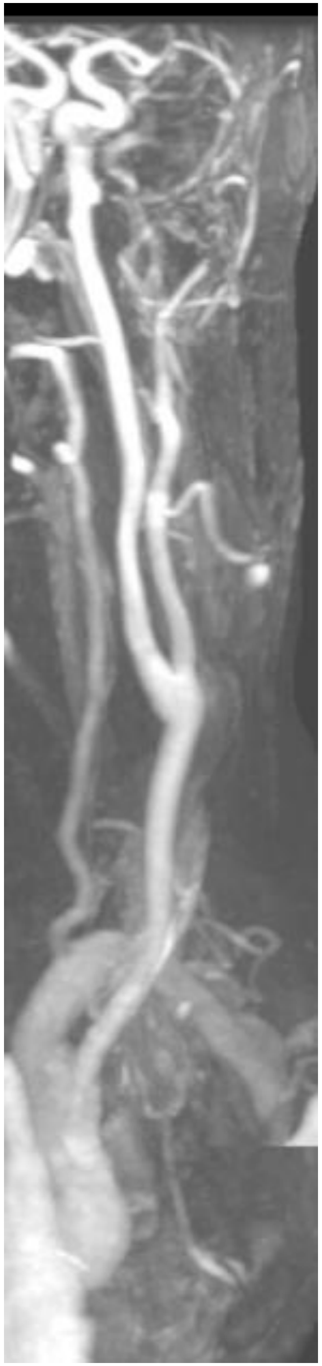

129e Score

0-30

31-50

51-70

>70

Near occlusion

Occluded

Quality

1

2

3

4

5

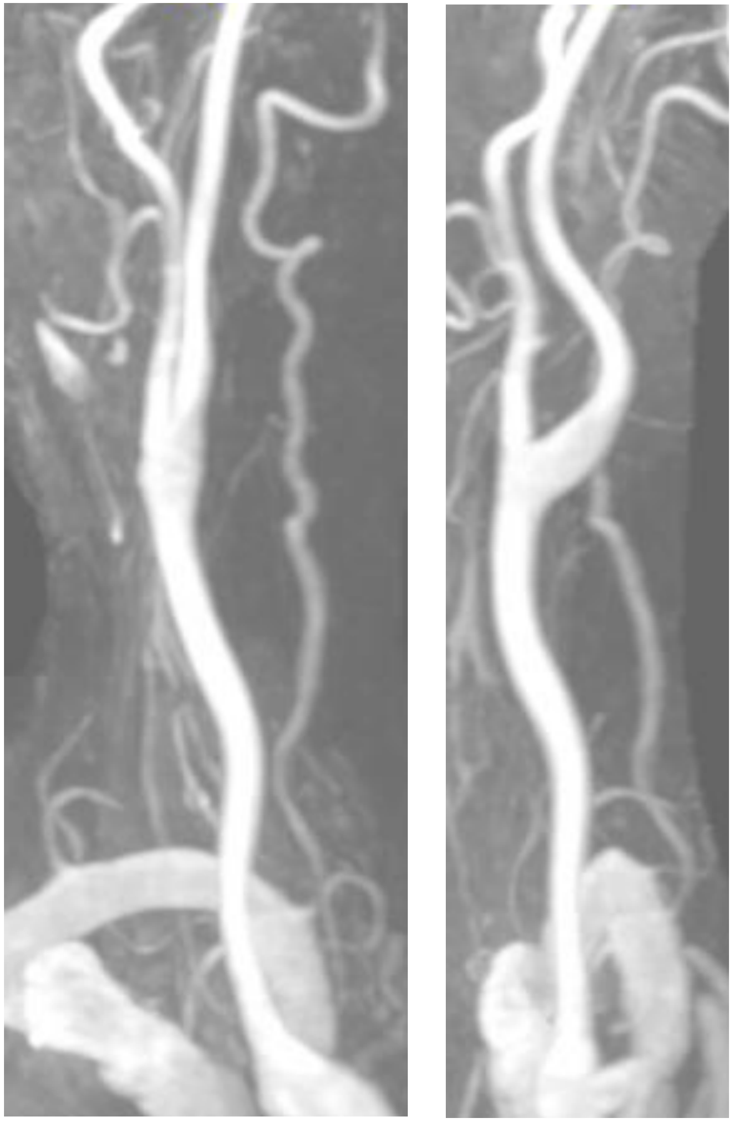

130d Score

0-30

31-50

51-70

>70

Near occlusion

Occluded

Quality

1

2

3

4

5

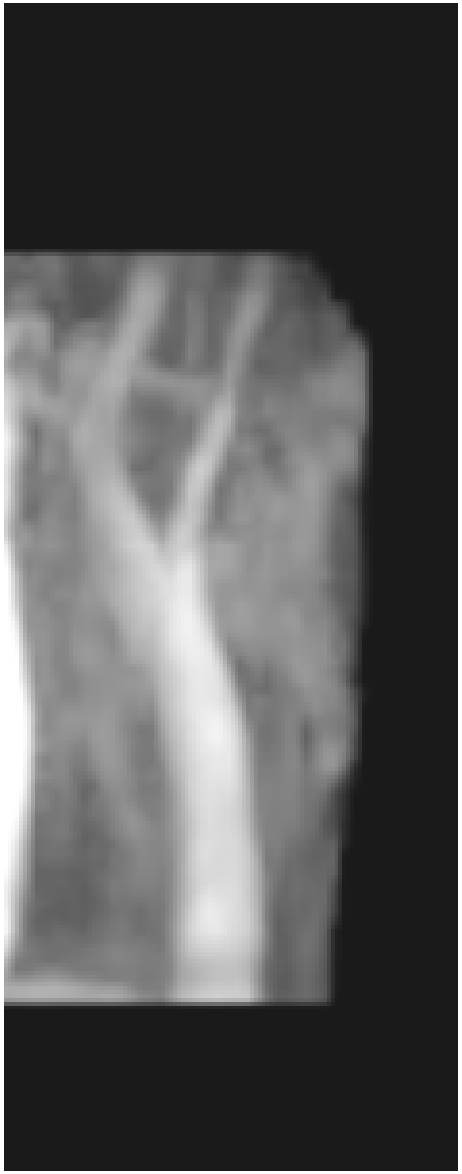

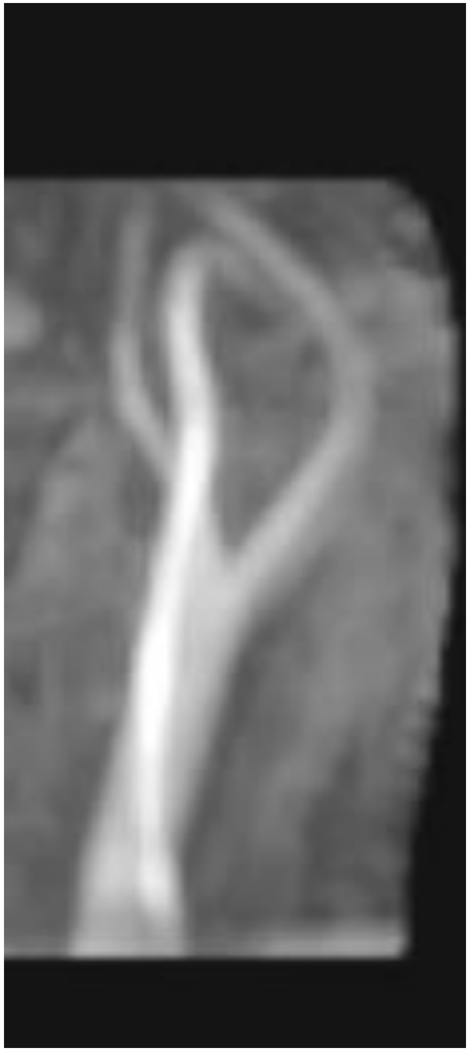

131c Score

0-30

31-50

51-70

>70

Near occlusion

Occluded

Quality

1

2

3

4

5

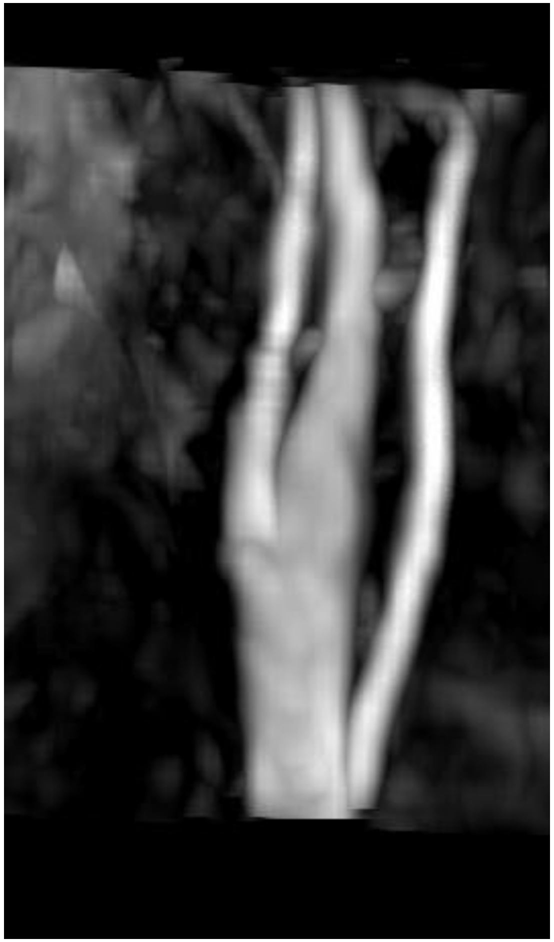

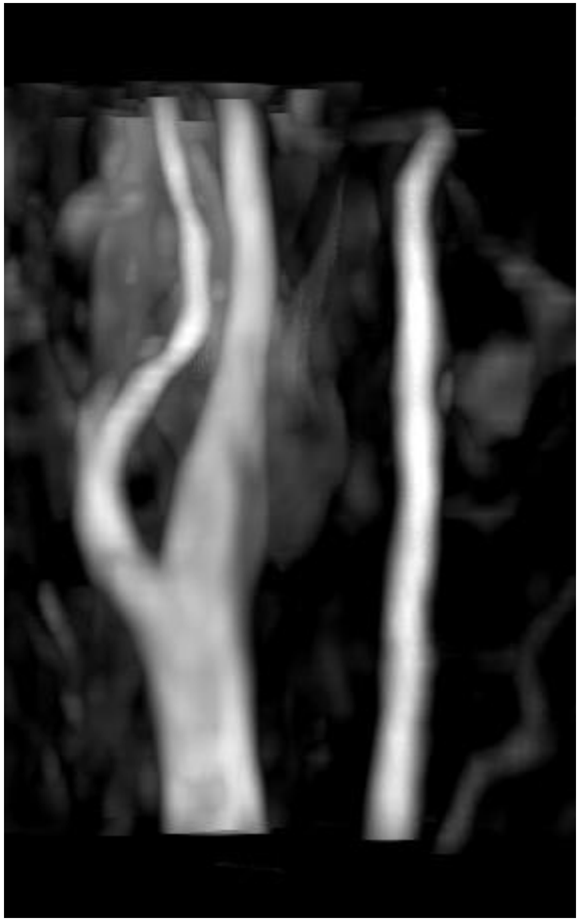

132b Score

0-30

31-50

51-70

>70

Near occlusion

Occluded

Quality

1

2

3

4

5

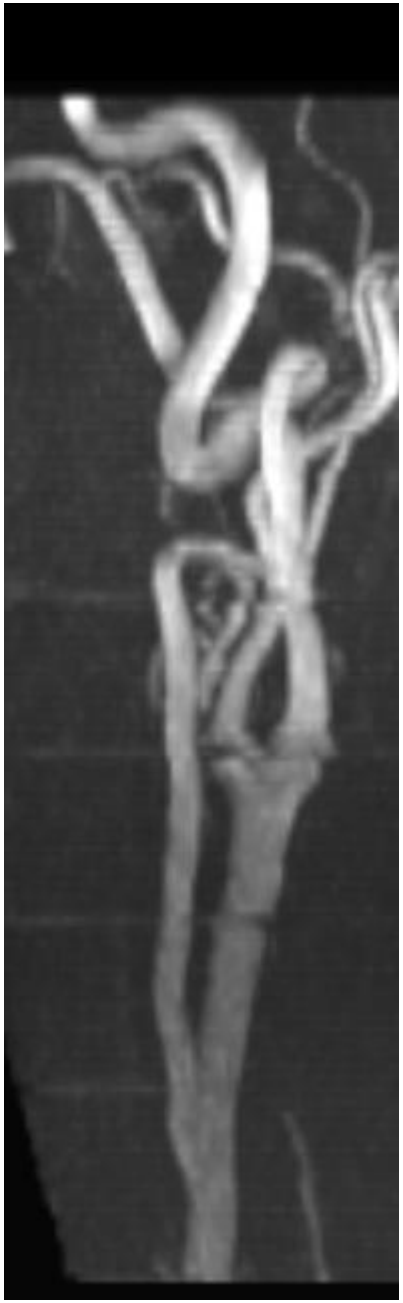

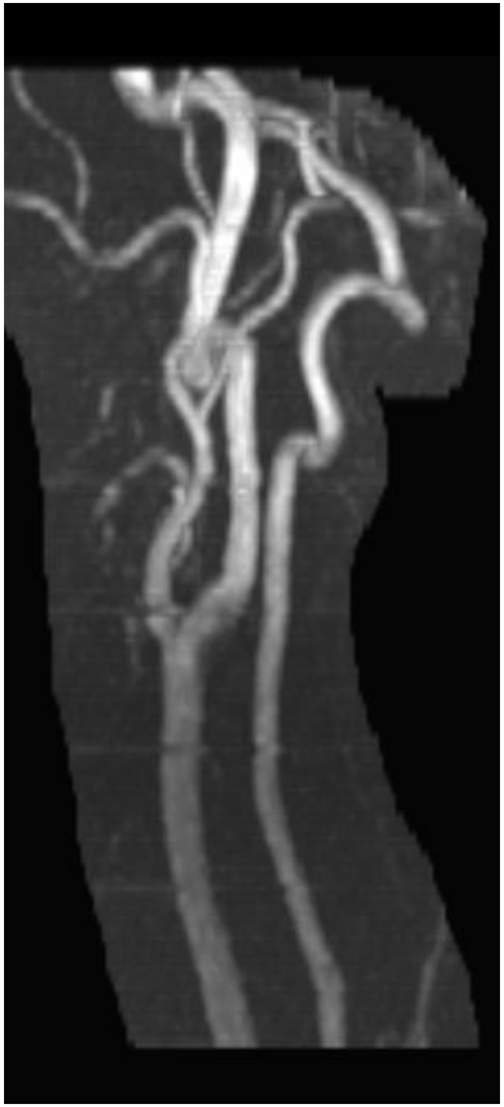

133a Score

0-30

31-50

51-70

>70

Near occlusion

Occluded

Quality

1

2

3

4

5

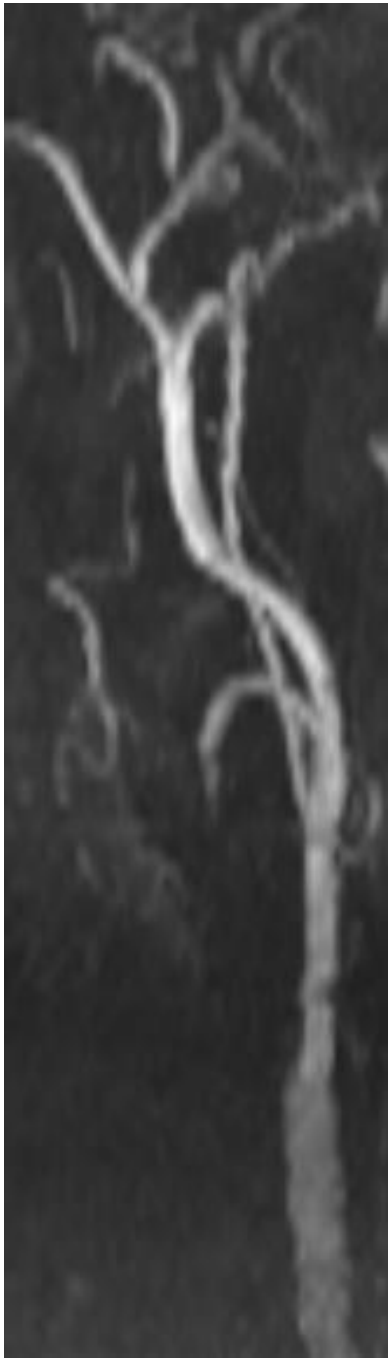

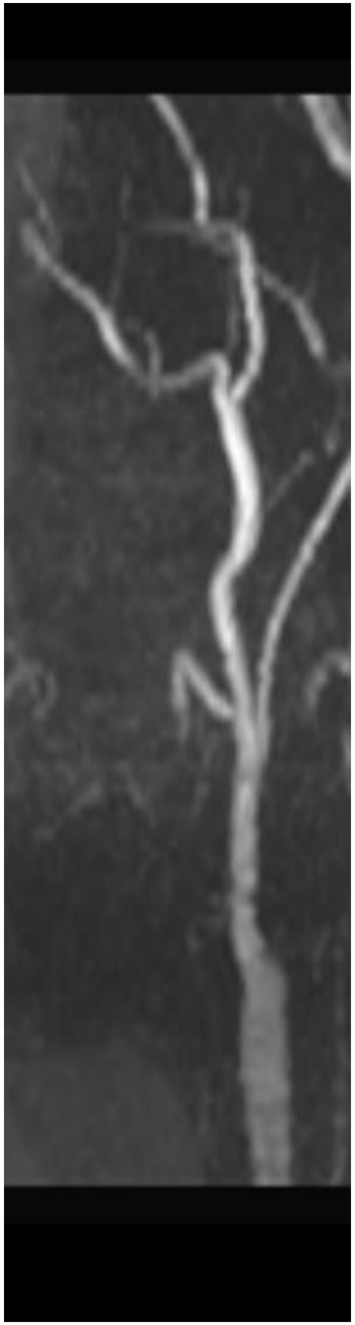

133f Score

0-30

31-50

51-70

>70

Near occlusion

Occluded

Quality

1

2

3

4

5

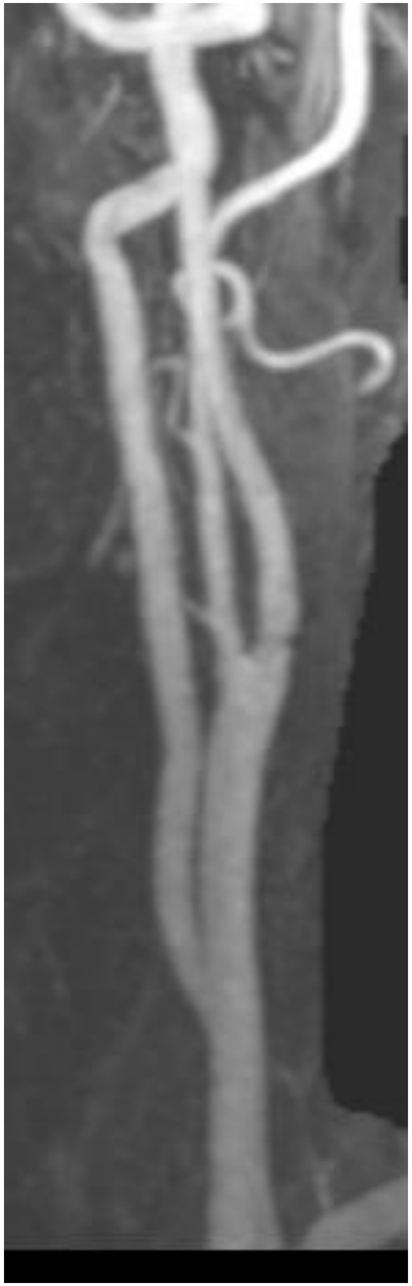

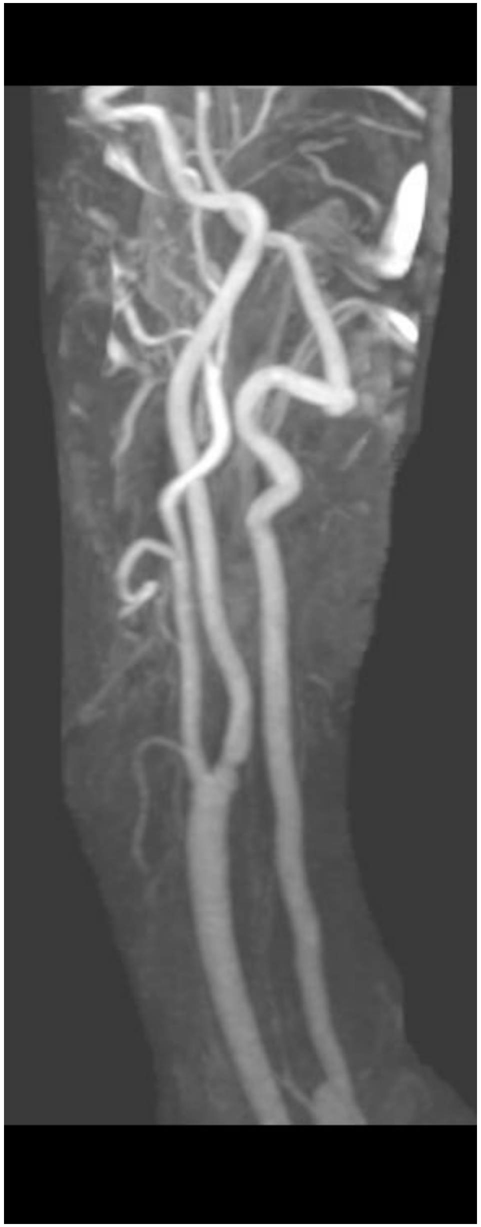

134e Score

0-30

31-50

51-70

>70

Near occlusion

Occluded

Quality

1

2

3

4

5

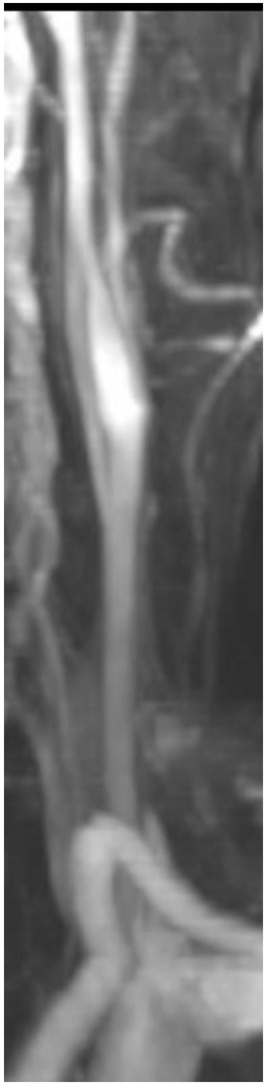

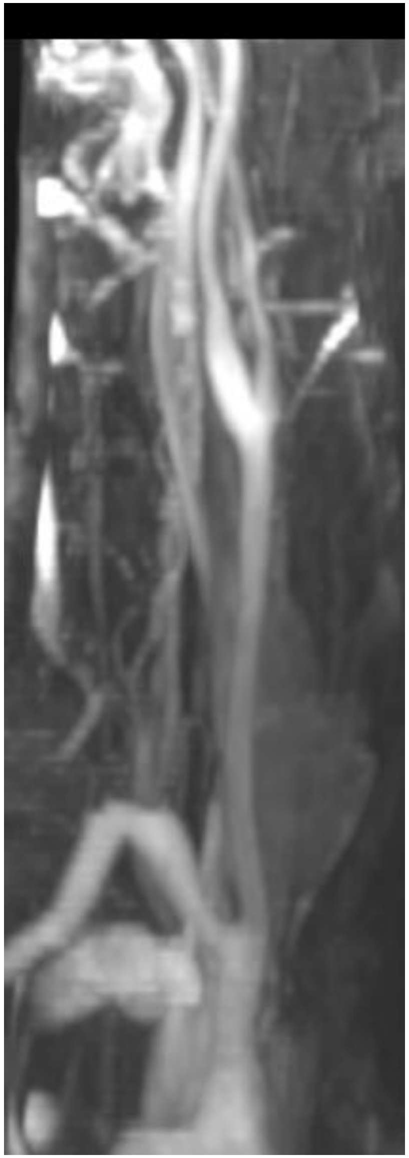

135d Score

0-30

31-50

51-70

>70

Near occlusion

Occluded

Quality

1

2

3

4

5

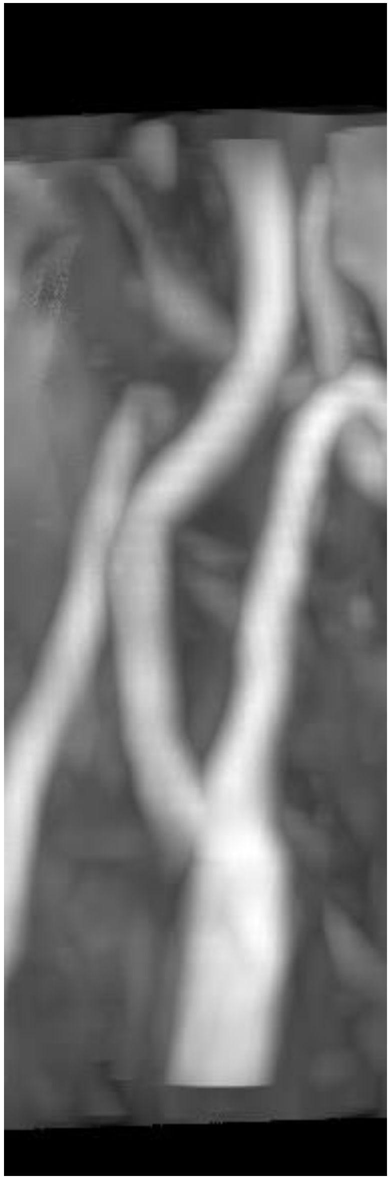

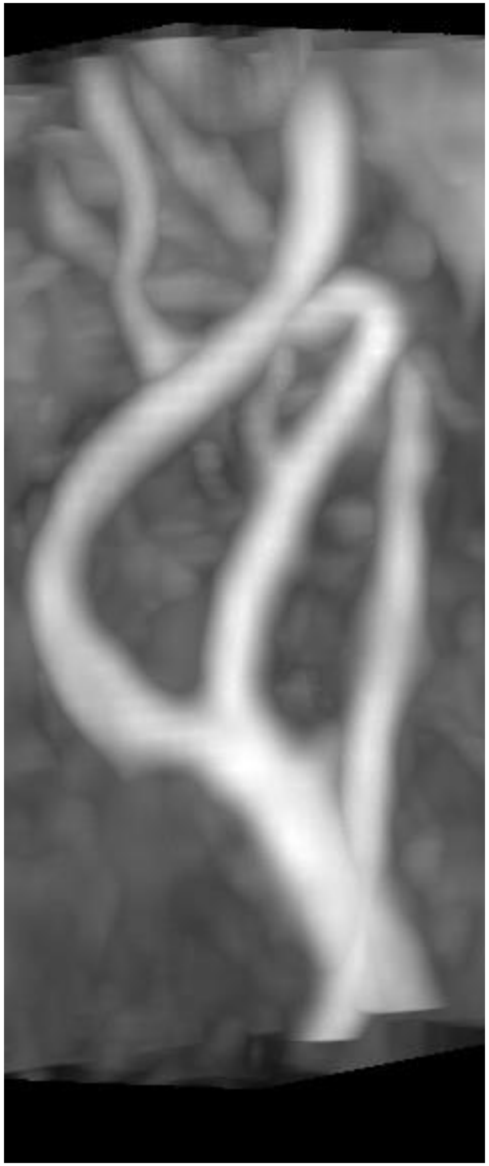

136c Score

0-30

31-50

51-70

>70

Near occlusion

Occluded

Quality

1

2

3

4

5

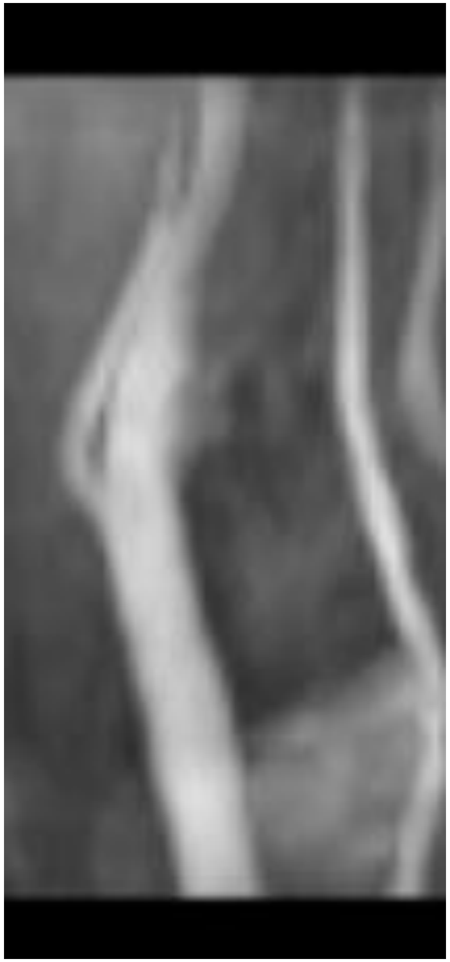

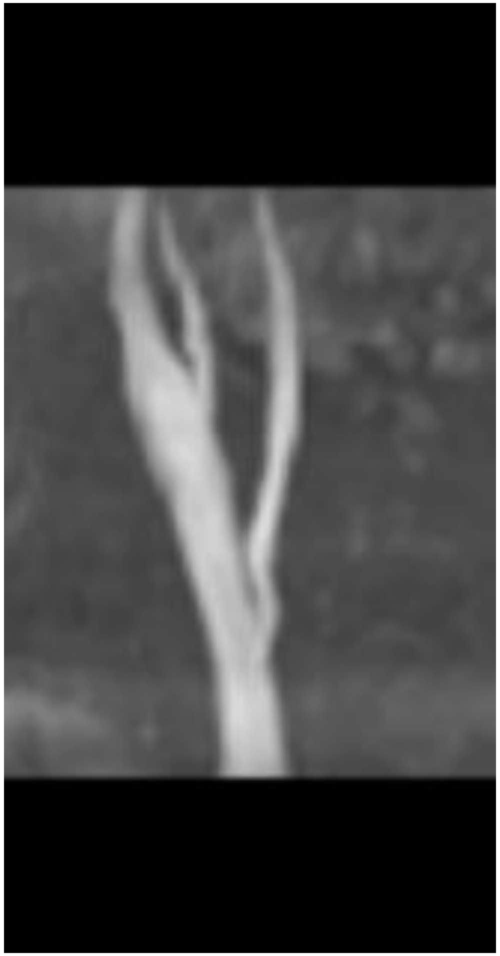

137b Score

0-30

31-50

51-70

>70

Near occlusion

Occluded

Quality

1

2

3

4

5

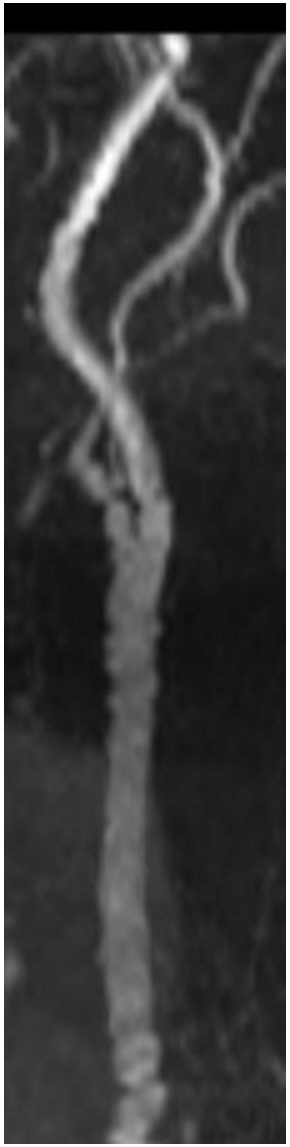

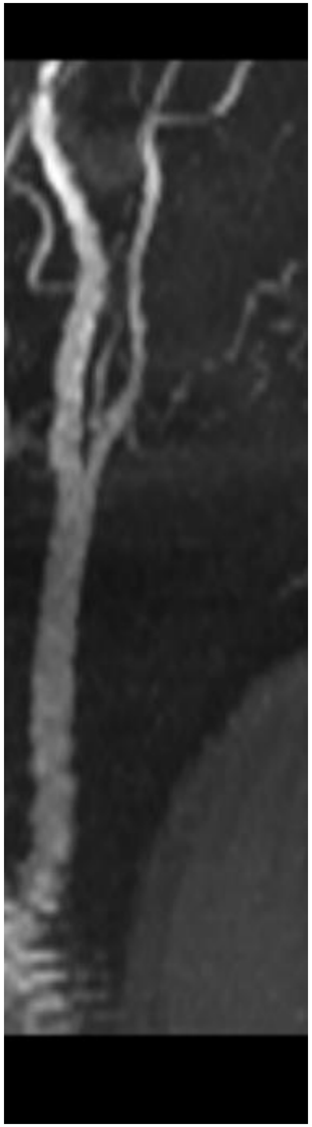

138a Score

0-30

31-50

51-70

>70

Near occlusion

Occluded

Quality

1

2

3

4

5

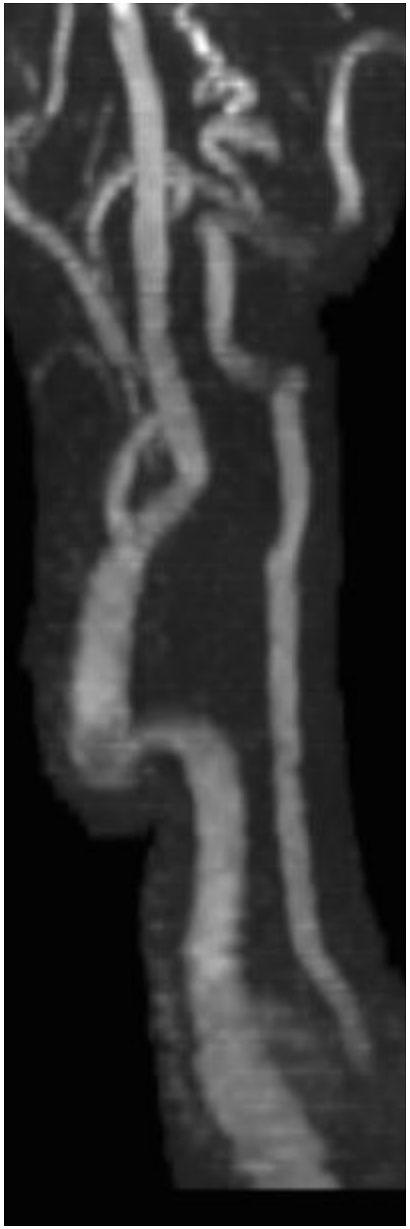

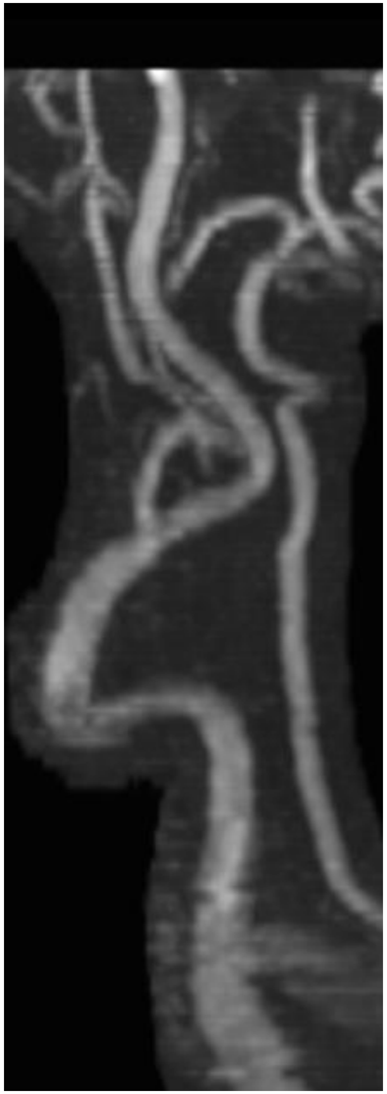

138f Score

0-30

31-50

51-70

>70

Near occlusion

Occluded

Quality

1

2

3

4

5

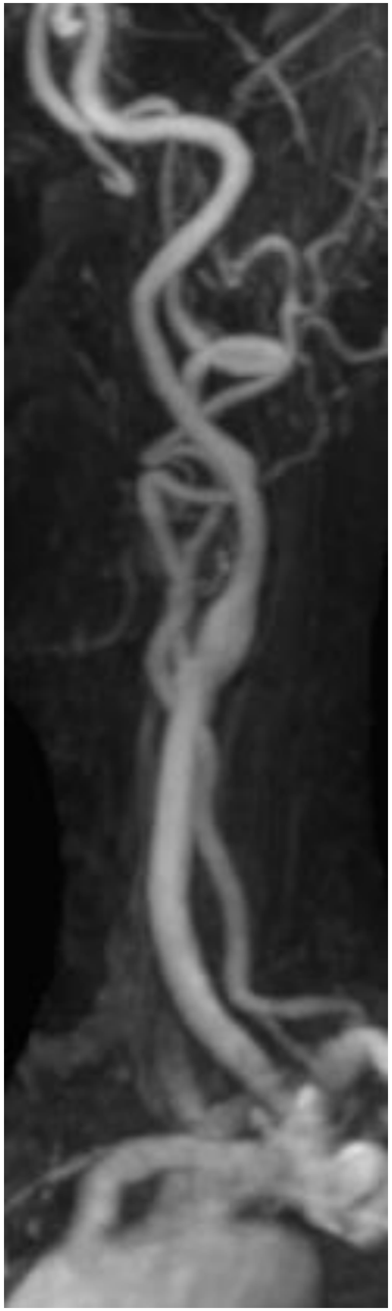

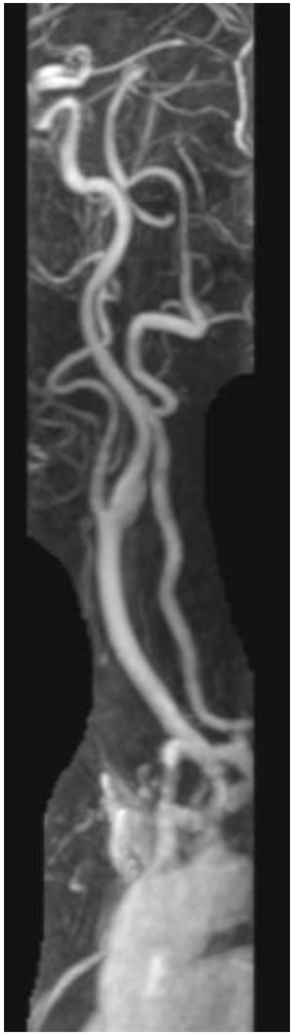

139e Score

0-30

31-50

51-70

>70

Near occlusion

Occluded

Quality

1

2

3

4

5

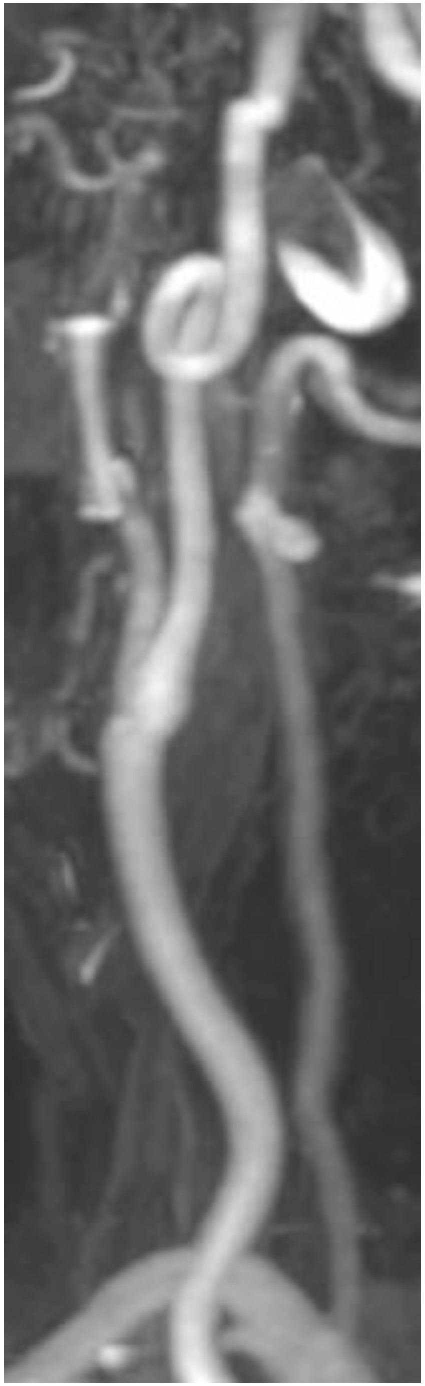

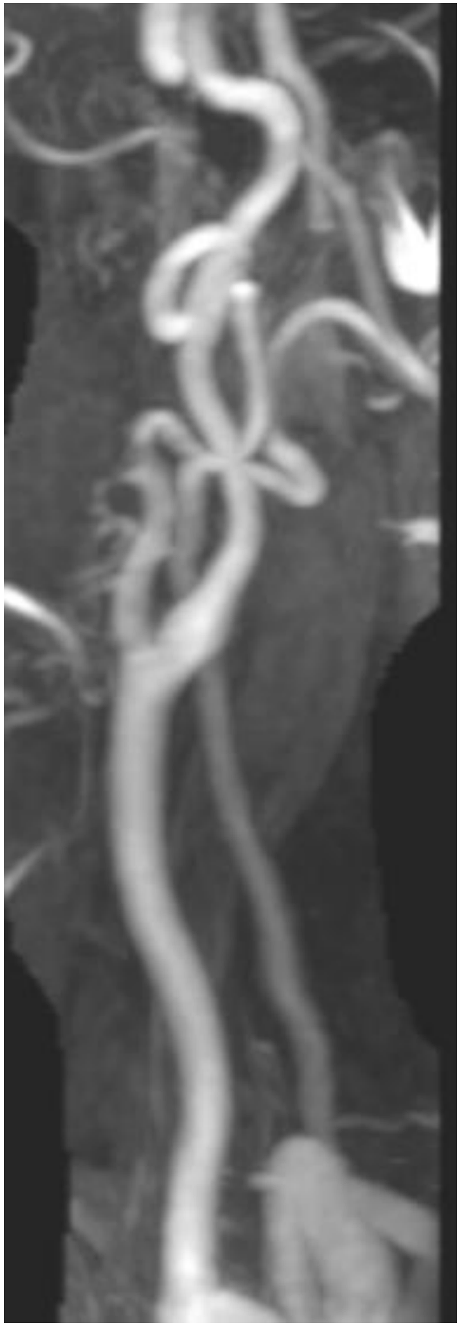

140d Score

0-30

31-50

51-70

>70

Near occlusion

Occluded

Quality

1

2

3

4

5

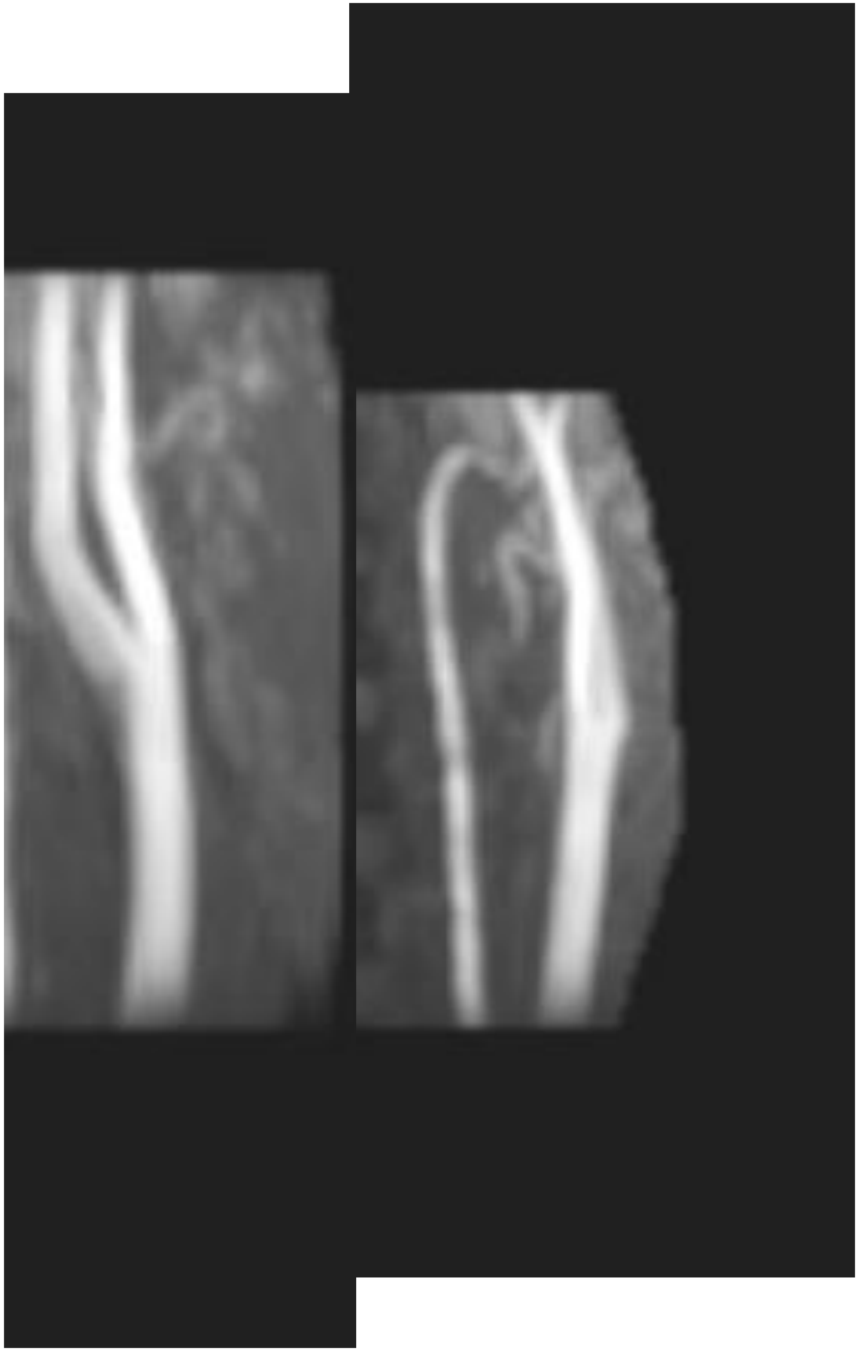

141c Score

0-30

31-50

51-70

>70

Near occlusion

Occluded

Quality

1

2

3

4

5

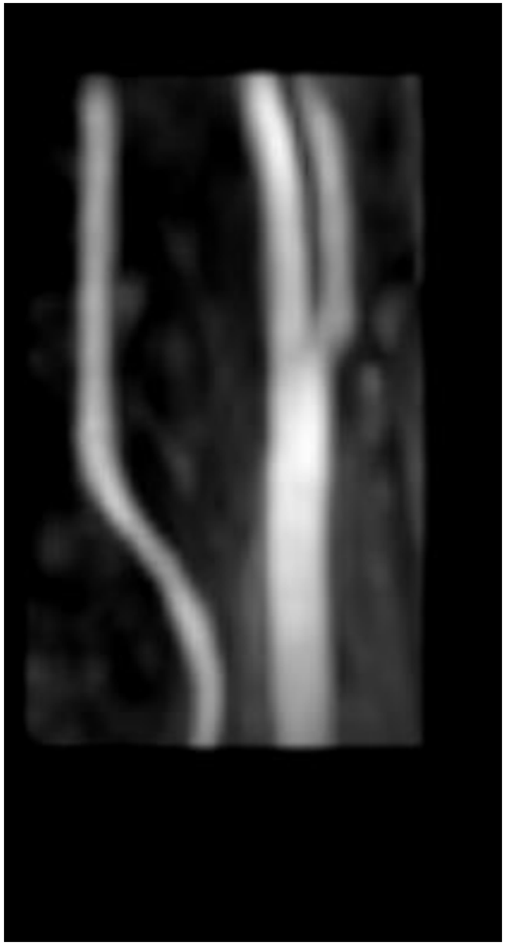

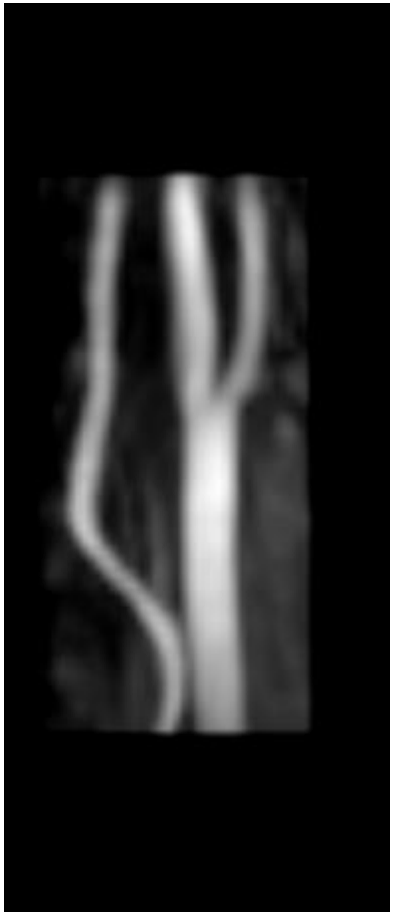

142b Score

0-30

31-50

51-70

>70

Near occlusion

Occluded

Quality

1

2

3

4

5

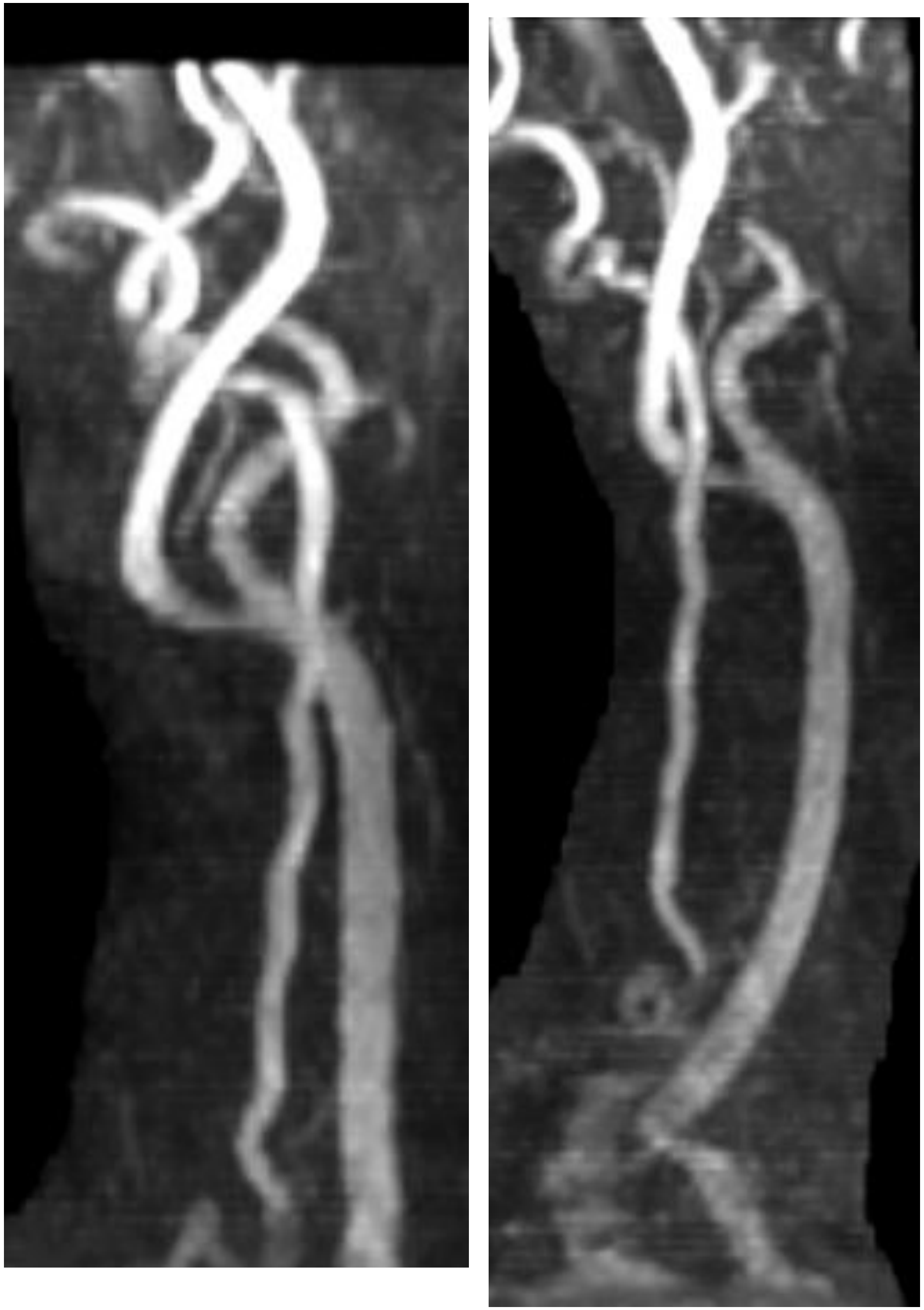

143a Score

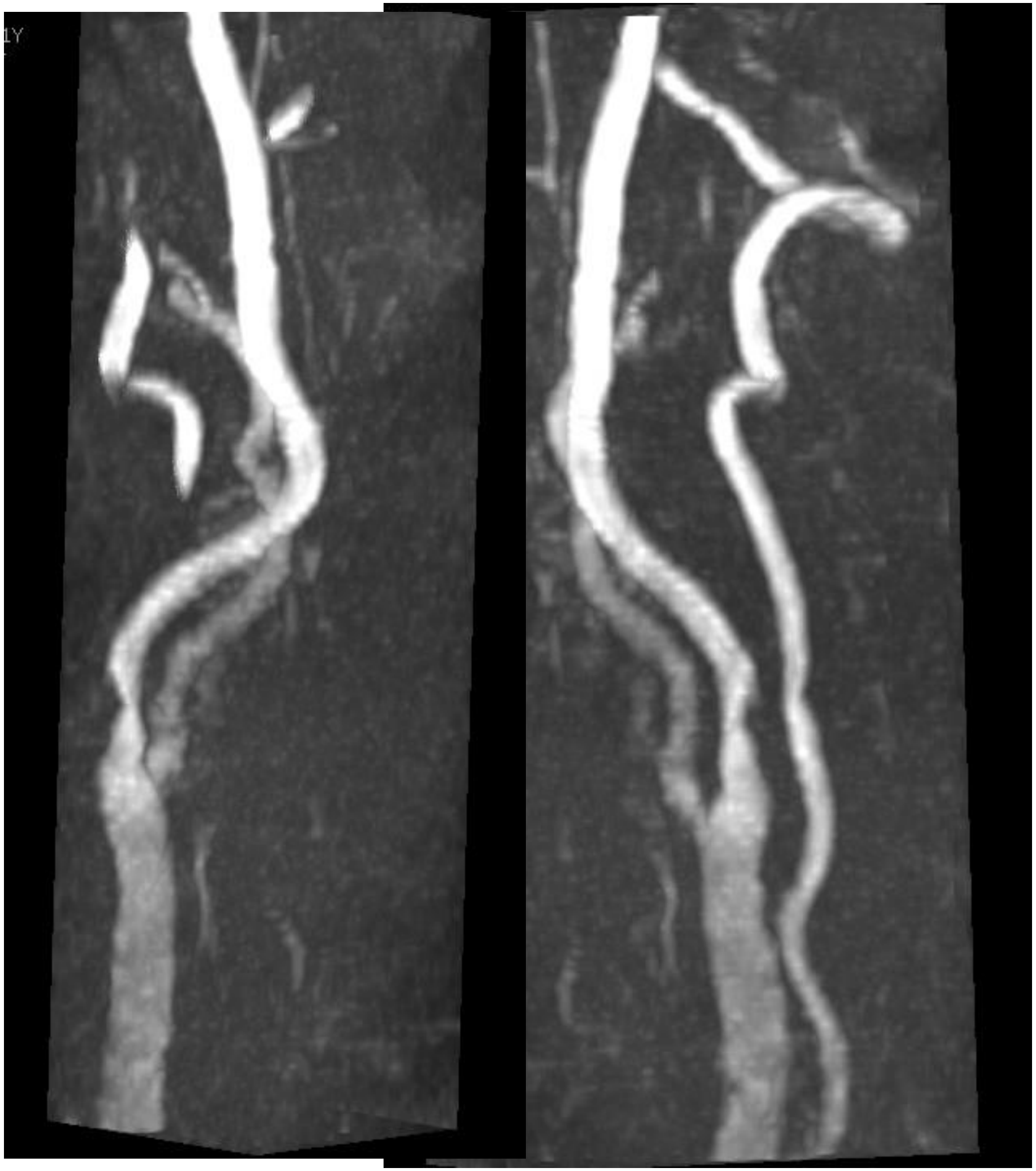

0-30

31-50

51-70

>70

Near occlusion

Occluded

Quality

1

2

3

4

5

143f Score

0-30

31-50

51-70

>70

Near occlusion

Occluded

Quality

1

2

3

4

5

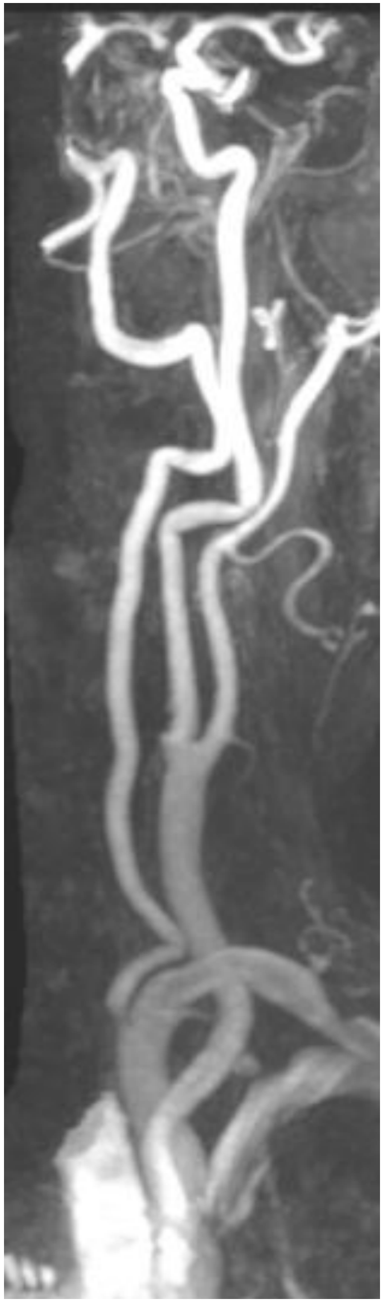

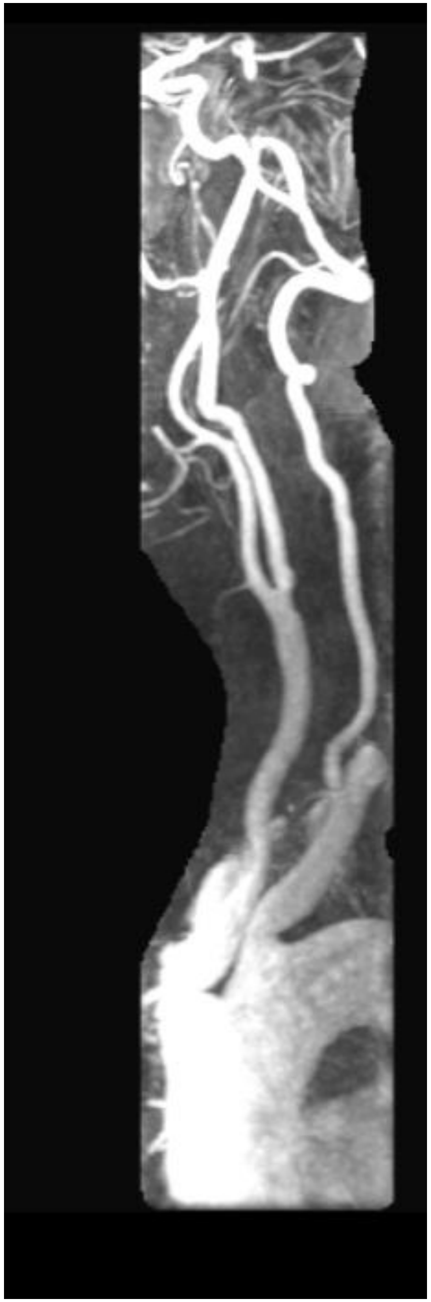

144e Score

0-30

31-50

51-70

>70

Near occlusion

Occluded

Quality

1

2

3

4

5

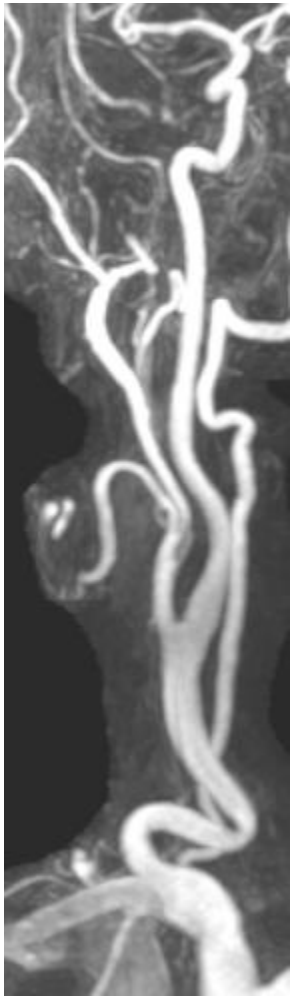

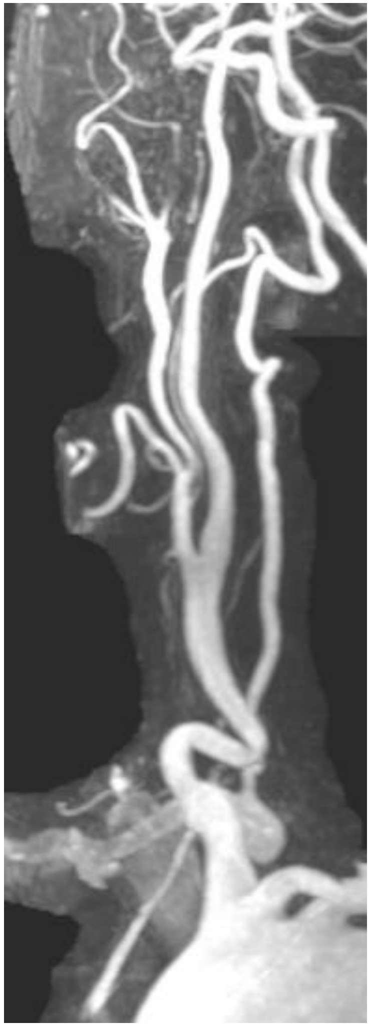

145d Score

0-30

31-50

51-70

>70

Near occlusion

Occluded

Quality

1

2

3

4

5

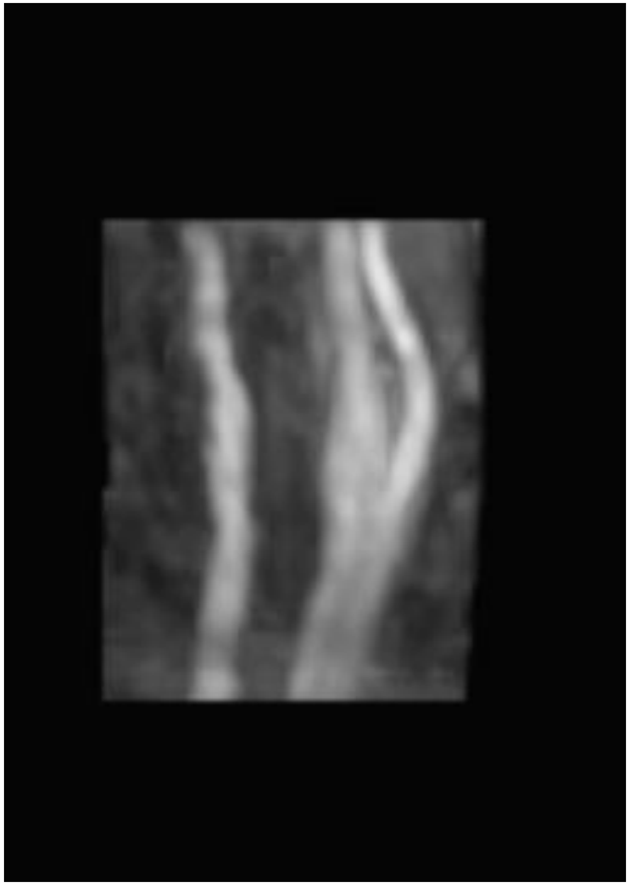

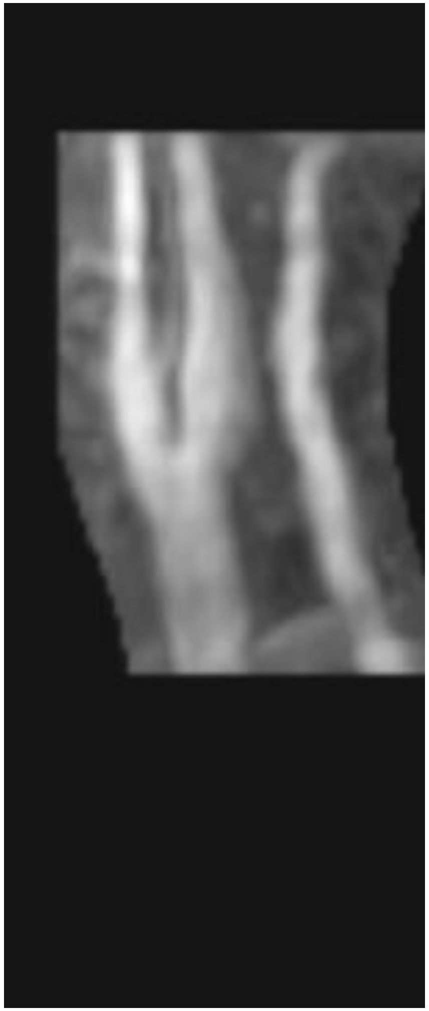

146c Score

0-30

31-50

51-70

>70

Near occlusion

Occluded

Quality

1

2

3

4

5

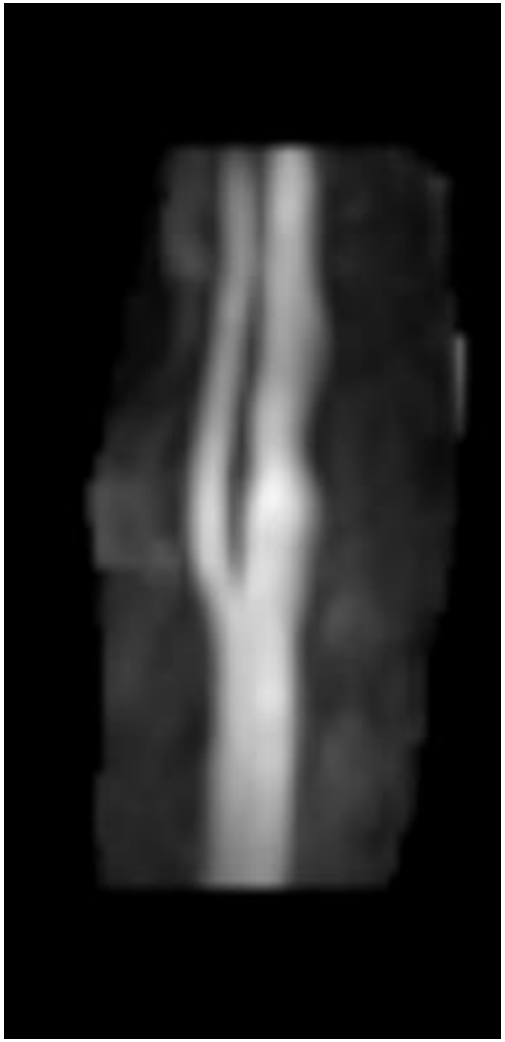

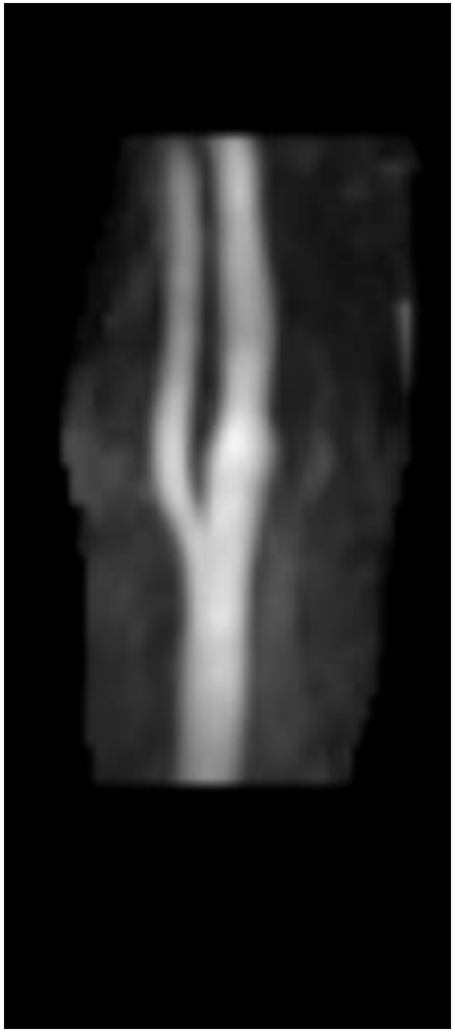

147b Score

0-30

31-50

51-70

>70

Near occlusion

Occluded

Quality

1

2

3

4

5

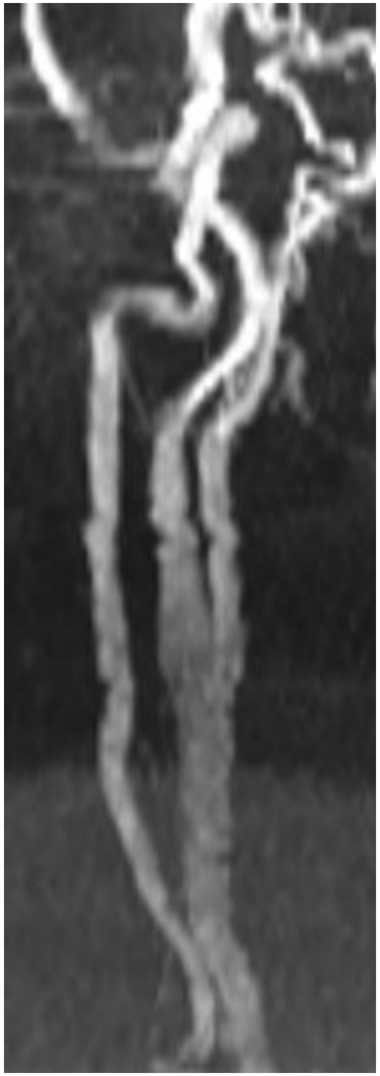

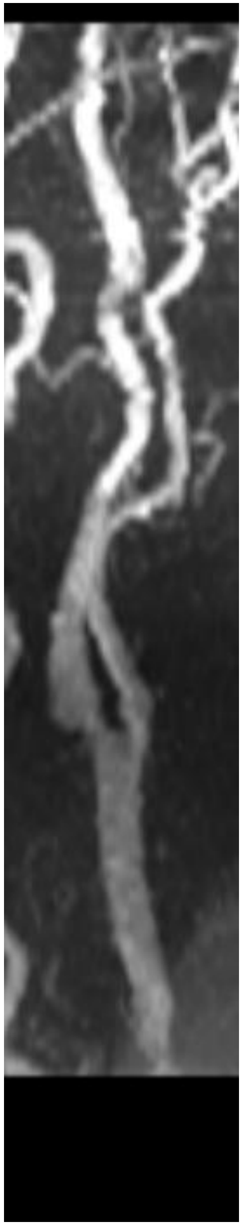

148a Score

0-30

31-50

51-70

>70

Near occlusion

Occluded

Quality

1

2

3

4

5

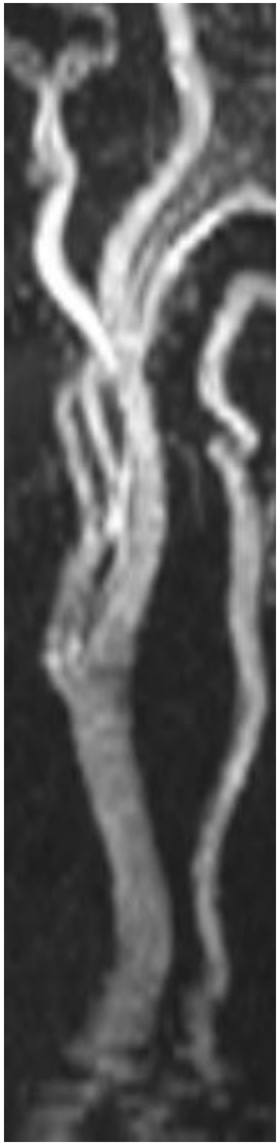

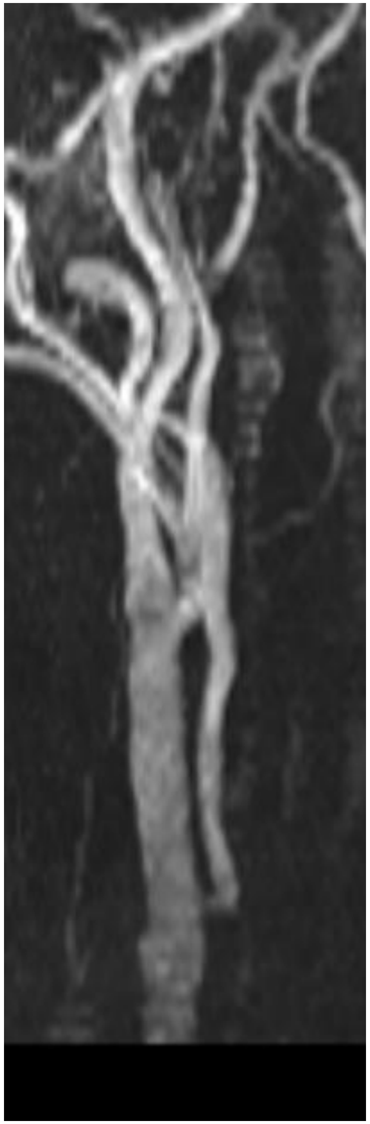

148f Score

0-30

31-50

51-70

>70

Near occlusion

Occluded

Quality

1

2

3

4

5

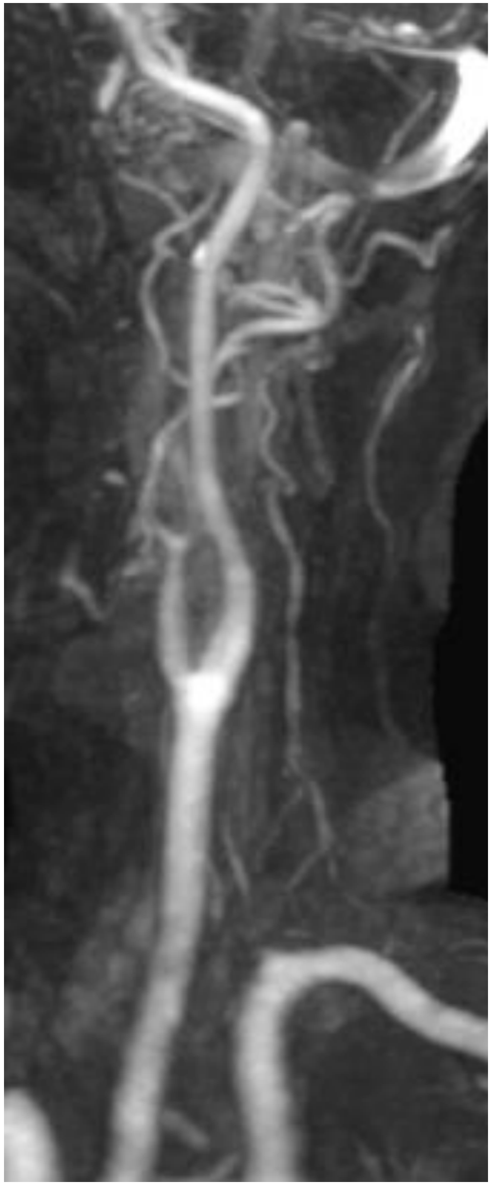

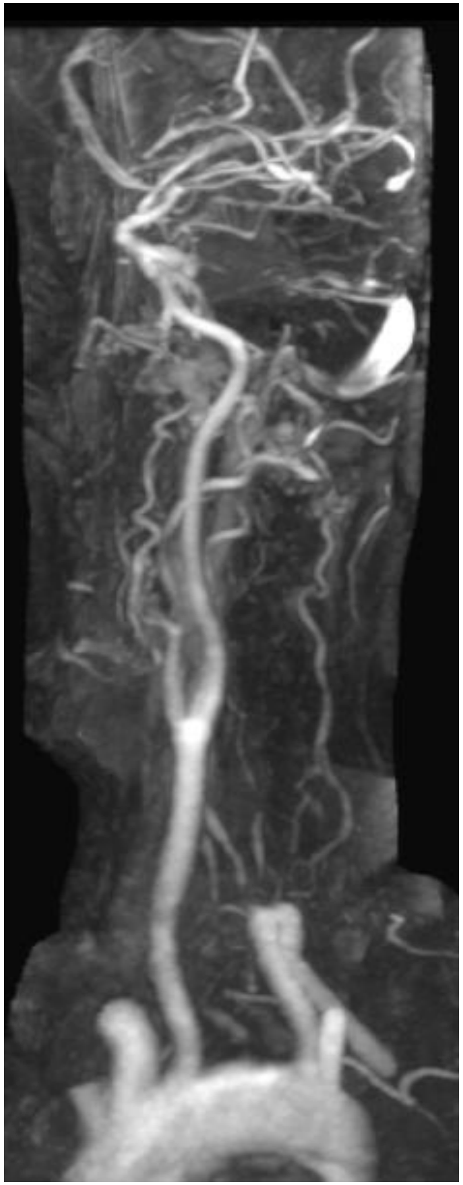

149e Score

0-30

31-50

51-70

>70

Near occlusion

Occluded

Quality

1

2

3

4

5

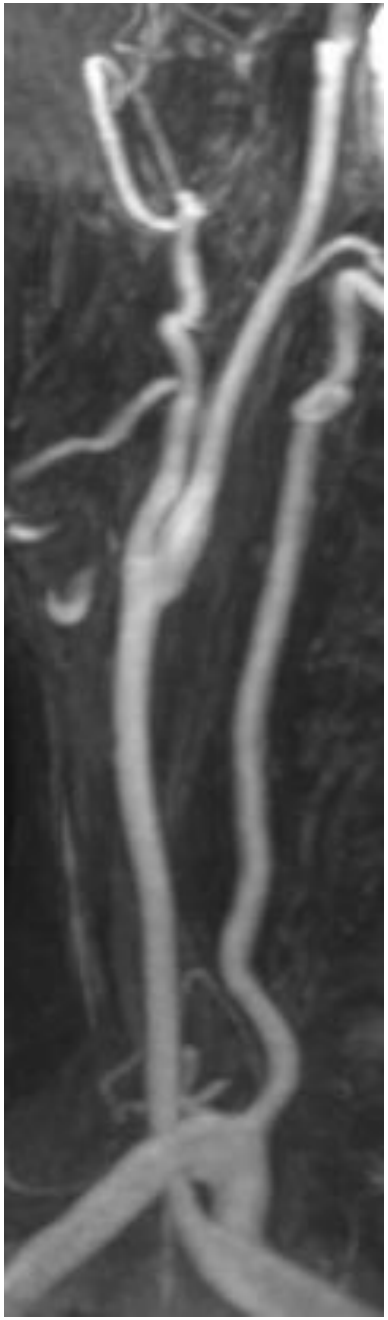

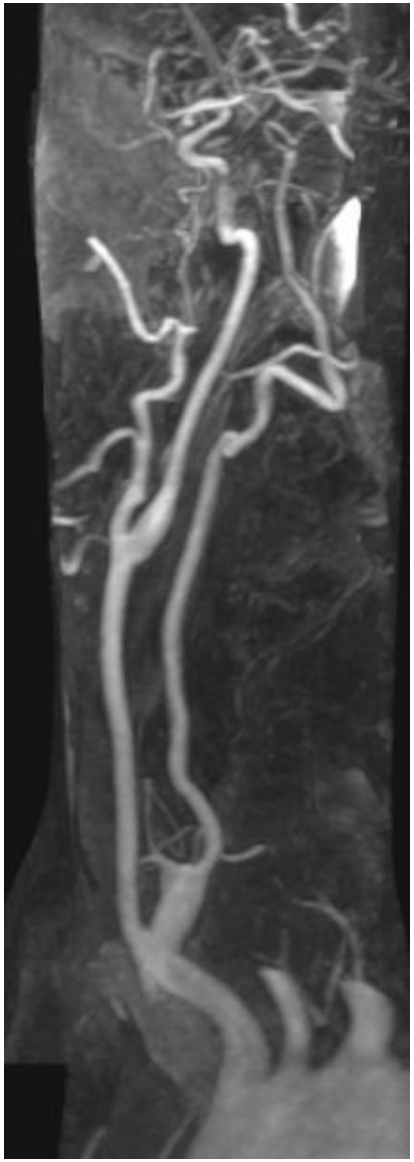

150d Score

0-30

31-50

51-70

>70

Near occlusion

Occluded

Quality

1

2

3

4

5

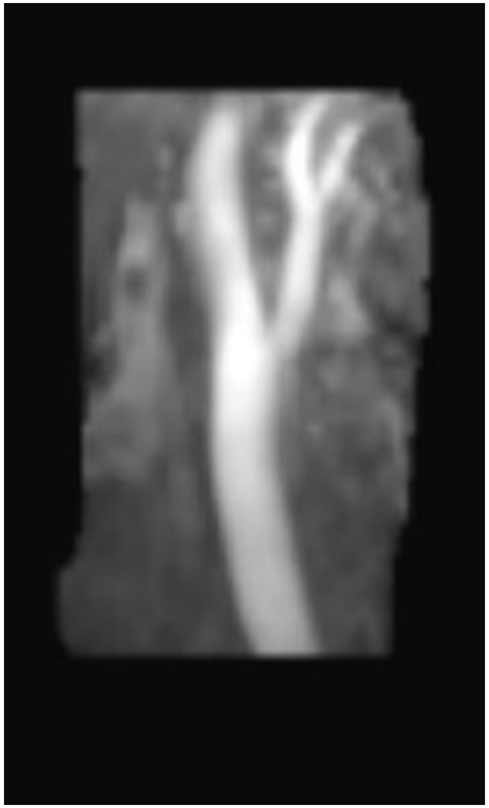

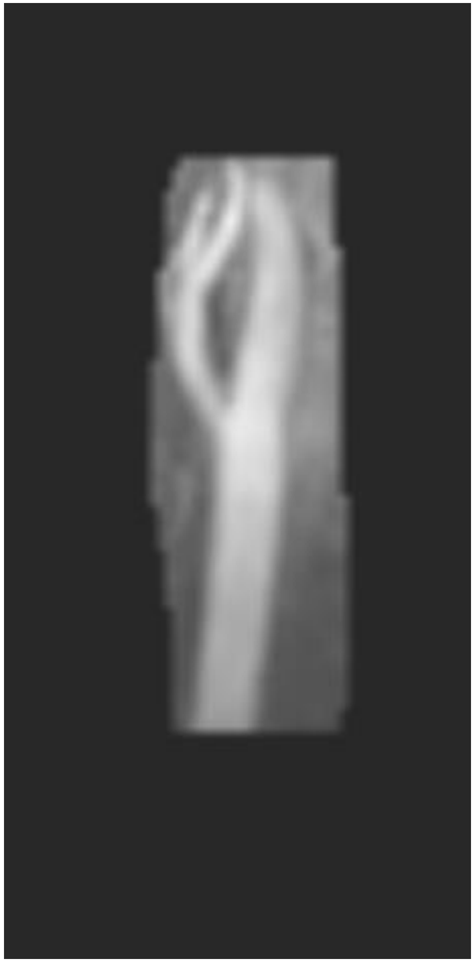

151c Score

0-30

31-50

51-70

>70

Near occlusion

Occluded

Quality

1

2

3

4

5

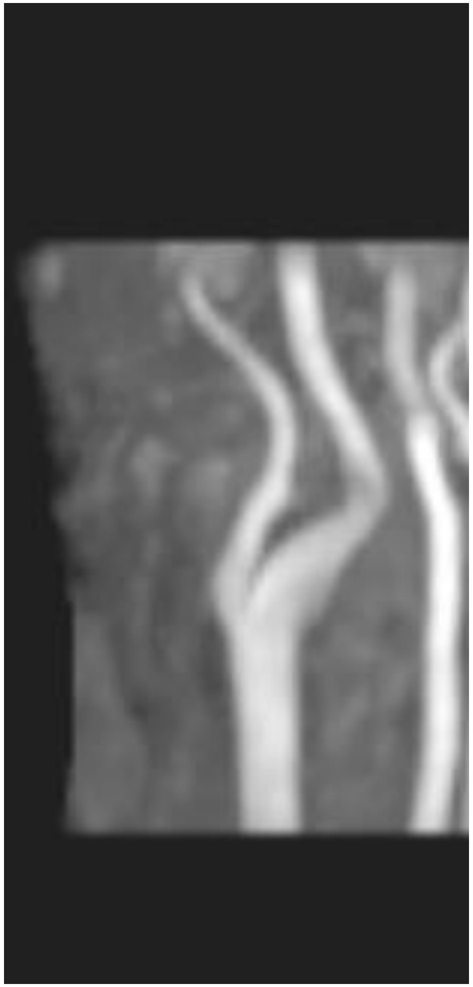

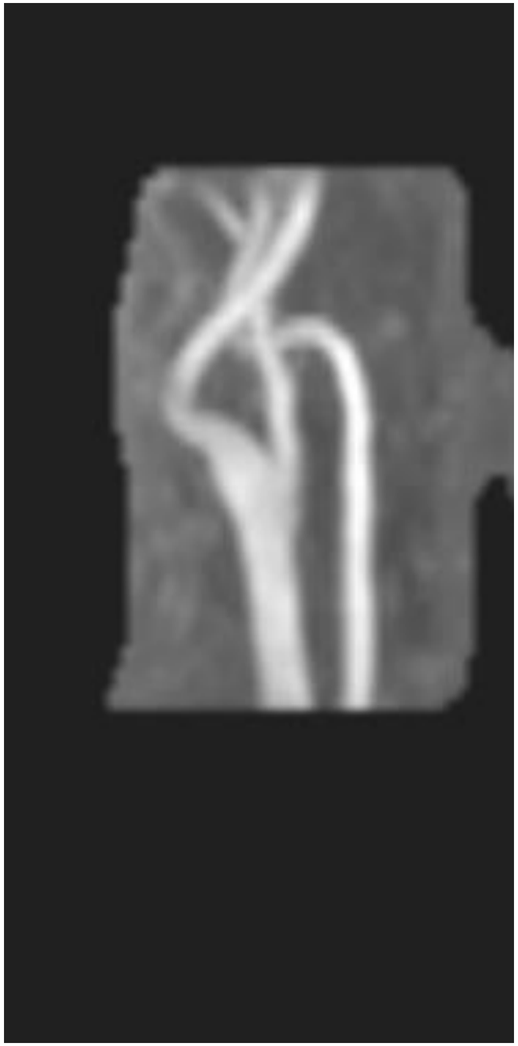

152b Score

0-30

31-50

51-70

>70

Near occlusion

Occluded

Quality

1

2

3

4

5

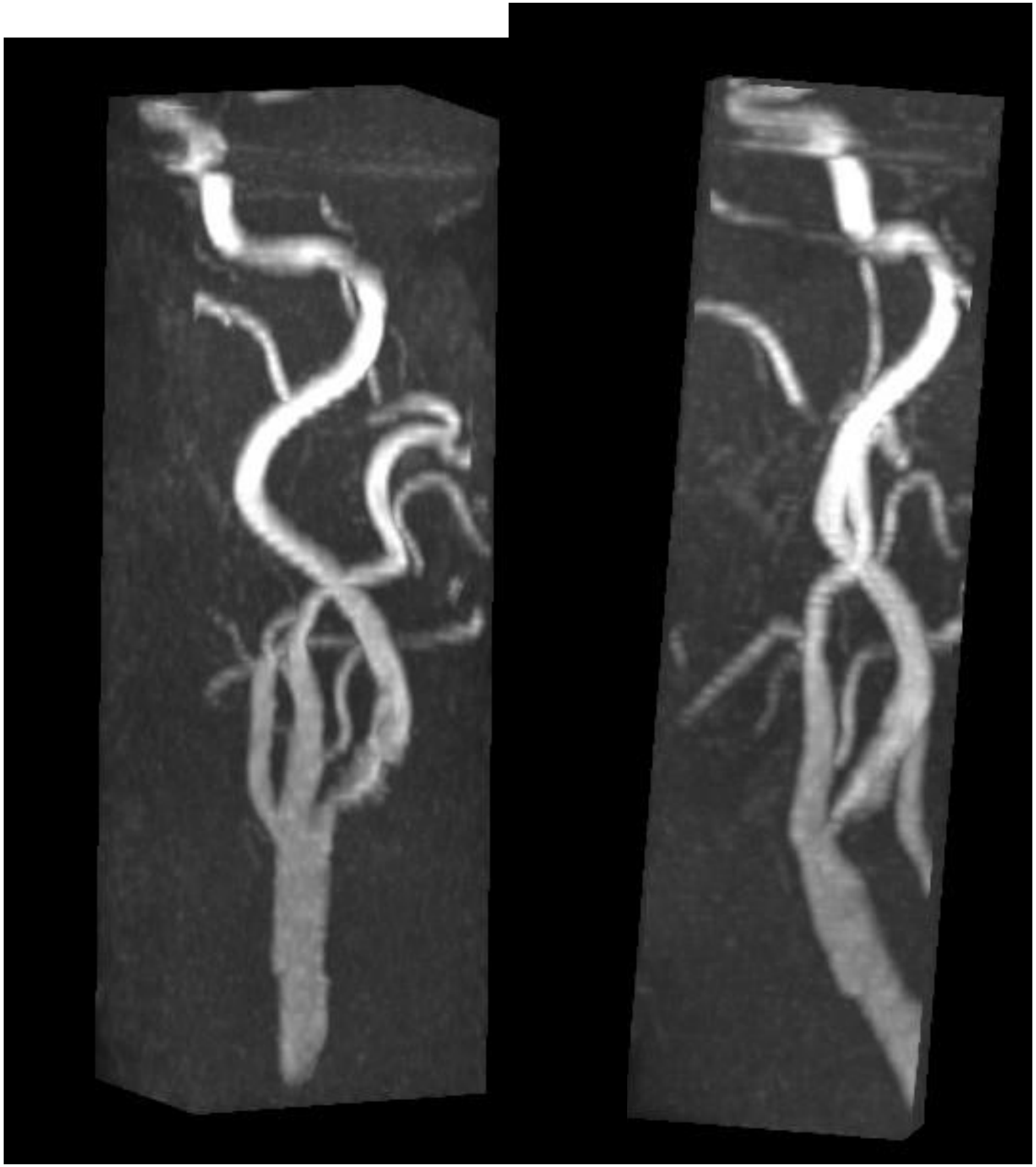

153a Score

0-30

31-50

51-70

>70

Near occlusion

Occluded

Quality

1

2

3

4

5

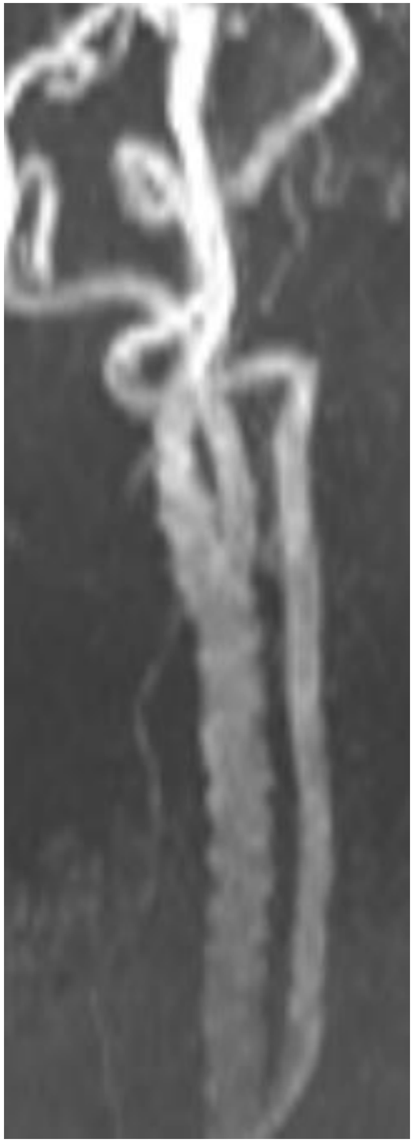

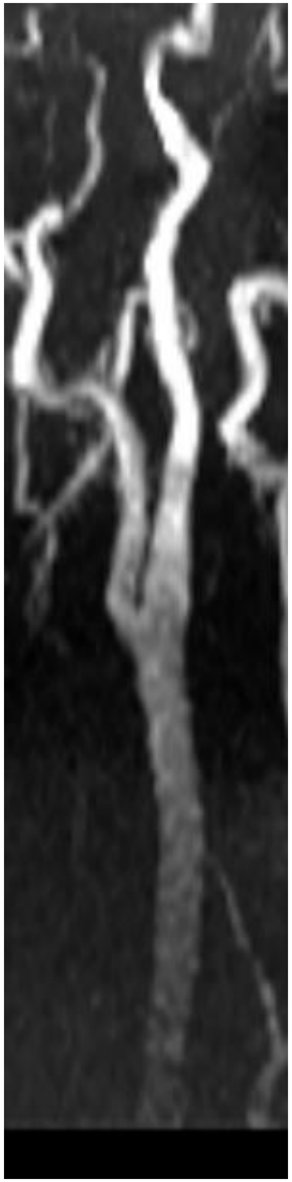

153f Score

0-30

31-50

51-70

>70

Near occlusion

Occluded

Quality

1

2

3

4

5

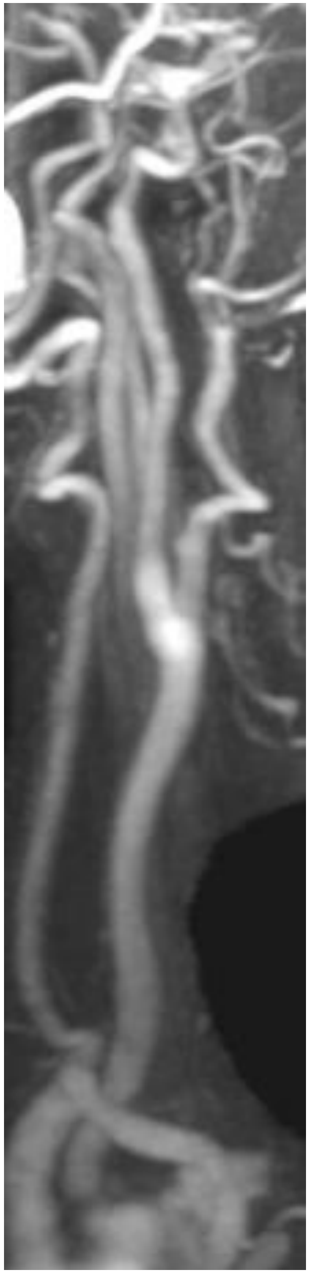

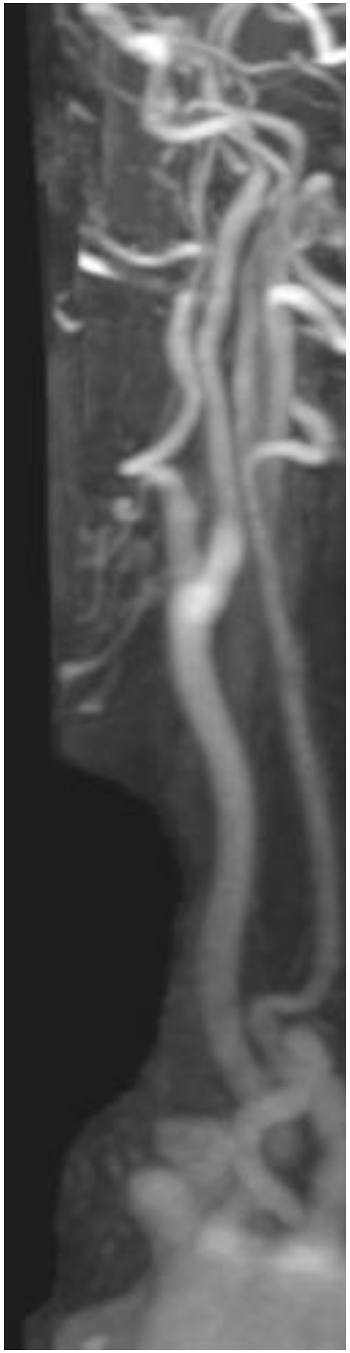

154e Score

0-30

31-50

51-70

>70

Near occlusion

Occluded

Quality

1

2

3

4

5

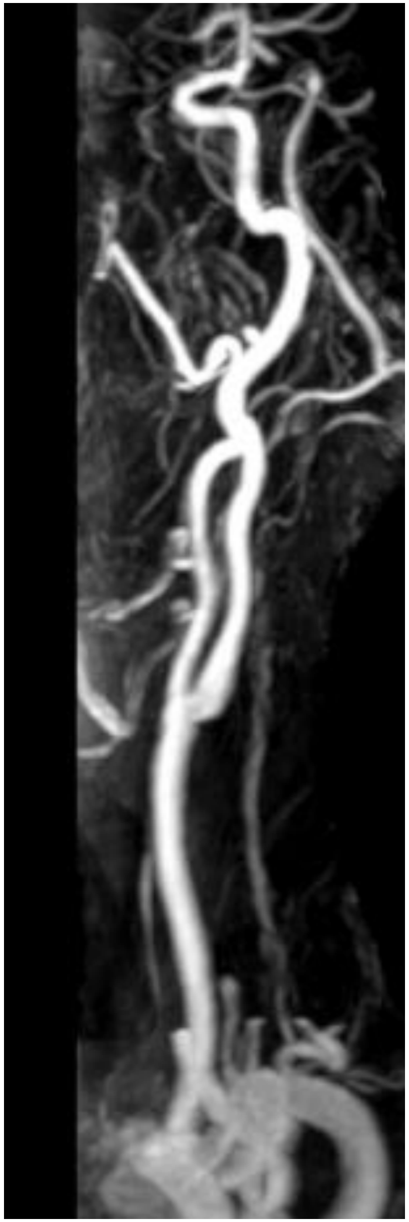

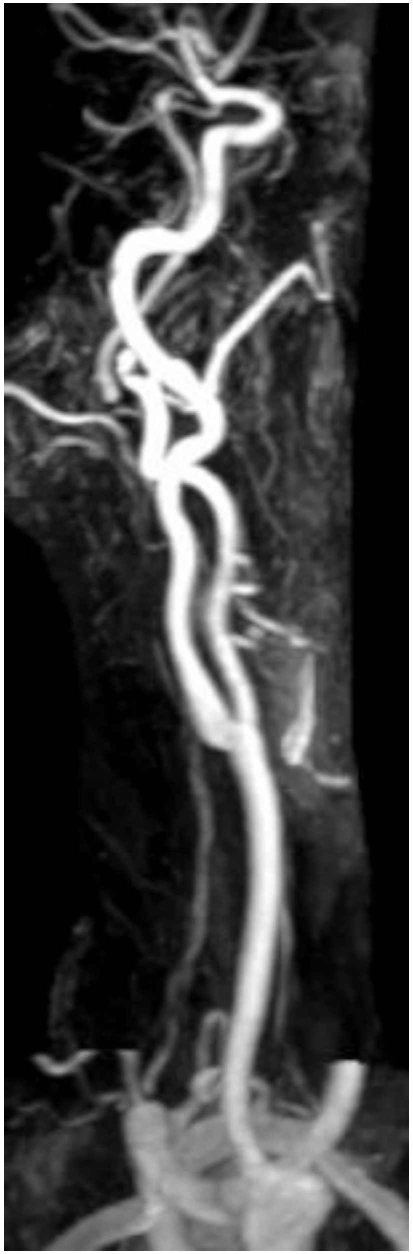

155d Score

0-30

31-50

51-70

>70

Near occlusion

Occluded

Quality

1

2

3

4

5

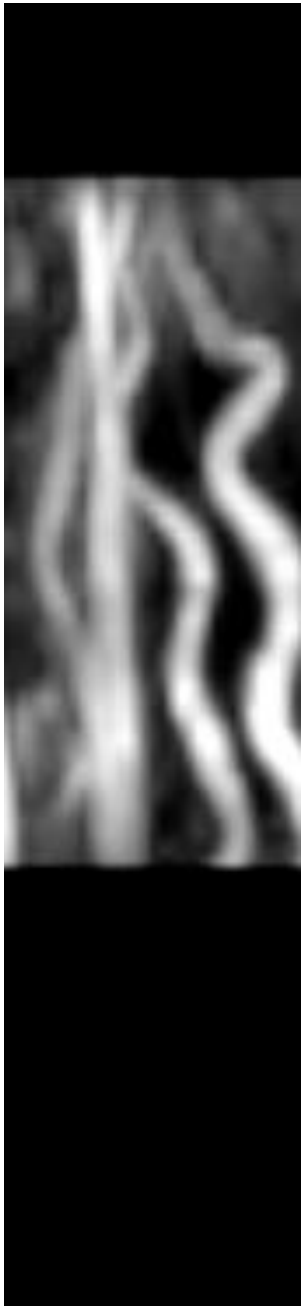

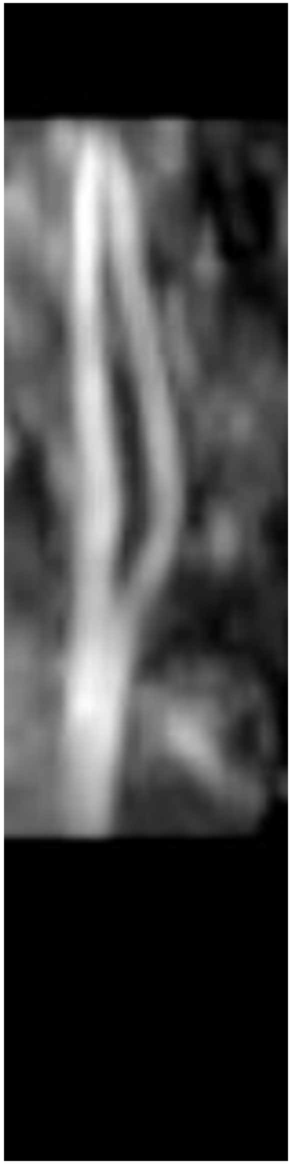

156c Score

0-30

31-50

51-70

>70

Near occlusion

Occluded

Quality

1

2

3

4

5

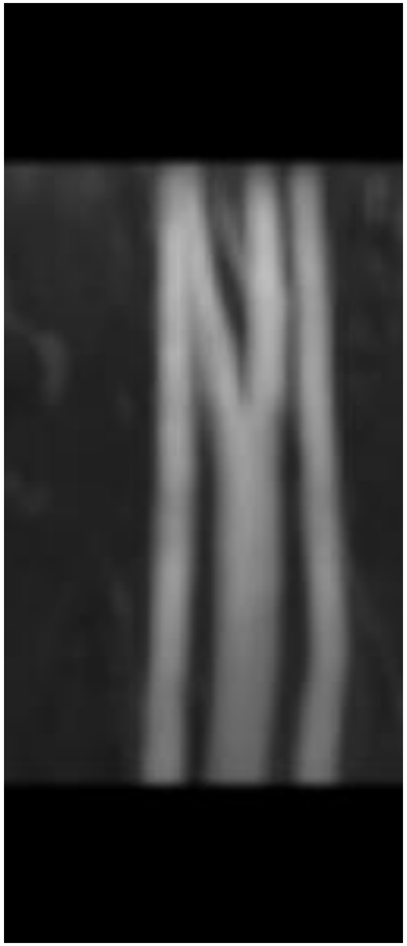

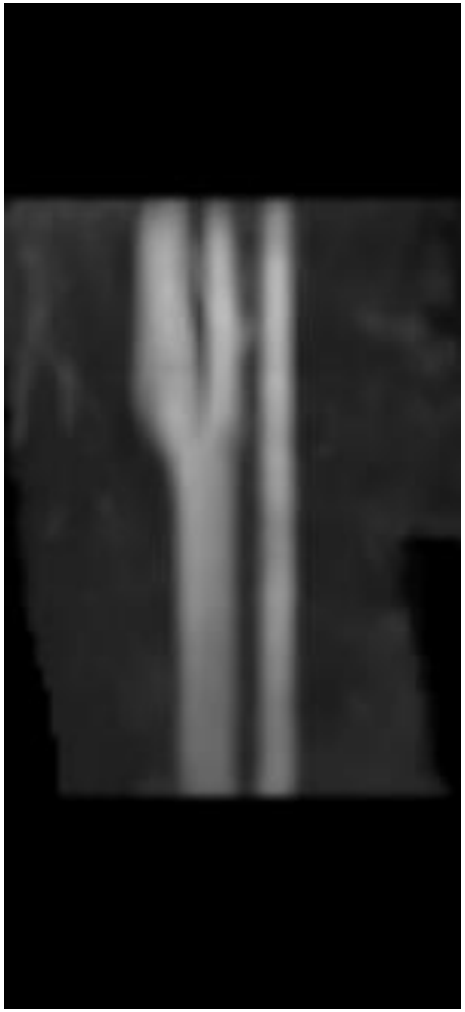

157b Score

0-30

31-50

51-70

>70

Near occlusion

Occluded

Quality

1

2

3

4

5

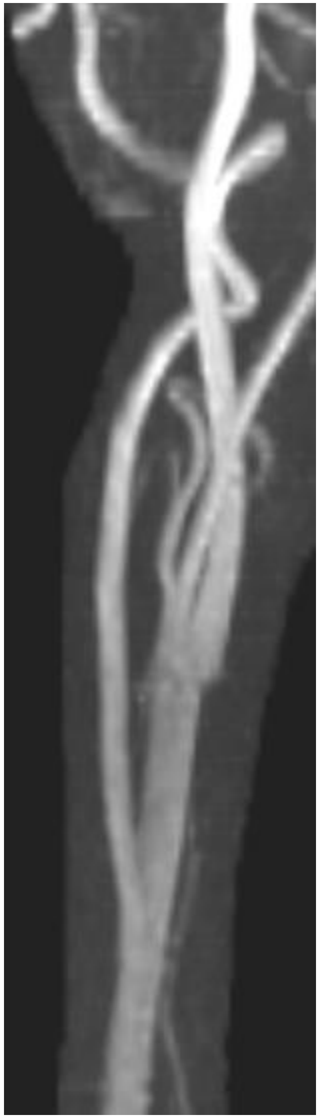

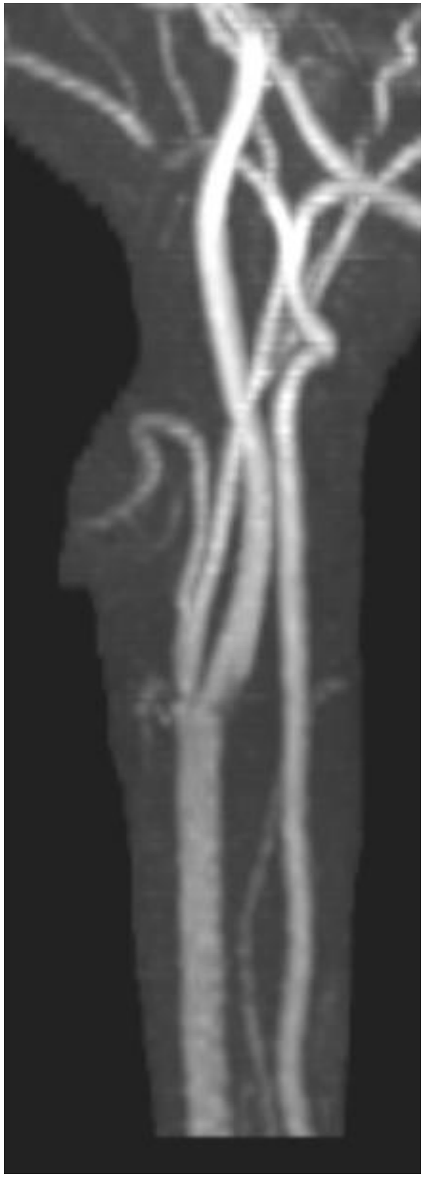

158a Score

0-30

31-50

51-70

>70

Near occlusion

Occluded

Quality

1

2

3

4

5

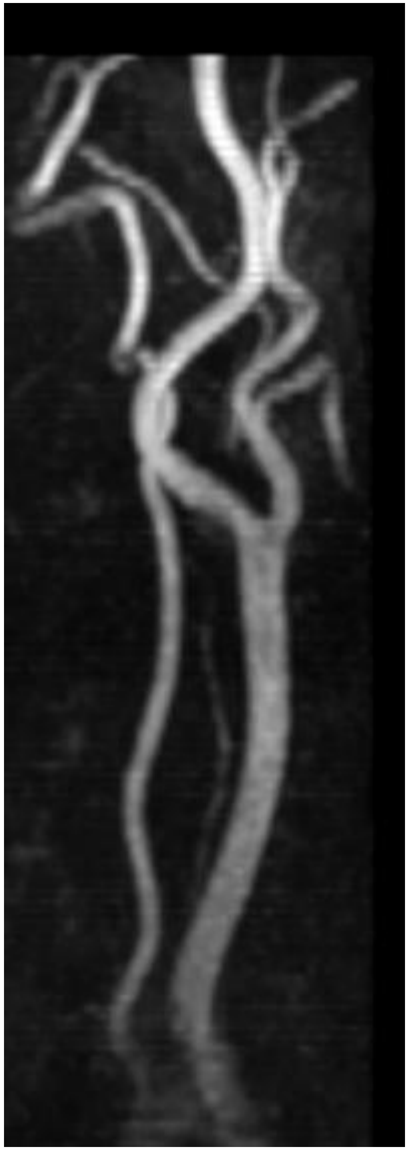

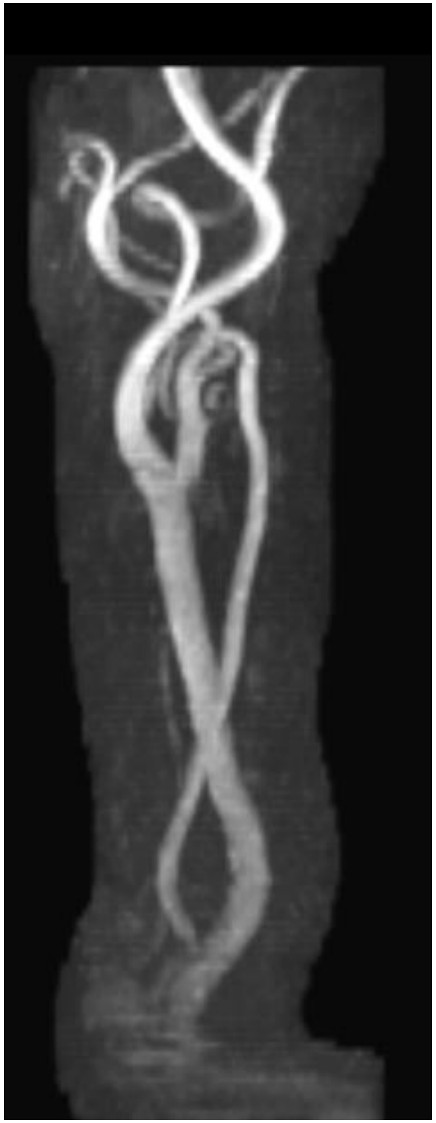

158f Score

0-30

31-50

51-70

>70

Near occlusion

Occluded

Quality

1

2

3

4

5

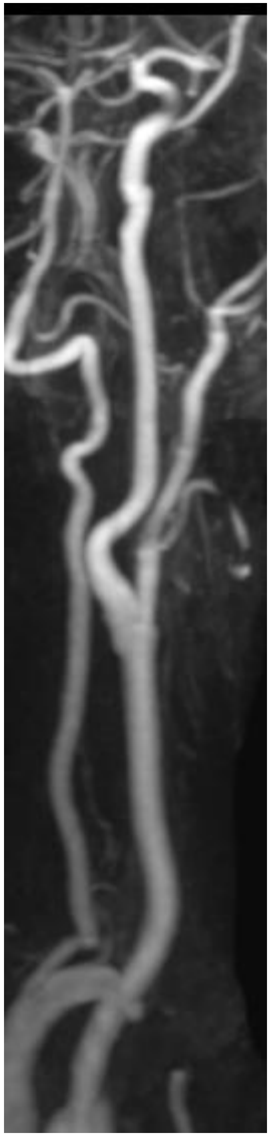

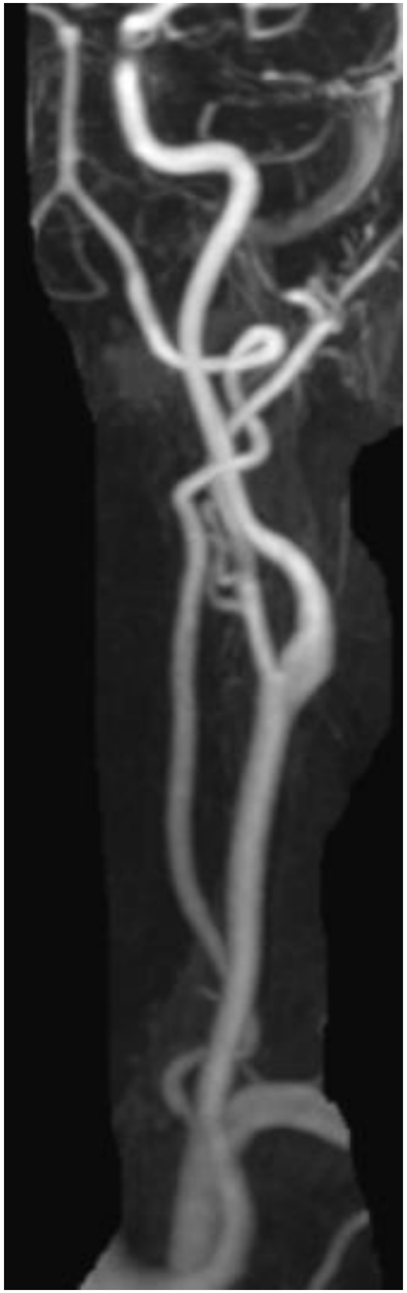

159e Score

0-30

31-50

51-70

>70

Near occlusion

Occluded

Quality

1

2

3

4

5

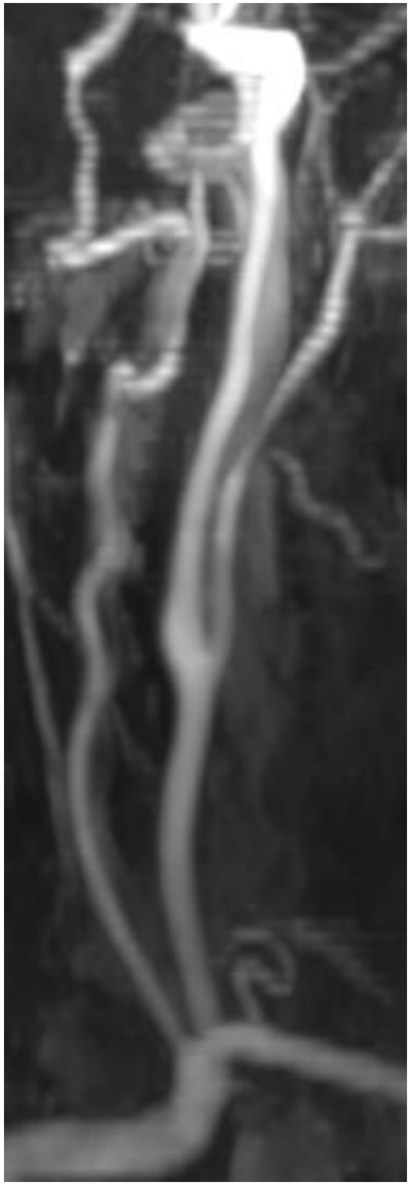

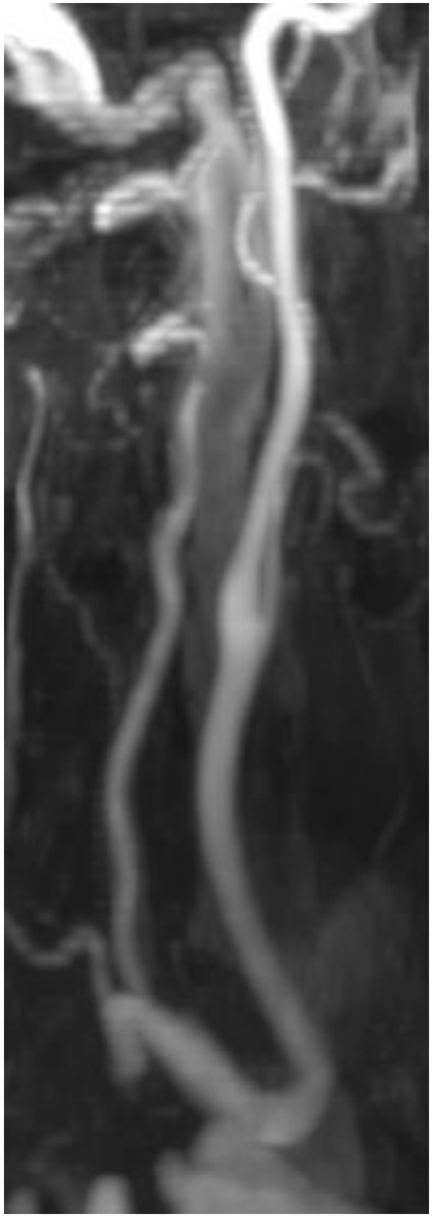

160d Score

0-30

31-50

51-70

>70

Near occlusion

Occluded

Quality

1

2

3

4

5

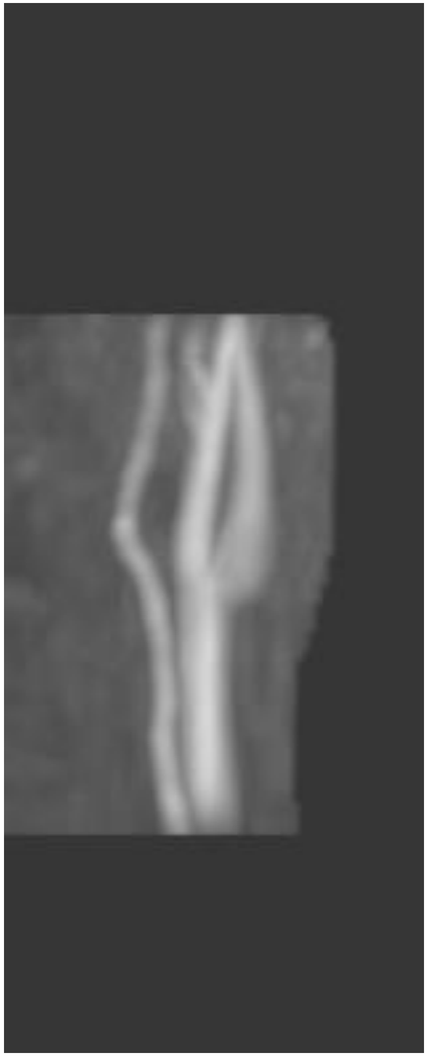

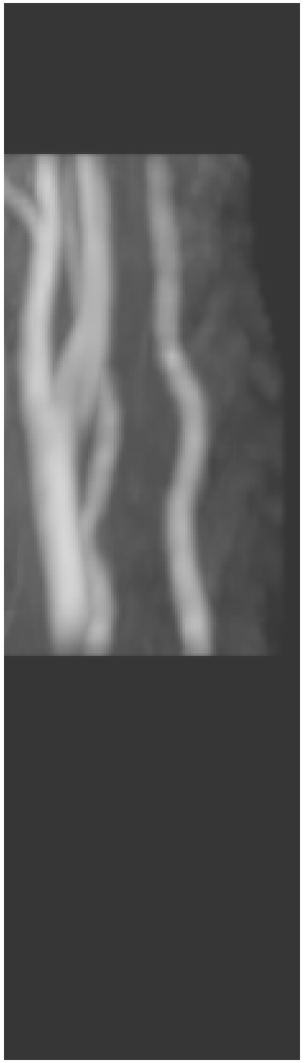

161c Score

0-30

31-50

51-70

>70

Near occlusion

Occluded

Quality

1

2

3

4

5

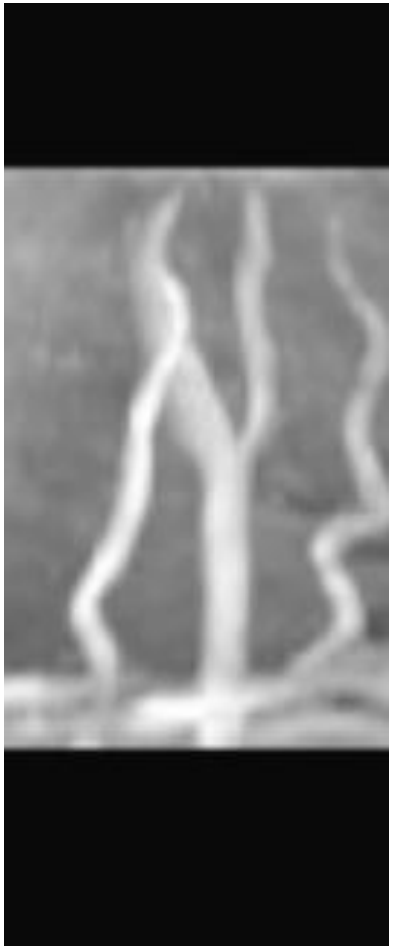

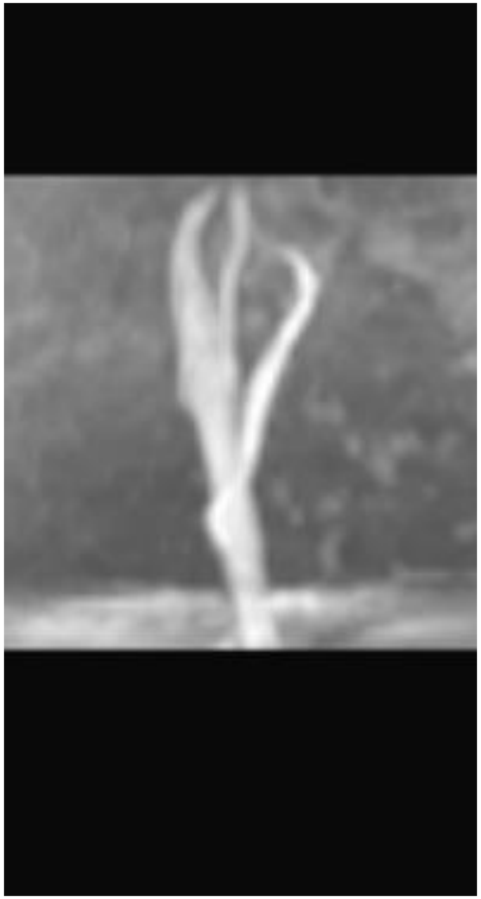

162b Score

0-30

31-50

51-70

>70

Near occlusion

Occluded

Quality

1

2

3

4

5

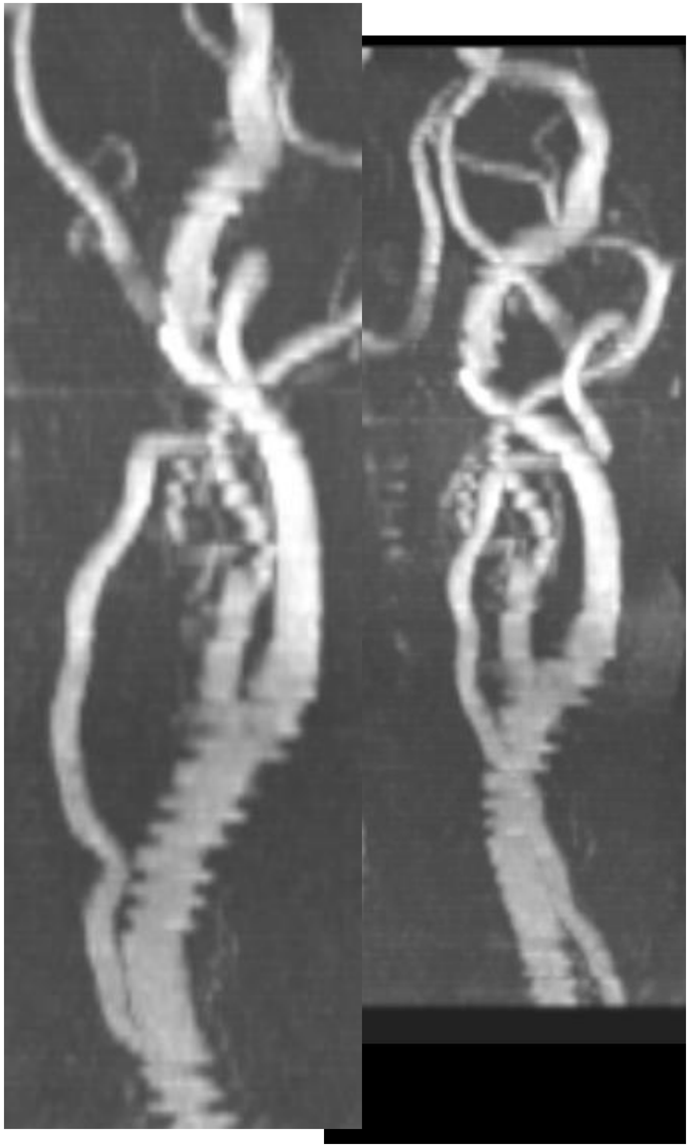

163a Score

0-30

31-50

51-70

>70

Near occlusion

Occluded

Quality

1

2

3

4

5

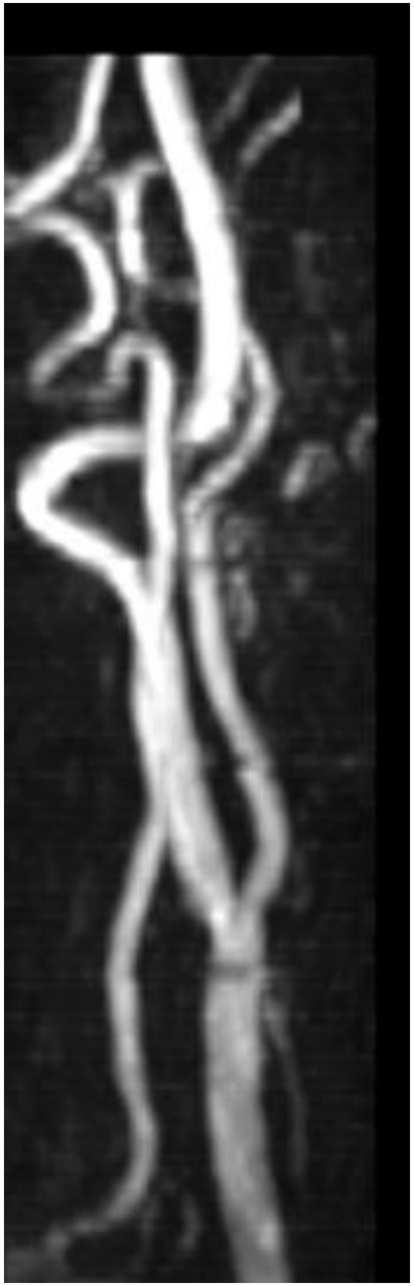

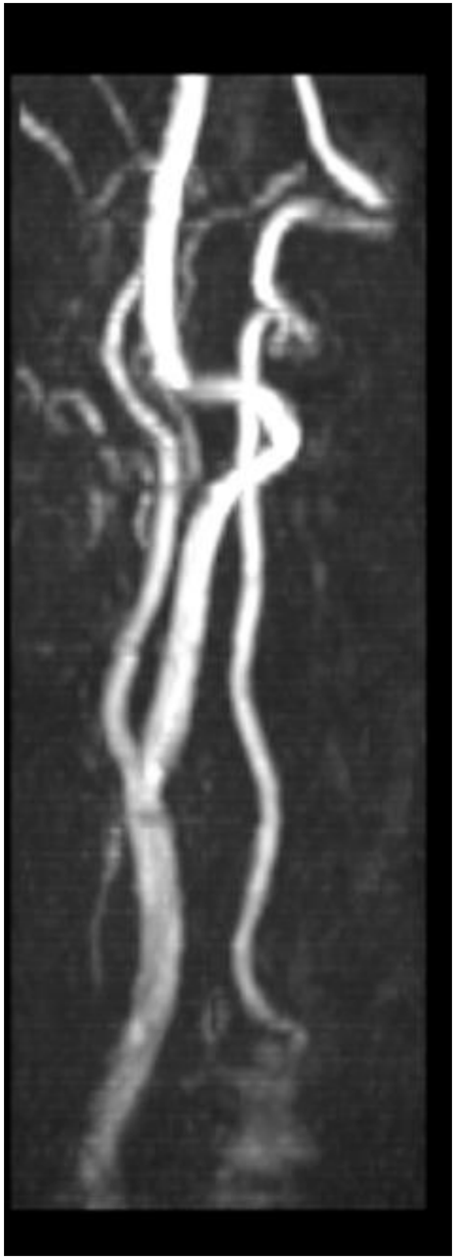

163f Score

0-30

31-50

51-70

>70

Near occlusion

Occluded

Quality

1

2

3

4

5

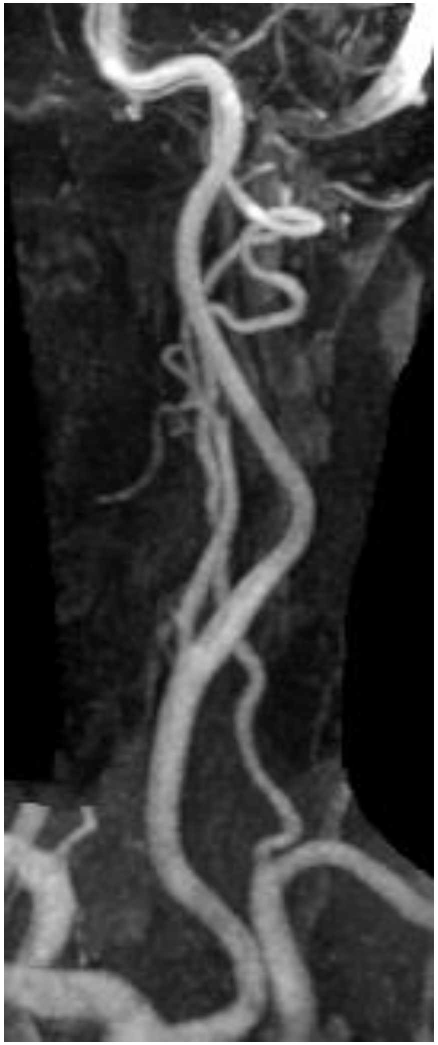

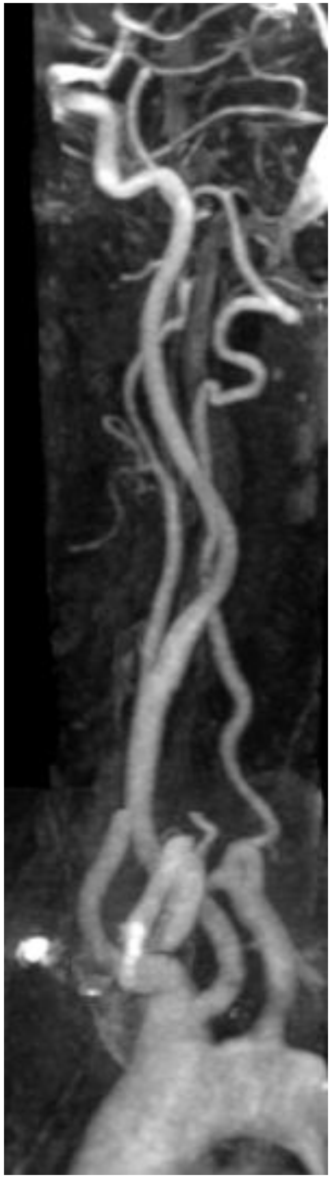

164e Score

0-30

31-50

51-70

>70

Near occlusion

Occluded

Quality

1

2

3

4

5

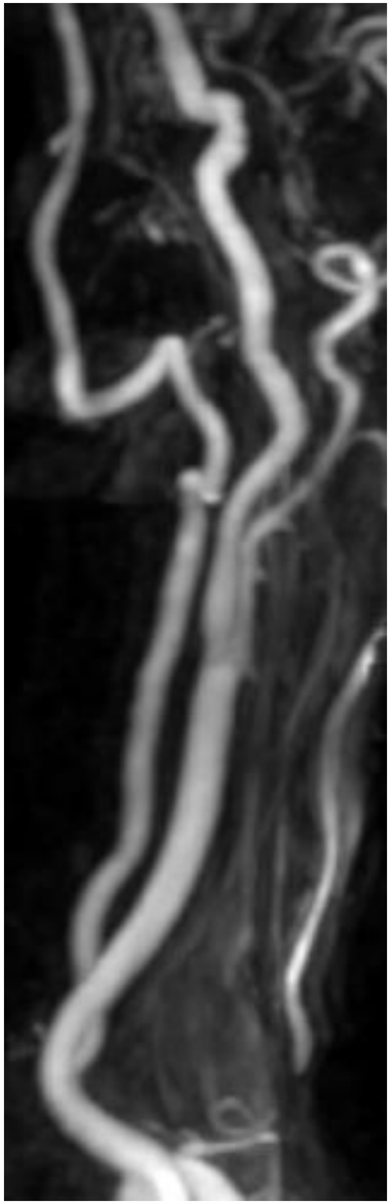

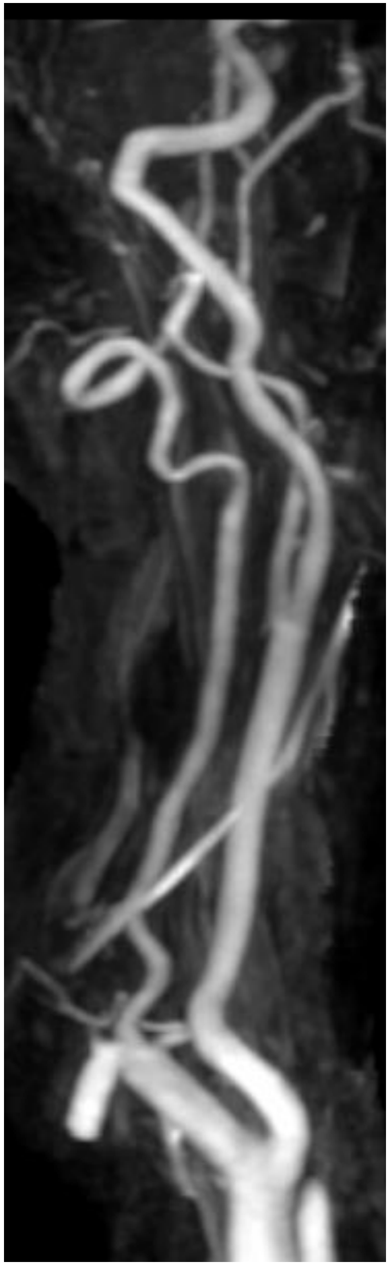

165d Score

0-30

31-50

51-70

>70

Near occlusion

Occluded

Quality

1

2

3

4

5

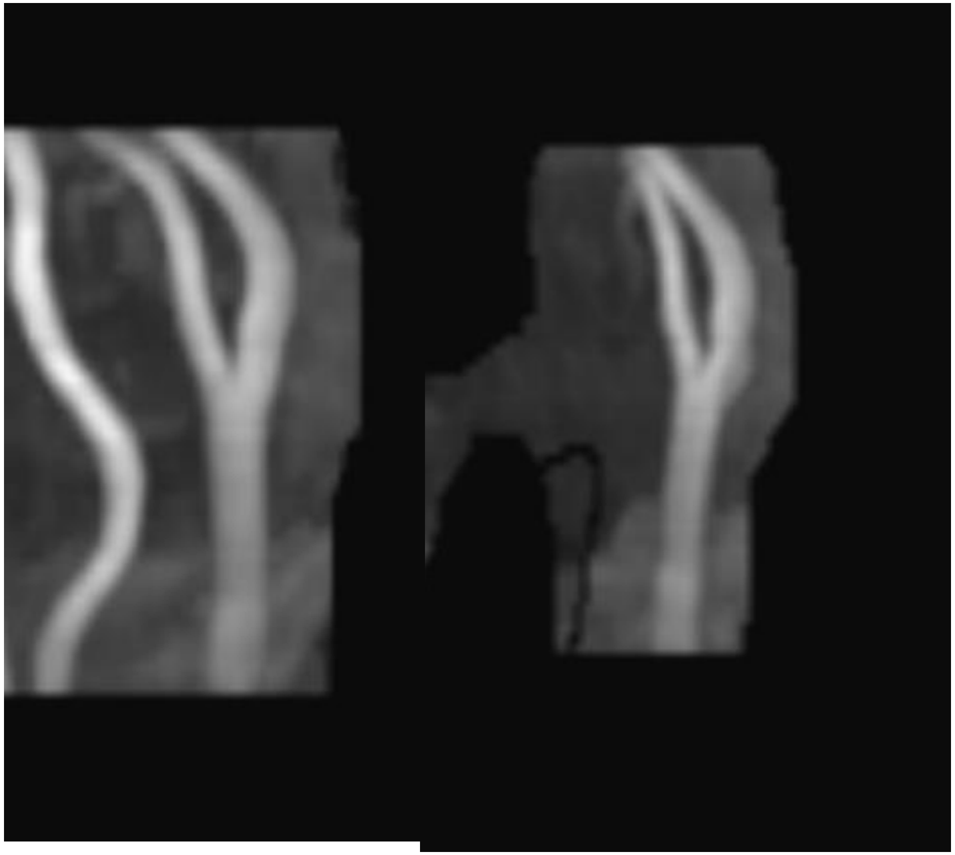

166c Score

0-30

31-50

51-70

>70

Near occlusion

Occluded

Quality

1

2

3

4

5

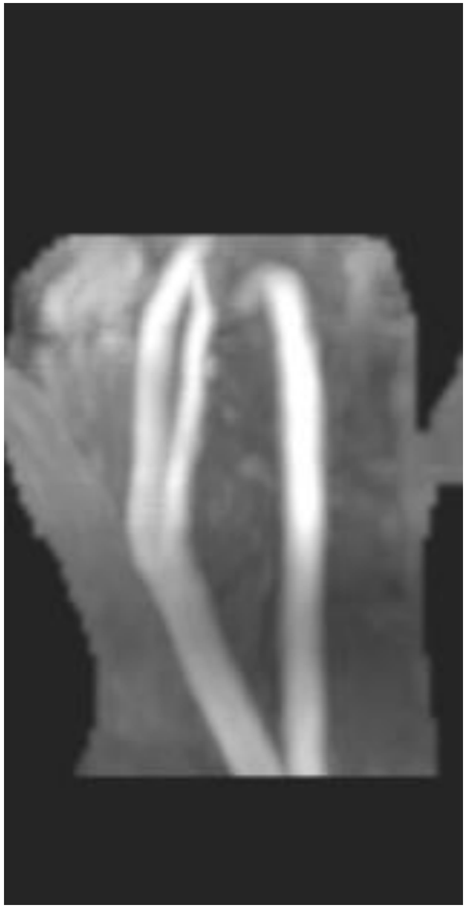

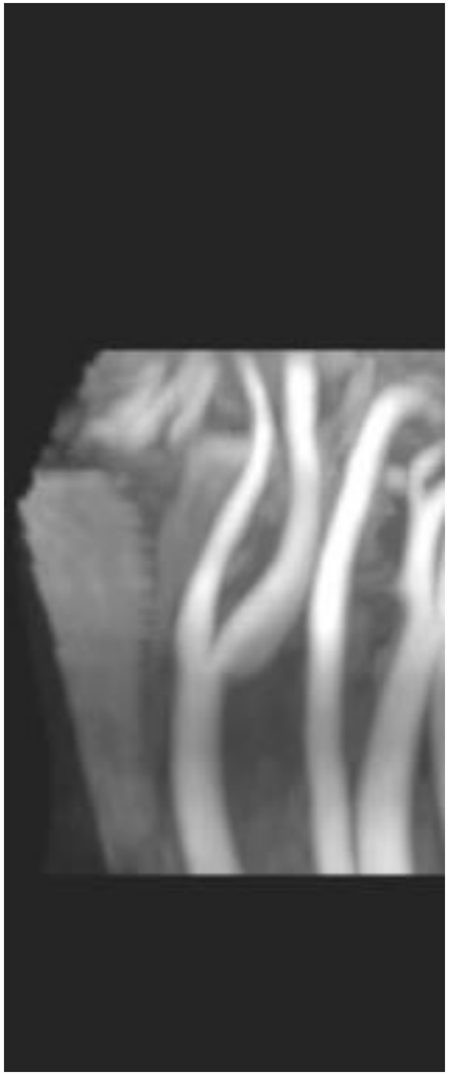

167b Score

0-30

31-50

51-70

>70

Near occlusion

Occluded

Quality

1

2

3

4

5

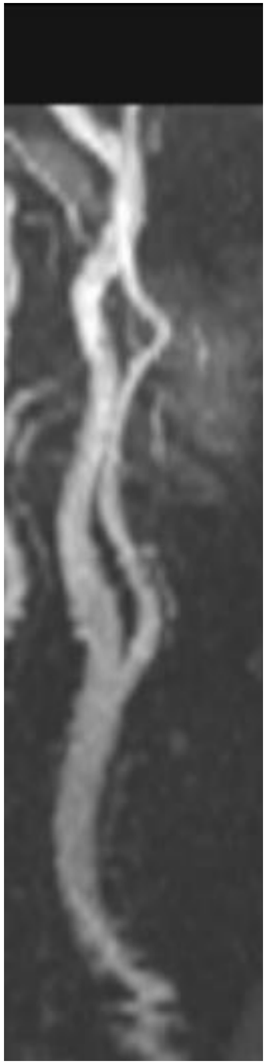

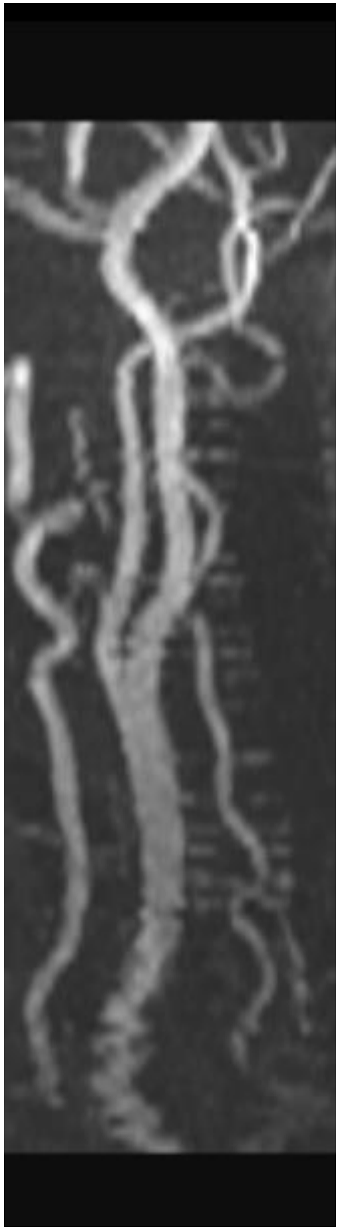

168a Score

0-30

31-50

51-70

>70

Near occlusion

Occluded

Quality

1

2

3

4

5

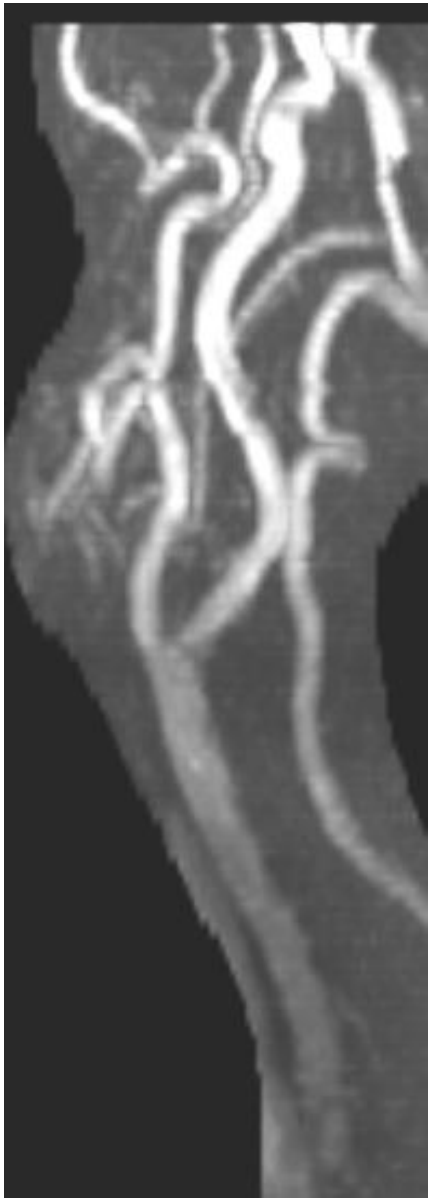

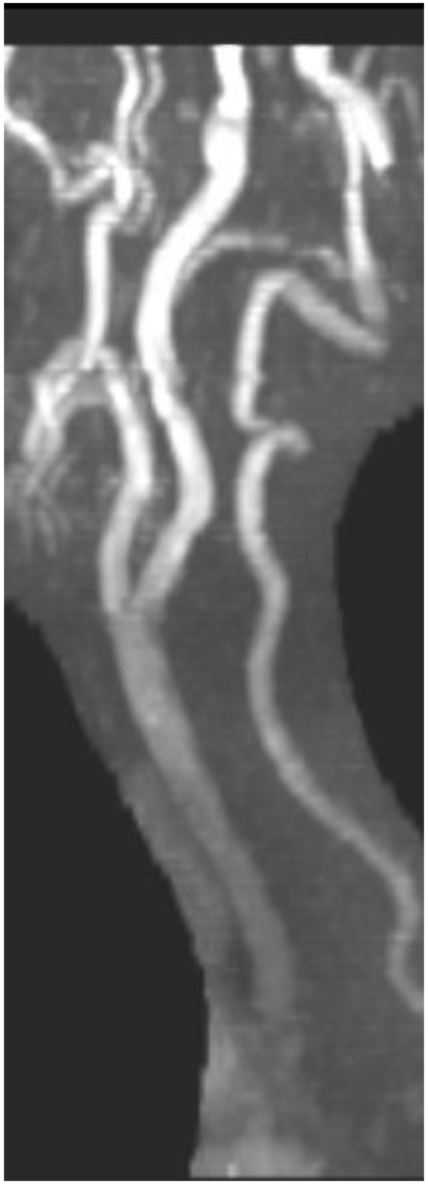

168f Score

0-30

31-50

51-70

>70

Near occlusion

Occluded

Quality

1

2

3

4

5

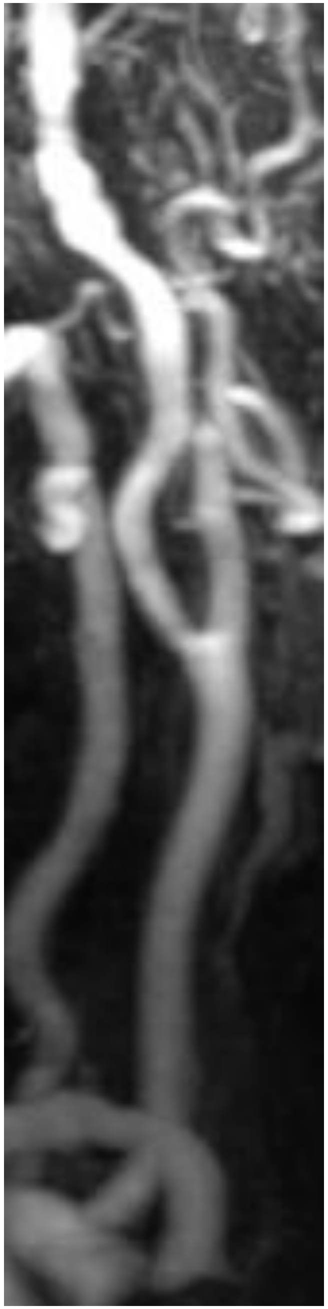

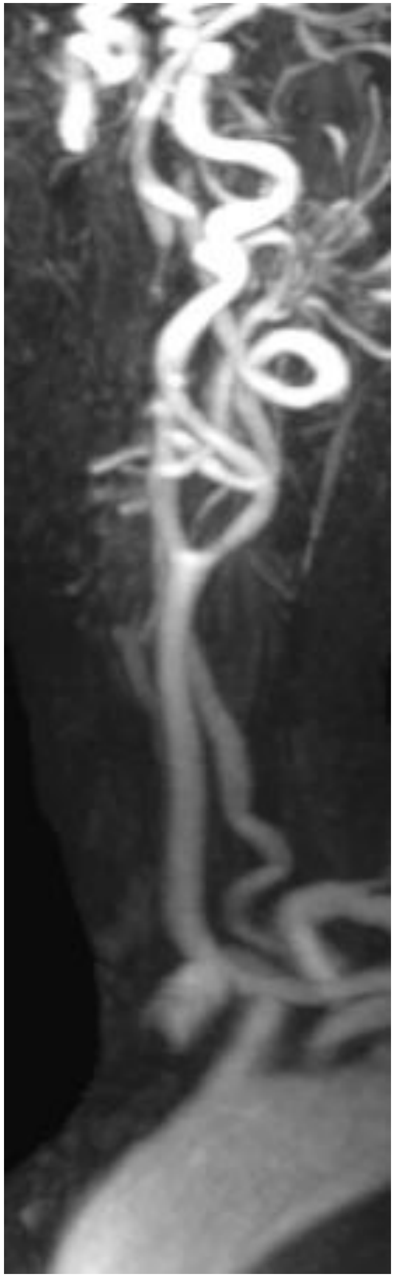

169e Score

0-30

31-50

51-70

>70

Near occlusion

Occluded

Quality

1

2

3

4

5

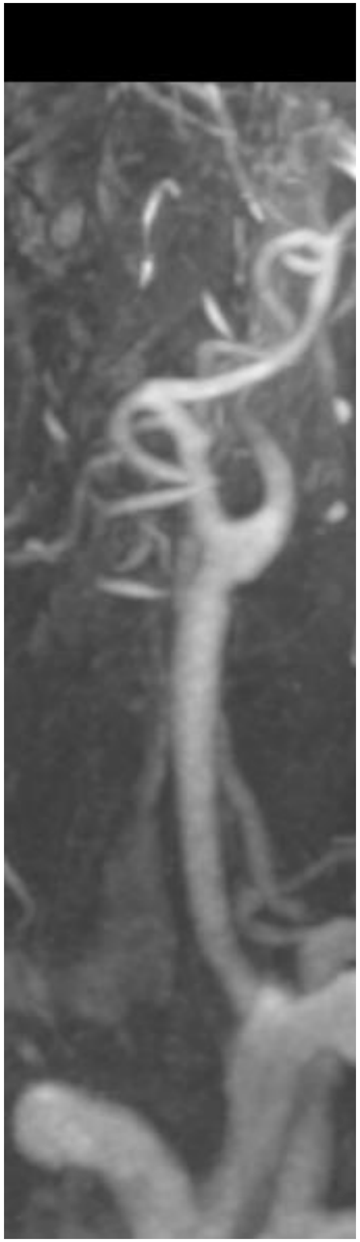

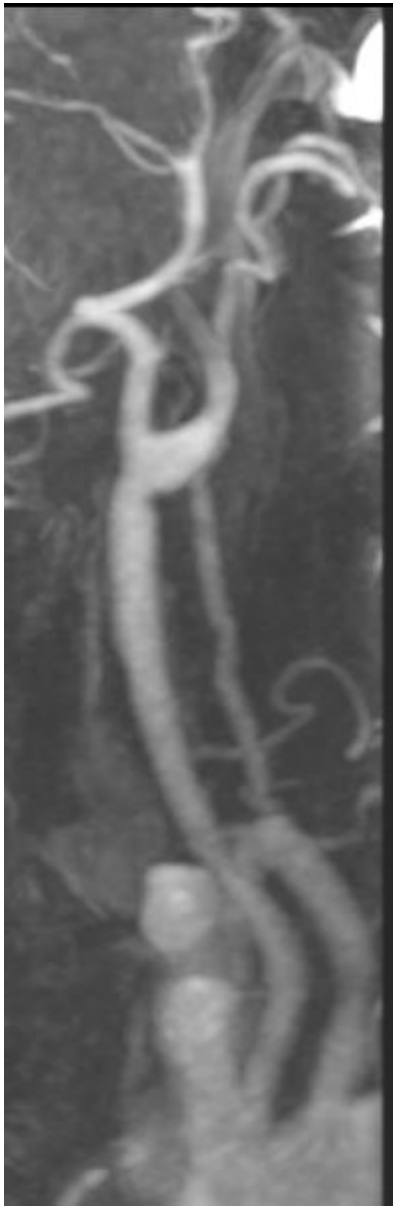

170d Score

0-30

31-50

51-70

>70

Near occlusion

Occluded

Quality

1

2

3

4

5

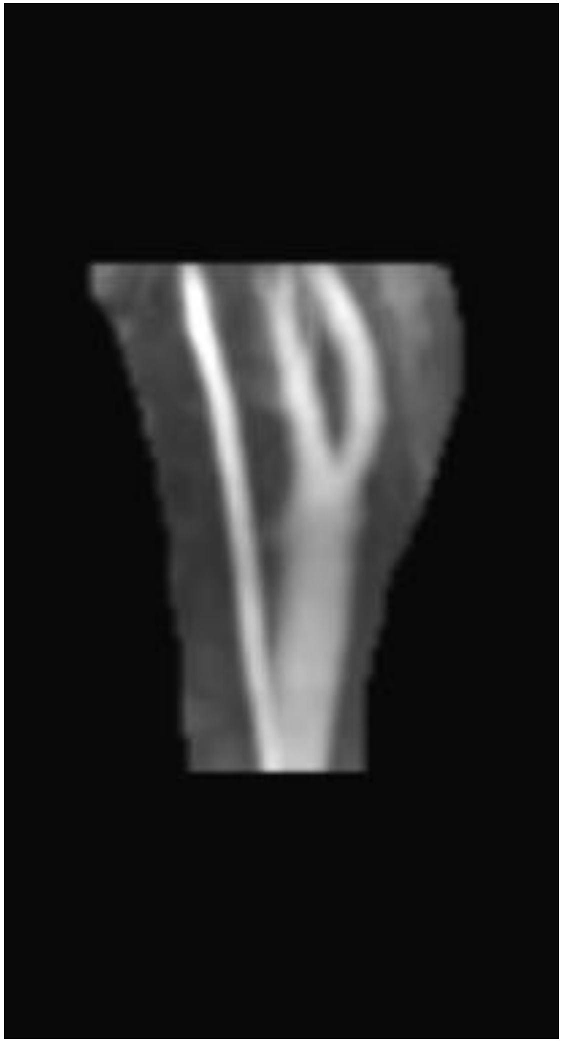

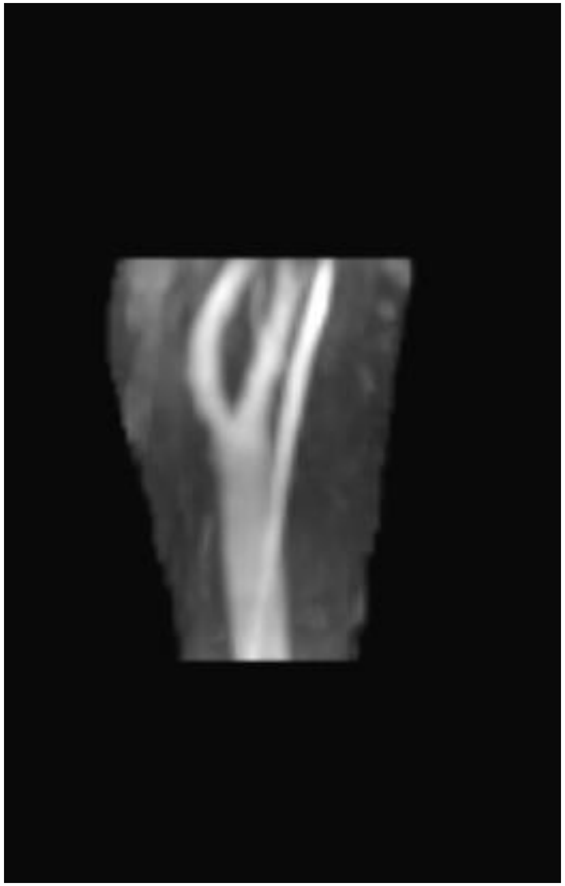

171c Score

0-30

31-50

51-70

>70

Near occlusion

Occluded

Quality

1

2

3

4

5

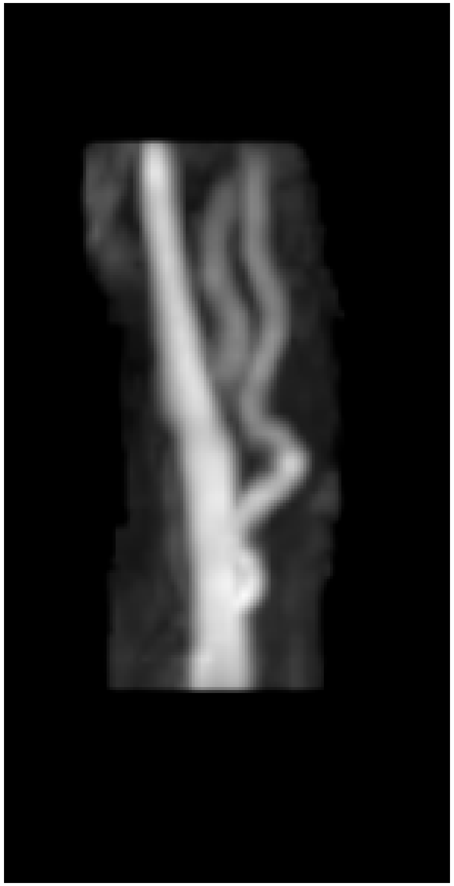

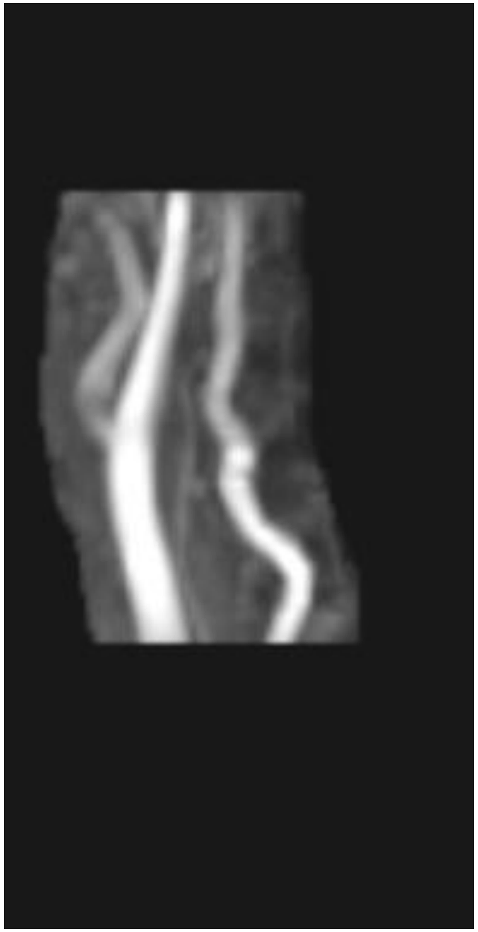

172b Score

0-30

31-50

51-70

>70

Near occlusion

Occluded

Quality

1

2

3

4

5

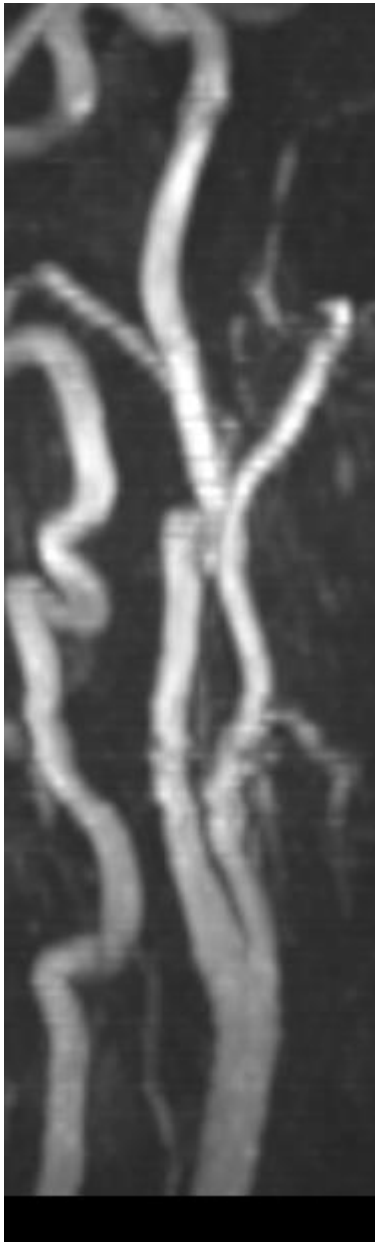

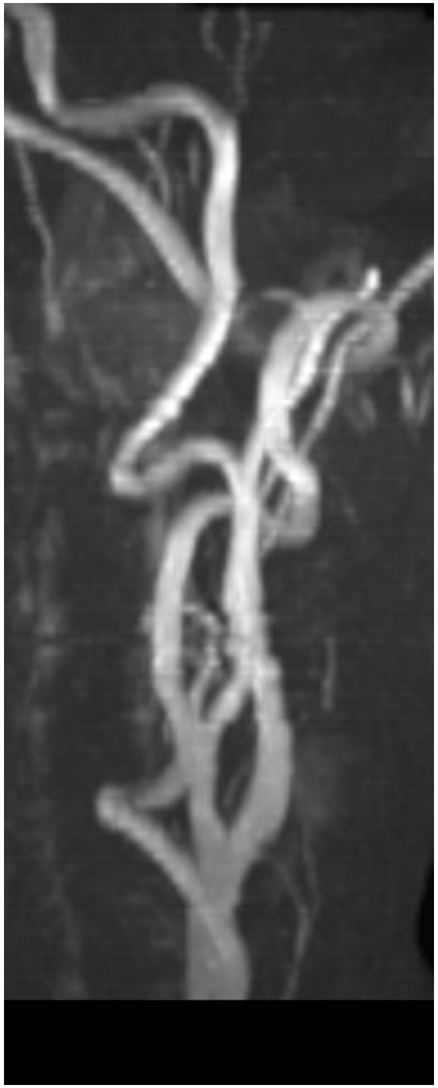

173a Score

0-30

31-50

51-70

>70

Near occlusion

Occluded

Quality

1

2

3

4

5

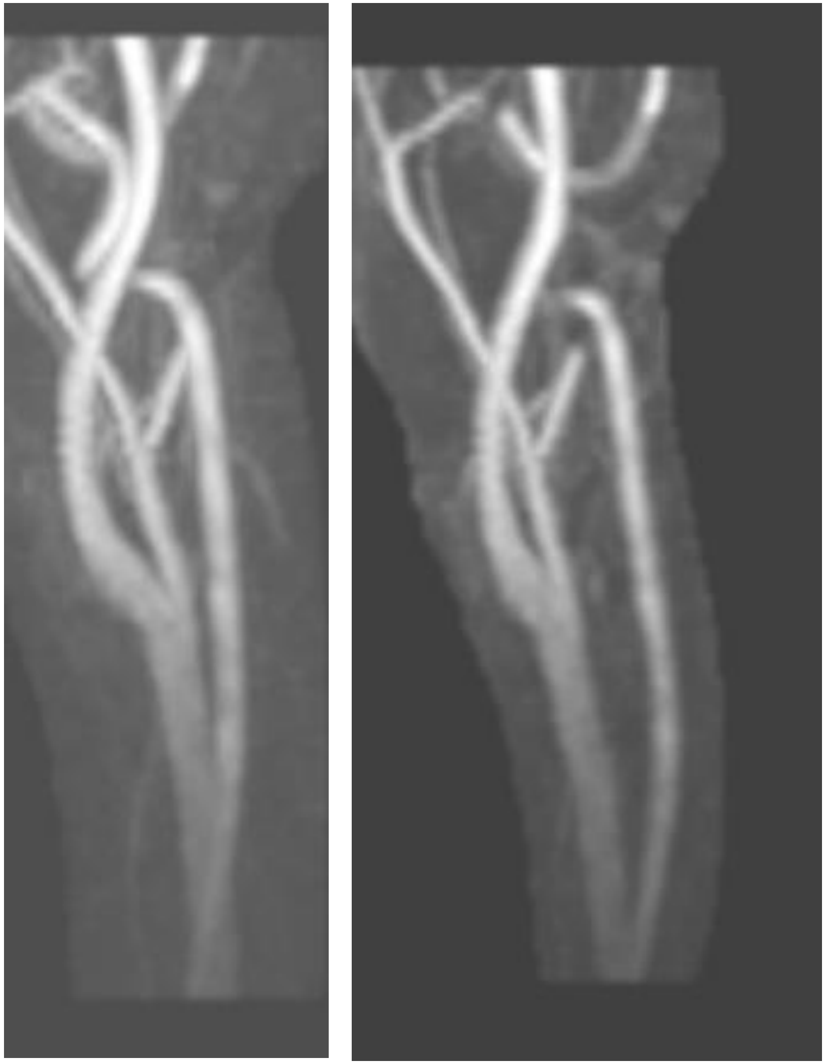

173f Score

0-30

31-50

51-70

>70

Near occlusion

Occluded

Quality

1

2

3

4

5

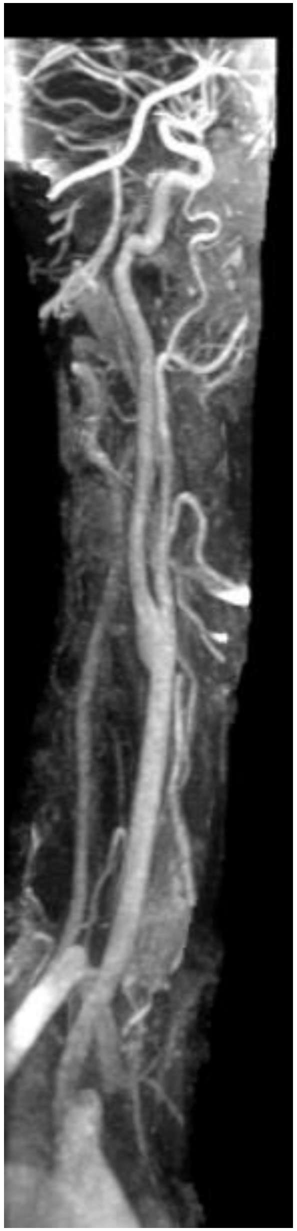

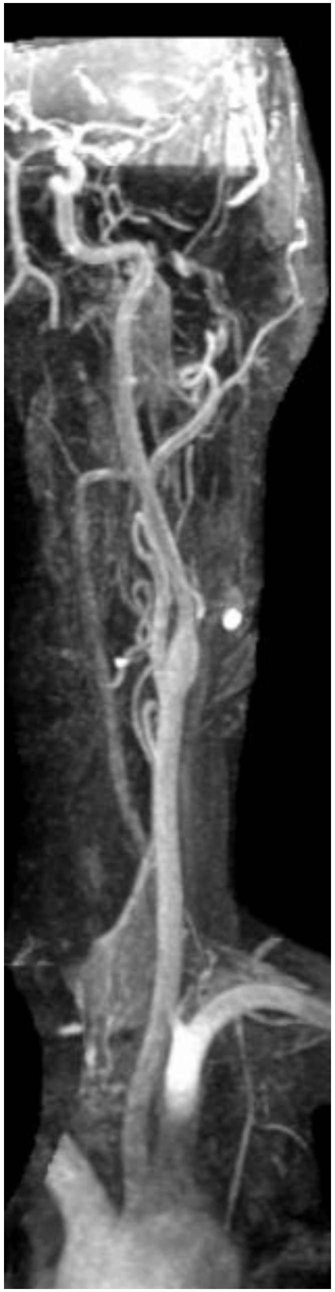

174e Score

0-30

31-50

51-70

>70

Near occlusion

Occluded

Quality

1

2

3

4

5

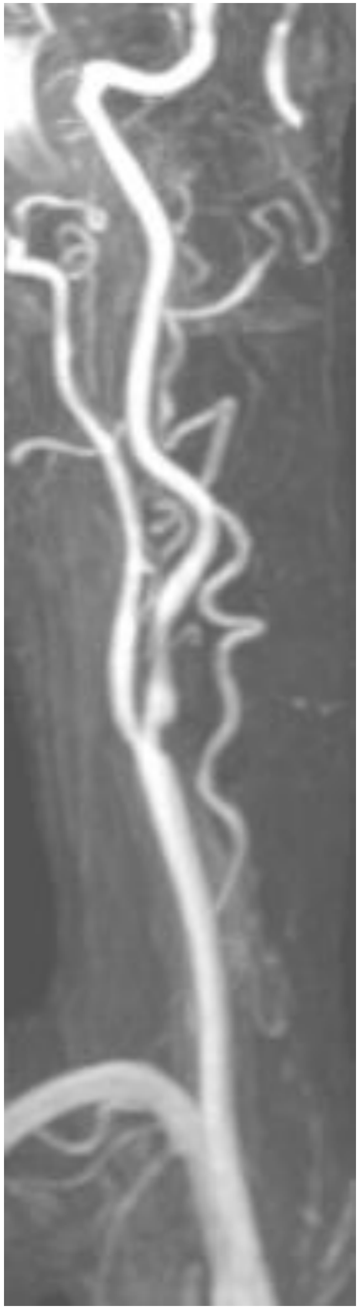

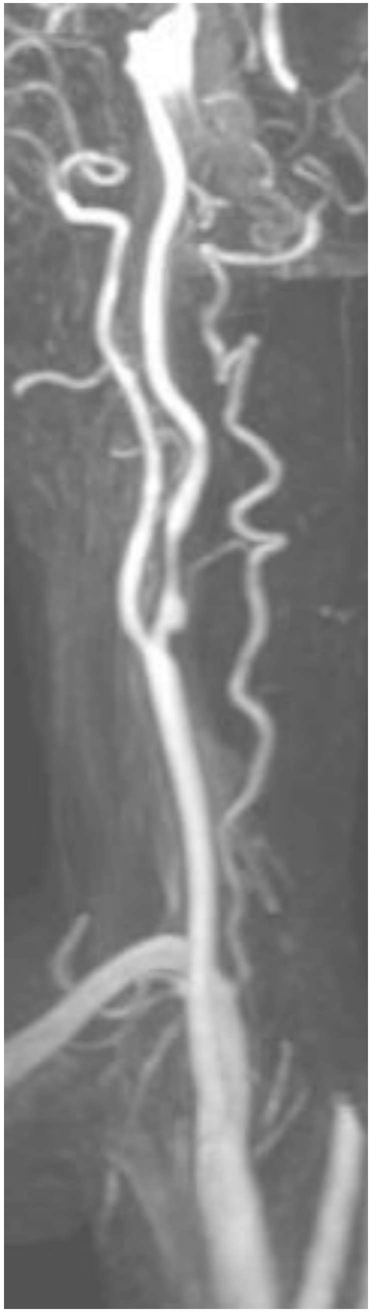

175d Score

0-30

31-50

51-70

>70

Near occlusion

Occluded

Quality

1

2

3

4

5

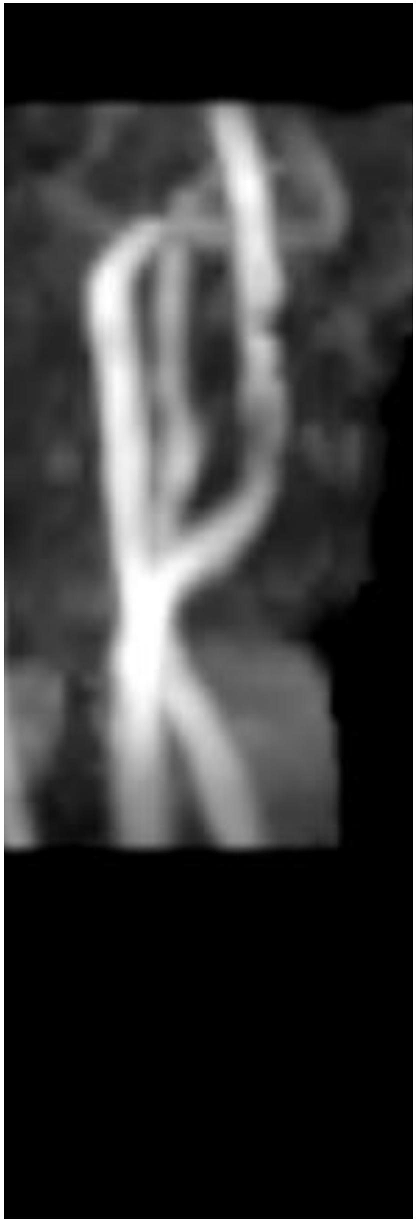

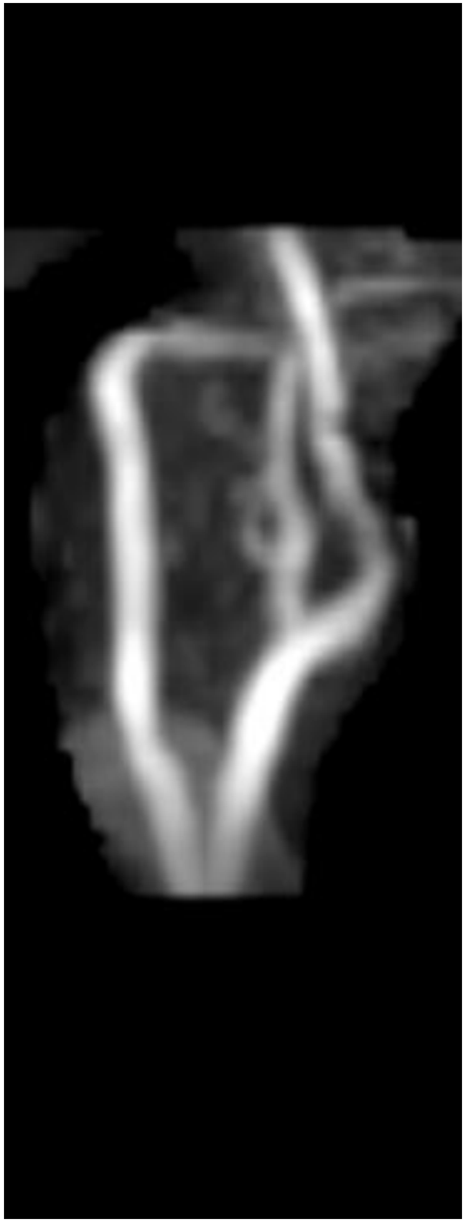

176c Score

0-30

31-50

51-70

>70

Near occlusion

Occluded

Quality

1

2

3

4

5

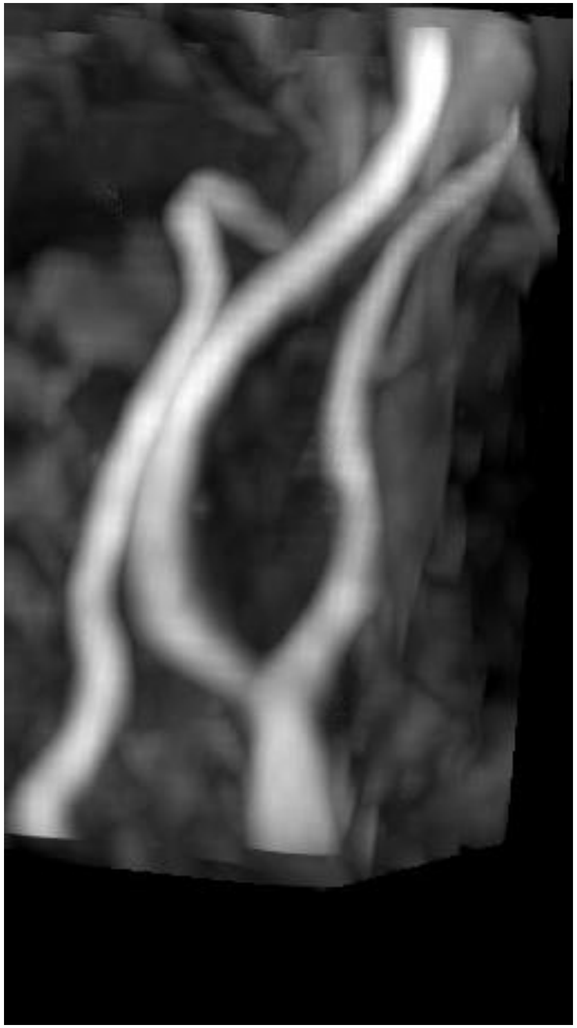

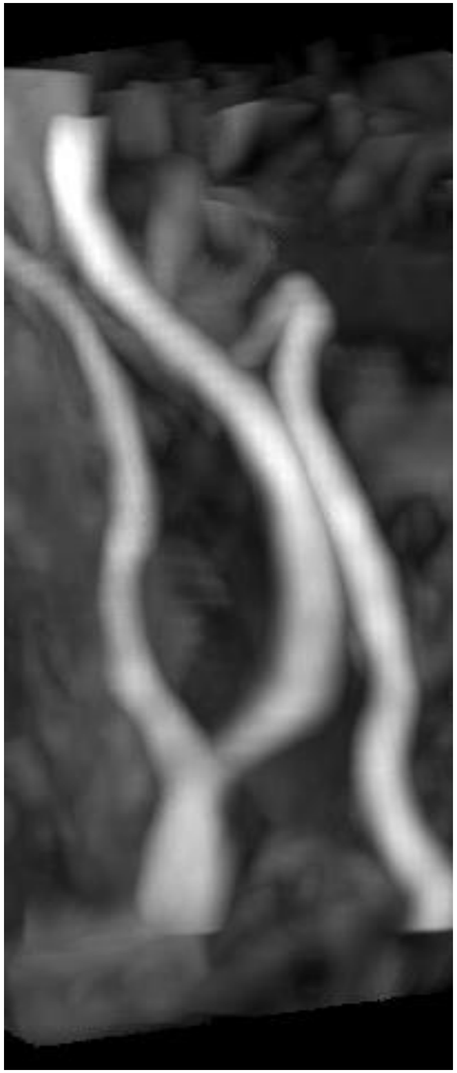

177b Score

0-30

31-50

51-70

>70

Near occlusion

Occluded

Quality

1

2

3

4

5

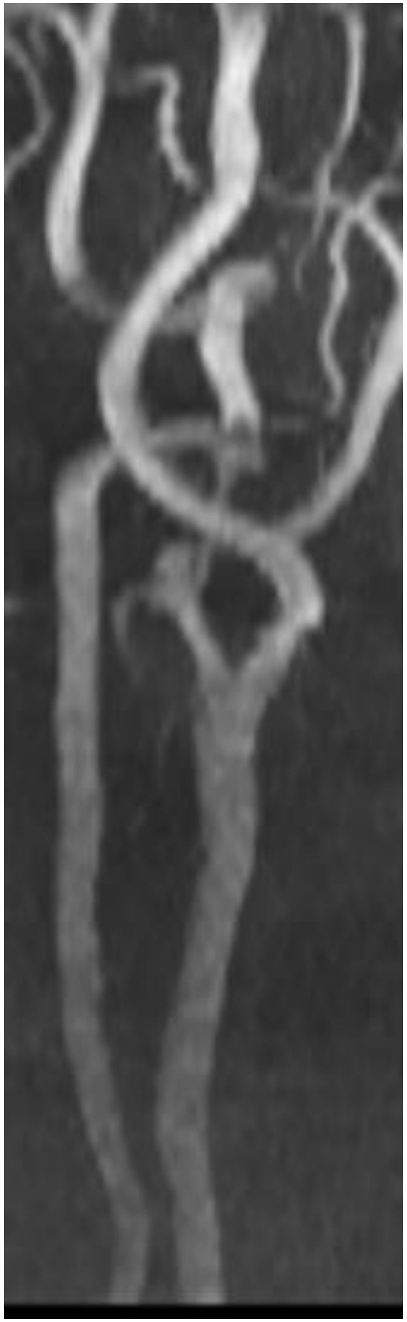

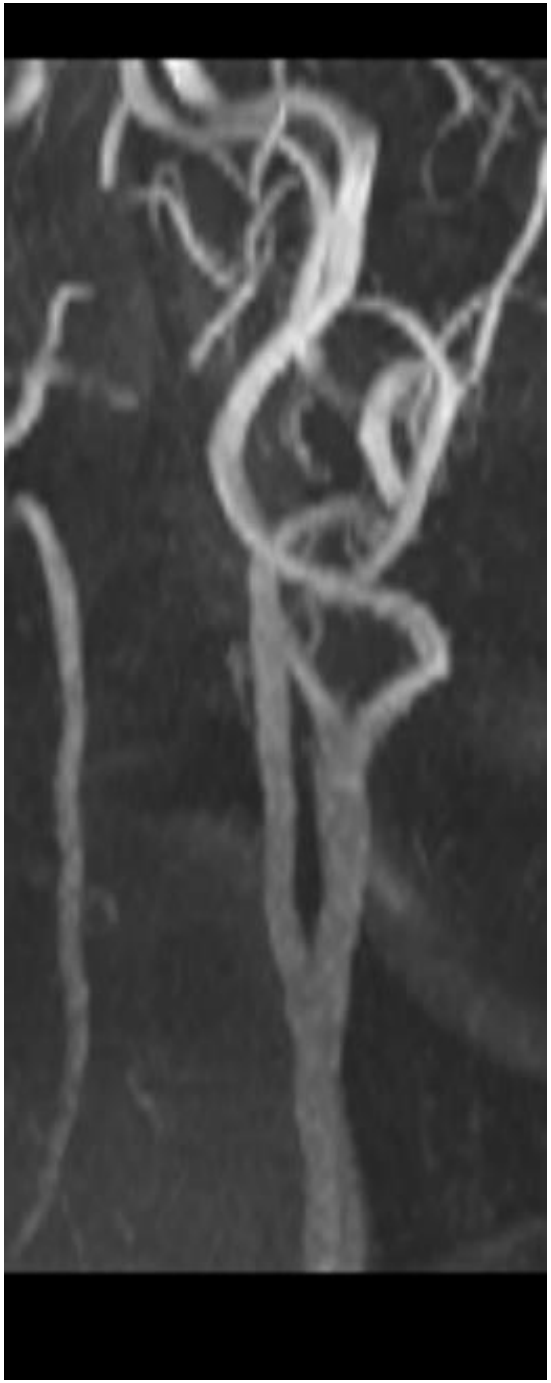

178a Score

0-30

31-50

51-70

>70

Near occlusion

Occluded

Quality

1

2

3

4

5

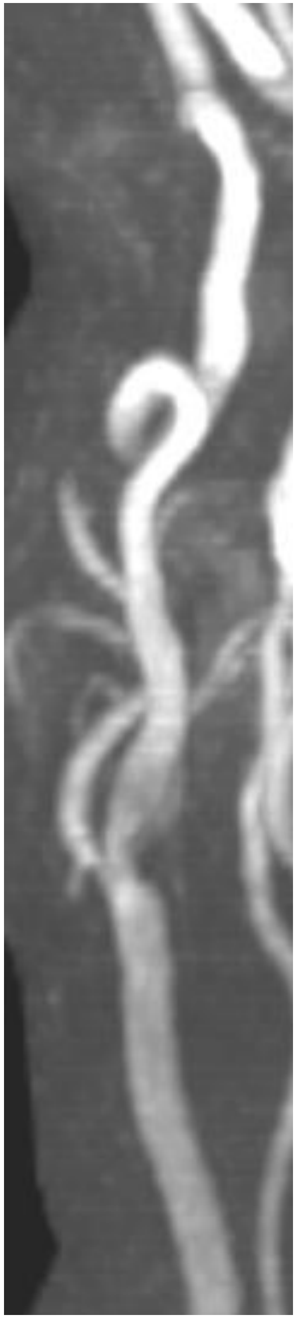

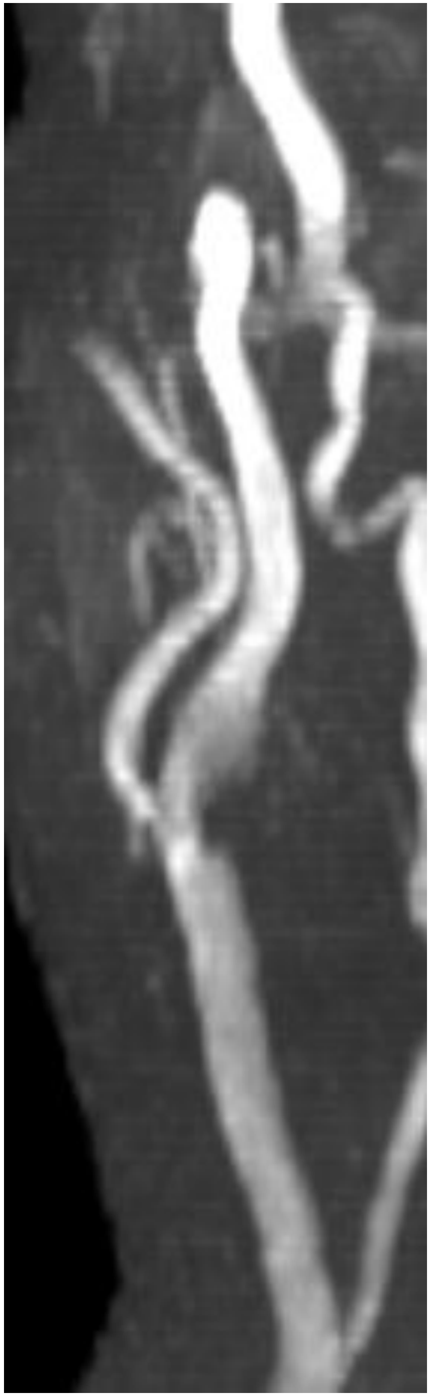

178f Score

0-30

31-50

51-70

>70

Near occlusion

Occluded

Quality

1

2

3

4

5

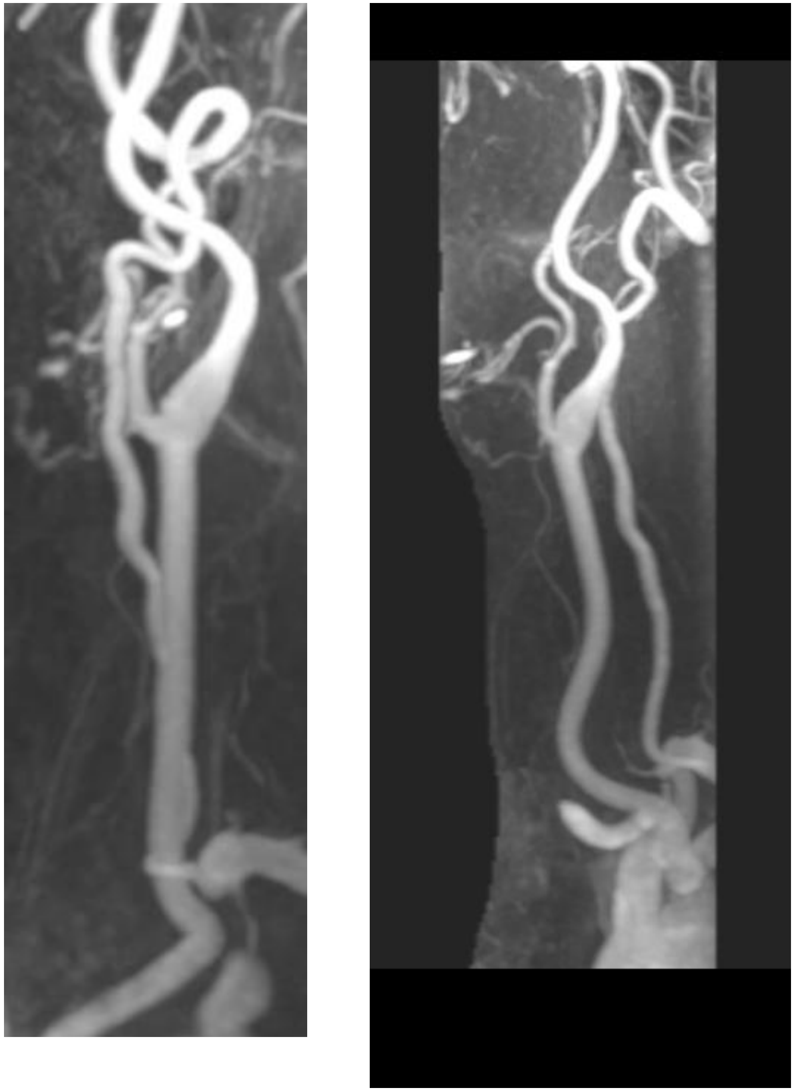

179e Score

0-30

31-50

51-70

>70

Near occlusion

Occluded

Quality

1

2

3

4

5

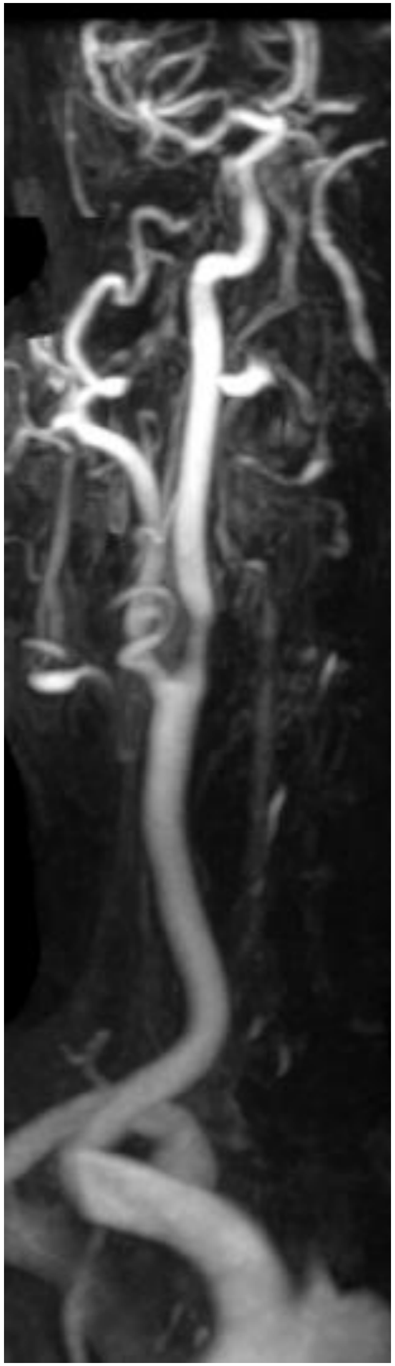

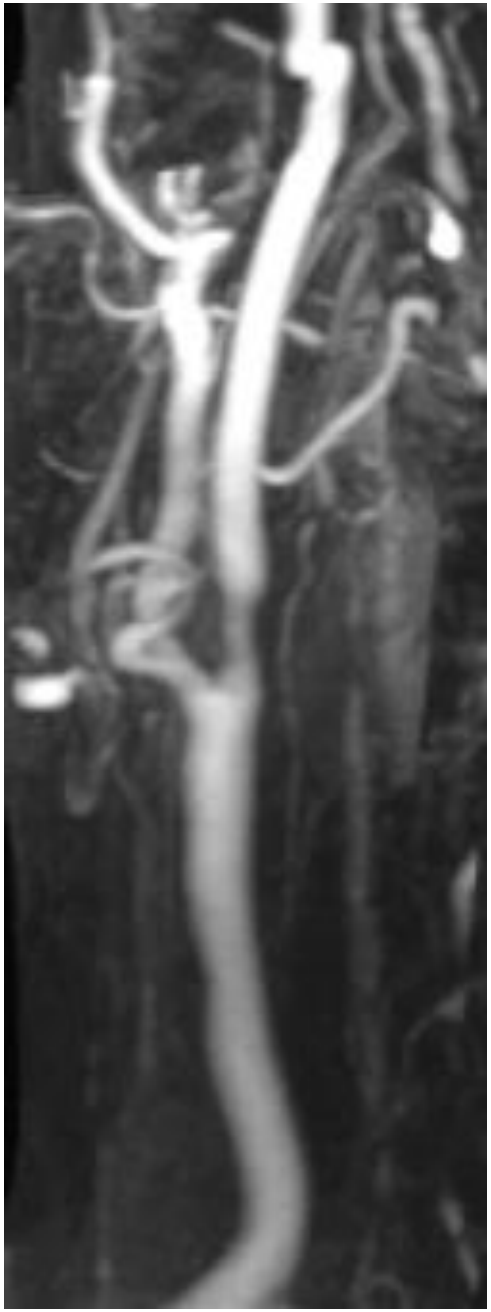

180d Score

0-30

31-50

51-70

>70

Near occlusion

Occluded

Quality

1

2

3

4

5

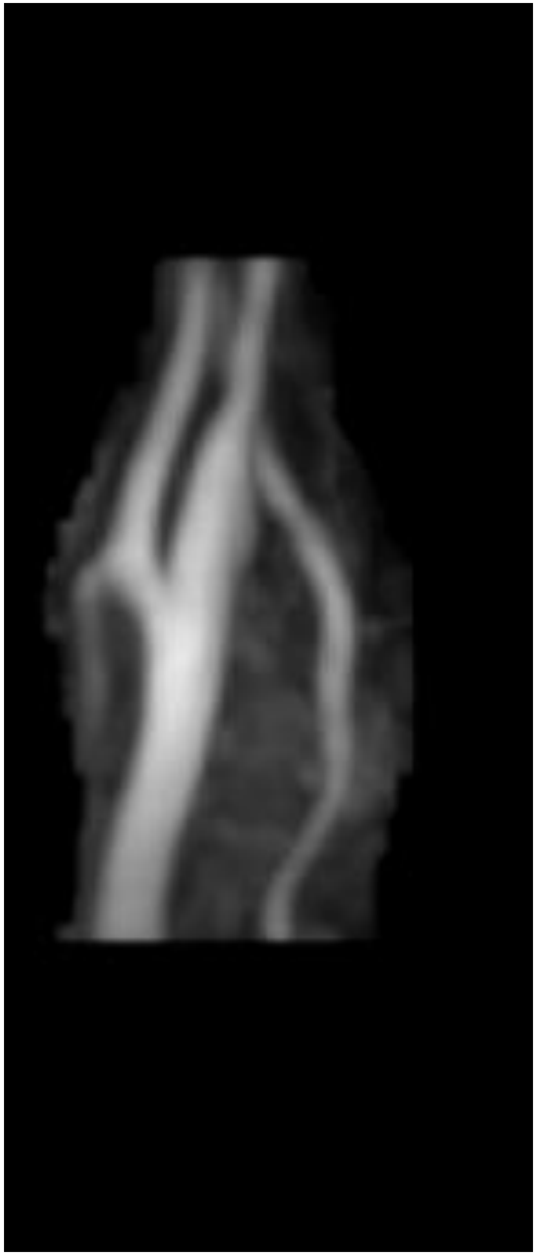

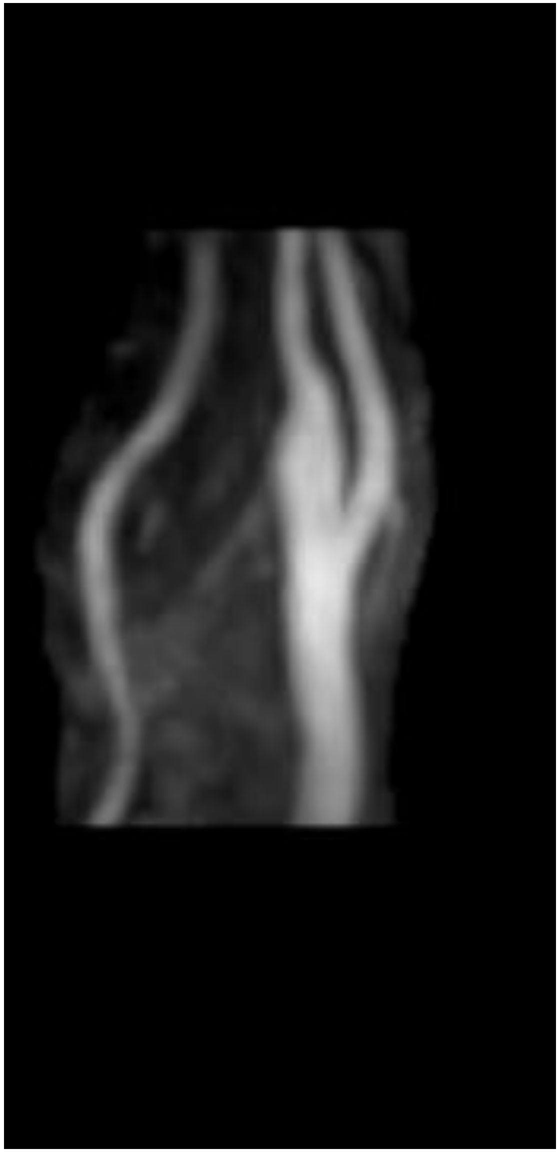

181c Score

0-30

31-50

51-70

>70

Near occlusion

Occluded

Quality

1

2

3

4

5

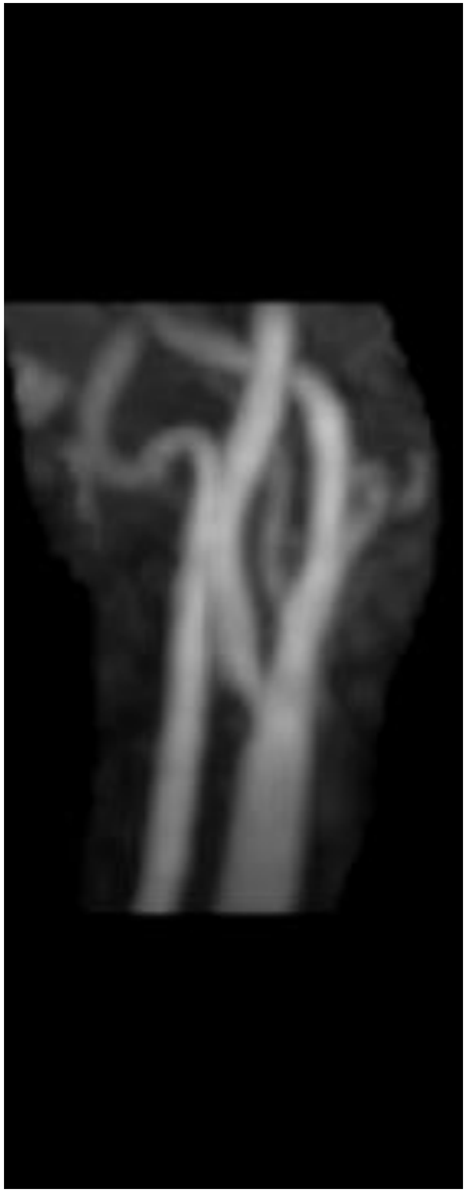

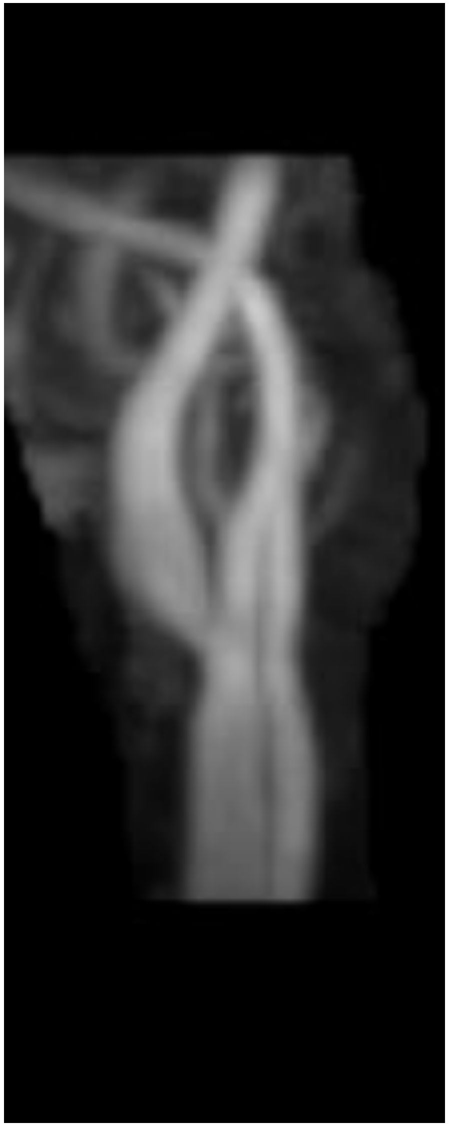

182b Score

0-30

31-50

51-70

>70

Near occlusion

Occluded

Quality

1

2

3

4

5

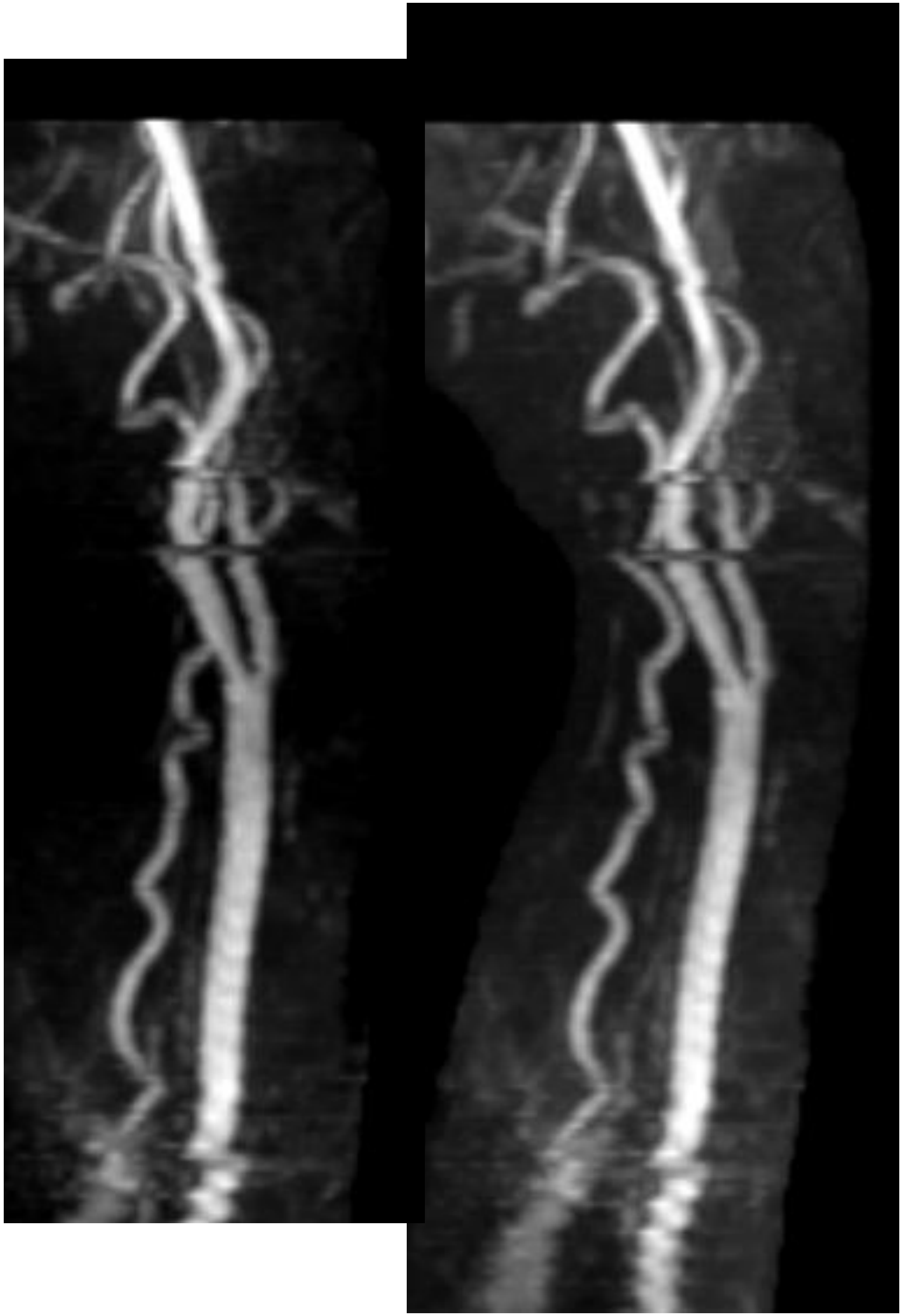

183a Score

0-30

31-50

51-70

>70

Near occlusion

Occluded

Quality

1

2

3

4

5

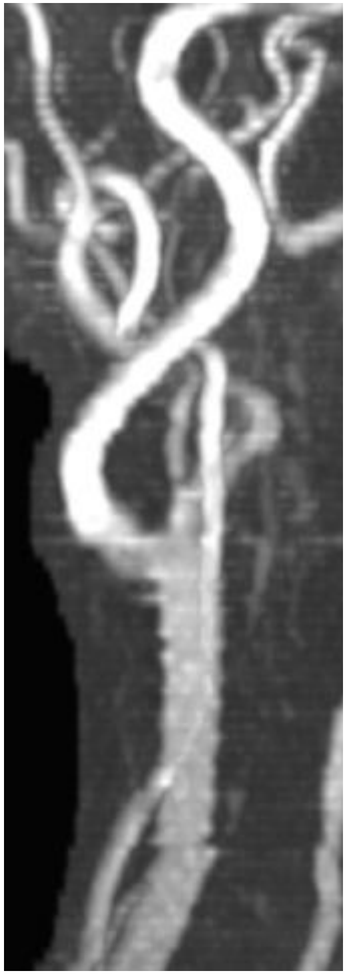

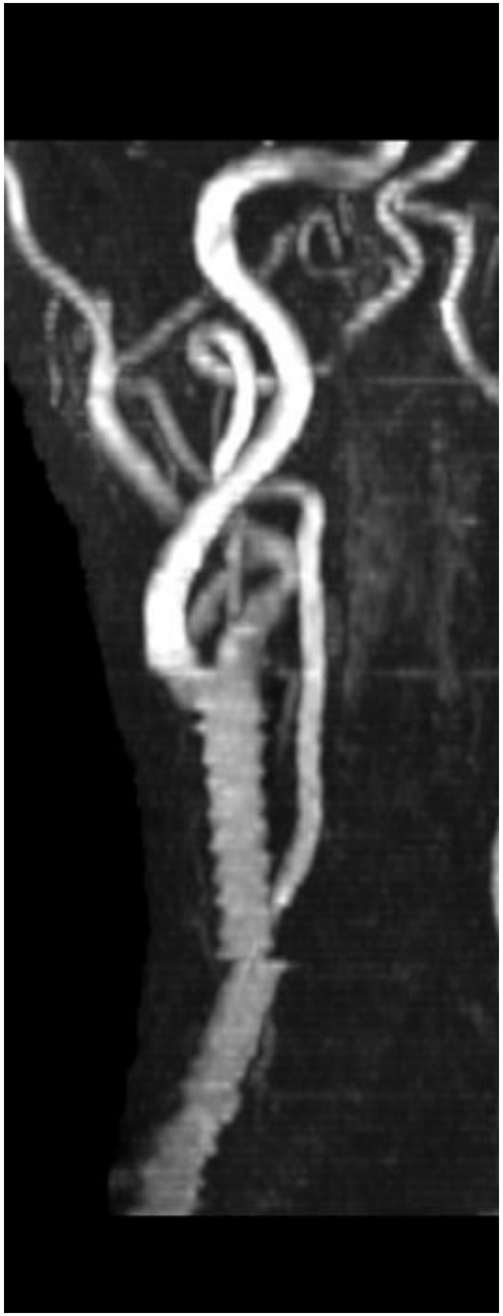

183f Score

0-30

31-50

51-70

>70

Near occlusion

Occluded

Quality

1

2

3

4

5

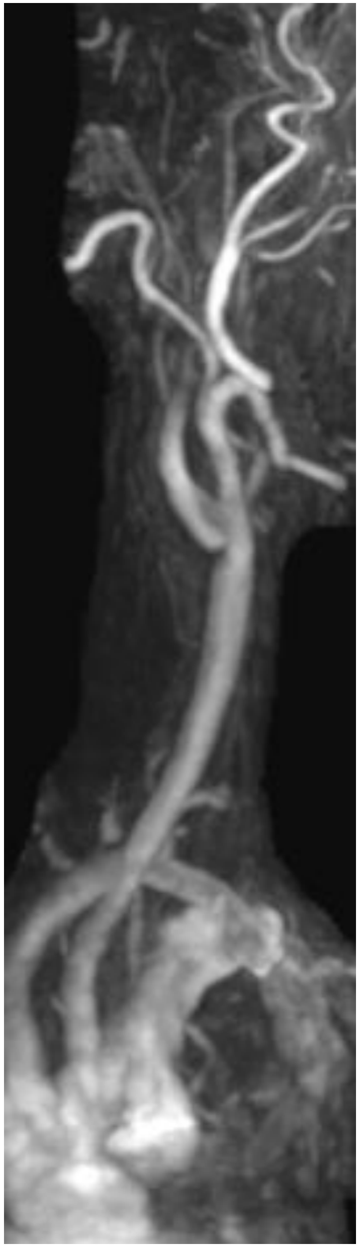

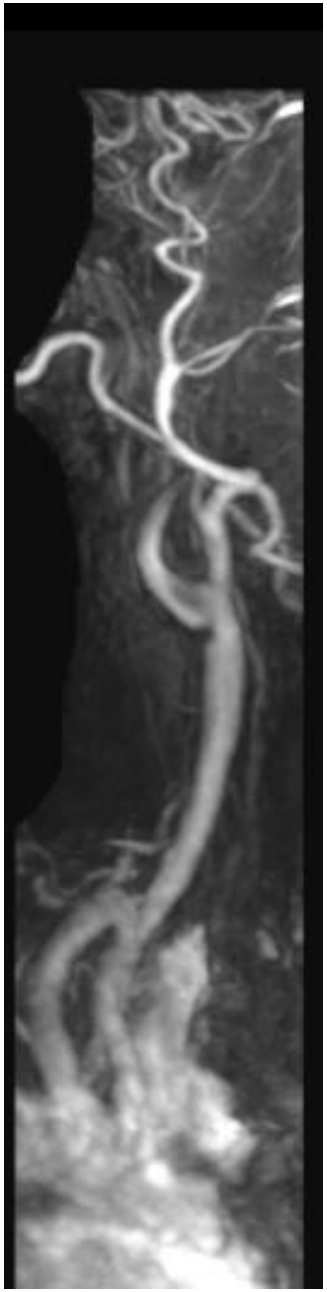

184e Score

0-30

31-50

51-70

>70

Near occlusion

Occluded

Quality

1

2

3

4

5

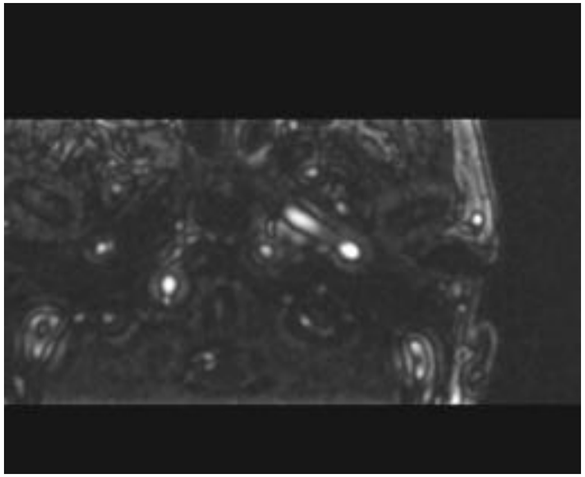

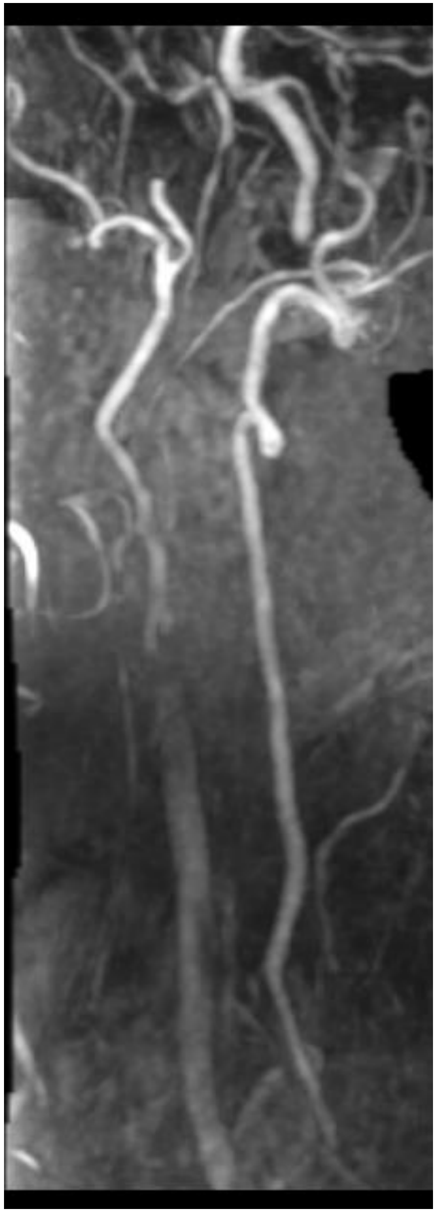

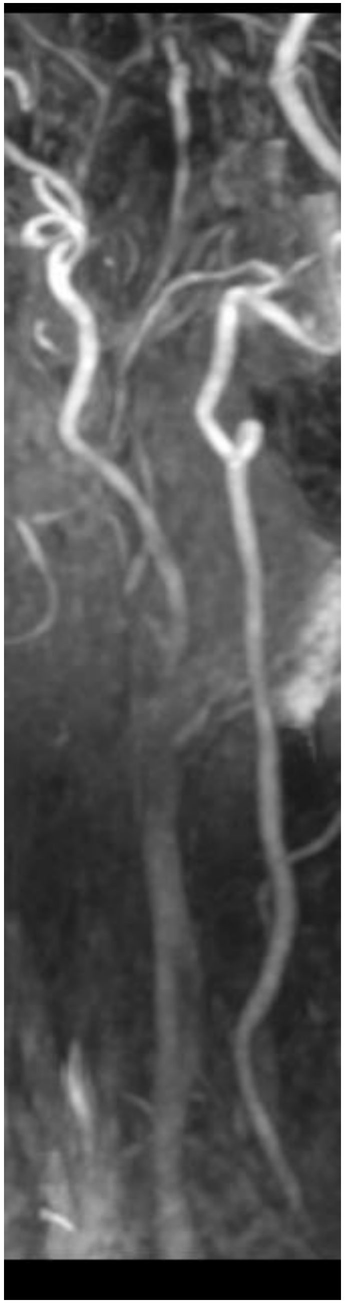

185d Score

0-30

31-50

51-70

>70

Near occlusion

Occluded

Quality

1

2

3

4

5

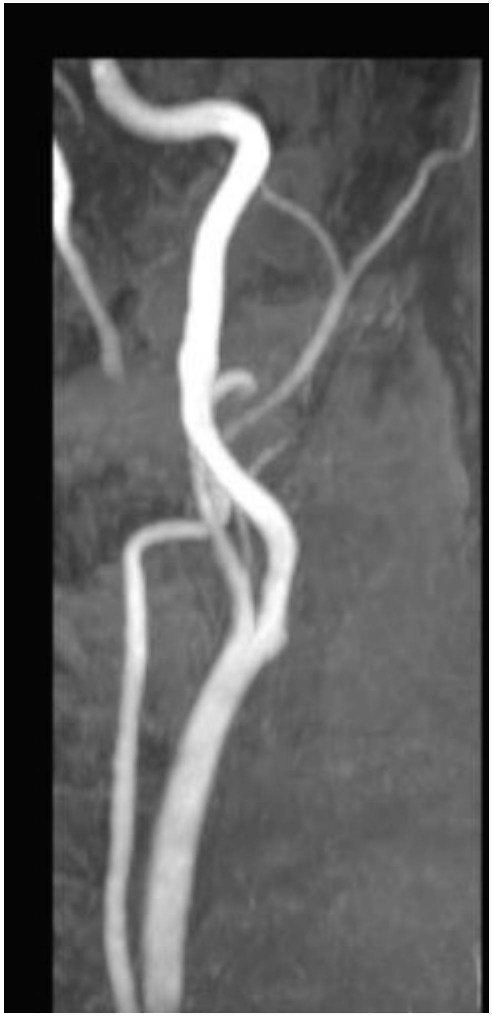

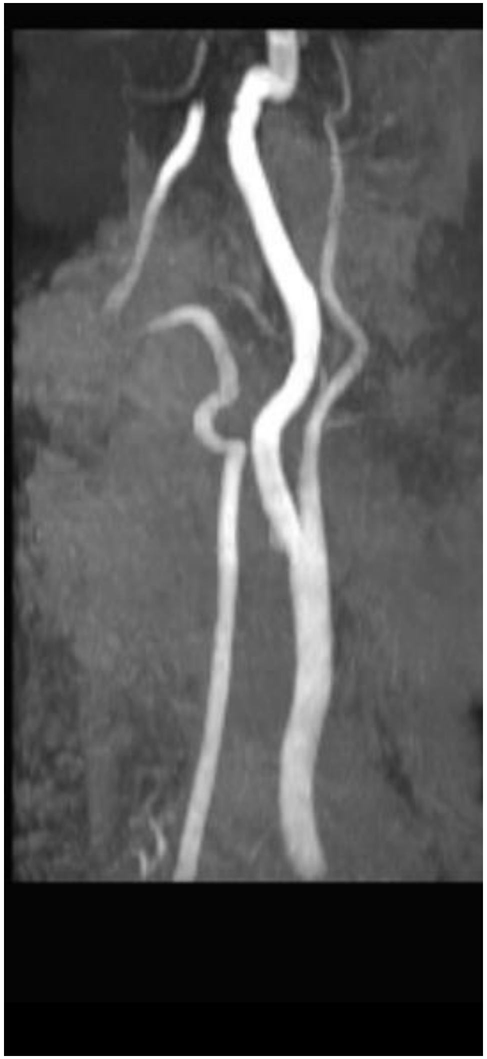

186c Score

0-30

31-50

51-70

>70

Near occlusion

Occluded

Quality

1

2

3

4

5

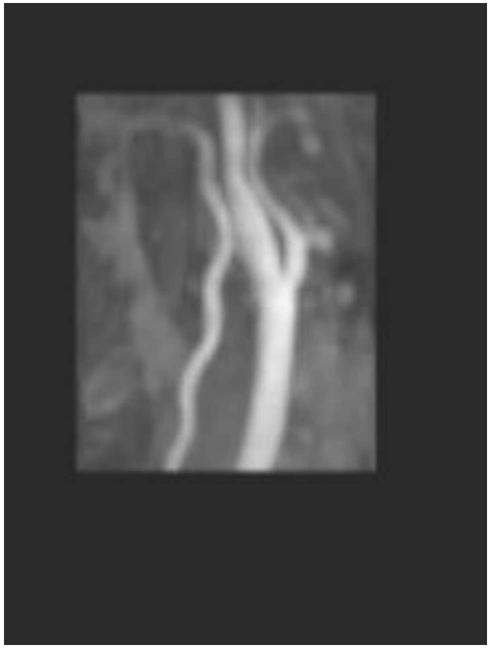

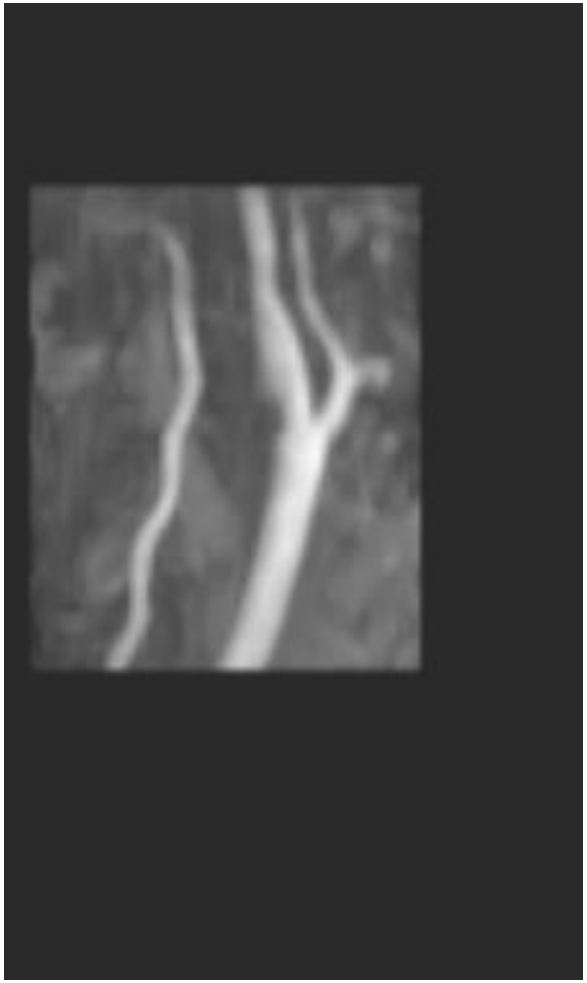

187b Score

0-30

31-50

51-70

>70

Near occlusion

Occluded

Quality

1

2

3

4

5

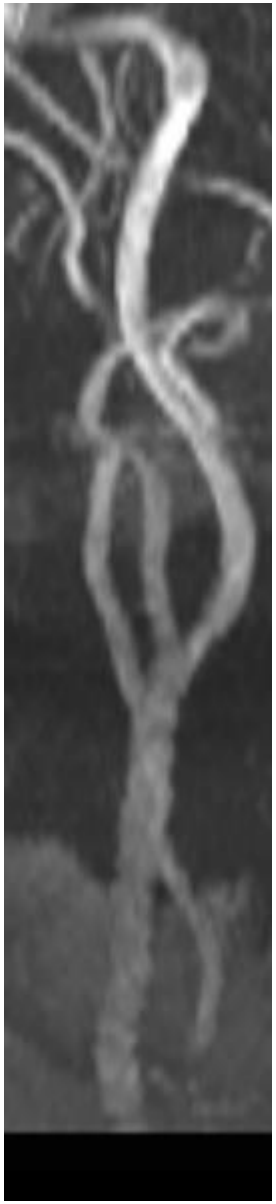

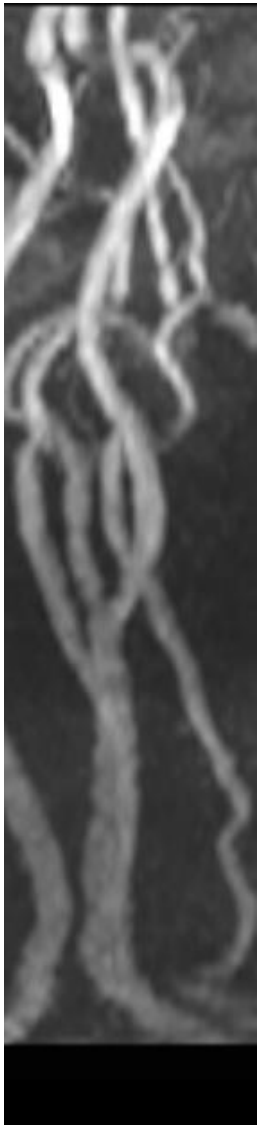

188a Score

0-30

31-50

51-70

>70

Near occlusion

Occluded

Quality

1

2

3

4

5

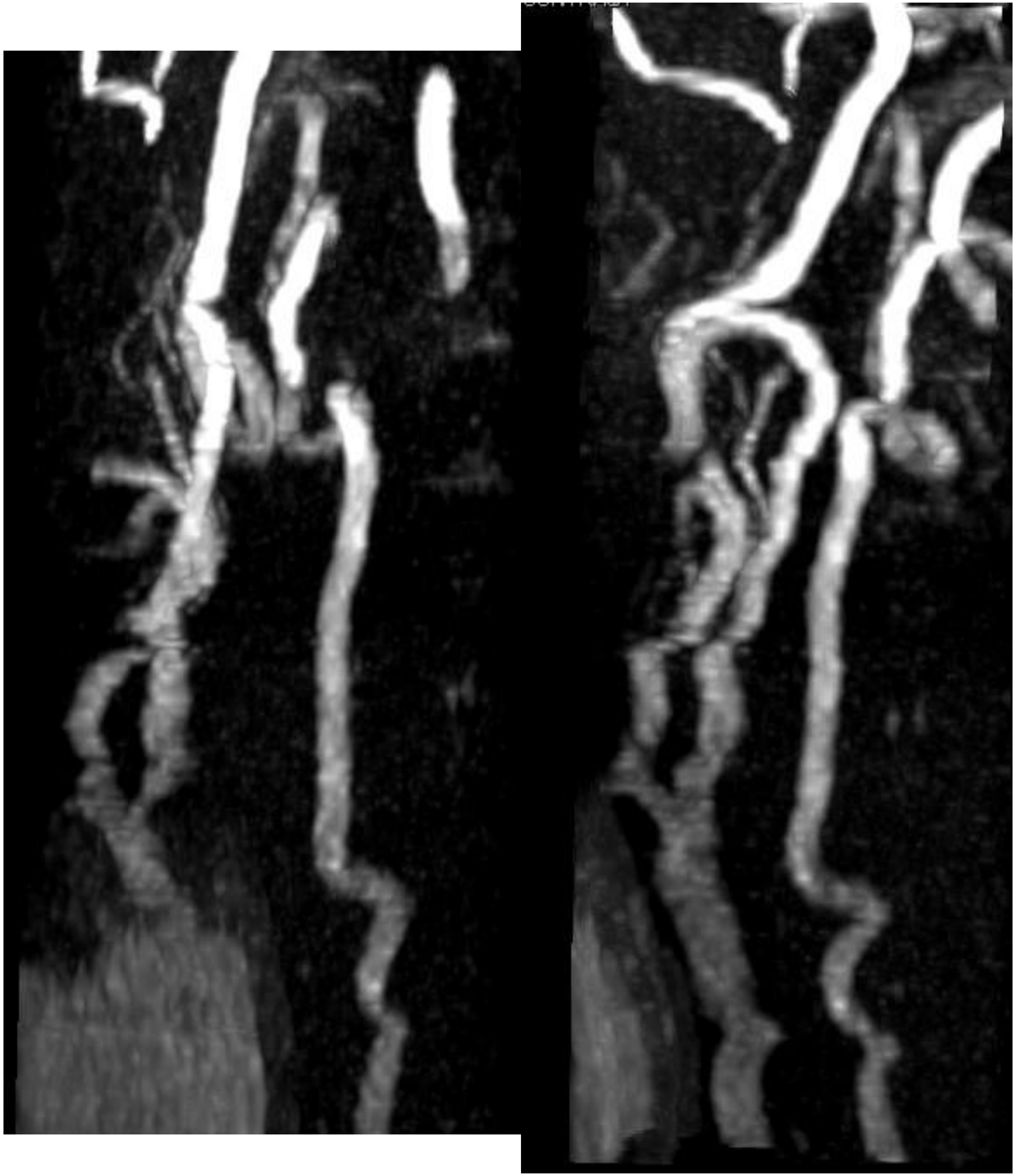

188f Score

0-30

31-50

51-70

>70

Near occlusion

Occluded

Quality

1

2

3

4

5

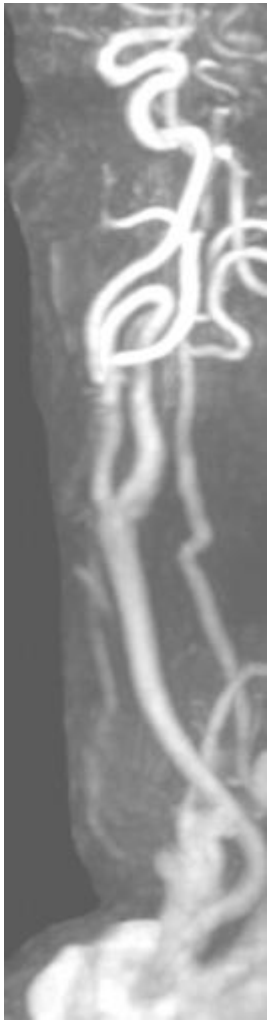

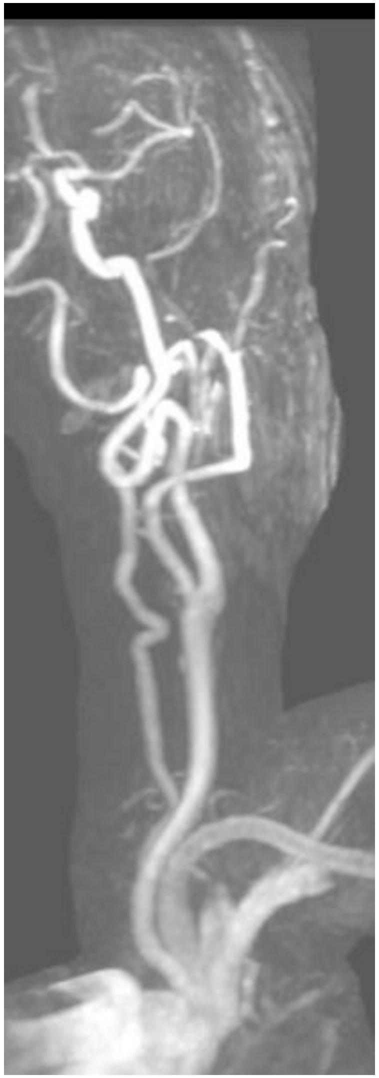

189e Score

0-30

31-50

51-70

>70

Near occlusion

Occluded

Quality

1

2

3

4

5

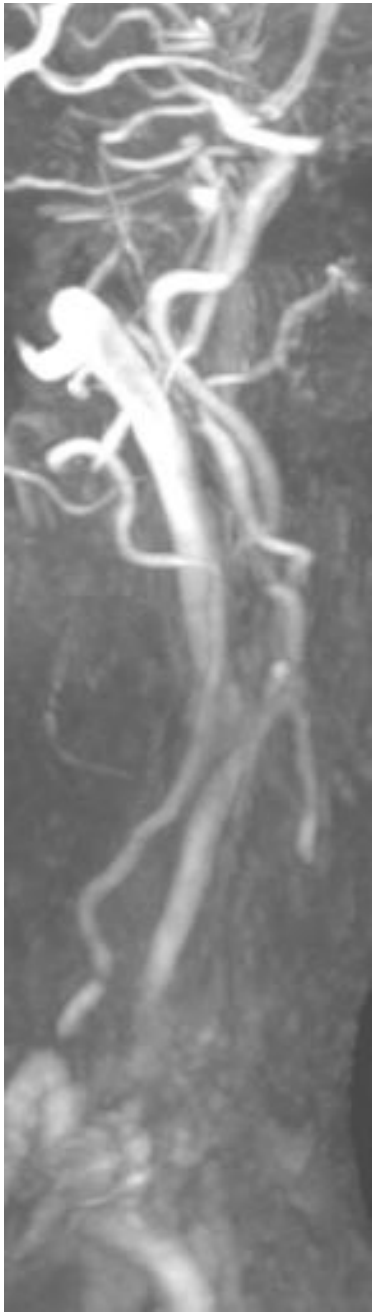

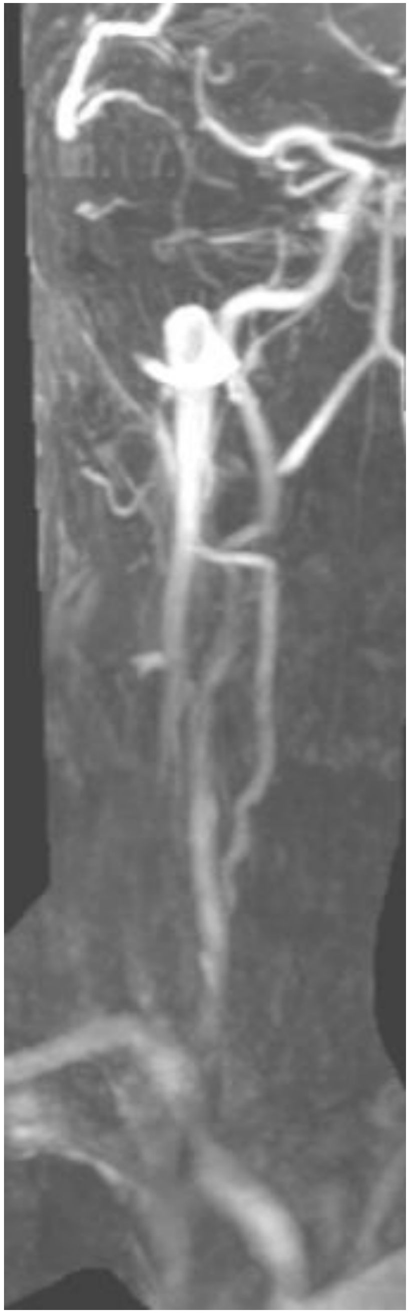

190d Score

0-30

31-50

51-70

>70

Near occlusion

Occluded

Quality

1

2

3

4

5

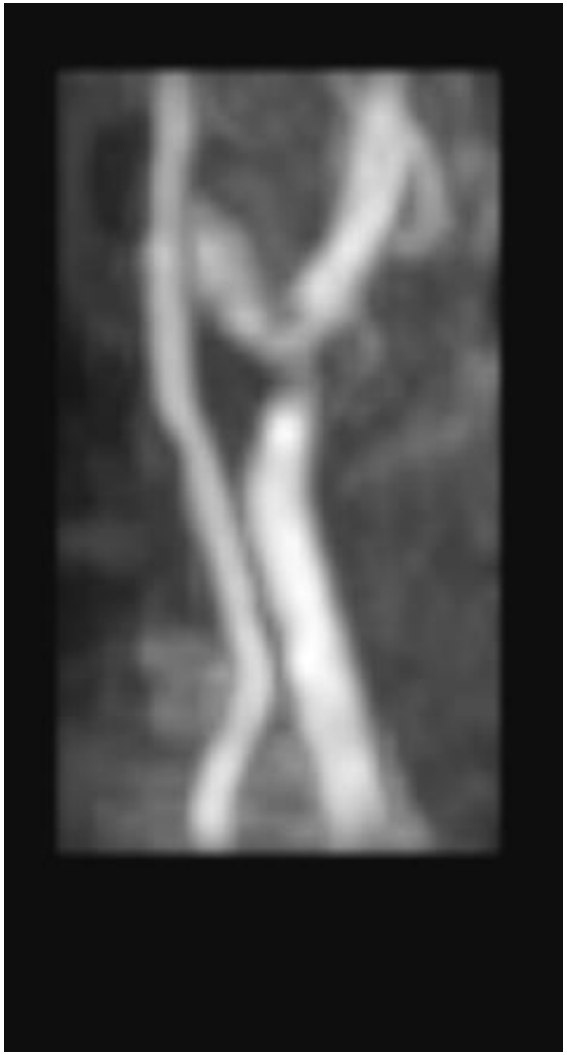

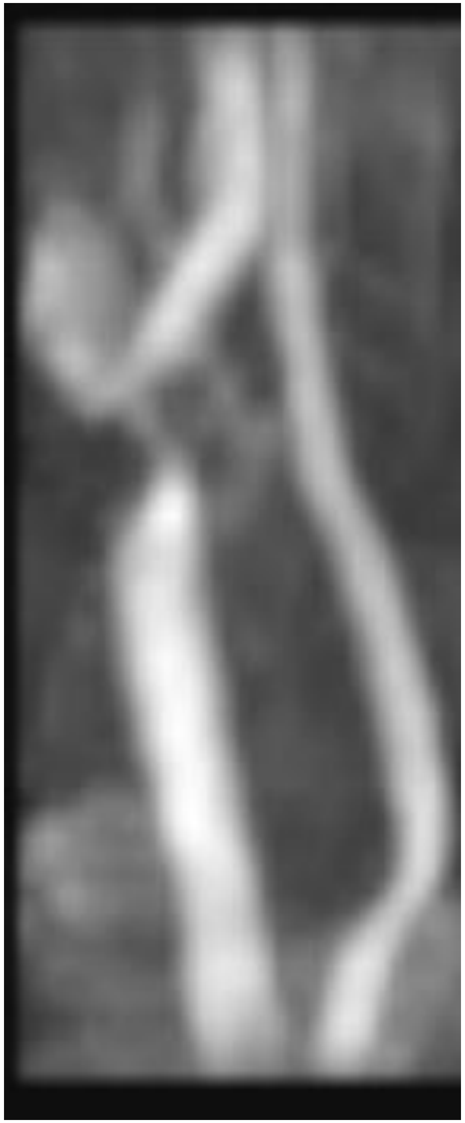

191c Score

0-30

31-50

51-70

>70

Near occlusion

Occluded

Quality

1

2

3

4

5

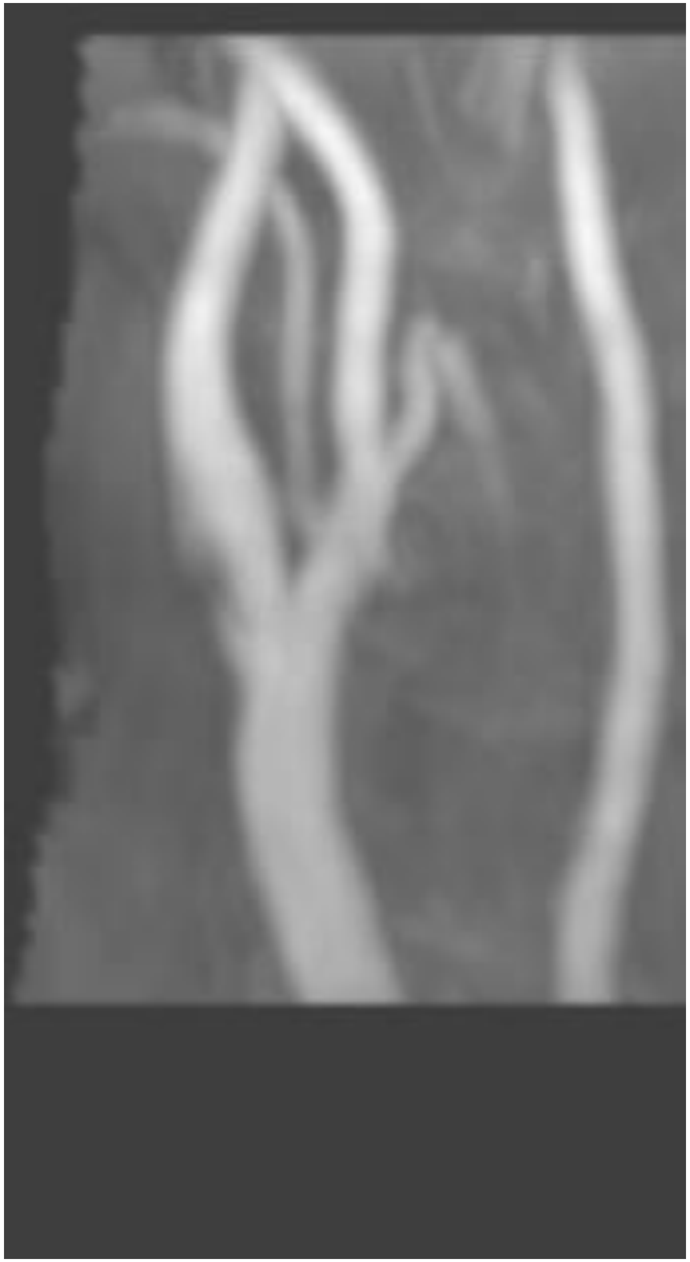

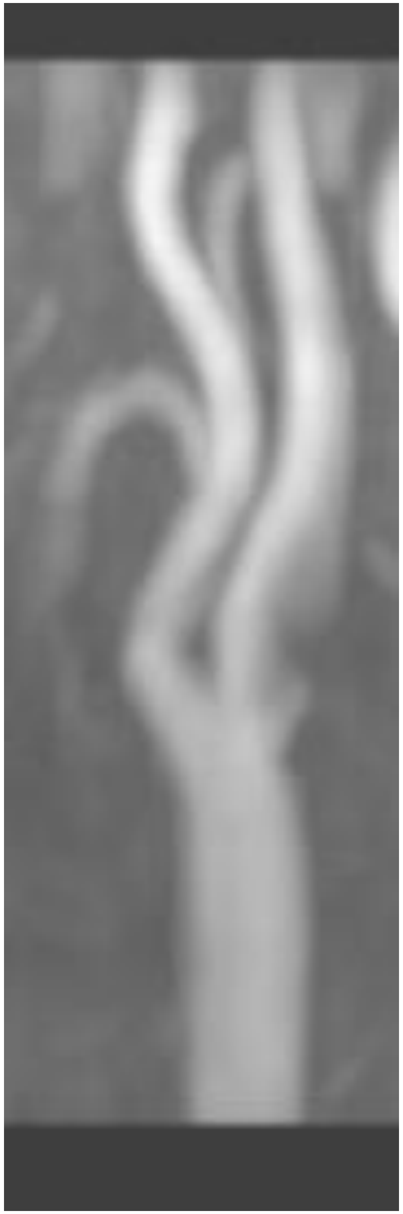

192b Score

0-30

31-50

51-70

>70

Near occlusion

Occluded

Quality

1

2

3

4

5

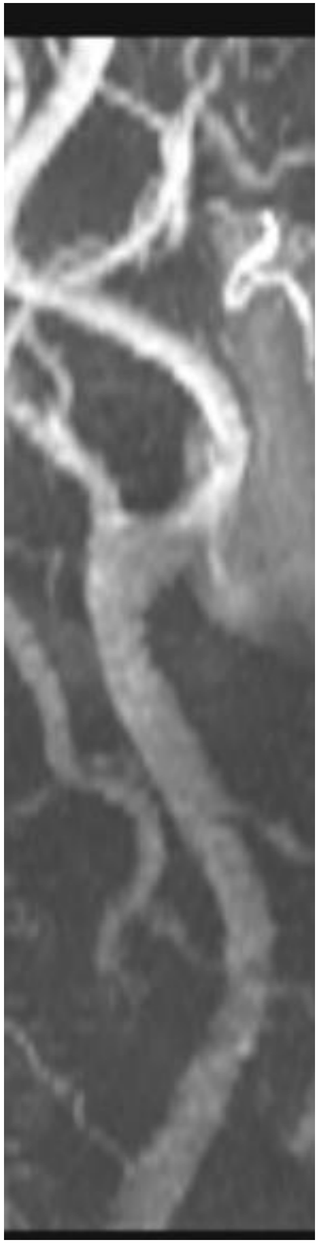

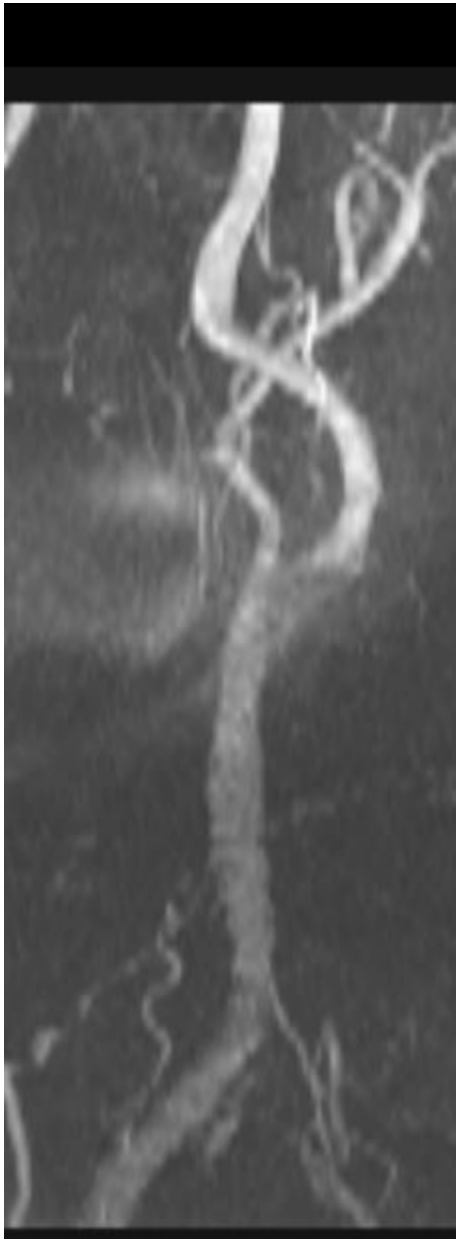

193a Score

0-30

31-50

51-70

>70

Near occlusion

Occluded

Quality

1

2

3

4

5

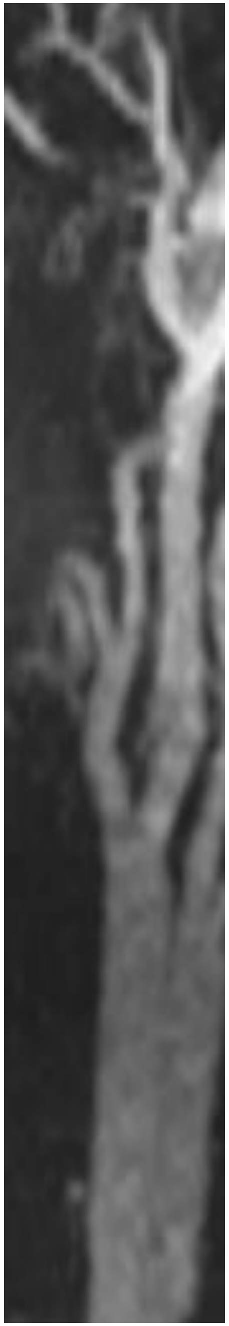

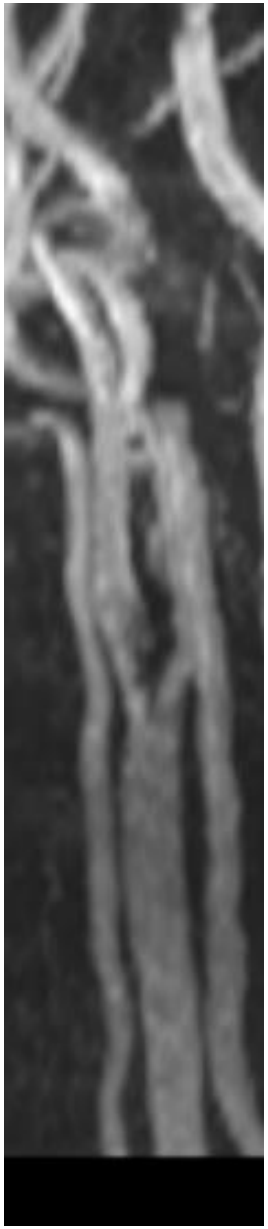

193f Score

0-30

31-50

51-70

>70

Near occlusion

Occluded

Quality

1

2

3

4

5

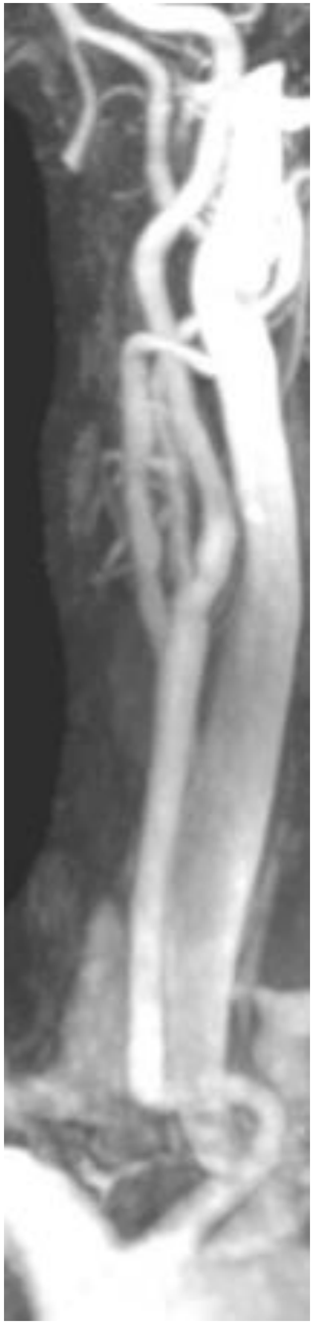

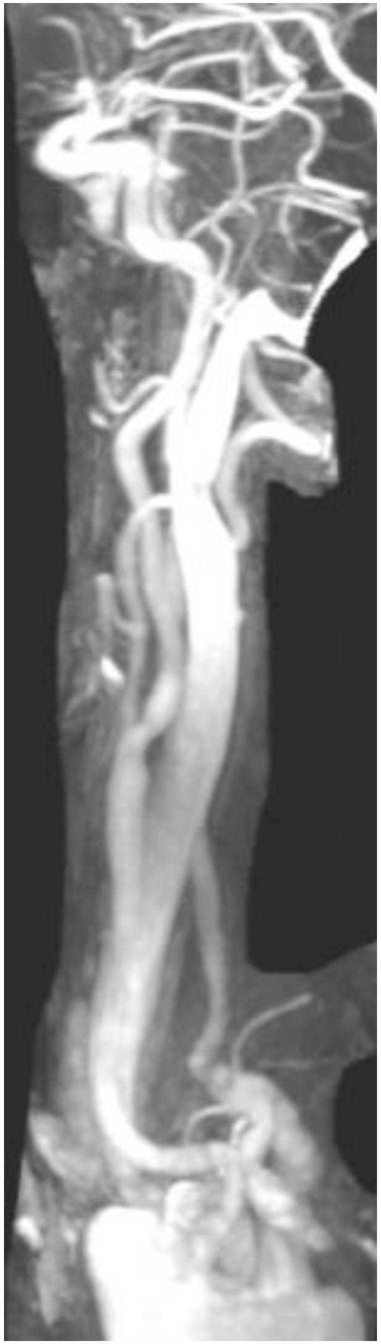

194e Score

0-30

31-50

51-70

>70

Near occlusion

Occluded

Quality

1

2

3

4

5

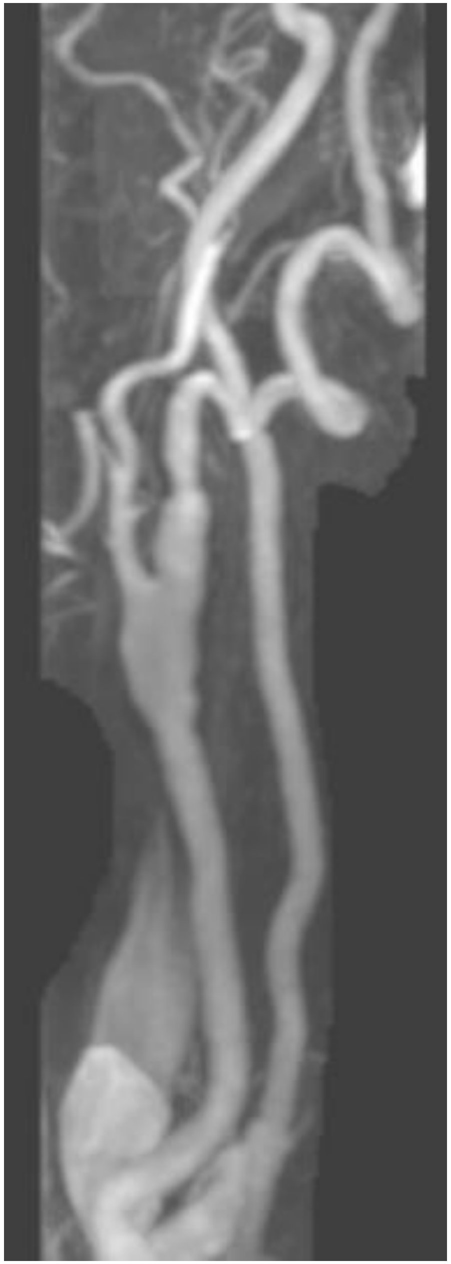

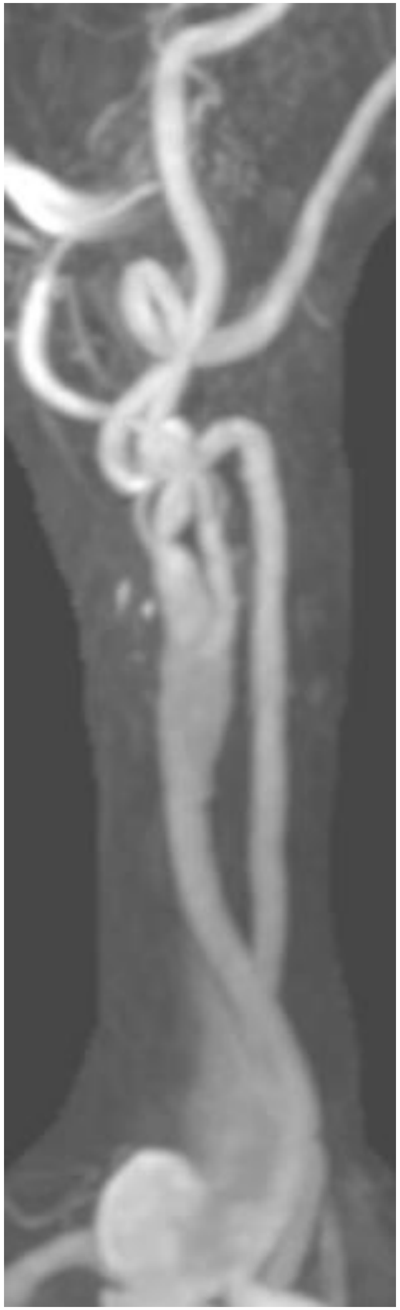

195d Score

0-30

31-50

51-70

>70

Near occlusion

Occluded

Quality

1

2

3

4

5

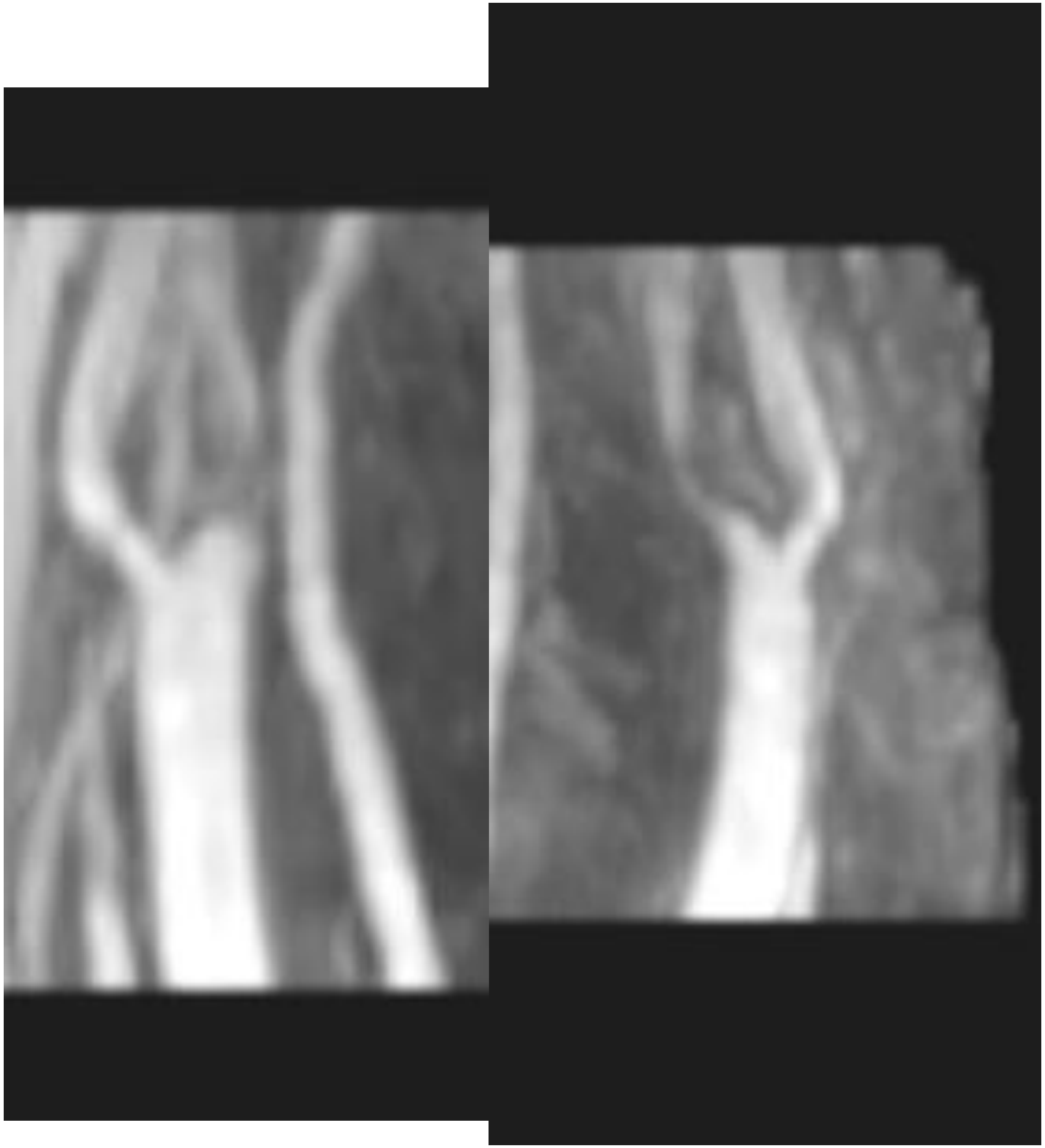

196c Score

0-30

31-50

51-70

>70

Near occlusion

Occluded

Quality

1

2

3

4

5

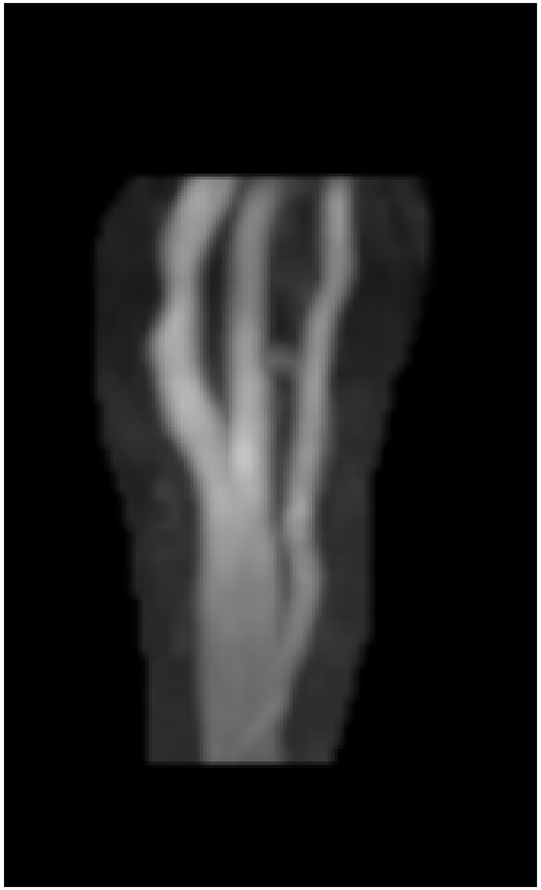

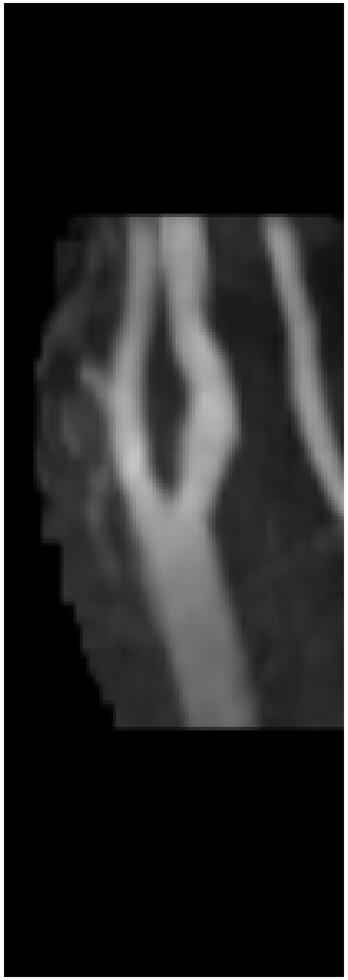

197b Score

0-30

31-50

51-70

>70

Near occlusion

Occluded

Quality

1

2

3

4

5

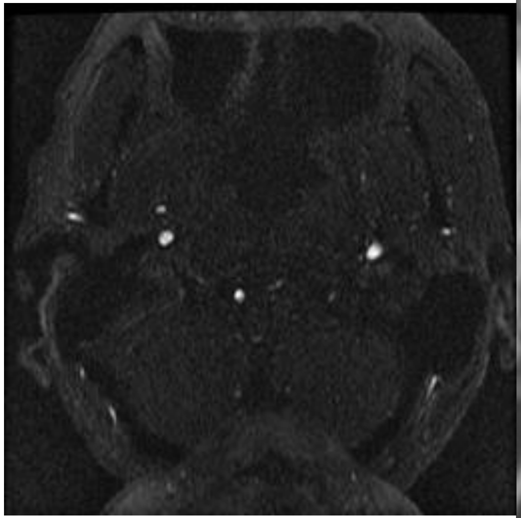

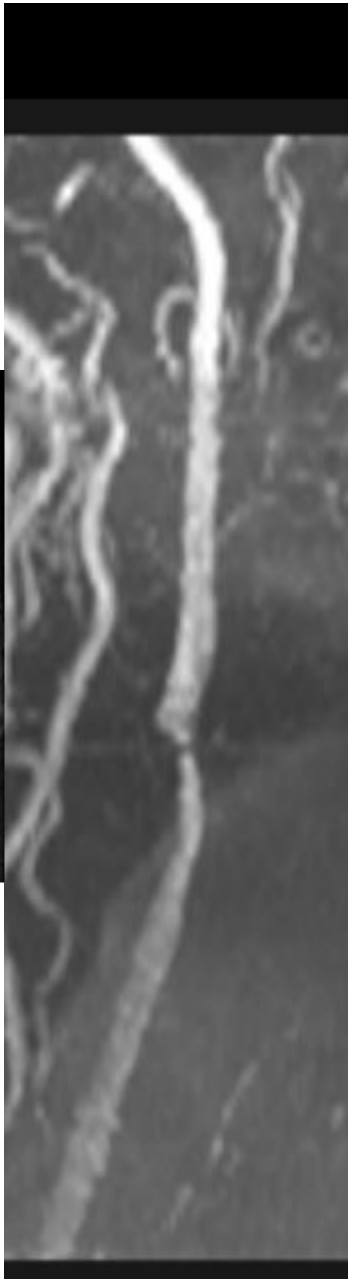

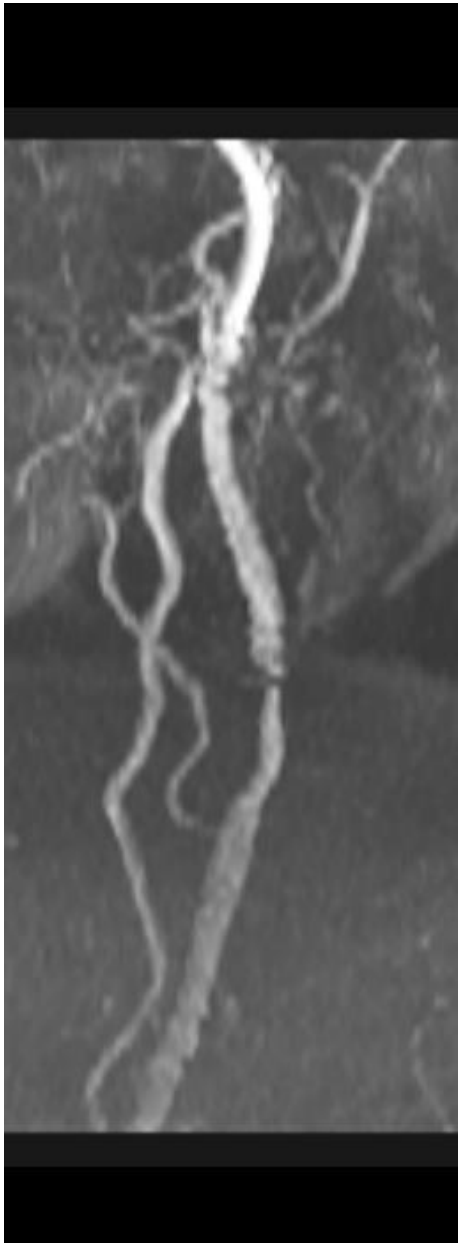

198a Score

0-30

31-50

51-70

>70

Near occlusion

Occluded

Quality

1

2

3

4

5

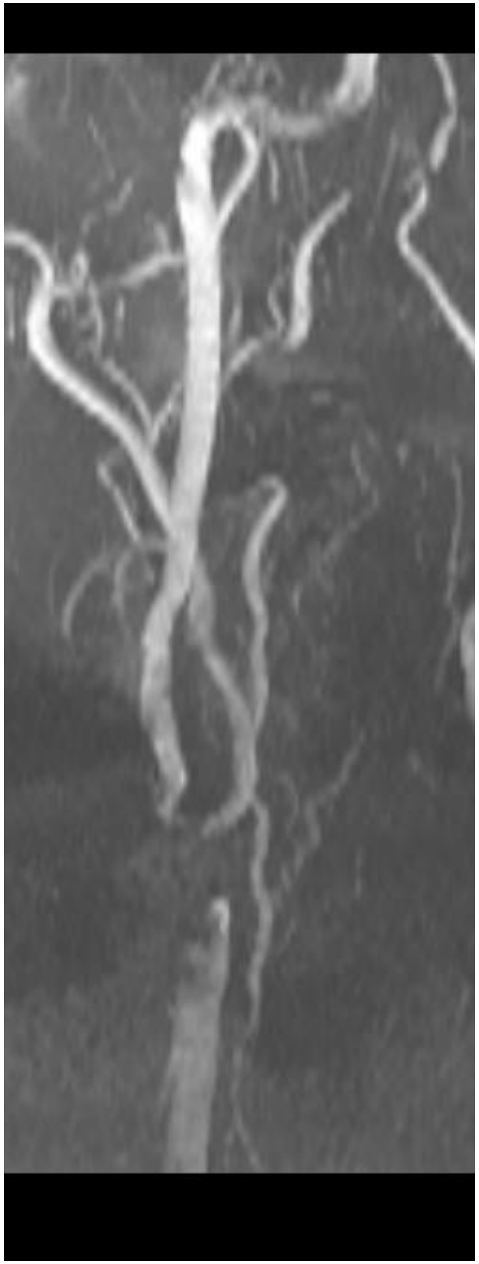

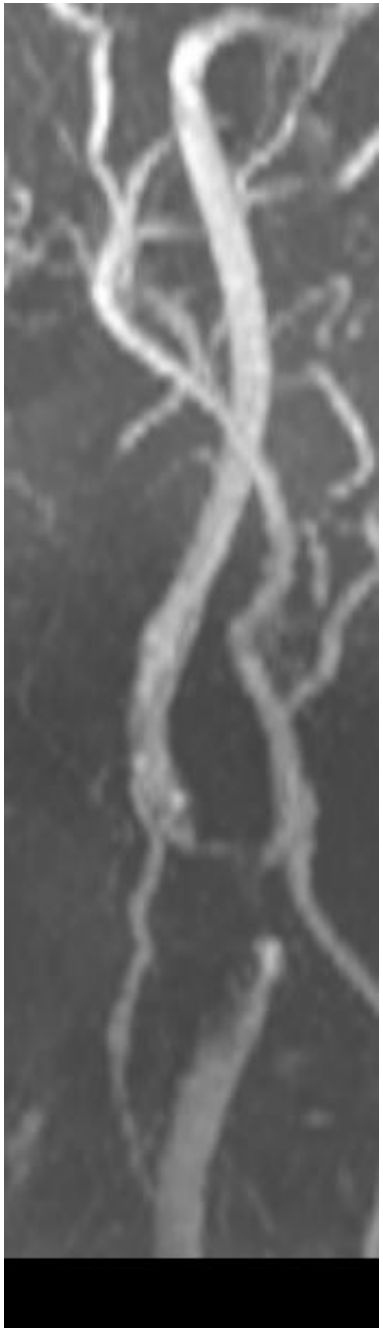

198f Score

0-30

31-50

51-70

>70

Near occlusion

Occluded

Quality

1

2

3

4

5

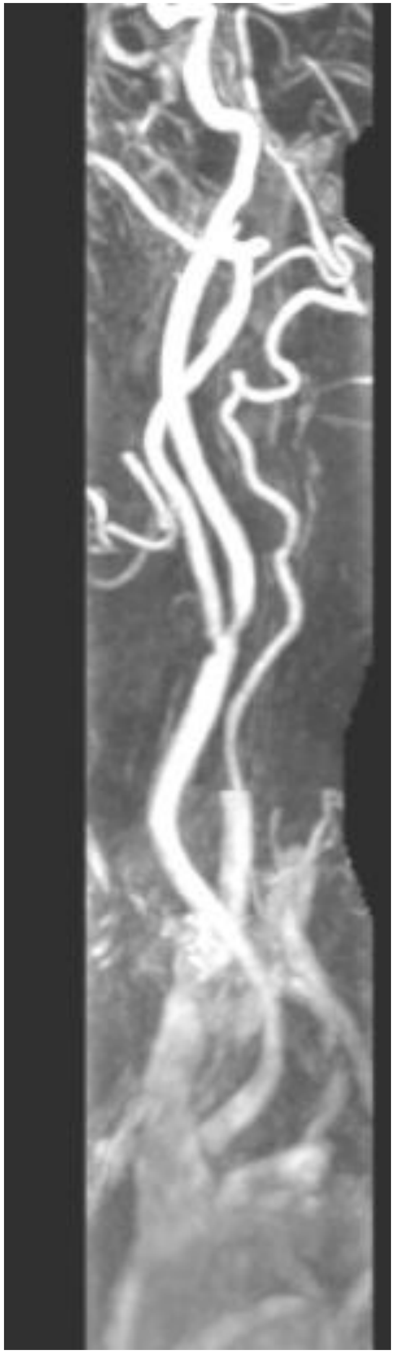

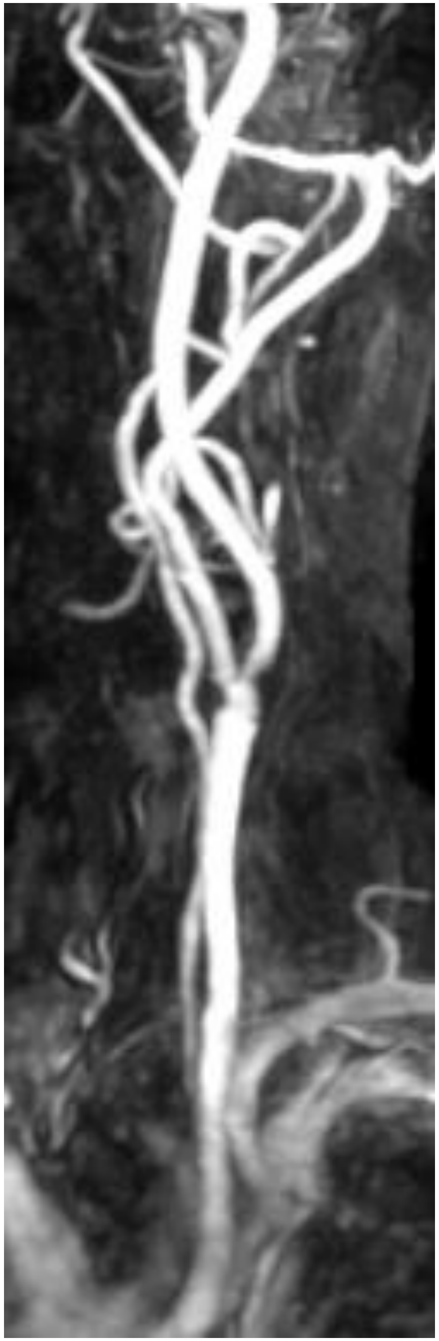

199e Score

0-30

31-50

51-70

>70

Near occlusion

Occluded

Quality

1

2

3

4

5

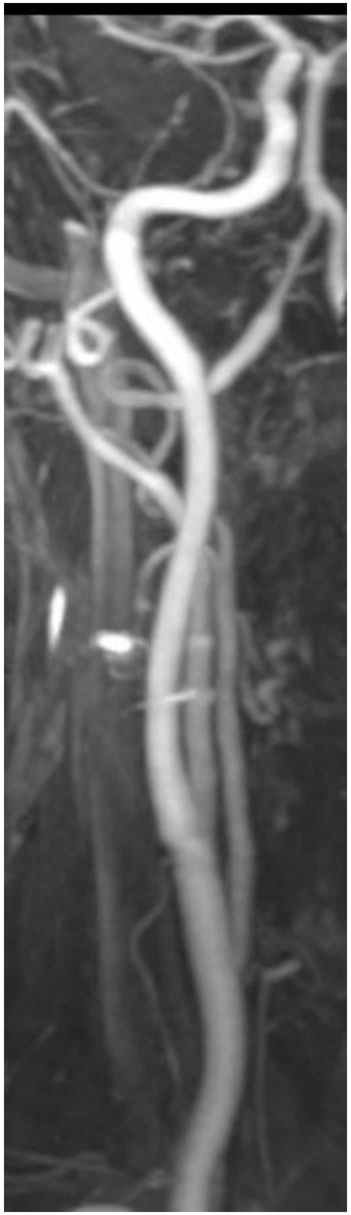

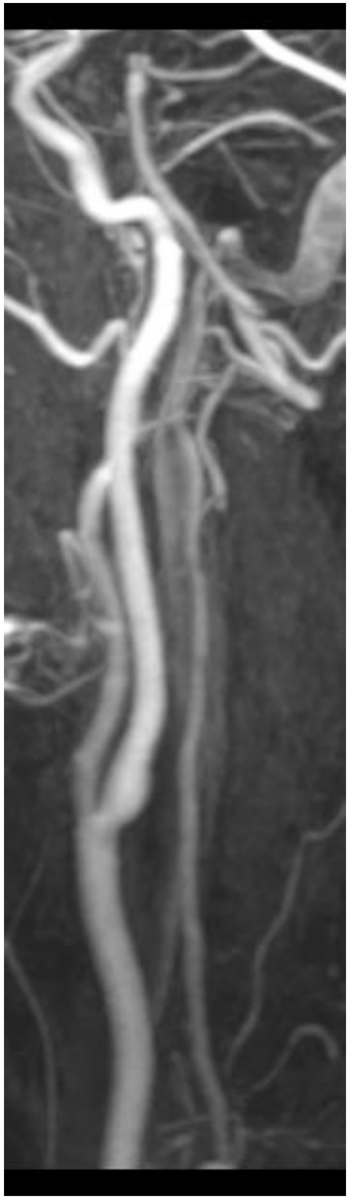

200d Score

0-30

31-50

51-70

>70

Near occlusion

Occluded

Quality

1

2

3

4

5

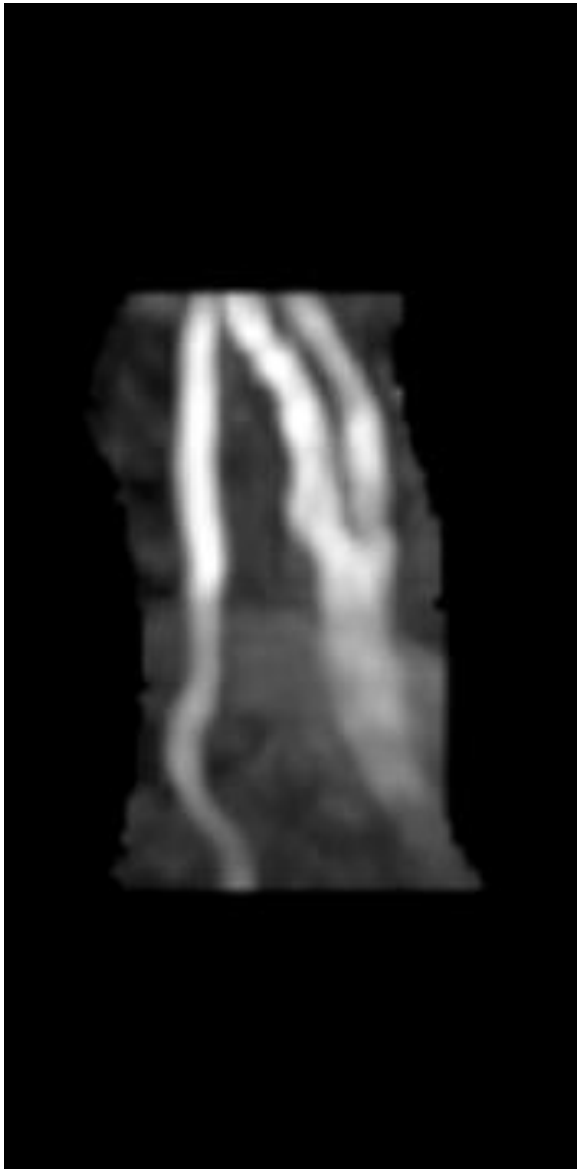

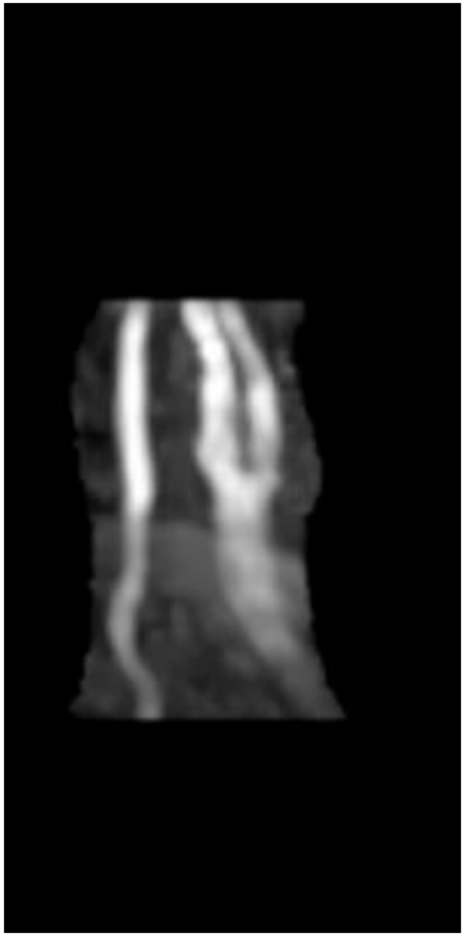

Supplement: S3 File — (PDF) [file pone.0237856.s005.pdf]
