# Supplementary material for: Multiple reader comparison of 2D TOF, 3D TOF, and CEMRA in screening of the carotid bifurcations: Time to reconsider routine contrast use?
Source: PLoS One. 2020 Sep 2;15(9):e0237856. doi: 10.1371/journal.pone.0237856 (PMC7467222; doi:10.1371/journal.pone.0237856)

1d Score

0-30

31-50

51-70

>70

Near occlusion

Occluded

Quality

1

2

3

4

5

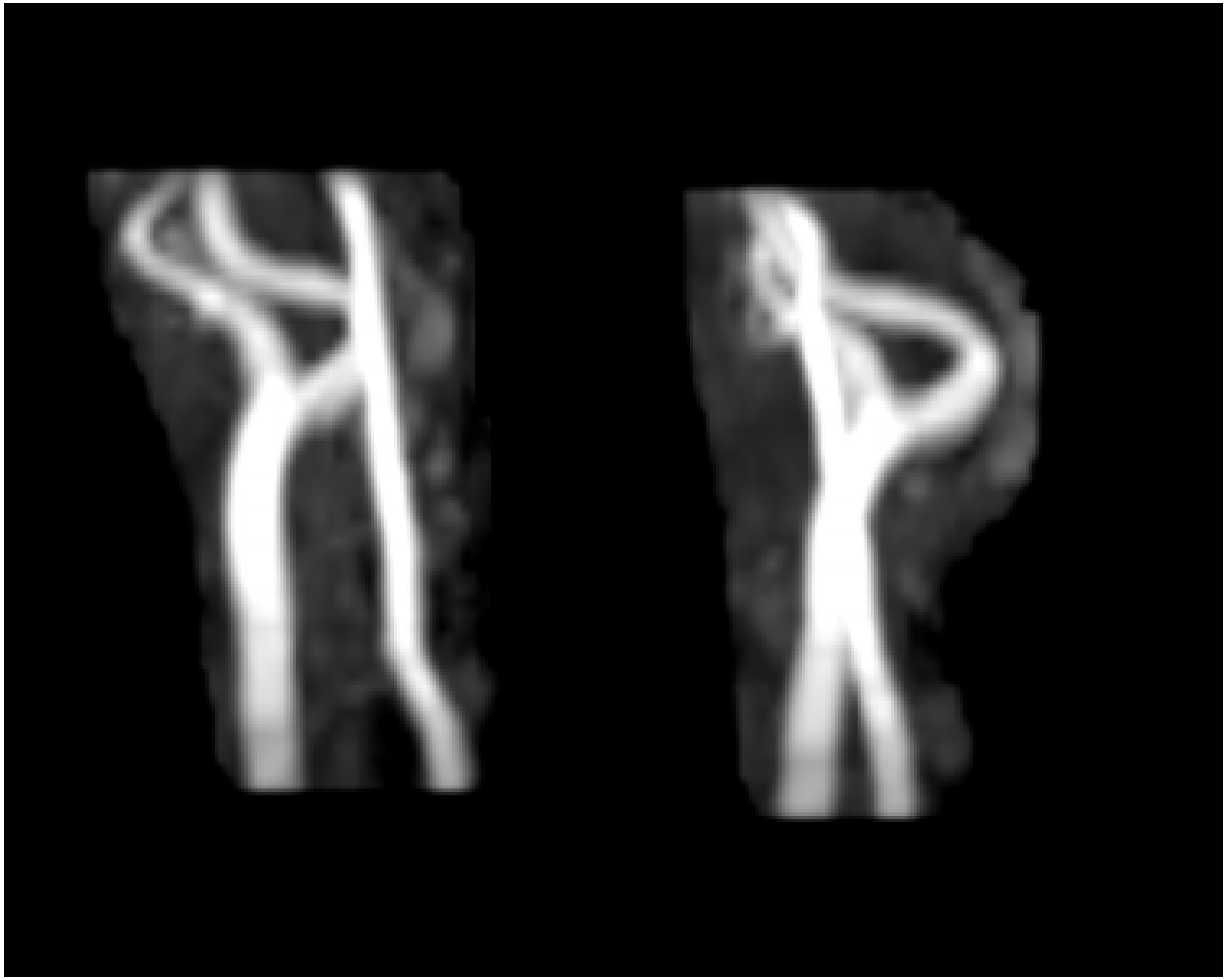

2c Score

0-30

31-50

51-70

>70

Near occlusion

Occluded

Quality

1

2

3

4

5

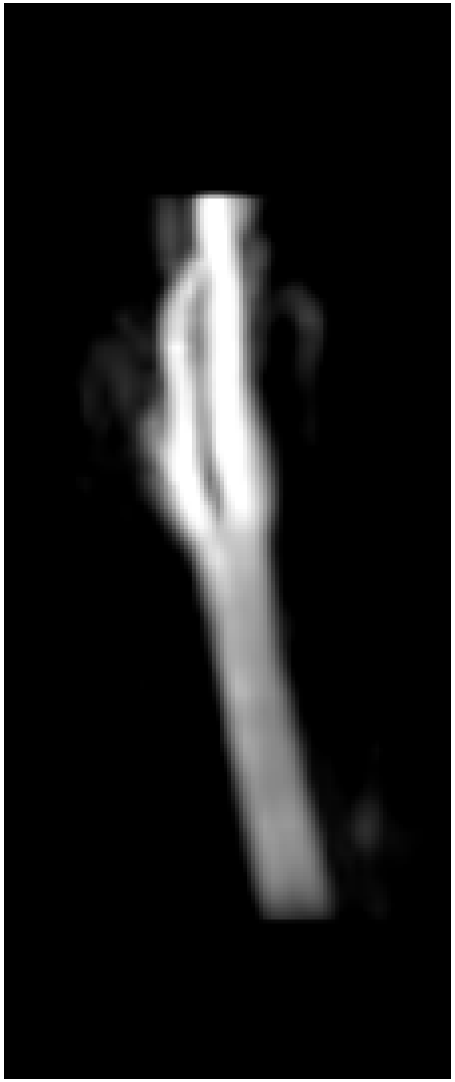

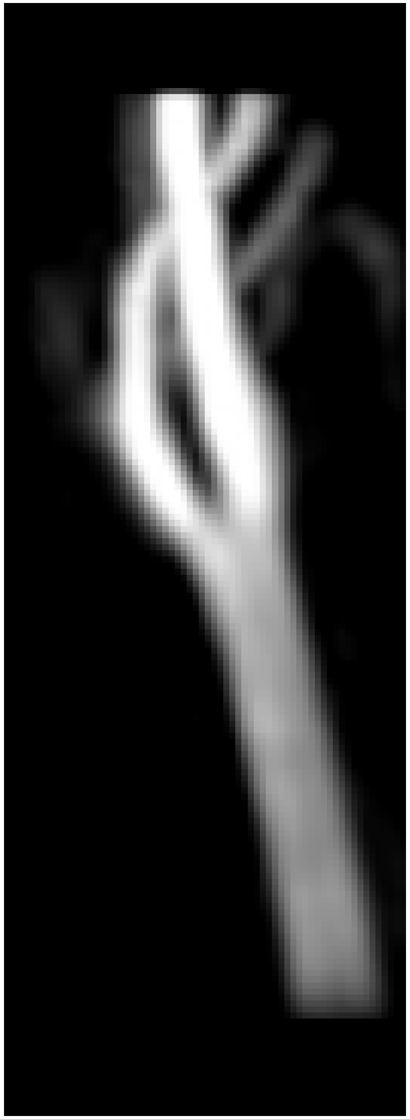

3b Score

0-30

31-50

51-70

>70

Near occlusion

Occluded

Quality

1

2

3

4

5

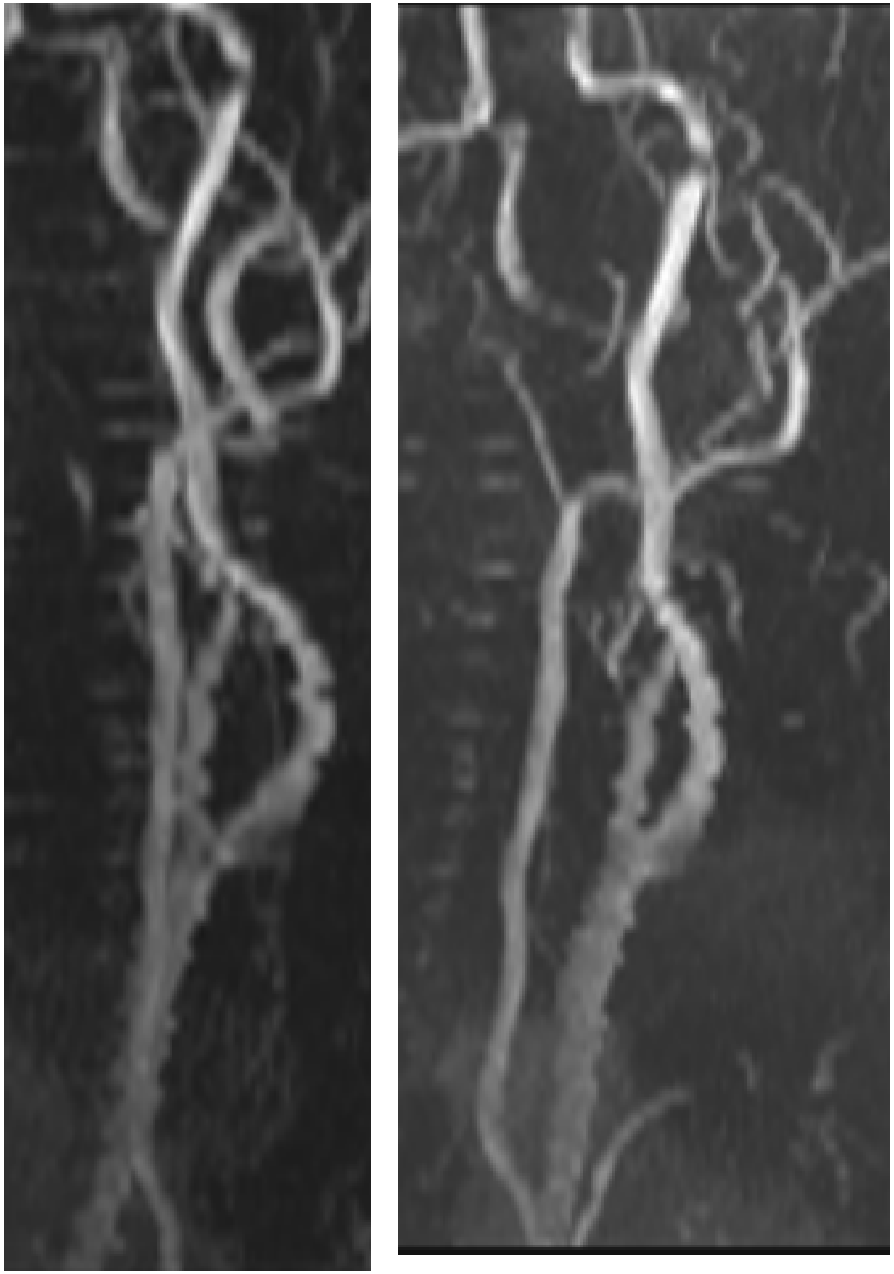

4a Score

0-30

31-50

51-70

>70

Near occlusion

Occluded

Quality

1

2

3

4

5

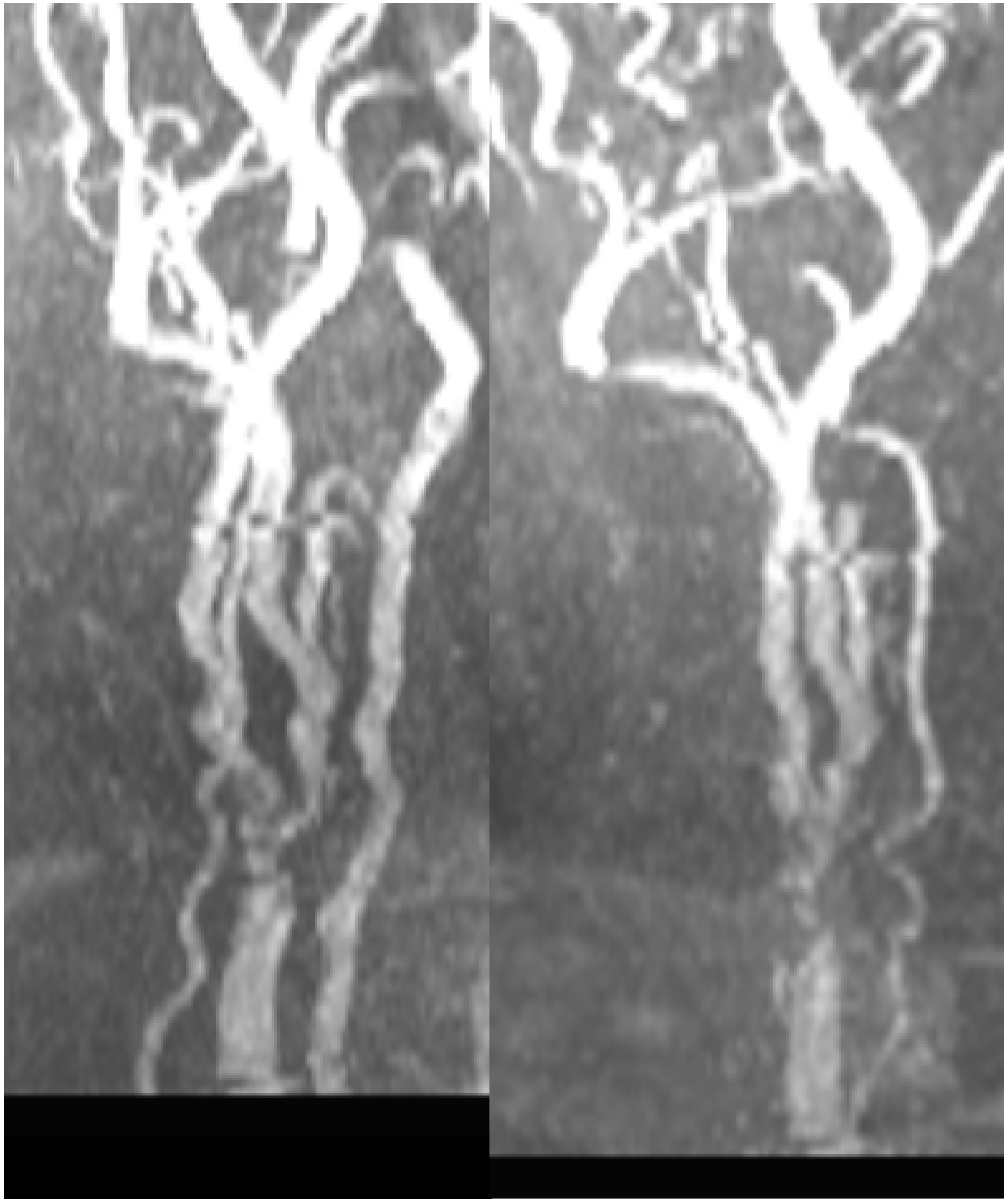

4f Score

0-30

31-50

51-70

>70

Near occlusion

Occluded

Quality

1

2

3

4

5

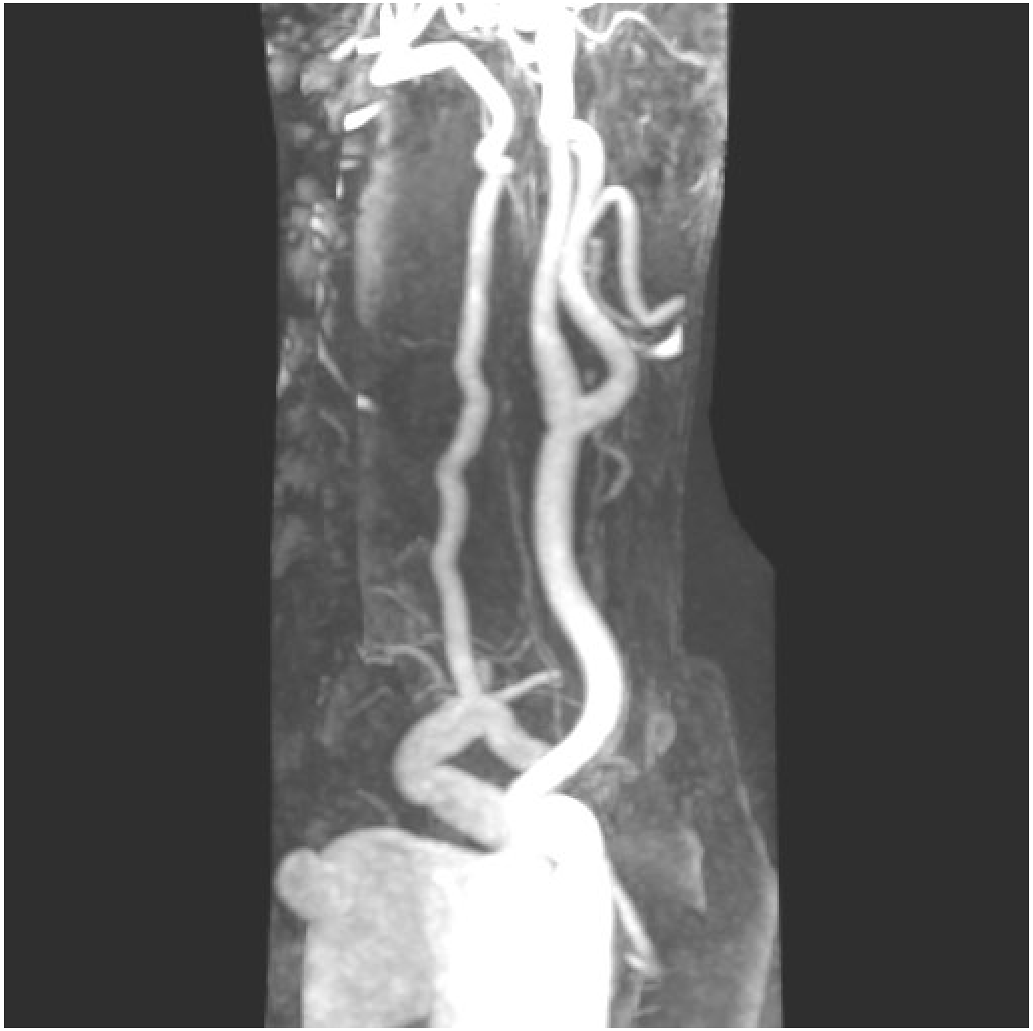

5e Score

0-30

31-50

51-70

>70

Near occlusion

Occluded

Quality

1

2

3

4

5

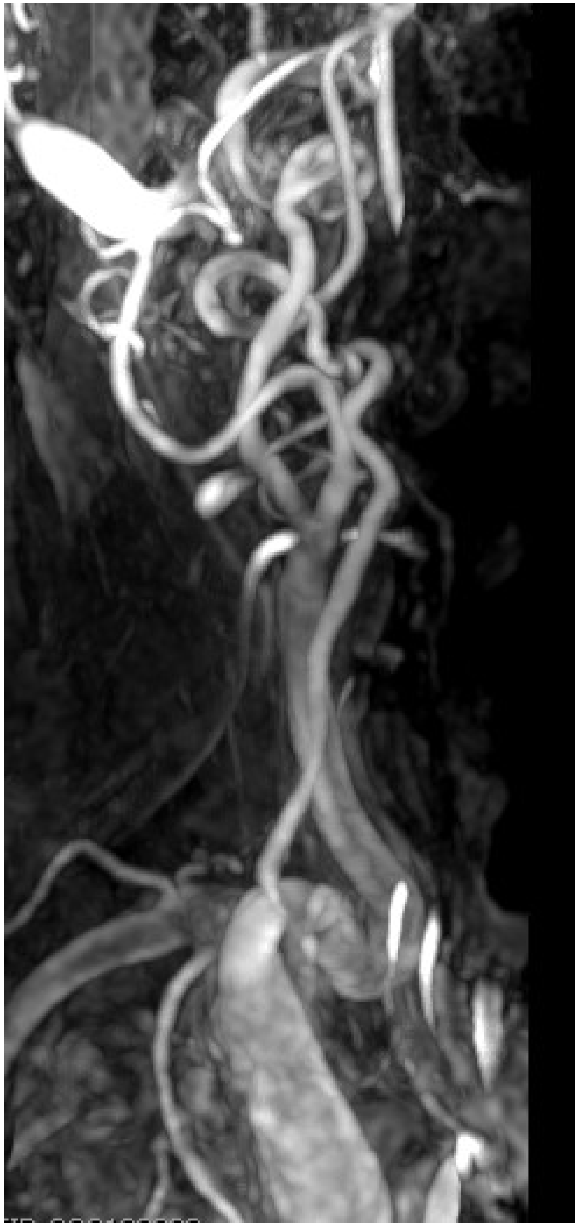

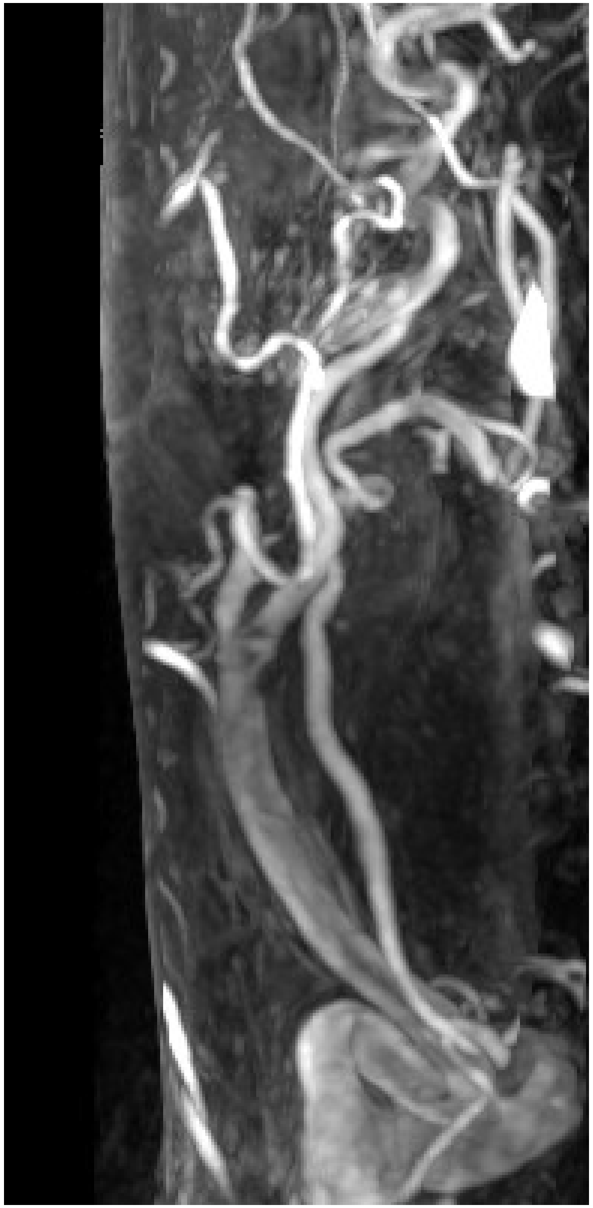

6d Score

0-30

31-50

51-70

>70

Near occlusion

Occluded

Quality

1

2

3

4

5

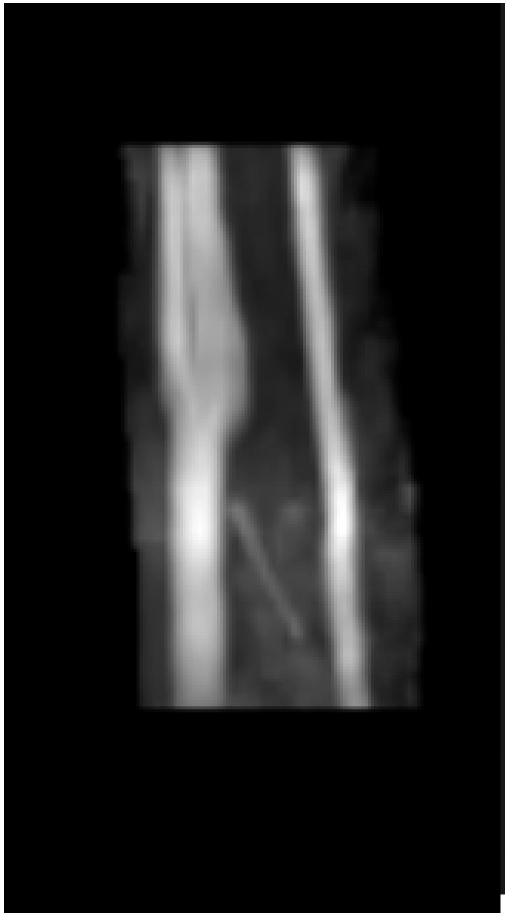

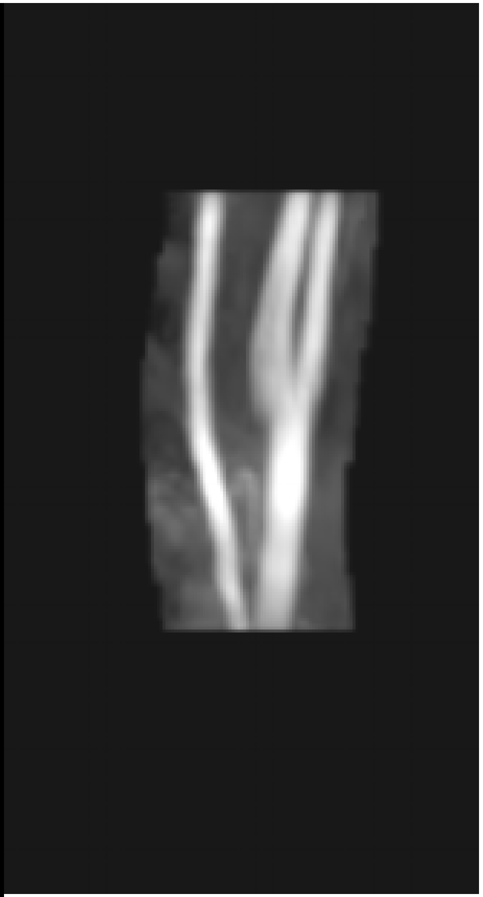

7c Score

0-30

31-50

51-70

>70

Near occlusion

Occluded

Quality

1

2

3

4

5

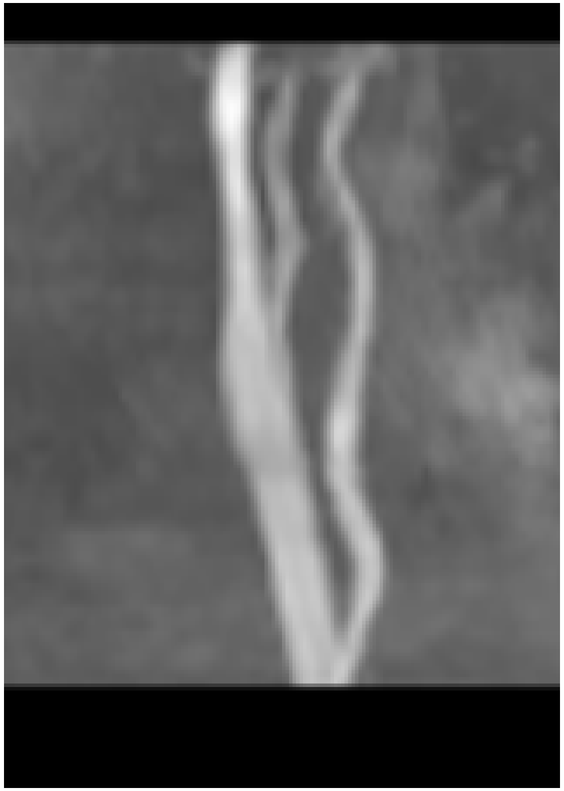

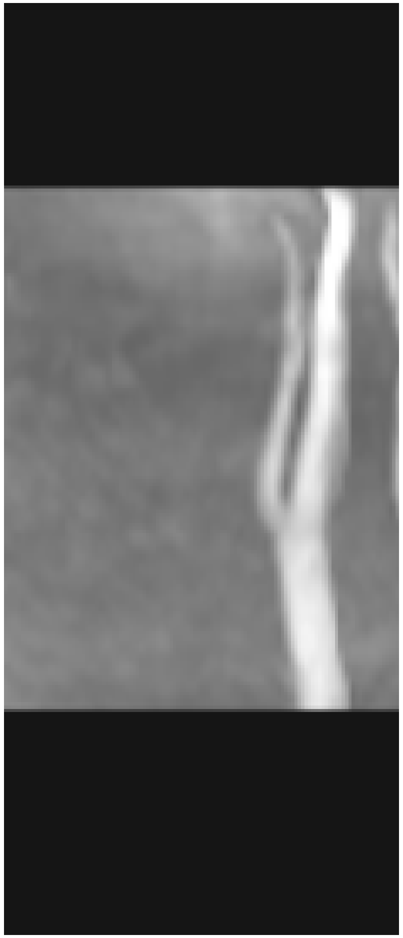

8b Score

0-30

31-50

51-70

>70

Near occlusion

Occluded

Quality

1

2

3

4

5

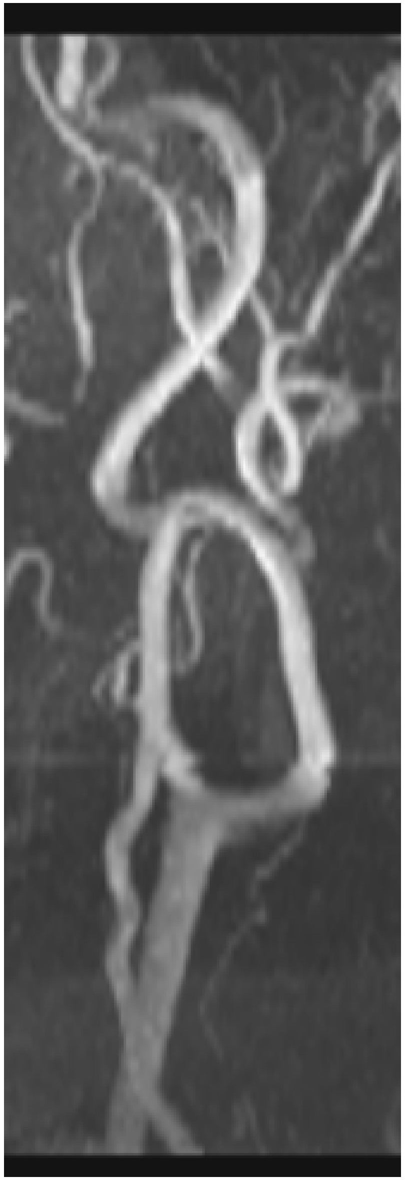

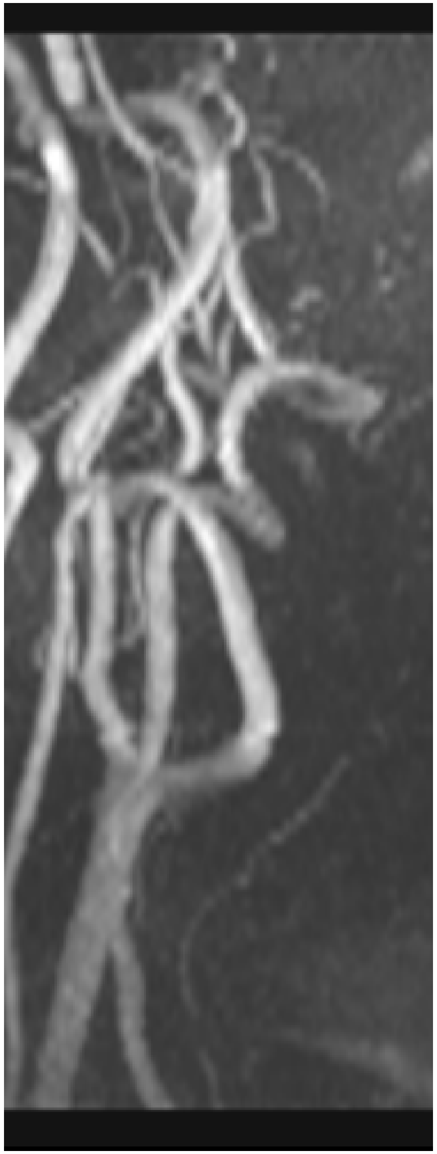

9a Score

0-30

31-50

51-70

>70

Near occlusion

Occluded

Quality

1

2

3

4

5

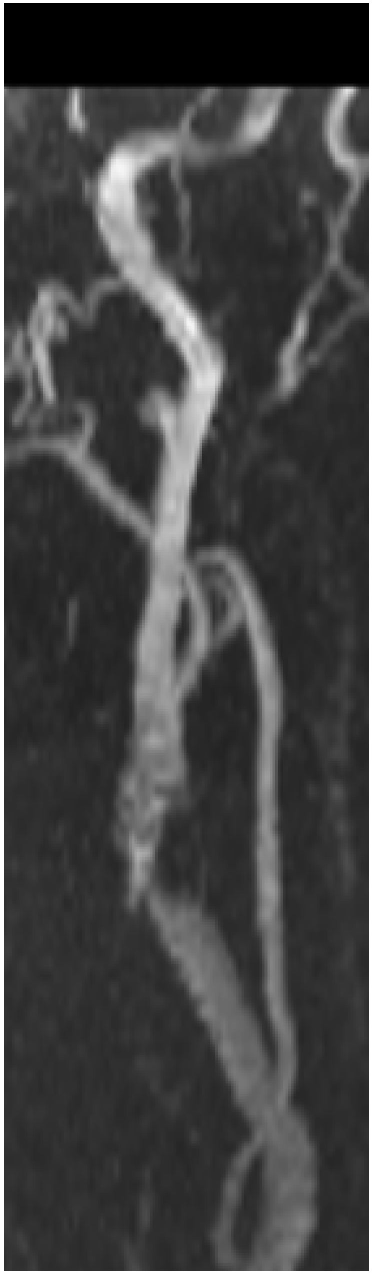

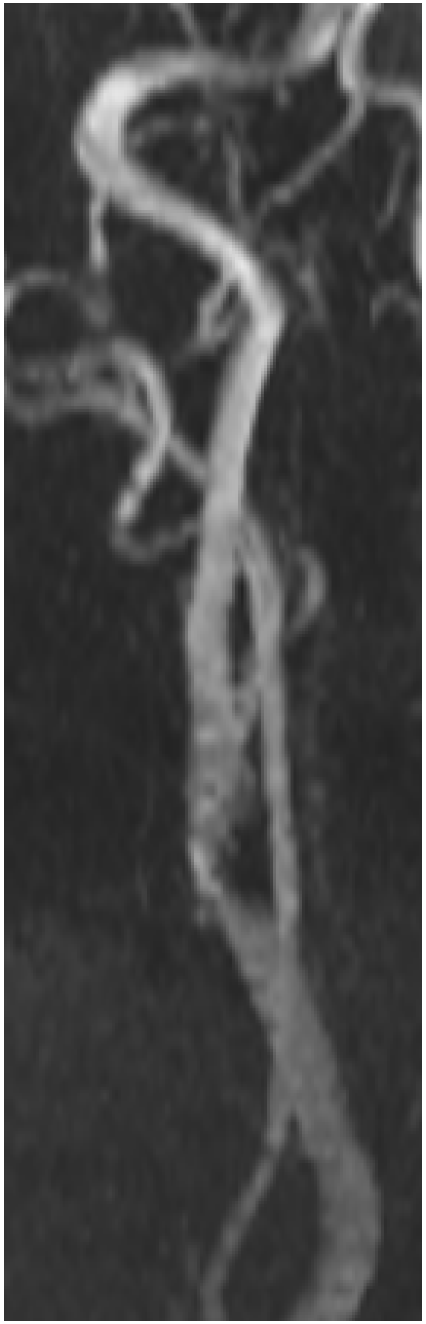

9f Score

0-30

31-50

51-70

>70

Near occlusion

Occluded

Quality

1

2

3

4

5

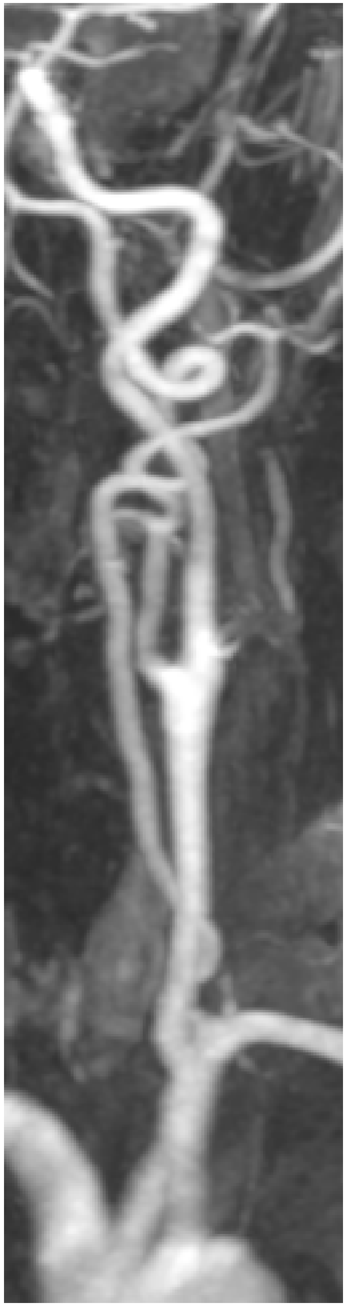

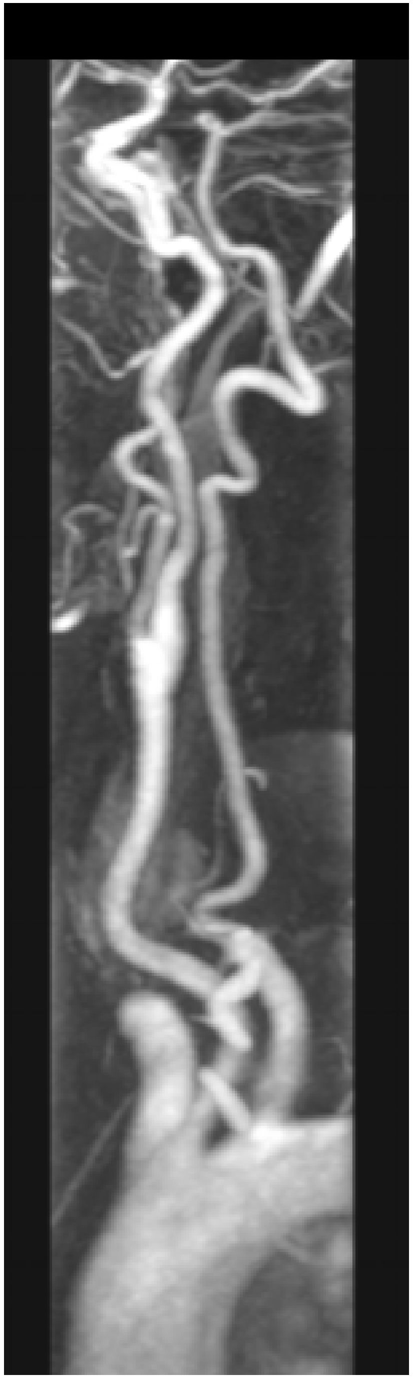

10e Score

0-30

31-50

51-70

>70

Near occlusion

Occluded

Quality

1

2

3

4

5

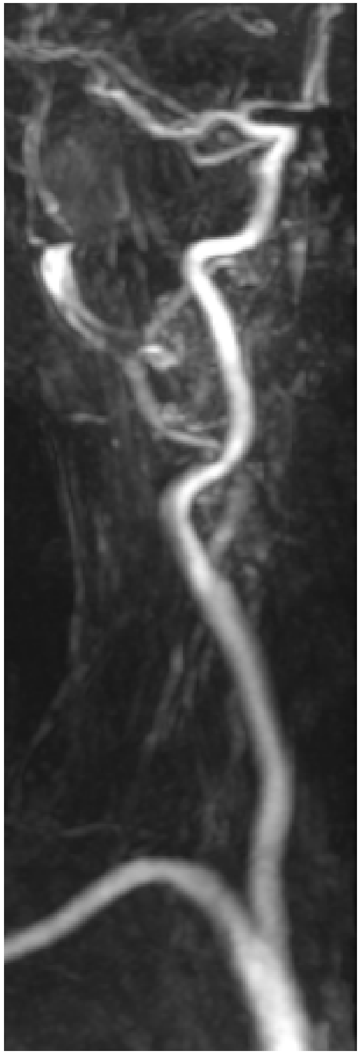

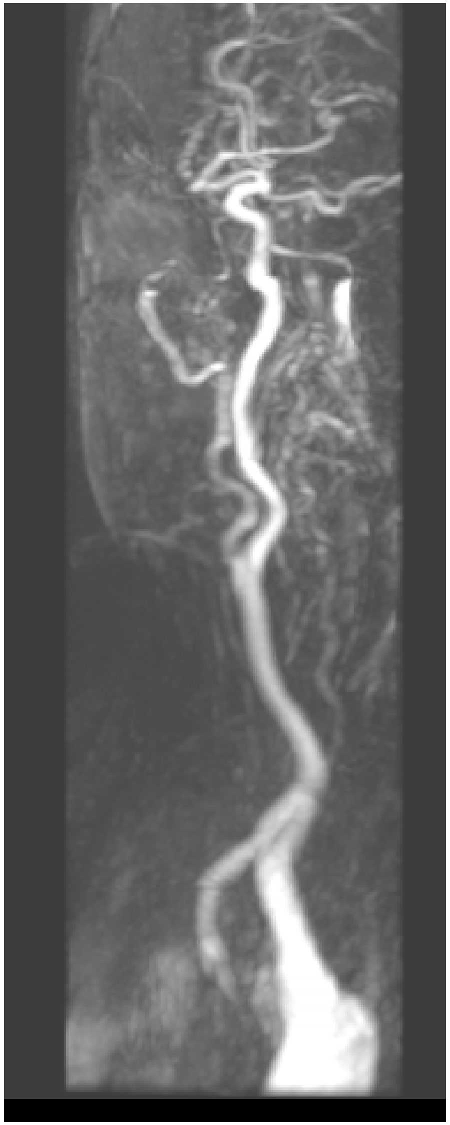

11d Score

0-30

31-50

51-70

>70

Near occlusion

Occluded

Quality

1

2

3

4

5

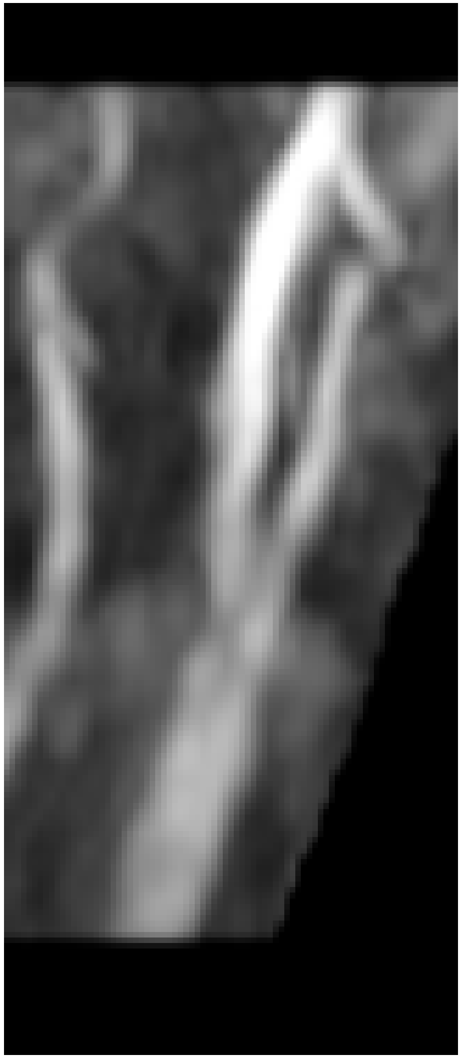

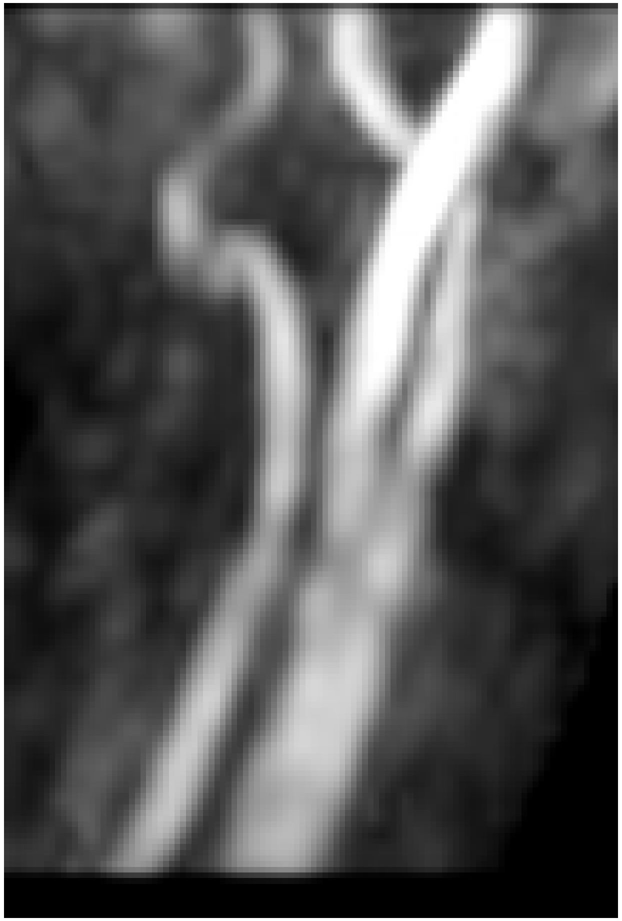

12c Score

0-30

31-50

51-70

>70

Near occlusion

Occluded

Quality

1

2

3

4

5

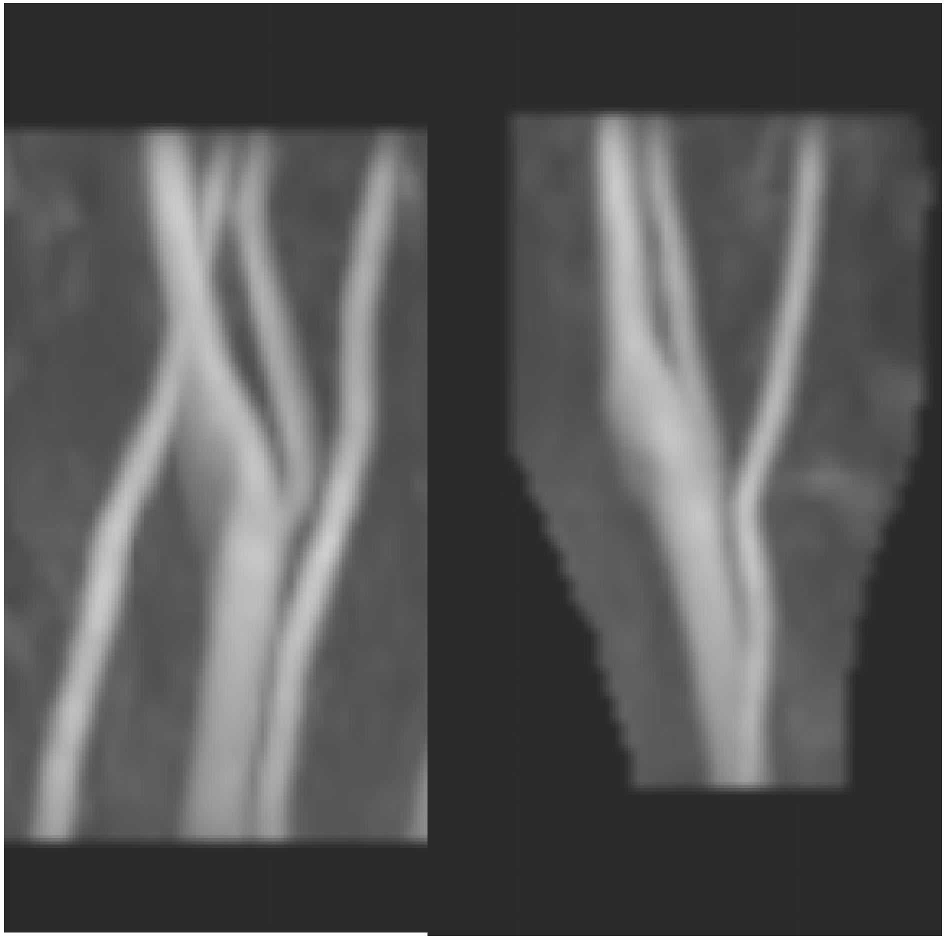

13b Score

0-30

31-50

51-70

>70

Near occlusion

Occluded

Quality

1

2

3

4

5

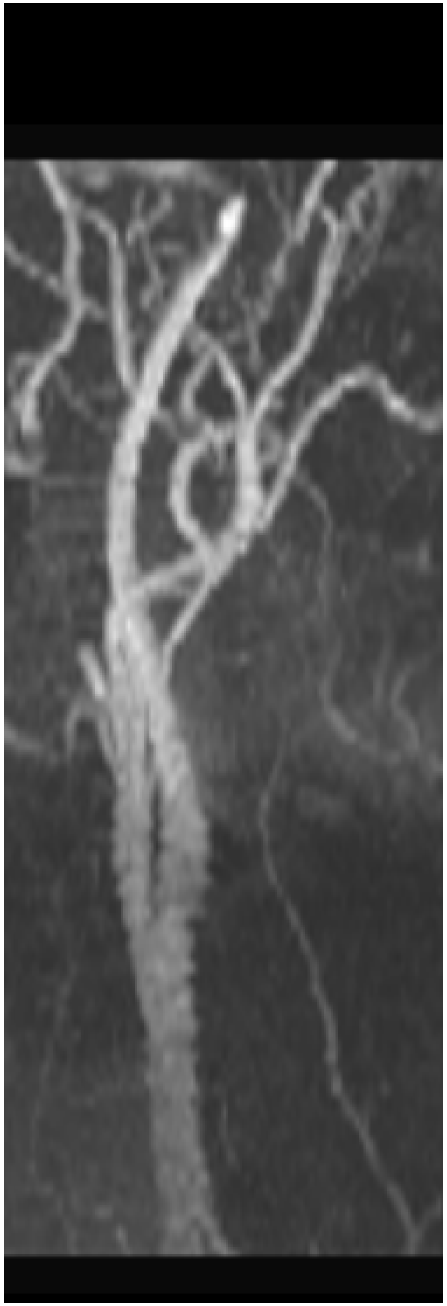

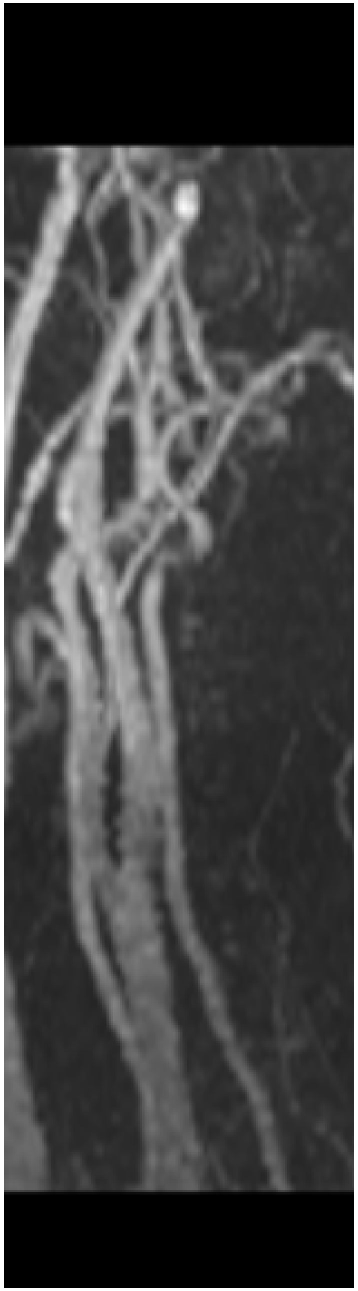

14a Score

0-30

31-50

51-70

>70

Near occlusion

Occluded

Quality

1

2

3

4

5

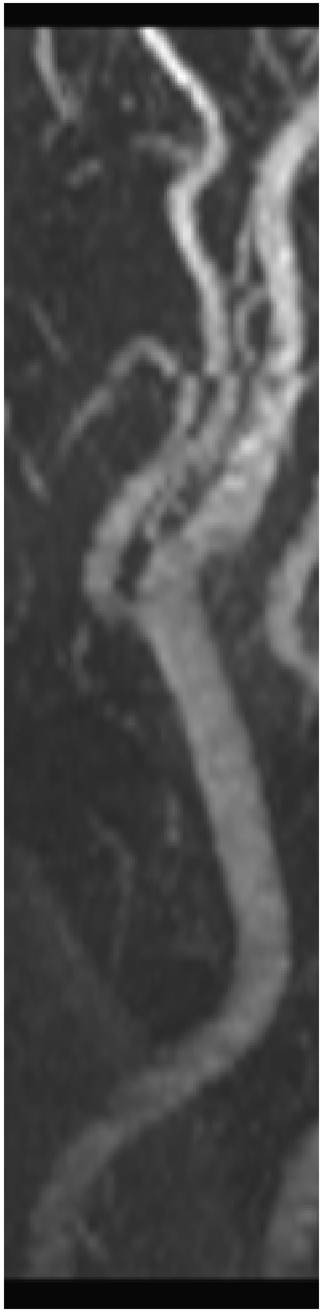

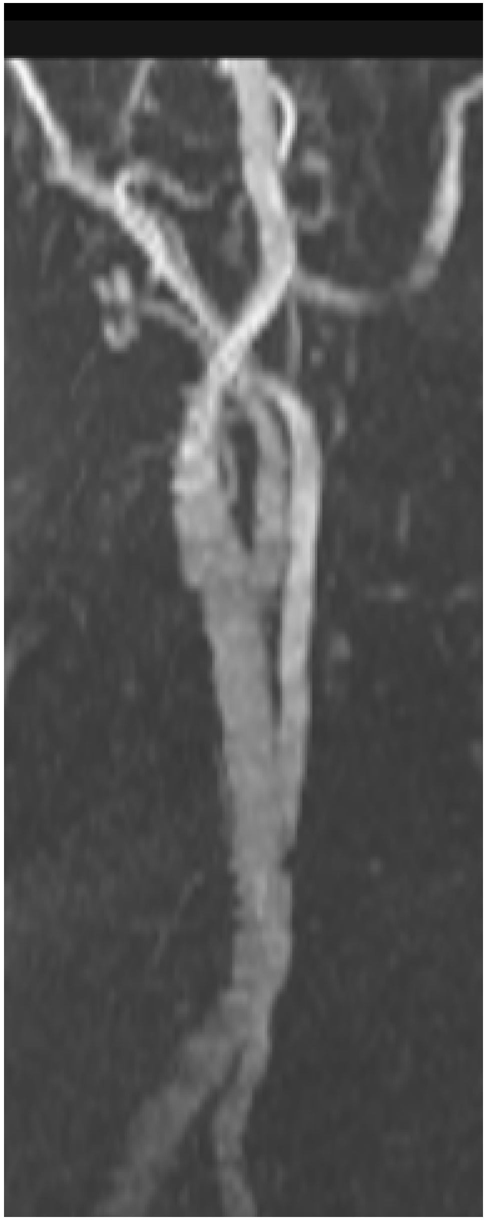

14f Score

0-30

31-50

51-70

>70

Near occlusion

Occluded

Quality

1

2

3

4

5

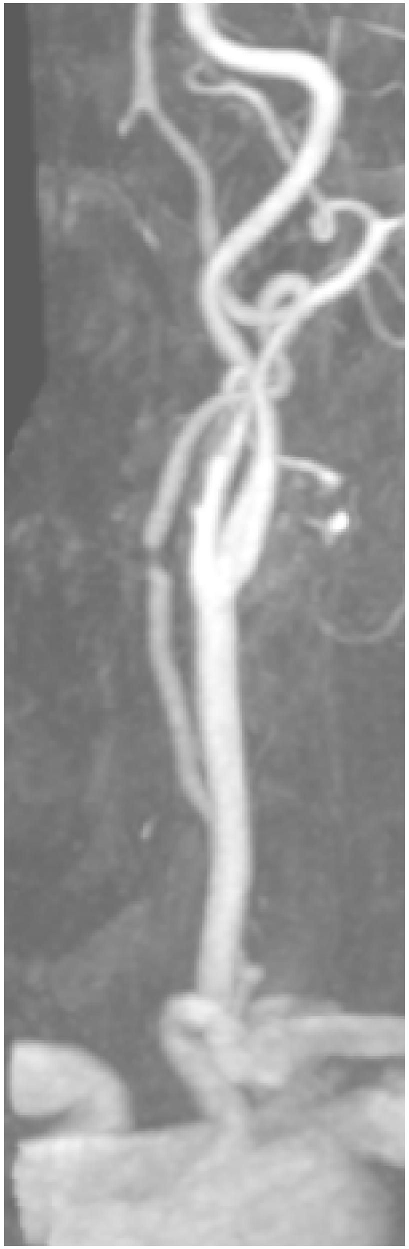

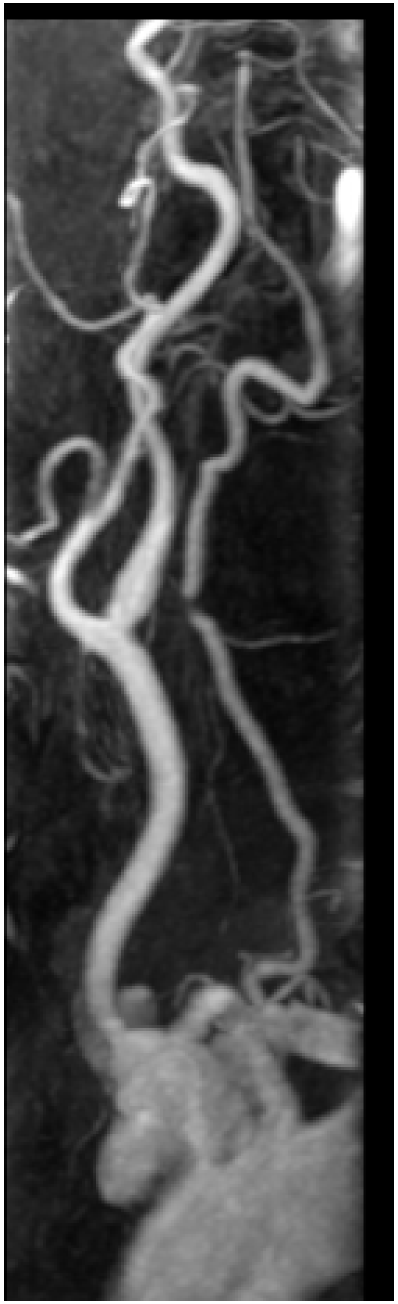

15e Score

0-30

31-50

51-70

>70

Near occlusion

Occluded

Quality

1

2

3

4

5

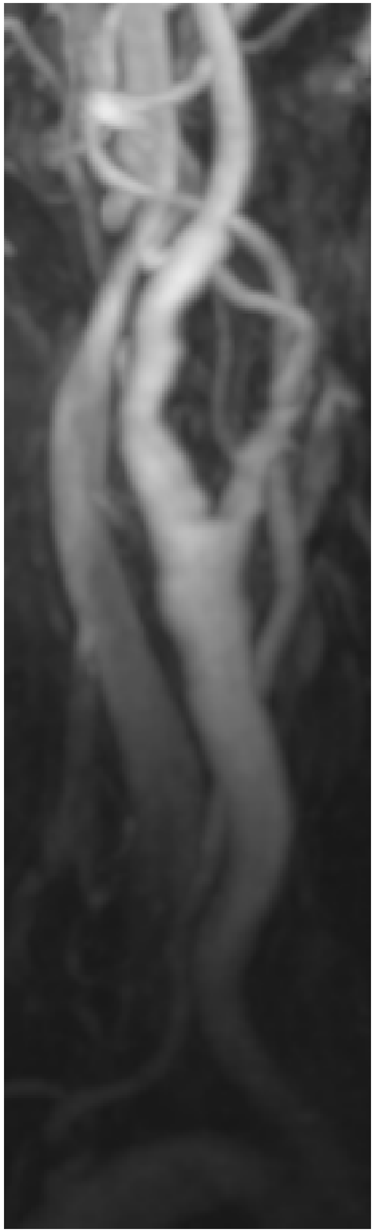

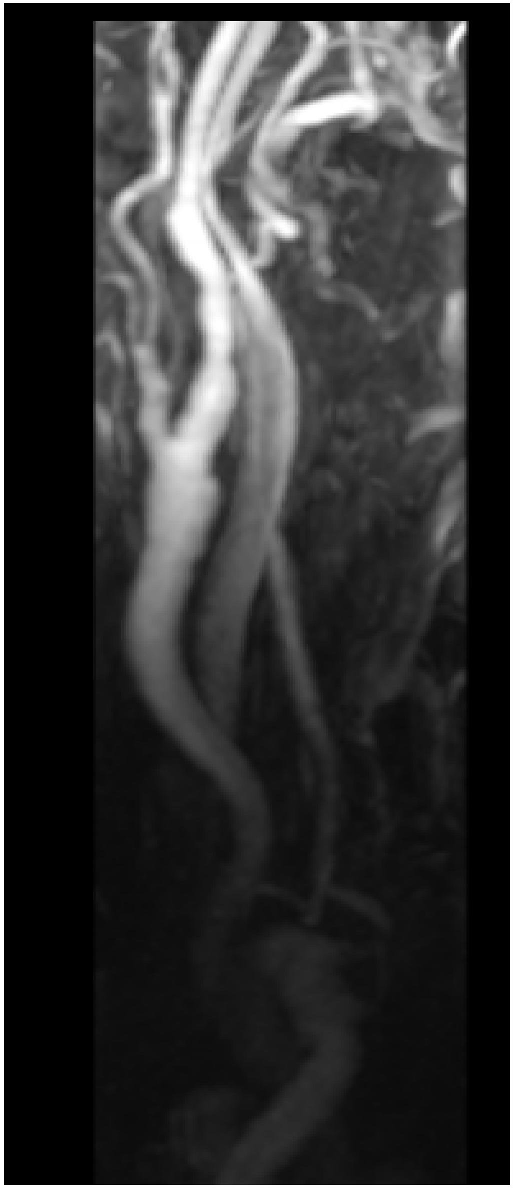

16d Score

0-30

31-50

51-70

>70

Near occlusion

Occluded

Quality

1

2

3

4

5

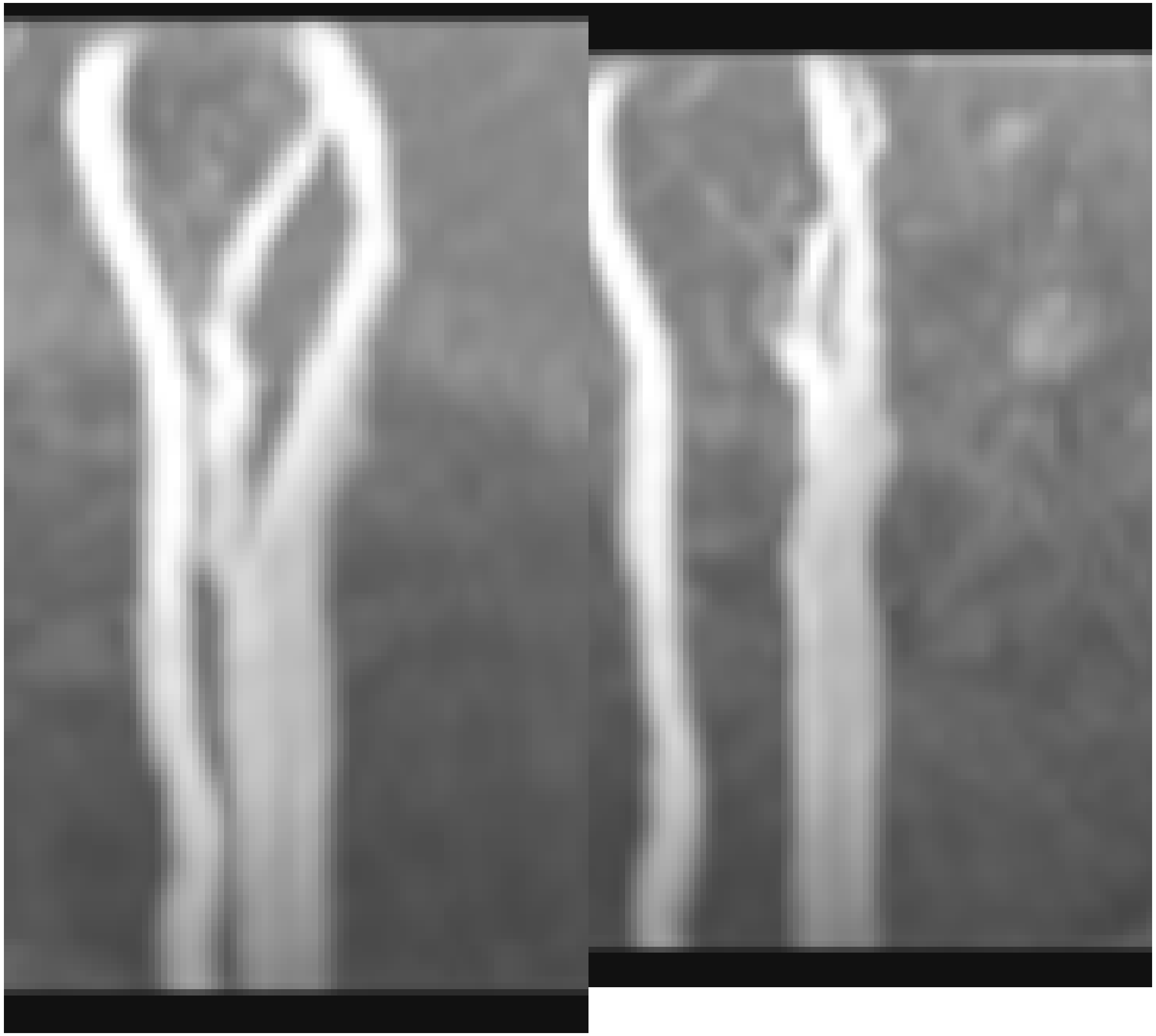

17c Score

0-30

31-50

51-70

>70

Near occlusion

Occluded

Quality

1

2

3

4

5

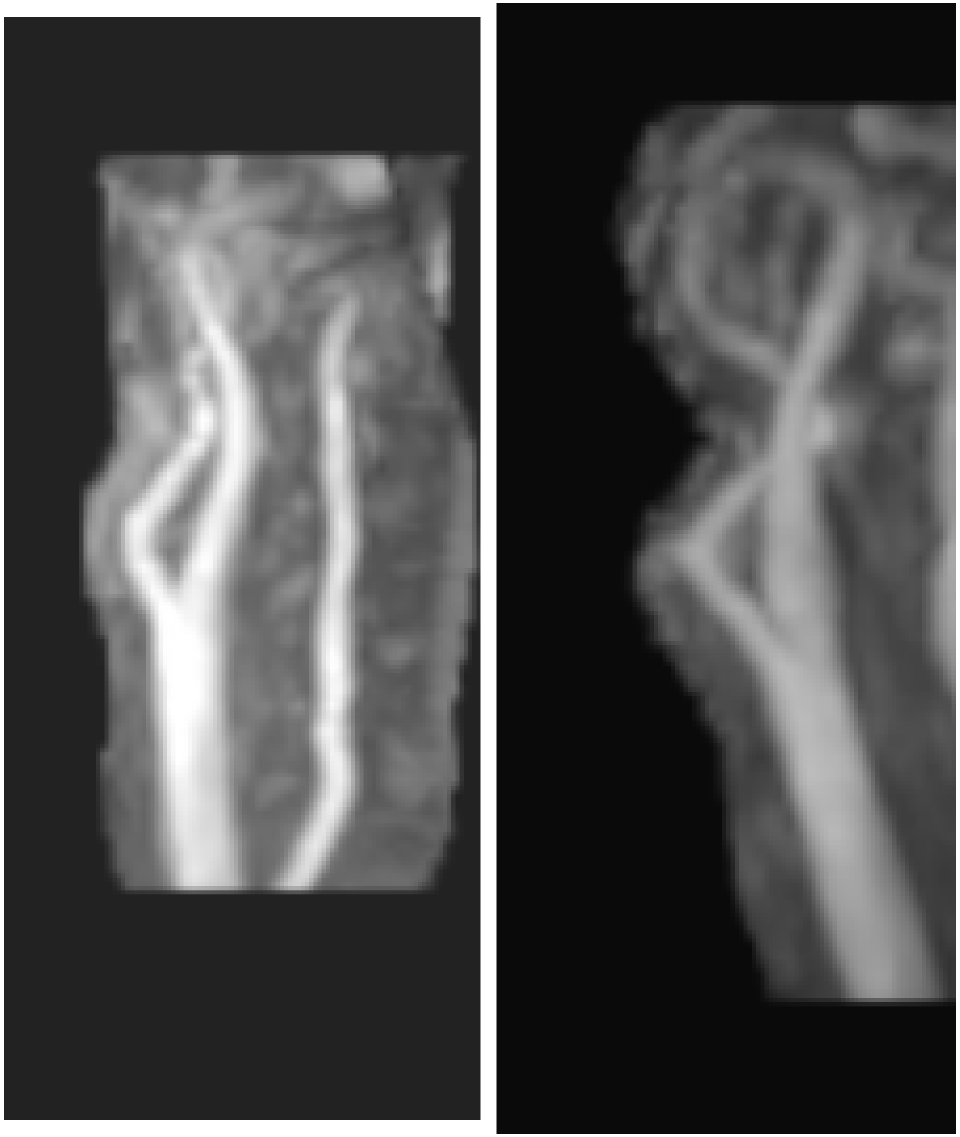

18b Score

0-30

31-50

51-70

>70

Near occlusion

Occluded

Quality

1

2

3

4

5

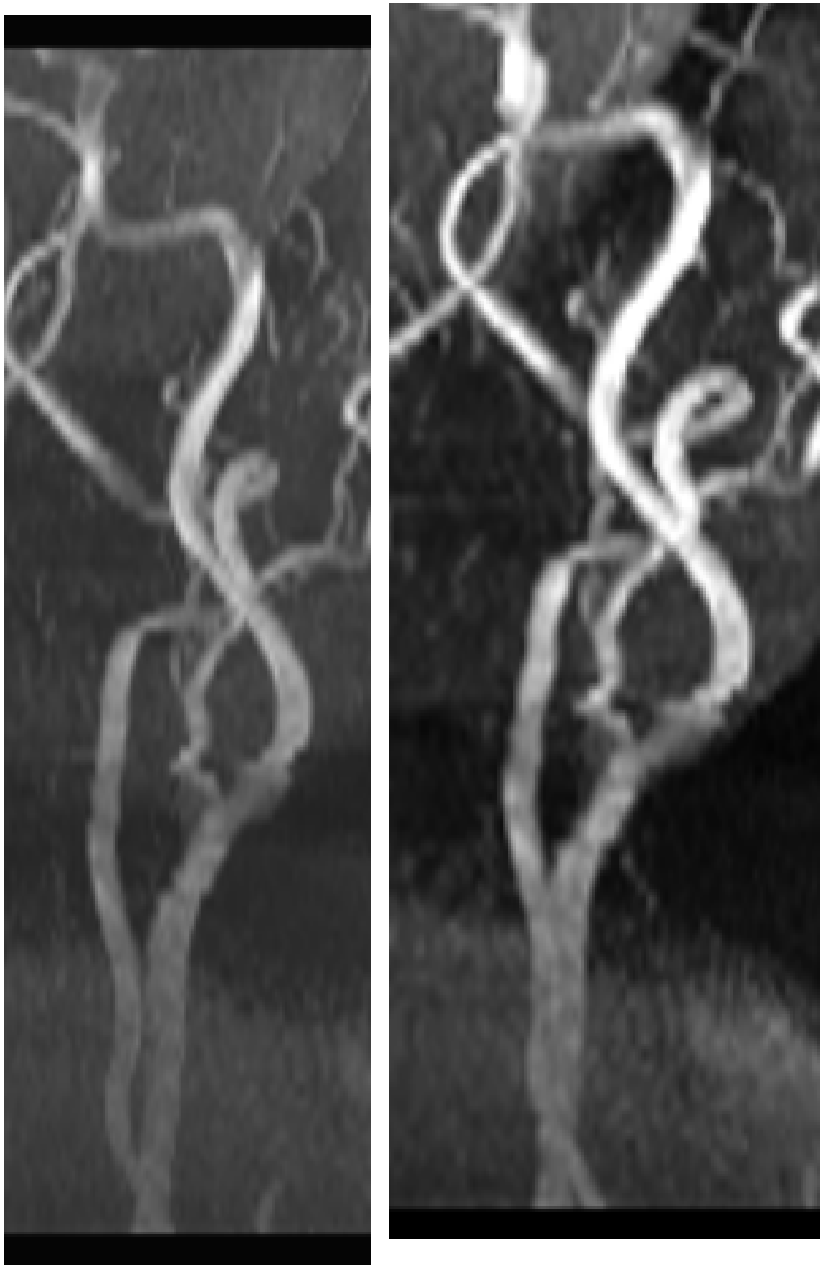

19a Score

0-30

31-50

51-70

>70

Near occlusion

Occluded

Quality

1

2

3

4

5

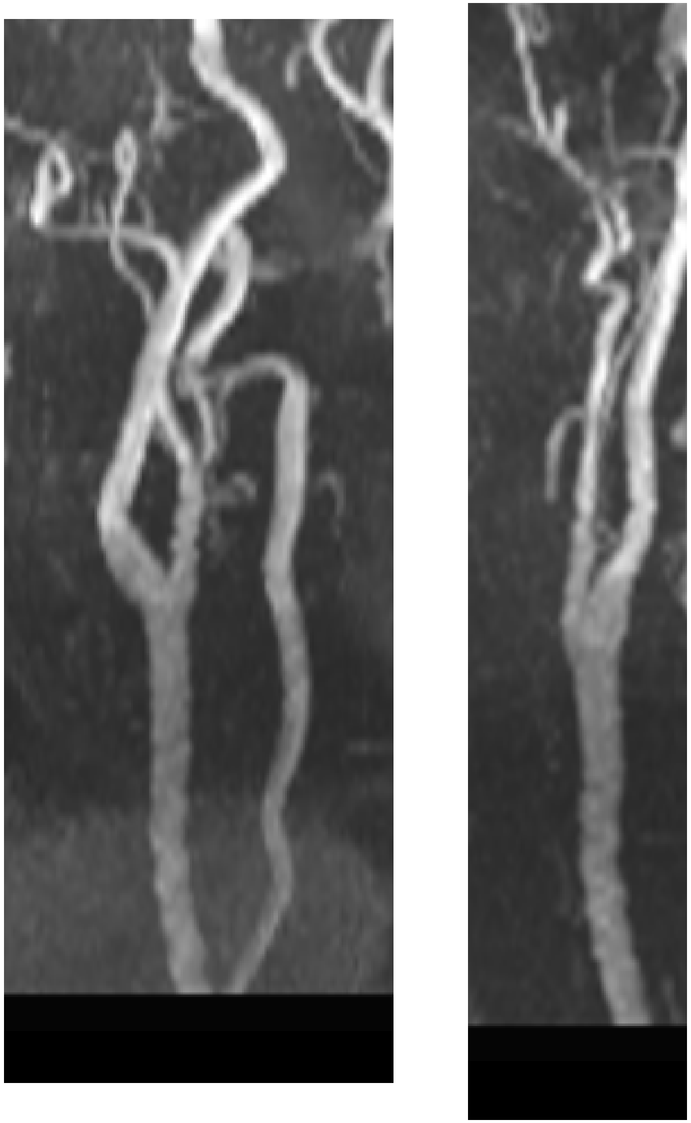

19f Score

0-30

31-50

51-70

>70

Near occlusion

Occluded

Quality

1

2

3

4

5

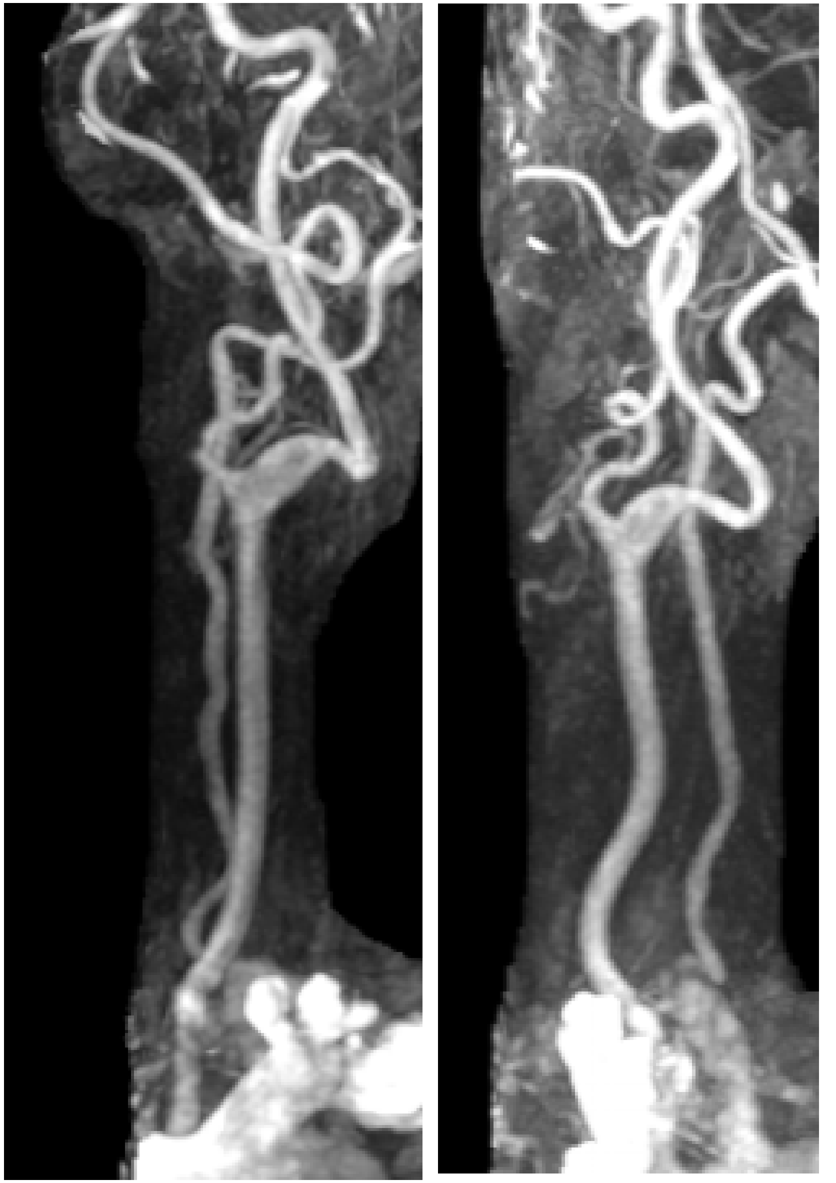

20e Score

0-30

31-50

51-70

>70

Near occlusion

Occluded

Quality

1

2

3

4

5

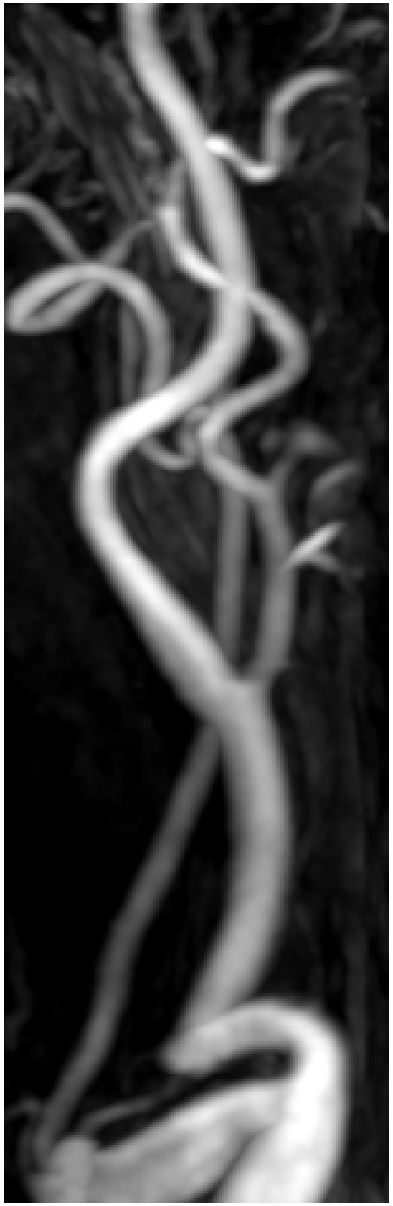

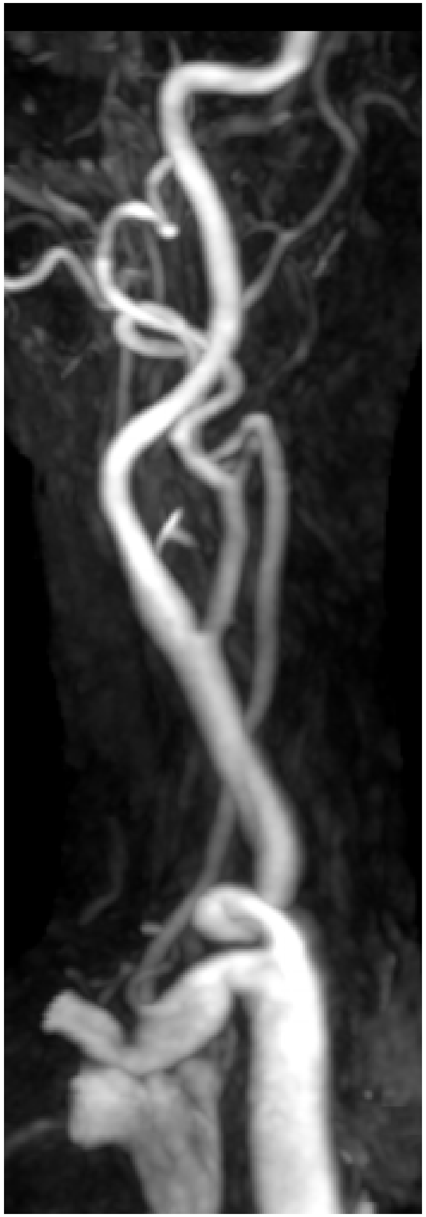

21d Score

0-30

31-50

51-70

>70

Near occlusion

Occluded

Quality

1

2

3

4

5

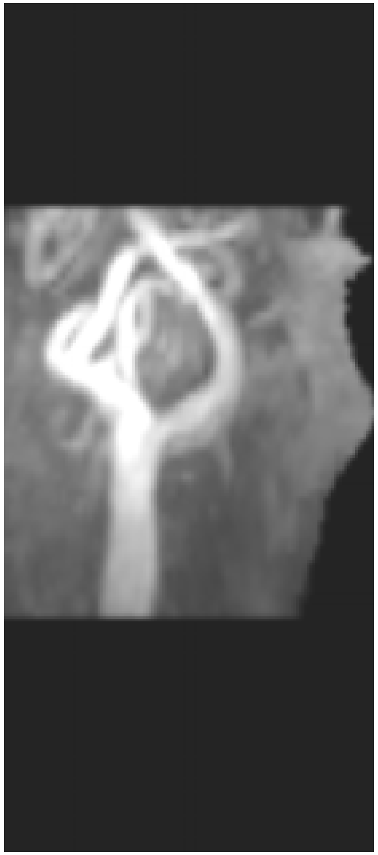

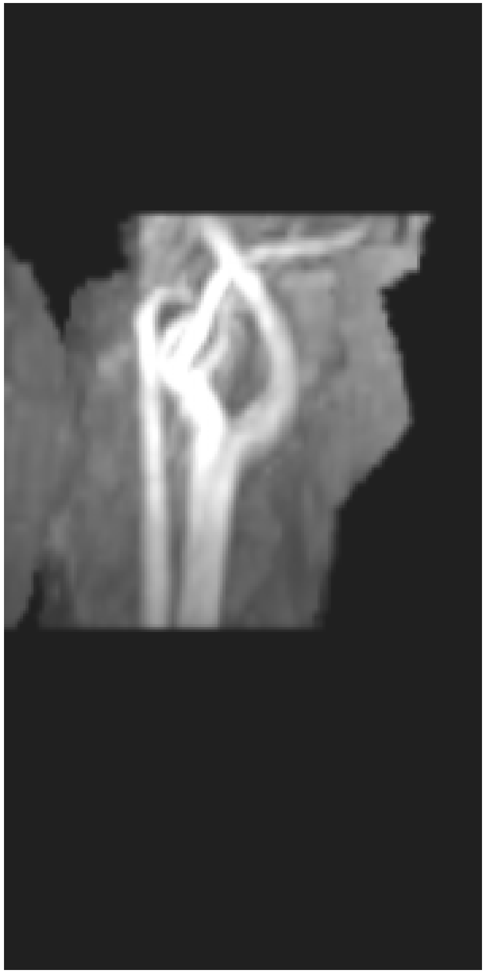

22c Score

0-30

31-50

51-70

>70

Near occlusion

Occluded

Quality

1

2

3

4

5

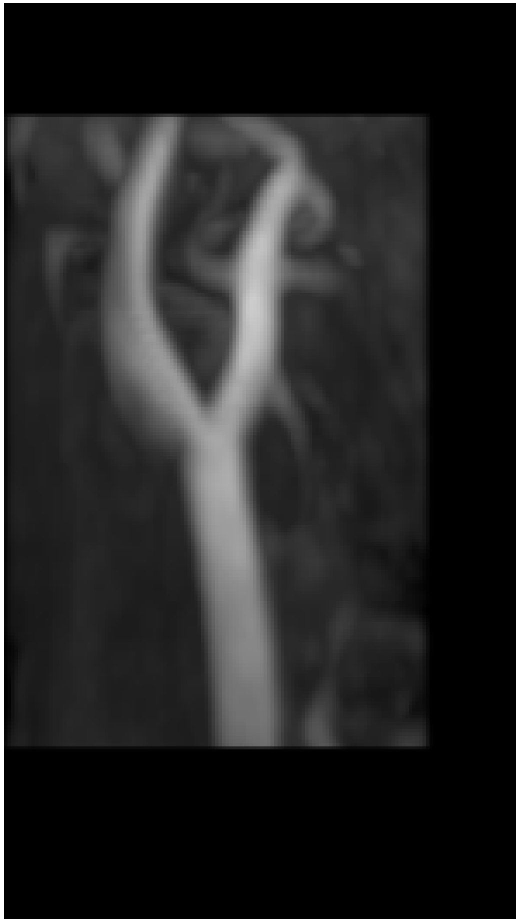

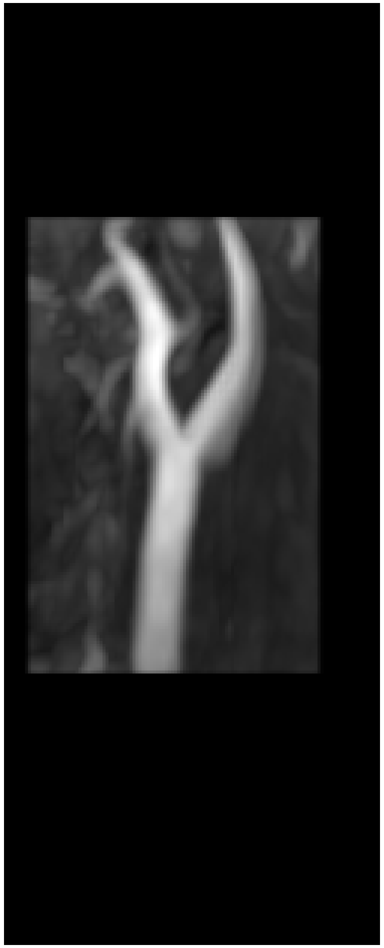

23b Score

0-30

31-50

51-70

>70

Near occlusion

Occluded

Quality

1

2

3

4

5

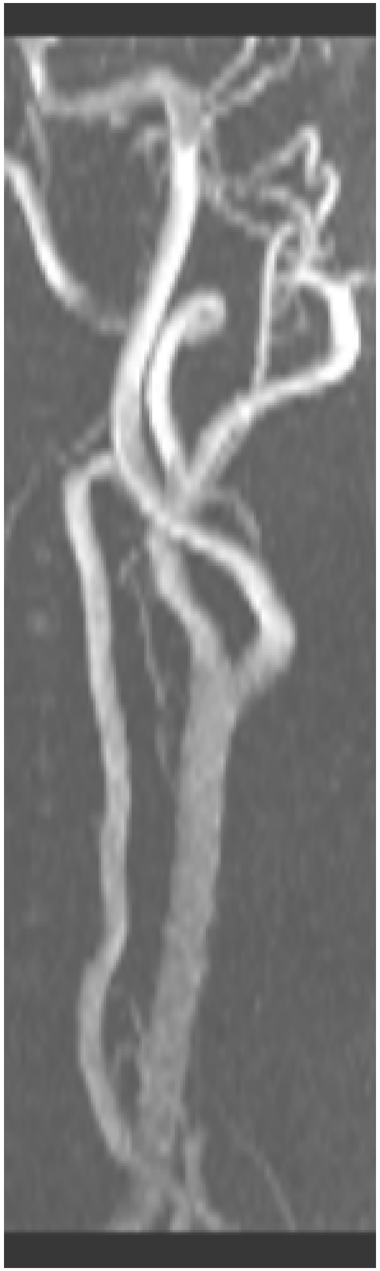

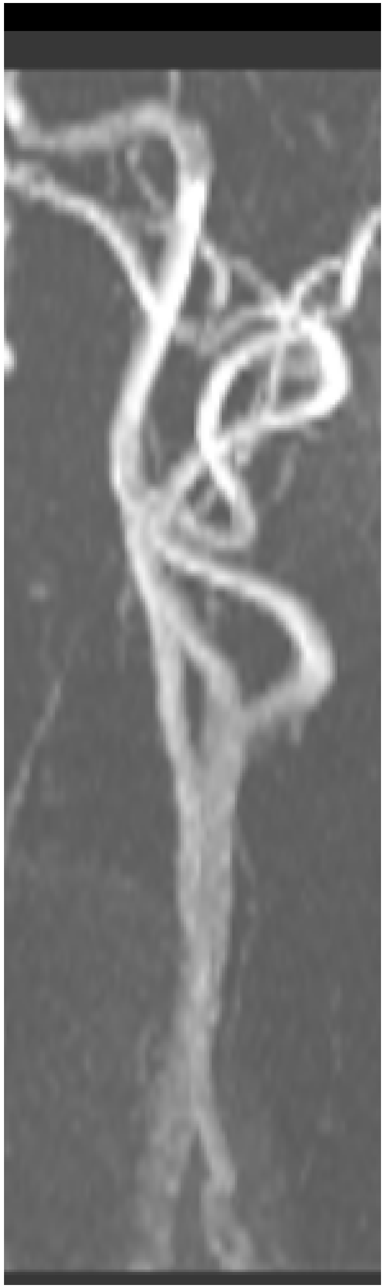

24a Score

0-30

31-50

51-70

>70

Near occlusion

Occluded

Quality

1

2

3

4

5

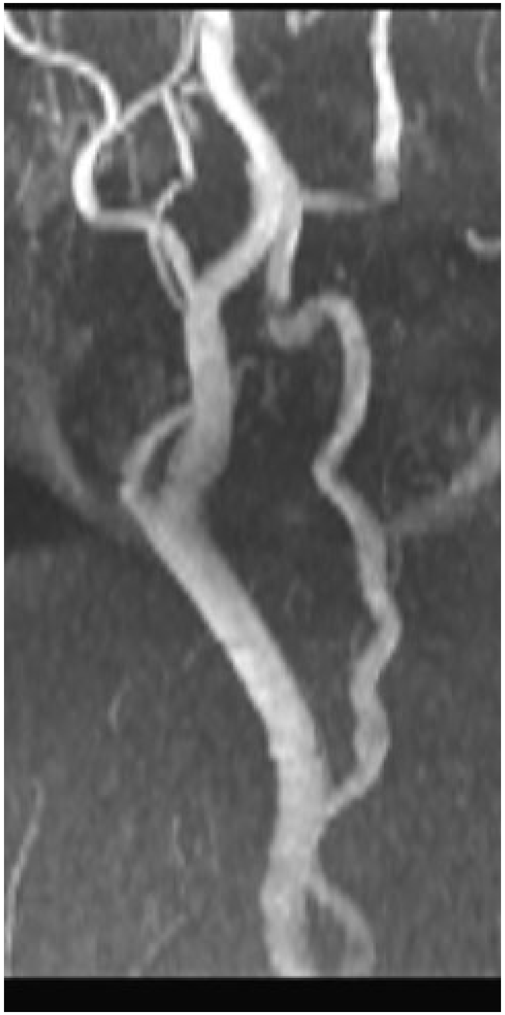

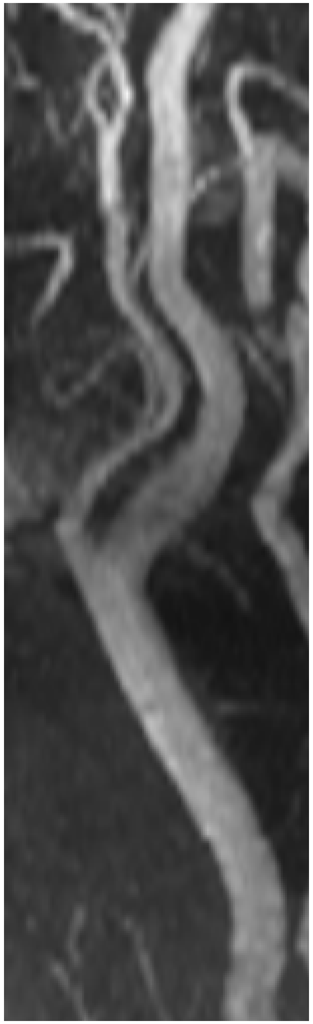

24f Score

0-30

31-50

51-70

>70

Near occlusion

Occluded

Quality

1

2

3

4

5

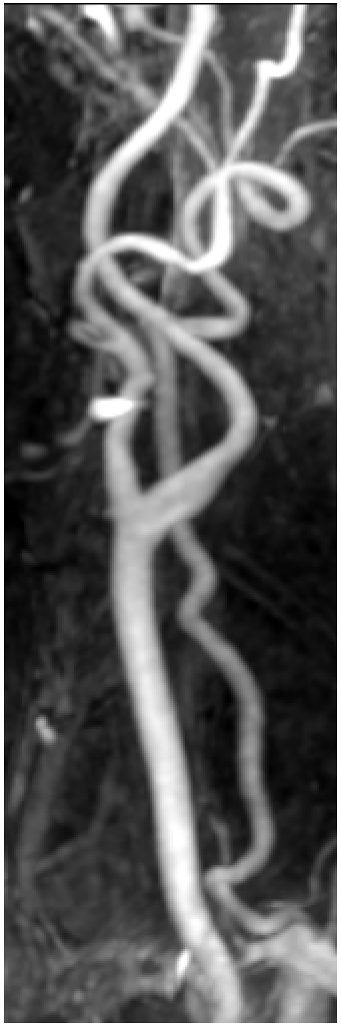

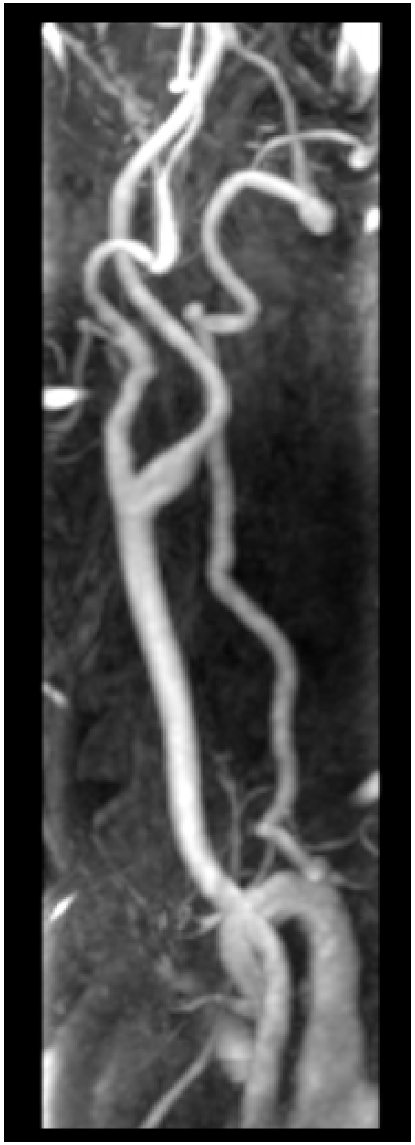

25e Score

0-30

31-50

51-70

>70

Near occlusion

Occluded

Quality

1

2

3

4

5

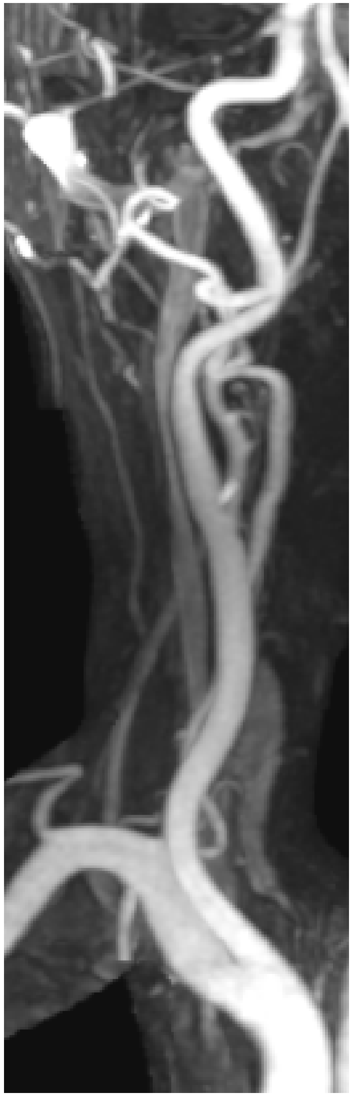

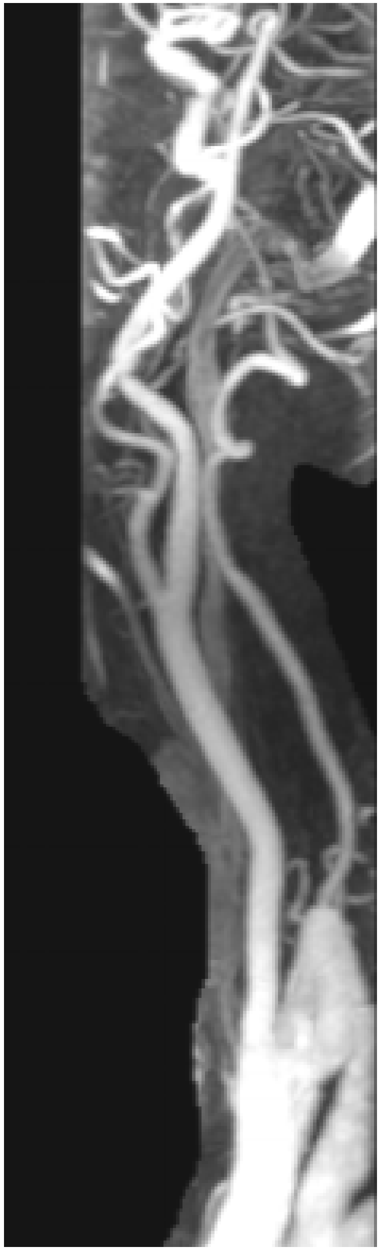

26d Score

0-30

31-50

51-70

>70

Near occlusion

Occluded

Quality

1

2

3

4

5

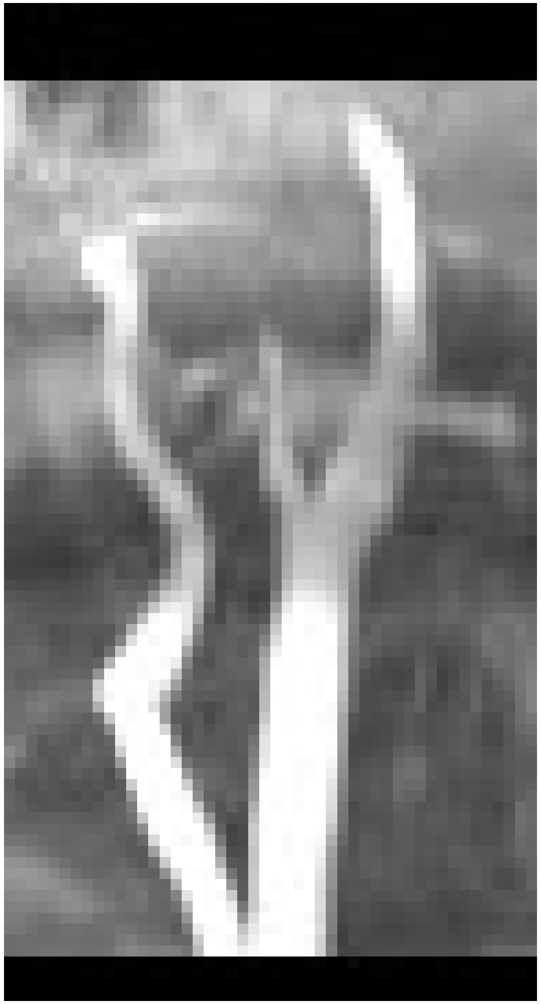

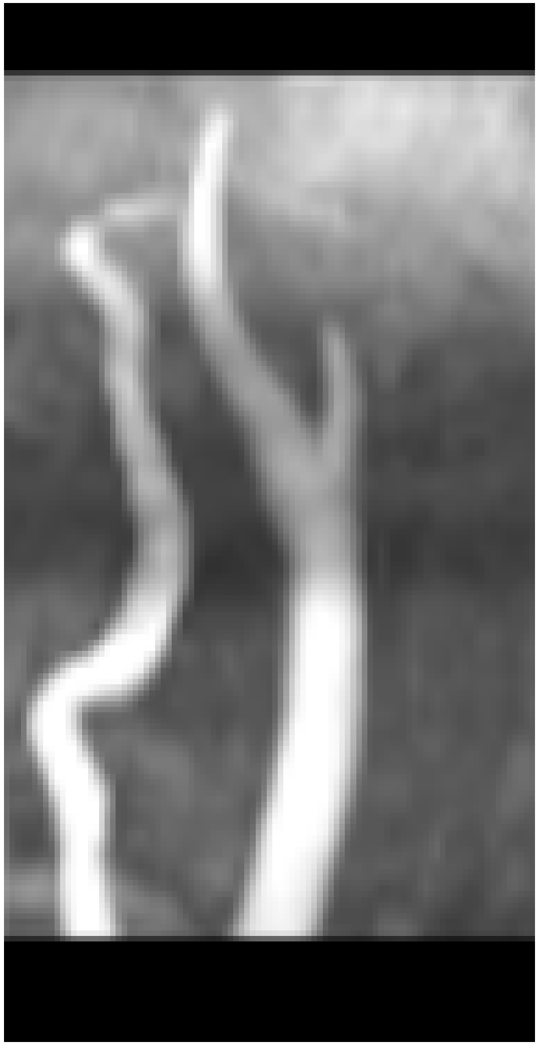

27c Score

0-30

31-50

51-70

>70

Near occlusion

Occluded

Quality

1

2

3

4

5

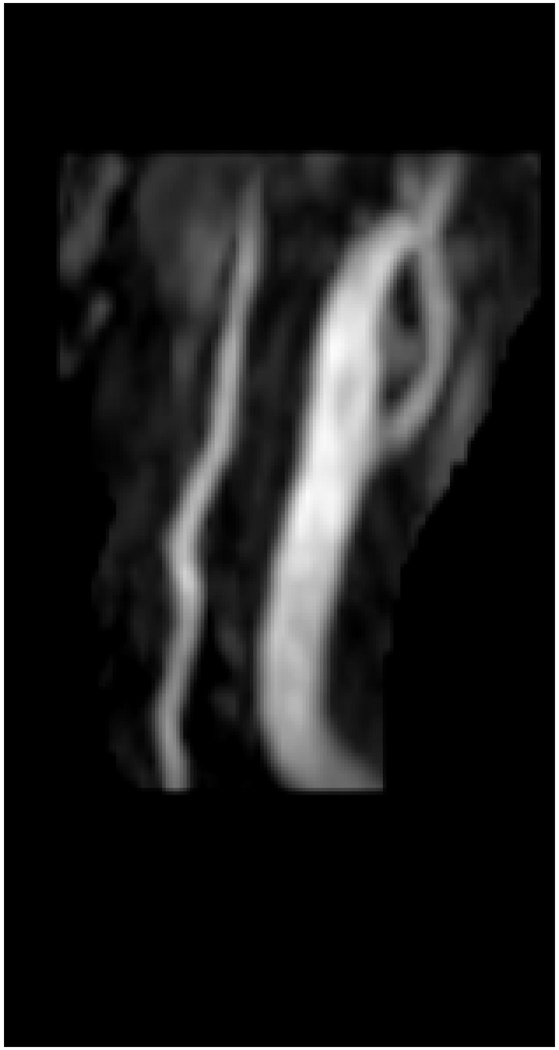

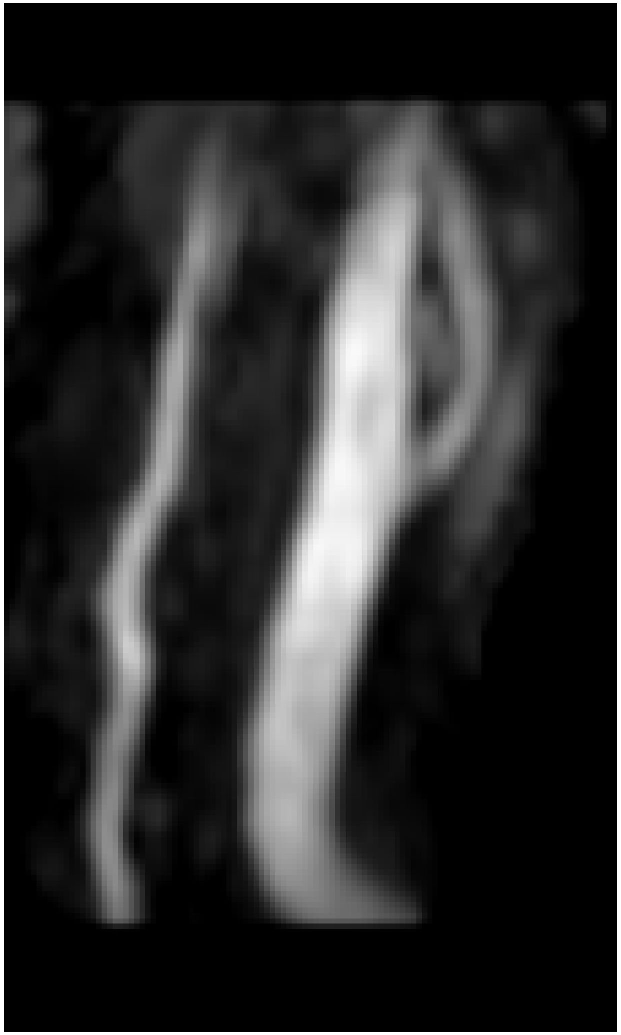

28b Score

0-30

31-50

51-70

>70

Near occlusion

Occluded

Quality

1

2

3

4

5

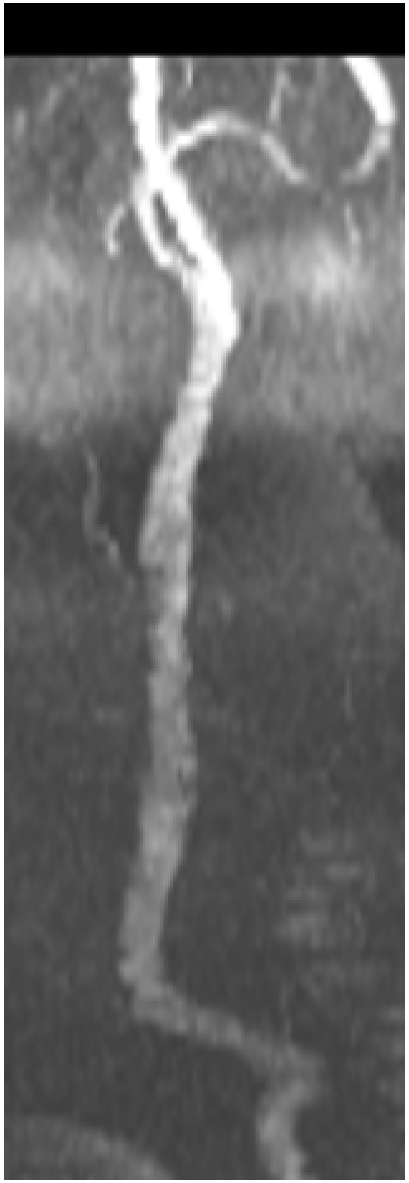

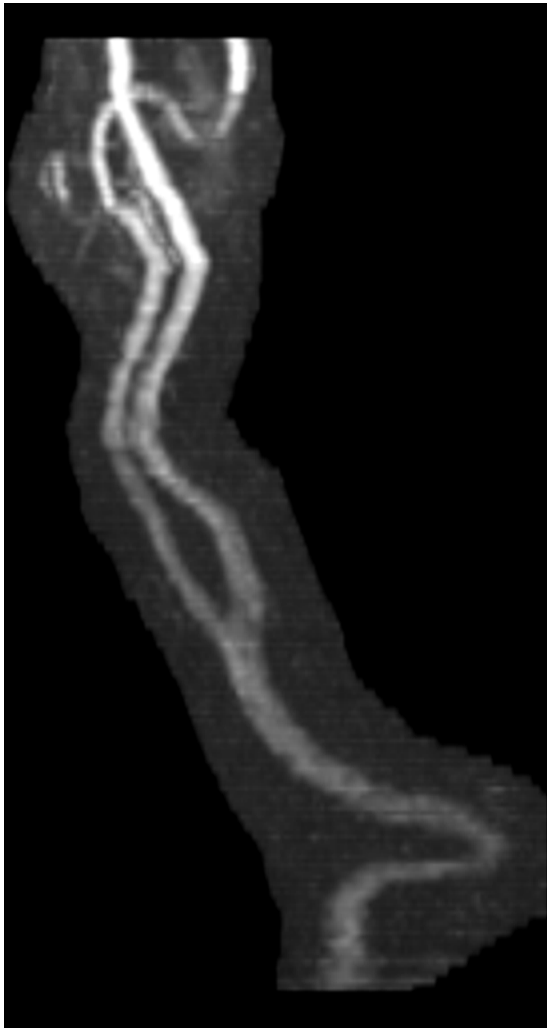

29a Score

0-30

31-50

51-70

>70

Near occlusion

Occluded

Quality

1

2

3

4

5

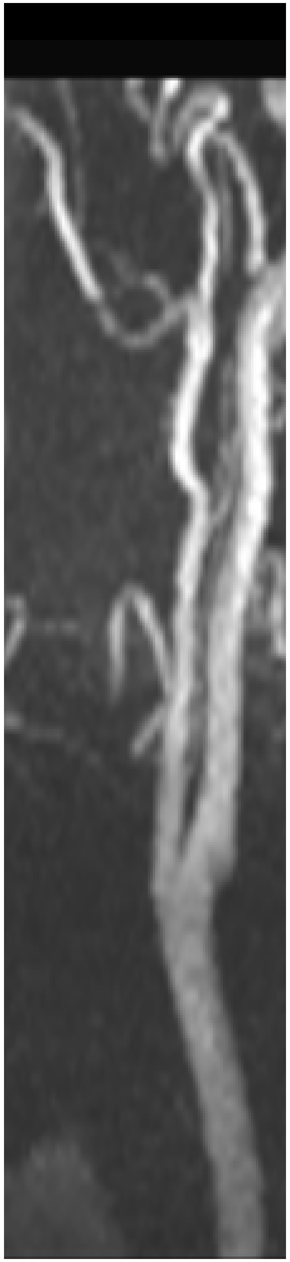

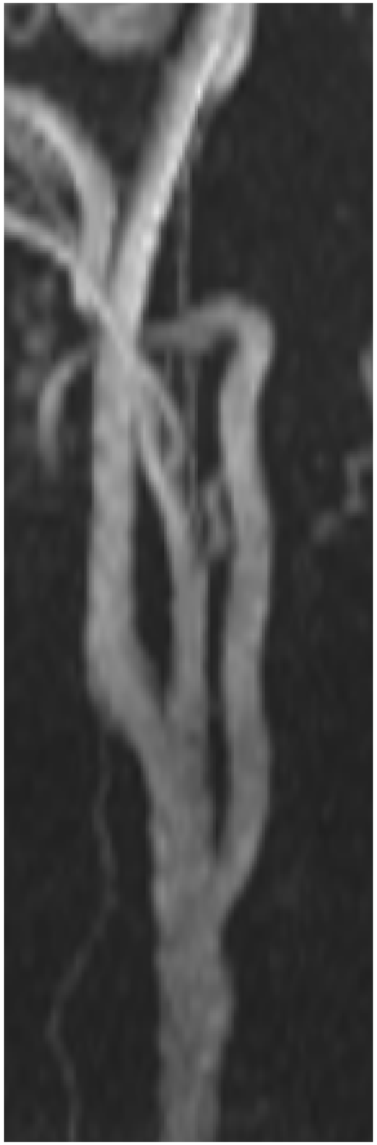

29f Score

0-30

31-50

51-70

>70

Near occlusion

Occluded

Quality

1

2

3

4

5

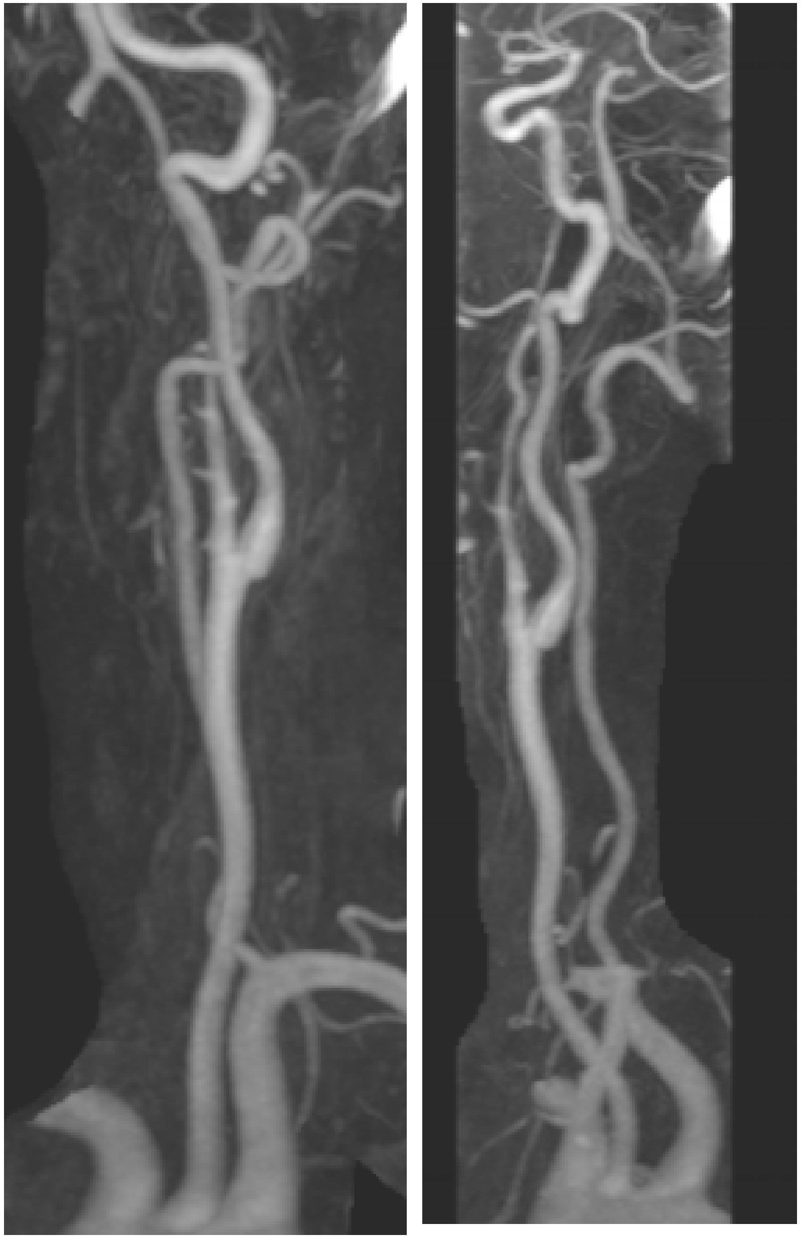

30e Score

0-30

31-50

51-70

>70

Near occlusion

Occluded

Quality

1

2

3

4

5

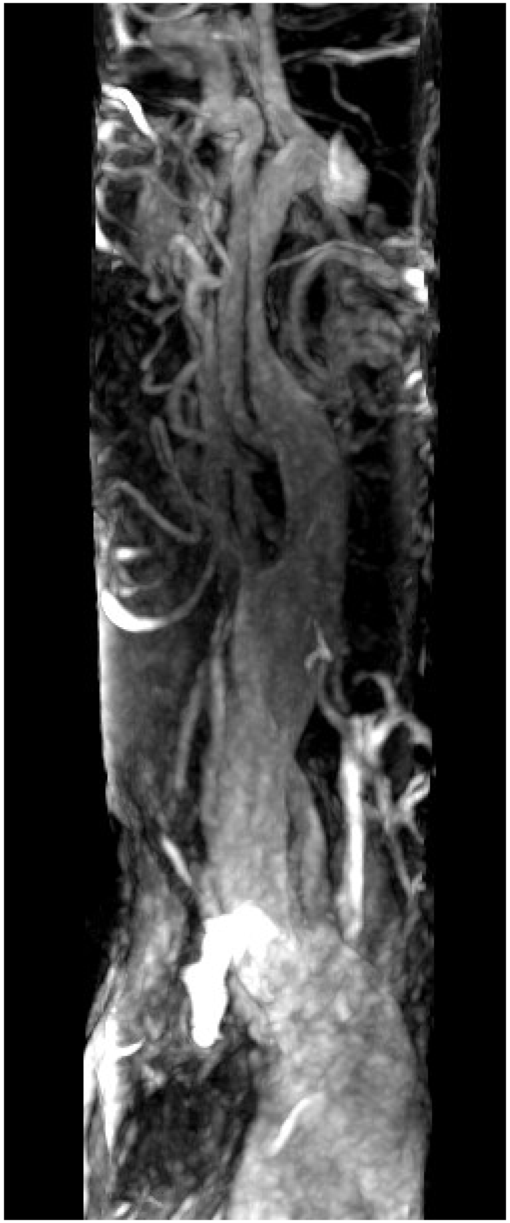

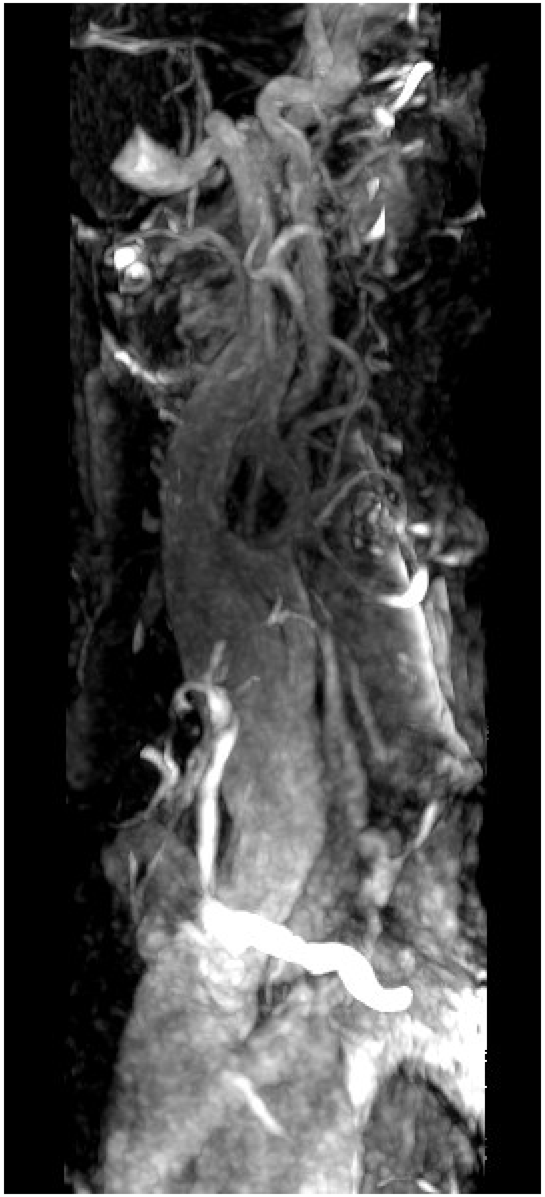

31d Score

0-30

31-50

51-70

>70

Near occlusion

Occluded

Quality

1

2

3

4

5

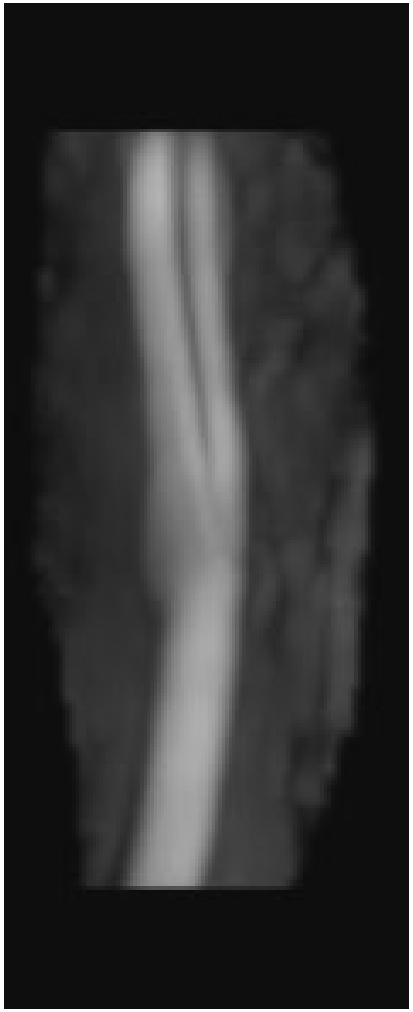

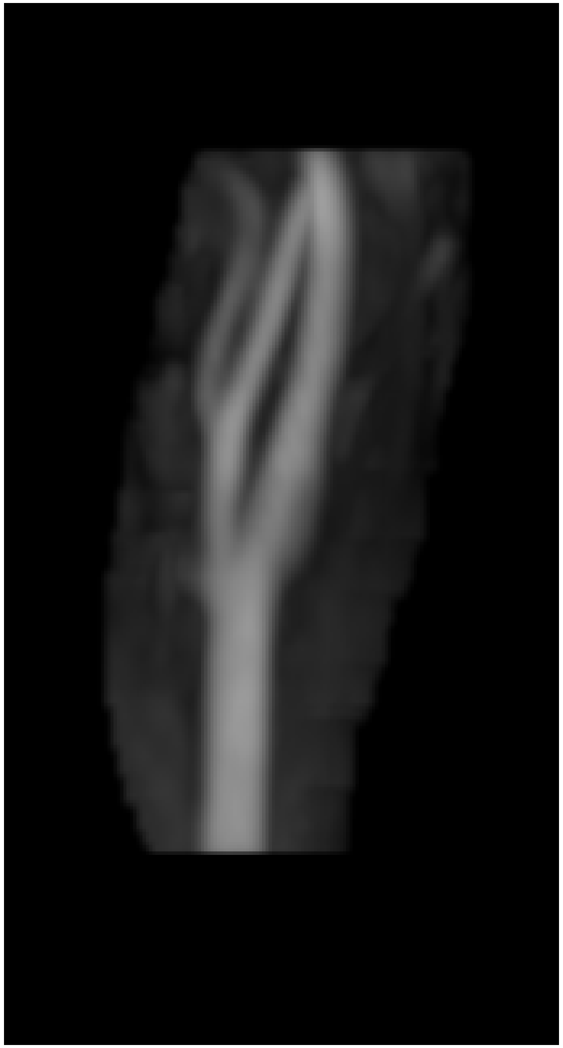

32c Score

0-30

31-50

51-70

>70

Near occlusion

Occluded

Quality

1

2

3

4

5

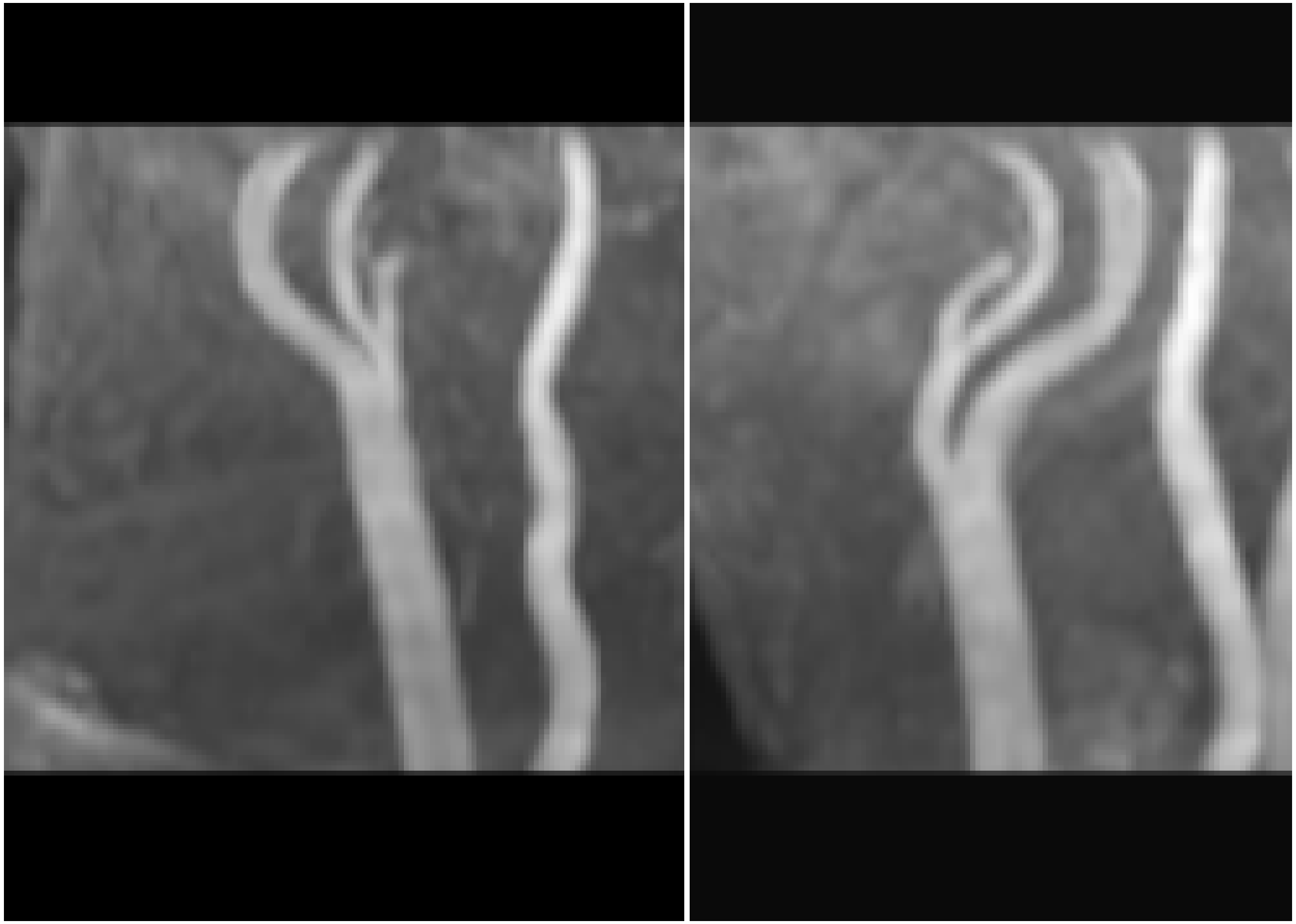

33b Score

0-30

31-50

51-70

>70

Near occlusion

Occluded

Quality

1

2

3

4

5

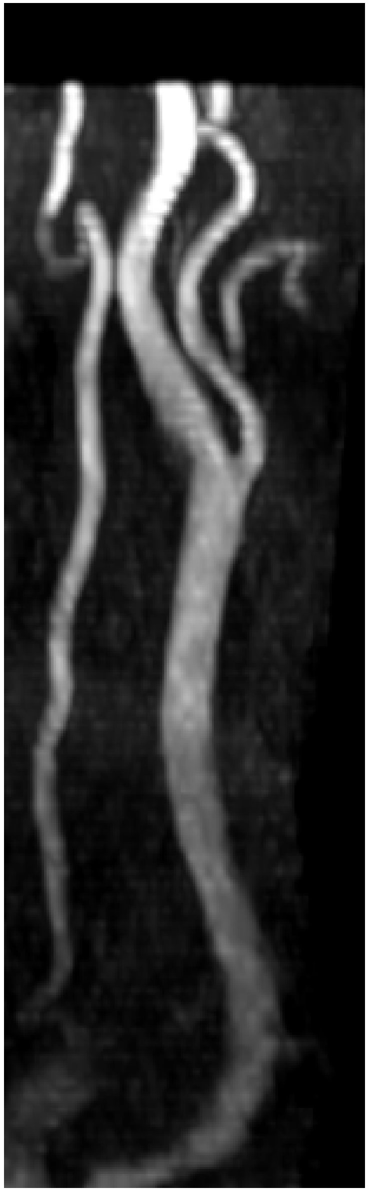

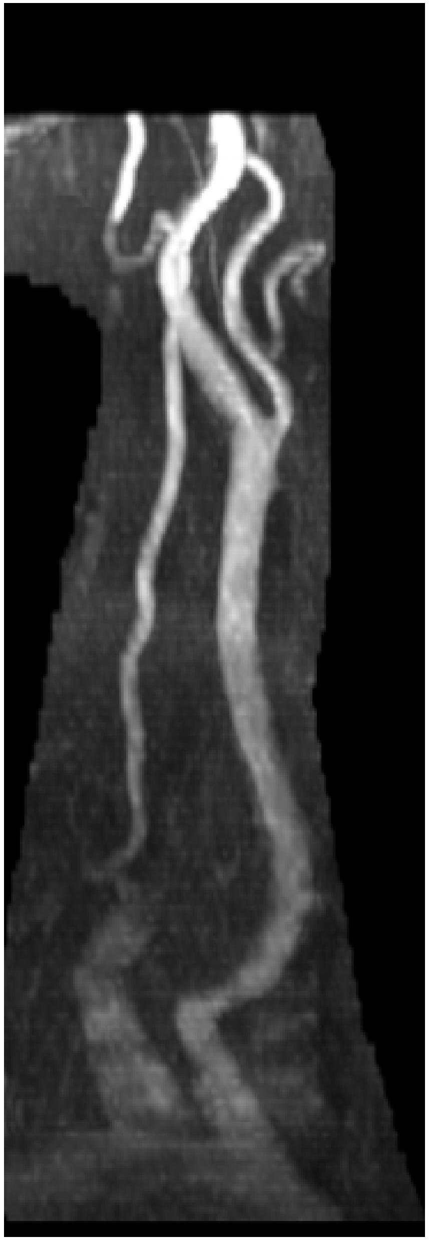

34a Score

0-30

31-50

51-70

>70

Near occlusion

Occluded

Quality

1

2

3

4

5

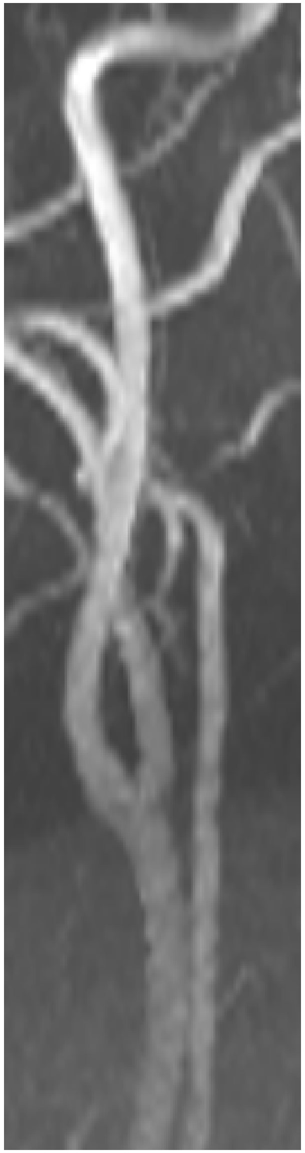

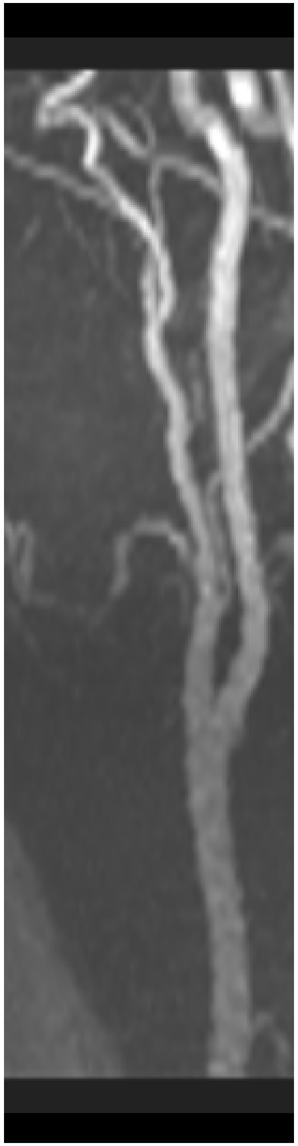

34f Score

0-30

31-50

51-70

>70

Near occlusion

Occluded

Quality

1

2

3

4

5

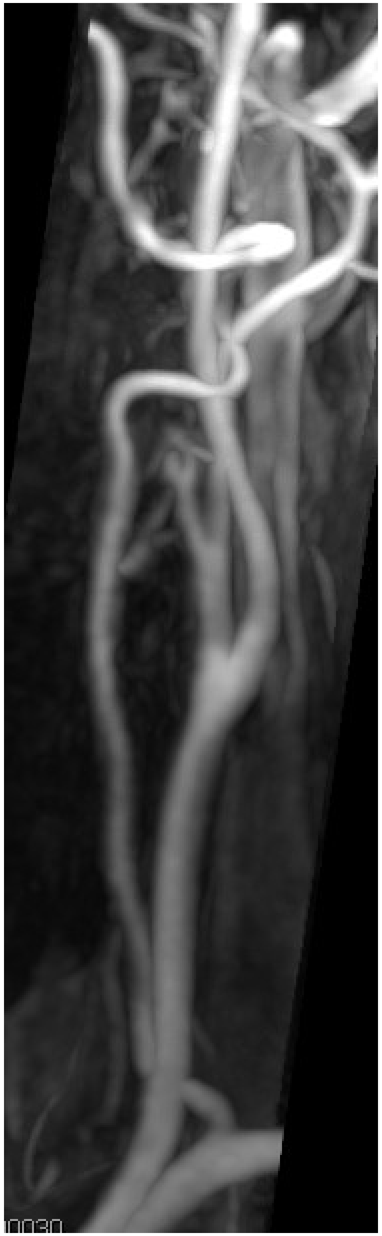

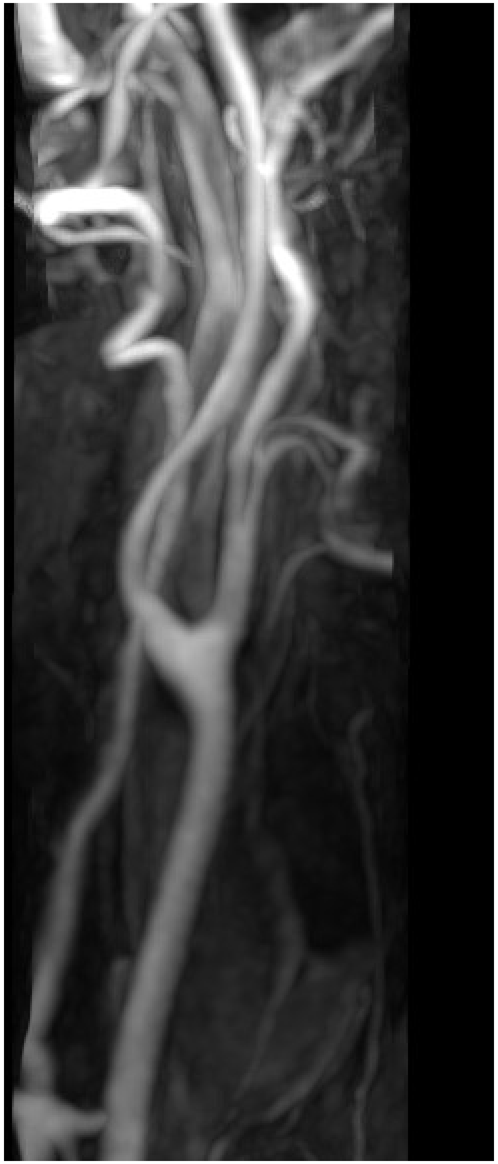

35e Score

0-30

31-50

51-70

>70

Near occlusion

Occluded

Quality

1

2

3

4

5

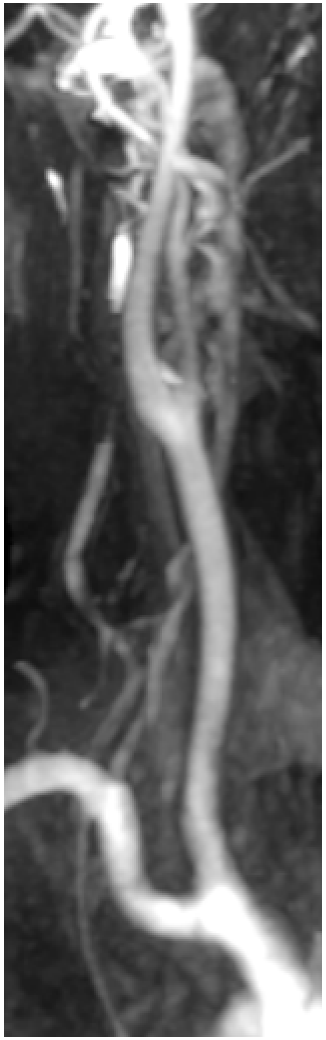

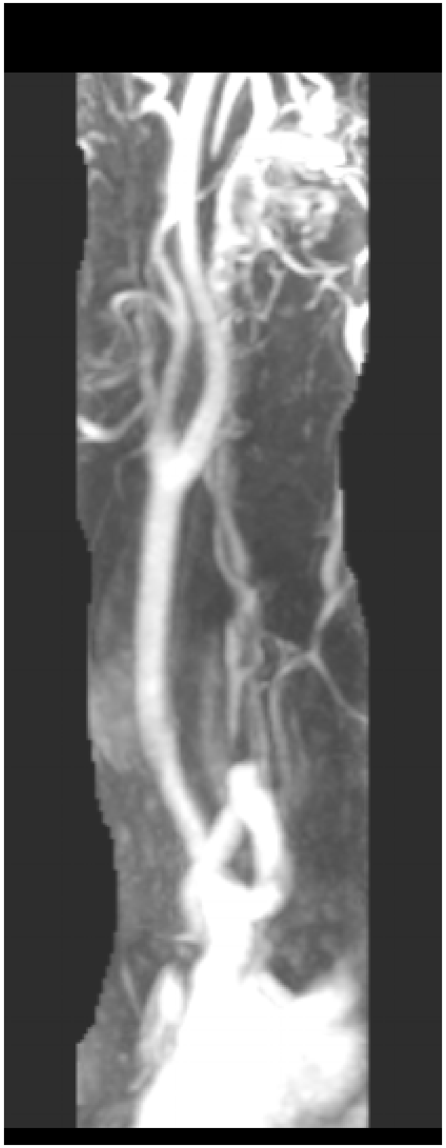

36d Score (LEFT)

0-30

31-50

51-70

>70

Near occlusion

Occluded

Quality

1

2

3

4

5

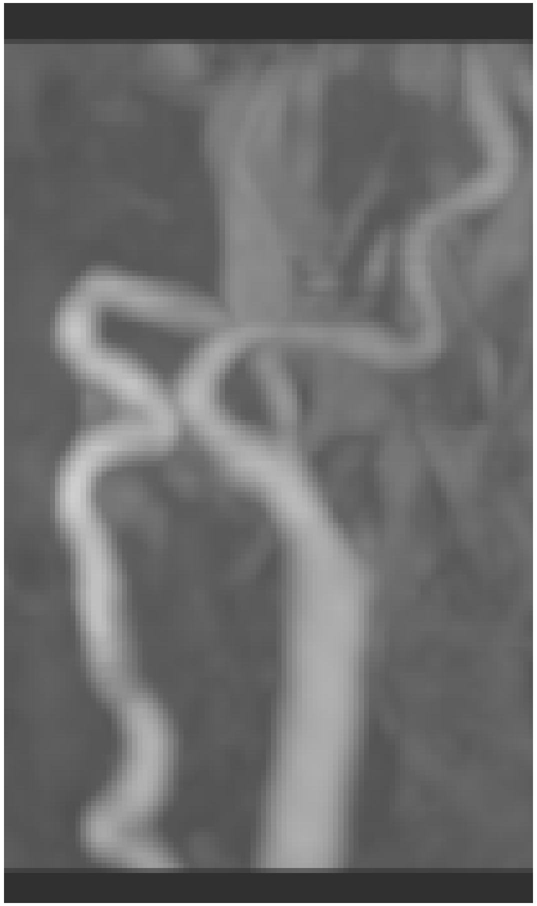

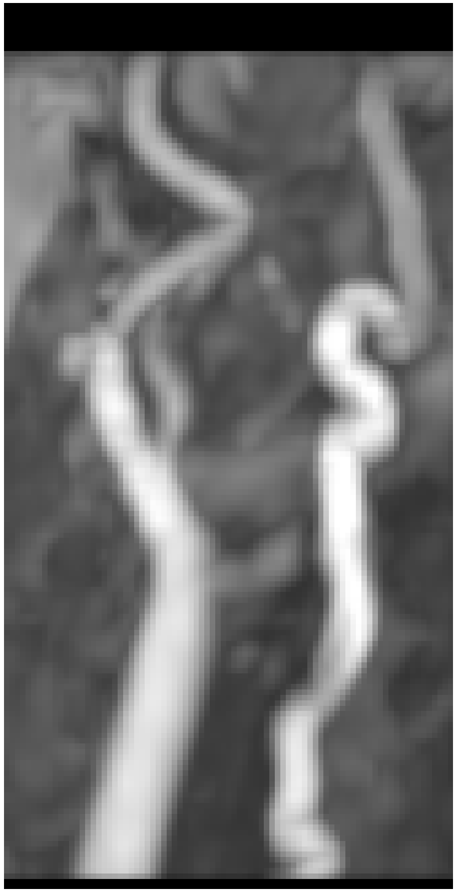

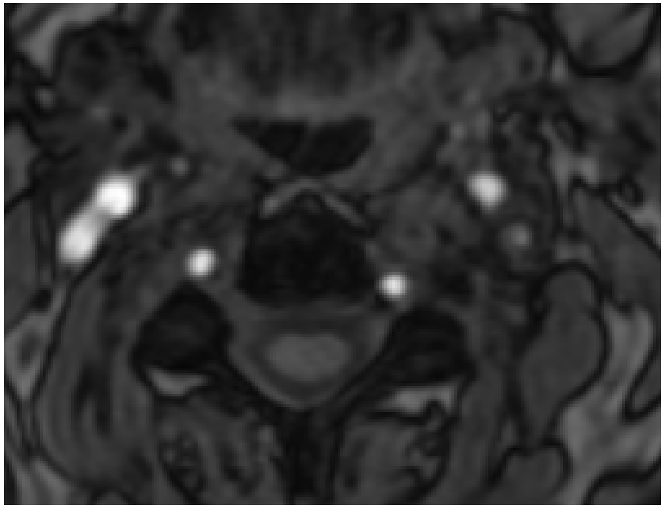

37c Score

0-30

31-50

51-70

>70

Near occlusion

Occluded

Quality

1

2

3

4

5

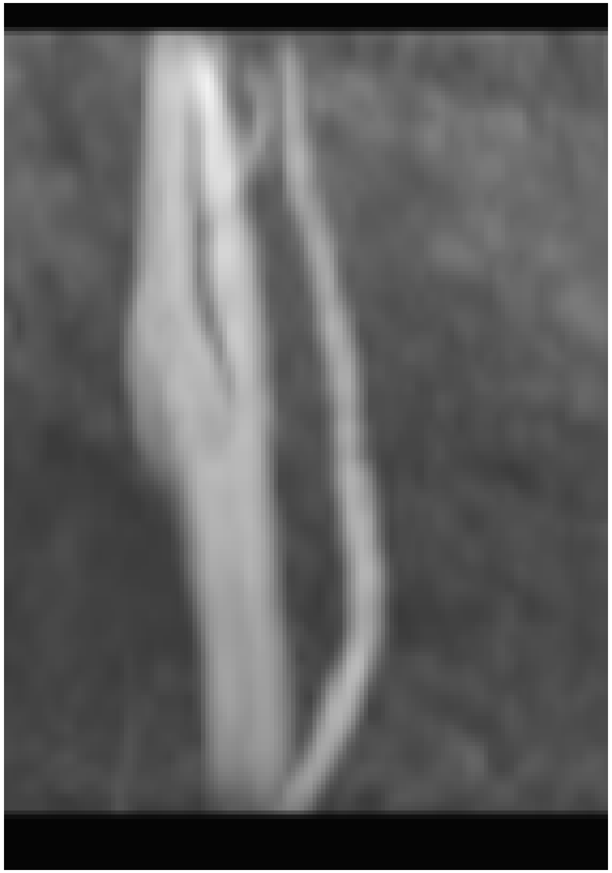

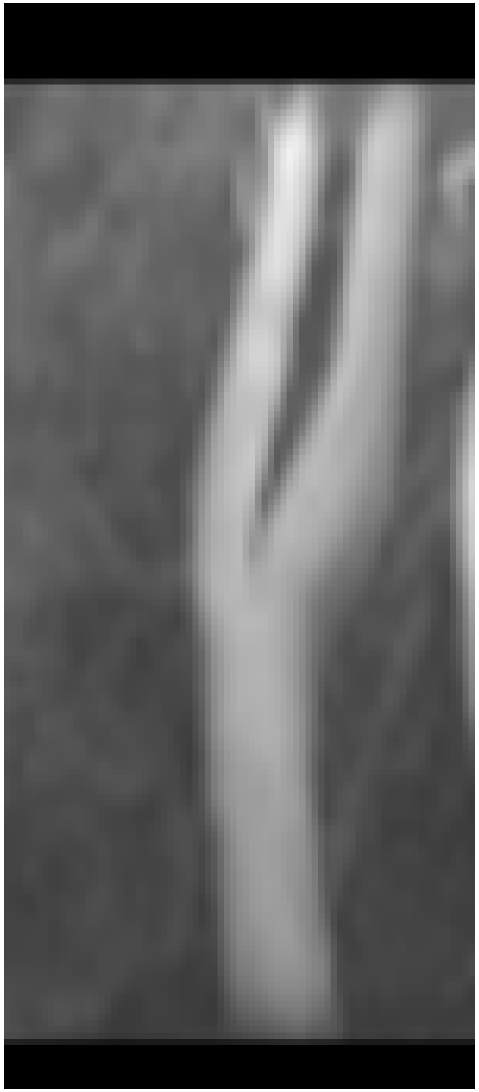

38b Score

0-30

31-50

51-70

>70

Near occlusion

Occluded

Quality

1

2

3

4

5

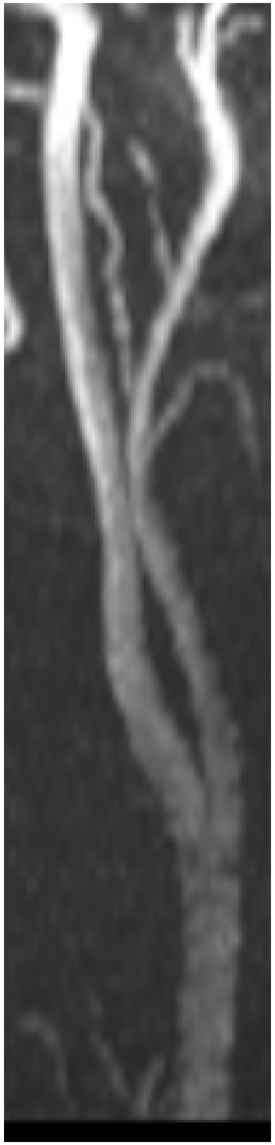

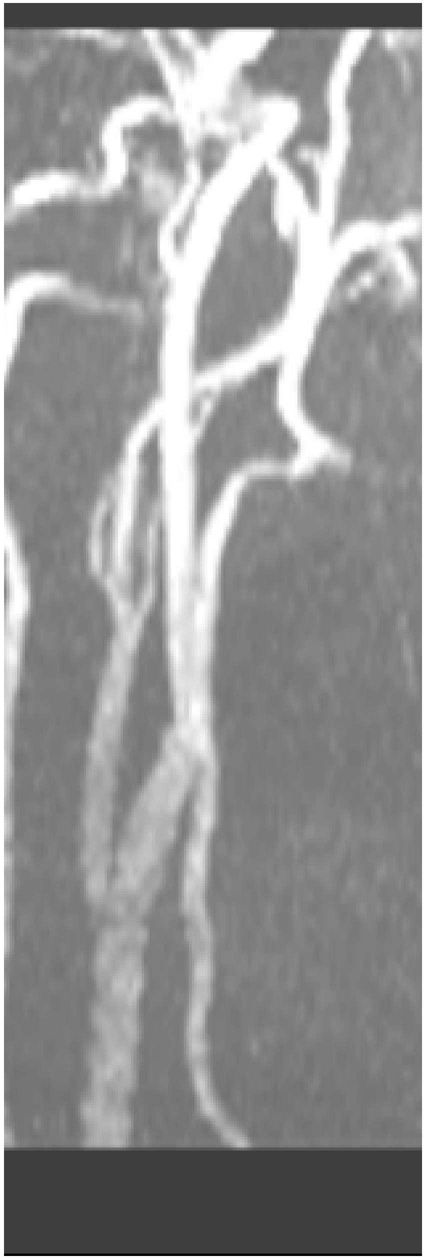

39a Score

0-30

31-50

51-70

>70

Near occlusion

Occluded

Quality

1

2

3

4

5

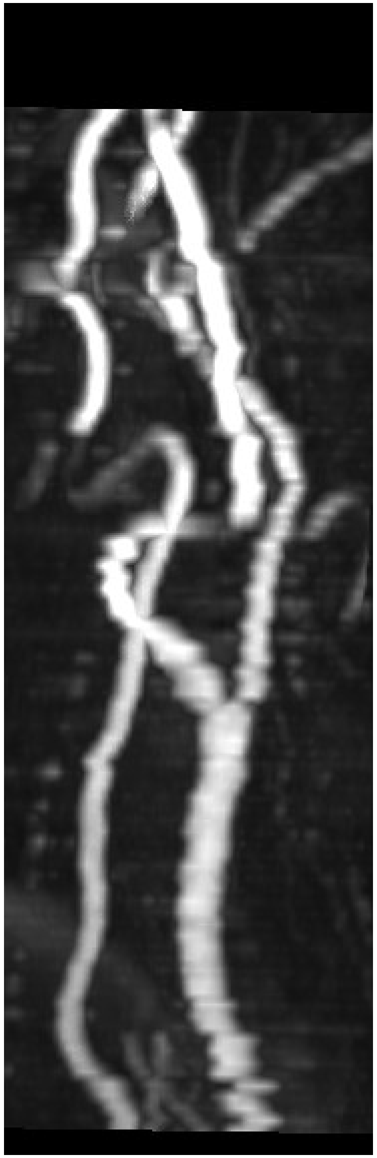

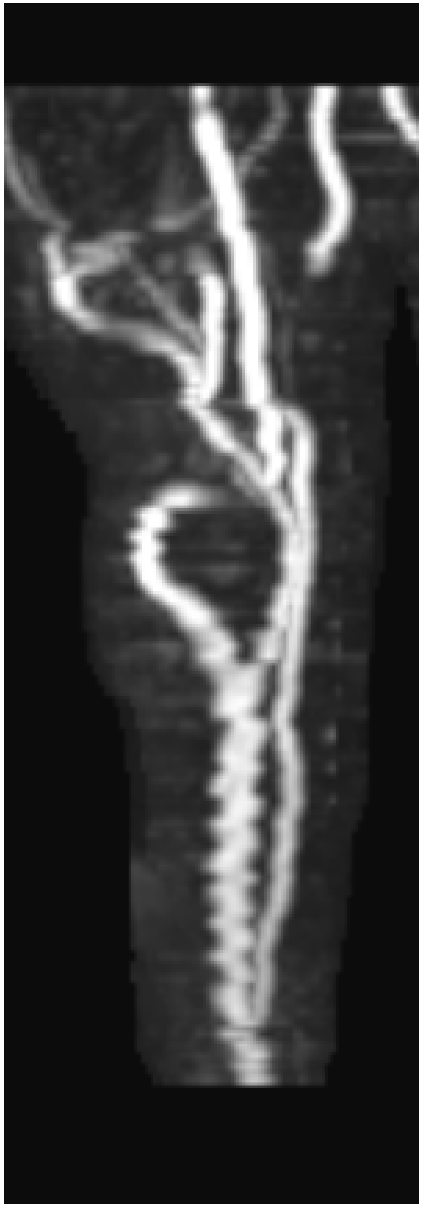

39f Score

0-30

31-50

51-70

>70

Near occlusion

Occluded

Quality

1

2

3

4

5

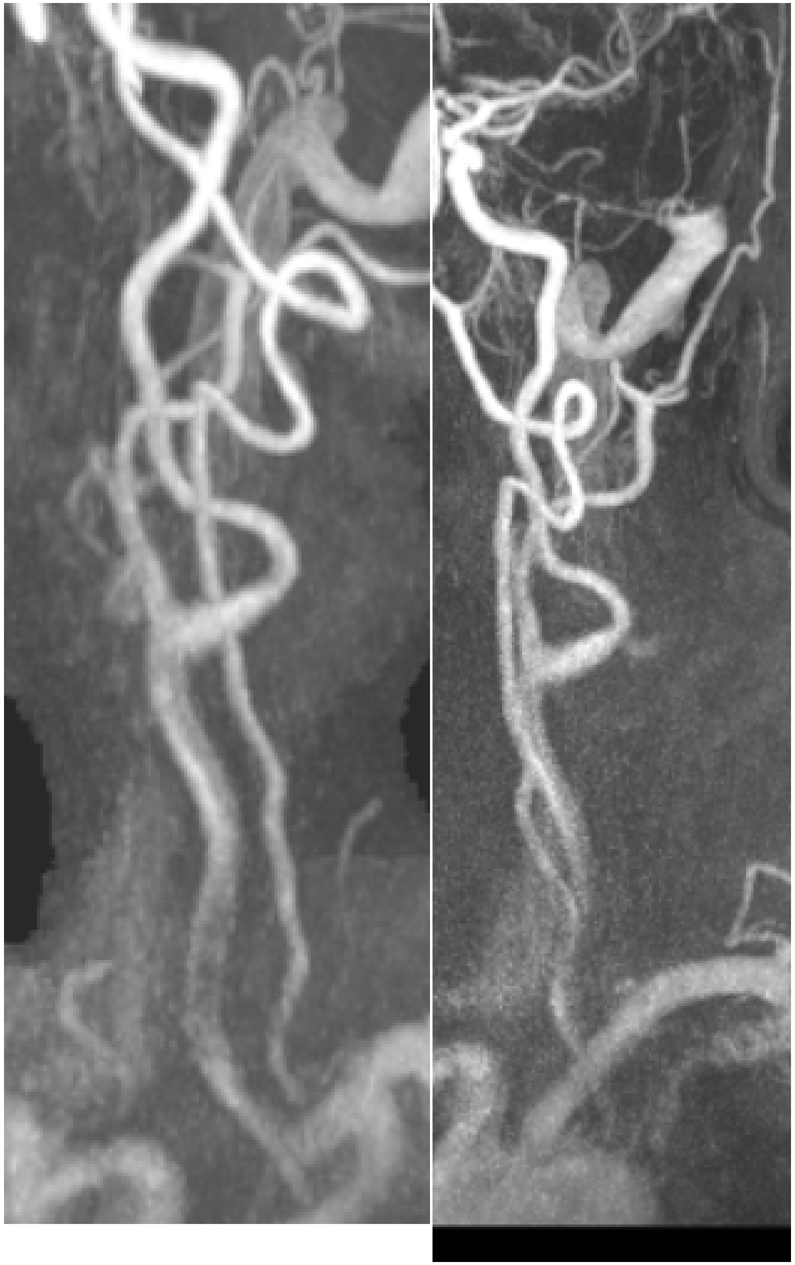

40e Score

0-30

31-50

51-70

>70

Near occlusion

Occluded

Quality

1

2

3

4

5

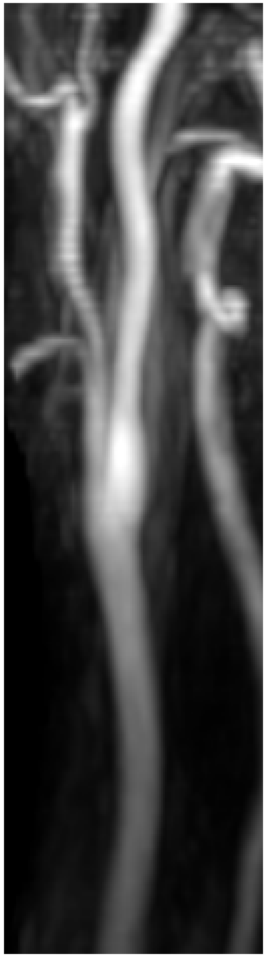

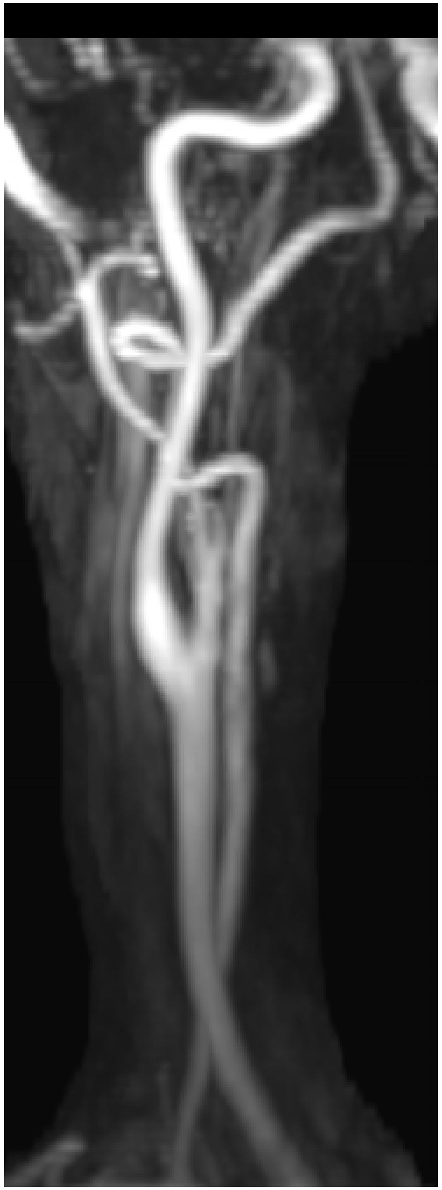

41d Score

0-30

31-50

51-70

>70

Near occlusion

Occluded

Quality

1

2

3

4

5

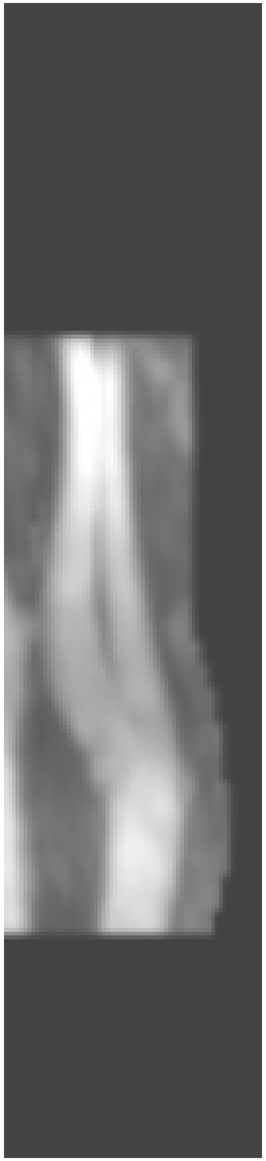

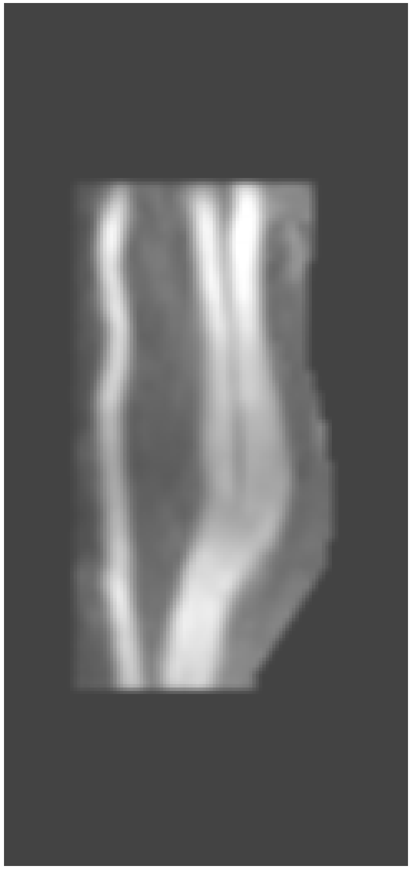

42c Score

0-30

31-50

51-70

>70

Near occlusion

Occluded

Quality

1

2

3

4

5

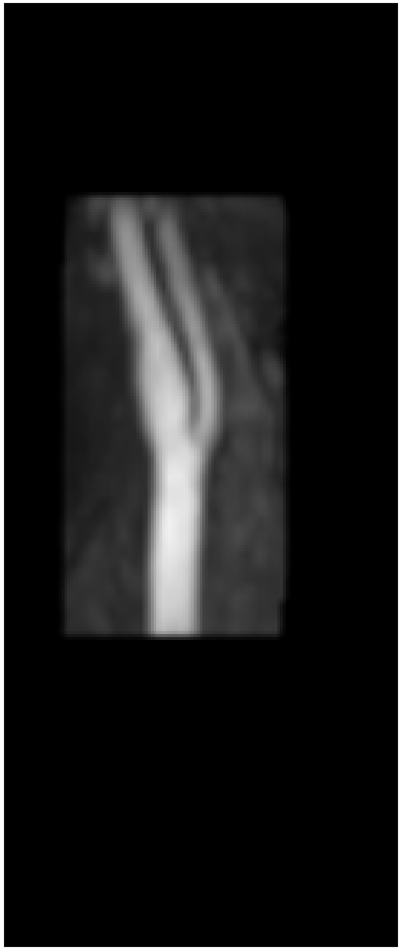

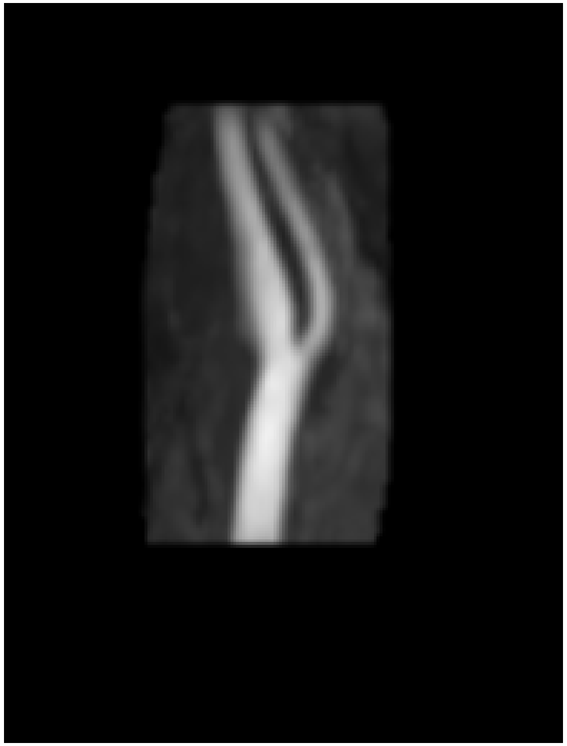

43b Score

0-30

31-50

51-70

>70

Near occlusion

Occluded

Quality

1

2

3

4

5

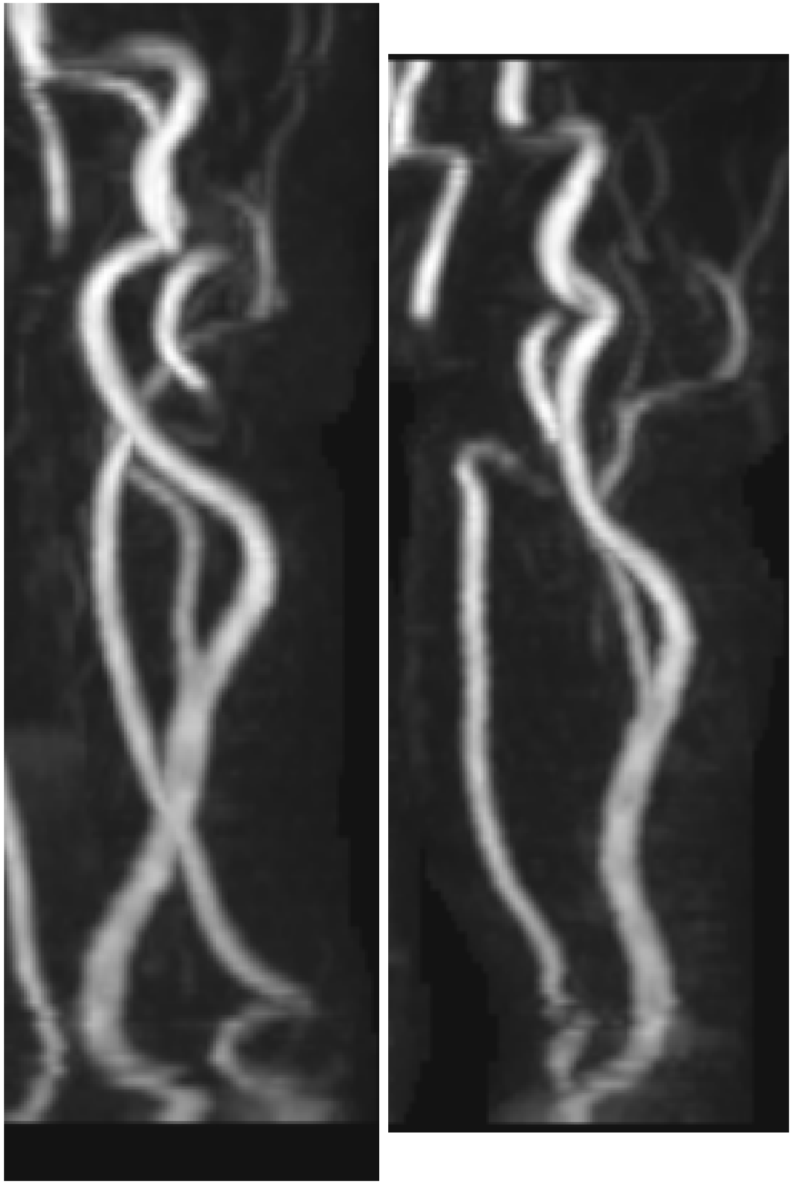

44a Score

0-30

31-50

51-70

>70

Near occlusion

Occluded

Quality

1

2

3

4

5

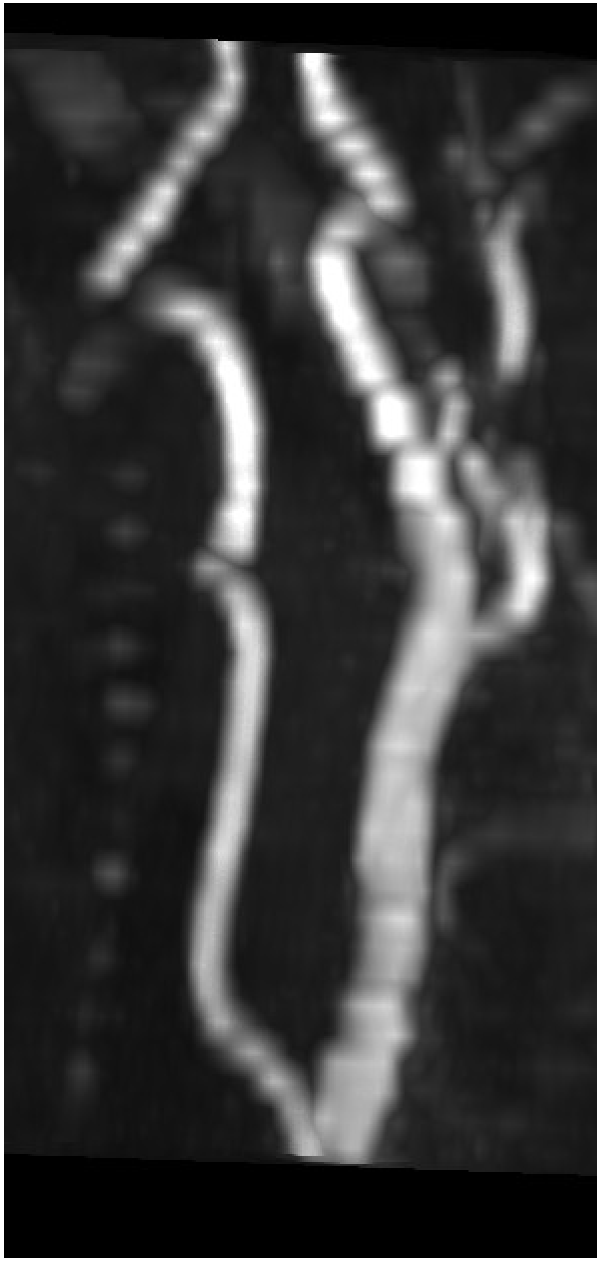

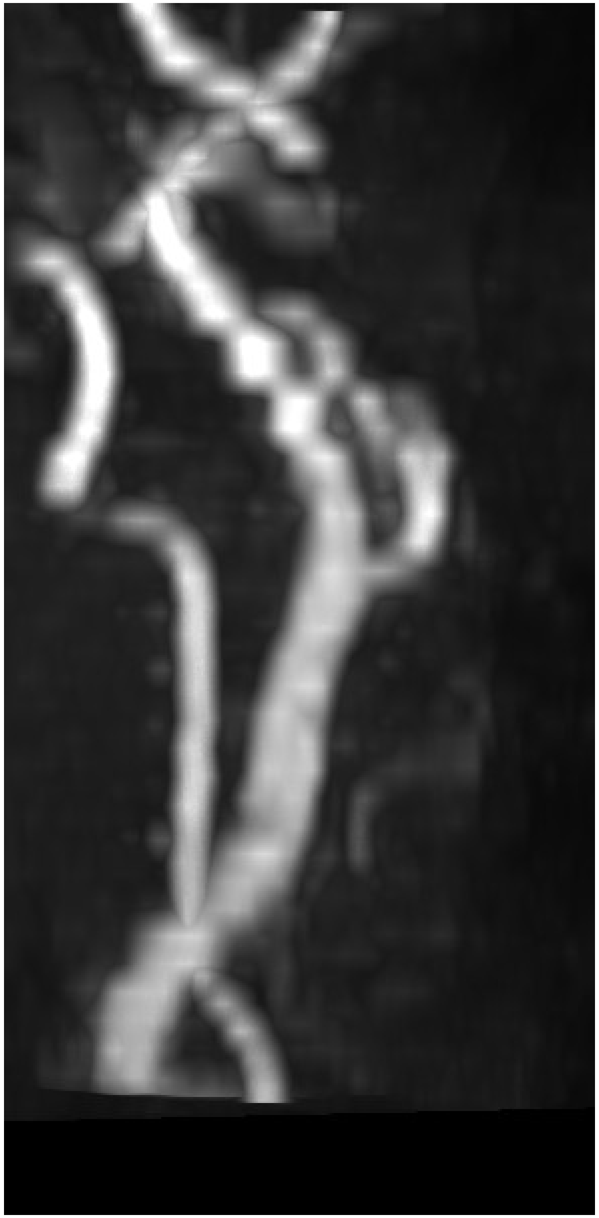

44f Score

0-30

31-50

51-70

>70

Near occlusion

Occluded

Quality

1

2

3

4

5

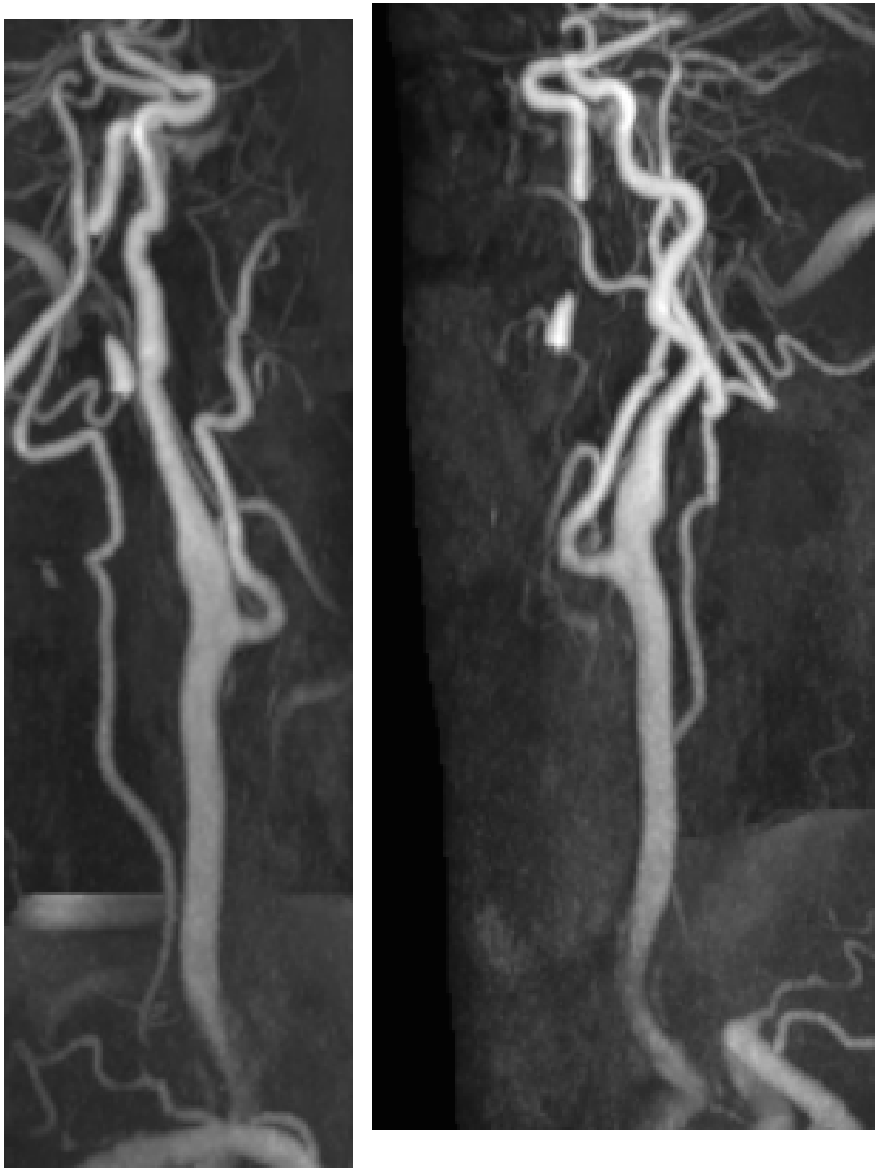

45e Score

0-30

31-50

51-70

>70

Near occlusion

Occluded

Quality

1

2

3

4

5

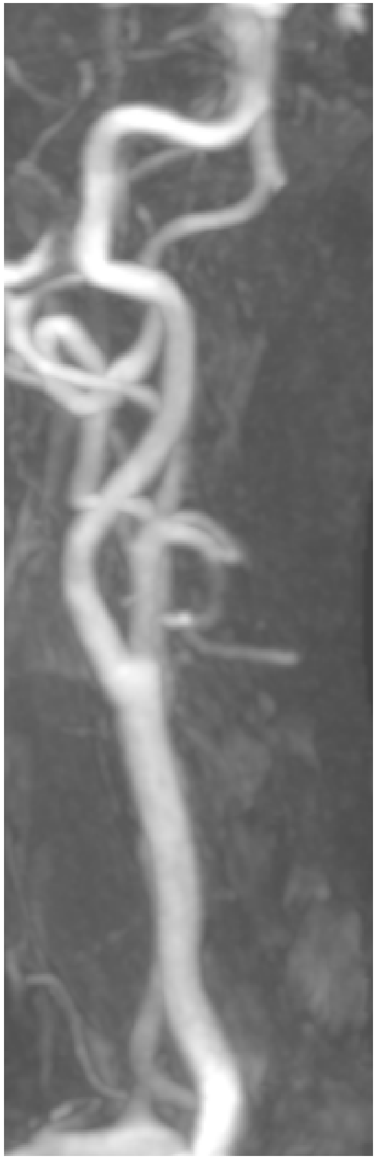

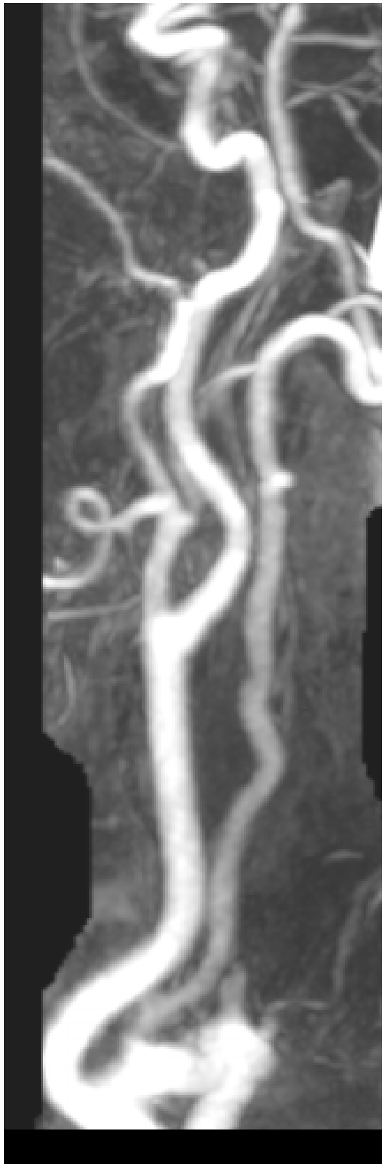

46d Score

0-30

31-50

51-70

>70

Near occlusion

Occluded

Quality

1

2

3

4

5

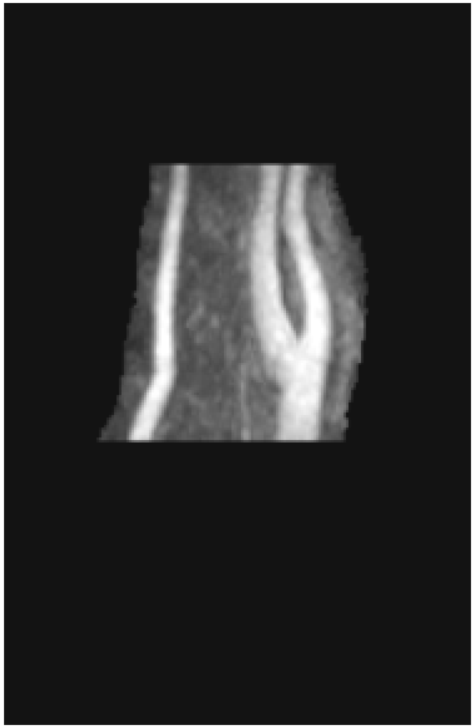

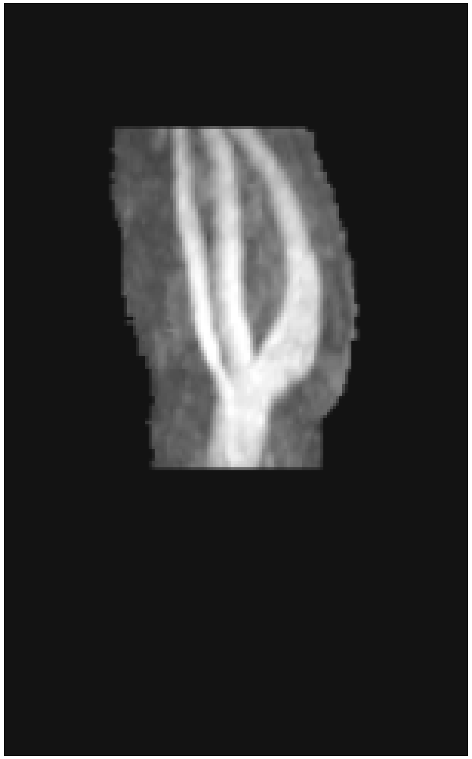

47c Score

0-30

31-50

51-70

>70

Near occlusion

Occluded

Quality

1

2

3

4

5

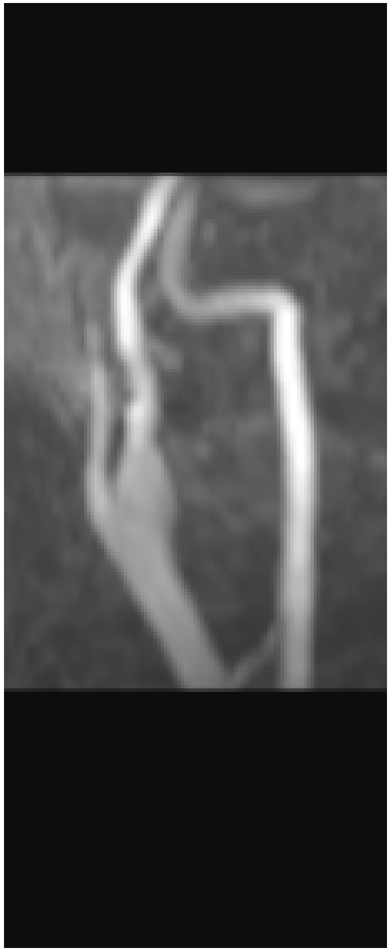

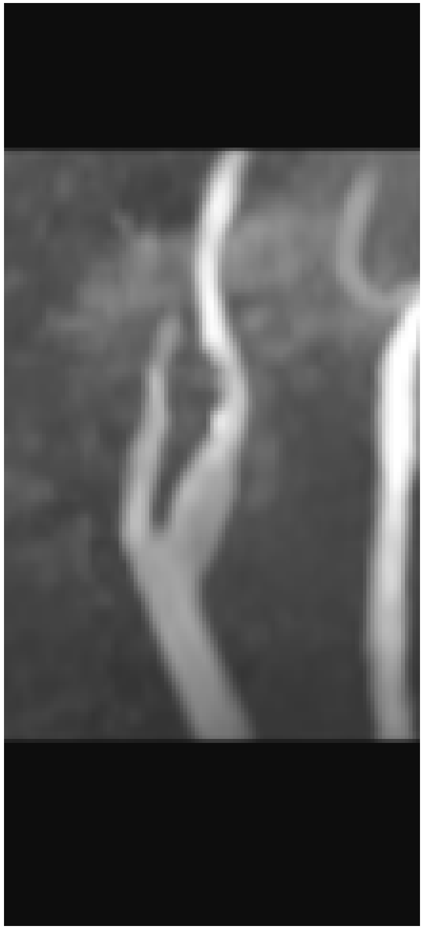

48b Score

0-30

31-50

51-70

>70

Near occlusion

Occluded

Quality

1

2

3

4

5

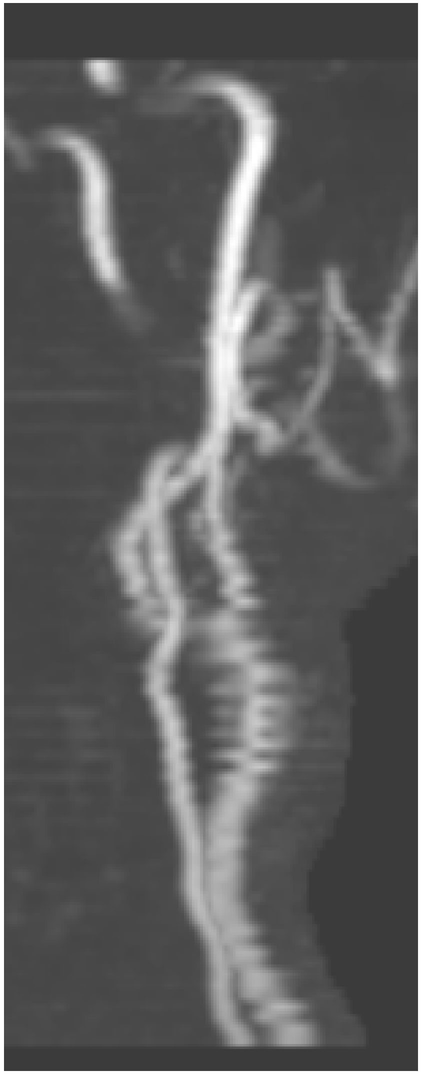

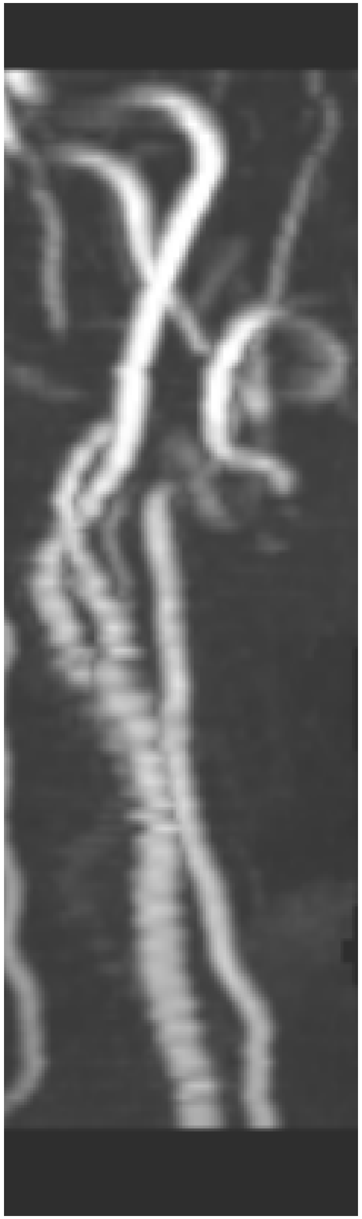

49a Score

0-30

31-50

51-70

>70

Near occlusion

Occluded

Quality

1

2

3

4

5

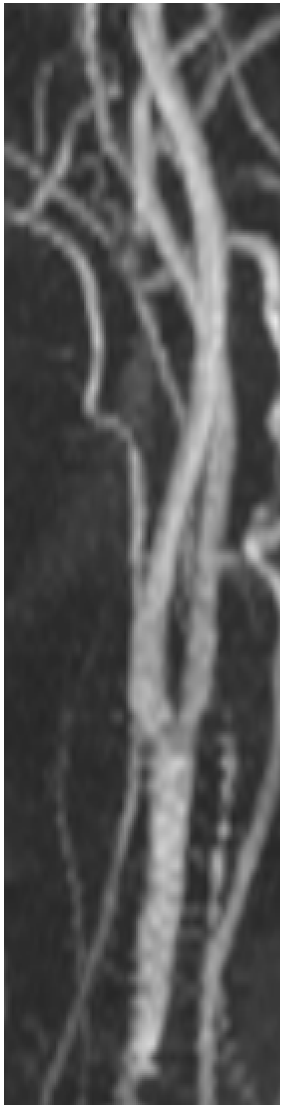

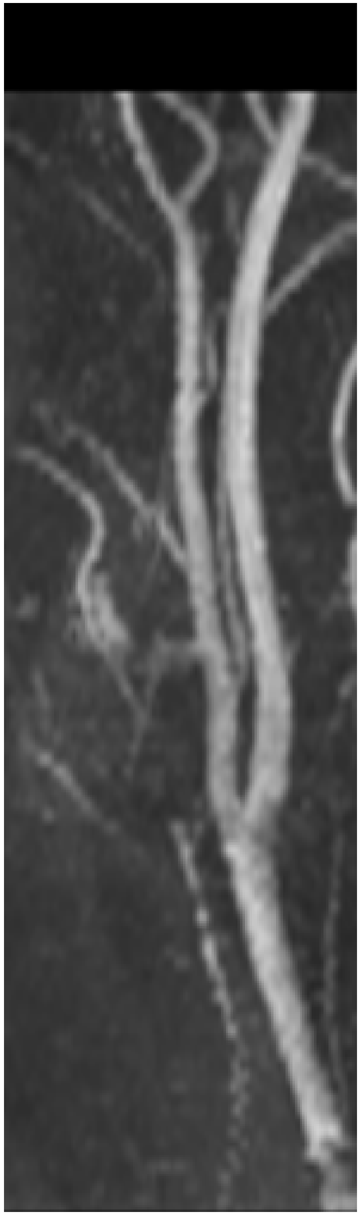

49 Score

0-30

31-50

51-70

>70

Near occlusion

Occluded

Quality

1

2

3

4

5

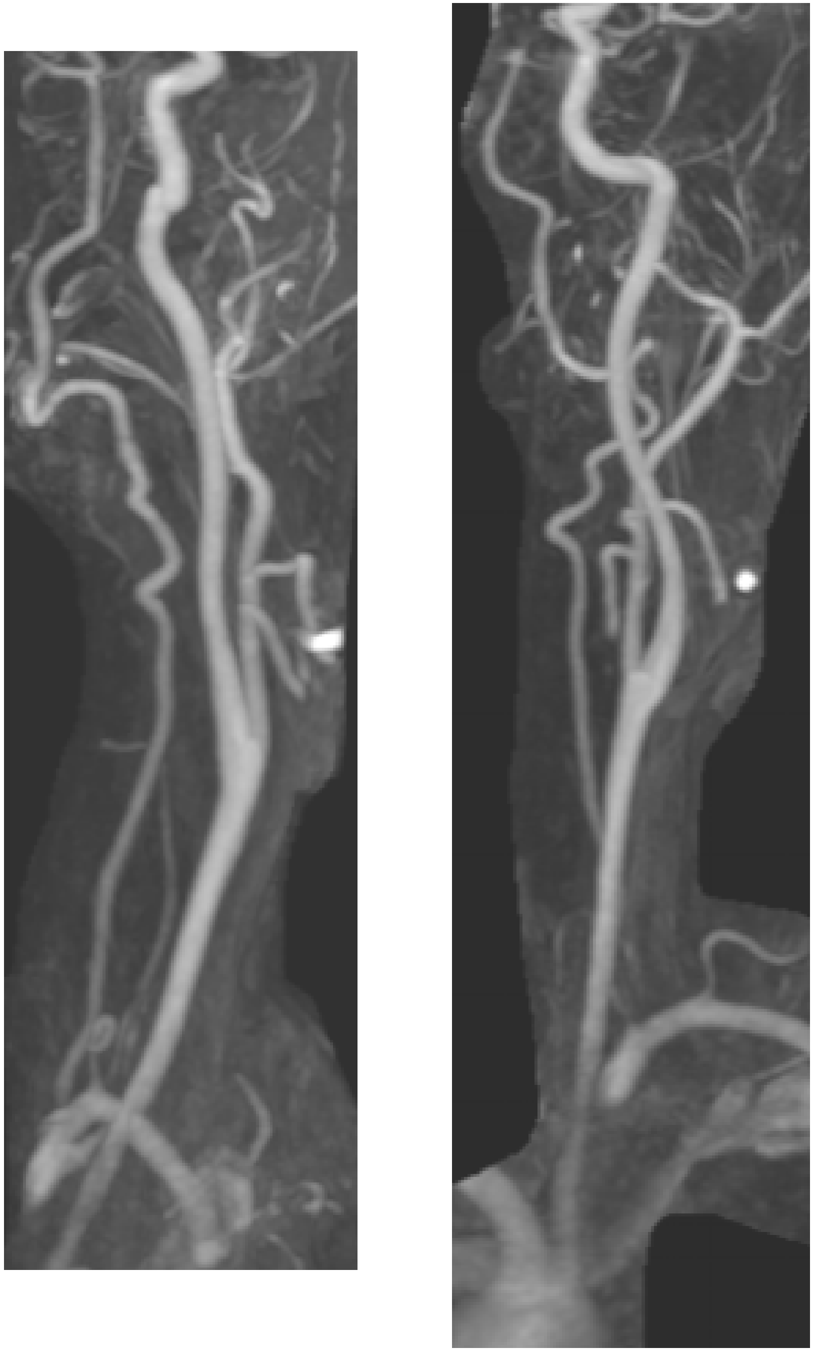

50e Score

0-30

31-50

51-70

>70

Near occlusion

Occluded

Quality

1

2

3

4

5

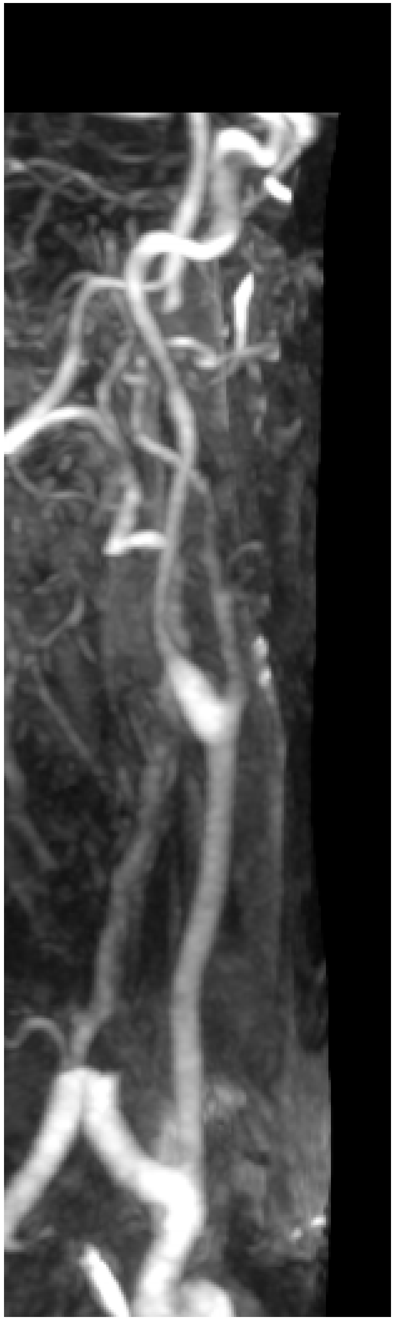

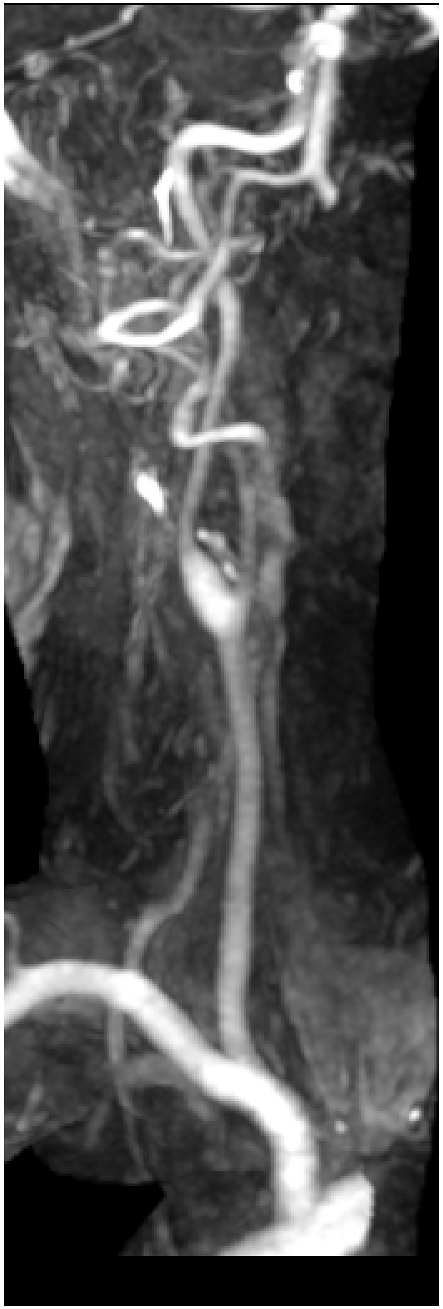

51d Score

0-30

31-50

51-70

>70

Near occlusion

Occluded

Quality

1

2

3

4

5

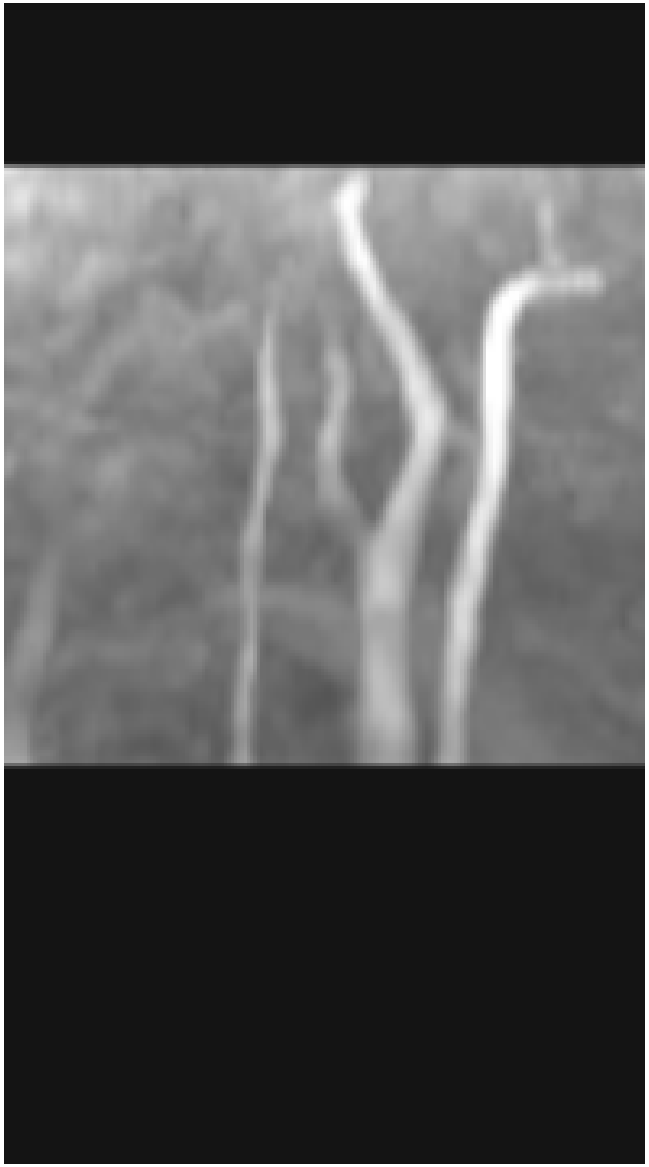

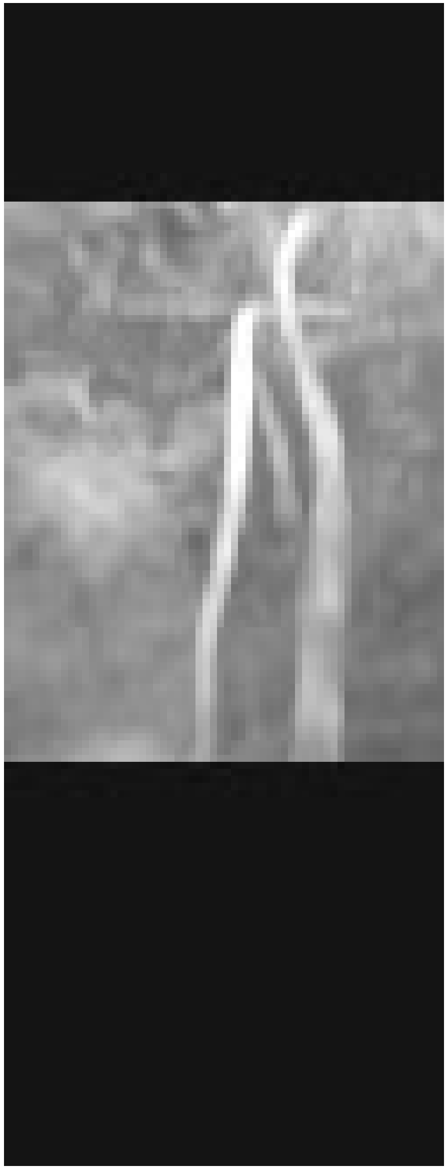

52c Score

0-30

31-50

51-70

>70

Near occlusion

Occluded

Quality

1

2

3

4

5

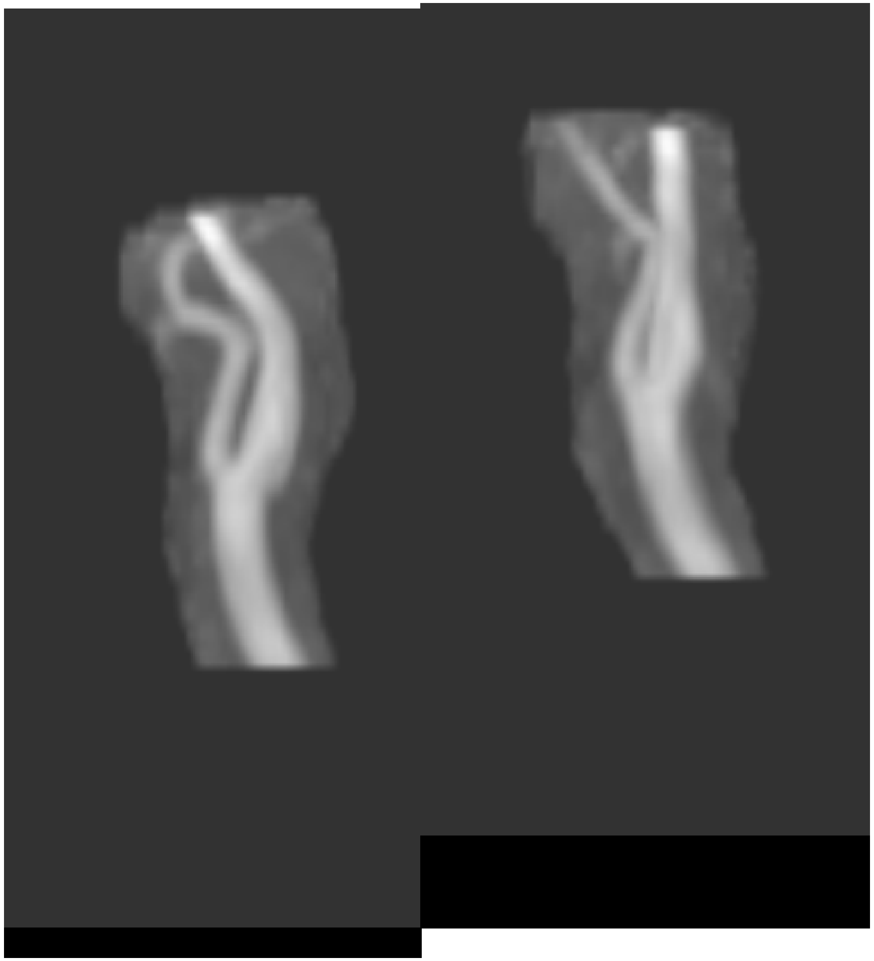

53b Score

0-30

31-50

51-70

>70

Near occlusion

Occluded

Quality

1

2

3

4

5

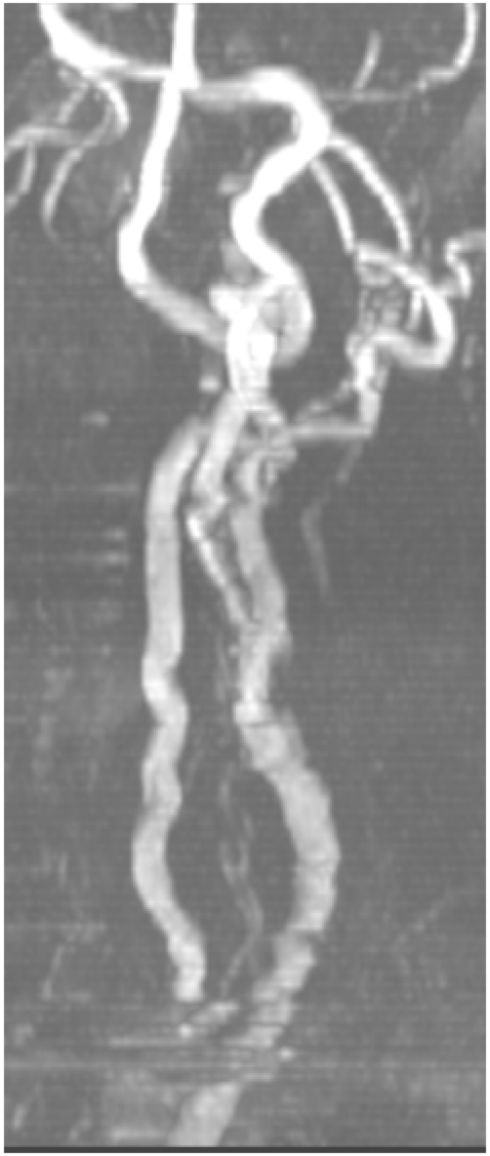

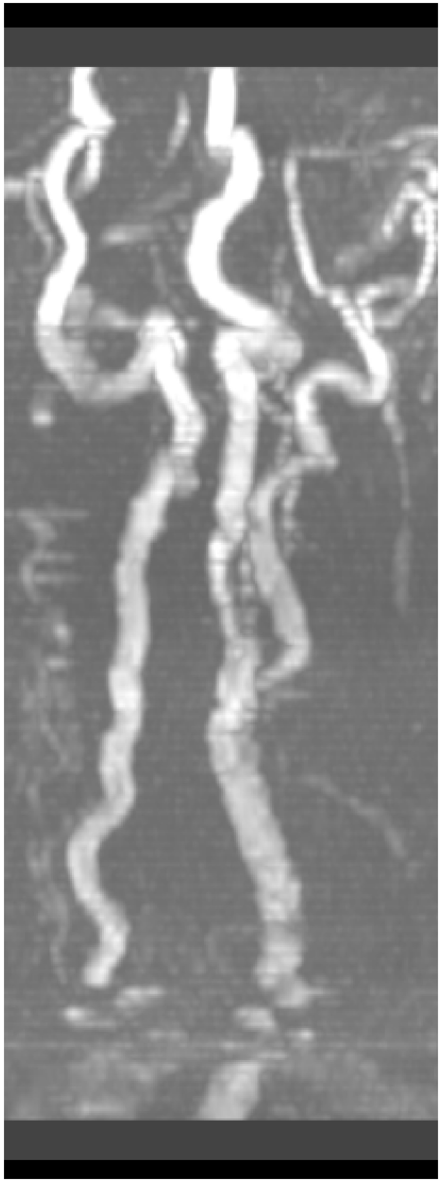

54a Score

0-30

31-50

51-70

>70

Near occlusion

Occluded

Quality

1

2

3

4

5

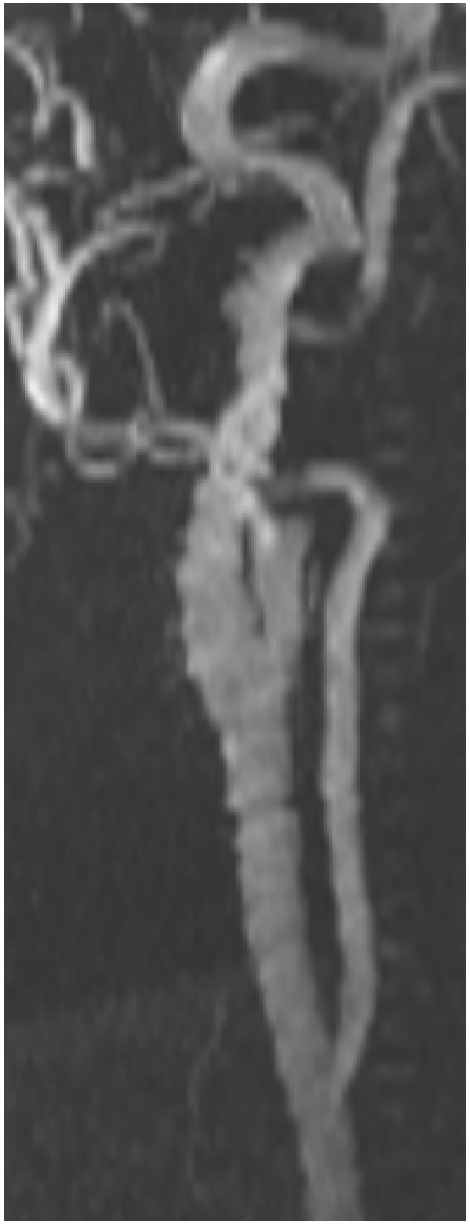

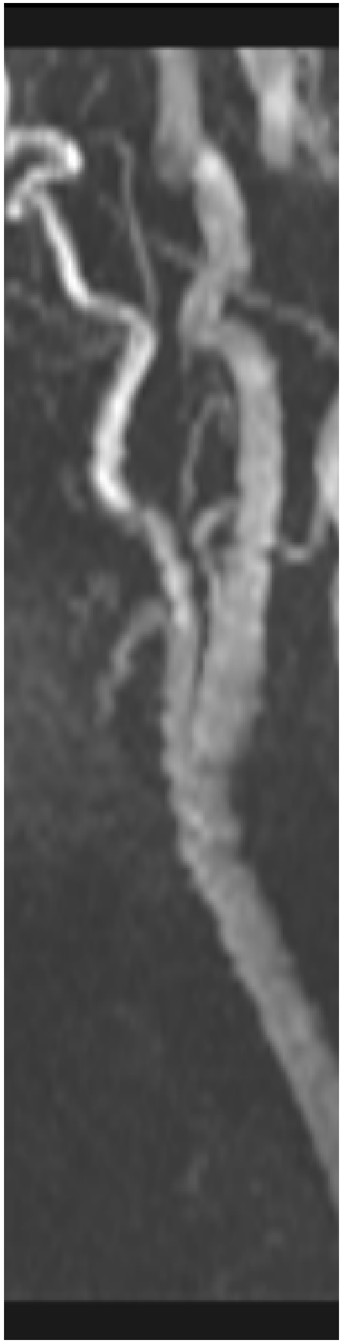

54f Score

0-30

31-50

51-70

>70

Near occlusion

Occluded

Quality

1

2

3

4

5

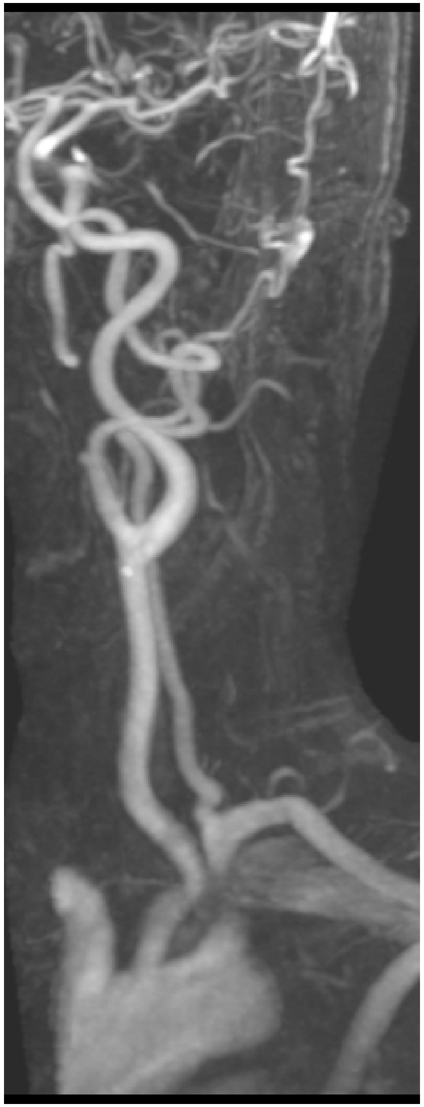

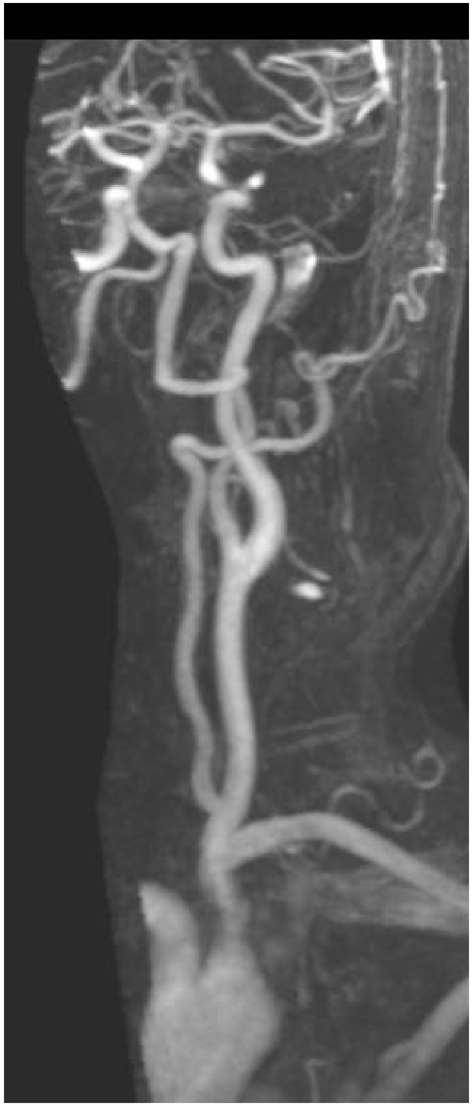

55e Score

0-30

31-50

51-70

>70

Near occlusion

Occluded

Quality

1

2

3

4

5

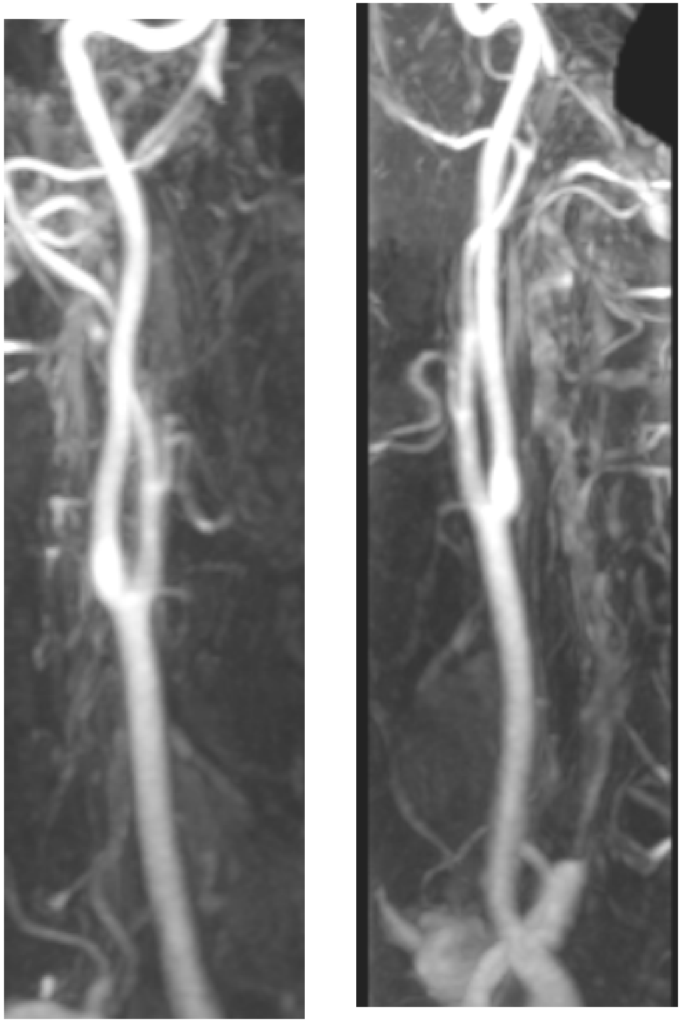

56d Score

0-30

31-50

51-70

>70

Near occlusion

Occluded

Quality

1

2

3

4

5

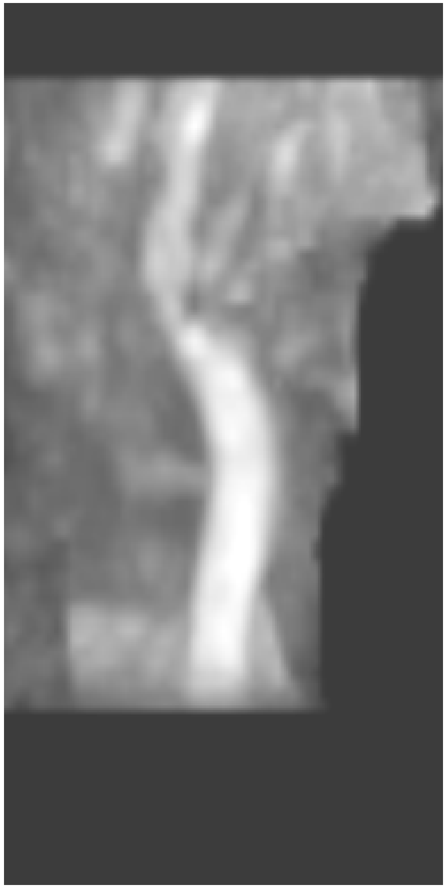

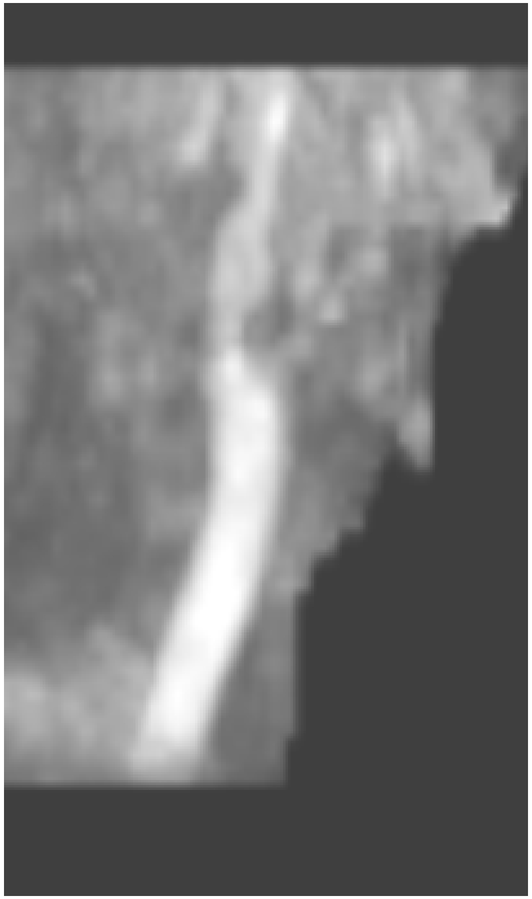

57c Score

0-30

31-50

51-70

>70

Near occlusion

Occluded

Quality

1

2

3

4

5

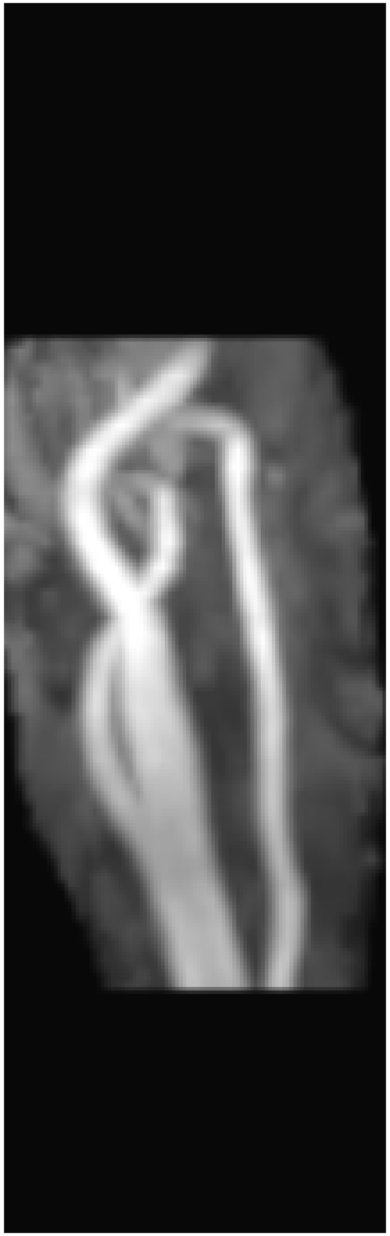

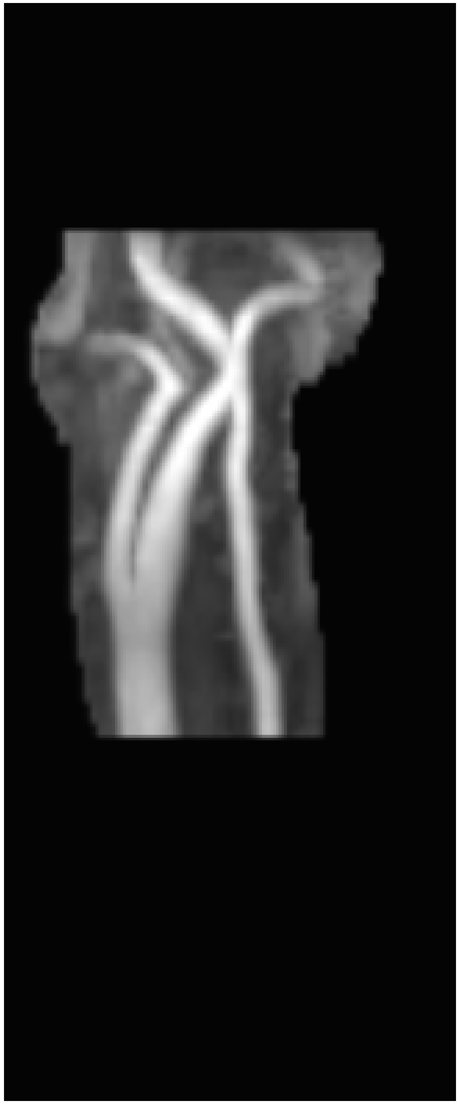

58b Score

0-30

31-50

51-70

>70

Near occlusion

Occluded

Quality

1

2

3

4

5

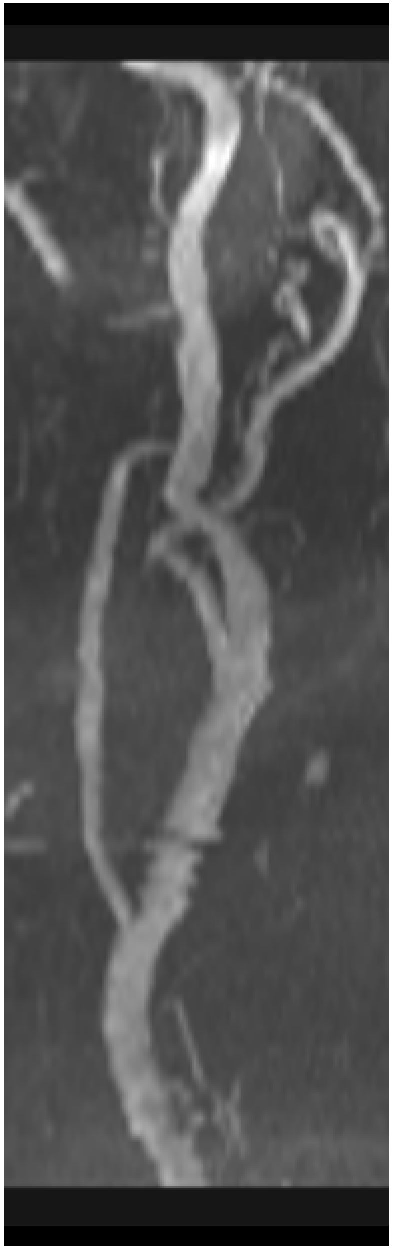

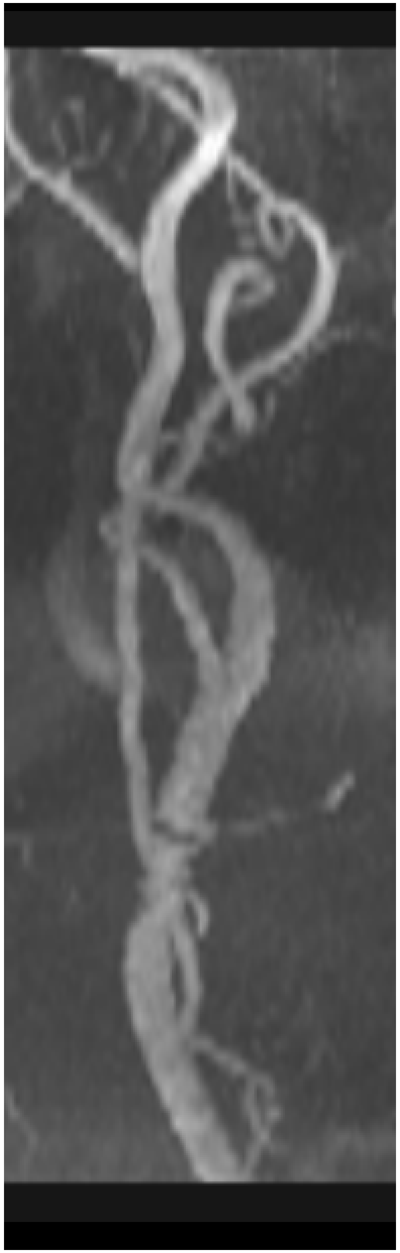

59a Score

0-30

31-50

51-70

>70

Near occlusion

Occluded

Quality

1

2

3

4

5

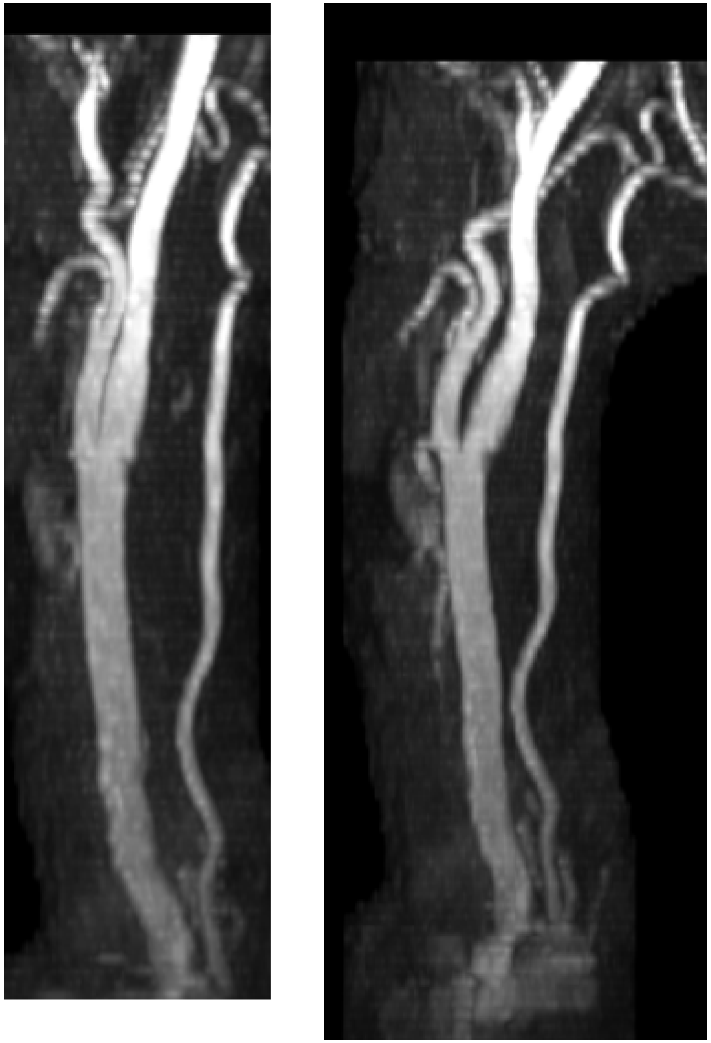

59f Score

0-30

31-50

51-70

>70

Near occlusion

Occluded

Quality

1

2

3

4

5

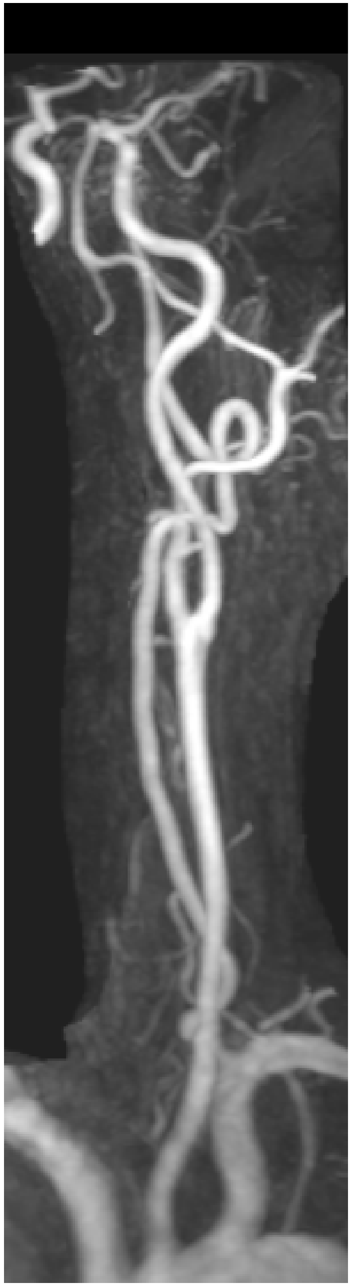

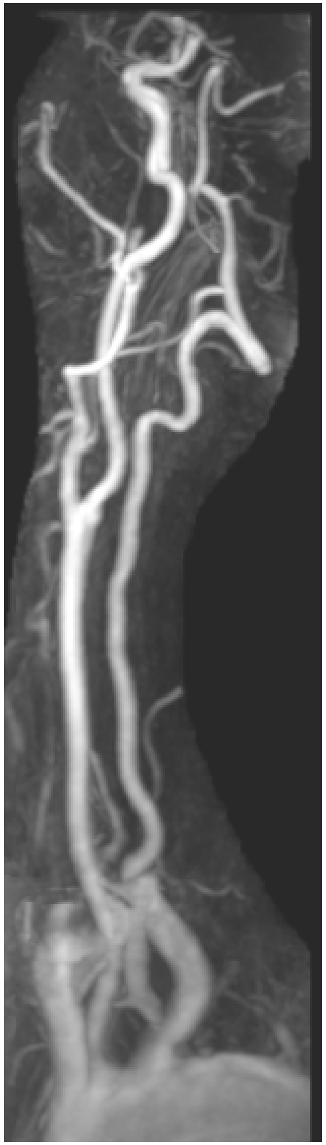

60e Score

0-30

31-50

51-70

>70

Near occlusion

Occluded

Quality

1

2

3

4

5

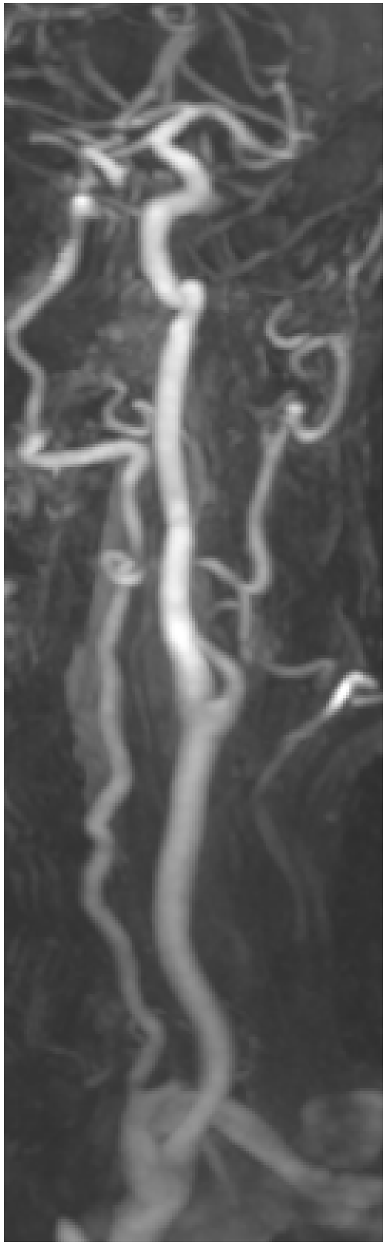

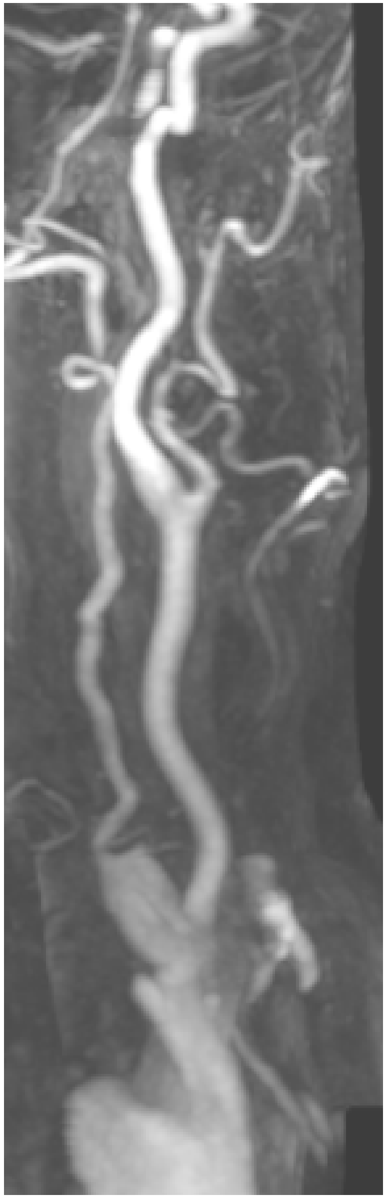

61d Score

0-30

31-50

51-70

>70

Near occlusion

Occluded

Quality

1

2

3

4

5

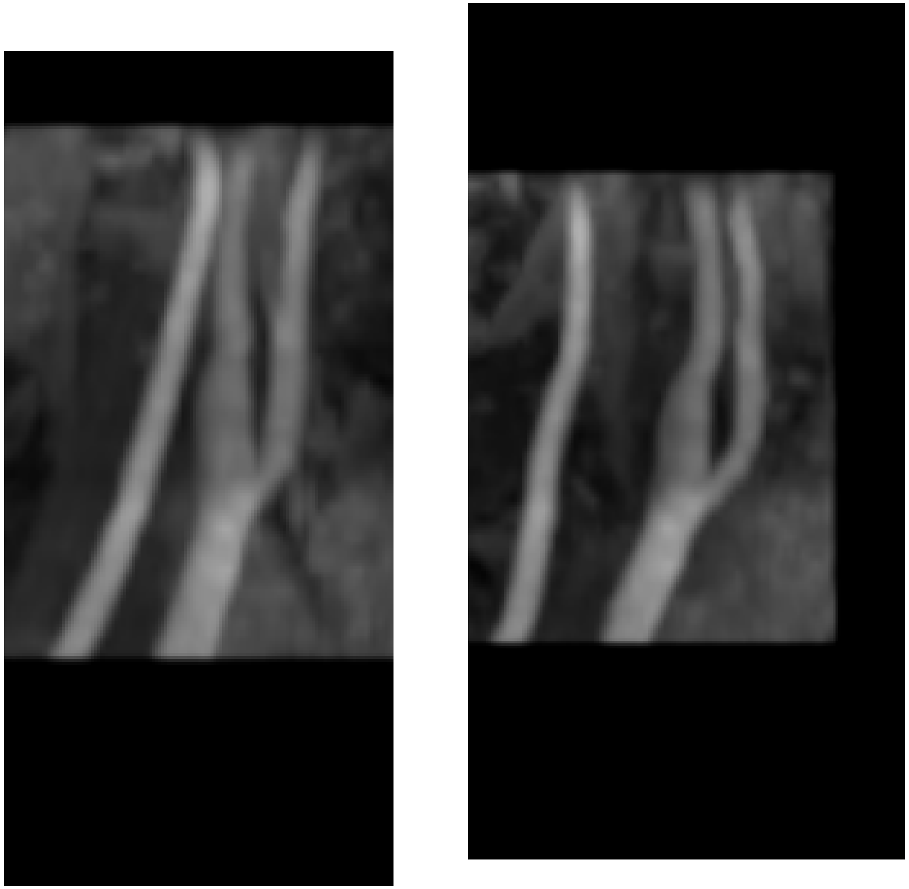

62c Score

0-30

31-50

51-70

>70

Near occlusion

Occluded

Quality

1

2

3

4

5

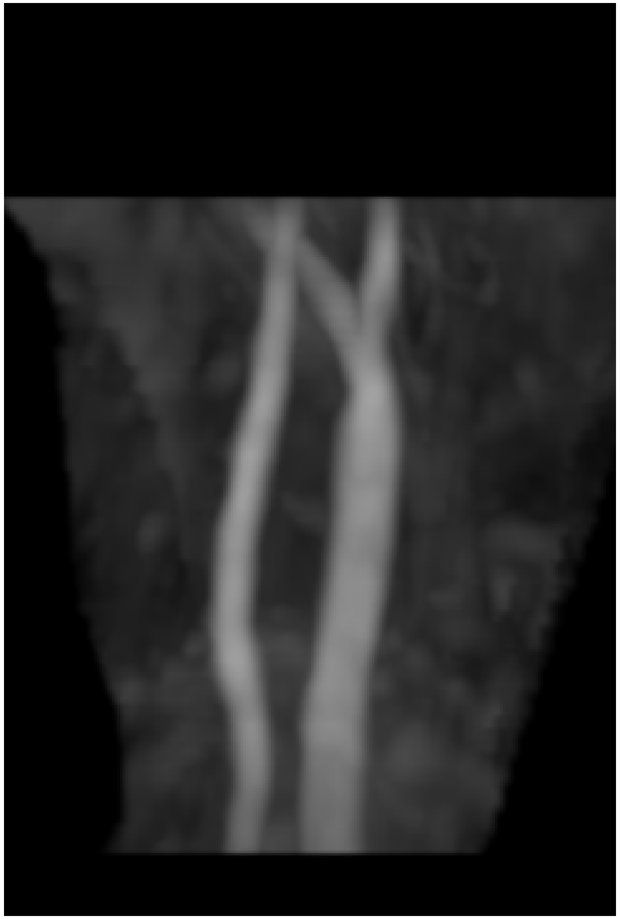

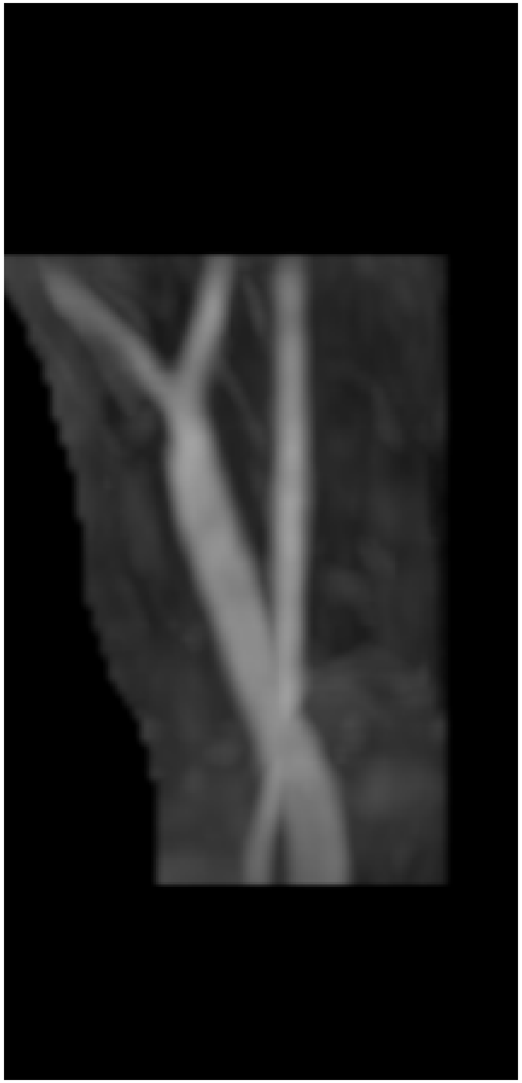

63b Score

0-30

31-50

51-70

>70

Near occlusion

Occluded

Quality

1

2

3

4

5

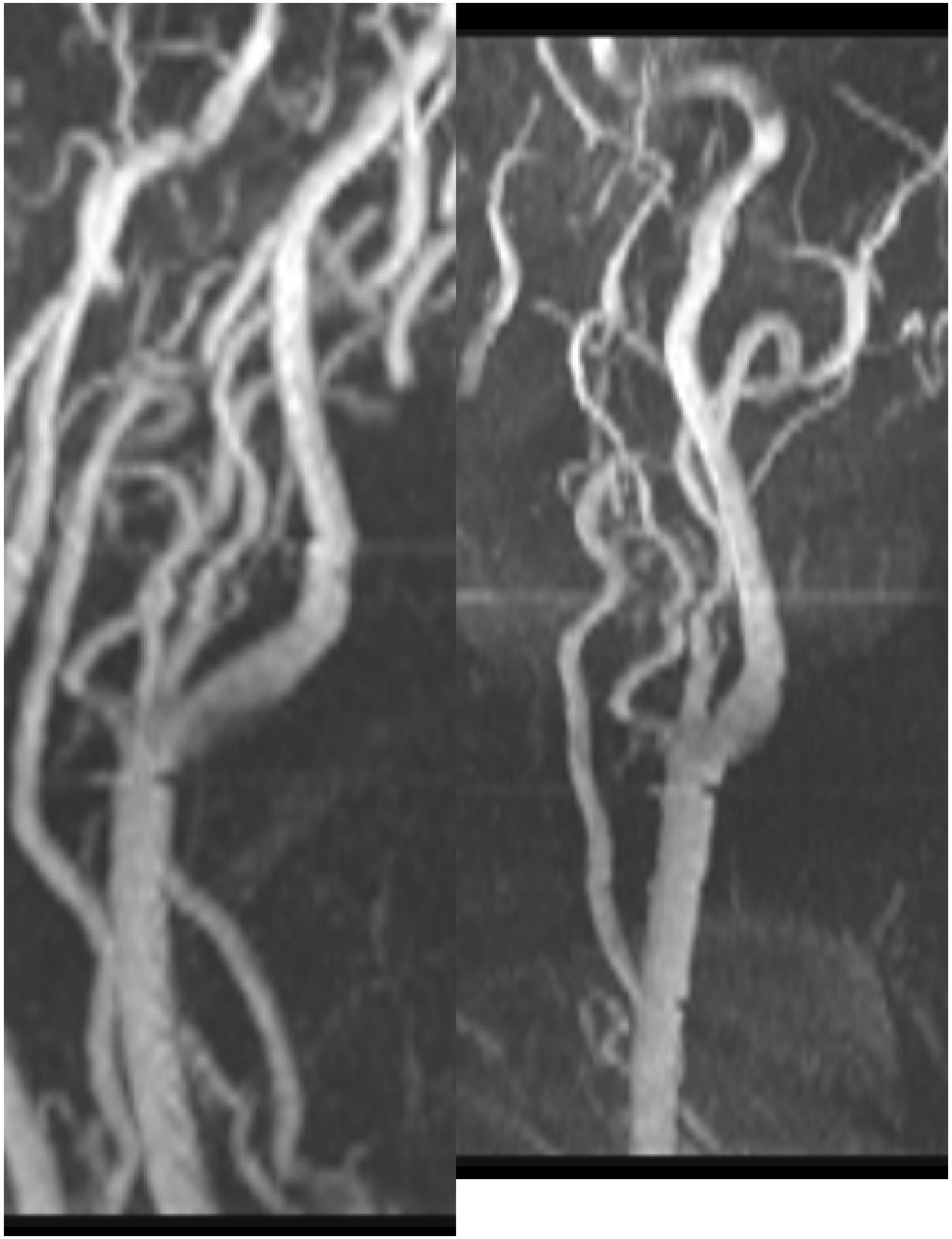

64a Score

0-30

31-50

51-70

>70

Near occlusion

Occluded

Quality

1

2

3

4

5

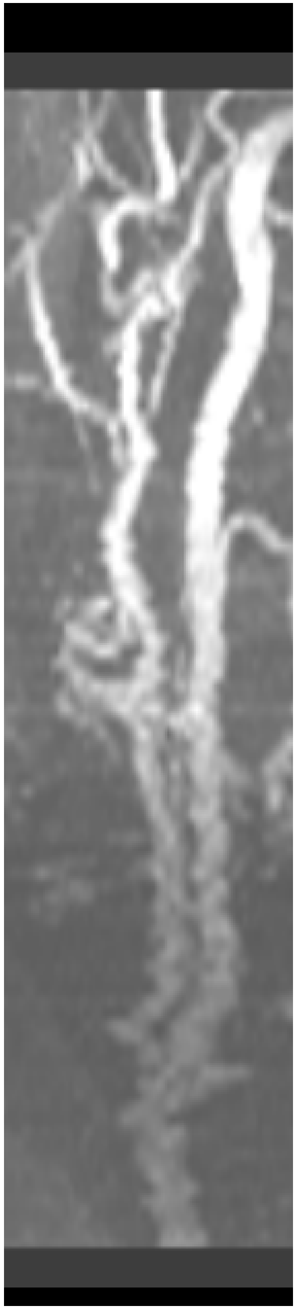

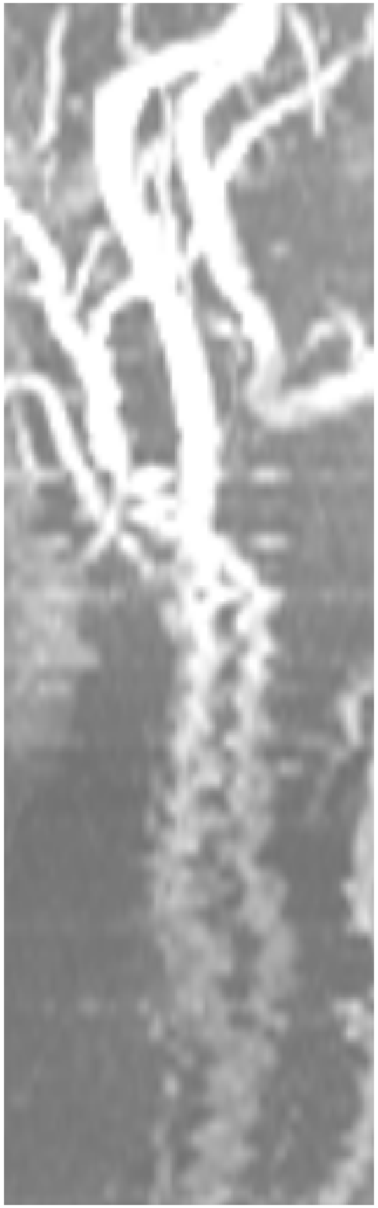

64f Score

0-30

31-50

51-70

>70

Near occlusion

Occluded

Quality

1

2

3

4

5

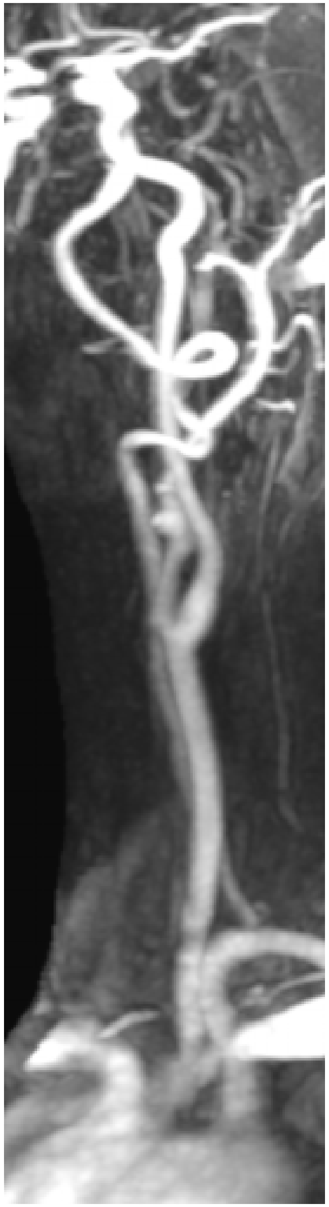

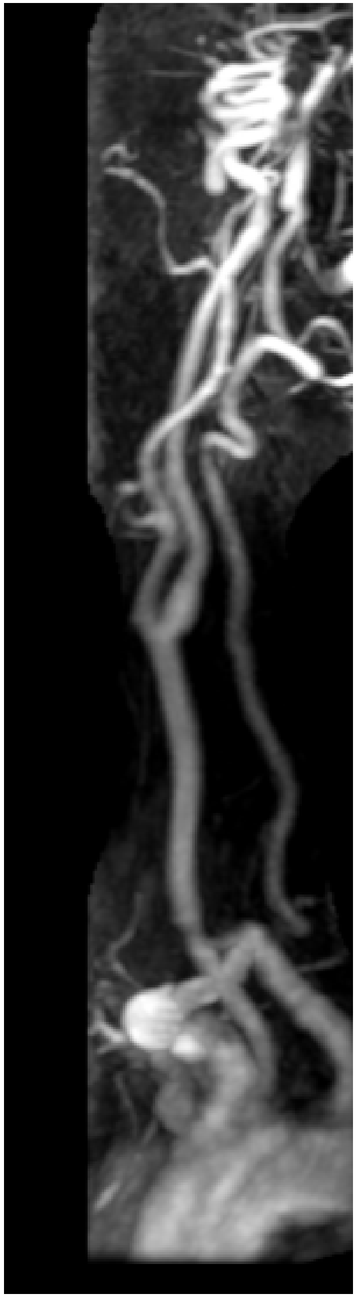

65e Score

0-30

31-50

51-70

>70

Near occlusion

Occluded

Quality

1

2

3

4

5

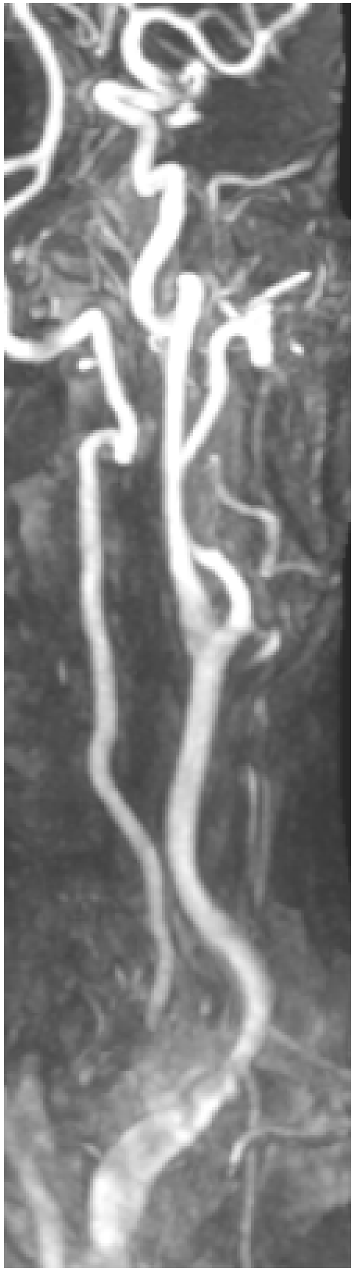

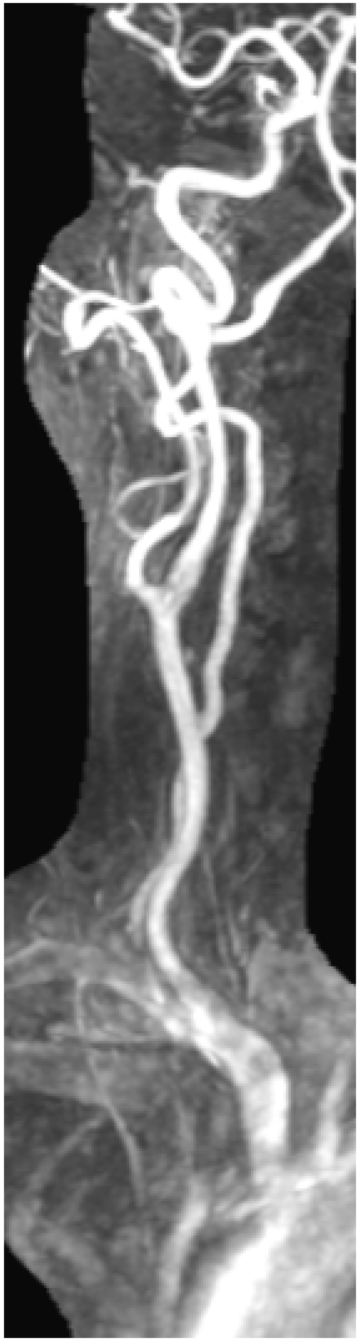

66d Score

0-30

31-50

51-70

>70

Near occlusion

Occluded

Quality

1

2

3

4

5

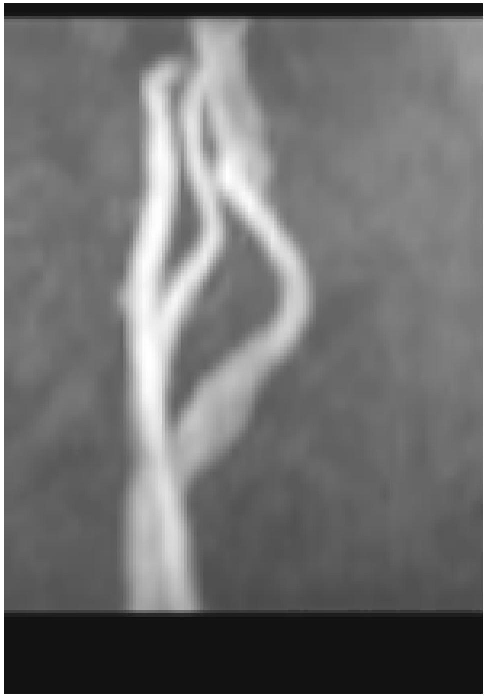

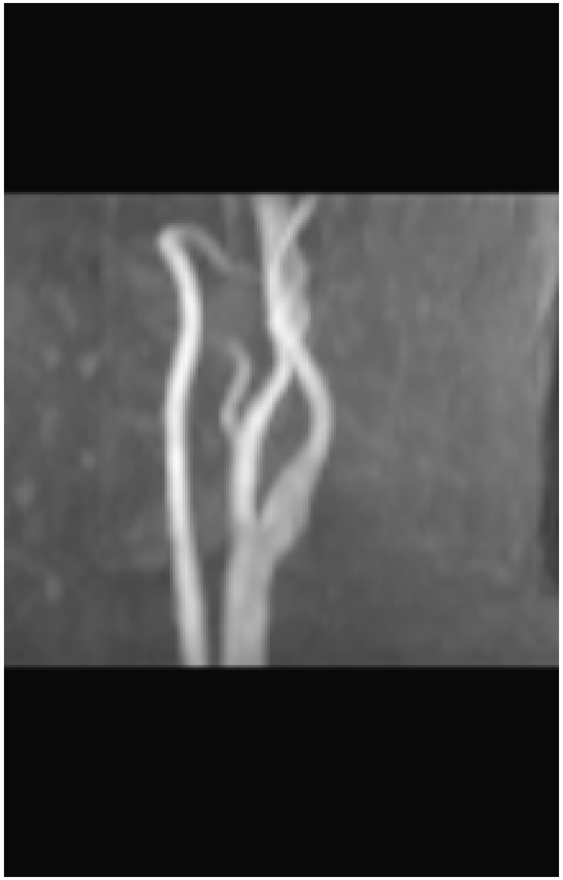

67c Score

0-30

31-50

51-70

>70

Near occlusion

Occluded

Quality

1

2

3

4

5

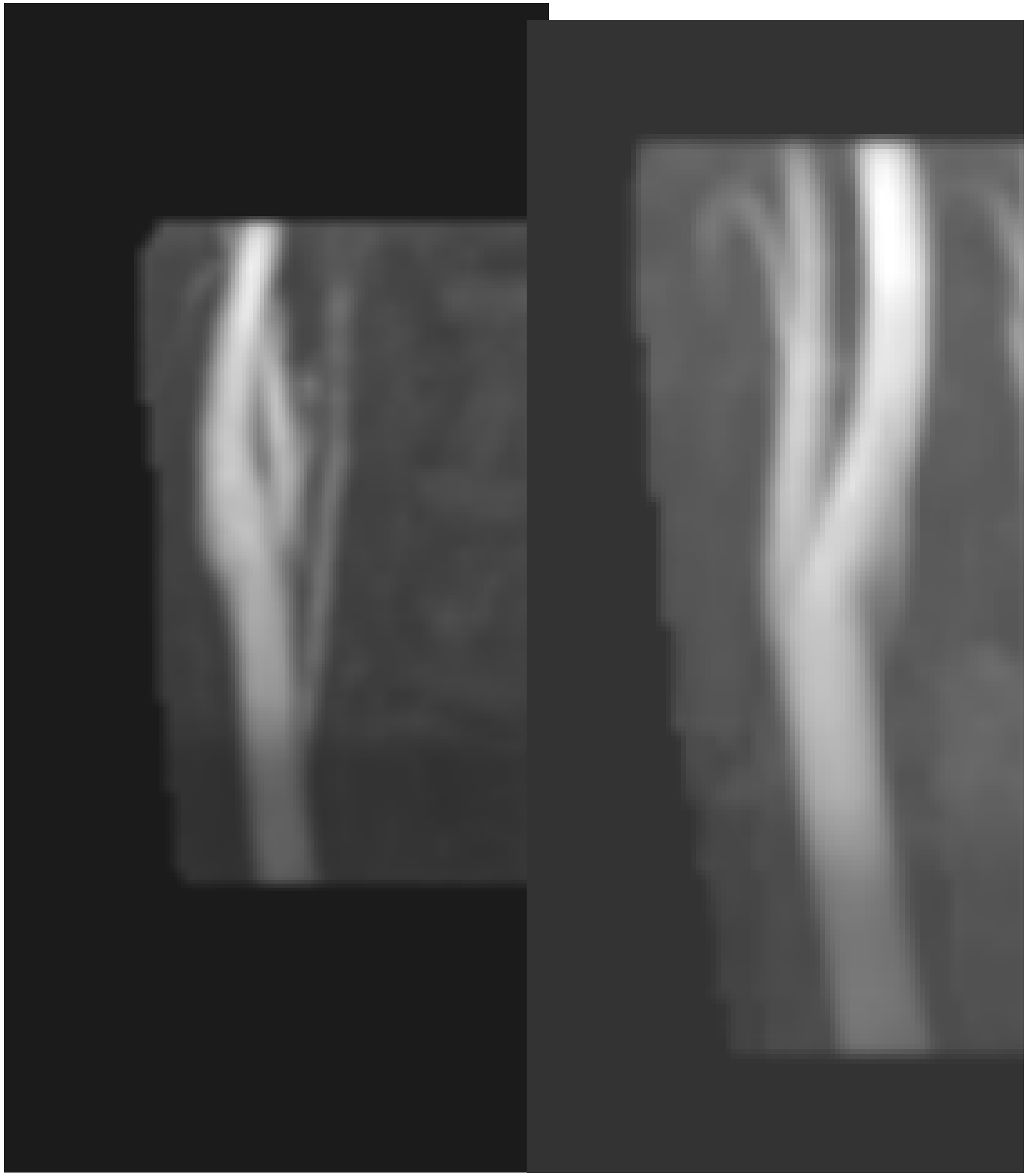

68b Score

0-30

31-50

51-70

>70

Near occlusion

Occluded

Quality

1

2

3

4

5

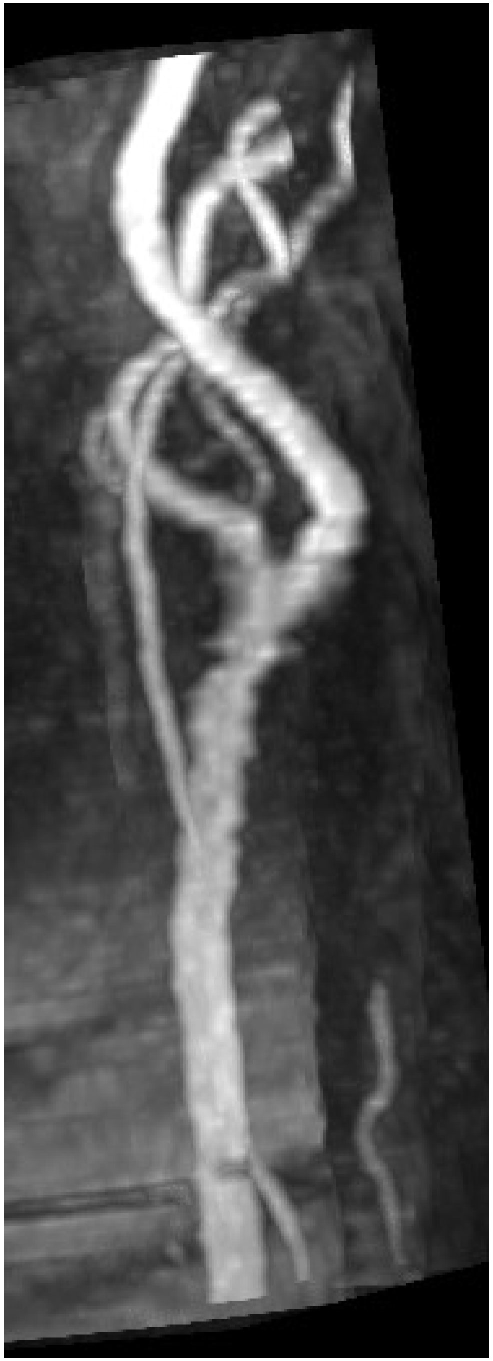

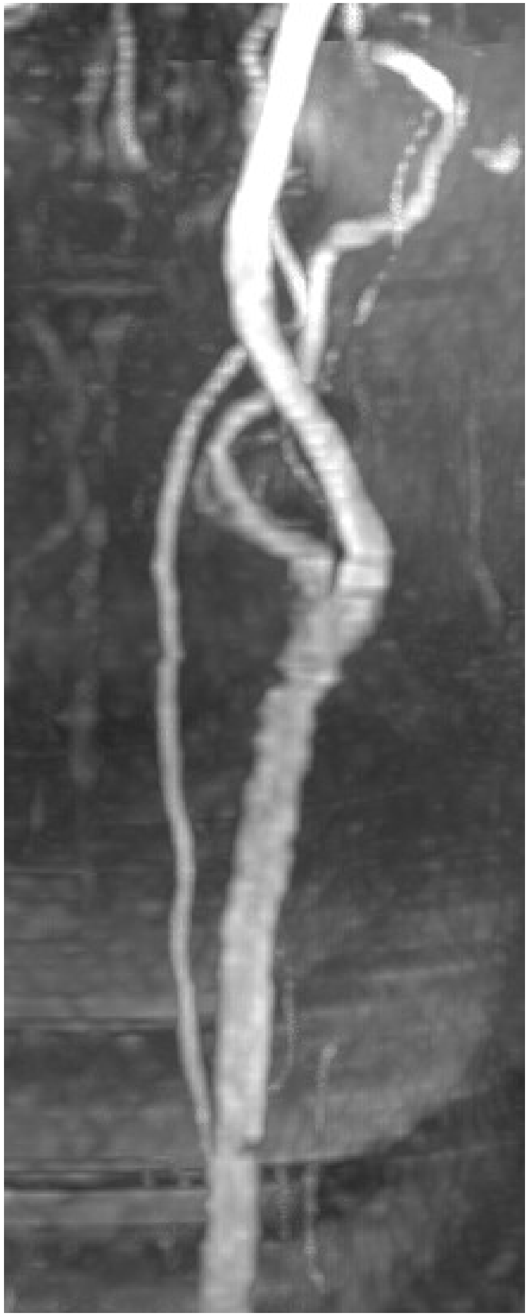

69a Score

0-30

31-50

51-70

>70

Near occlusion

Occluded

Quality

1

2

3

4

5

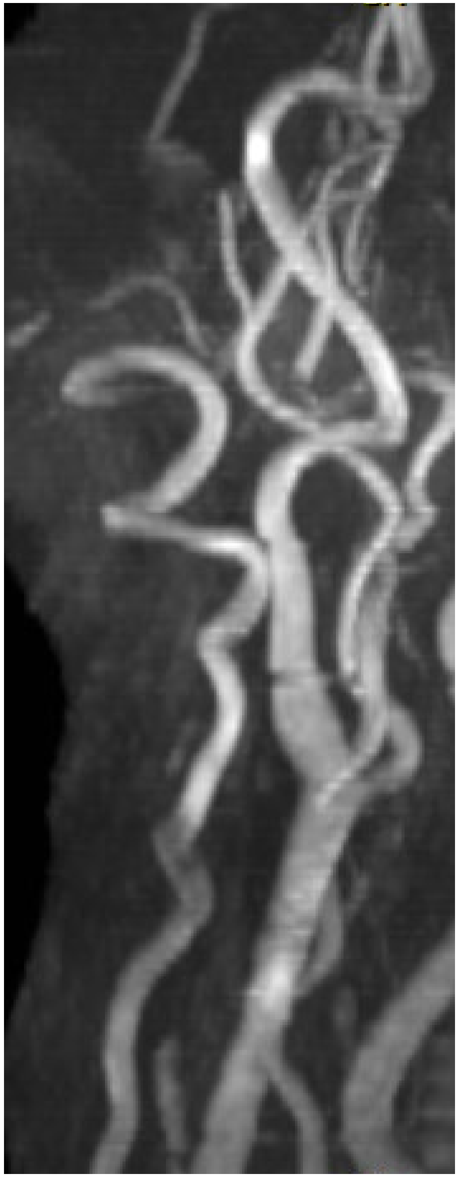

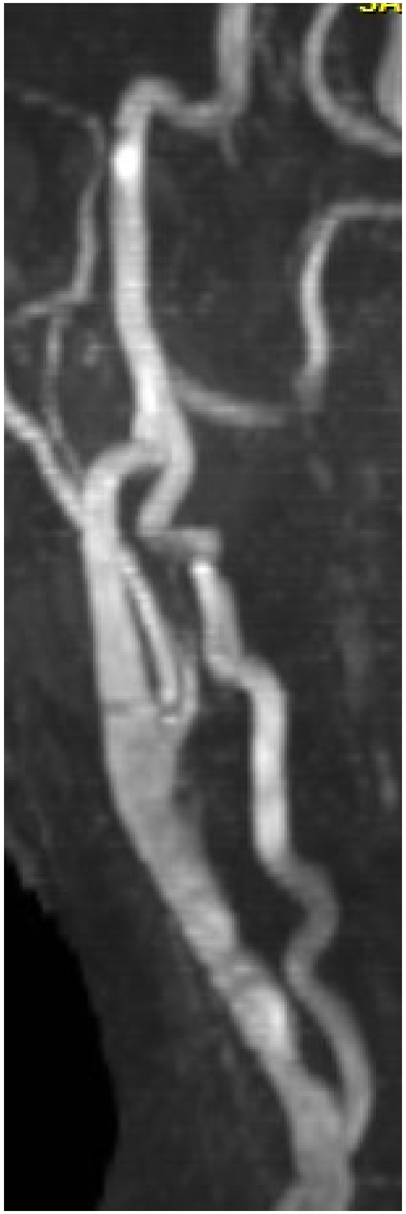

69f Score

0-30

31-50

51-70

>70

Near occlusion

Occluded

Quality

1

2

3

4

5

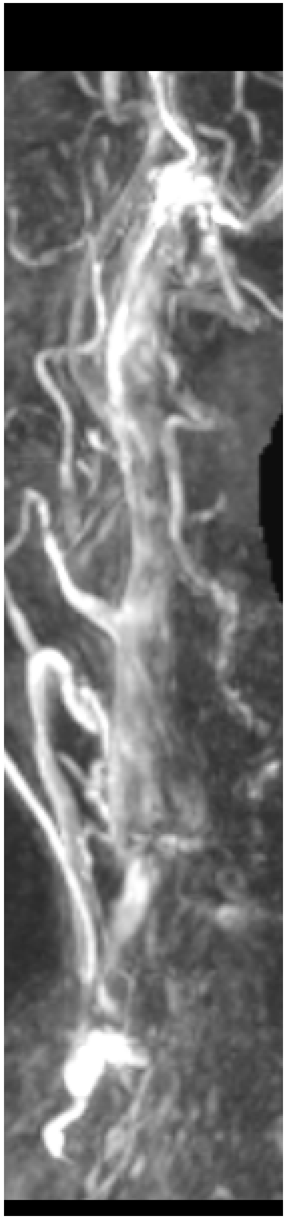

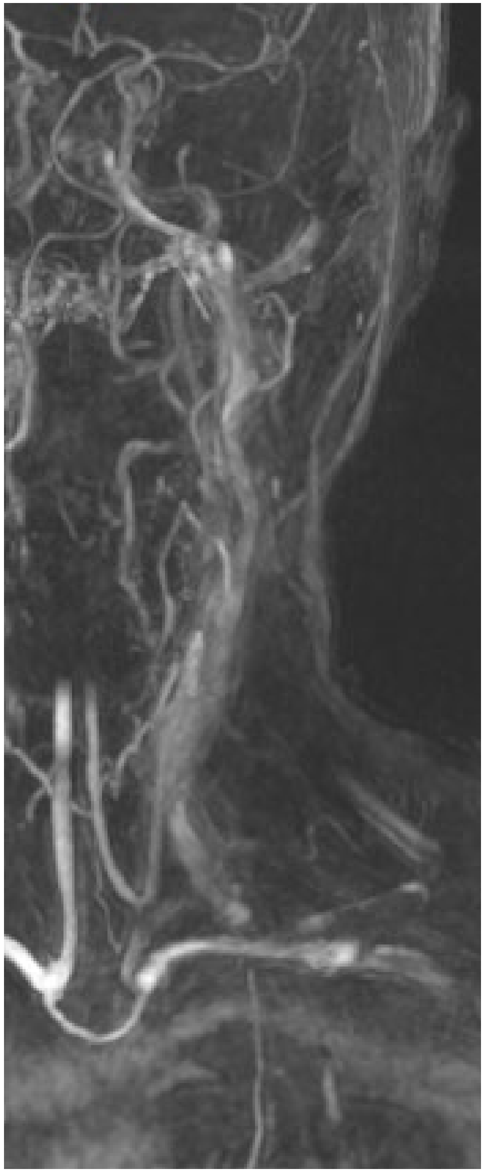

70e Score

0-30

31-50

51-70

>70

Near occlusion

Occluded

Quality

1

2

3

4

5

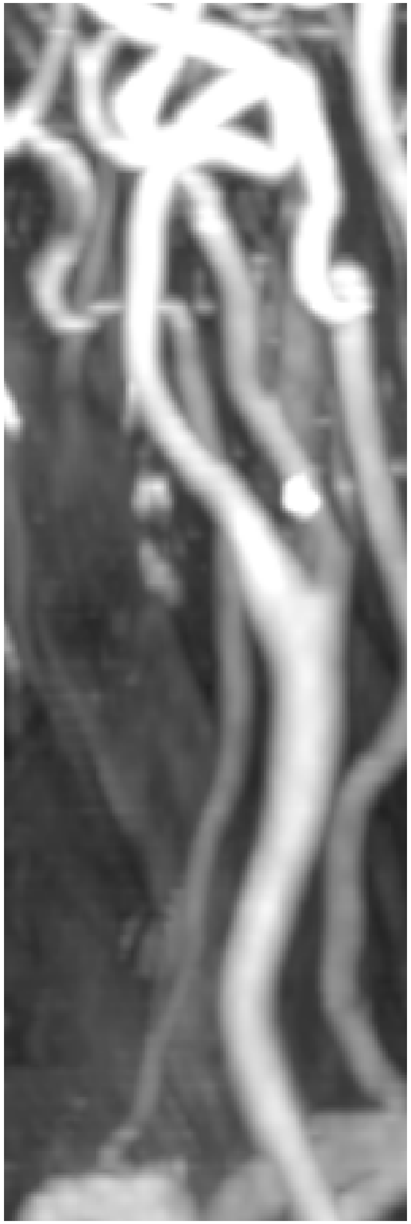

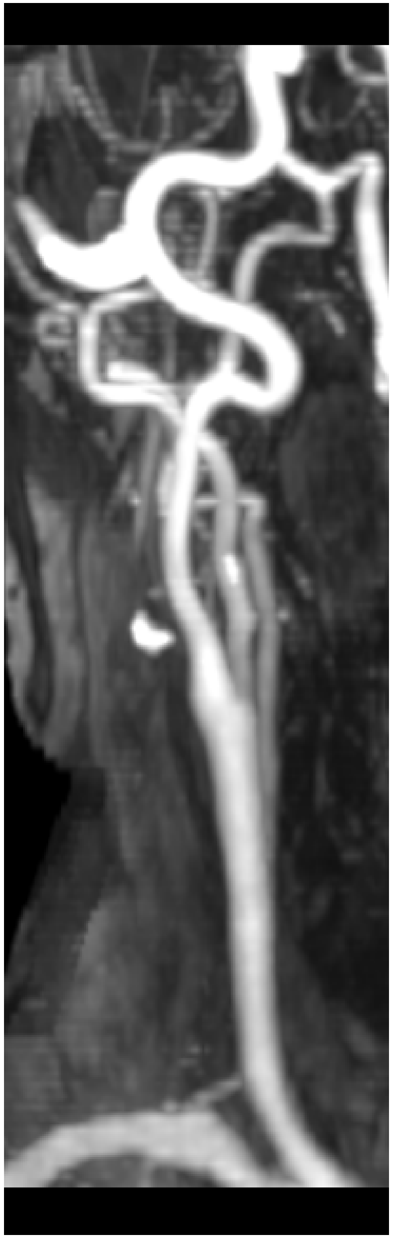

71d Score

0-30

31-50

51-70

>70

Near occlusion

Occluded

Quality

1

2

3

4

5

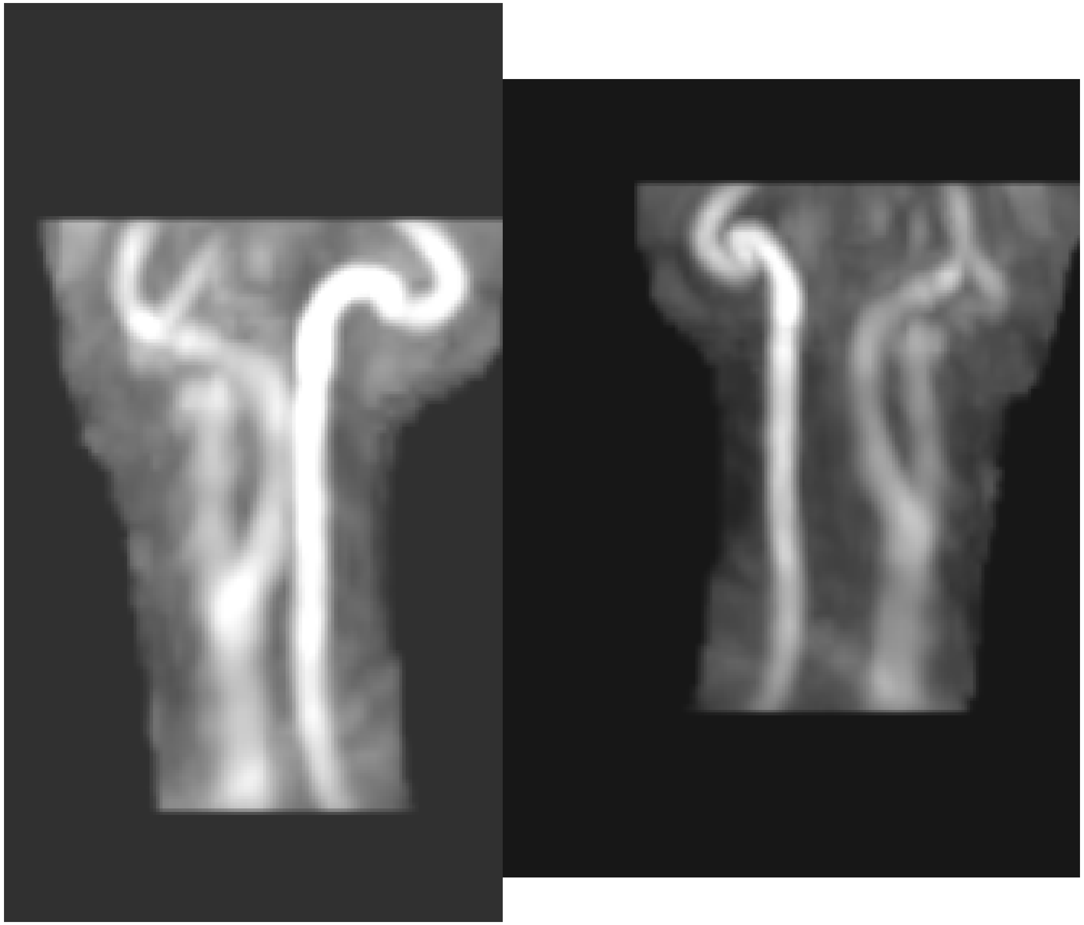

72c Score

0-30

31-50

51-70

>70

Near occlusion

Occluded

Quality

1

2

3

4

5

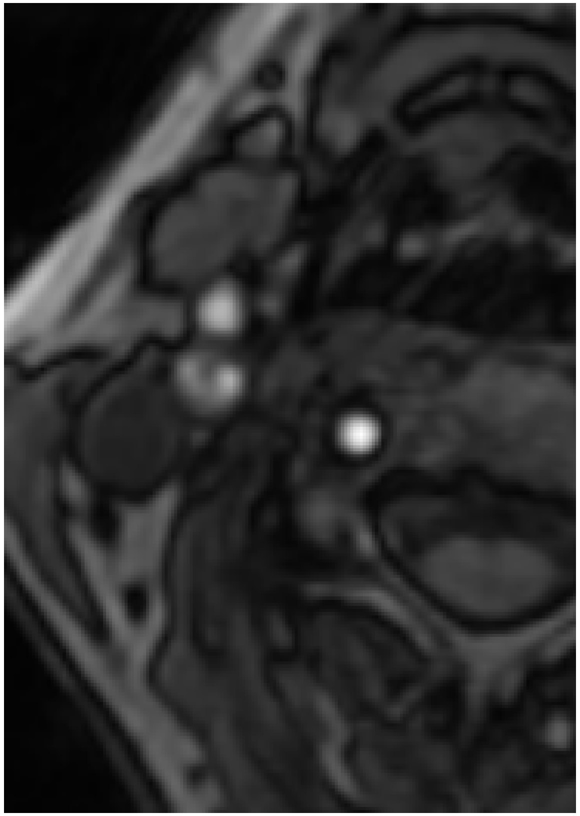

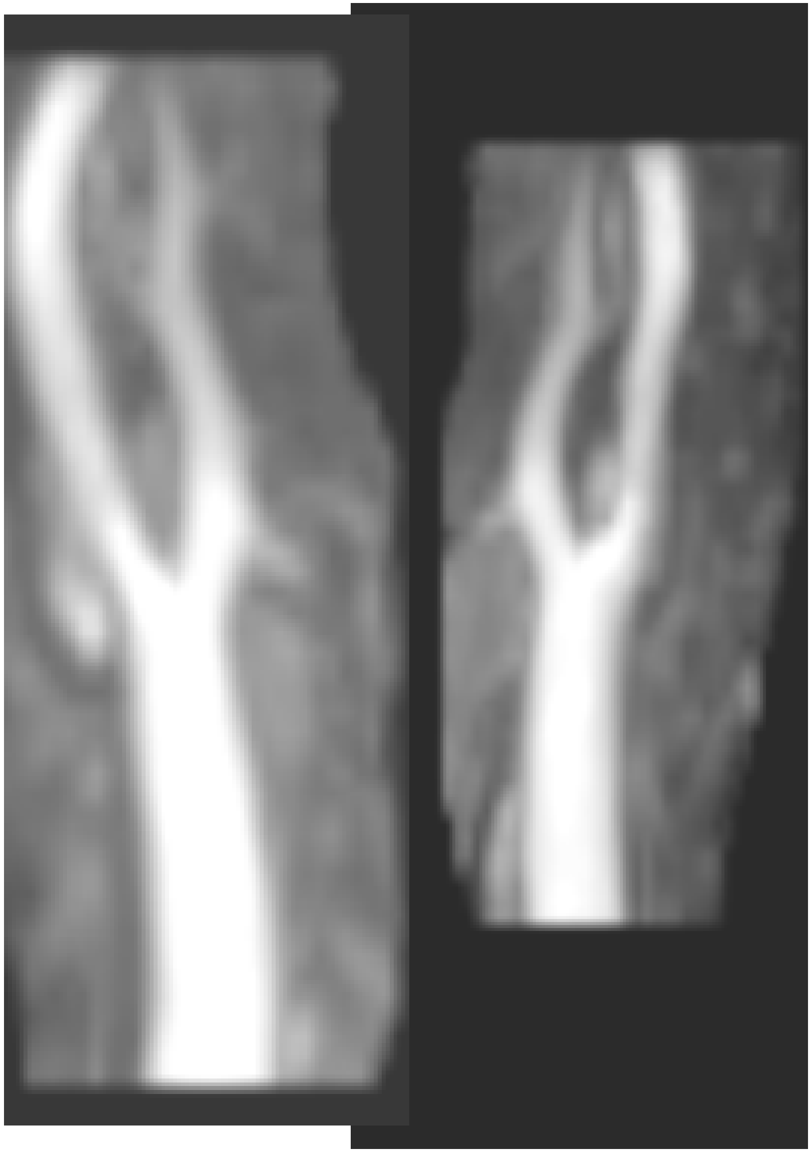

73b Score

0-30

31-50

51-70

>70

Near occlusion

Occluded

Quality

1

2

3

4

5

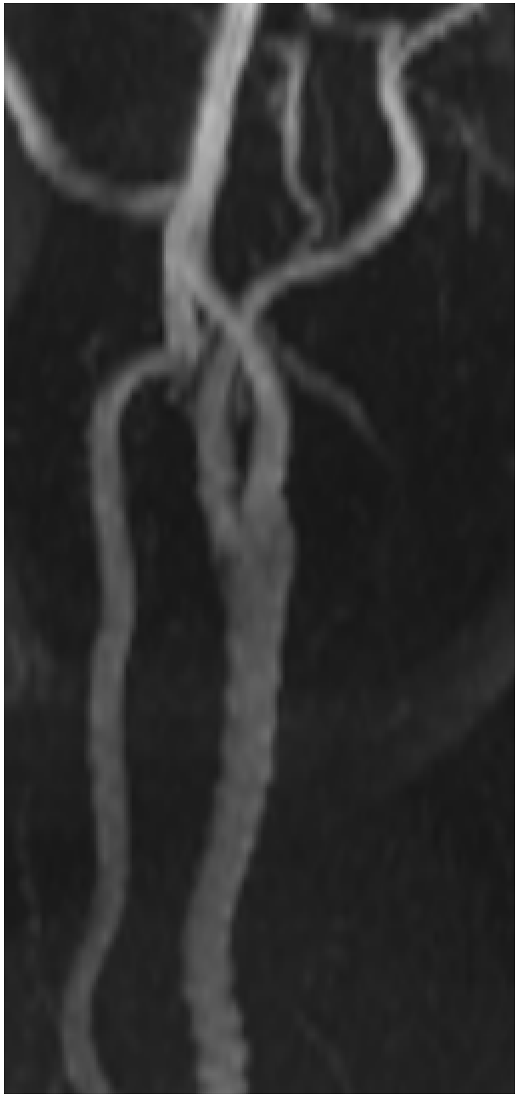

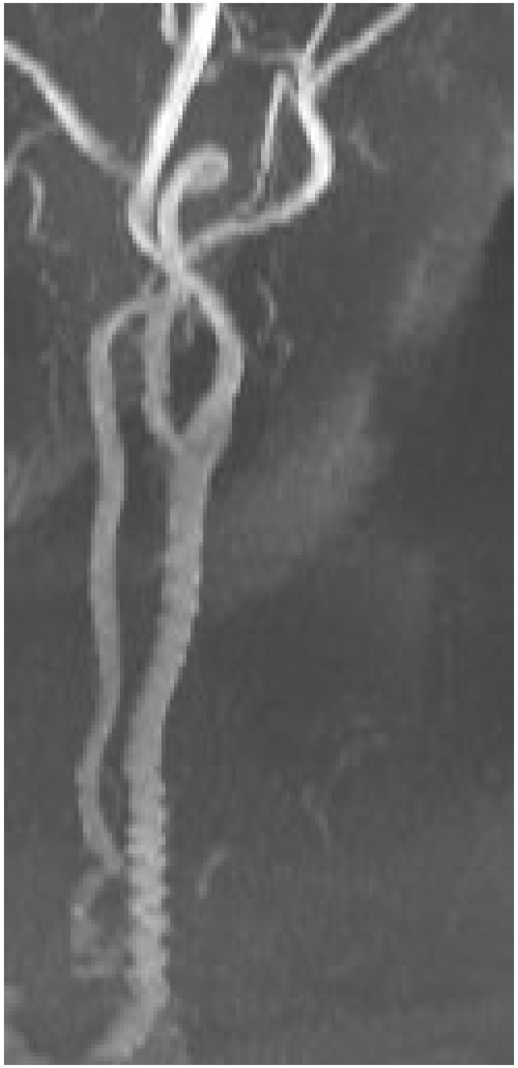

74a Score

0-30

31-50

51-70

>70

Near occlusion

Occluded

Quality

1

2

3

4

5

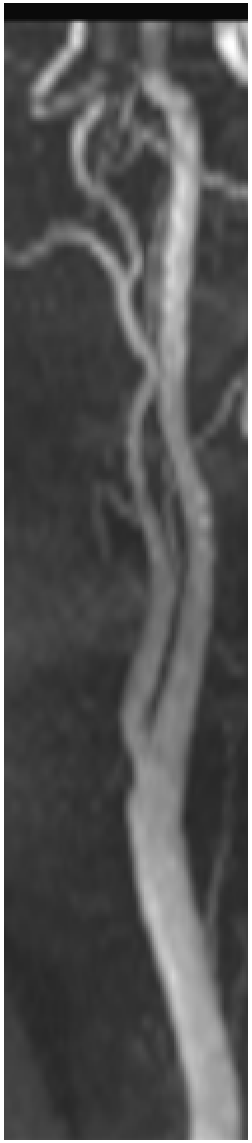

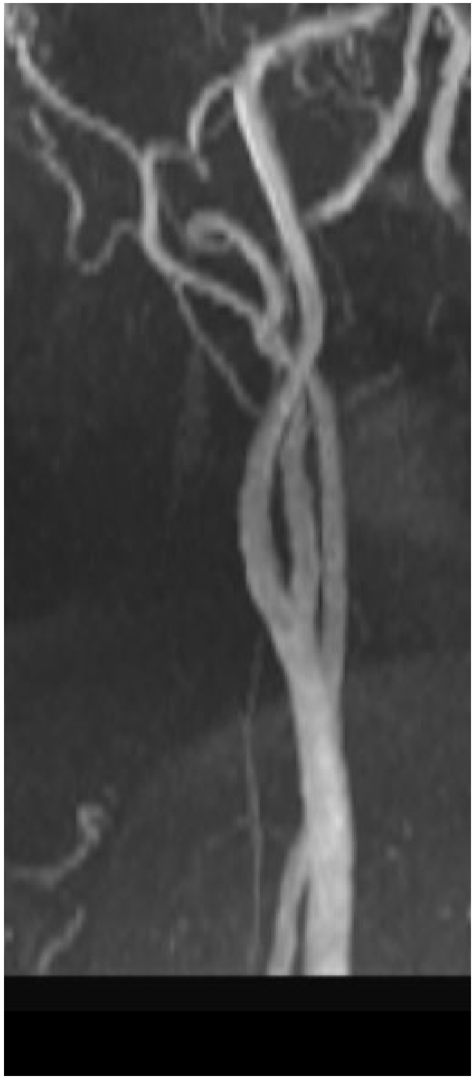

74f Score

0-30

31-50

51-70

>70

Near occlusion

Occluded

Quality

1

2

3

4

5

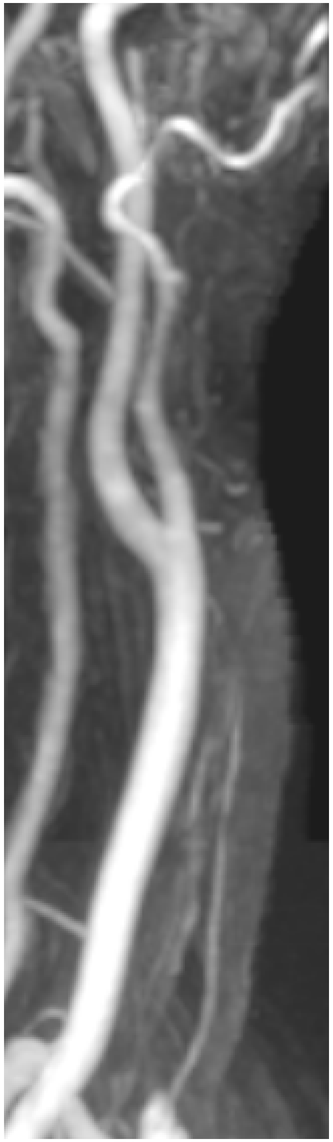

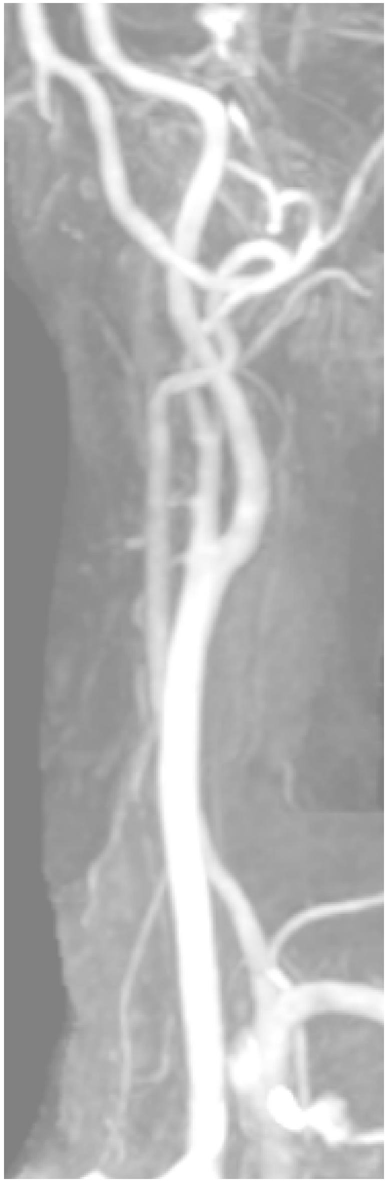

75e Score

0-30

31-50

51-70

>70

Near occlusion

Occluded

Quality

1

2

3

4

5

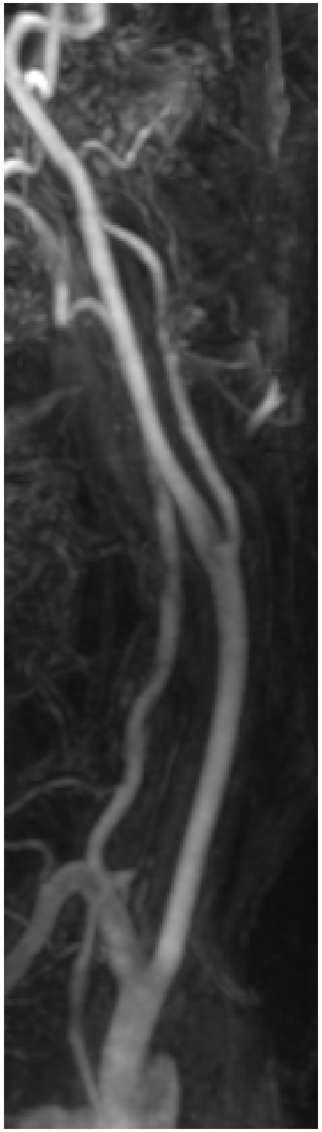

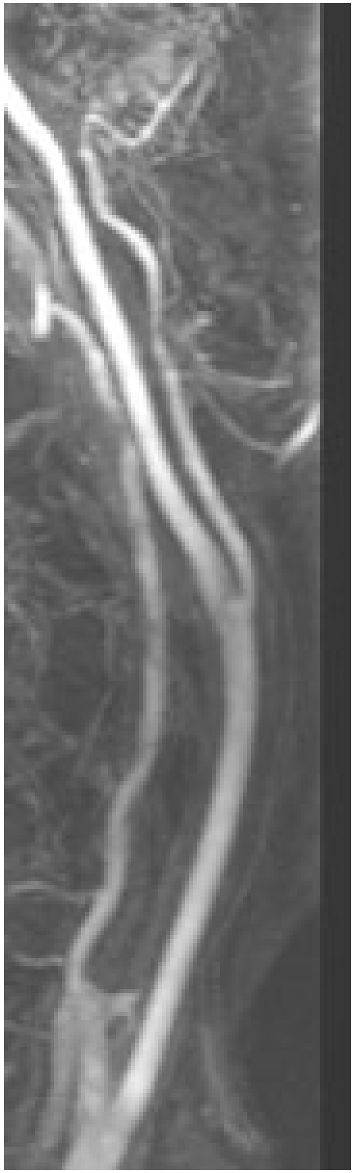

76d Score

0-30

31-50

51-70

>70

Near occlusion

Occluded

Quality

1

2

3

4

5

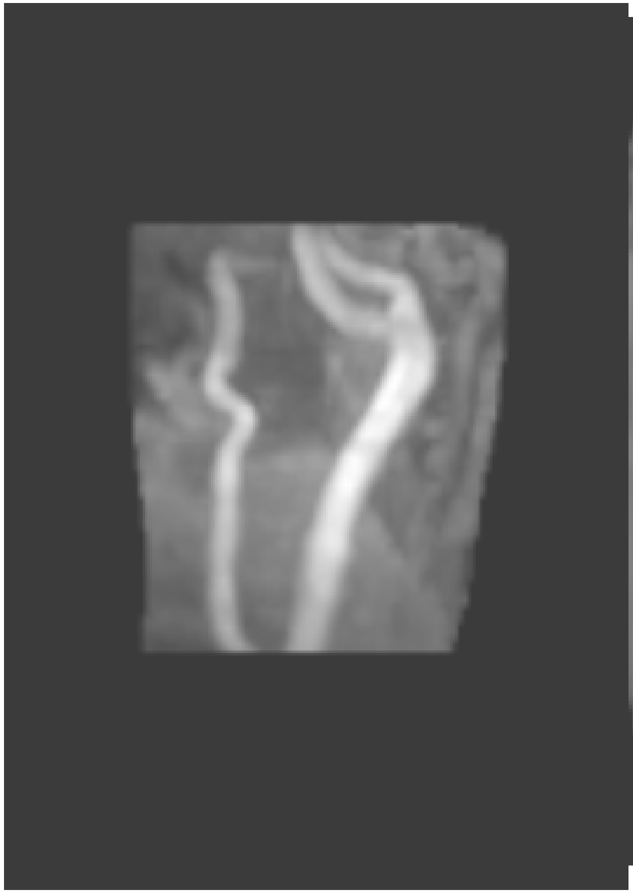

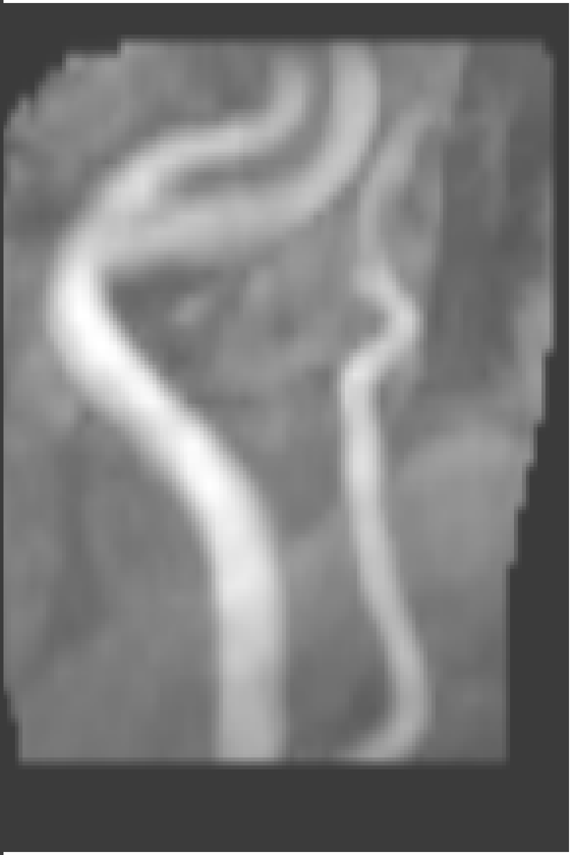

77c Score

0-30

31-50

51-70

>70

Near occlusion

Occluded

Quality

1

2

3

4

5

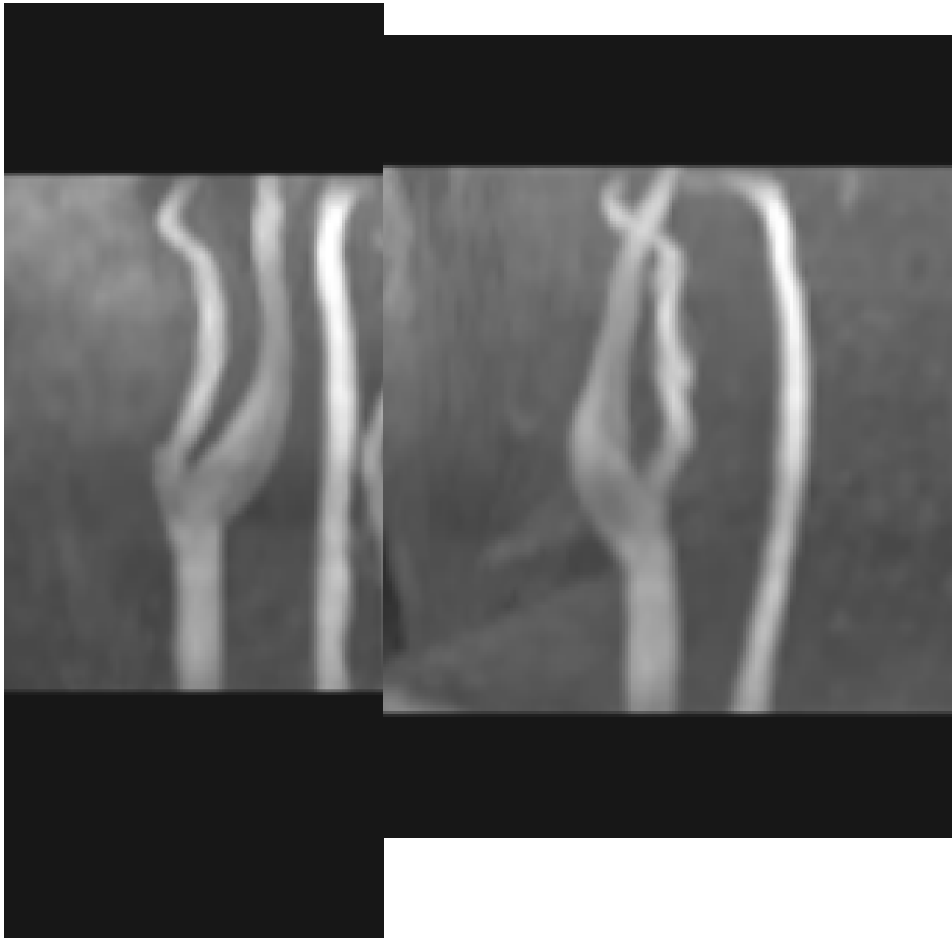

78b Score

0-30

31-50

51-70

>70

Near occlusion

Occluded

Quality

1

2

3

4

5

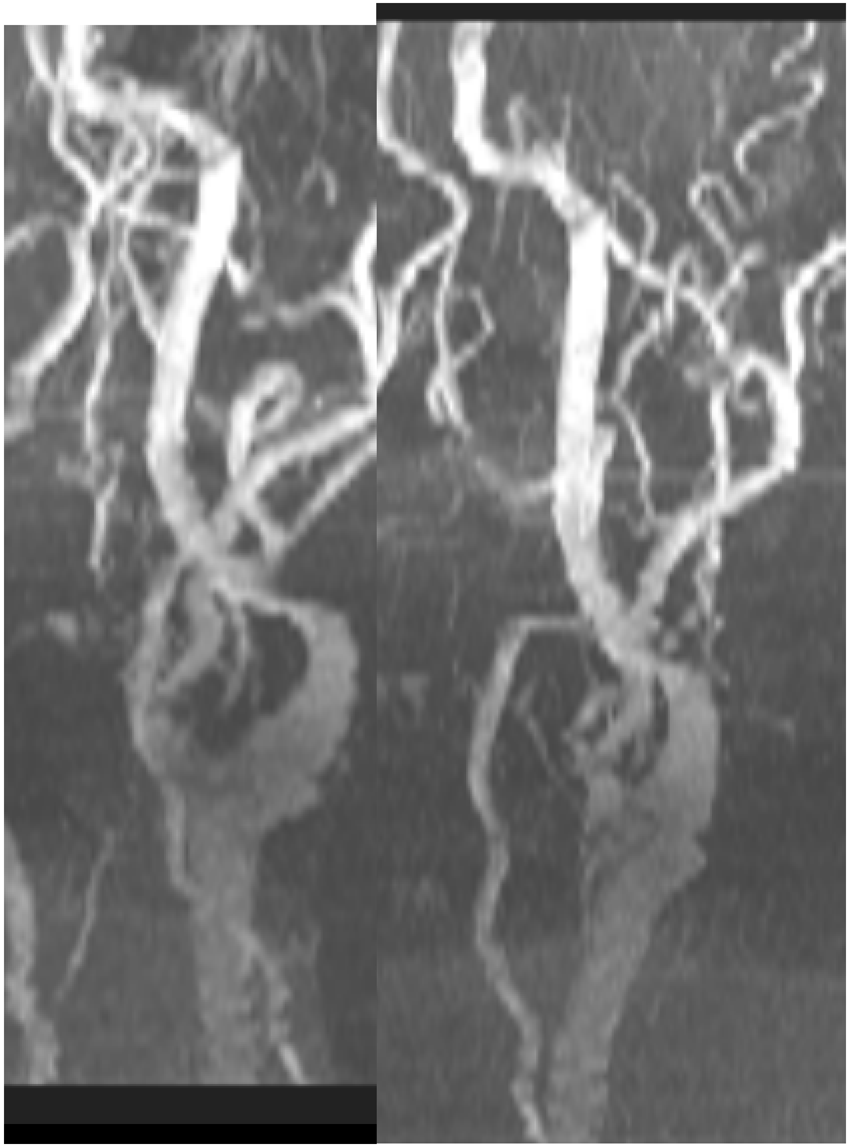

79a Score

0-30

31-50

51-70

>70

Near occlusion

Occluded

Quality

1

2

3

4

5

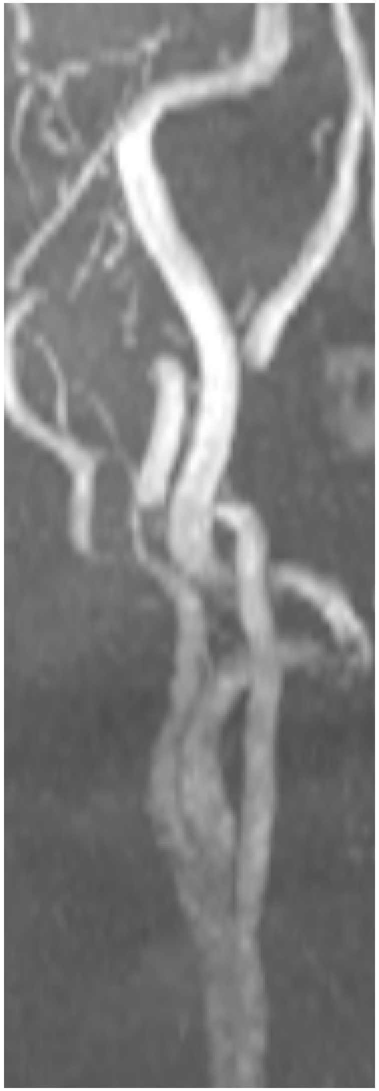

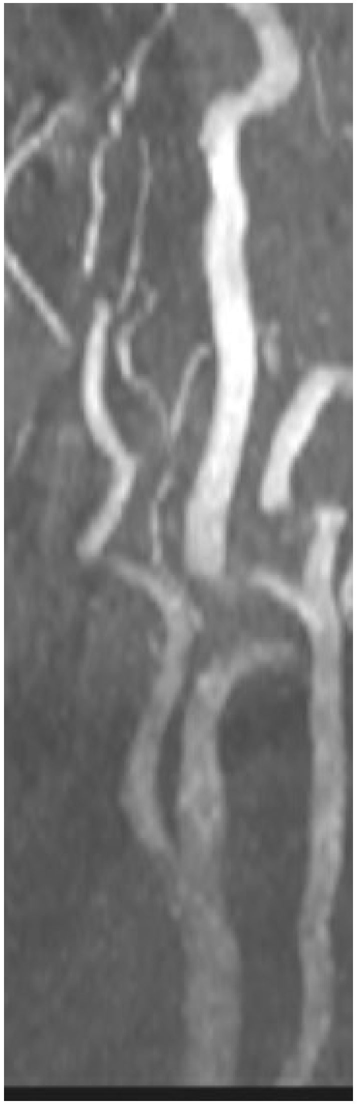

79f Score

0-30

31-50

51-70

>70

Near occlusion

Occluded

Quality

1

2

3

4

5

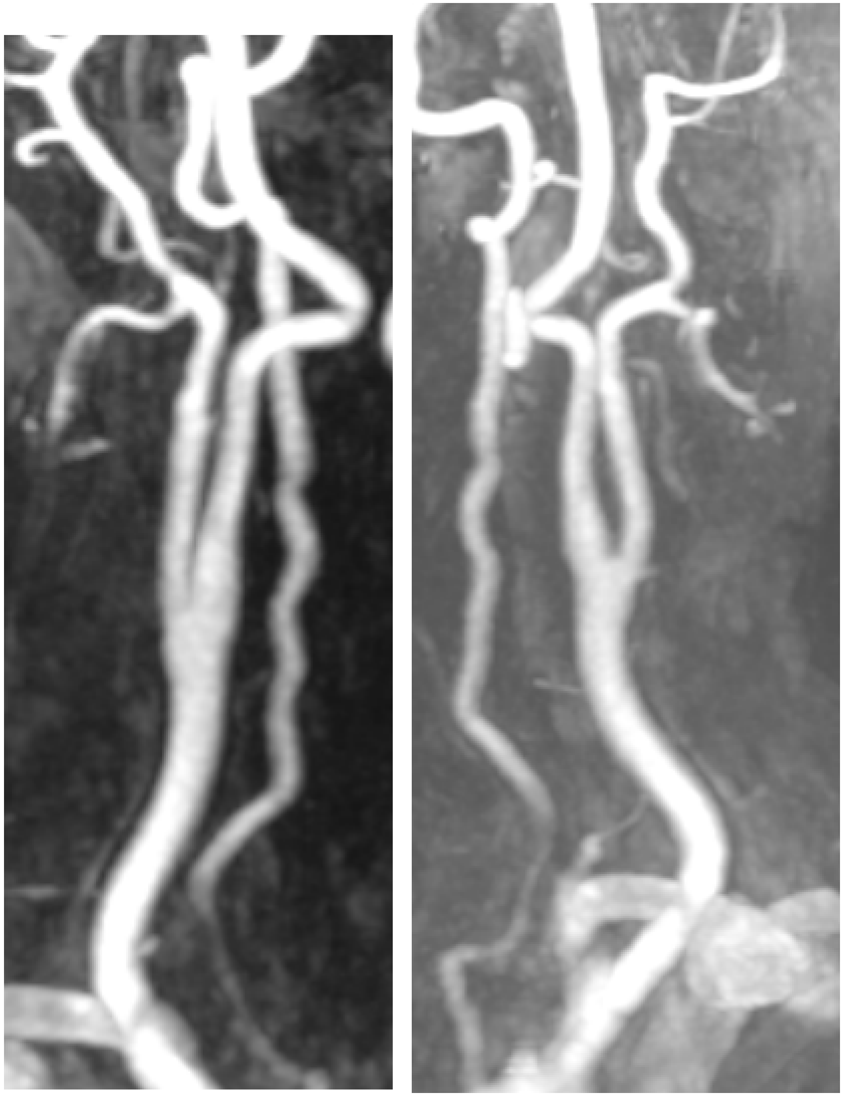

80e Score

0-30

31-50

51-70

>70

Near occlusion

Occluded

Quality

1

2

3

4

5

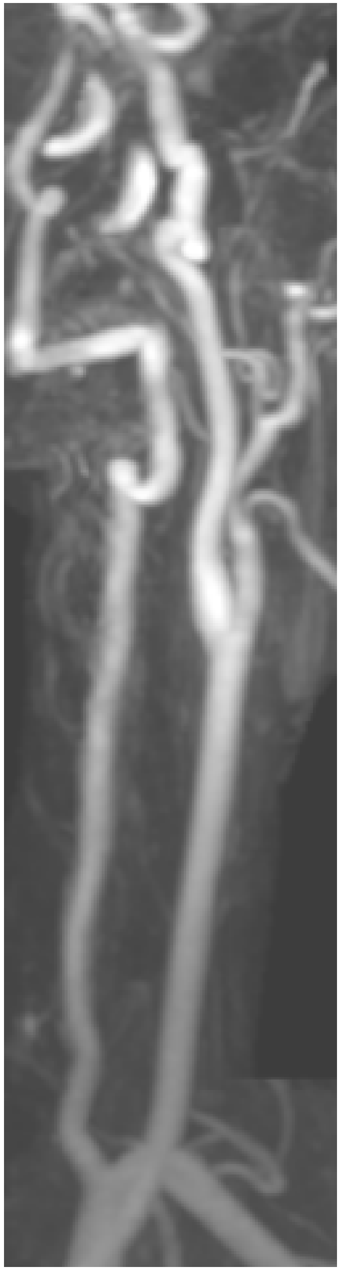

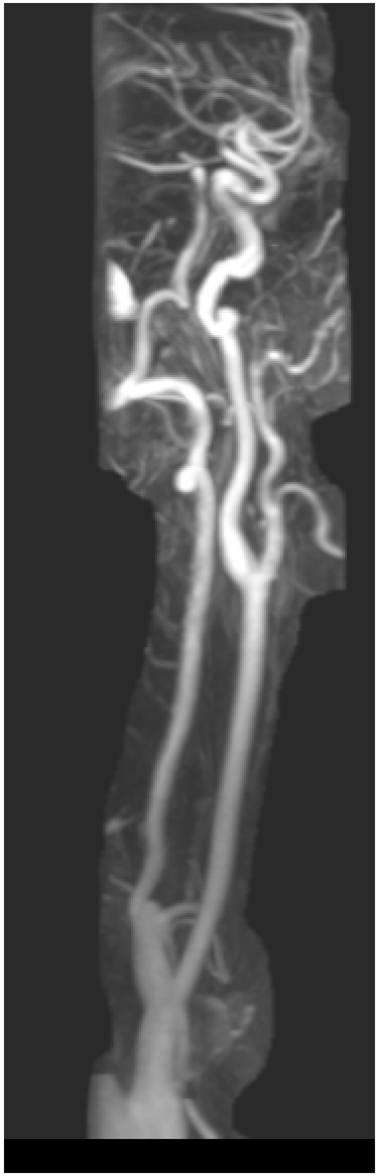

81d Score

0-30

31-50

51-70

>70

Near occlusion

Occluded

Quality

1

2

3

4

5

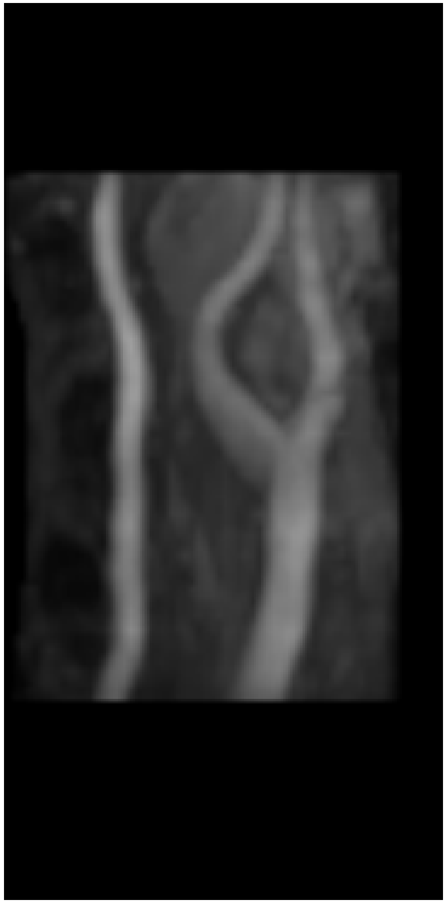

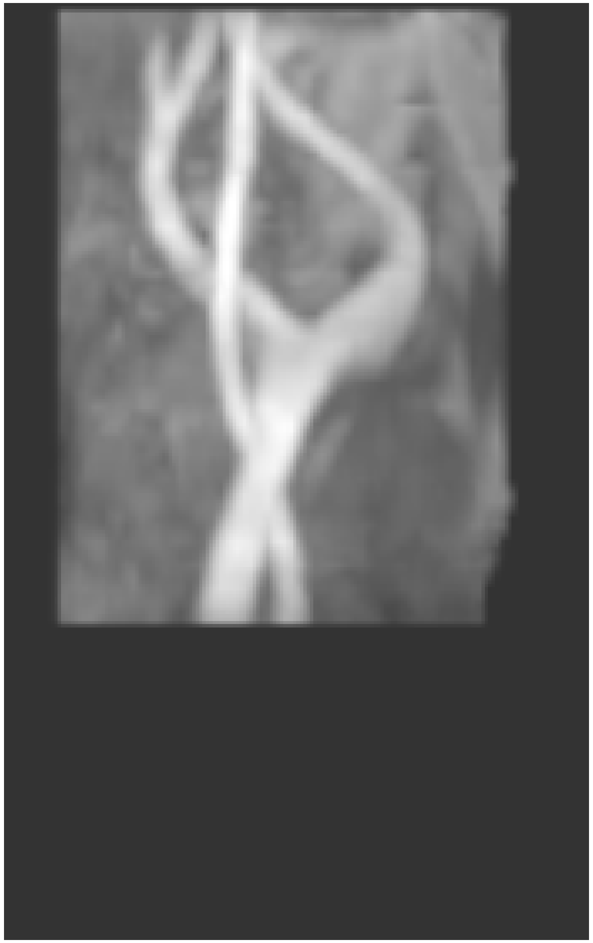

82c Score

0-30

31-50

51-70

>70

Near occlusion

Occluded

Quality

1

2

3

4

5

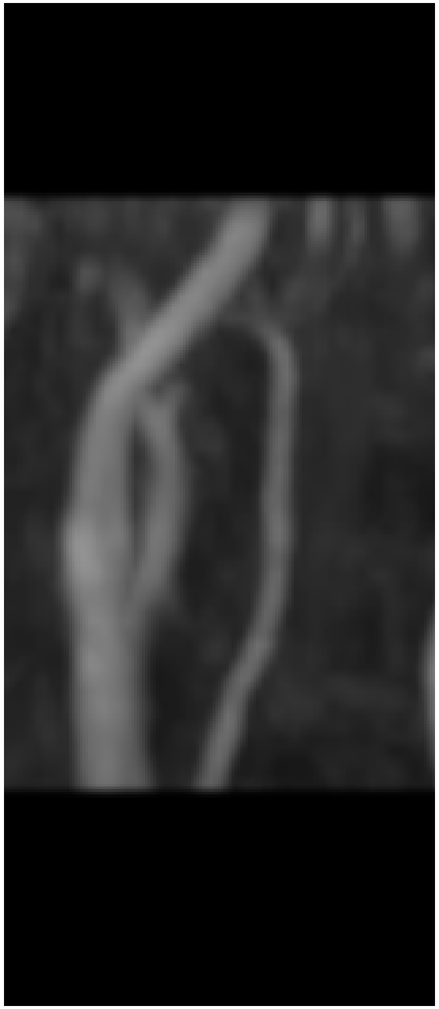

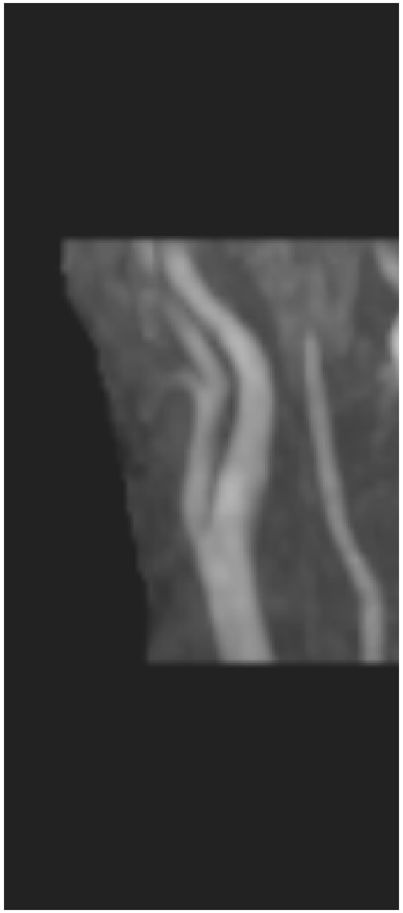

83b Score

0-30

31-50

51-70

>70

Near occlusion

Occluded

Quality

1

2

3

4

5

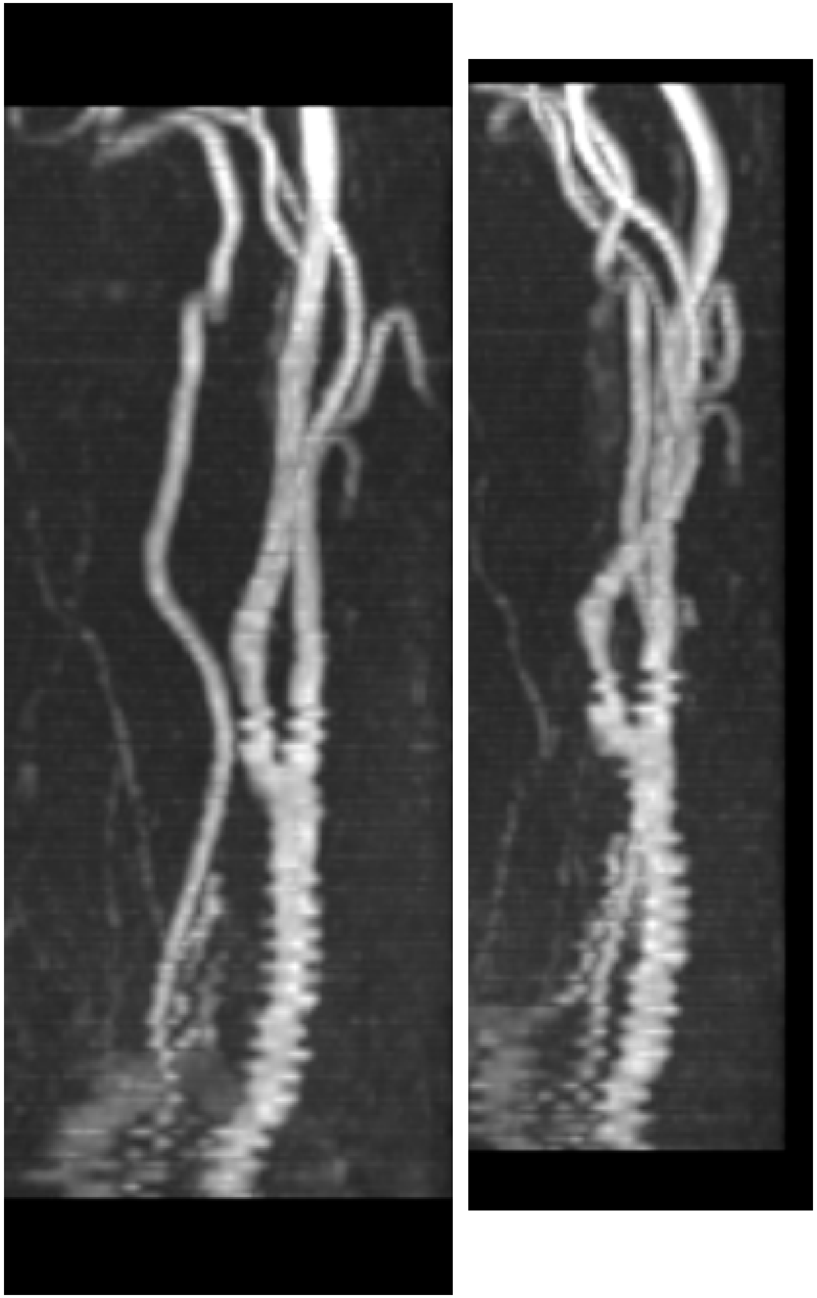

84a Score

0-30

31-50

51-70

>70

Near occlusion

Occluded

Quality

1

2

3

4

5

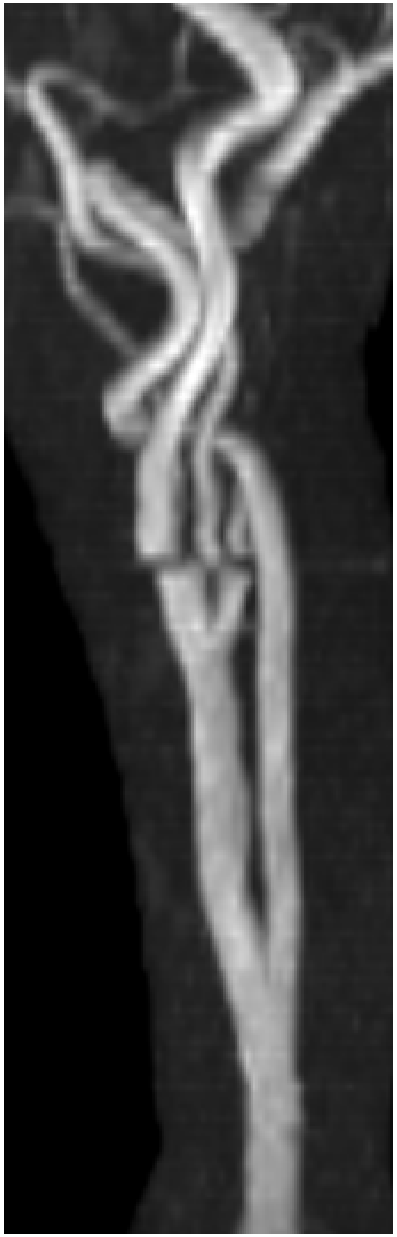

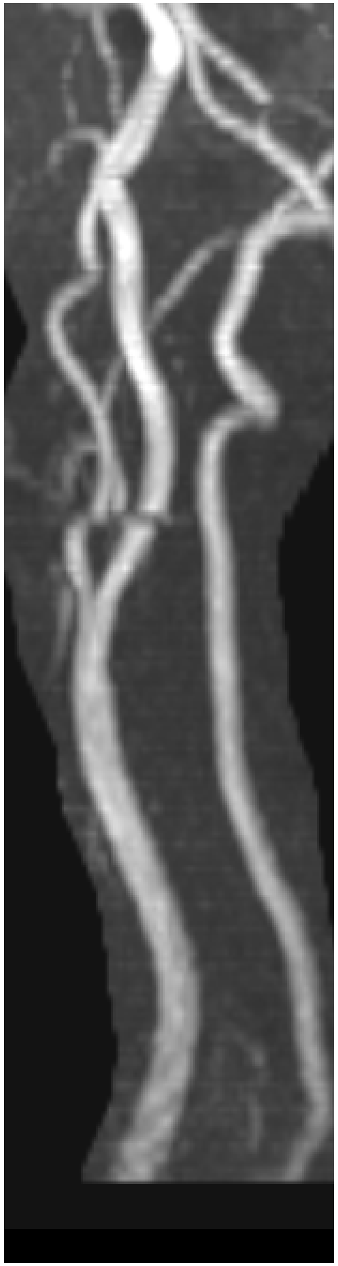

84f Score

0-30

31-50

51-70

>70

Near occlusion

Occluded

Quality

1

2

3

4

5

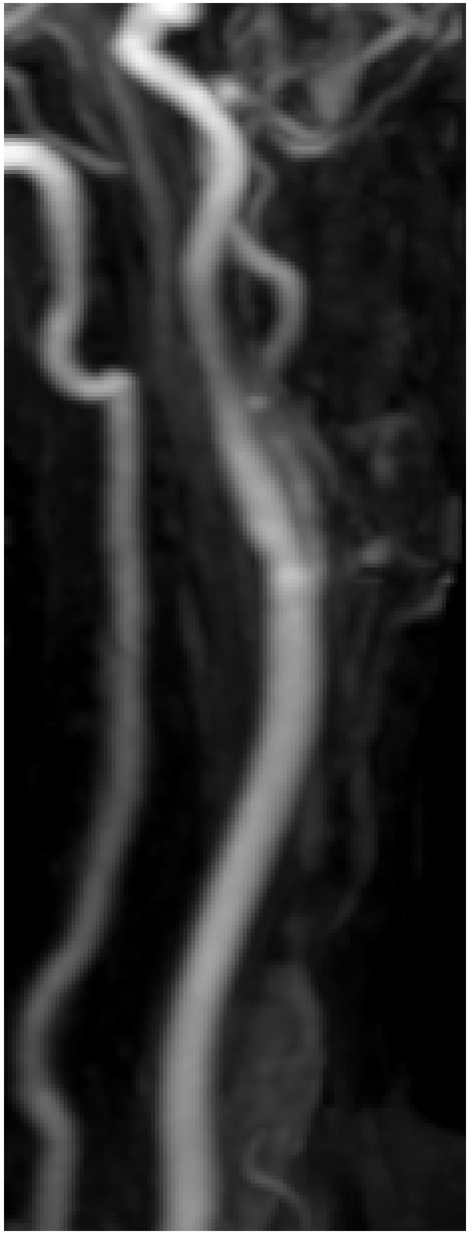

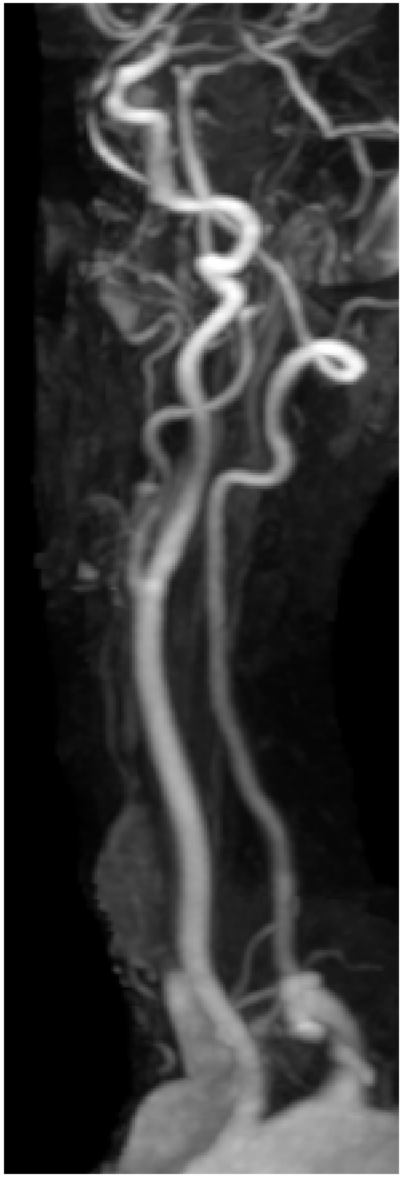

85e Score

0-30

31-50

51-70

>70

Near occlusion

Occluded

Quality

1

2

3

4

5

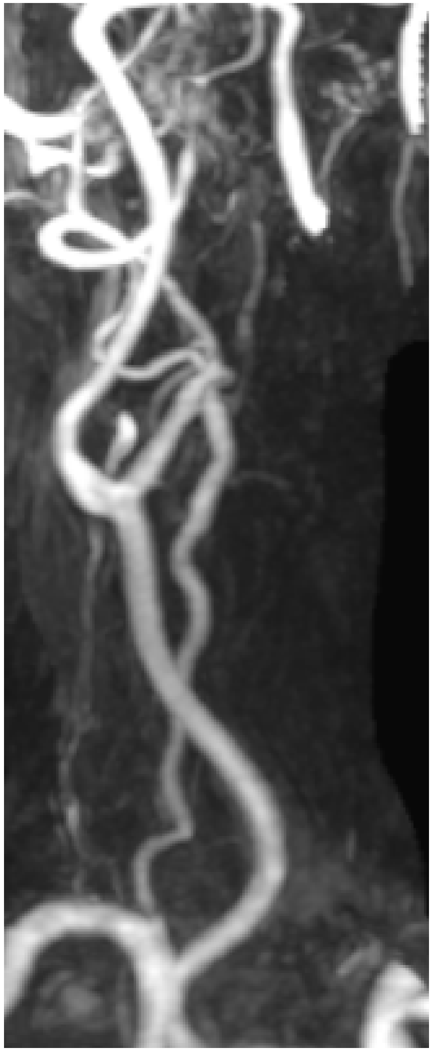

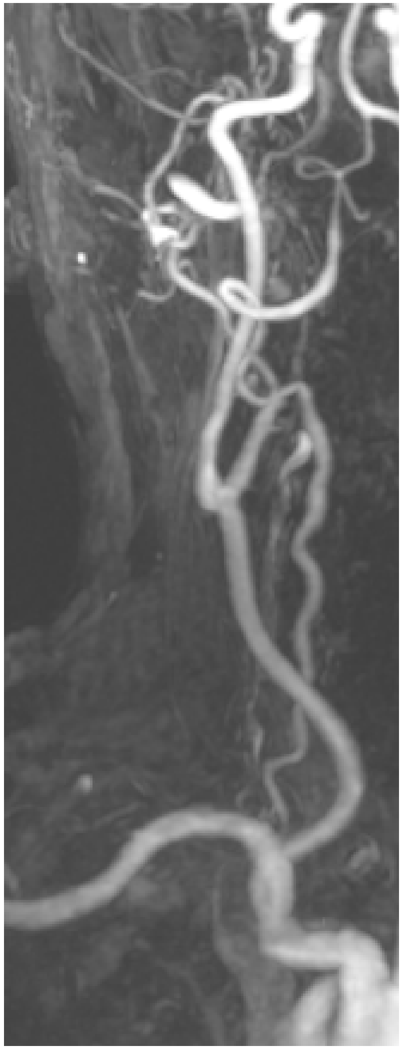

86d Score

0-30

31-50

51-70

>70

Near occlusion

Occluded

Quality

1

2

3

4

5

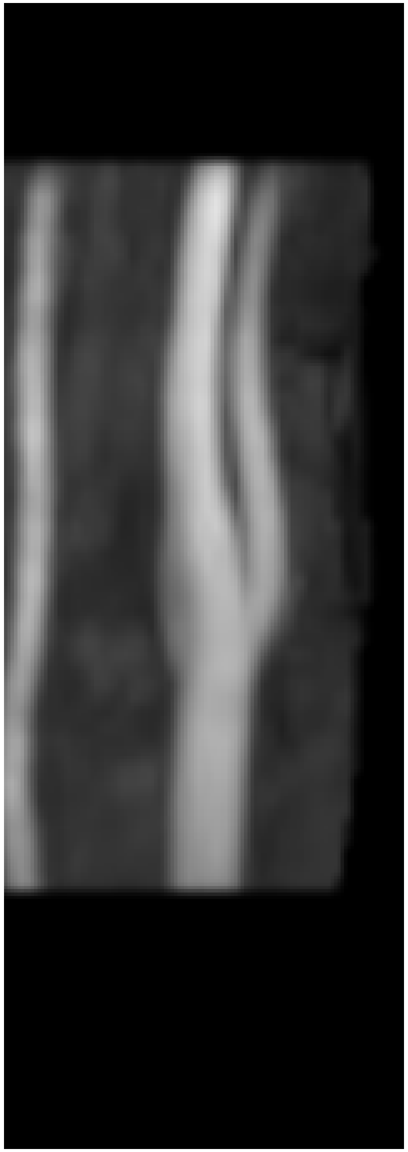

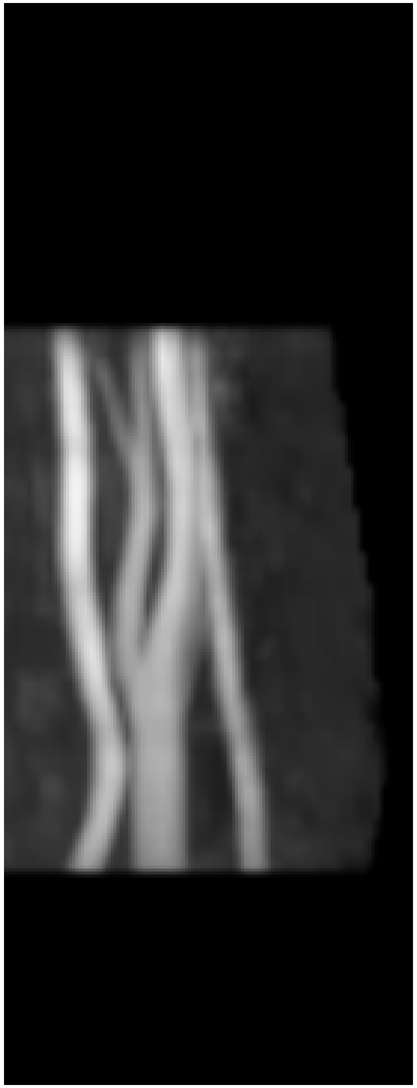

87c Score

0-30

31-50

51-70

>70

Near occlusion

Occluded

Quality

1

2

3

4

5

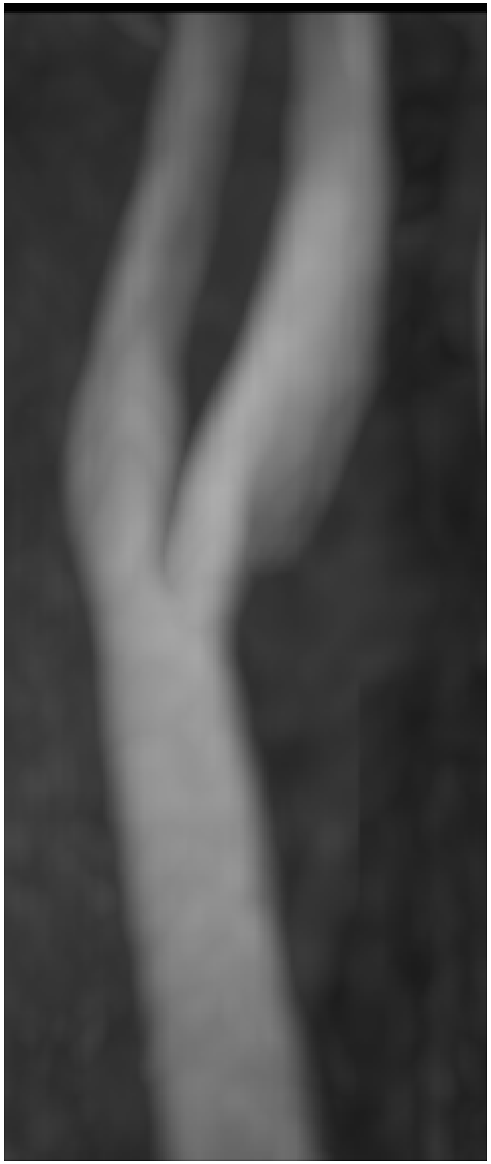

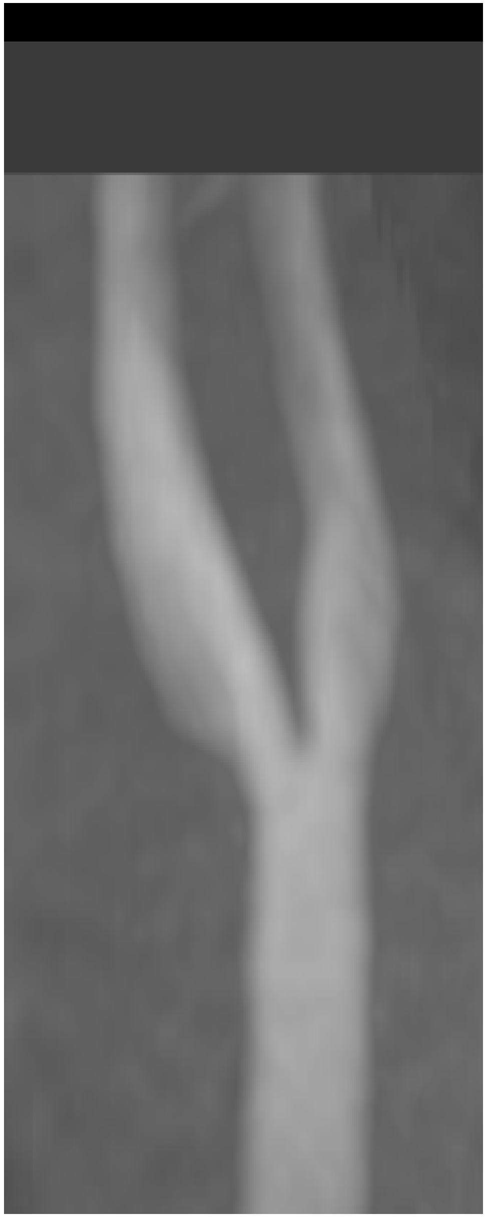

88b Score

0-30

31-50

51-70

>70

Near occlusion

Occluded

Quality

1

2

3

4

5

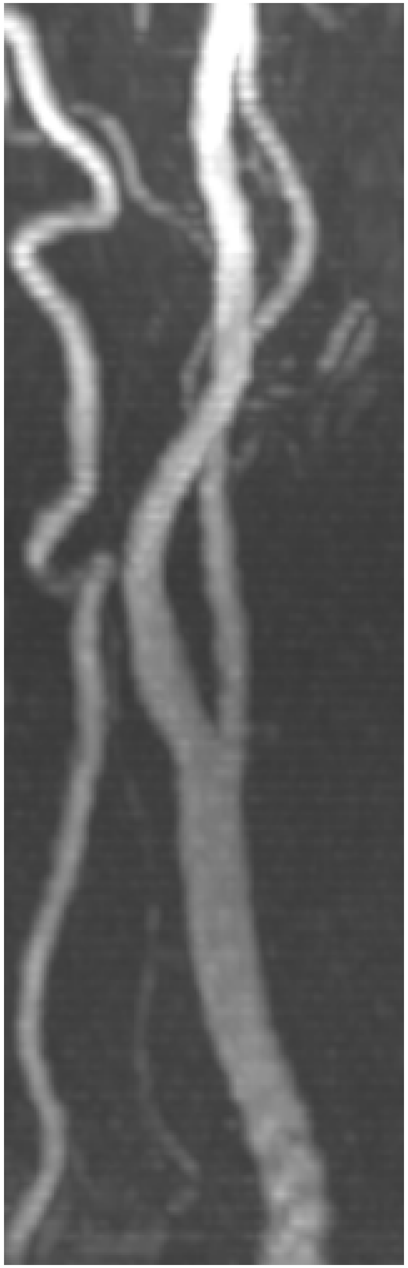

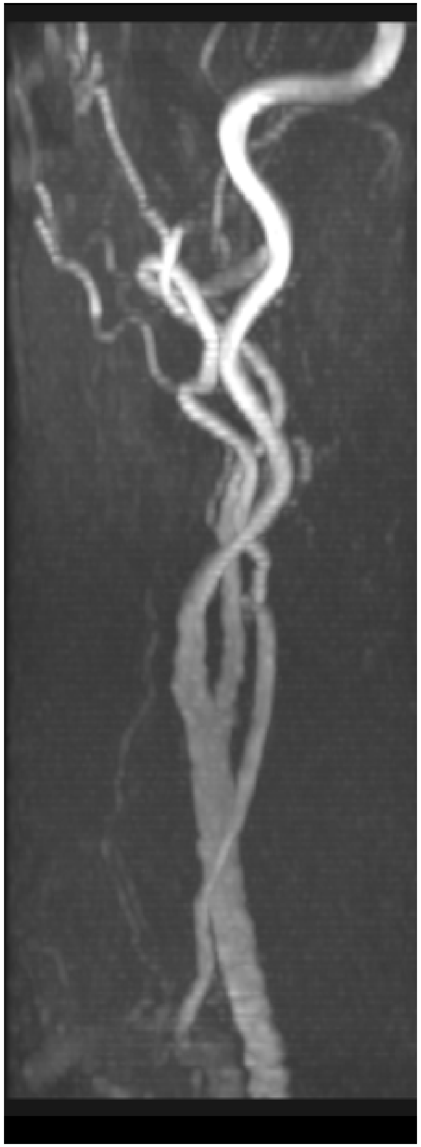

89a Score

0-30

31-50

51-70

>70

Near occlusion

Occluded

Quality

1

2

3

4

5

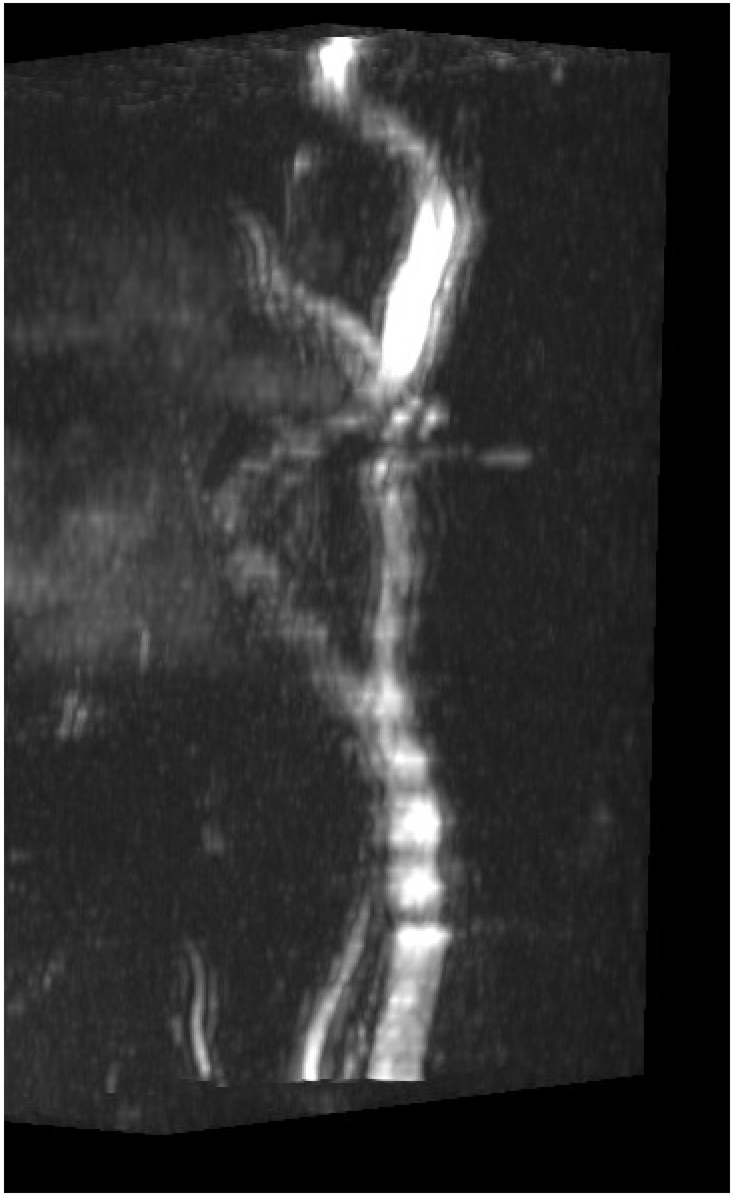

89f Score

0-30

31-50

51-70

>70

Near occlusion

Occluded

Quality

1

2

3

4

5

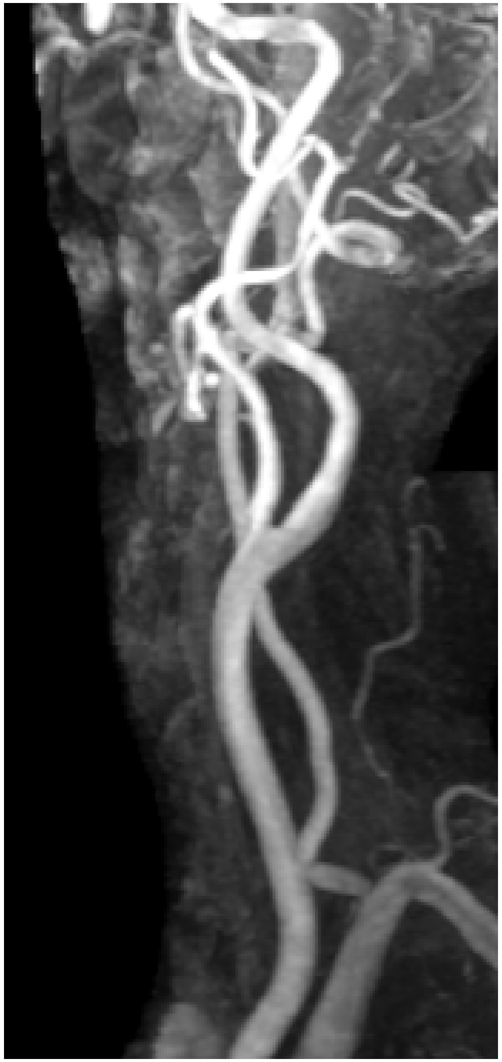

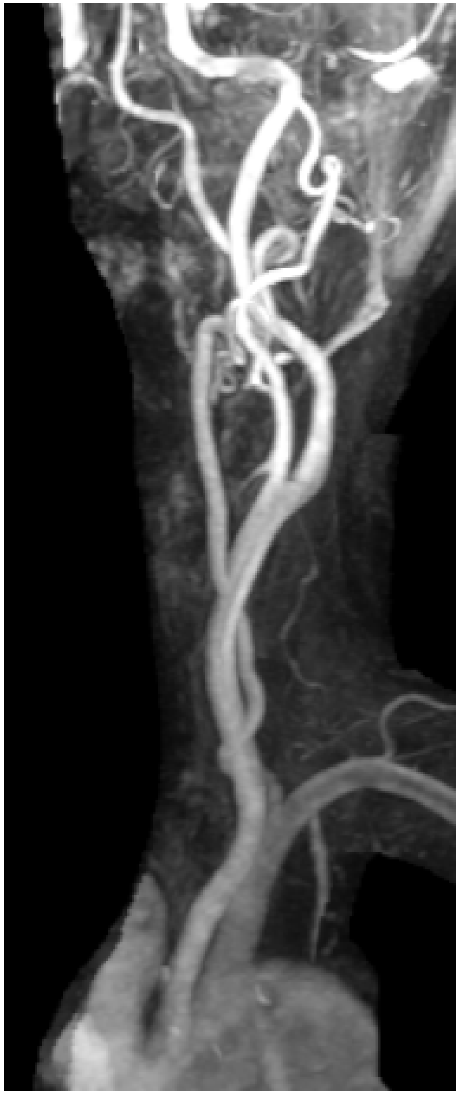

90e Score

0-30

31-50

51-70

>70

Near occlusion

Occluded

Quality

1

2

3

4

5

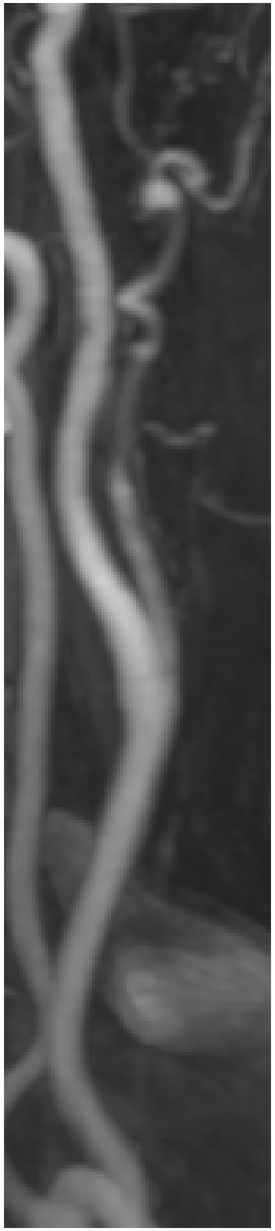

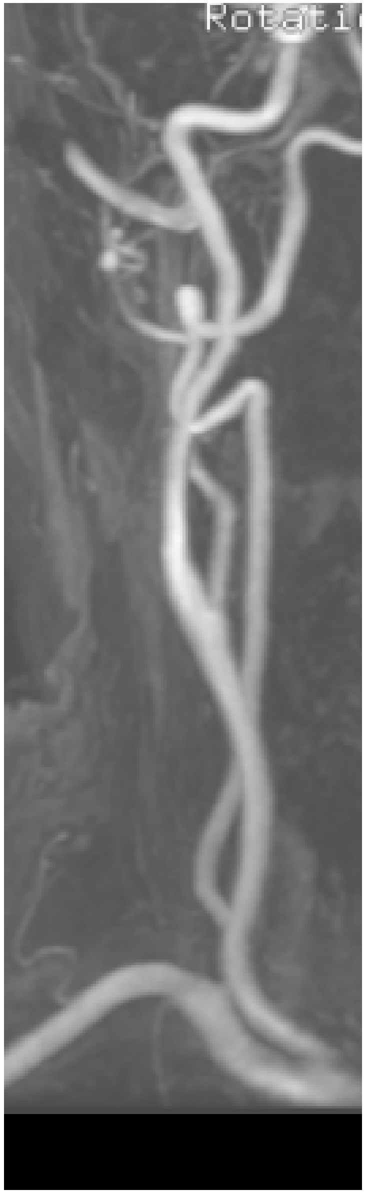

91d Score

0-30

31-50

51-70

>70

Near occlusion

Occluded

Quality

1

2

3

4

5

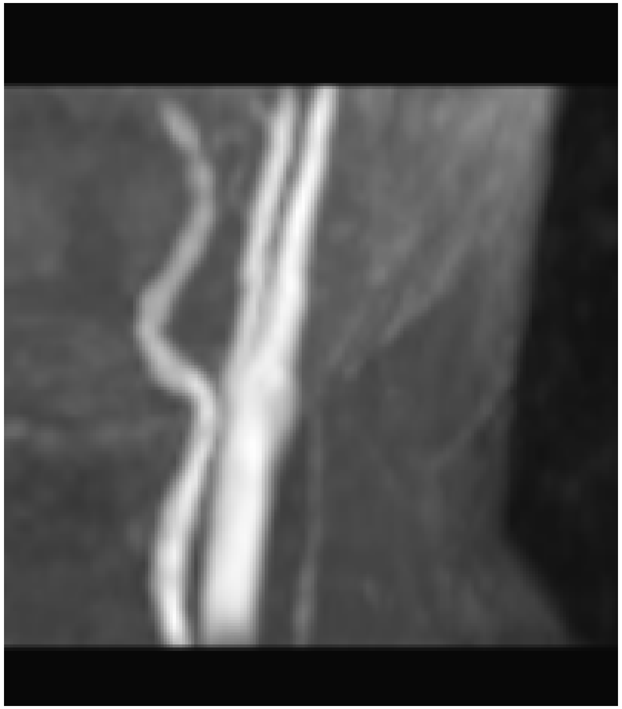

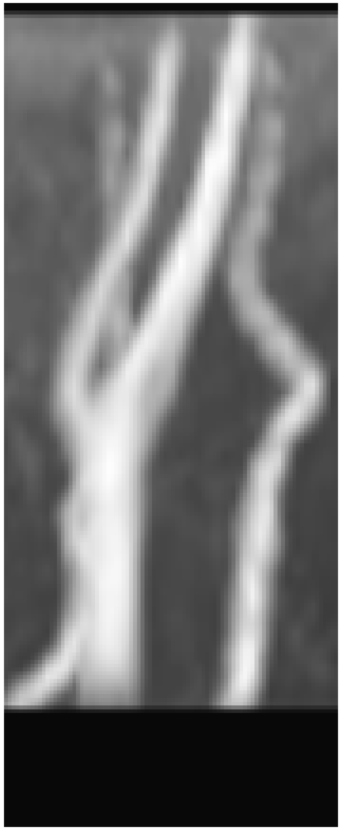

92c Score

0-30

31-50

51-70

>70

Near occlusion

Occluded

Quality

1

2

3

4

5

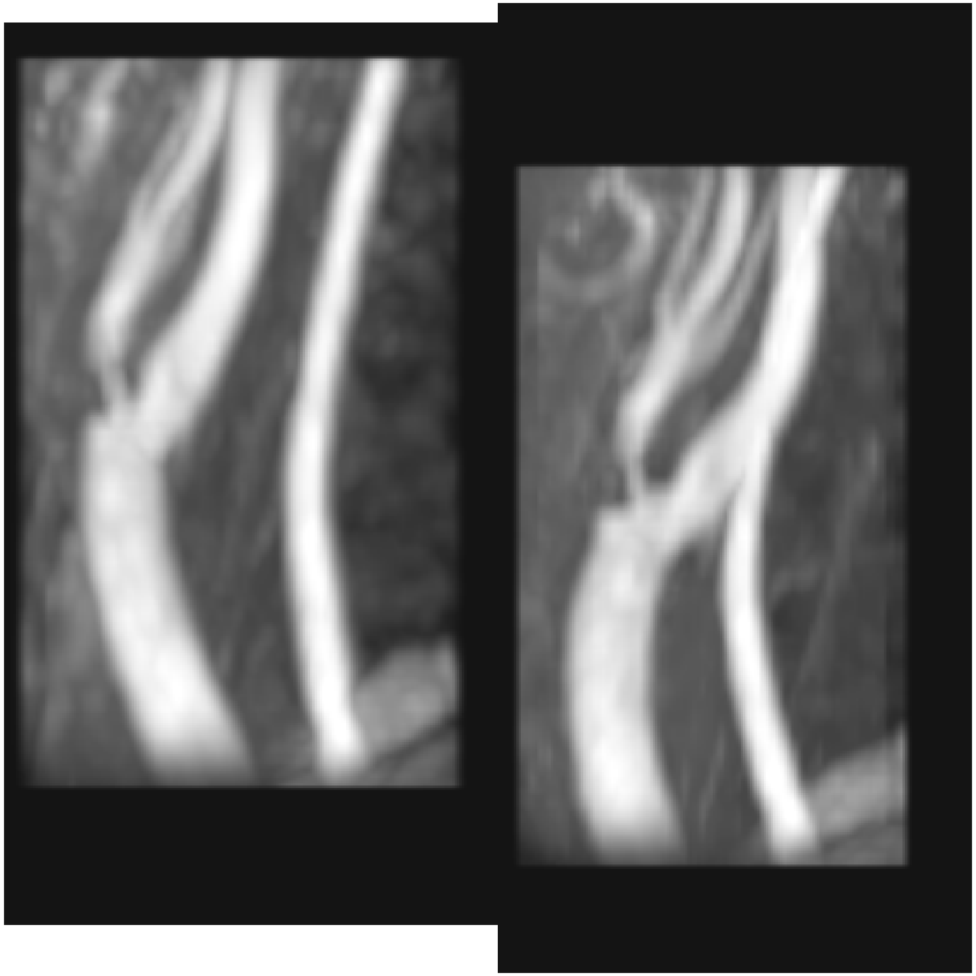

93b Score

0-30

31-50

51-70

>70

Near occlusion

Occluded

Quality

1

2

3

4

5

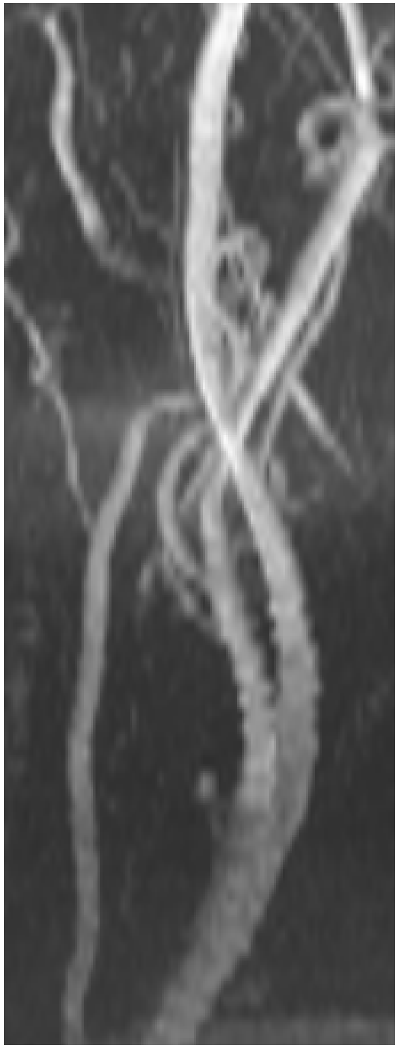

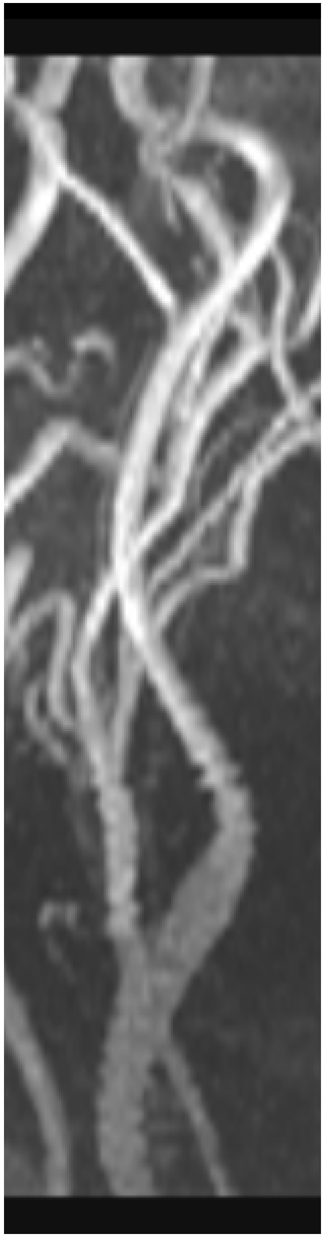

94a Score

0-30

31-50

51-70

>70

Near occlusion

Occluded

Quality

1

2

3

4

5

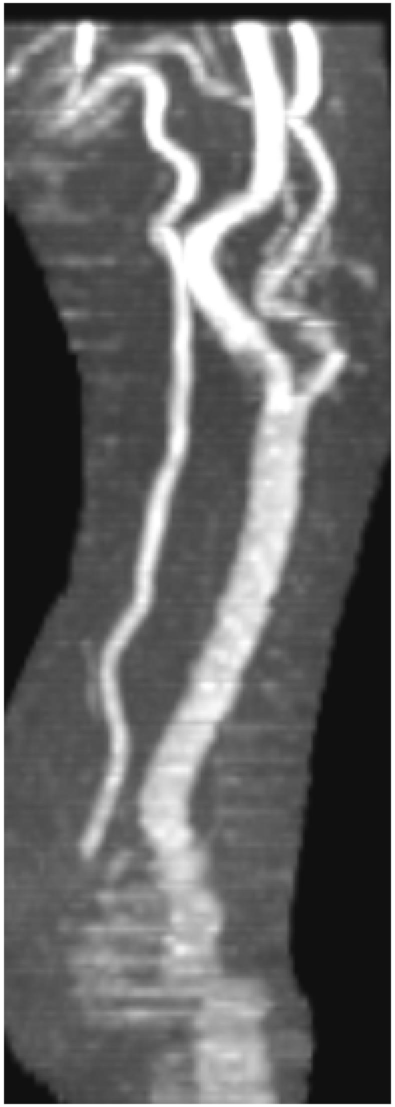

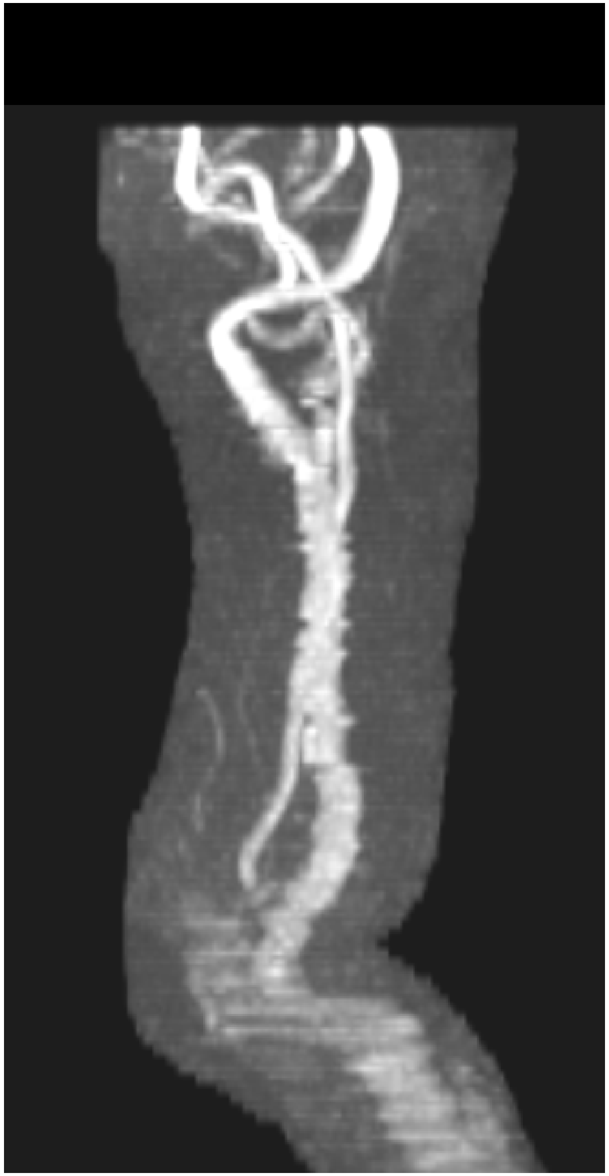

94f Score

0-30

31-50

51-70

>70

Near occlusion

Occluded

Quality

1

2

3

4

5

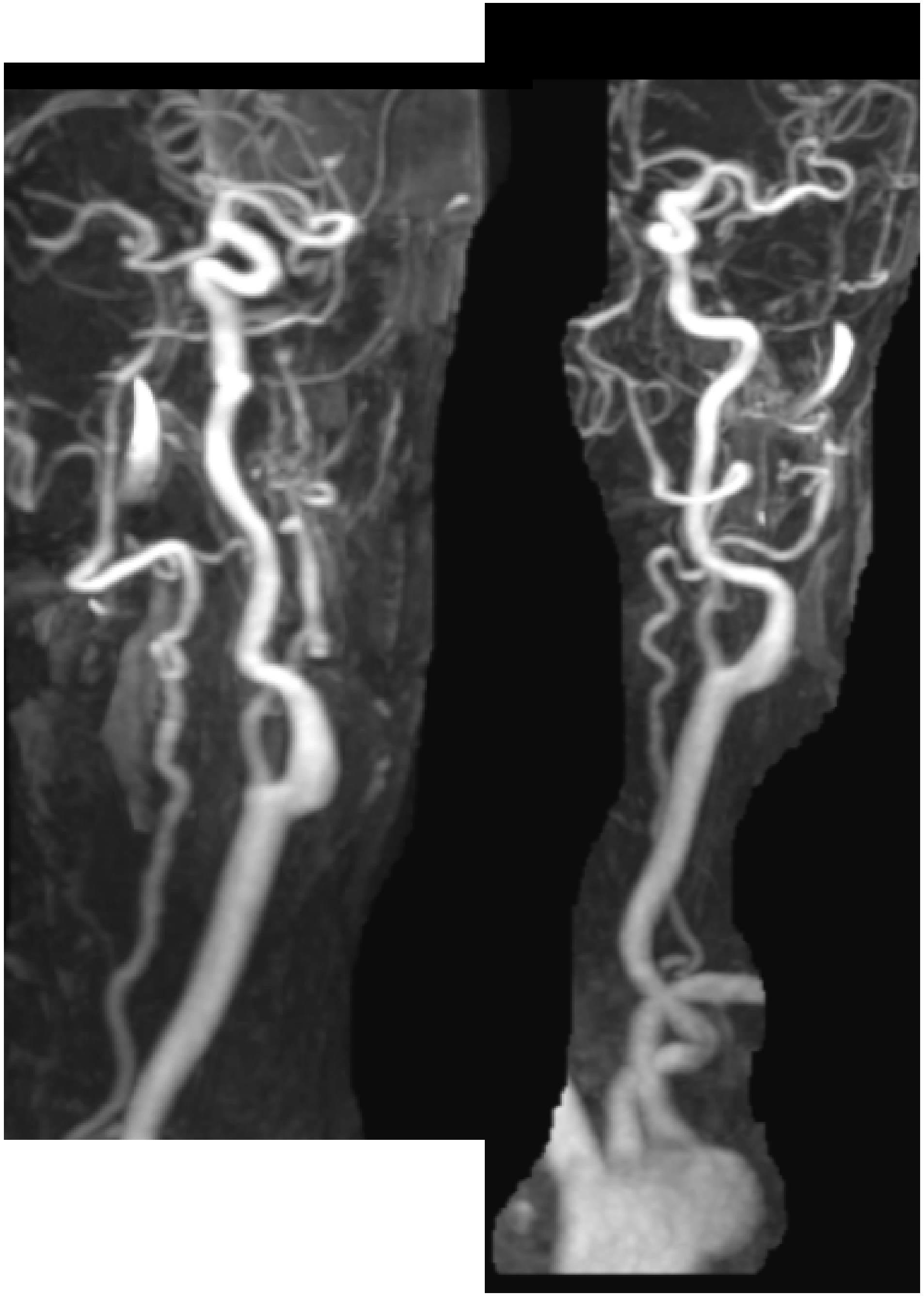

95e Score

0-30

31-50

51-70

>70

Near occlusion

Occluded

Quality

1

2

3

4

5

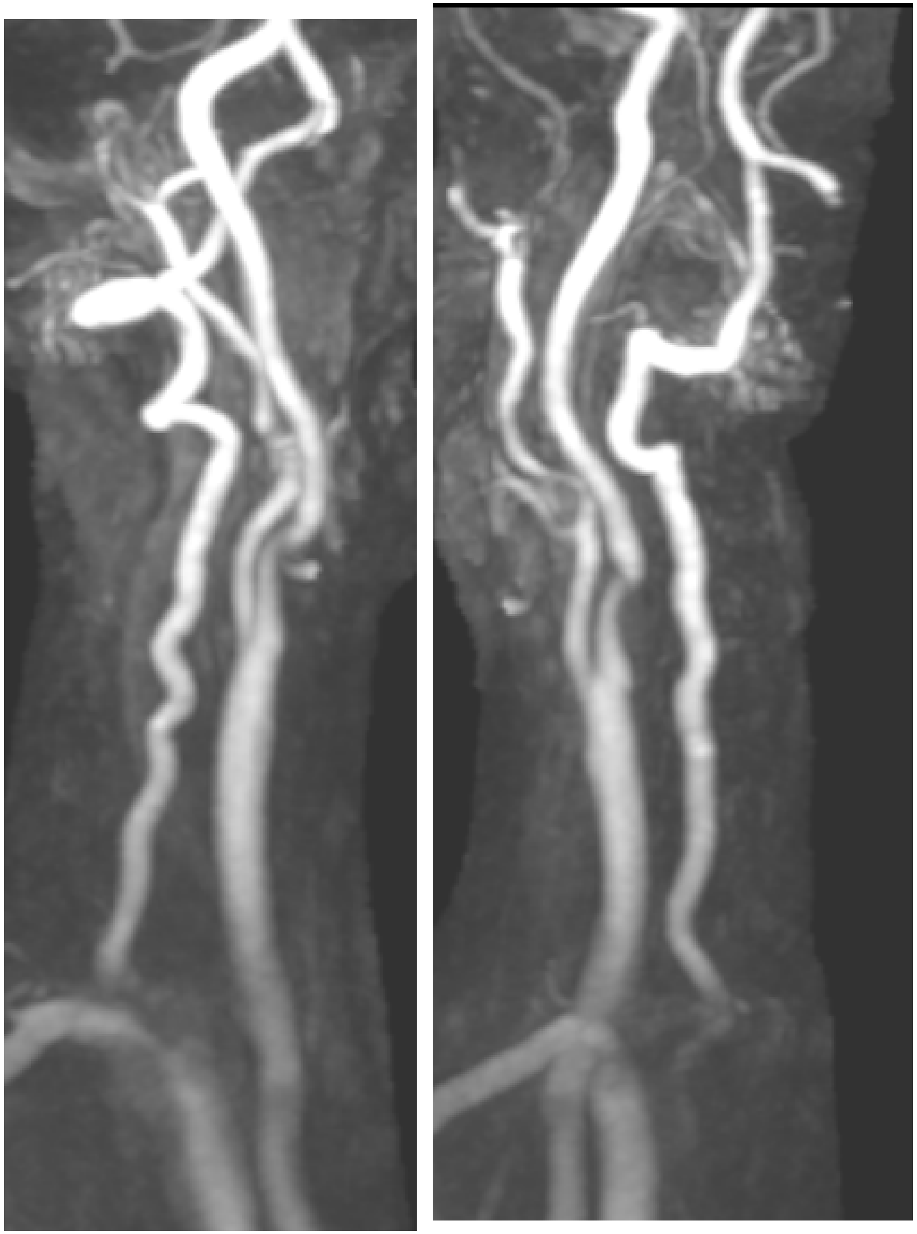

96d Score

0-30

31-50

51-70

>70

Near occlusion

Occluded

Quality

1

2

3

4

5

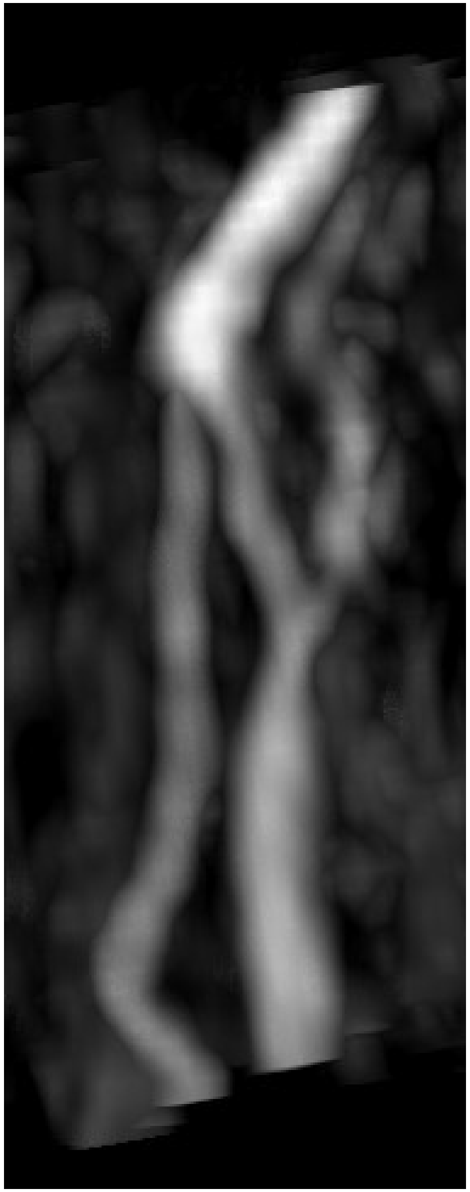

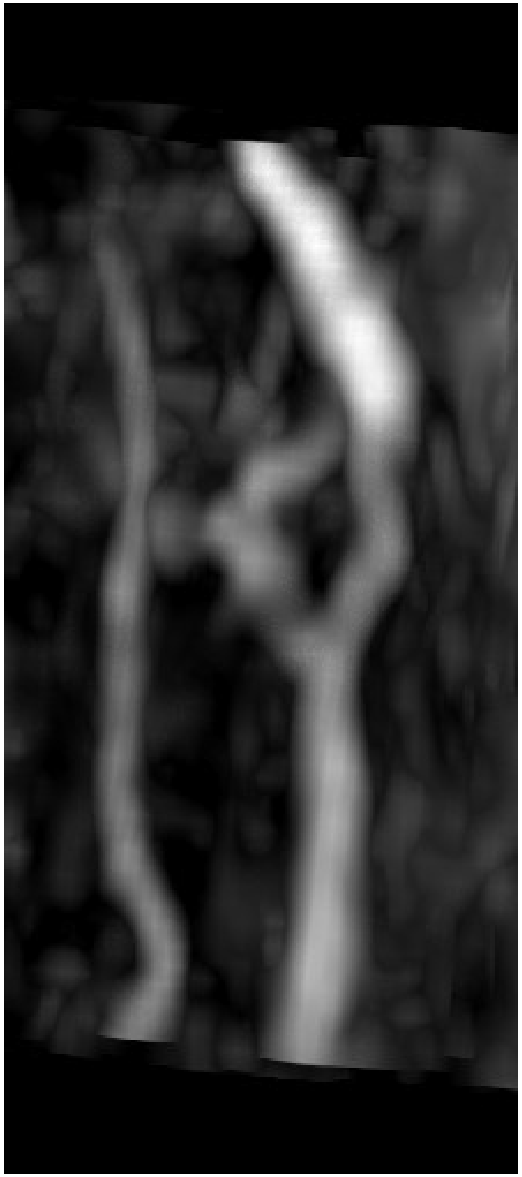

97c Score

0-30

31-50

51-70

>70

Near occlusion

Occluded

Quality

1

2

3

4

5

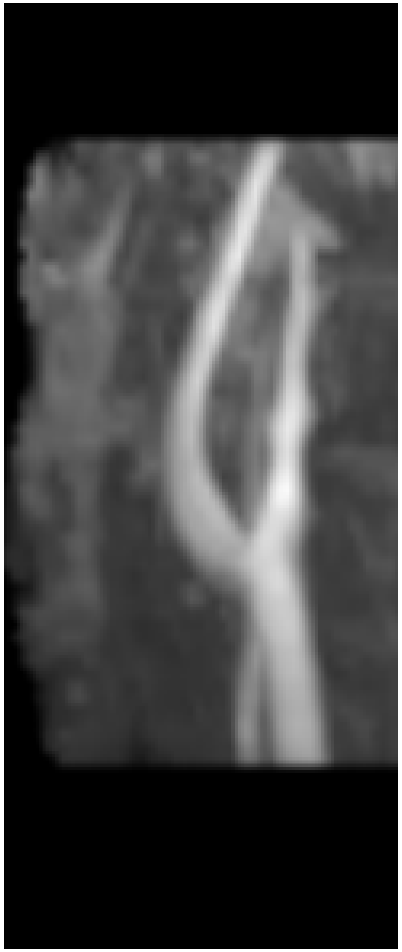

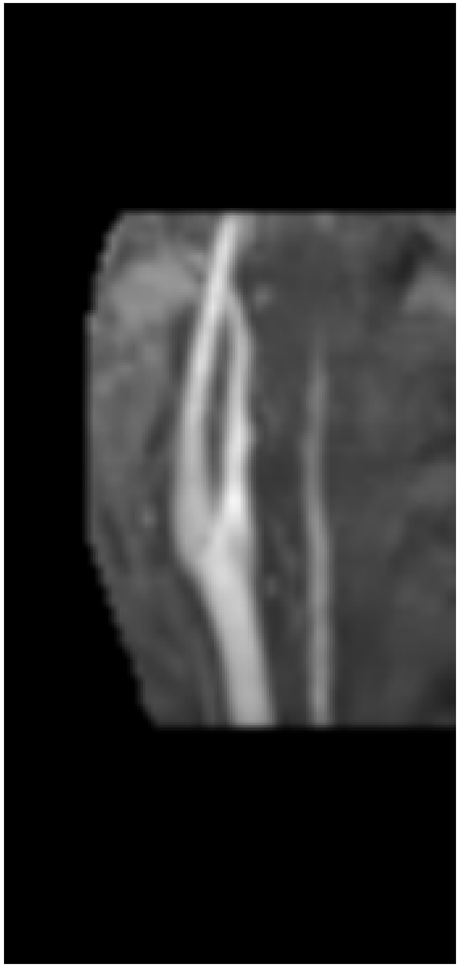

98b Score

0-30

31-50

51-70

>70

Near occlusion

Occluded

Quality

1

2

3

4

5

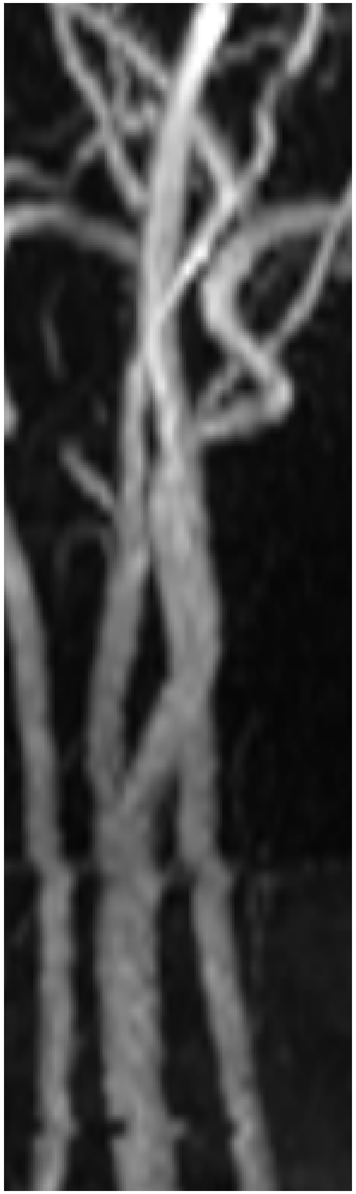

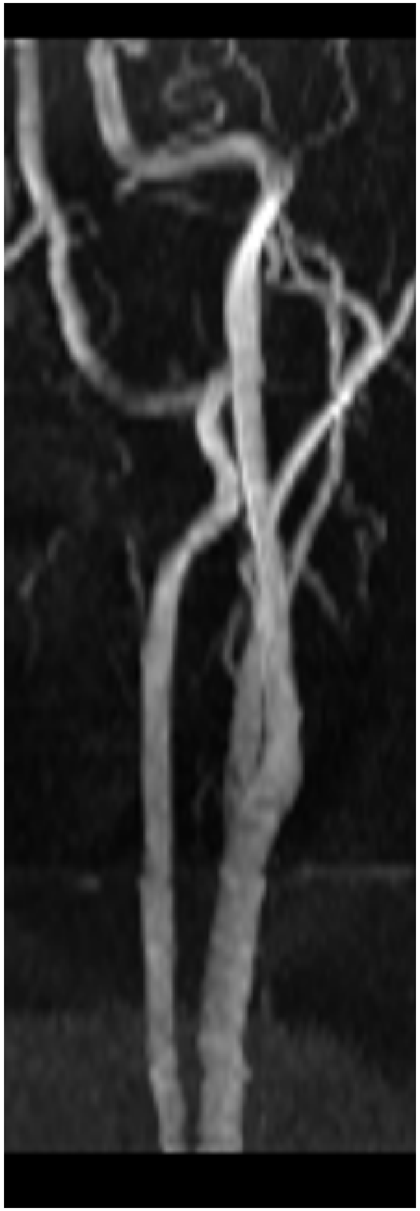

99a Score

0-30

31-50

51-70

>70

Near occlusion

Occluded

Quality

1

2

3

4

5

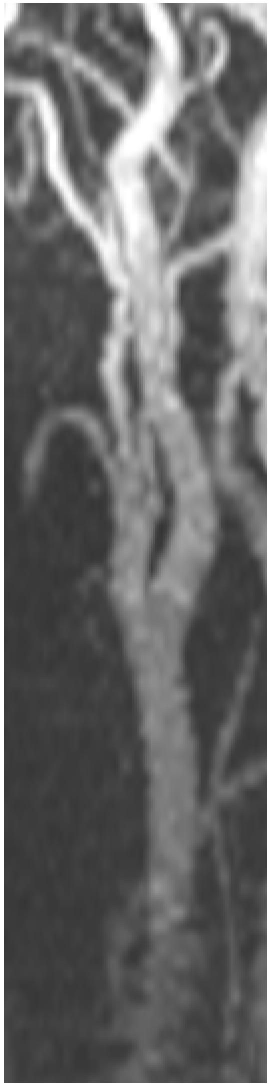

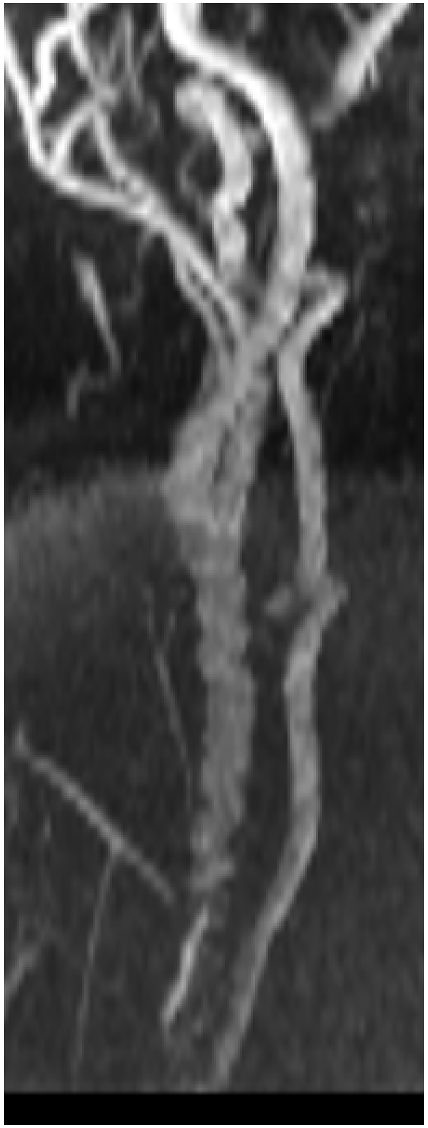

99f Score

0-30

31-50

51-70

>70

Near occlusion

Occluded

Quality

1

2

3

4

5

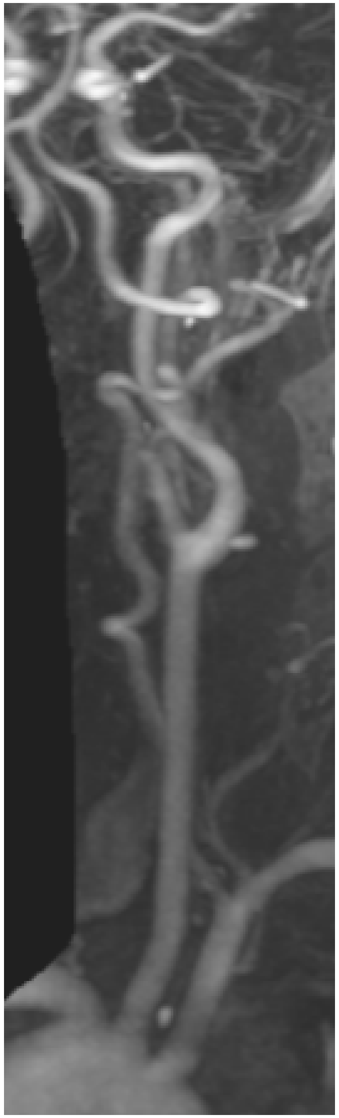

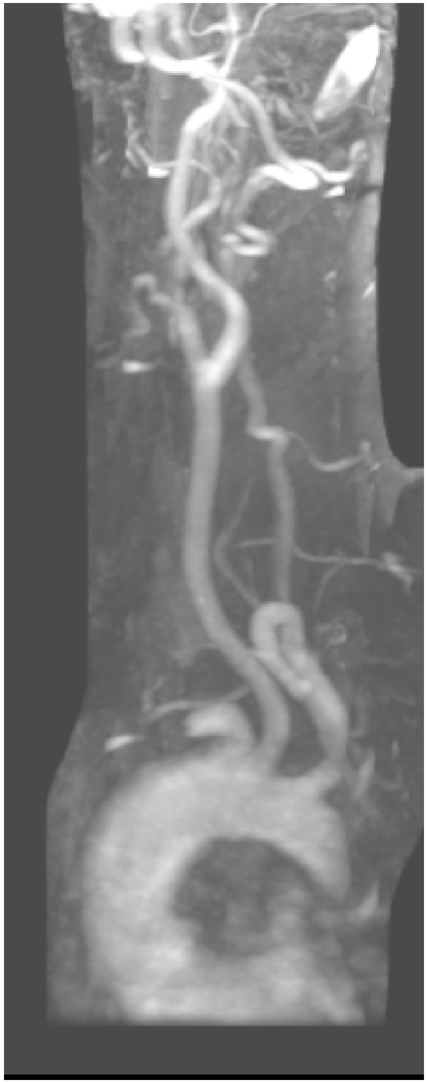

100e Score

0-30

31-50

51-70

>70

Near occlusion

Occluded

Quality

1

2

3

4

5

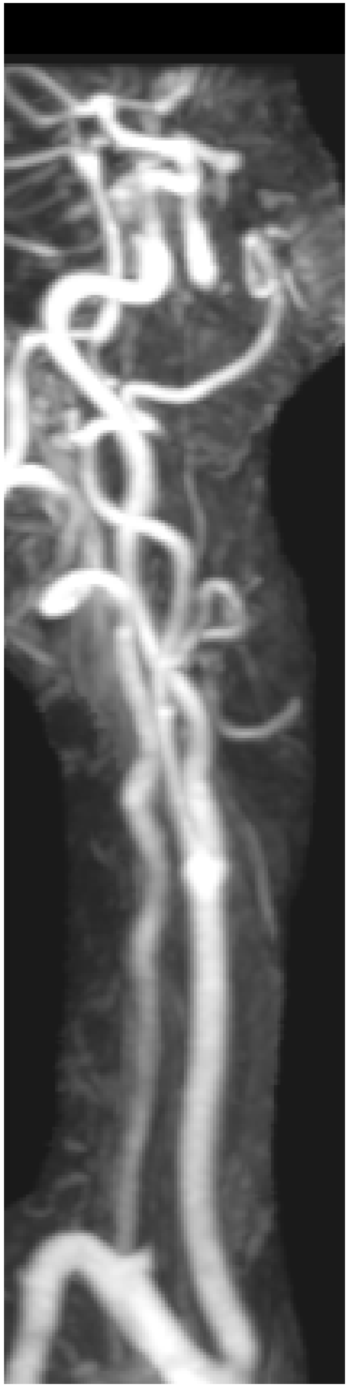

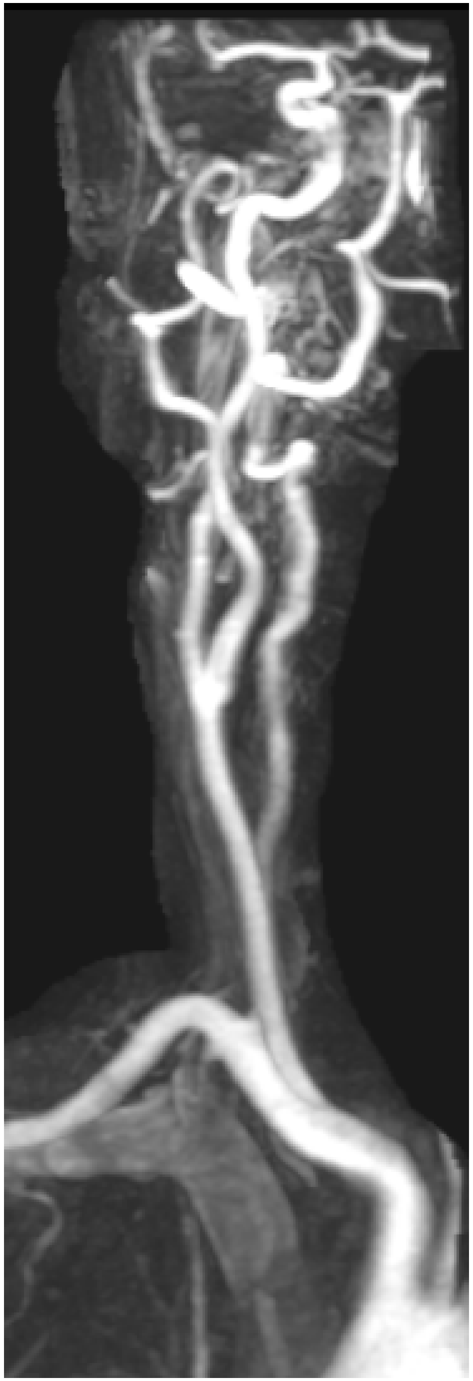

101d Score

0-30

31-50

51-70

>70

Near occlusion

Occluded

Quality

1

2

3

4

5

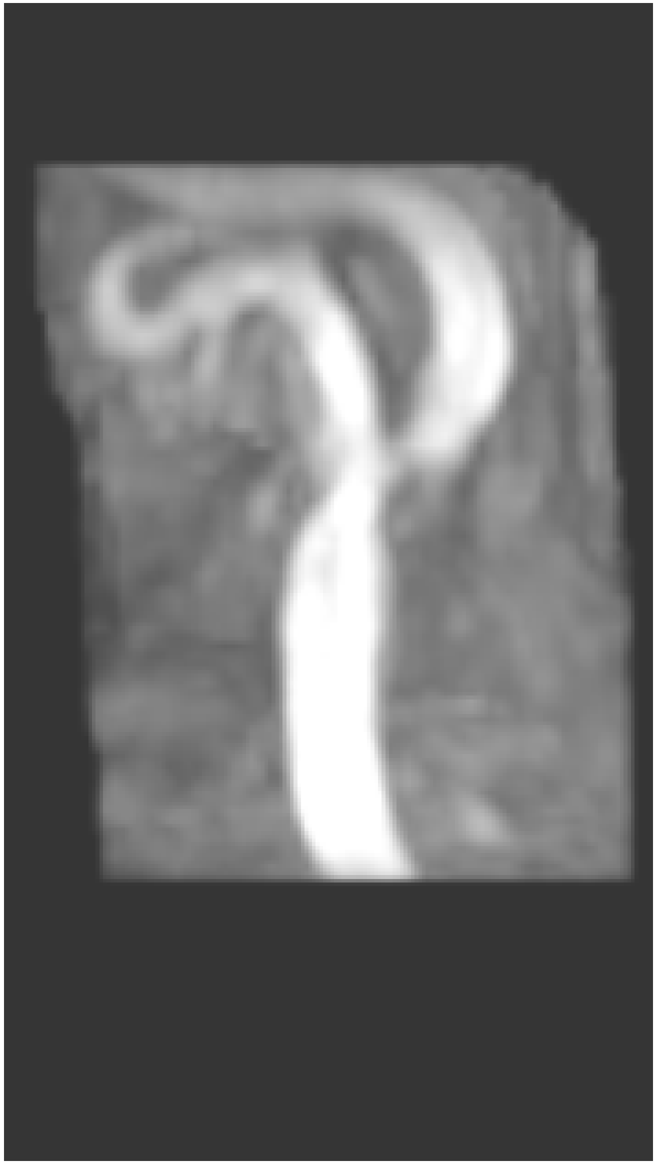

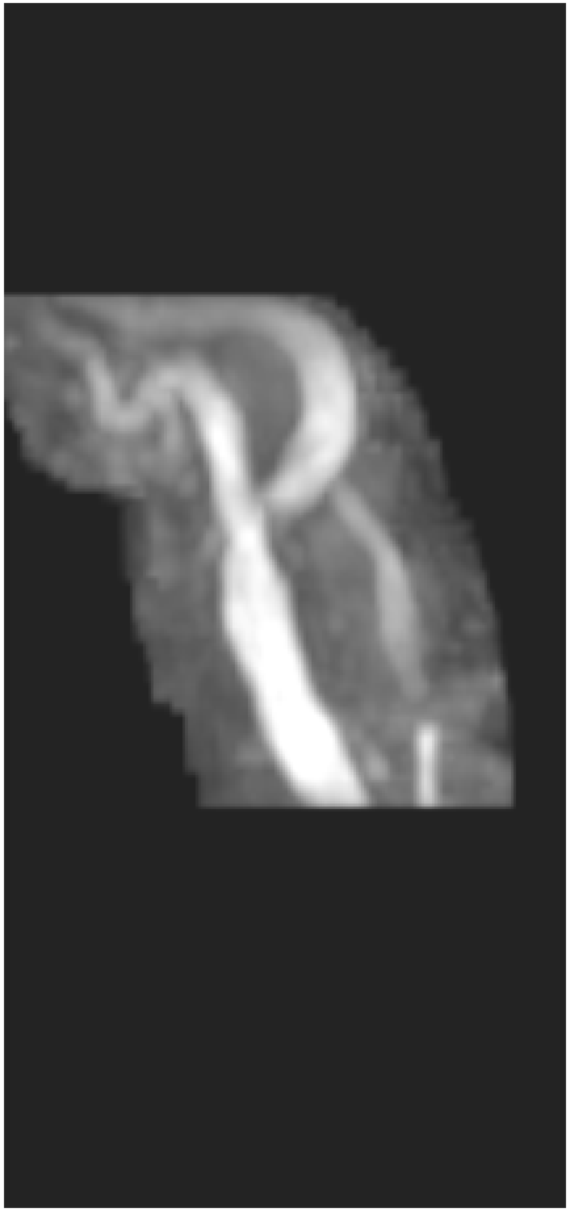

102c Score

0-30

31-50

51-70

>70

Near occlusion

Occluded

Quality

1

2

3

4

5

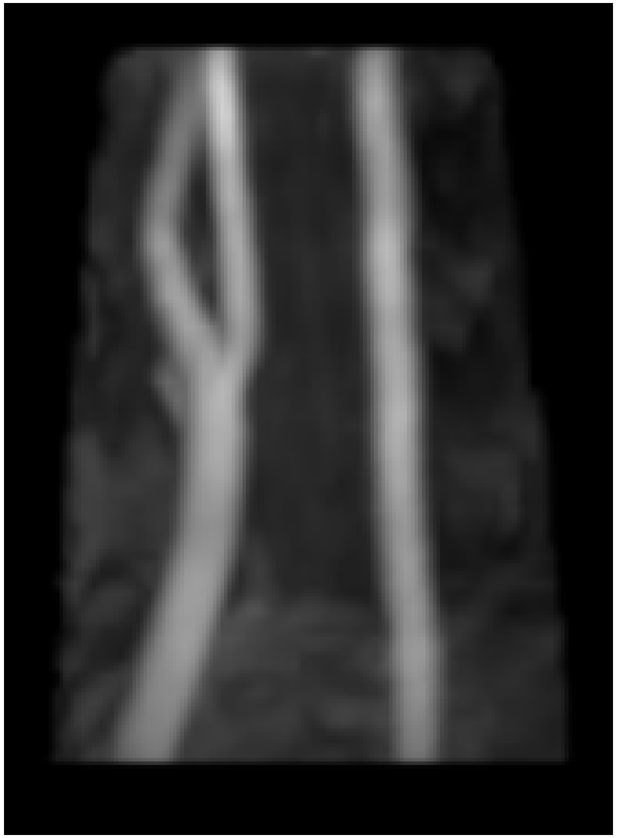

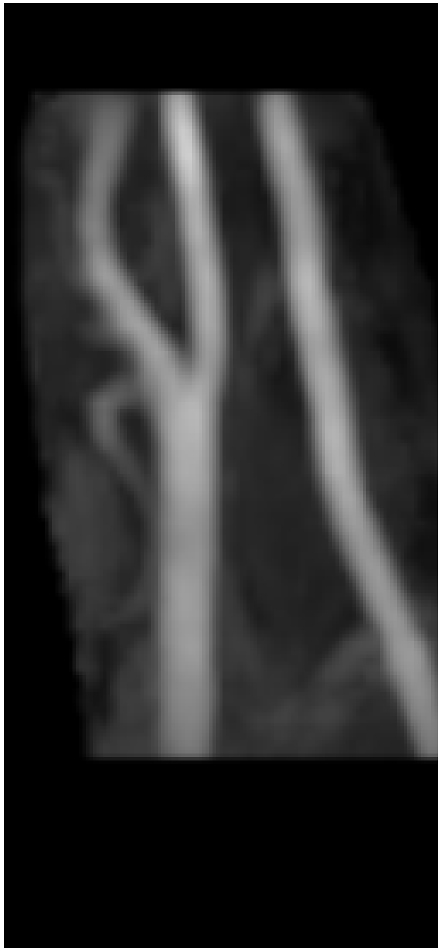

103b Score

0-30

31-50

51-70

>70

Near occlusion

Occluded

Quality

1

2

3

4

5

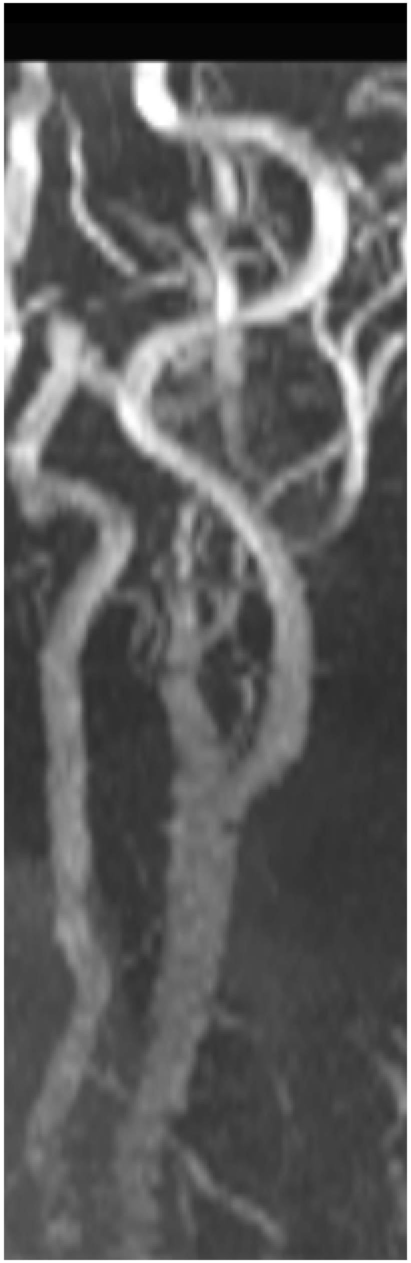

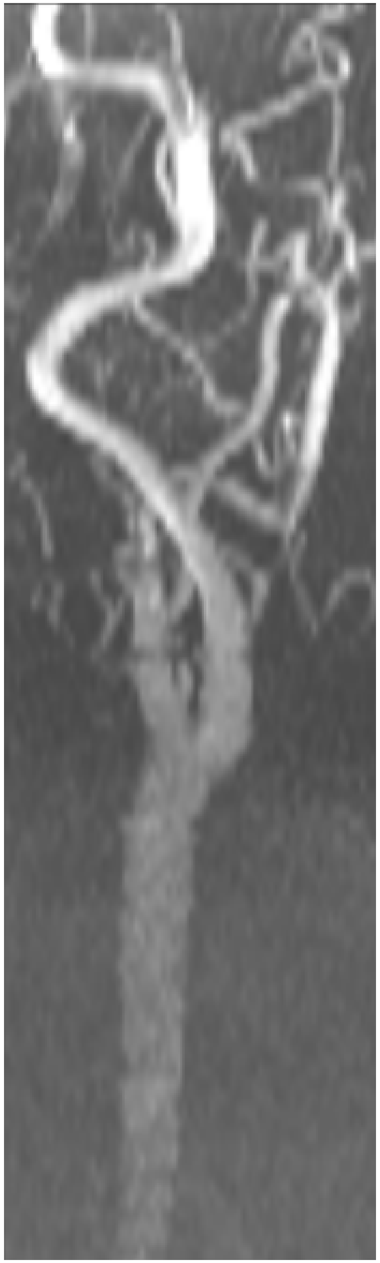

104a Score

0-30

31-50

51-70

>70

Near occlusion

Occluded

Quality

1

2

3

4

5

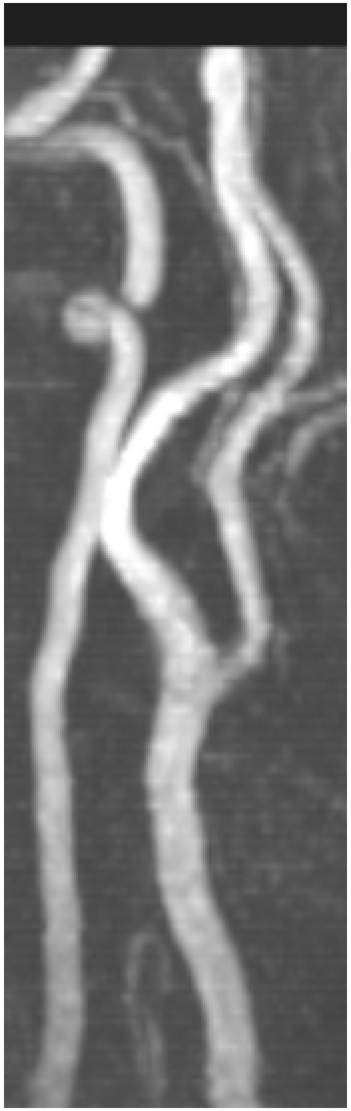

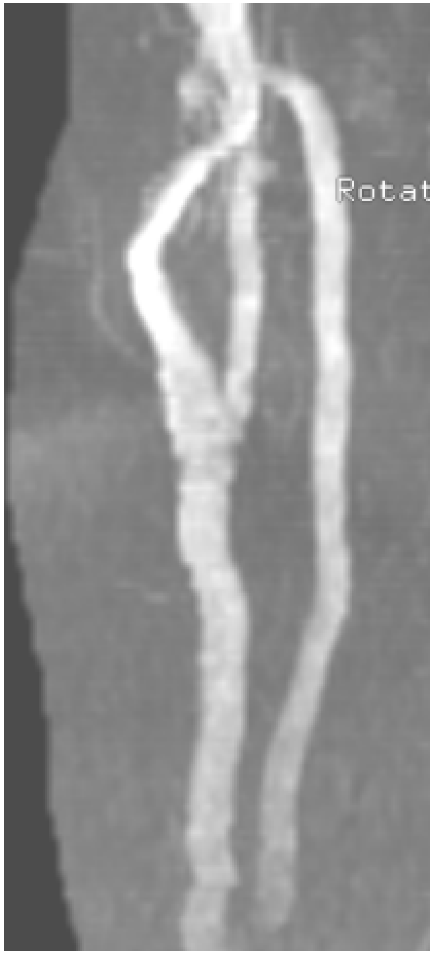

104f Score

0-30

31-50

51-70

>70

Near occlusion

Occluded

Quality

1

2

3

4

5

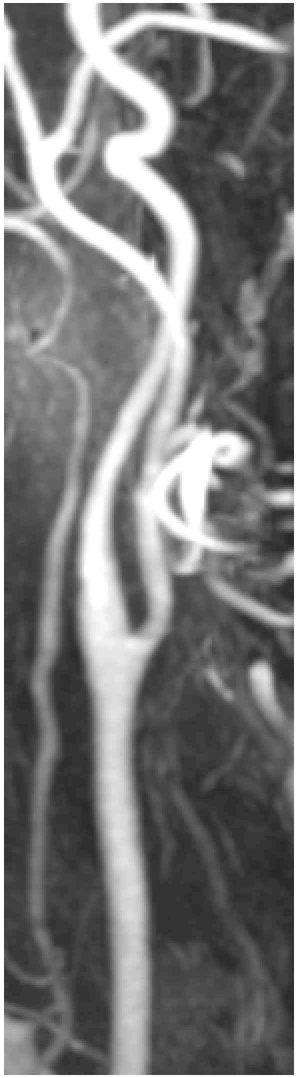

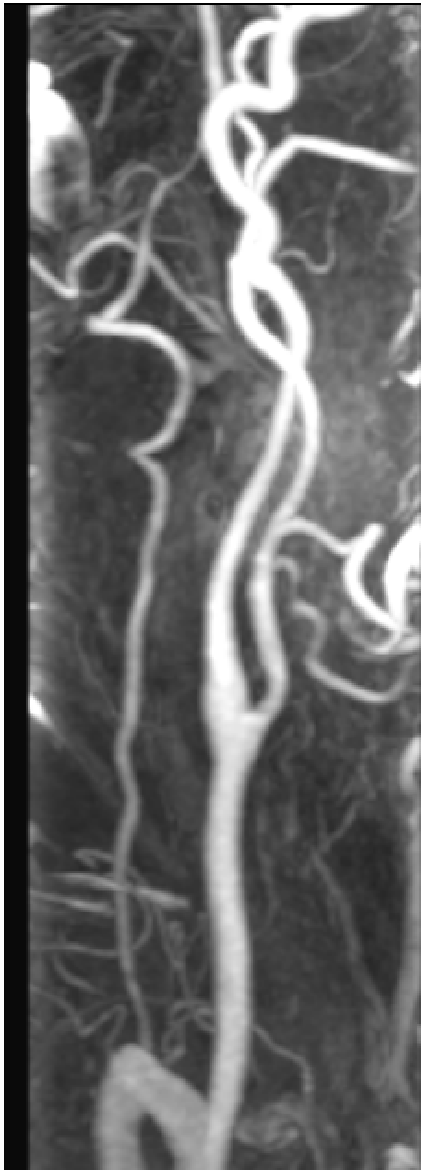

105e Score

0-30

31-50

51-70

>70

Near occlusion

Occluded

Quality

1

2

3

4

5

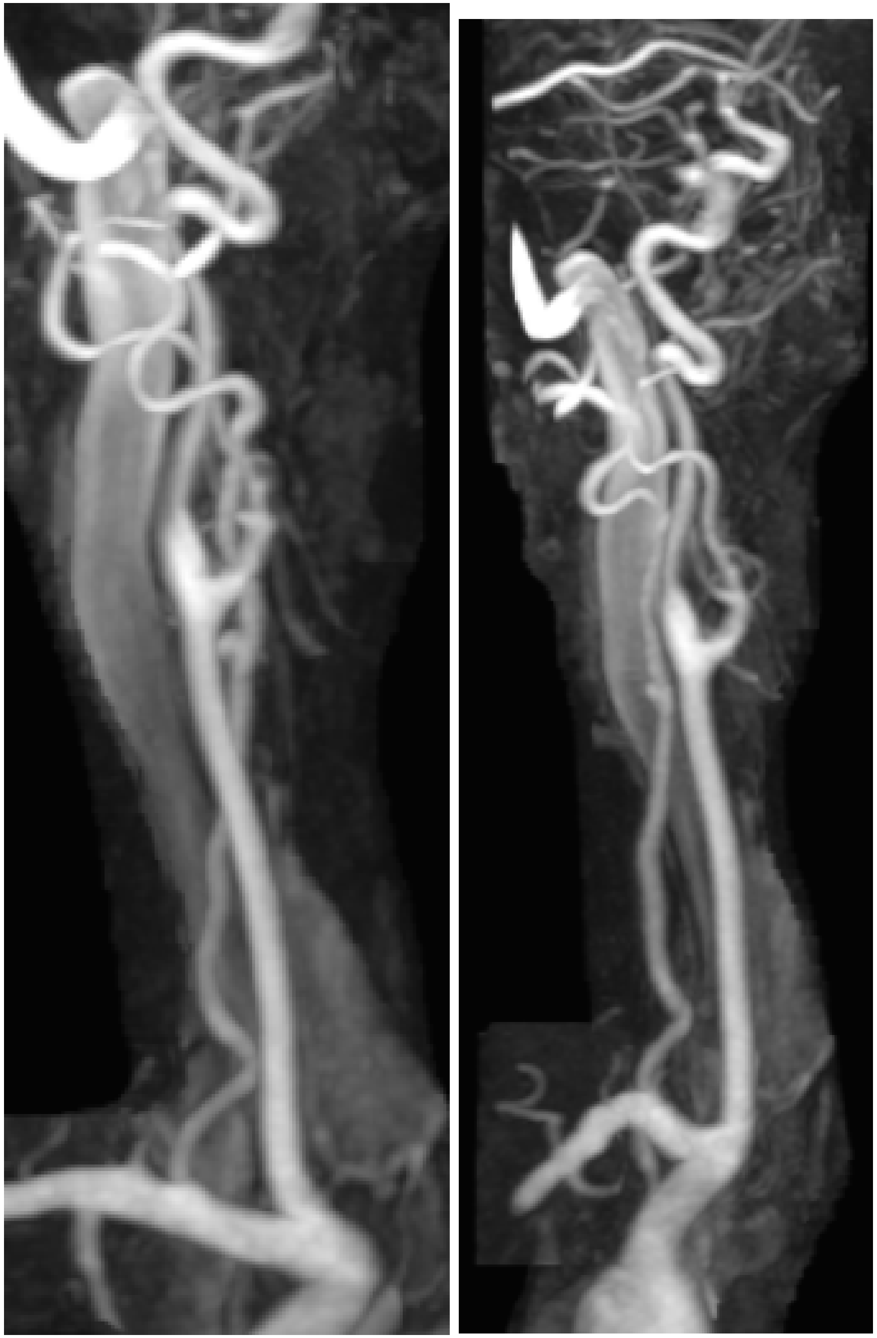

106d Score

0-30

31-50

51-70

>70

Near occlusion

Occluded

Quality

1

2

3

4

5

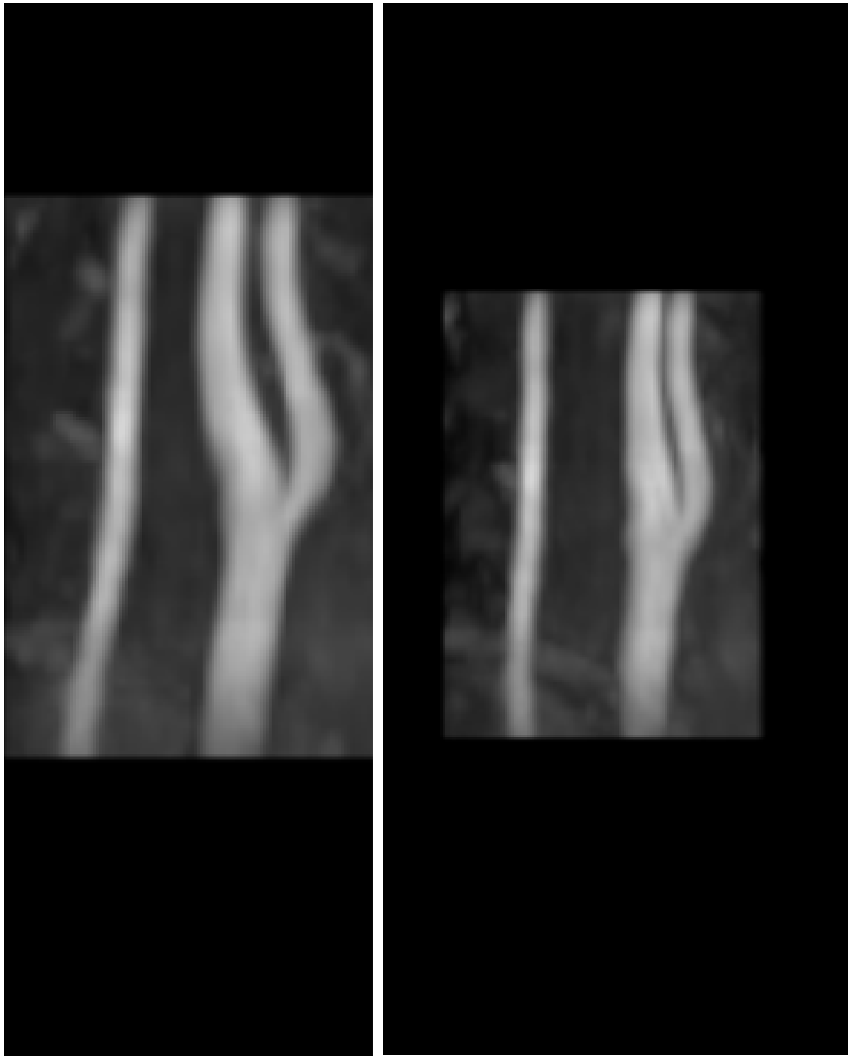

107c Score

0-30

31-50

51-70

>70

Near occlusion

Occluded

Quality

1

2

3

4

5

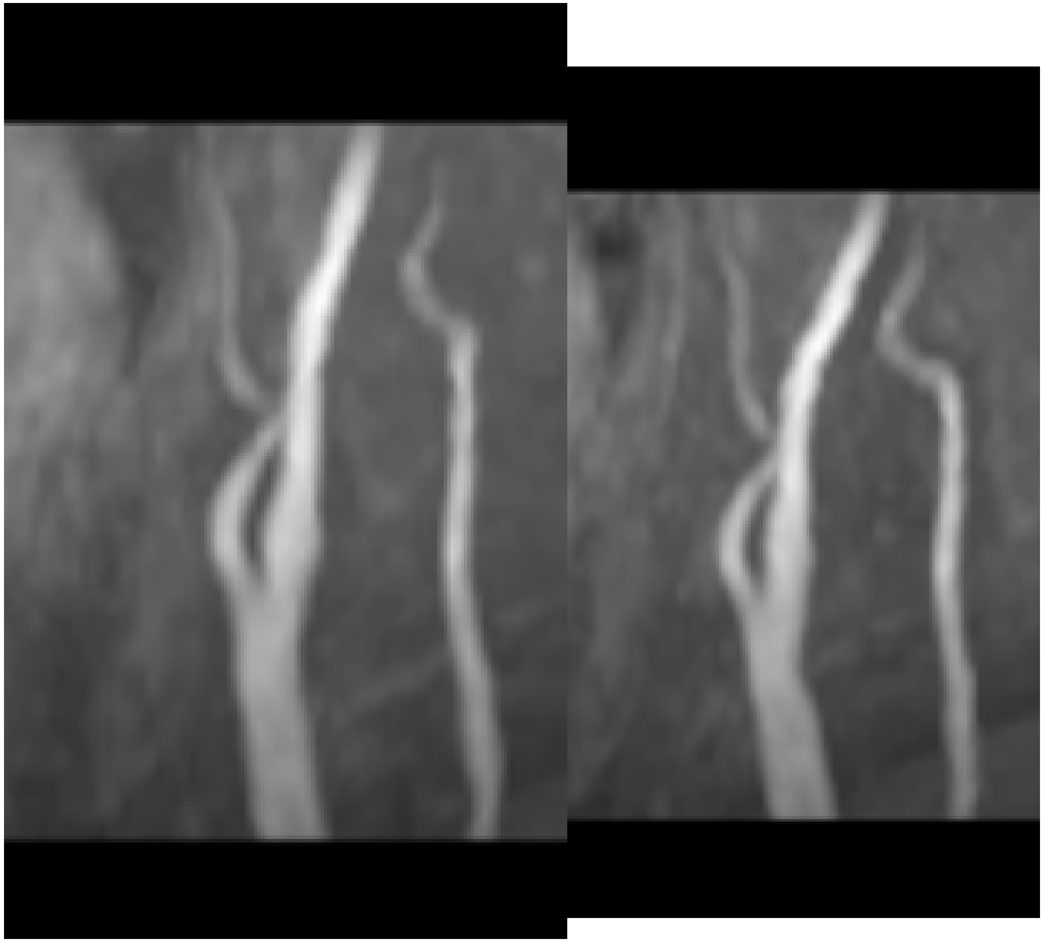

108b Score

0-30

31-50

51-70

>70

Near occlusion

Occluded

Quality

1

2

3

4

5

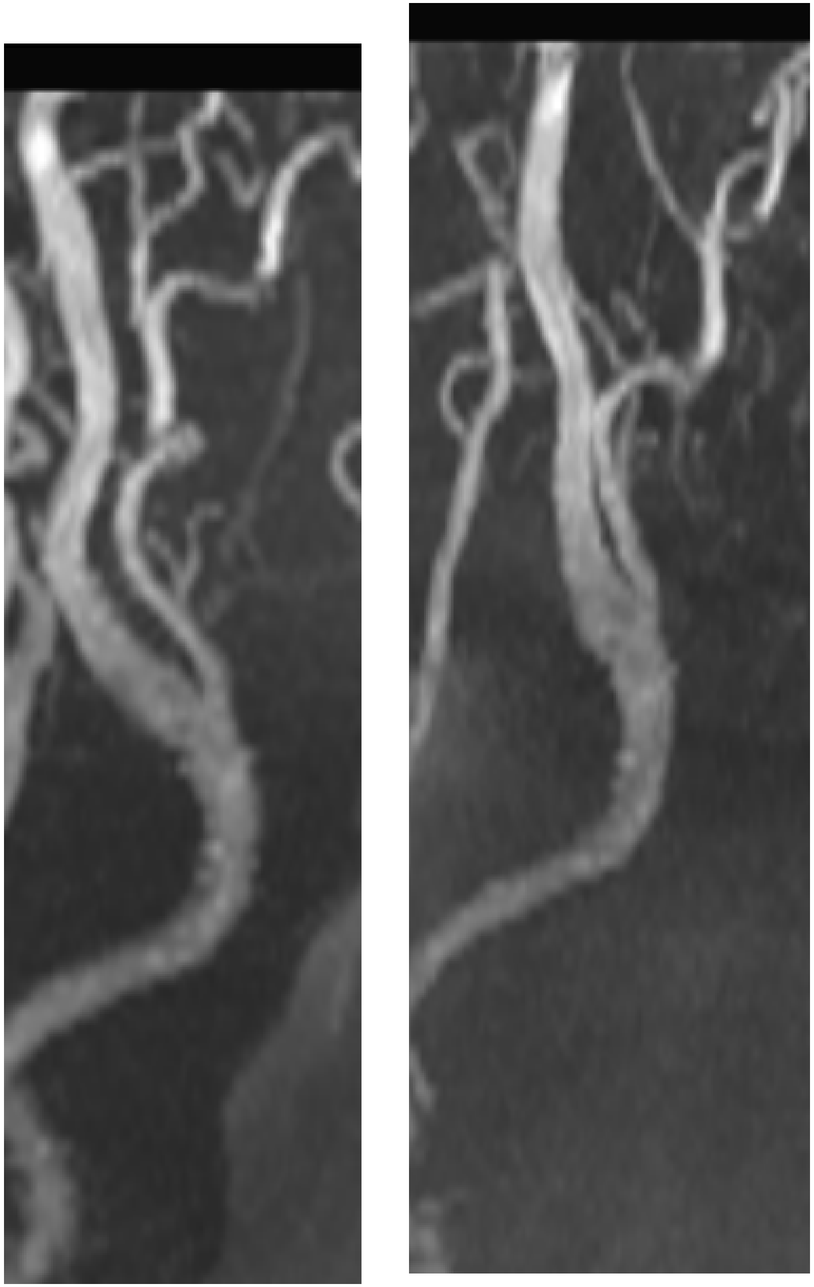

109a Score

0-30

31-50

51-70

>70

Near occlusion

Occluded

Quality

1

2

3

4

5

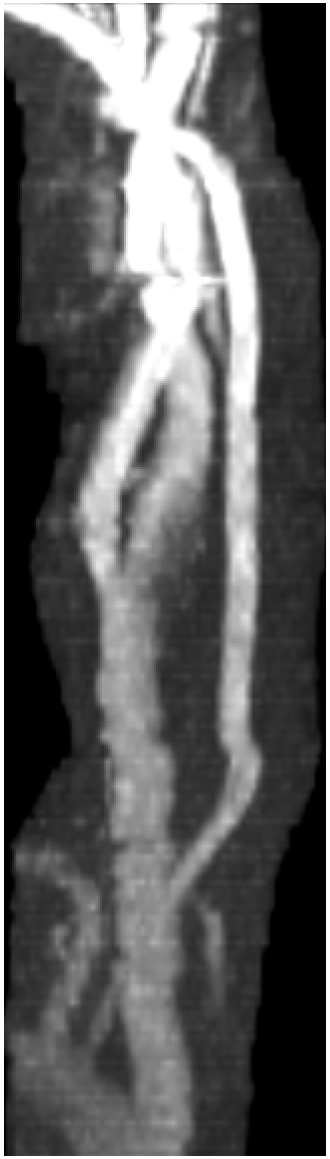

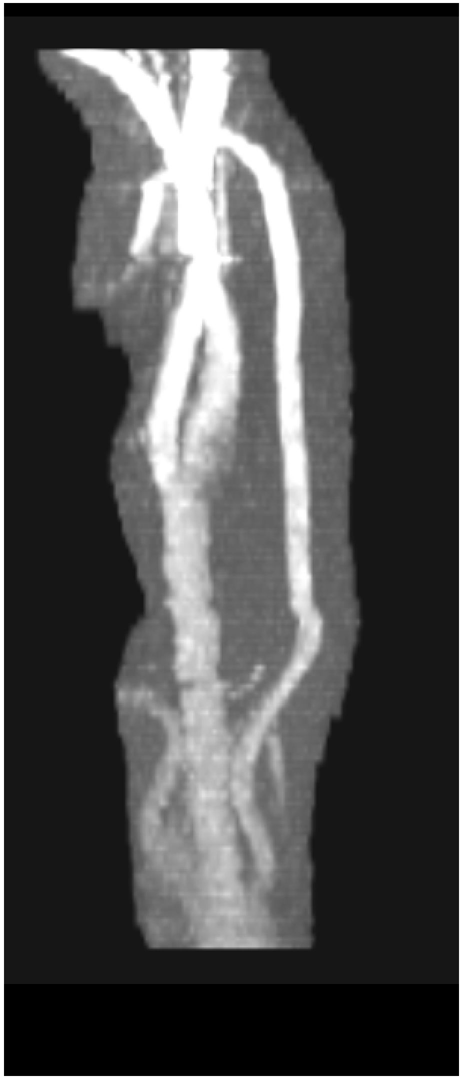

109f Score

0-30

31-50

51-70

>70

Near occlusion

Occluded

Quality

1

2

3

4

5

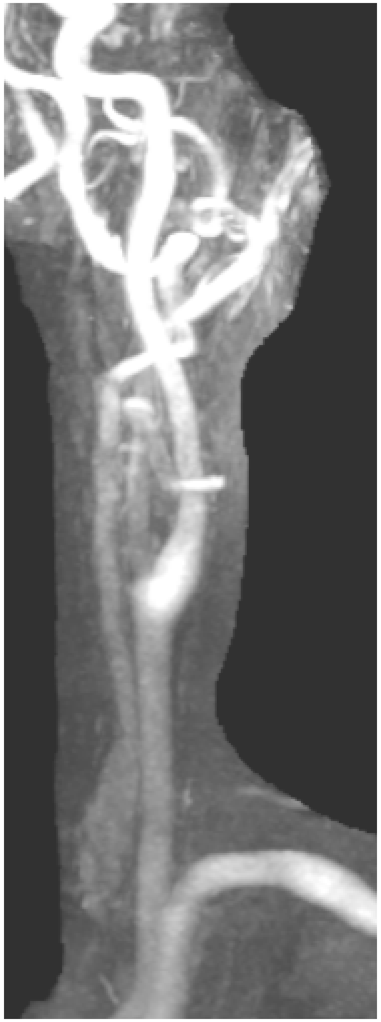

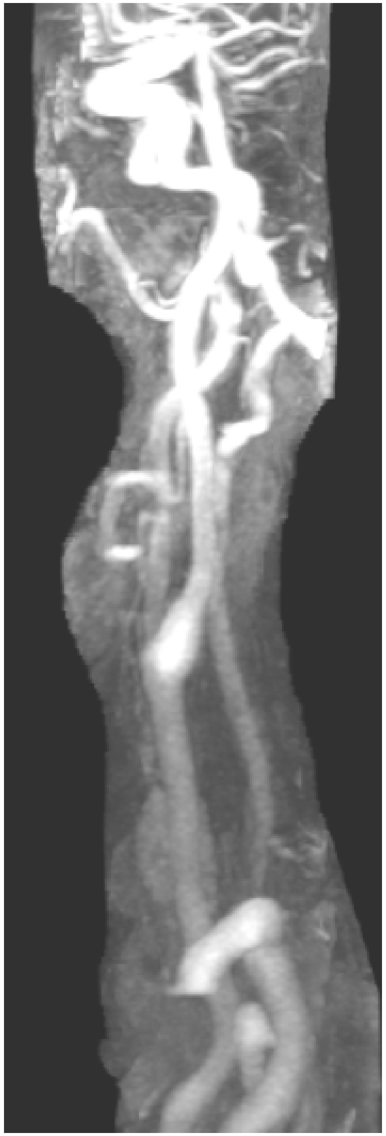

110e Score

0-30

31-50

51-70

>70

Near occlusion

Occluded

Quality

1

2

3

4

5

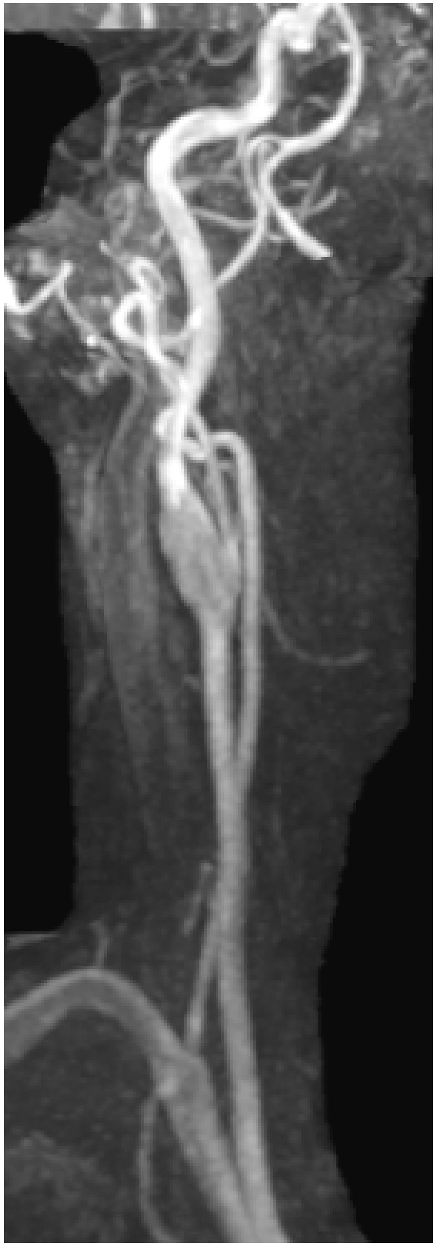

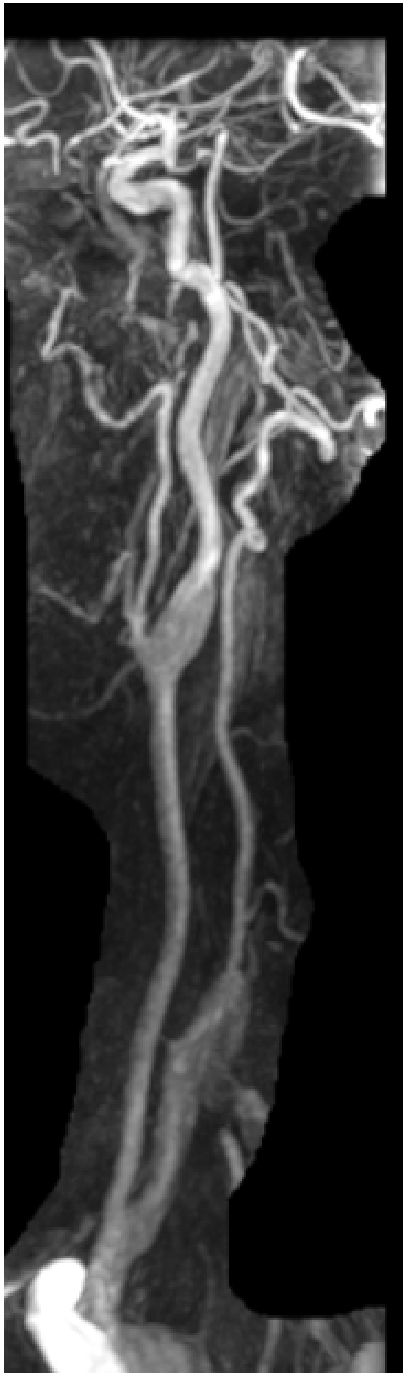

111d Score

0-30

31-50

51-70

>70

Near occlusion

Occluded

Quality

1

2

3

4

5

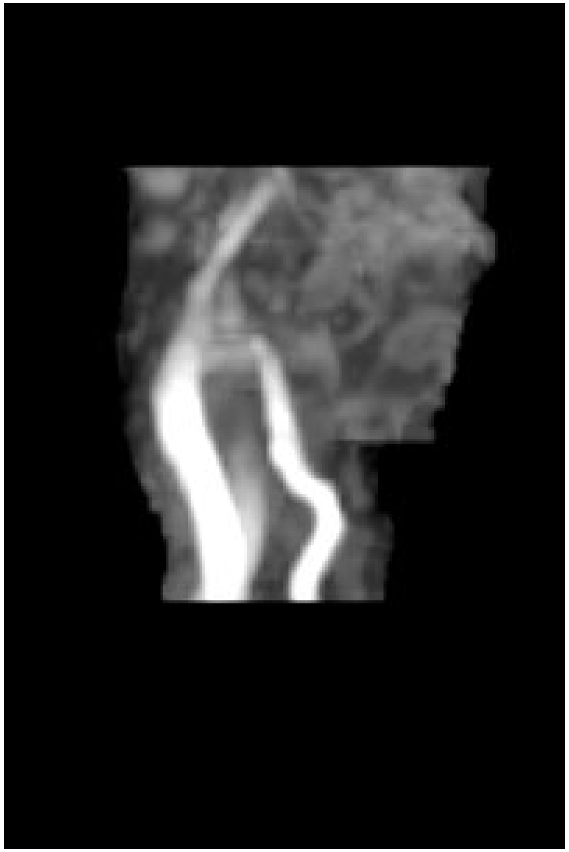

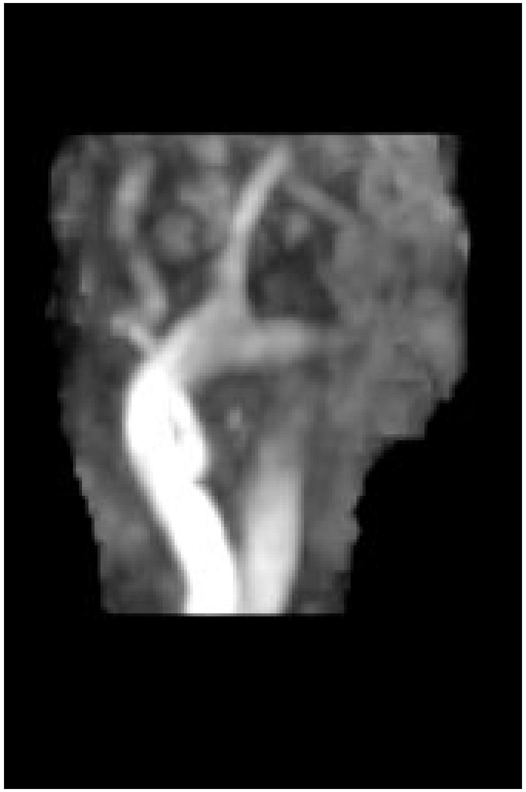

112c Score

0-30

31-50

51-70

>70

Near occlusion

Occluded

Quality

1

2

3

4

5

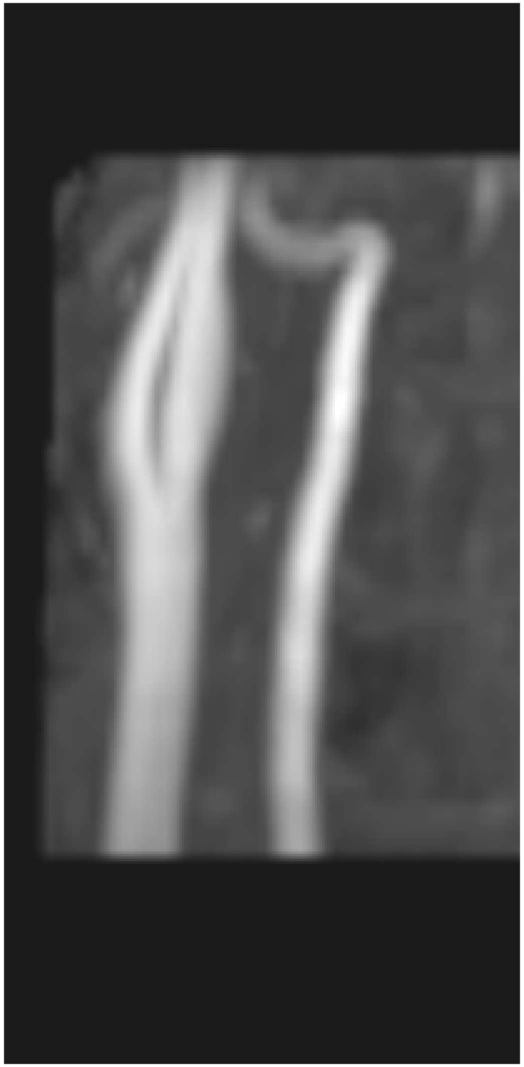

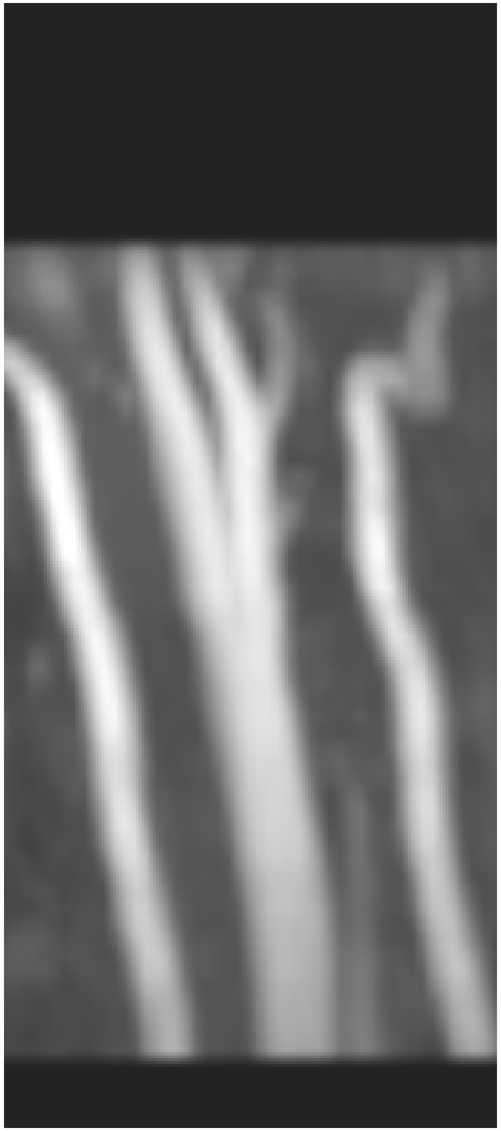

113b Score

0-30

31-50

51-70

>70

Near occlusion

Occluded

Quality

1

2

3

4

5

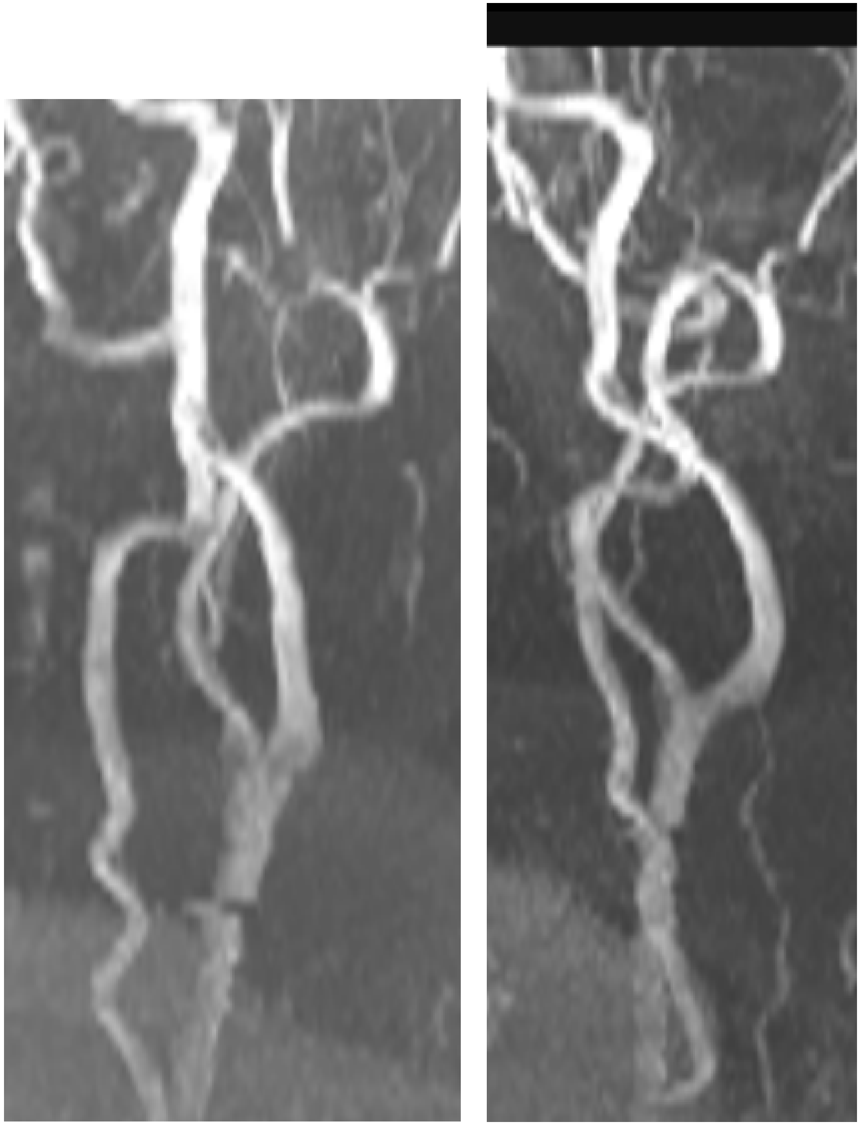

114a Score

0-30

31-50

51-70

>70

Near occlusion

Occluded

Quality

1

2

3

4

5

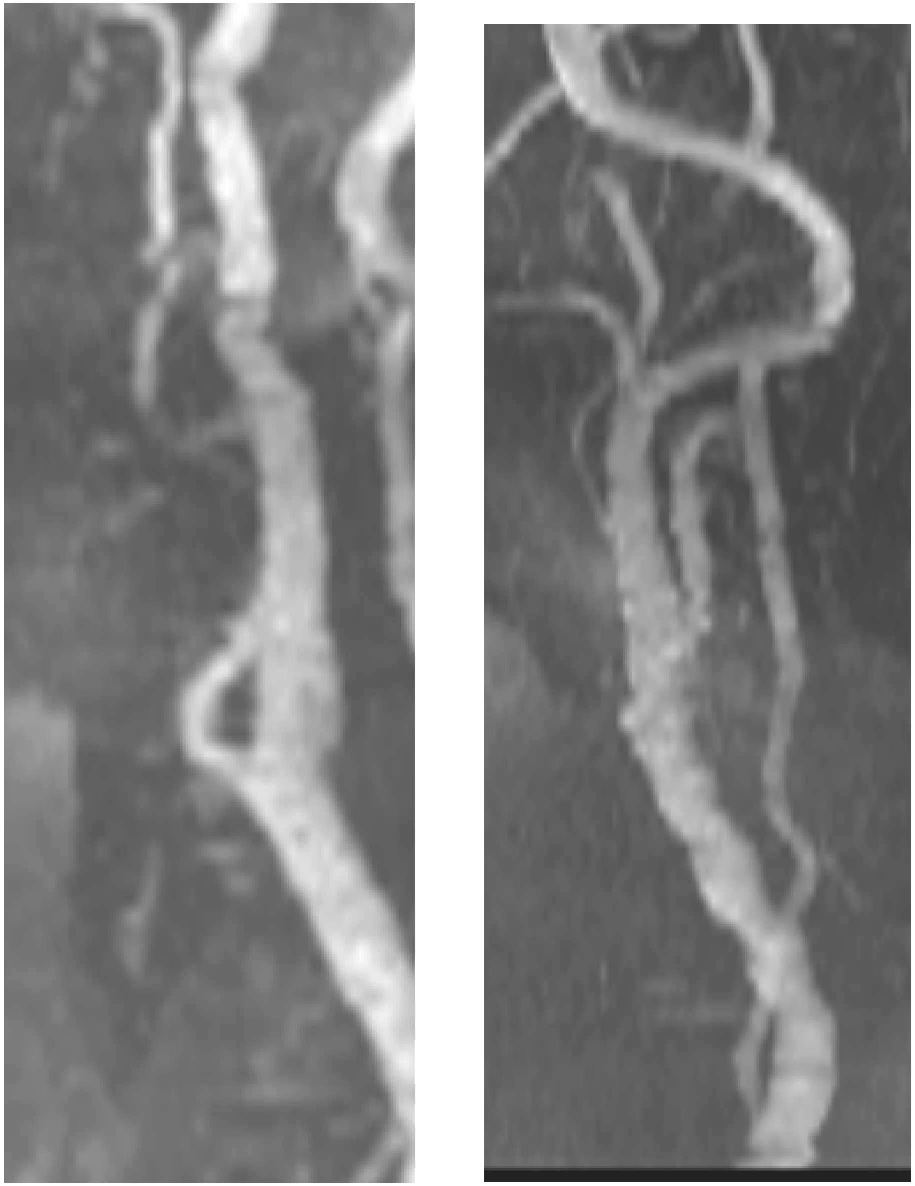

114f Score

0-30

31-50

51-70

>70

Near occlusion

Occluded

Quality

1

2

3

4

5

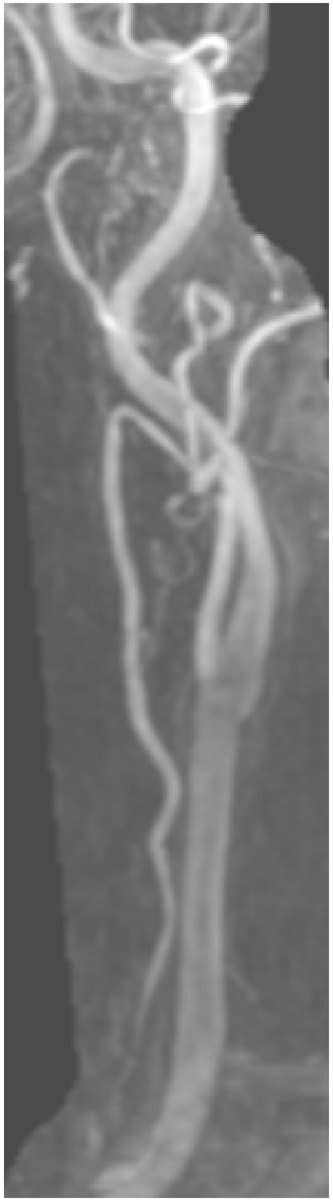

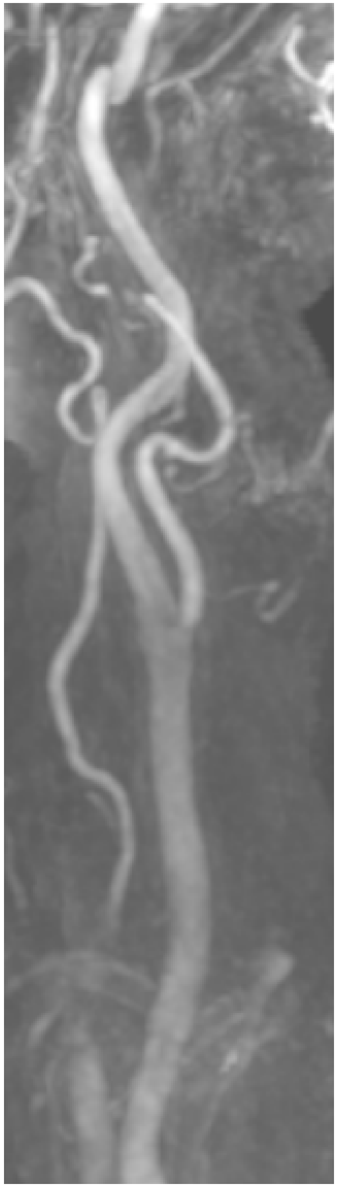

115e Score

0-30

31-50

51-70

>70

Near occlusion

Occluded

Quality

1

2

3

4

5

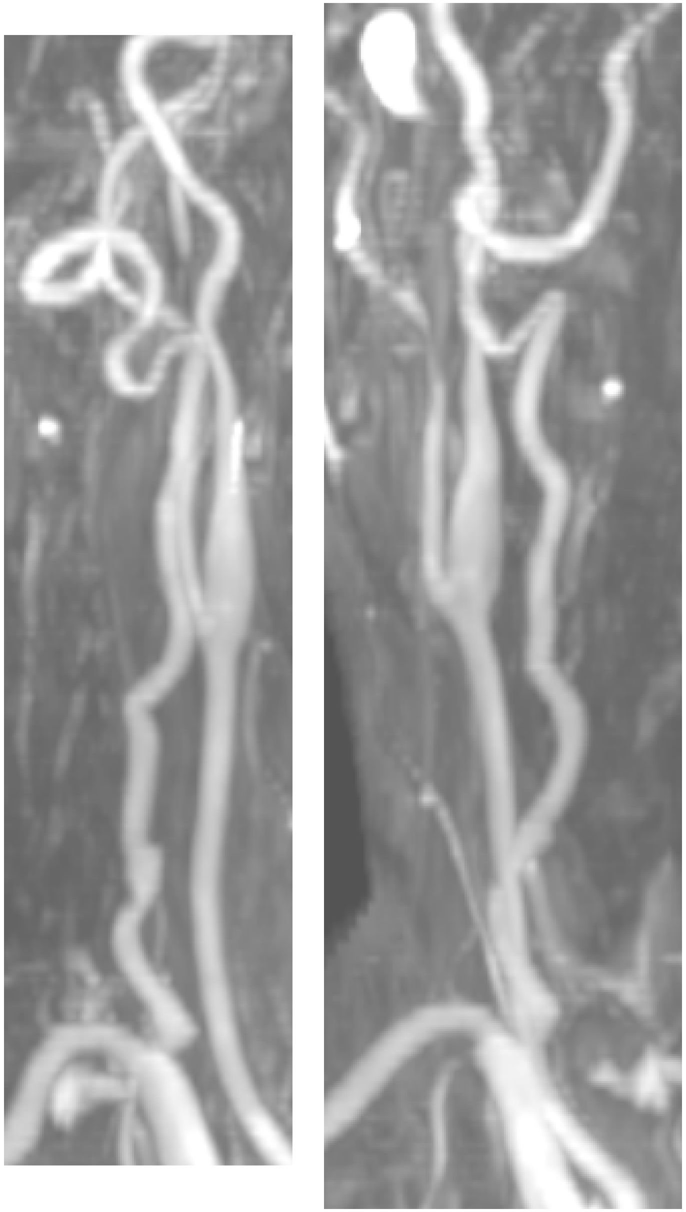

116d Score

0-30

31-50

51-70

>70

Near occlusion

Occluded

Quality

1

2

3

4

5

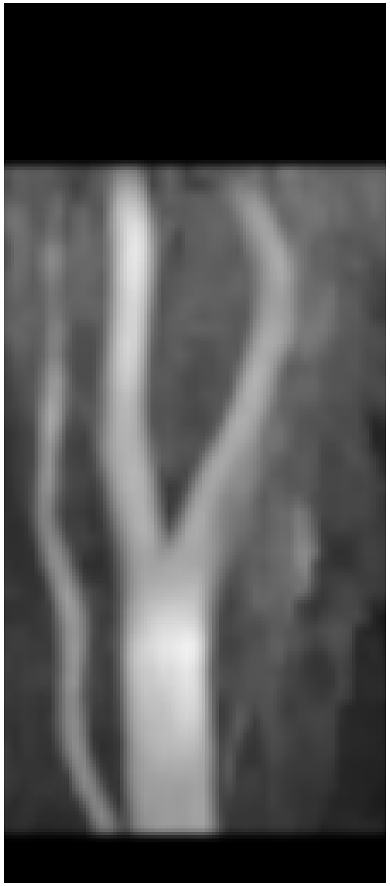

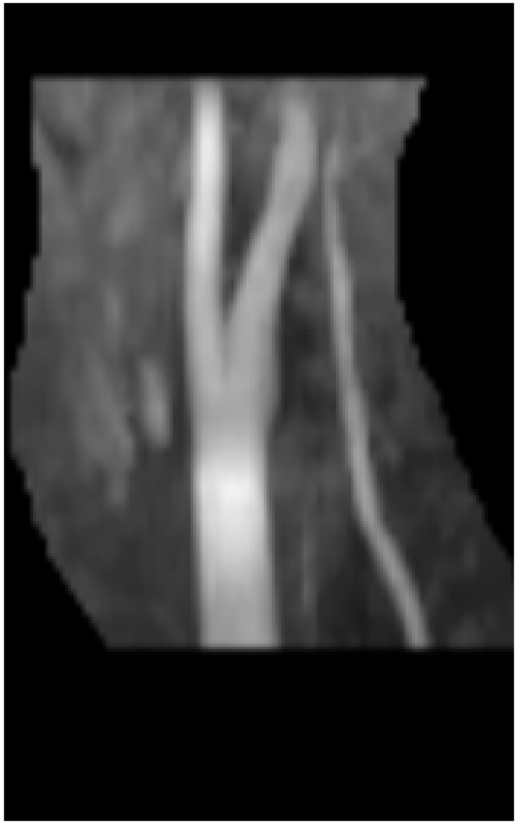

117c Score

0-30

31-50

51-70

>70

Near occlusion

Occluded

Quality

1

2

3

4

5

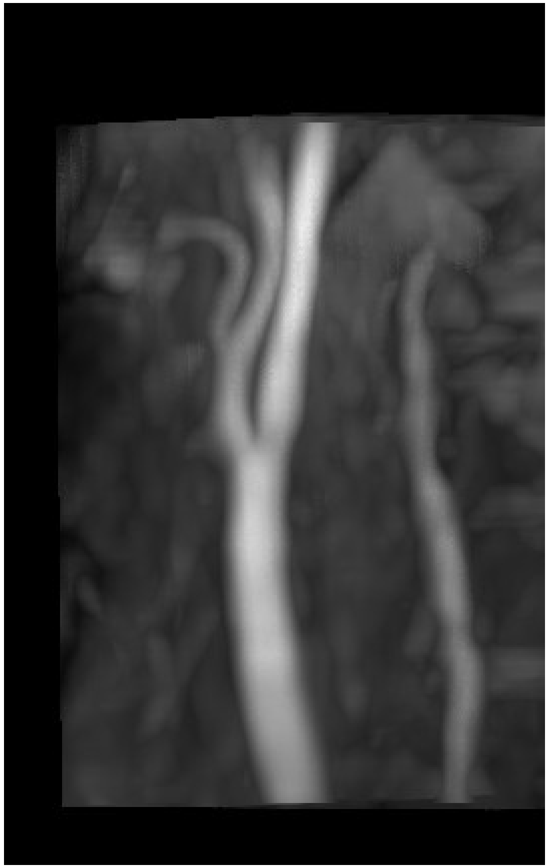

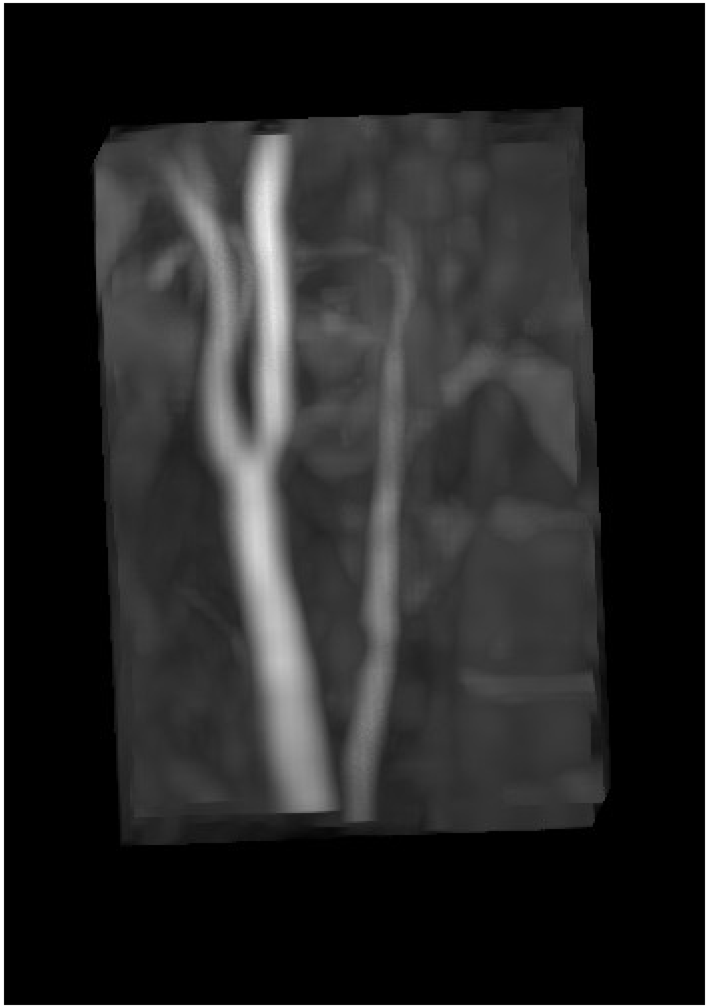

118b Score

0-30

31-50

51-70

>70

Near occlusion

Occluded

Quality

1

2

3

4

5

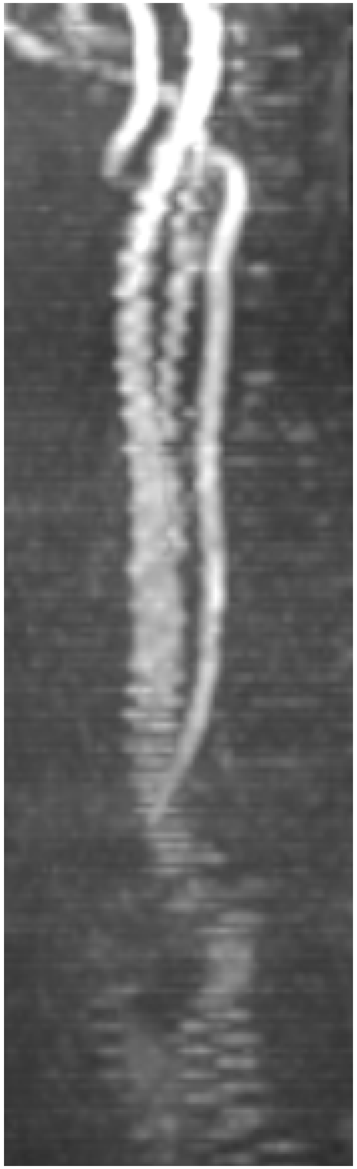

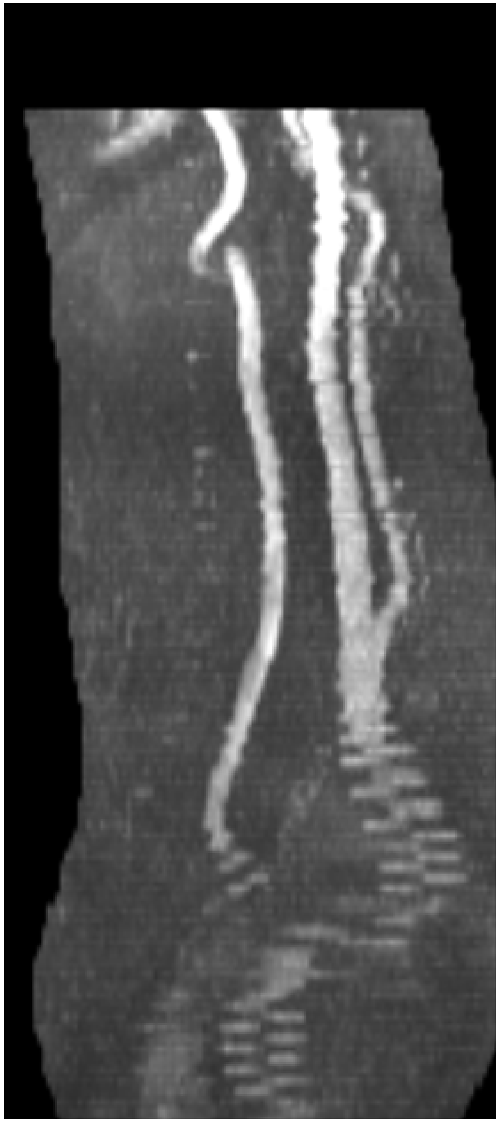

119a Score

0-30

31-50

51-70

>70

Near occlusion

Occluded

Quality

1

2

3

4

5

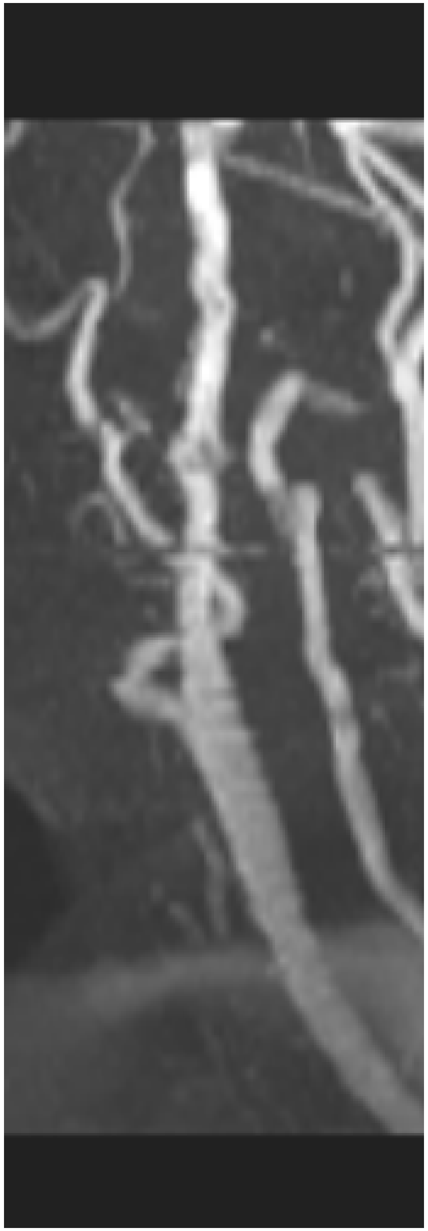

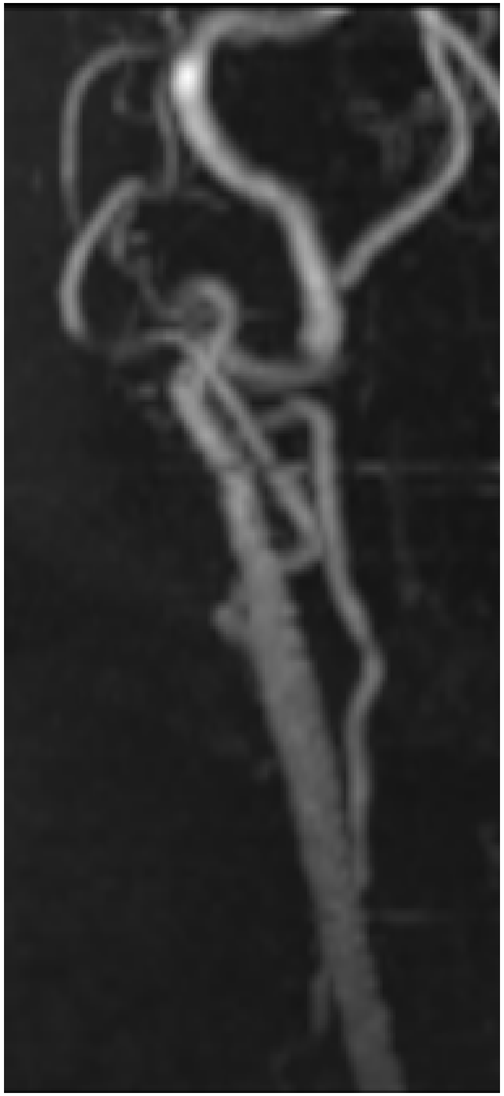

119f Score

0-30

31-50

51-70

>70

Near occlusion

Occluded

Quality

1

2

3

4

5

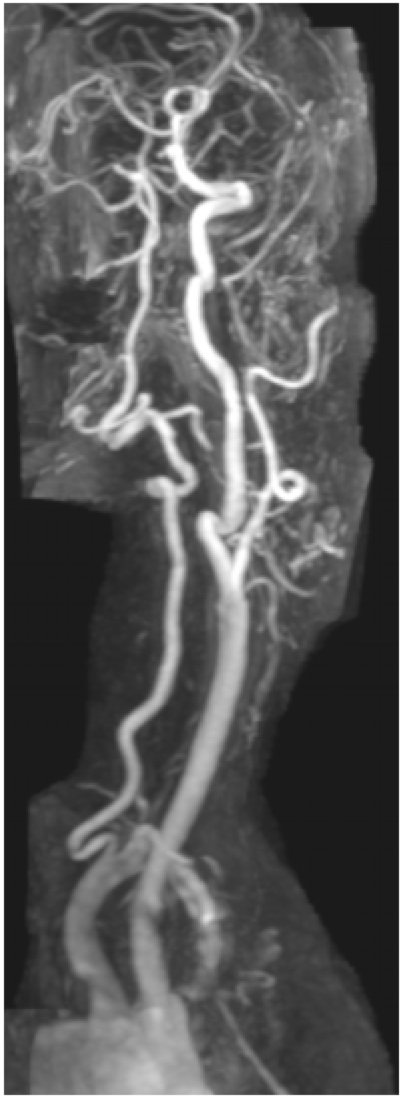

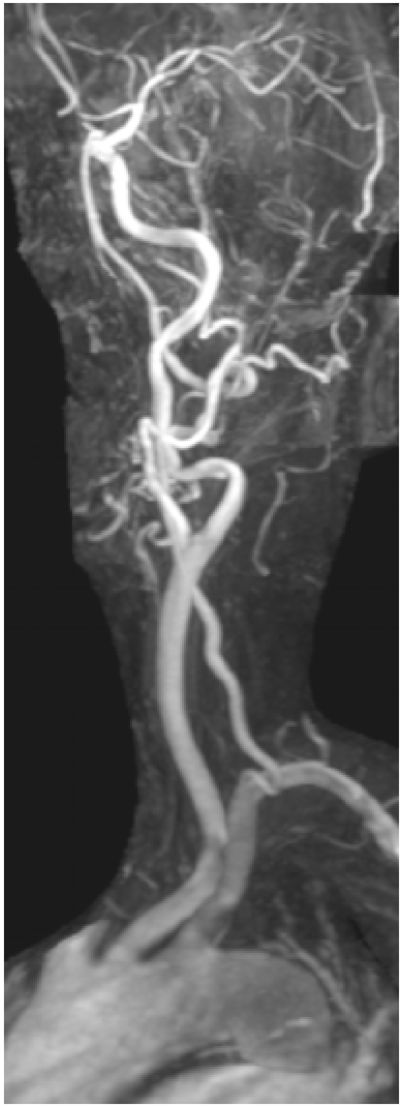

120e Score

0-30

31-50

51-70

>70

Near occlusion

Occluded

Quality

1

2

3

4

5

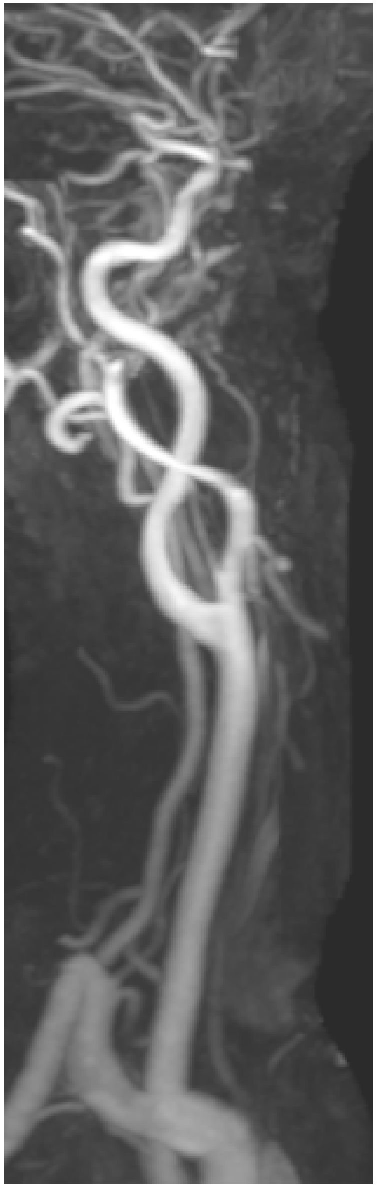

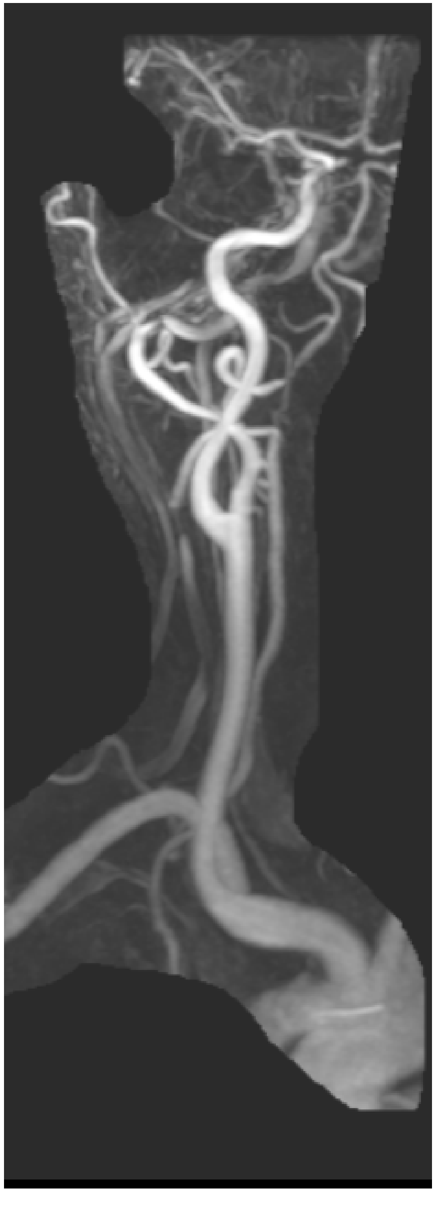

121d Score

0-30

31-50

51-70

>70

Near occlusion

Occluded

Quality

1

2

3

4

5

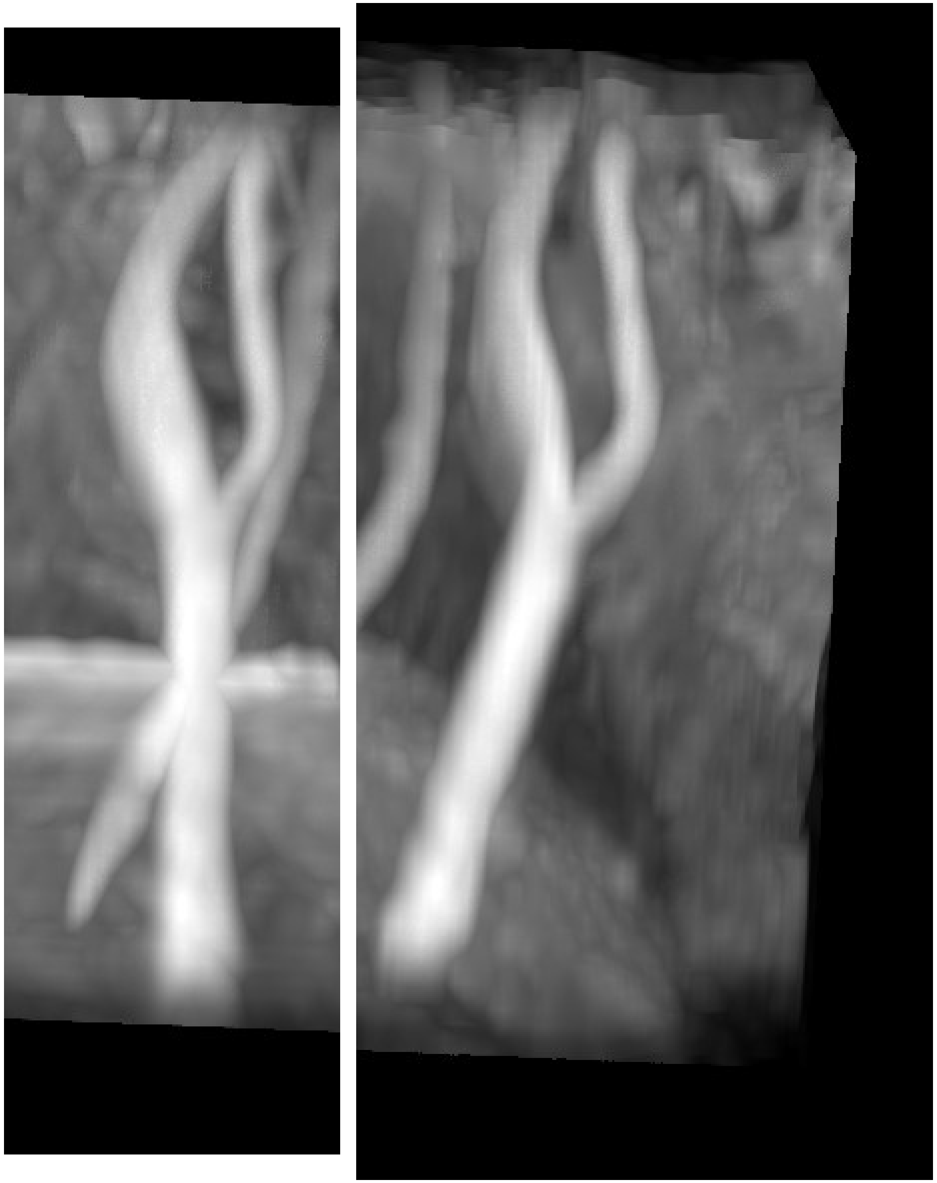

122c Score

0-30

31-50

51-70

>70

Near occlusion

Occluded

Quality

1

2

3

4

5

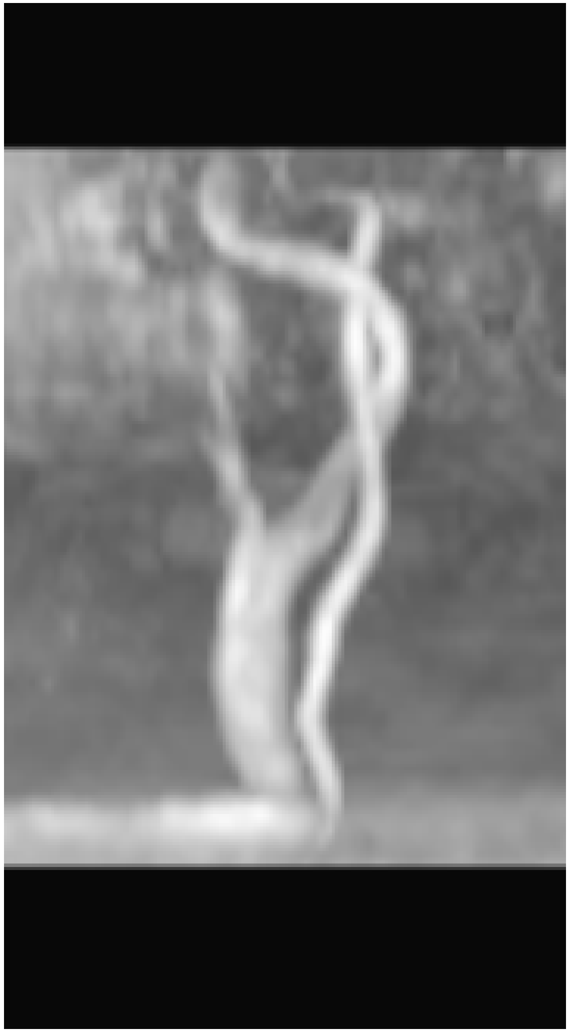

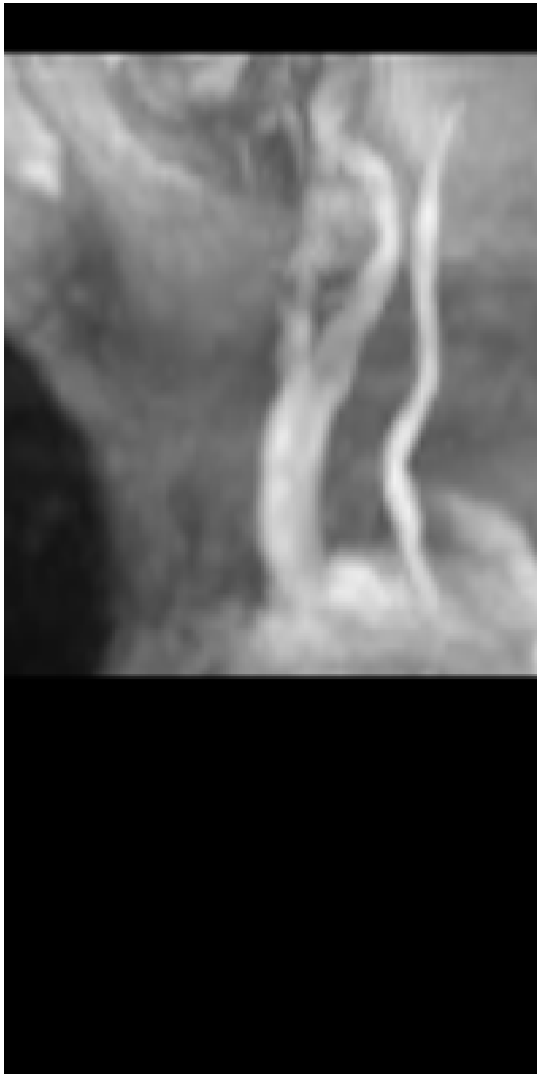

123b Score

0-30

31-50

51-70

>70

Near occlusion

Occluded

Quality

1

2

3

4

5

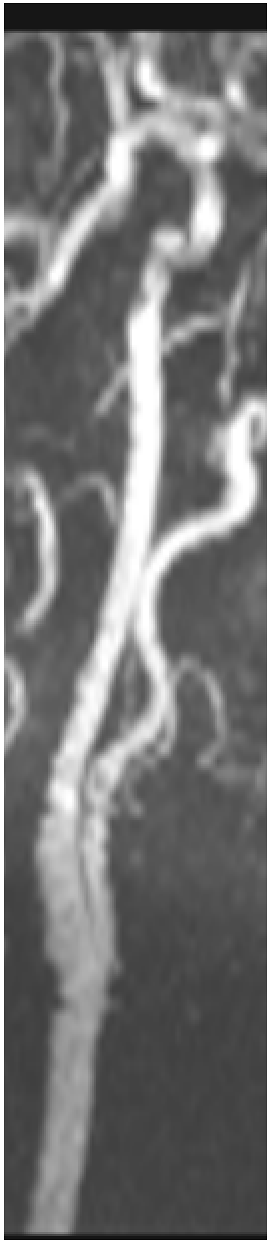

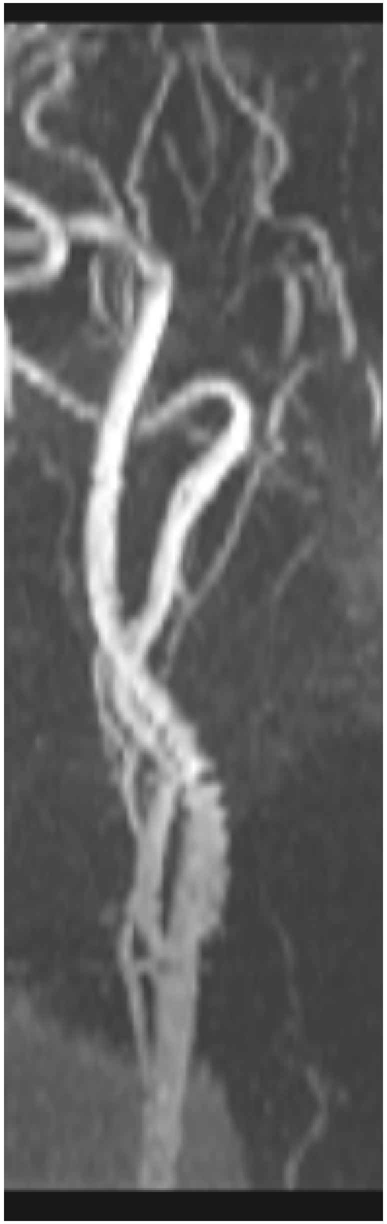

124a Score

0-30

31-50

51-70

>70

Near occlusion

Occluded

Quality

1

2

3

4

5

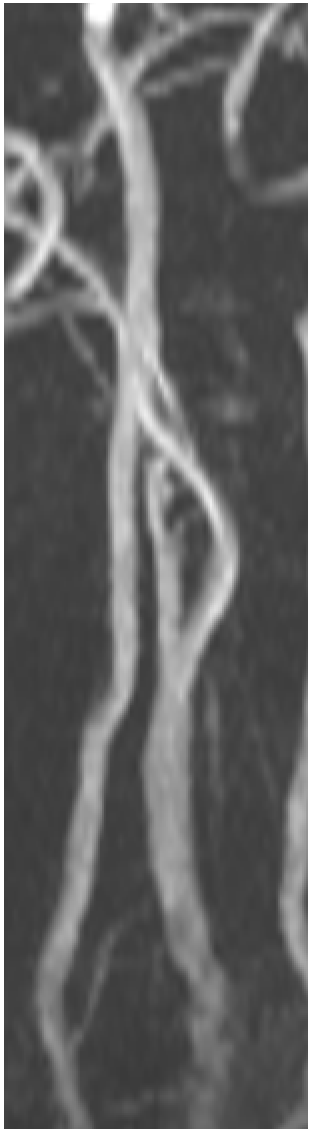

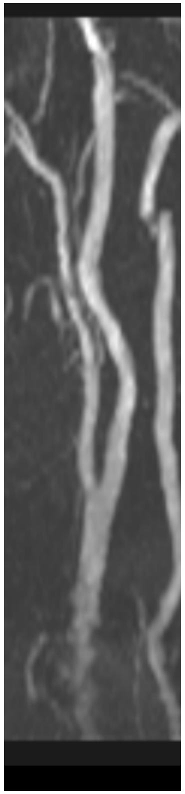

124f Score

0-30

31-50

51-70

>70

Near occlusion

Occluded

Quality

1

2

3

4

5

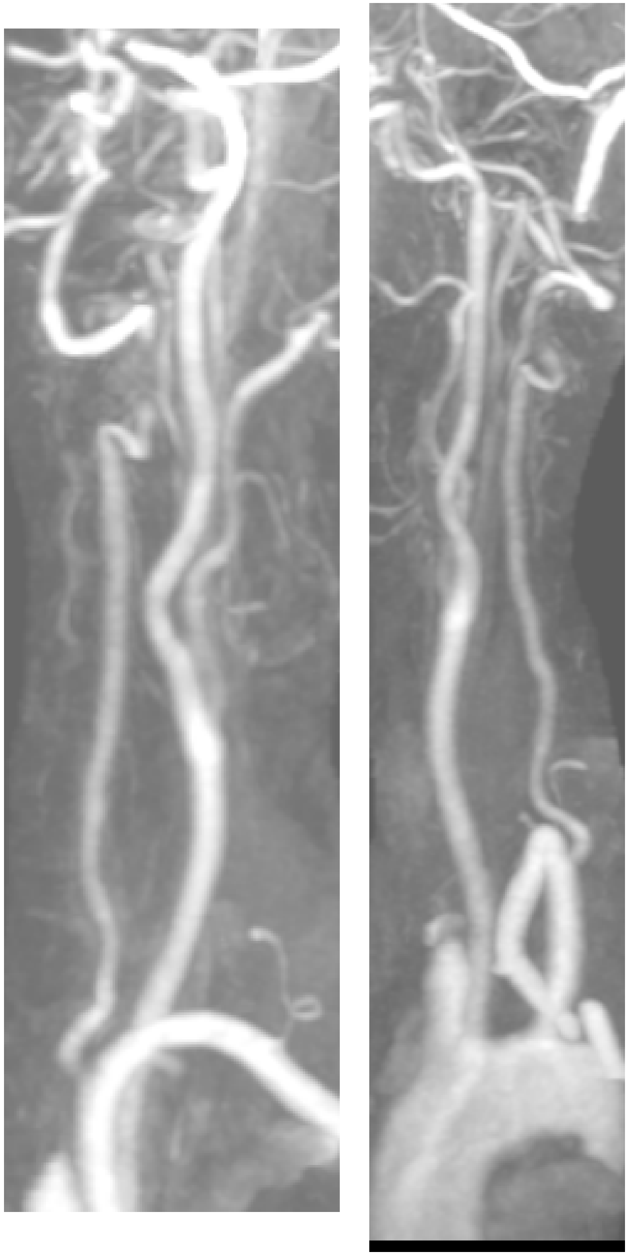

125e Score

0-30

31-50

51-70

>70

Near occlusion

Occluded

Quality

1

2

3

4

5

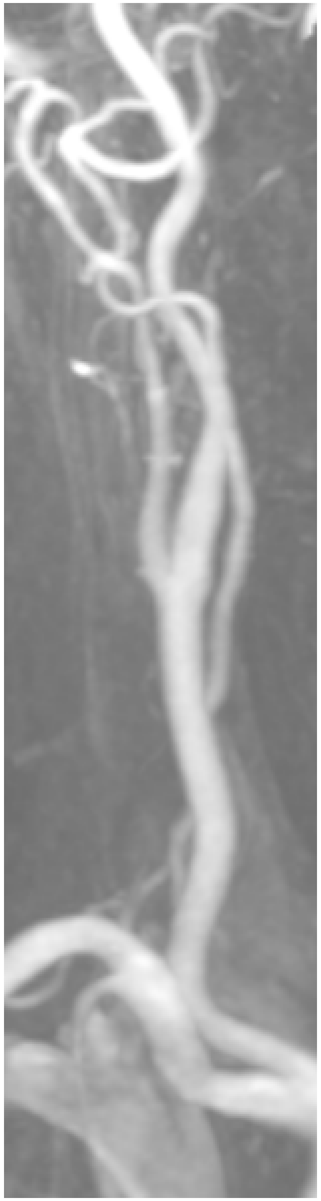

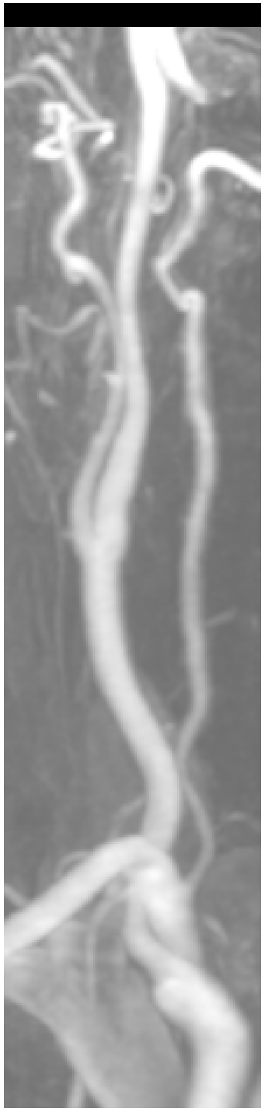

126d Score

0-30

31-50

51-70

>70

Near occlusion

Occluded

Quality

1

2

3

4

5

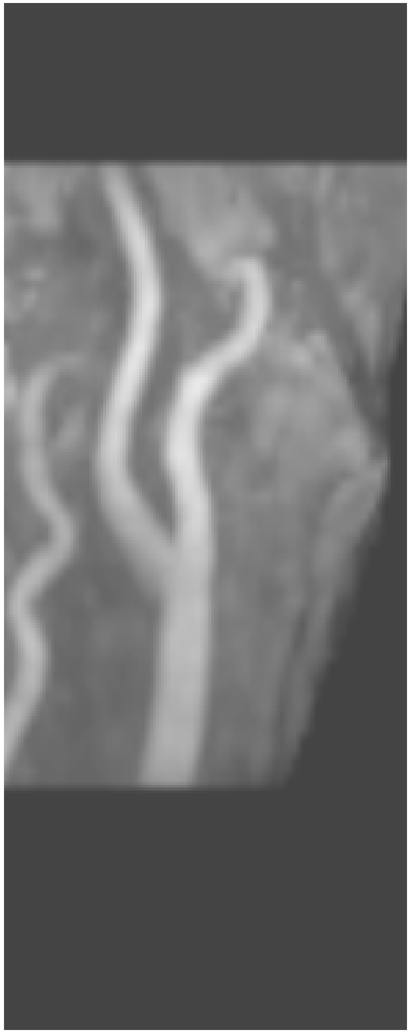

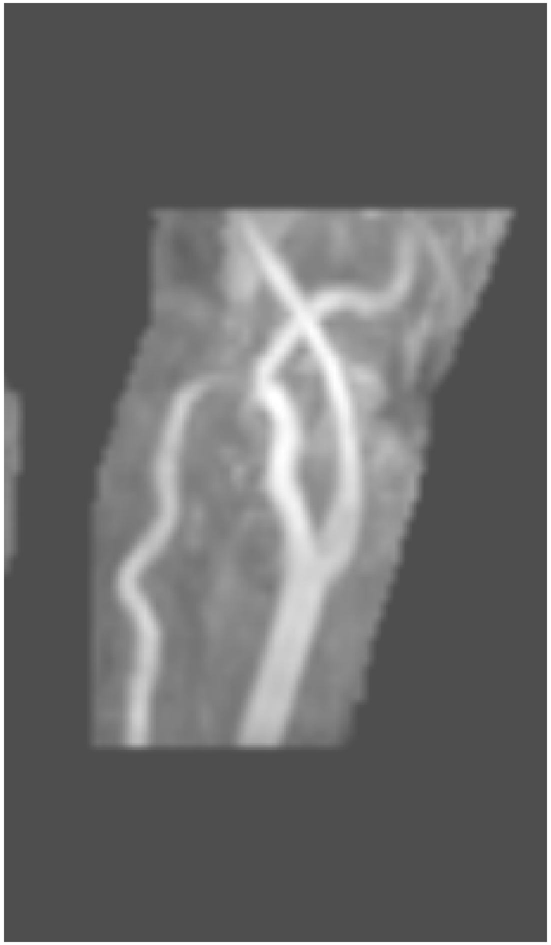

127c Score

0-30

31-50

51-70

>70

Near occlusion

Occluded

Quality

1

2

3

4

5

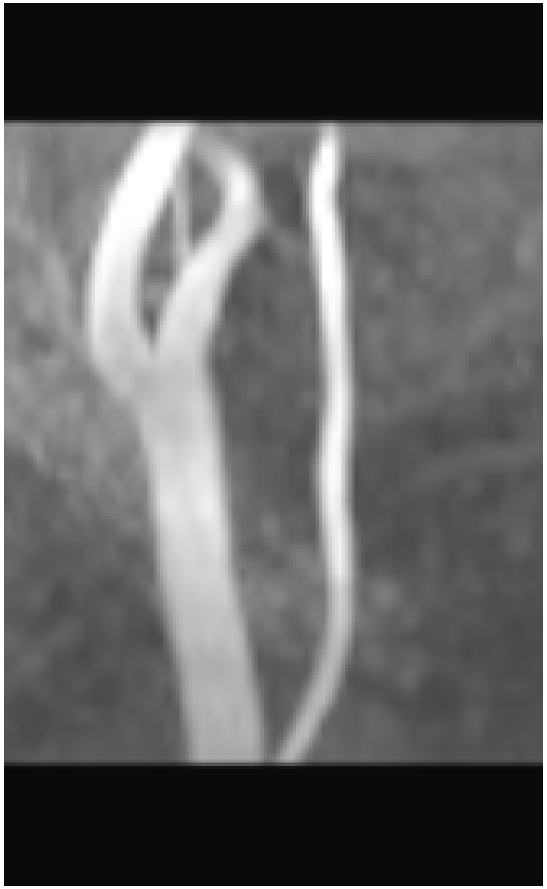

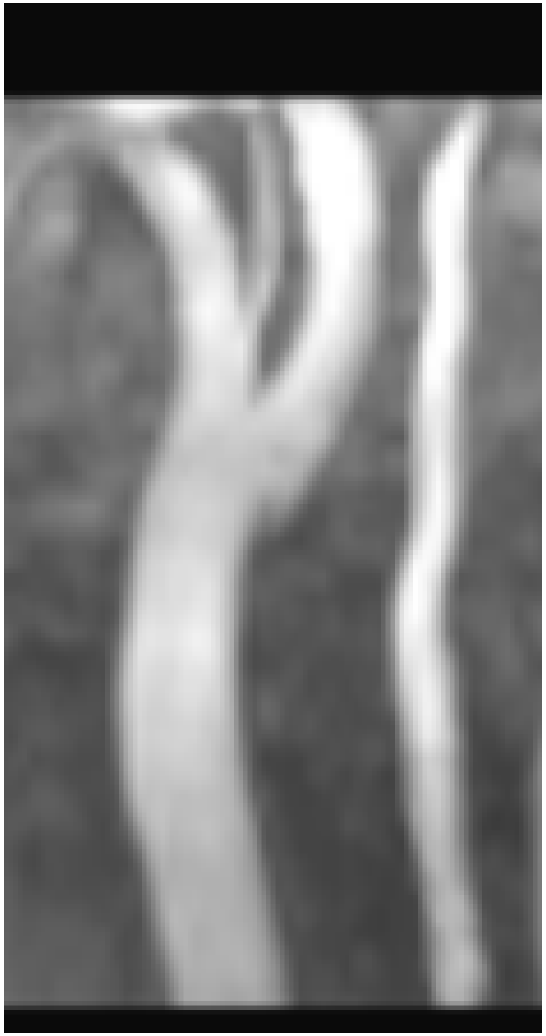

128b Score

0-30

31-50

51-70

>70

Near occlusion

Occluded

Quality

1

2

3

4

5

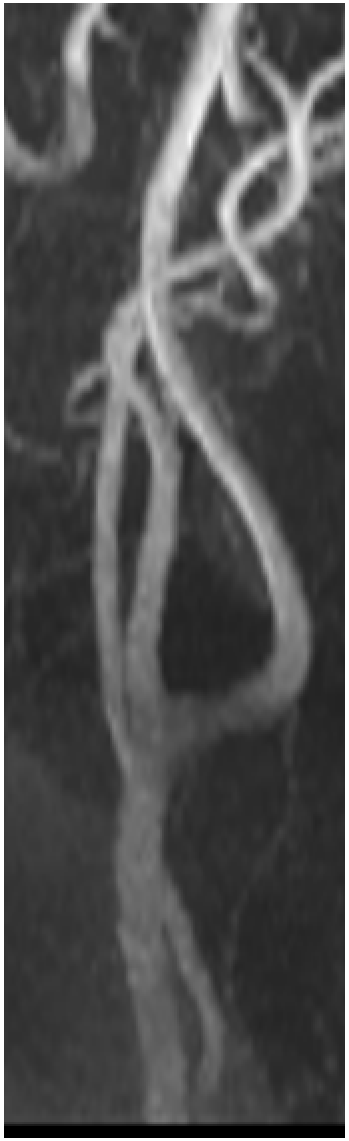

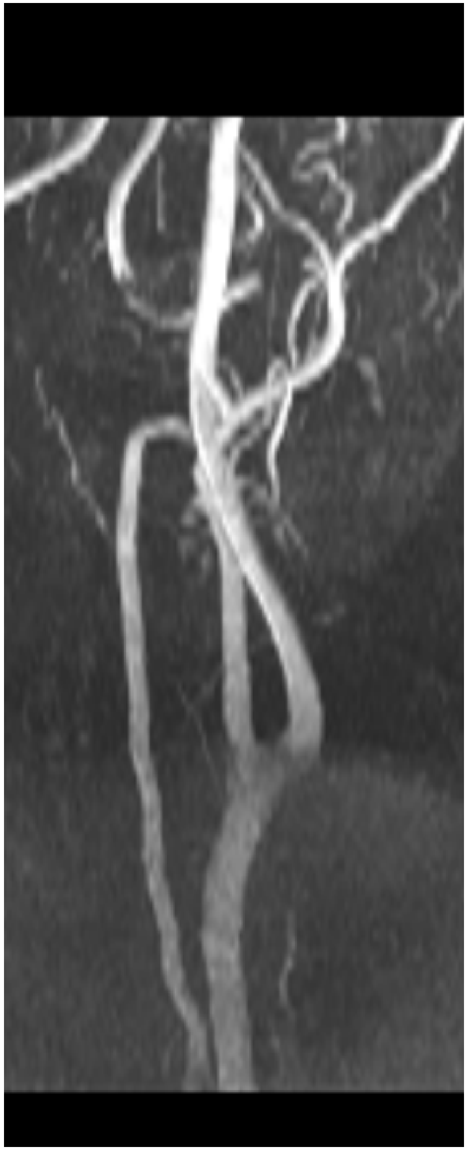

129a Score

0-30

31-50

51-70

>70

Near occlusion

Occluded

Quality

1

2

3

4

5

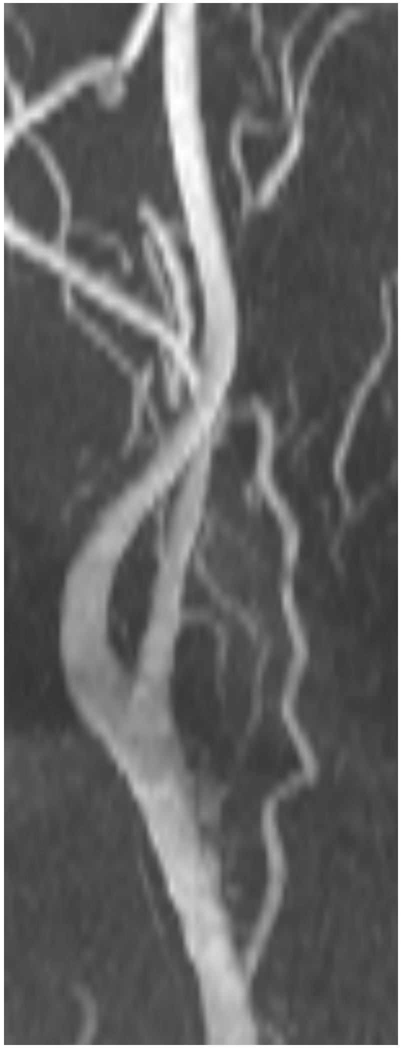

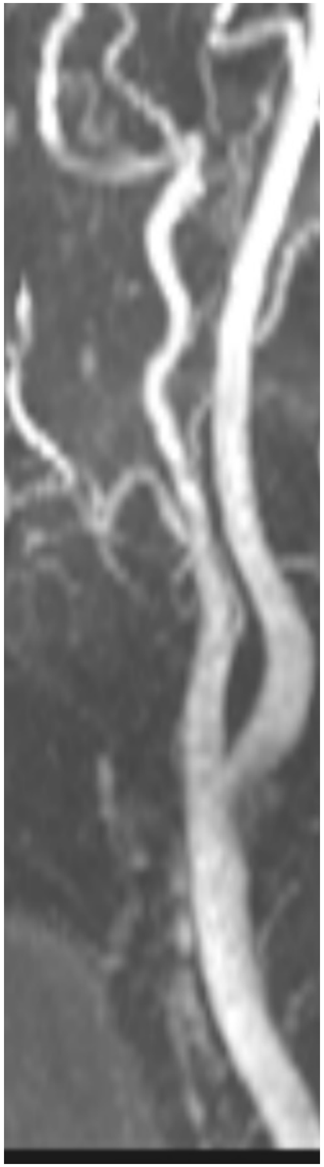

129f Score

0-30

31-50

51-70

>70

Near occlusion

Occluded

Quality

1

2

3

4

5

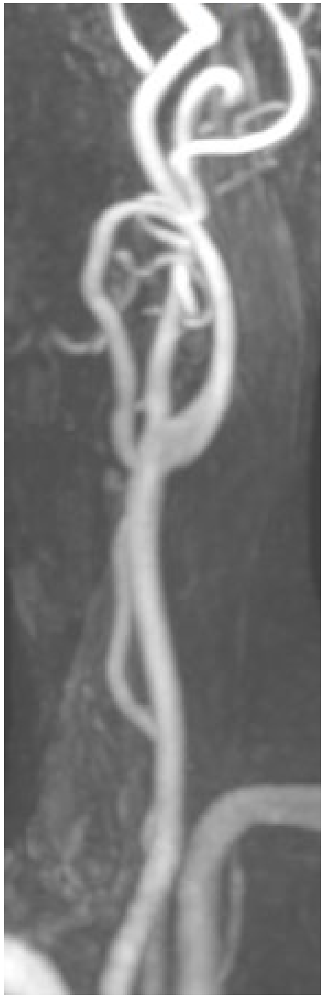

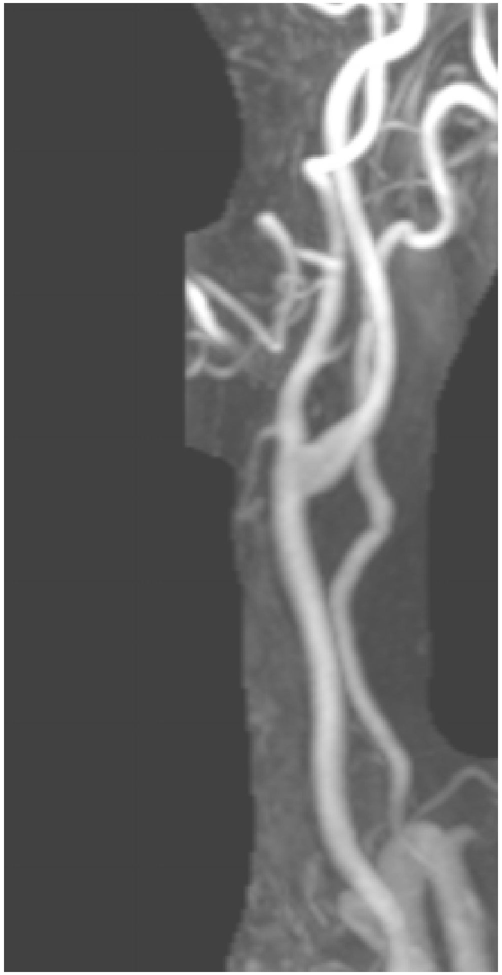

130e Score

0-30

31-50

51-70

>70

Near occlusion

Occluded

Quality

1

2

3

4

5

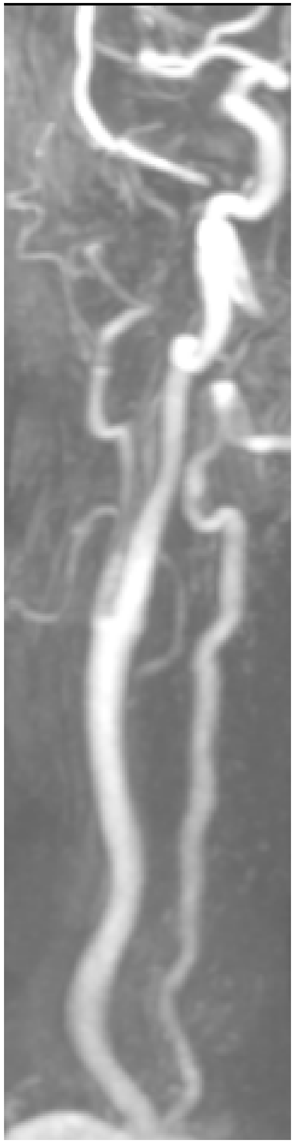

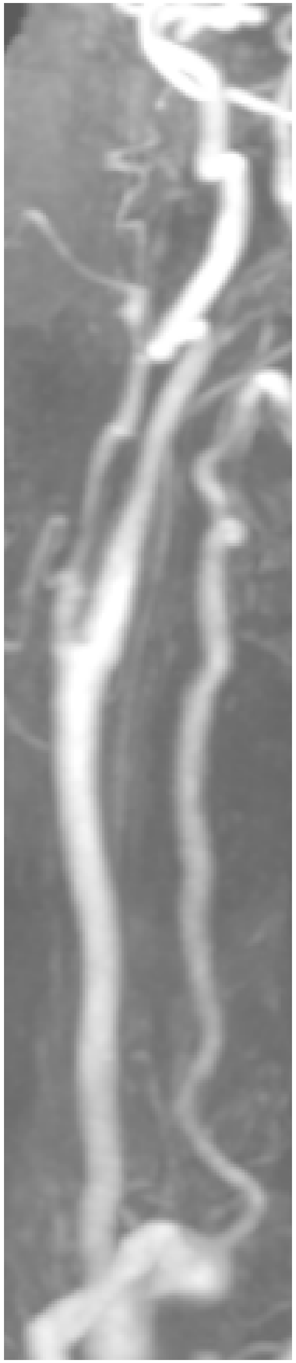

131d Score

0-30

31-50

51-70

>70

Near occlusion

Occluded

Quality

1

2

3

4

5

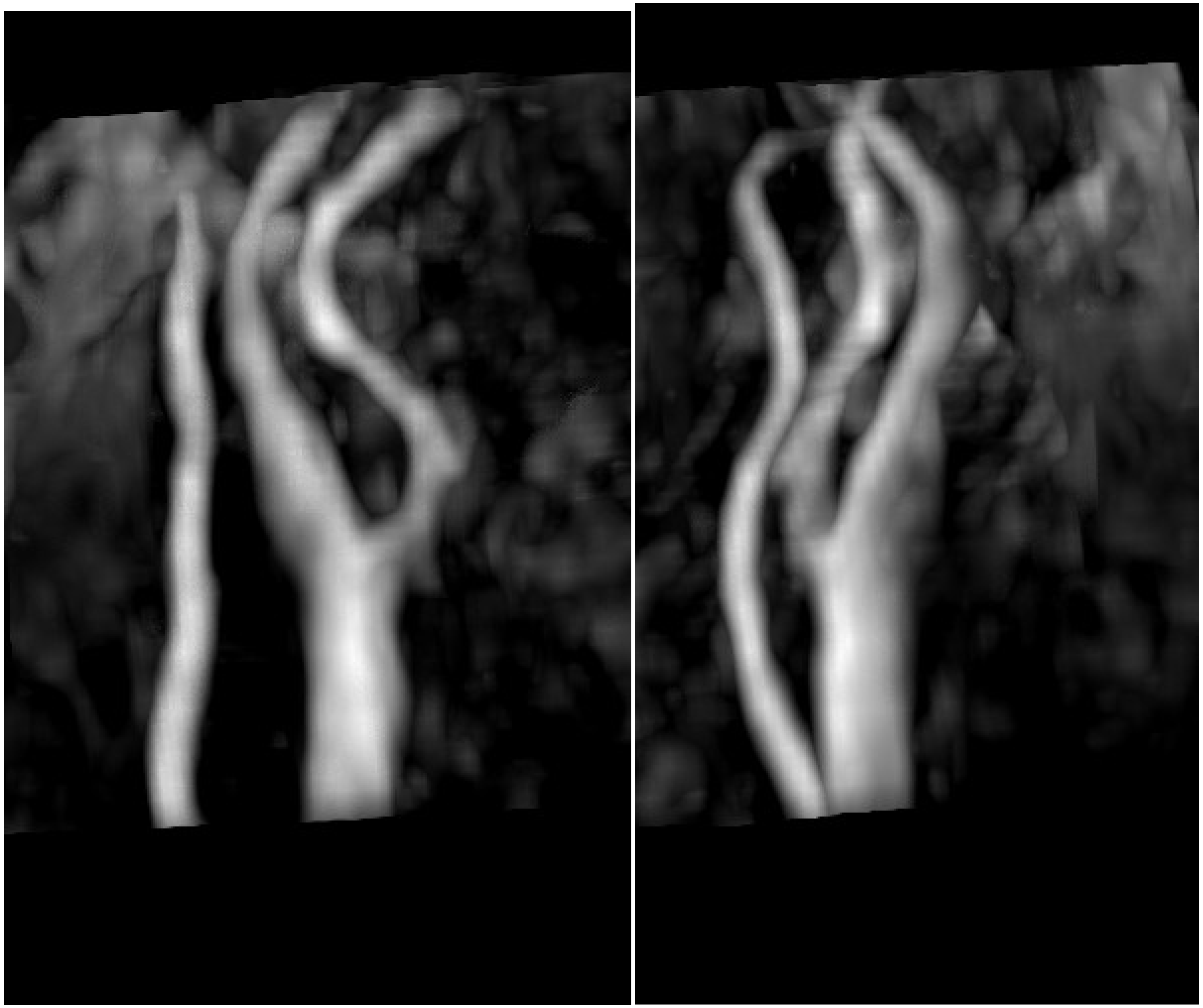

132c Score

0-30

31-50

51-70

>70

Near occlusion

Occluded

Quality

1

2

3

4

5

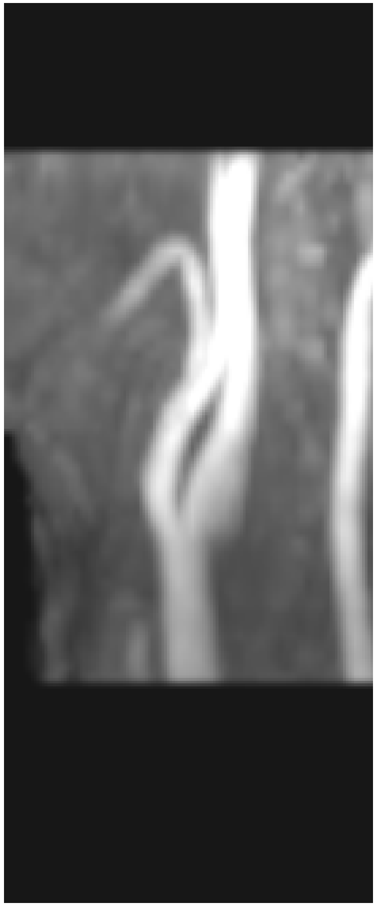

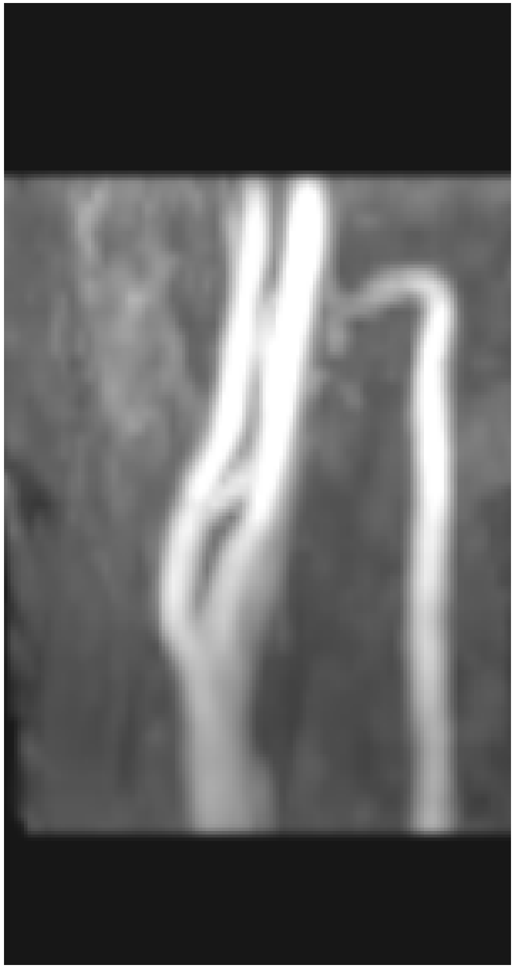

133b Score

0-30

31-50

51-70

>70

Near occlusion

Occluded

Quality

1

2

3

4

5

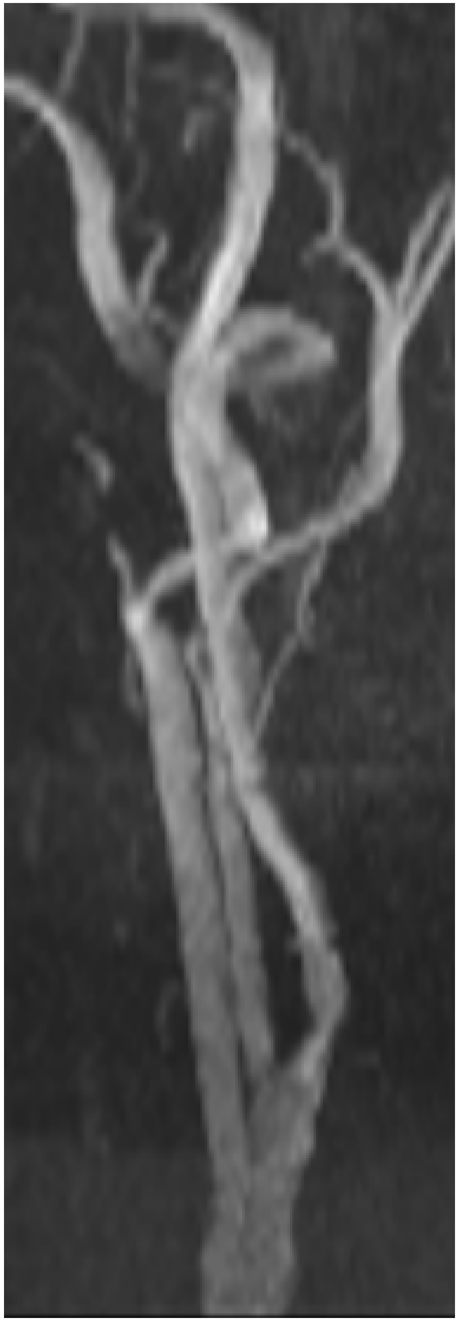

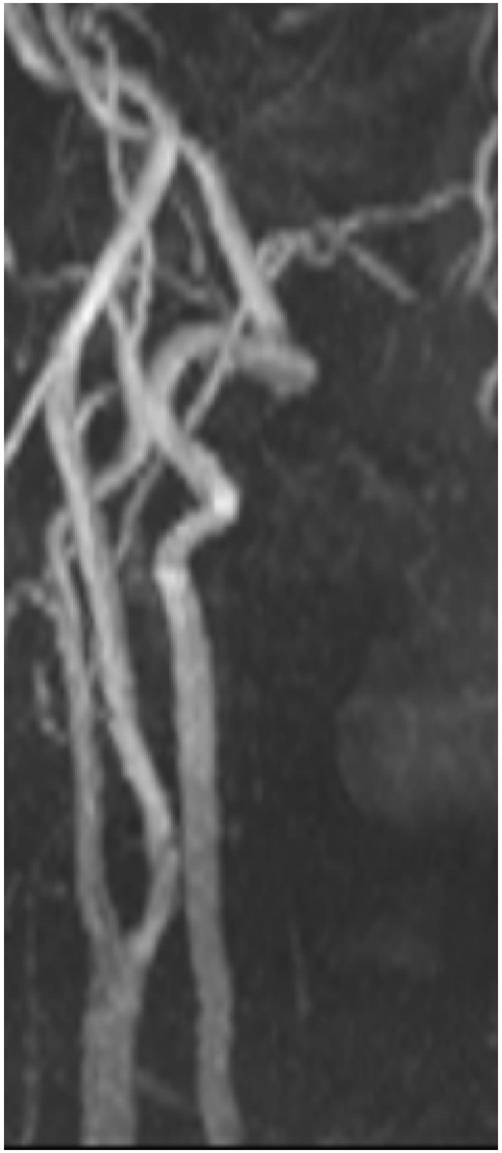

134a Score

0-30

31-50

51-70

>70

Near occlusion

Occluded

Quality

1

2

3

4

5

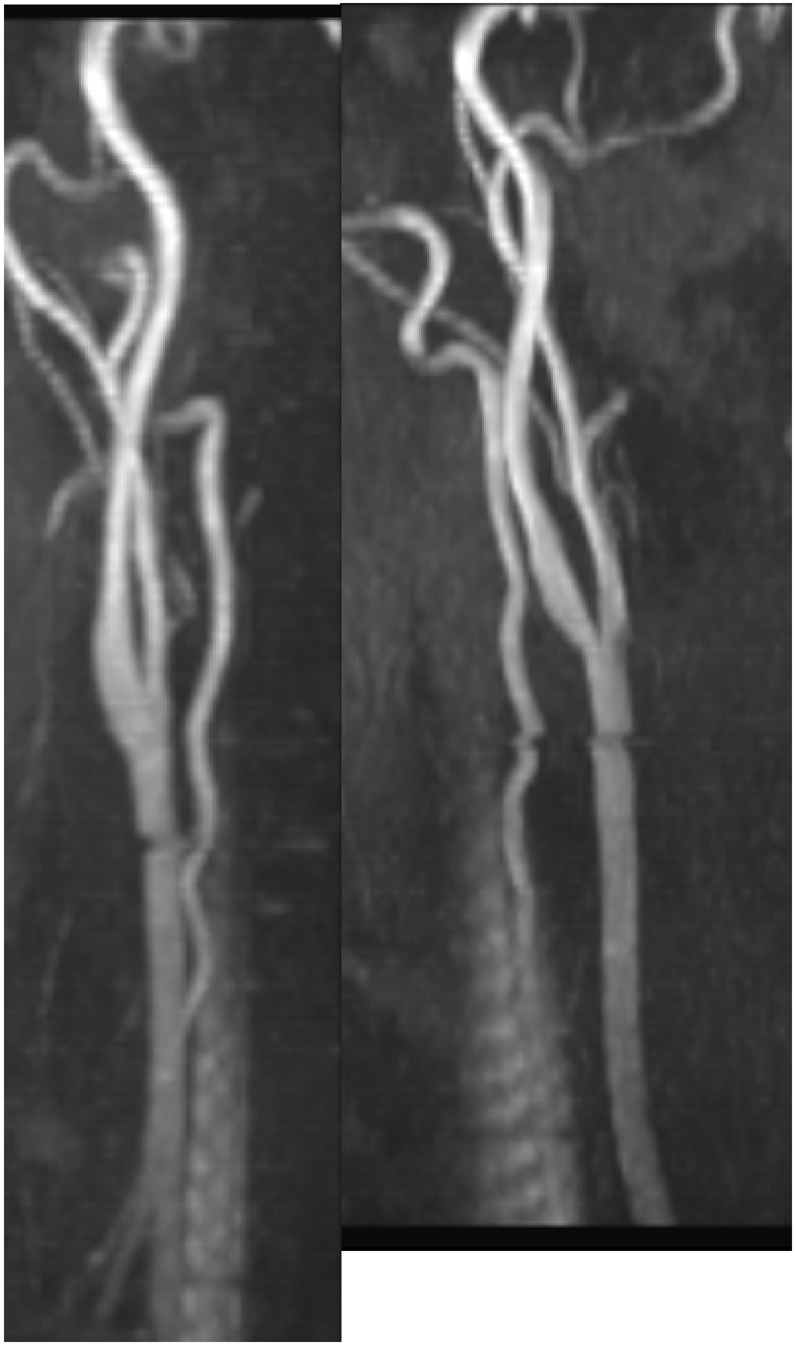

134f Score

0-30

31-50

51-70

>70

Near occlusion

Occluded

Quality

1

2

3

4

5

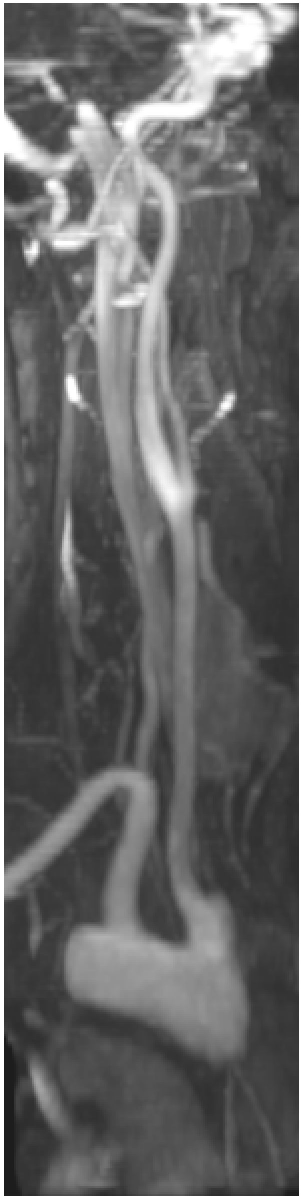

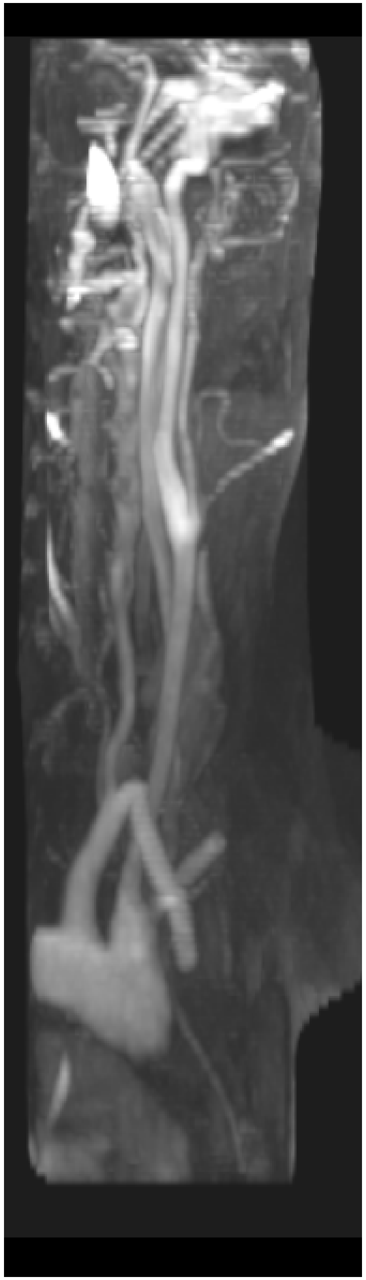

135e Score

0-30

31-50

51-70

>70

Near occlusion

Occluded

Quality

1

2

3

4

5

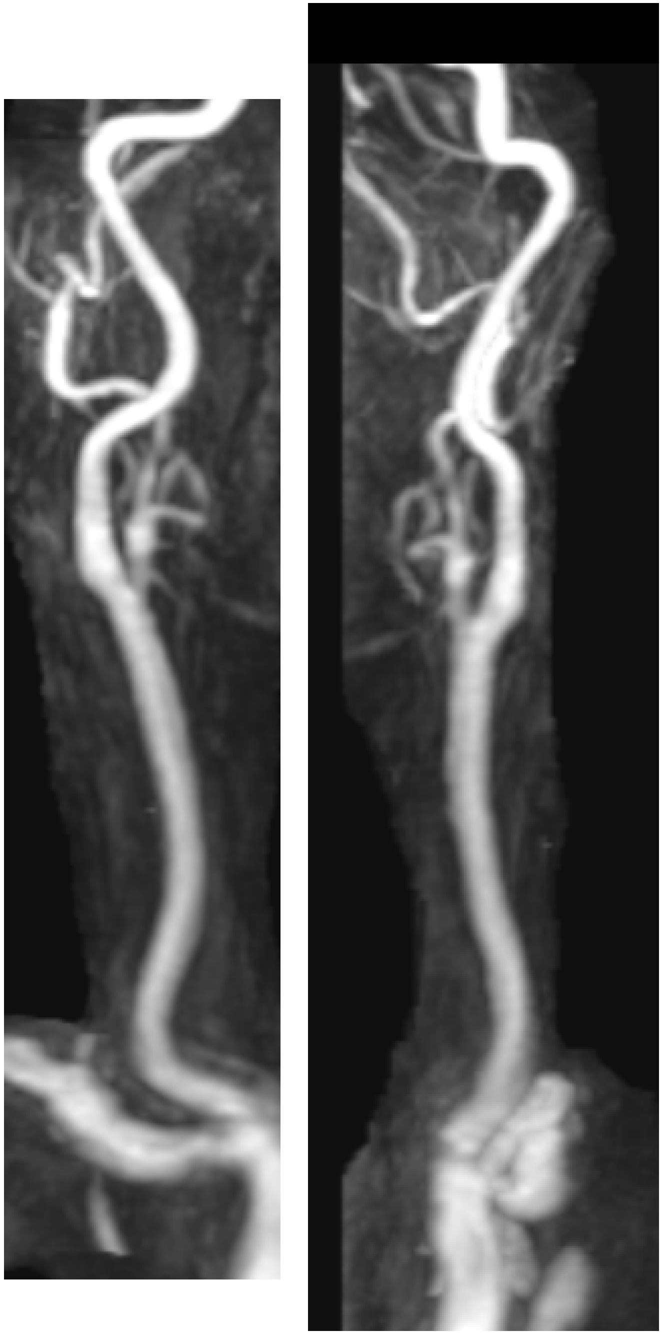

136d Score

0-30

31-50

51-70

>70

Near occlusion

Occluded

Quality

1

2

3

4

5

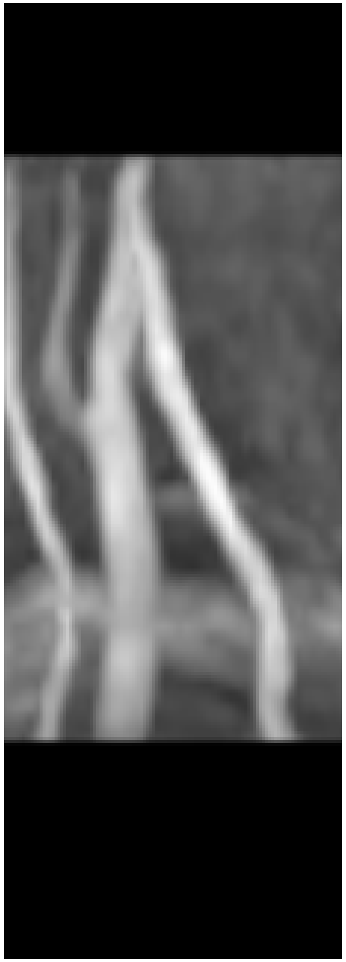

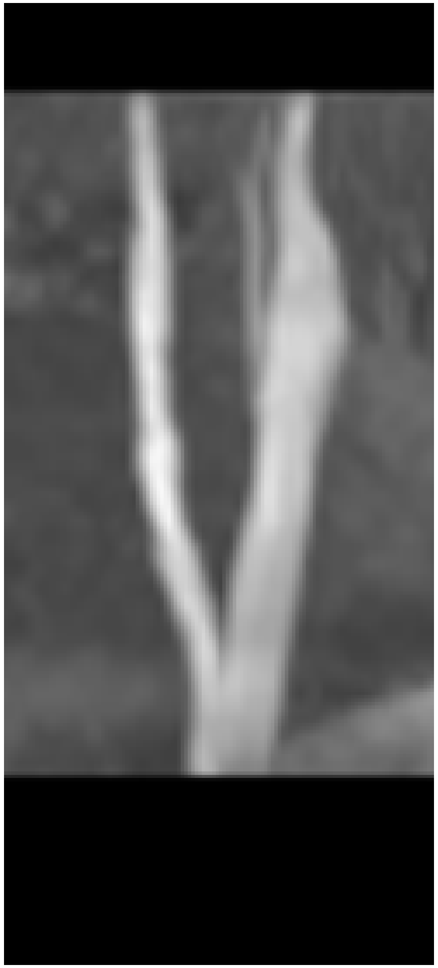

137c Score

0-30

31-50

51-70

>70

Near occlusion

Occluded

Quality

1

2

3

4

5

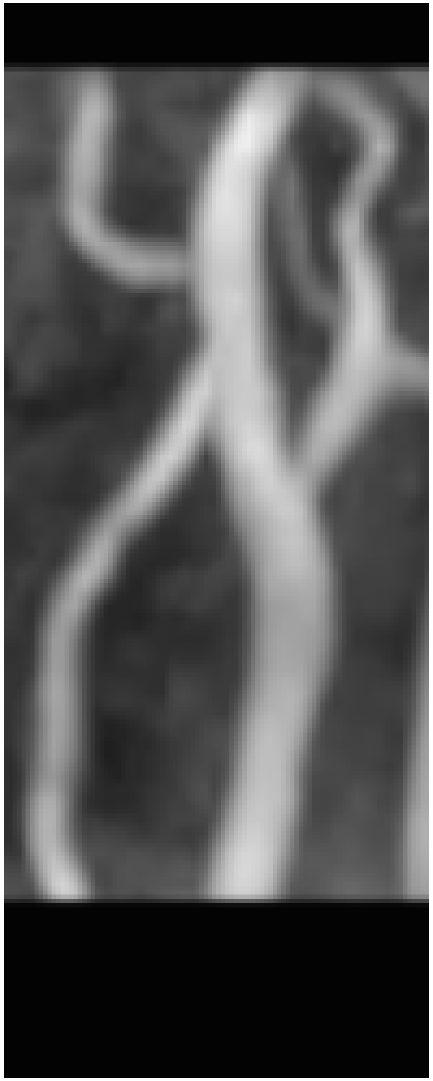

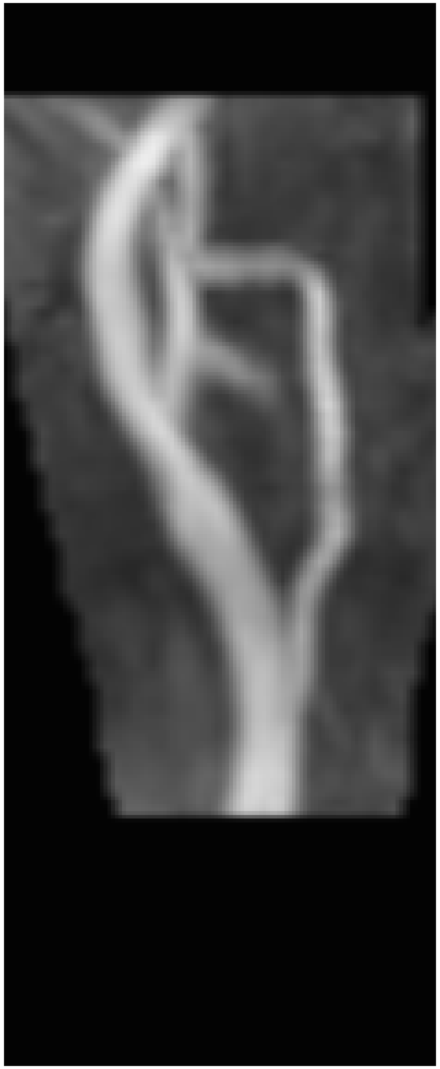

138b Score

0-30

31-50

51-70

>70

Near occlusion

Occluded

Quality

1

2

3

4

5

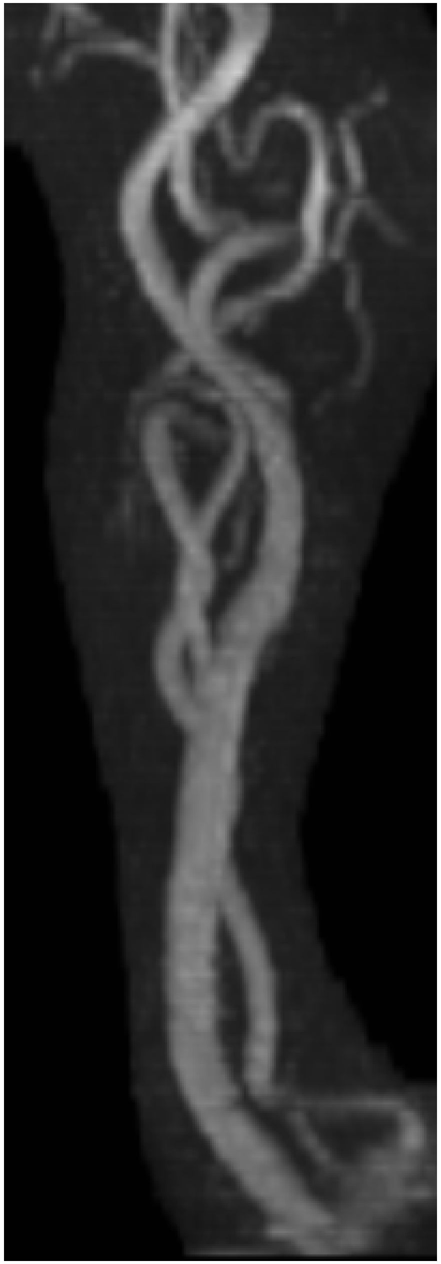

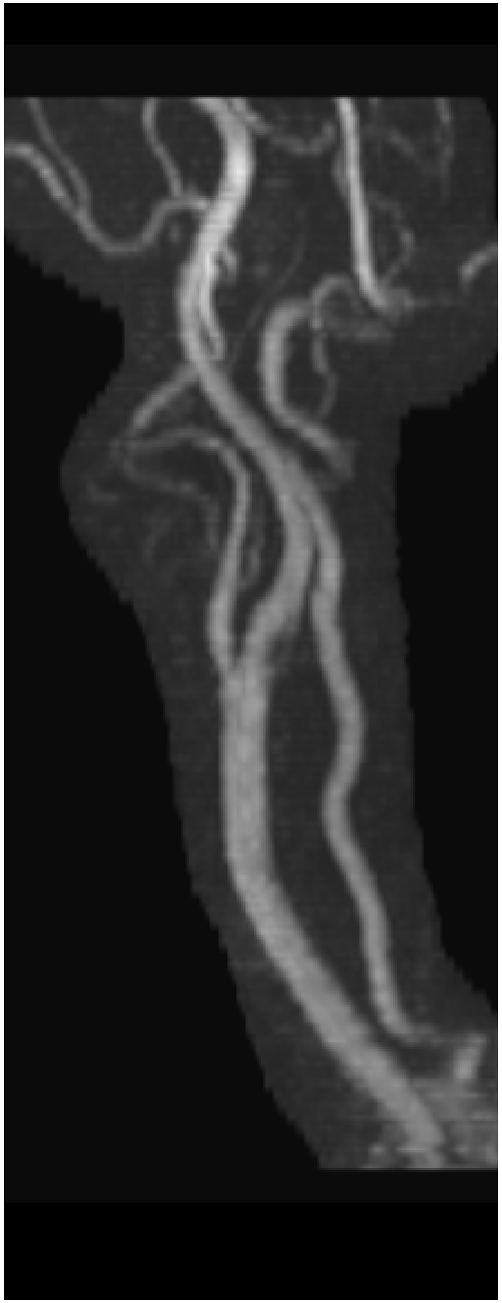

139a Score

0-30

31-50

51-70

>70

Near occlusion

Occluded

Quality

1

2

3

4

5

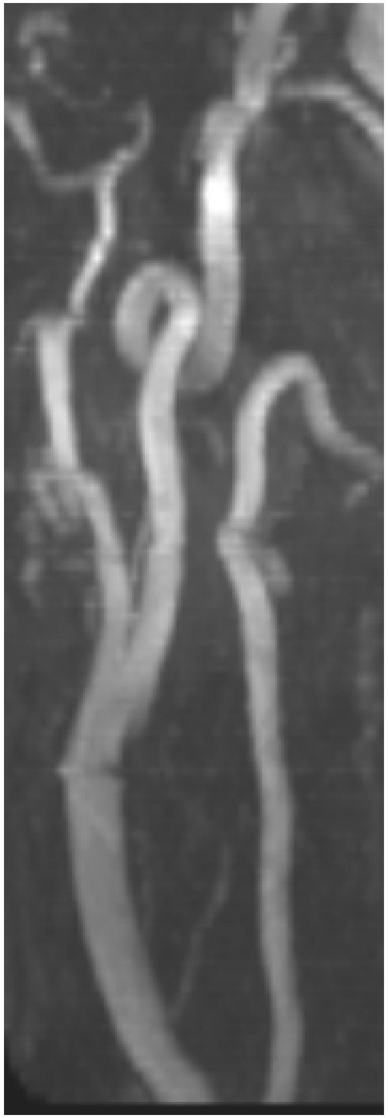

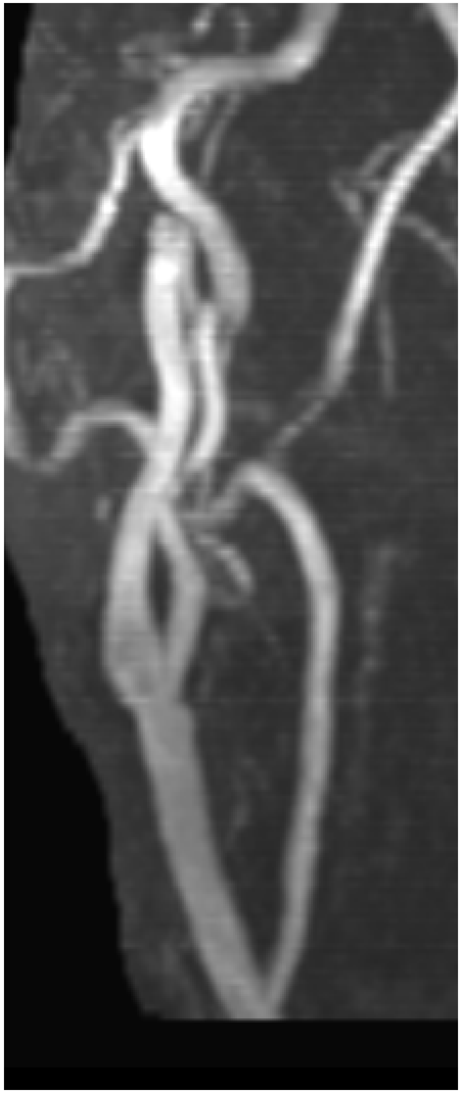

139f Score

0-30

31-50

51-70

>70

Near occlusion

Occluded

Quality

1

2

3

4

5

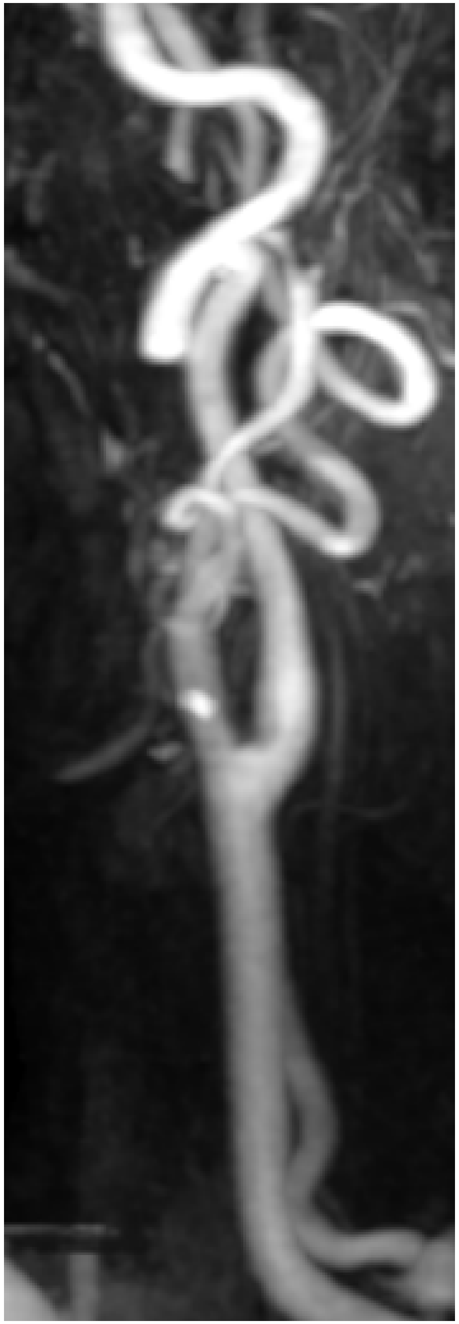

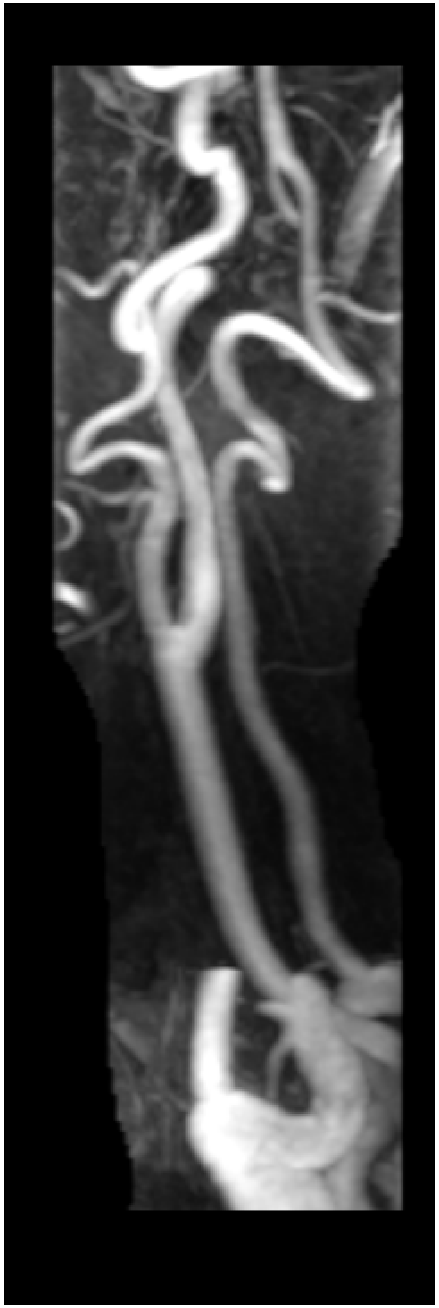

140e Score

0-30

31-50

51-70

>70

Near occlusion

Occluded

Quality

1

2

3

4

5

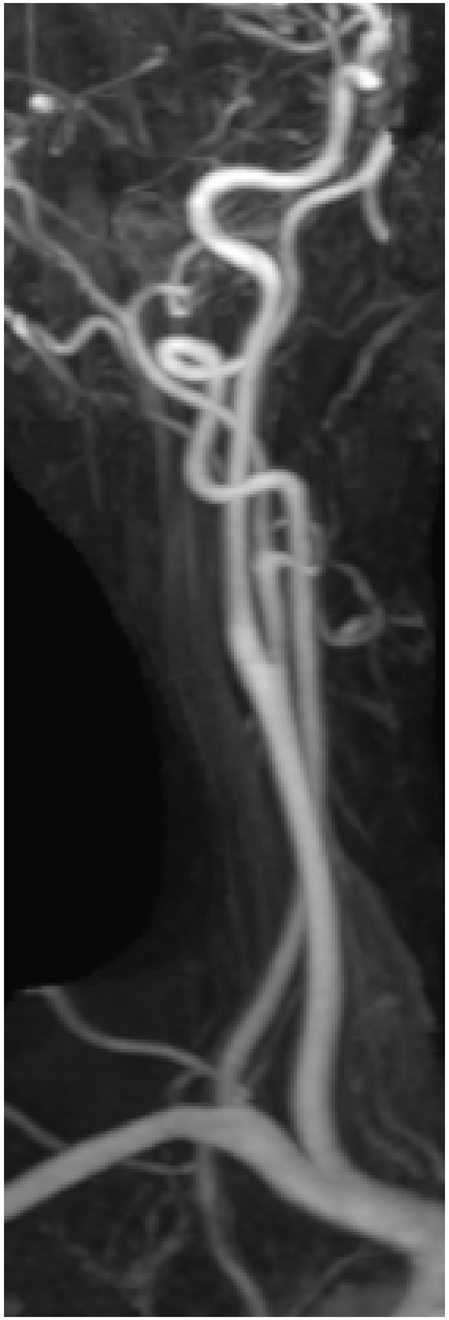

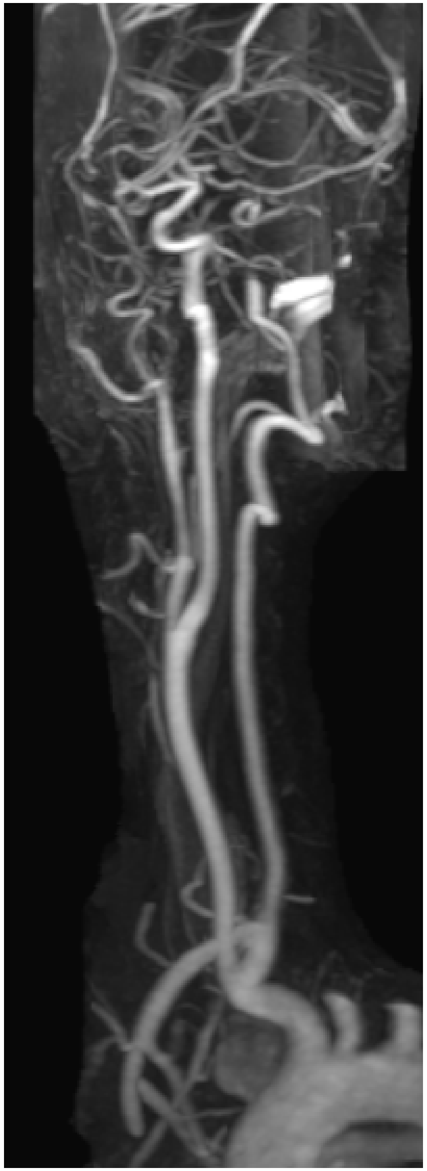

141d Score

0-30

31-50

51-70

>70

Near occlusion

Occluded

Quality

1

2

3

4

5

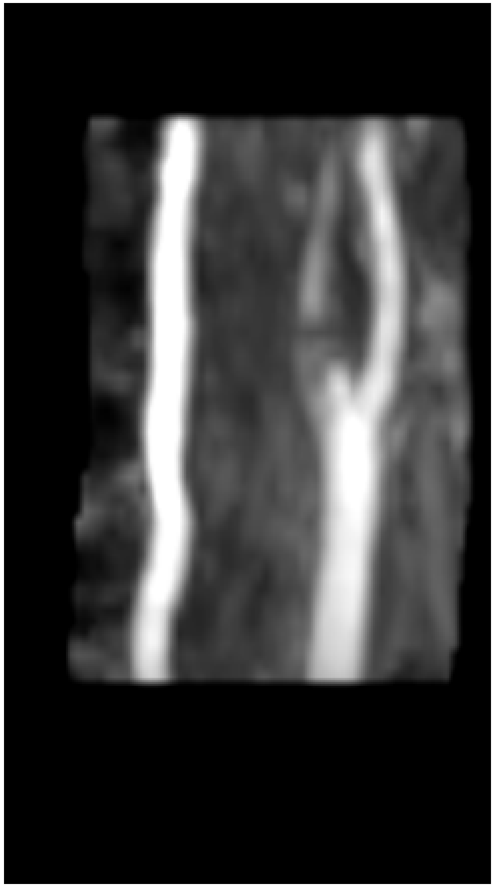

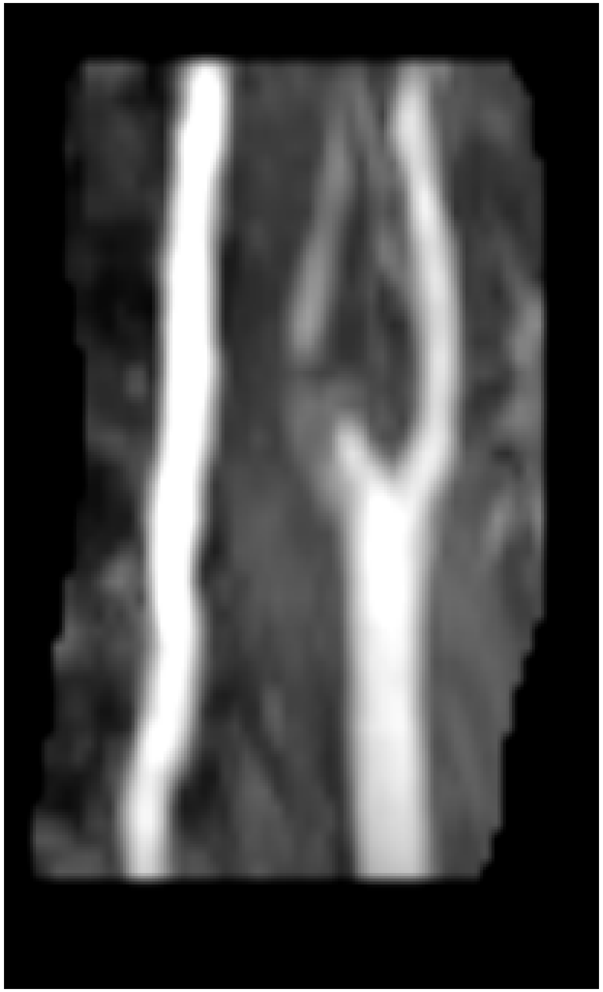

142c Score

0-30

31-50

51-70

>70

Near occlusion

Occluded

Quality

1

2

3

4

5

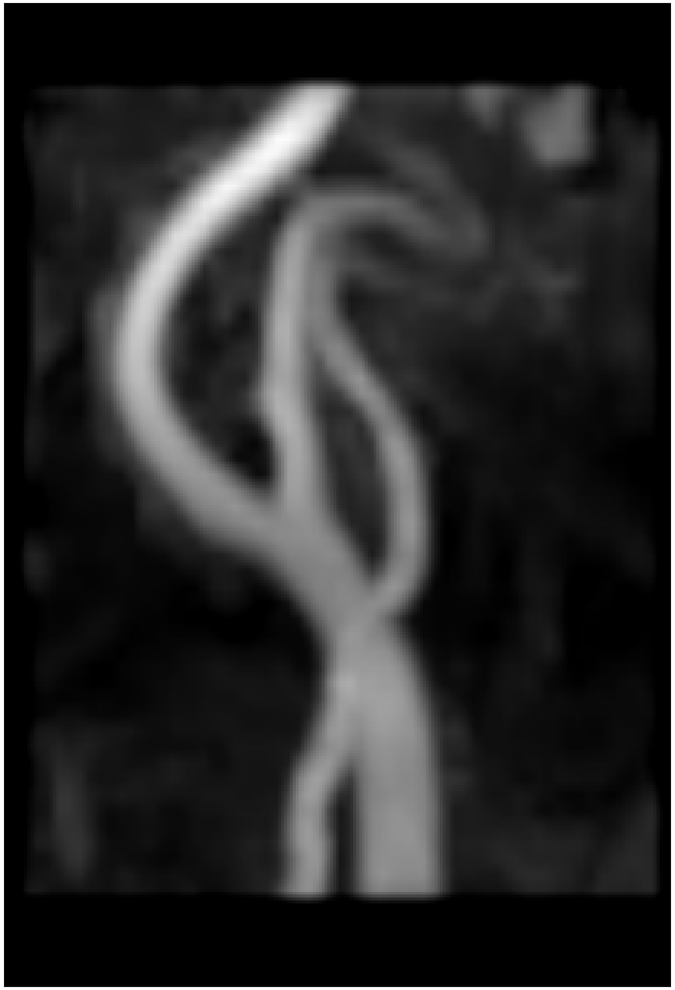

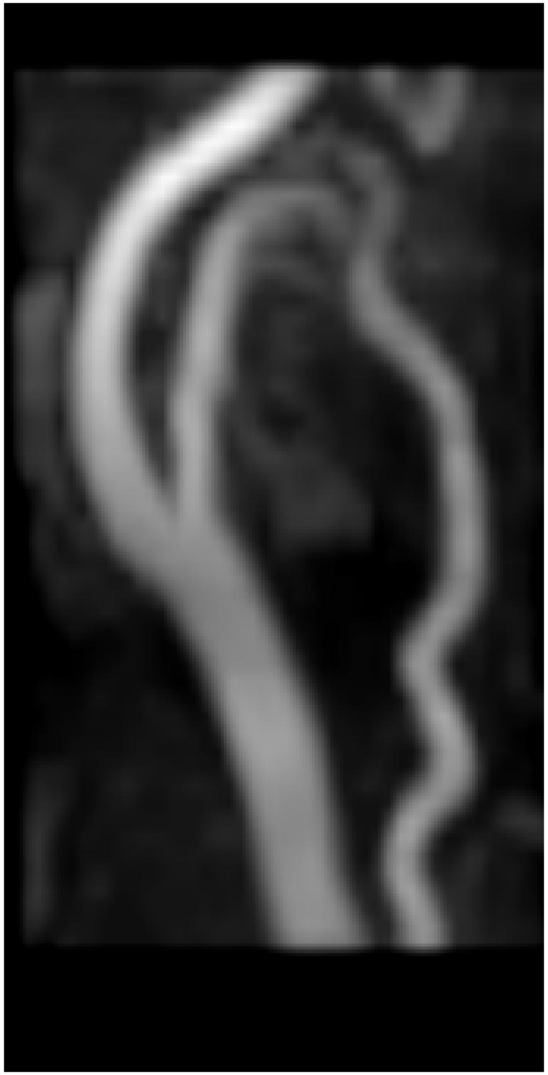

143b Score

0-30

31-50

51-70

>70

Near occlusion

Occluded

Quality

1

2

3

4

5

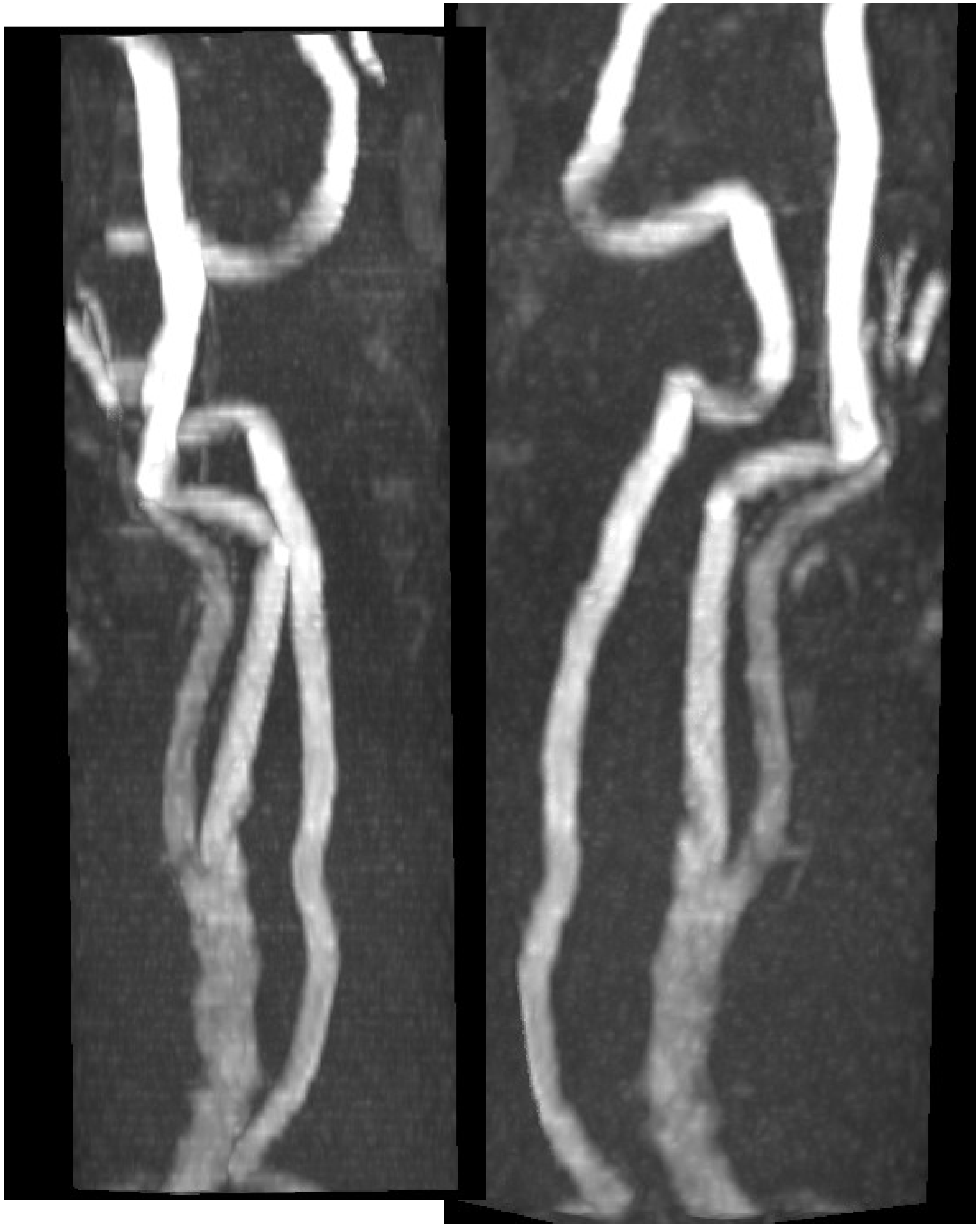

144a Score

0-30

31-50

51-70

>70

Near occlusion

Occluded

Quality

1

2

3

4

5

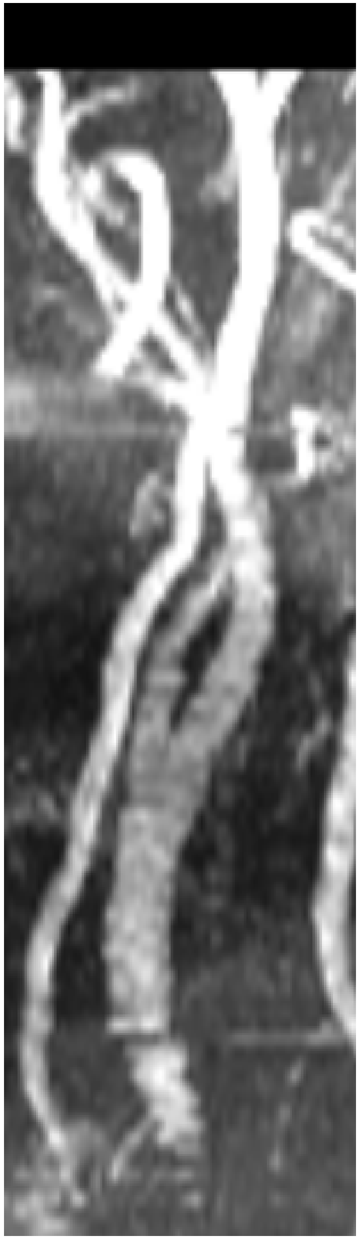

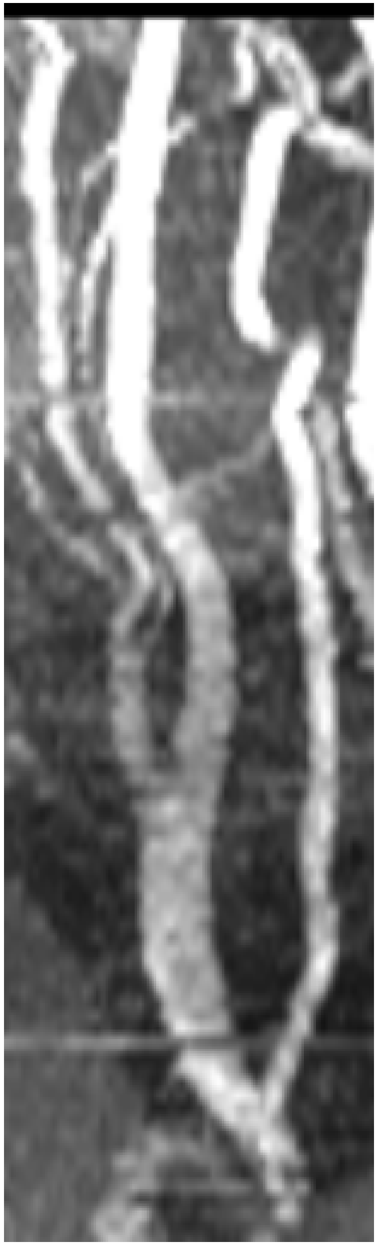

144f Score

0-30

31-50

51-70

>70

Near occlusion

Occluded

Quality

1

2

3

4

5

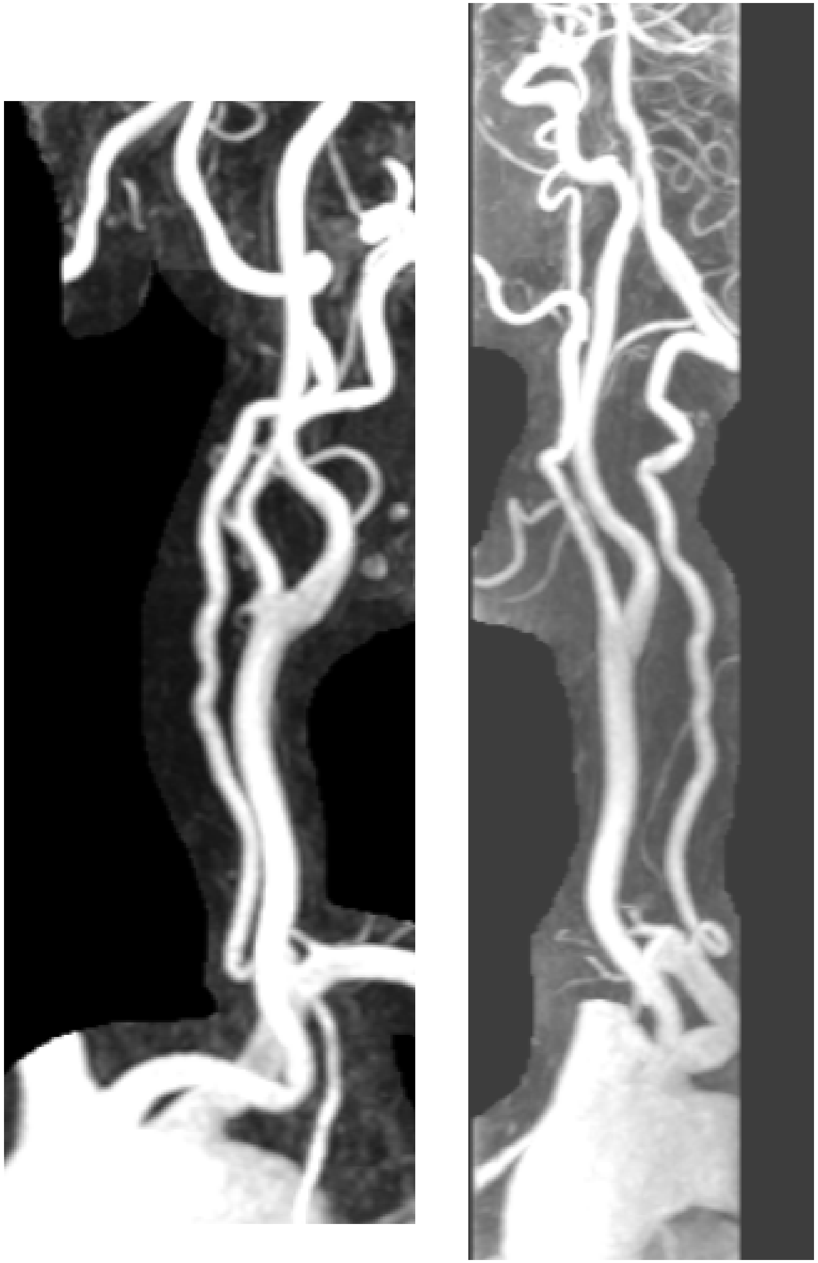

145e Score

0-30

31-50

51-70

>70

Near occlusion

Occluded

Quality

1

2

3

4

5

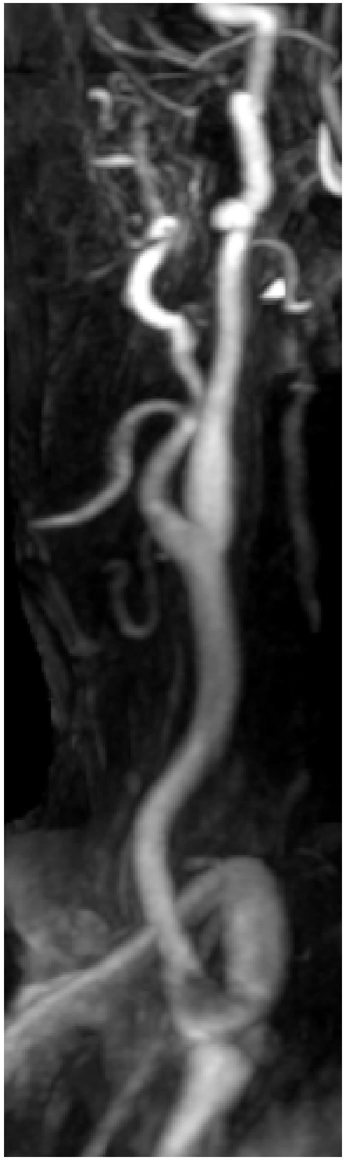

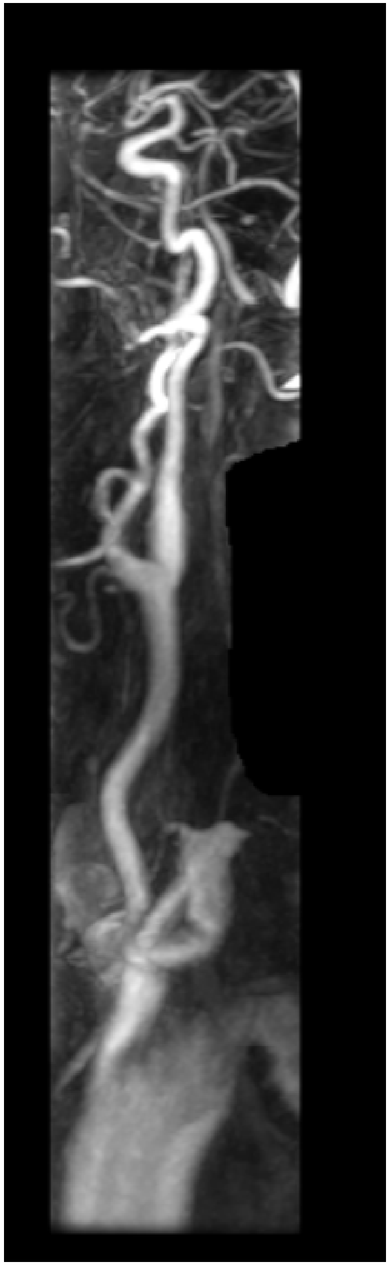

146d Score (left)

0-30

31-50

51-70

>70

Near occlusion

Occluded

Quality

1

2

3

4

5

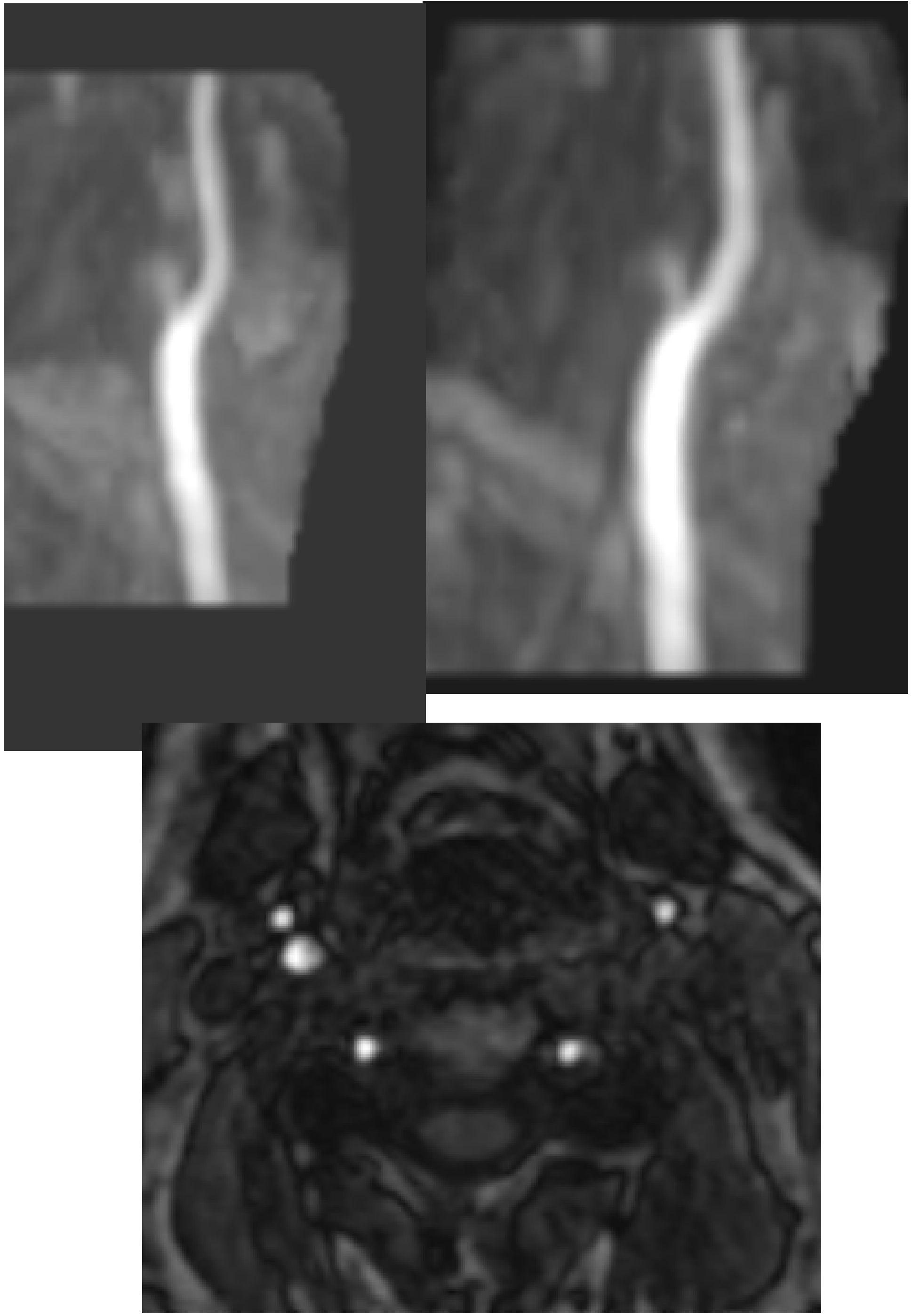

147c Score

0-30

31-50

51-70

>70

Near occlusion

Occluded

Quality

1

2

3

4

5

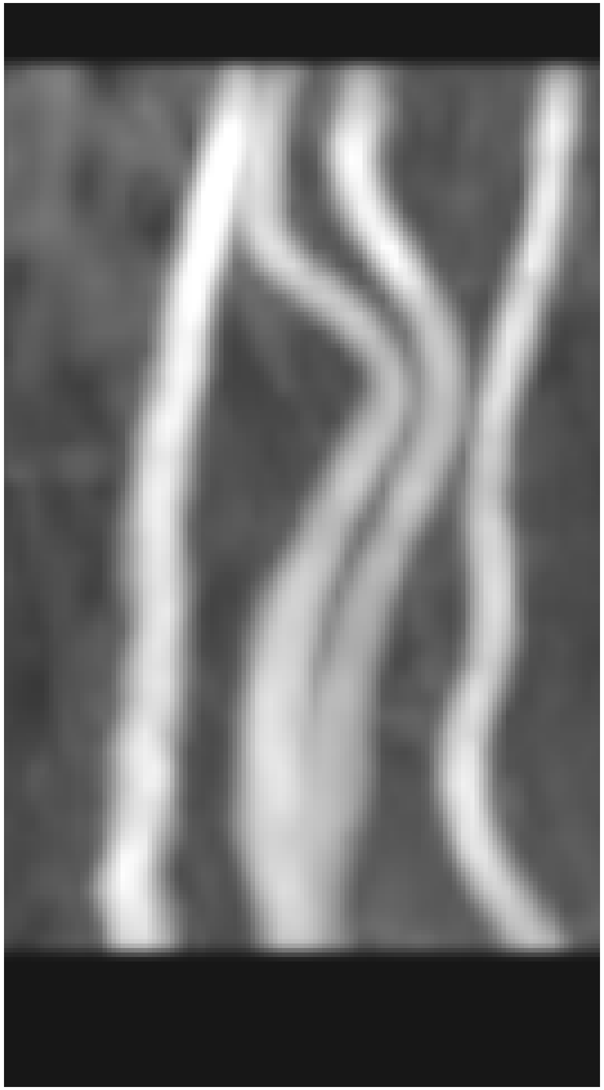

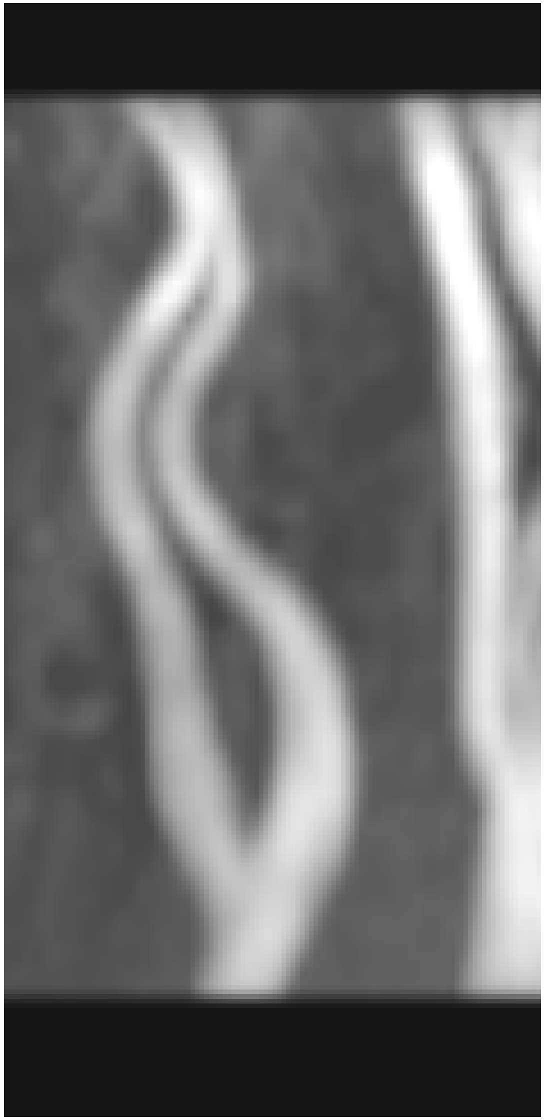

148b Score

0-30

31-50

51-70

>70

Near occlusion

Occluded

Quality

1

2

3

4

5

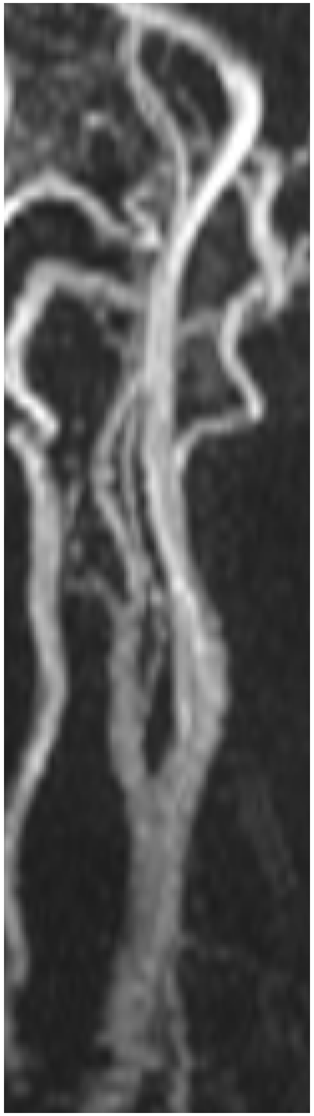

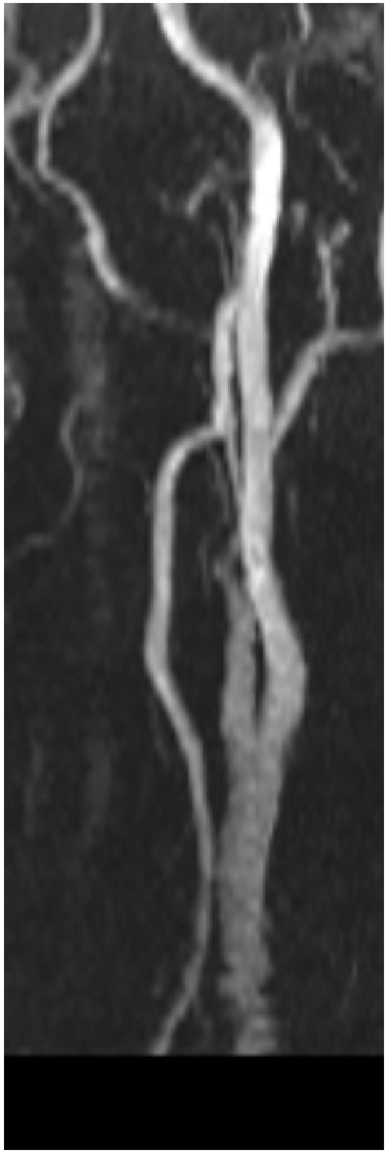

149a Score

0-30

31-50

51-70

>70

Near occlusion

Occluded

Quality

1

2

3

4

5

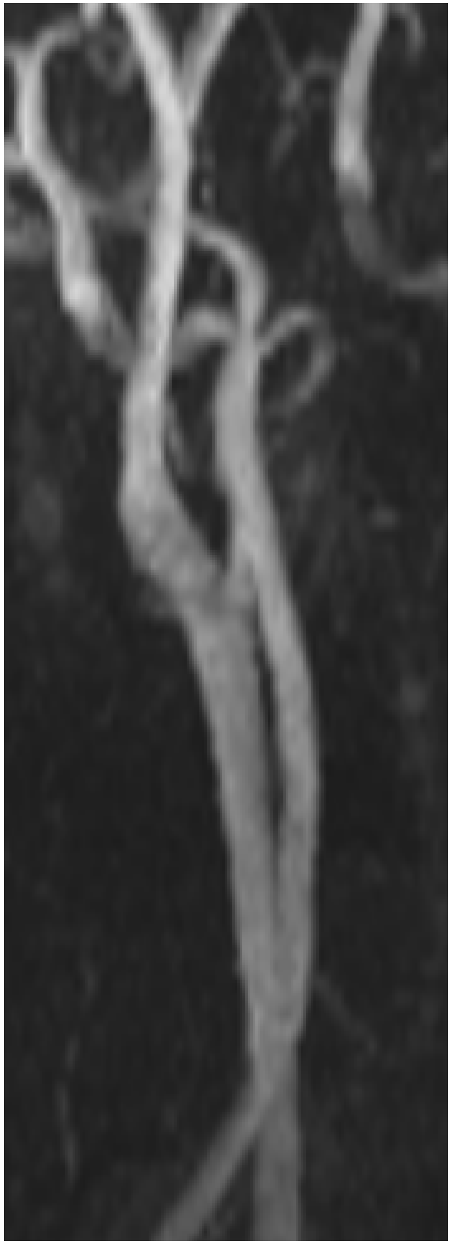

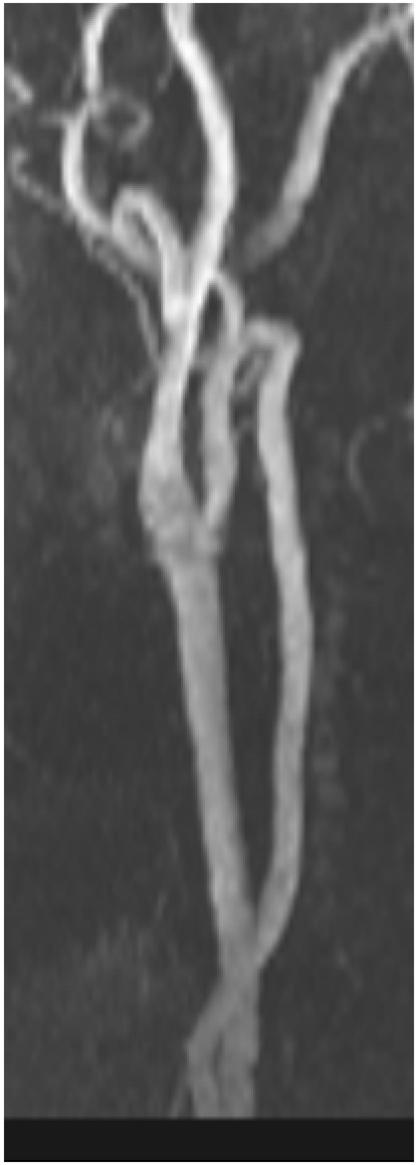

149f Score

0-30

31-50

51-70

>70

Near occlusion

Occluded

Quality

1

2

3

4

5

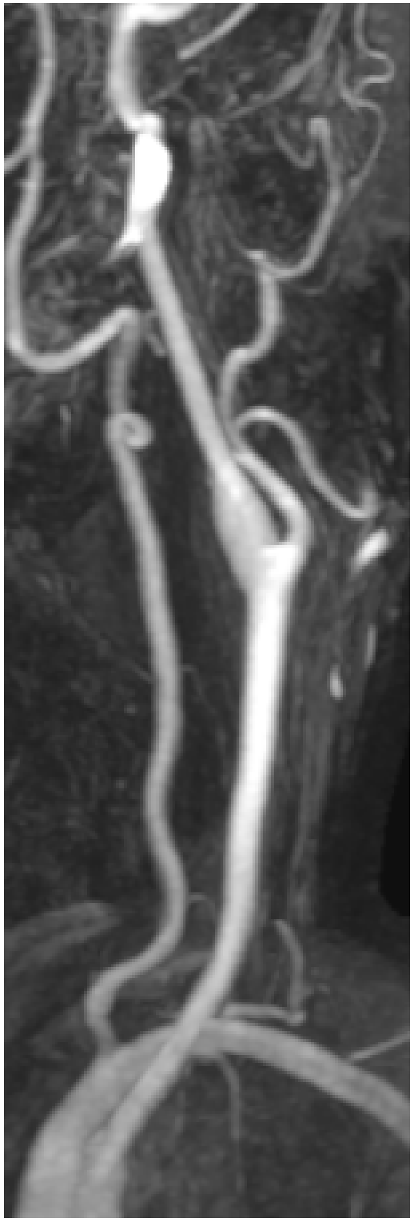

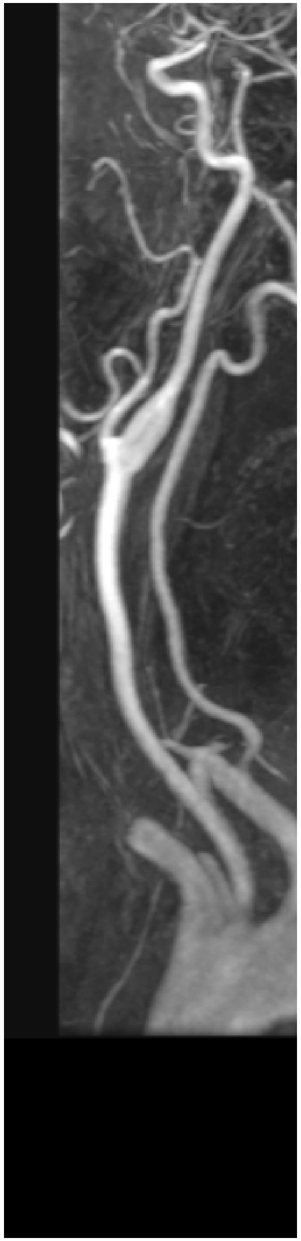

150e Score

0-30

31-50

51-70

>70

Near occlusion

Occluded

Quality

1

2

3

4

5

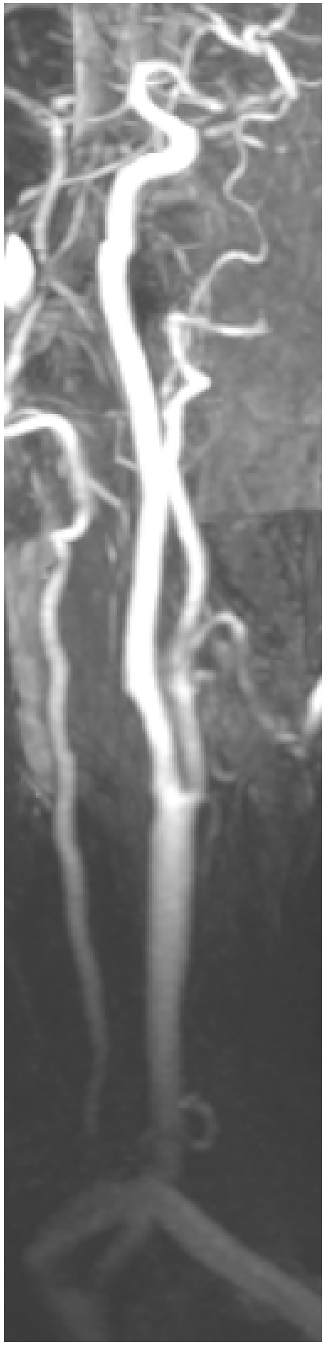

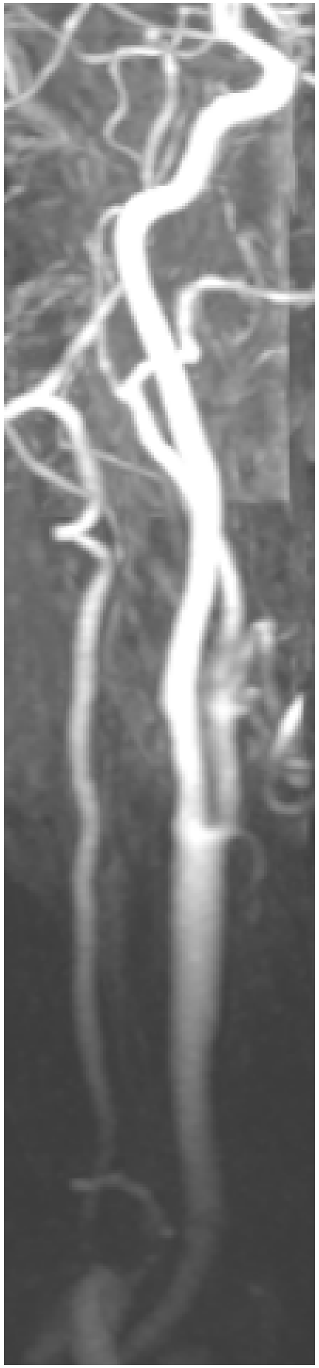

151d Score

0-30

31-50

51-70

>70

Near occlusion

Occluded

Quality

1

2

3

4

5

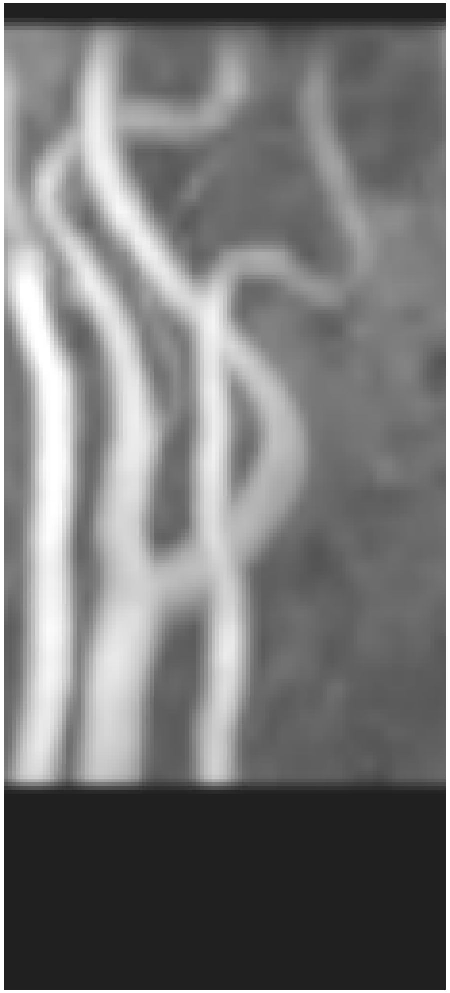

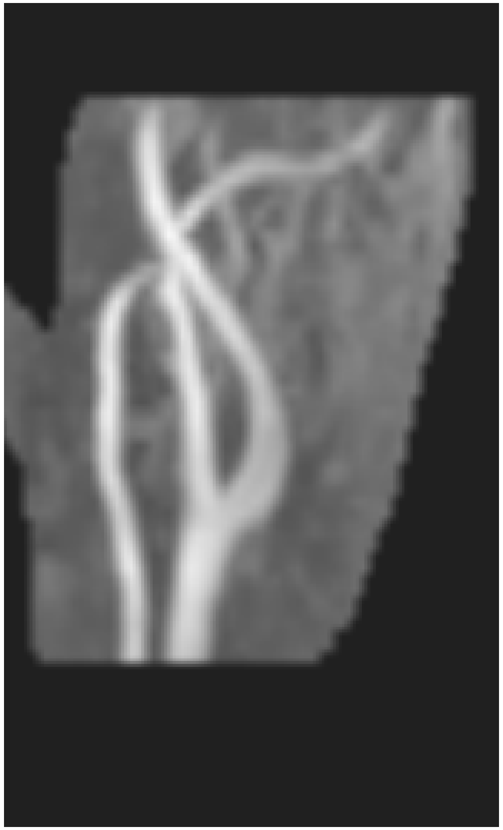

152c Score

0-30

31-50

51-70

>70

Near occlusion

Occluded

Quality

1

2

3

4

5

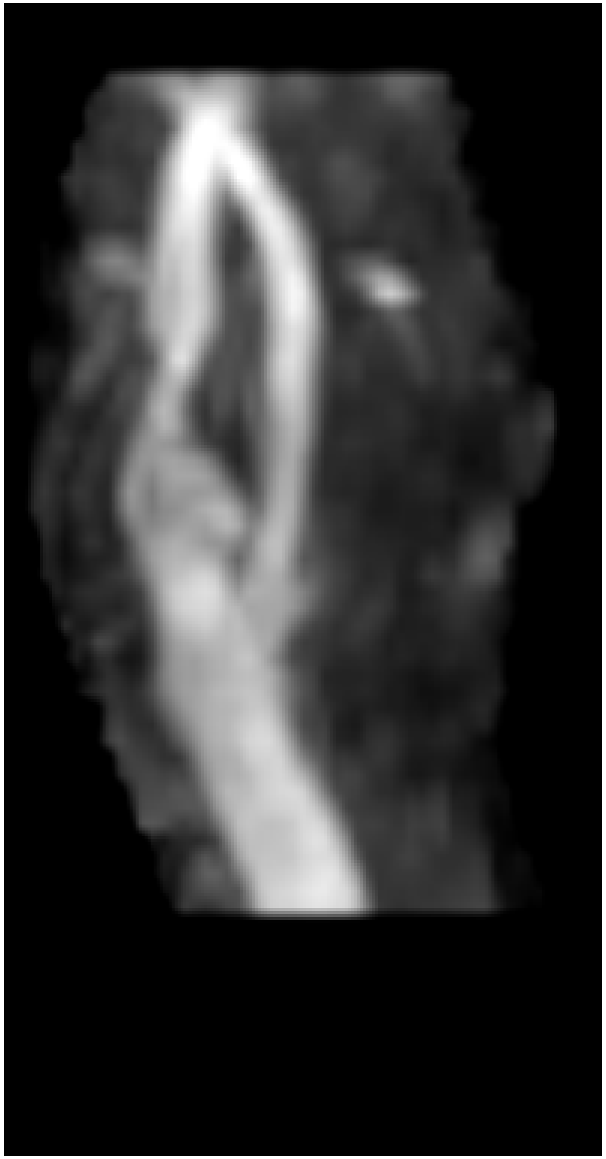

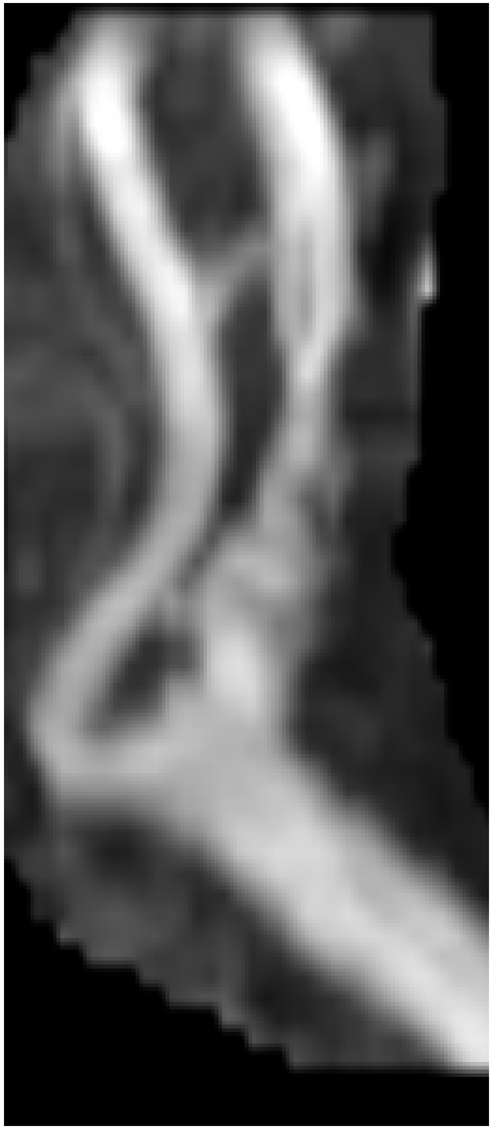

153b Score

0-30

31-50

51-70

>70

Near occlusion

Occluded

Quality

1

2

3

4

5

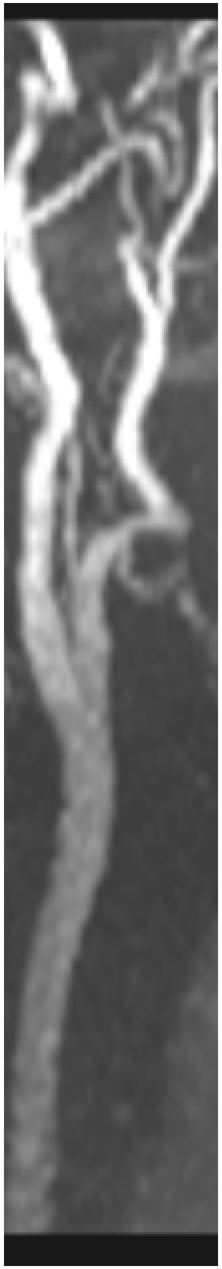

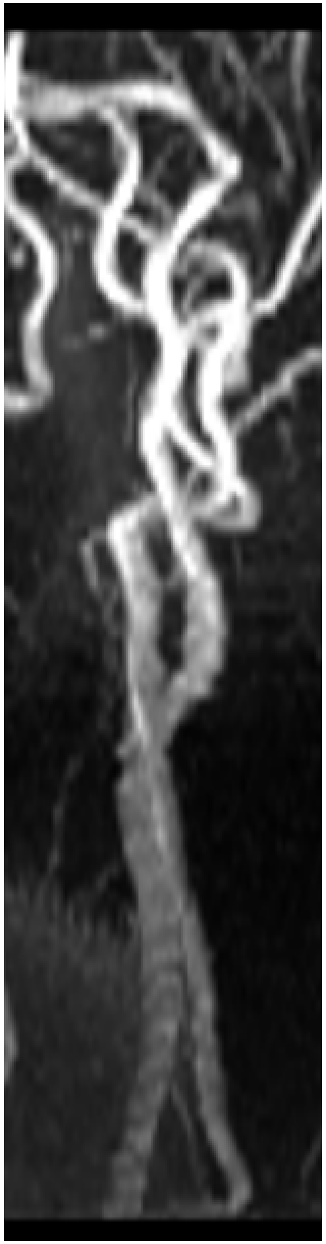

154a Score

0-30

31-50

51-70

>70

Near occlusion

Occluded

Quality

1

2

3

4

5

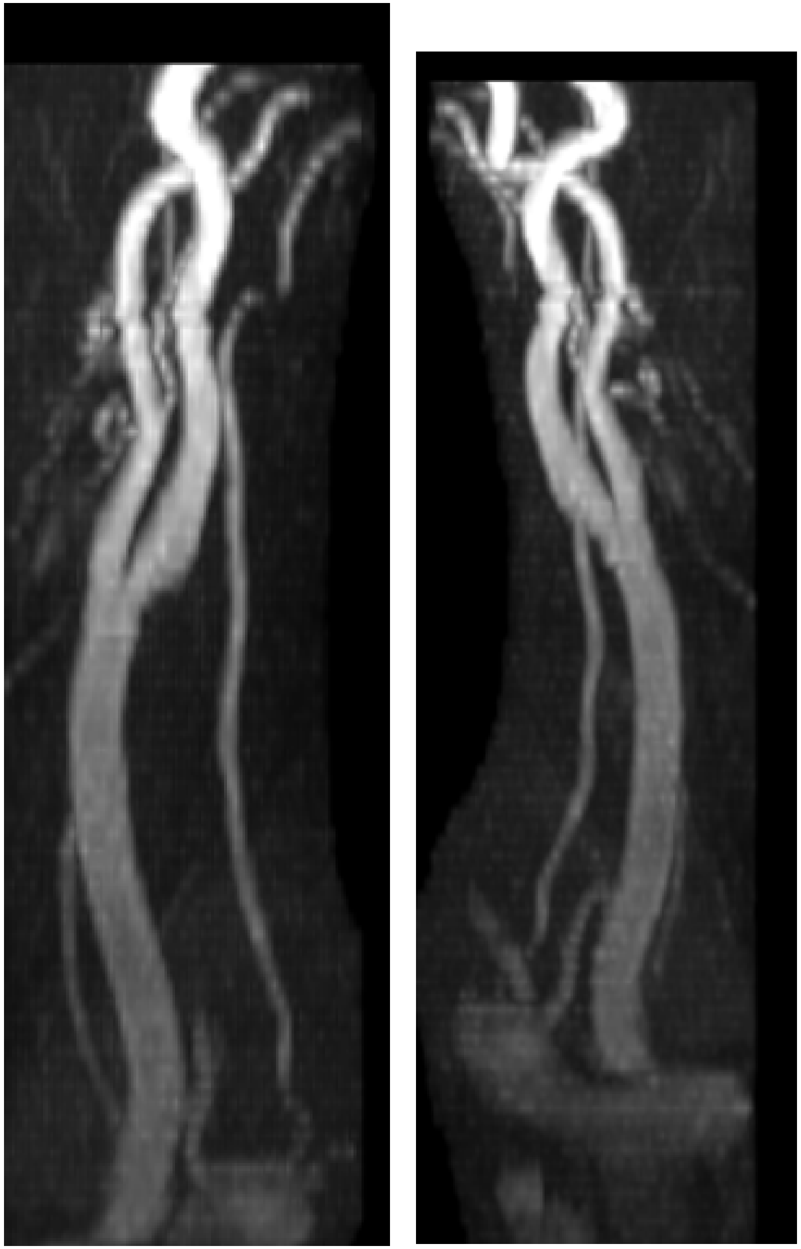

154f Score

0-30

31-50

51-70

>70

Near occlusion

Occluded

Quality

1

2

3

4

5

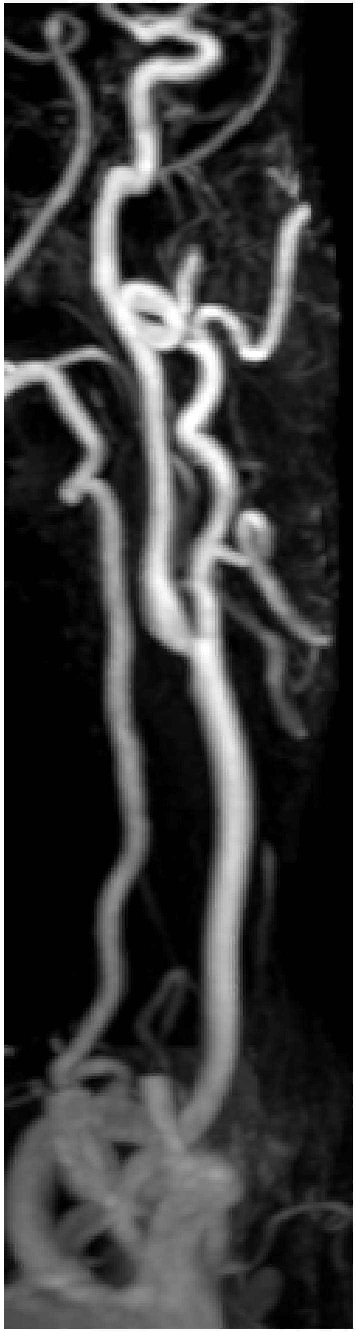

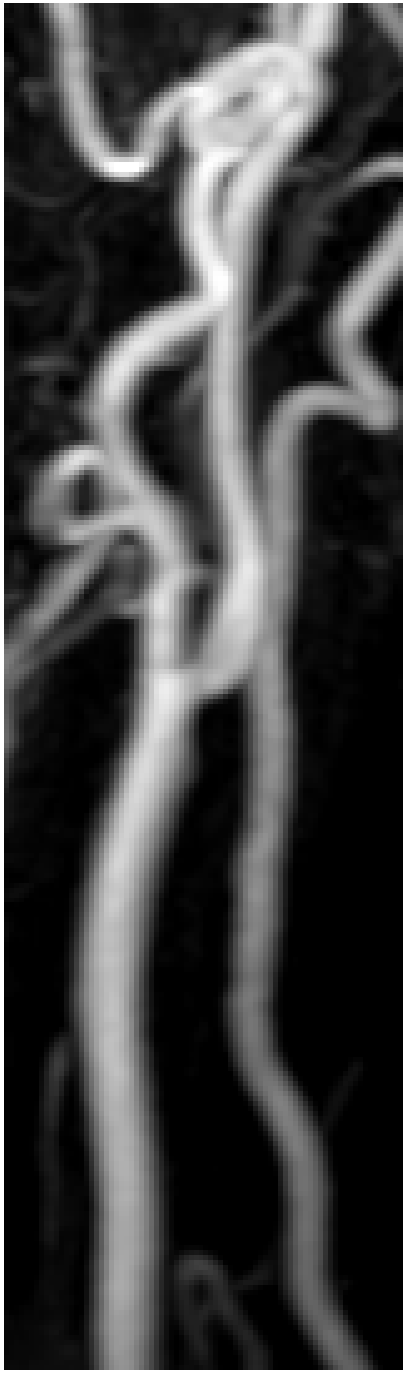

155e Score

0-30

31-50

51-70

>70

Near occlusion

Occluded

Quality

1

2

3

4

5

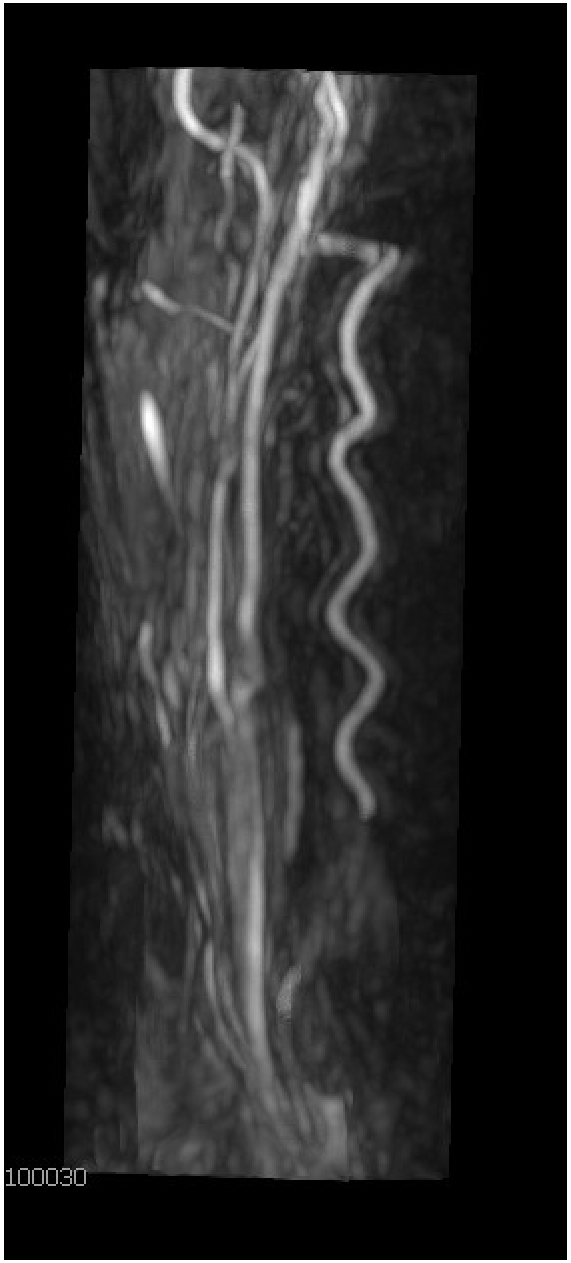

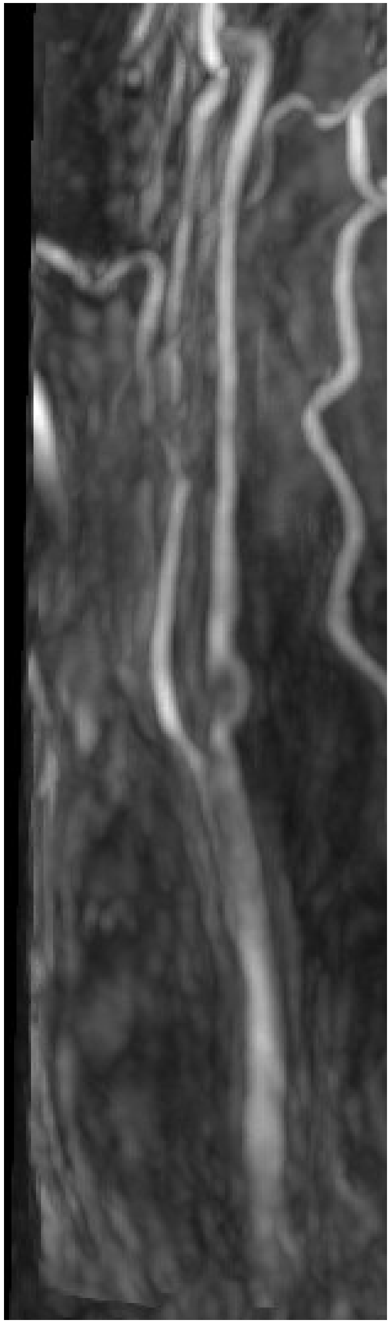

156d Score

0-30

31-50

51-70

>70

Near occlusion

Occluded

Quality

1

2

3

4

5

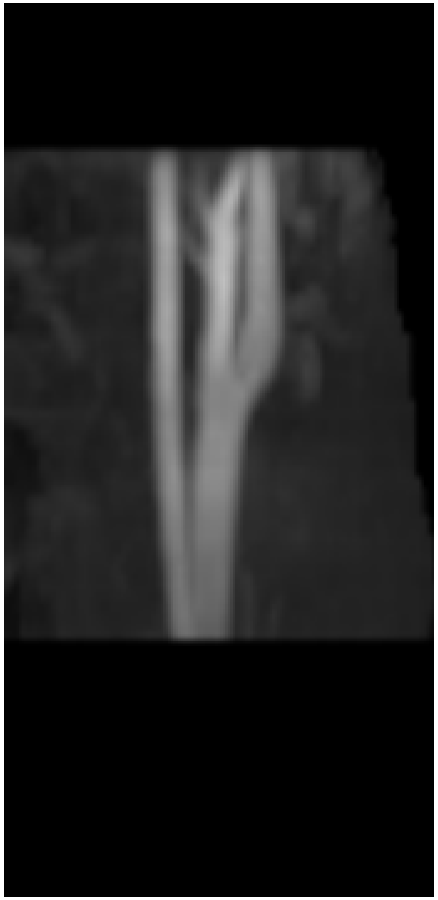

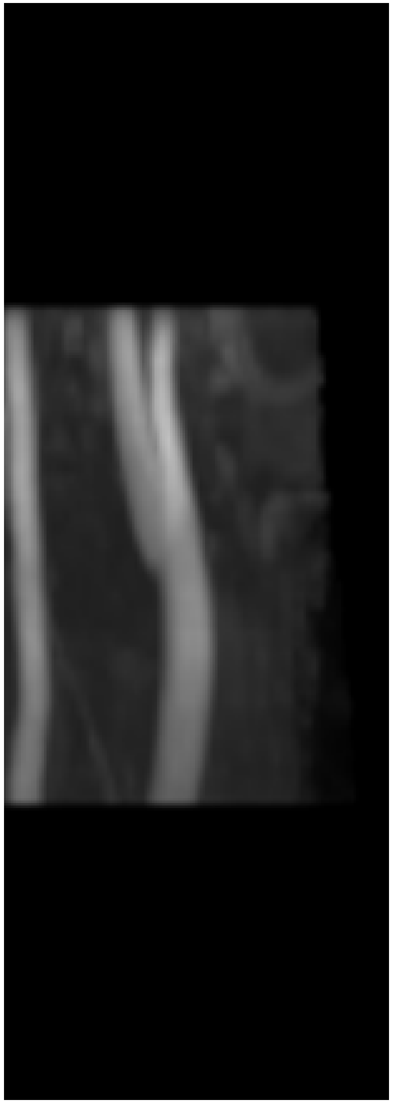

157c Score

0-30

31-50

51-70

>70

Near occlusion

Occluded

Quality

1

2

3

4

5

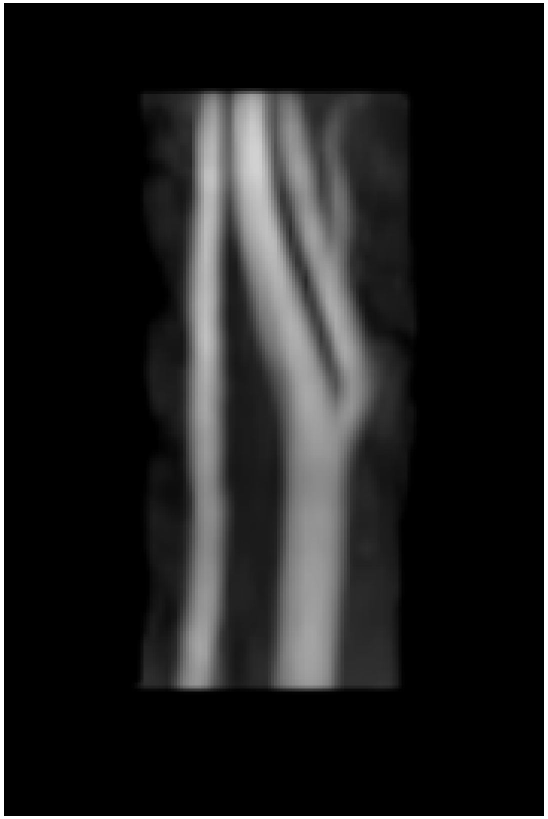

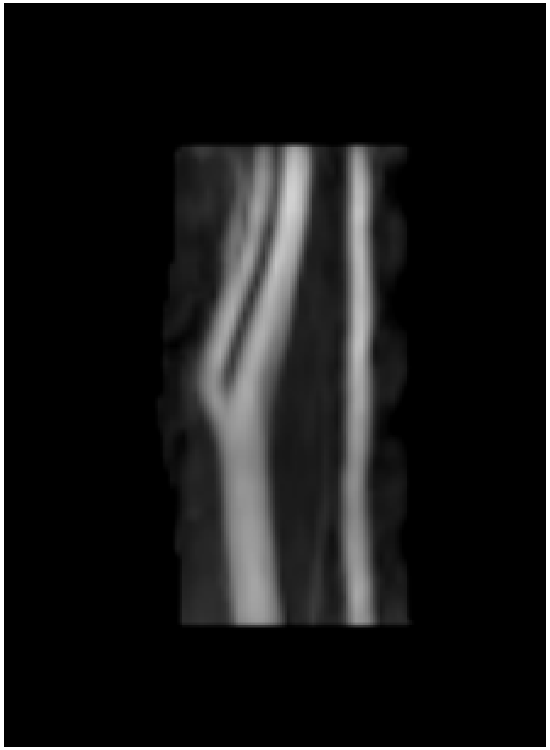

158b Score

0-30

31-50

51-70

>70

Near occlusion

Occluded

Quality

1

2

3

4

5

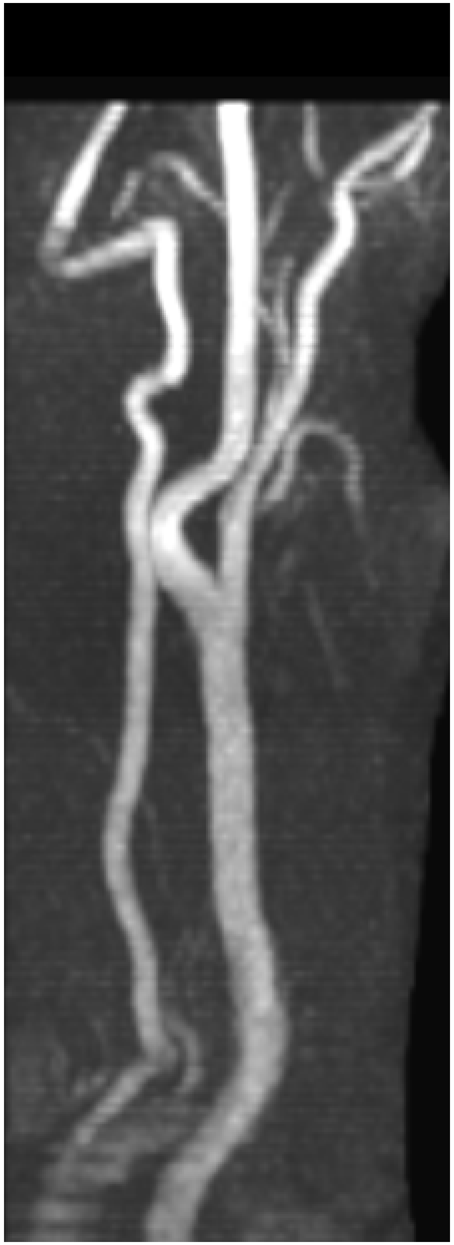

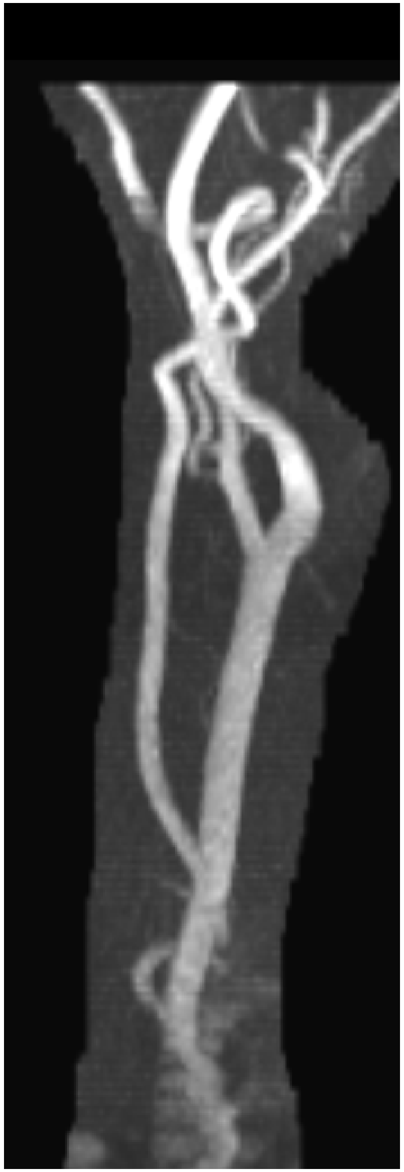

159a Score

0-30

31-50

51-70

>70

Near occlusion

Occluded

Quality

1

2

3

4

5

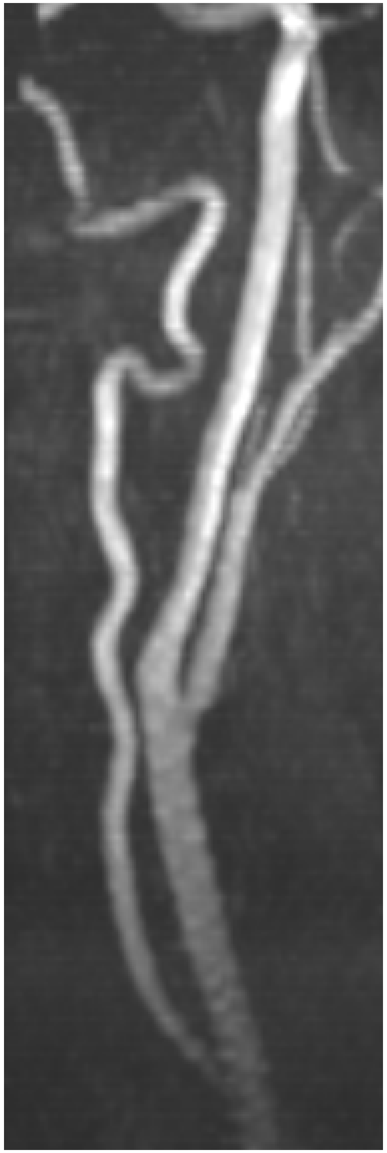

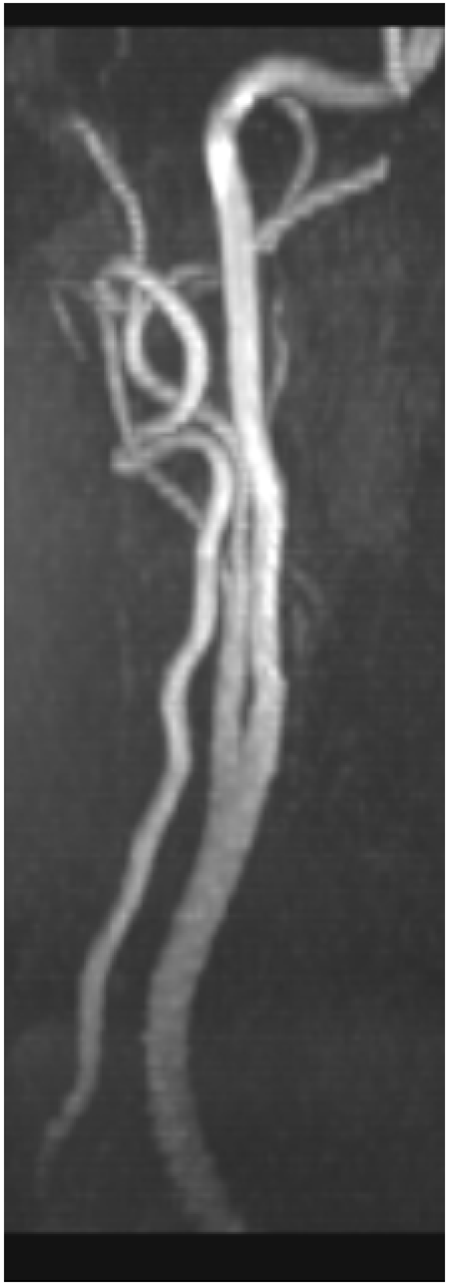

159f Score

0-30

31-50

51-70

>70

Near occlusion

Occluded

Quality

1

2

3

4

5

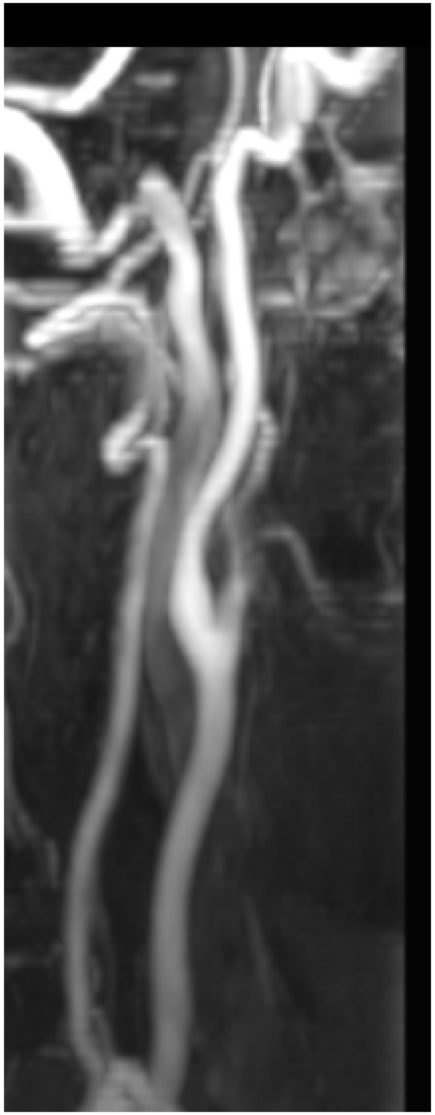

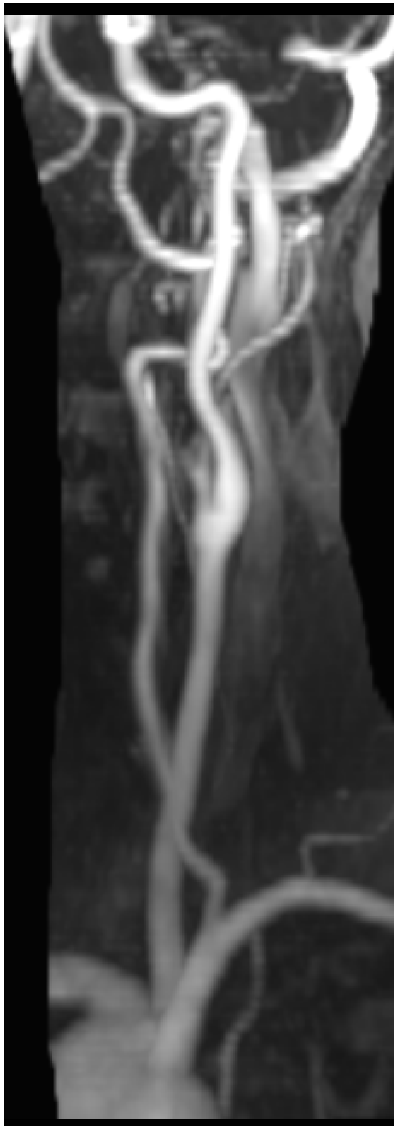

160e Score

0-30

31-50

51-70

>70

Near occlusion

Occluded

Quality

1

2

3

4

5

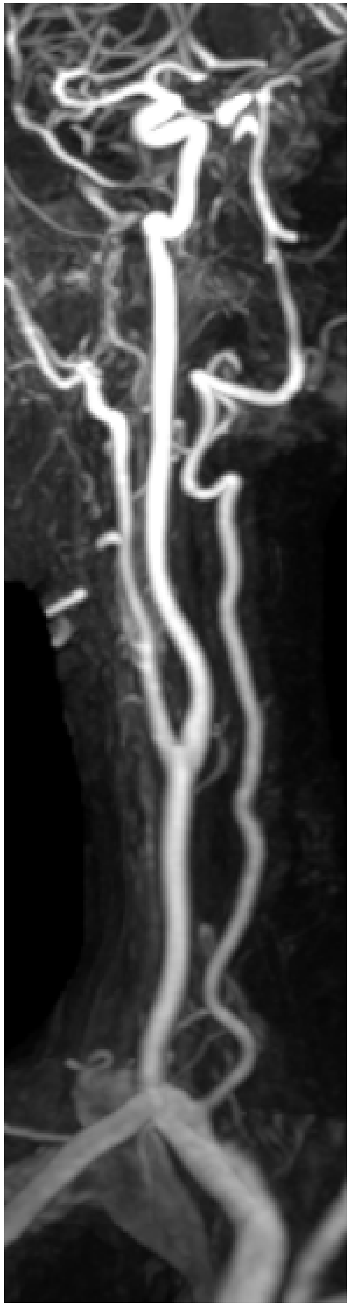

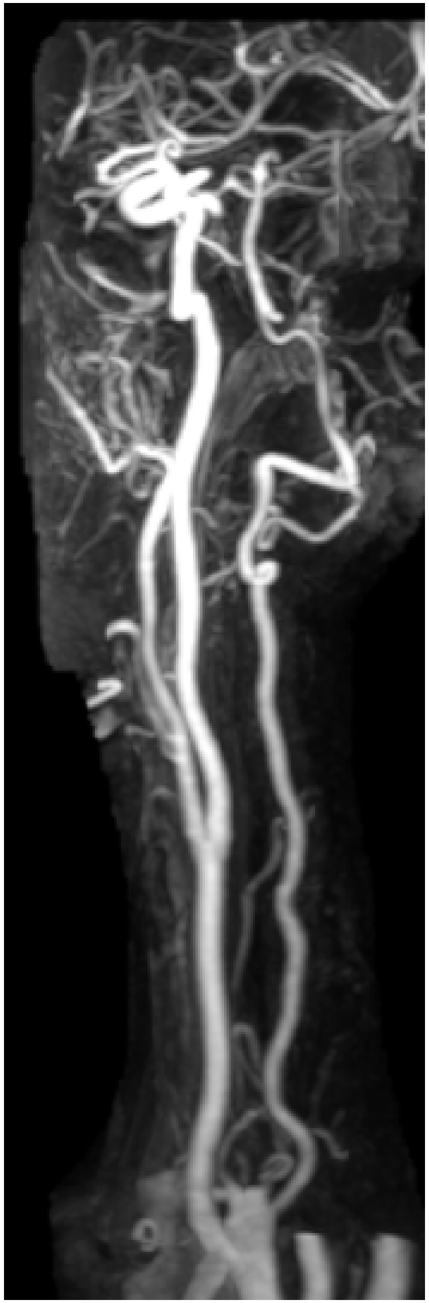

161d Score

0-30

31-50

51-70

>70

Near occlusion

Occluded

Quality

1

2

3

4

5

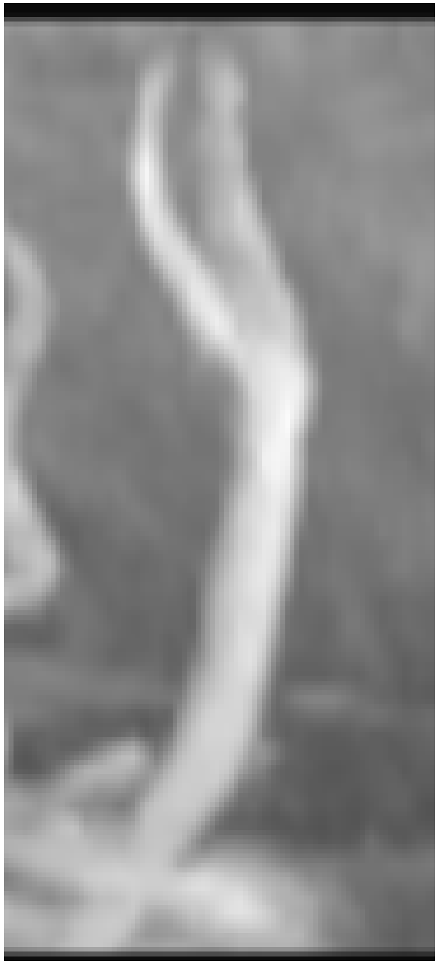

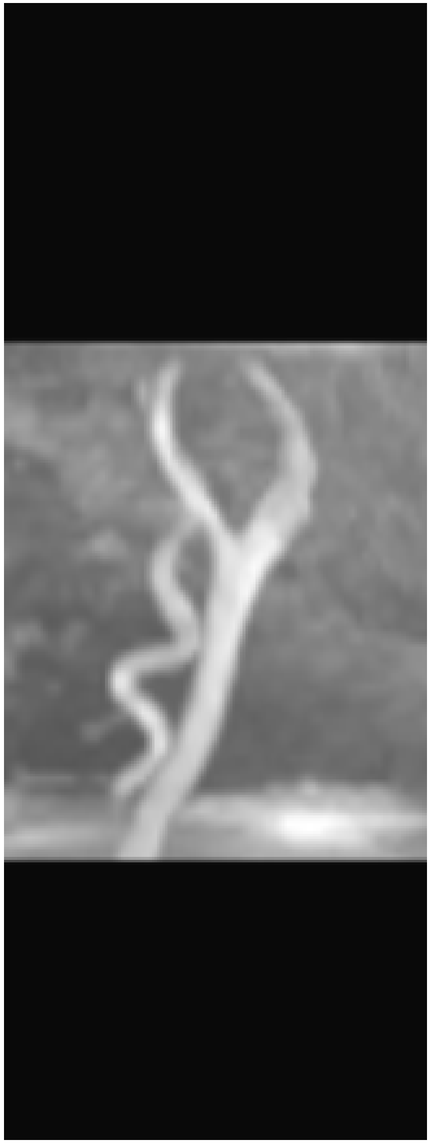

162c Score

0-30

31-50

51-70

>70

Near occlusion

Occluded

Quality

1

2

3

4

5

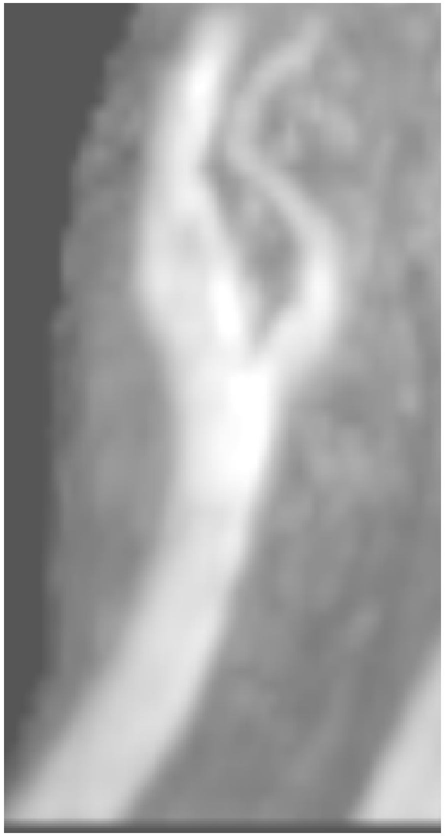

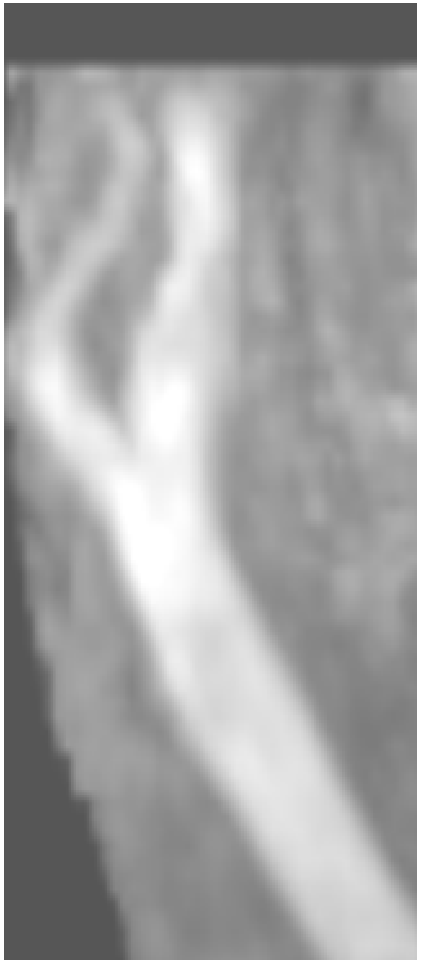

163b Score

0-30

31-50

51-70

>70

Near occlusion

Occluded

Quality

1

2

3

4

5

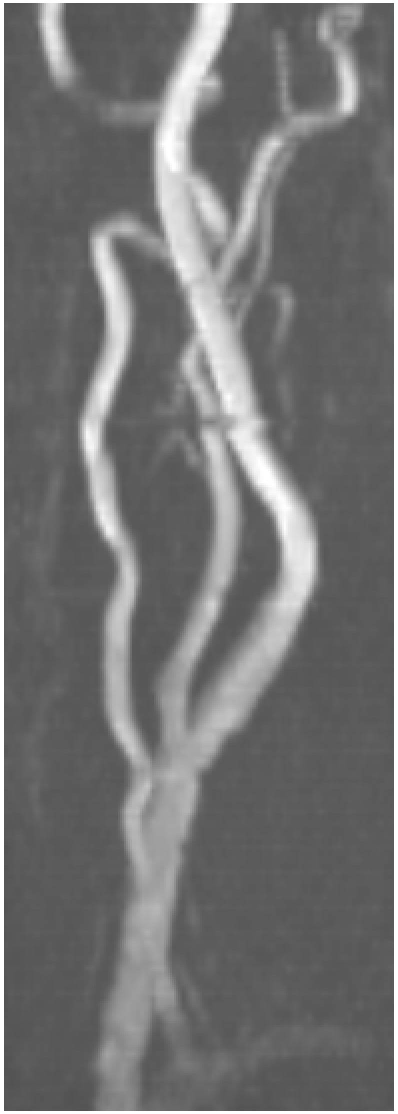

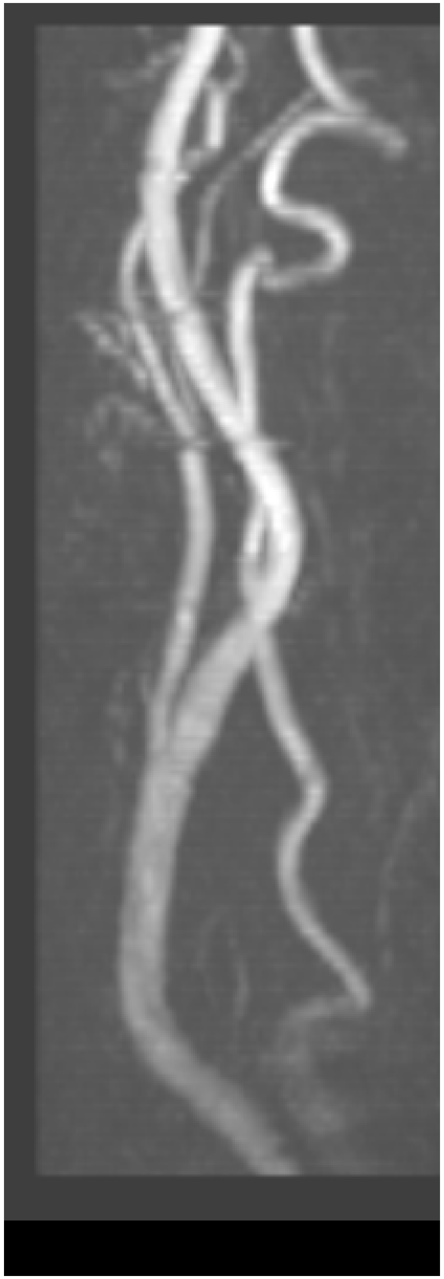

164a Score

0-30

31-50

51-70

>70

Near occlusion

Occluded

Quality

1

2

3

4

5

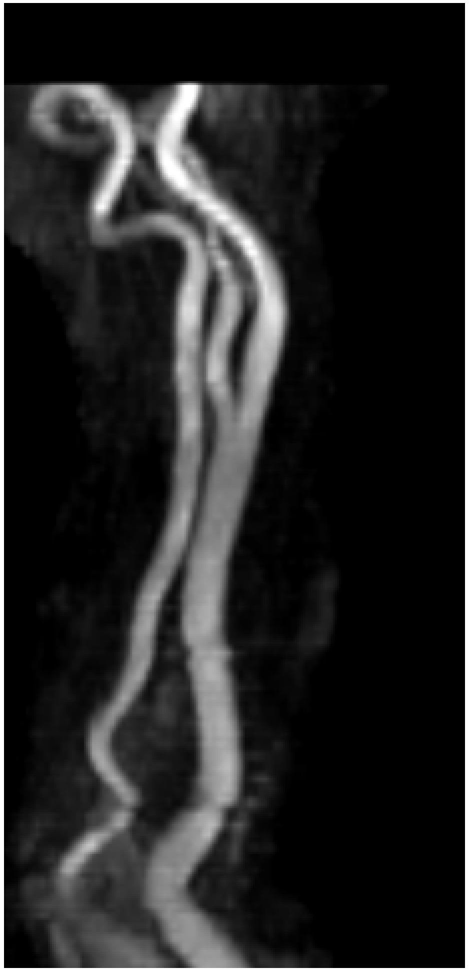

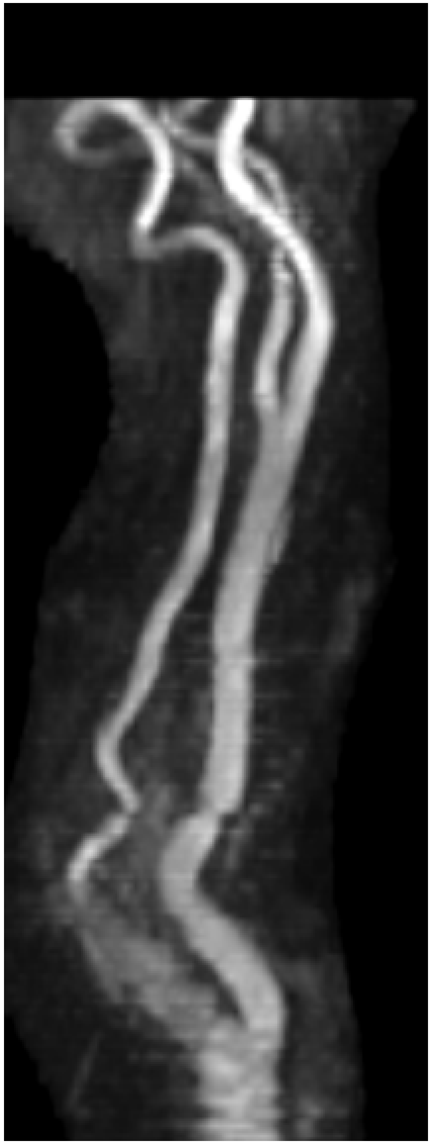

164f Score

0-30

31-50

51-70

>70

Near occlusion

Occluded

Quality

1

2

3

4

5

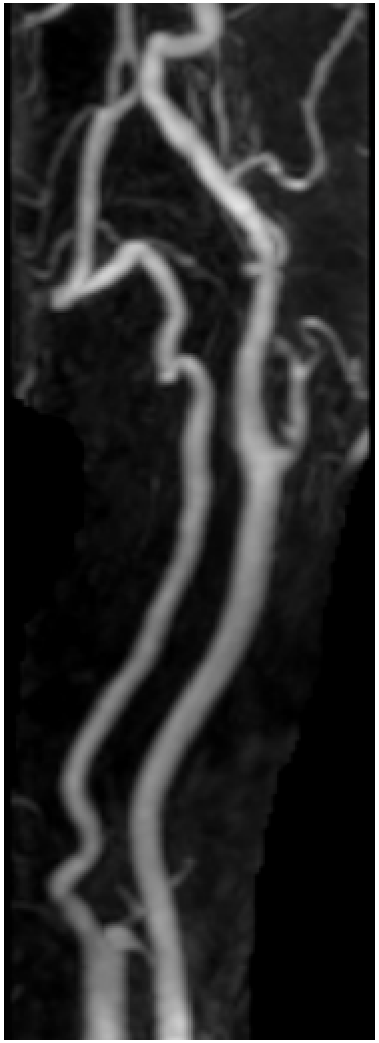

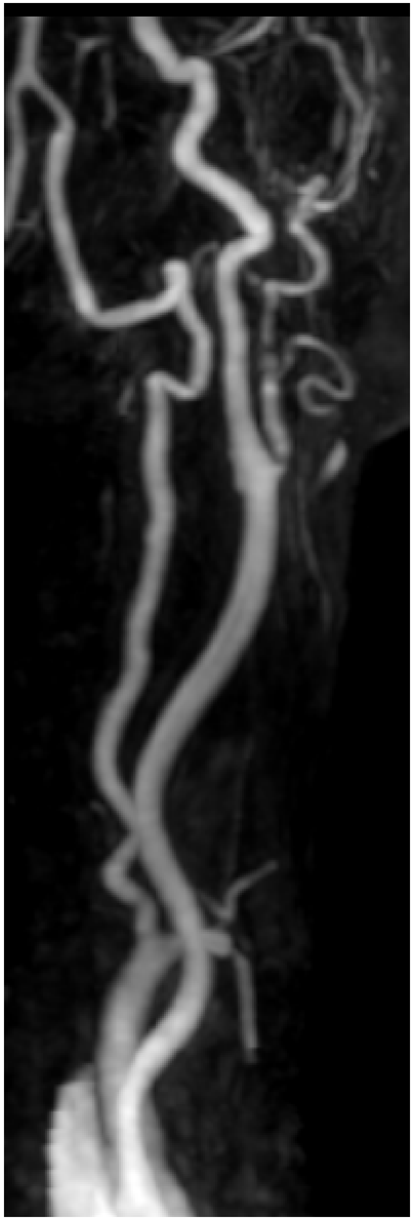

165e Score

0-30

31-50

51-70

>70

Near occlusion

Occluded

Quality

1

2

3

4

5

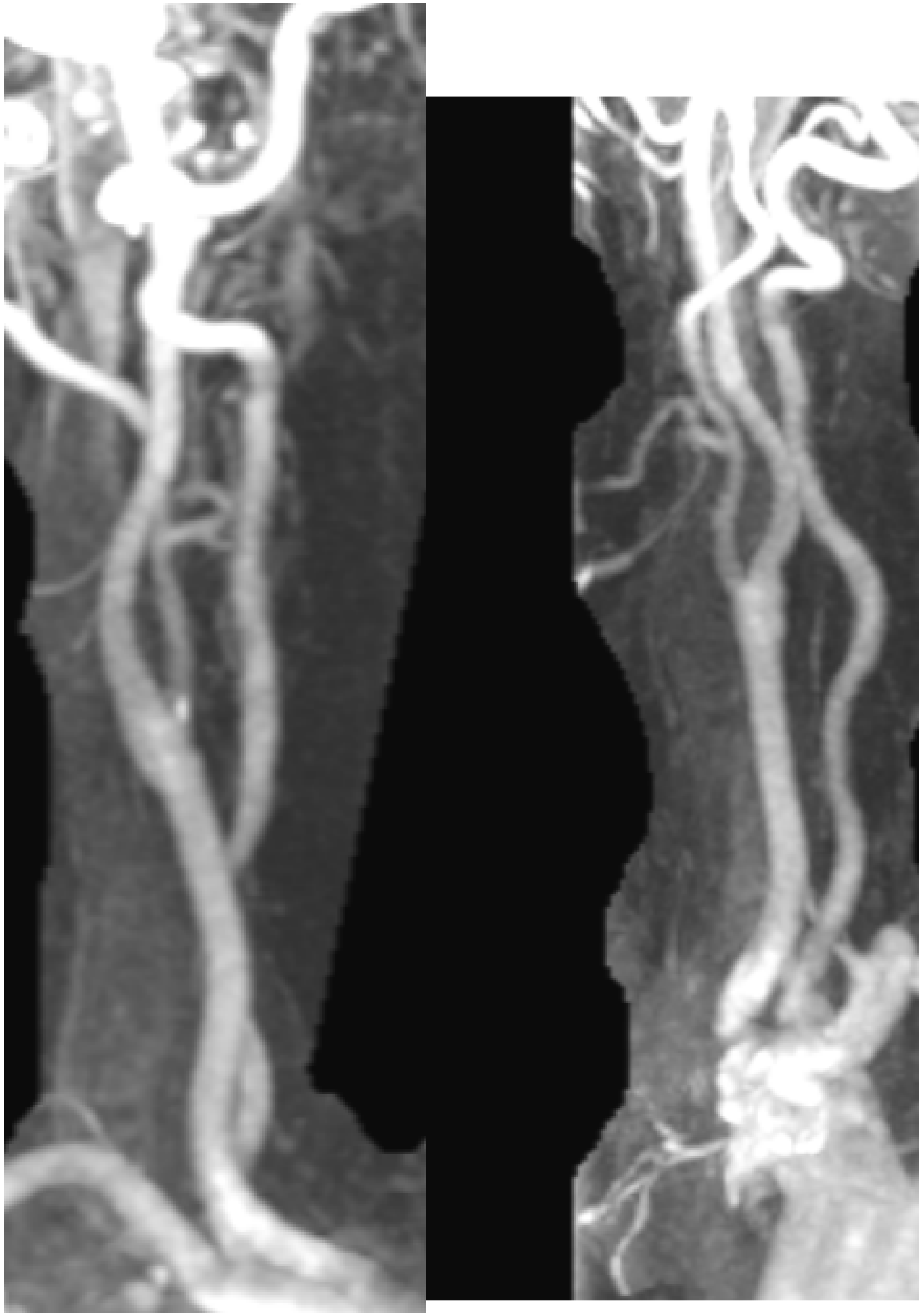

166d Score

0-30

31-50

51-70

>70

Near occlusion

Occluded

Quality

1

2

3

4

5

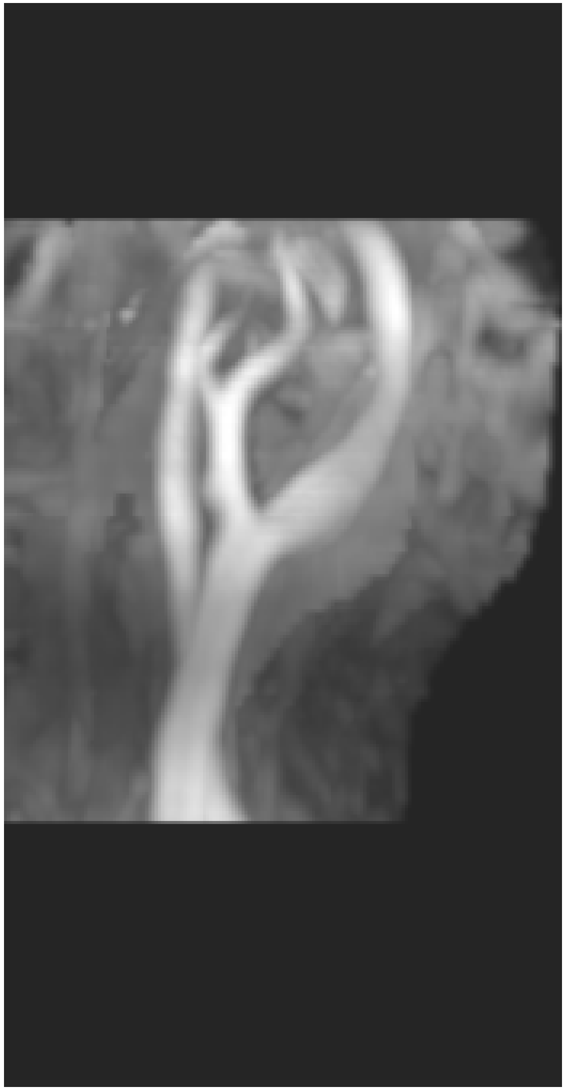

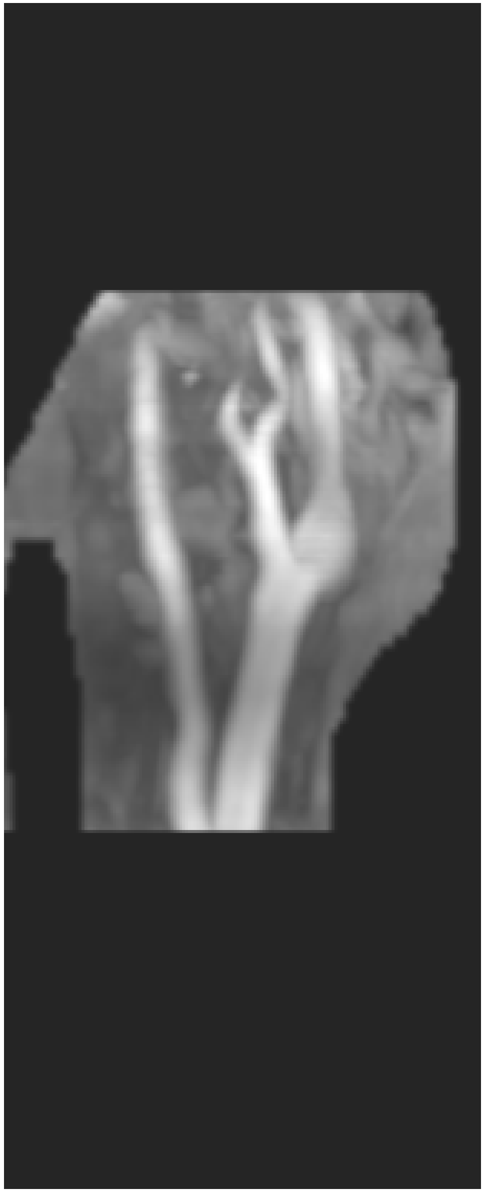

167c Score

0-30

31-50

51-70

>70

Near occlusion

Occluded

Quality

1

2

3

4

5

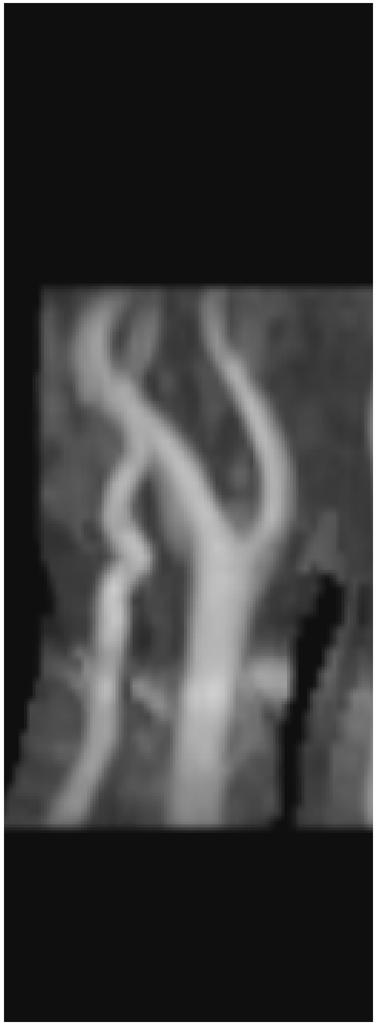

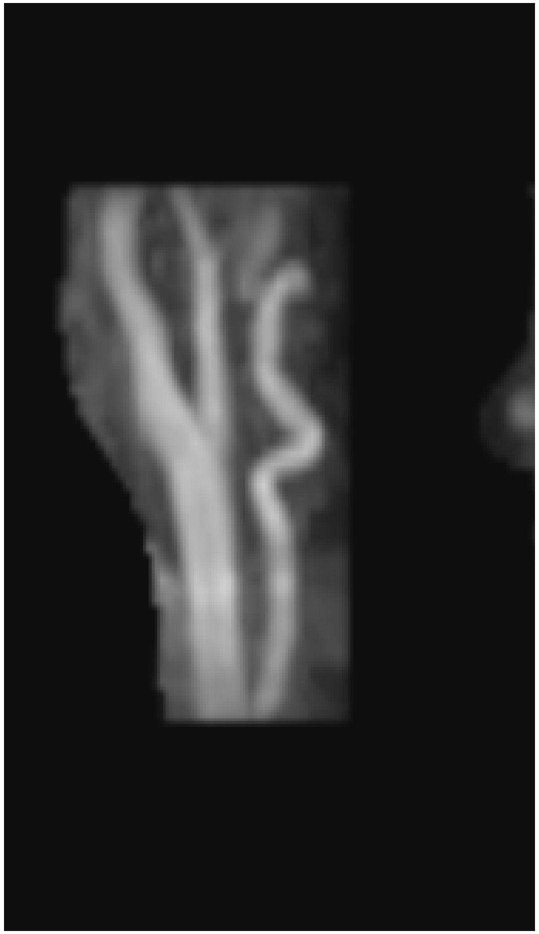

168b Score

0-30

31-50

51-70

>70

Near occlusion

Occluded

Quality

1

2

3

4

5

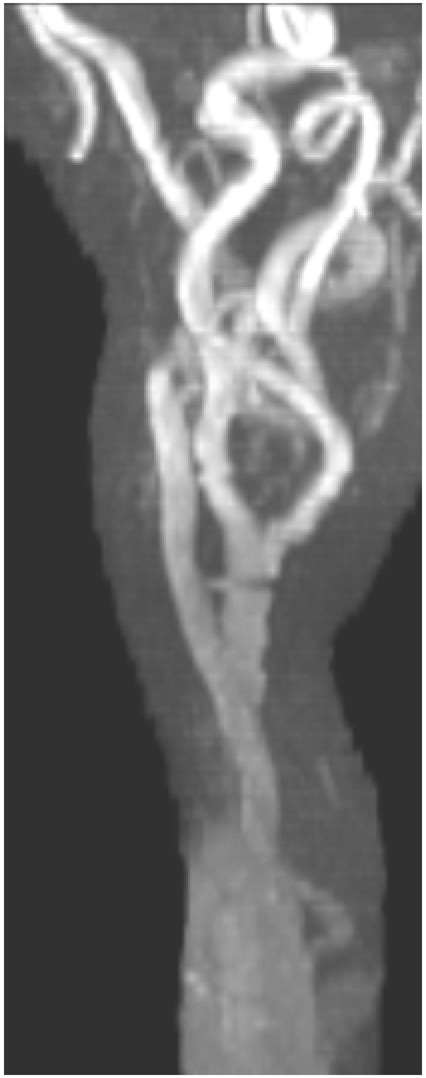

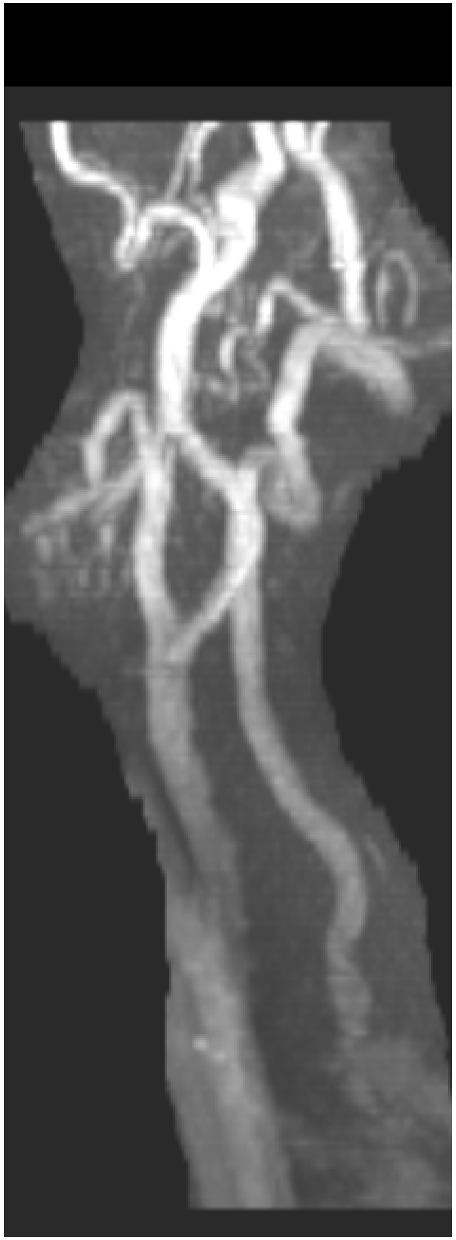

169a Score

0-30

31-50

51-70

>70

Near occlusion

Occluded

Quality

1

2

3

4

5

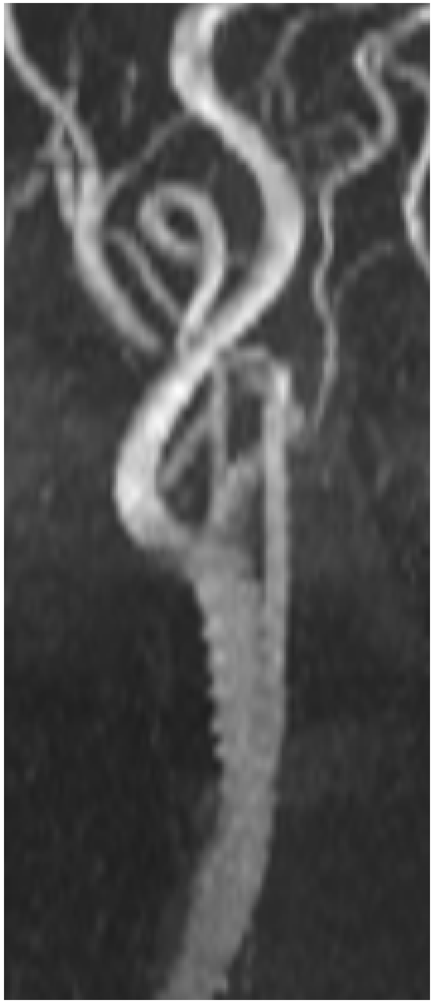

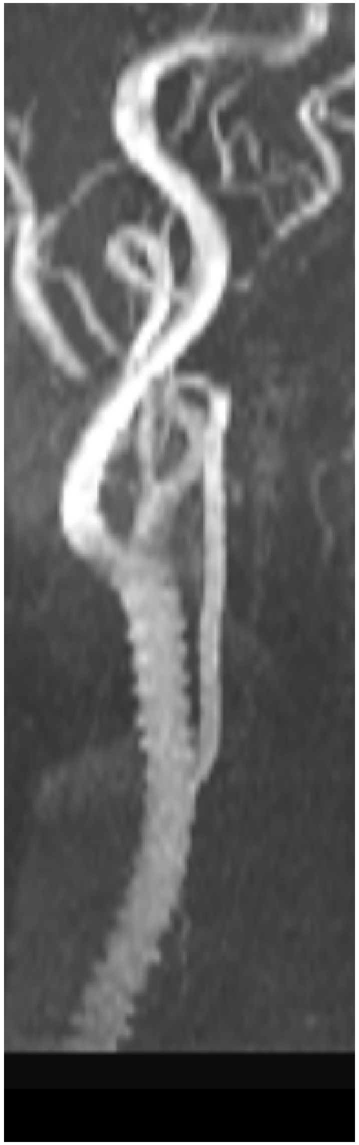

169f Score (left)

0-30

31-50

51-70

>70

Near occlusion

Occluded

Quality

1

2

3

4

5

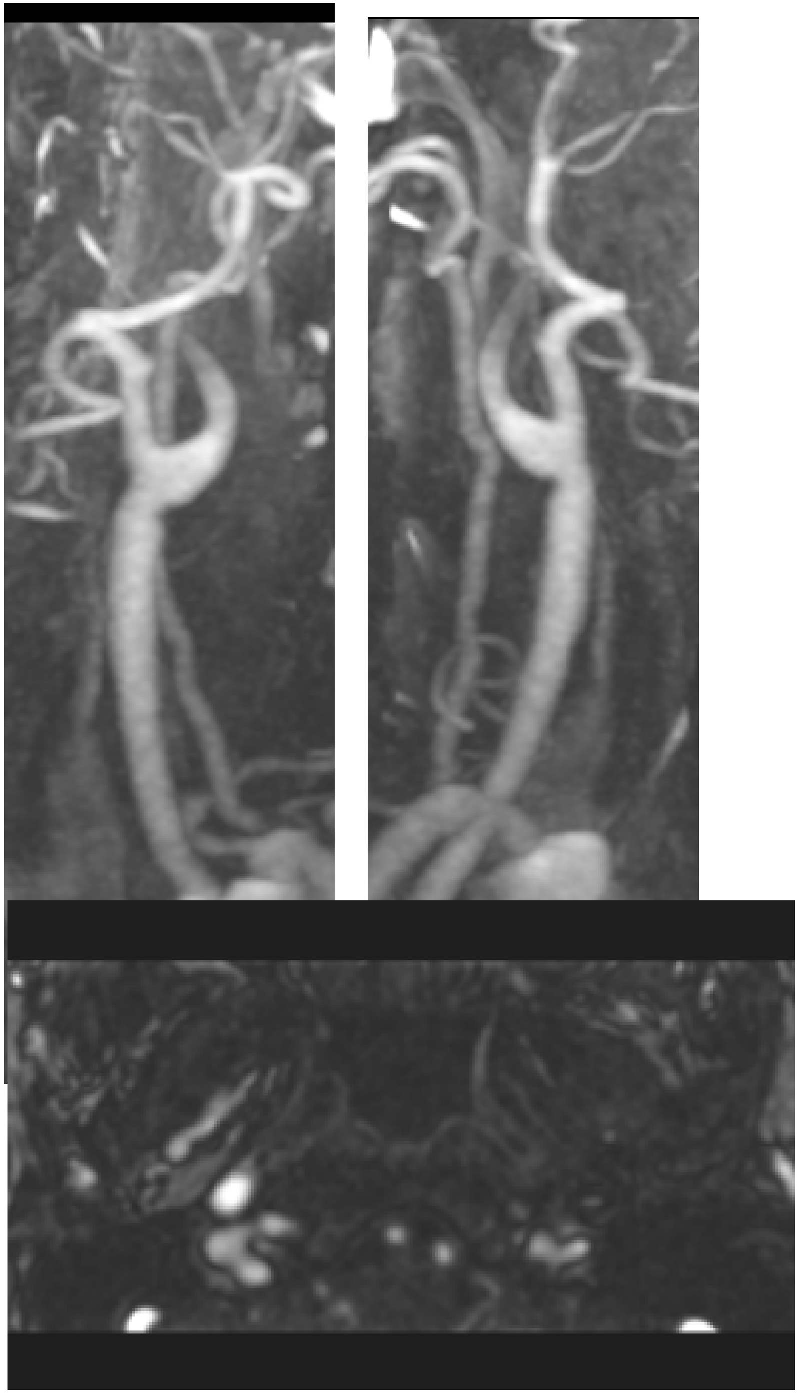

170e Score

0-30

31-50

51-70

>70

Near occlusion

Occluded

Quality

1

2

3

4

5

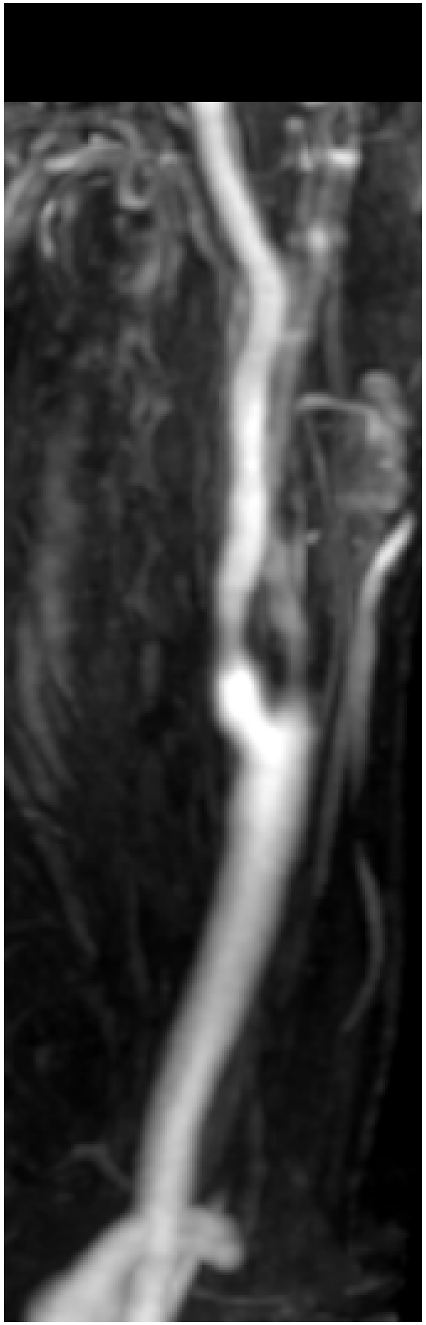

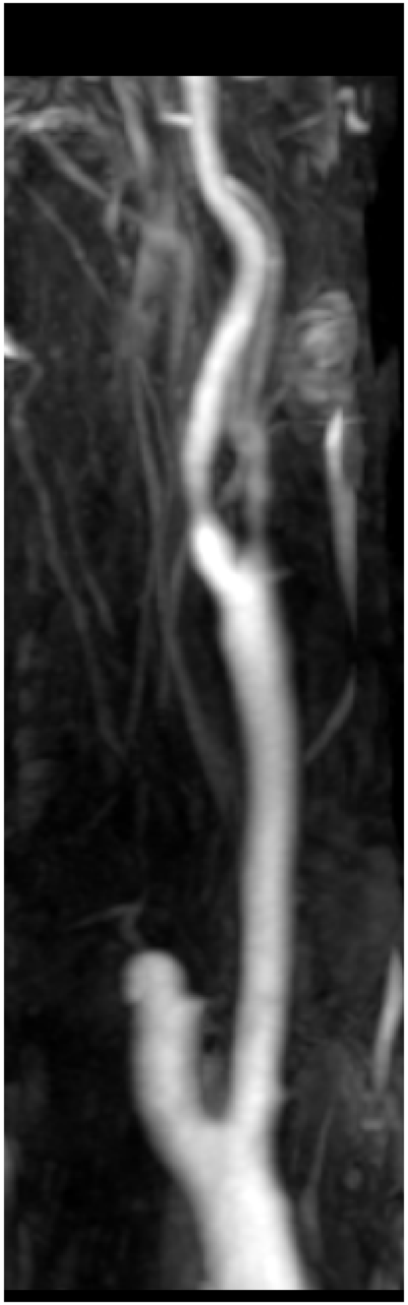

171d Score

0-30

31-50

51-70

>70

Near occlusion

Occluded

Quality

1

2

3

4

5

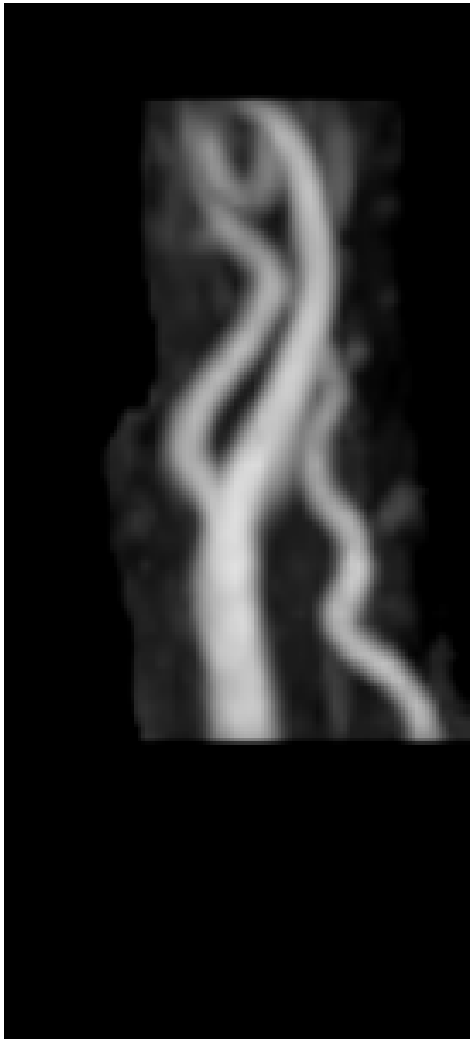

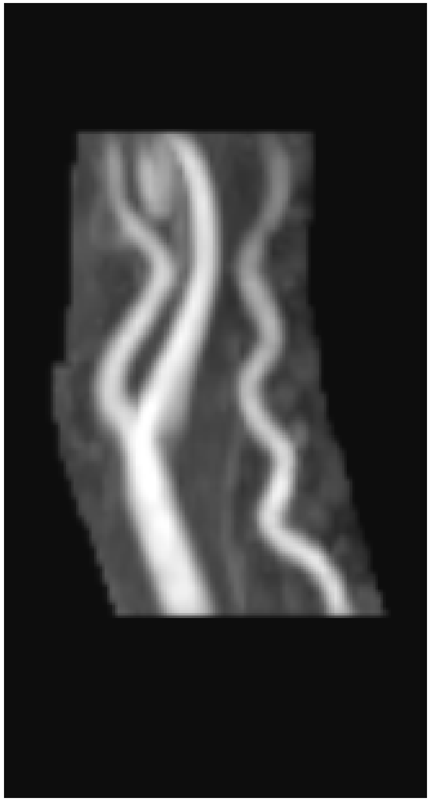

172c Score

0-30

31-50

51-70

>70

Near occlusion

Occluded

Quality

1

2

3

4

5

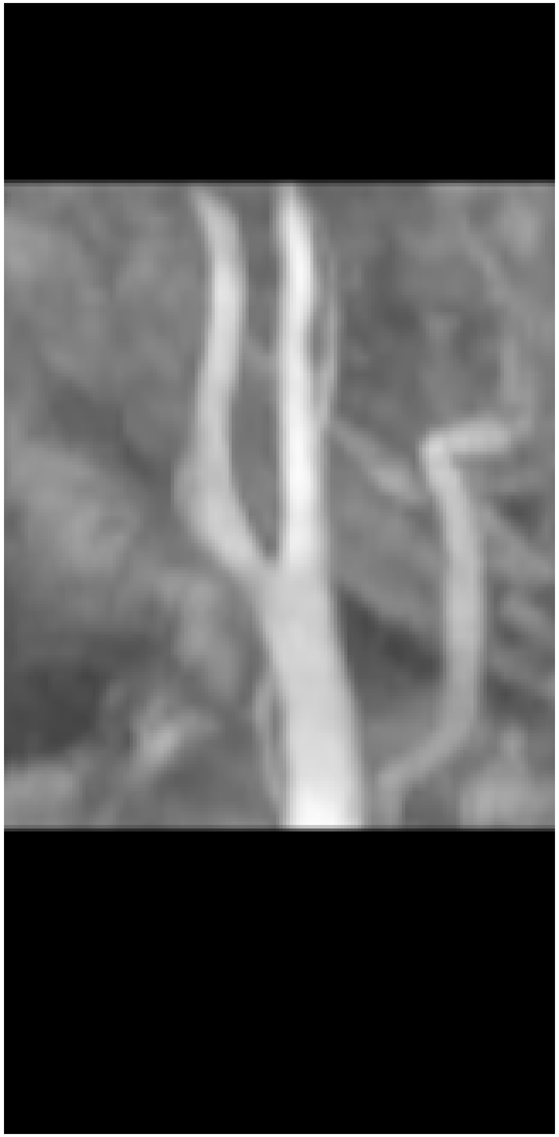

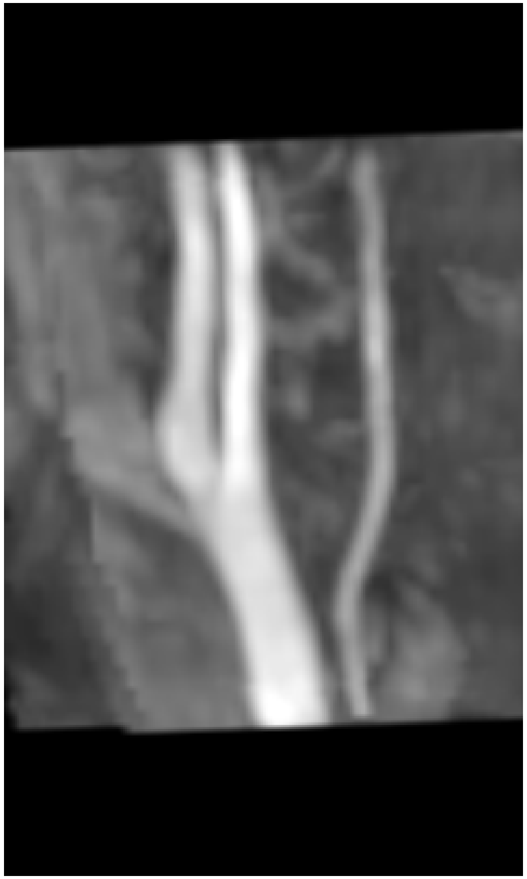

173b Score

0-30

31-50

51-70

>70

Near occlusion

Occluded

Quality

1

2

3

4

5

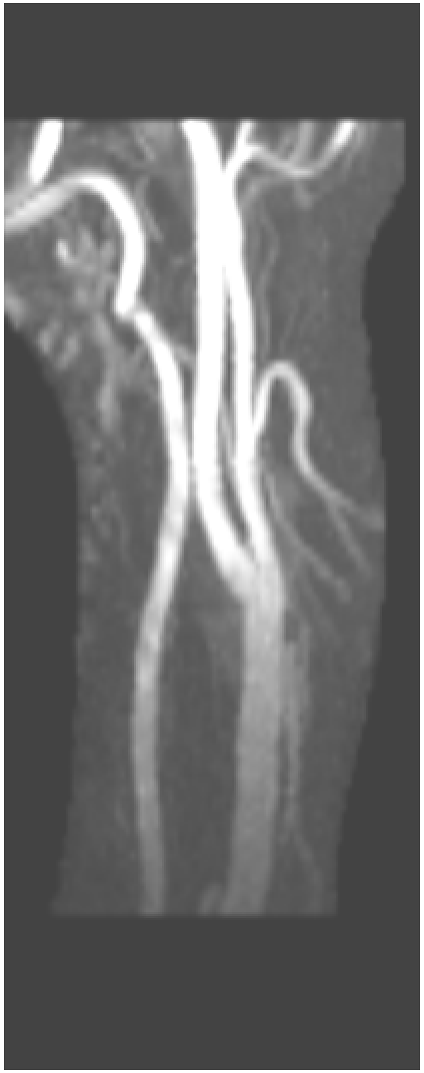

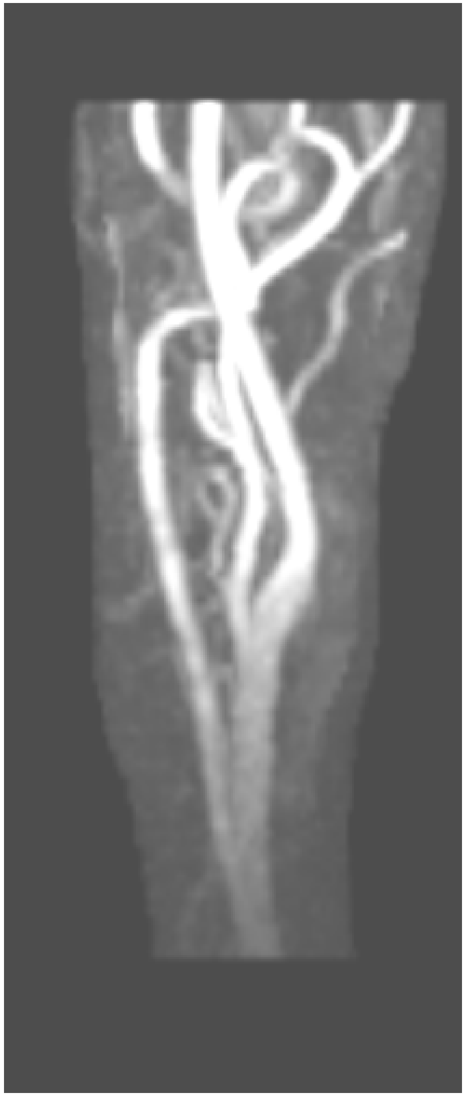

174a Score

0-30

31-50

51-70

>70

Near occlusion

Occluded

Quality

1

2

3

4

5

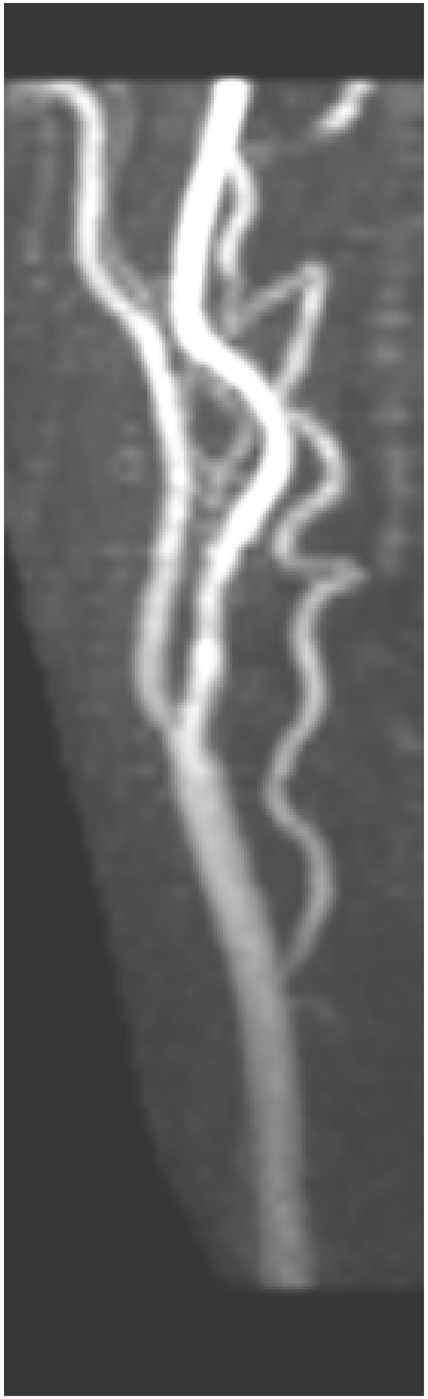

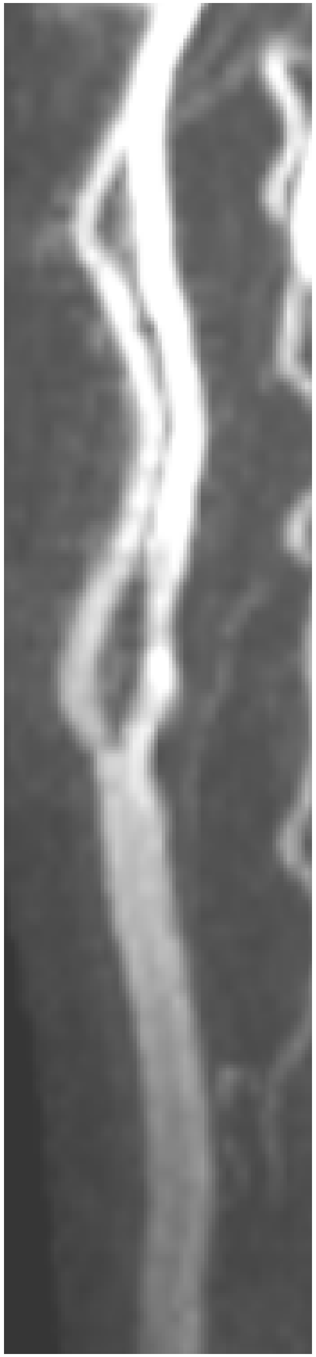

174f Score

0-30

31-50

51-70

>70

Near occlusion

Occluded

Quality

1

2

3

4

5

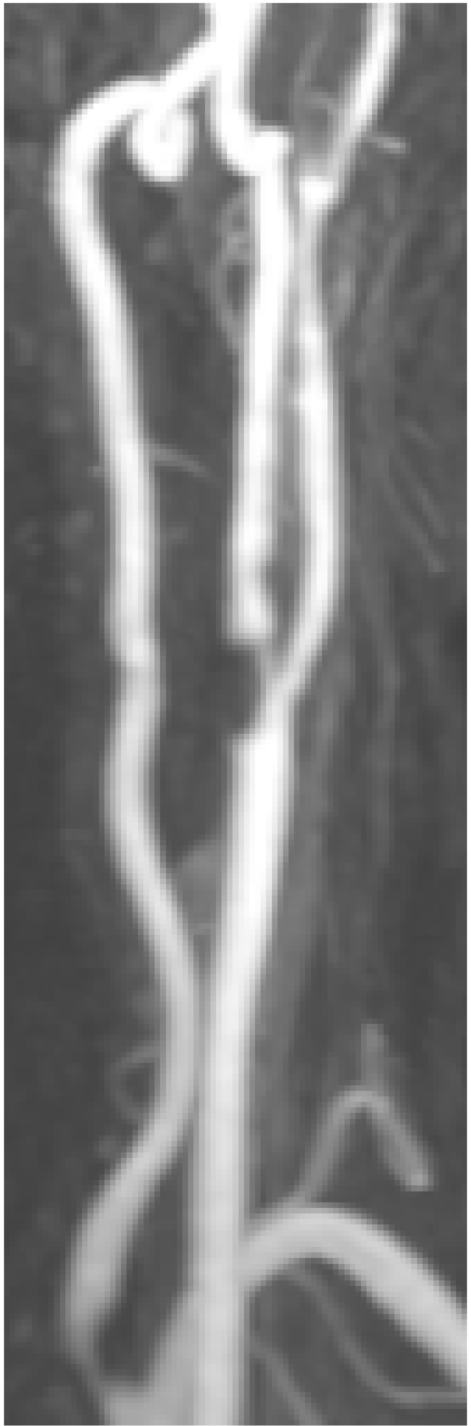

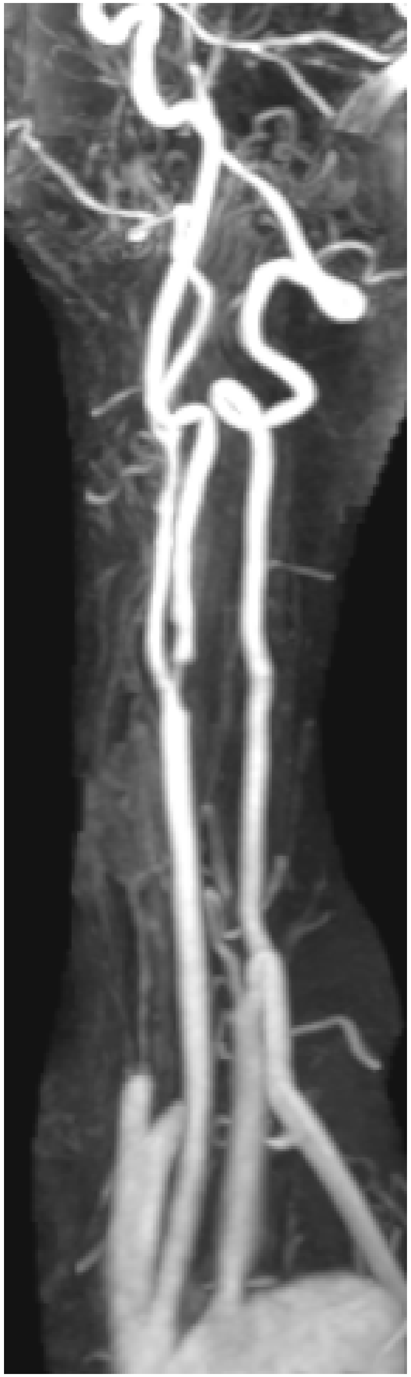

175e Score

0-30

31-50

51-70

>70

Near occlusion

Occluded

Quality

1

2

3

4

5

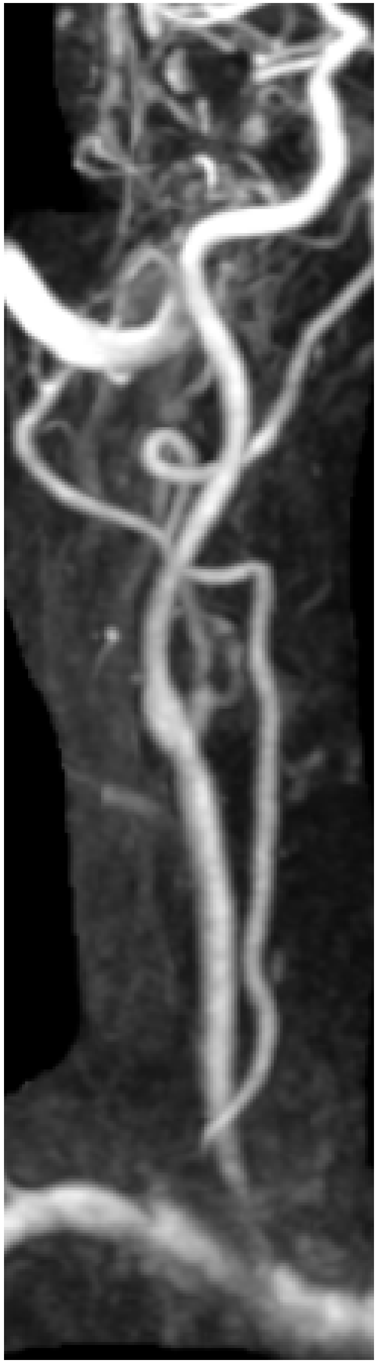

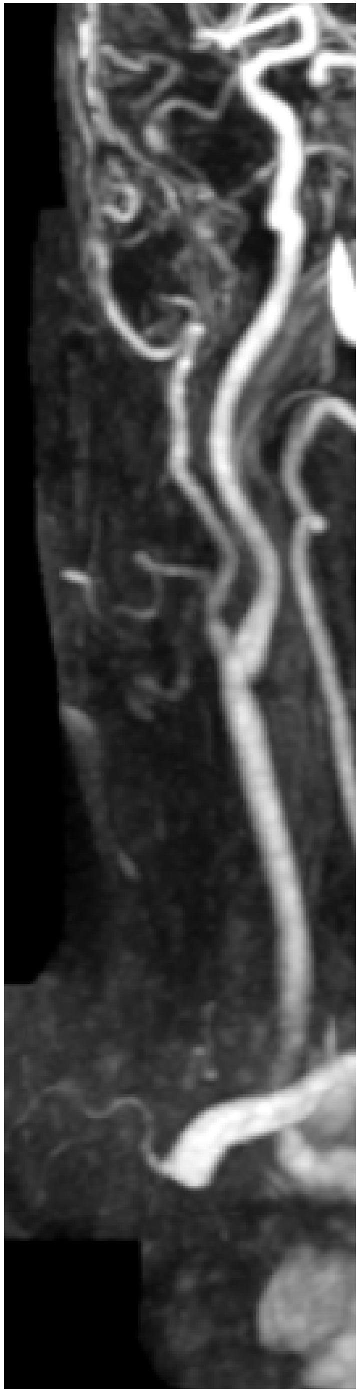

176d Score

0-30

31-50

51-70

>70

Near occlusion

Occluded

Quality

1

2

3

4

5

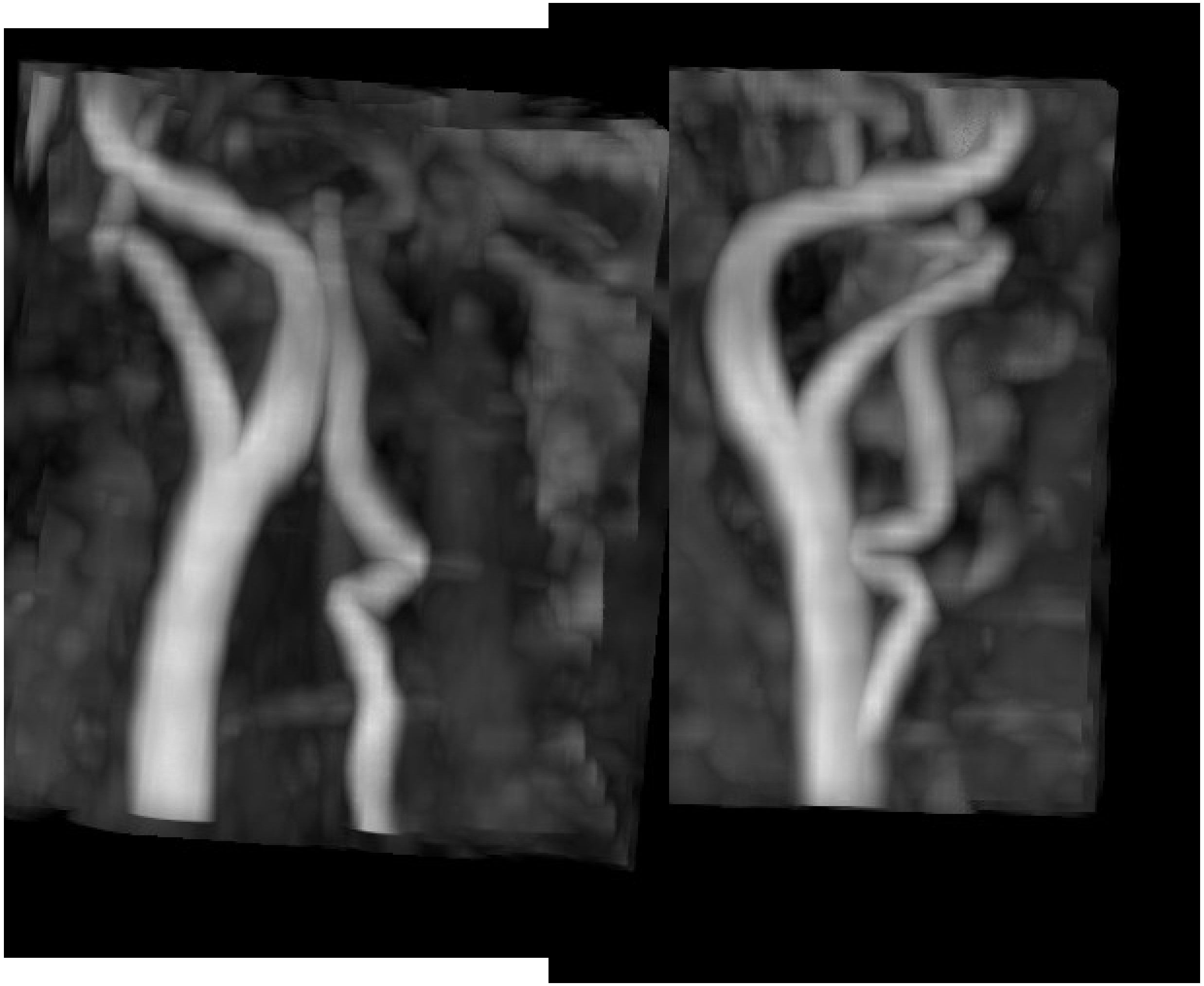

177c Score

0-30

31-50

51-70

>70

Near occlusion

Occluded

Quality

1

2

3

4

5

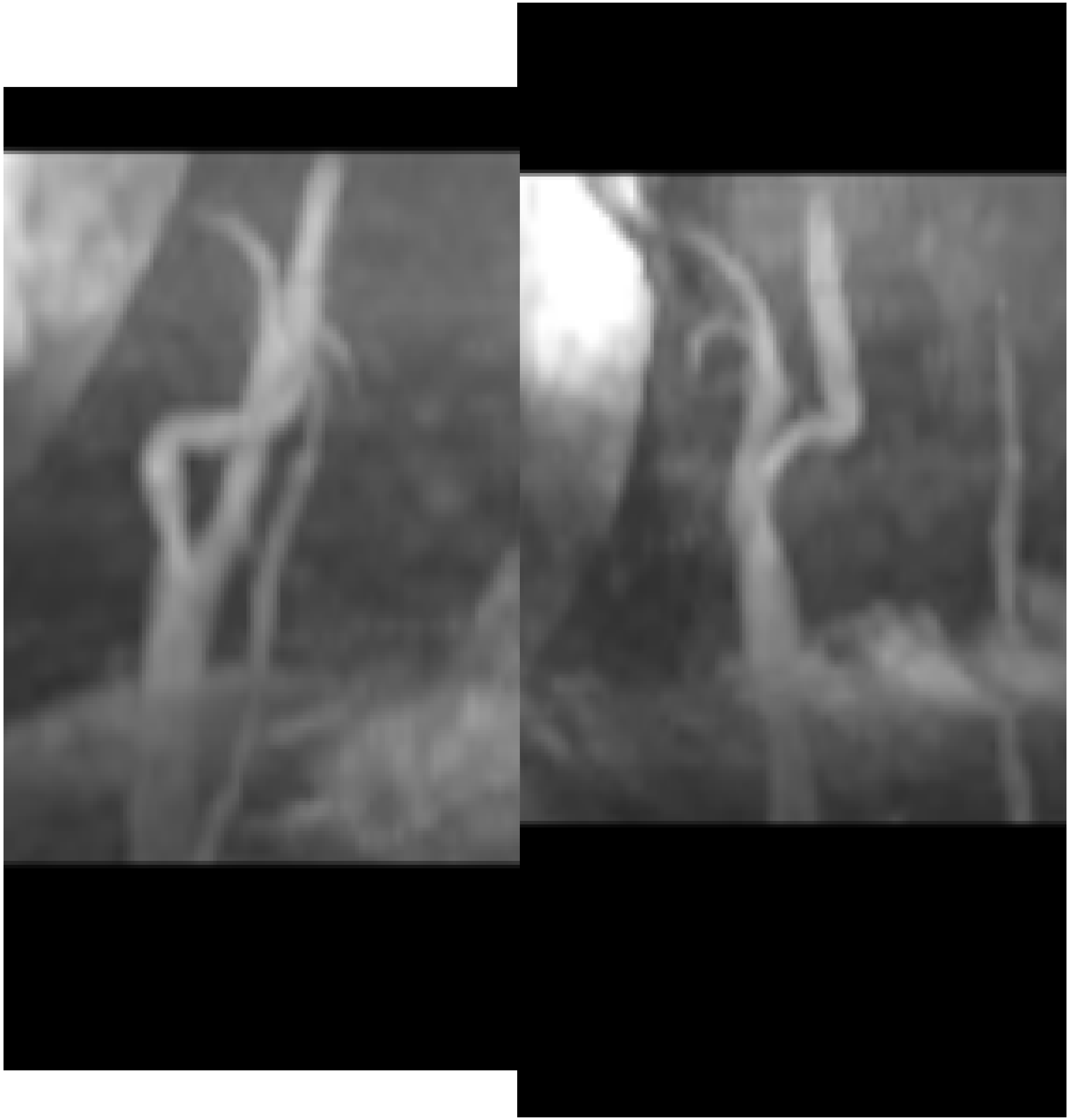

178b Score

0-30

31-50

51-70

>70

Near occlusion

Occluded

Quality

1

2

3

4

5

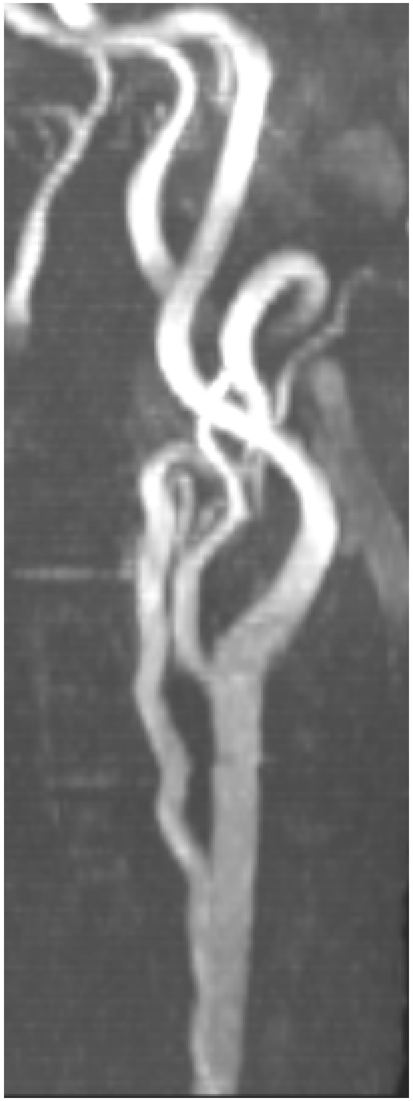

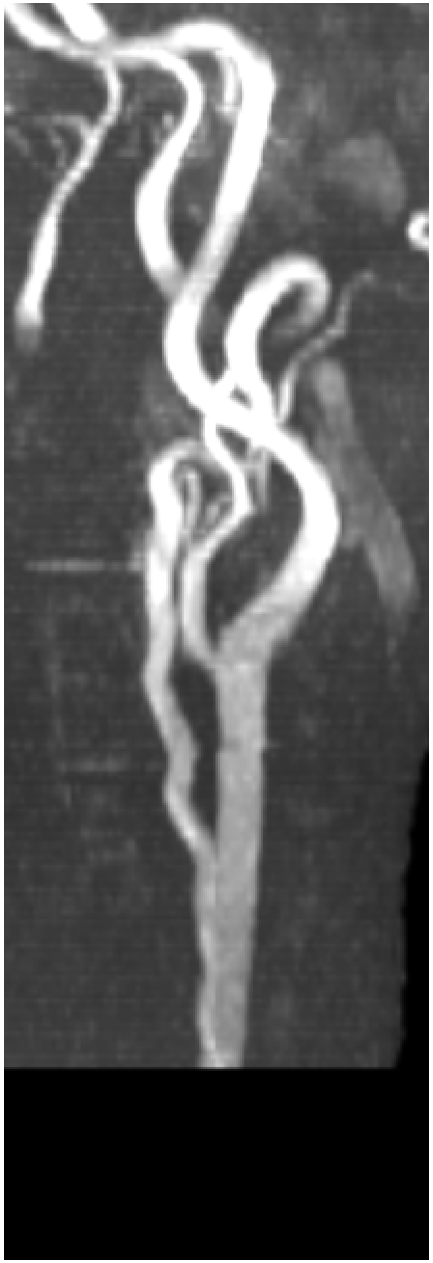

179a Score

0-30

31-50

51-70

>70

Near occlusion

Occluded

Quality

1

2

3

4

5

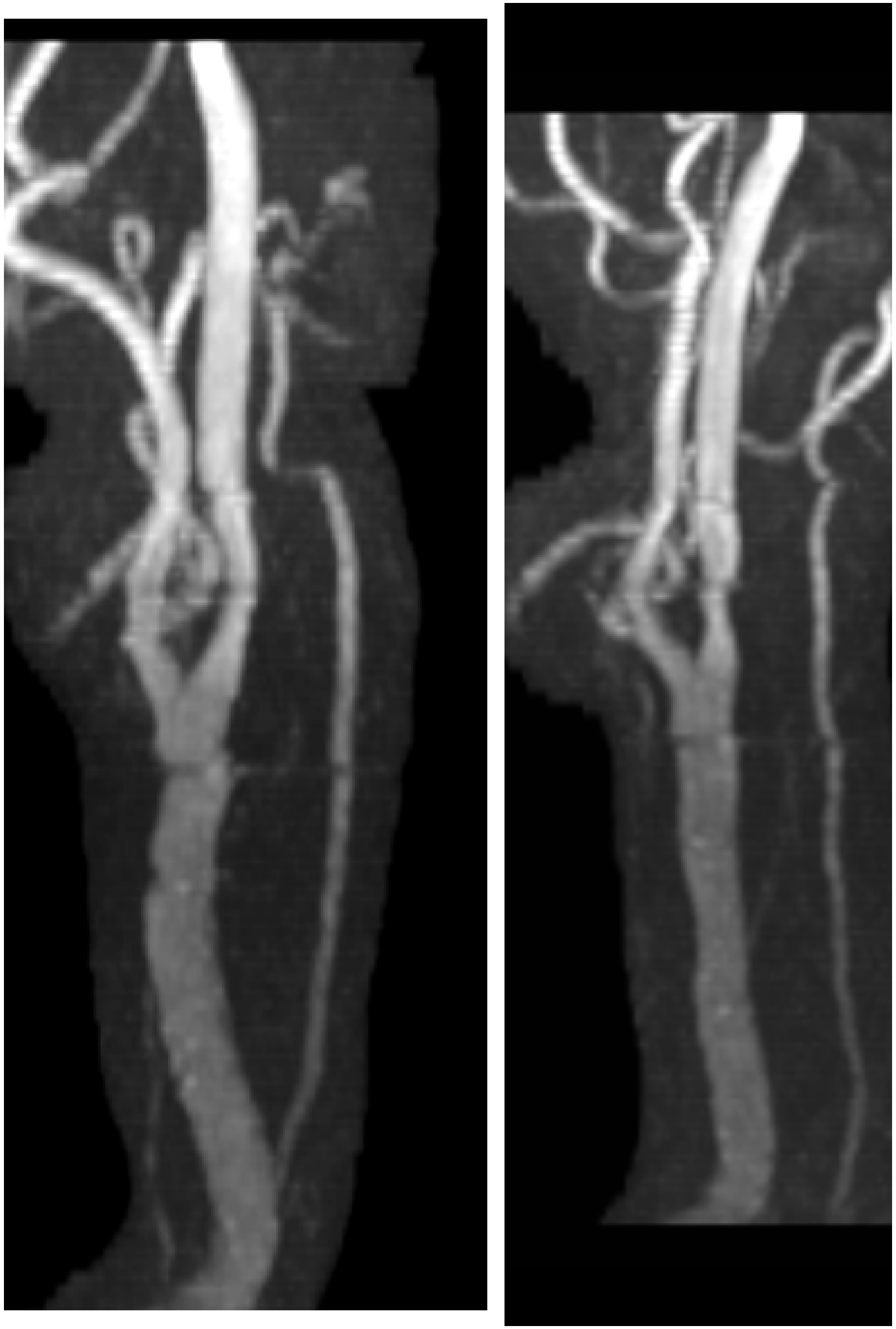

179f Score

0-30

31-50

51-70

>70

Near occlusion

Occluded

Quality

1

2

3

4

5

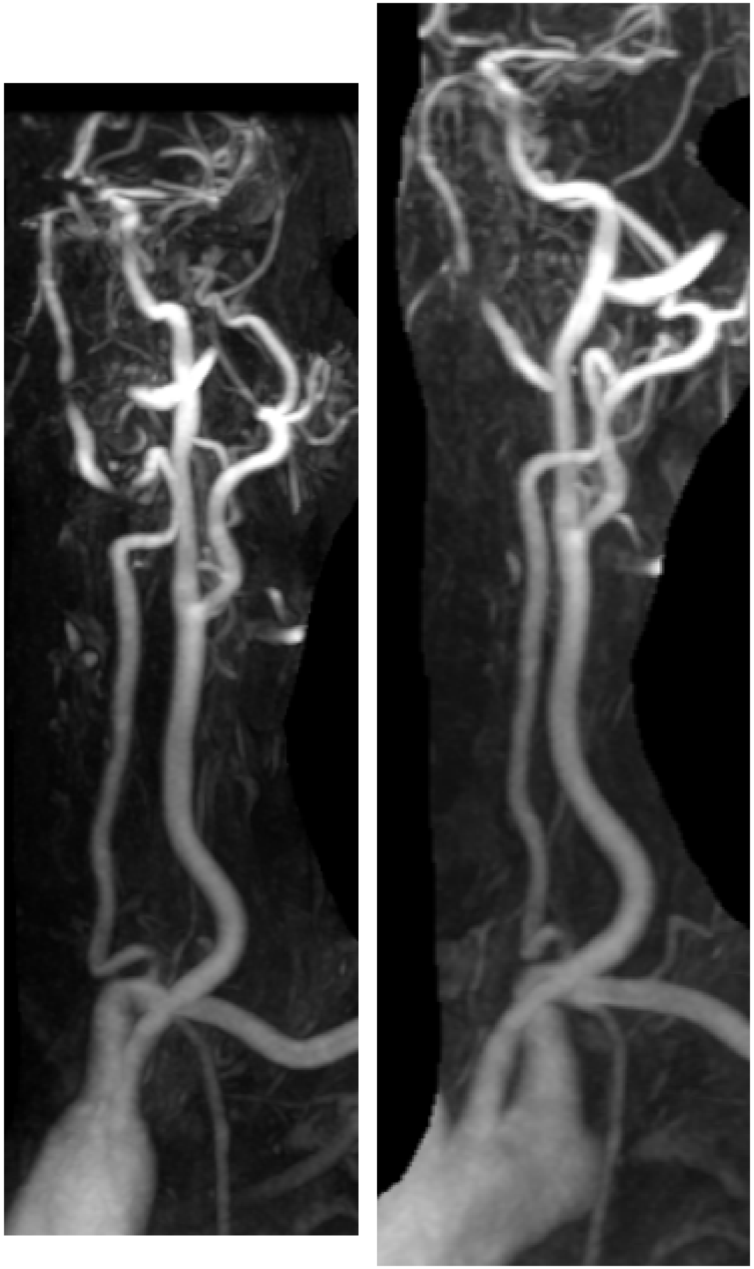

180e Score

0-30

31-50

51-70

>70

Near occlusion

Occluded

Quality

1

2

3

4

5

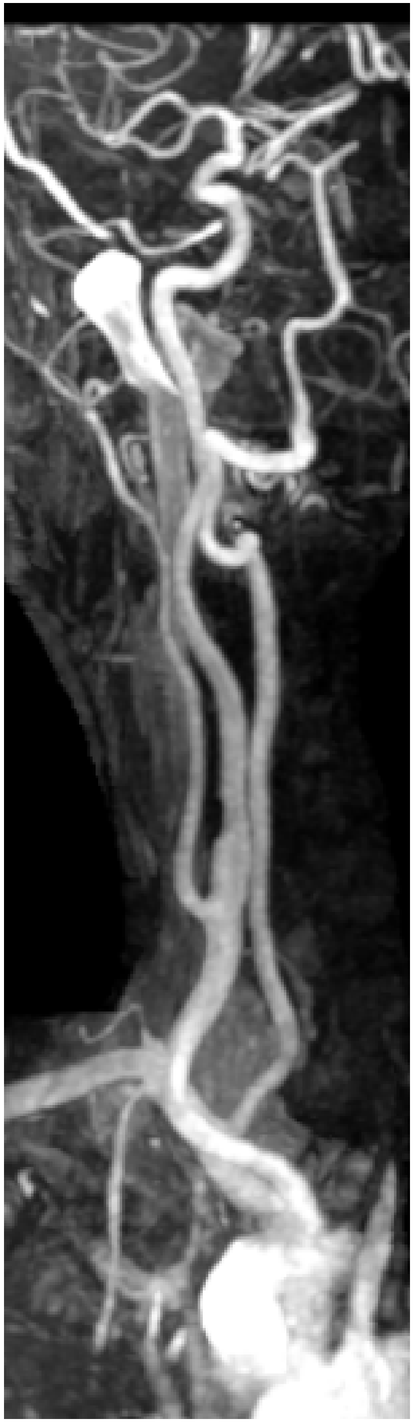

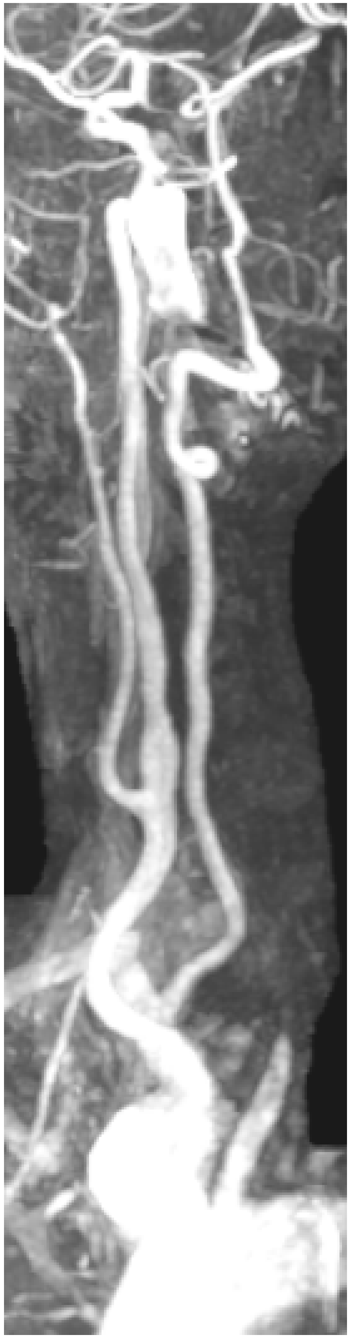

181d Score

0-30

31-50

51-70

>70

Near occlusion

Occluded

Quality

1

2

3

4

5

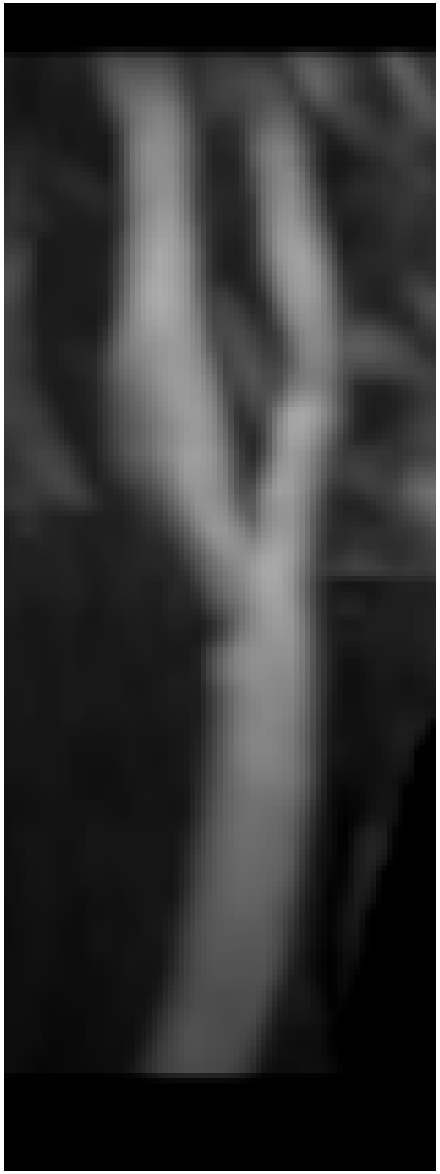

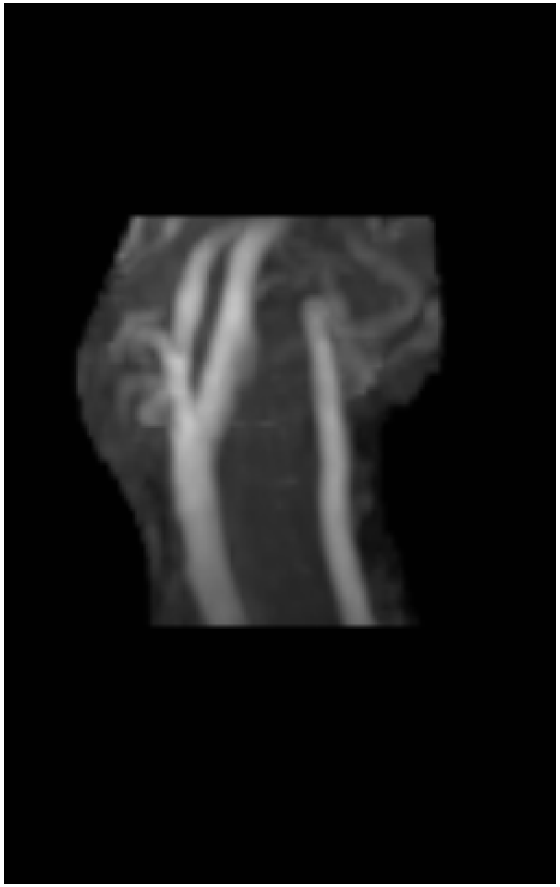

182c Score

0-30

31-50

51-70

>70

Near occlusion

Occluded

Quality

1

2

3

4

5

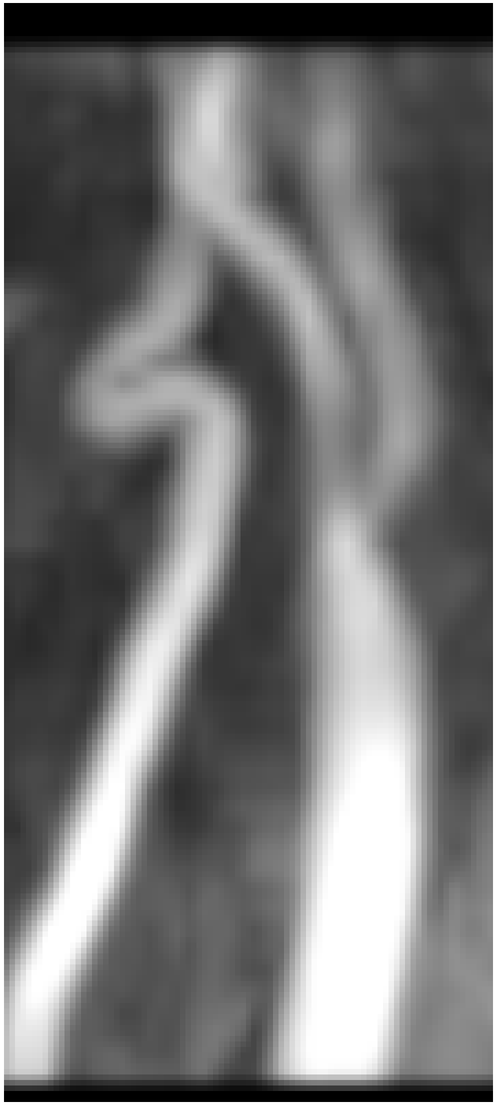

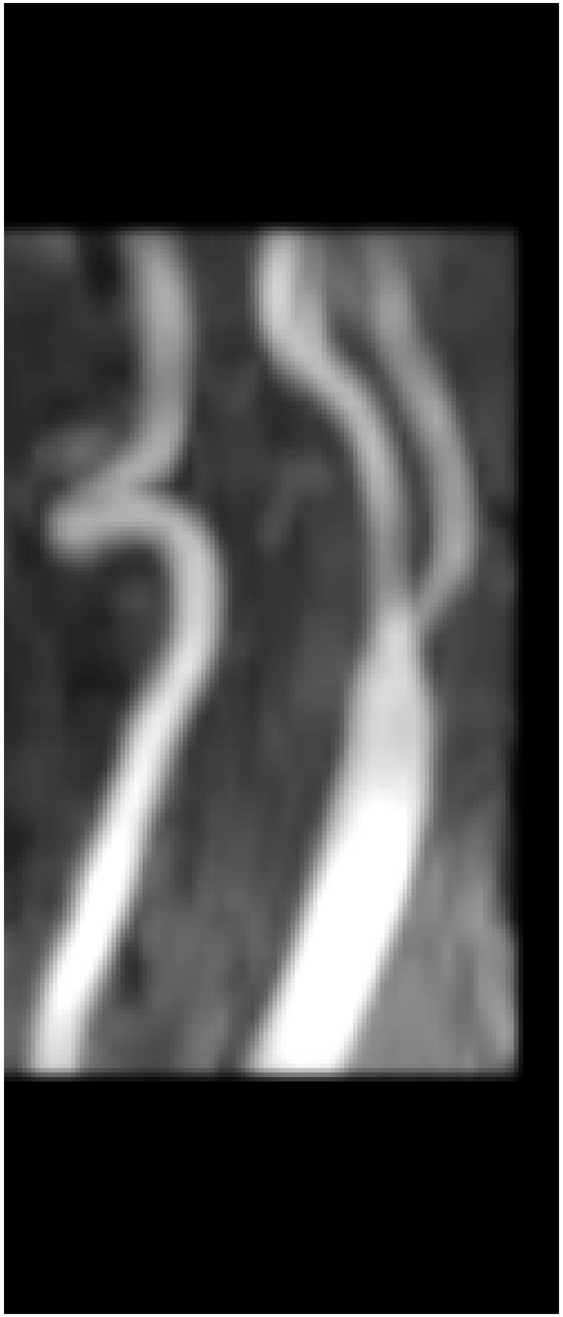

183b Score (left)

0-30

31-50

51-70

>70

Near occlusion

Occluded

Quality

1

2

3

4

5

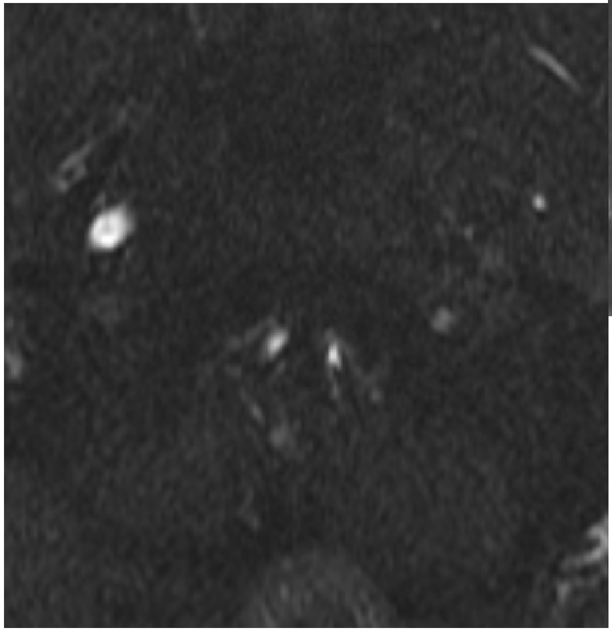

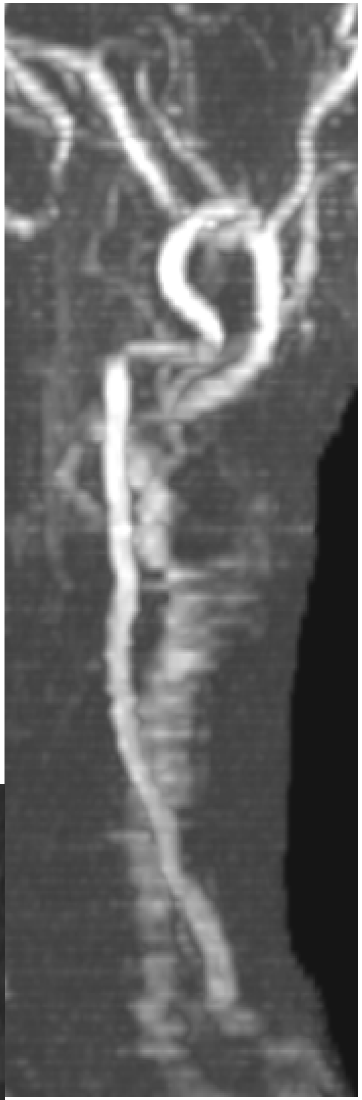

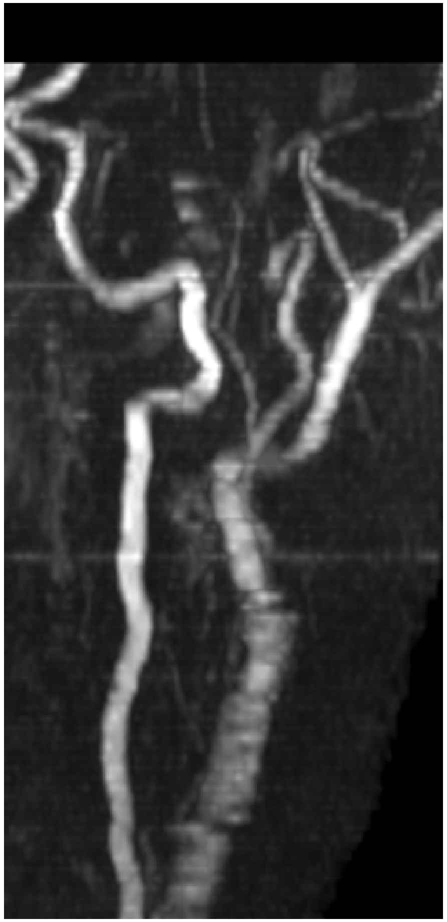

184a Score (right)

0-30

31-50

51-70

>70

Near occlusion

Occluded

Quality

1

2

3

4

5

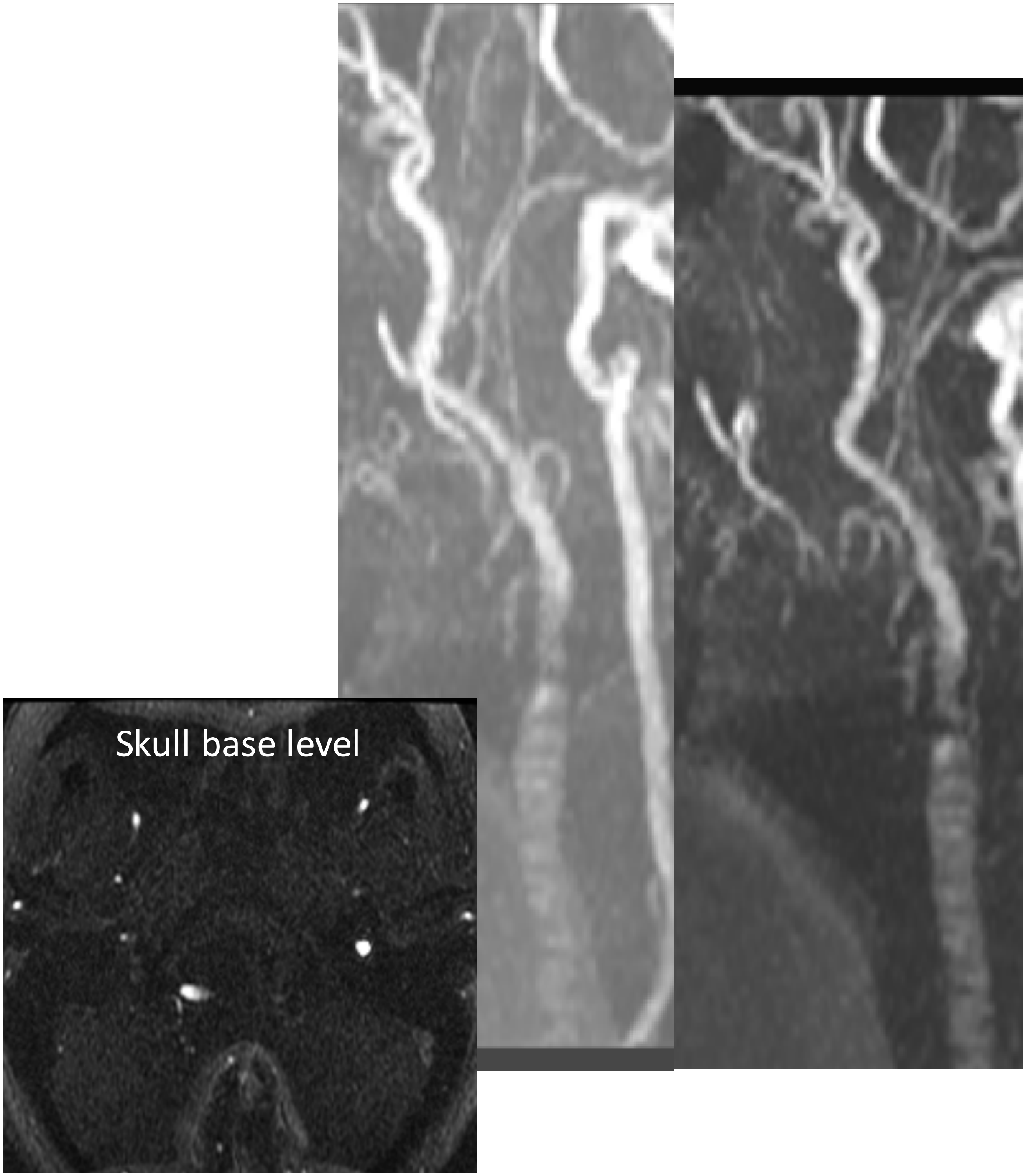

184f Score

0-30

31-50

51-70

>70

Near occlusion

Occluded

Quality

1

2

3

4

5

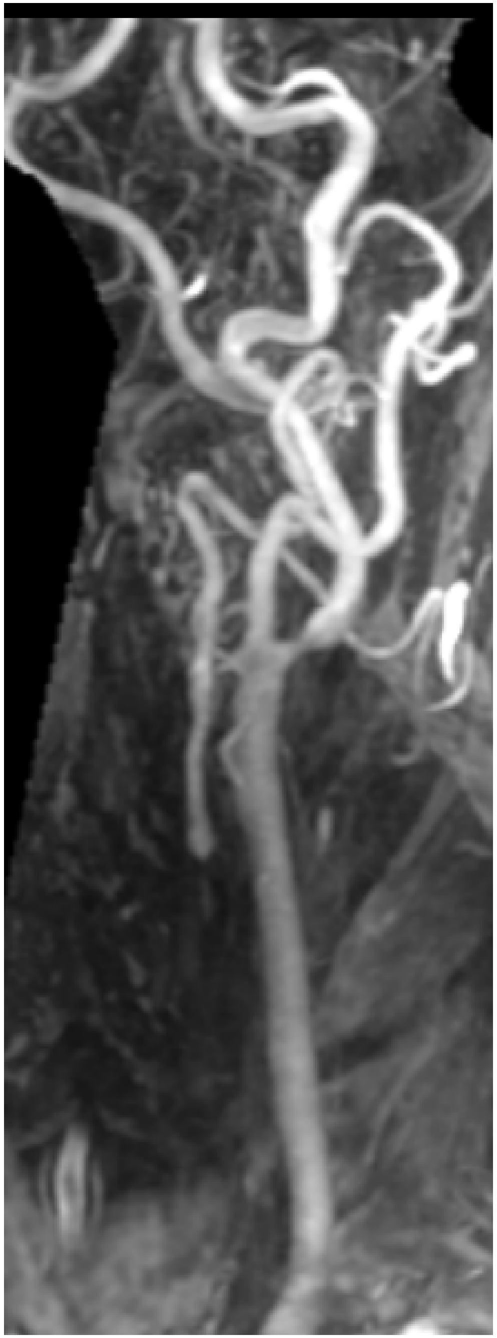

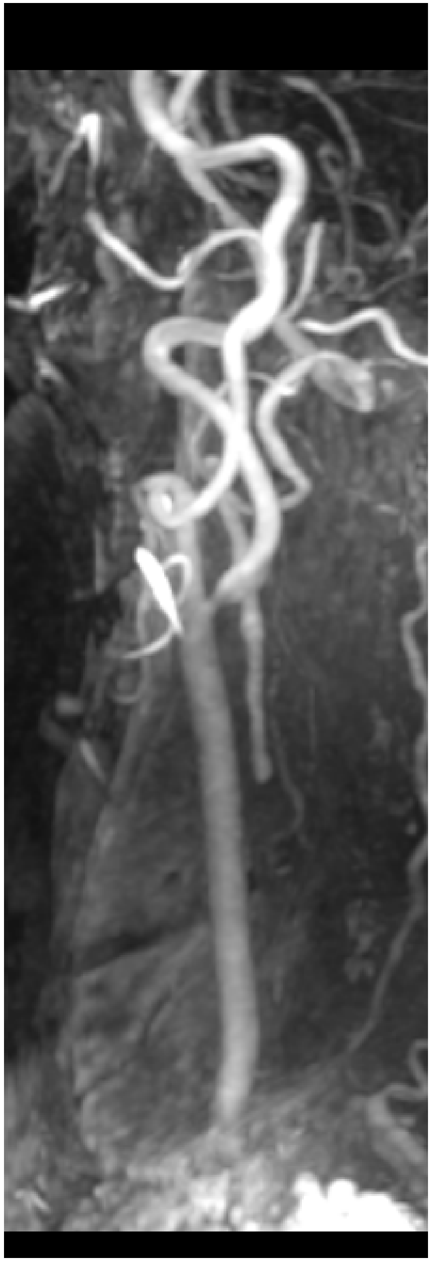

185e Score

0-30

31-50

51-70

>70

Near occlusion

Occluded

Quality

1

2

3

4

5

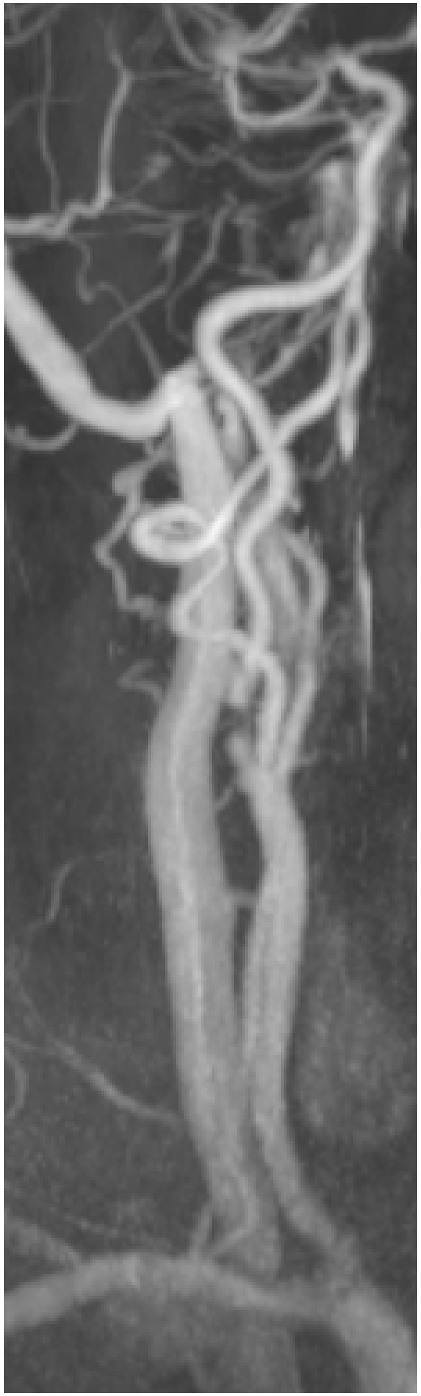

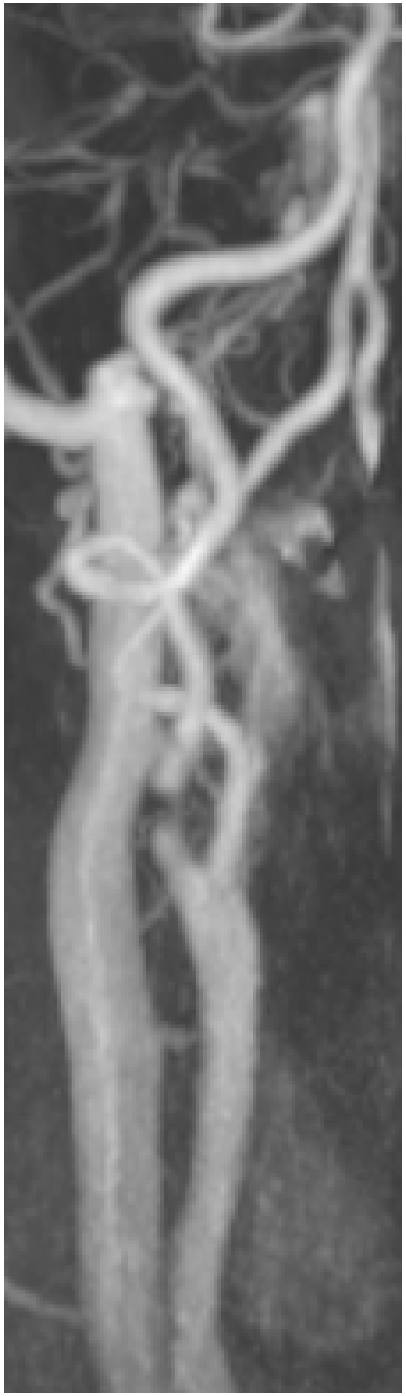

186d Score

0-30

31-50

51-70

>70

Near occlusion

Occluded

Quality

1

2

3

4

5

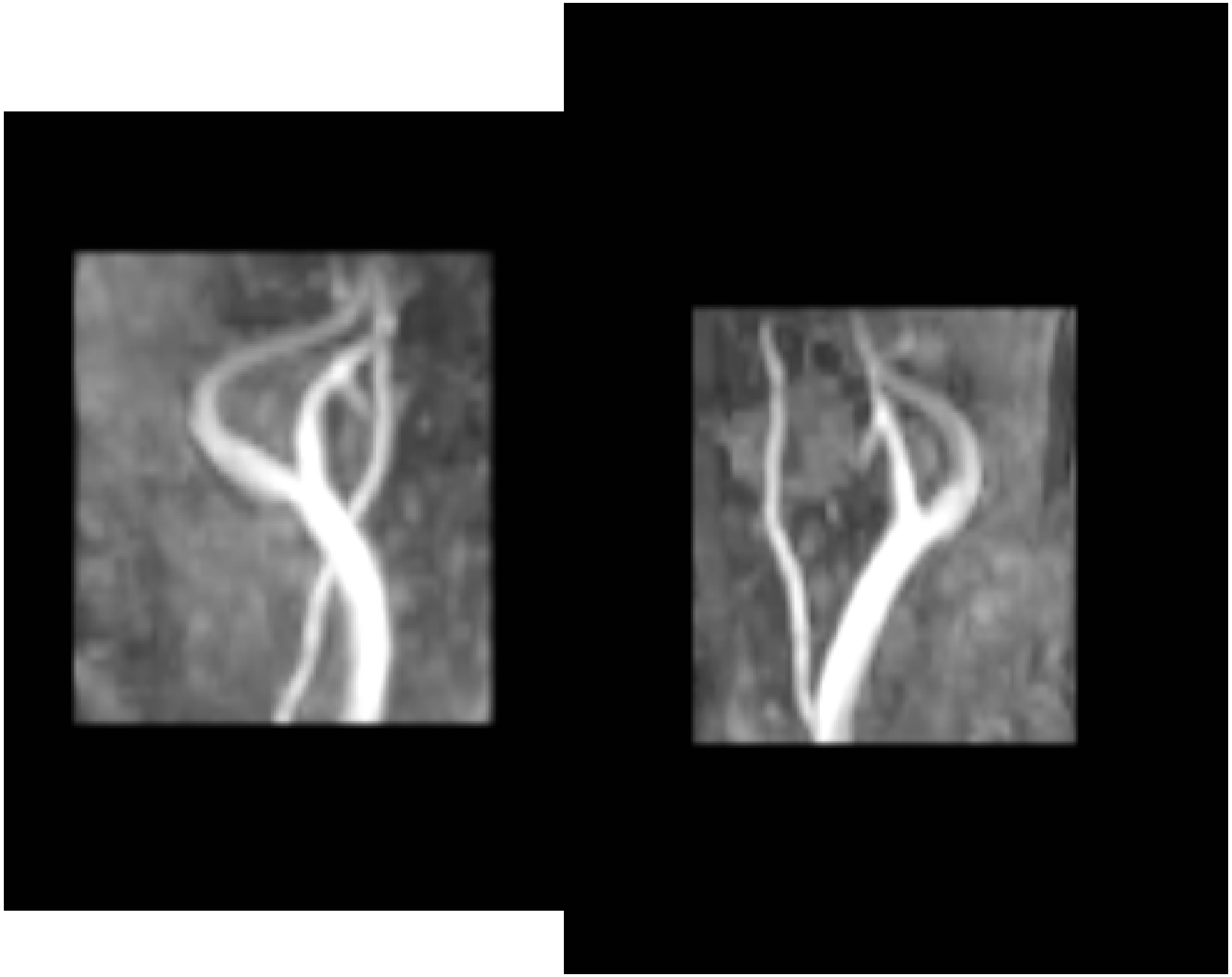

187c Score

0-30

31-50

51-70

>70

Near occlusion

Occluded

Quality

1

2

3

4

5

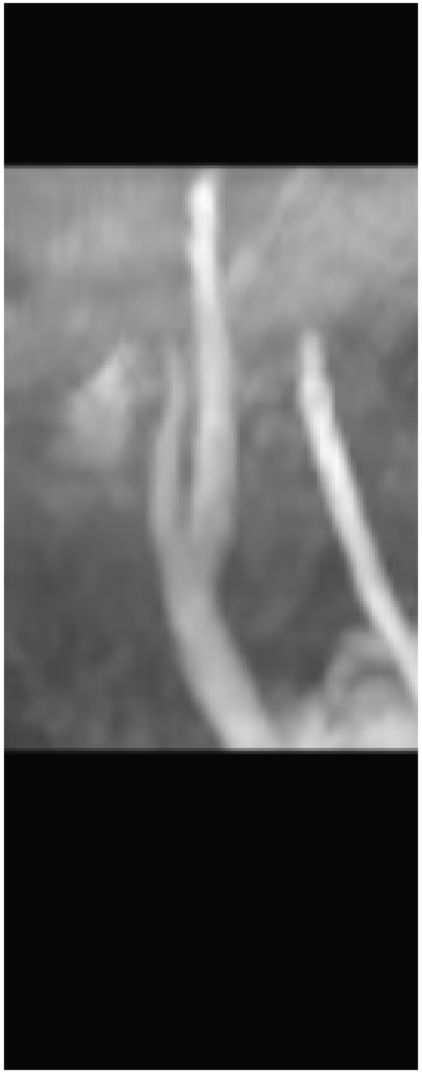

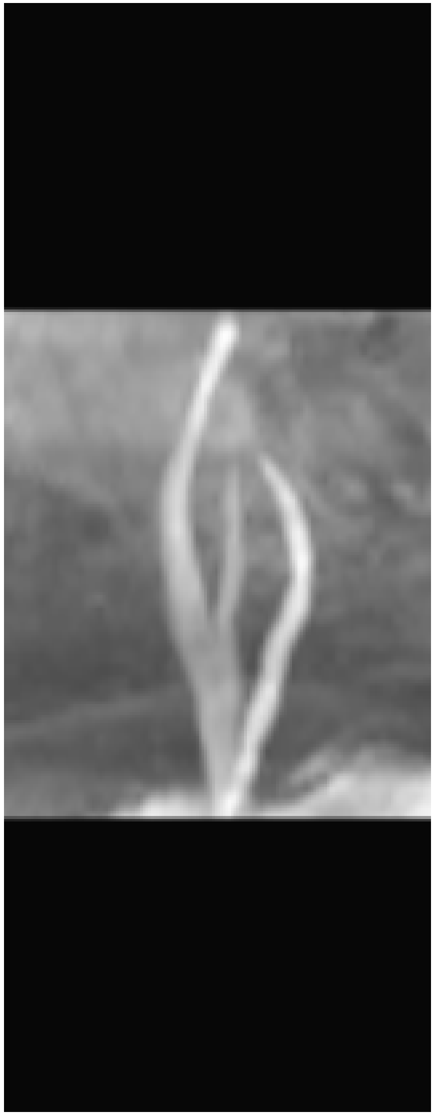

188b Score

0-30

31-50

51-70

>70

Near occlusion

Occluded

Quality

1

2

3

4

5

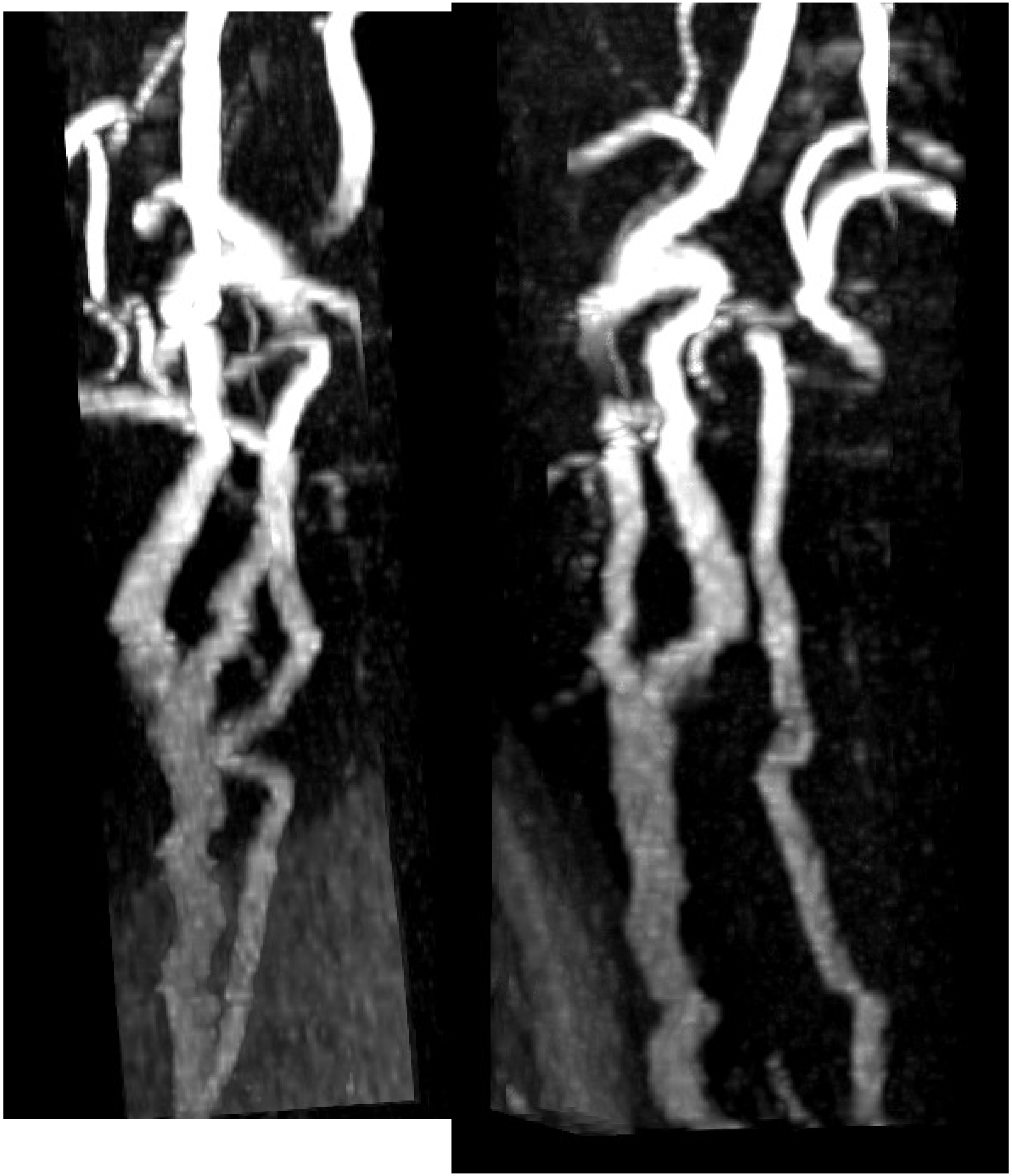

189a Score

0-30

31-50

51-70

>70

Near occlusion

Occluded

Quality

1

2

3

4

5

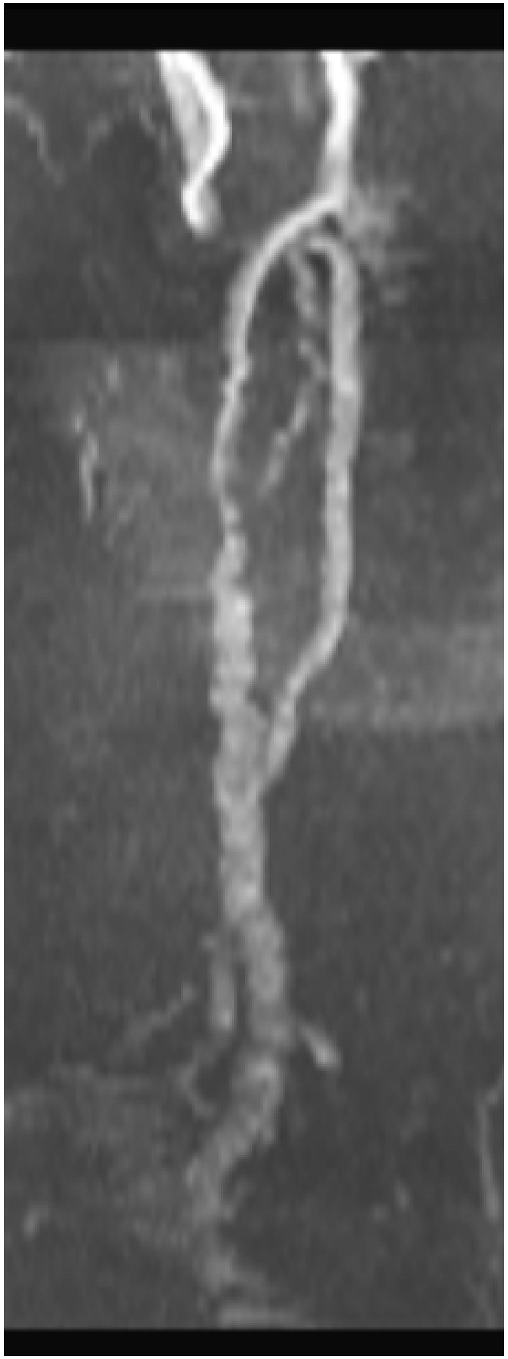

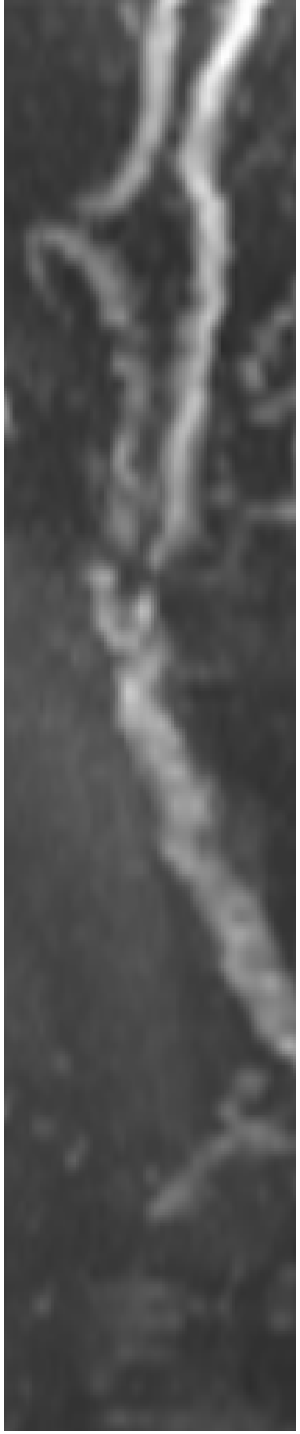

189f Score

0-30

31-50

51-70

>70

Near occlusion

Occluded

Quality

1

2

3

4

5

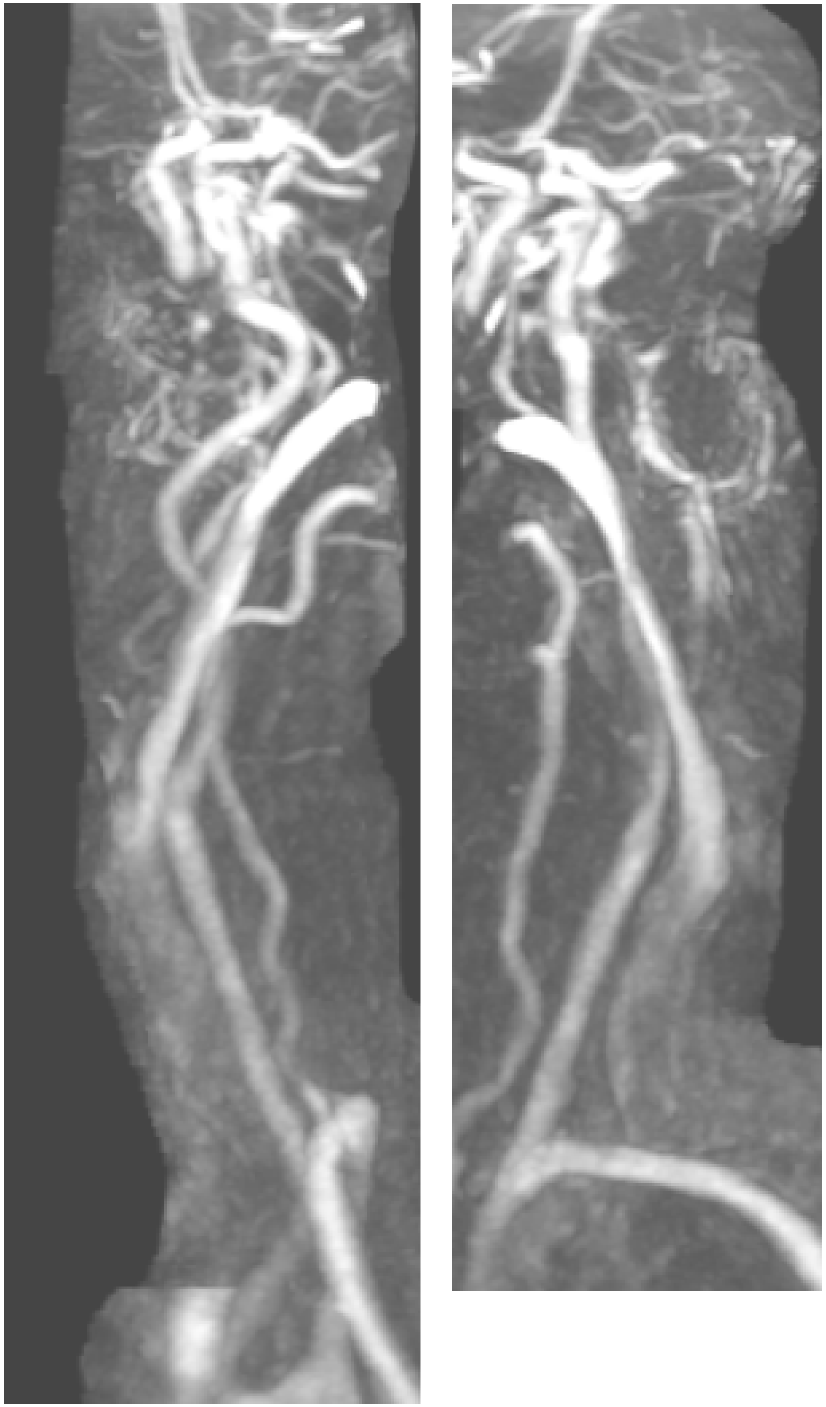

190e Score

0-30

31-50

51-70

>70

Near occlusion

Occluded

Quality

1

2

3

4

5

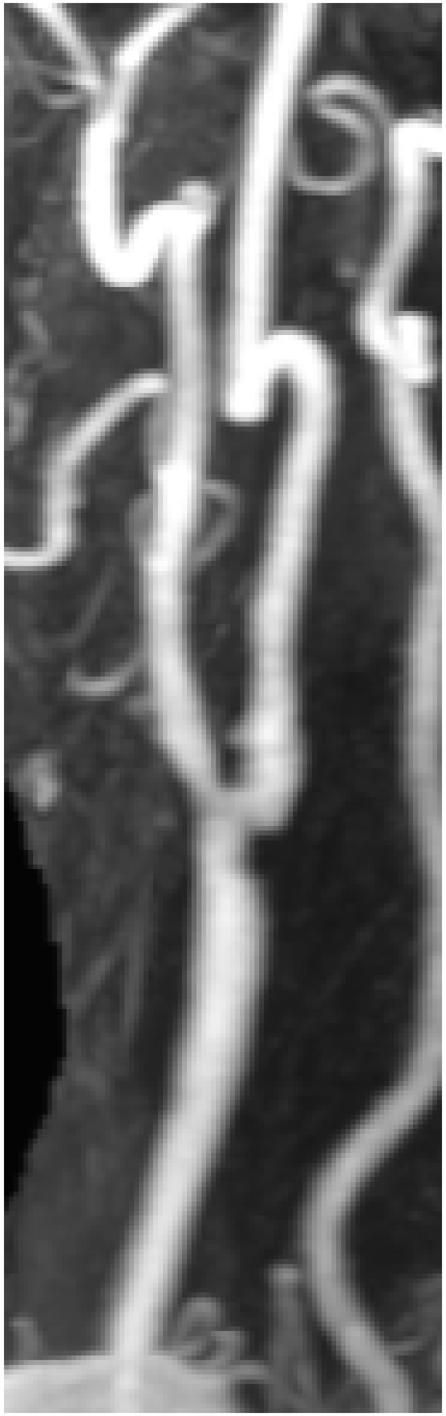

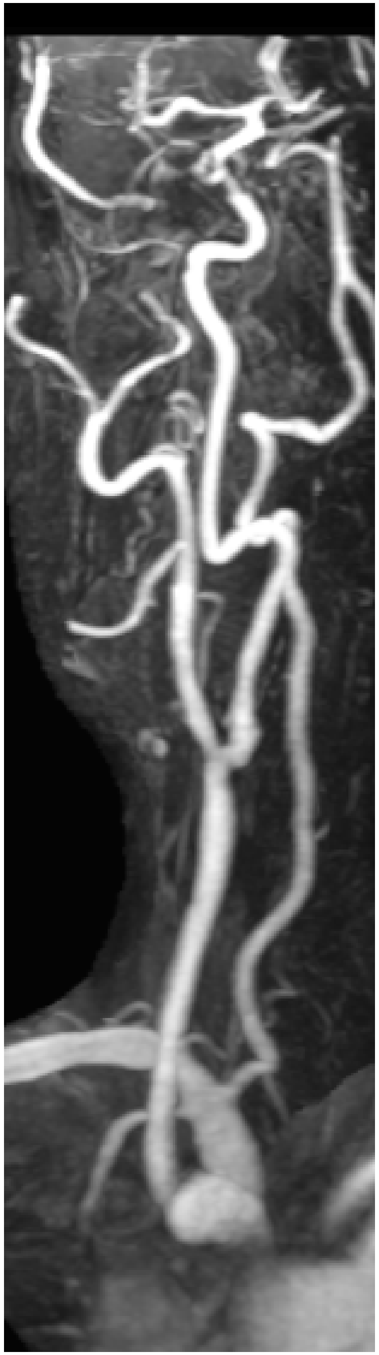

191d Score

0-30

31-50

51-70

>70

Near occlusion

Occluded

Quality

1

2

3

4

5

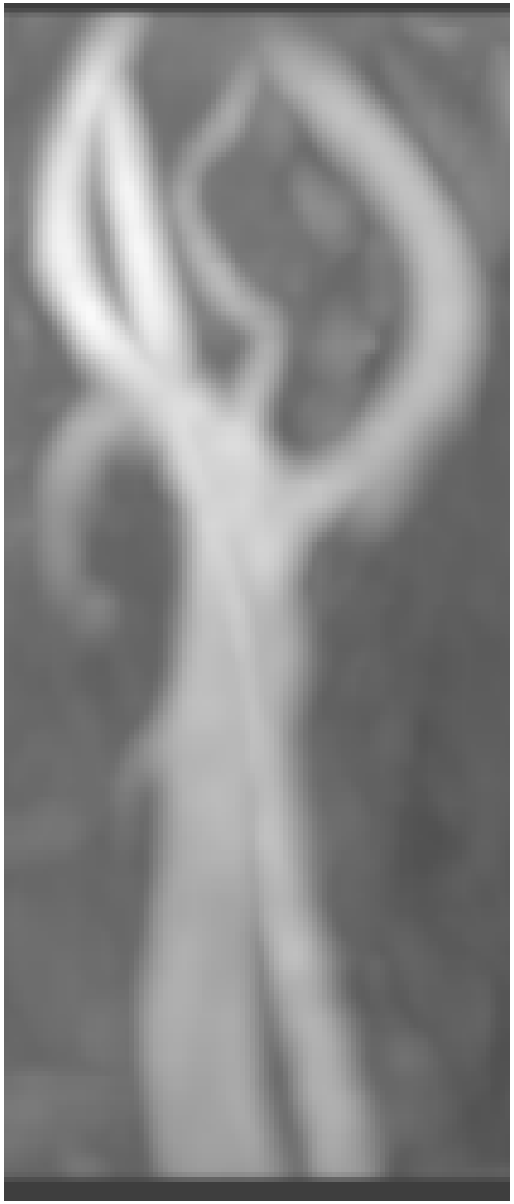

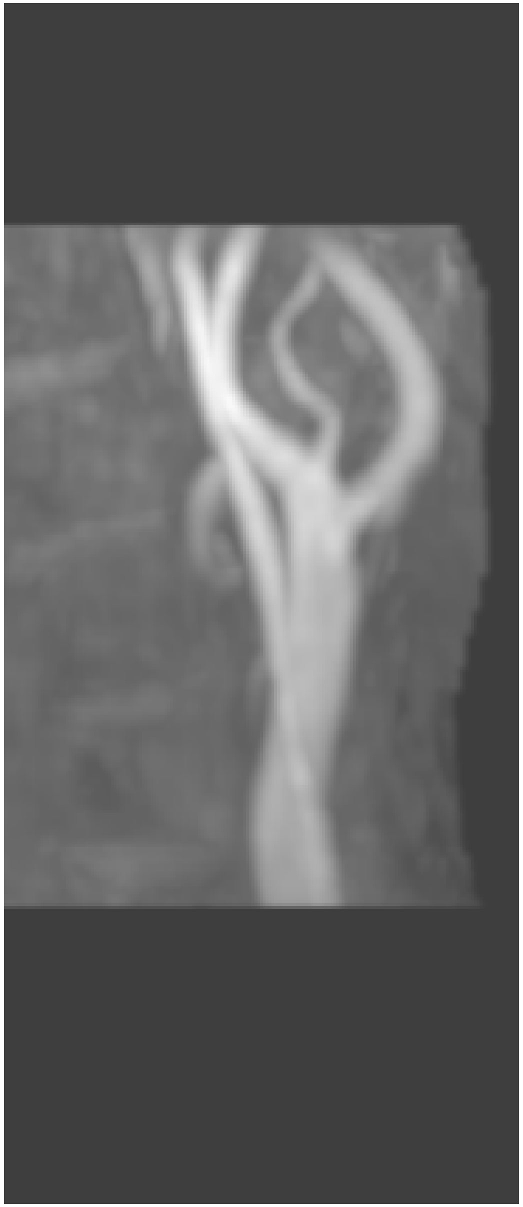

192c Score

0-30

31-50

51-70

>70

Near occlusion

Occluded

Quality

1

2

3

4

5

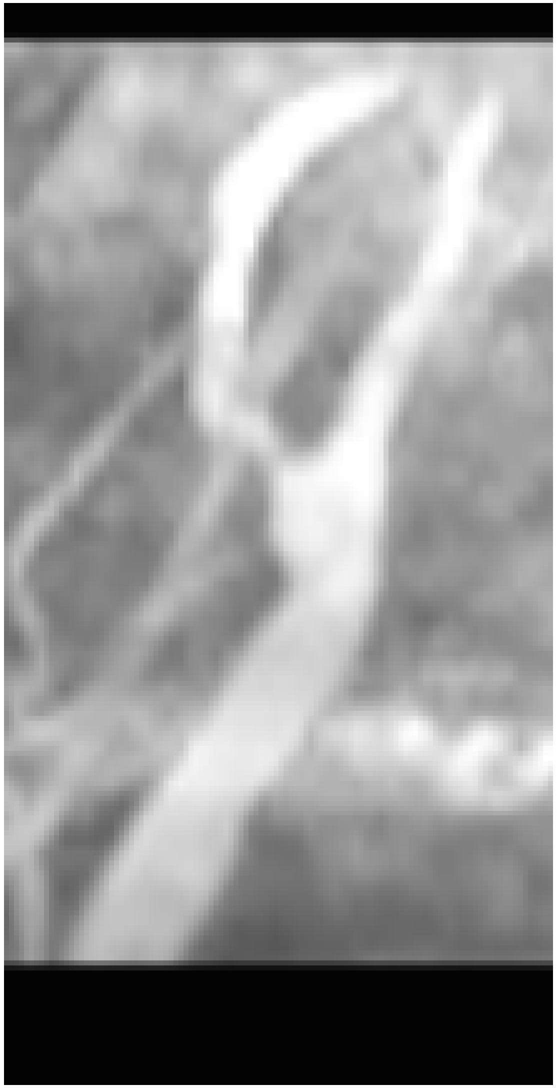

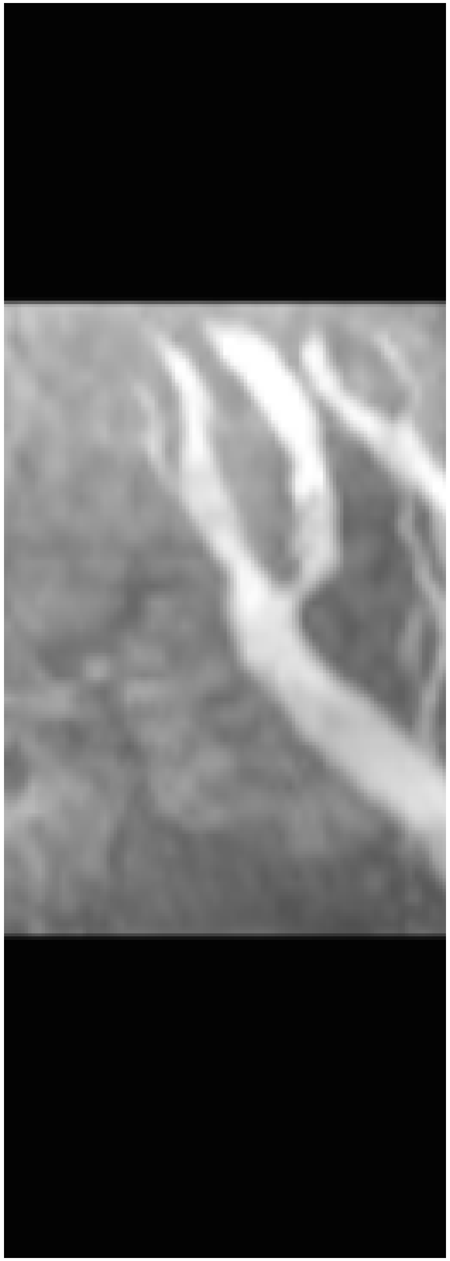

193b Score

0-30

31-50

51-70

>70

Near occlusion

Occluded

Quality

1

2

3

4

5

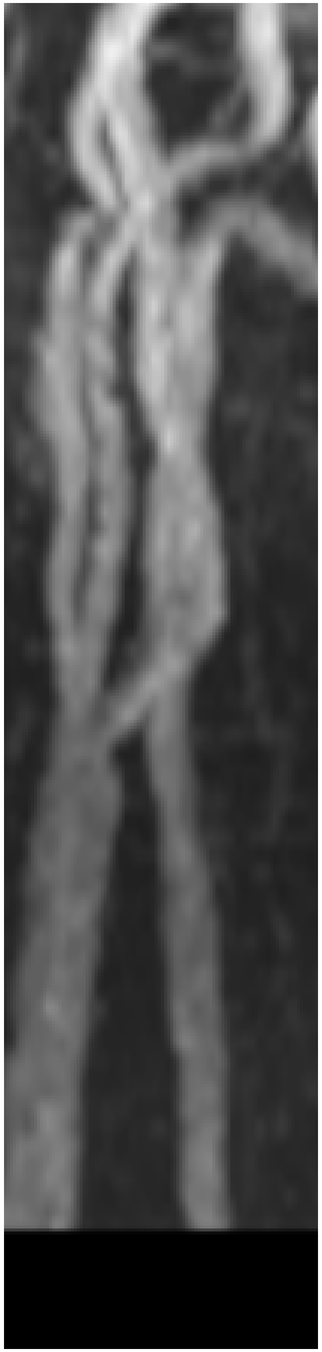

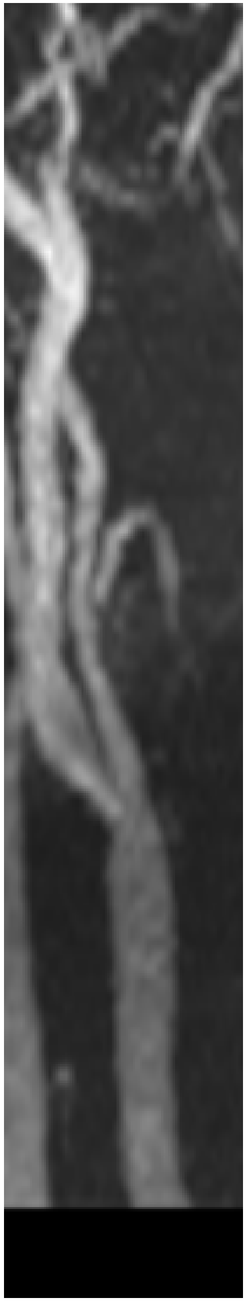

194a Score

0-30

31-50

51-70

>70

Near occlusion

Occluded

Quality

1

2

3

4

5

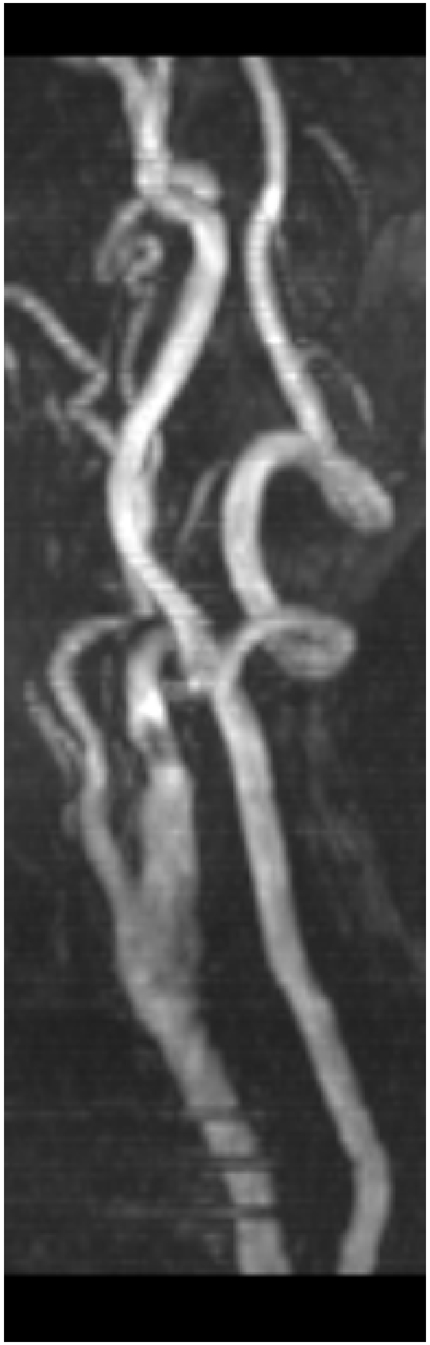

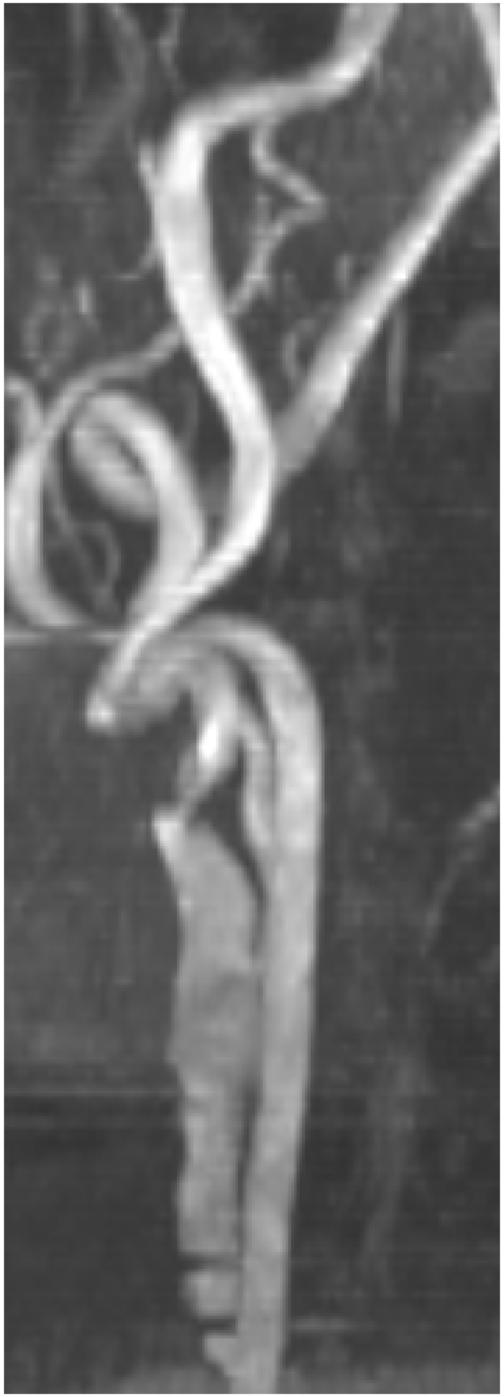

194f Score

0-30

31-50

51-70

>70

Near occlusion

Occluded

Quality

1

2

3

4

5

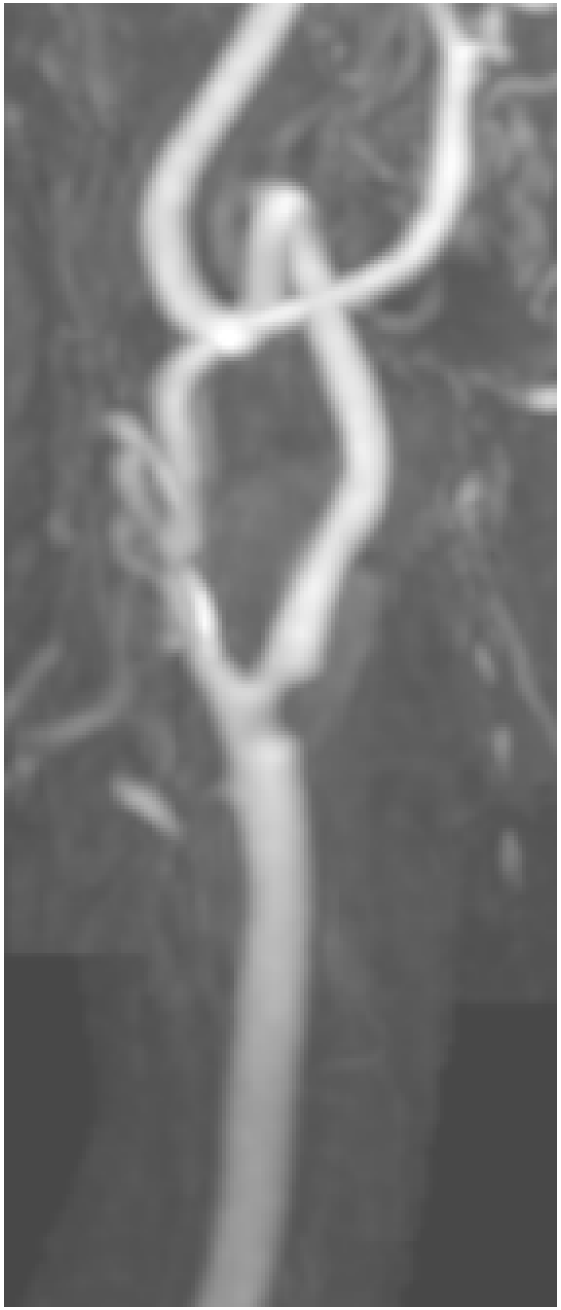

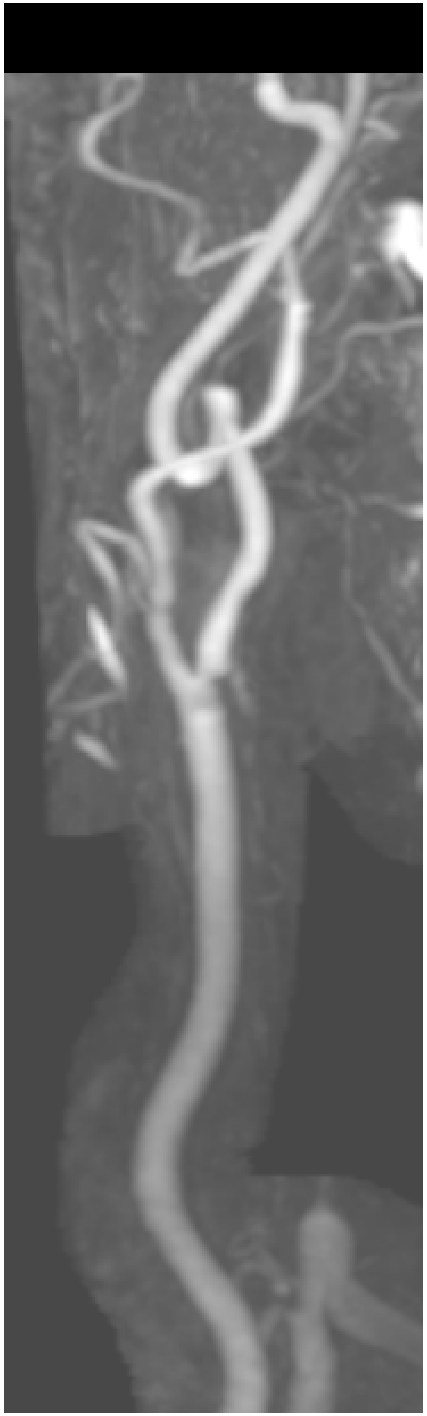

195e Score

0-30

31-50

51-70

>70

Near occlusion

Occluded

Quality

1

2

3

4

5

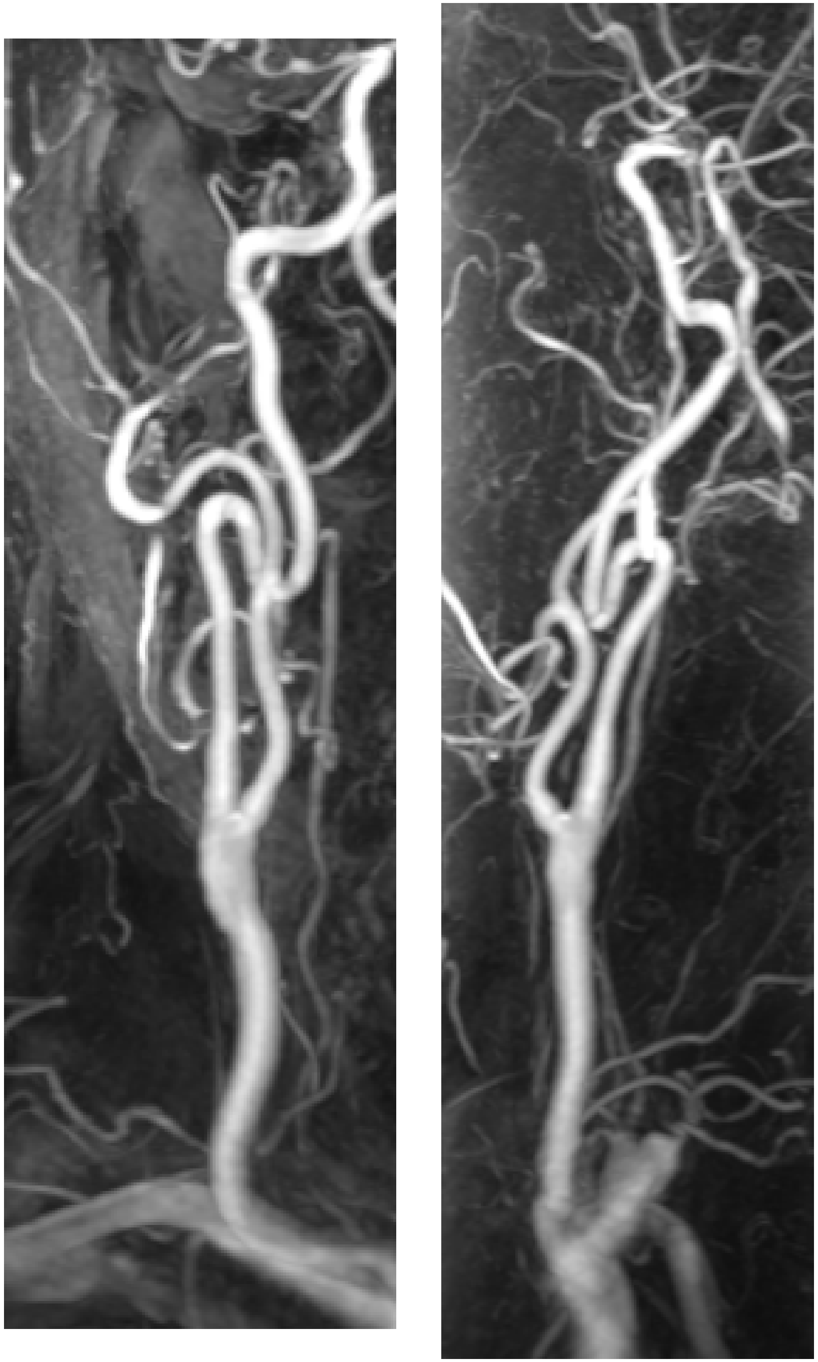

196d Score

0-30

31-50

51-70

>70

Near occlusion

Occluded

Quality

1

2

3

4

5

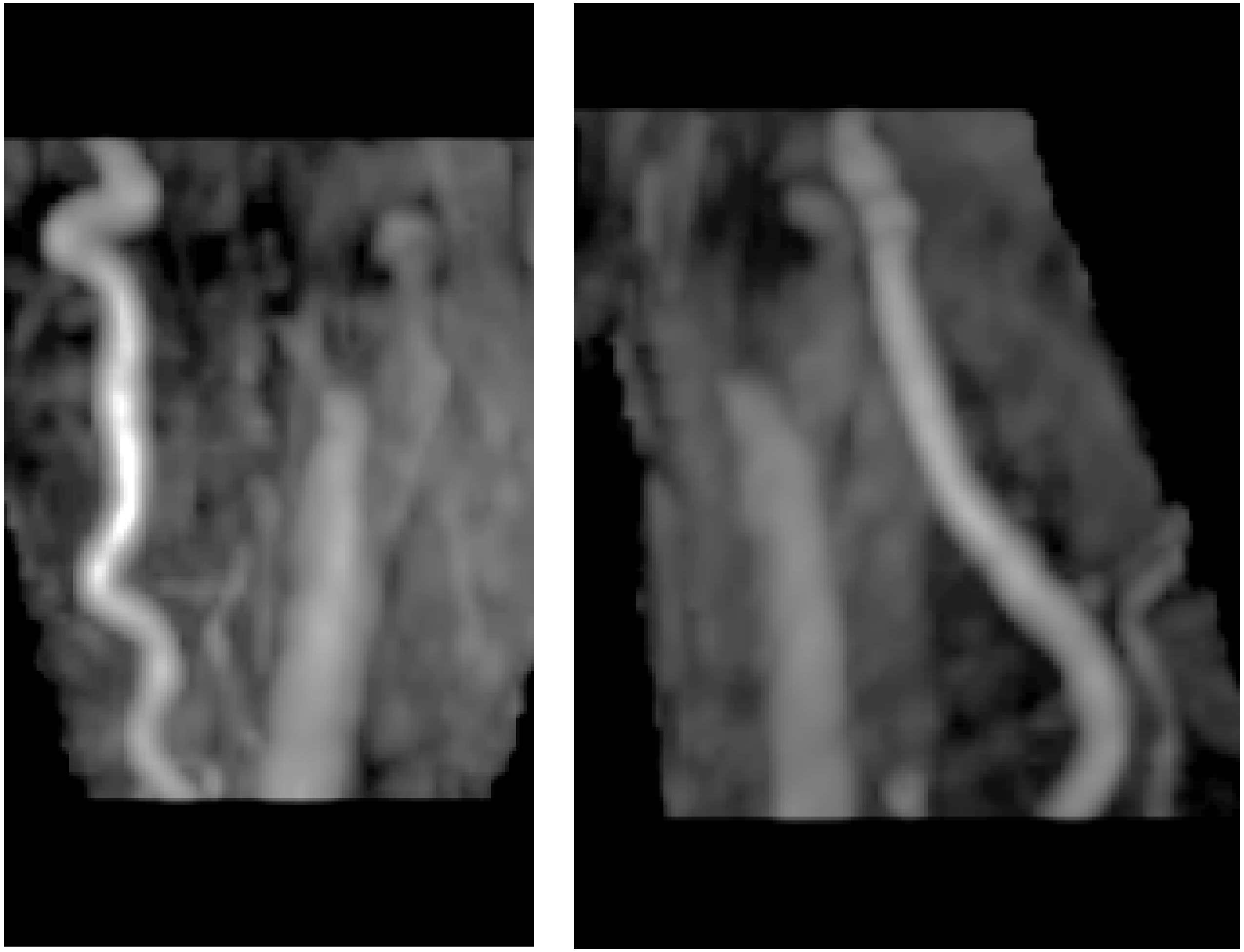

197c Score

0-30

31-50

51-70

>70

Near occlusion

Occluded

Quality

1

2

3

4

5

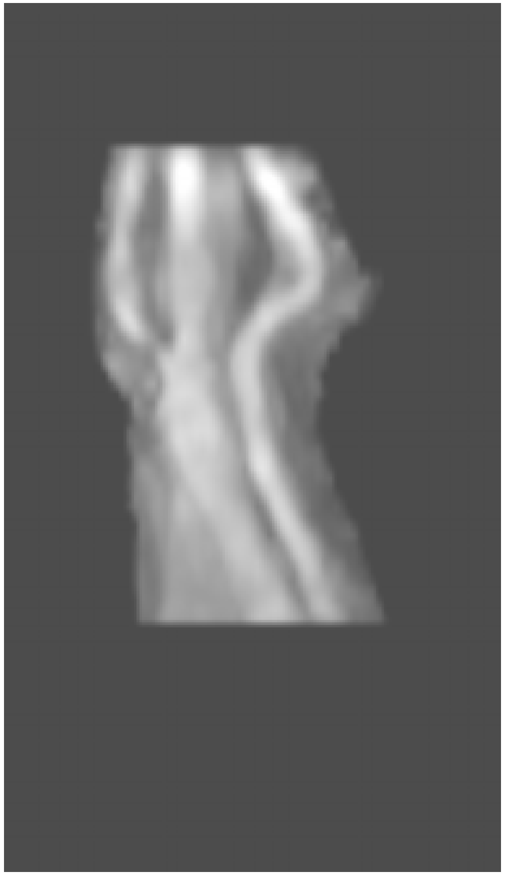

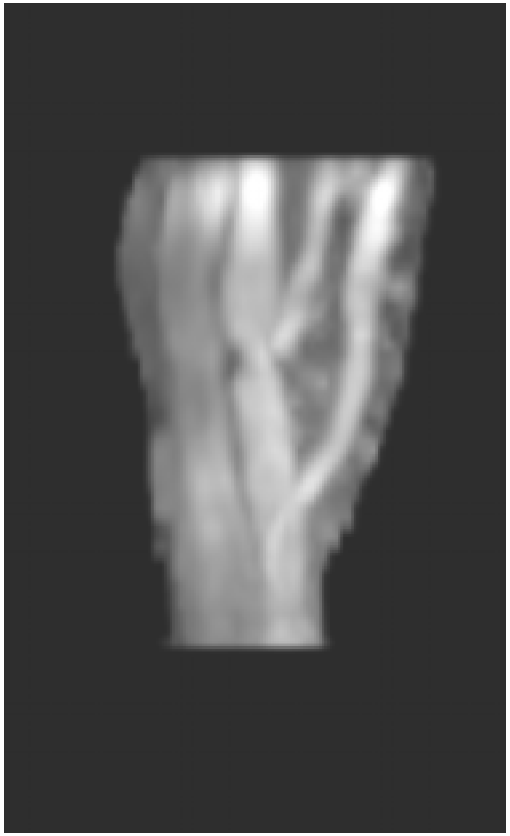

198b Score

0-30

31-50

51-70

>70

Near occlusion

Occluded

Quality

1

2

3

4

5

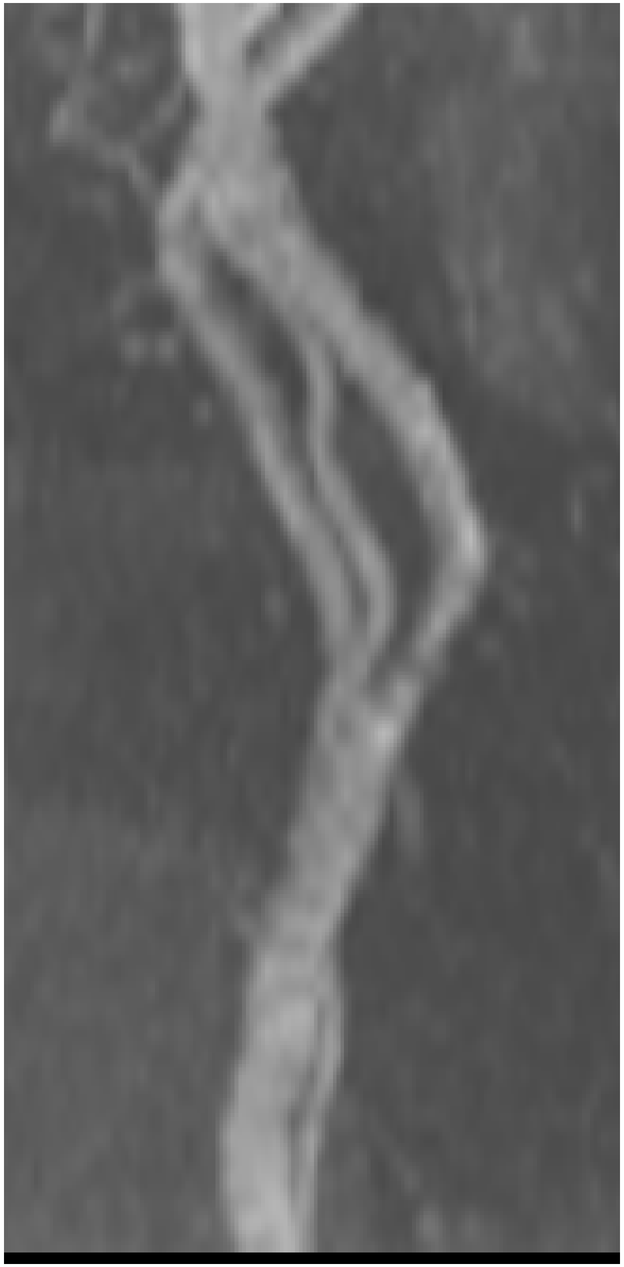

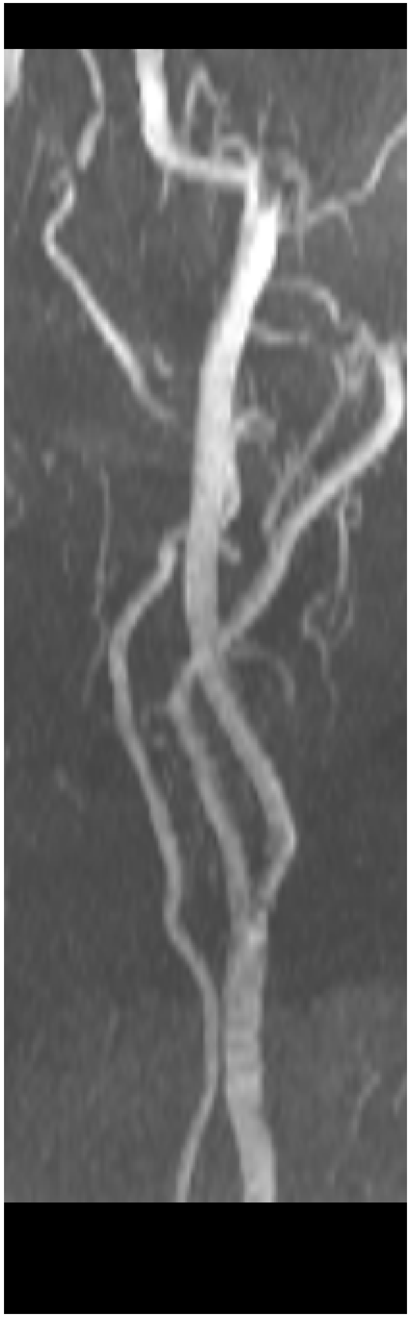

199a Score

0-30

31-50

51-70

>70

Near occlusion

Occluded

Quality

1

2

3

4

5

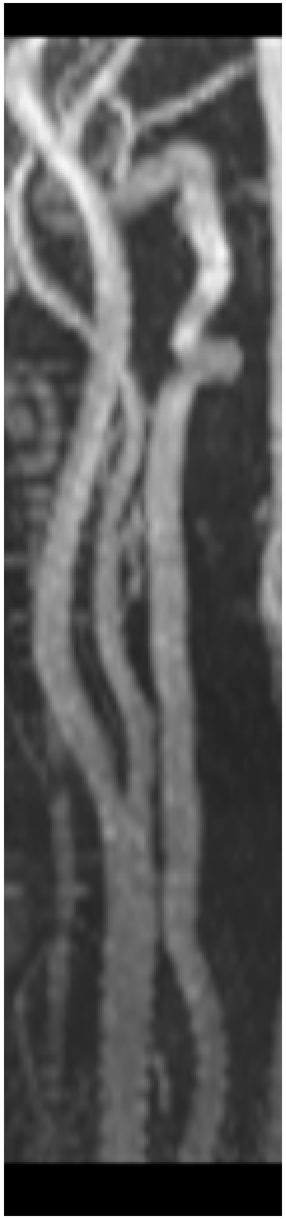

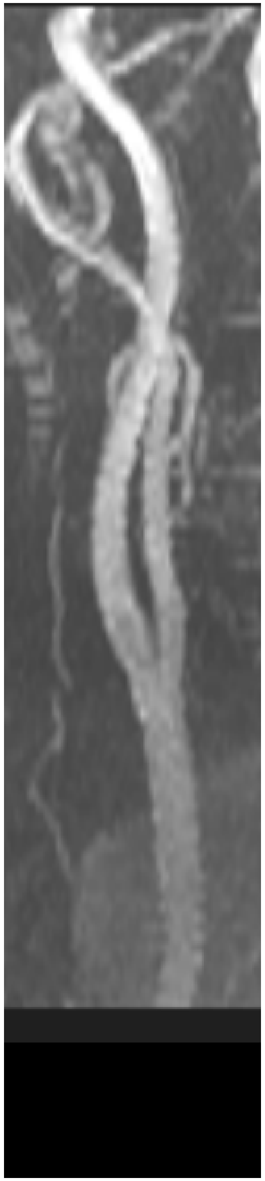

199f Score

0-30

31-50

51-70

>70

Near occlusion

Occluded

Quality

1

2

3

4

5

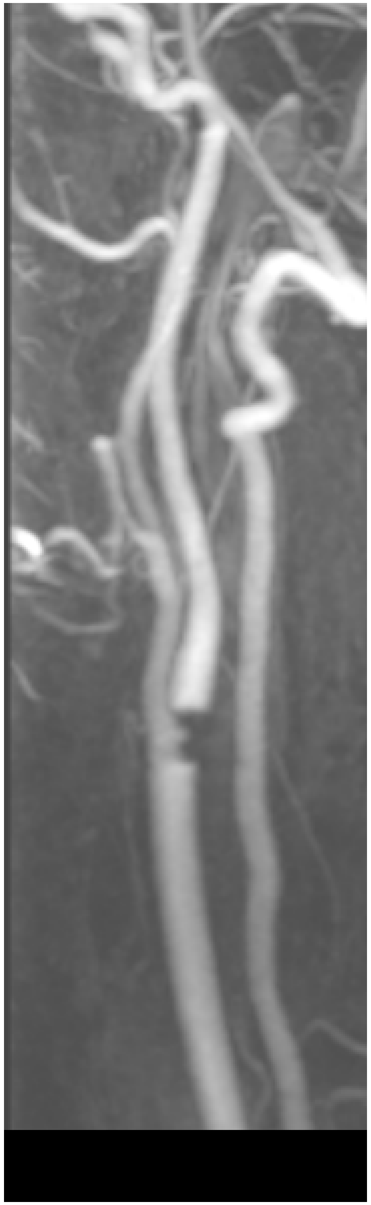

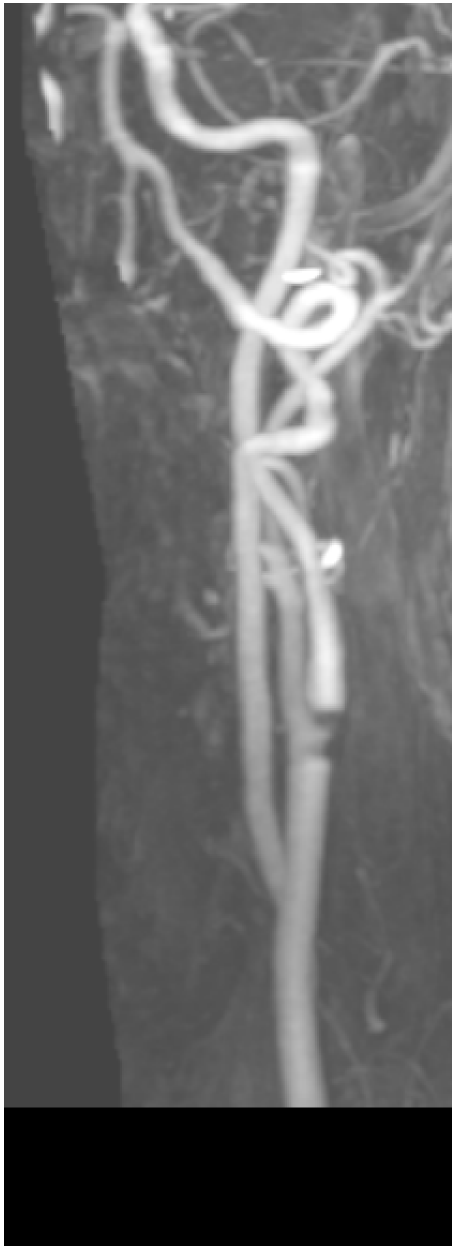

200e Score

0-30

31-50

51-70

>70

Near occlusion

Occluded

Quality

1

2

3

4

5

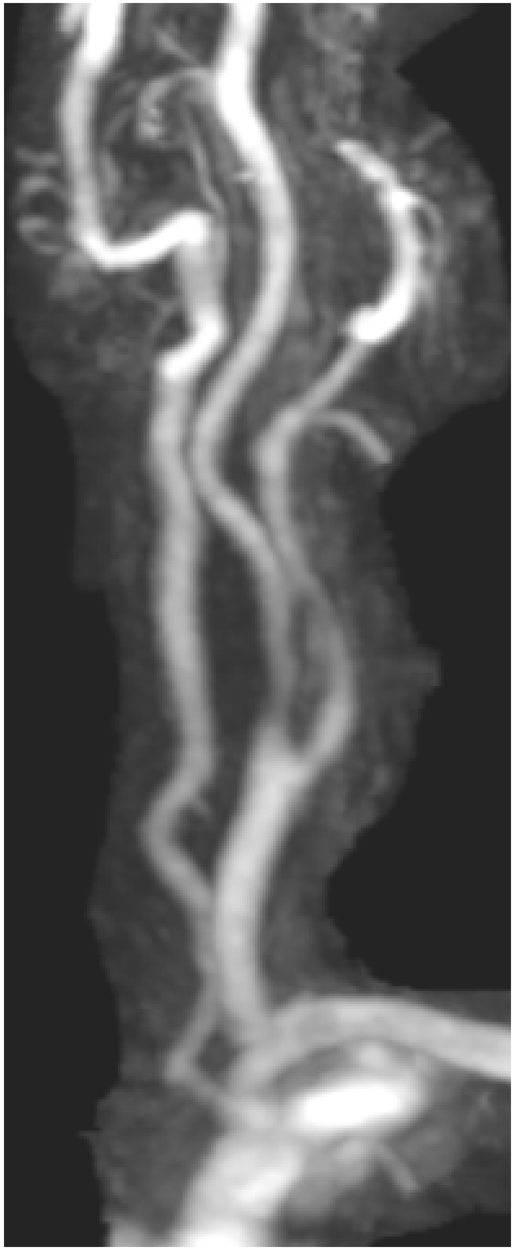

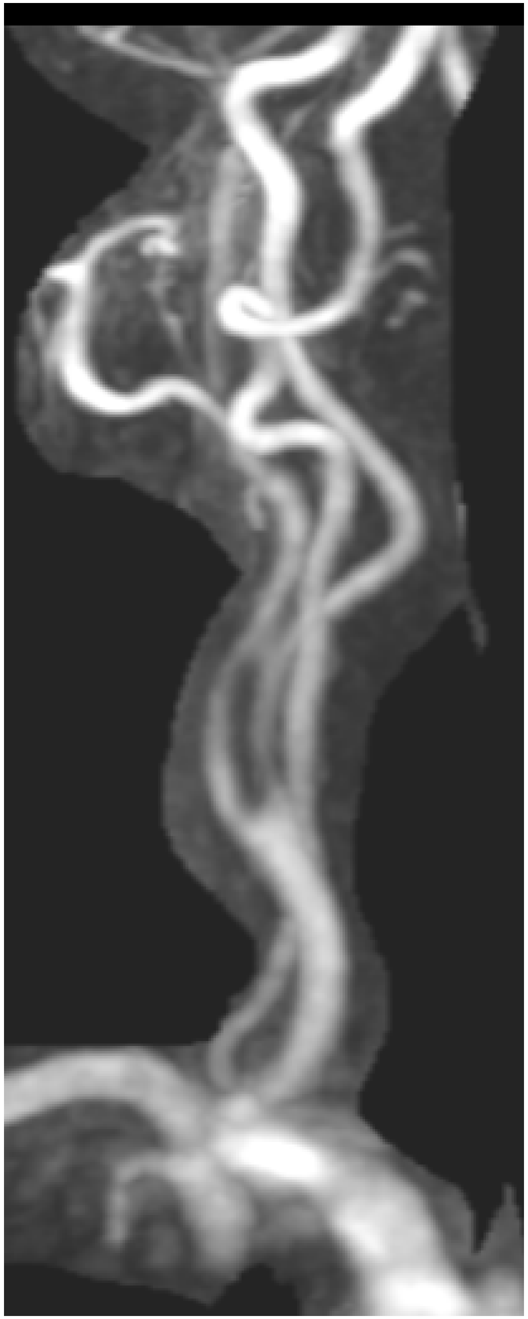

Supplement: S4 File — (PDF) [file pone.0237856.s006.pdf]
