# Supplementary material for: Multiple reader comparison of 2D TOF, 3D TOF, and CEMRA in screening of the carotid bifurcations: Time to reconsider routine contrast use?
Source: PLoS One. 2020 Sep 2;15(9):e0237856. doi: 10.1371/journal.pone.0237856 (PMC7467222; doi:10.1371/journal.pone.0237856)

1e Score

0-30

31-50

51-70

>70

Near occlusion

Occluded

Quality

1

2

3

4

5

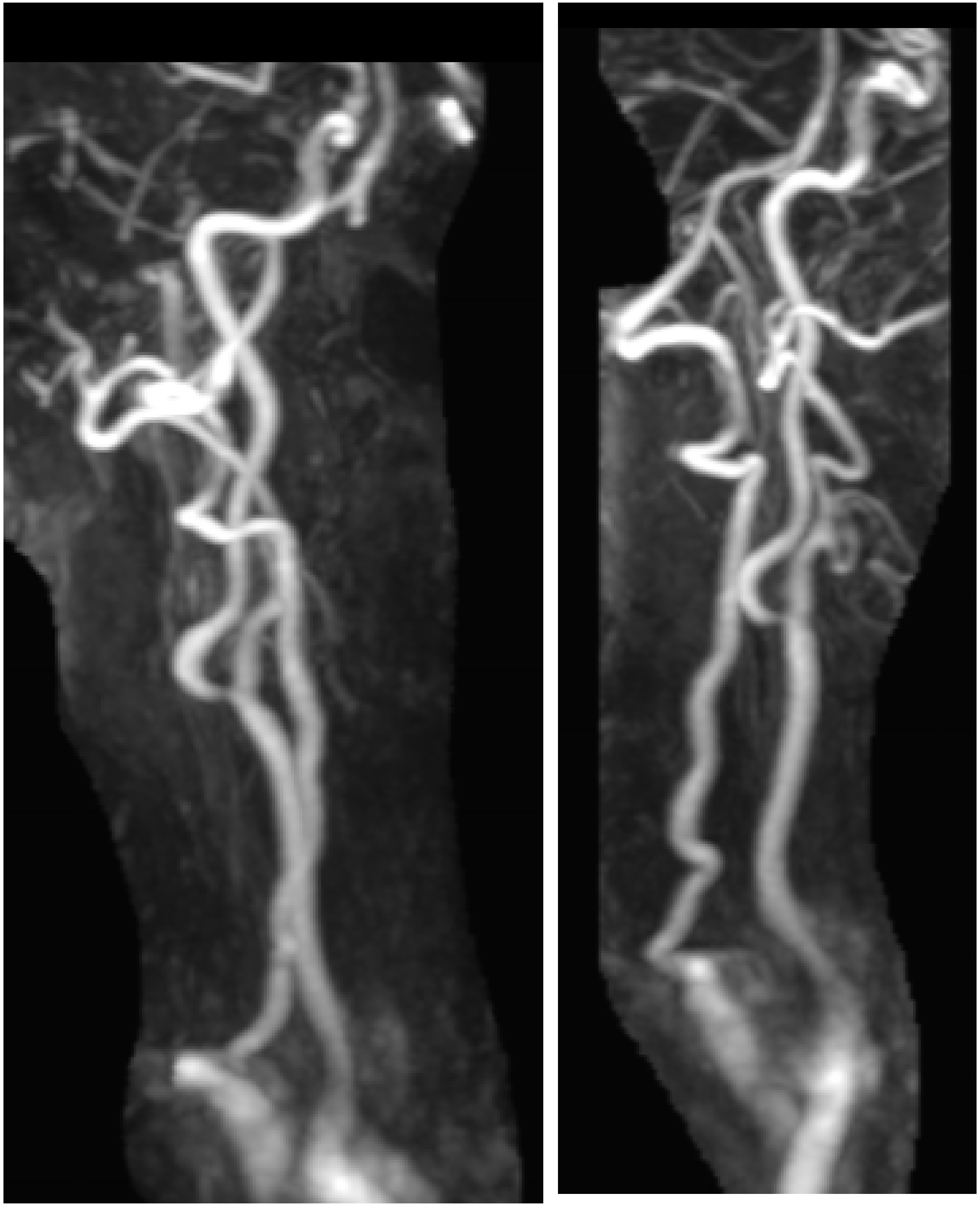

2d Score

0-30

31-50

51-70

>70

Near occlusion

Occluded

Quality

1

2

3

4

5

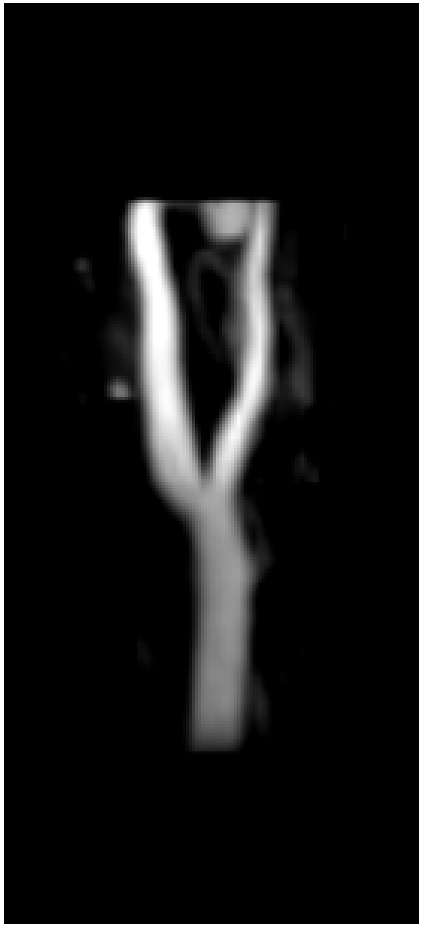

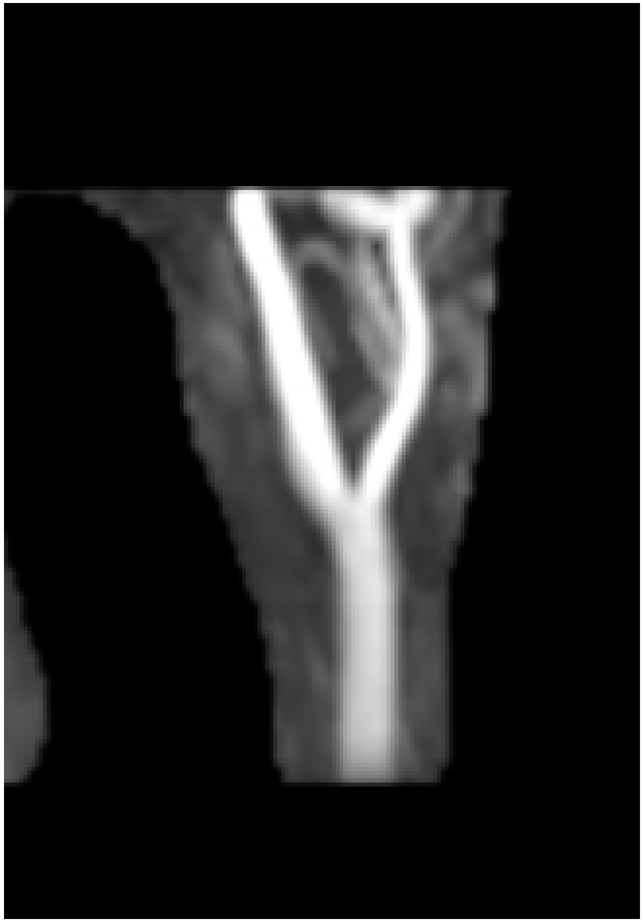

3c Score

0-30

31-50

51-70

>70

Near occlusion

Occluded

Quality

1

2

3

4

5

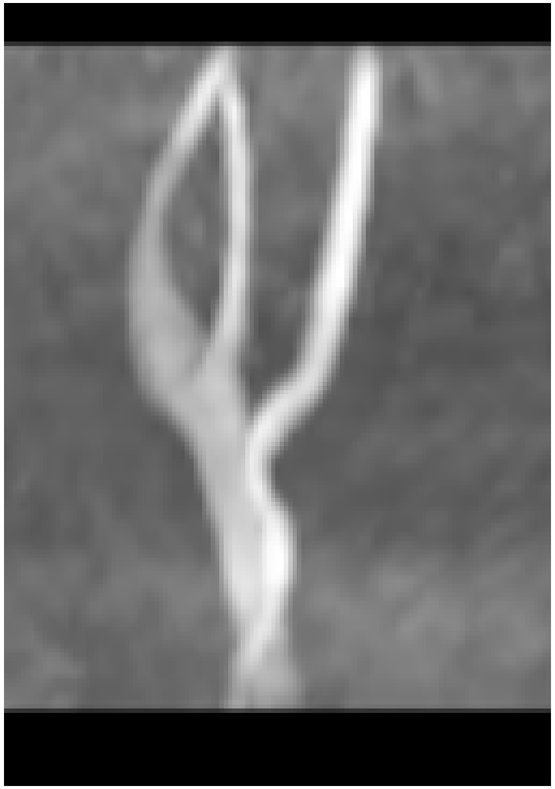

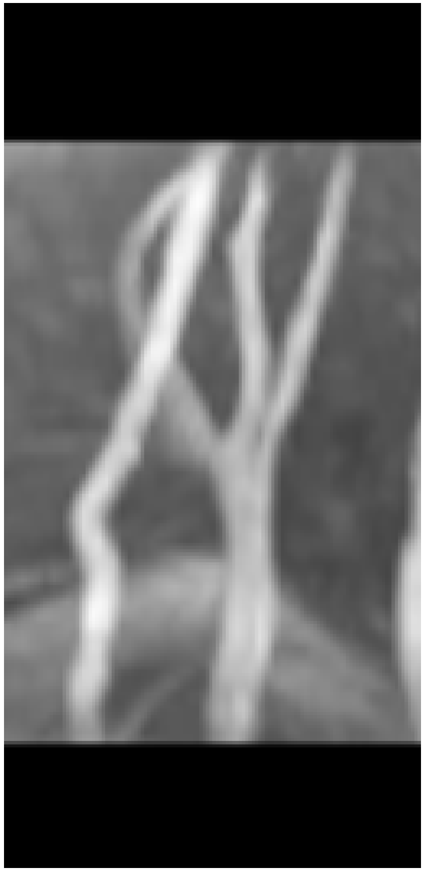

4b Score

0-30

31-50

51-70

>70

Near occlusion

Occluded

Quality

1

2

3

4

5

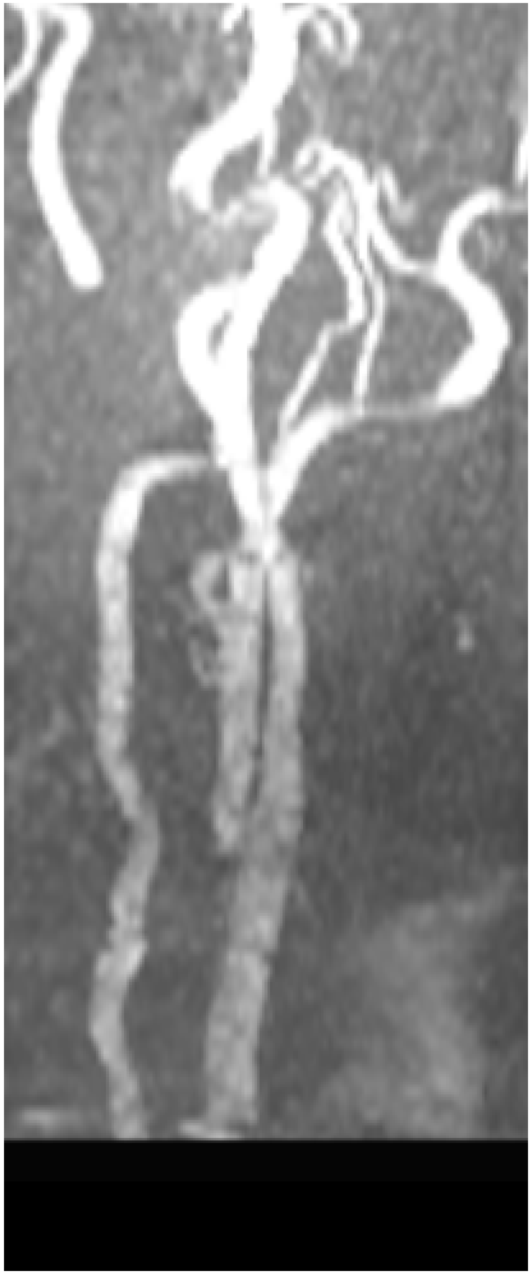

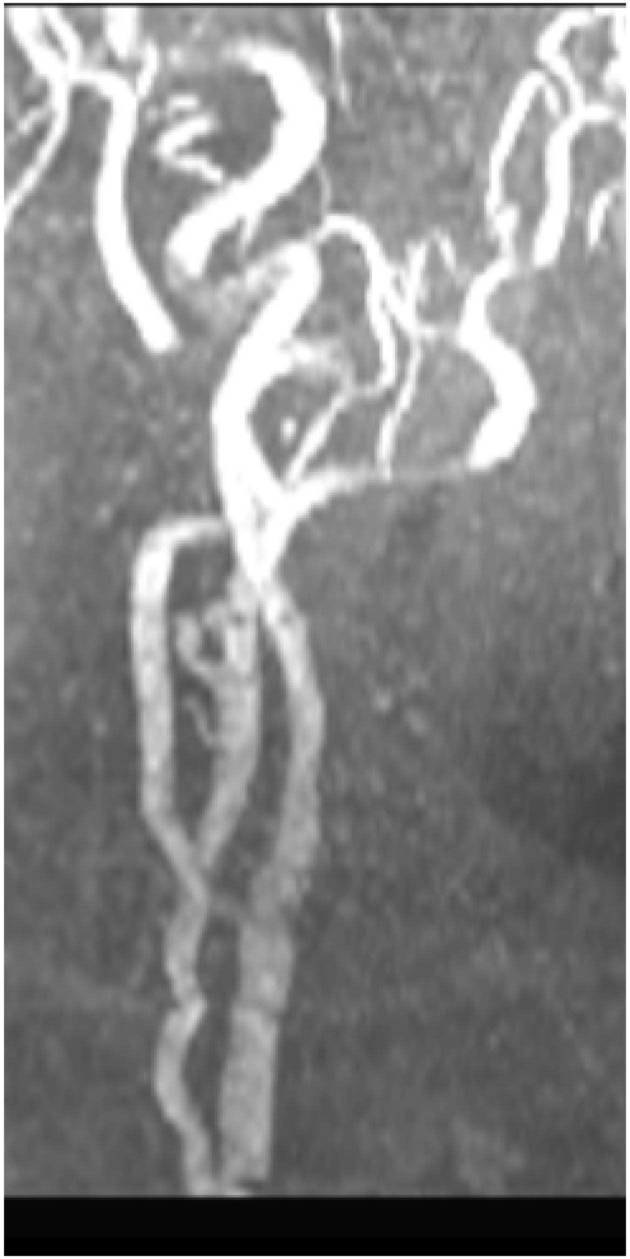

5a Score

0-30

31-50

51-70

>70

Near occlusion

Occluded

Quality

1

2

3

4

5

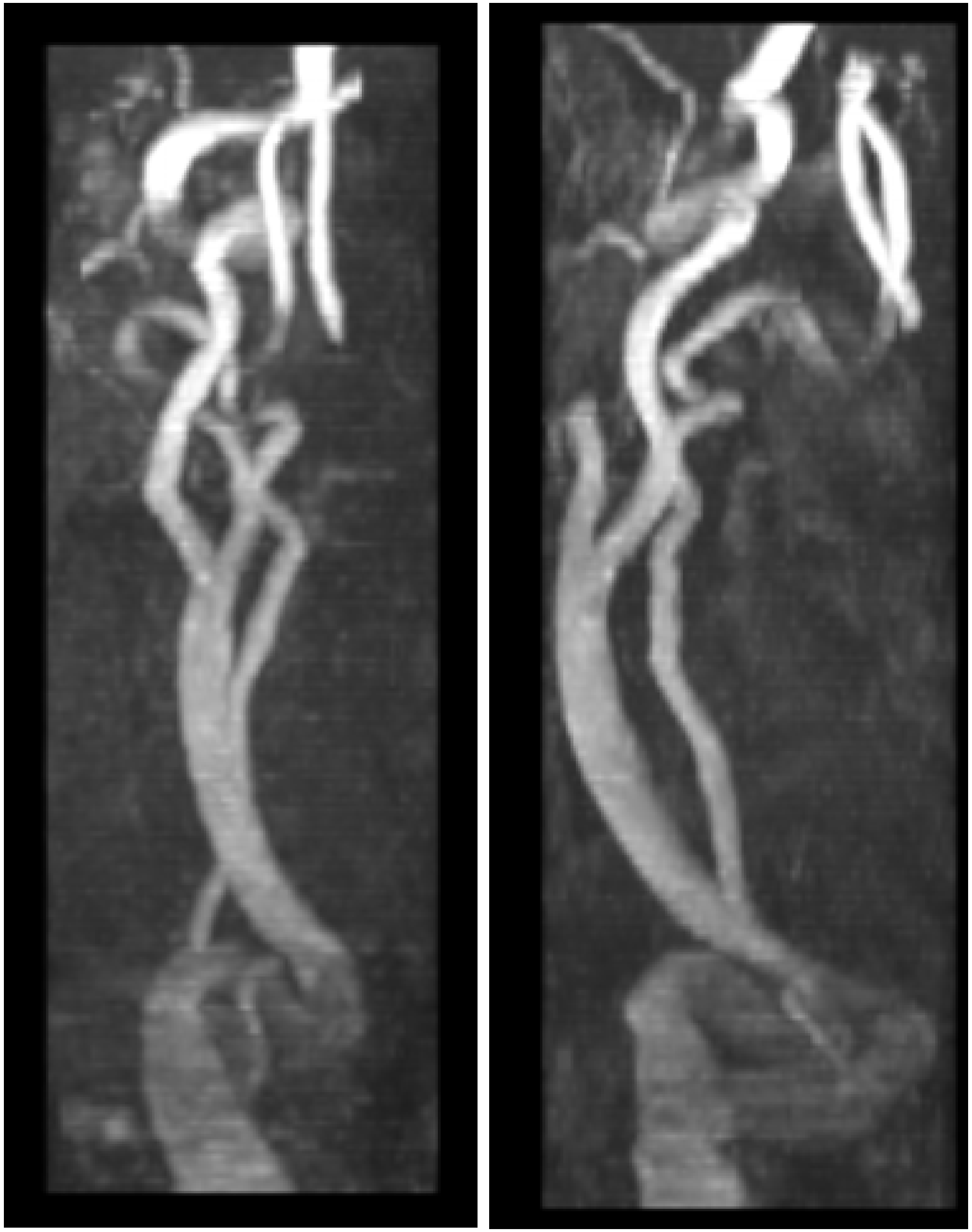

5f Score

0-30

31-50

51-70

>70

Near occlusion

Occluded

Quality

1

2

3

4

5

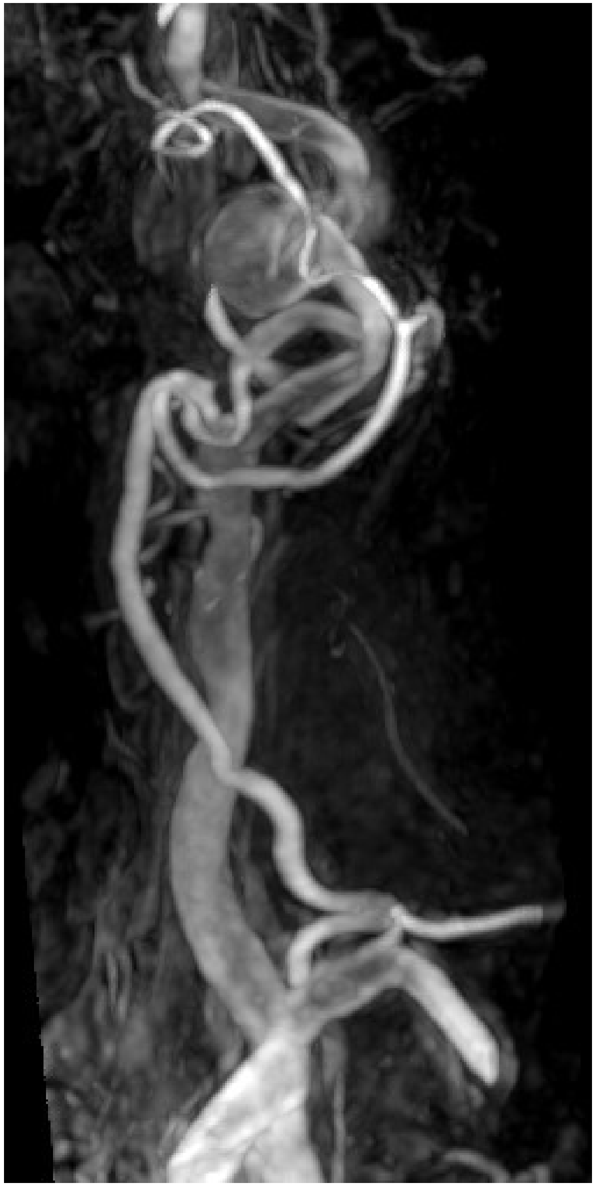

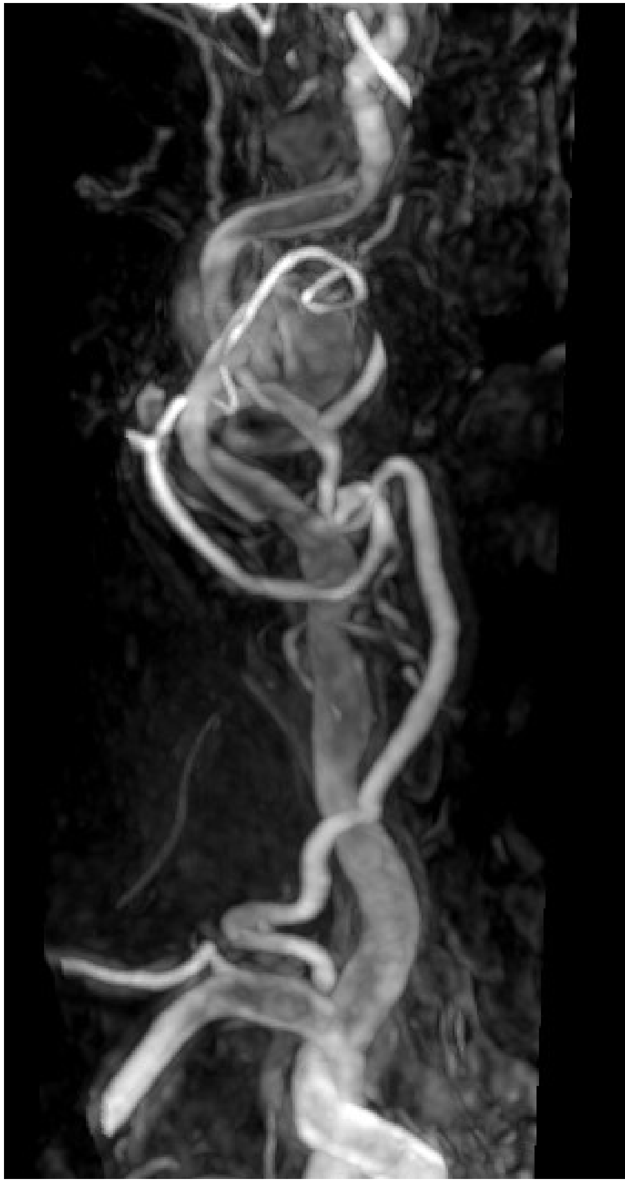

6e Score

0-30

31-50

51-70

>70

Near occlusion

Occluded

Quality

1

2

3

4

5

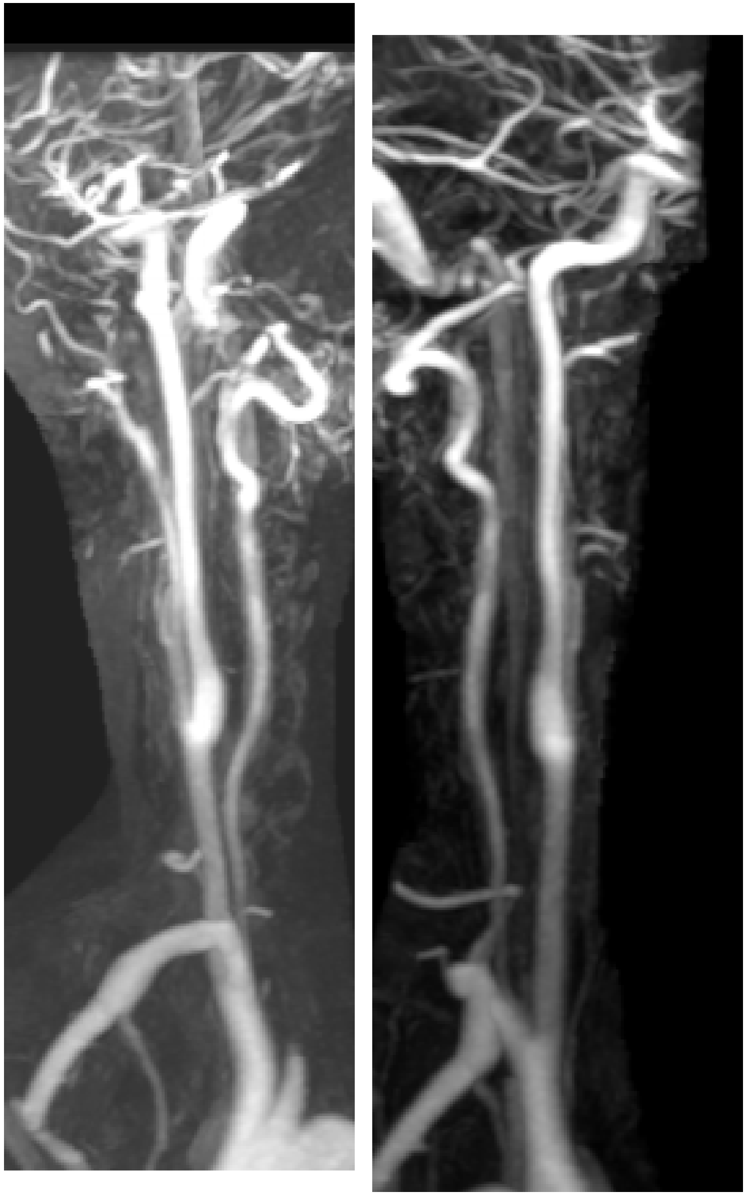

7d Score

0-30

31-50

51-70

>70

Near occlusion

Occluded

Quality

1

2

3

4

5

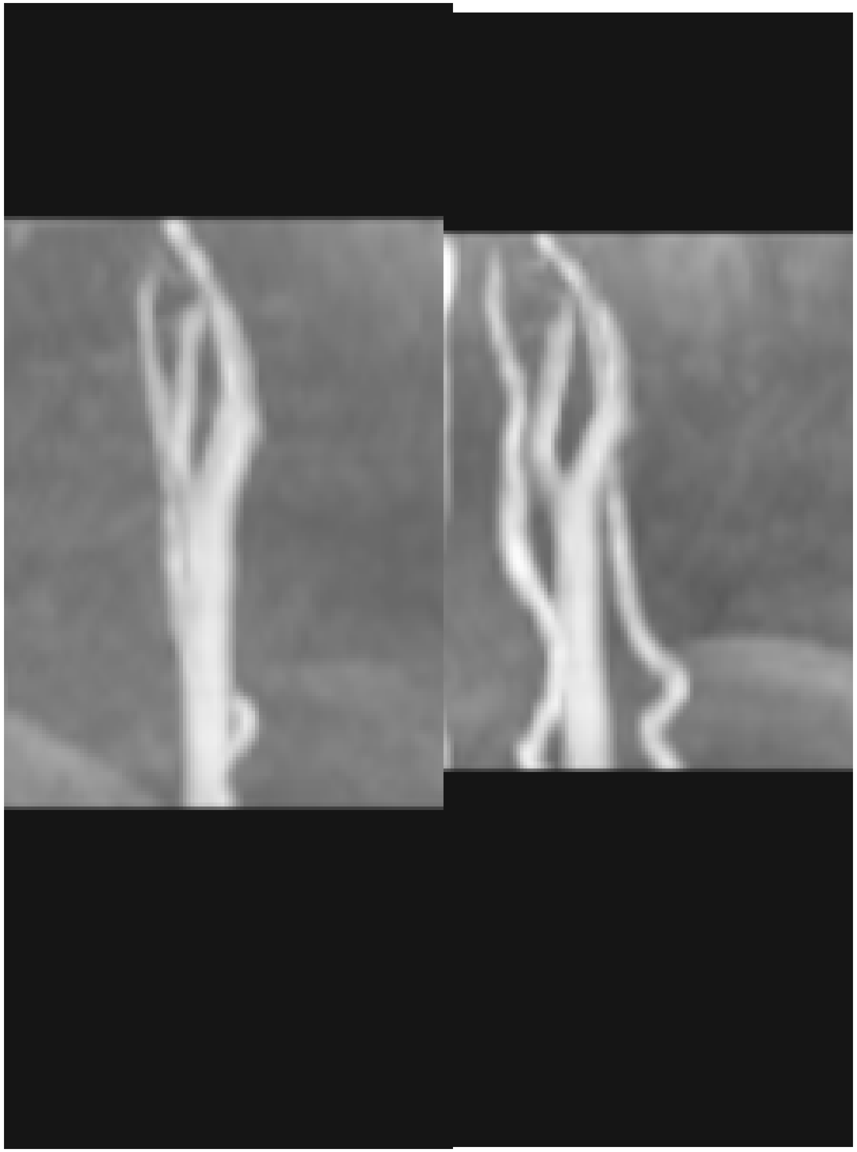

8c Score

0-30

31-50

51-70

>70

Near occlusion

Occluded

Quality

1

2

3

4

5

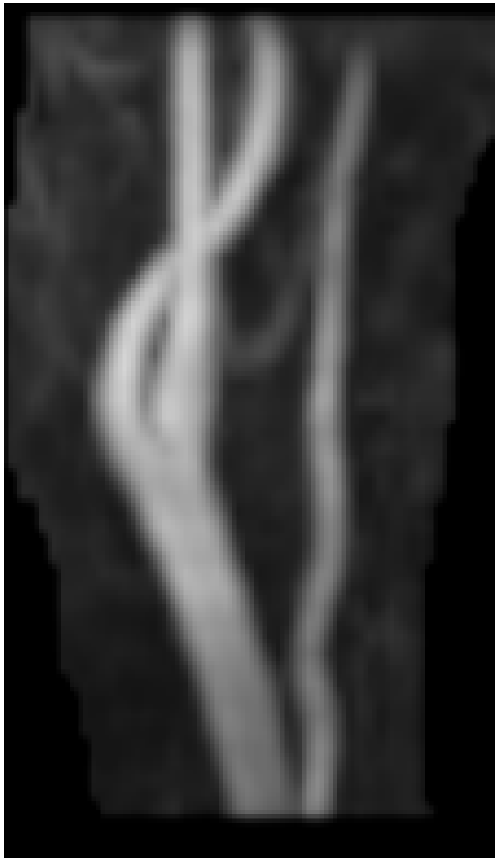

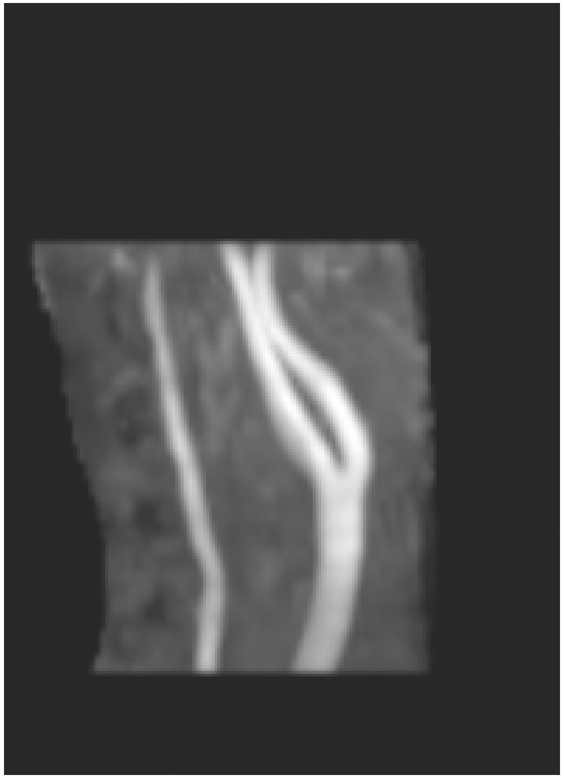

9b Score

0-30

31-50

51-70

>70

Near occlusion

Occluded

Quality

1

2

3

4

5

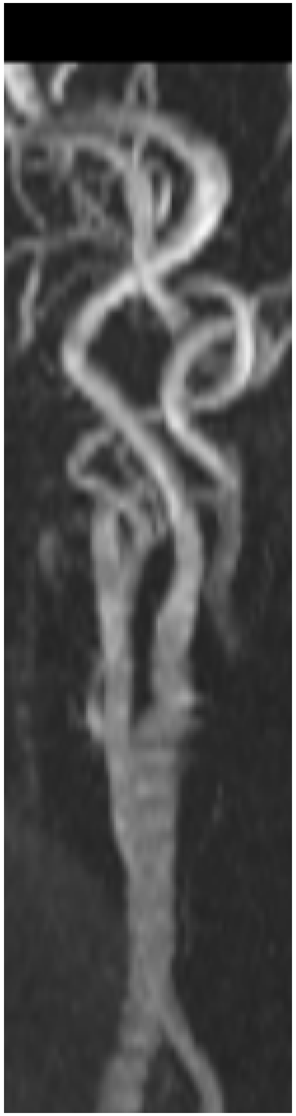

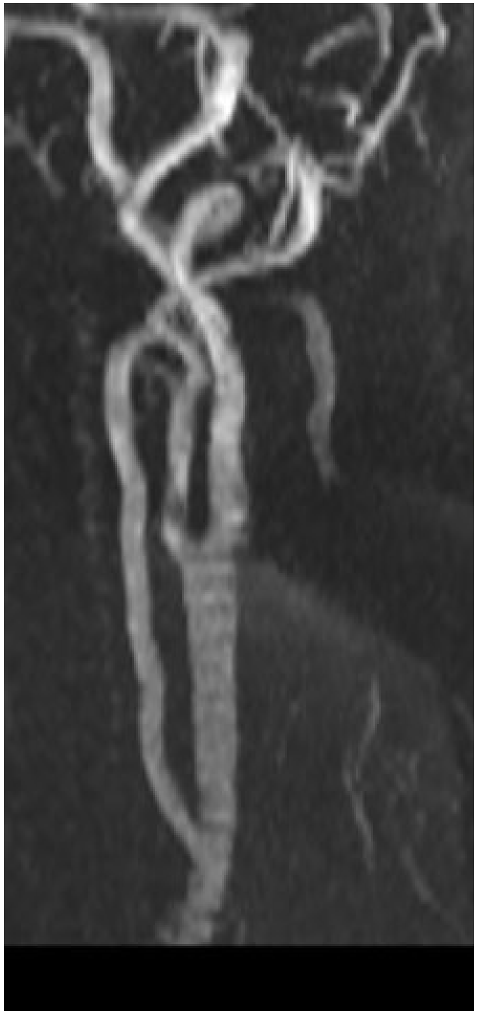

10 a Score

0-30

31-50

51-70

>70

Near occlusion

Occluded

Quality

1

2

3

4

5

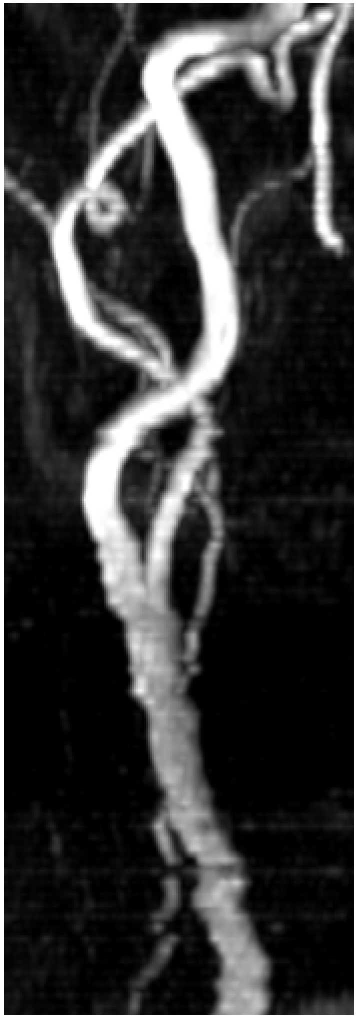

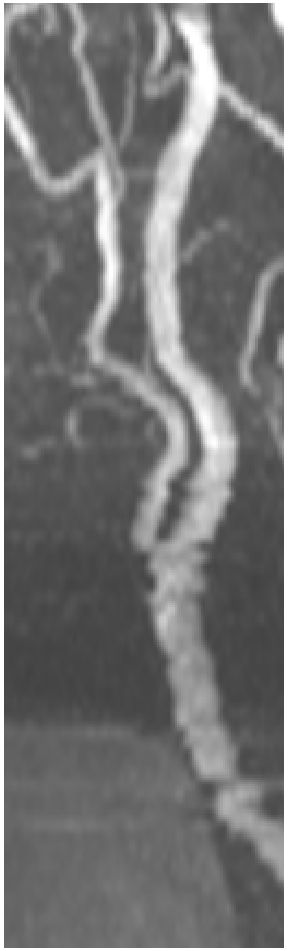

10f Score

0-30

31-50

51-70

>70

Near occlusion

Occluded

Quality

1

2

3

4

5

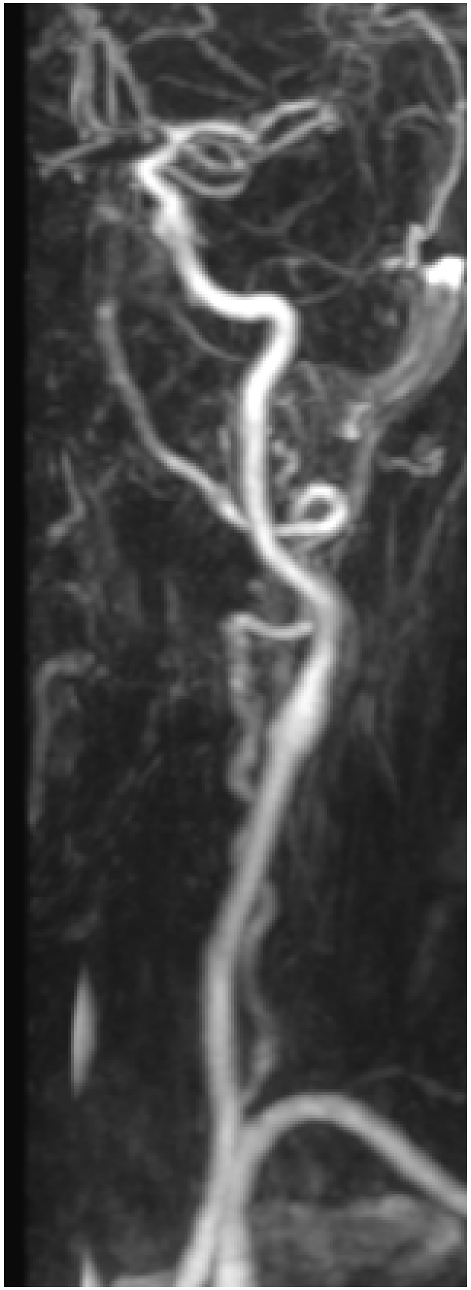

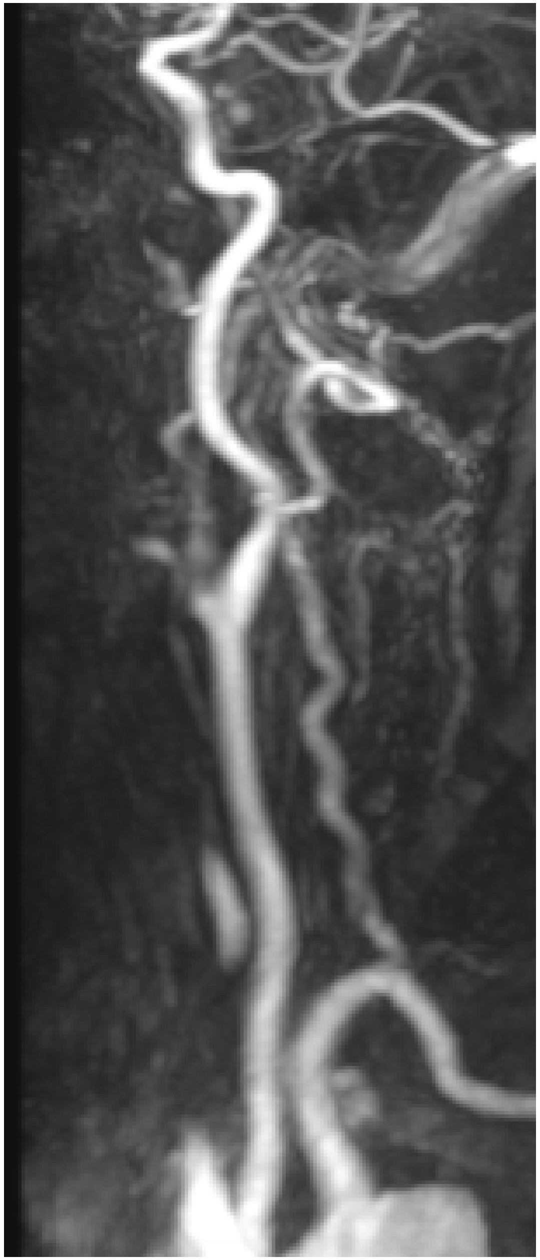

11e Score

0-30

31-50

51-70

>70

Near occlusion

Occluded

Quality

1

2

3

4

5

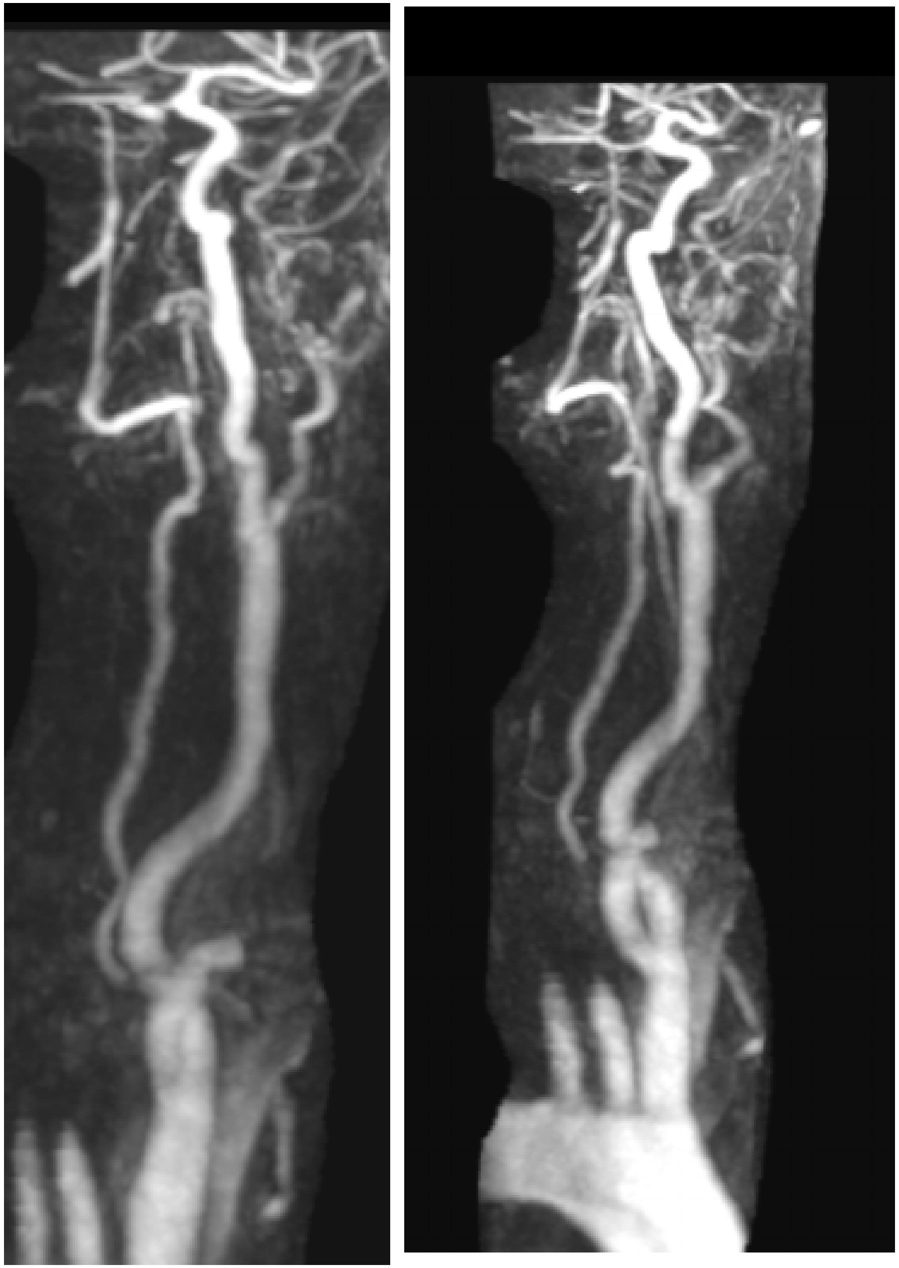

12d Score

0-30

31-50

51-70

>70

Near occlusion

Occluded

Quality

1

2

3

4

5

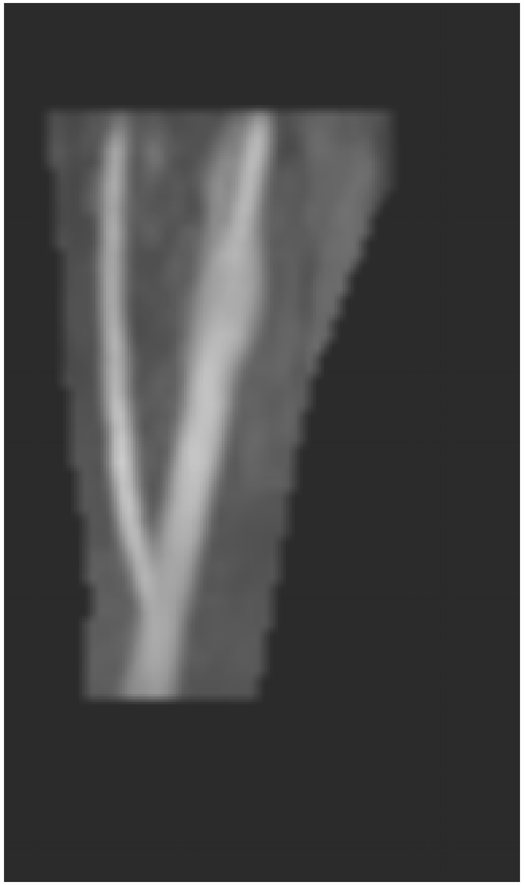

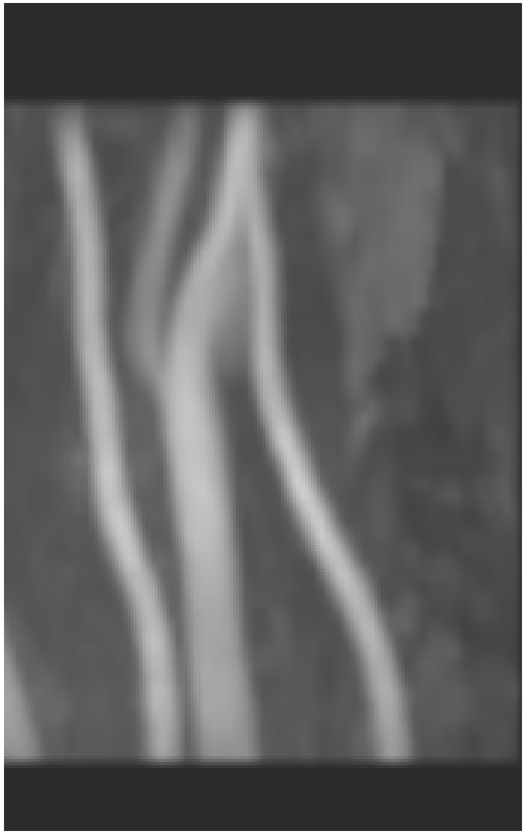

13c Score

0-30

31-50

51-70

>70

Near occlusion

Occluded

Quality

1

2

3

4

5

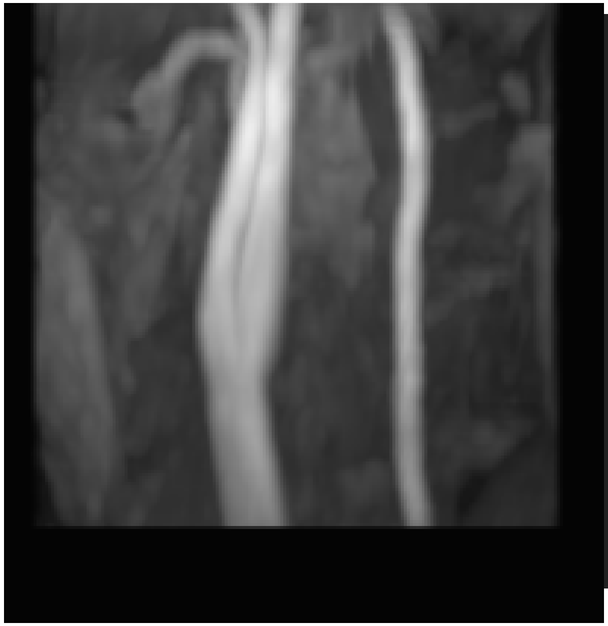

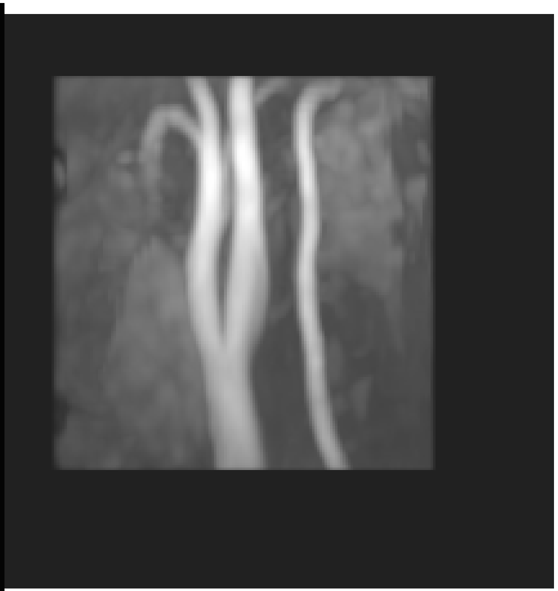

14b Score

0-30

31-50

51-70

>70

Near occlusion

Occluded

Quality

1

2

3

4

5

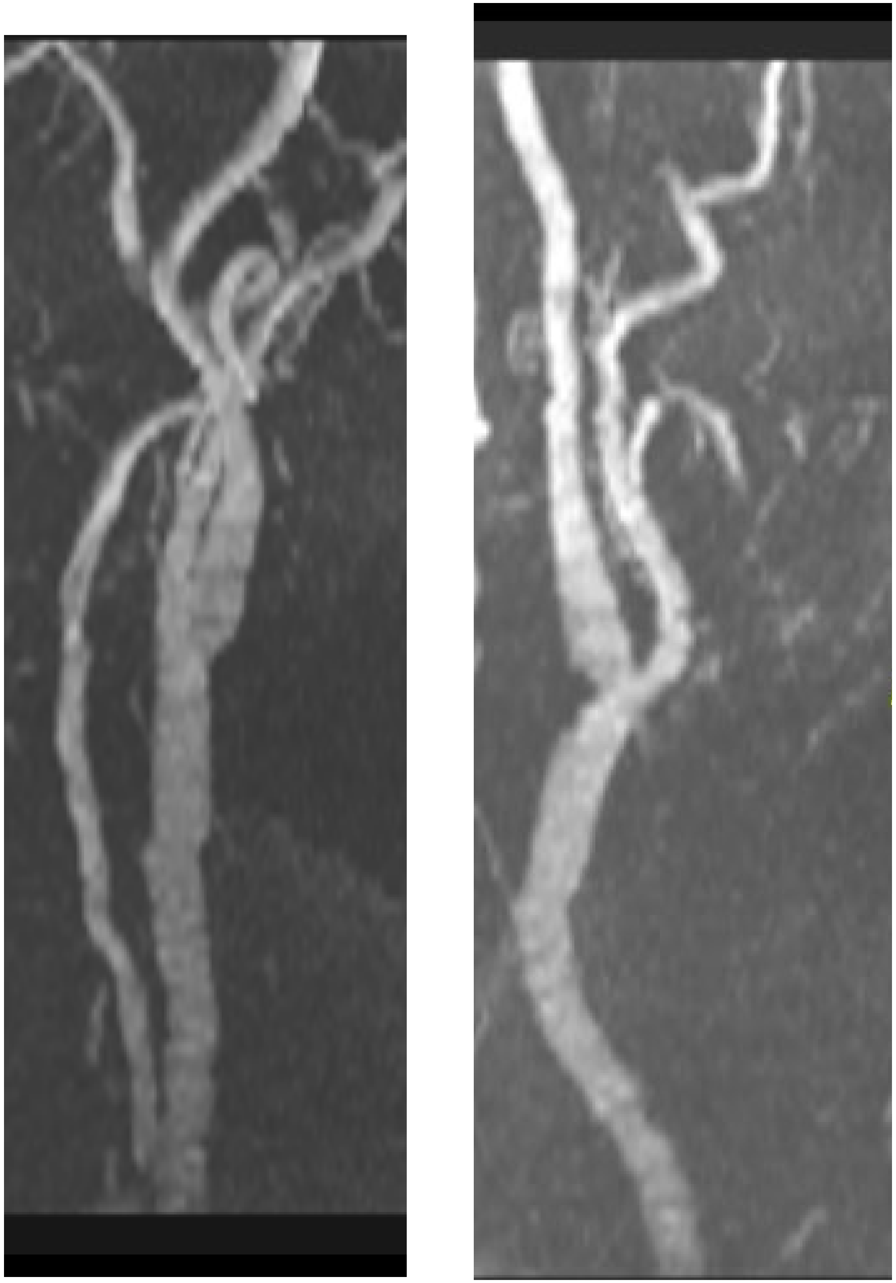

15a Score

0-30

31-50

51-70

>70

Near occlusion

Occluded

Quality

1

2

3

4

5

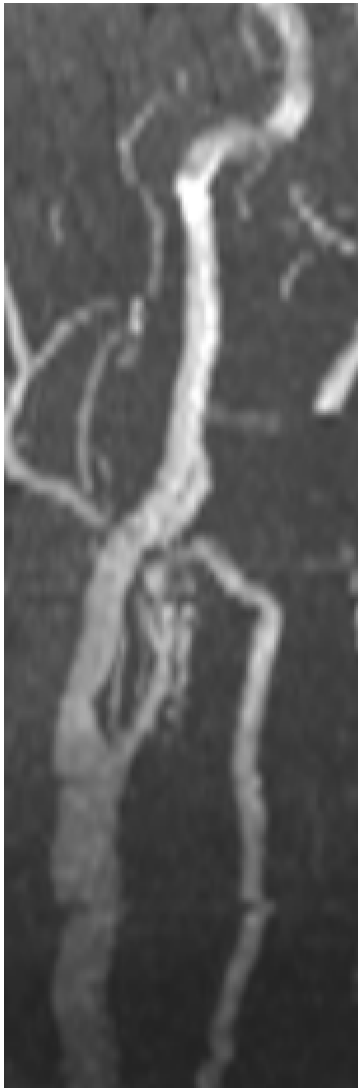

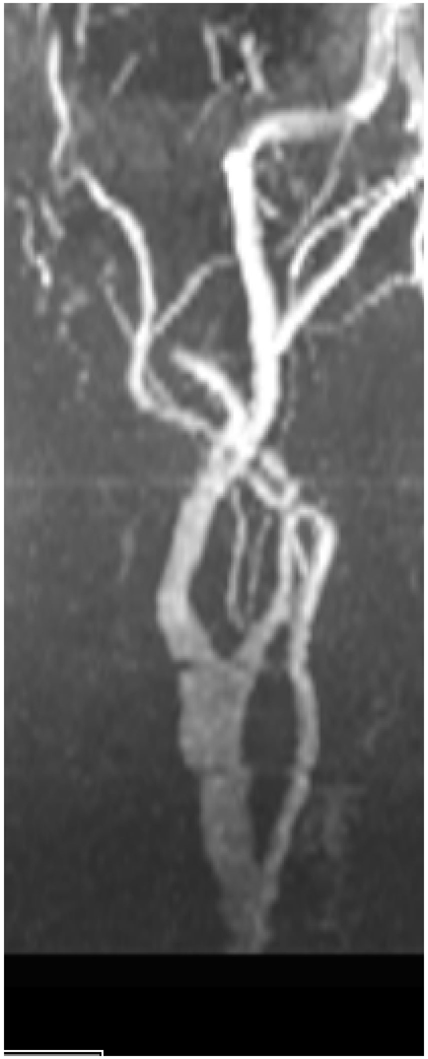

15f Score

0-30

31-50

51-70

>70

Near occlusion

Occluded

Quality

1

2

3

4

5

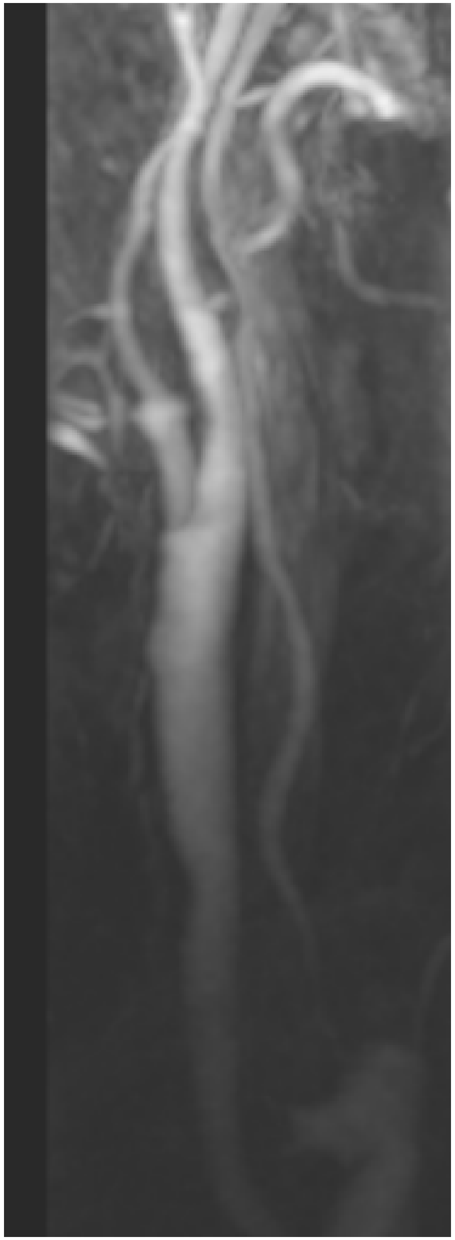

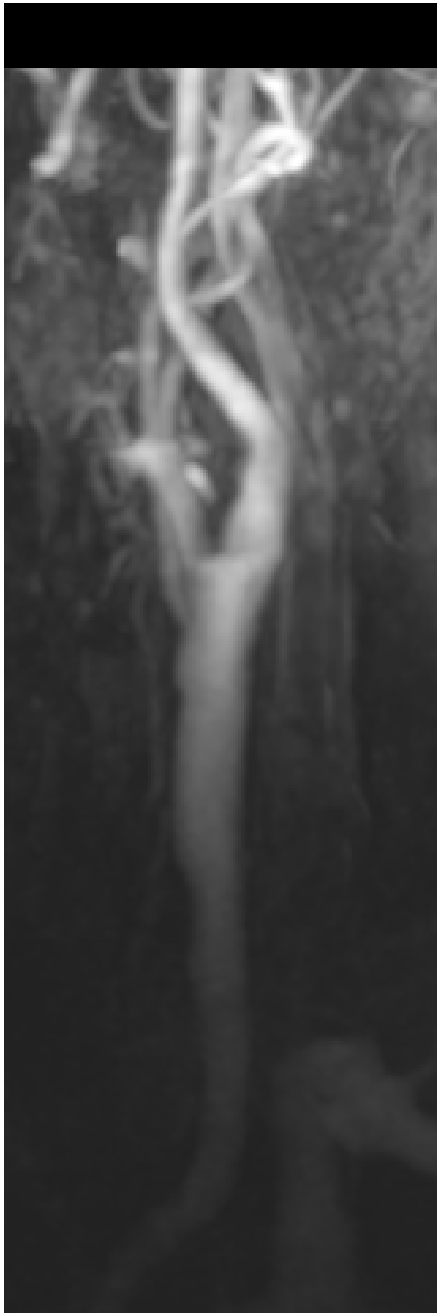

16e Score

0-30

31-50

51-70

>70

Near occlusion

Occluded

Quality

1

2

3

4

5

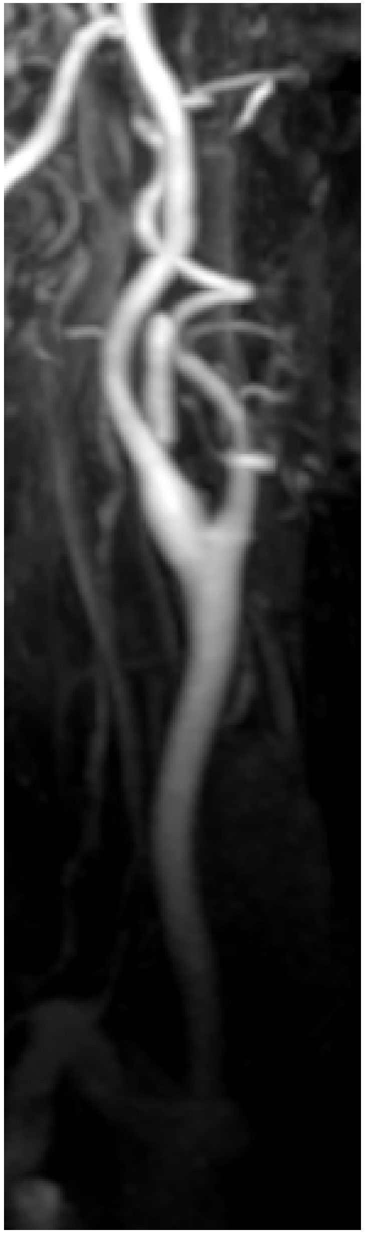

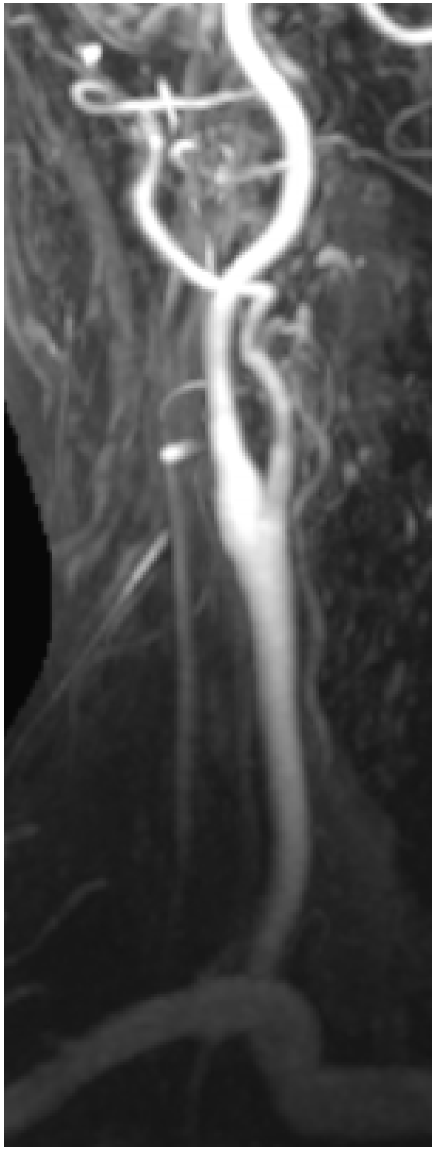

17d Score

0-30

31-50

51-70

>70

Near occlusion

Occluded

Quality

1

2

3

4

5

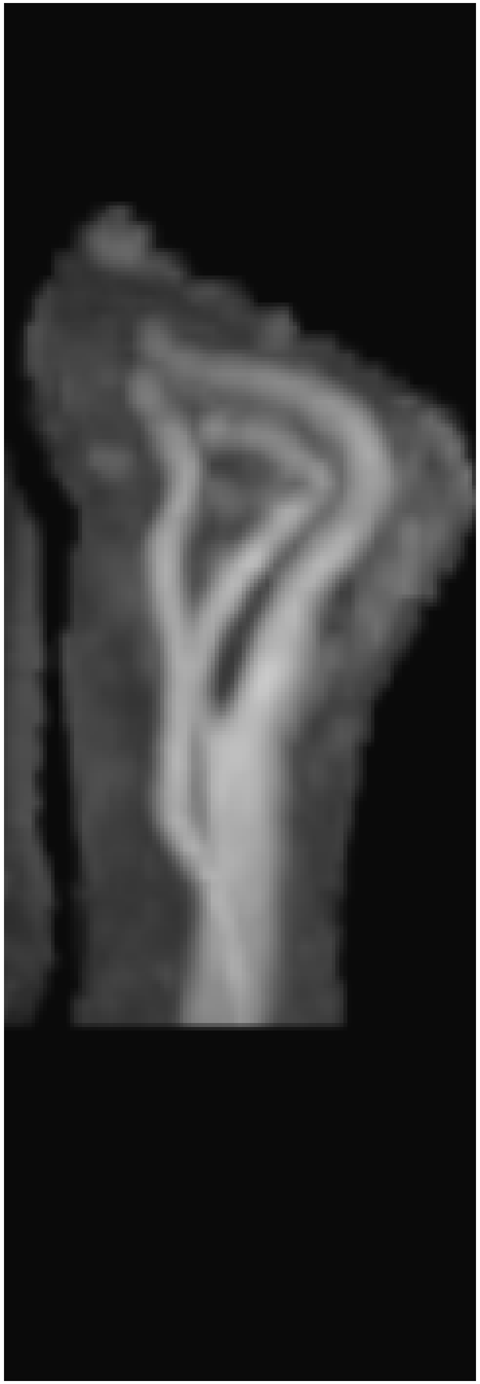

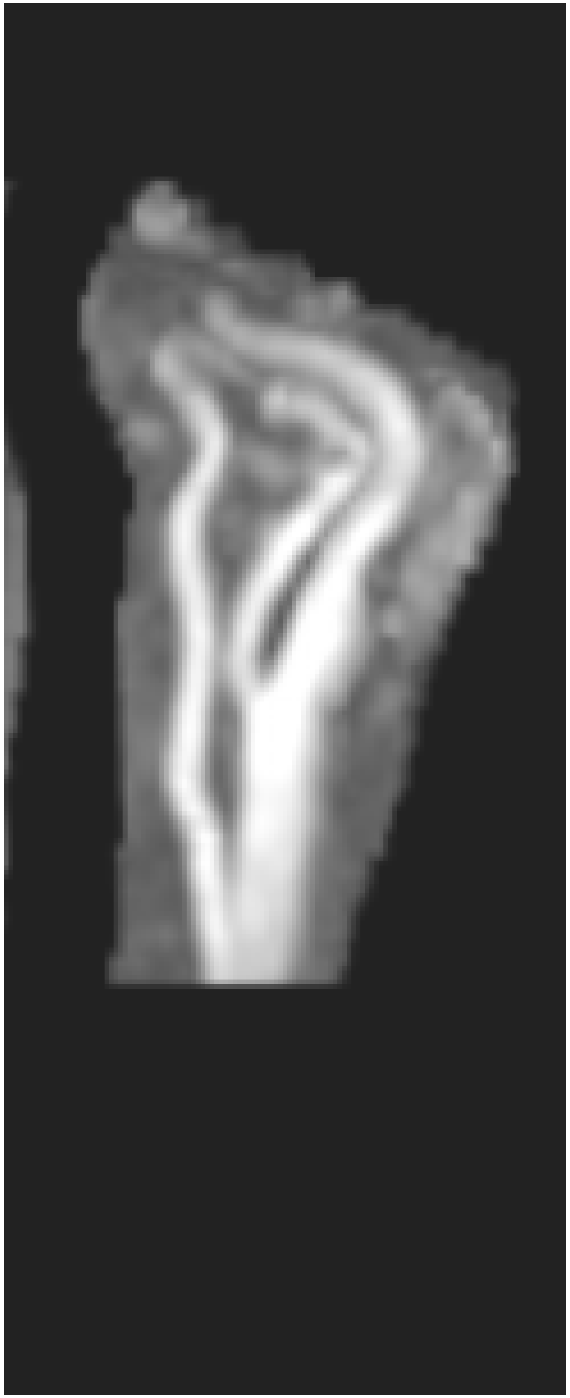

18c Score

0-30

31-50

51-70

>70

Near occlusion

Occluded

Quality

1

2

3

4

5

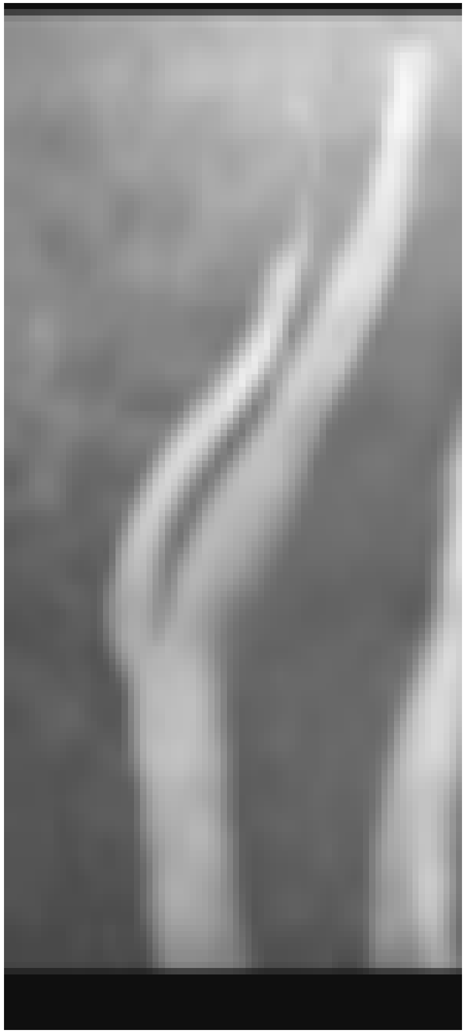

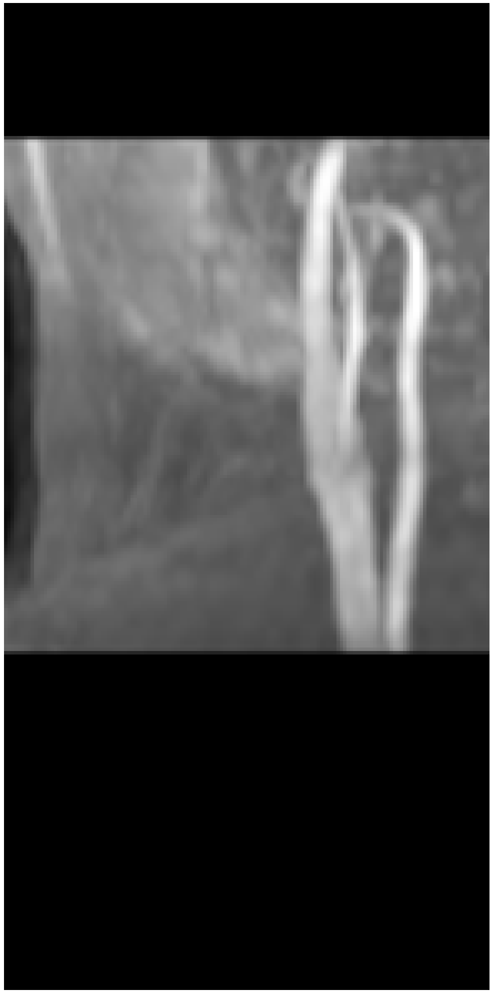

19b Score

0-30

31-50

51-70

>70

Near occlusion

Occluded

Quality

1

2

3

4

5

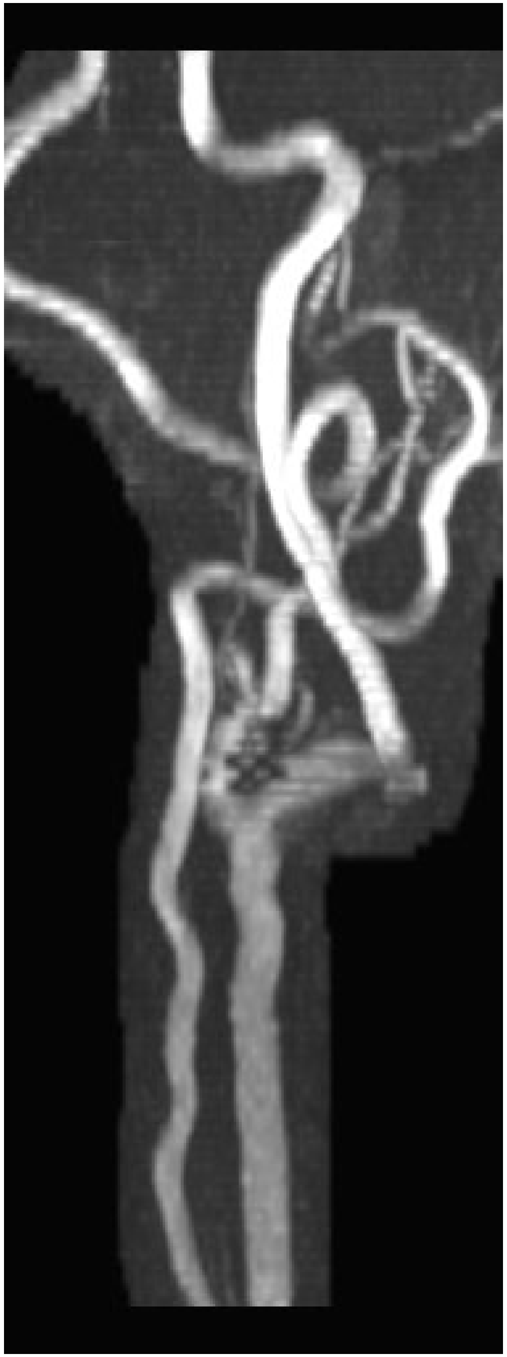

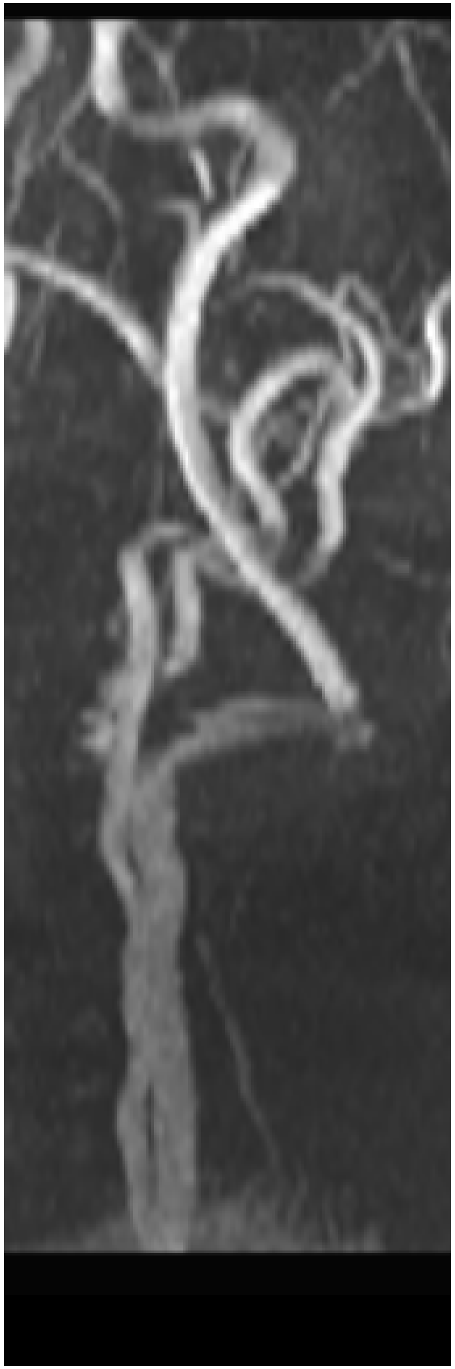

20a Score

0-30

31-50

51-70

>70

Near occlusion

Occluded

Quality

1

2

3

4

5

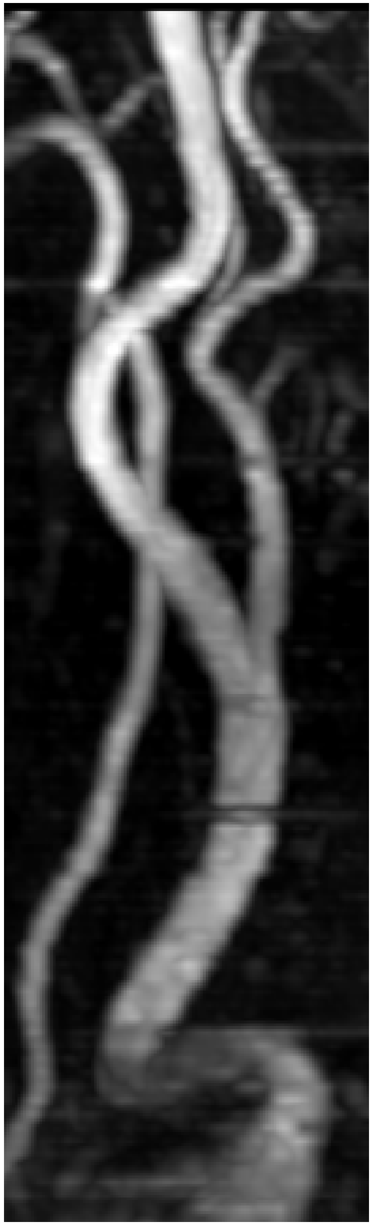

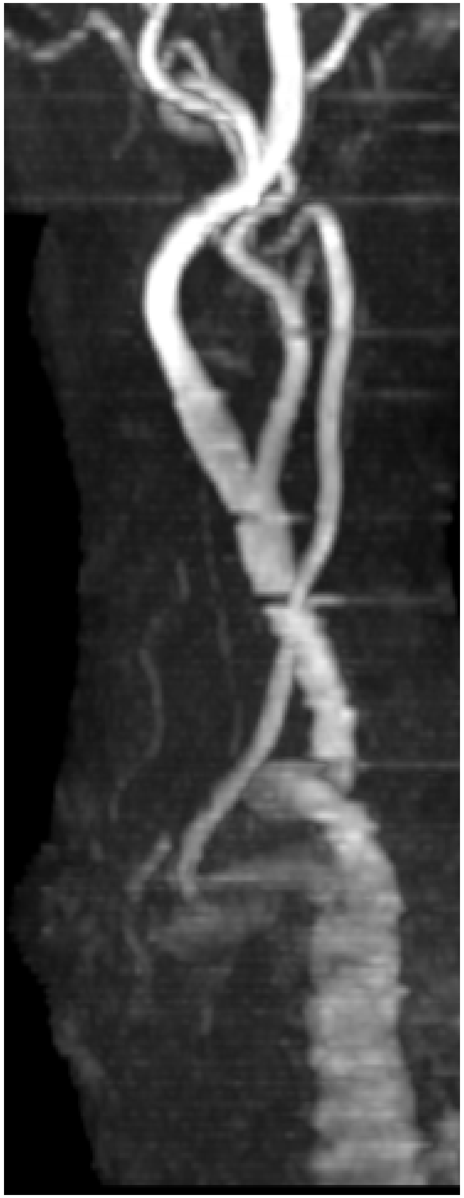

20f Score

0-30

31-50

51-70

>70

Near occlusion

Occluded

Quality

1

2

3

4

5

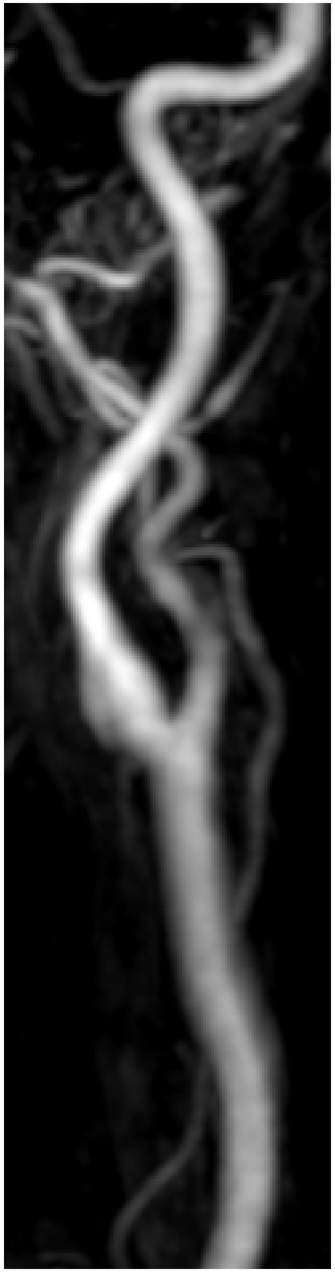

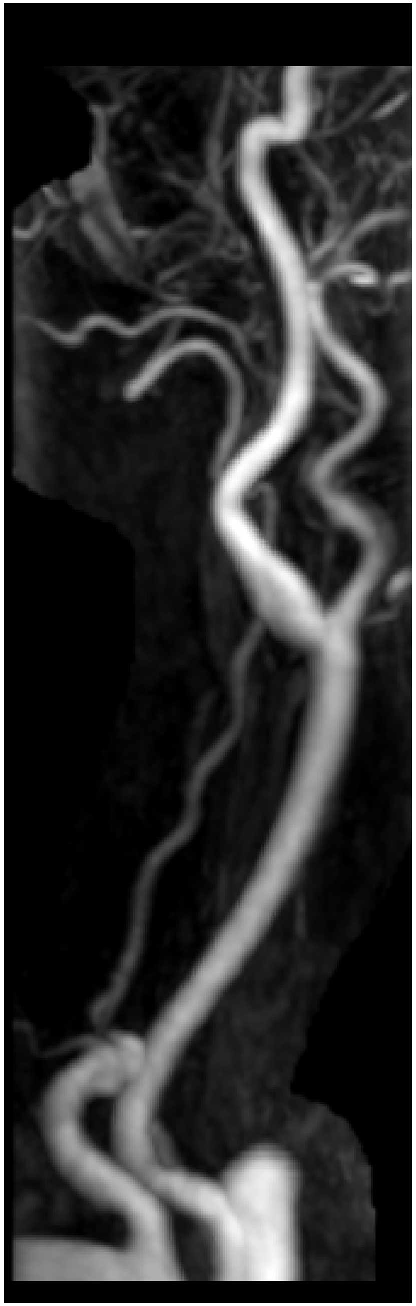

21e Score

0-30

31-50

51-70

>70

Near occlusion

Occluded

Quality

1

2

3

4

5

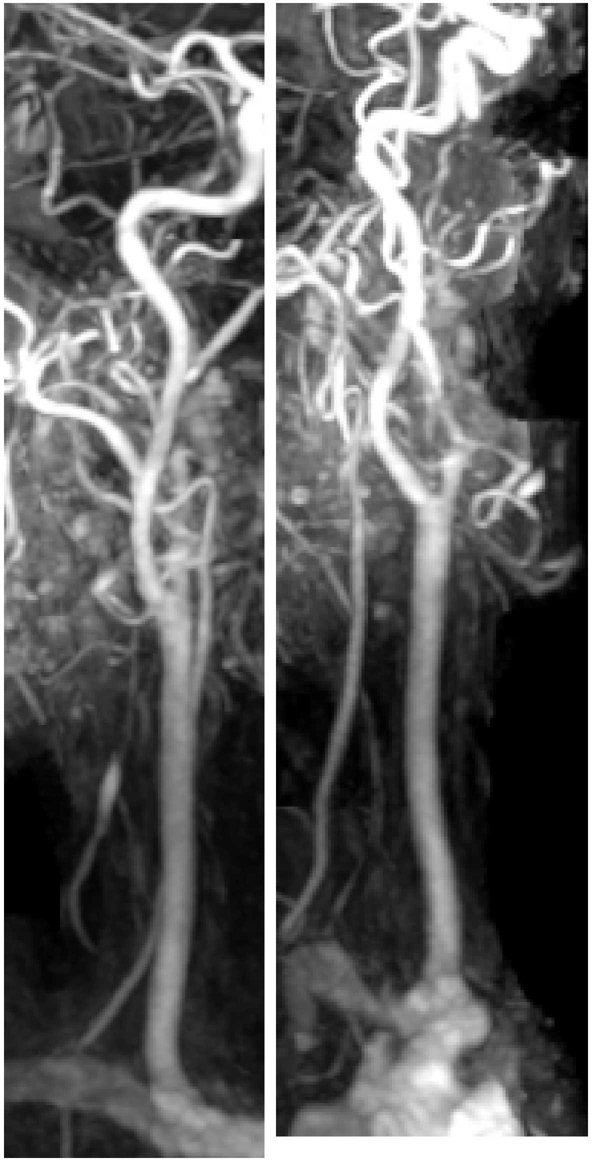

22d Score

0-30

31-50

51-70

>70

Near occlusion

Occluded

Quality

1

2

3

4

5

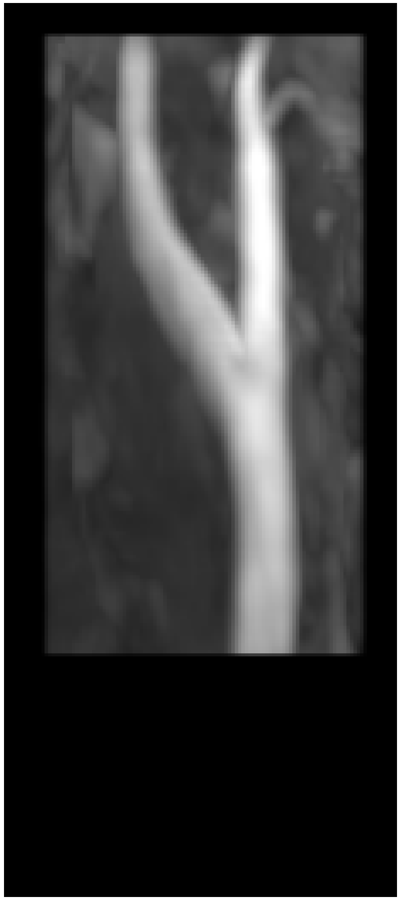

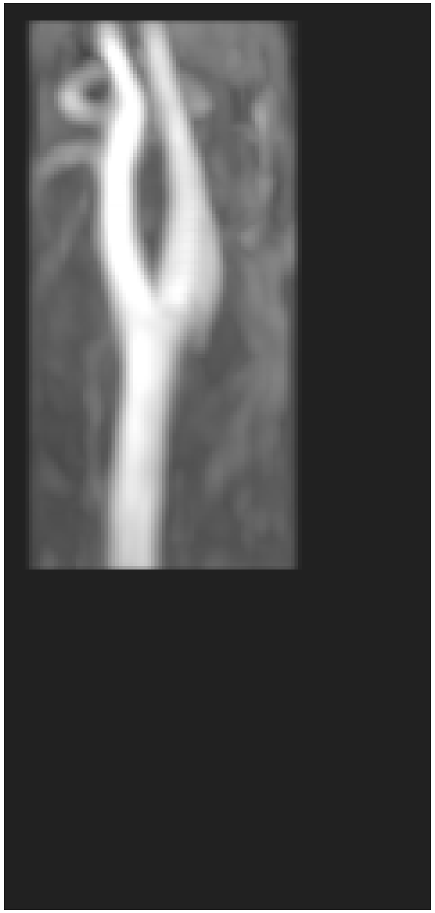

23c Score

0-30

31-50

51-70

>70

Near occlusion

Occluded

Quality

1

2

3

4

5

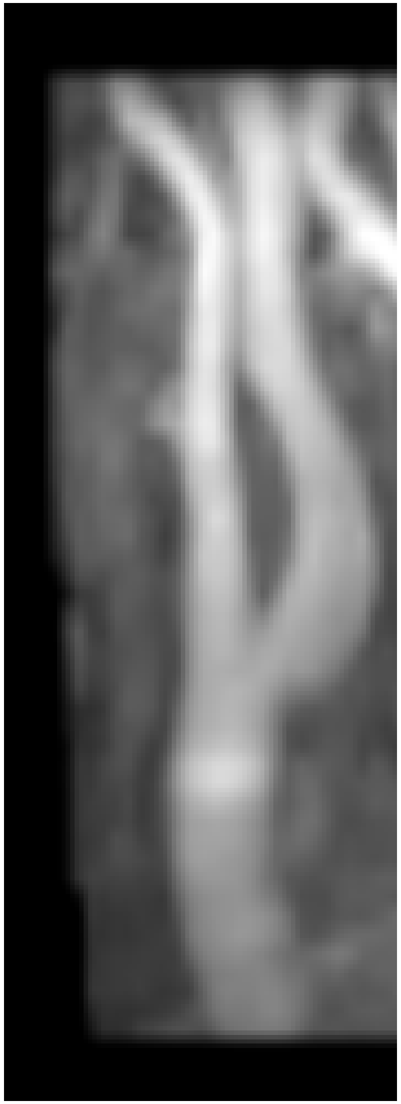

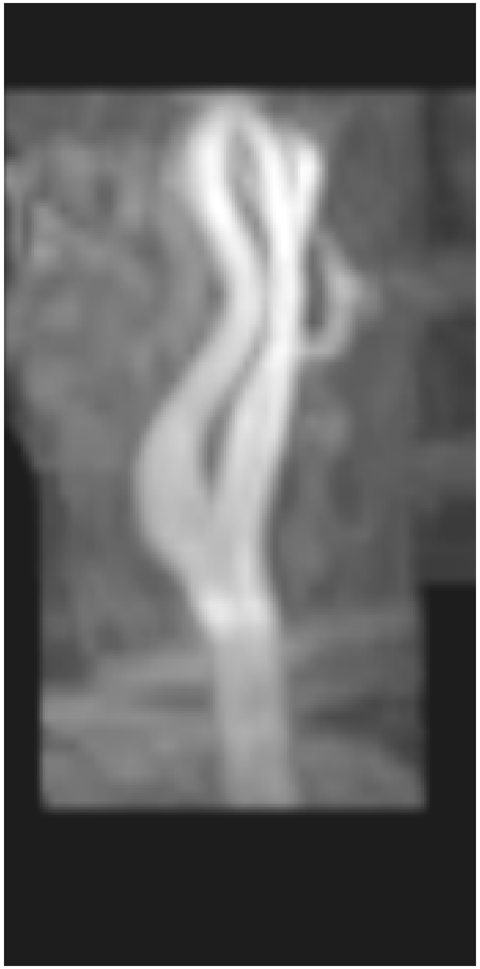

24b Score

0-30

31-50

51-70

>70

Near occlusion

Occluded

Quality

1

2

3

4

5

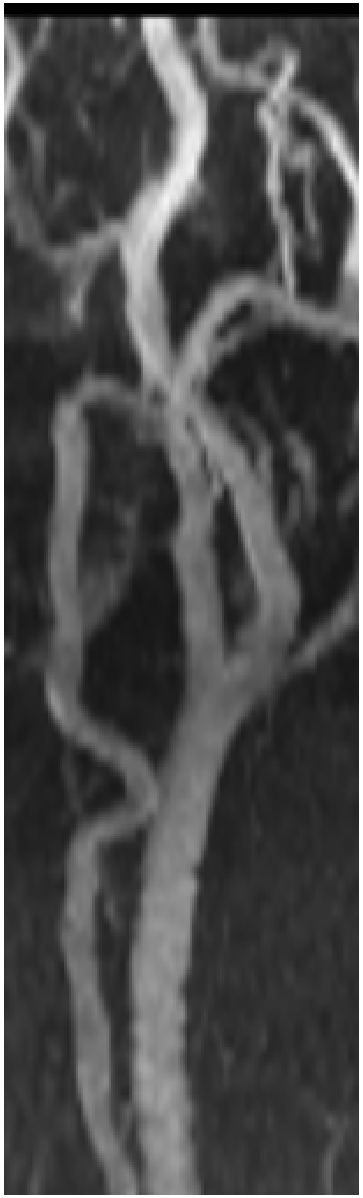

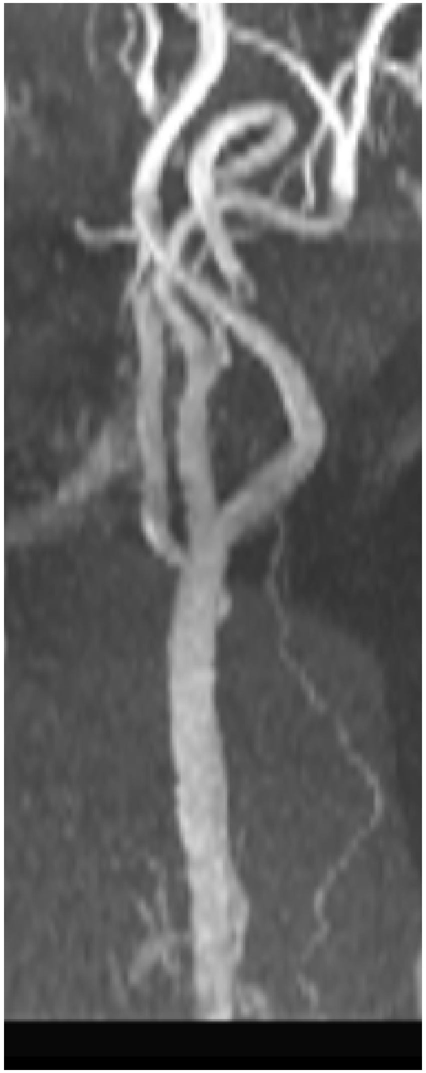

25a Score

0-30

31-50

51-70

>70

Near occlusion

Occluded

Quality

1

2

3

4

5

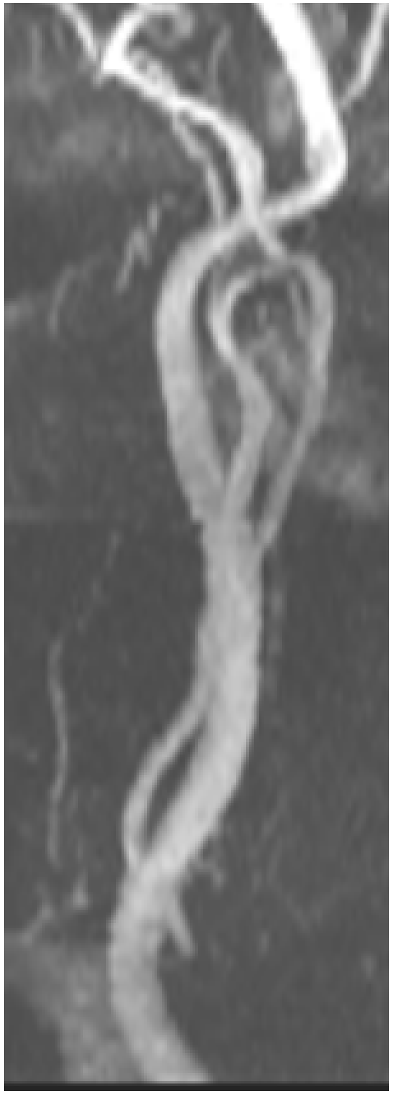

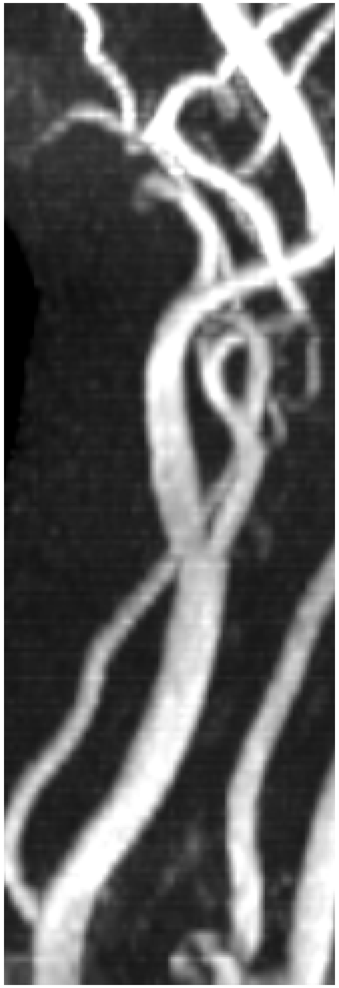

25f Score

0-30

31-50

51-70

>70

Near occlusion

Occluded

Quality

1

2

3

4

5

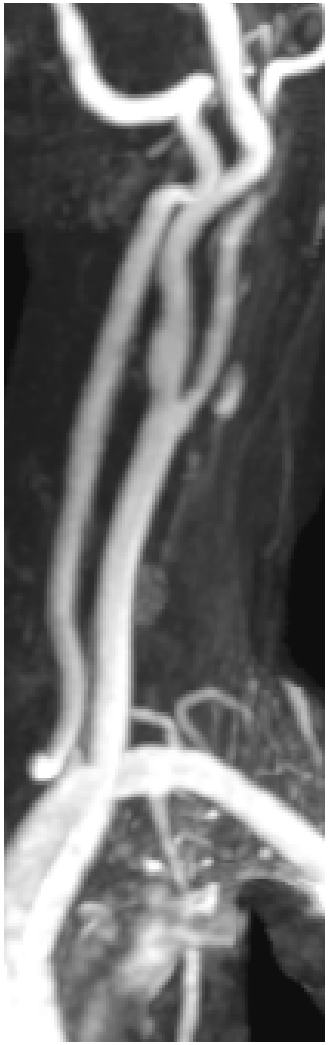

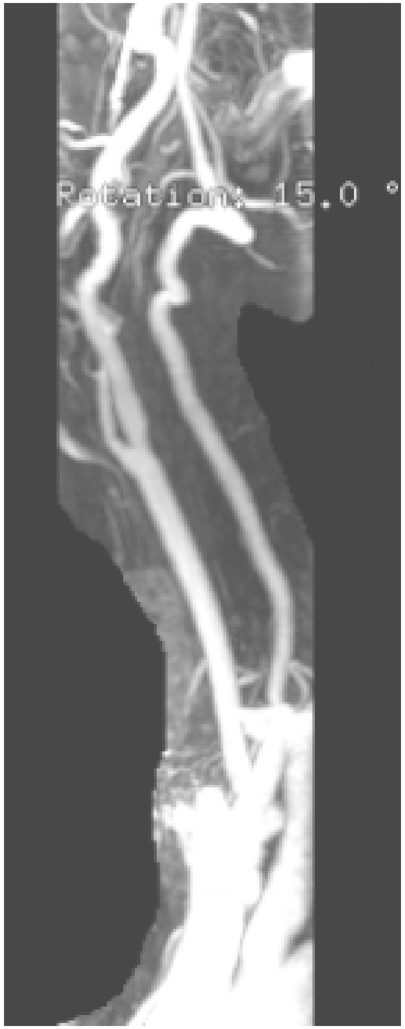

26e Score

0-30

31-50

51-70

>70

Near occlusion

Occluded

Quality

1

2

3

4

5

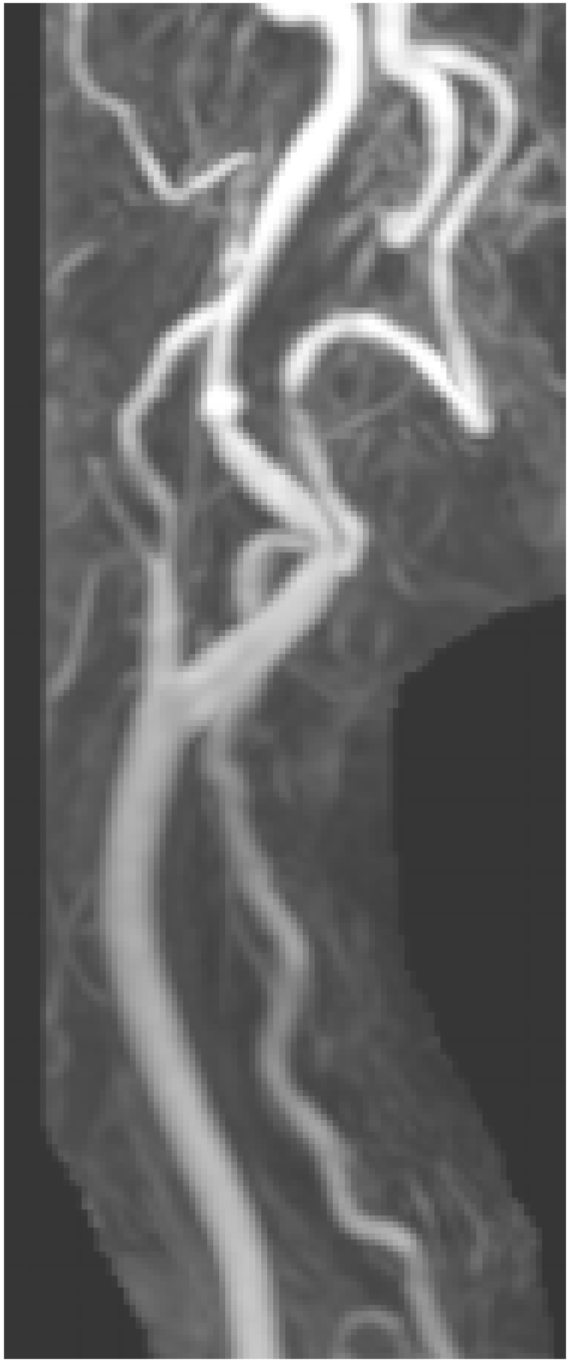

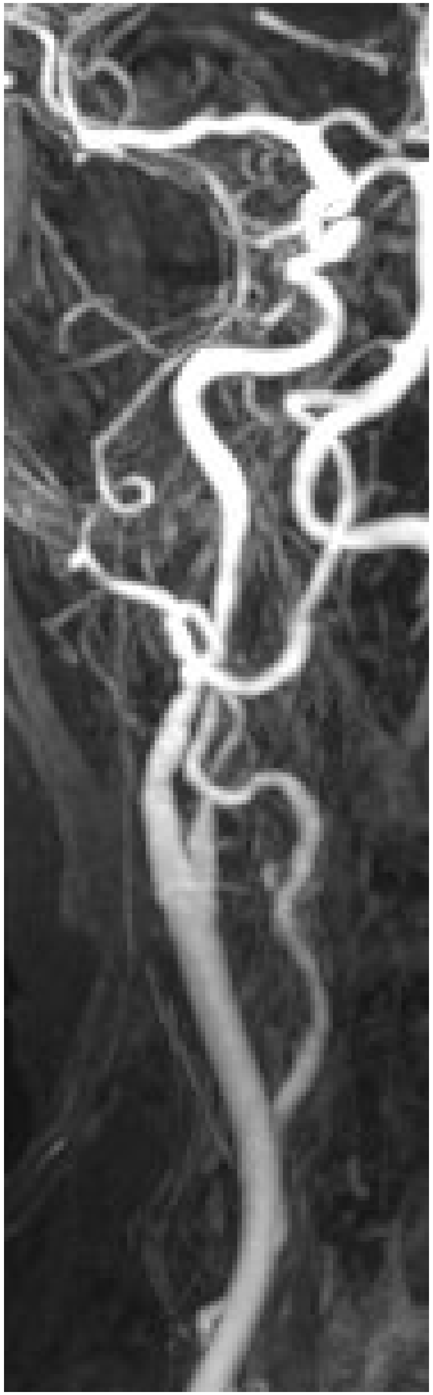

27d Score

0-30

31-50

51-70

>70

Near occlusion

Occluded

Quality

1

2

3

4

5

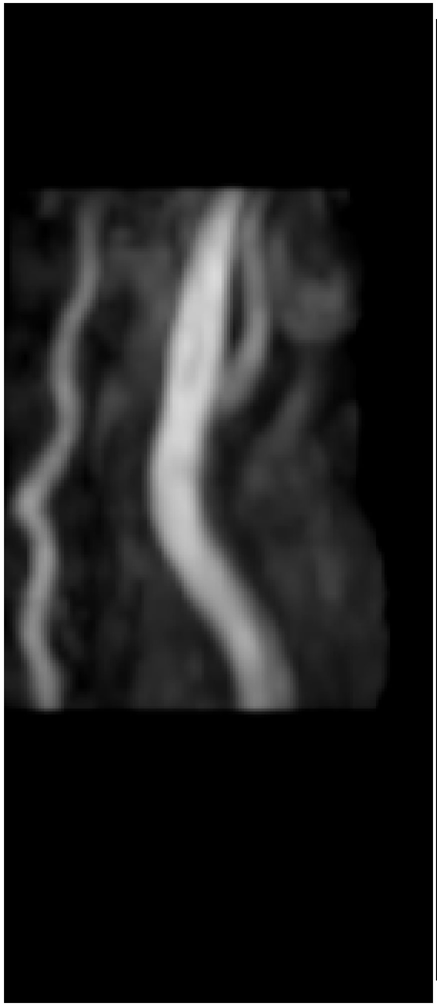

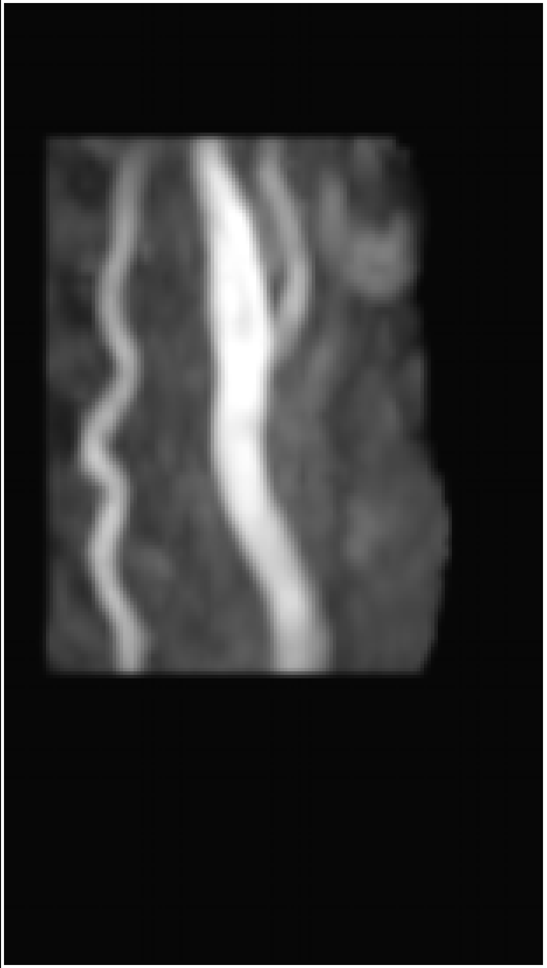

28c Score

0-30

31-50

51-70

>70

Near occlusion

Occluded

Quality

1

2

3

4

5

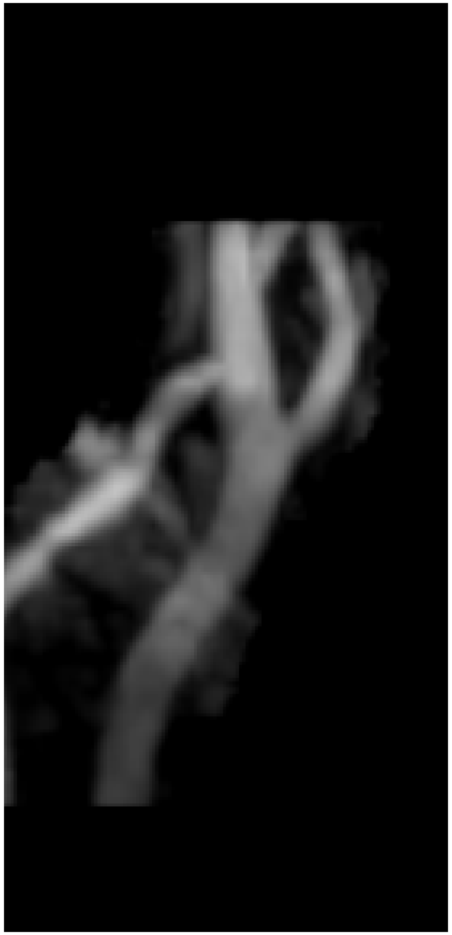

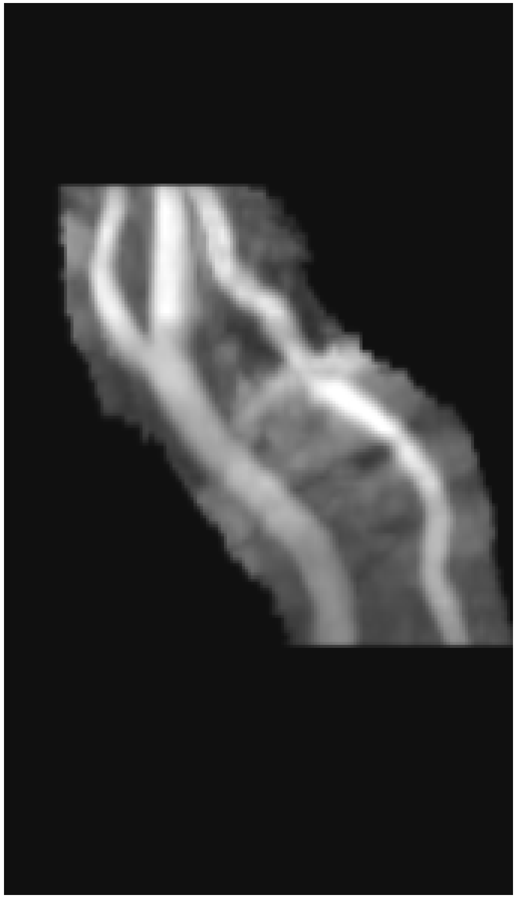

29b Score

0-30

31-50

51-70

>70

Near occlusion

Occluded

Quality

1

2

3

4

5

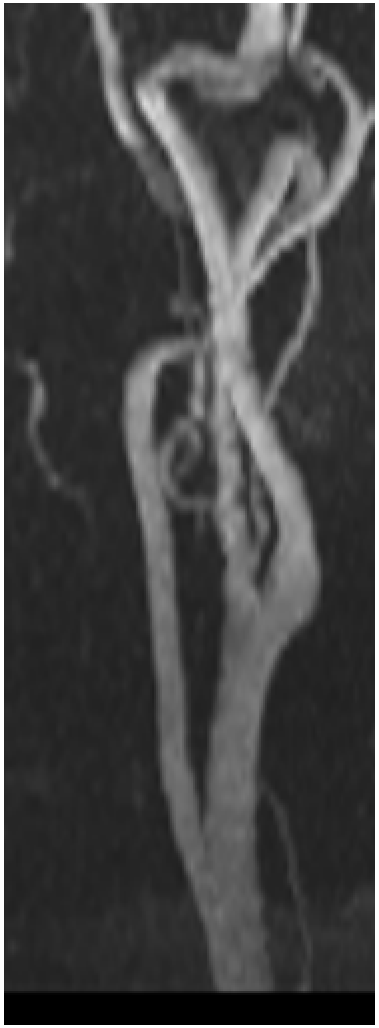

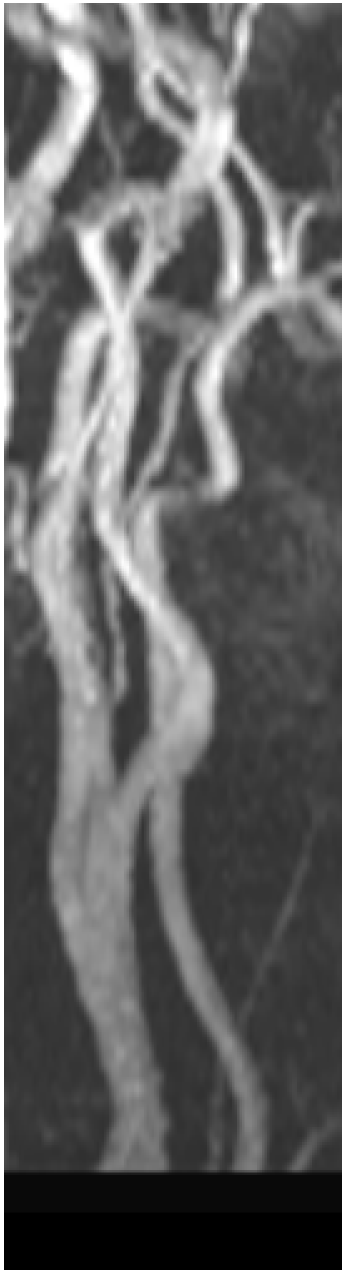

30a Score

0-30

31-50

51-70

>70

Near occlusion

Occluded

Quality

1

2

3

4

5

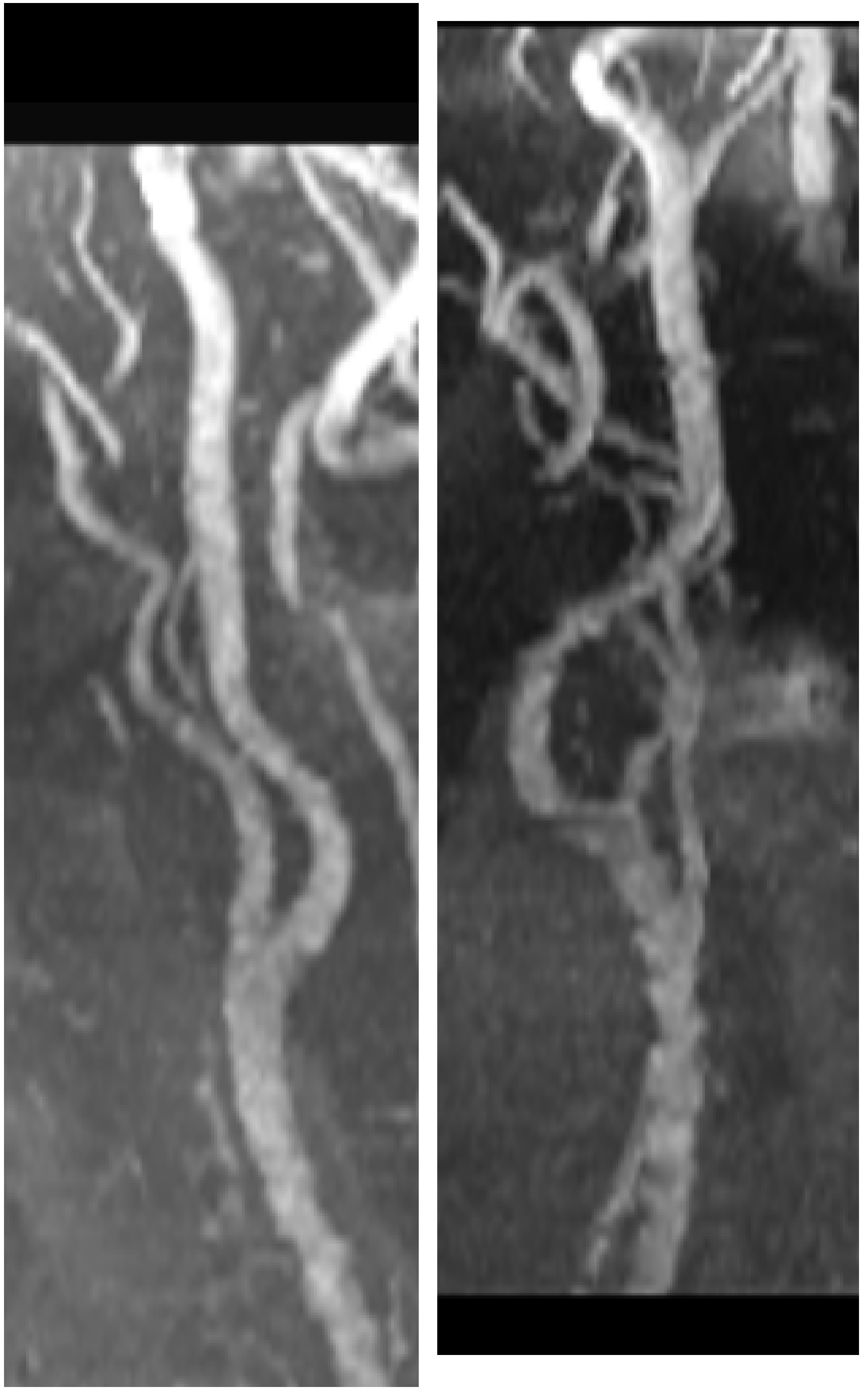

30f Score

0-30

31-50

51-70

>70

Near occlusion

Occluded

Quality

1

2

3

4

5

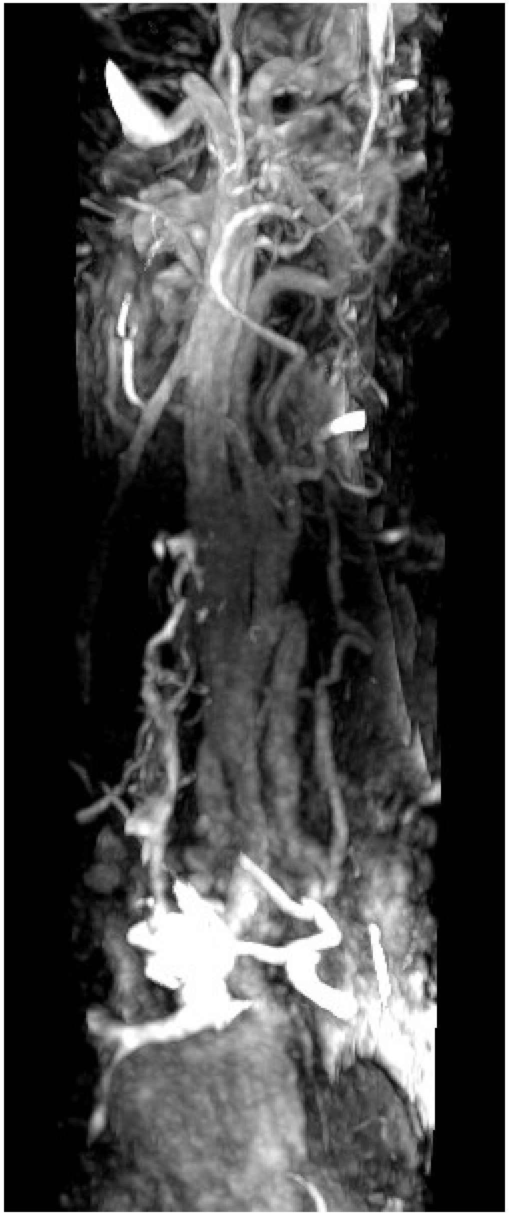

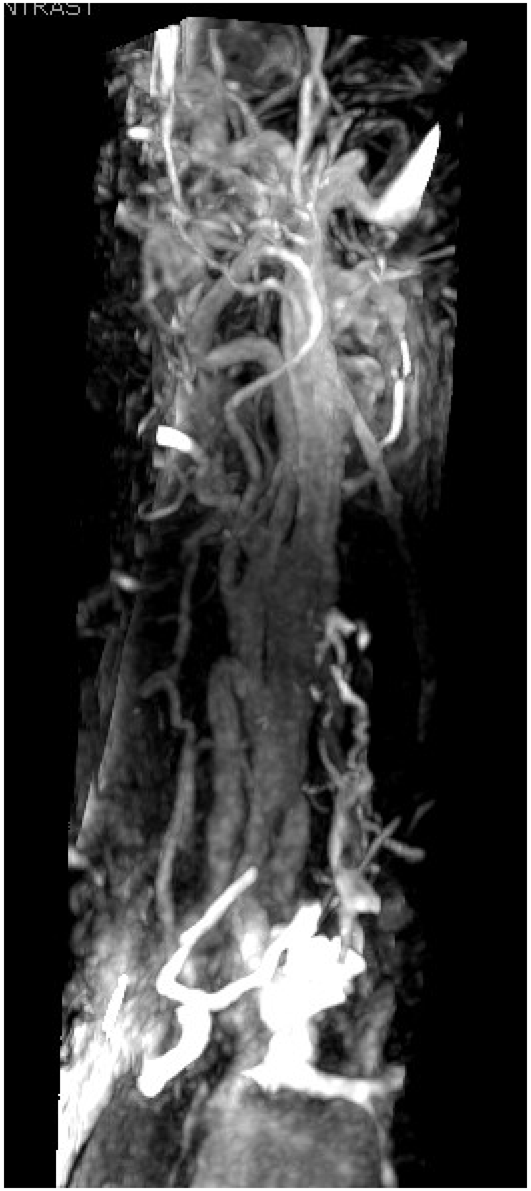

31e Score

0-30

31-50

51-70

>70

Near occlusion

Occluded

Quality

1

2

3

4

5

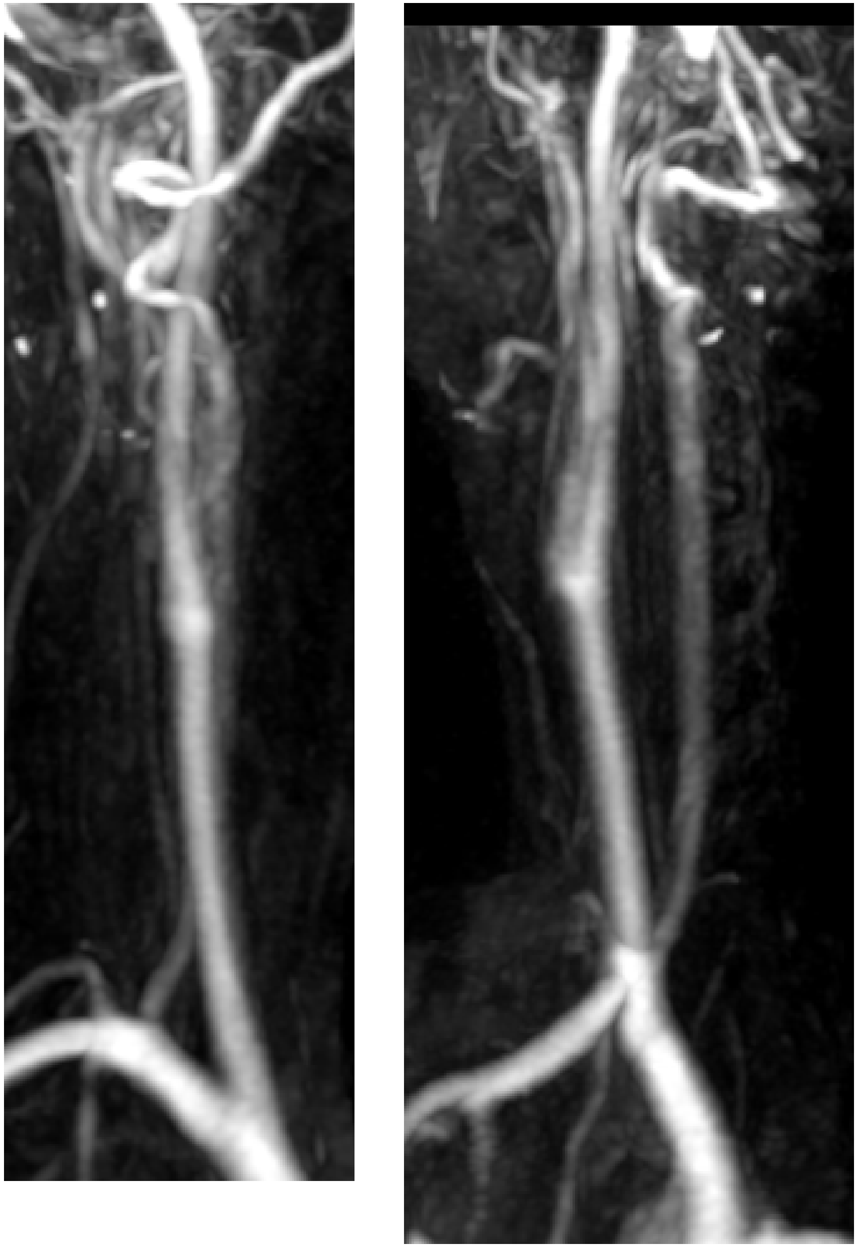

32d Score

0-30

31-50

51-70

>70

Near occlusion

Occluded

Quality

1

2

3

4

5

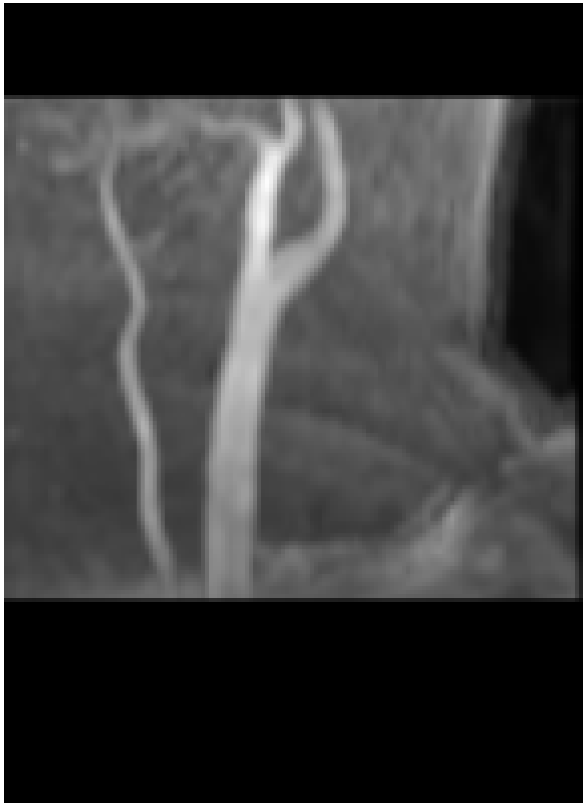

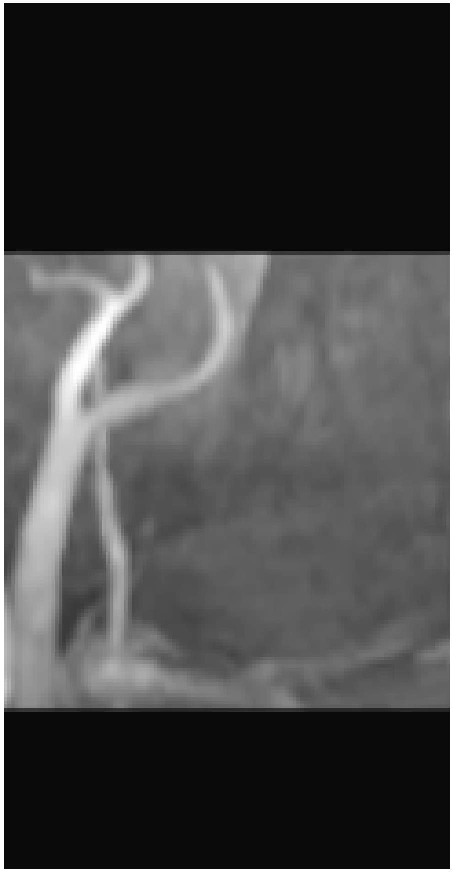

33c Score

0-30

31-50

51-70

>70

Near occlusion

Occluded

Quality

1

2

3

4

5

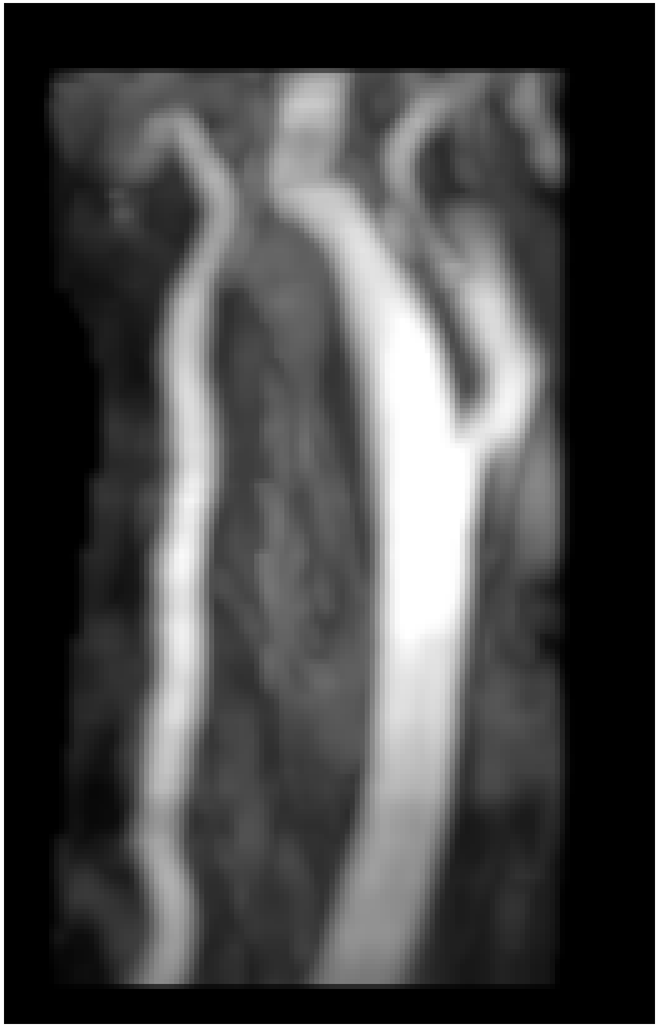

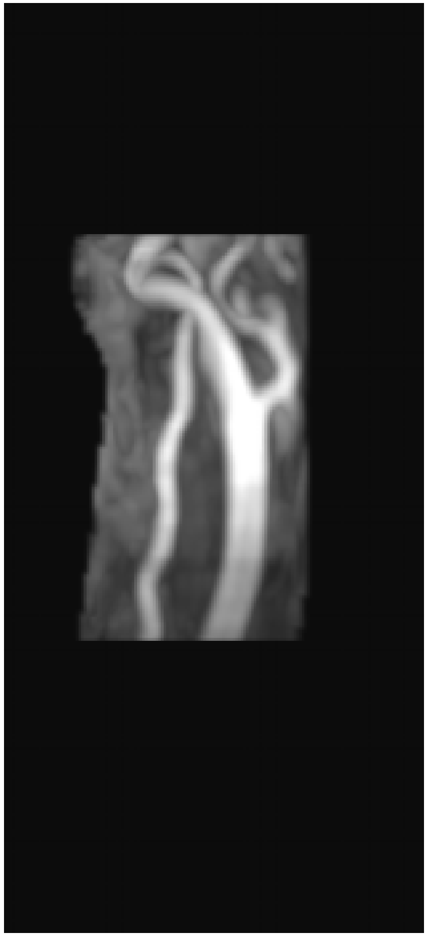

34b Score

0-30

31-50

51-70

>70

Near occlusion

Occluded

Quality

1

2

3

4

5

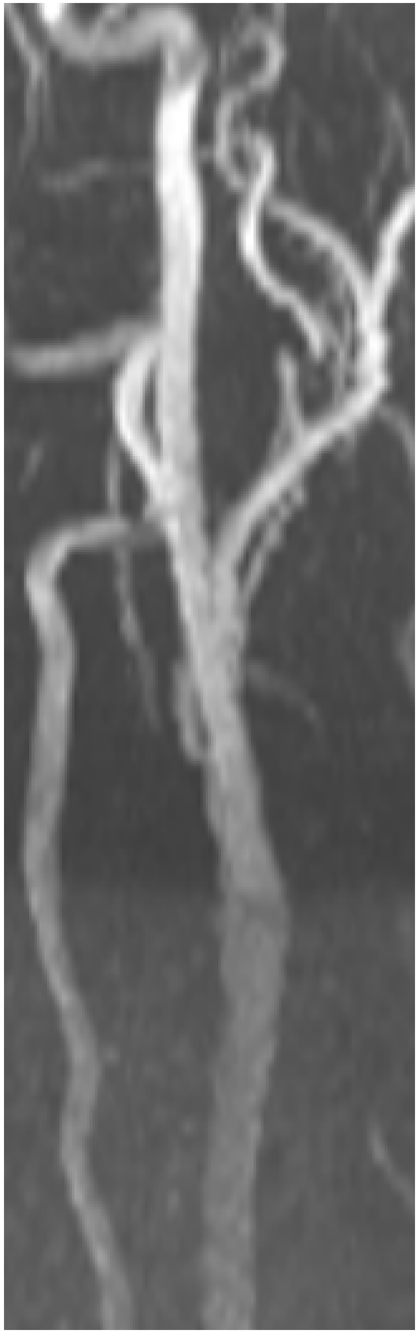

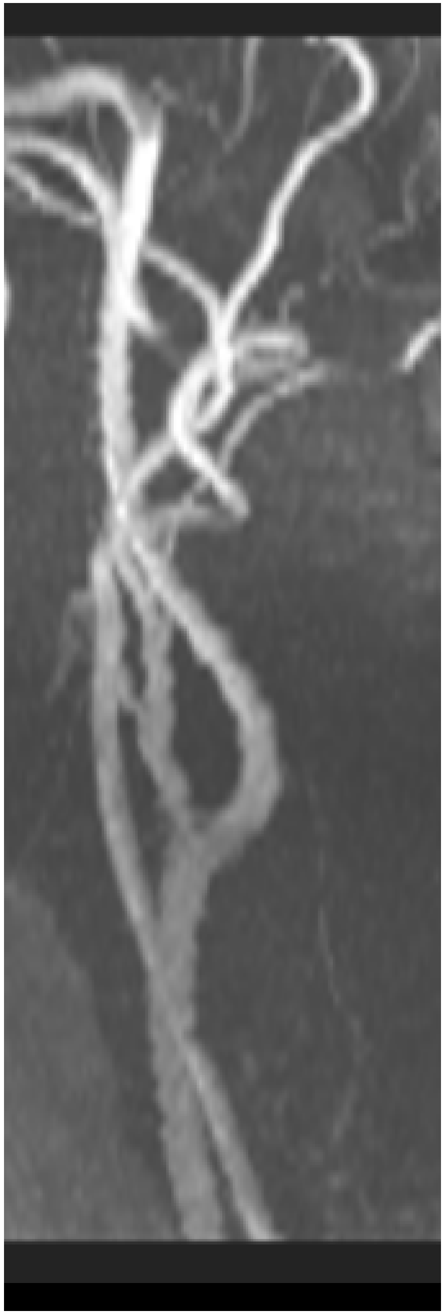

35a Score

0-30

31-50

51-70

>70

Near occlusion

Occluded

Quality

1

2

3

4

5

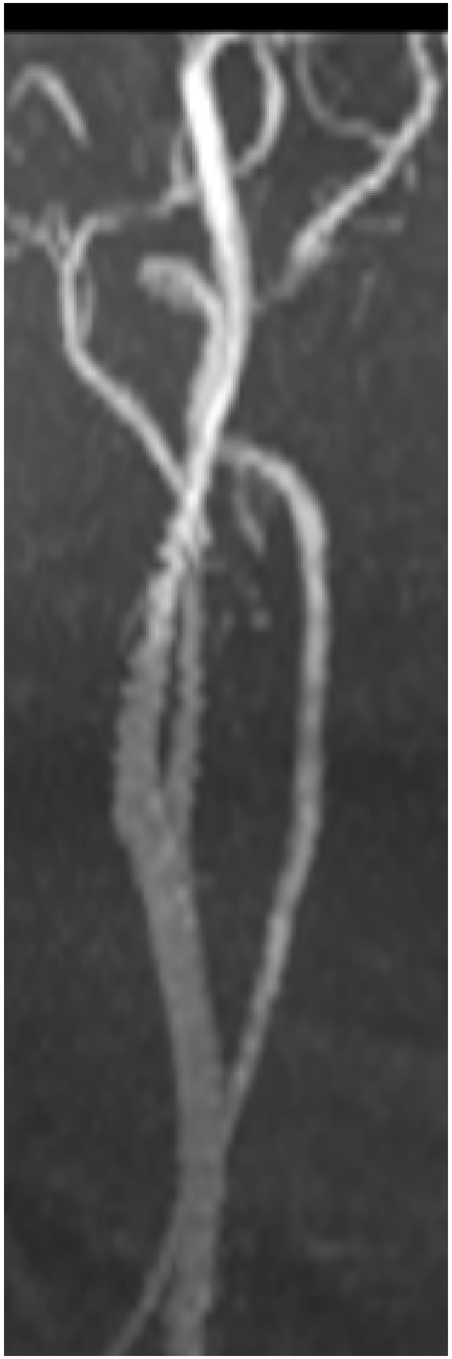

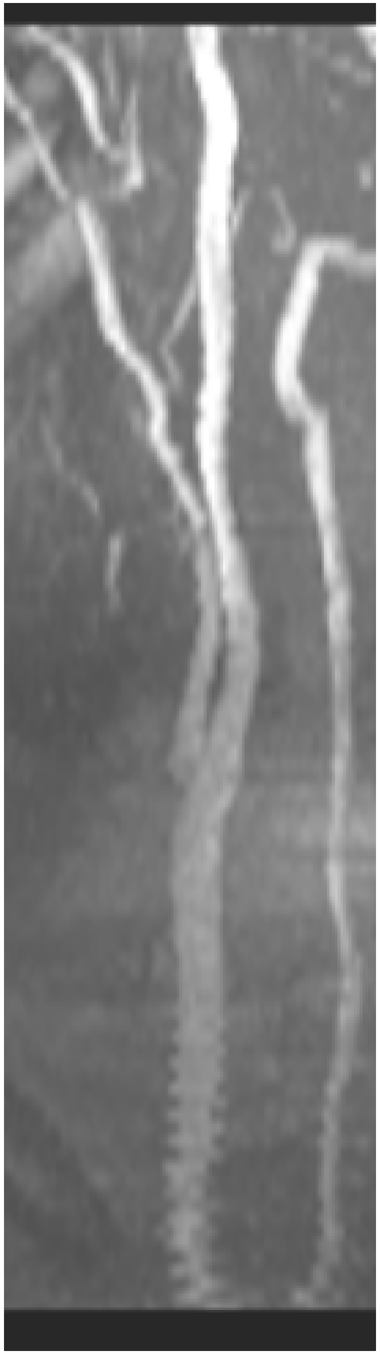

35f Score

0-30

31-50

51-70

>70

Near occlusion

Occluded

Quality

1

2

3

4

5

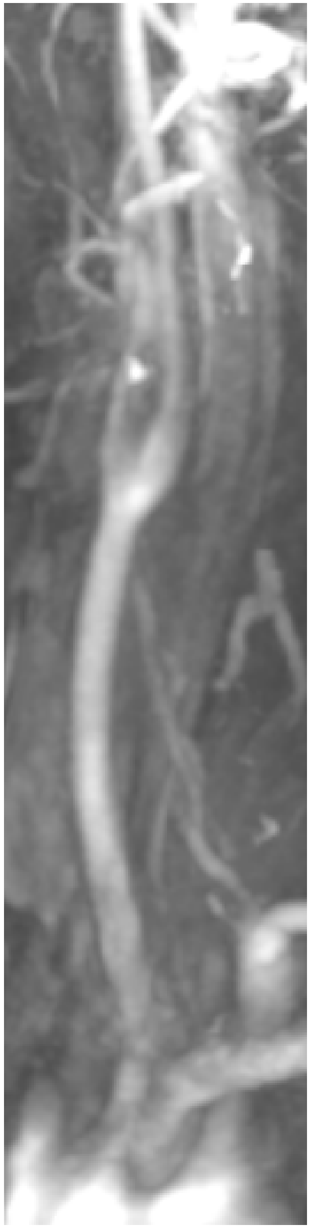

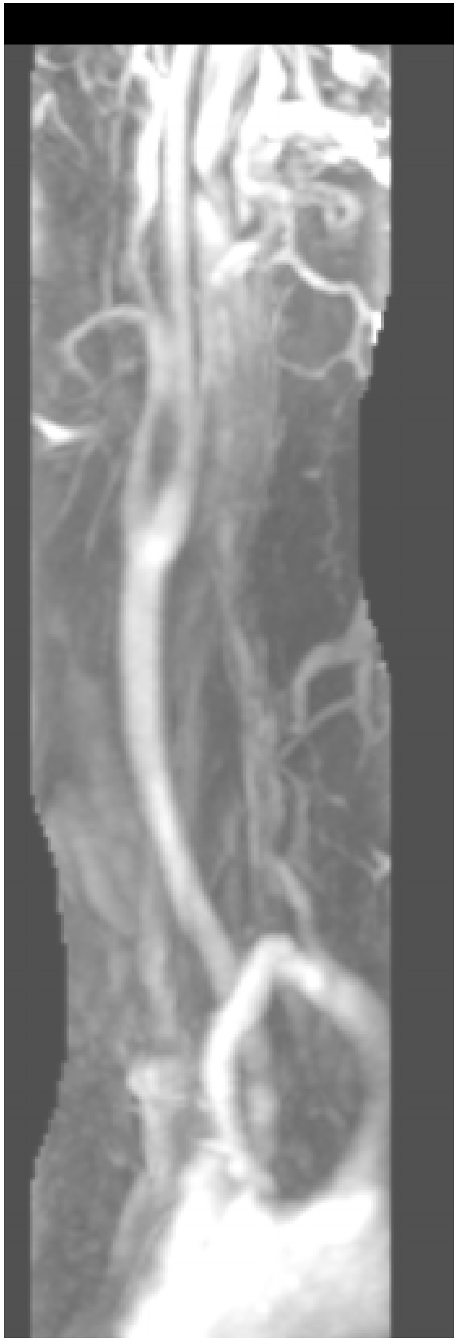

36e Score

0-30

31-50

51-70

>70

Near occlusion

Occluded

Quality

1

2

3

4

5

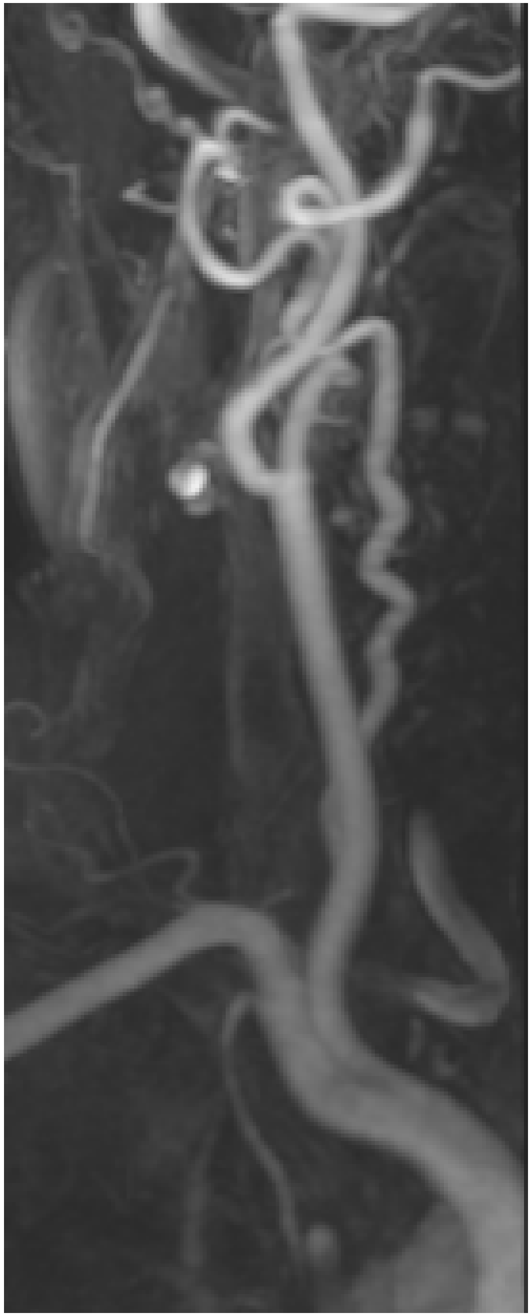

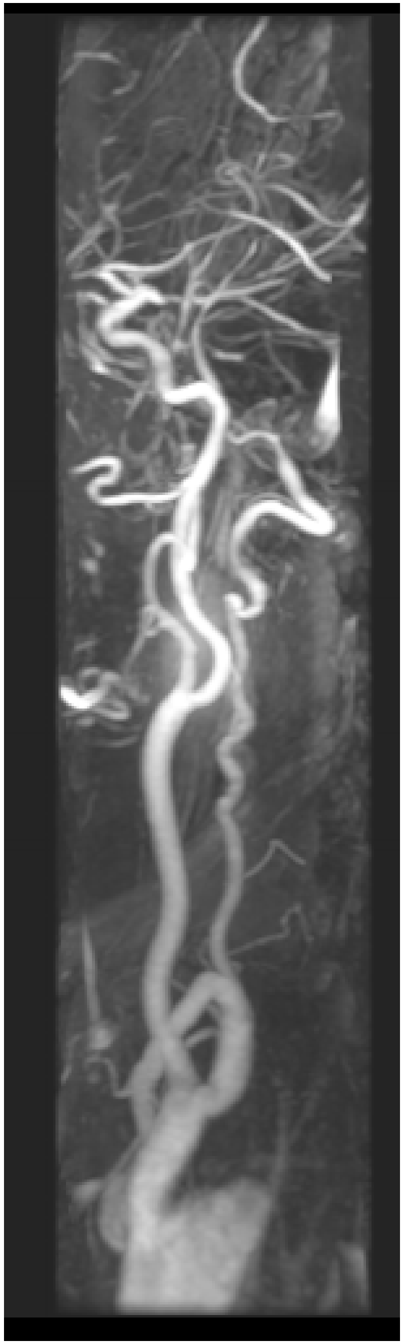

37d Score

0-30

31-50

51-70

>70

Near occlusion

Occluded

Quality

1

2

3

4

5

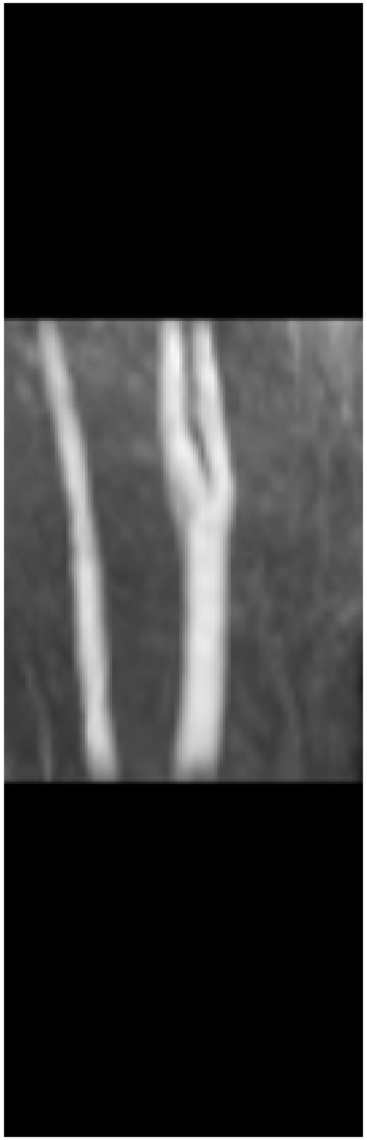

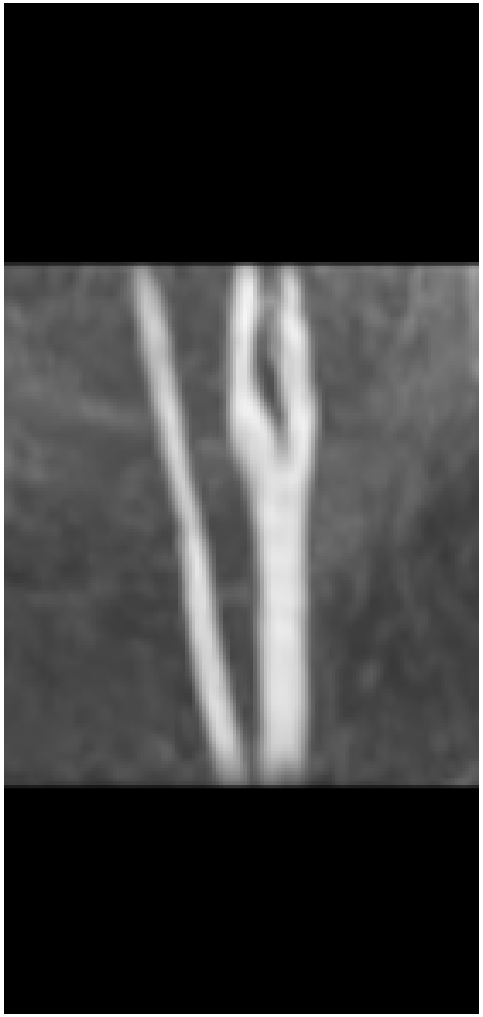

38c Score

0-30

31-50

51-70

>70

Near occlusion

Occluded

Quality

1

2

3

4

5

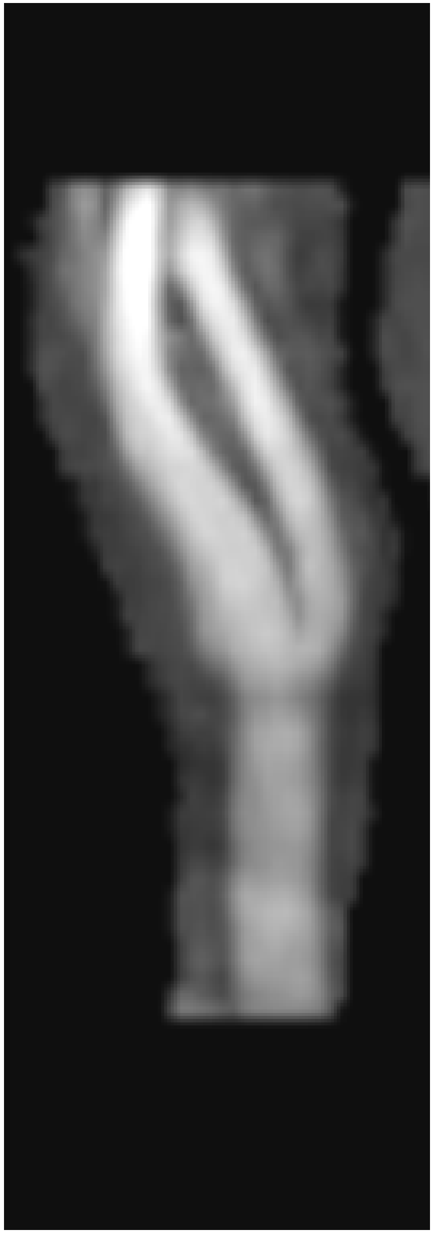

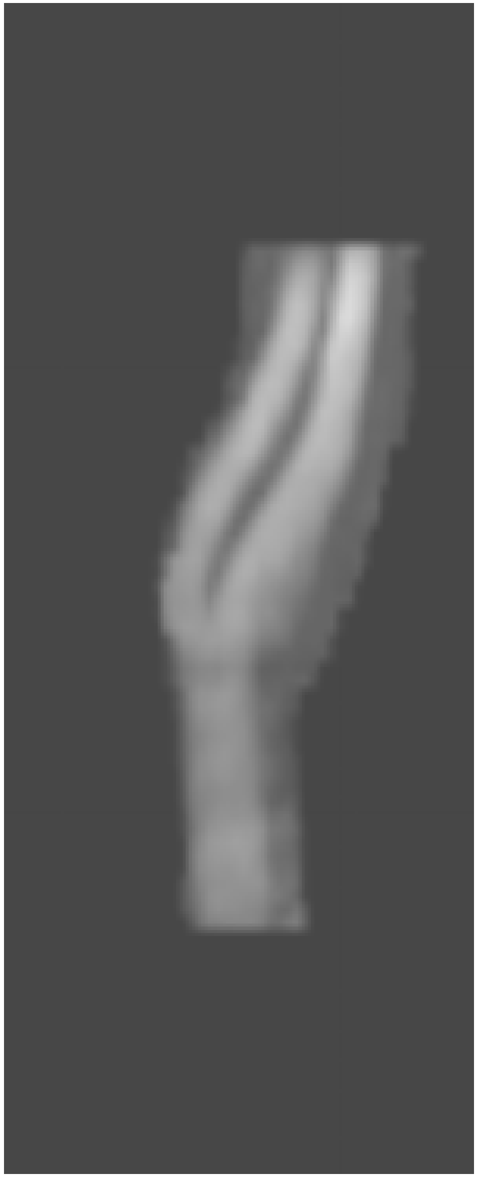

39b Score

0-30

31-50

51-70

>70

Near occlusion

Occluded

Quality

1

2

3

4

5

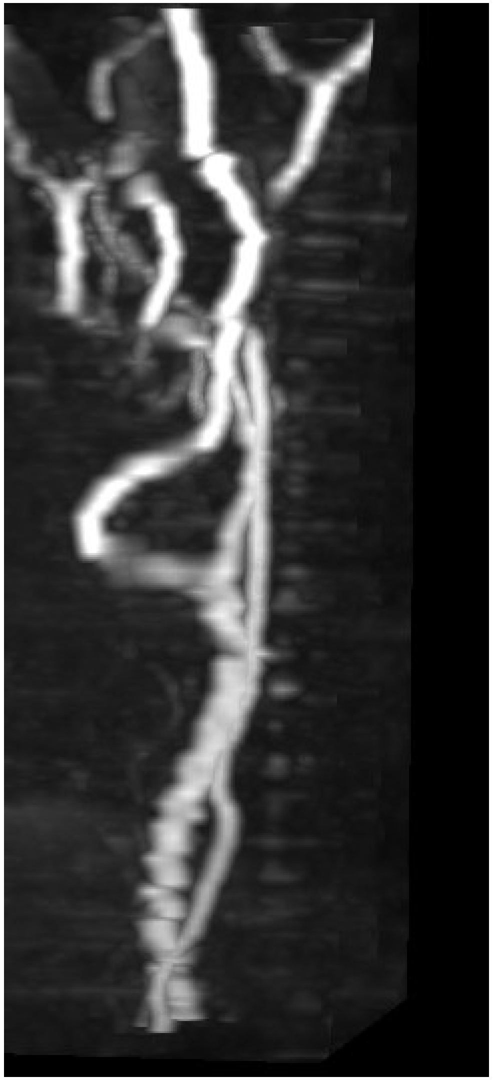

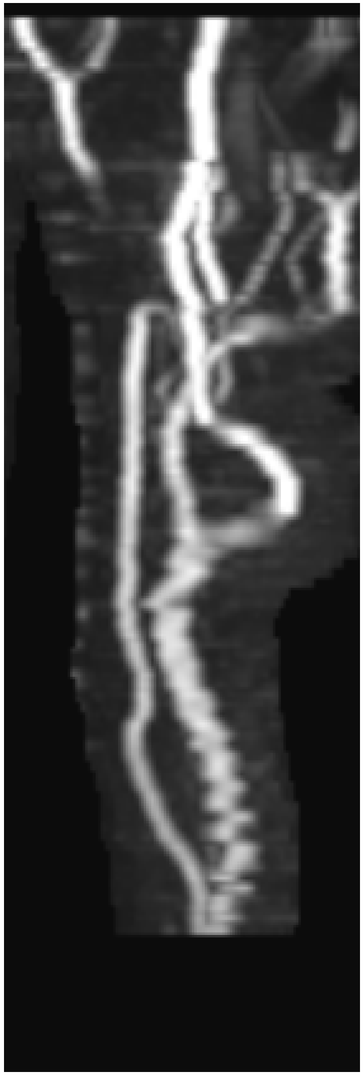

40a Score

0-30

31-50

51-70

>70

Near occlusion

Occluded

Quality

1

2

3

4

5

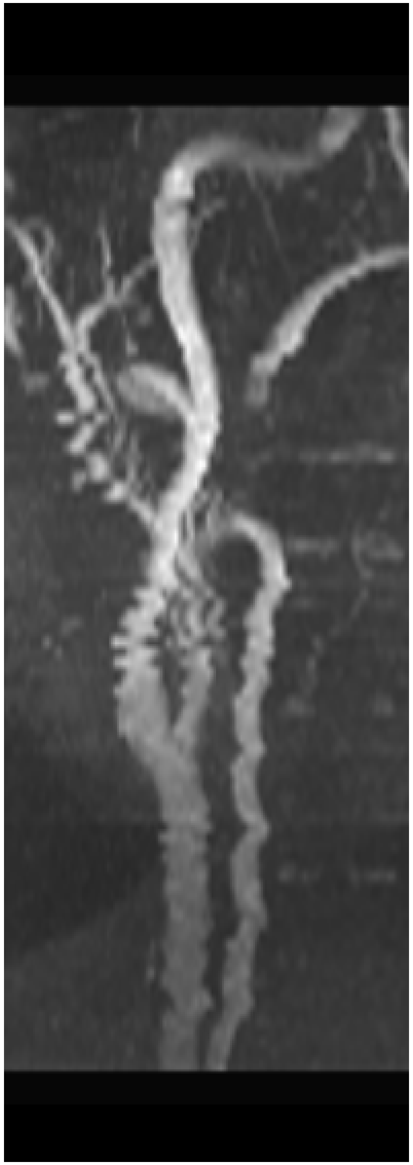

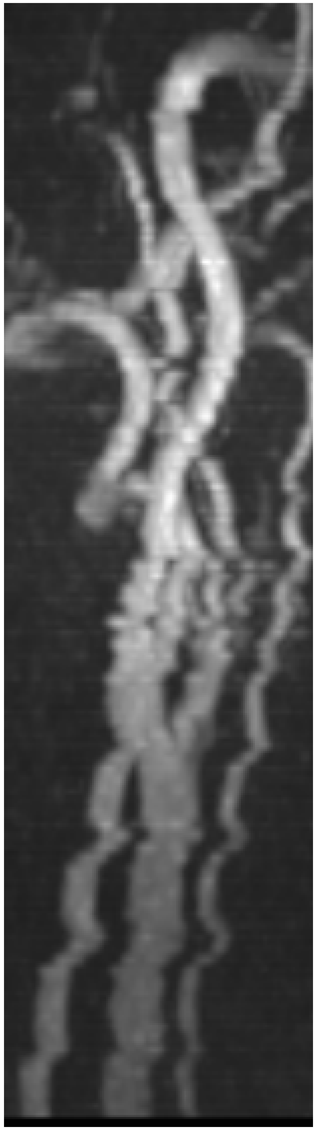

40f Score

0-30

31-50

51-70

>70

Near occlusion

Occluded

Quality

1

2

3

4

5

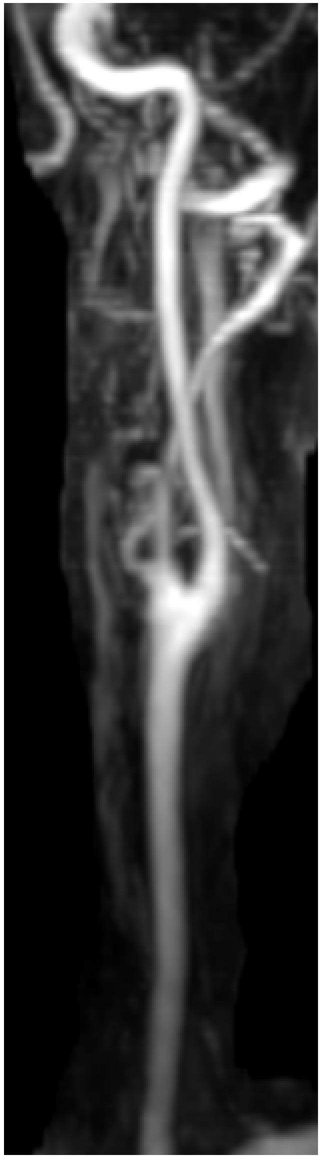

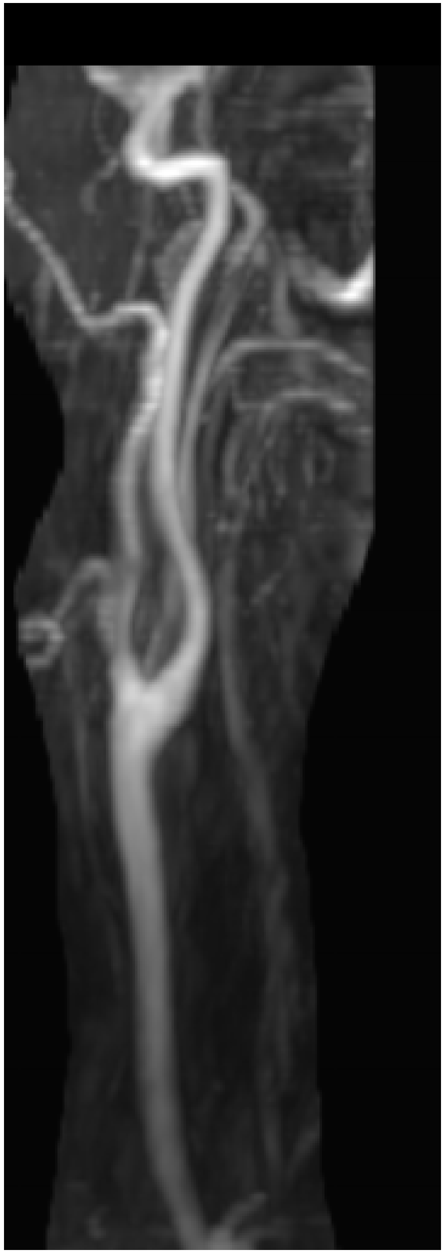

41e Score

0-30

31-50

51-70

>70

Near occlusion

Occluded

Quality

1

2

3

4

5

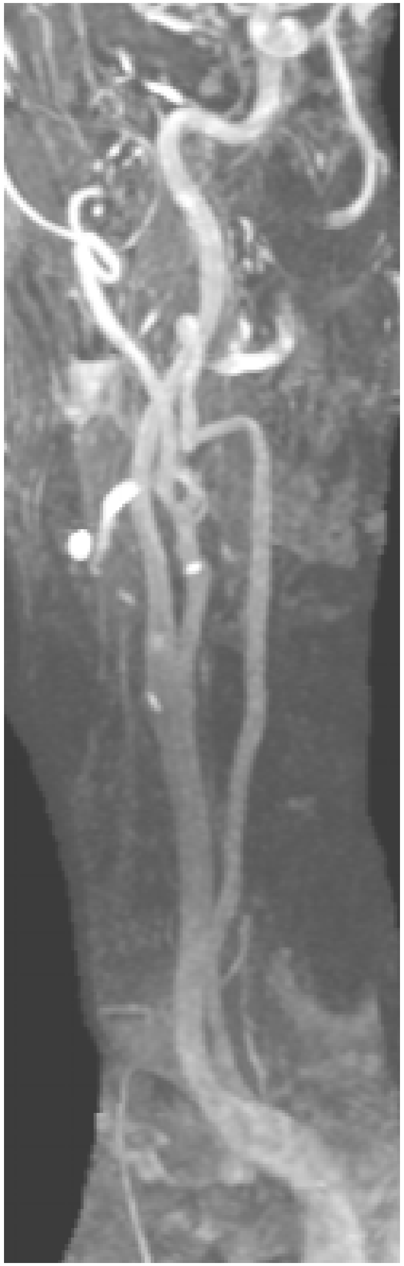

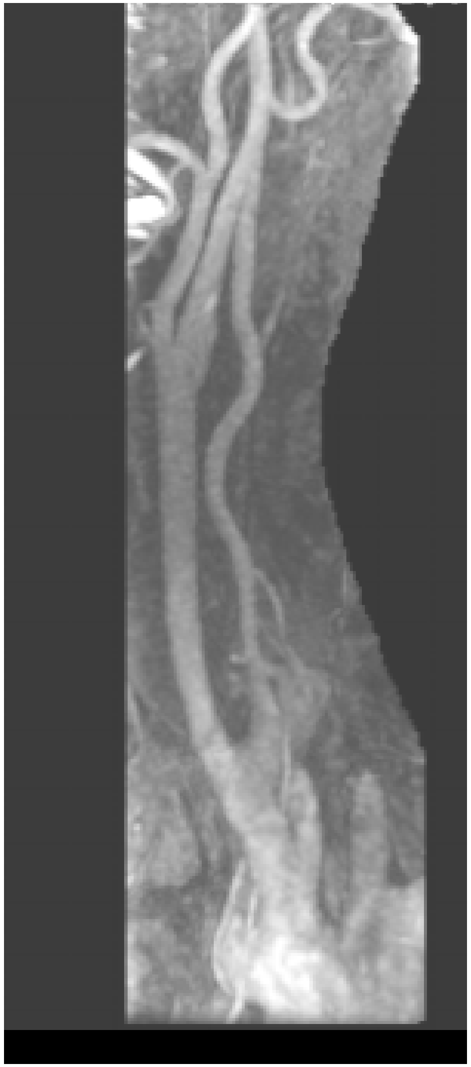

42d Score

0-30

31-50

51-70

>70

Near occlusion

Occluded

Quality

1

2

3

4

5

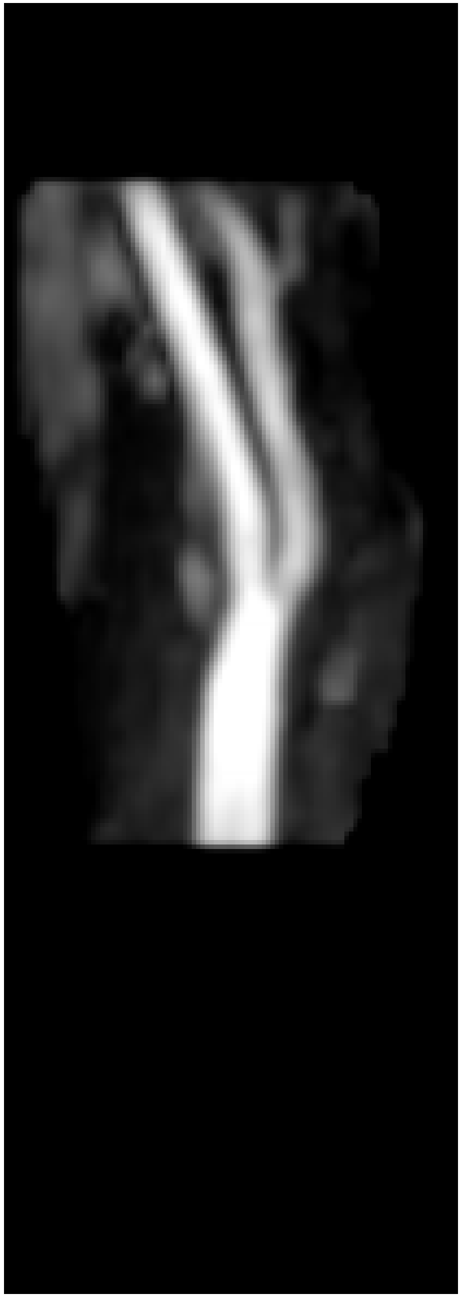

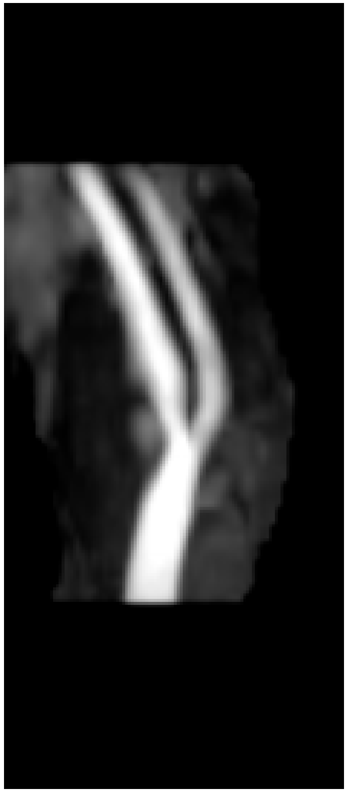

43c Score

0-30

31-50

51-70

>70

Near occlusion

Occluded

Quality

1

2

3

4

5

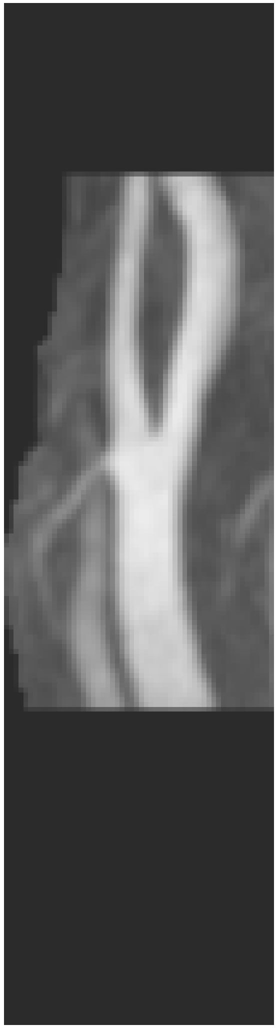

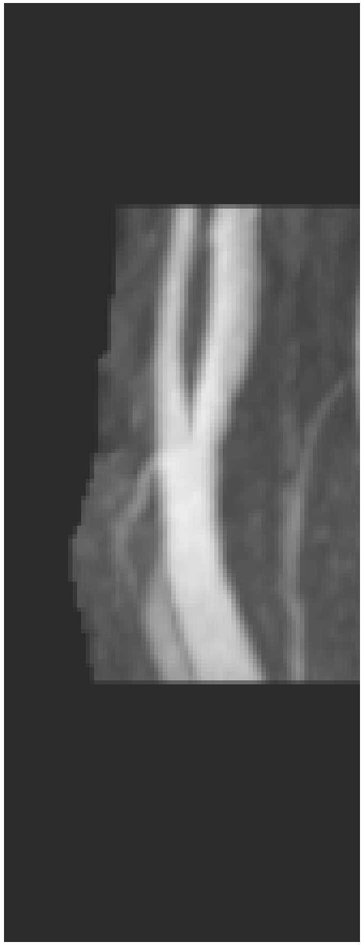

44b Score

0-30

31-50

51-70

>70

Near occlusion

Occluded

Quality

1

2

3

4

5

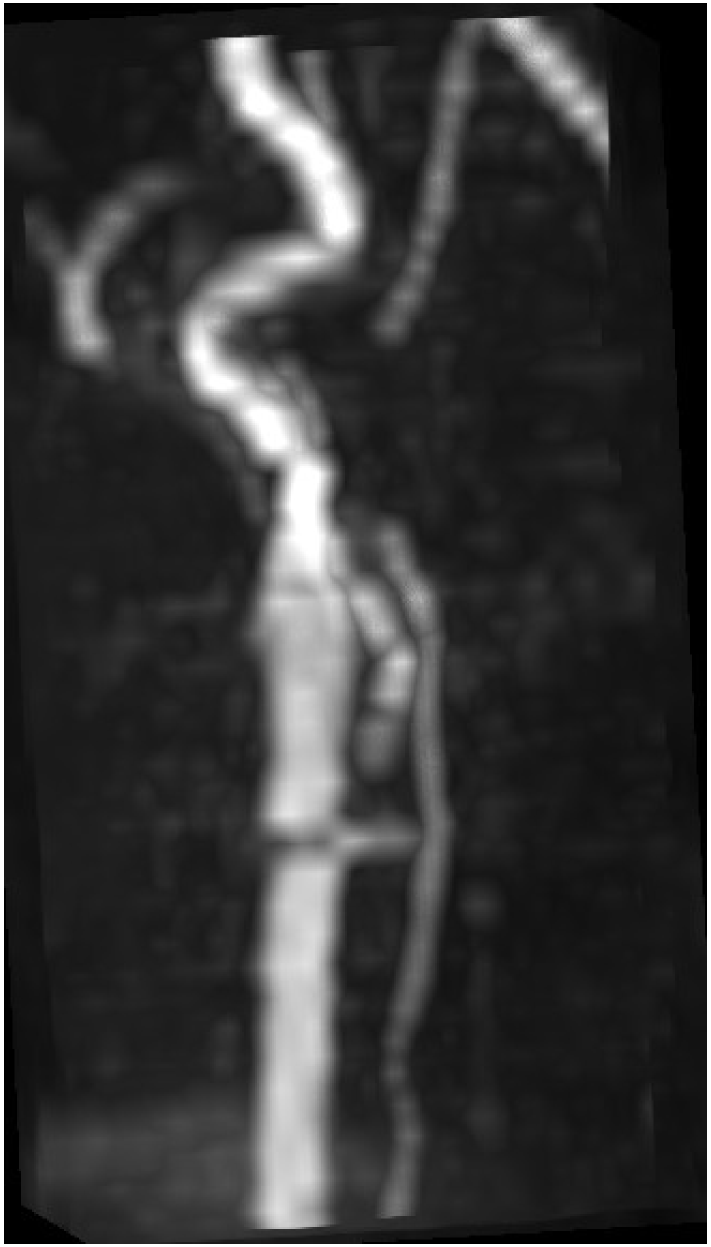

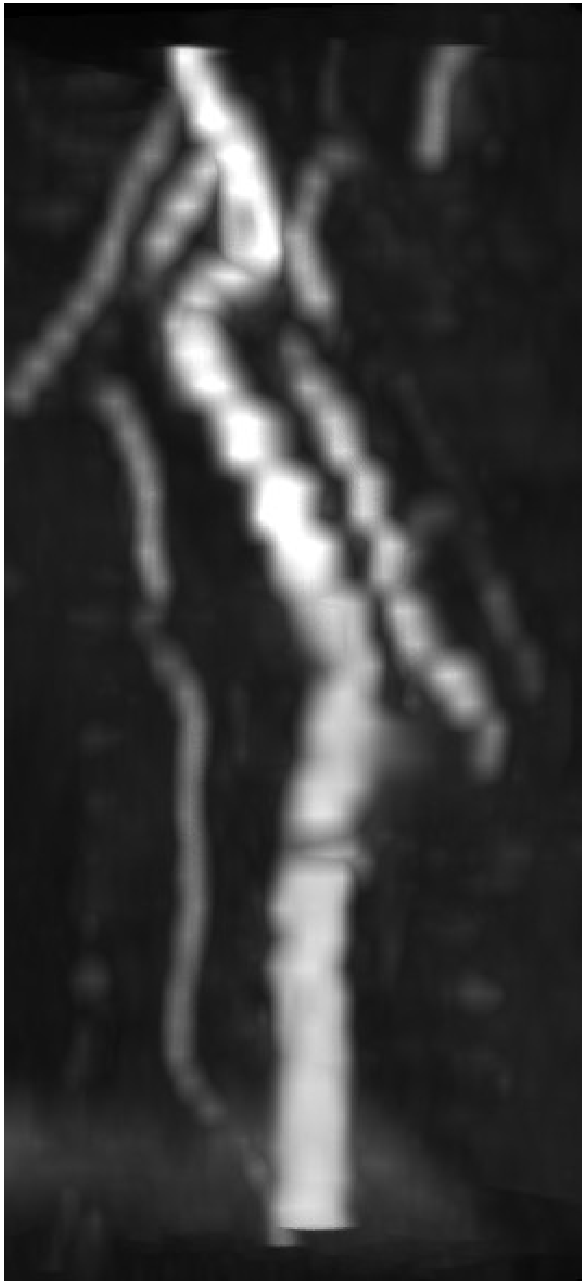

45a Score

0-30

31-50

51-70

>70

Near occlusion

Occluded

Quality

1

2

3

4

5

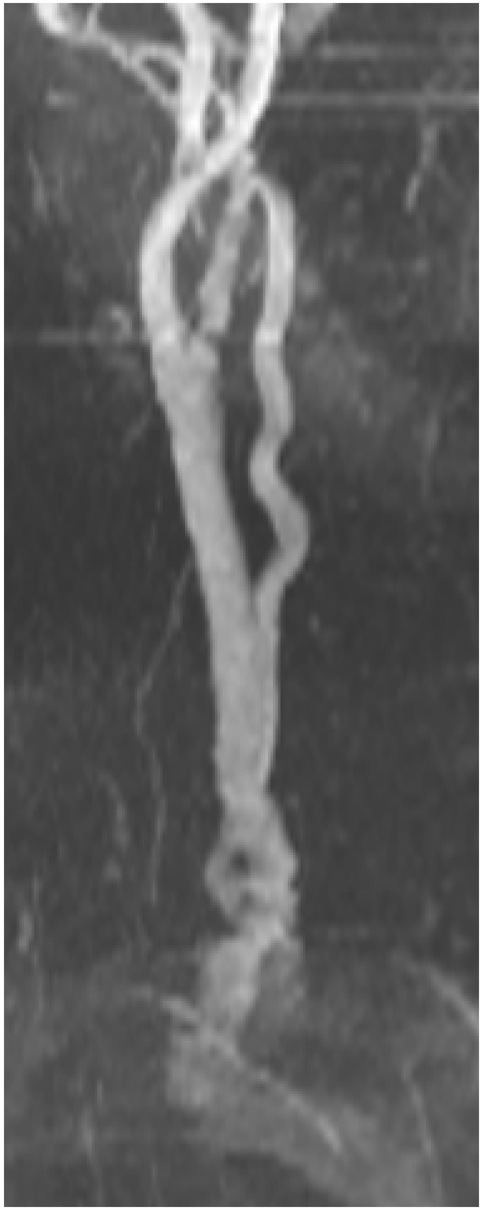

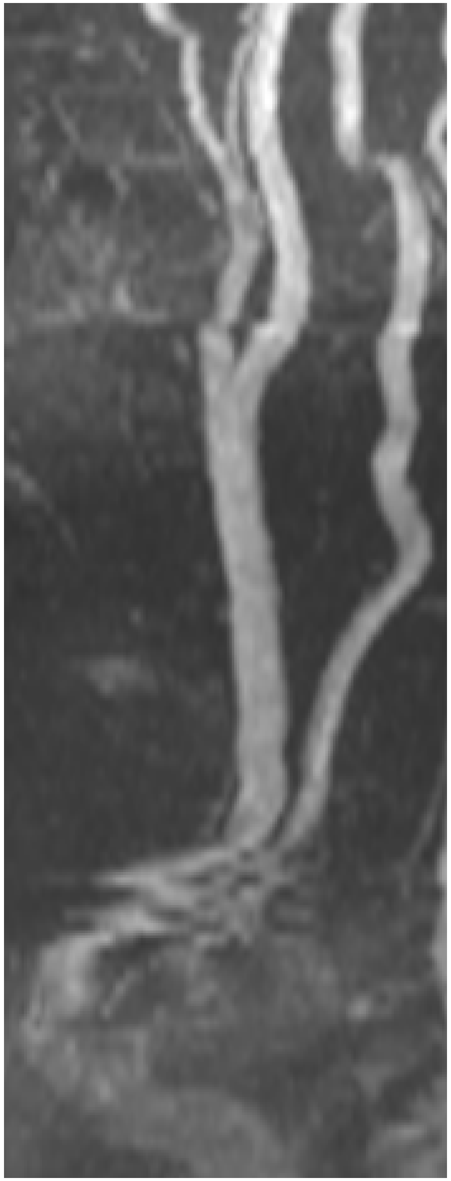

45f Score

0-30

31-50

51-70

>70

Near occlusion

Occluded

Quality

1

2

3

4

5

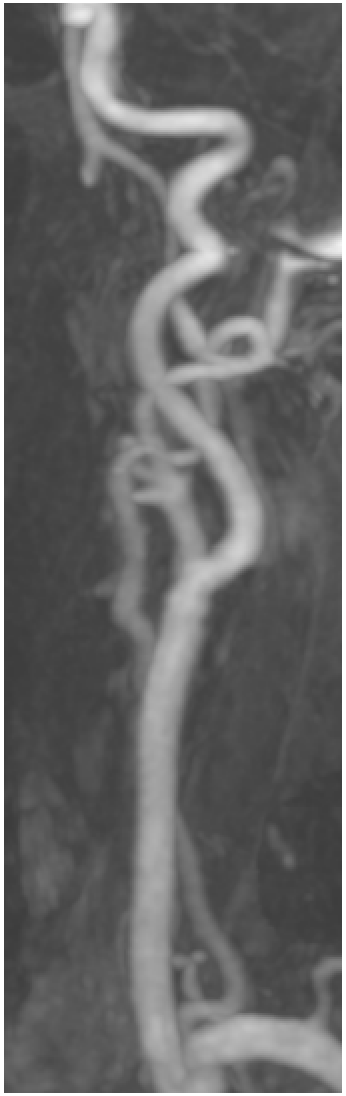

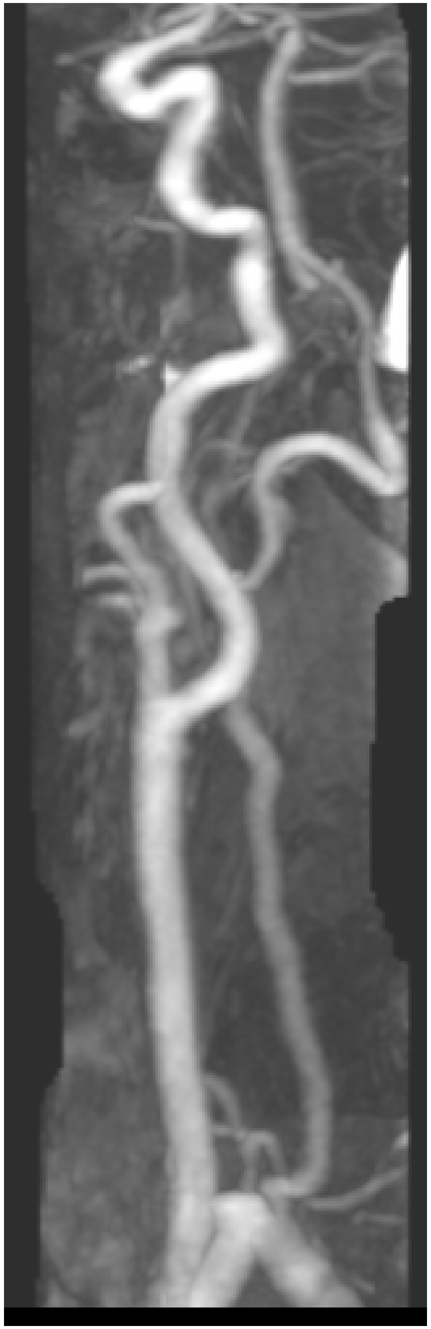

46e Score

0-30

31-50

51-70

>70

Near occlusion

Occluded

Quality

1

2

3

4

5

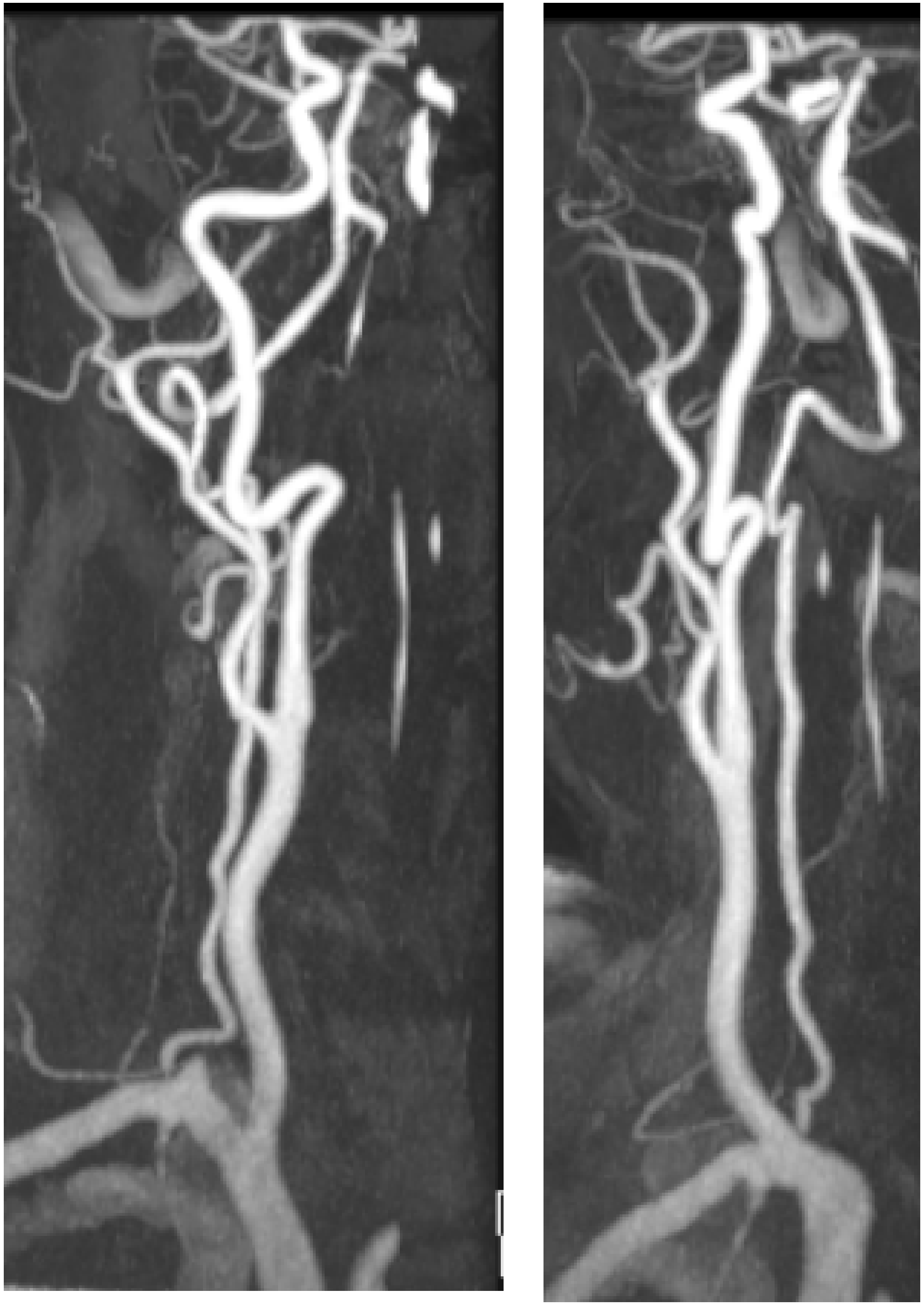

47d Score

0-30

31-50

51-70

>70

Near occlusion

Occluded

Quality

1

2

3

4

5

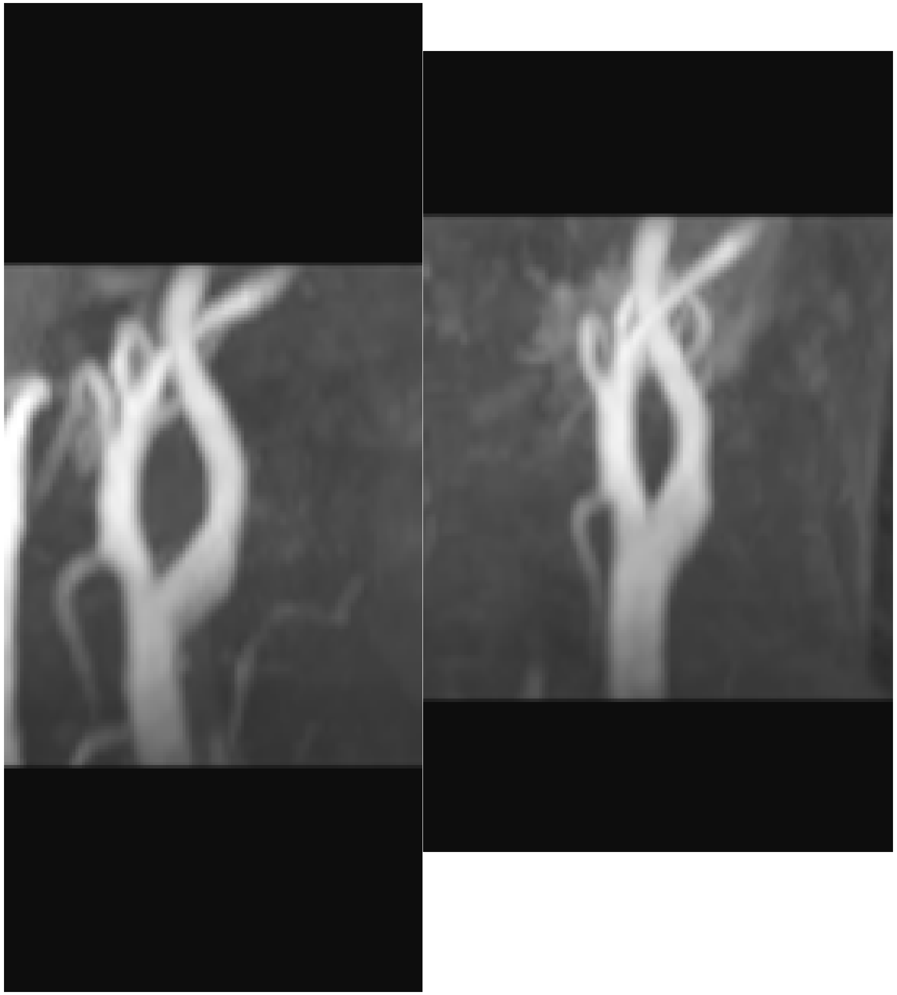

48c Score

0-30

31-50

51-70

>70

Near occlusion

Occluded

Quality

1

2

3

4

5

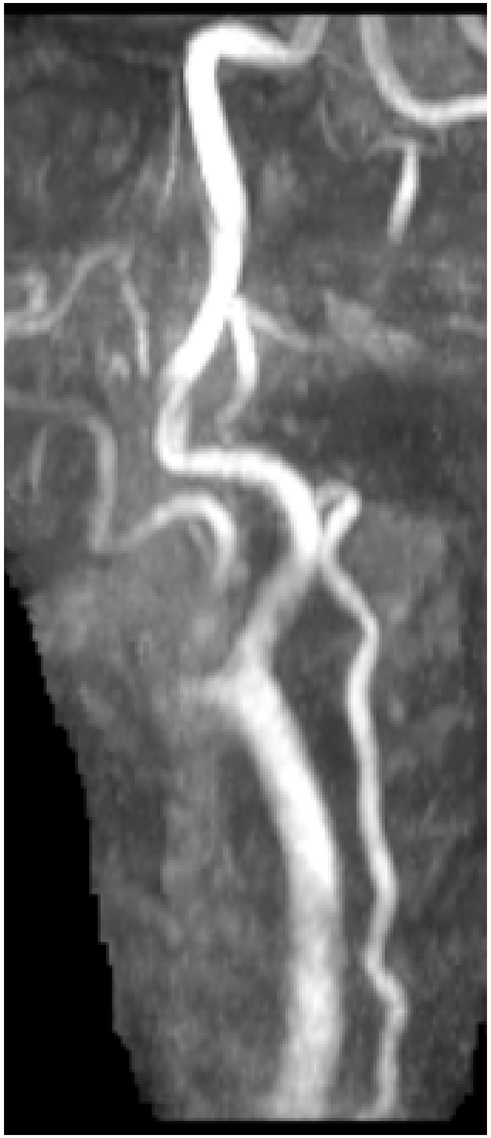

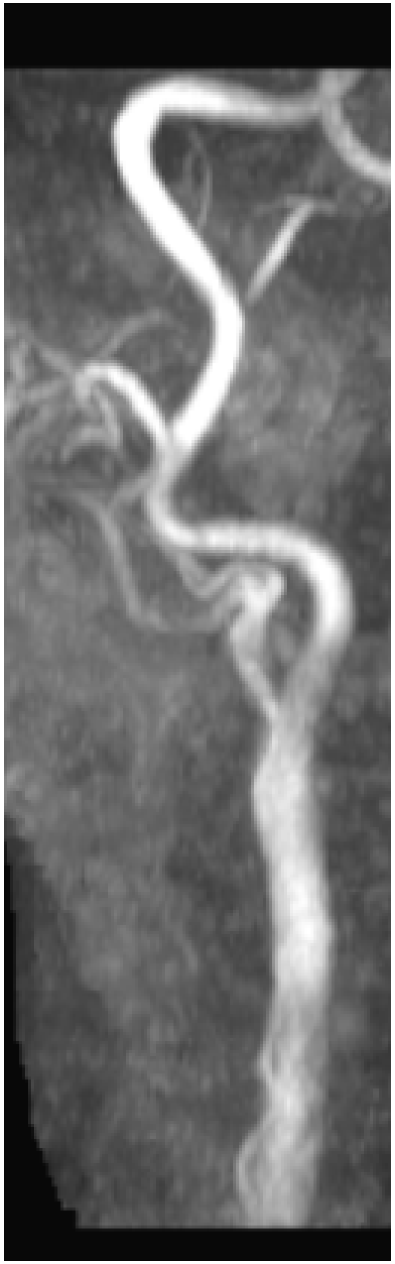

49b Score

0-30

31-50

51-70

>70

Near occlusion

Occluded

Quality

1

2

3

4

5

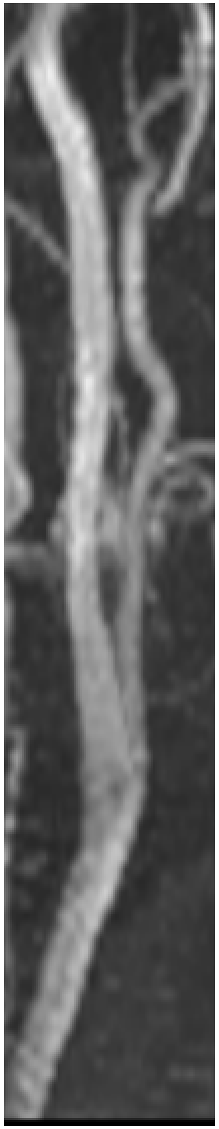

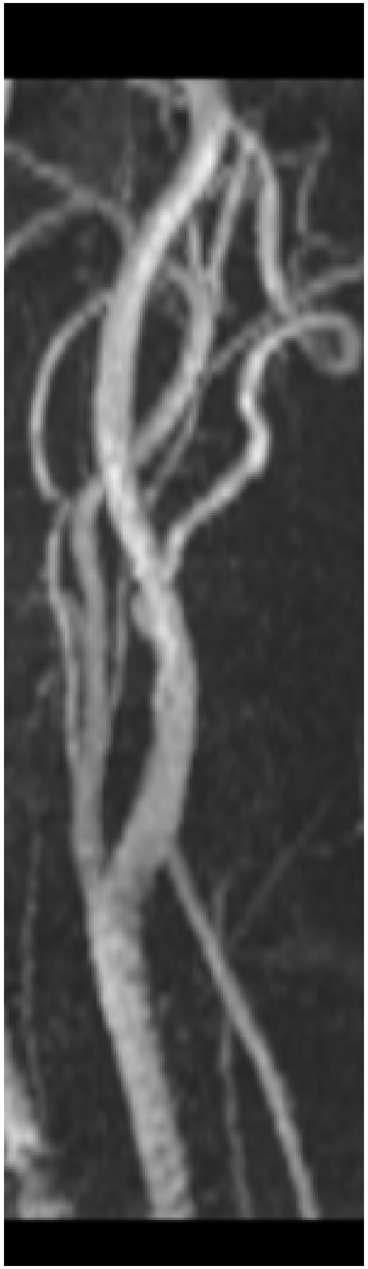

50a Score

0-30

31-50

51-70

>70

Near occlusion

Occluded

Quality

1

2

3

4

5

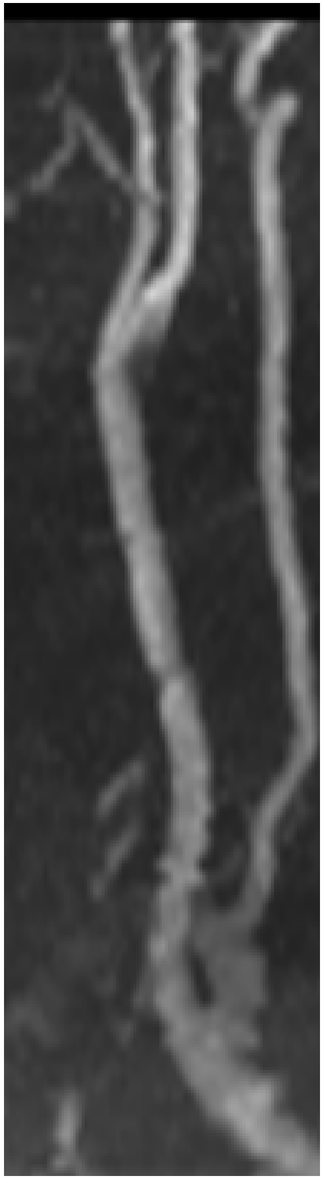

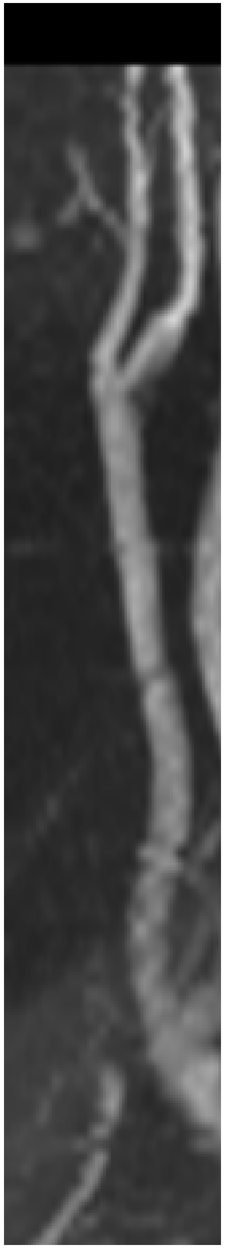

50f Score

0-30

31-50

51-70

>70

Near occlusion

Occluded

Quality

1

2

3

4

5

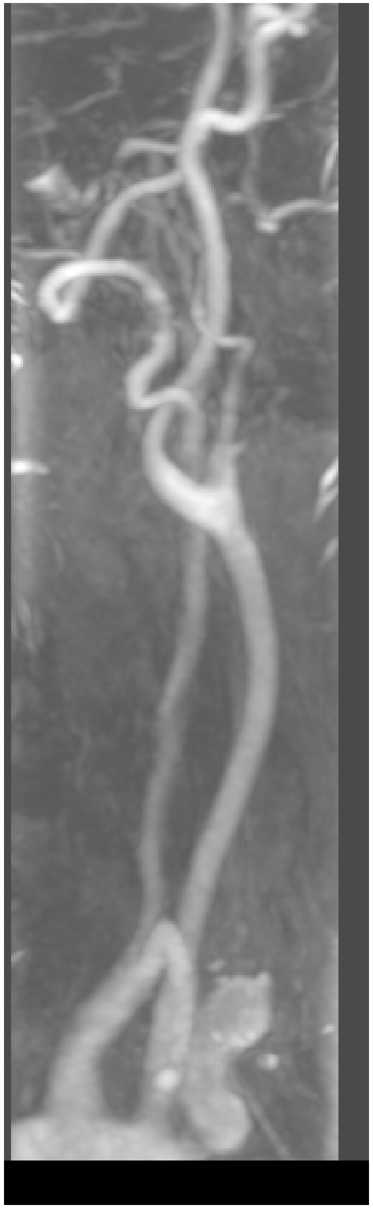

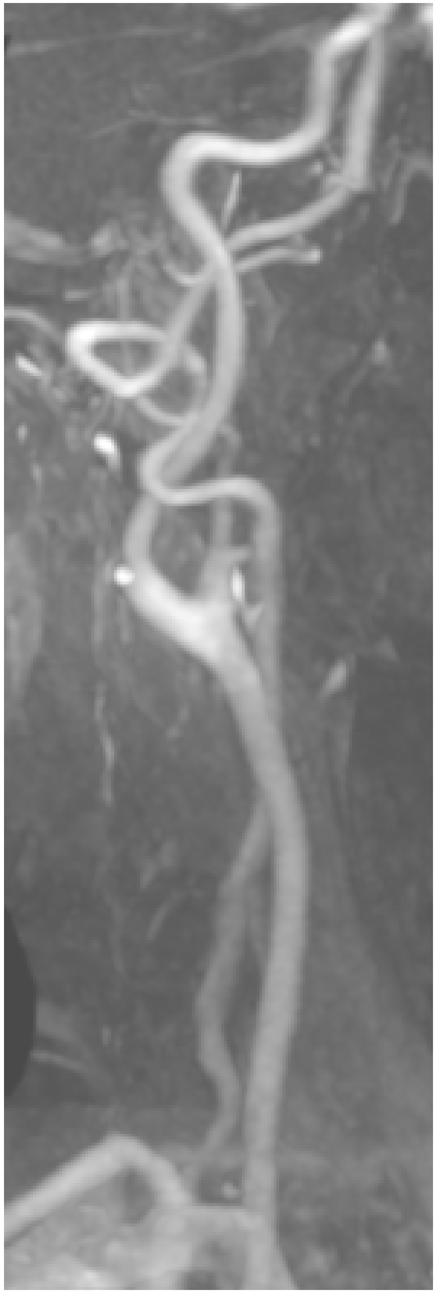

51e Score

0-30

31-50

51-70

>70

Near occlusion

Occluded

Quality

1

2

3

4

5

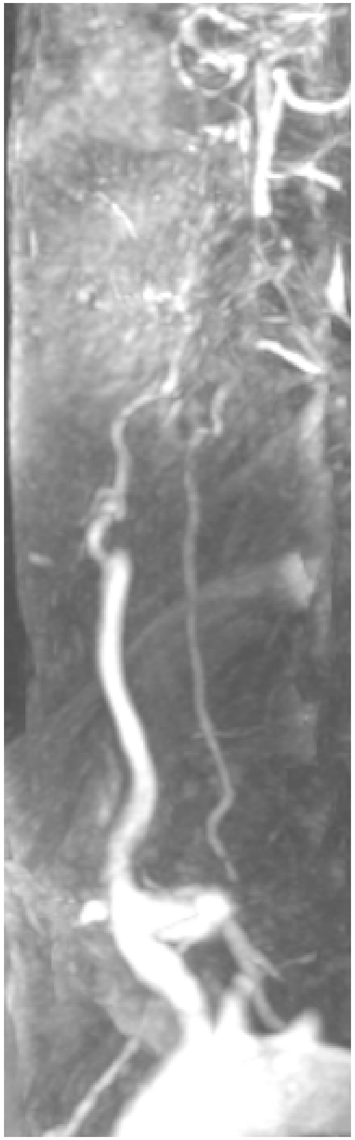

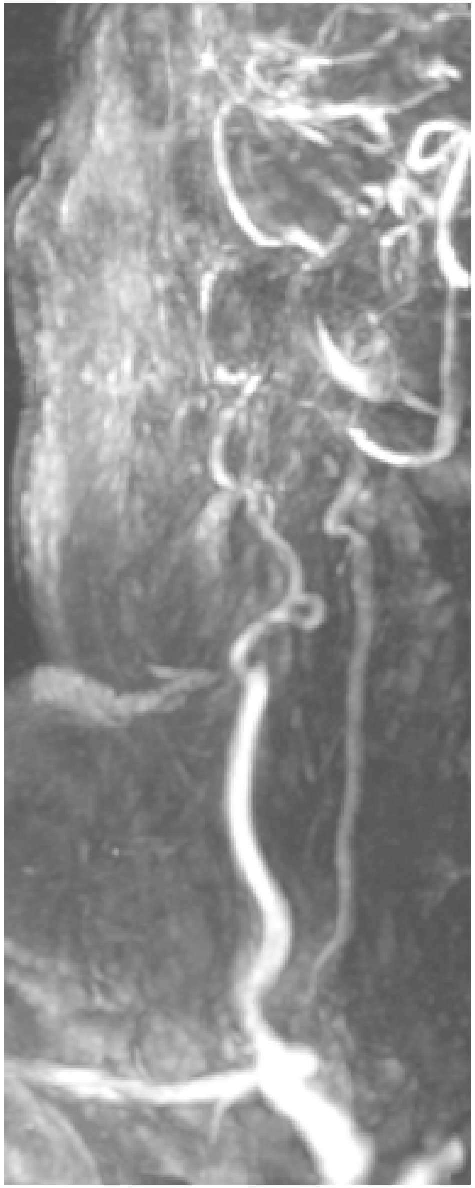

52d Score

0-30

31-50

51-70

>70

Near occlusion

Occluded

Quality

1

2

3

4

5

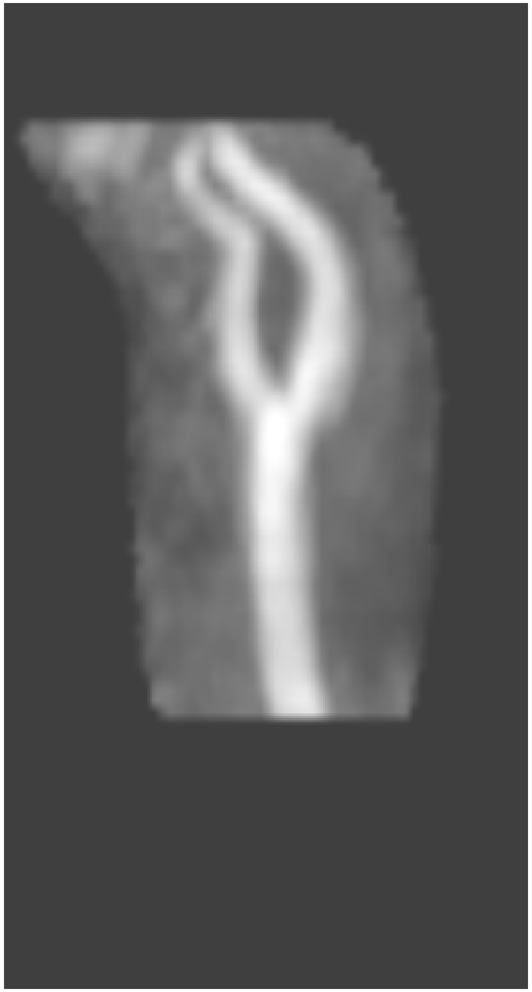

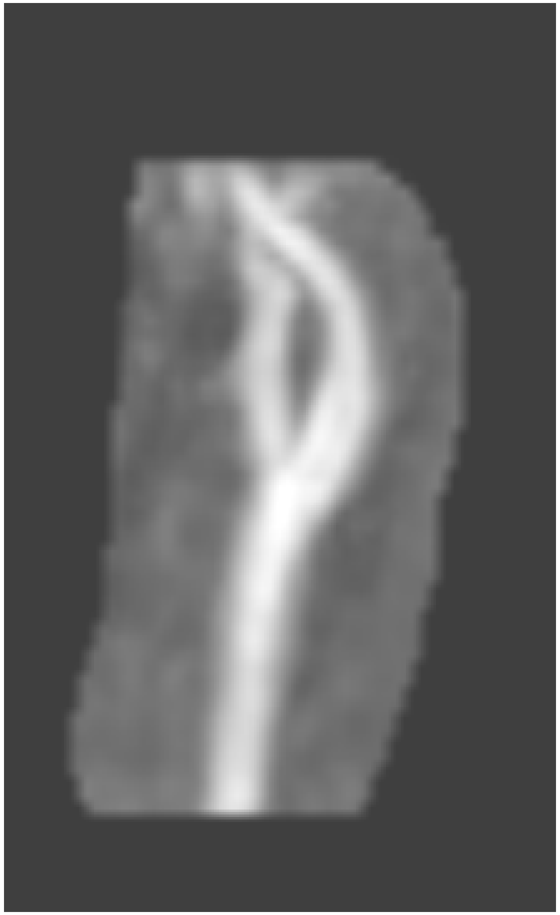

53c Score

0-30

31-50

51-70

>70

Near occlusion

Occluded

Quality

1

2

3

4

5

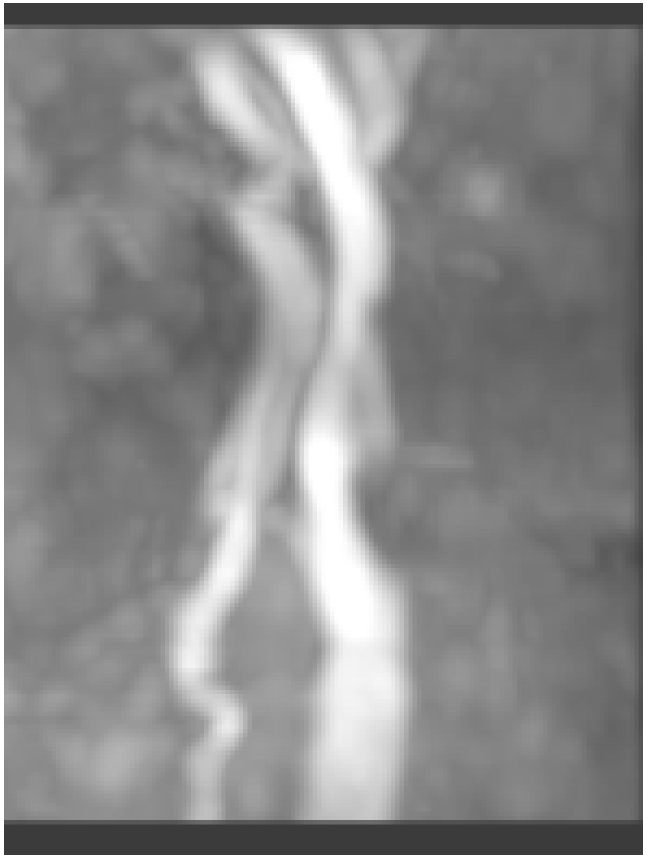

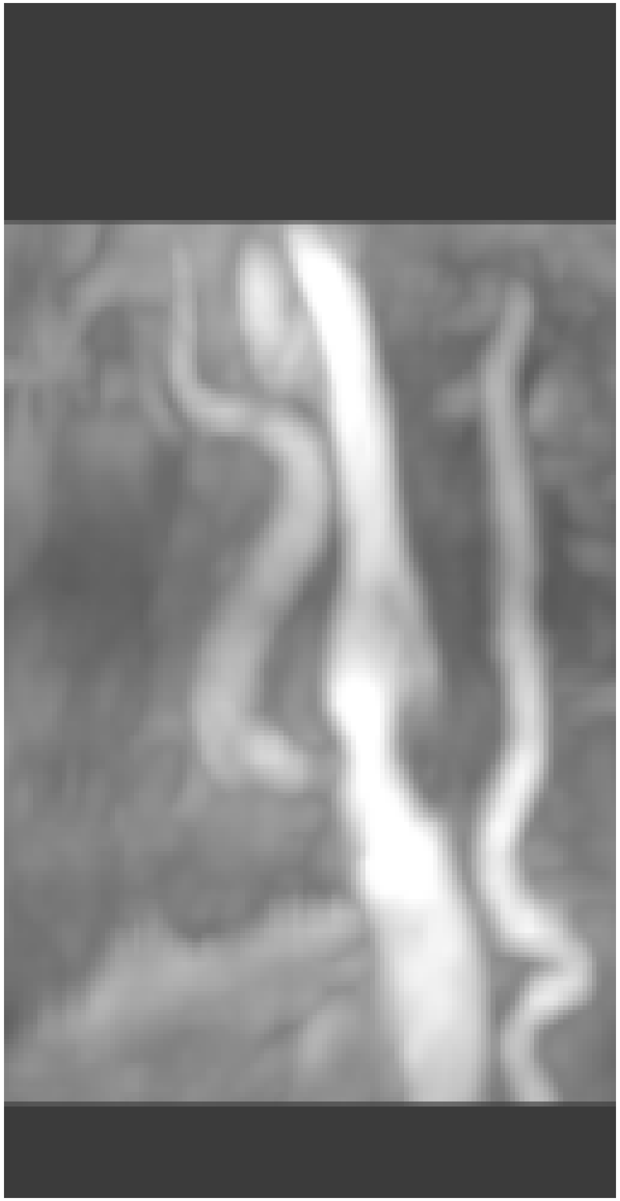

54b Score

0-30

31-50

51-70

>70

Near occlusion

Occluded

Quality

1

2

3

4

5

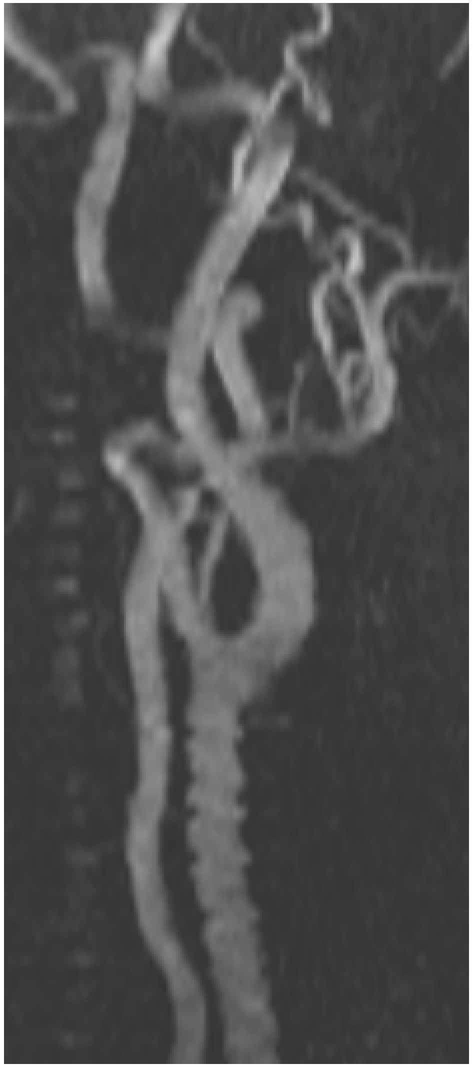

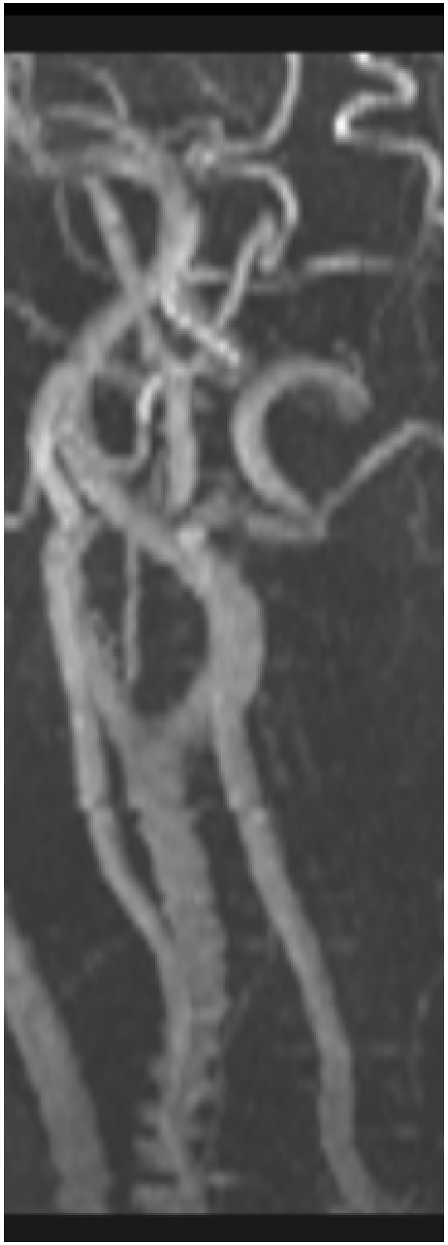

55a Score

0-30

31-50

51-70

>70

Near occlusion

Occluded

Quality

1

2

3

4

5

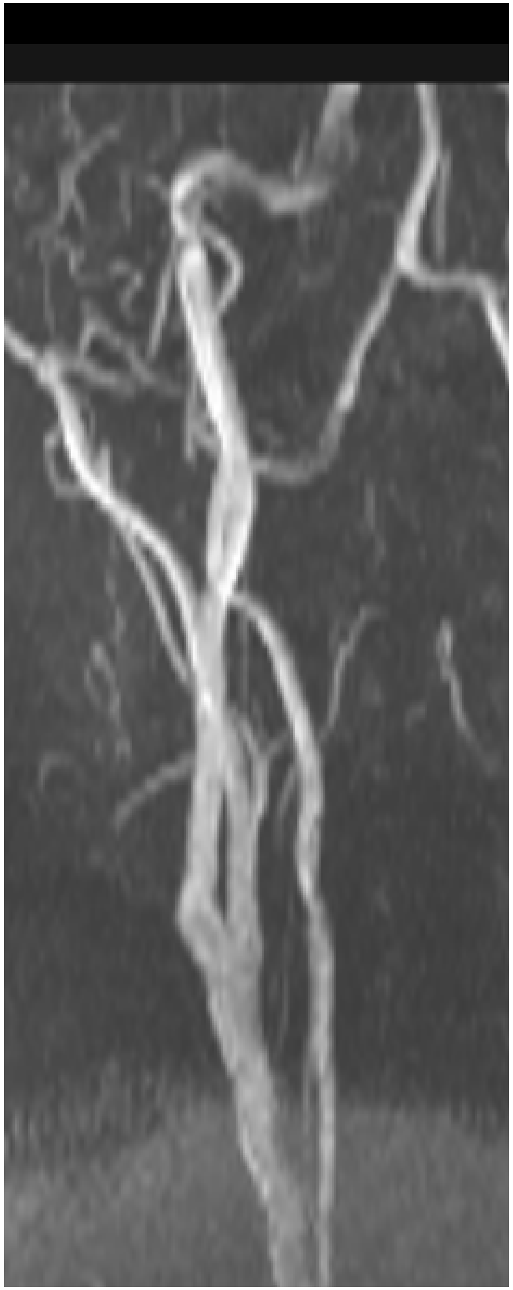

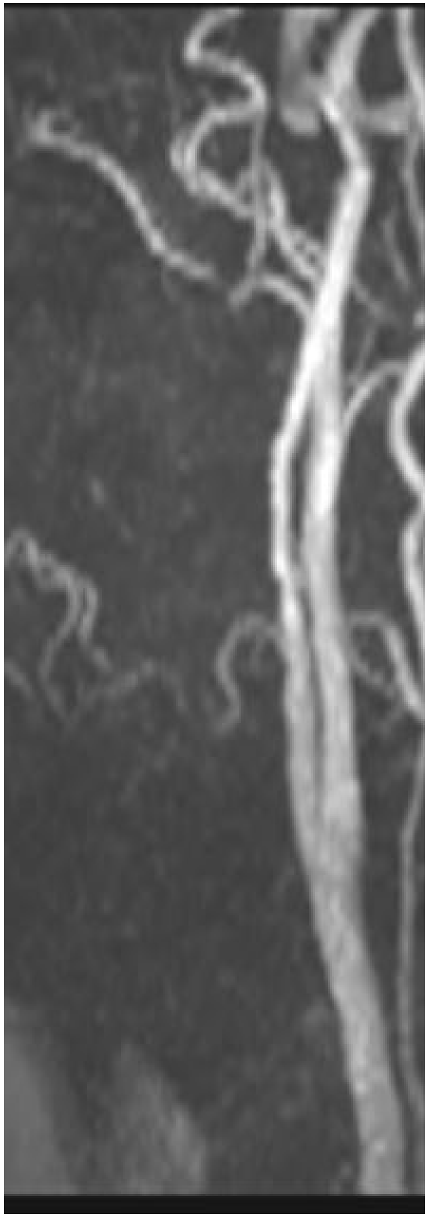

55f Score

0-30

31-50

51-70

>70

Near occlusion

Occluded

Quality

1

2

3

4

5

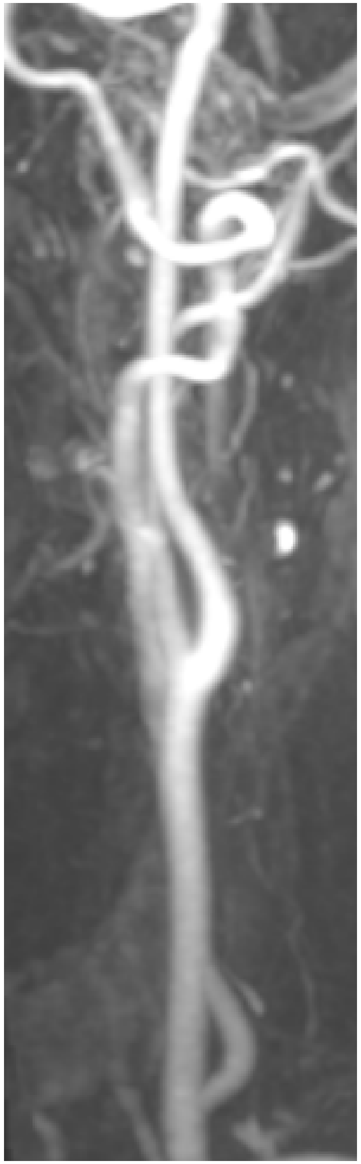

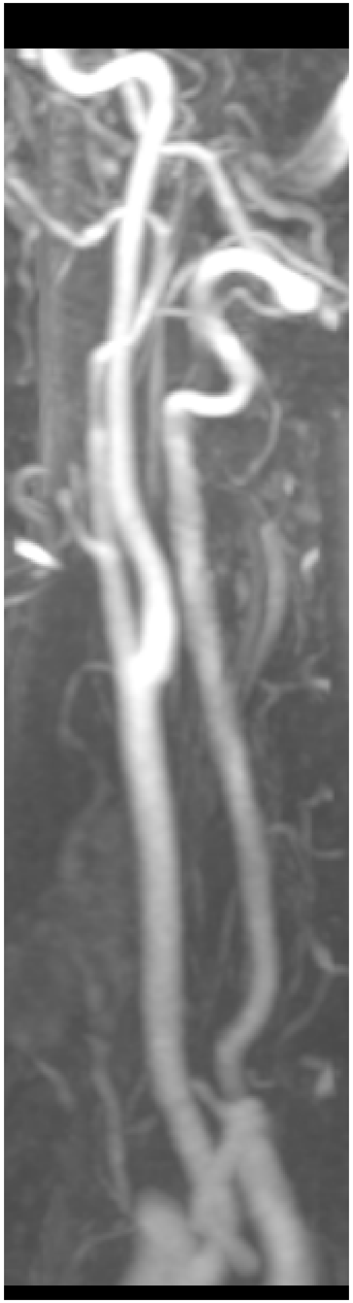

56e Score

0-30

31-50

51-70

>70

Near occlusion

Occluded

Quality

1

2

3

4

5

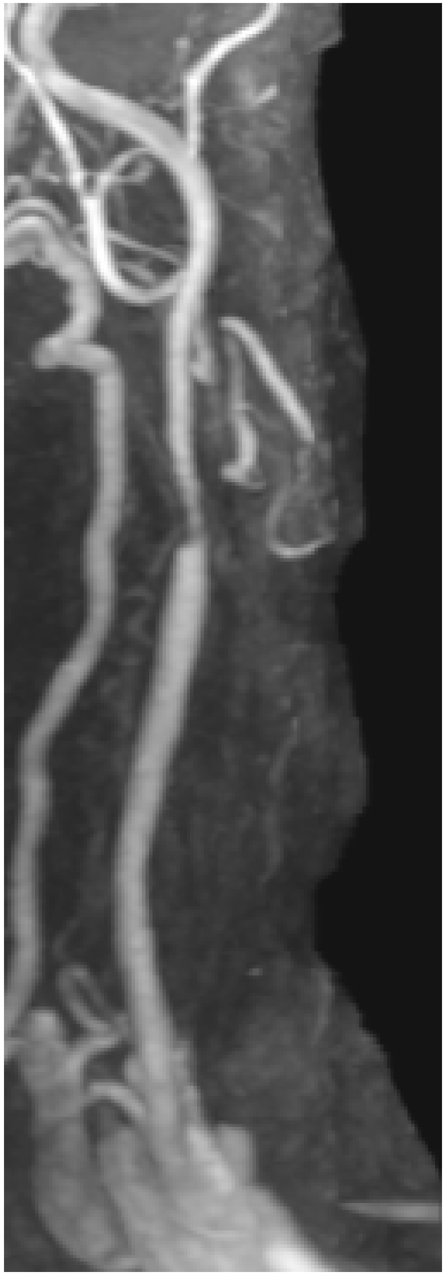

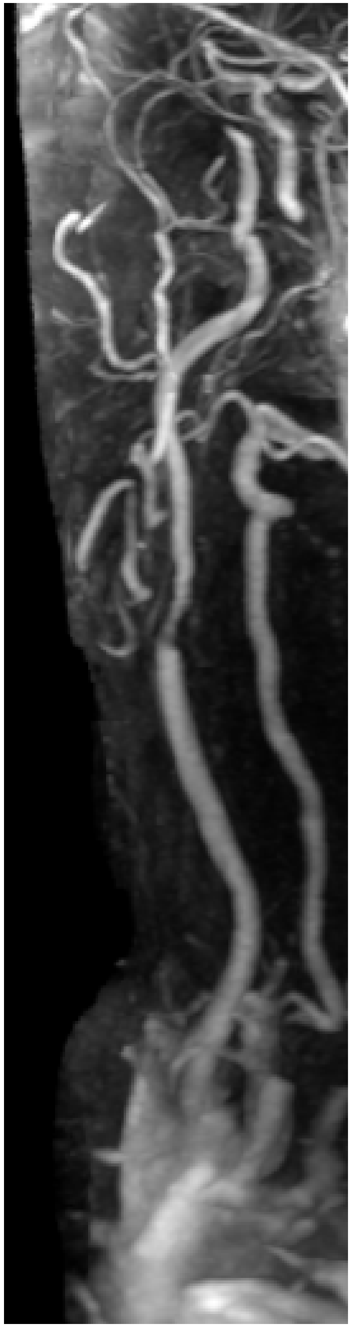

57d Score

0-30

31-50

51-70

>70

Near occlusion

Occluded

Quality

1

2

3

4

5

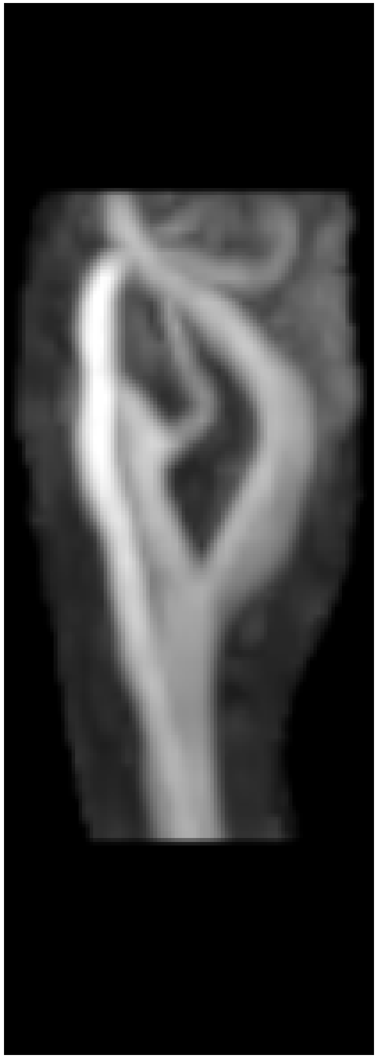

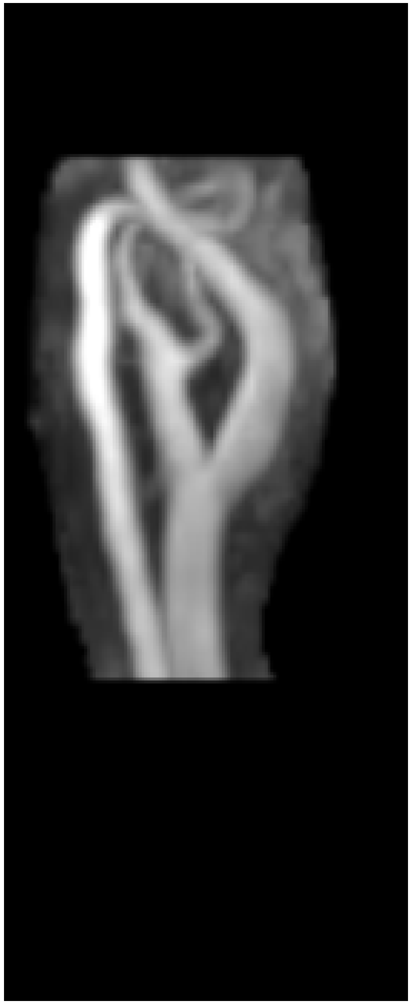

58c Score

0-30

31-50

51-70

>70

Near occlusion

Occluded

Quality

1

2

3

4

5

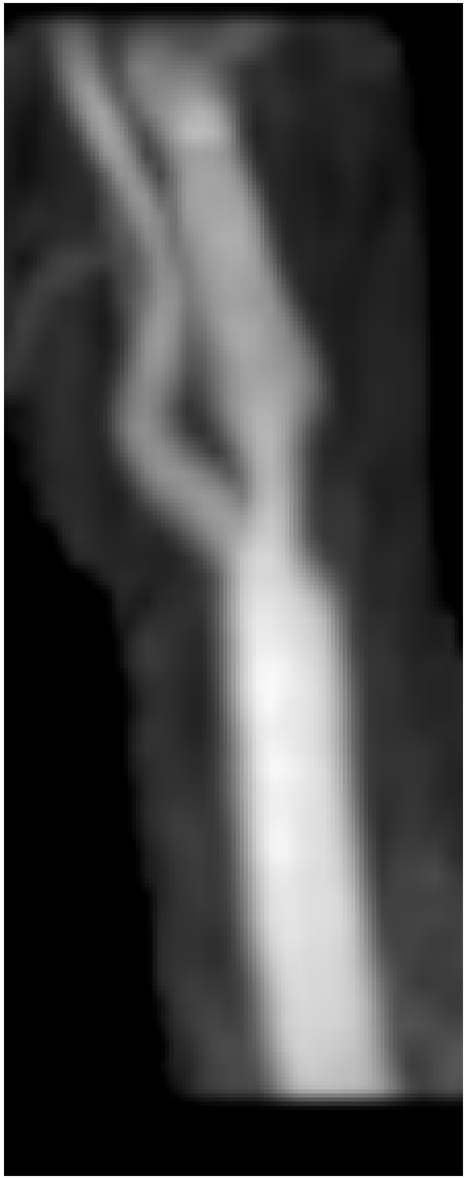

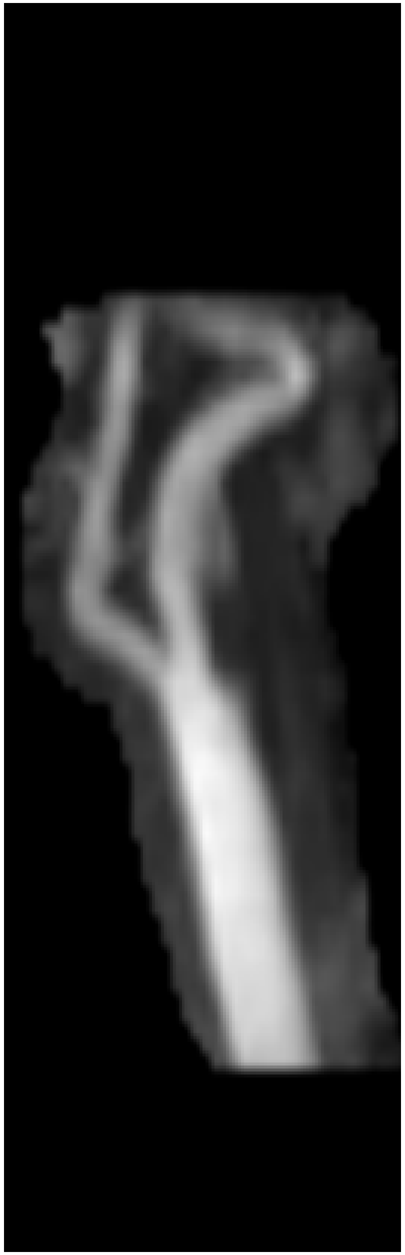

59b Score

0-30

31-50

51-70

>70

Near occlusion

Occluded

Quality

1

2

3

4

5

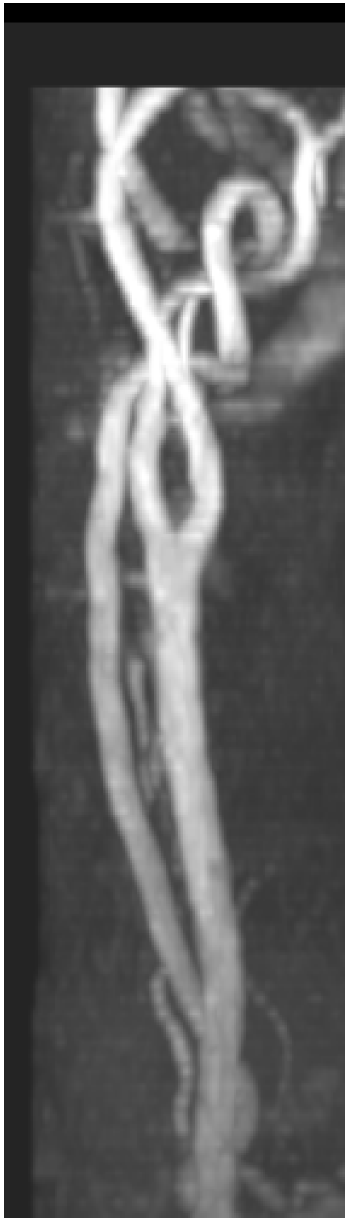

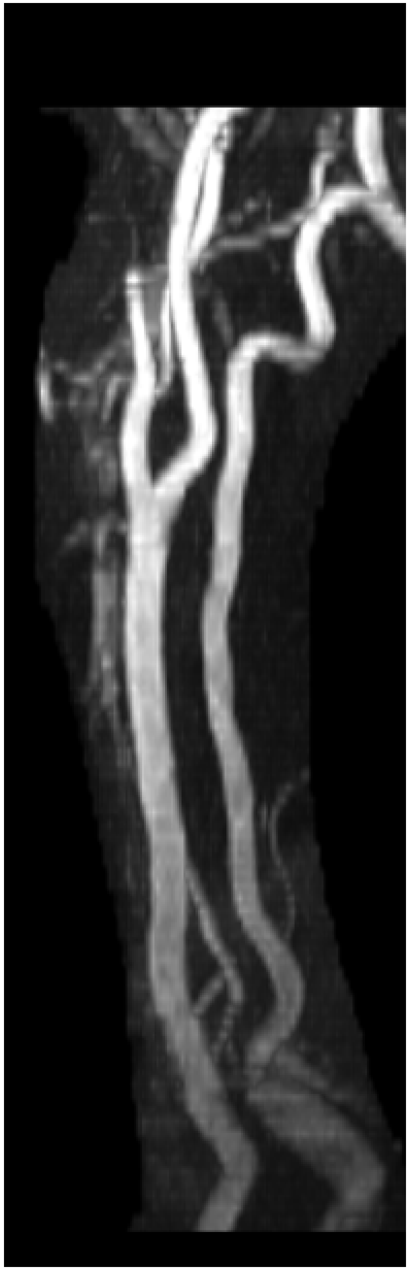

60a Score

0-30

31-50

51-70

>70

Near occlusion

Occluded

Quality

1

2

3

4

5

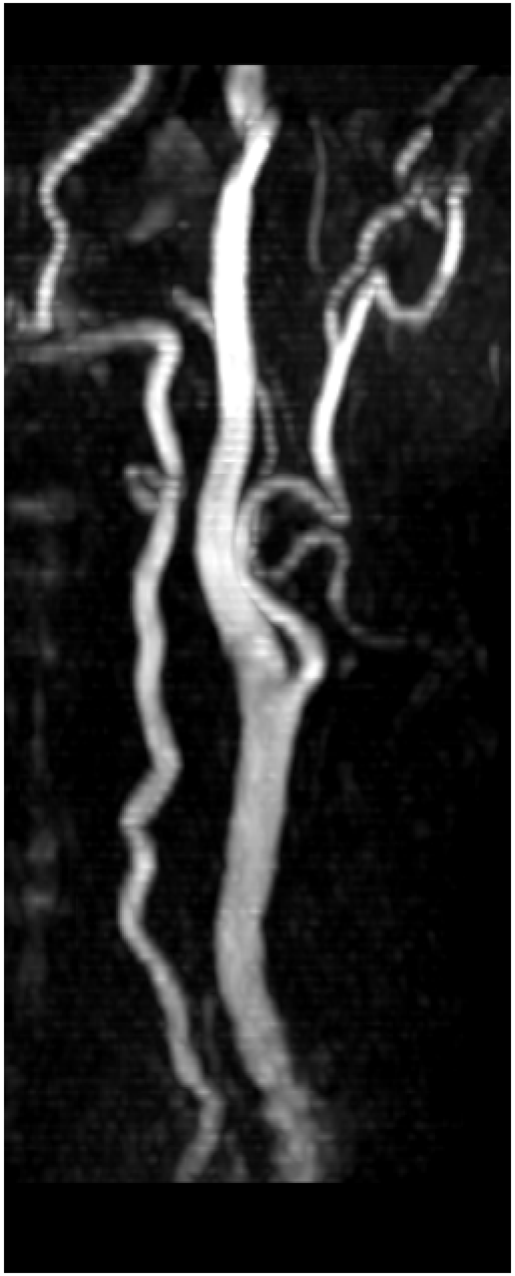

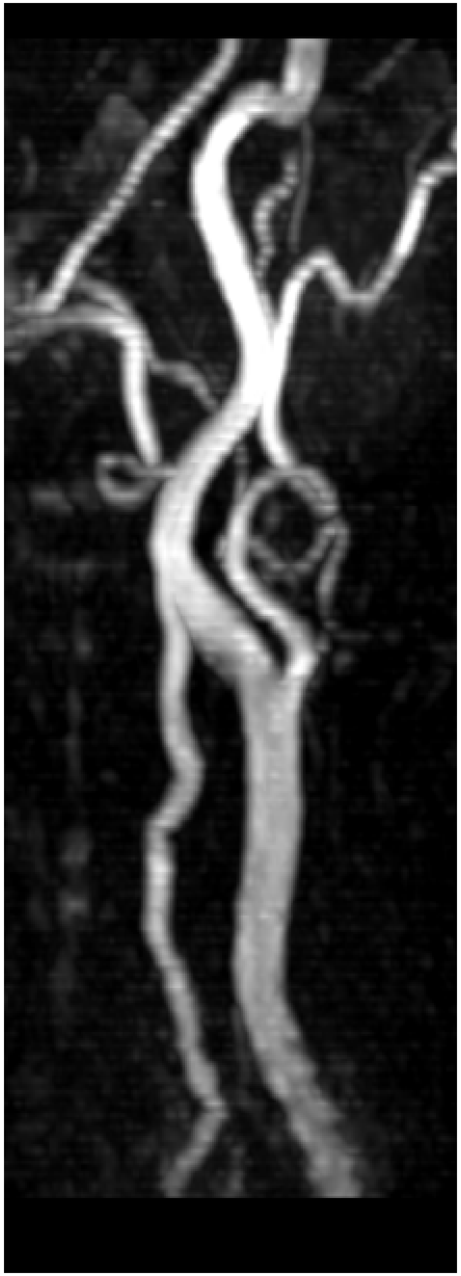

60f Score

0-30

31-50

51-70

>70

Near occlusion

Occluded

Quality

1

2

3

4

5

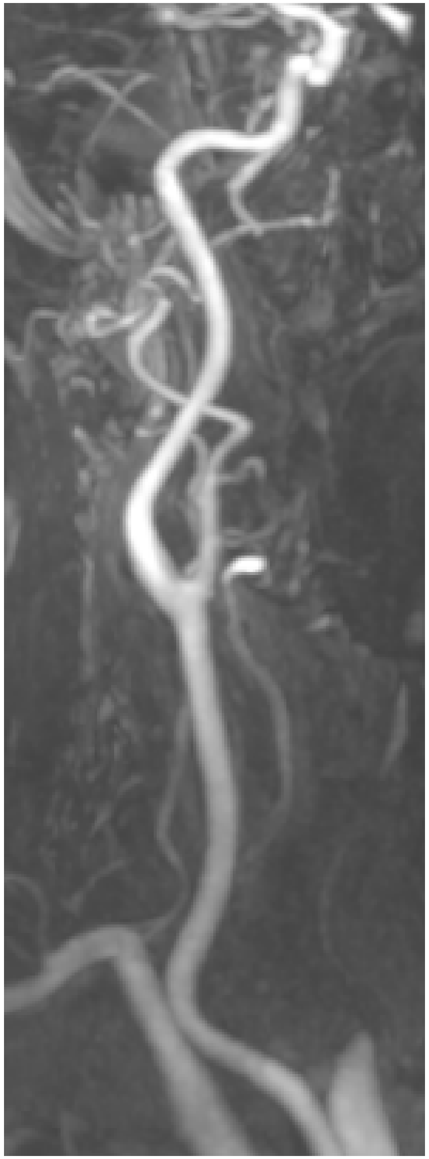

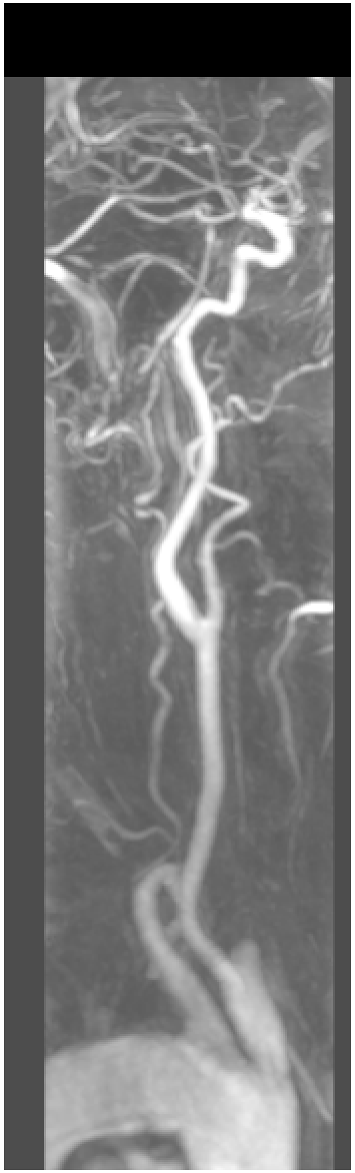

61e Score

0-30

31-50

51-70

>70

Near occlusion

Occluded

Quality

1

2

3

4

5

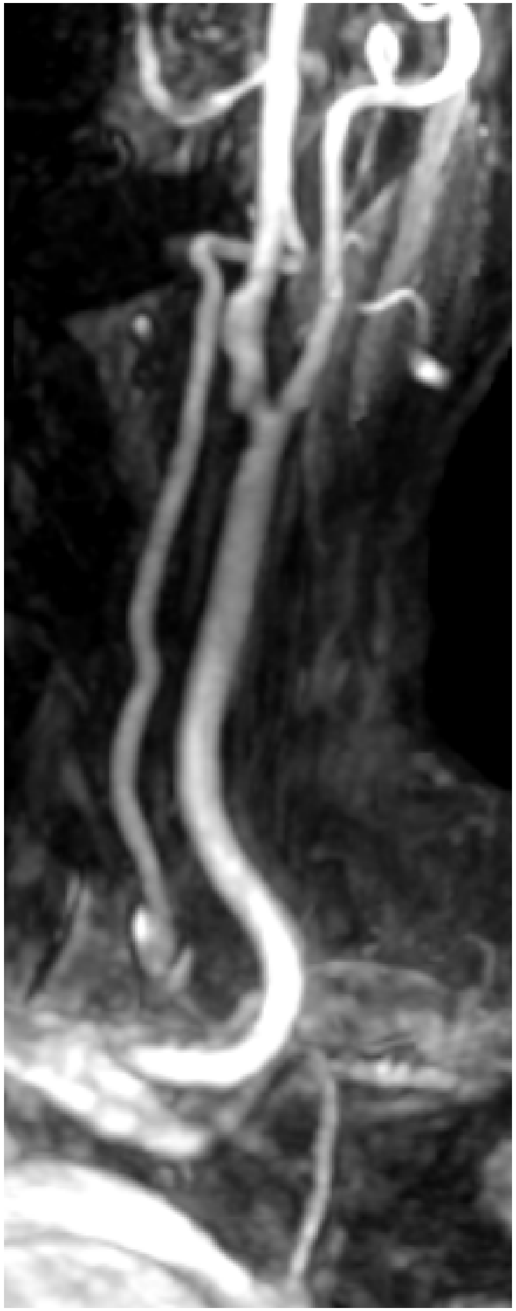

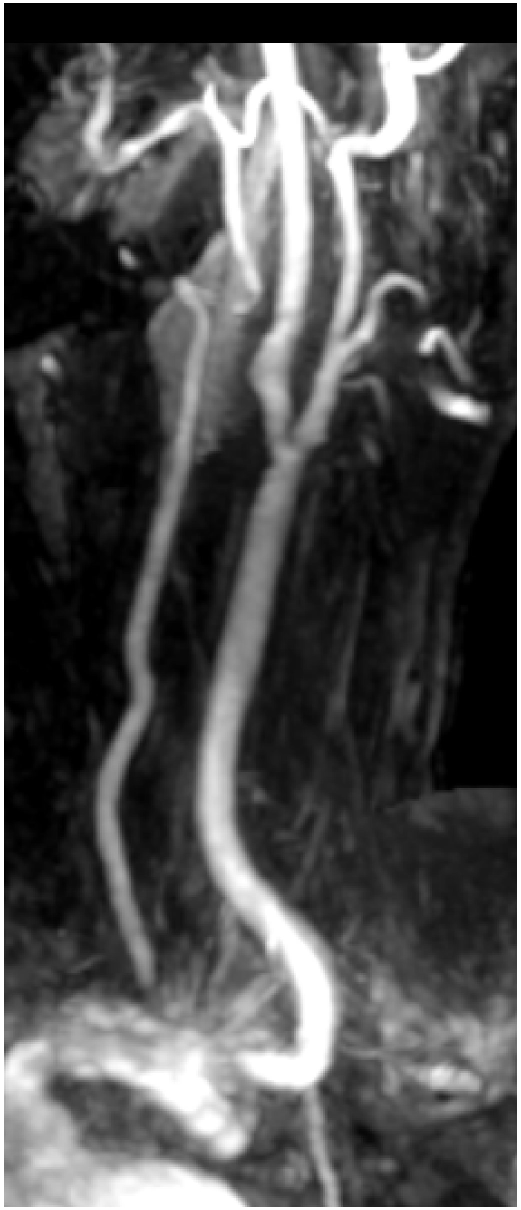

62d Score

0-30

31-50

51-70

>70

Near occlusion

Occluded

Quality

1

2

3

4

5

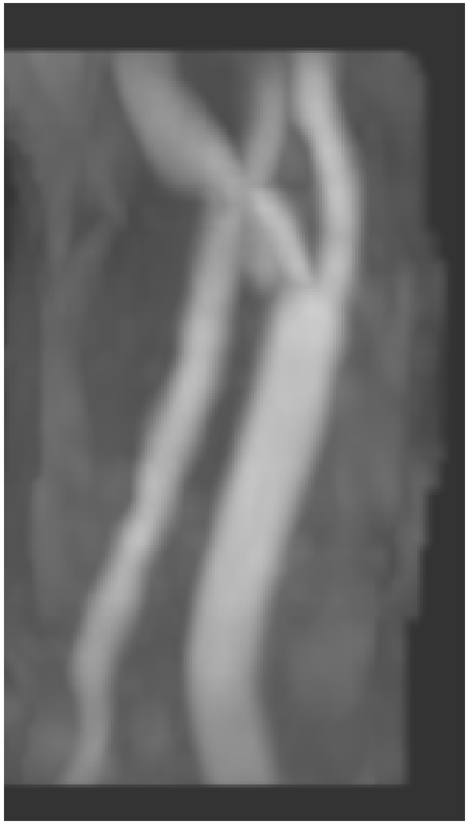

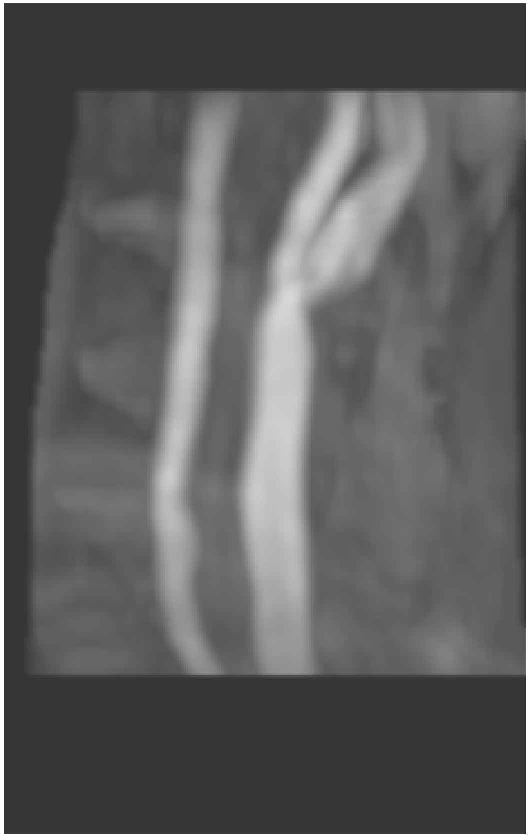

63c Score

0-30

31-50

51-70

>70

Near occlusion

Occluded

Quality

1

2

3

4

5

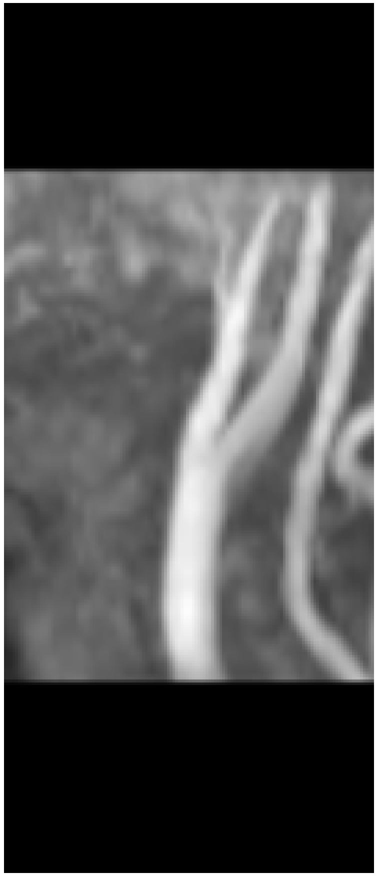

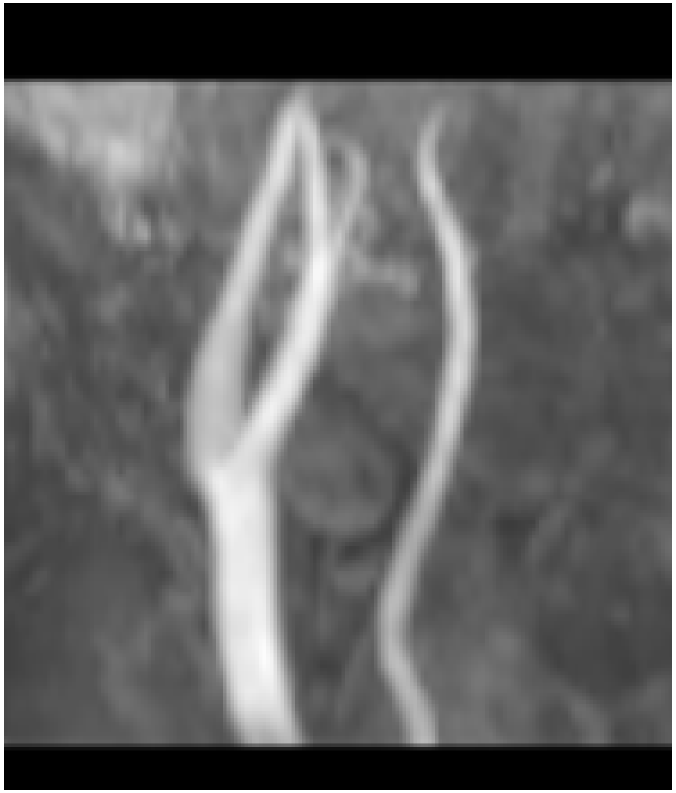

64b Score

0-30

31-50

51-70

>70

Near occlusion

Occluded

Quality

1

2

3

4

5

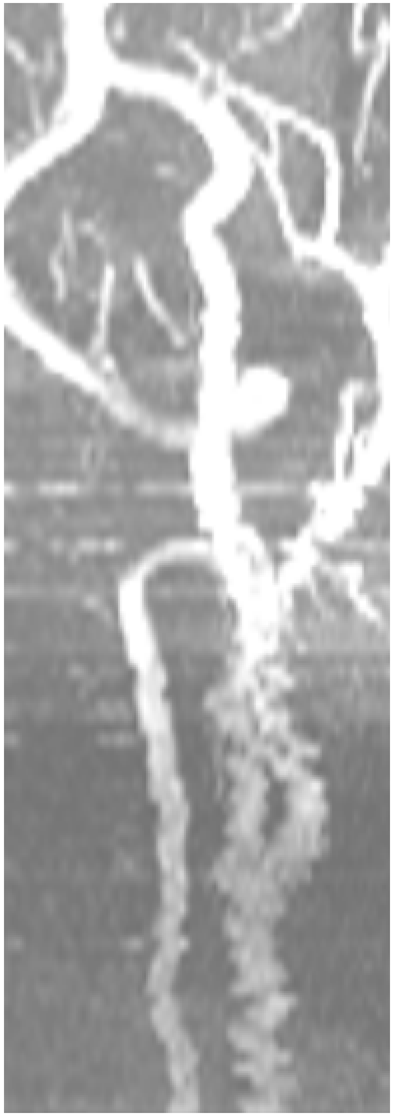

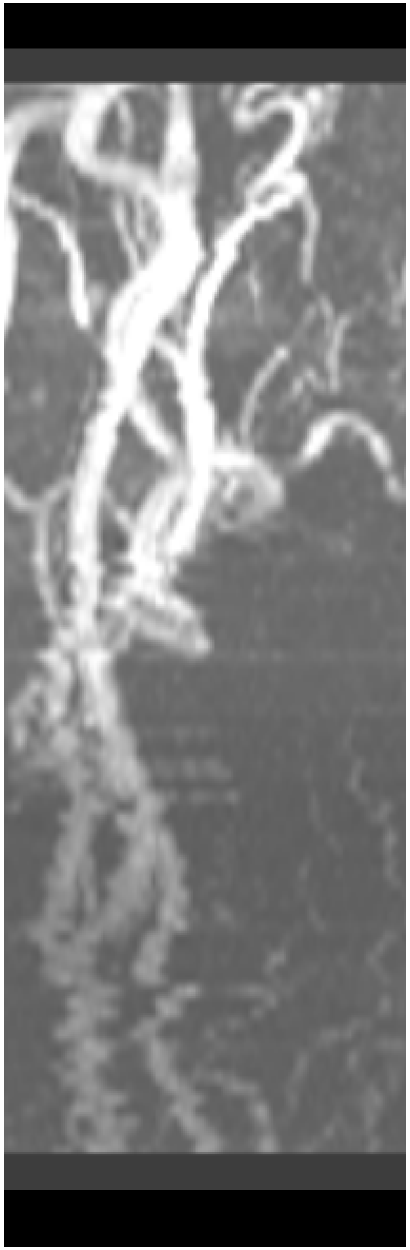

65a Score

0-30

31-50

51-70

>70

Near occlusion

Occluded

Quality

1

2

3

4

5

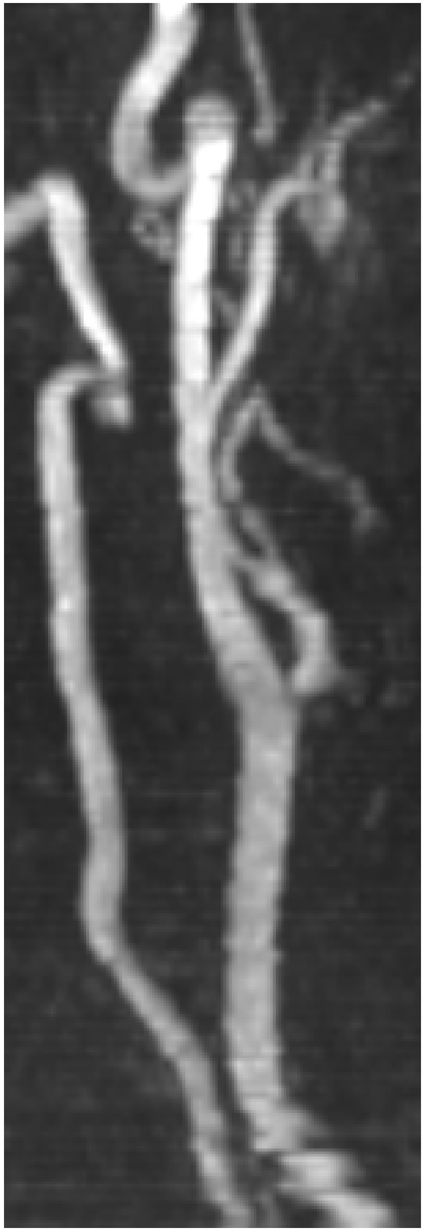

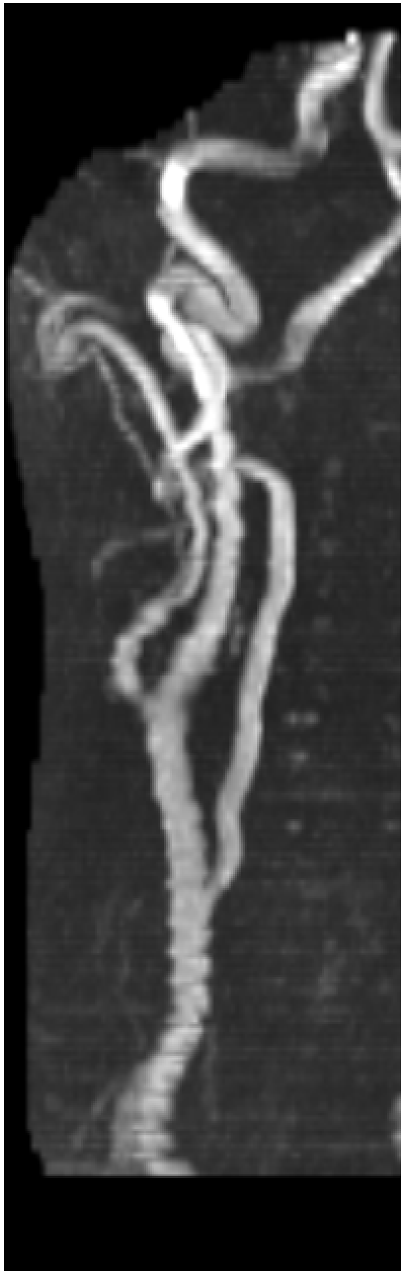

65f Score

0-30

31-50

51-70

>70

Near occlusion

Occluded

Quality

1

2

3

4

5

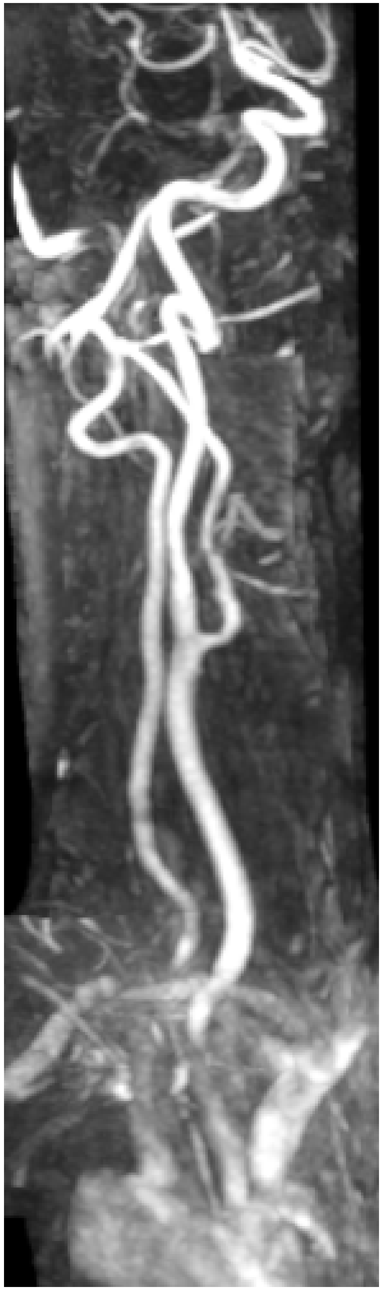

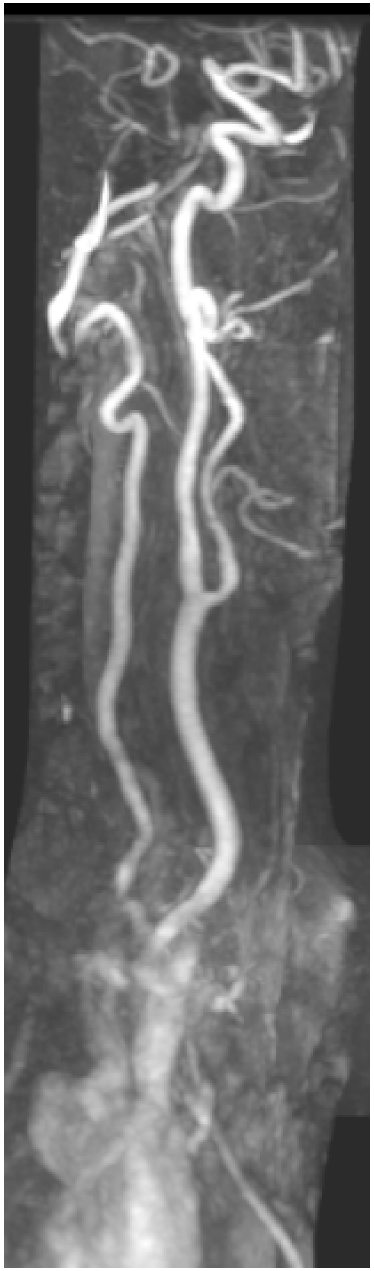

66e Score

0-30

31-50

51-70

>70

Near occlusion

Occluded

Quality

1

2

3

4

5

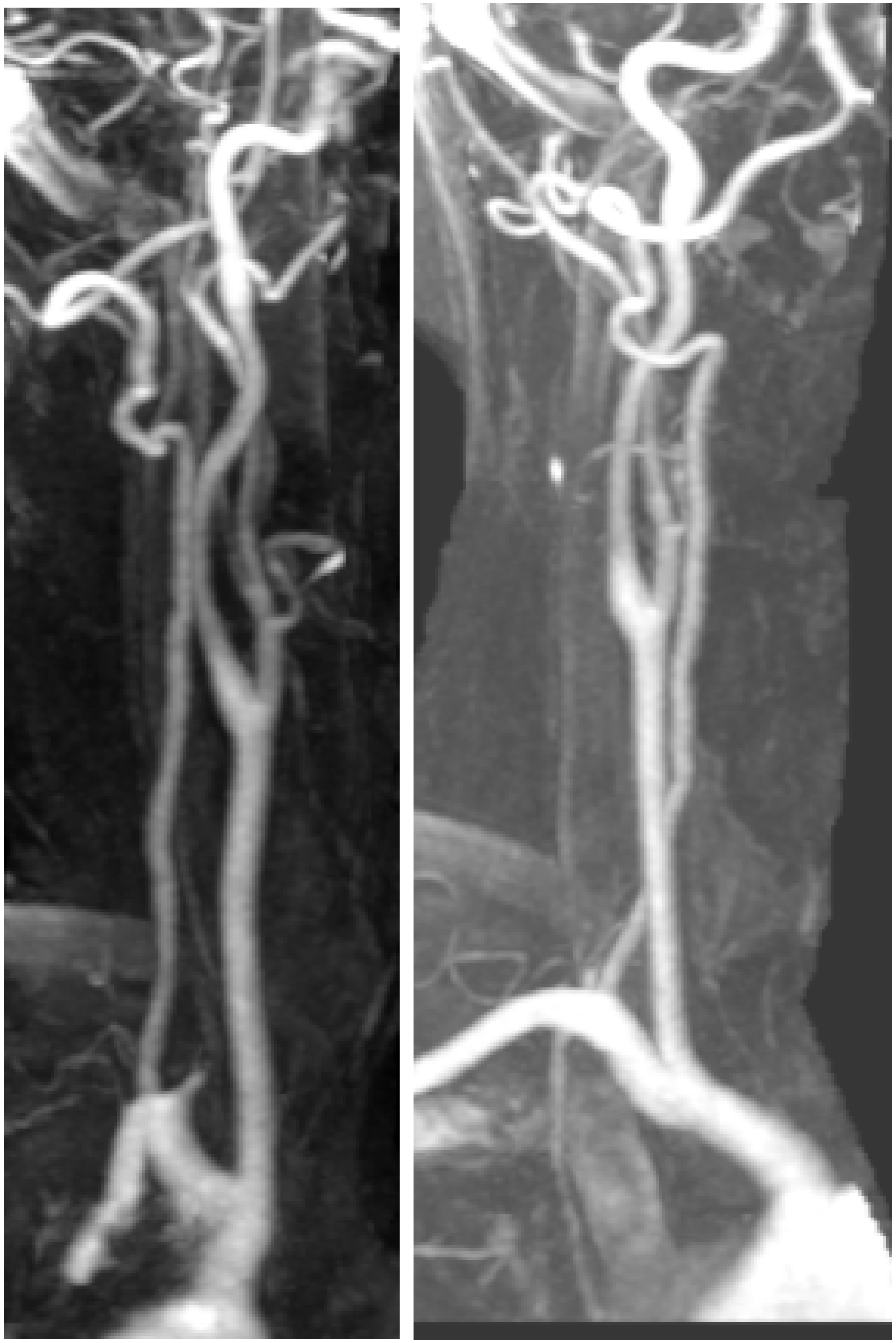

67d Score

0-30

31-50

51-70

>70

Near occlusion

Occluded

Quality

1

2

3

4

5

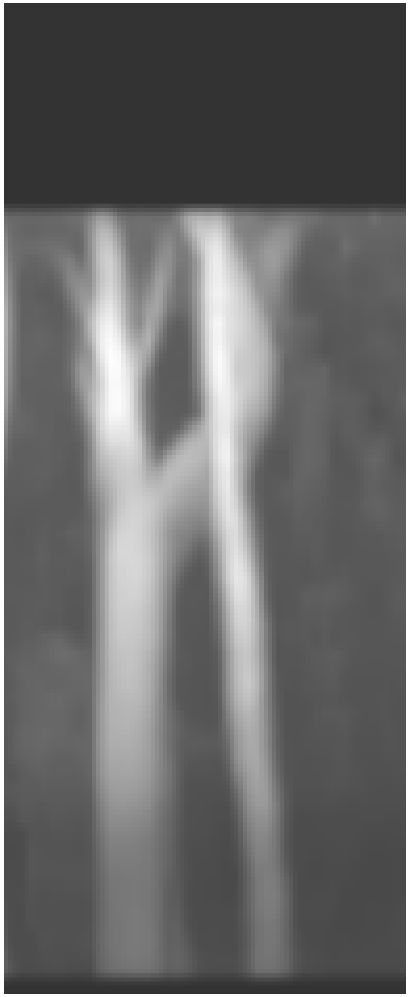

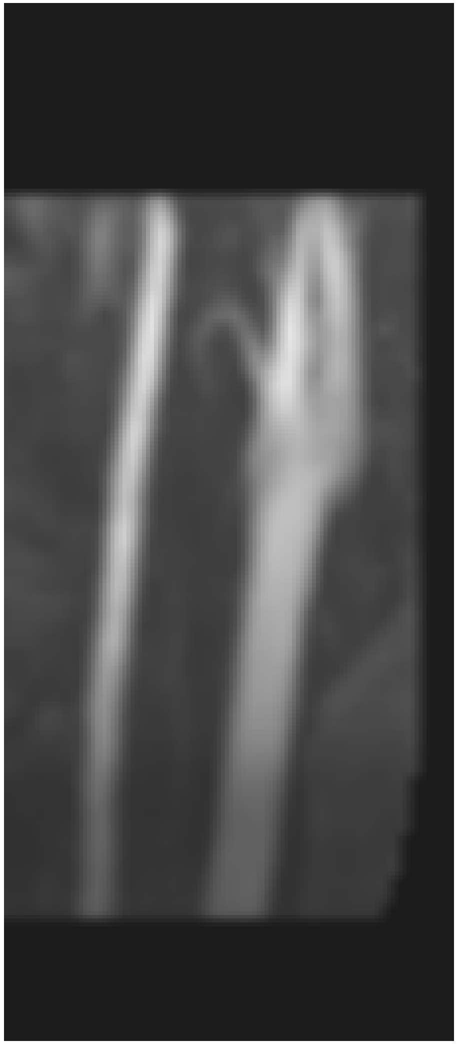

68c Score

0-30

31-50

51-70

>70

Near occlusion

Occluded

Quality

1

2

3

4

5

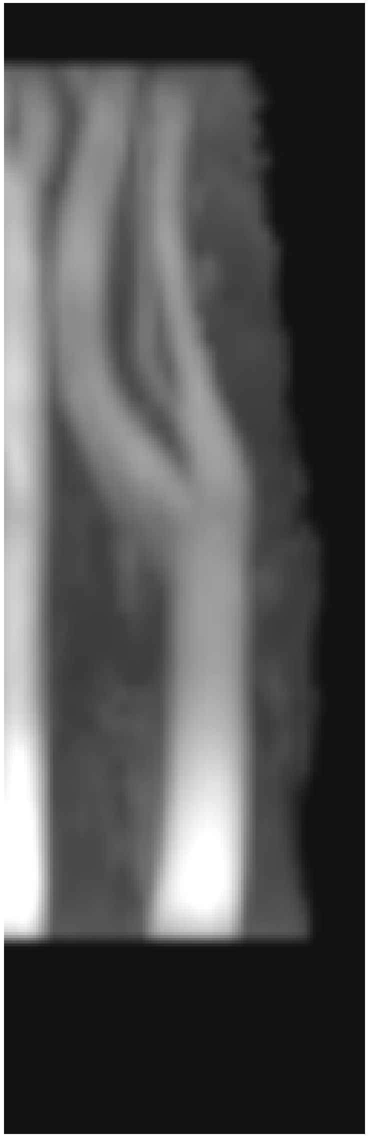

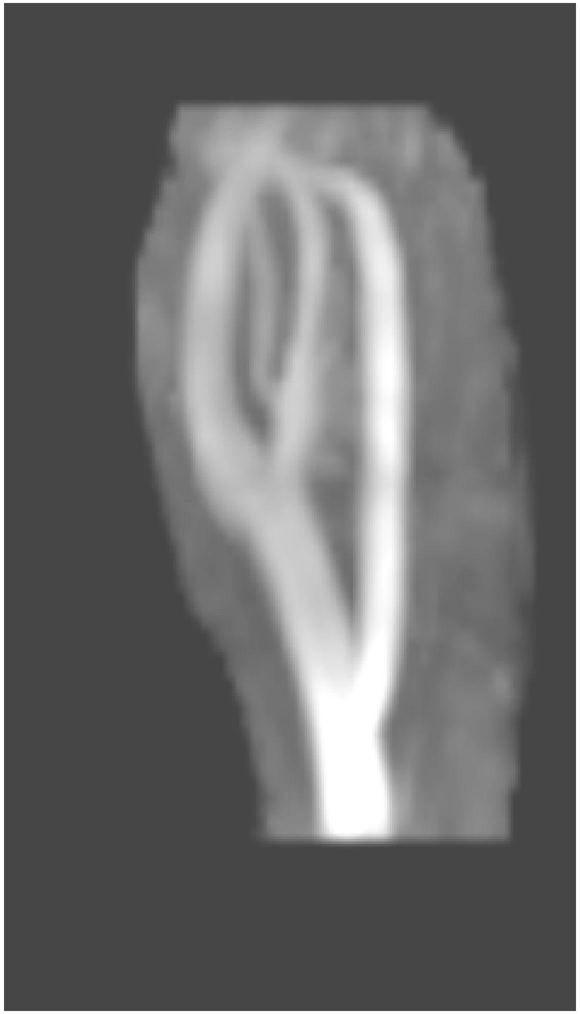

69b Score

0-30

31-50

51-70

>70

Near occlusion

Occluded

Quality

1

2

3

4

5

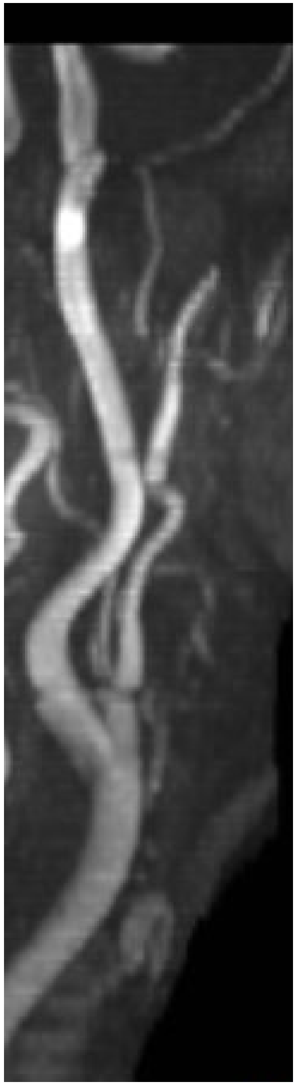

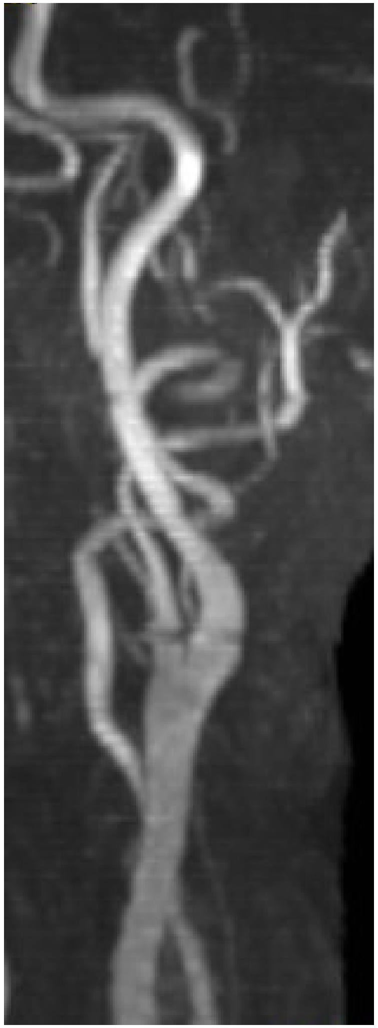

70a Score

0-30

31-50

51-70

>70

Near occlusion

Occluded

Quality

1

2

3

4

5

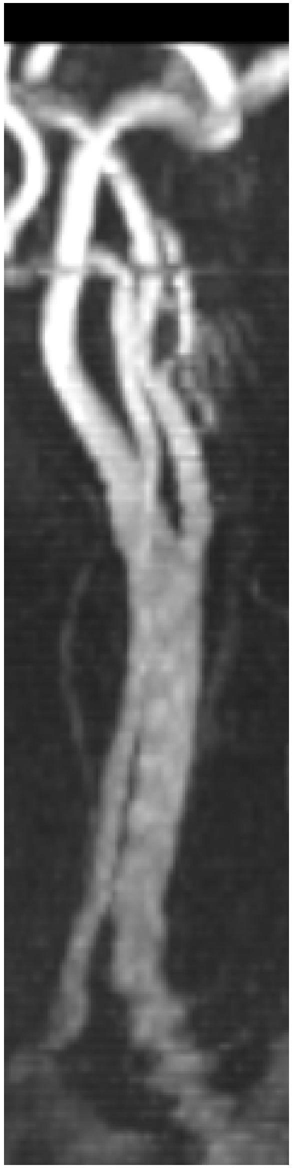

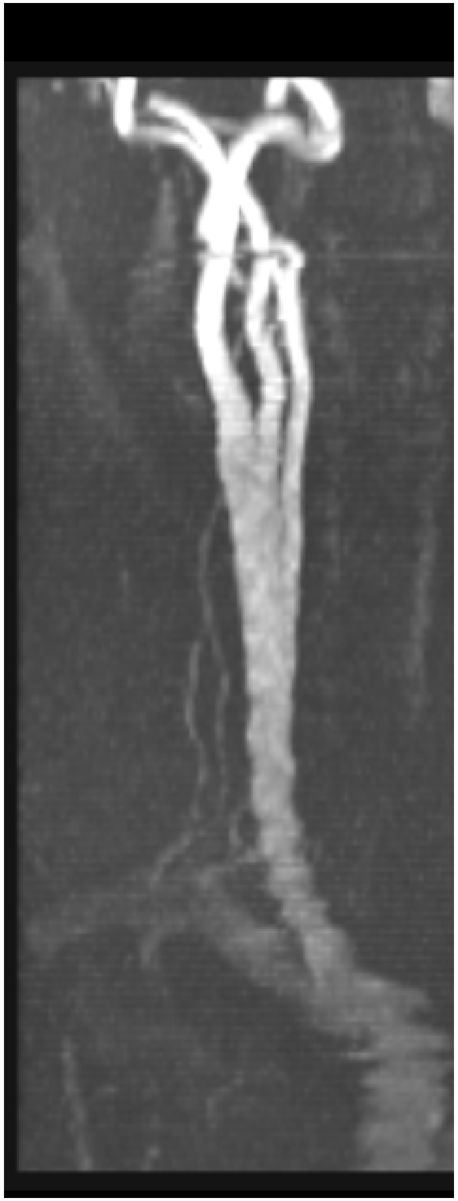

70f Score

0-30

31-50

51-70

>70

Near occlusion

Occluded

Quality

1

2

3

4

5

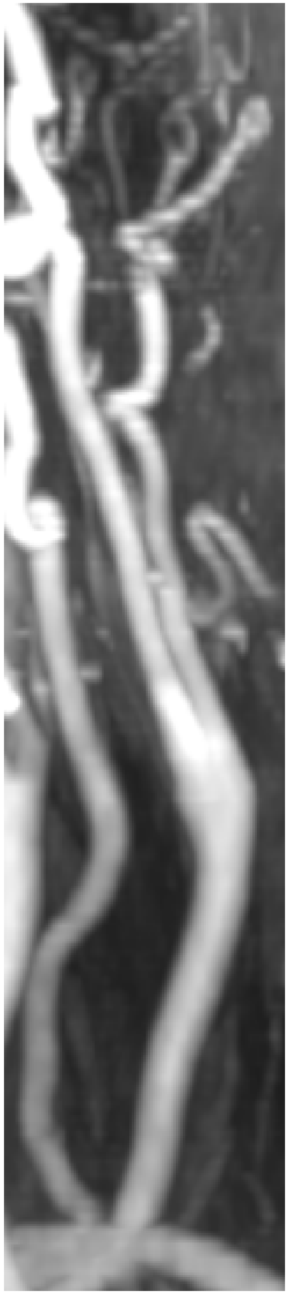

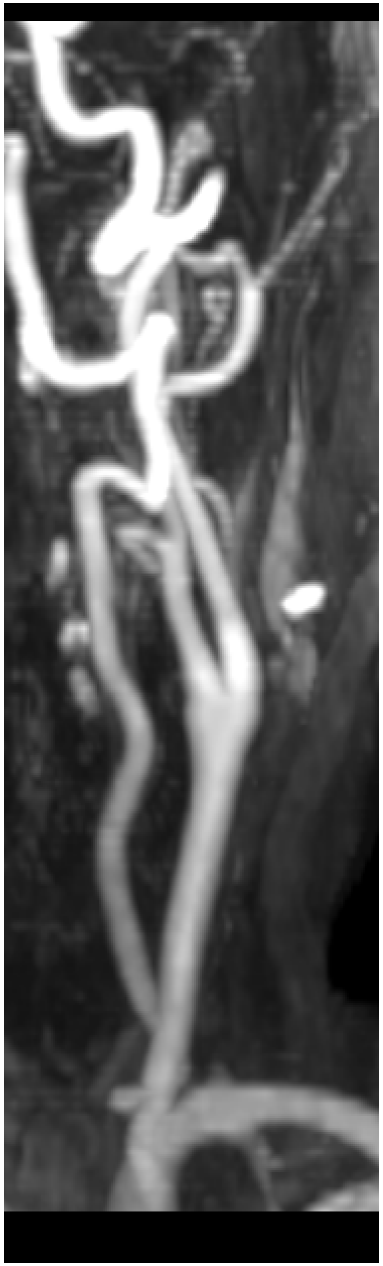

71e Score

0-30

31-50

51-70

>70

Near occlusion

Occluded

Quality

1

2

3

4

5

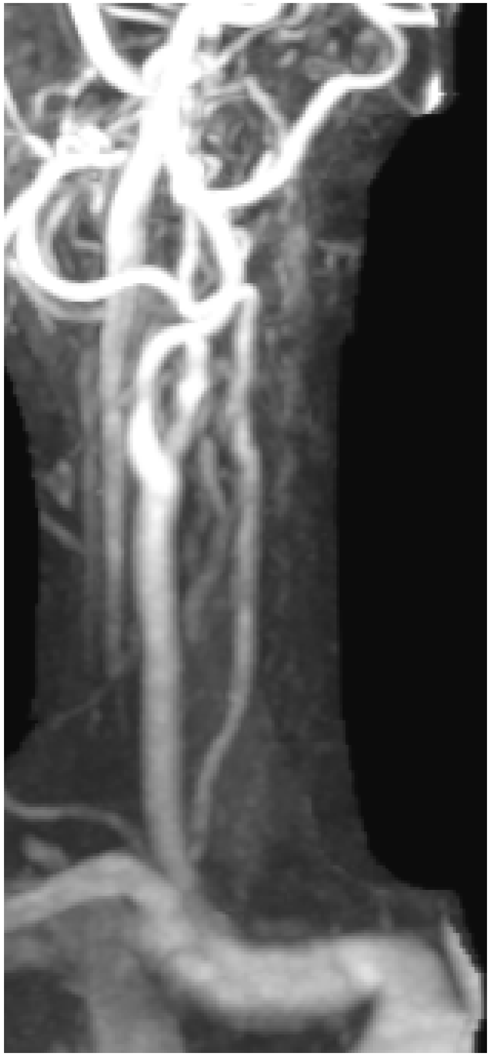

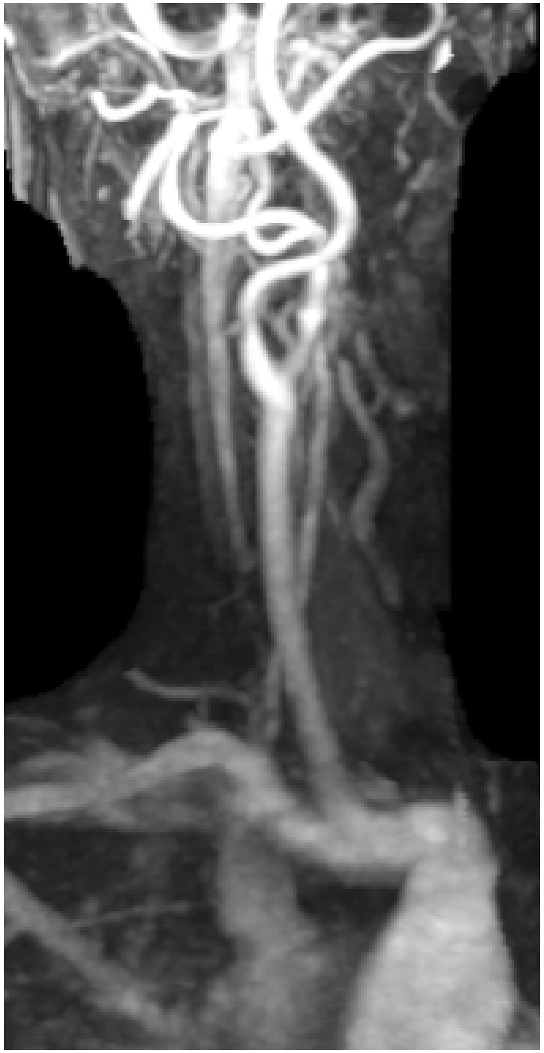

72d Score

0-30

31-50

51-70

>70

Near occlusion

Occluded

Quality

1

2

3

4

5

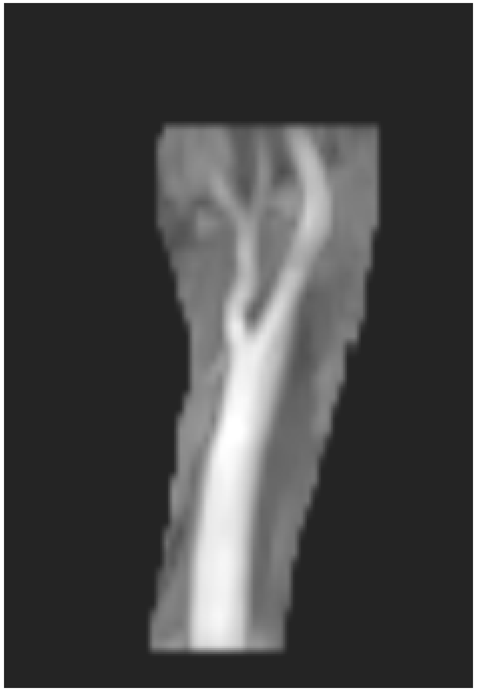

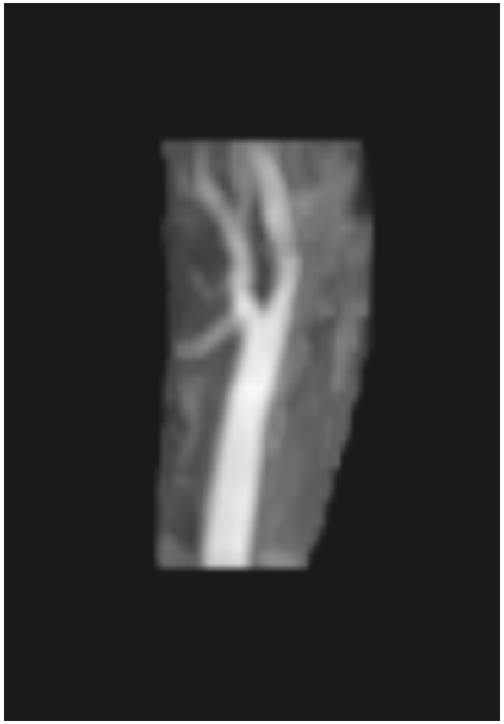

73c Score

0-30

31-50

51-70

>70

Near occlusion

Occluded

Quality

1

2

3

4

5

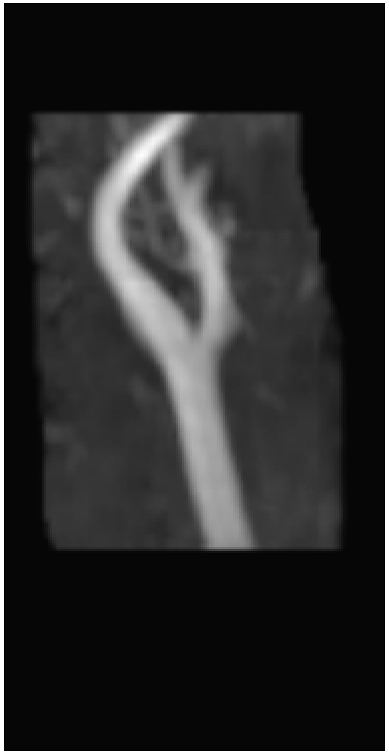

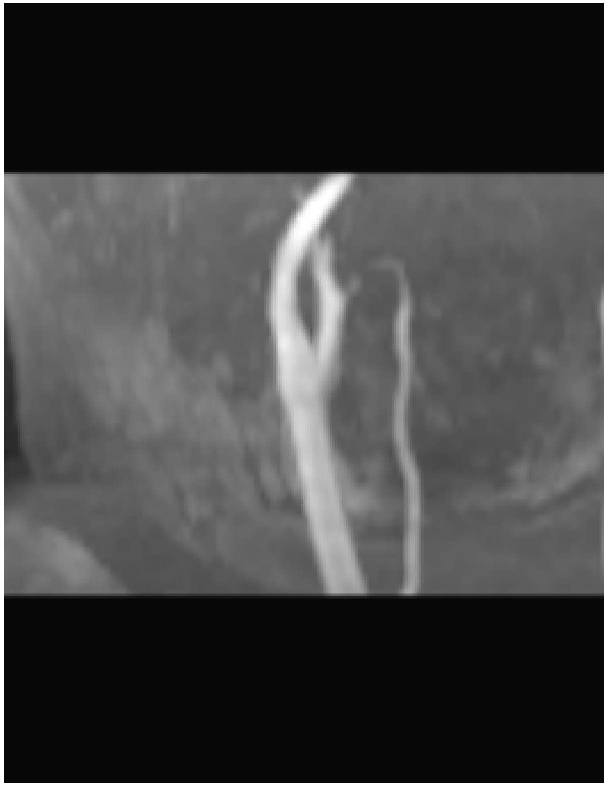

74b Score

0-30

31-50

51-70

>70

Near occlusion

Occluded

Quality

1

2

3

4

5

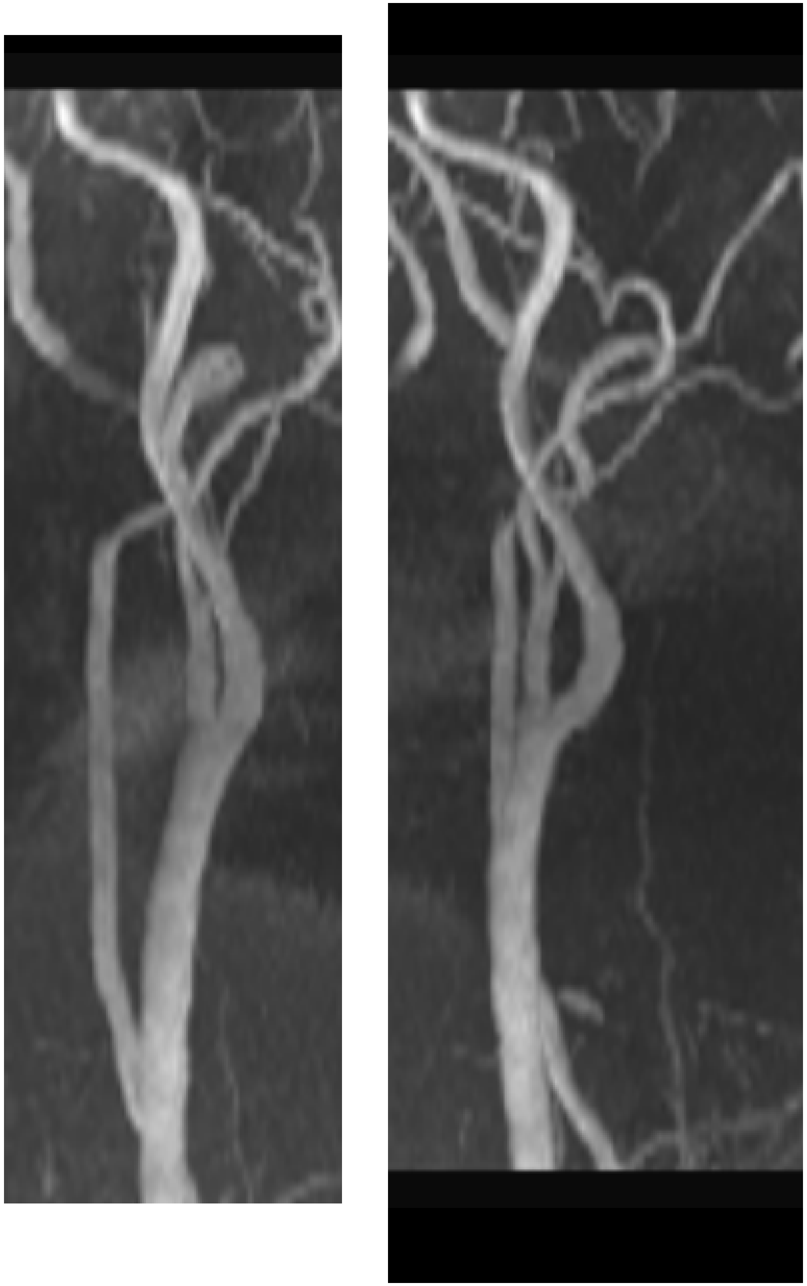

75a Score

0-30

31-50

51-70

>70

Near occlusion

Occluded

Quality

1

2

3

4

5

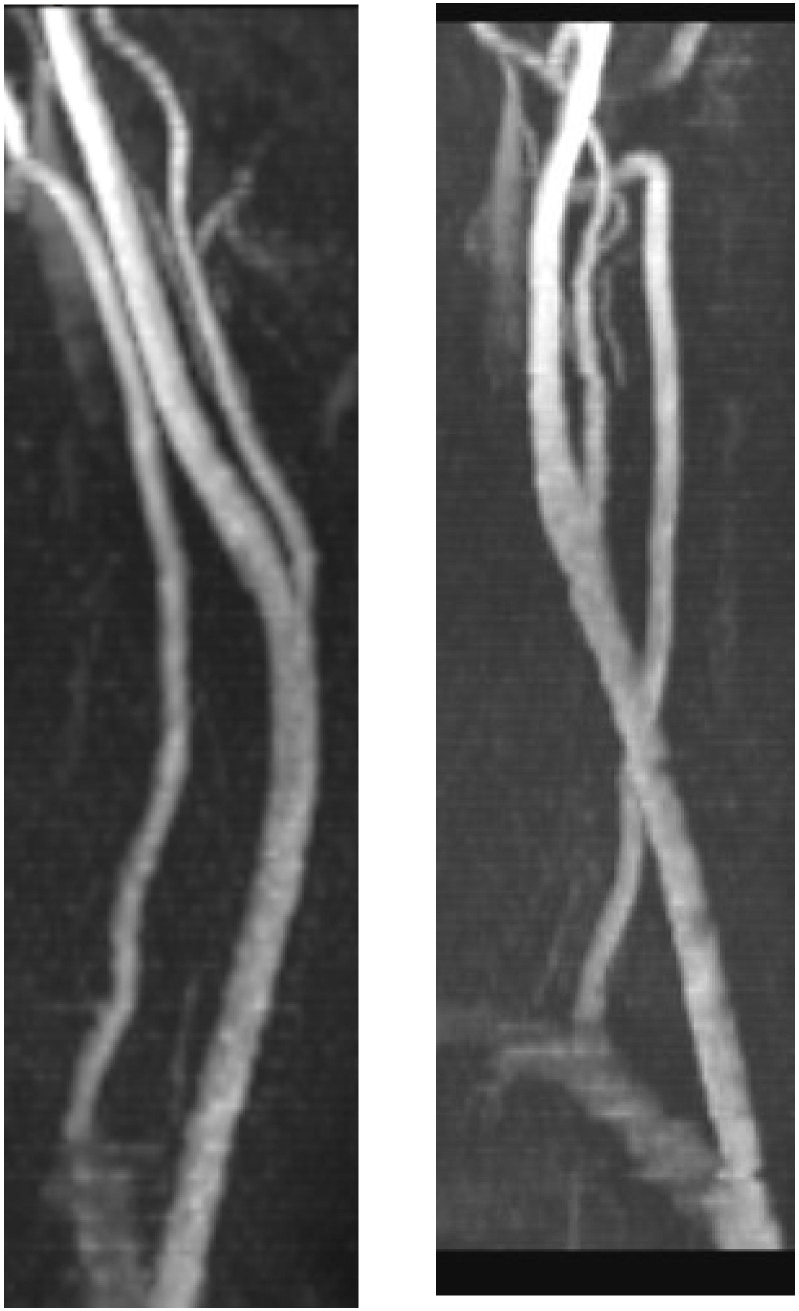

75f Score

0-30

31-50

51-70

>70

Near occlusion

Occluded

Quality

1

2

3

4

5

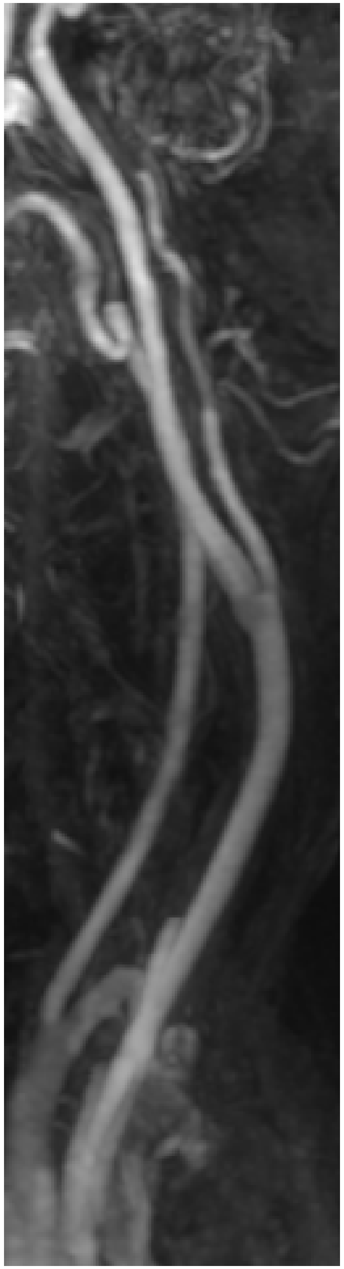

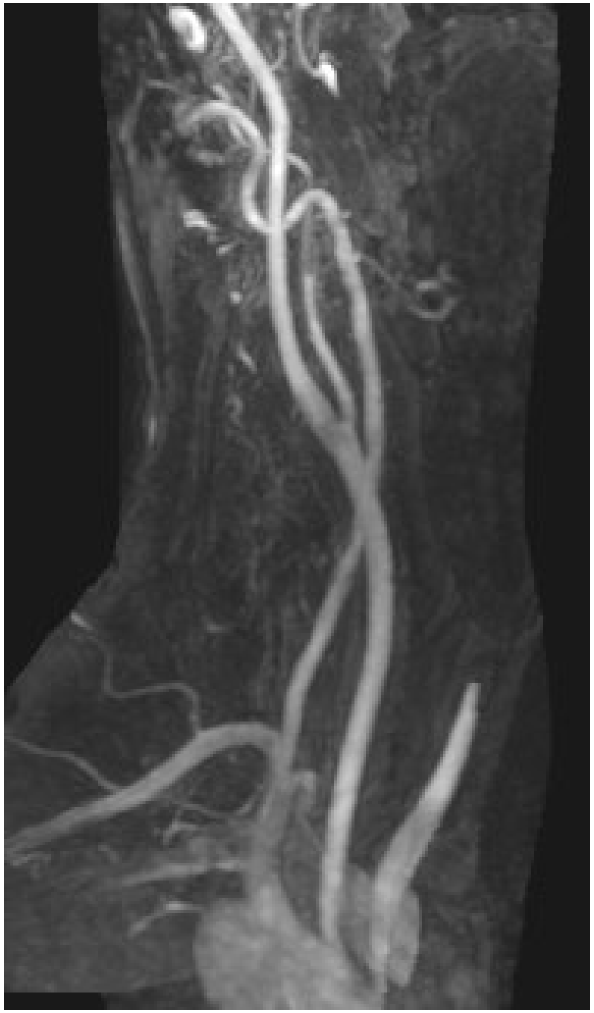

76e Score

0-30

31-50

51-70

>70

Near occlusion

Occluded

Quality

1

2

3

4

5

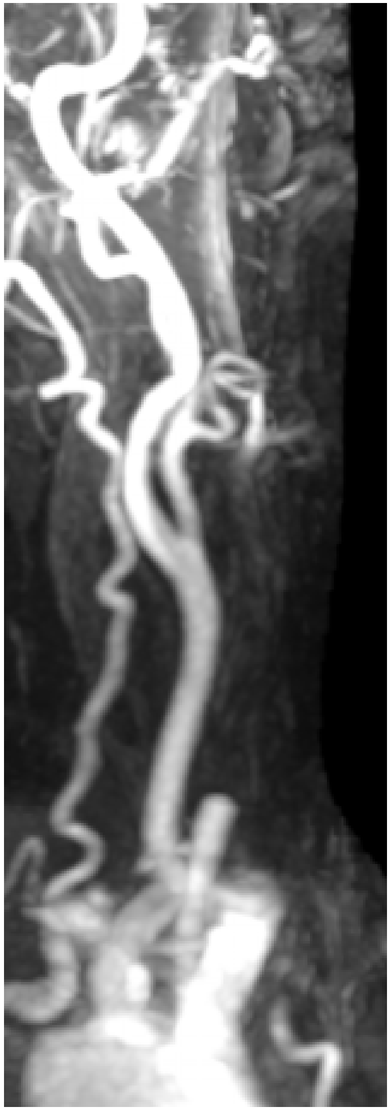

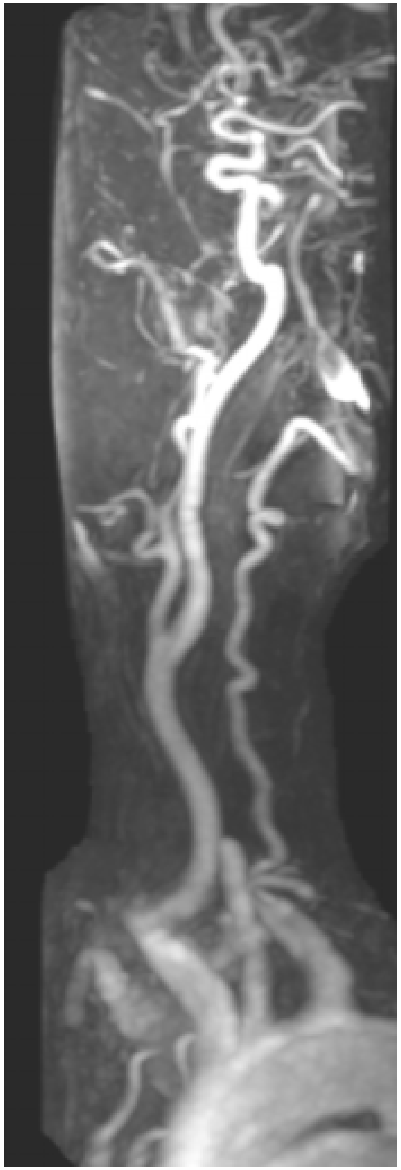

77d Score

0-30

31-50

51-70

>70

Near occlusion

Occluded

Quality

1

2

3

4

5

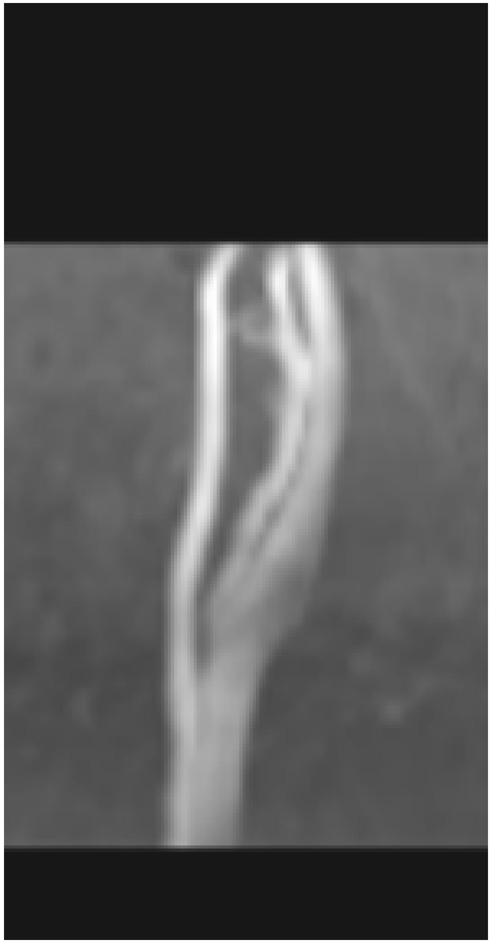

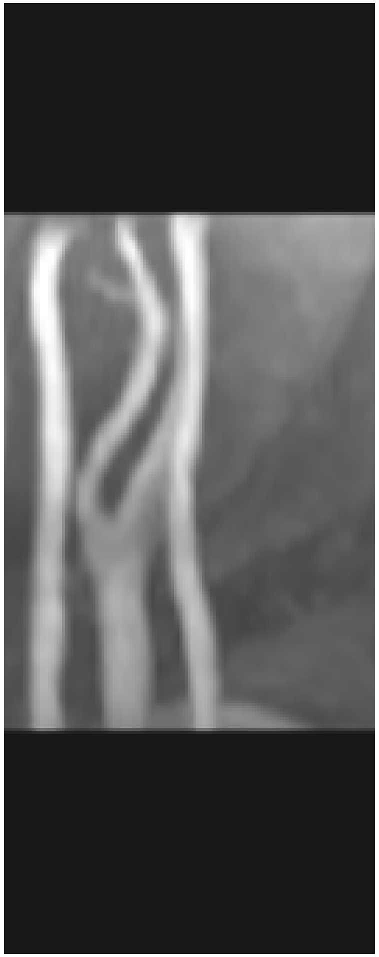

78c Score

0-30

31-50

51-70

>70

Near occlusion

Occluded

Quality

1

2

3

4

5

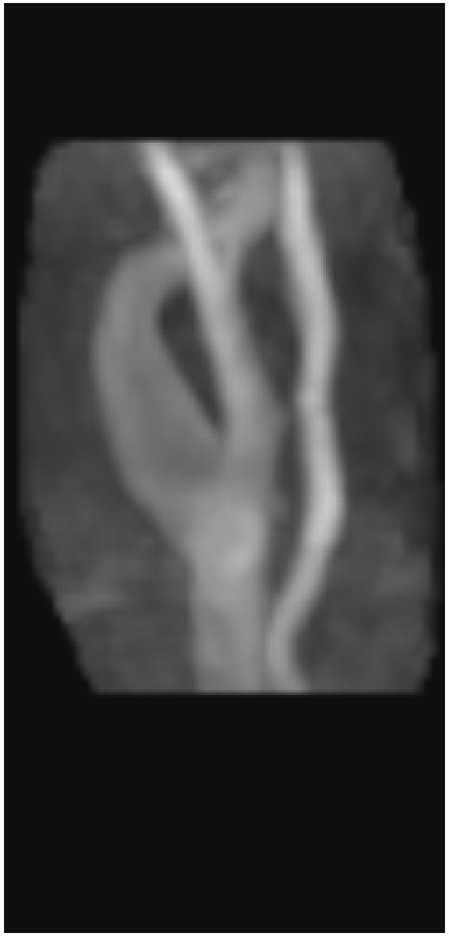

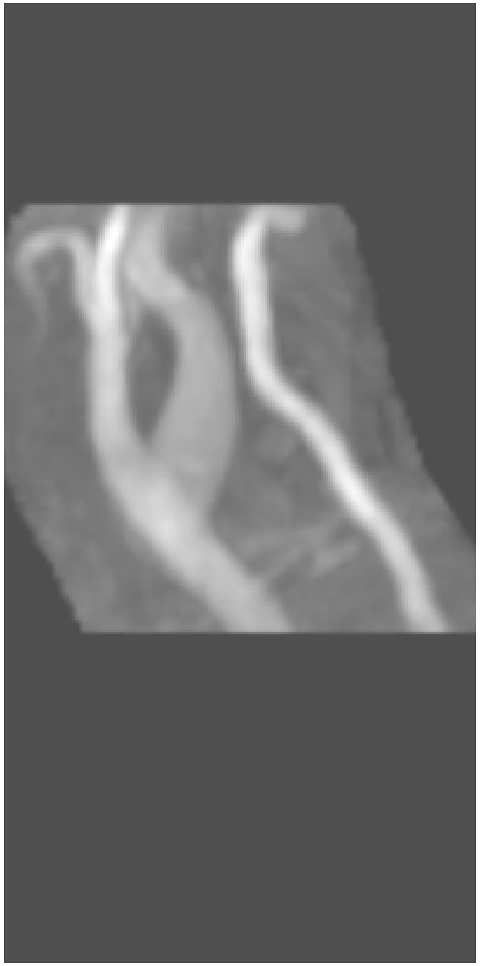

79b Score

0-30

31-50

51-70

>70

Near occlusion

Occluded

Quality

1

2

3

4

5

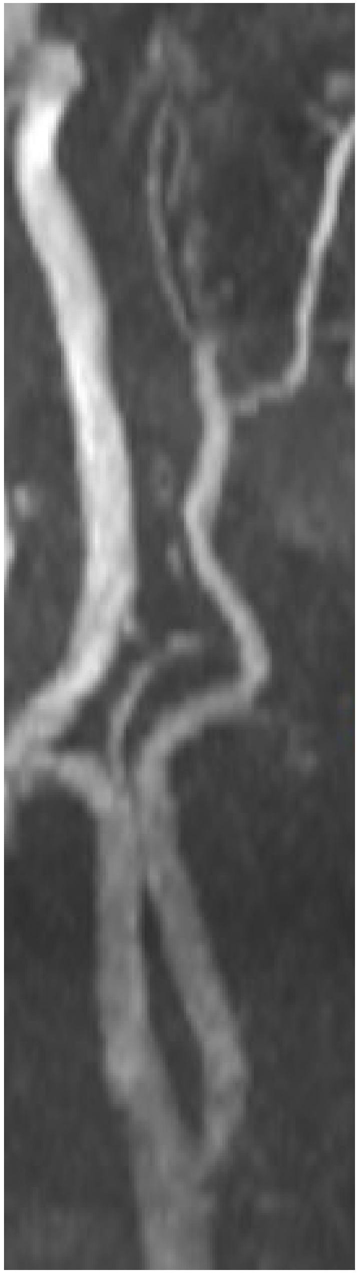

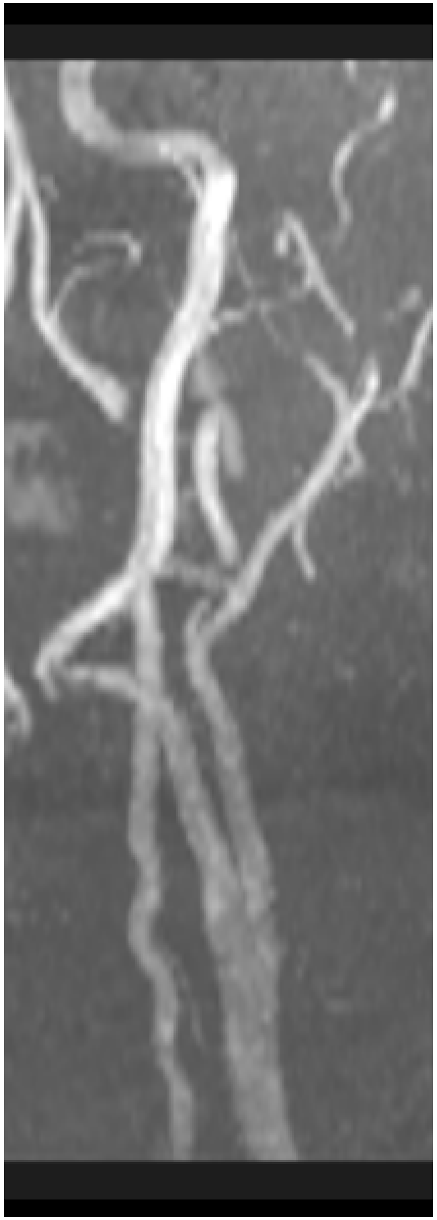

80a Score

0-30

31-50

51-70

>70

Near occlusion

Occluded

Quality

1

2

3

4

5

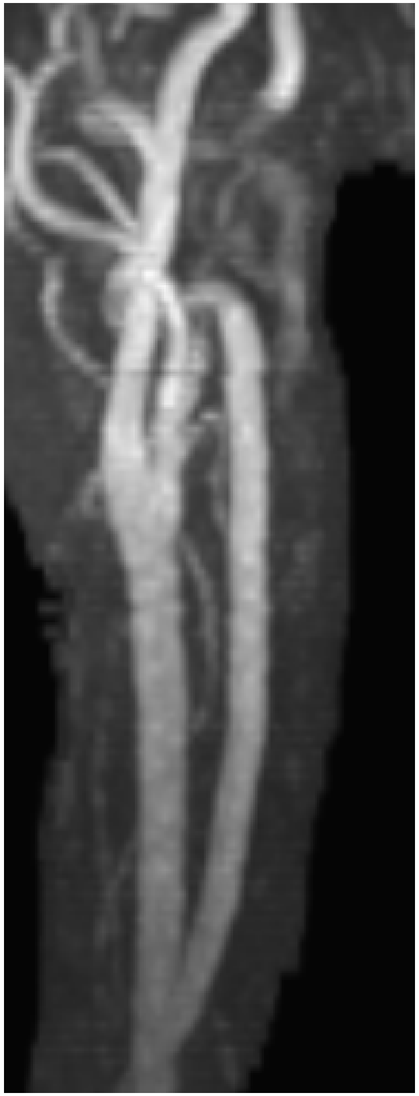

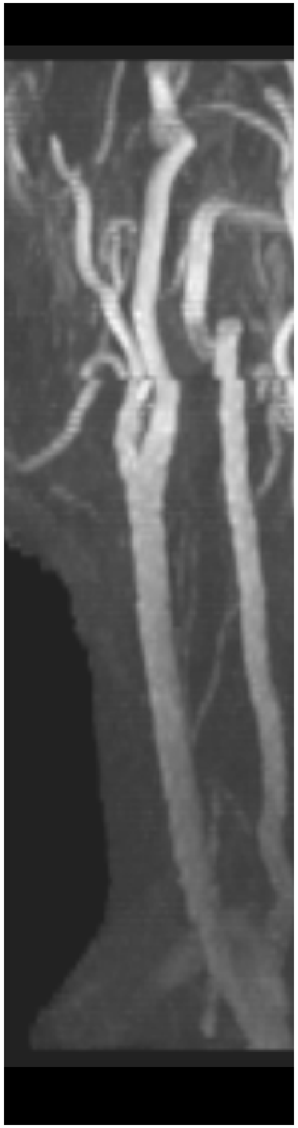

80f Score

0-30

31-50

51-70

>70

Near occlusion

Occluded

Quality

1

2

3

4

5

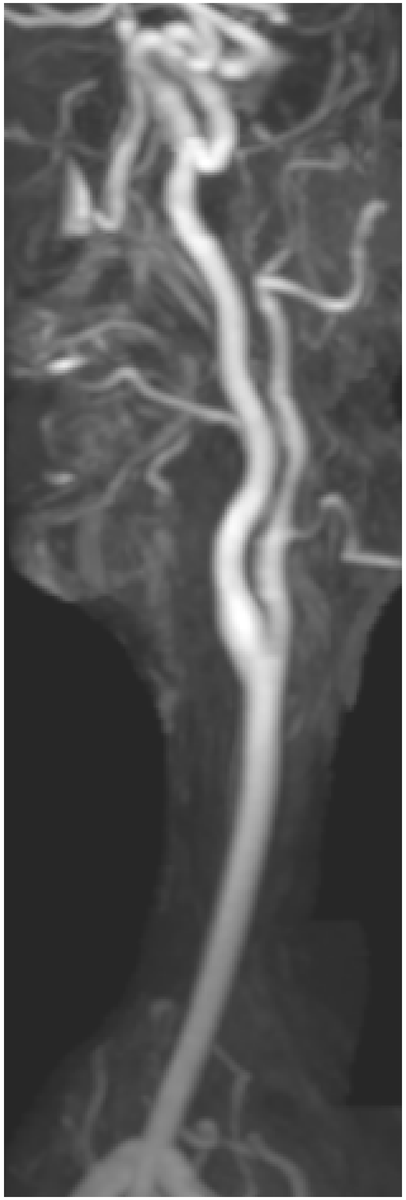

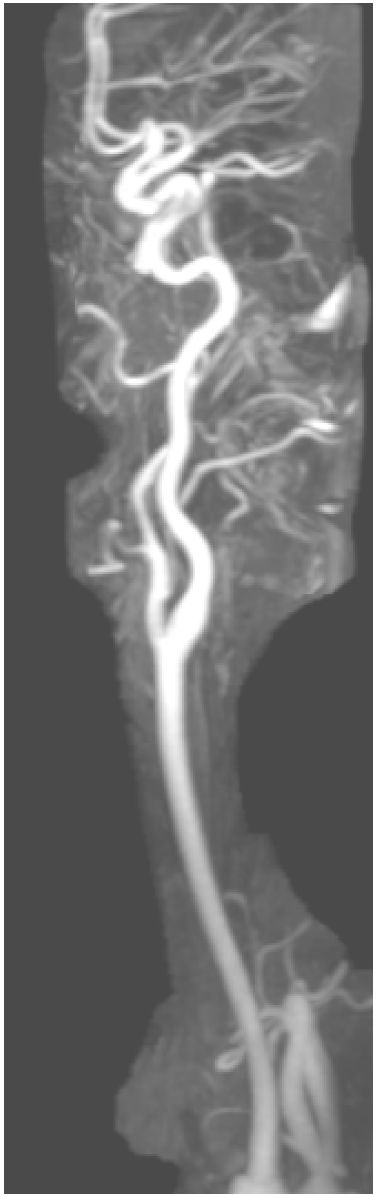

81e Score

0-30

31-50

51-70

>70

Near occlusion

Occluded

Quality

1

2

3

4

5

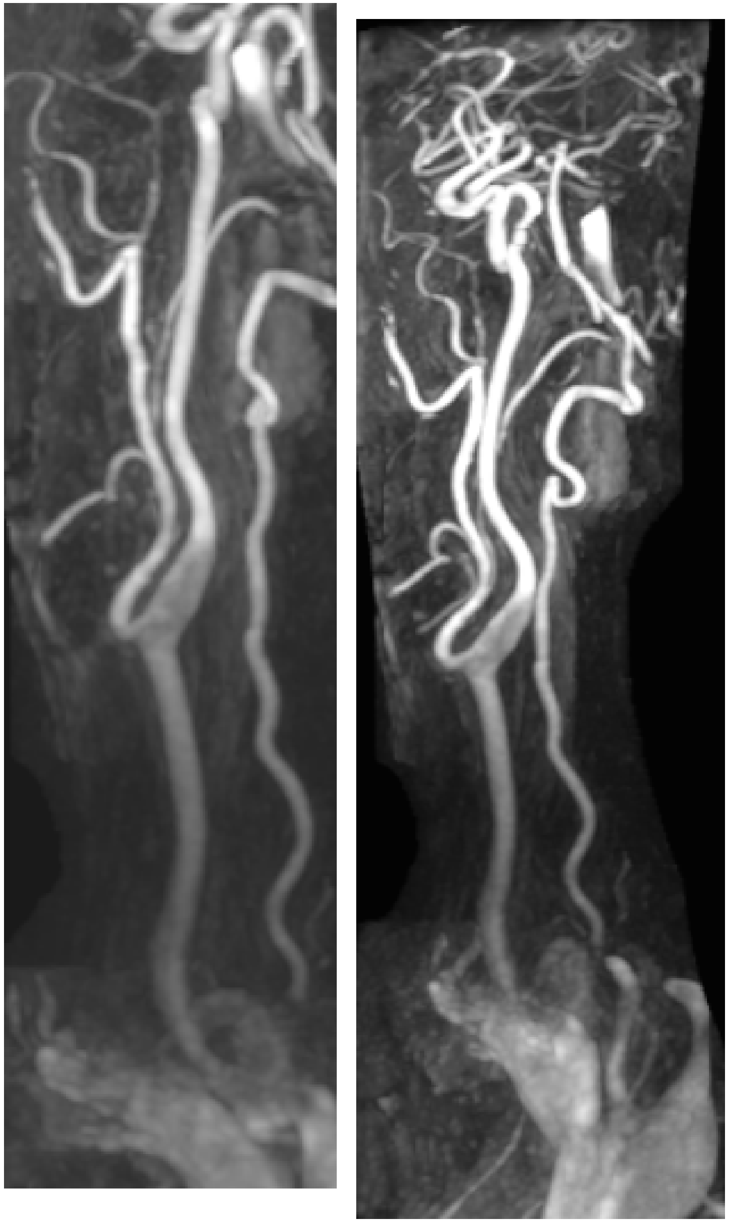

82d Score

0-30

31-50

51-70

>70

Near occlusion

Occluded

Quality

1

2

3

4

5

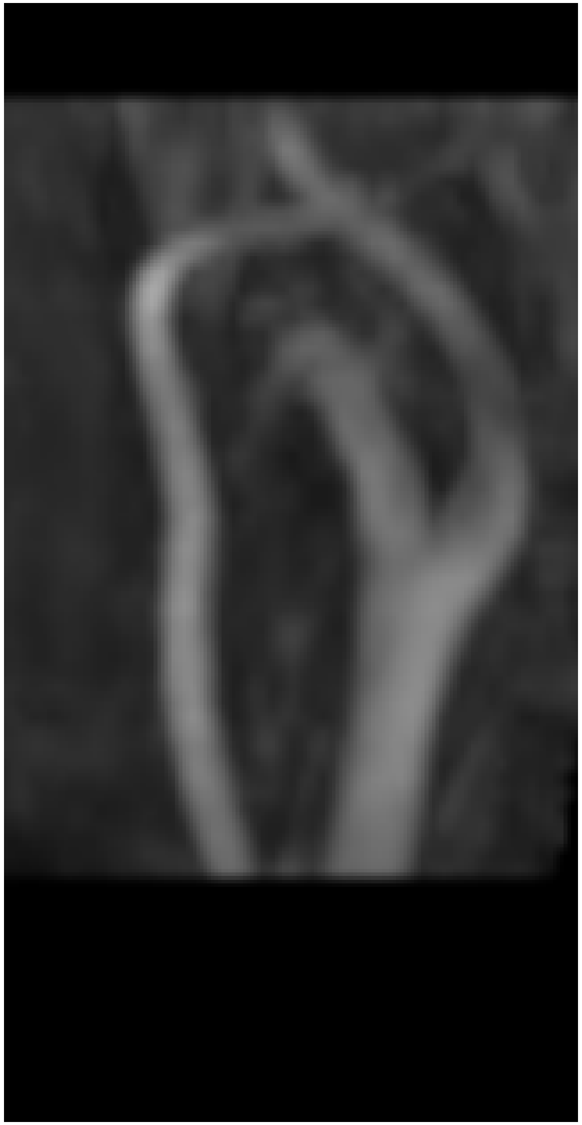

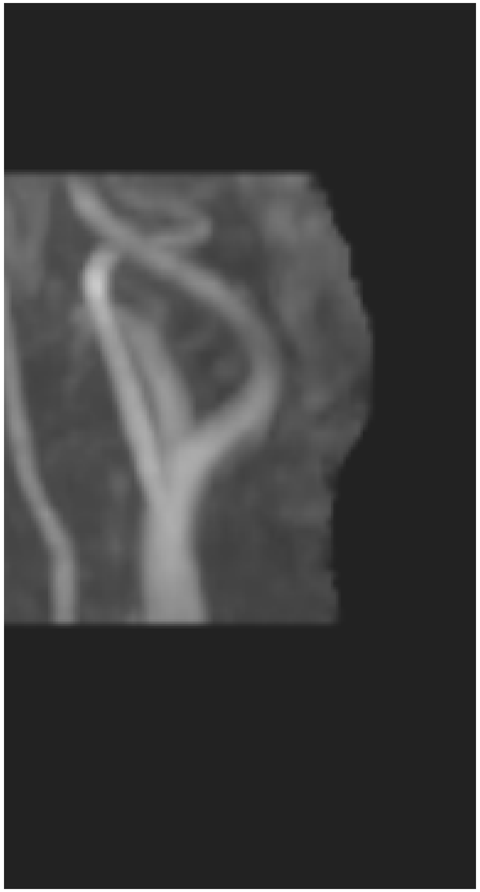

83c Score

0-30

31-50

51-70

>70

Near occlusion

Occluded

Quality

1

2

3

4

5

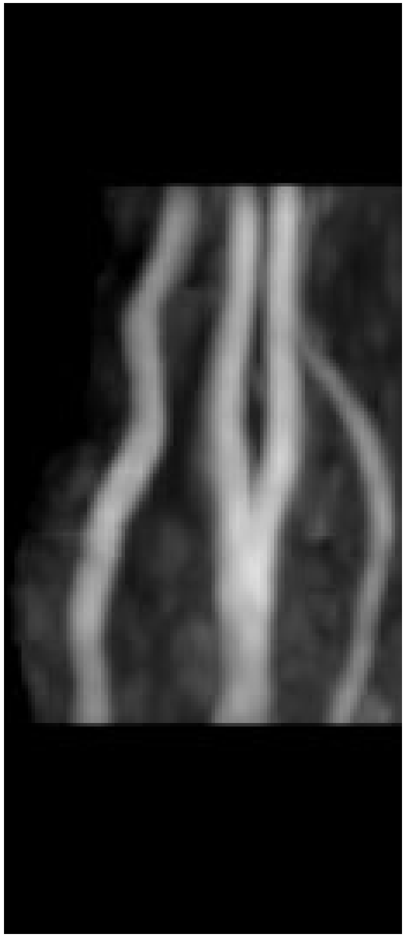

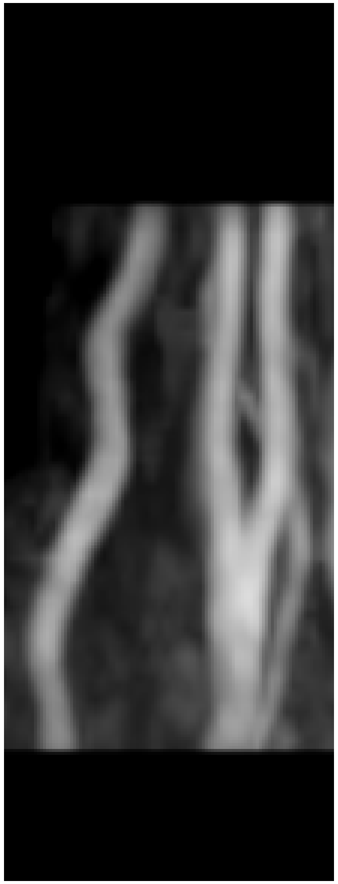

84b Score

0-30

31-50

51-70

>70

Near occlusion

Occluded

Quality

1

2

3

4

5

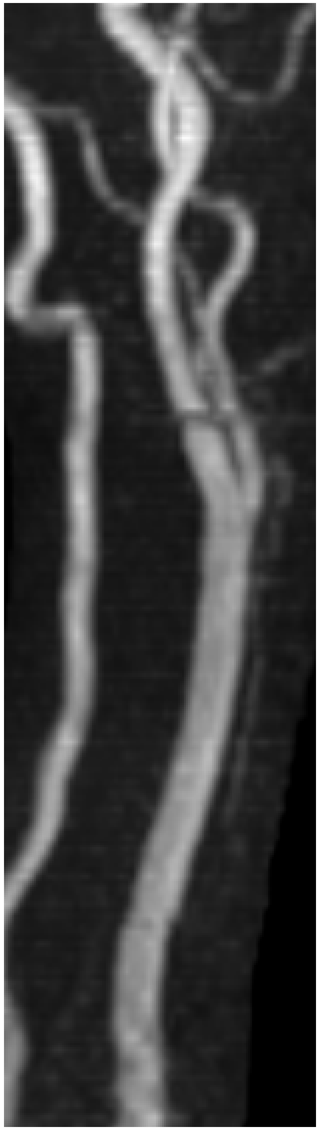

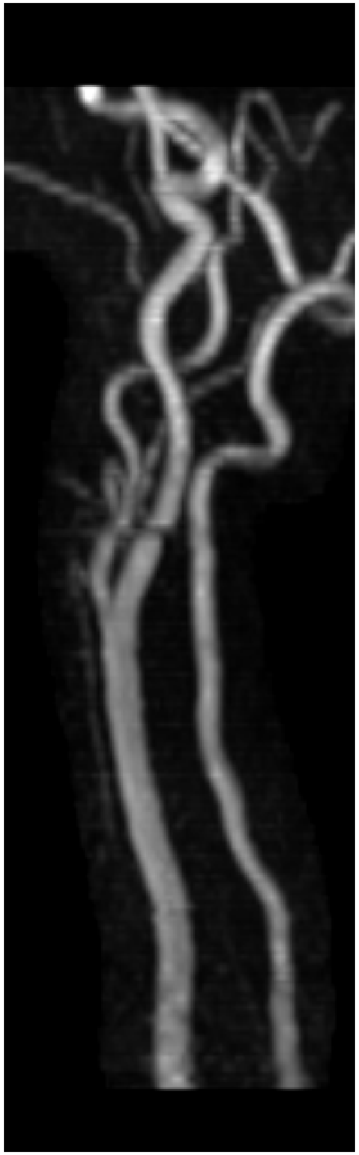

85a Score

0-30

31-50

51-70

>70

Near occlusion

Occluded

Quality

1

2

3

4

5

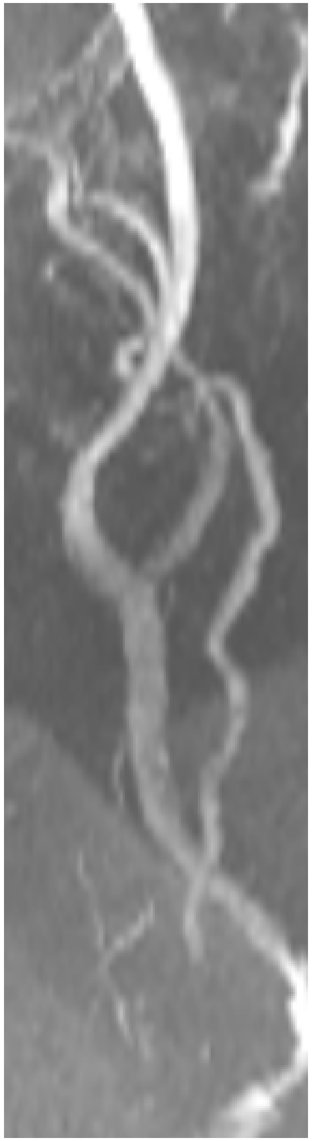

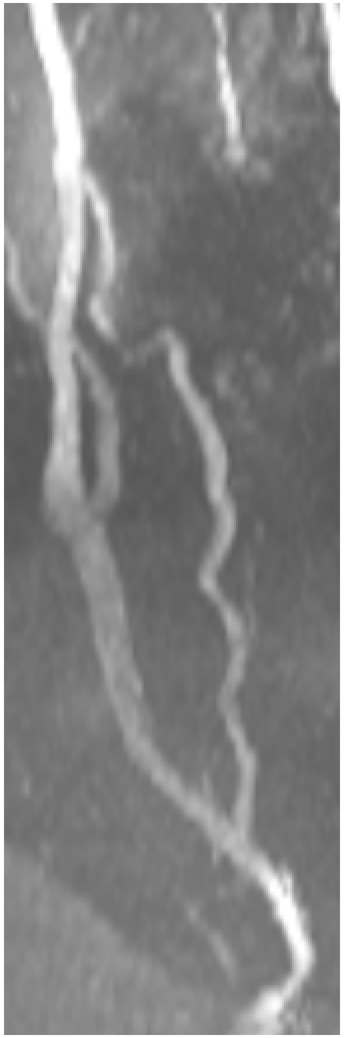

85f Score

0-30

31-50

51-70

>70

Near occlusion

Occluded

Quality

1

2

3

4

5

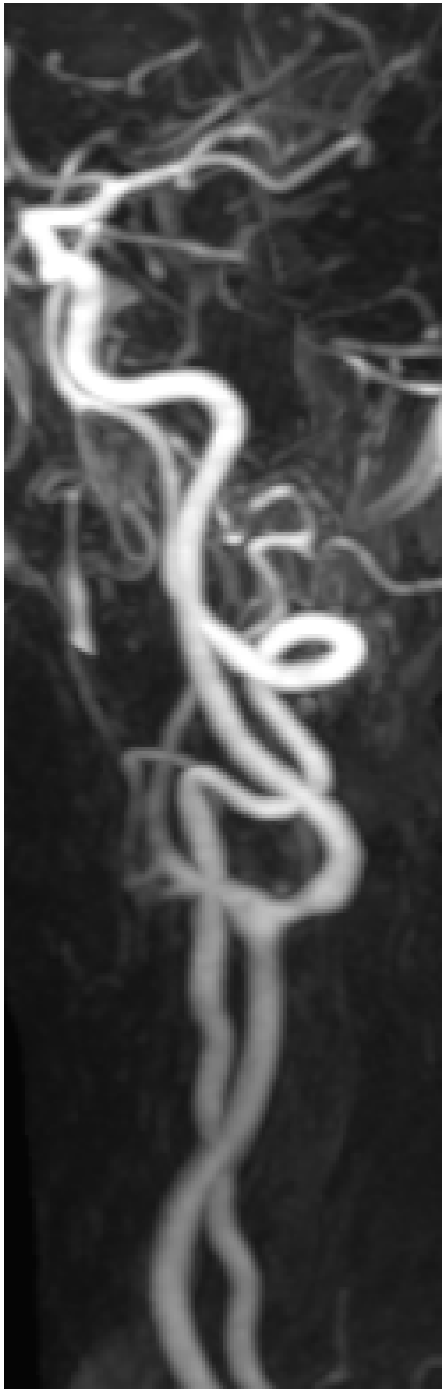

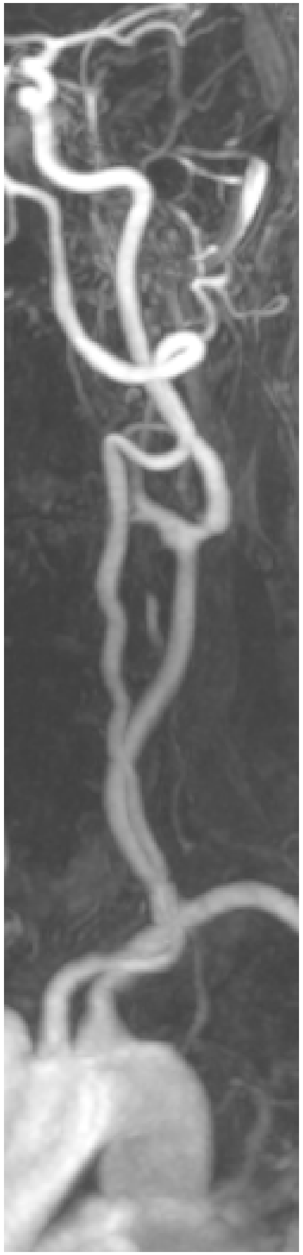

86e Score

0-30

31-50

51-70

>70

Near occlusion

Occluded

Quality

1

2

3

4

5

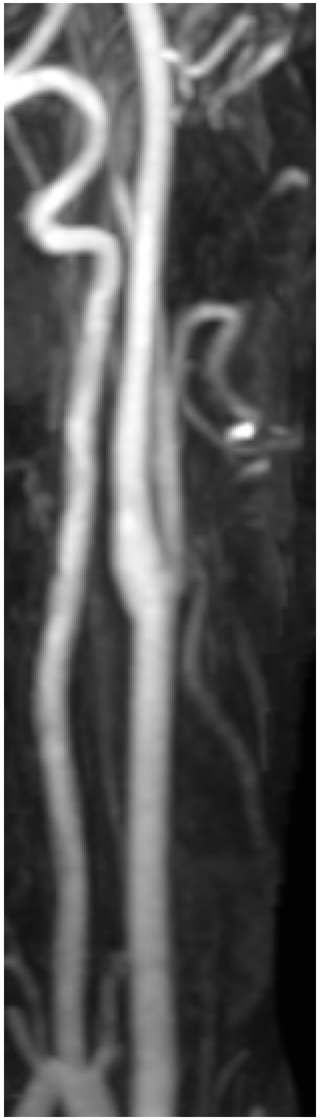

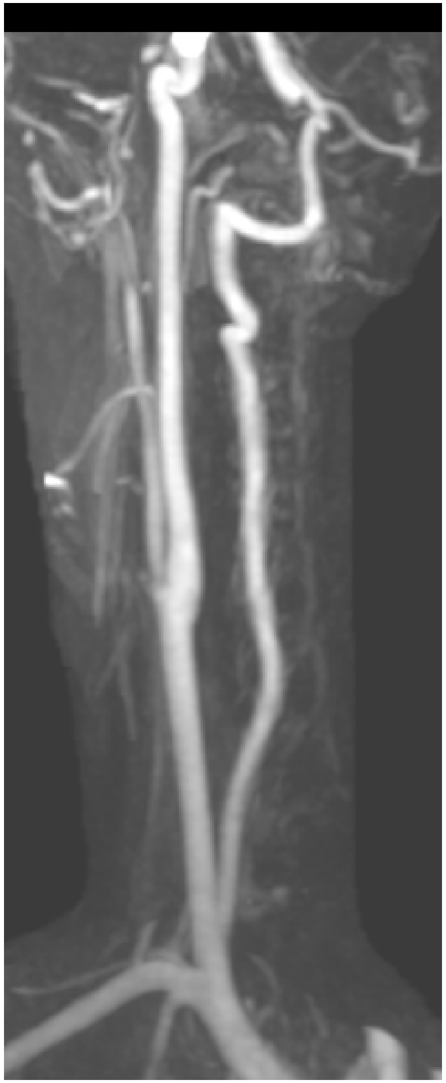

87d Score

0-30

31-50

51-70

>70

Near occlusion

Occluded

Quality

1

2

3

4

5

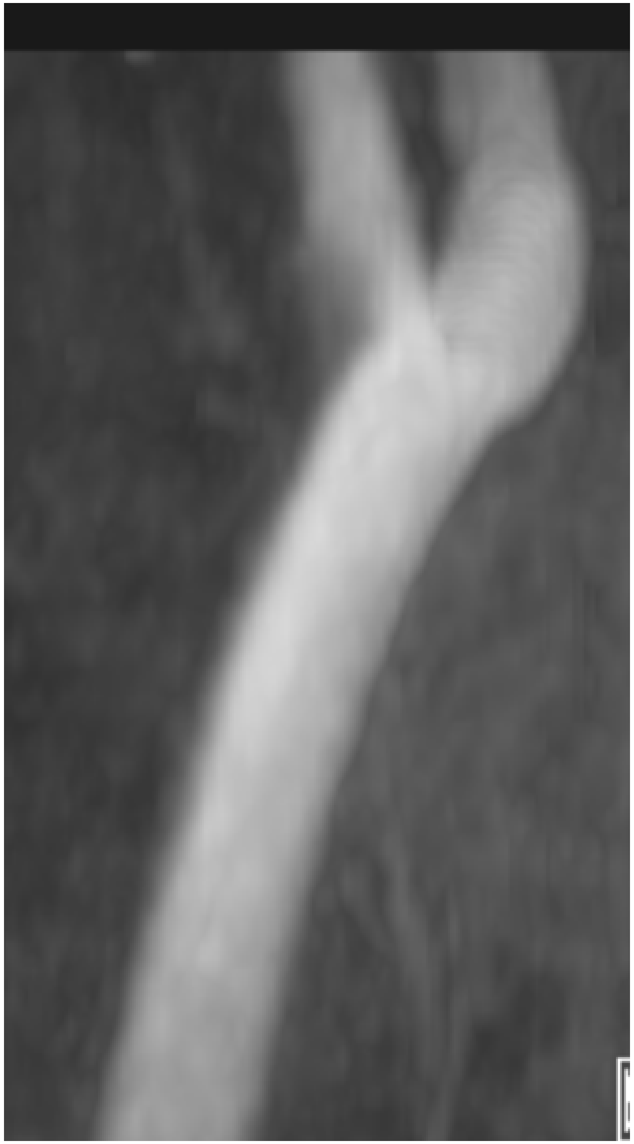

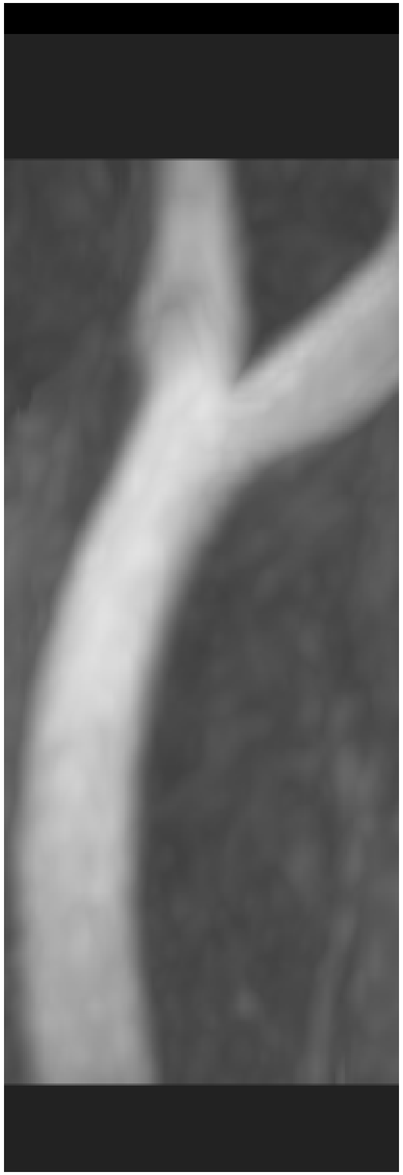

88c Score

0-30

31-50

51-70

>70

Near occlusion

Occluded

Quality

1

2

3

4

5

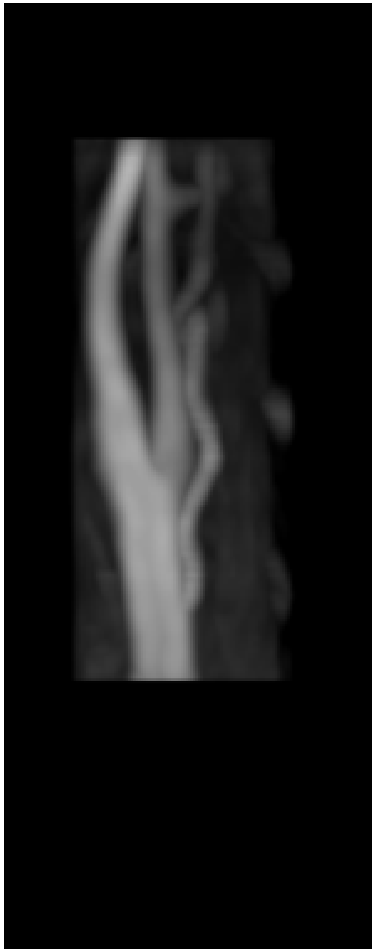

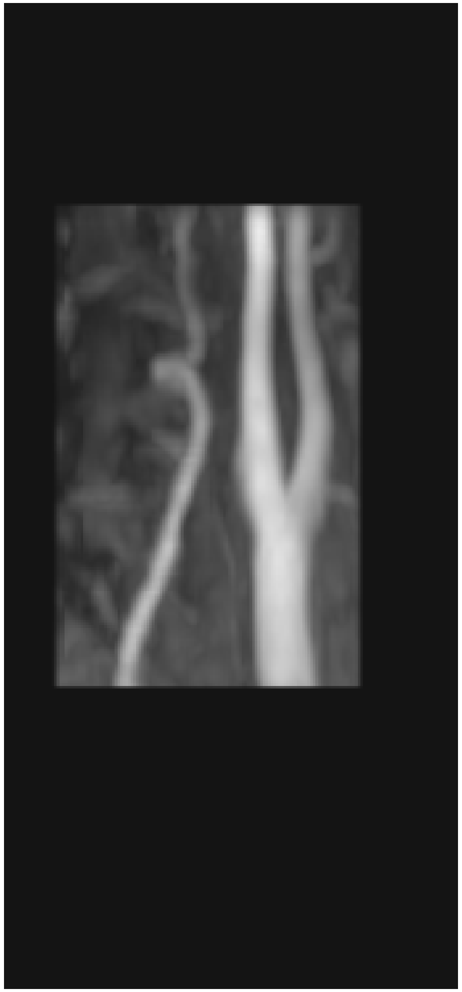

89b Score

0-30

31-50

51-70

>70

Near occlusion

Occluded

Quality

1

2

3

4

5

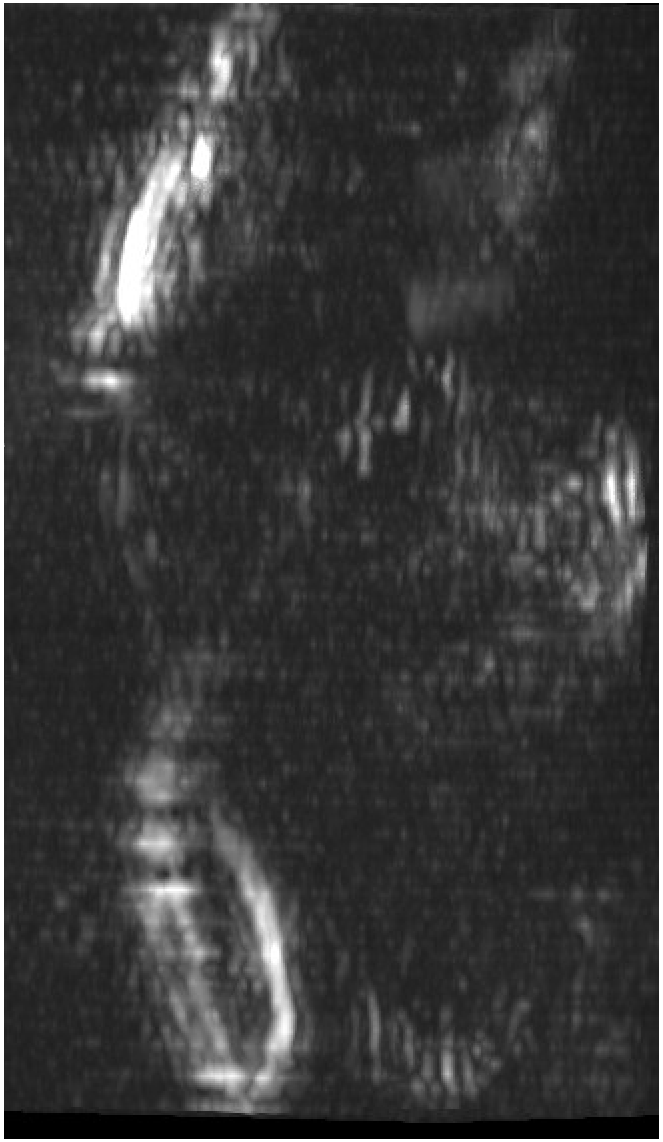

90a Score

0-30

31-50

51-70

>70

Near occlusion

Occluded

Quality

1

2

3

4

5

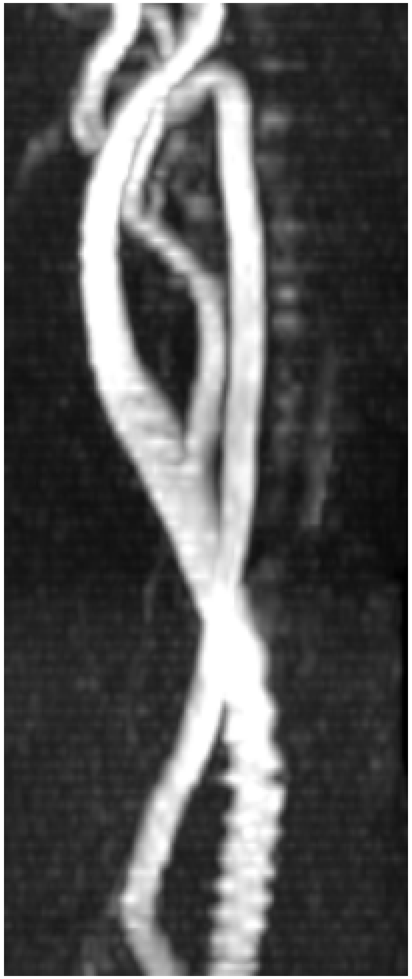

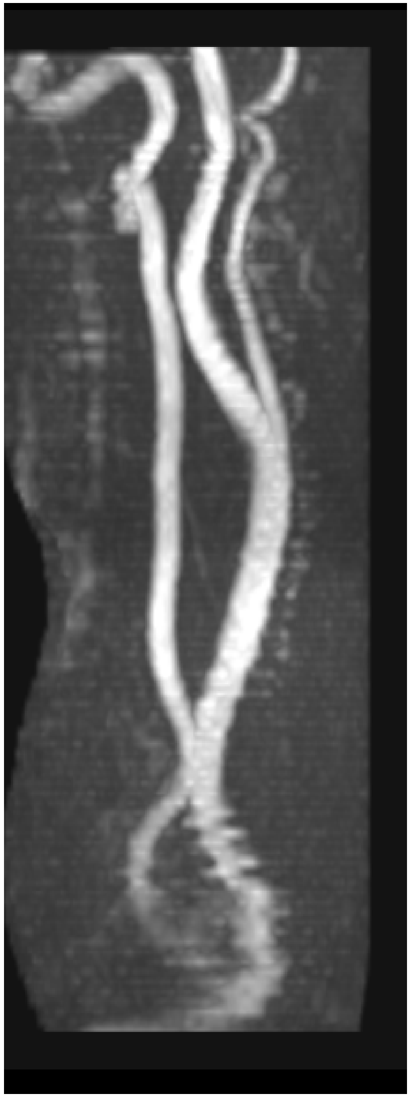

90f Score

0-30

31-50

51-70

>70

Near occlusion

Occluded

Quality

1

2

3

4

5

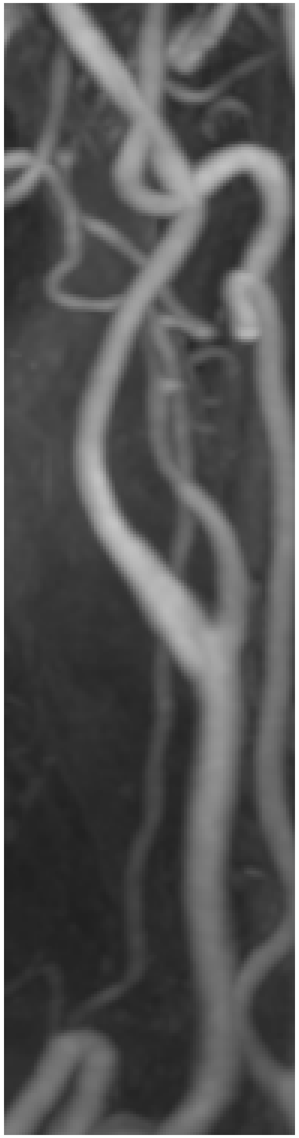

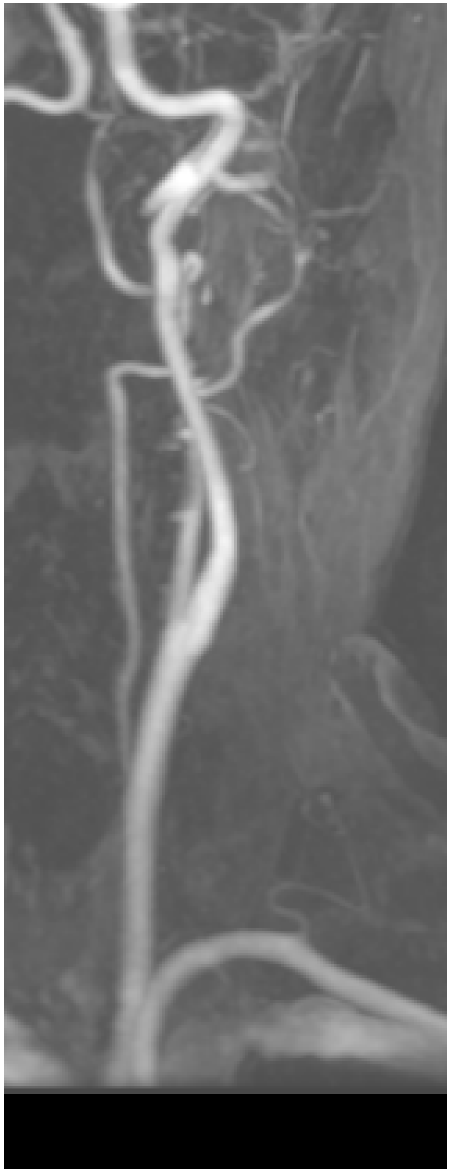

91e Score

0-30

31-50

51-70

>70

Near occlusion

Occluded

Quality

1

2

3

4

5

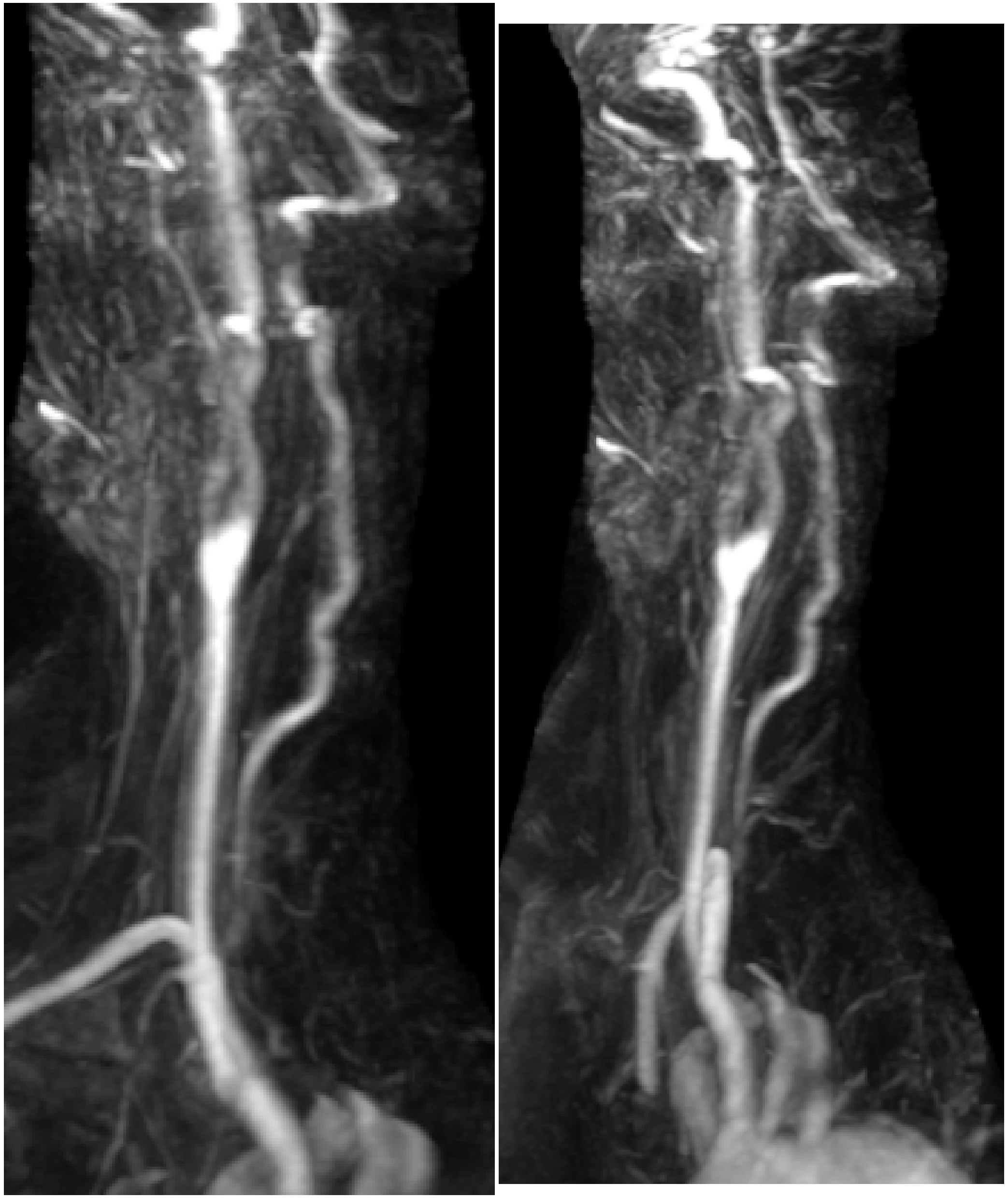

92d Score

0-30

31-50

51-70

>70

Near occlusion

Occluded

Quality

1

2

3

4

5

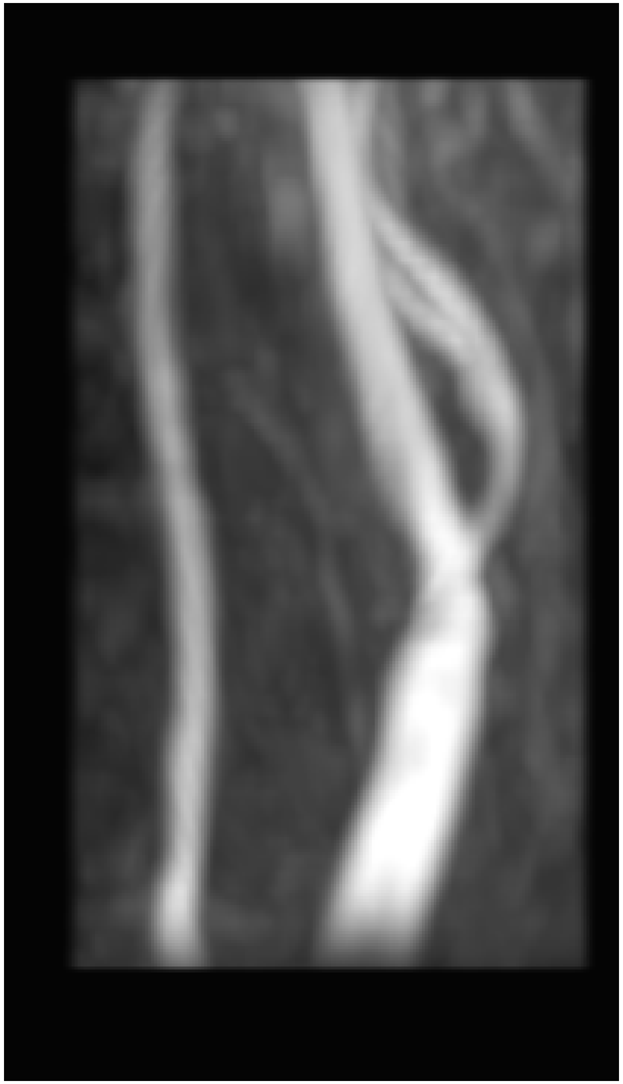

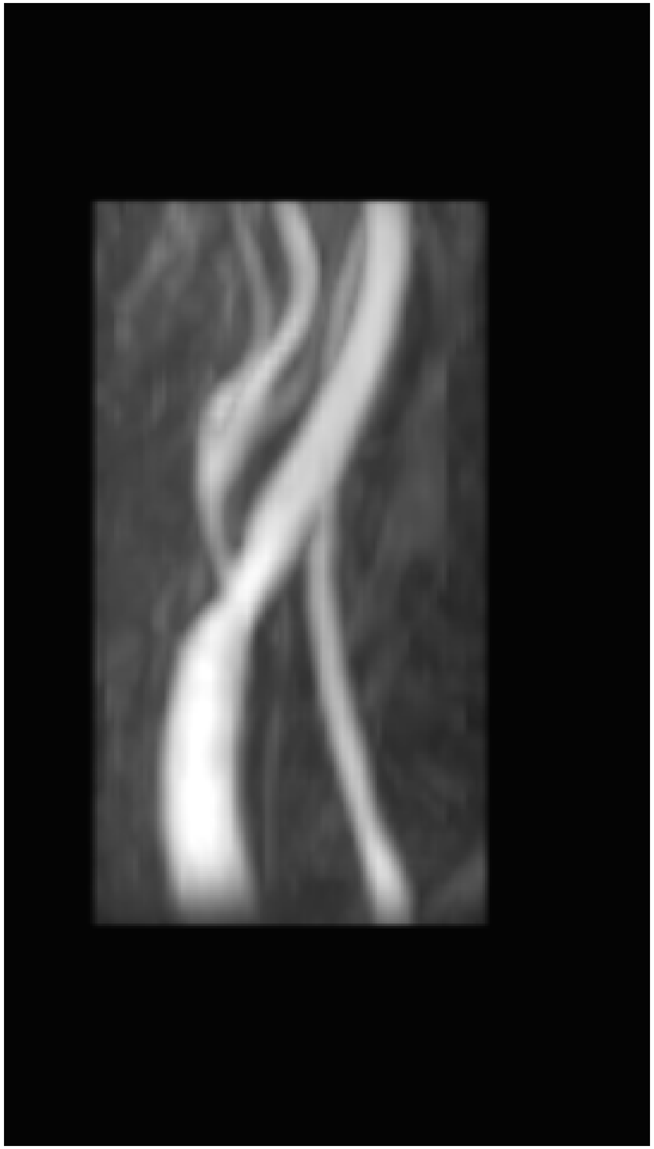

93c Score

0-30

31-50

51-70

>70

Near occlusion

Occluded

Quality

1

2

3

4

5

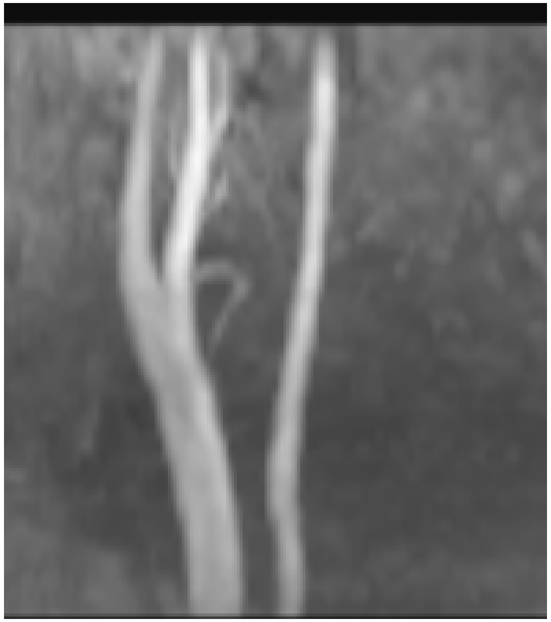

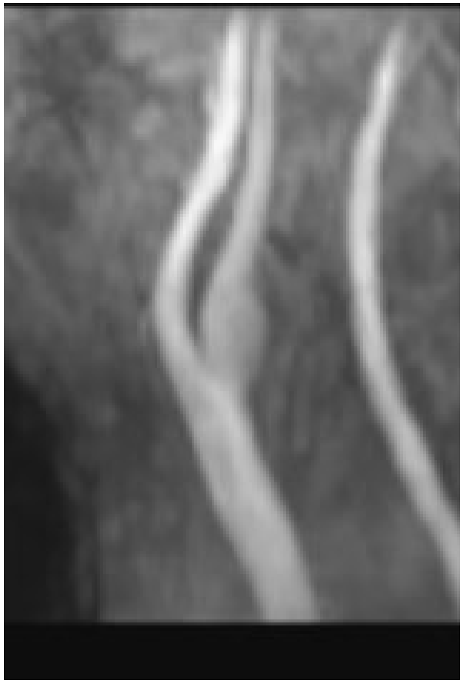

94b Score

0-30

31-50

51-70

>70

Near occlusion

Occluded

Quality

1

2

3

4

5

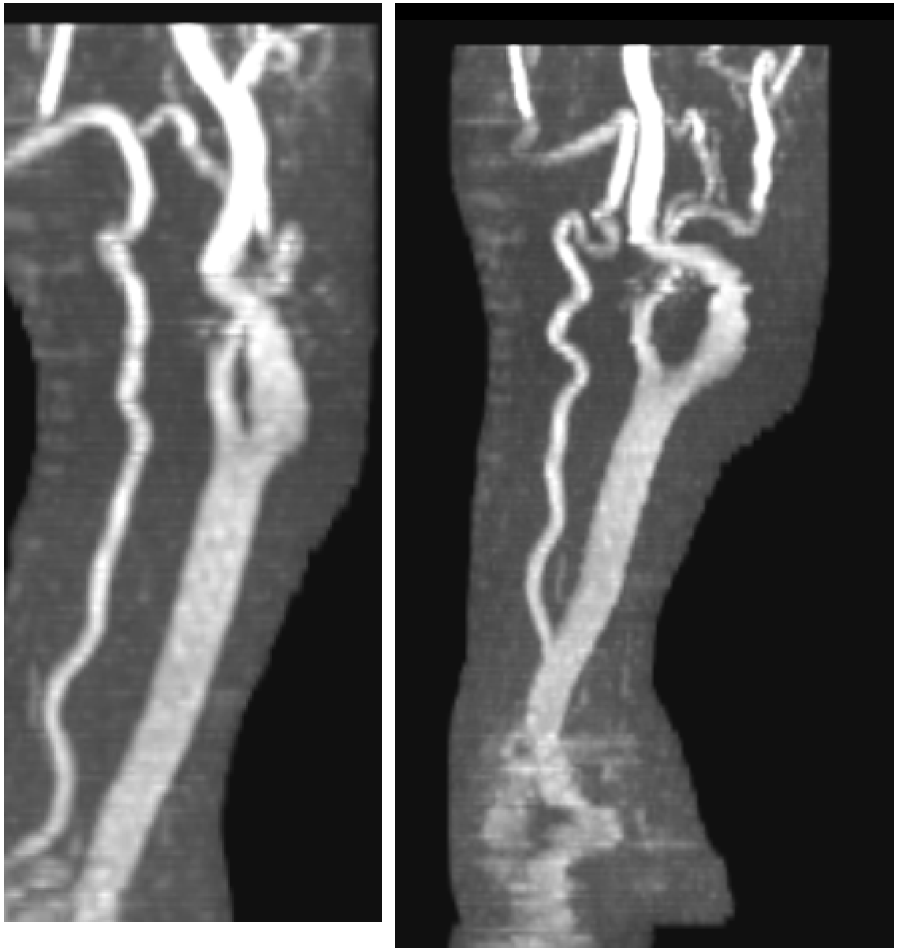

95a Score

0-30

31-50

51-70

>70

Near occlusion

Occluded

Quality

1

2

3

4

5

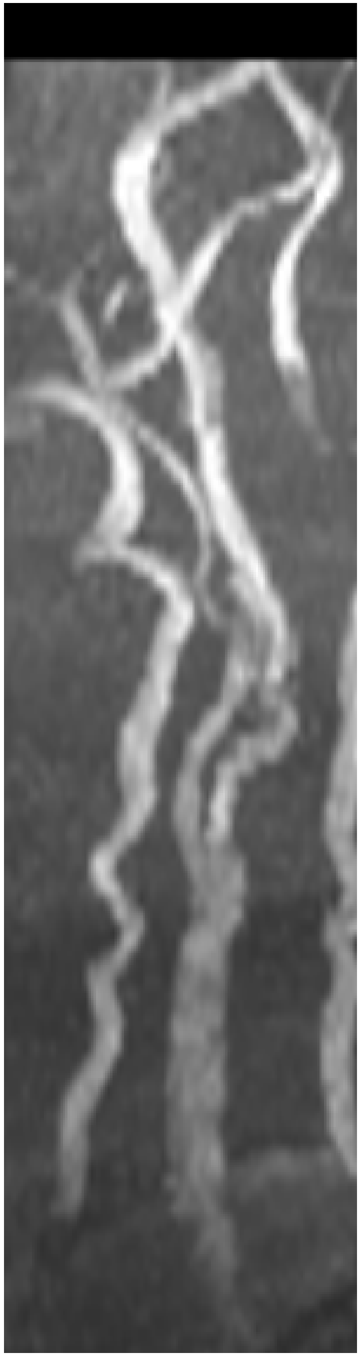

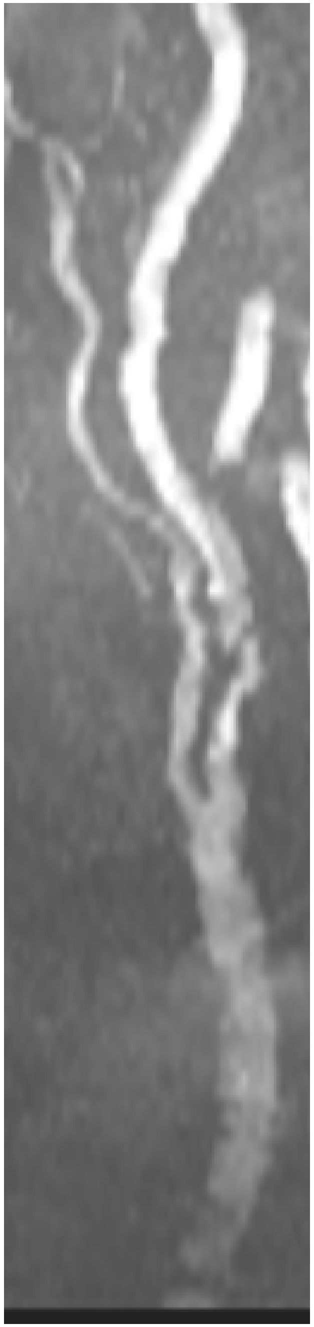

95f Score

0-30

31-50

51-70

>70

Near occlusion

Occluded

Quality

1

2

3

4

5

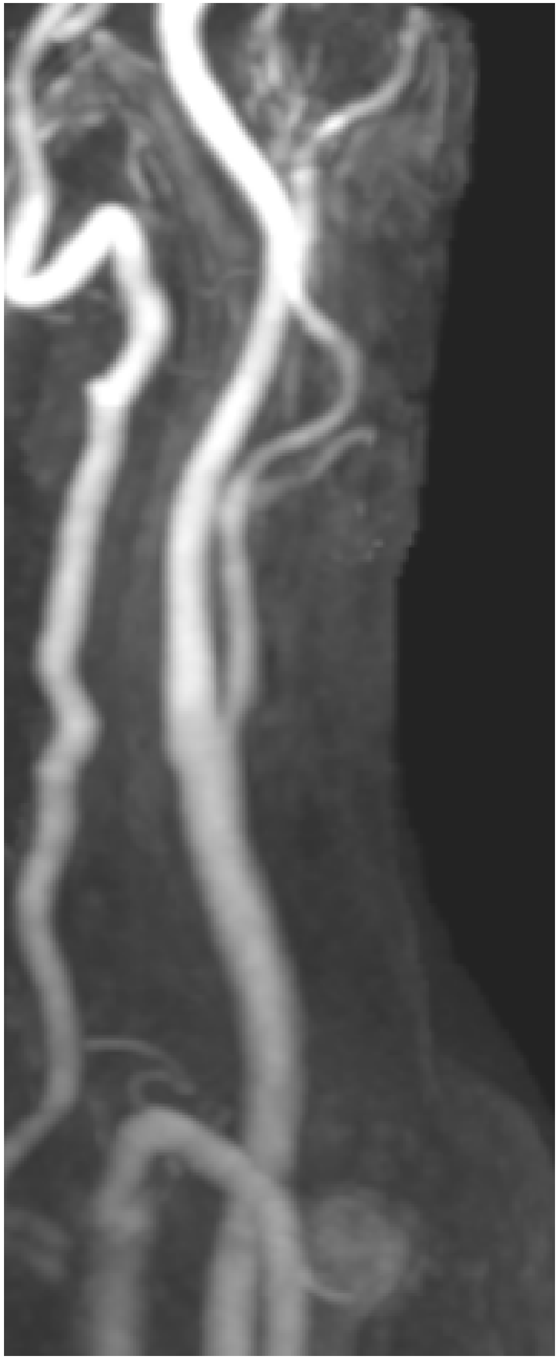

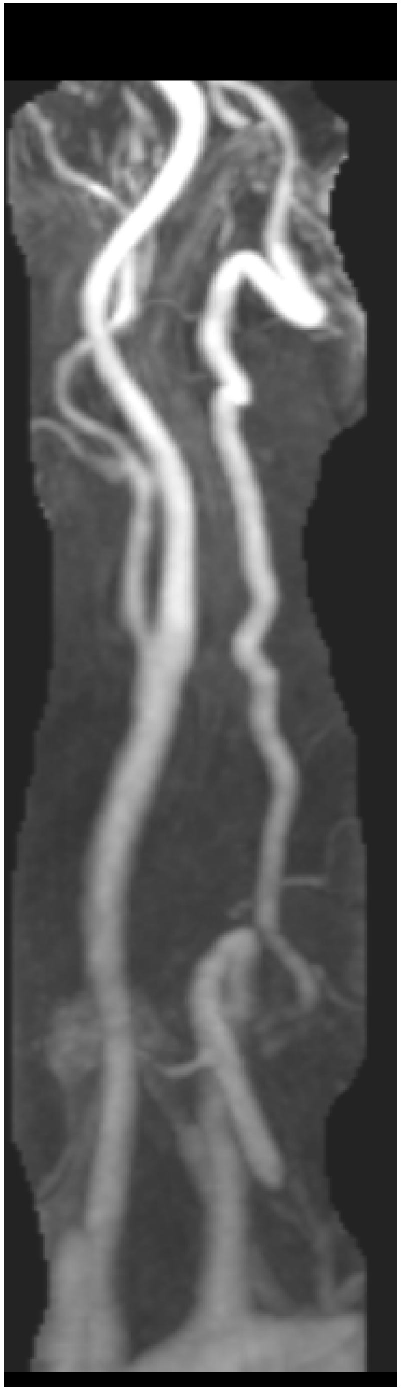

96e Score

0-30

31-50

51-70

>70

Near occlusion

Occluded

Quality

1

2

3

4

5

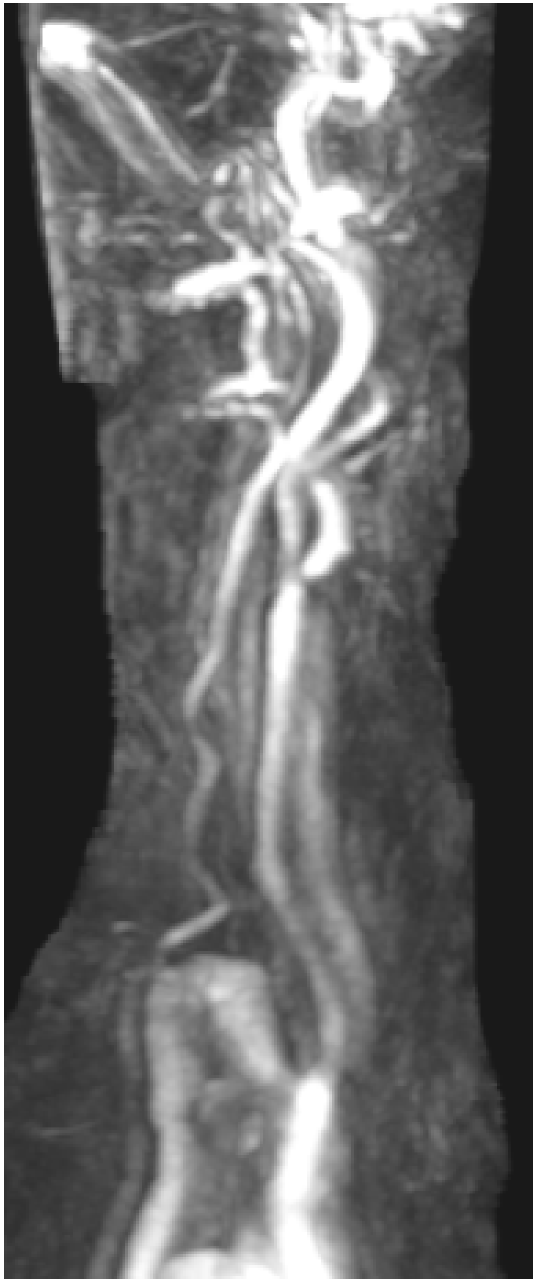

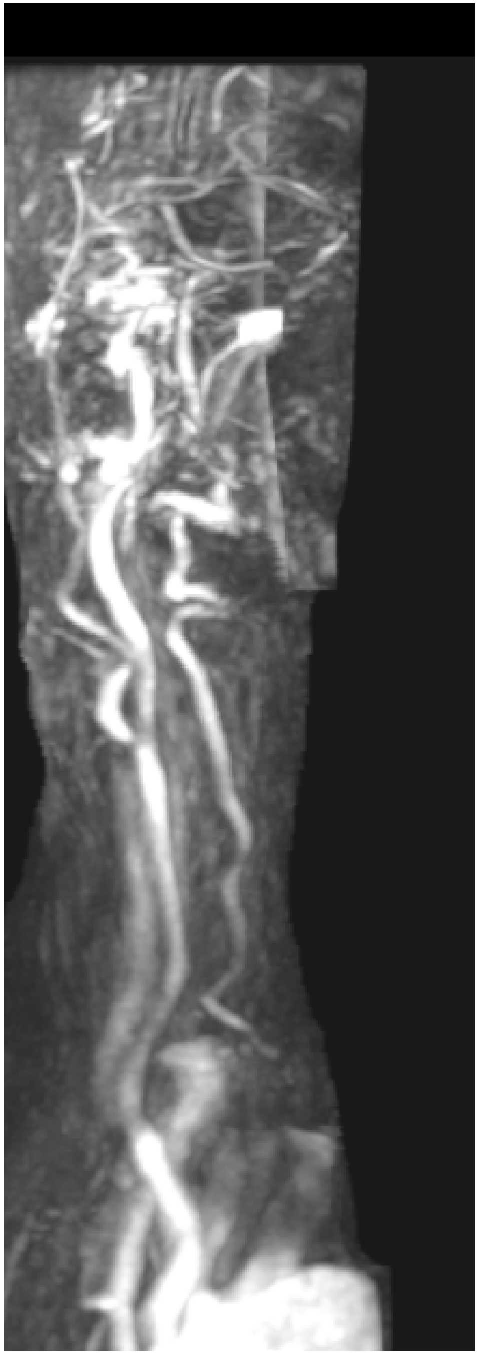

97d Score

0-30

31-50

51-70

>70

Near occlusion

Occluded

Quality

1

2

3

4

5

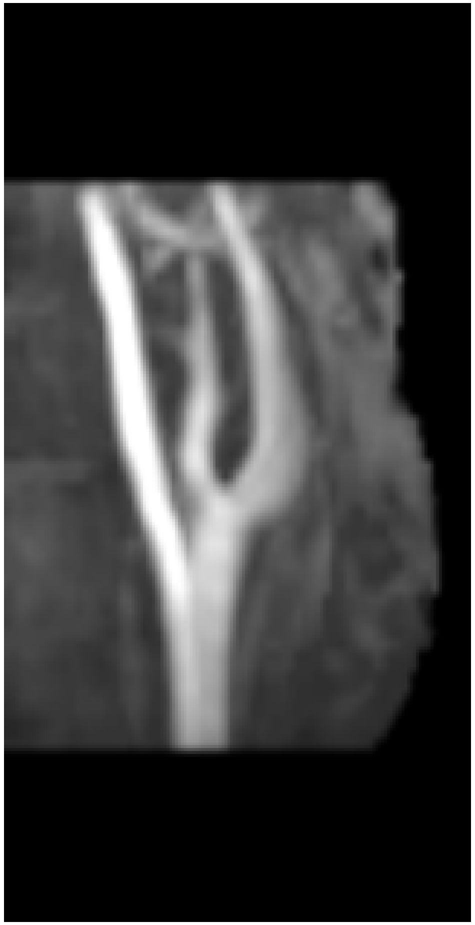

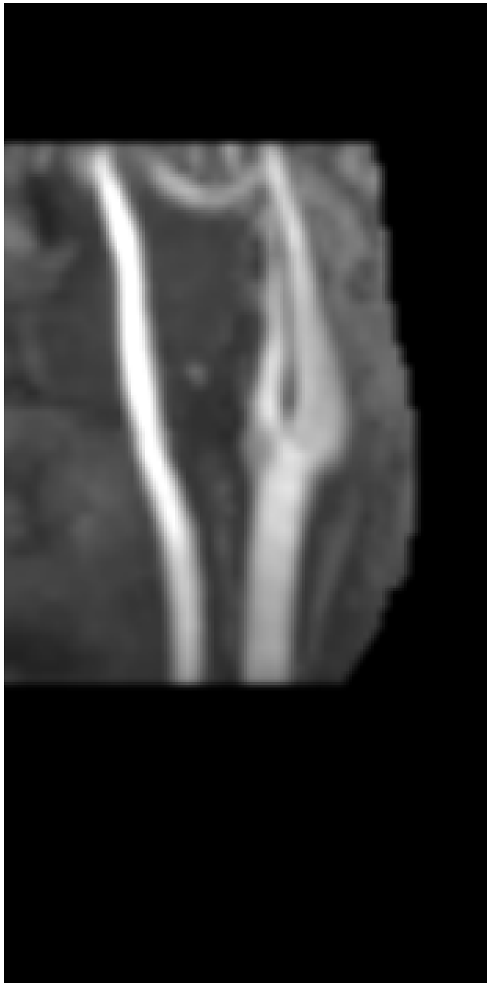

98c Score

0-30

31-50

51-70

>70

Near occlusion

Occluded

Quality

1

2

3

4

5

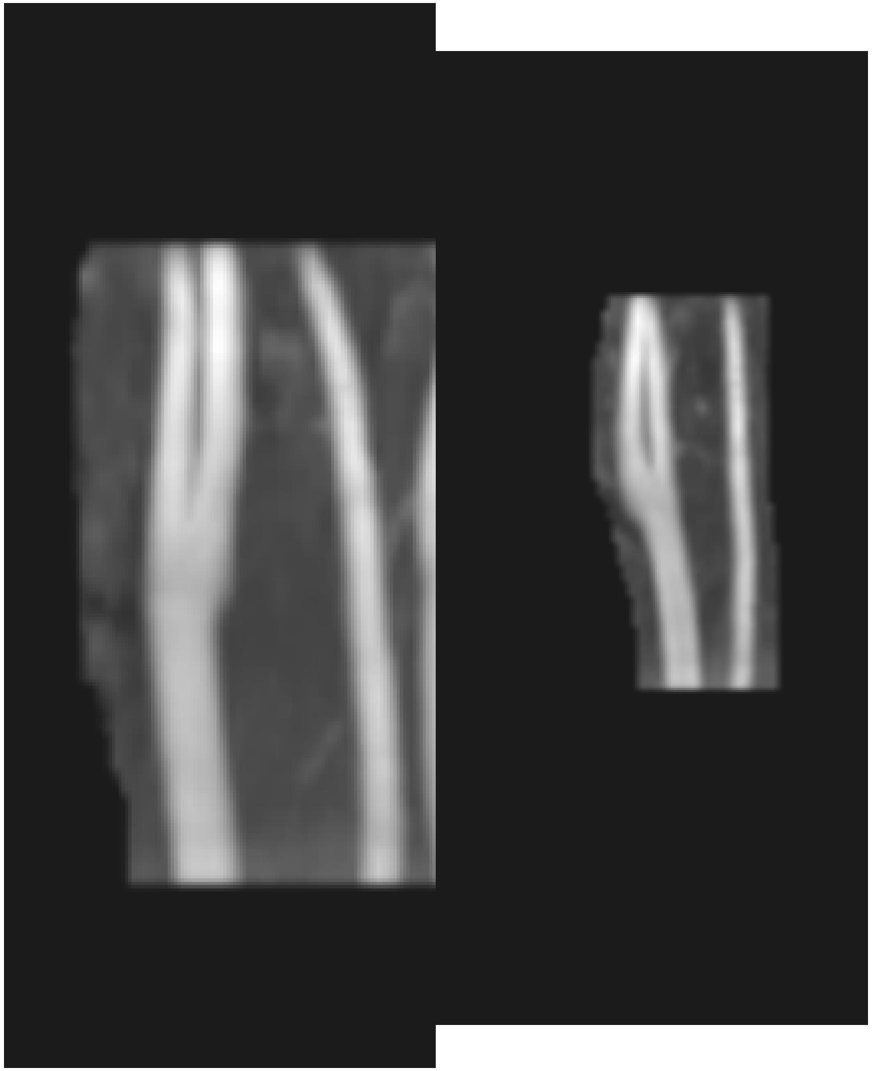

99b Score

0-30

31-50

51-70

>70

Near occlusion

Occluded

Quality

1

2

3

4

5

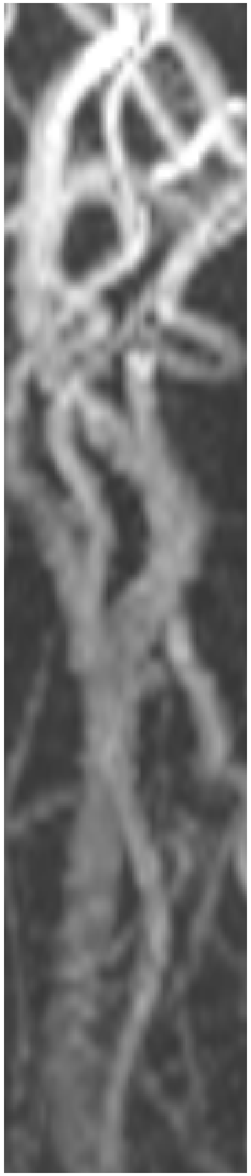

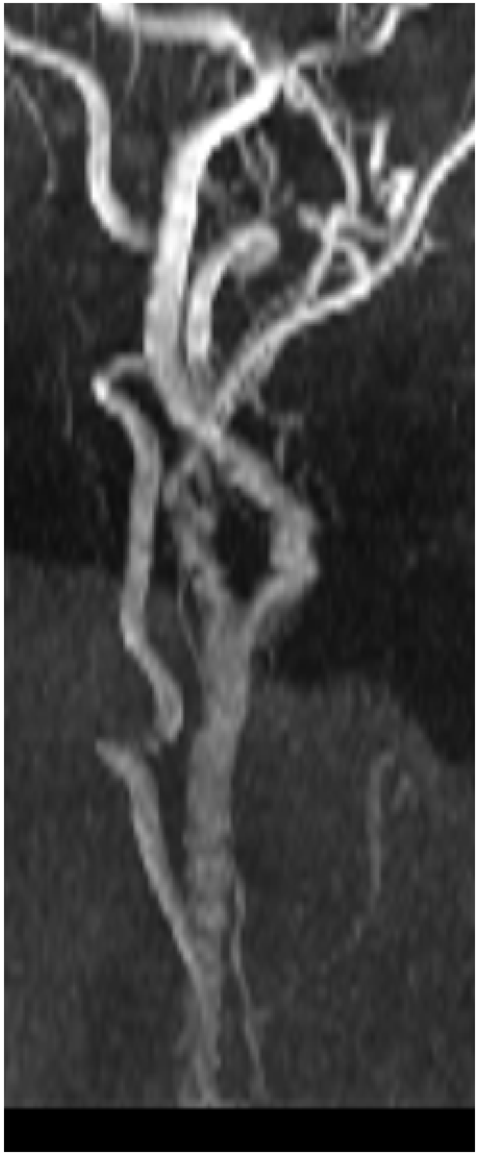

100a Score

0-30

31-50

51-70

>70

Near occlusion

Occluded

Quality

1

2

3

4

5

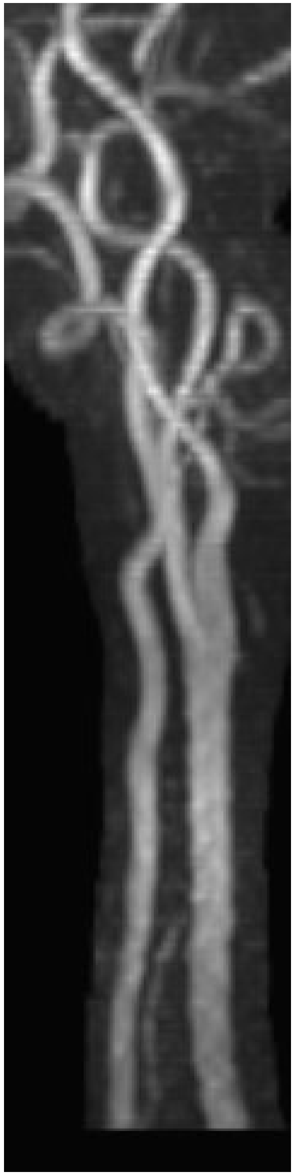

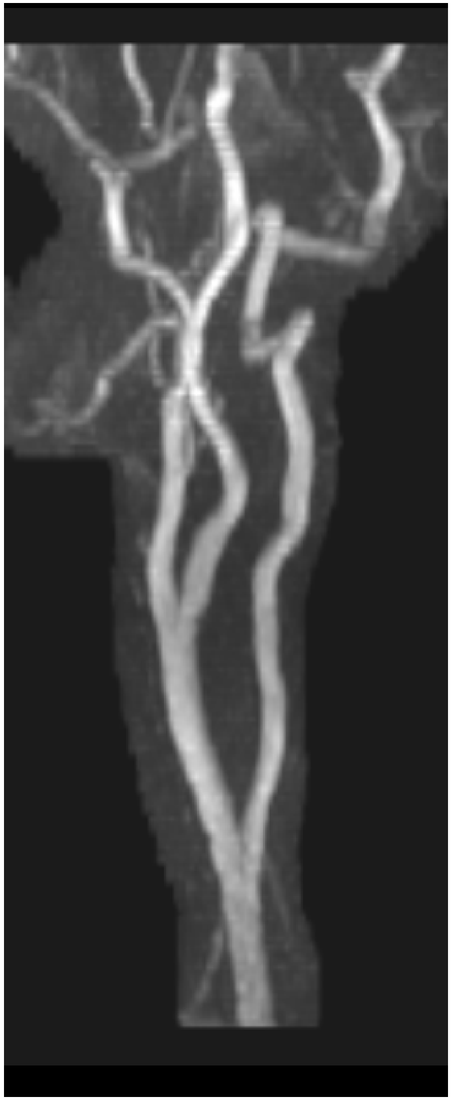

100f Score

0-30

31-50

51-70

>70

Near occlusion

Occluded

Quality

1

2

3

4

5

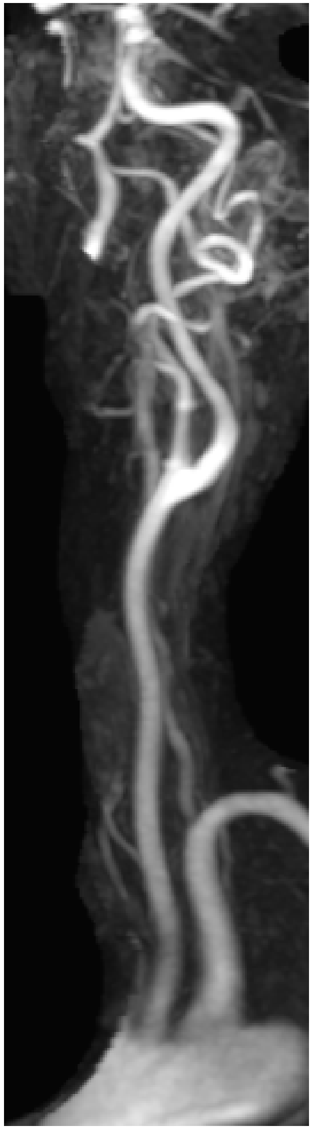

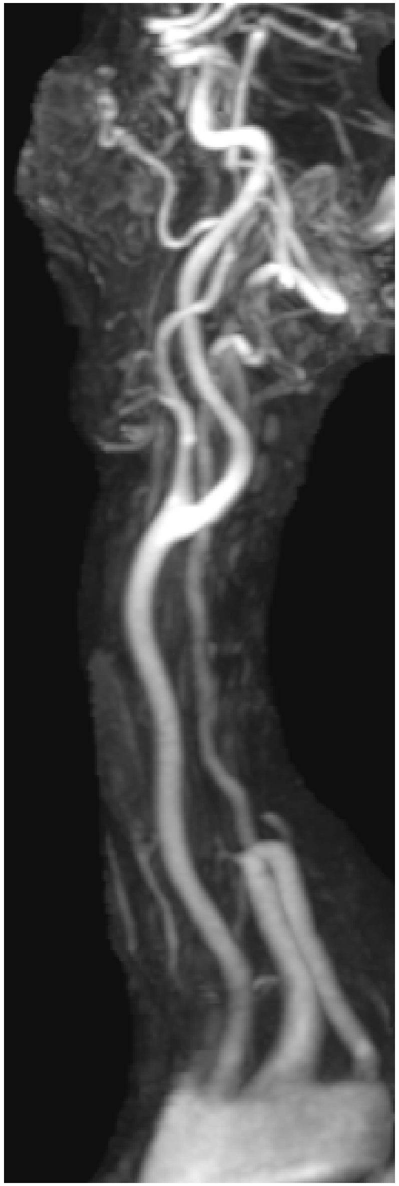

101e Score

0-30

31-50

51-70

>70

Near occlusion

Occluded

Quality

1

2

3

4

5

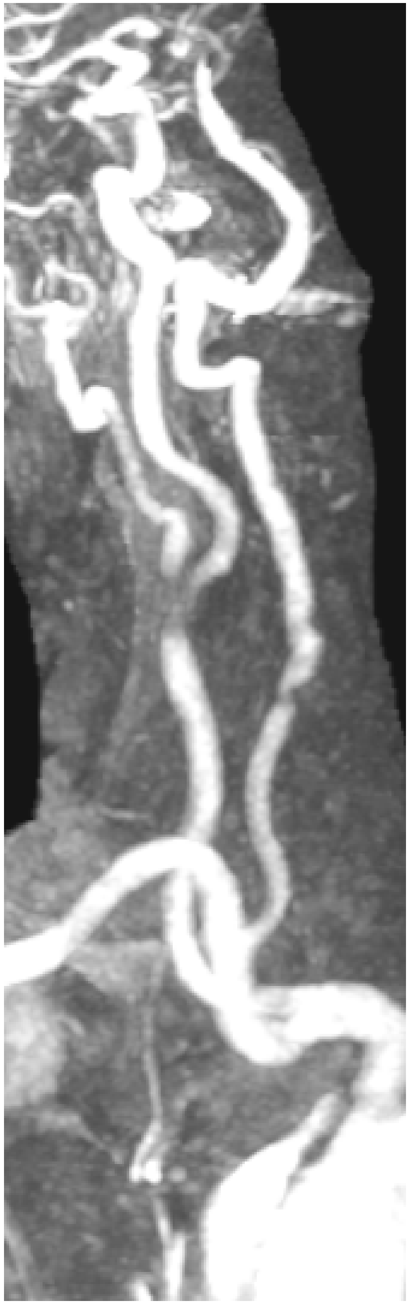

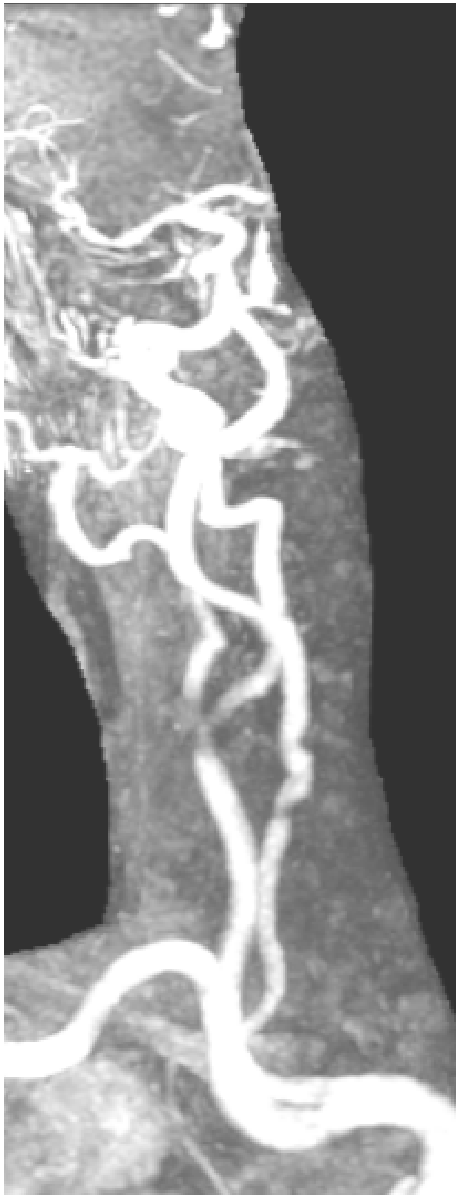

102d Score

0-30

31-50

51-70

>70

Near occlusion

Occluded

Quality

1

2

3

4

5

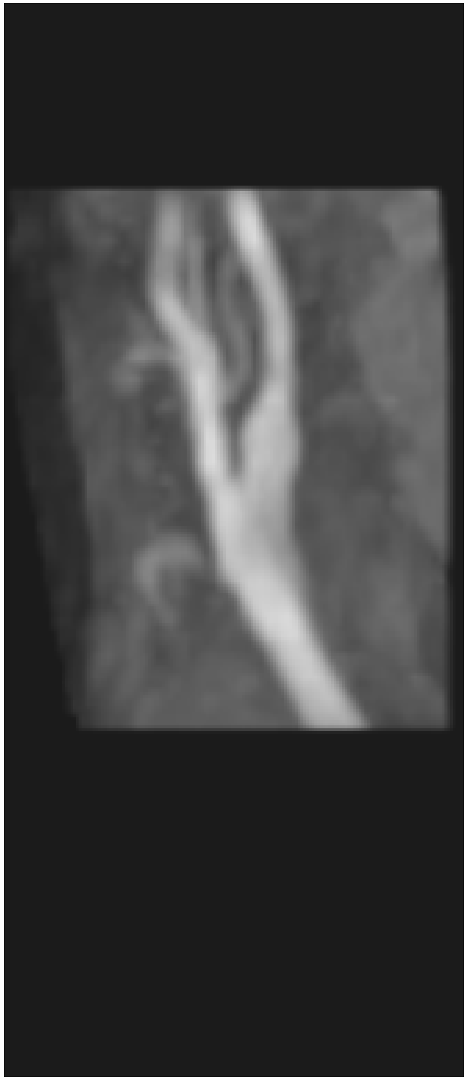

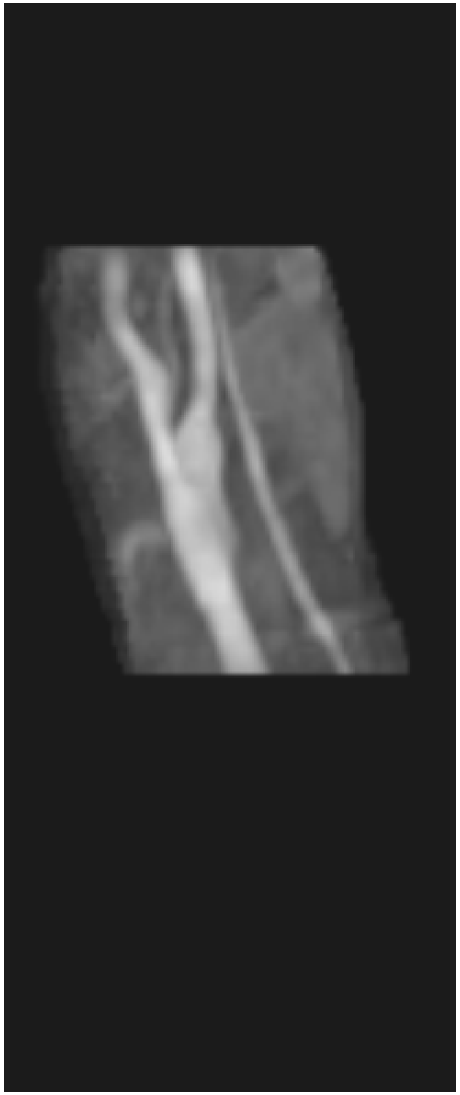

103c Score

0-30

31-50

51-70

>70

Near occlusion

Occluded

Quality

1

2

3

4

5

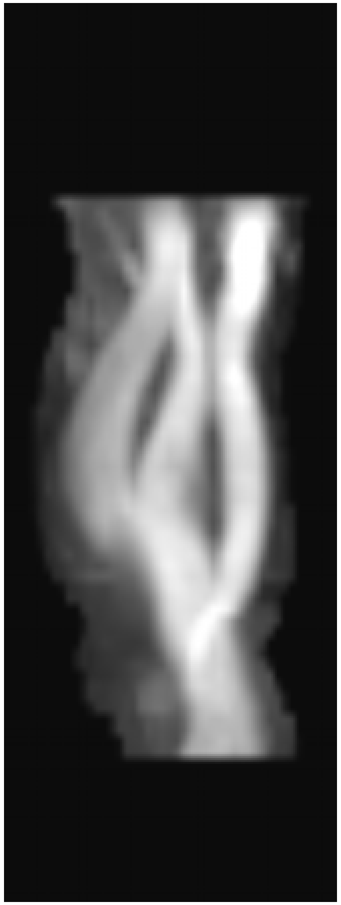

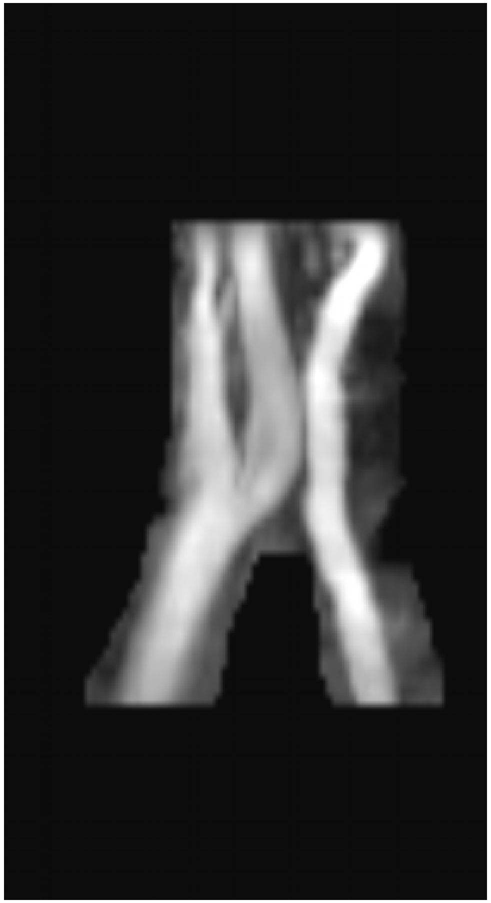

104b Score

0-30

31-50

51-70

>70

Near occlusion

Occluded

Quality

1

2

3

4

5

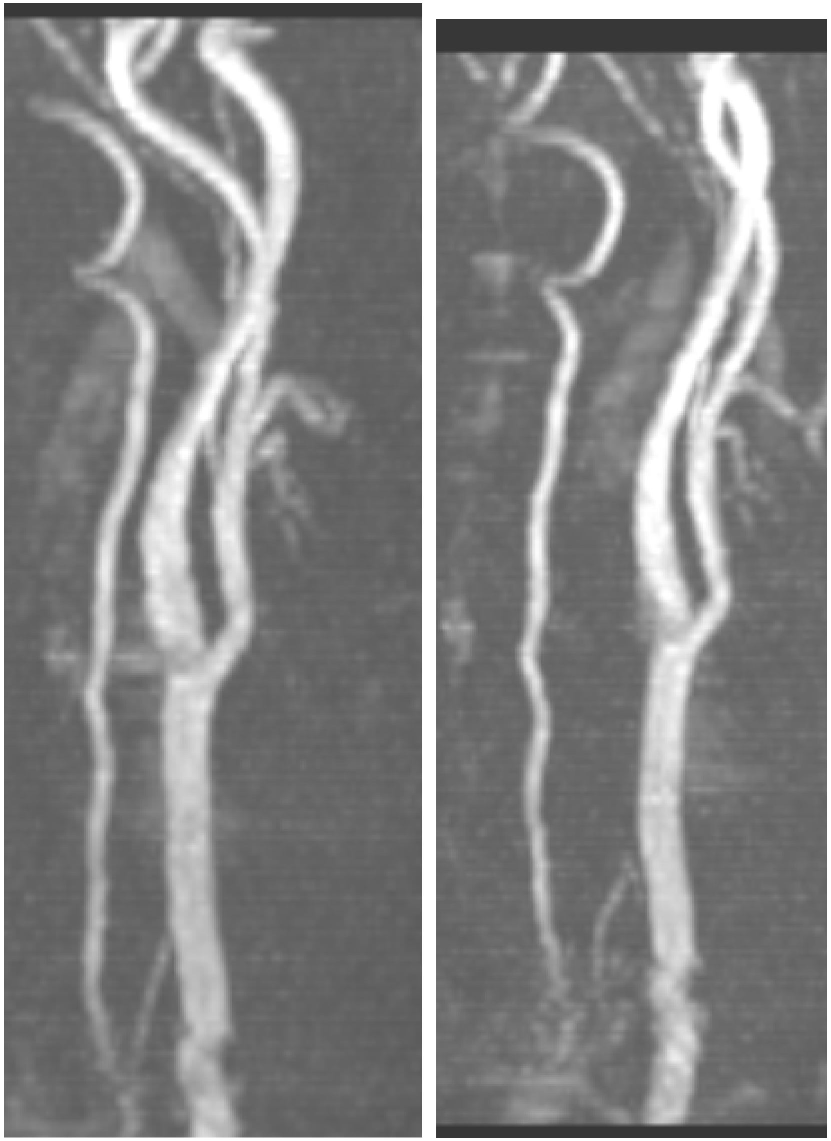

105a Score

0-30

31-50

51-70

>70

Near occlusion

Occluded

Quality

1

2

3

4

5

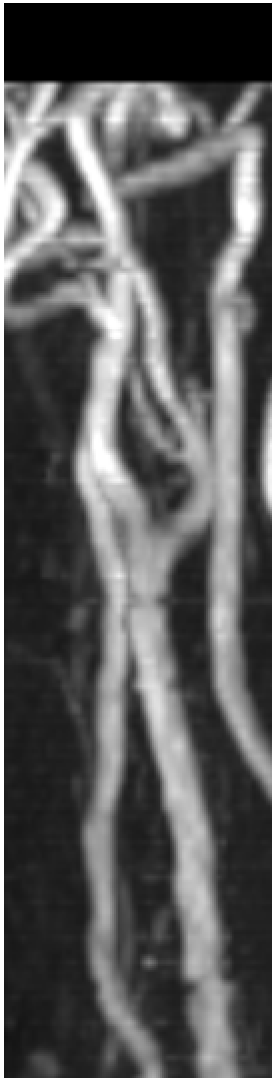

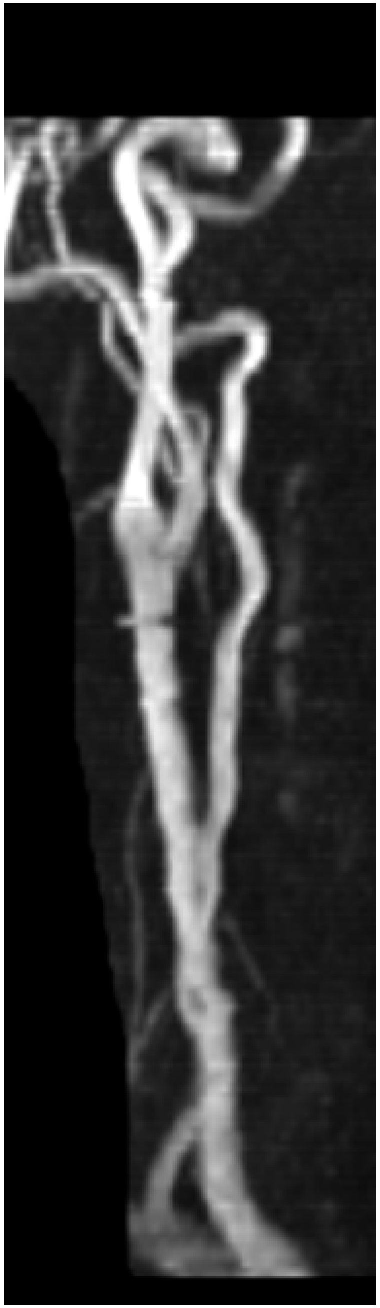

105f Score

0-30

31-50

51-70

>70

Near occlusion

Occluded

Quality

1

2

3

4

5

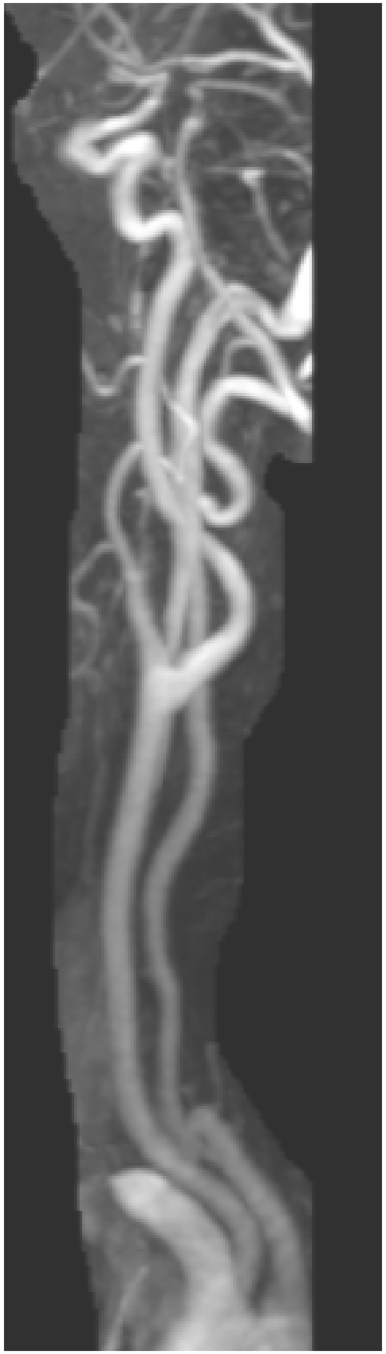

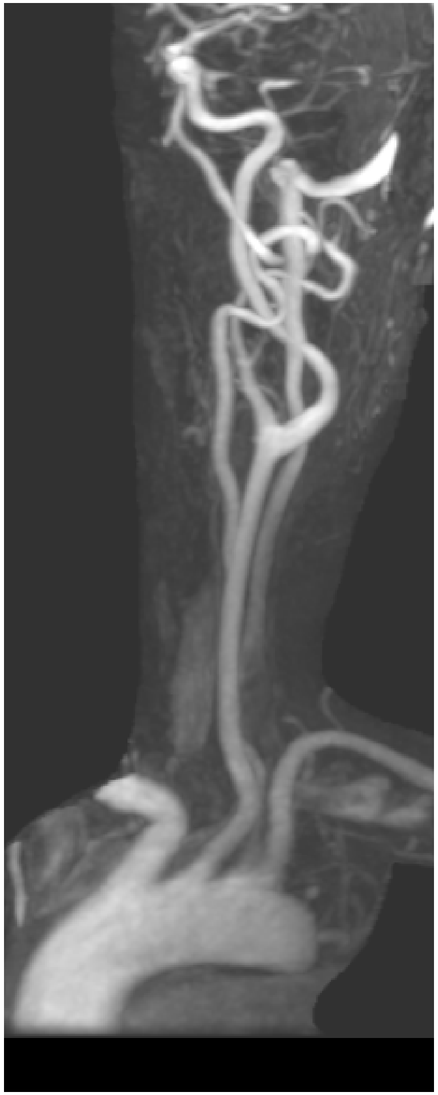

106e Score

0-30

31-50

51-70

>70

Near occlusion

Occluded

Quality

1

2

3

4

5

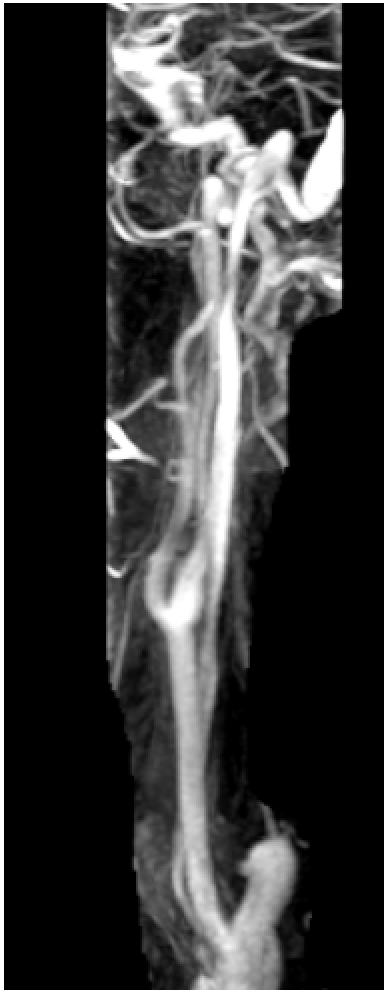

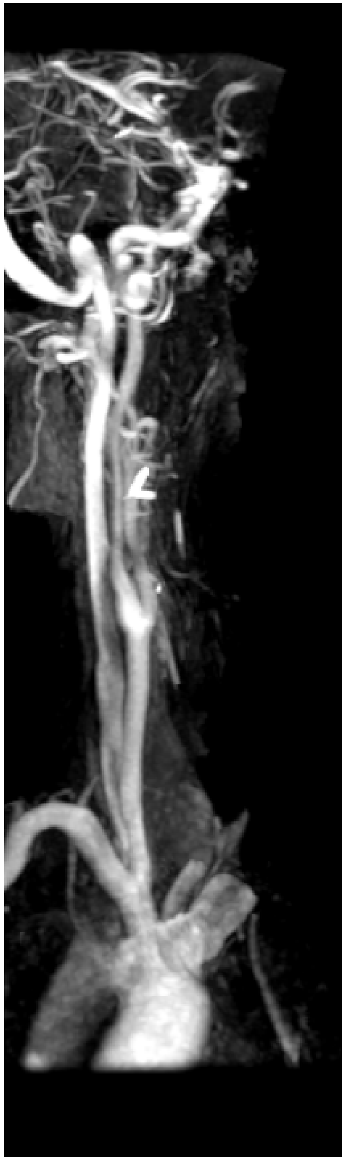

107d Score

0-30

31-50

51-70

>70

Near occlusion

Occluded

Quality

1

2

3

4

5

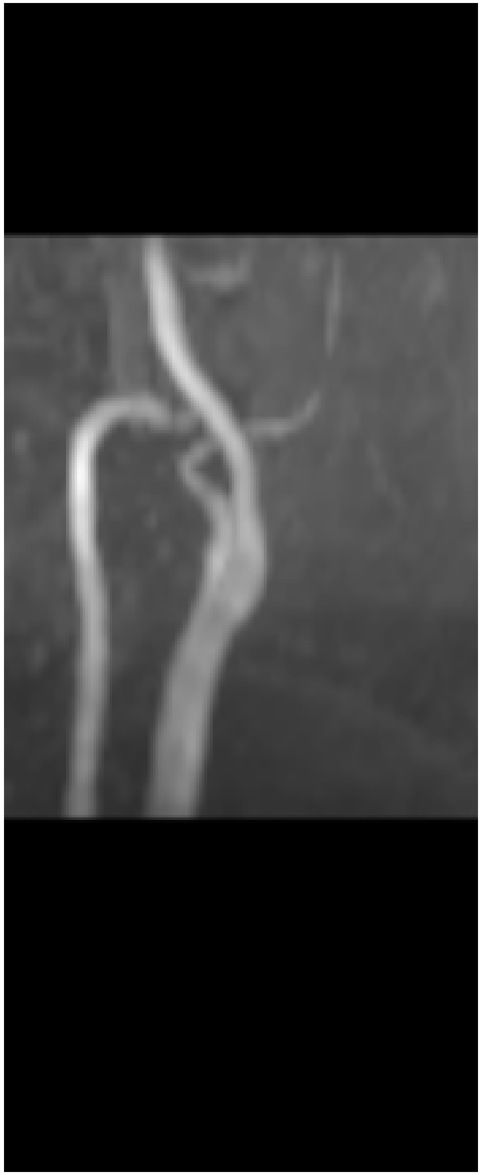

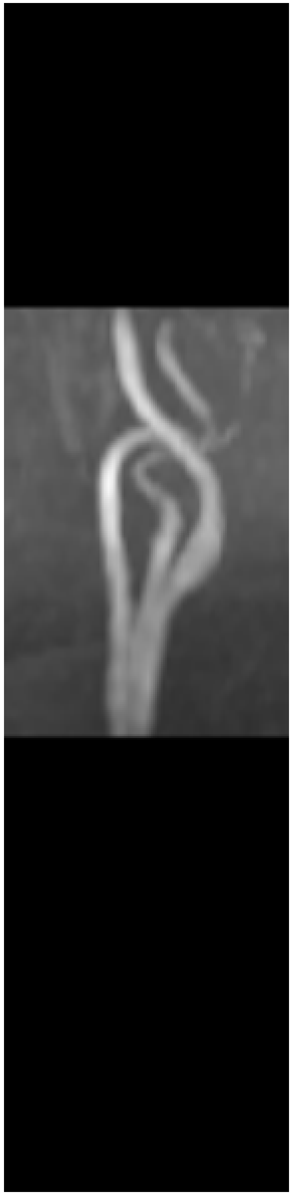

108c Score

0-30

31-50

51-70

>70

Near occlusion

Occluded

Quality

1

2

3

4

5

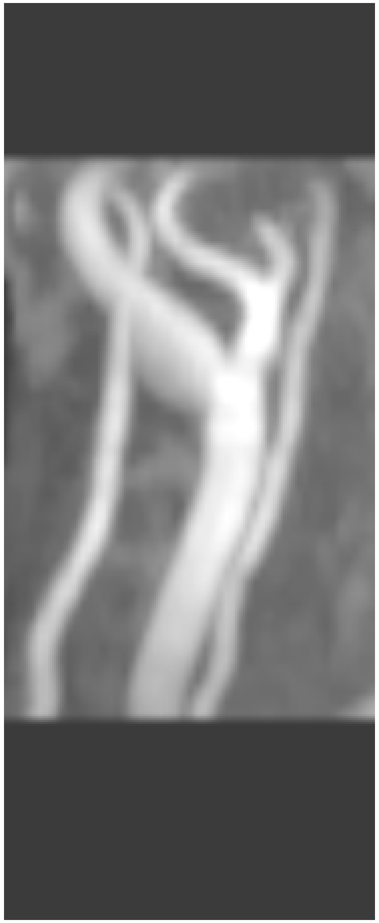

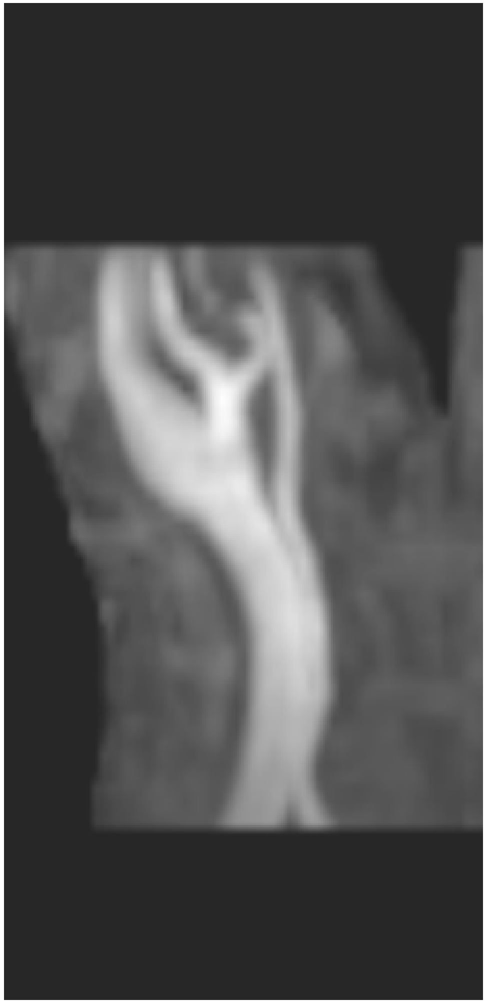

109b Score

0-30

31-50

51-70

>70

Near occlusion

Occluded

Quality

1

2

3

4

5

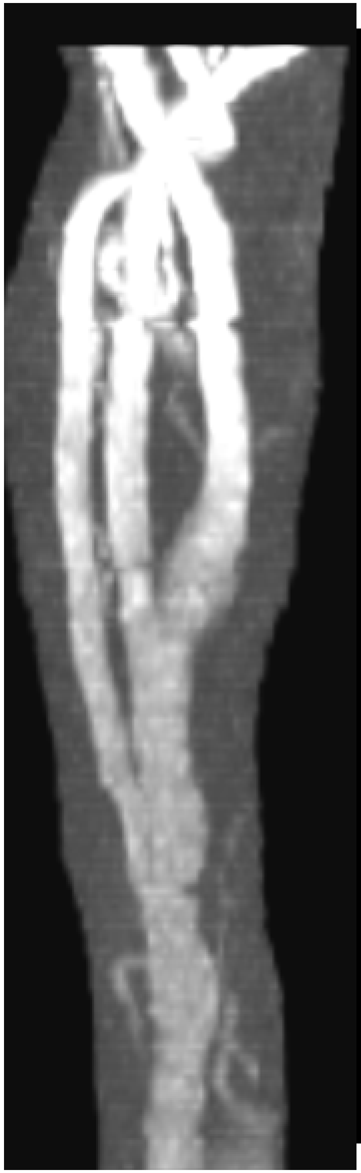

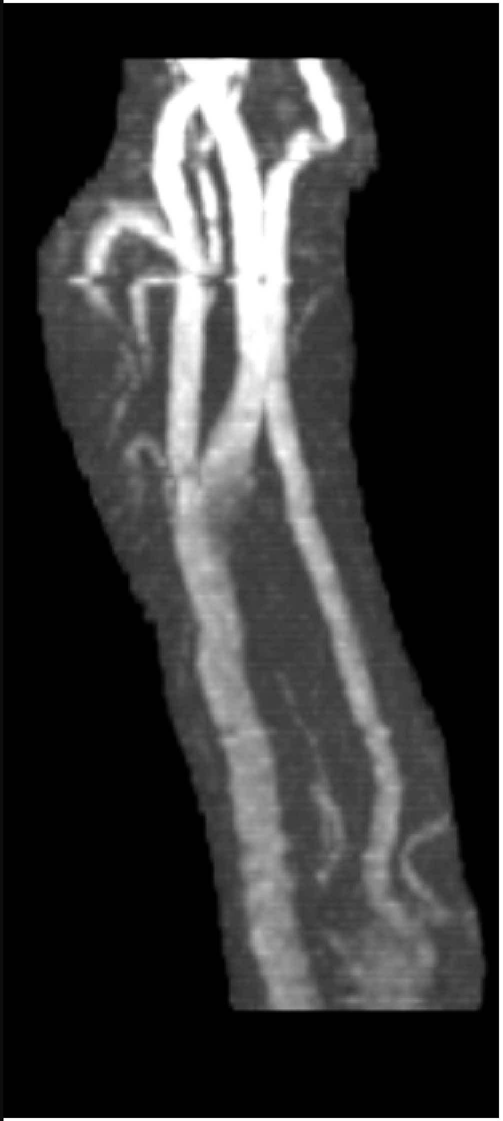

110a Score

0-30

31-50

51-70

>70

Near occlusion

Occluded

Quality

1

2

3

4

5

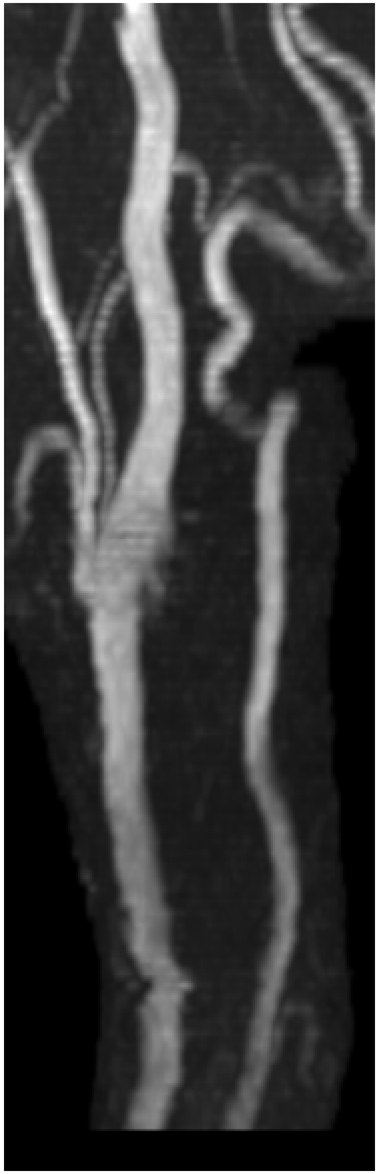

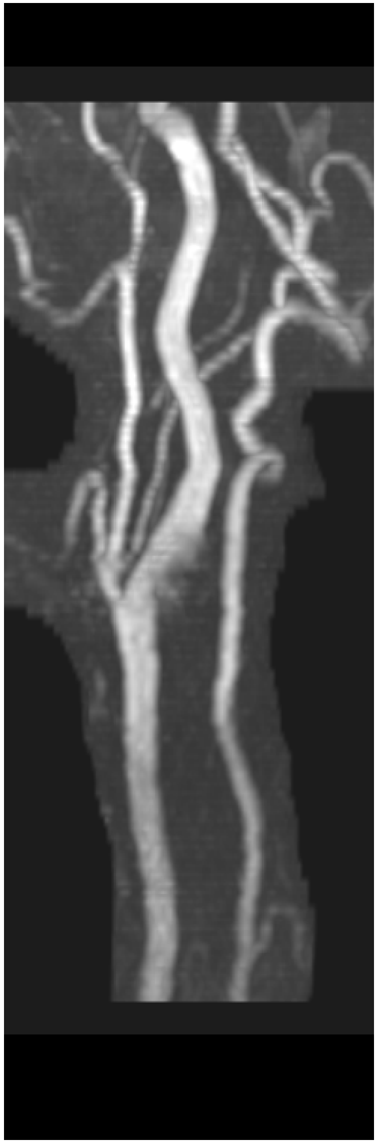

110f Score

0-30

31-50

51-70

>70

Near occlusion

Occluded

Quality

1

2

3

4

5

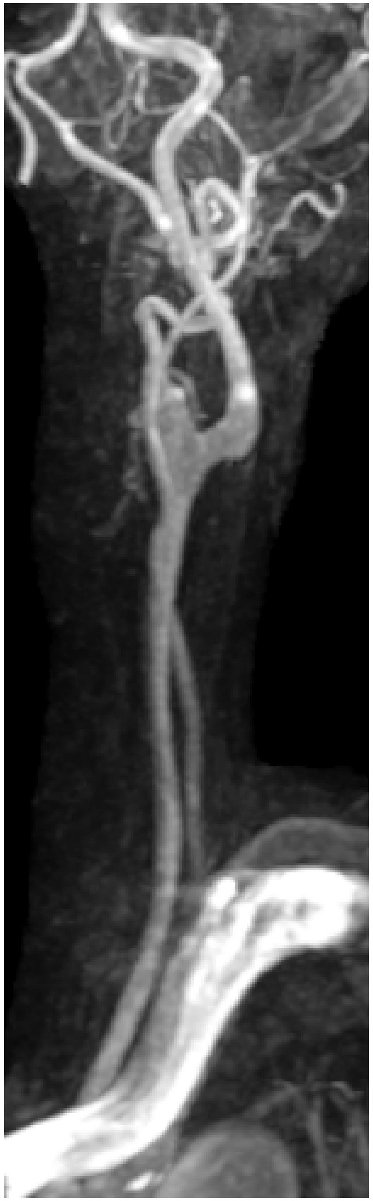

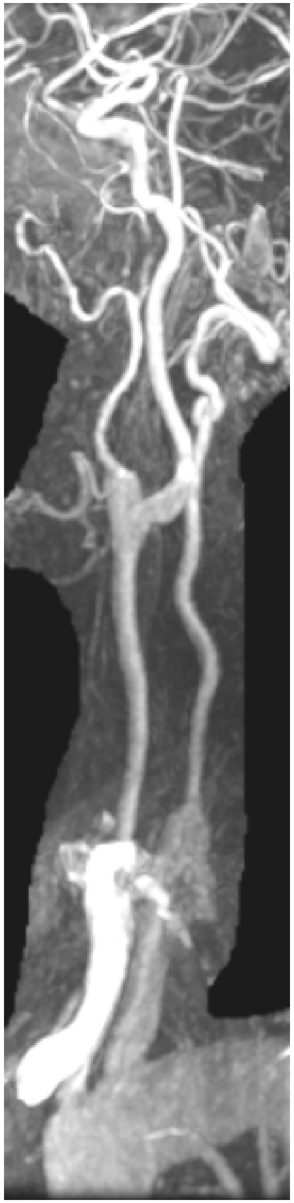

111e Score

0-30

31-50

51-70

>70

Near occlusion

Occluded

Quality

1

2

3

4

5

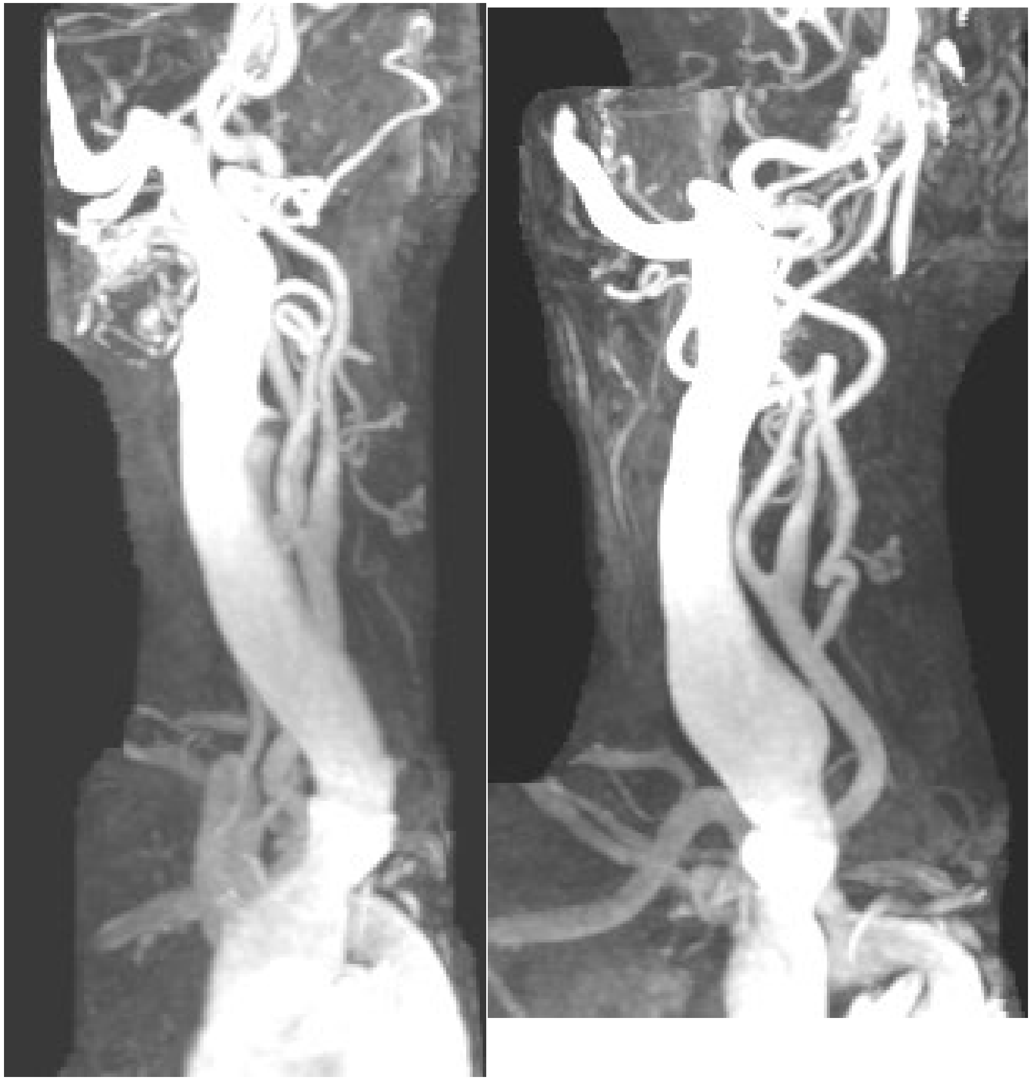

112d Score

0-30

31-50

51-70

>70

Near occlusion

Occluded

Quality

1

2

3

4

5

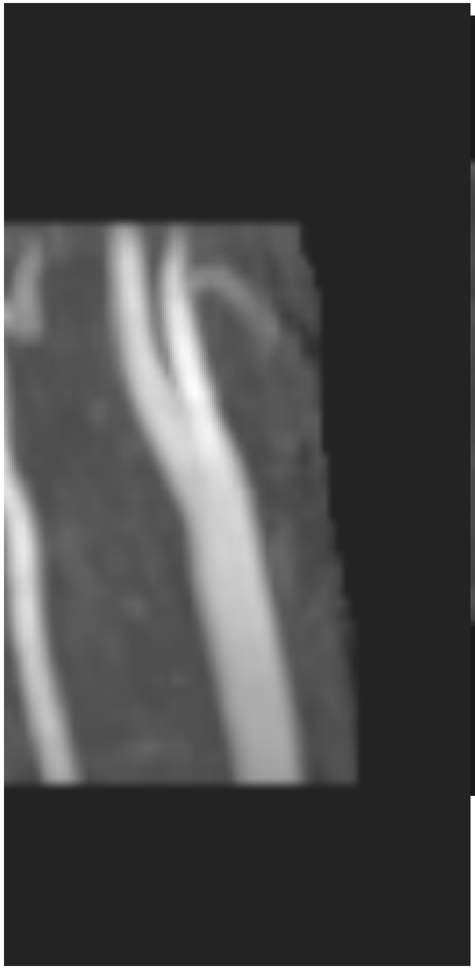

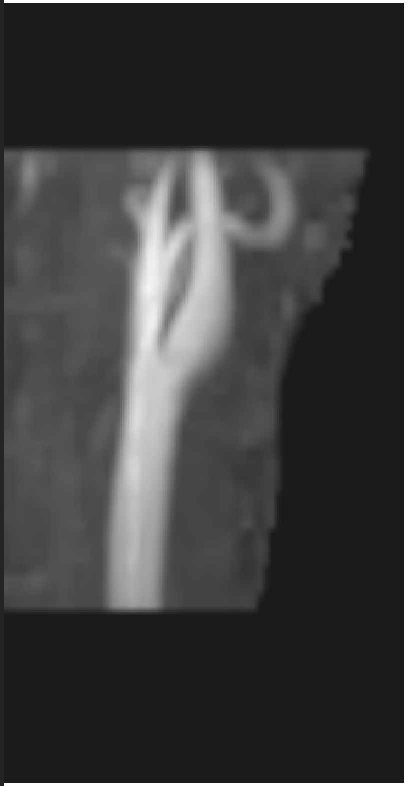

113c Score

0-30

31-50

51-70

>70

Near occlusion

Occluded

Quality

1

2

3

4

5

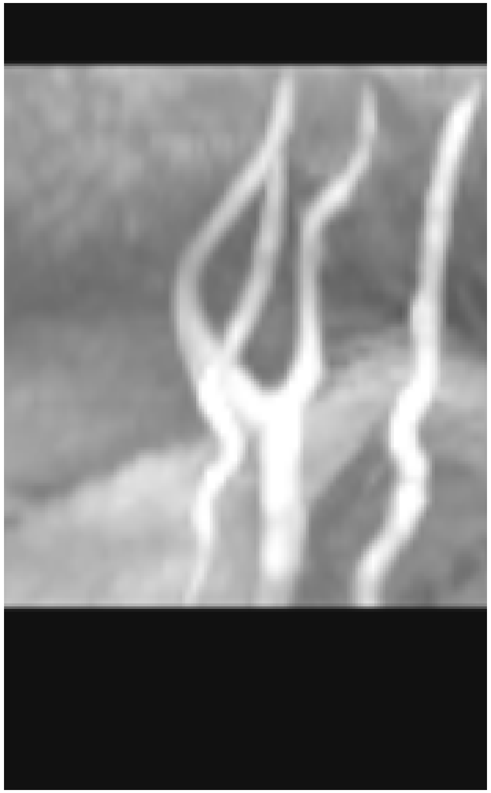

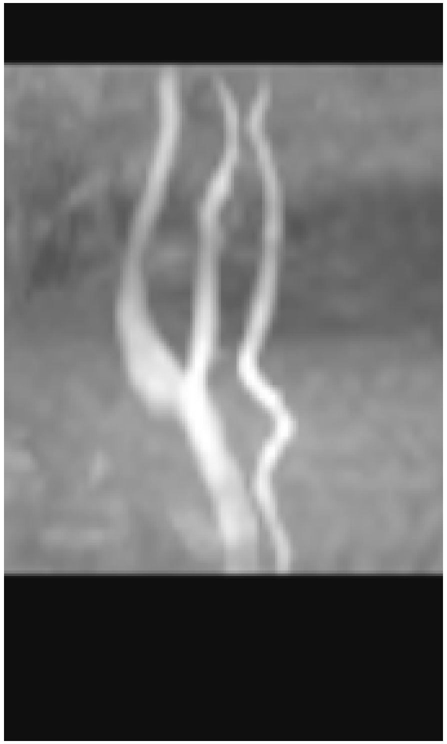

114b Score

0-30

31-50

51-70

>70

Near occlusion

Occluded

Quality

1

2

3

4

5

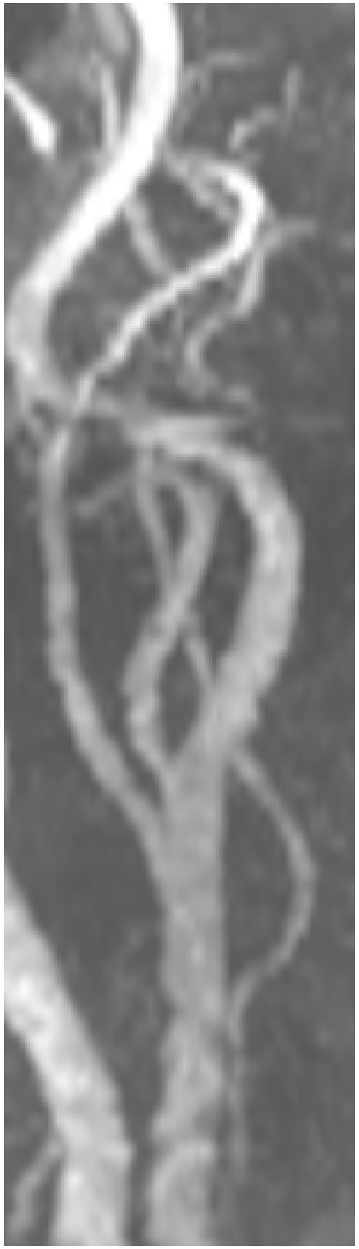

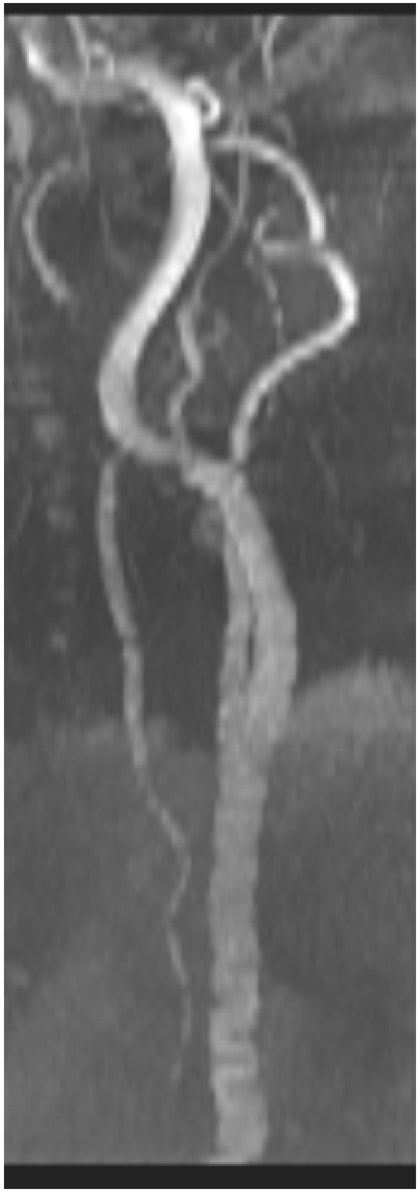

115a Score

0-30

31-50

51-70

>70

Near occlusion

Occluded

Quality

1

2

3

4

5

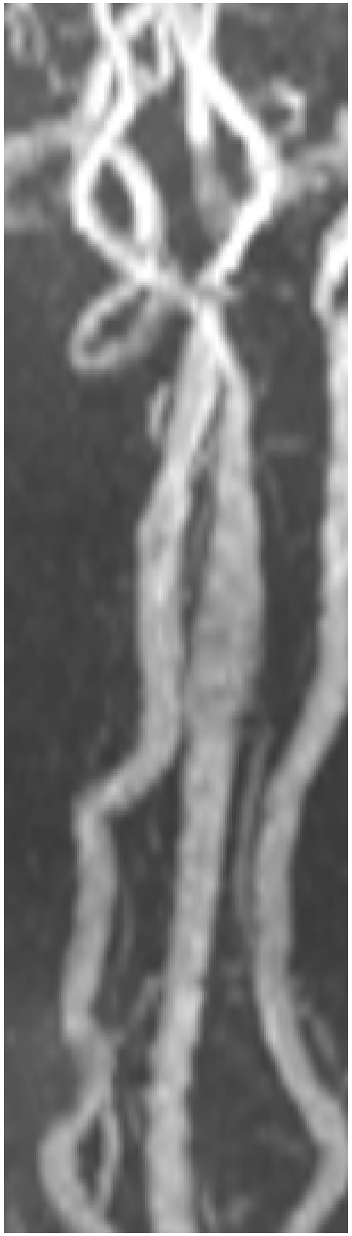

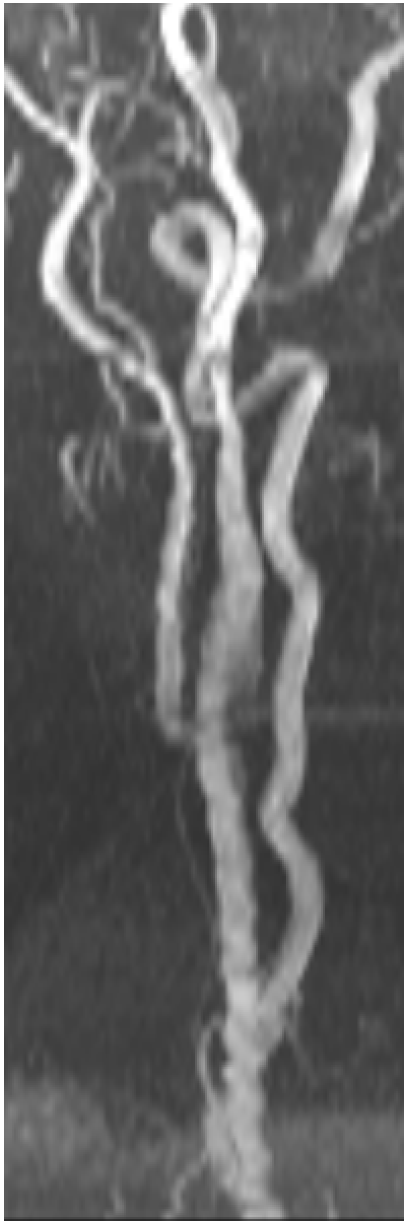

115f Score

0-30

31-50

51-70

>70

Near occlusion

Occluded

Quality

1

2

3

4

5

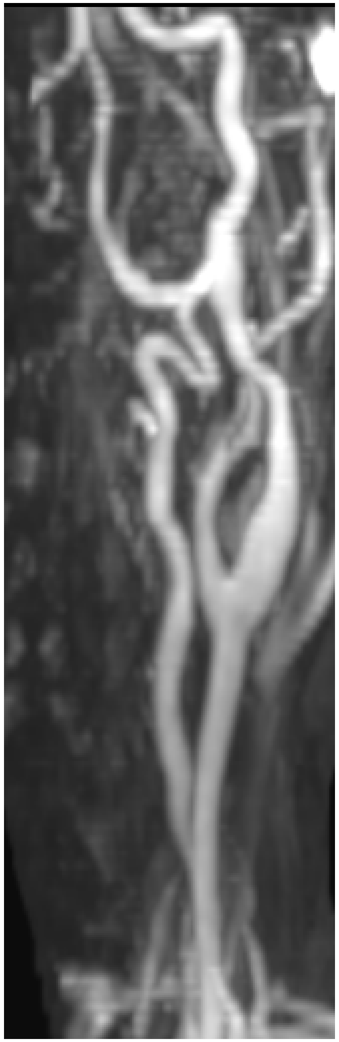

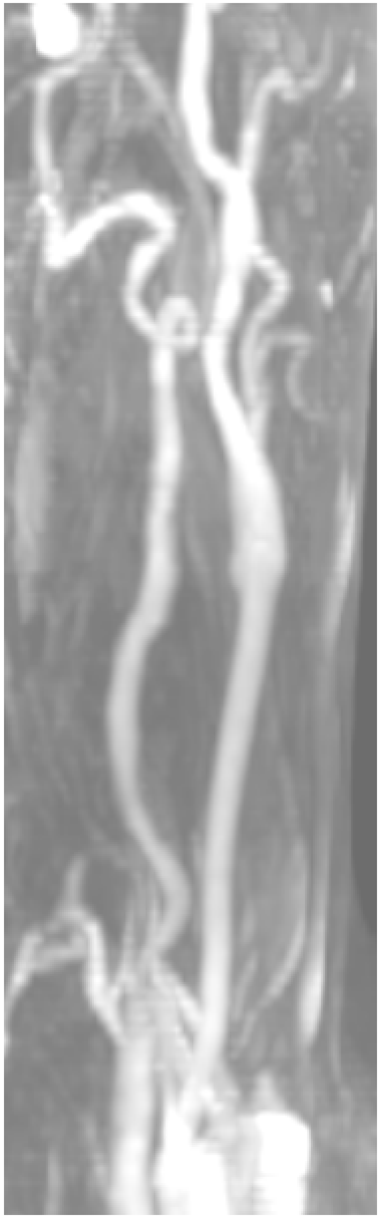

116e Score

0-30

31-50

51-70

>70

Near occlusion

Occluded

Quality

1

2

3

4

5

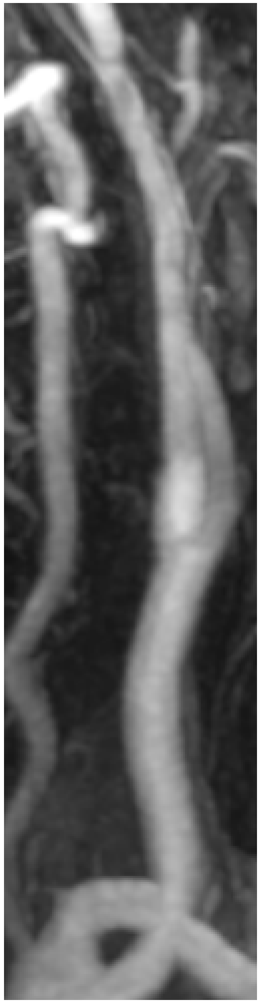

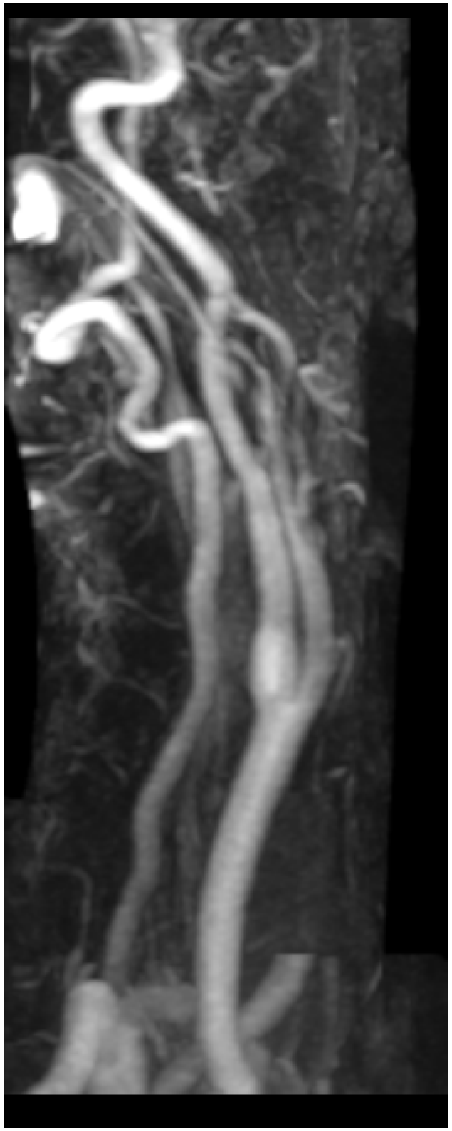

117d Score

0-30

31-50

51-70

>70

Near occlusion

Occluded

Quality

1

2

3

4

5

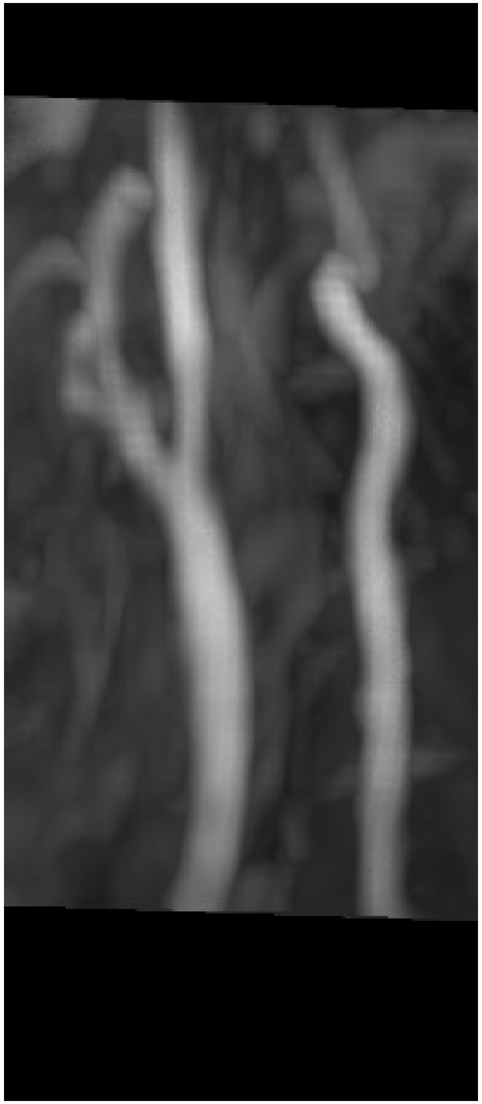

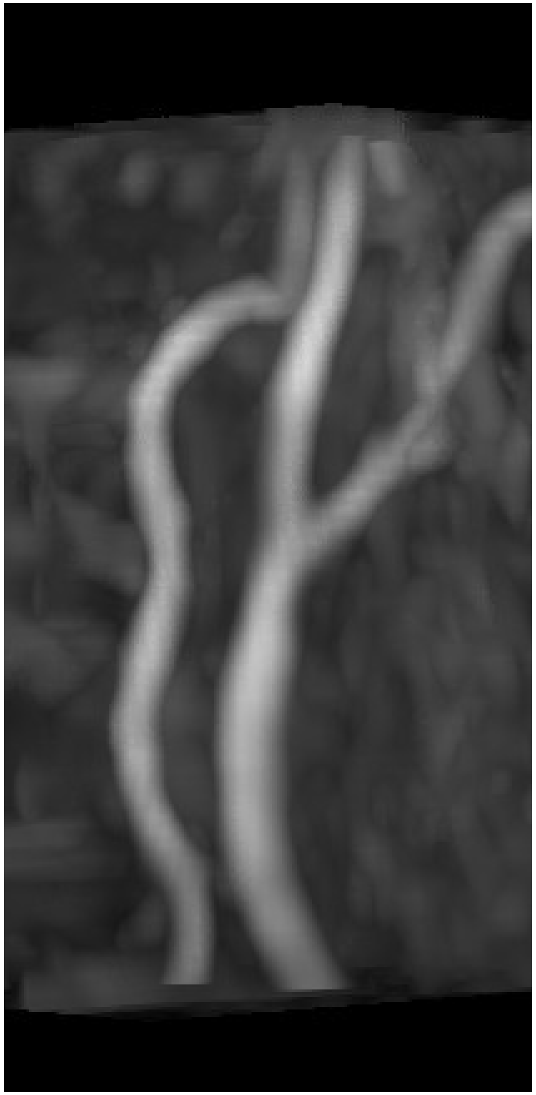

118c Score

0-30

31-50

51-70

>70

Near occlusion

Occluded

Quality

1

2

3

4

5

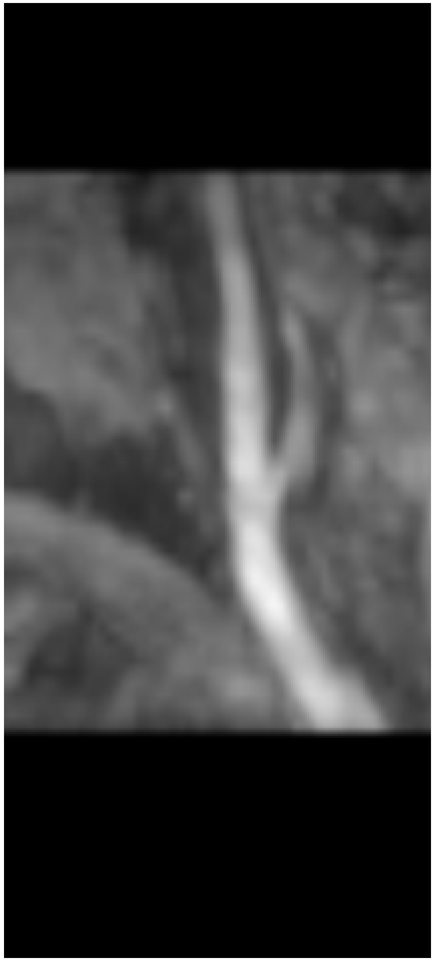

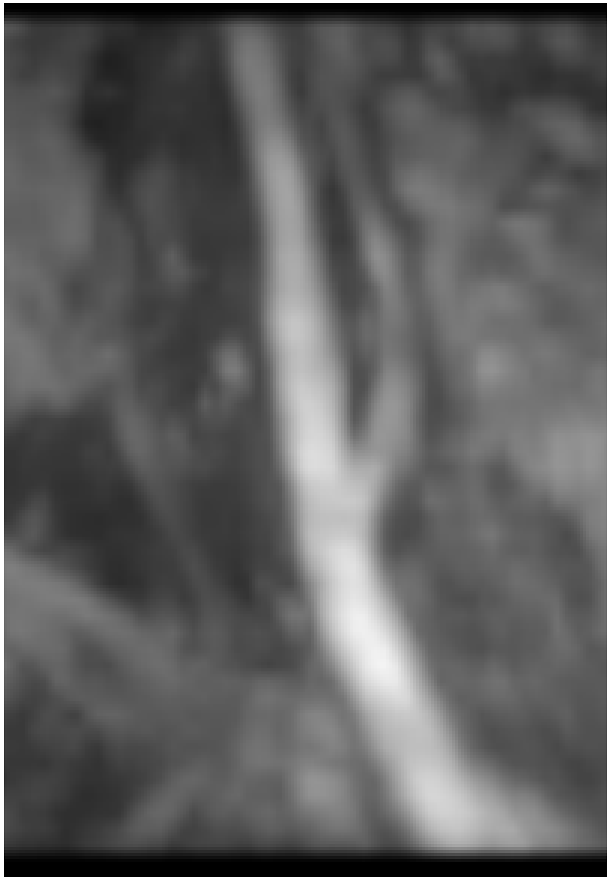

119b Score

0-30

31-50

51-70

>70

Near occlusion

Occluded

Quality

1

2

3

4

5

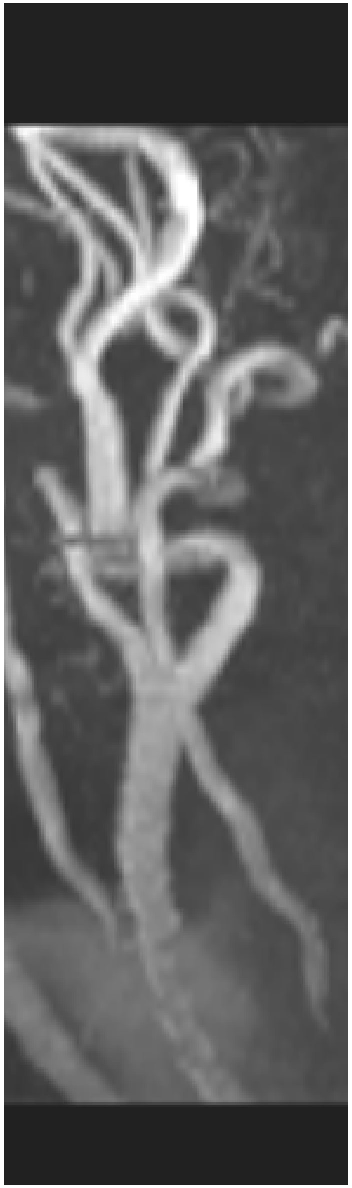

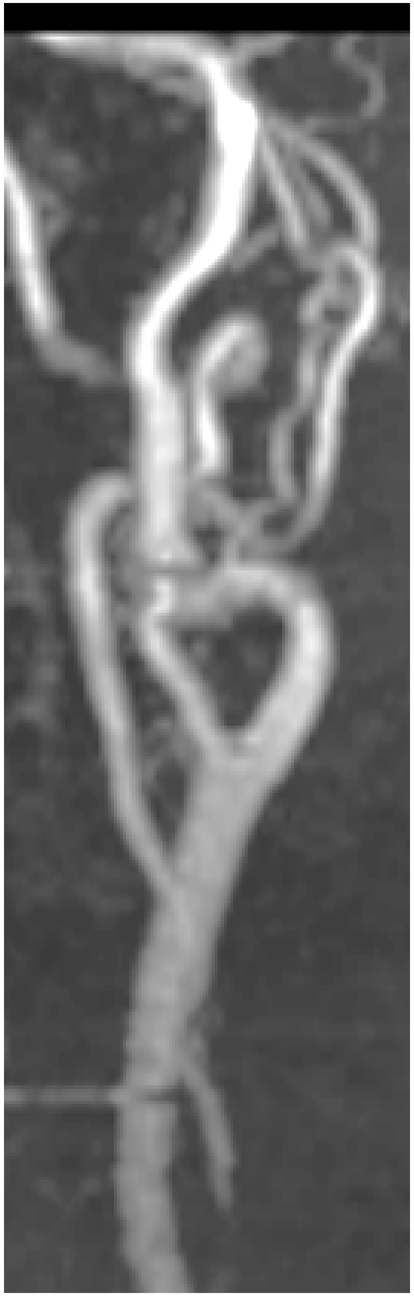

120a Score

0-30

31-50

51-70

>70

Near occlusion

Occluded

Quality

1

2

3

4

5

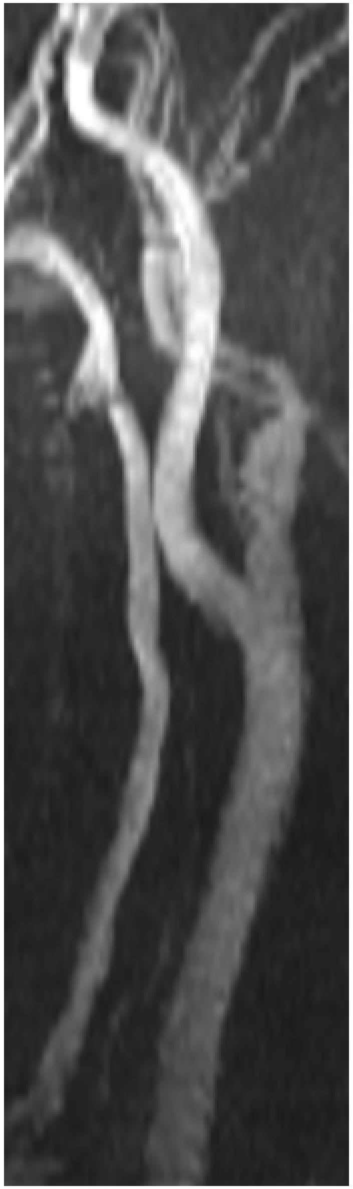

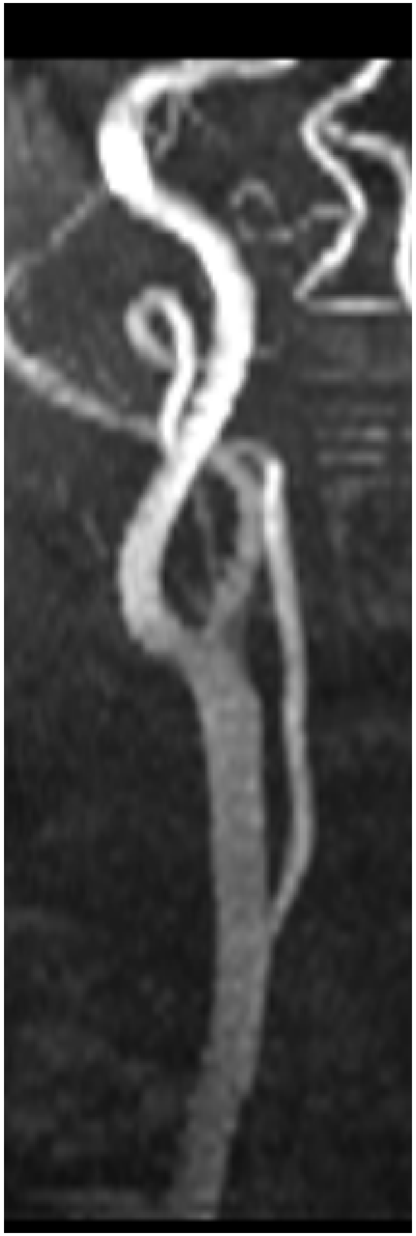

120f Score

0-30

31-50

51-70

>70

Near occlusion

Occluded

Quality

1

2

3

4

5

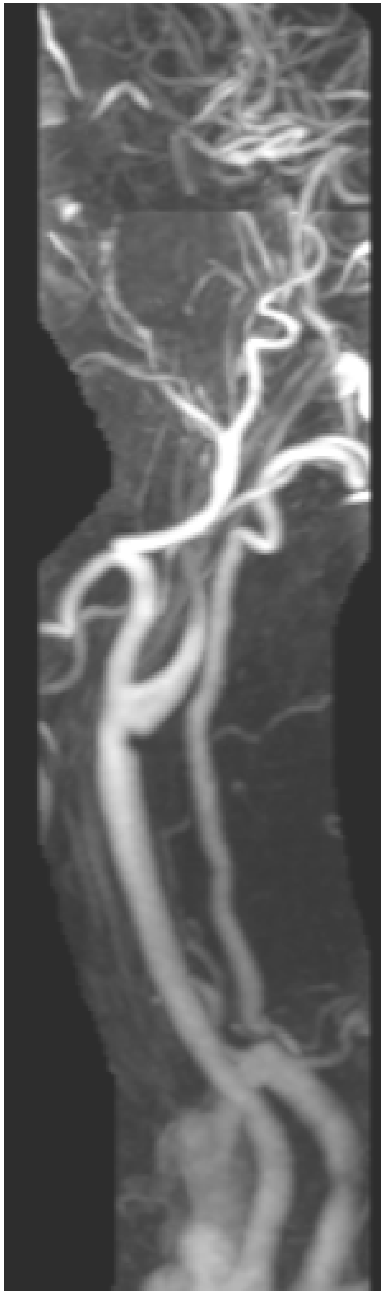

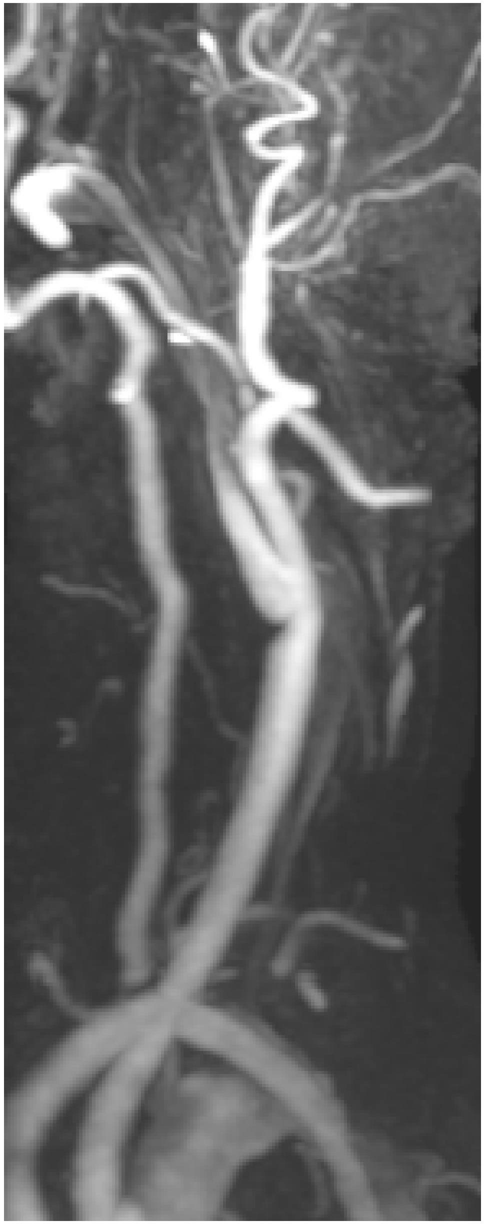

121e Score

0-30

31-50

51-70

>70

Near occlusion

Occluded

Quality

1

2

3

4

5

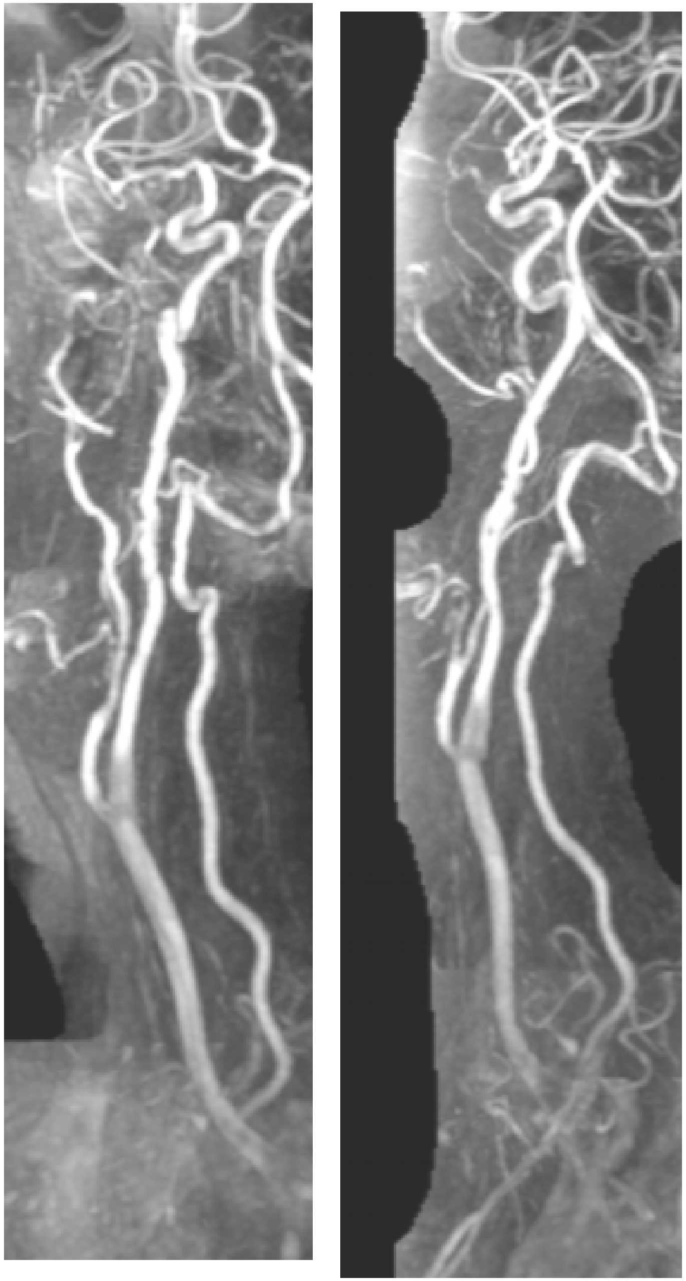

122d Score

0-30

31-50

51-70

>70

Near occlusion

Occluded

Quality

1

2

3

4

5

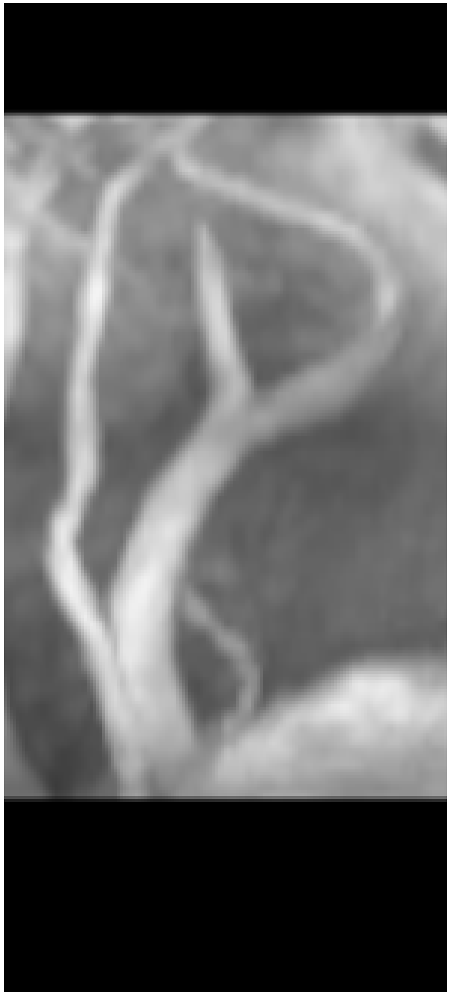

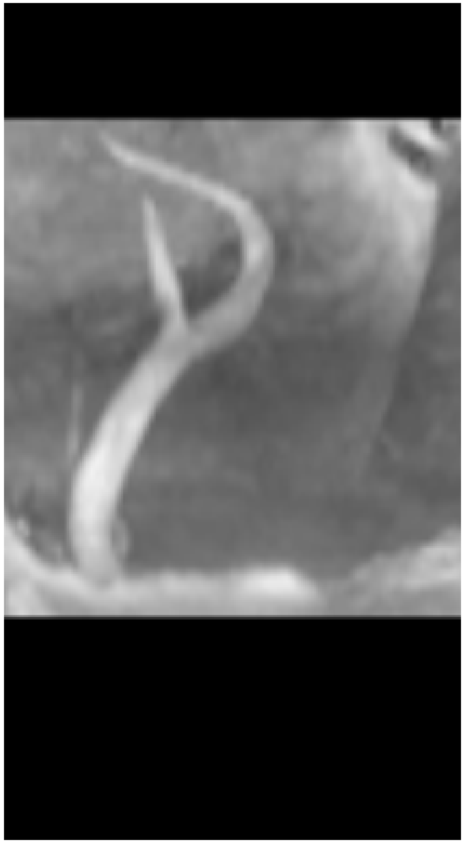

123c Score

0-30

31-50

51-70

>70

Near occlusion

Occluded

Quality

1

2

3

4

5

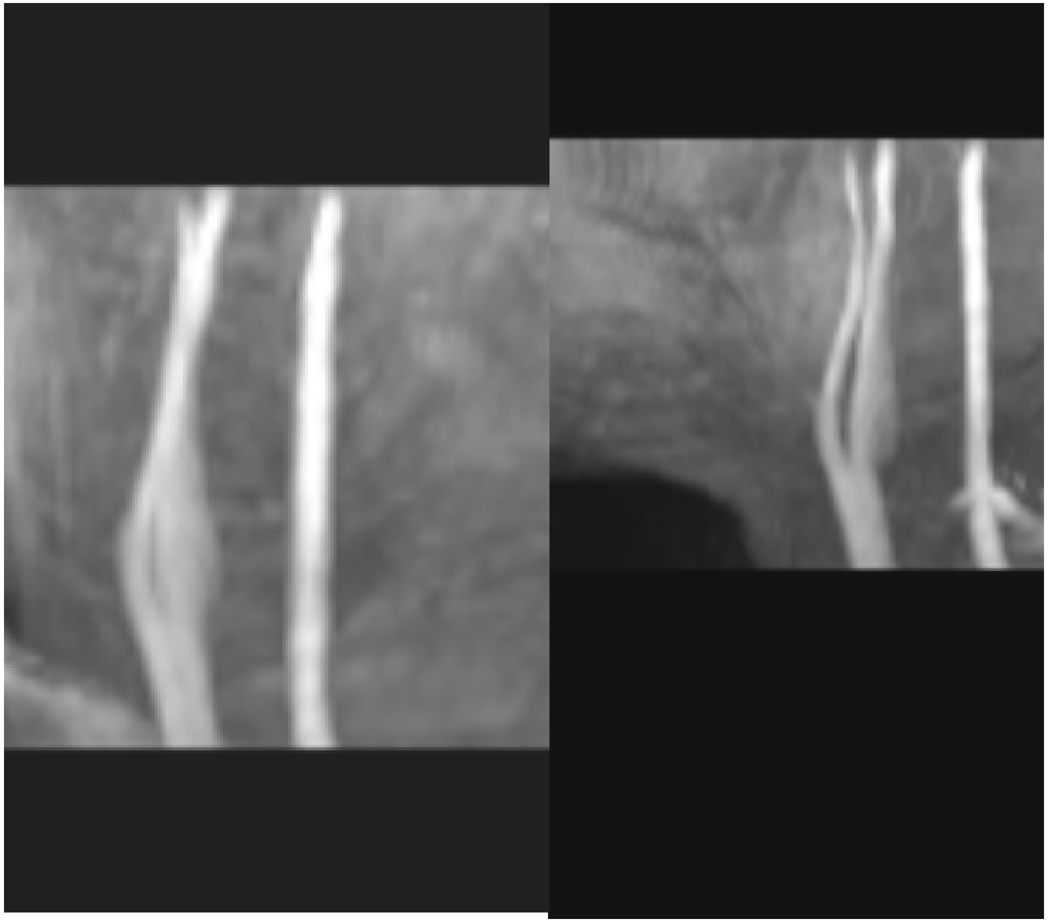

124b Score

0-30

31-50

51-70

>70

Near occlusion

Occluded

Quality

1

2

3

4

5

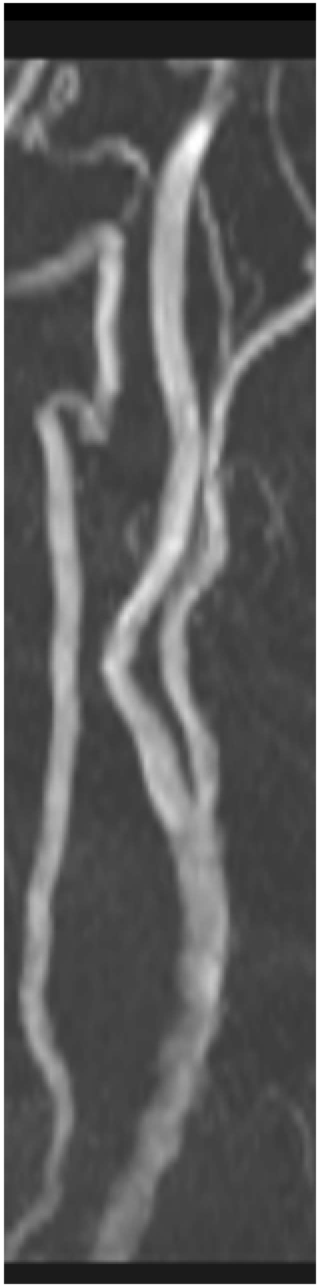

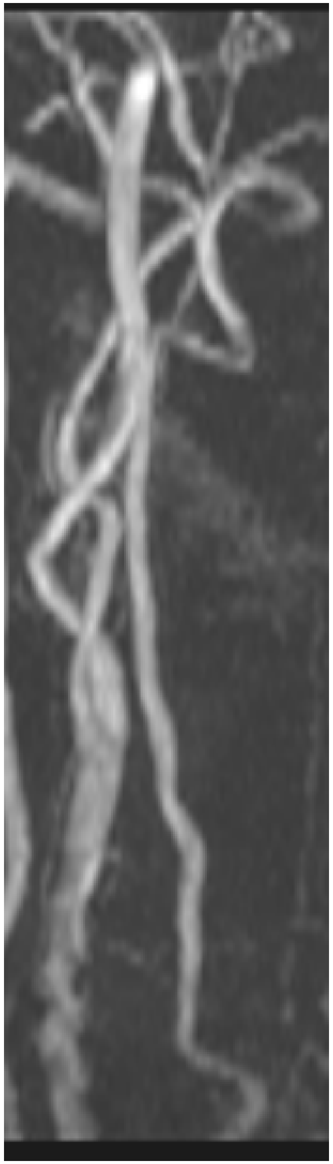

125a Score

0-30

31-50

51-70

>70

Near occlusion

Occluded

Quality

1

2

3

4

5

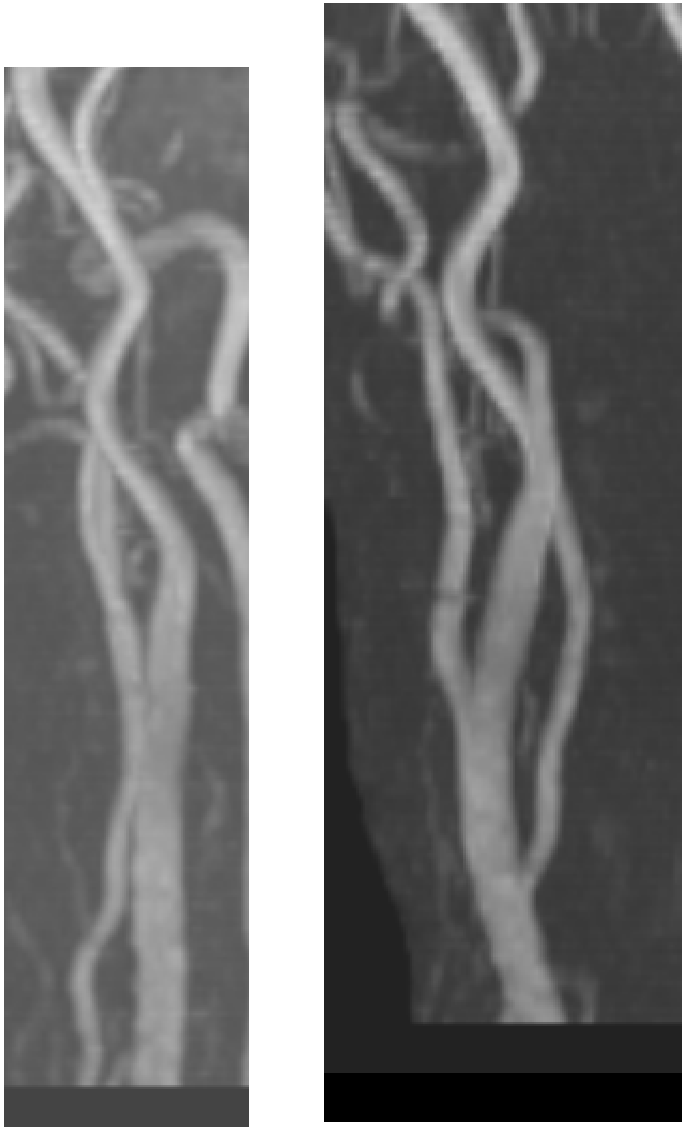

125f Score

0-30

31-50

51-70

>70

Near occlusion

Occluded

Quality

1

2

3

4

5

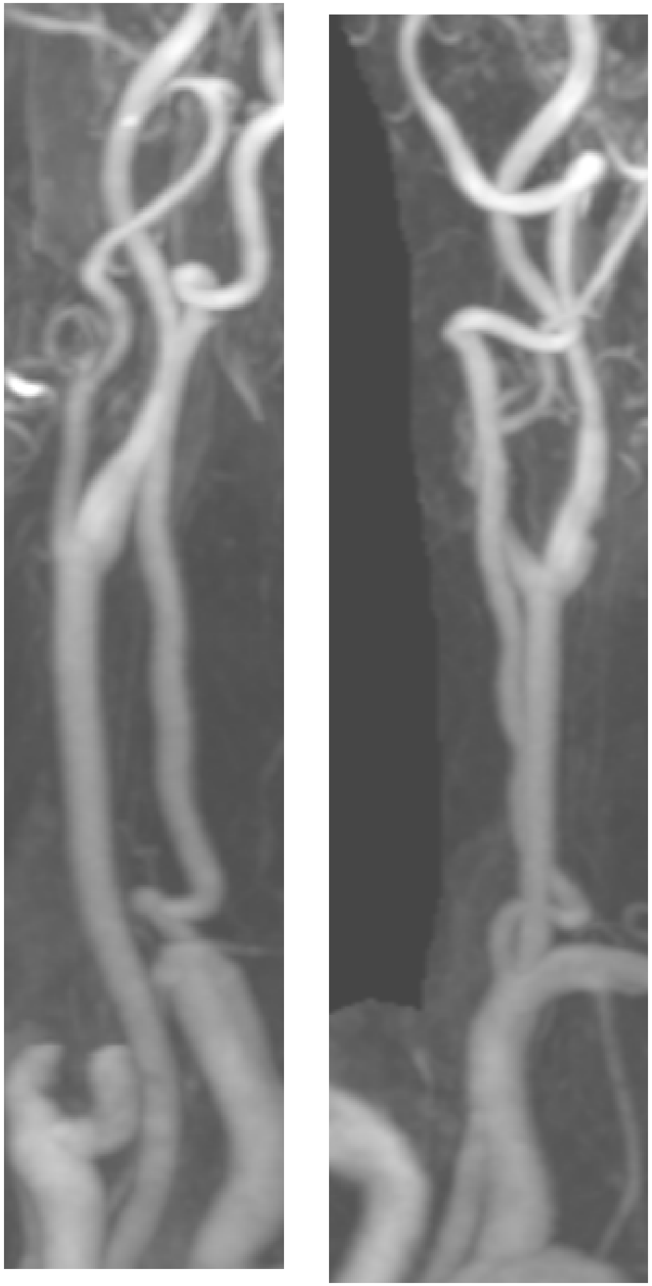

126e Score

0-30

31-50

51-70

>70

Near occlusion

Occluded

Quality

1

2

3

4

5

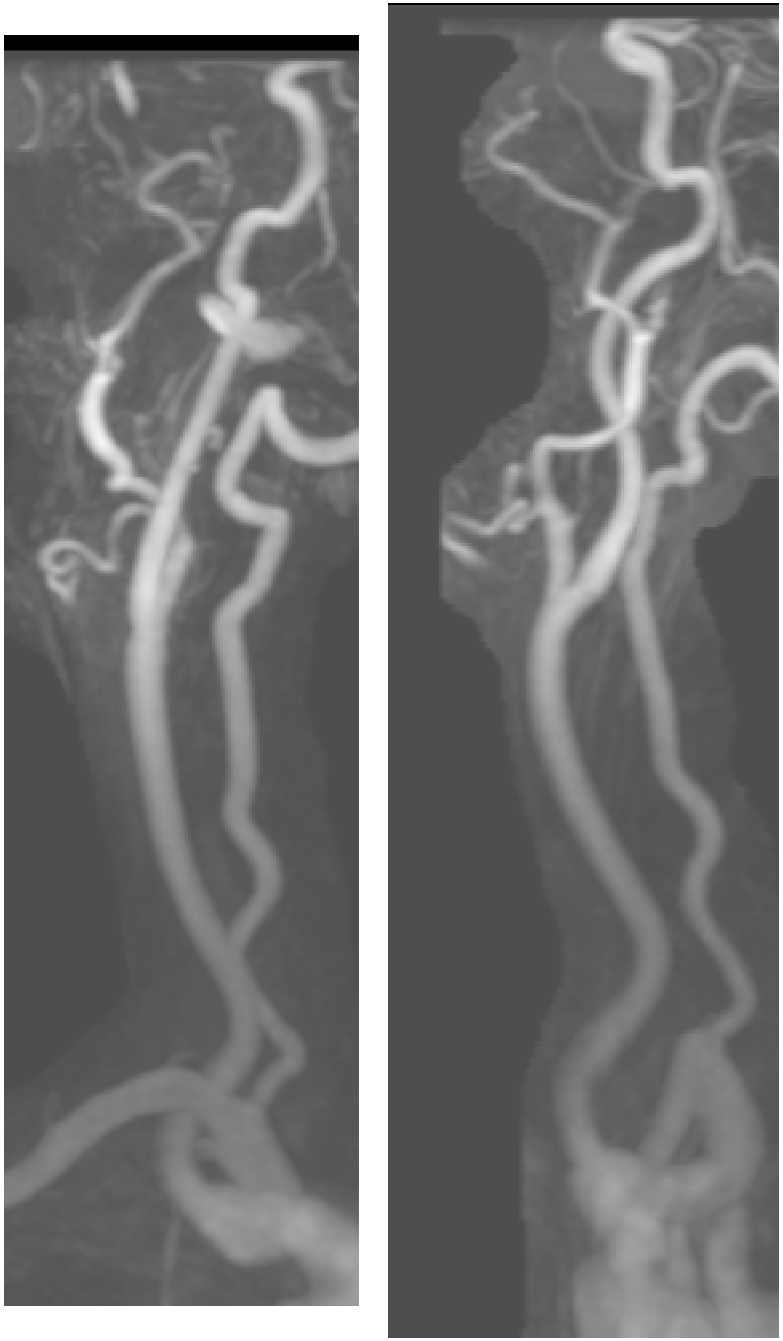

127d Score

0-30

31-50

51-70

>70

Near occlusion

Occluded

Quality

1

2

3

4

5

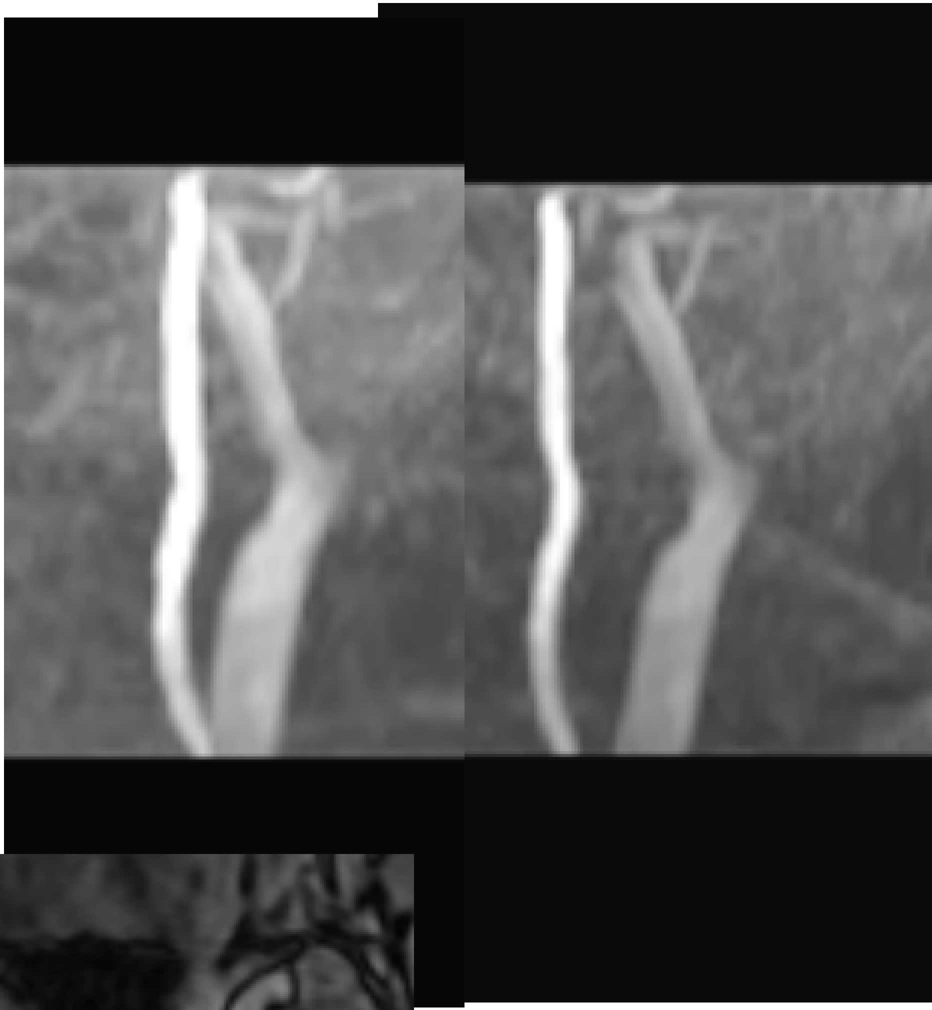

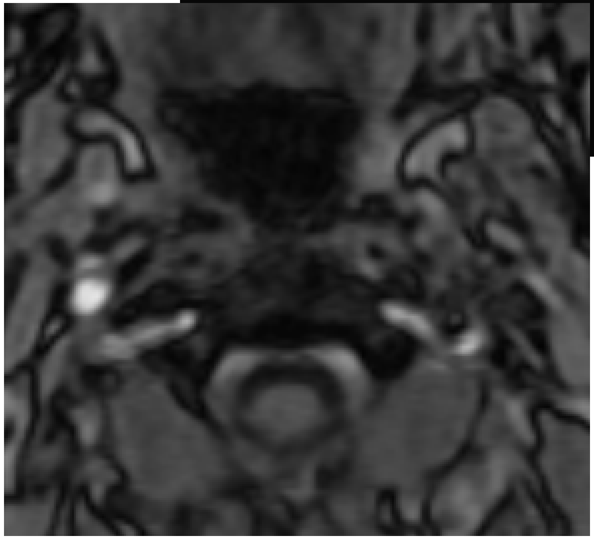

128c Score

0-30

31-50

51-70

>70

Near occlusion

Occluded

Quality

1

2

3

4

5

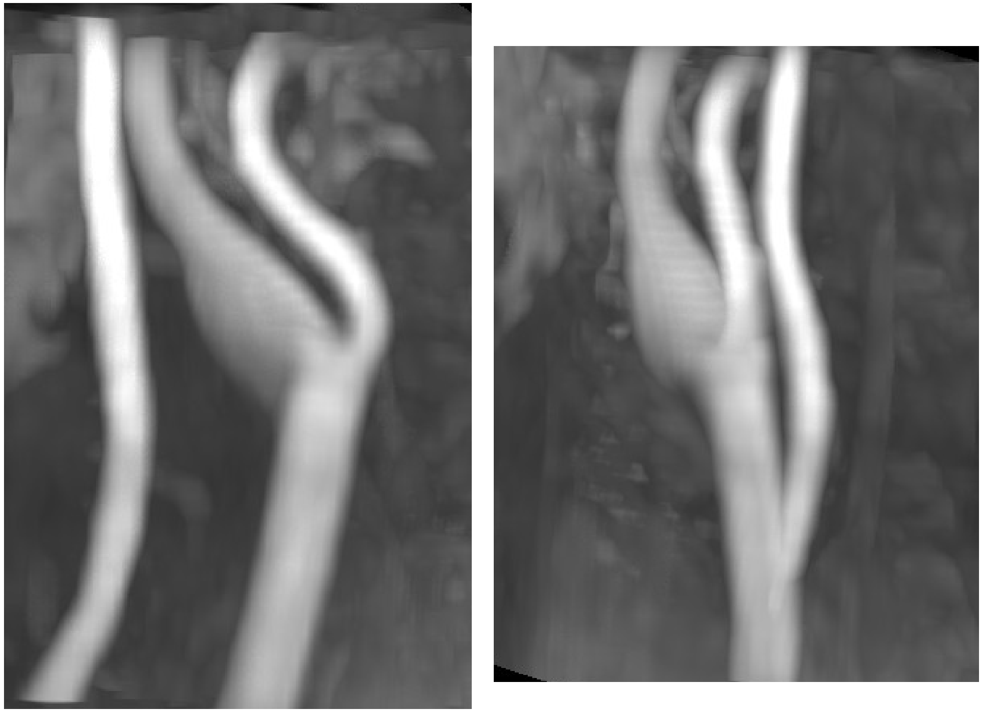

129b Score

0-30

31-50

51-70

>70

Near occlusion

Occluded

Quality

1

2

3

4

5

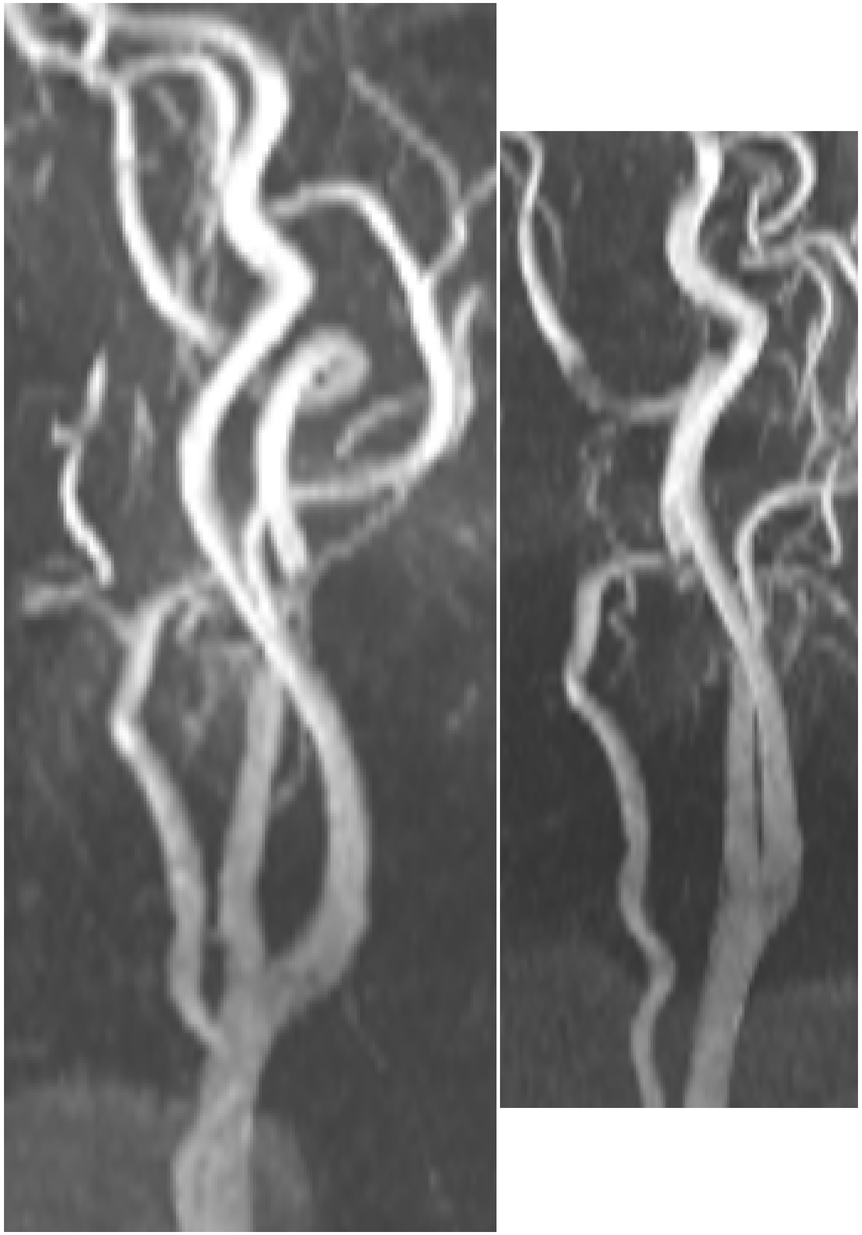

130a Score

0-30

31-50

51-70

>70

Near occlusion

Occluded

Quality

1

2

3

4

5

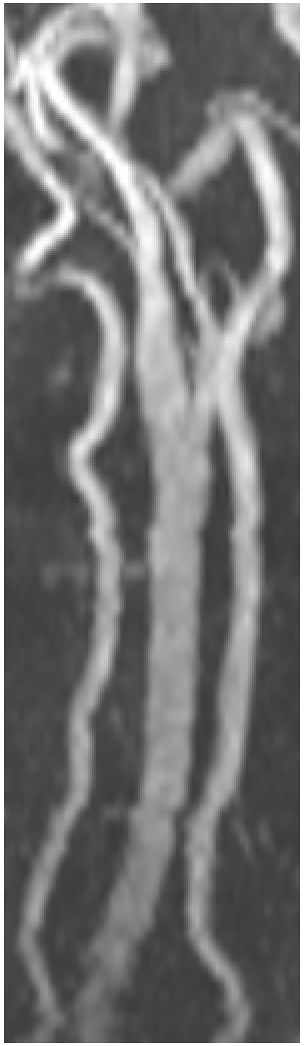

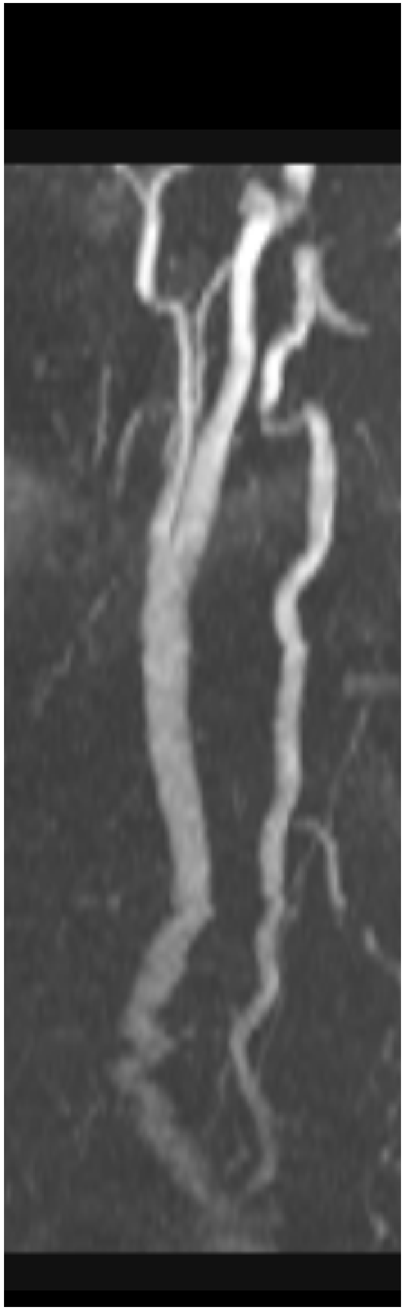

130f Score

0-30

31-50

51-70

>70

Near occlusion

Occluded

Quality

1

2

3

4

5

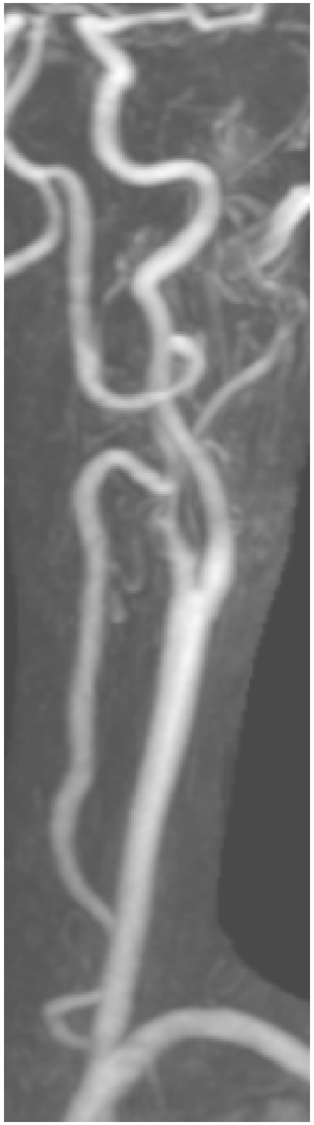

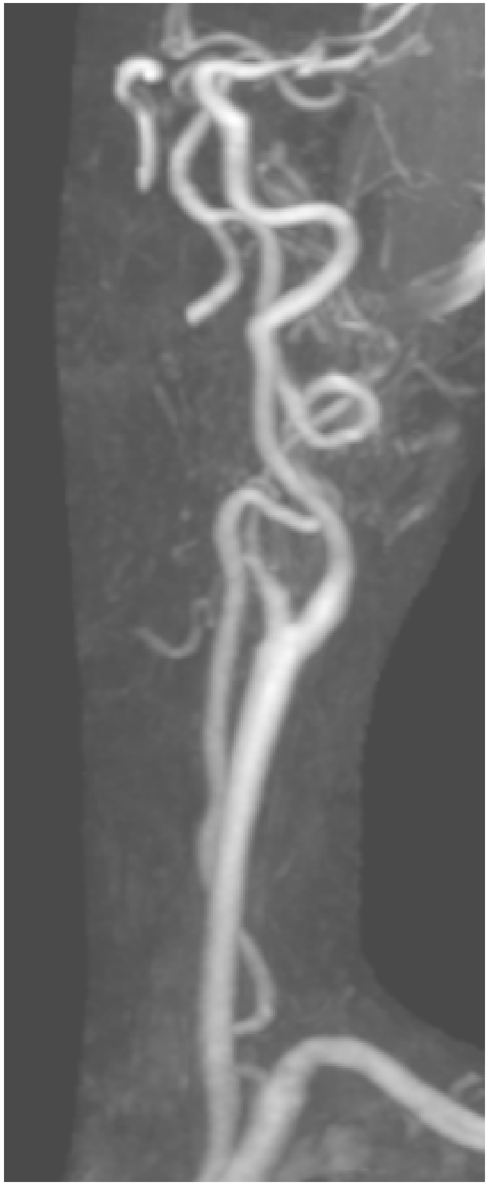

131e Score

0-30

31-50

51-70

>70

Near occlusion

Occluded

Quality

1

2

3

4

5

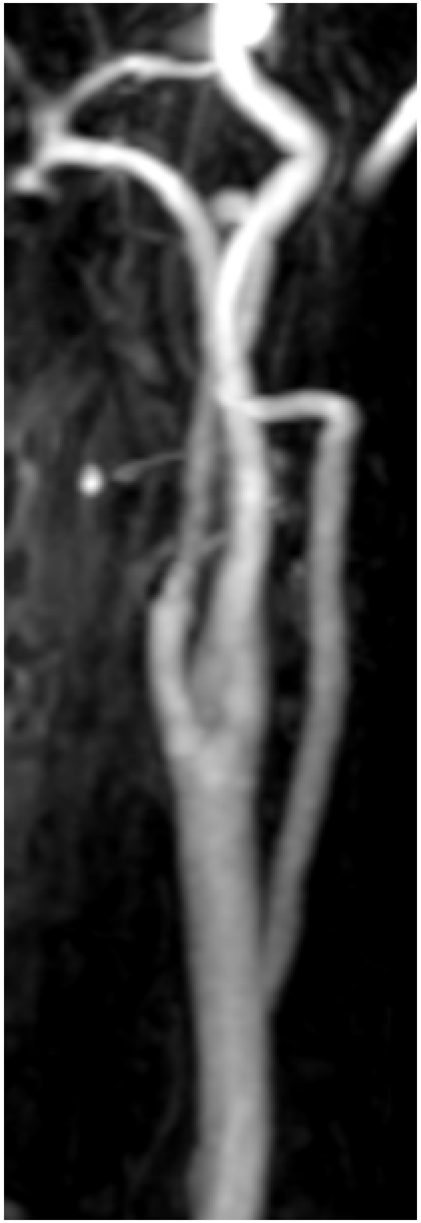

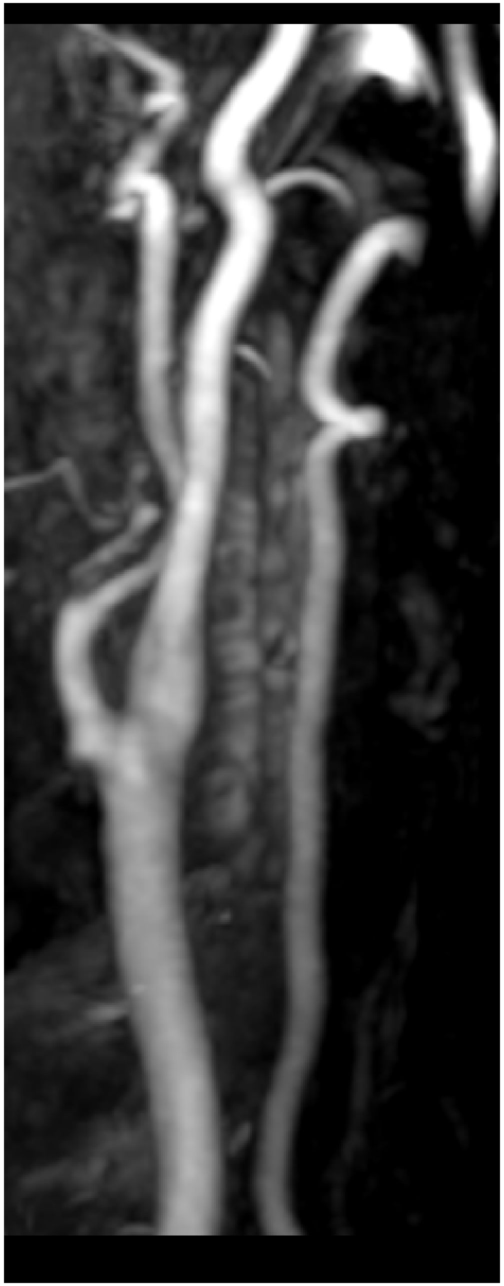

132d Score

0-30

31-50

51-70

>70

Near occlusion

Occluded

Quality

1

2

3

4

5

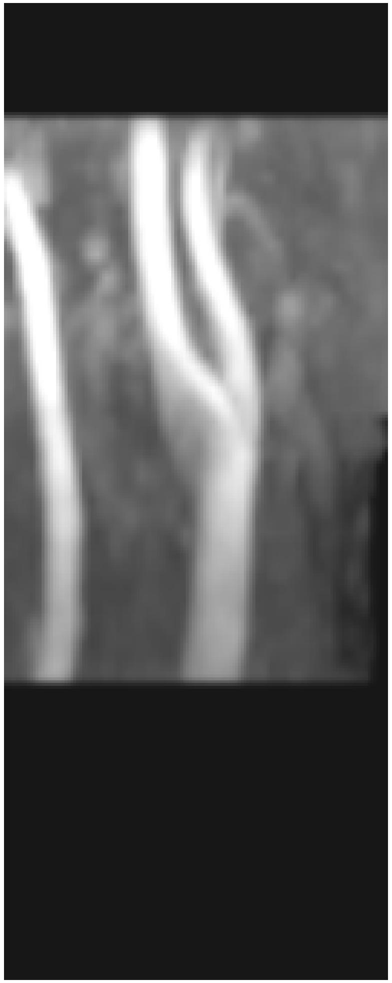

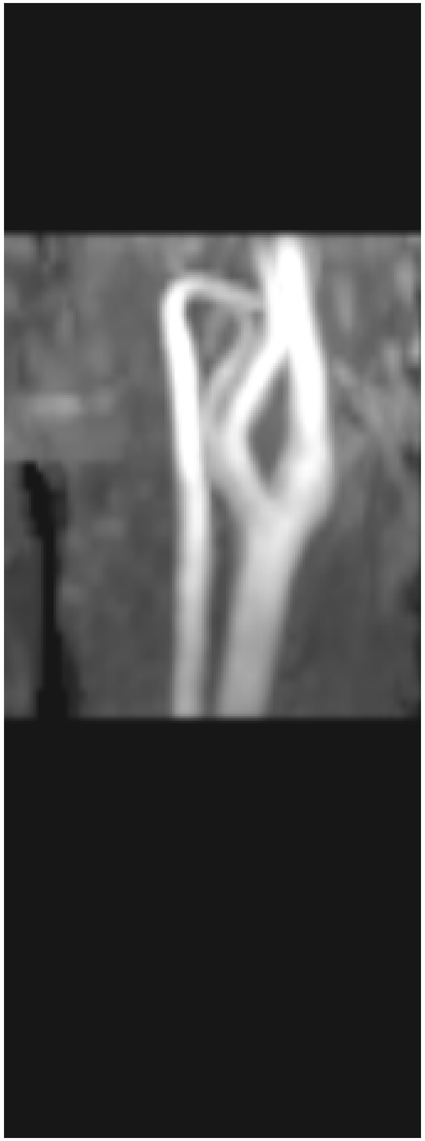

133c Score (right)

0-30

31-50

51-70

>70

Near occlusion

Occluded

Quality

1

2

3

4

5

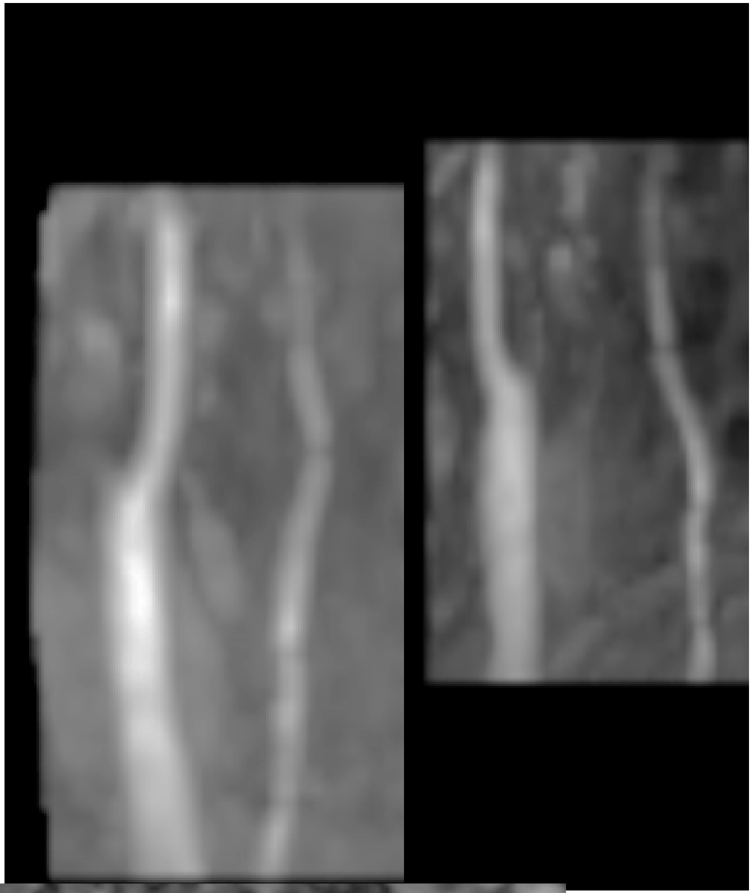

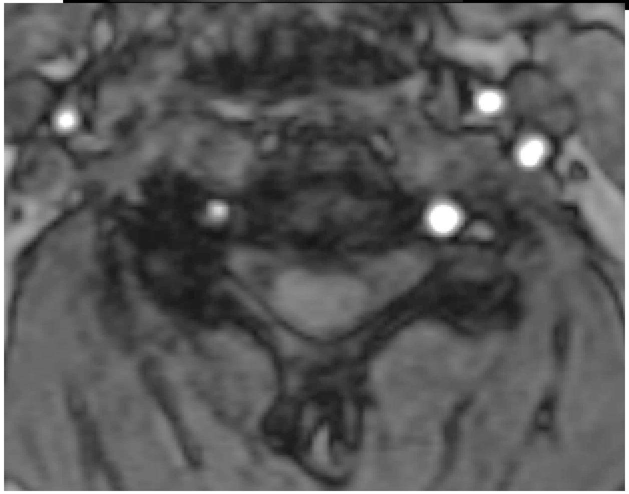

134b Score

0-30

31-50

51-70

>70

Near occlusion

Occluded

Quality

1

2

3

4

5

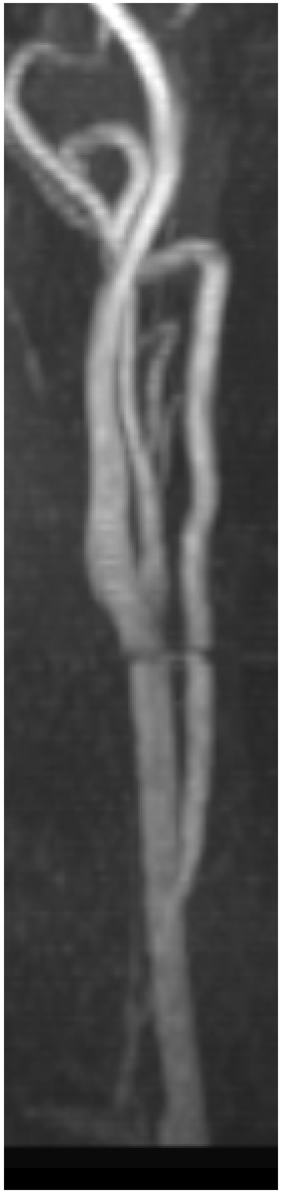

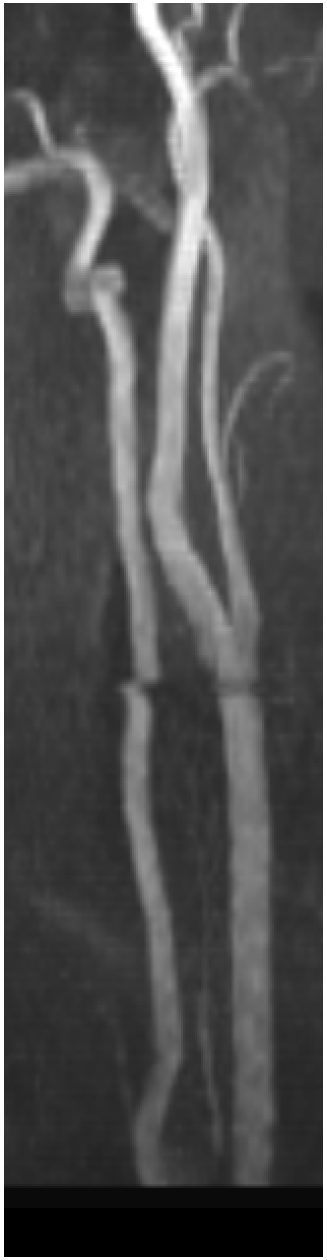

135a Score

0-30

31-50

51-70

>70

Near occlusion

Occluded

Quality

1

2

3

4

5

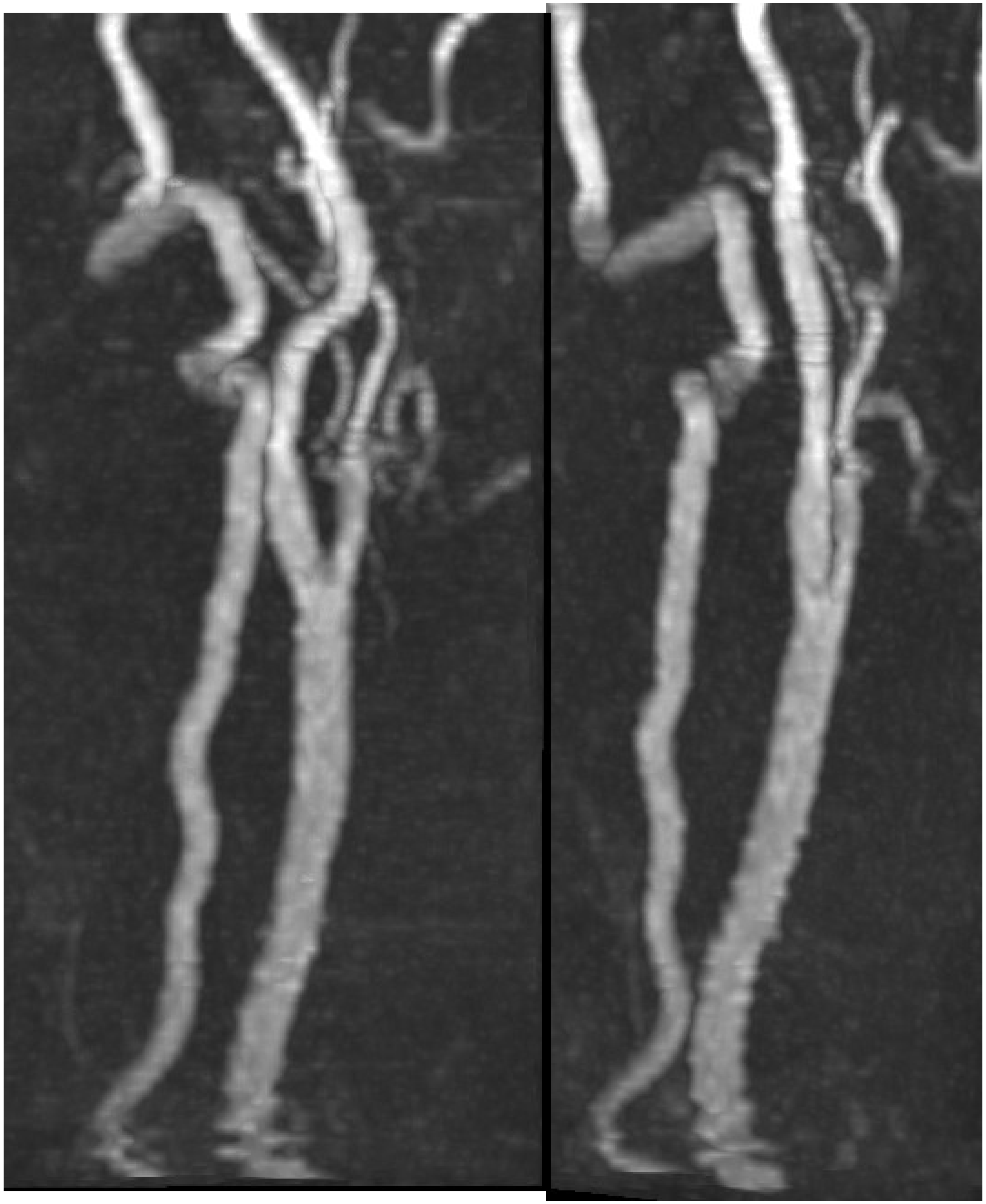

135f Score

0-30

31-50

51-70

>70

Near occlusion

Occluded

Quality

1

2

3

4

5

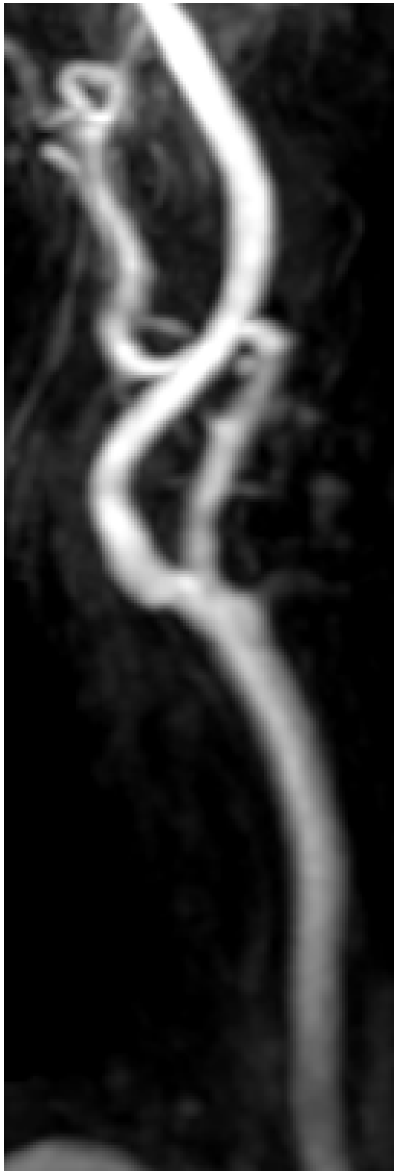

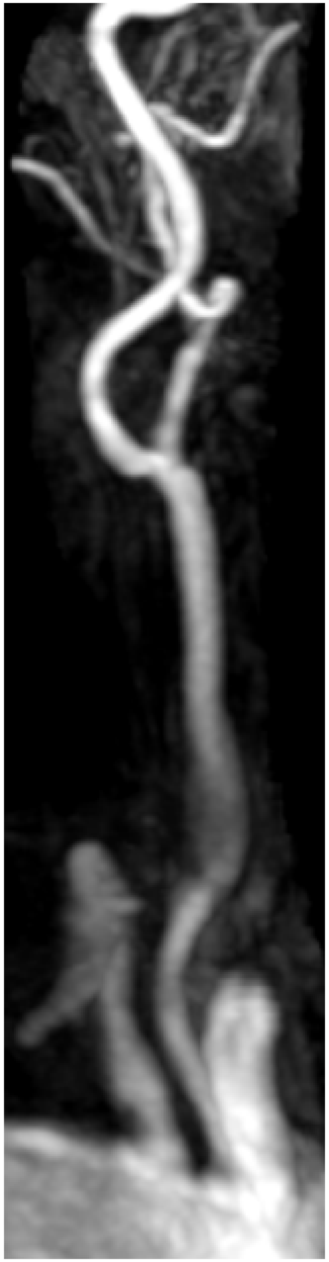

136e Score

0-30

31-50

51-70

>70

Near occlusion

Occluded

Quality

1

2

3

4

5

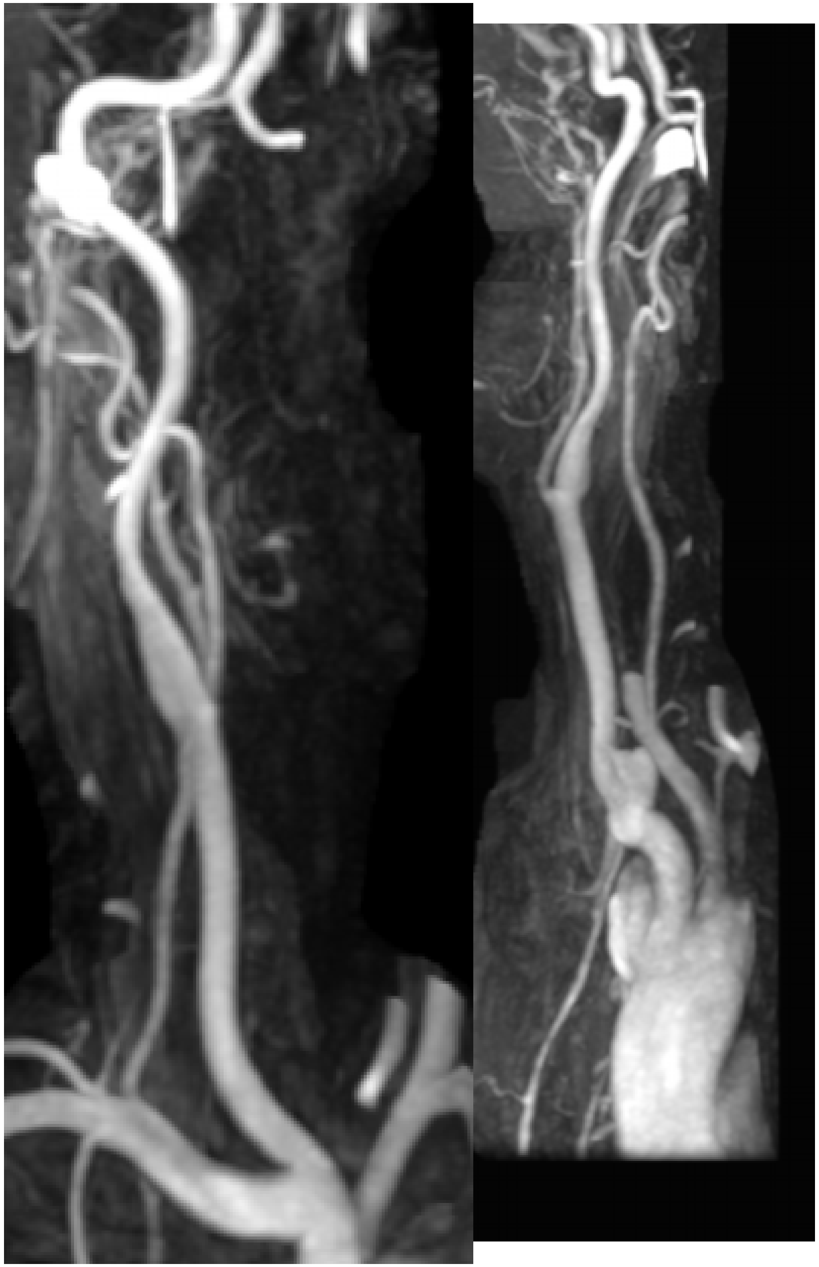

137d Score

0-30

31-50

51-70

>70

Near occlusion

Occluded

Quality

1

2

3

4

5

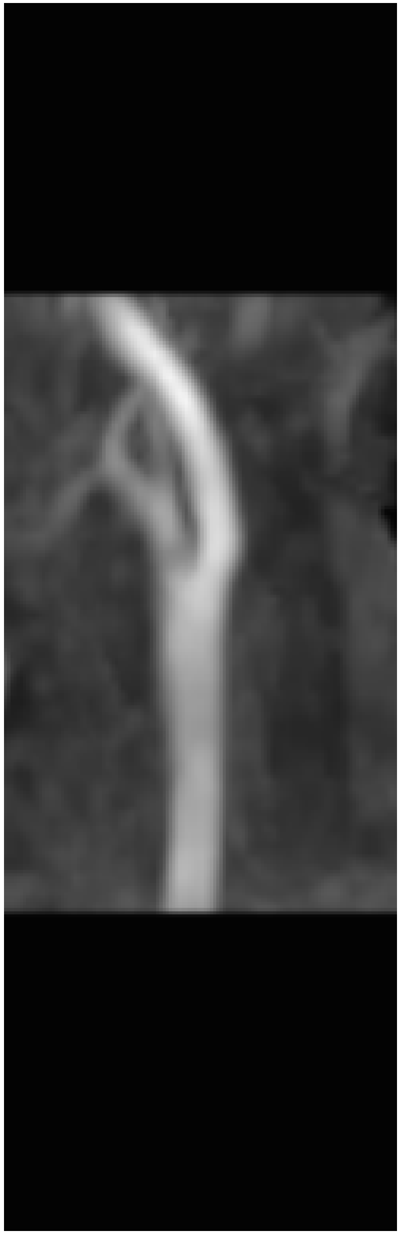

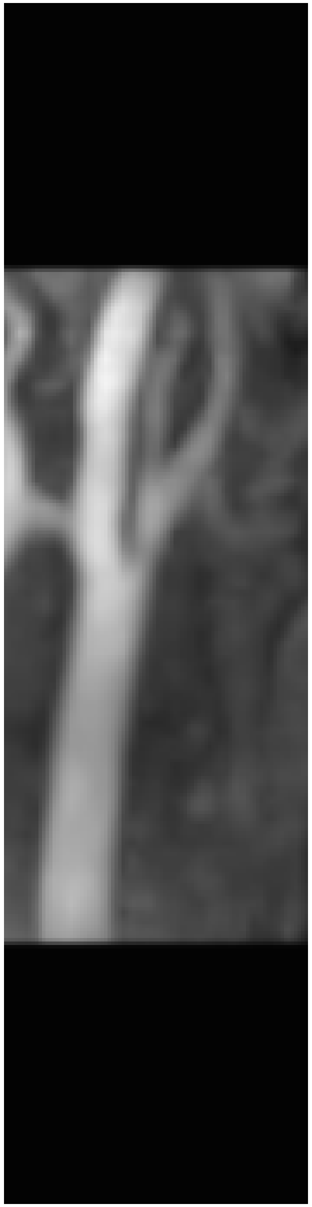

138c Score

0-30

31-50

51-70

>70

Near occlusion

Occluded

Quality

1

2

3

4

5

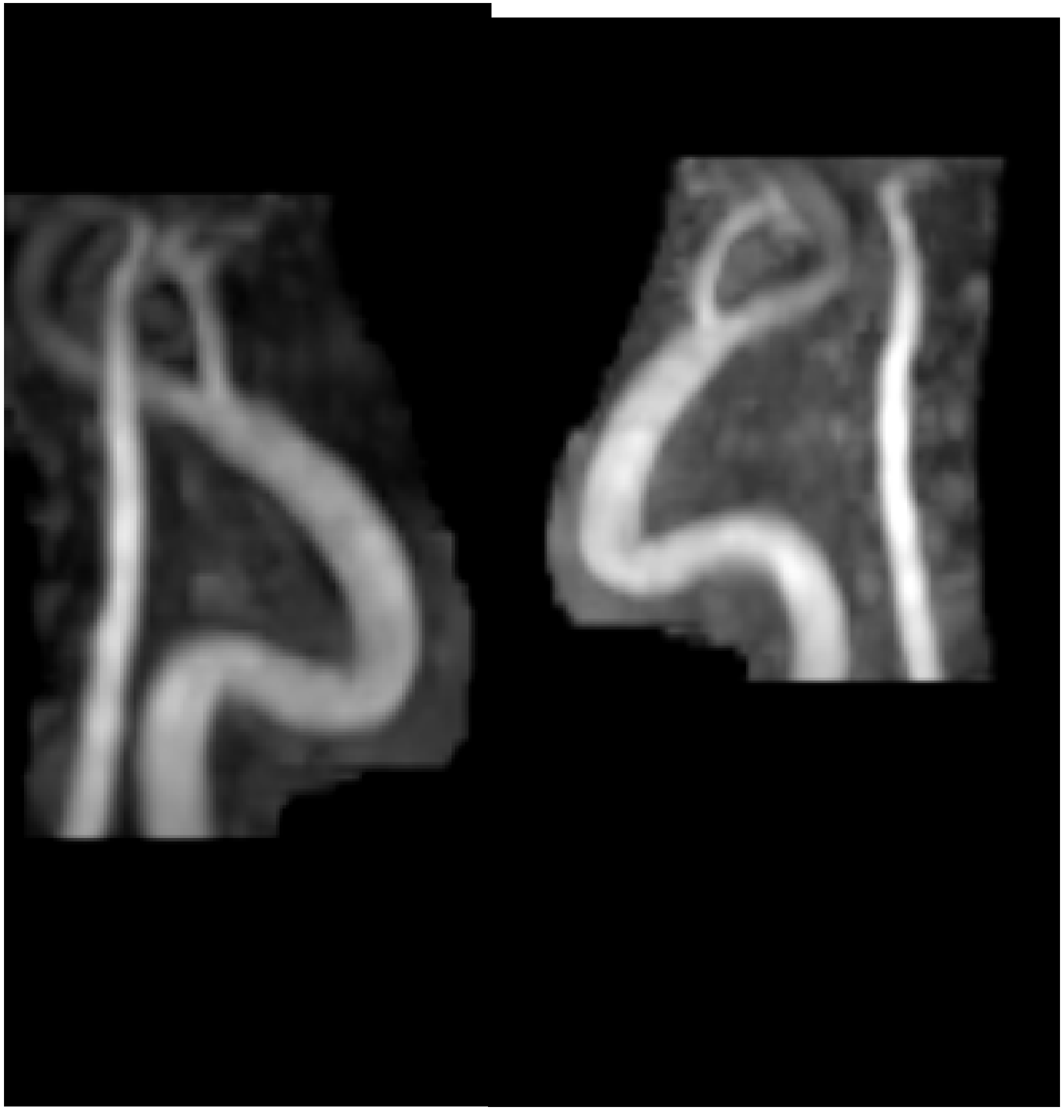

139b Score

0-30

31-50

51-70

>70

Near occlusion

Occluded

Quality

1

2

3

4

5

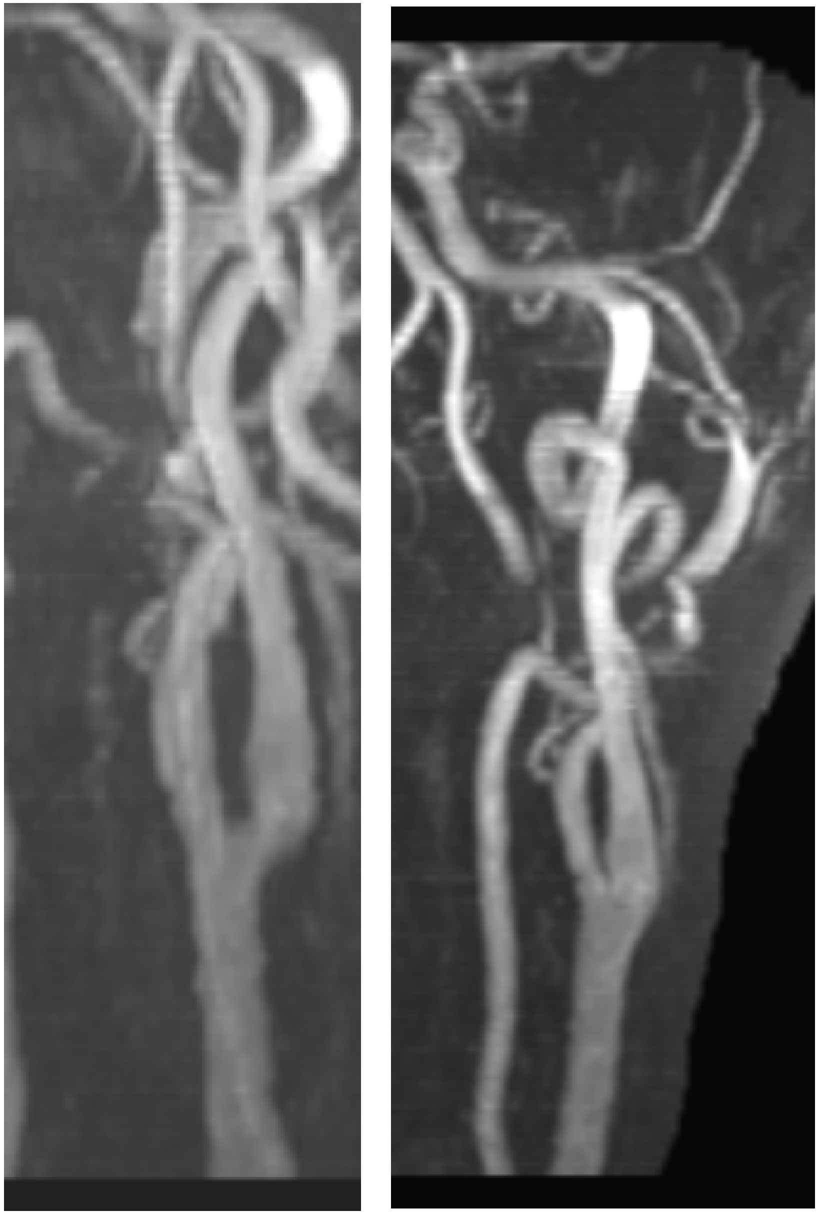

140a Score

0-30

31-50

51-70

>70

Near occlusion

Occluded

Quality

1

2

3

4

5

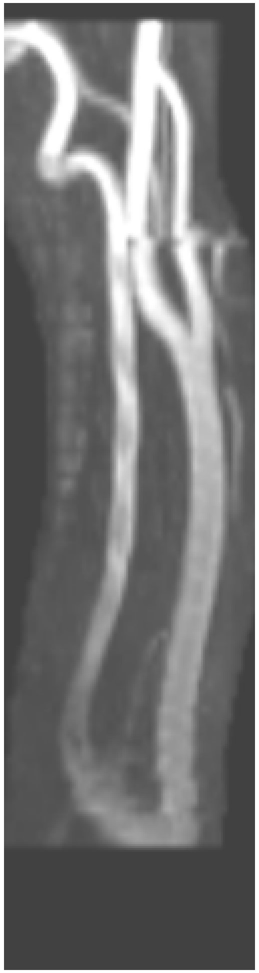

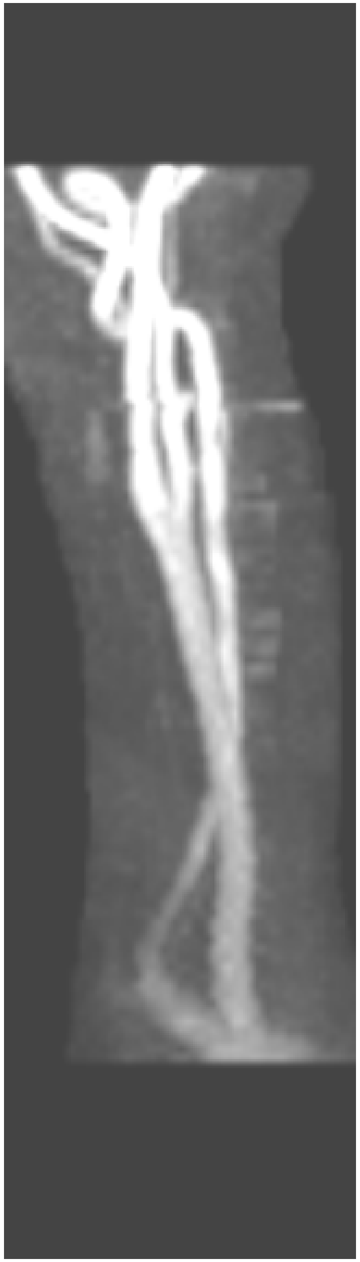

140f Score

0-30

31-50

51-70

>70

Near occlusion

Occluded

Quality

1

2

3

4

5

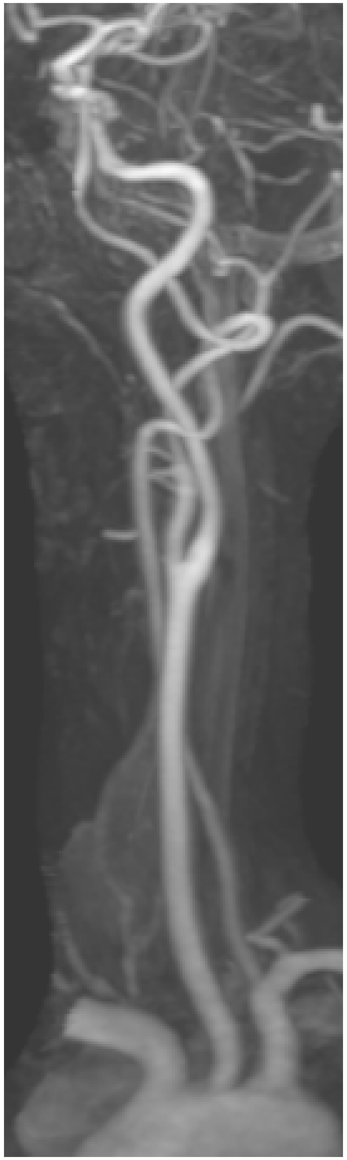

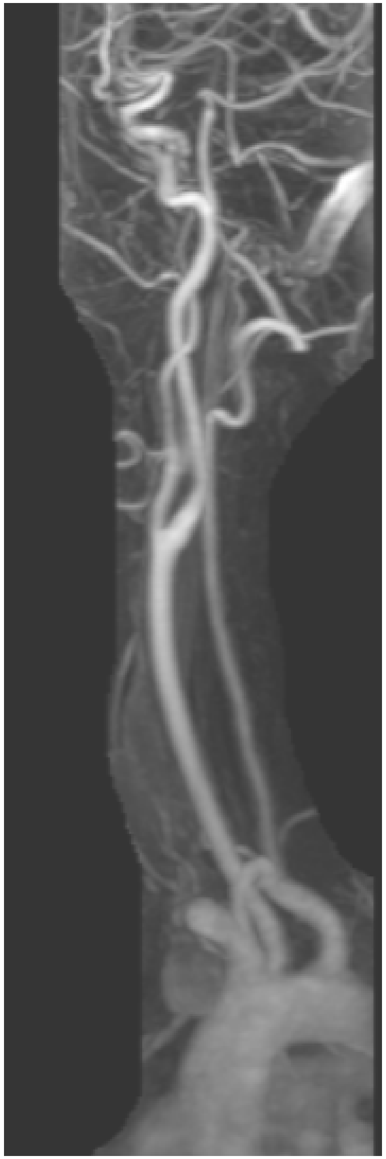

141e Score

0-30

31-50

51-70

>70

Near occlusion

Occluded

Quality

1

2

3

4

5

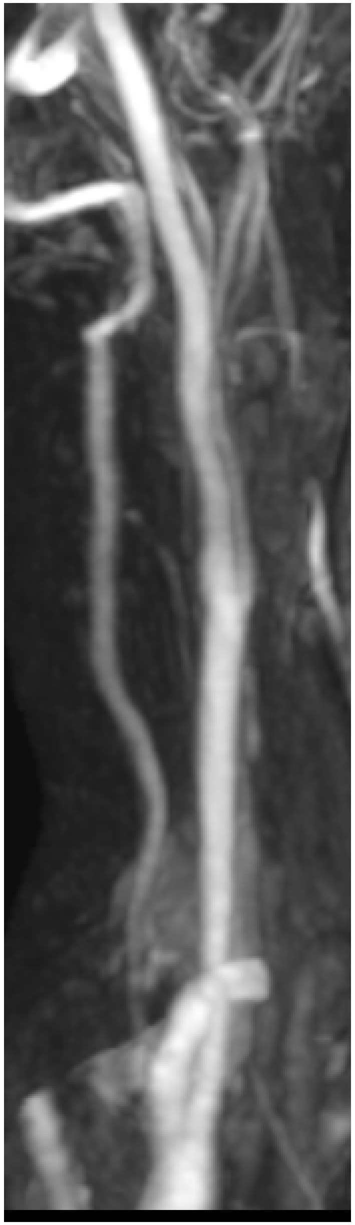

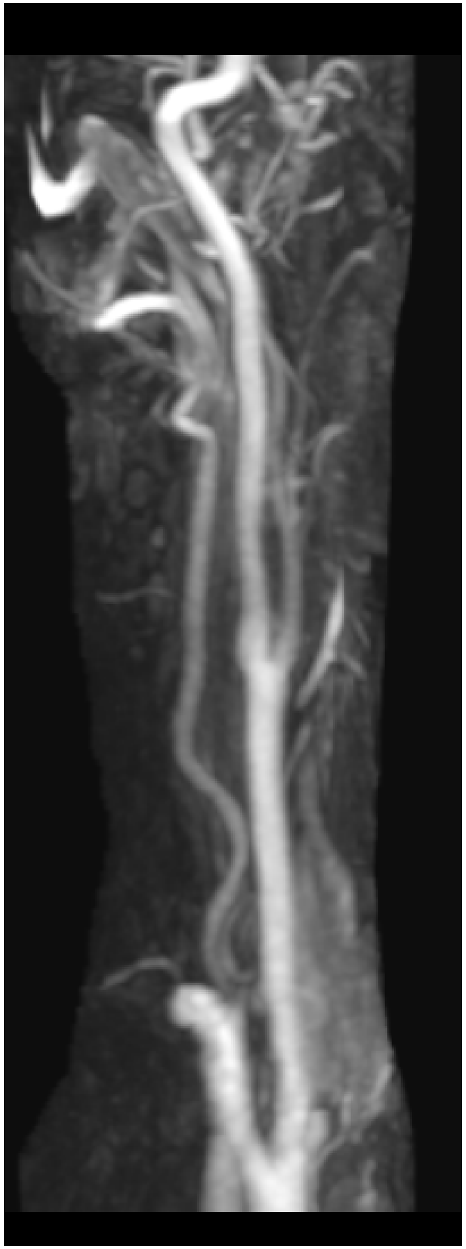

142d Score

0-30

31-50

51-70

>70

Near occlusion

Occluded

Quality

1

2

3

4

5

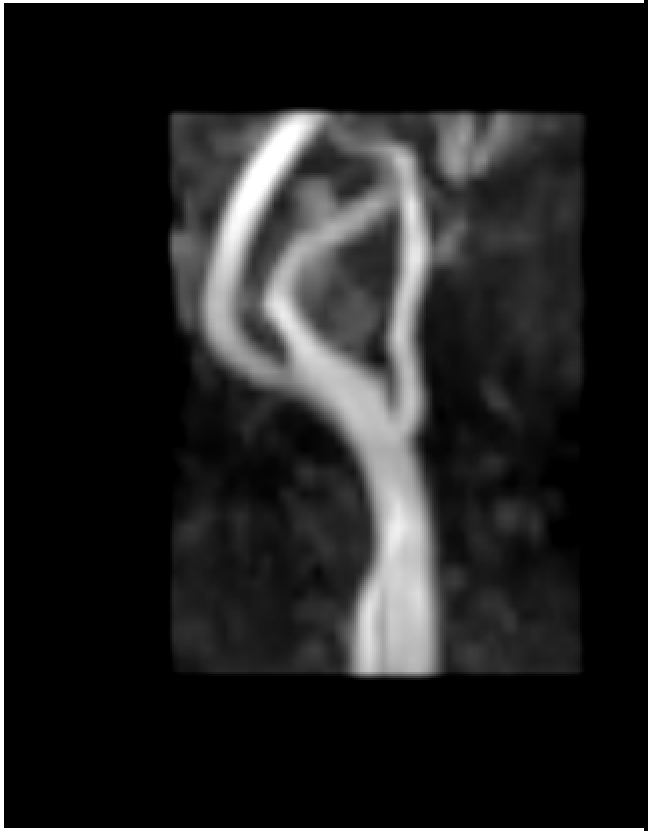
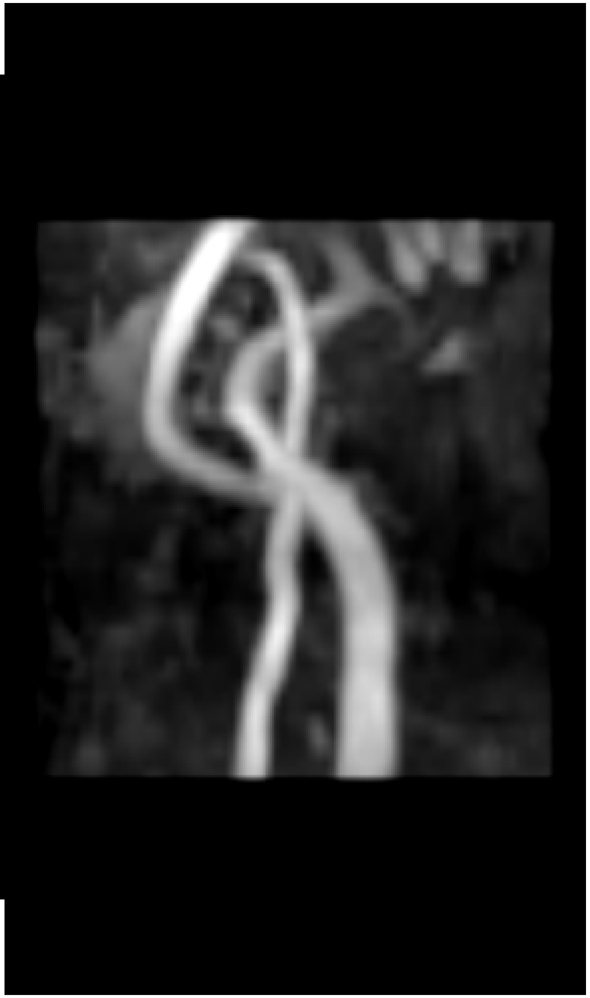

143c Score

0-30

31-50

51-70

>70

Near occlusion

Occluded

Quality

1

2

3

4

5

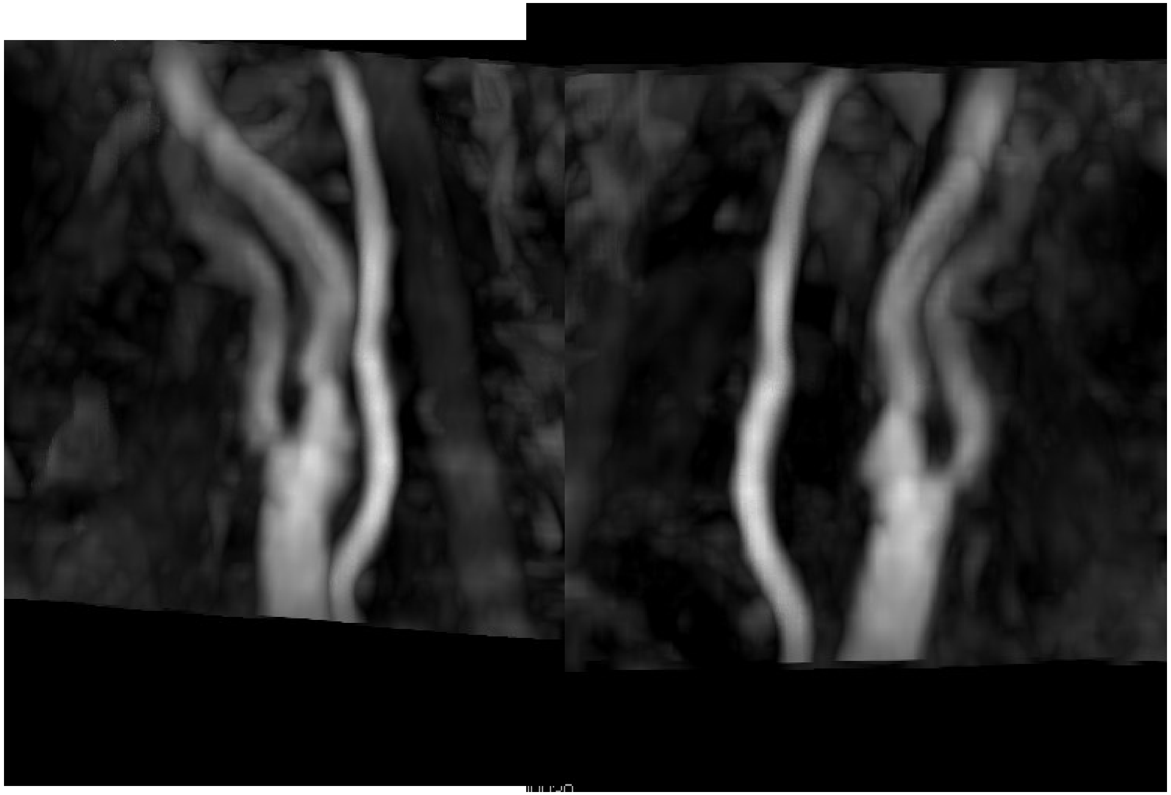

144b Score

0-30

31-50

51-70

>70

Near occlusion

Occluded

Quality

1

2

3

4

5

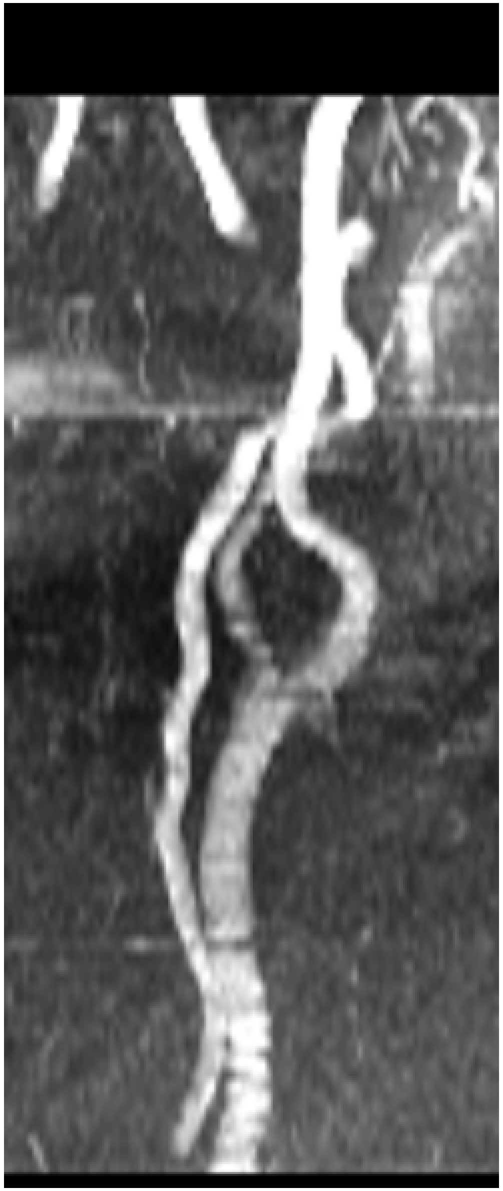

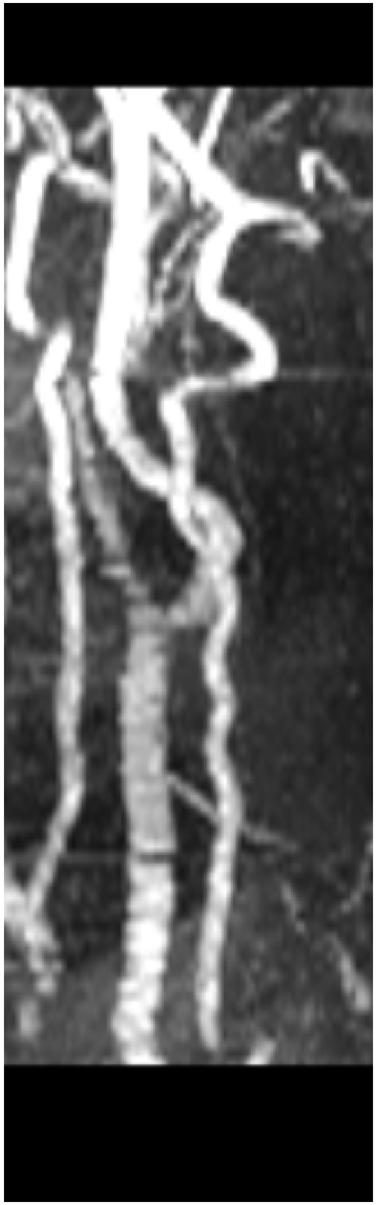

145a Score

0-30

31-50

51-70

>70

Near occlusion

Occluded

Quality

1

2

3

4

5

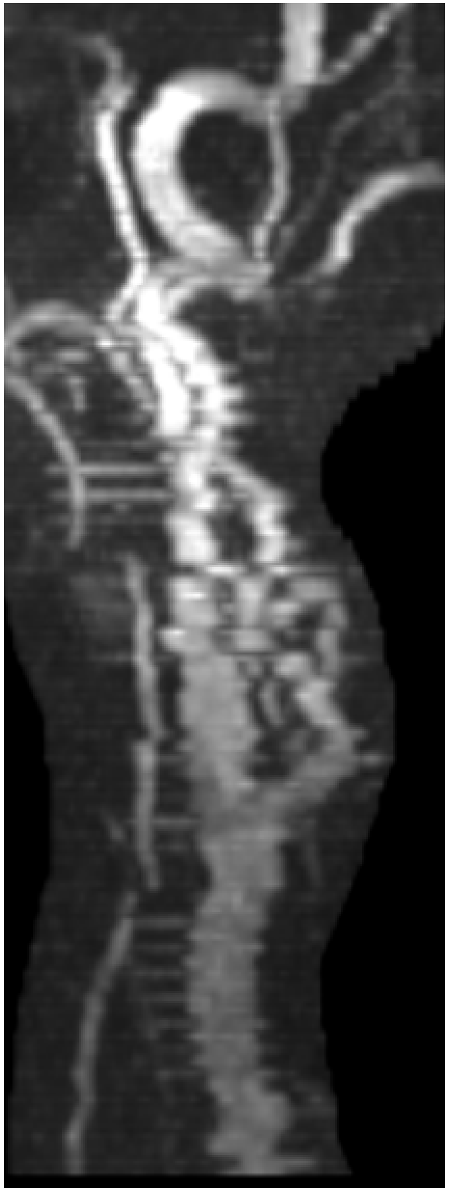

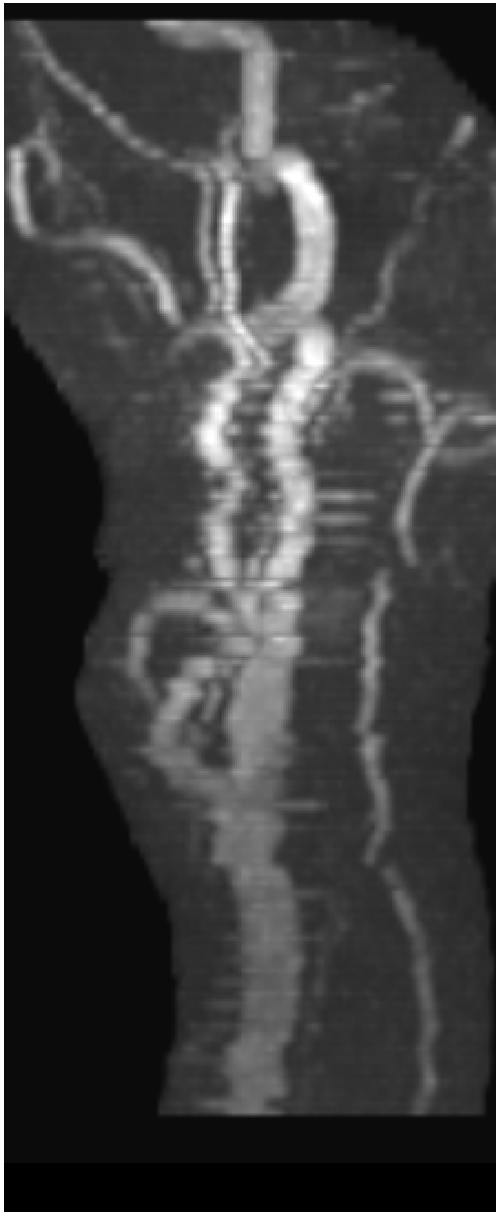

145f Score

0-30

31-50

51-70

>70

Near occlusion

Occluded

Quality

1

2

3

4

5

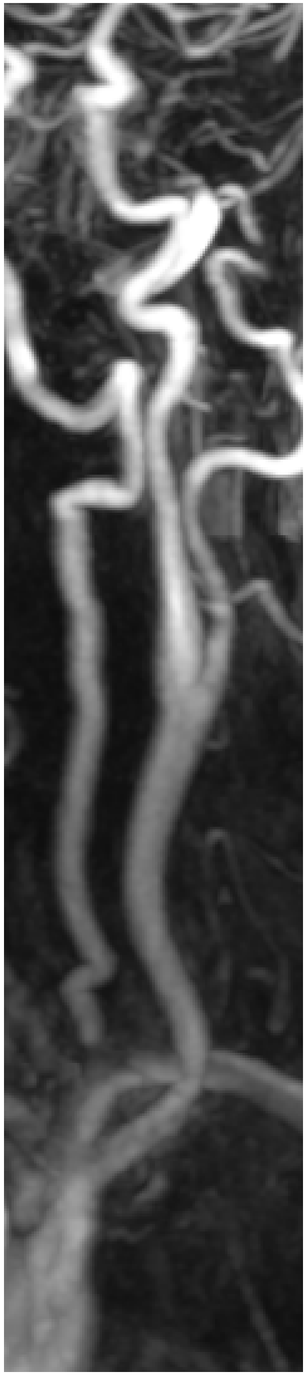

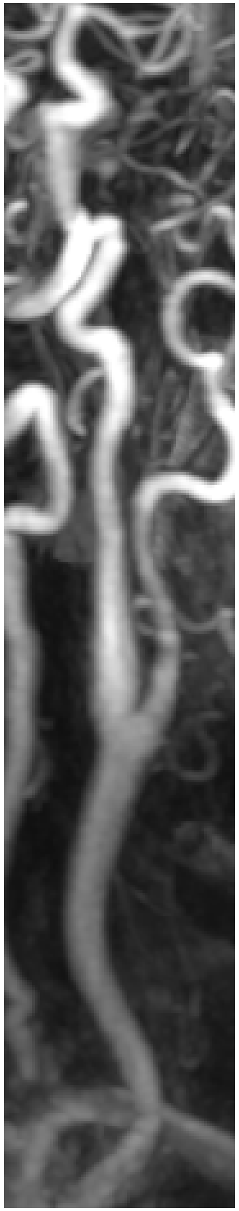

146e Score

0-30

31-50

51-70

>70

Near occlusion

Occluded

Quality

1

2

3

4

5

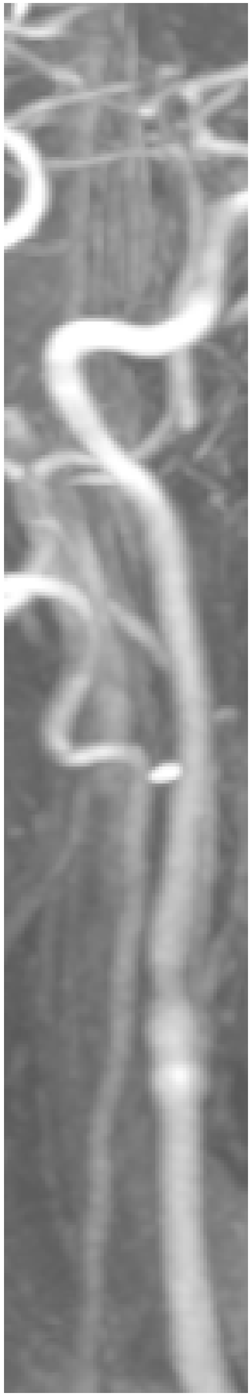

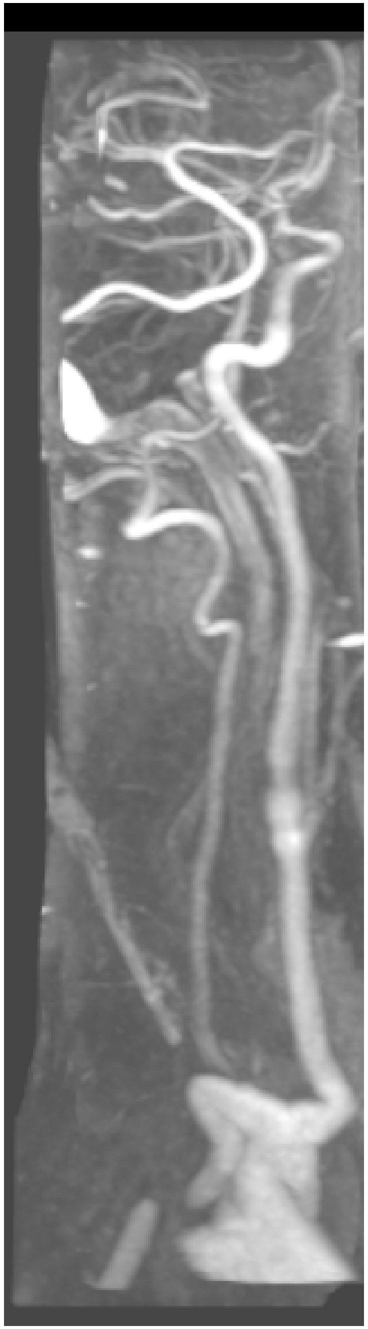

147d Score

0-30

31-50

51-70

>70

Near occlusion

Occluded

Quality

1

2

3

4

5

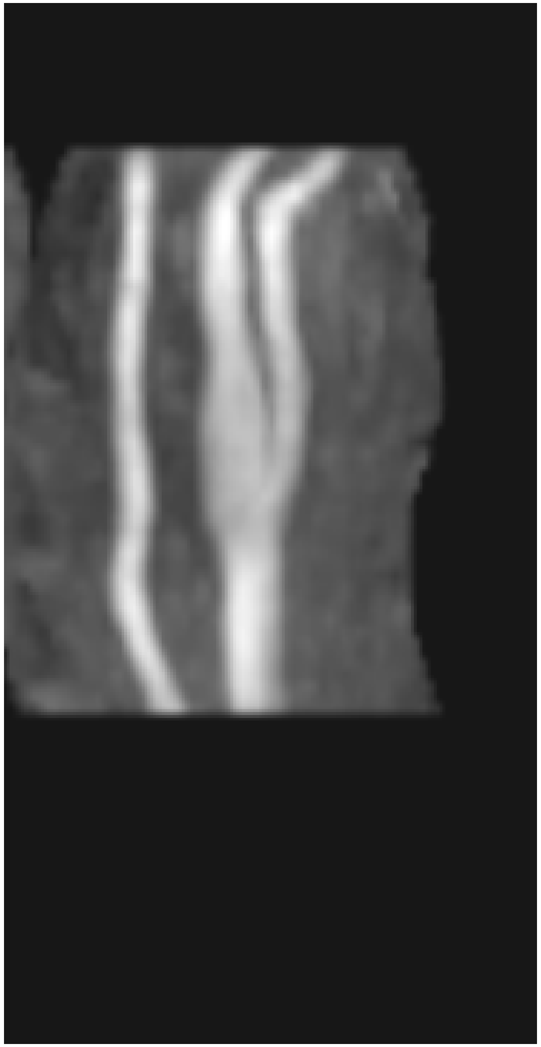

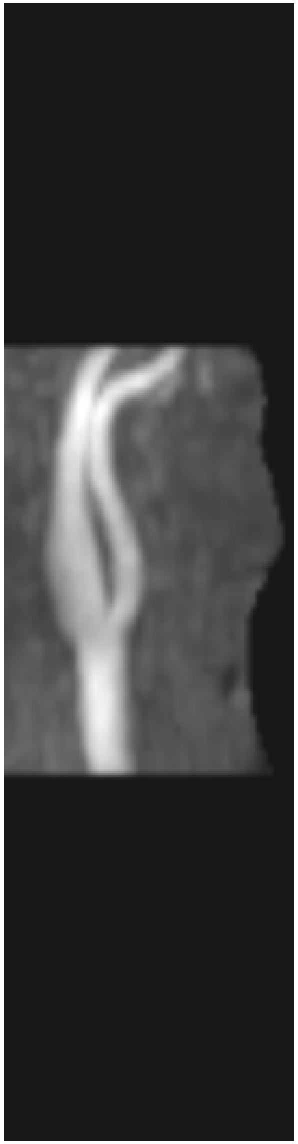

148c Score

0-30

31-50

51-70

>70

Near occlusion

Occluded

Quality

1

2

3

4

5

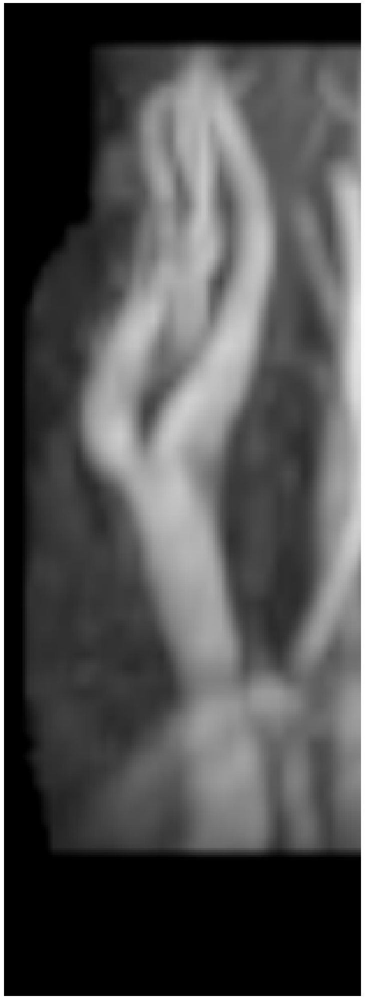

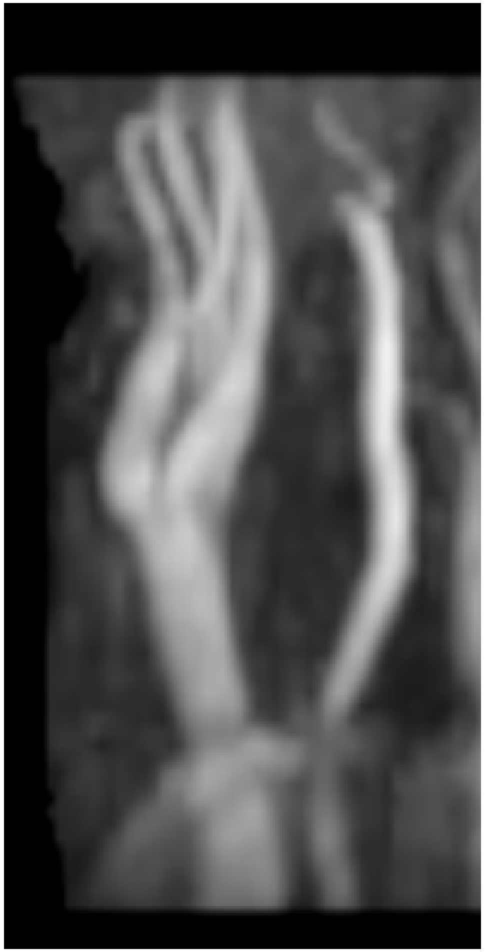

149b Score

0-30

31-50

51-70

>70

Near occlusion

Occluded

Quality

1

2

3

4

5

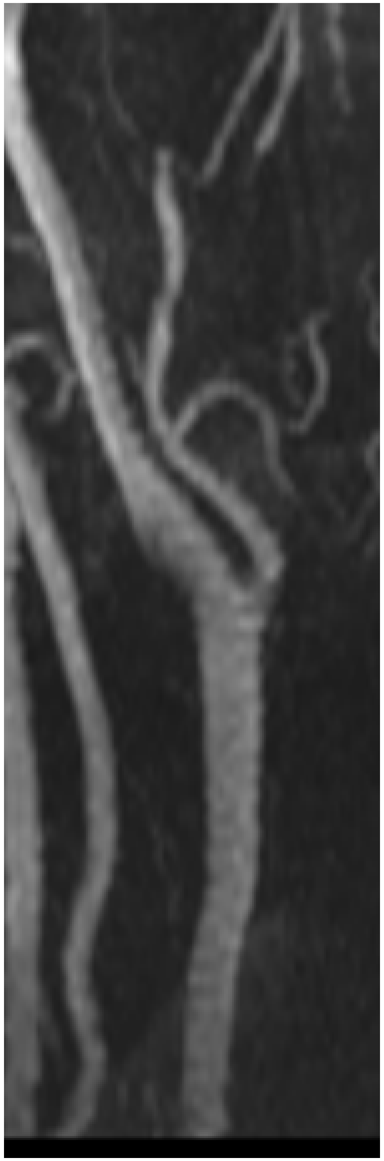

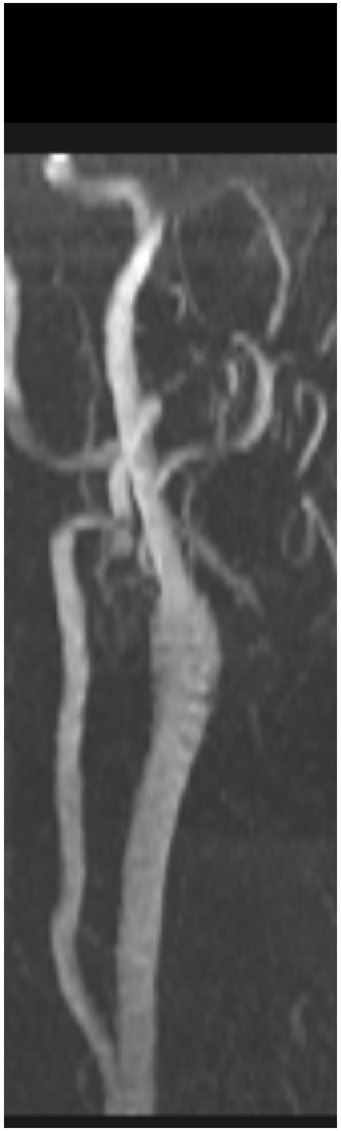

150a Score

0-30

31-50

51-70

>70

Near occlusion

Occluded

Quality

1

2

3

4

5

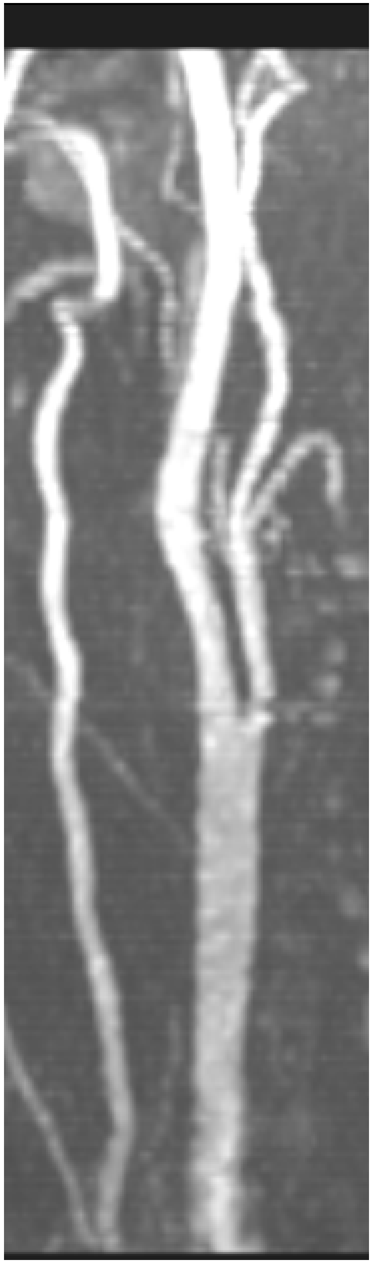

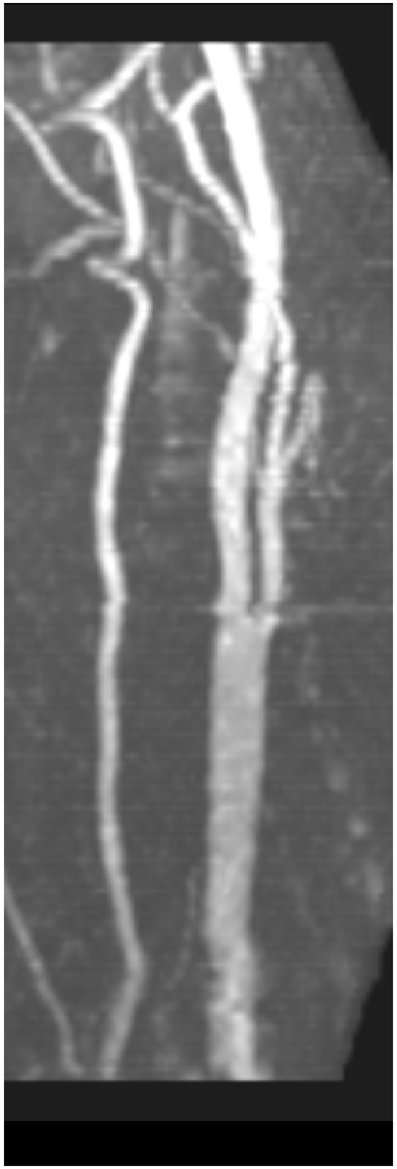

150f Score

0-30

31-50

51-70

>70

Near occlusion

Occluded

Quality

1

2

3

4

5

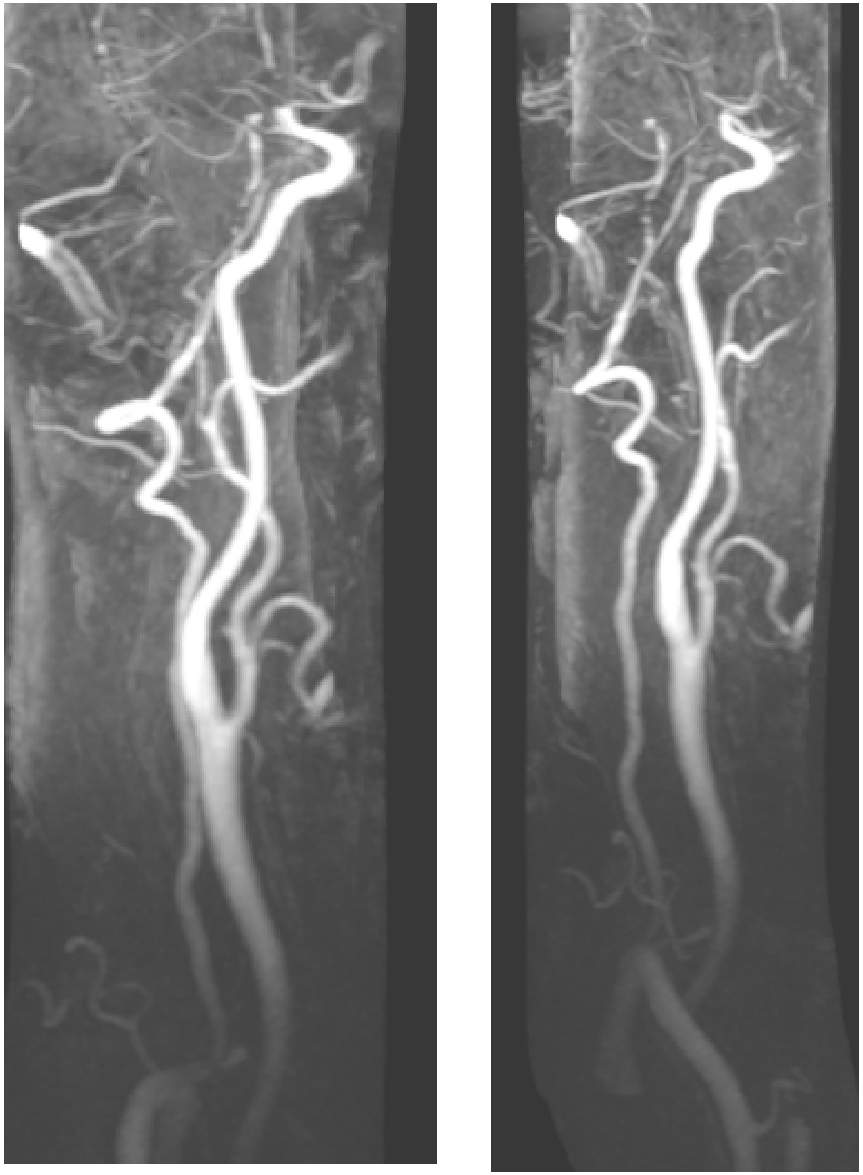

151e Score

0-30

31-50

51-70

>70

Near occlusion

Occluded

Quality

1

2

3

4

5

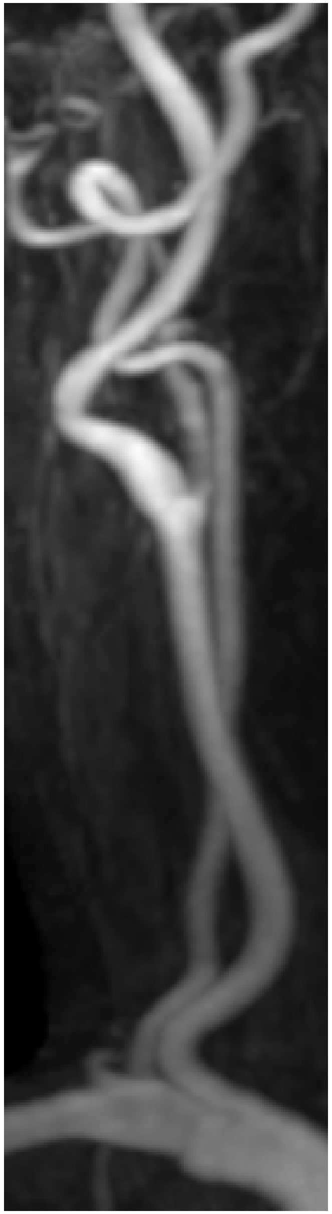

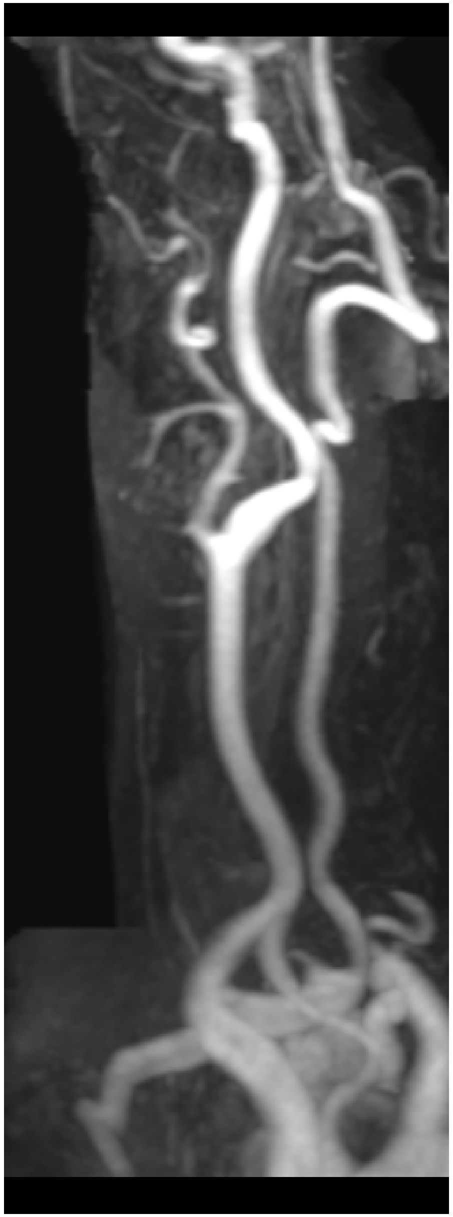

152d Score

0-30

31-50

51-70

>70

Near occlusion

Occluded

Quality

1

2

3

4

5

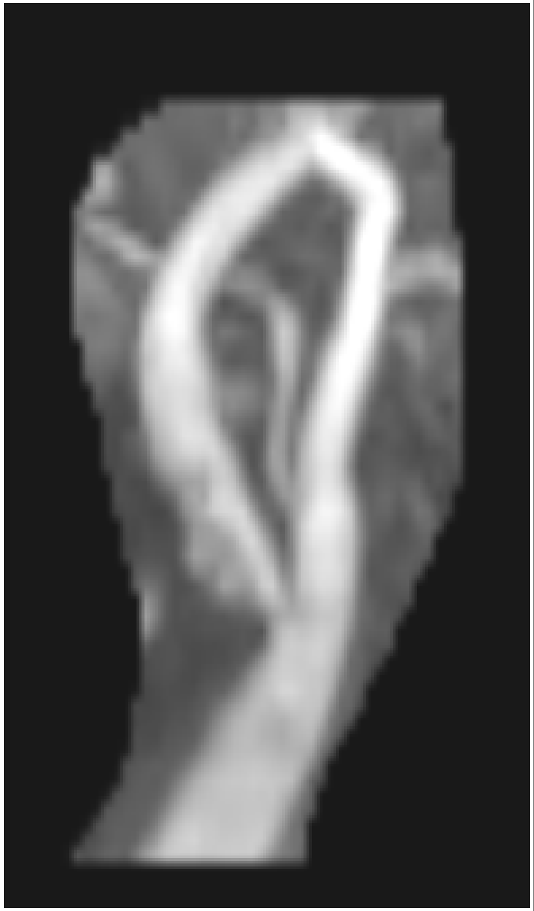

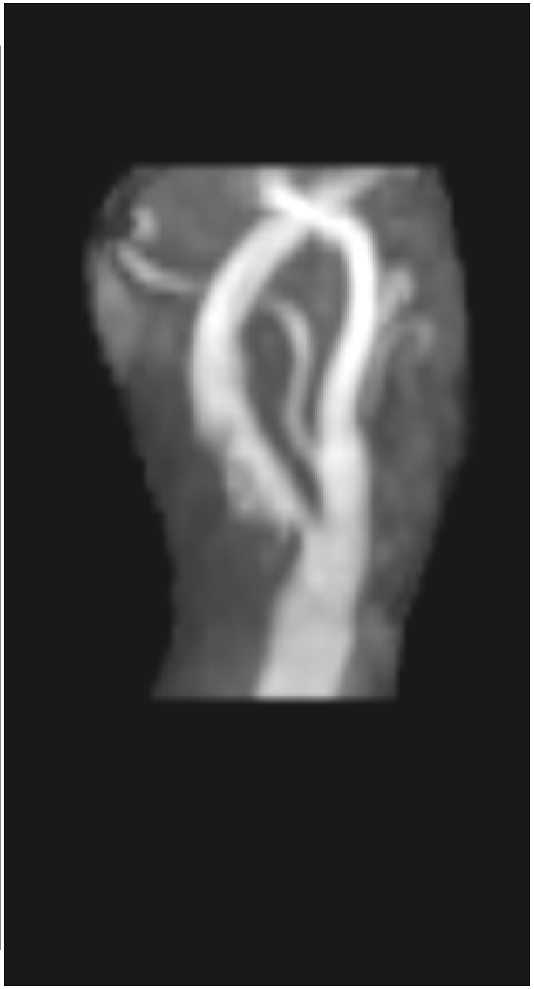

153c Score

0-30

31-50

51-70

>70

Near occlusion

Occluded

Quality

1

2

3

4

5

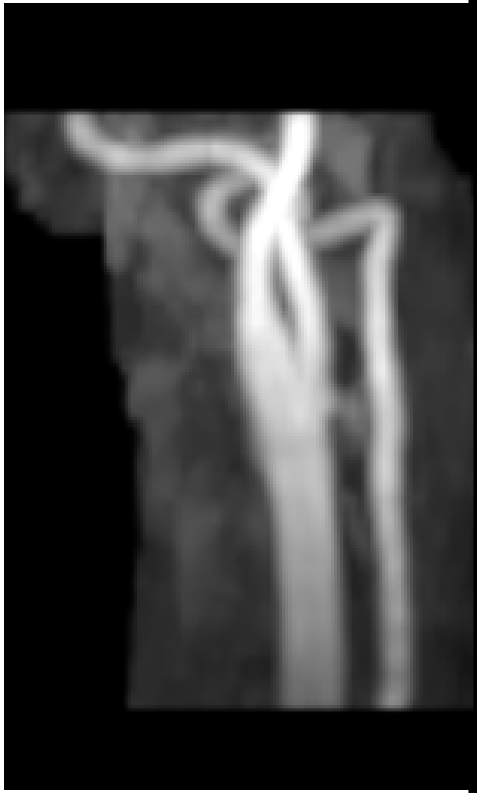

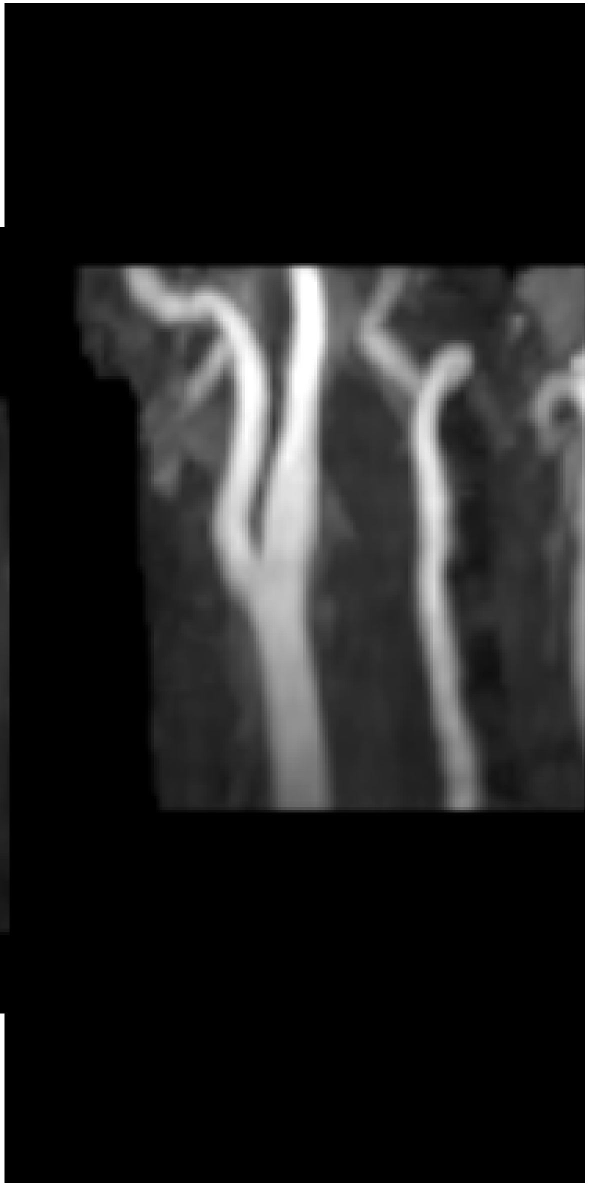

154b Score

0-30

31-50

51-70

>70

Near occlusion

Occluded

Quality

1

2

3

4

5

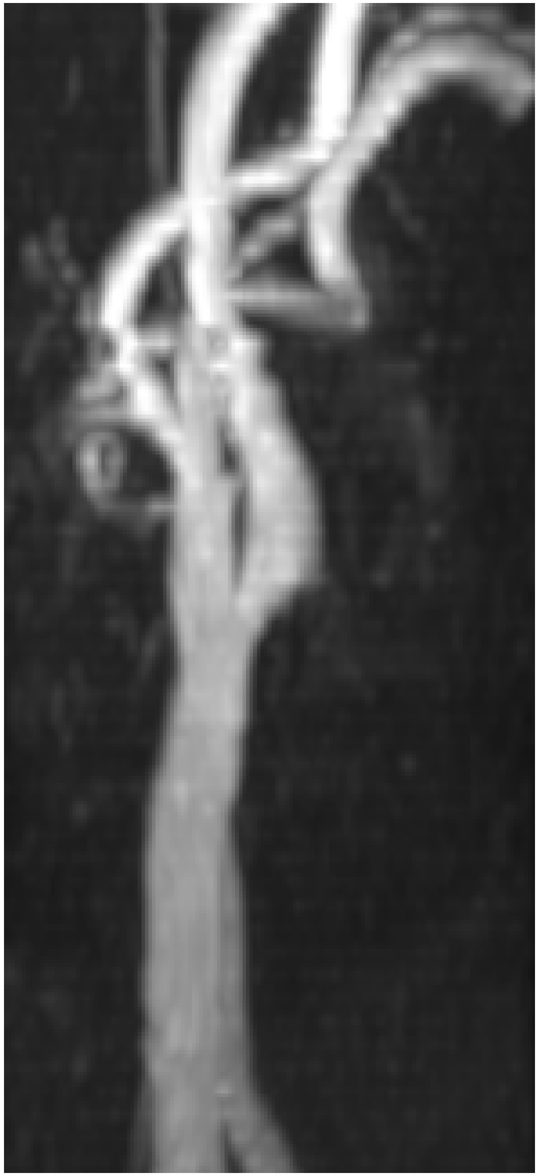

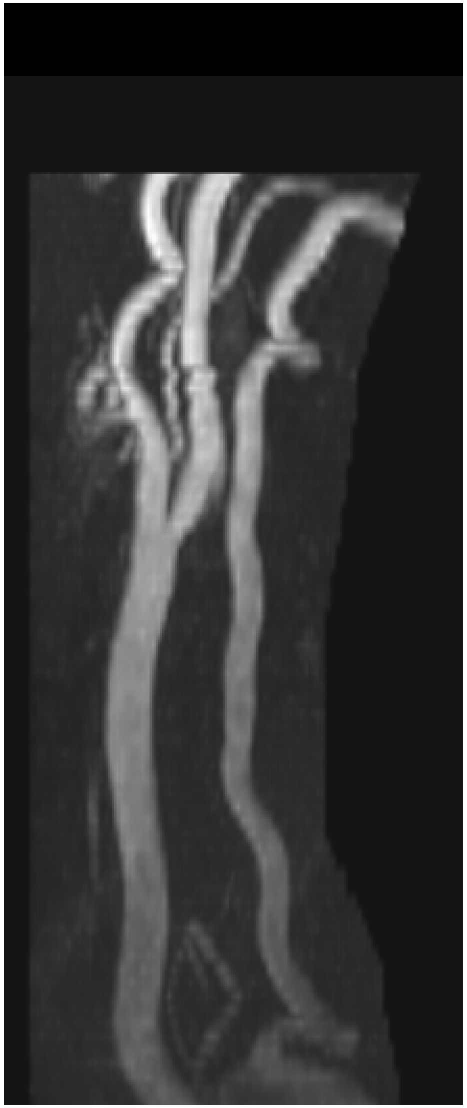

155a Score

0-30

31-50

51-70

>70

Near occlusion

Occluded

Quality

1

2

3

4

5

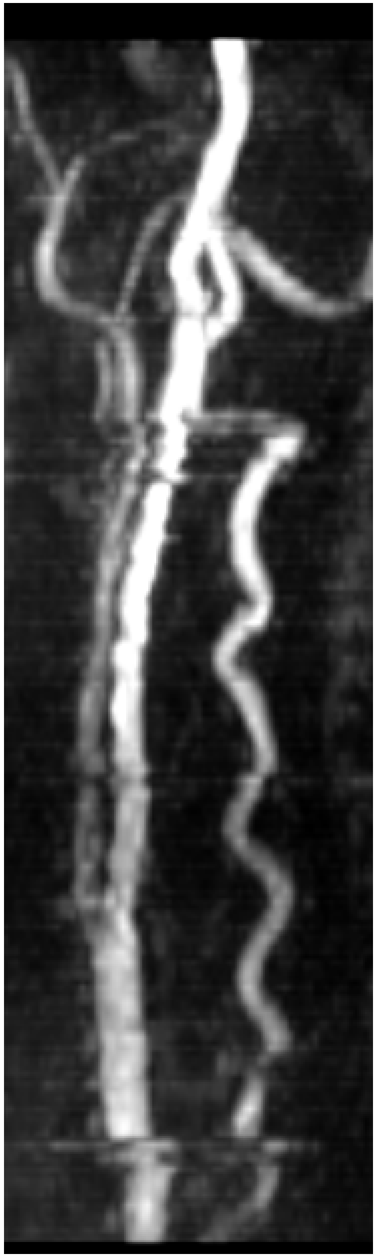

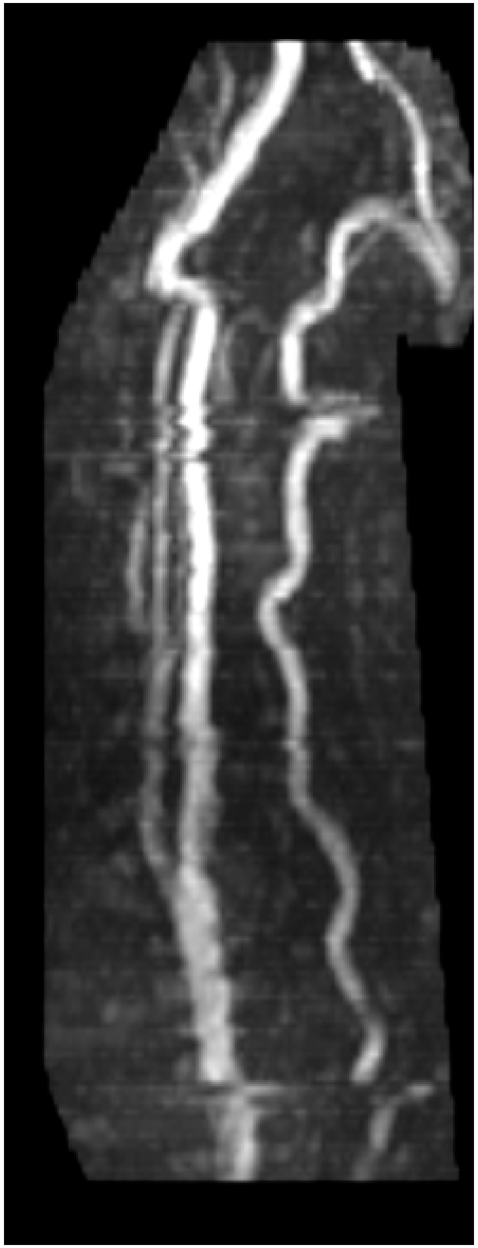

155f Score

0-30

31-50

51-70

>70

Near occlusion

Occluded

Quality

1

2

3

4

5

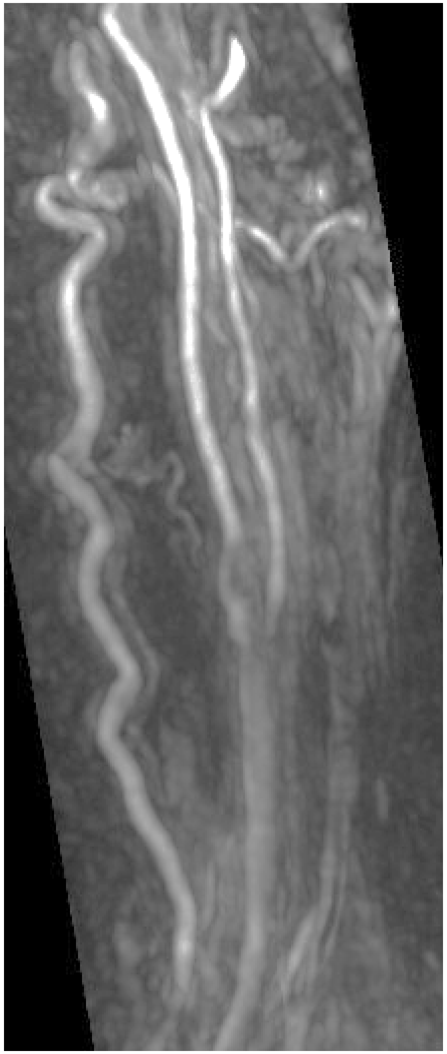

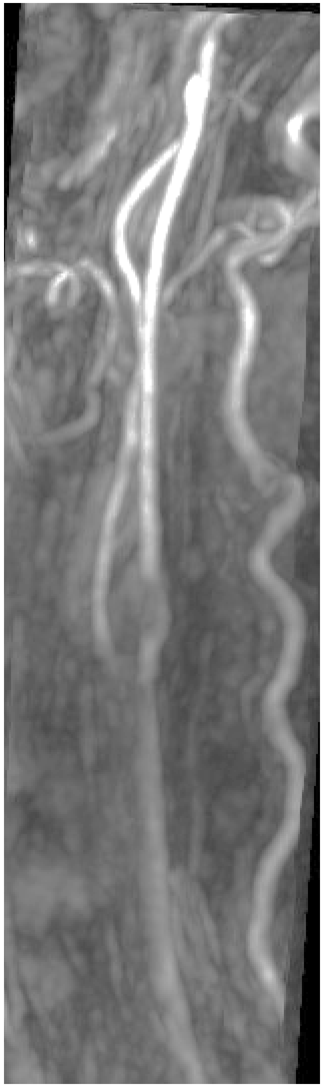

156e Score

0-30

31-50

51-70

>70

Near occlusion

Occluded

Quality

1

2

3

4

5

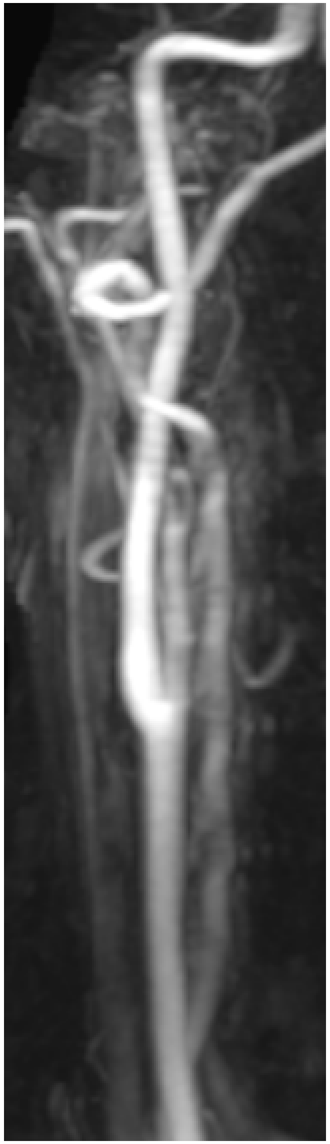

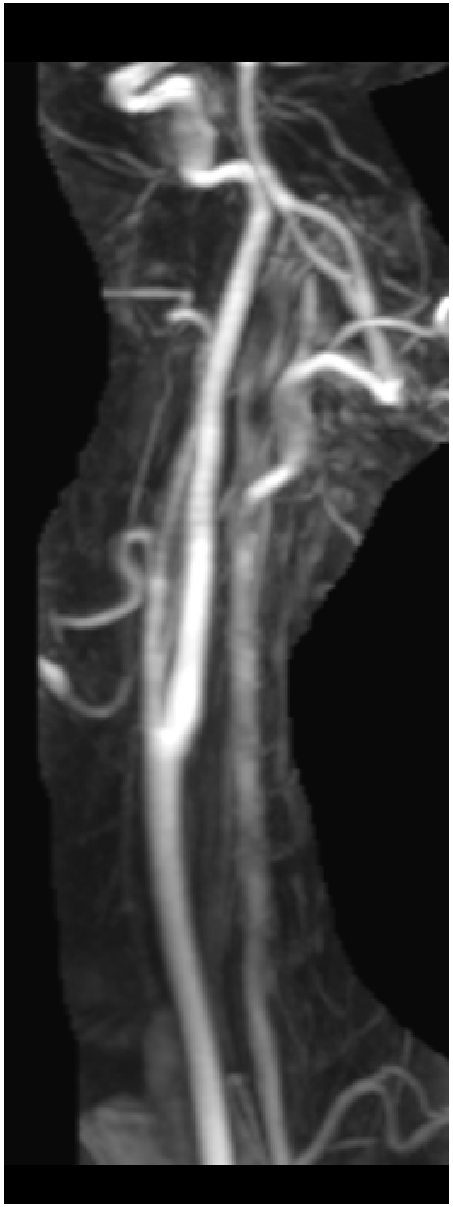

157d Score

0-30

31-50

51-70

>70

Near occlusion

Occluded

Quality

1

2

3

4

5

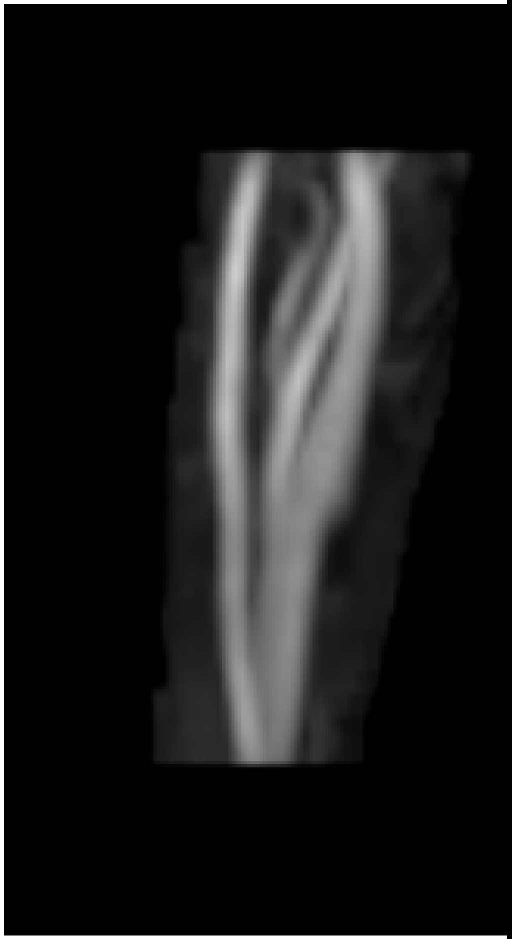

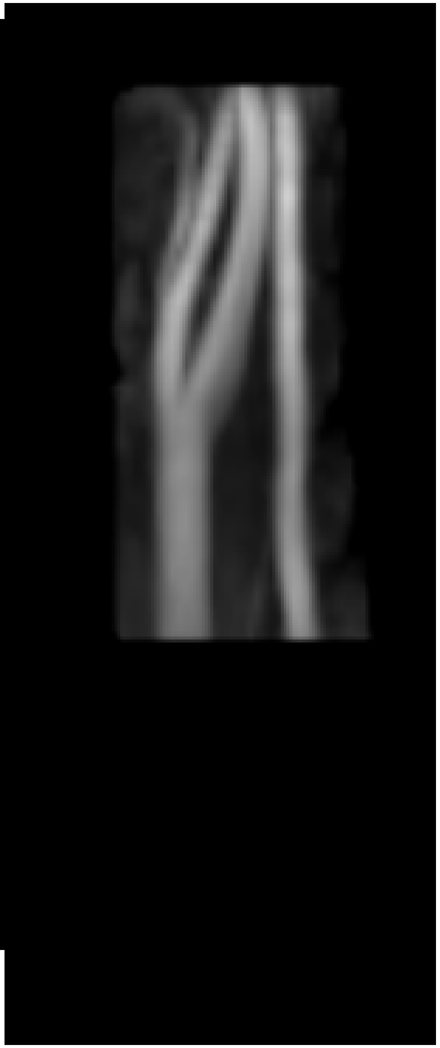

158c Score

0-30

31-50

51-70

>70

Near occlusion

Occluded

Quality

1

2

3

4

5

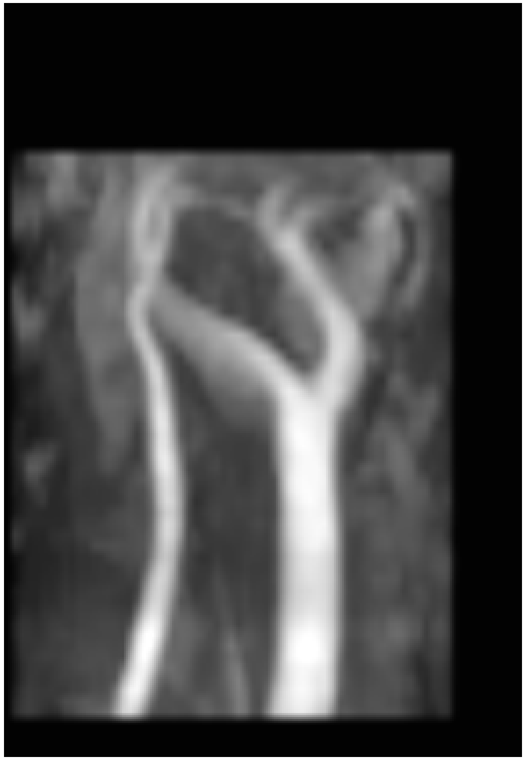

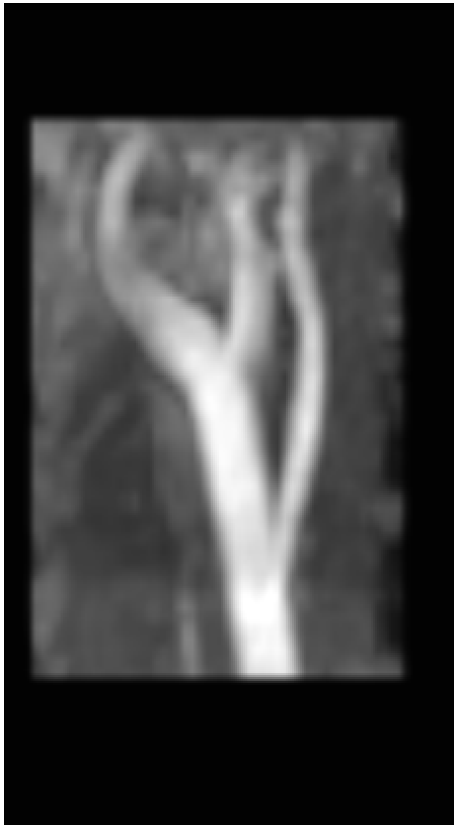

159b Score

0-30

31-50

51-70

>70

Near occlusion

Occluded

Quality

1

2

3

4

5

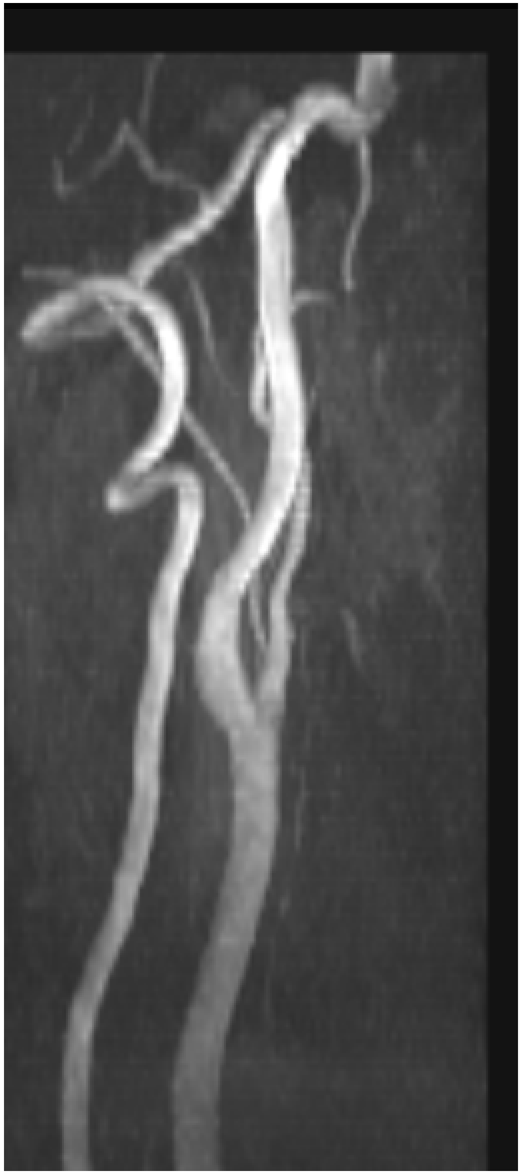

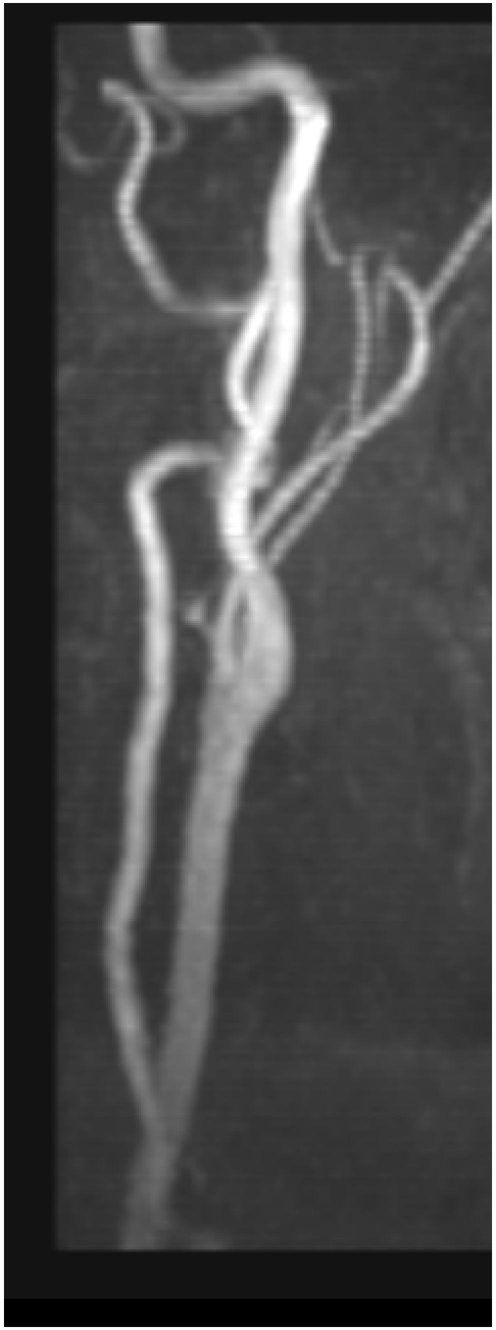

160a Score

0-30

31-50

51-70

>70

Near occlusion

Occluded

Quality

1

2

3

4

5

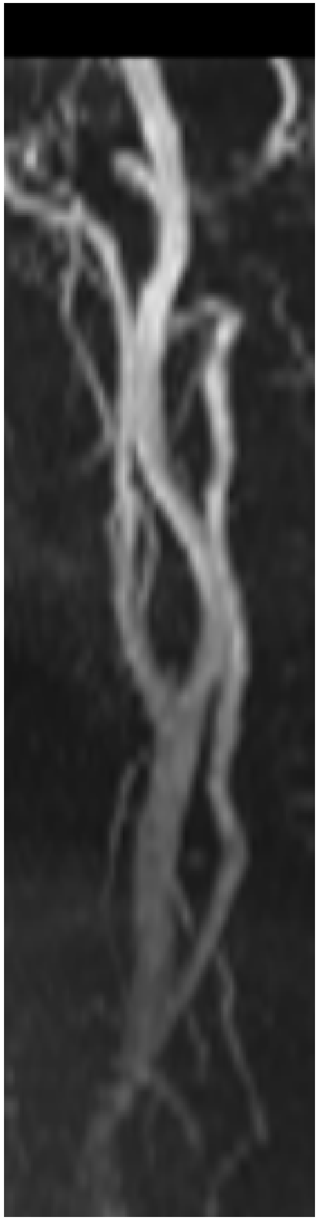

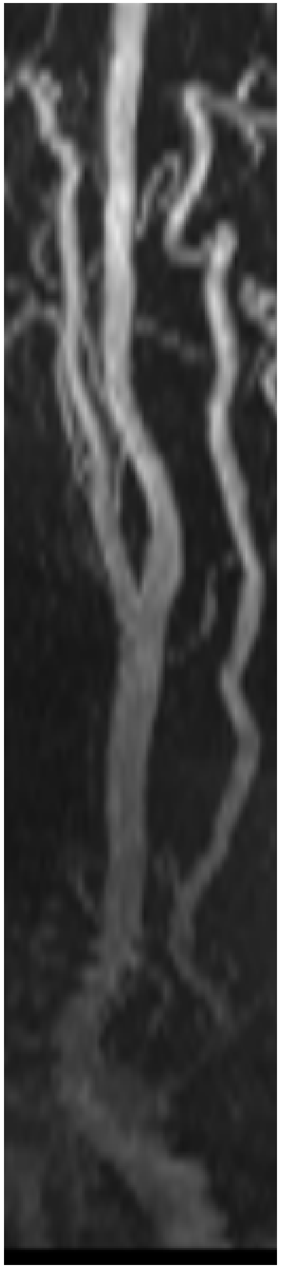

160f Score

0-30

31-50

51-70

>70

Near occlusion

Occluded

Quality

1

2

3

4

5

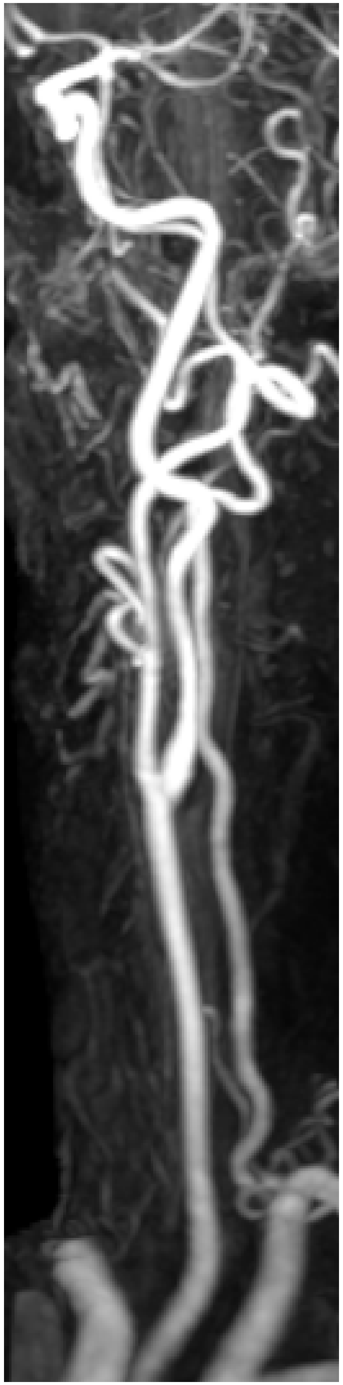

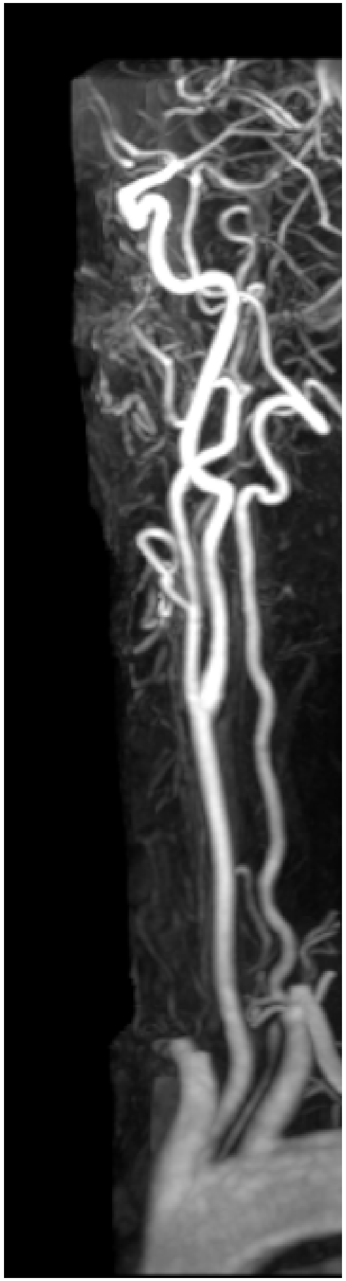

161e Score

0-30

31-50

51-70

>70

Near occlusion

Occluded

Quality

1

2

3

4

5

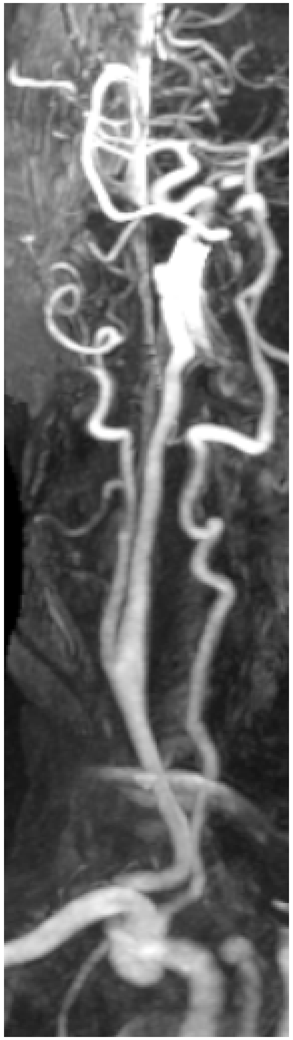

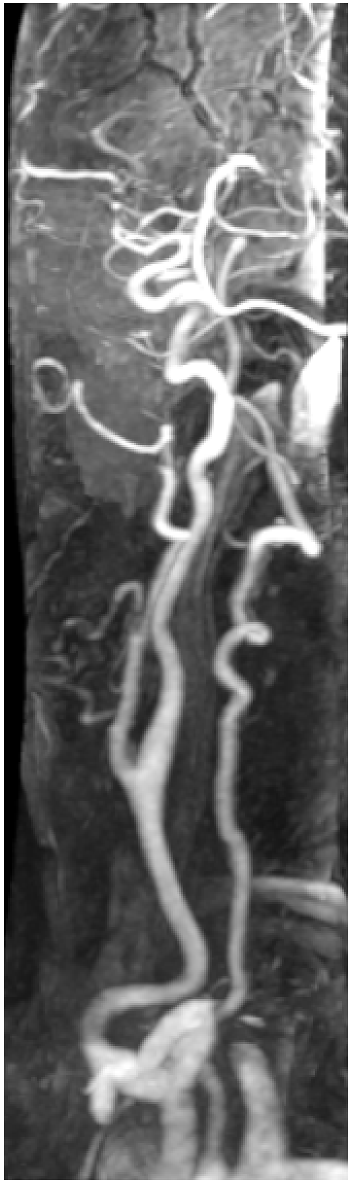

162d Score

0-30

31-50

51-70

>70

Near occlusion

Occluded

Quality

1

2

3

4

5

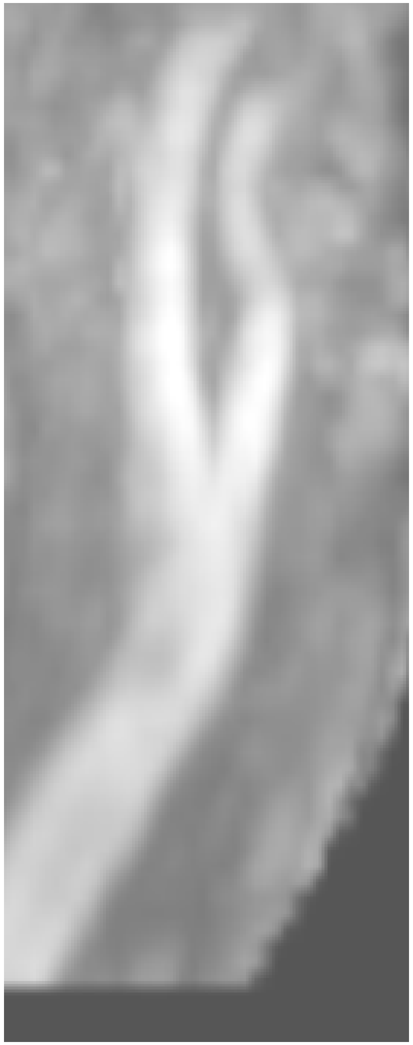

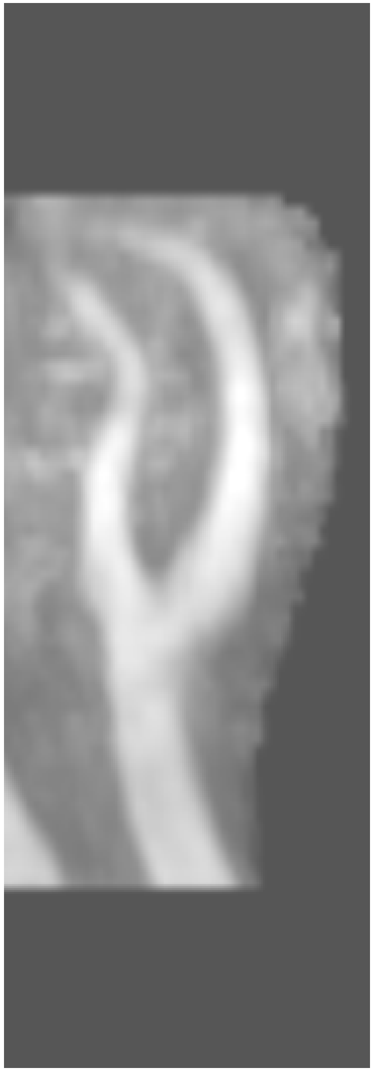

163c Score

0-30

31-50

51-70

>70

Near occlusion

Occluded

Quality

1

2

3

4

5

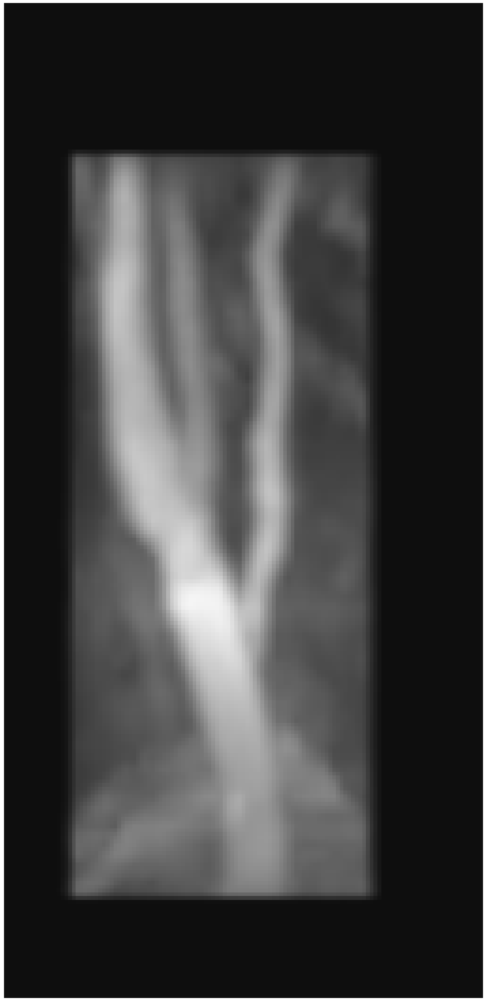

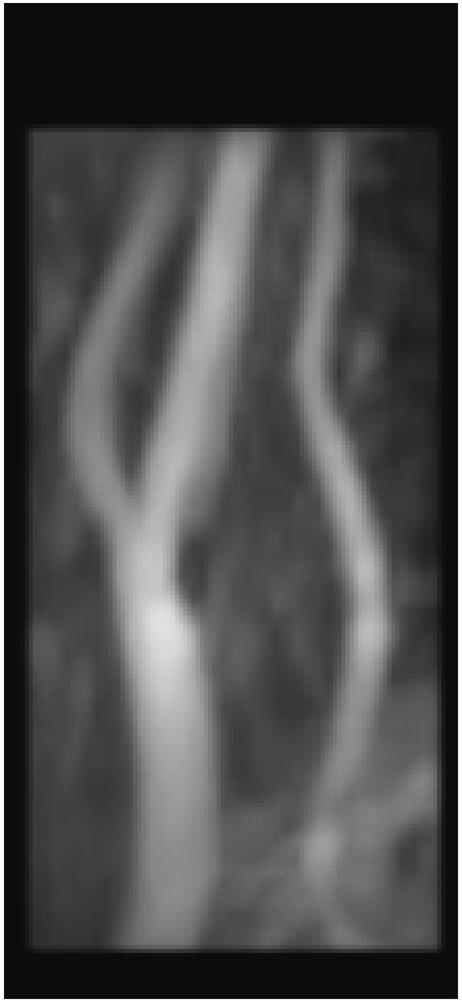

164b Score

0-30

31-50

51-70

>70

Near occlusion

Occluded

Quality

1

2

3

4

5

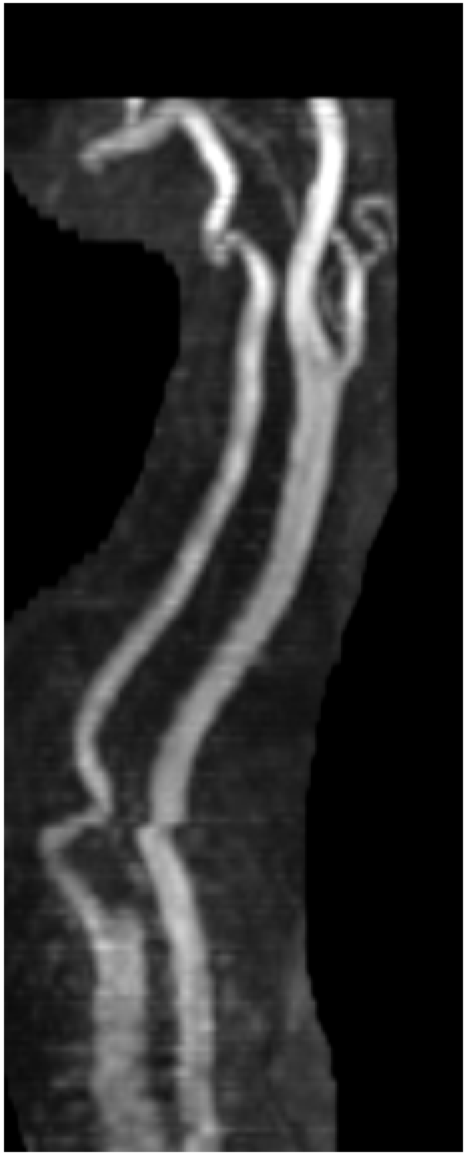

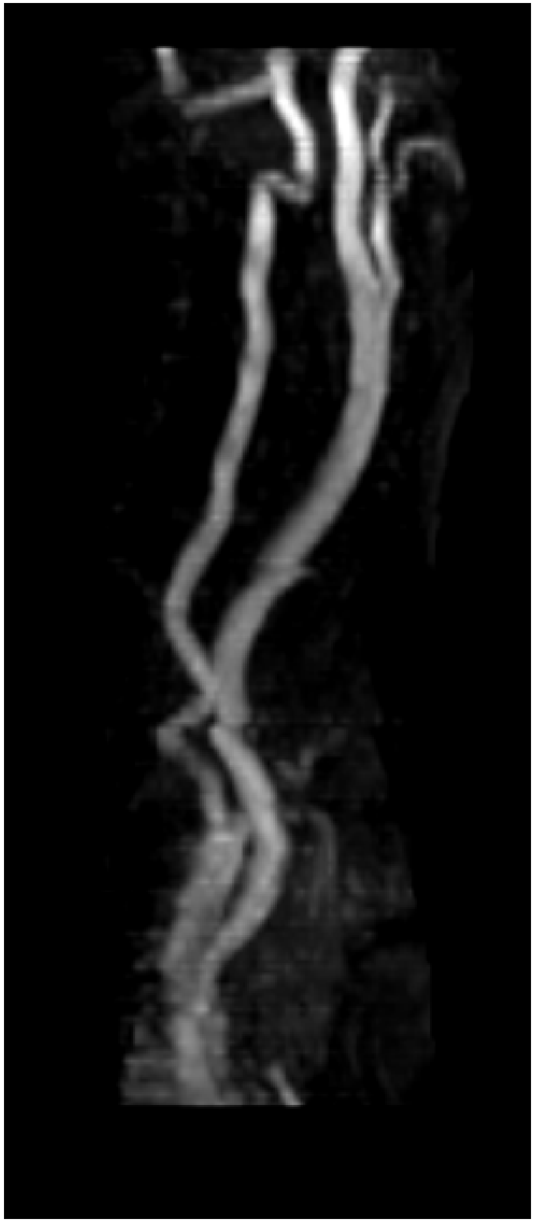

165a Score

0-30

31-50

51-70

>70

Near occlusion

Occluded

Quality

1

2

3

4

5

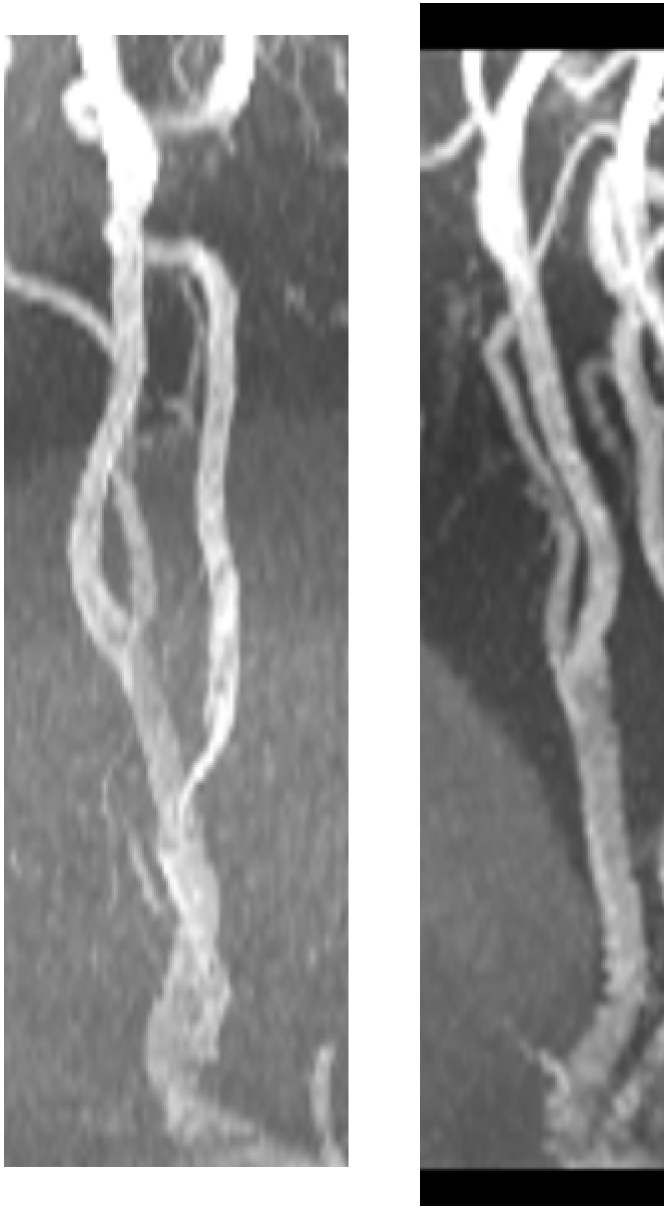

165f Score

0-30

31-50

51-70

>70

Near occlusion

Occluded

Quality

1

2

3

4

5

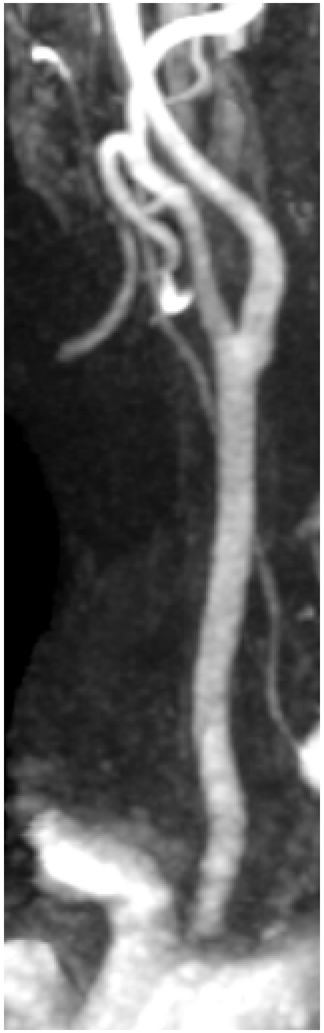

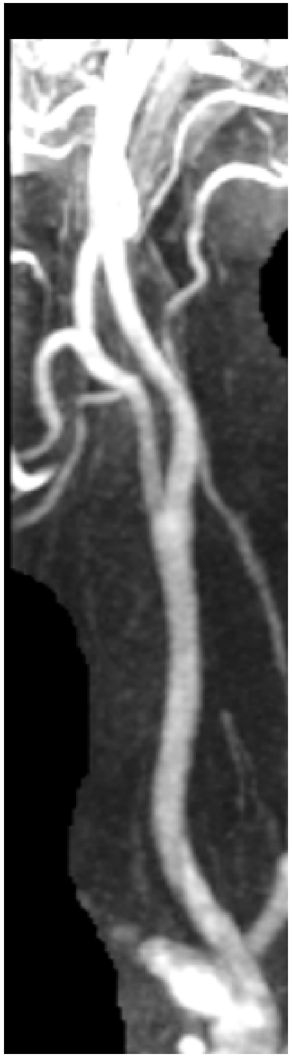

166e Score

0-30

31-50

51-70

>70

Near occlusion

Occluded

Quality

1

2

3

4

5

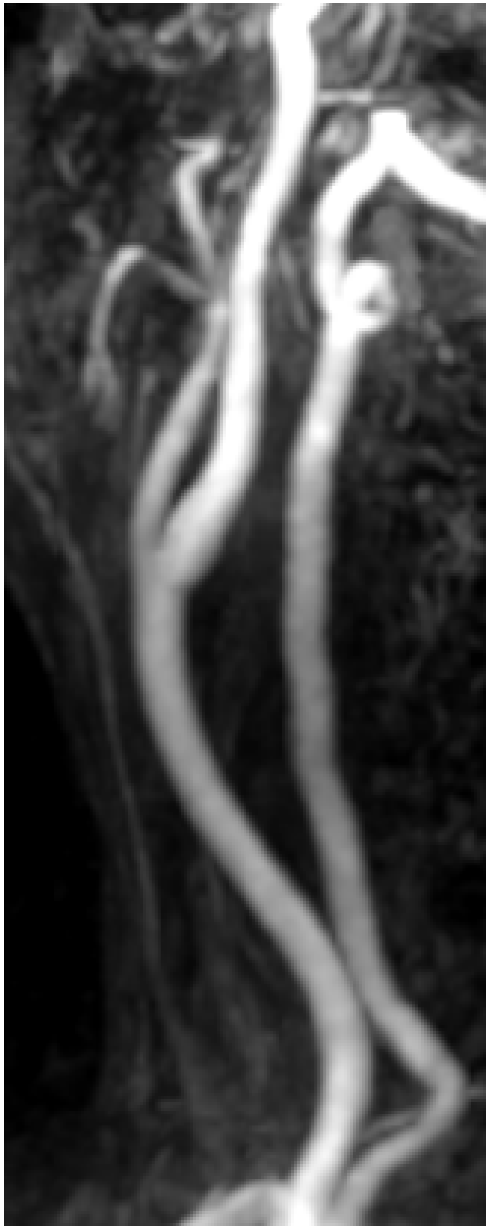

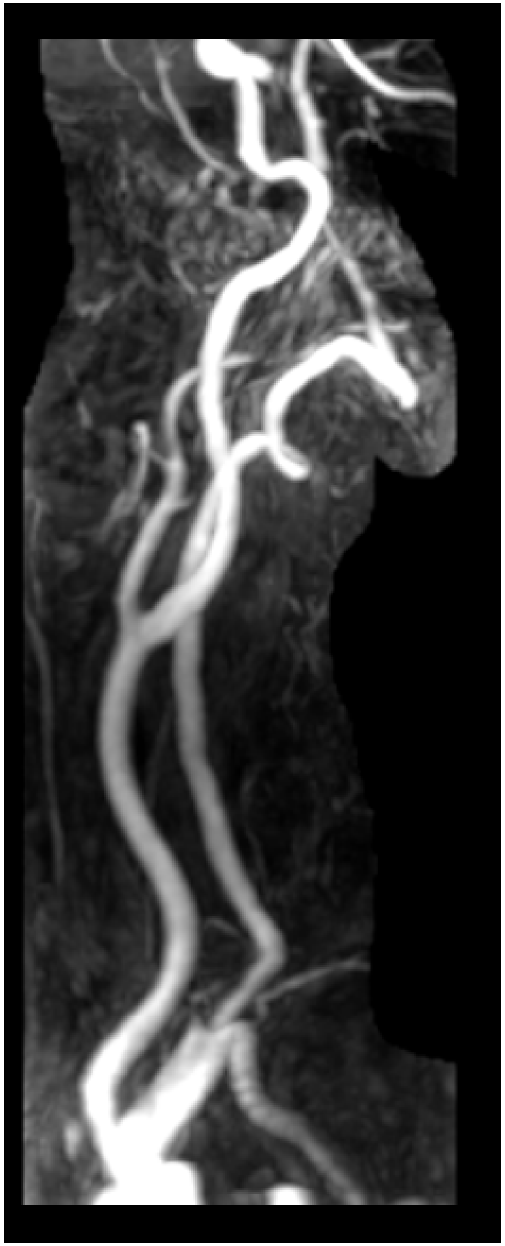

167d Score

0-30

31-50

51-70

>70

Near occlusion

Occluded

Quality

1

2

3

4

5

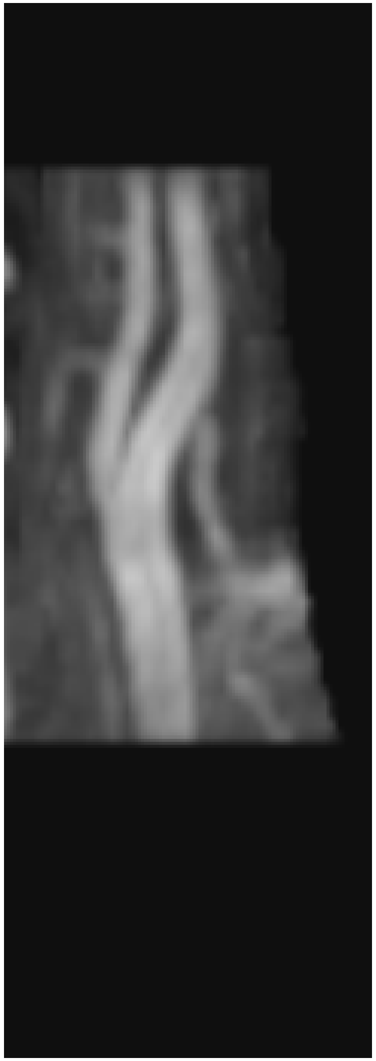

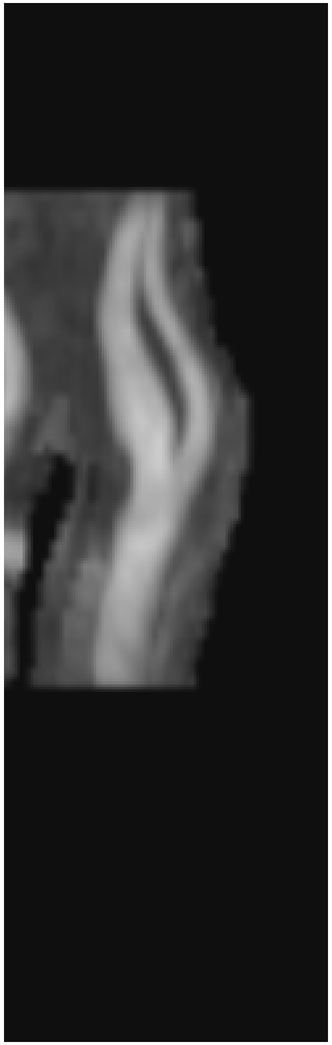

168c Score

0-30

31-50

51-70

>70

Near occlusion

Occluded

Quality

1

2

3

4

5

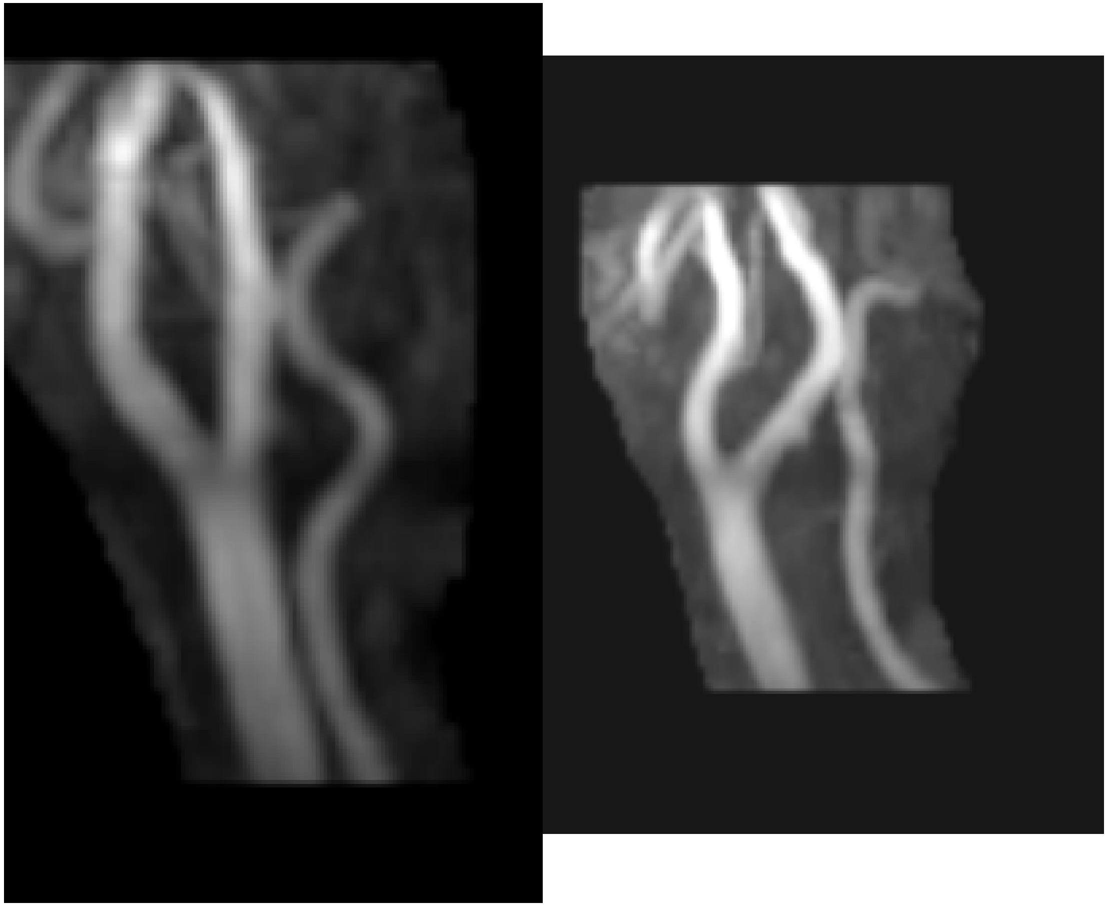

169b Score (left)

0-30

31-50

51-70

>70

Near occlusion

Occluded

Quality

1

2

3

4

5

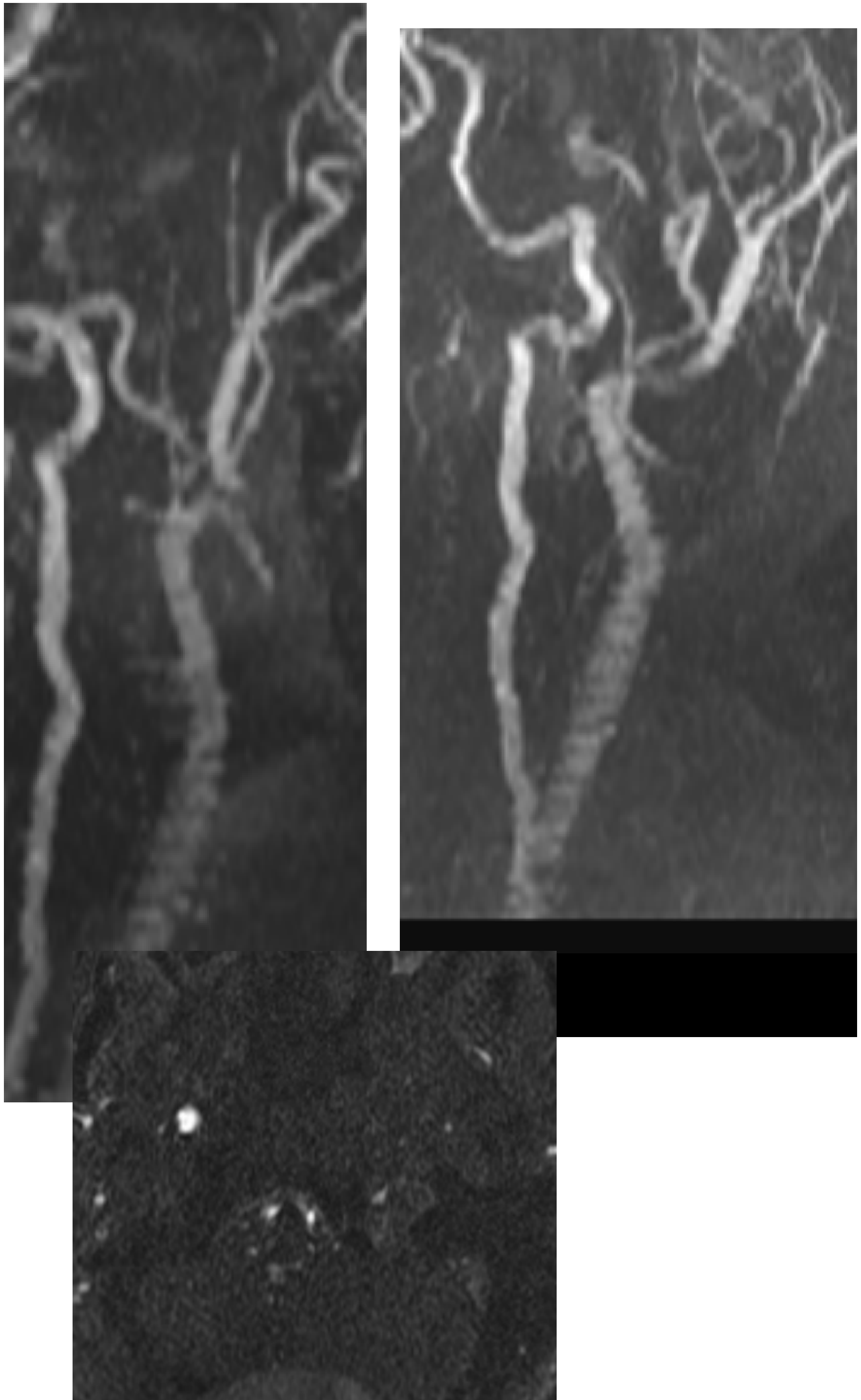

170a Score

0-30

31-50

51-70

>70

Near occlusion

Occluded

Quality

1

2

3

4

5

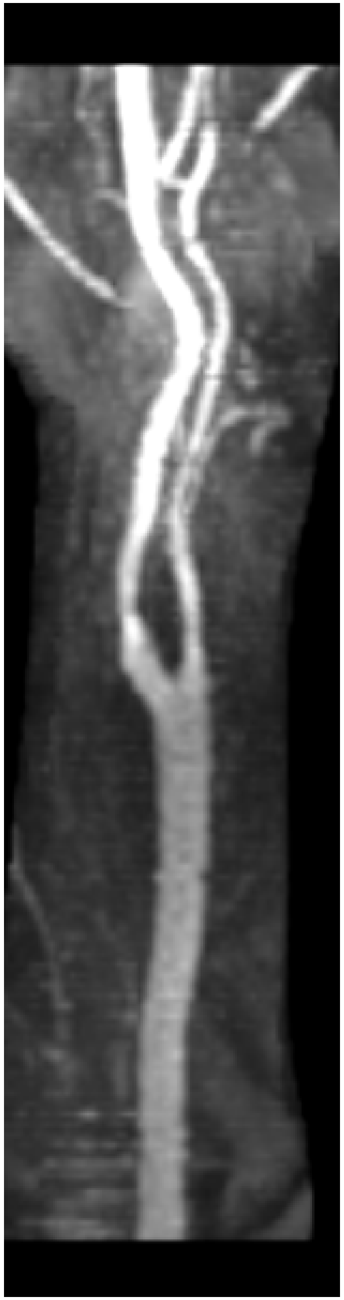

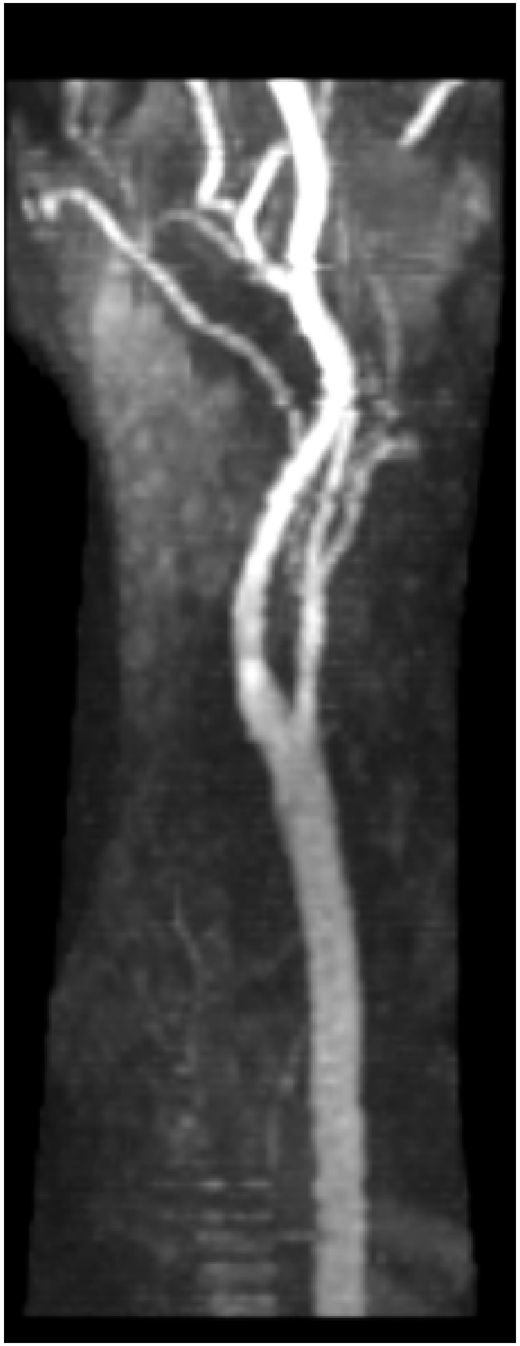

170f Score

0-30

31-50

51-70

>70

Near occlusion

Occluded

Quality

1

2

3

4

5

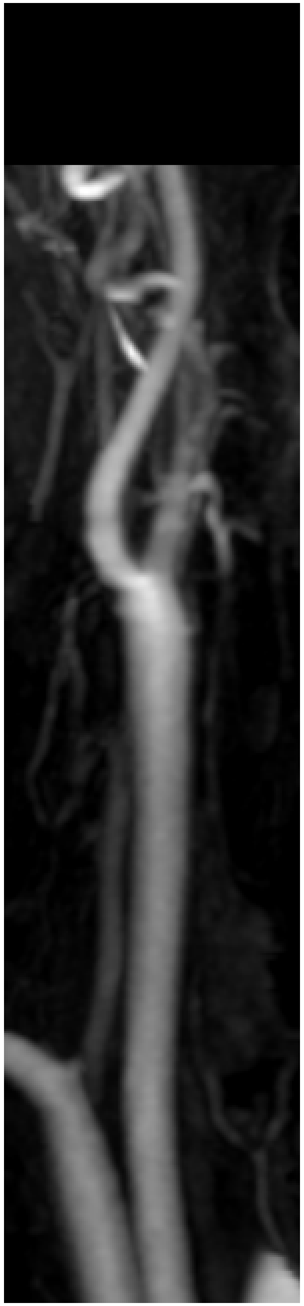

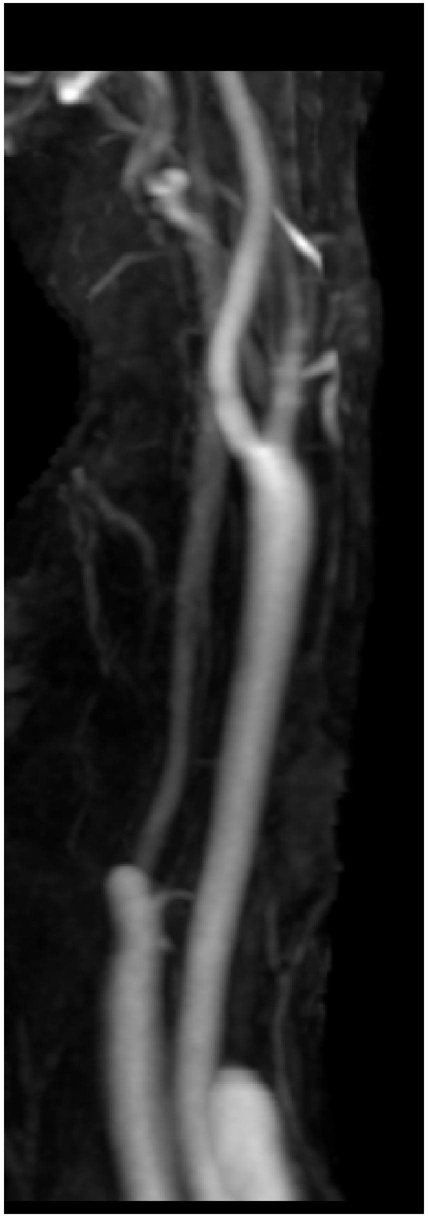

171e Score

0-30

31-50

51-70

>70

Near occlusion

Occluded

Quality

1

2

3

4

5

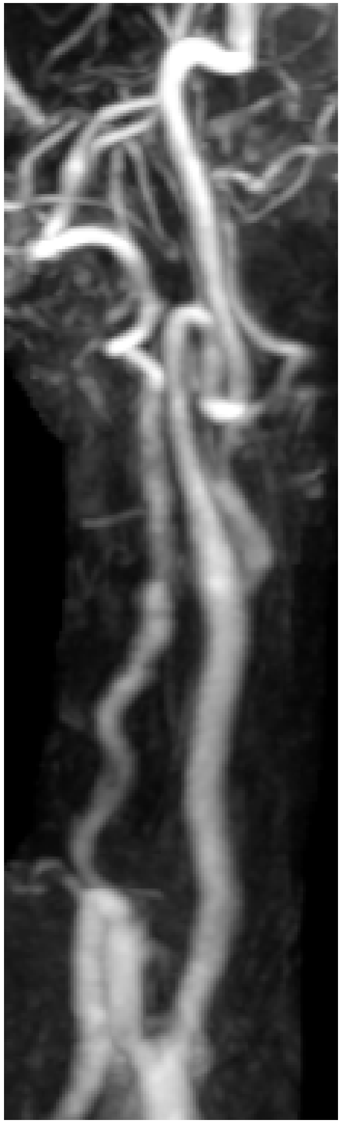

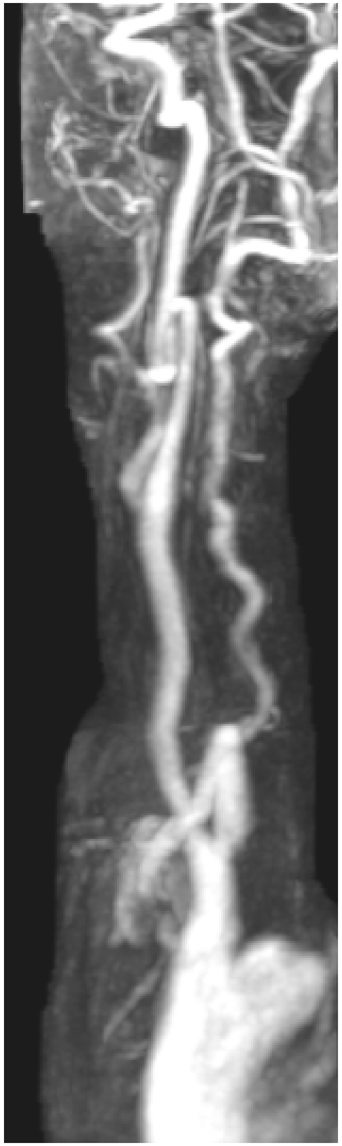

172d Score

0-30

31-50

51-70

>70

Near occlusion

Occluded

Quality

1

2

3

4

5

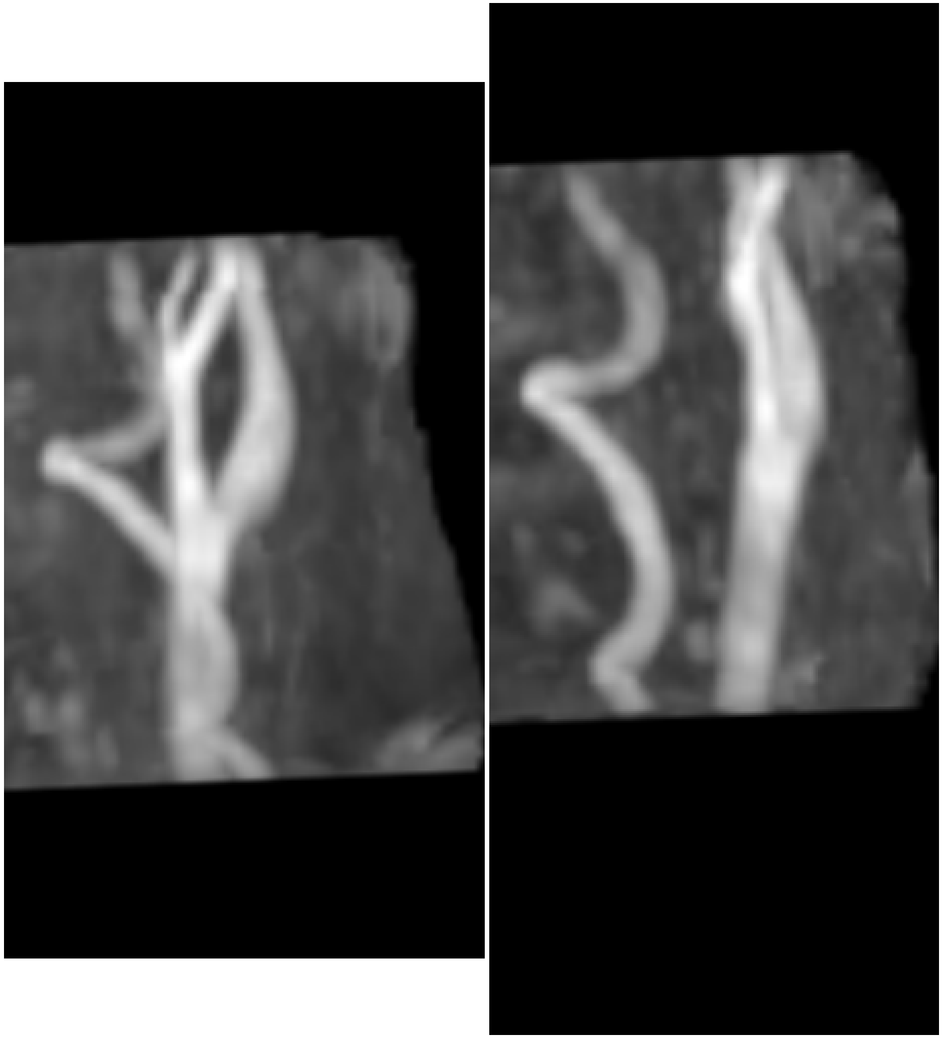

173c Score

0-30

31-50

51-70

>70

Near occlusion

Occluded

Quality

1

2

3

4

5

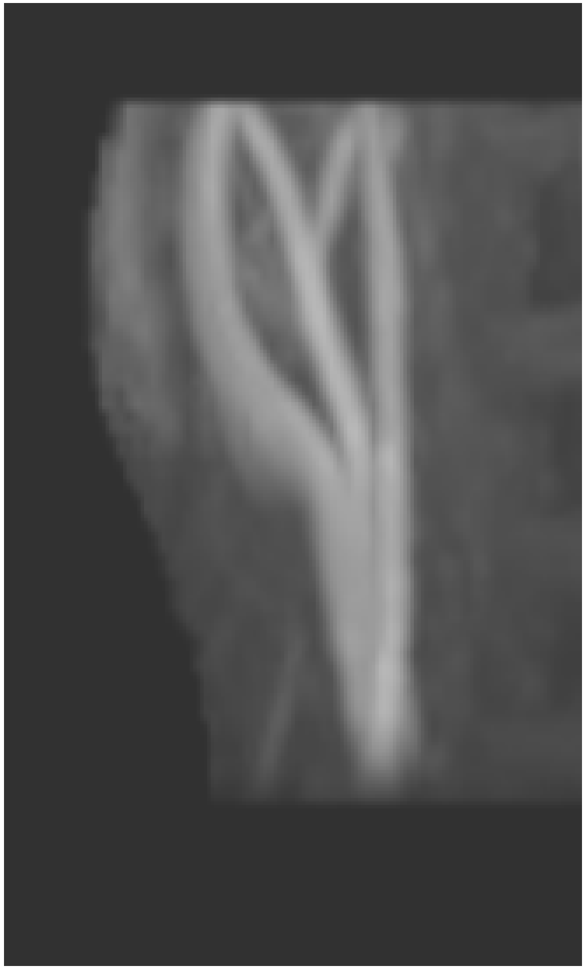

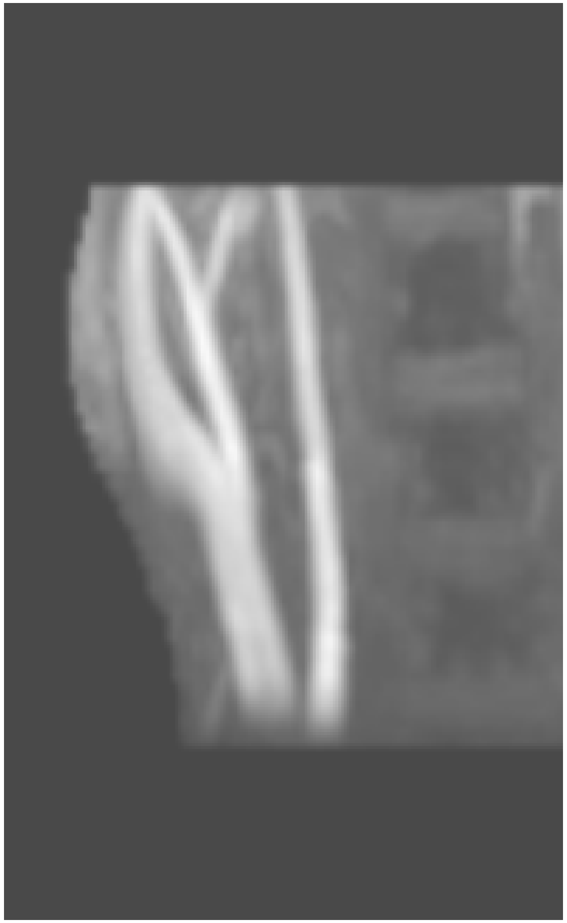

174b Score

0-30

31-50

51-70

>70

Near occlusion

Occluded

Quality

1

2

3

4

5

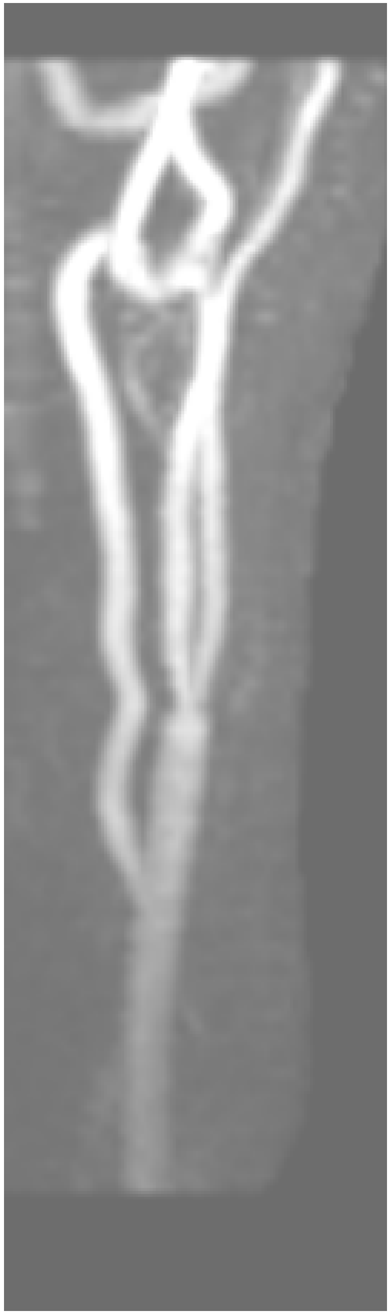

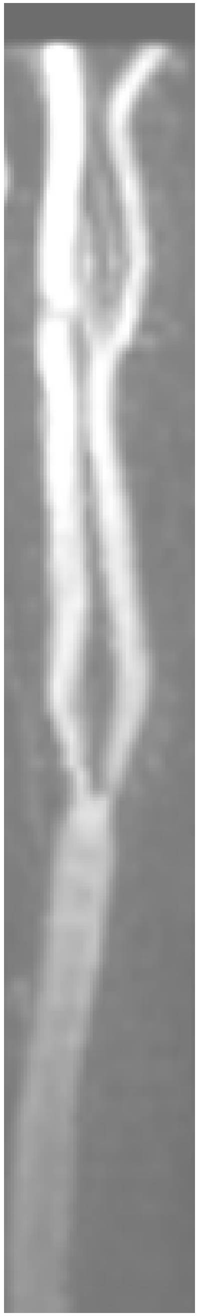

175a Score

0-30

31-50

51-70

>70

Near occlusion

Occluded

Quality

1

2

3

4

5

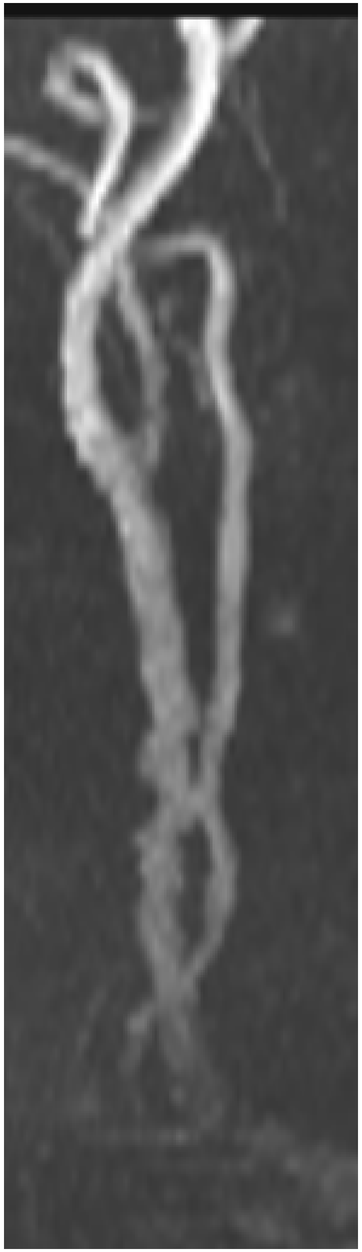

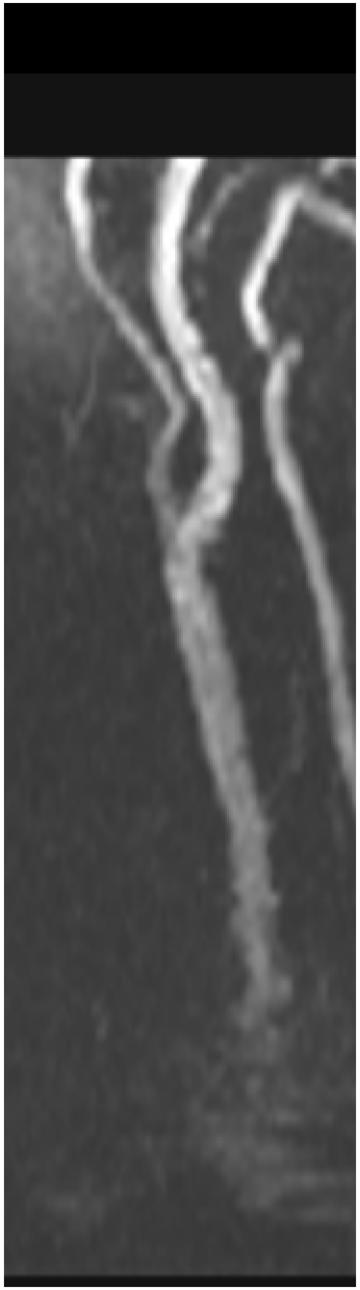

175f Score

0-30

31-50

51-70

>70

Near occlusion

Occluded

Quality

1

2

3

4

5

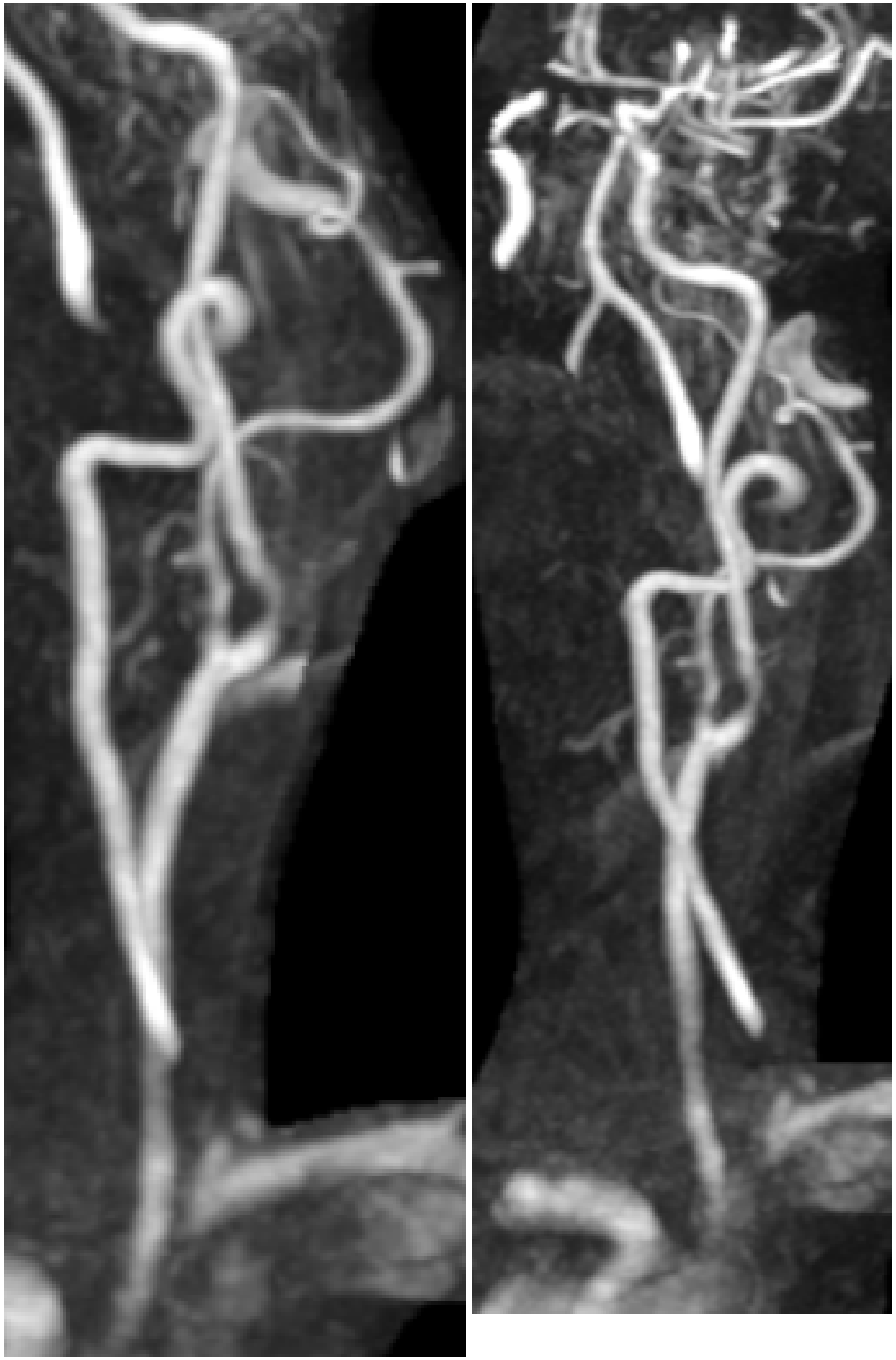

176e Score

0-30

31-50

51-70

>70

Near occlusion

Occluded

Quality

1

2

3

4

5

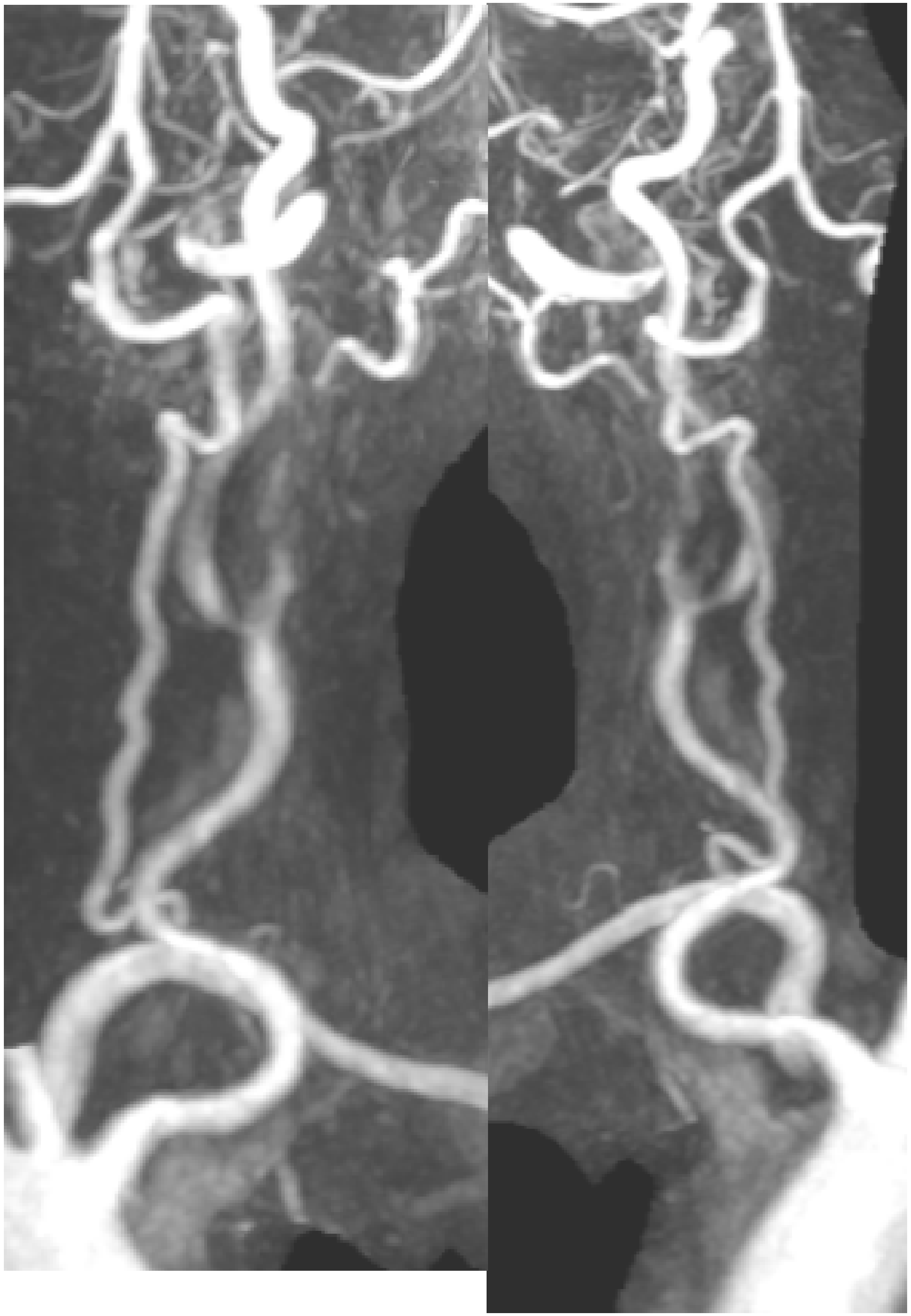

177d Score

0-30

31-50

51-70

>70

Near occlusion

Occluded

Quality

1

2

3

4

5

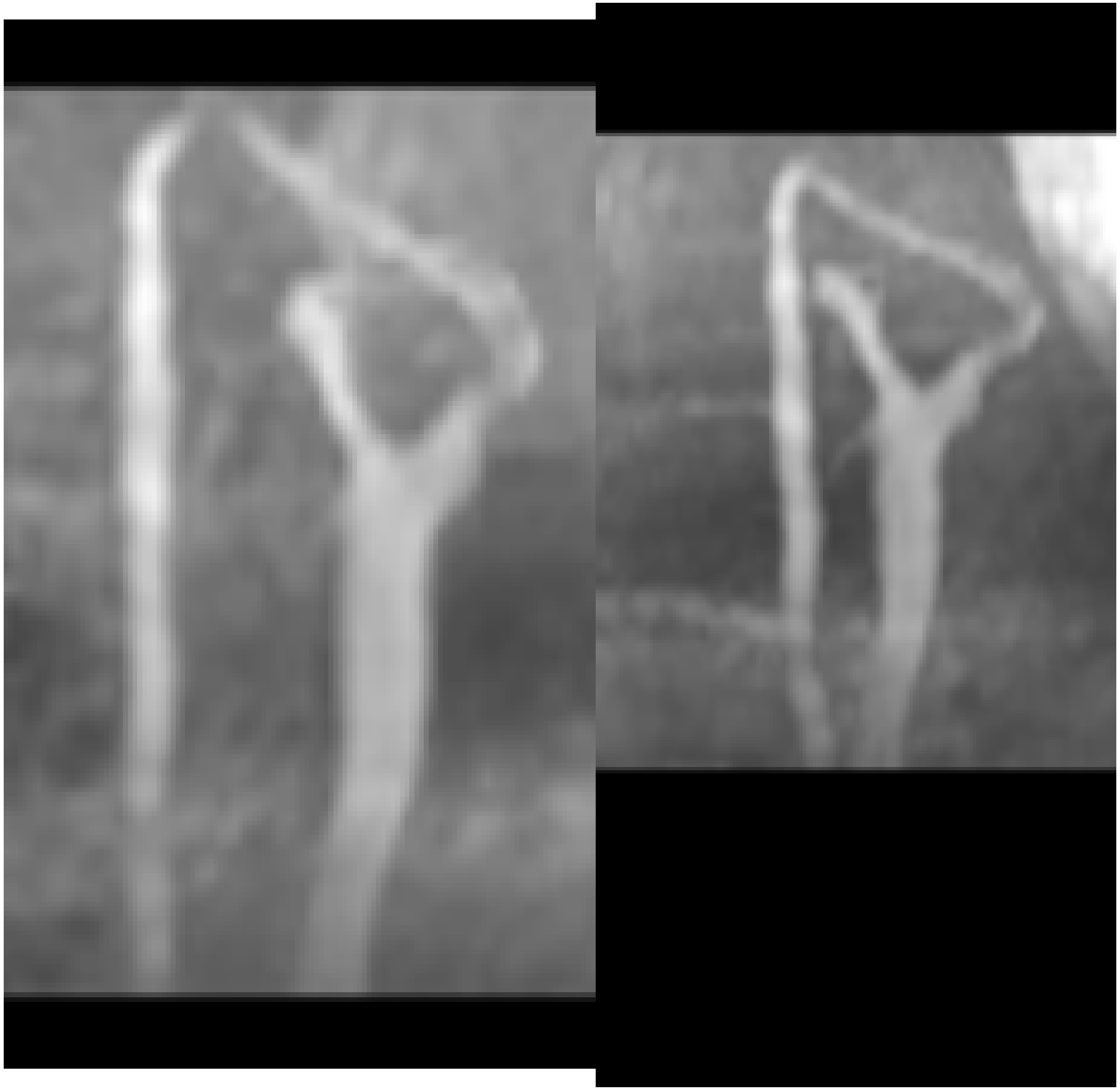

178c Score

0-30

31-50

51-70

>70

Near occlusion

Occluded

Quality

1

2

3

4

5

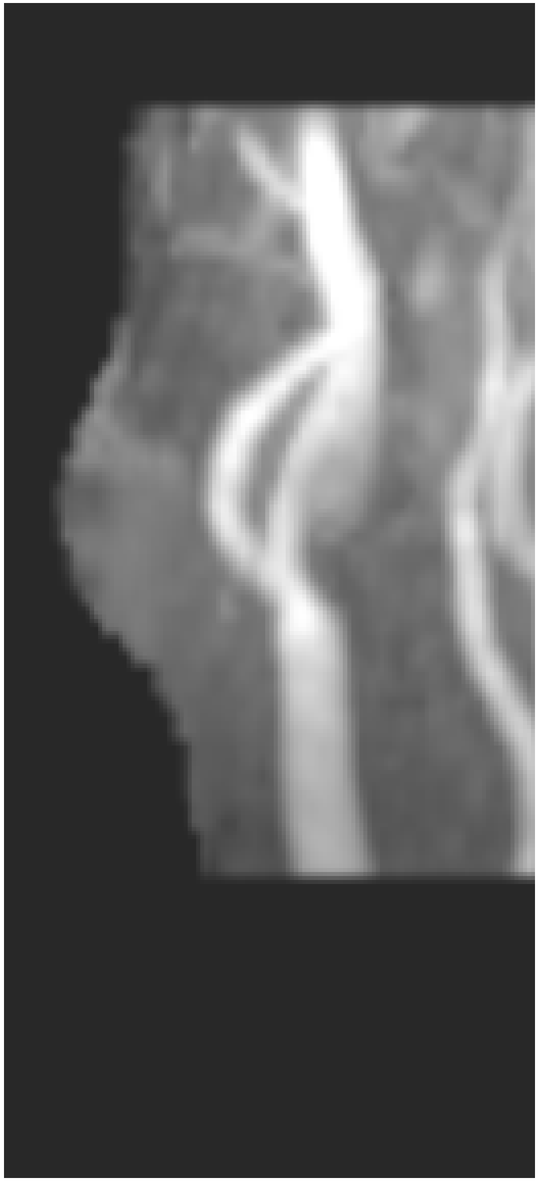

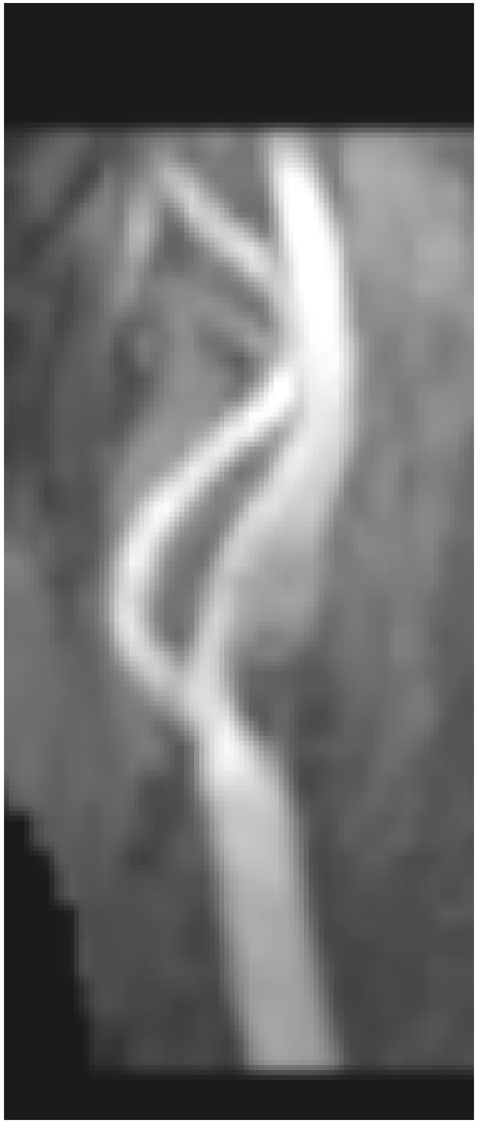

179b Score

0-30

31-50

51-70

>70

Near occlusion

Occluded

Quality

1

2

3

4

5

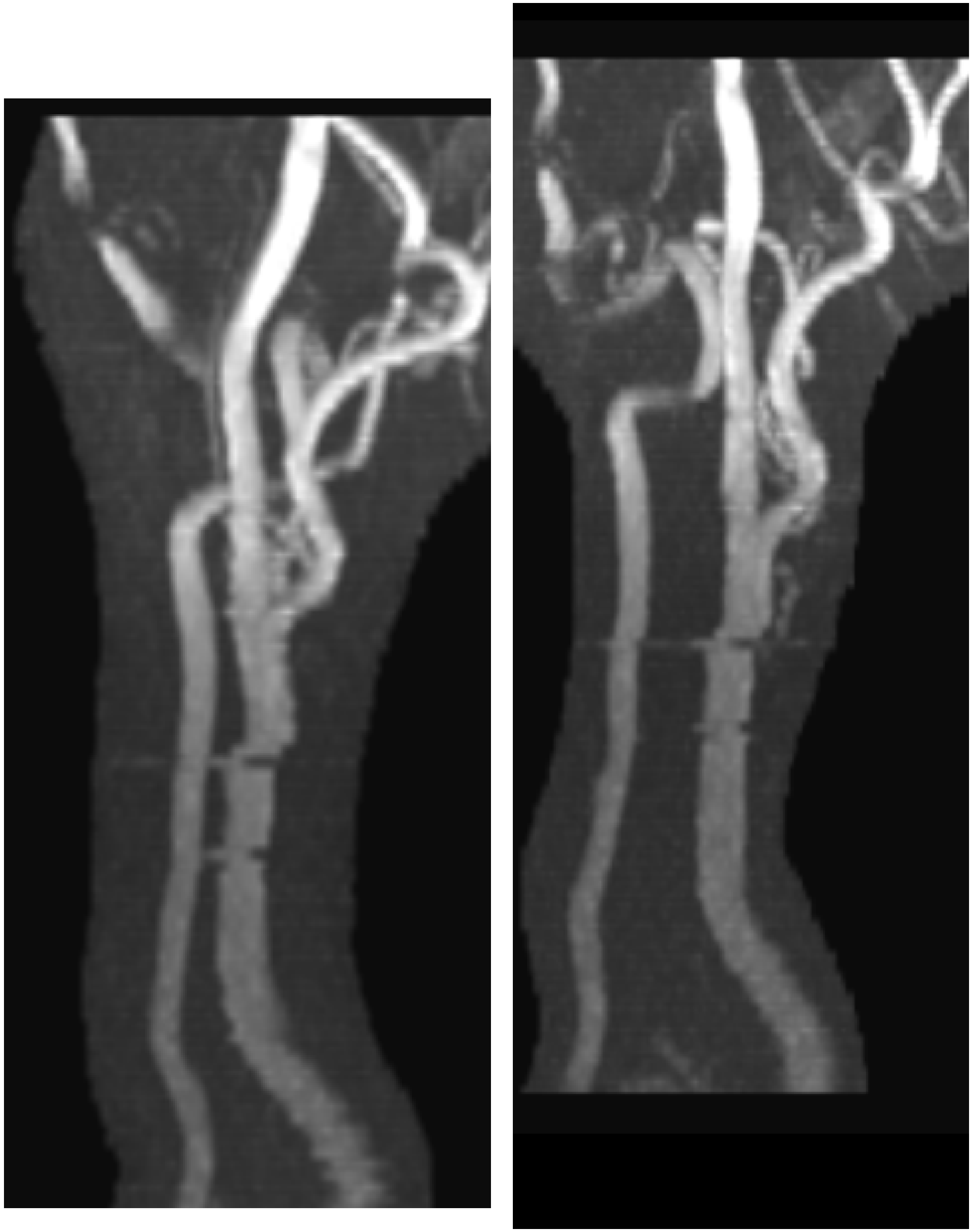

180a Score

0-30

31-50

51-70

>70

Near occlusion

Occluded

Quality

1

2

3

4

5

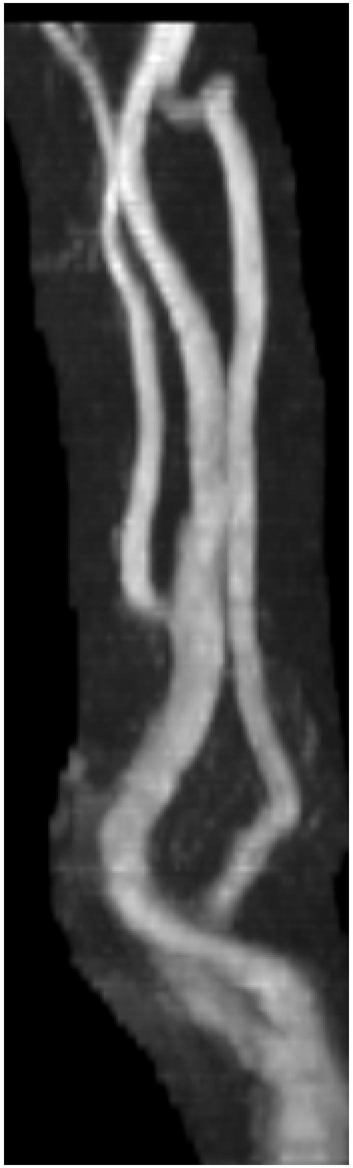

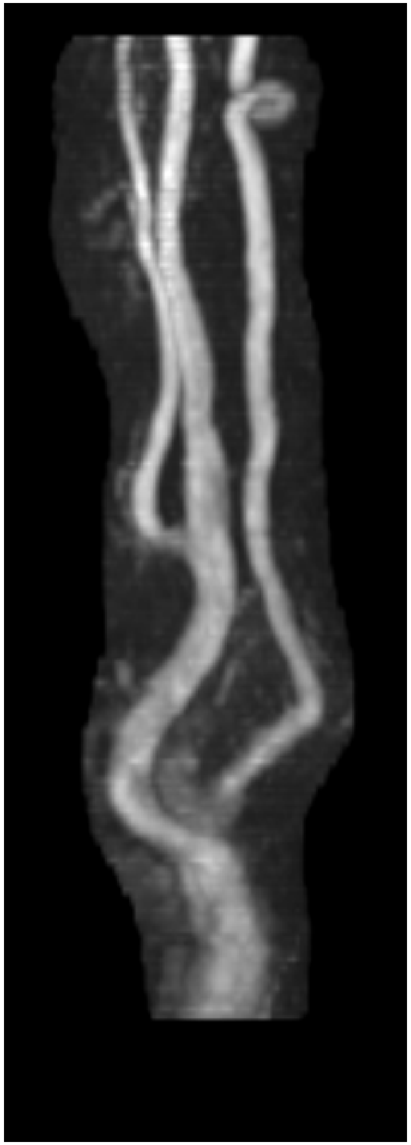

180f Score

0-30

31-50

51-70

>70

Near occlusion

Occluded

Quality

1

2

3

4

5

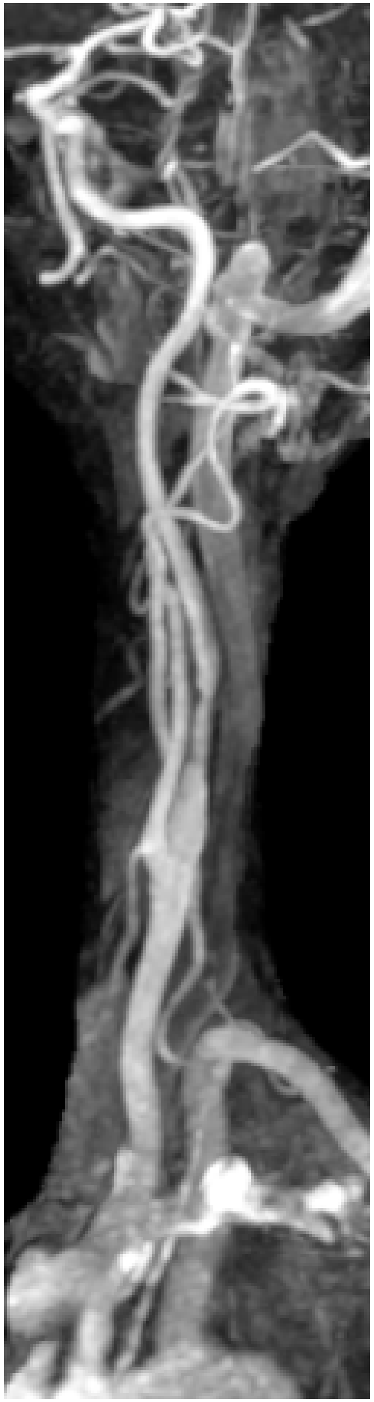

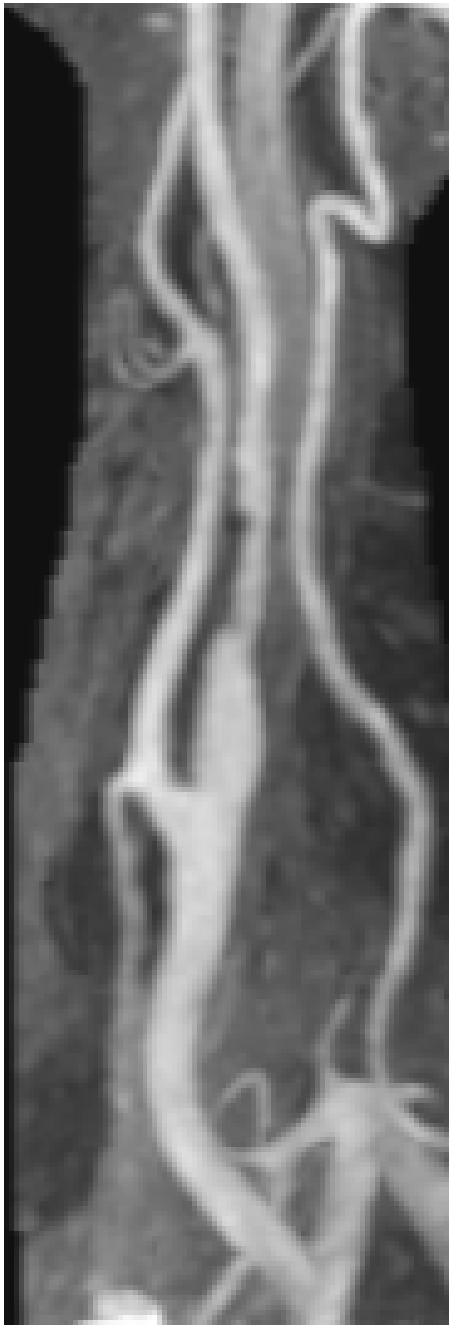

181e Score

0-30

31-50

51-70

>70

Near occlusion

Occluded

Quality

1

2

3

4

5

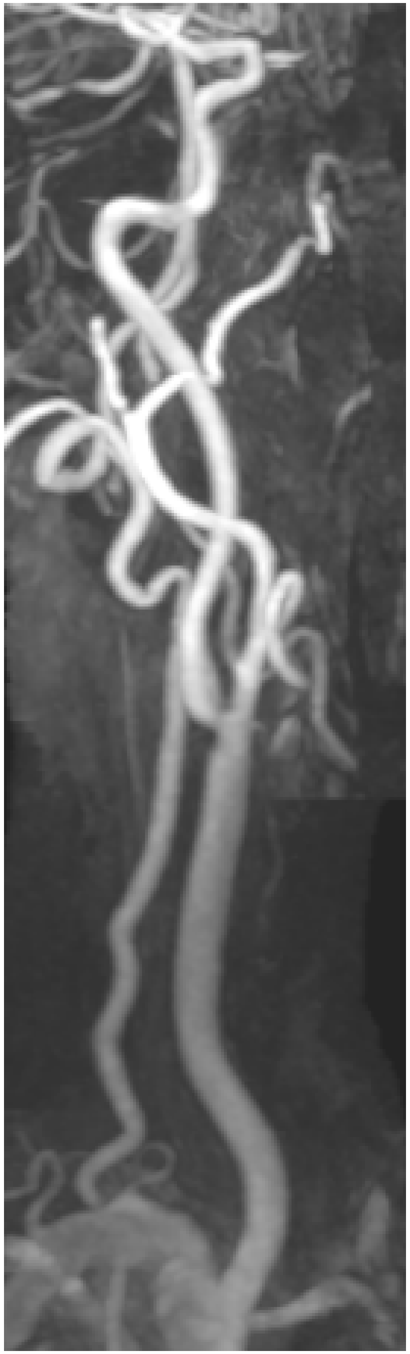

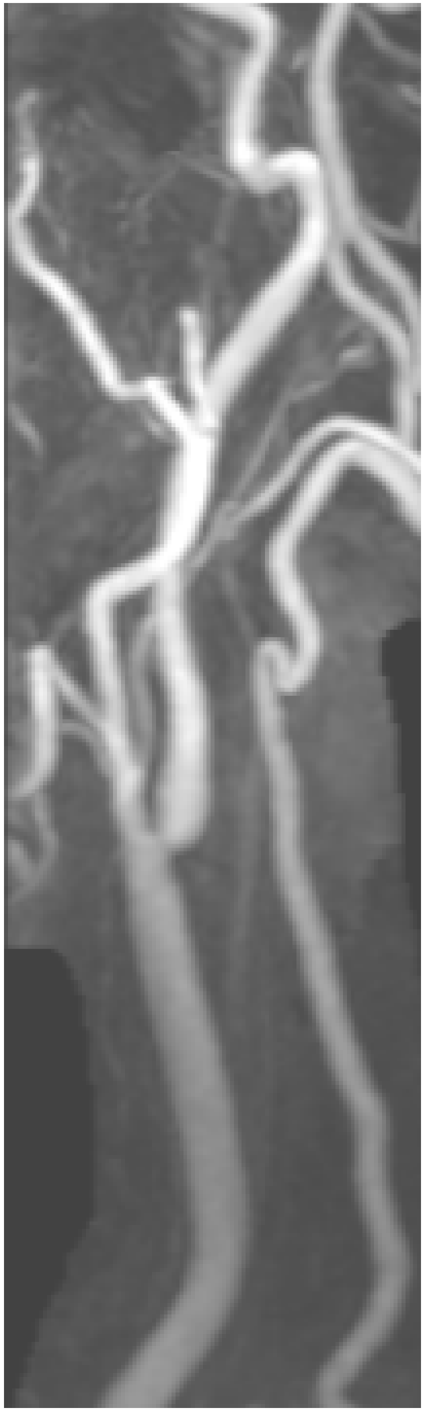

182d Score

0-30

31-50

51-70

>70

Near occlusion

Occluded

Quality

1

2

3

4

5

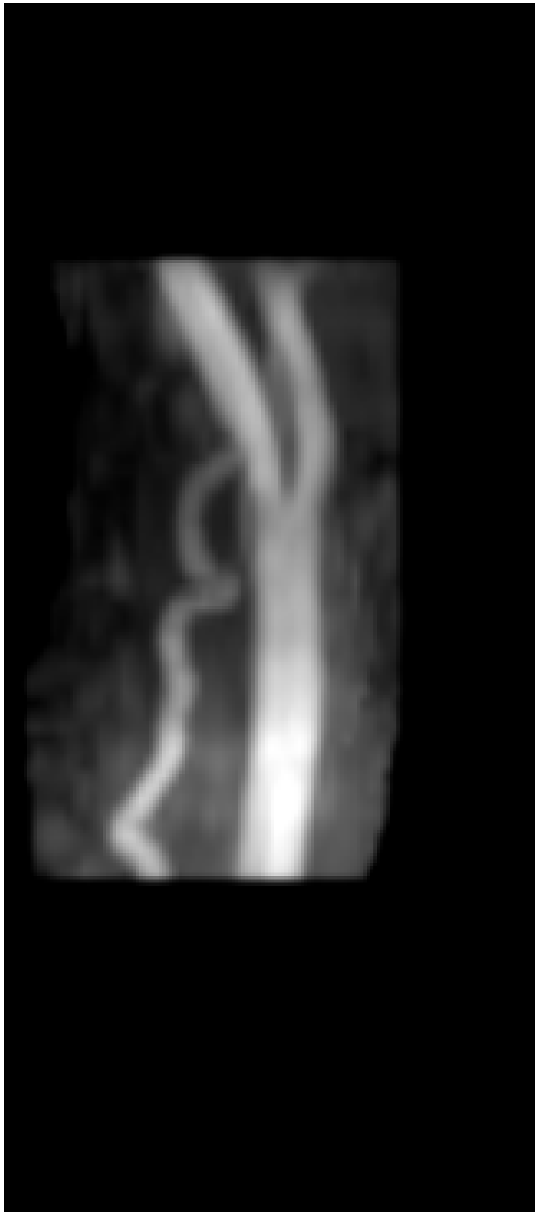

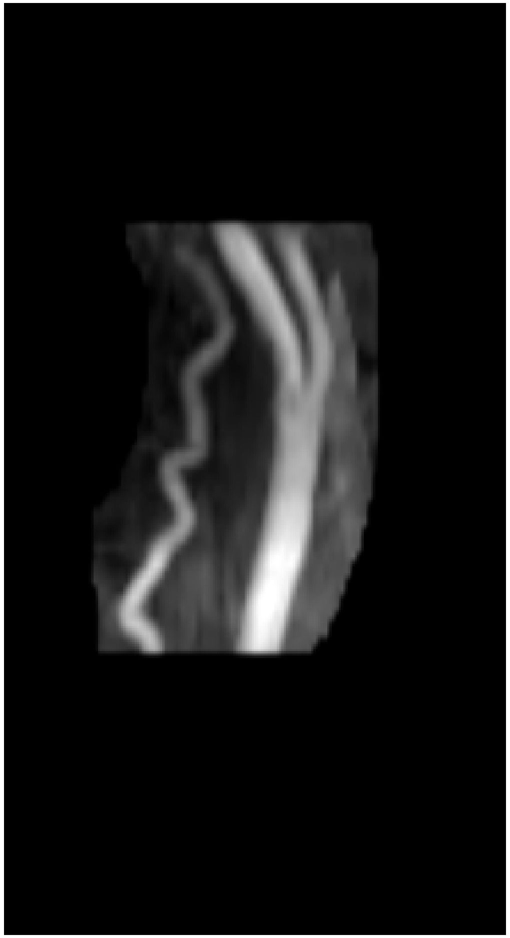

183c Score

0-30

31-50

51-70

>70

Near occlusion

Occluded

Quality

1

2

3

4

5

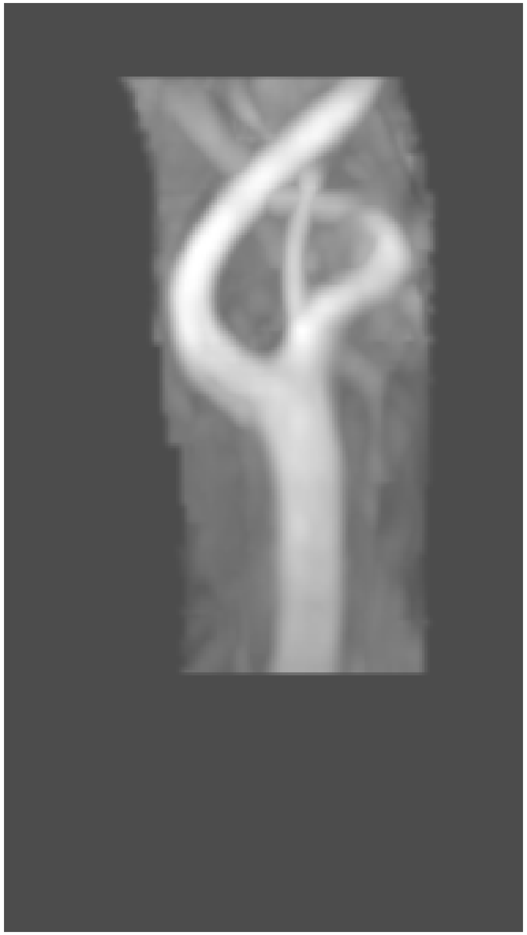

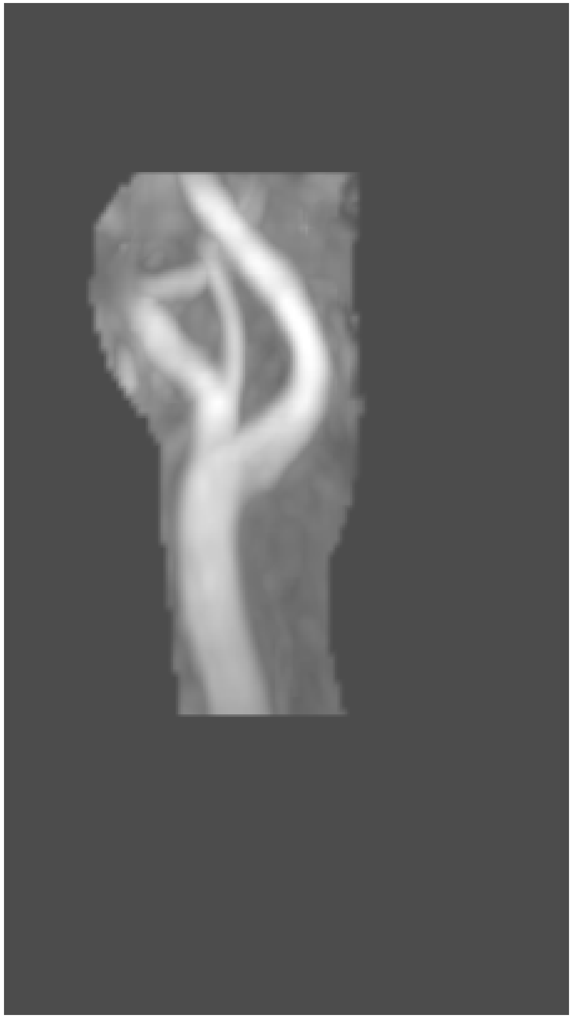

184b Score

0-30

31-50

51-70

>70

Near occlusion

Occluded

Quality

1

2

3

4

5

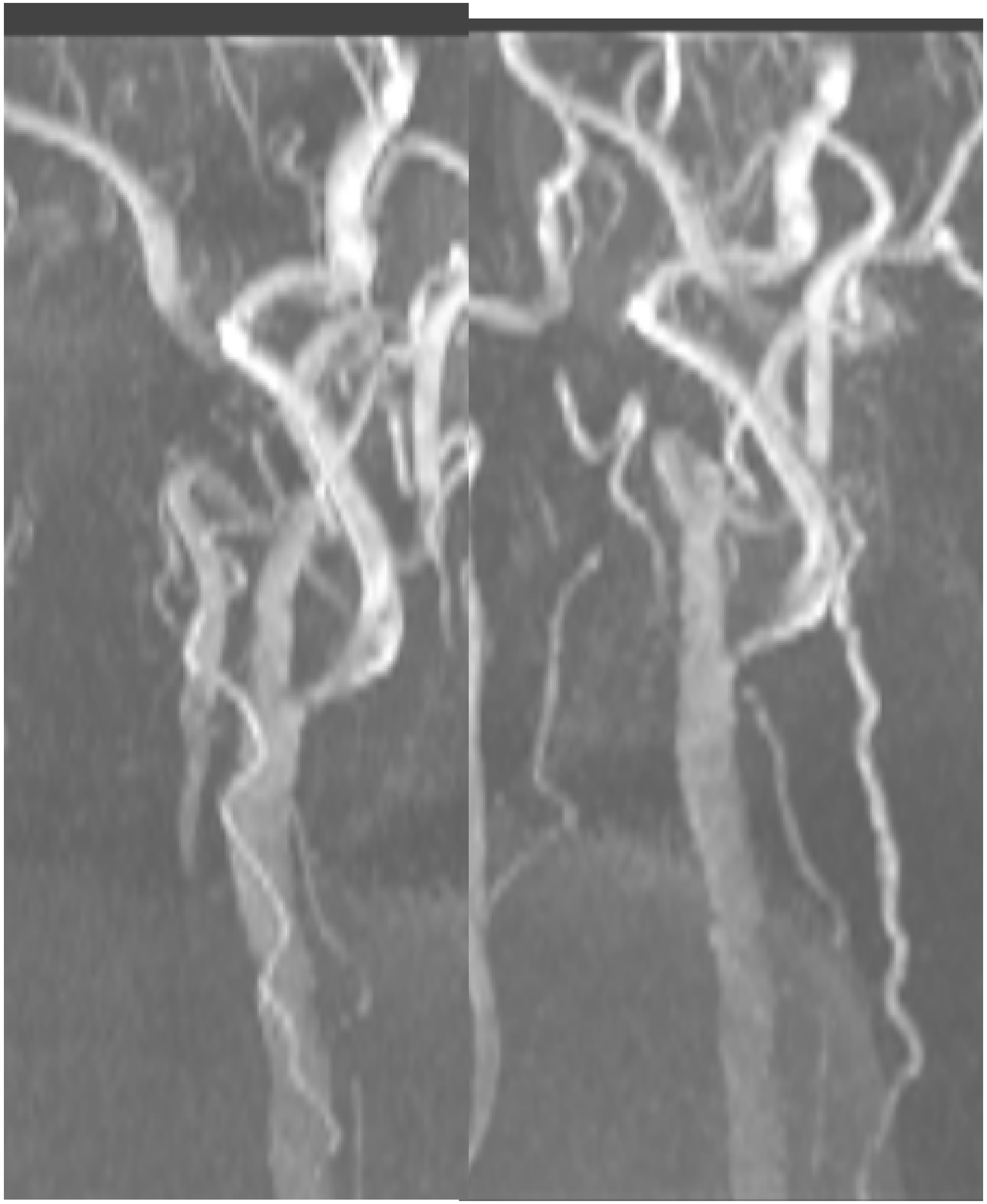

185a Score

0-30

31-50

51-70

>70

Near occlusion

Occluded

Quality

1

2

3

4

5

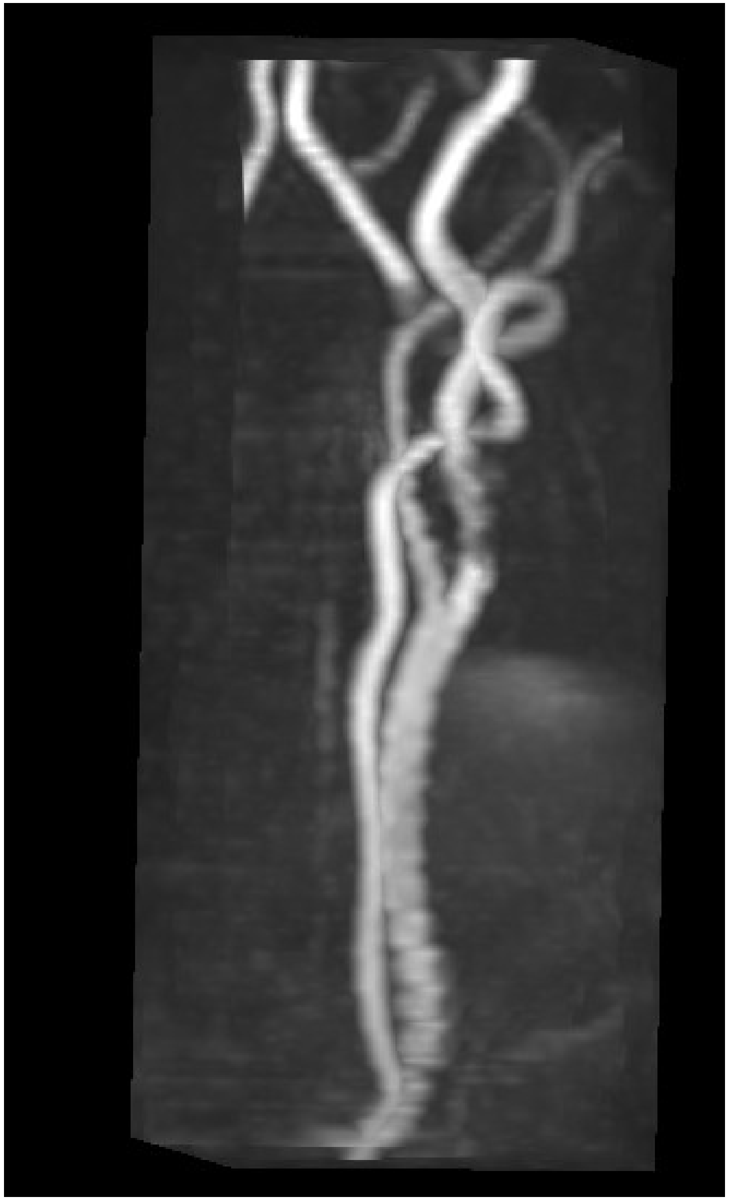

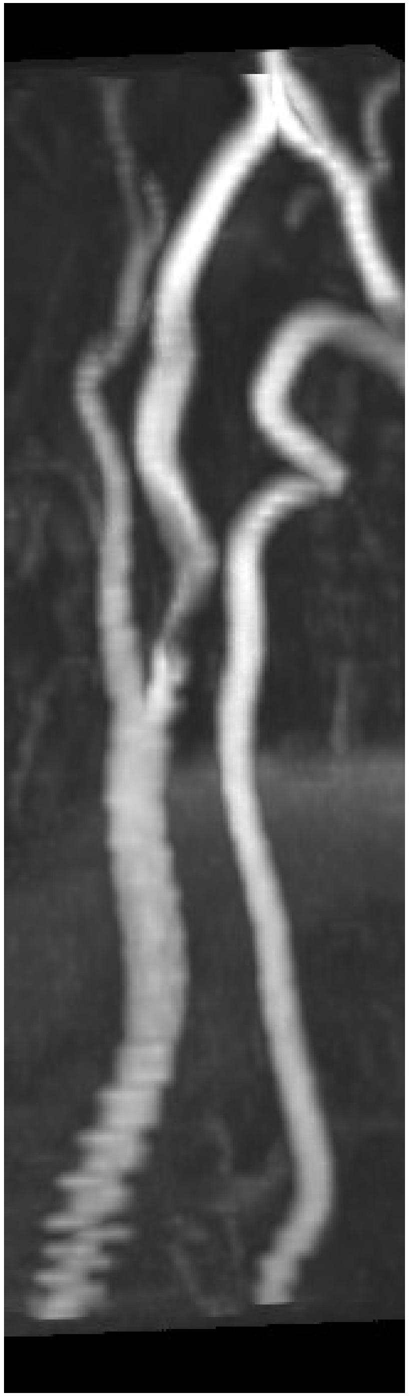

185f Score

0-30

31-50

51-70

>70

Near occlusion

Occluded

Quality

1

2

3

4

5

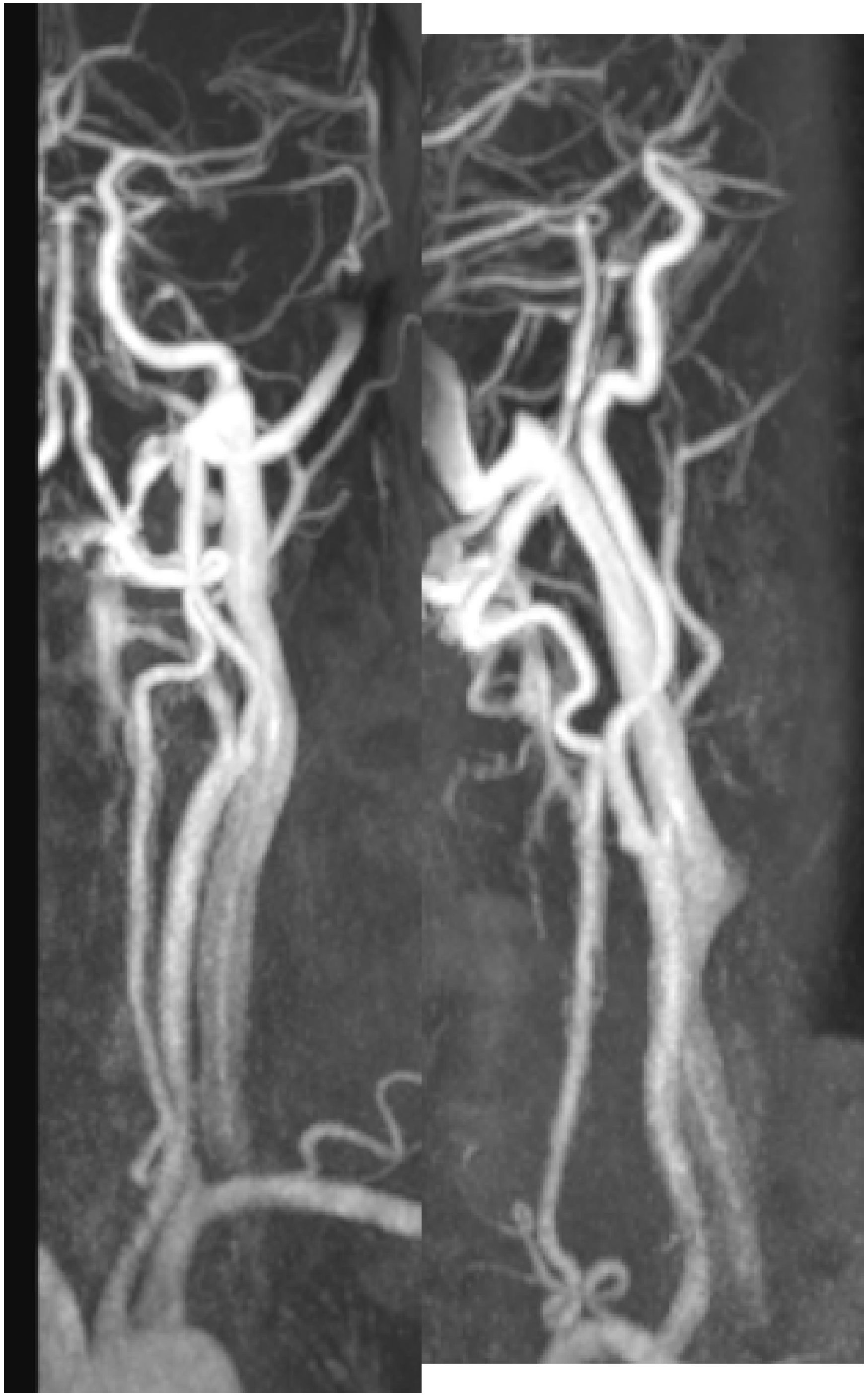

186e Score

0-30

31-50

51-70

>70

Near occlusion

Occluded

Quality

1

2

3

4

5

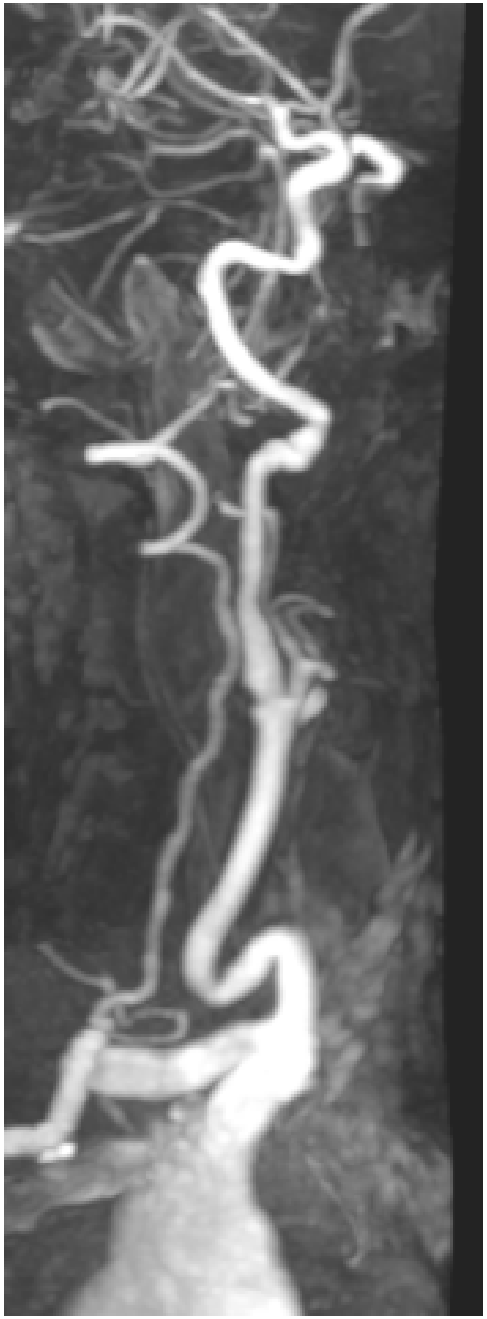

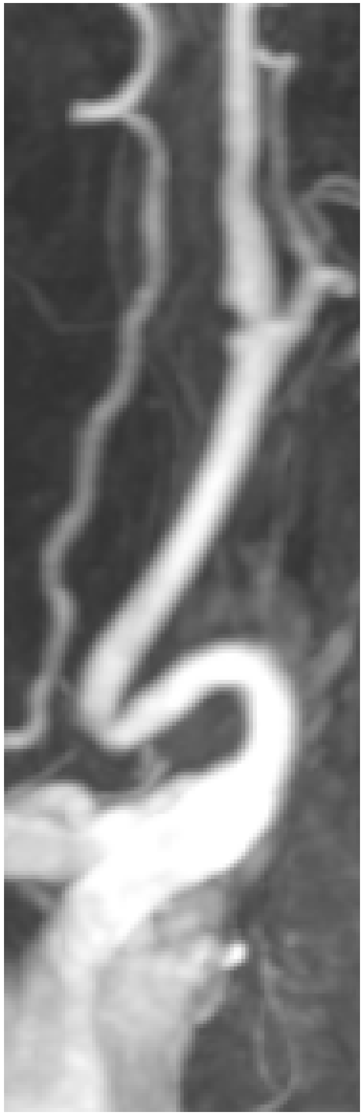

187d Score

0-30

31-50

51-70

>70

Near occlusion

Occluded

Quality

1

2

3

4

5

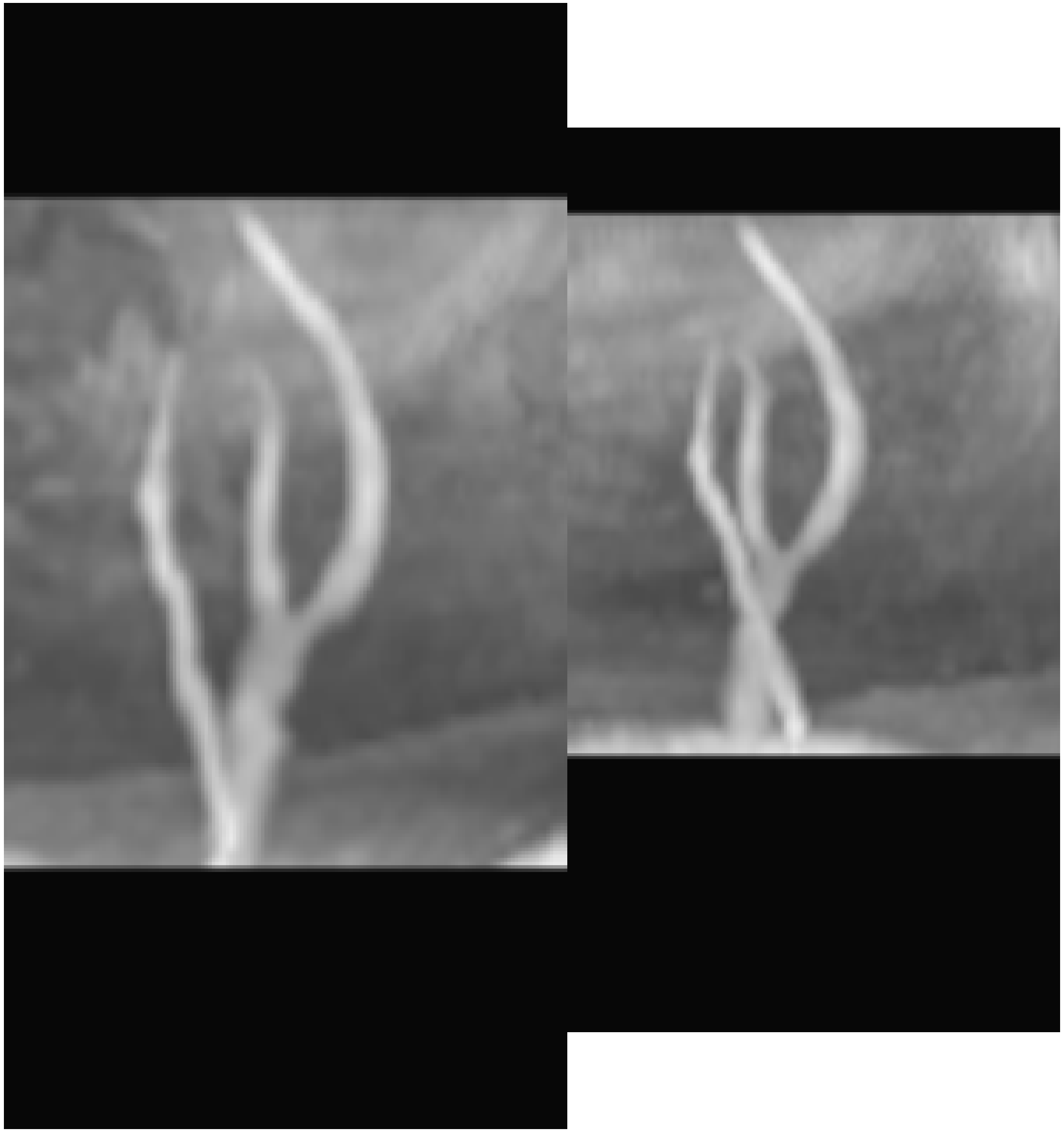

188c Score

0-30

31-50

51-70

>70

Near occlusion

Occluded

Quality

1

2

3

4

5

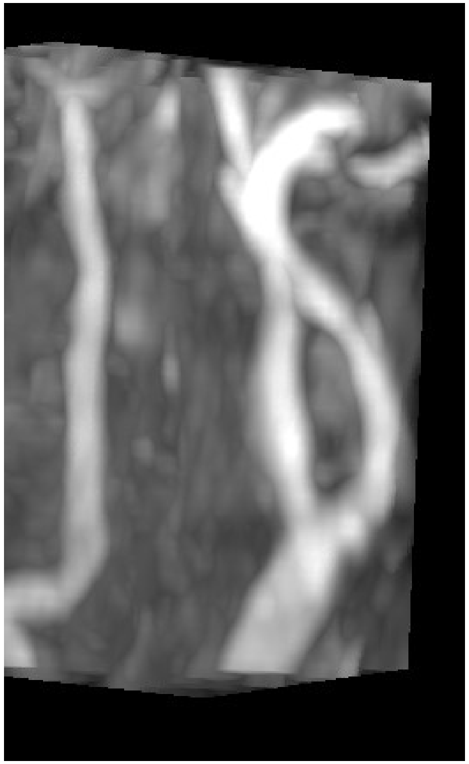

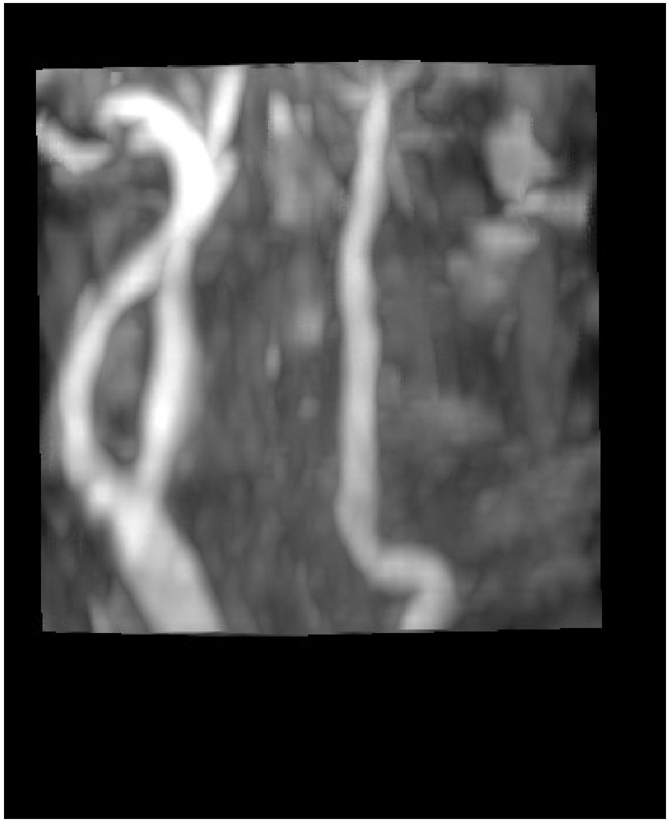

189b Score

0-30

31-50

51-70

>70

Near occlusion

Occluded

Quality

1

2

3

4

5

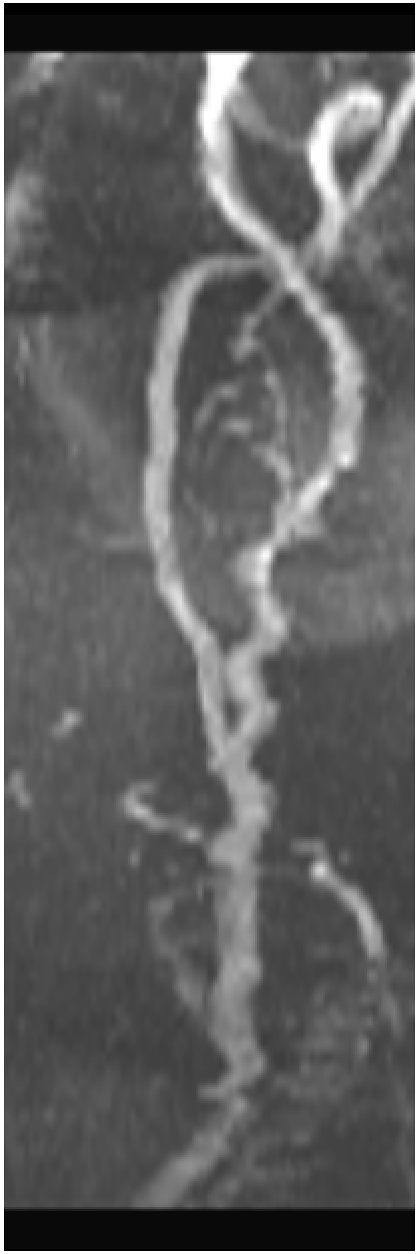

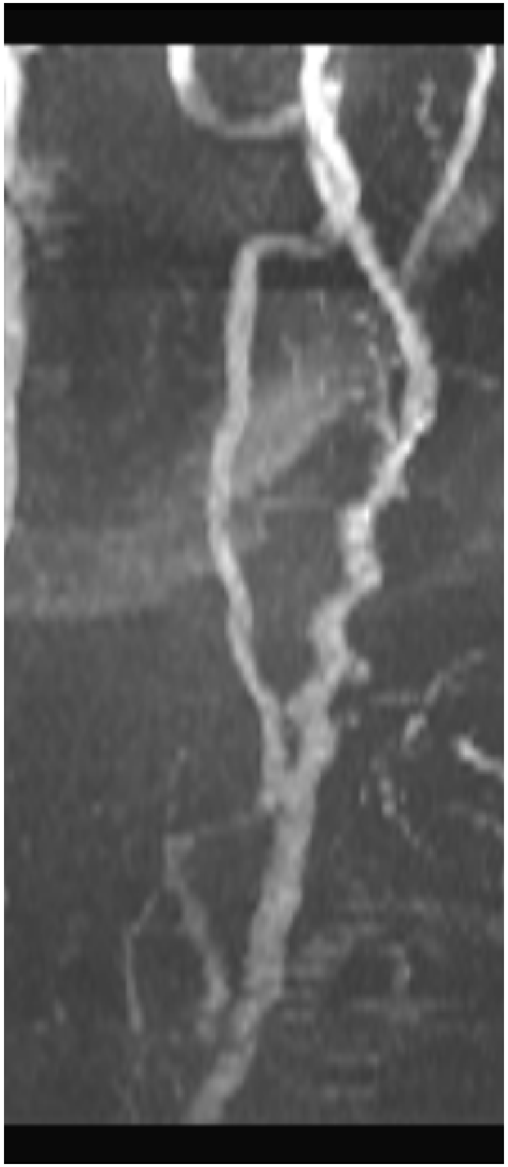

190a Score

0-30

31-50

51-70

>70

Near occlusion

Occluded

Quality

1

2

3

4

5

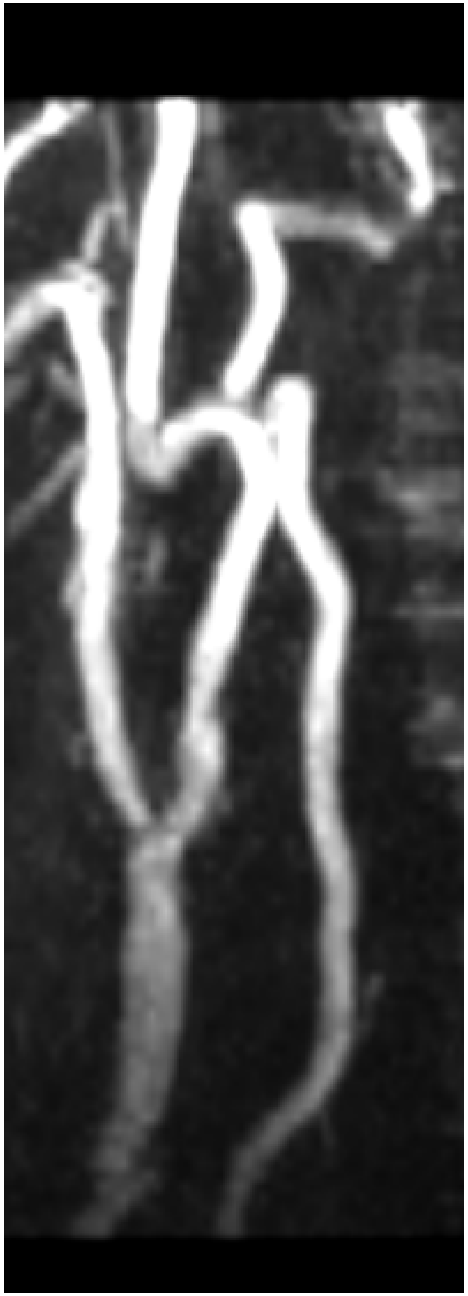

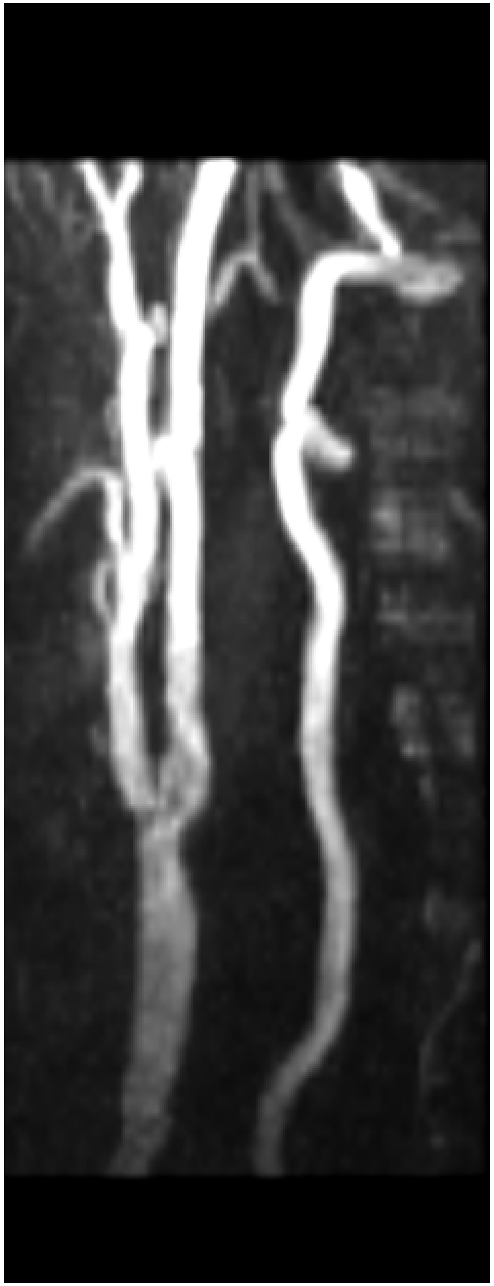

190f Score

0-30

31-50

51-70

>70

Near occlusion

Occluded

Quality

1

2

3

4

5

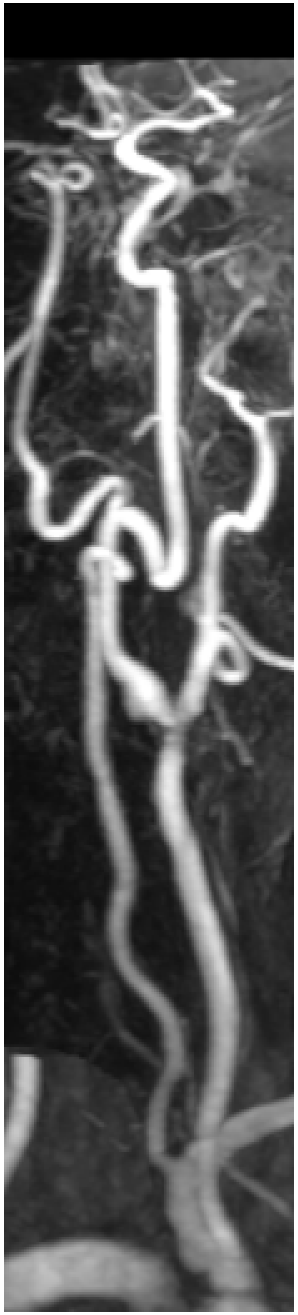

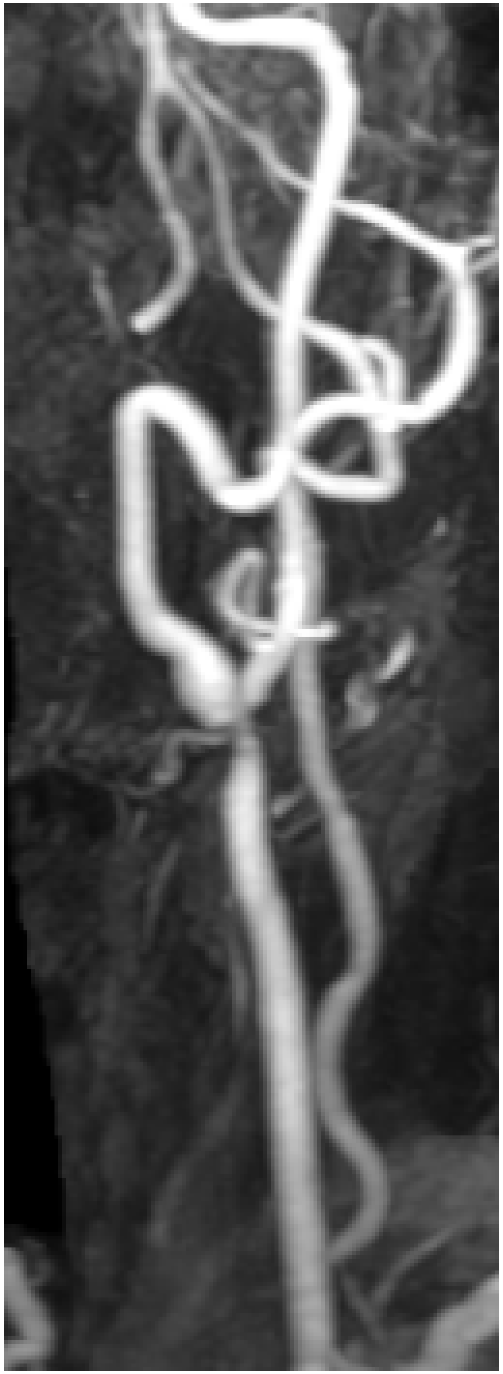

191e Score

0-30

31-50

51-70

>70

Near occlusion

Occluded

Quality

1

2

3

4

5

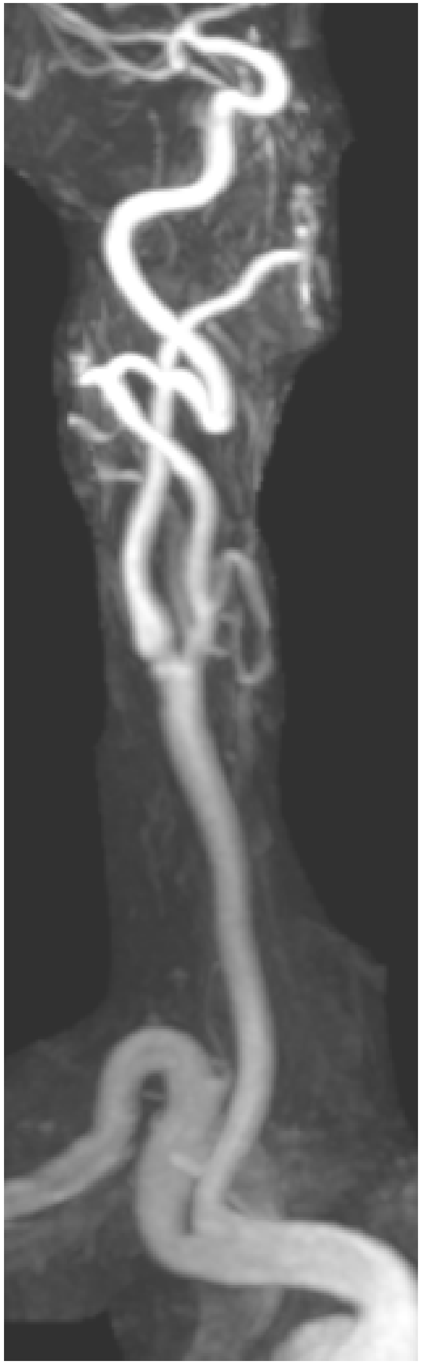

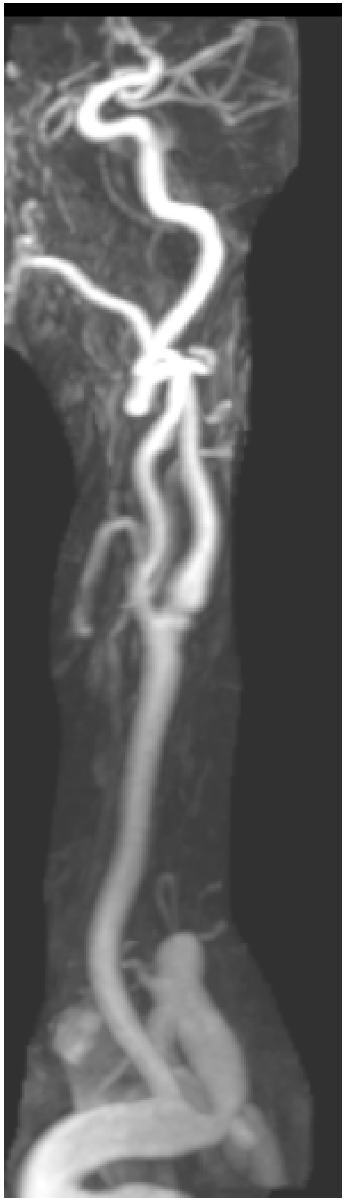

192d Score

0-30

31-50

51-70

>70

Near occlusion

Occluded

Quality

1

2

3

4

5

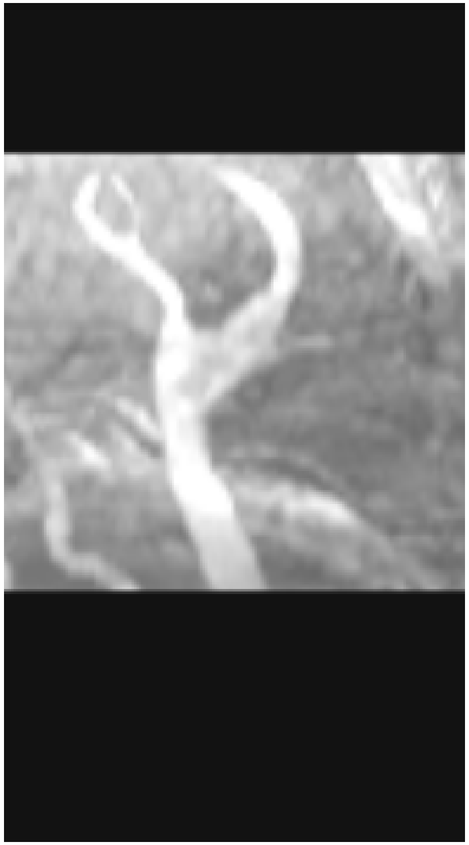

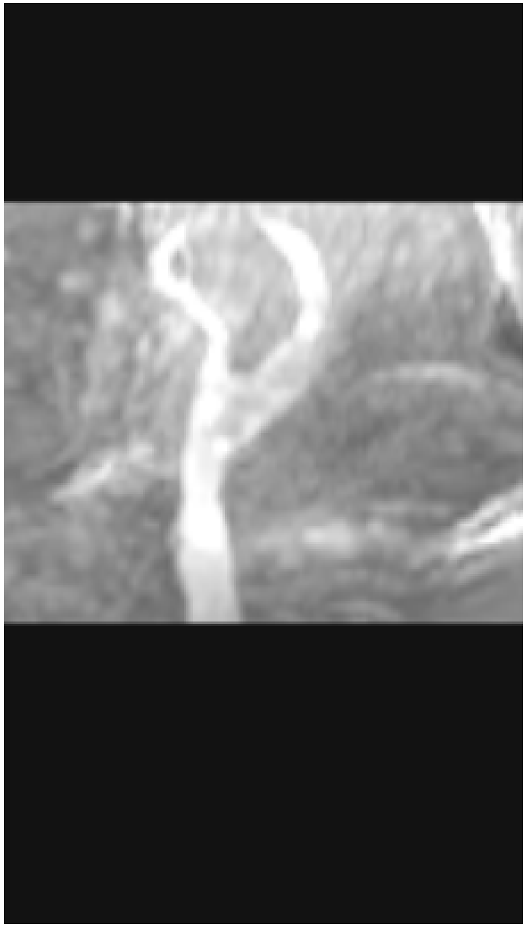

193c Score

0-30

31-50

51-70

>70

Near occlusion

Occluded

Quality

1

2

3

4

5

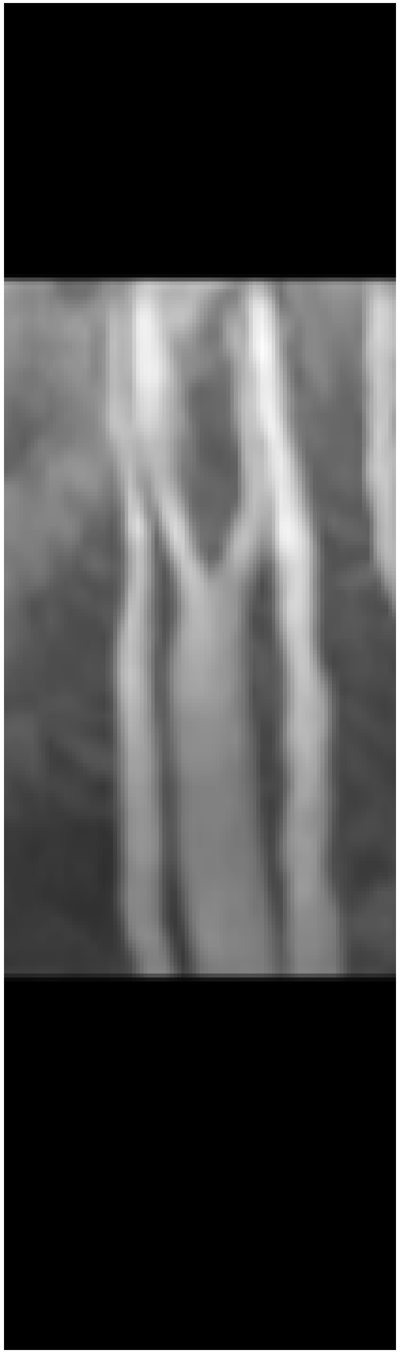

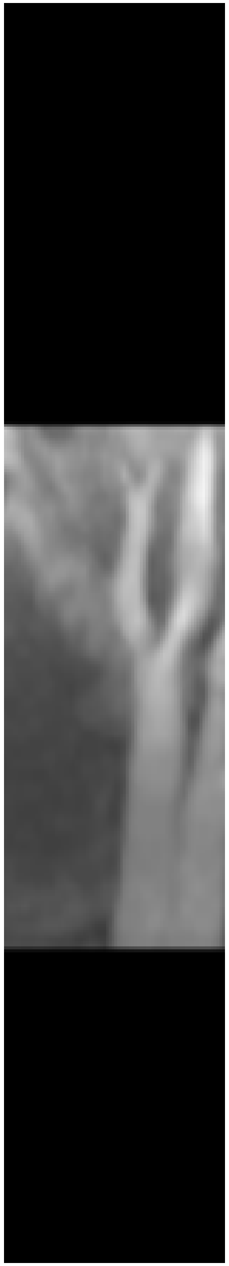

194b Score

0-30

31-50

51-70

>70

Near occlusion

Occluded

Quality

1

2

3

4

5

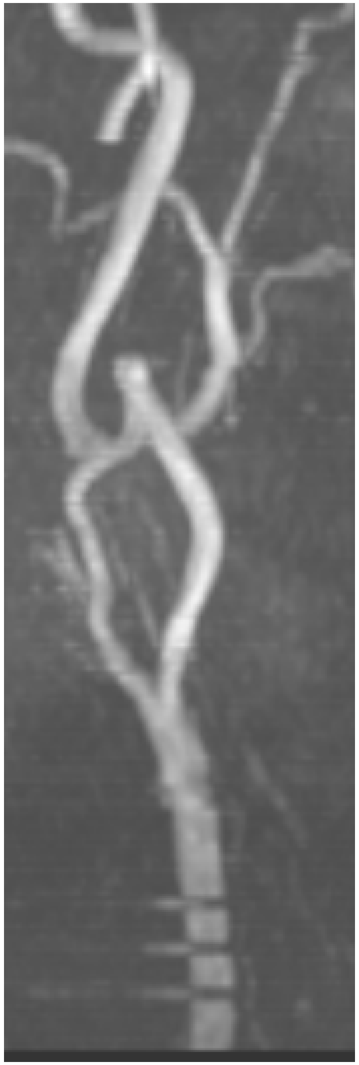

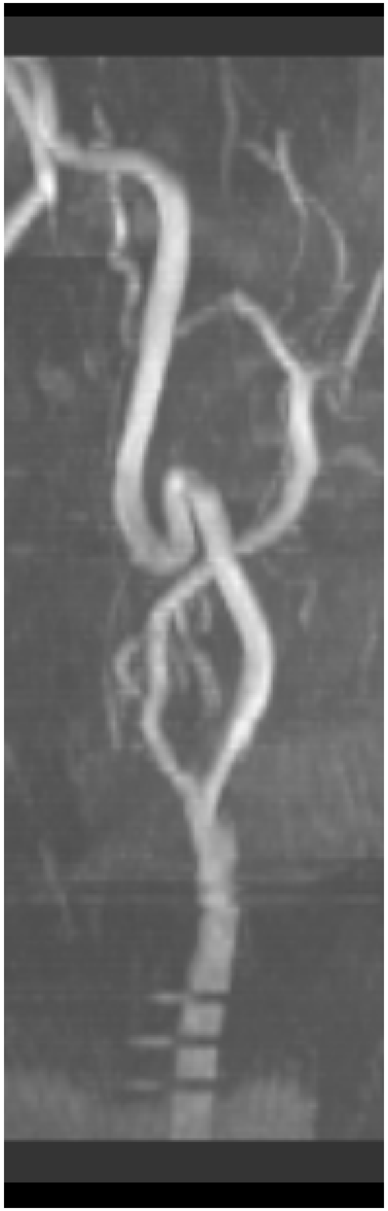

195a Score

0-30

31-50

51-70

>70

Near occlusion

Occluded

Quality

1

2

3

4

5

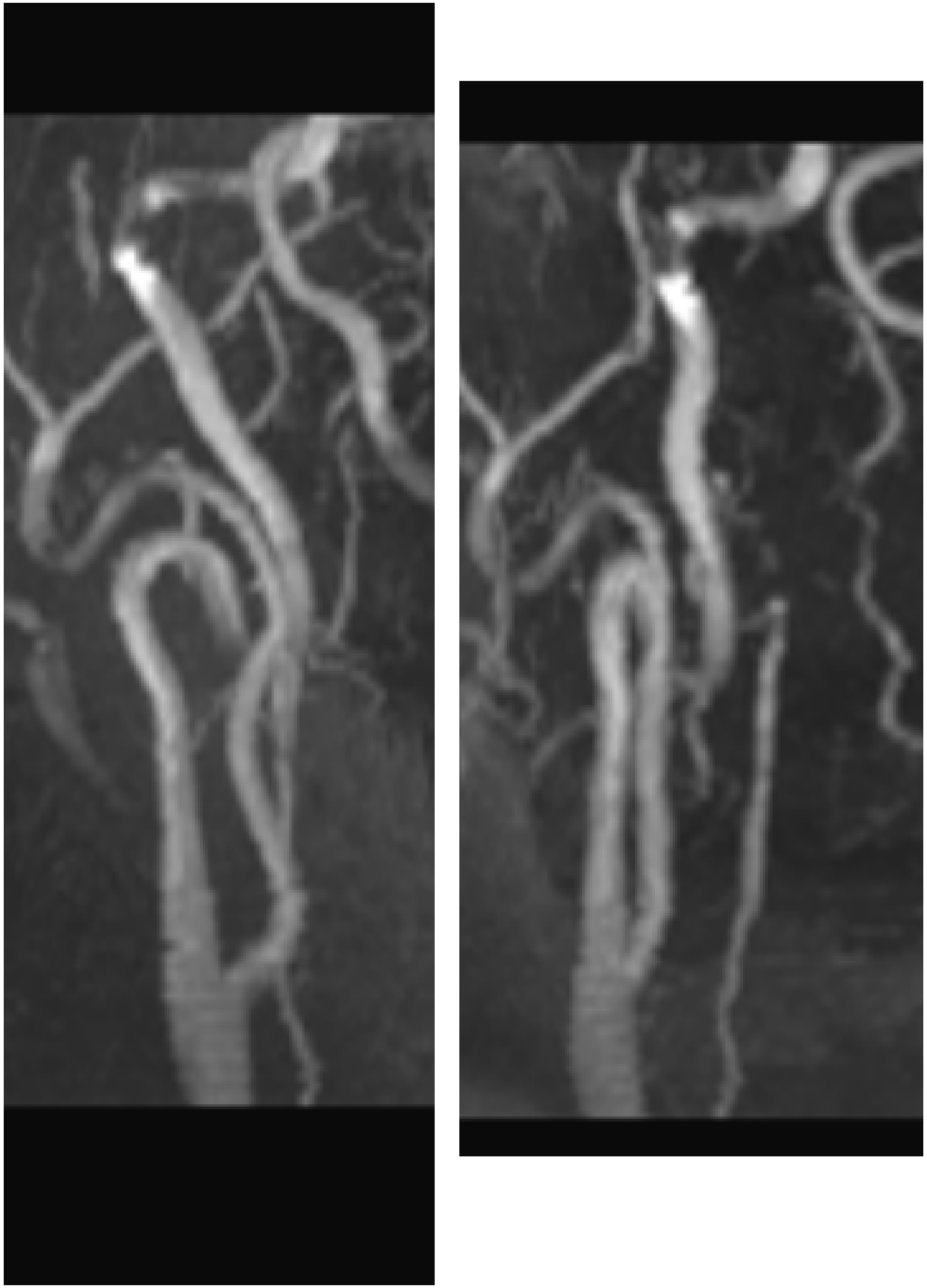

195f Score

0-30

31-50

51-70

>70

Near occlusion

Occluded

Quality

1

2

3

4

5

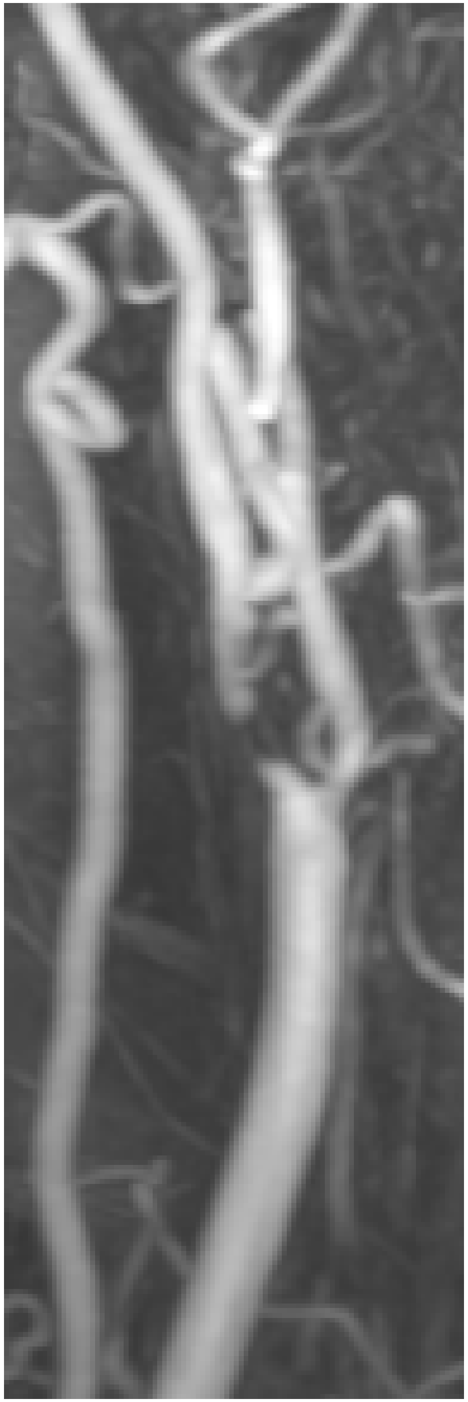

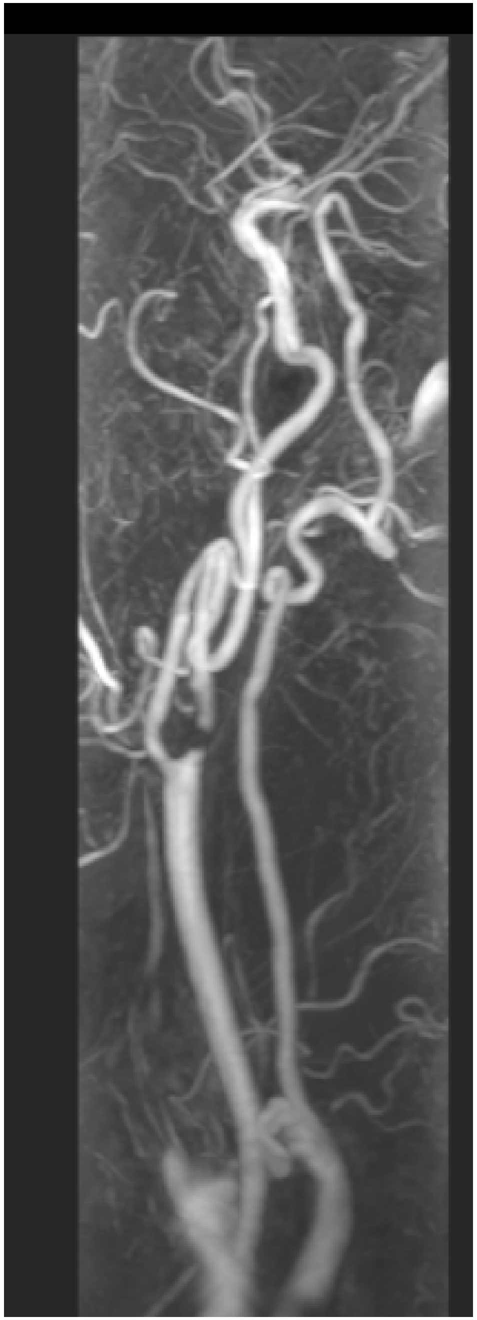

196e Score

0-30

31-50

51-70

>70

Near occlusion

Occluded

Quality

1

2

3

4

5

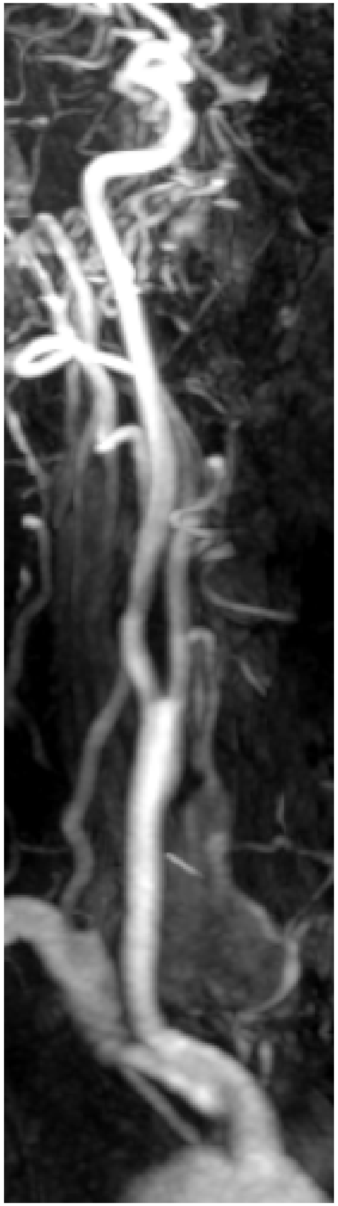

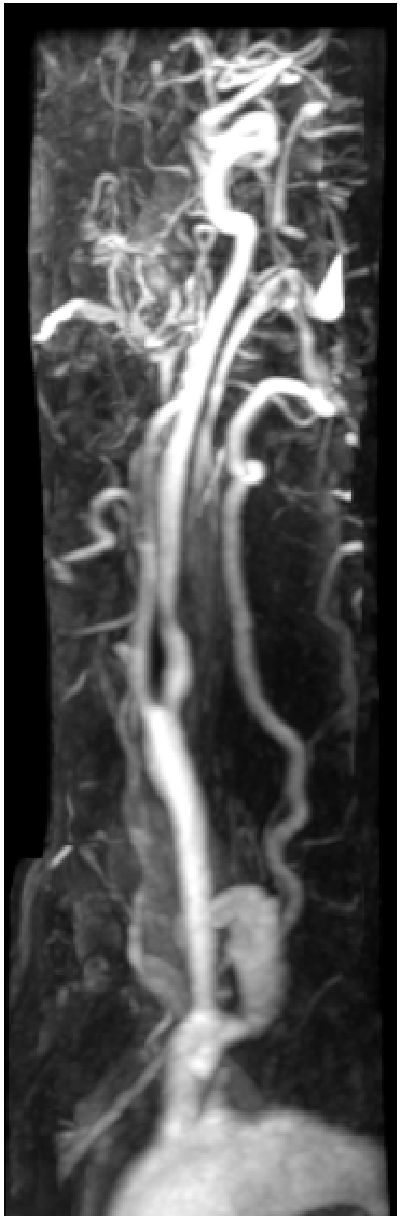

197d Score

0-30

31-50

51-70

>70

Near occlusion

Occluded

Quality

1

2

3

4

5

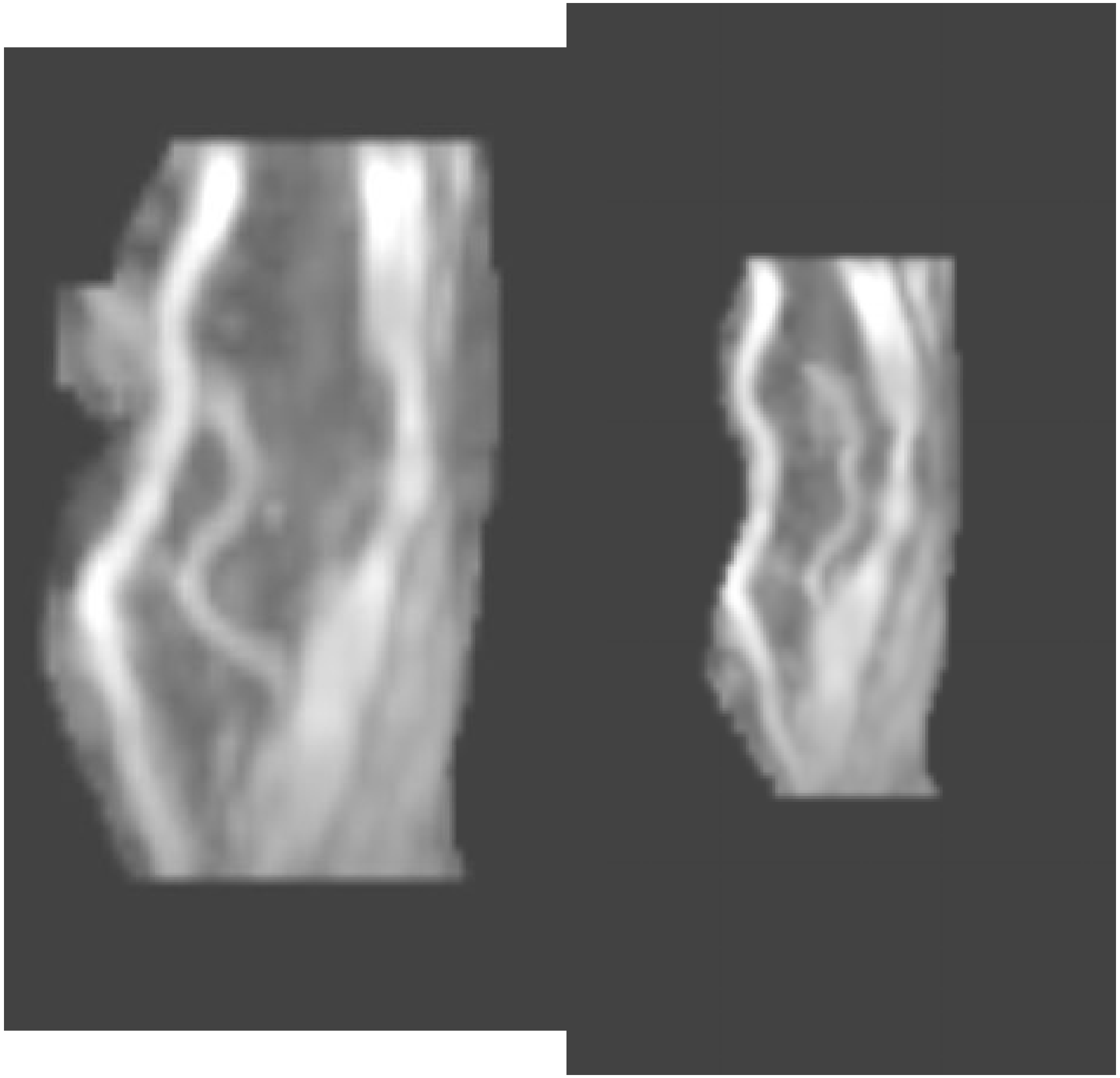

198c Score

0-30

31-50

51-70

>70

Near occlusion

Occluded

Quality

1

2

3

4

5

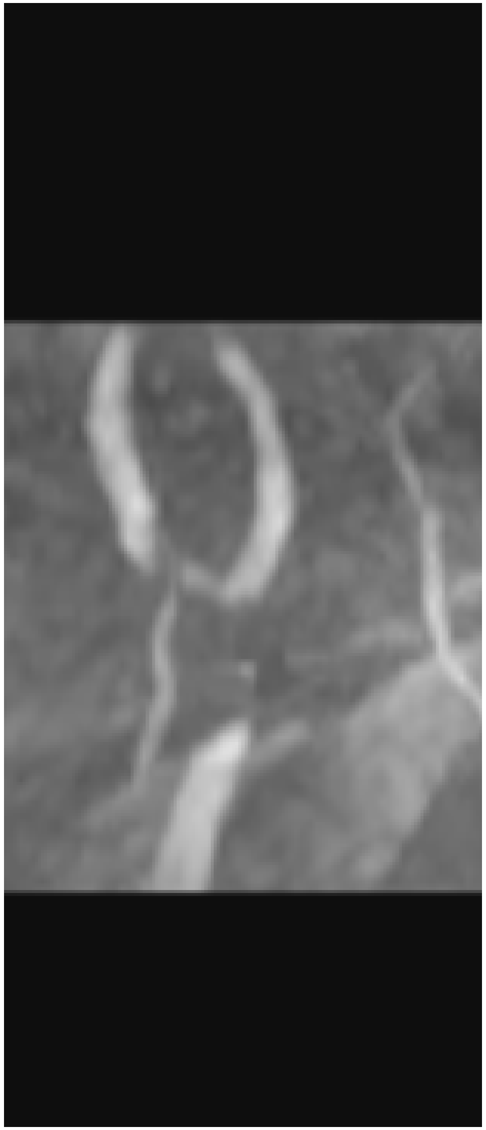

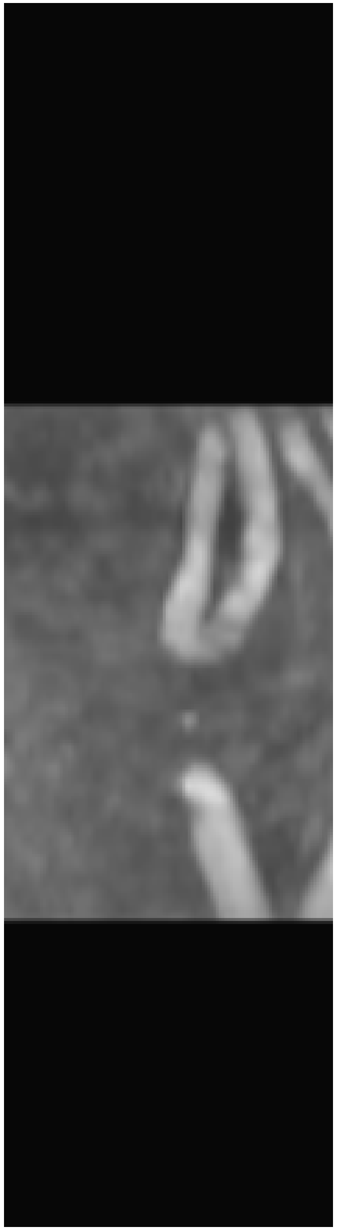

199b Score

0-30

31-50

51-70

>70

Near occlusion

Occluded

Quality

1

2

3

4

5

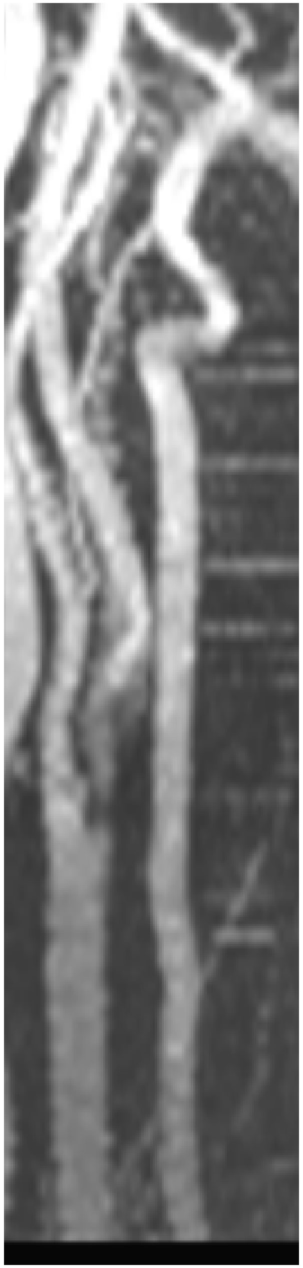

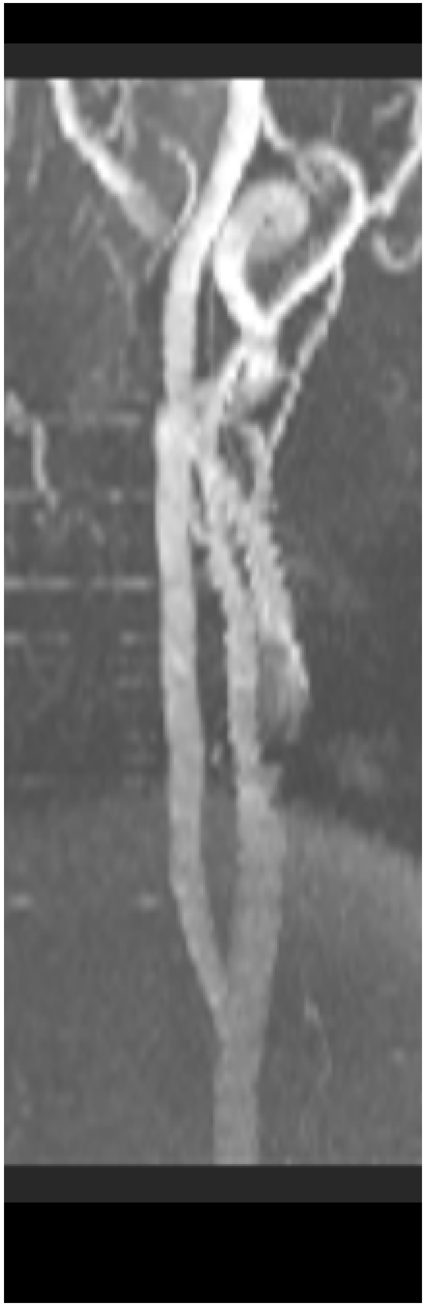

200a Score

0-30

31-50

51-70

>70

Near occlusion

Occluded

Quality

1

2

3

4

5

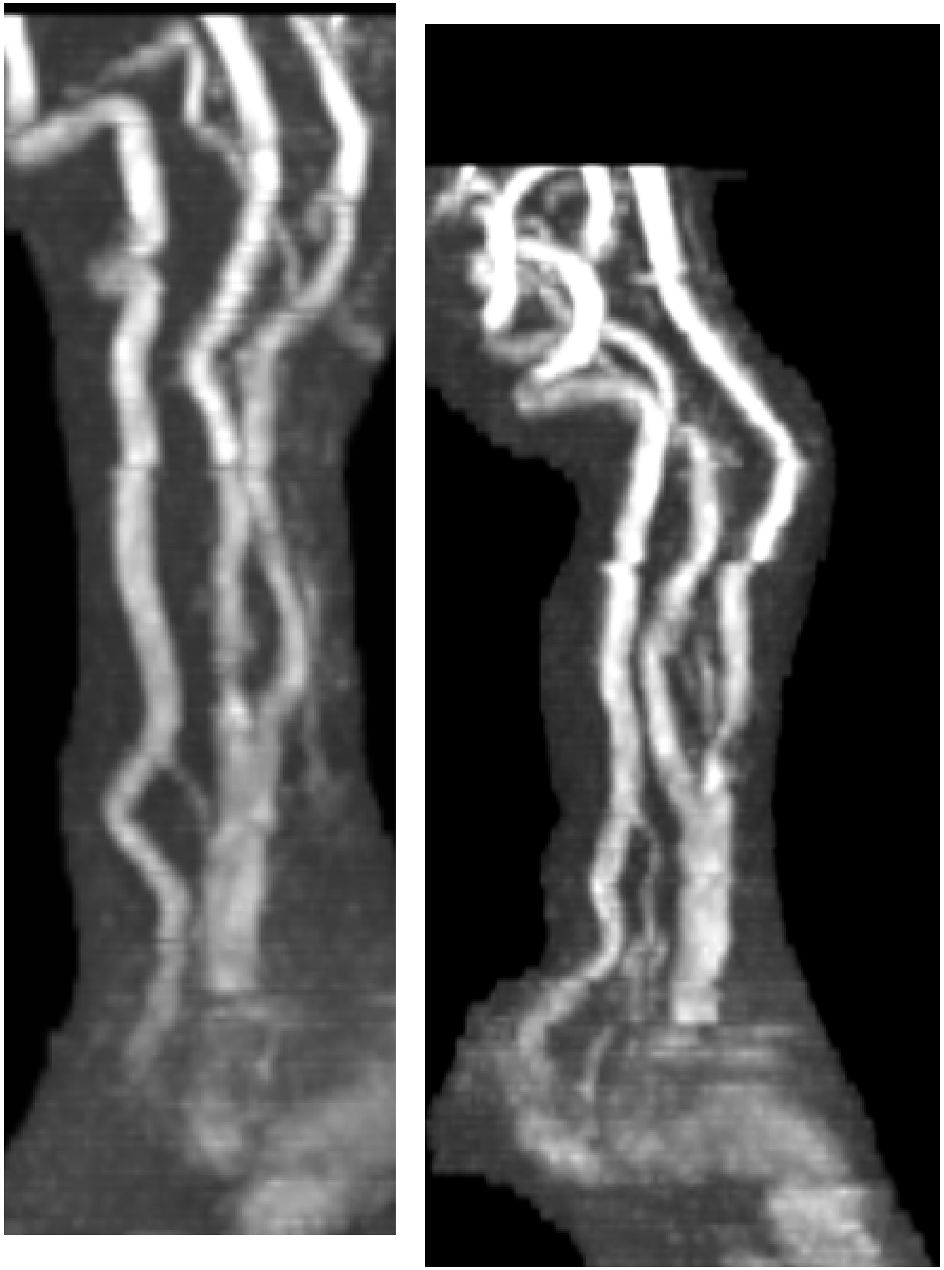

200f Score

0-30

31-50

51-70

>70

Near occlusion

Occluded

Quality

1

2

3

4

5

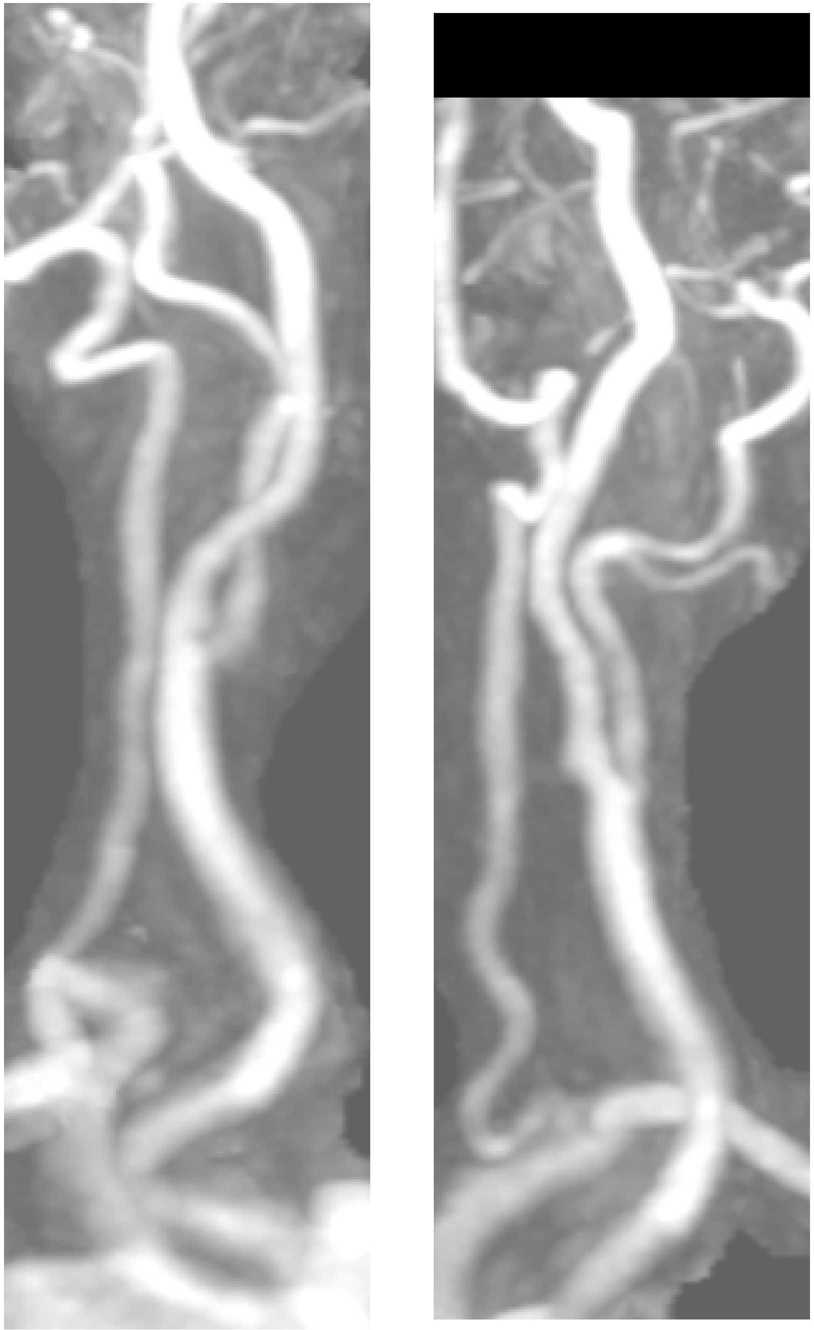

Supplement: S5 File — (PDF) [file pone.0237856.s007.pdf]
